# Supplementary material for: Importance of natural land cover for plant species’ conservation: A nationwide study in The Netherlands
Source: PLoS One. 2021 Nov 16;16(11):e0259255. doi: 10.1371/journal.pone.0259255 (PMC8594855; doi:10.1371/journal.pone.0259255)
Supplement: S1 Text — (DOCX) [file pone.0259255.s001.docx]

**S1 text**

**1. Sensitivity analysis for removing grids with more than 10% open water**

Since the land cover of open water does not support vascular plants, including cells with large amounts of open water may introduce a bias due to the species-area relationship. We therefore omitted cells with more than 10% open water, following as did [1,2]. We further used 850 species to check the effect of this omission on the occurrence frequency of species, which represents the occurrence probability of species to some extent, based on grid cells with land cover more than 90% and land cover equalling 100% (Equation 1-2). Results were shown in S1 Fig. Our sensitivity analysis showed that the average occurrence frequency from grid cells with land cover more than 90% are almost highly 1:1 linearly correlated to occurrence probability from grid cells with land cover equalling 100%. Based on sensitivity analysis and references above, we decided grid cells with more than 90% land cover are acceptable.

The average occurrence frequency of each species (${AOF}_{ij}$) either based on either grid cells with land cover more than 90% or land cover equalling 100% was calculated by

                                                               ${OF}_{ij}=N_{ij}/N_{i}$ Equation 1

${AOF}_{ij}=\sum_{i=0}^{i=n<=100} {OF}_{ij}/n$ Equation 2

Where, $N_{ij}$ is the number of grids with natural land cover (NLC) equalling *i* and occupied by species *j* and $N_{i}$ is the number of grids with NLC equalling *i*. ${OF}_{ij}$is the occurrence frequency of species *j* in all grids with NLC equalling *i*.

**2. Calculation of plant species richness responding to NLC**

*Predicted richness~NLC relationships.* Based on the response curve, we predicted the richness~NLC relationship. First, for each NLC, we calculated the number of species with occurrence probability above 80% and took it as the richness corresponding to the NLC. Further, we gave four different fitting models (null, linear, quadratic, exponential) and used AIC to choose the best fitting model with a significant *p*-value. Finally, the quadratic model was the best fitting model with a significant *p*-value.

*Richness~NLC relationships based on real observations.* First, we overlaid species occurrence data with grid cells with different NLCs. Then, we calculated the number of species occurring in each grid and analyzed the relationships between richness and NLC.

**3. Average marginal occurrence probability increase**

The average marginal occurrence probability change of all plant species with 1% NLC increase was calculated by

${OP}_{i}={\sum_{j=1}^{j=n} ({OP}_{ij}-{OP}_{(i-1)j})}/n$ Equation 4.

where ${OP}_{i}$ is the average marginal occurrence probability change, ${OP}_{ij}$ is the occurrence probability of species *j* at NLC *i*%, ${OP}_{(i-1)j}$ is the occurrence probability of species *j* at NLC (*i*-1)%. *n* is the number of species.

References

**1**. Rachelle E. Desrochers, Jeremy T. Kerr, David J. Currie. How, and how much, natural cover loss increases species richness. Global Ecology and Biogeography. 2011; 20:857–67. doi: 10.1111/j.1466-8238.2011.00658.x.

**2**. Clark CM, Simkin SM, Allen EB, Bowman WD, Belnap J, Brooks ML, et al. Potential vulnerability of 348 herbaceous species to atmospheric deposition of nitrogen and sulfur in the United States. Nat Plants. 2019; 5:697–705. doi: 10.1038/s41477-019-0442-8 PMID: 31263243.
