# Supplementary material for: Importance of natural land cover for plant species’ conservation: A nationwide study in The Netherlands
Source: PLoS One. 2021 Nov 16;16(11):e0259255. doi: 10.1371/journal.pone.0259255 (PMC8594855; doi:10.1371/journal.pone.0259255)
Supplement: S1 Table — (DOCX) [file pone.0259255.s002.docx]

**S1 Table. Land cover types in this research.**

| Rank | Classes in Marshall [1] | Merged classes in our study | |
| --- | --- | --- | --- |
|  |  | Natural/non-natural area | Natural forest/open |
| 1 | Heather | Natural area | Natural open |
| 2 | Urban green | Natural area | Natural open |
| 3 | Semi-natural forest | Natural area | Natural forest |
| 4 | Production forest | Natural area | Natural forest |
| 5 | Swamp peat | Natural area | Natural open |
| 6 | Semi-natural grassland | Natural area | Natural open |
| 7 | Dune | Natural area | Natural open |
| 8 | River swamp | Natural area | Natural open |
| 9 | Crop | Non-natural area |  |
| 10 | Fruit | Non-natural area |  |
| 11 | Pasture | Non-natural area |  |
| 12 | Urban grey | Non-natural area |  |
| 13 | Greenhouse horticulture | Non-natural area |  |
| 14 | Other agricultural uses | Non-natural area |  |
| 15 | Fresh water | exclude |  |
| 16 | Brackish water | exclude |  |

References

**1**. Marshall L. Wild bee diversity across space and time: the role of land use/land cover and climate. Presses universitaires de Namur; 2018.
