# Supplementary material for: Importance of natural land cover for plant species’ conservation: A nationwide study in The Netherlands
Source: PLoS One. 2021 Nov 16;16(11):e0259255. doi: 10.1371/journal.pone.0259255 (PMC8594855; doi:10.1371/journal.pone.0259255)
Supplement: S3 Table — (DOCX) [file pone.0259255.s004.docx]

**S3 Table. PCA for 19 bioclimatic variables.** The first five principal components explained more than 90% of the variability in the full suite of 19 bioclimatic variables.

|  | Variance | % of var. | Cumulative % of var. |
| --- | --- | --- | --- |
| Dim.1 | 8 | 42.1 | 42.1 |
| Dim.2 | 5.3 | 28.1 | 70.1 |
| Dim.3 | 2.4 | 12.9 | 83 |
| Dim.4 | 1.2 | 6.4 | 89.4 |
| Dim.5 | 0.7 | 3.9 | 93.3 |
| Dim.6 | 0.4 | 2 | 95.3 |
| Dim.7 | 0.3 | 1.7 | 97 |
| Dim.8 | 0.2 | 1 | 98 |
| Dim.9 | 0.1 | 0.7 | 98.7 |
| Dim.10 | 0.1 | 0.5 | 99.2 |
| Dim.11 | 0.1 | 0.3 | 99.5 |
| Dim.12 | 0 | 0.1 | 99.7 |
| Dim.13 | 0 | 0.1 | 99.8 |
| Dim.14 | 0 | 0.1 | 99.9 |
| Dim.15 | 0 | 0 | 99.9 |
| Dim.16 | 0 | 0 | 100 |
| Dim.17 | 0 | 0 | 100 |
| Dim.18 | 0 | 0 | 100 |
| Dim.19 | 0 | 0 | 100 |
