# Supplementary material for: Importance of natural land cover for plant species’ conservation: A nationwide study in The Netherlands
Source: PLoS One. 2021 Nov 16;16(11):e0259255. doi: 10.1371/journal.pone.0259255 (PMC8594855; doi:10.1371/journal.pone.0259255)
Supplement: S1 Fig — Each point indicates one plant species. The x axis means the average occurrence frequency based on all grid cells with 100% land cover. The y axis means the average occurrence frequency based on all grid cells with more than 90% land cover. (DOCX) [file pone.0259255.s006.docx]

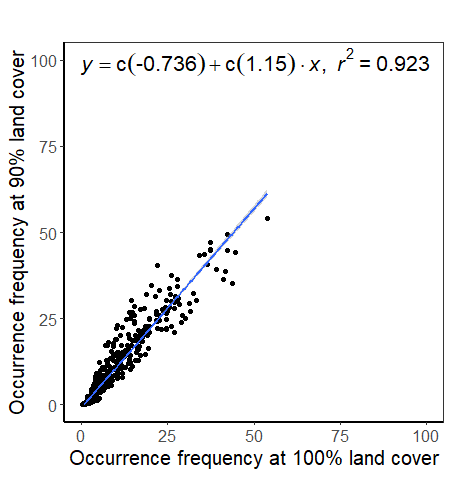


**S1 Fig. The average occurrence frequency based on grid cells with more than 90% land cover vs grid cells with 100% land cover.** Each point indicates one plant species. The x axis means the average occurrence frequency based on all grid cells with 100% land cover. The y axis means the average occurrence frequency based on all grid cells with more than 90% land cover.
