# Supplementary figures and images for: Importance of natural land cover for plant species’ conservation: A nationwide study in The Netherlands
Source: PLoS One. 2021 Nov 16;16(11):e0259255. doi: 10.1371/journal.pone.0259255 (PMC8594855; doi:10.1371/journal.pone.0259255)

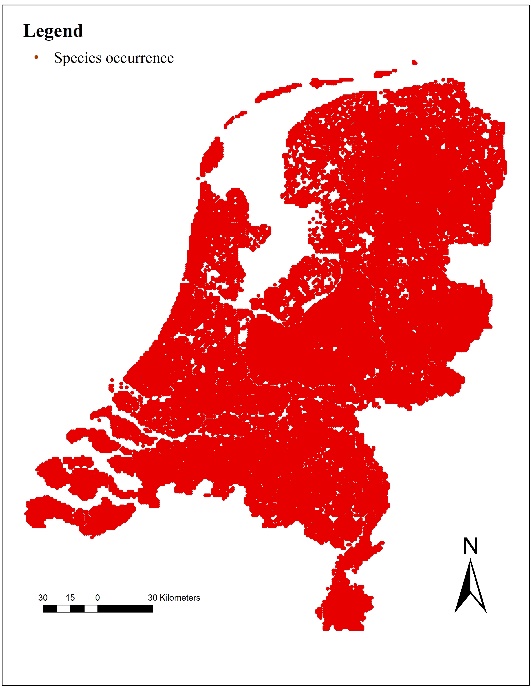


**S2 Fig. 4 773 313 observations of plant species.**

Supplement: S2 Fig — (DOCX) [file pone.0259255.s007.docx]
