# Supplementary material for: Importance of natural land cover for plant species’ conservation: A nationwide study in The Netherlands
Source: PLoS One. 2021 Nov 16;16(11):e0259255. doi: 10.1371/journal.pone.0259255 (PMC8594855; doi:10.1371/journal.pone.0259255)
Supplement: S4 Fig — 2 species with a significantly neutral (without a relationship, named ‘None’) response were omitted as not preferring any NLC. a-h, Response curves of plant species to NLC. k-l, Summary statistics of preferred NLCs. a, Summary of all 230 threatened plants responding to NLC (with percentages in parentheses). b-d, Response curves of species with increasing (each red line indicates one species) (b), unimodal (each blue line indicates one species) (c) and decreasing (each green line indicates one species) (d) relations with NLC. The dark black line is the average response curve of each species group. The yellow line indicates the standardized proportion of grids with different NLCs in the Netherlands. Both the occurrence probability and the standardized proportion (percentage) range from 0% to 100% and are indicated by the y axis. e-h, Species with unimodal shapes are split into four categories based on their preferred NLCs (e, species with preferred NLCs ranging from 0–25%; f, species with preferred NLCs ranging from 25–50%; g, species with preferred NLCs ranging from 50–75%; h, species with preferred NLCs ranging from 75–100%). i-j, Statistics of preferred NLCs of all 230 threatened plant species (i, summary of 230 plant species’ preferred NLCs; j, percentage of species in different categories, including 0%, 0–25%, 25–50%, 50–75%, 75–100% and 100%). k-l, Statistics of preferred NLCs of species with unimodal shapes (k, summary of plant species’ preferred NLCs; l, histogram of preferred NLCs of plant species with unimodal relations with NLC). All mean values are means ± SE. (DOCX) [file pone.0259255.s009.docx]

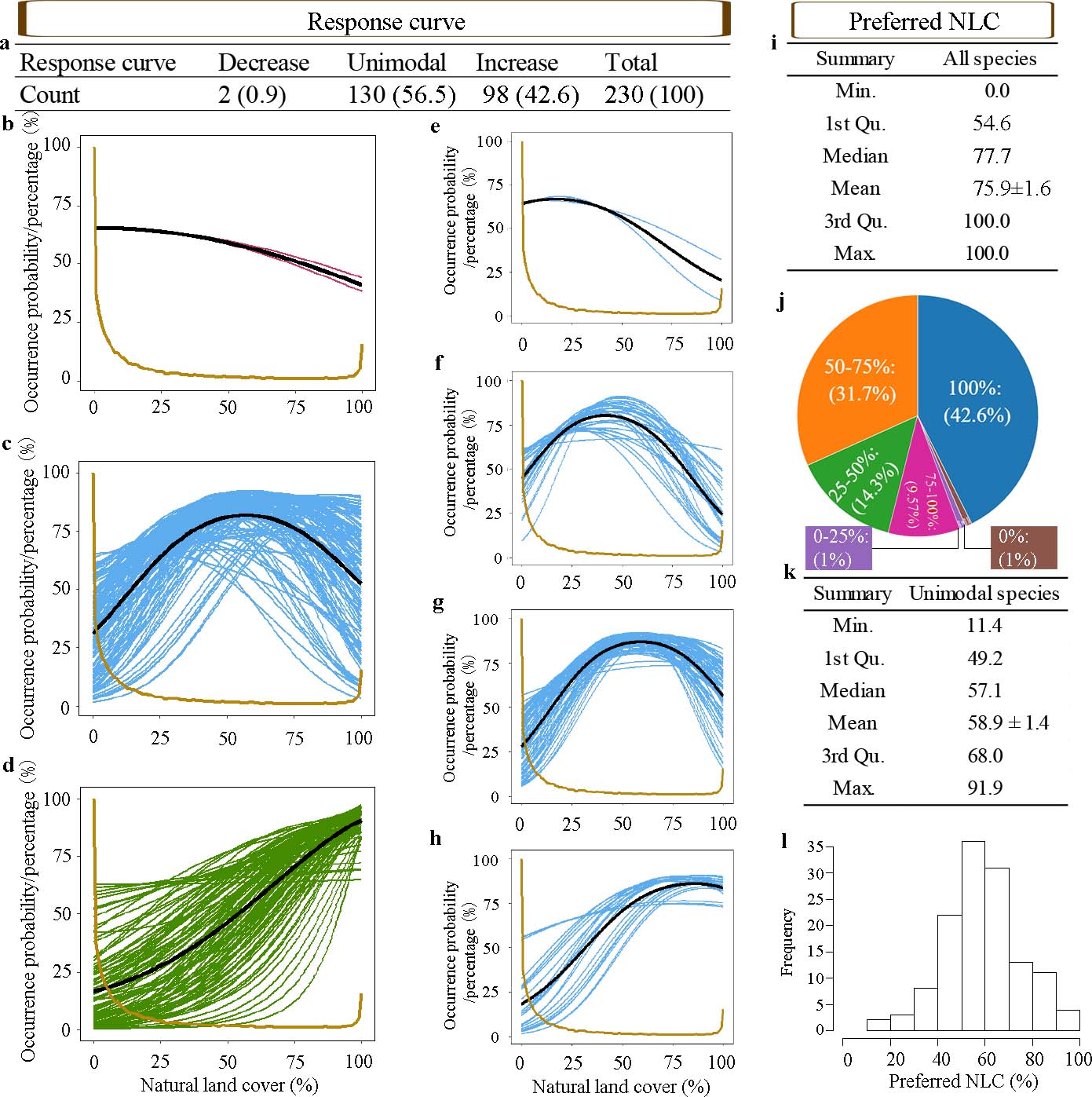


**S4 Fig. 230 threatened plant species responding to natural land cover (NLC).** 2 species with a significantly neutral (without a relationship, named ‘None’) response were omitted as not preferring any NLC. **a-h**, Response curves of plant species to NLC. **k-l**, Summary statistics of preferred NLCs. **a**, Summary of all 230 threatened plants responding to NLC (with percentages in parentheses). **b-d**, Response curves of species with increasing (each red line indicates one species) (**b**), unimodal (each blue line indicates one species) (**c**) and decreasing (each green line indicates one species) (**d**) relations with NLC. The dark black line is the average response curve of each species group. The yellow line indicates the standardized proportion of grids with different NLCs in the Netherlands. Both the occurrence probability and the standardized proportion (percentage) range from 0% to 100% and are indicated by the y axis. **e-h**, Species with unimodal shapes are split into four categories based on their preferred NLCs (**e**, species with preferred NLCs ranging from 0-25%; **f**, species with preferred NLCs ranging from 25-50%; **g**, species with preferred NLCs ranging from 50-75%; **h**, species with preferred NLCs ranging from 75-100%). **i-j**, Statistics of preferred NLCs of all 230 threatened plant species (**i**, summary of 230 plant species’ preferred NLCs; **j**, percentage of species in different categories, including 0%, 0-25%, 25-50%, 50-75%, 75-100% and 100%). **k-l**, Statistics of preferred NLCs of species with unimodal shapes (**k**, summary of plant species’ preferred NLCs; **l**, histogram of preferred NLCs of plant species with unimodal relations with NLC). All mean values are means ± SE.
