# Supplementary material for: Importance of natural land cover for plant species’ conservation: A nationwide study in The Netherlands
Source: PLoS One. 2021 Nov 16;16(11):e0259255. doi: 10.1371/journal.pone.0259255 (PMC8594855; doi:10.1371/journal.pone.0259255)
Supplement: S5 Fig — 24 species with ‘U-shaped’ responses were omitted as not ecologically realistic, and 6 species with a significantly neutral (without a relationship, named ‘None’) response were omitted as not preferring any NLC-F. a-h, Response curves of plant species to NLC-F. k-l, Summary statistics of preferred NLC-Fs. a, Summary of all 1 091 native plants responding to NLC-F (with percentages in parentheses). b-d, Response curves of species with decreasing (each red line indicates one species) (b), unimodal (each blue line indicates one species) (c) and increasing (each green line indicates one species) (d) relations with NLC-F. The dark black line is the average response curve of each species group. The yellow line indicates the standardized proportion of grids with different NLC-Fs in the Netherlands. Both the occurrence probability and the standardized proportion (percentage) range from 0% to 100% and are indicated by the y axis. e-h, Species with unimodal shapes are split into four categories based on their preferred NLC-Fs (e, species with preferred NLC-Fs ranging from 0–25%; f, species with preferred NLC-Fs ranging from 25–50%; g, species with preferred NLC-Fs ranging from 50–75%; h, species with preferred NLC-Fs ranging from 75–100%). i-j, Statistics of preferred NLC-Fs of all 1 091 native plant species (i, summary of 1 091 plant species’ preferred NLC-Fs; j, percentage of species in different categories, including 0%, 0–25%, 25–50%, 50–75%, 75–100% and 100%). k-l, Statistics of preferred NLC-Fs of species with unimodal shapes (k, summary of plant species’ preferred NLC-Fs; l, histogram of preferred NLC-Fs of plant species with unimodal relations with NLC-Fs). All mean values are means ± SE. (DOCX) [file pone.0259255.s010.docx]

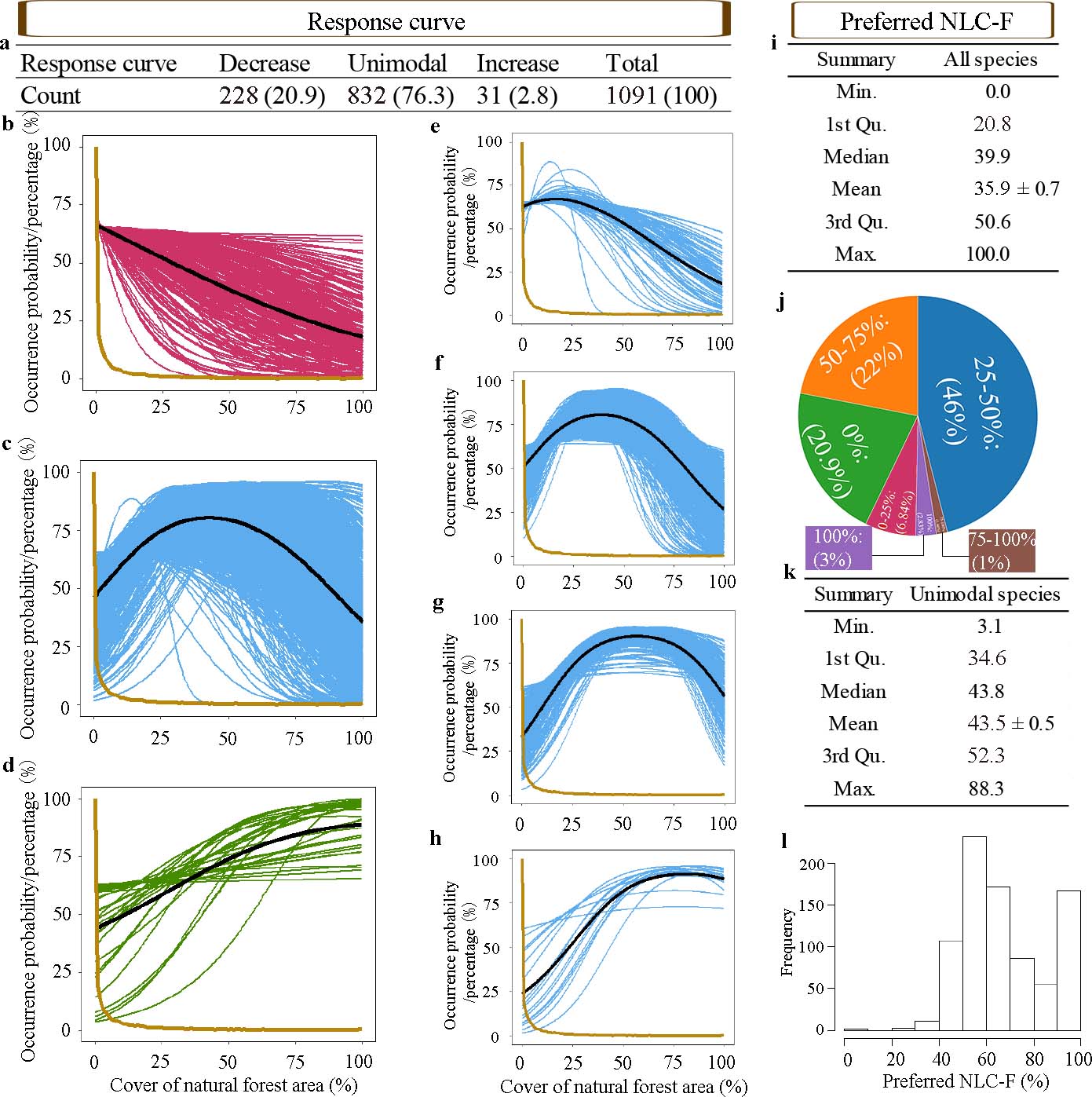


**S5 Fig. 1 091 native plant species responding to the cover of natural forest area (NLC-F).** 24 species with ‘U-shaped’ responses were omitted as not ecologically realistic, and 6 species with a significantly neutral (without a relationship, named ‘None’) response were omitted as not preferring any NLC-F. **a-h**, Response curves of plant species to NLC-F. **k-l**, Summary statistics of preferred NLC-Fs. **a**, Summary of all 1 091 native plants responding to NLC-F (with percentages in parentheses). **b-d**, Response curves of species with decreasing (each red line indicates one species) (**b**), unimodal (each blue line indicates one species) (**c**) and increasing (each green line indicates one species) (**d**) relations with NLC-F. The dark black line is the average response curve of each species group. The yellow line indicates the standardized proportion of grids with different NLC-Fs in the Netherlands. Both the occurrence probability and the standardized proportion (percentage) range from 0% to 100% and are indicated by the y axis. **e-h**, Species with unimodal shapes are split into four categories based on their preferred NLC-Fs (**e**, species with preferred NLC-Fs ranging from 0-25%; **f**, species with preferred NLC-Fs ranging from 25-50%; **g**, species with preferred NLC-Fs ranging from 50-75%; **h**, species with preferred NLC-Fs ranging from 75-100%). **i-j**, Statistics of preferred NLC-Fs of all 1 091 native plant species (**i**, summary of 1 091 plant species’ preferred NLC-Fs; **j**, percentage of species in different categories, including 0%, 0-25%, 25-50%, 50-75%, 75-100% and 100%). **k-l**, Statistics of preferred NLC-Fs of species with unimodal shapes (**k**, summary of plant species’ preferred NLC-Fs; **l**, histogram of preferred NLC-Fs of plant species with unimodal relations with NLC-Fs). All mean values are means ± SE.
