# Supplementary material for: Importance of natural land cover for plant species’ conservation: A nationwide study in The Netherlands
Source: PLoS One. 2021 Nov 16;16(11):e0259255. doi: 10.1371/journal.pone.0259255 (PMC8594855; doi:10.1371/journal.pone.0259255)
Supplement: S7 Fig — a, Two-dimensional plot indicating the preferred NLC-F and NLC-O of each species. Each dot indicates one species. Red dots indicate threatened species and blue dots indicate not threatened species. b, Number of dots within each subgroup along the x axis (0%, 0–25%, 25–50%, 50–75%, 75–100% and 100%). c, Number of dots within each subgroup along the y axis (0%, 0–25%, 25–50%, 50–75%, 75–100% and 100%). (DOCX) [file pone.0259255.s012.docx]

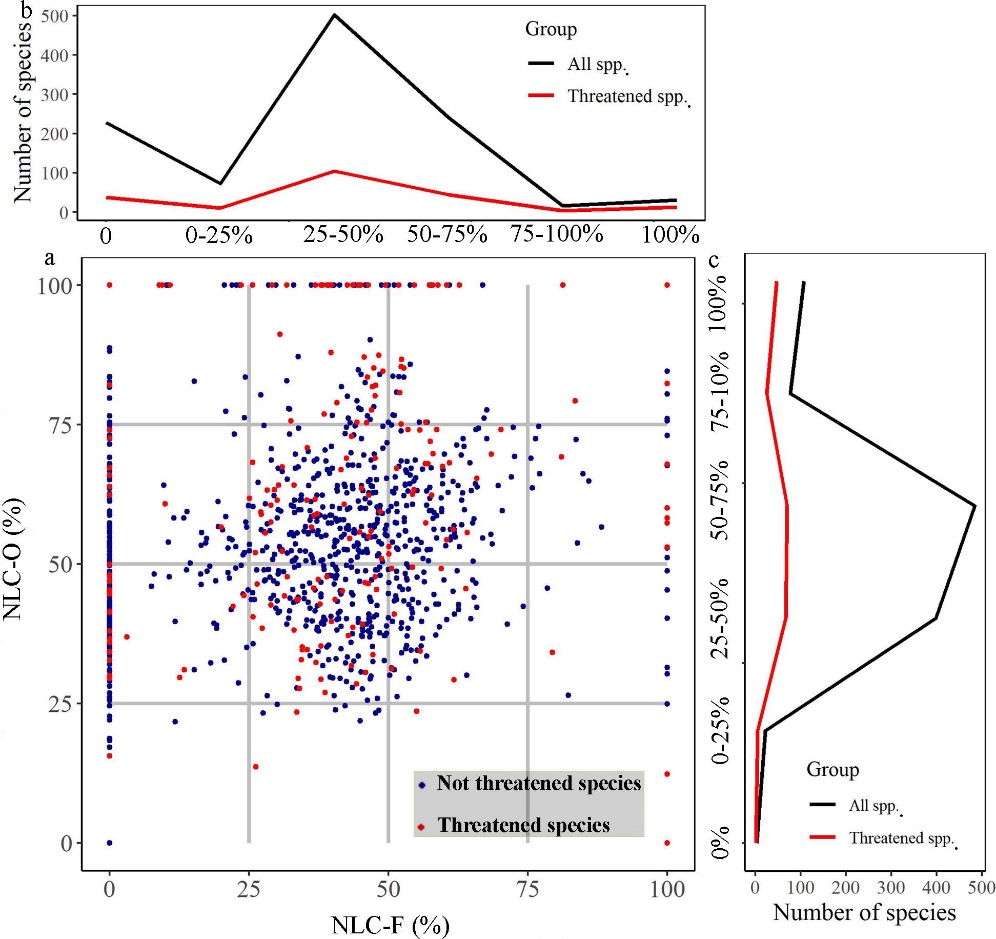


**S7 Fig. Preferred NLC-F and NLC-O of native plant species, which are classified into threatened and not threatened species.** **a**, Two-dimensional plot indicating the preferred NLC-F and NLC-O of each species. Each dot indicates one species. Red dots indicate threatened species and blue dots indicate not threatened species. **b**, Number of dots within each subgroup along the x axis (0%, 0-25%, 25-50%, 50-75%, 75-100% and 100%). **c**, Number of dots within each subgroup along the y axis (0%, 0-25%, 25-50%, 50-75%, 75-100% and 100%).
