# Supplementary material for: Importance of natural land cover for plant species’ conservation: A nationwide study in The Netherlands
Source: PLoS One. 2021 Nov 16;16(11):e0259255. doi: 10.1371/journal.pone.0259255 (PMC8594855; doi:10.1371/journal.pone.0259255)
Supplement: S9 Fig — a1-a3, Differences of preferred NLC within groups of threatened status, rarity and origin. b1-b3, Differences of preferred NLC-F within groups of threatened status, rarity and origin. c1-c3, Differences of preferred NLC-O within groups of threatened status, rarity and origin. Preferred NLC, NLC-F and NLC-O mean the preferences for natural land cover (preferred NLC), the cover of natural forest area (preferred NLC-F) and the cover for natural open area (preferred NLC-O). (DOCX) [file pone.0259255.s014.docx]

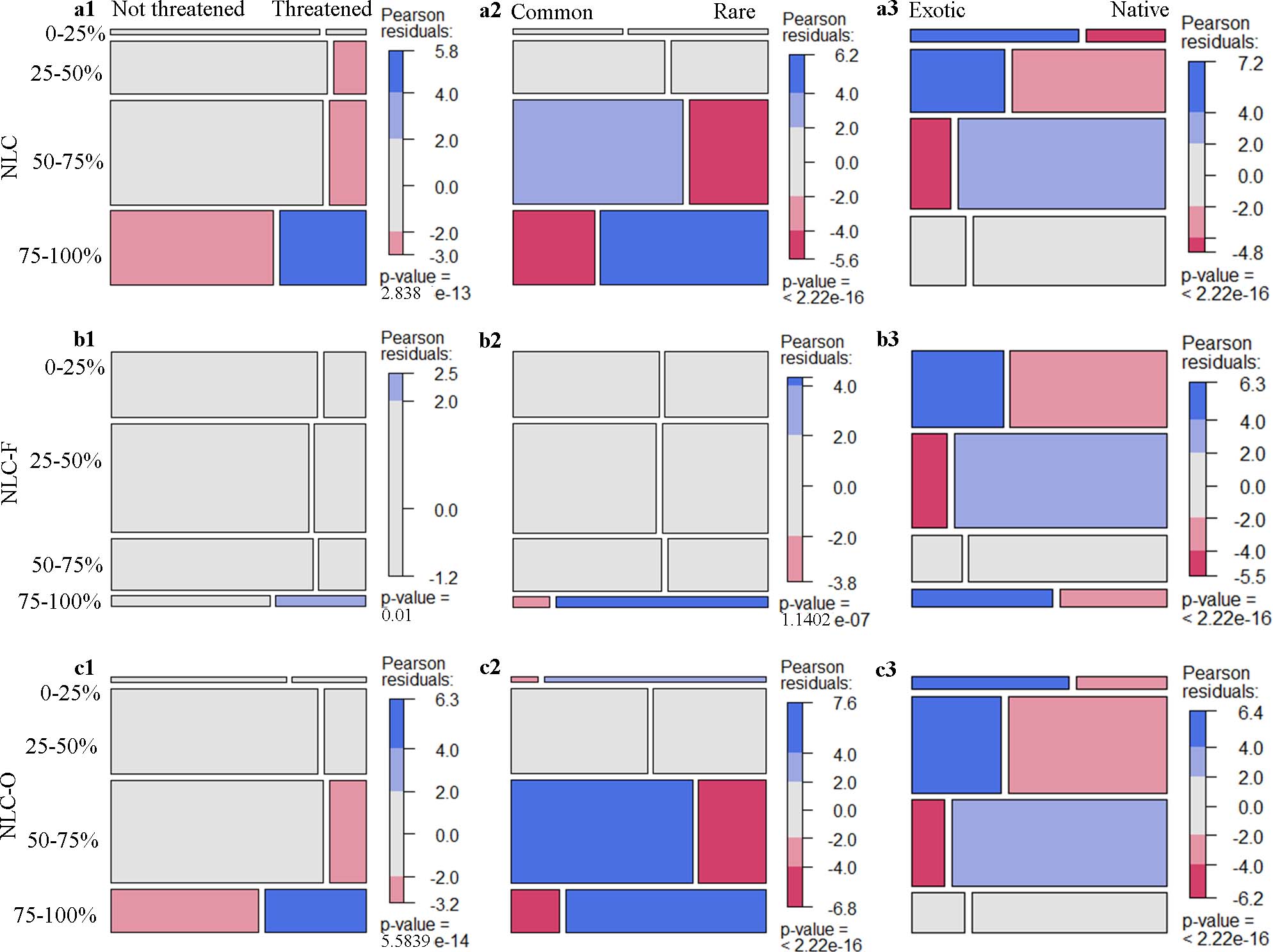


**S9 Fig. Contingency Analysis on whether threatened status, rarity and origin affect the preferred NLC, NLC-F and NLC-O.** **a1-a3**, Differences of preferred NLC within groups of threatened status, rarity and origin. **b1-b3**, Differences of preferred NLC-F within groups of threatened status, rarity and origin. **c1-c3**, Differences of preferred NLC-O within groups of threatened status, rarity and origin. Preferred NLC, NLC-F and NLC-O mean the preferences for natural land cover (preferred NLC), the cover of natural forest area (preferred NLC-F) and the cover for natural open area (preferred NLC-O).
