# Supplementary material for: Importance of natural land cover for plant species’ conservation: A nationwide study in The Netherlands
Source: PLoS One. 2021 Nov 16;16(11):e0259255. doi: 10.1371/journal.pone.0259255 (PMC8594855; doi:10.1371/journal.pone.0259255)
Supplement: S11 Fig — a, All native species vs exotic species. b, Threatened species vs not threatened species. c, Rare species vs common species. d, Rare species vs common species, but rare species are classified into three categories (i.e. very rare, rare, rather rare) according to the Red List of Vascular Plants of the Netherlands [1]. Lines represent the best-fit regressions. The average marginal occurrence probability change means the average occurrence probability change with 1% natural land cover increase, e.g. natural land cover at 1x1 km resolution increases from 10% to 11%). (DOCX) [file pone.0259255.s016.docx]

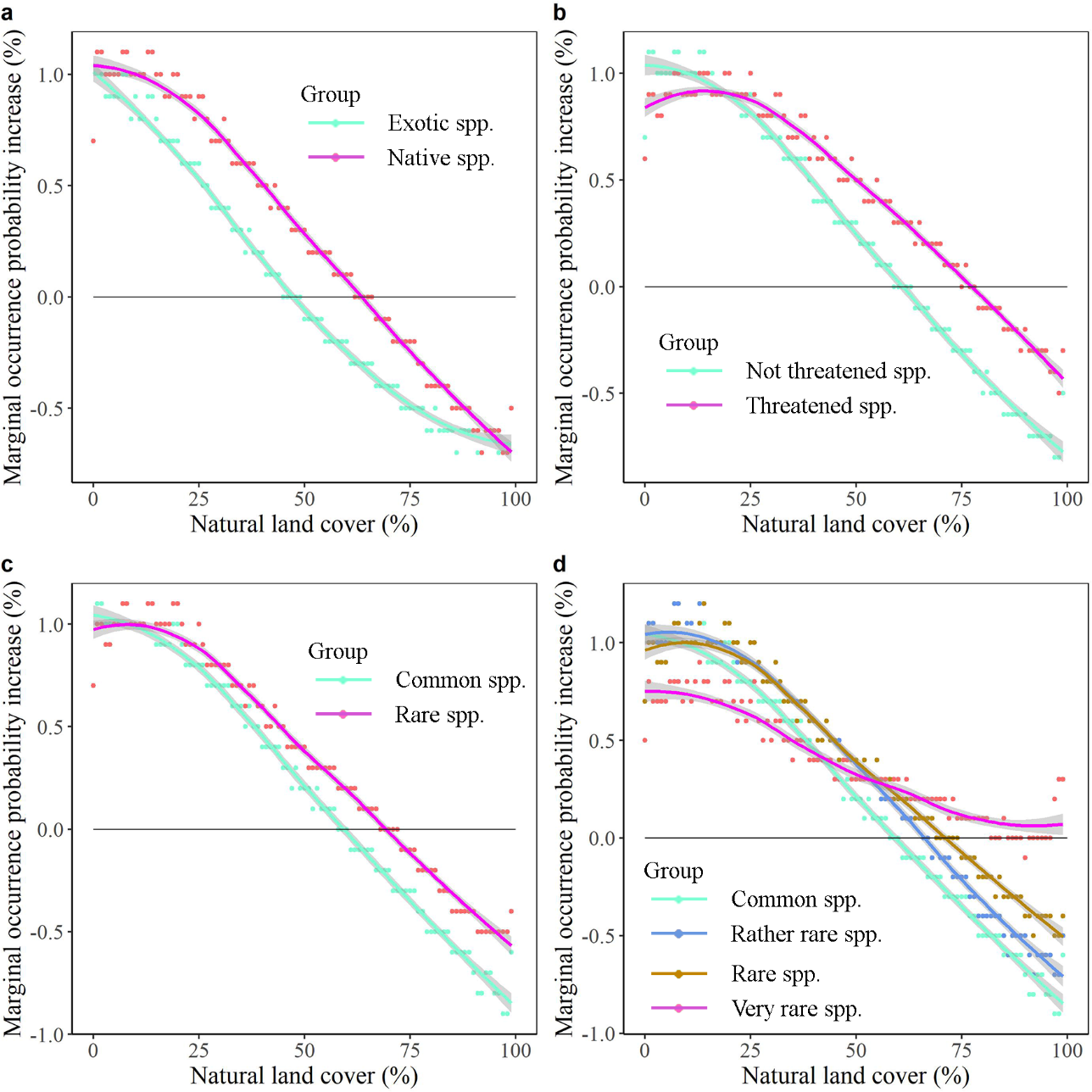


**S11 Fig. The average marginal occurrence probability change of different species groups.** a, All native species vs exotic species. b, Threatened species vs not threatened species. c, Rare species vs common species. d, Rare species vs common species, but rare species are classified into three categories (i.e. very rare, rare, rather rare) according to the Red List of Vascular Plants of the Netherlands [1]. Lines represent the best-fit regressions. The average marginal occurrence probability change means the average occurrence probability change with 1% natural land cover increase, e.g. natural land cover at 1x1 km resolution increases from 10% to 11%)

References

**1**. Sparrius, L. B., Odé, B., & Beringen, R. Basisrapport Rode Lijst Vaatplanten 2012 volgens Nederlandse en IUCN-criteria. FLORON Rapport. 2014; 57.
