# Supplementary material for: Importance of natural land cover for plant species’ conservation: A nationwide study in The Netherlands
Source: PLoS One. 2021 Nov 16;16(11):e0259255. doi: 10.1371/journal.pone.0259255 (PMC8594855; doi:10.1371/journal.pone.0259255)
Supplement: S12 Fig — a, The occurrence probability of 1 122 native plant species responding to NLC. Each grey line indicates one species, and the dark line means the average response of all 1 122 native species. b, Predicted relationships of richness responding to NLC, with a best fitting quadratic model. c, Relationships of plant species richness responding to NLC based on observations, with a best fitting quadratic model. Lines represent the best-fit regressions. (DOCX) [file pone.0259255.s017.docx]

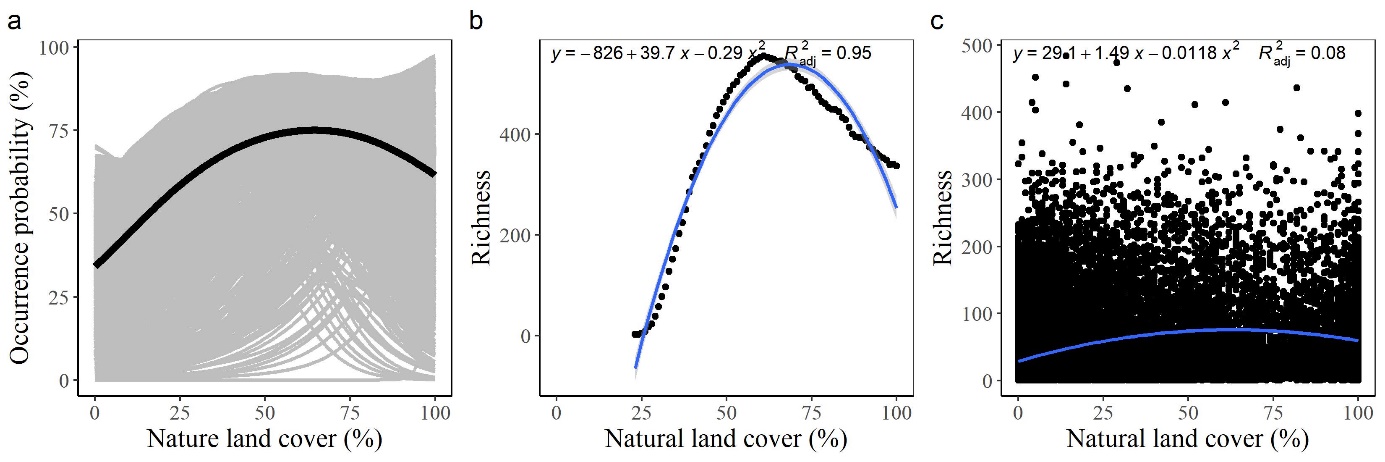


**S12 Fig. Plants responding to natural land cover (NLC) at species-level and community-level.** **a**, The occurrence probability of 1 122 native plant species responding to NLC. Each grey line indicates one species, and the dark line means the average response of all 1 122 native species. **b**, Predicted relationships of richness responding to NLC, with a best fitting quadratic model. **c**, Relationships of plant species richness responding to NLC based on observations, with a best fitting quadratic model. Lines represent the best-fit regressions.
