# Supplementary material for: Importance of natural land cover for plant species’ conservation: A nationwide study in The Netherlands
Source: PLoS One. 2021 Nov 16;16(11):e0259255. doi: 10.1371/journal.pone.0259255 (PMC8594855; doi:10.1371/journal.pone.0259255)
Supplement: S13 Fig — (DOCX) [file pone.0259255.s018.docx]

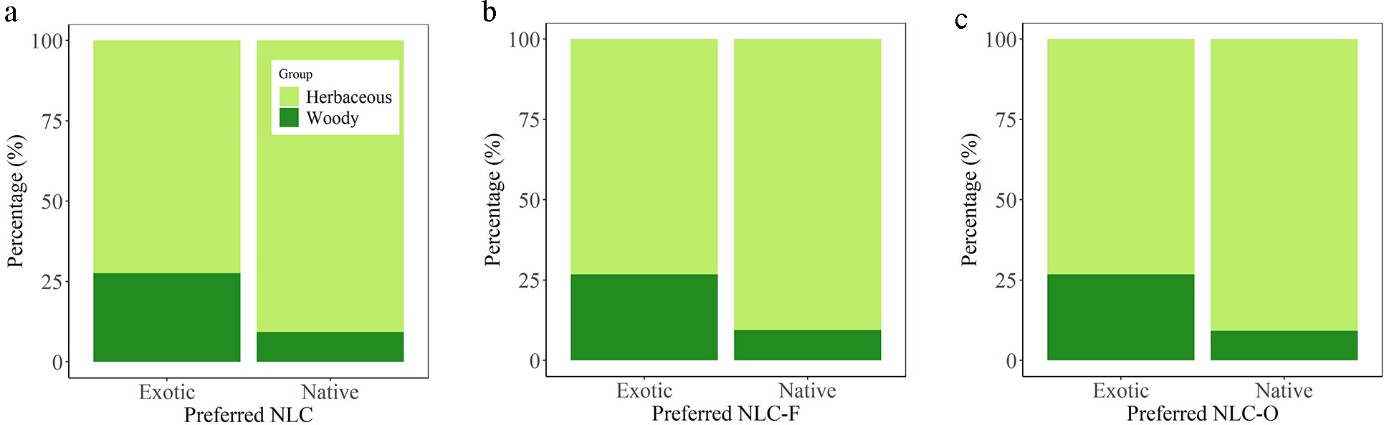


**S13 Fig.** **The percentage of woody and herbaceous species within each species category (native species vs exotic species).**
