# Supplementary material for: Importance of natural land cover for plant species’ conservation: A nationwide study in The Netherlands
Source: PLoS One. 2021 Nov 16;16(11):e0259255. doi: 10.1371/journal.pone.0259255 (PMC8594855; doi:10.1371/journal.pone.0259255)
Supplement: S3 Fig — (PDF) [file pone.0259255.s008.pdf]

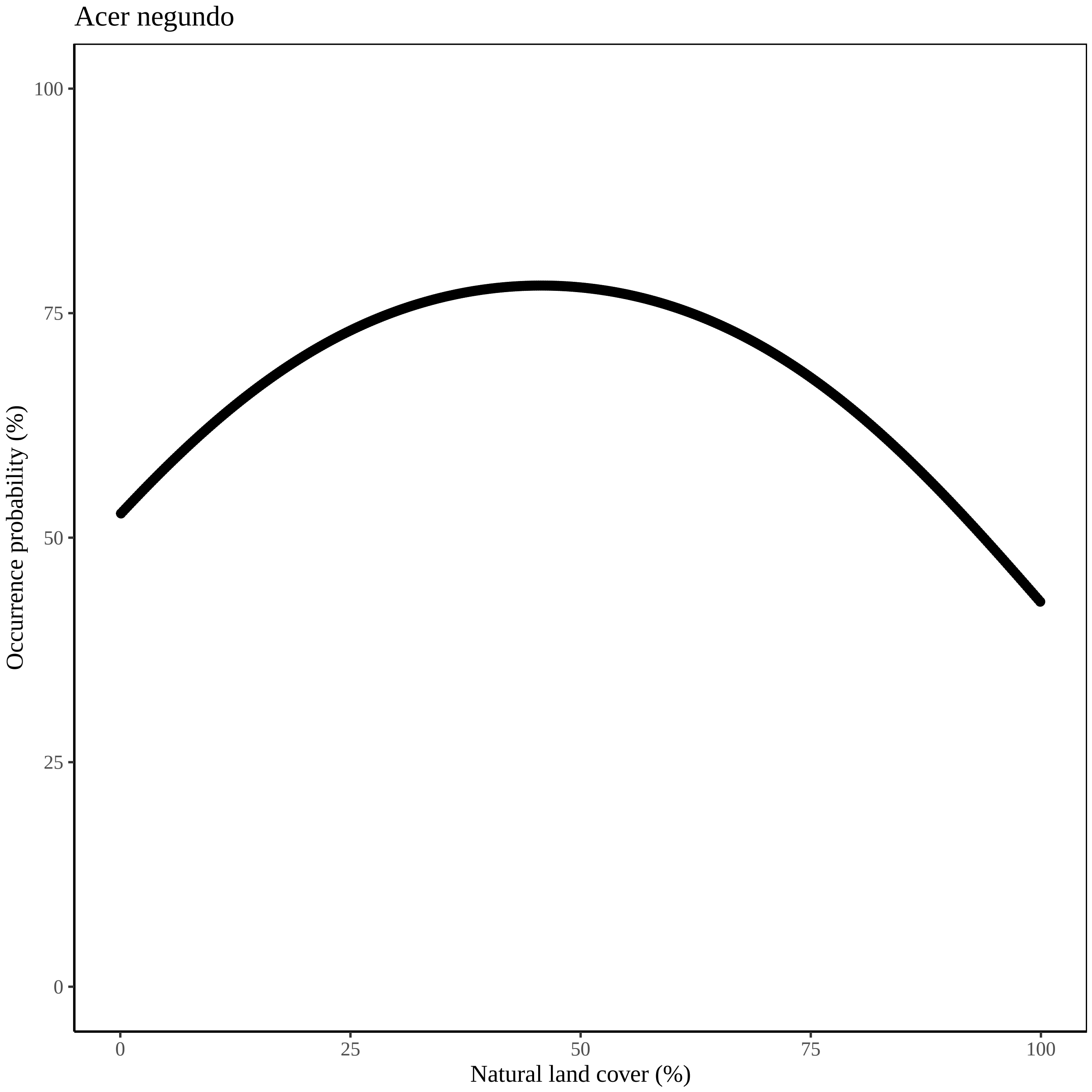

Acer negundo
100
75
50
25
0
Occurrence probability (%)
0
25
50
75
100
Natural land cover (%)

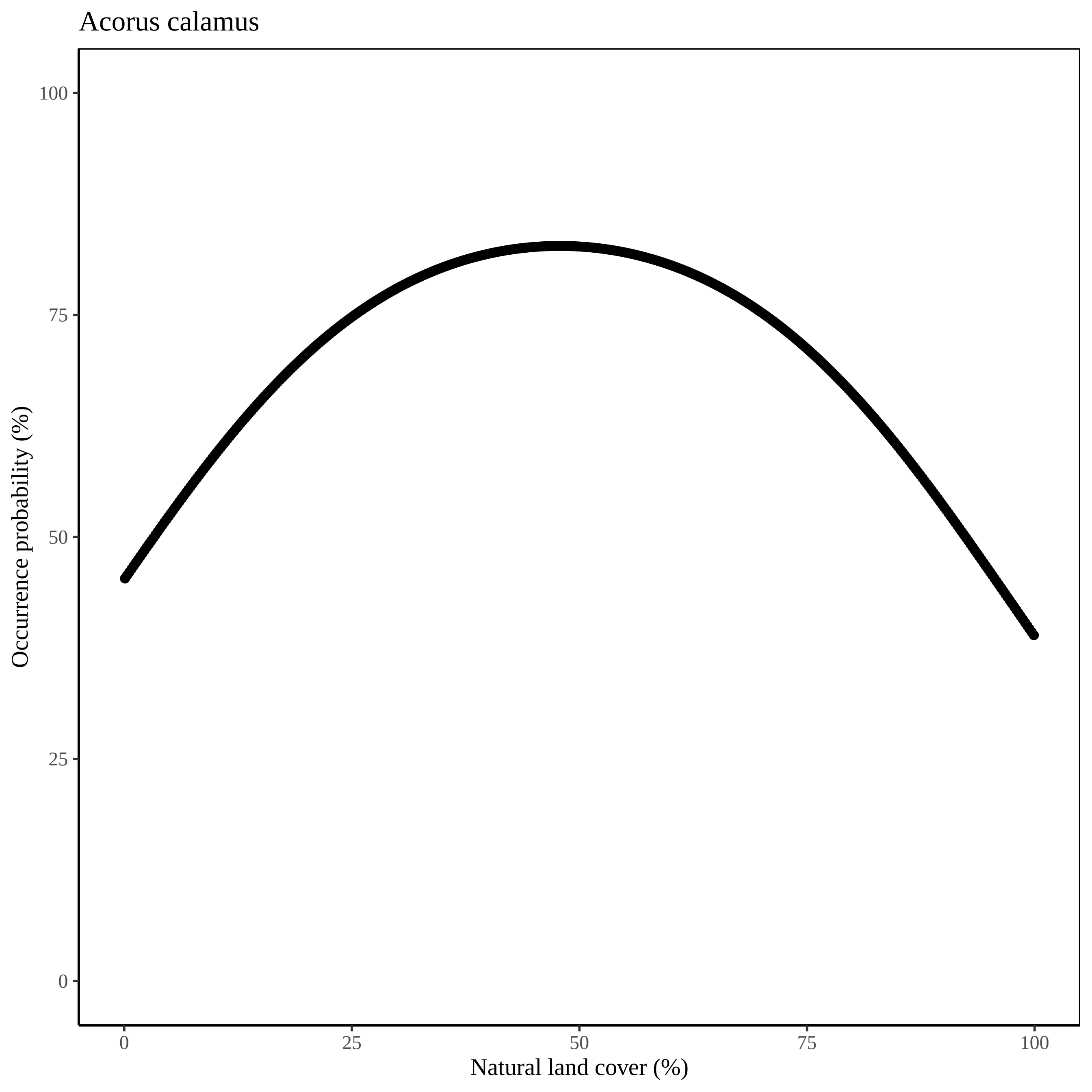
Acorus calamus
Occurrence probability (%)
100
75
50
25
0
0
25
50
75
100
Natural land cover (%)

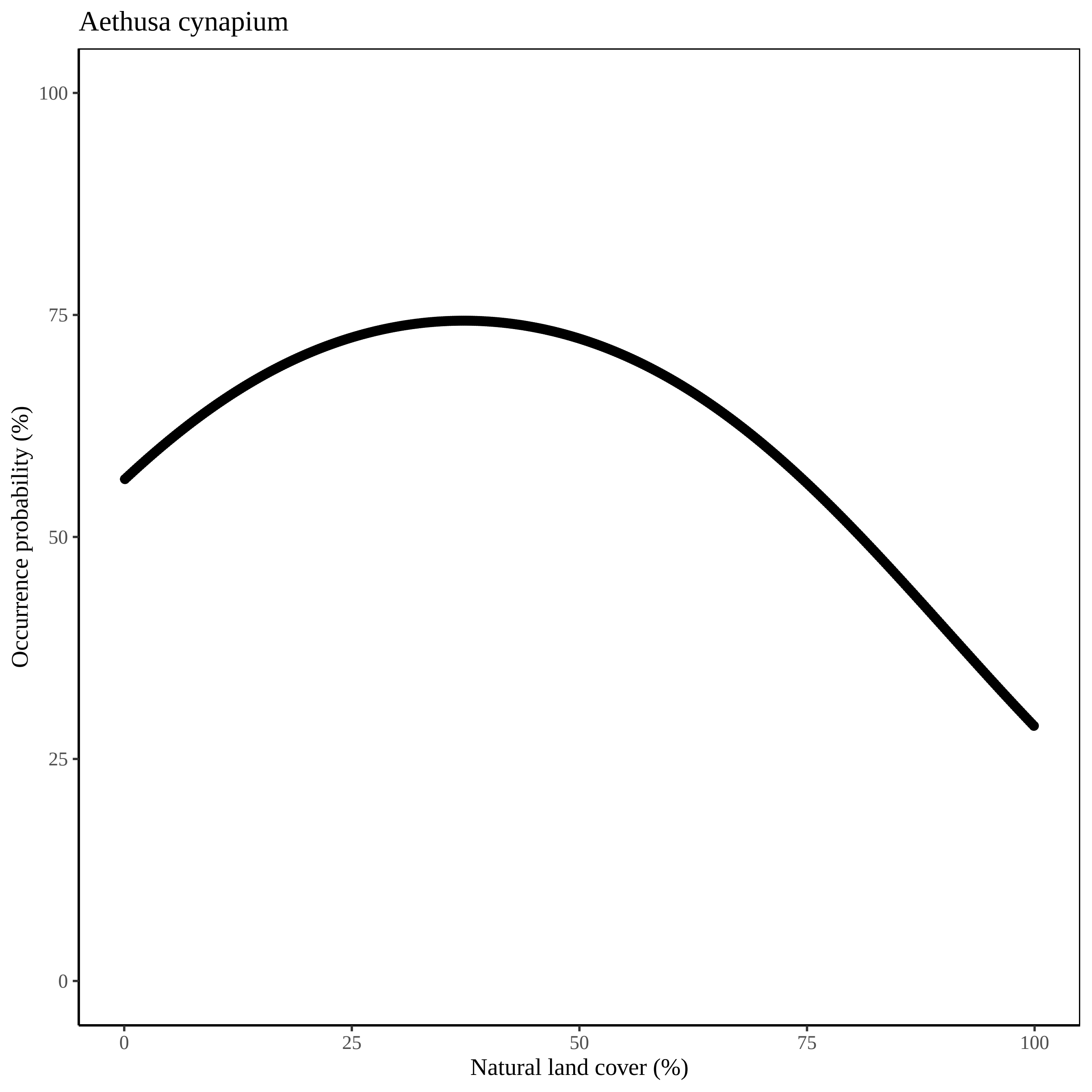
Aethusa cynapium
Occurrence probability (%)
Natural land cover (%)
0
25
50
75
100

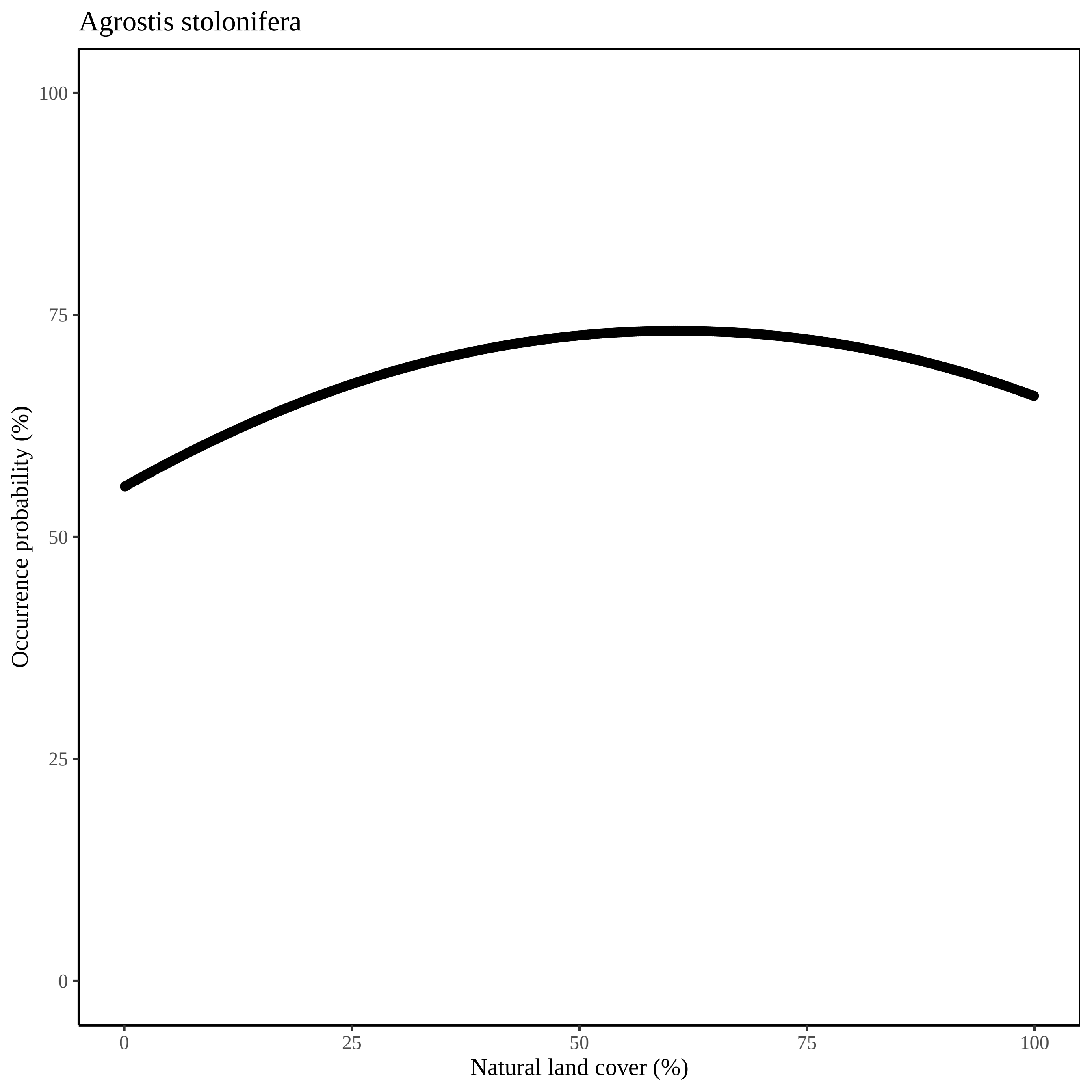
Agrostis stolonifera
100
75
50
25
0
Occurrence probability (%)
0
25
50
75
100
Natural land cover (%)

Alisma gramineum

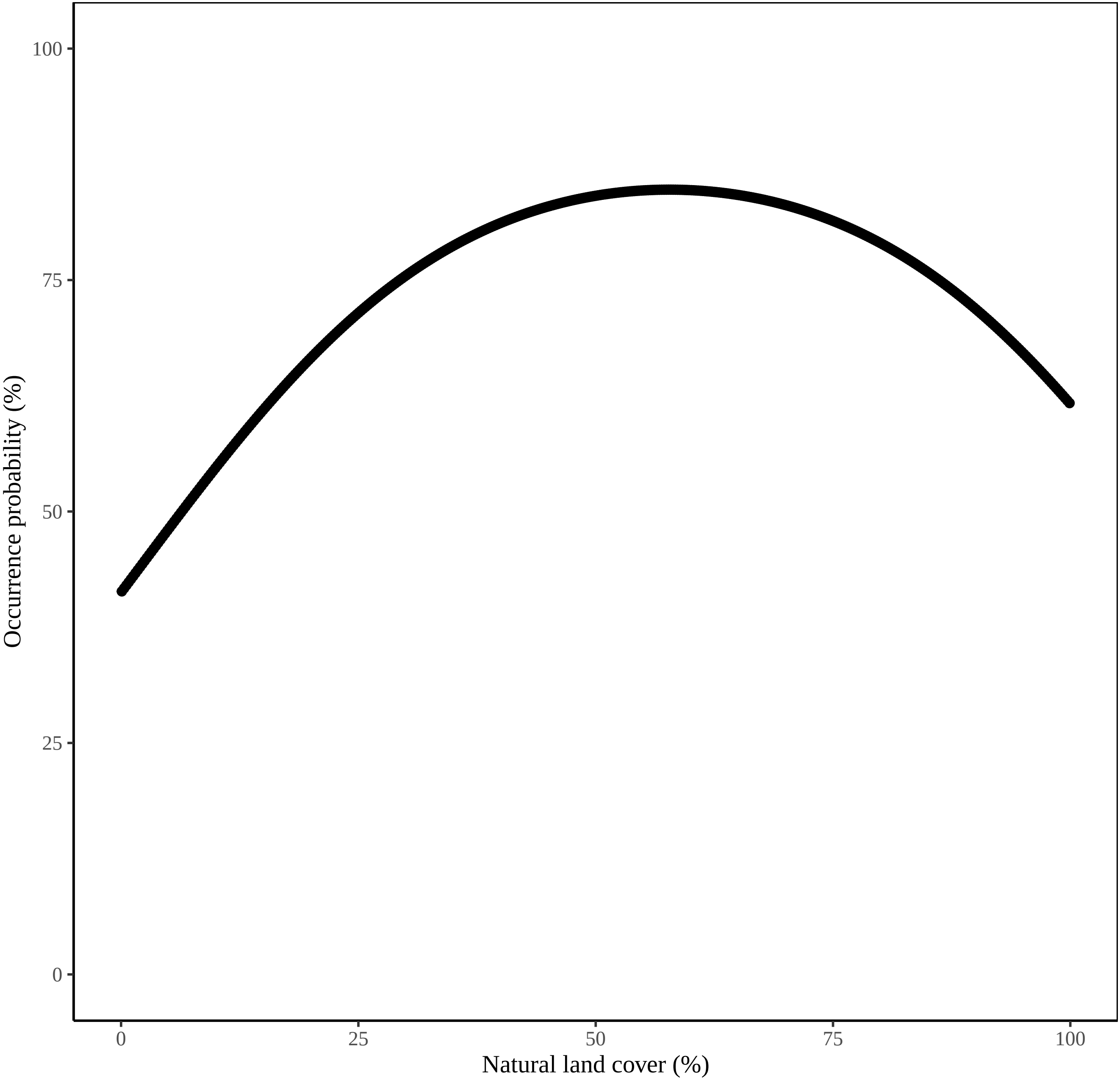

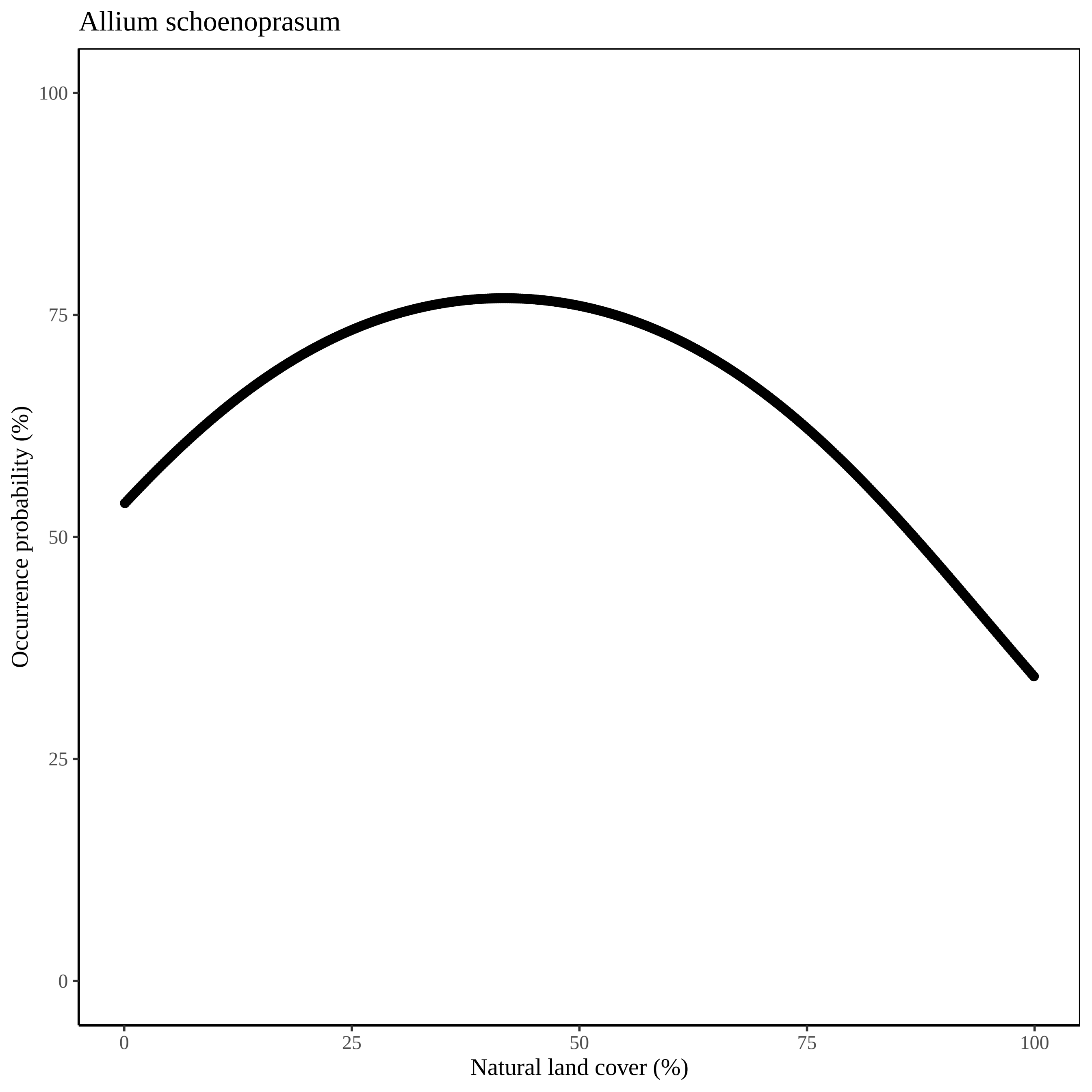
Allium schoenoprasum
Occurrence probability (%)
100
75
50
25
0
0
25
50
75
100
Natural land cover (%)

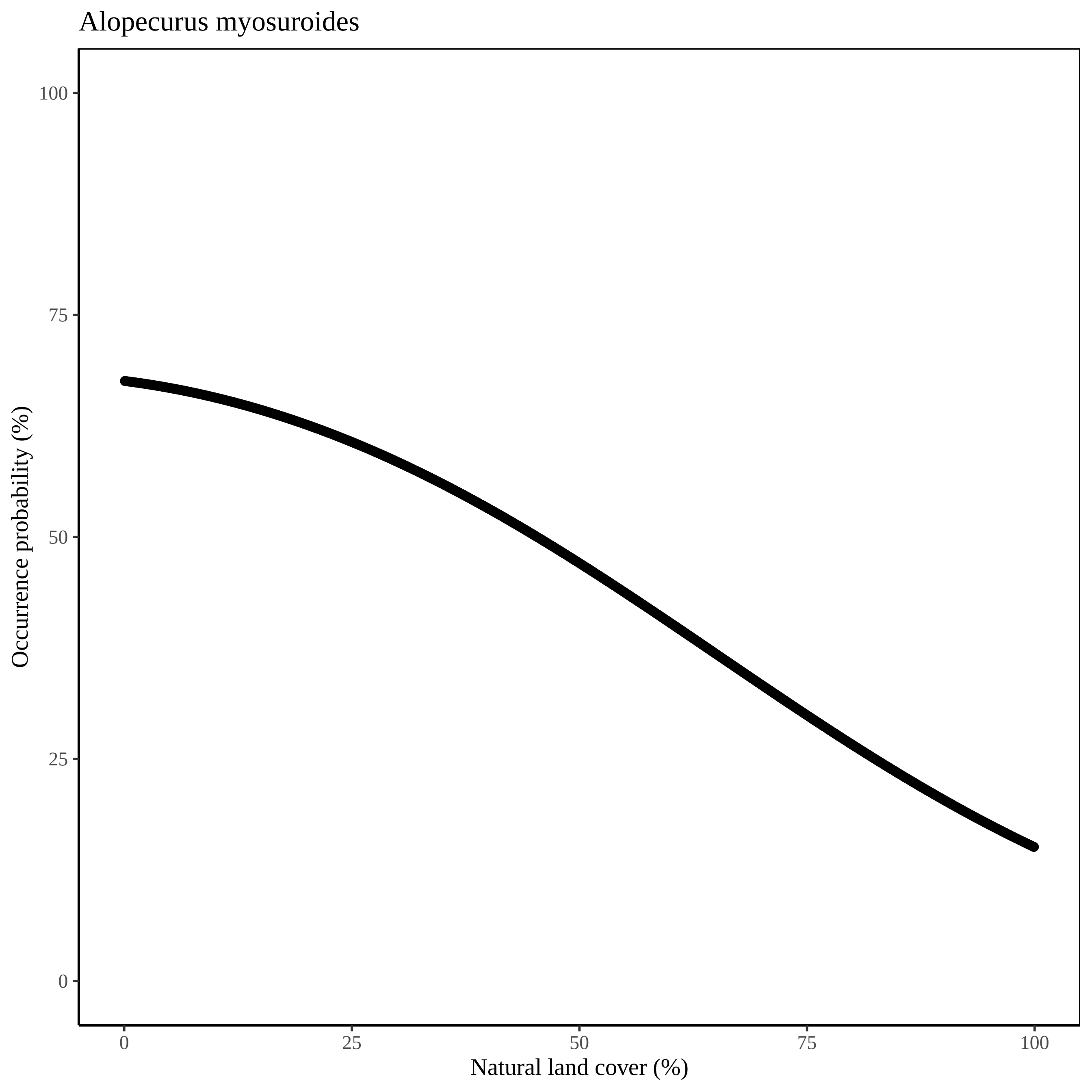
Alopecurus myosuroides
Occurrence probability (%)
100
75
50
25
0
0
25
50
75
100
Natural land cover (%)

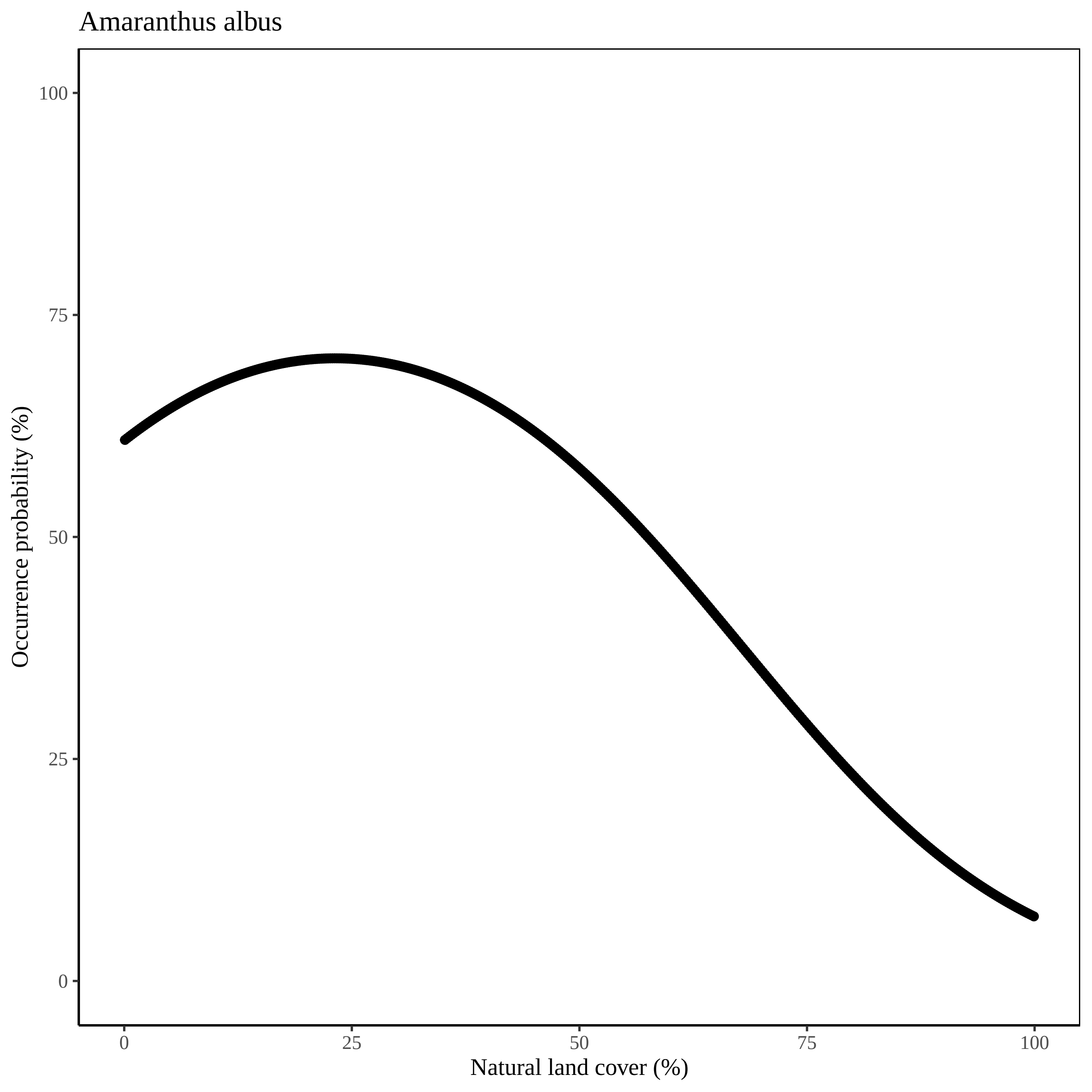

Amaranthus albus
Occurrence probability (%)
100
75
50
25
0
0
25
50
75
100
Natural land cover (%)

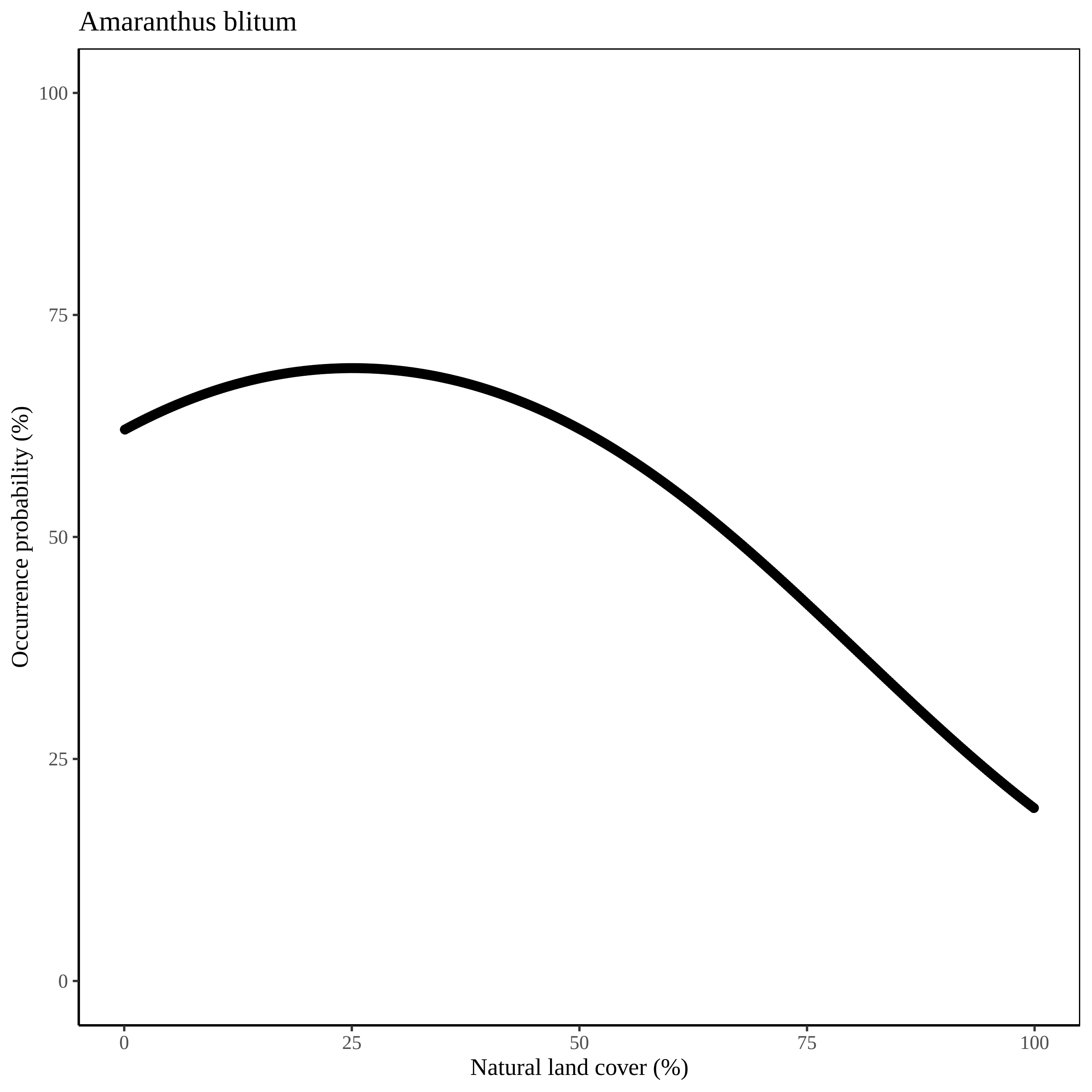

Amaranthus blitum
Occurrence probability (%)
100
75
50
25
0
0
25
50
75
100
Natural land cover (%)

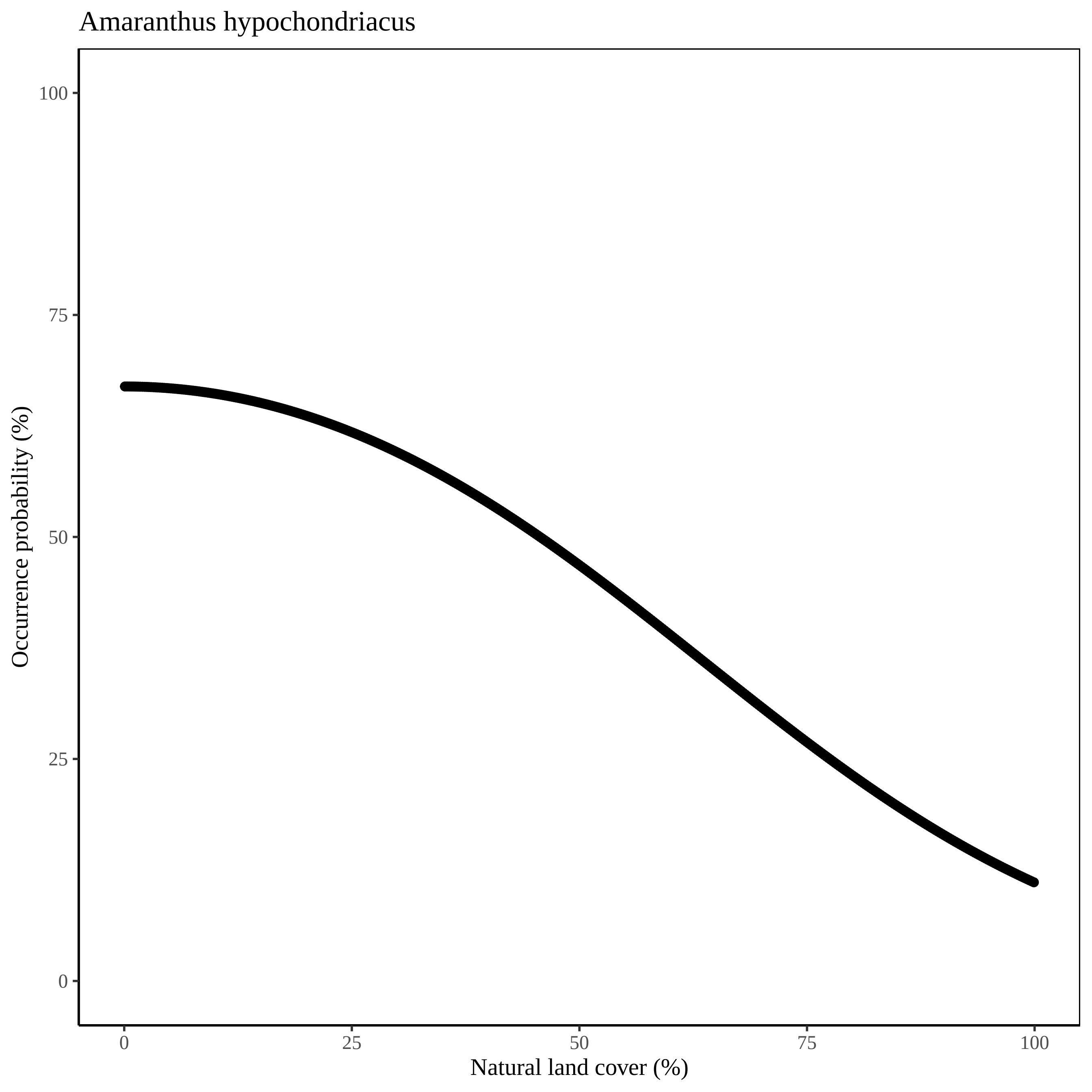

Amaranthus hypochondriacus
Occurrence probability (%)
100
75
50
25
0
0
25
50
75
100
Natural land cover (%)

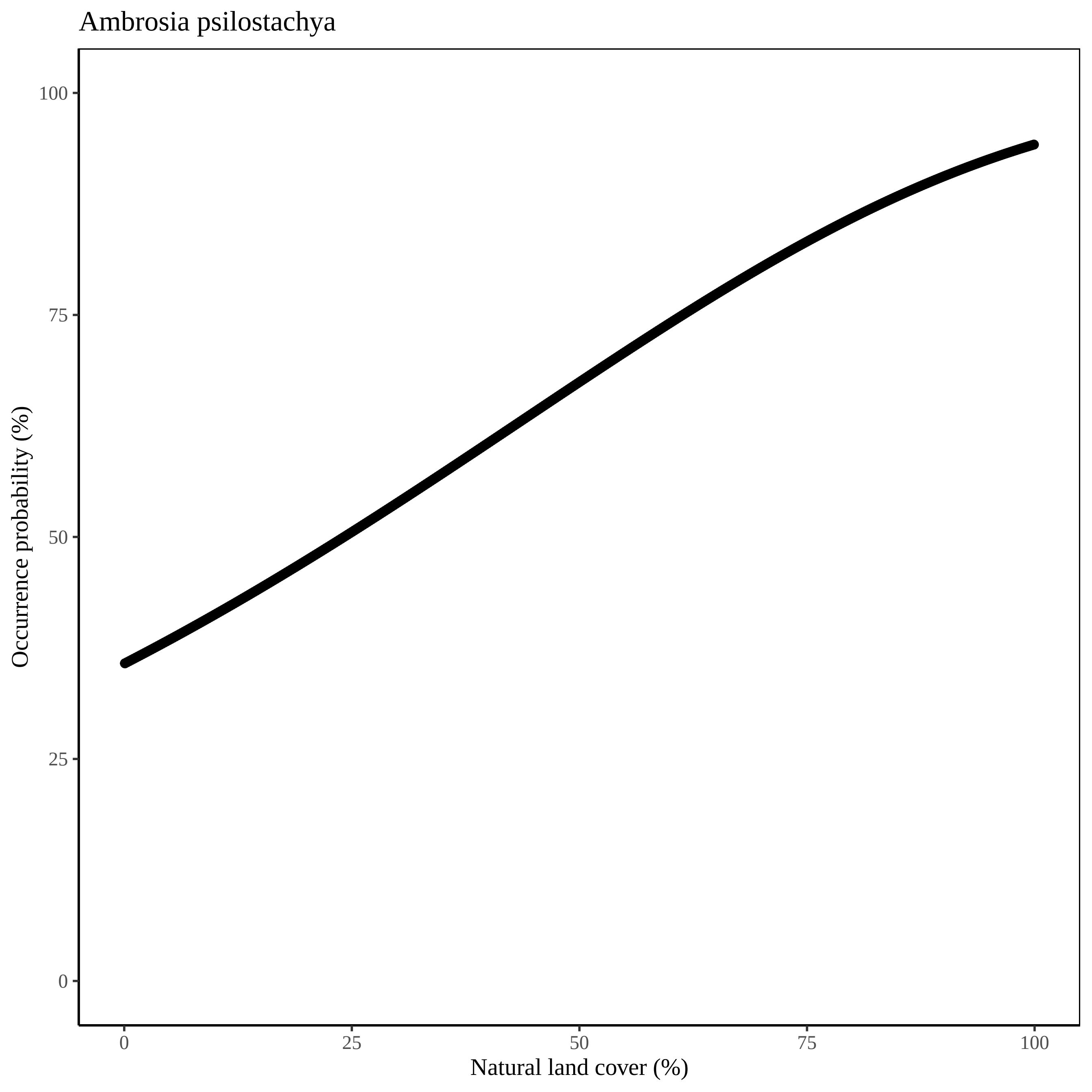
Ambrosia psilostachya
Occurrence probability (%)
100
75
50
25
0
0
25
50
75
100
Natural land cover (%)

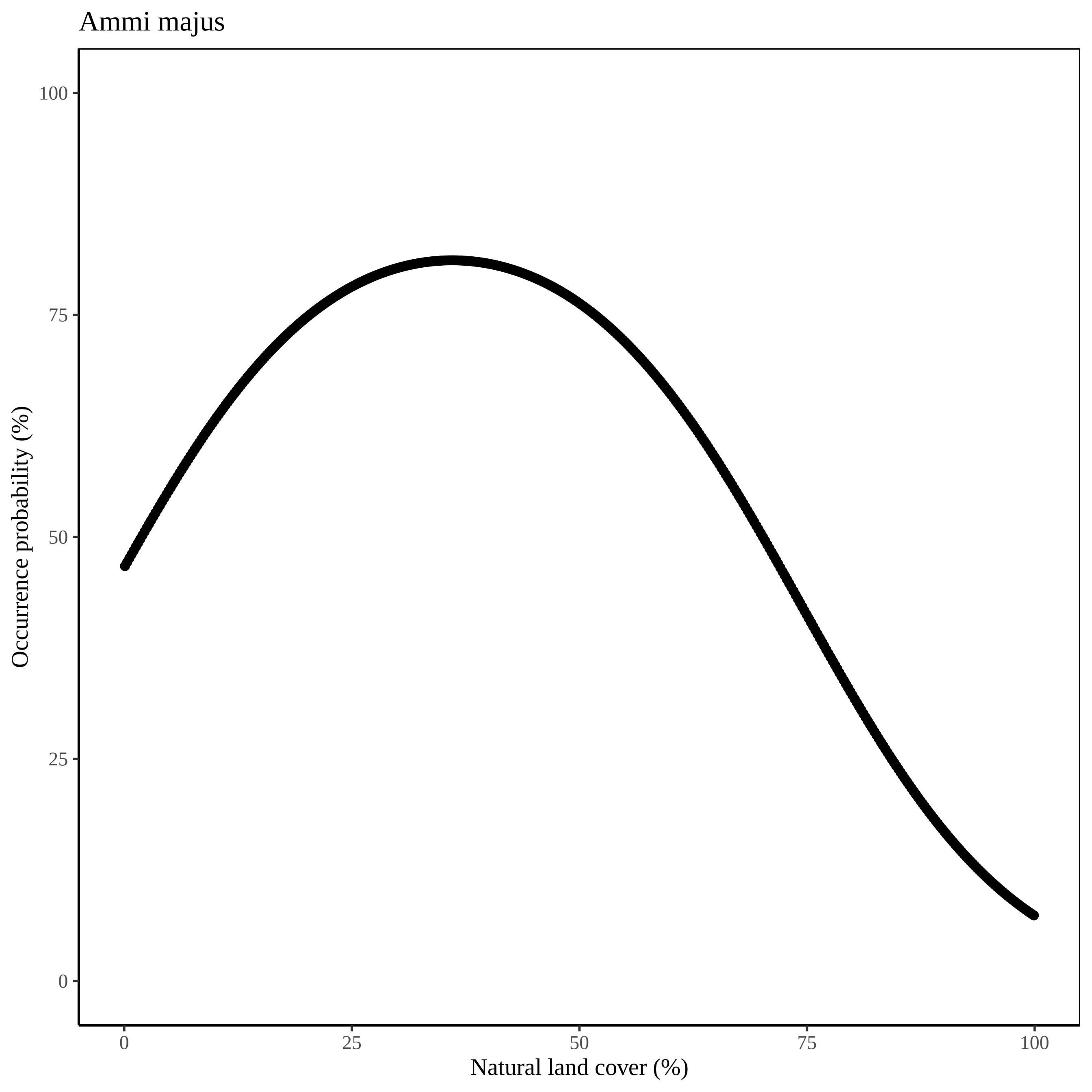

Ammi majus
Occurrence probability (%)
100
75
50
25
0
0
25
50
75
100
Natural land cover (%)

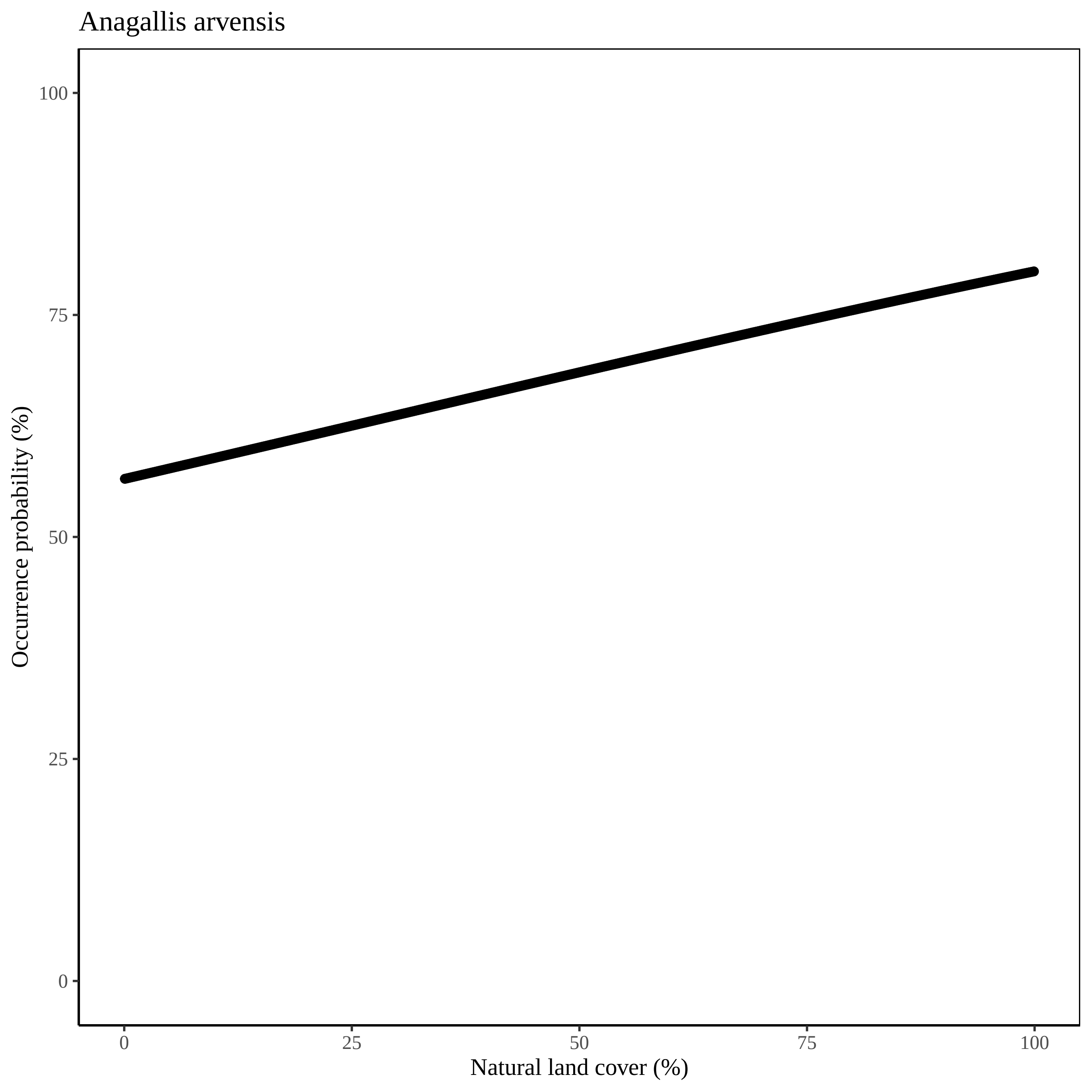
Anagallis arvensis
Occurrence probability (%)
100
75
50
25
0
0
25
50
75
100
Natural land cover (%)

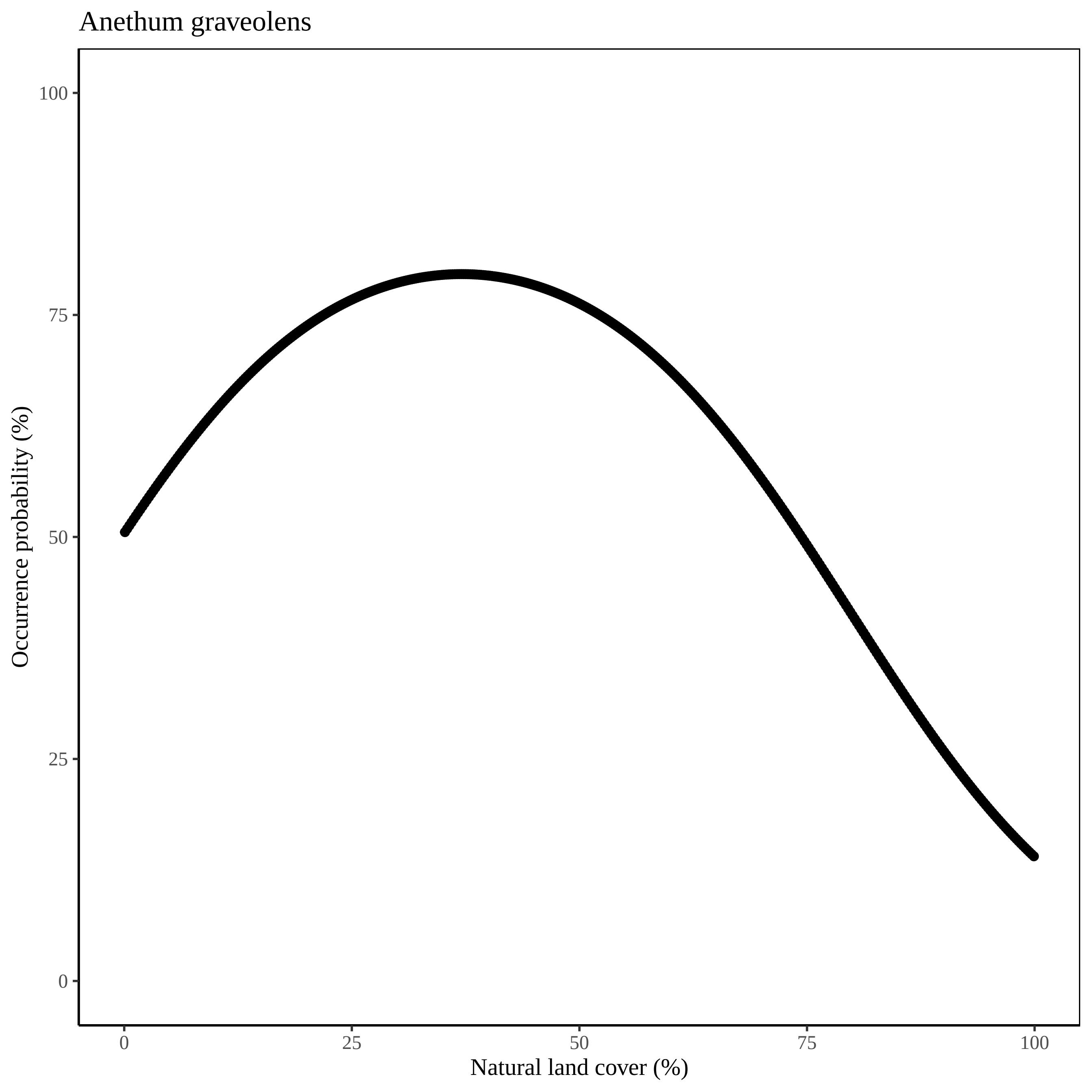

Anethum graveolens
Occurrence probability (%)
100
75
50
25
0
0
25
50
75
100
Natural land cover (%)

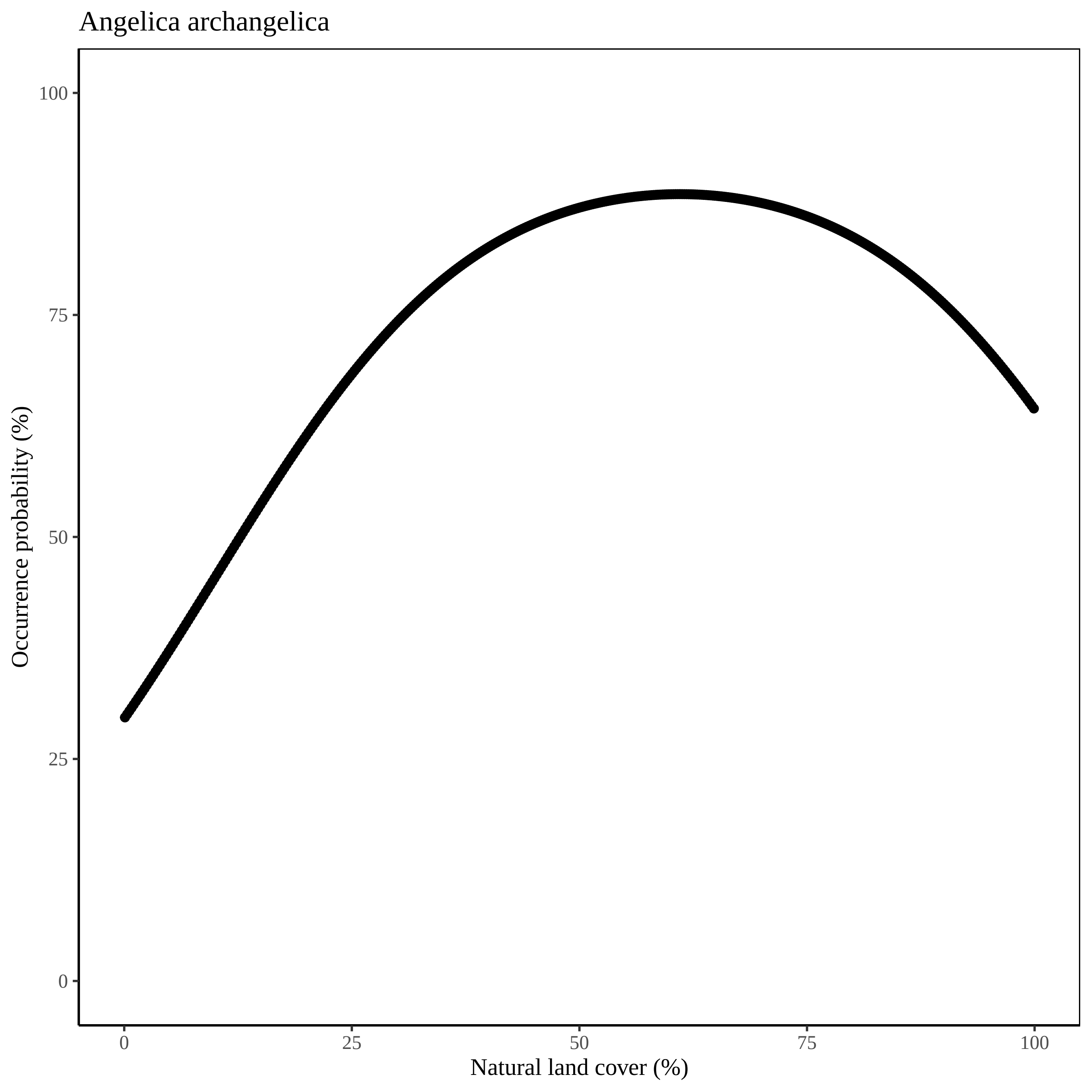
Angelica archangelica
Occurrence probability (%)
100
75
50
25
0
0
25
50
75
100
Natural land cover (%)

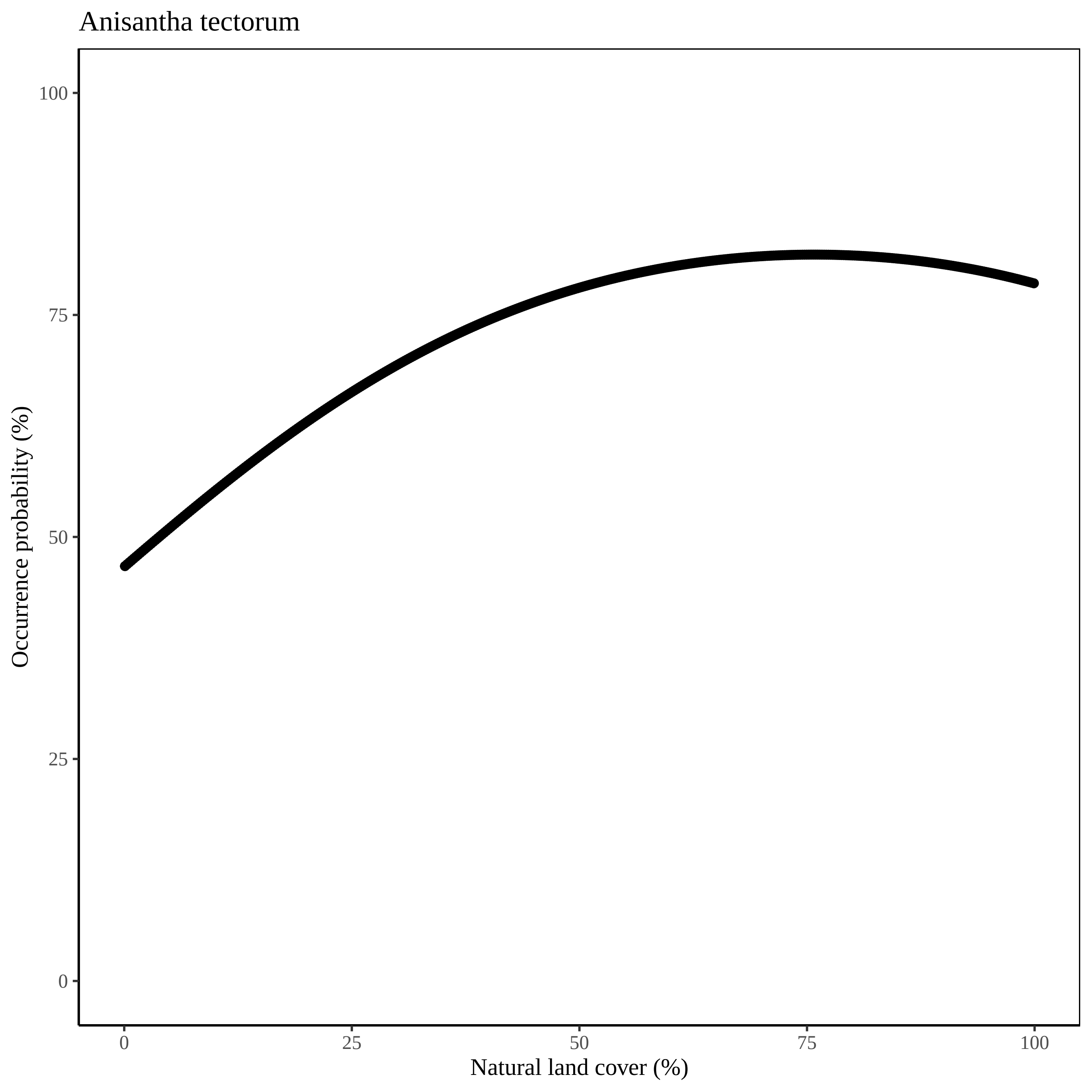
Anisantha tectorum
Occurrence probability (%)
100
75
50
25
0
0
25
50
75
100
Natural land cover (%)

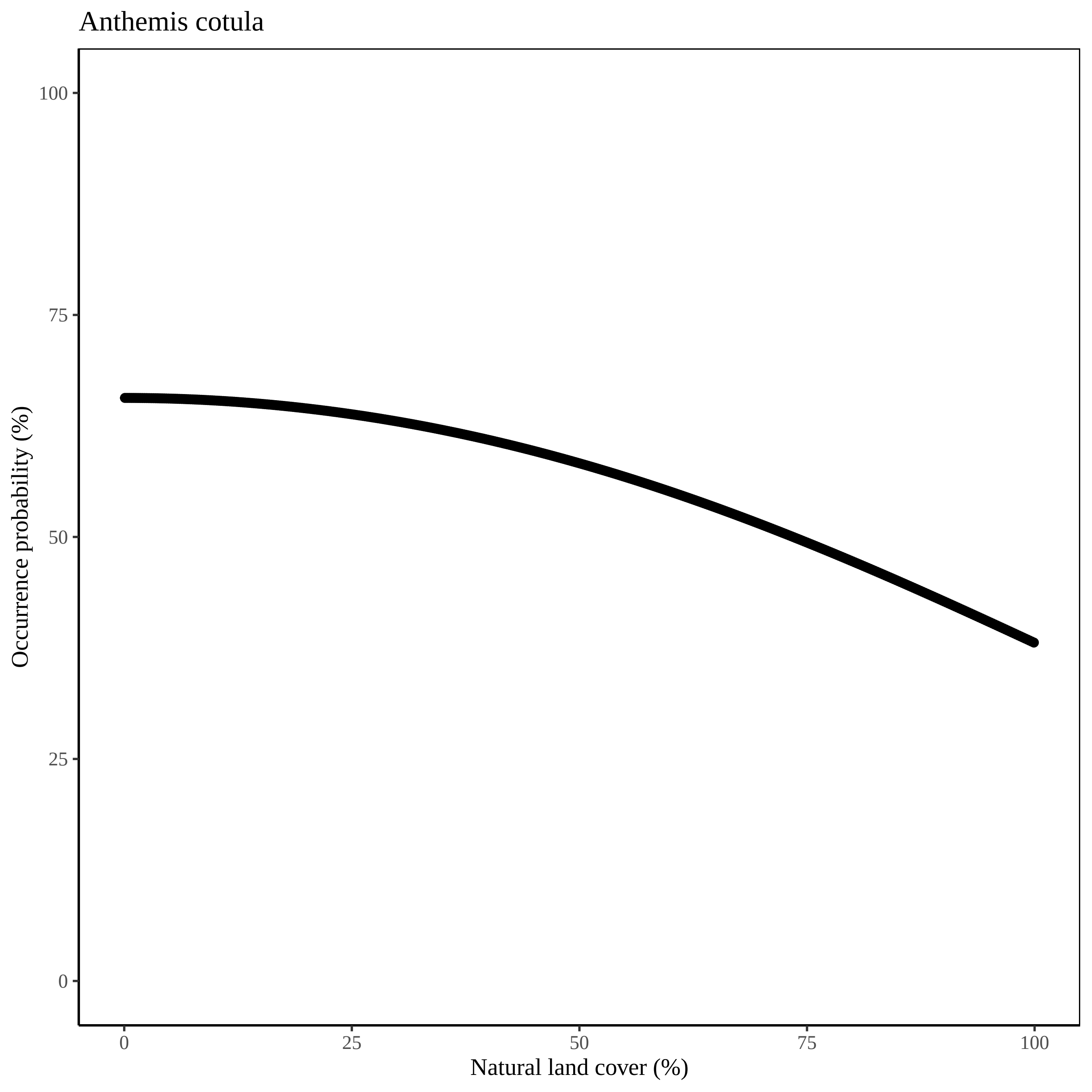

Anthemis cotula
100
75
50
25
0
Occurrence probability (%)
0
25
50
75
100
Natural land cover (%)

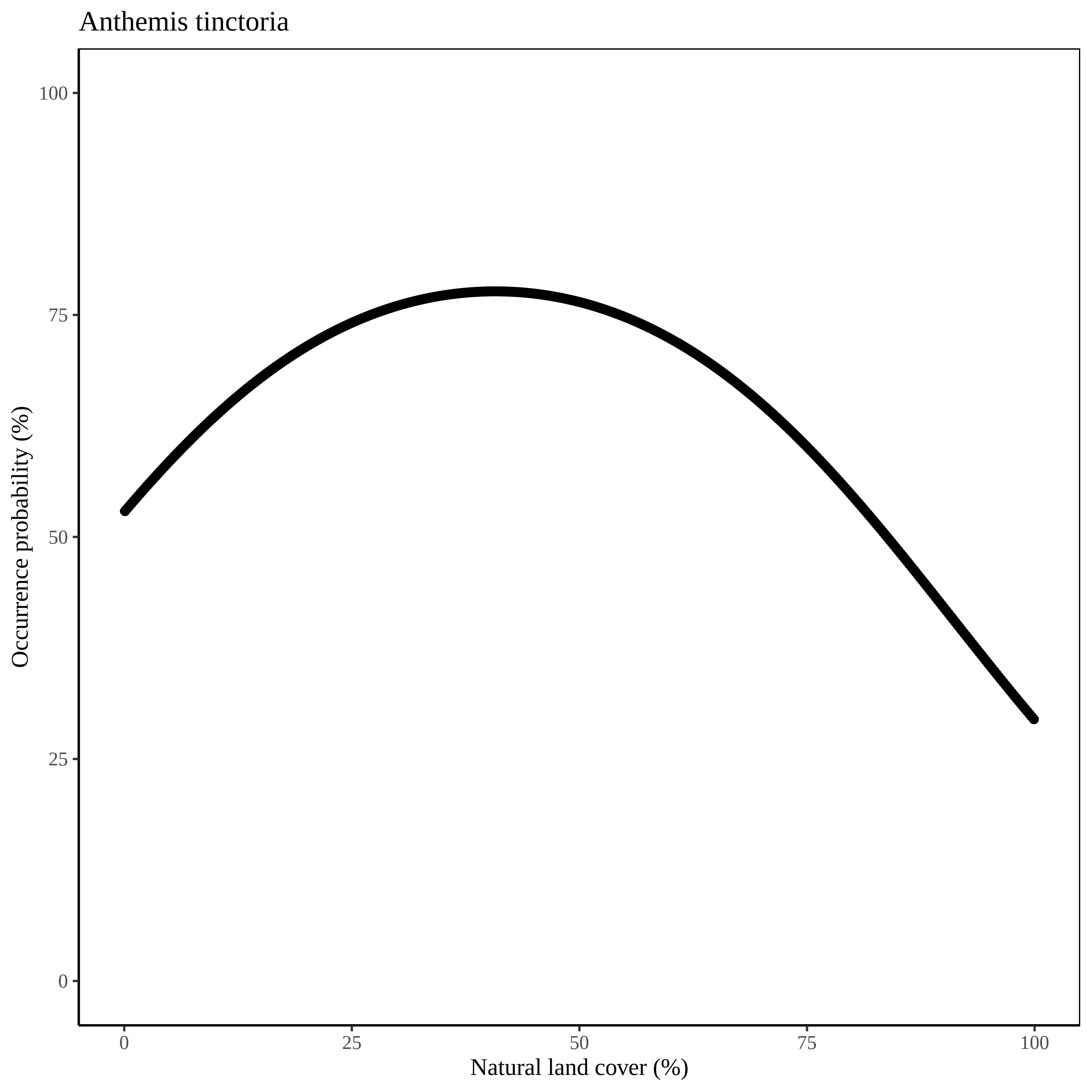
Anthemis tinctoria
Occurrence probability (%)
100
75
50
25
0
0
25
50
75
100
Natural land cover (%)

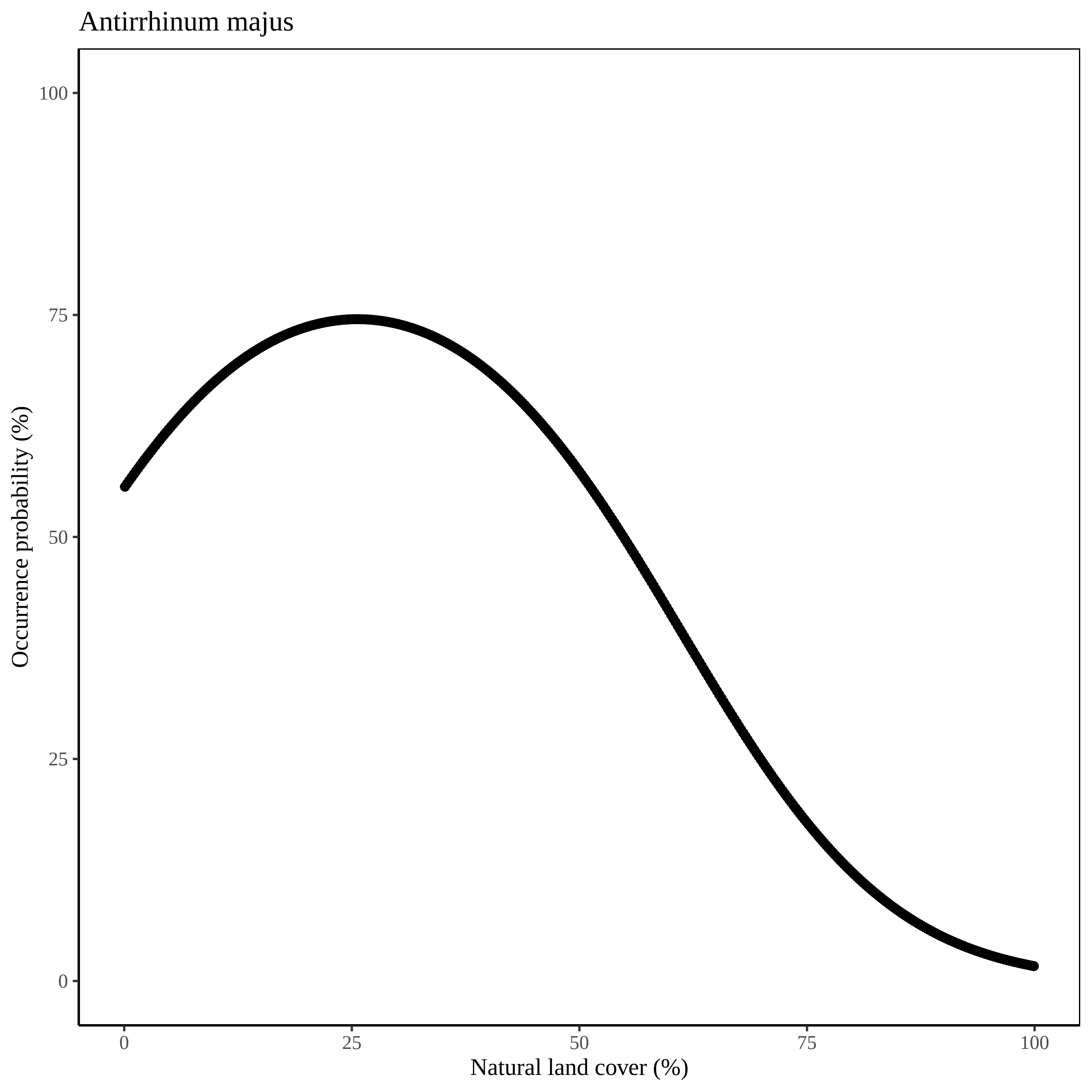
Antirrhinum majus
Occurrence probability (%)
100
75
50
25
0
0
25
50
75
100
Natural land cover (%)

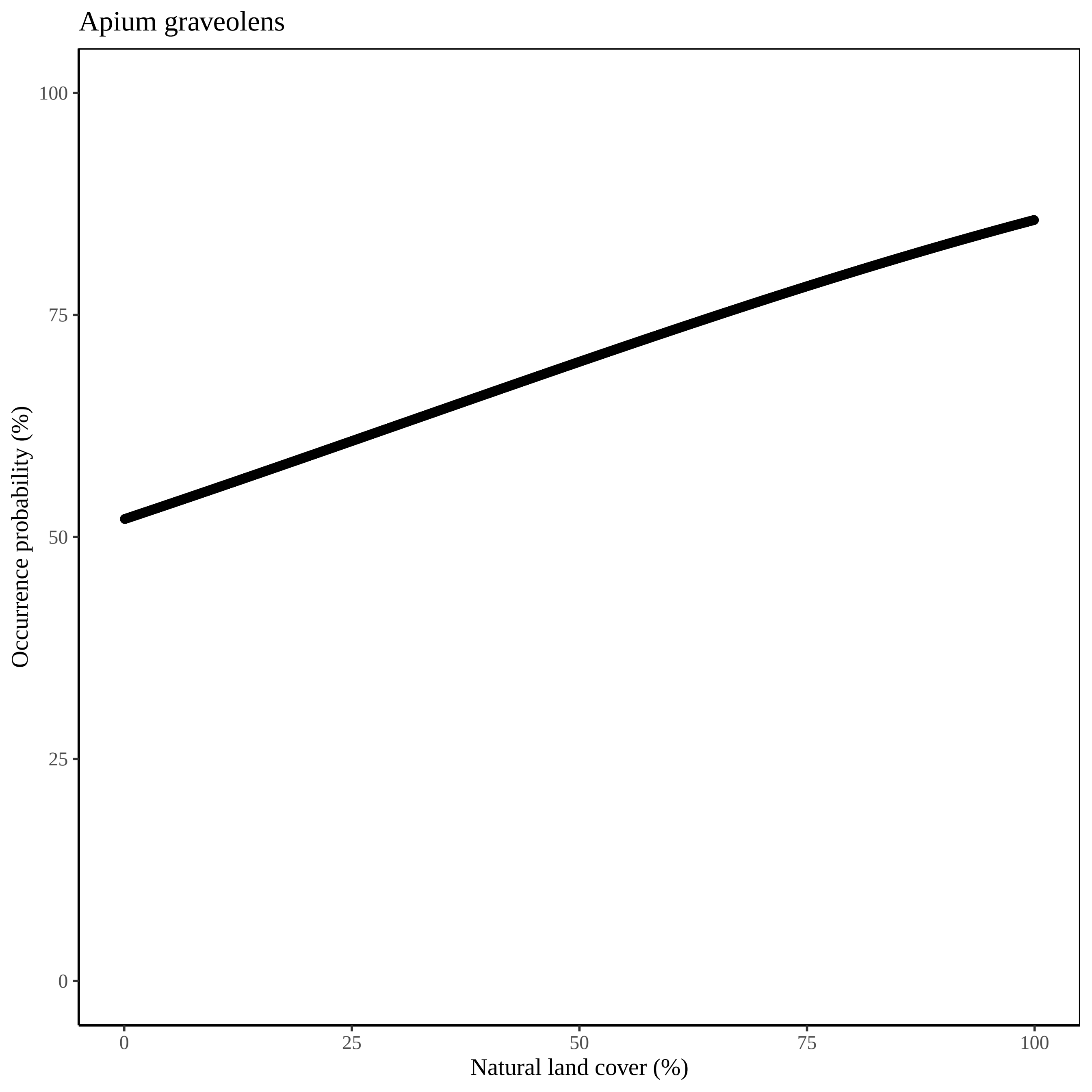
Apium graveolens
Occurrence probability (%)
100
75
50
25
0
Natural land cover (%)
0
25
50
75
100

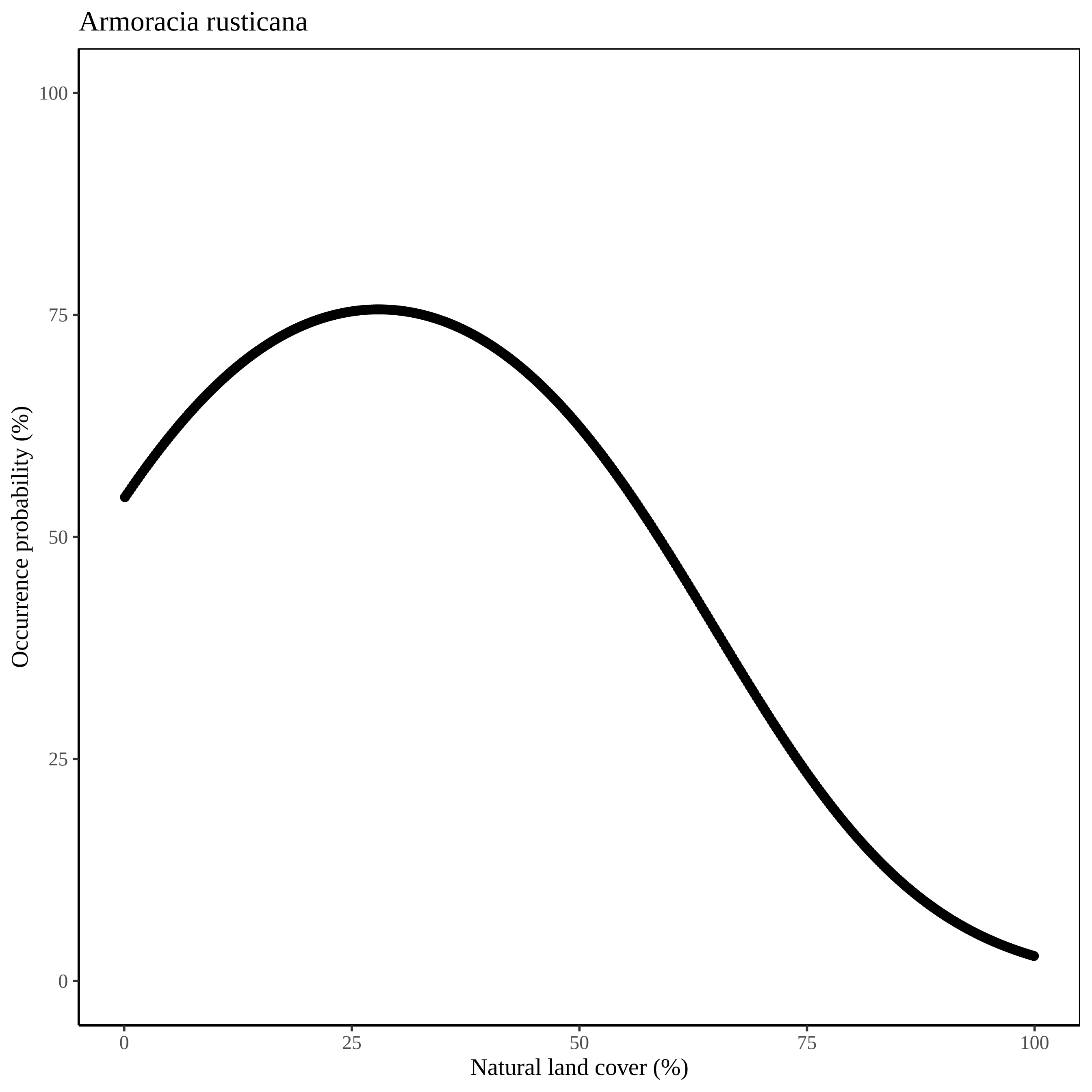

Armoracia rusticana
Occurrence probability (%)
100
75
50
25
0
0
25
50
75
100
Natural land cover (%)

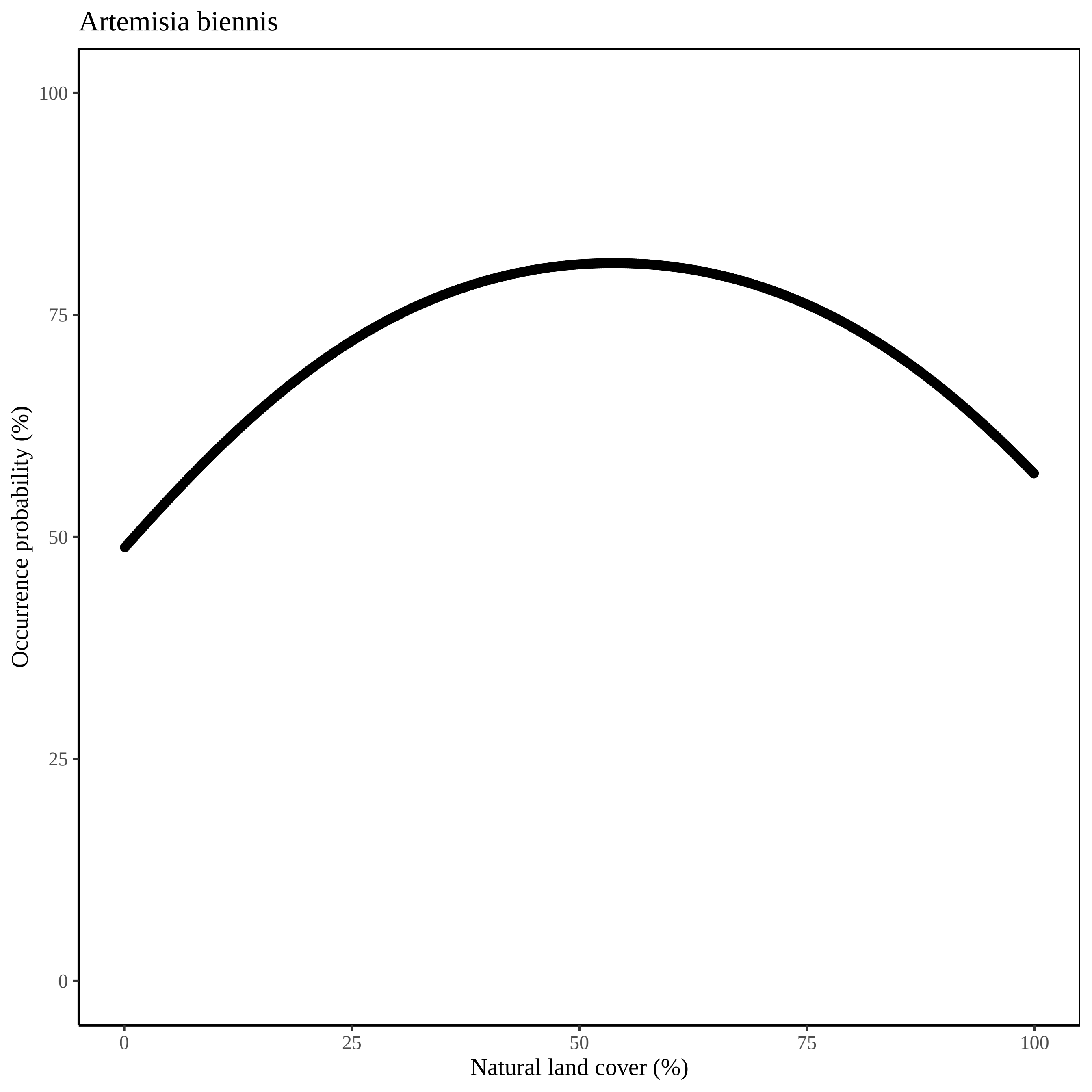
Artemisia biennis
100
75
50
25
0
Occurrence probability (%)
0
25
50
75
100
Natural land cover (%)

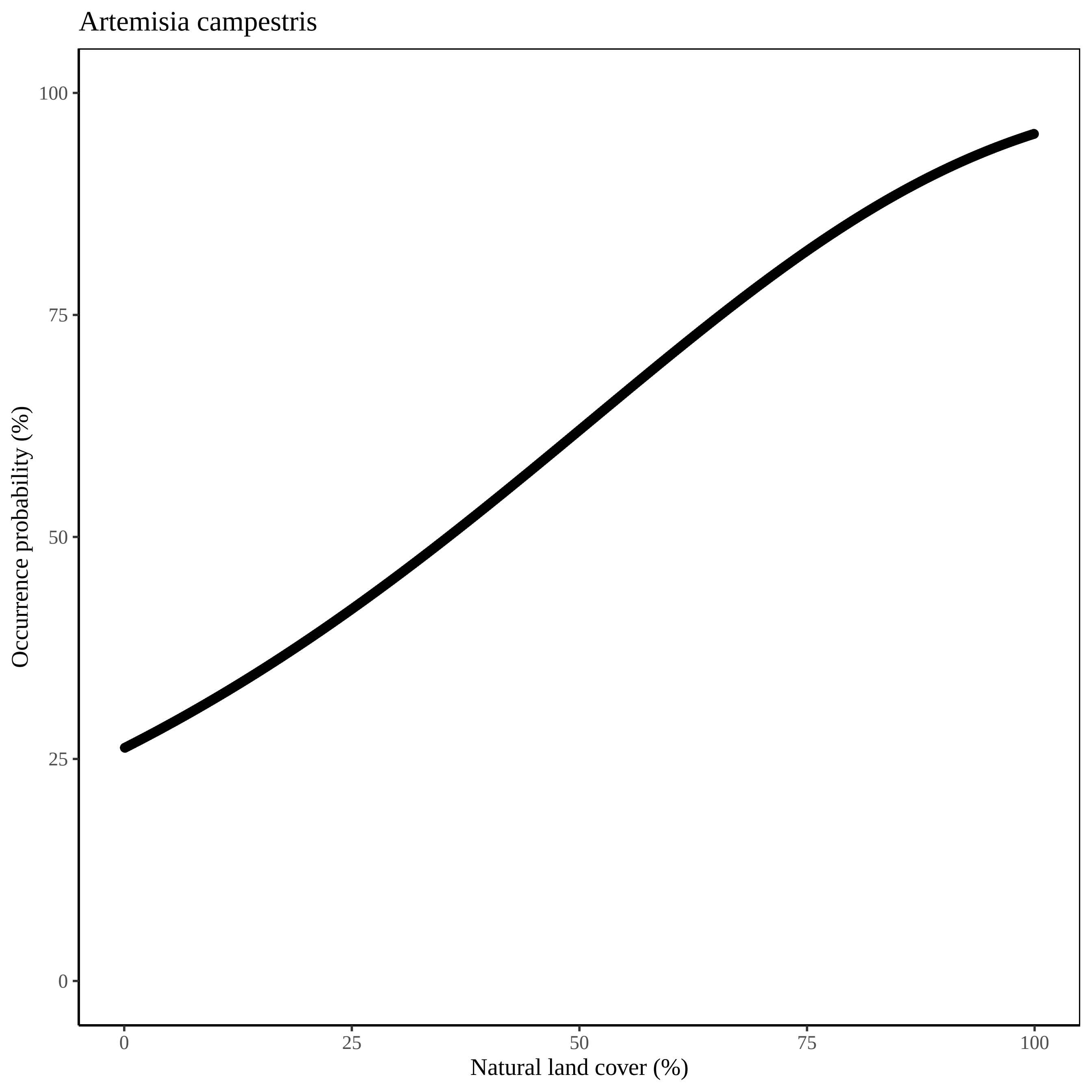
Artemisia campestris
Occurrence probability (%)
100
75
50
25
0
0
25
50
75
100
Natural land cover (%)

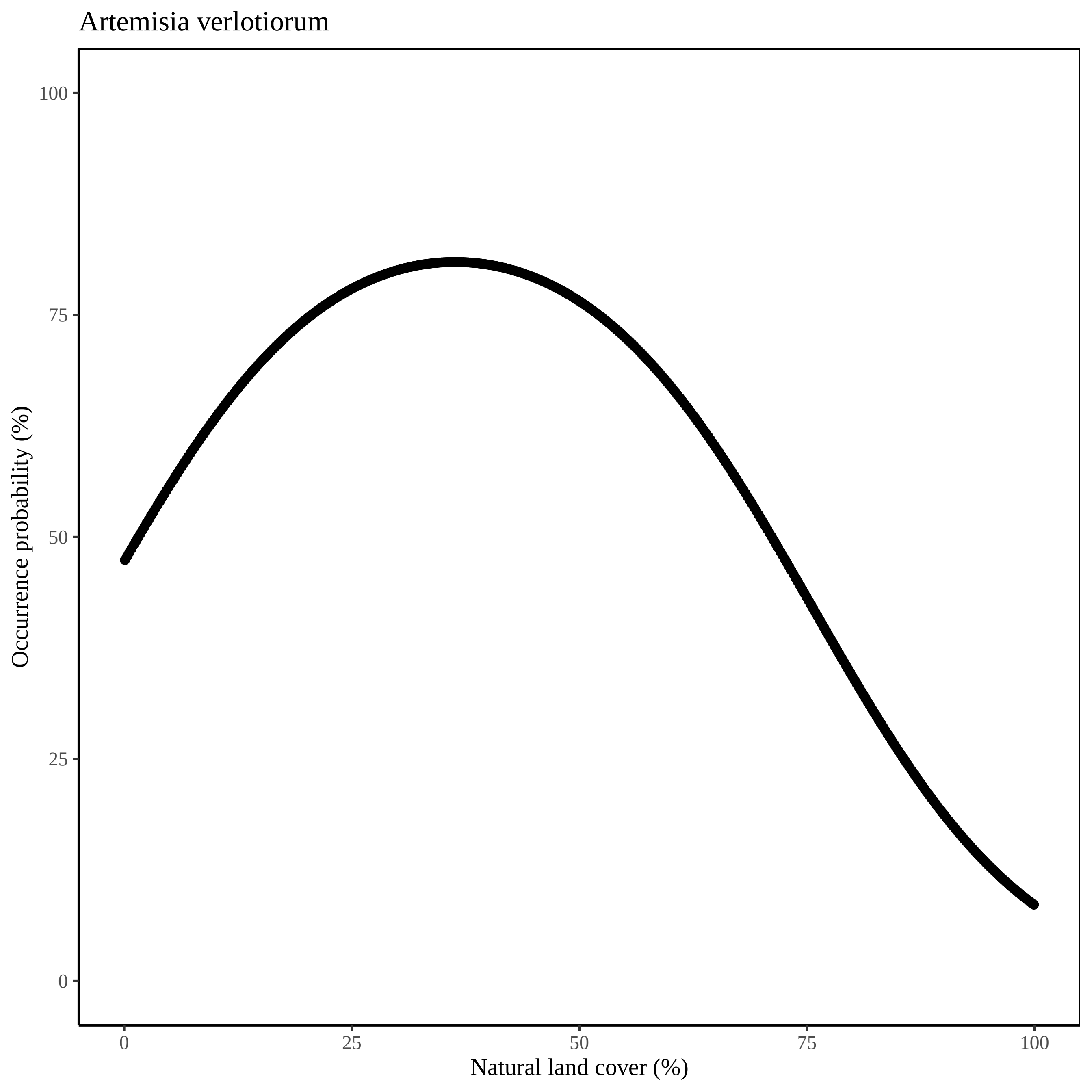
Artemisia verlotiorum
Occurrence probability (%)
100
75
50
25
0
0
25
50
75
100
Natural land cover (%)

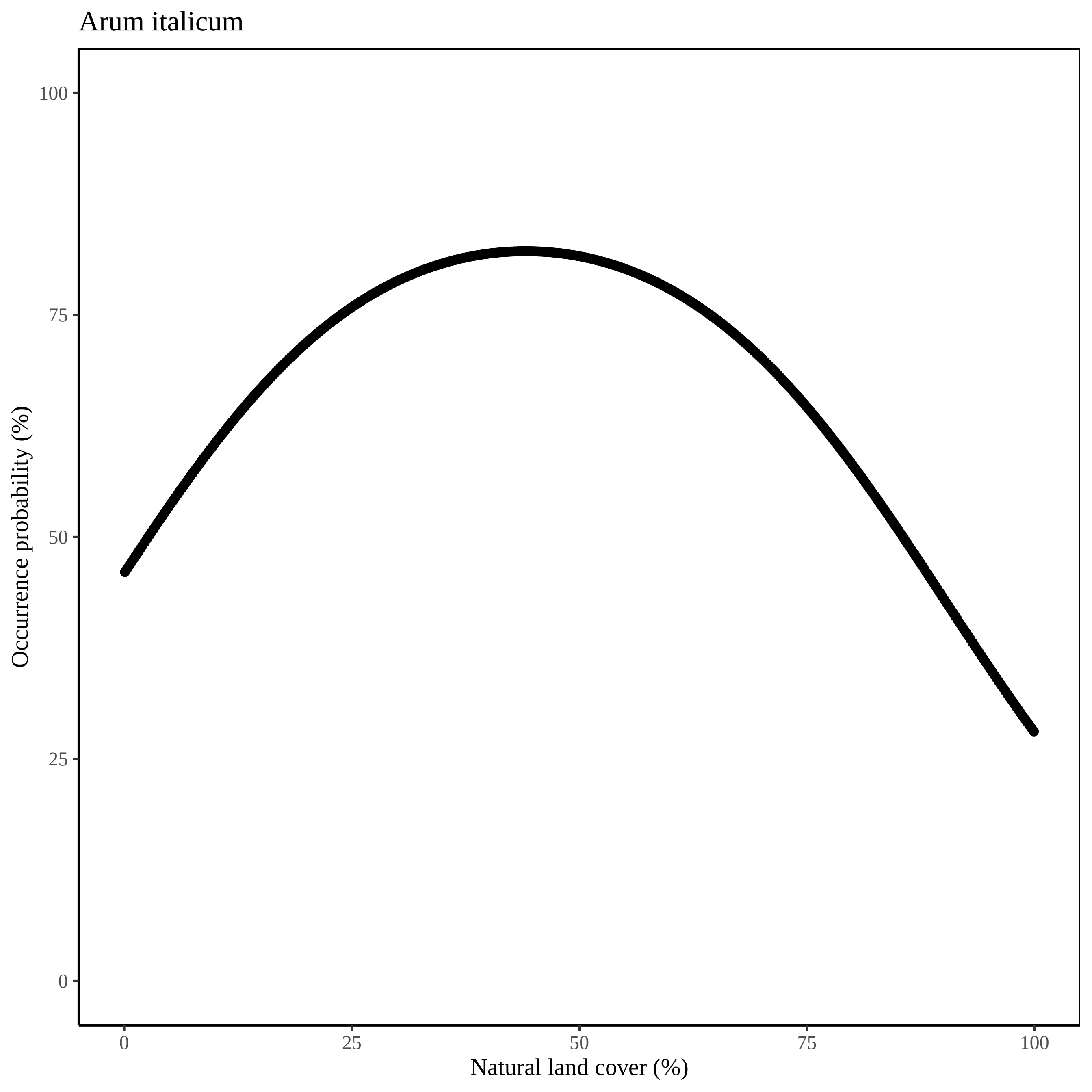
Arum italicum
Occurrence probability (%)
100
75
50
25
0
0
25
50
75
100
Natural land cover (%)

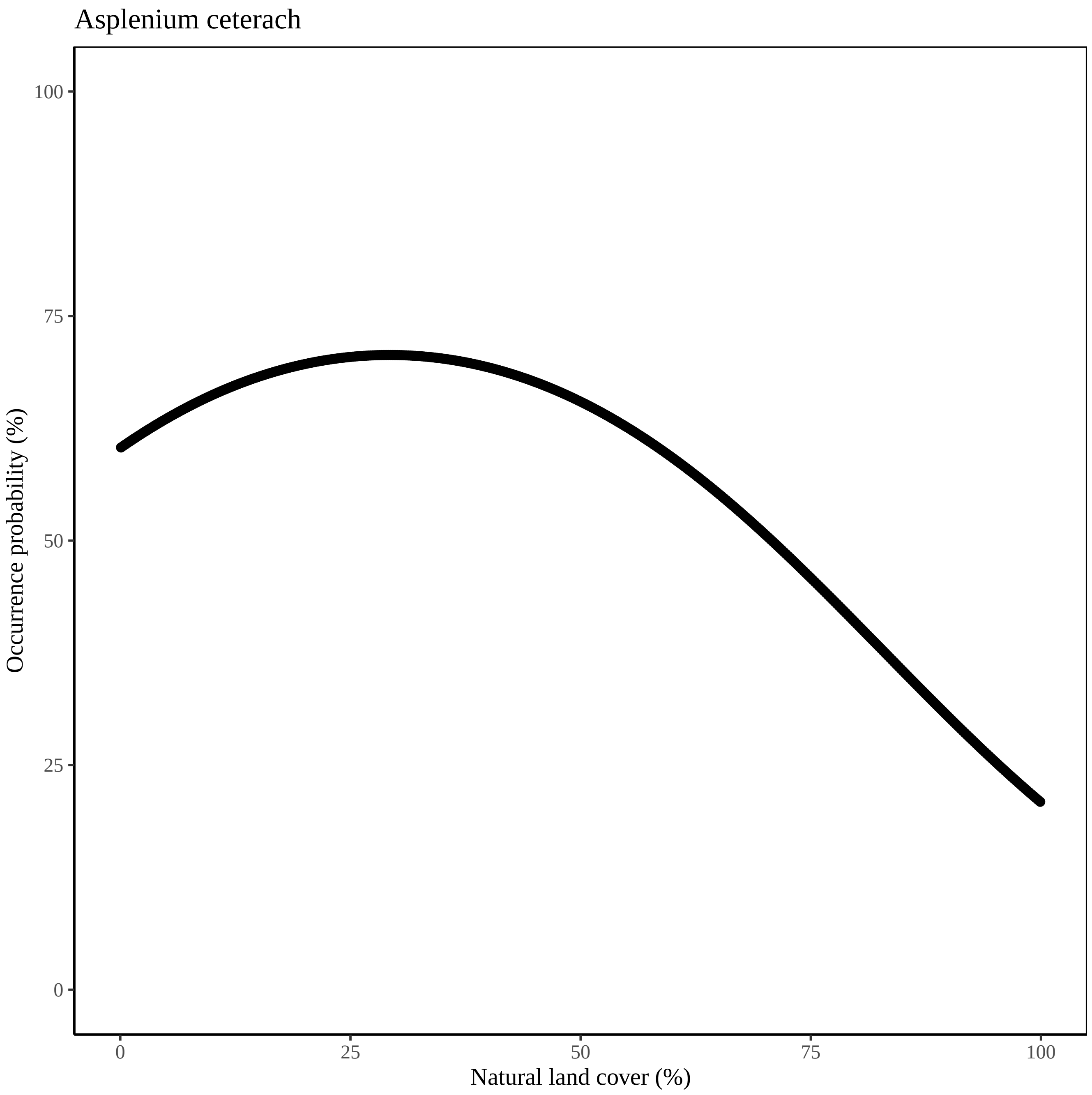
Asplenium ceterach
Occurrence probability (%)
100
75
50
25
0
0
25
50
75
100
Natural land cover (%)

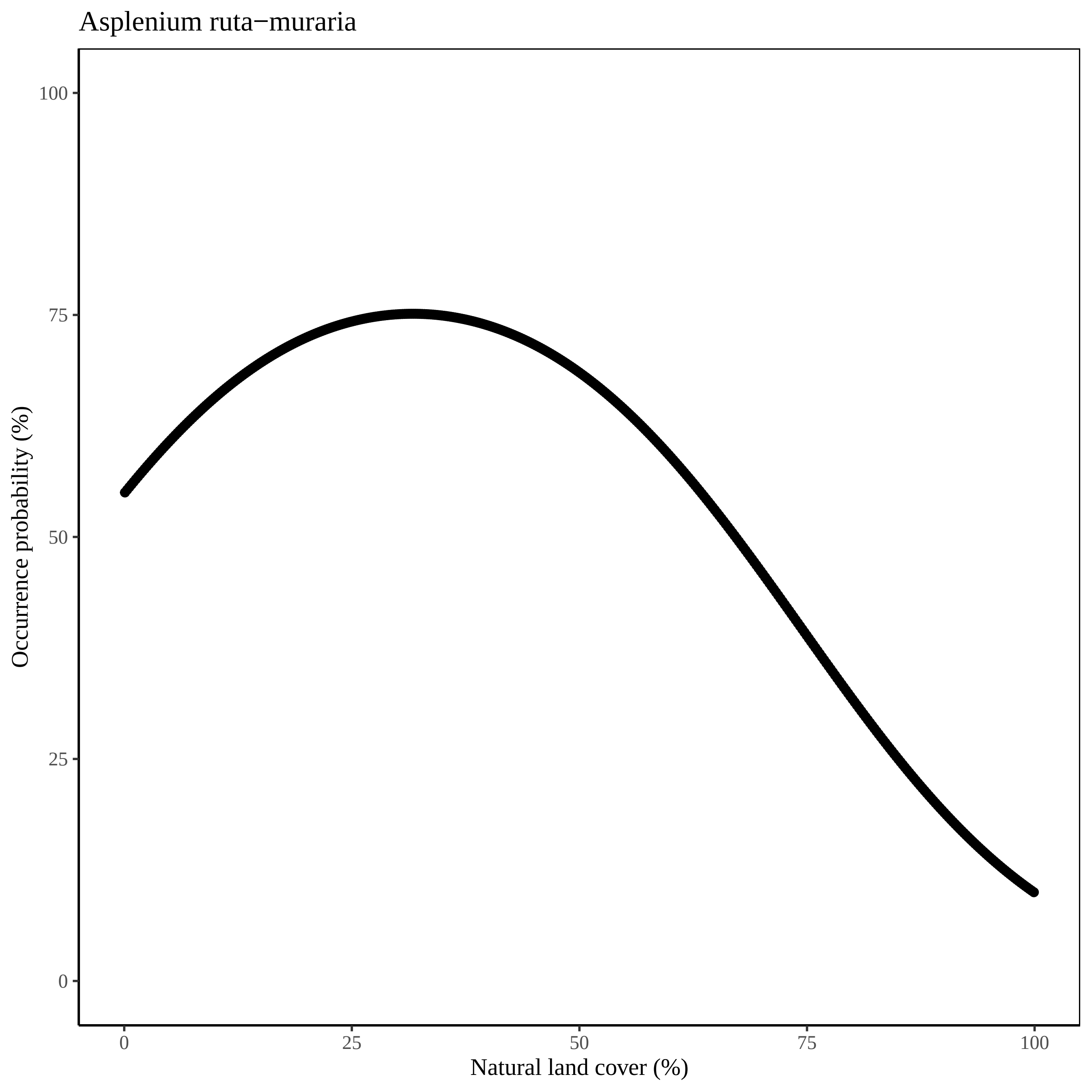

Asplenium ruta−muraria
Occurrence probability (%)
100
75
50
25
0
0
25
50
75
100
Natural land cover (%)

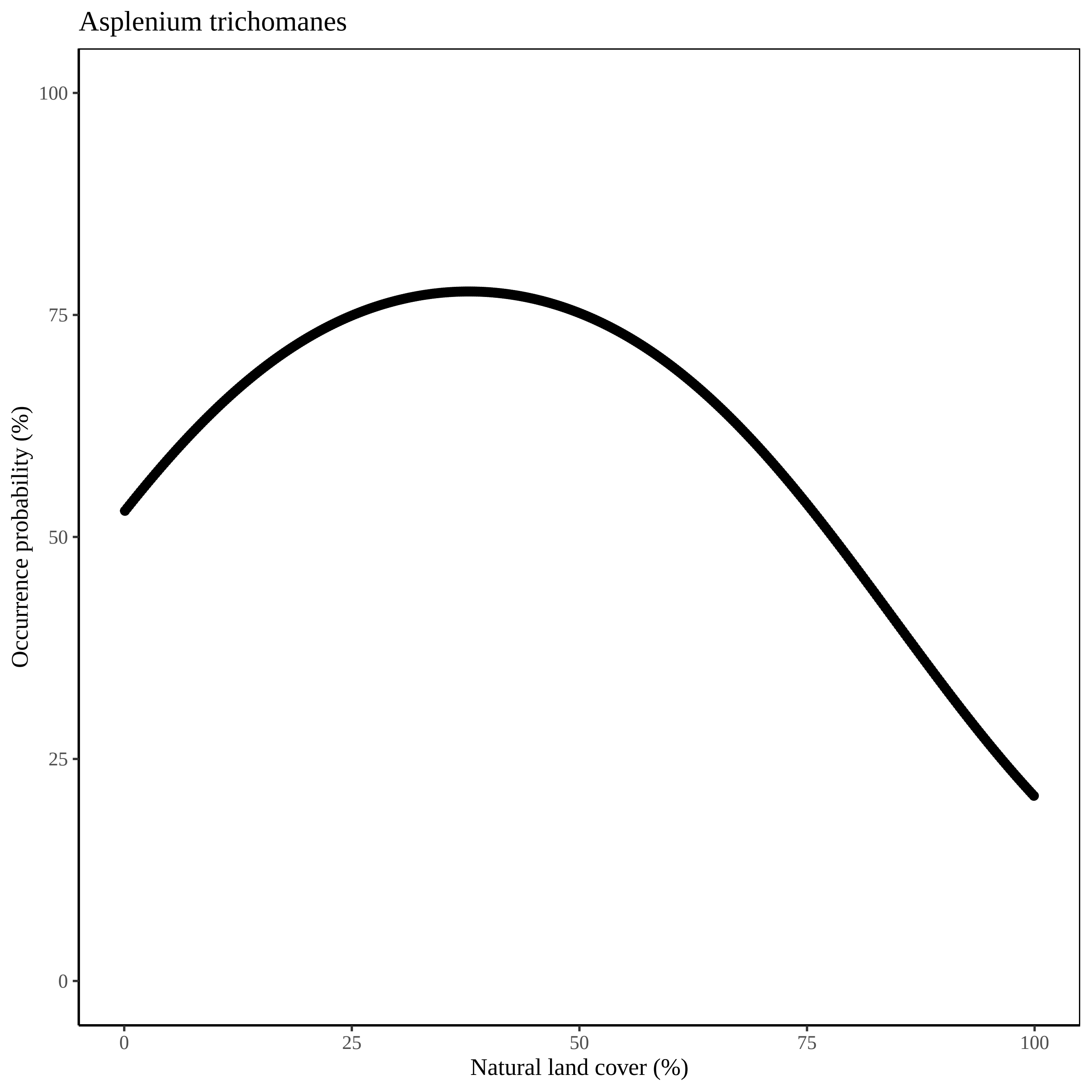
Asplenium trichomanes
Occurrence probability (%)
100
75
50
25
0
0
25
50
75
100
Natural land cover (%)

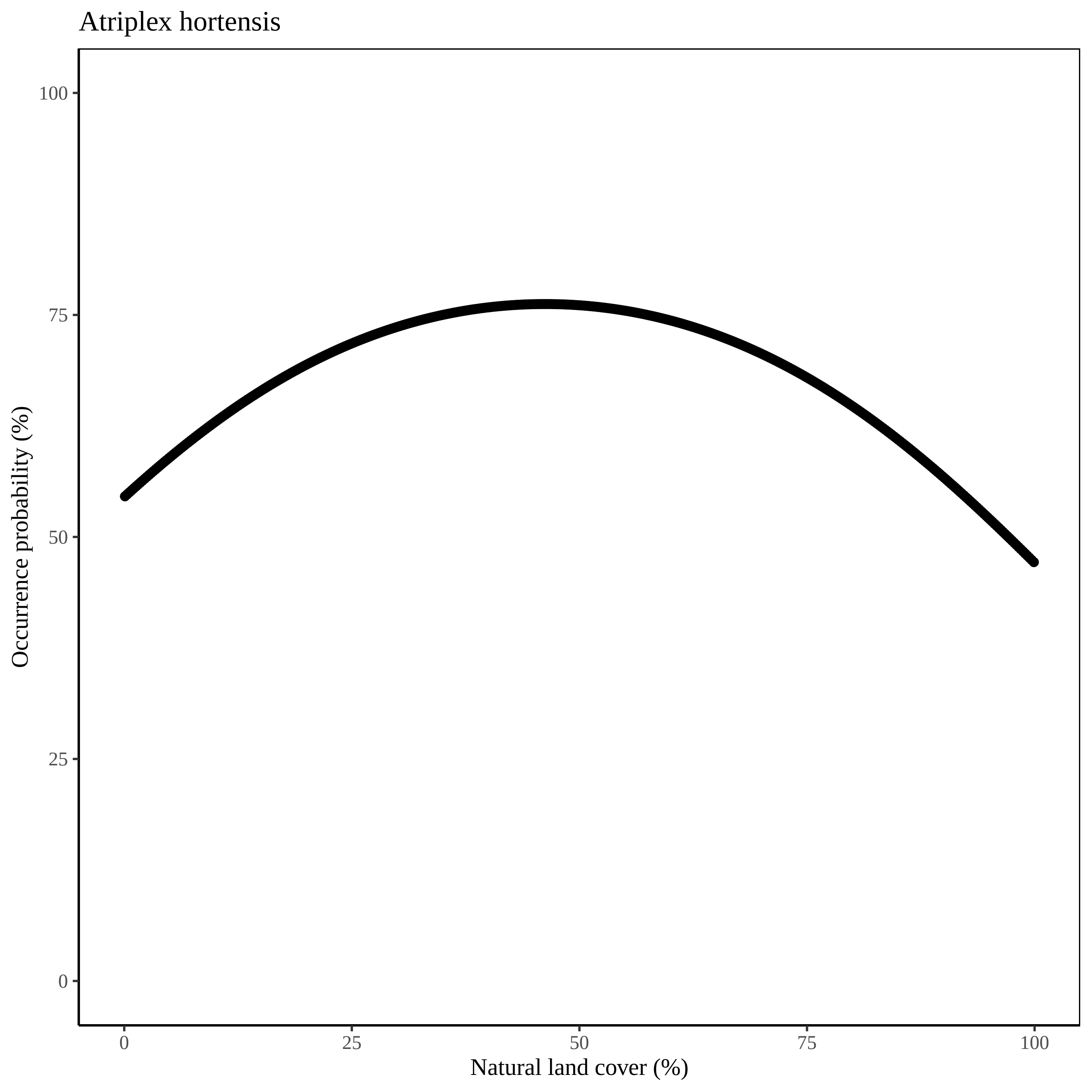
Atriplex hortensis
Occurrence probability (%)
100
75
50
25
0
0
25
50
75
100
Natural land cover (%)

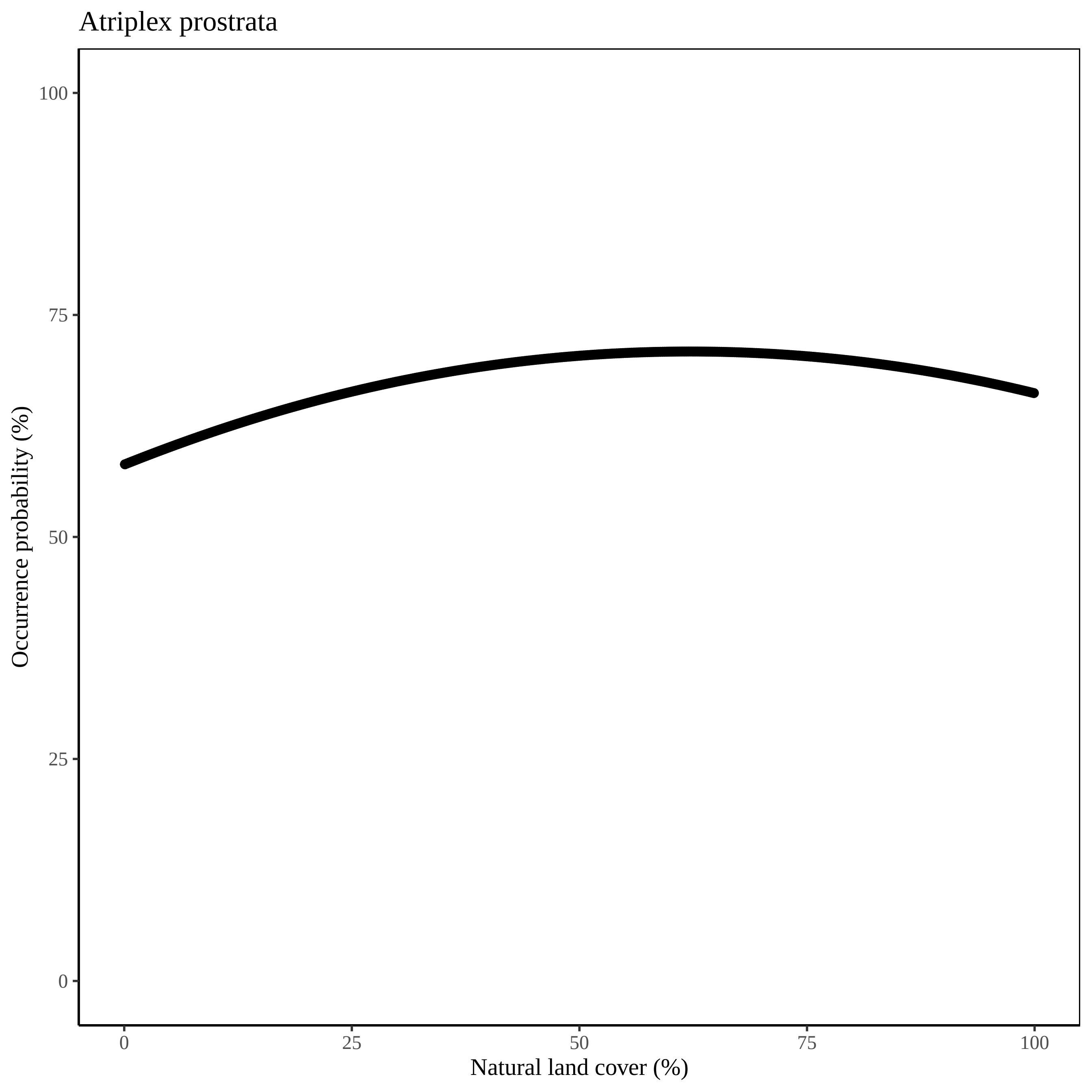

Atriplex prostrata
Occurrence probability (%)
100
75
50
25
0
0
25
50
75
100
Natural land cover (%)

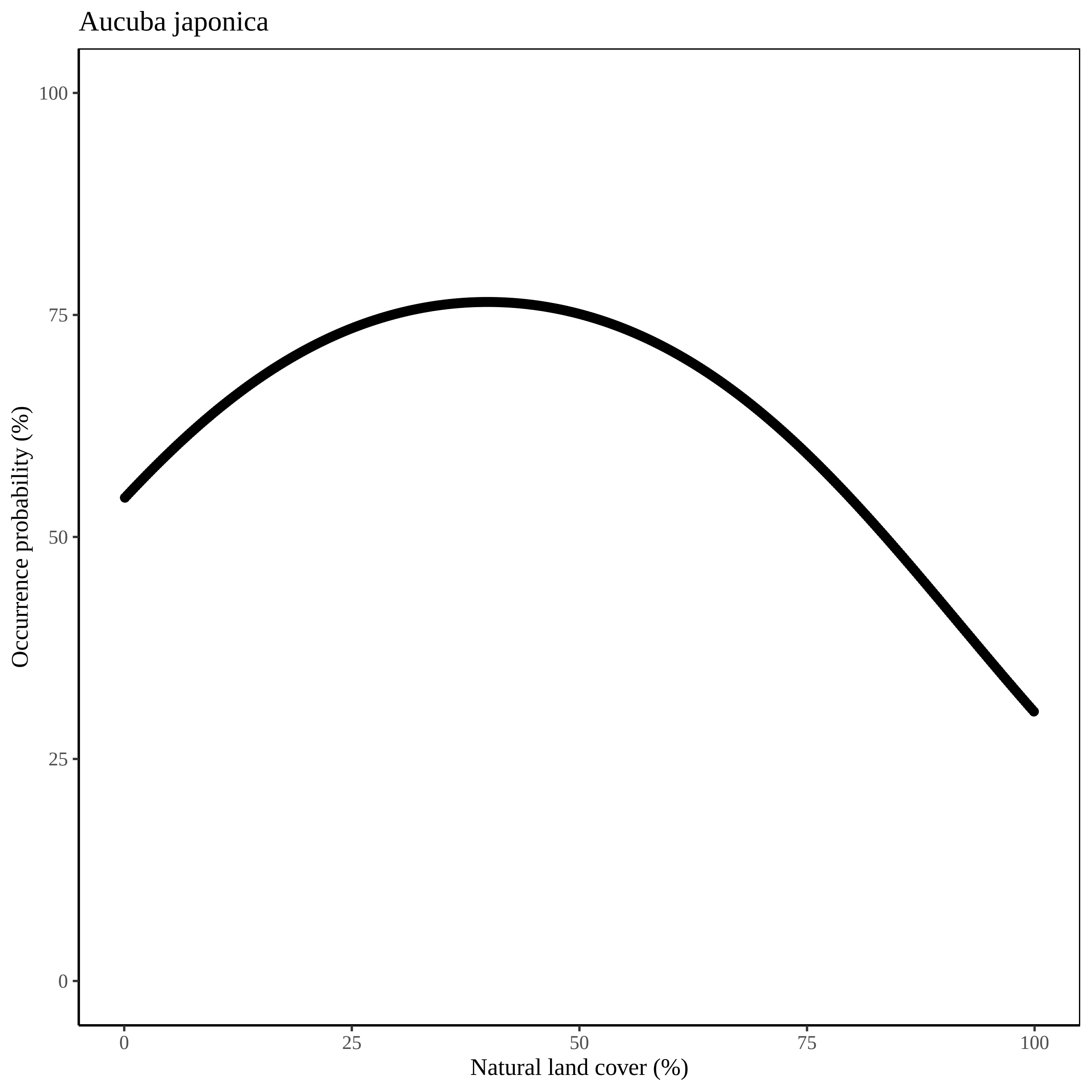

Aucuba japonica
100
75
50
25
0
Occurrence probability (%)
0
25
50
75
100
Natural land cover (%)

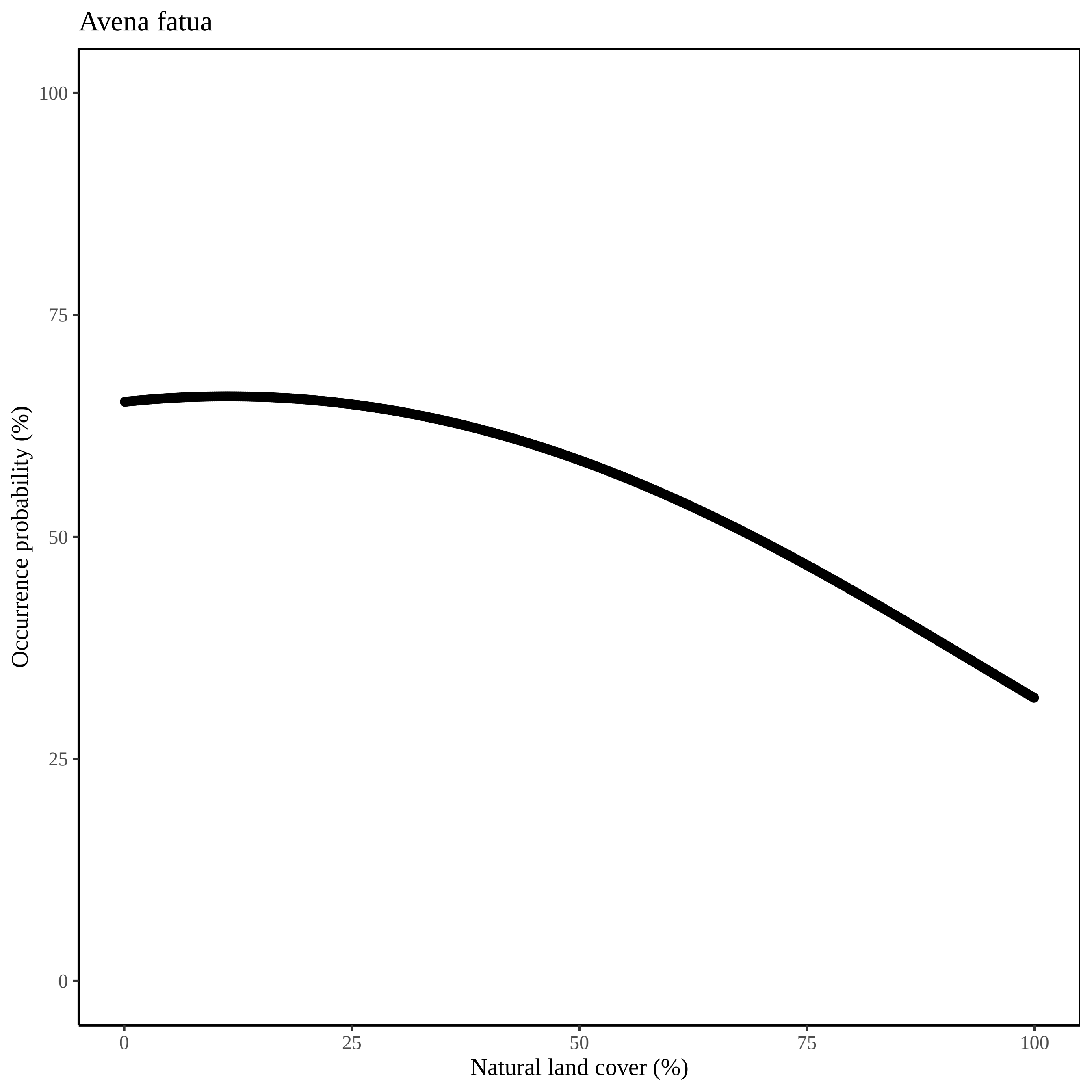

Avena fatua
Occurrence probability (%)
100
75
50
25
0
0
25
50
75
100
Natural land cover (%)

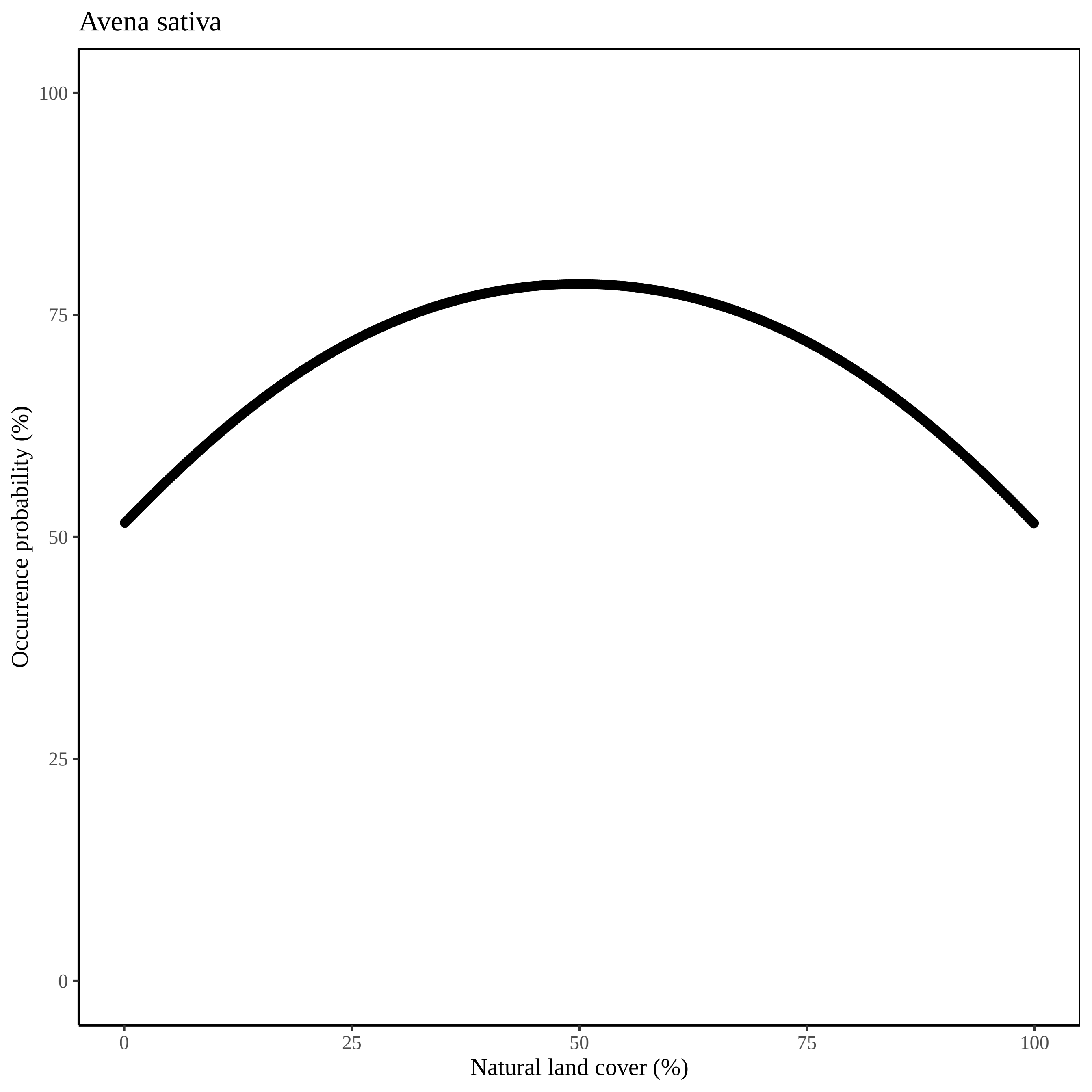
Avena sativa
Occurrence probability (%)
100
75
50
25
0
0
25
50
75
100
Natural land cover (%)

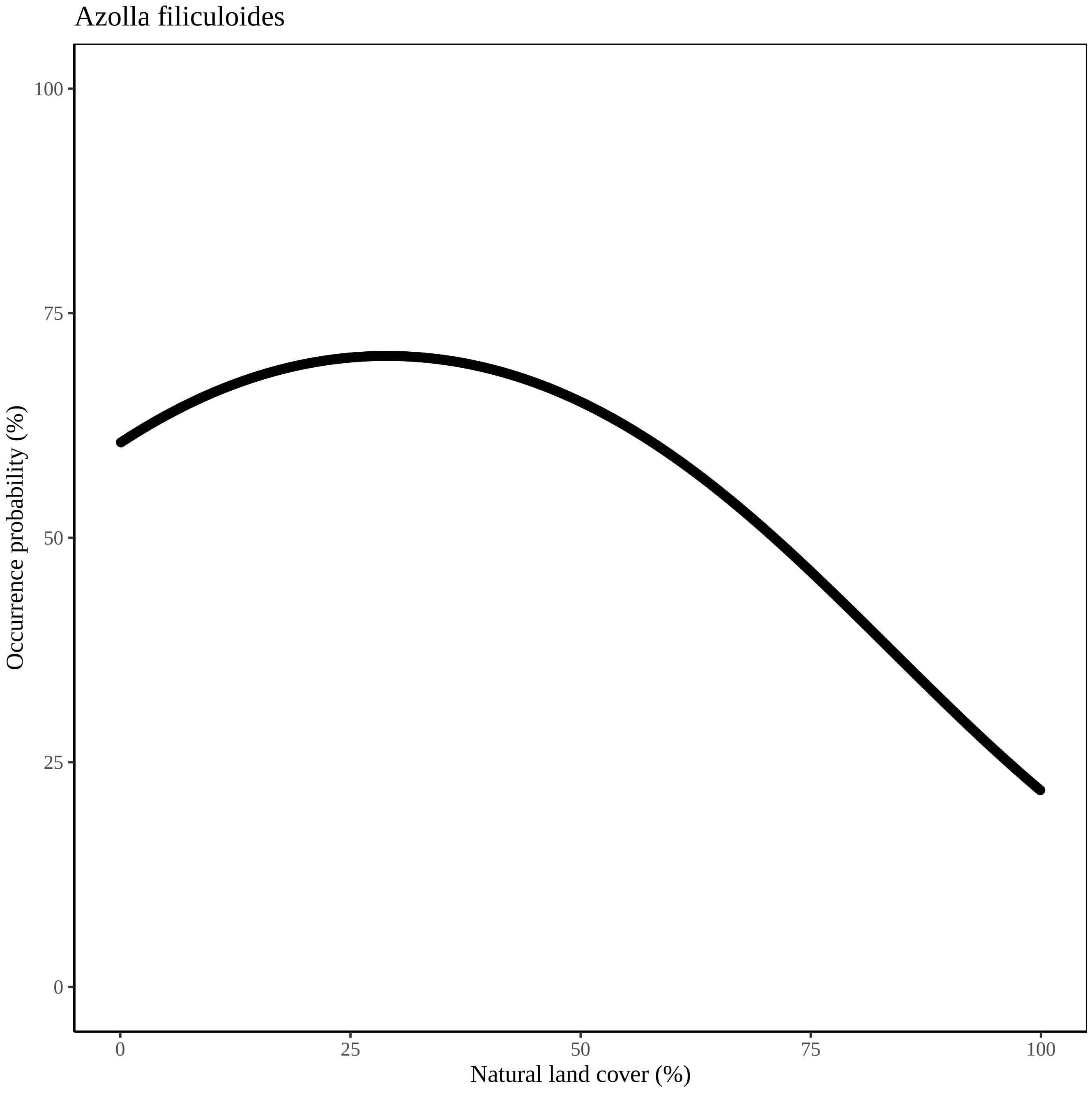
Azolla filiculoides
Occurrence probability (%)
100
75
50
25
0
0
25
50
75
100
Natural land cover (%)

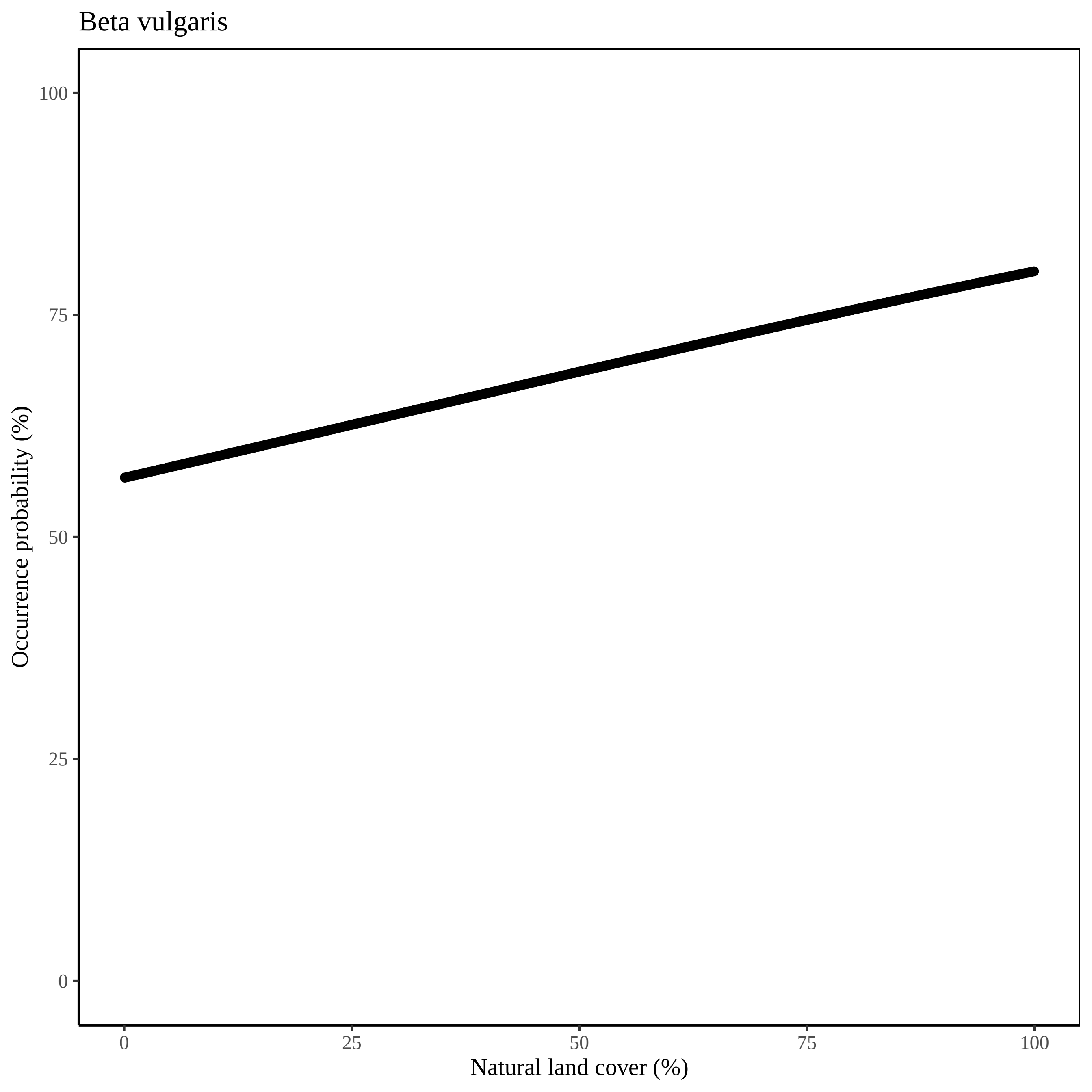

Beta vulgaris
Occurrence probability (%)
100
75
50
25
0
0
25
50
75
100
Natural land cover (%)

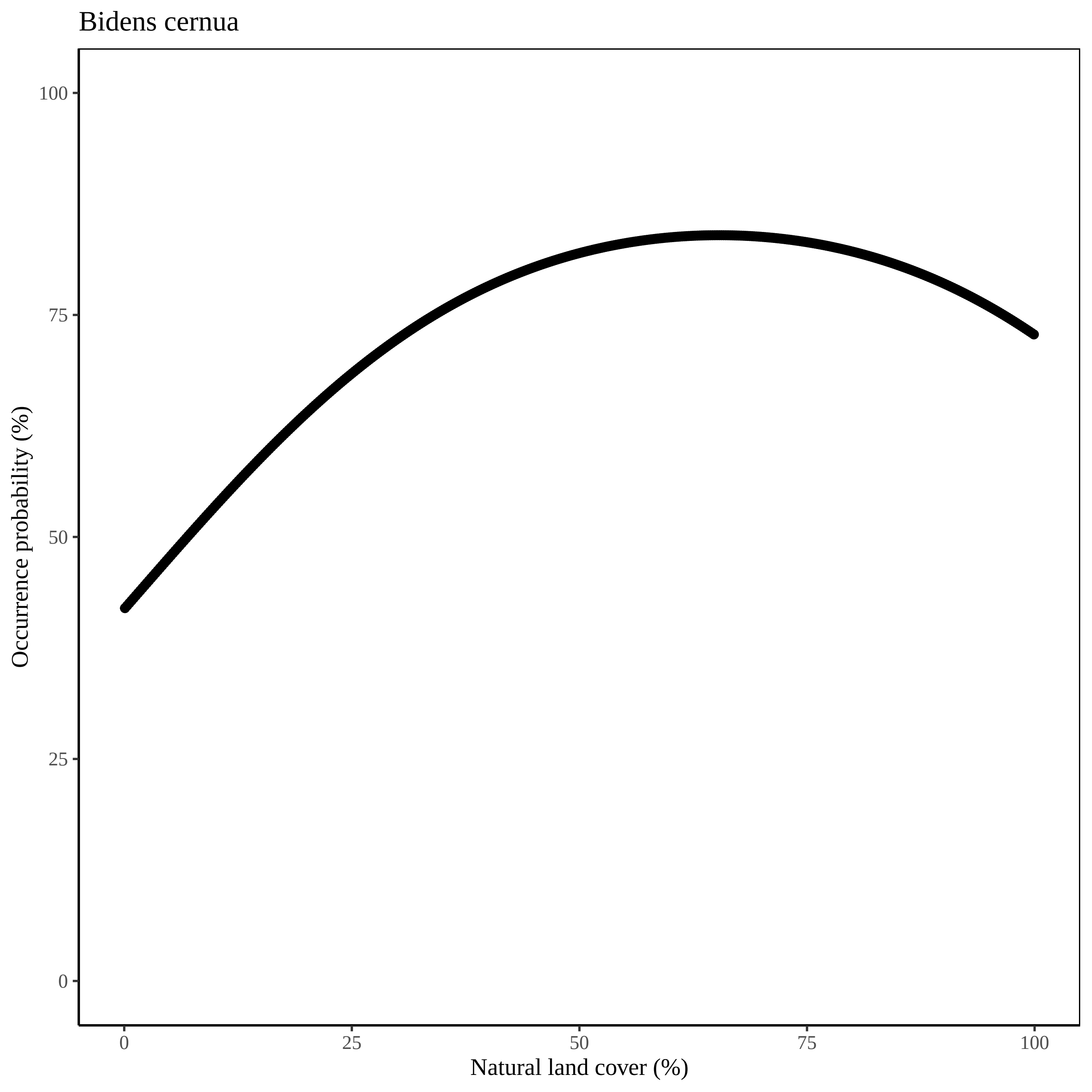
Bidens cernua
Occurrence probability (%)
100
75
50
25
0
0
25
50
75
100
Natural land cover (%)

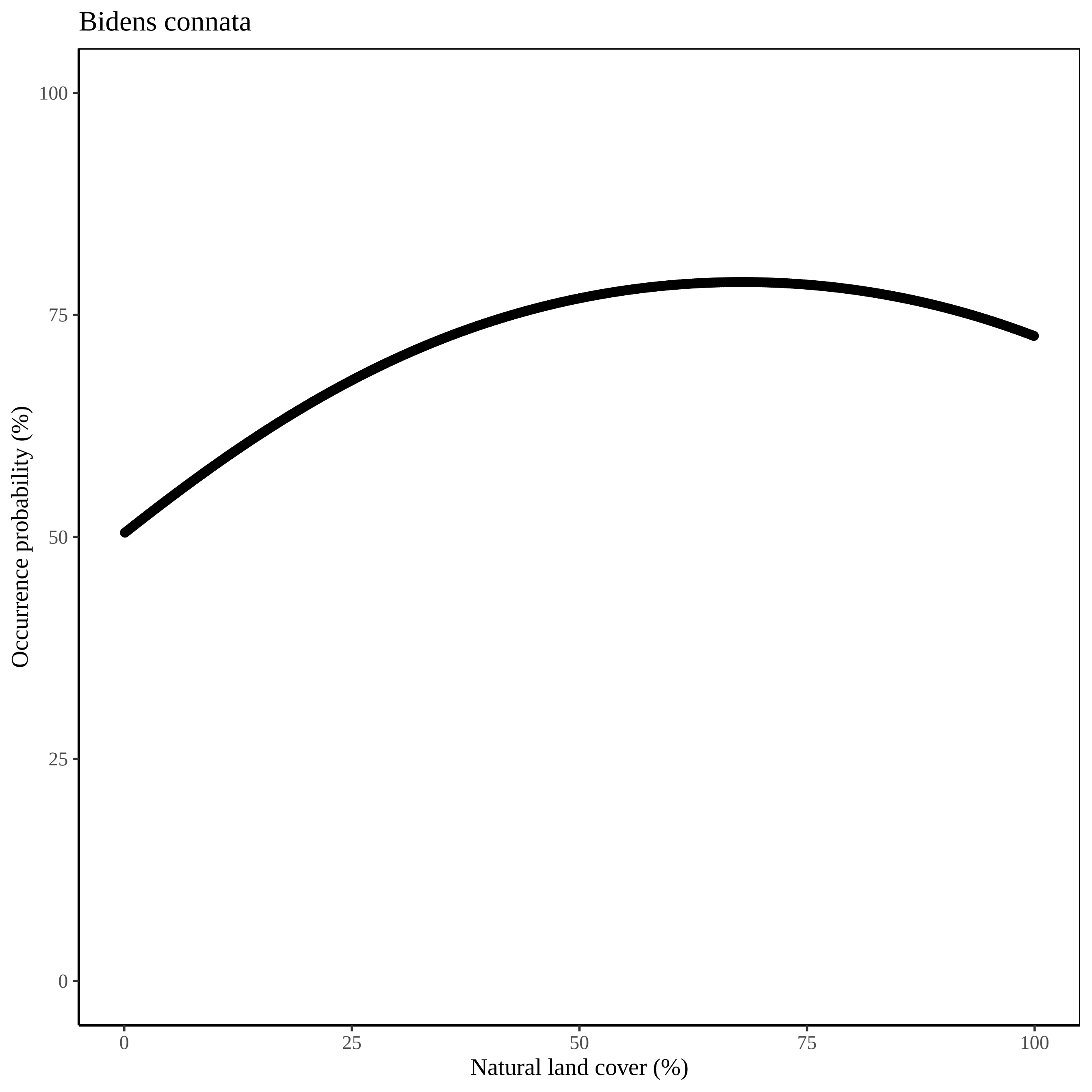
Bidens connata
Occurrence probability (%)
0
25
50
75
100
Natural land cover (%)
0
25
50
75
100

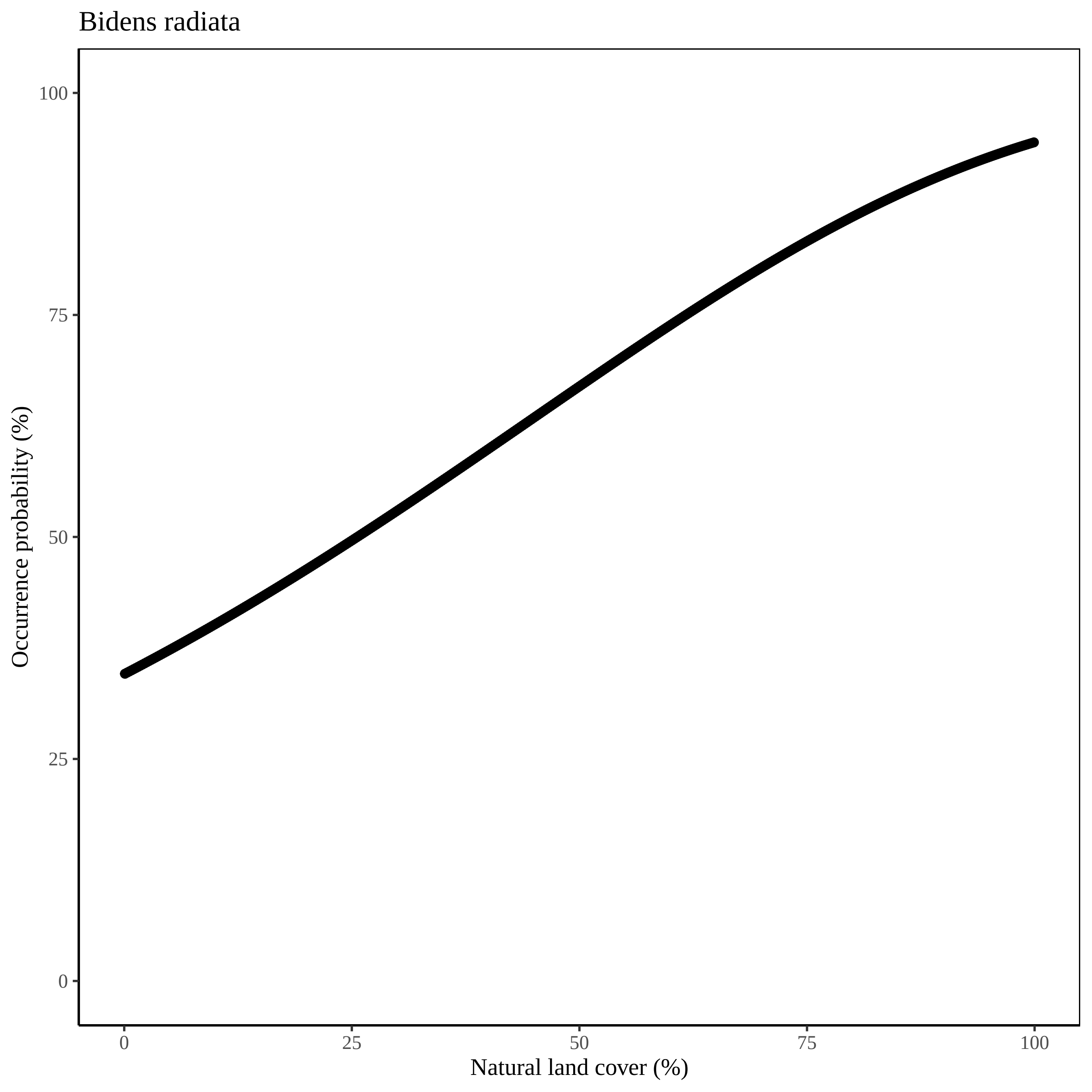

Bidens radiata
100
75
50
25
0
0
25
50
75
100
Occurrence probability (%)
Natural land cover (%)

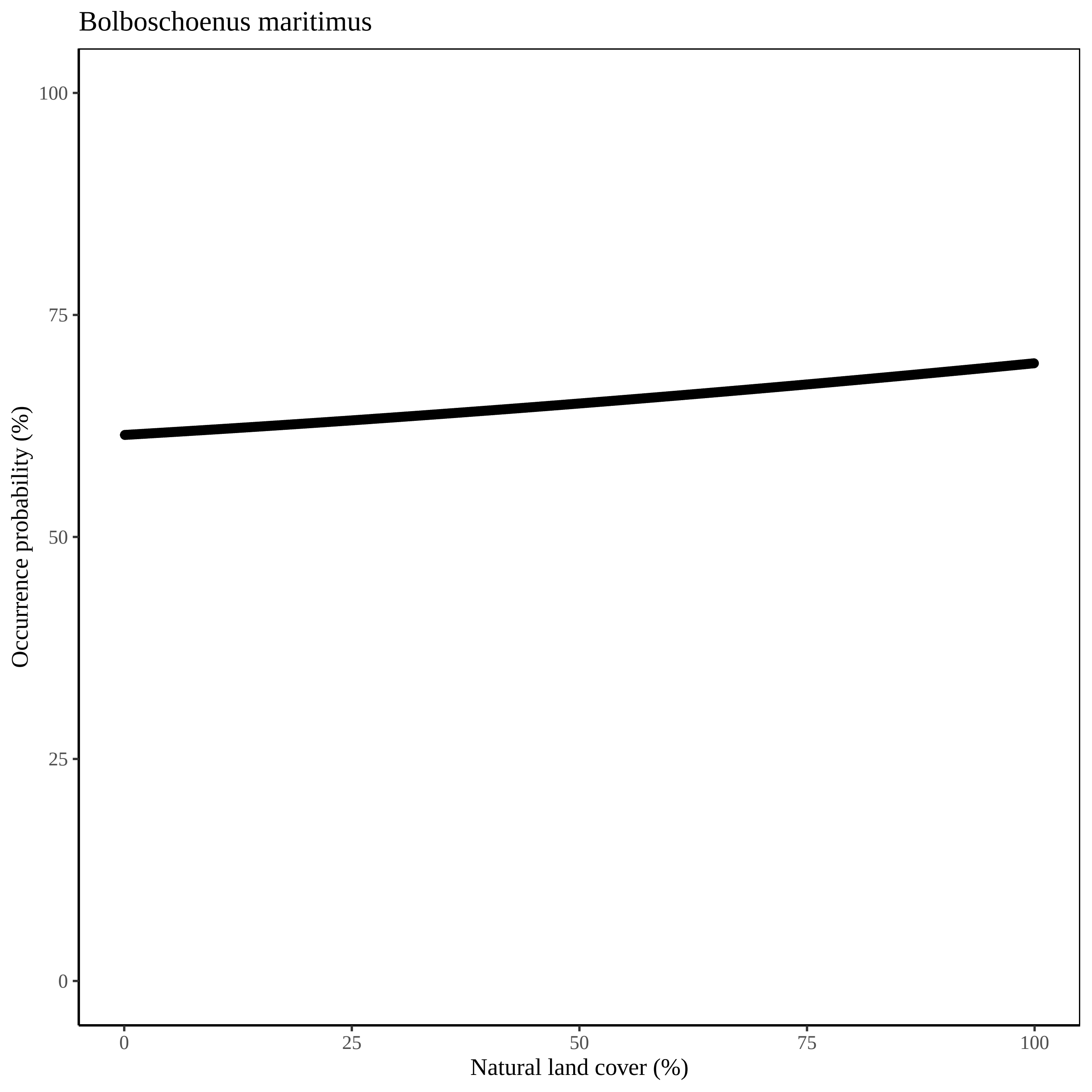

Bolboschoenus maritimus
Occurrence probability (%)
100
75
50
25
0
0
25
50
75
100
Natural land cover (%)

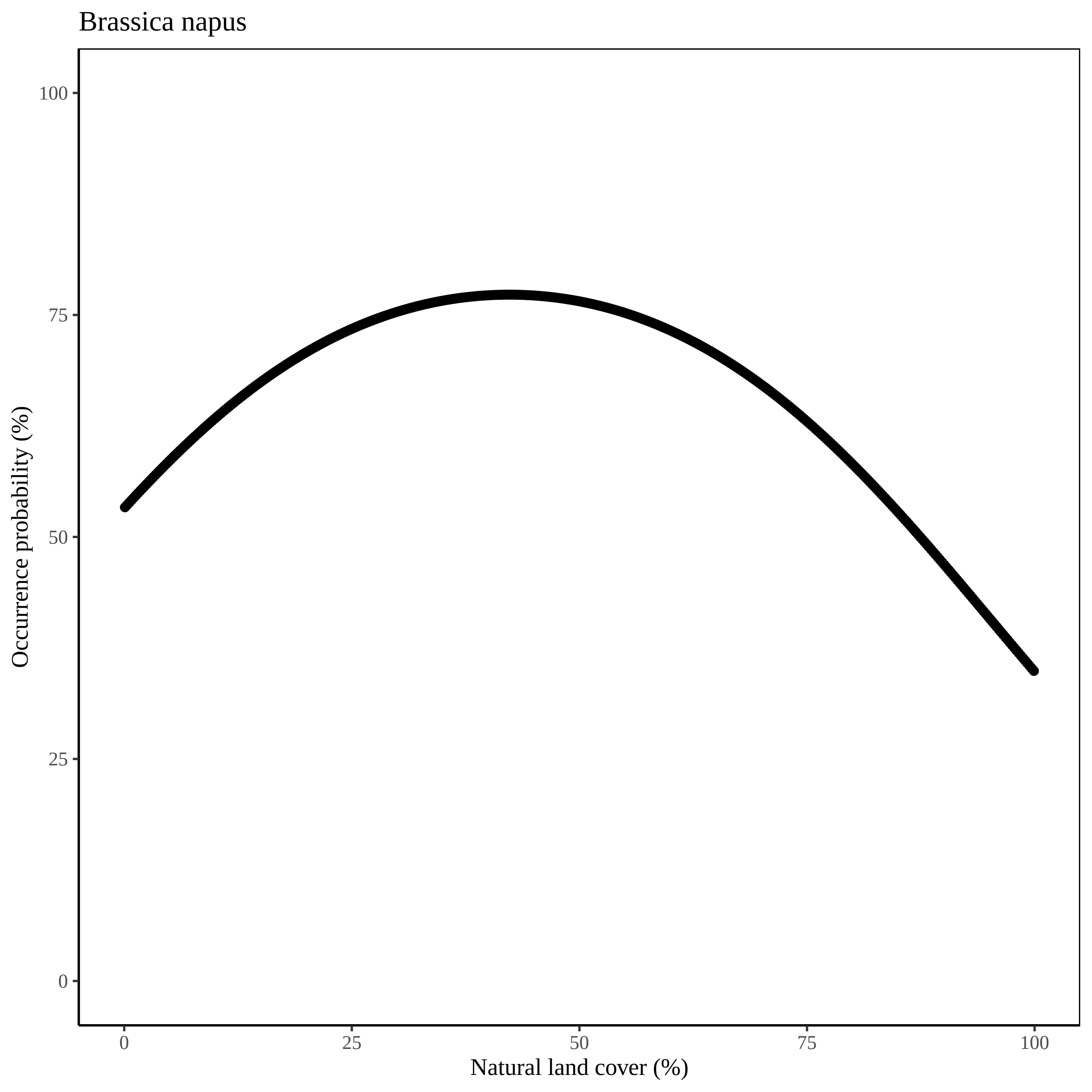
Brassica napus
Occurrence probability (%)
100
75
50
25
0
0
25
50
75
100
Natural land cover (%)

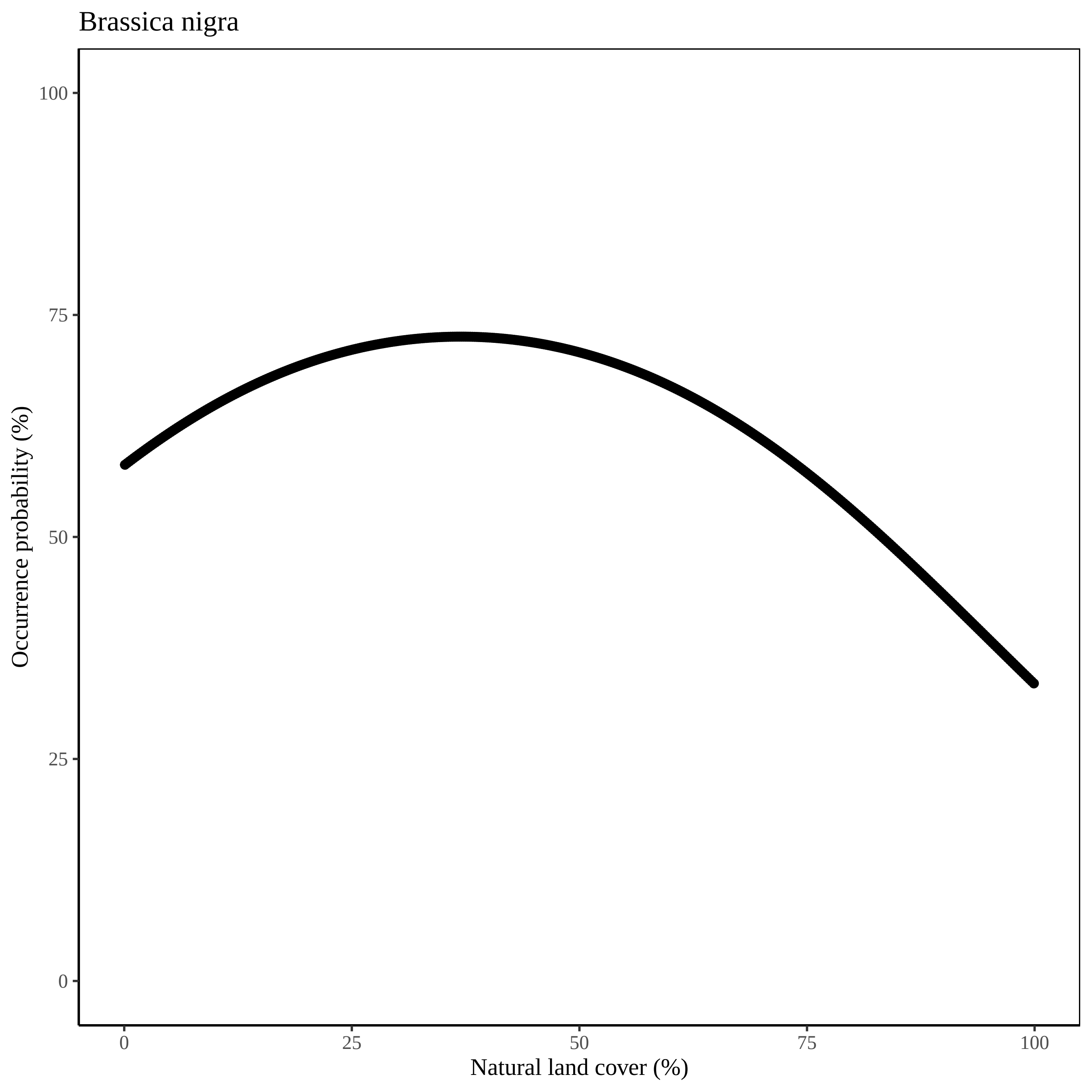

Brassica nigra
Occurrence probability (%)
100
75
50
25
0
0
25
50
75
100
Natural land cover (%)

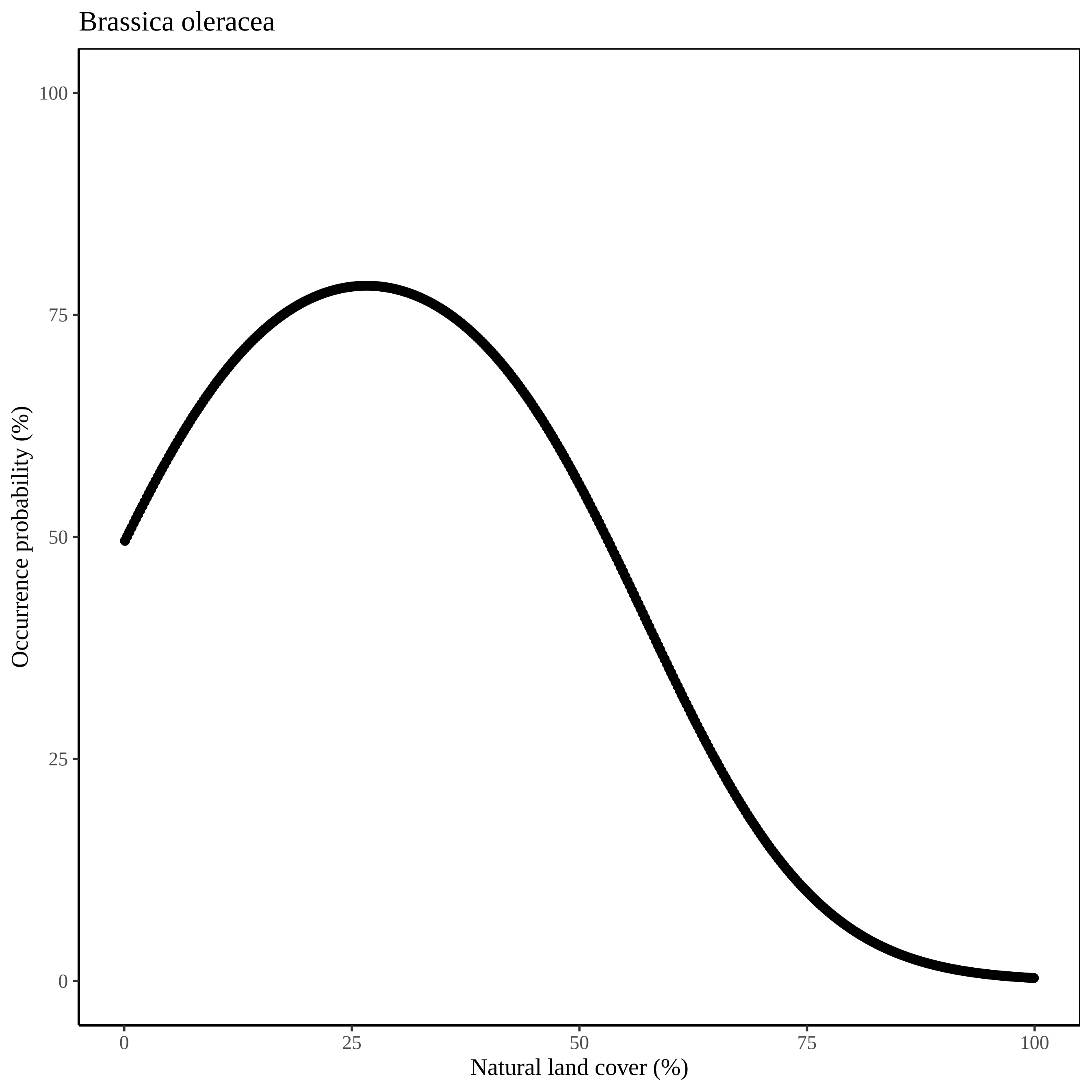
Brassica oleracea
100
75
50
25
0
Occurrence probability (%)
0
25
50
75
100
Natural land cover (%)

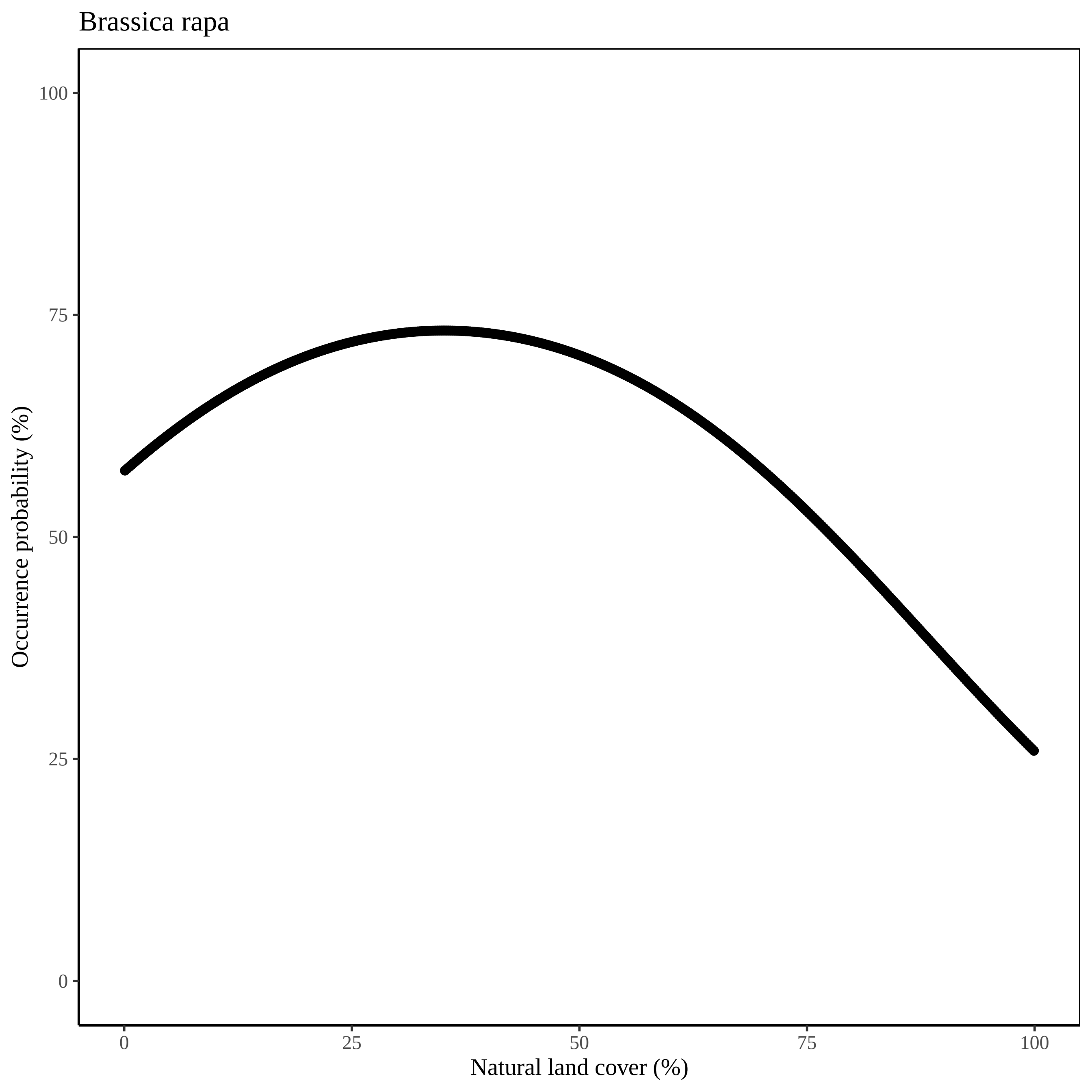

Brassica rapa
Occurrence probability (%)
100
75
50
25
0
0
25
50
75
100
Natural land cover (%)

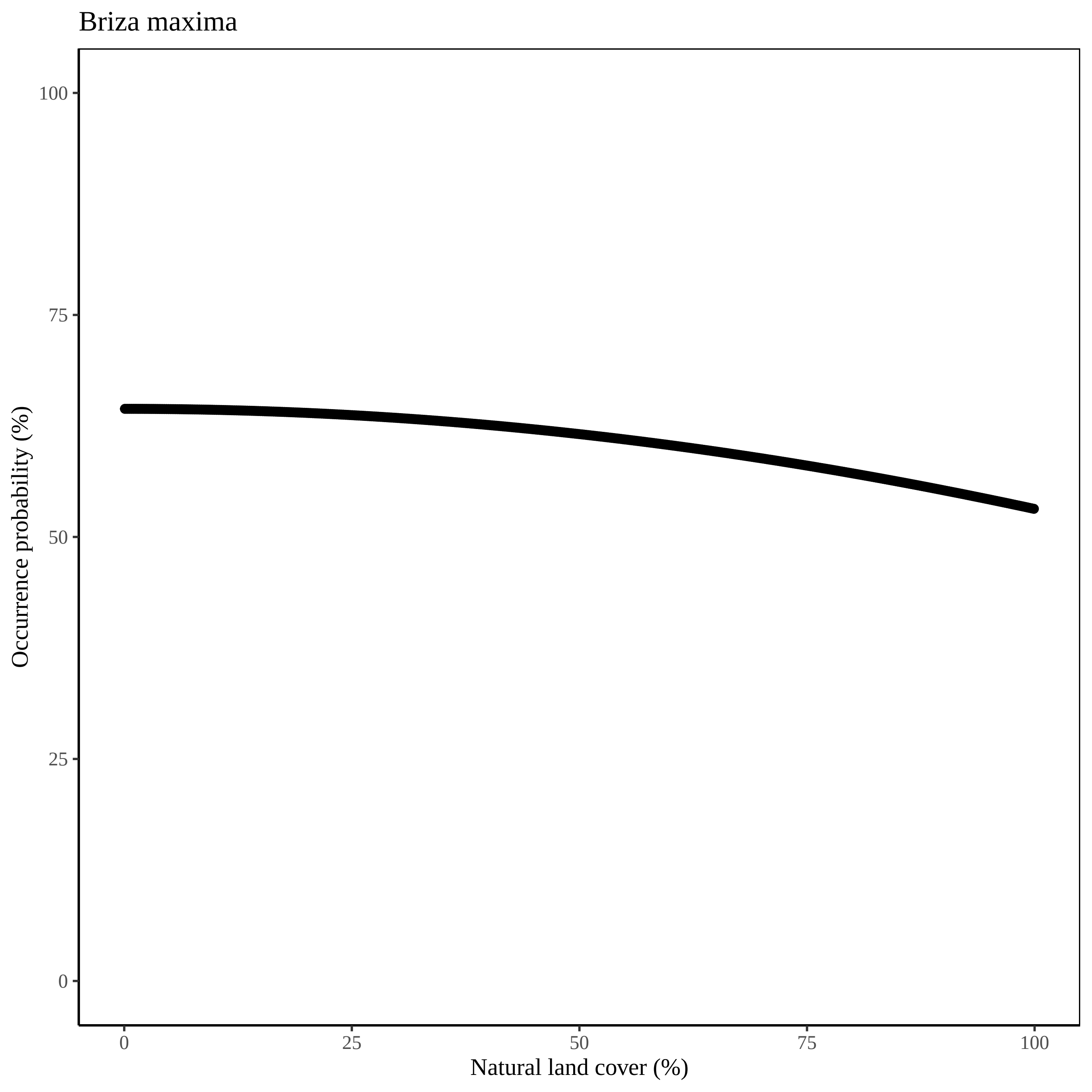
Briza maxima
Occurrence probability (%)
100
75
50
25
0
0
25
50
75
100
Natural land cover (%)

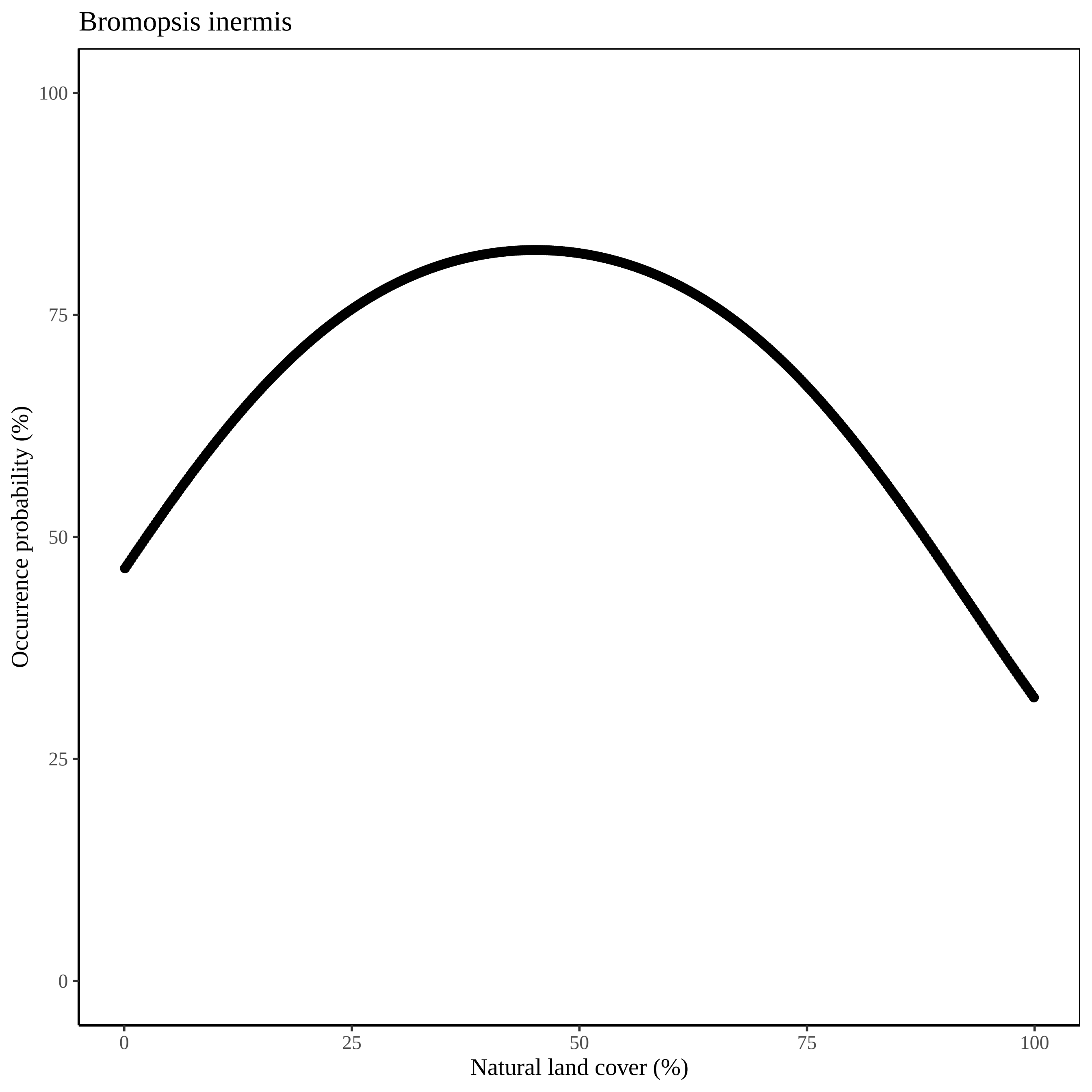
Bromopsis inermis
100
75
50
25
0
Occurrence probability (%)
0
25
50
75
100
Natural land cover (%)

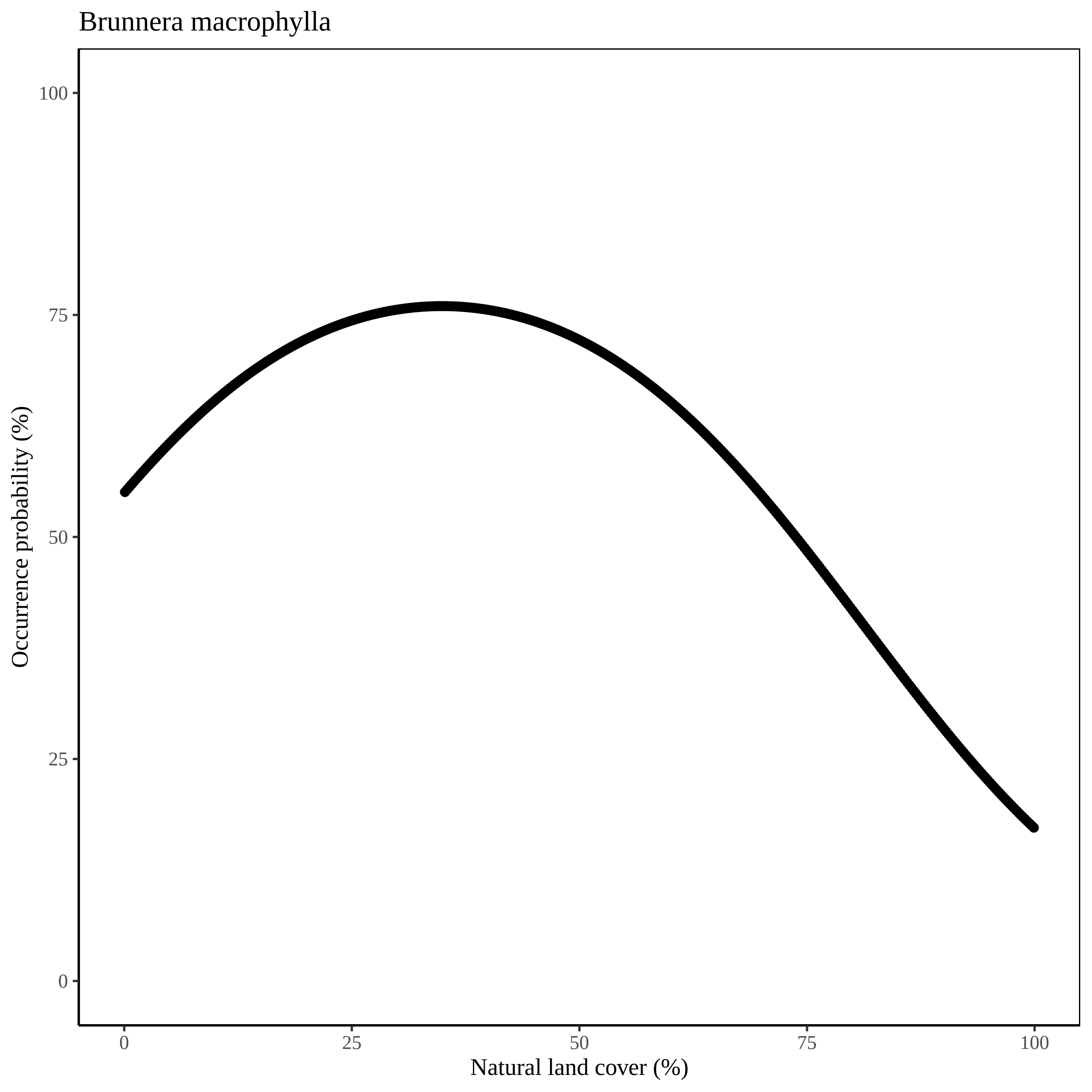
Brunnera macrophylla
Occurrence probability (%)
100
75
50
25
0
0
25
50
75
100
Natural land cover (%)

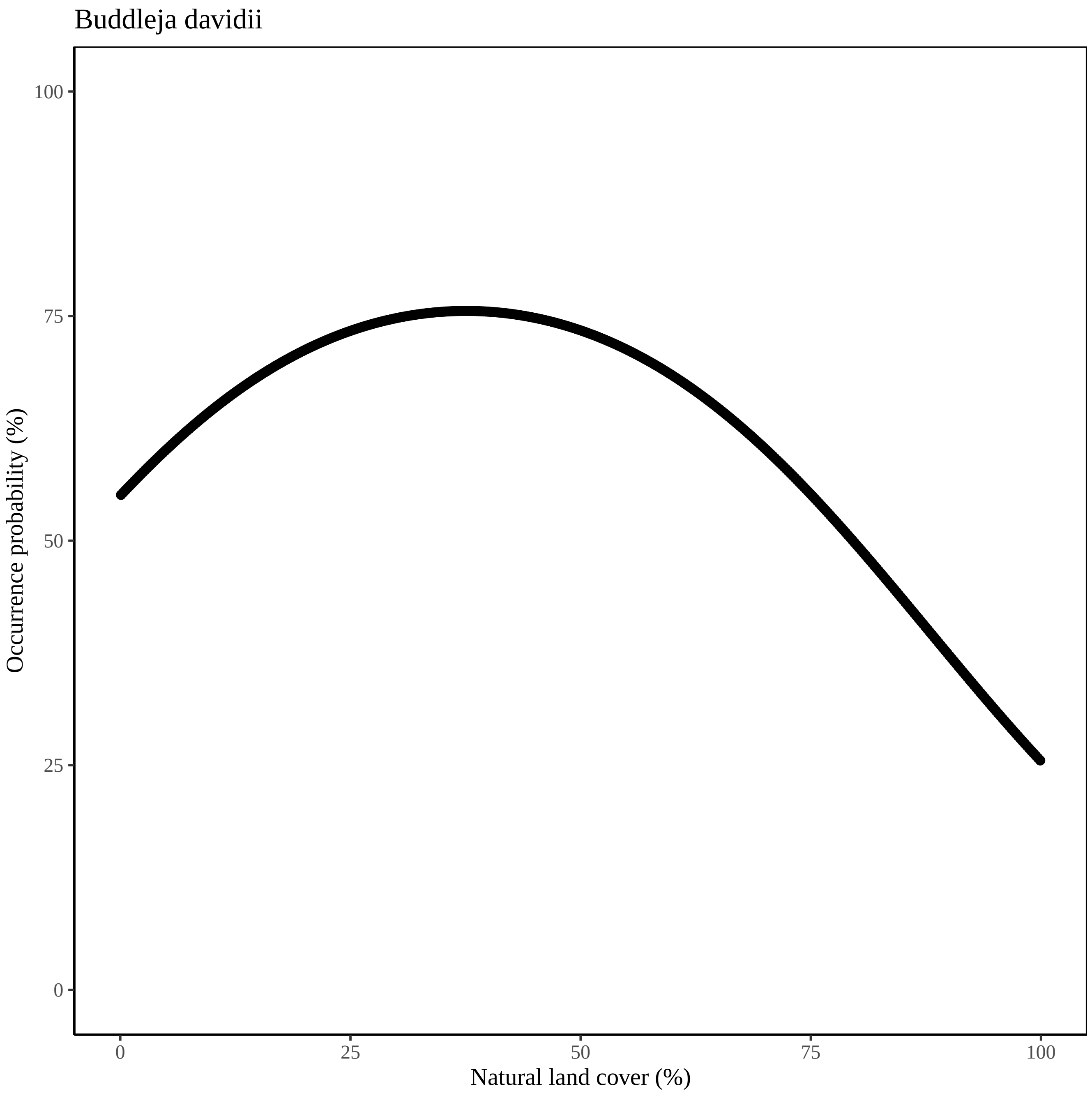
Buddleja davidii
Occurrence probability (%)
100
75
50
25
0
0
25
50
75
100
Natural land cover (%)

Bupleurum tenuissimum

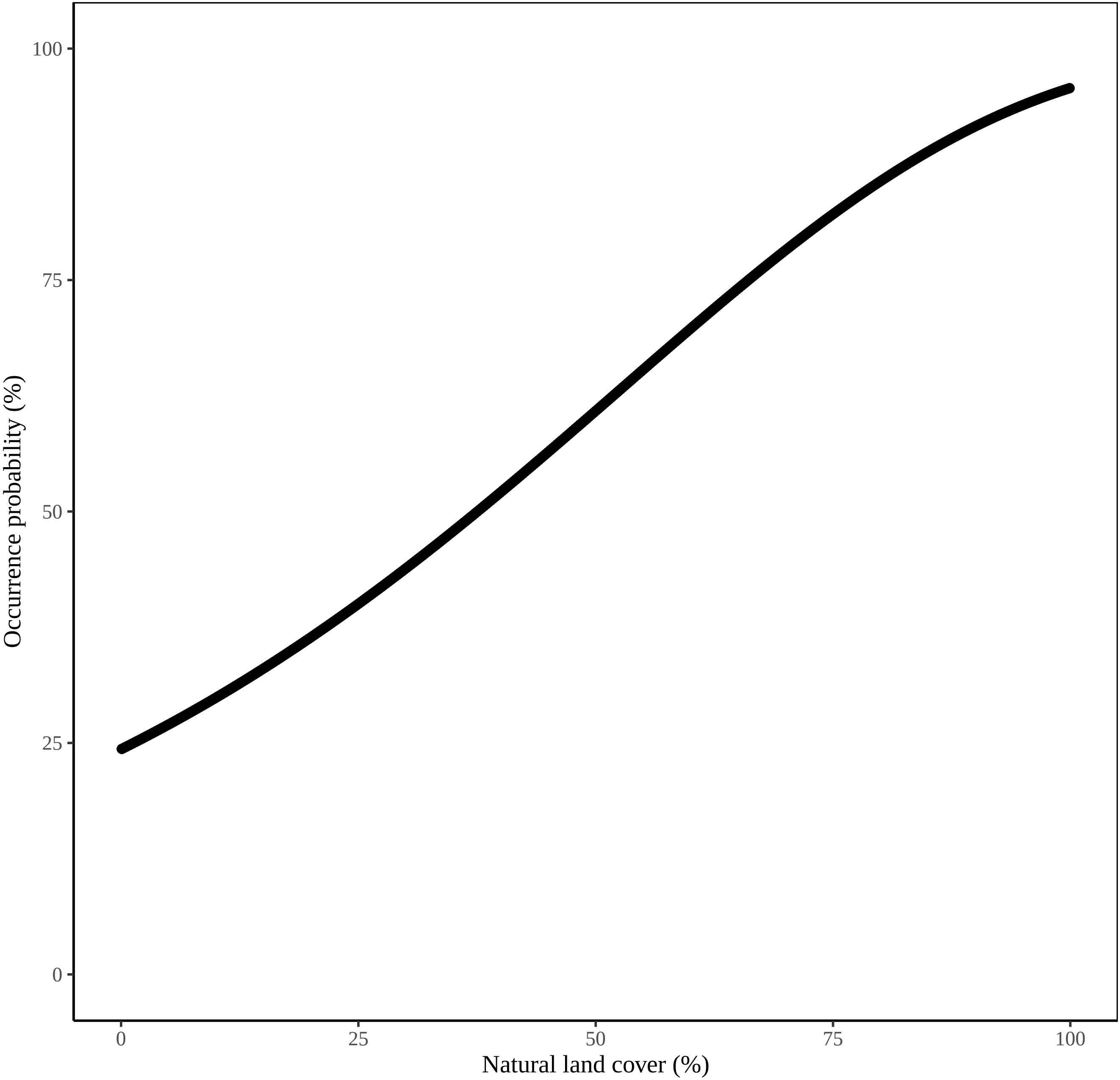

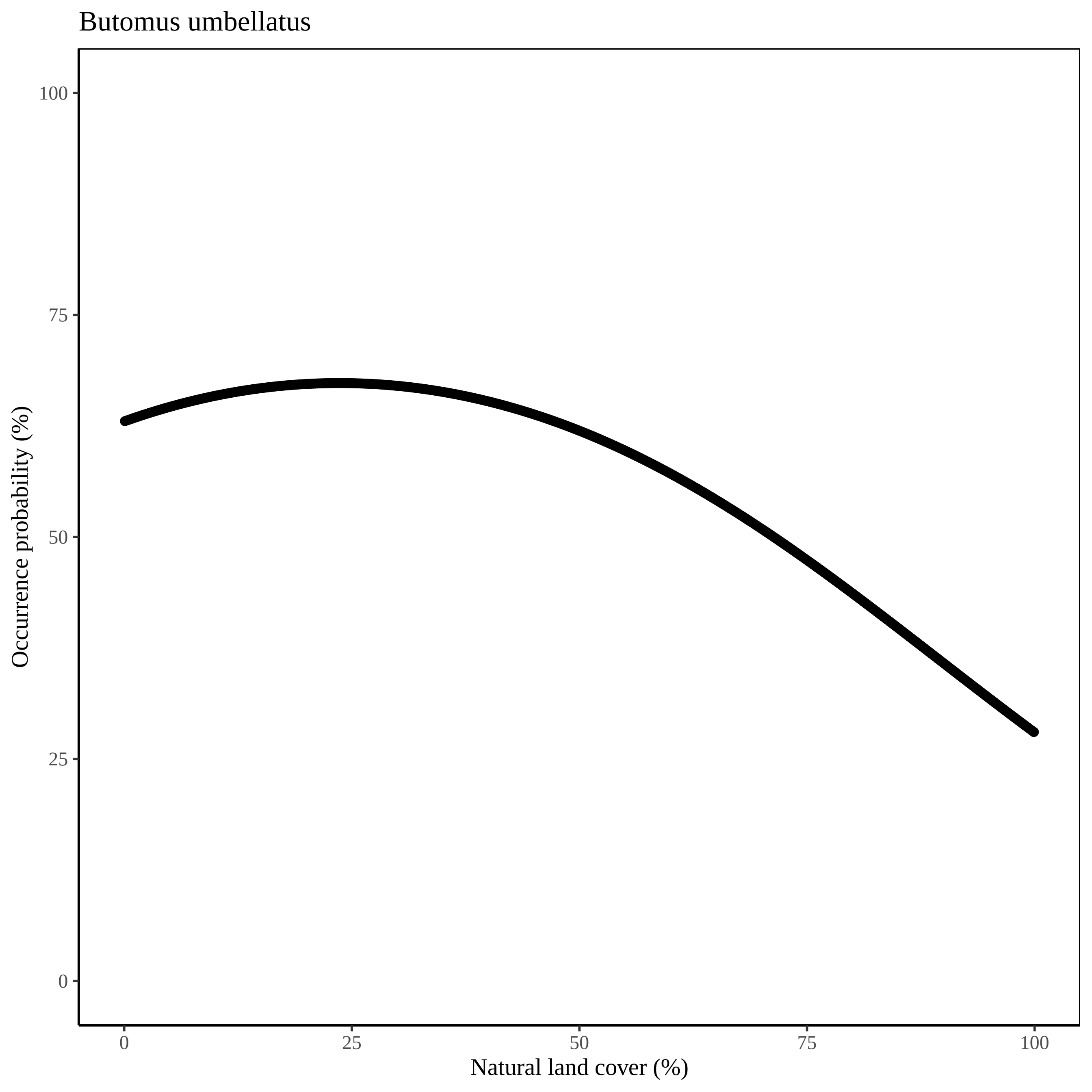
Butomus umbellatus
Occurrence probability (%)
100
75
50
25
0
0
25
50
75
100
Natural land cover (%)

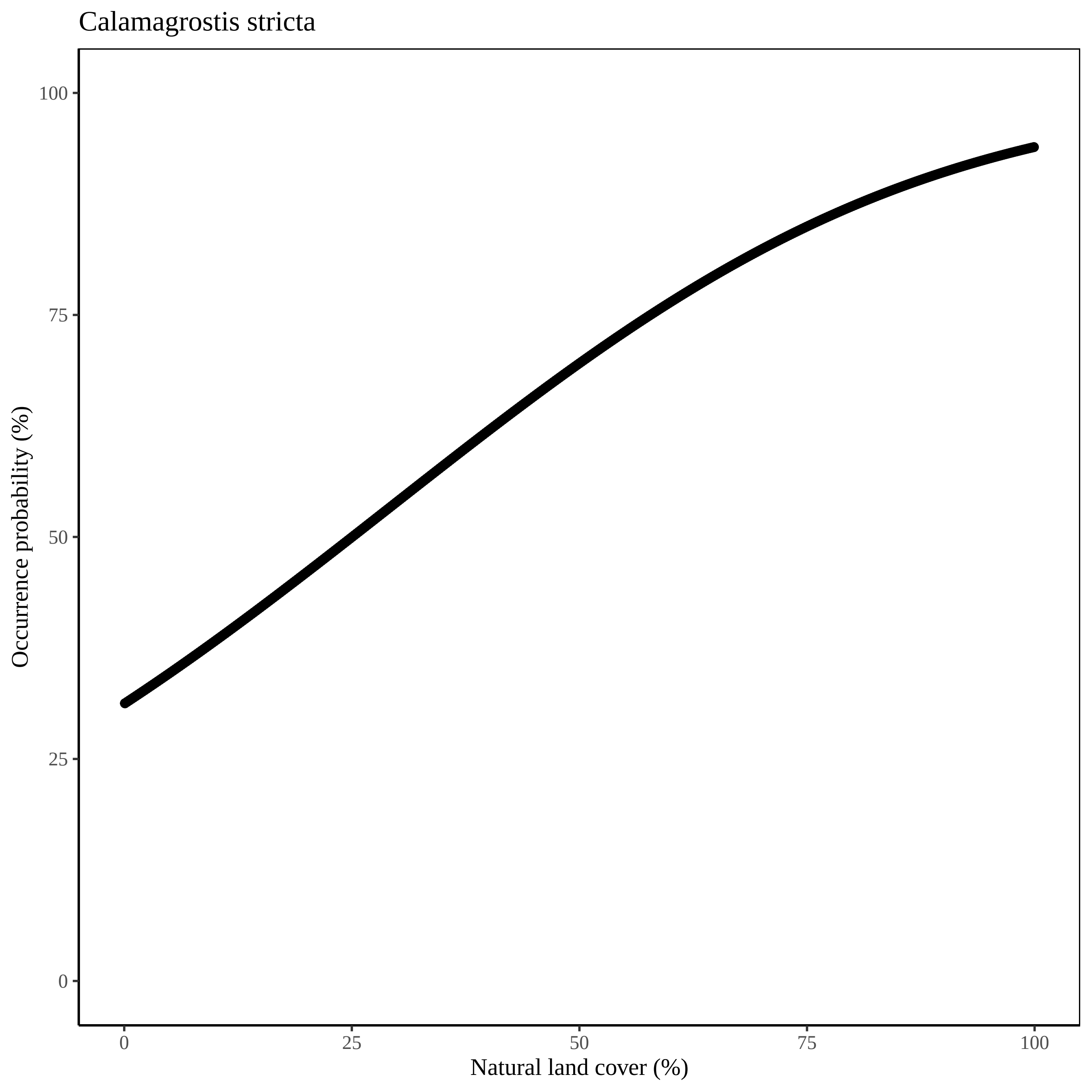
Calamagrostis stricta
Occurrence probability (%)
100
75
50
25
0
0
25
50
75
100
Natural land cover (%)

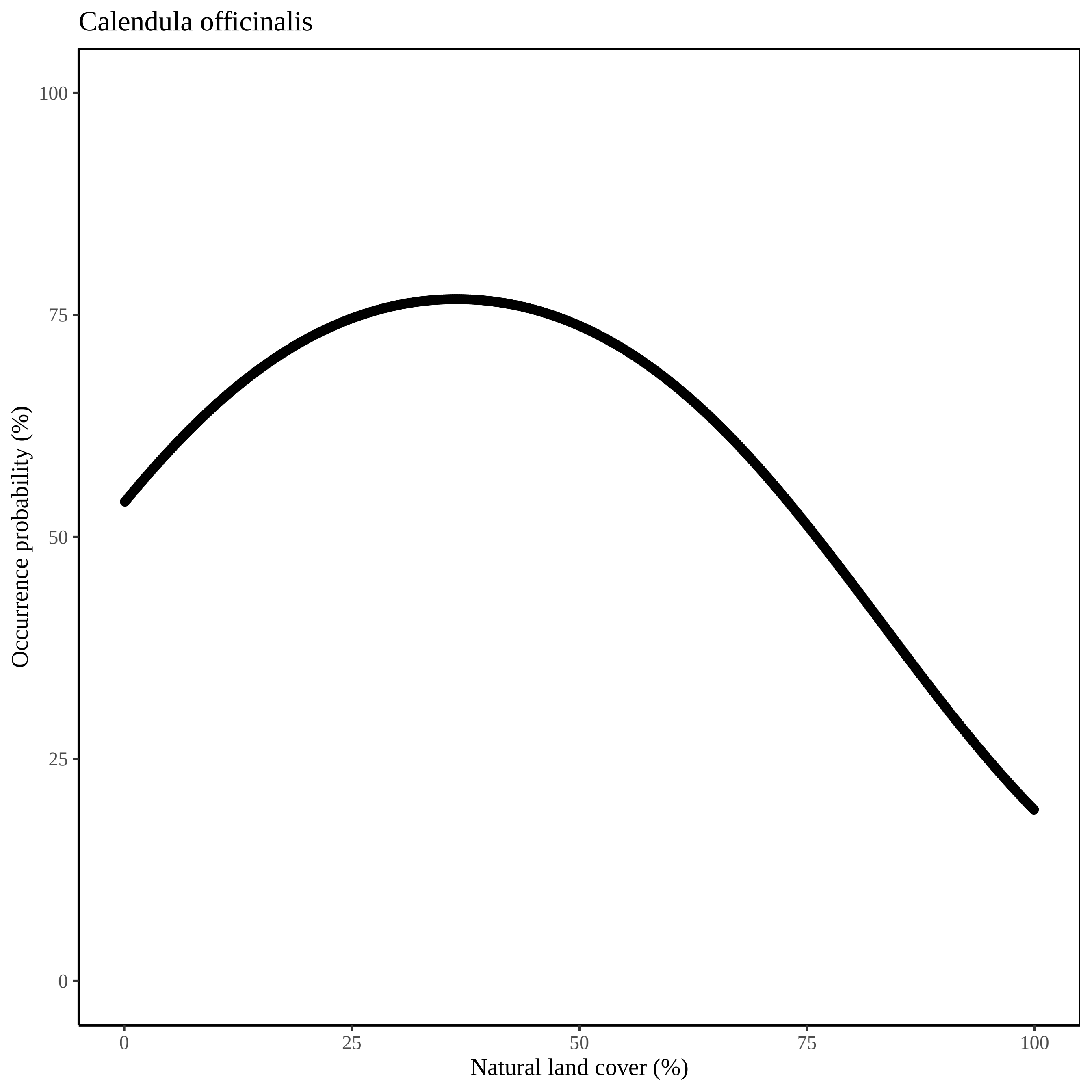

Calendula officinalis
Occurrence probability (%)
Natural land cover (%)
0
25
50
75
100

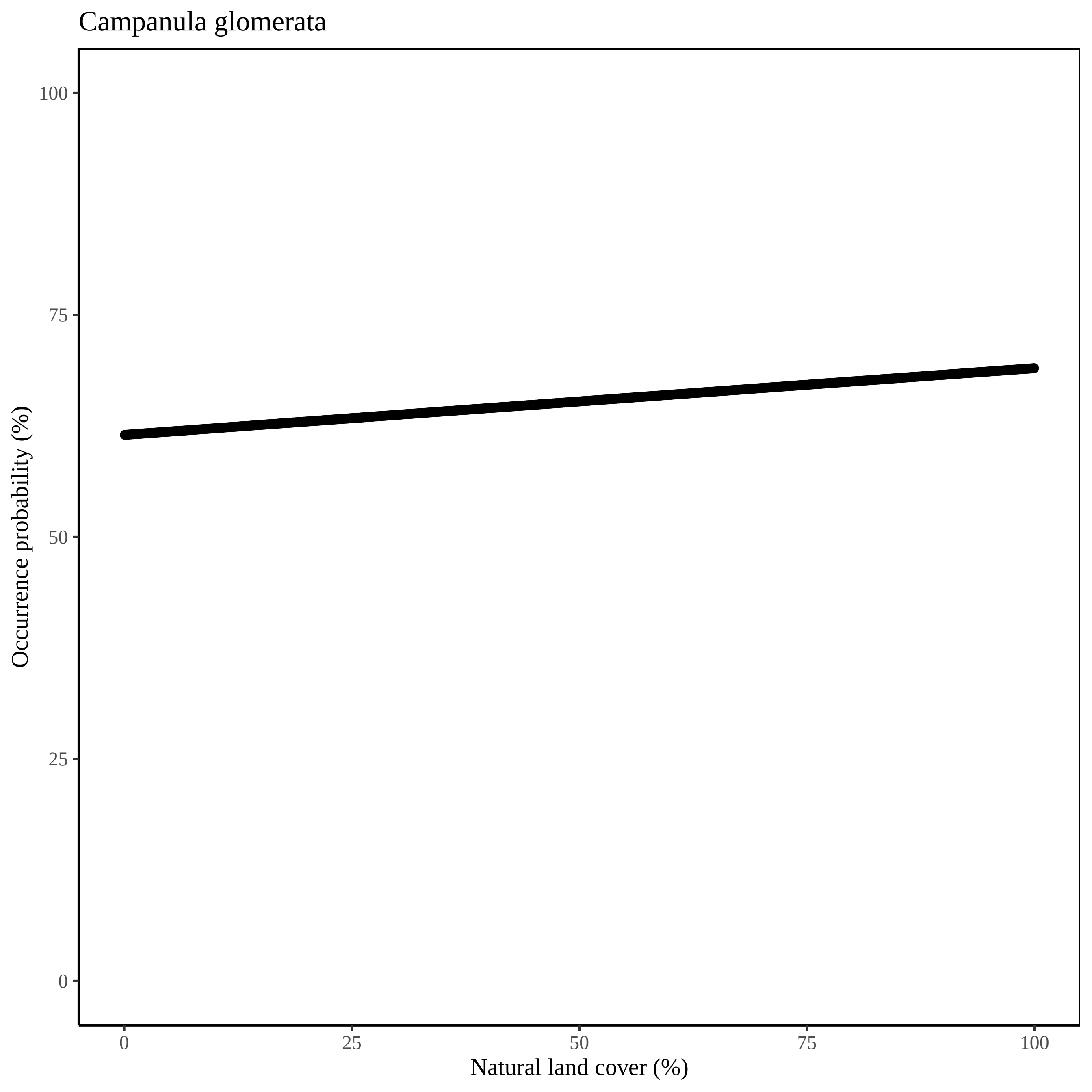

Campanula glomerata
Occurrence probability (%)
100
75
50
25
0
0
25
50
75
100
Natural land cover (%)

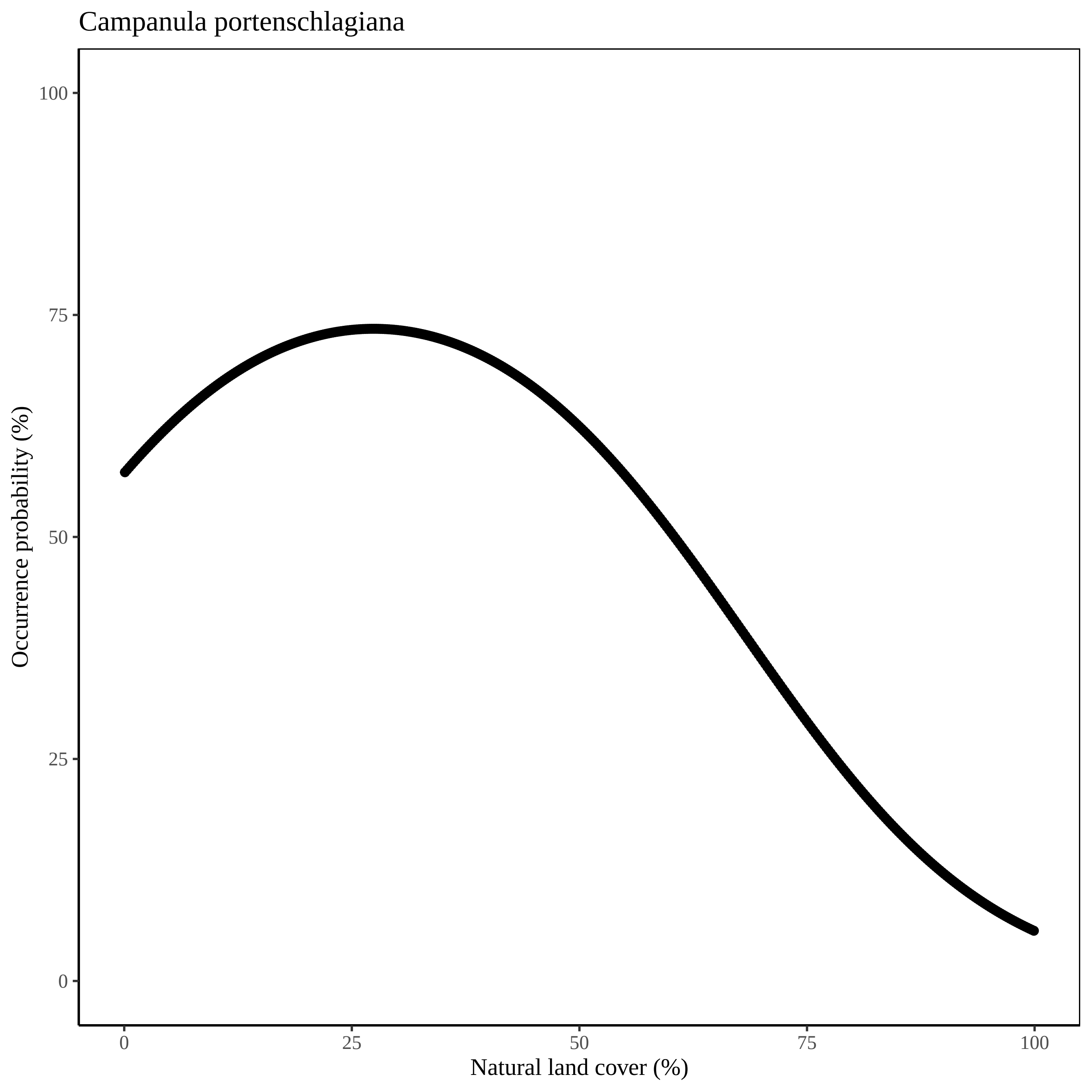

Campanula portenschlagiana
Occurrence probability (%)
100
75
50
25
0
0
25
50
75
100
Natural land cover (%)

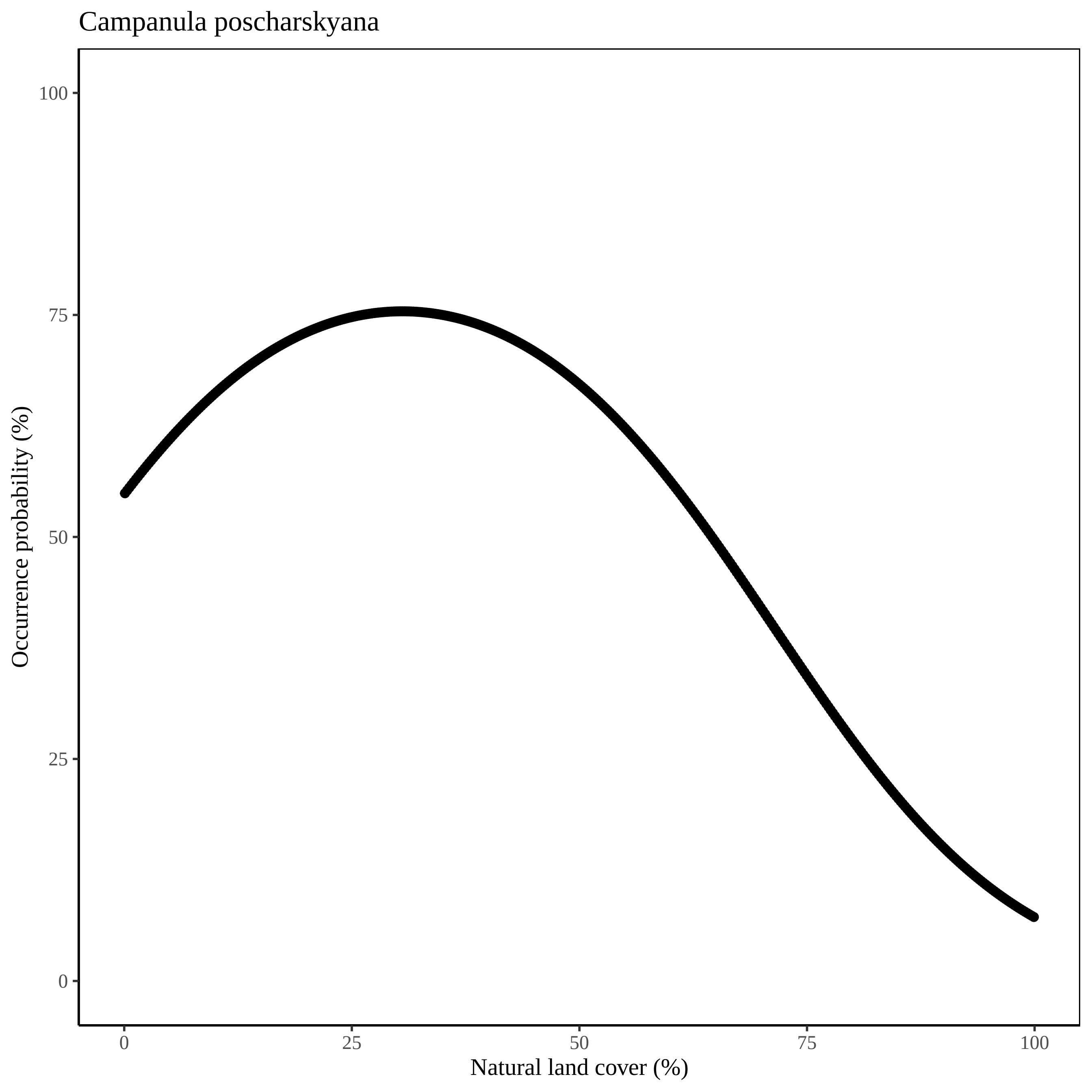

Campanula poscharskyana
Occurrence probability (%)
100
75
50
25
0
0
25
50
75
100
Natural land cover (%)

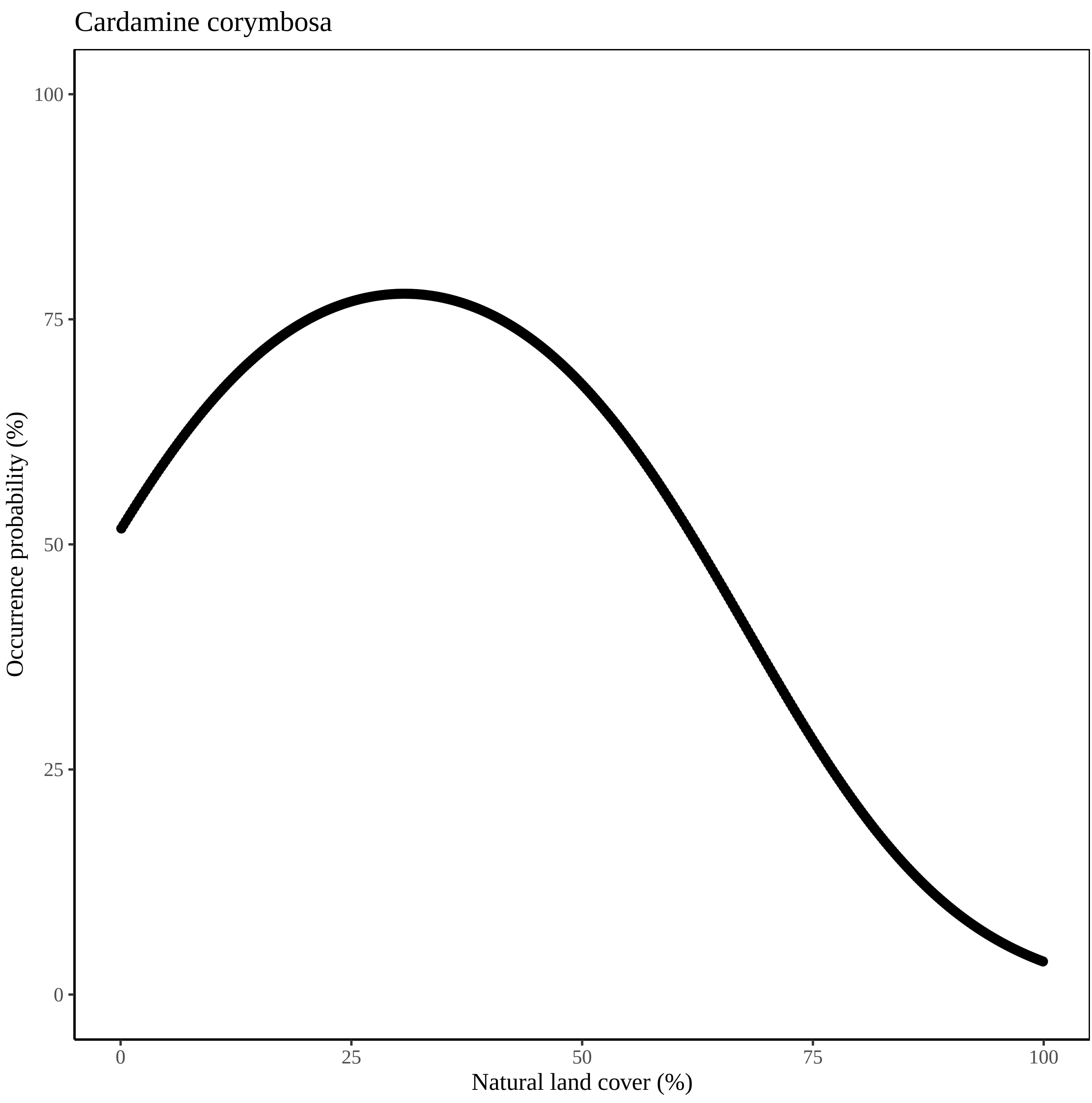
Cardamine corymbosa
Occurrence probability (%)
100
75
50
25
0
0
25
50
75
100
Natural land cover (%)

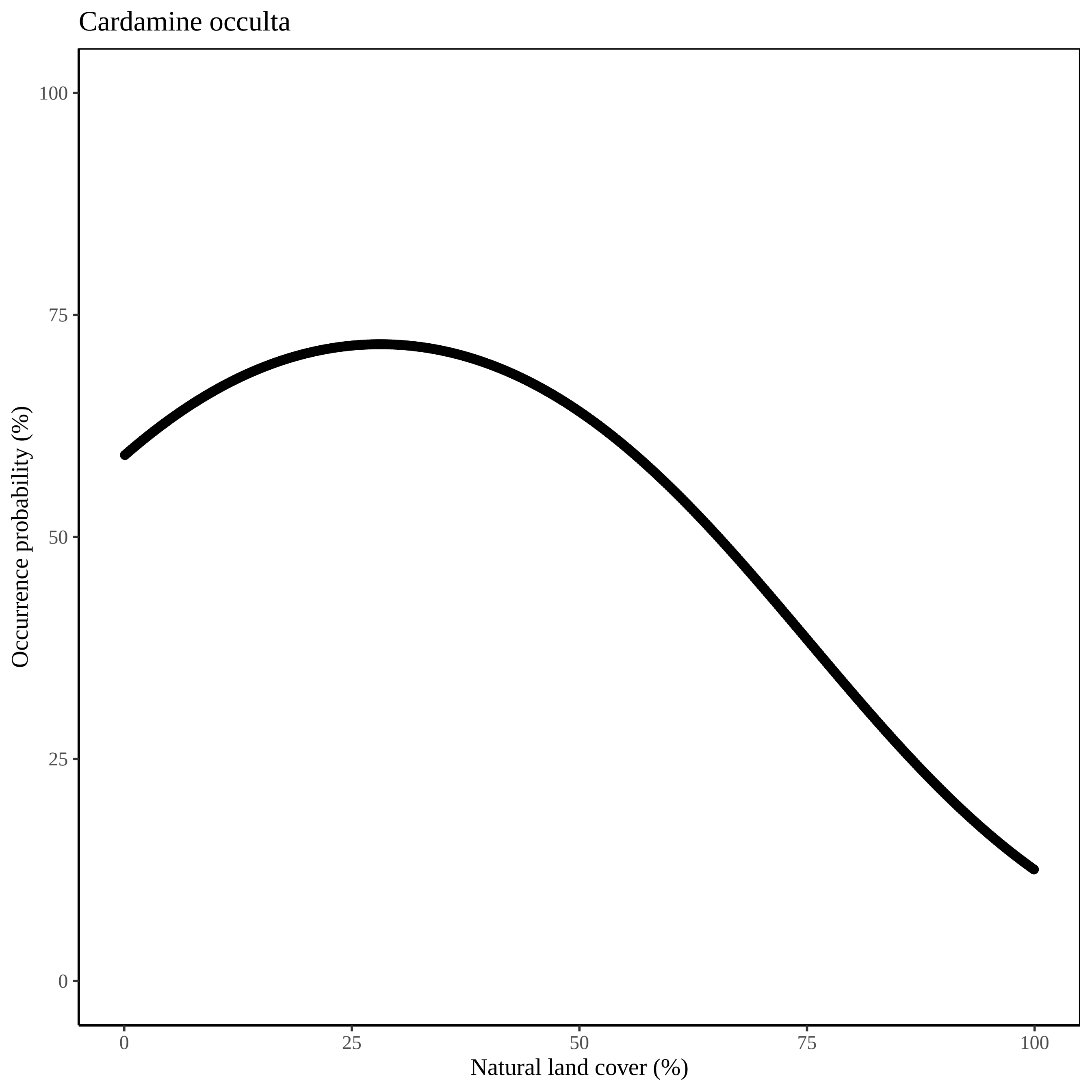

Cardamine occulta
Occurrence probability (%)
100
75
50
25
0
0
25
50
75
100
Natural land cover (%)

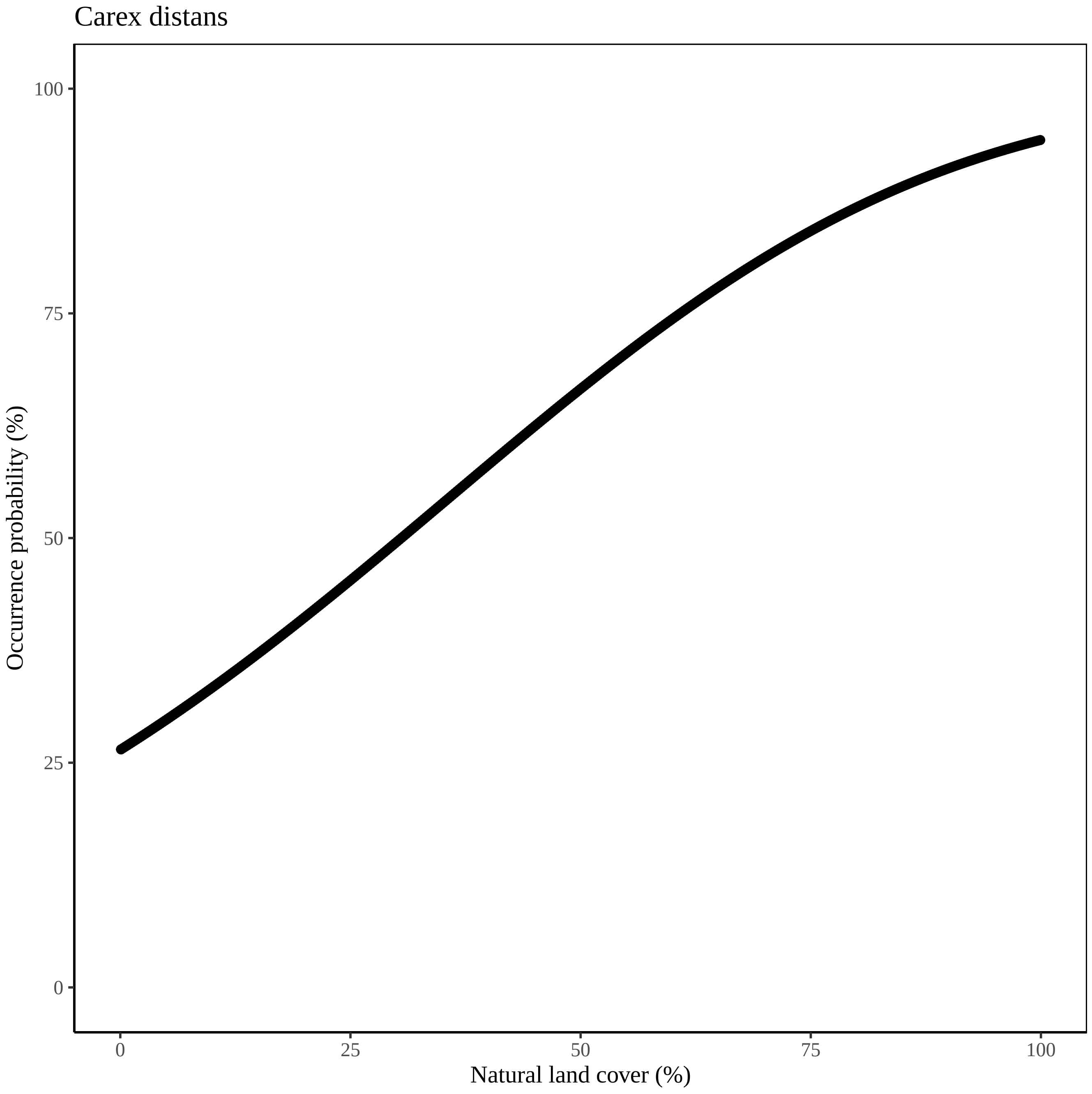

Carex distans
Occurrence probability (%)
100
75
50
25
0
0
25
50
75
100
Natural land cover (%)

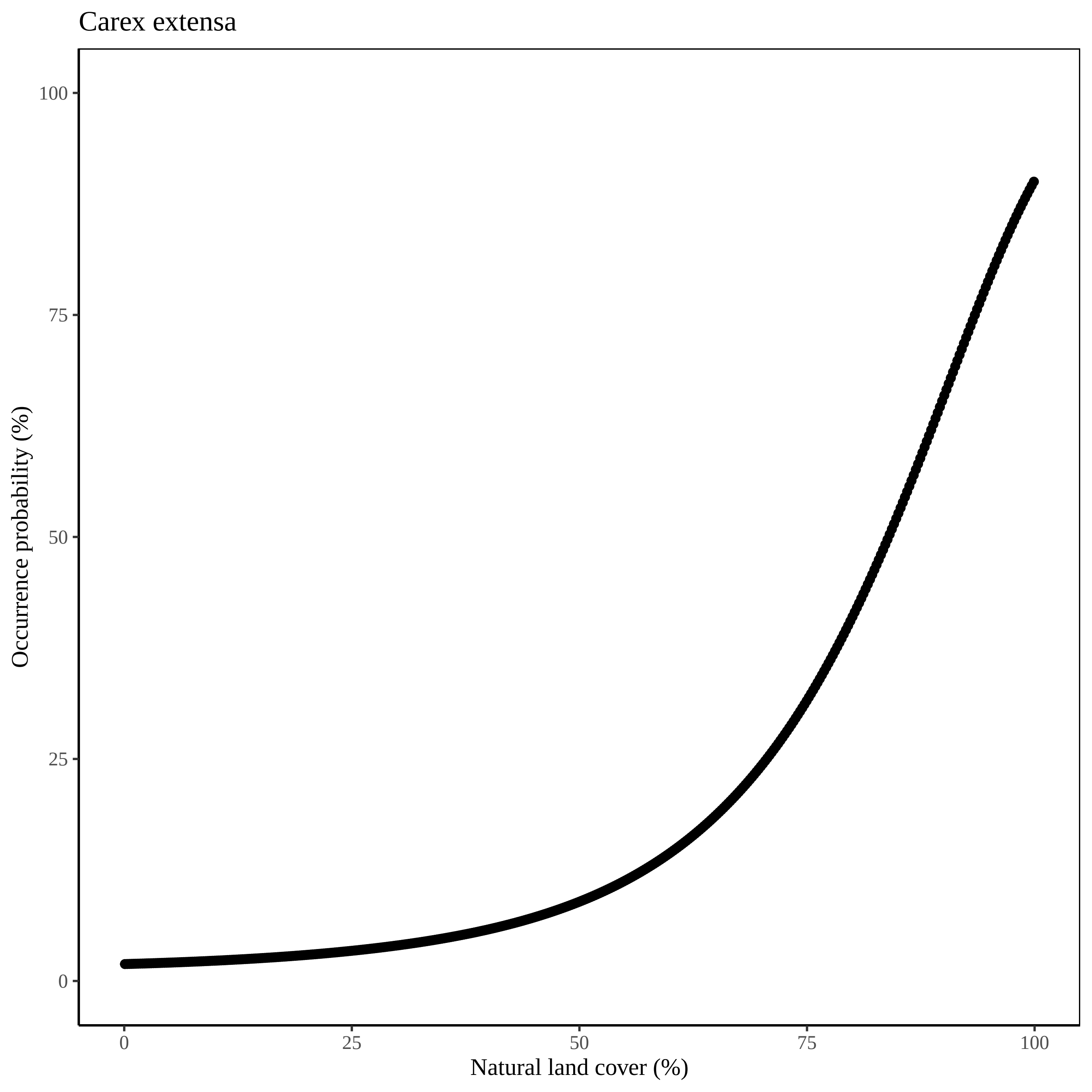

Carex extensa
Occurrence probability (%)
100
75
50
25
0
0
25
50
75
100
Natural land cover (%)

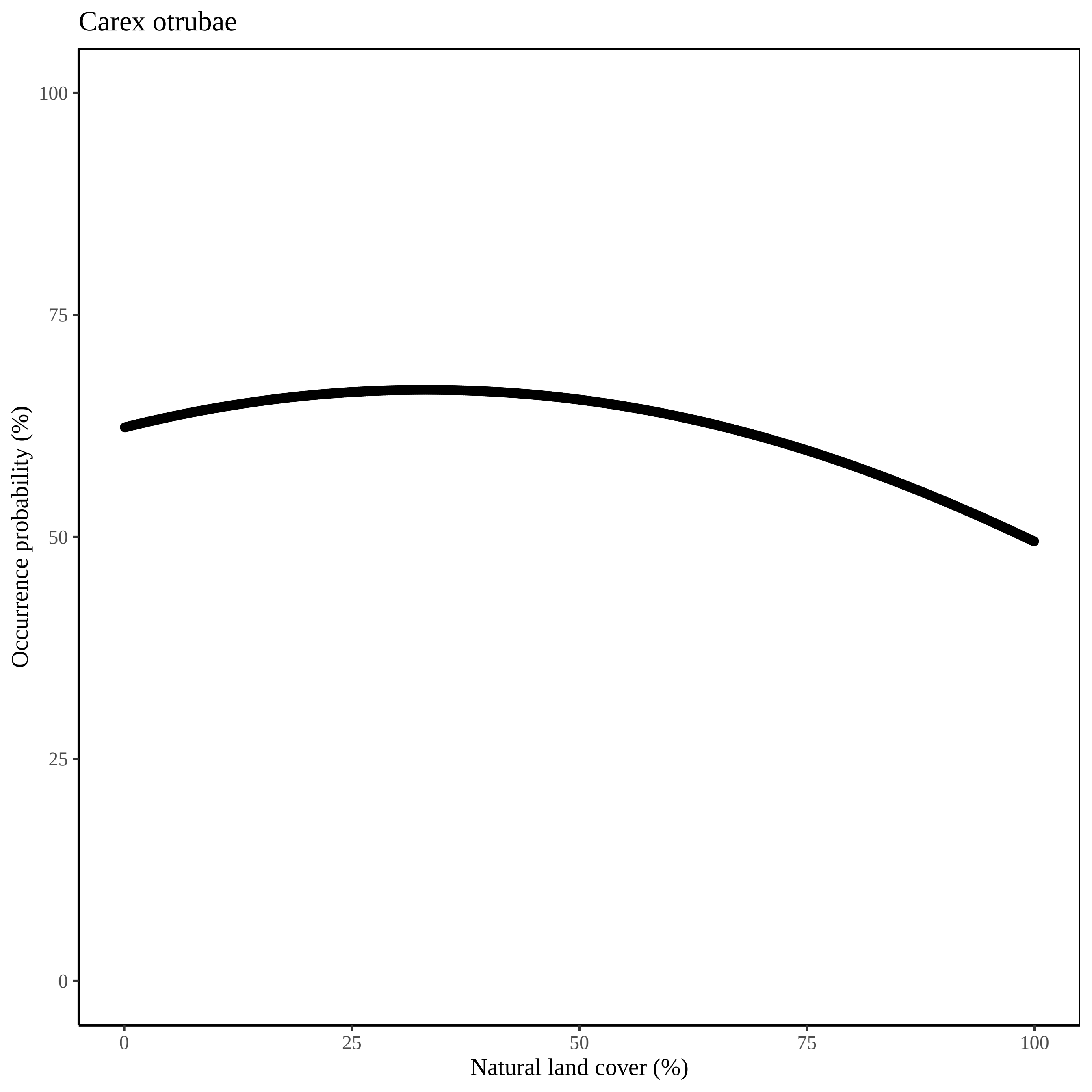
Carex otrubae
Occurrence probability (%)
100
75
50
25
0
0
25
50
75
100
Natural land cover (%)

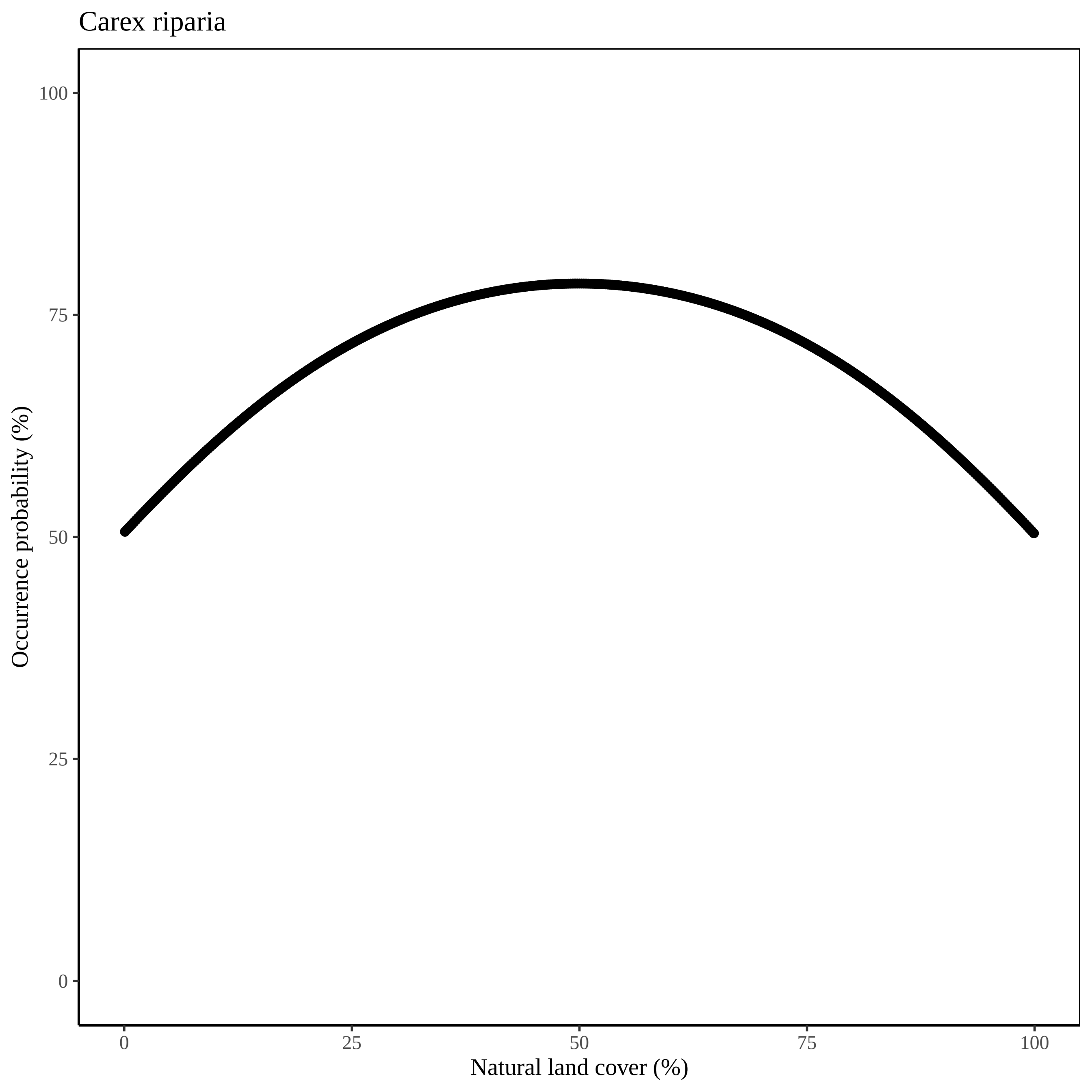

Carex riparia
100
75
50
25
0
Occurrence probability (%)
0
25
50
75
100
Natural land cover (%)

Carum carvi

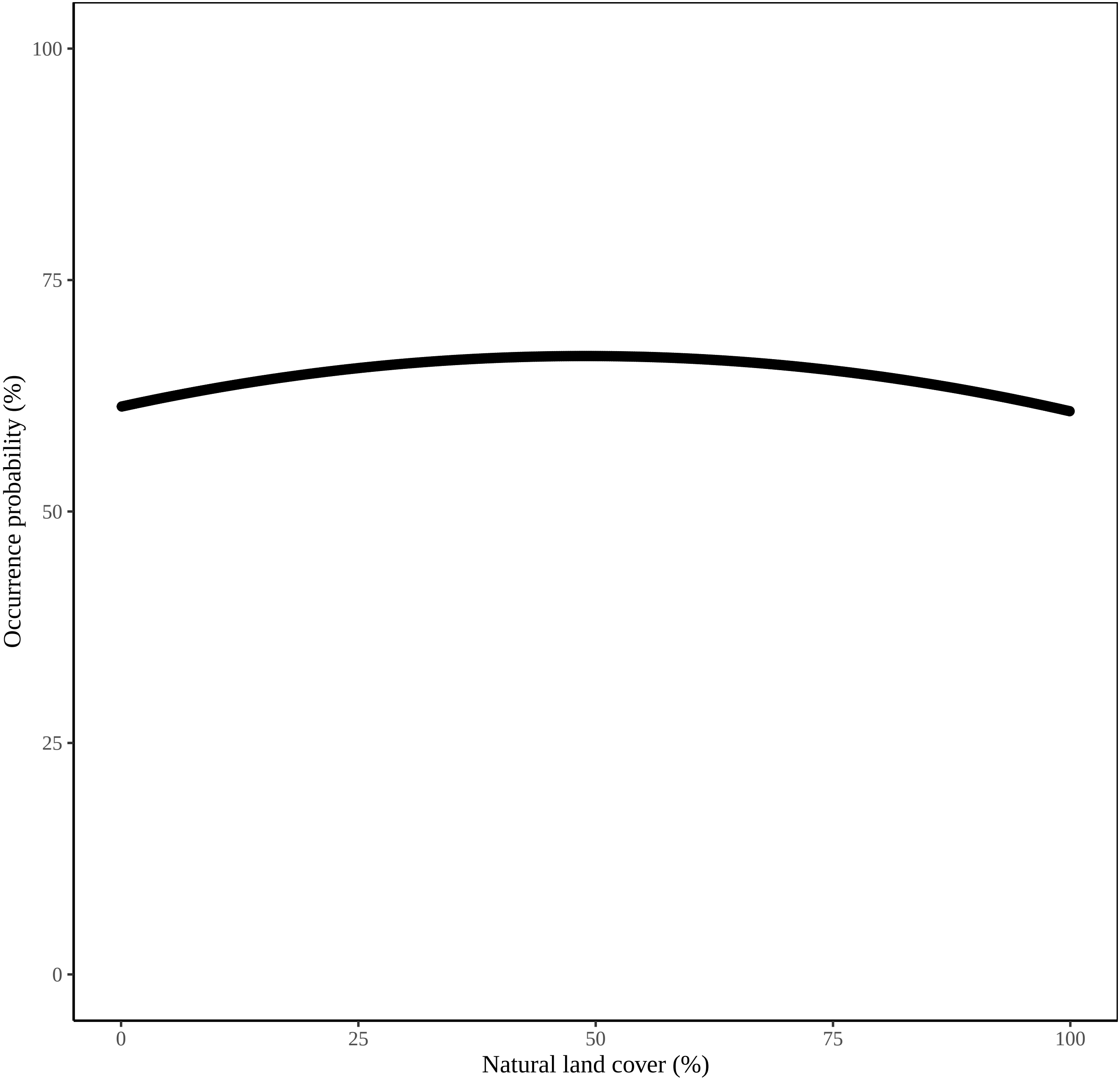

100
75
50
25
0
Occurrence probability (%)
0
25
50
75
100
Natural land cover (%)

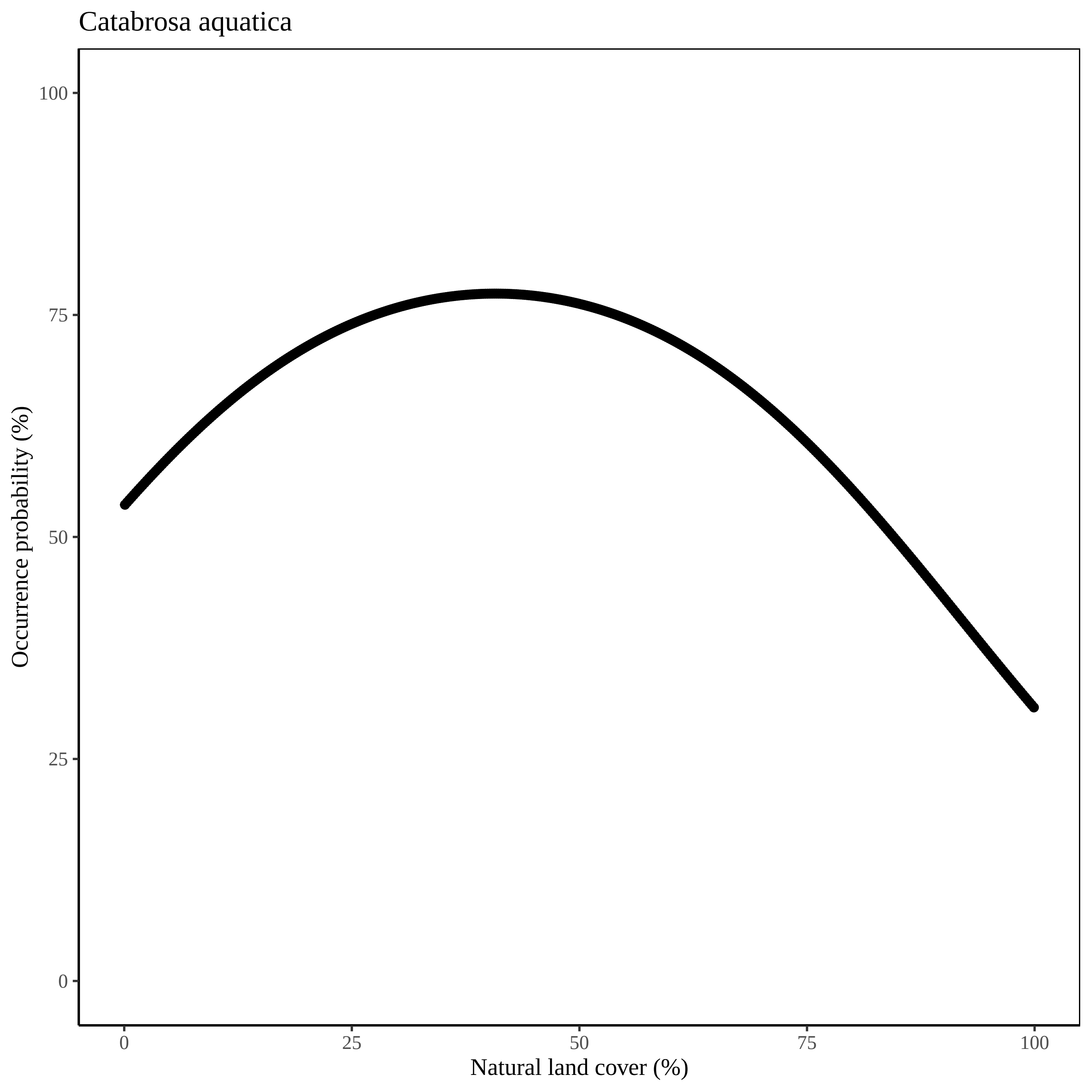

Catabrosa aquatica
Occurrence probability (%)
100
75
50
25
0
0
25
50
75
100
Natural land cover (%)

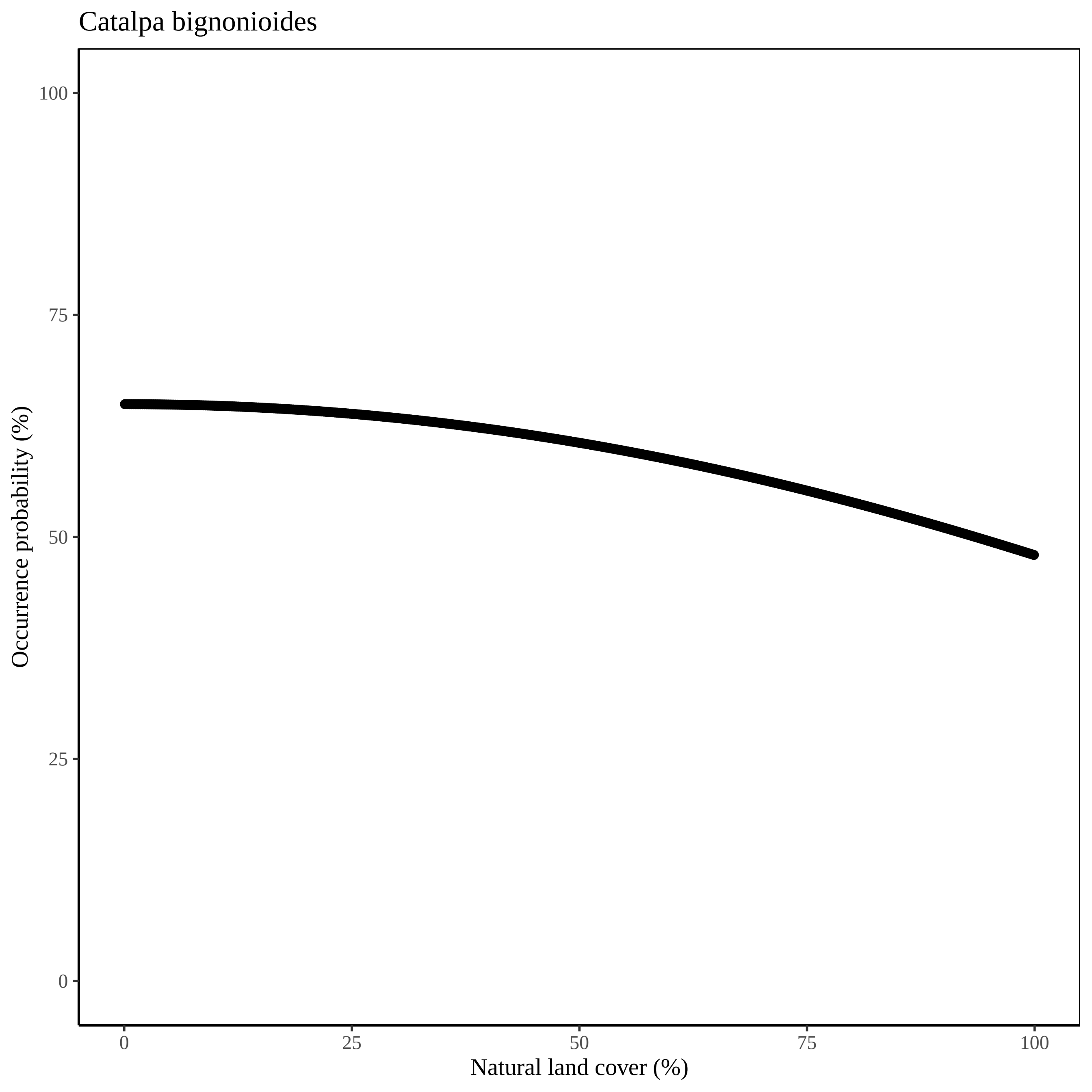

Catalpa bignonioides
Occurrence probability (%)
100
75
50
25
0
0
25
50
75
100
Natural land cover (%)

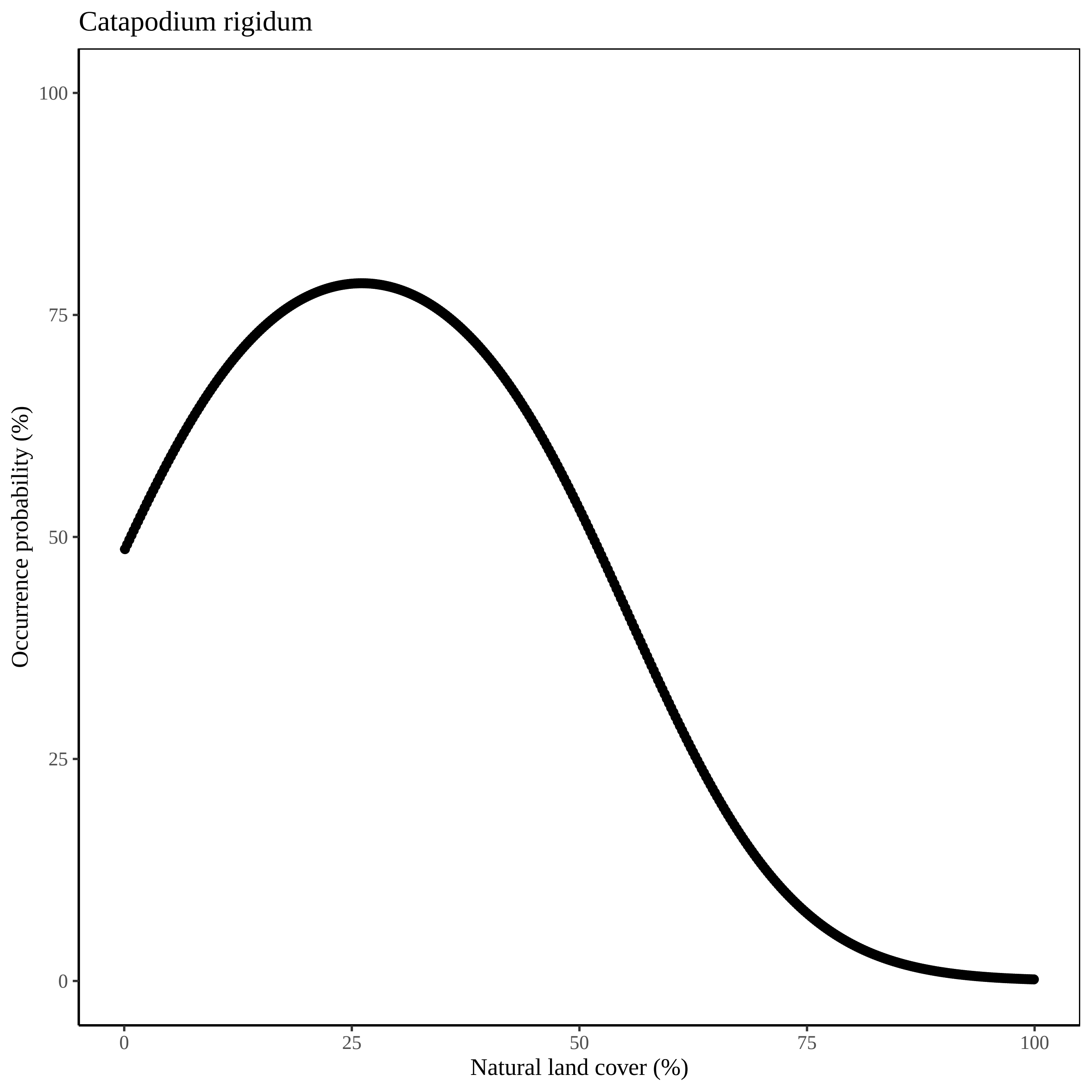
Catapodium rigidum
Occurrence probability (%)
100
75
50
25
0
0
25
50
75
100
Natural land cover (%)

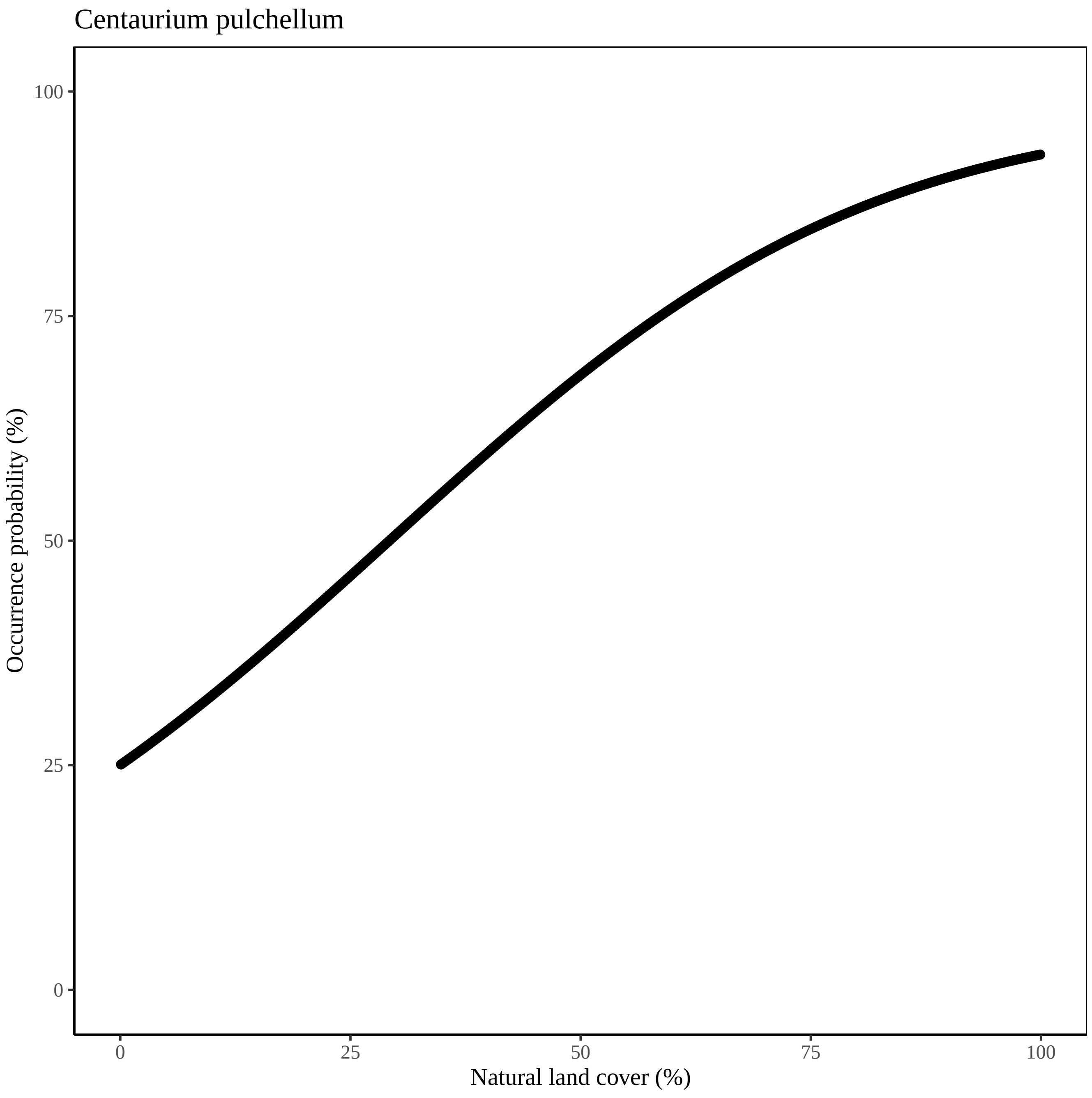
Centaurium pulchellum
Occurrence probability (%)
100
75
50
25
0
0
25
50
75
100
Natural land cover (%)

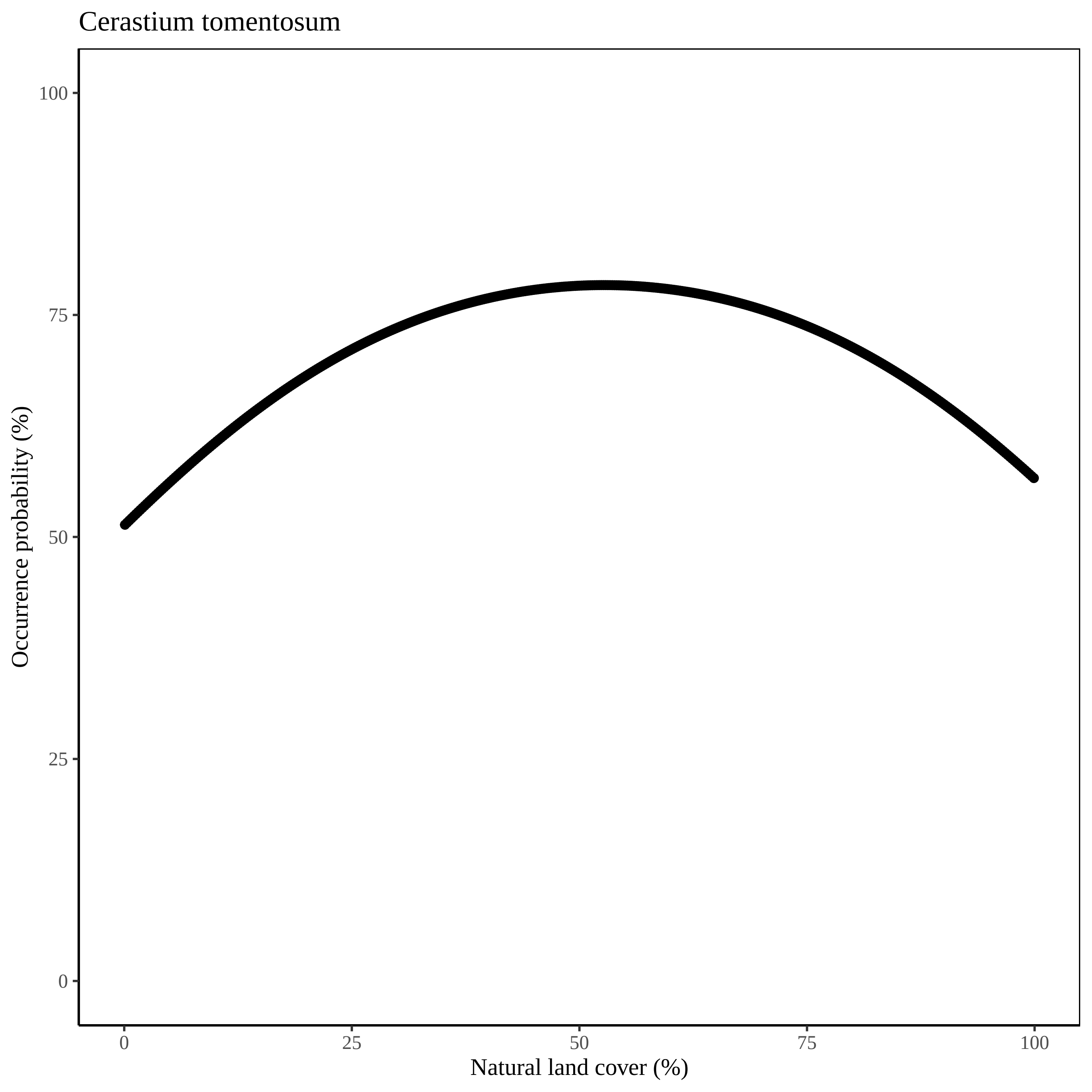
Cerastium tomentosum
100
75
50
25
0
Occurrence probability (%)
0
25
50
75
100
Natural land cover (%)

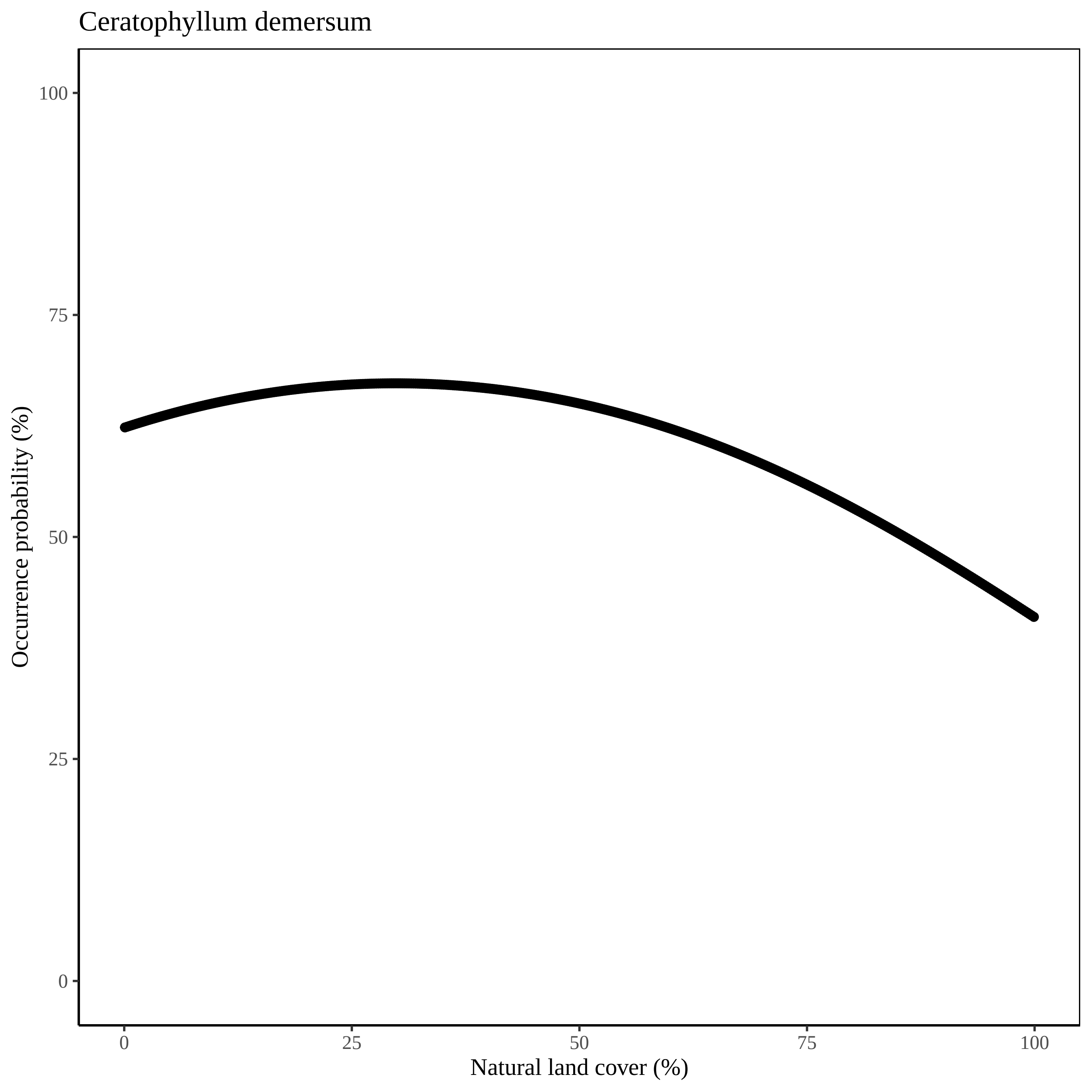

Ceratophyllum demersum
Occurrence probability (%)
100
75
50
25
0
0
25
50
75
100
Natural land cover (%)

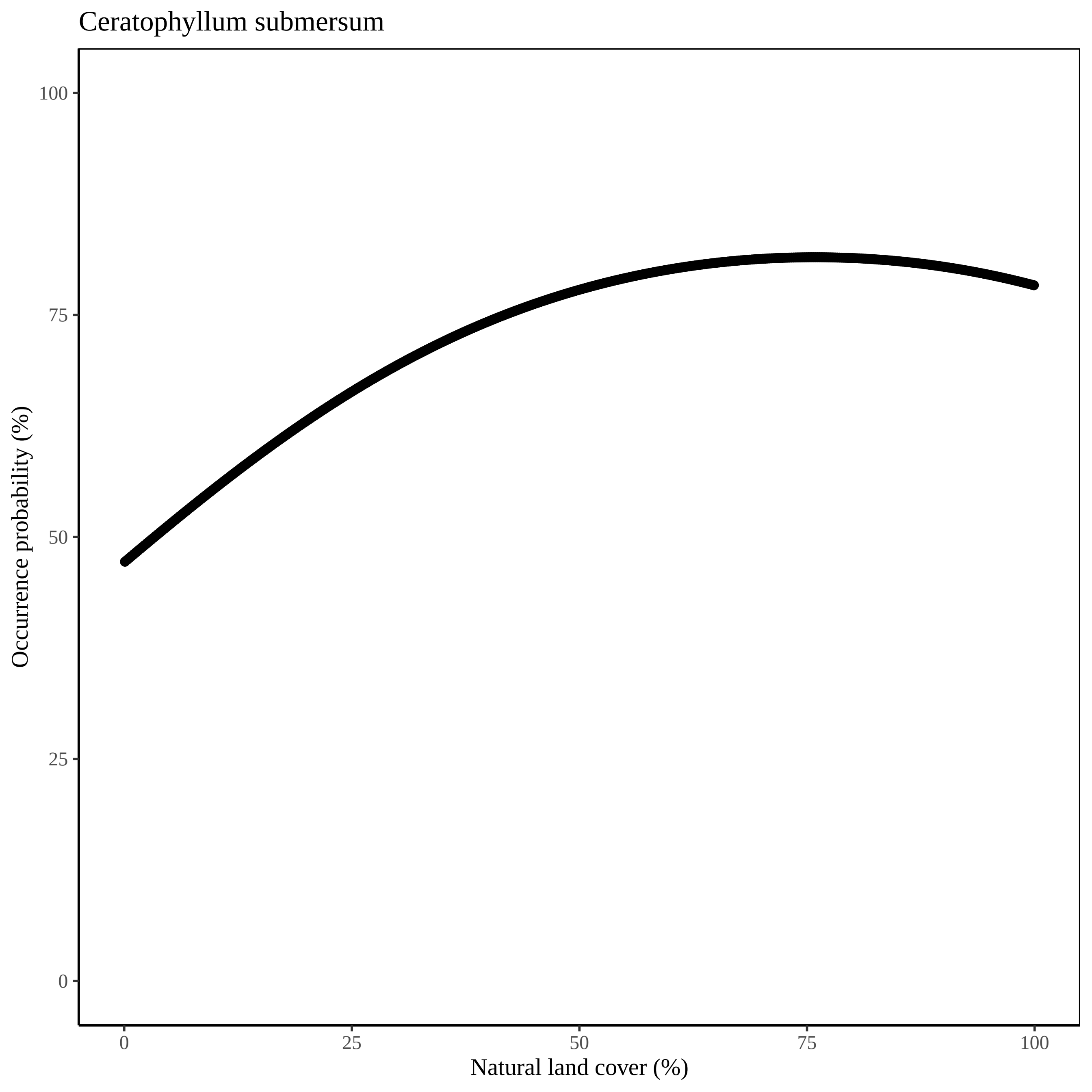

Ceratophyllum submersum
100
75
50
25
0
Occurrence probability (%)
0
25
50
75
100
Natural land cover (%)

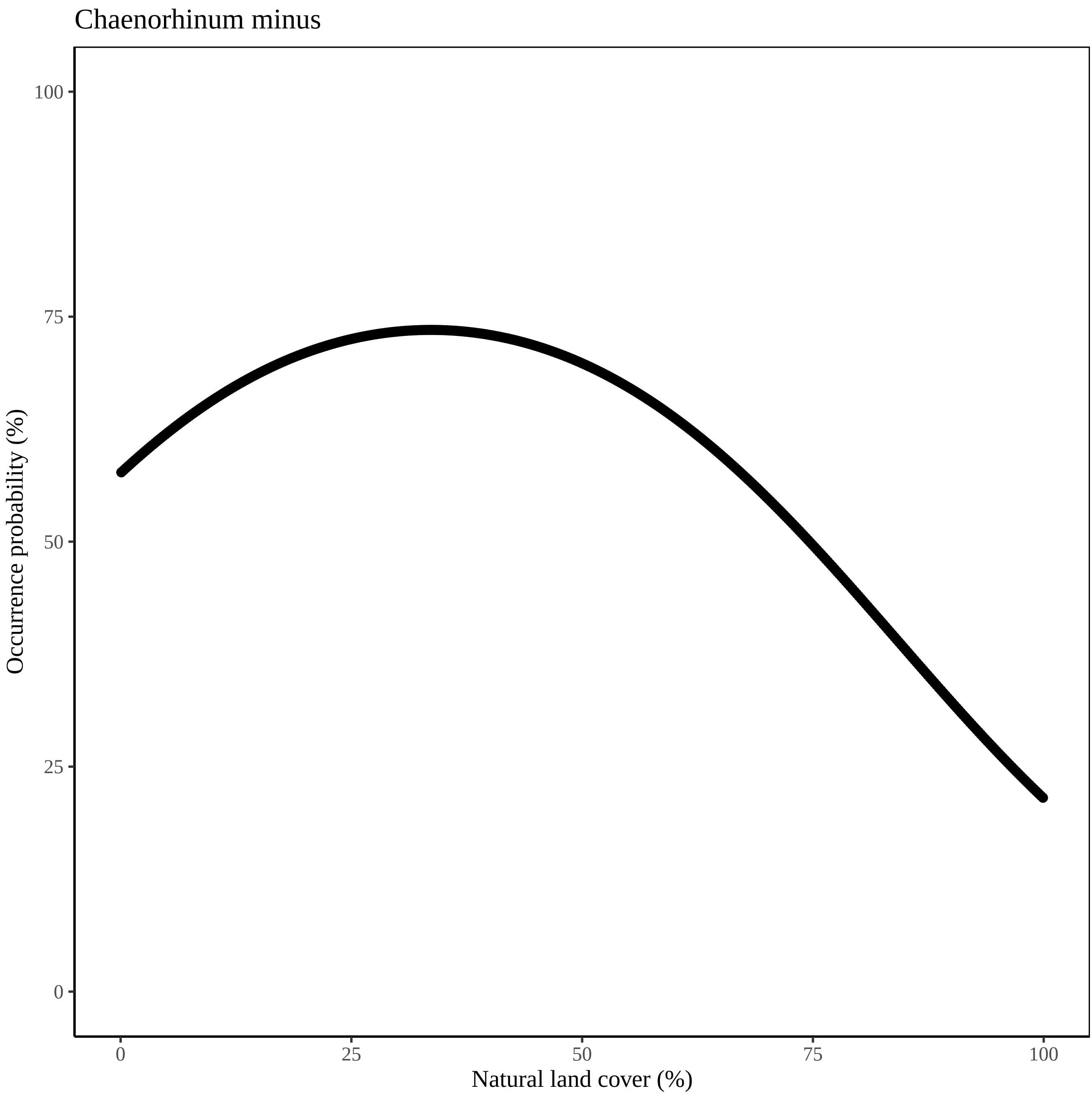
Chaenorhinum minus
Occurrence probability (%)
100
75
50
25
0
0
25
50
75
100
Natural land cover (%)

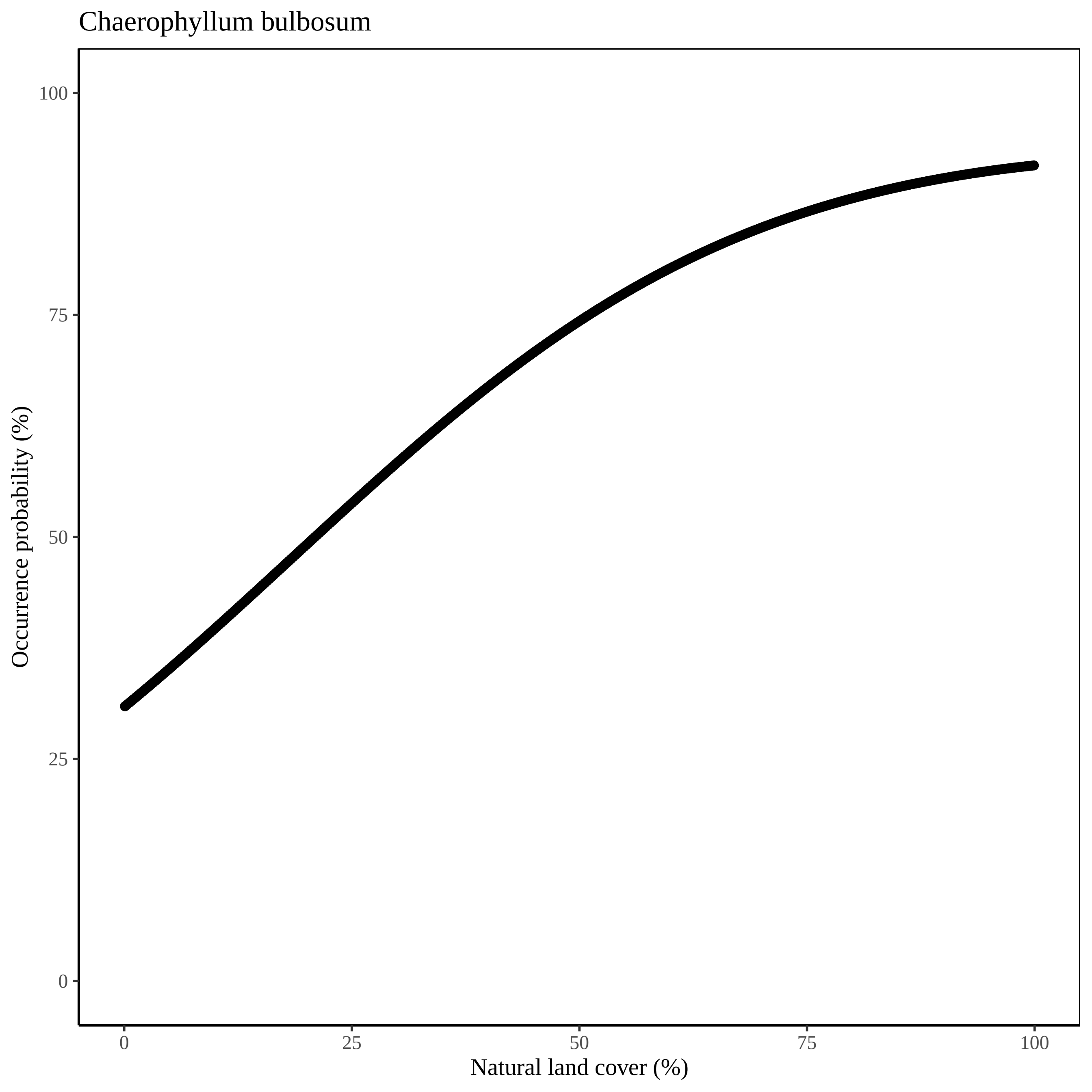
Chaerophyllum bulbosum
Occurrence probability (%)
100
75
50
25
0
0
25
50
75
100
Natural land cover (%)

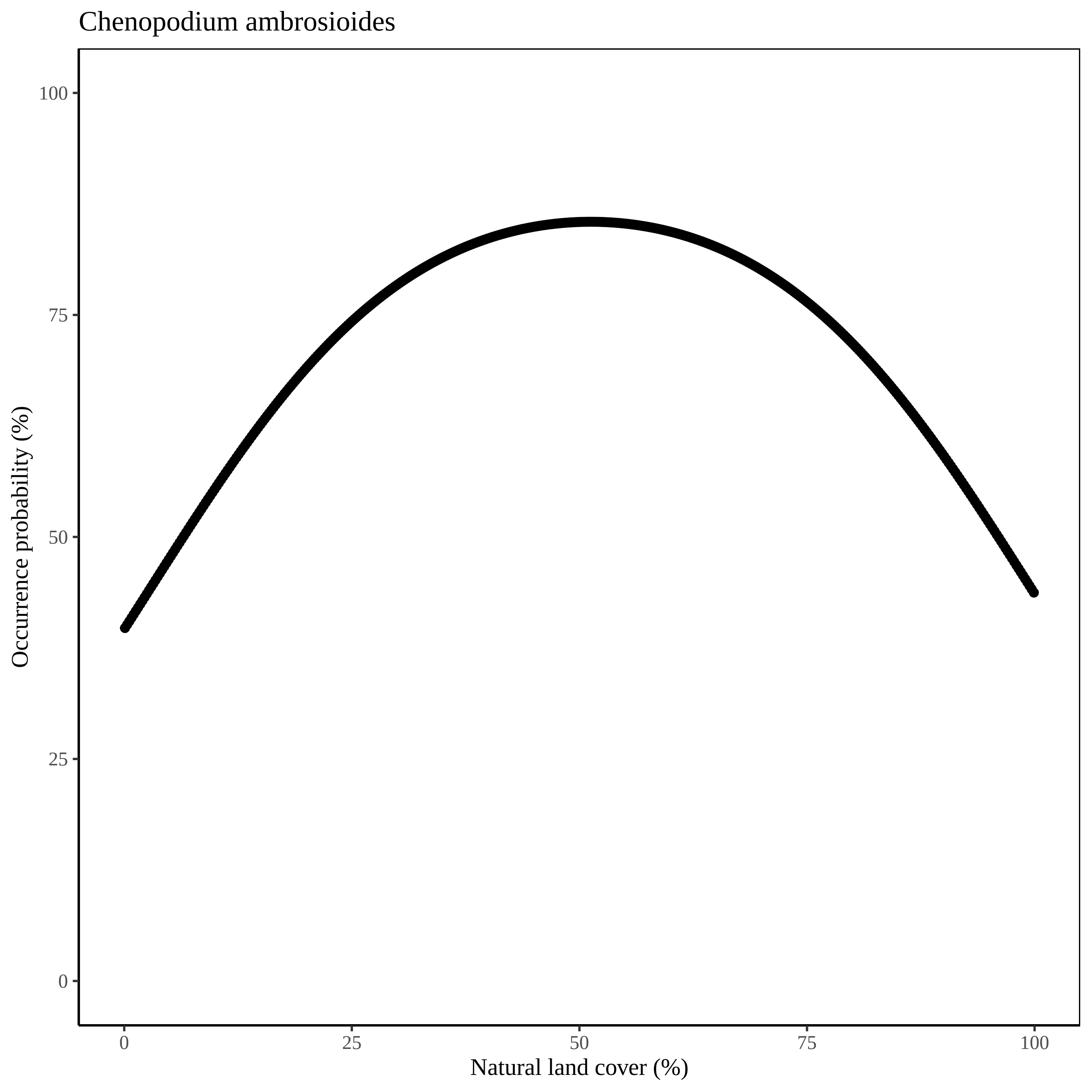
Chenopodium ambrosioides
Occurrence probability (%)
100
75
50
25
0
0
25
50
75
100
Natural land cover (%)

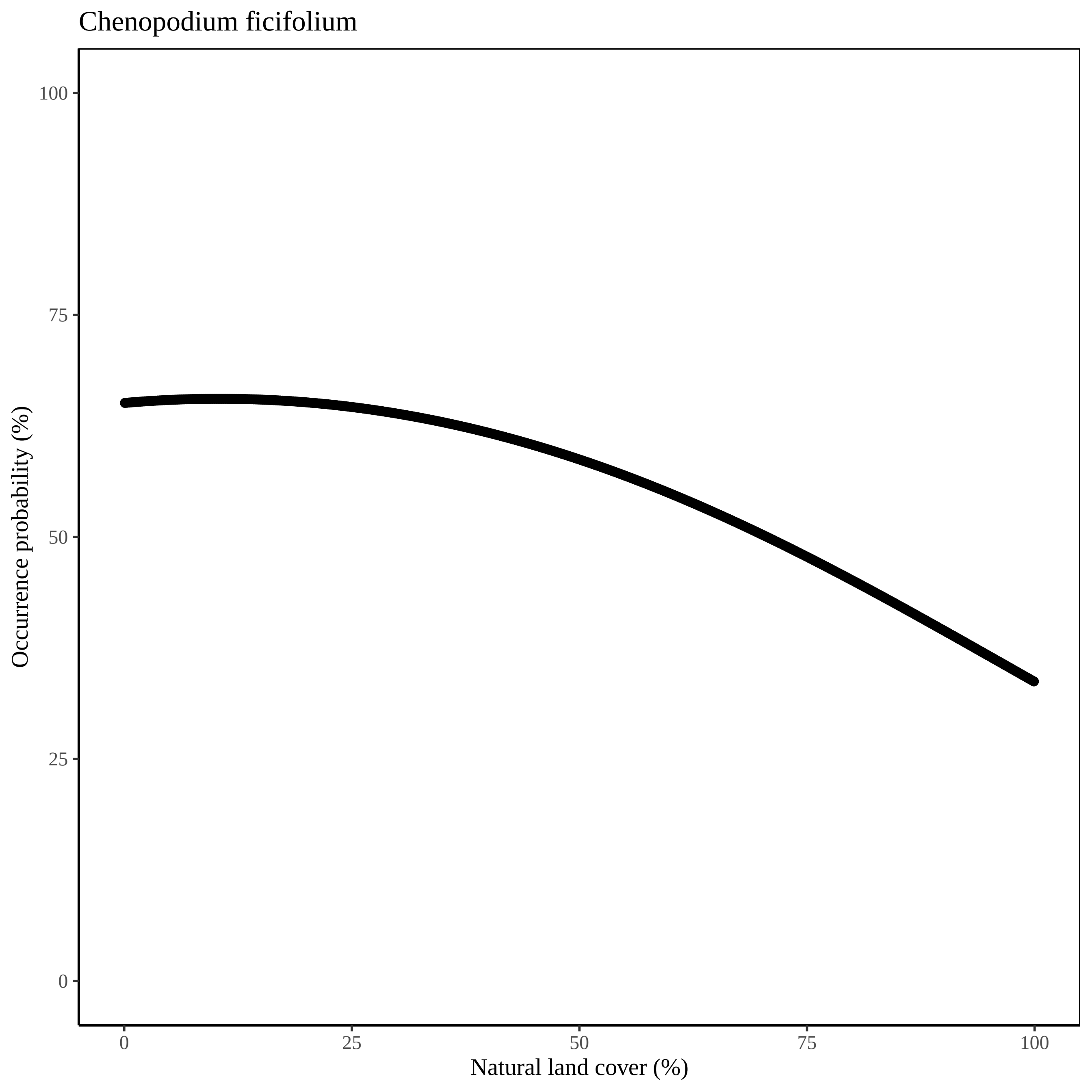
Chenopodium ficifolium
Occurrence probability (%)
100
75
50
25
0
0
25
50
75
100
Natural land cover (%)

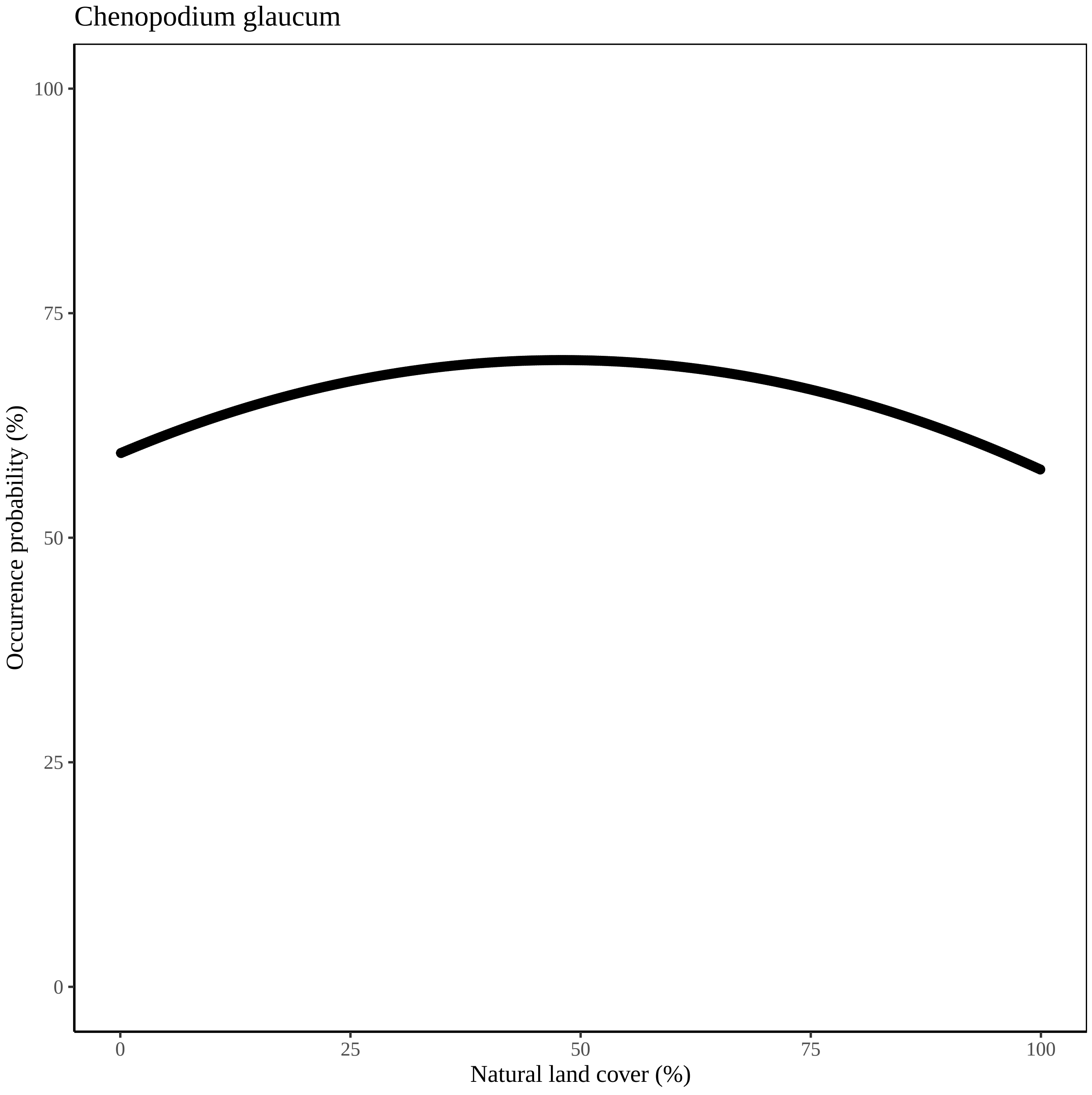
Chenopodium glaucum
Occurrence probability (%)
100
75
50
25
0
0
25
50
75
100
Natural land cover (%)

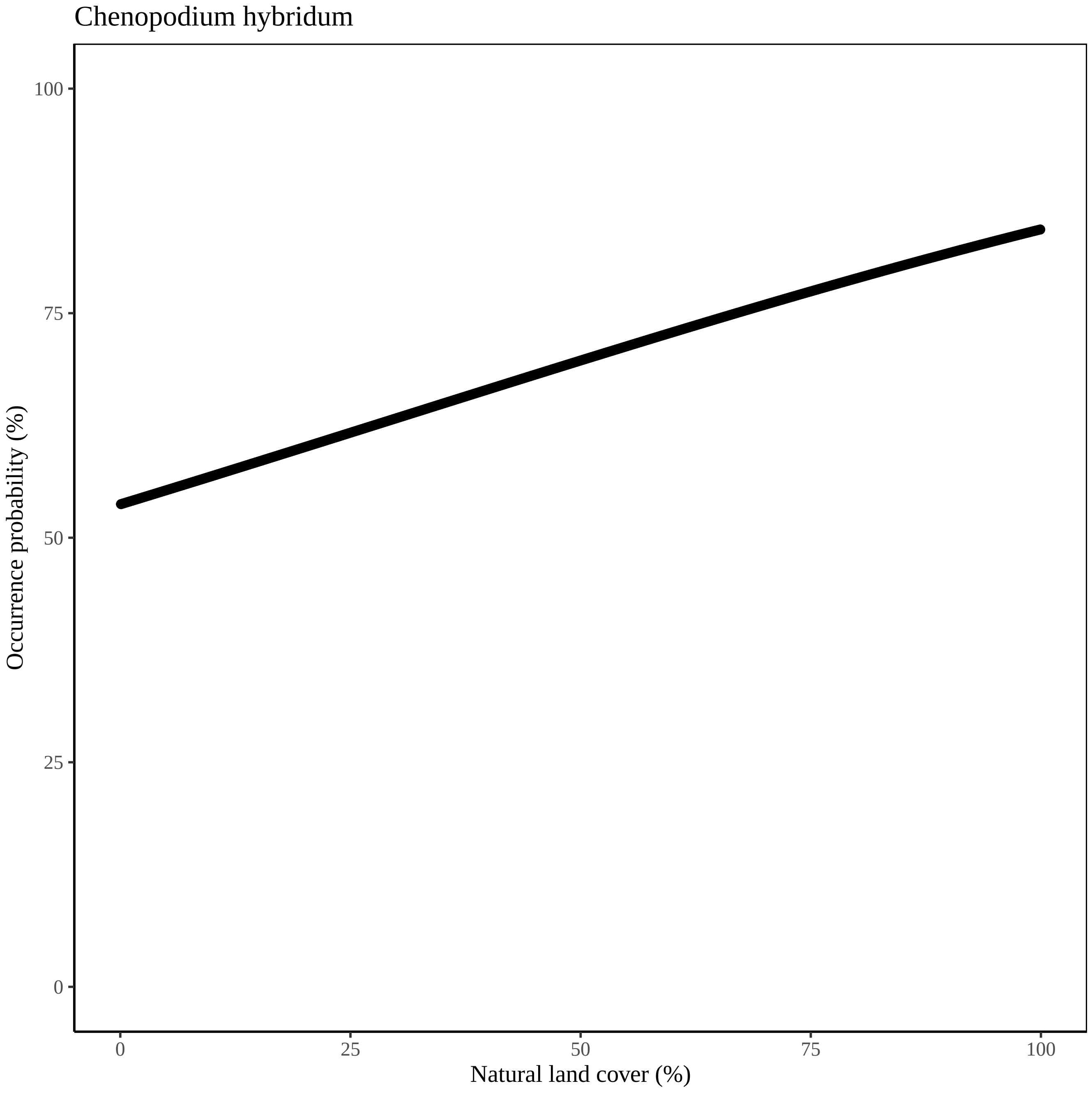

Chenopodium hybridum
100
75
50
25
0
Occurrence probability (%)
0
25
50
75
100
Natural land cover (%)

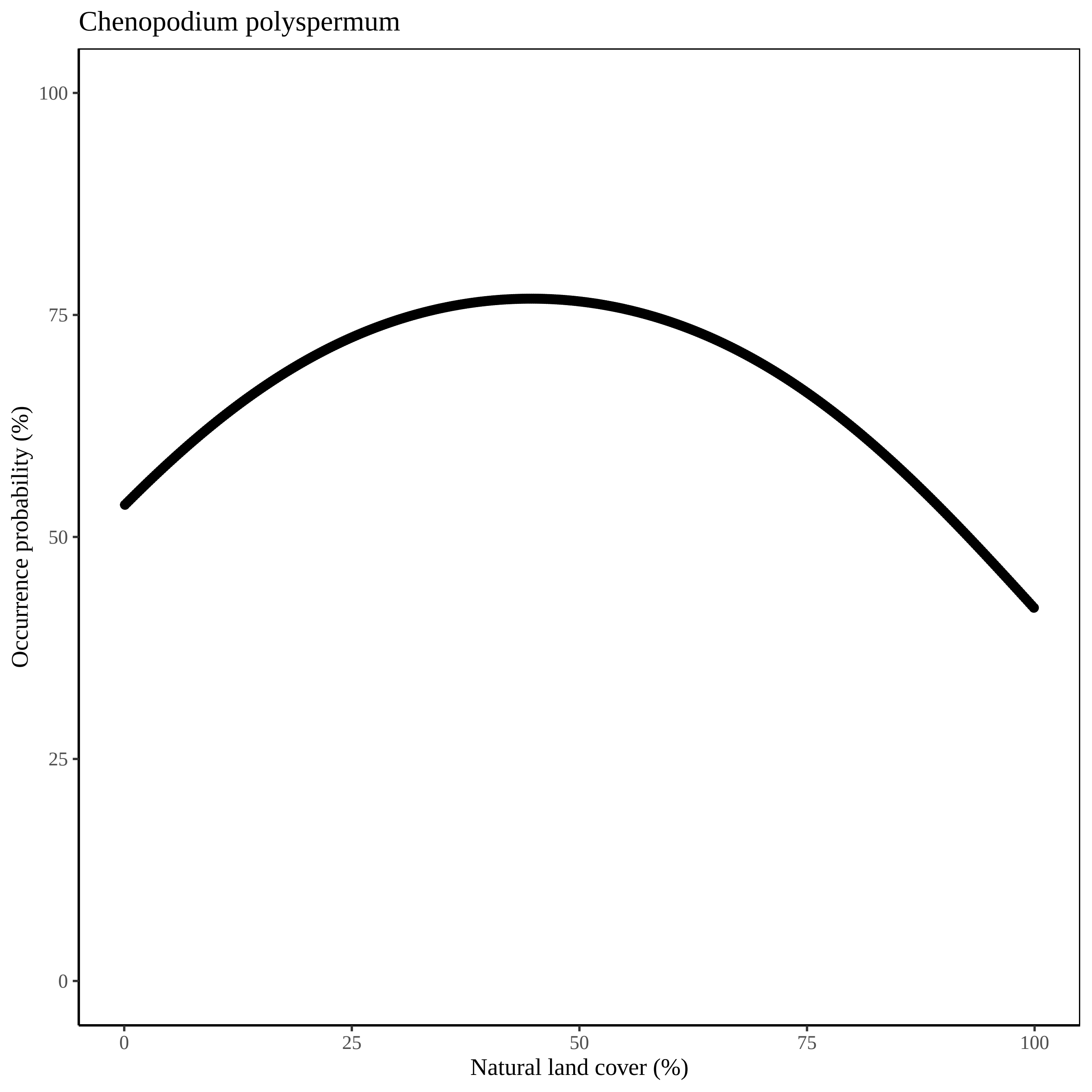

Chenopodium polyspermum
Occurrence probability (%)
100
75
50
25
0
0
25
50
75
100
Natural land cover (%)

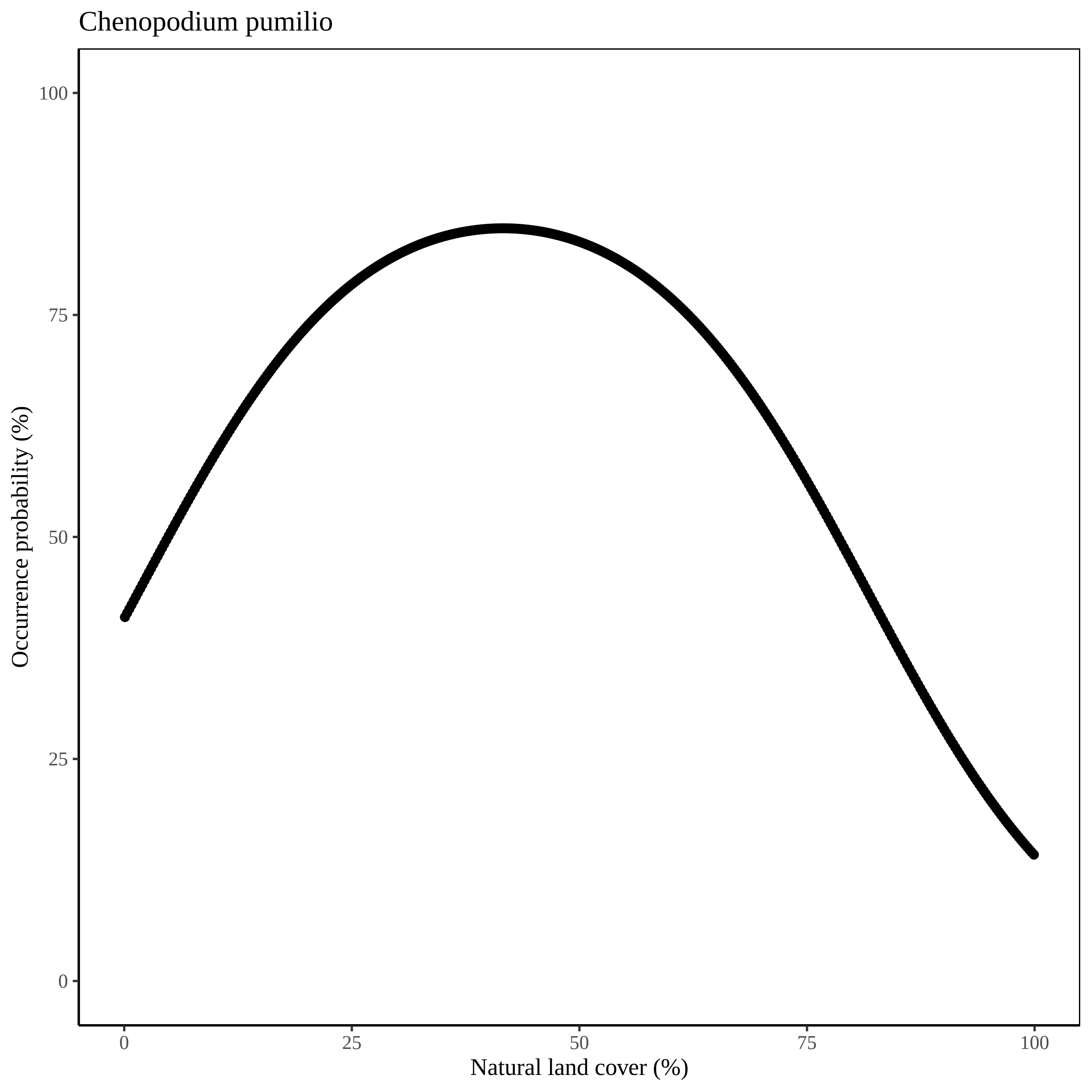
Chenopodium pumilio
Occurrence probability (%)
100
75
50
25
0
0
25
50
75
100
Natural land cover (%)

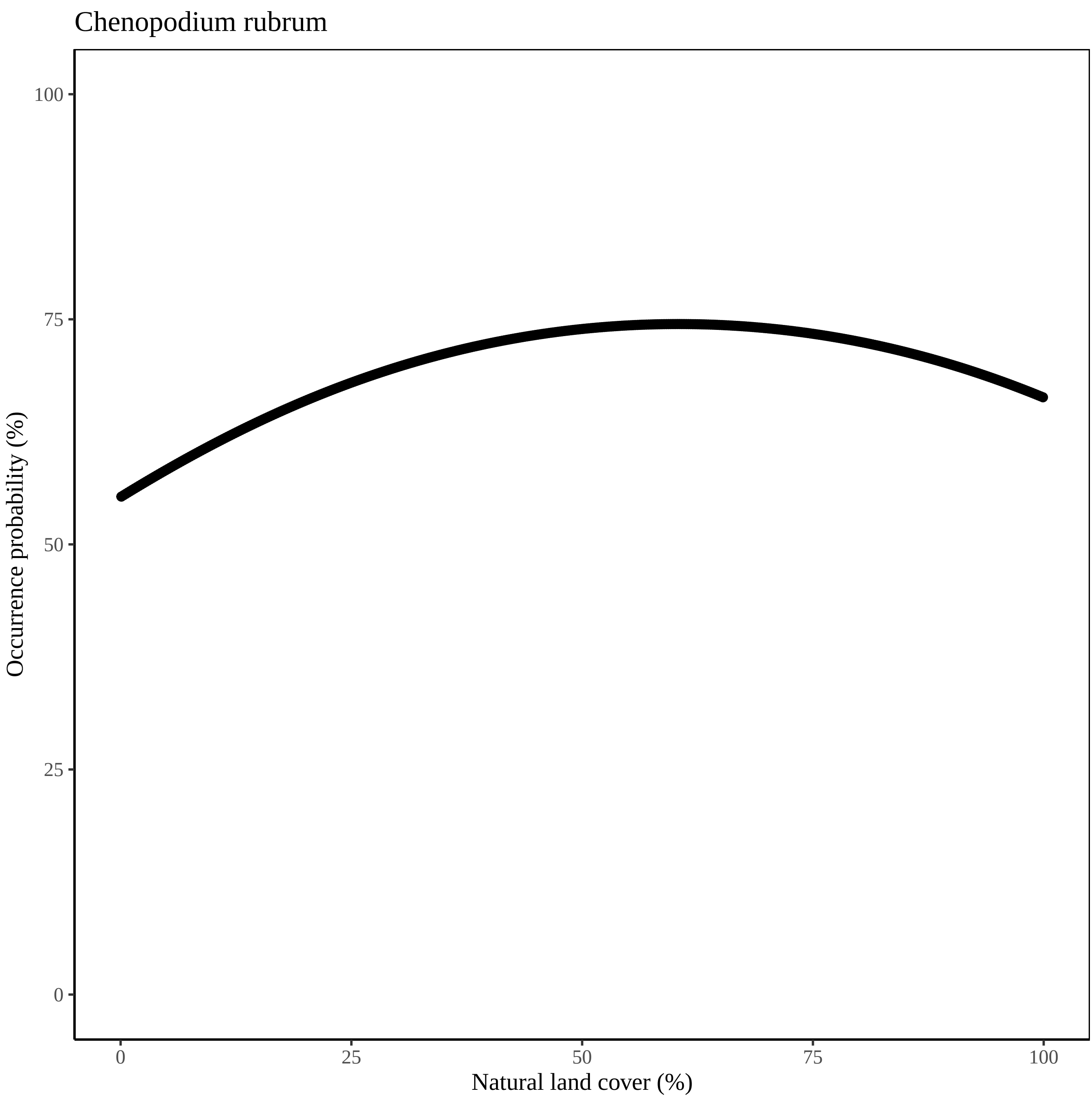
Chenopodium rubrum
Occurrence probability (%)
100
75
50
25
0
0
25
50
75
100
Natural land cover (%)

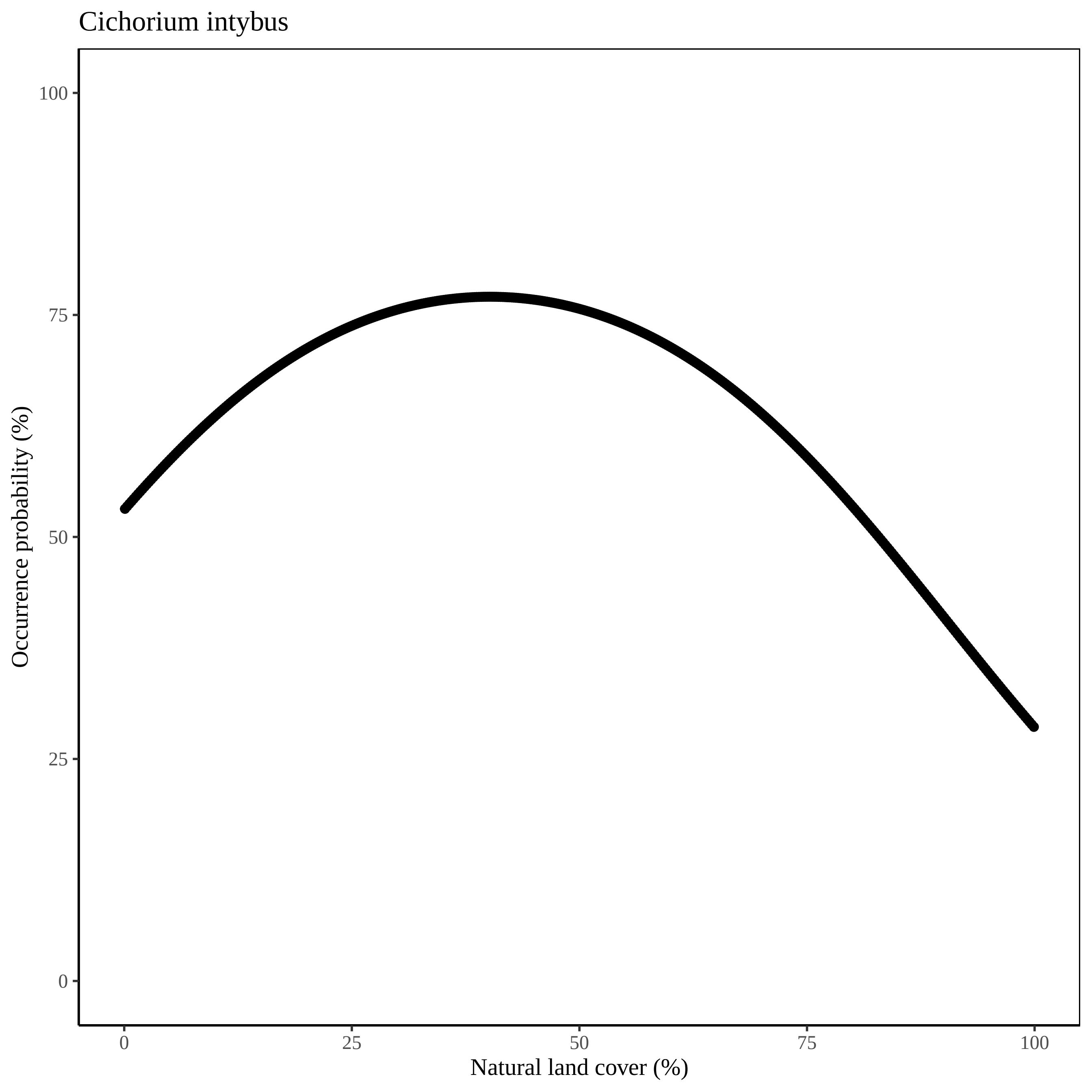

Cichorium intybus
100
75
50
25
0
Occurrence probability (%)
0
25
50
75
100
Natural land cover (%)

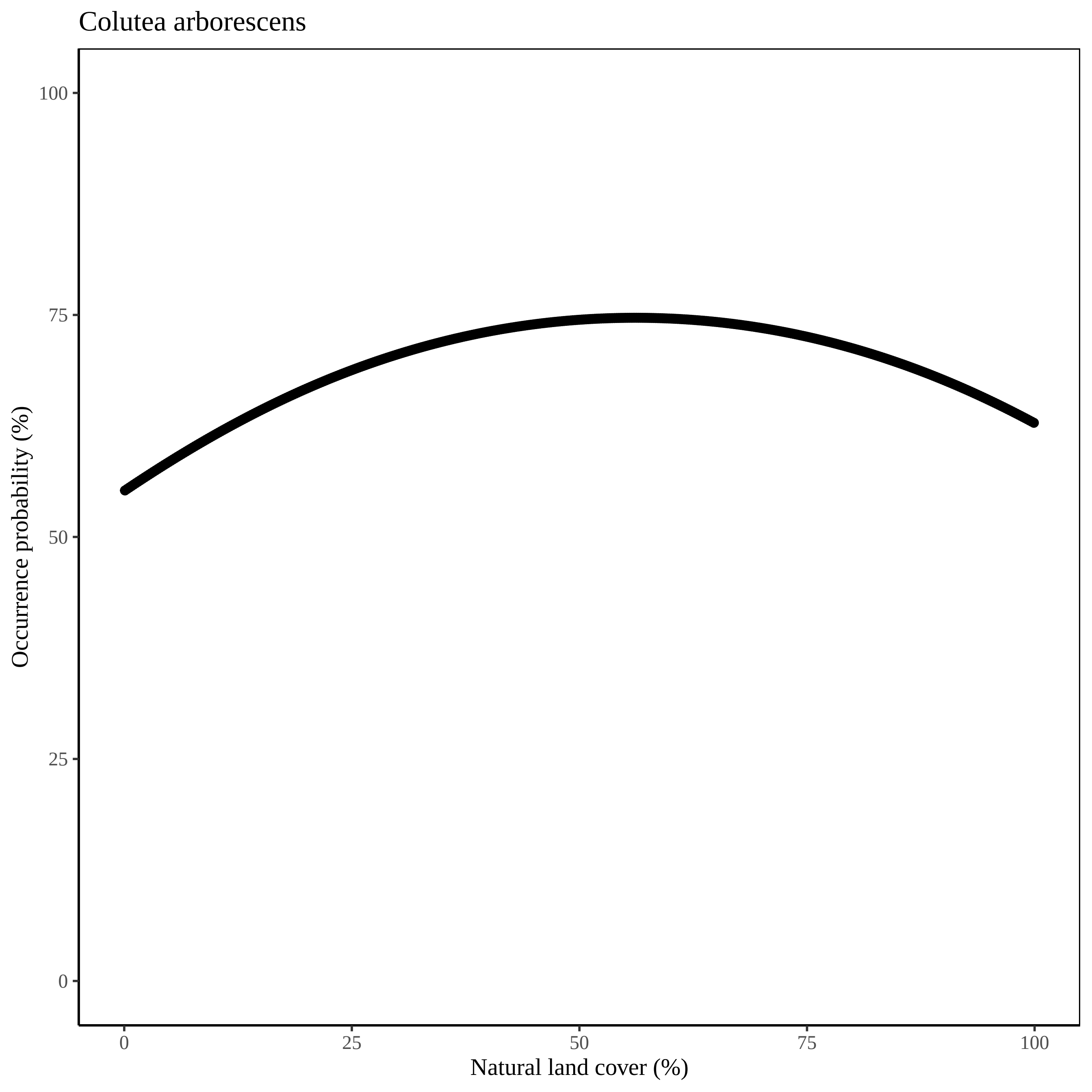

Colutea arborescens
Occurrence probability (%)
100
75
50
25
0
0
25
50
75
100
Natural land cover (%)

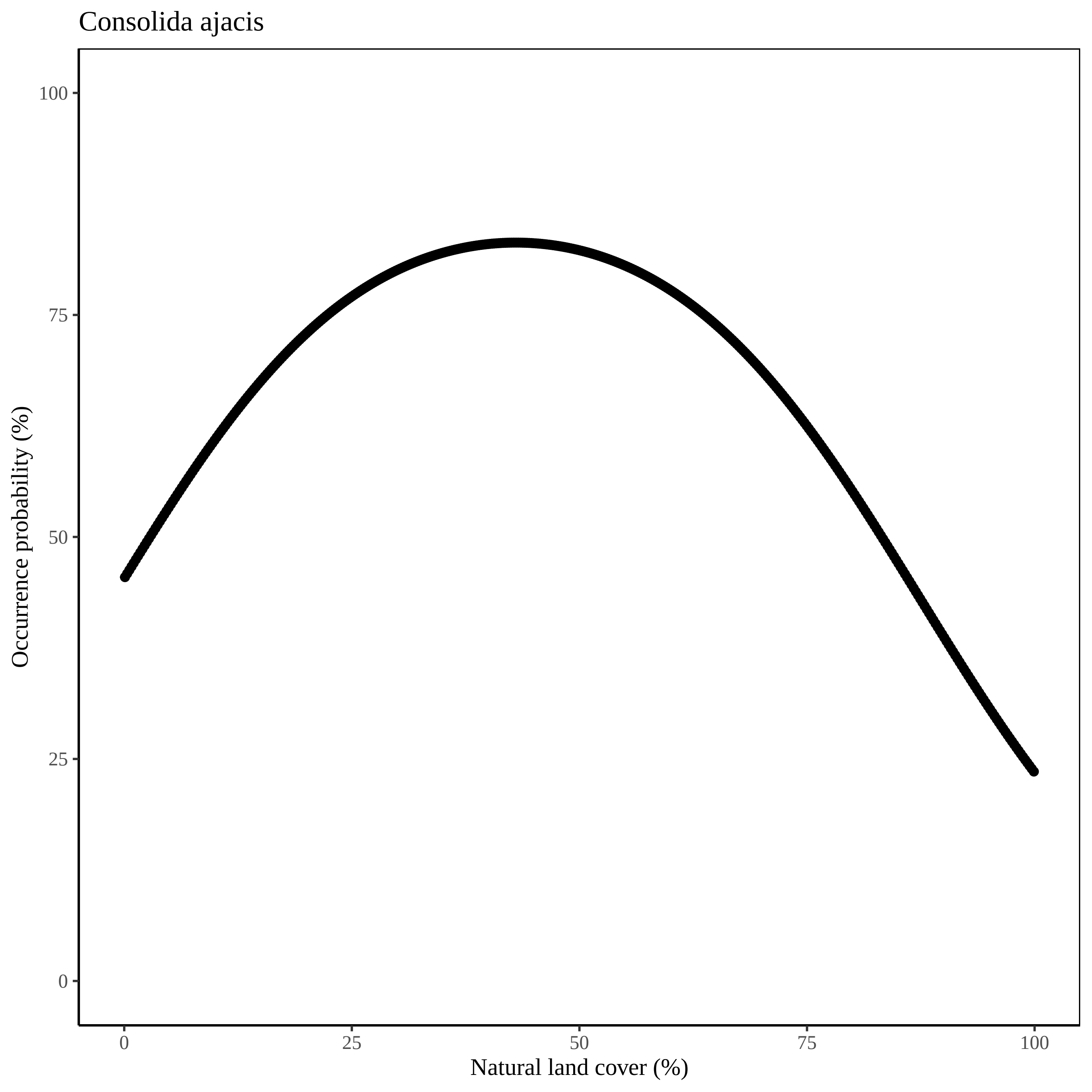

Consolida ajacis
Occurrence probability (%)
100
75
50
25
0
0
25
50
75
100
Natural land cover (%)

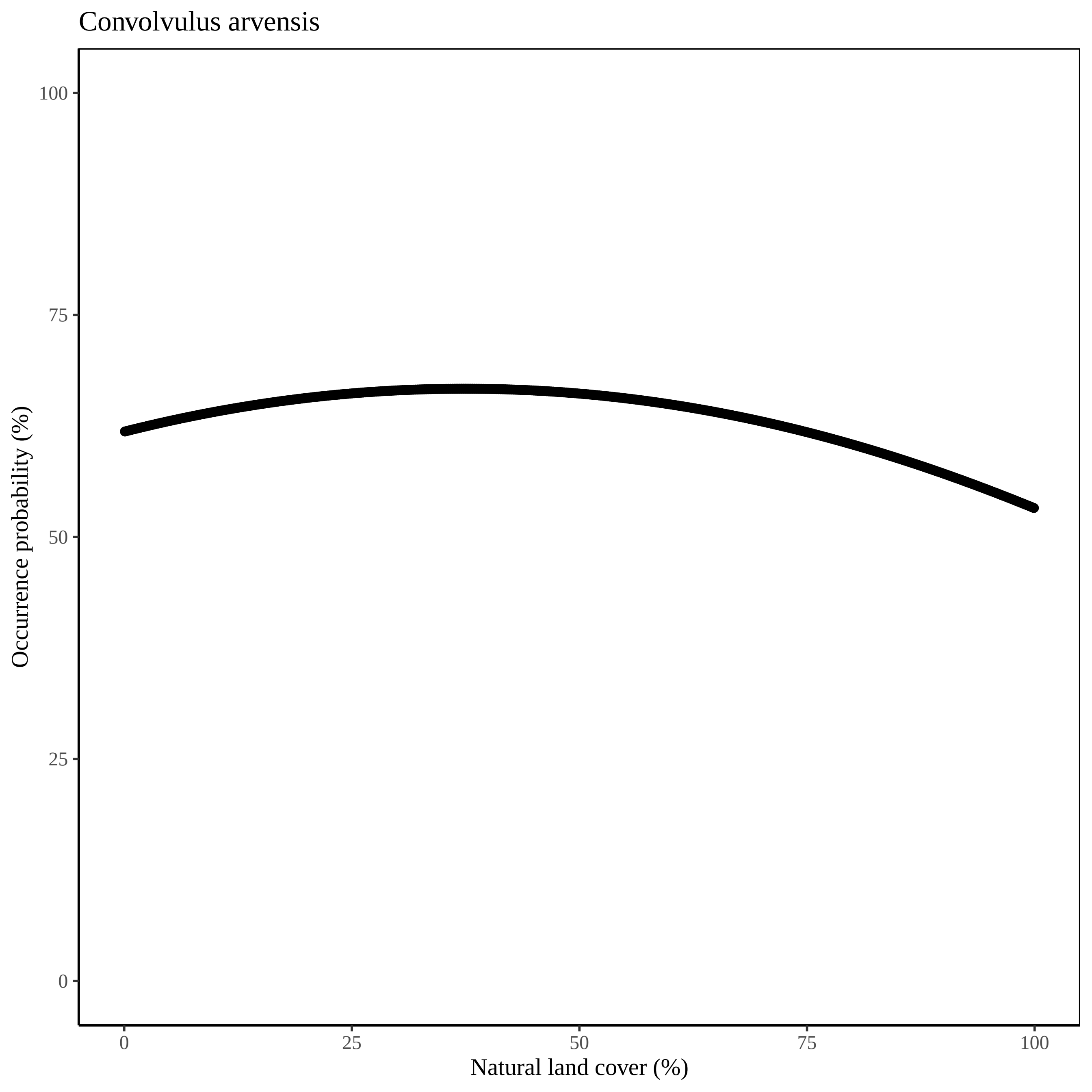
Convolvulus arvensis
Occurrence probability (%)
100
75
50
25
0
0
25
50
75
100
Natural land cover (%)

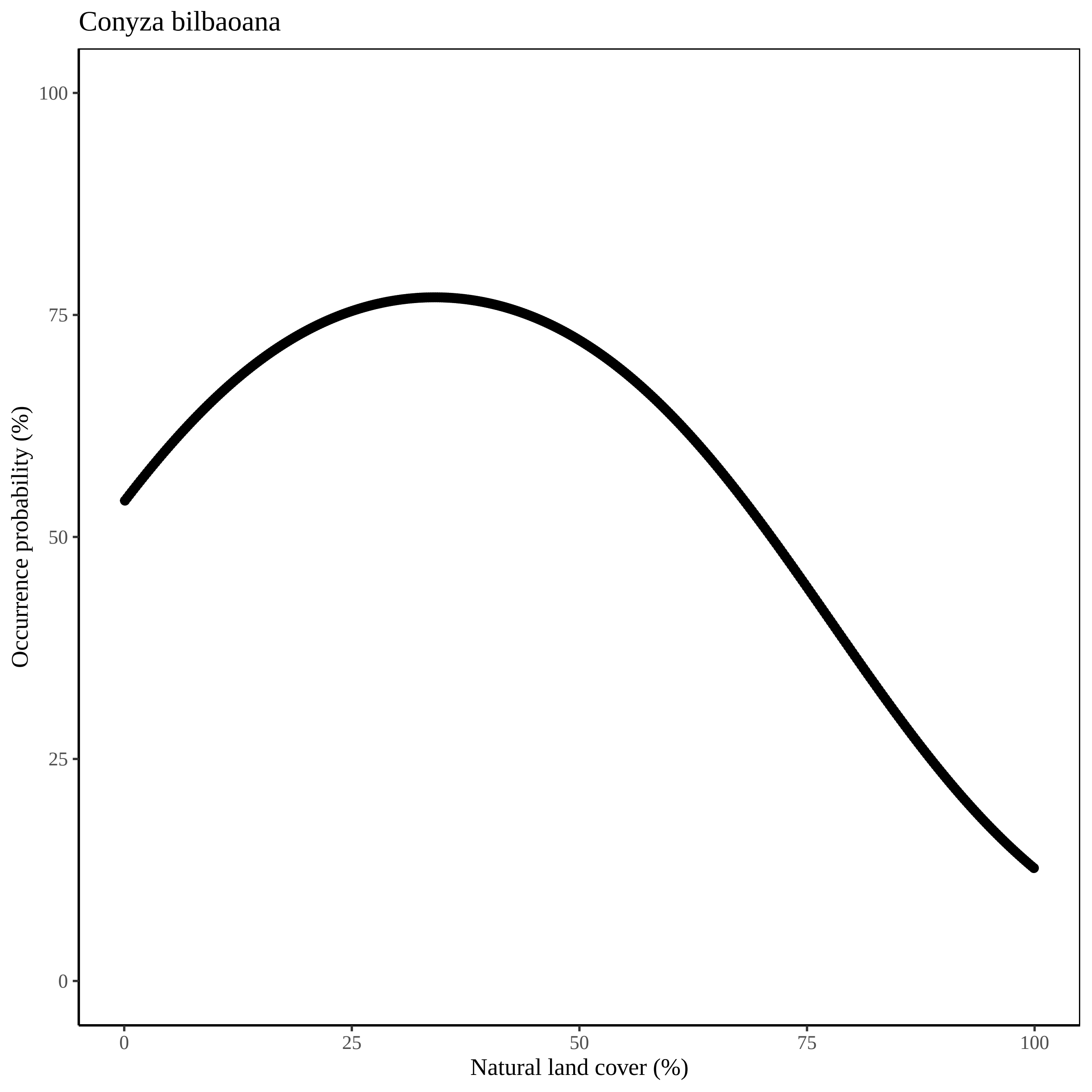
Conyza bilbaoana
Occurrence probability (%)
100
75
50
25
0
0
25
50
75
100
Natural land cover (%)

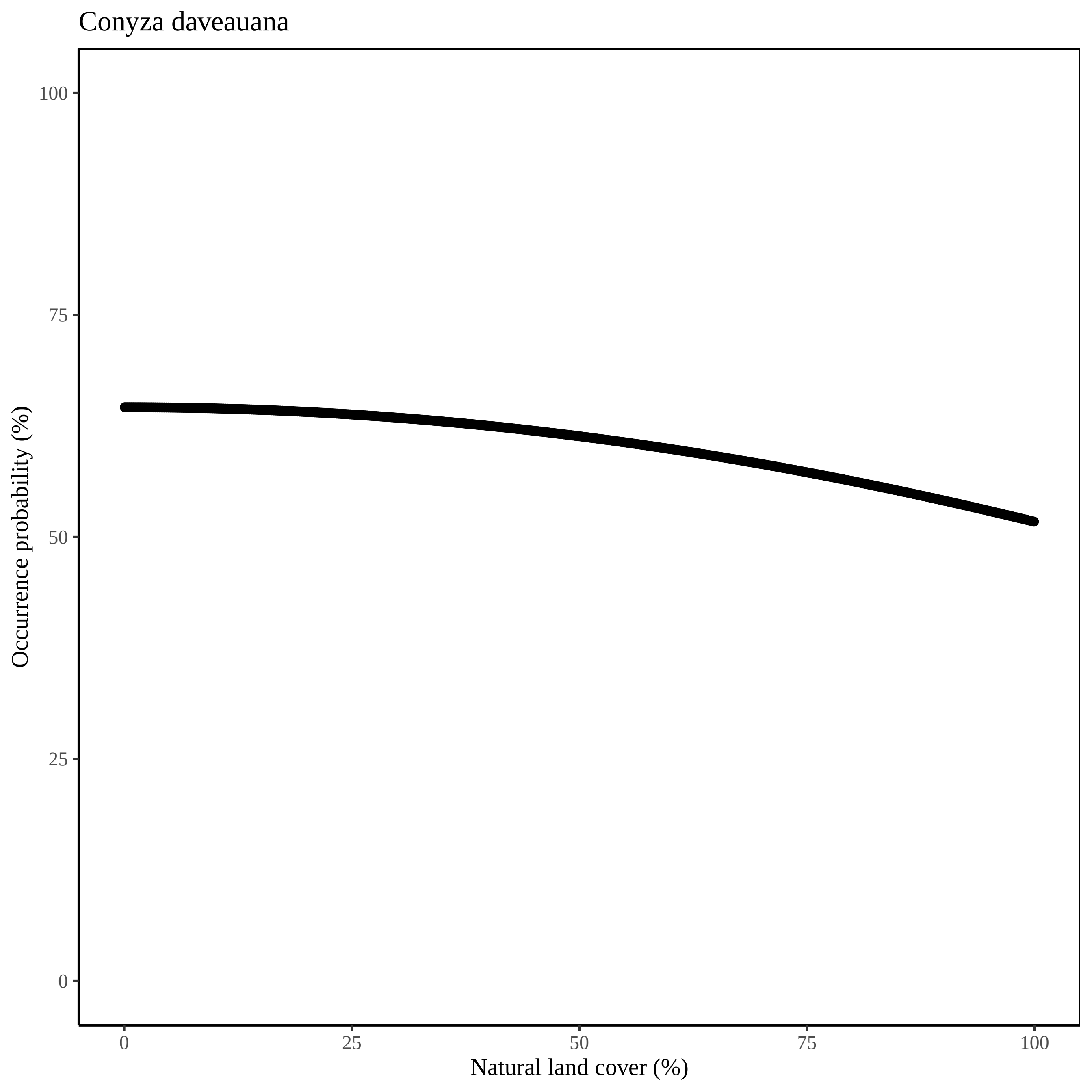

Conyza daveauana
Occurrence probability (%)
100
75
50
25
0
0
25
50
75
100
Natural land cover (%)

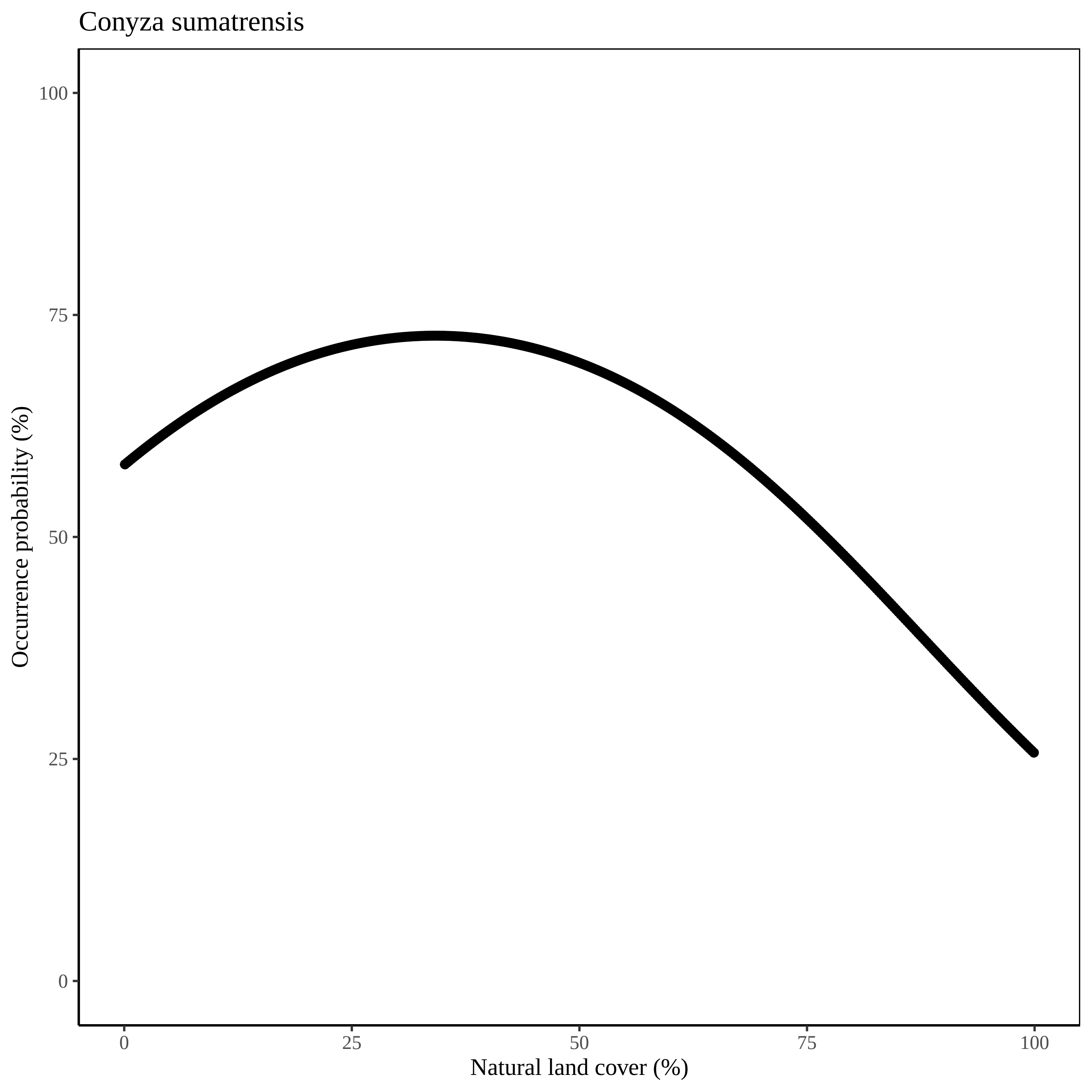
Conyza sumatrensis
Occurrence probability (%)
100
75
50
25
0
0
25
50
75
100
Natural land cover (%)

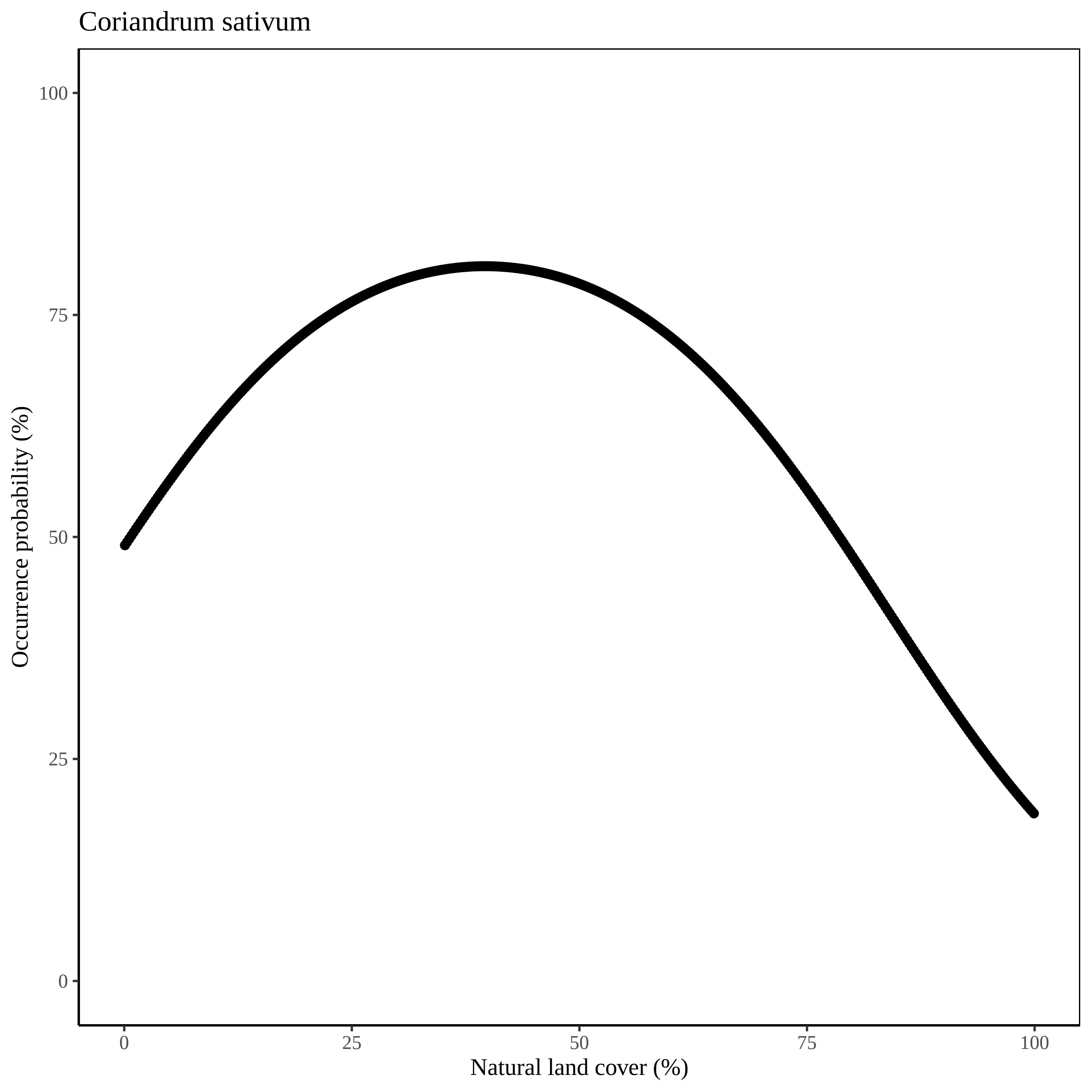

Coriandrum sativum
Occurrence probability (%)
100
75
50
25
0
0
25
50
75
100
Natural land cover (%)

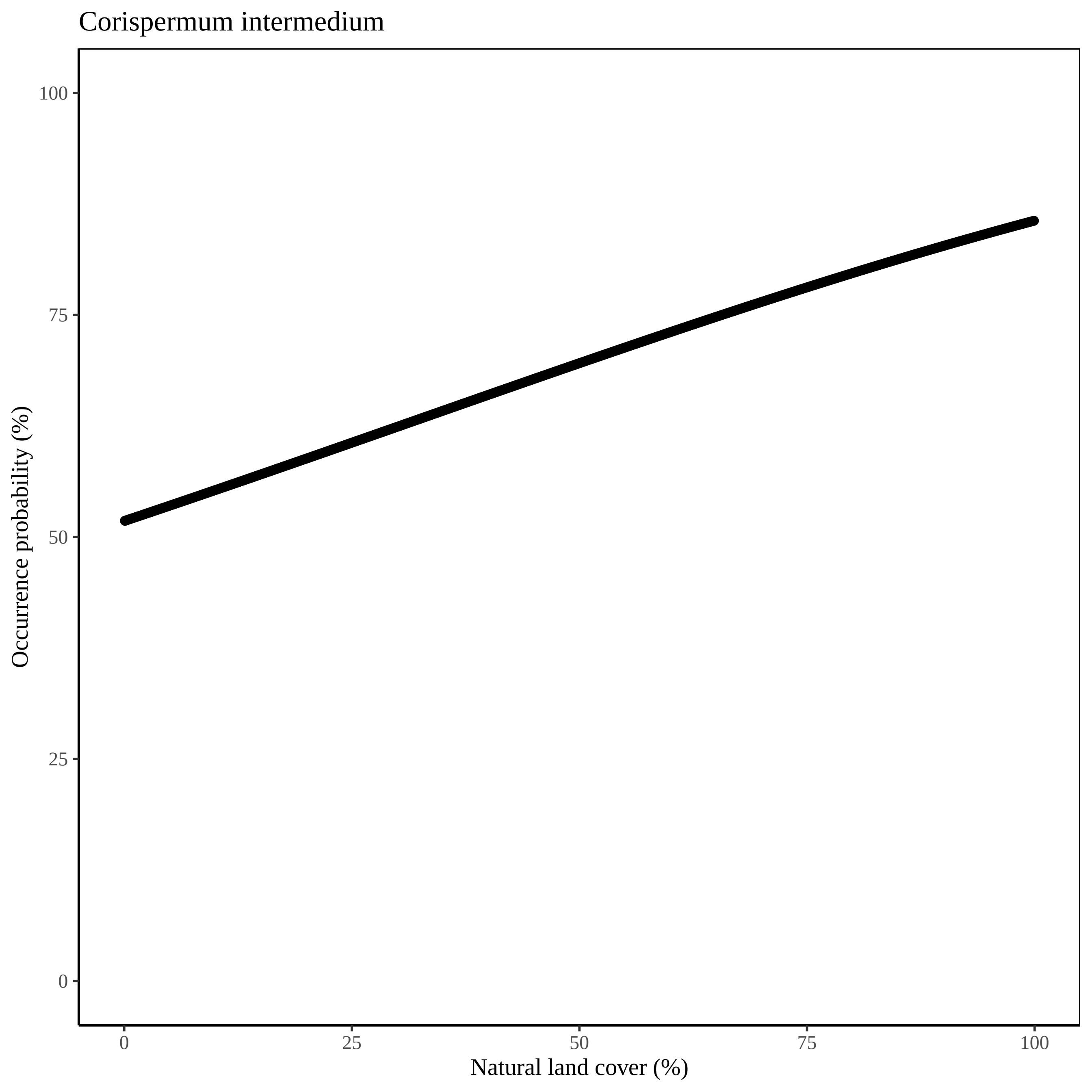

Corispermum intermedium
Occurrence probability (%)
100
75
50
25
0
0
25
50
75
100
Natural land cover (%)

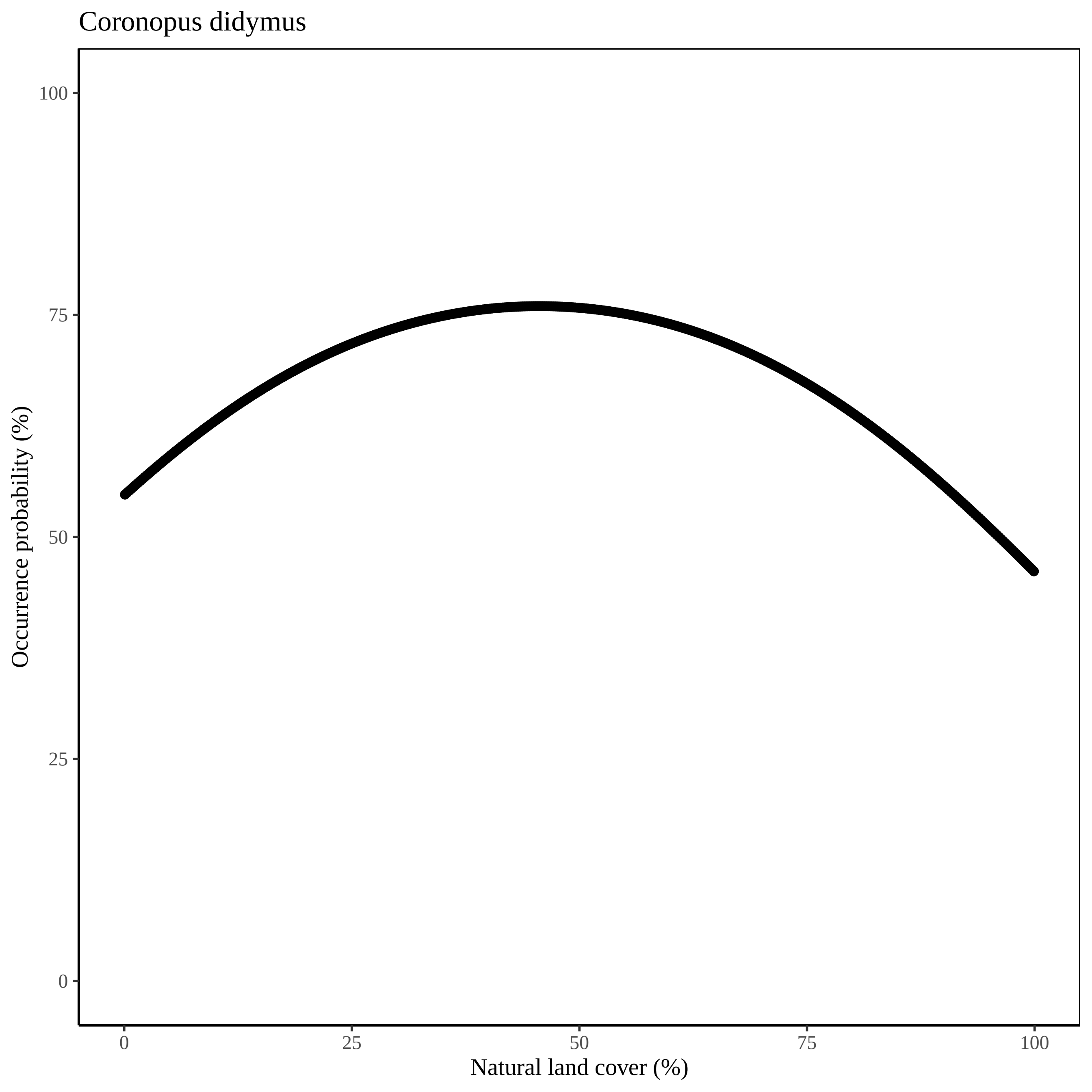

Coronopus didymus
Occurrence probability (%)
100
75
50
25
0
0
25
50
75
100
Natural land cover (%)

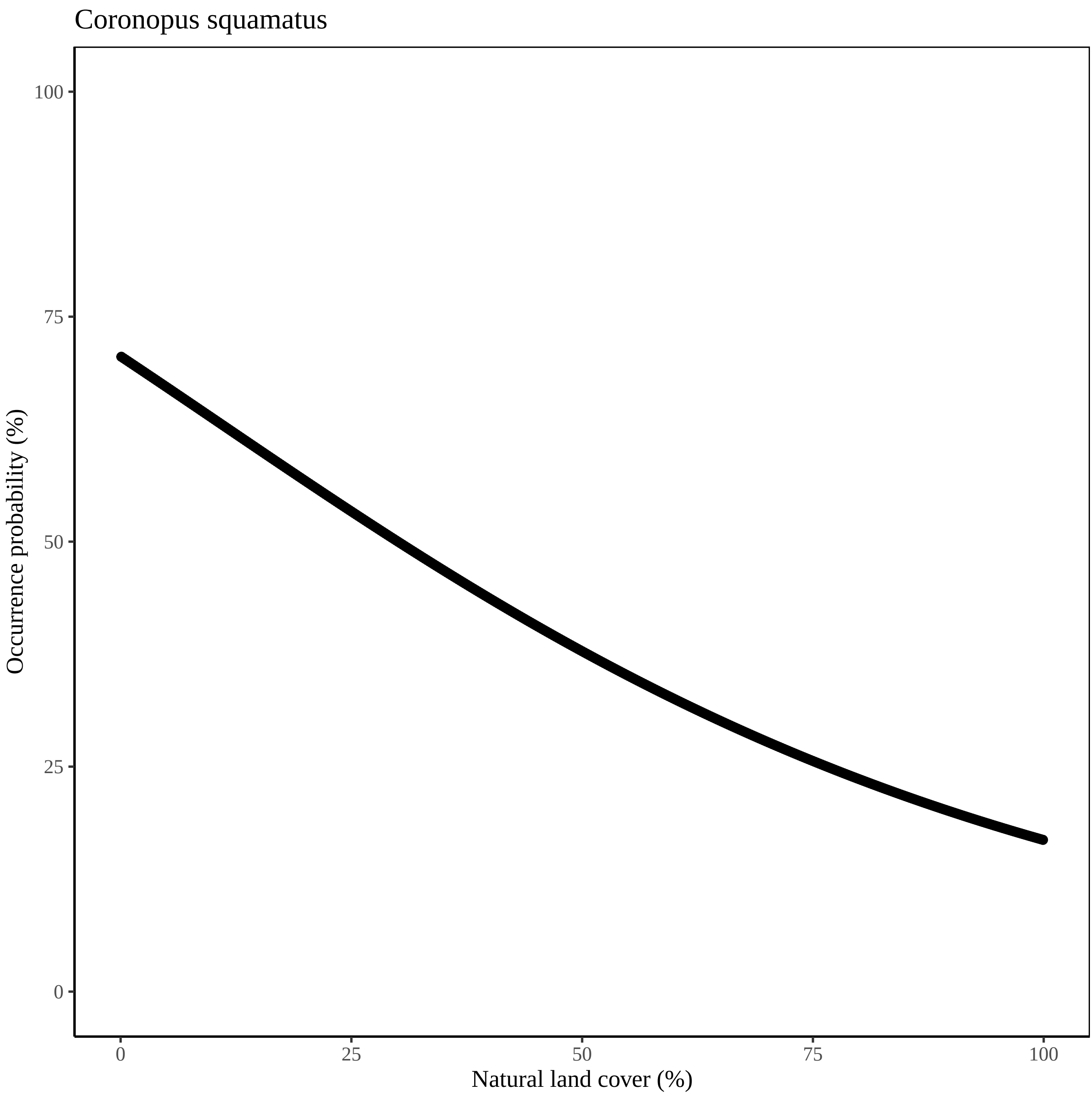
Coronopus squamatus
Occurrence probability (%)
100
75
50
25
0
0
25
50
75
100
Natural land cover (%)

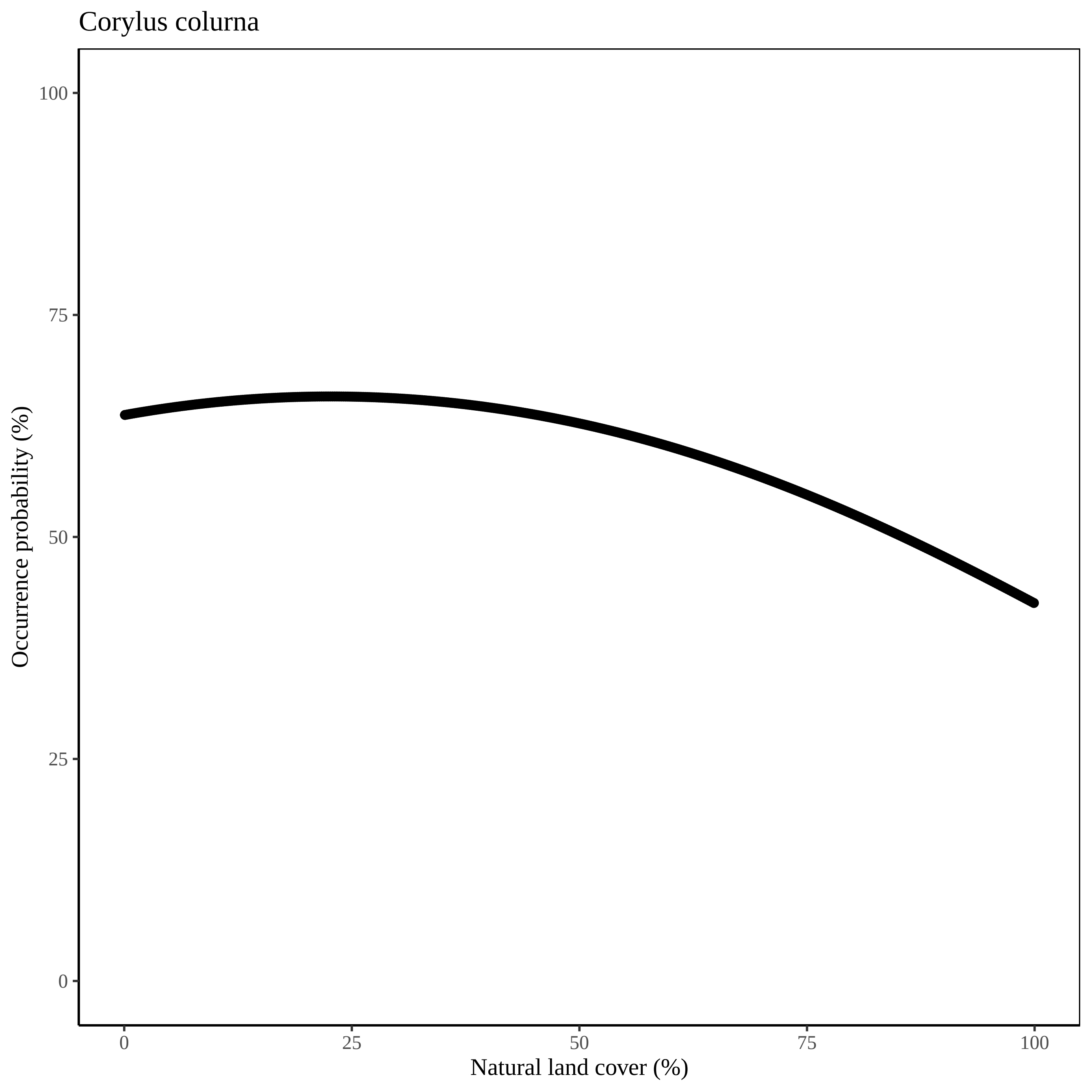
Corylus colurna
Occurrence probability (%)
100
75
50
25
0
0
25
50
75
100
Natural land cover (%)

Crambe maritima

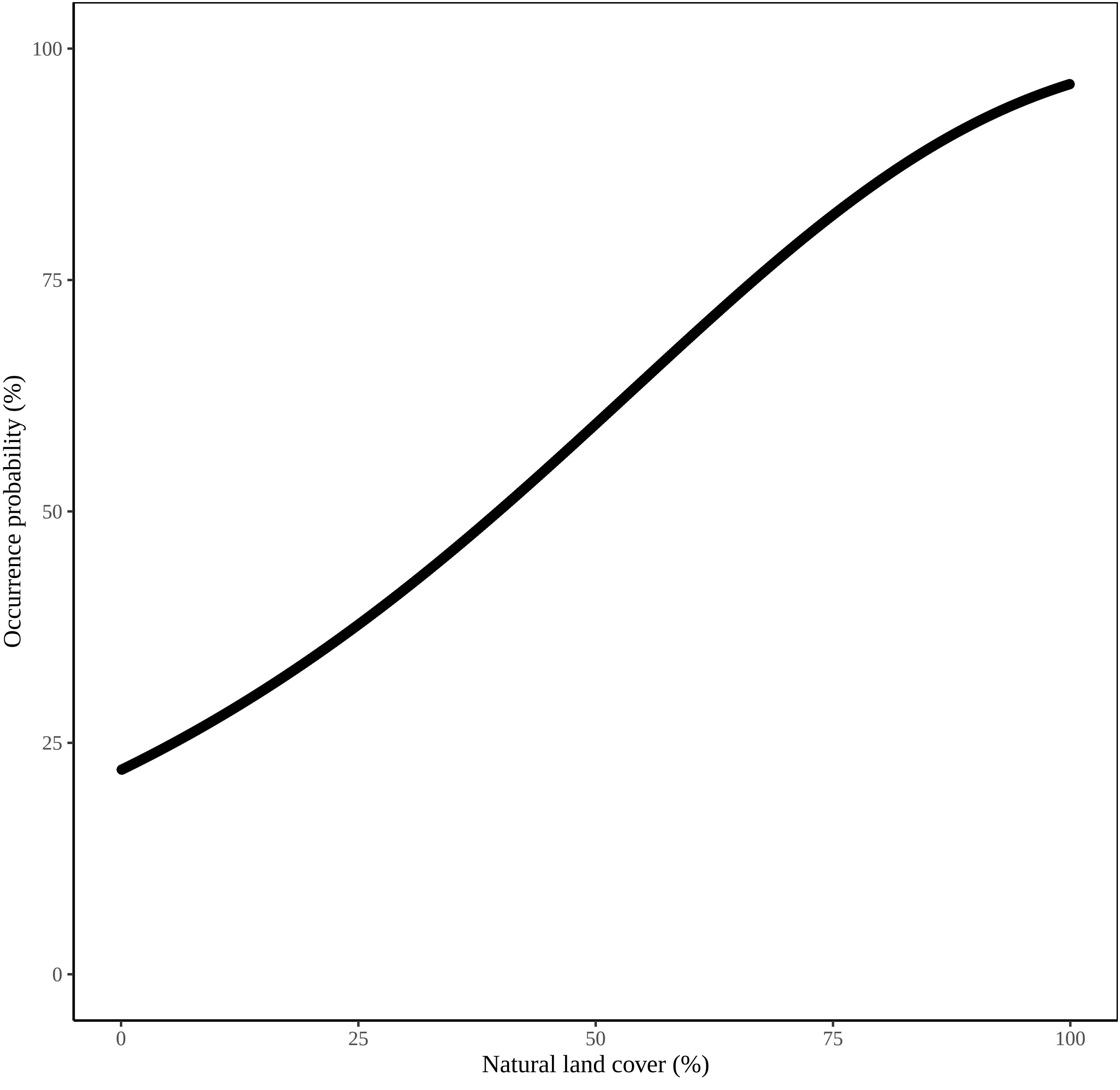

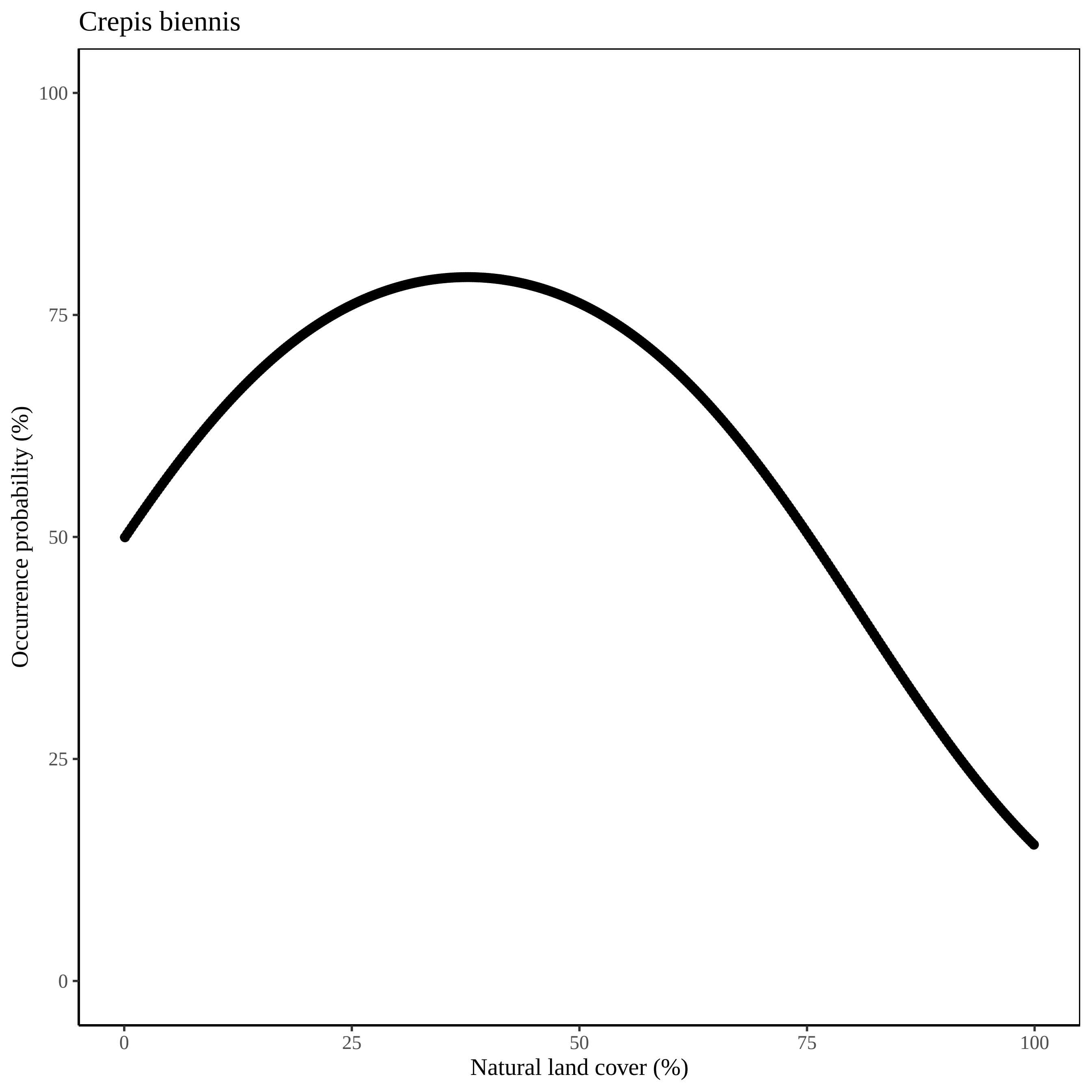

Crepis biennis
Occurrence probability (%)
100
75
50
25
0
0
25
50
75
100
Natural land cover (%)

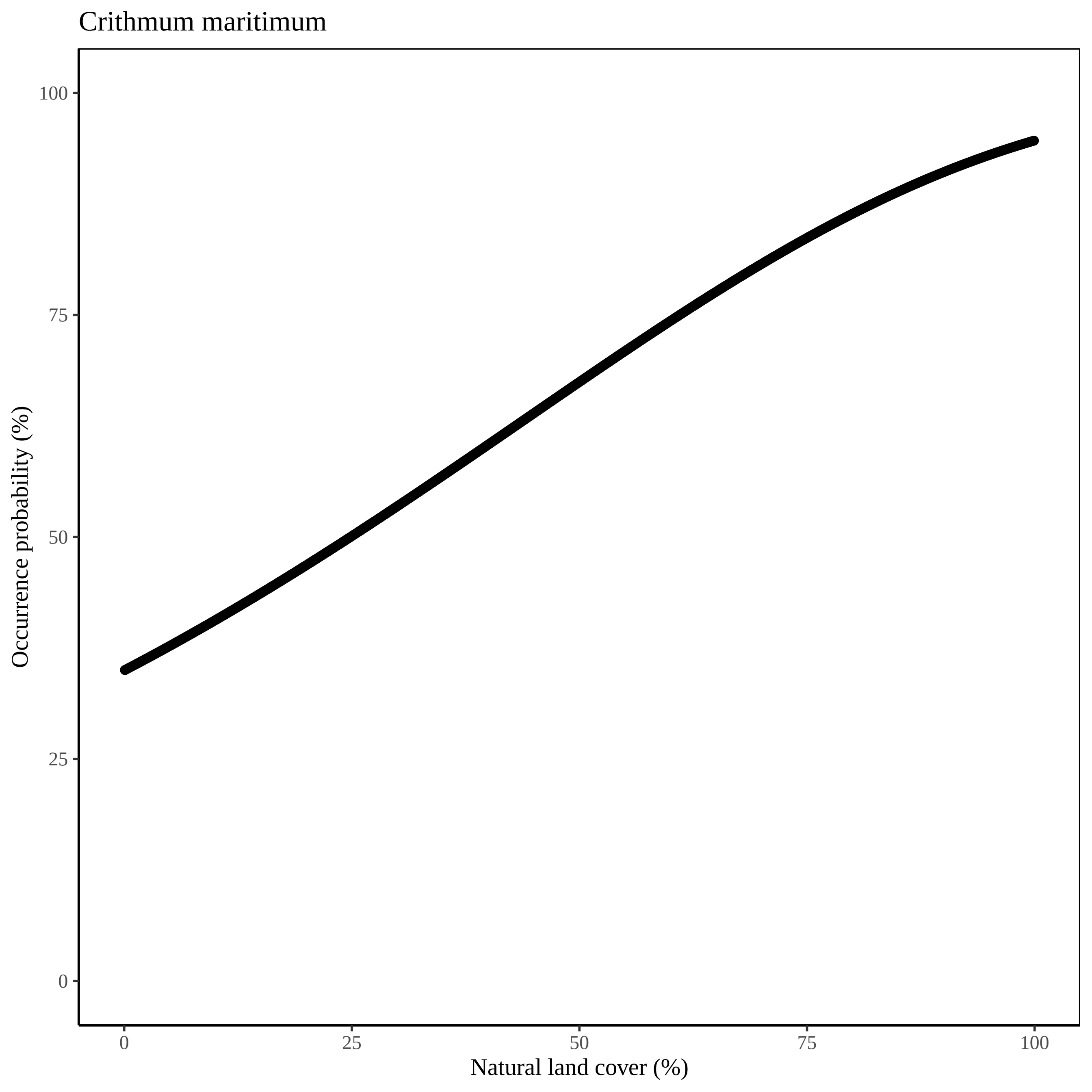
Crithmum maritimum
Occurrence probability (%)
100
75
50
25
0
0
25
50
75
100
Natural land cover (%)

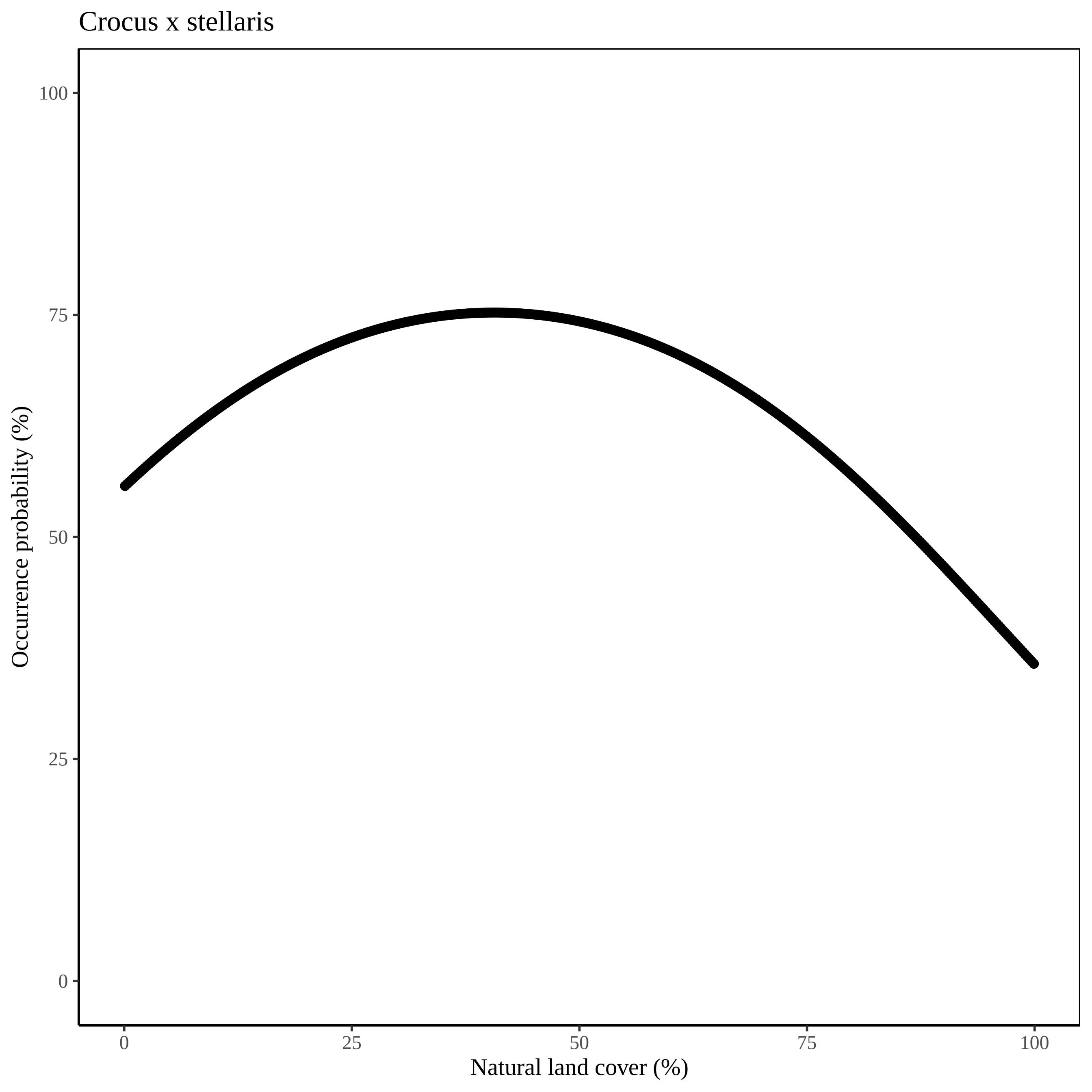
Crocus x stellaris
Occurrence probability (%)
100
75
50
25
0
0
25
50
75
100
Natural land cover (%)

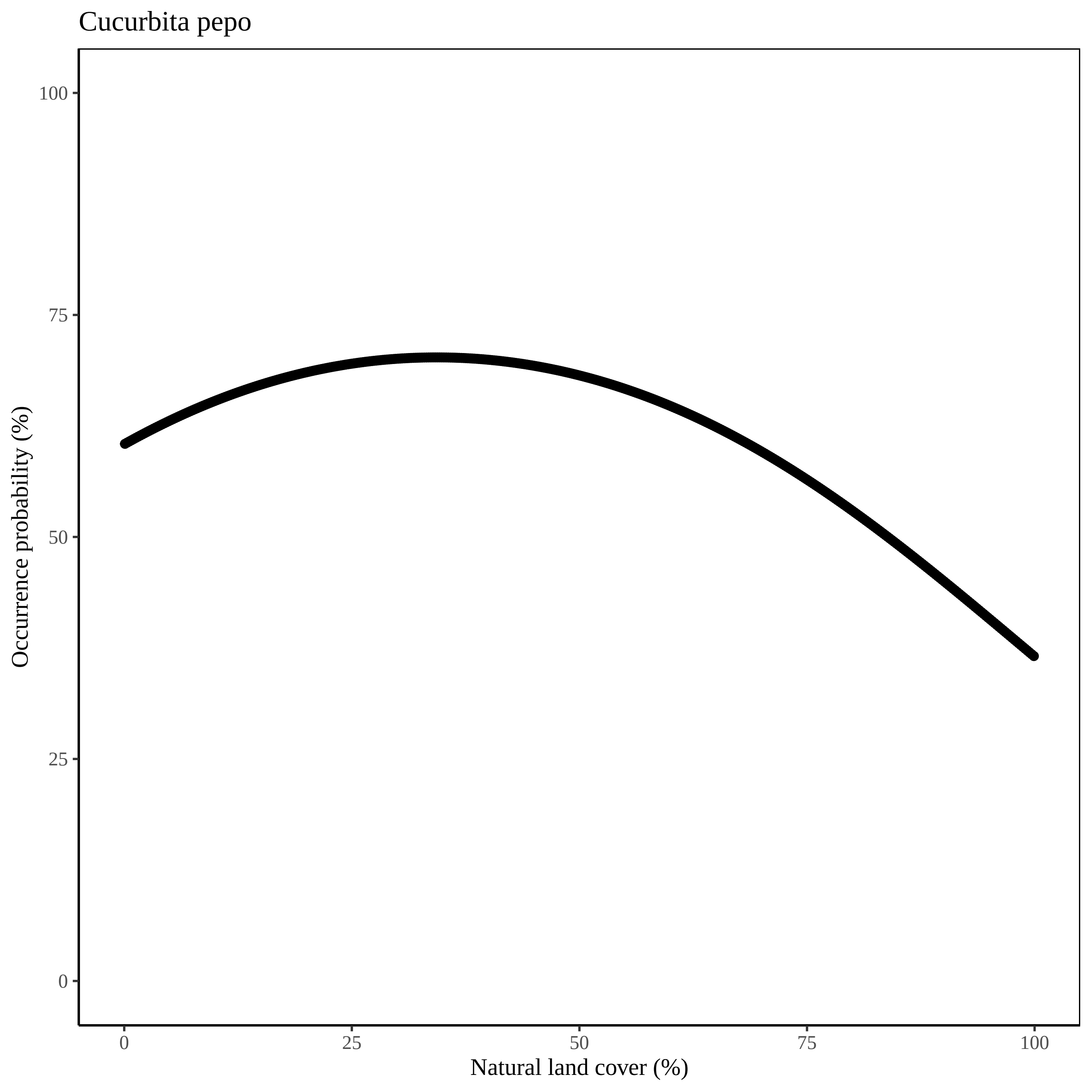

Cucurbita pepo
Occurrence probability (%)
100
75
50
25
0
0
25
50
75
100
Natural land cover (%)

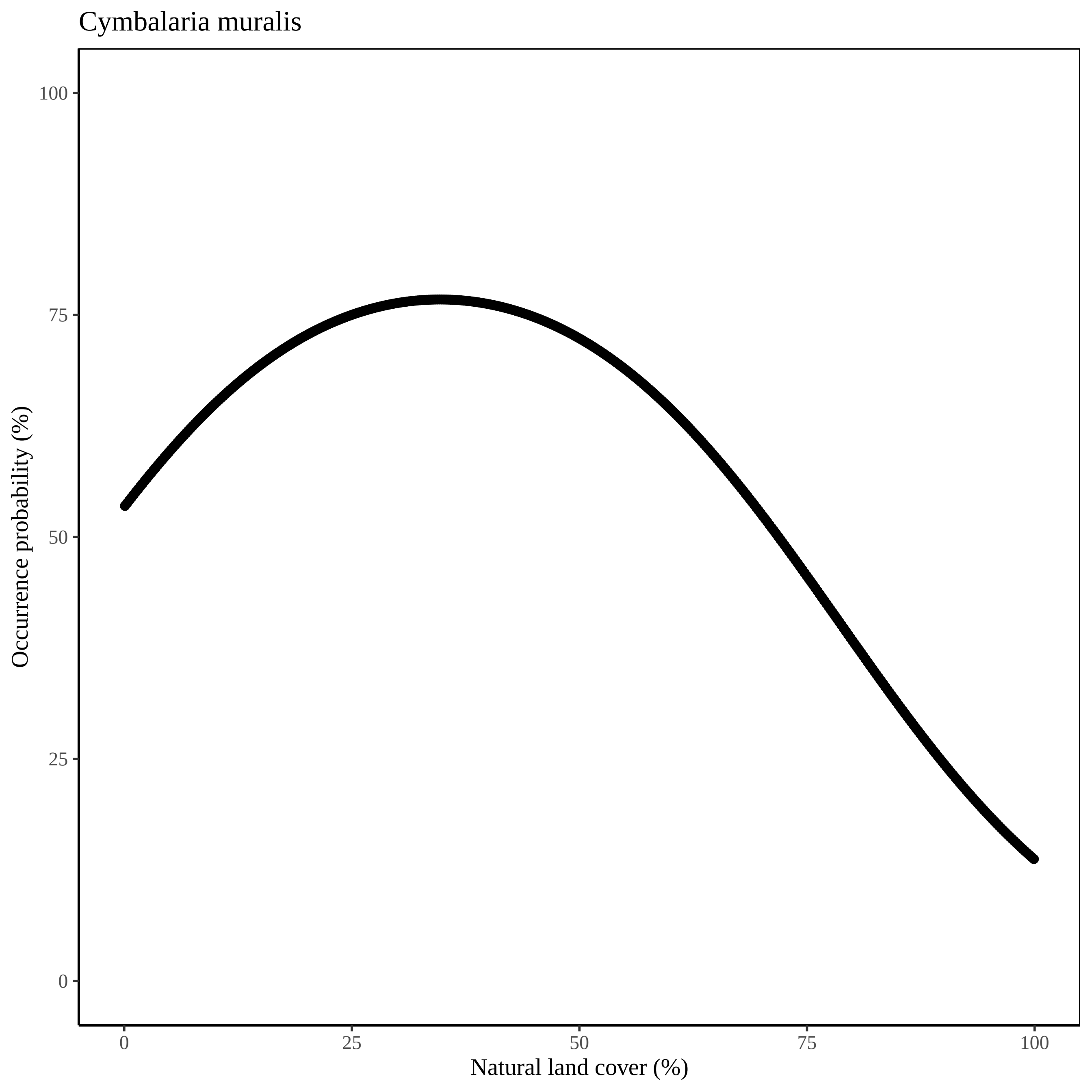
Cymbalaria muralis
Occurrence probability (%)
100
75
50
25
0
0
25
50
75
100
Natural land cover (%)

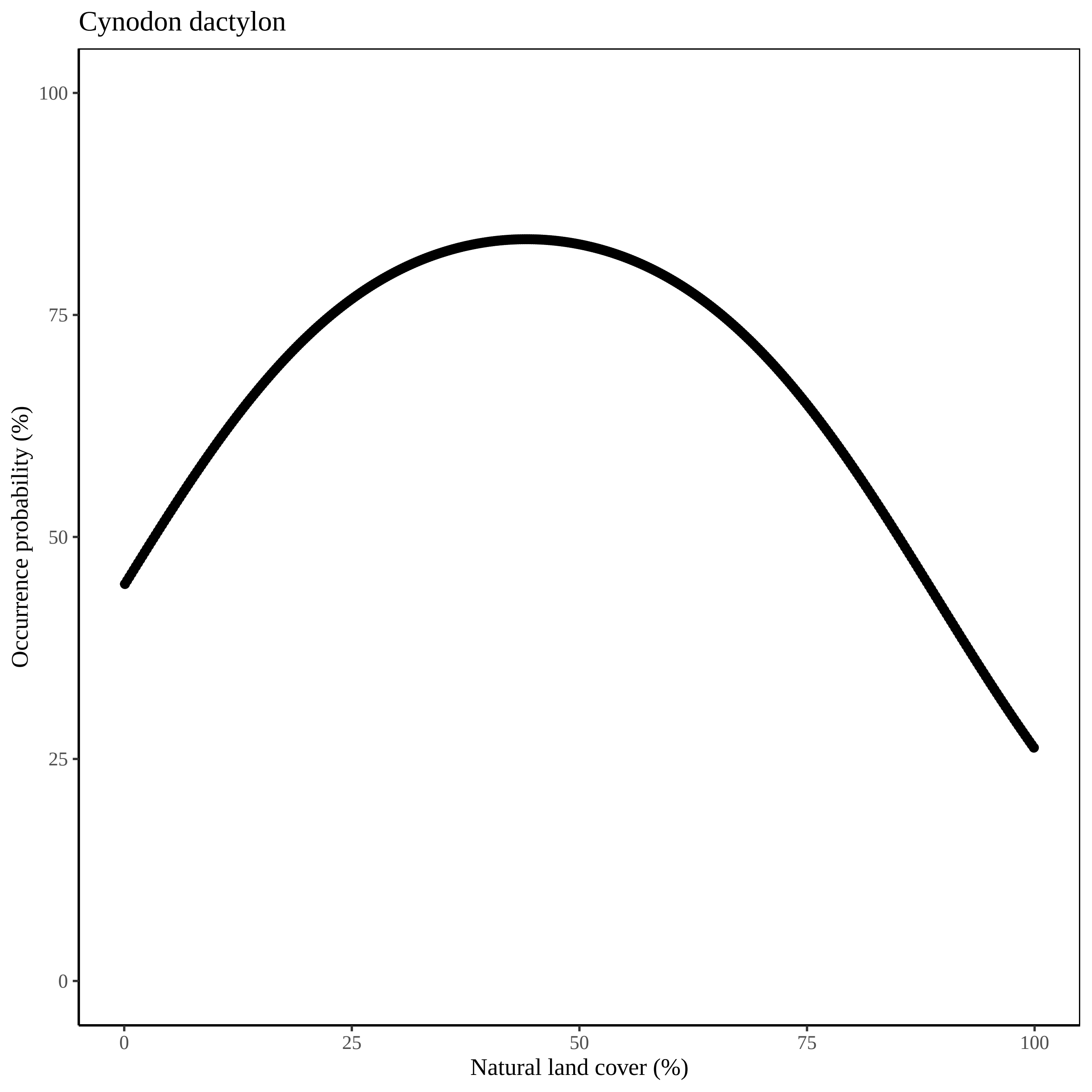
Cynodon dactylon
Occurrence probability (%)
100
75
50
25
0
0
25
50
75
100
Natural land cover (%)

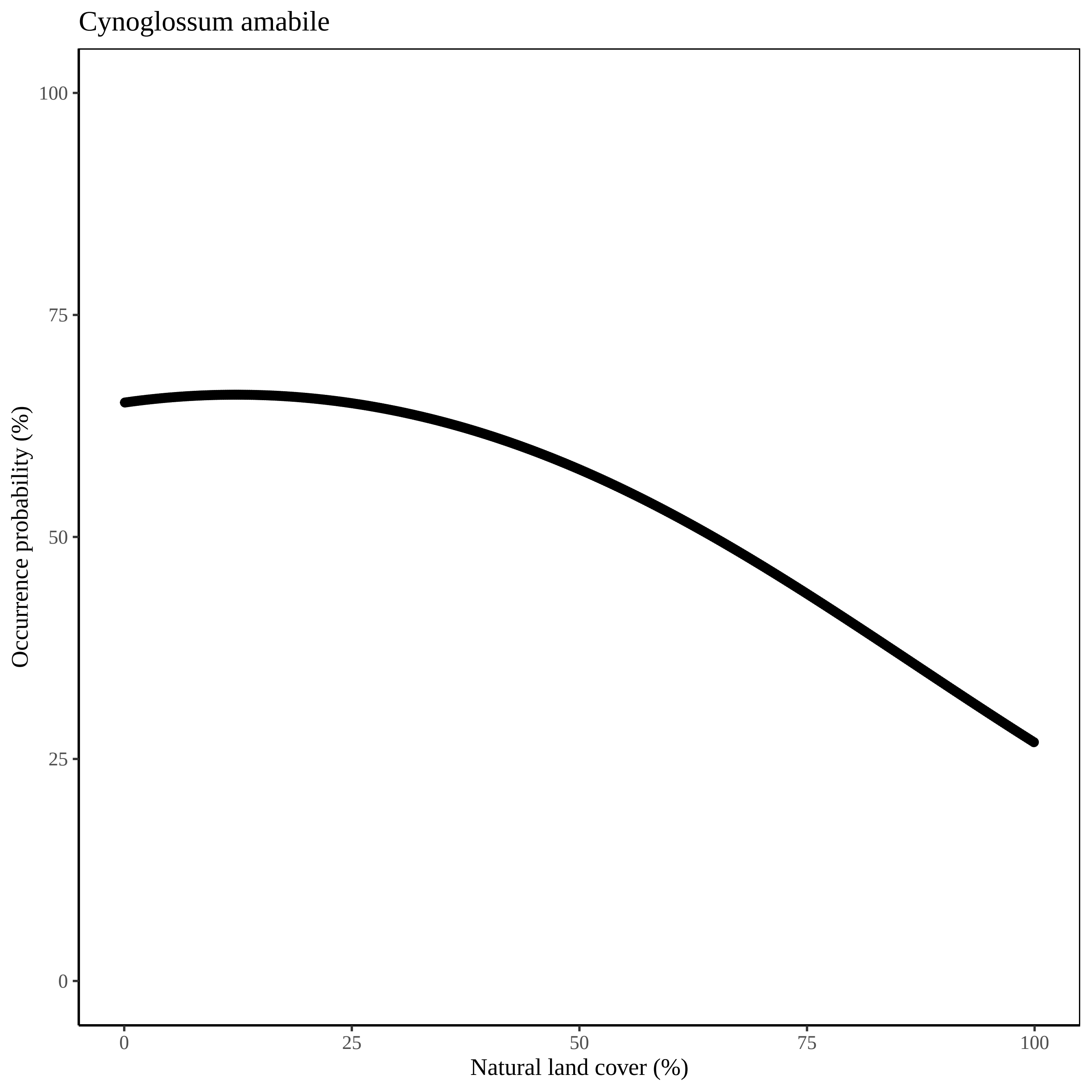
Cynoglossum amabile
Occurrence probability (%)
100
75
50
25
0
0
25
50
75
100
Natural land cover (%)

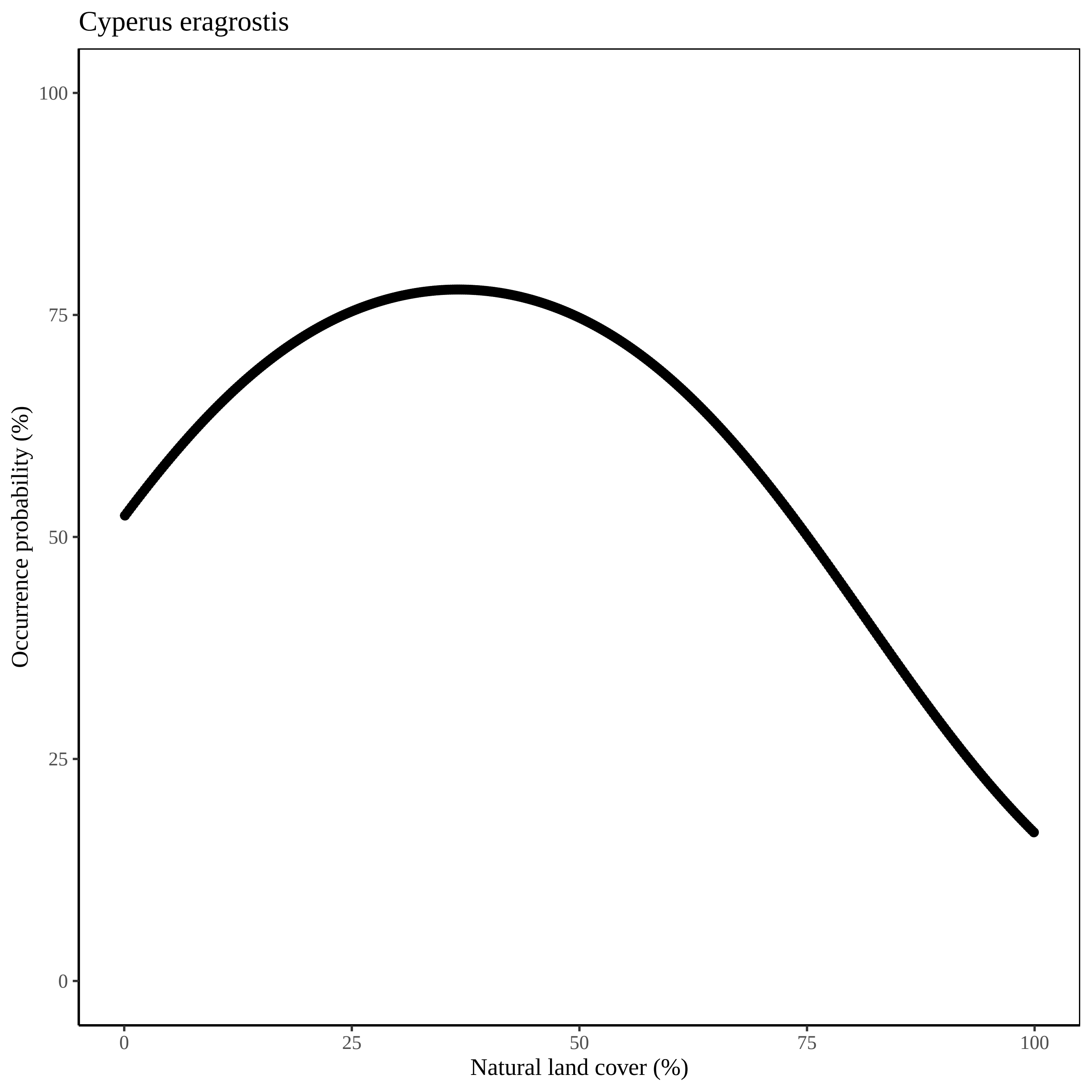

Cyperus eragrostis
Occurrence probability (%)
100
75
50
25
0
0
25
50
75
100
Natural land cover (%)

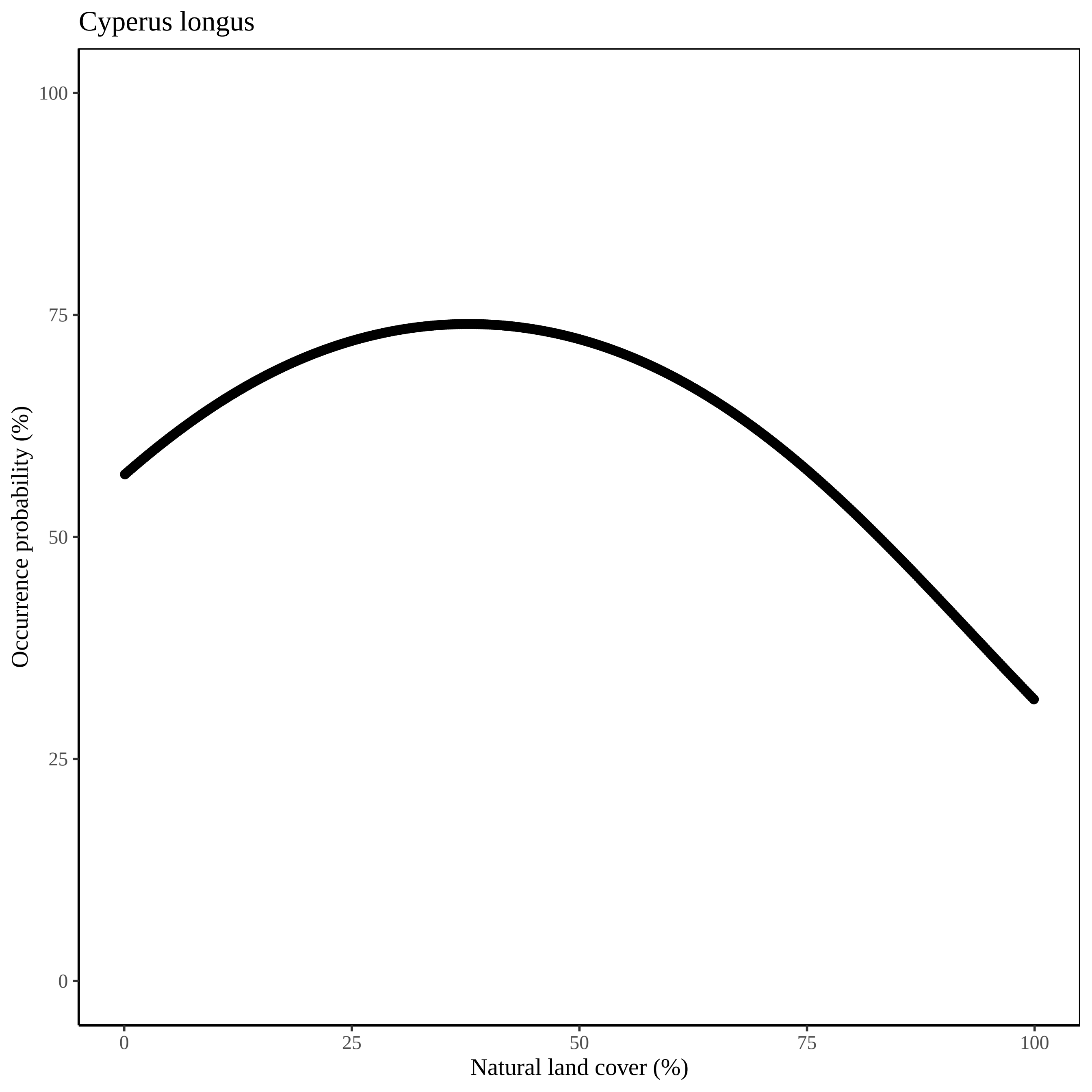
Cyperus longus
Occurrence probability (%)
100
75
50
25
0
0
25
50
75
100
Natural land cover (%)

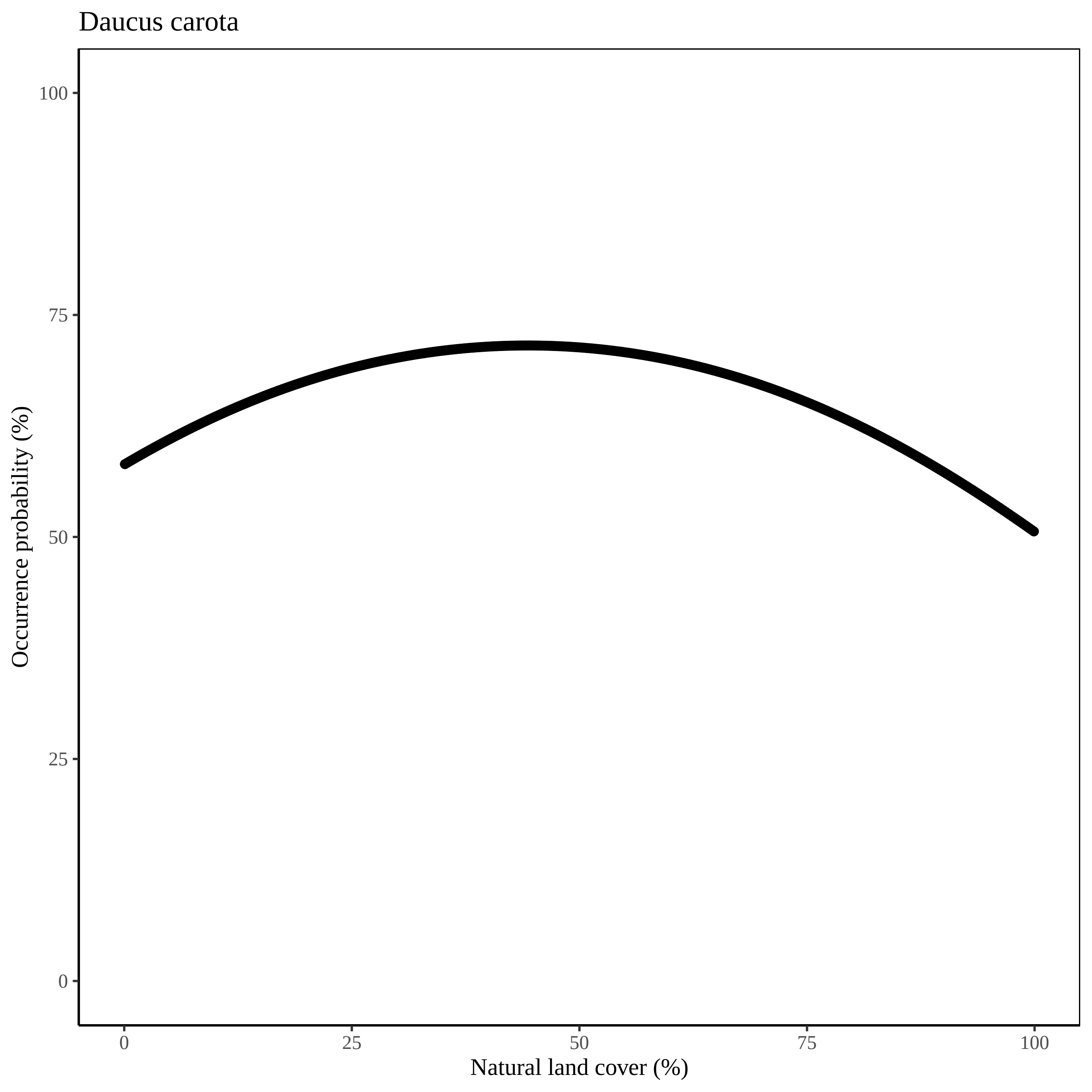
Daucus carota
Occurrence probability (%)
100
75
50
25
0
0
25
50
75
100
Natural land cover (%)

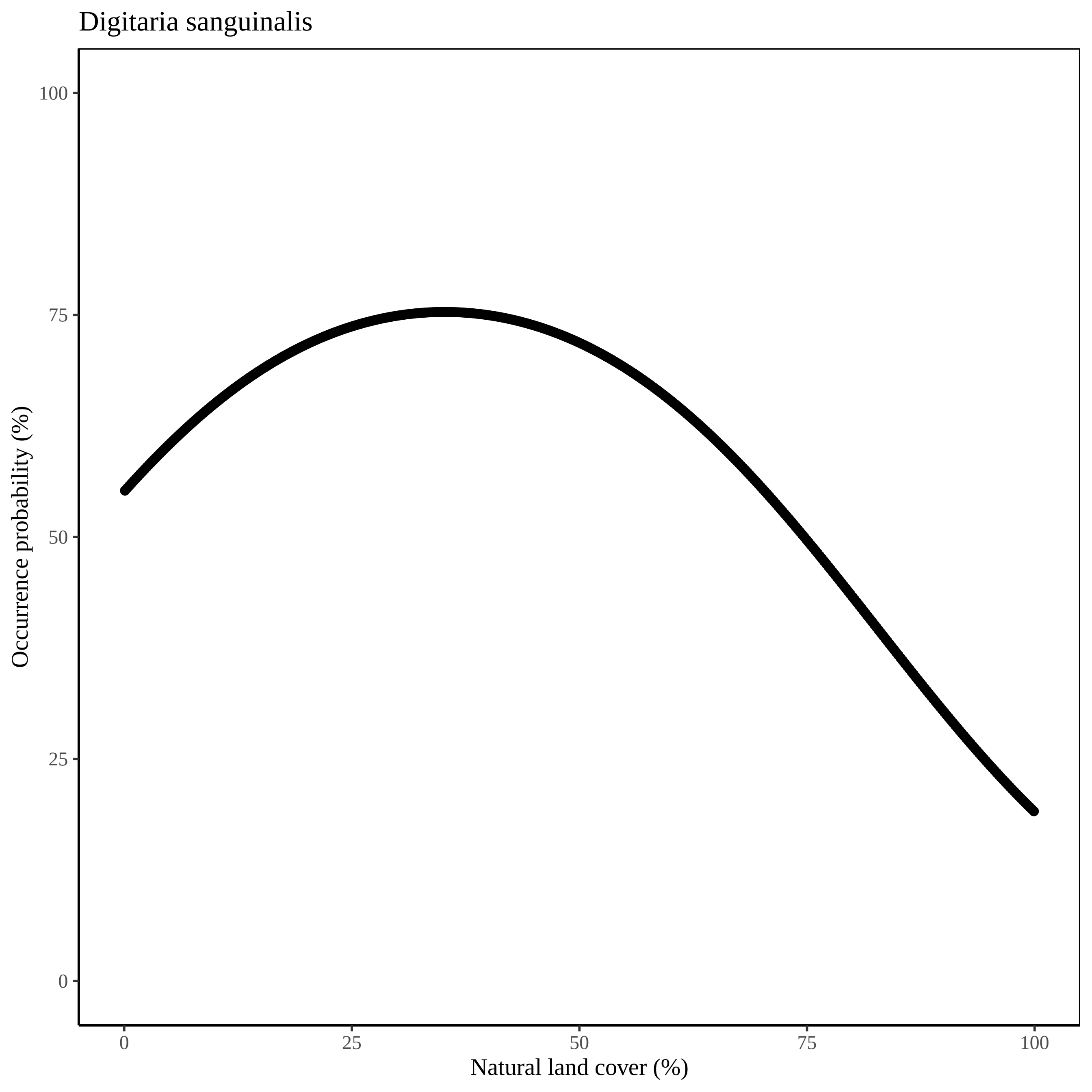
Digitaria sanguinalis
Occurrence probability (%)
100
75
50
25
0
0
25
50
75
100
Natural land cover (%)

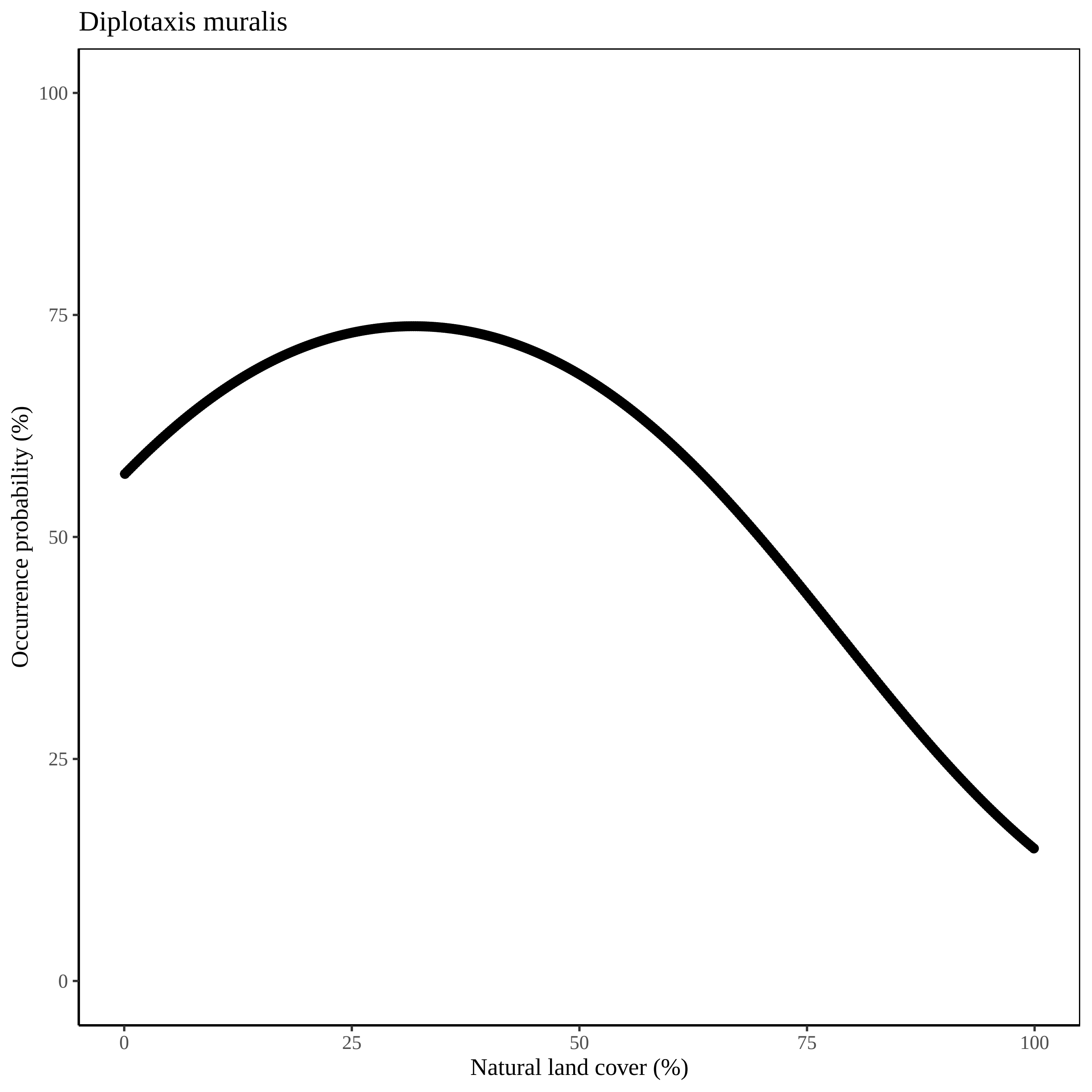

Diplotaxis muralis
Occurrence probability (%)
100
75
50
25
0
0
25
50
75
100
Natural land cover (%)

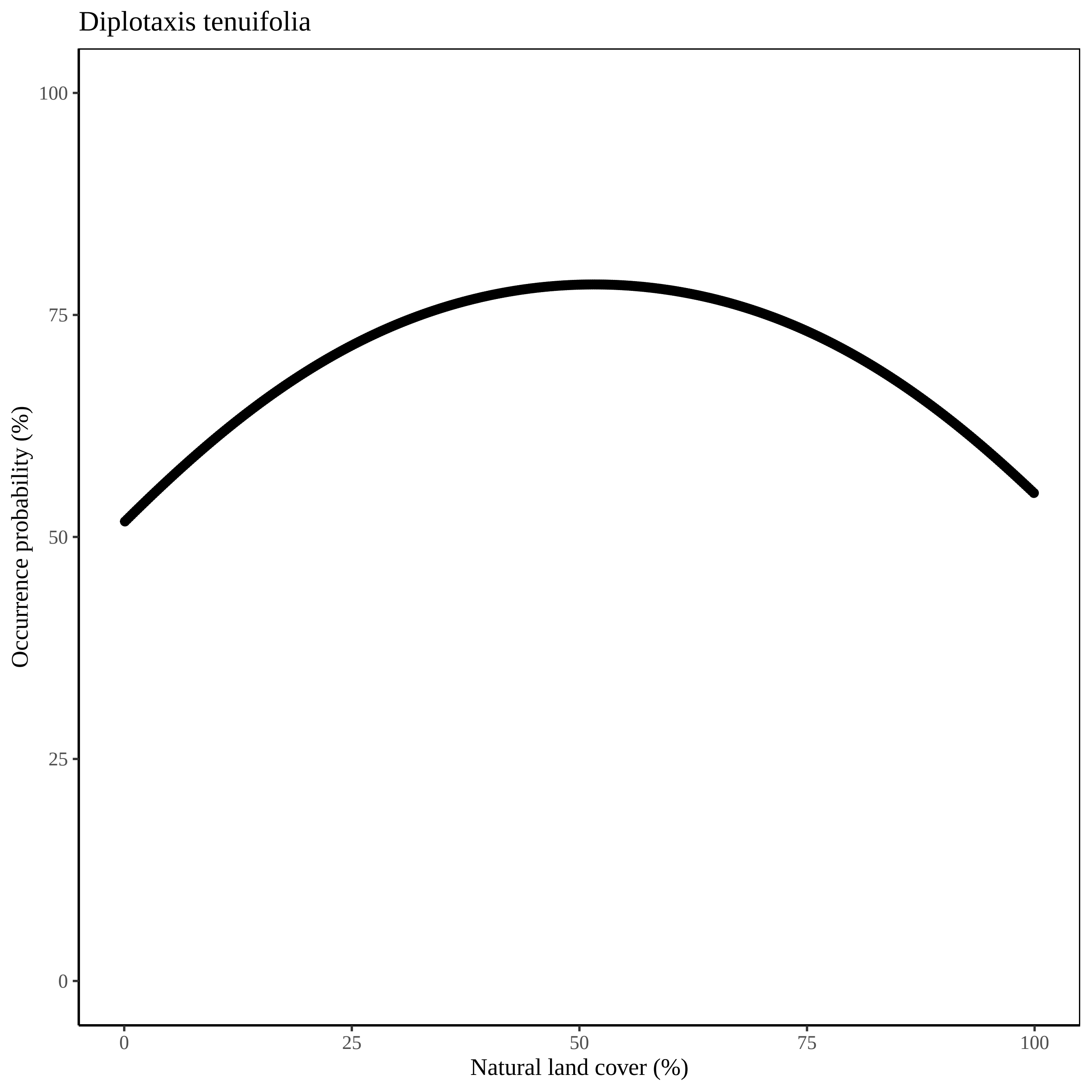
Diplotaxis tenuifolia
Occurrence probability (%)
100
75
50
25
0
0
25
50
75
100
Natural land cover (%)

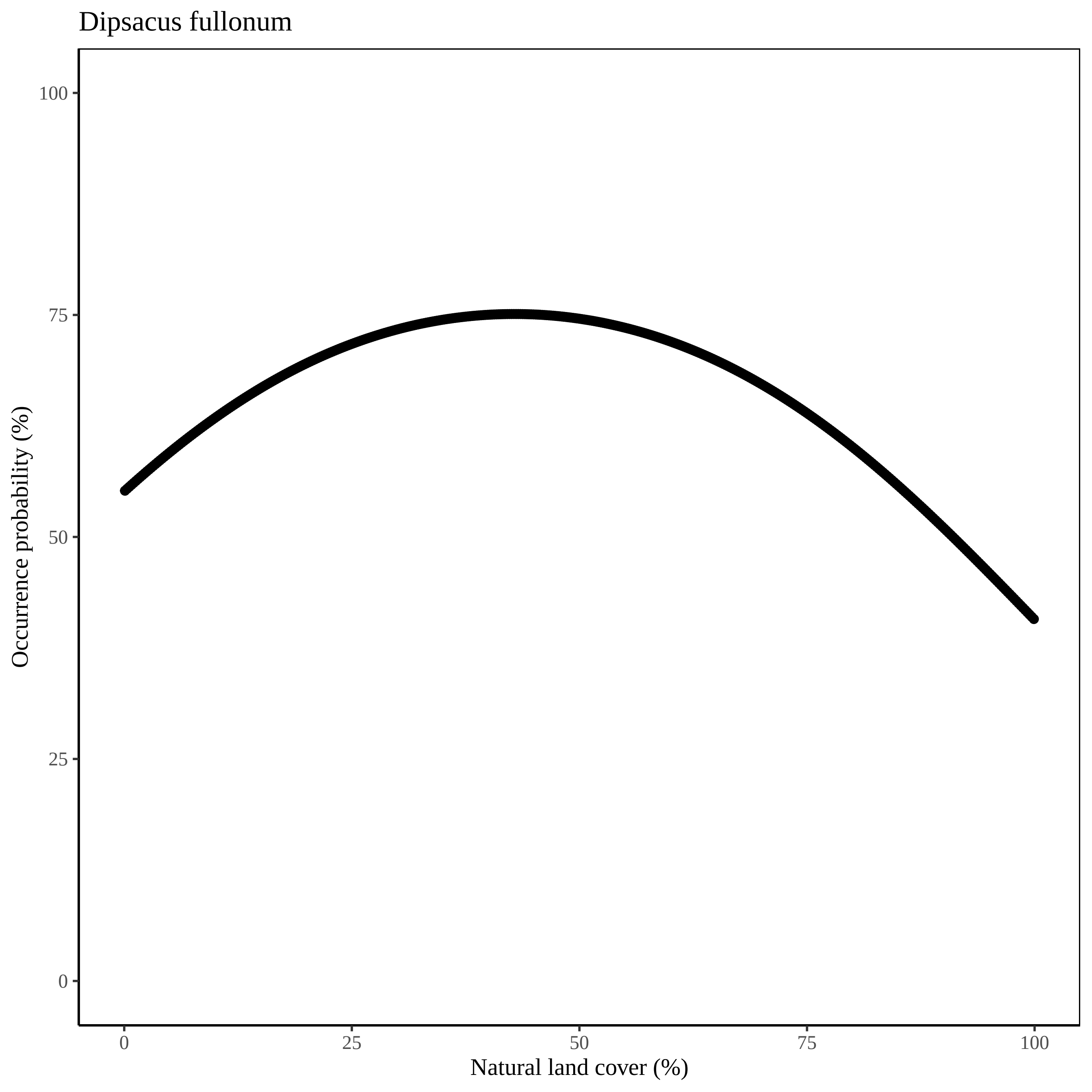

Dipsacus fullonum
100
75
50
25
0
Occurrence probability (%)
0
25
50
75
100
Natural land cover (%)

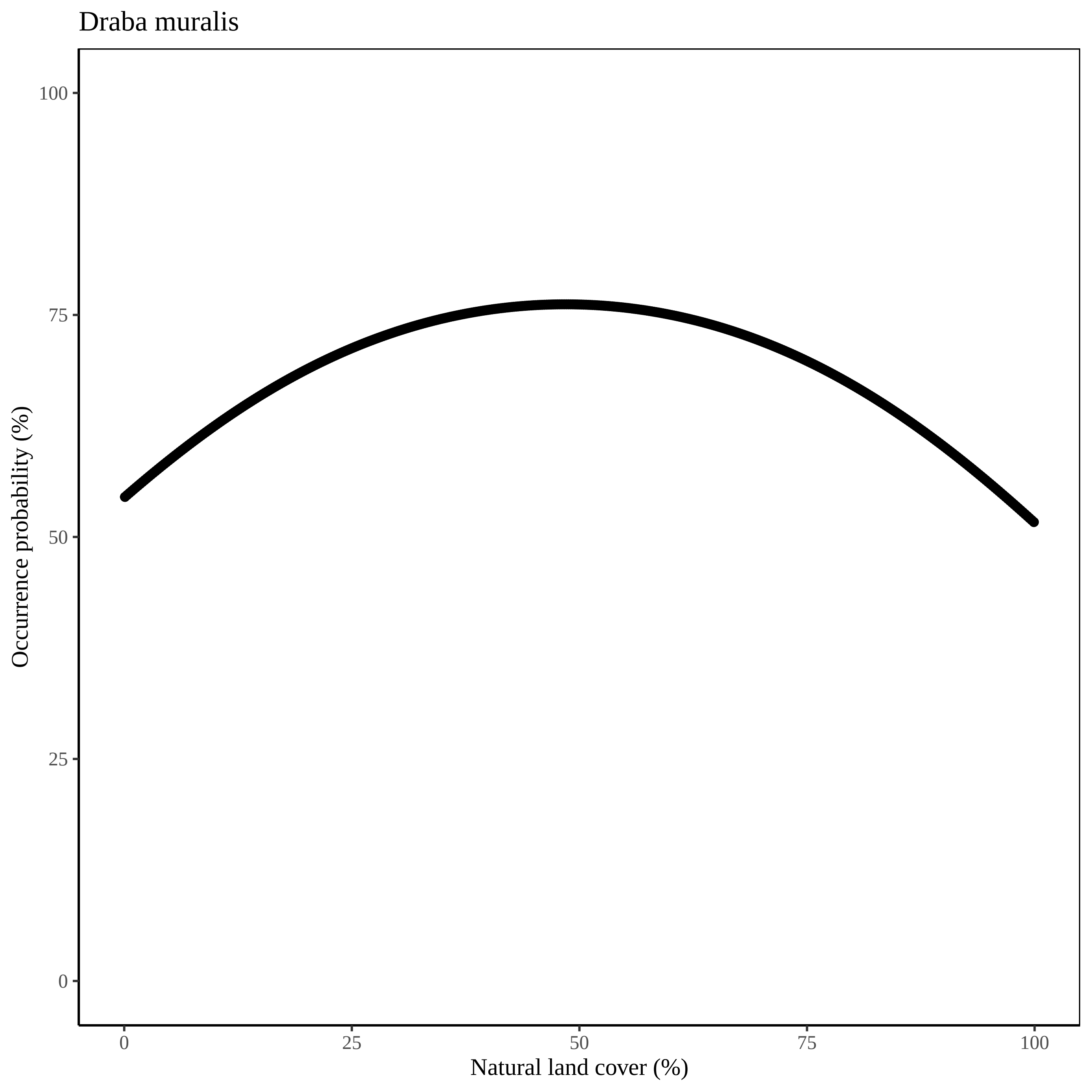

Draba muralis
Occurrence probability (%)
100
75
50
25
0
0
25
50
75
100
Natural land cover (%)

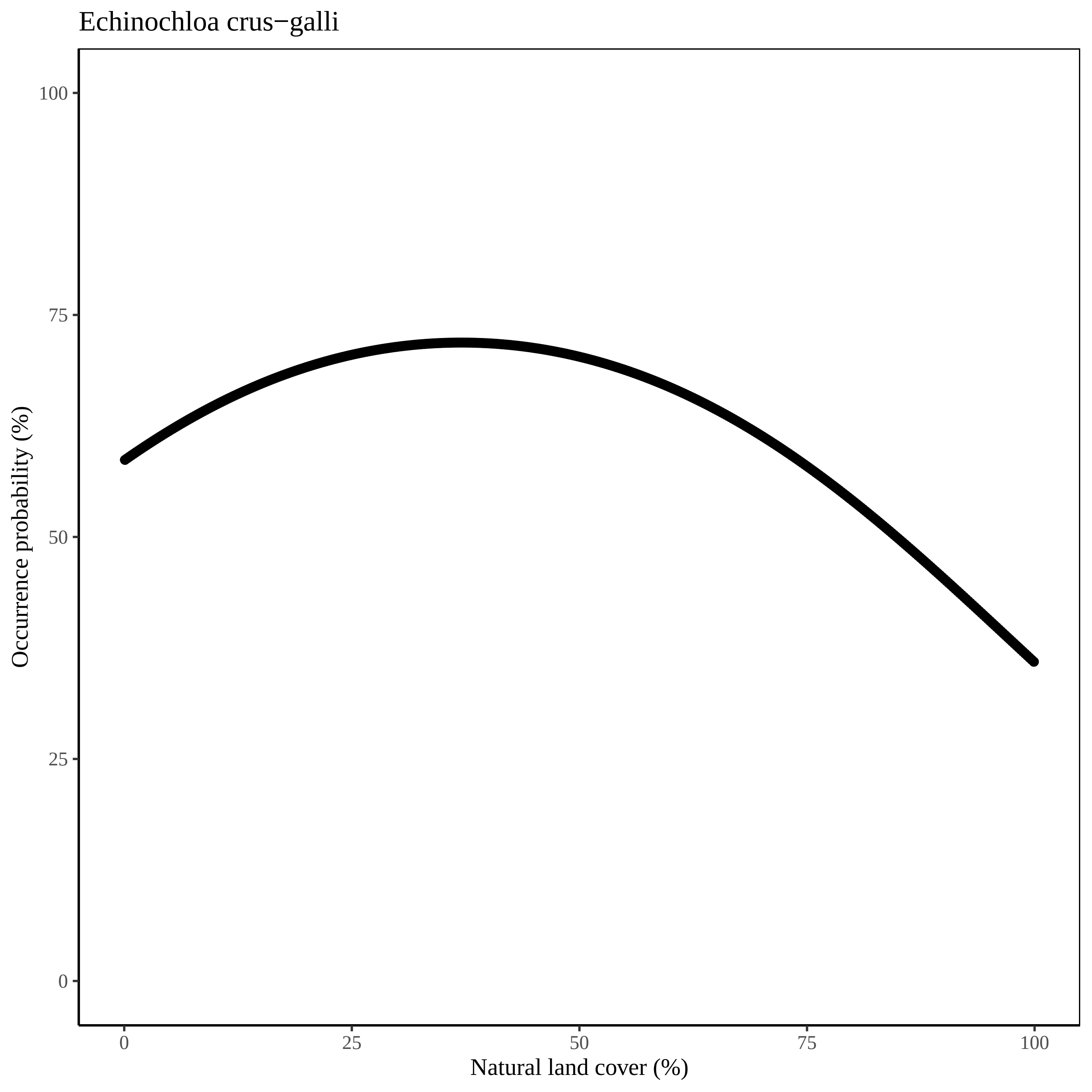
Echinochloa crus−galli
Occurrence probability (%)
100
75
50
25
0
0
25
50
75
100
Natural land cover (%)

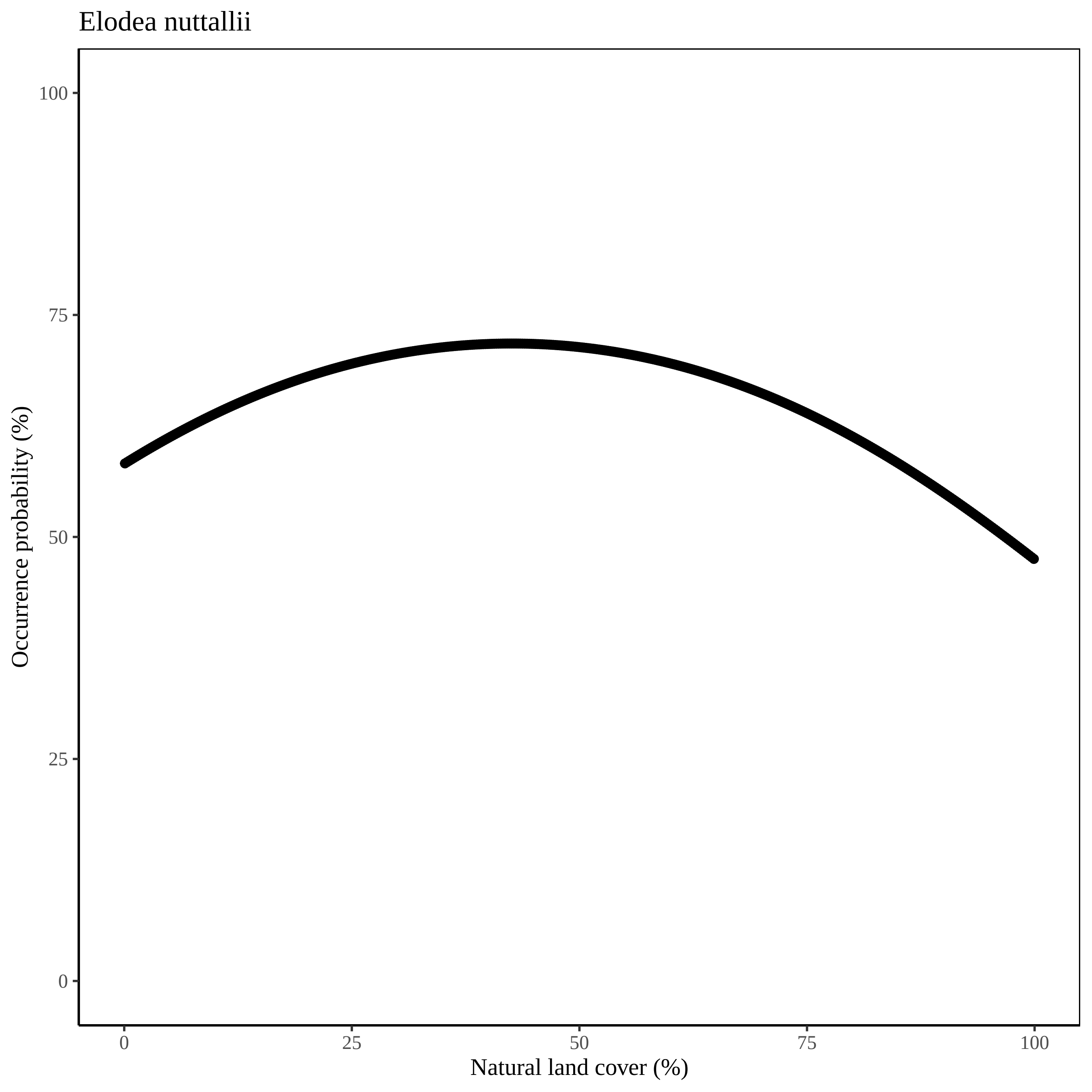

Elodea nuttallii
100
75
50
25
0
Occurrence probability (%)
0
25
50
75
100
Natural land cover (%)

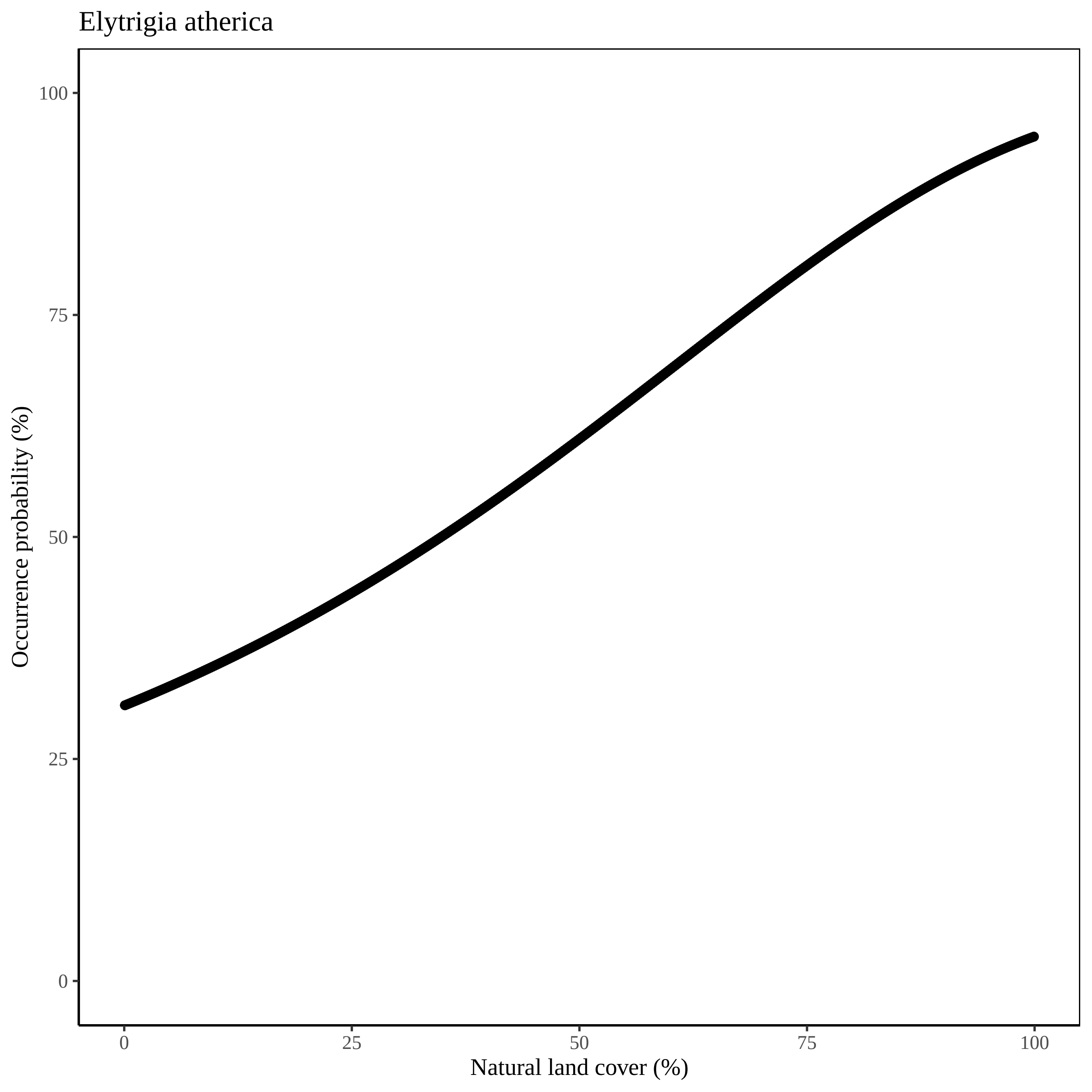

Elytrigia atherica
Occurrence probability (%)
100
75
50
25
0
0
25
50
75
100
Natural land cover (%)

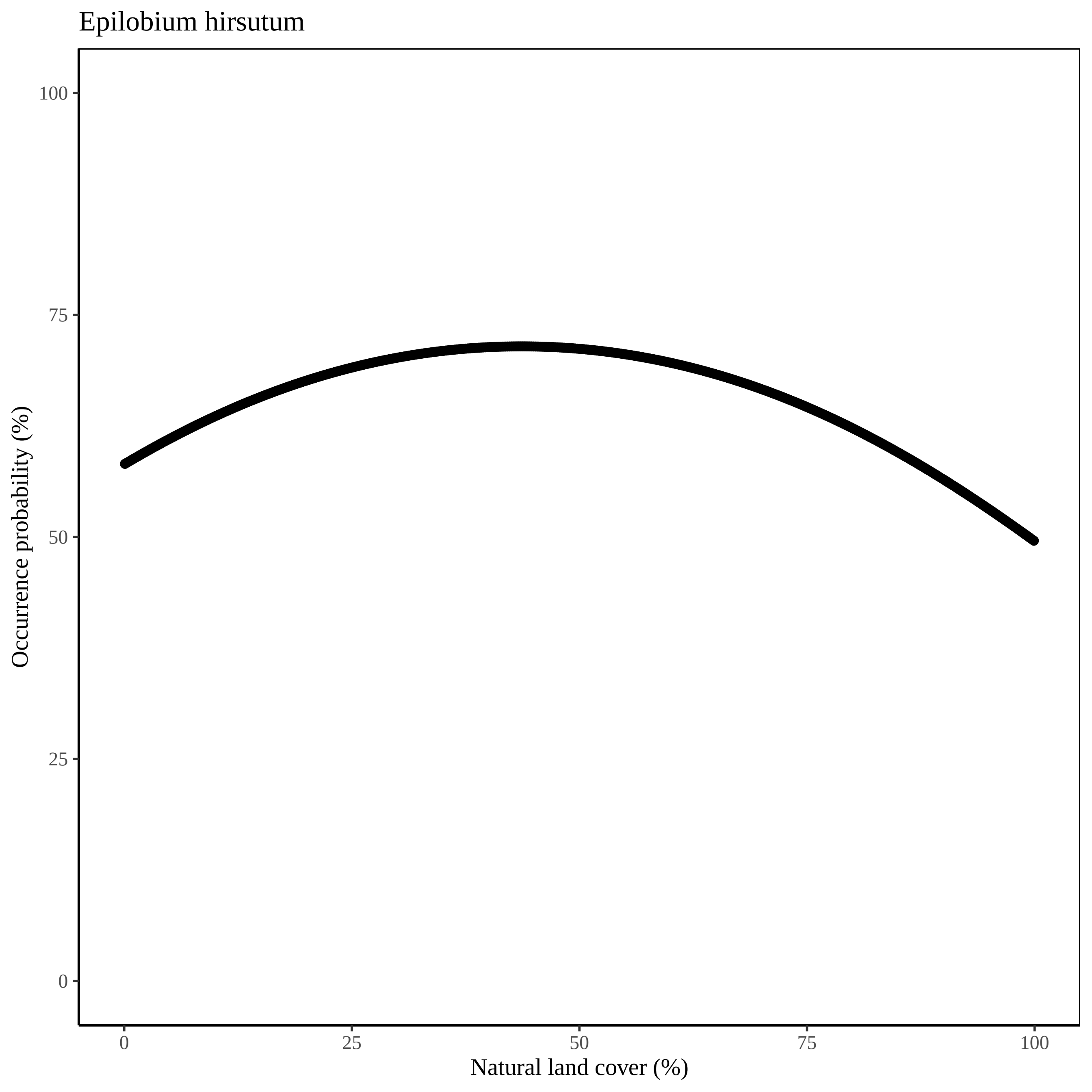

Epilobium hirsutum
Occurrence probability (%)
100
75
50
25
0
0
25
50
75
100
Natural land cover (%)

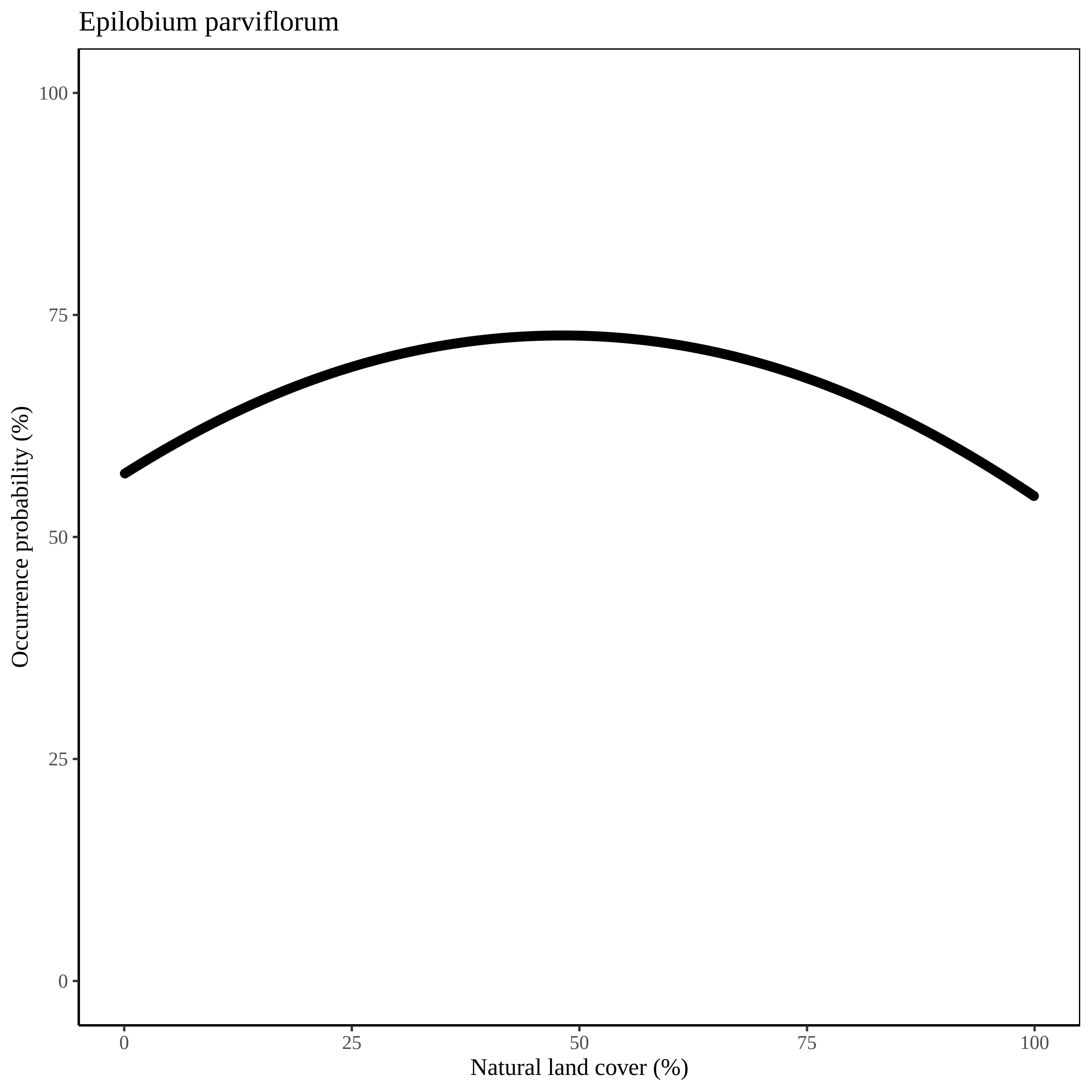

Epilobium parviflorum
Occurrence probability (%)
100
75
50
25
0
0
25
50
75
100
Natural land cover (%)

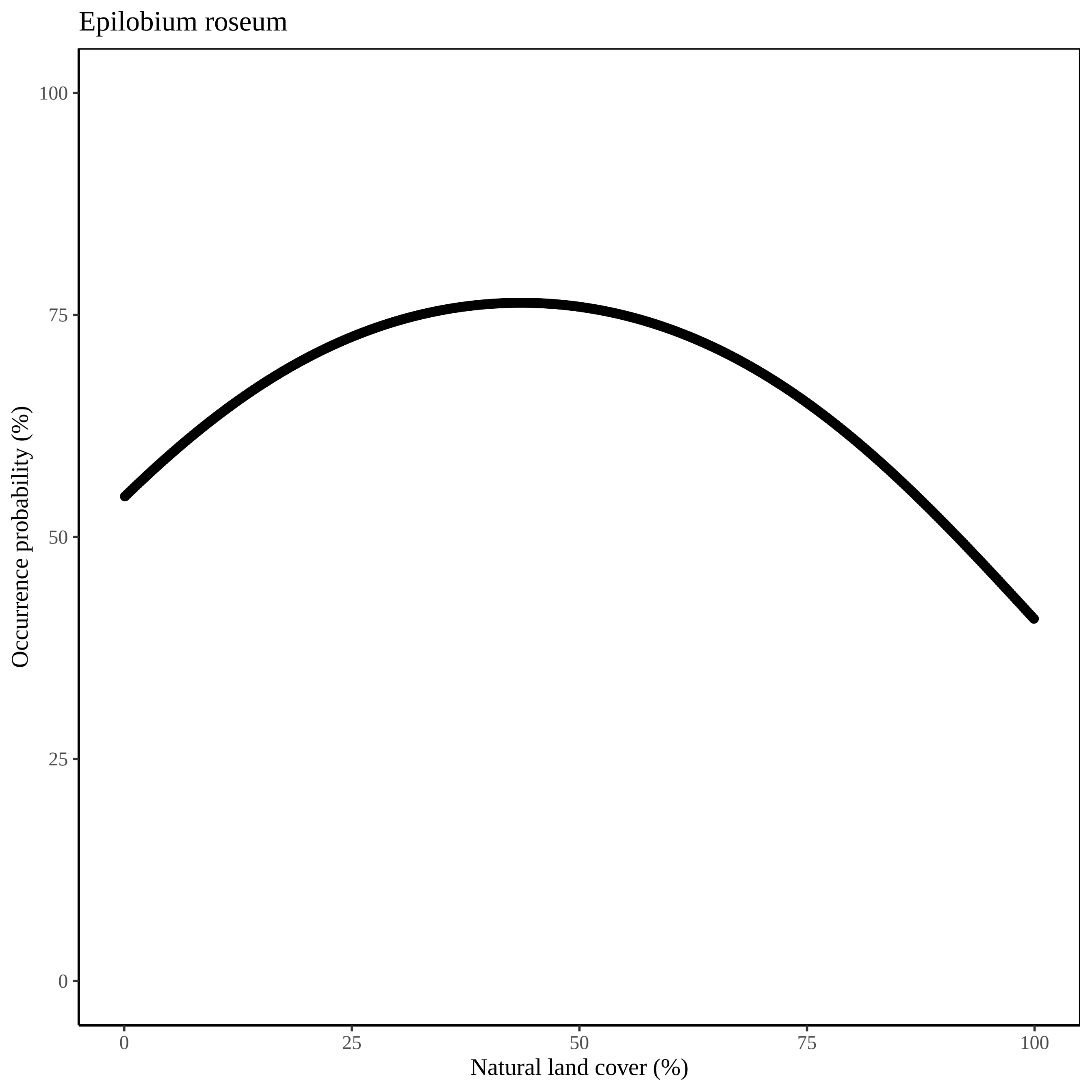

Epilobium roseum
Occurrence probability (%)
100
75
50
25
0
0
25
50
75
100
Natural land cover (%)

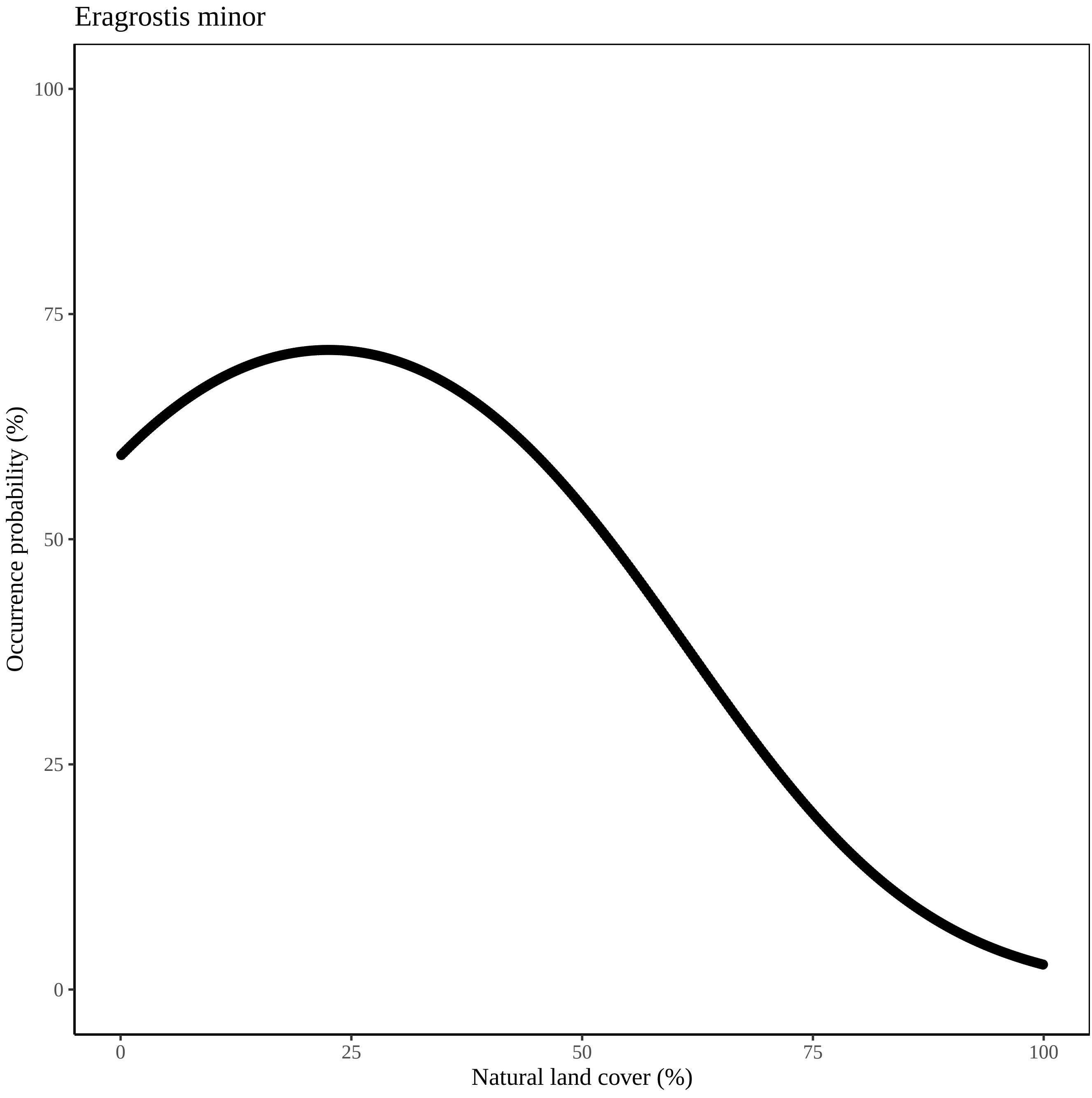

Eragrostis minor
Occurrence probability (%)
100
75
50
25
0
0
25
50
75
100
Natural land cover (%)

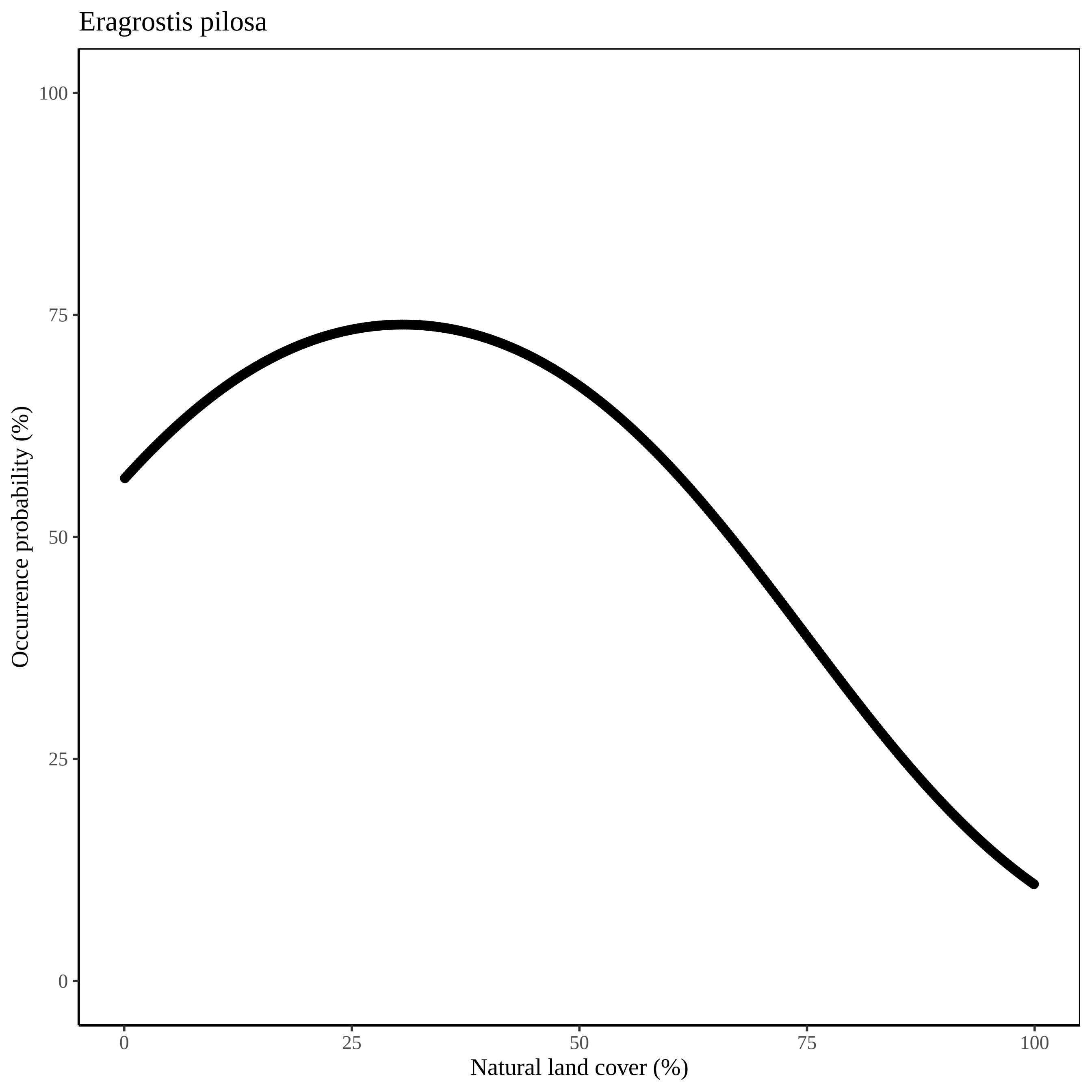

Eragrostis pilosa
100
75
50
25
0
Occurrence probability (%)
0
25
50
75
100
Natural land cover (%)

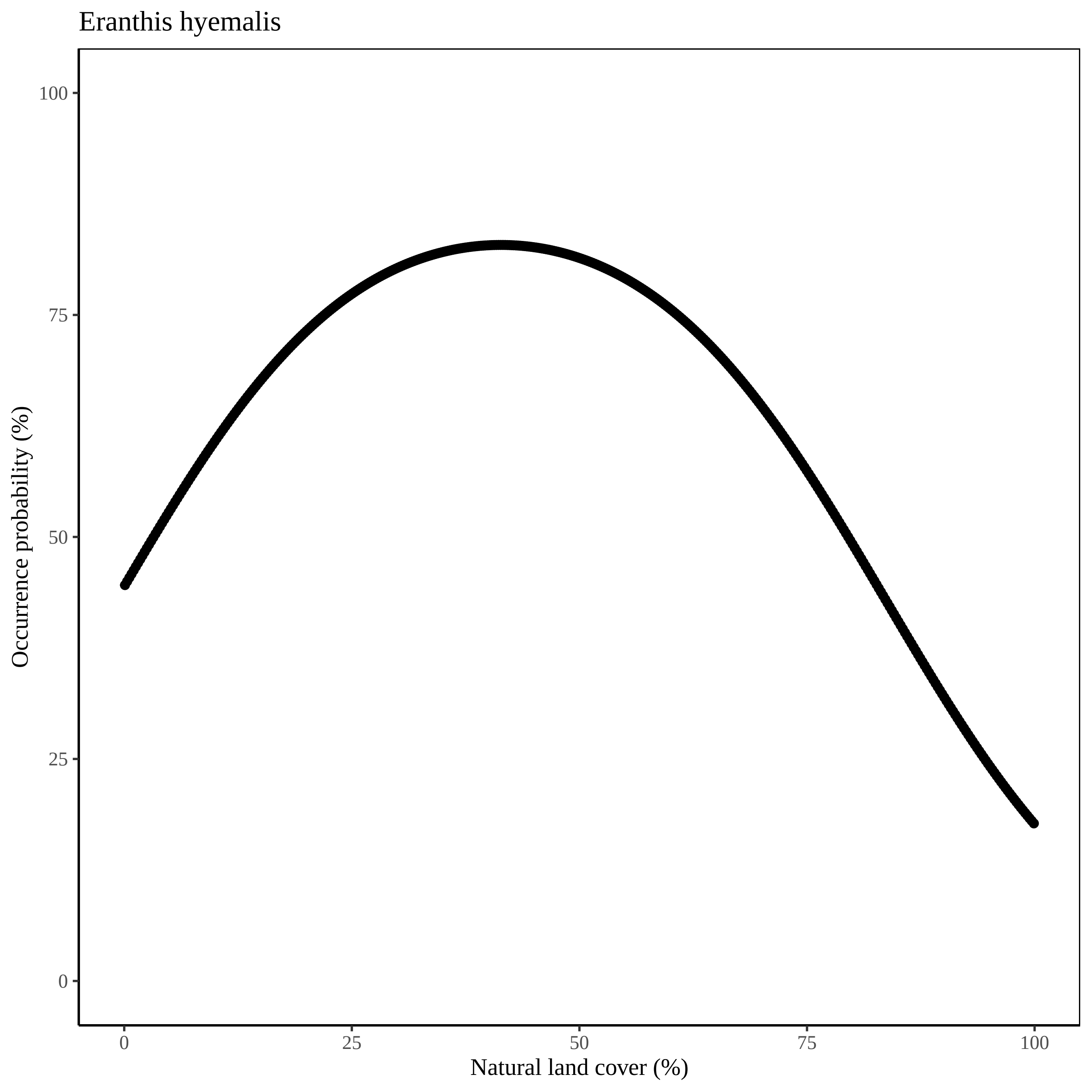
Eranthis hyemalis
Occurrence probability (%)
100
75
50
25
0
0
25
50
75
100
Natural land cover (%)

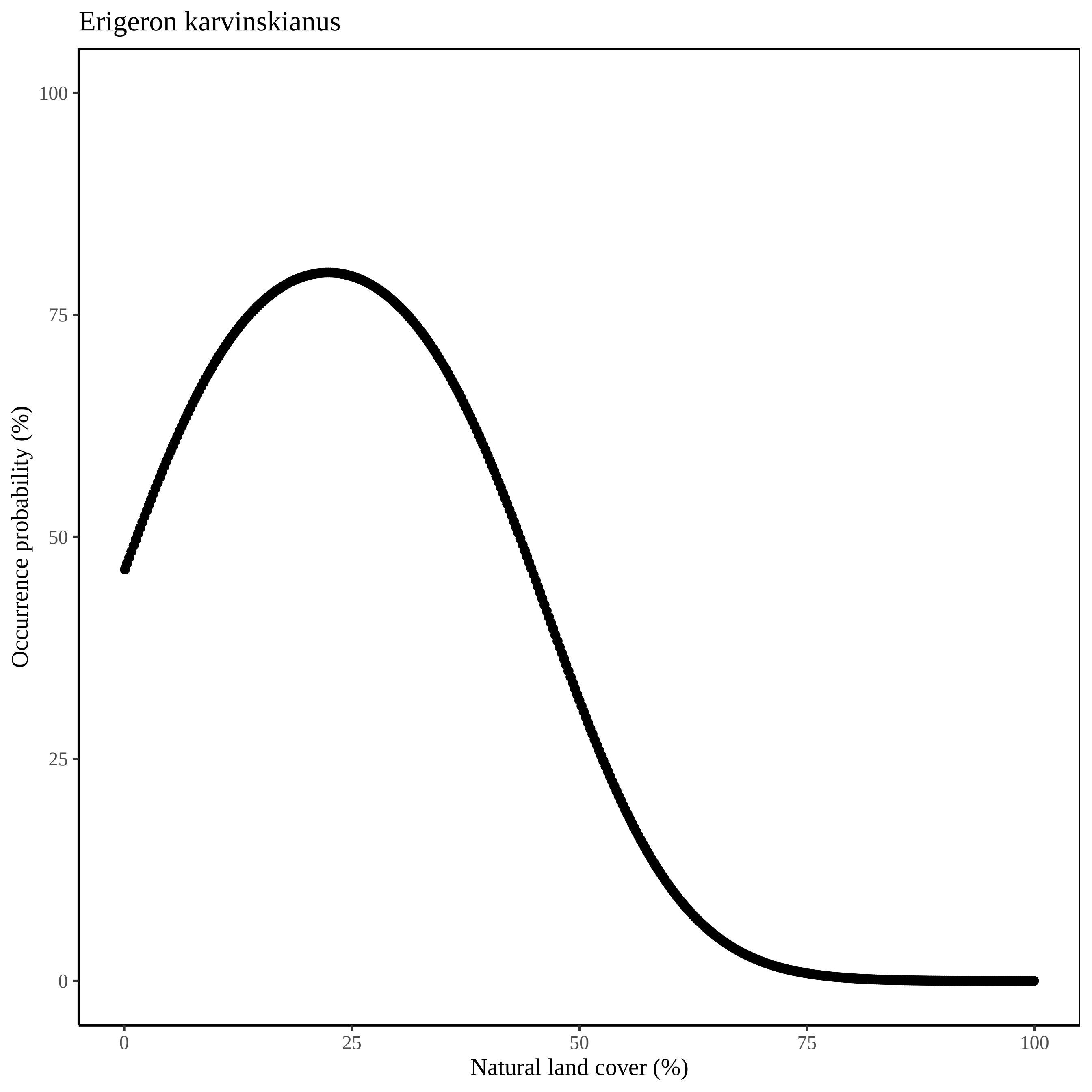

Erigeron karvinskianus
Occurrence probability (%)
100
75
50
25
0
0
25
50
75
100
Natural land cover (%)

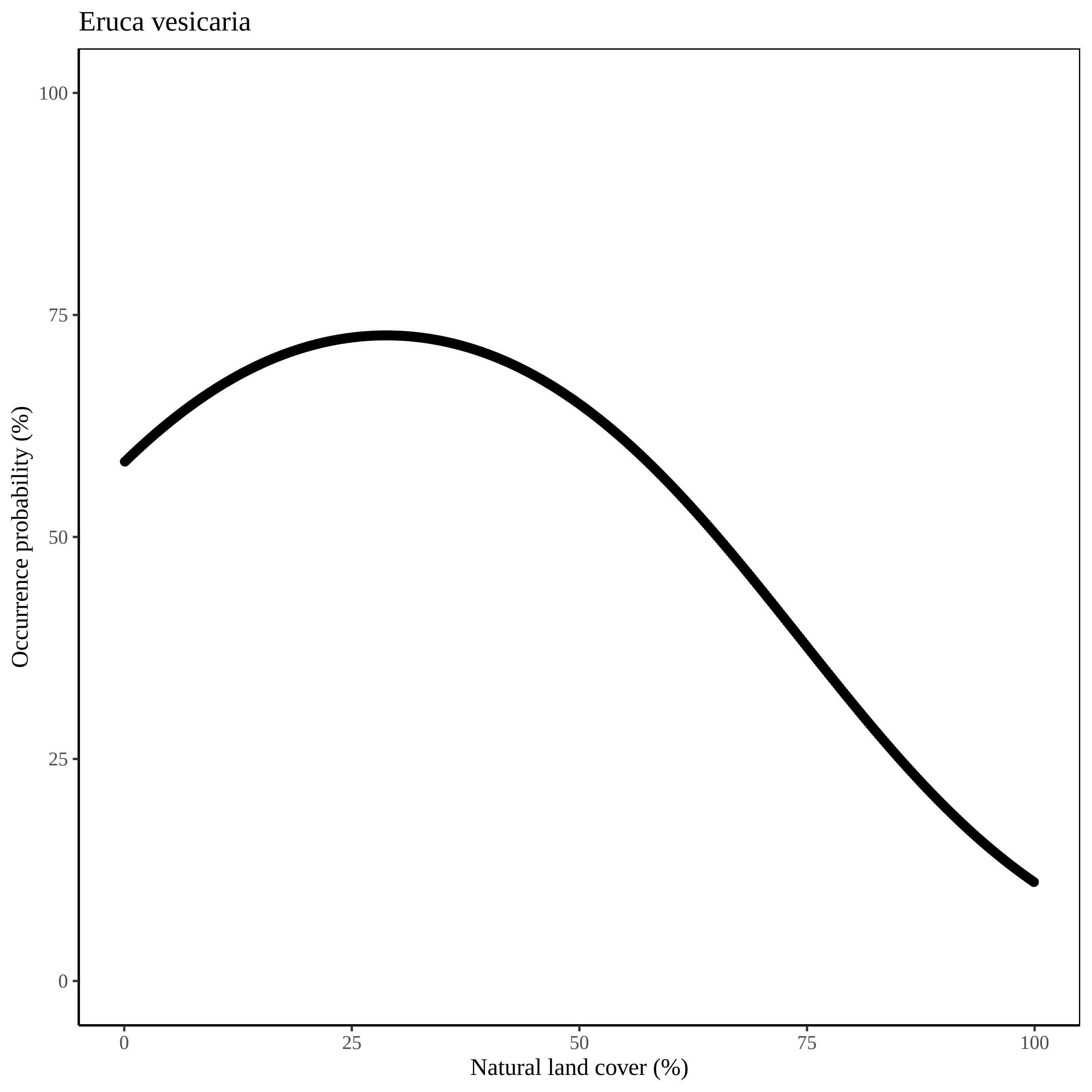

Eruca vesicaria
Occurrence probability (%)
100
75
50
25
0
0
25
50
75
100
Natural land cover (%)

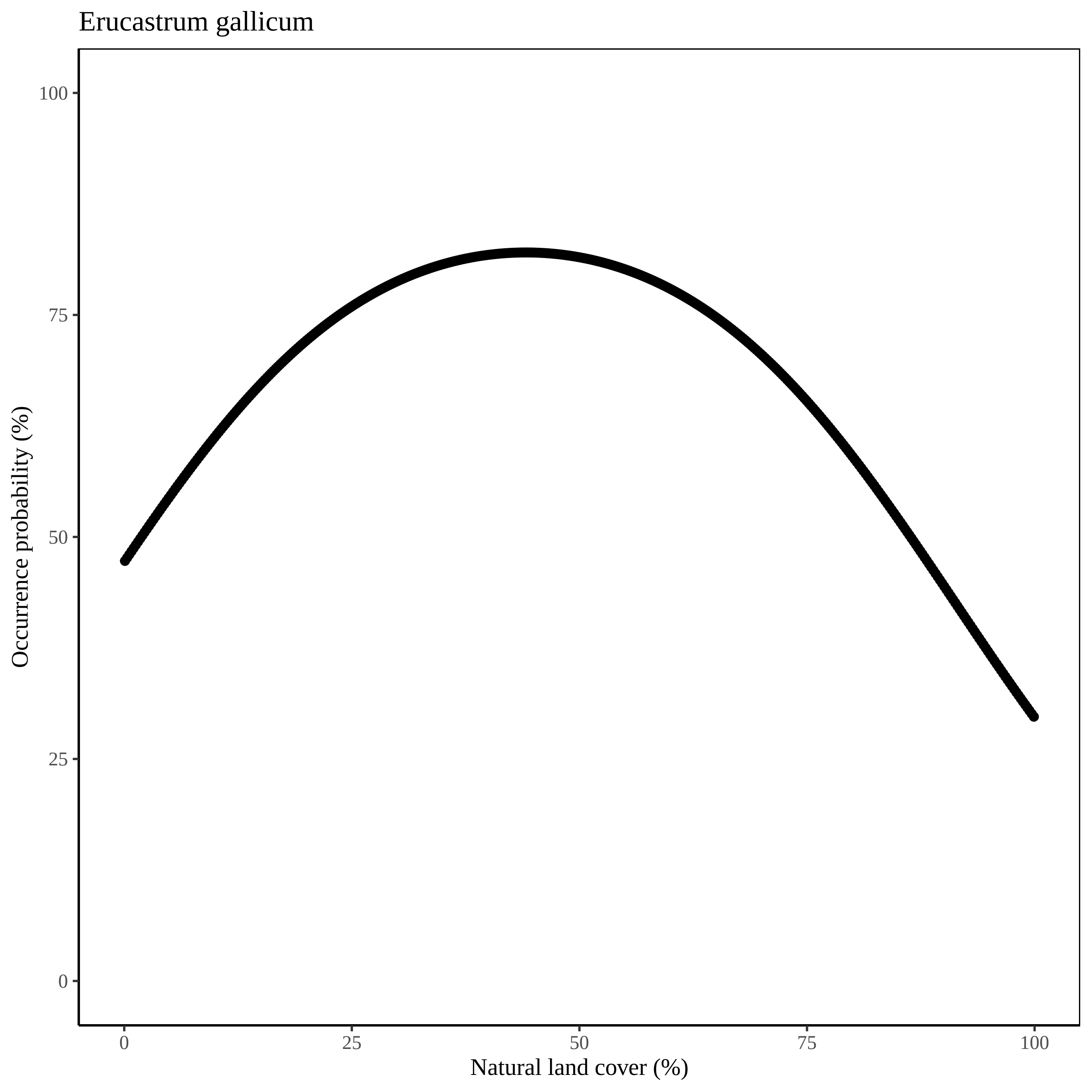

Erucastrum gallicum
Occurrence probability (%)
100
75
50
25
0
0
25
50
75
100
Natural land cover (%)

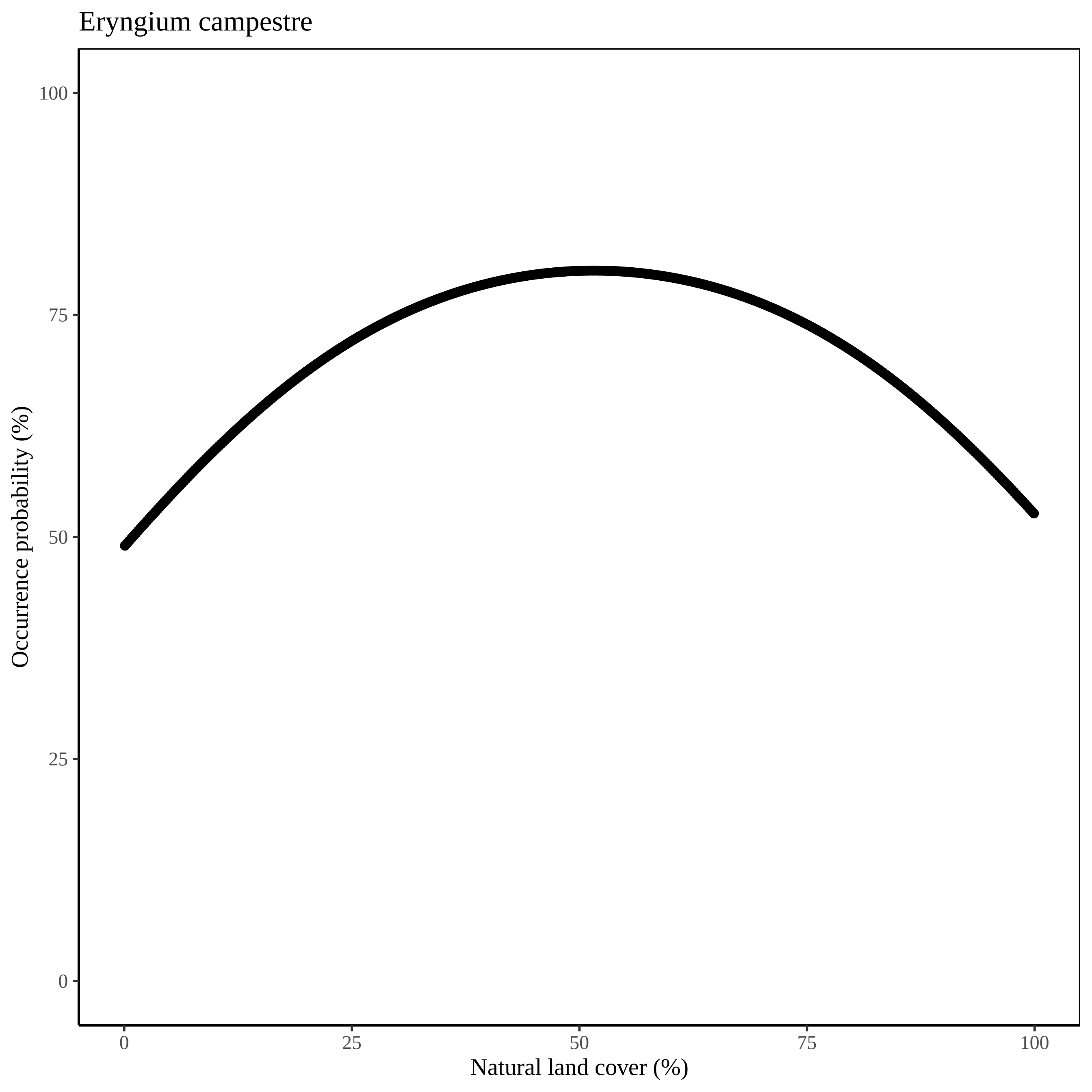

Eryngium campestre
Occurrence probability (%)
100
75
50
25
0
0
25
50
75
100
Natural land cover (%)

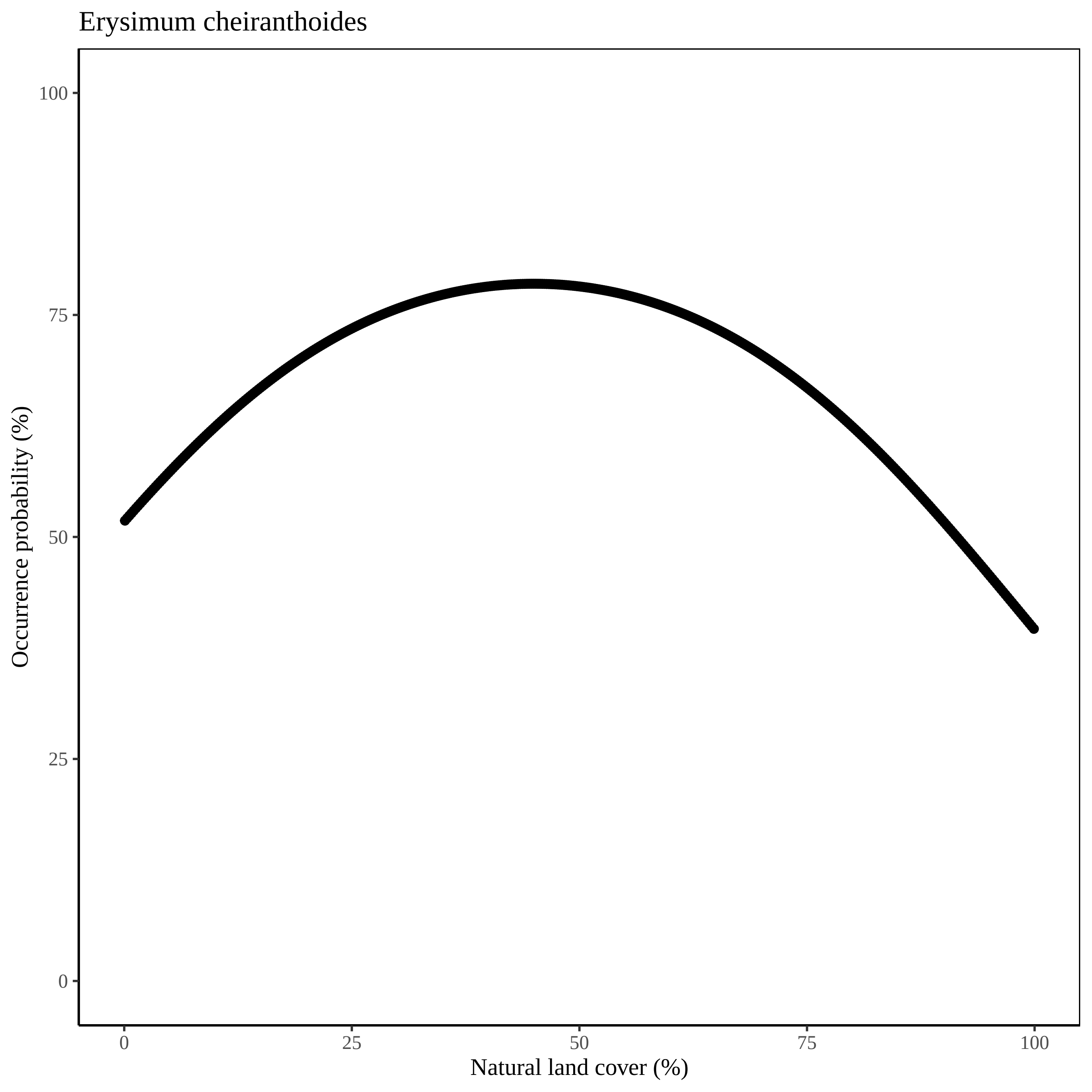

Erysimum cheiranthoides
Occurrence probability (%)
Natural land cover (%)
0
25
50
75
100
0
25
50
75
100

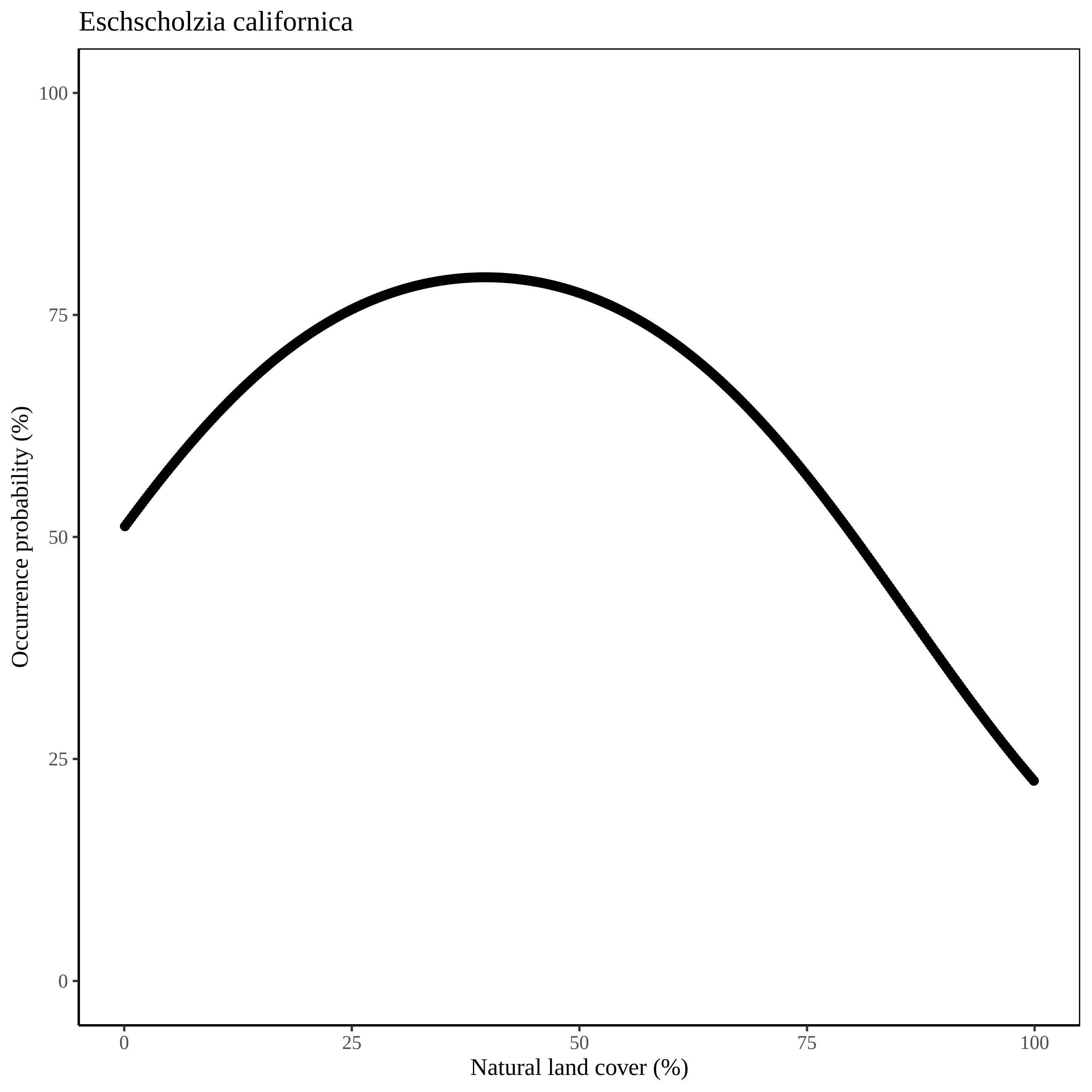

Eschscholzia californica
Occurrence probability (%)
100
75
50
25
0
0
25
50
75
100
Natural land cover (%)

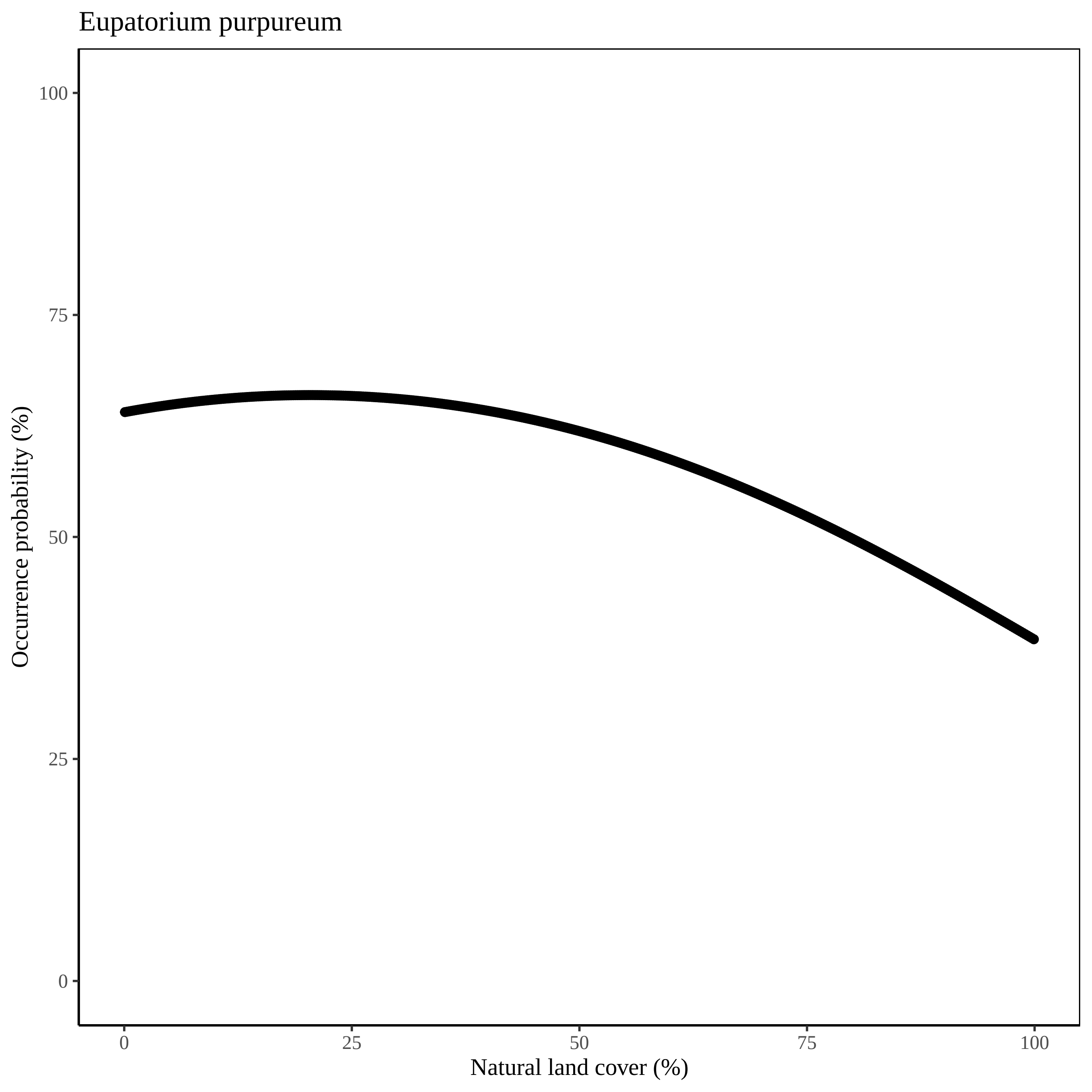

Eupatorium purpureum
100
75
50
25
0
Occurrence probability (%)
0
25
50
75
100
Natural land cover (%)

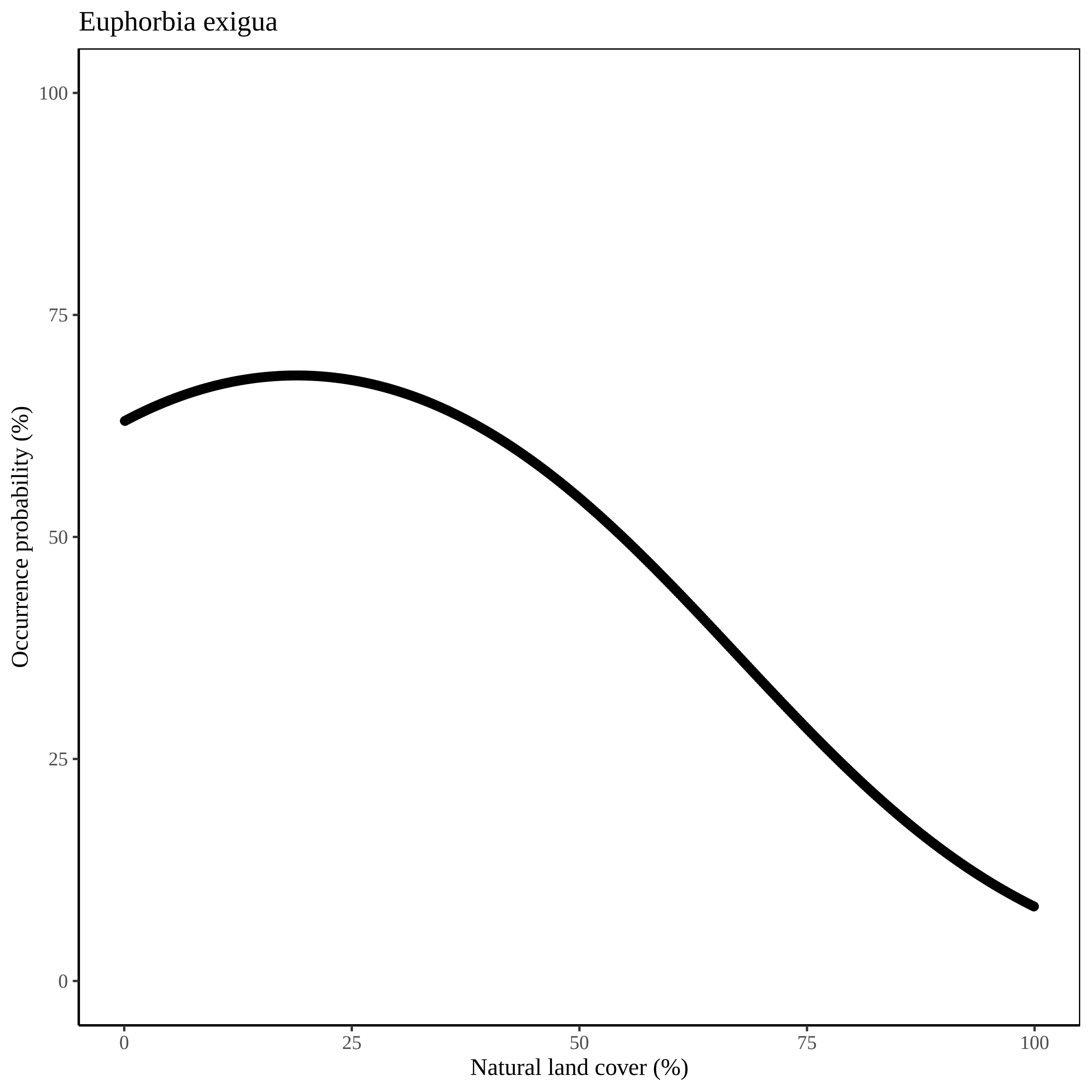
Euphorbia exigua
Occurrence probability (%)
100
75
50
25
0
0
25
50
75
100
Natural land cover (%)

Euphorbia helioscopia

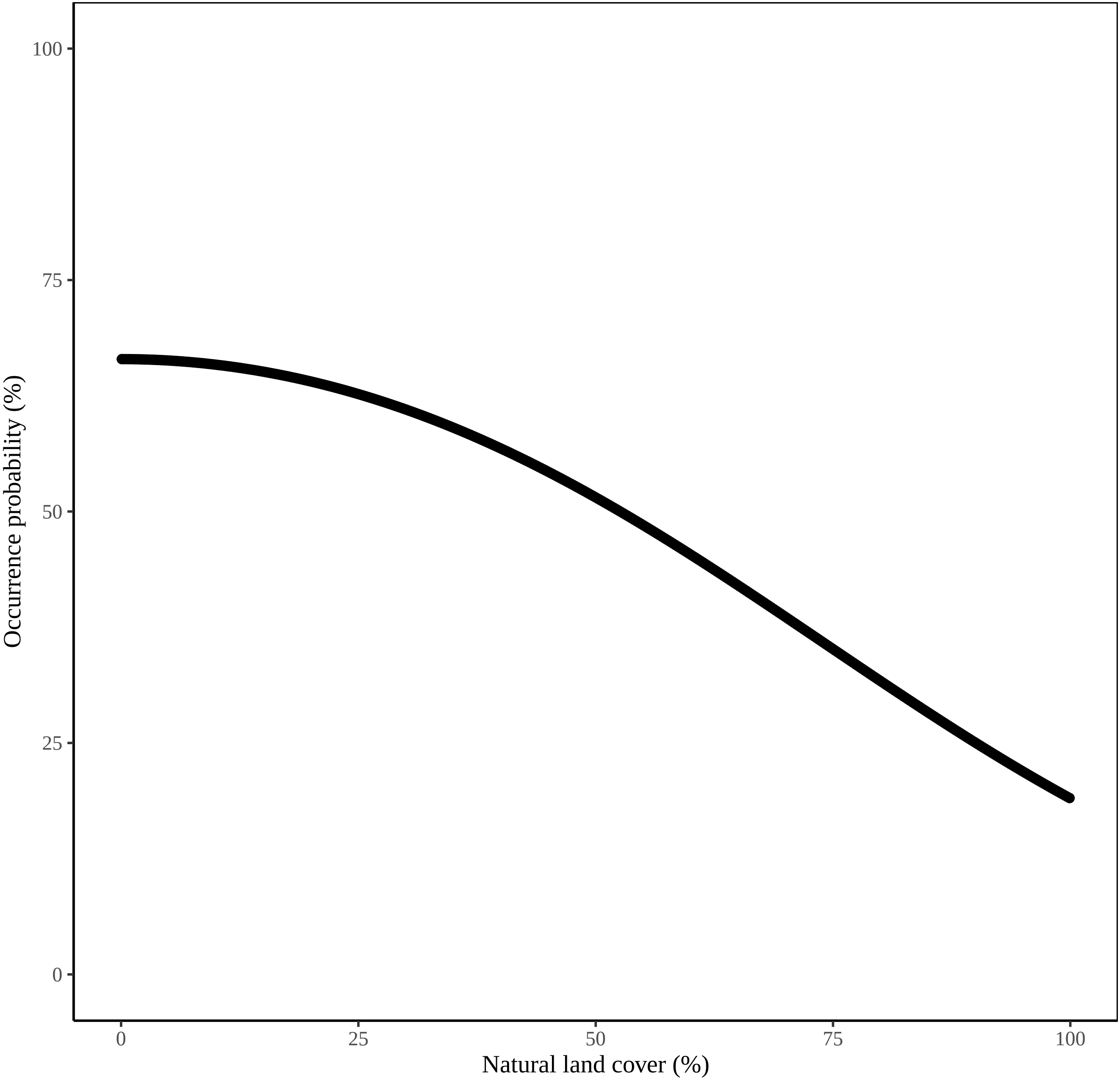

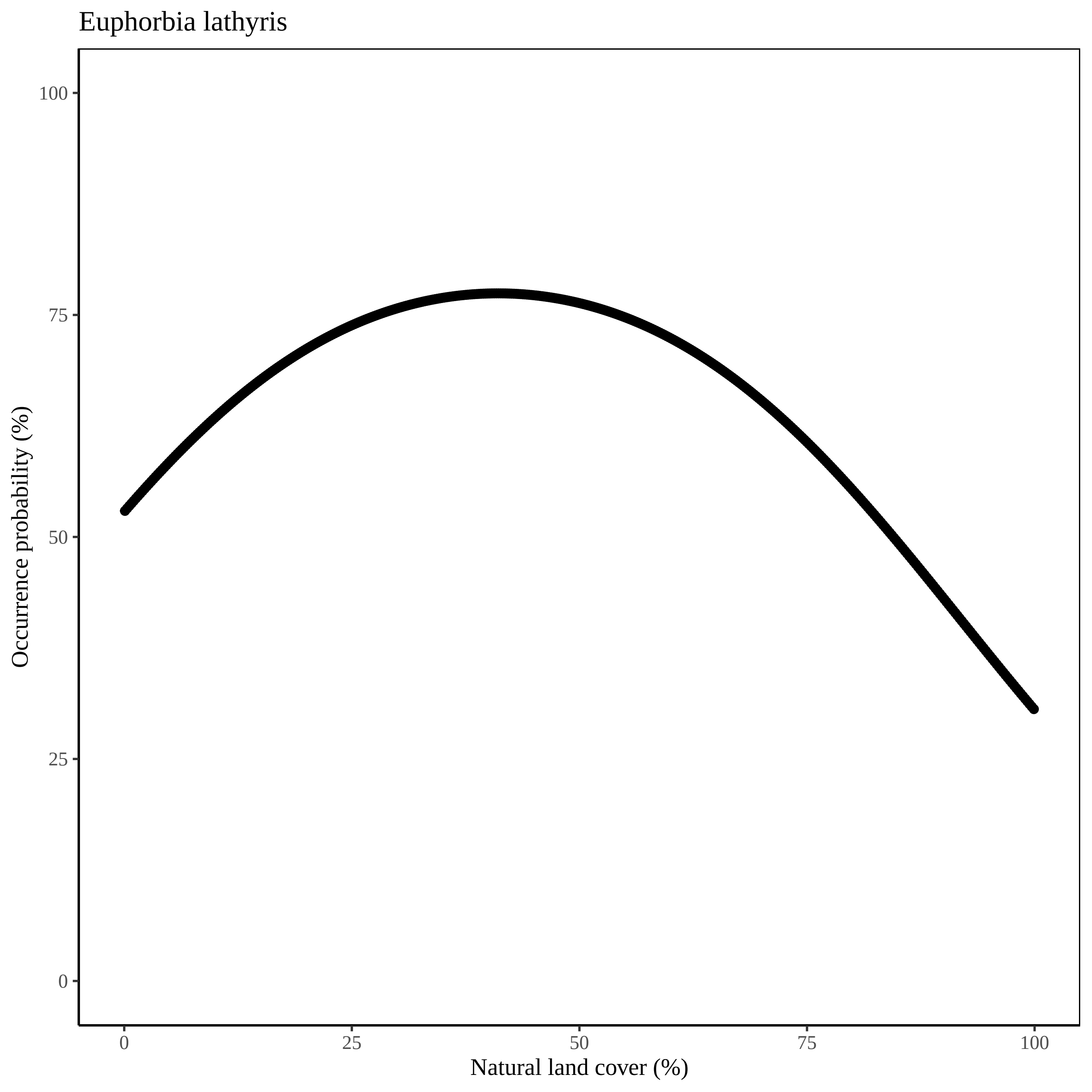

Euphorbia lathyris
Occurrence probability (%)
100
75
50
25
0
0
25
50
75
100
Natural land cover (%)

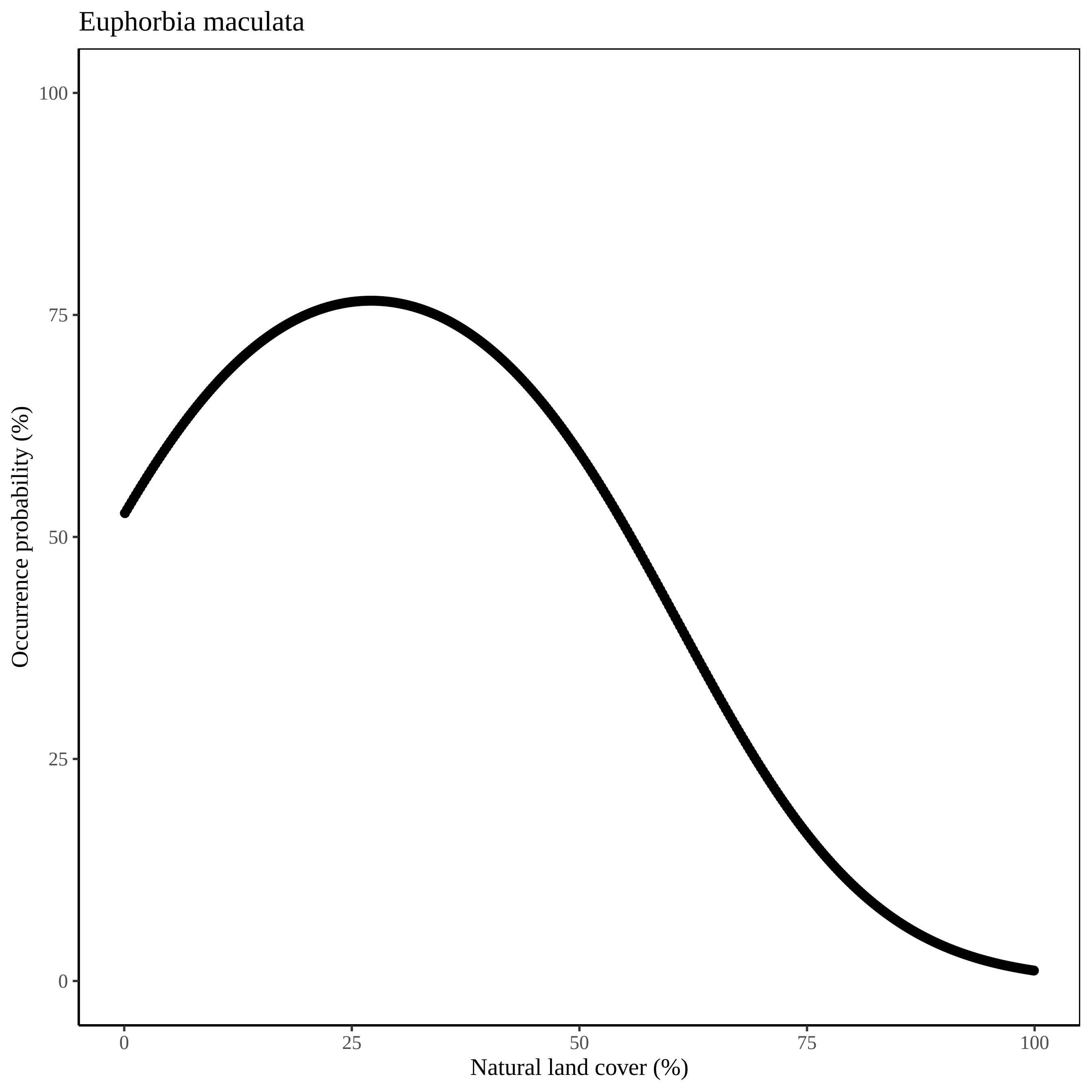

Euphorbia maculata
100
75
50
25
0
Occurrence probability (%)
0
25
50
75
100
Natural land cover (%)

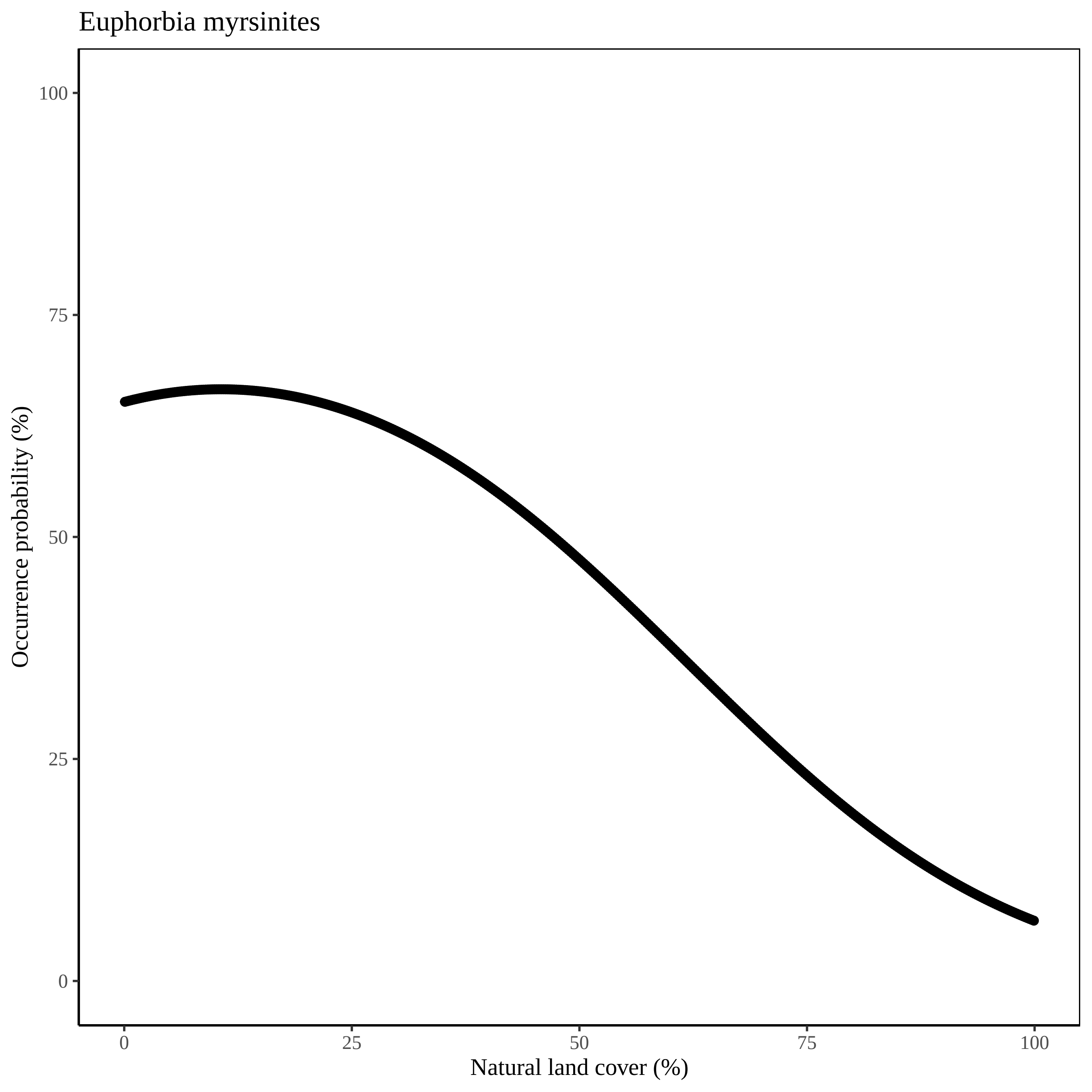
Euphorbia myrsinites
Occurrence probability (%)
100
75
50
25
0
0
25
50
75
100
Natural land cover (%)

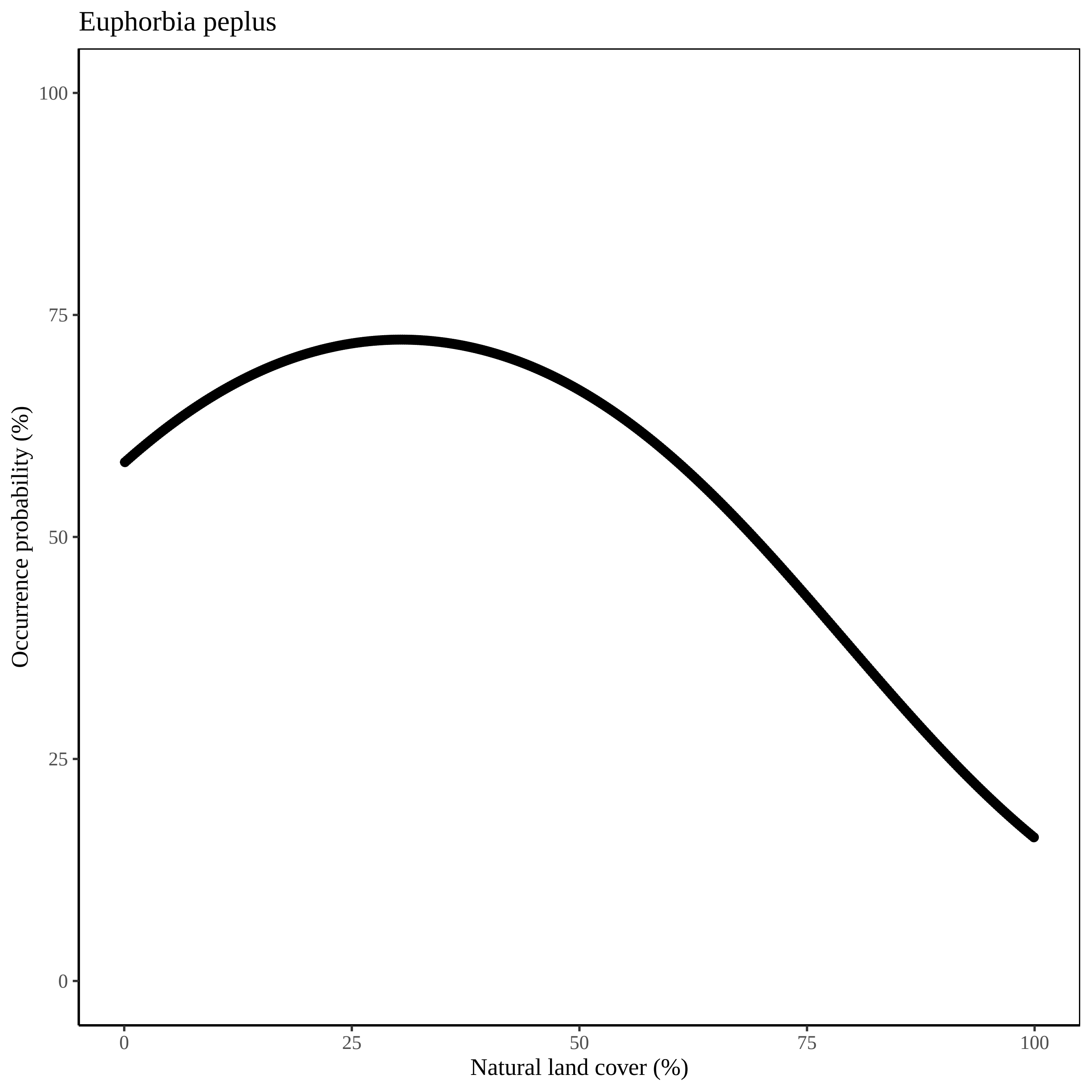
Euphorbia peplus
Occurrence probability (%)
100
75
50
25
0
0
25
50
75
100
Natural land cover (%)

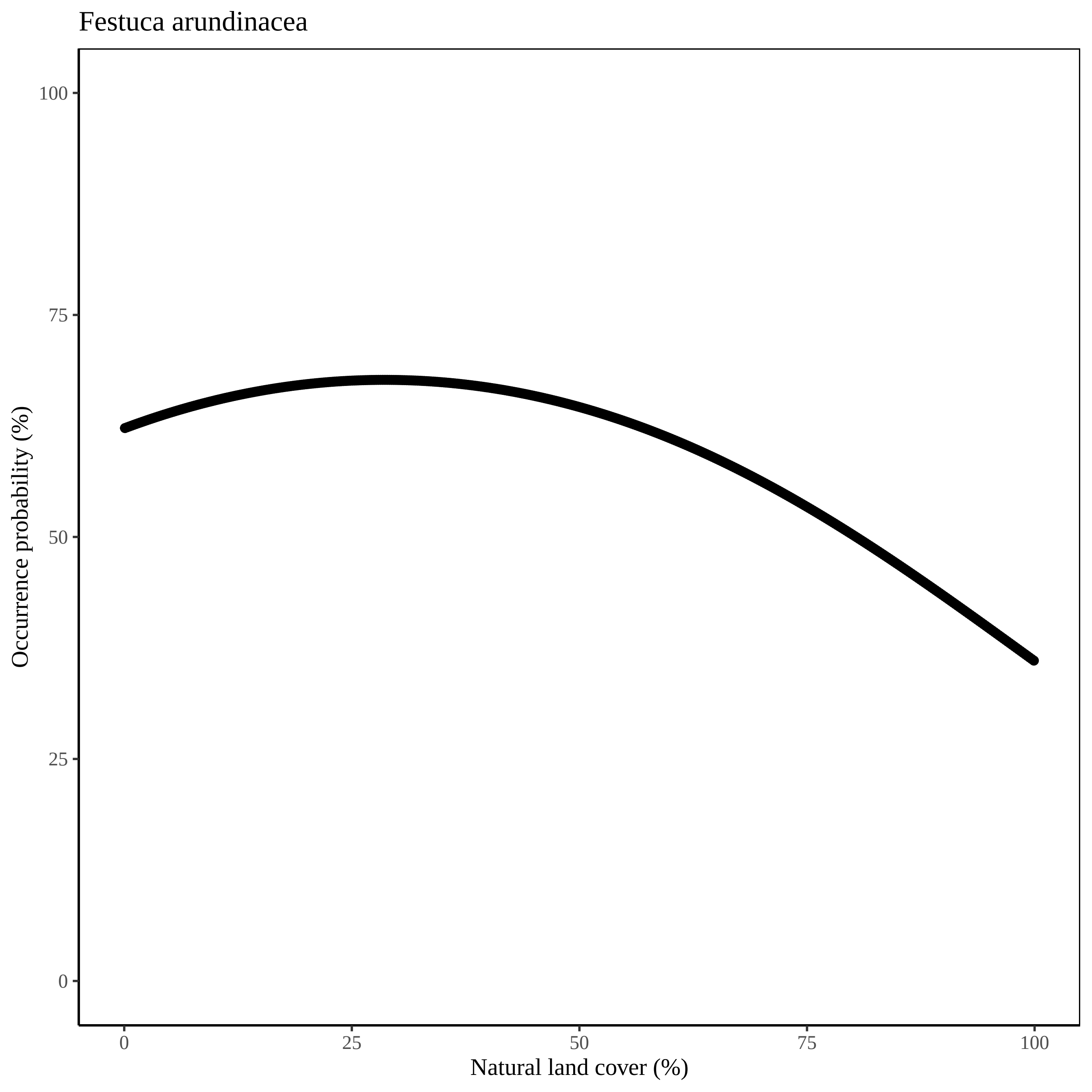
Festuca arundinacea
Occurrence probability (%)
100
75
50
25
0
0
25
50
75
100
Natural land cover (%)

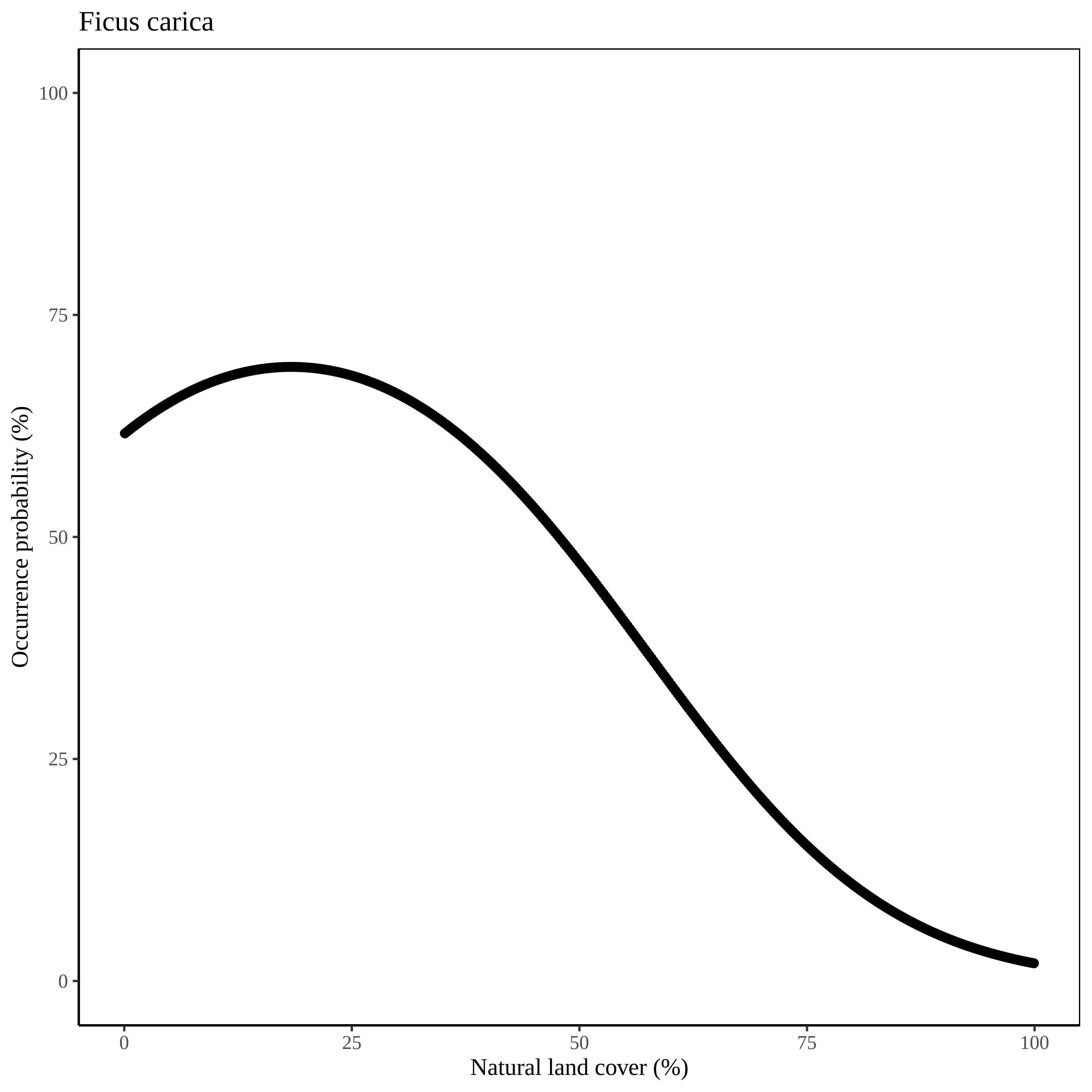
Ficus carica
Occurrence probability (%)
100
75
50
25
0
0
25
50
75
100
Natural land cover (%)

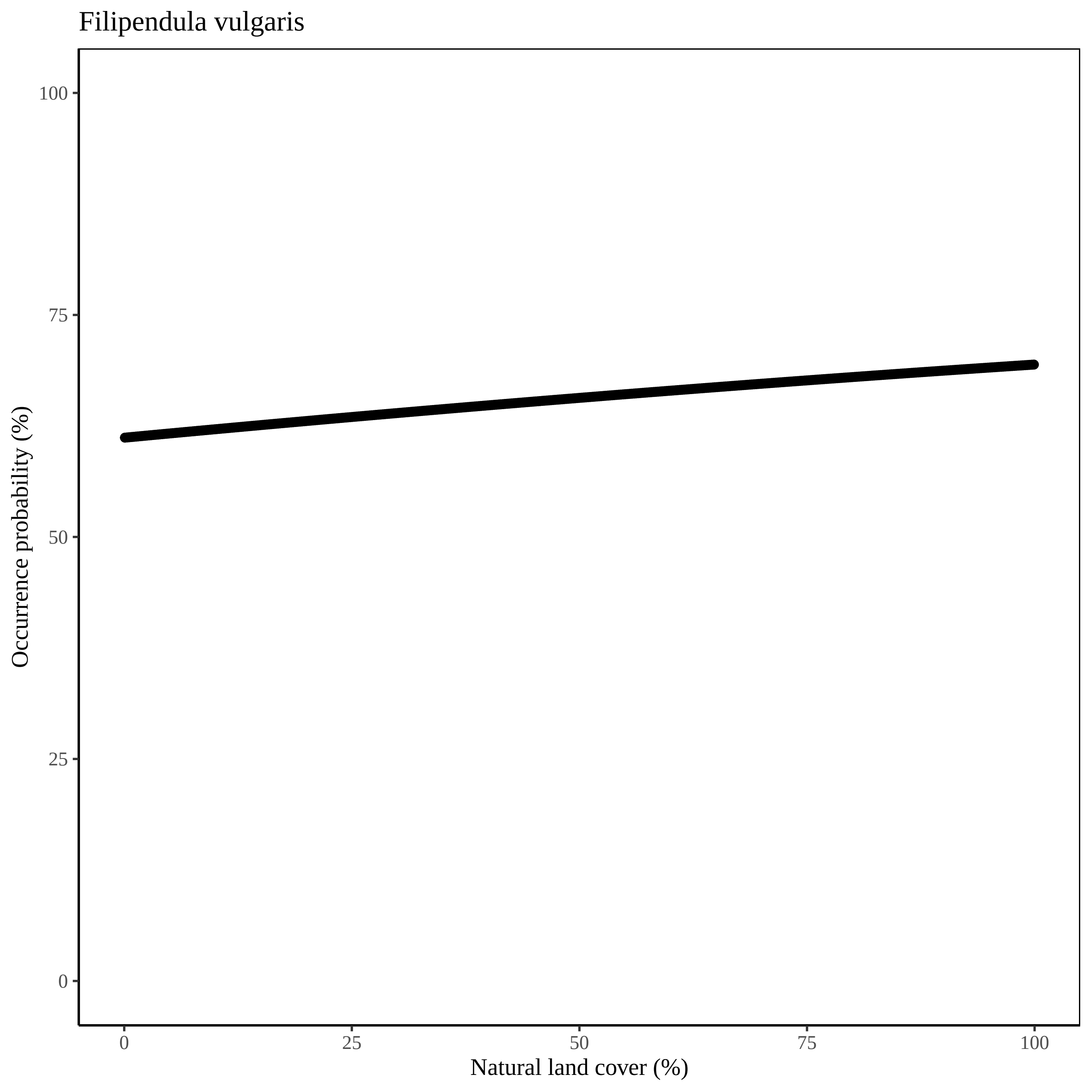

Filipendula vulgaris
Occurrence probability (%)
100
75
50
25
0
0
25
50
75
100
Natural land cover (%)

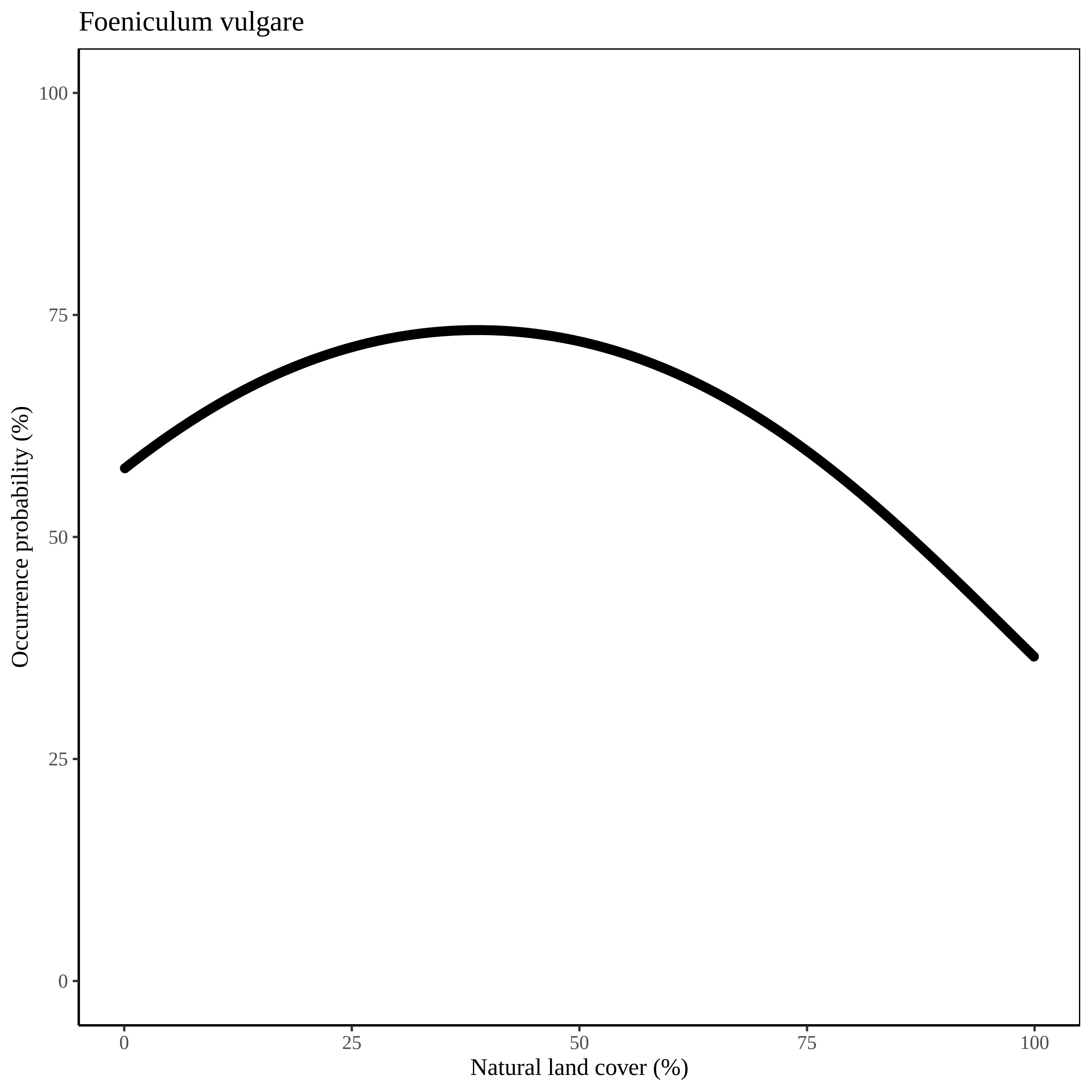
Foeniculum vulgare
Occurrence probability (%)
100
75
50
25
0
0
25
50
75
100
Natural land cover (%)

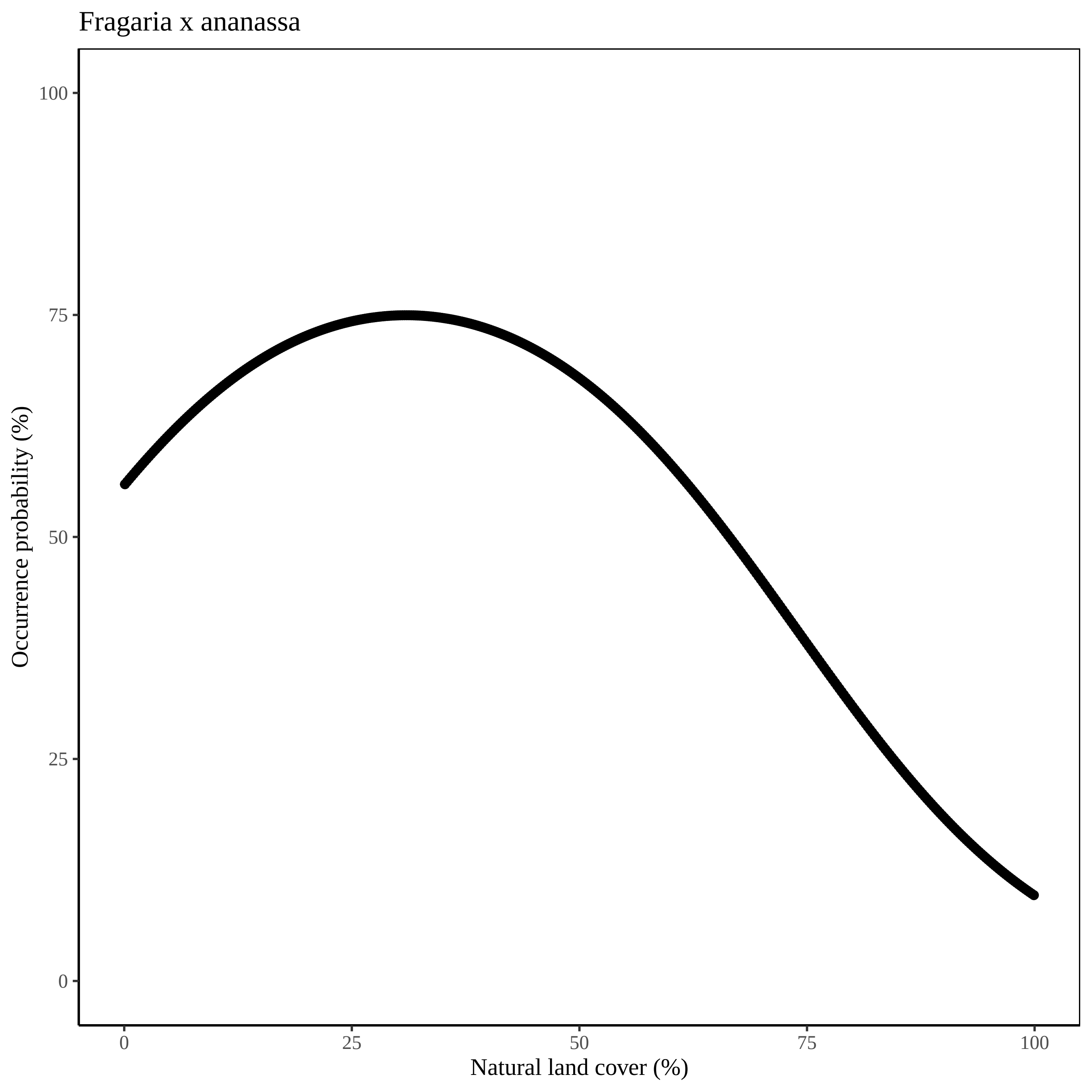

Fragaria x ananassa
Occurrence probability (%)
100
75
50
25
0
0
25
50
75
100
Natural land cover (%)

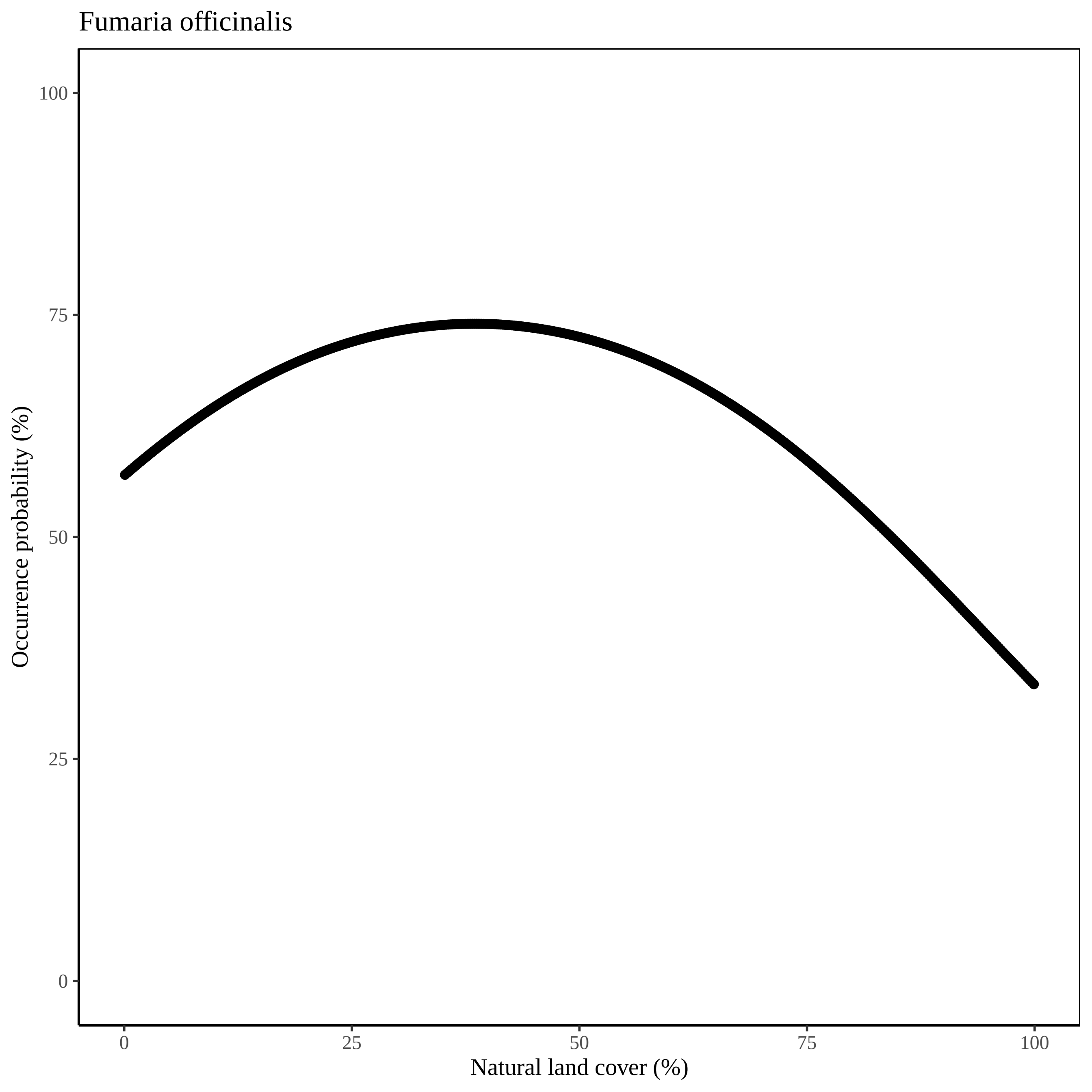

Fumaria officinalis
100
75
50
25
0
Occurrence probability (%)
0
25
50
75
100
Natural land cover (%)

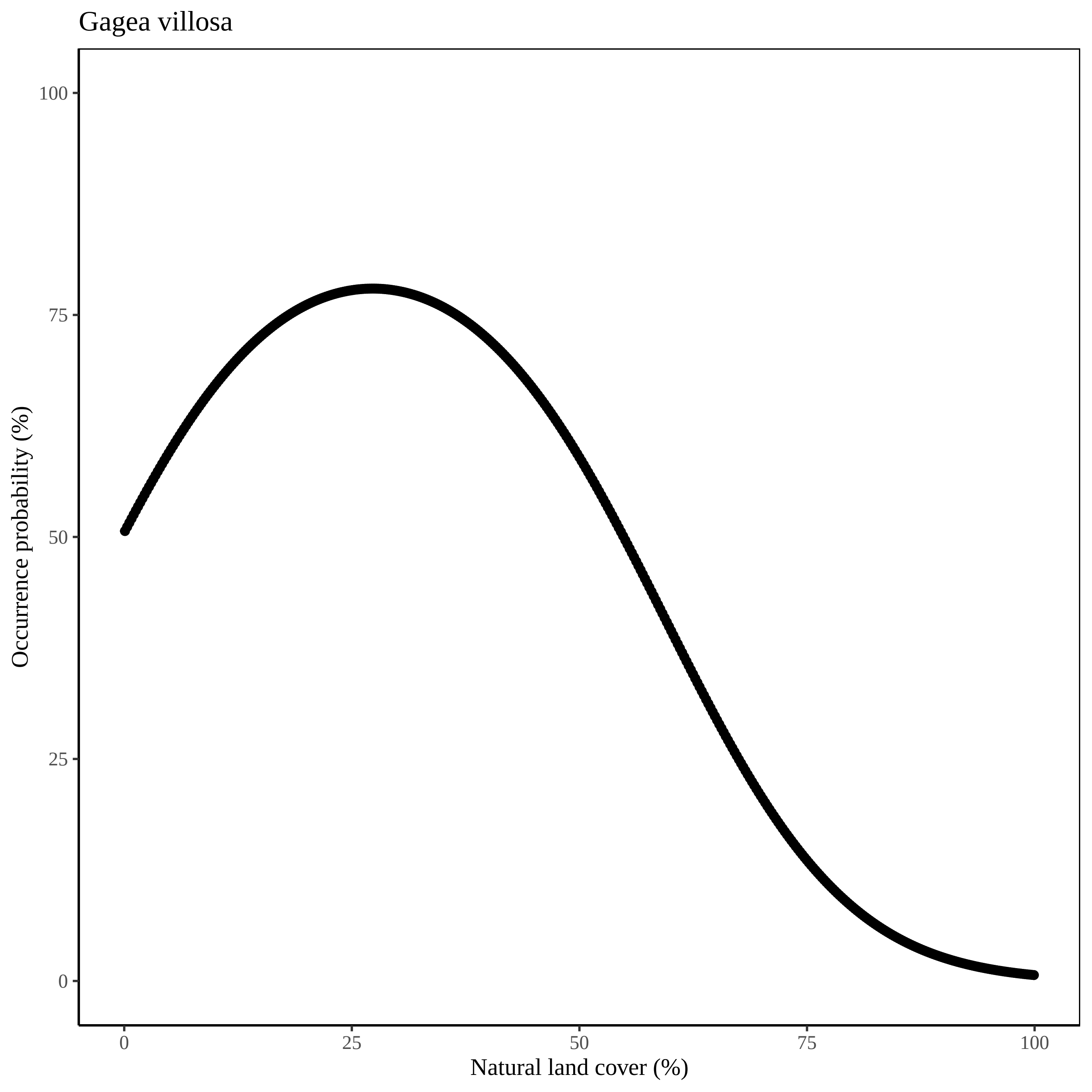
Gagea villosa
100
75
50
25
0
Occurrence probability (%)
0
25
50
75
100
Natural land cover (%)

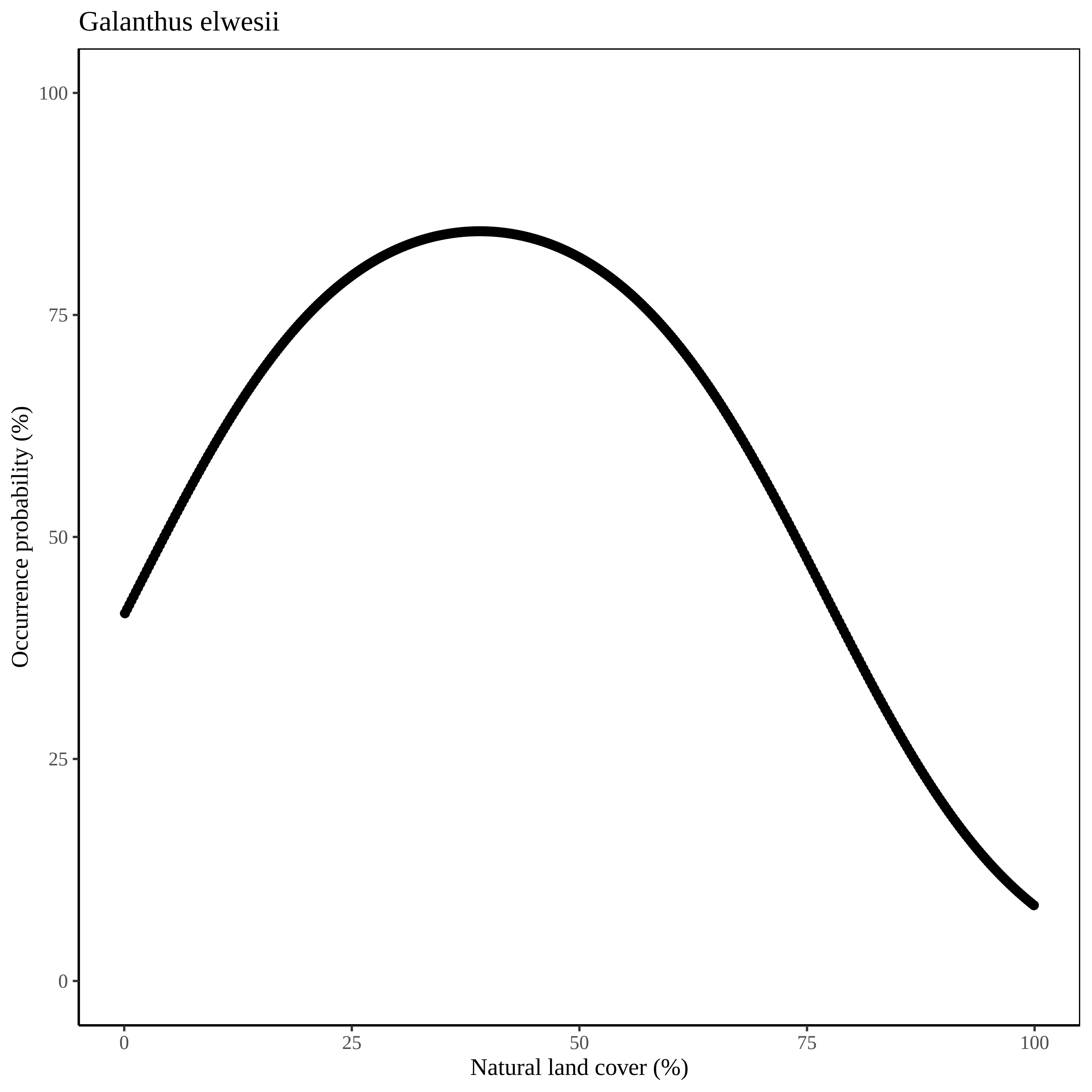

Galanthus elwesii
100
75
50
25
0
Occurrence probability (%)
0
25
50
75
100
Natural land cover (%)

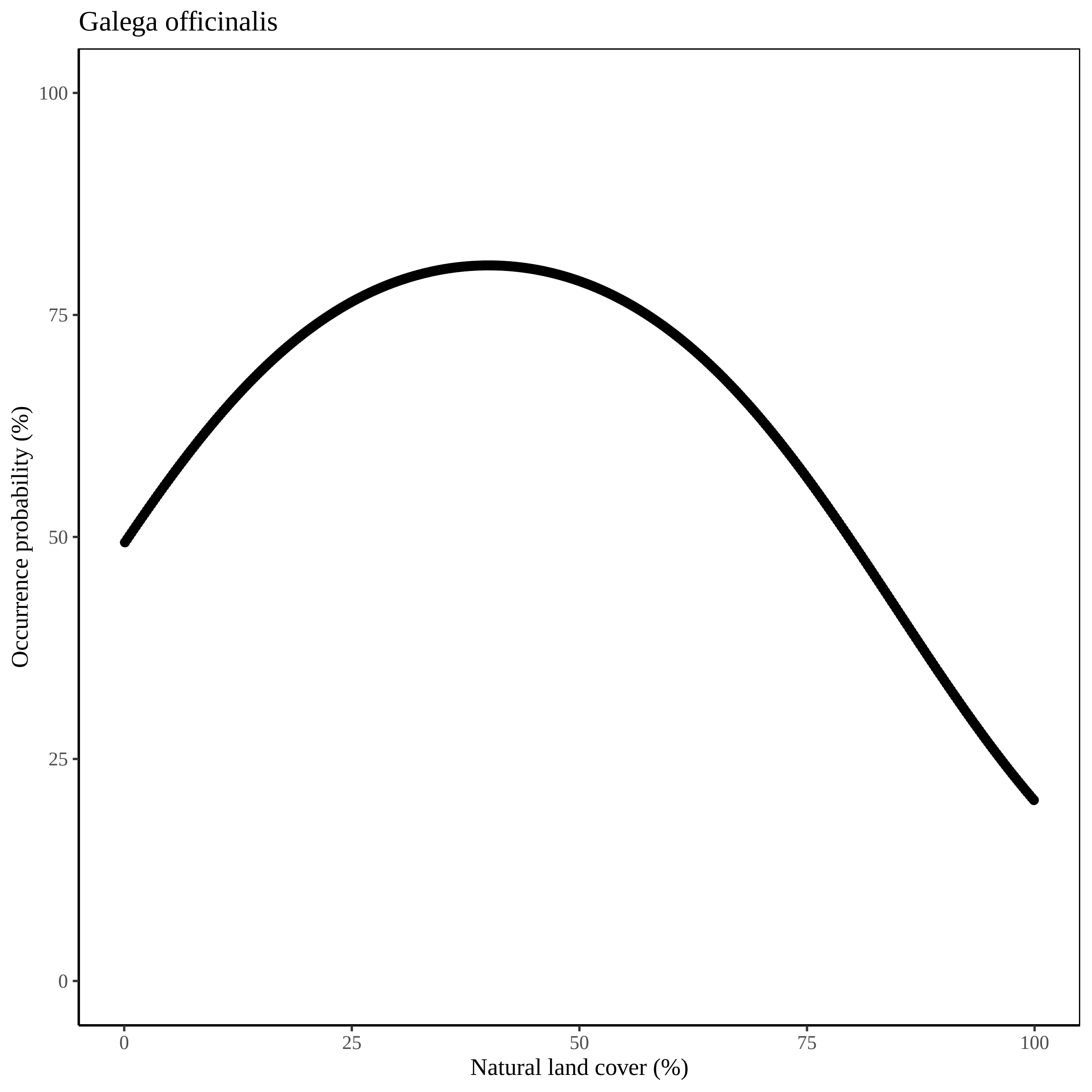
Galega officinalis
Occurrence probability (%)
100
75
50
25
0
0
25
50
75
100
Natural land cover (%)

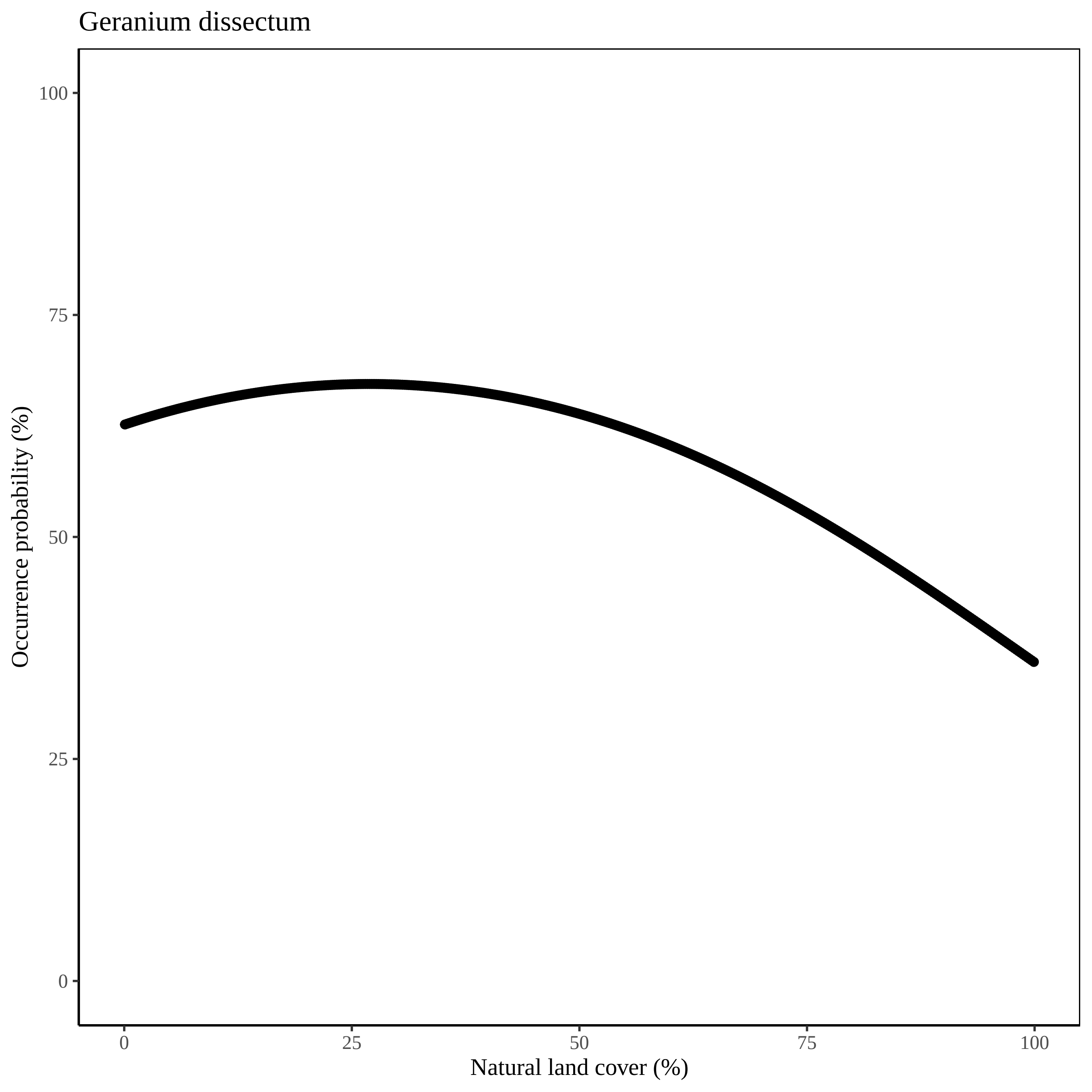
Geranium dissectum
Occurrence probability (%)
0
25
50
75
100
0
25
50
75
100
Natural land cover (%)

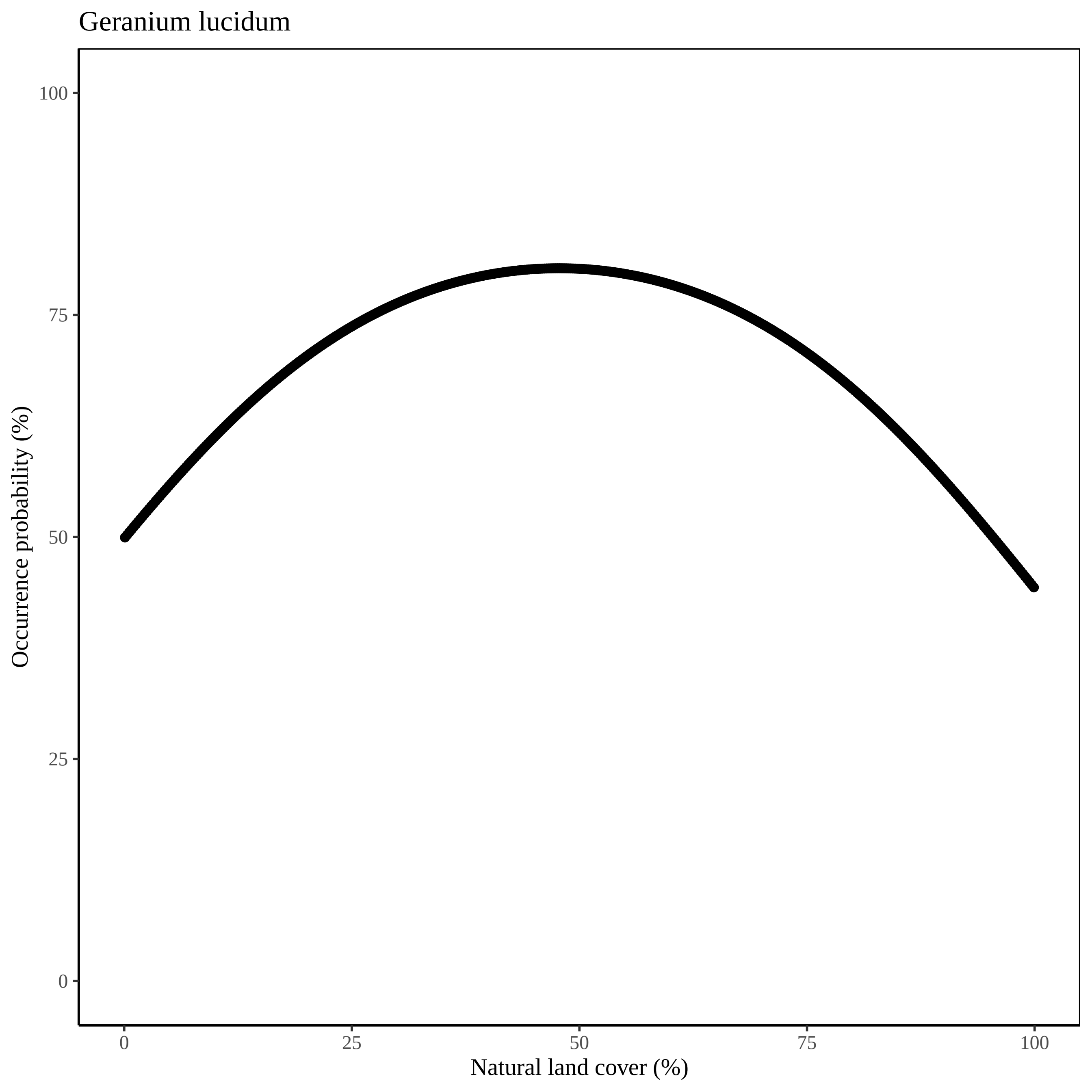

Geranium lucidum
Occurrence probability (%)
100
75
50
25
0
0
25
50
75
100
Natural land cover (%)

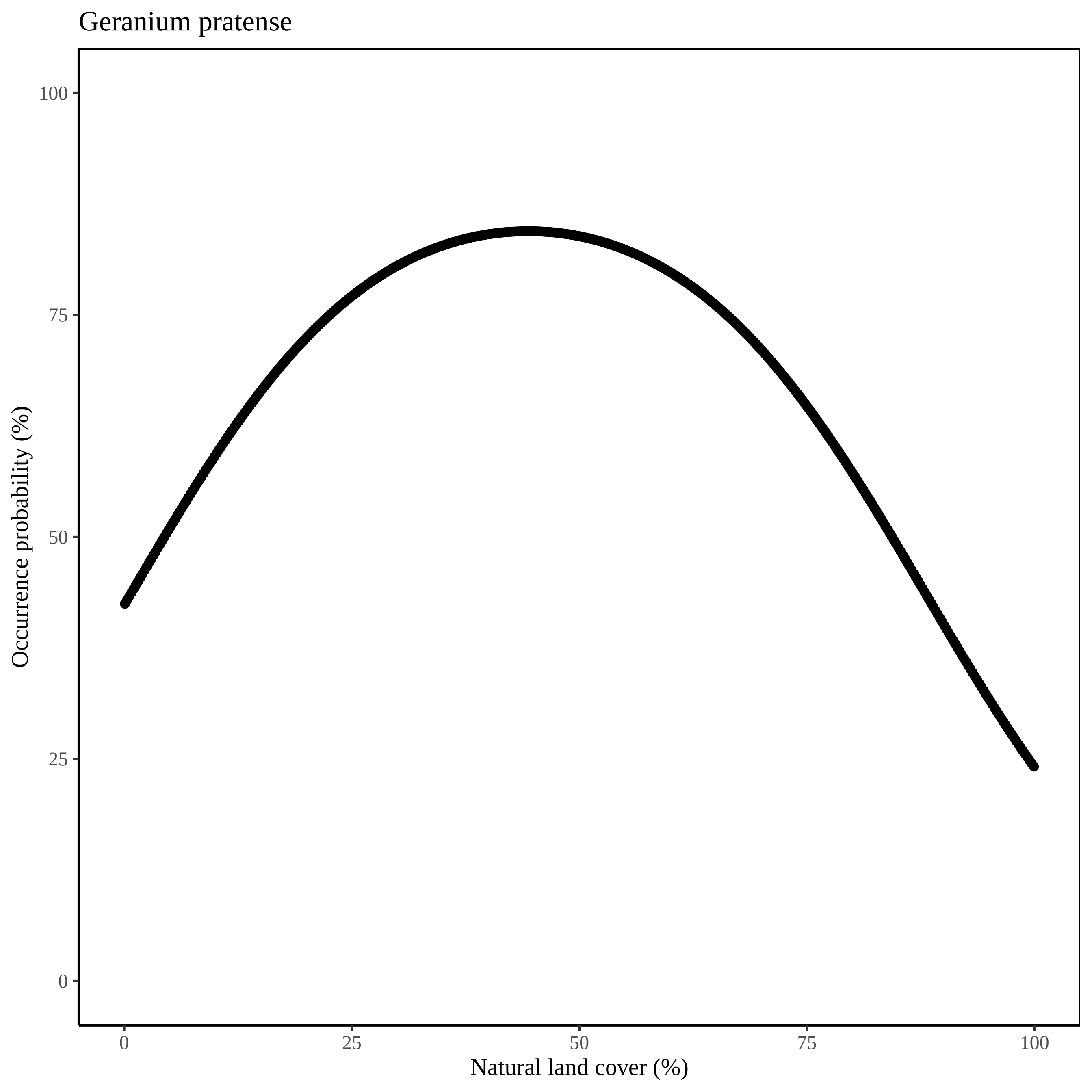

Geranium pratense
Occurrence probability (%)
100
75
50
25
0
0
25
50
75
100
Natural land cover (%)

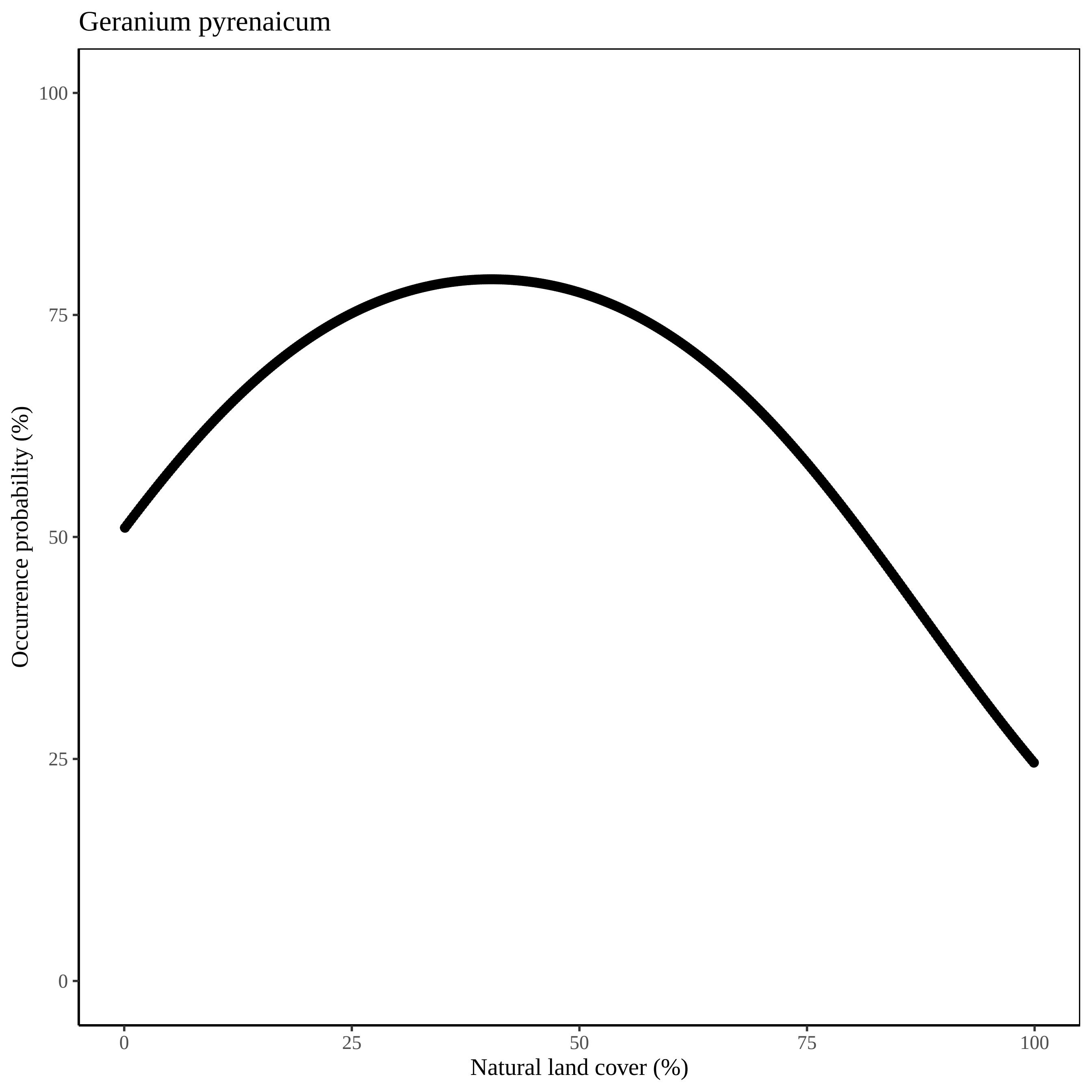
Geranium pyrenaicum
100
75
50
25
0
Occurrence probability (%)
0
25
50
75
100
Natural land cover (%)

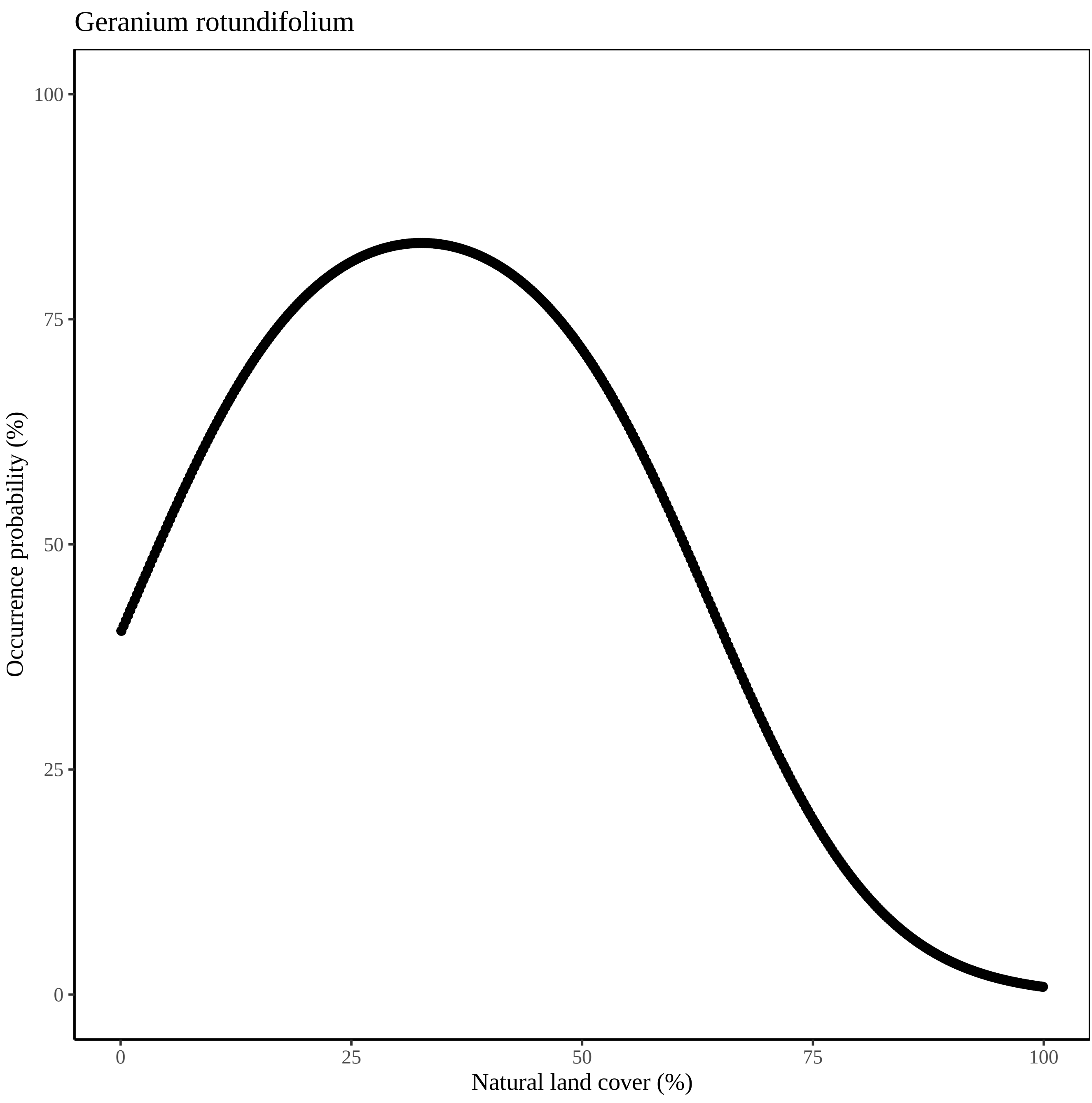

Geranium rotundifolium
Occurrence probability (%)
100
75
50
25
0
0
25
50
75
100
Natural land cover (%)

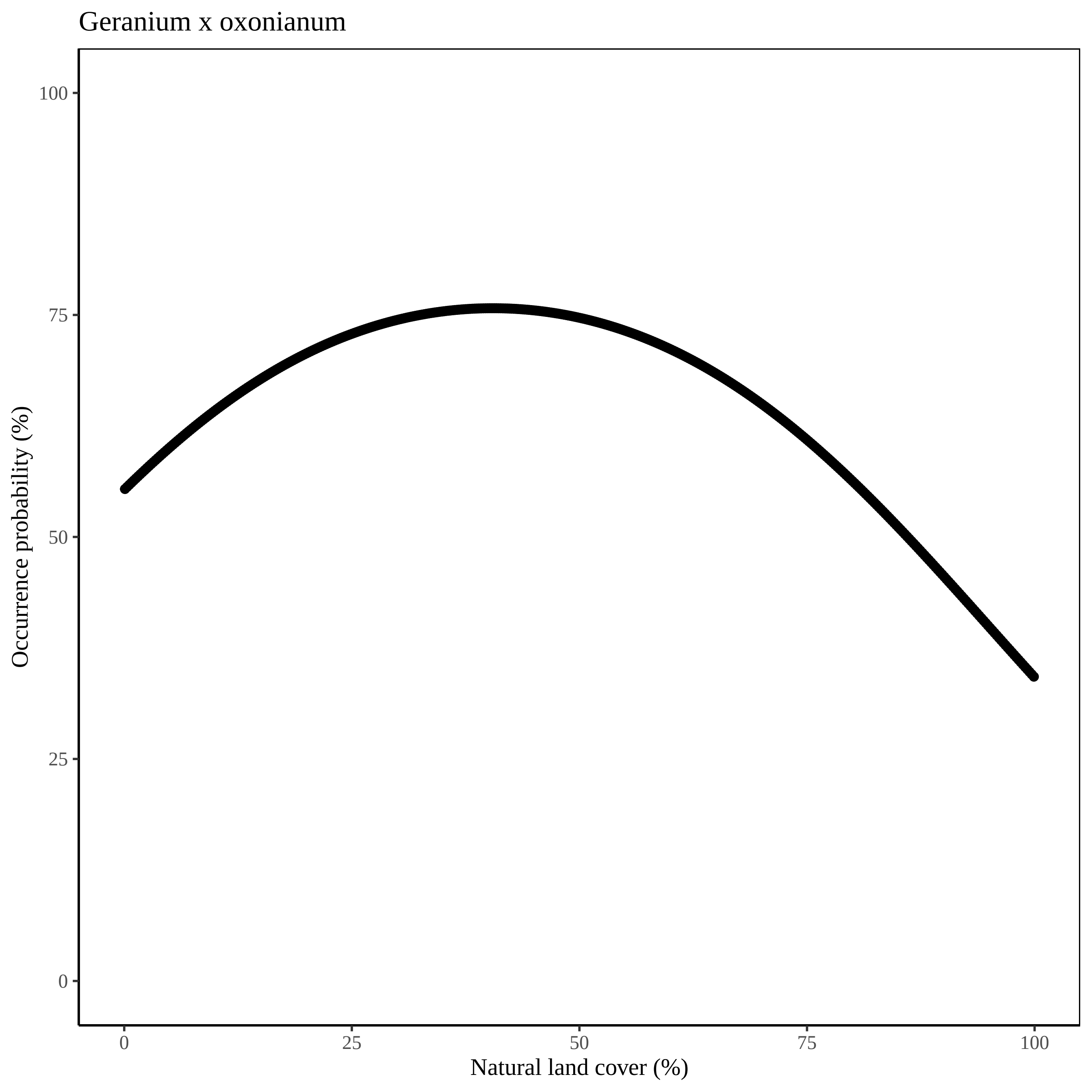

Geranium x oxonianum
Occurrence probability (%)
100
75
50
25
0
0
25
50
75
100
Natural land cover (%)

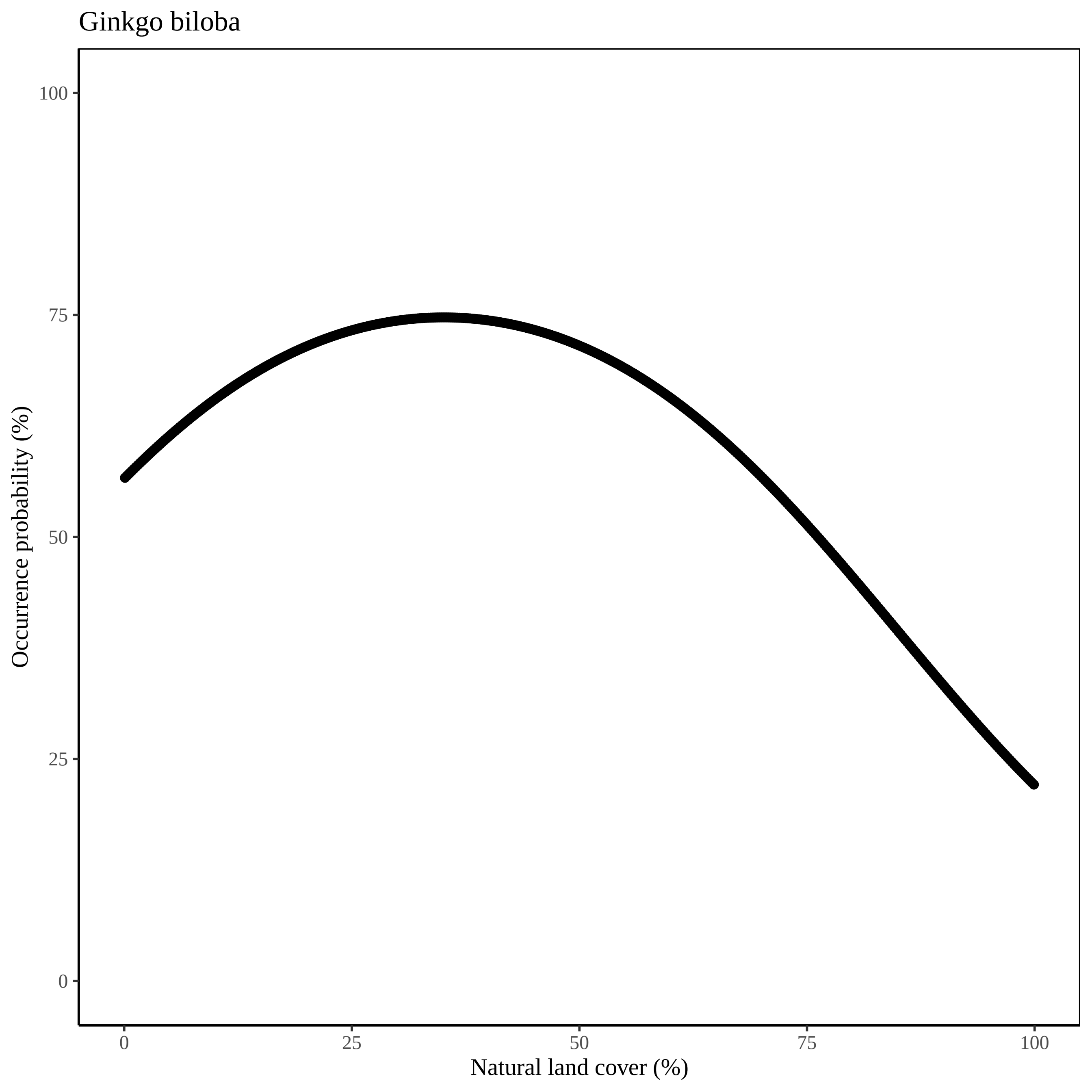
Ginkgo biloba
Occurrence probability (%)
100
75
50
25
0
0
25
50
75
100
Natural land cover (%)

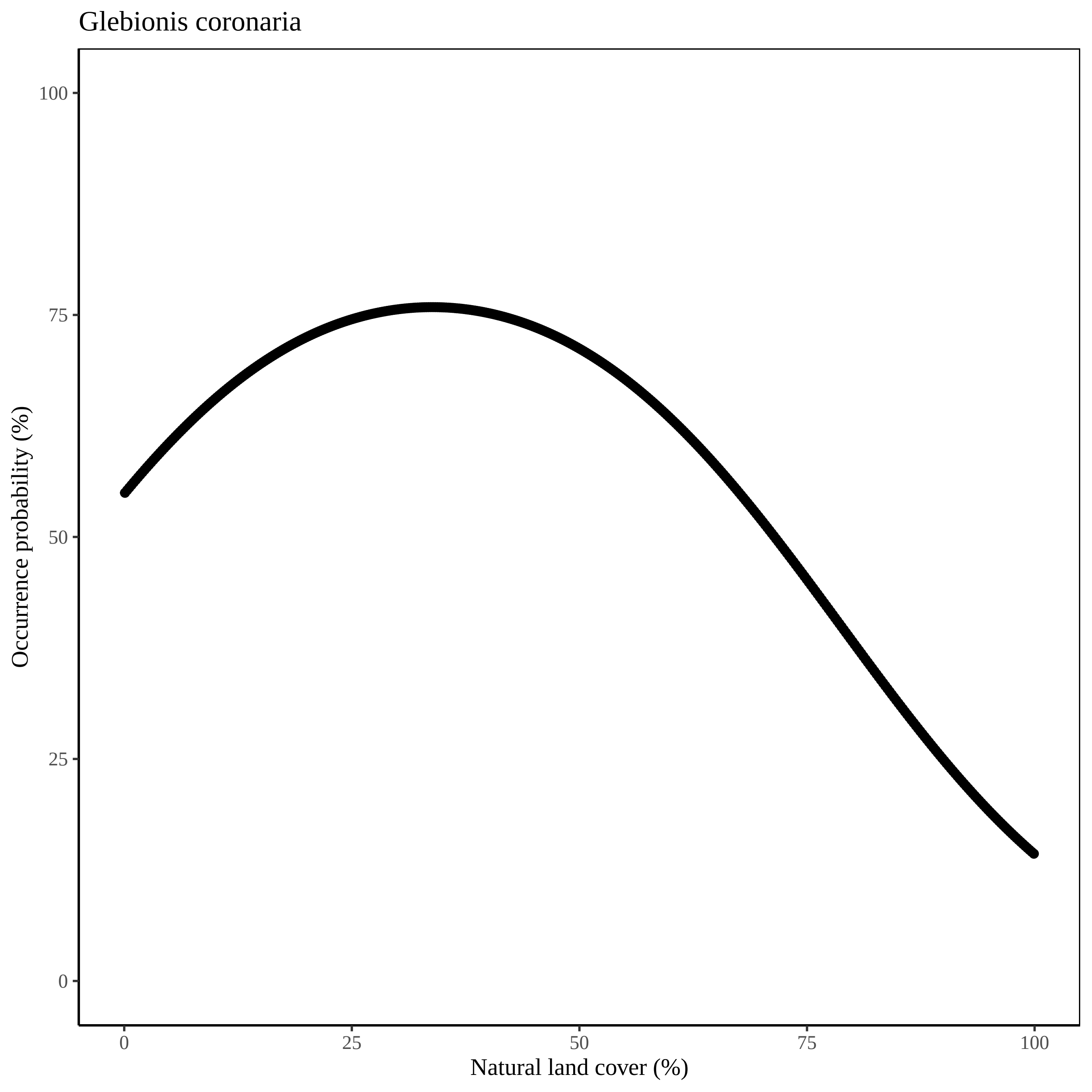
Glebionis coronaria
Occurrence probability (%)
100
75
50
25
0
0
25
50
75
100
Natural land cover (%)

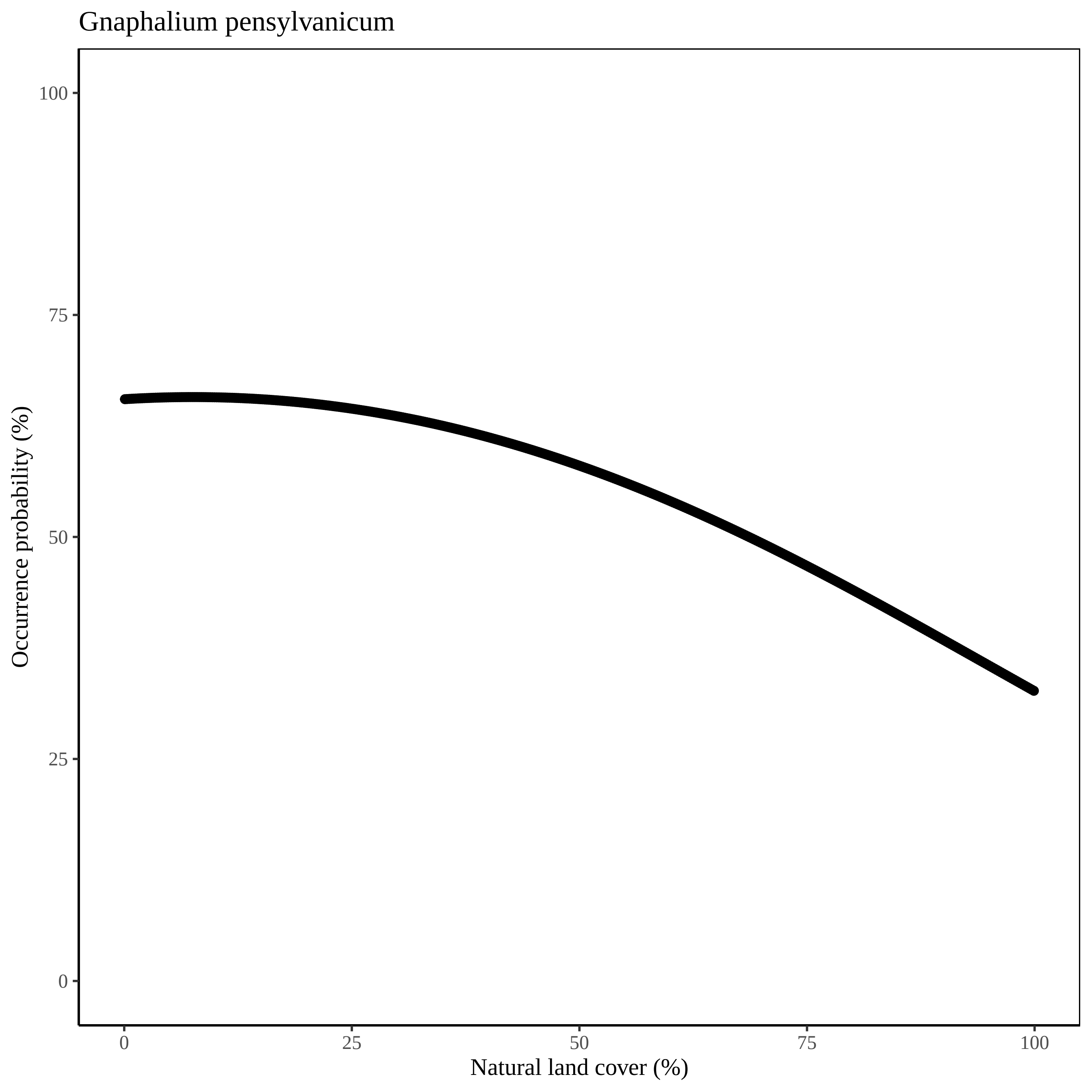
Gnaphalium pensylvanicum
Occurrence probability (%)
100
75
50
25
0
Natural land cover (%)
0
25
50
75
100

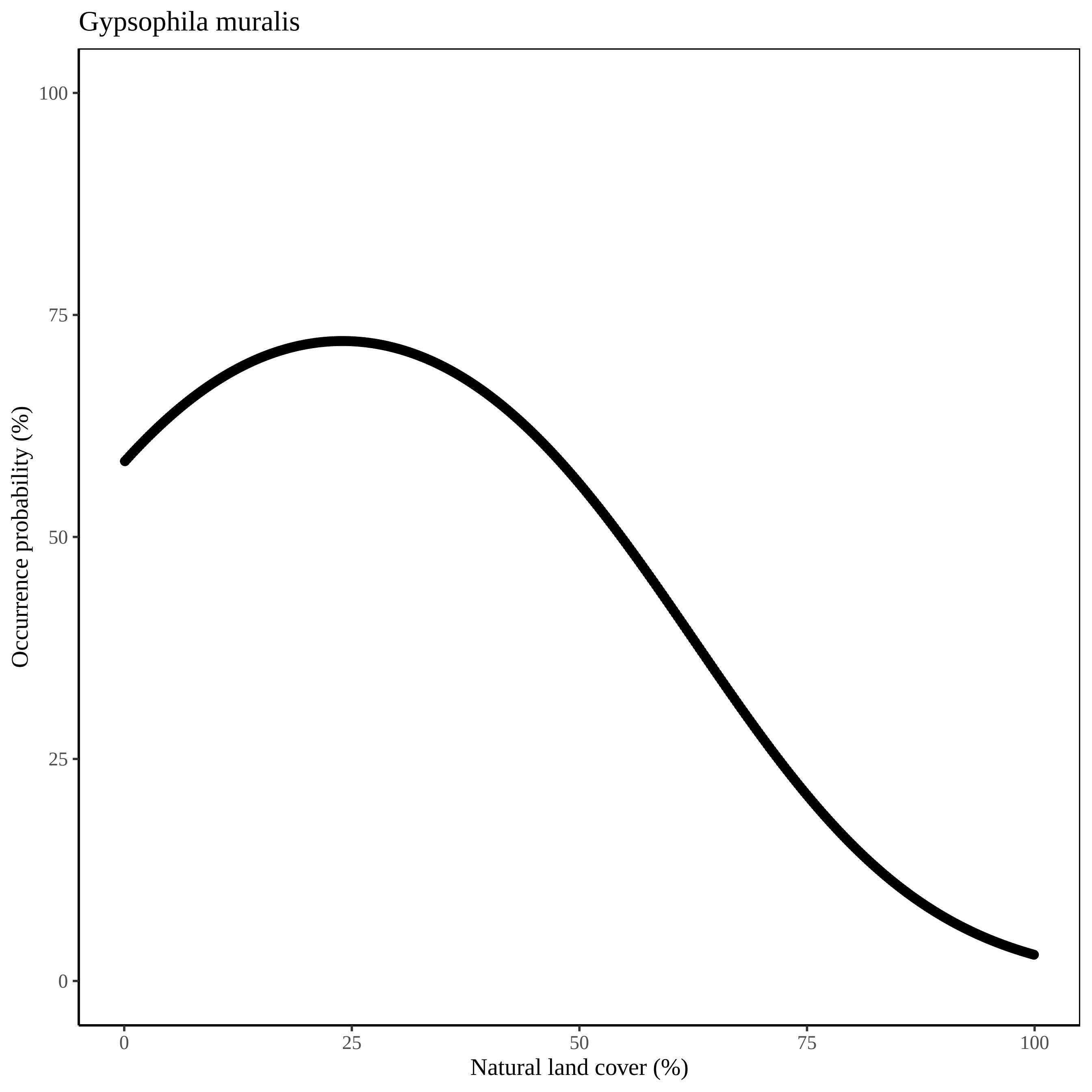

Gypsophila muralis
Occurrence probability (%)
100
75
50
25
0
0
25
50
75
100
Natural land cover (%)

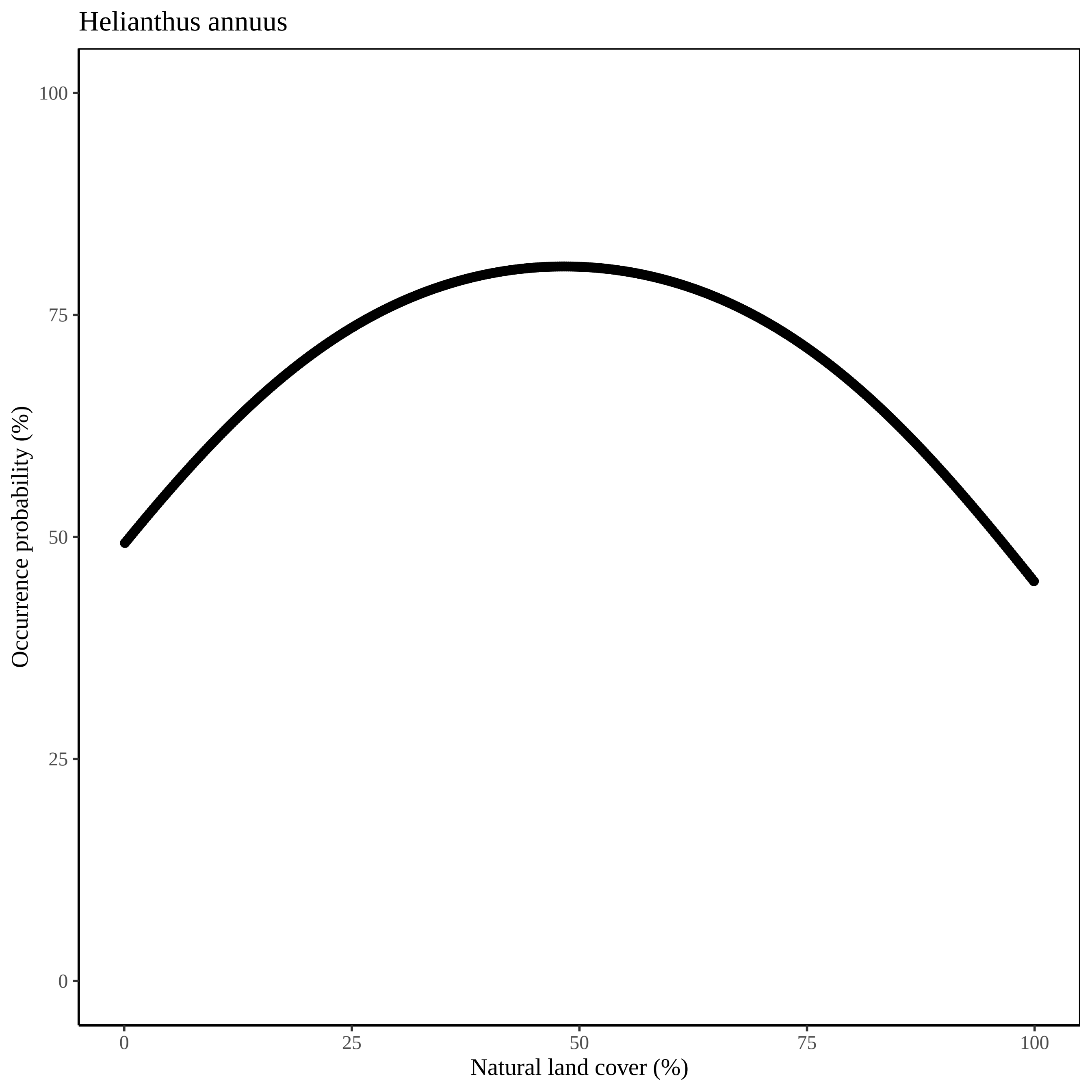
Helianthus annuus
Occurrence probability (%)
100
75
50
25
0
0
25
50
75
100
Natural land cover (%)

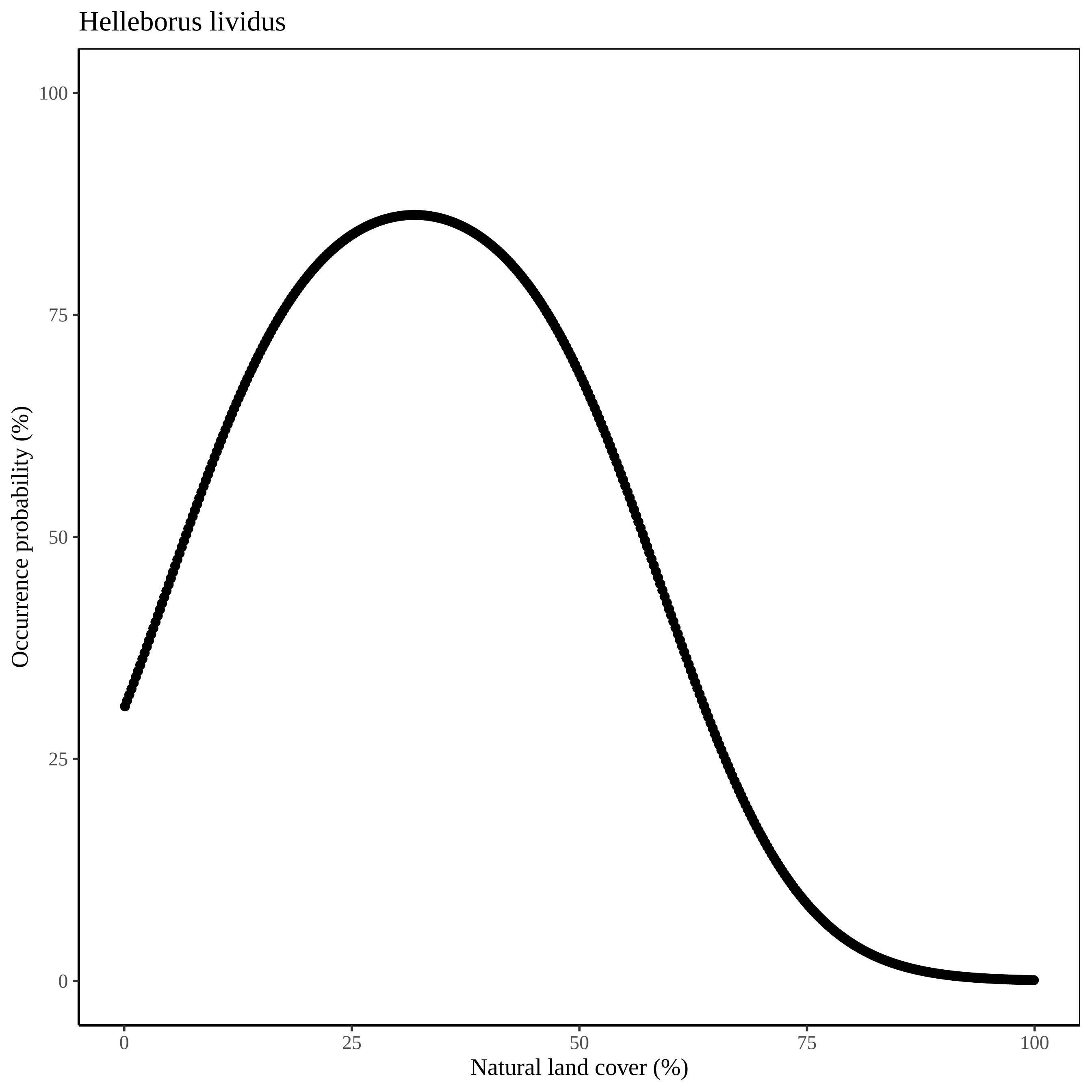
Helleborus lividus
Occurrence probability (%)
100
75
50
25
0
0
25
50
75
100
Natural land cover (%)

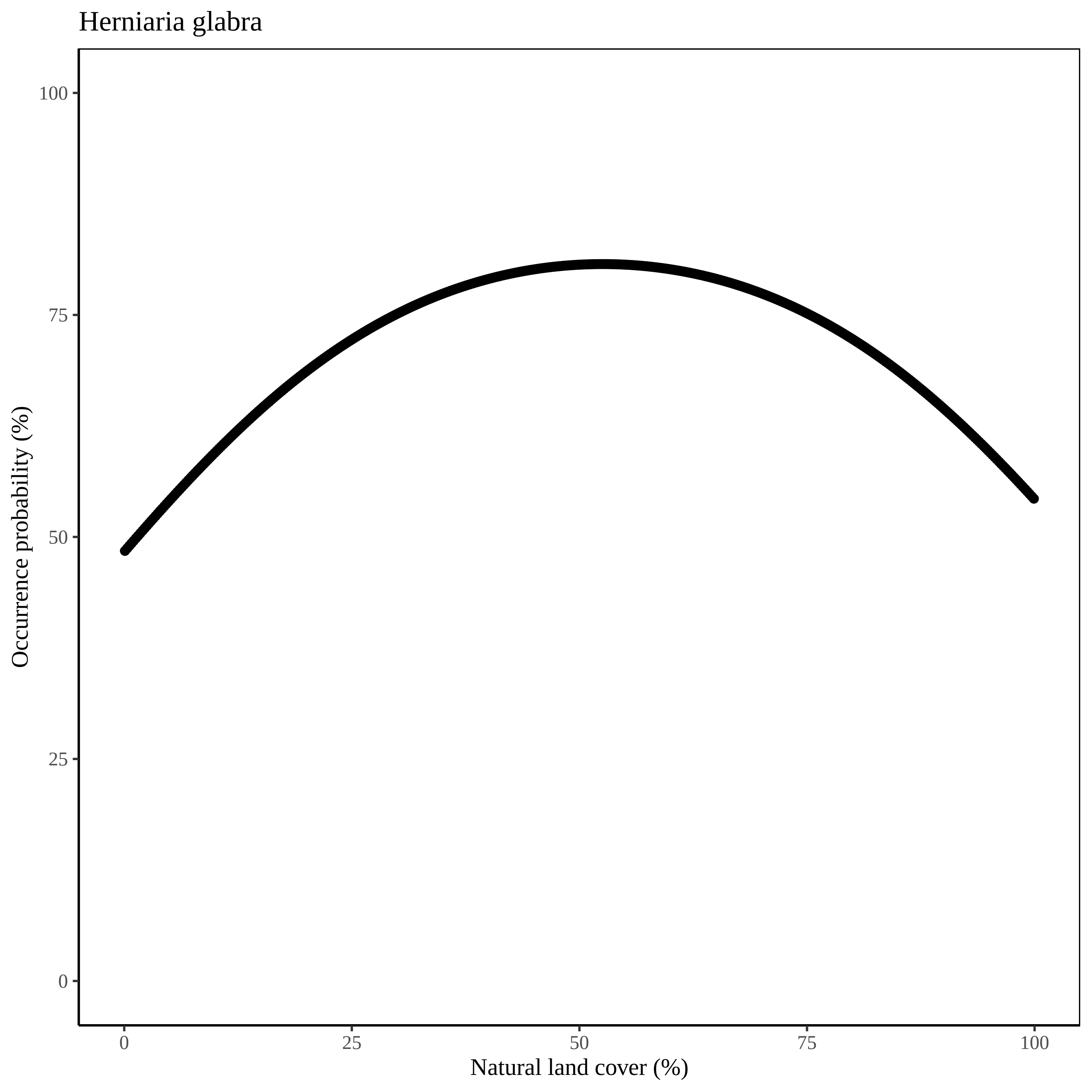
Herniaria glabra
Occurrence probability (%)
100
75
50
25
0
0
25
50
75
100
Natural land cover (%)

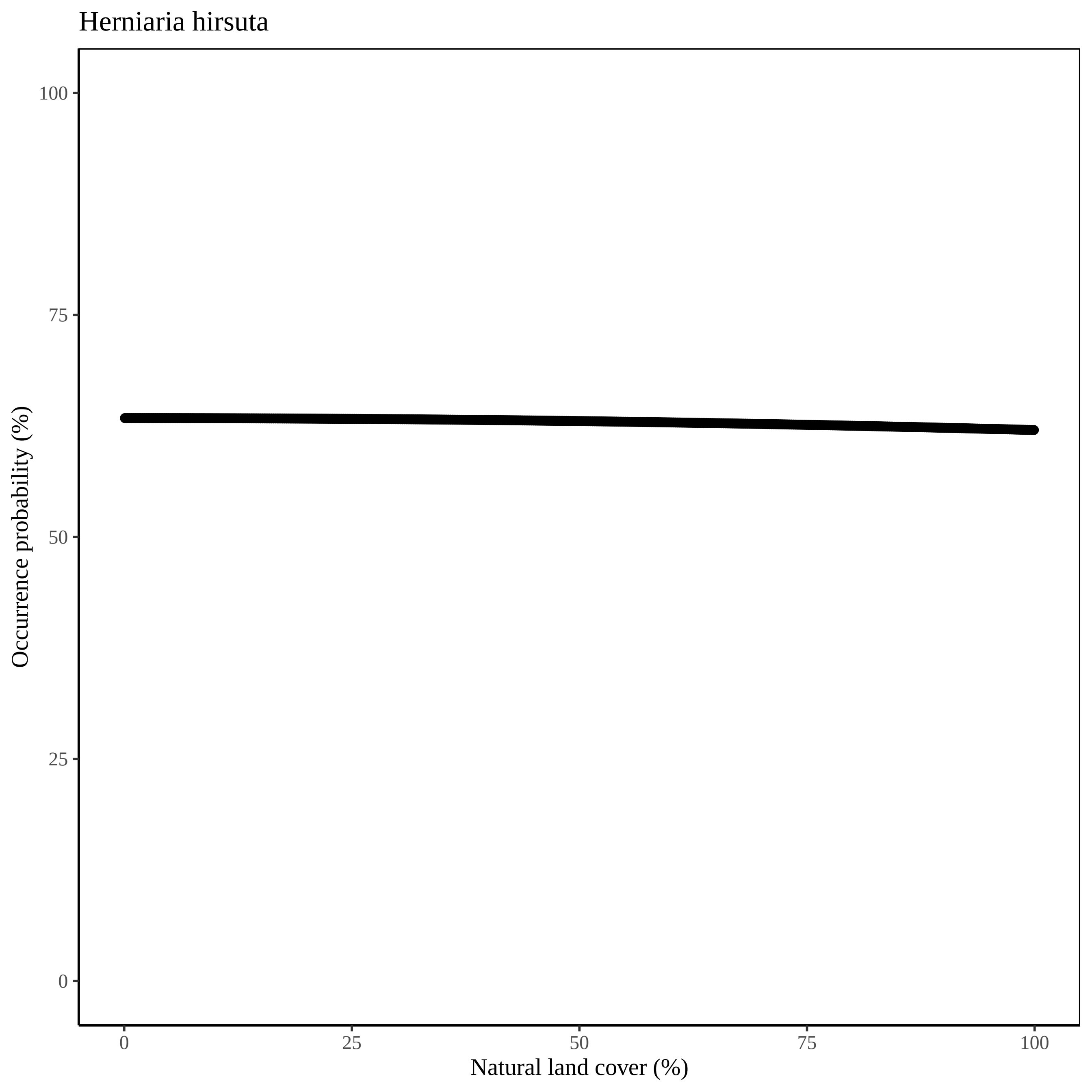

Herniaria hirsuta
Occurrence probability (%)
100
75
50
25
0
0
25
50
75
100
Natural land cover (%)

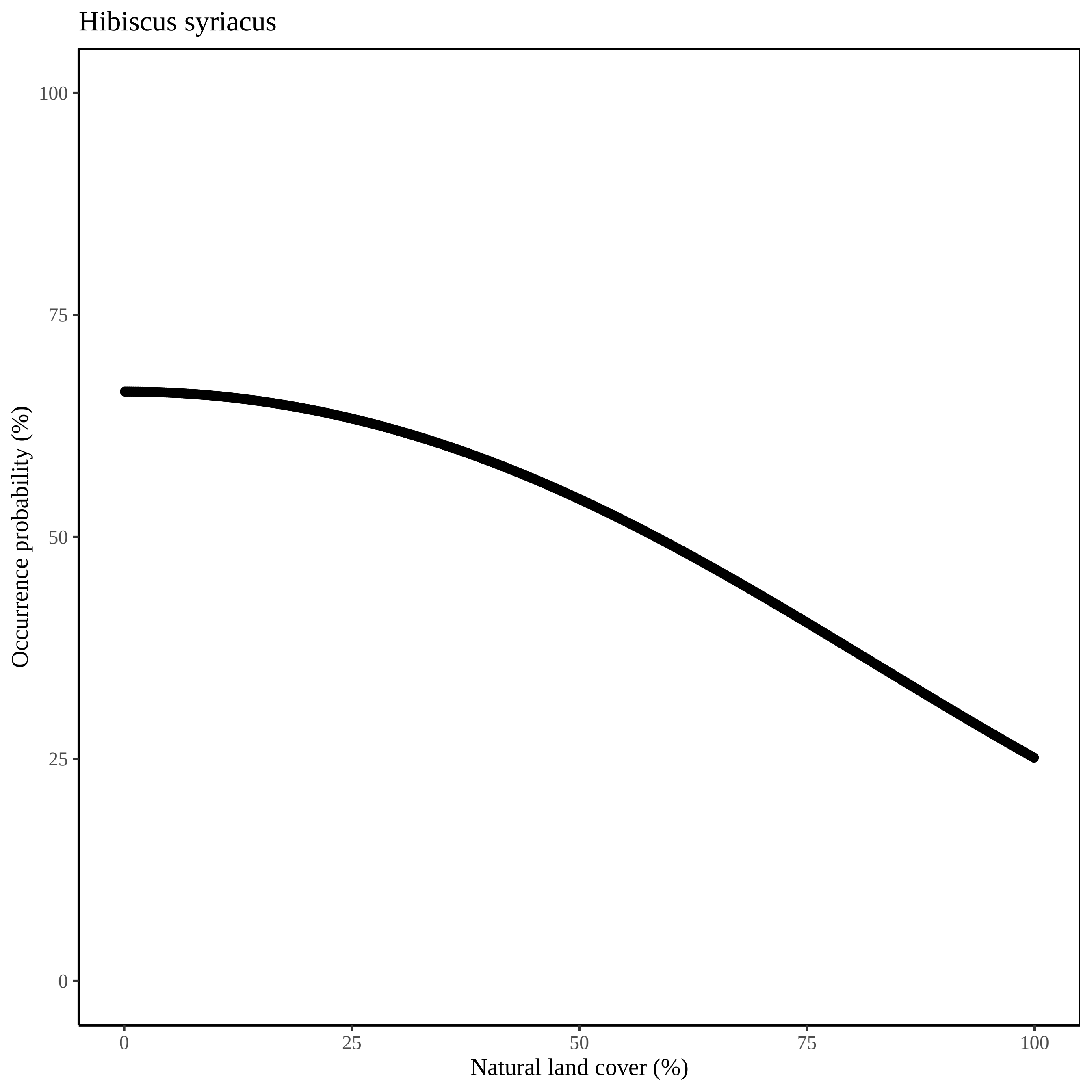
Hibiscus syriacus
Occurrence probability (%)
100
75
50
25
0
0
25
50
75
100
Natural land cover (%)

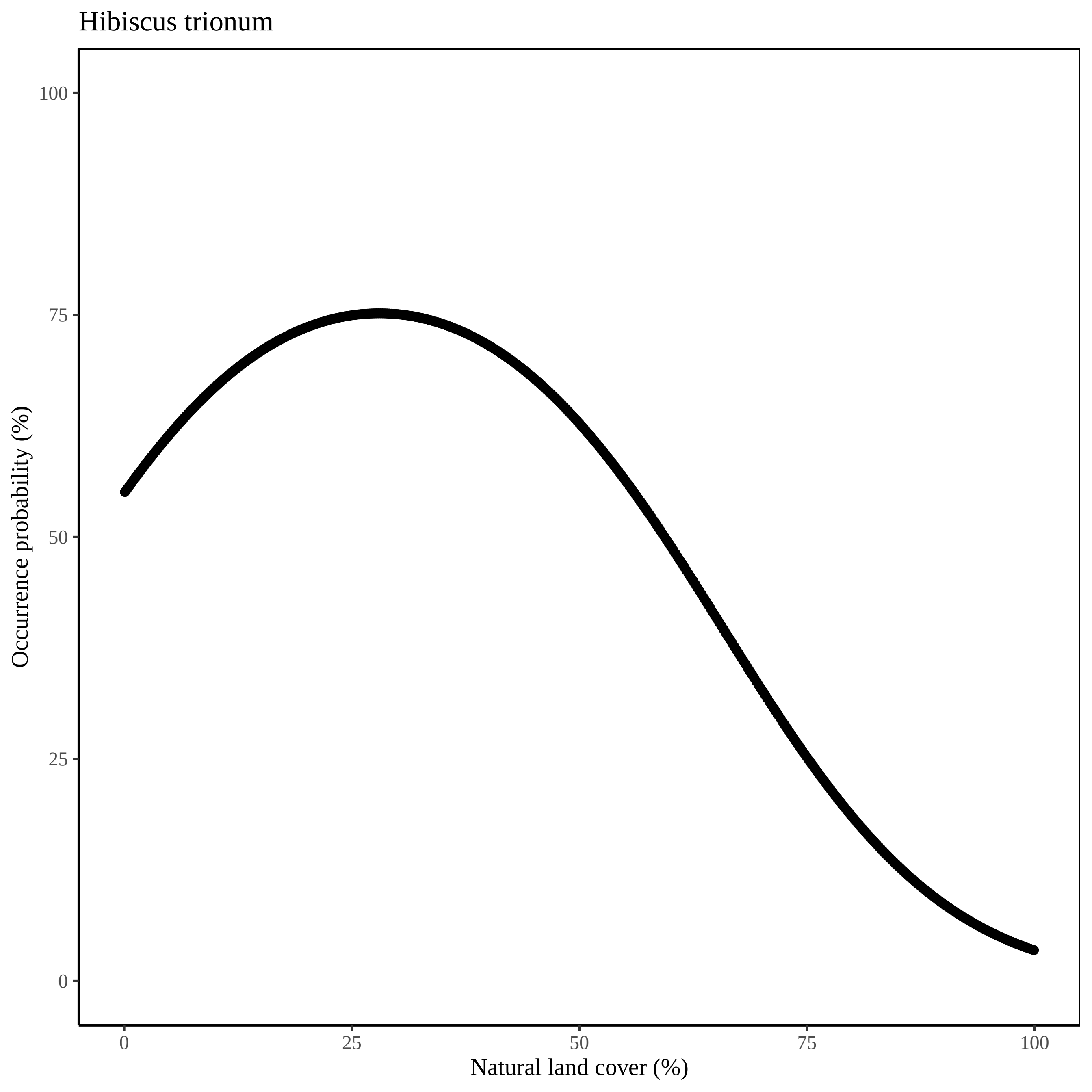

Hibiscus trionum
100
75
50
25
0
Occurrence probability (%)
0
25
50
75
100
Natural land cover (%)

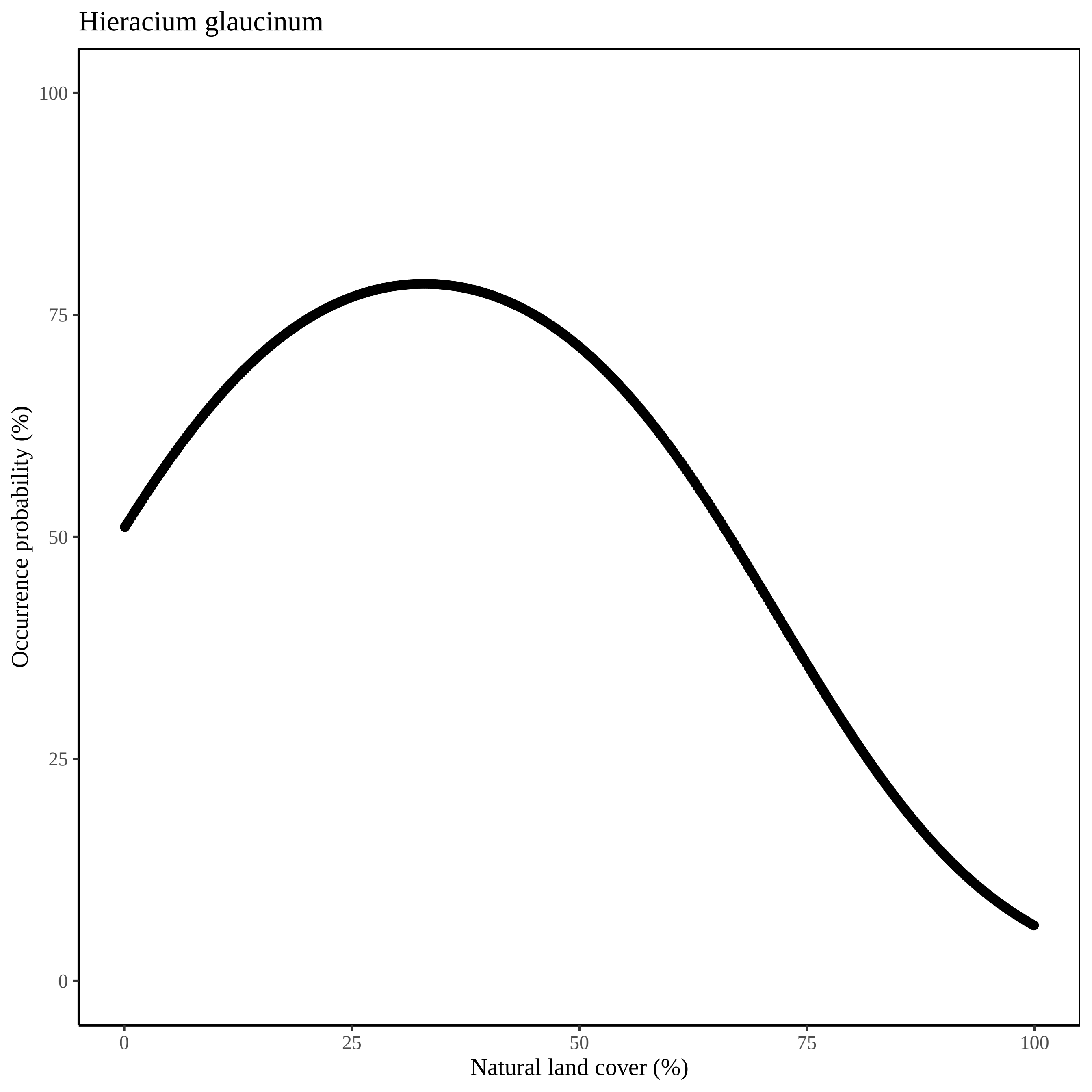
Hieracium glaucinum
Occurrence probability (%)
100
75
50
25
0
0
25
50
75
100
Natural land cover (%)

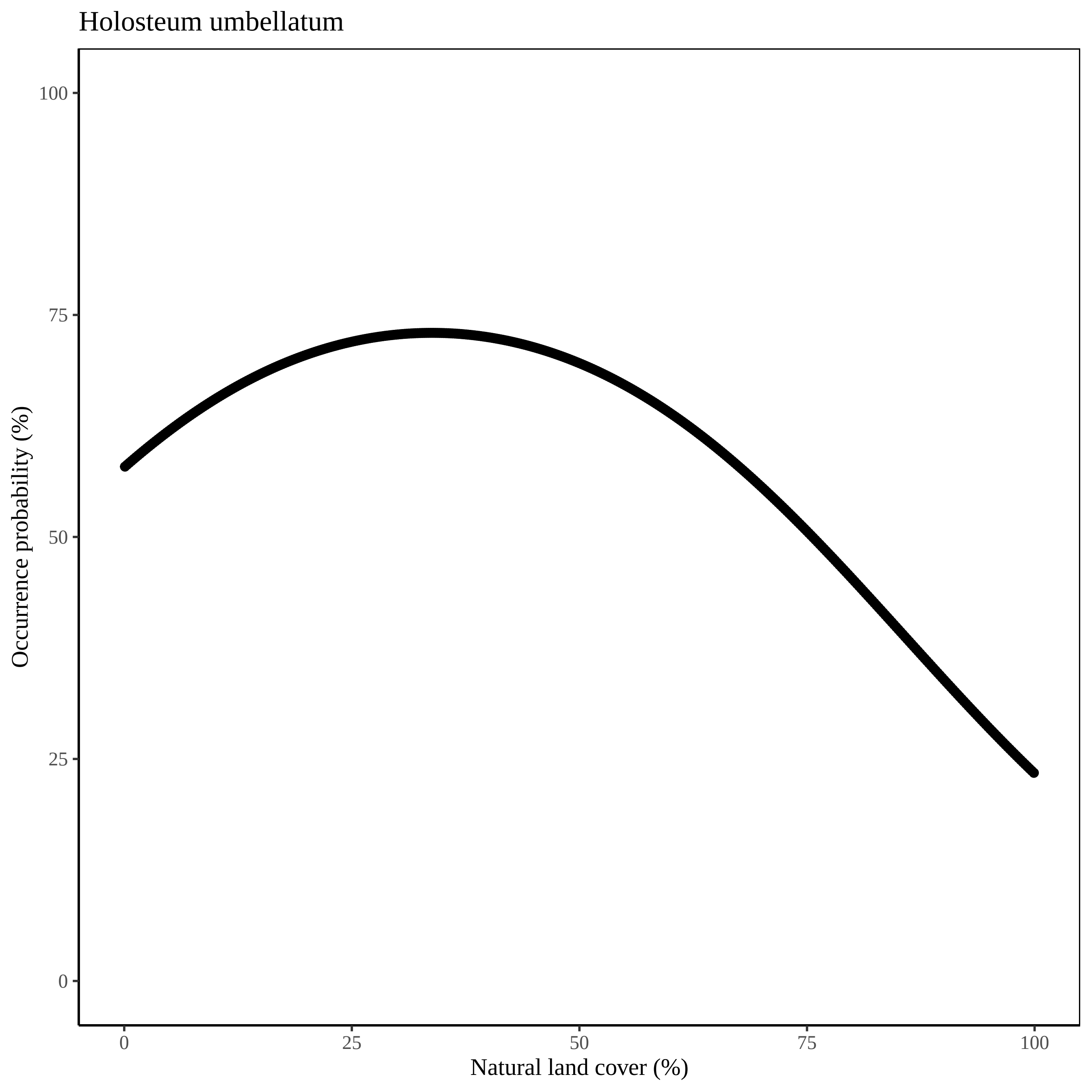
Holosteum umbellatum
Occurrence probability (%)
100
75
50
25
0
0
25
50
75
100
Natural land cover (%)

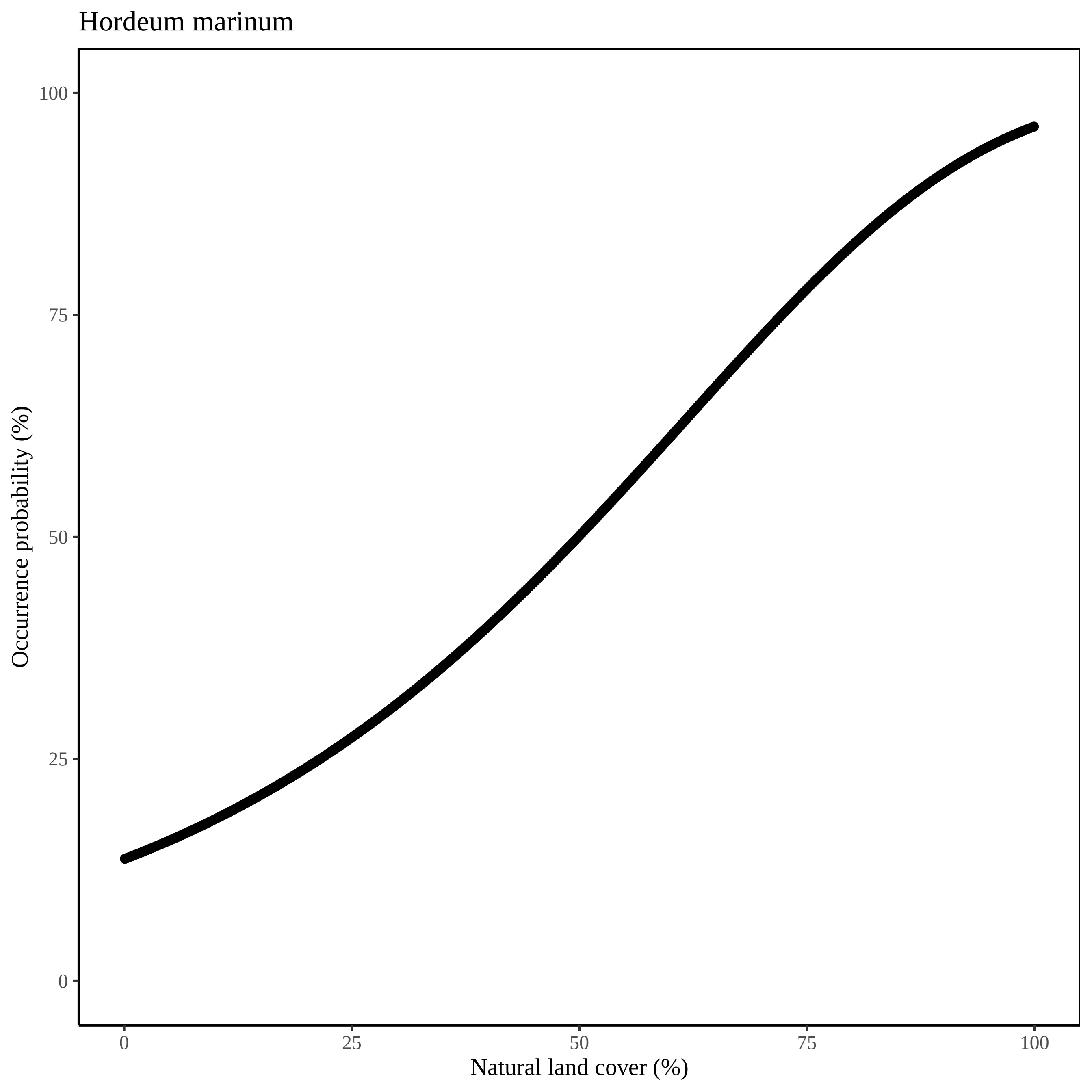
Hordeum marinum
Occurrence probability (%)
100
75
50
25
0
0
25
50
75
100
Natural land cover (%)

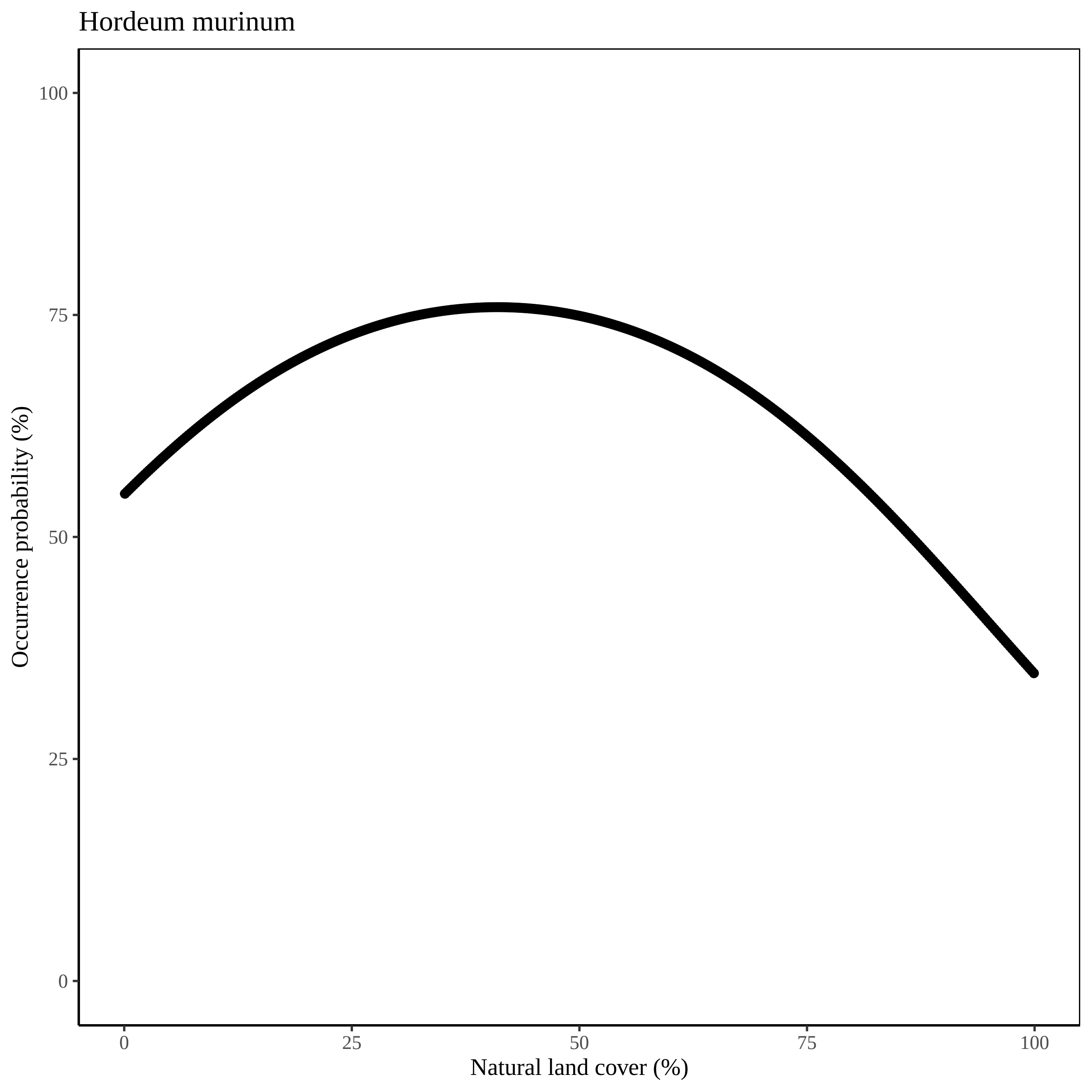
Hordeum murinum
Occurrence probability (%)
100
75
50
25
0
0
25
50
75
100
Natural land cover (%)

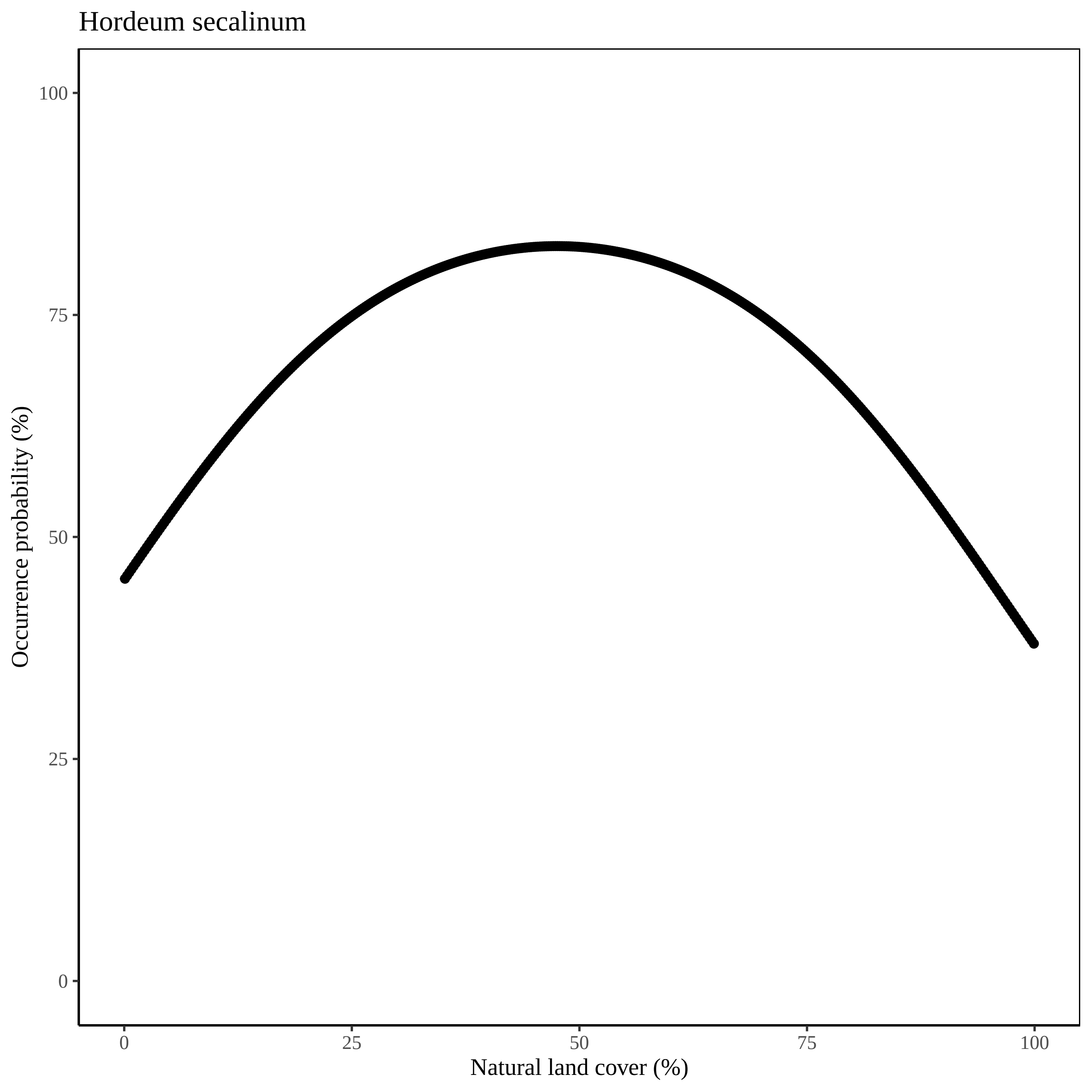
Hordeum secalinum
Occurrence probability (%)
100
75
50
25
0
0
25
50
75
100
Natural land cover (%)

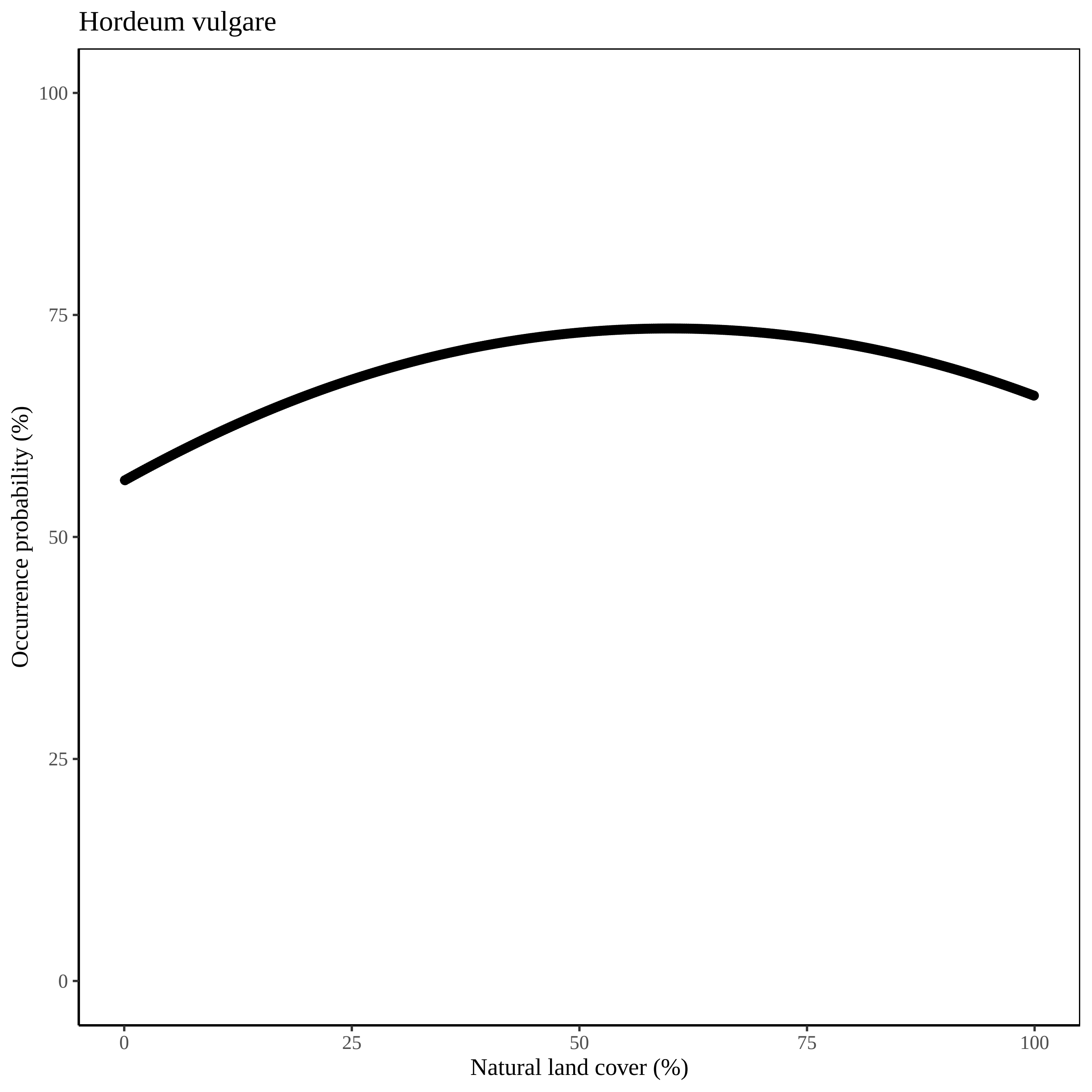

Hordeum vulgare
Occurrence probability (%)
100
75
50
25
0
0
25
50
75
100
Natural land cover (%)

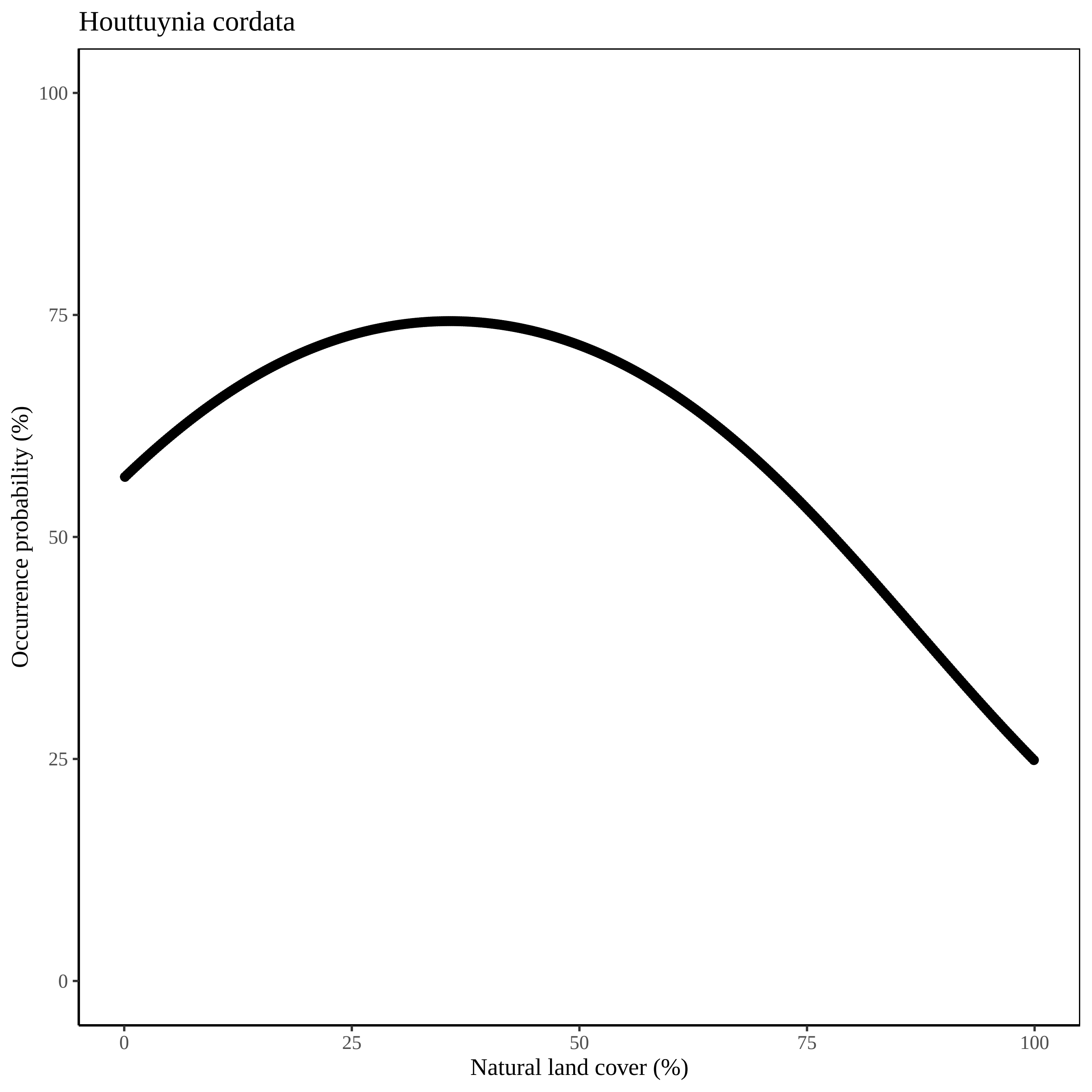

Houttuynia cordata
Occurrence probability (%)
100
75
50
25
0
0
25
50
75
100
Natural land cover (%)

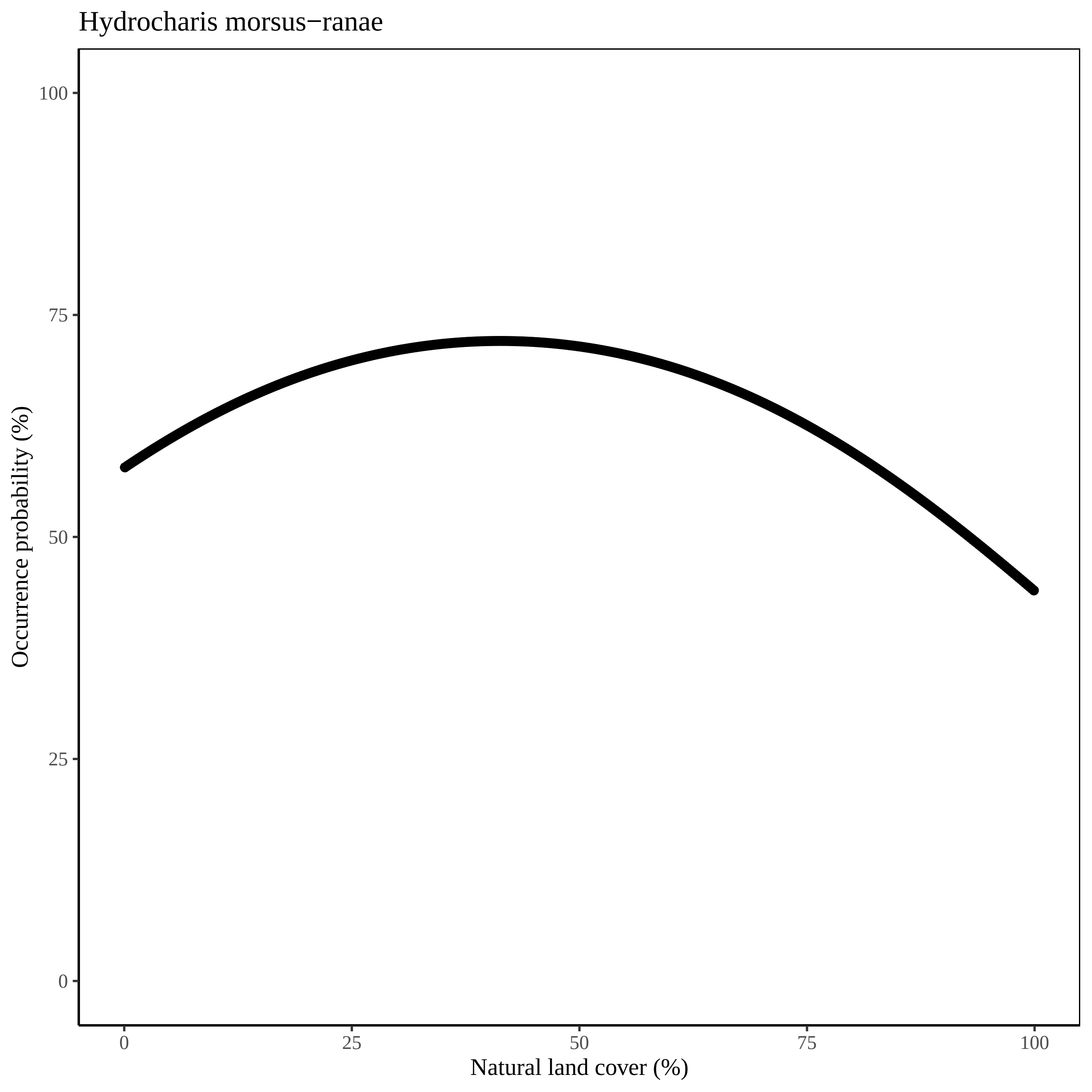
Hydrocharis morsus−ranae
Occurrence probability (%)
100
75
50
25
0
0
25
50
75
100
Natural land cover (%)

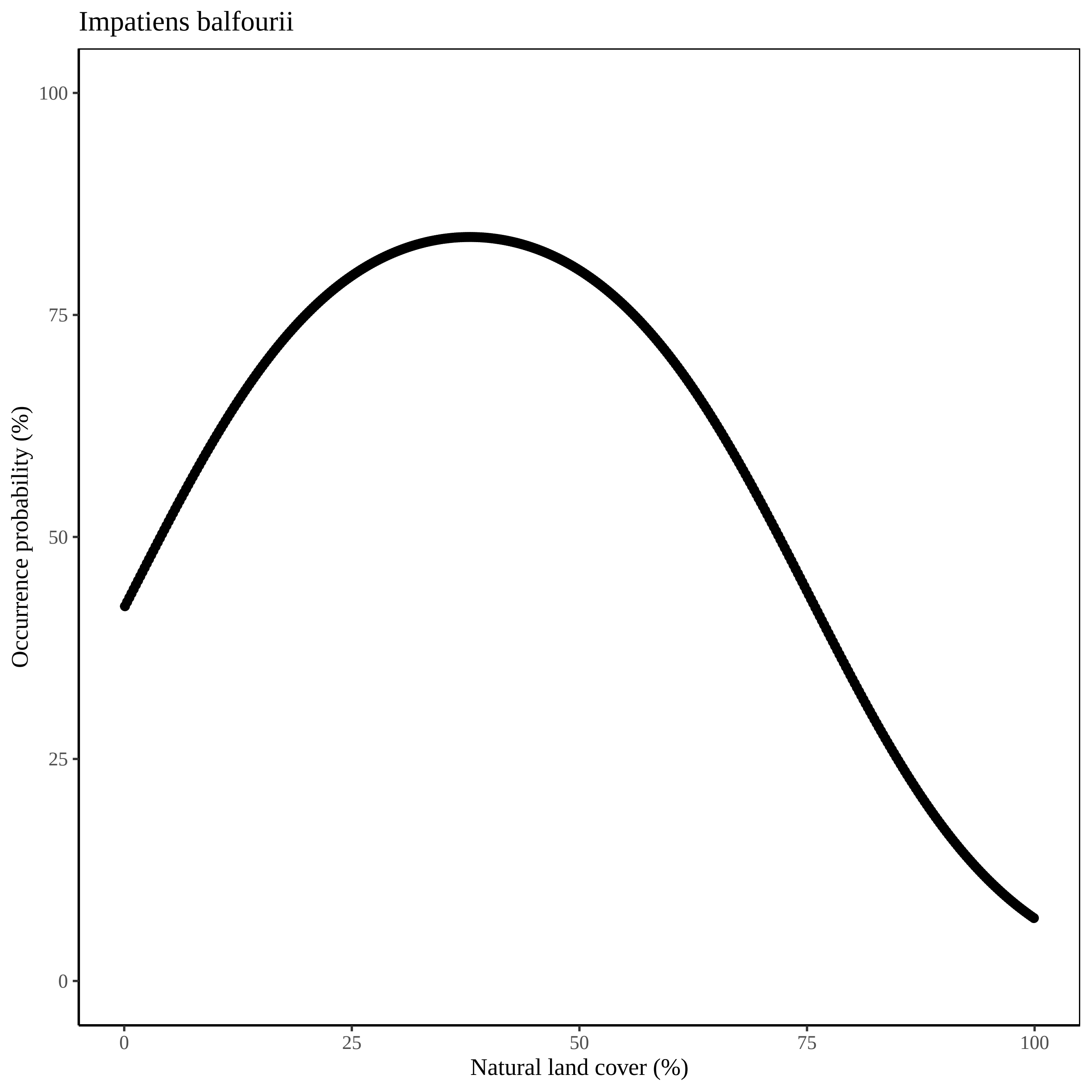
Impatiens balfourii
Occurrence probability (%)
100
75
50
25
0
0
25
50
75
100
Natural land cover (%)

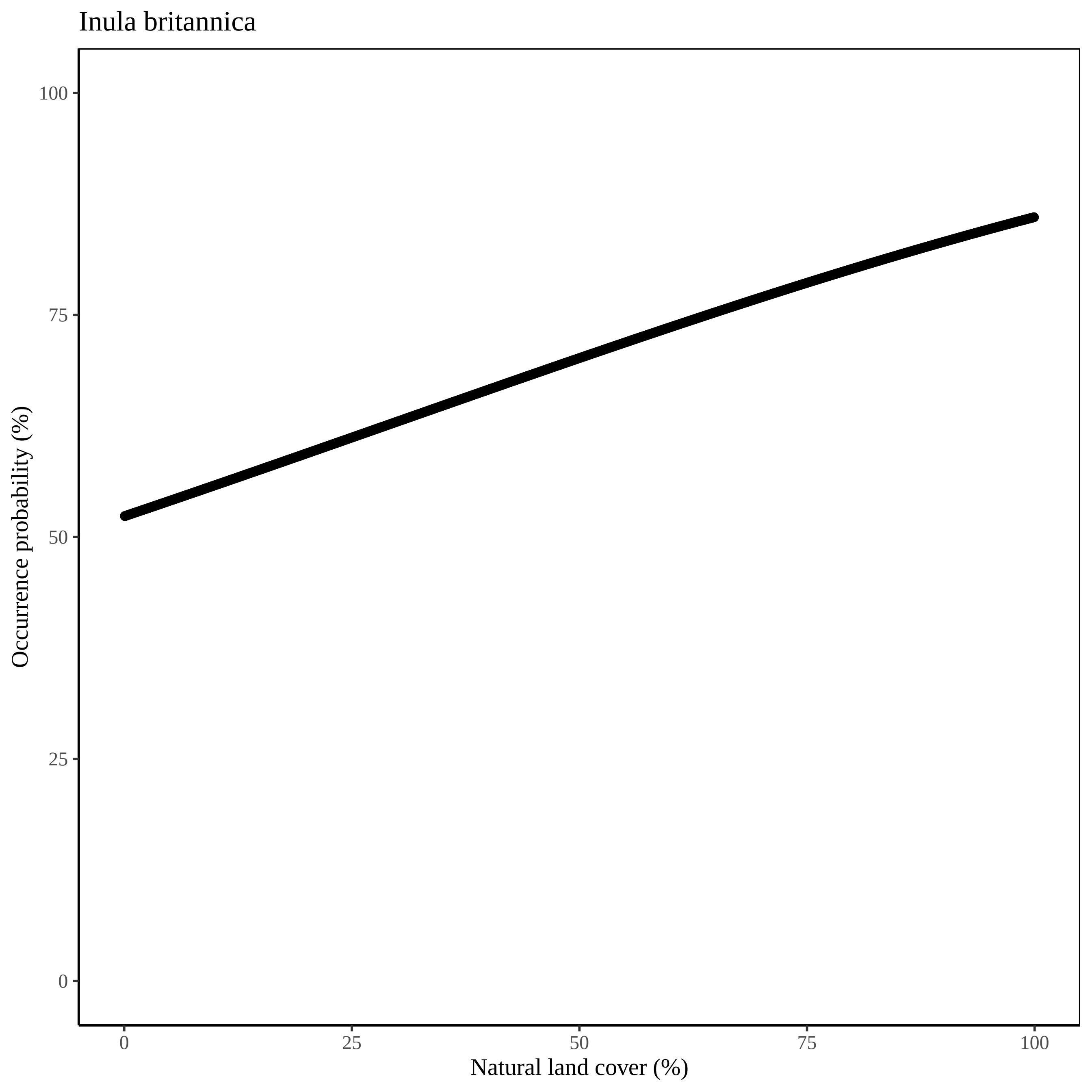

Inula britannica
Occurrence probability (%)
100
75
50
25
0
0
25
50
75
100
Natural land cover (%)

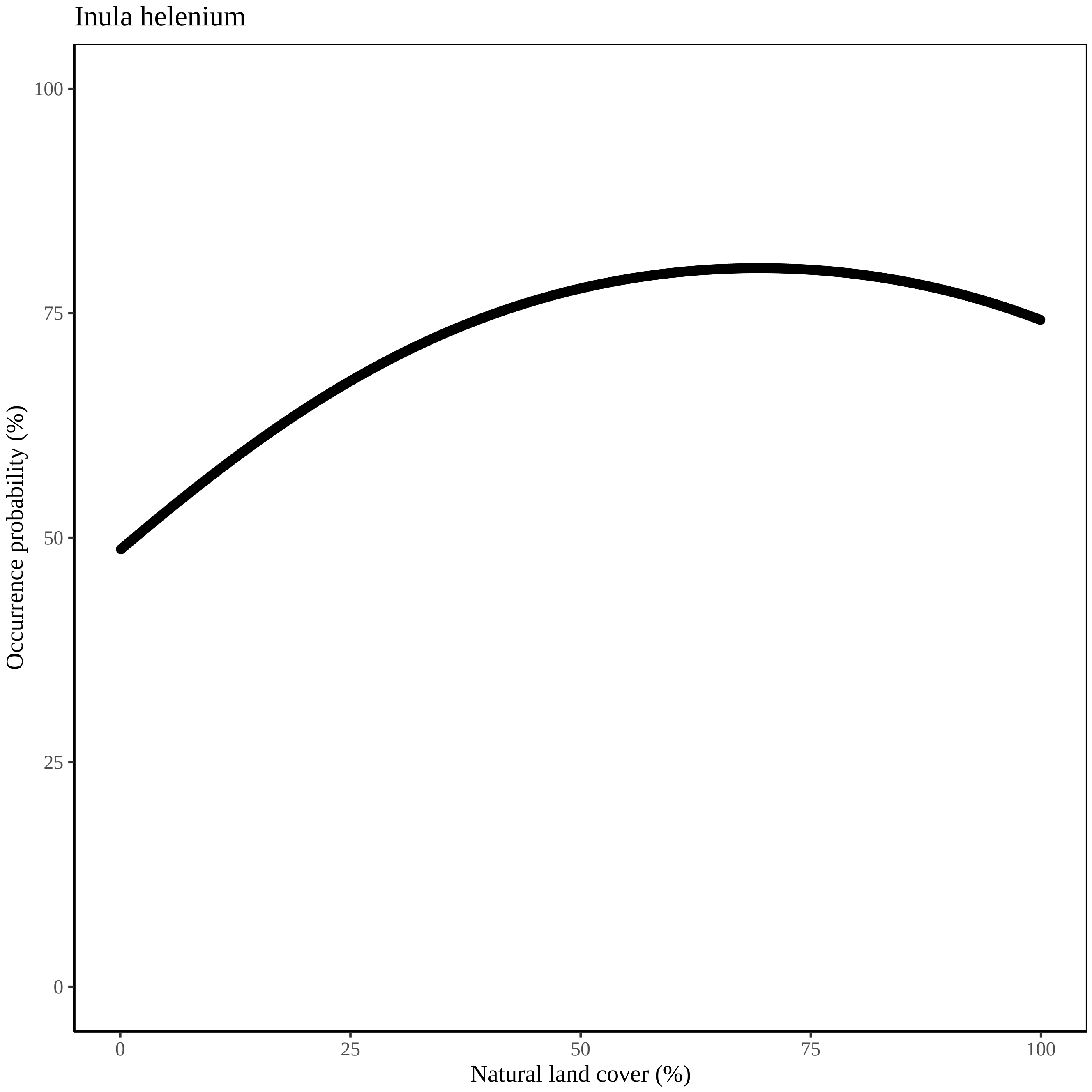

Inula helenium
Occurrence probability (%)
100
75
50
25
0
0
25
50
75
100
Natural land cover (%)

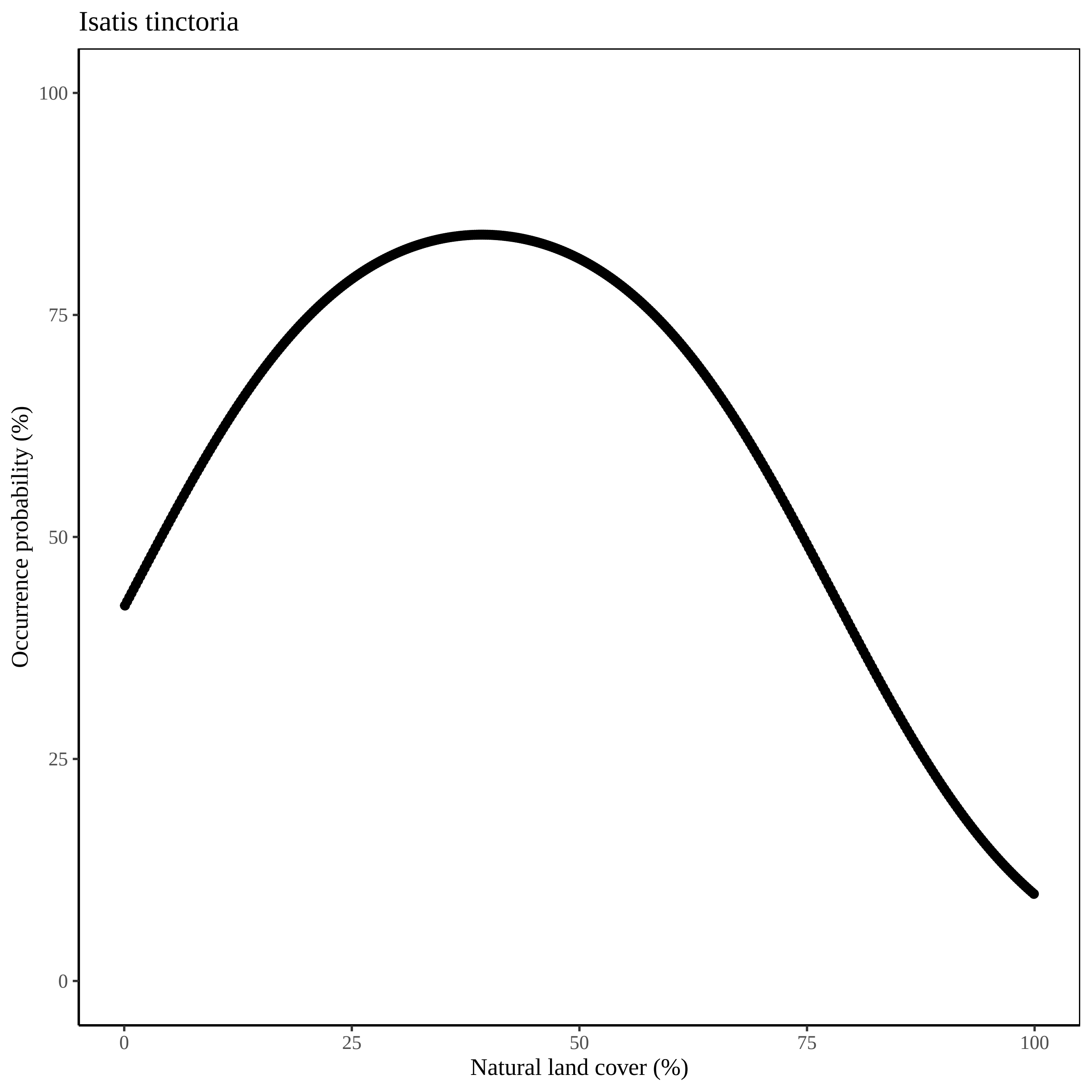

Isatis tinctoria
100
75
50
25
0
Occurrence probability (%)
0
25
50
75
100
Natural land cover (%)

Jacobaea erucifolia

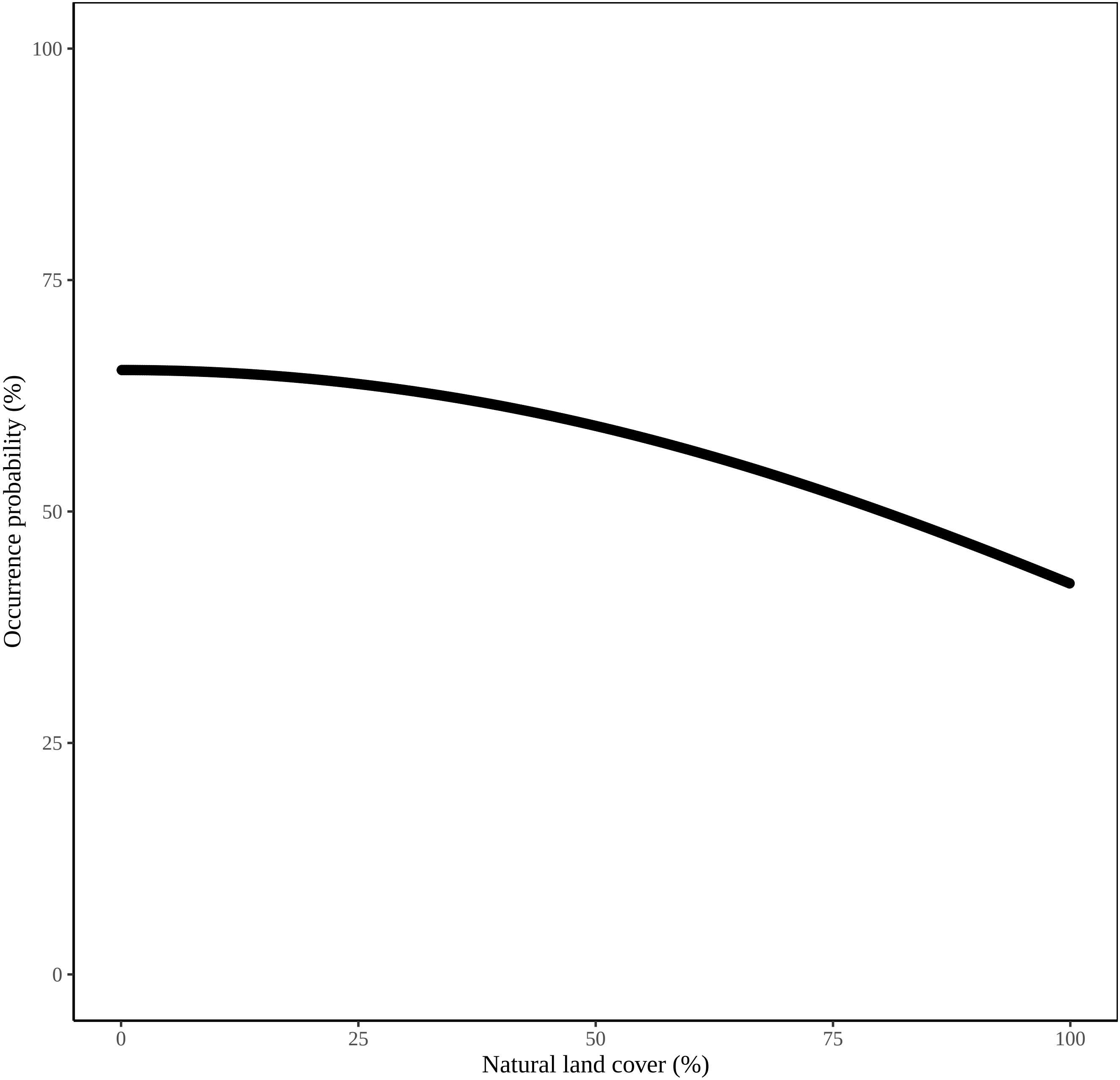

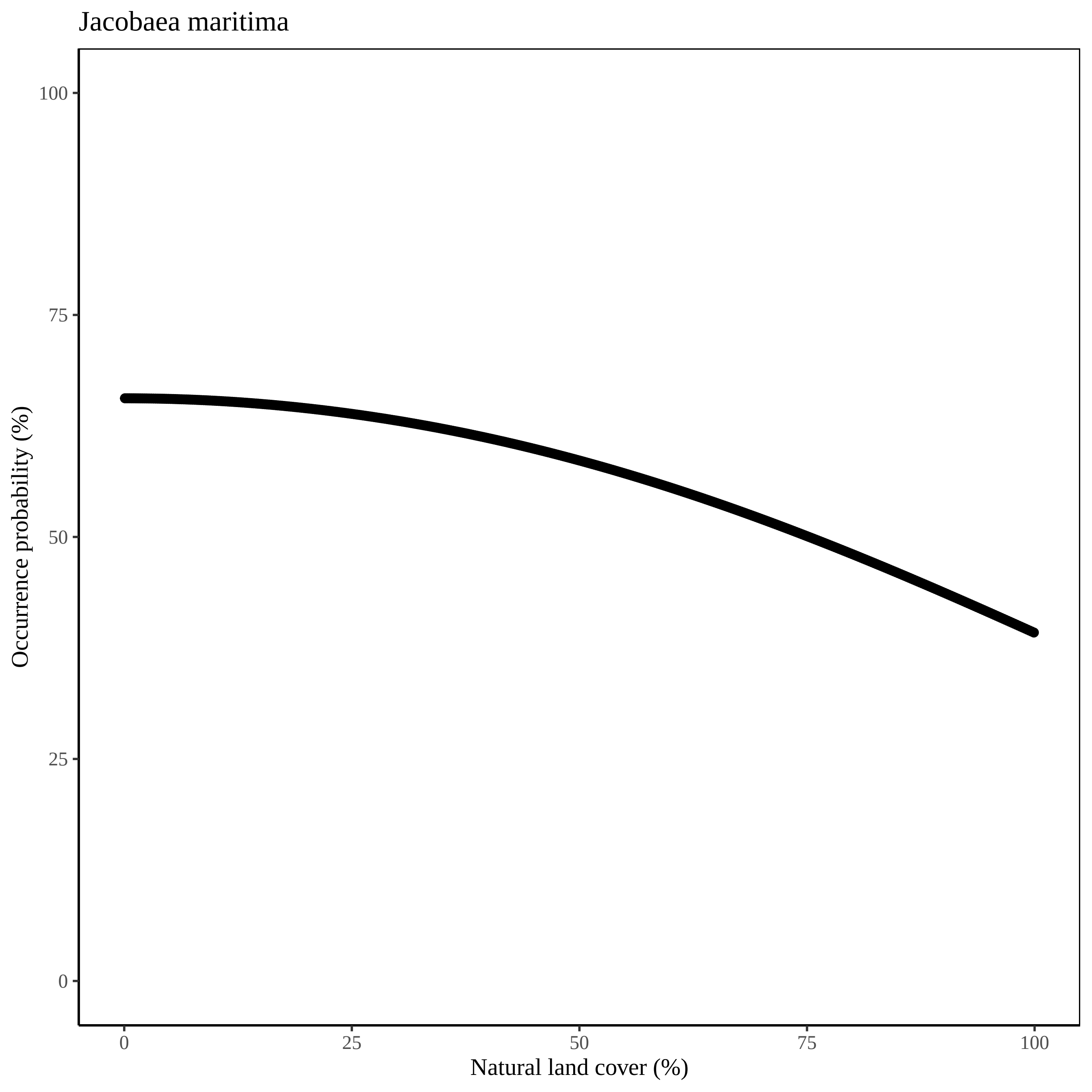
Jacobaea maritima
Occurrence probability (%)
100
75
50
25
0
0
25
50
75
100
Natural land cover (%)

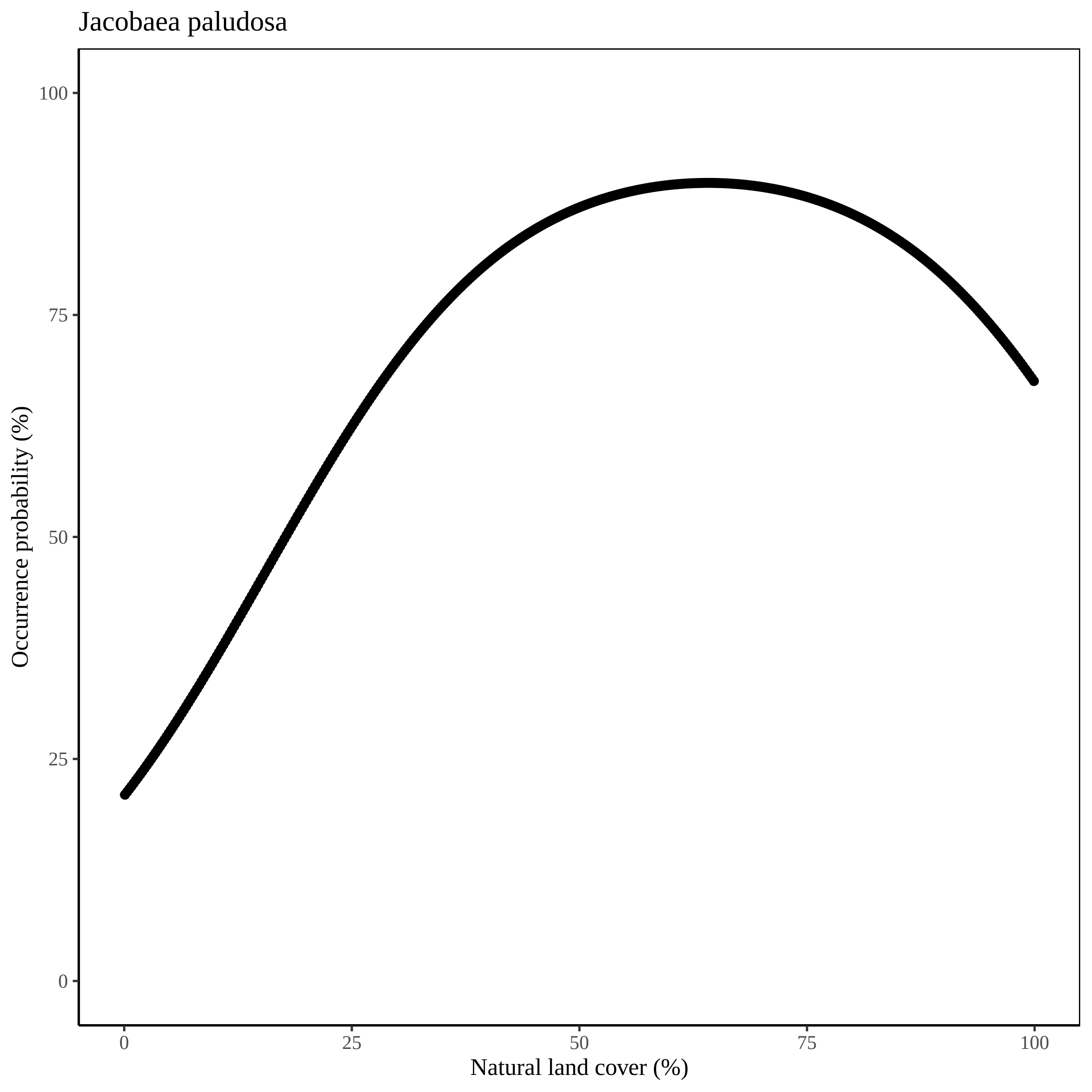
Jacobaea paludosa
Occurrence probability (%)
100
75
50
25
0
0
25
50
75
100
Natural land cover (%)

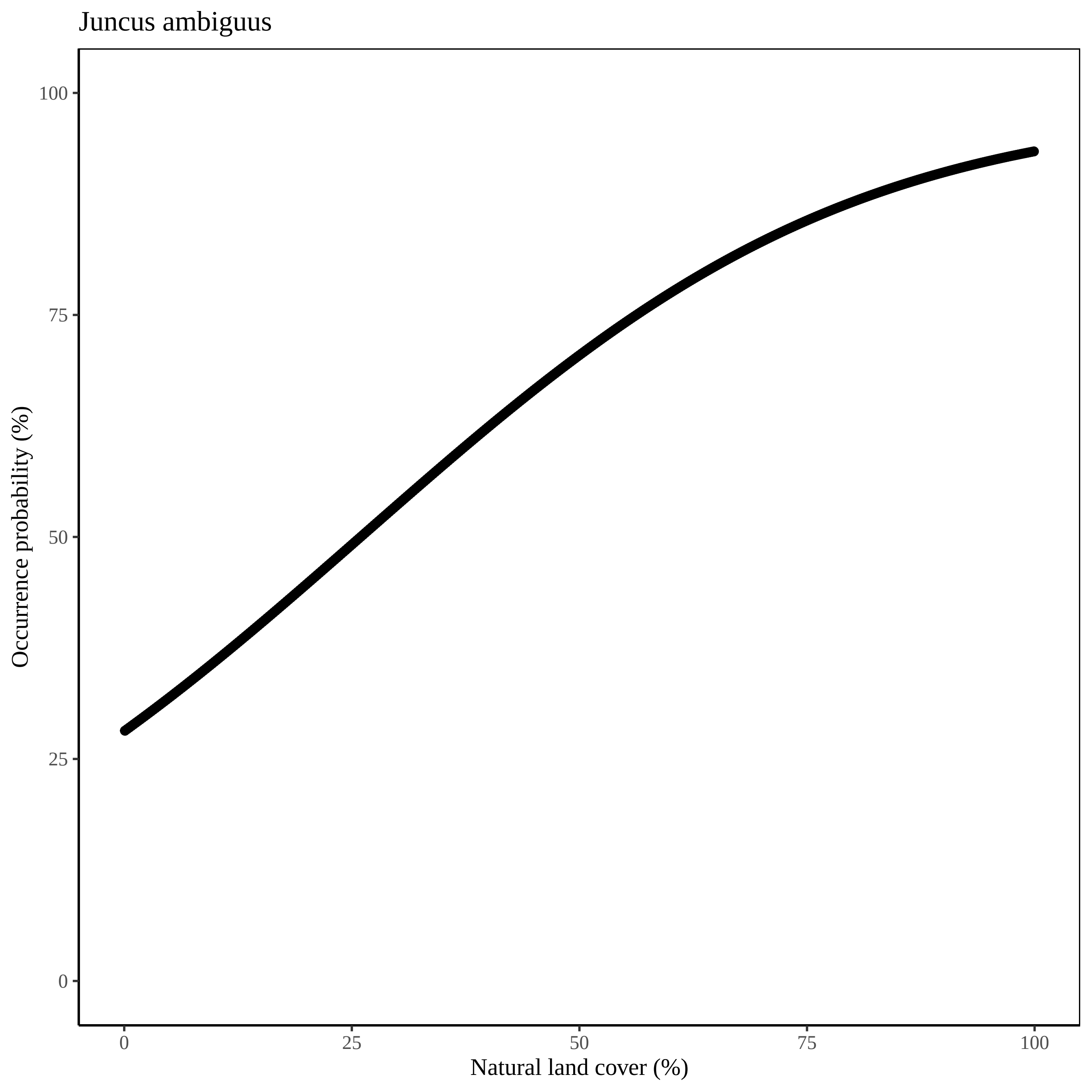

Juncus ambiguus
Occurrence probability (%)
100
75
50
25
0
0
25
50
75
100
Natural land cover (%)

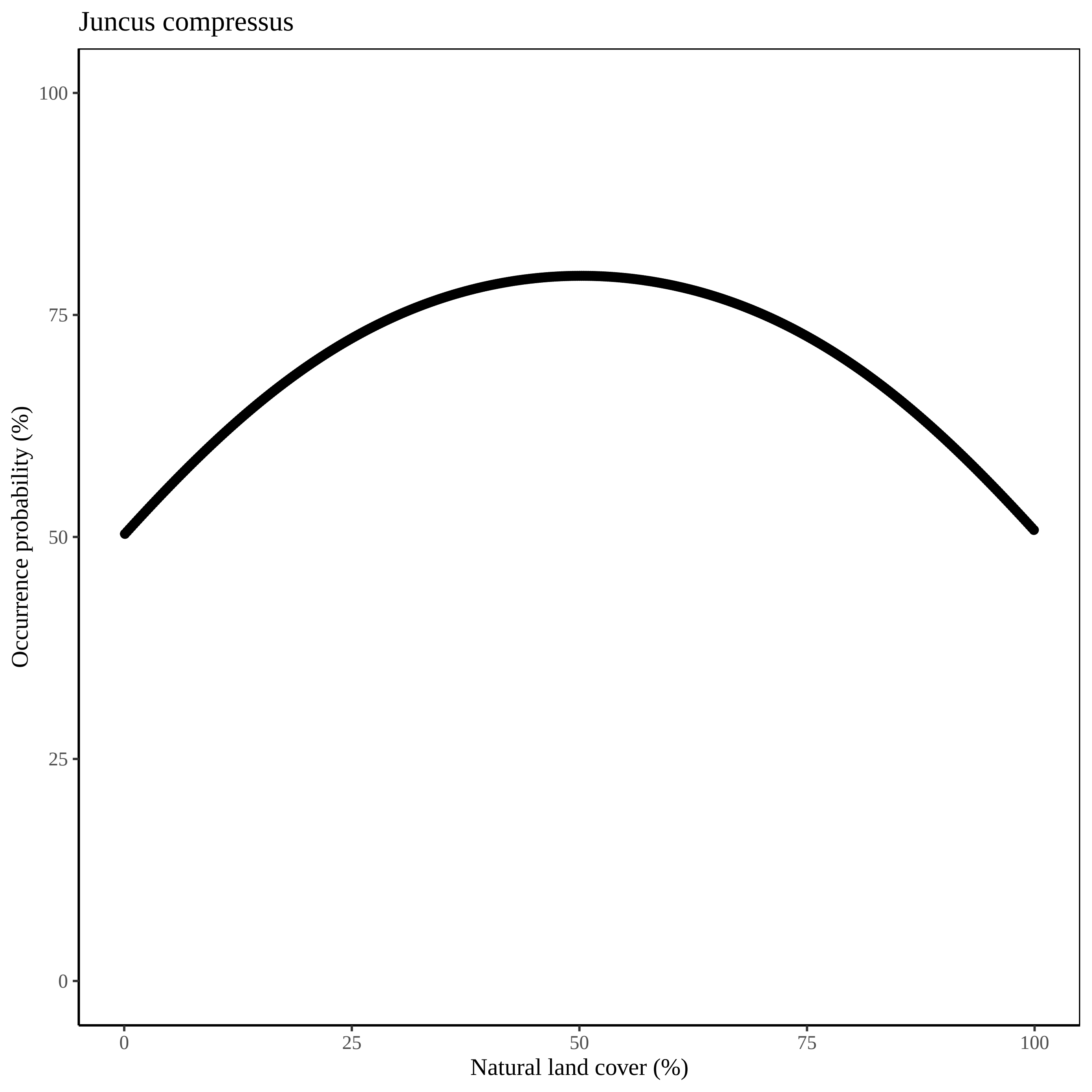

Juncus compressus
100
75
50
25
0
Occurrence probability (%)
0
25
50
75
100
Natural land cover (%)

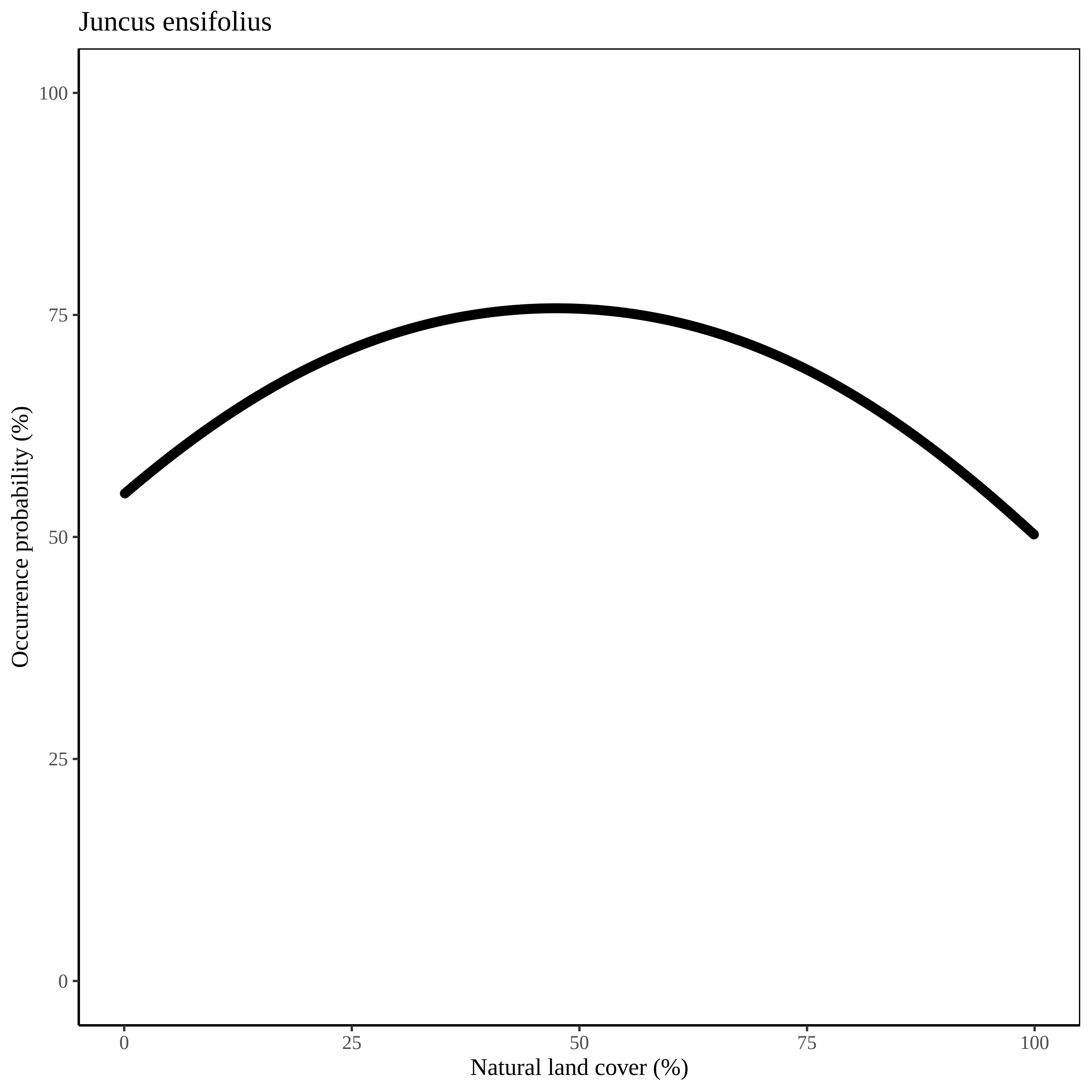
Juncus ensifolius
Occurrence probability (%)
100
75
50
25
0
0
25
50
75
100
Natural land cover (%)

Juncus gerardii

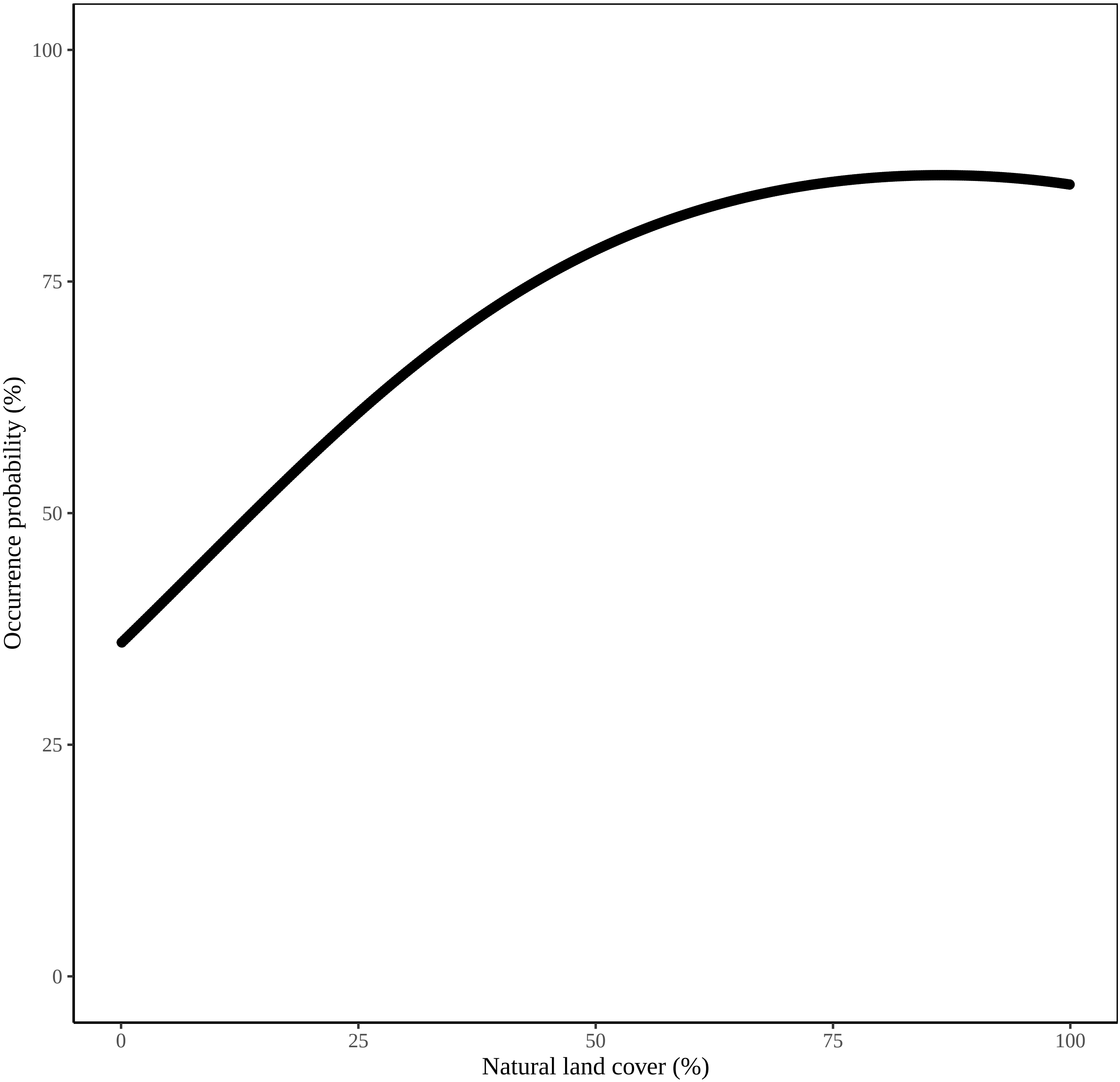

Juncus inflexus

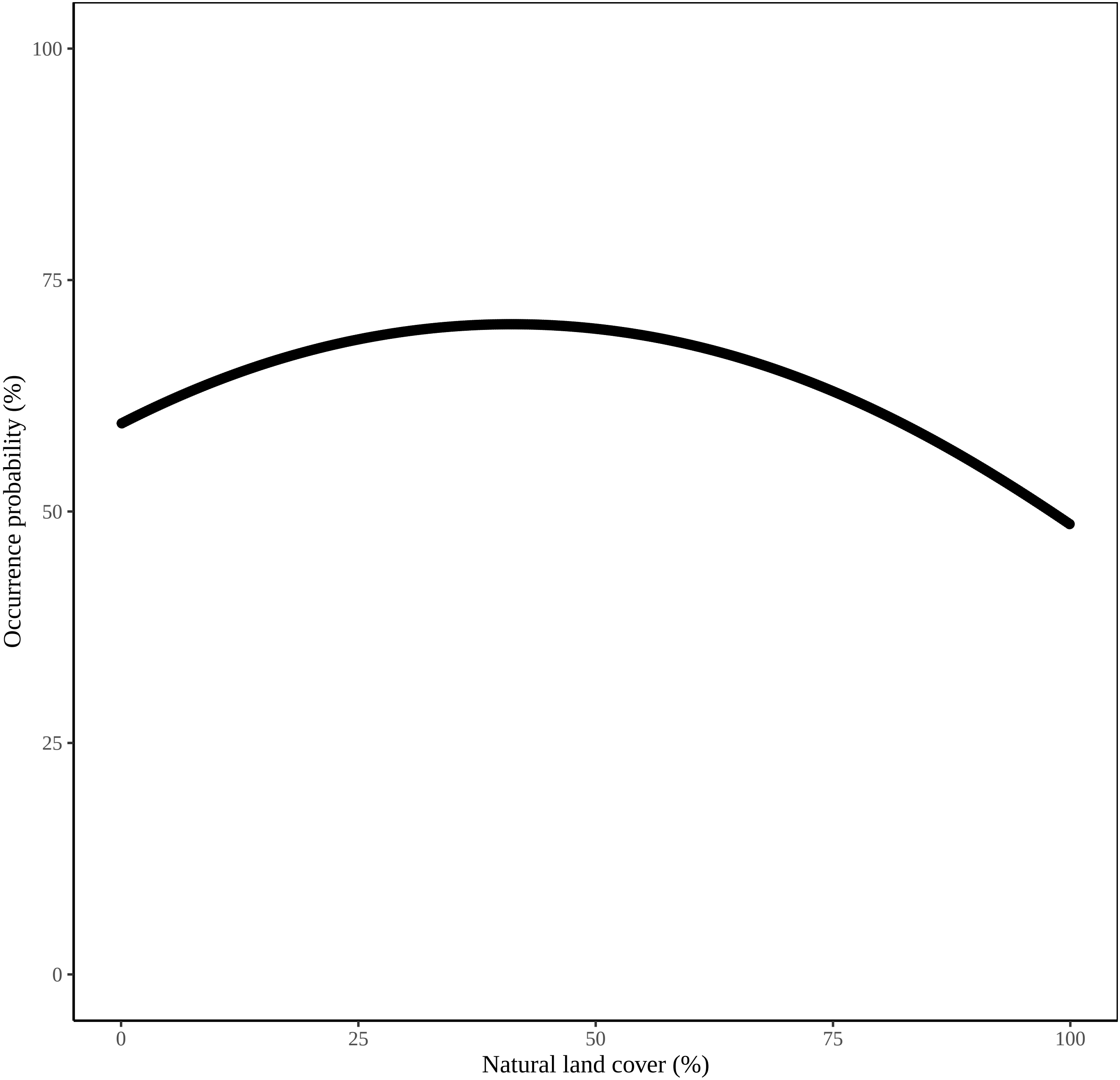

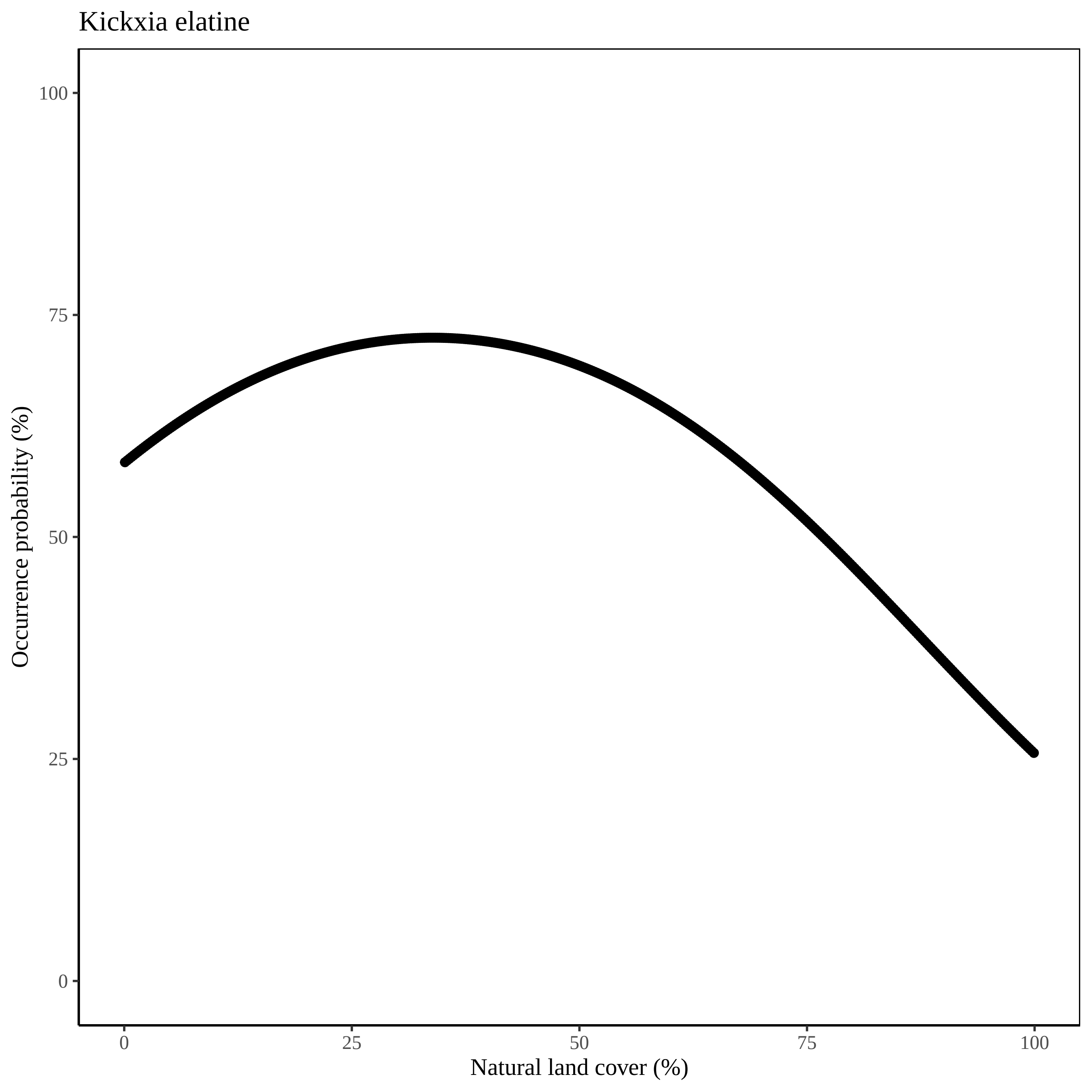

Kickxia elatine
Occurrence probability (%)
100
75
50
25
0
0
25
50
75
100
Natural land cover (%)

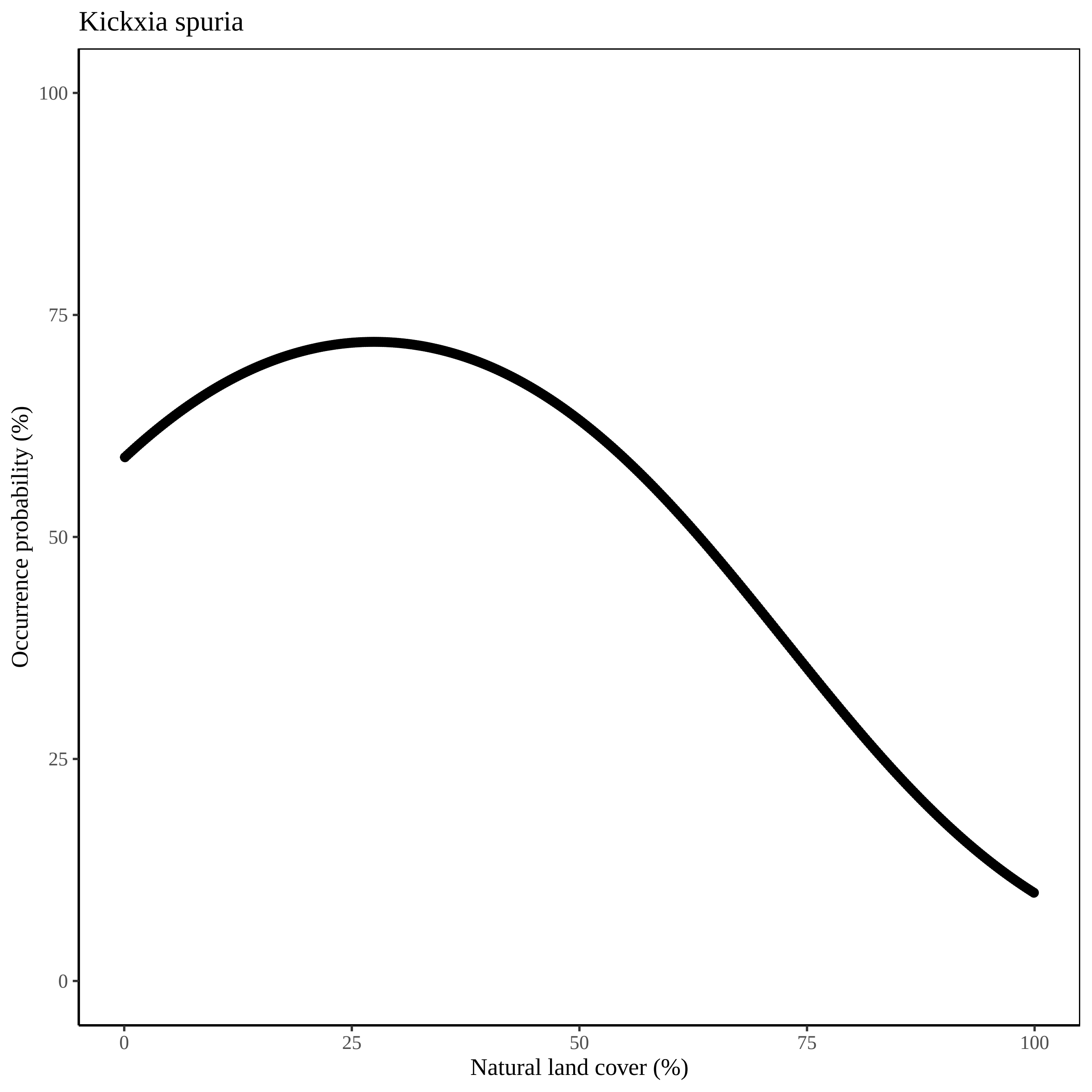
Kickxia spuria
Occurrence probability (%)
100
75
50
25
0
0
25
50
75
100
Natural land cover (%)

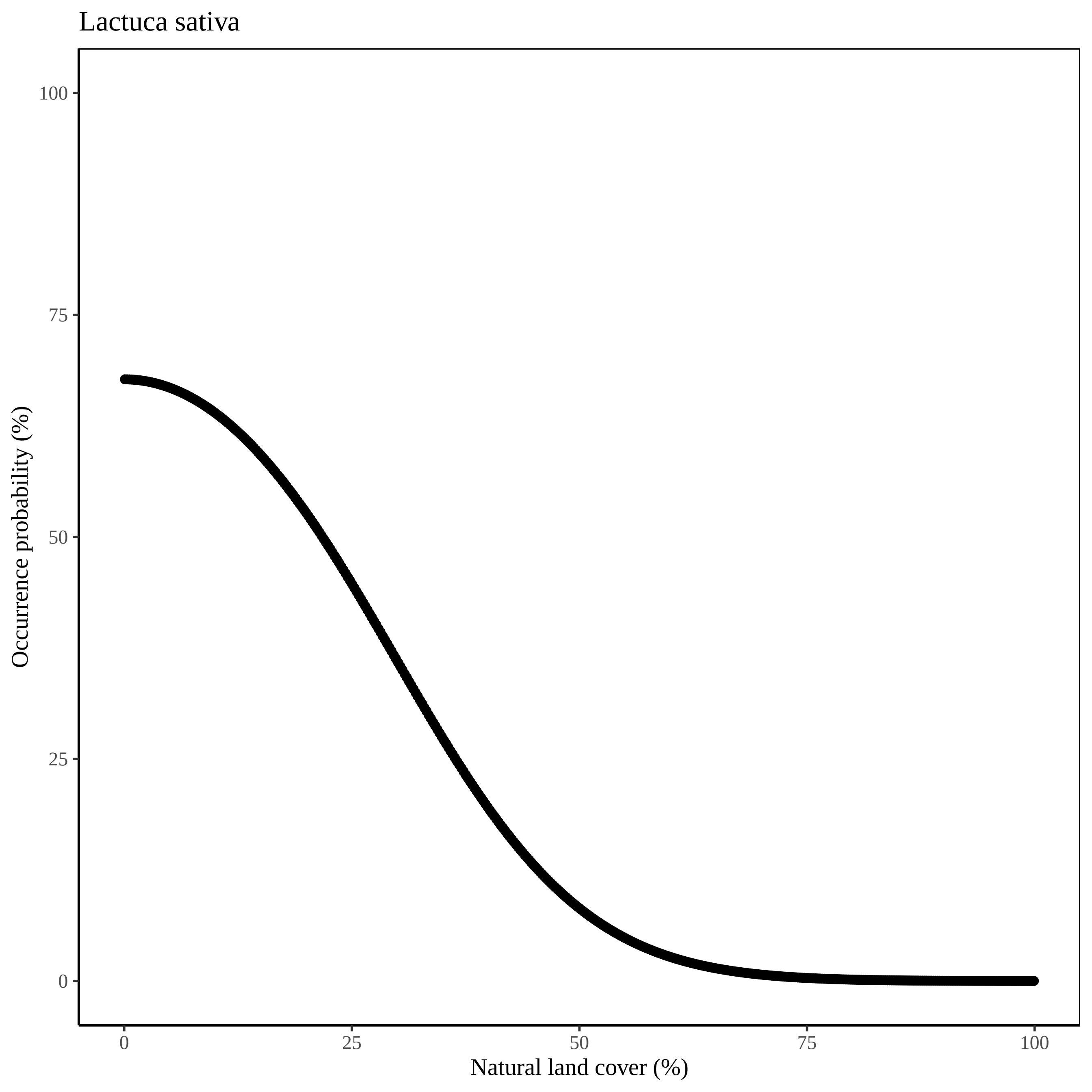
Lactuca sativa
Occurrence probability (%)
100
75
50
25
0
0
25
50
75
100
Natural land cover (%)

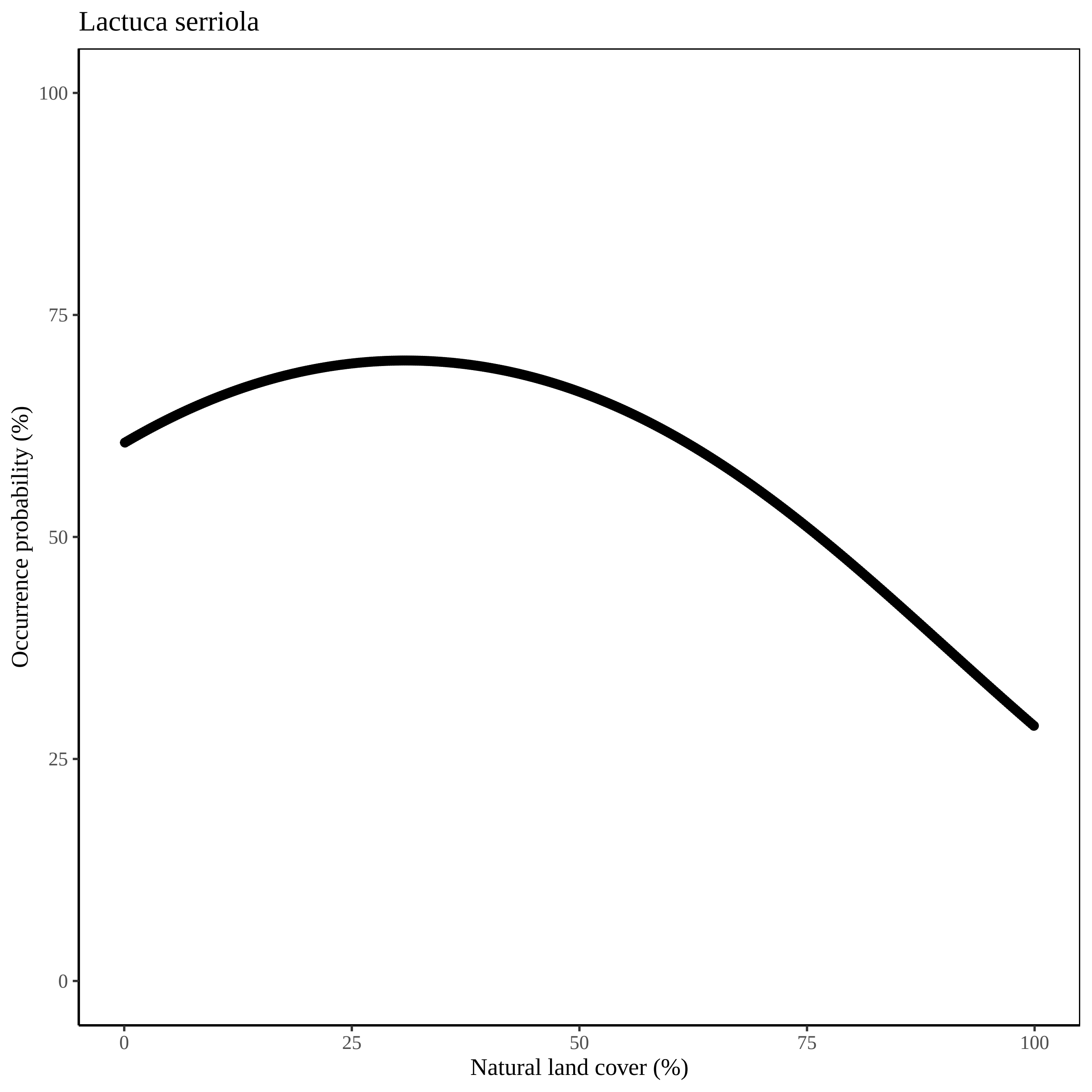

Lactuca serriola
Occurrence probability (%)
100
75
50
25
0
0
25
50
75
100
Natural land cover (%)

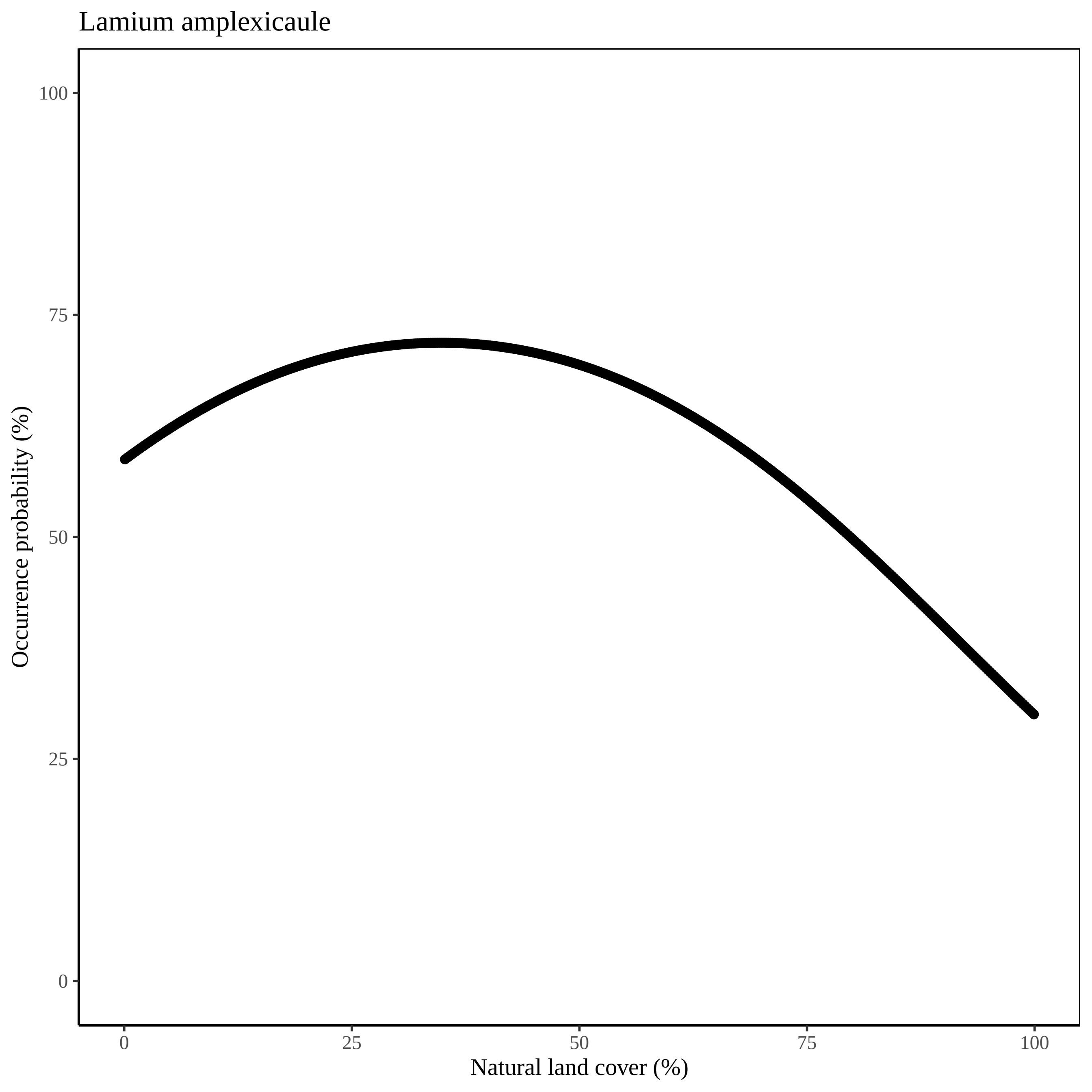

Lamium amplexicaule
Occurrence probability (%)
100
75
50
25
0
0
25
50
75
100
Natural land cover (%)

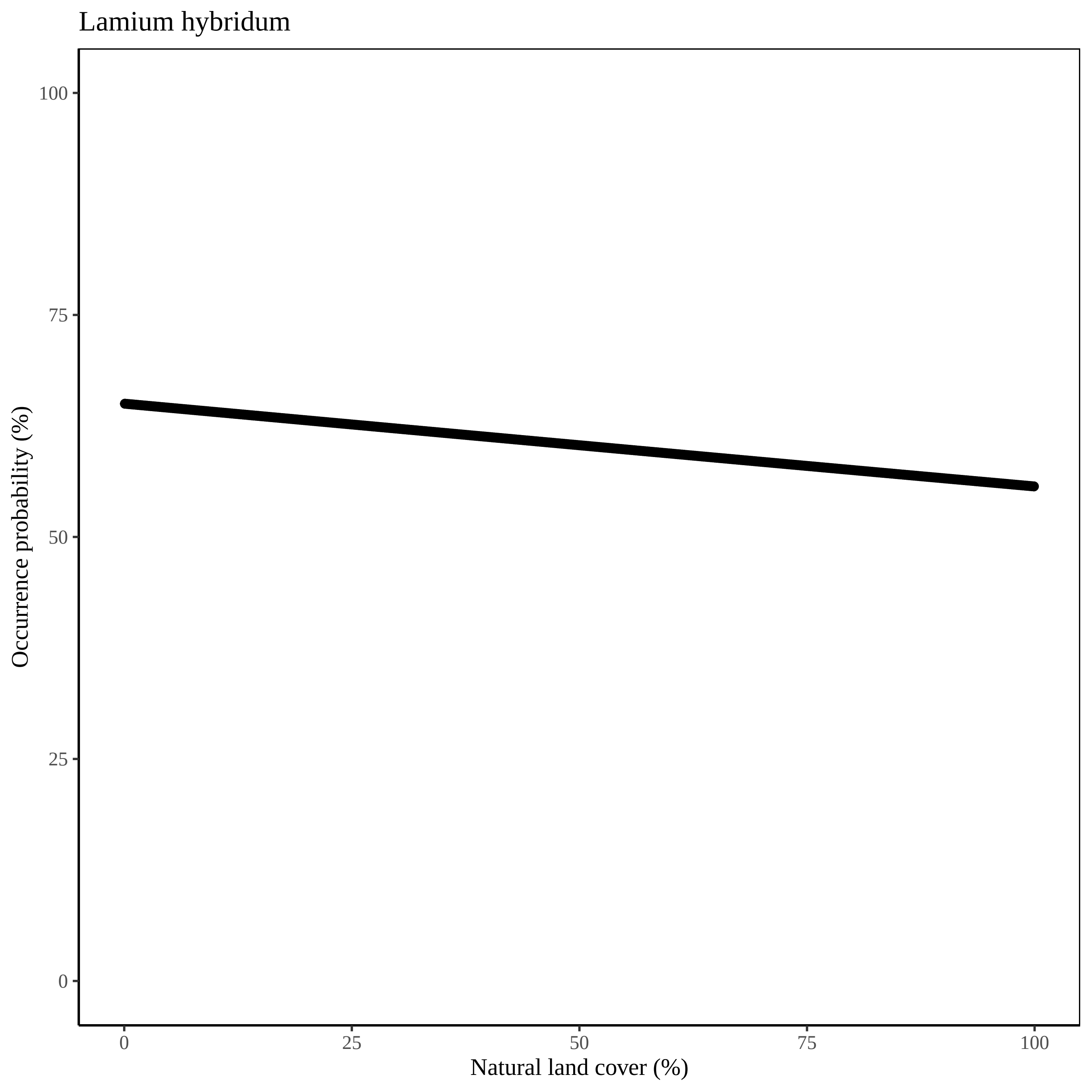
Lamium hybridum
Occurrence probability (%)
100
75
50
25
0
0
25
50
75
100
Natural land cover (%)

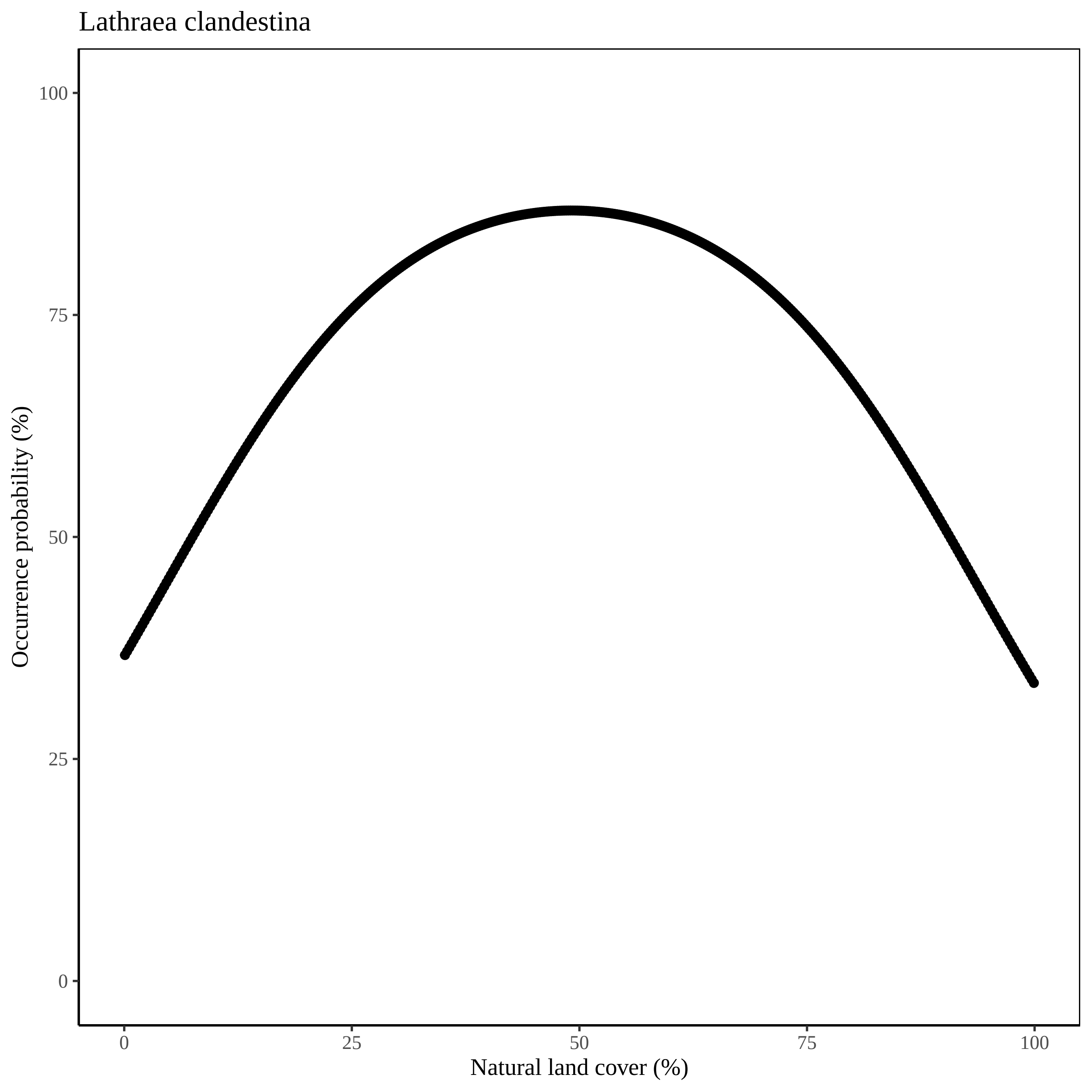
Lathraea clandestina
100
75
50
25
0
Occurrence probability (%)
0
25
50
75
100
Natural land cover (%)

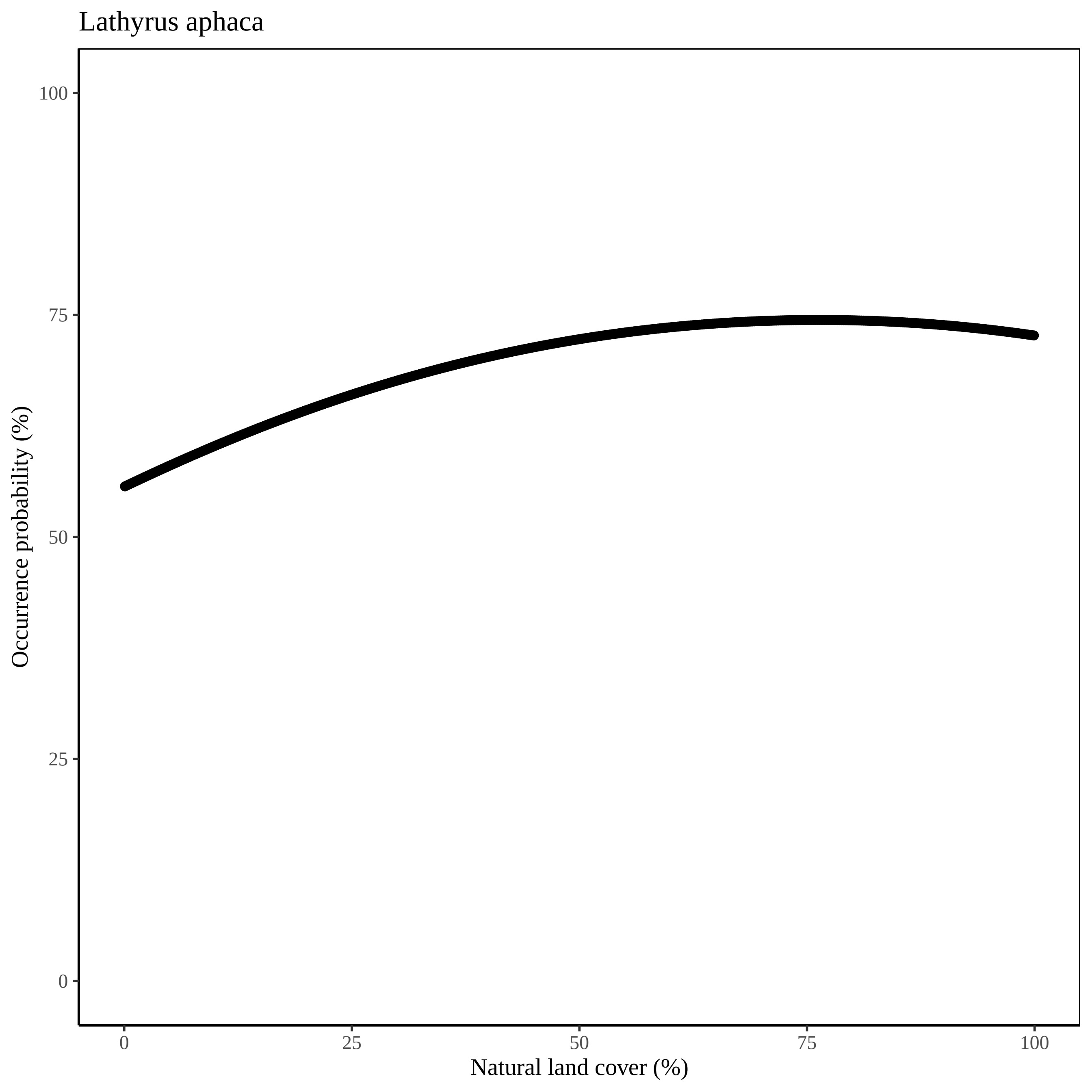
Lathyrus aphaca
Occurrence probability (%)
100
75
50
25
0
0
25
50
75
100
Natural land cover (%)

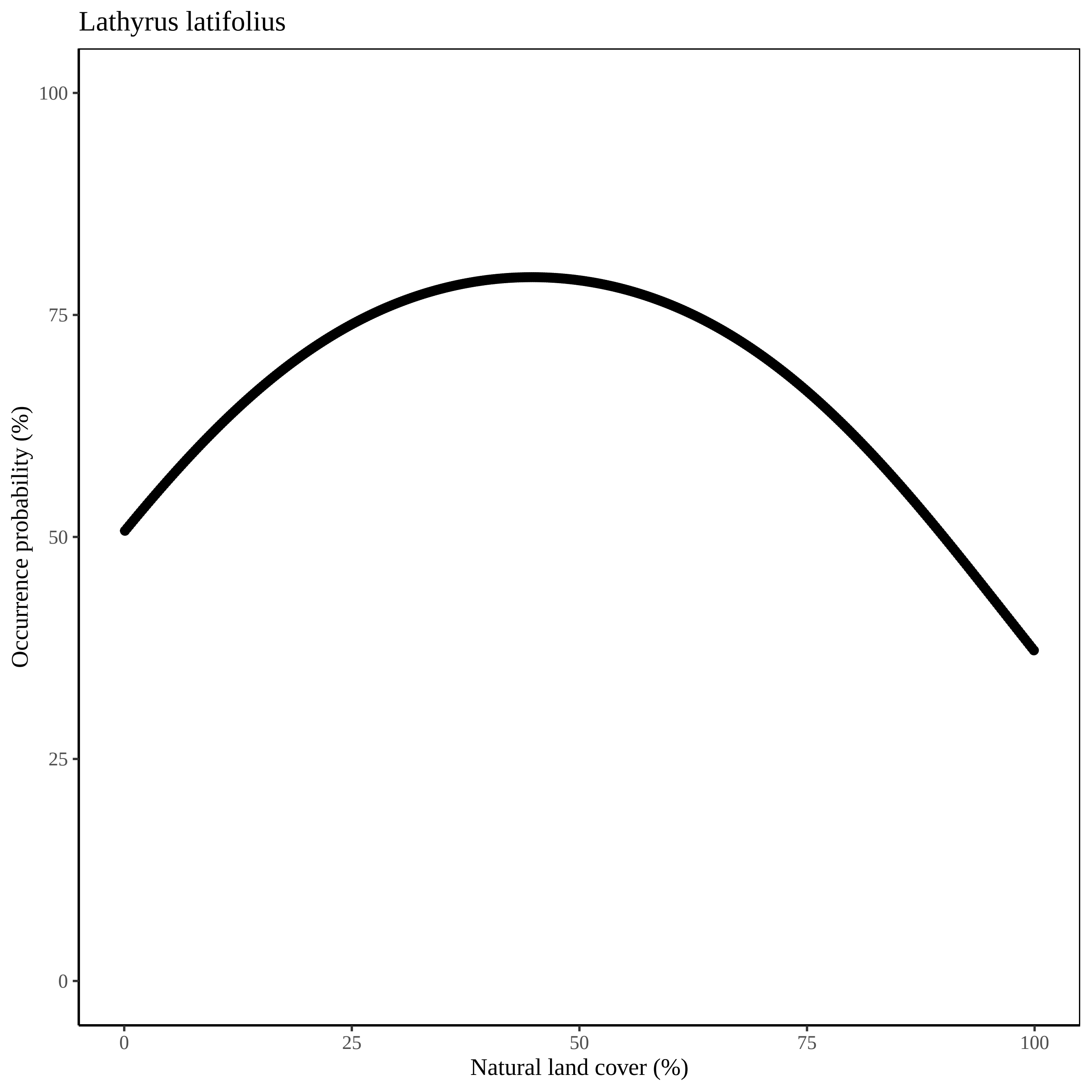

Lathyrus latifolius
Occurrence probability (%)
100
75
50
25
0
0
25
50
75
100
Natural land cover (%)

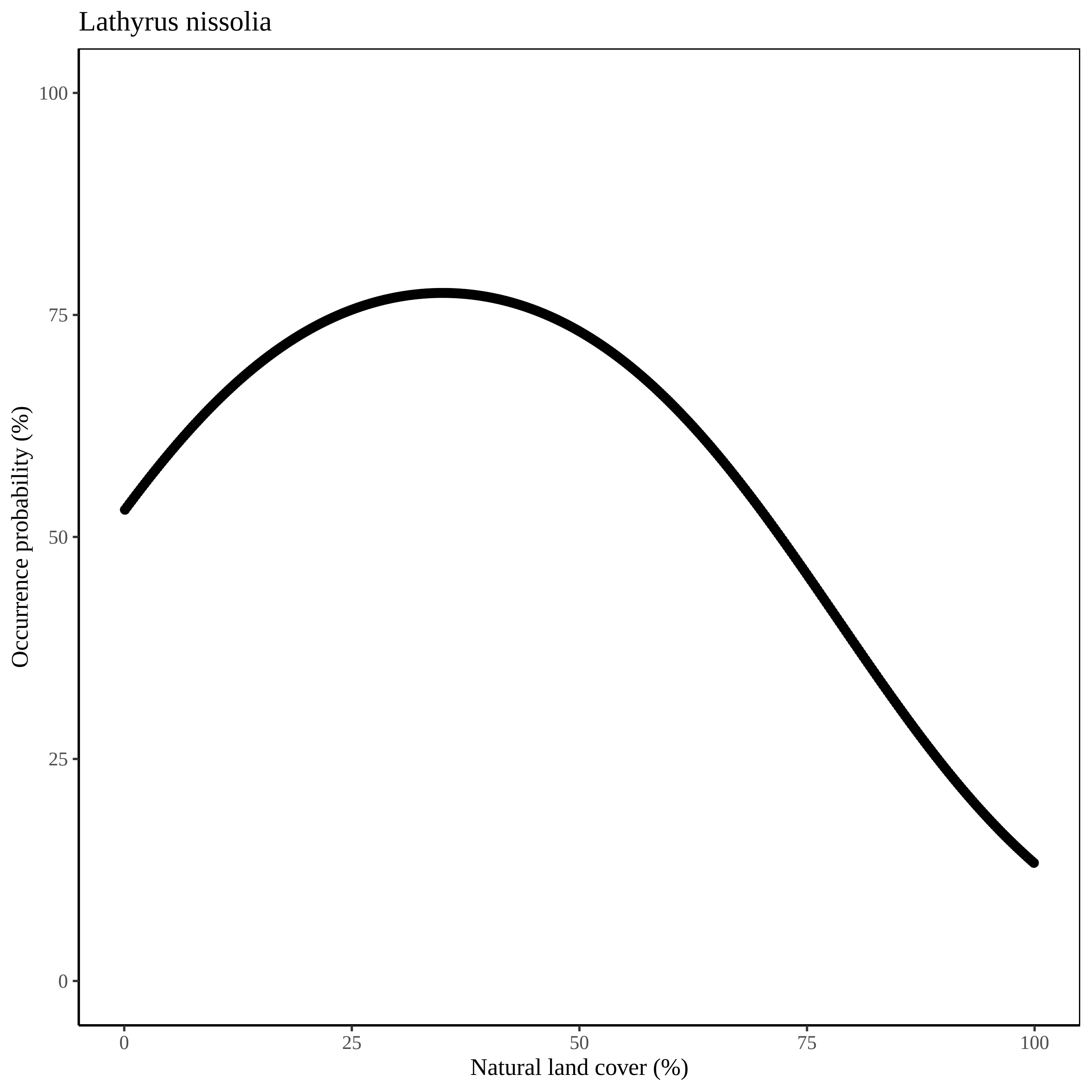
Lathyrus nissolia
Occurrence probability (%)
100
75
50
25
0
0
25
50
75
100
Natural land cover (%)

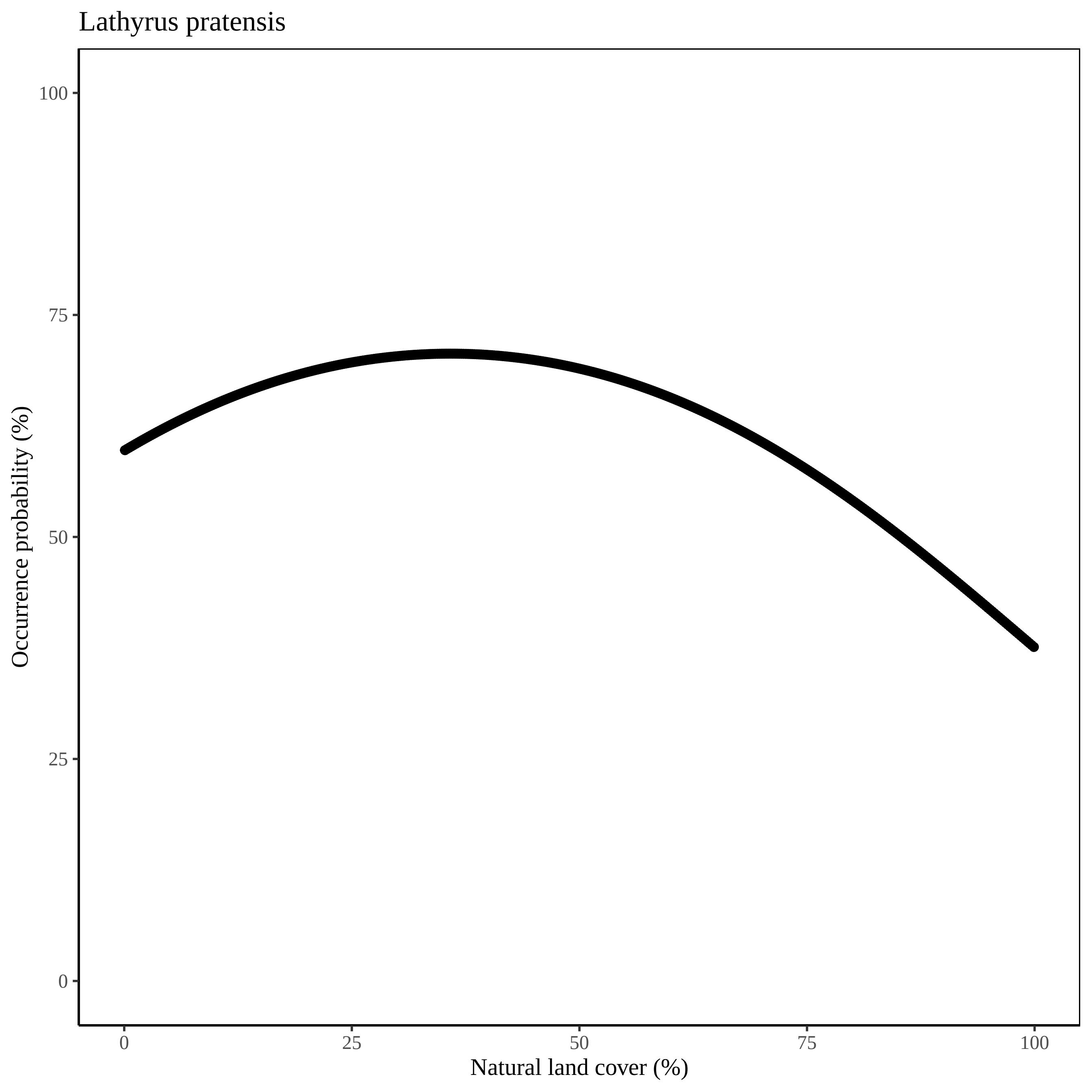

Lathyrus pratensis
100
75
50
25
0
Occurrence probability (%)
0
25
50
75
100
Natural land cover (%)

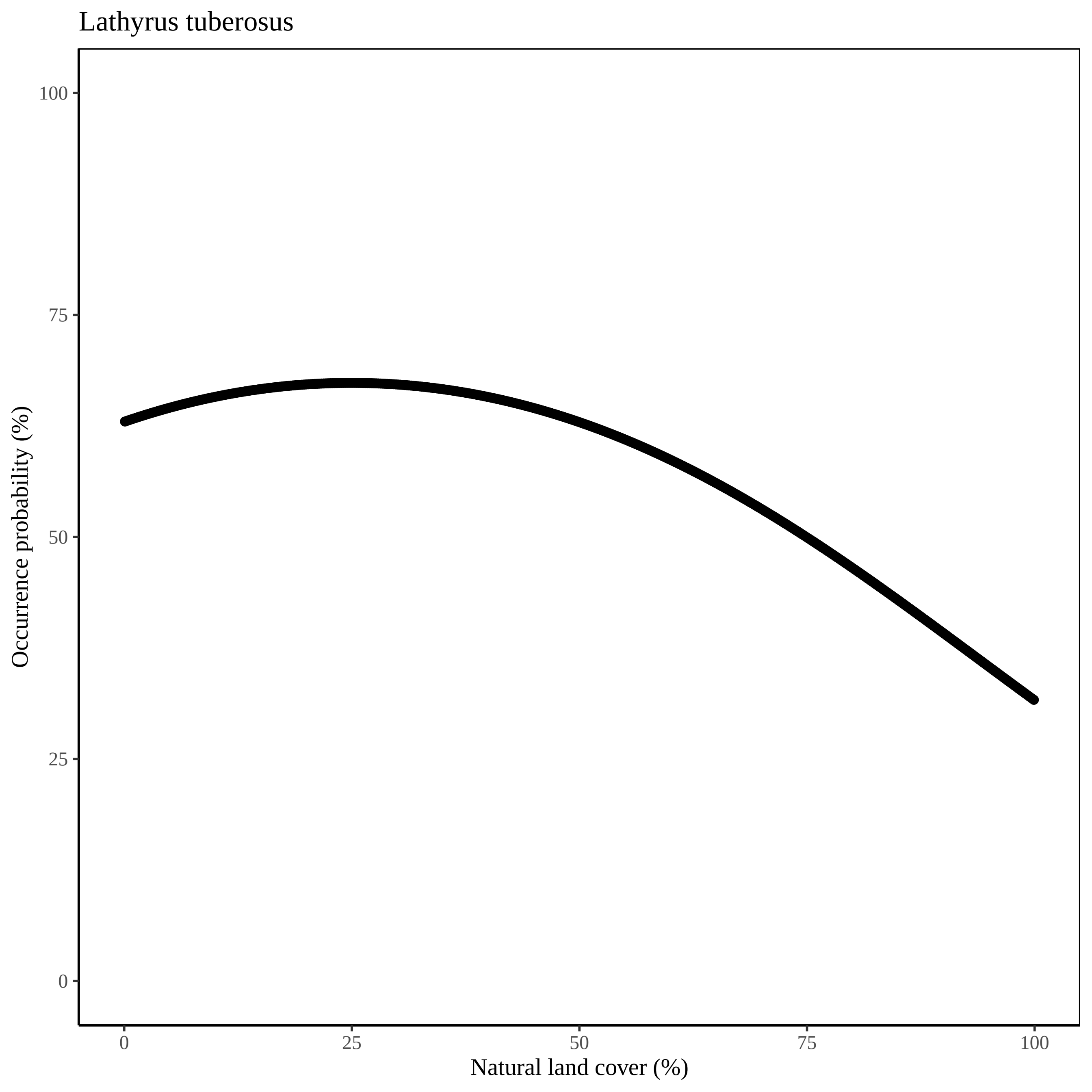

Lathyrus tuberosus
Occurrence probability (%)
100
75
50
25
0
0
25
50
75
100
Natural land cover (%)

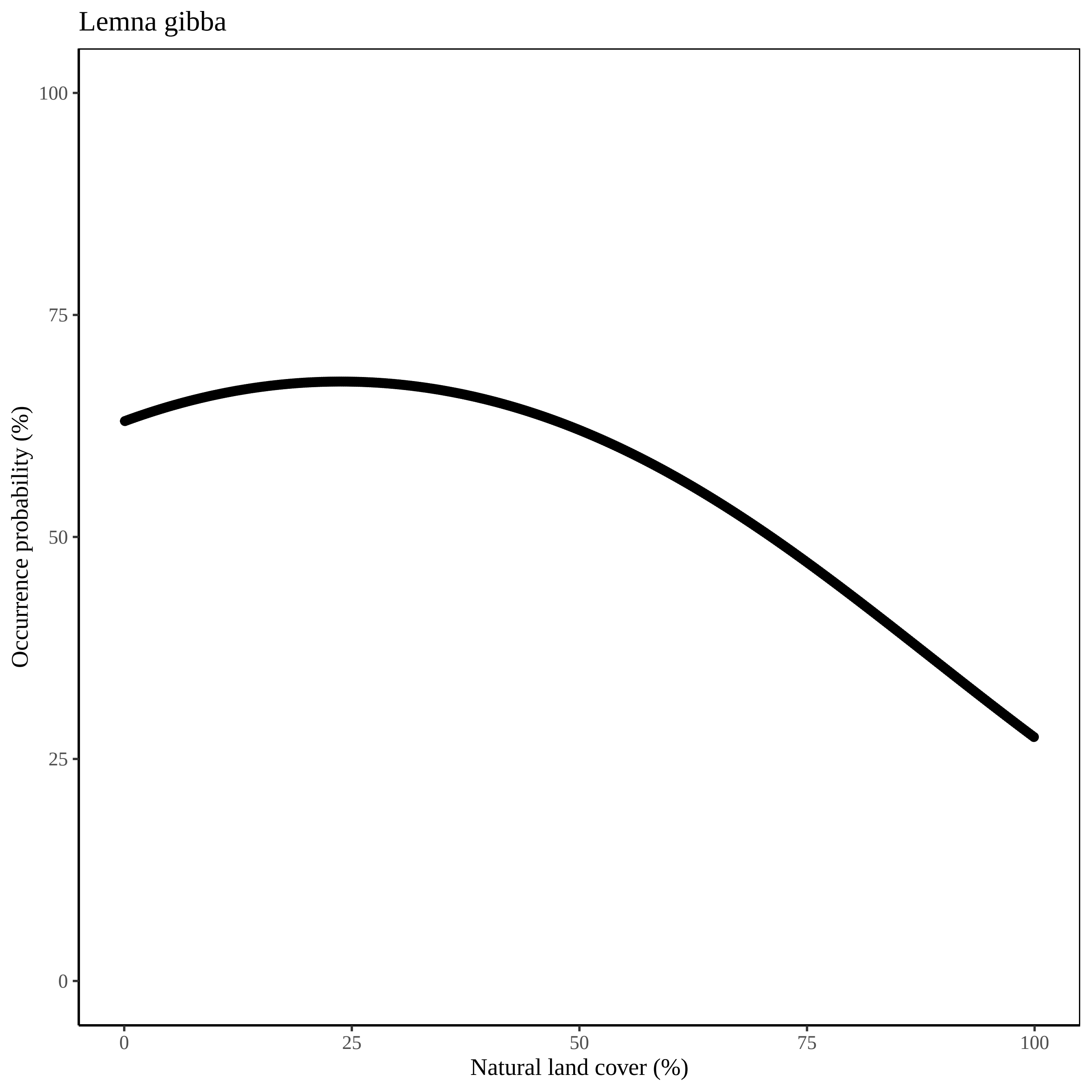
Lemna gibba
Occurrence probability (%)
100
75
50
25
0
Natural land cover (%)
0
25
50
75
100

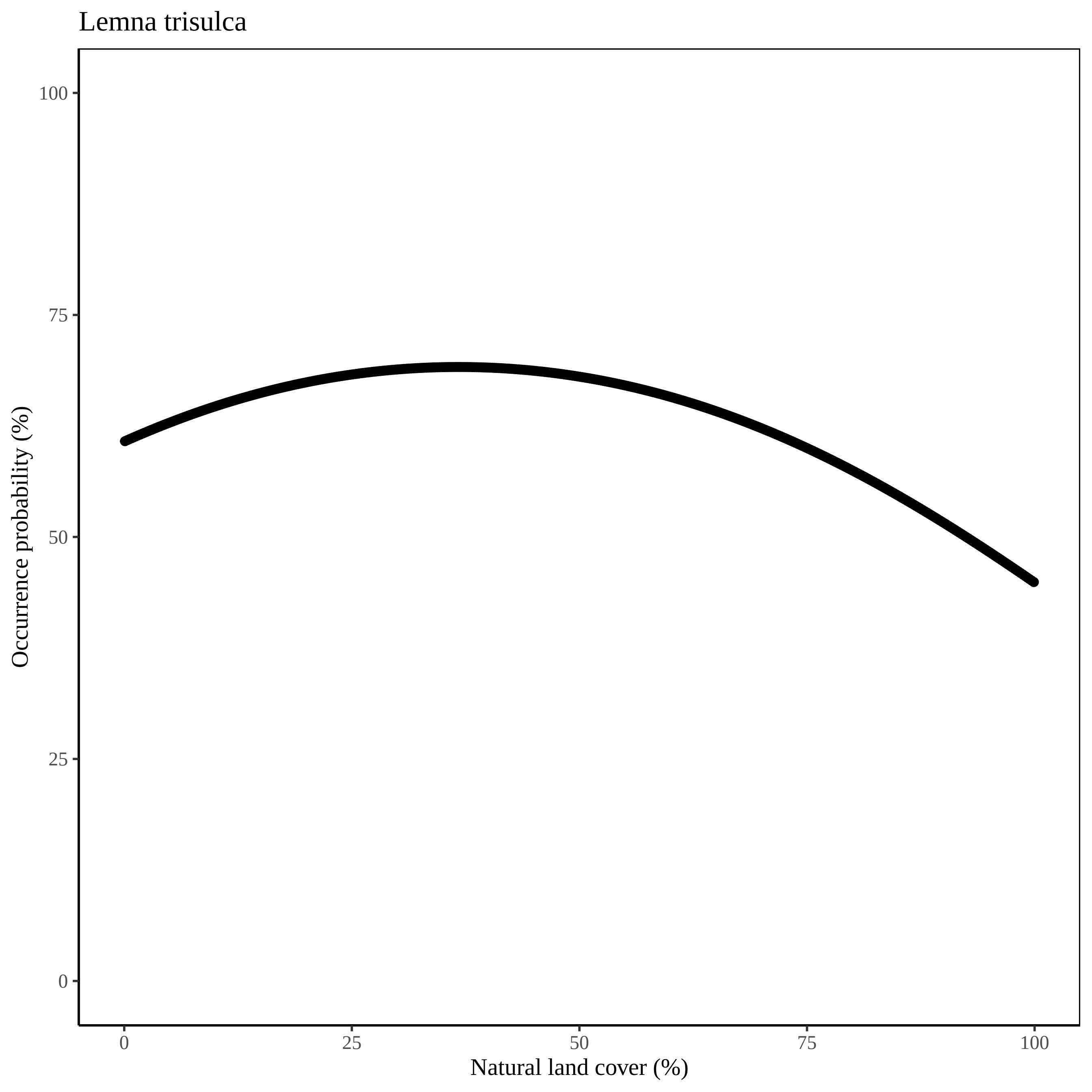
Lemna trisulca
Occurrence probability (%)
100
75
50
25
0
0
25
50
75
100
Natural land cover (%)

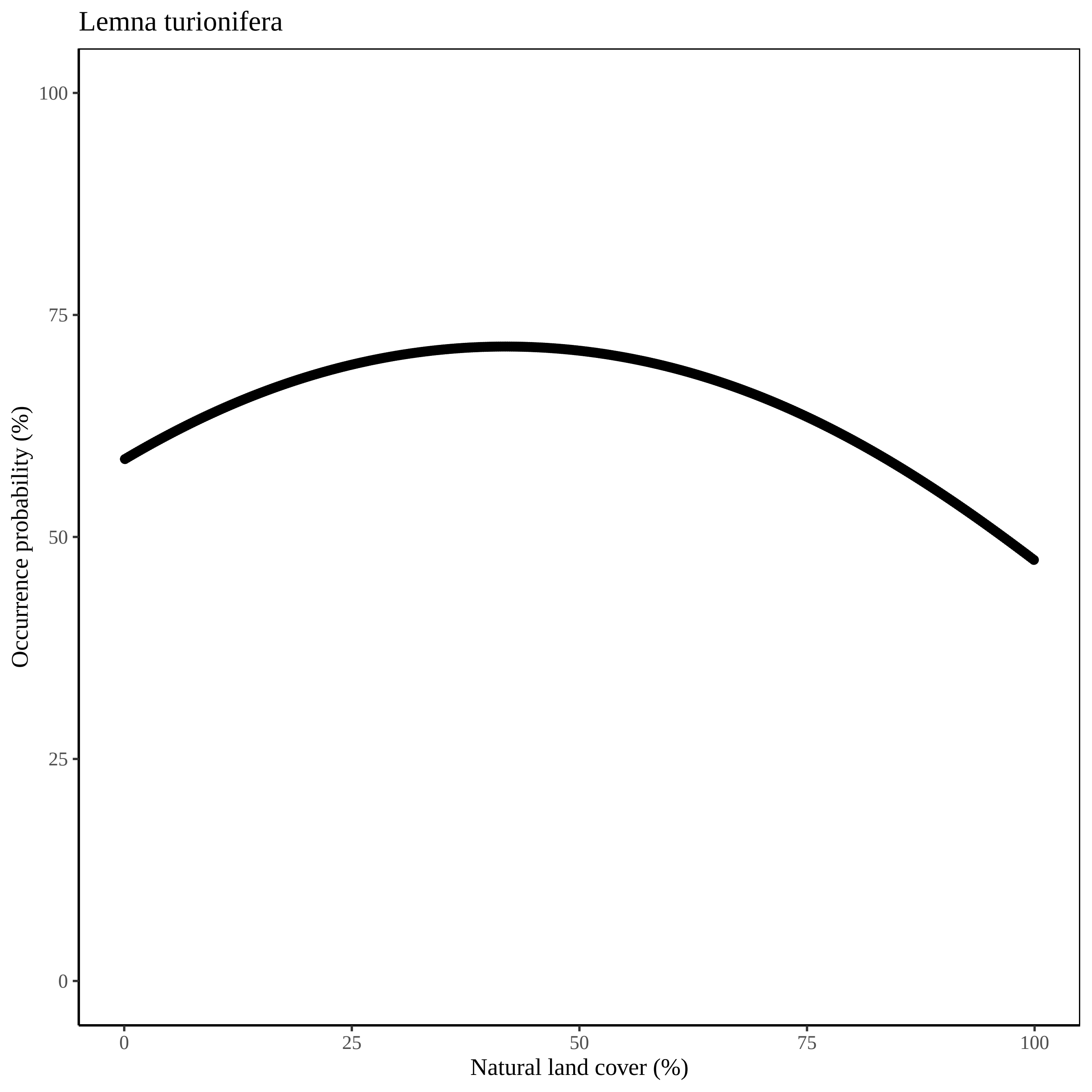
Lemna turionifera
Occurrence probability (%)
100
75
50
25
0
0
25
50
75
100
Natural land cover (%)

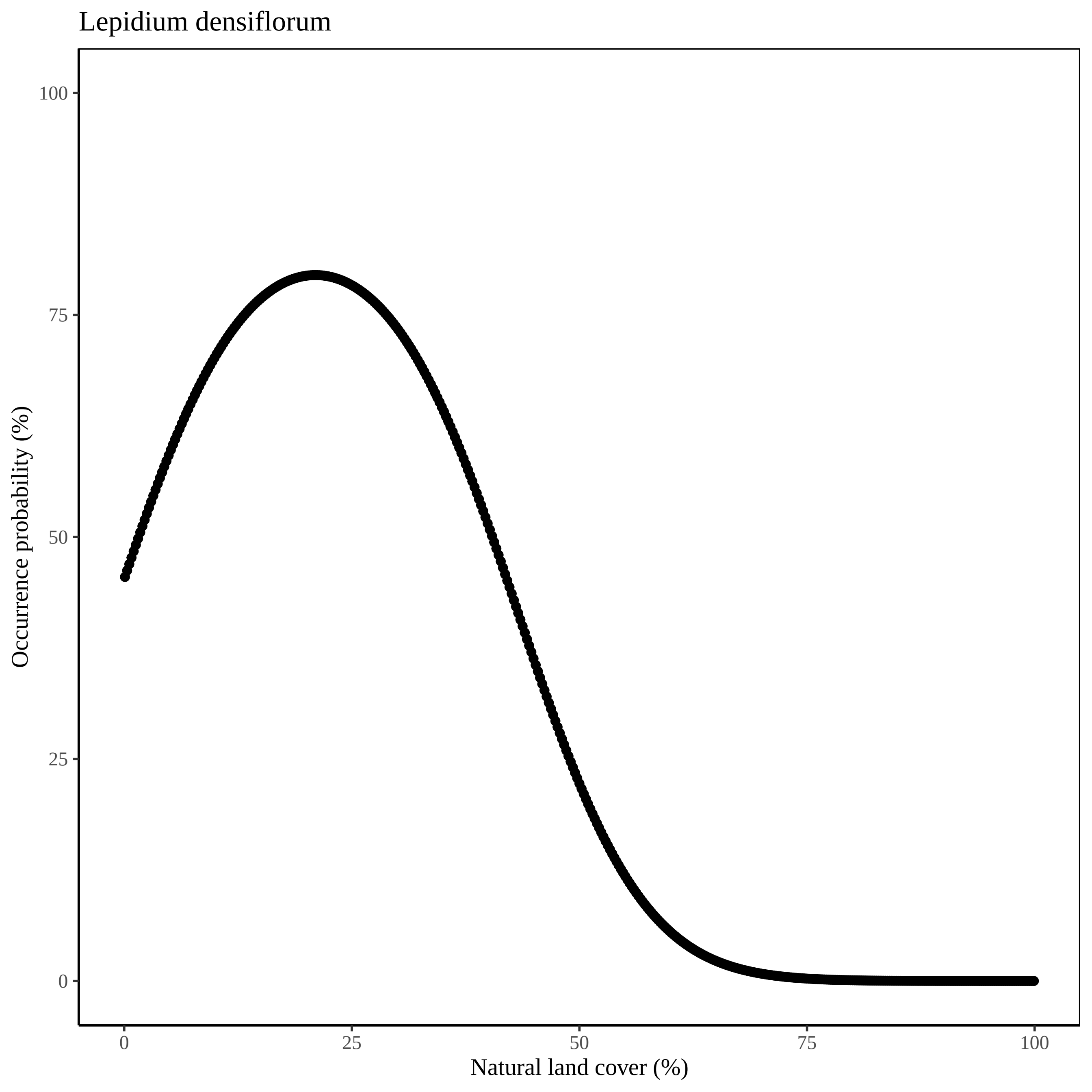

Lepidium densiflorum
Occurrence probability (%)
100
75
50
25
0
Natural land cover (%)
0
25
50
75
100

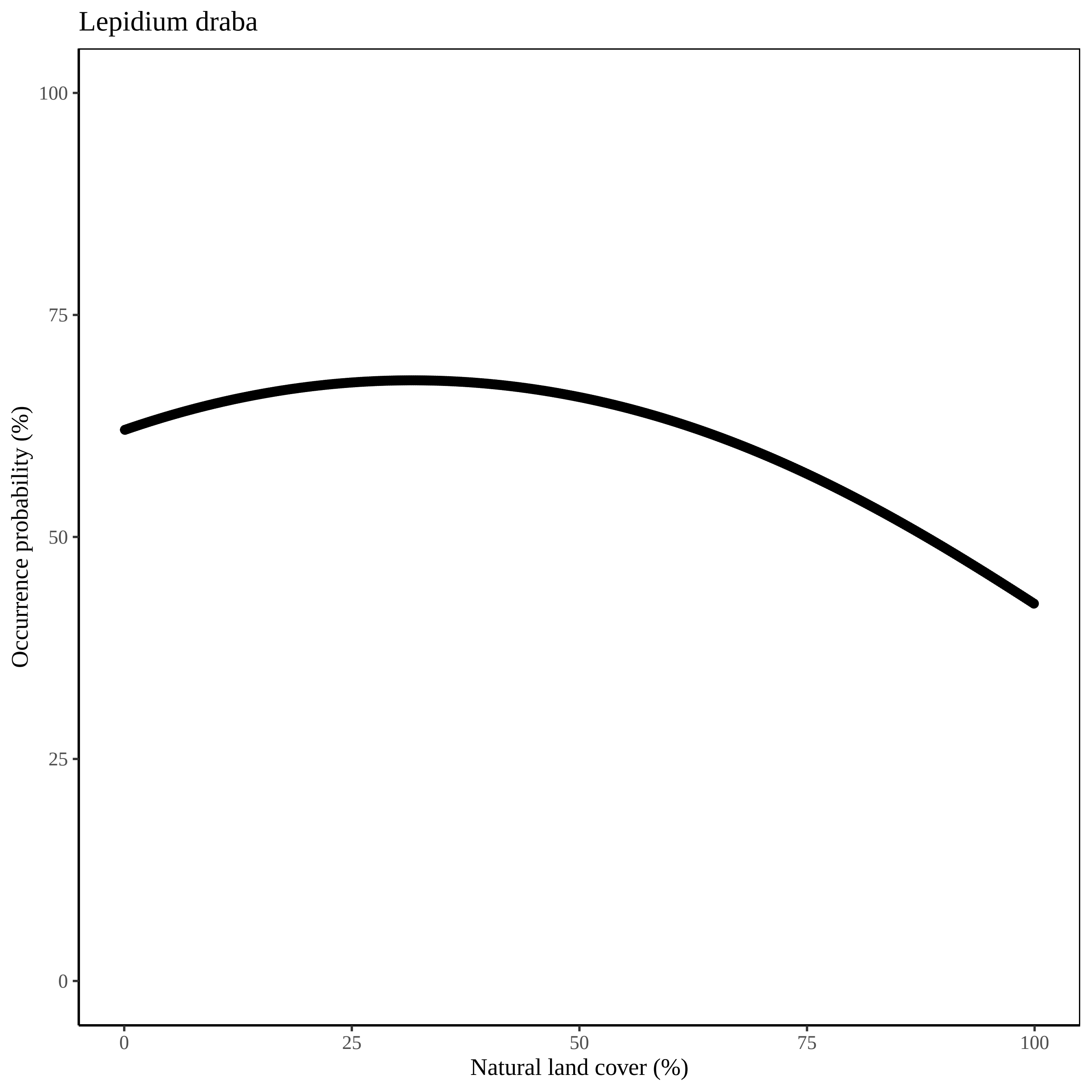
Lepidium draba
Occurrence probability (%)
100
75
50
25
0
0
25
50
75
100
Natural land cover (%)

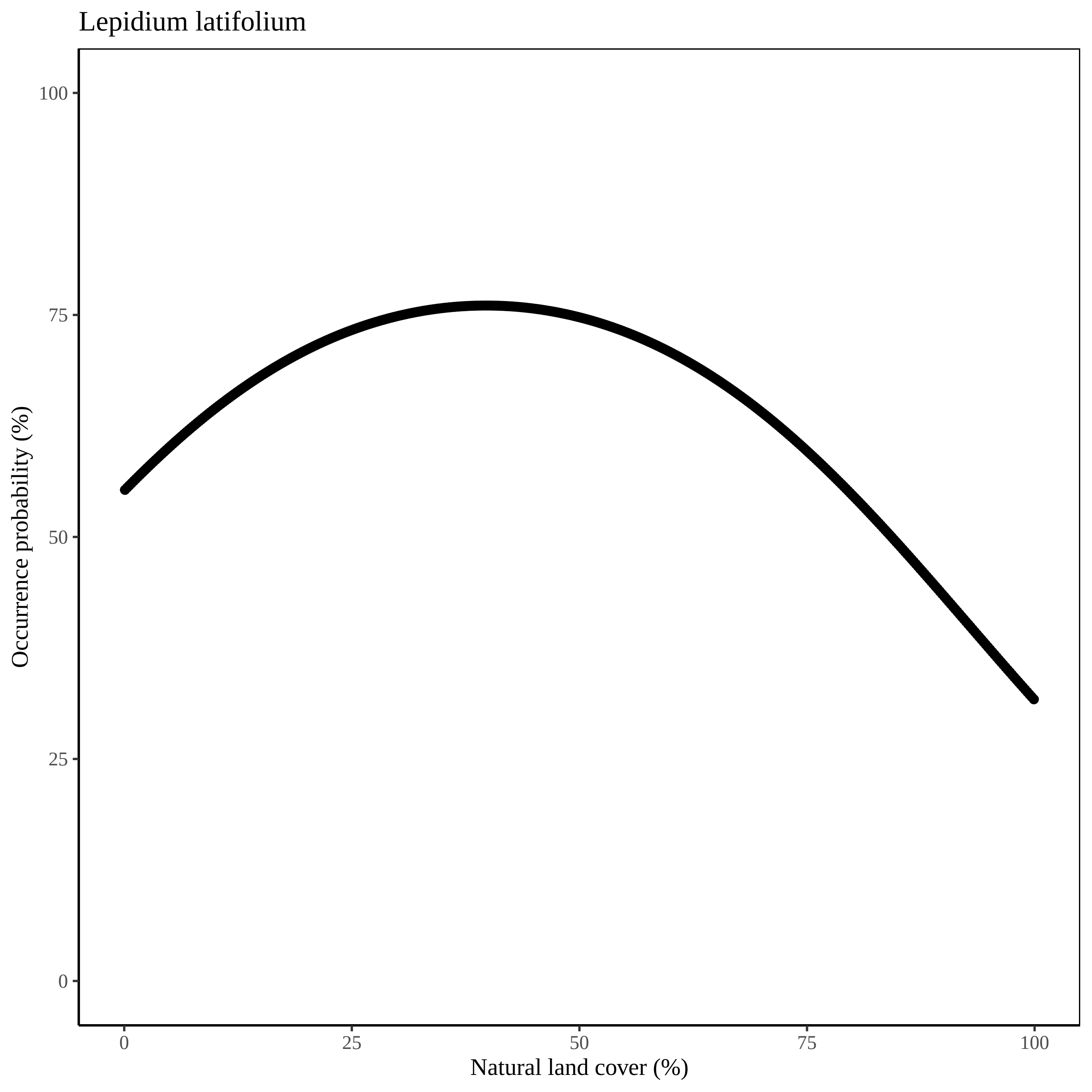

Lepidium latifolium
Occurrence probability (%)
100
75
50
25
0
0
25
50
75
100
Natural land cover (%)

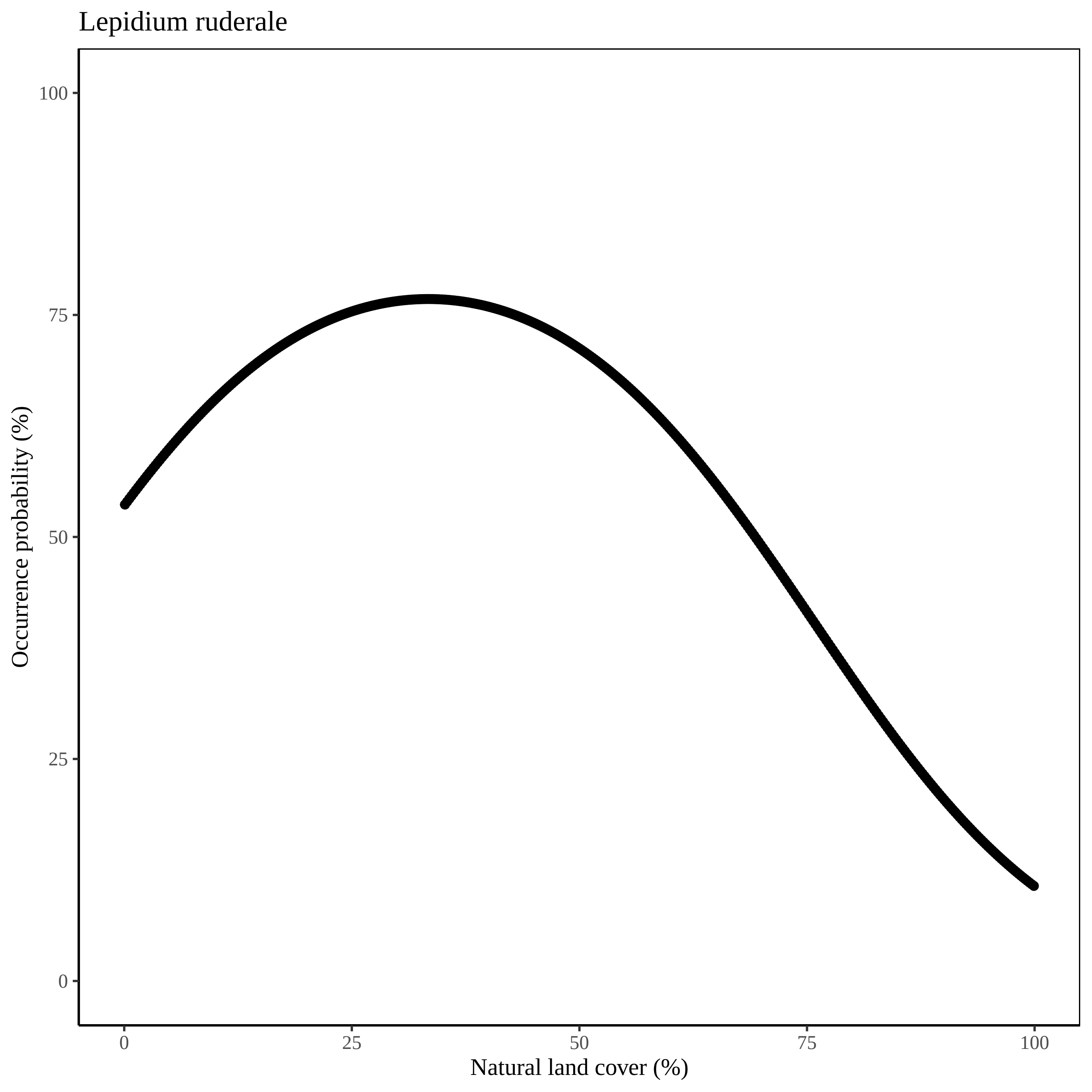
Lepidium ruderale
100
75
50
25
0
Occurrence probability (%)
0
25
50
75
100
Natural land cover (%)

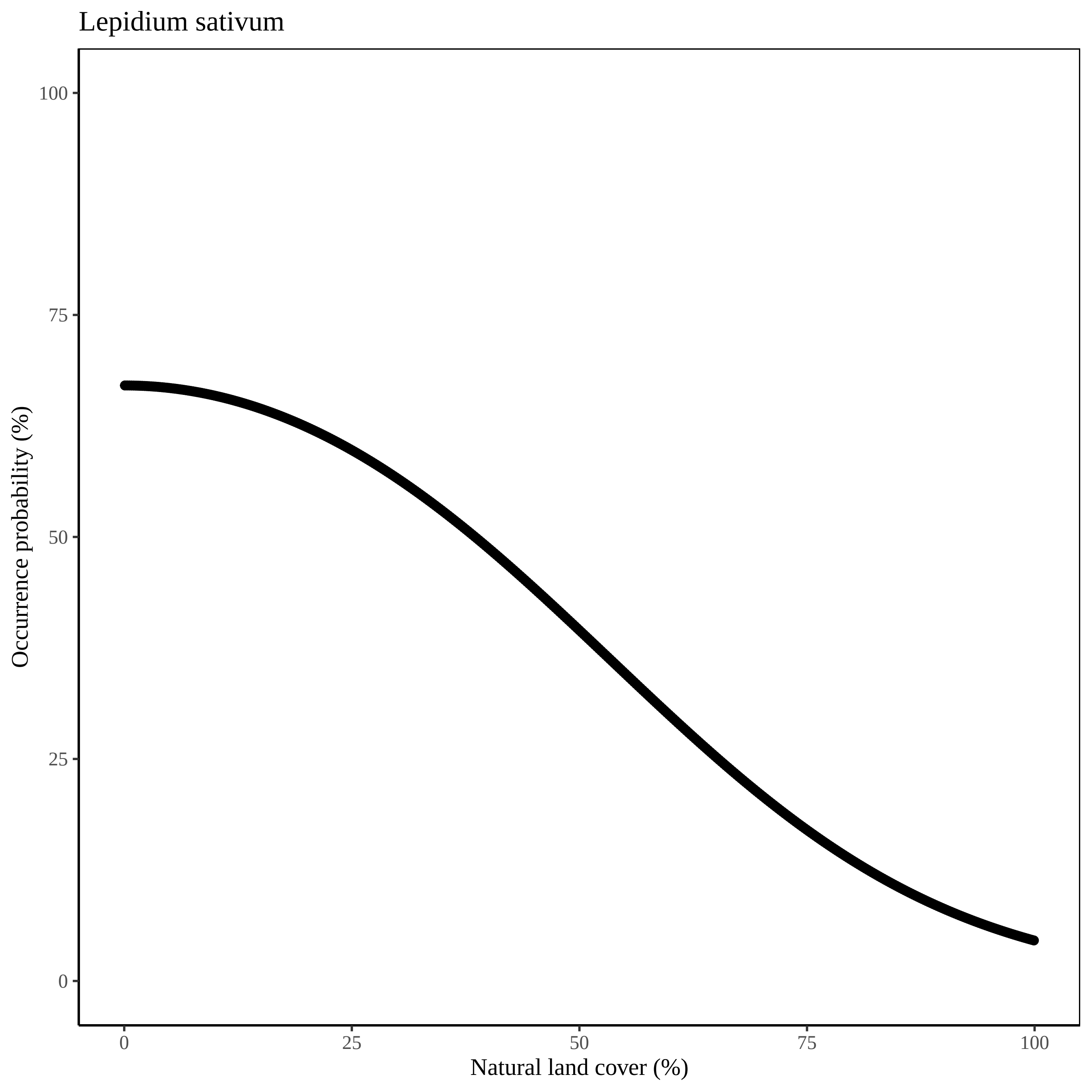
Lepidium sativum
Occurrence probability (%)
100
75
50
25
0
0
25
50
75
100
Natural land cover (%)

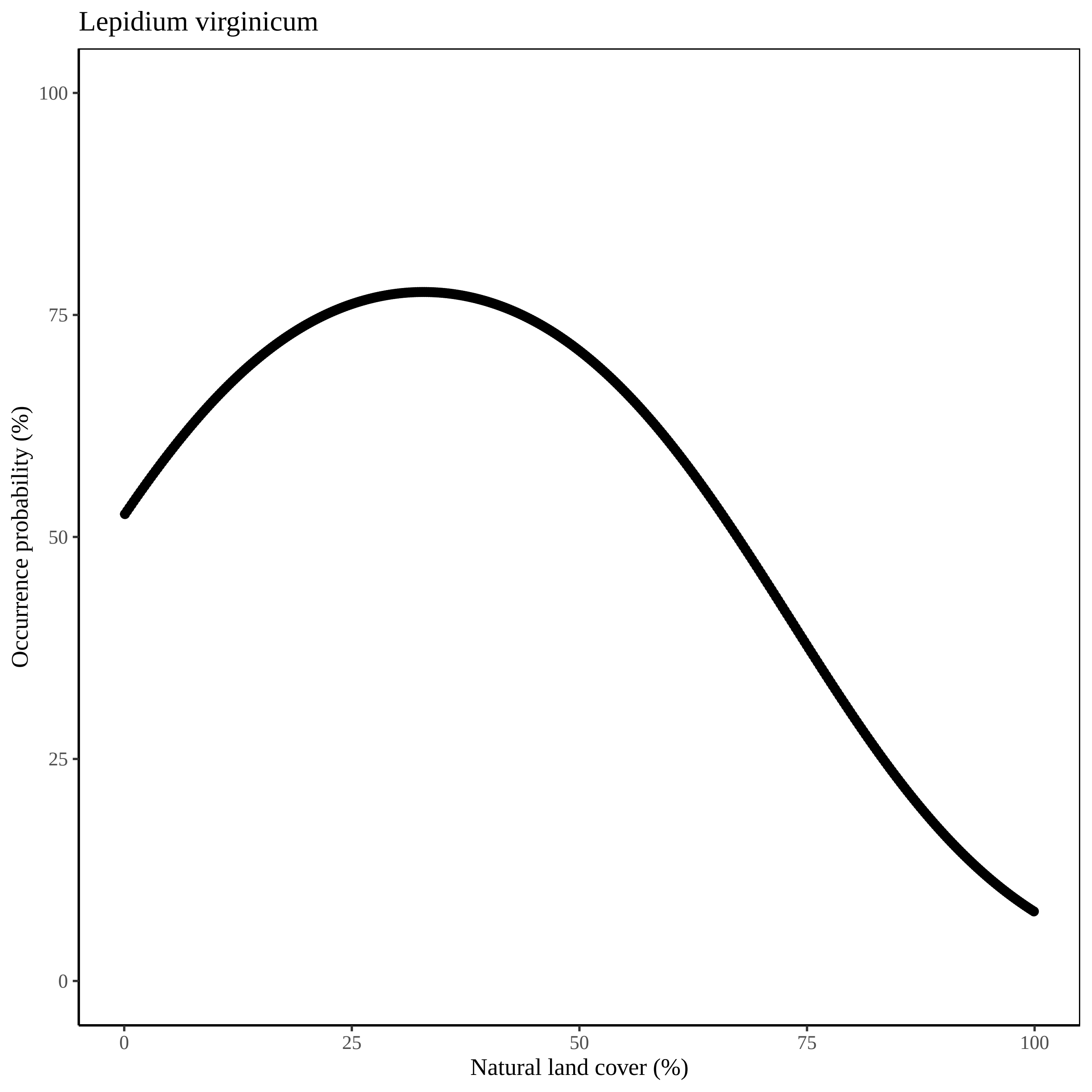
Lepidium virginicum
Occurrence probability (%)
100
75
50
25
0
0
25
50
75
100
Natural land cover (%)

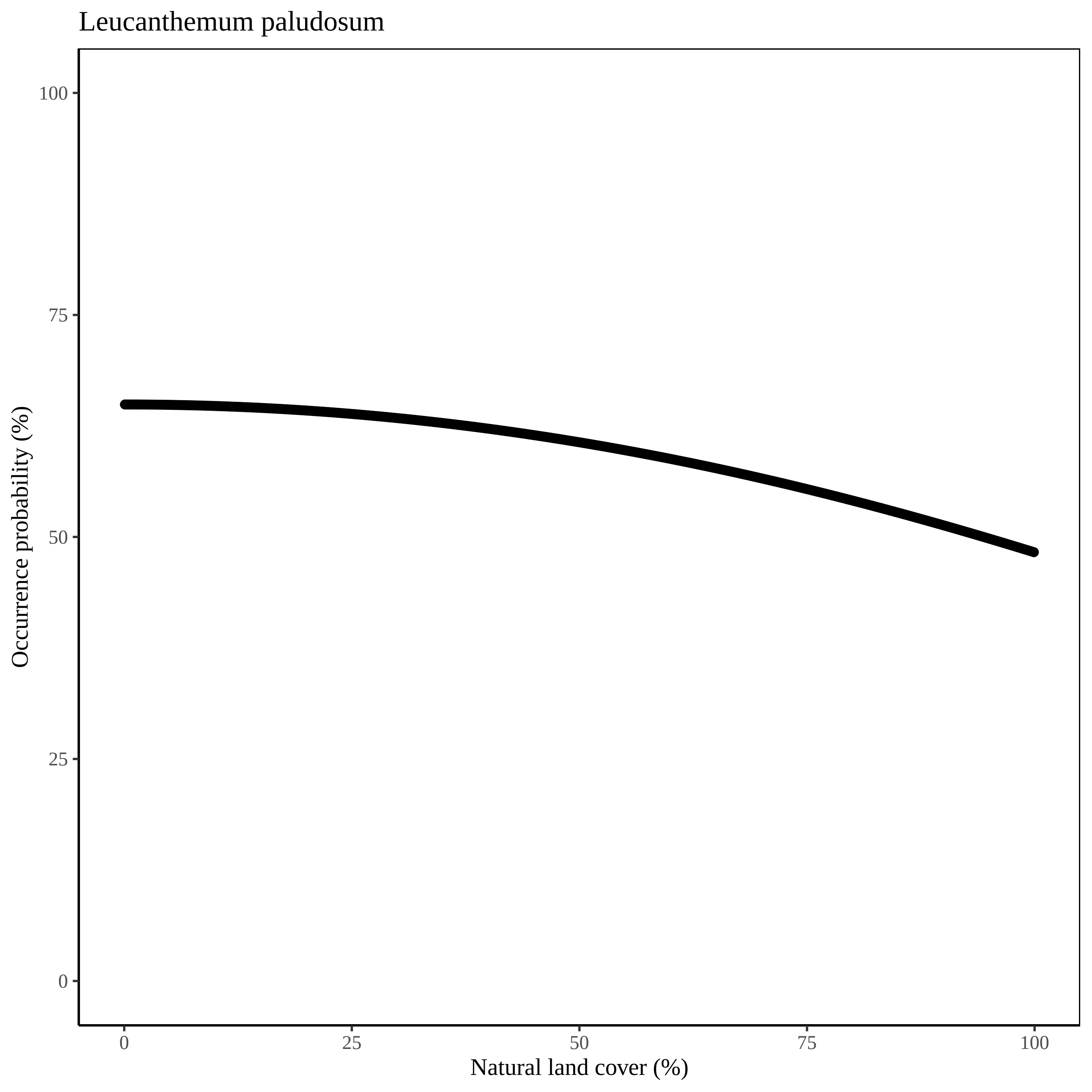

Leucanthemum paludosum
100
75
50
25
0
Occurrence probability (%)
0
25
50
75
100
Natural land cover (%)

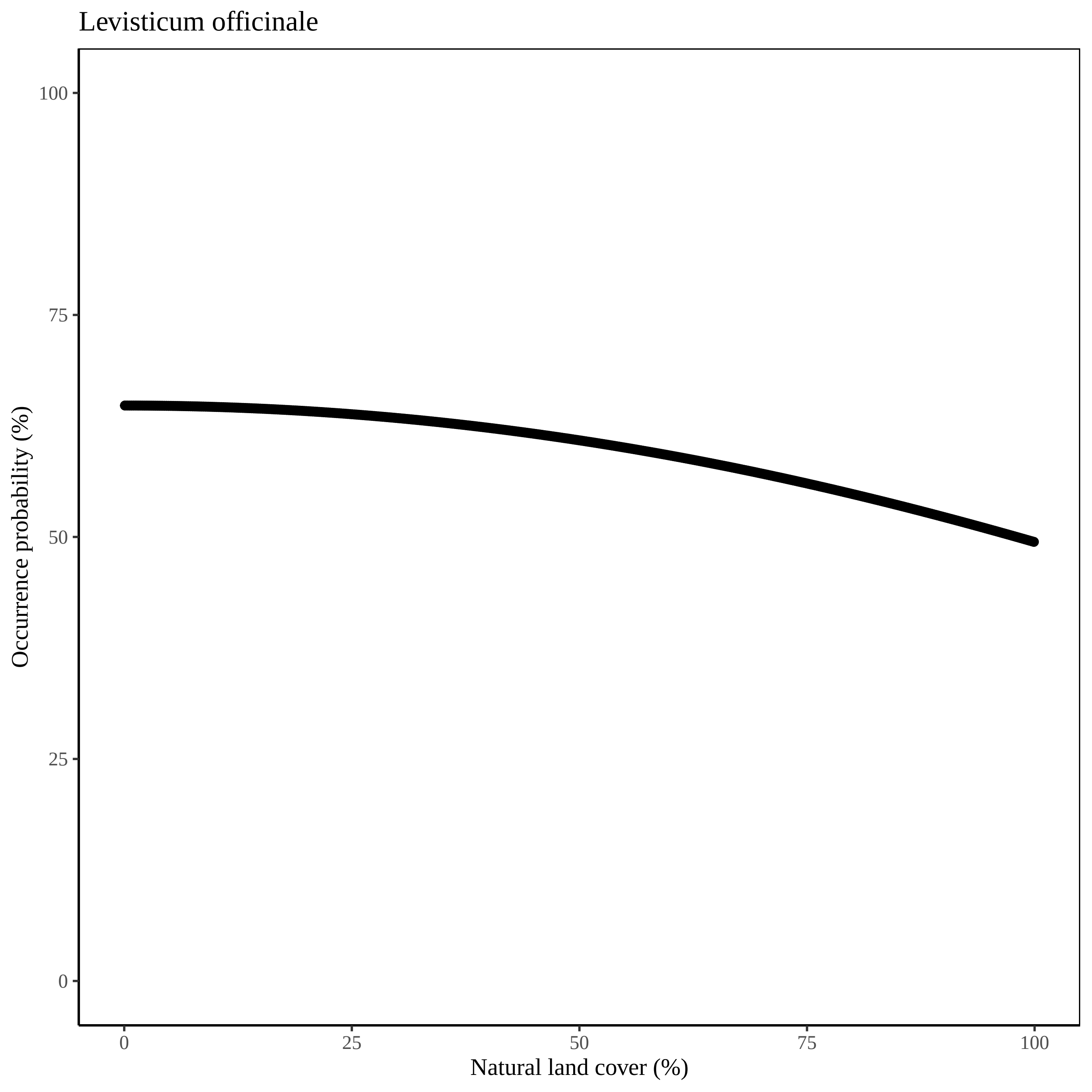

Levisticum officinale
Occurrence probability (%)
100
75
50
25
0
0
25
50
75
100
Natural land cover (%)

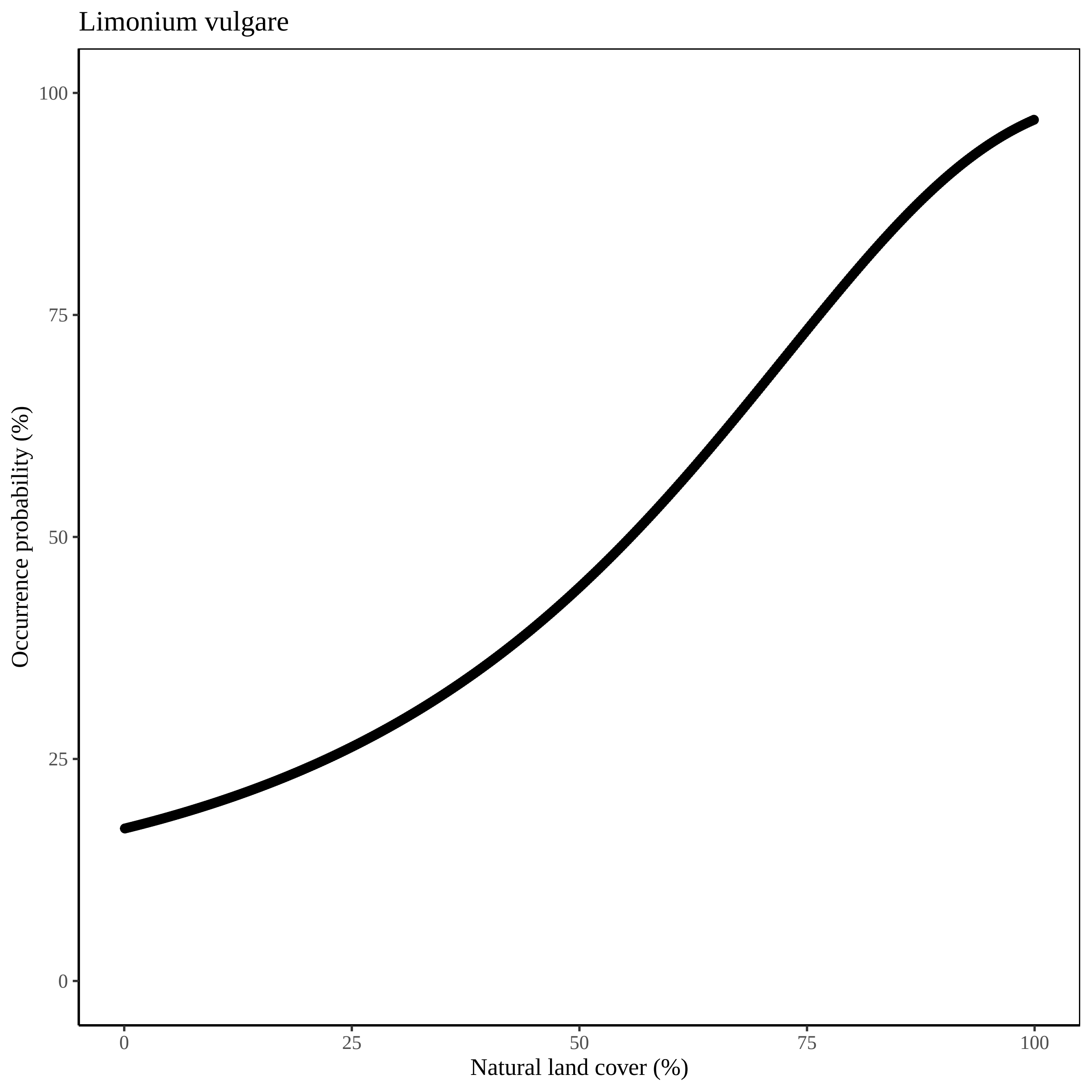
Limonium vulgare
Occurrence probability (%)
100
75
50
25
0
0
25
50
75
100
Natural land cover (%)

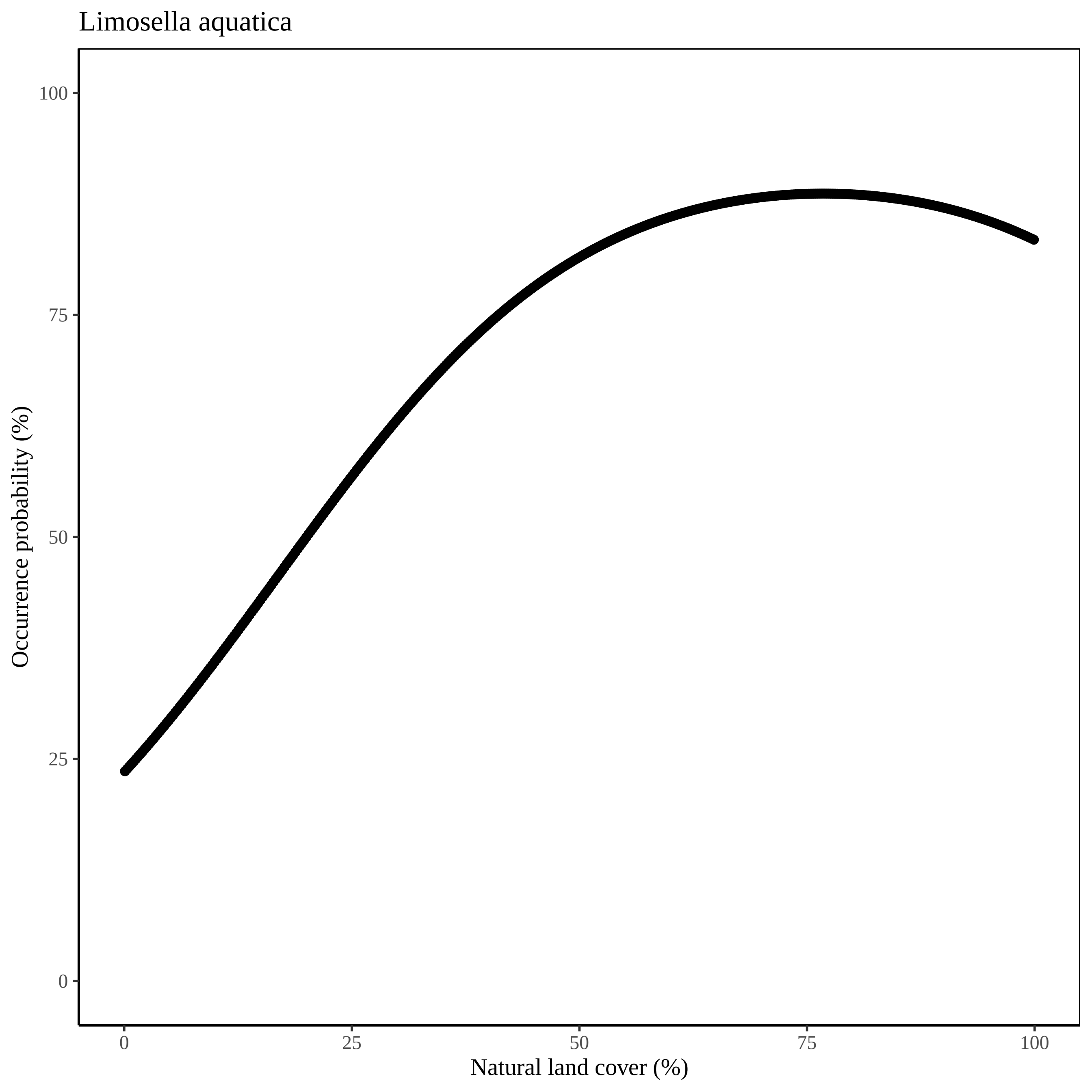

Limosella aquatica
100
75
50
25
0
Occurrence probability (%)
0
25
50
75
100
Natural land cover (%)

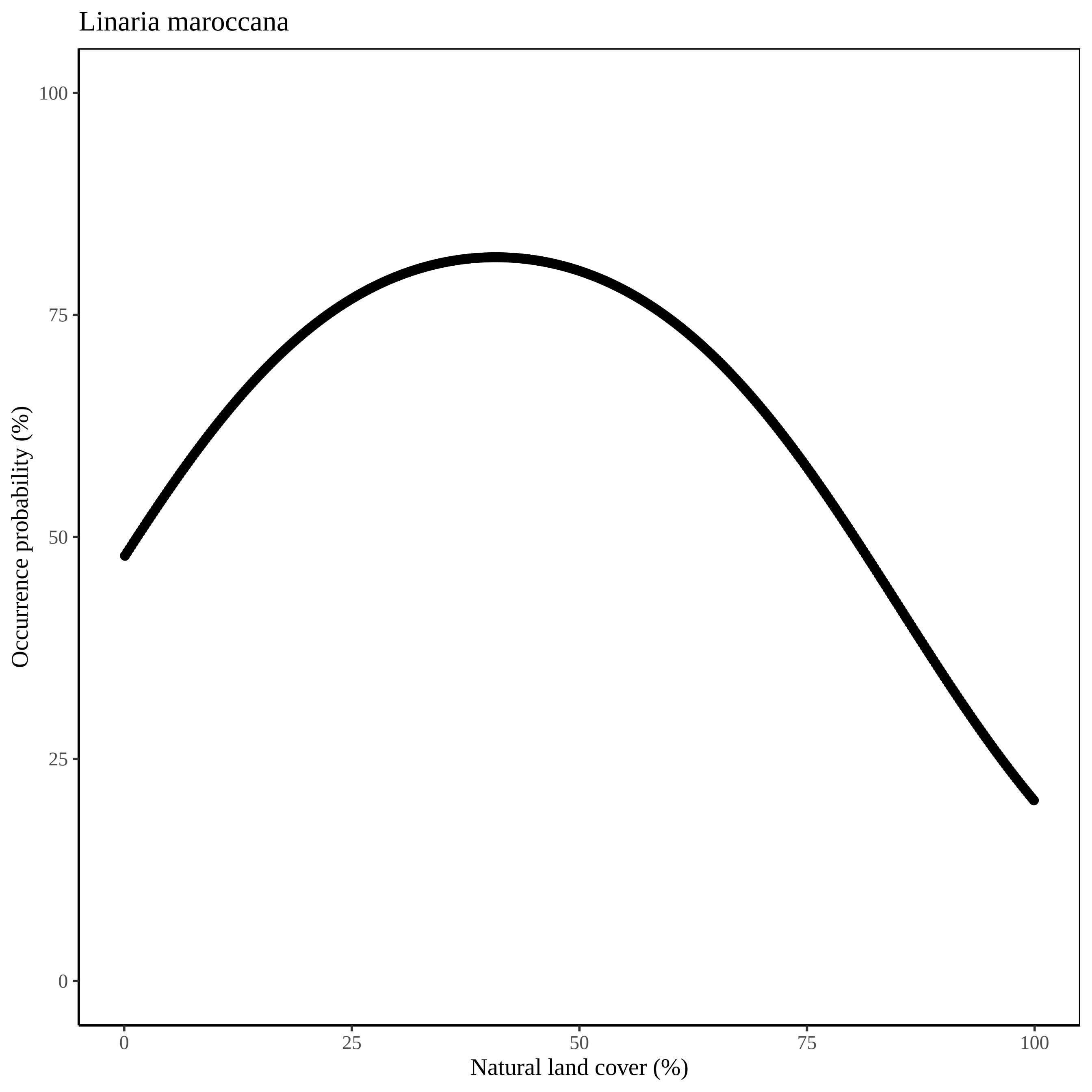
Linaria maroccana
Occurrence probability (%)
100
75
50
25
0
Natural land cover (%)
0
25
50
75
100

Lobelia erinus

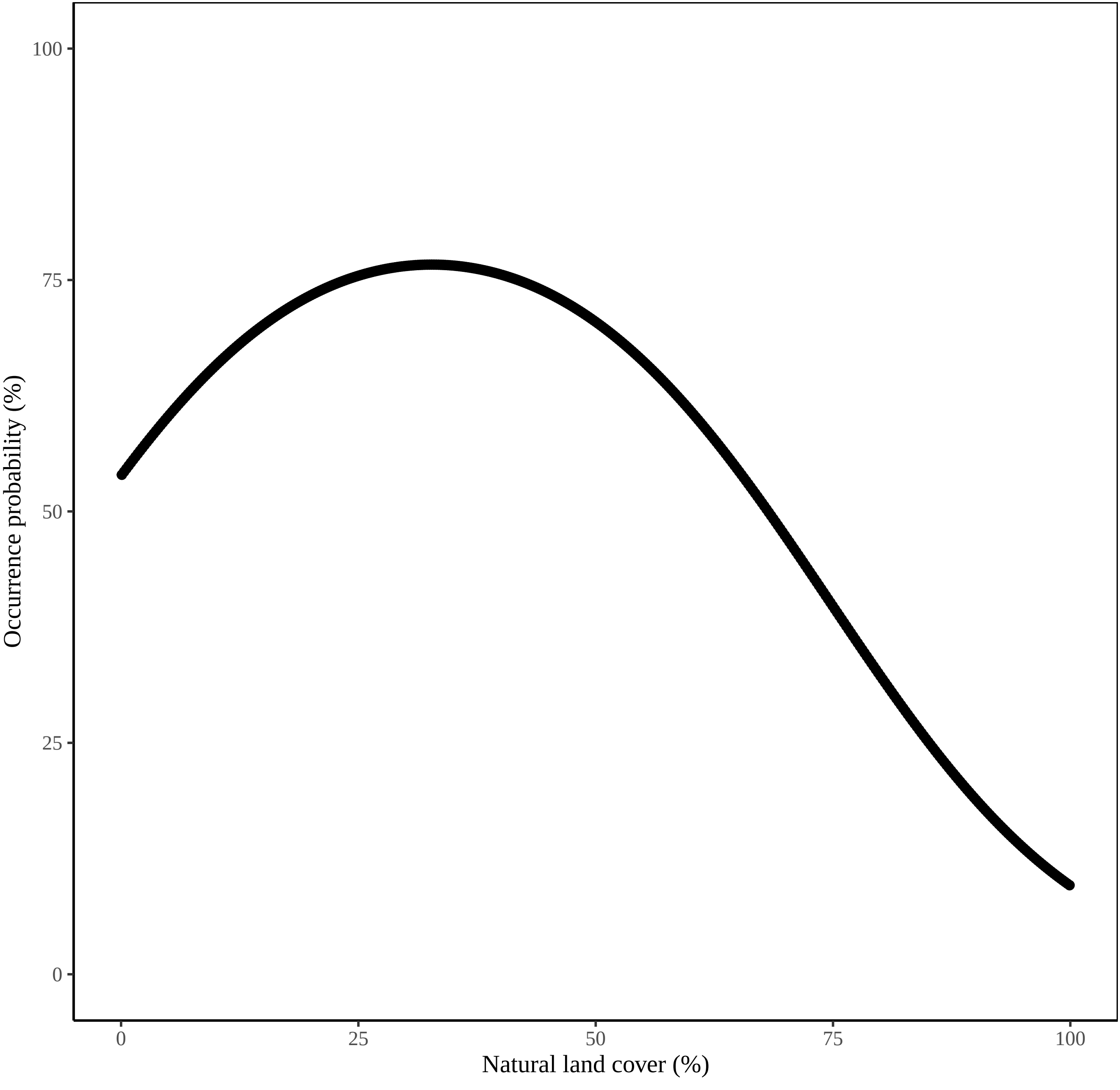

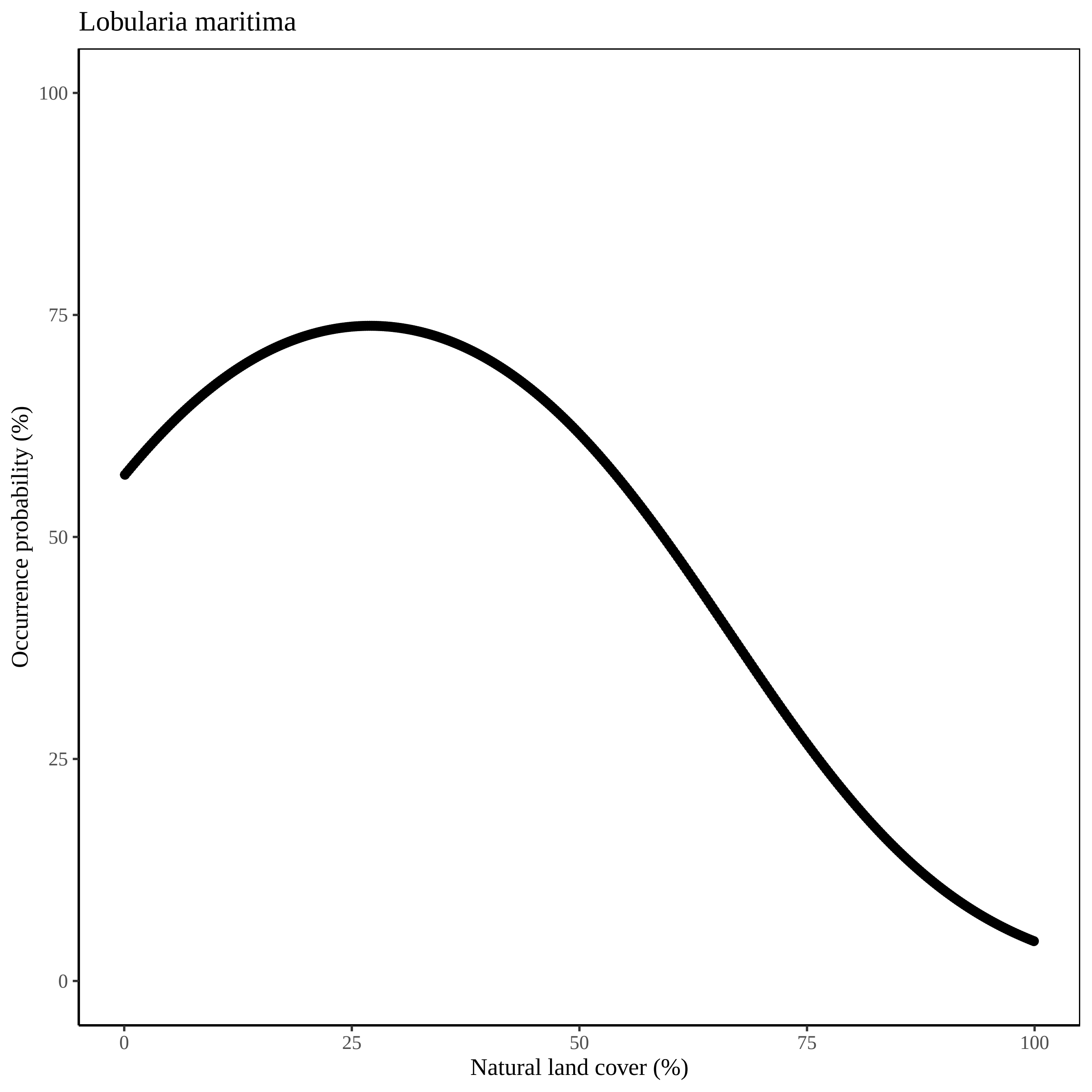

Lobularia maritima
Occurrence probability (%)
100
75
50
25
0
0
25
50
75
100
Natural land cover (%)

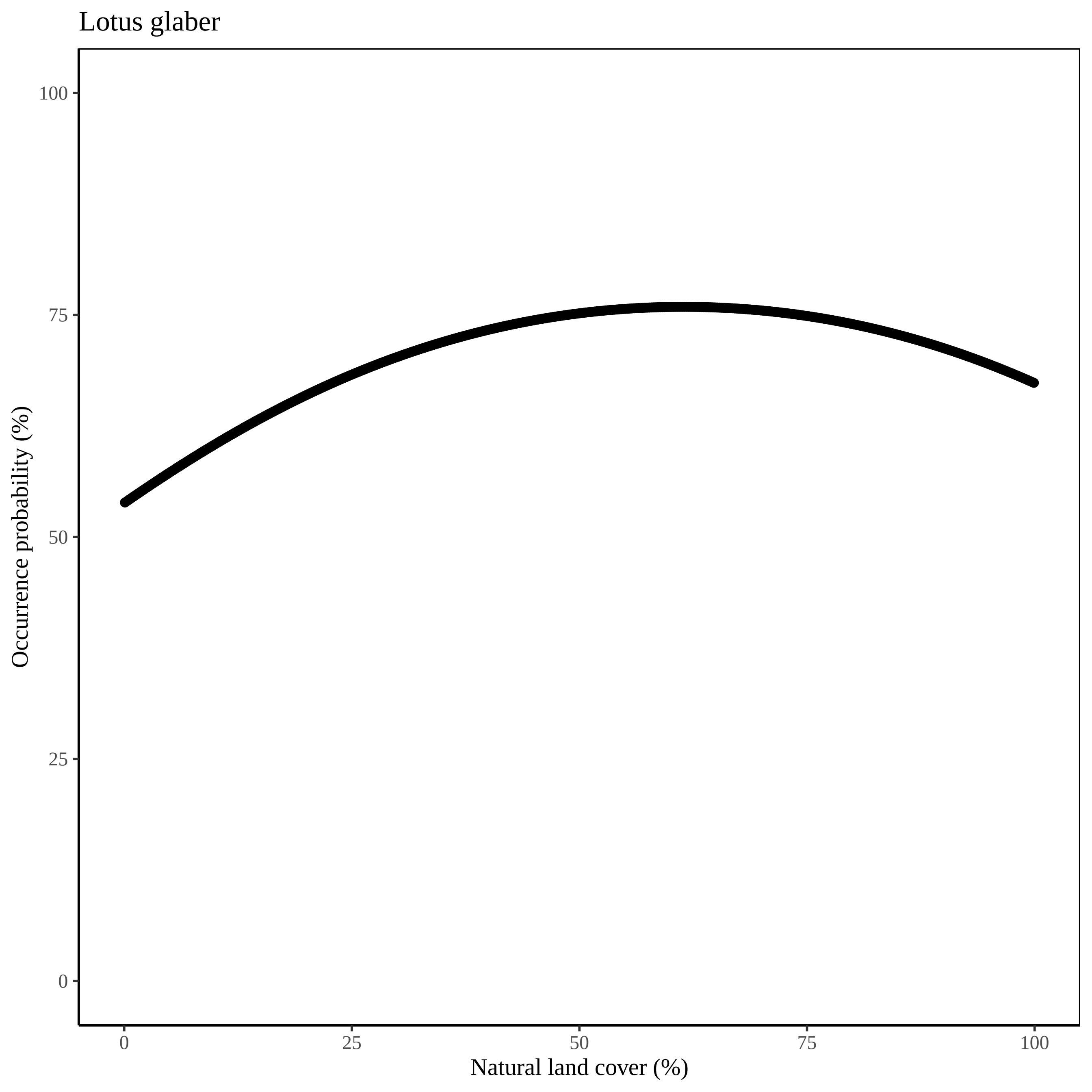

Lotus glaber
Occurrence probability (%)
100
75
50
25
0
0
25
50
75
100
Natural land cover (%)

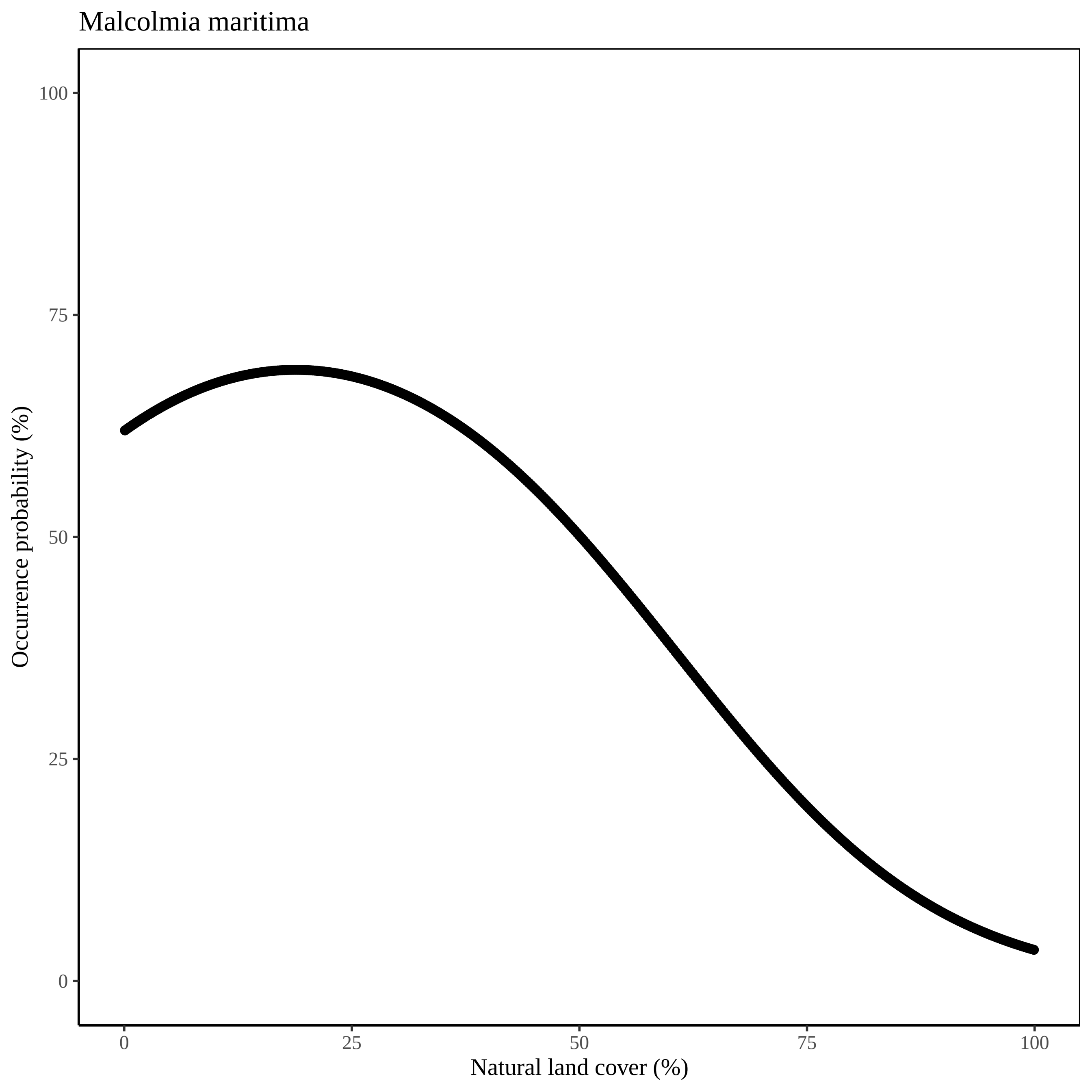

Malcolmia maritima
Occurrence probability (%)
0
25
50
75
100
Natural land cover (%)
0
25
50
75
100

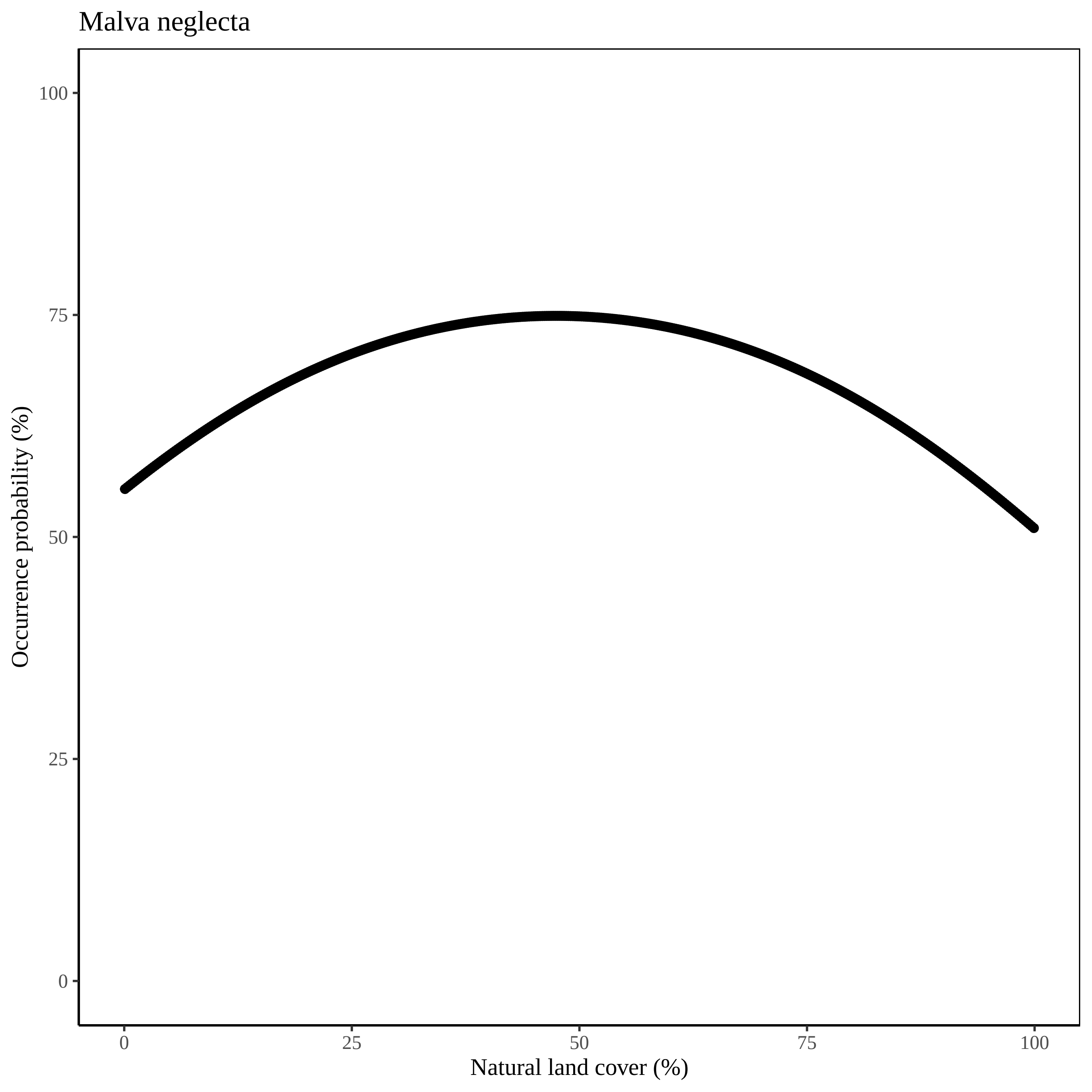

Malva neglecta
100
75
50
25
0
Occurrence probability (%)
0
25
50
75
100
Natural land cover (%)

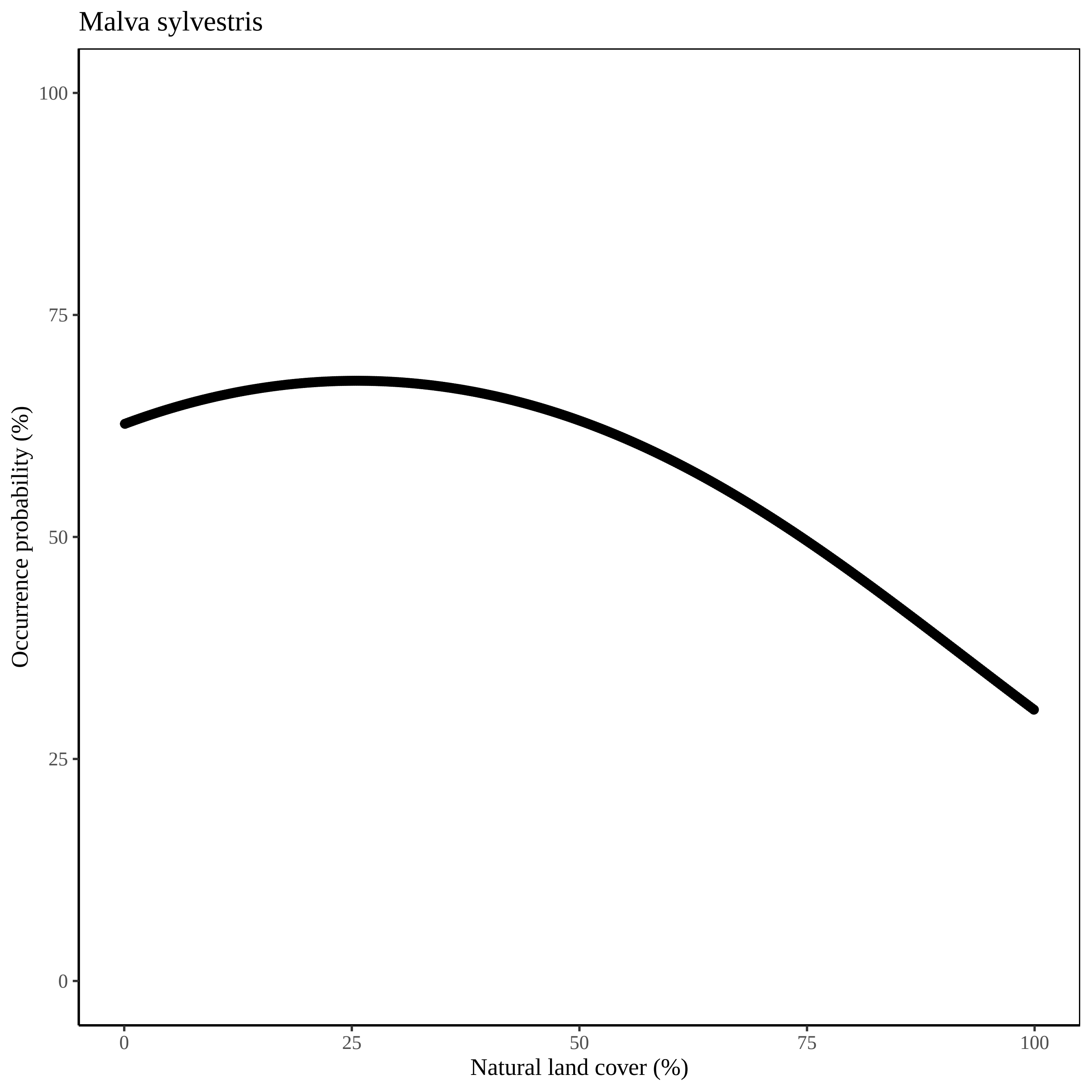

Malva sylvestris
100
75
50
25
0
Occurrence probability (%)
0
25
50
75
100
Natural land cover (%)

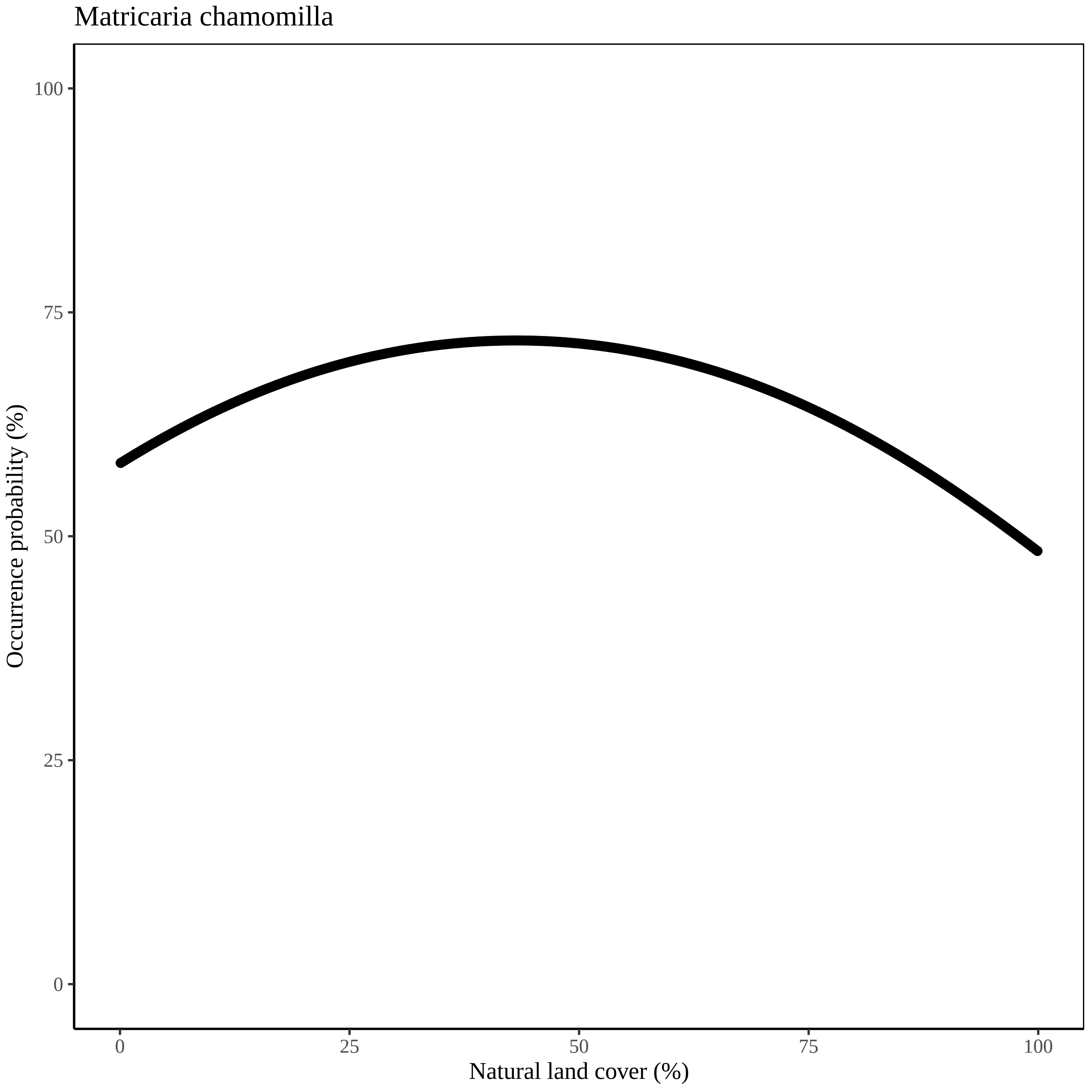
Matricaria chamomilla
Occurrence probability (%)
100
75
50
25
0
0
25
50
75
100
Natural land cover (%)

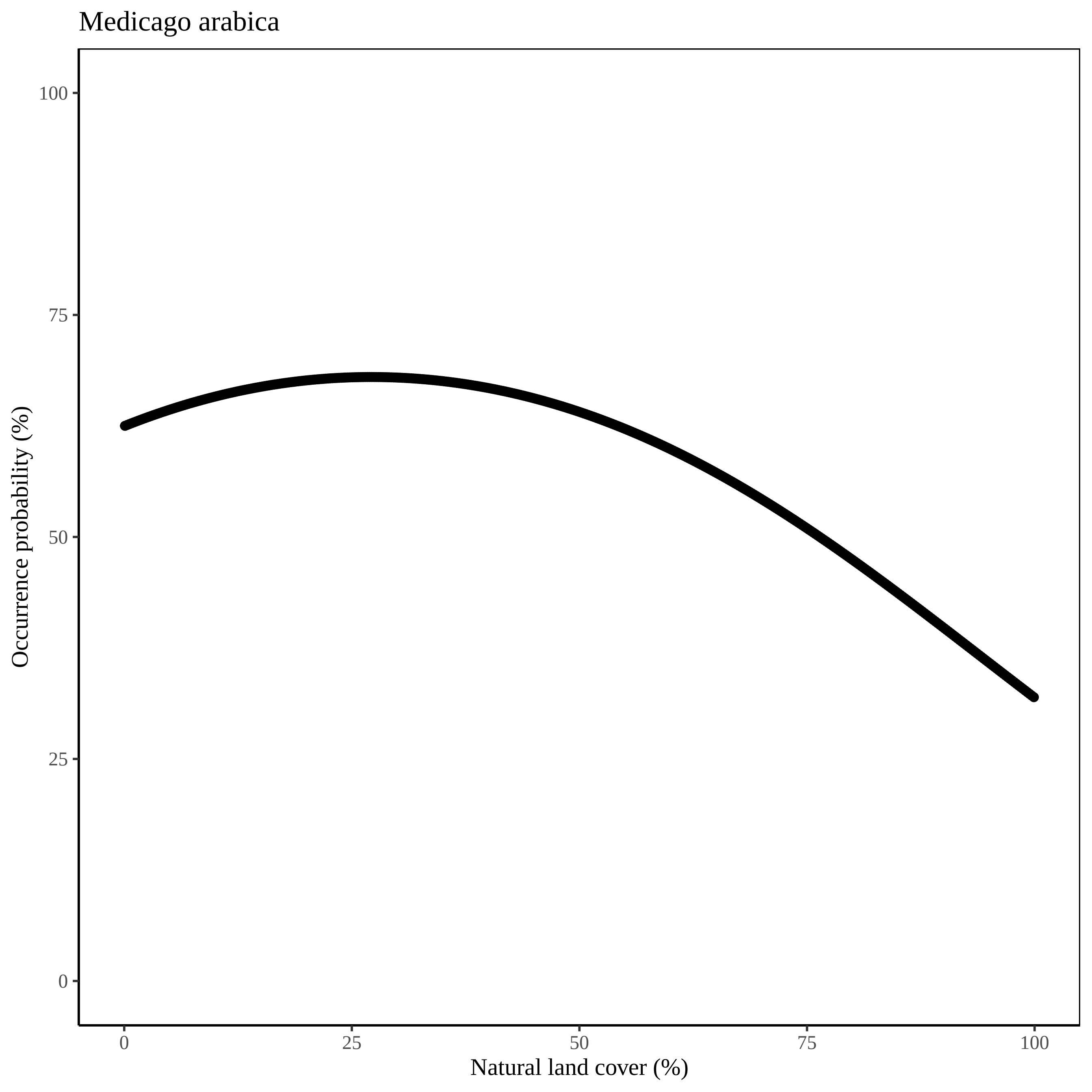
Medicago arabica
Occurrence probability (%)
100
75
50
25
0
0
25
50
75
100
Natural land cover (%)

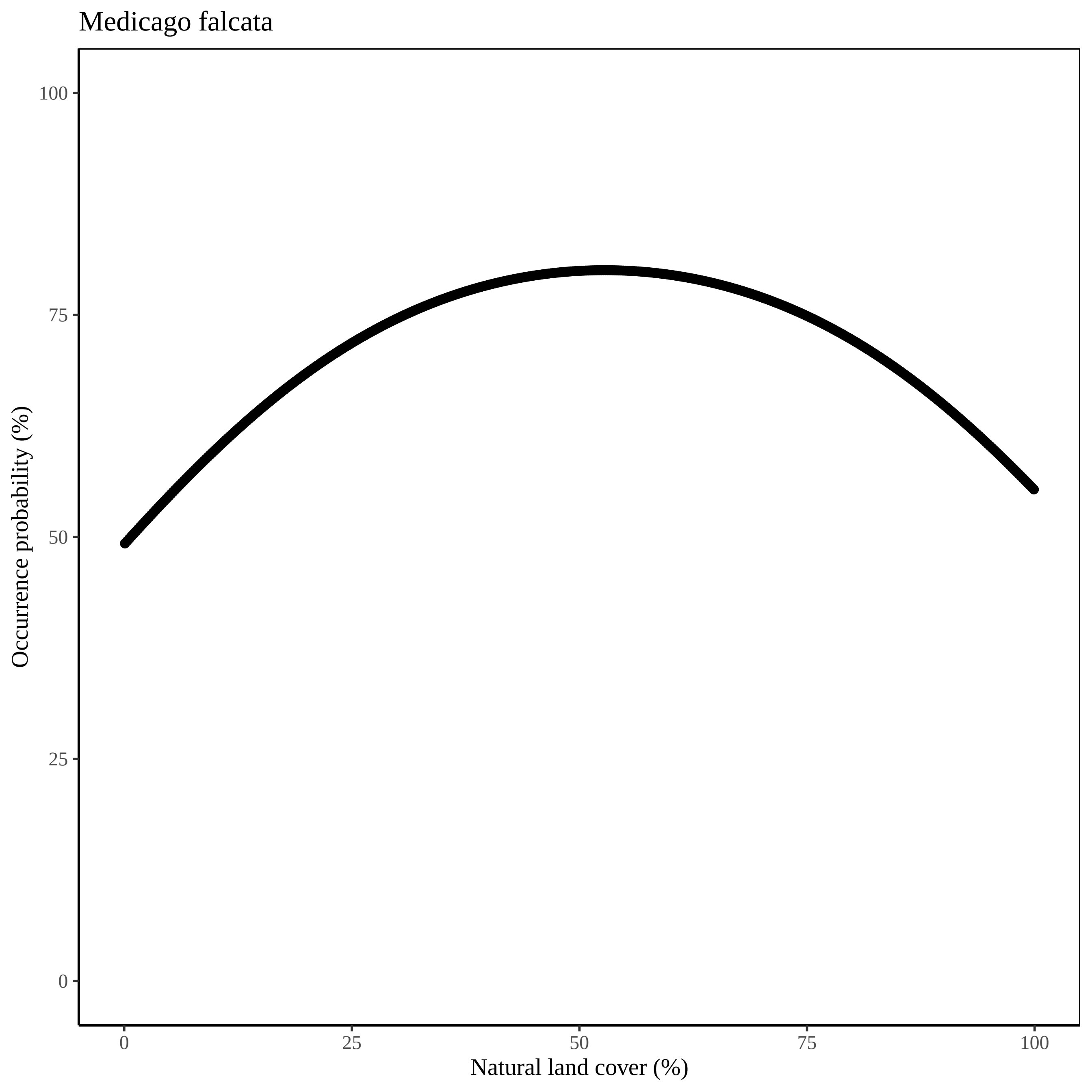
Medicago falcata
Occurrence probability (%)
100
75
50
25
0
0
25
50
75
100
Natural land cover (%)

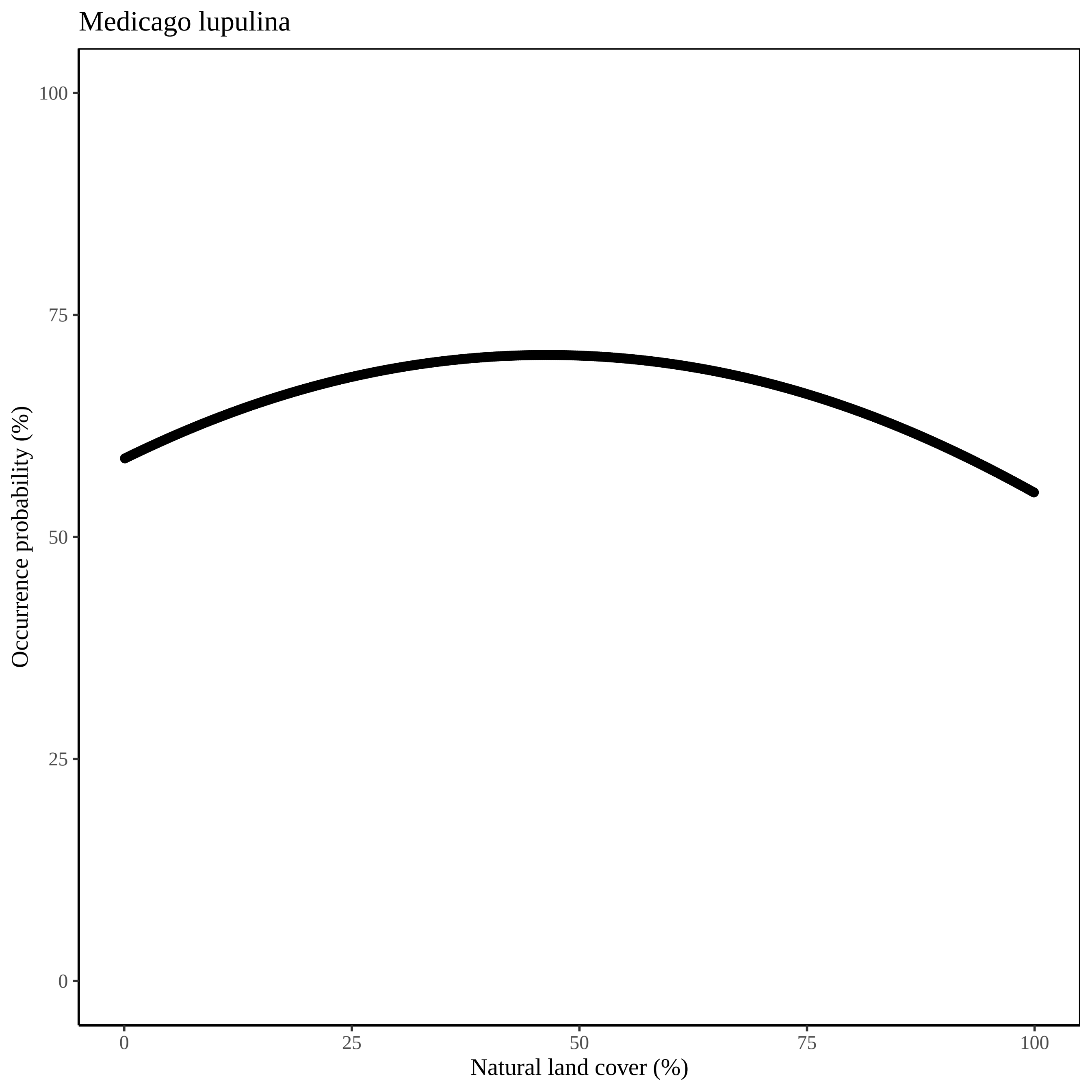
Medicago lupulina
100
75
50
25
0
Occurrence probability (%)
0
25
50
75
100
Natural land cover (%)

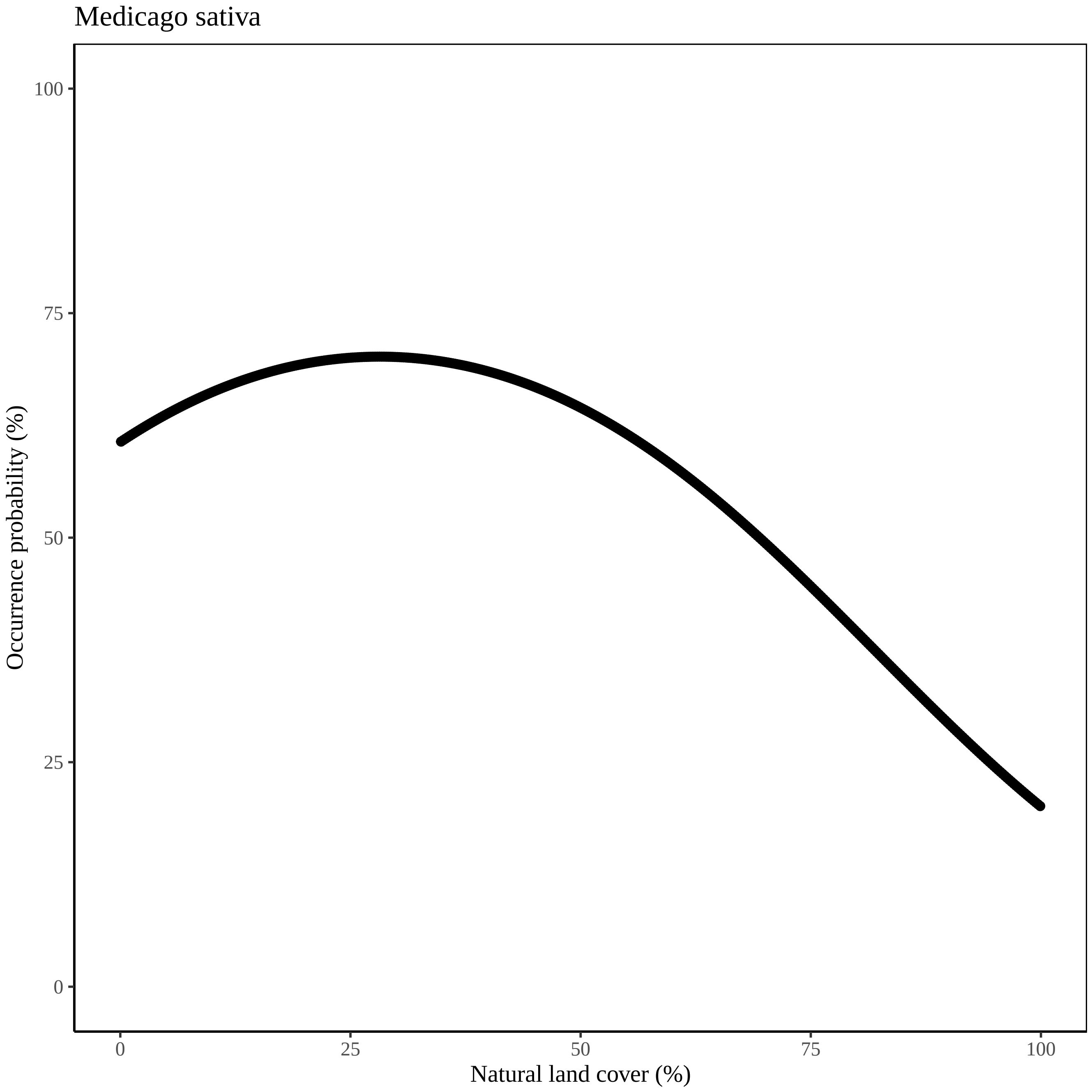

Medicago sativa
Occurrence probability (%)
100
75
50
25
0
0
25
50
75
100
Natural land cover (%)

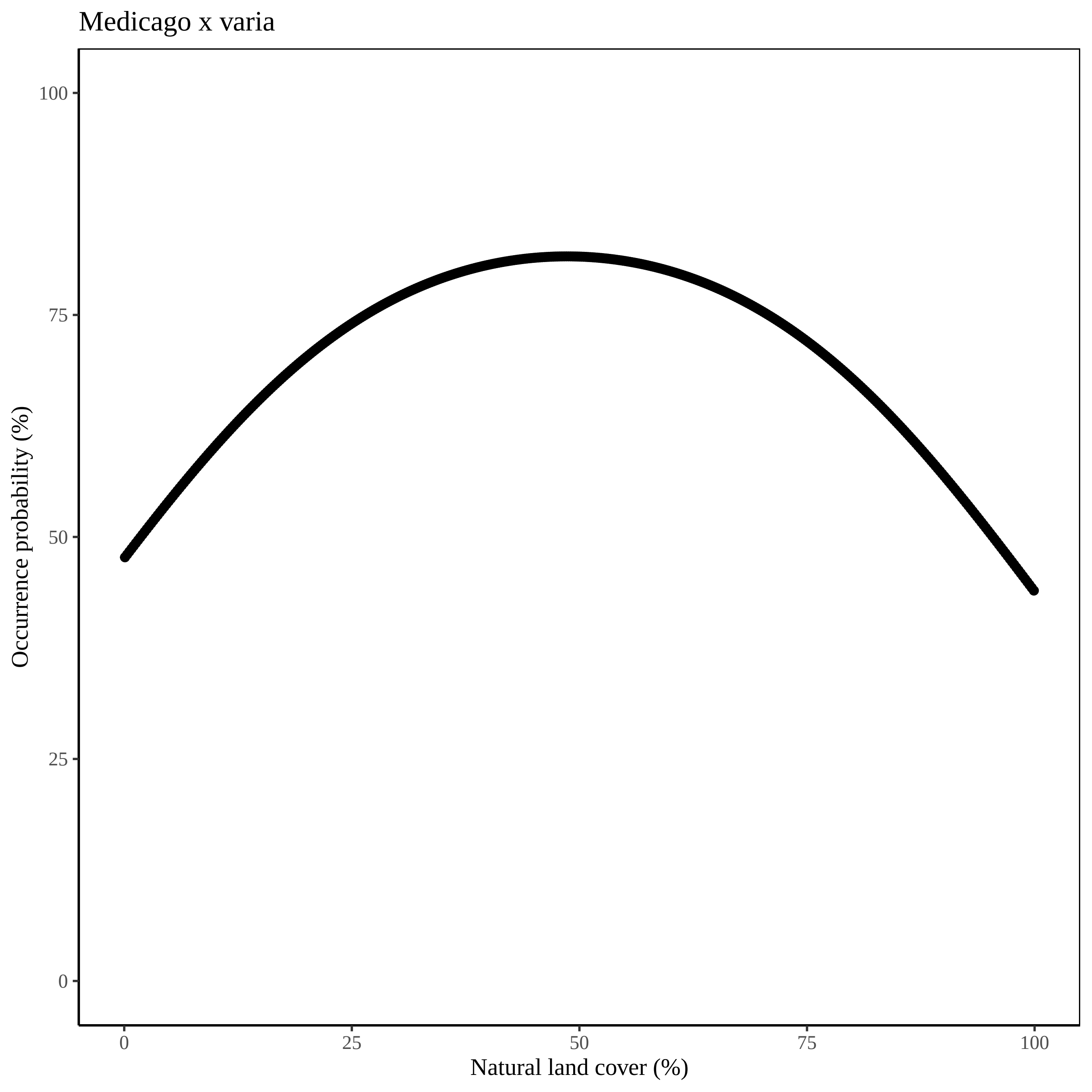
Medicago x varia
Occurrence probability (%)
100
75
50
25
0
0
25
50
75
100
Natural land cover (%)

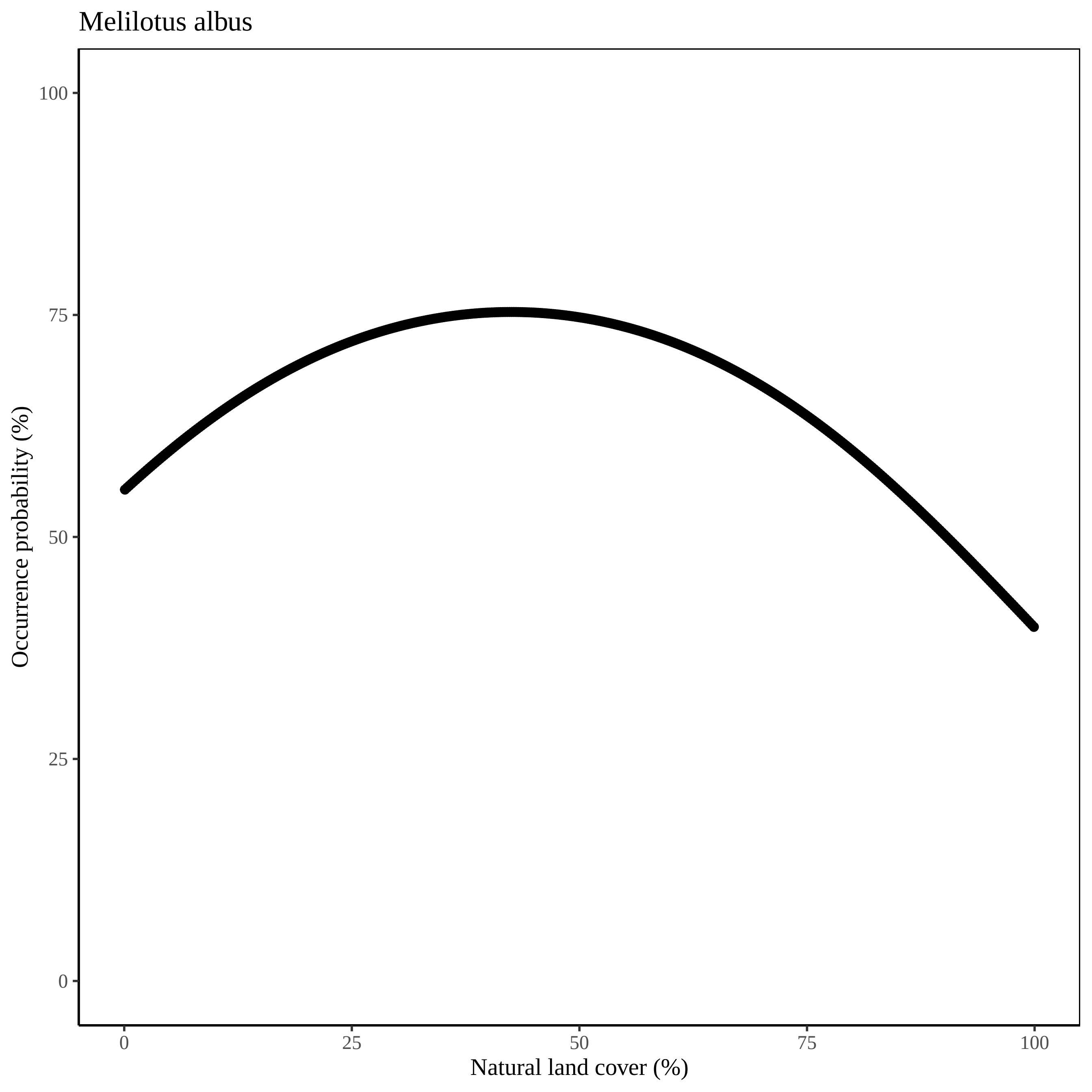

Melilotus albus
Occurrence probability (%)
100
75
50
25
0
0
25
50
75
100
Natural land cover (%)

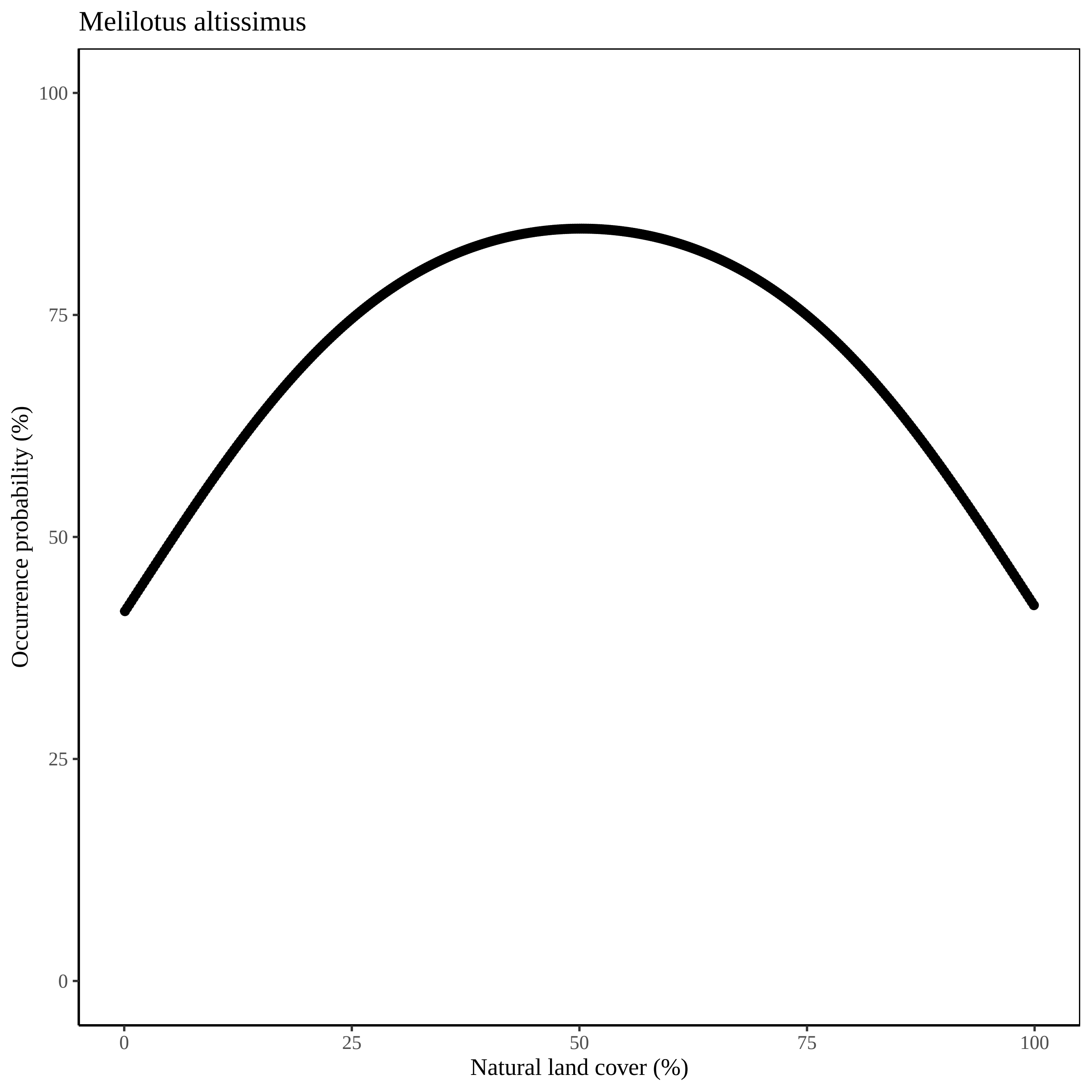

Melilotus altissimus
Occurrence probability (%)
100
75
50
25
0
0
25
50
75
100
Natural land cover (%)

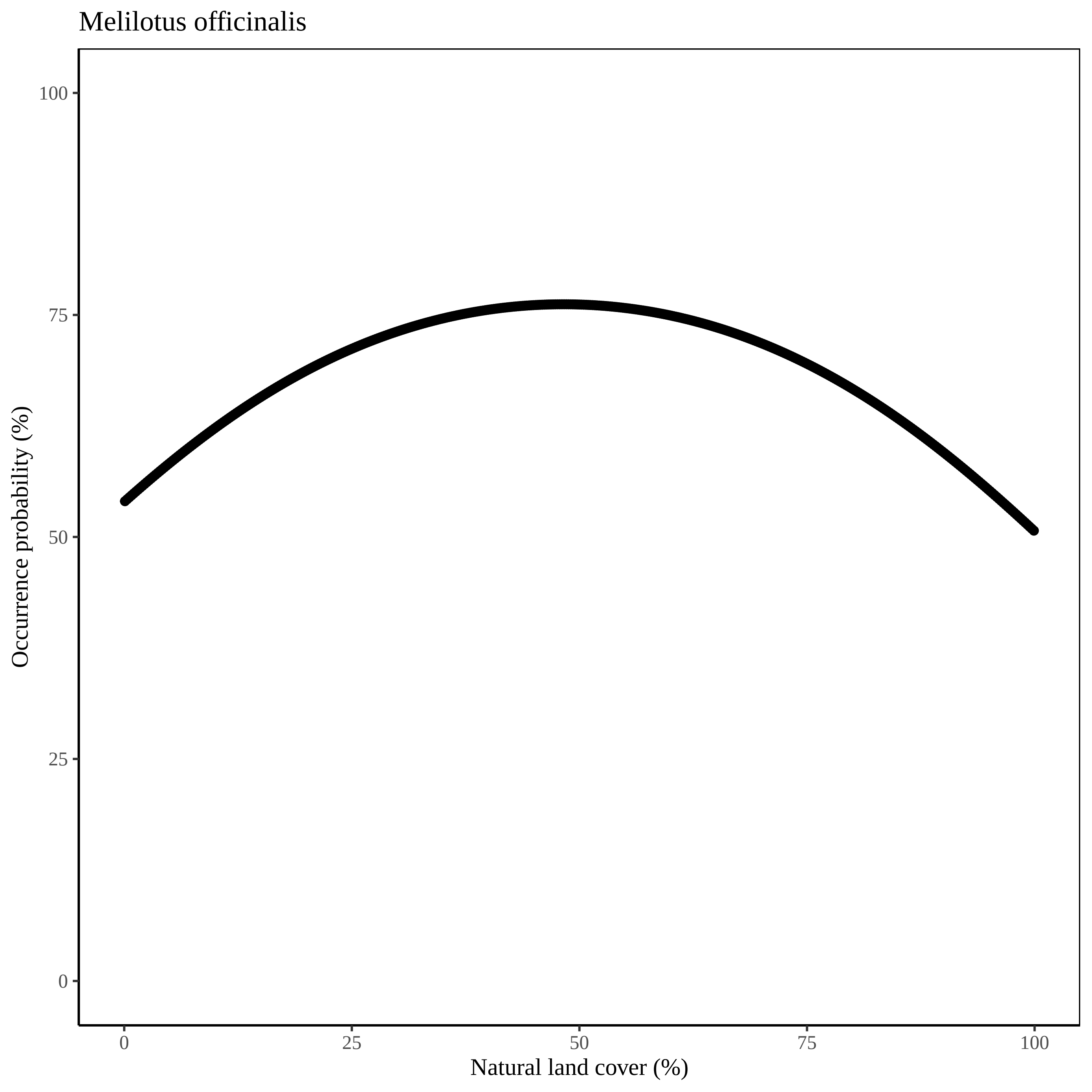
Melilotus officinalis
Occurrence probability (%)
100
75
50
25
0
0
25
50
75
100
Natural land cover (%)

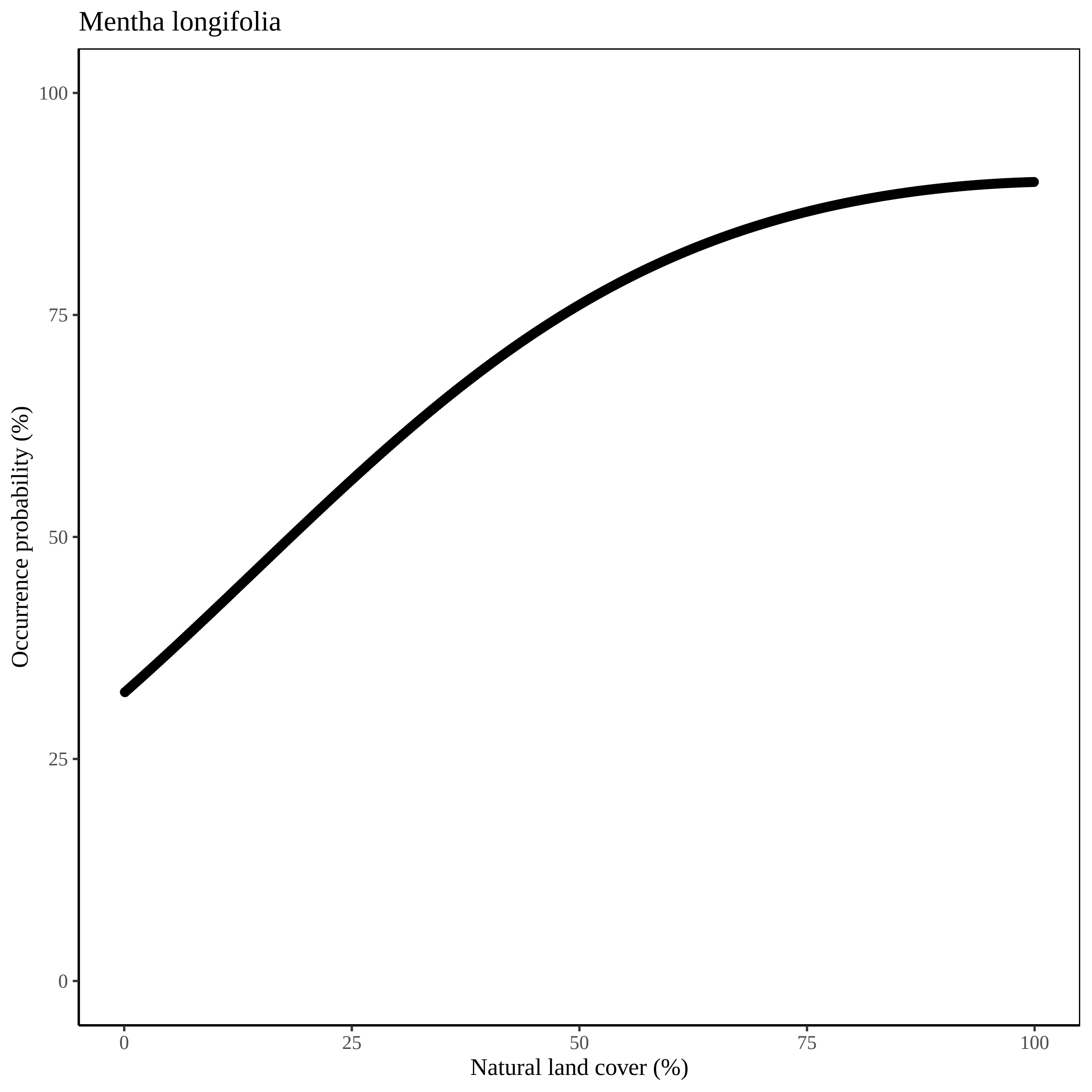
Mentha longifolia
Occurrence probability (%)
0
25
50
75
100
Natural land cover (%)
0
25
50
75
100

Mentha pulegium

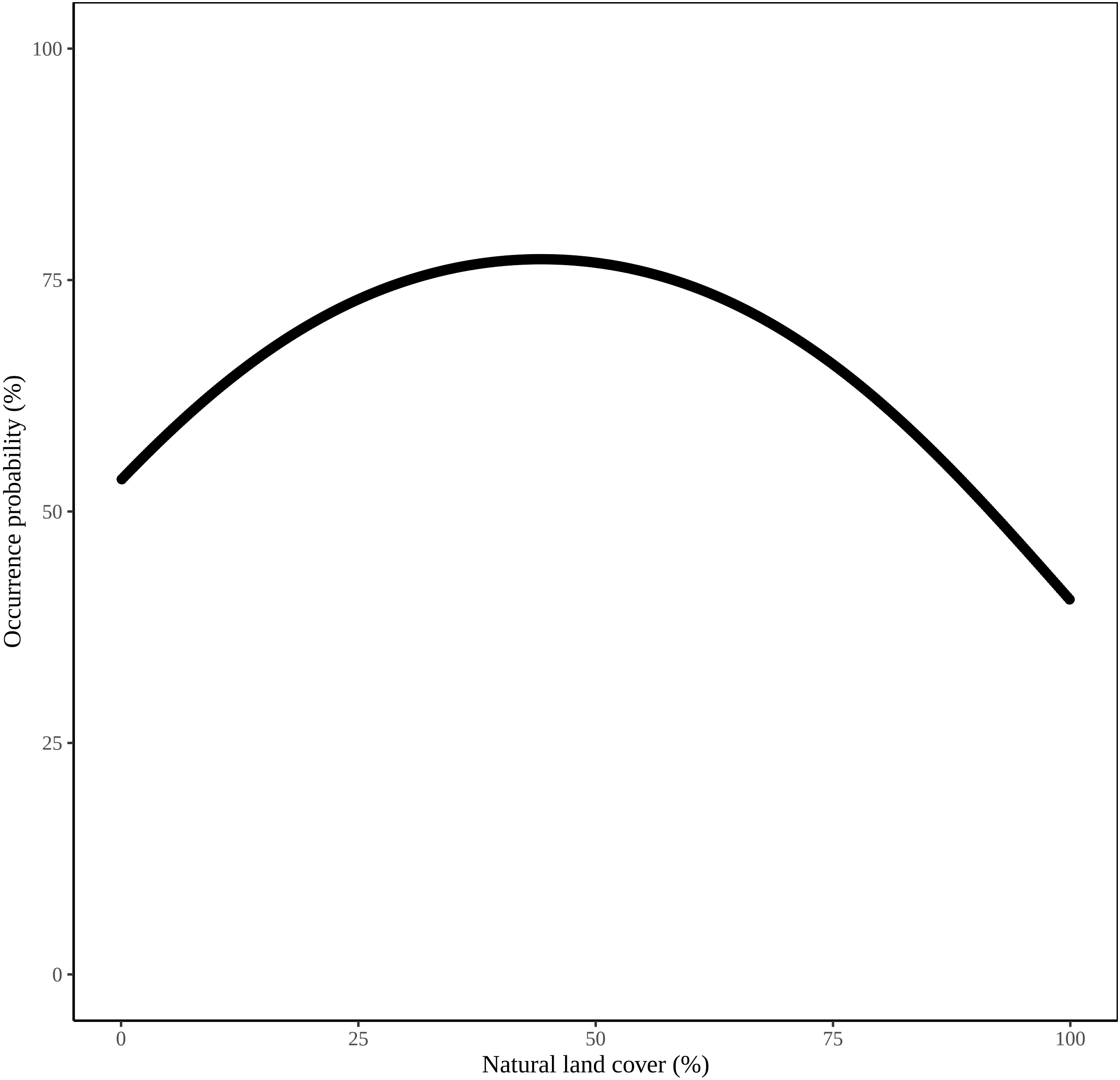

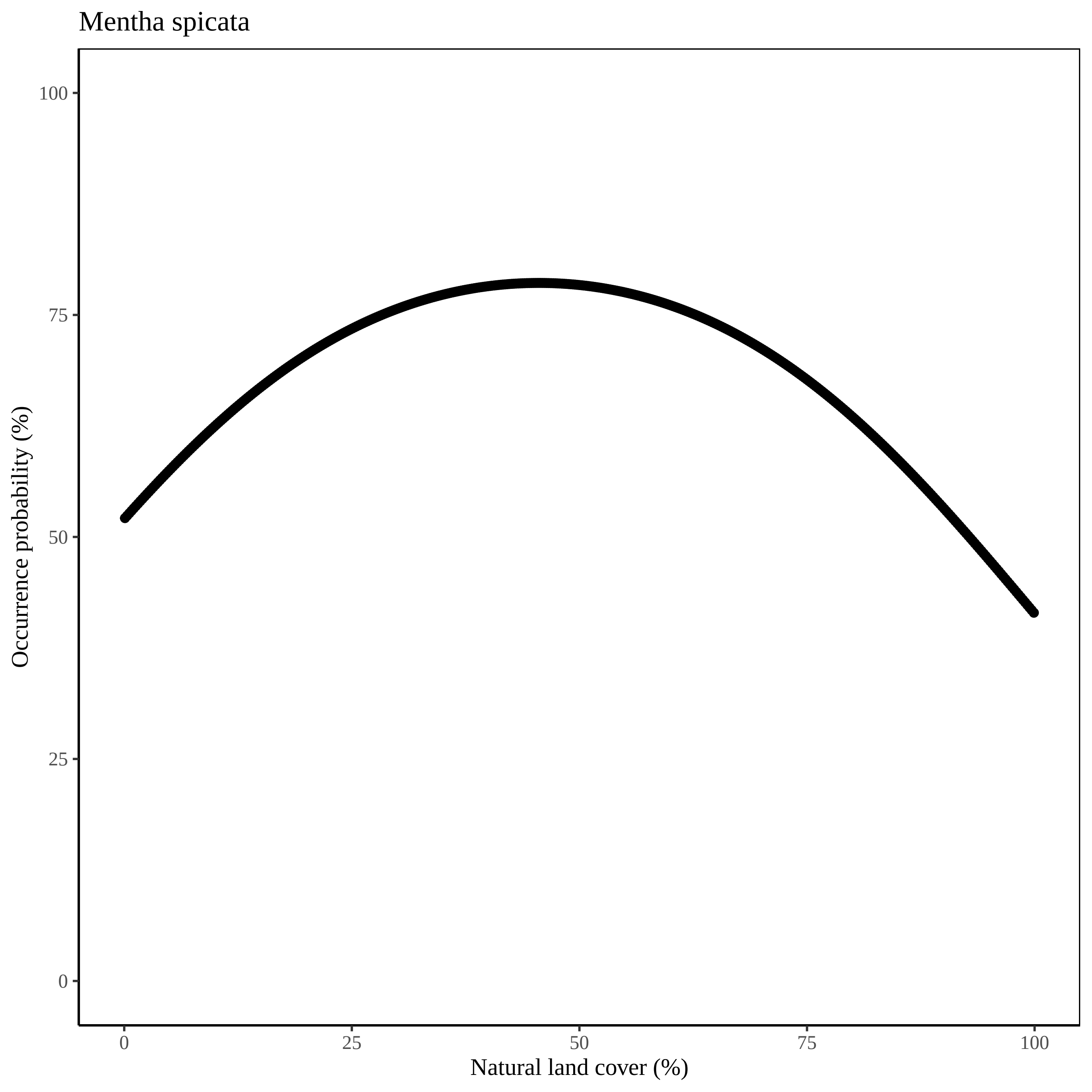

Mentha spicata
Occurrence probability (%)
100
75
50
25
0
0
25
50
75
100
Natural land cover (%)

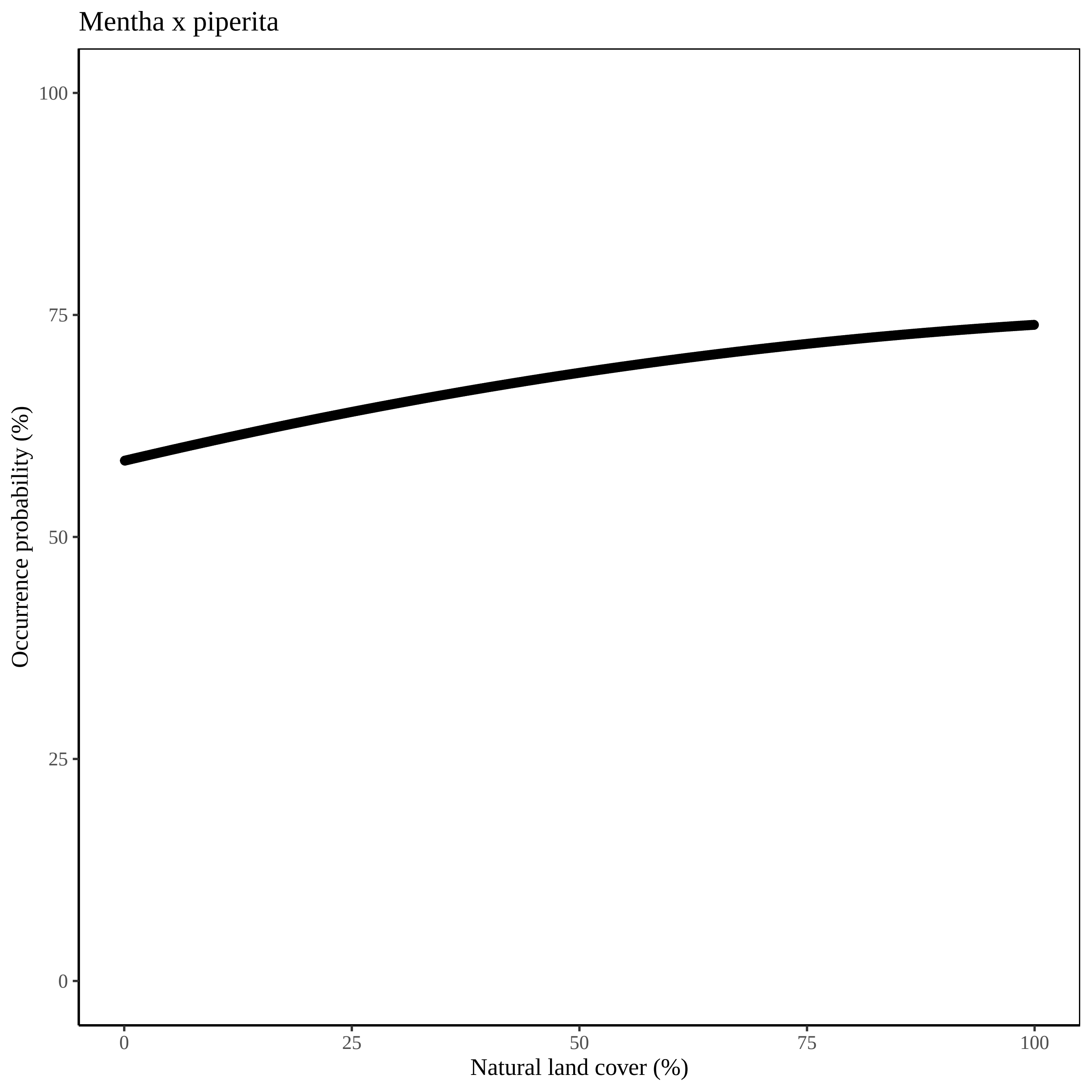
Mentha x piperita
Occurrence probability (%)
100
75
50
25
0
0
25
50
75
100
Natural land cover (%)

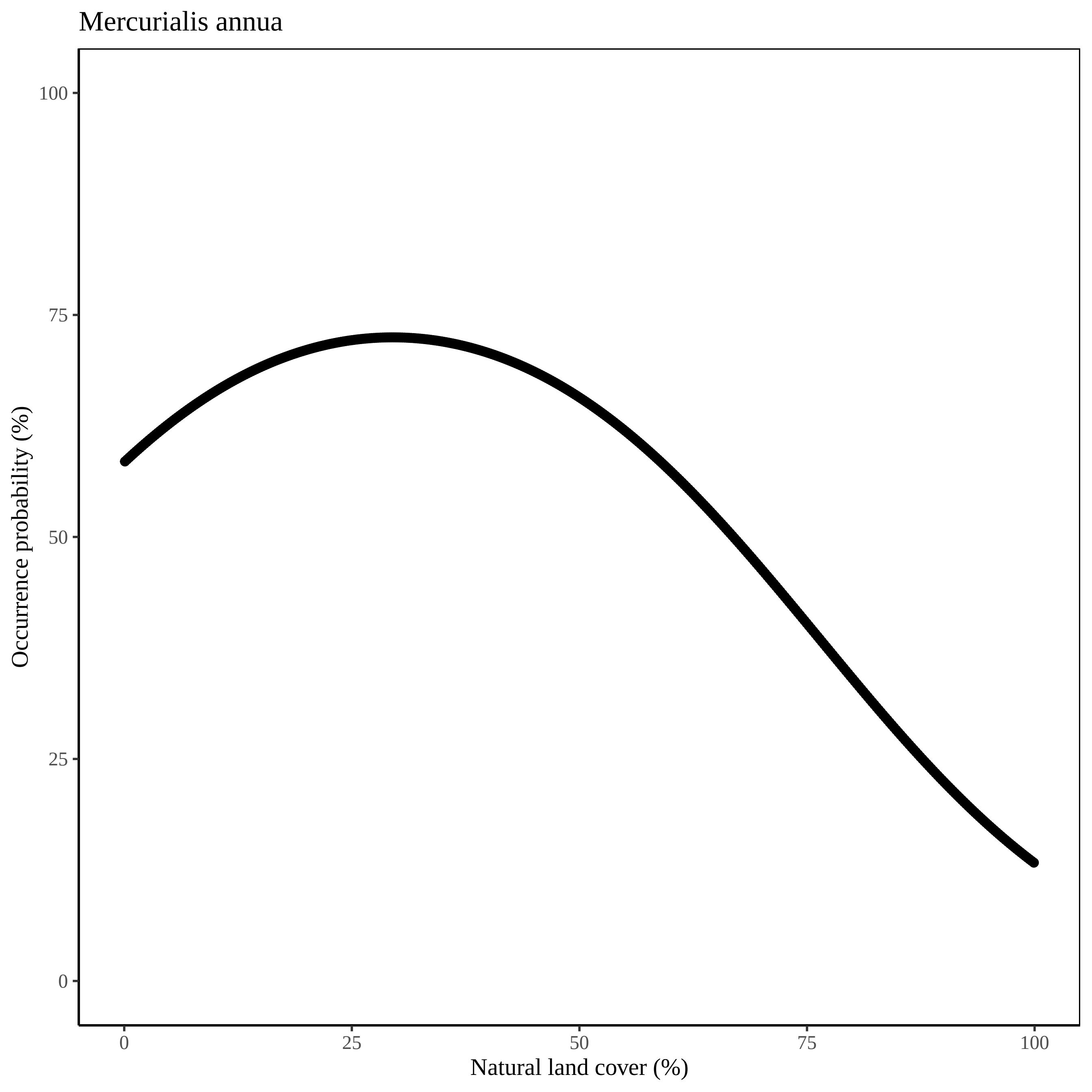
Mercurialis annua
Occurrence probability (%)
100
75
50
25
0
0
25
50
75
100
Natural land cover (%)

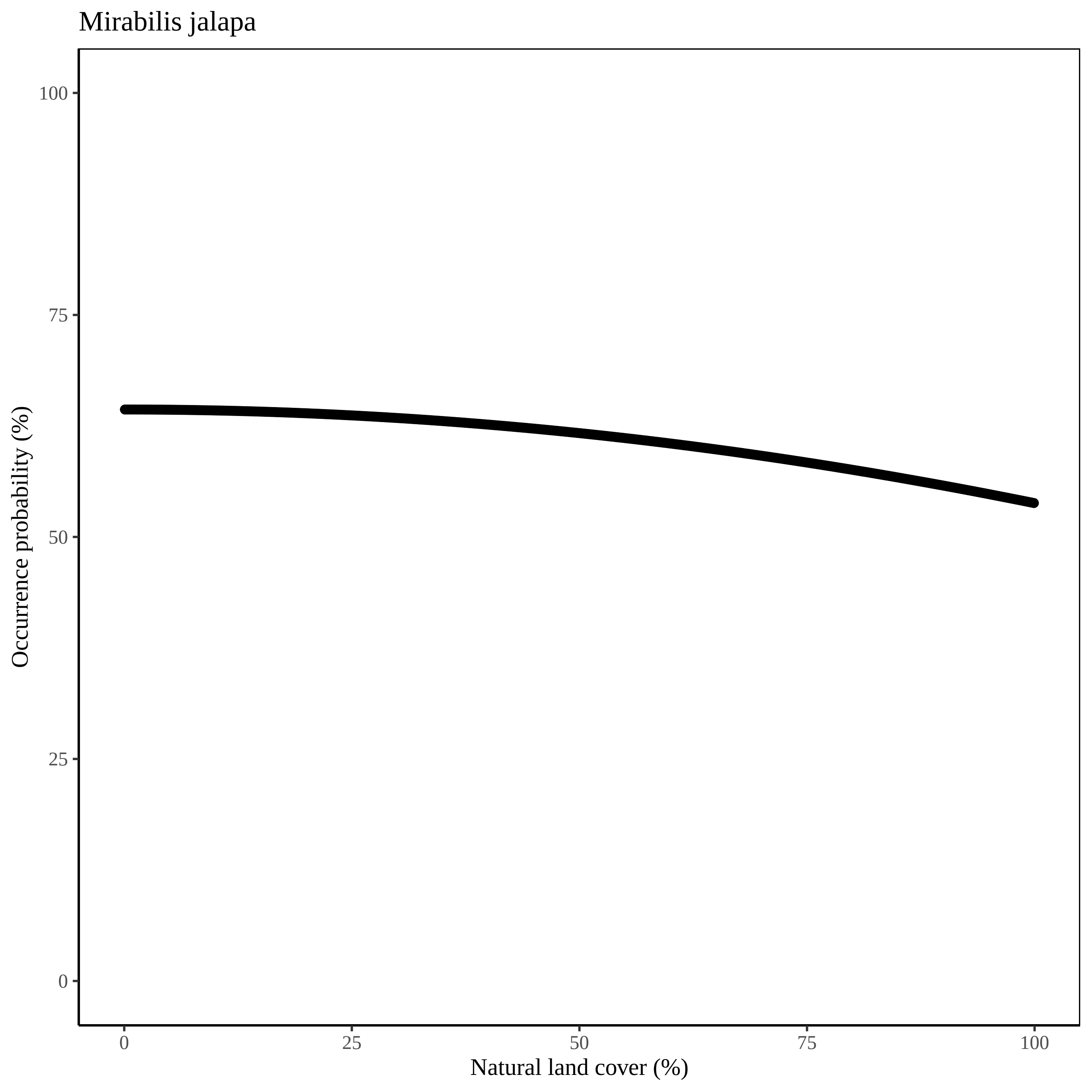

Mirabilis jalapa
Occurrence probability (%)
100
75
50
25
0
0
25
50
75
100
Natural land cover (%)

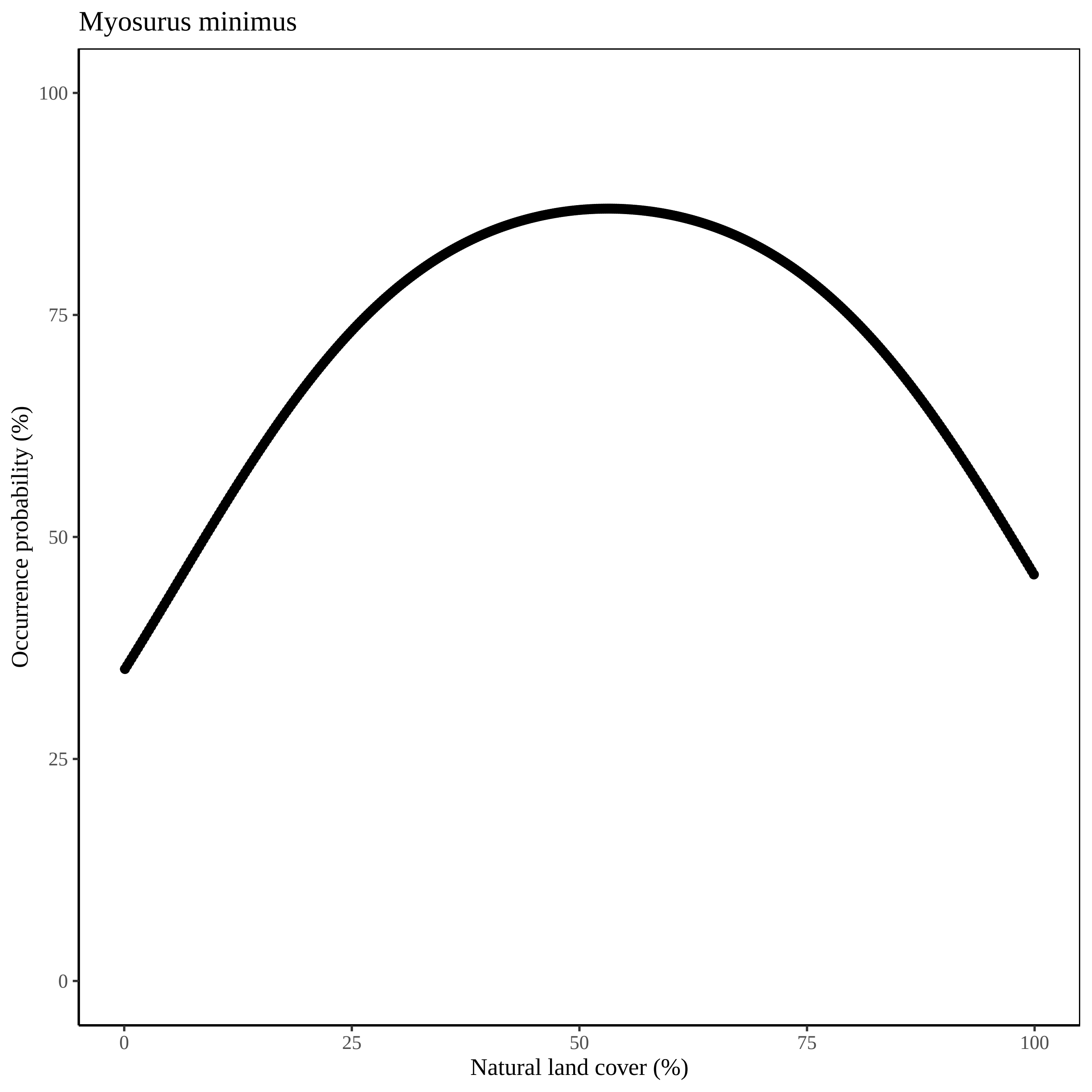
Myosurus minimus
Occurrence probability (%)
100
75
50
25
0
0
25
50
75
100
Natural land cover (%)

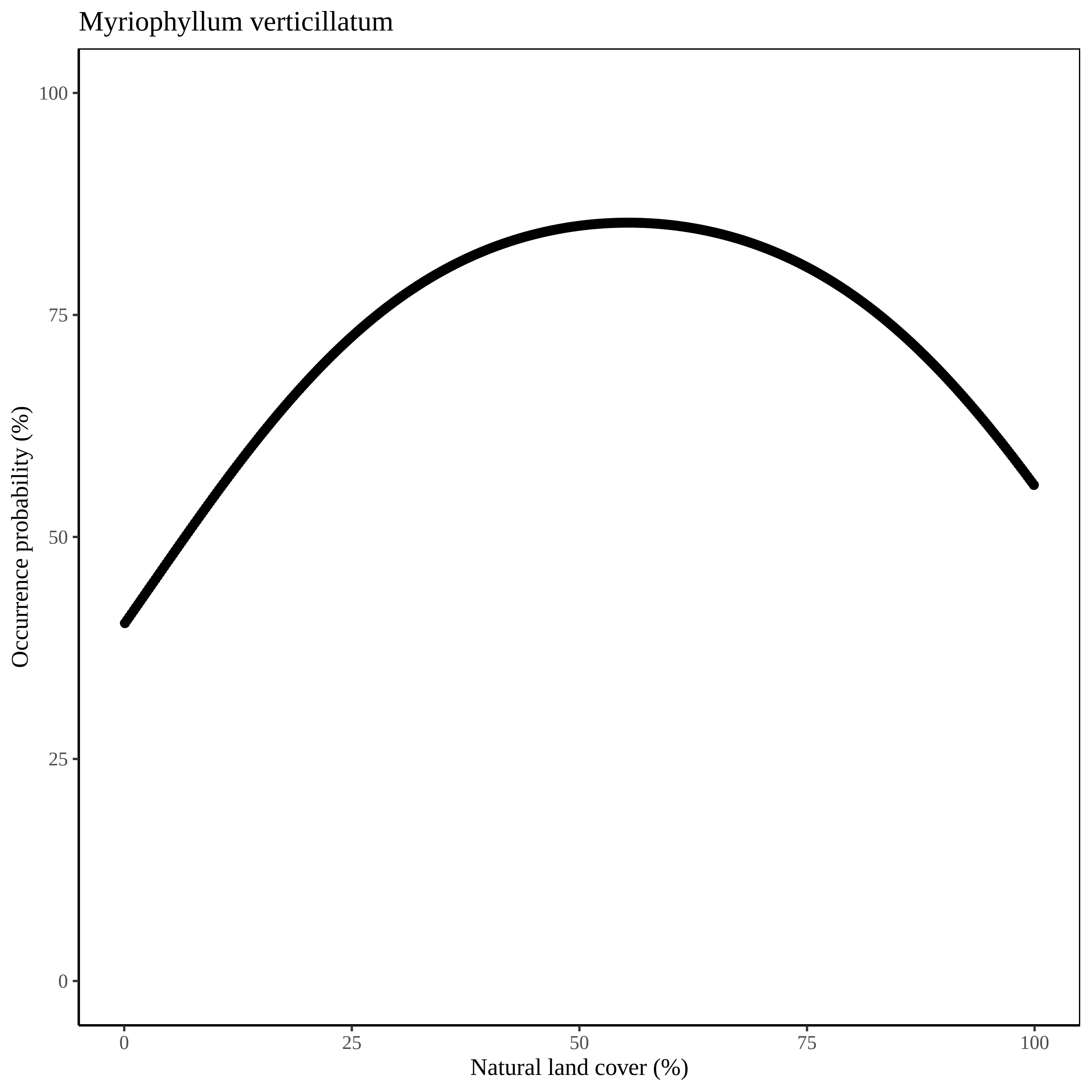
Myriophyllum verticillatum
Occurrence probability (%)
100
75
50
25
0
0
25
50
75
100
Natural land cover (%)

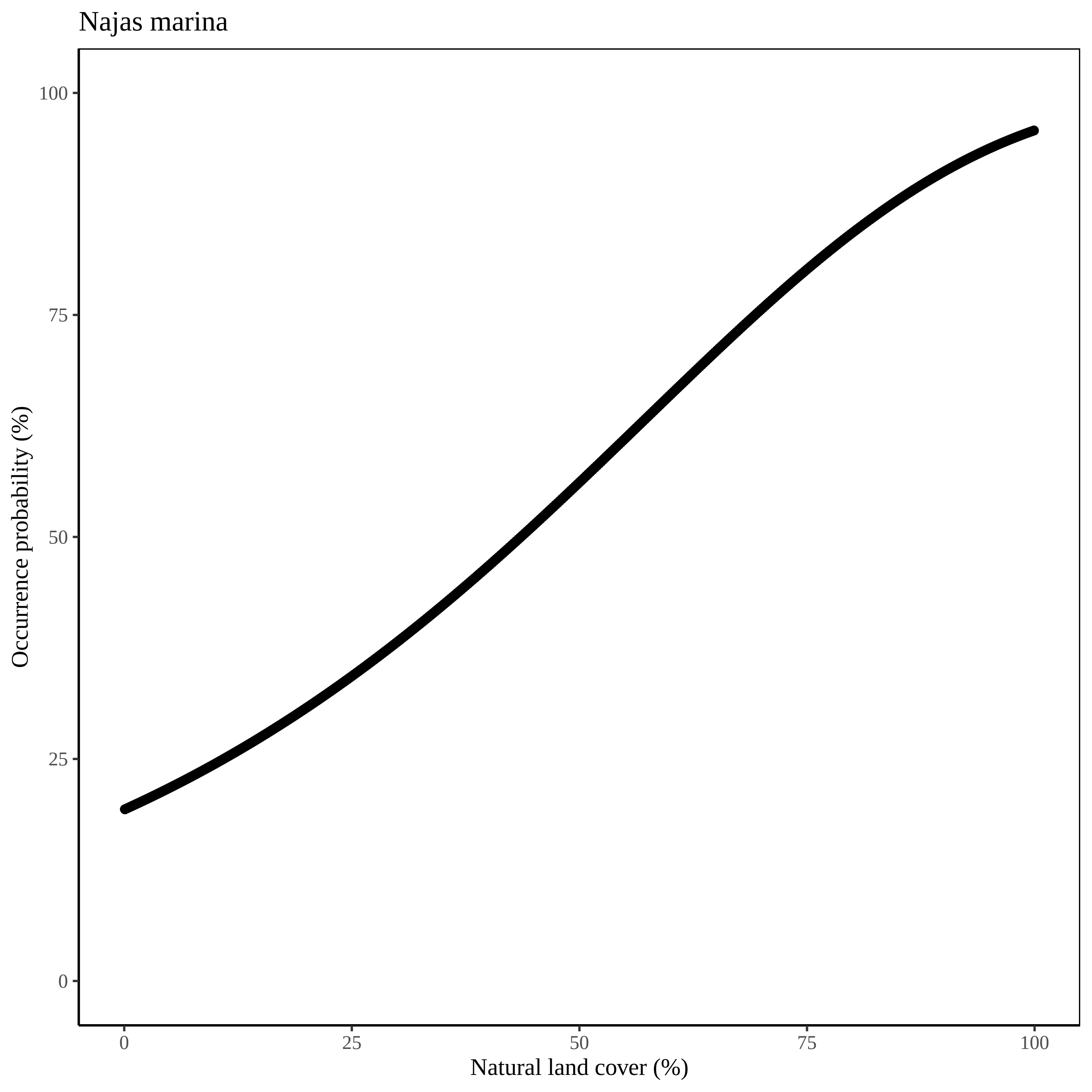

Najas marina
Occurrence probability (%)
100
75
50
25
0
0
25
50
75
100
Natural land cover (%)

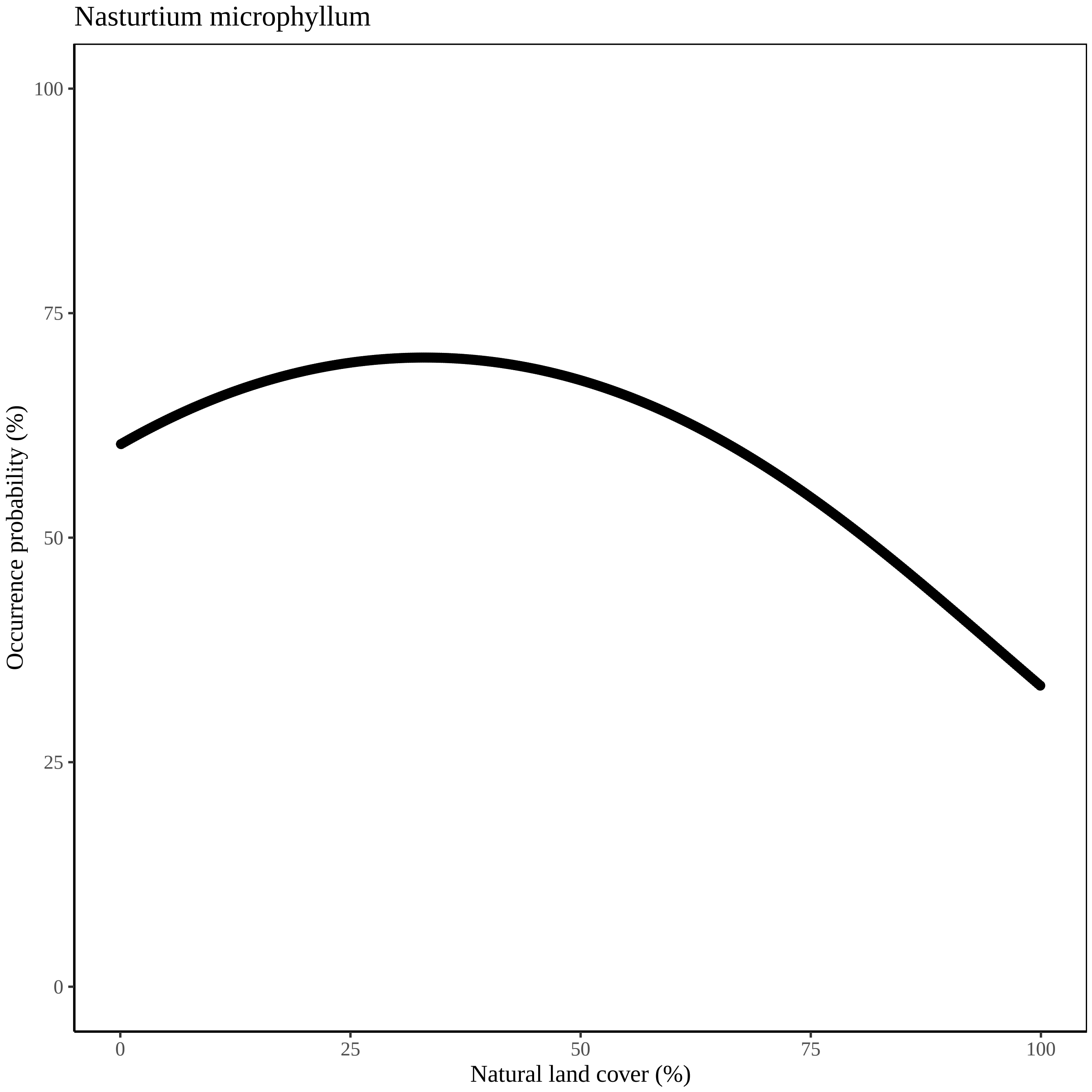
Nasturtium microphyllum
Occurrence probability (%)
100
75
50
25
0
0
25
50
75
100
Natural land cover (%)

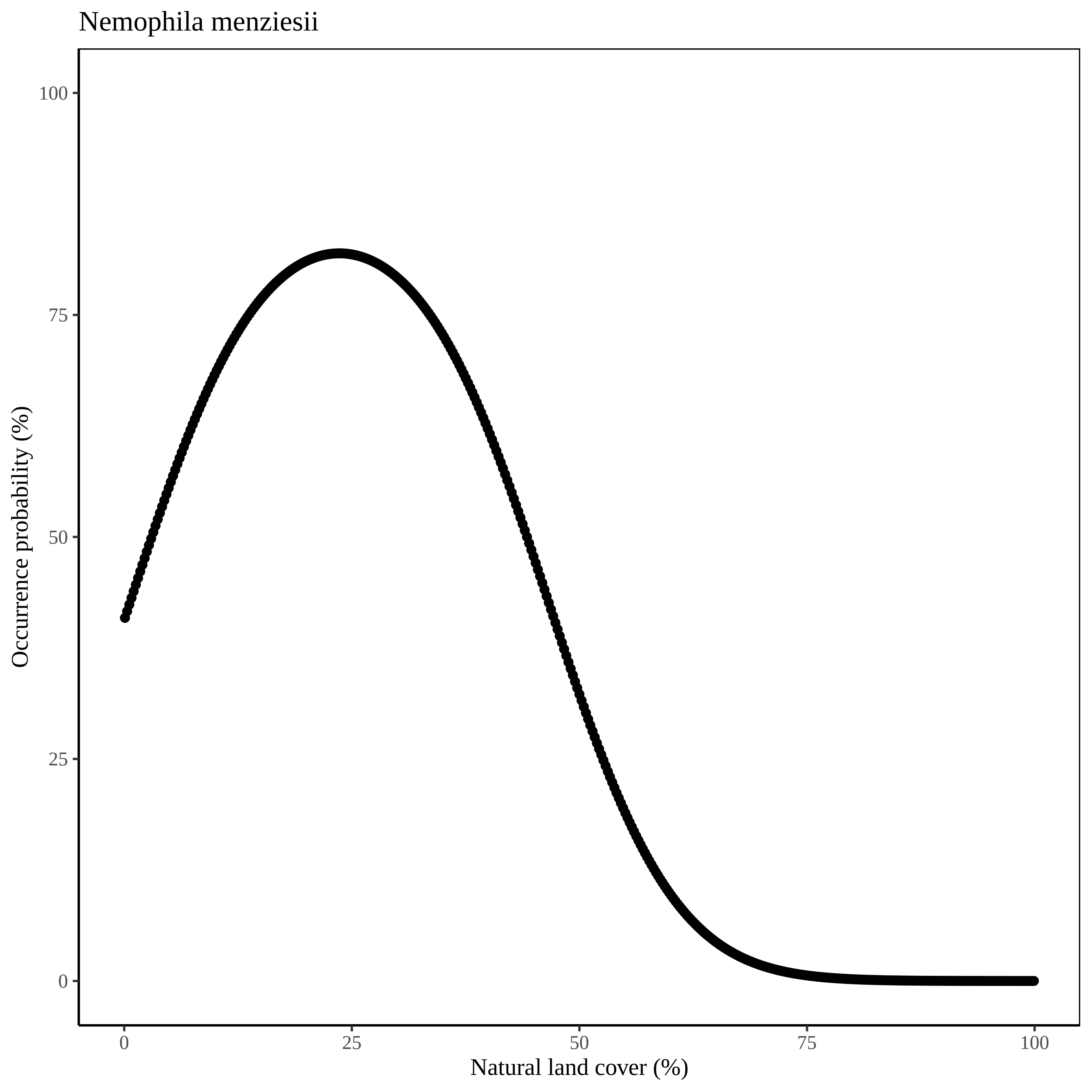
Nemophila menziesii
Occurrence probability (%)
100
75
50
25
0
0
25
50
75
100
Natural land cover (%)

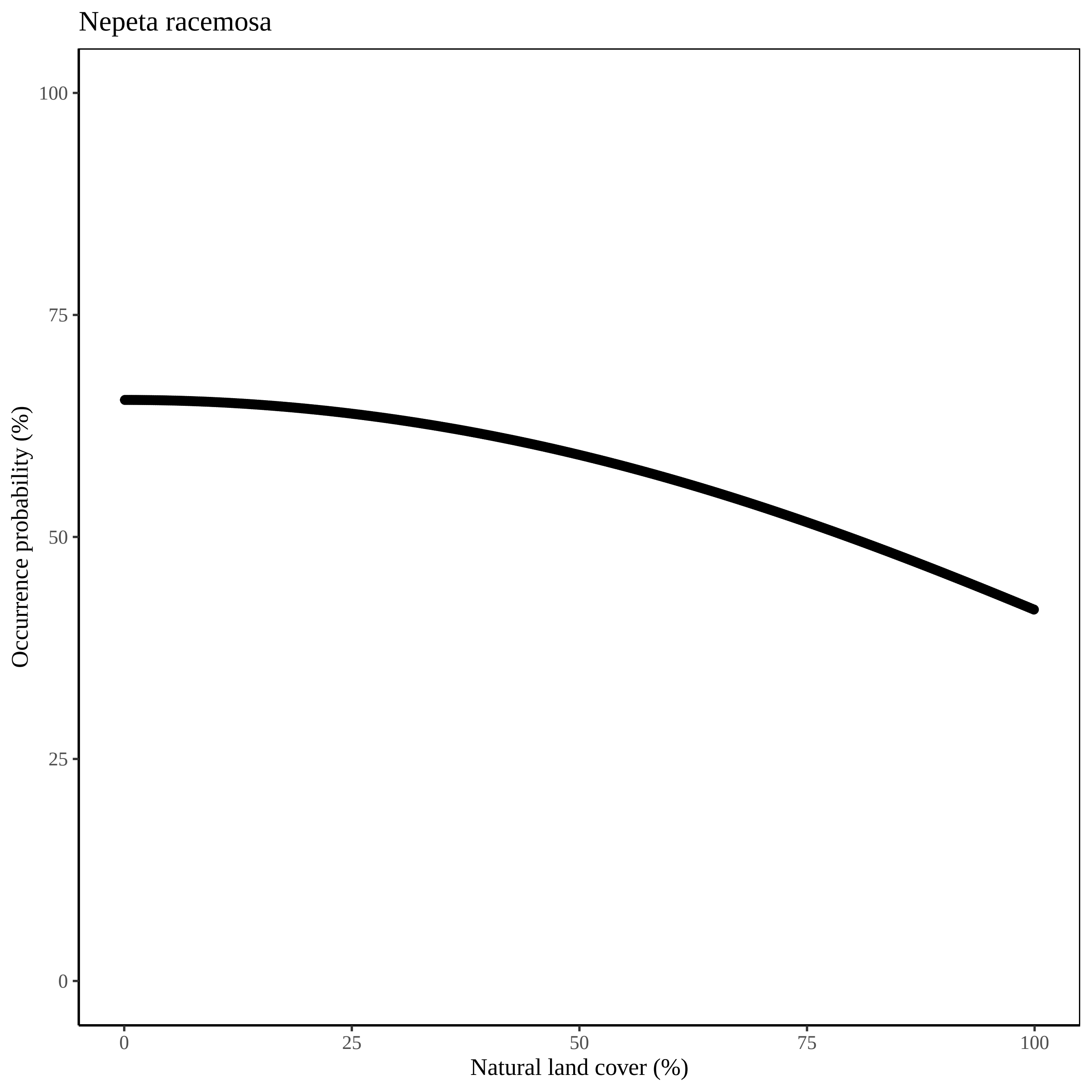

Nepeta racemosa
Occurrence probability (%)
100
75
50
25
0
0
25
50
75
100
Natural land cover (%)

Nepeta x faassenii

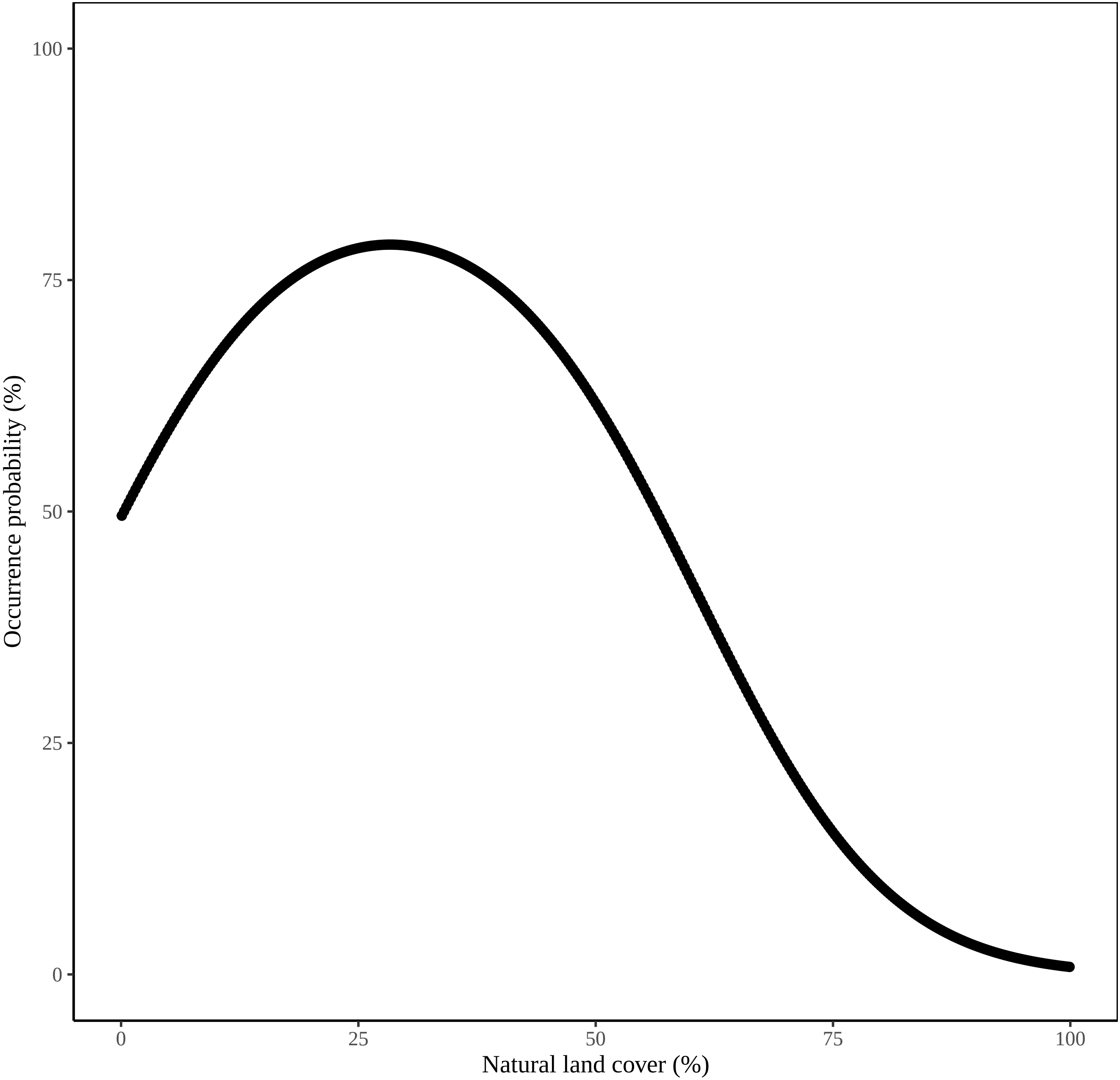

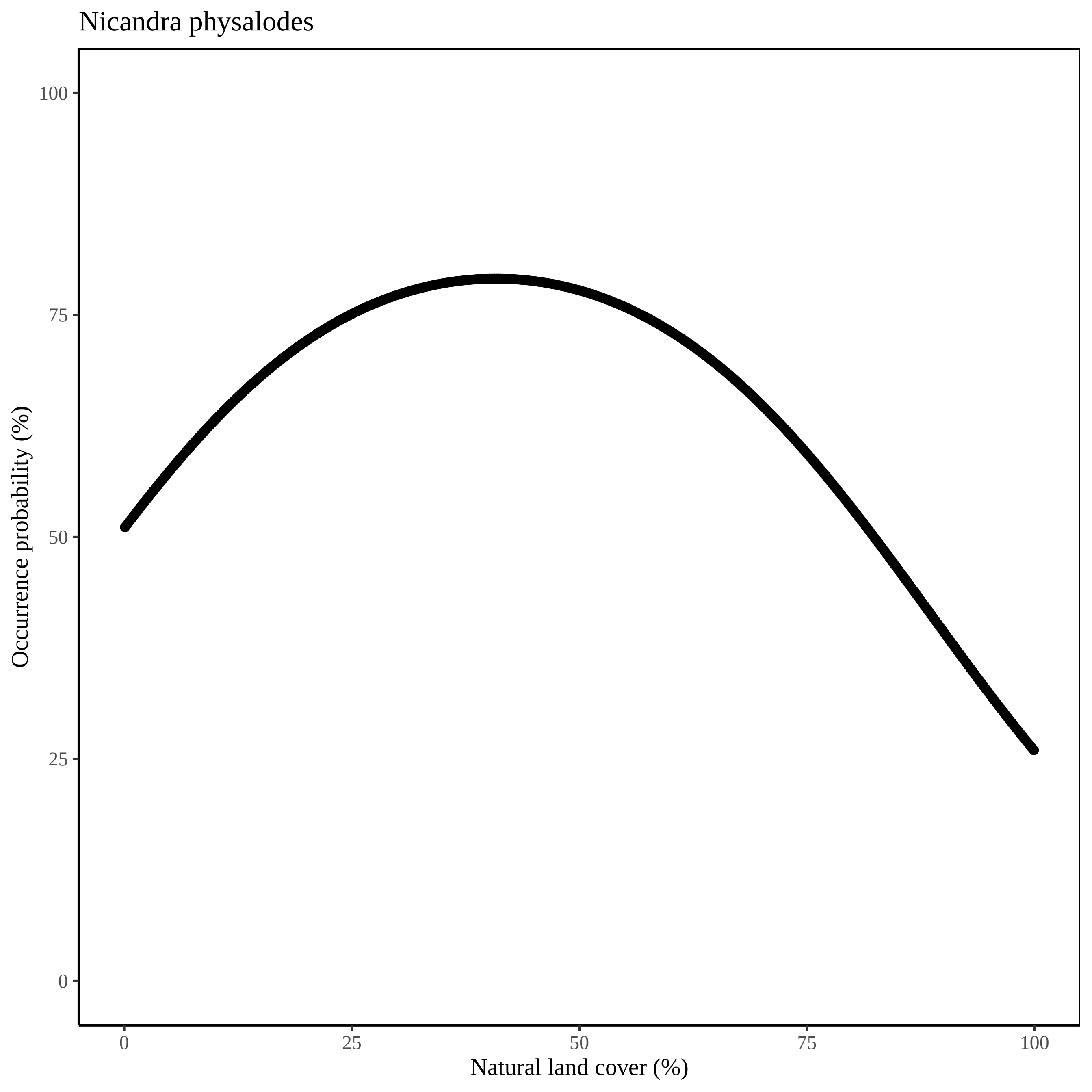
Nicandra physalodes
Occurrence probability (%)
100
75
50
25
0
0
25
50
75
100
Natural land cover (%)

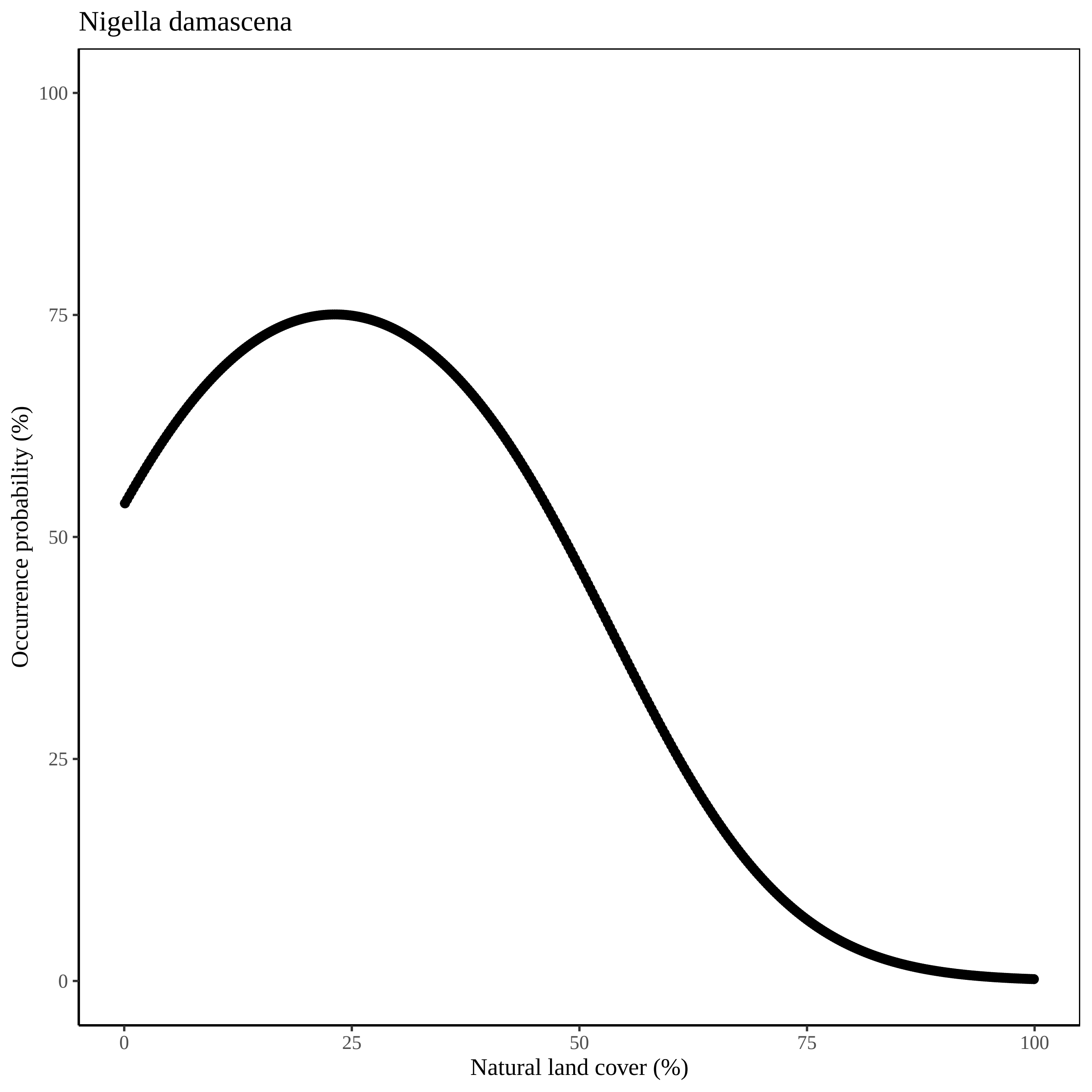
Nigella damascena
100
75
50
25
0
0
25
50
75
100
Occurrence probability (%)
Natural land cover (%)

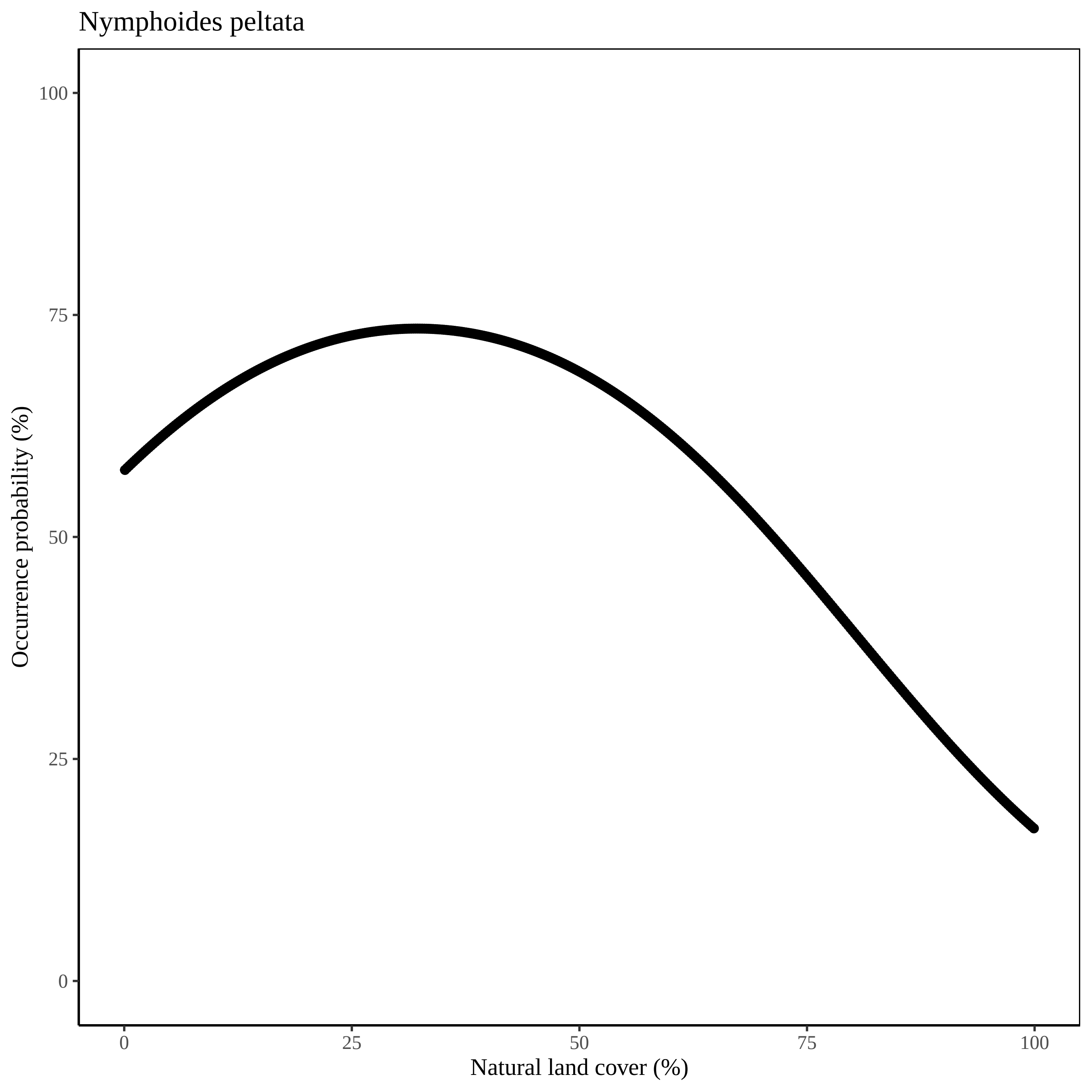

Nymphoides peltata
Occurrence probability (%)
100
75
50
25
0
0
25
50
75
100
Natural land cover (%)

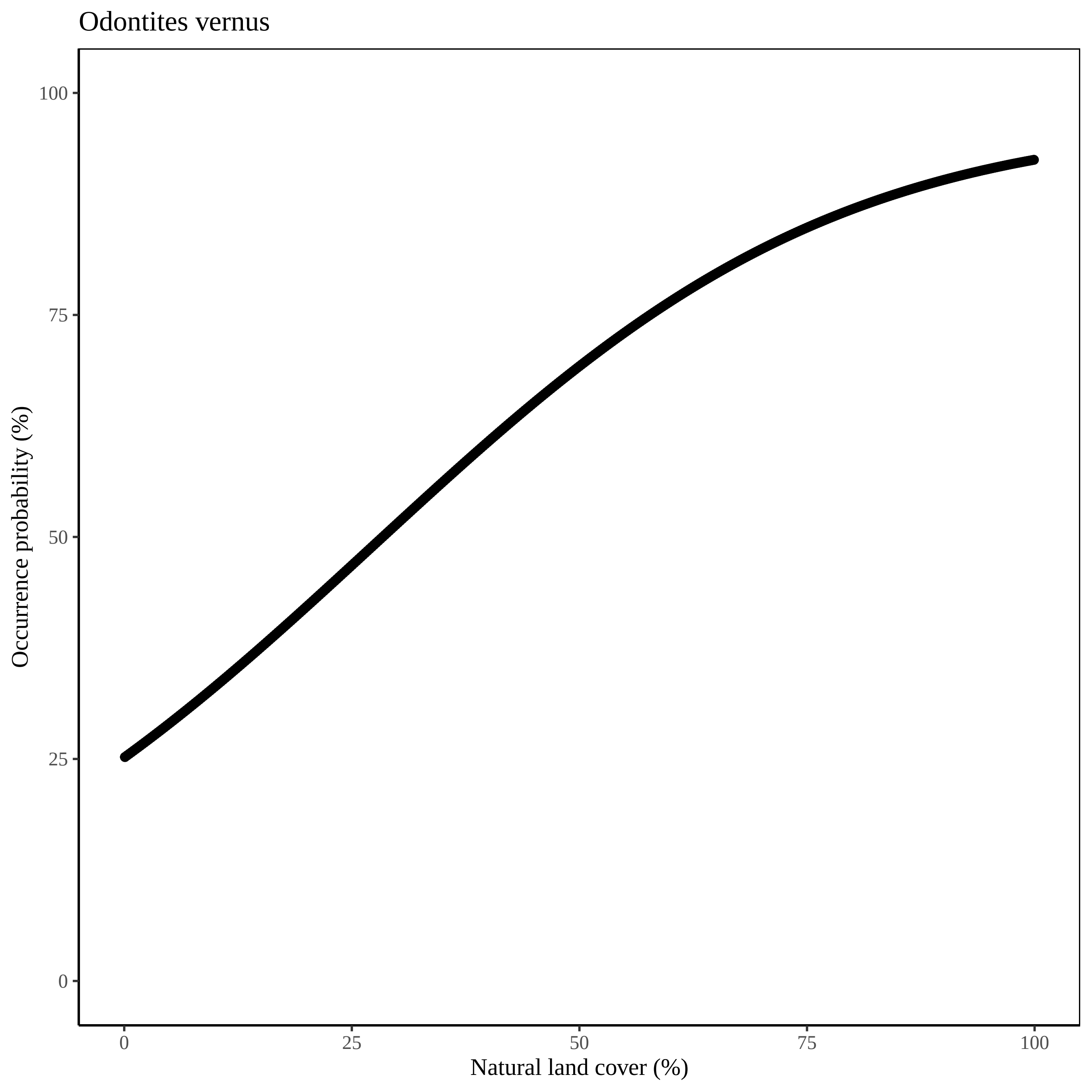
Odontites vernus
Occurrence probability (%)
100
75
50
25
0
0
25
50
75
100
Natural land cover (%)

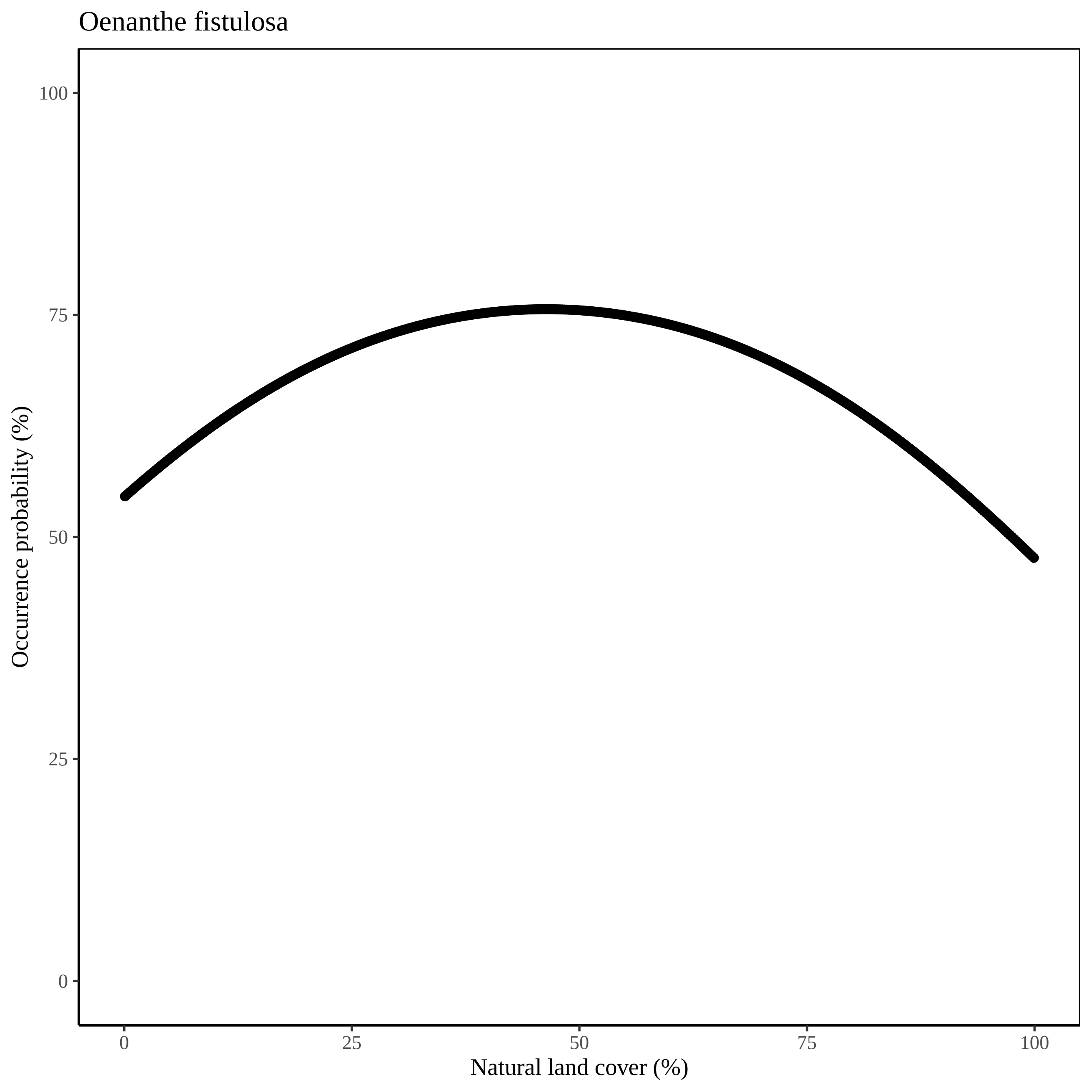
Oenanthe fistulosa
Occurrence probability (%)
100
75
50
25
0
0
25
50
75
100
Natural land cover (%)

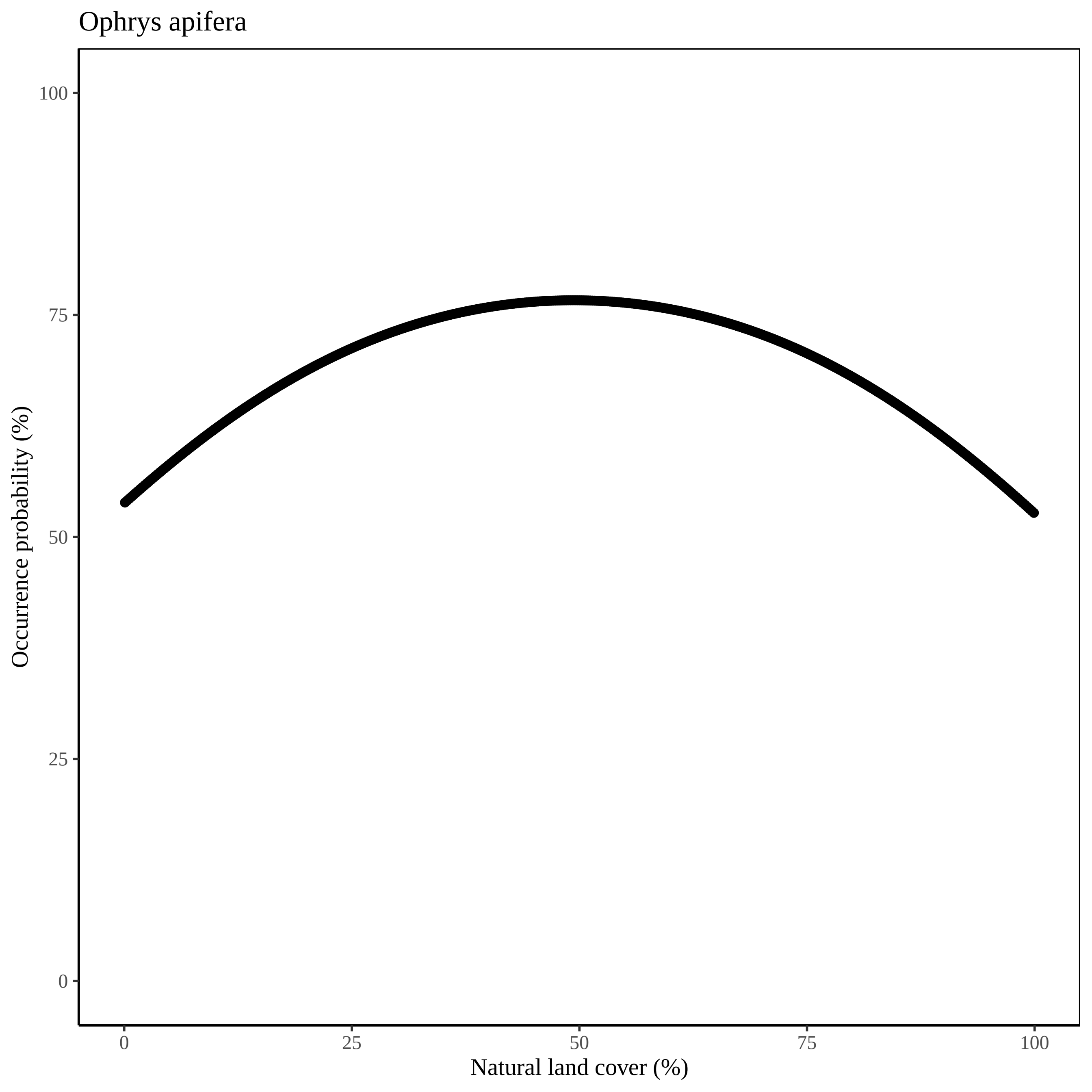

Ophrys apifera
Occurrence probability (%)
100
75
50
25
0
0
25
50
75
100
Natural land cover (%)

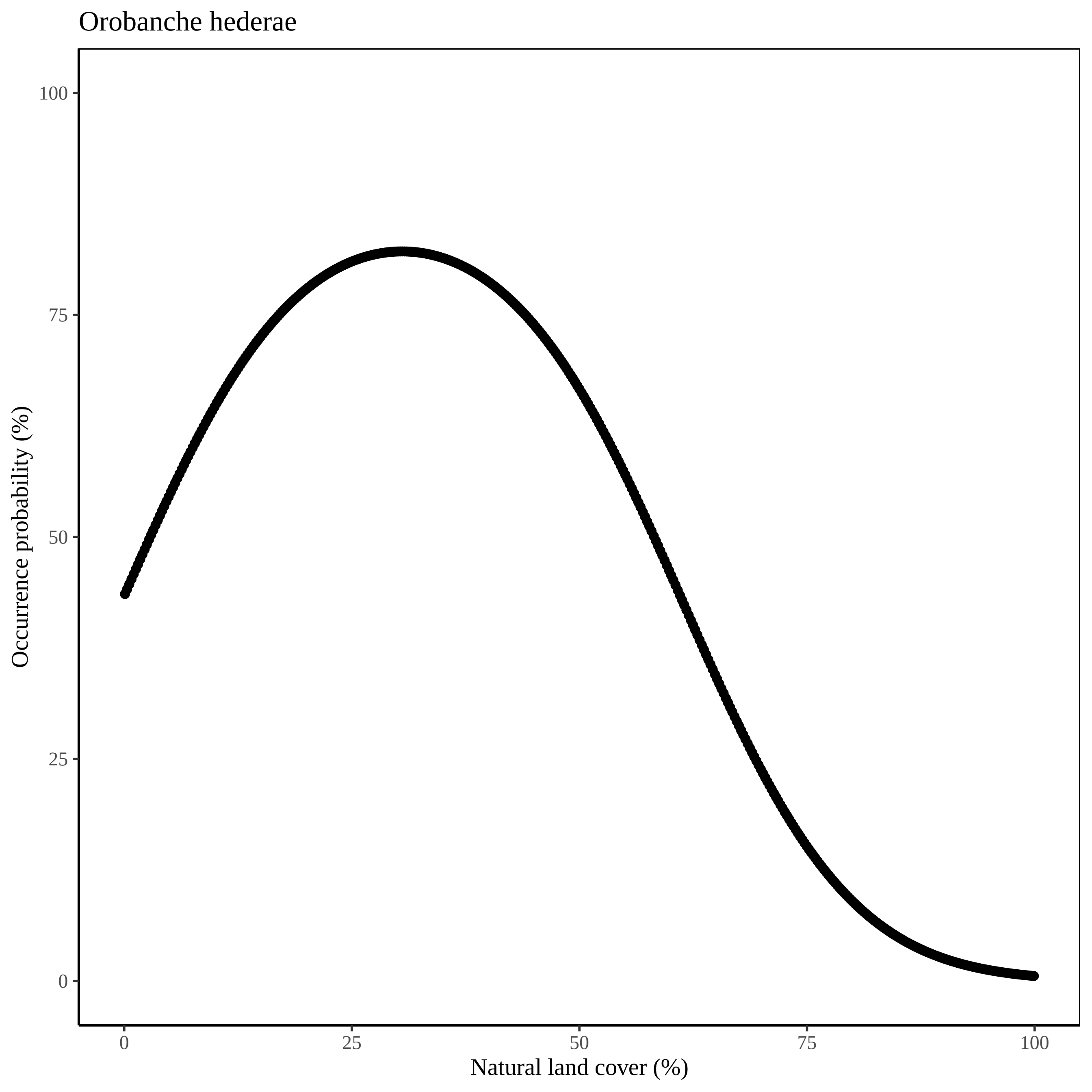
Orobanche hederae
Occurrence probability (%)
100
75
50
25
0
0
25
50
75
100
Natural land cover (%)

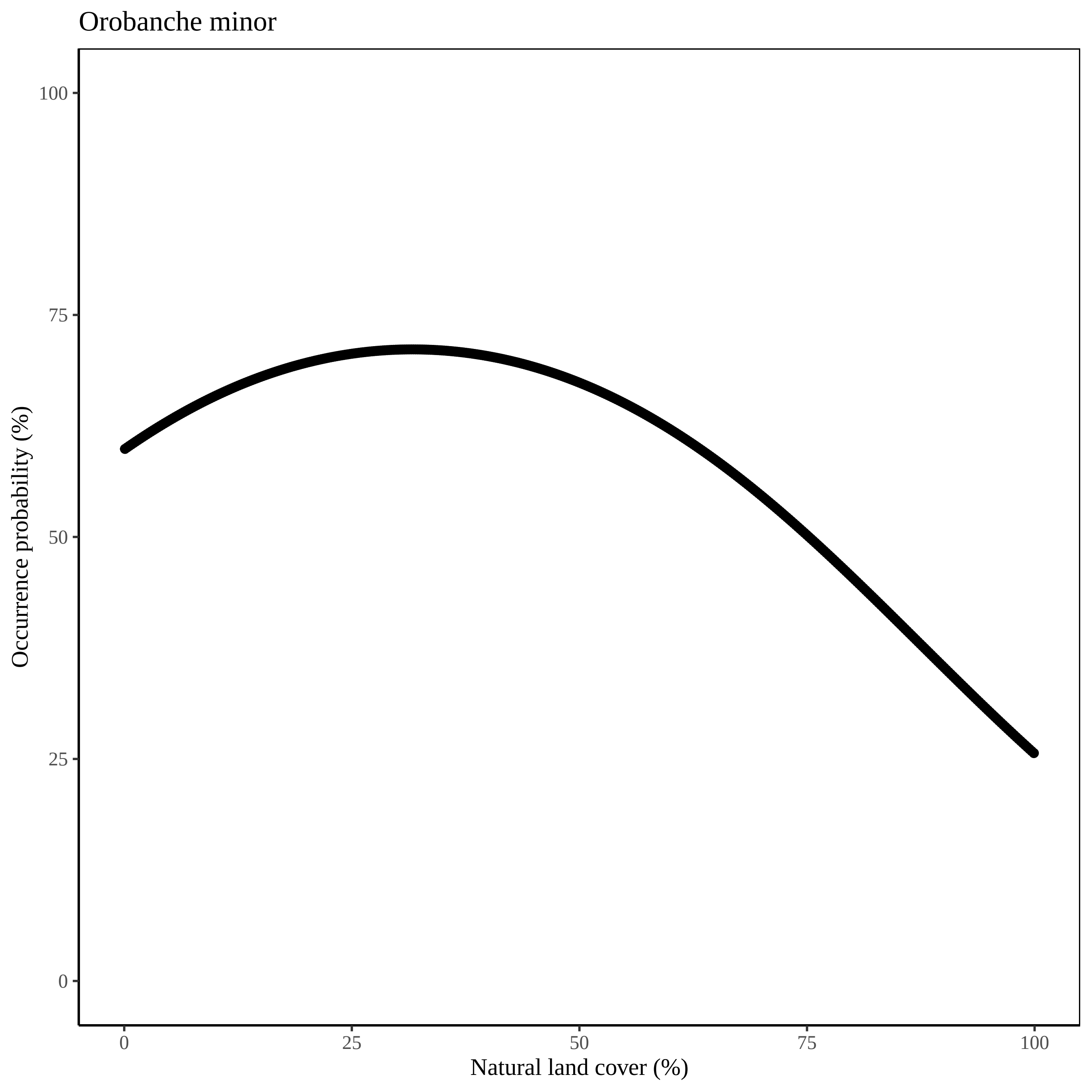
Orobanche minor
Occurrence probability (%)
100
75
50
25
0
0
25
50
75
100
Natural land cover (%)

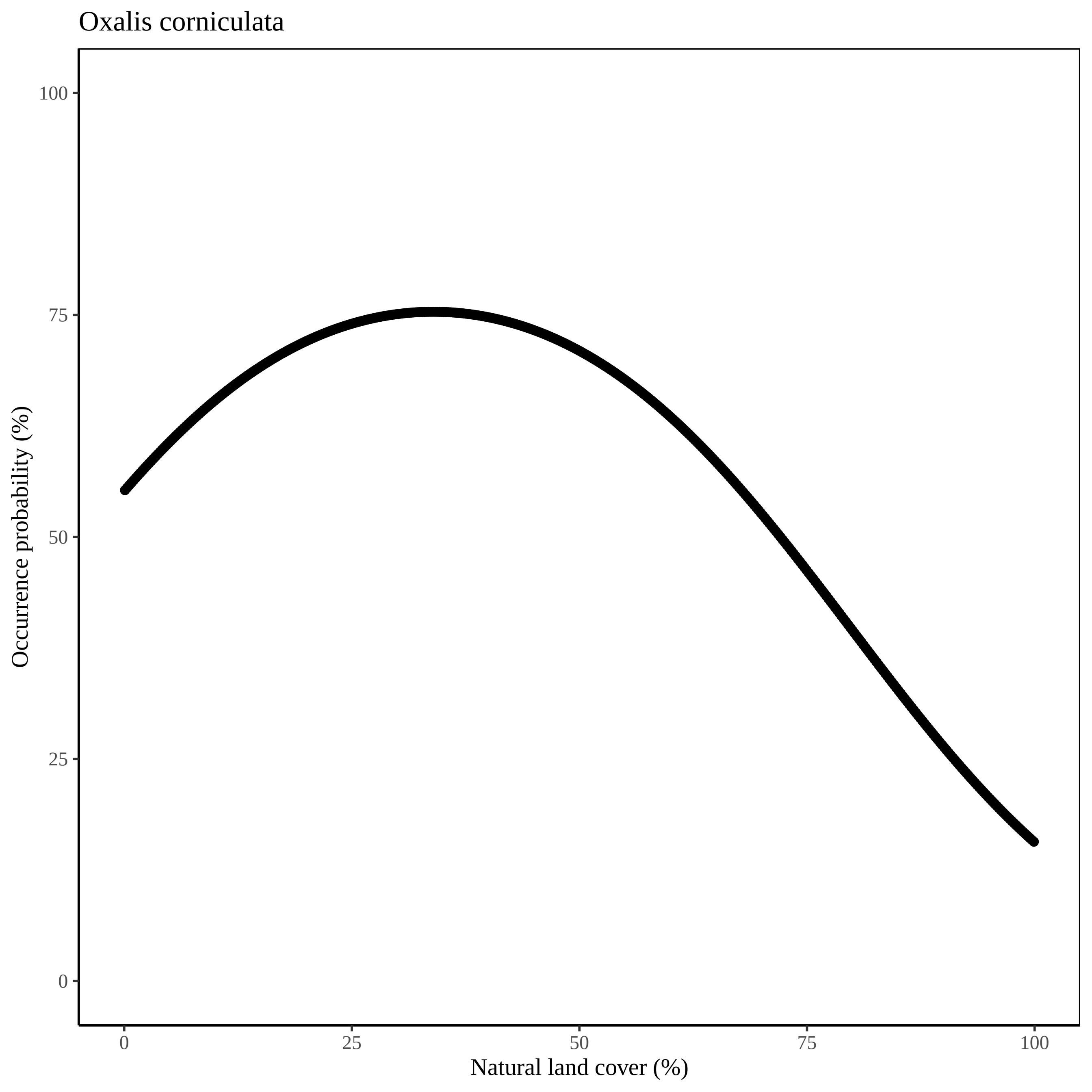
Oxalis corniculata
Occurrence probability (%)
100
75
50
25
0
0
25
50
75
100
Natural land cover (%)

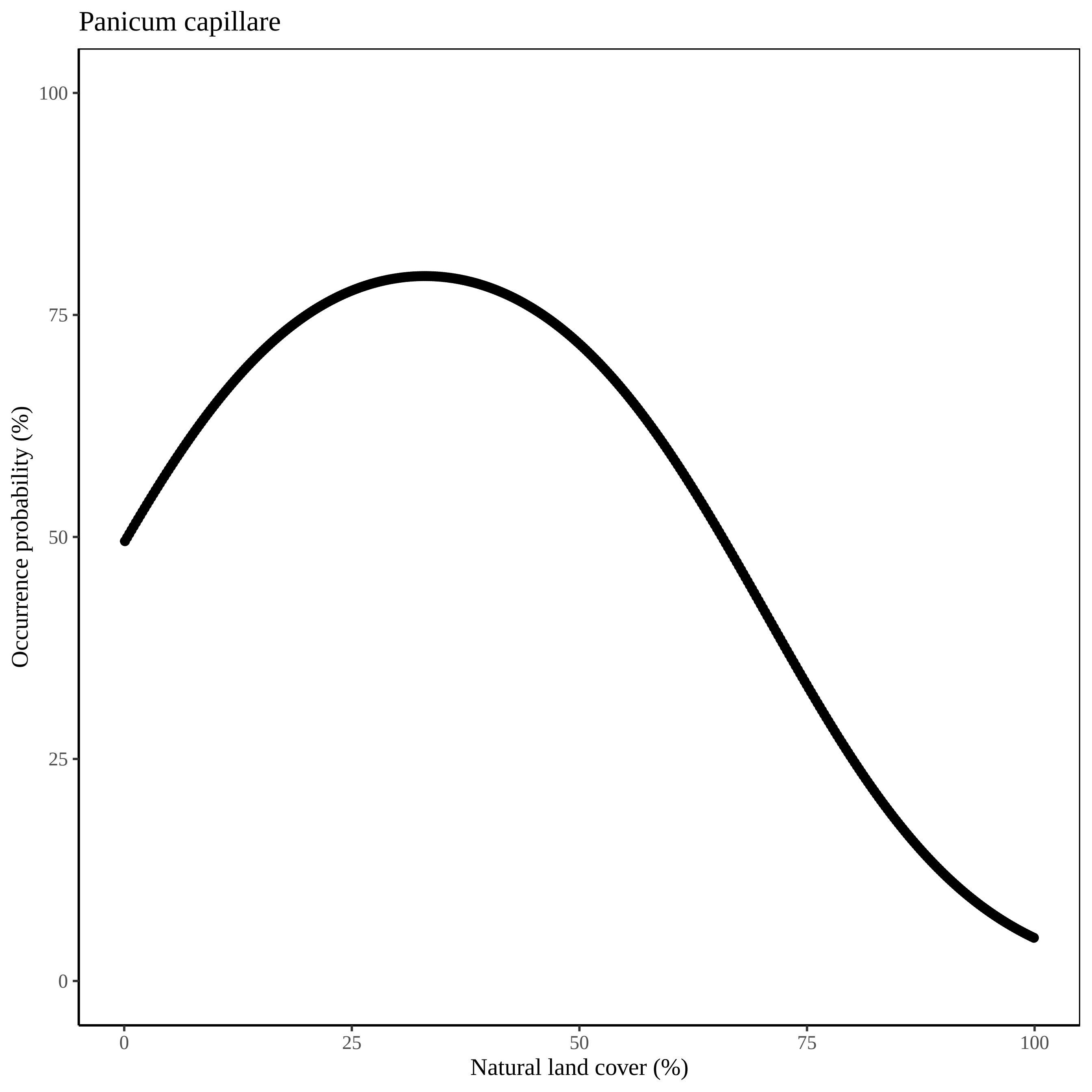

Panicum capillare
Occurrence probability (%)
100
75
50
25
0
0
25
50
75
100
Natural land cover (%)

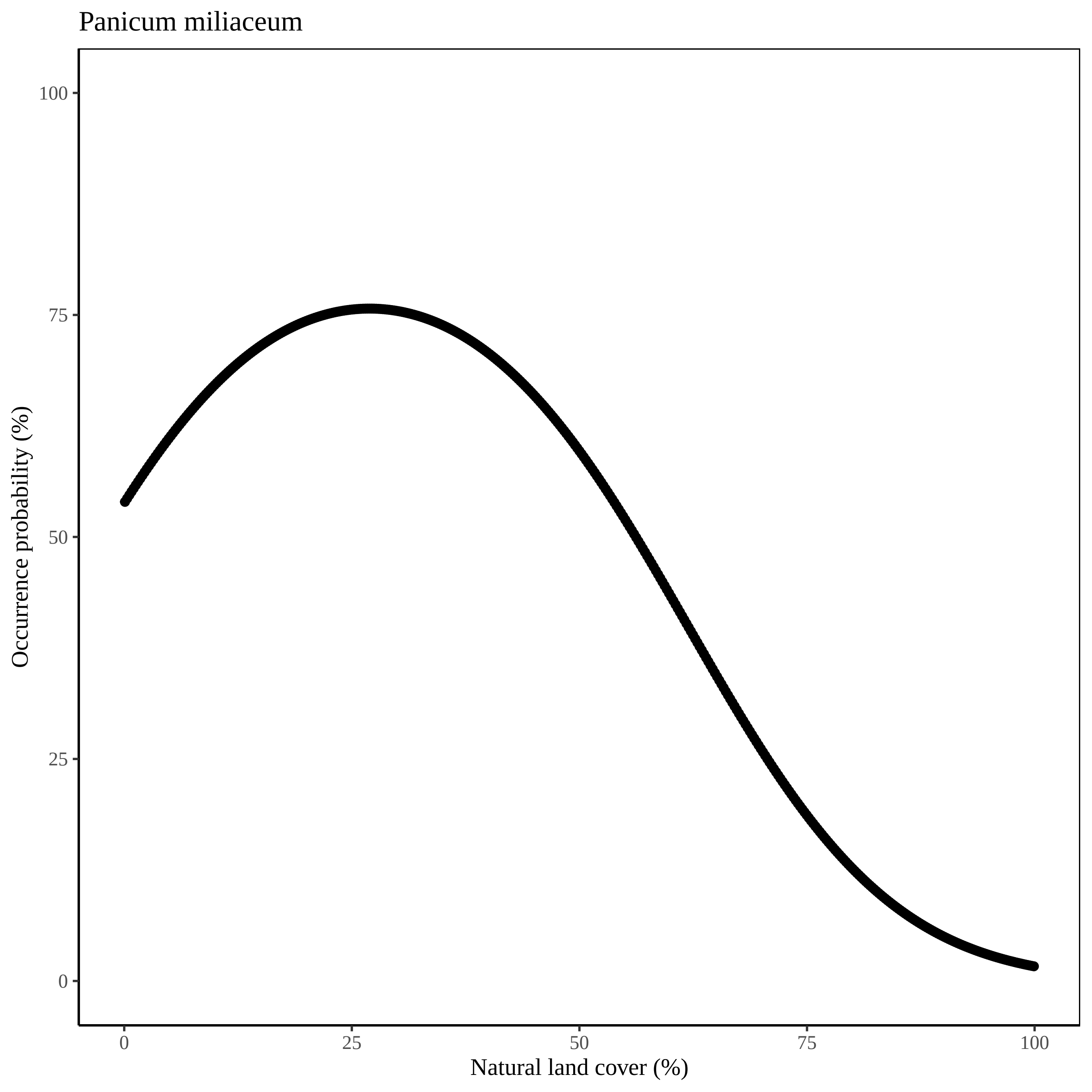
Panicum miliaceum
Occurrence probability (%)
100
75
50
25
0
0
25
50
75
100
Natural land cover (%)

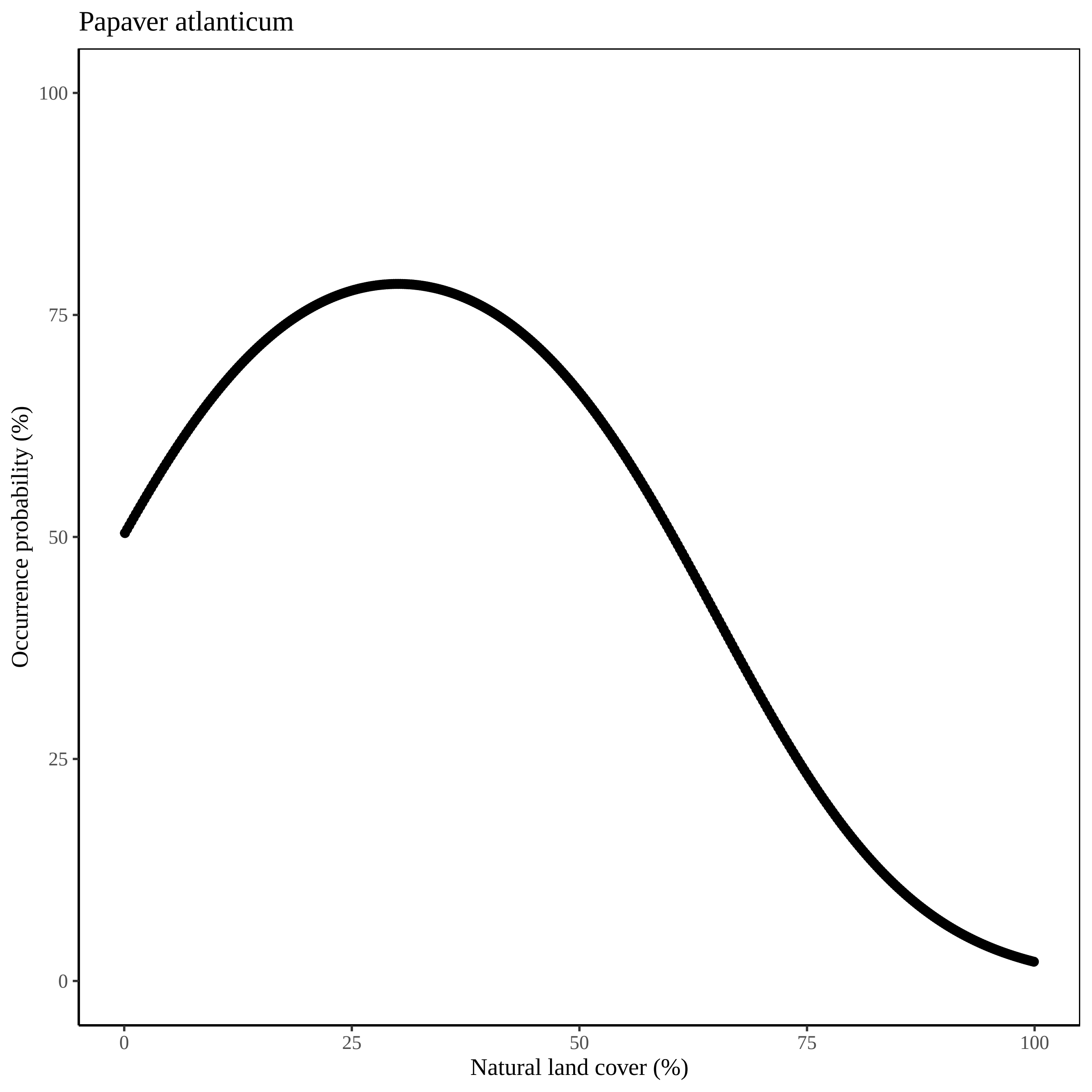

Papaver atlanticum
Occurrence probability (%)
100
75
50
25
0
0
25
50
75
100
Natural land cover (%)

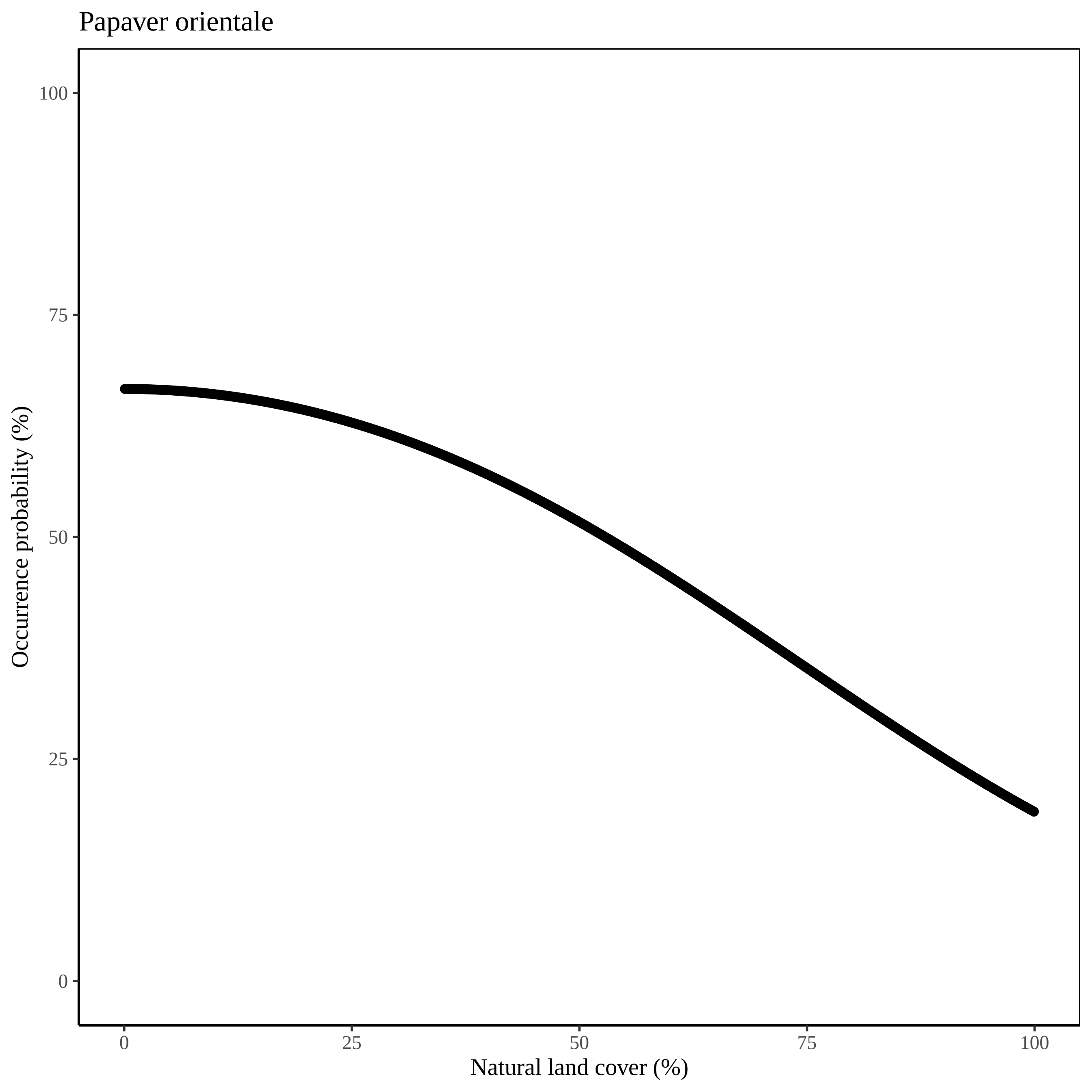
Papaver orientale
100
75
50
25
0
Occurrence probability (%)
0
25
50
75
100
Natural land cover (%)

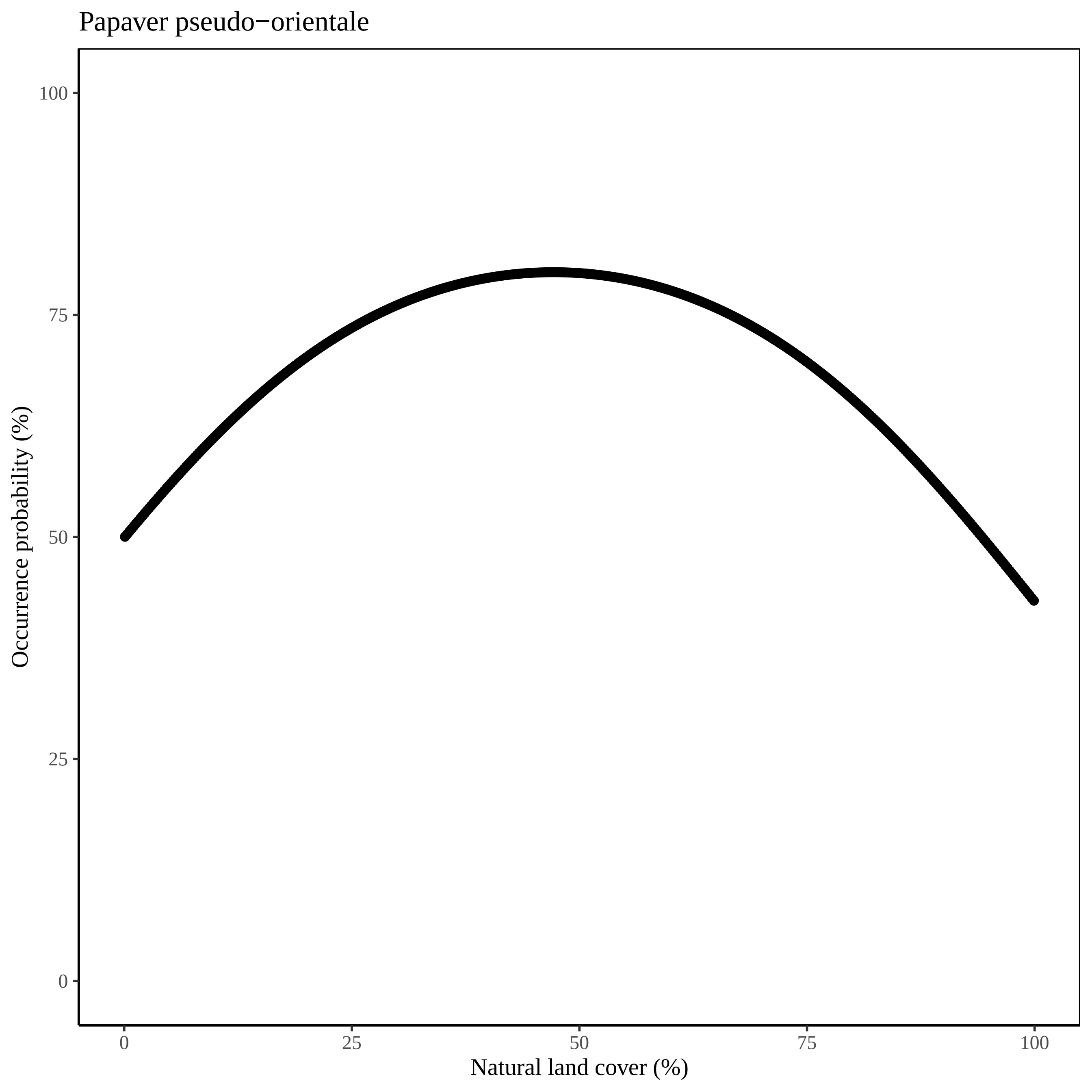

Papaver pseudo−orientale
Occurrence probability (%)
100
75
50
25
0
0
25
50
75
100
Natural land cover (%)

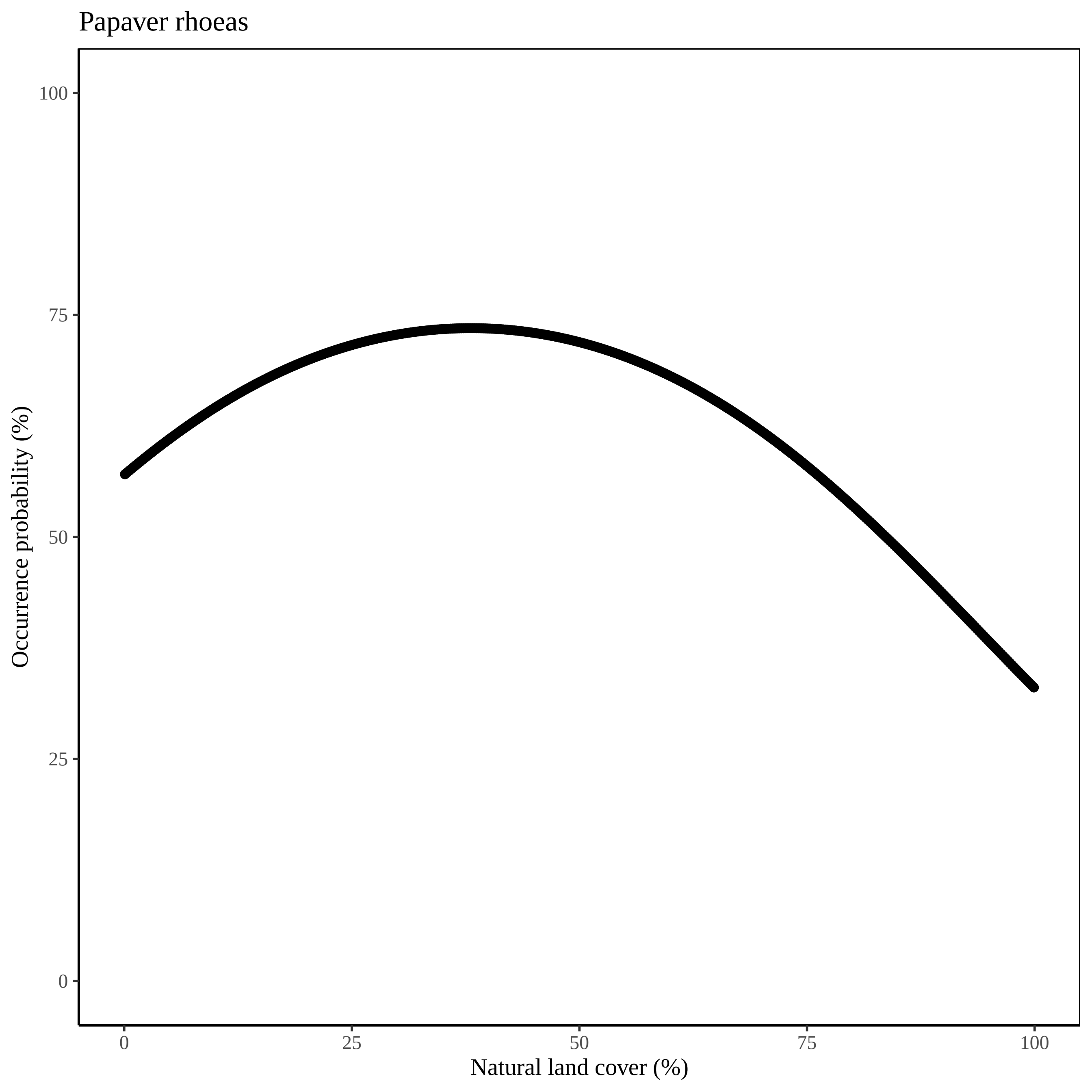

Papaver rhoeas
100
75
50
25
0
Occurrence probability (%)
0
25
50
75
100
Natural land cover (%)

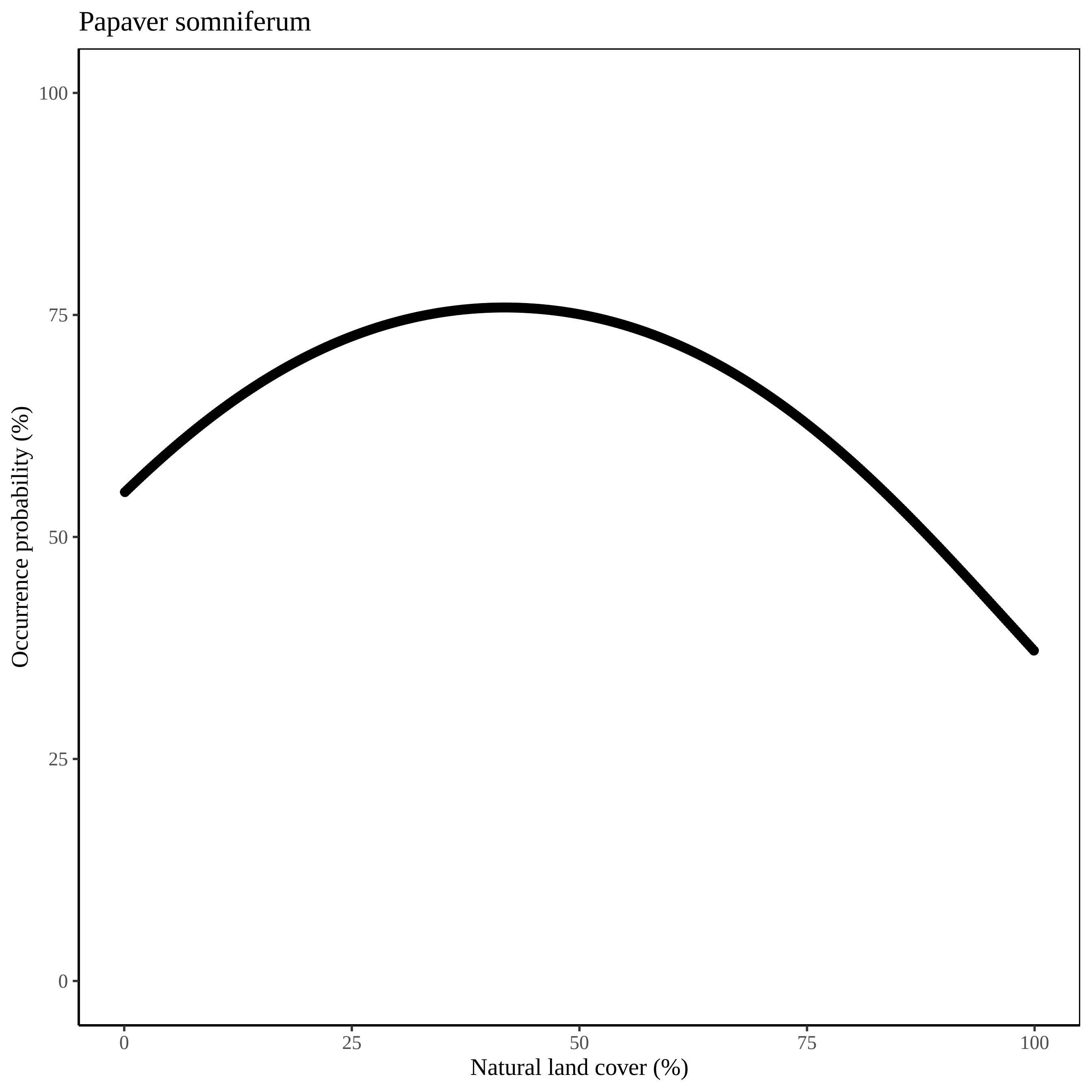
Papaver somniferum
Occurrence probability (%)
100
75
50
25
0
0
25
50
75
100
Natural land cover (%)

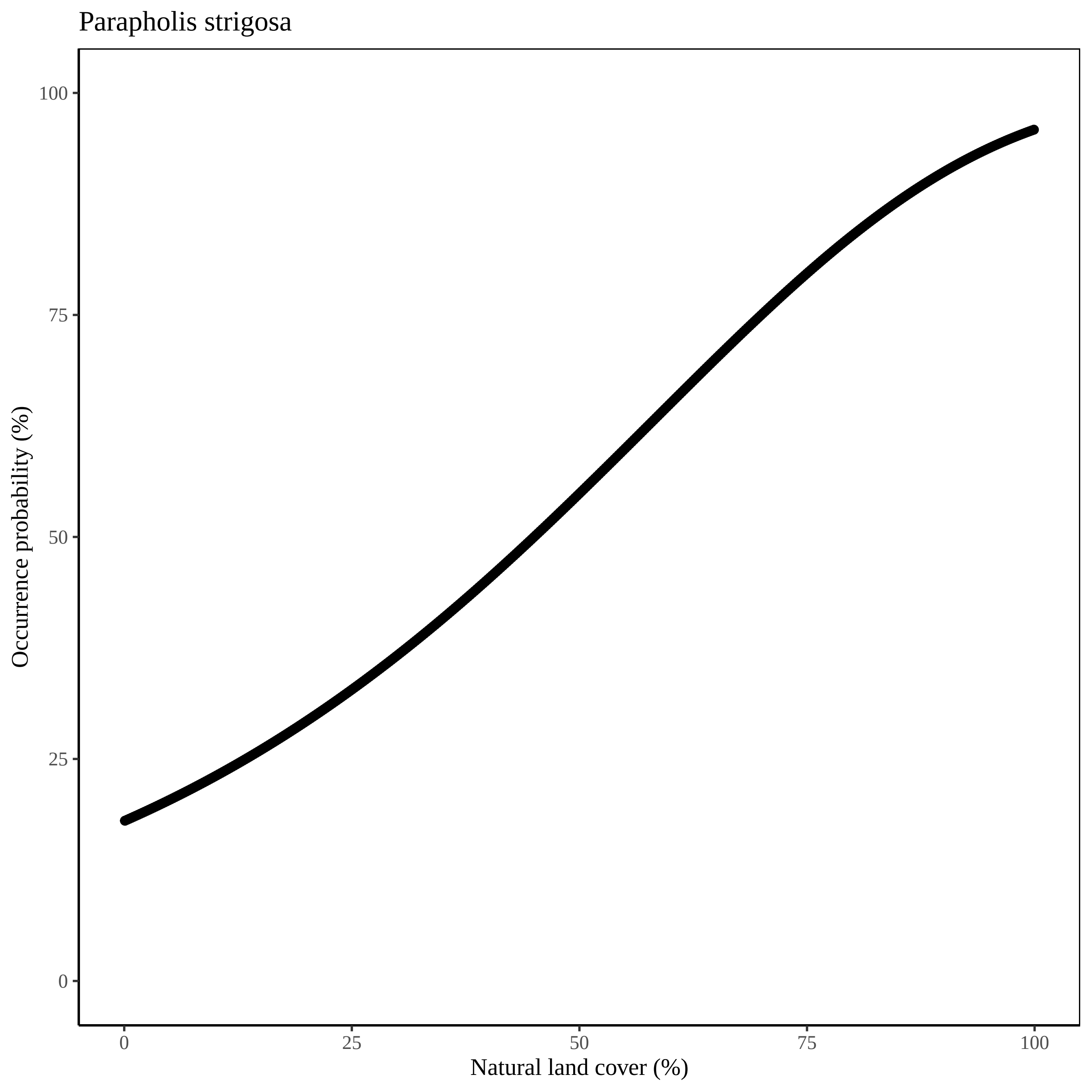

Parapholis strigosa
Occurrence probability (%)
100
75
50
25
0
0
25
50
75
100
Natural land cover (%)

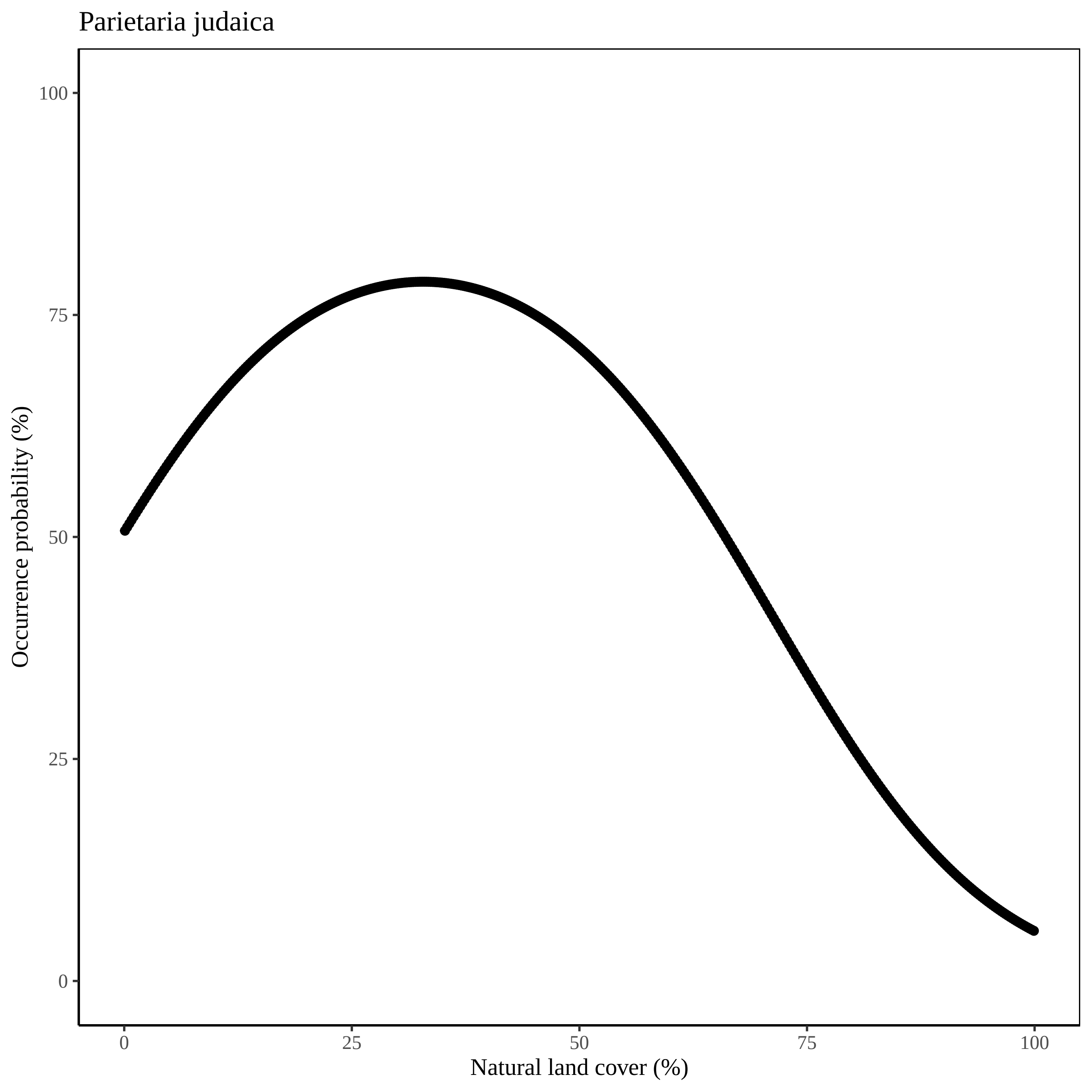
Parietaria judaica
Occurrence probability (%)
100
75
50
25
0
0
25
50
75
100
Natural land cover (%)

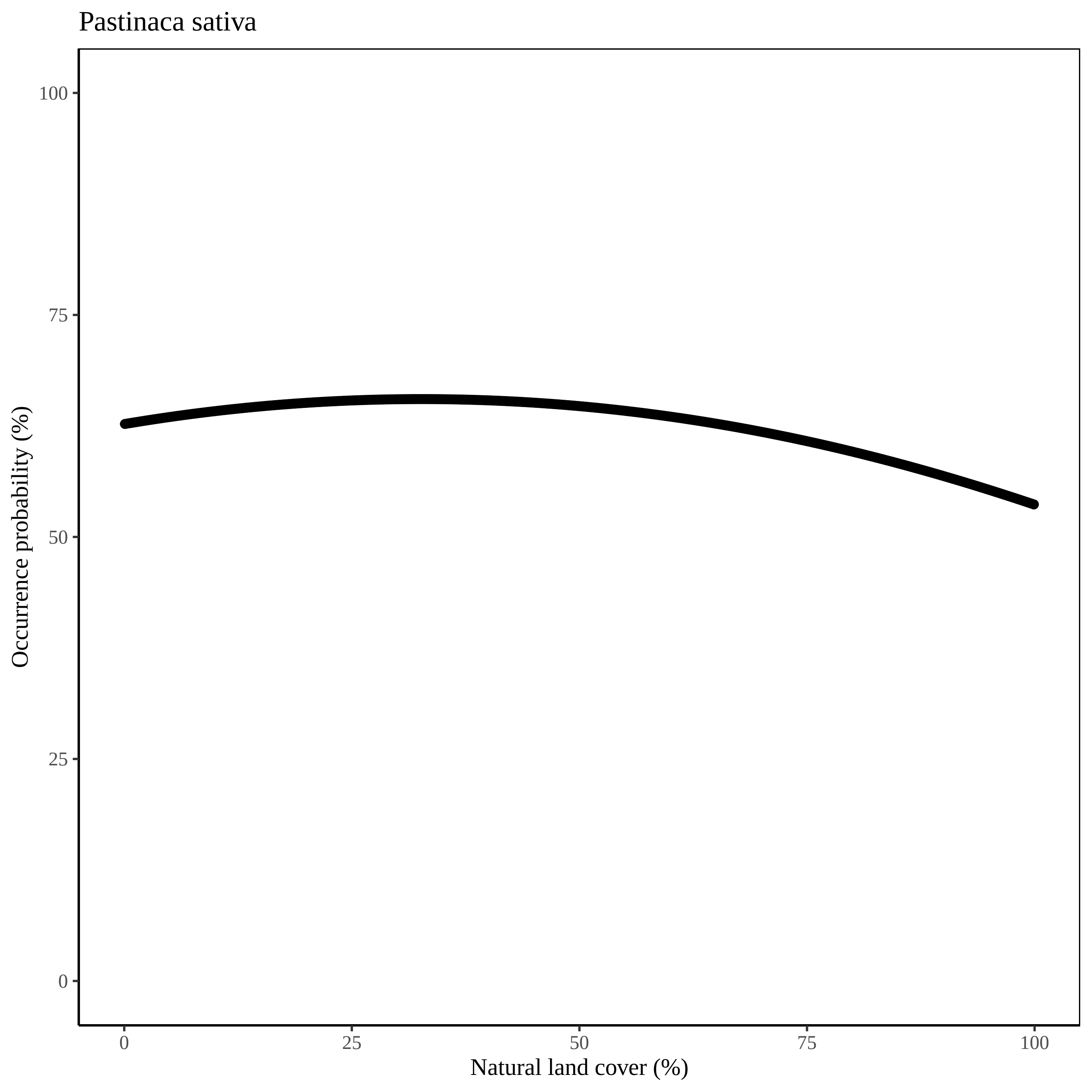
Pastinaca sativa
Occurrence probability (%)
100
75
50
25
0
0
25
50
75
100
Natural land cover (%)

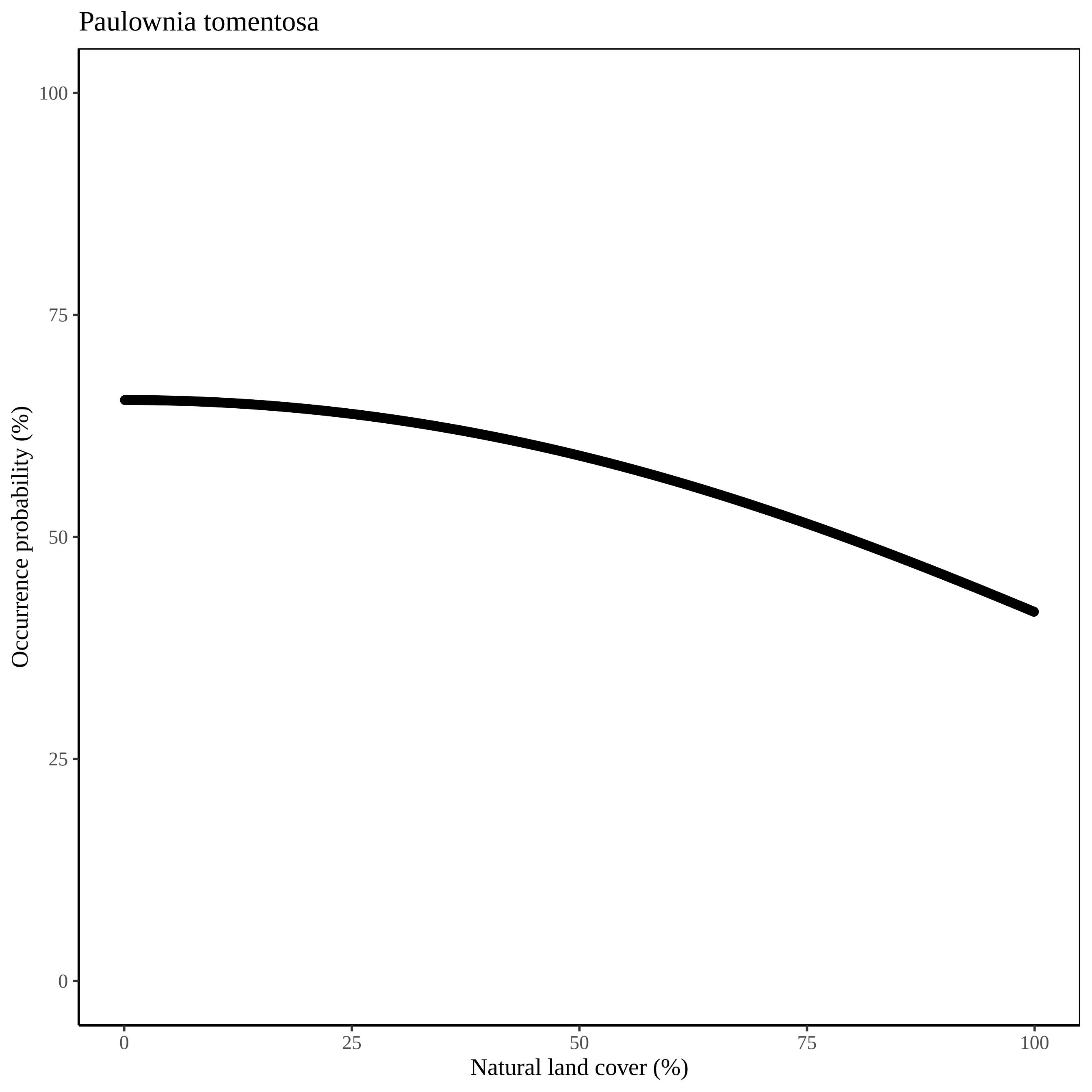

Paulownia tomentosa
Occurrence probability (%)
100
75
50
25
0
0
25
50
75
100
Natural land cover (%)

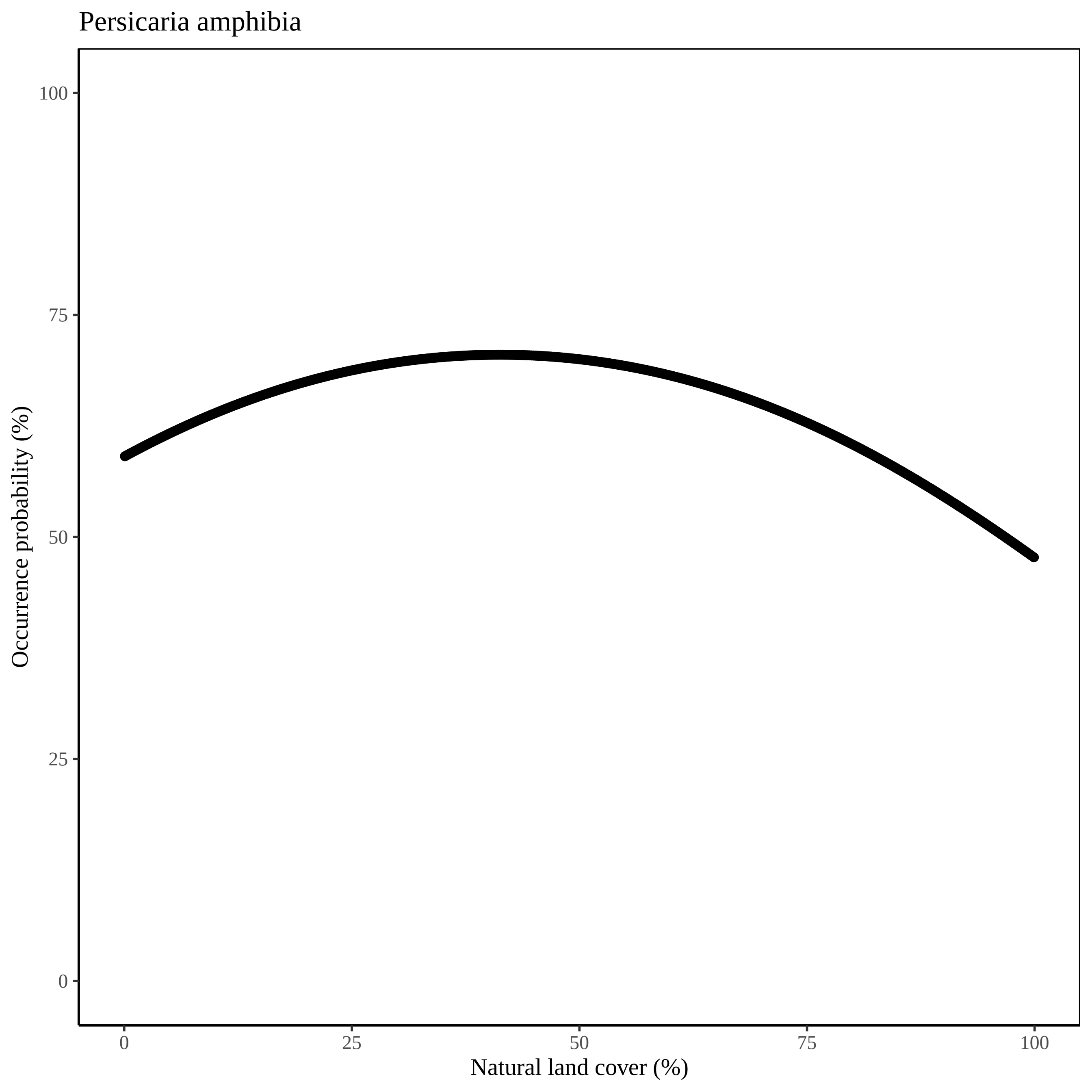
Persicaria amphibia
Occurrence probability (%)
100
75
50
25
0
0
25
50
75
100
Natural land cover (%)

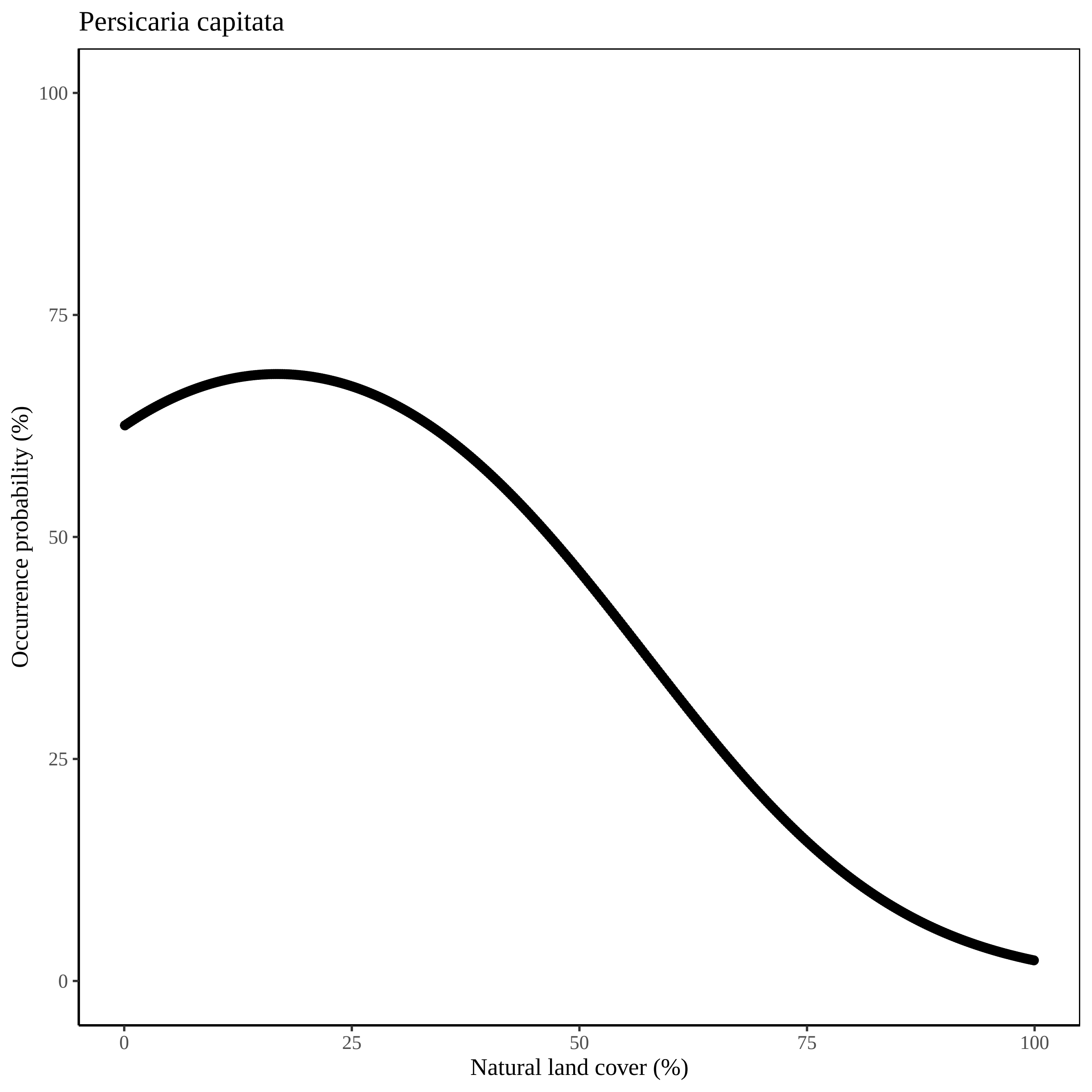

Persicaria capitata
Occurrence probability (%)
100
75
50
25
0
0
25
50
75
100
Natural land cover (%)

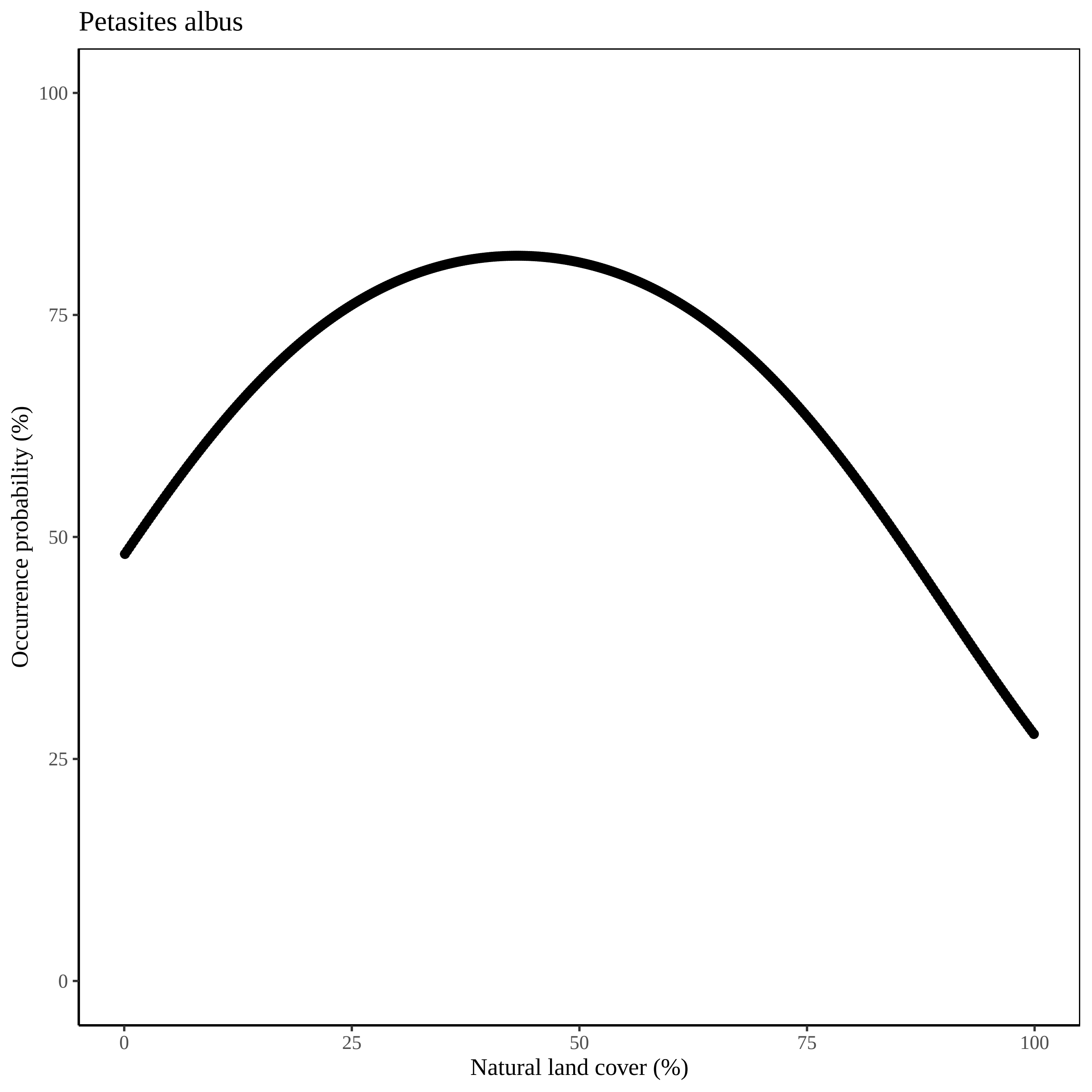

Petasites albus
100
75
50
25
0
Occurrence probability (%)
0
25
50
75
100
Natural land cover (%)

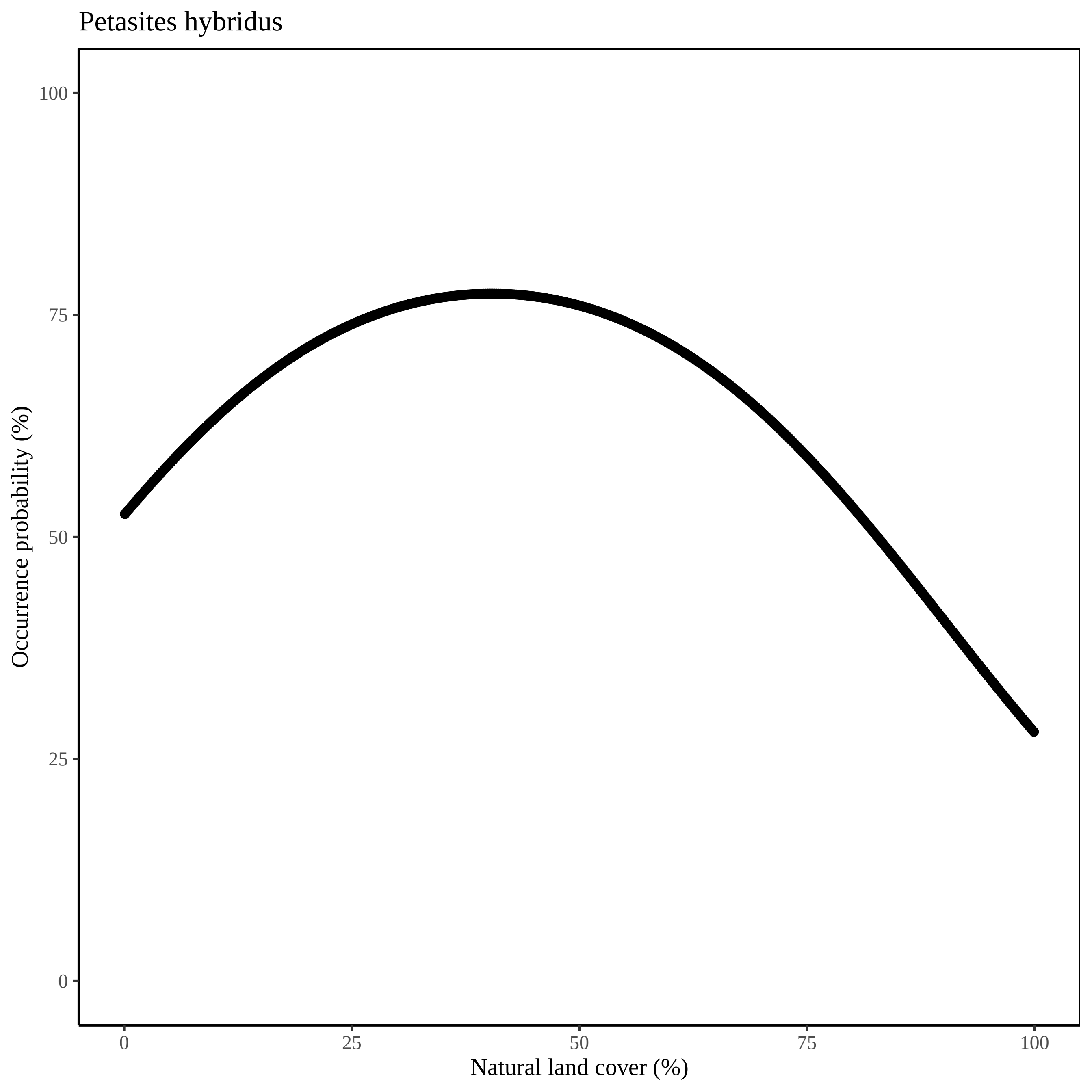
Petasites hybridus
Occurrence probability (%)
Natural land cover (%)
0
25
50
75
100
0
25
50
75
100

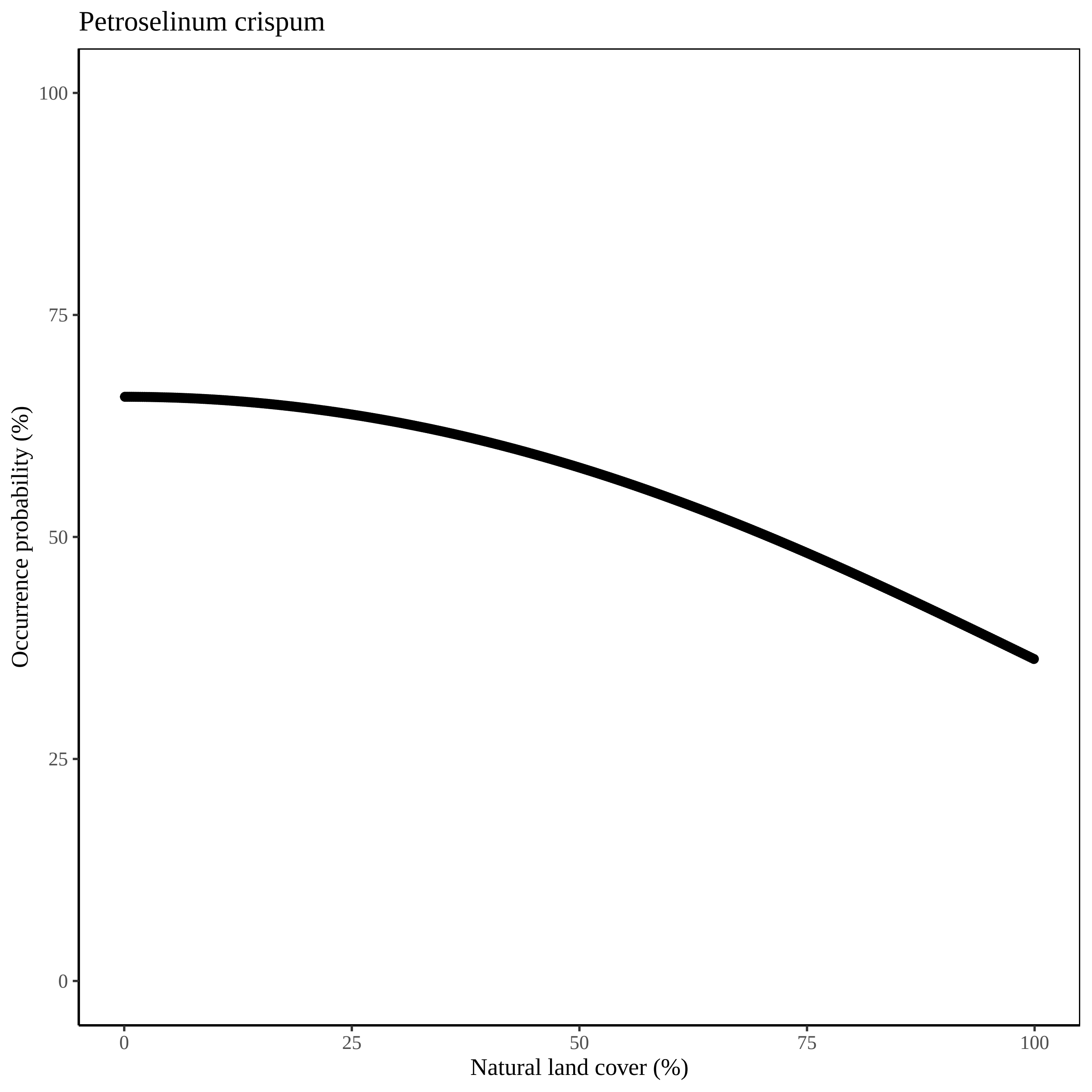
Petroselinum crispum
Occurrence probability (%)
100
75
50
25
0
0
25
50
75
100
Natural land cover (%)

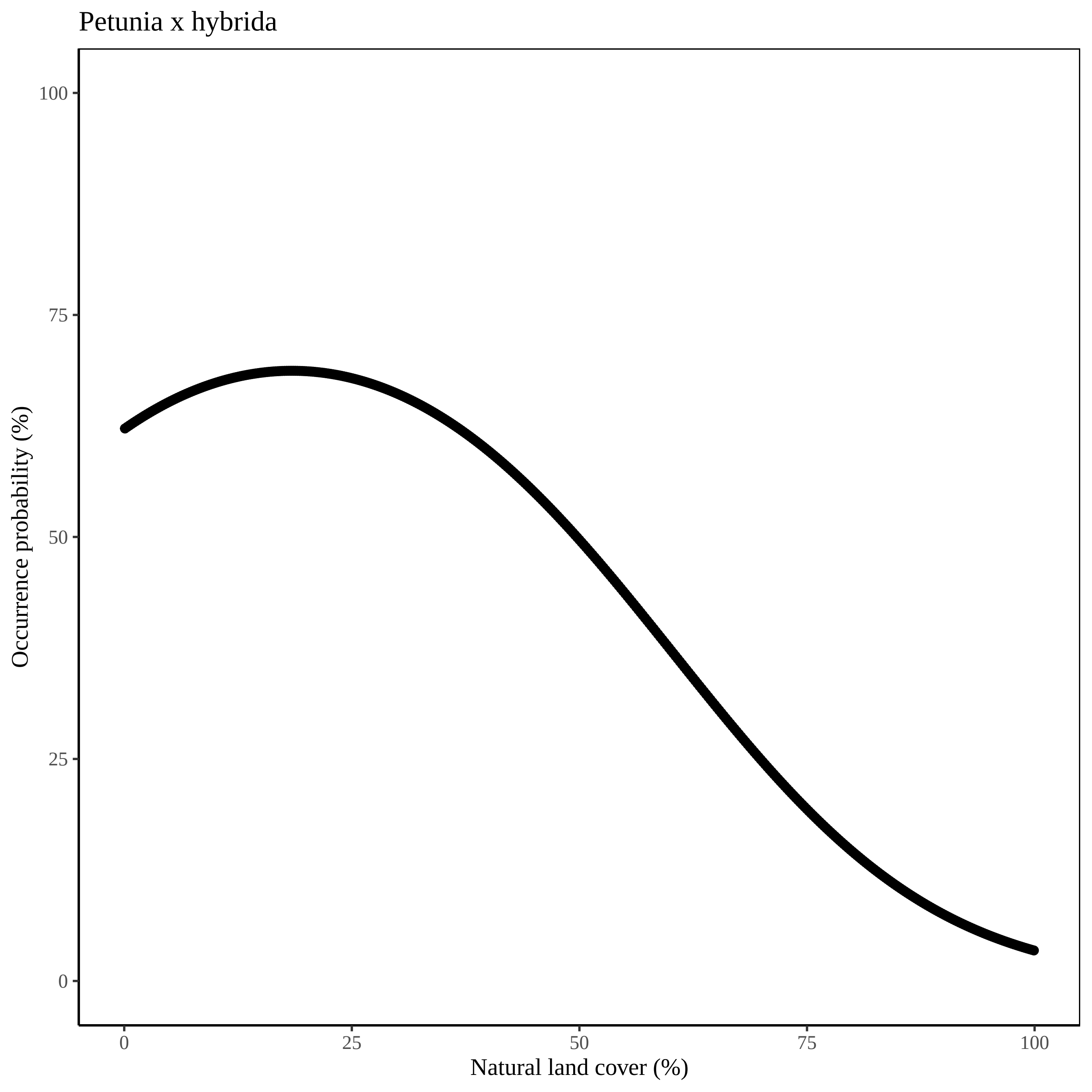
Petunia x hybrida
Occurrence probability (%)
100
75
50
25
0
0
25
50
75
100
Natural land cover (%)

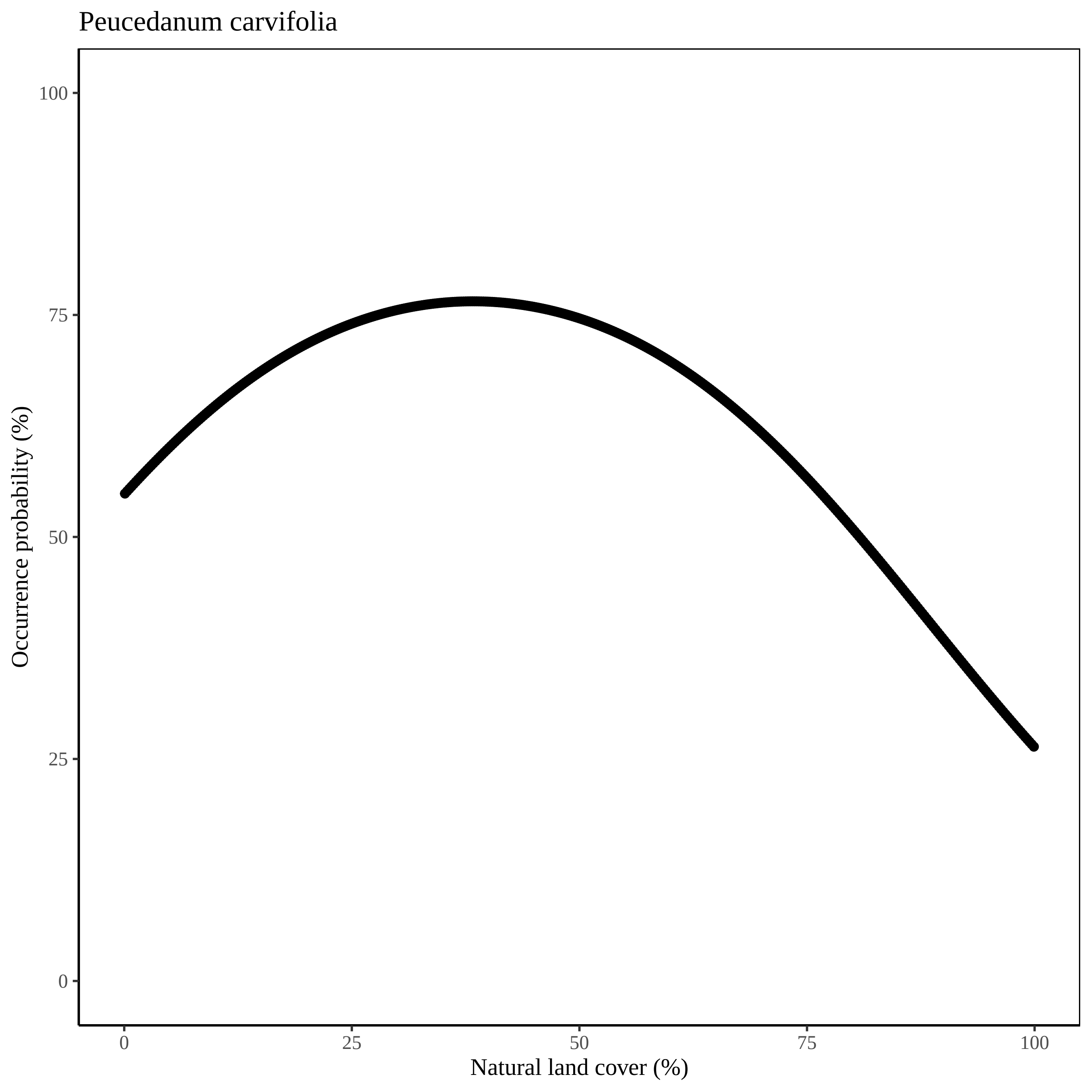
Peucedanum carvifolia
Occurrence probability (%)
100
75
50
25
0
0
25
50
75
100
Natural land cover (%)

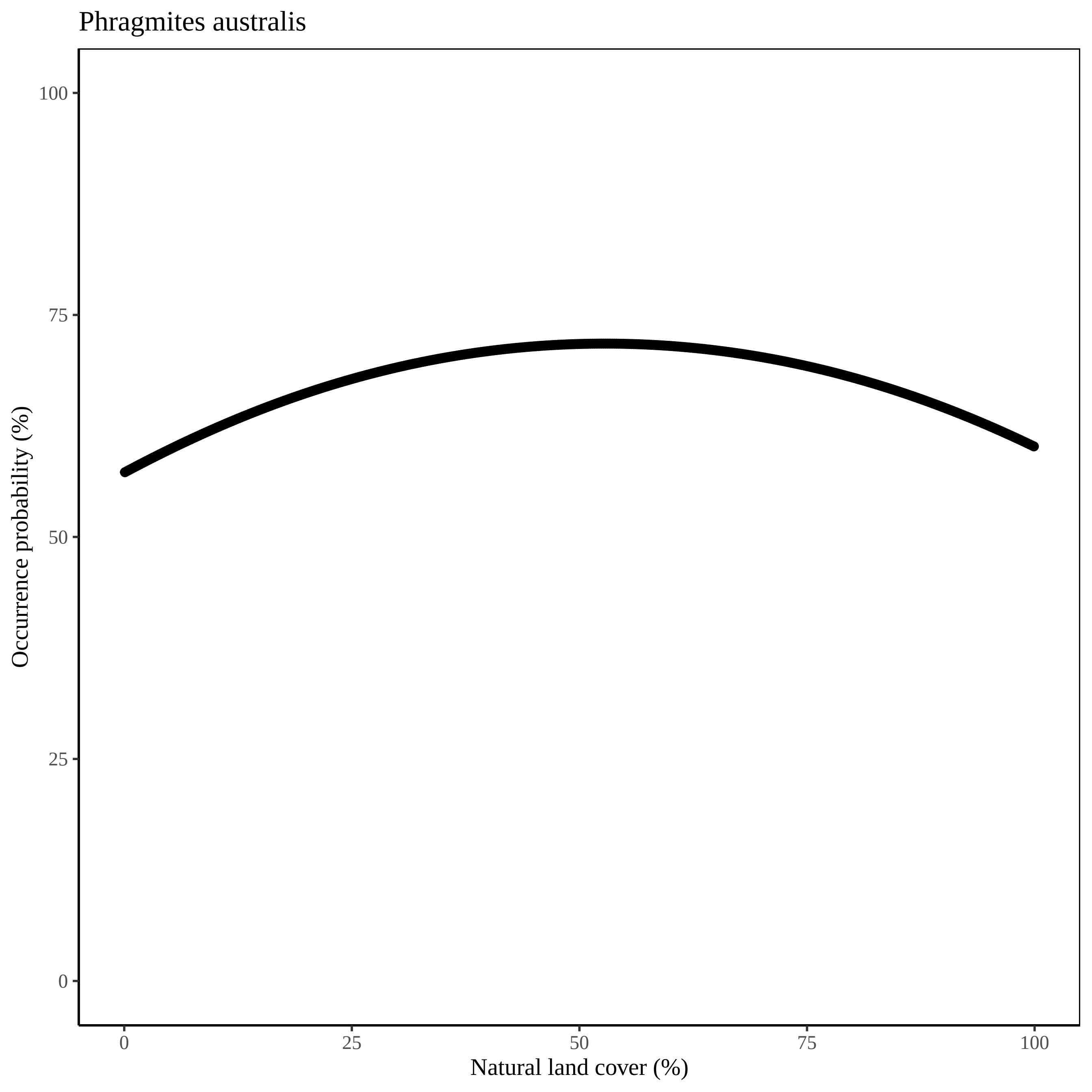

Phragmites australis
100
75
50
25
0
Occurrence probability (%)
0
25
50
75
100
Natural land cover (%)

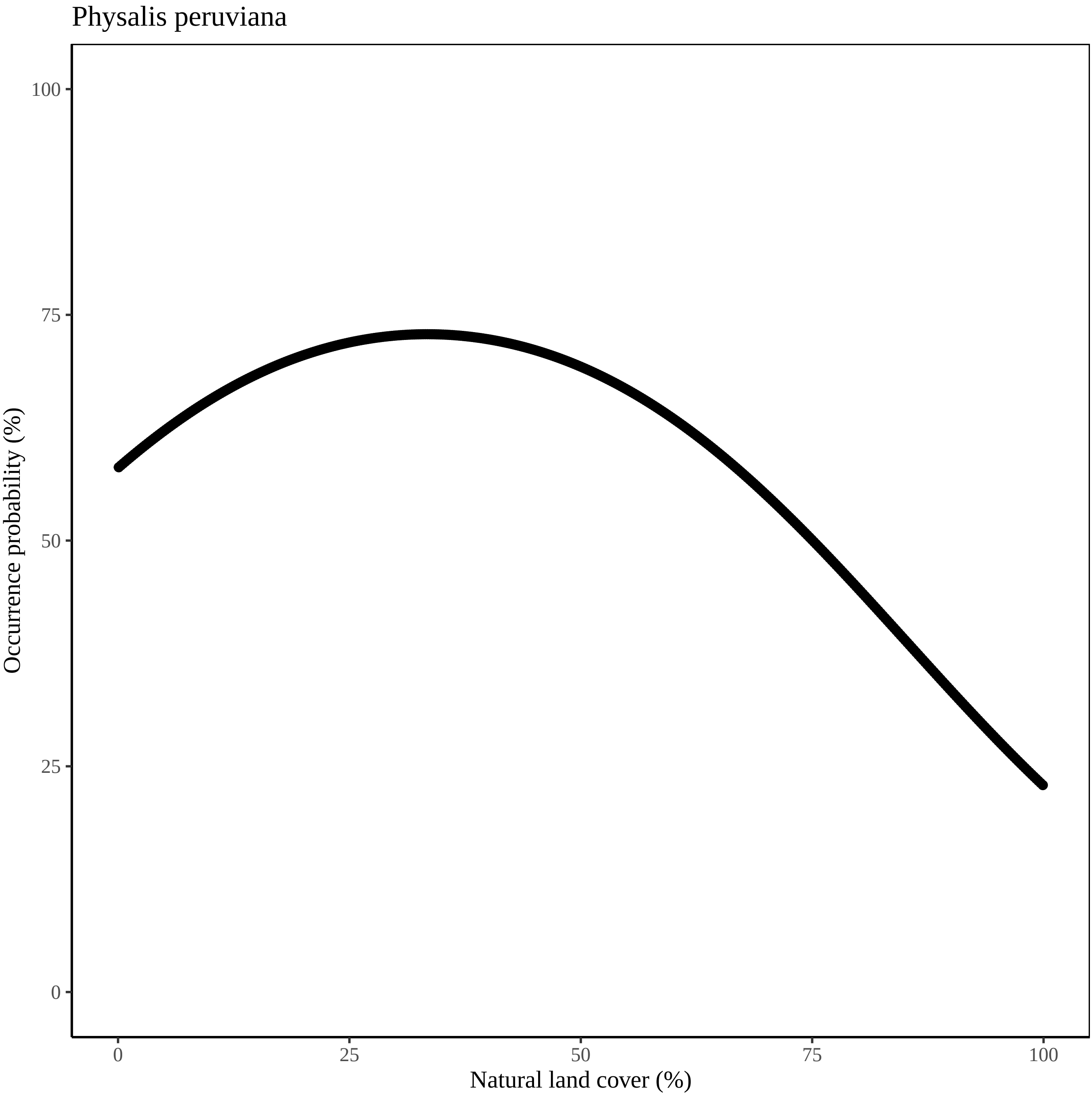

Physalis peruviana
Occurrence probability (%)
100
75
50
25
0
0
25
50
75
100
Natural land cover (%)

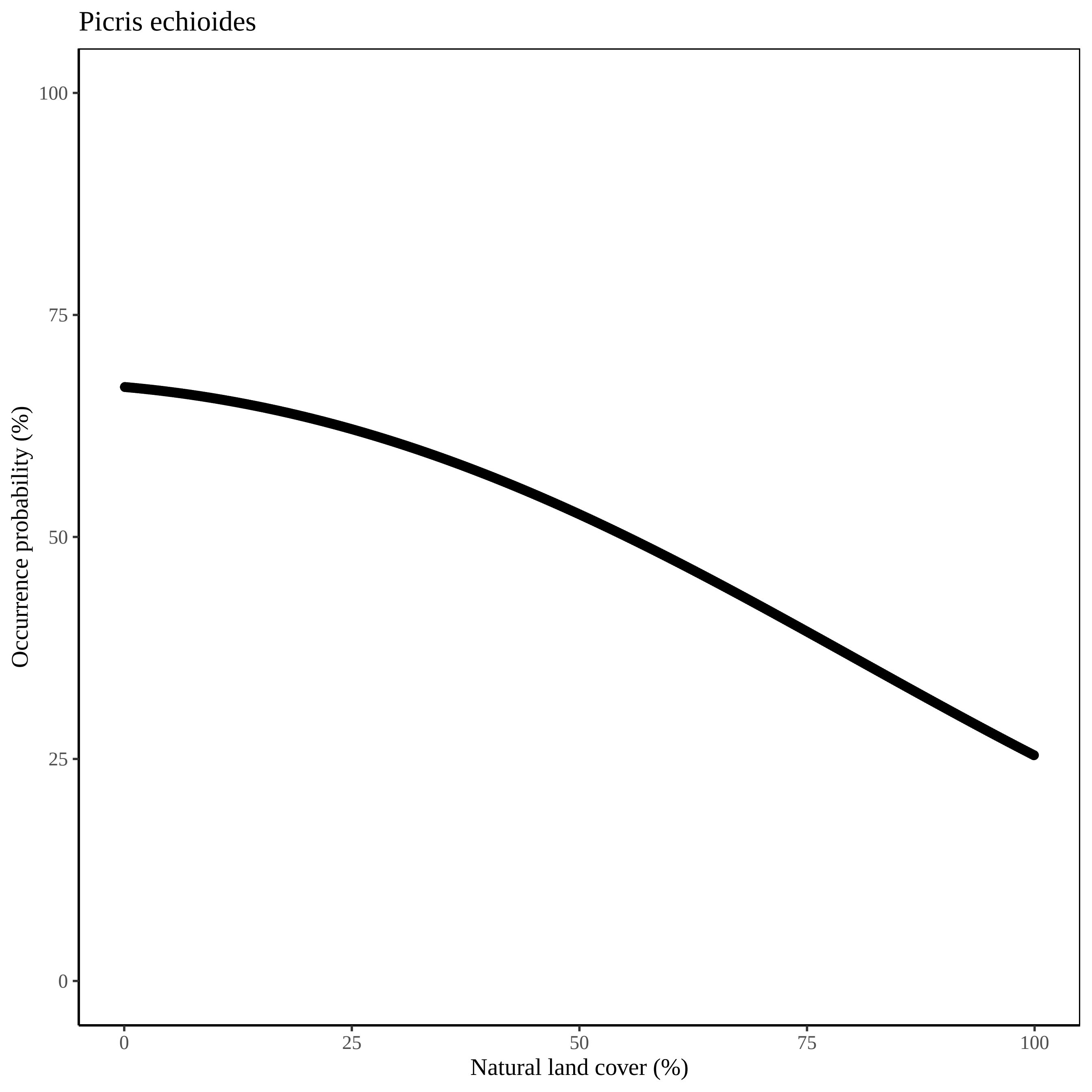
Picris echioides
Occurrence probability (%)
100
75
50
25
0
0
25
50
75
100
Natural land cover (%)

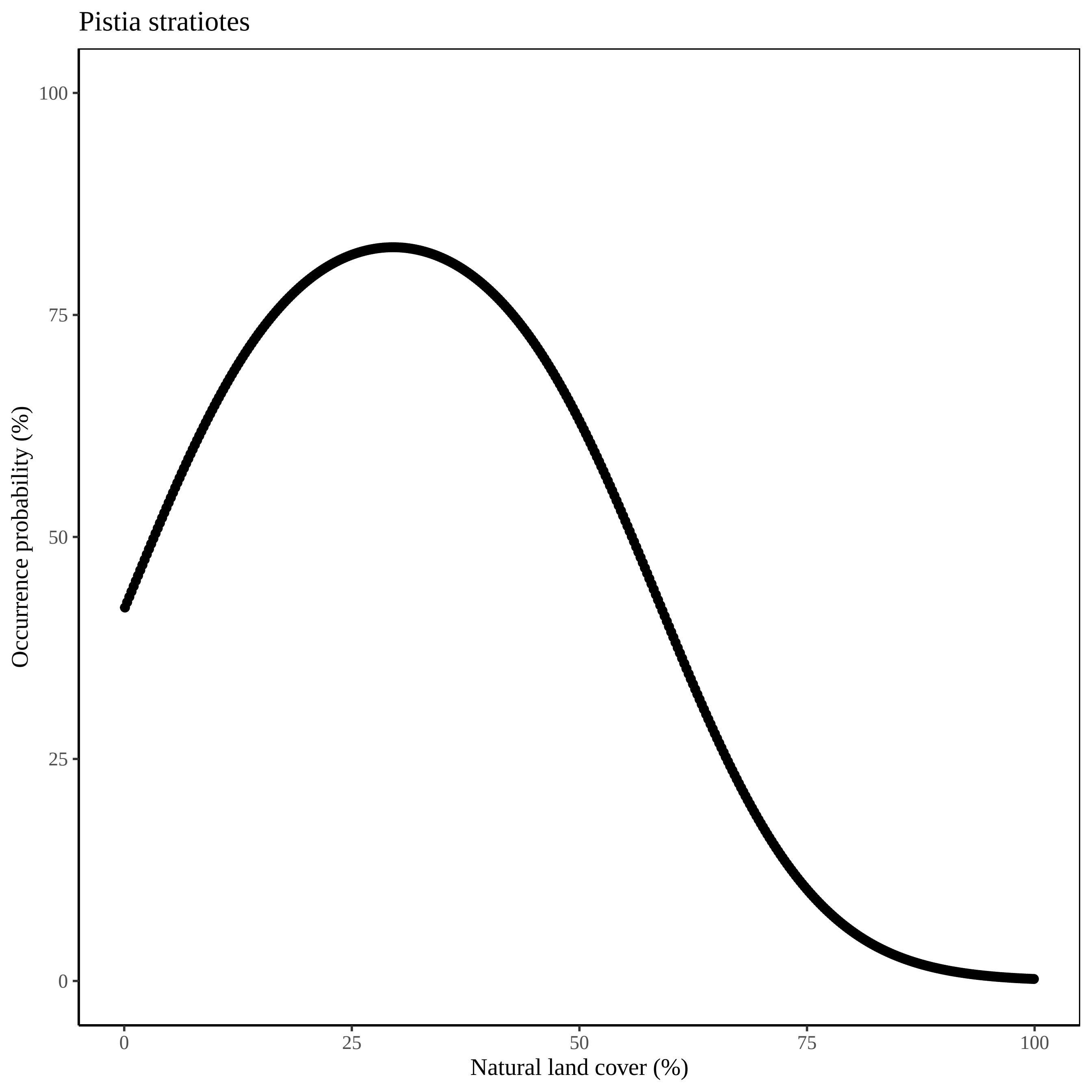

Pistia stratiotes
100
75
50
25
0
Occurrence probability (%)
0
25
50
75
100
Natural land cover (%)

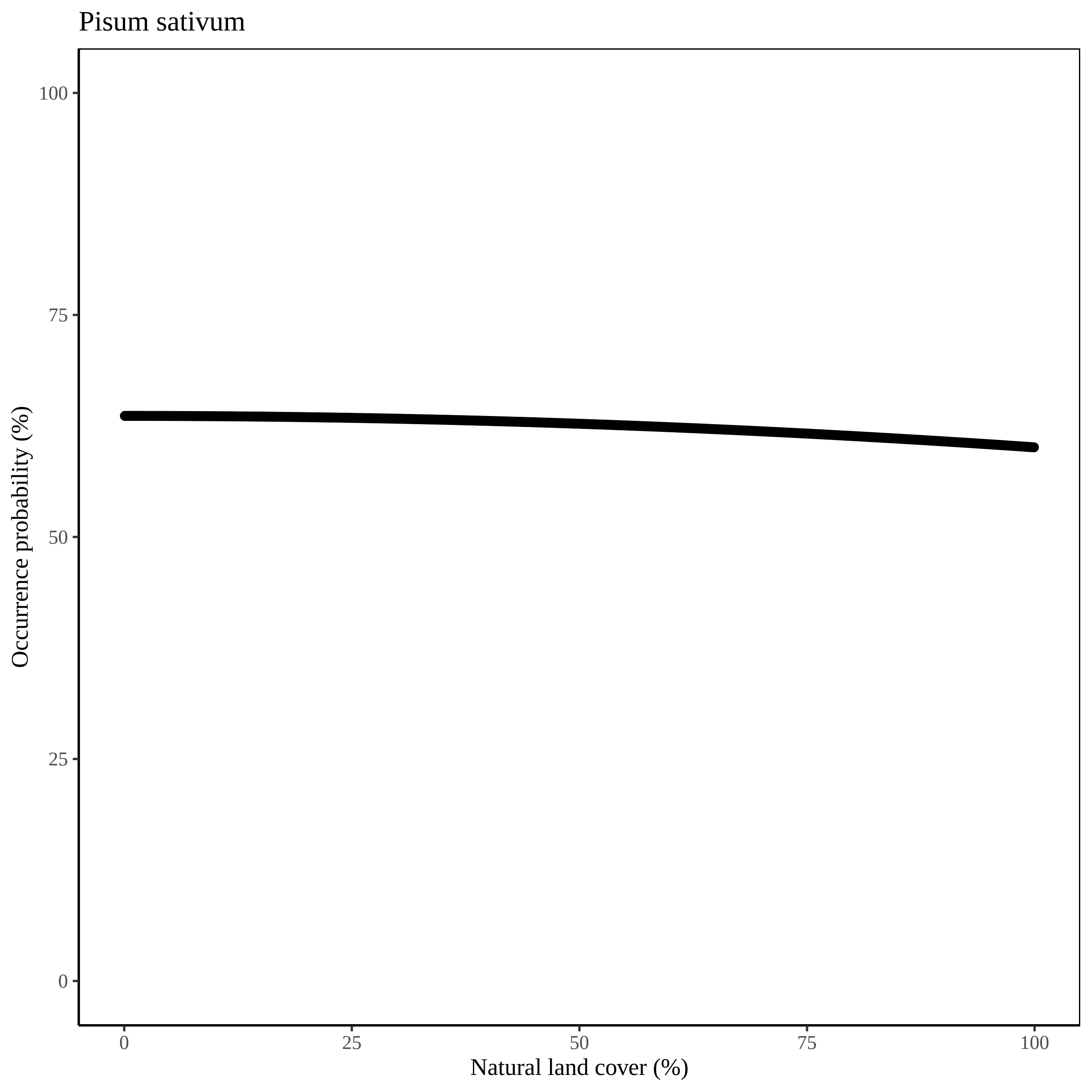
Pisum sativum
Occurrence probability (%)
Natural land cover (%)
0
25
50
75
100
0
25
50
75
100

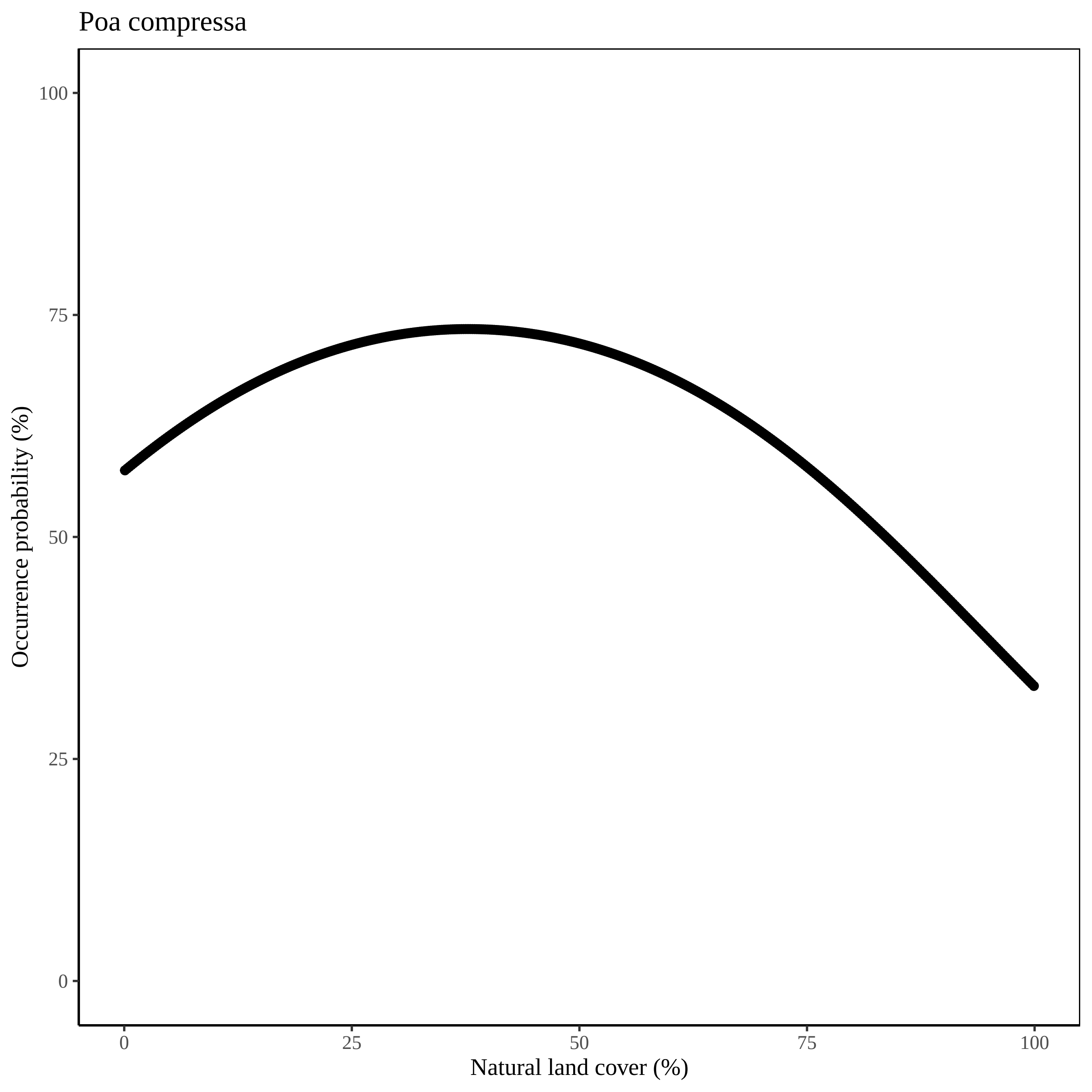

Poa compressa
100
75
50
25
0
Occurrence probability (%)
0
25
50
75
100
Natural land cover (%)

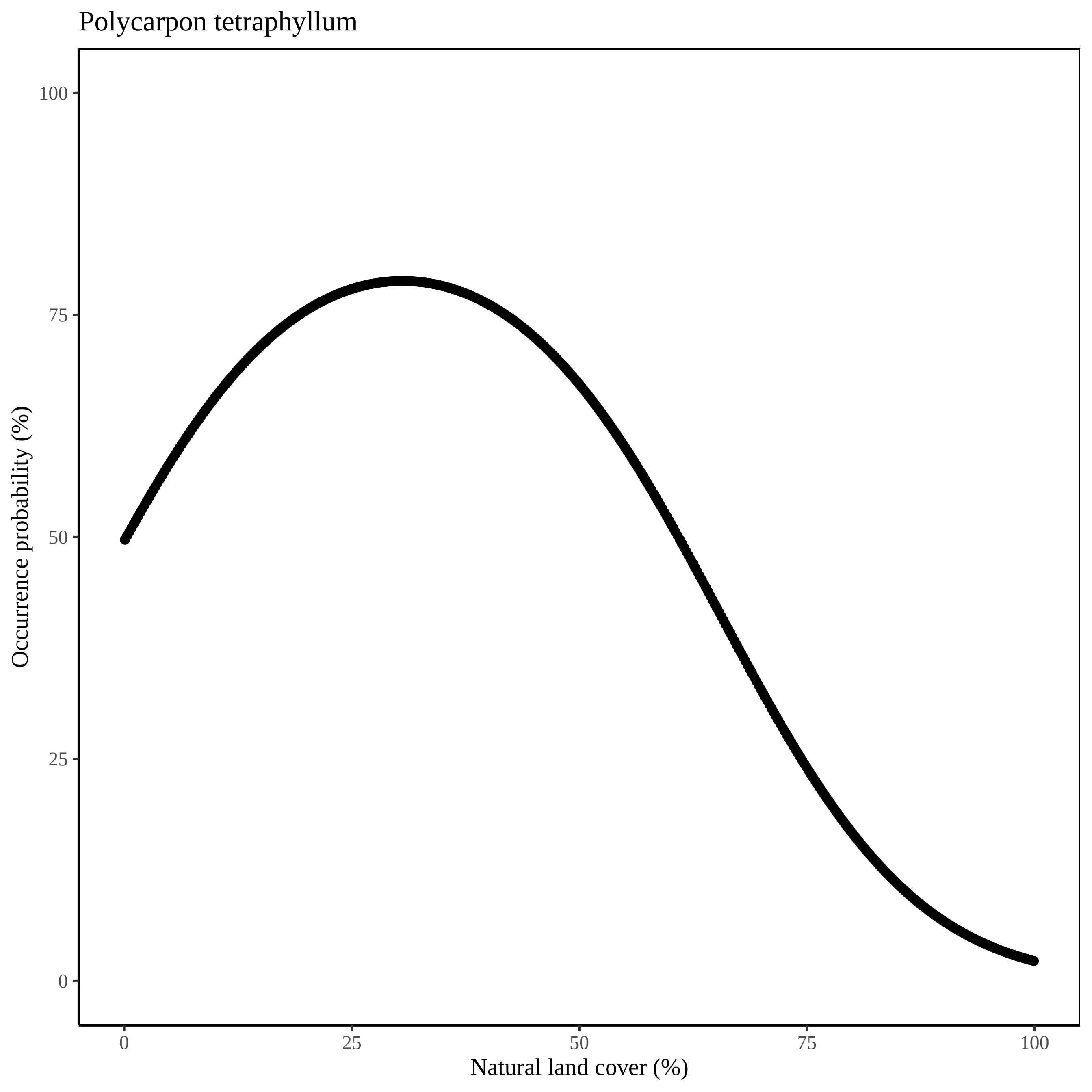

Polycarpon tetraphyllum
Occurrence probability (%)
100
75
50
25
0
0
25
50
75
100
Natural land cover (%)

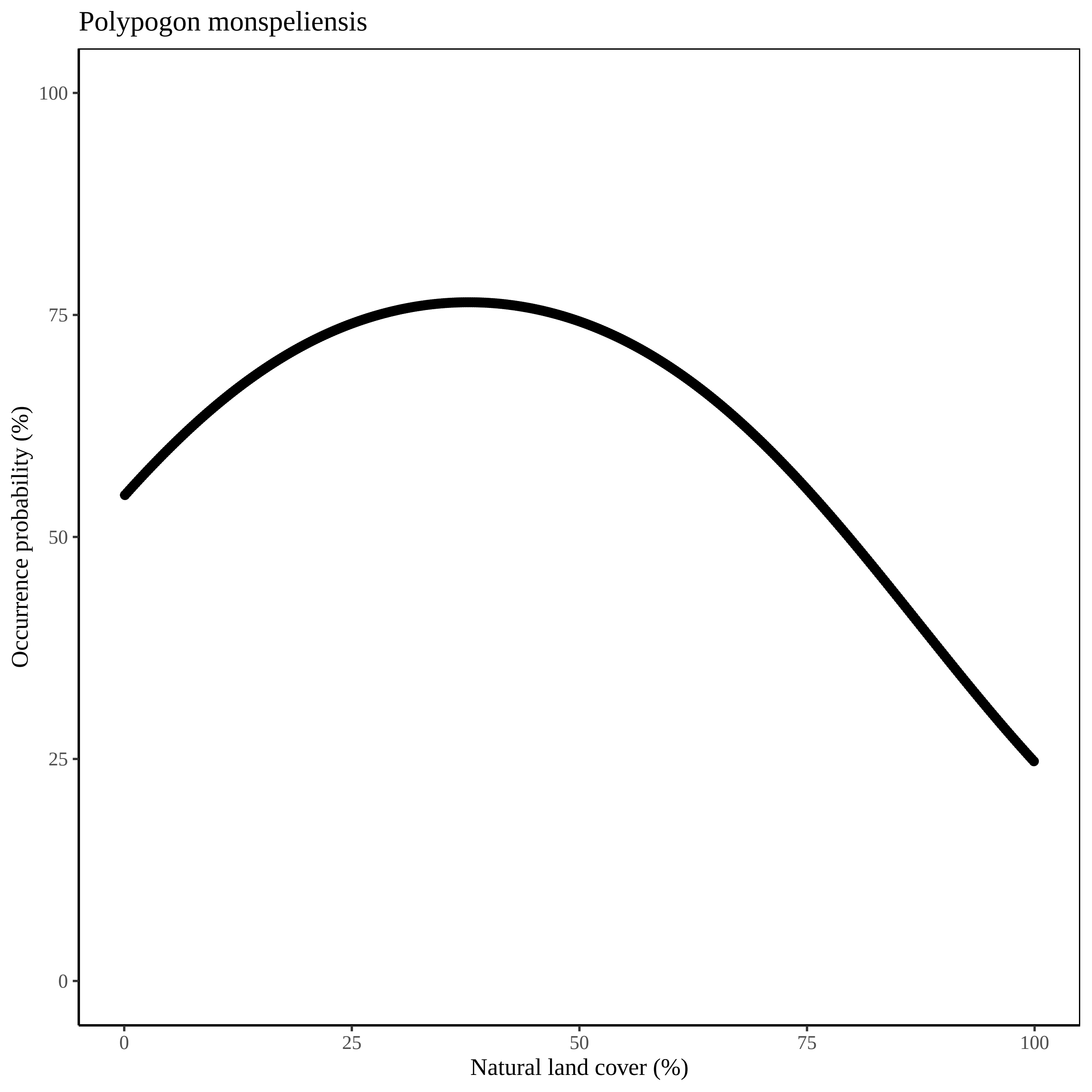
Polypogon monspeliensis
Occurrence probability (%)
100
75
50
25
0
0
25
50
75
100
Natural land cover (%)

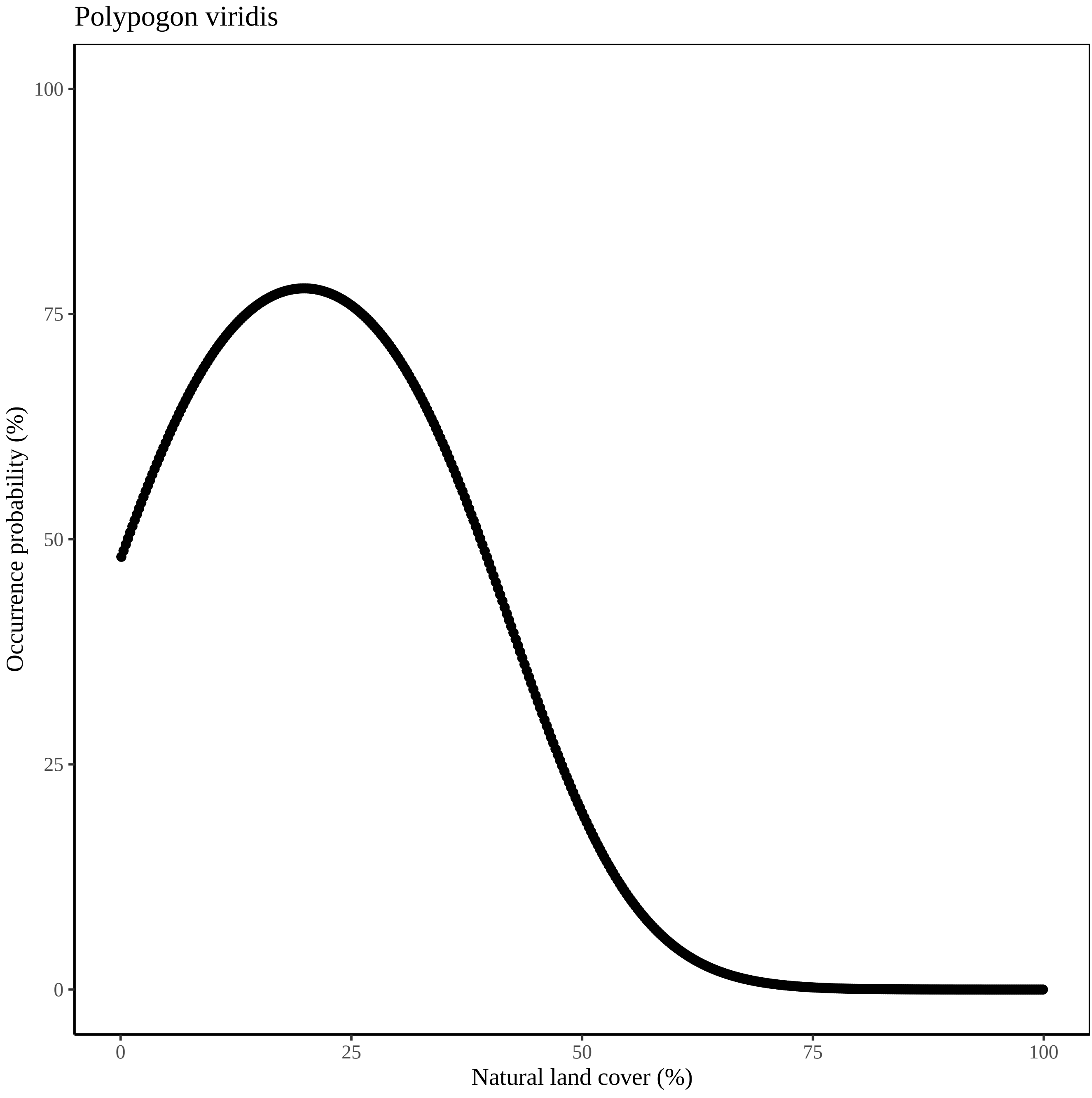

Polypogon viridis
Occurrence probability (%)
100
75
50
25
0
0
25
50
75
100
Natural land cover (%)

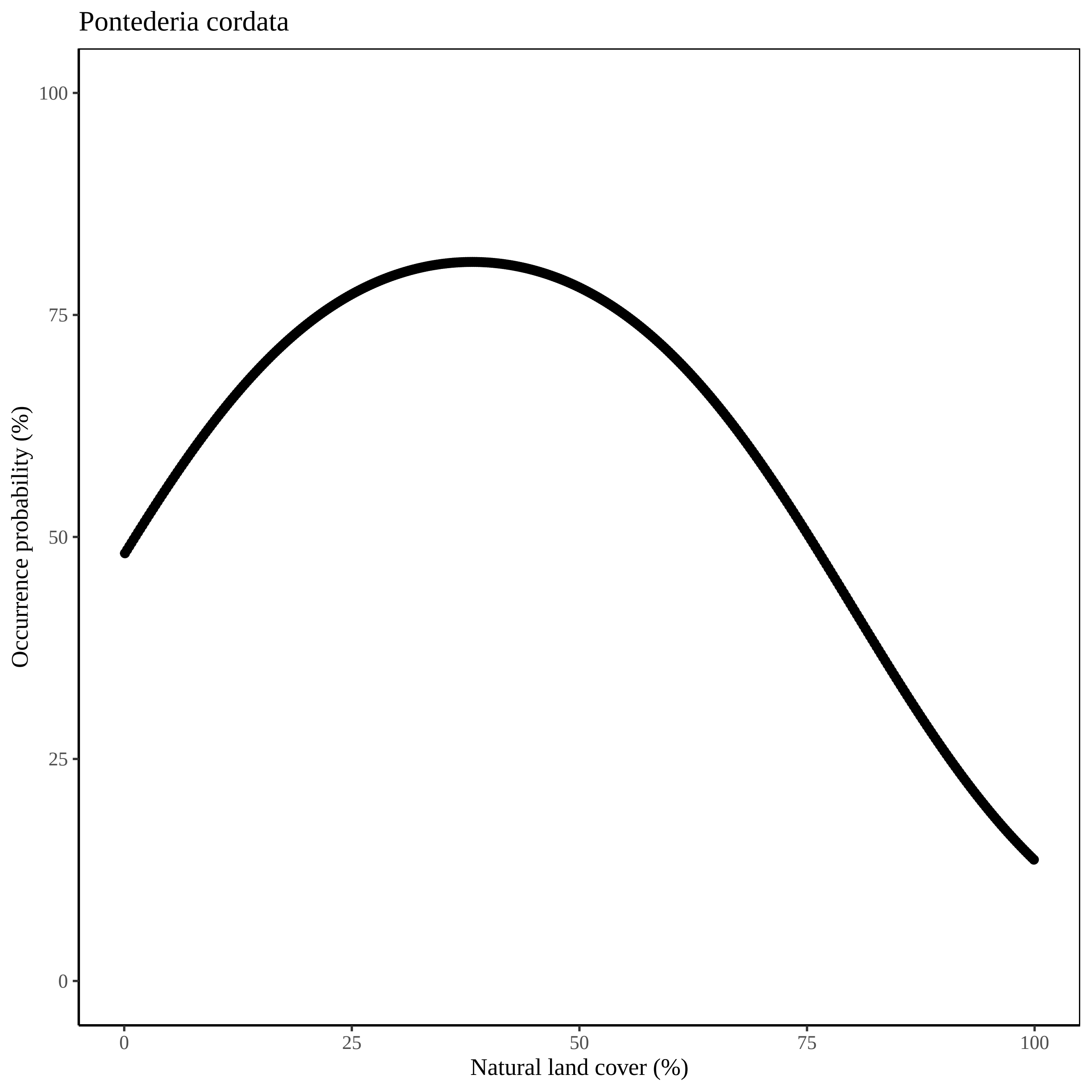
Pontederia cordata
100
75
50
25
0
Occurrence probability (%)
0
25
50
75
100
Natural land cover (%)

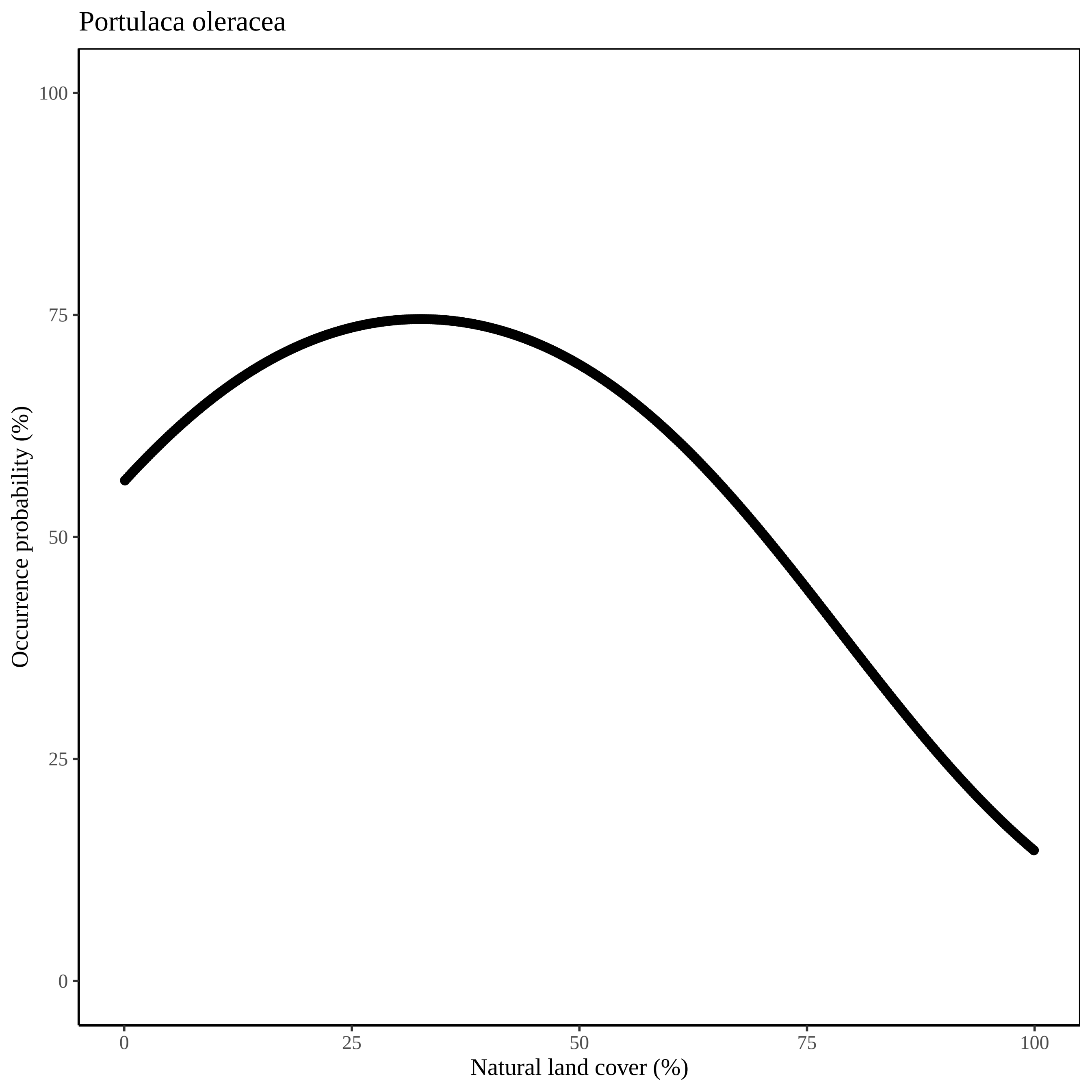
Portulaca oleracea
Occurrence probability (%)
100
75
50
25
0
0
25
50
75
100
Natural land cover (%)

Potamogeton compressus

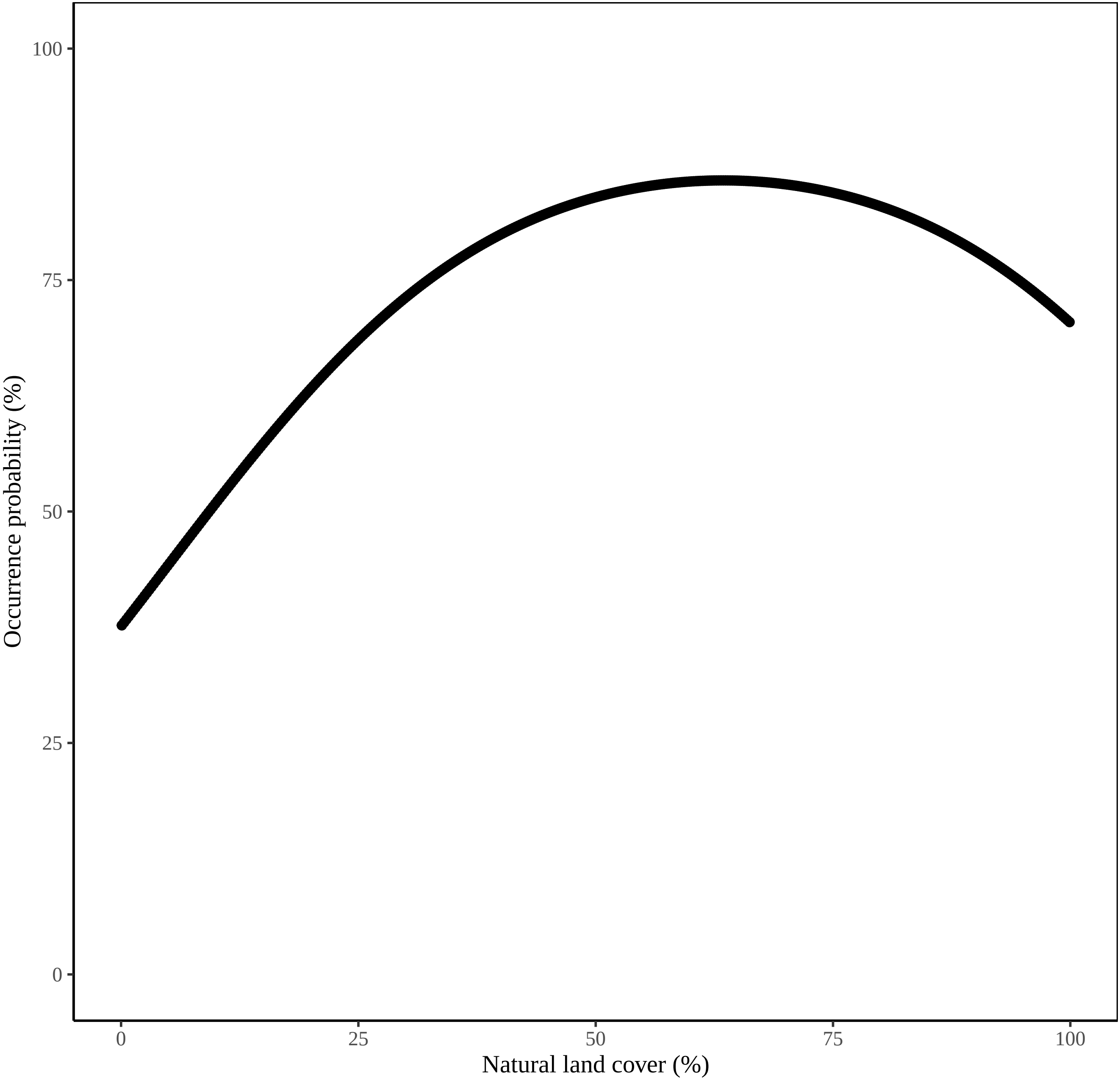

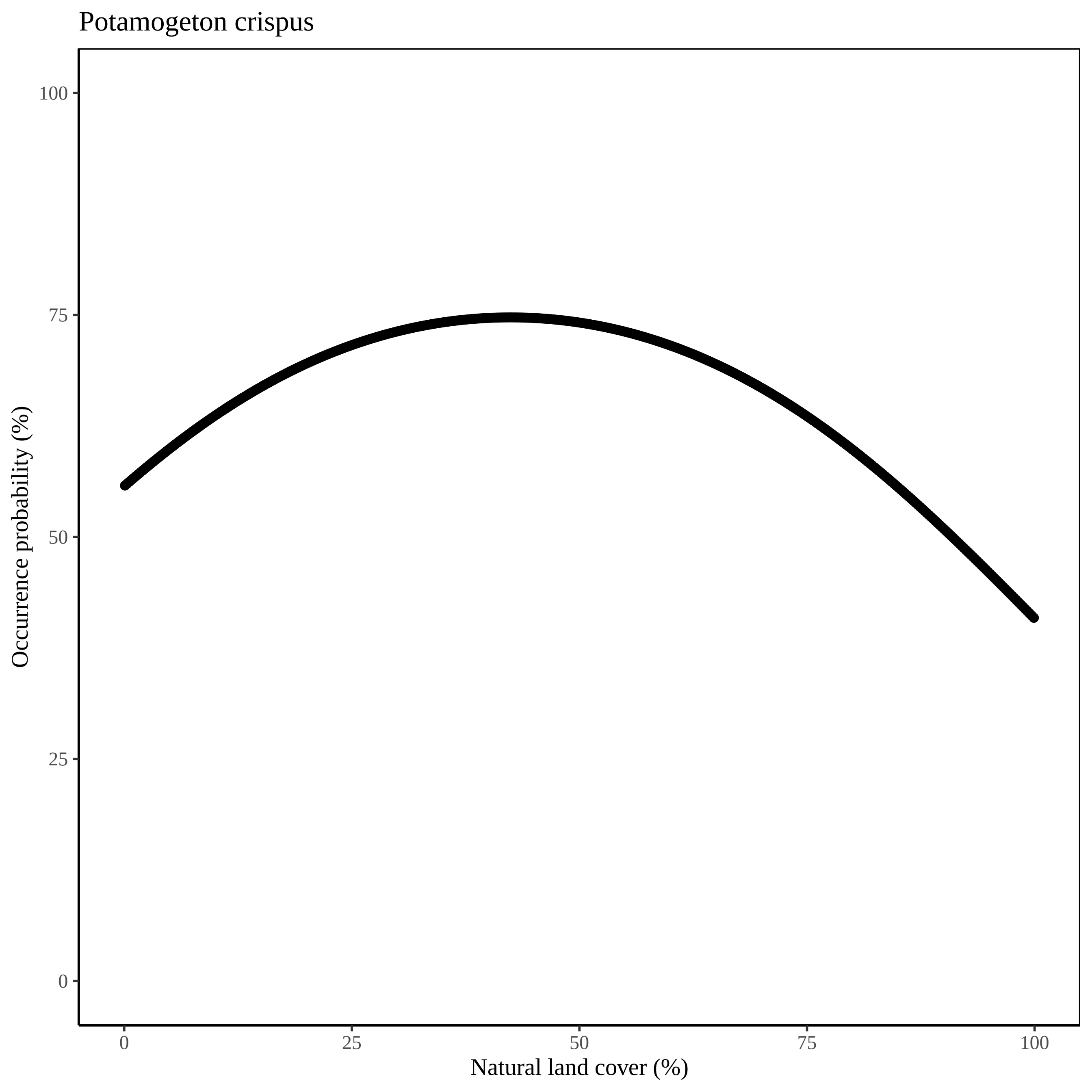

Potamogeton crispus
Occurrence probability (%)
100
75
50
25
0
0
25
50
75
100
Natural land cover (%)

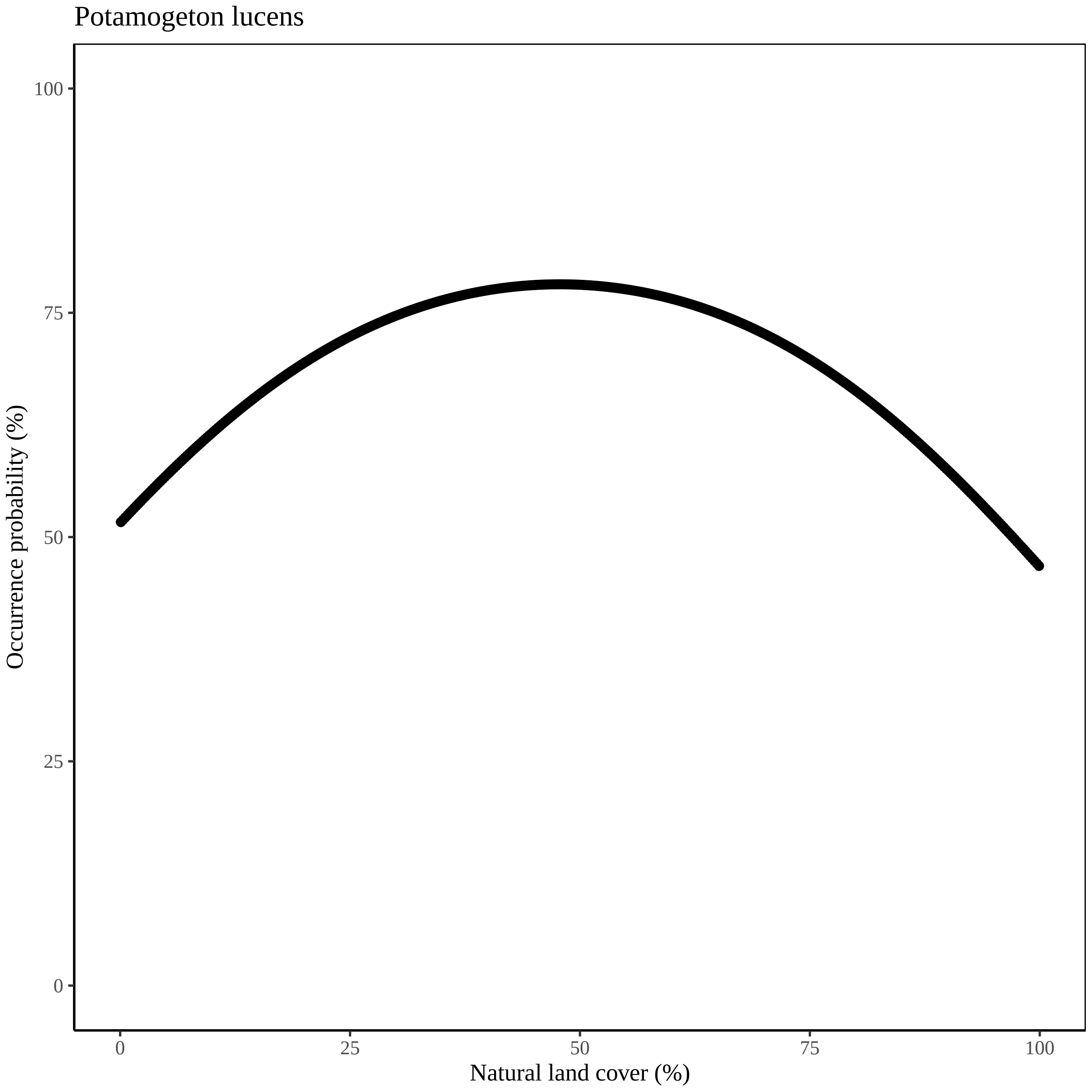
Potamogeton lucens
Occurrence probability (%)
100
75
50
25
0
0
25
50
75
100
Natural land cover (%)

Potamogeton mucronatus

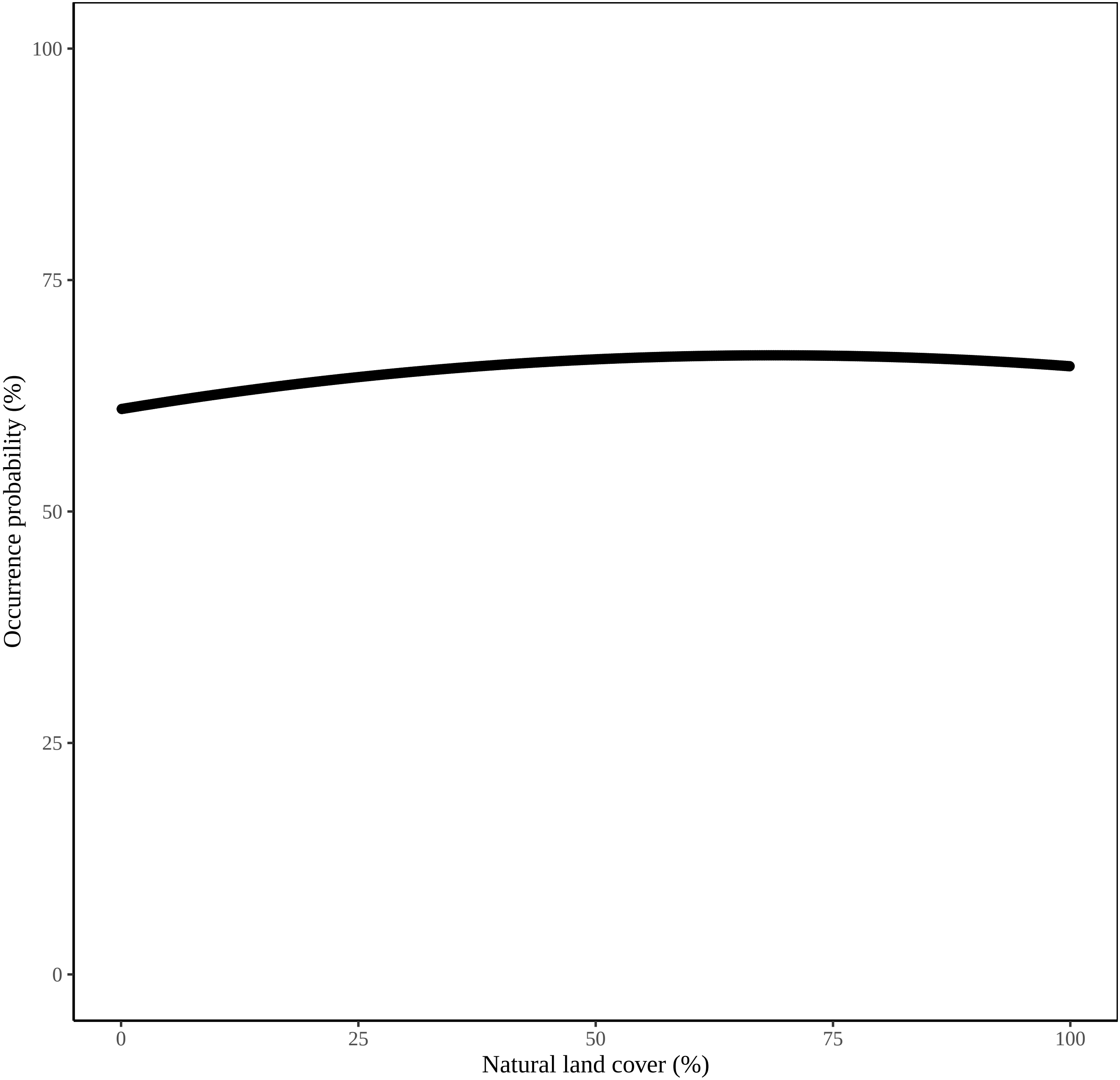

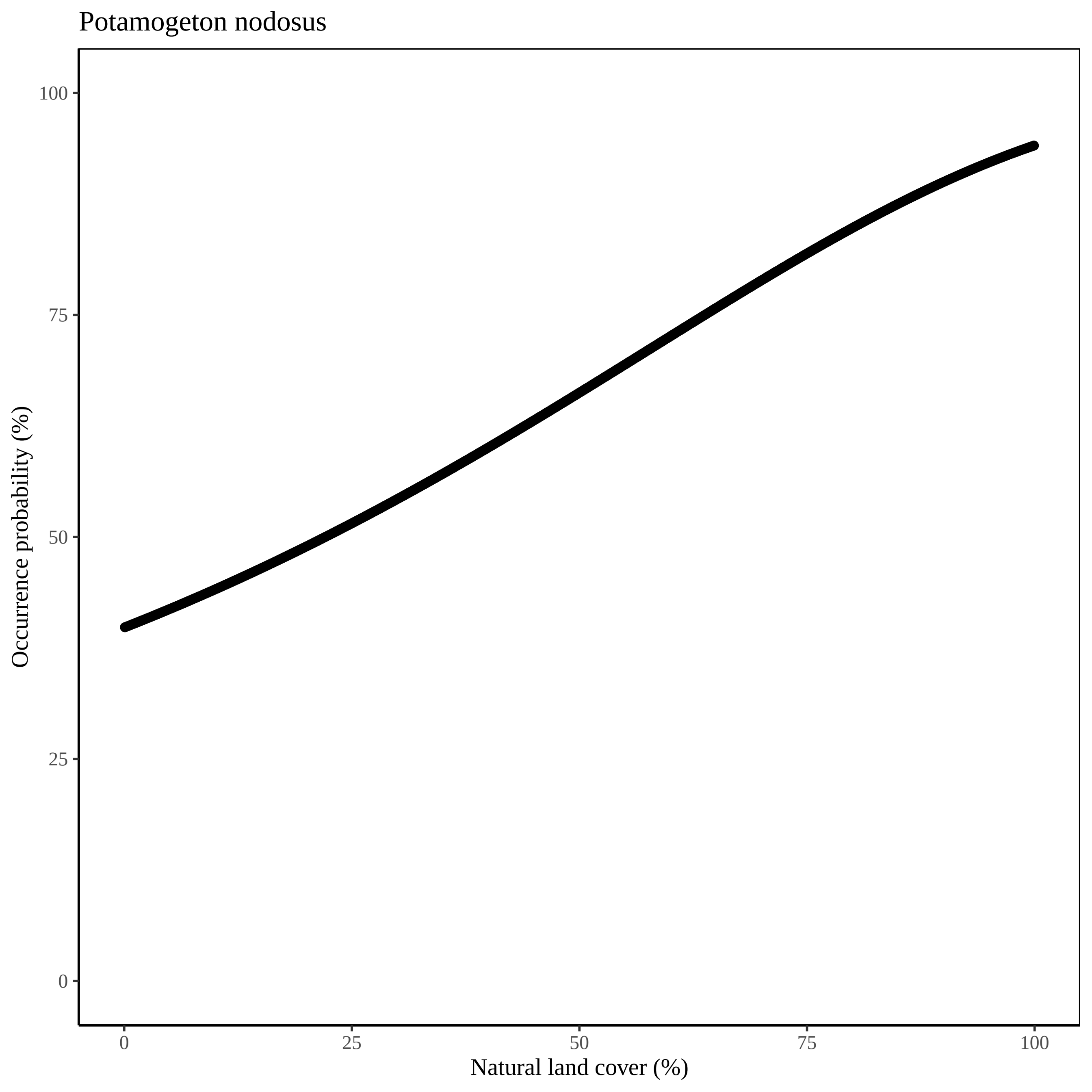

Potamogeton nodosus
Occurrence probability (%)
100
75
50
25
0
0
25
50
75
100
Natural land cover (%)

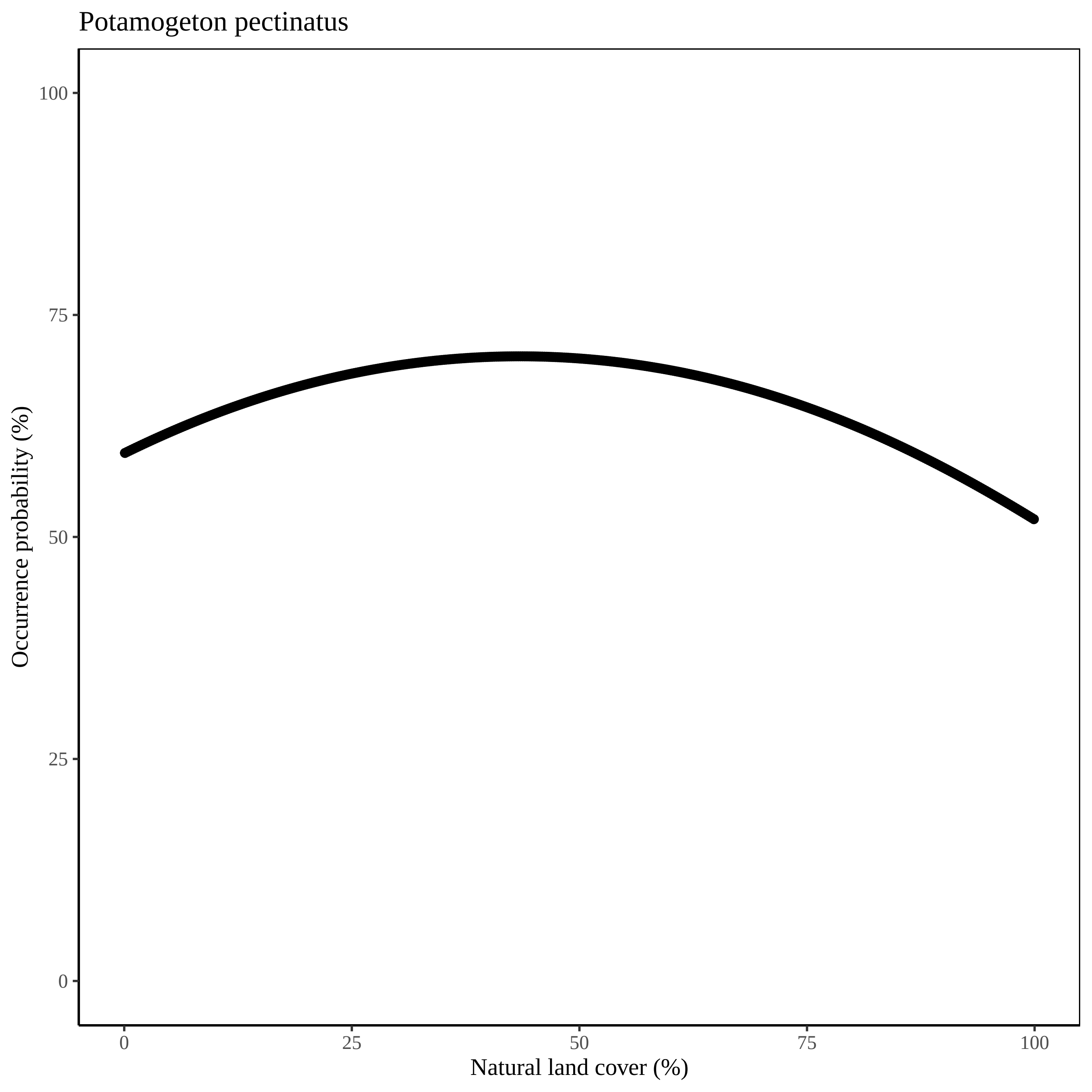

Potamogeton pectinatus
100
75
50
25
0
Occurrence probability (%)
0
25
50
75
100
Natural land cover (%)

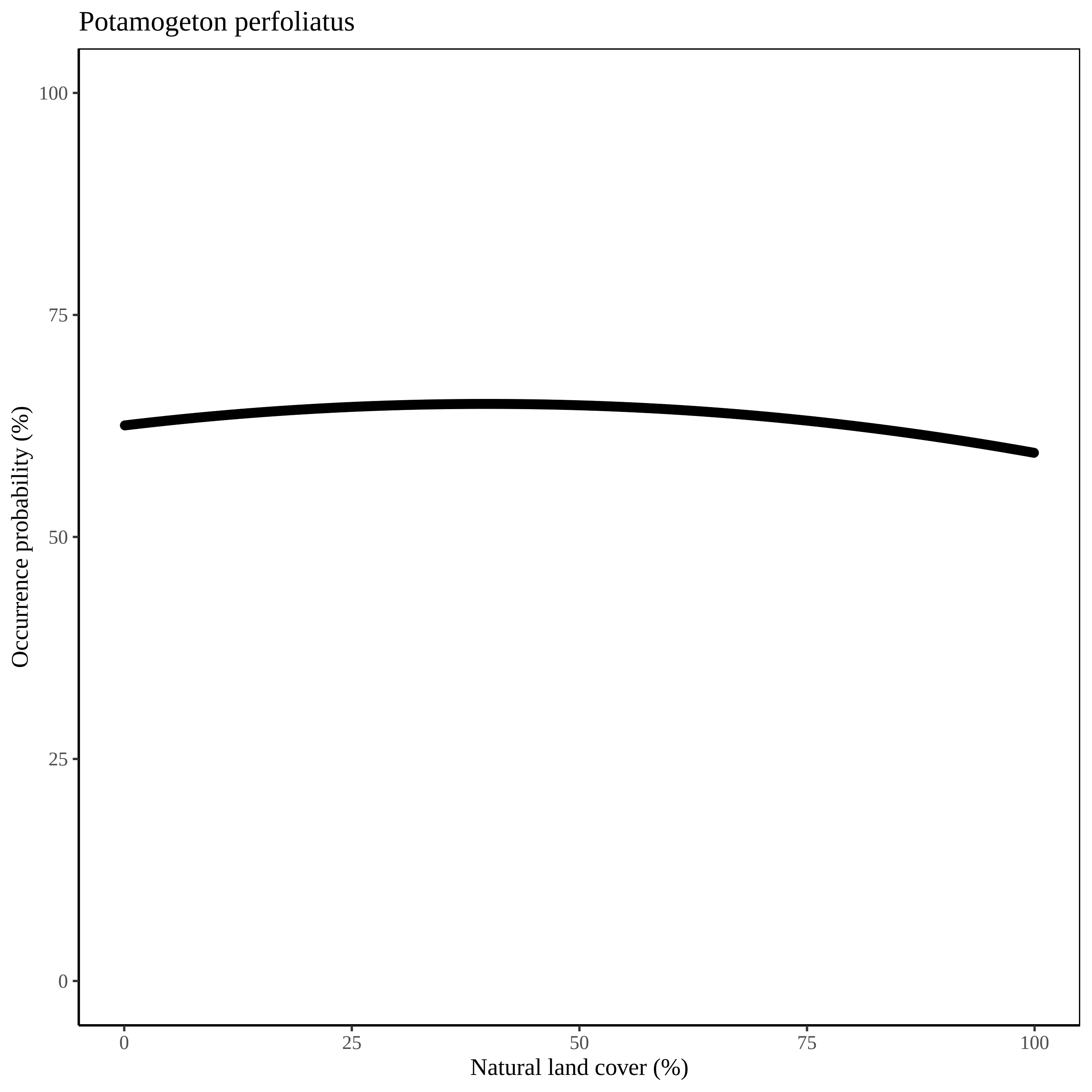

Potamogeton perfoliatus
Occurrence probability (%)
100
75
50
25
0
0
25
50
75
100
Natural land cover (%)

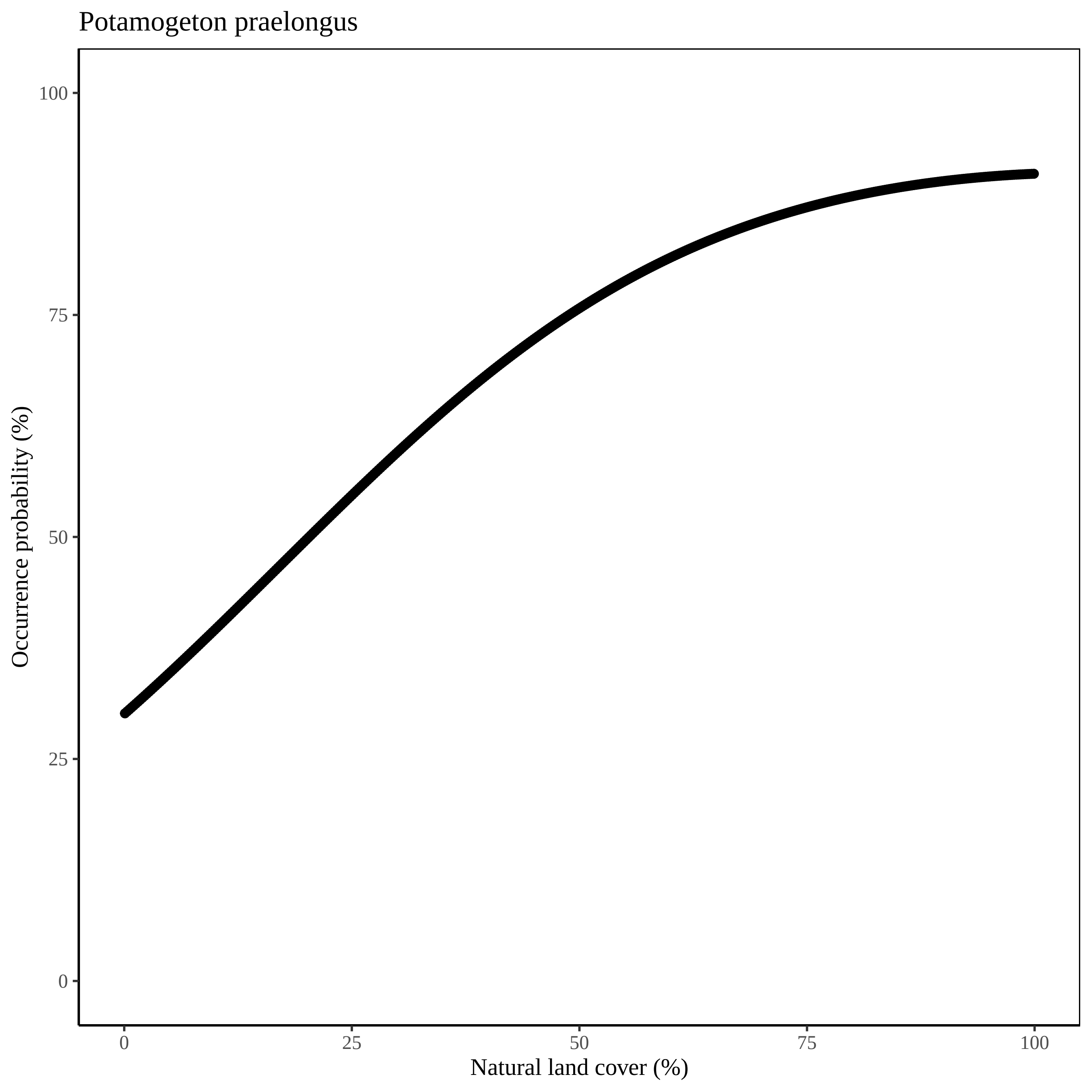

Potamogeton praelongus
Occurrence probability (%)
100
75
50
25
0
0
25
50
75
100
Natural land cover (%)

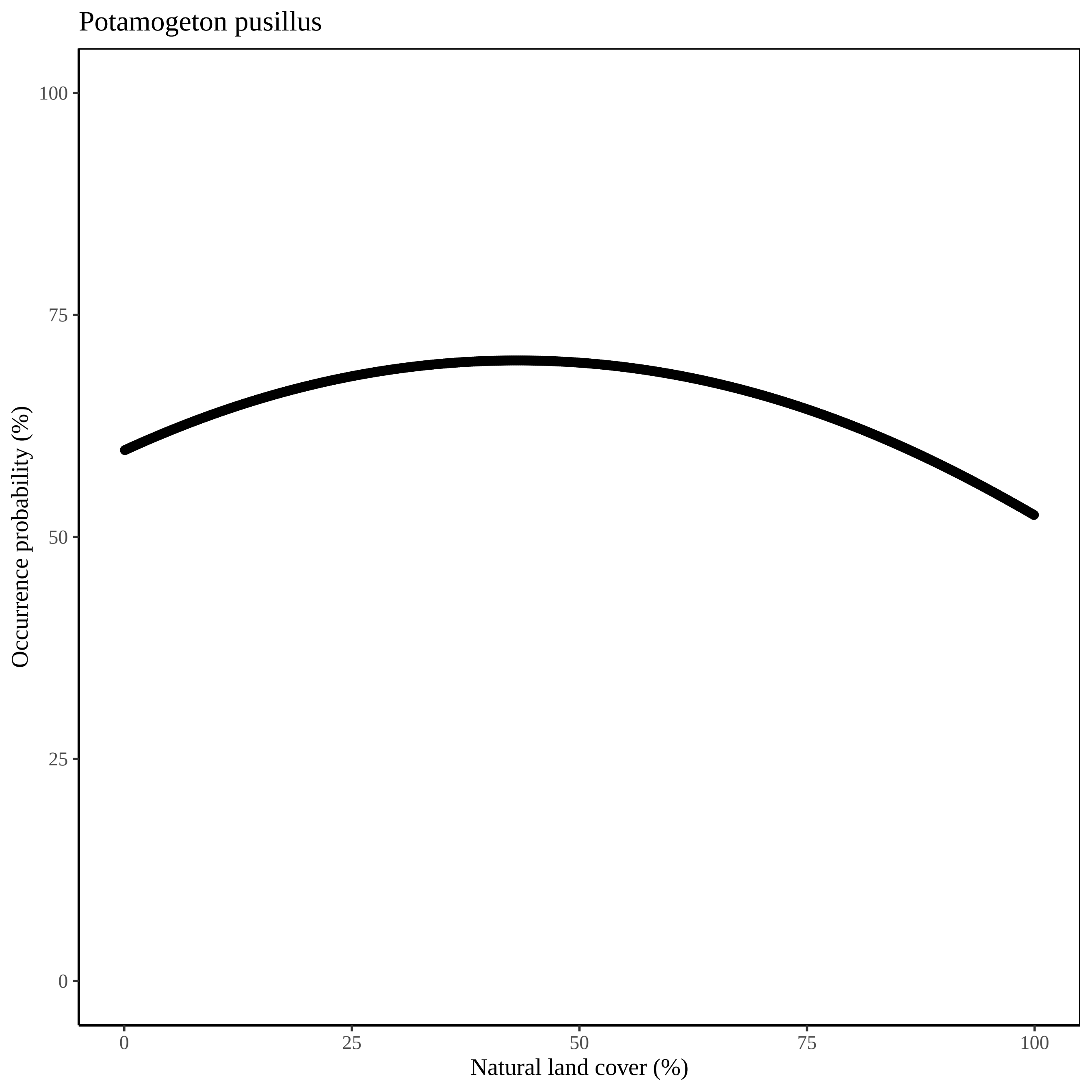

Potamogeton pusillus
Occurrence probability (%)
100
75
50
25
0
0
25
50
75
100
Natural land cover (%)

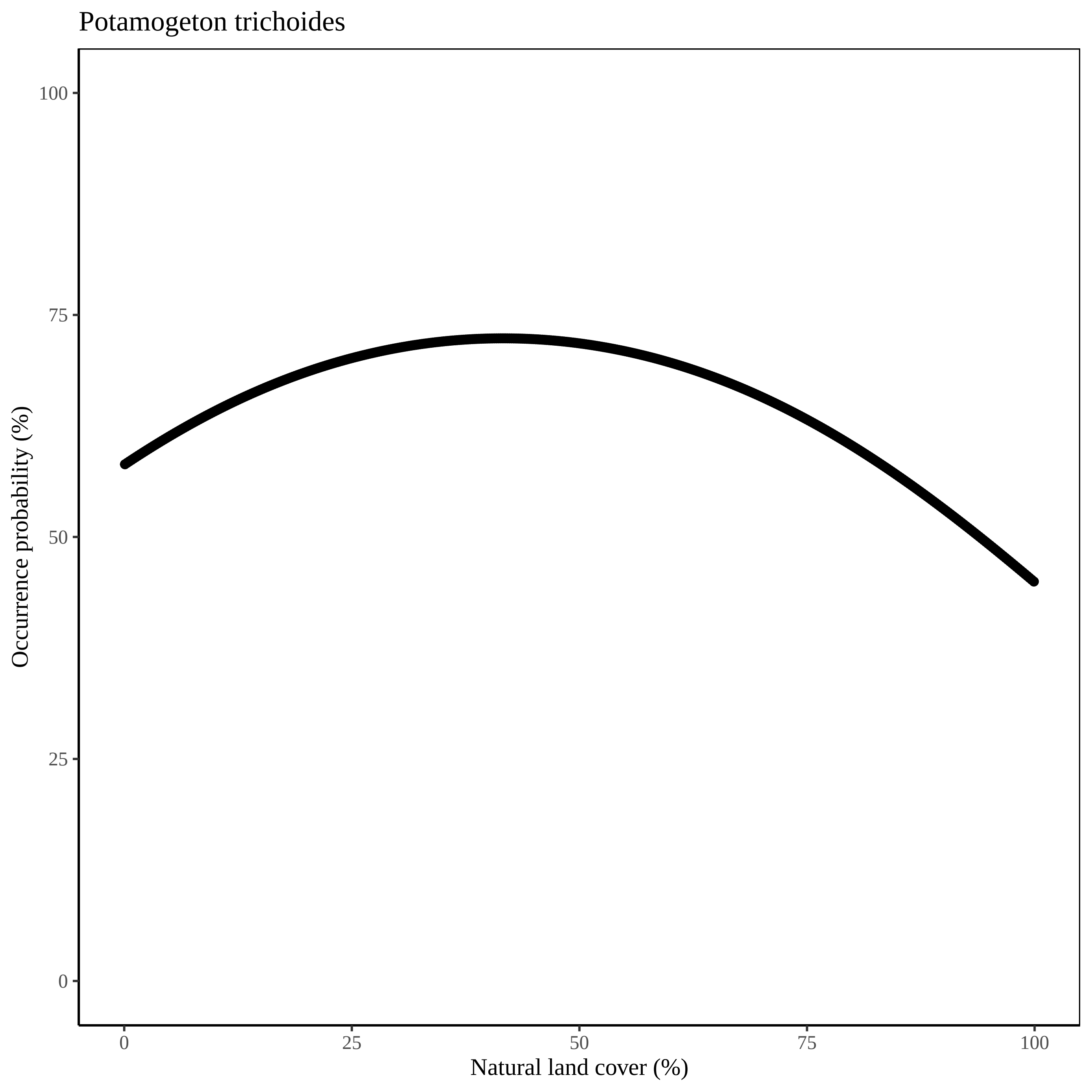

Potamogeton trichoides
Occurrence probability (%)
100
75
50
25
0
0
25
50
75
100
Natural land cover (%)

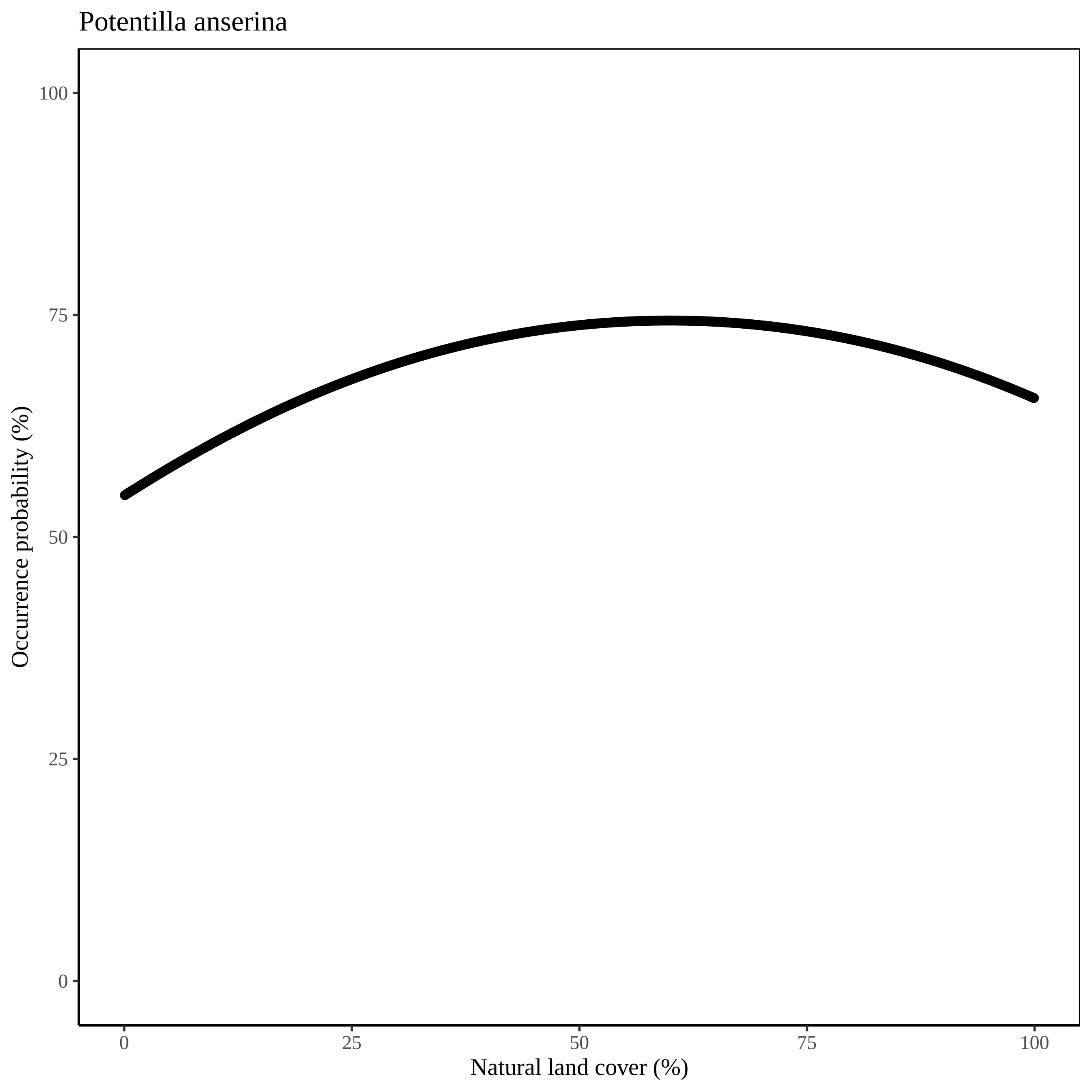

Potentilla anserina
100
75
50
25
0
Occurrence probability (%)
0
25
50
75
100
Natural land cover (%)

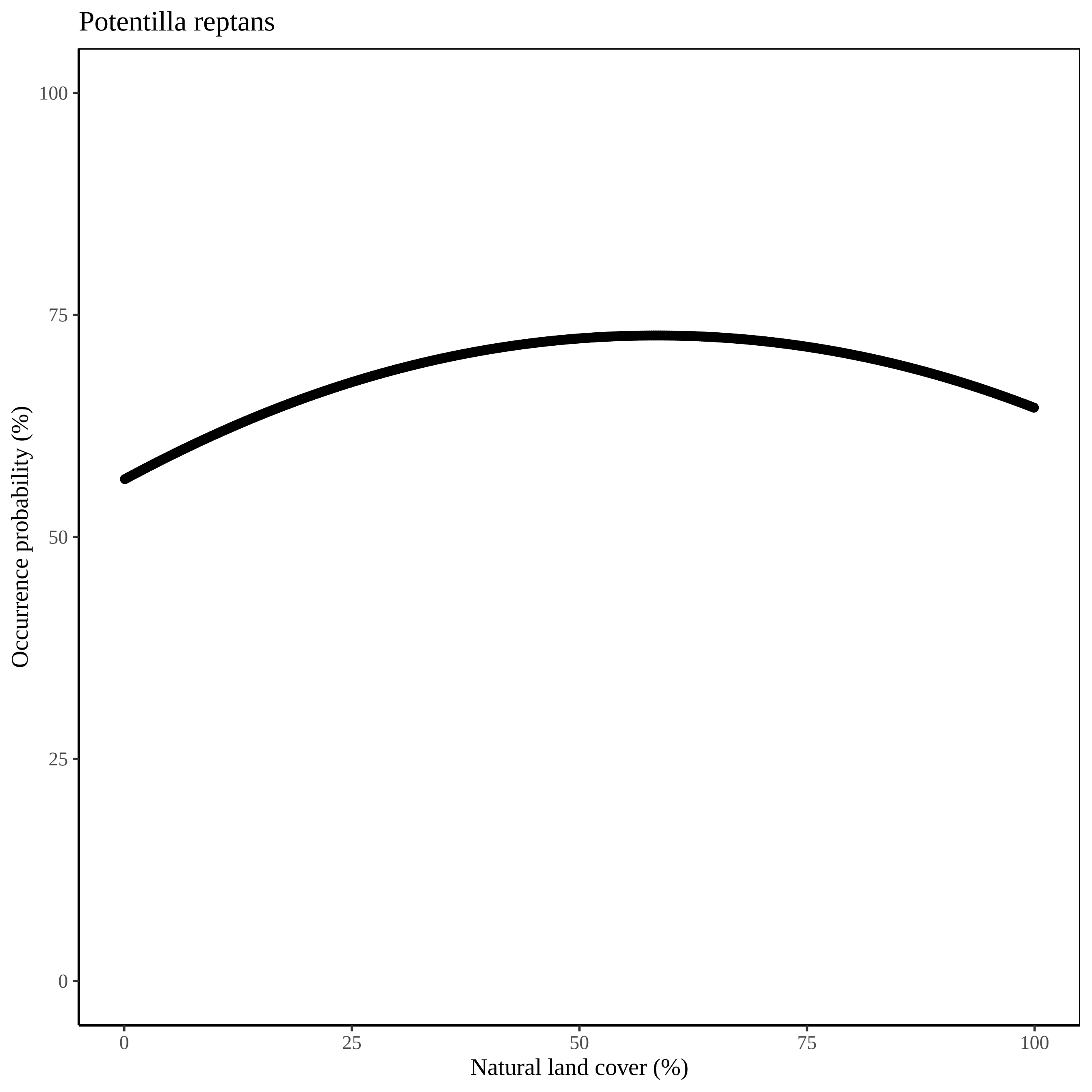

Potentilla reptans
Occurrence probability (%)
100
75
50
25
0
0
25
50
75
100
Natural land cover (%)

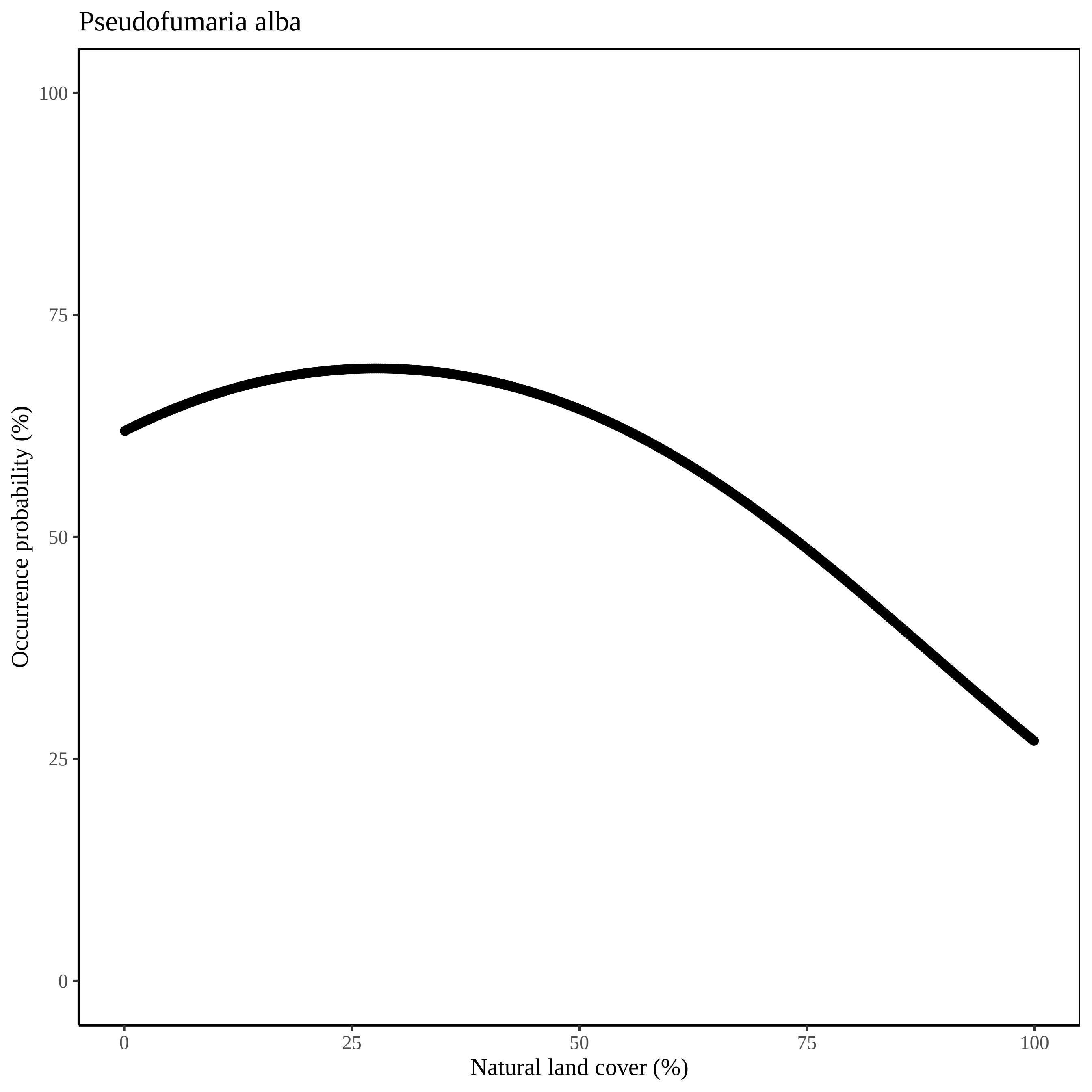

Pseudofumaria alba
Occurrence probability (%)
100
75
50
25
0
0
25
50
75
100
Natural land cover (%)

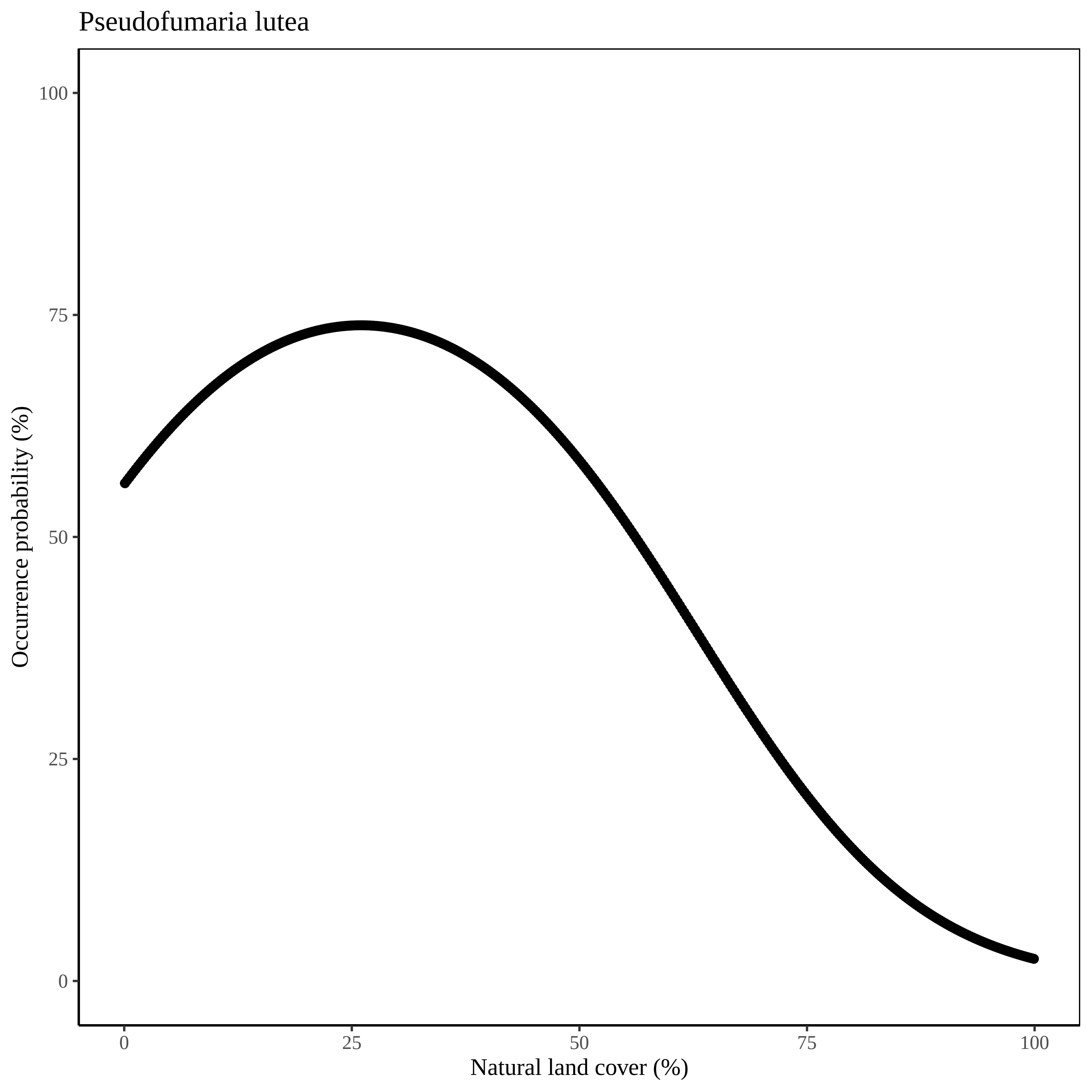

Pseudofumaria lutea
Occurrence probability (%)
100
75
50
25
0
0
25
50
75
100
Natural land cover (%)

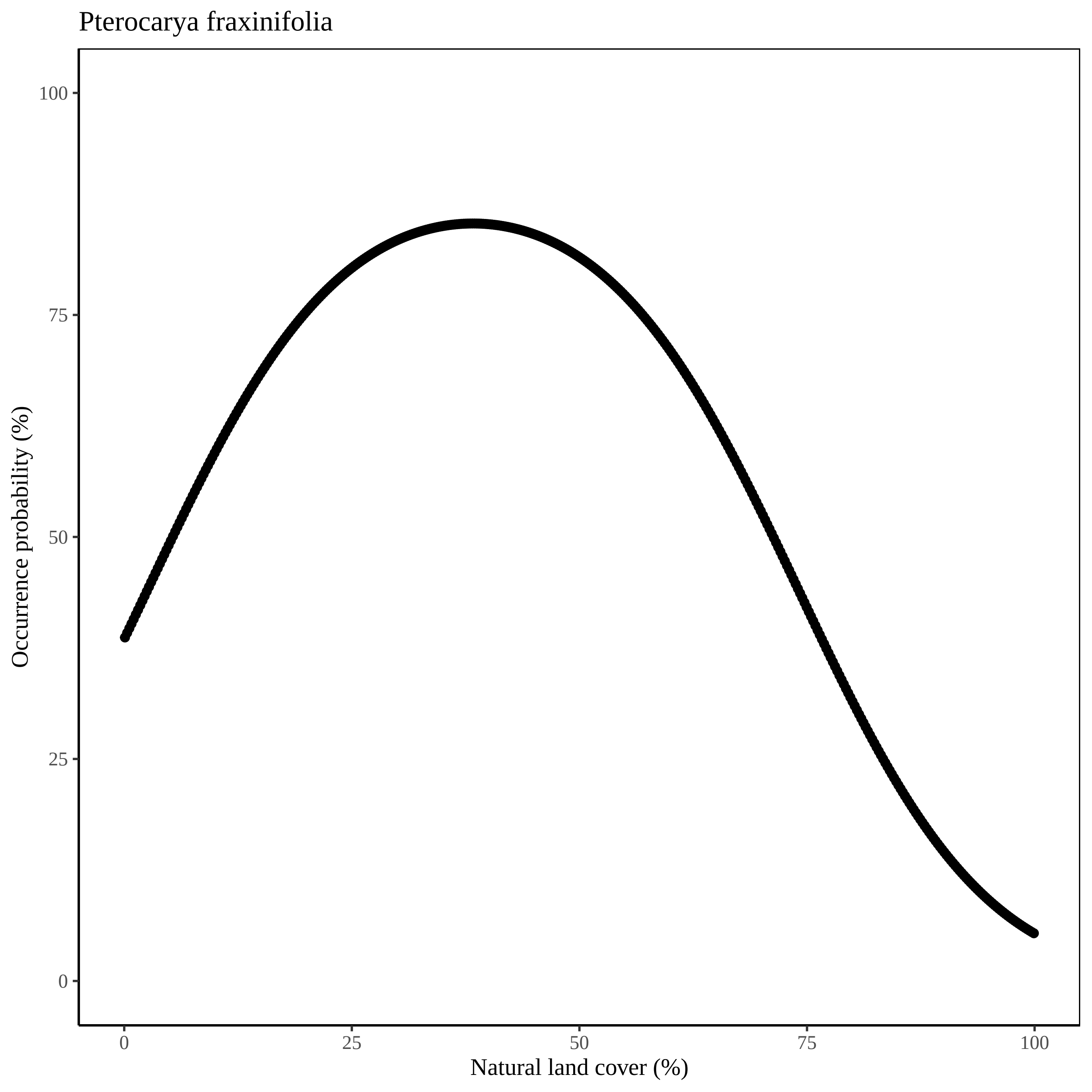
Pterocarya fraxinifolia
Occurrence probability (%)
100
75
50
25
0
0
25
50
75
100
Natural land cover (%)

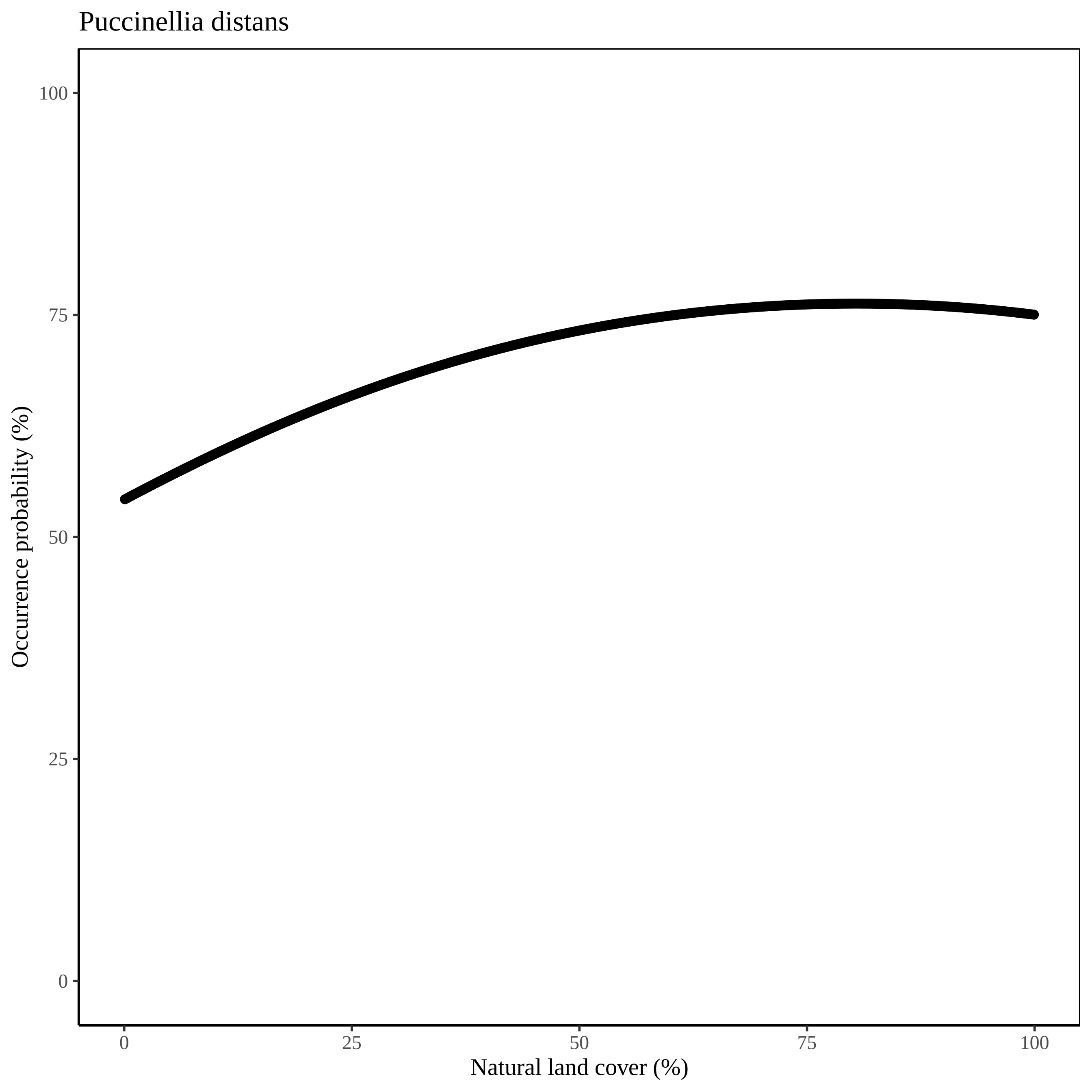

Puccinellia distans
Occurrence probability (%)
100
75
50
25
0
0
25
50
75
100
Natural land cover (%)

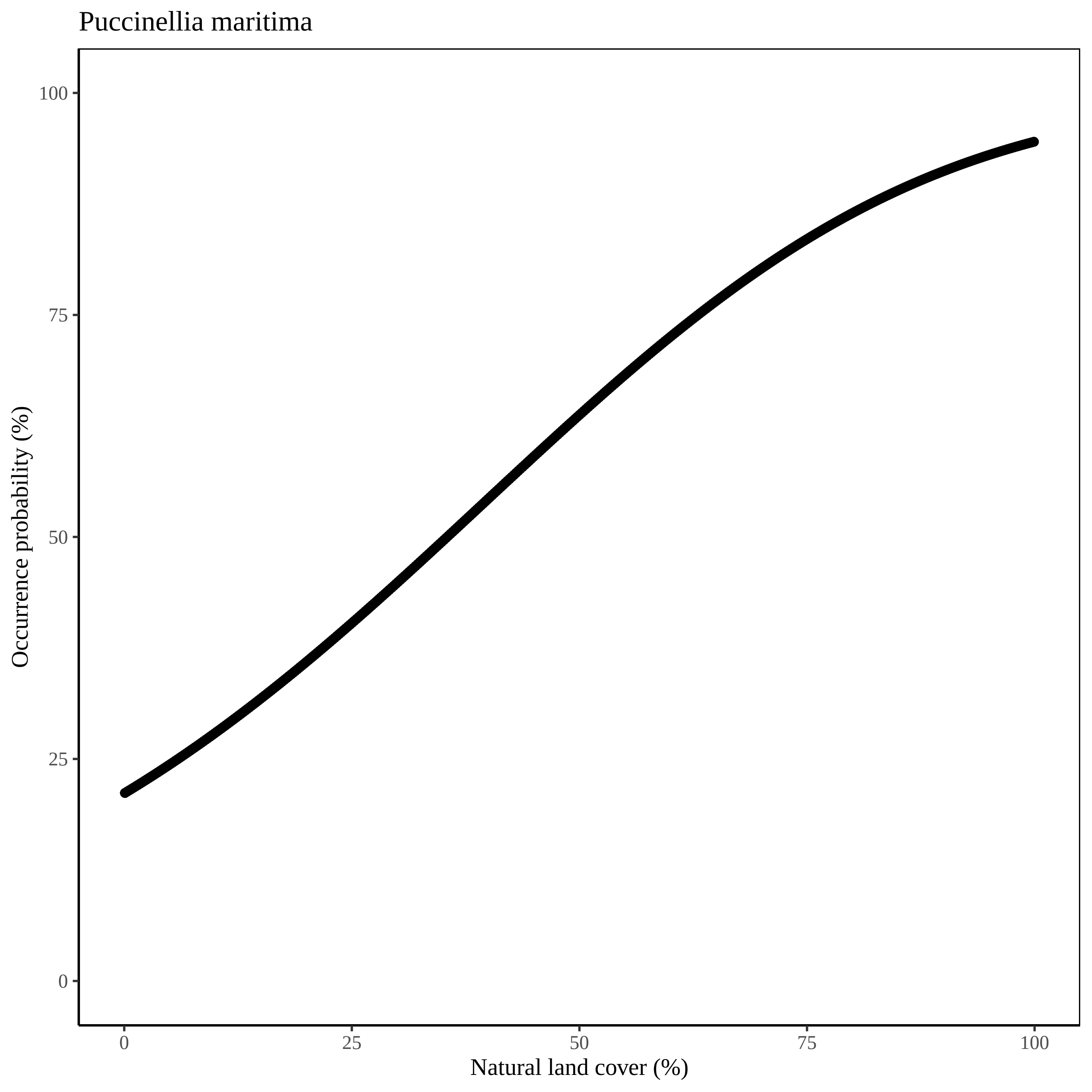

Puccinellia maritima
Occurrence probability (%)
100
75
50
25
0
0
25
50
75
100
Natural land cover (%)

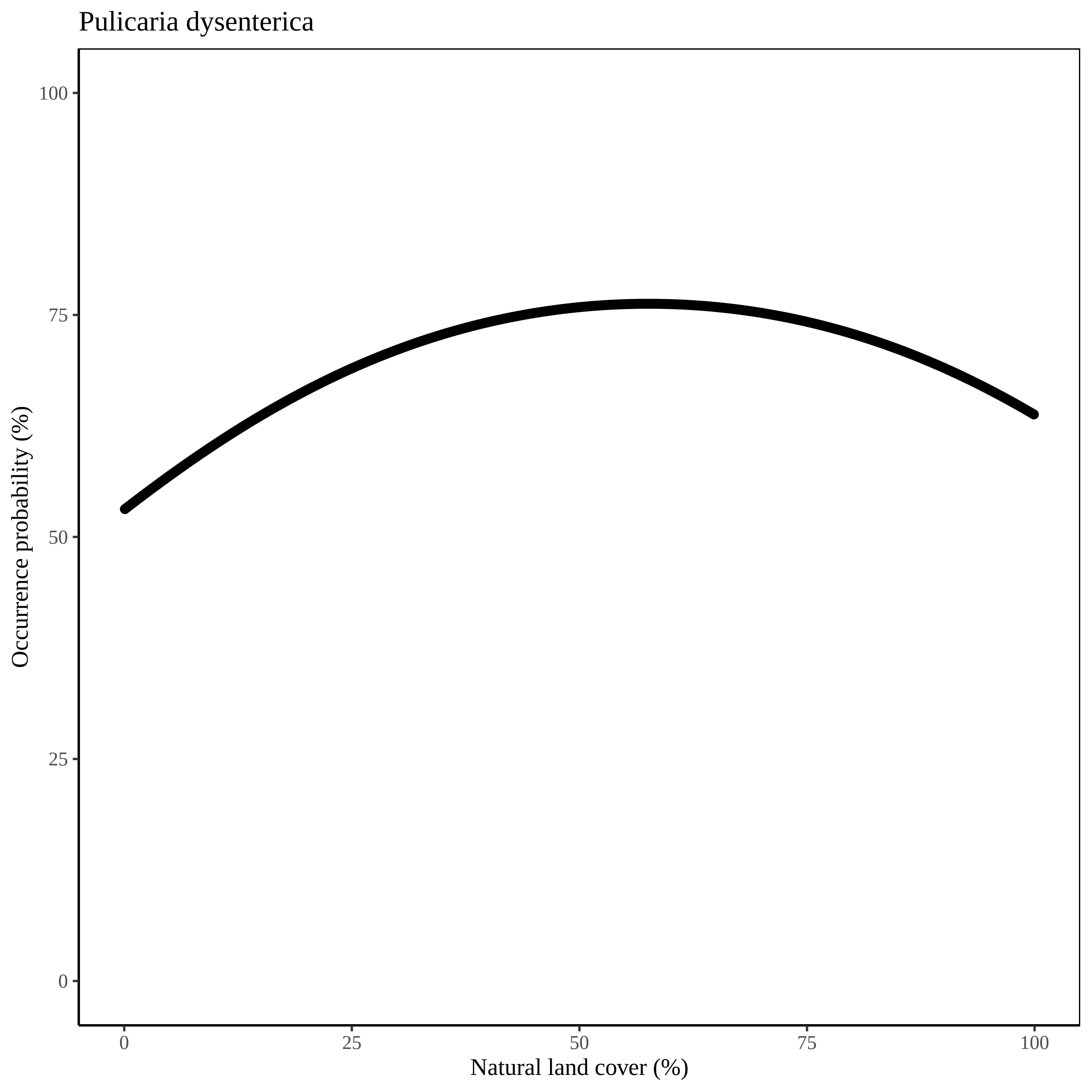

Pulicaria dysenterica
Occurrence probability (%)
100
75
50
25
0
0
25
50
75
100
Natural land cover (%)

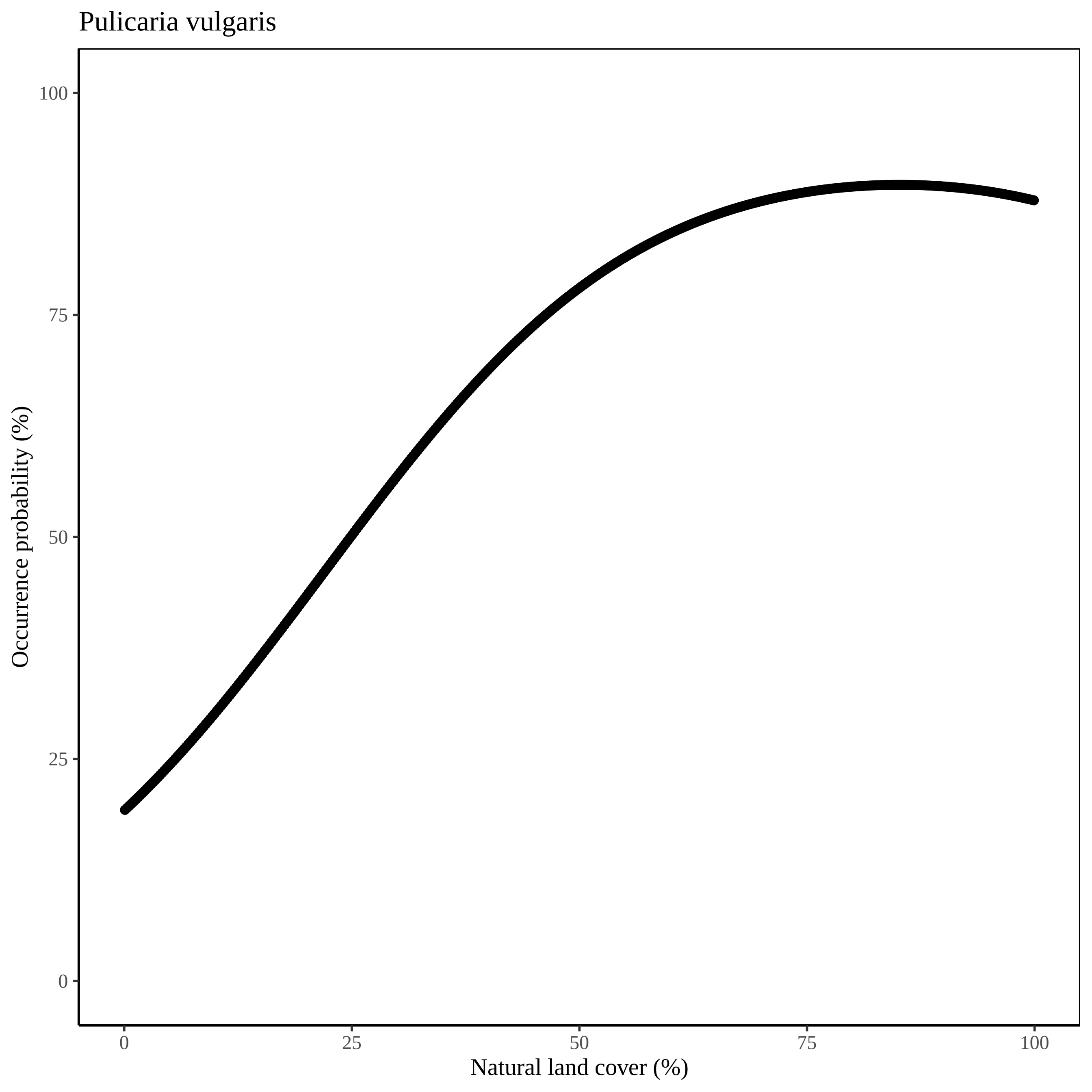

Pulicaria vulgaris
Occurrence probability (%)
0
25
50
75
100
Natural land cover (%)
0
25
50
75
100

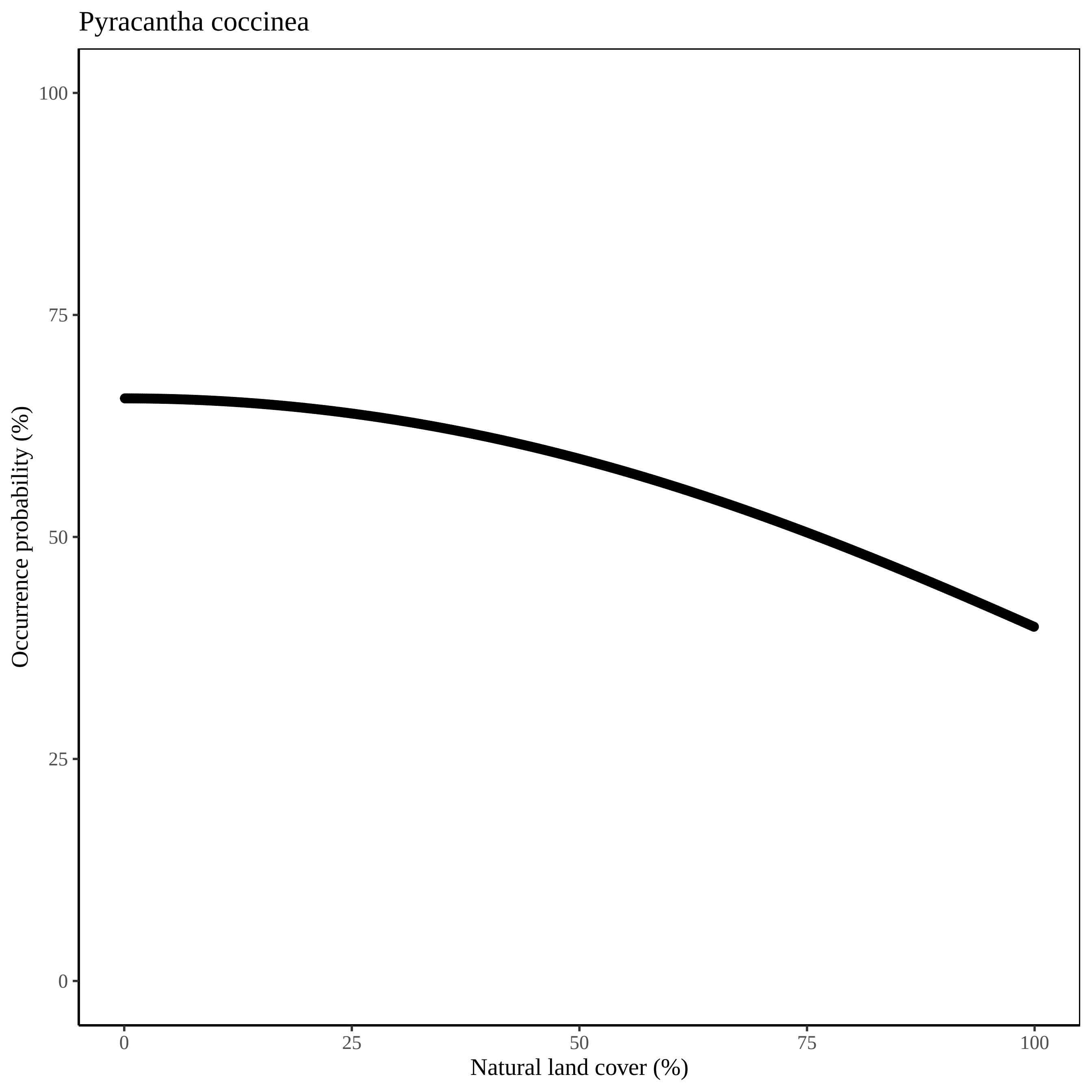

Pyracantha coccinea
Occurrence probability (%)
100
75
50
25
0
0
25
50
75
100
Natural land cover (%)

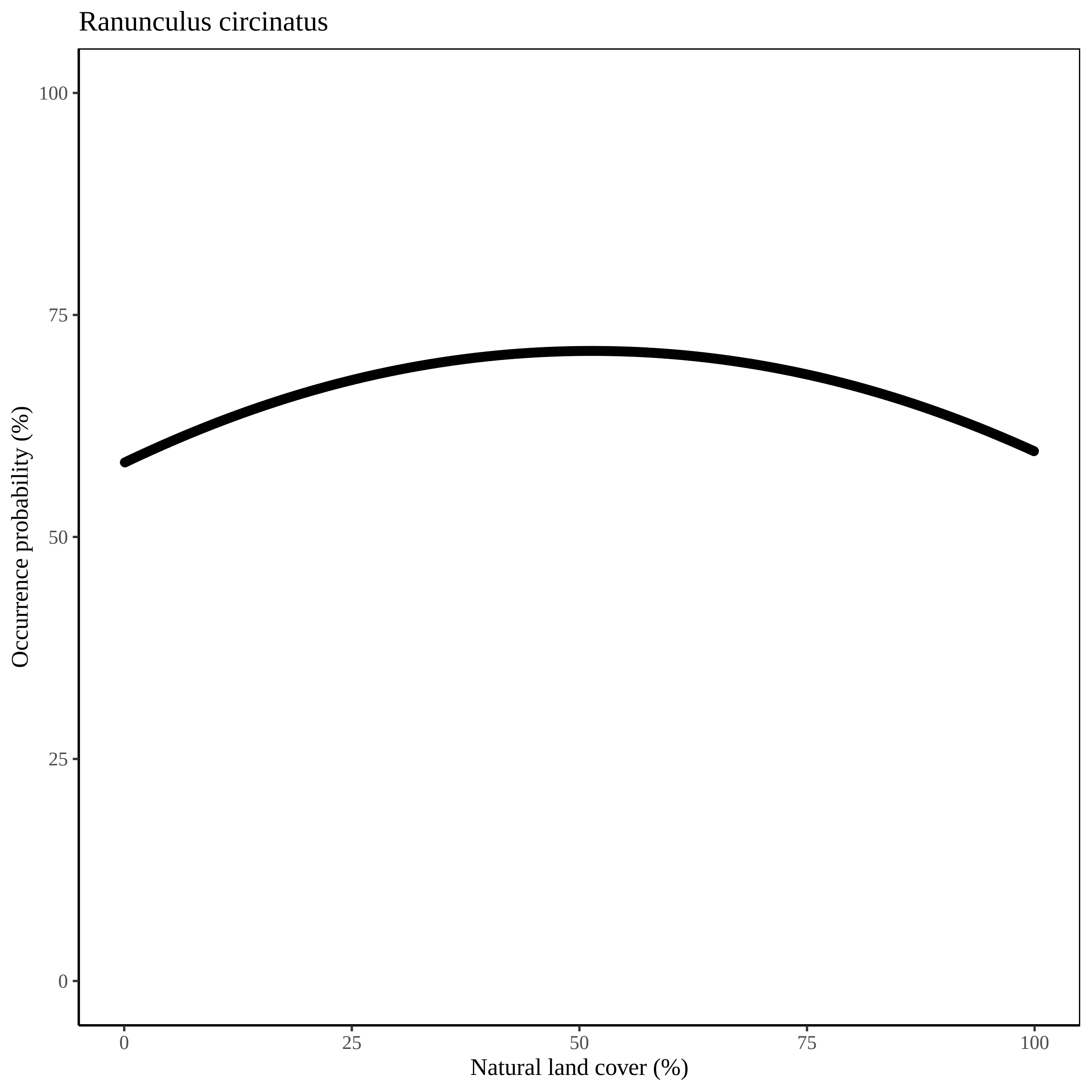

Ranunculus circinatus
Occurrence probability (%)
Natural land cover (%)
0
25
50
75
100

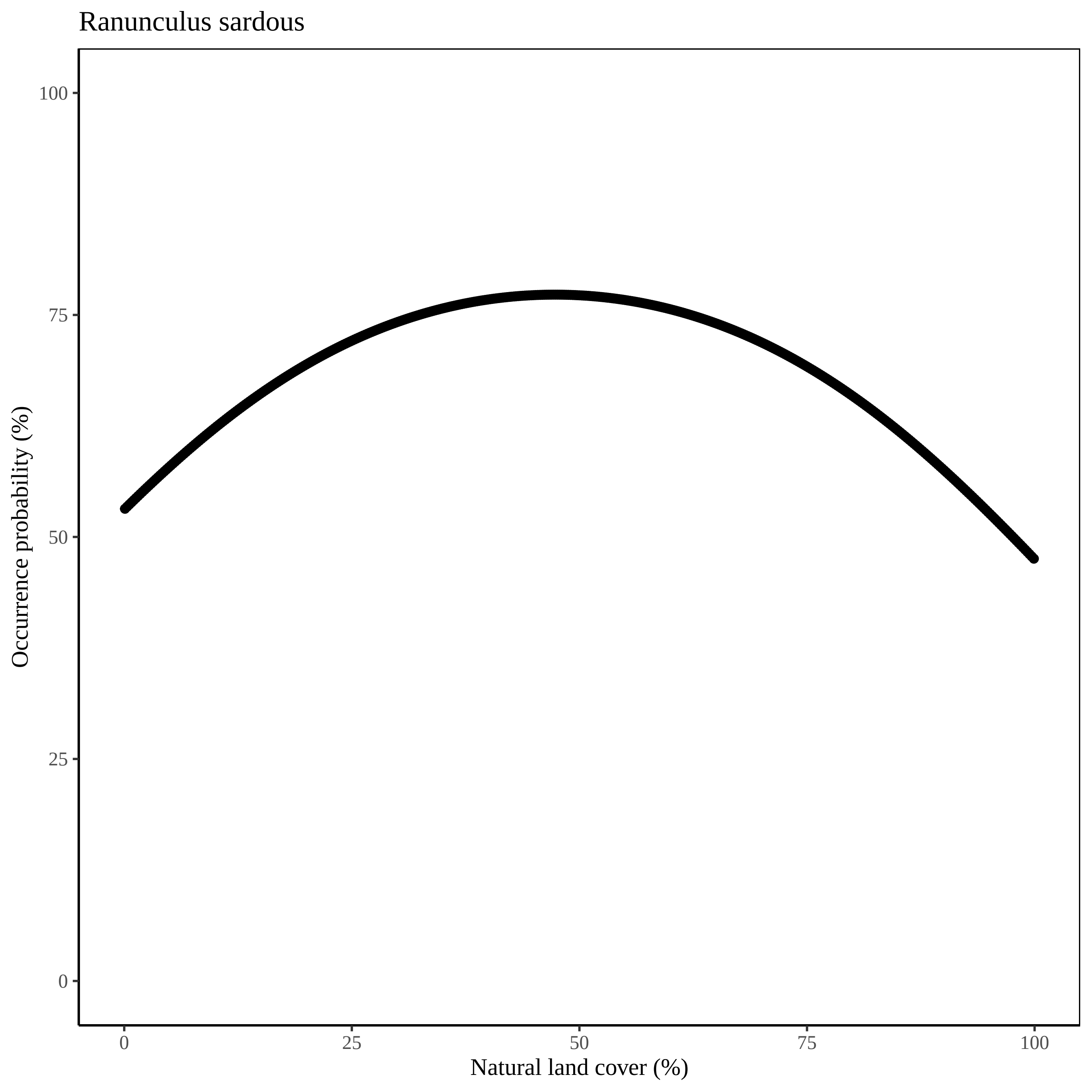
Ranunculus sardous
Occurrence probability (%)
100
75
50
25
0
0
25
50
75
100
Natural land cover (%)

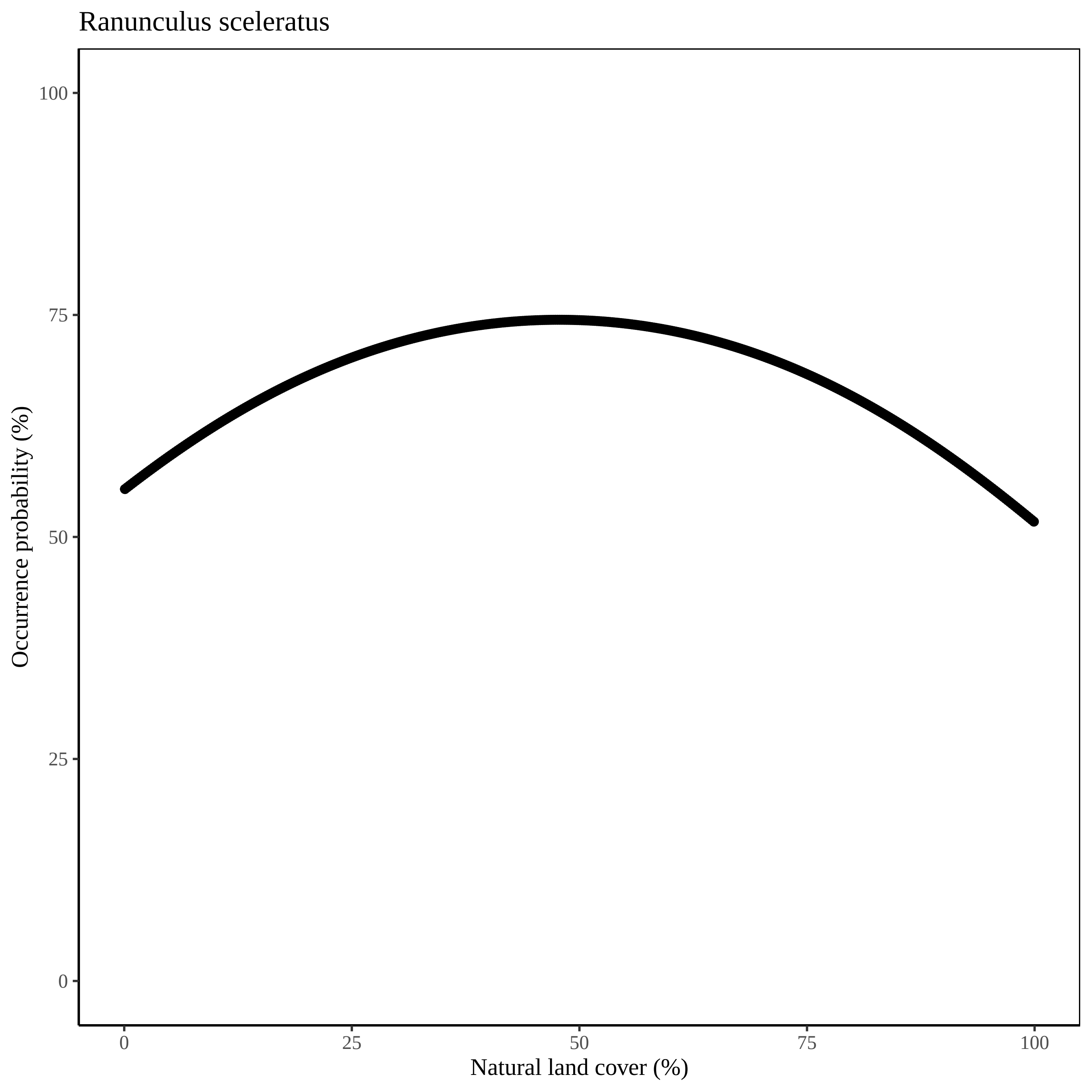
Ranunculus sceleratus
Occurrence probability (%)
Natural land cover (%)
0
25
50
75
100

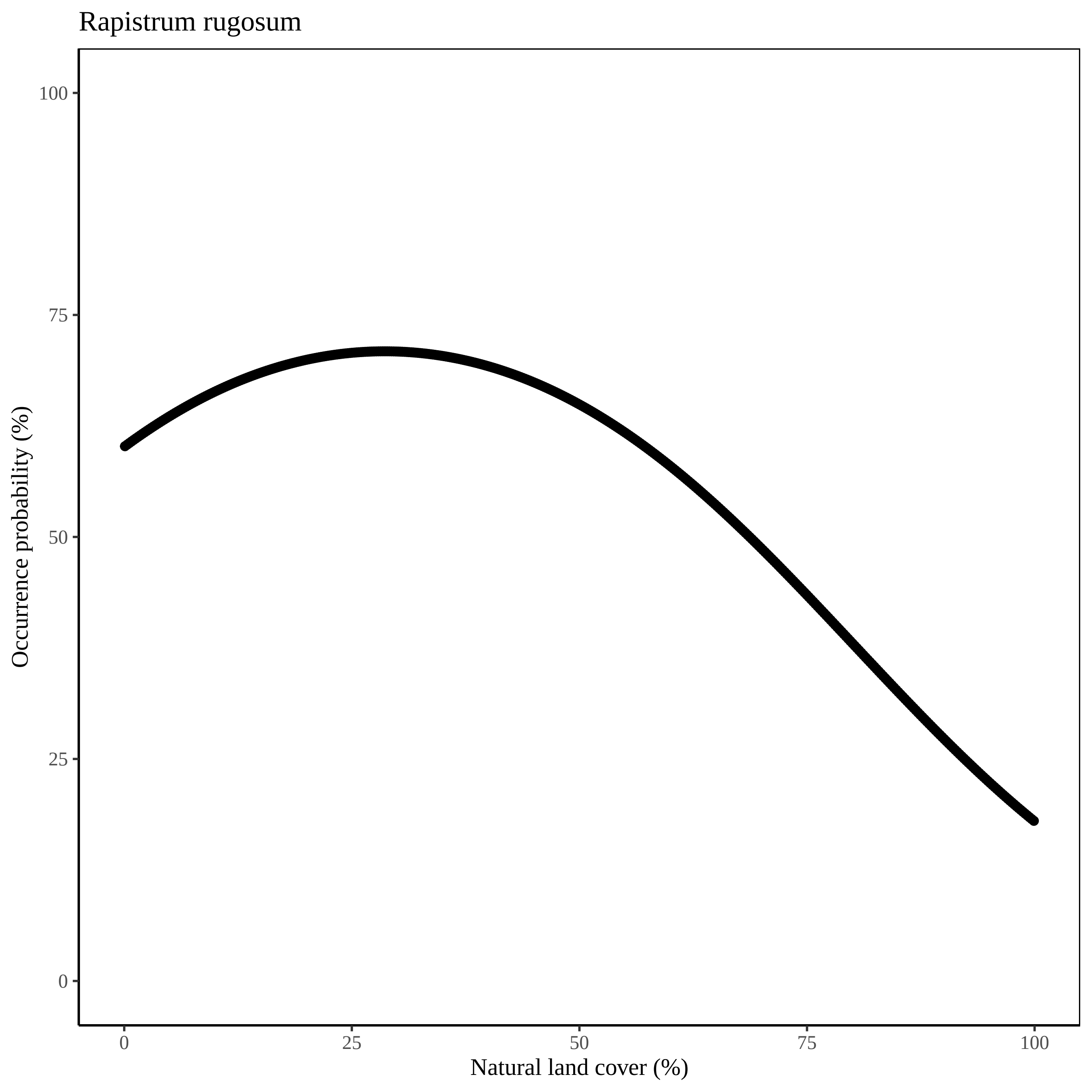
Rapistrum rugosum
100
75
50
25
0
Occurrence probability (%)
0
25
50
75
100
Natural land cover (%)

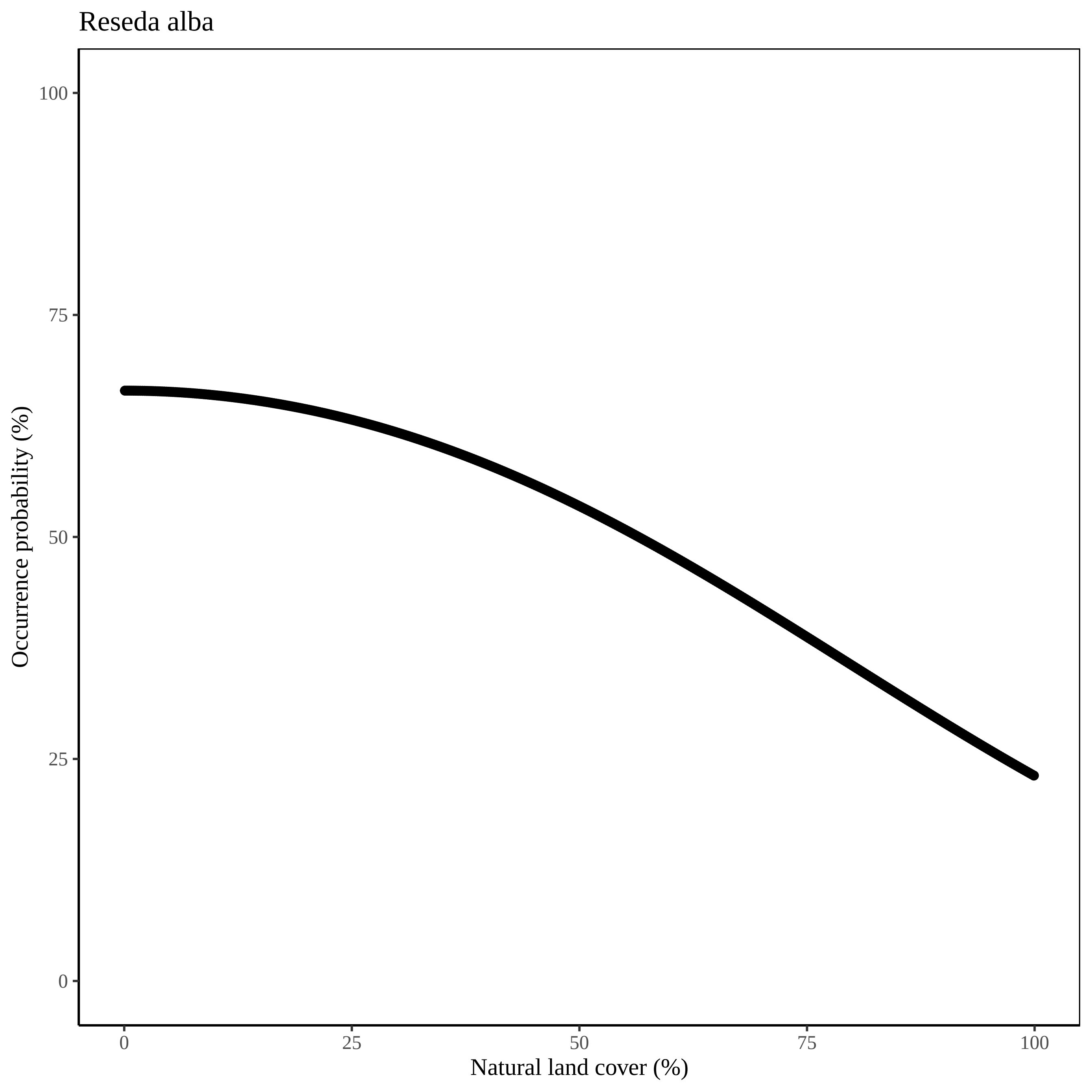

Reseda alba
100
75
50
25
0
Occurrence probability (%)
0
25
50
75
100
Natural land cover (%)

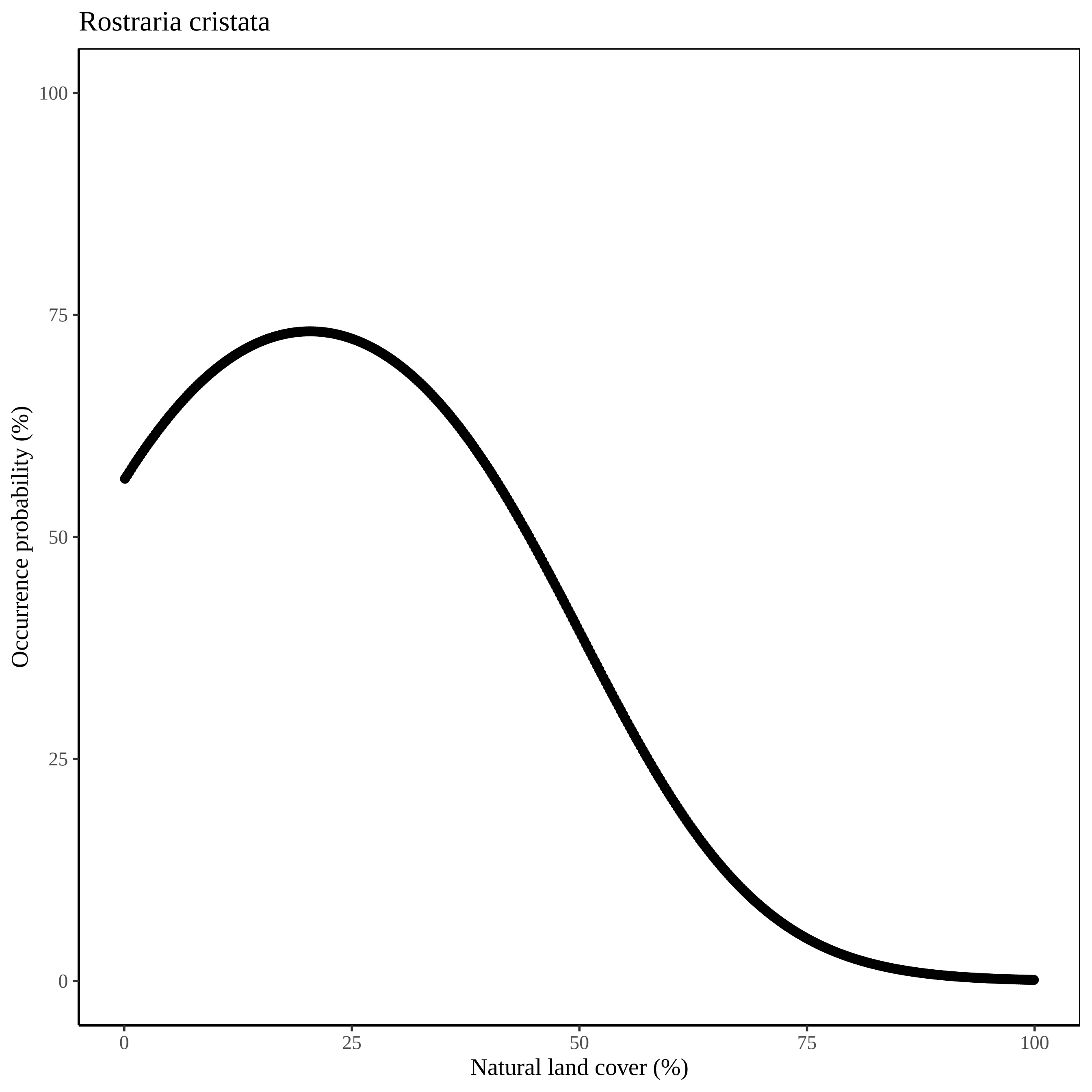

Rostraria cristata
Occurrence probability (%)
100
75
50
25
0
0
25
50
75
100
Natural land cover (%)

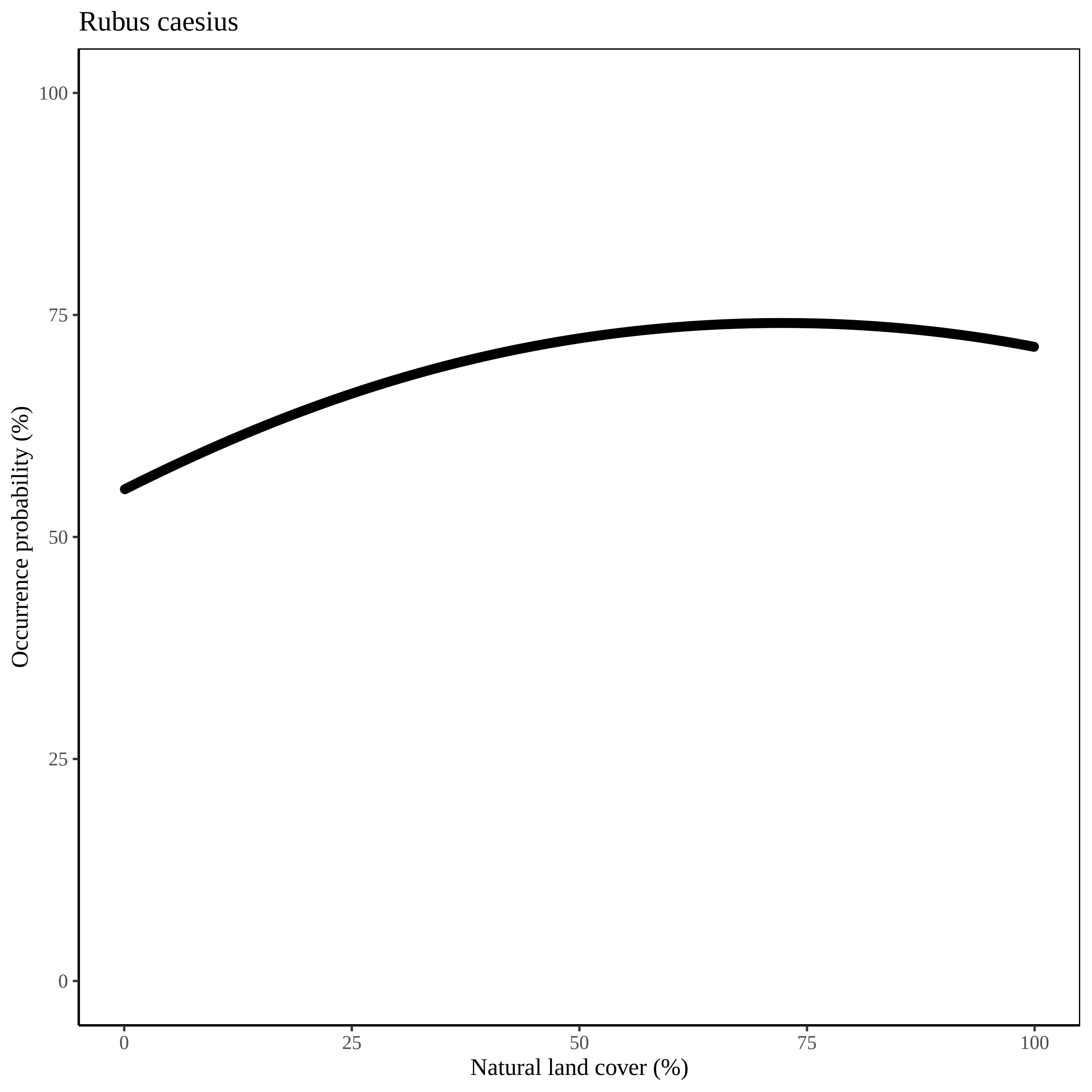

Rubus caesius
Occurrence probability (%)
100
75
50
25
0
0
25
50
75
100
Natural land cover (%)

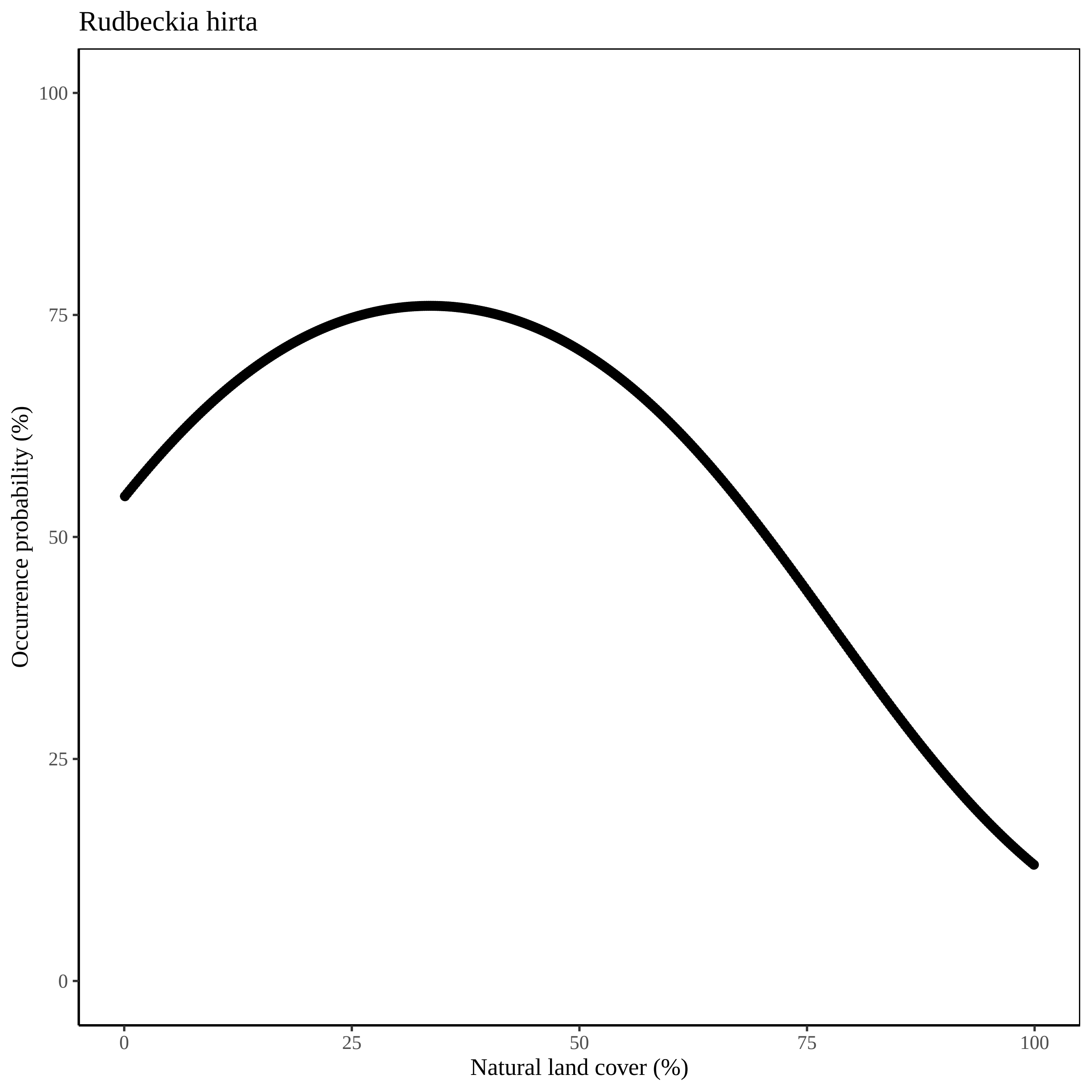
Rudbeckia hirta
Occurrence probability (%)
100
75
50
25
0
0
25
50
75
100
Natural land cover (%)

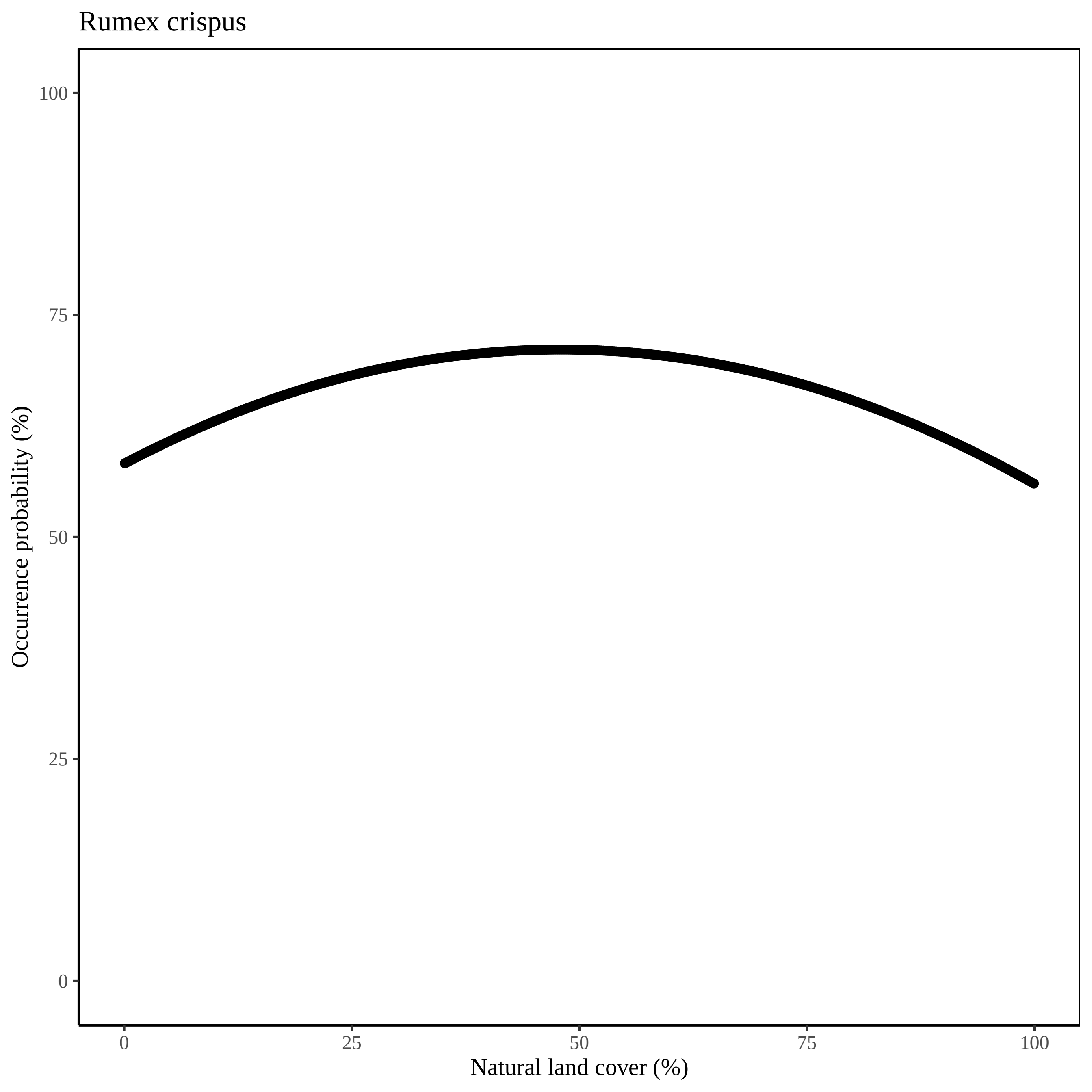

Rumex crispus
Occurrence probability (%)
100
75
50
25
0
Natural land cover (%)
0
25
50
75
100

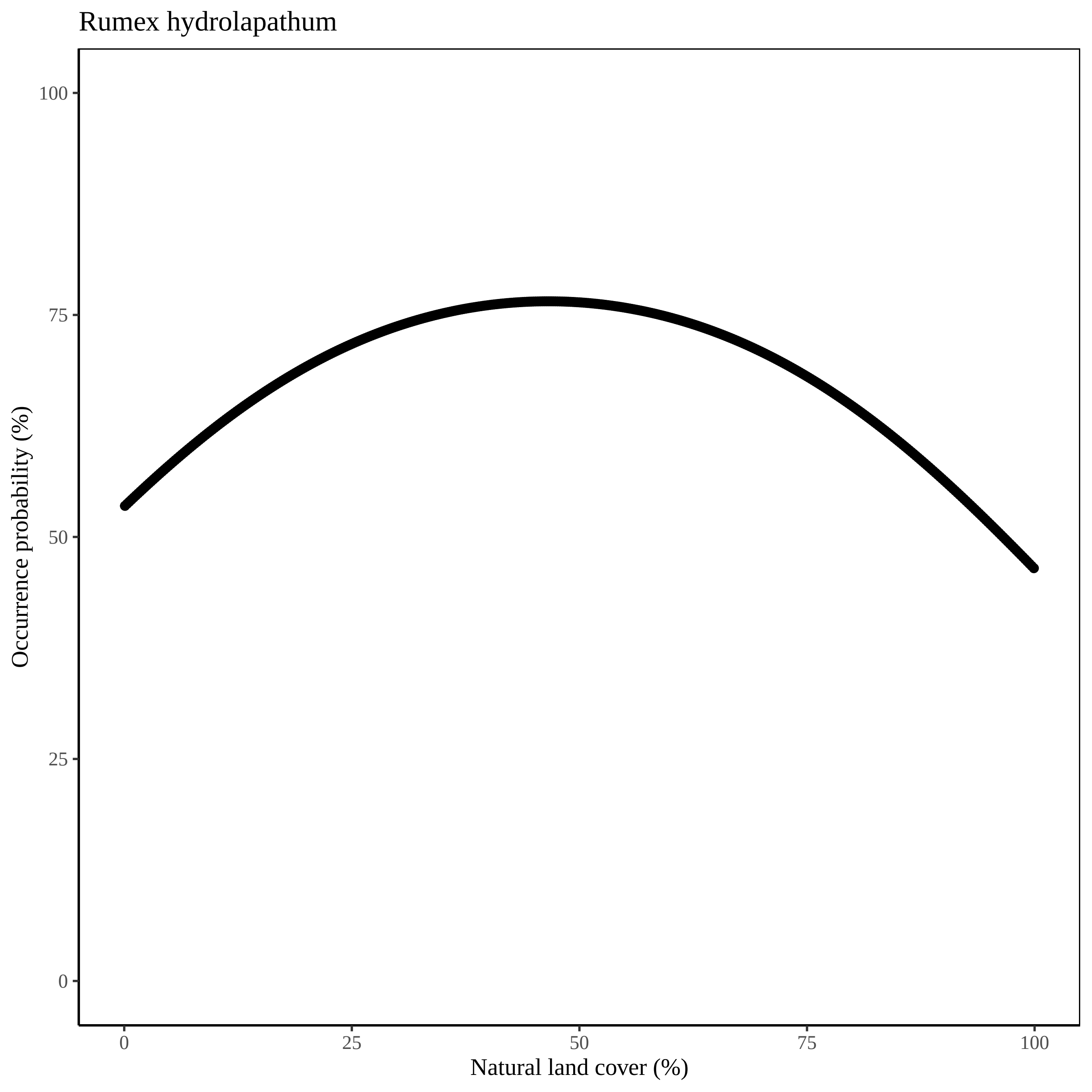

Rumex hydrolapathum
100
75
50
25
0
Occurrence probability (%)
0
25
50
75
100
Natural land cover (%)

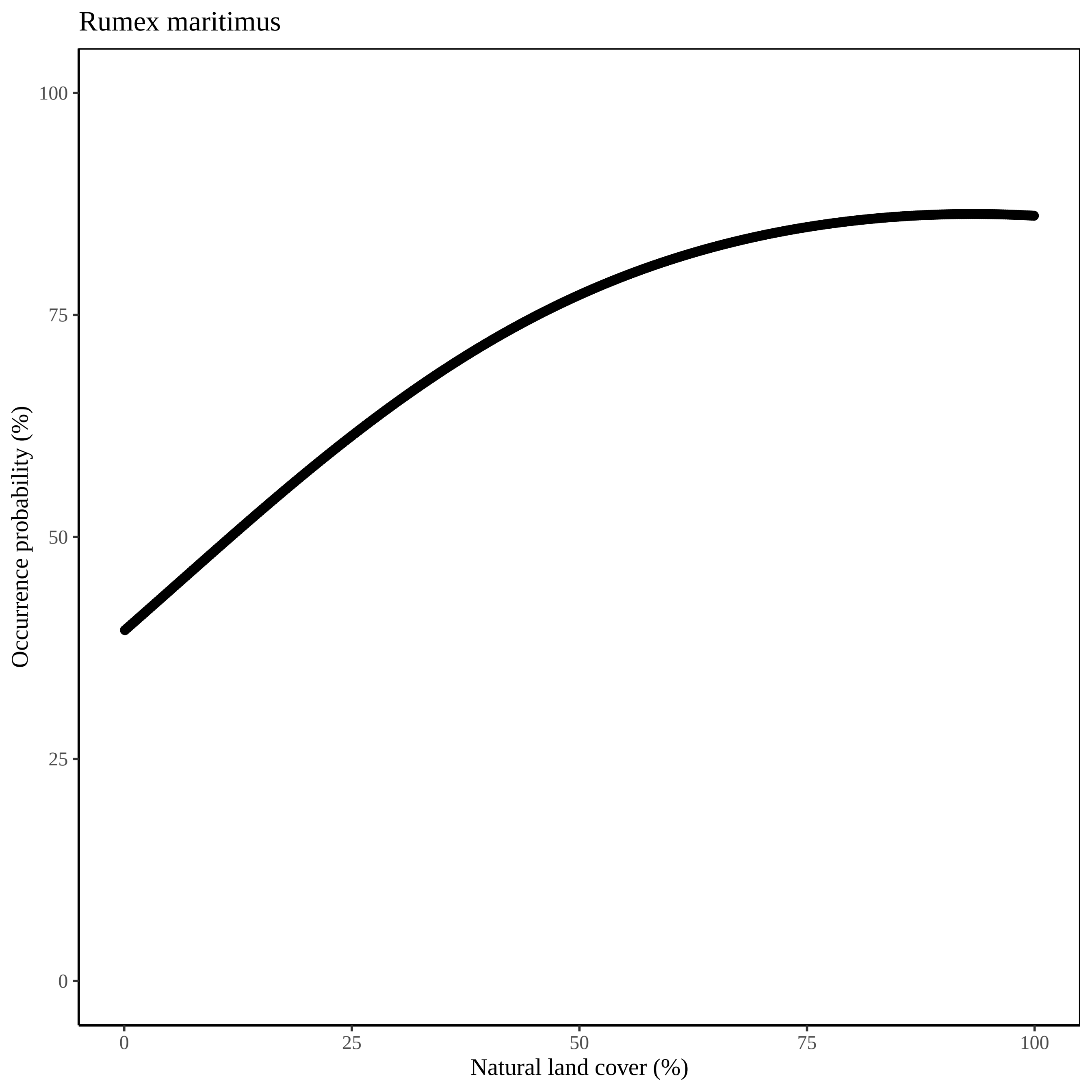
Rumex maritimus
Occurrence probability (%)
100
75
50
25
0
0
25
50
75
100
Natural land cover (%)

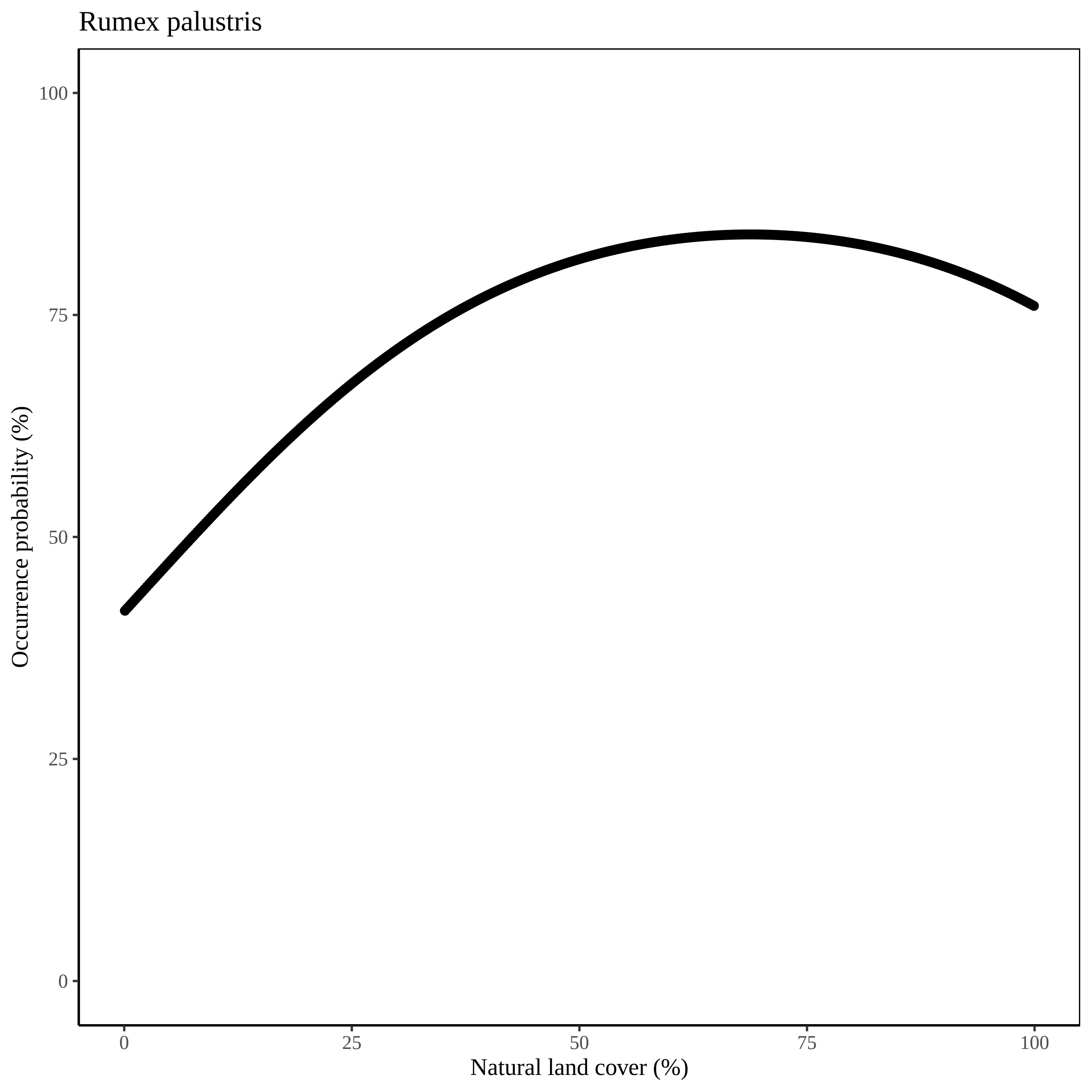

Rumex palustris
Occurrence probability (%)
100
75
50
25
0
0
25
50
75
100
Natural land cover (%)

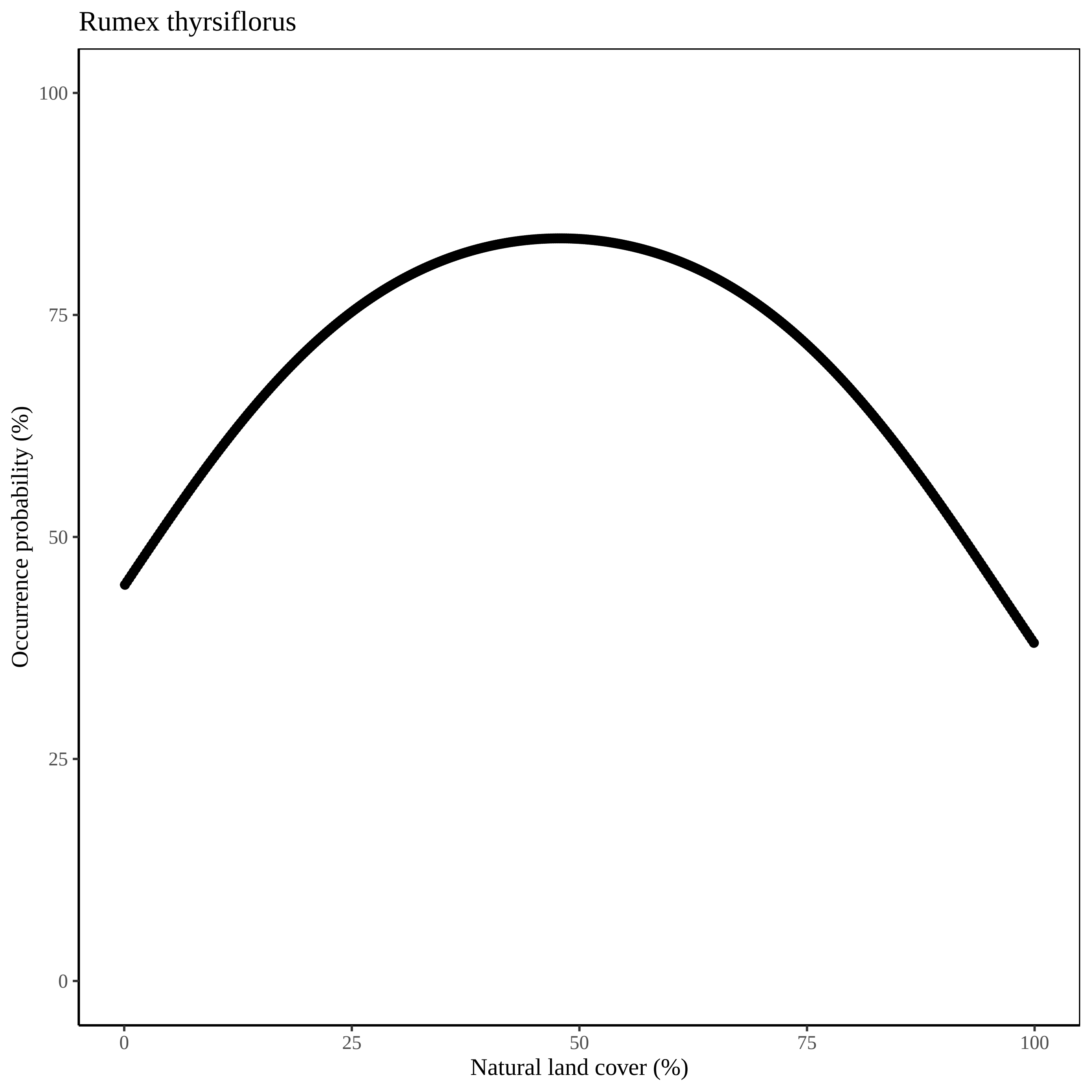

Rumex thyrsiflorus
100
75
50
25
0
Occurrence probability (%)
0
25
50
75
100
Natural land cover (%)

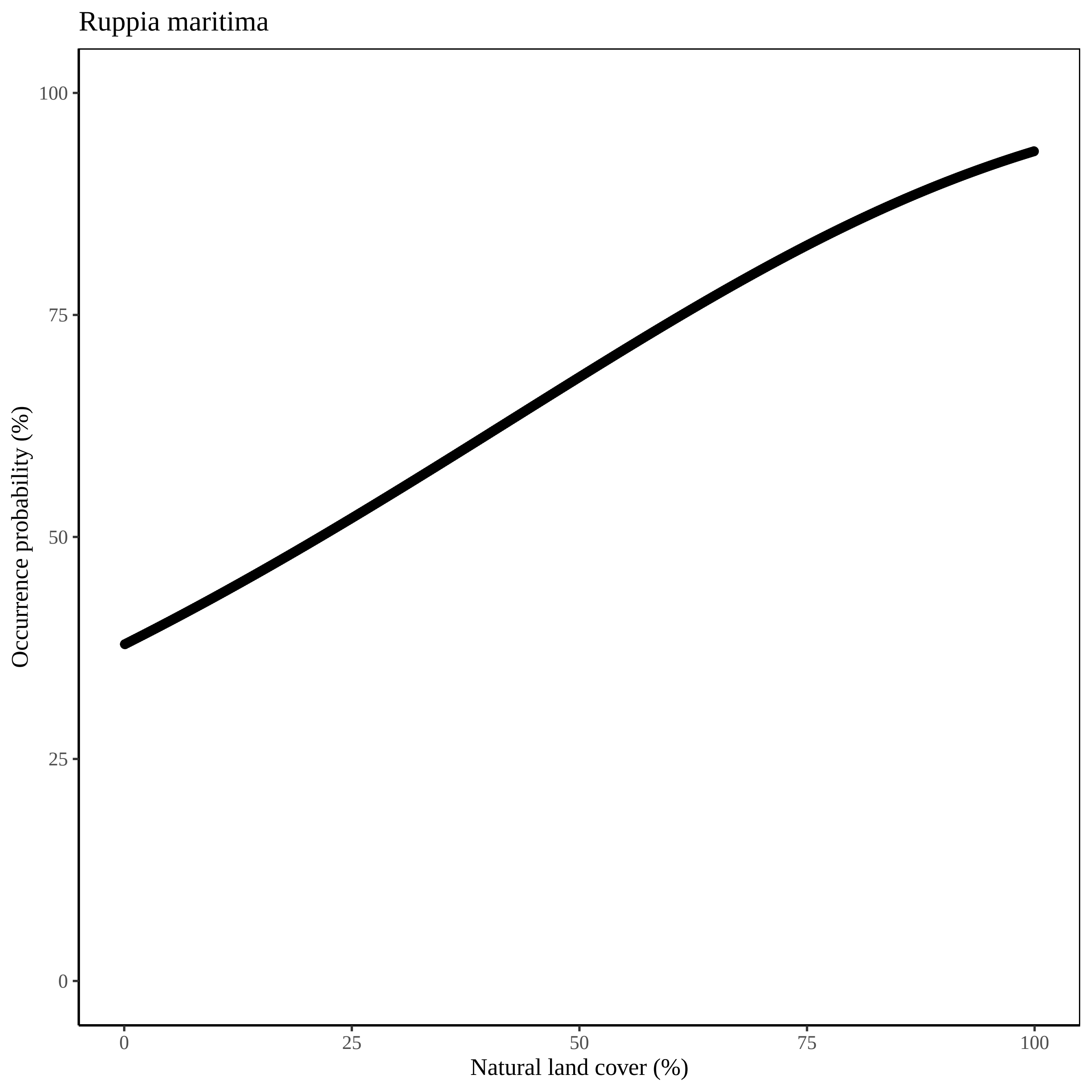

Ruppia maritima
100
75
50
25
0
Occurrence probability (%)
0
25
50
75
100
Natural land cover (%)

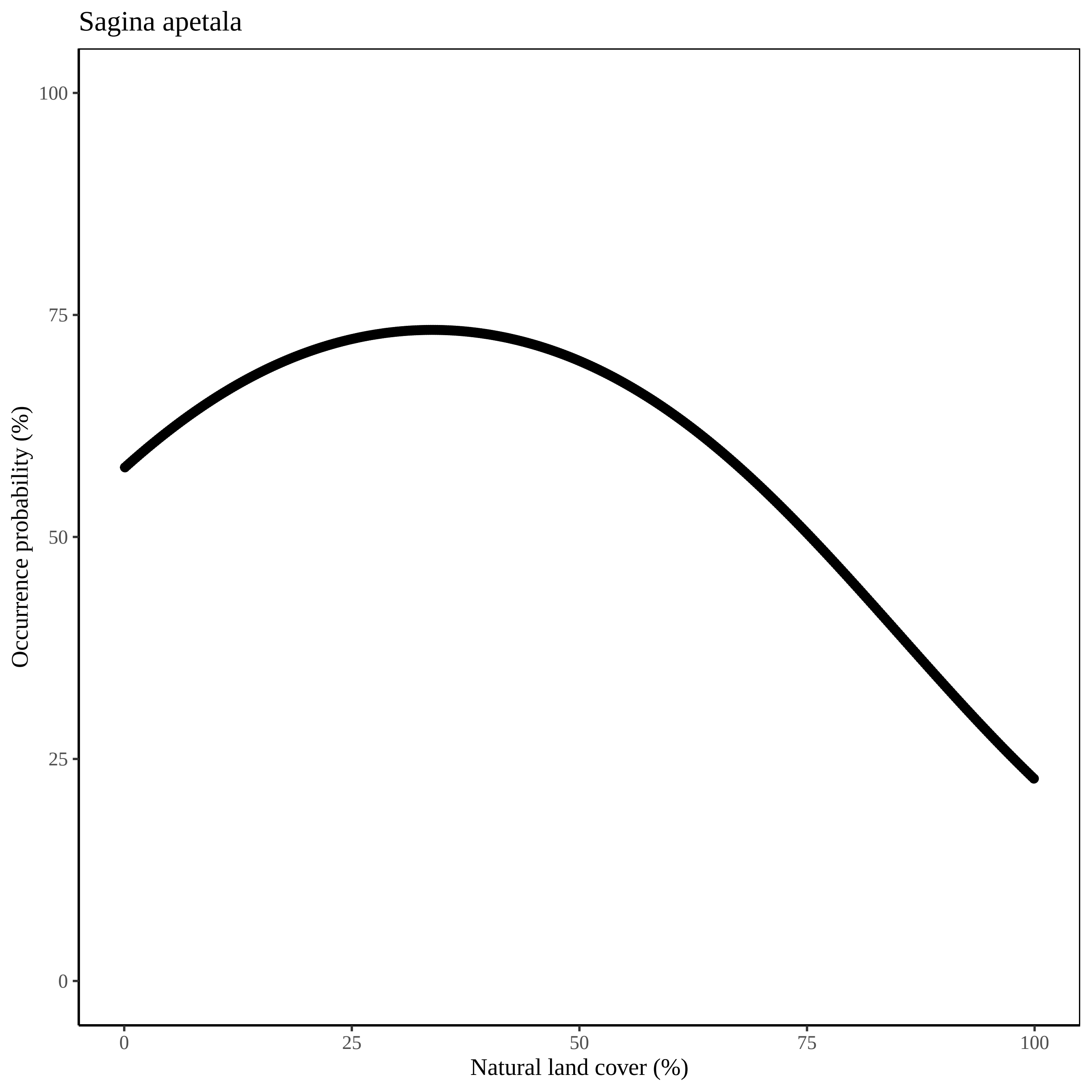

Sagina apetala
Occurrence probability (%)
100
75
50
25
0
0
25
50
75
100
Natural land cover (%)

Sagina maritima

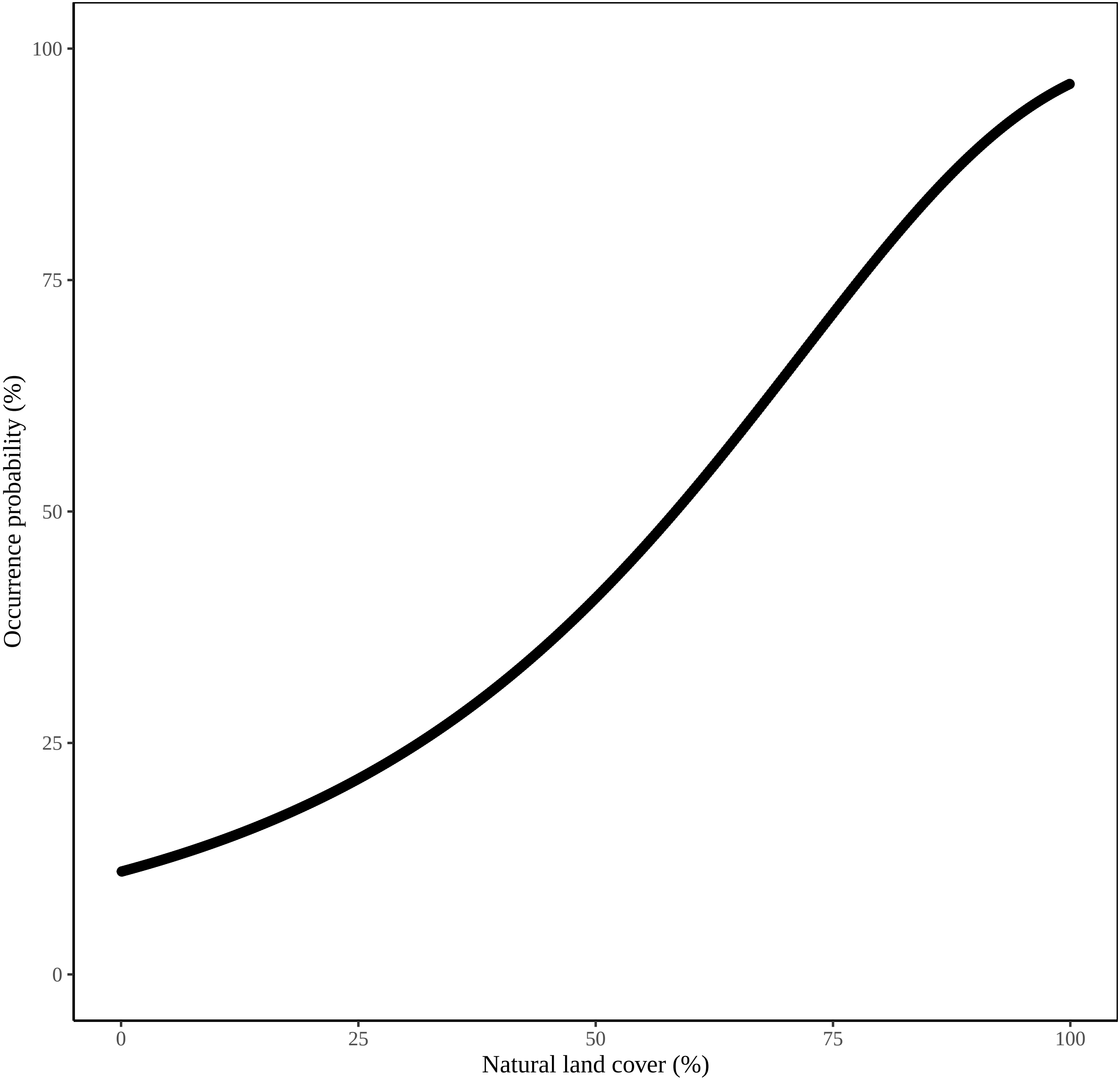

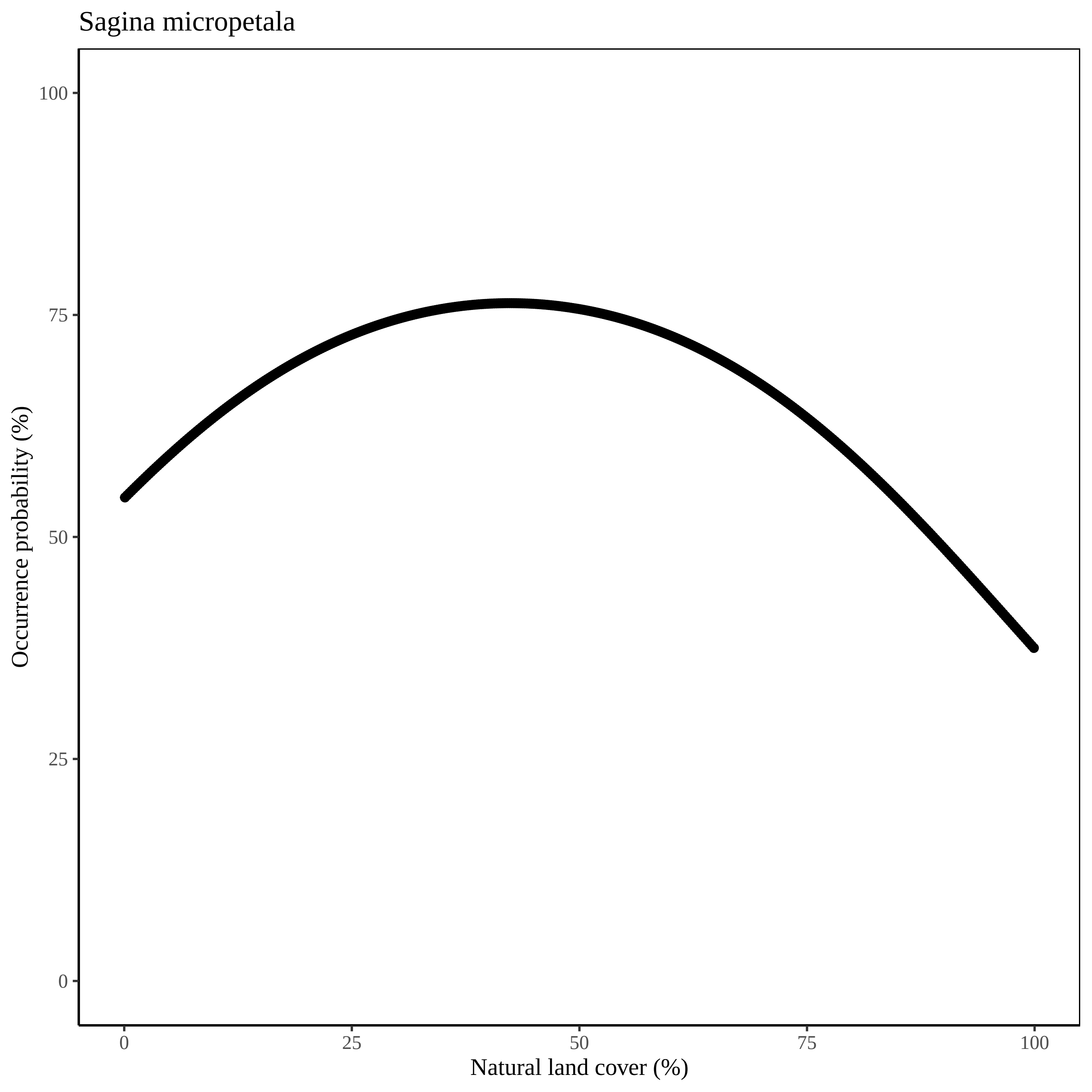
Sagina micropetala
Occurrence probability (%)
100
75
50
25
0
Natural land cover (%)
0
25
50
75
100

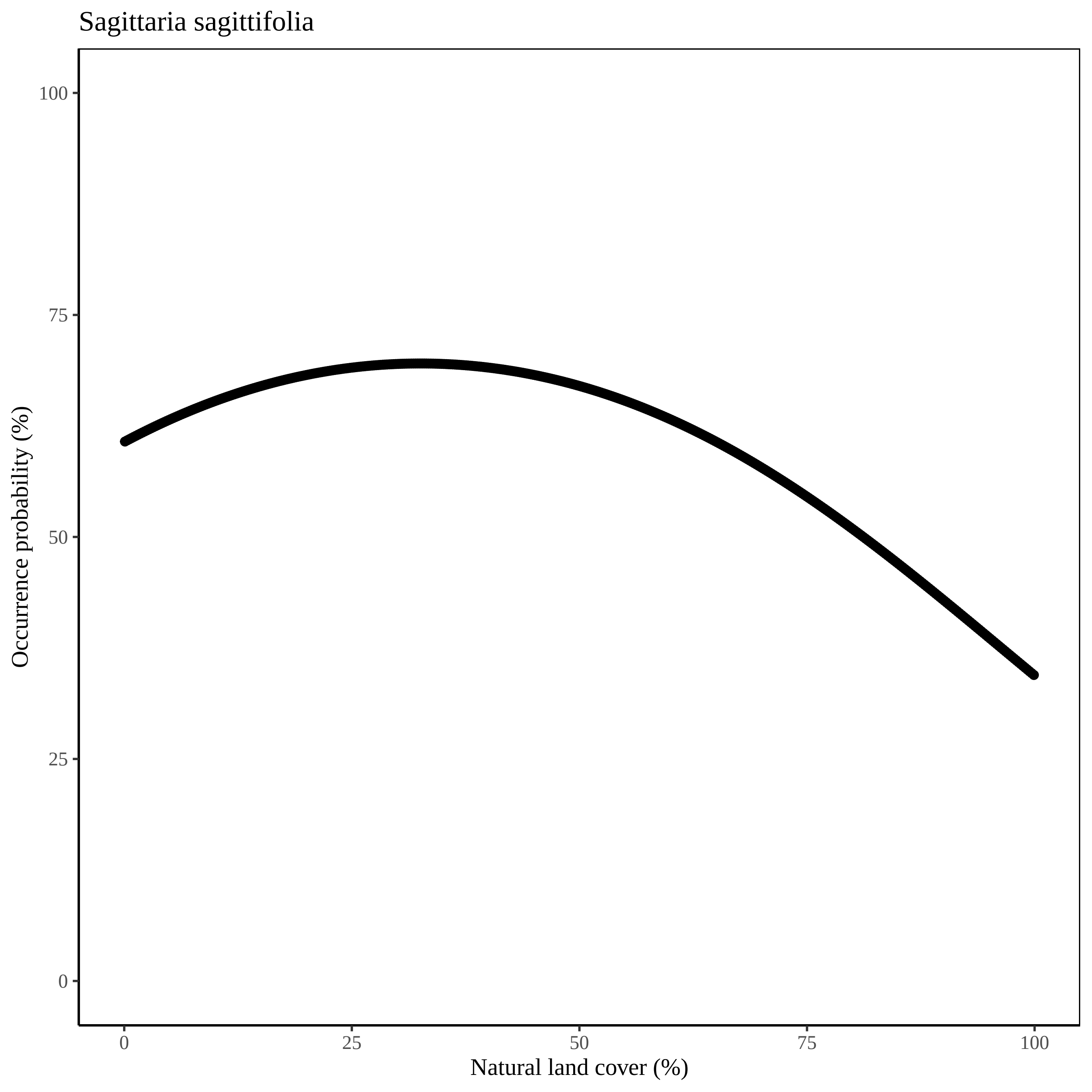
Sagittaria sagittifolia
100
75
50
25
0
Occurrence probability (%)
0
25
50
75
100
Natural land cover (%)

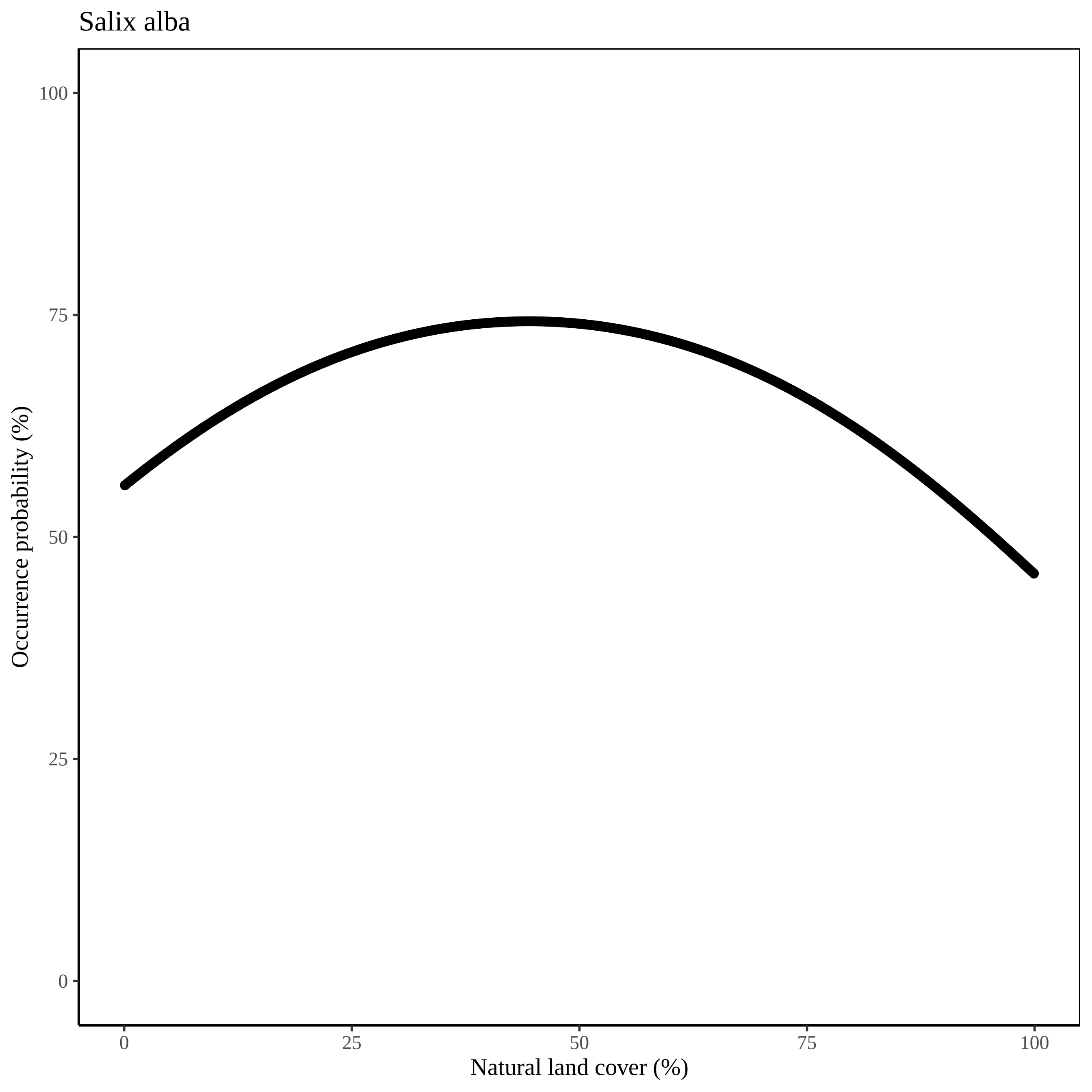
Salix alba
Occurrence probability (%)
100
75
50
25
0
0
25
50
75
100
Natural land cover (%)

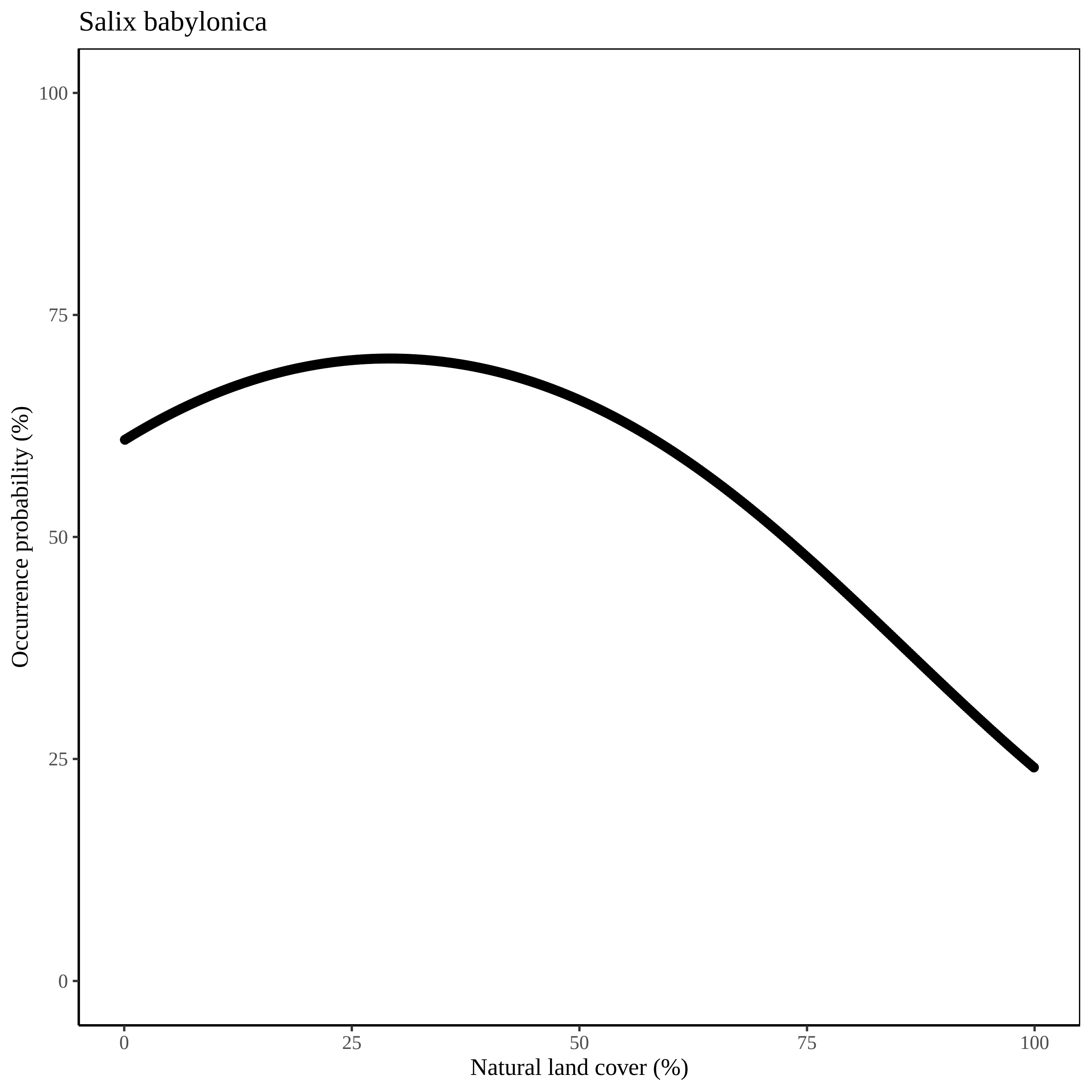

Salix babylonica
Occurrence probability (%)
100
75
50
25
0
0
25
50
75
100
Natural land cover (%)

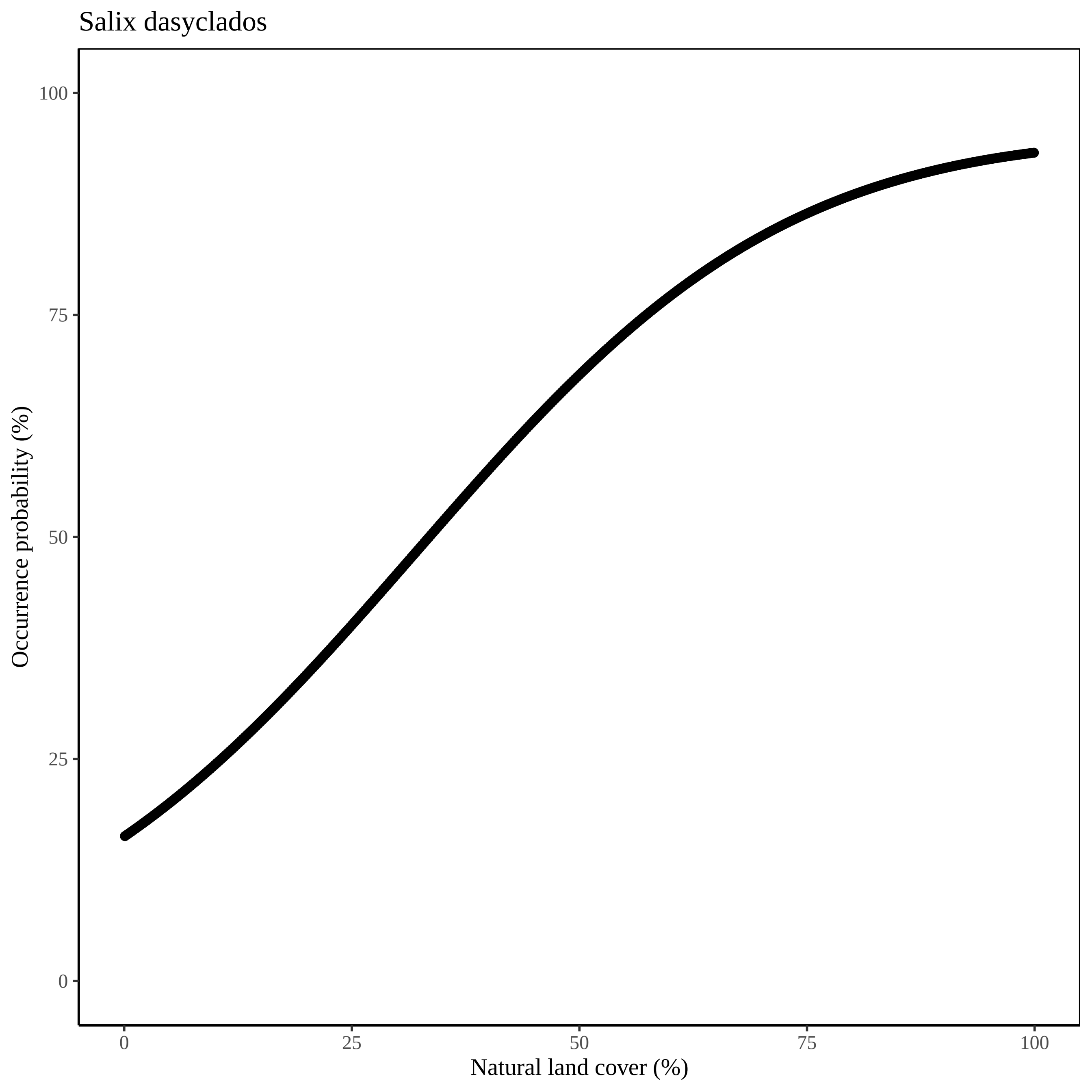
Salix dasyclados
Occurrence probability (%)
100
75
50
25
0
0
25
50
75
100
Natural land cover (%)

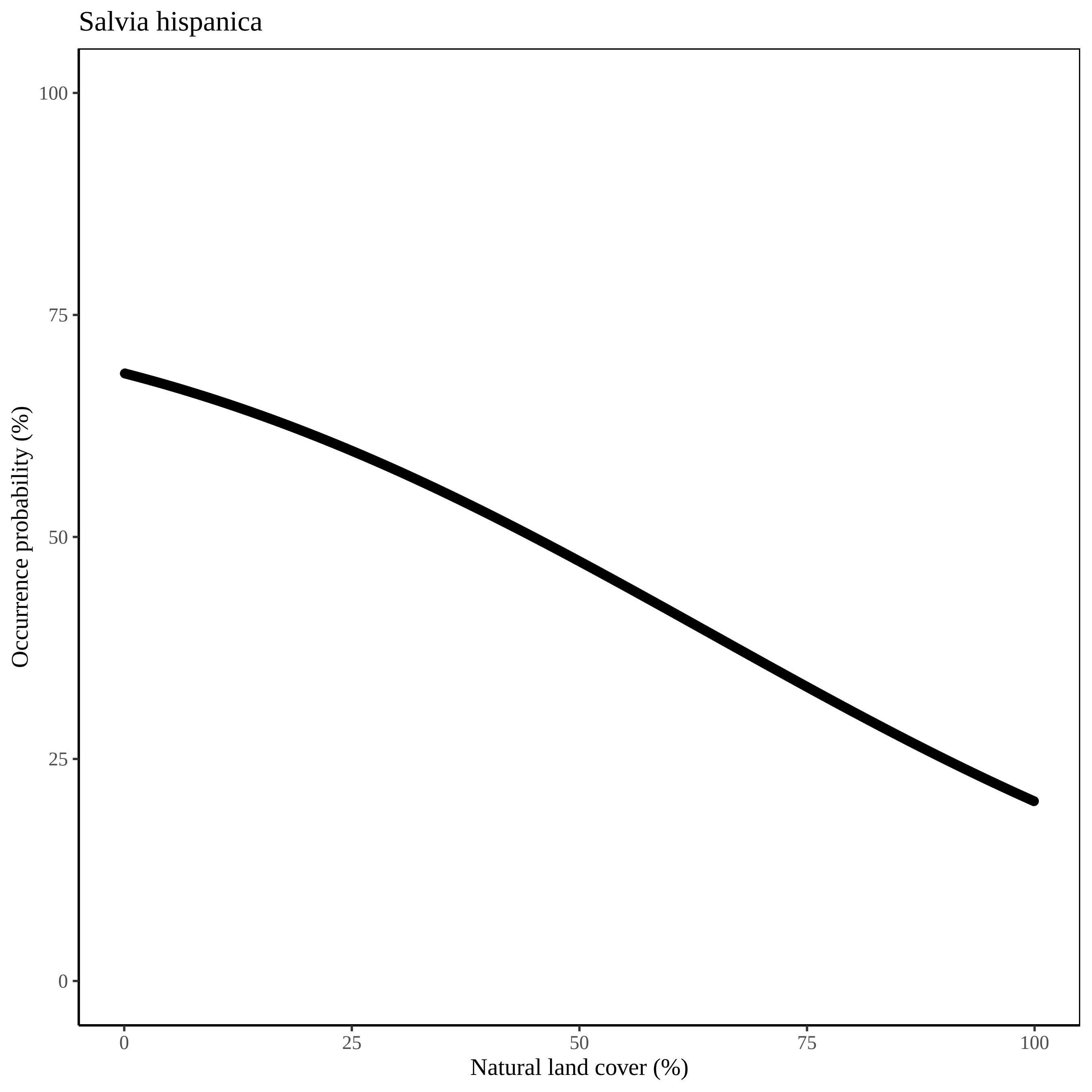

Salvia hispanica
Occurrence probability (%)
Natural land cover (%)
0
25
50
75
100

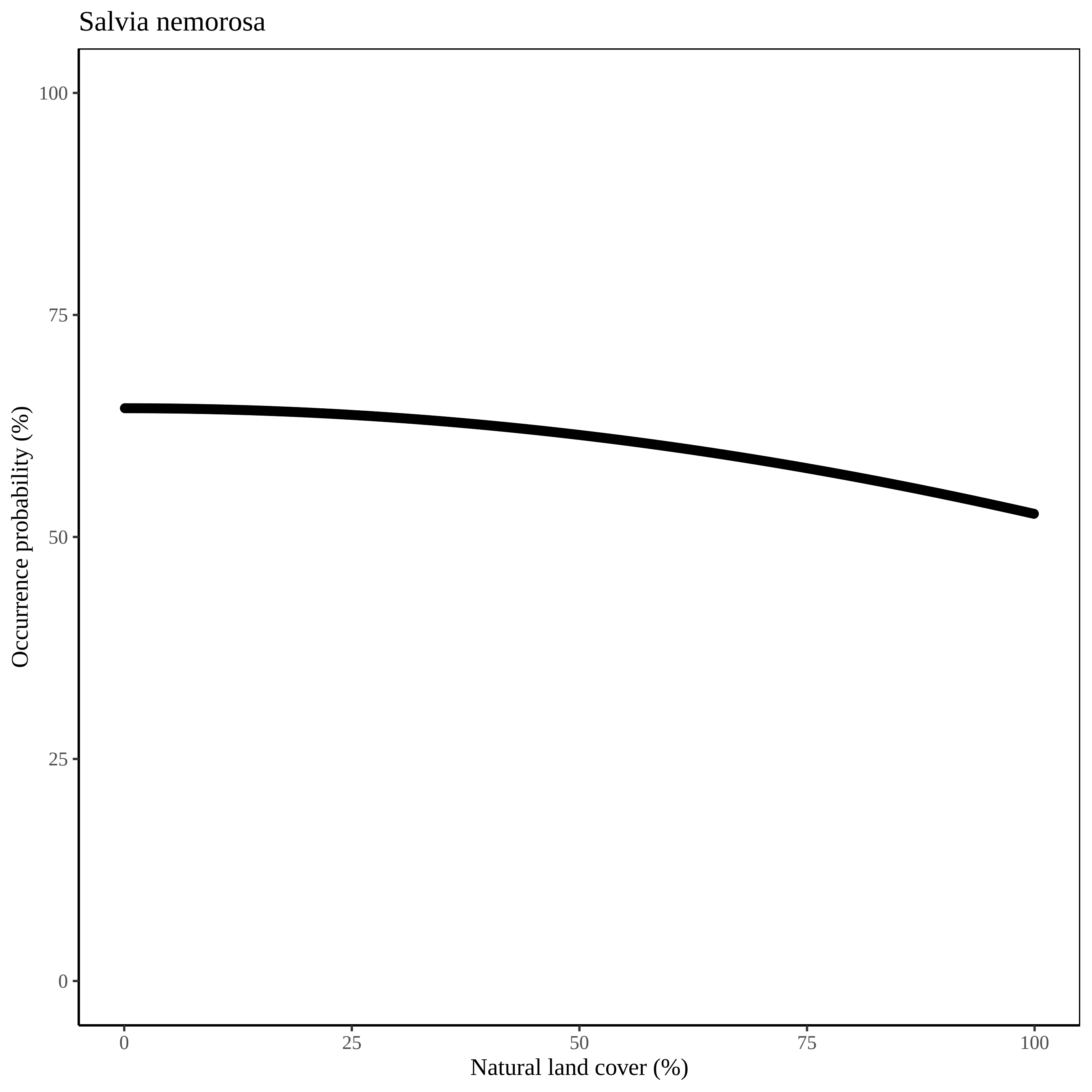

Salvia nemorosa
Occurrence probability (%)
100
75
50
25
0
0
25
50
75
100
Natural land cover (%)

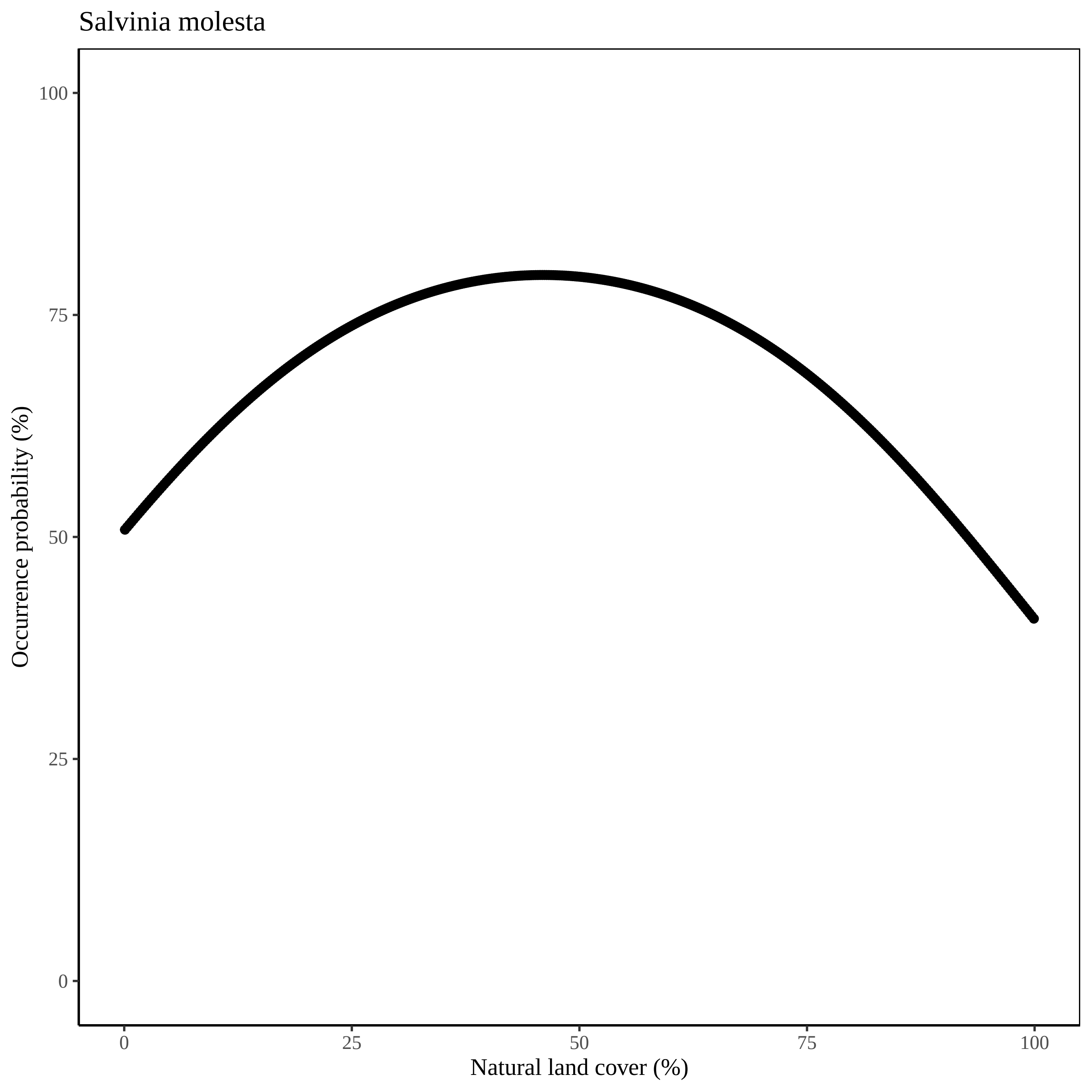
Salvinia molesta
Occurrence probability (%)
100
75
50
25
0
0
25
50
75
100
Natural land cover (%)

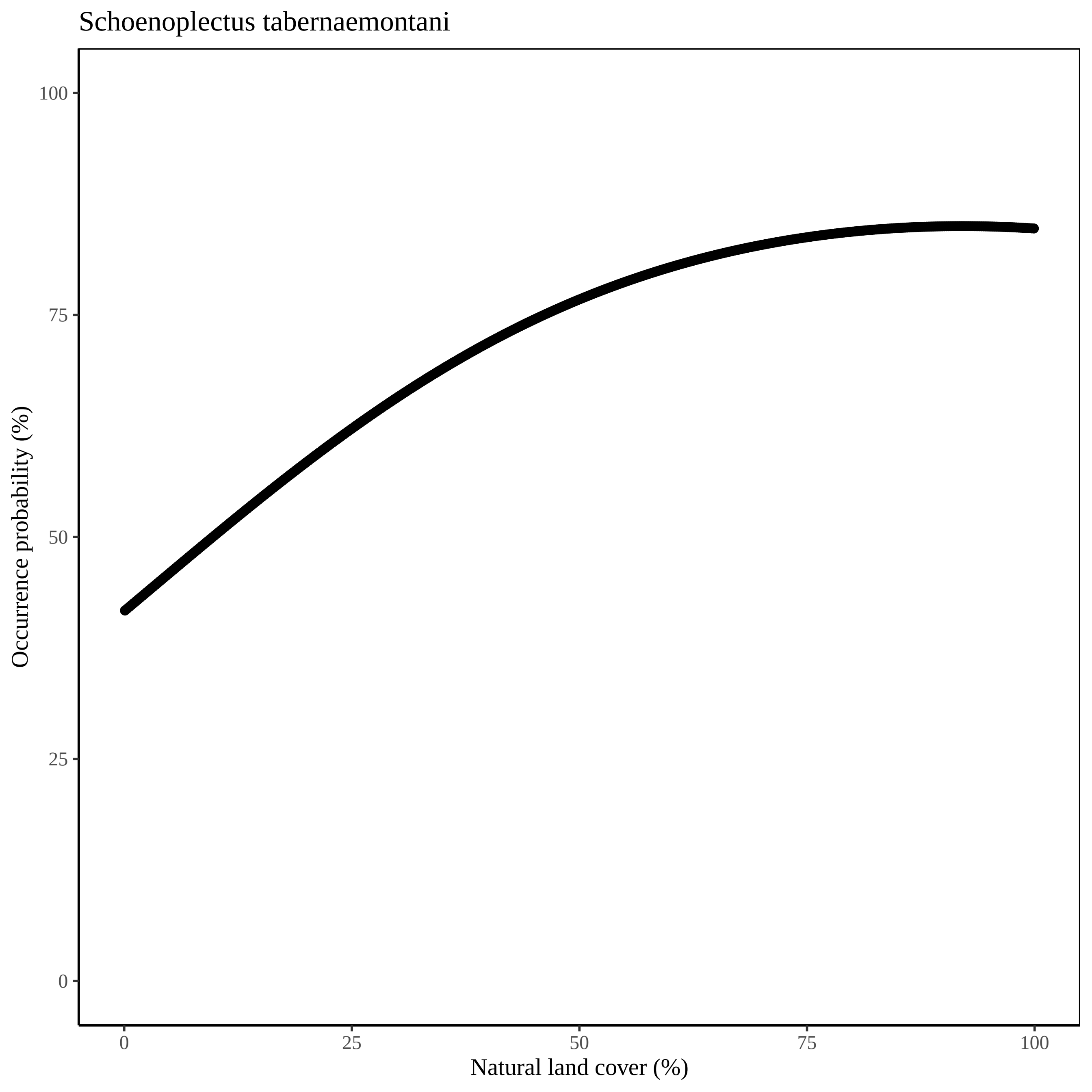

Schoenoplectus tabernaemontani
100
75
50
25
0
Occurrence probability (%)
0
25
50
75
100
Natural land cover (%)

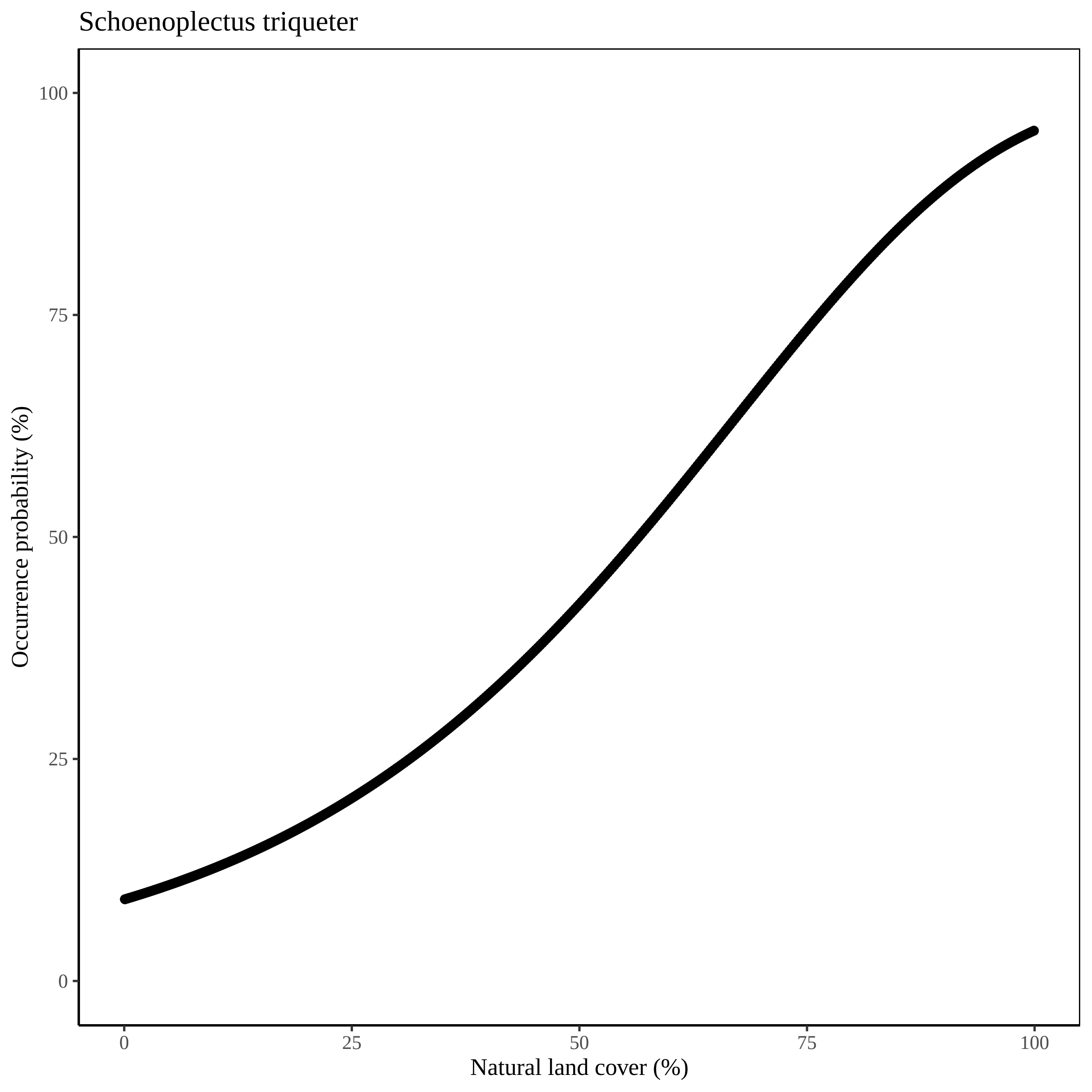
Schoenoplectus triqueter
Occurrence probability (%)
100
75
50
25
0
0
25
50
75
100
Natural land cover (%)

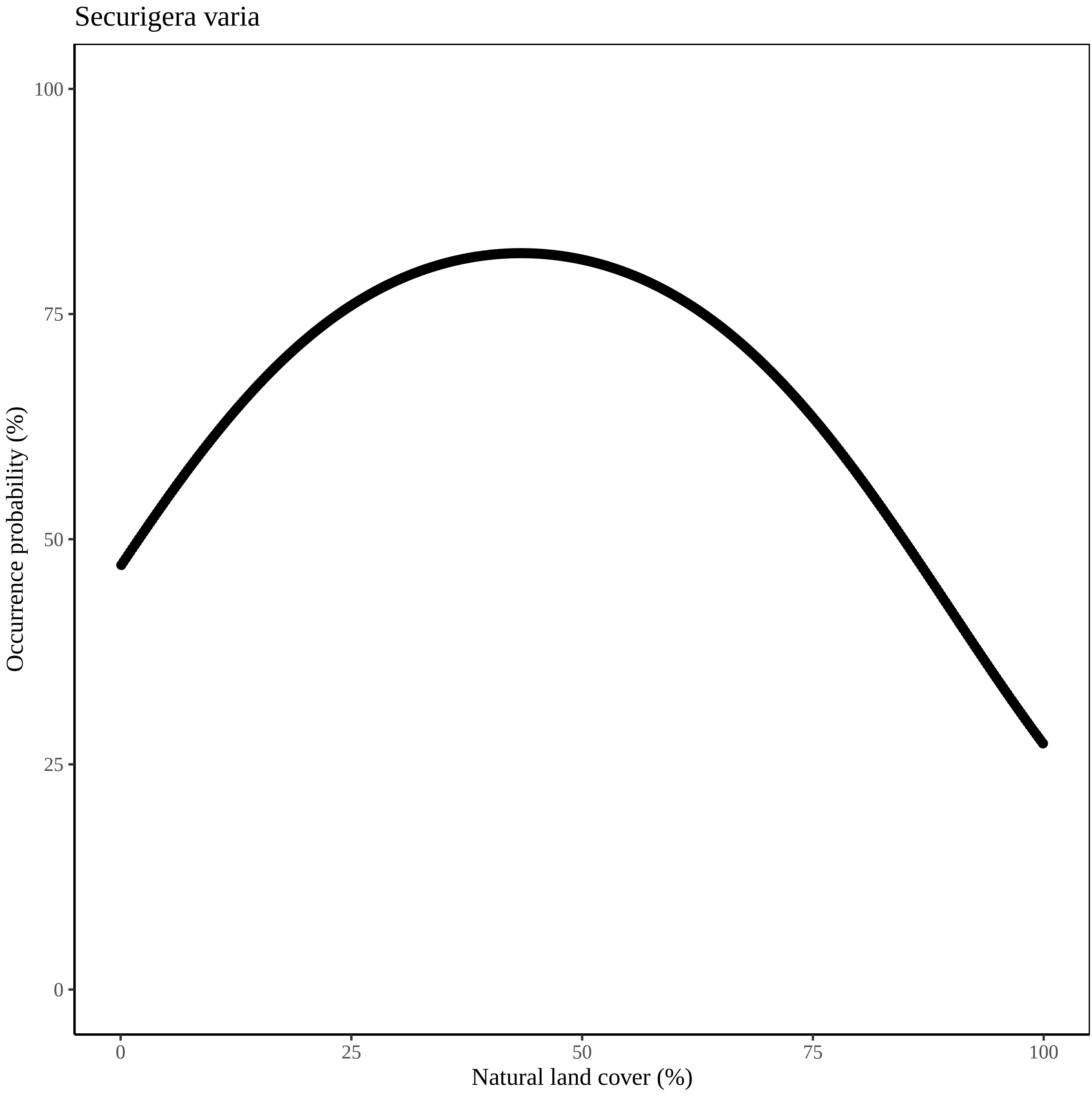

Securigera varia
100
75
50
25
0
Occurrence probability (%)
0
25
50
75
100
Natural land cover (%)

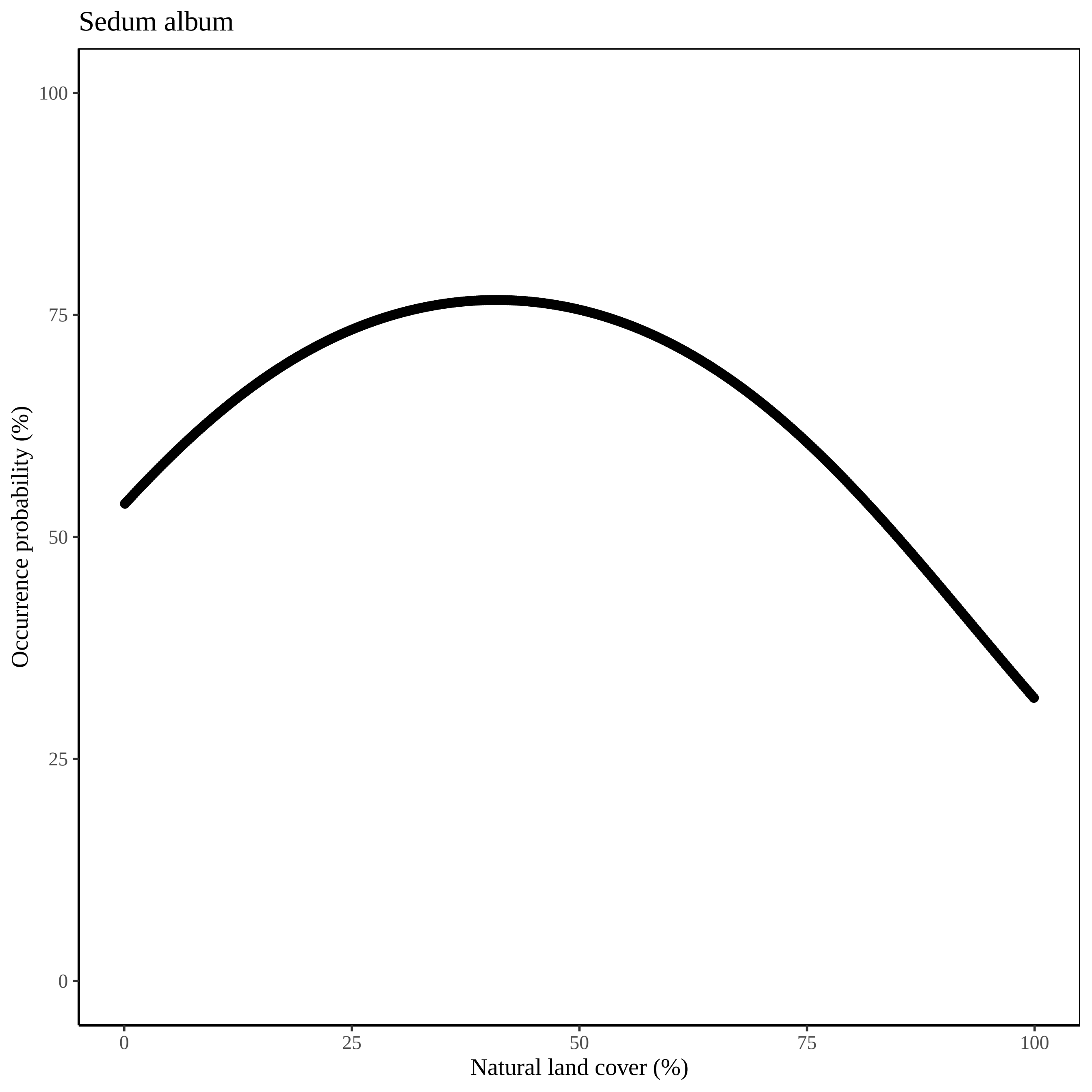

Sedum album
Occurrence probability (%)
100
75
50
25
0
0
25
50
75
100
Natural land cover (%)

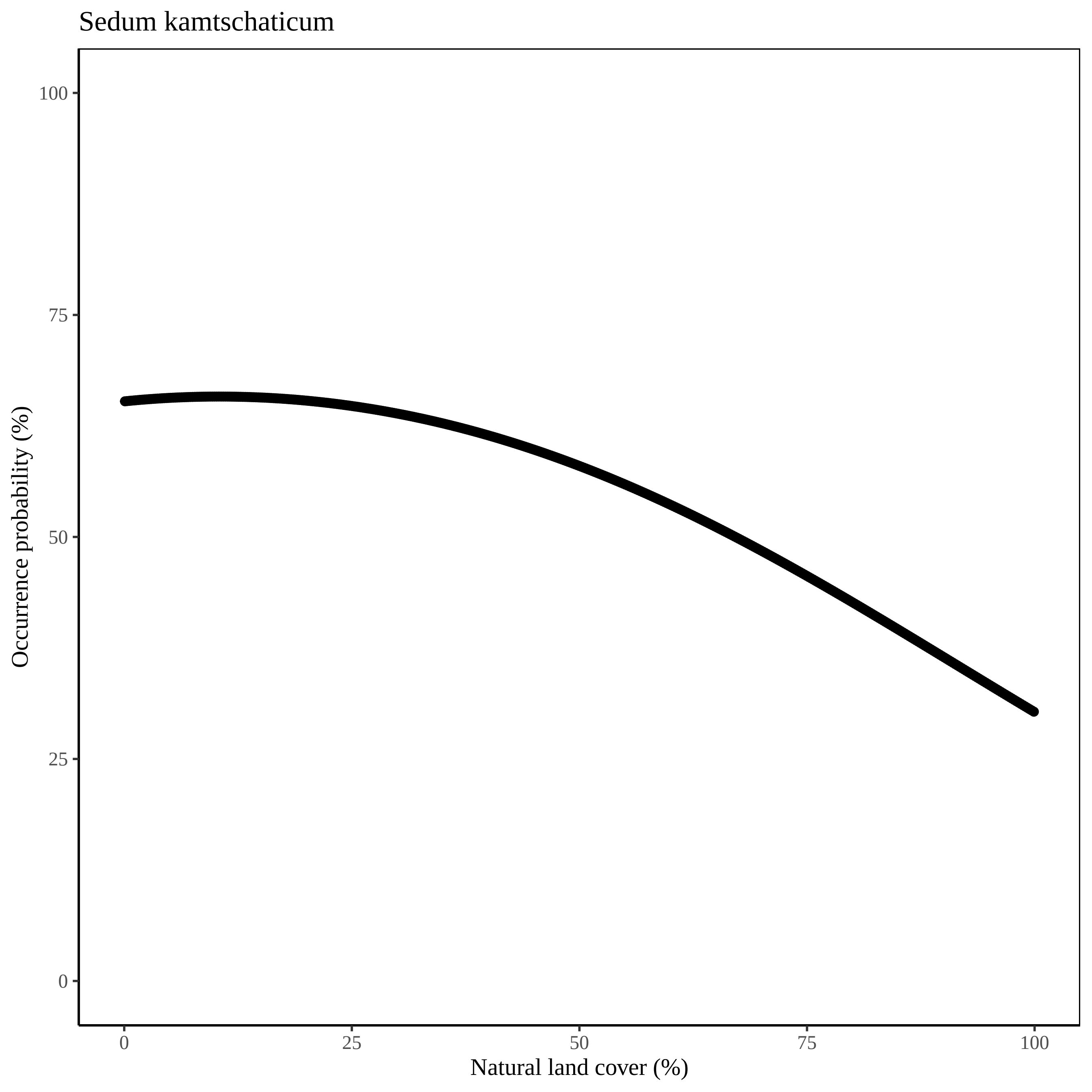
Sedum kamtschaticum
100
75
50
25
0
Occurrence probability (%)
0
25
50
75
100
Natural land cover (%)

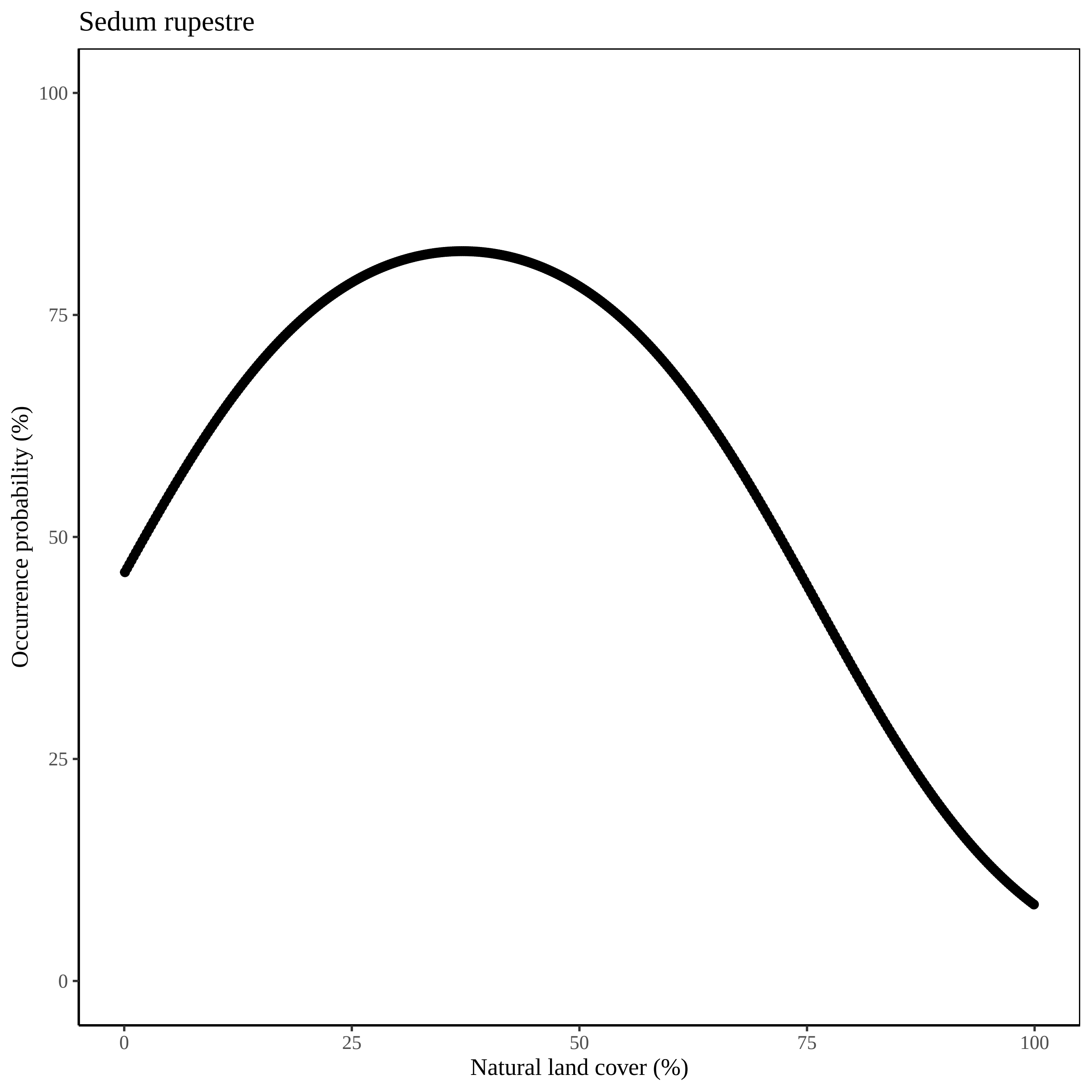

Sedum rupestre
Occurrence probability (%)
100
75
50
25
0
0
25
50
75
100
Natural land cover (%)

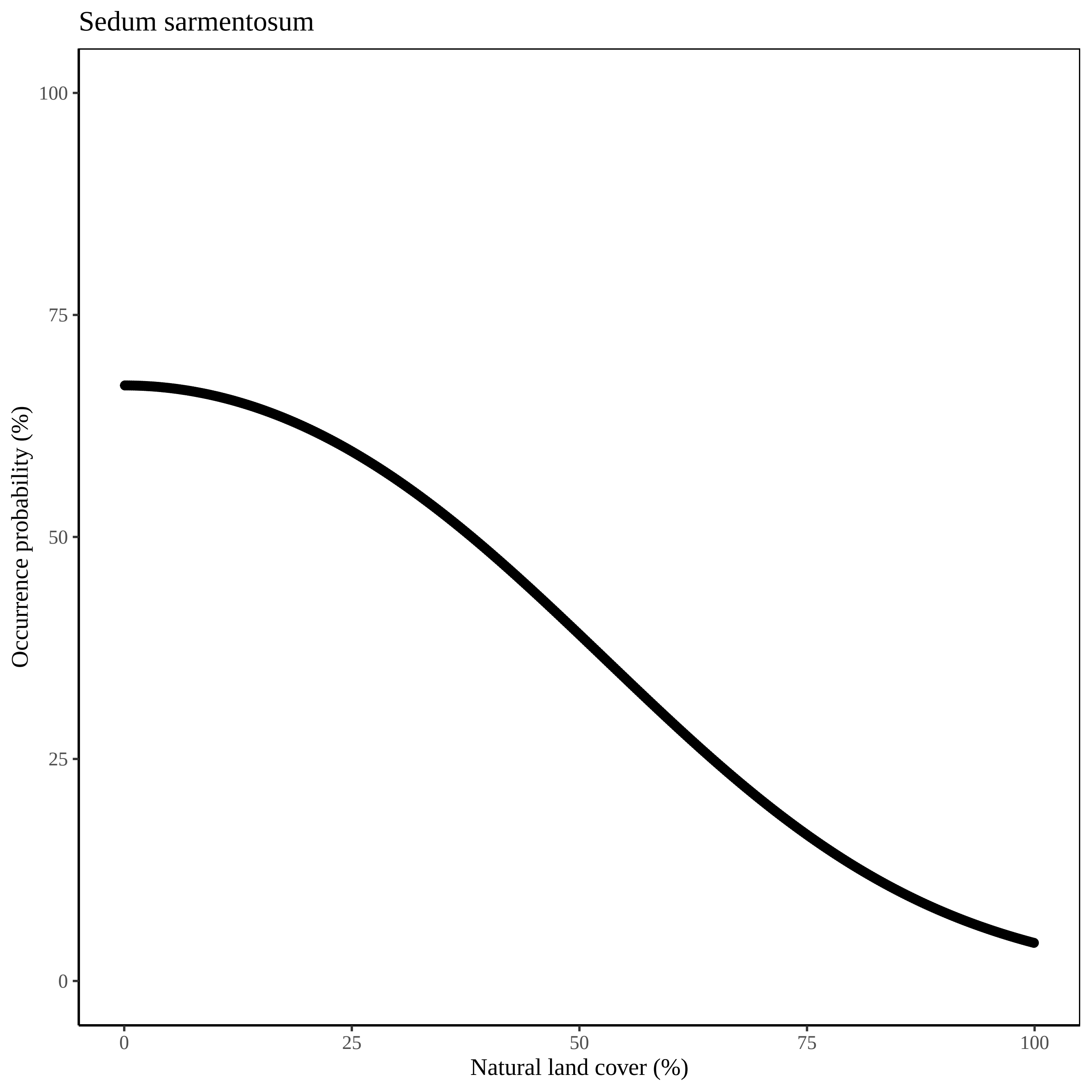

Sedum sarmentosum
100
75
50
25
0
Occurrence probability (%)
0
25
50
75
100
Natural land cover (%)

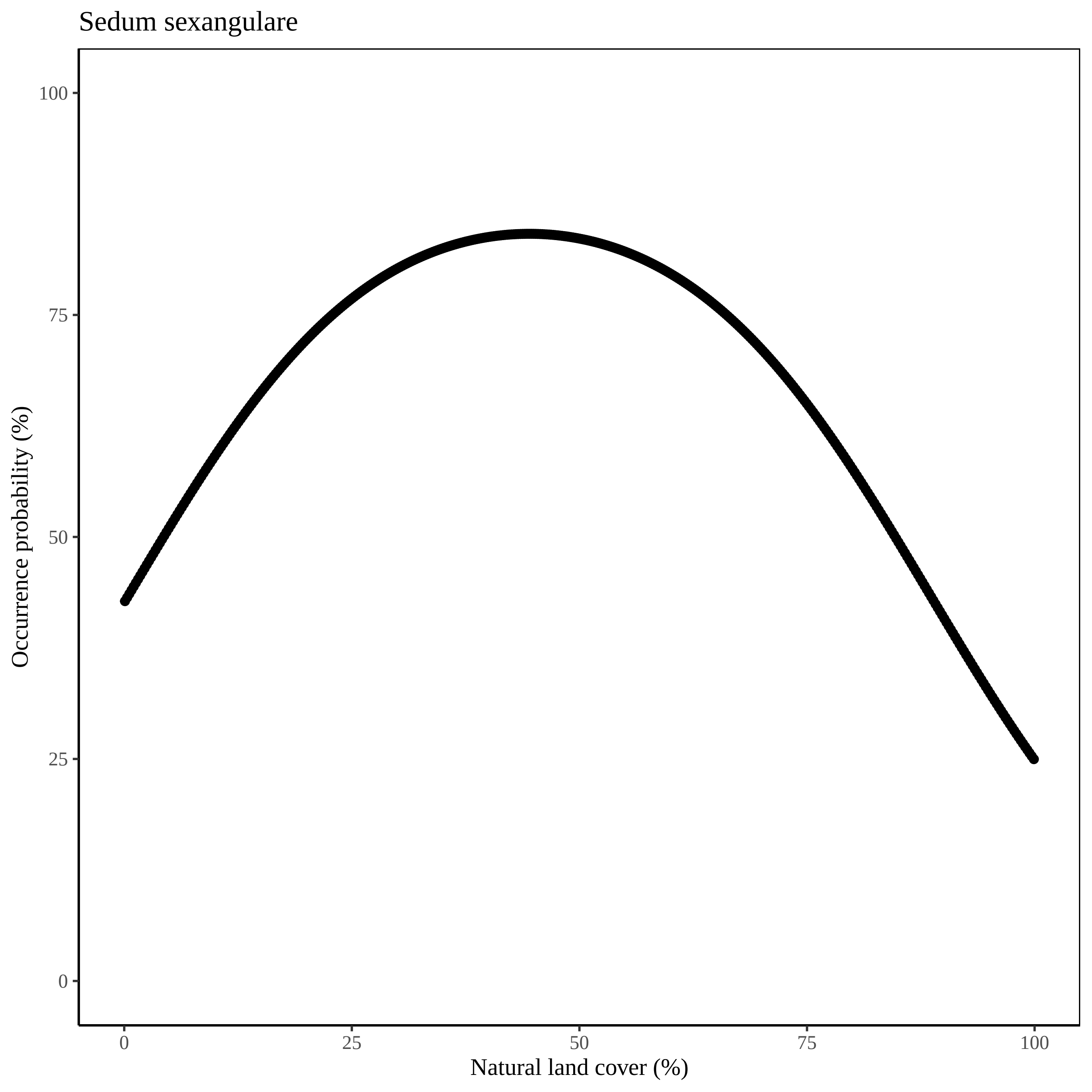

Sedum sexangulare
Occurrence probability (%)
100
75
50
25
0
0
25
50
75
100
Natural land cover (%)

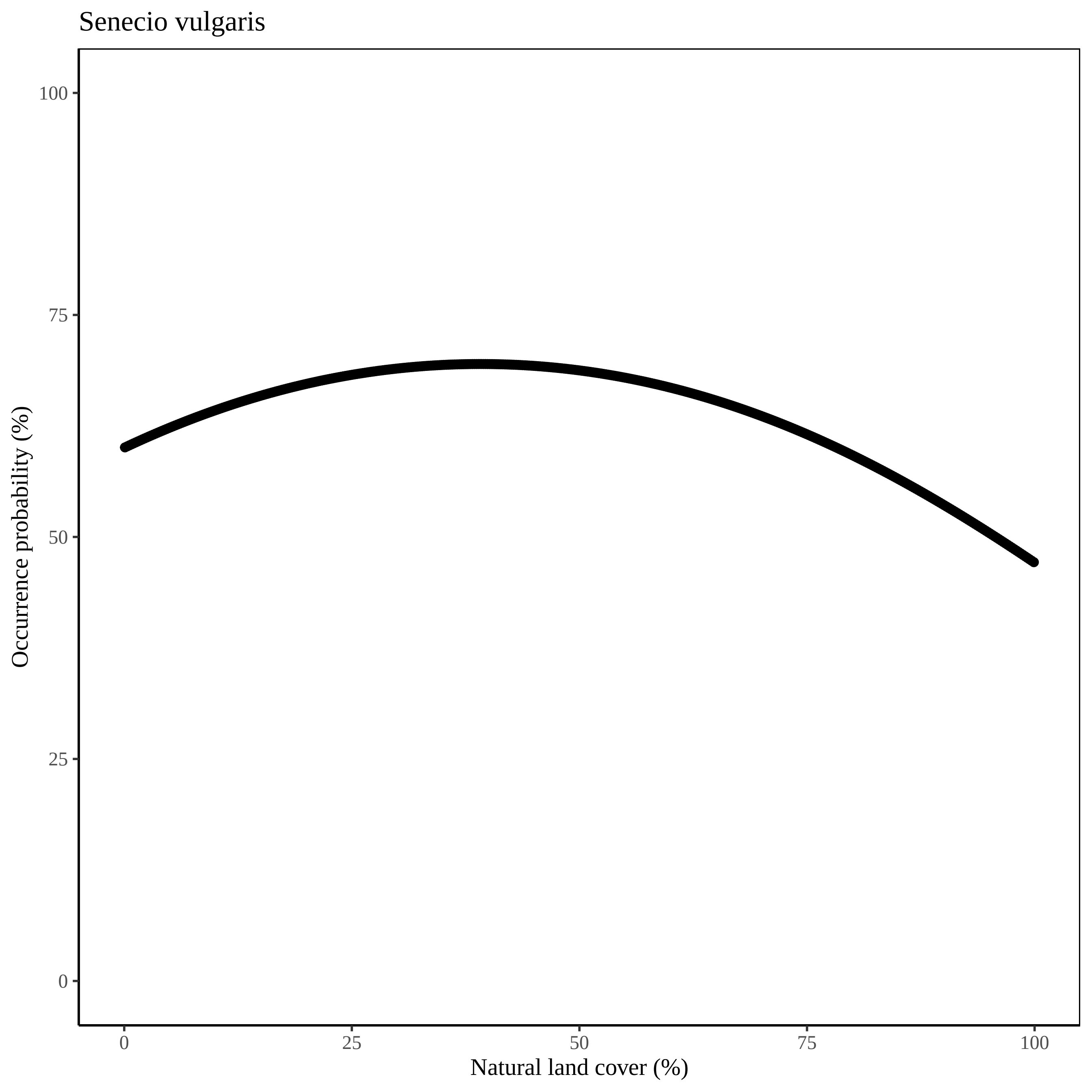
Senecio vulgaris
Occurrence probability (%)
100
75
50
25
0
0
25
50
75
100
Natural land cover (%)

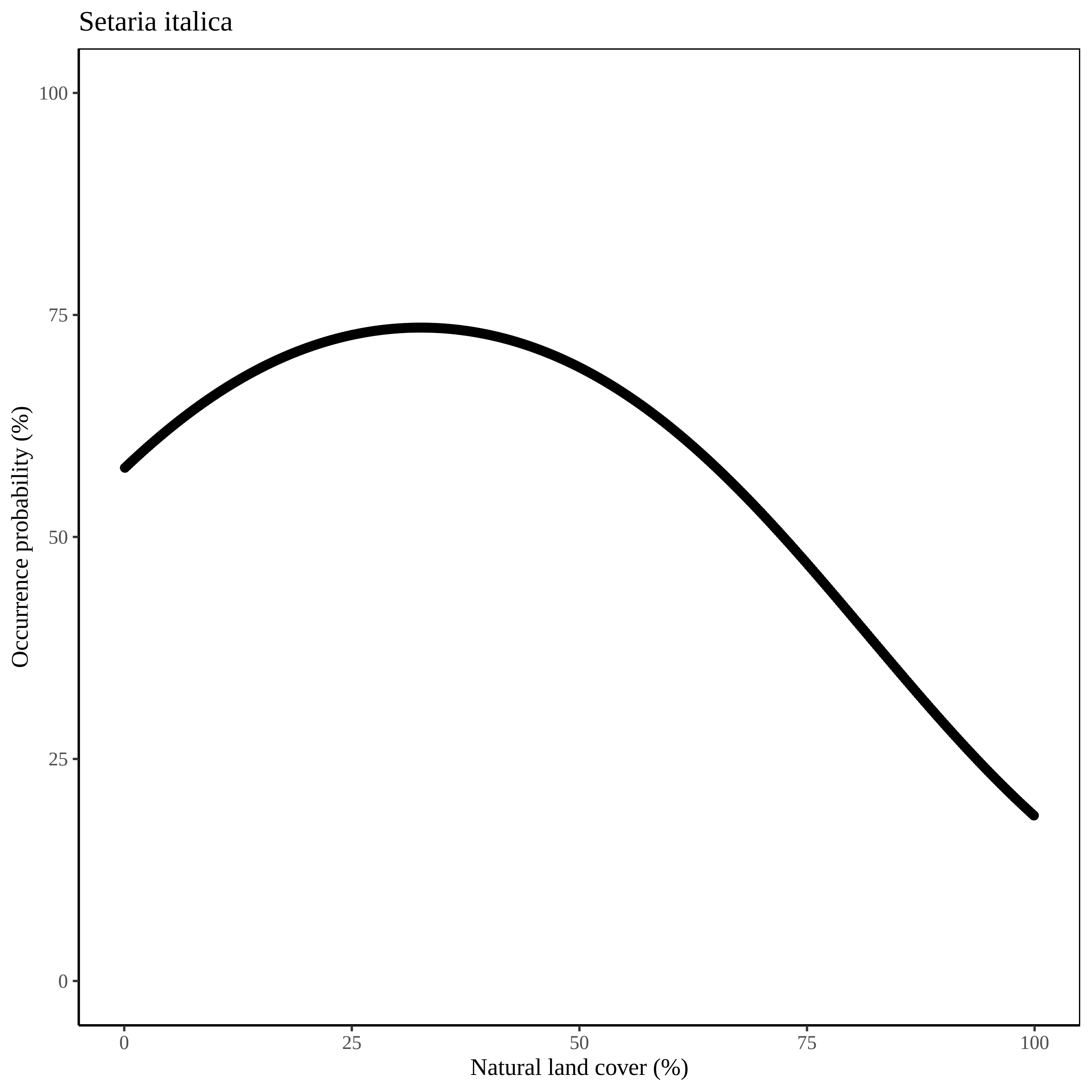

Setaria italica
Occurrence probability (%)
100
75
50
25
0
0
25
50
75
100
Natural land cover (%)

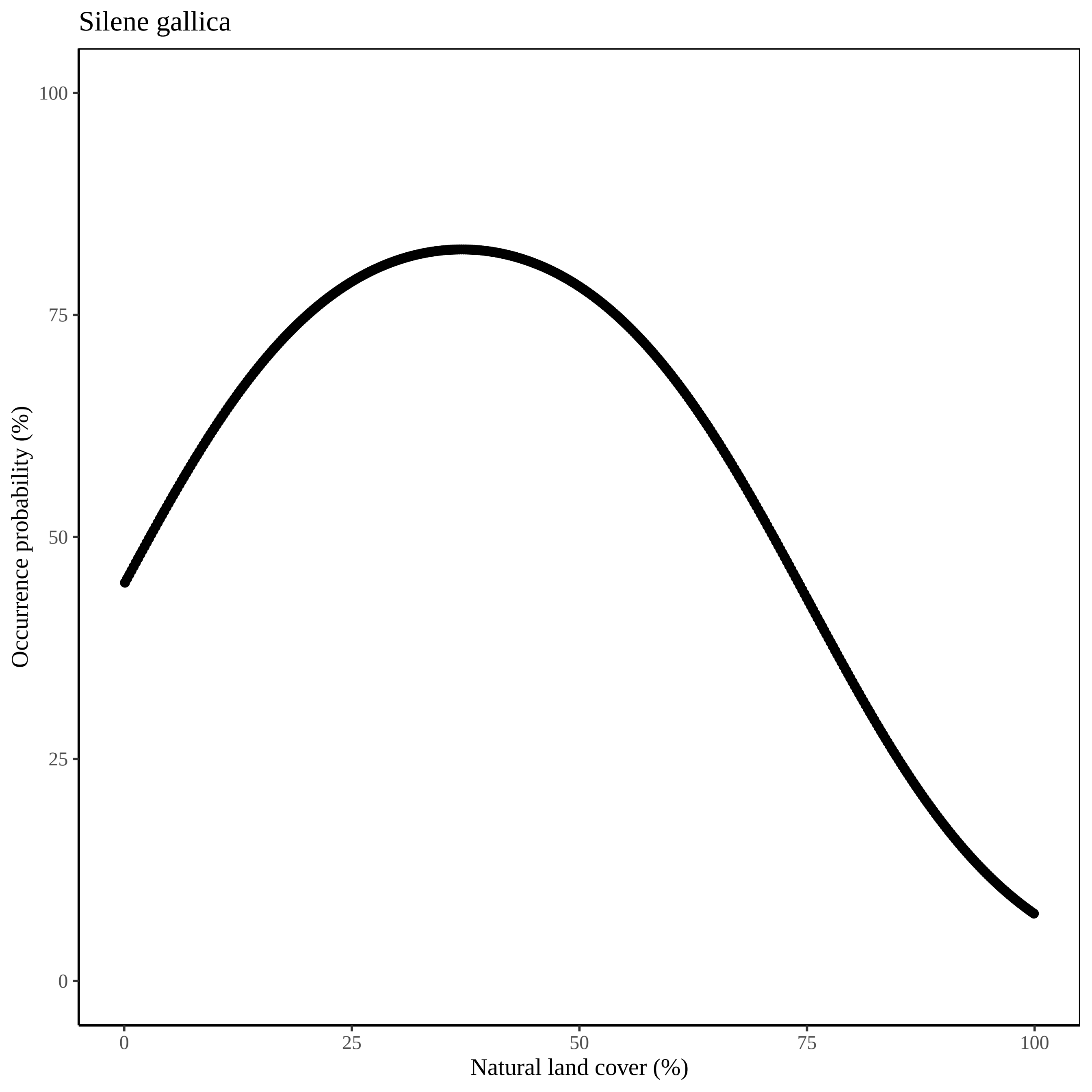

Silene gallica
100
75
50
25
0
Occurrence probability (%)
0
25
50
75
100
Natural land cover (%)

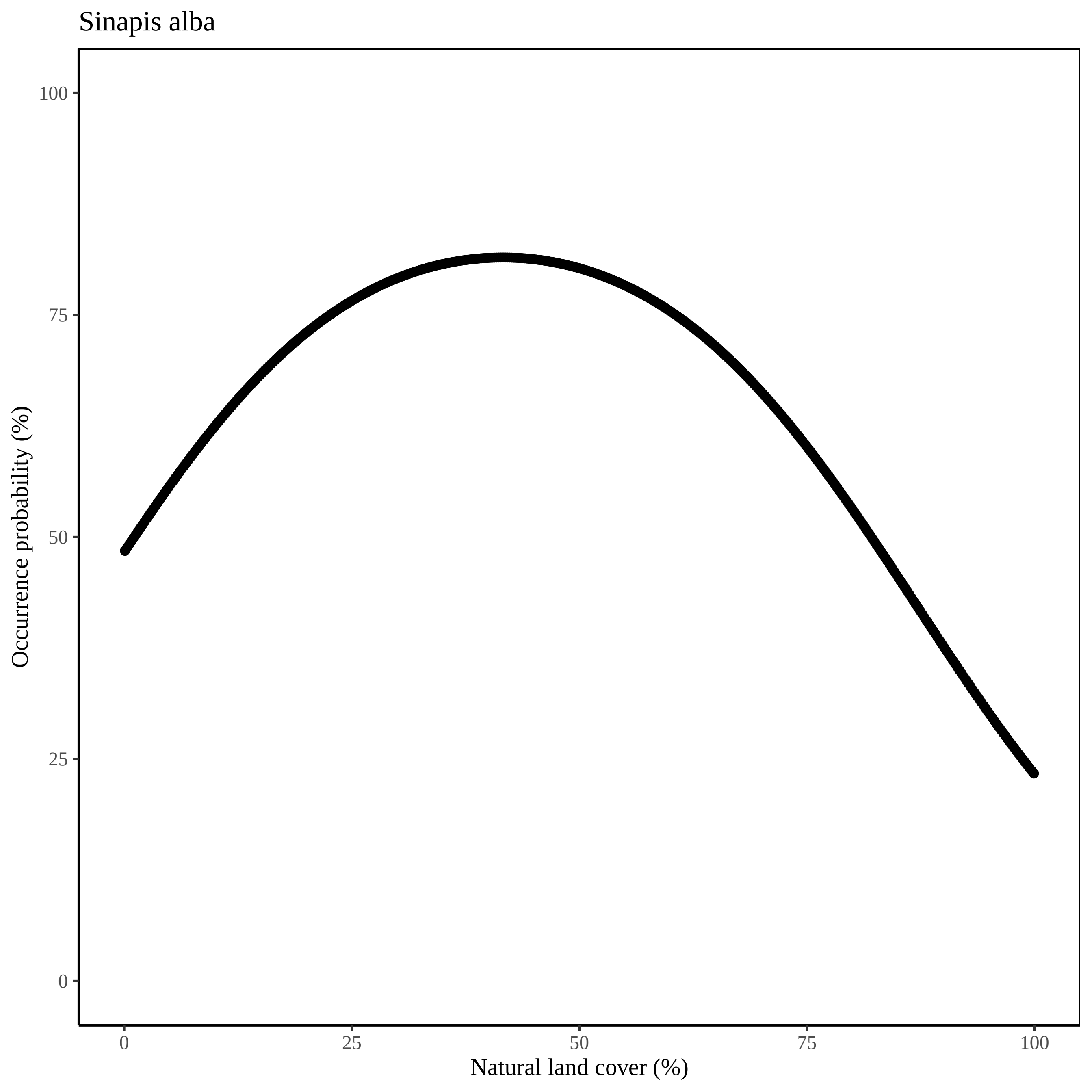
Sinapis alba
100
75
50
25
0
Occurrence probability (%)
0
25
50
75
100
Natural land cover (%)

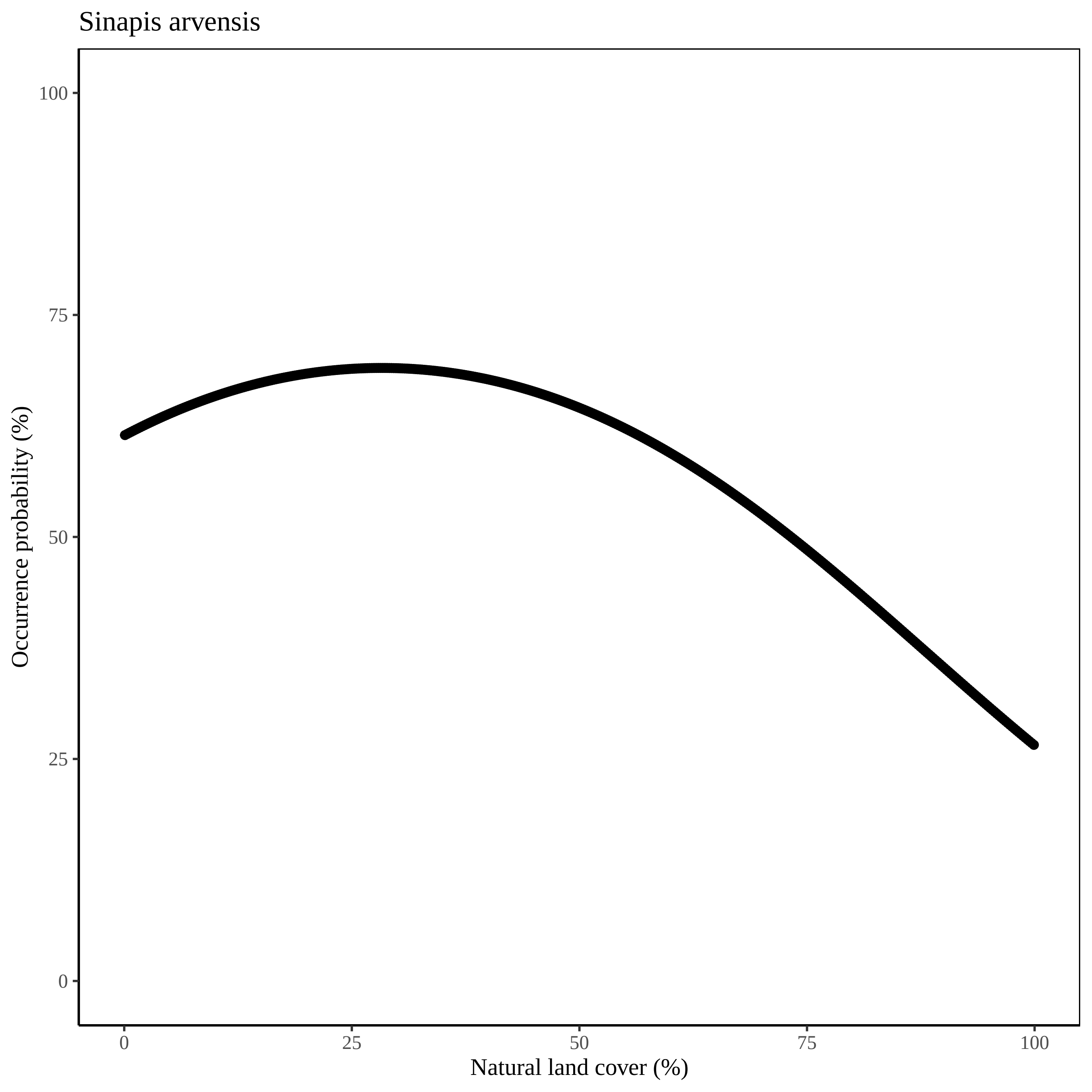

Sinapis arvensis
100
75
50
25
0
Occurrence probability (%)
0
25
50
75
100
Natural land cover (%)

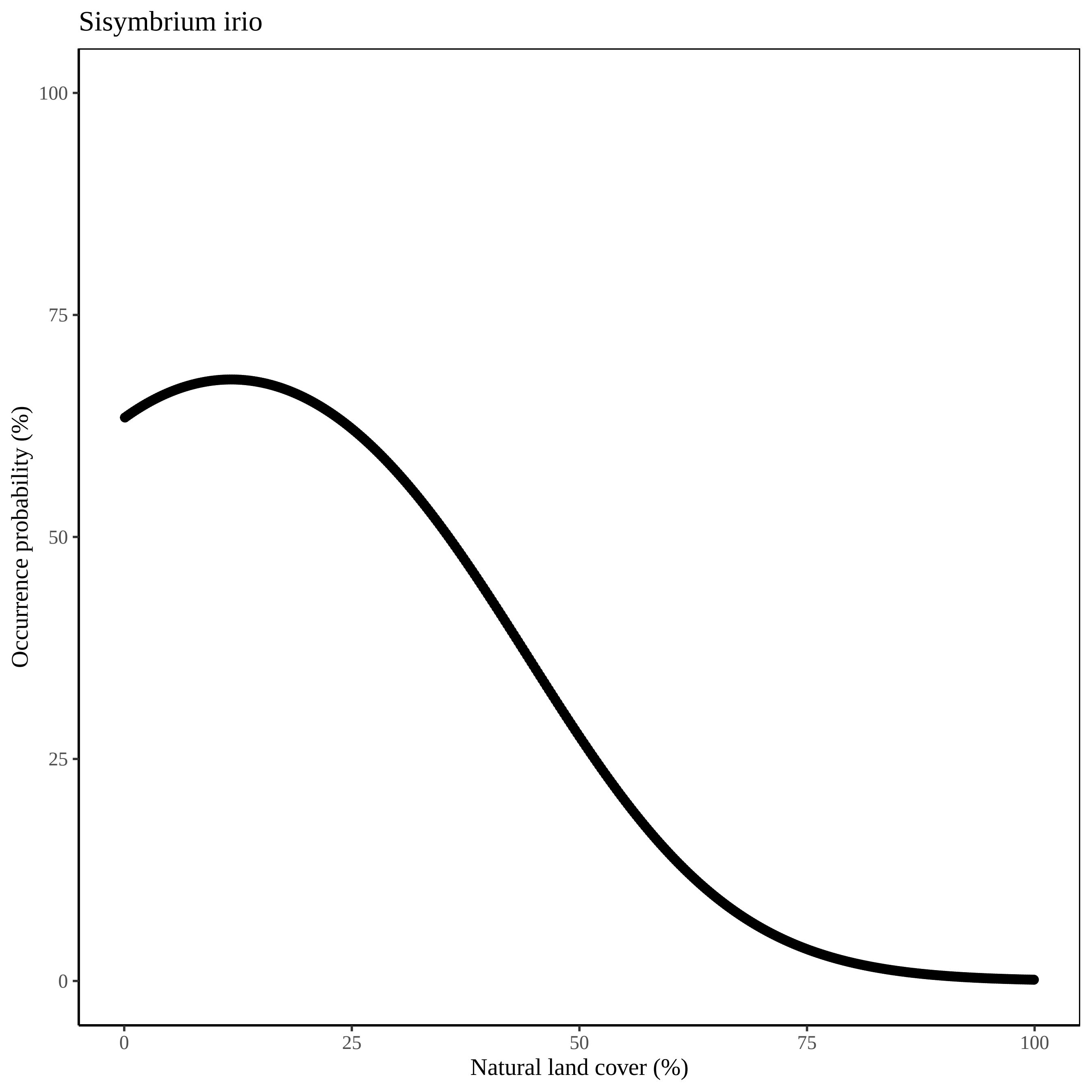

Sisymbrium irio
100
75
50
25
0
Occurrence probability (%)
0
25
50
75
100
Natural land cover (%)

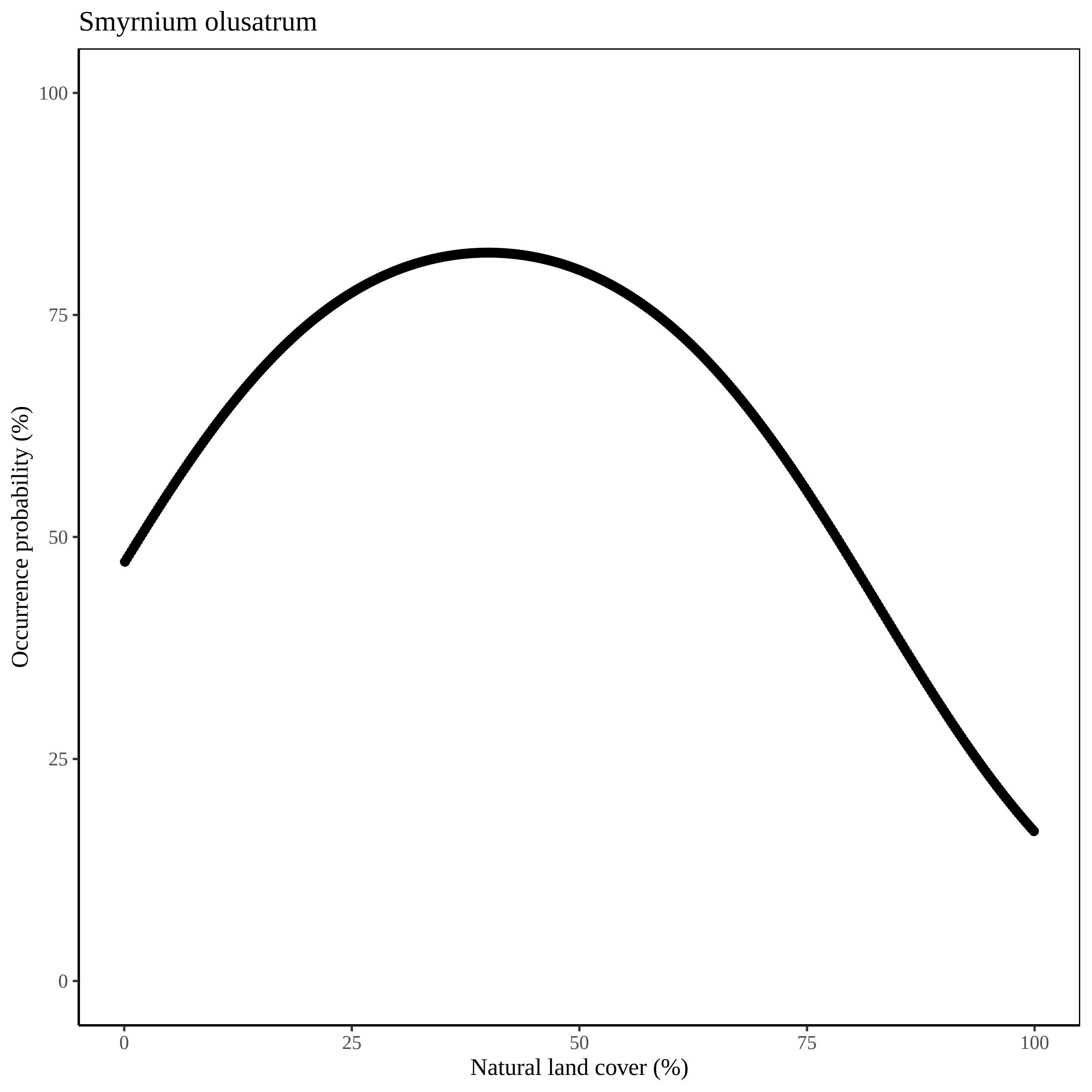

Smyrnium olusatrum
Occurrence probability (%)
100
75
50
25
0
0
25
50
75
100
Natural land cover (%)

Solanum tuberosum

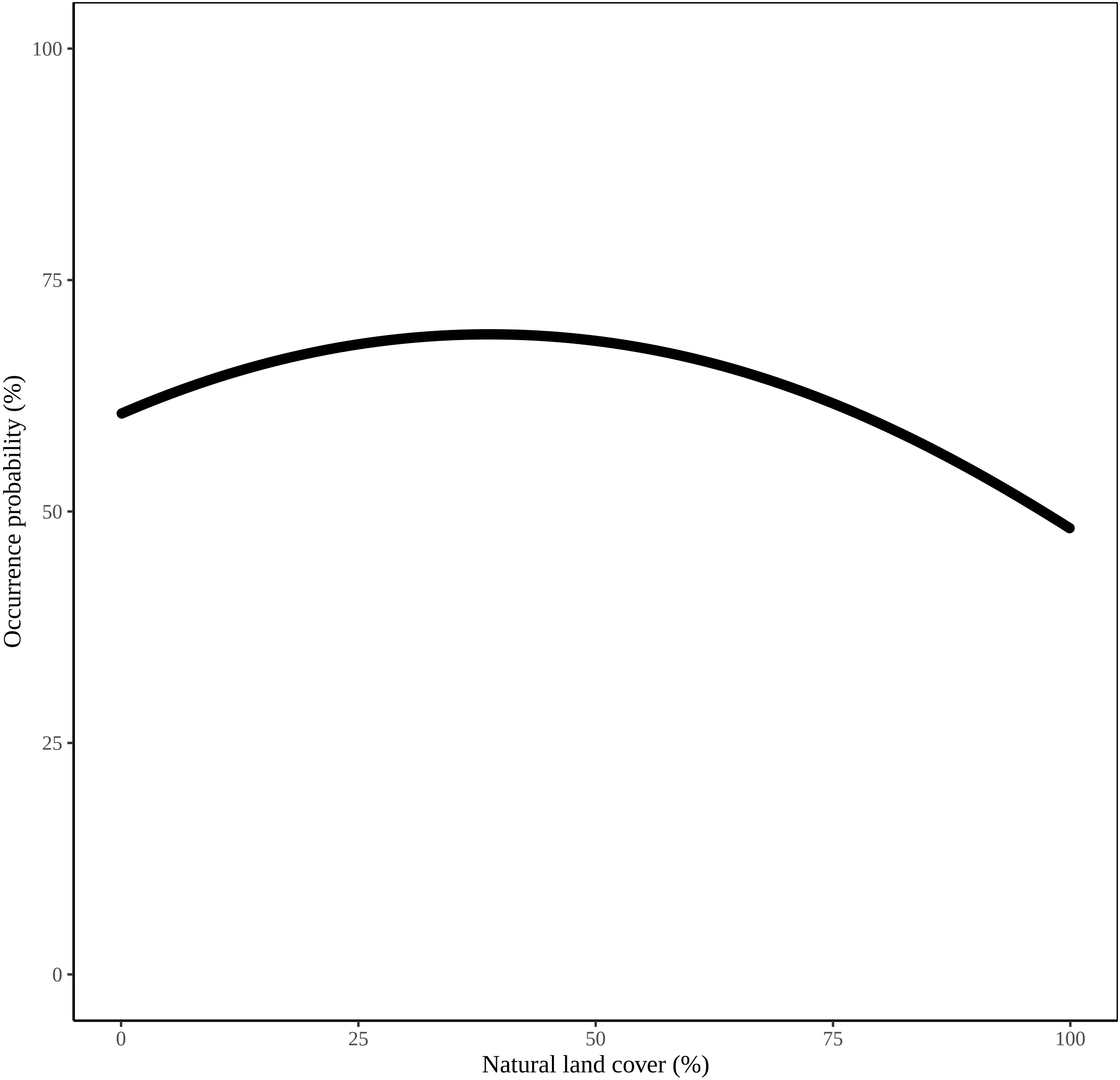

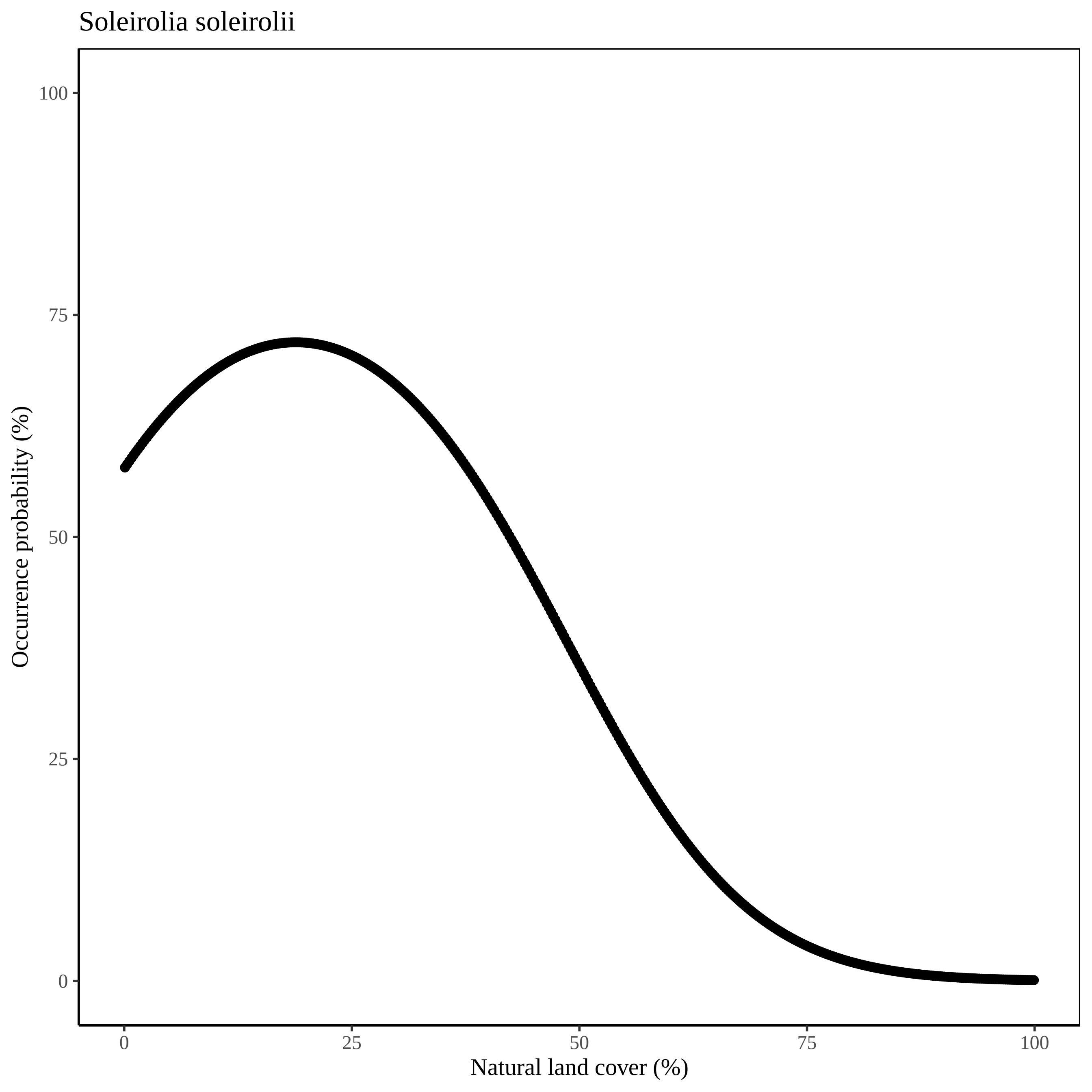
Soleirolia soleirolii
Occurrence probability (%)
0
25
50
75
100
Natural land cover (%)
0
25
50
75
100

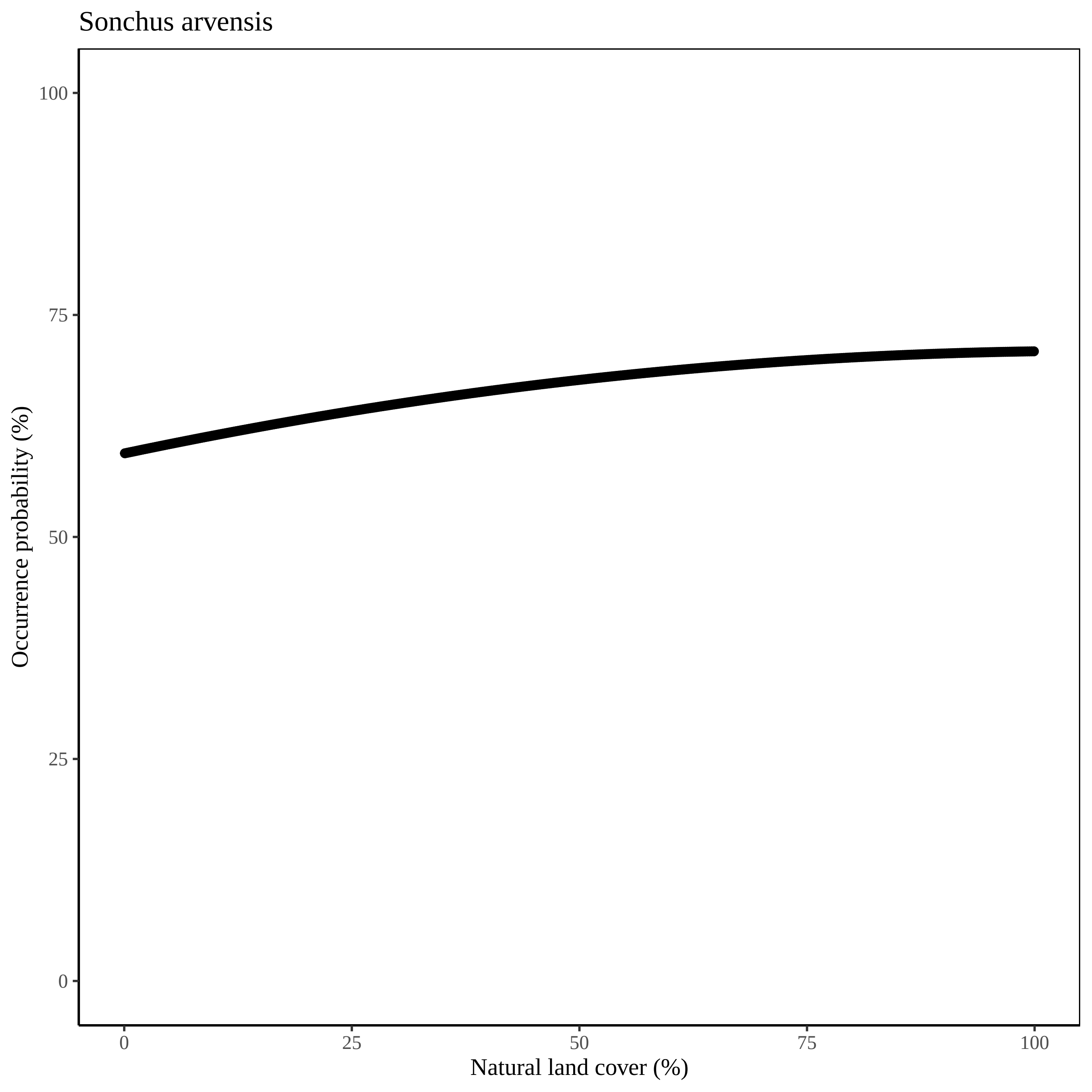

Sonchus arvensis
Occurrence probability (%)
100
75
50
25
0
0
25
50
75
100
Natural land cover (%)

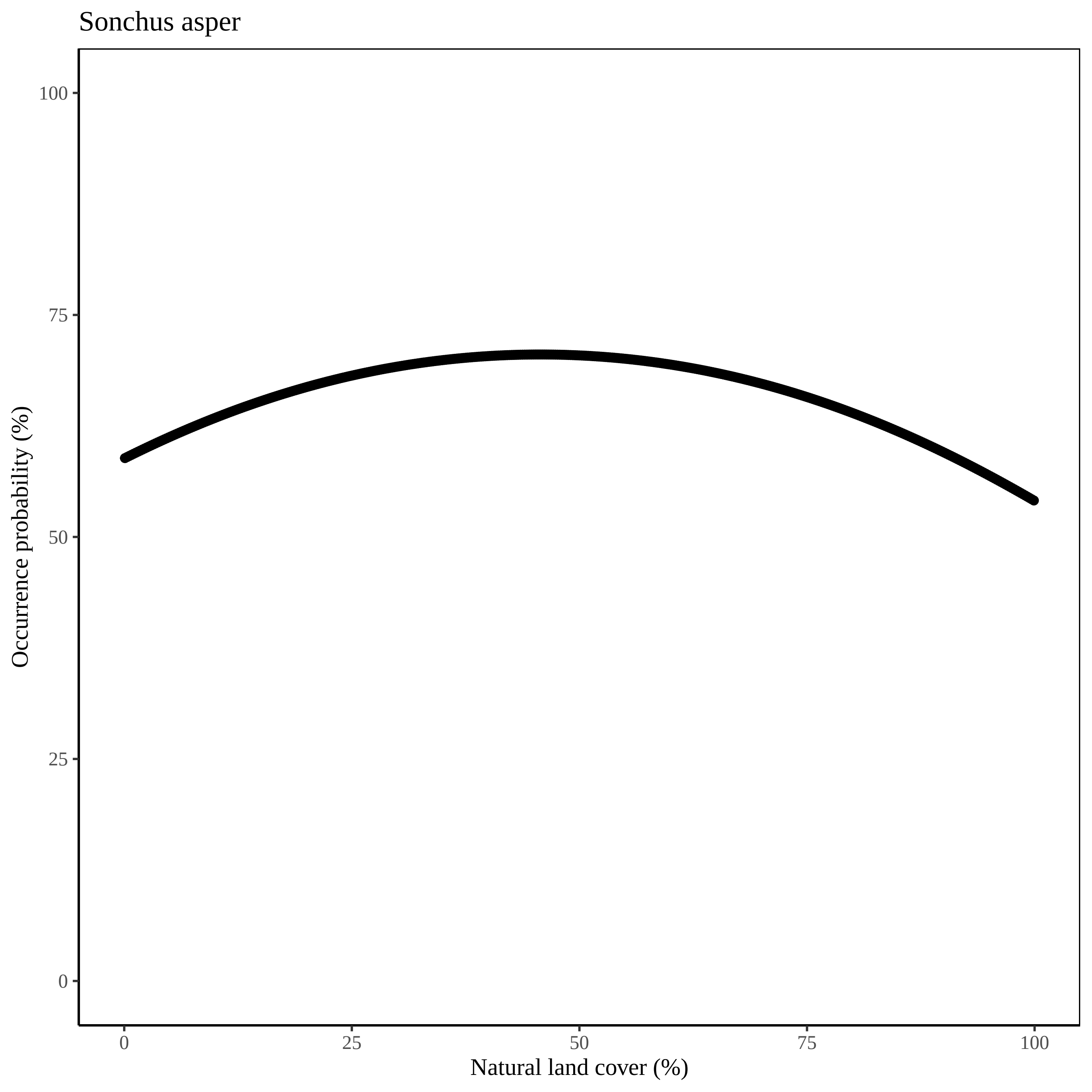

Sonchus asper
100
75
50
25
0
Occurrence probability (%)
0
25
50
75
100
Natural land cover (%)

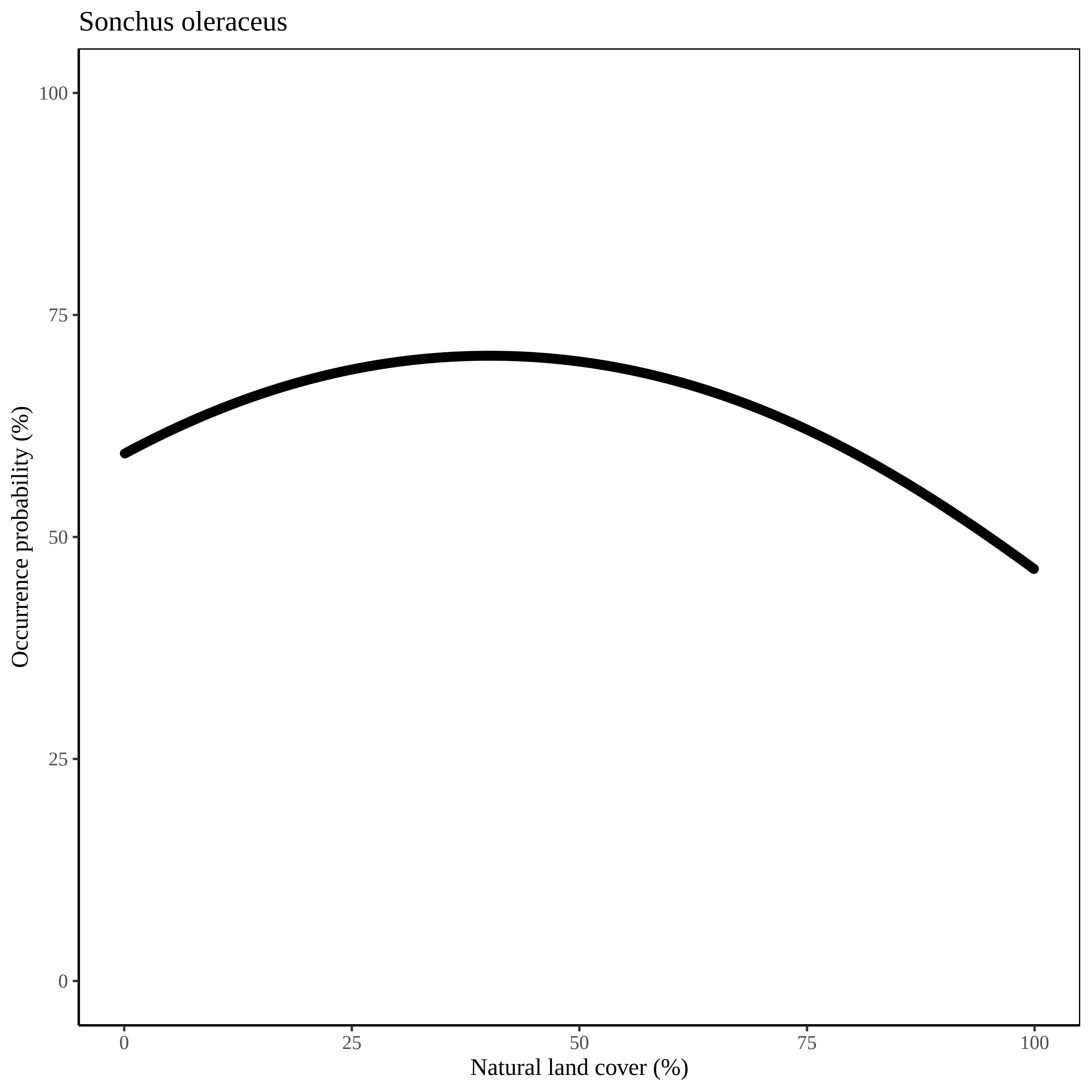

Sonchus oleraceus
Occurrence probability (%)
100
75
50
25
0
0
25
50
75
100
Natural land cover (%)

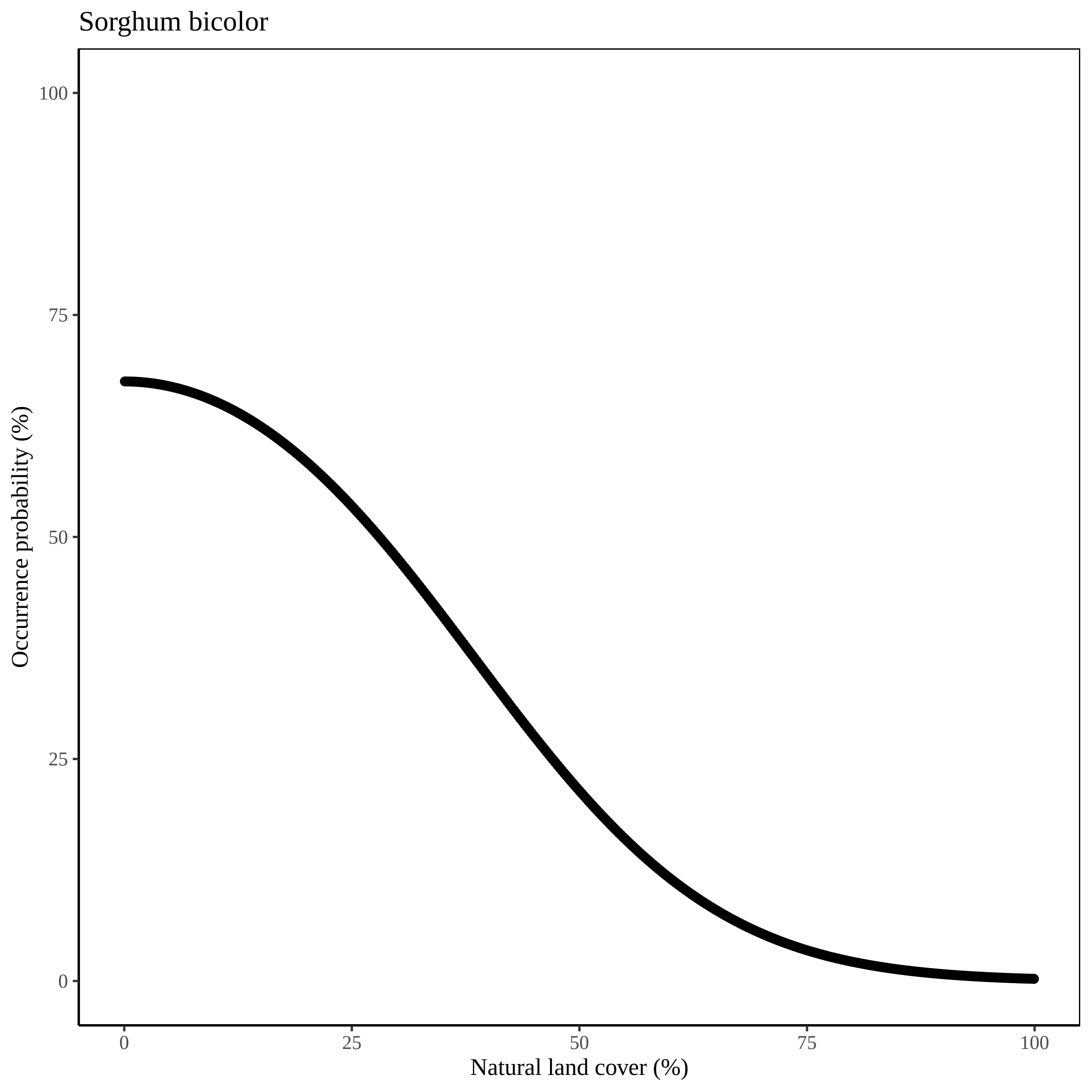

Sorghum bicolor
100
75
50
25
0
Occurrence probability (%)
0
25
50
75
100
Natural land cover (%)

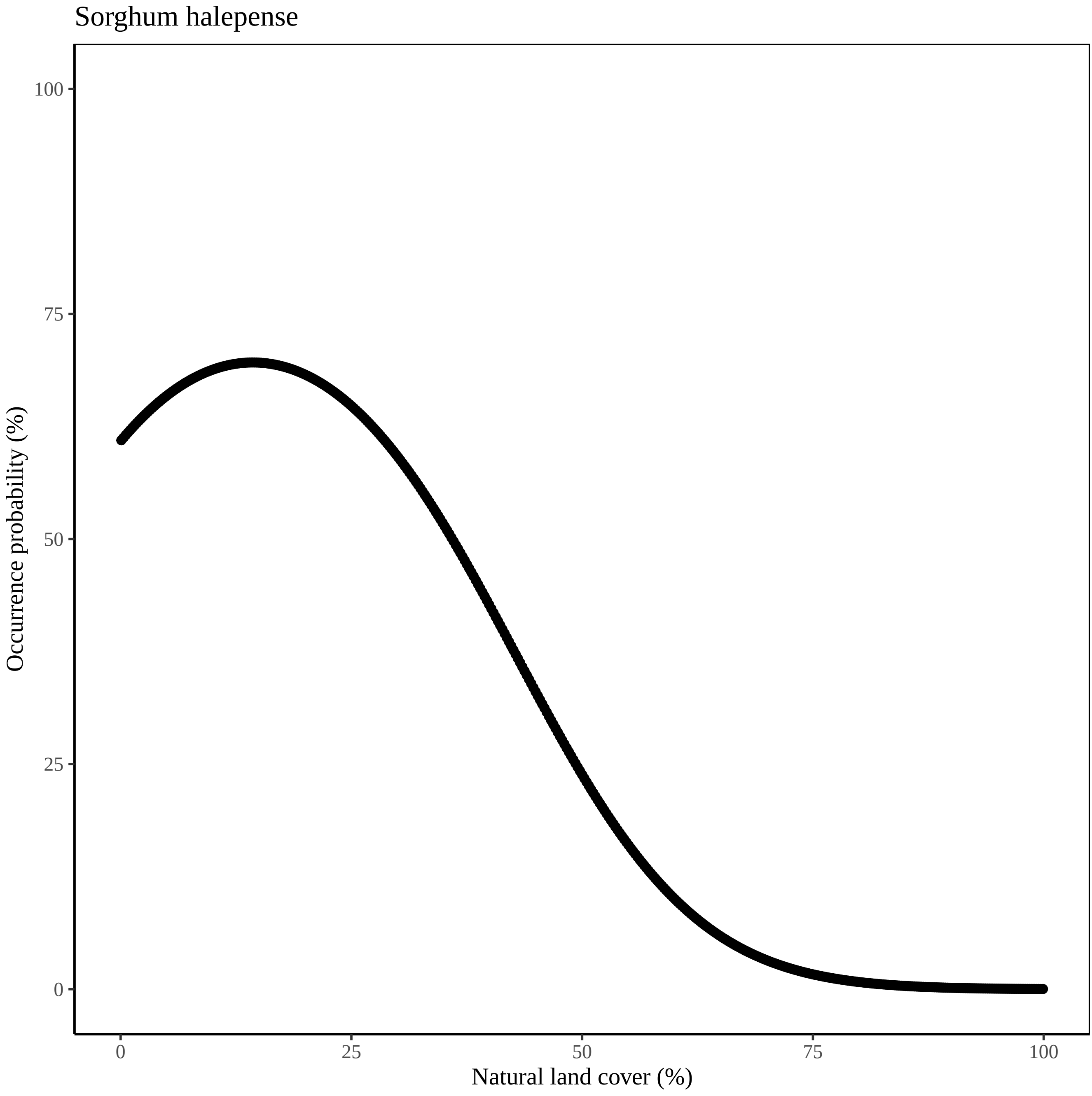
Sorghum halepense
Occurrence probability (%)
100
75
50
25
0
0
25
50
75
100
Natural land cover (%)

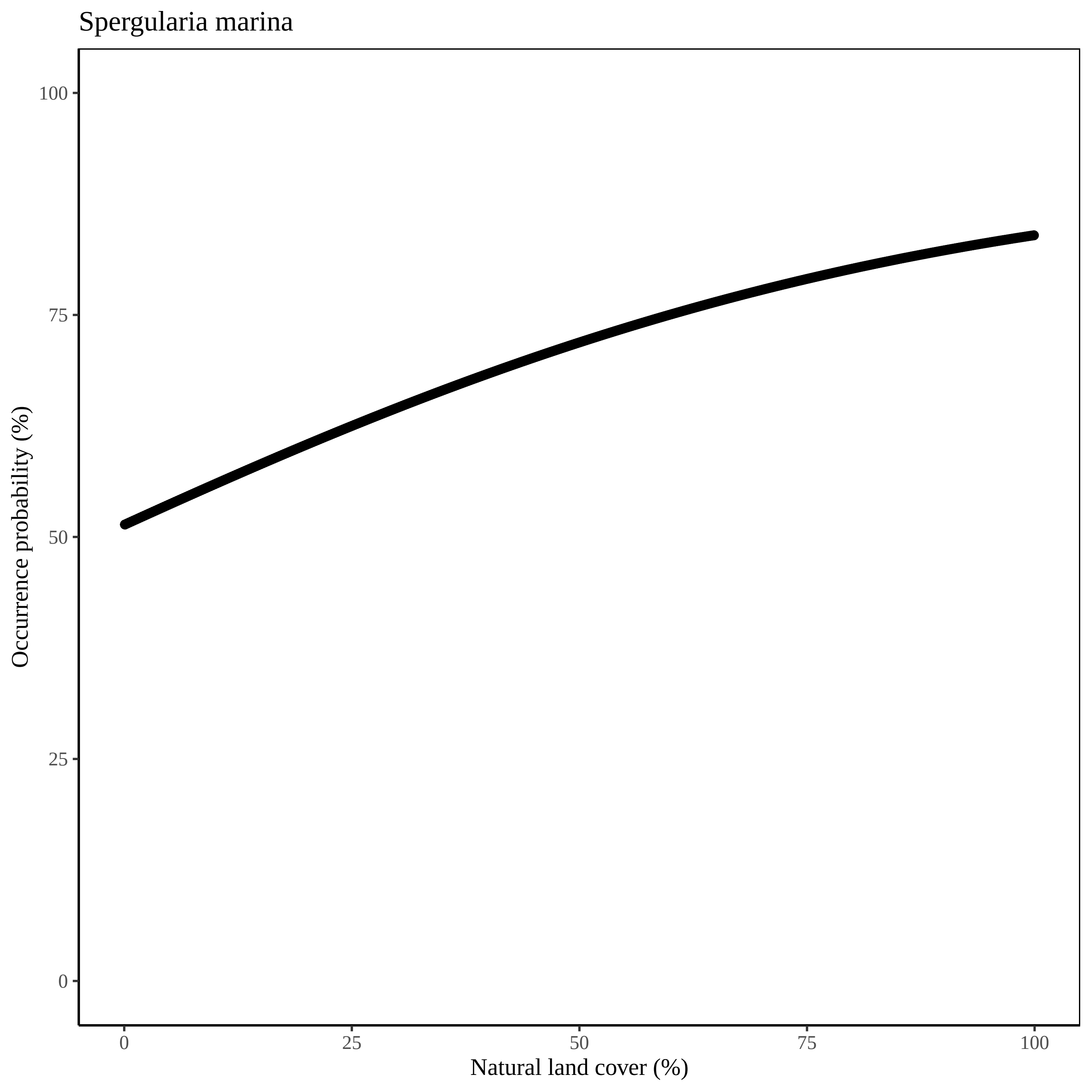
Spergularia marina
Occurrence probability (%)
100
75
50
25
0
0
25
50
75
100
Natural land cover (%)

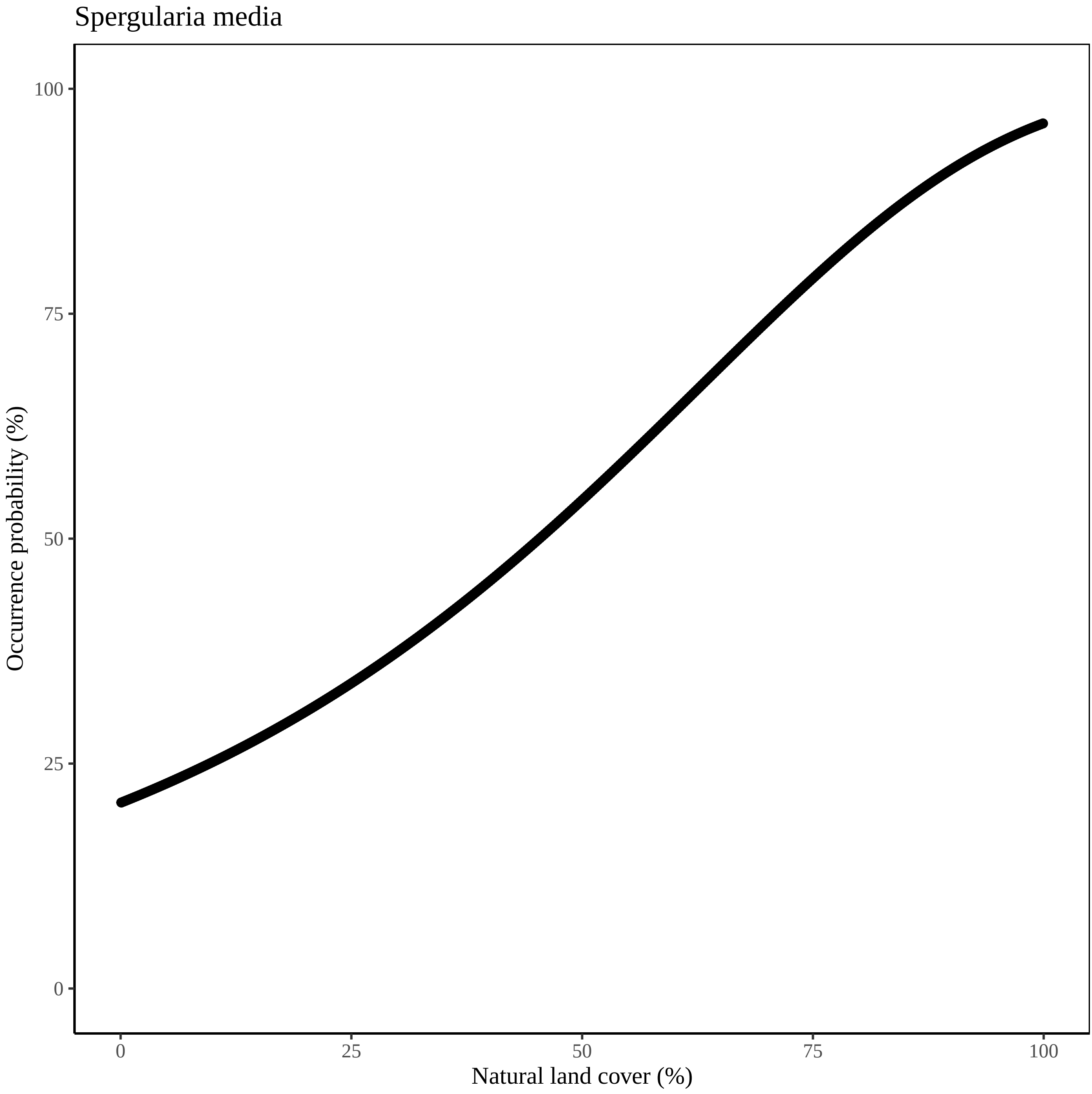
Spergularia media
Occurrence probability (%)
100
75
50
25
0
0
25
50
75
100
Natural land cover (%)

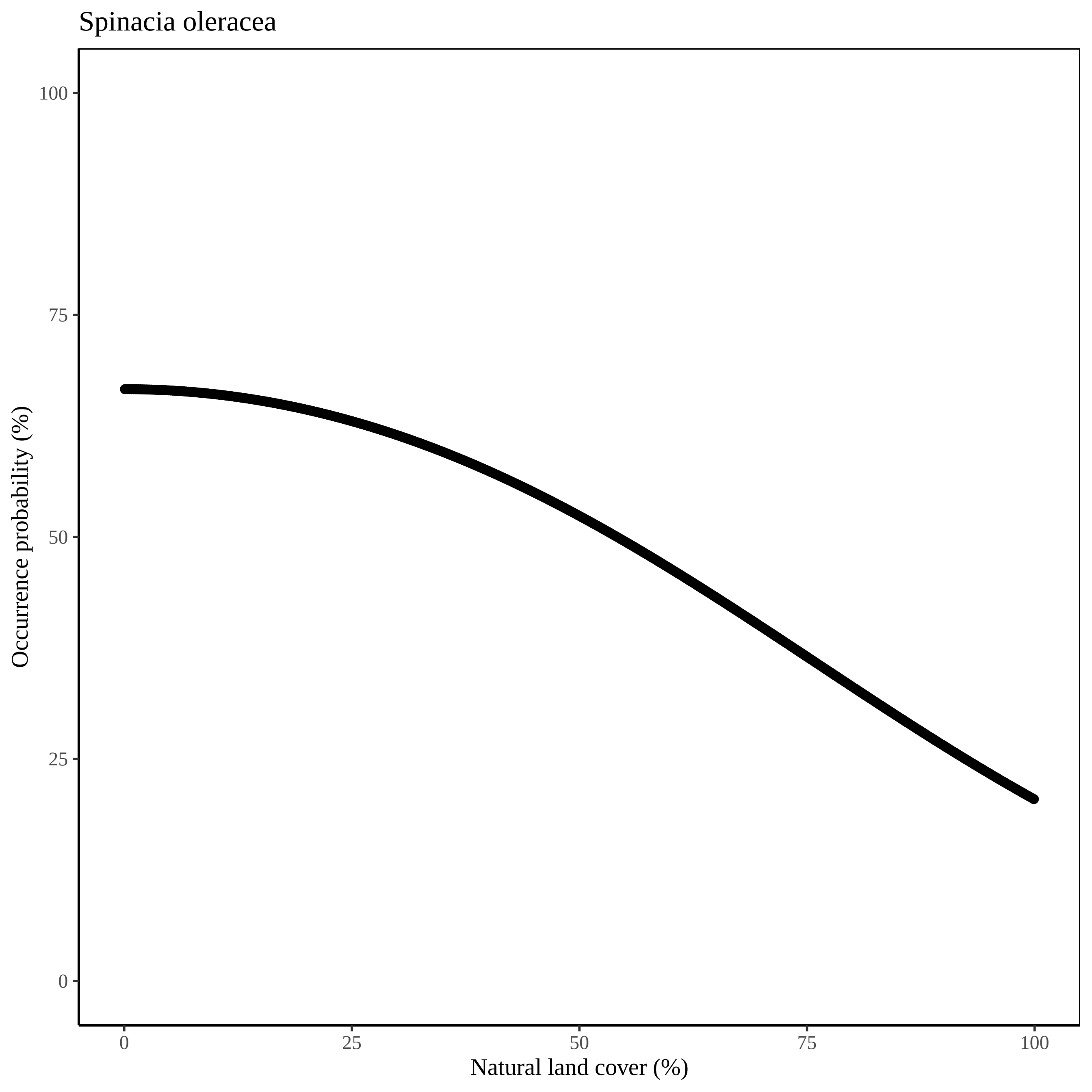

Spinacia oleracea
100
75
50
25
0
Occurrence probability (%)
0
25
50
75
100
Natural land cover (%)

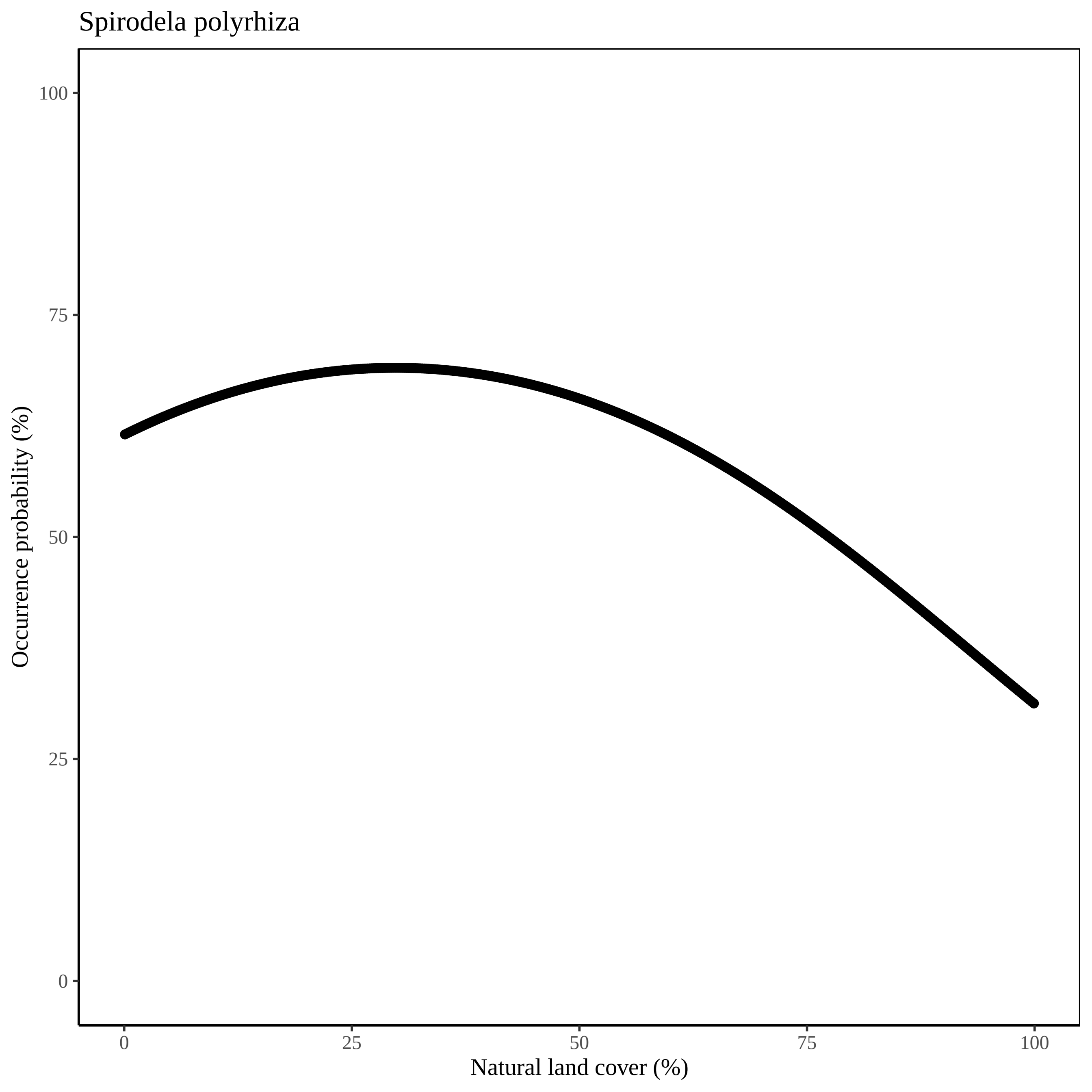
Spirodela polyrhiza
Occurrence probability (%)
100
75
50
25
0
0
25
50
75
100
Natural land cover (%)

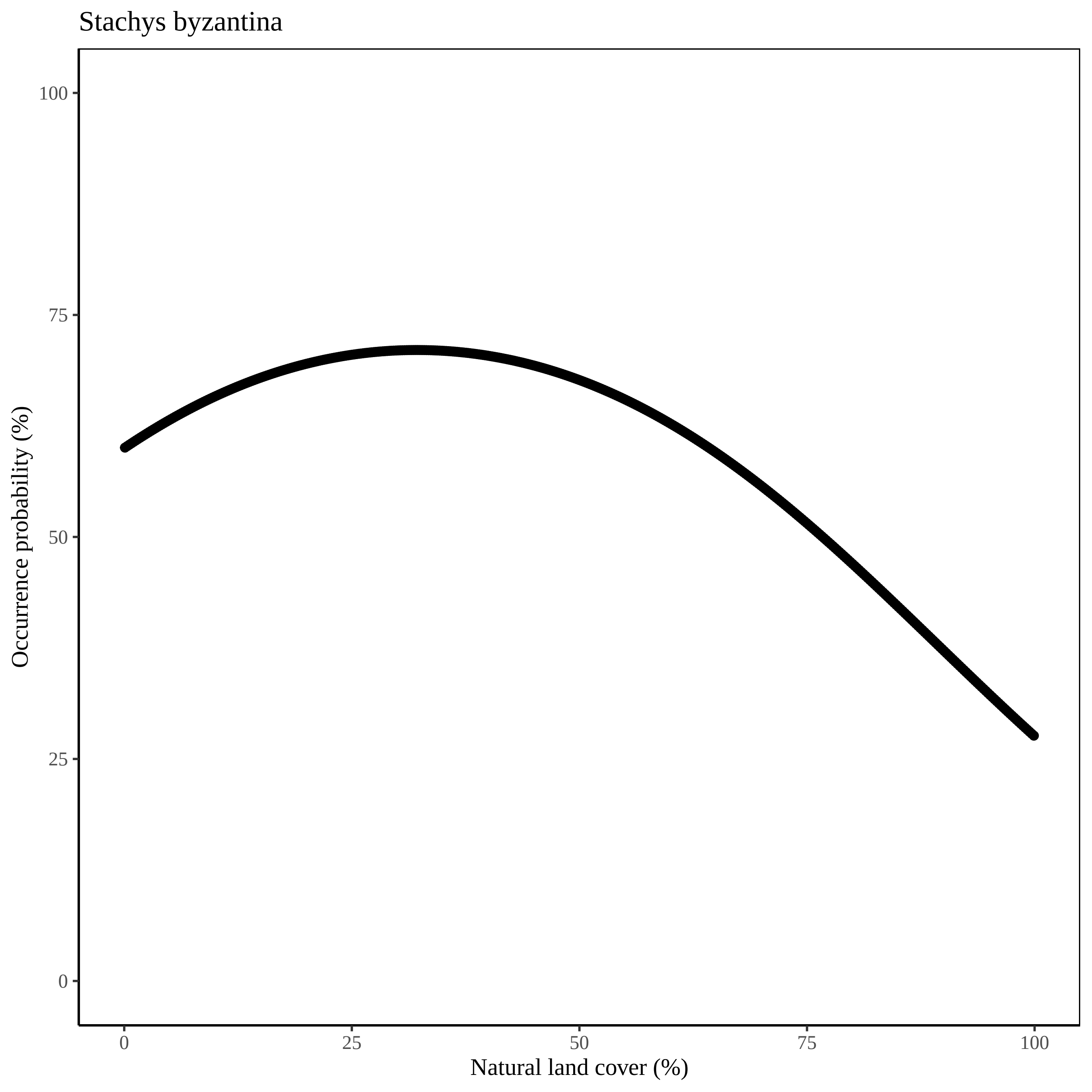

Stachys byzantina
Occurrence probability (%)
100
75
50
25
0
0
25
50
75
100
Natural land cover (%)

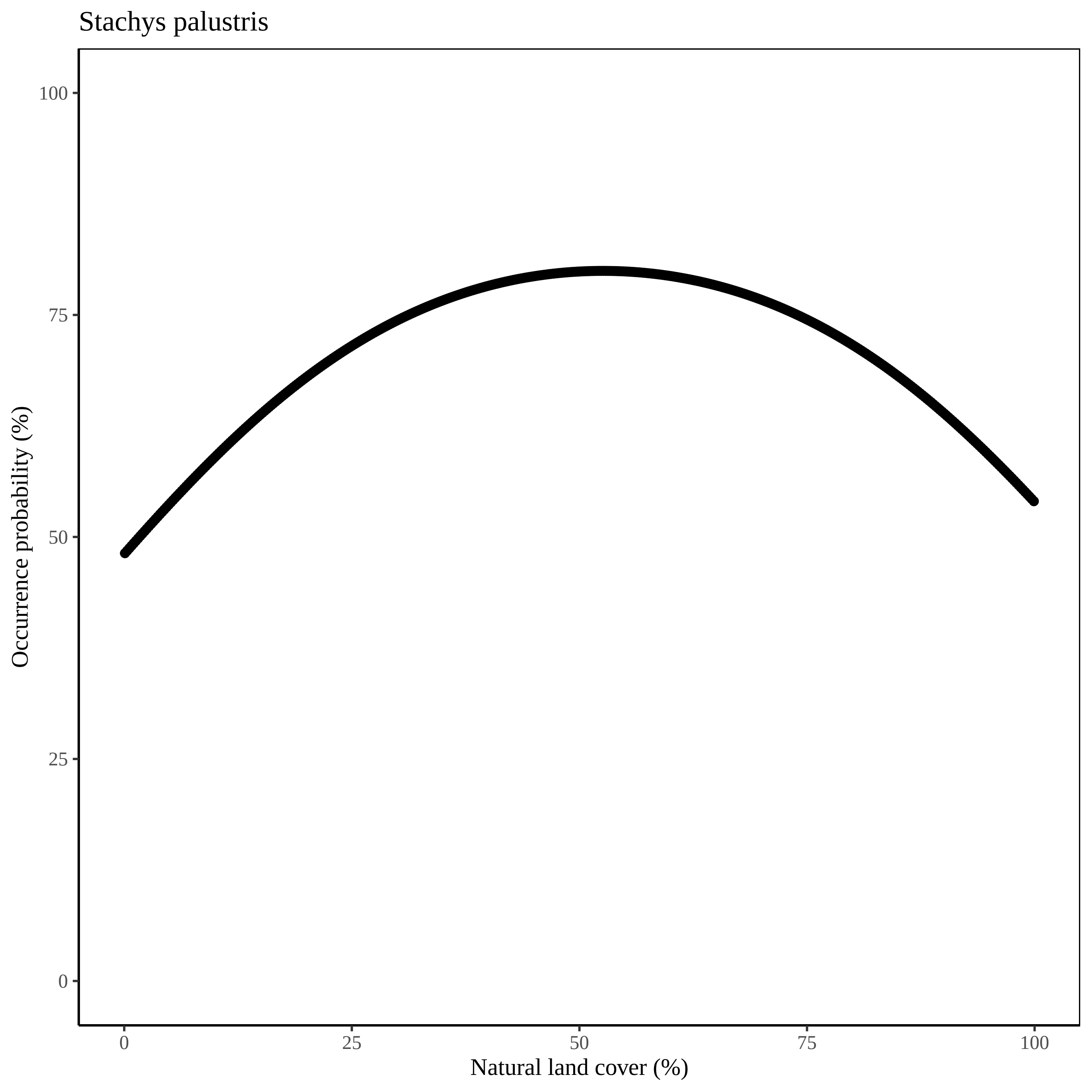

Stachys palustris
Occurrence probability (%)
100
75
50
25
0
0
25
50
75
100
Natural land cover (%)

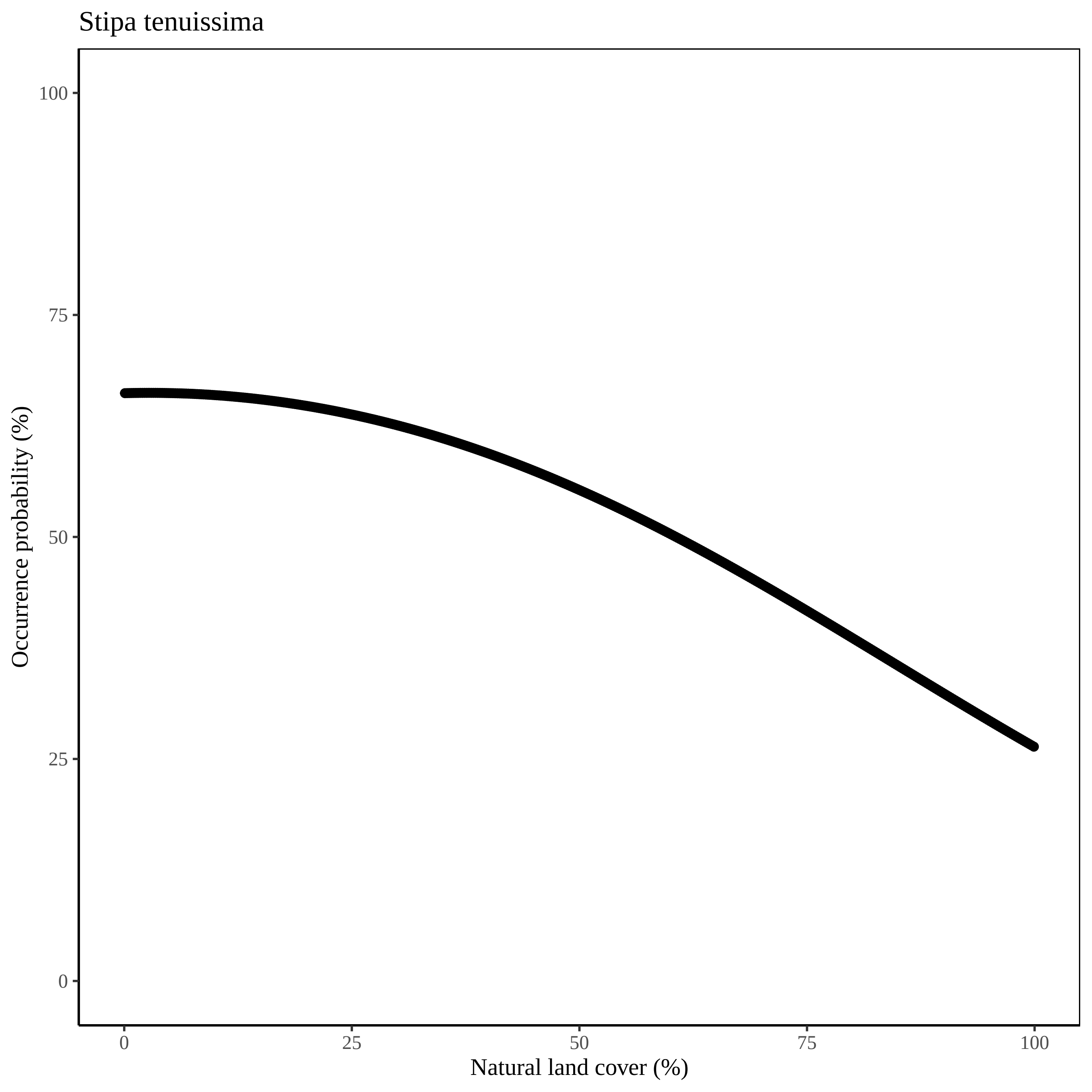
Stipa tenuissima
100
75
50
25
0
Occurrence probability (%)
0
25
50
75
100
Natural land cover (%)

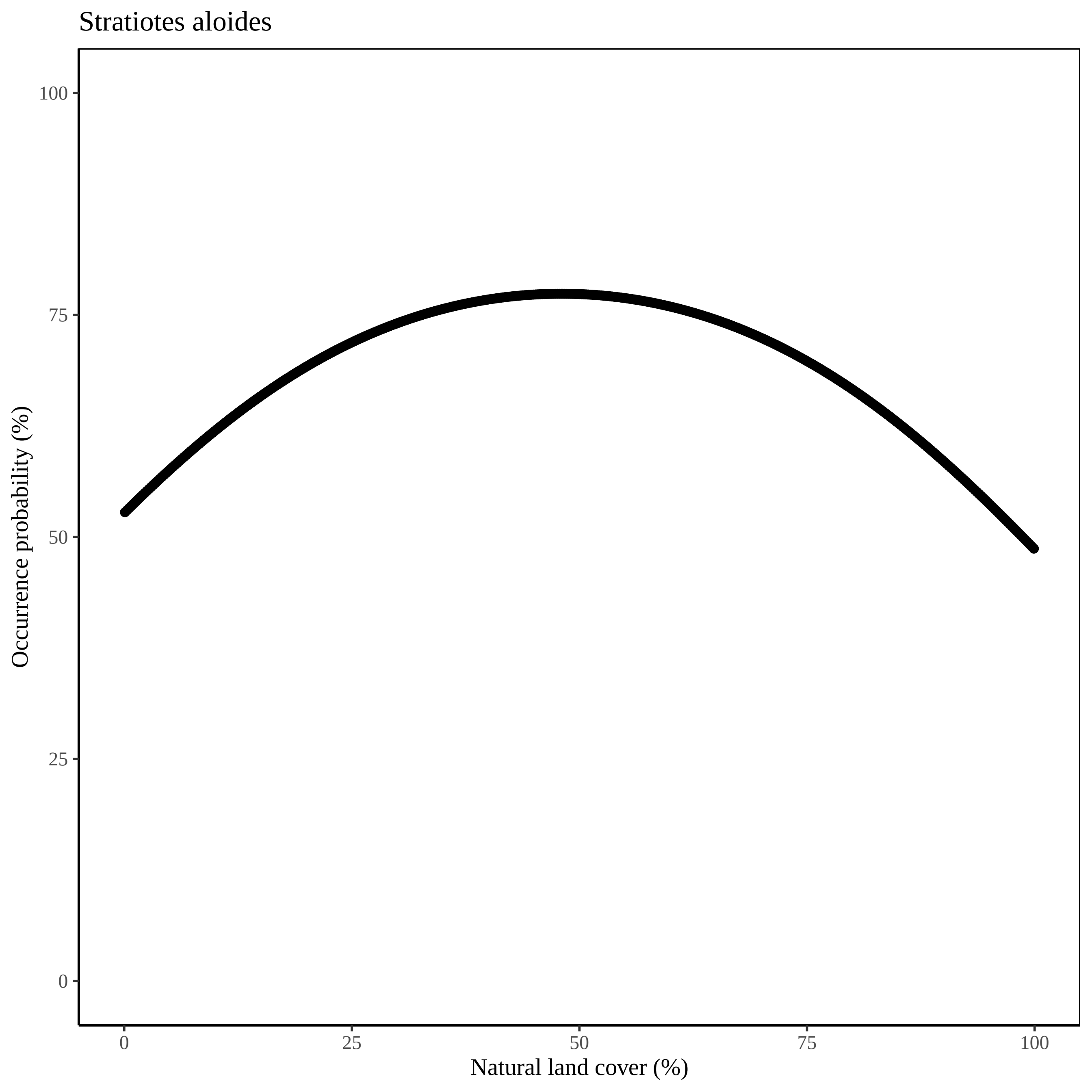

Stratiotes aloides
Occurrence probability (%)
100
75
50
25
0
0
25
50
75
100
Natural land cover (%)

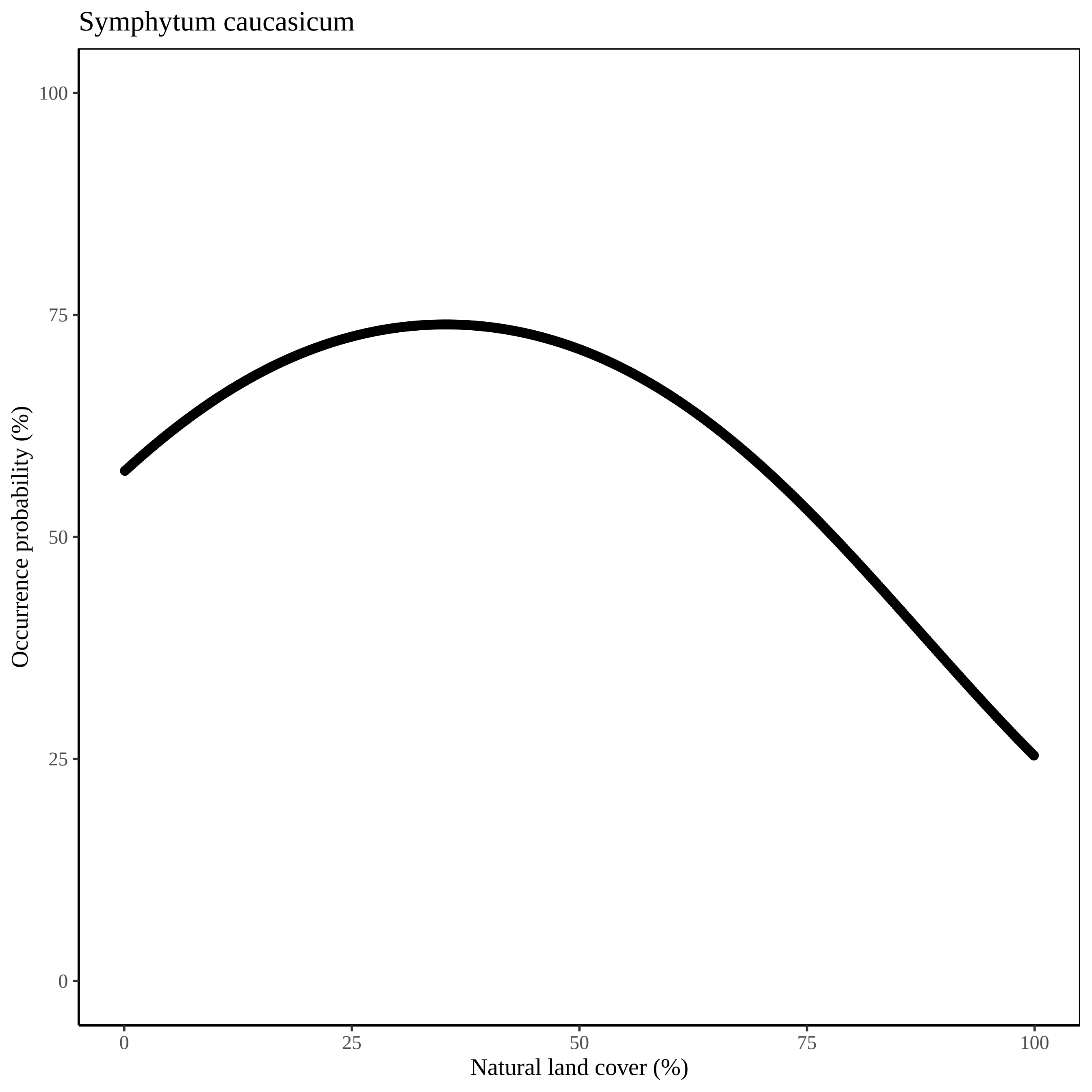
Symphytum caucasicum
Occurrence probability (%)
100
75
50
25
0
0
25
50
75
100
Natural land cover (%)

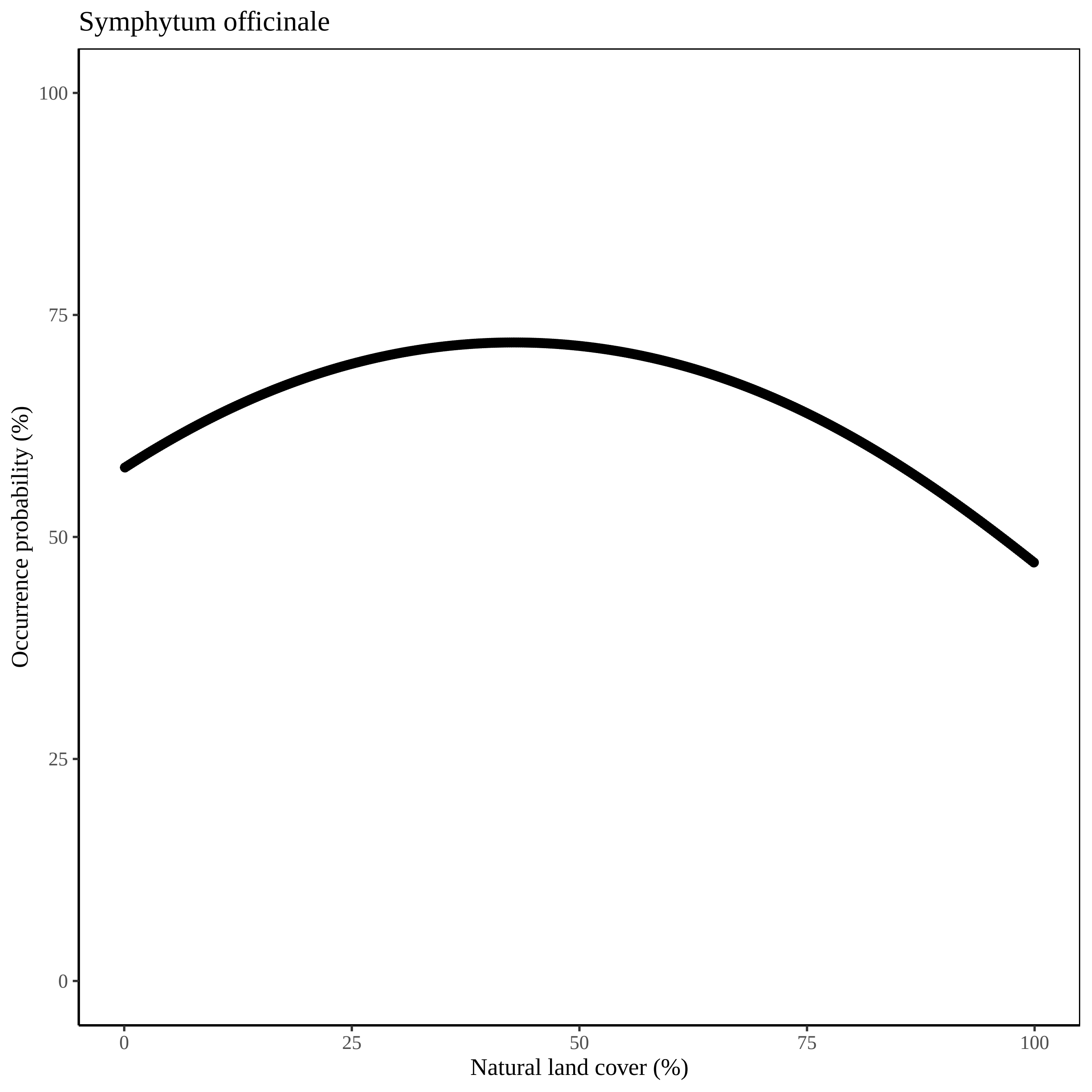
Symphytum officinale
Occurrence probability (%)
100
75
50
25
0
0
25
50
75
100
Natural land cover (%)

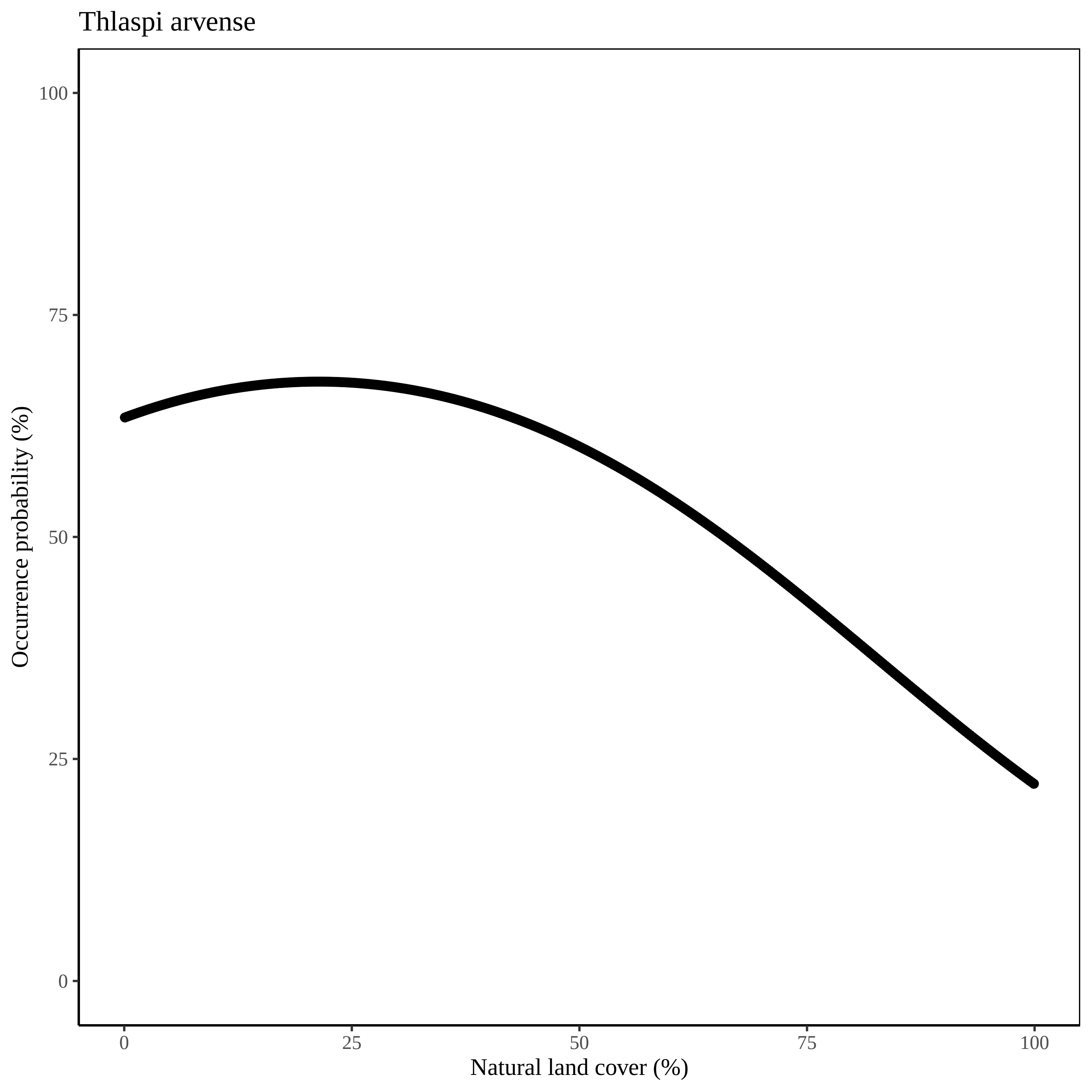

Thlaspi arvense
Occurrence probability (%)
100
75
50
25
0
0
25
50
75
100
Natural land cover (%)

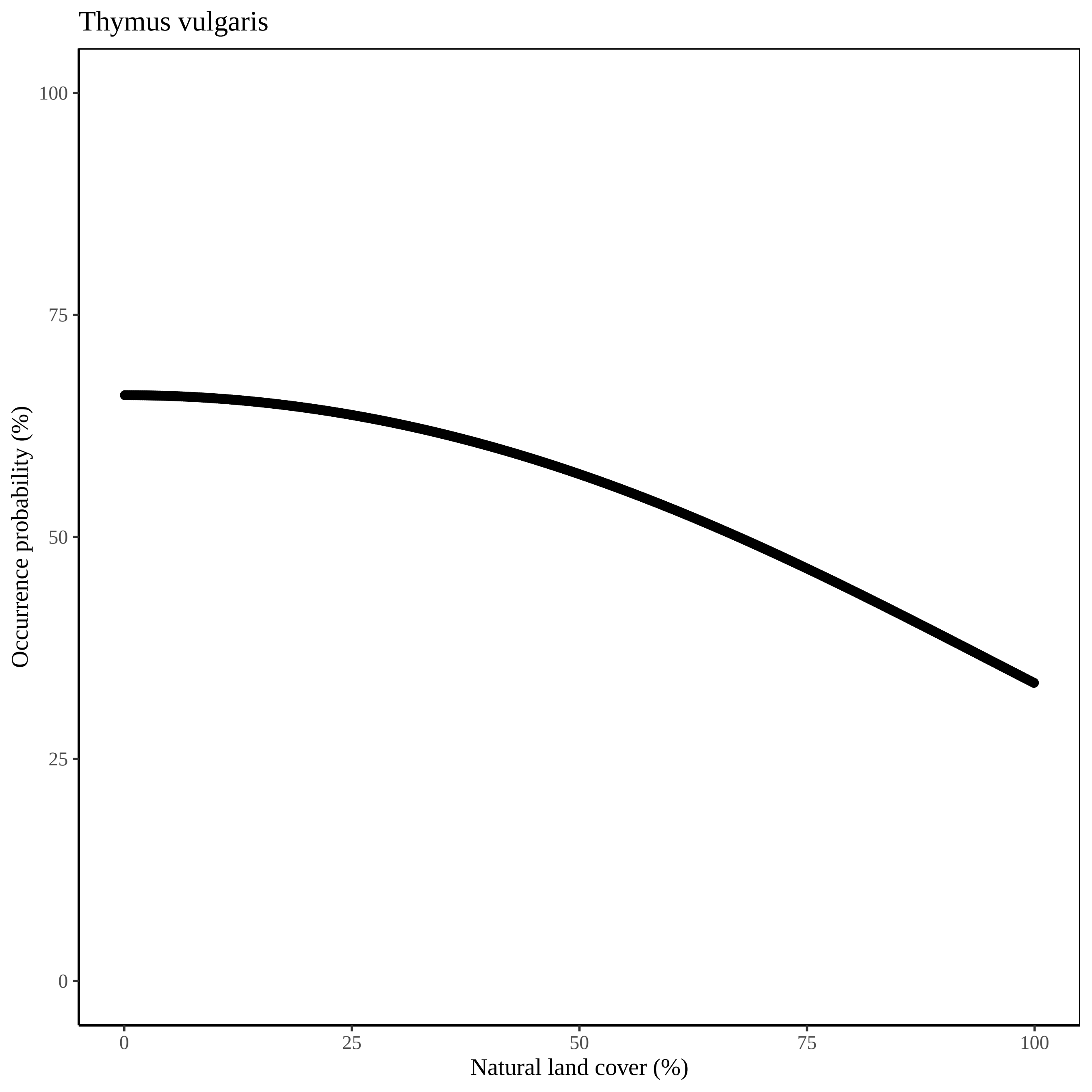
Thymus vulgaris
Occurrence probability (%)
100
75
50
25
0
0
25
50
75
100
Natural land cover (%)

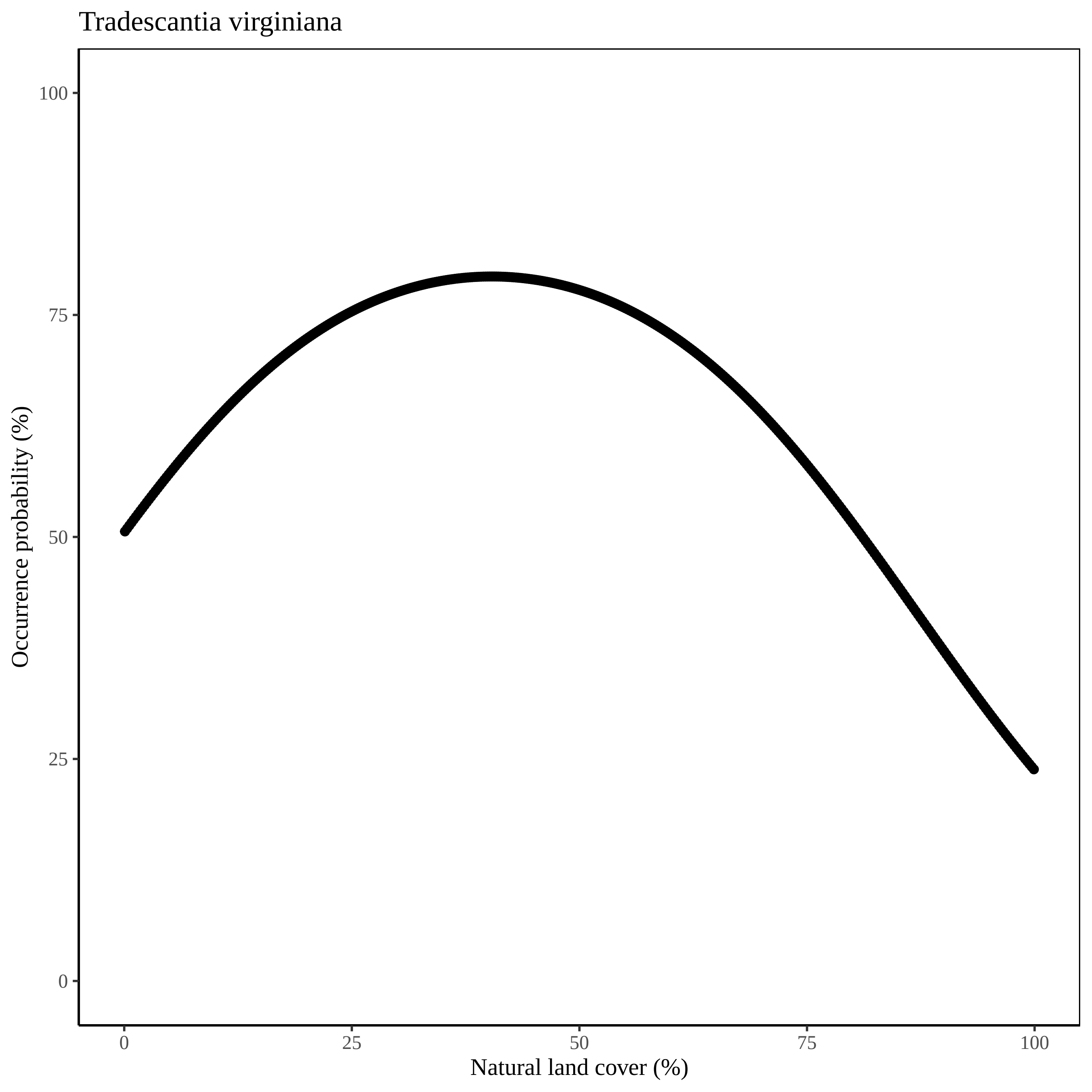

Tradescantia virginiana
Occurrence probability (%)
100
75
50
25
0
0
25
50
75
100
Natural land cover (%)

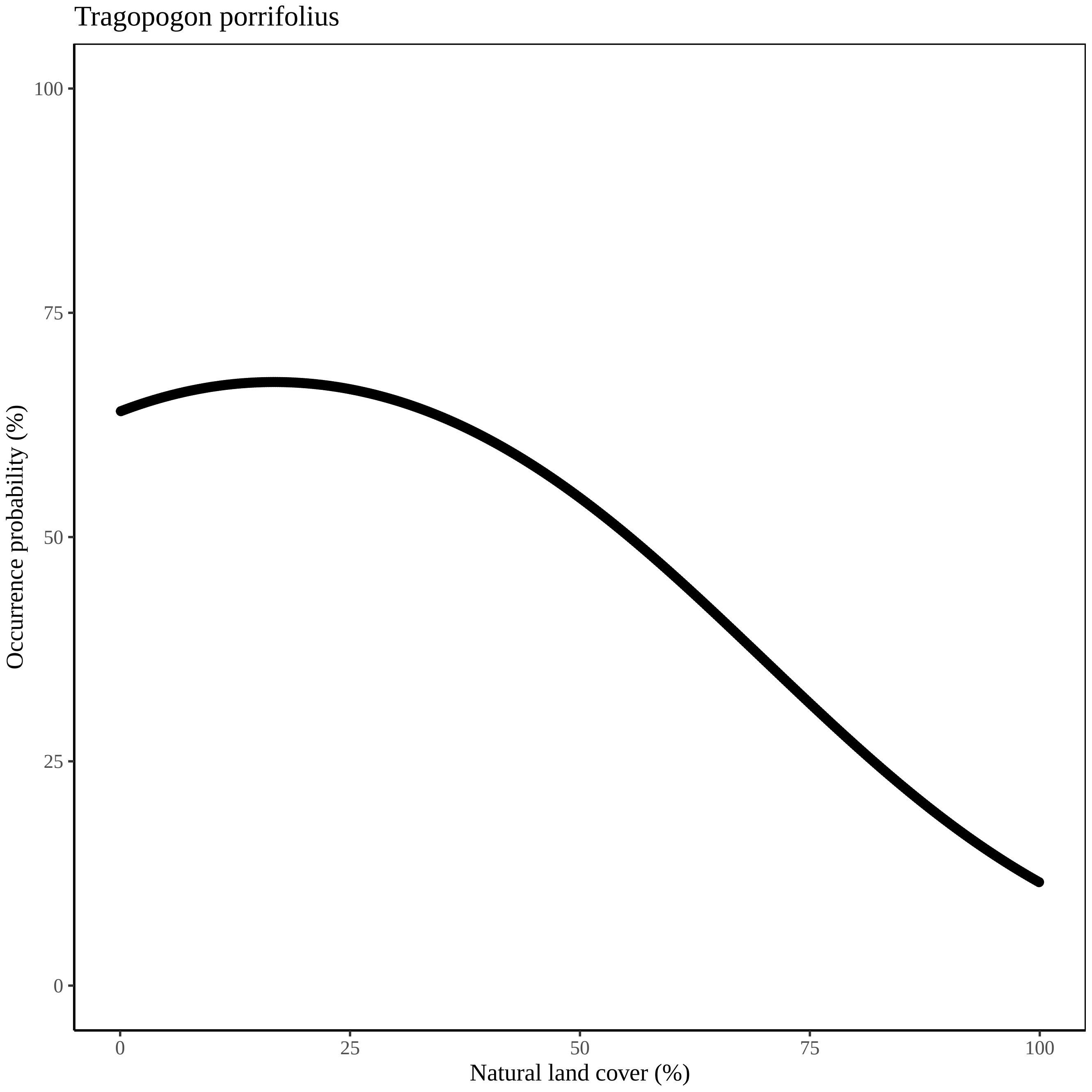

Tragopogon porrifolius
100
75
50
25
0
Occurrence probability (%)
0
25
50
75
100
Natural land cover (%)

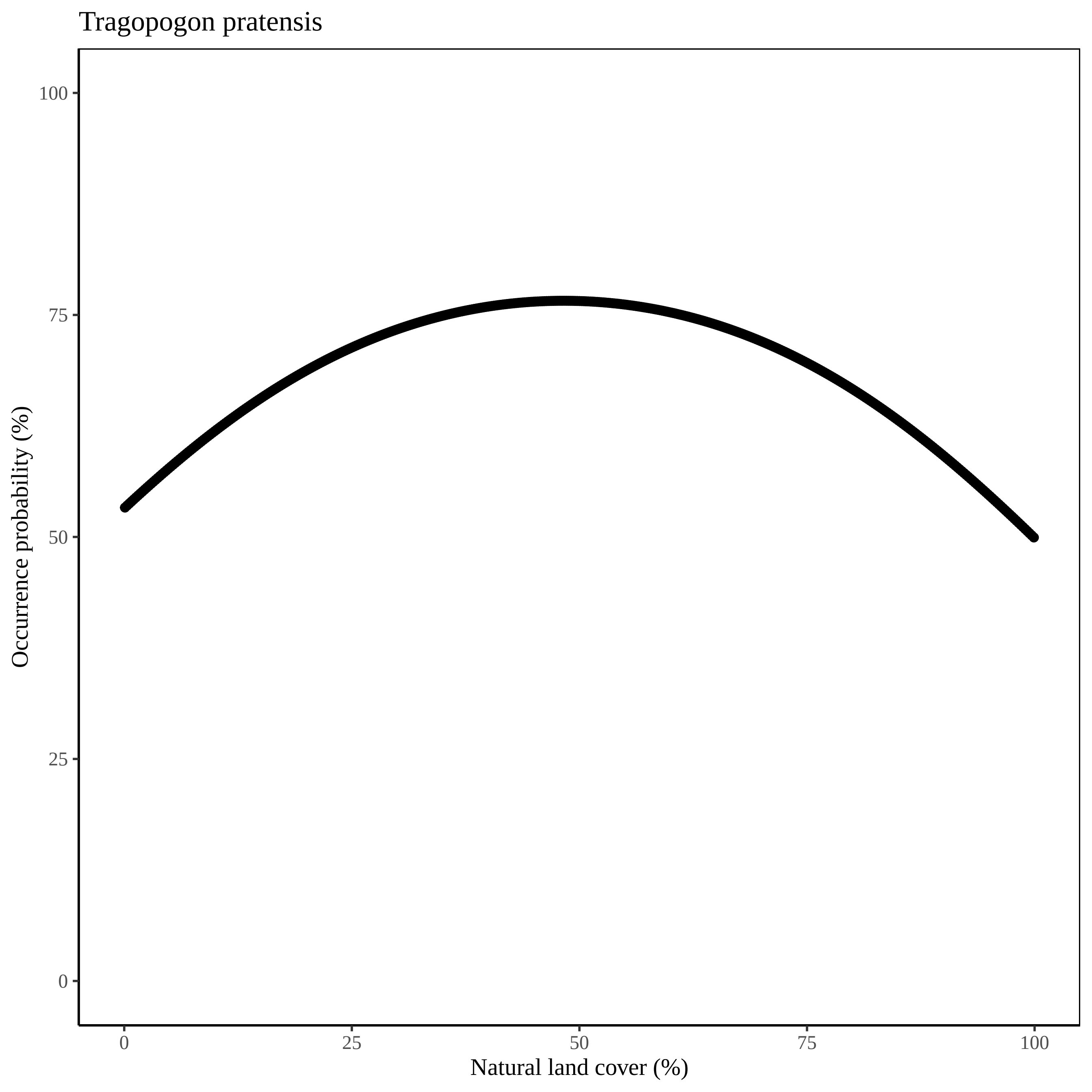
Tragopogon pratensis
Occurrence probability (%)
0
25
50
75
100
Natural land cover (%)
0
25
50
75
100

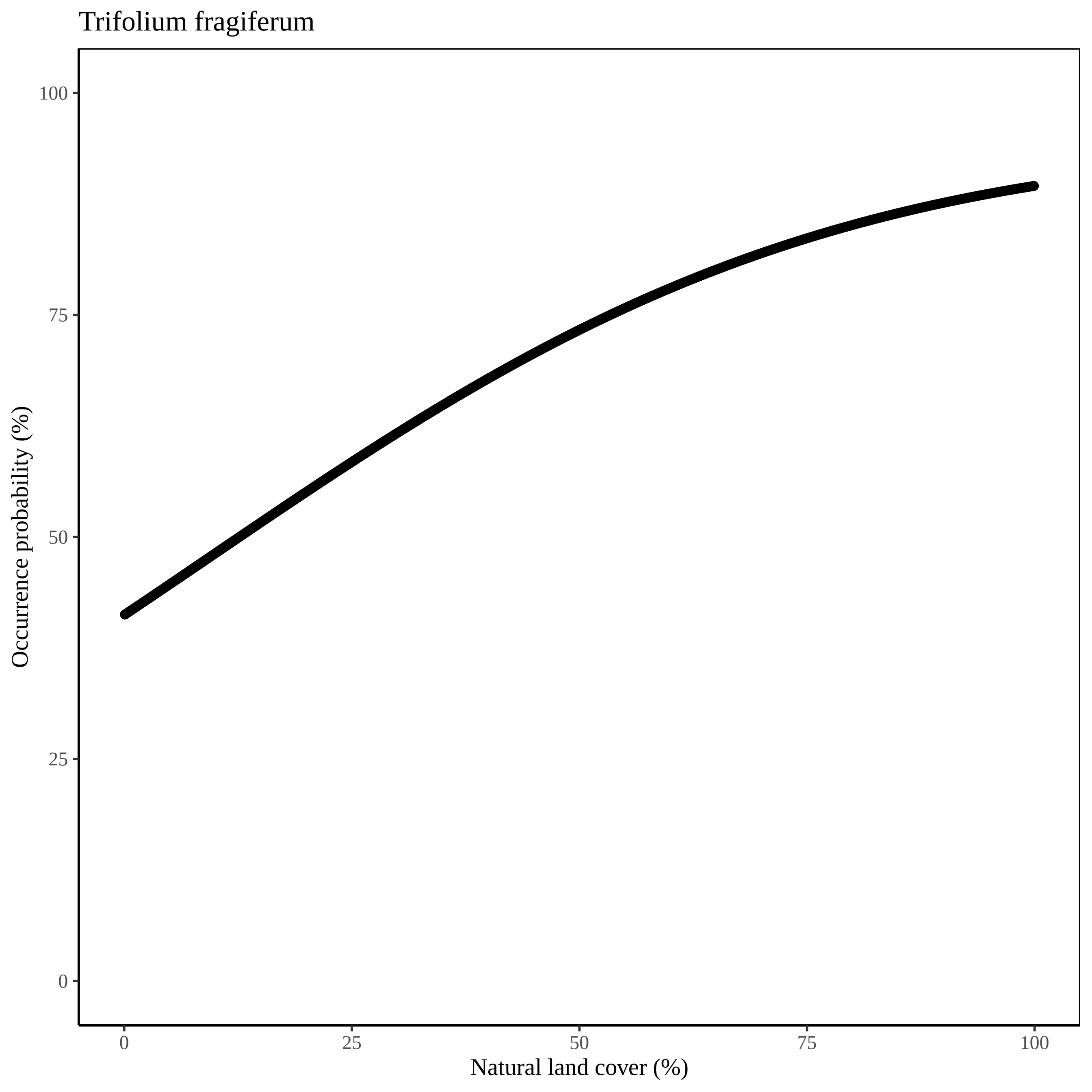

Trifolium fragiferum
Occurrence probability (%)
100
75
50
25
0
0
25
50
75
100
Natural land cover (%)

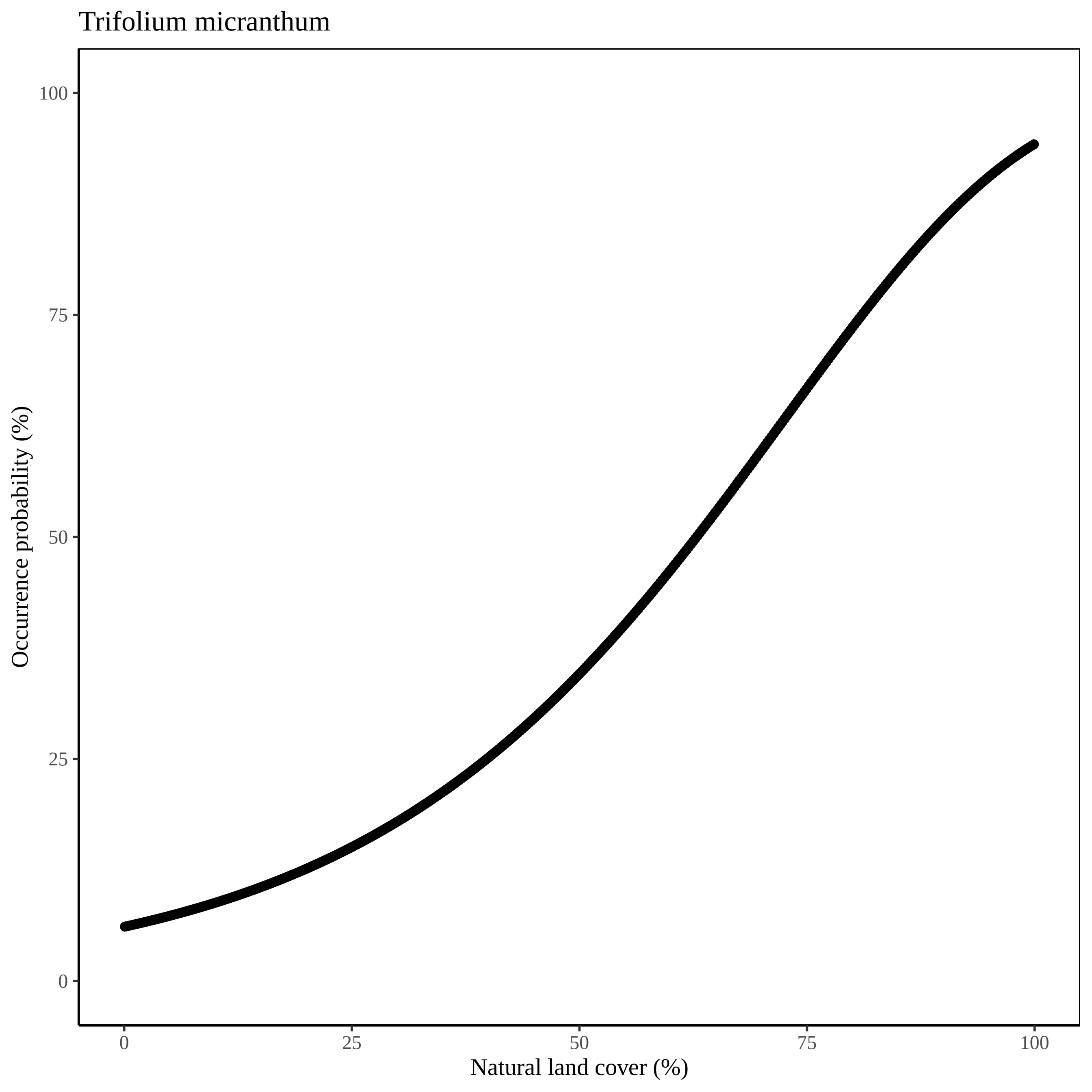

Trifolium micranthum
Occurrence probability (%)
100
75
50
25
0
0
25
50
75
100
Natural land cover (%)

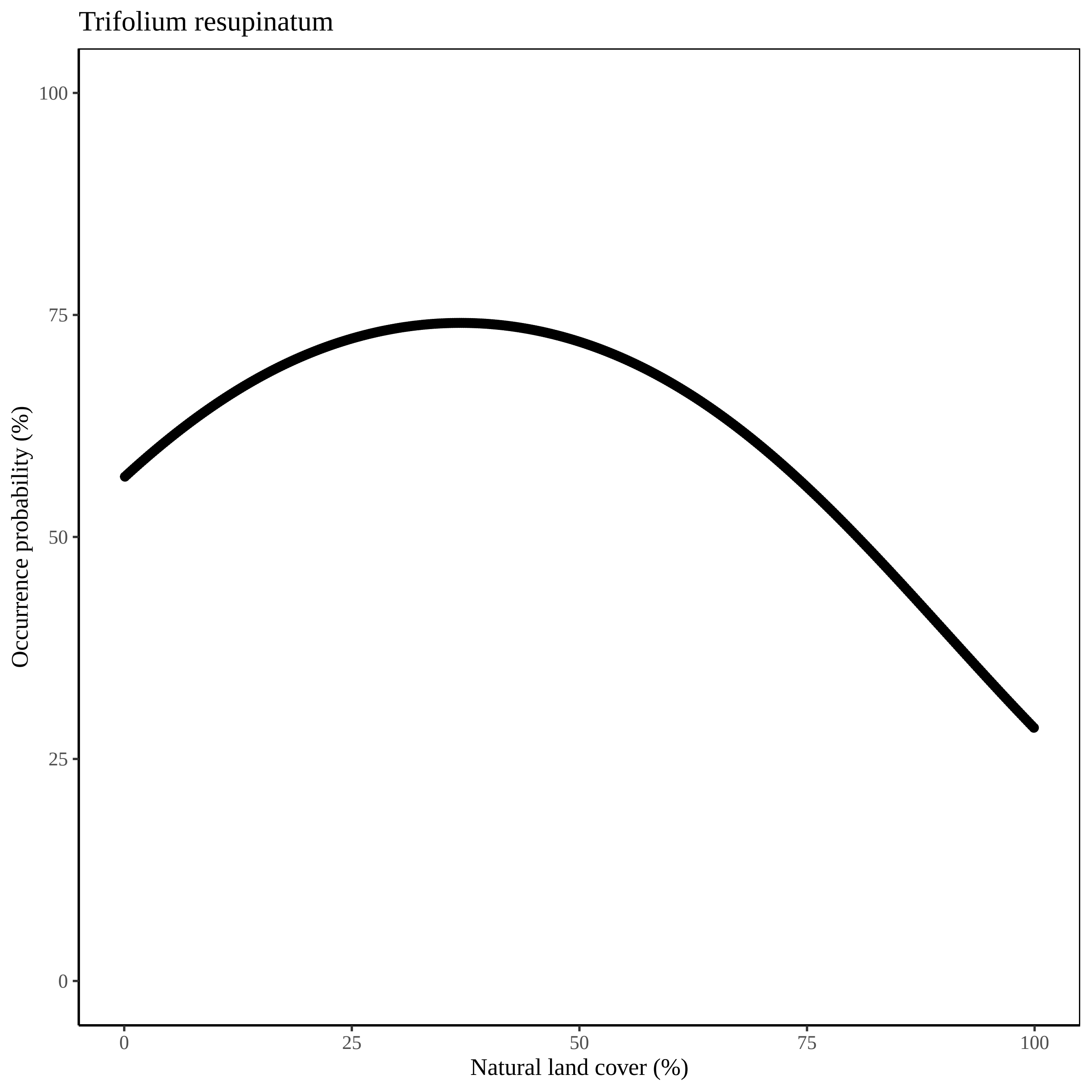
Trifolium resupinatum
Occurrence probability (%)
100
75
50
25
0
0
25
50
75
100
Natural land cover (%)

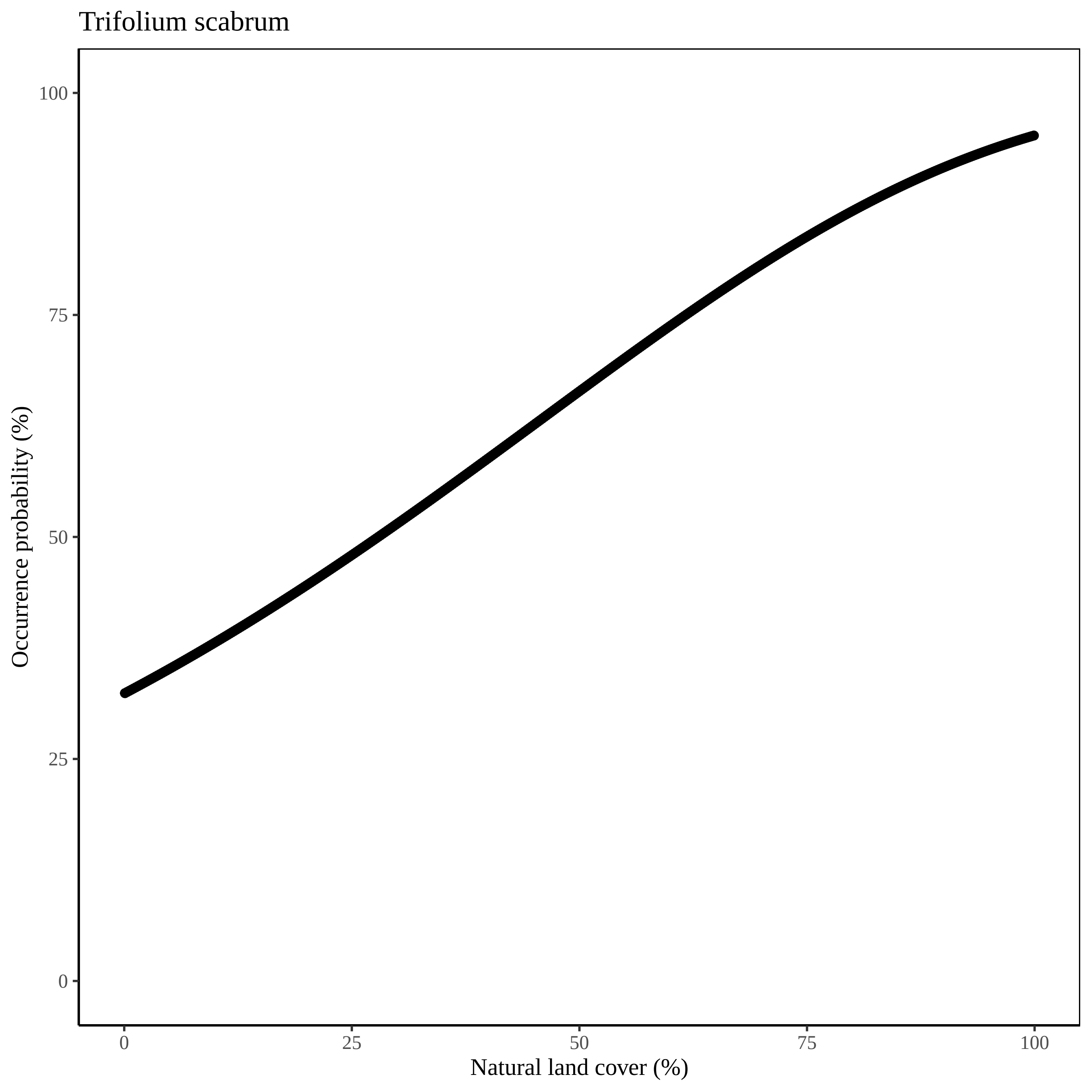
Trifolium scabrum
Occurrence probability (%)
100
75
50
25
0
0
25
50
75
100
Natural land cover (%)

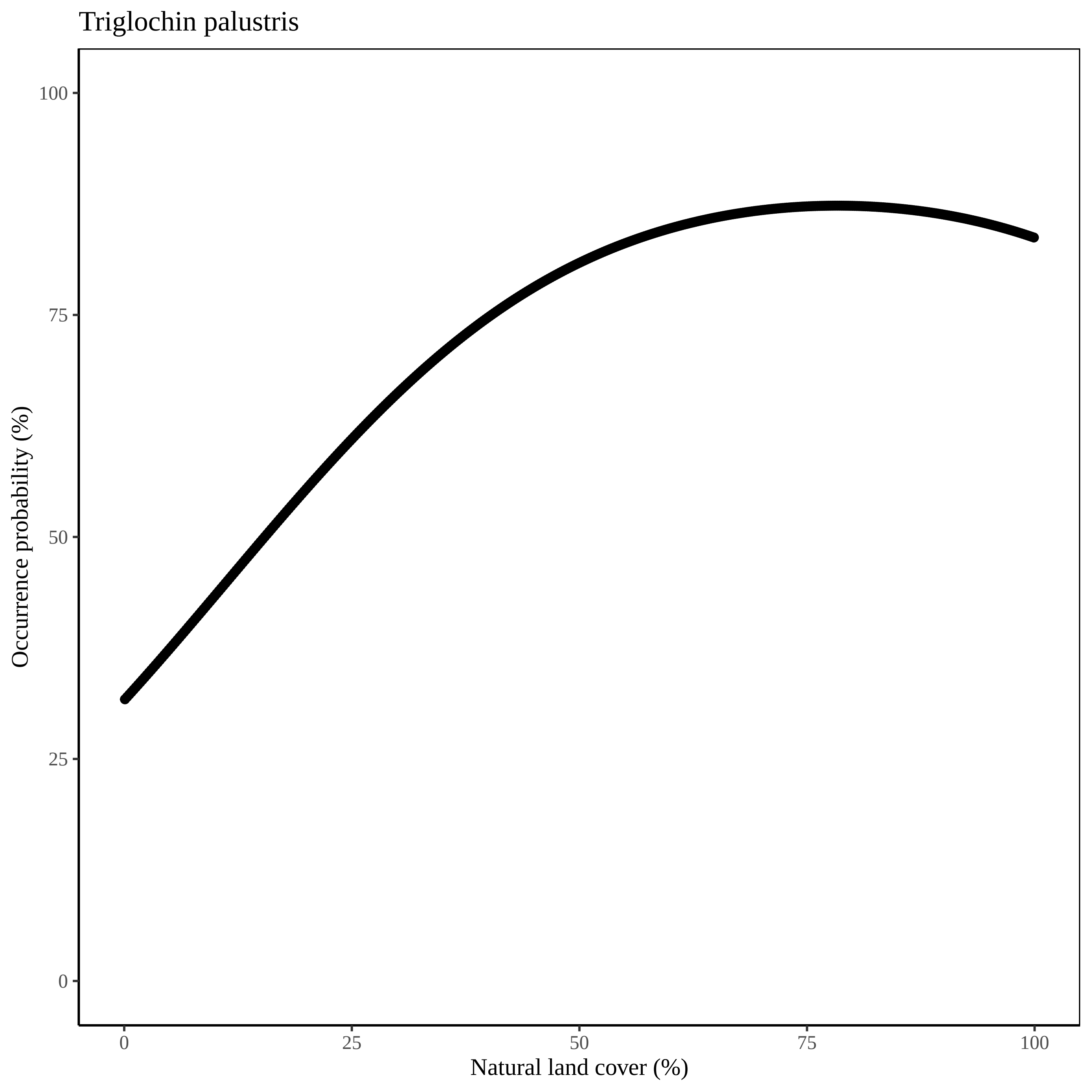
Triglochin palustris
Occurrence probability (%)
100
75
50
25
0
0
25
50
75
100
Natural land cover (%)

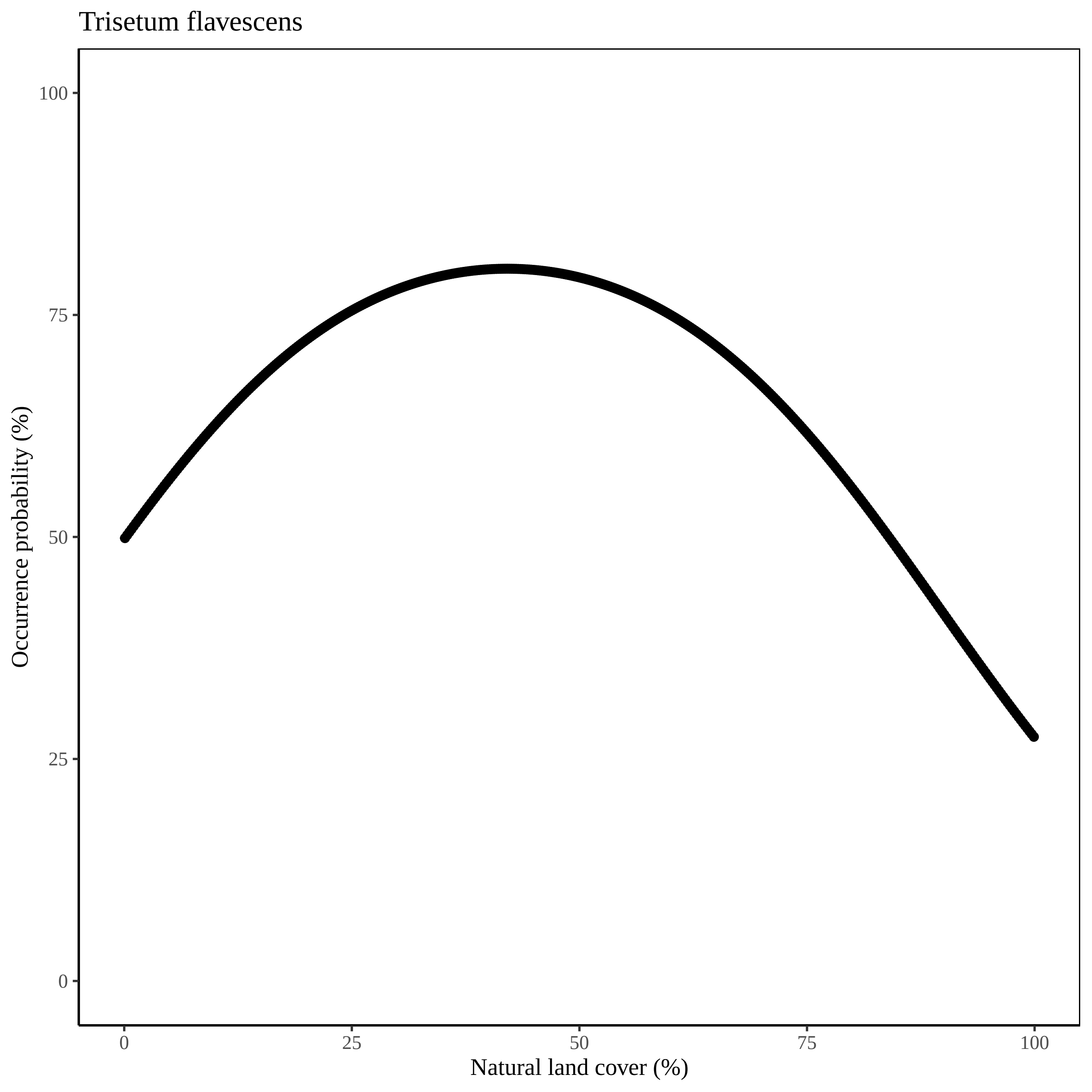
Trisetum flavescens
Occurrence probability (%)
100
75
50
25
0
0
25
50
75
100
Natural land cover (%)

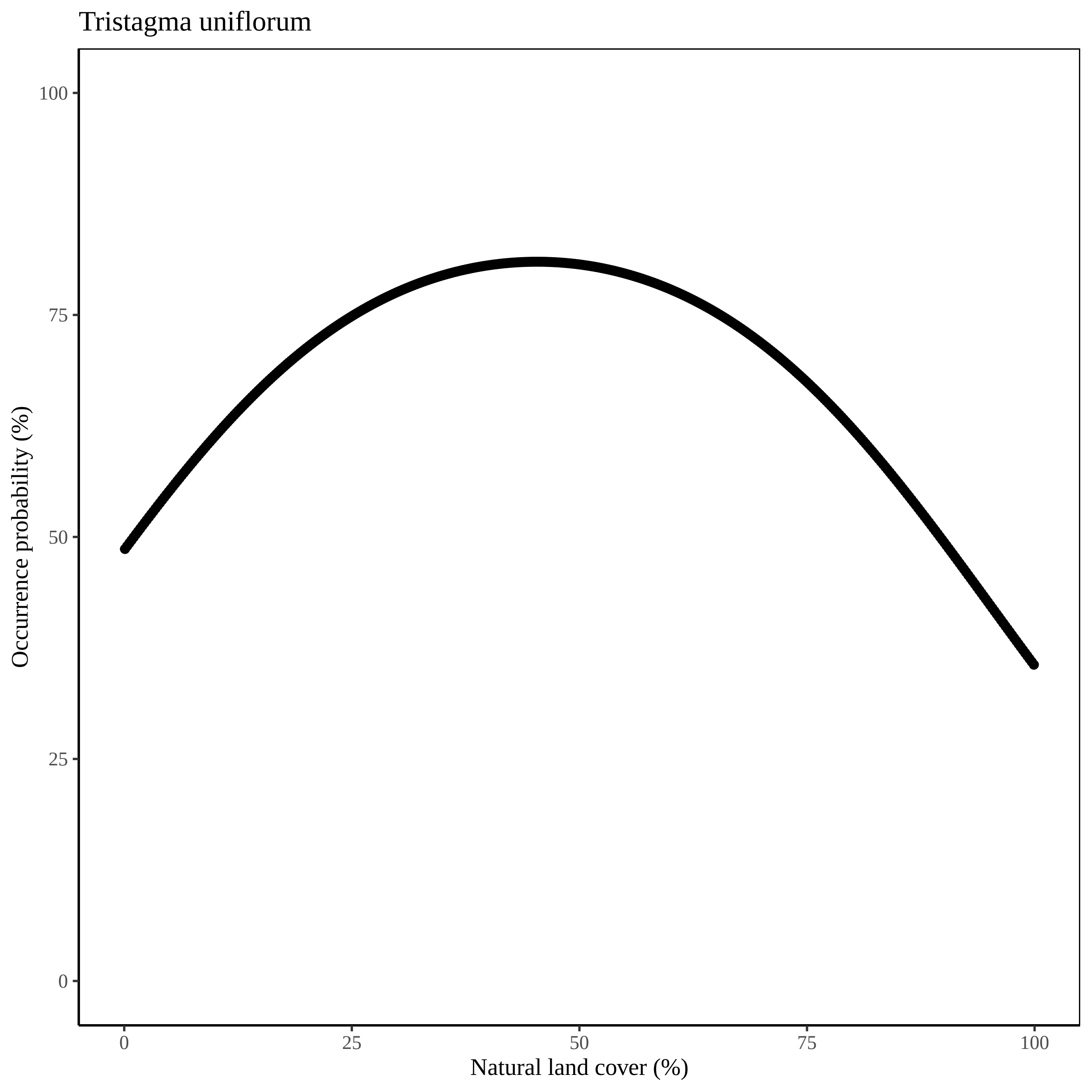

Tristagma uniflorum
Occurrence probability (%)
Natural land cover (%)
0
25
50
75
100
0
25
50
75
100

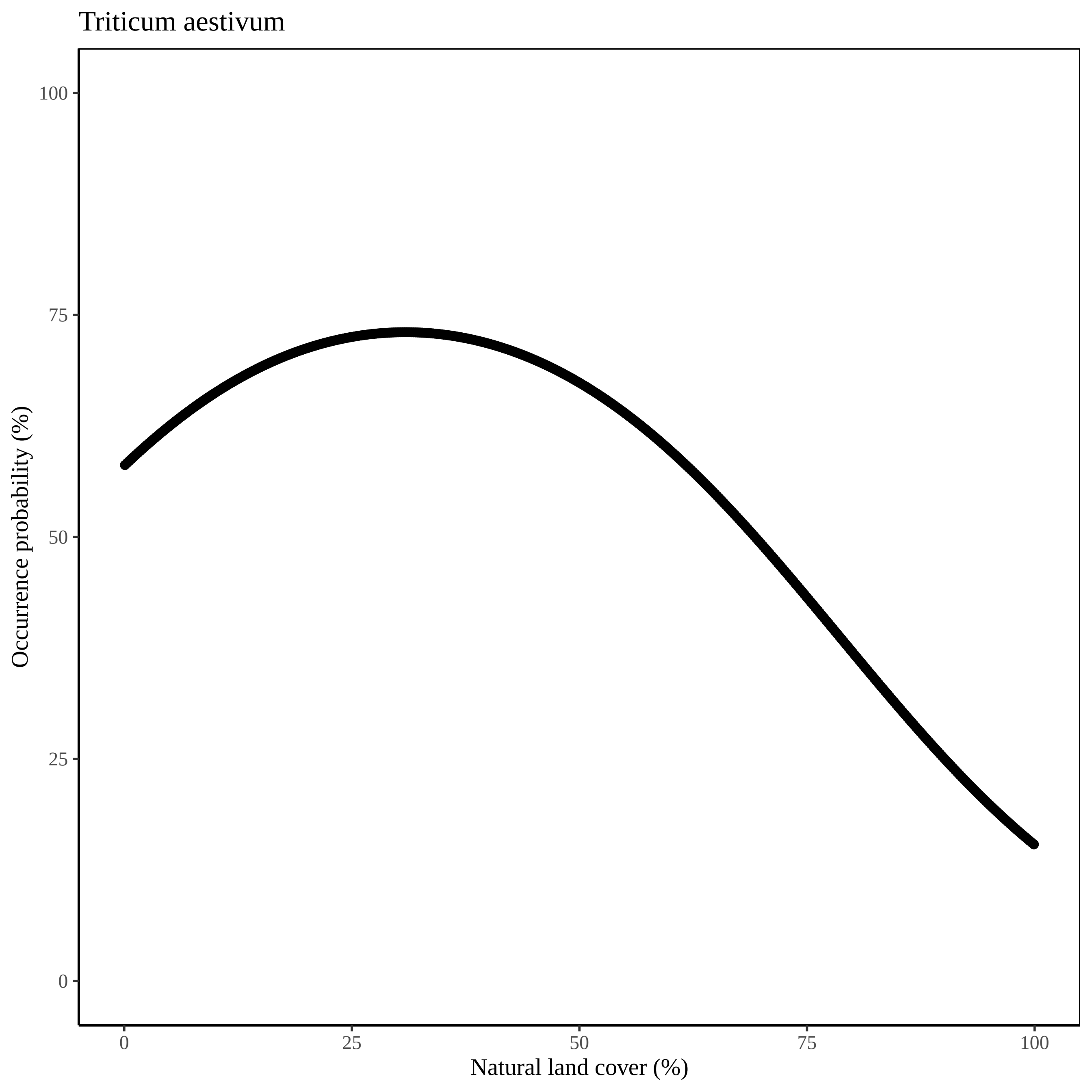

Triticum aestivum
Occurrence probability (%)
100
75
50
25
0
0
25
50
75
100
Natural land cover (%)

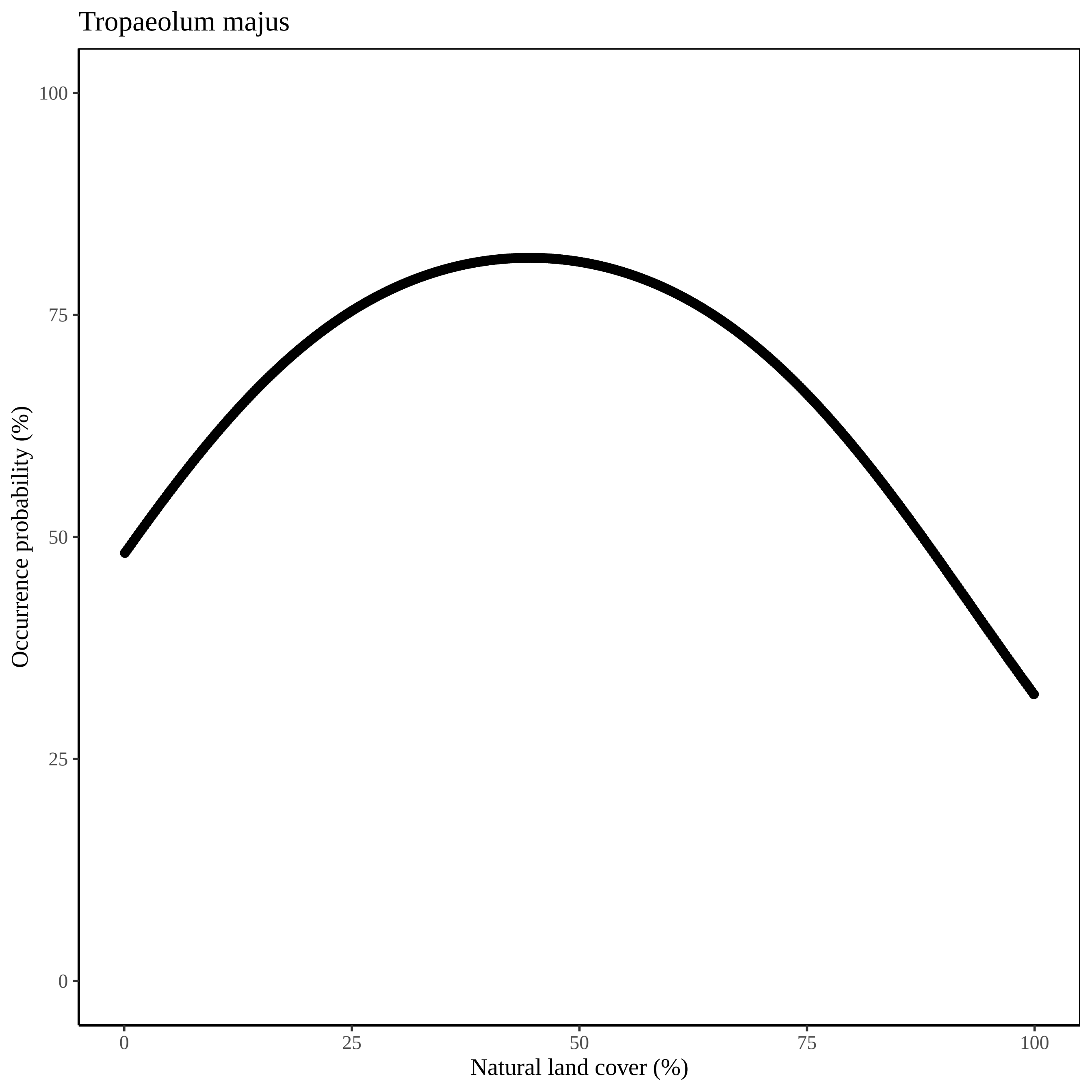

Tropaeolum majus
Occurrence probability (%)
100
75
50
25
0
0
25
50
75
100
Natural land cover (%)

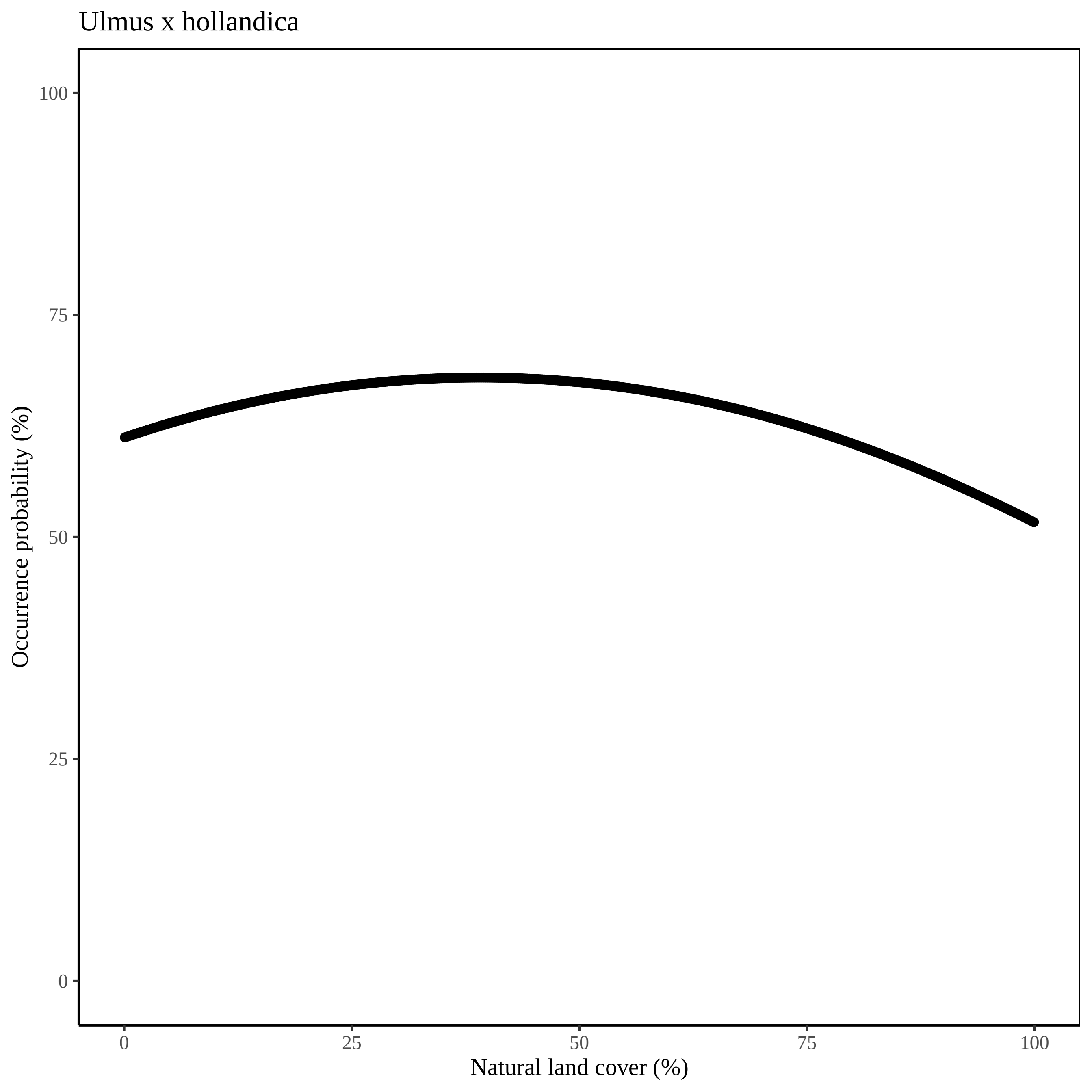
Ulmus x hollandica
Occurrence probability (%)
100
75
50
25
0
0
25
50
75
100
Natural land cover (%)

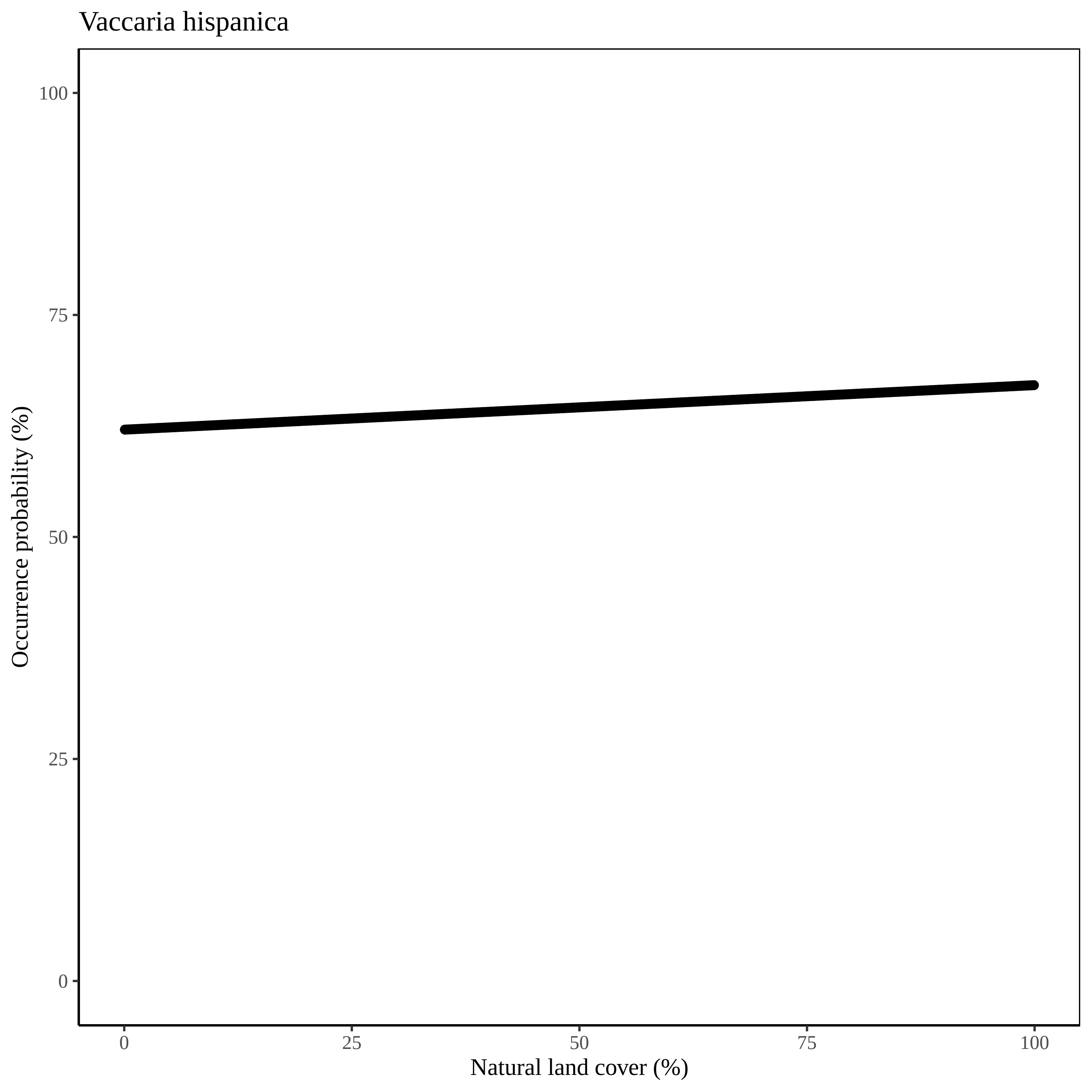

Vaccaria hispanica
100
75
50
25
0
Occurrence probability (%)
0
25
50
75
100
Natural land cover (%)

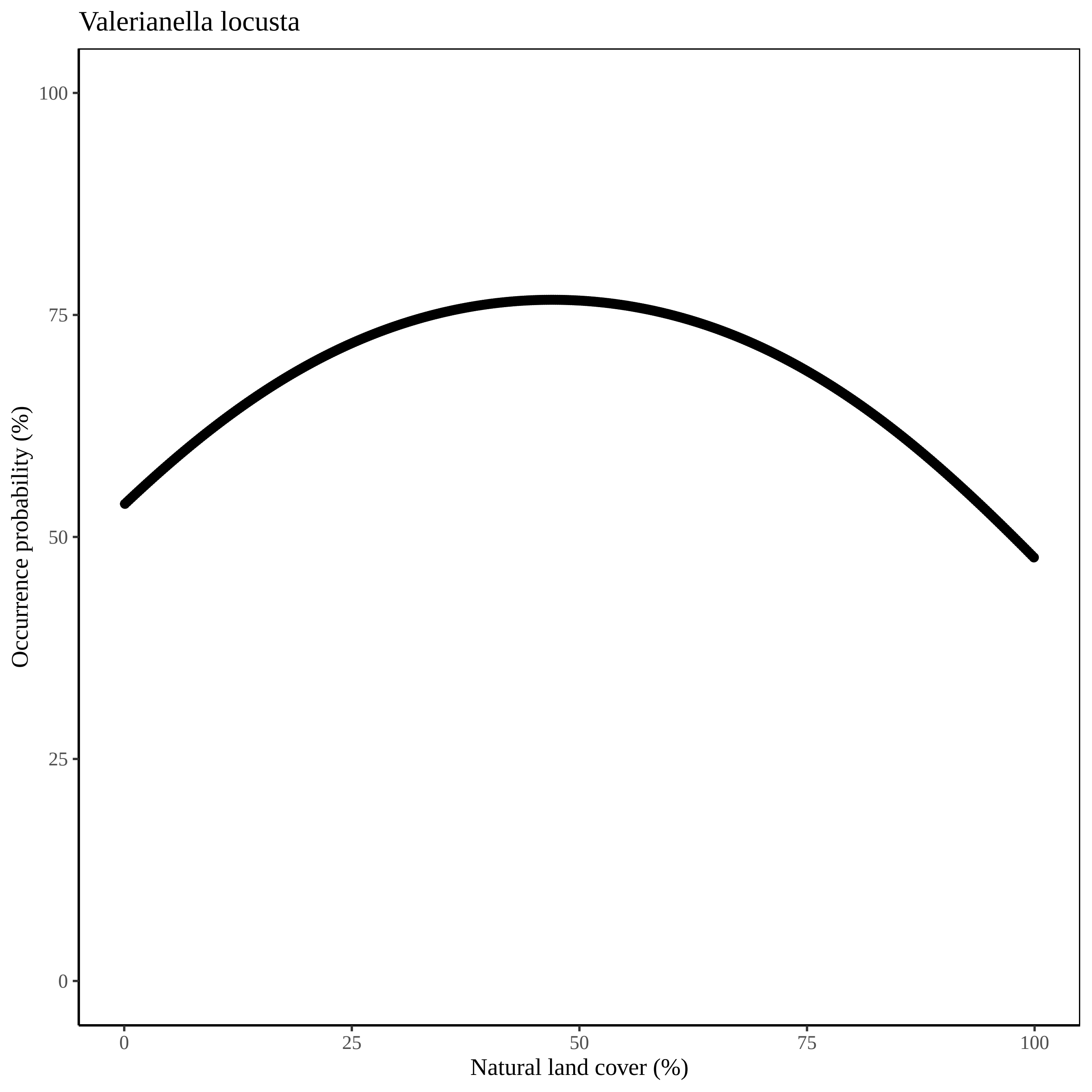

Valerianella locusta
Occurrence probability (%)
100
75
50
25
0
0
25
50
75
100
Natural land cover (%)

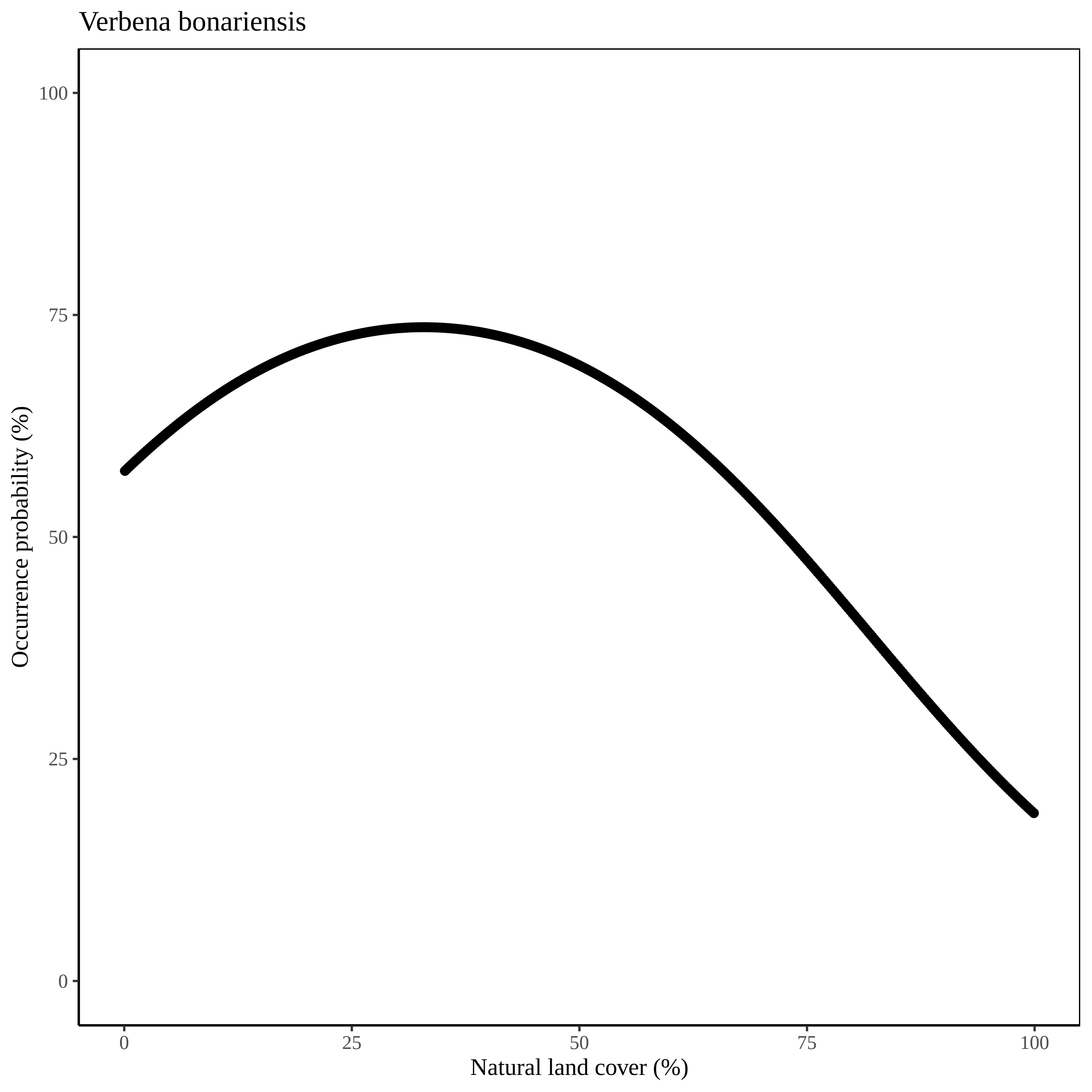
Verbena bonariensis
Occurrence probability (%)
100
75
50
25
0
0
25
50
75
100
Natural land cover (%)

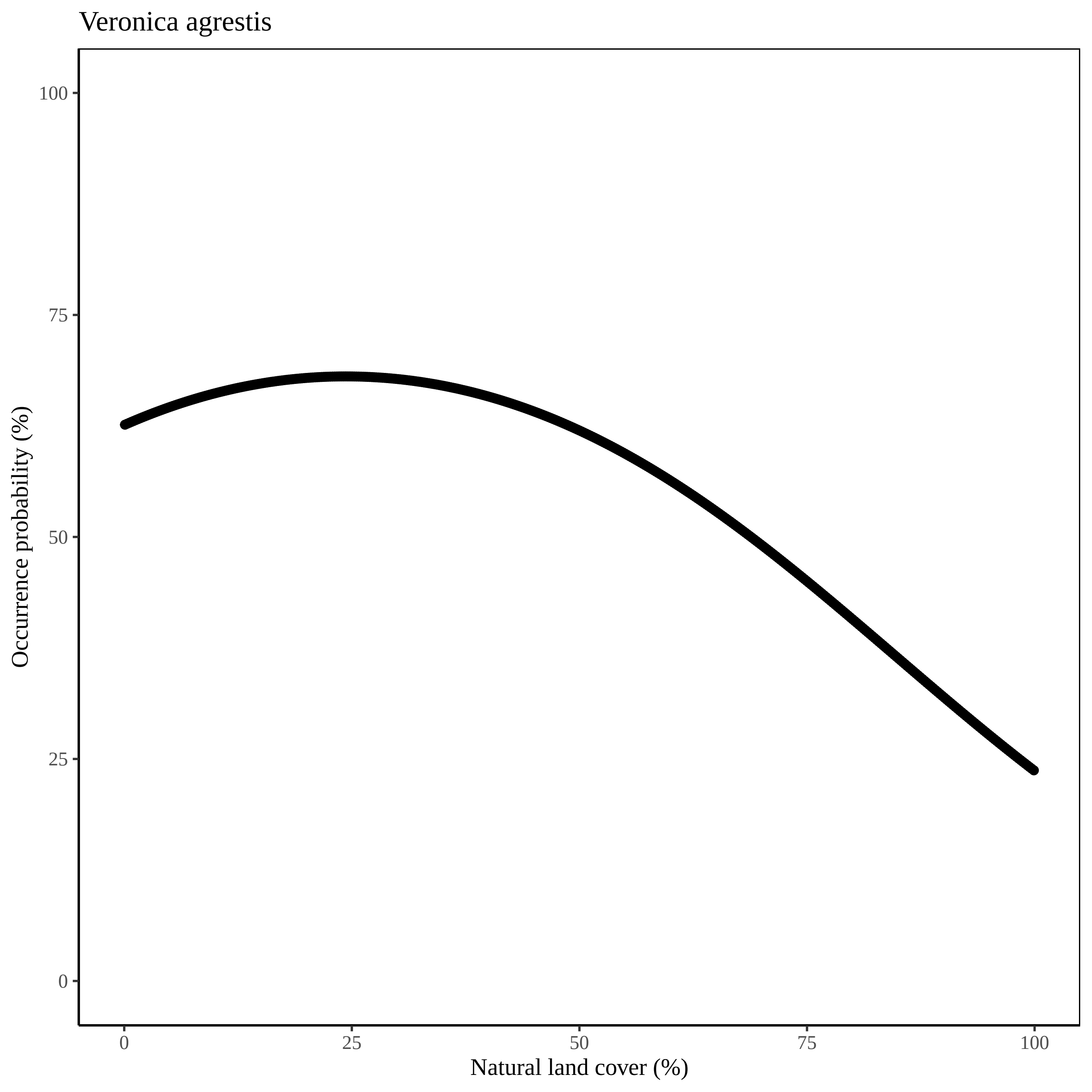

Veronica agrestis
100
75
50
25
0
0
25
50
75
100
Occurrence probability (%)
Natural land cover (%)

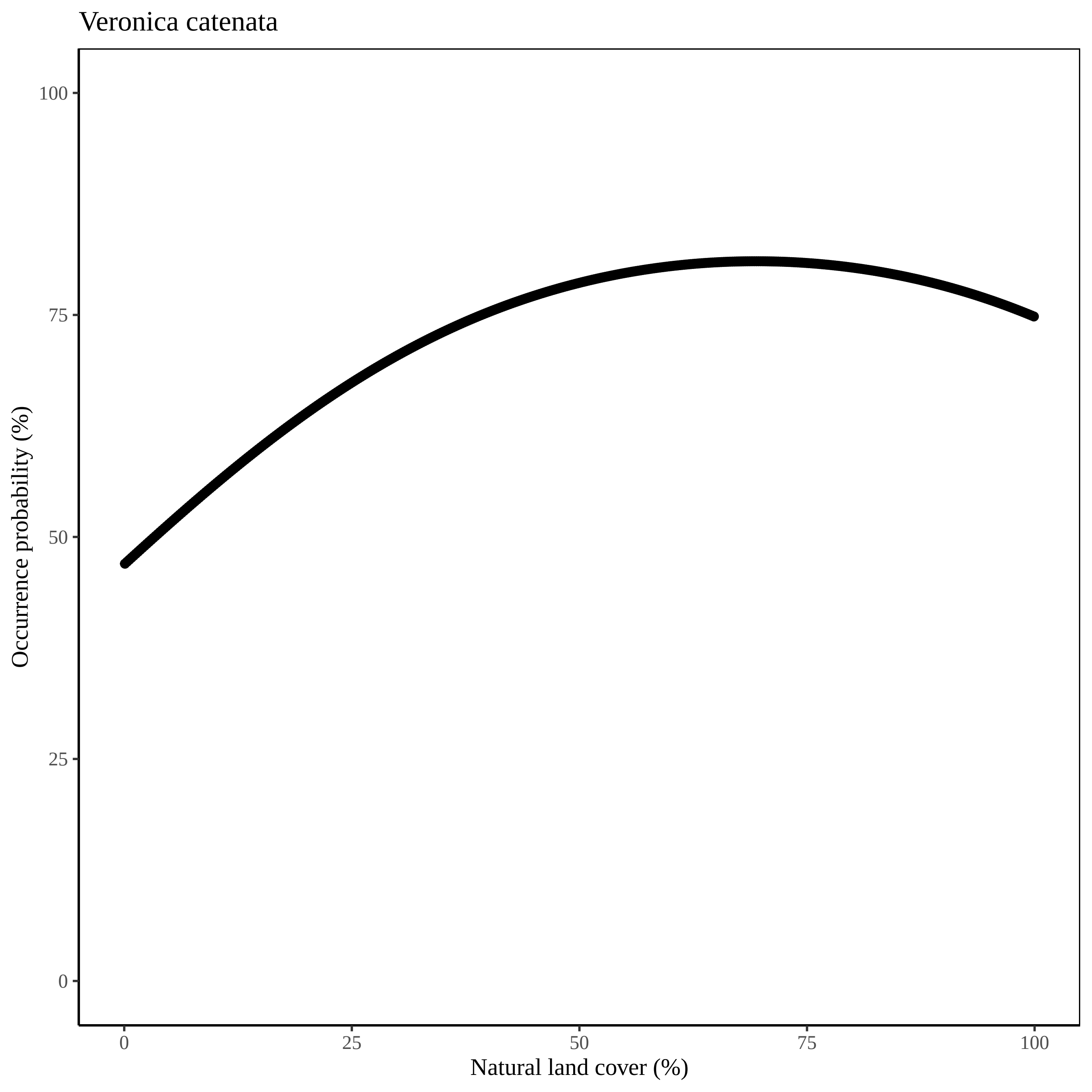

Veronica catenata
Occurrence probability (%)
100
75
50
25
0
0
25
50
75
100
Natural land cover (%)

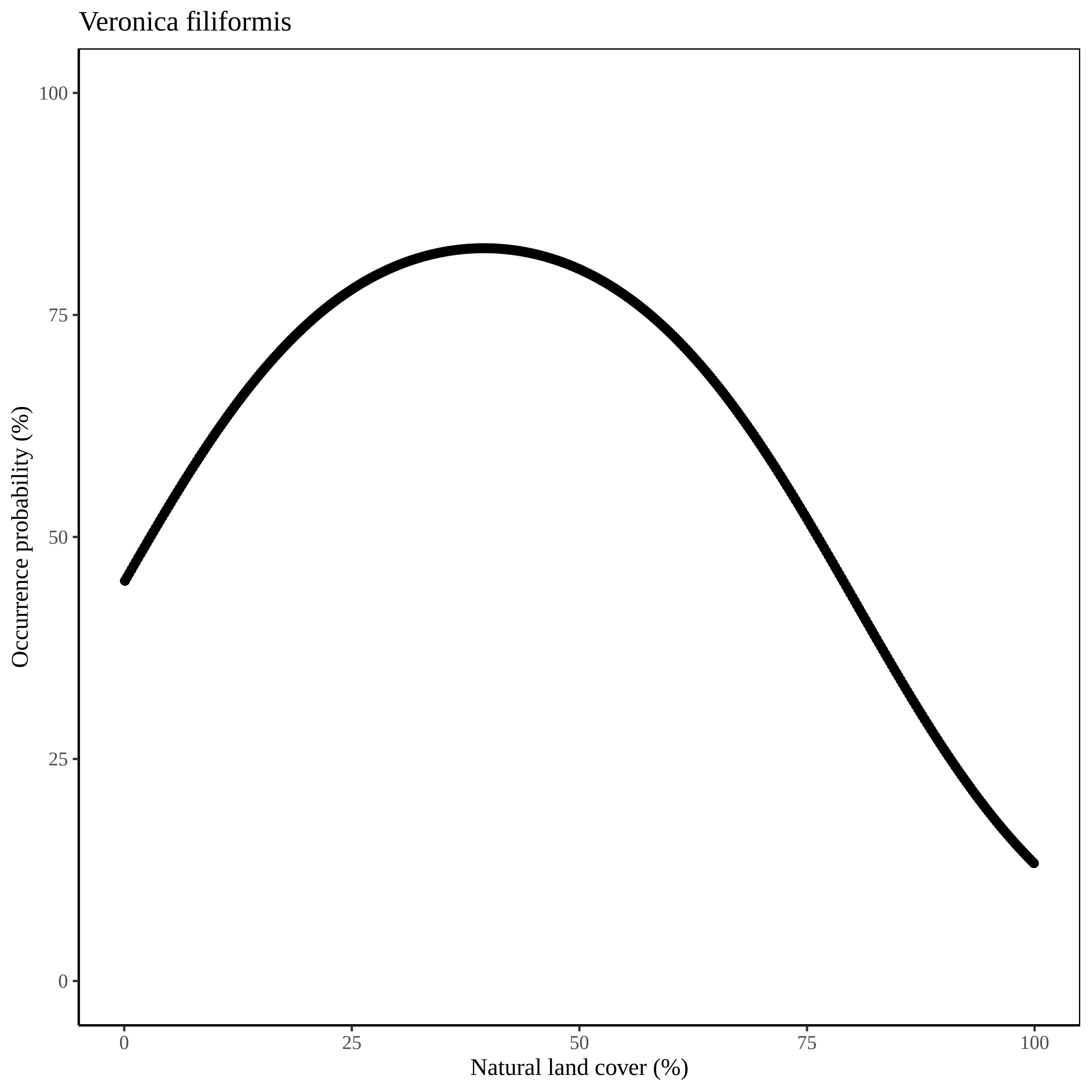
Veronica filiformis
Occurrence probability (%)
100
75
50
25
0
0
25
50
75
100
Natural land cover (%)

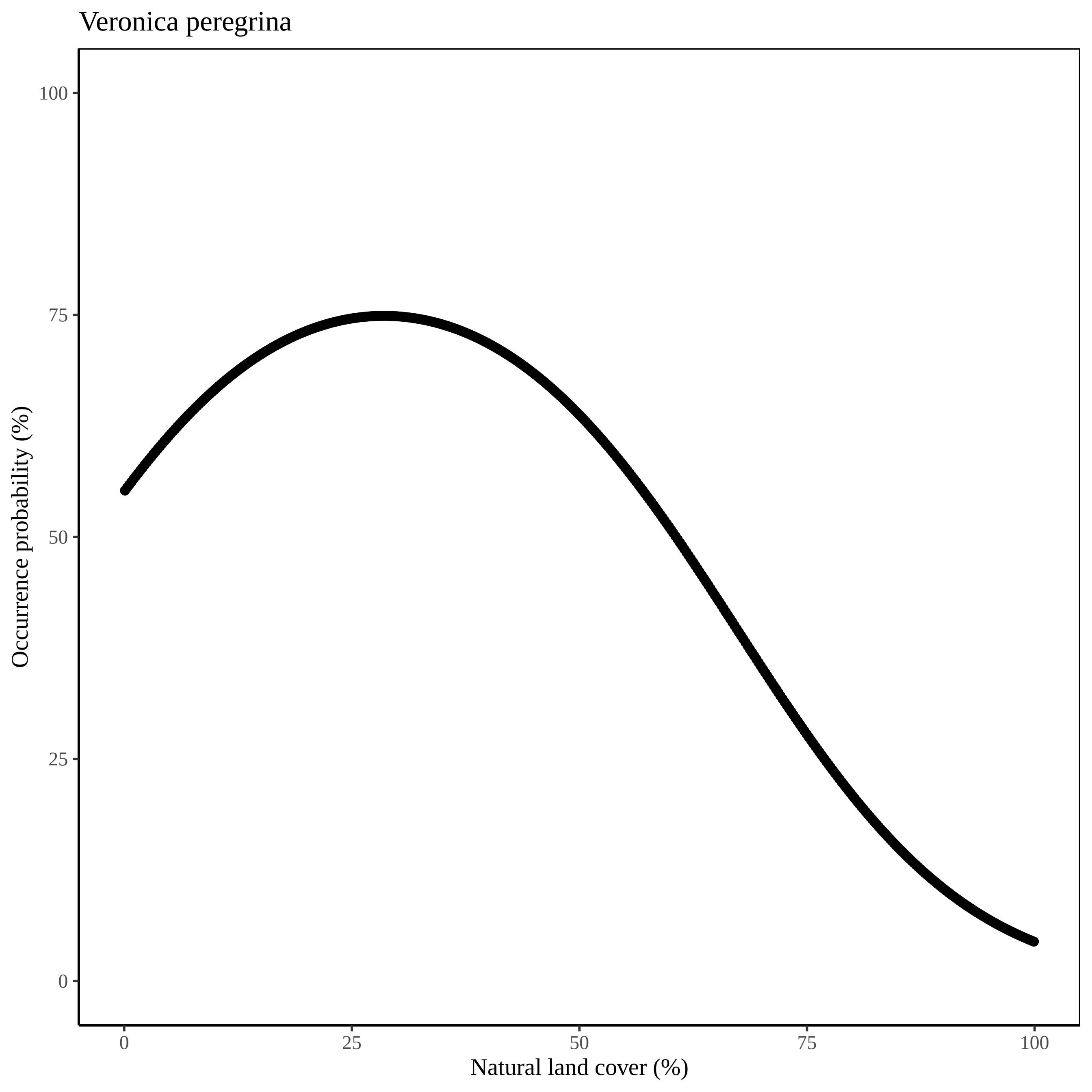

Veronica peregrina
Occurrence probability (%)
100
75
50
25
0
0
25
50
75
100
Natural land cover (%)

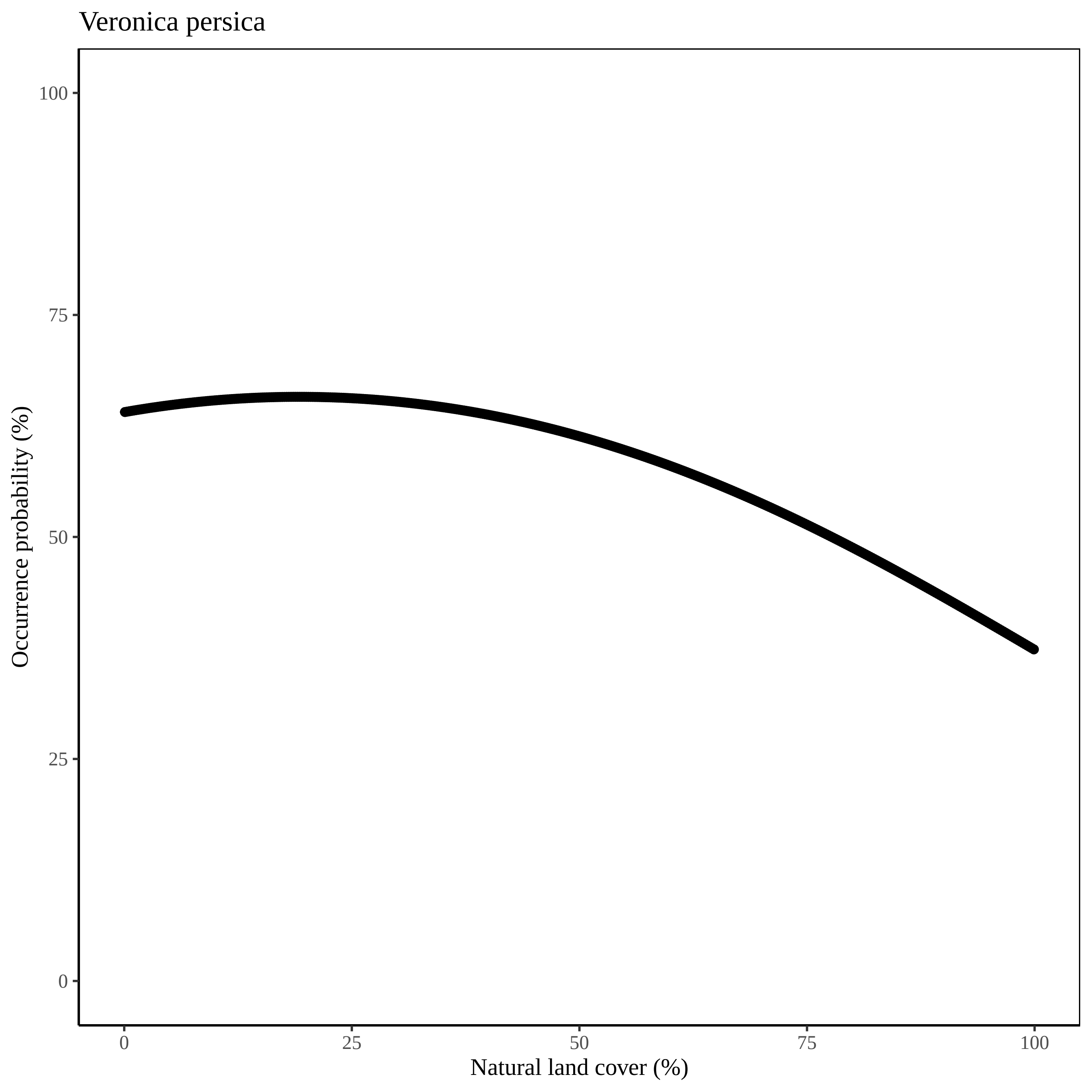
Veronica persica
Occurrence probability (%)
100
75
50
25
0
0
25
50
75
100
Natural land cover (%)

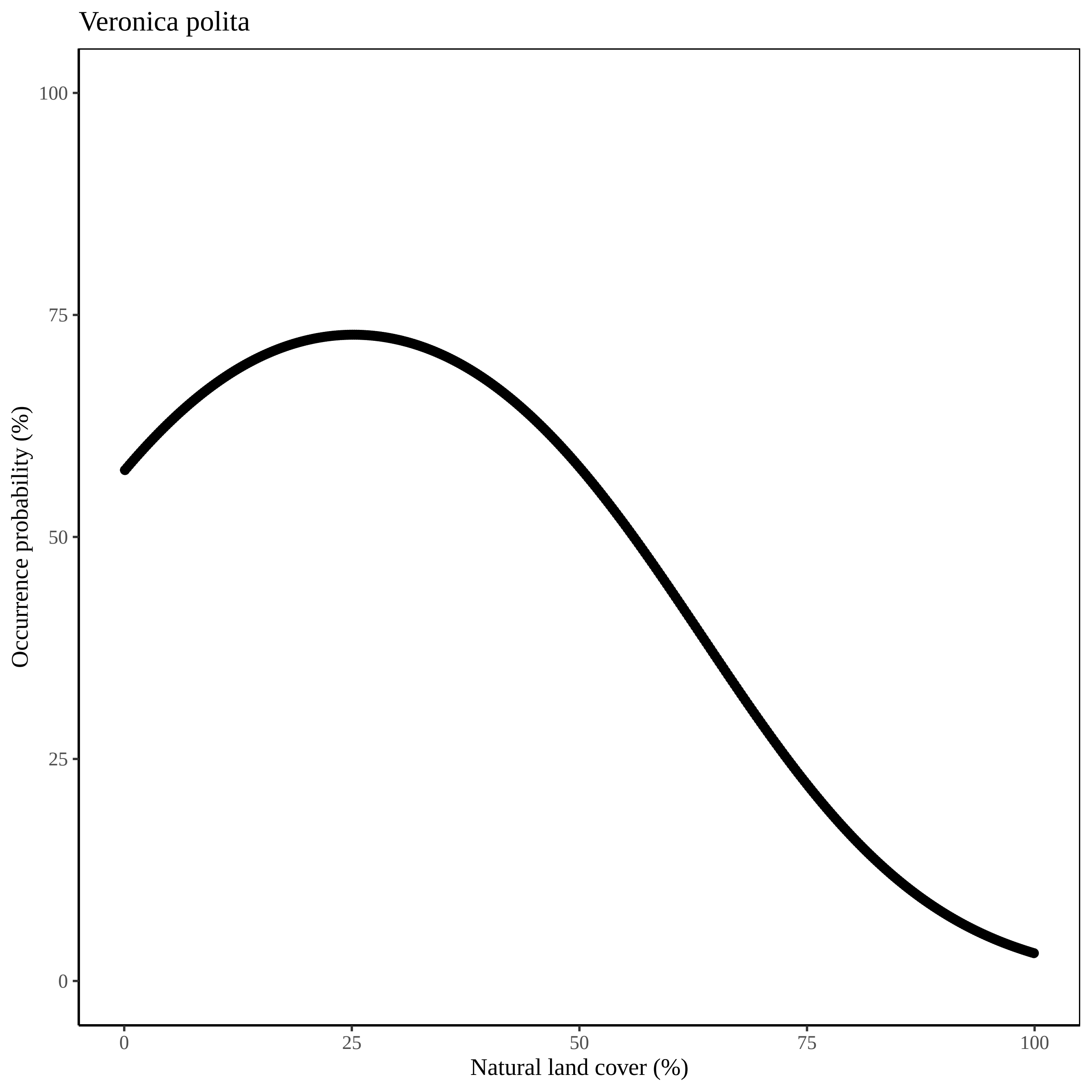
Veronica polita
Occurrence probability (%)
100
75
50
25
0
0
25
50
75
100
Natural land cover (%)

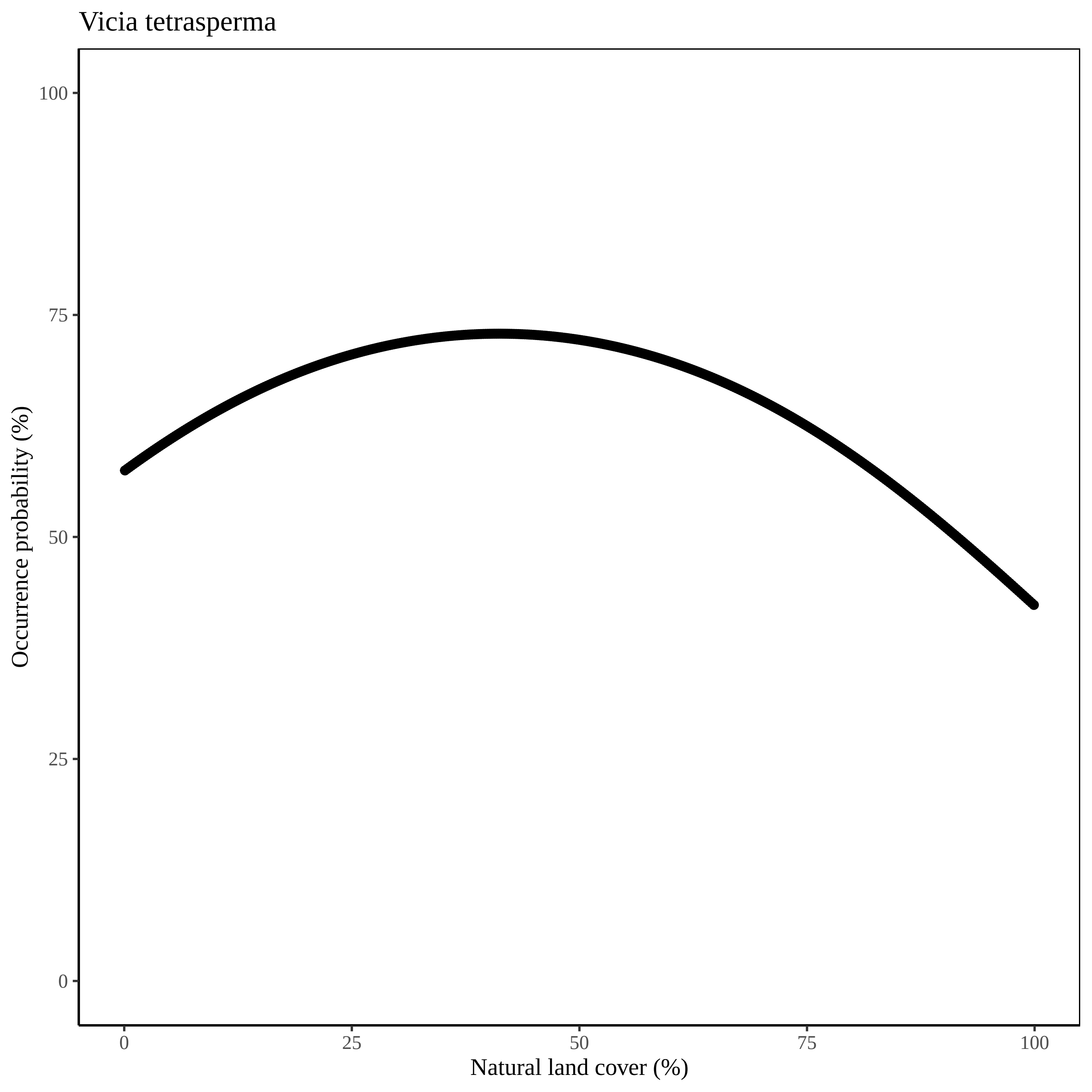

Vicia tetrasperma
Occurrence probability (%)
100
75
50
25
0
0
25
50
75
100
Natural land cover (%)

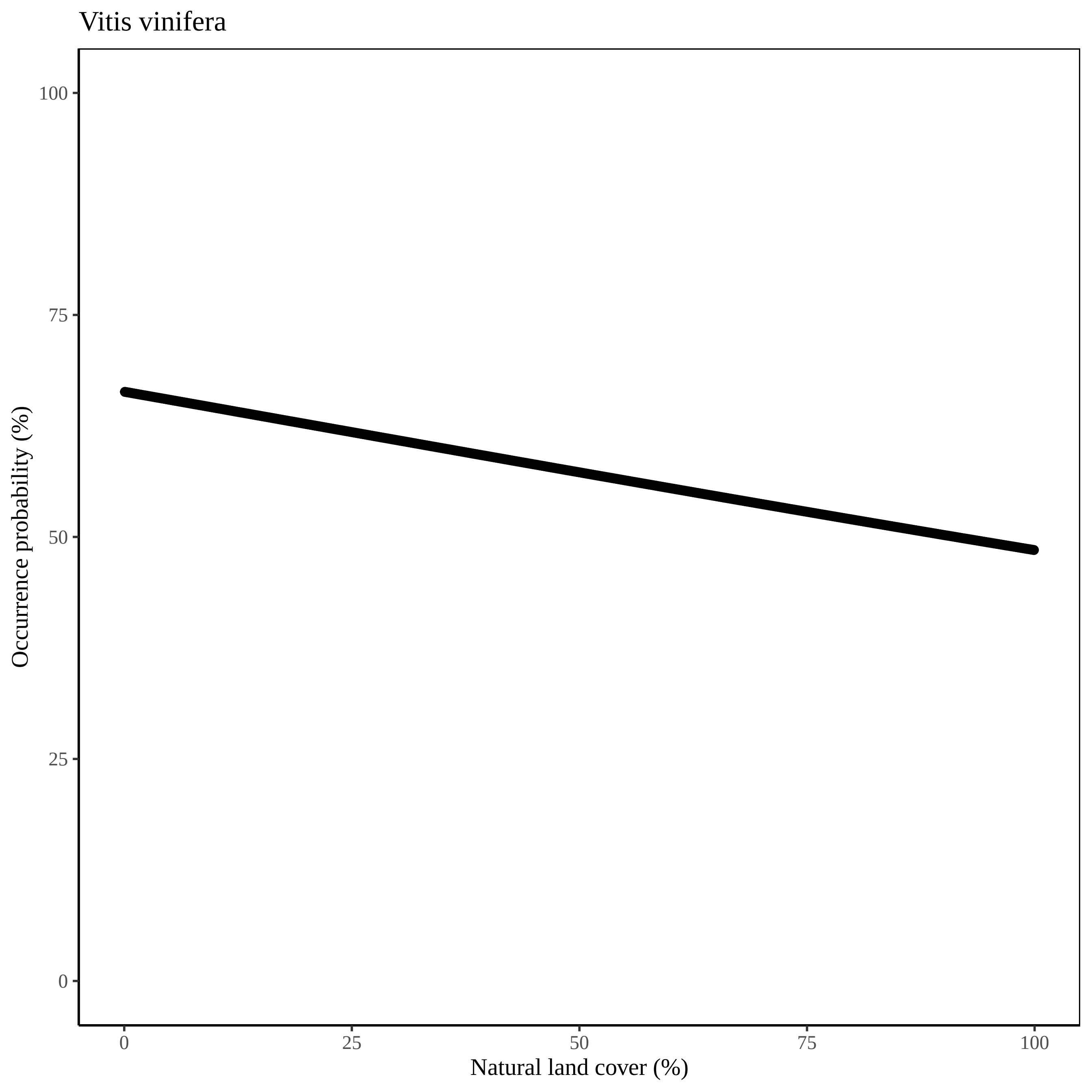
Vitis vinifera
Occurrence probability (%)
100
75
50
25
0
0
25
50
75
100
Natural land cover (%)

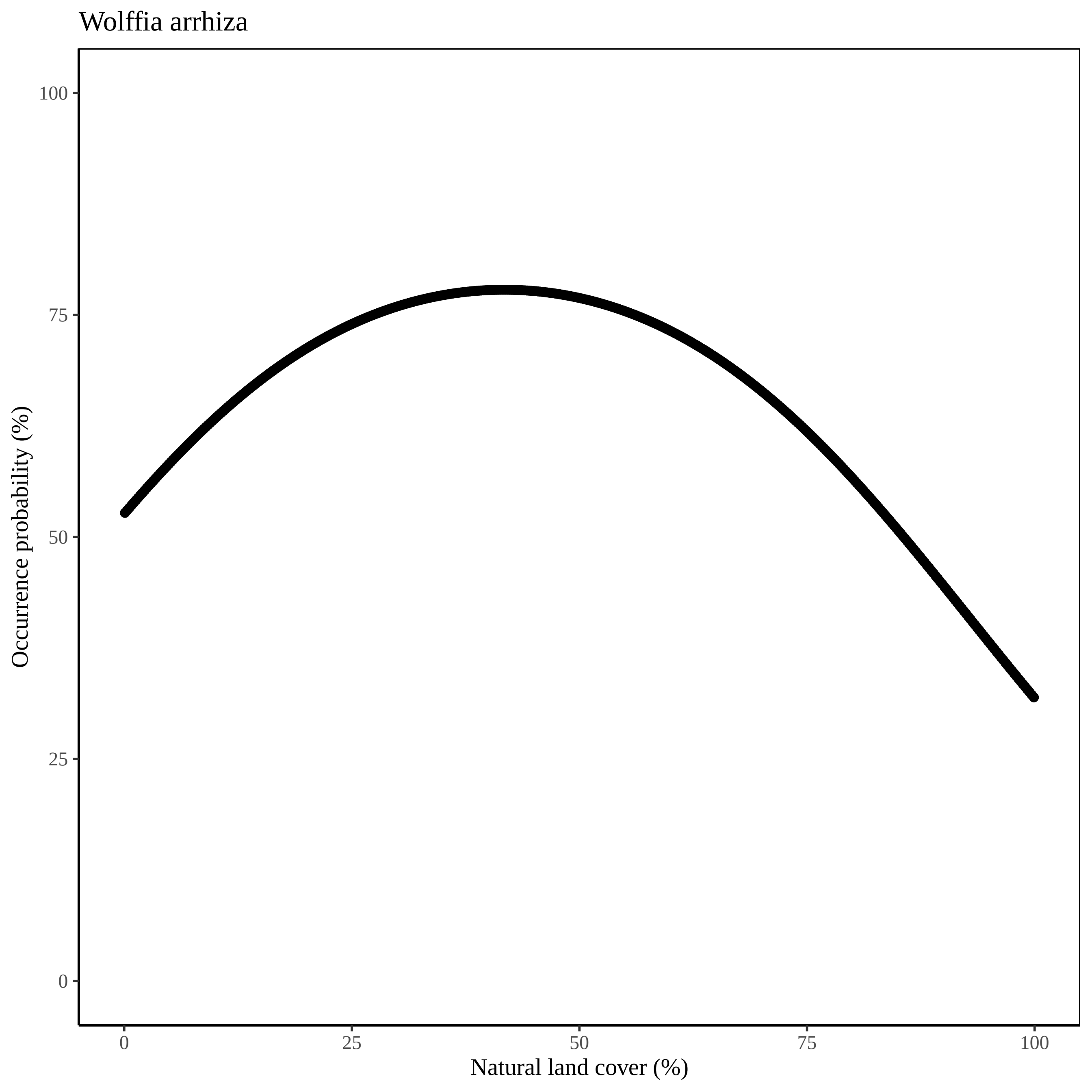
Wolffia arrhiza
Occurrence probability (%)
100
75
50
25
0
0
25
50
75
100
Natural land cover (%)

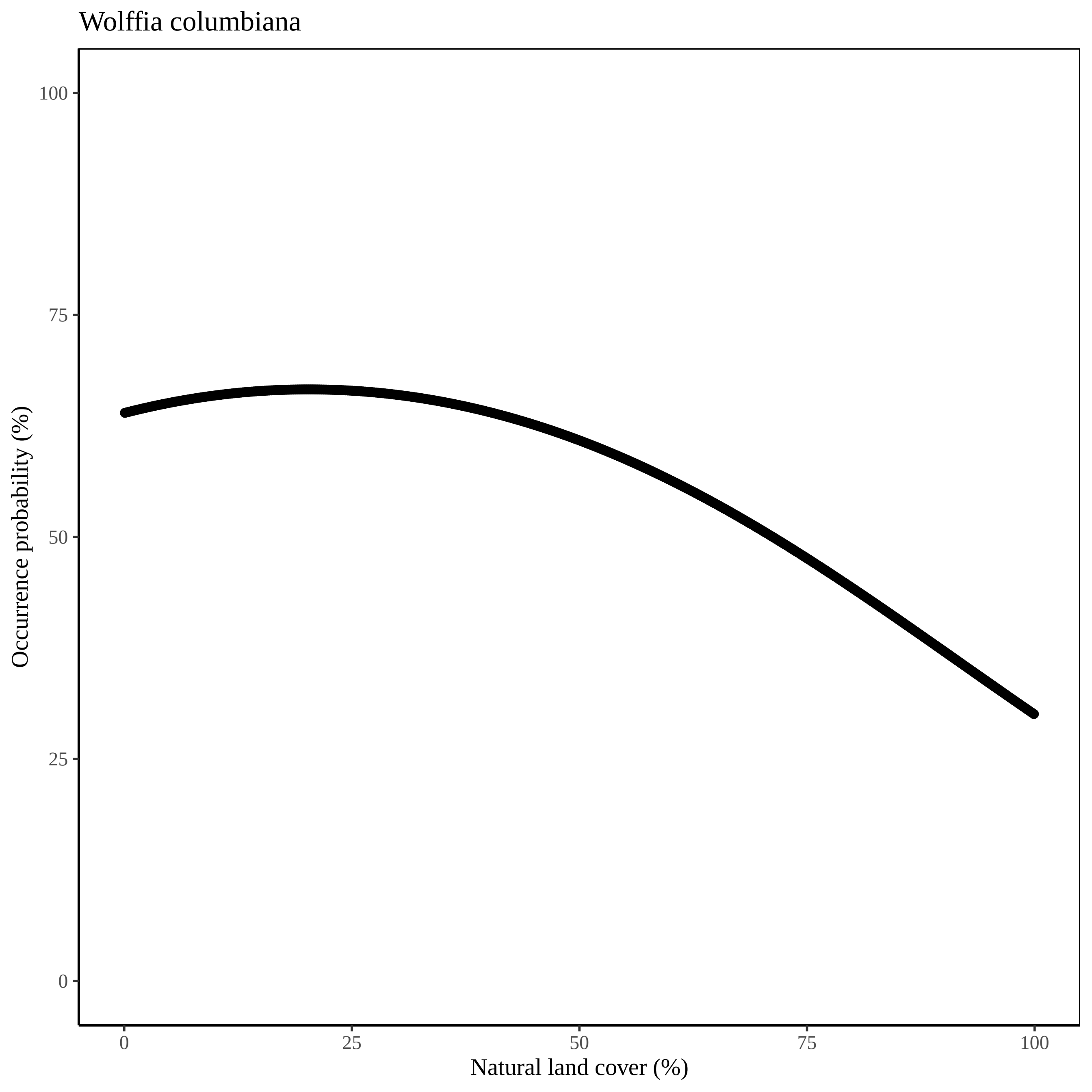
Wolffia columbiana
Occurrence probability (%)
100
75
50
25
0
0
25
50
75
100
Natural land cover (%)

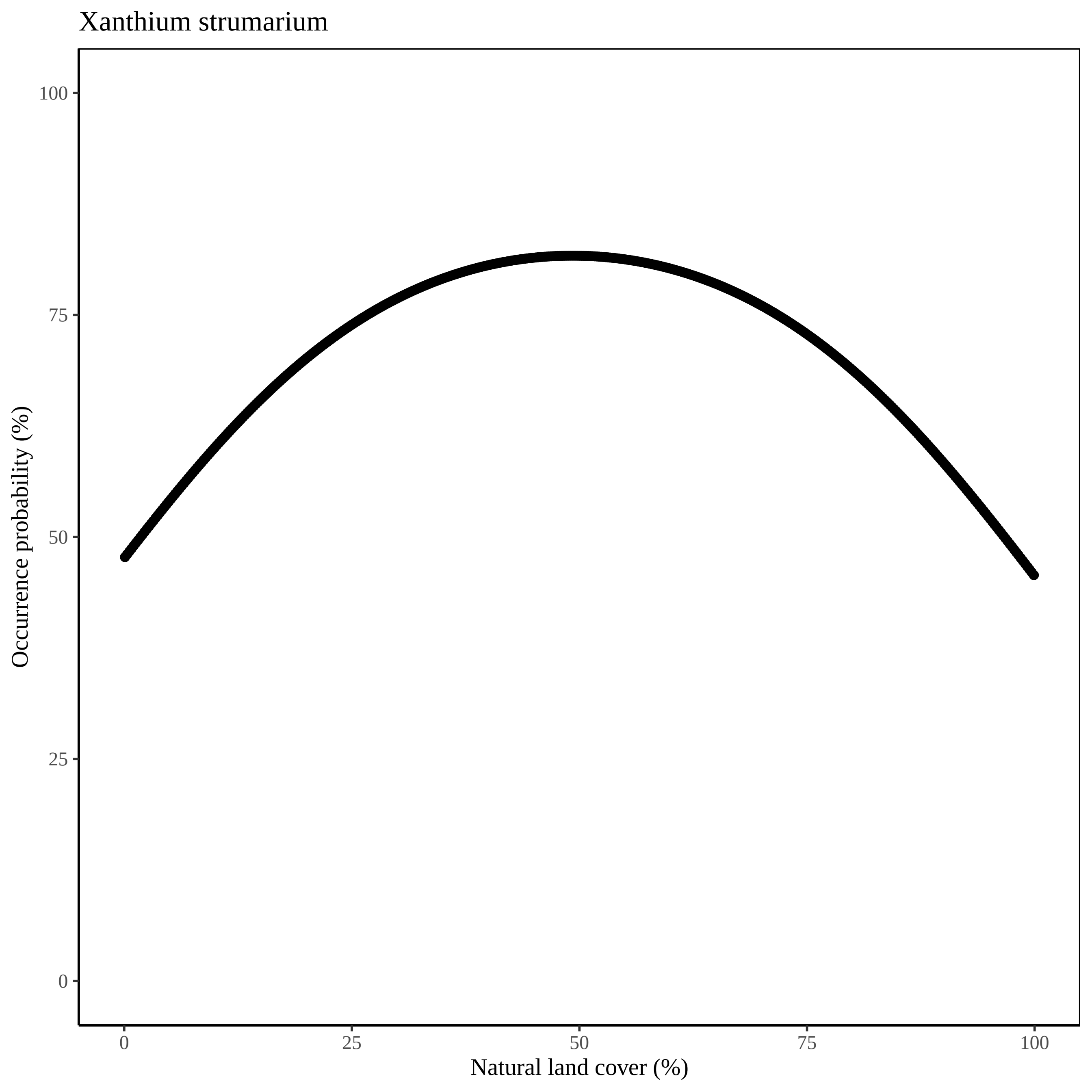
Xanthium strumarium
Occurrence probability (%)
100
75
50
25
0
0
25
50
75
100
Natural land cover (%)

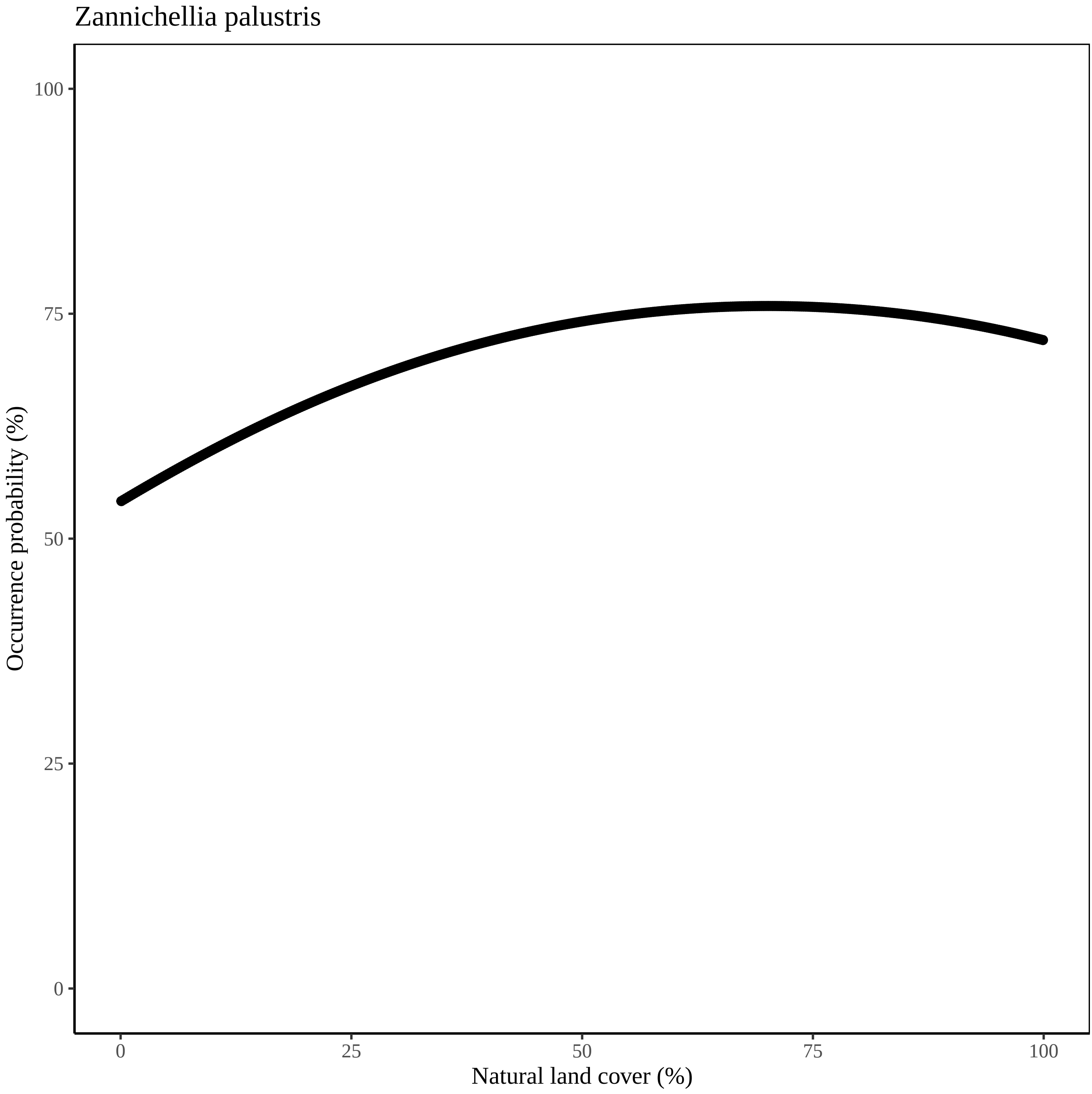

Zannichellia palustris
Occurrence probability (%)
100
75
50
25
0
0
25
50
75
100
Natural land cover (%)

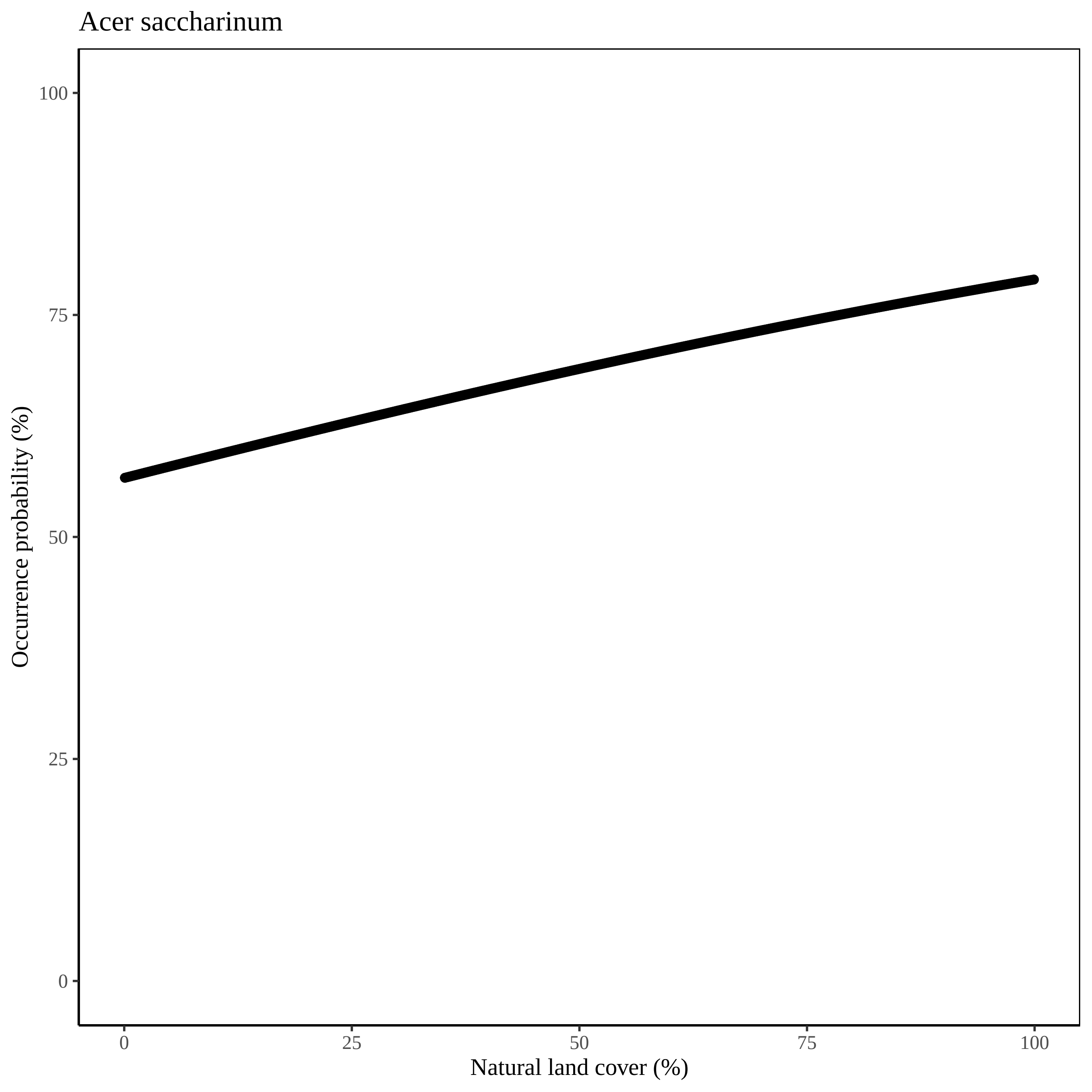

Acer saccharinum
Occurrence probability (%)
100
75
50
25
0
0
25
50
75
100
Natural land cover (%)

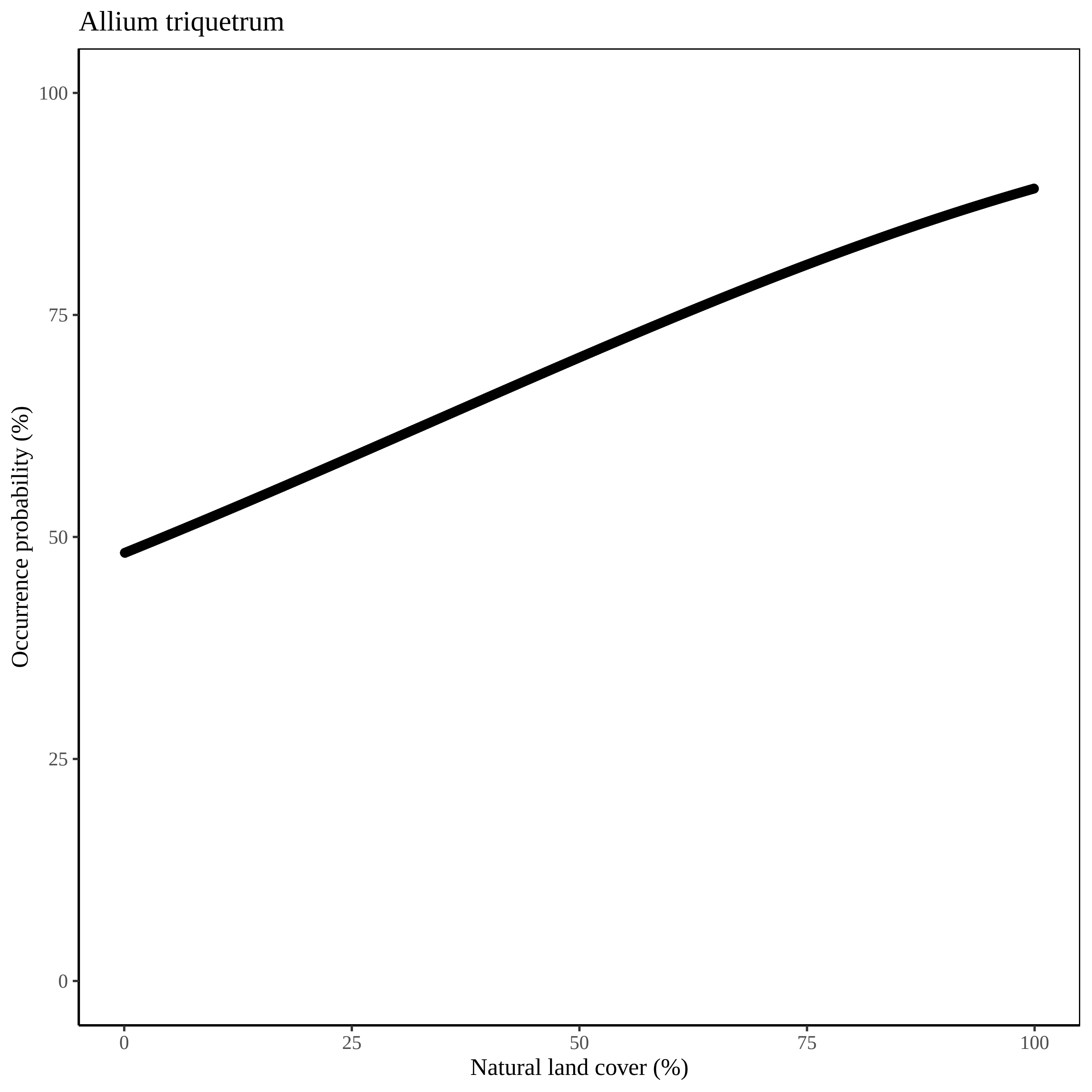

Allium triquetrum
Occurrence probability (%)
100
75
50
25
0
Natural land cover (%)
0
25
50
75
100

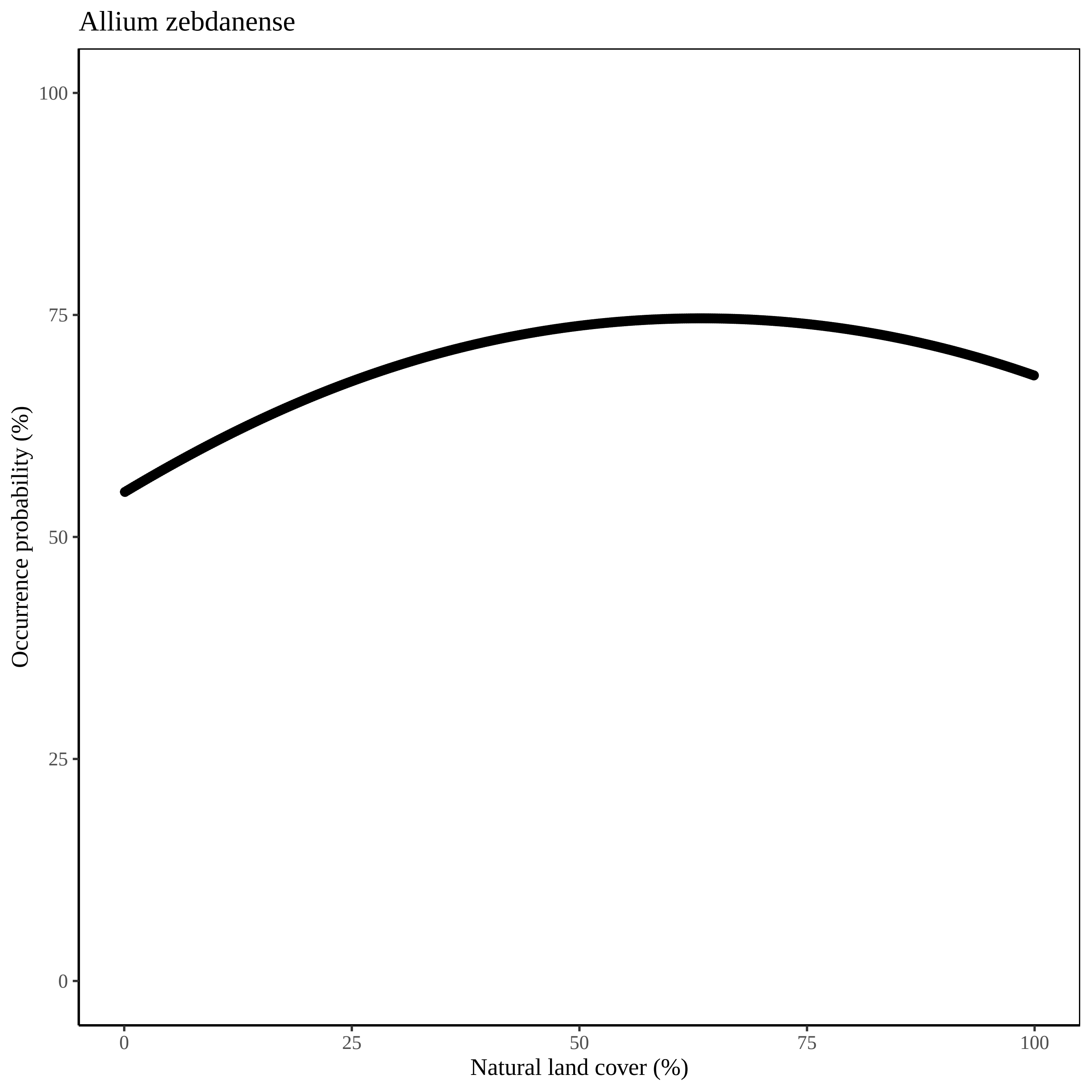
Allium zebdanense
Occurrence probability (%)
100
75
50
25
0
0
25
50
75
100
Natural land cover (%)

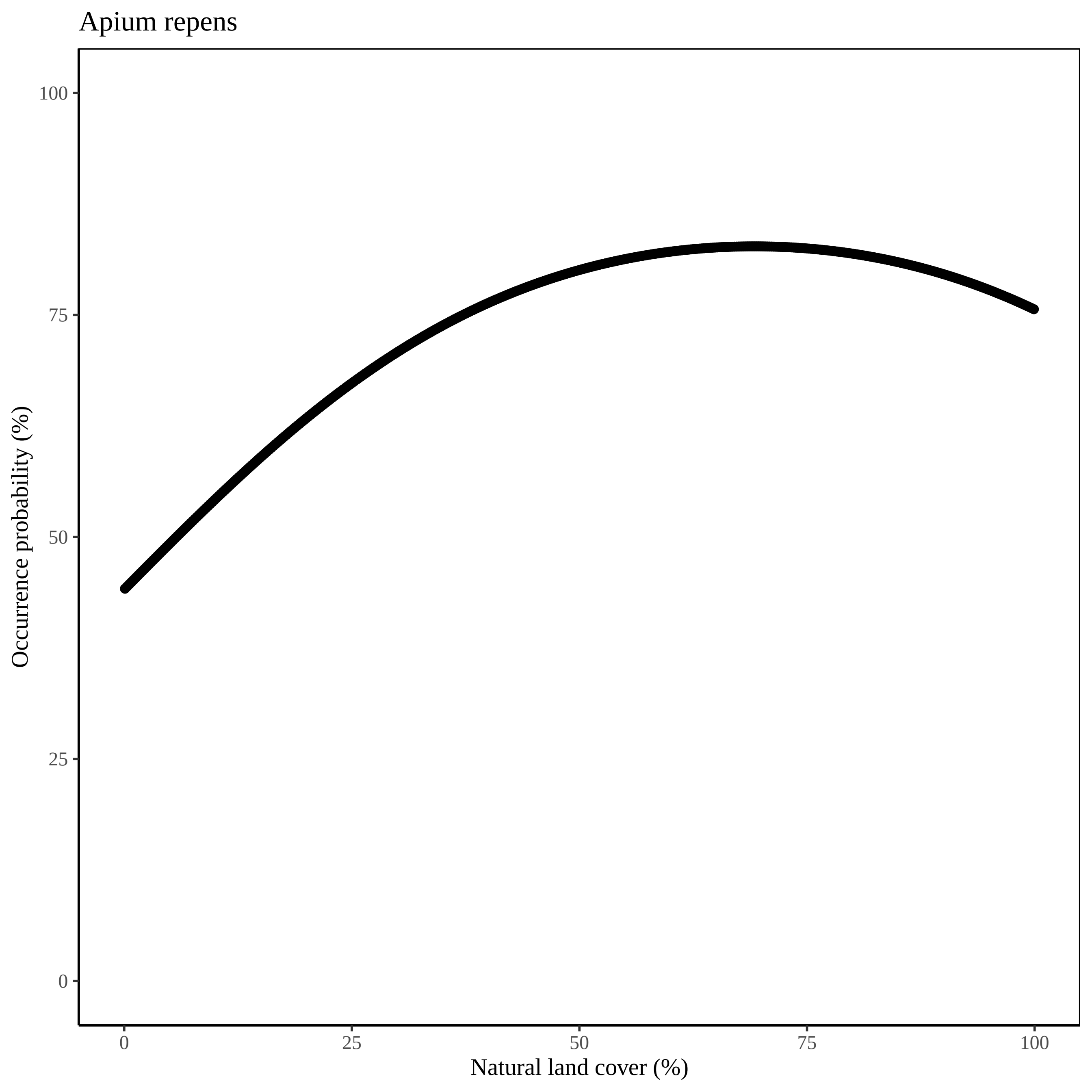
Apium repens
Occurrence probability (%)
100
75
50
25
0
0
25
50
75
100
Natural land cover (%)

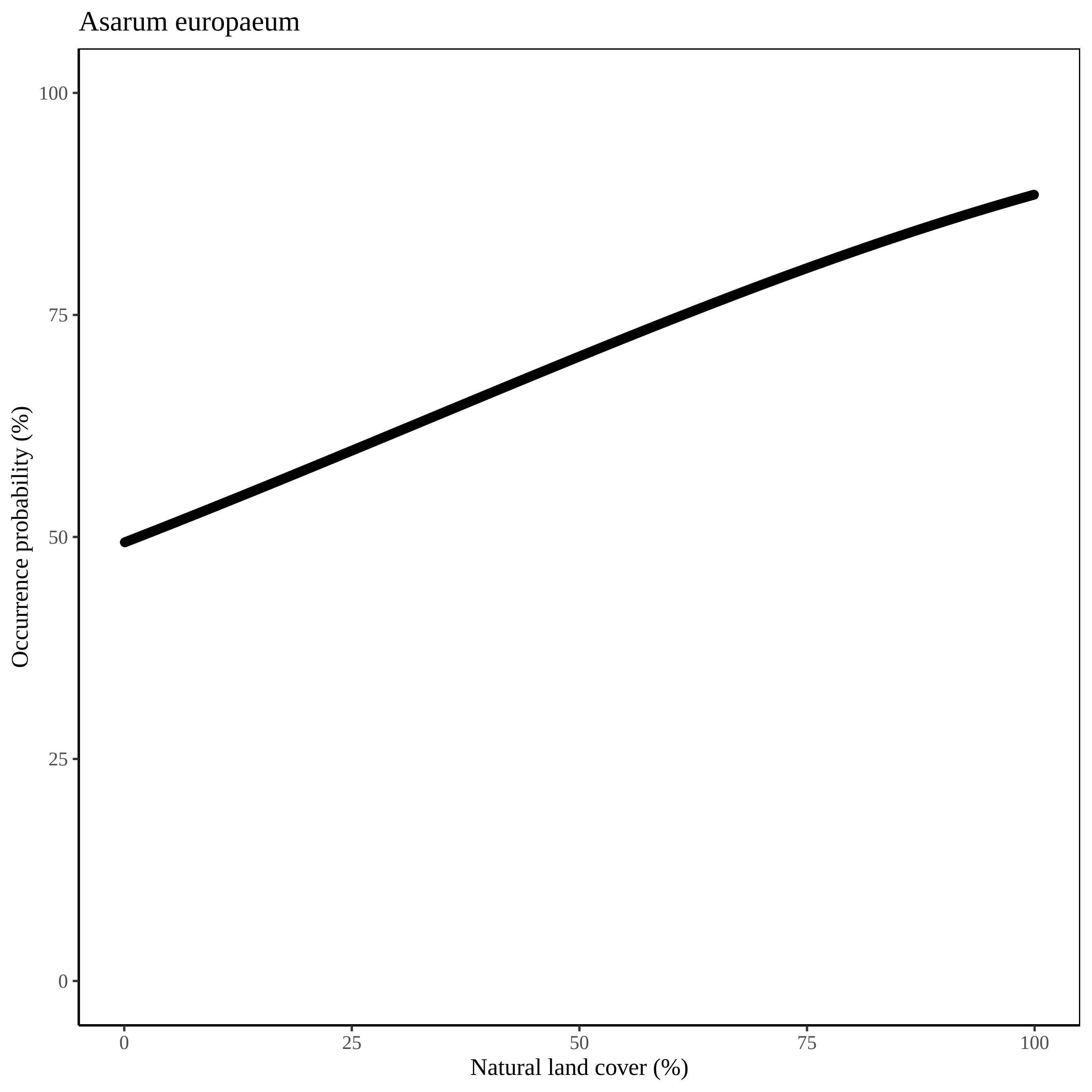
Asarum europaeum
Occurrence probability (%)
100
75
50
25
0
0
25
50
75
100
Natural land cover (%)

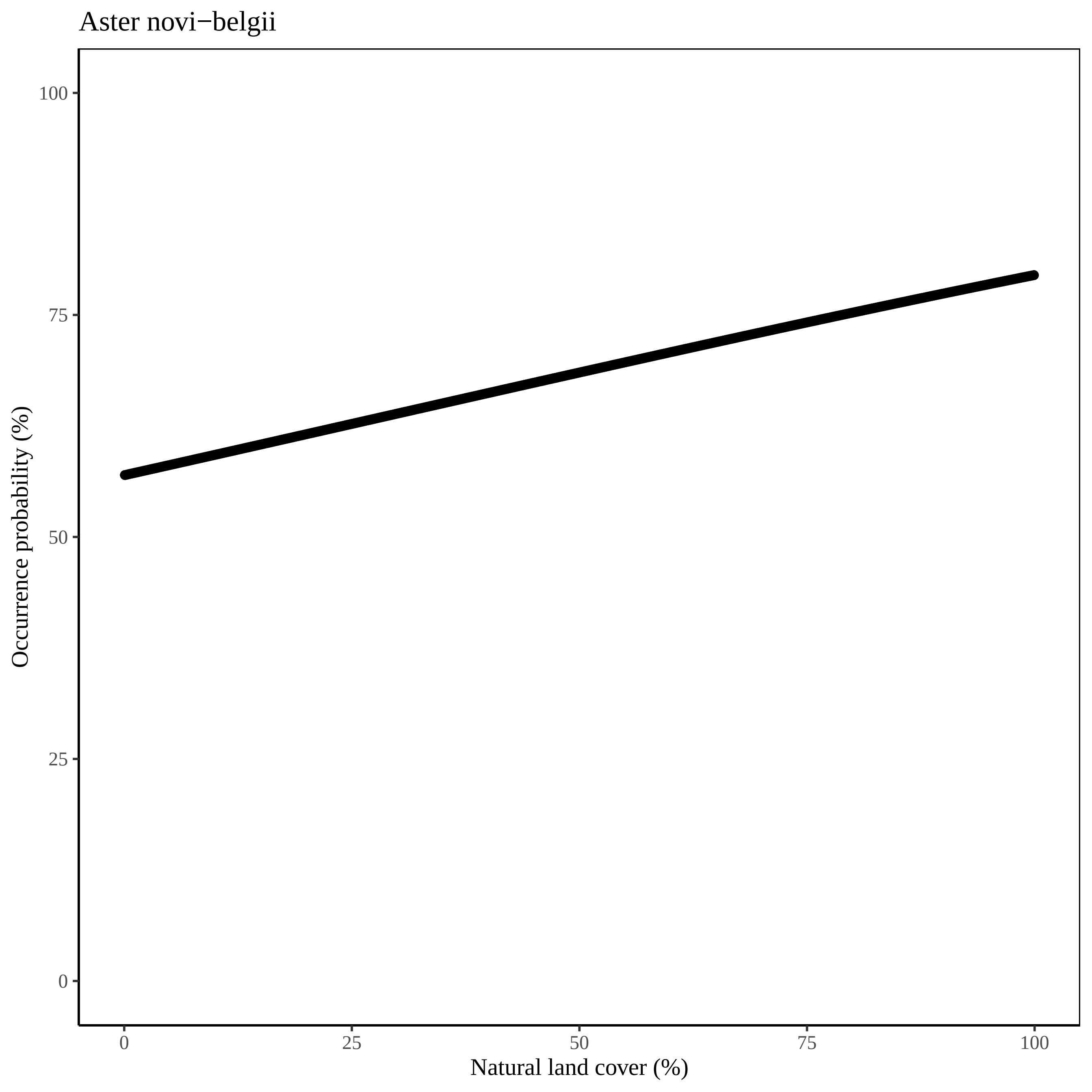
Aster novi−belgii
Occurrence probability (%)
100
75
50
25
0
0
25
50
75
100
Natural land cover (%)

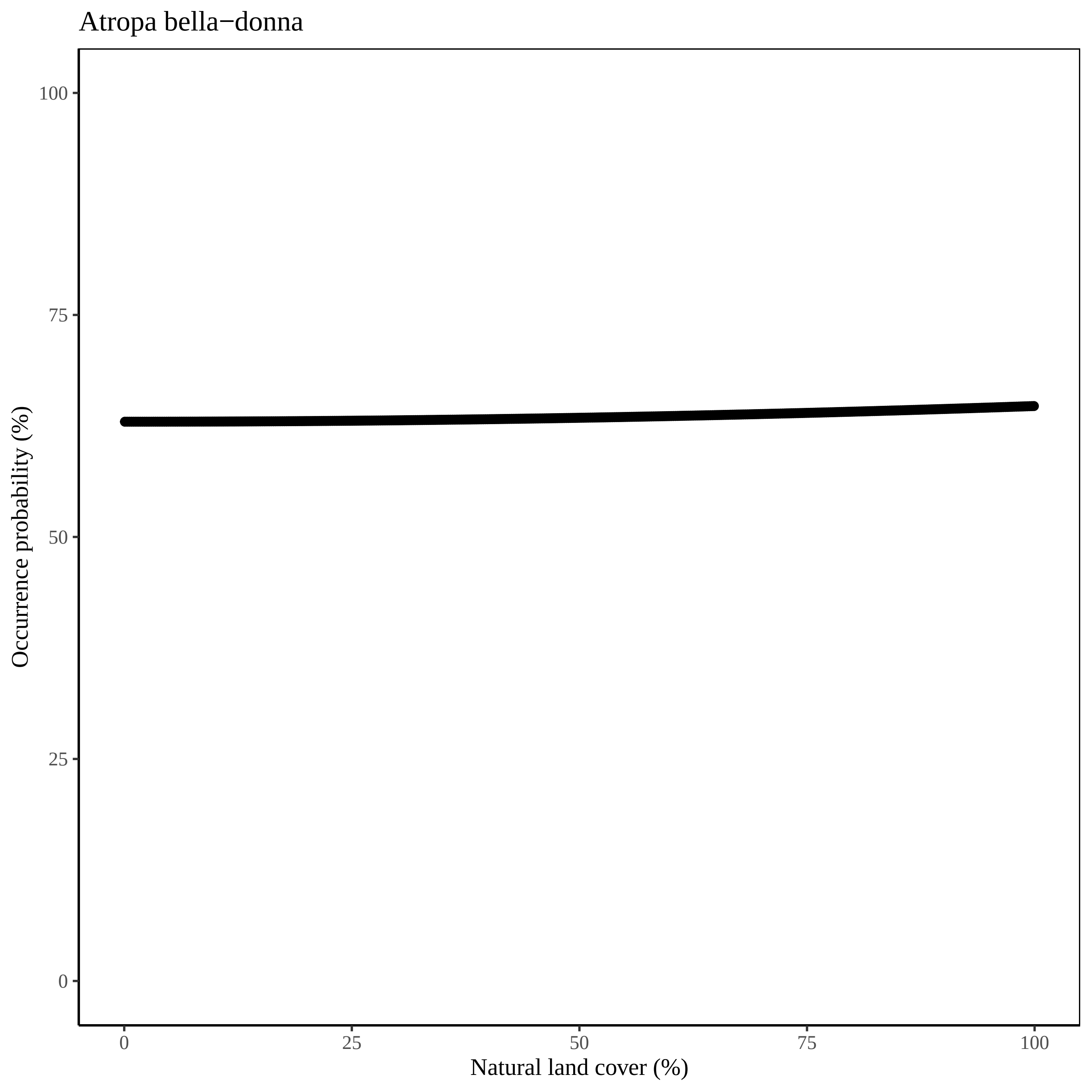
Atropa bella−donna
Occurrence probability (%)
100
75
50
25
0
0
25
50
75
100
Natural land cover (%)

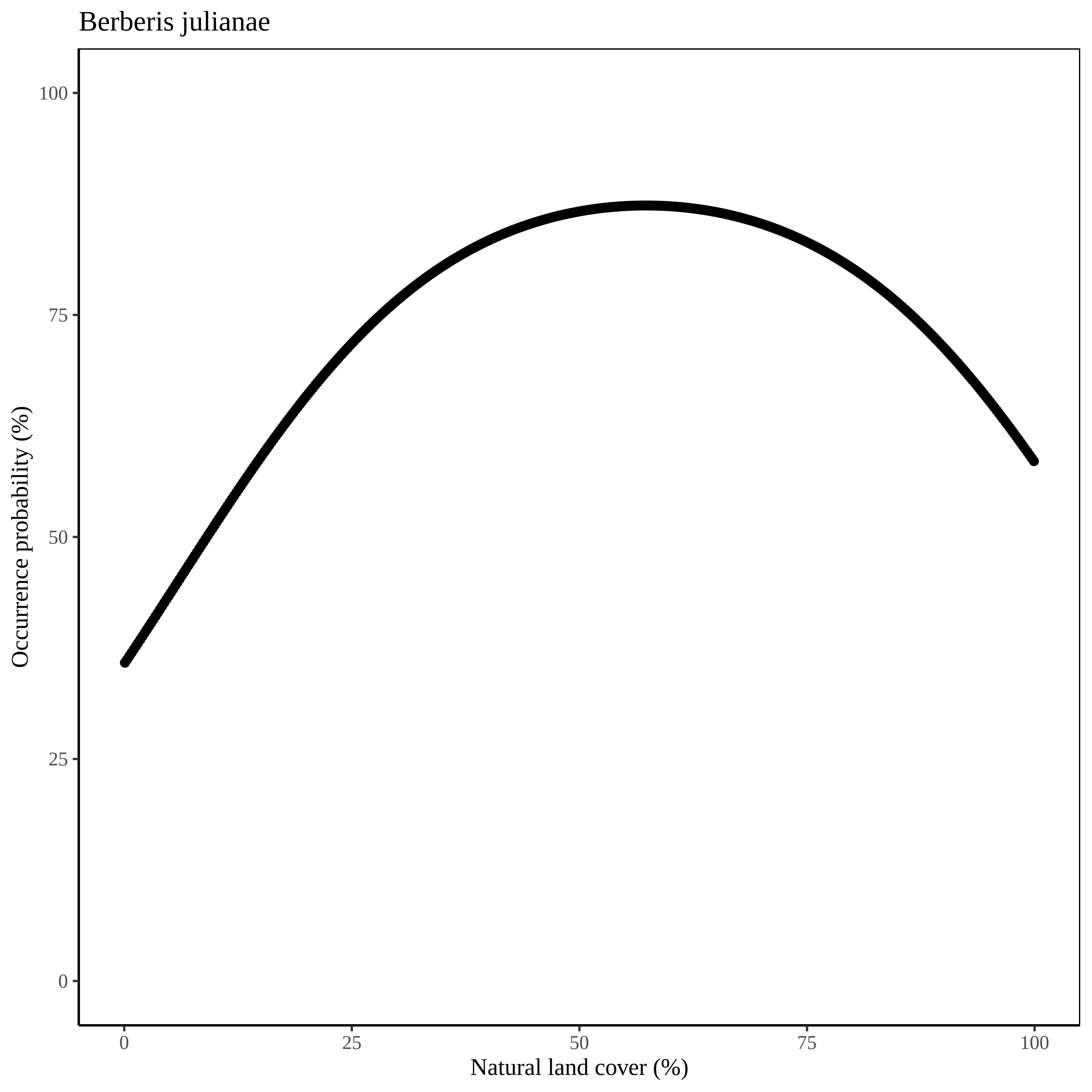
Berberis julianae
Occurrence probability (%)
100
75
50
25
0
0
25
50
75
100
Natural land cover (%)

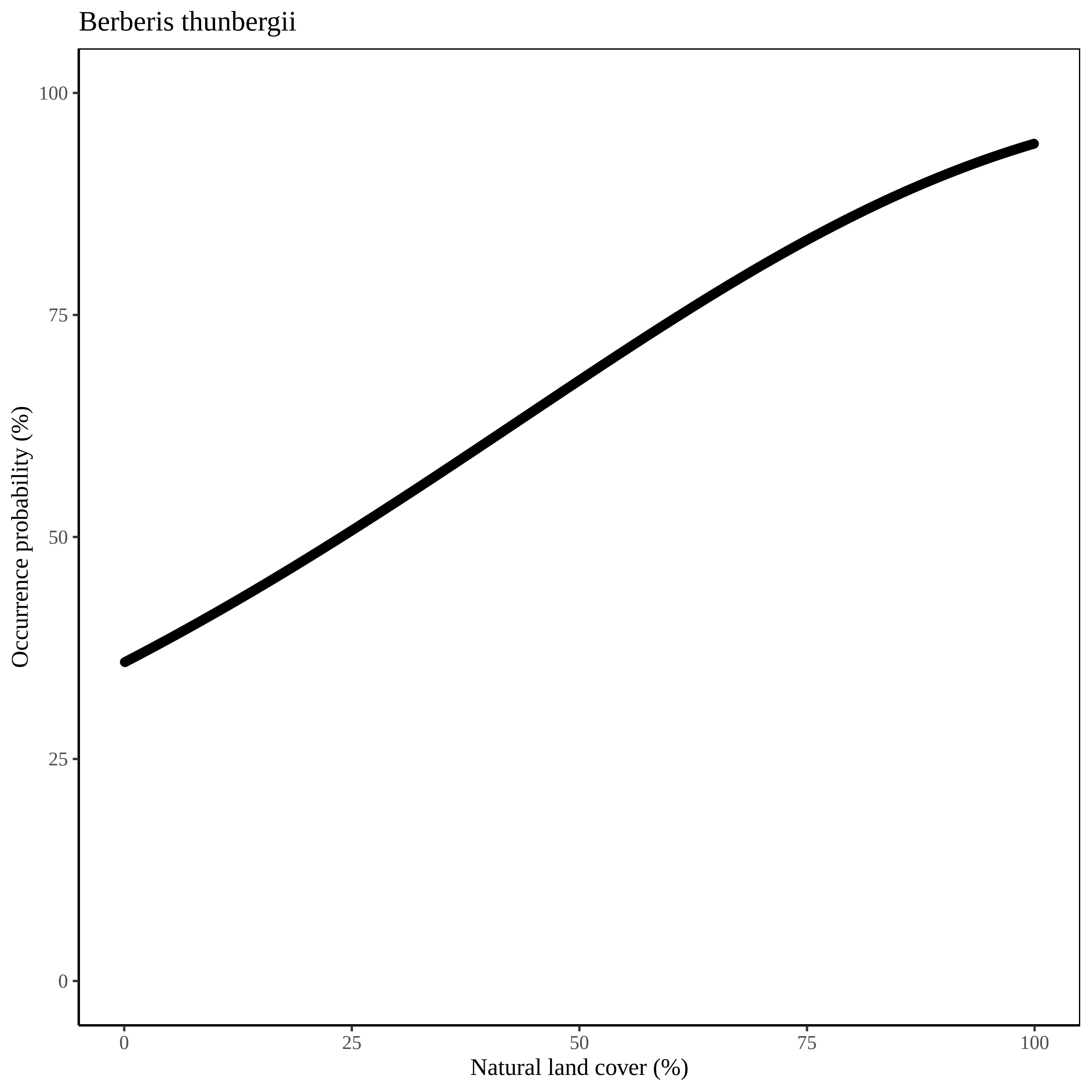

Berberis thunbergii
100
75
50
25
0
Occurrence probability (%)
0
25
50
75
100
Natural land cover (%)

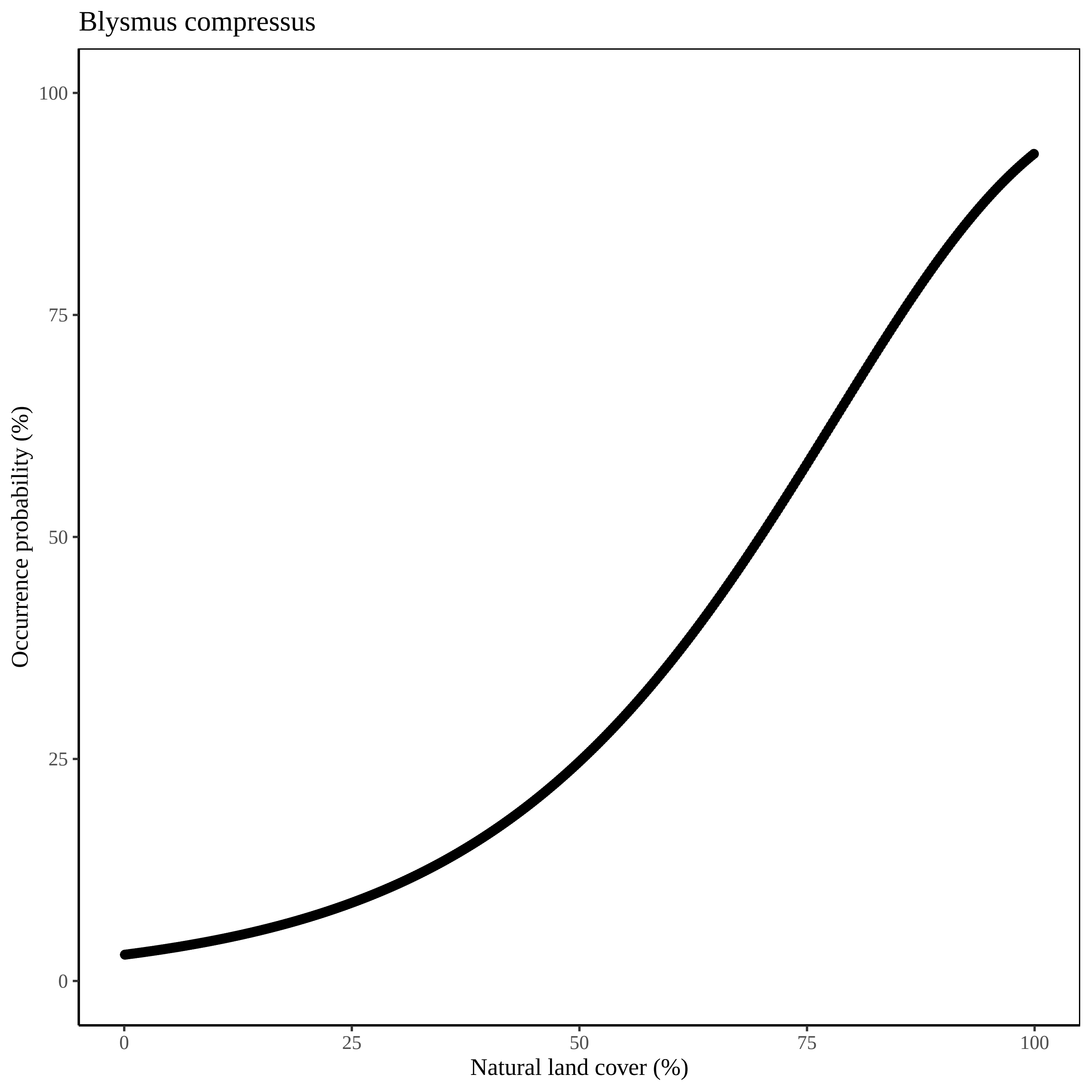
Blysmus compressus
Occurrence probability (%)
100
75
50
25
0
0
25
50
75
100
Natural land cover (%)

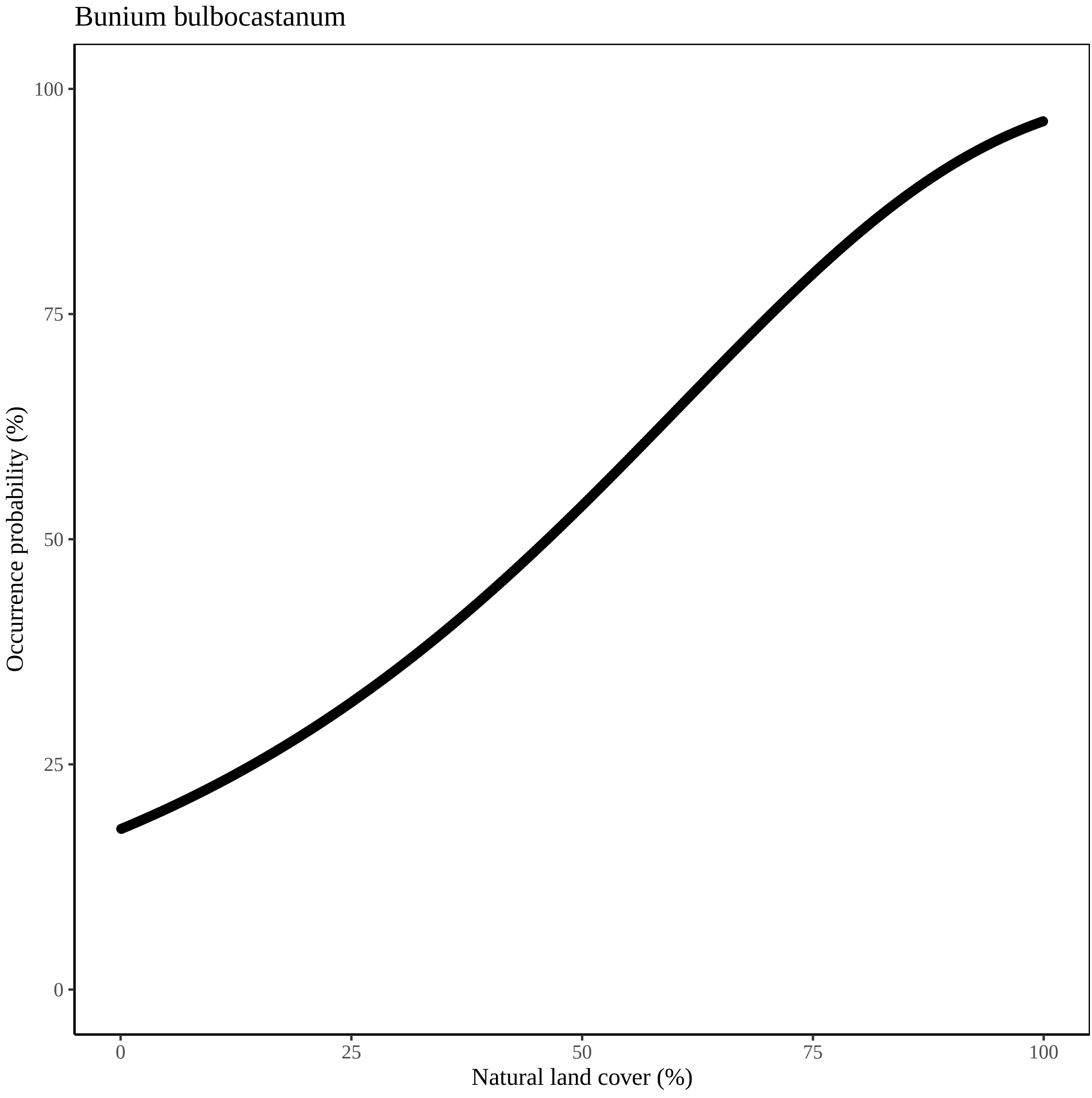
Bunium bulbocastanum
Occurrence probability (%)
100
75
50
25
0
0
25
50
75
100
Natural land cover (%)

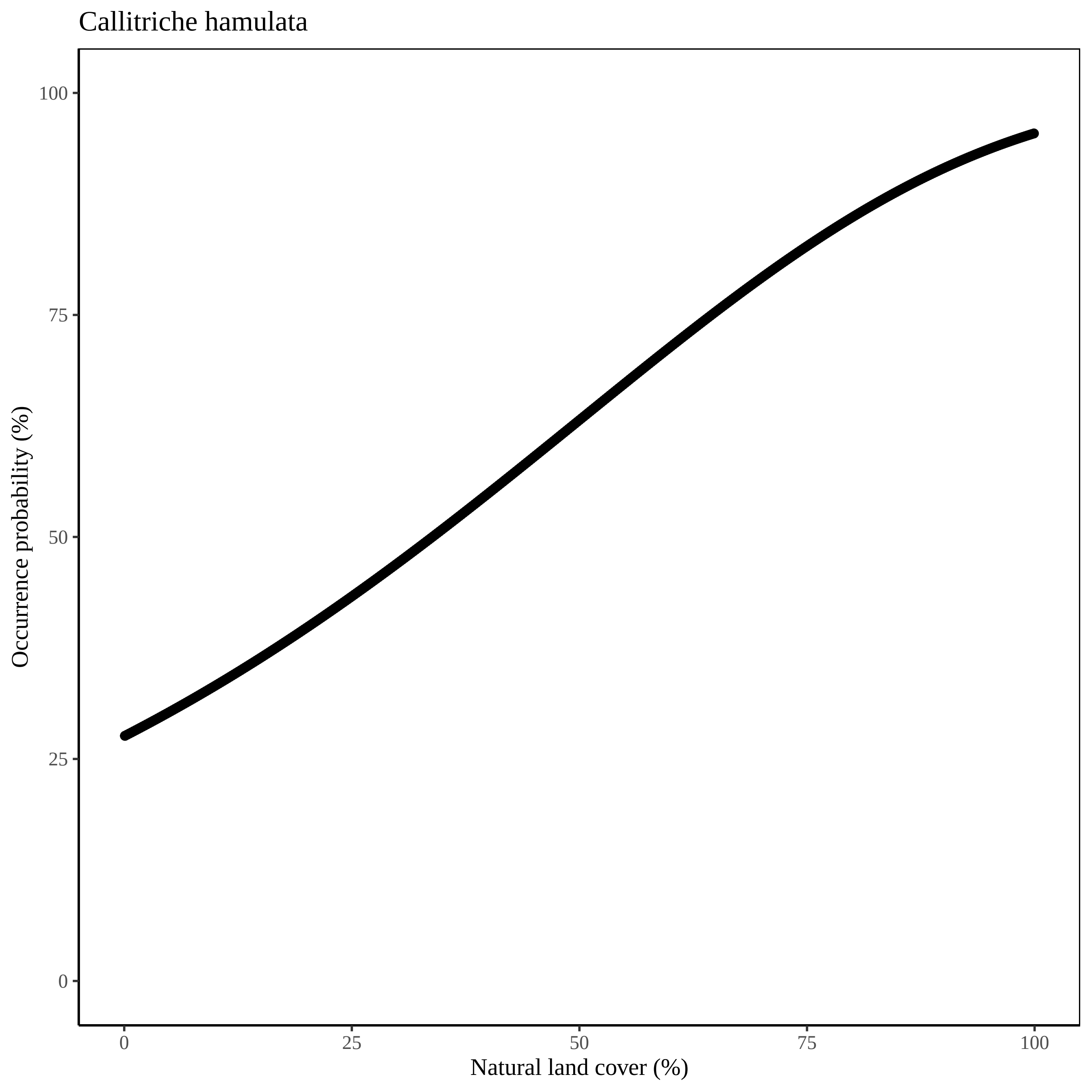
Callitriche hamulata
Occurrence probability (%)
100
75
50
25
0
0
25
50
75
100
Natural land cover (%)

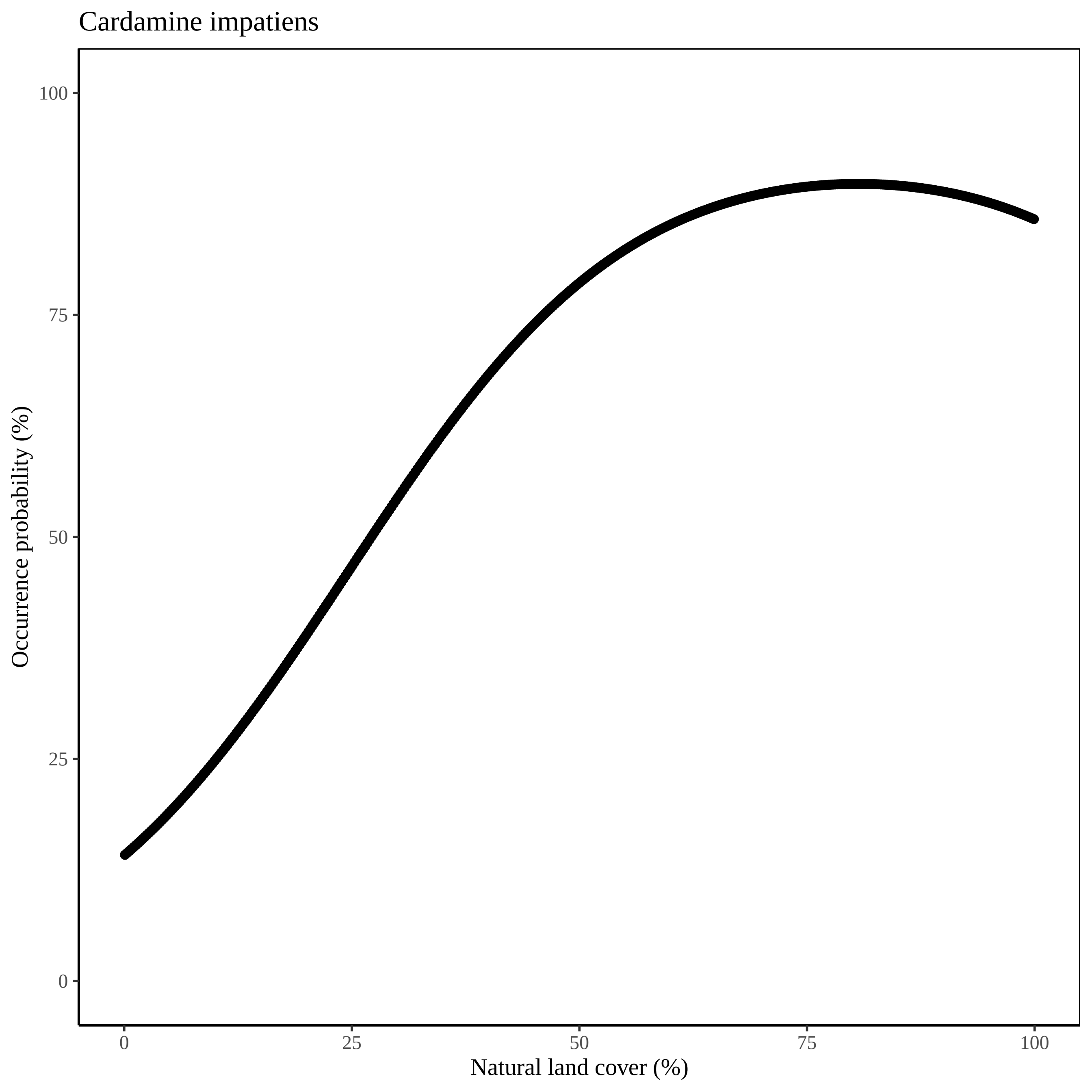
Cardamine impatiens
Occurrence probability (%)
100
75
50
25
0
0
25
50
75
100
Natural land cover (%)

Carex brizoides

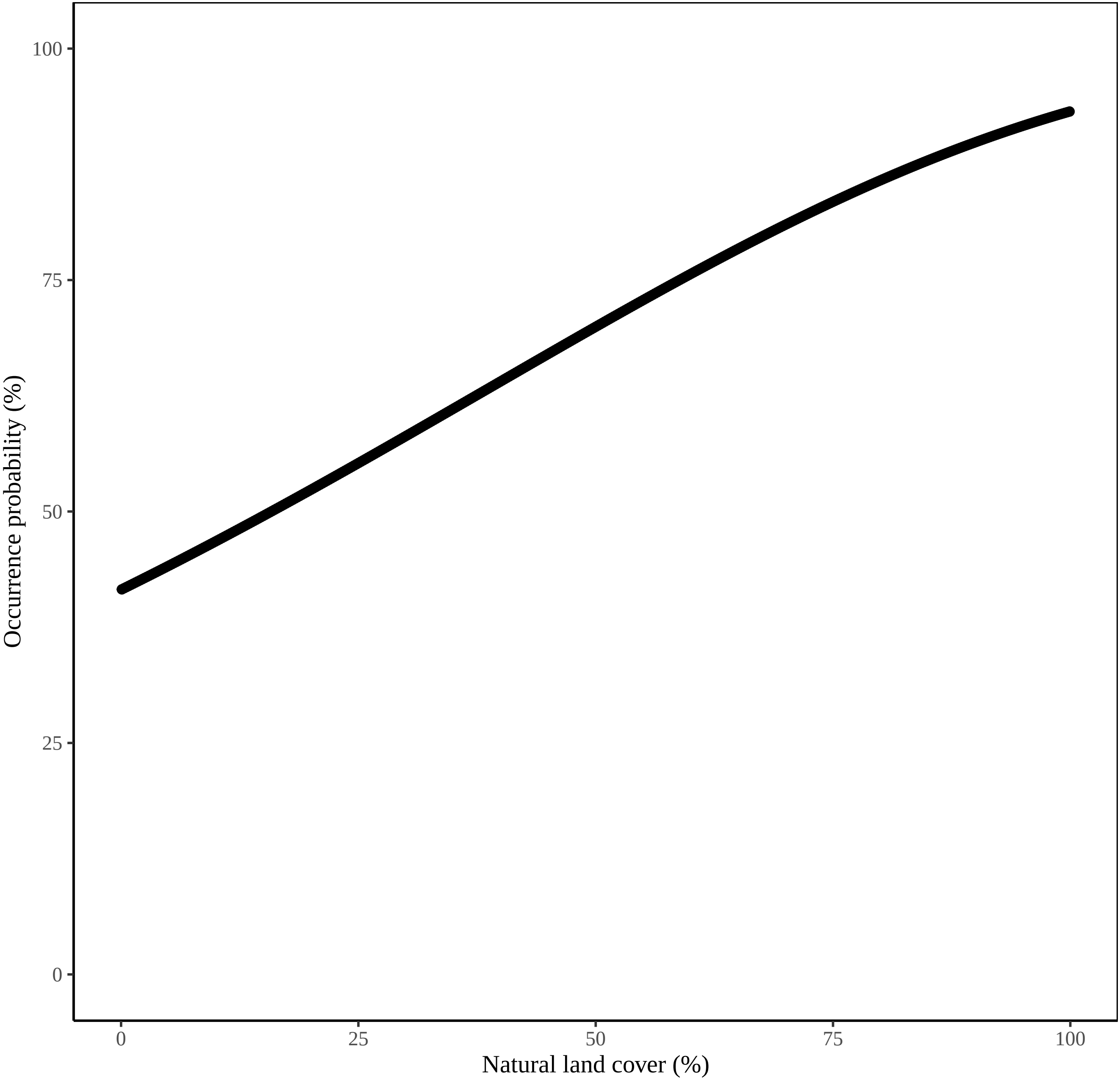

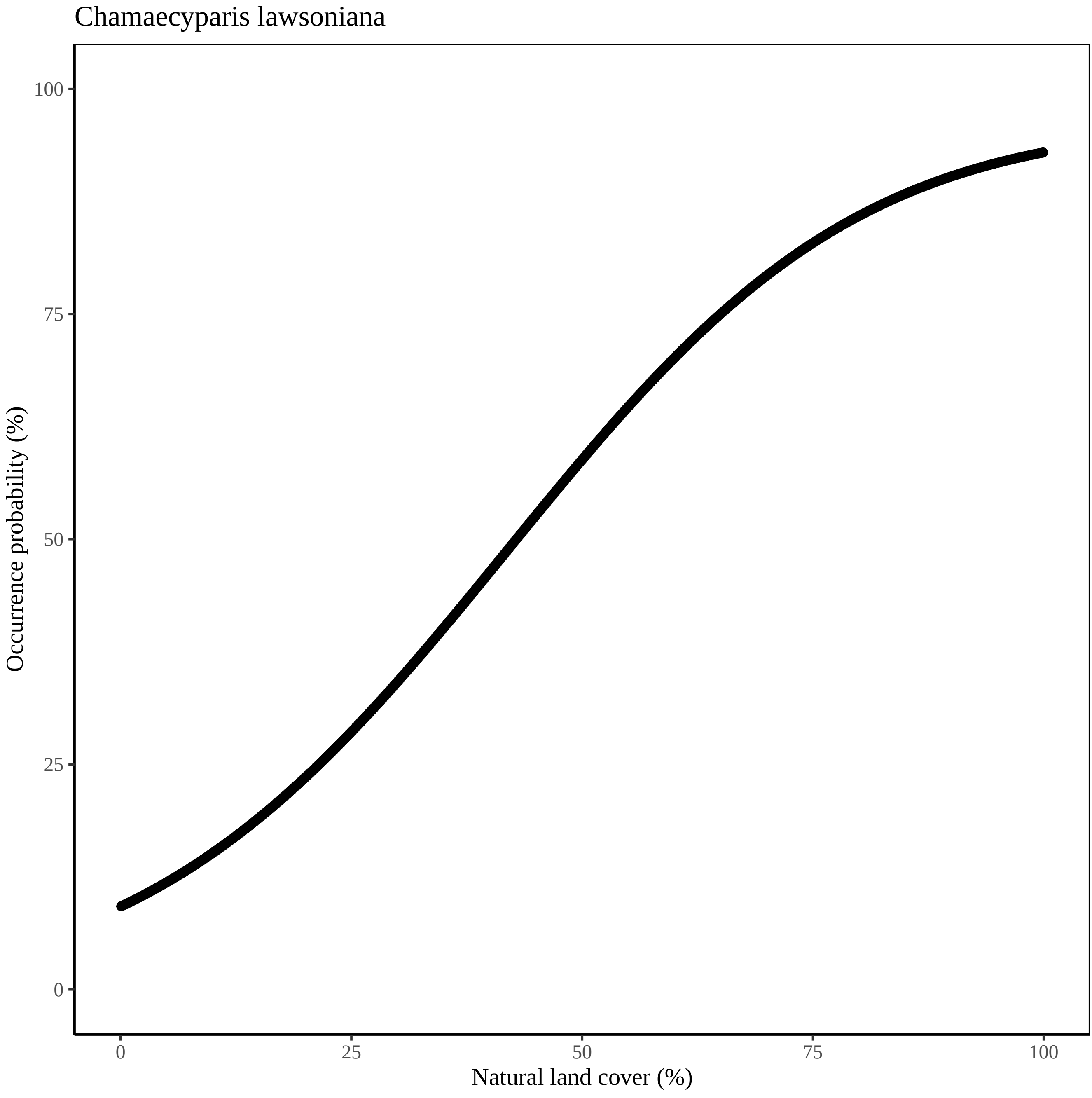

Chamaecyparis lawsoniana
Occurrence probability (%)
100
75
50
25
0
0
25
50
75
100
Natural land cover (%)

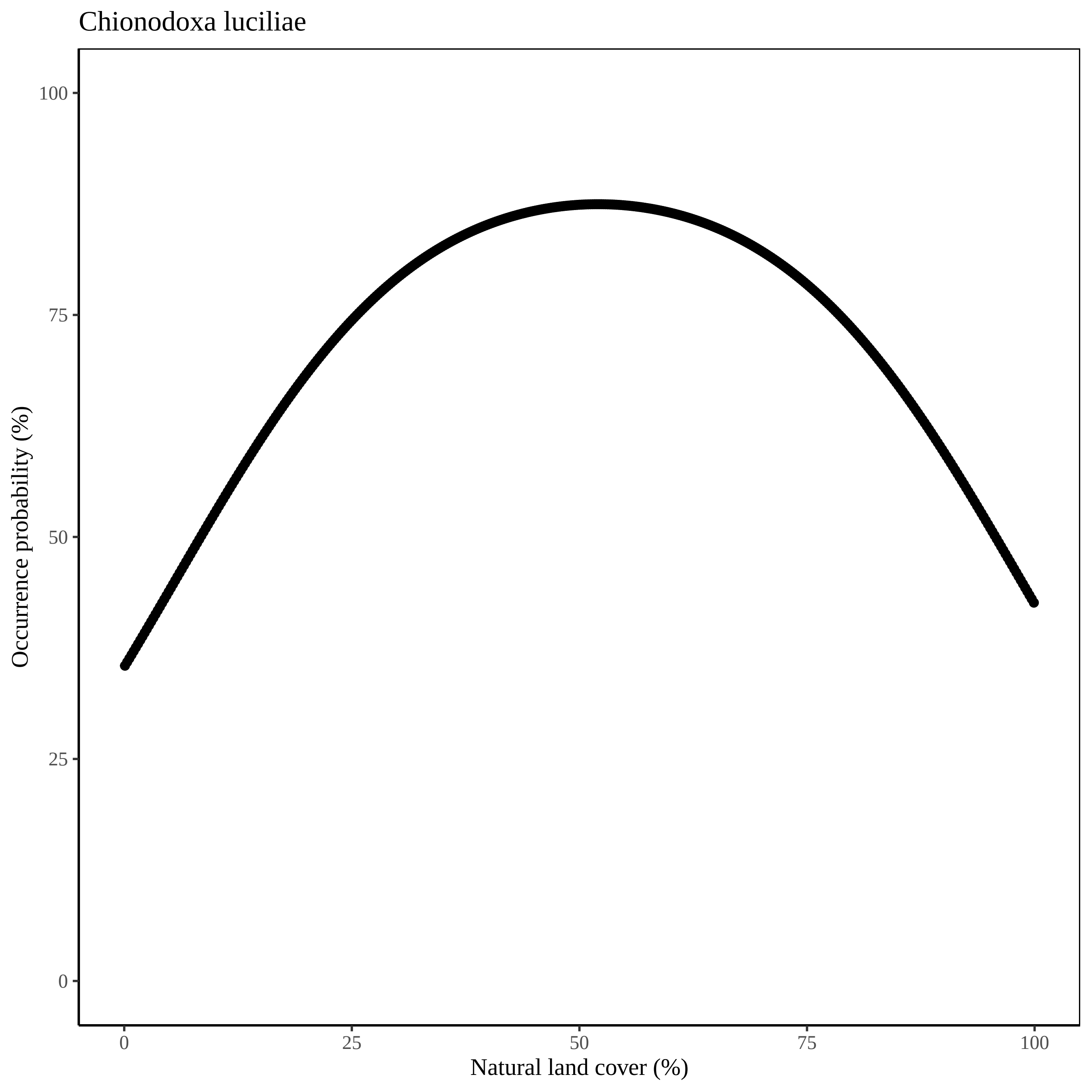
Chionodoxa luciliae
Occurrence probability (%)
100
75
50
25
0
0
25
50
75
100
Natural land cover (%)

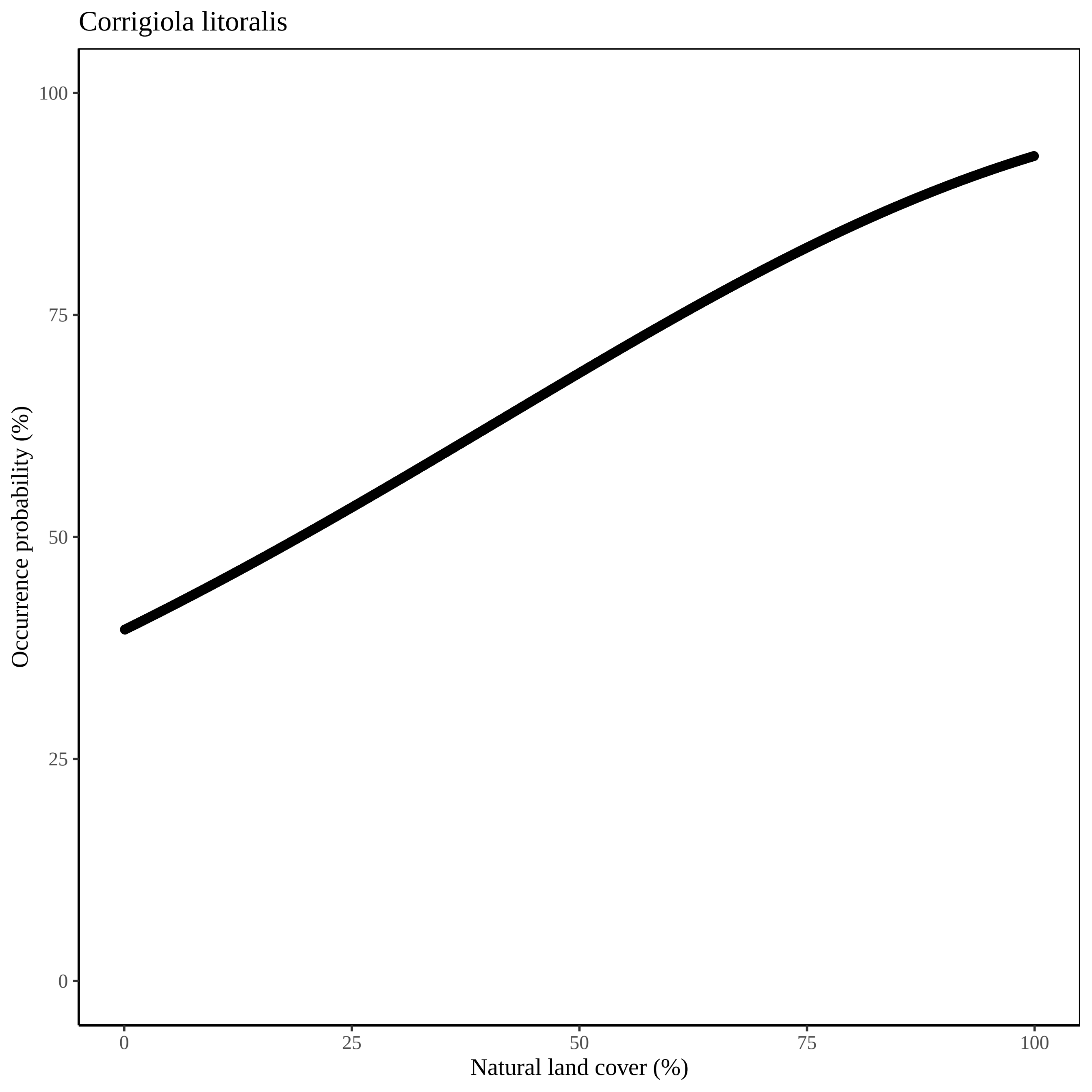

Corrigiola litoralis
Occurrence probability (%)
100
75
50
25
0
0
25
50
75
100
Natural land cover (%)

Cotoneaster divaricatus

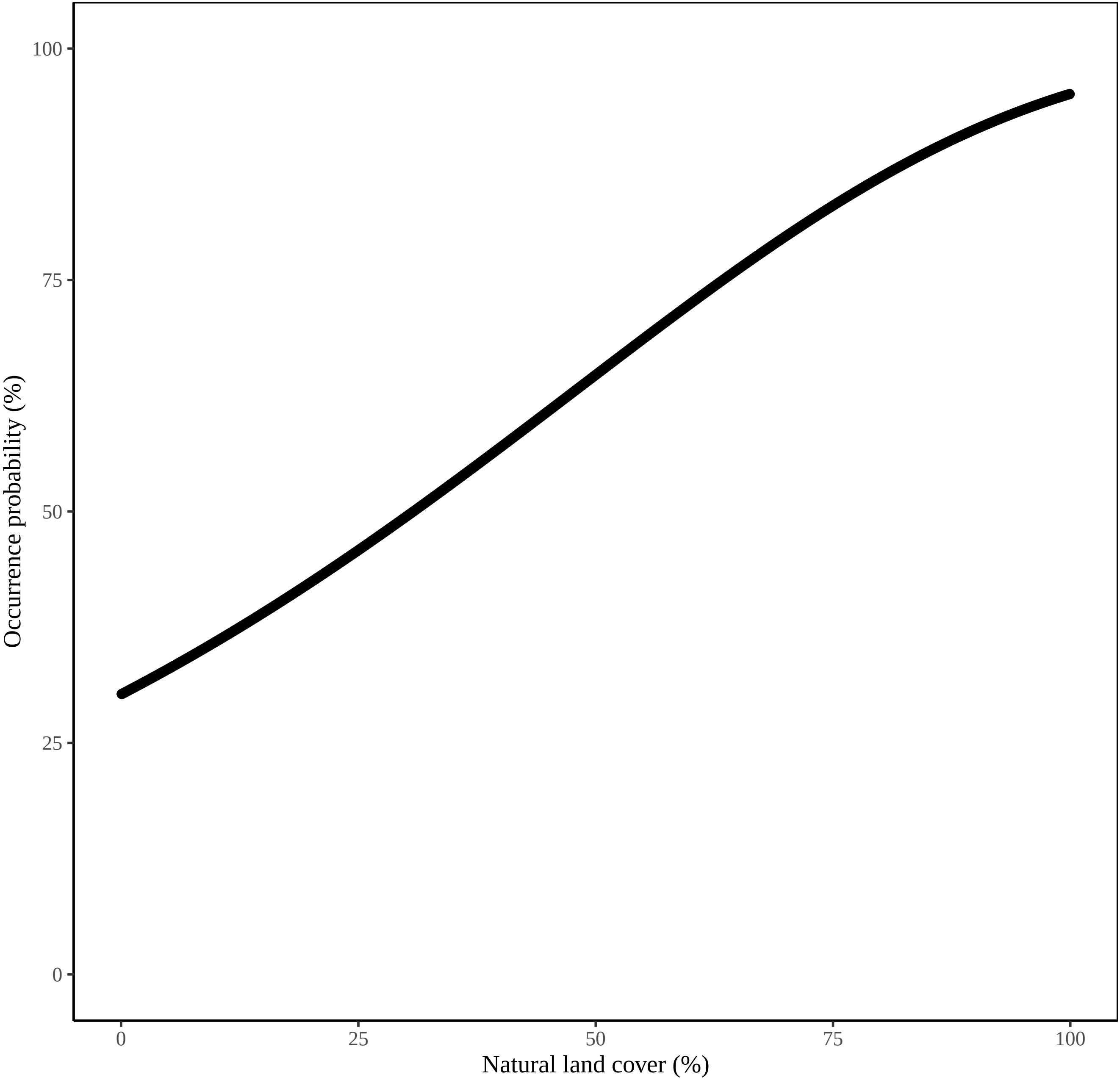

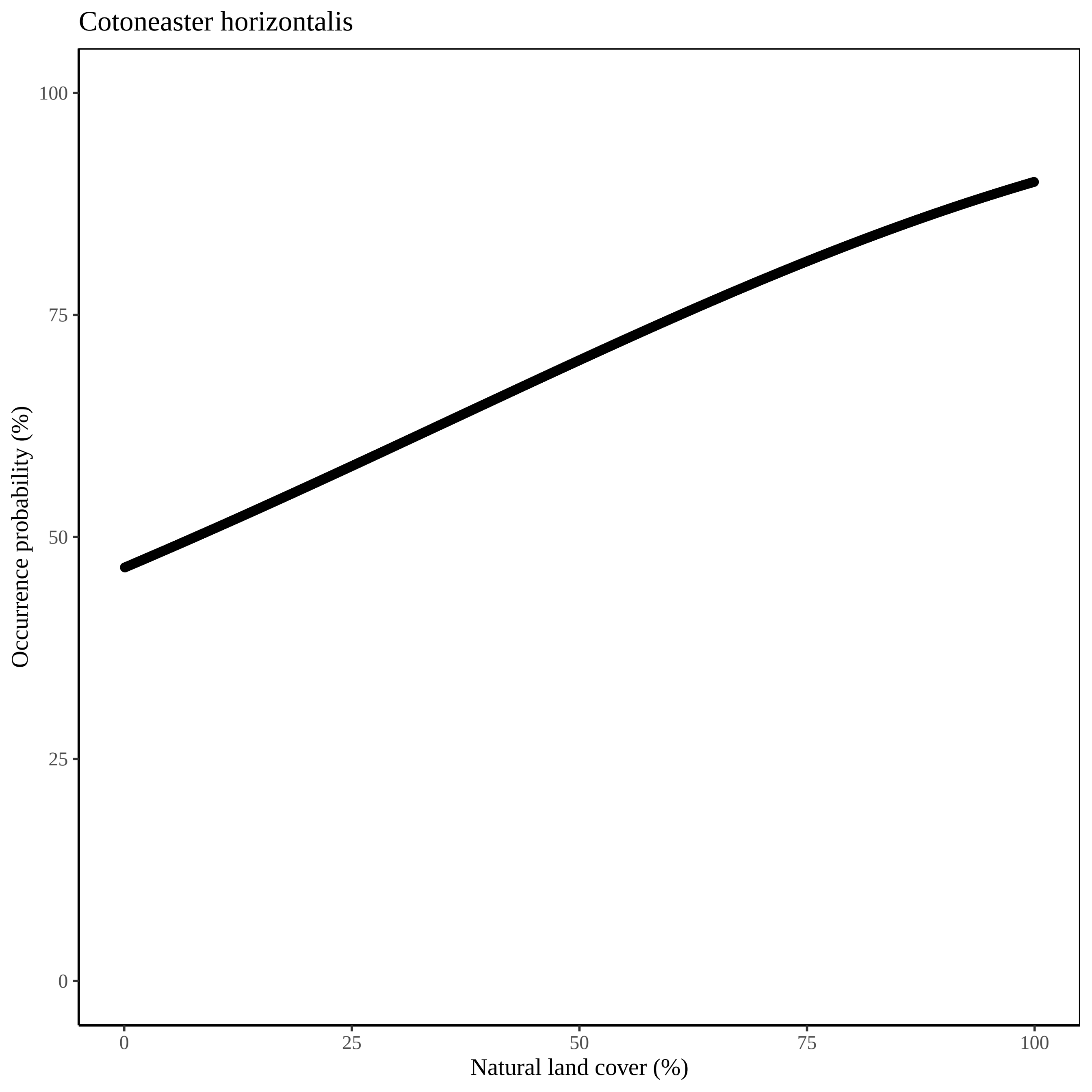
Cotoneaster horizontalis
100
75
50
25
0
Occurrence probability (%)
0
25
50
75
100
Natural land cover (%)

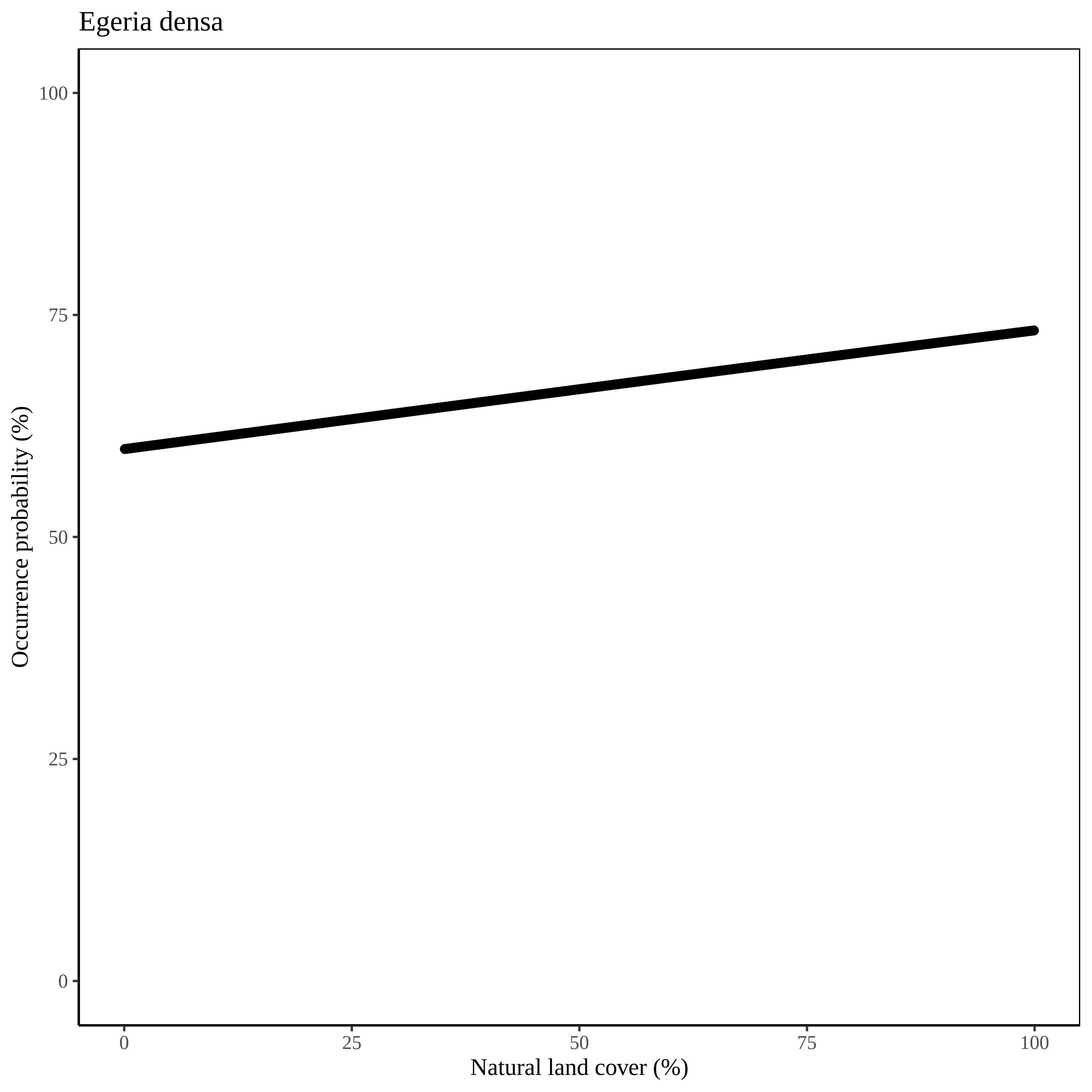
Egeria densa
Occurrence probability (%)
100
75
50
25
0
0
25
50
75
100
Natural land cover (%)

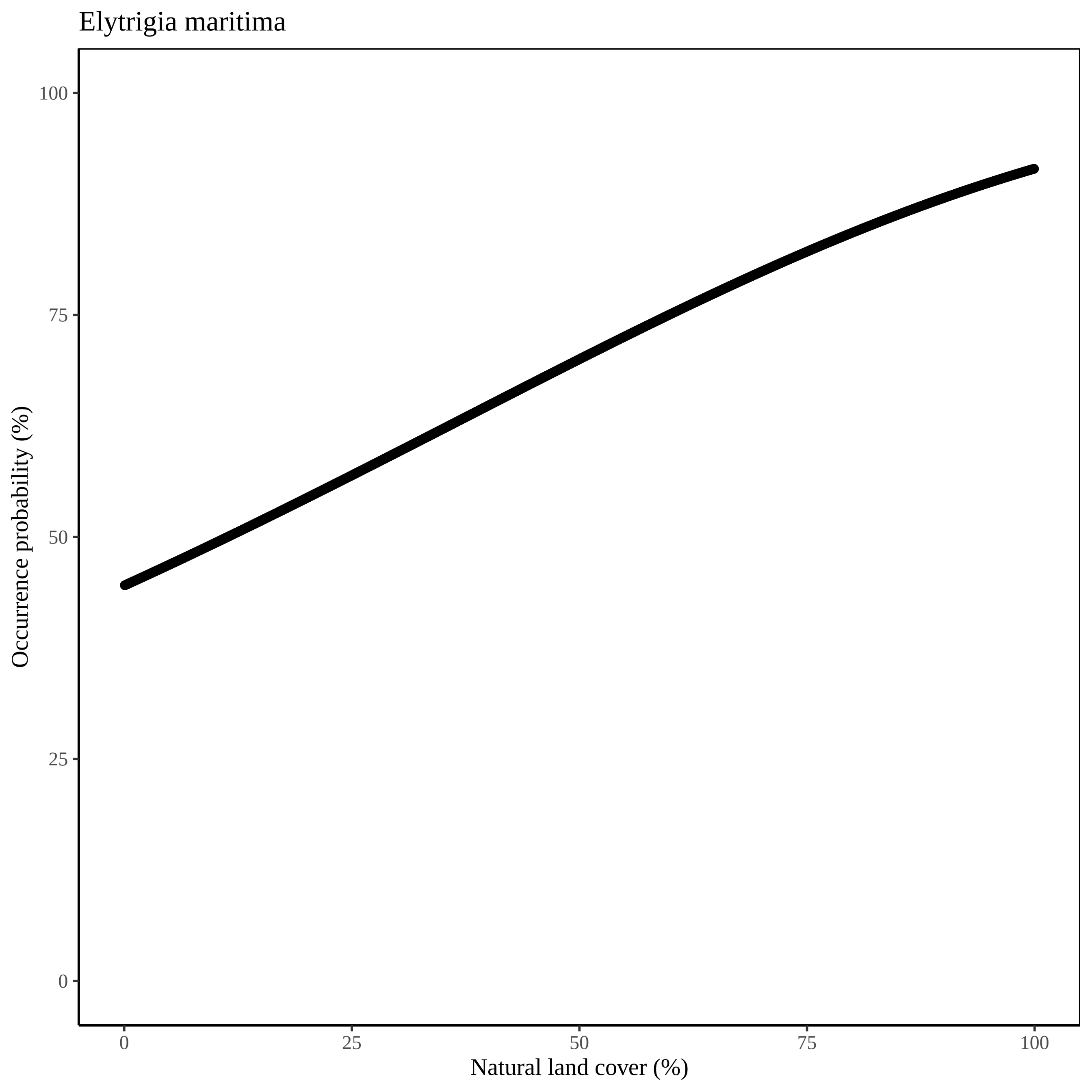
Elytrigia maritima
Occurrence probability (%)
100
75
50
25
0
0
25
50
75
100
Natural land cover (%)

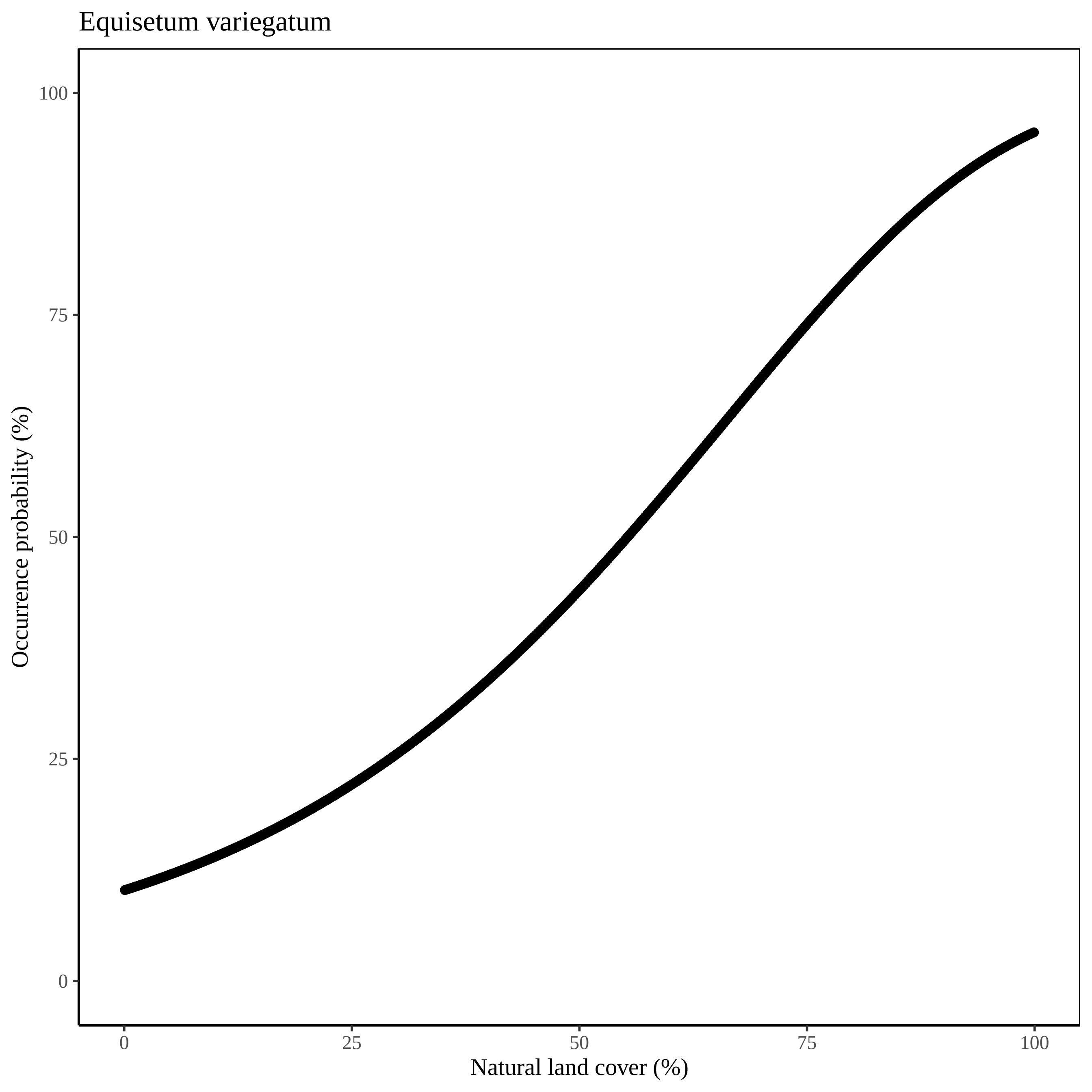
Equisetum variegatum
Occurrence probability (%)
100
75
50
25
0
0
25
50
75
100
Natural land cover (%)

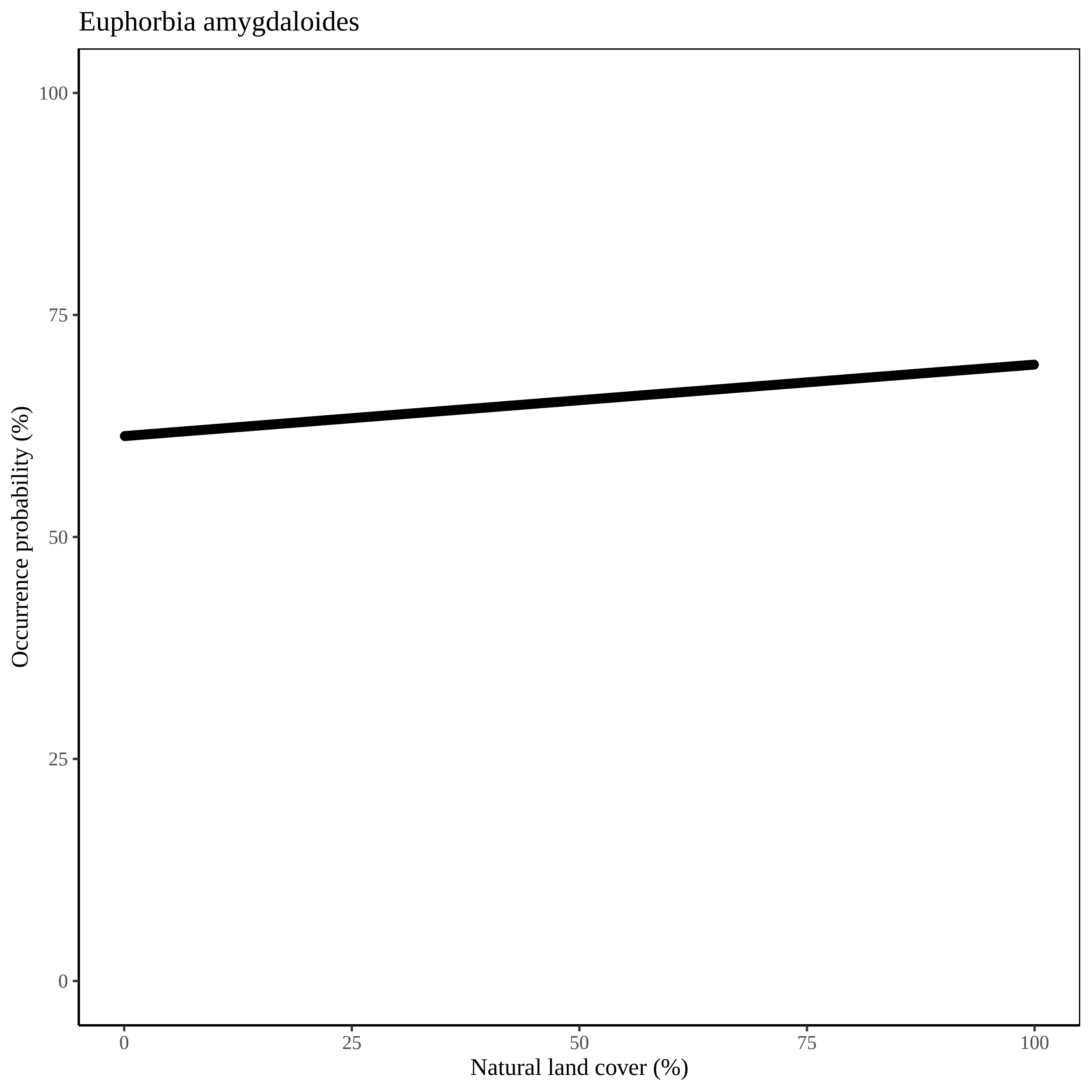

Euphorbia amygdaloides
Occurrence probability (%)
100
75
50
25
0
0
25
50
75
100
Natural land cover (%)

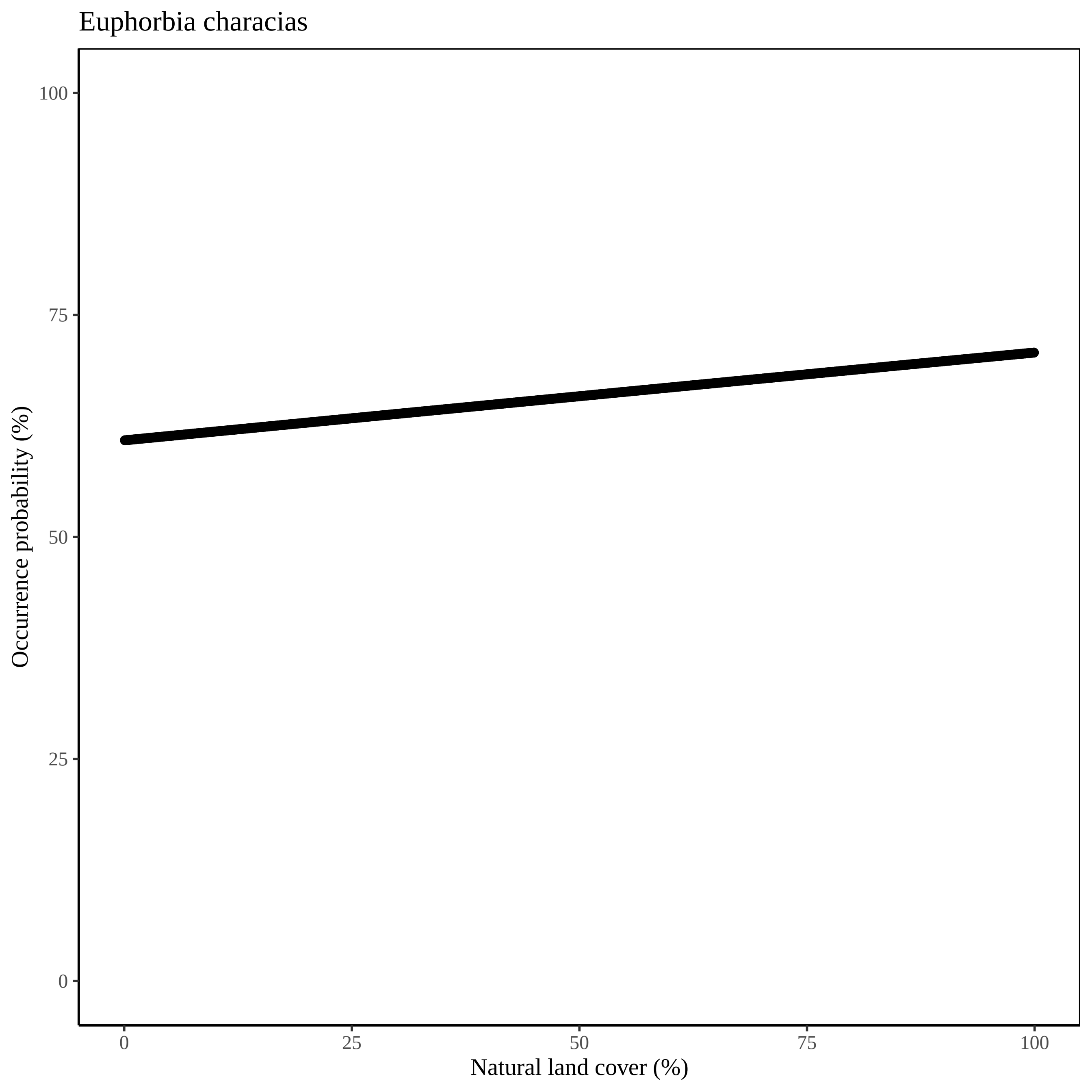
Euphorbia characias
Occurrence probability (%)
100
75
50
25
0
0
25
50
75
100
Natural land cover (%)

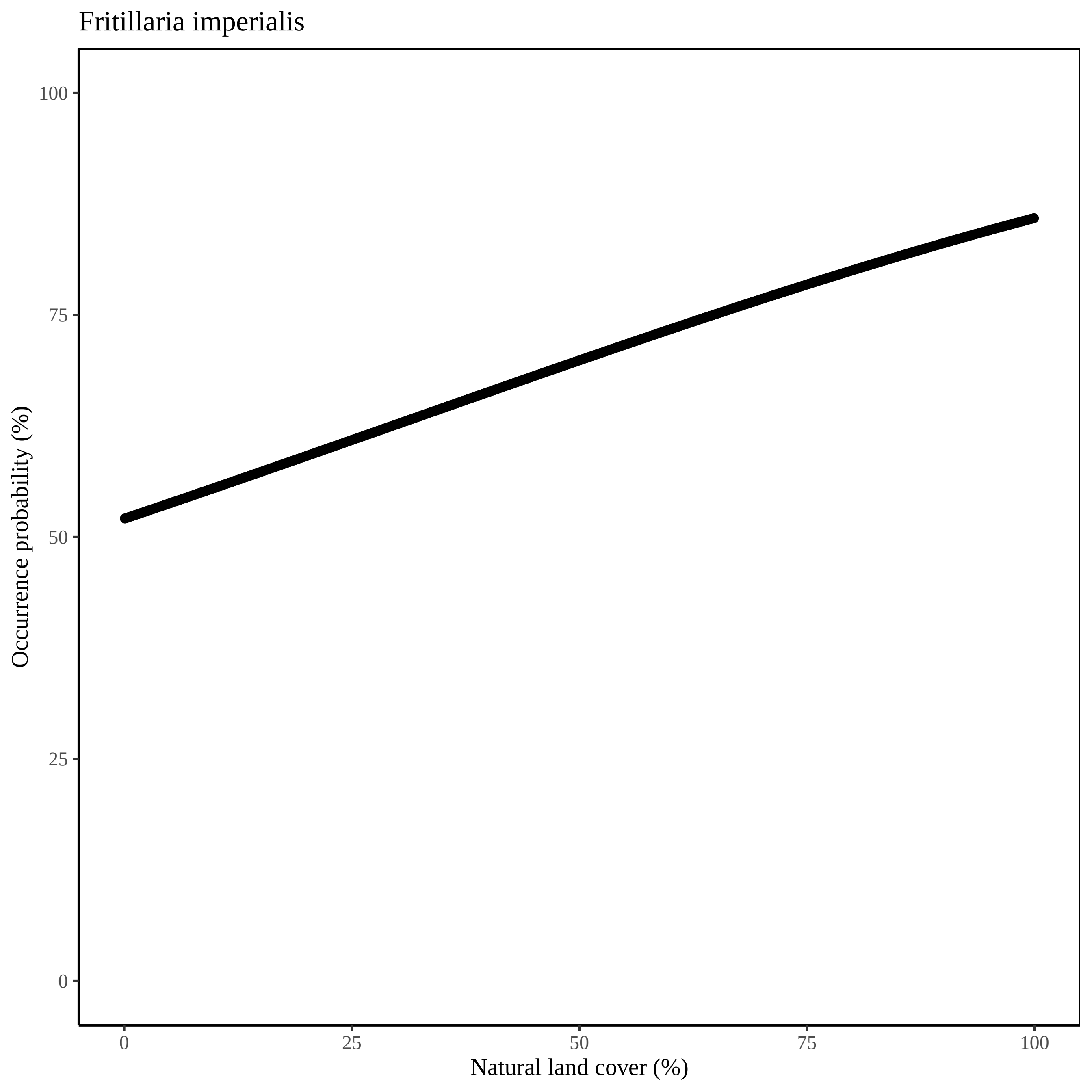

Fritillaria imperialis
Occurrence probability (%)
100
75
50
25
0
0
25
50
75
100
Natural land cover (%)

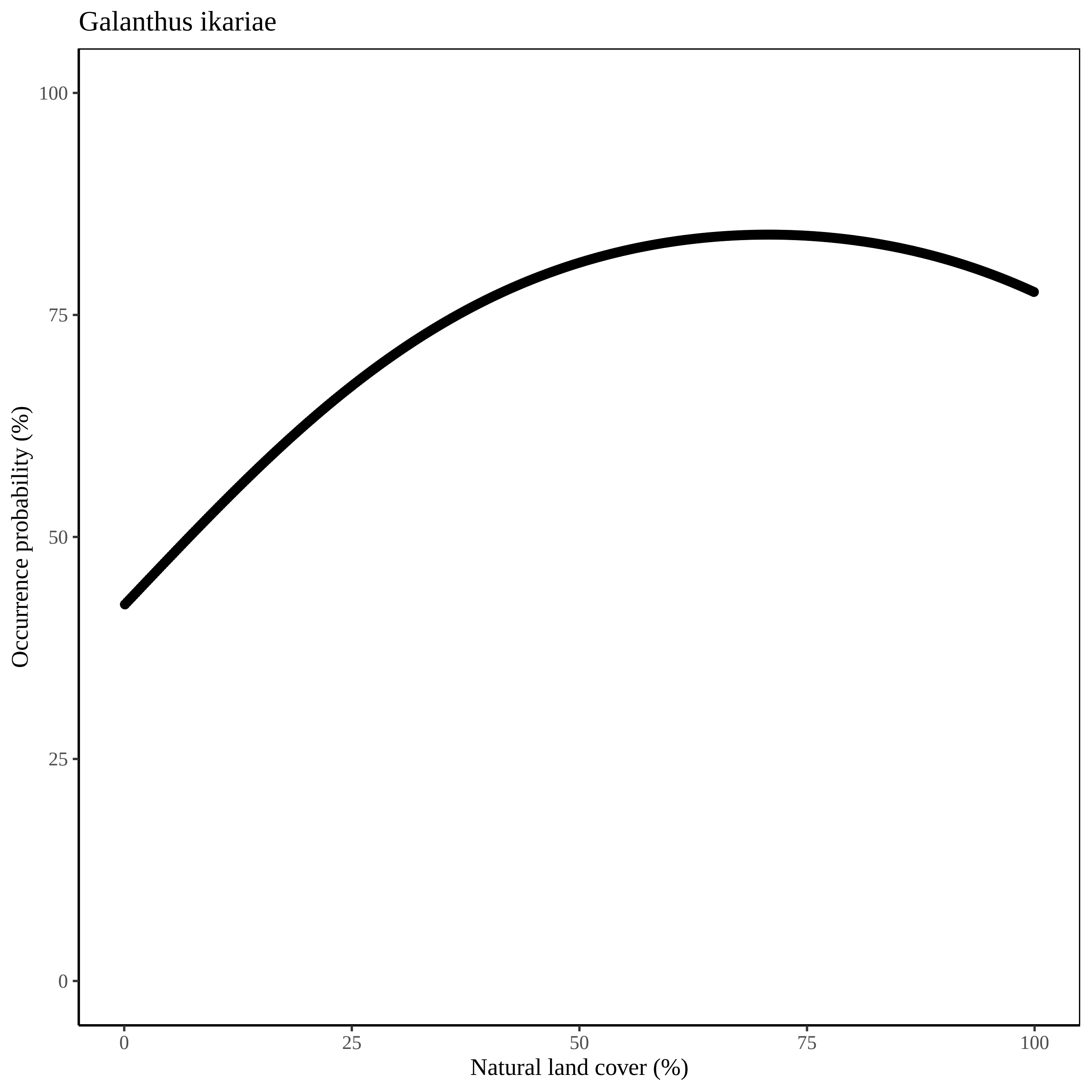

Galanthus ikariae
Occurrence probability (%)
100
75
50
25
0
0
25
50
75
100
Natural land cover (%)

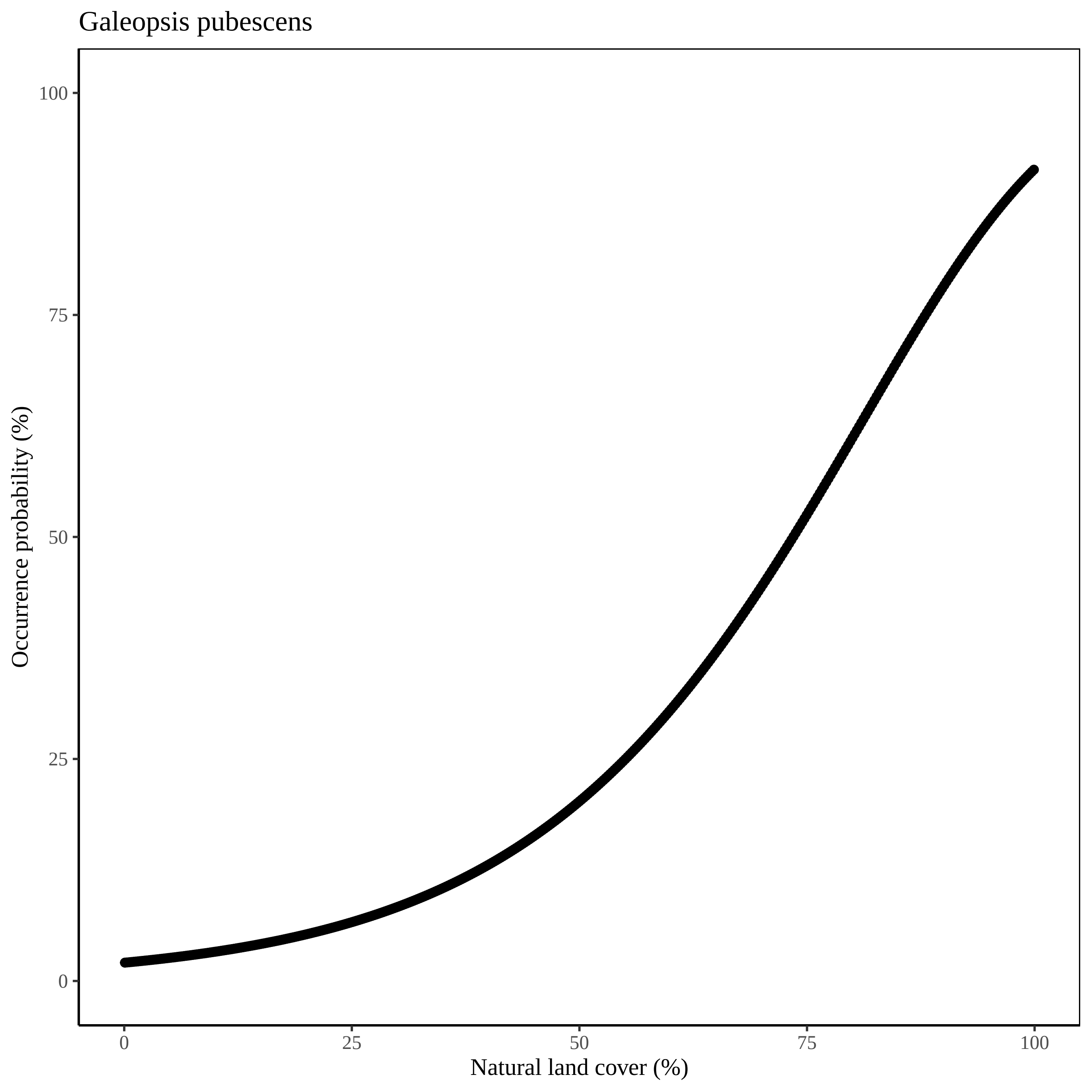
Galeopsis pubescens
Occurrence probability (%)
100
75
50
25
0
0
25
50
75
100
Natural land cover (%)

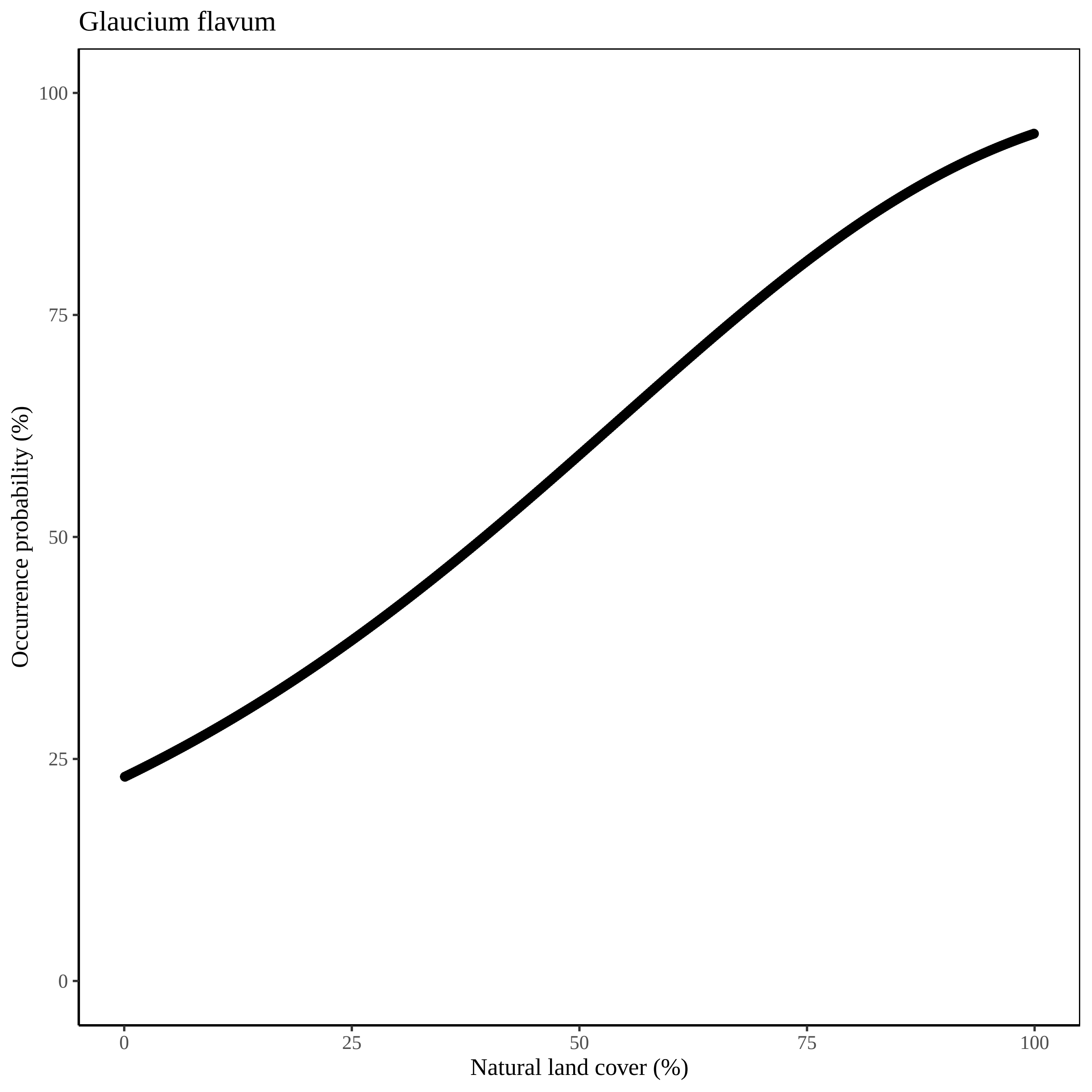
Glaucium flavum
Occurrence probability (%)
100
75
50
25
0
0
25
50
75
100
Natural land cover (%)

Gymnocarpium dryopteris

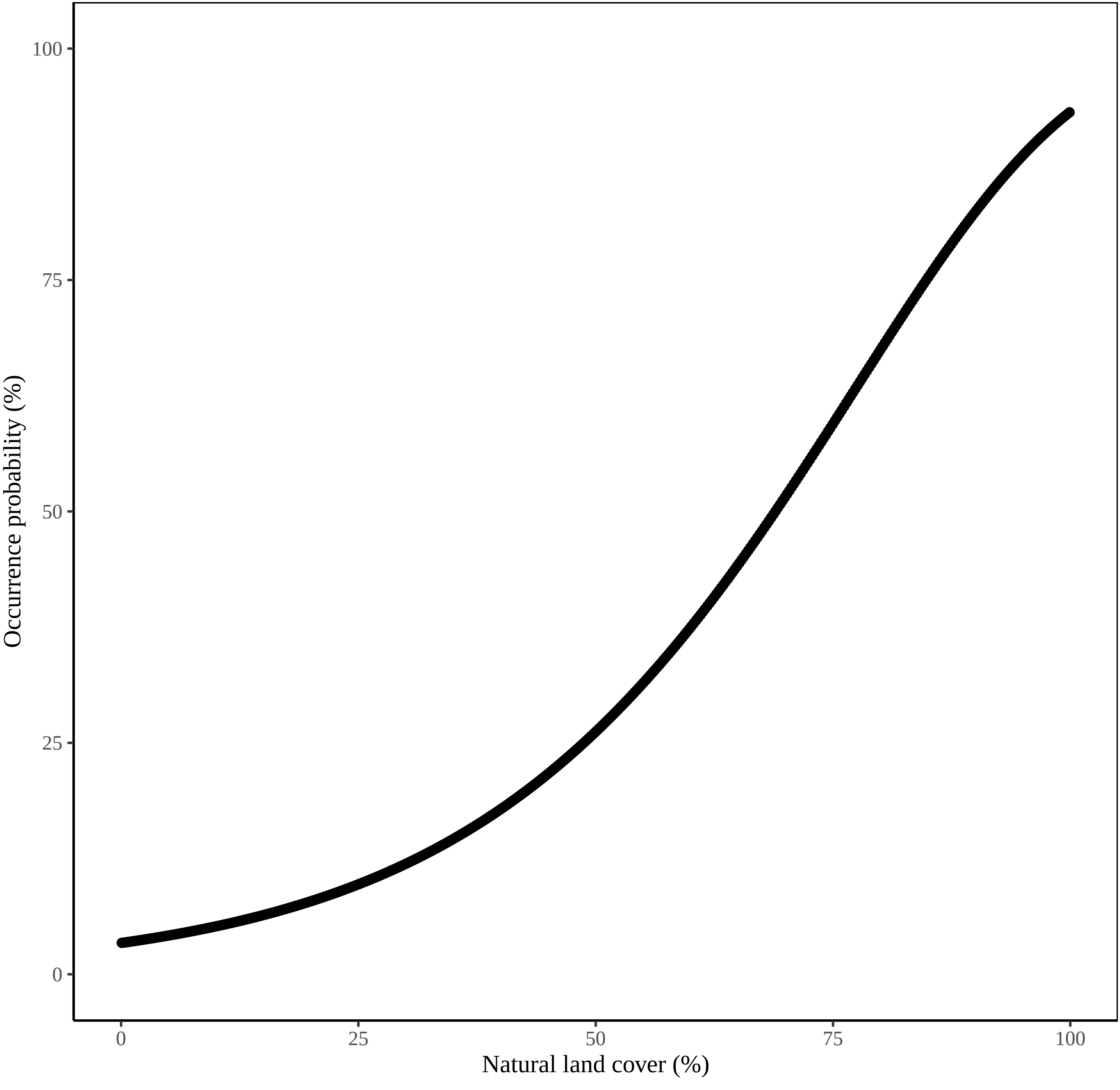

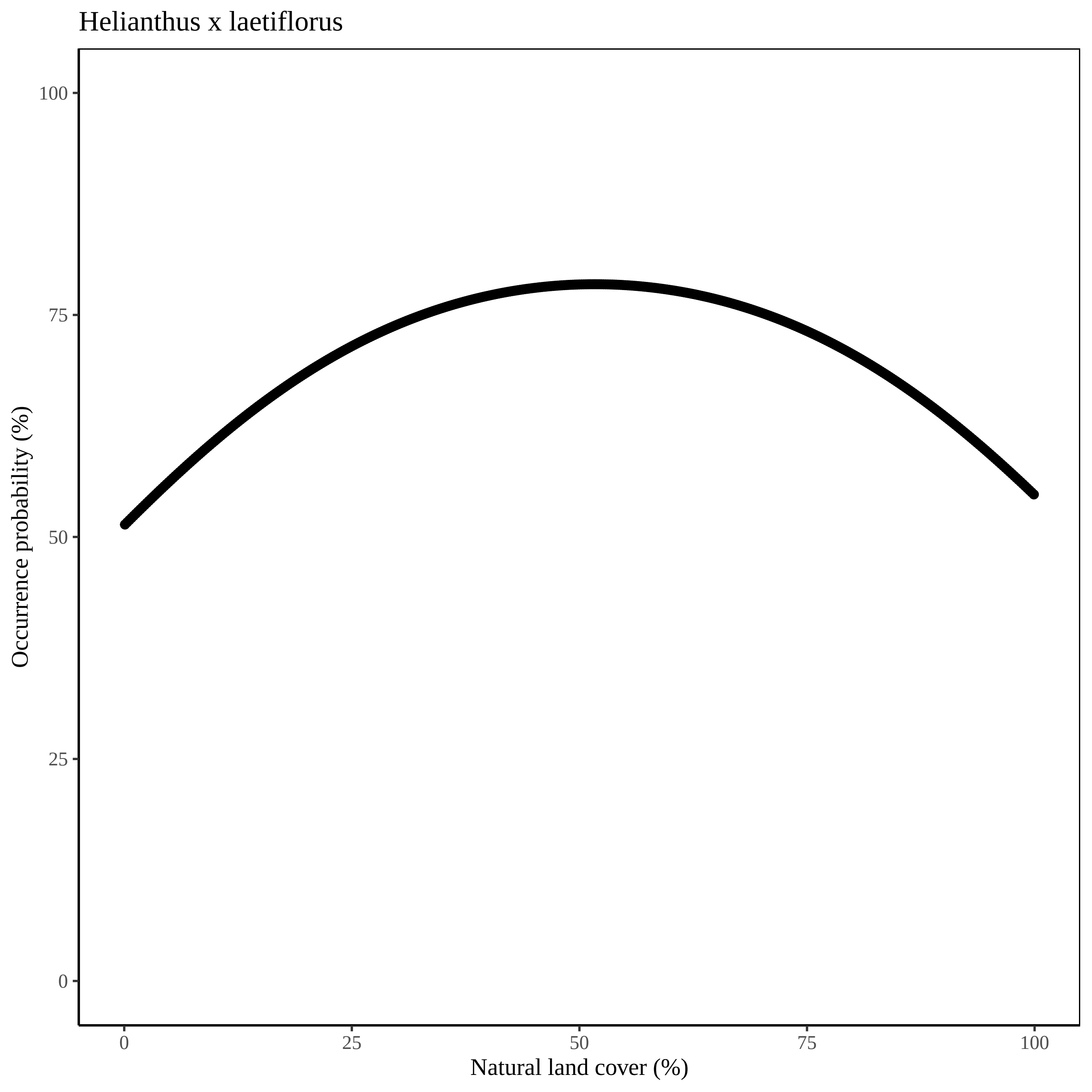
Helianthus x laetiflorus
Occurrence probability (%)
100
75
50
25
0
0
25
50
75
100
Natural land cover (%)

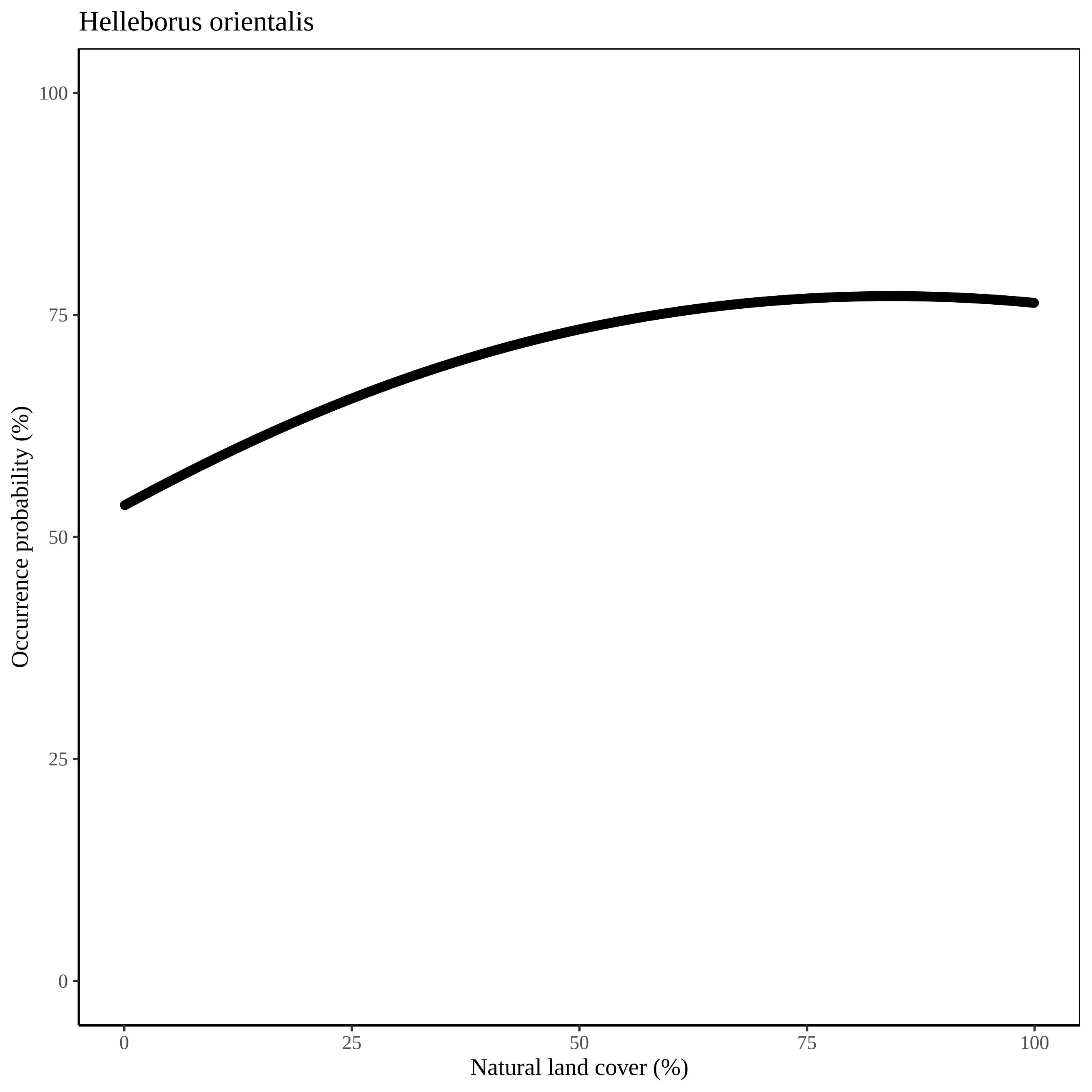

Helleborus orientalis
Occurrence probability (%)
100
75
50
25
0
0
25
50
75
100
Natural land cover (%)

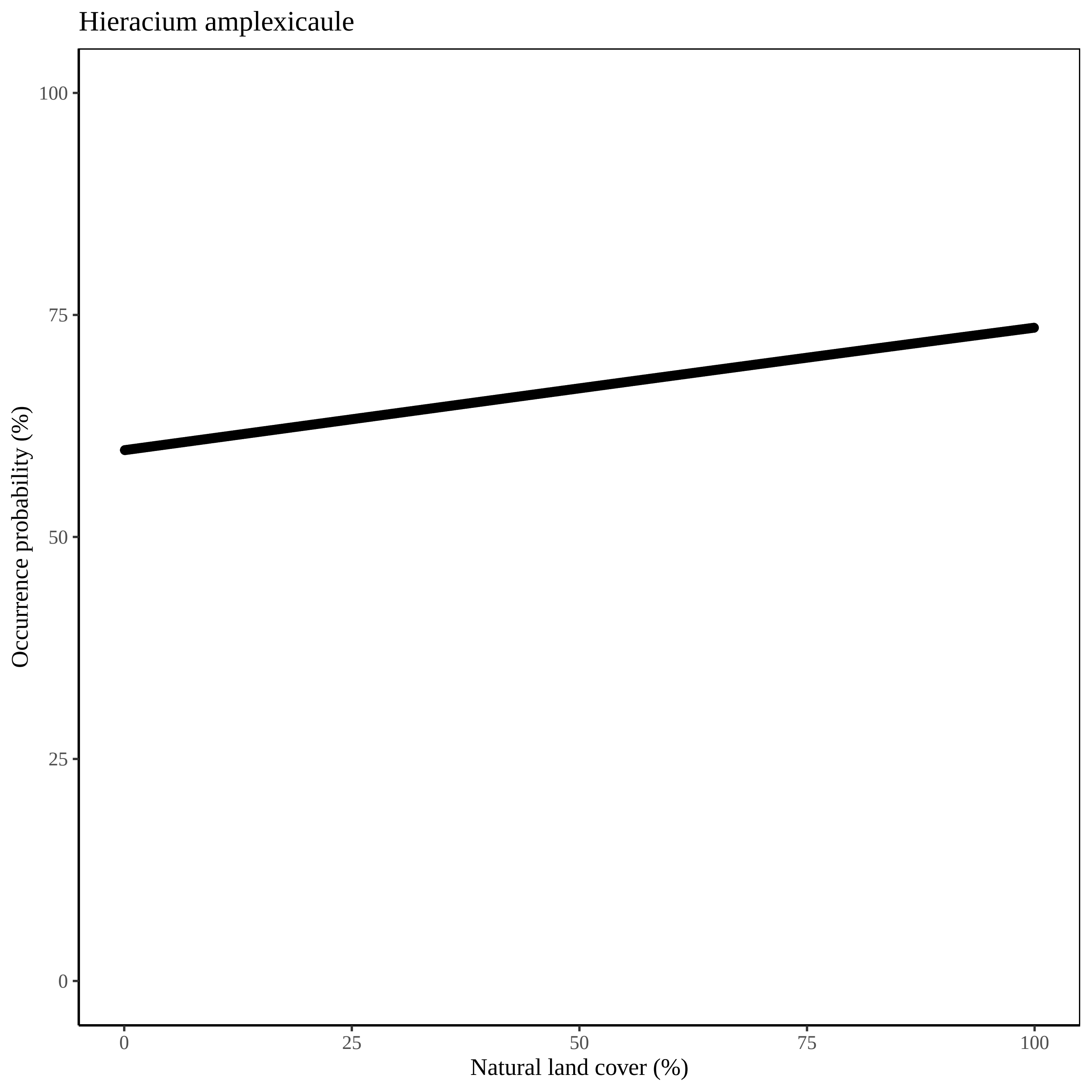

Hieracium amplexicaule
Occurrence probability (%)
100
75
50
25
0
0
25
50
75
100
Natural land cover (%)

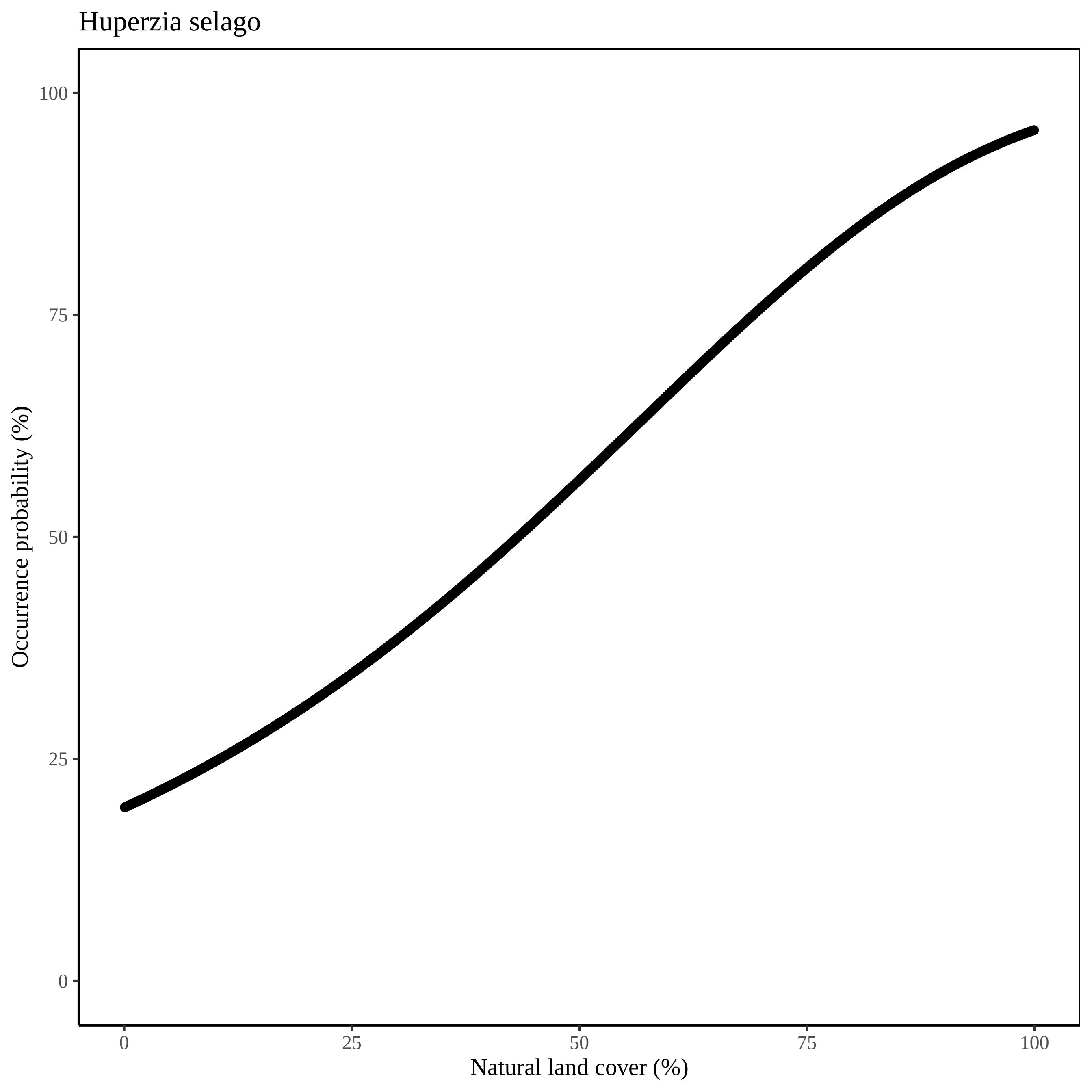
Huperzia selago
Occurrence probability (%)
100
75
50
25
0
0
25
50
75
100
Natural land cover (%)

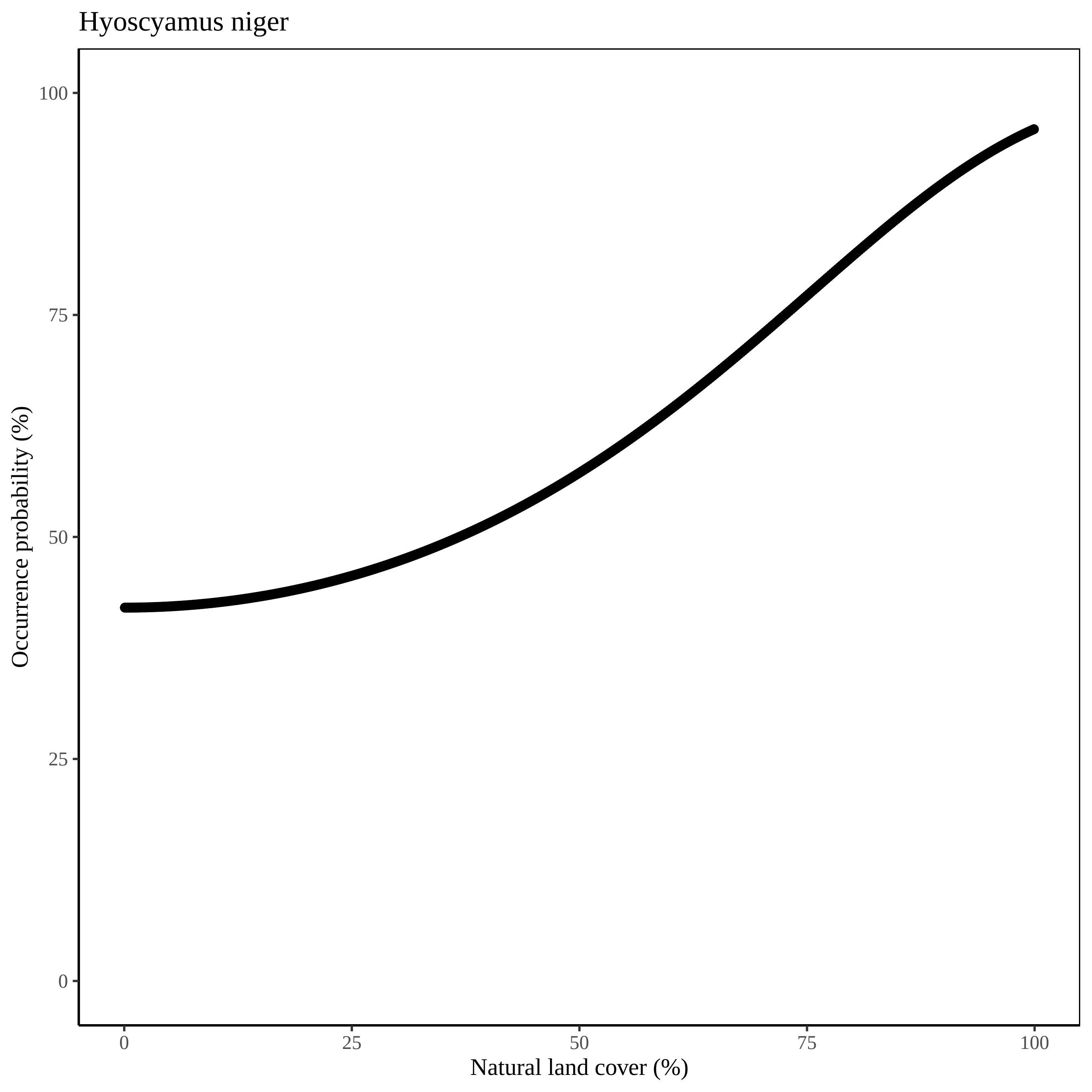
Hyoscyamus niger
Occurrence probability (%)
100
75
50
25
0
0
25
50
75
100
Natural land cover (%)

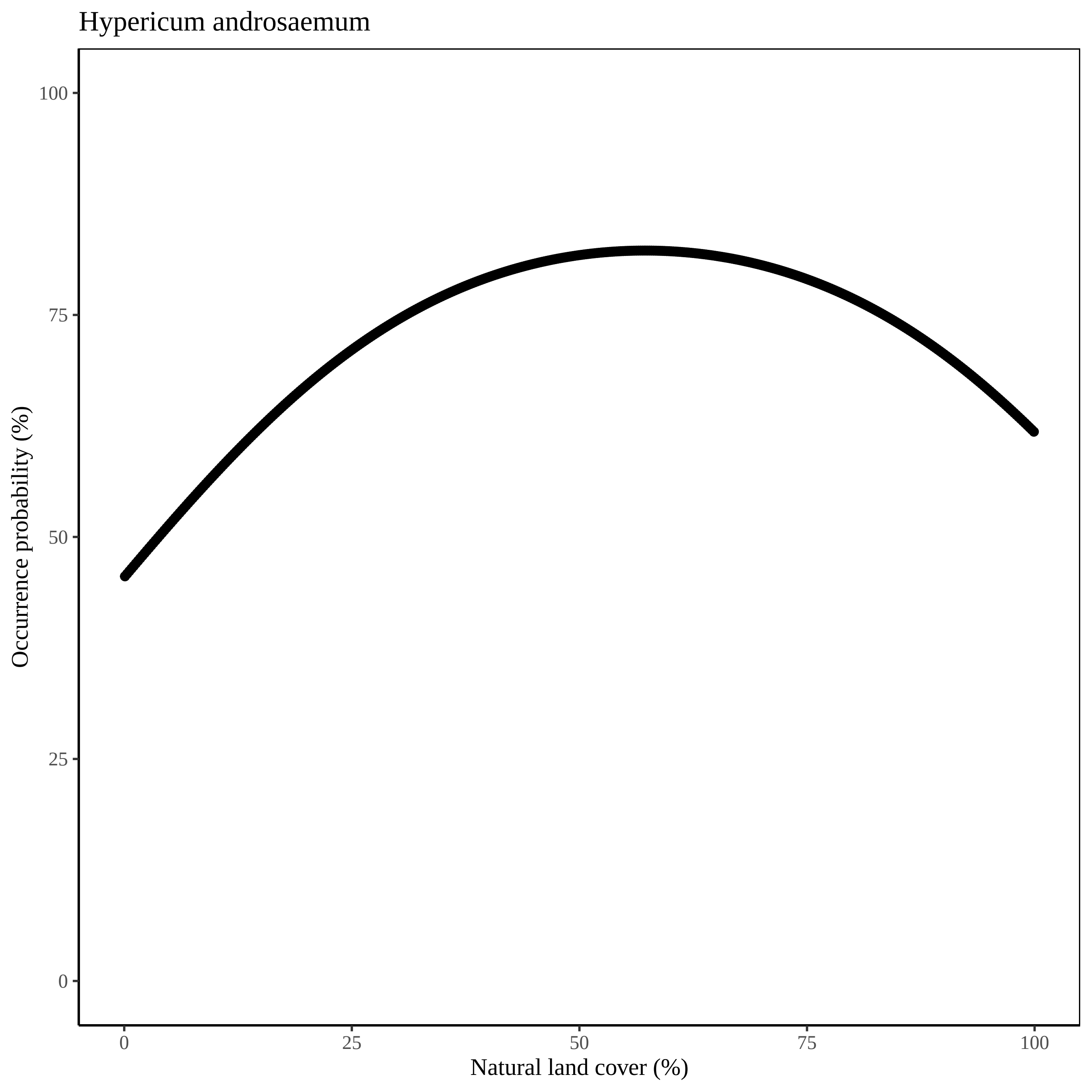
Hypericum androsaemum
Occurrence probability (%)
100
75
50
25
0
0
25
50
75
100
Natural land cover (%)

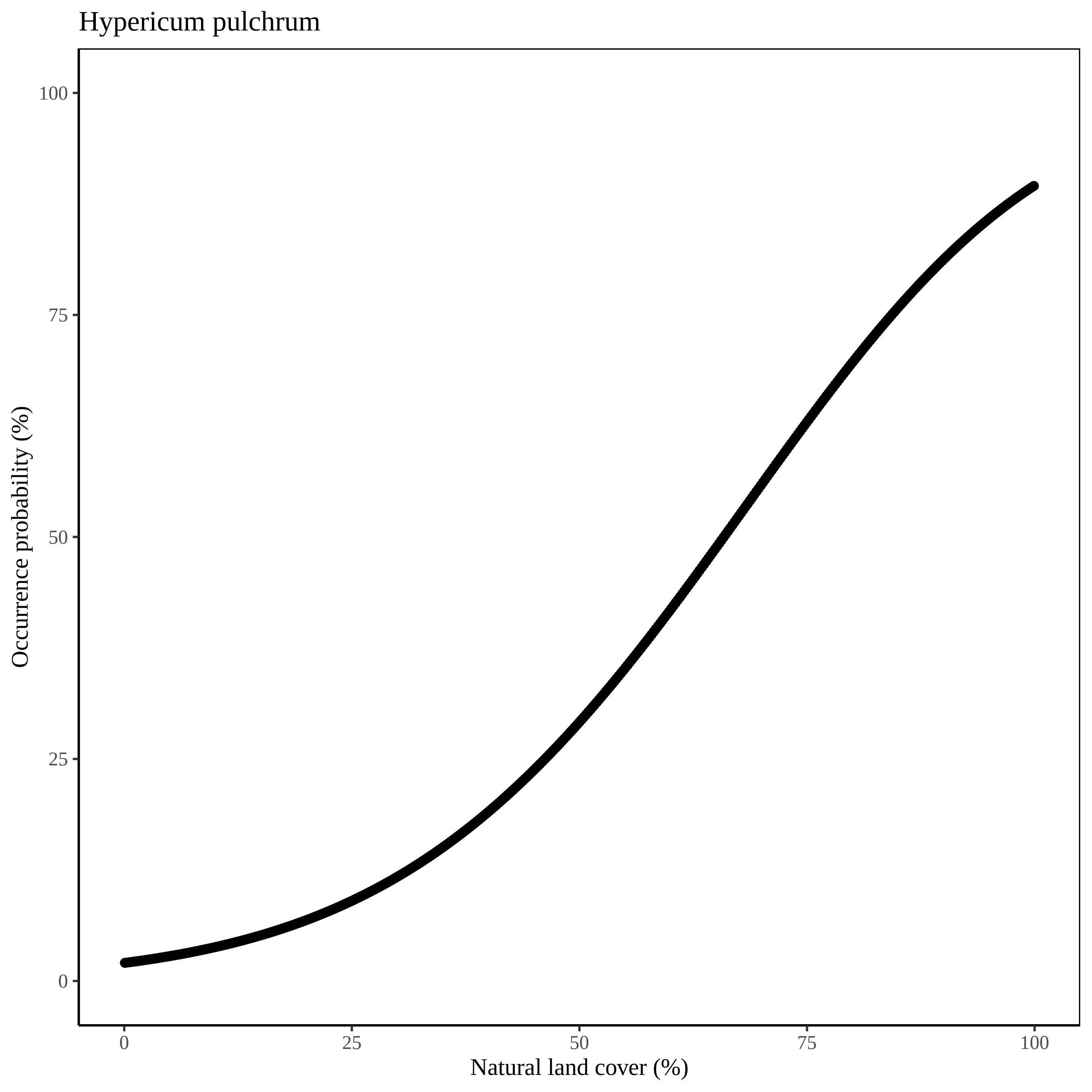
Hypericum pulchrum
Occurrence probability (%)
100
75
50
25
0
0
25
50
75
100
Natural land cover (%)

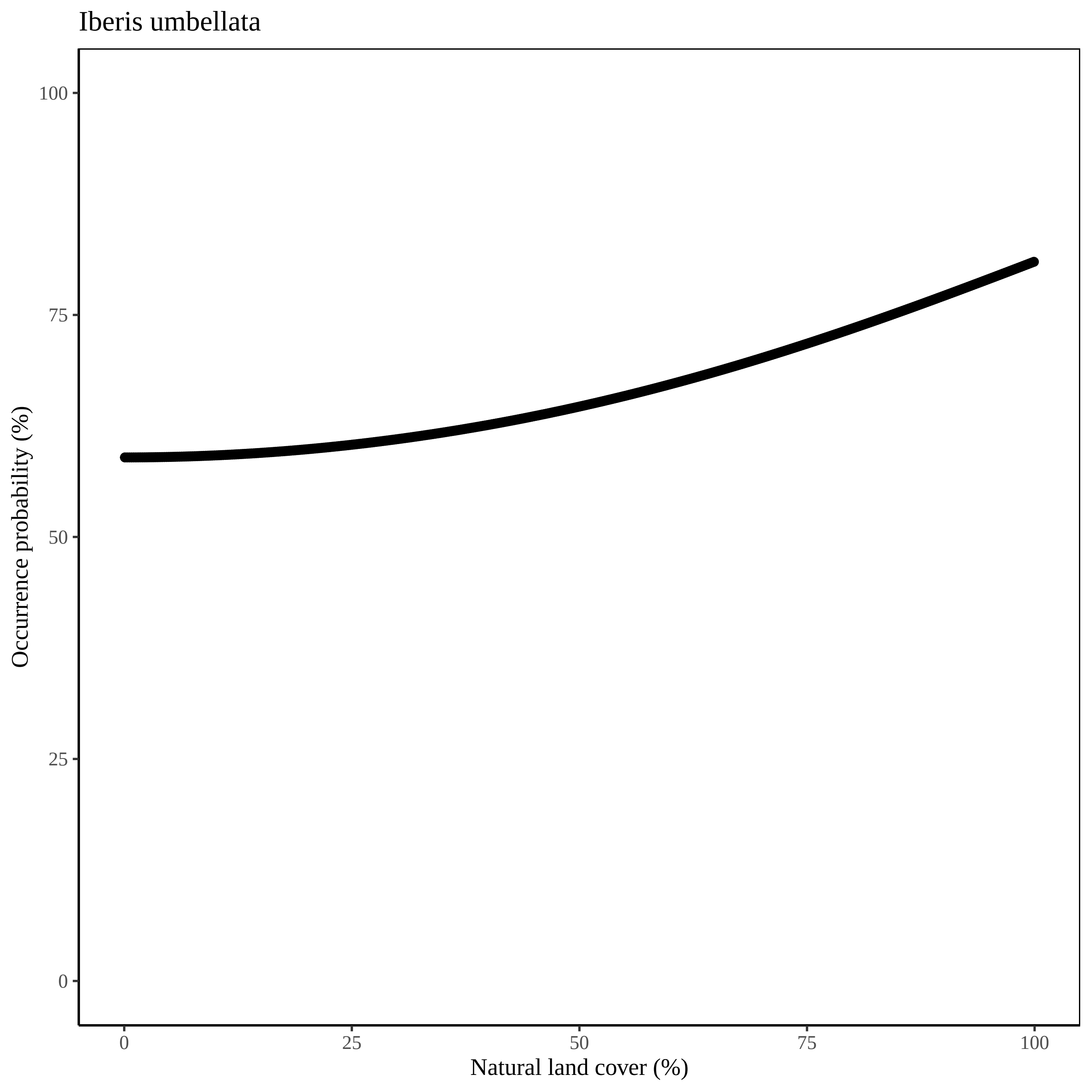

Iberis umbellata
Occurrence probability (%)
100
75
50
25
0
0
25
50
75
100
Natural land cover (%)

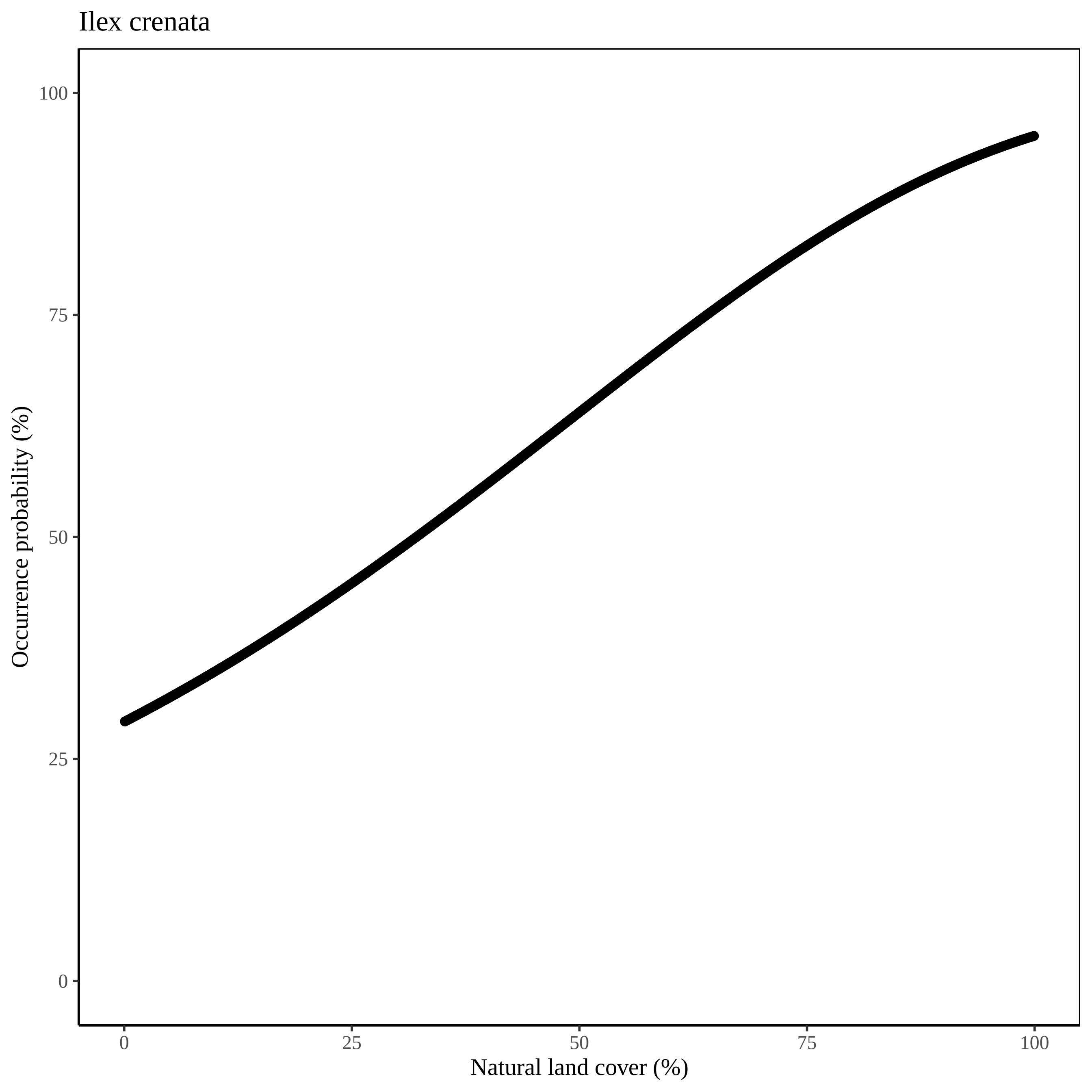
Ilex crenata
Occurrence probability (%)
100
75
50
25
0
0
25
50
75
100
Natural land cover (%)

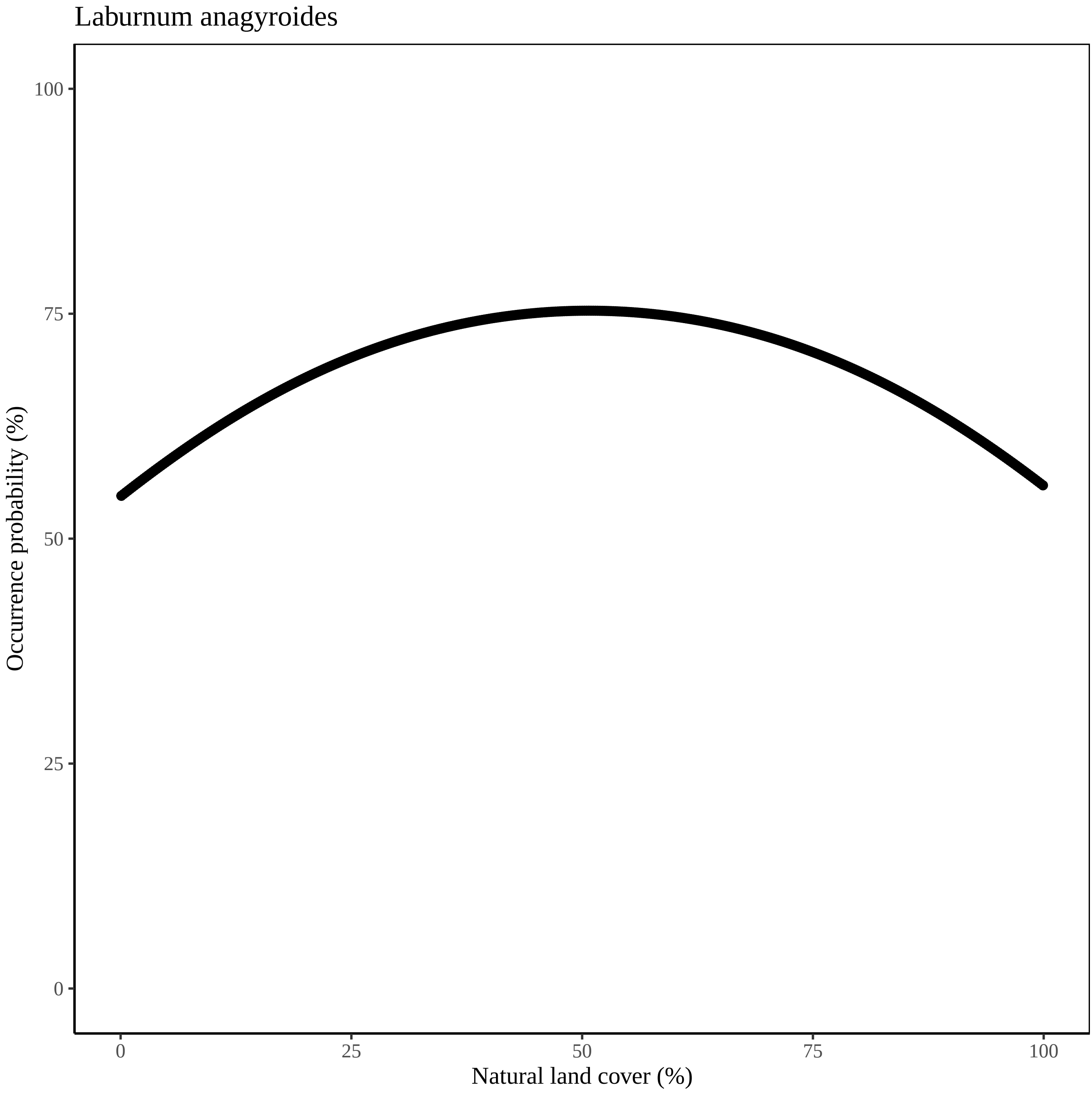
Laburnum anagyroides
Occurrence probability (%)
100
75
50
25
0
0
25
50
75
100
Natural land cover (%)

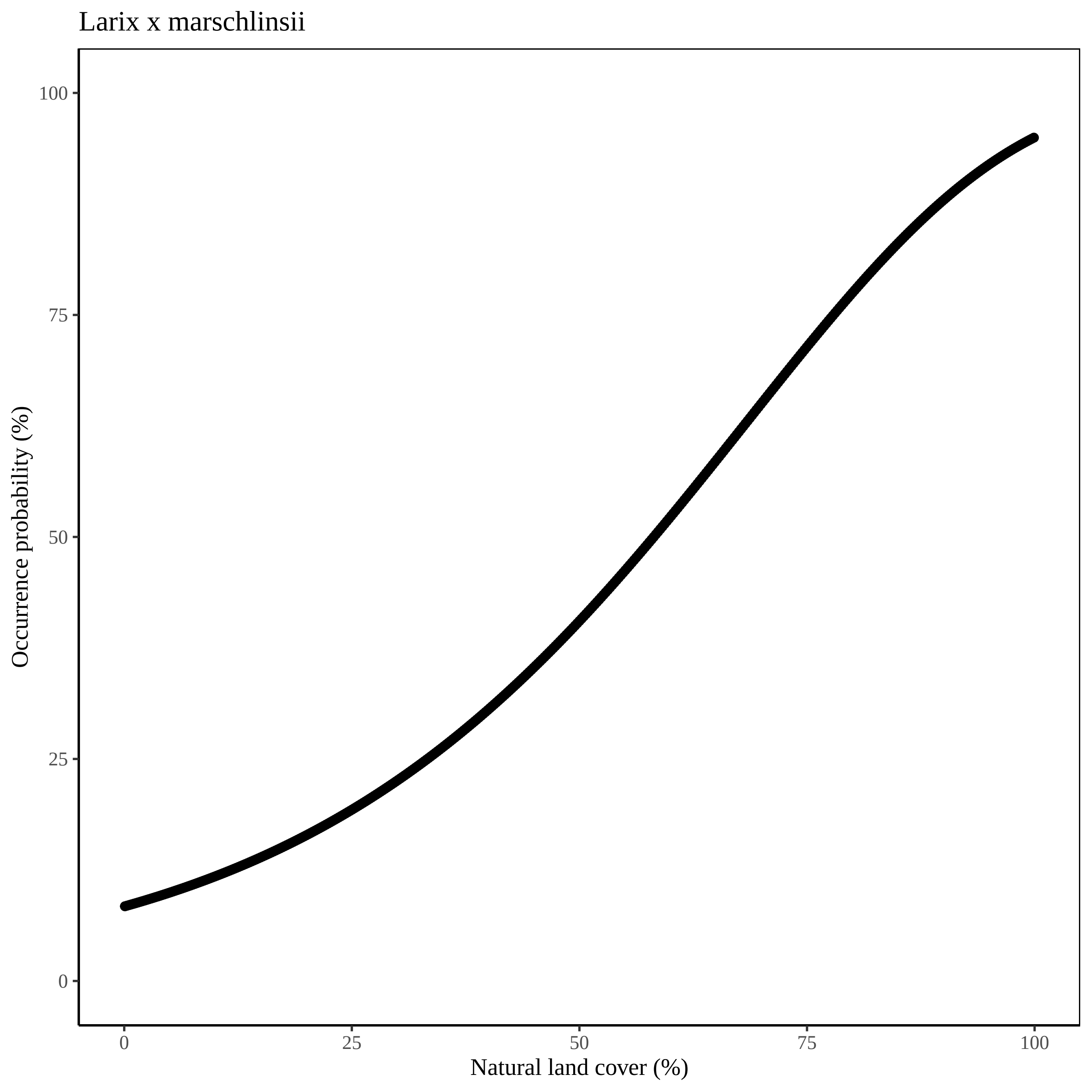
Larix x marschlinsii
Occurrence probability (%)
100
75
50
25
0
0
25
50
75
100
Natural land cover (%)

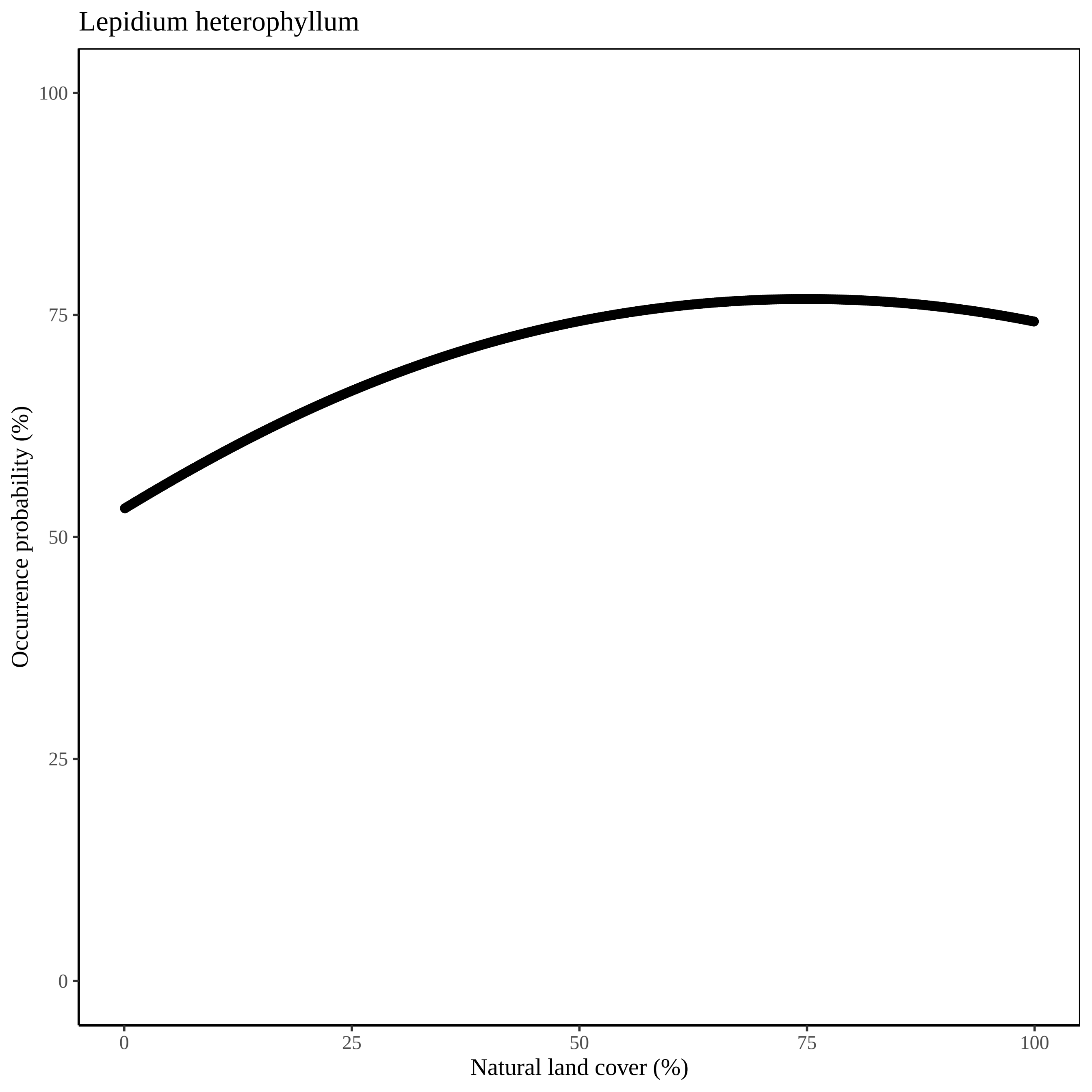
Lepidium heterophyllum
Occurrence probability (%)
100
75
50
25
0
0
25
50
75
100
Natural land cover (%)

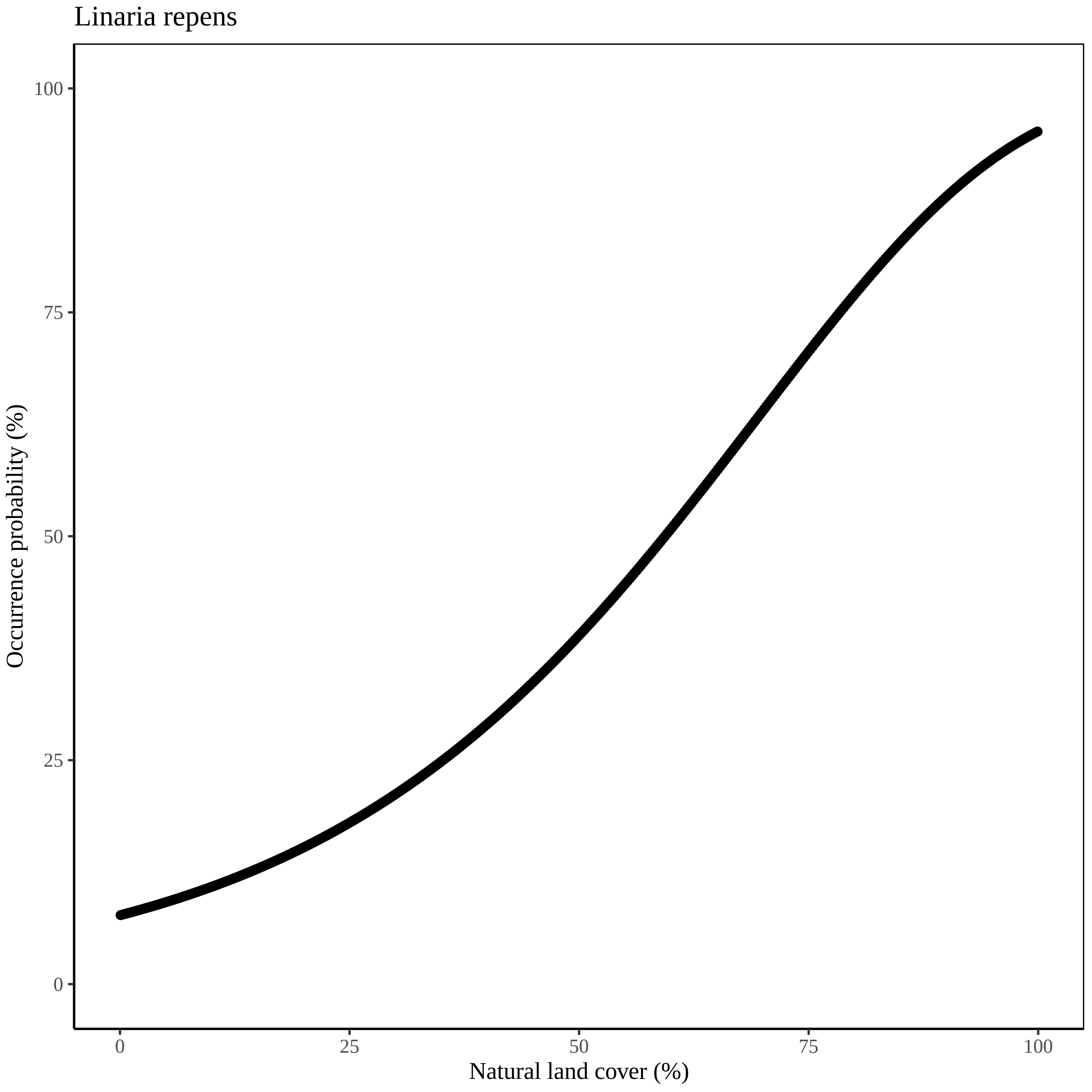
Linaria repens
100
75
50
25
0
Occurrence probability (%)
0
25
50
75
100
Natural land cover (%)

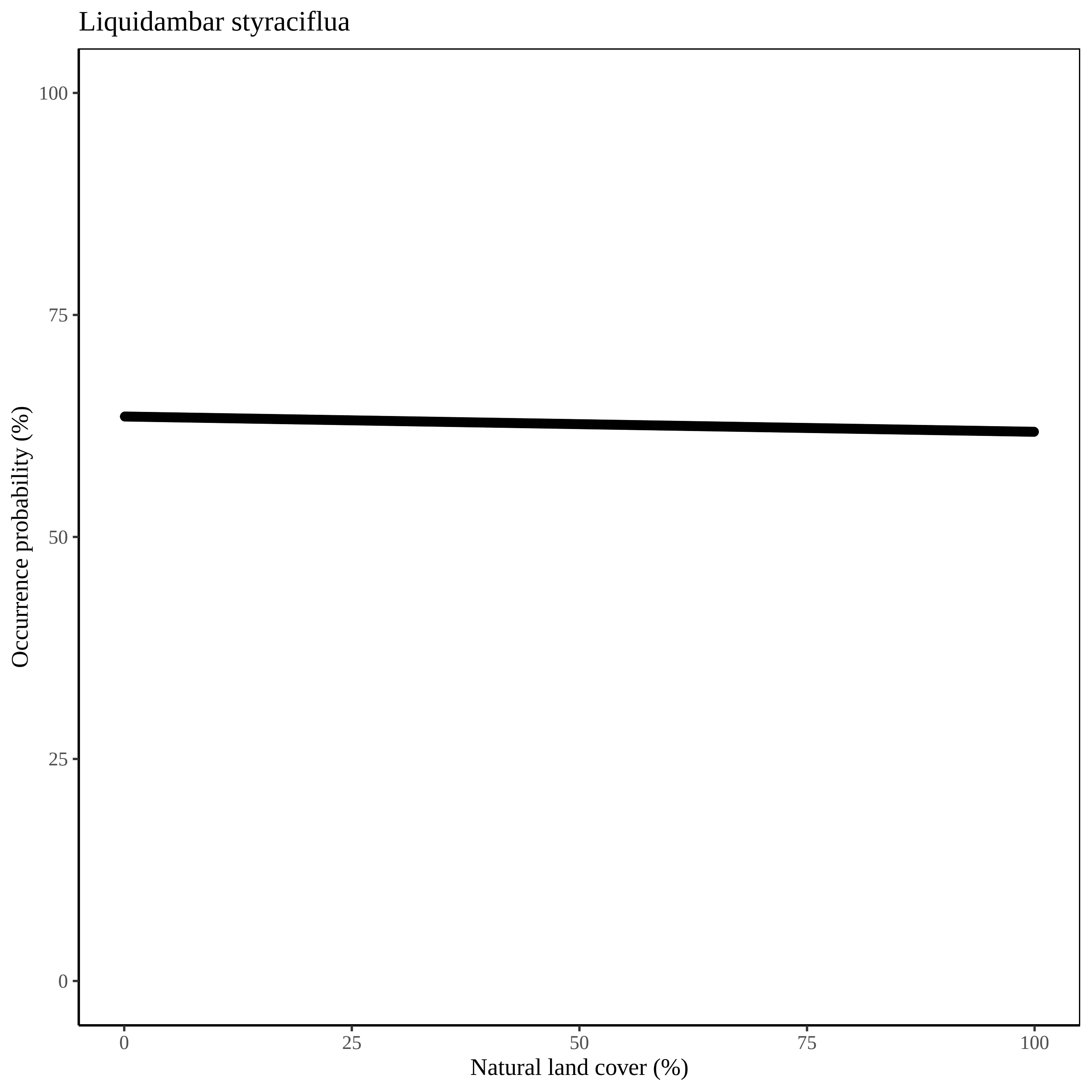
Liquidambar styraciflua
Occurrence probability (%)
100
75
50
25
0
0
25
50
75
100
Natural land cover (%)

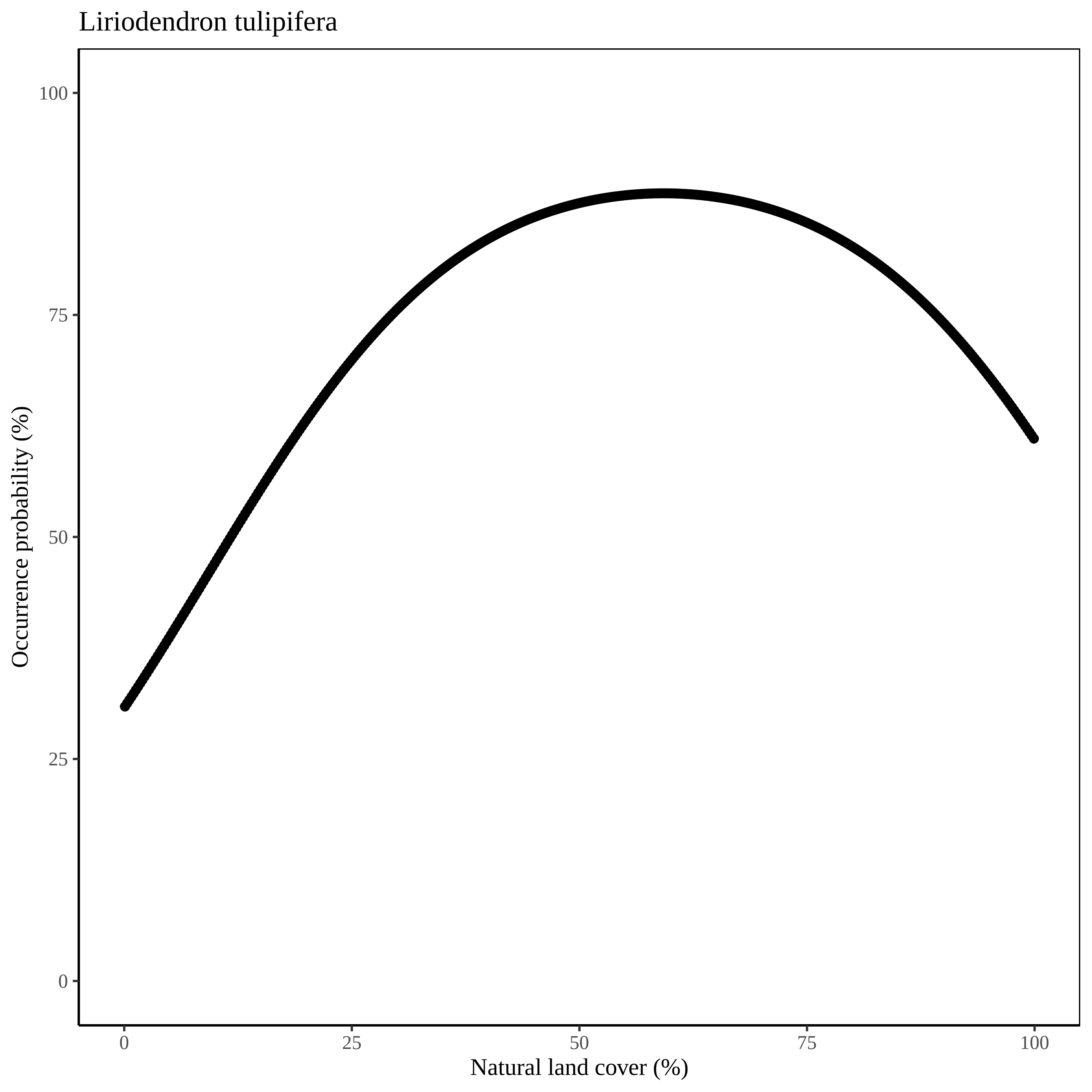
Liriodendron tulipifera
Occurrence probability (%)
100
75
50
25
0
0
25
50
75
100
Natural land cover (%)

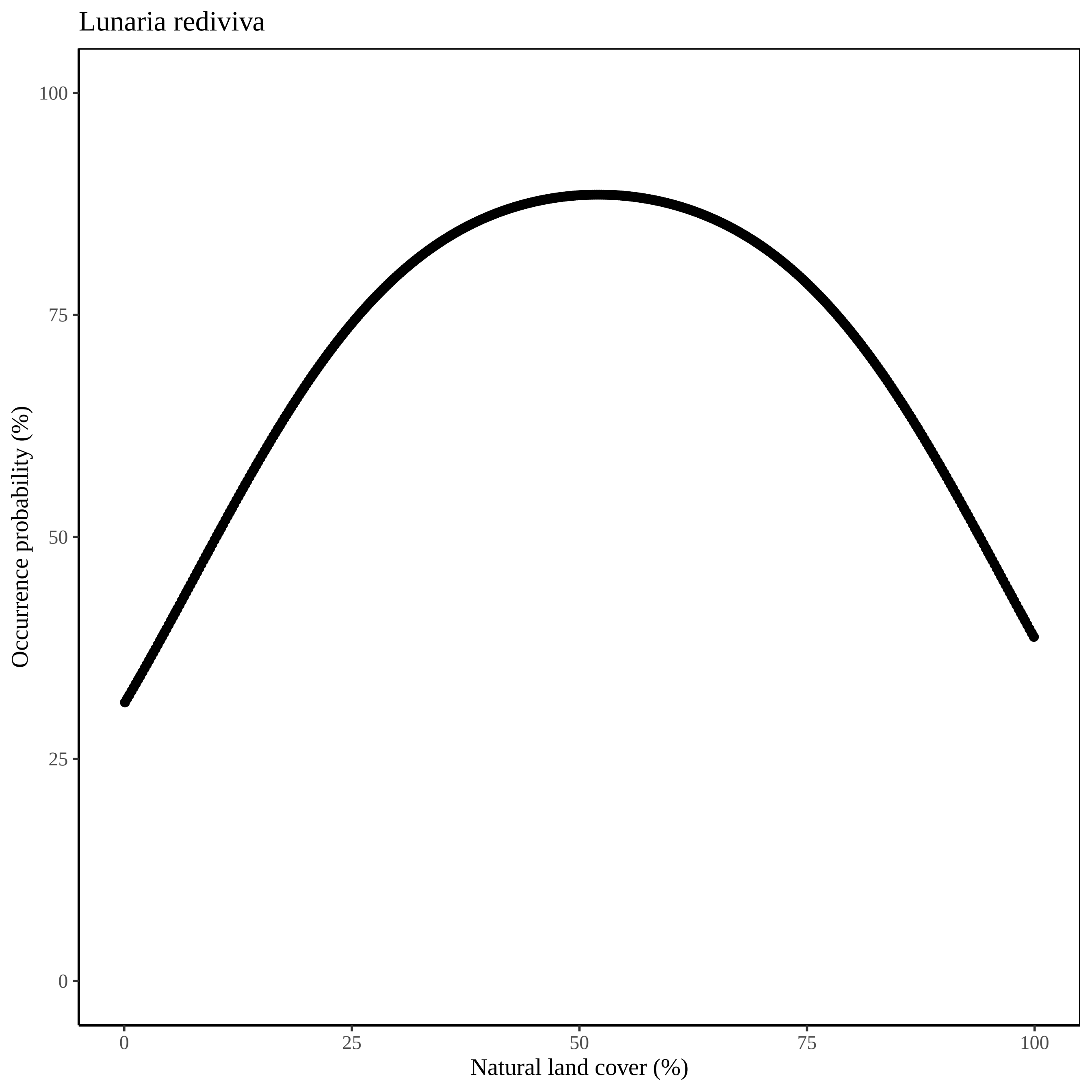

Lunaria rediviva
Occurrence probability (%)
100
75
50
25
0
Natural land cover (%)
0
25
50
75
100

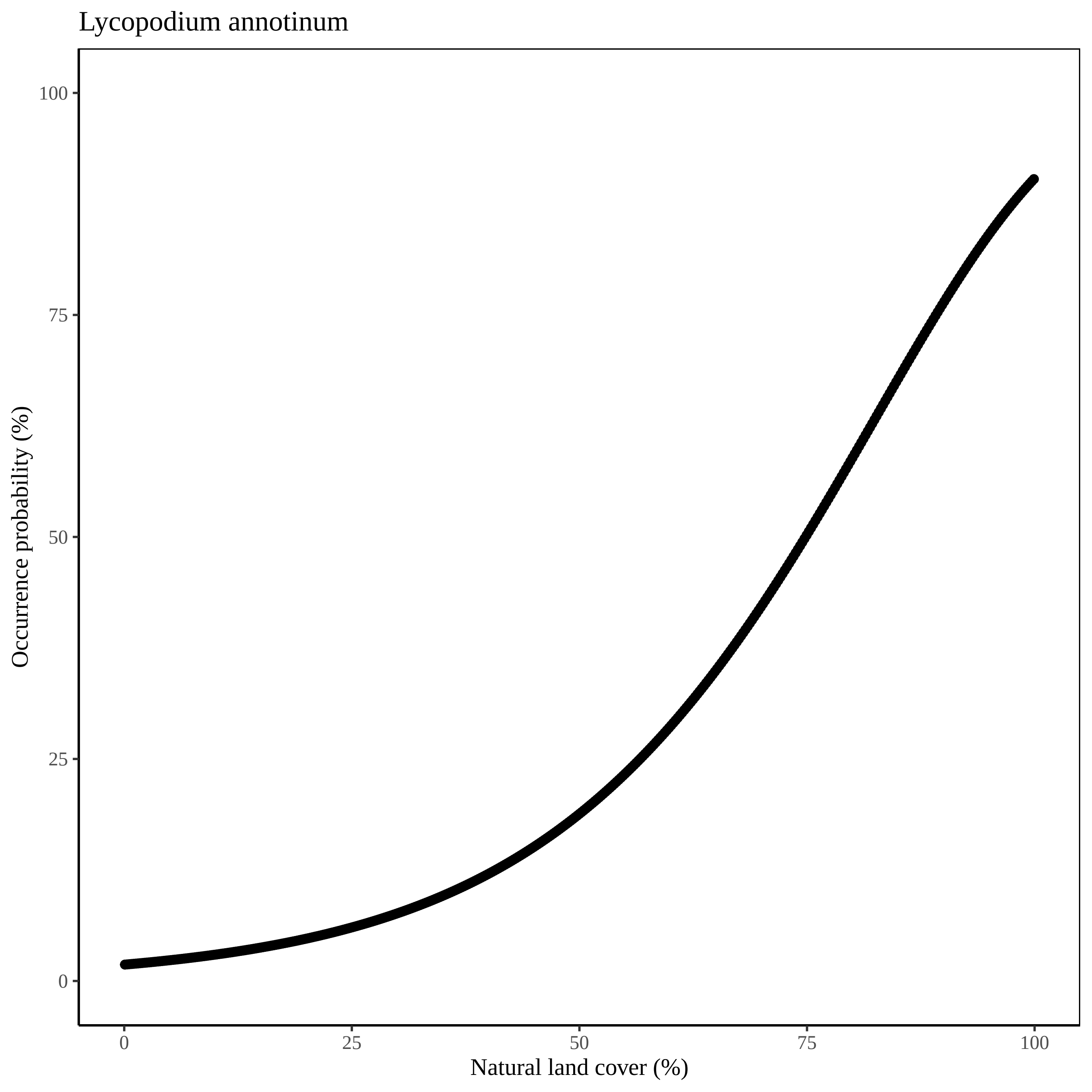
Lycopodium annotinum
Occurrence probability (%)
100
75
50
25
0
0
25
50
75
100
Natural land cover (%)

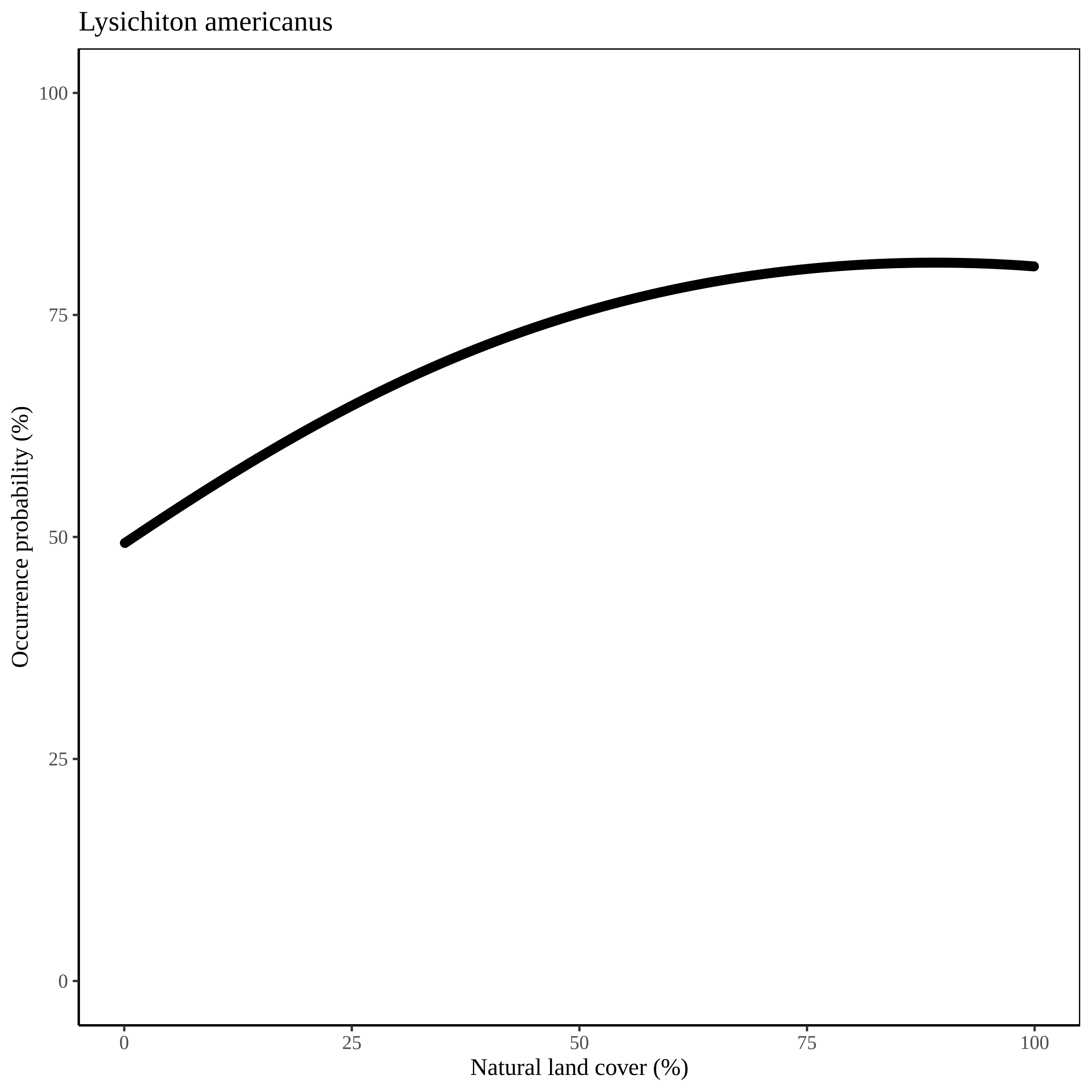

Lysichiton americanus
Occurrence probability (%)
100
75
50
25
0
0
25
50
75
100
Natural land cover (%)

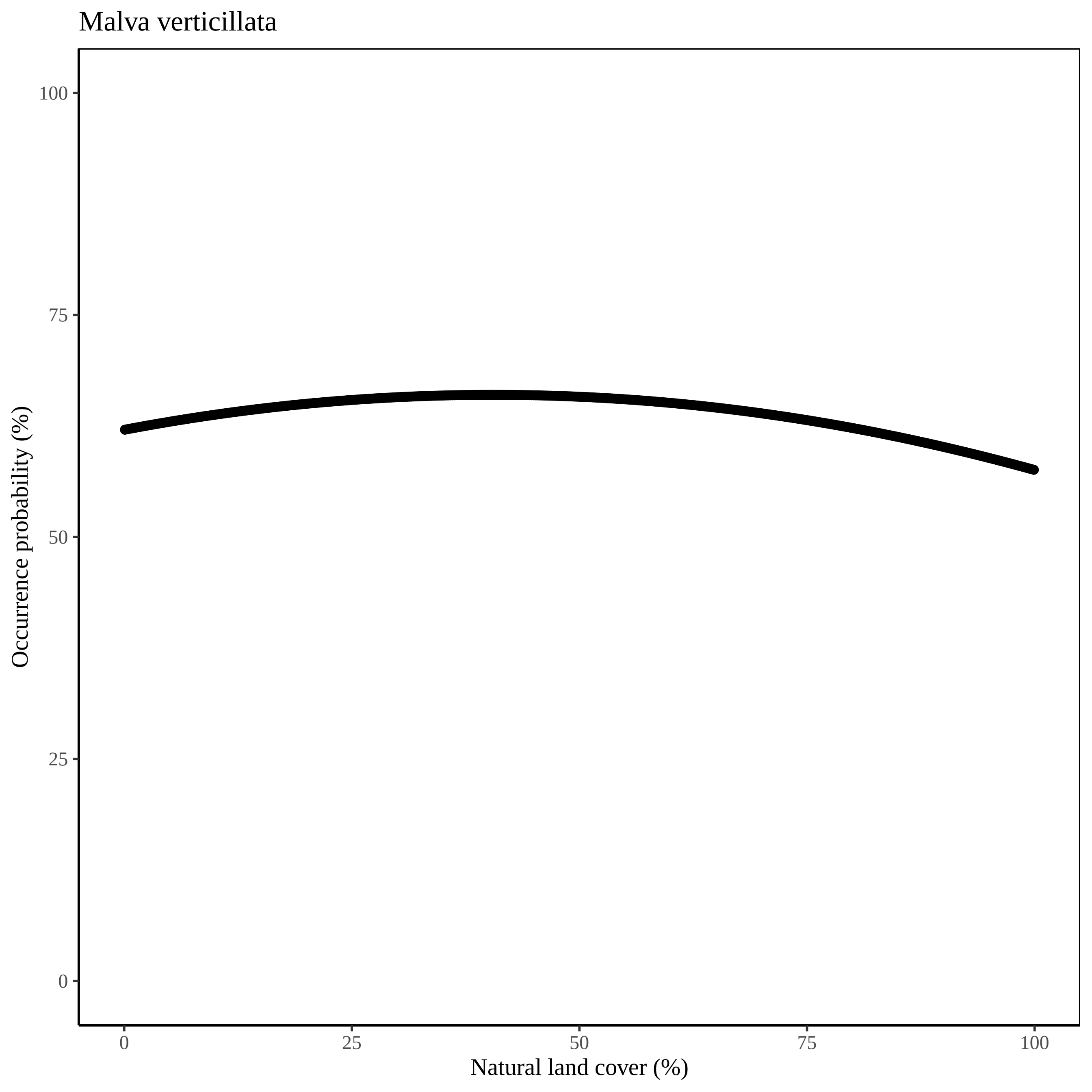

Malva verticillata
100
75
50
25
0
Occurrence probability (%)
0
25
50
75
100
Natural land cover (%)

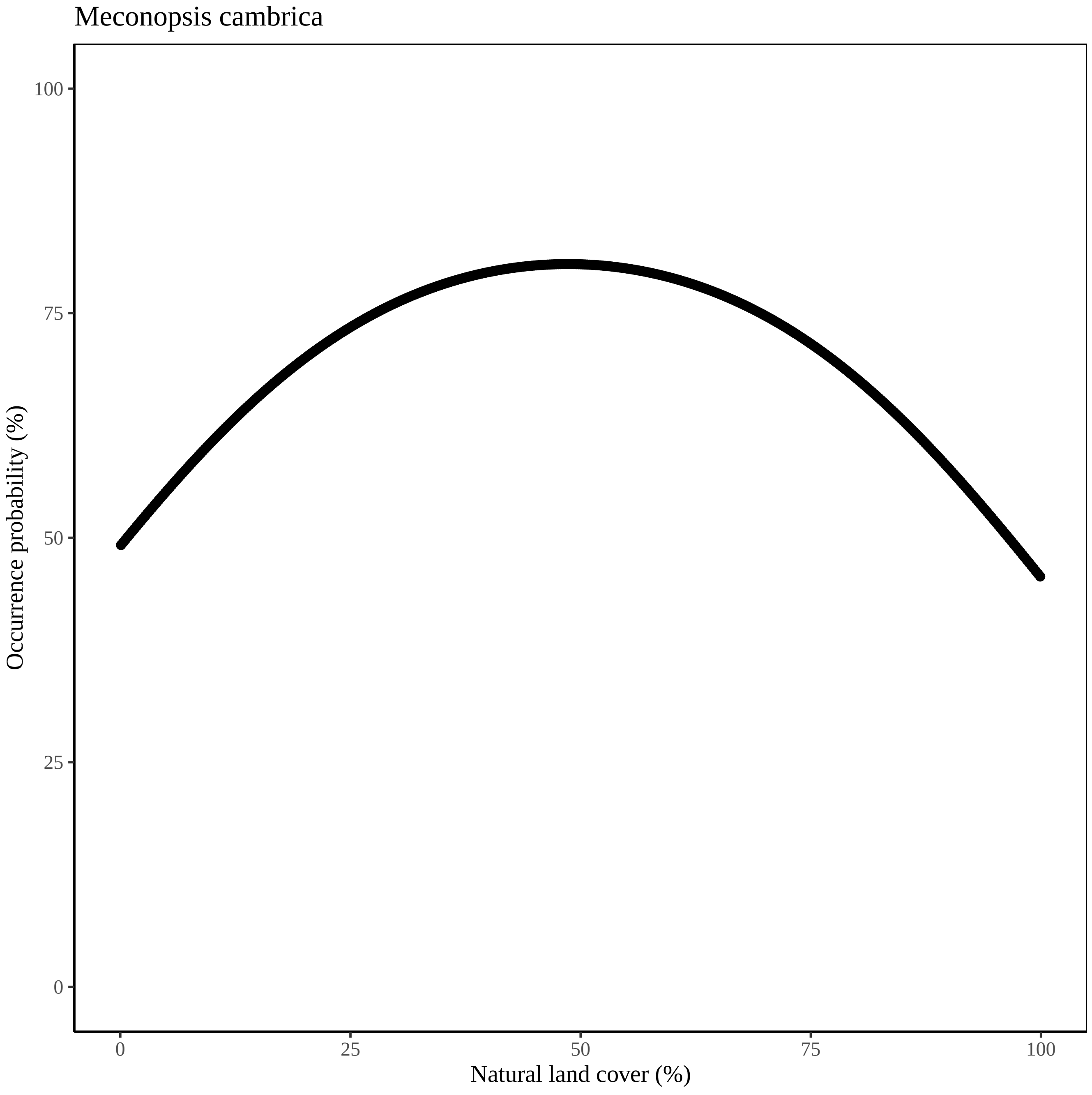

Meconopsis cambrica
Occurrence probability (%)
100
75
50
25
0
0
25
50
75
100
Natural land cover (%)

Mibora minima

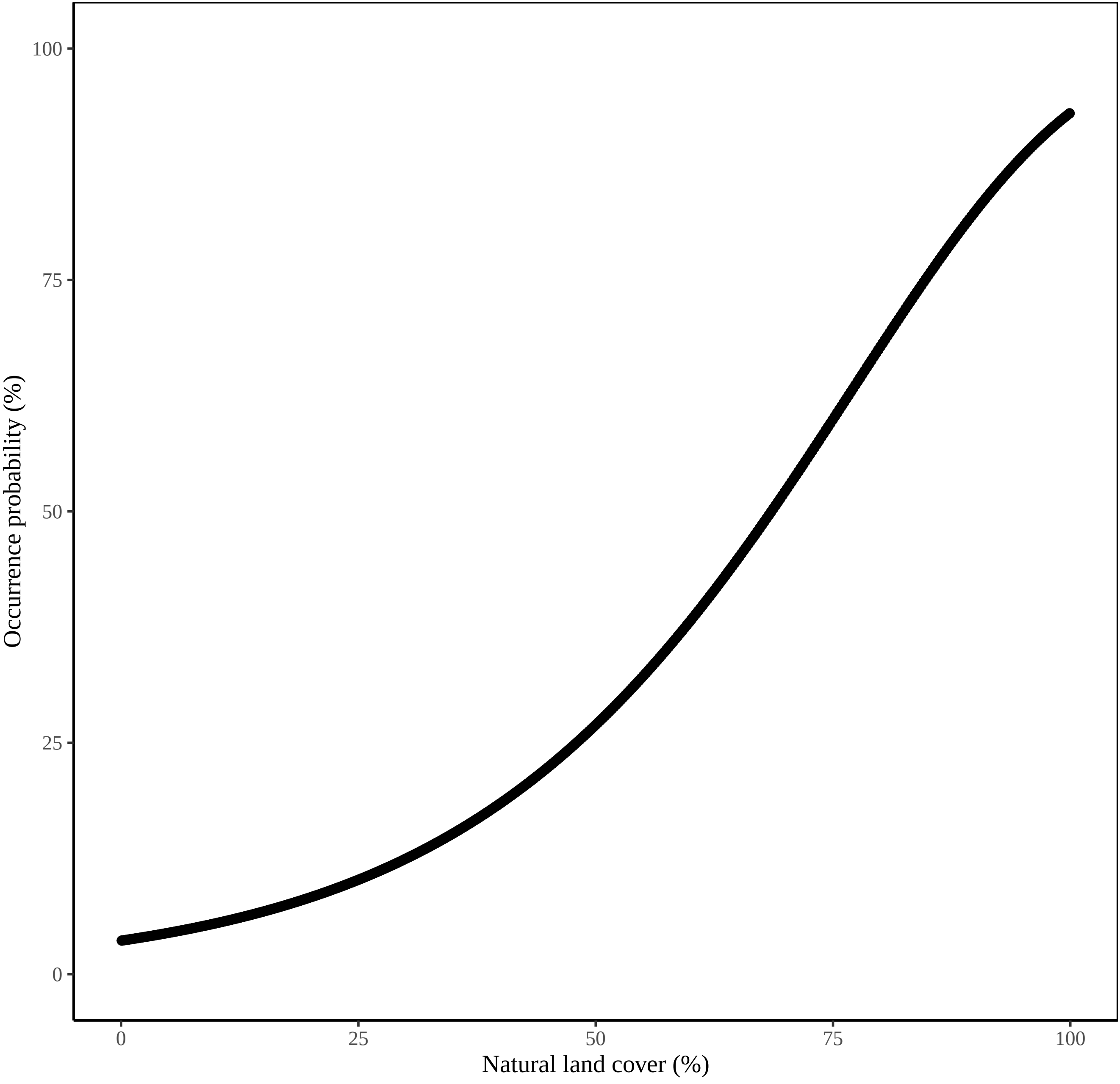

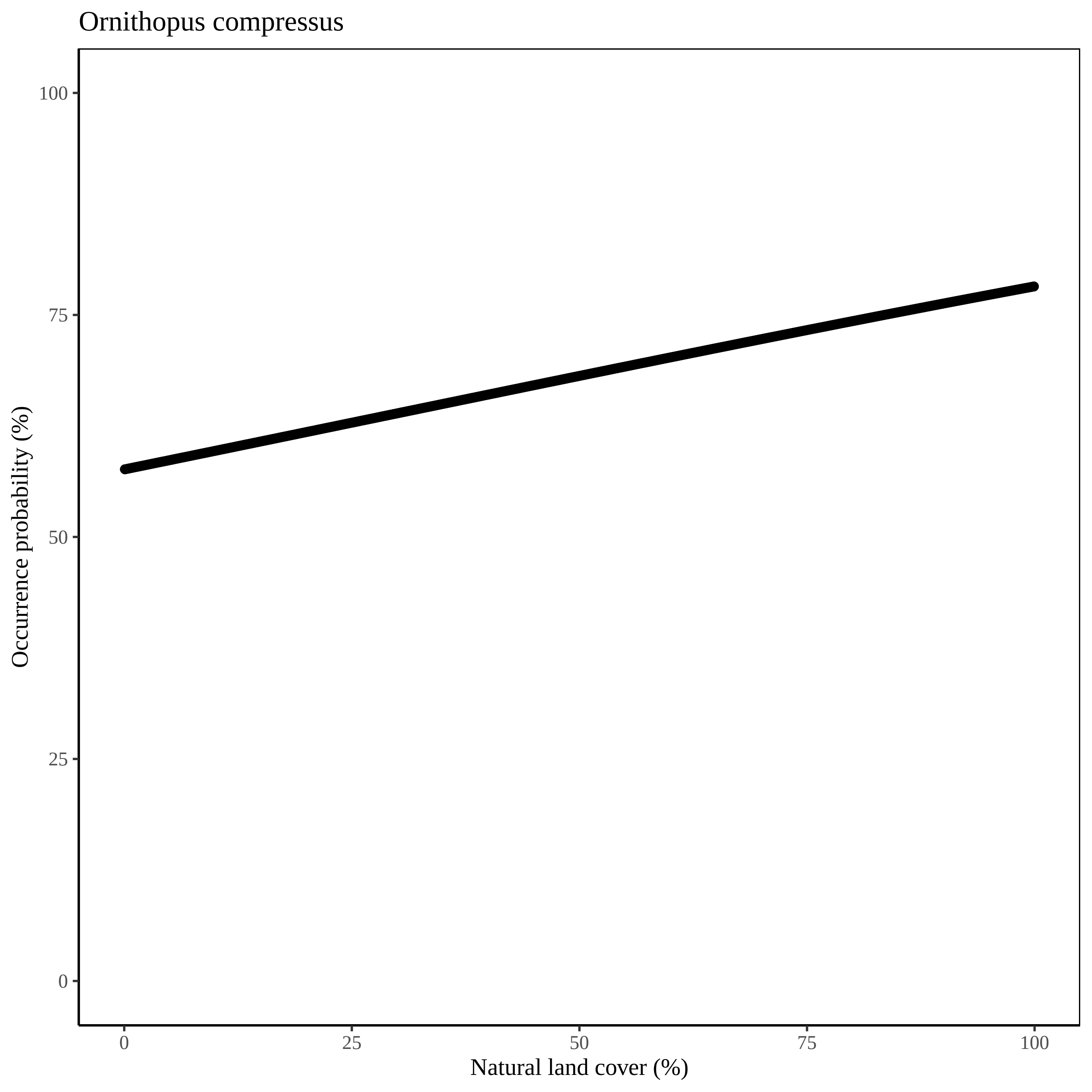
Ornithopus compressus
Occurrence probability (%)
100
75
50
25
0
0
25
50
75
100
Natural land cover (%)

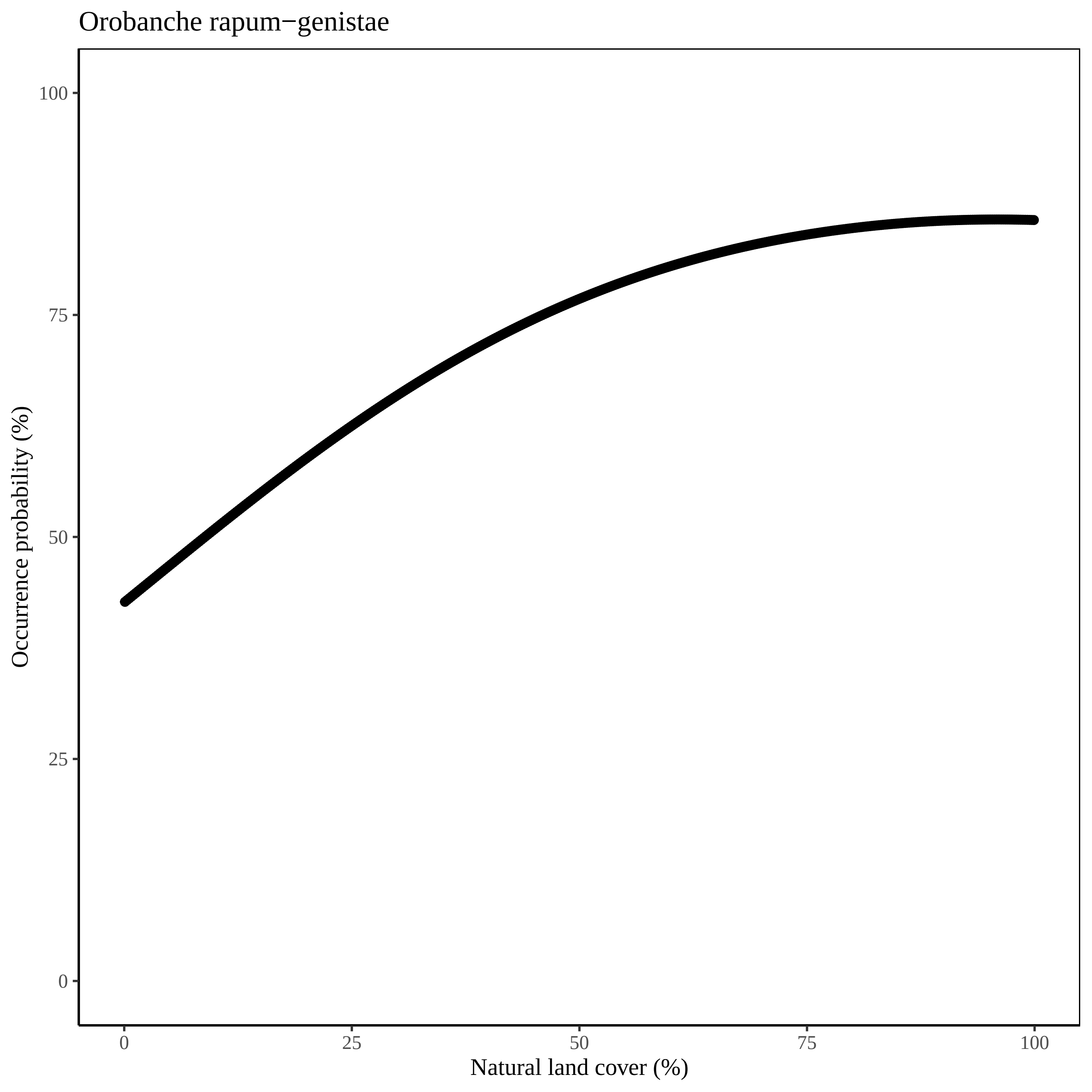

Orobanche rapum−genistae
Occurrence probability (%)
Natural land cover (%)
0
25
50
75
100

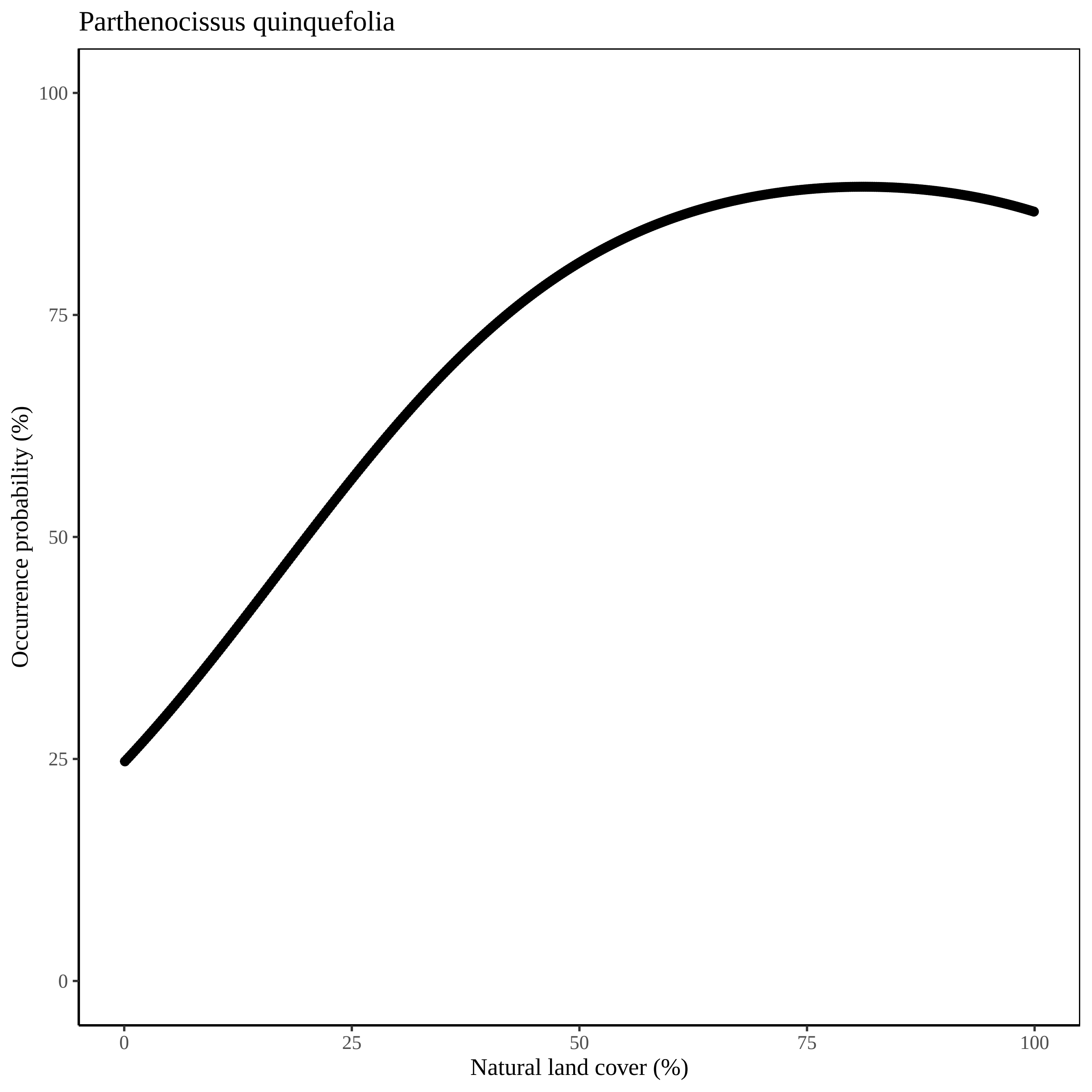
Parthenocissus quinquefolia
Occurrence probability (%)
100
75
50
25
0
0
25
50
75
100
Natural land cover (%)

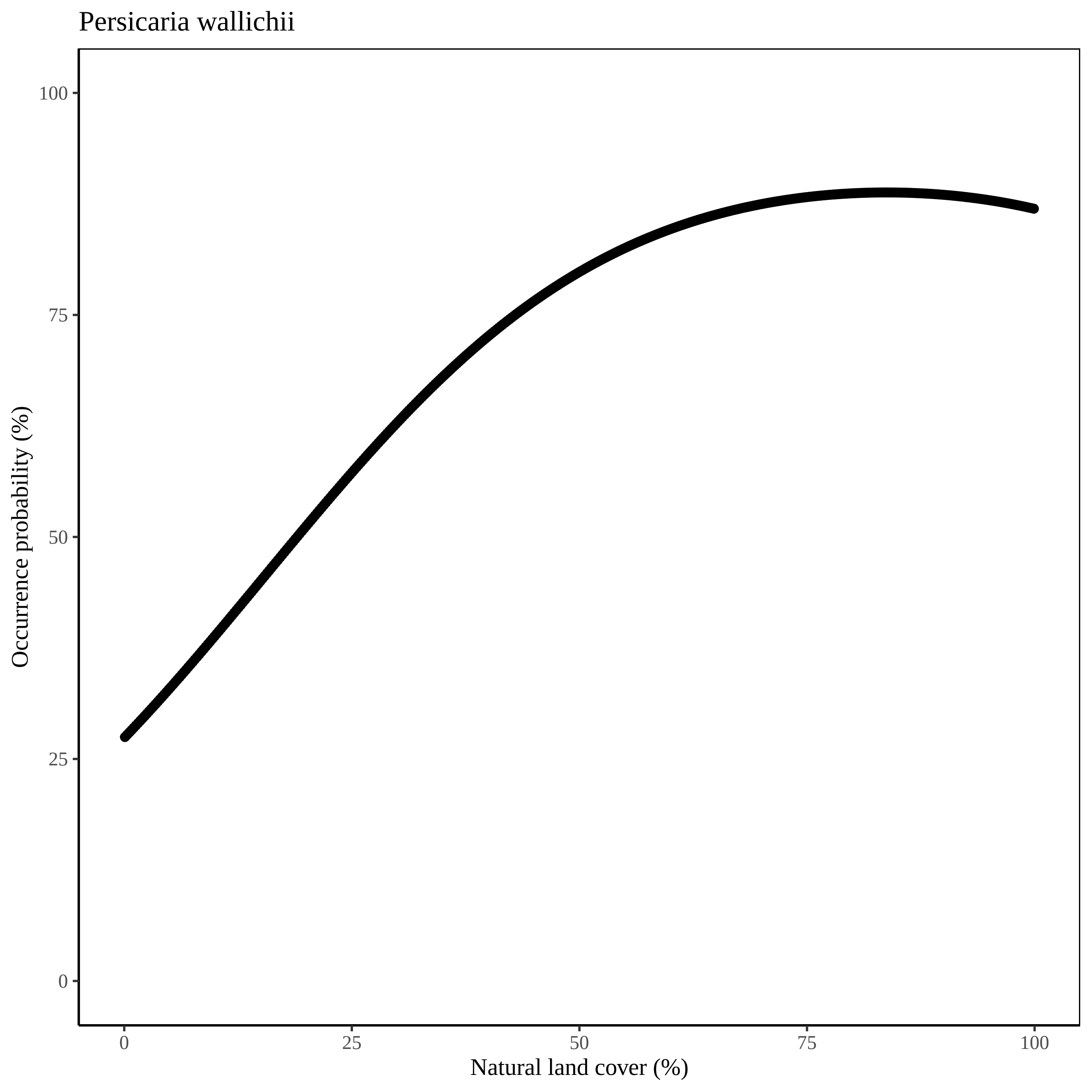

Persicaria wallichii
100
75
50
25
0
0
25
50
75
100
Occurrence probability (%)
Natural land cover (%)

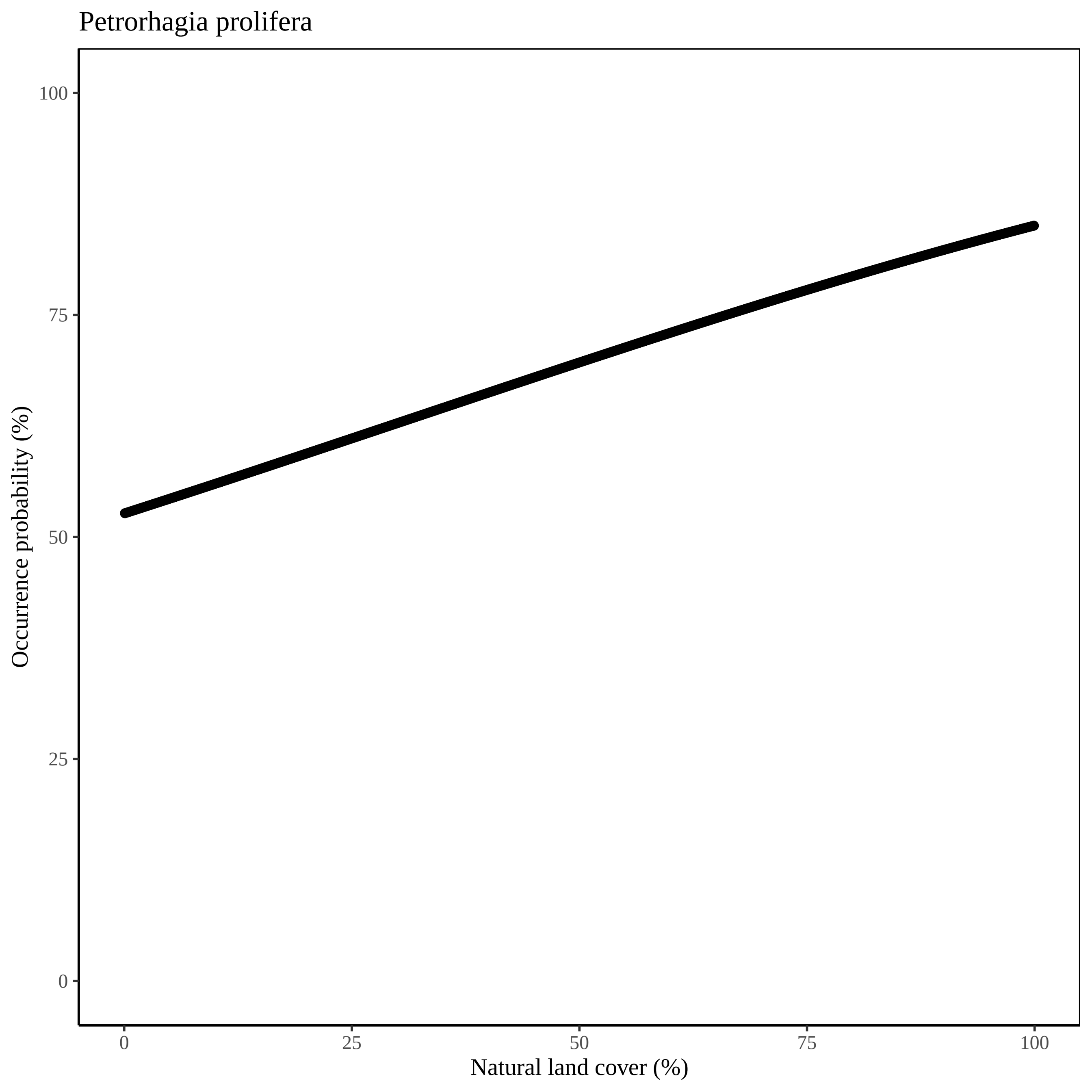
Petrorhagia prolifera
Occurrence probability (%)
100
75
50
25
0
0
25
50
75
100
Natural land cover (%)

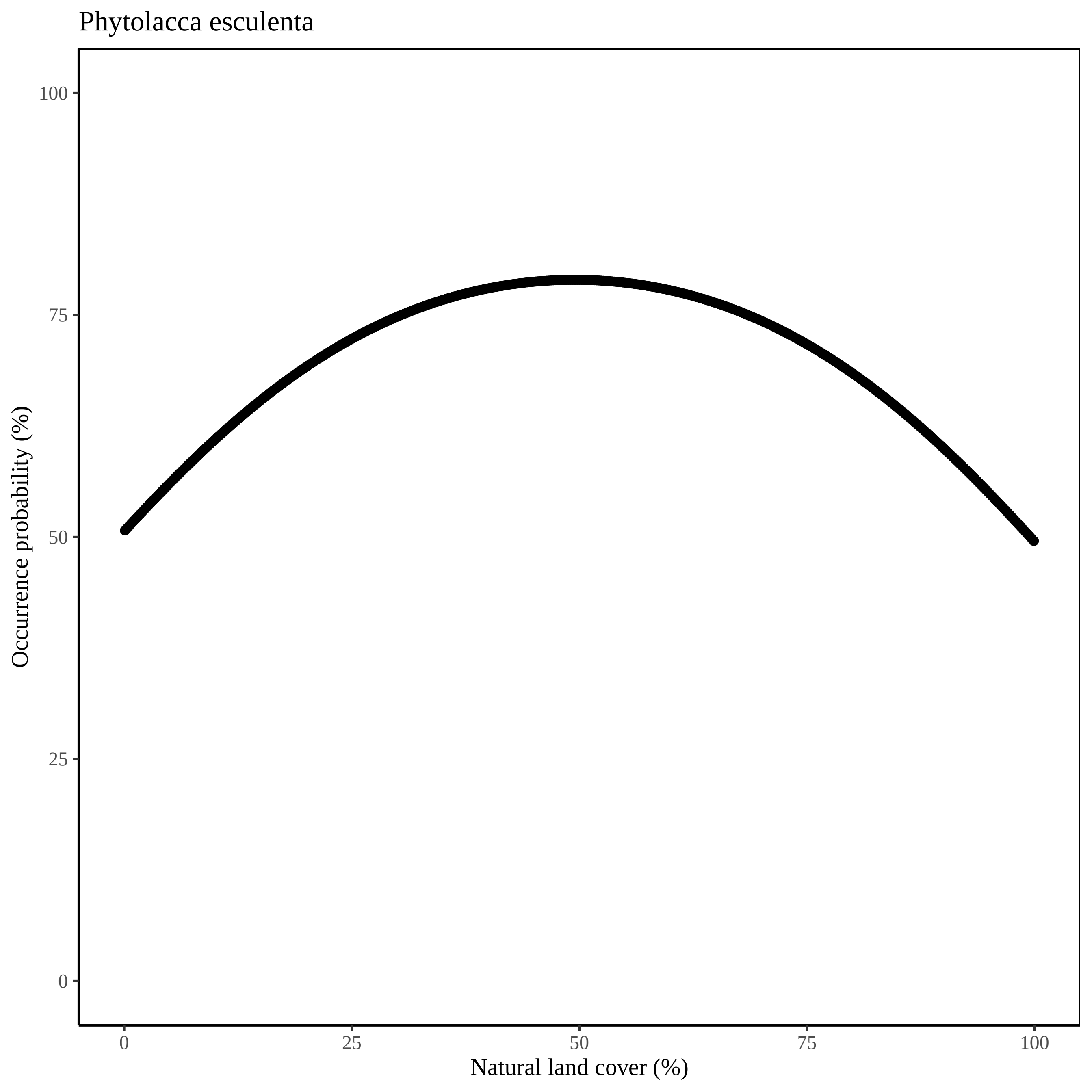
Phytolacca esculenta
Occurrence probability (%)
0
25
50
75
100
Natural land cover (%)

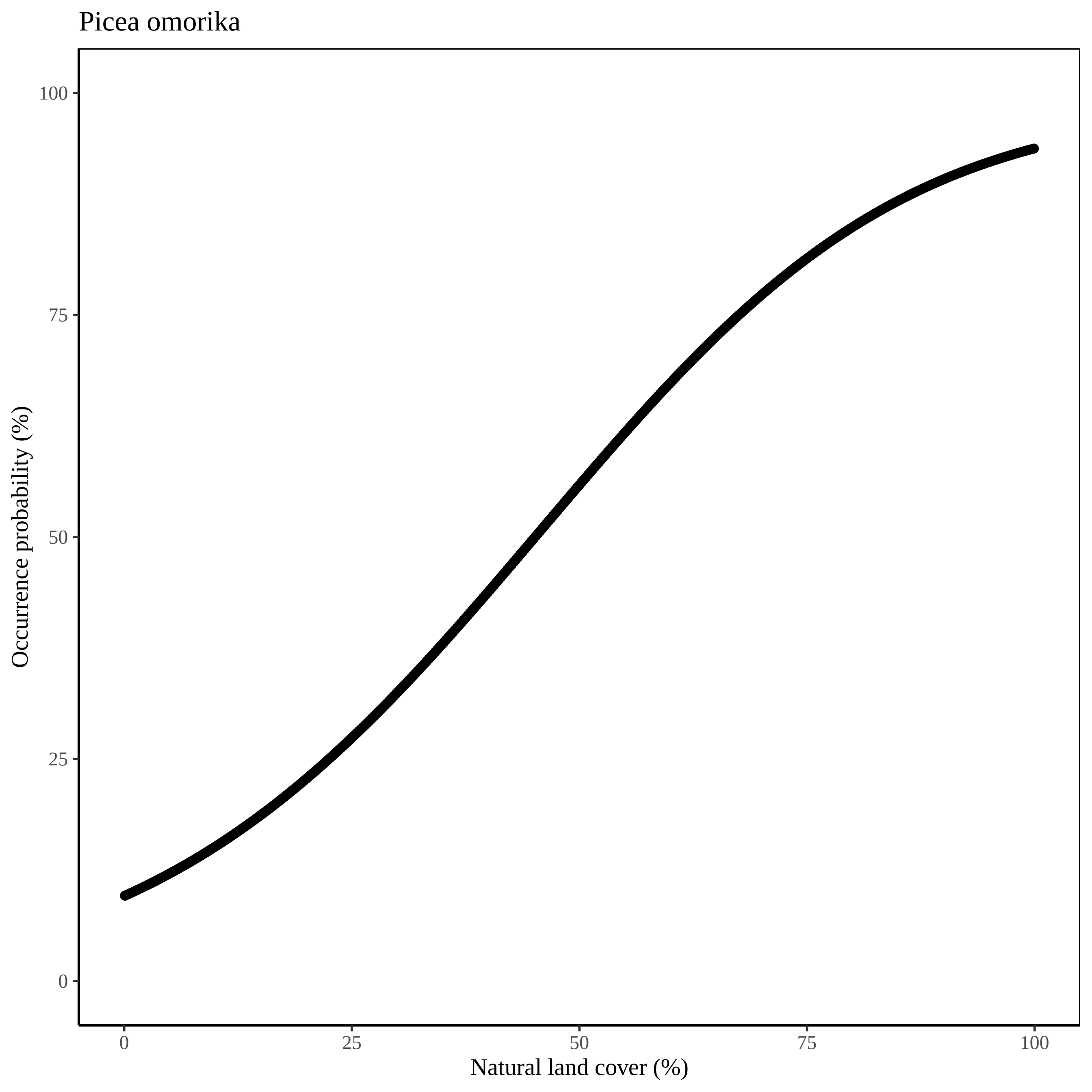
Picea omorika
Occurrence probability (%)
100
75
50
25
0
0
25
50
75
100
Natural land cover (%)

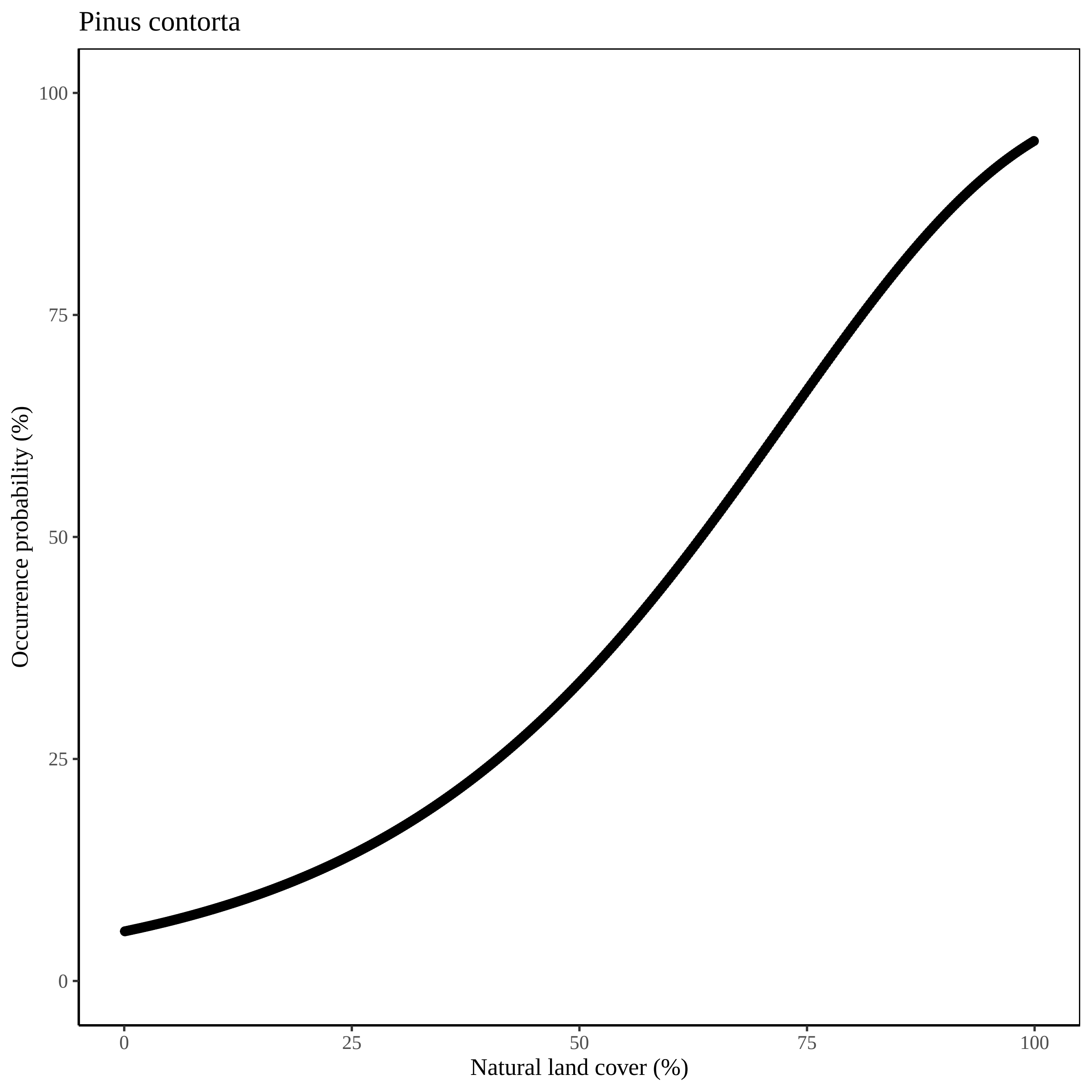

Pinus contorta
Occurrence probability (%)
100
75
50
25
0
0
25
50
75
100
Natural land cover (%)

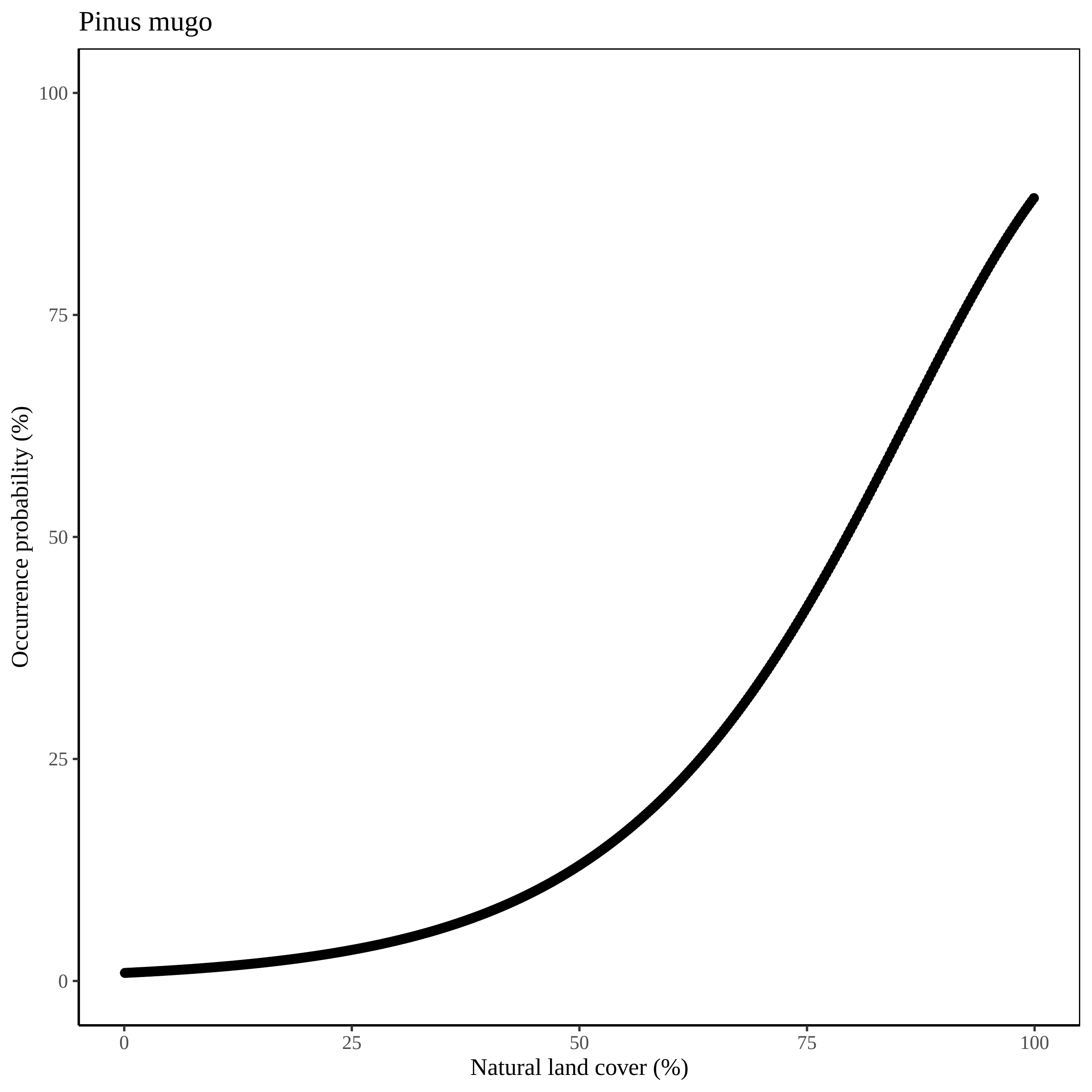

Pinus mugo
100
75
50
25
0
0
25
50
75
100
Occurrence probability (%)
Natural land cover (%)

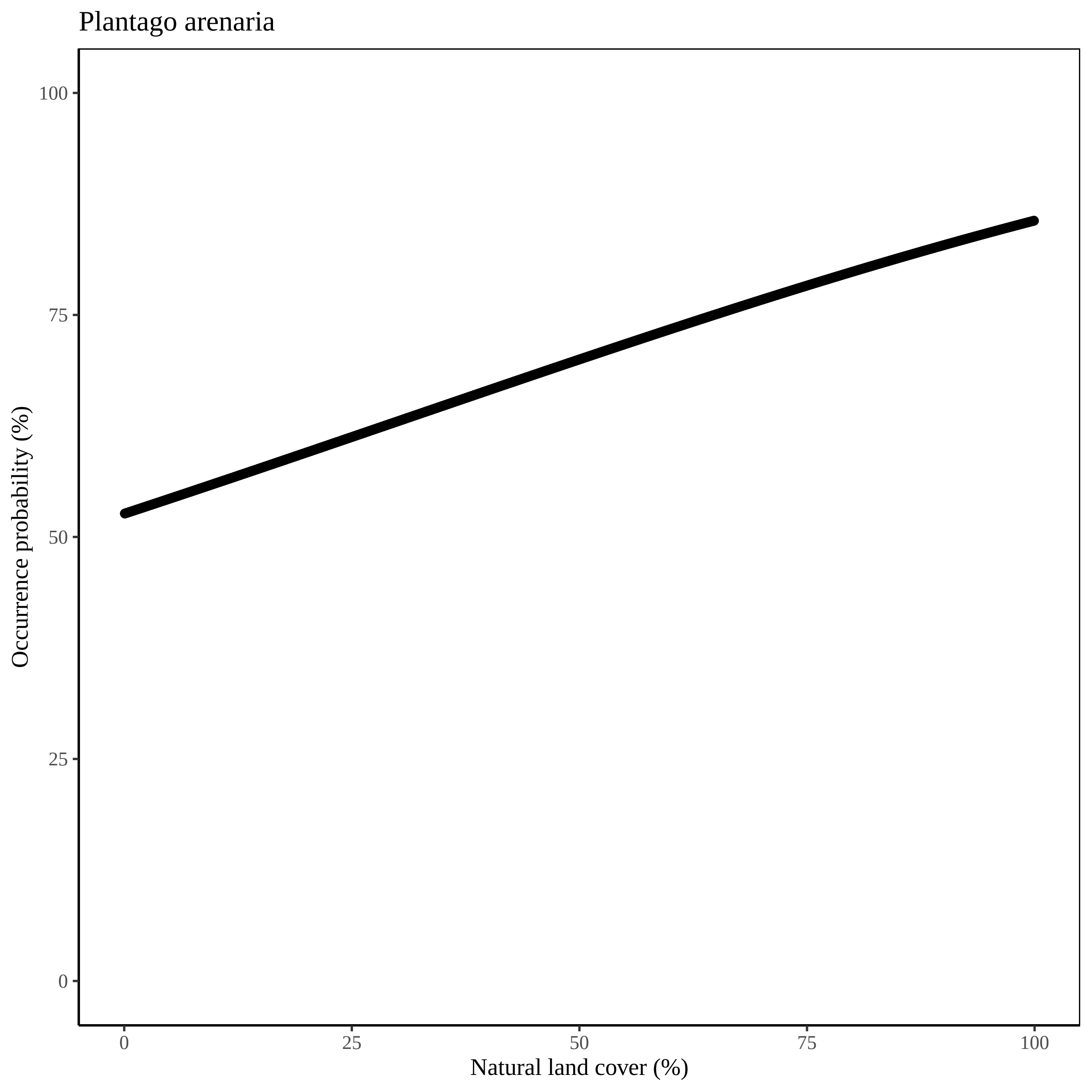

Plantago arenaria
100
75
50
25
0
Occurrence probability (%)
0
25
50
75
100
Natural land cover (%)

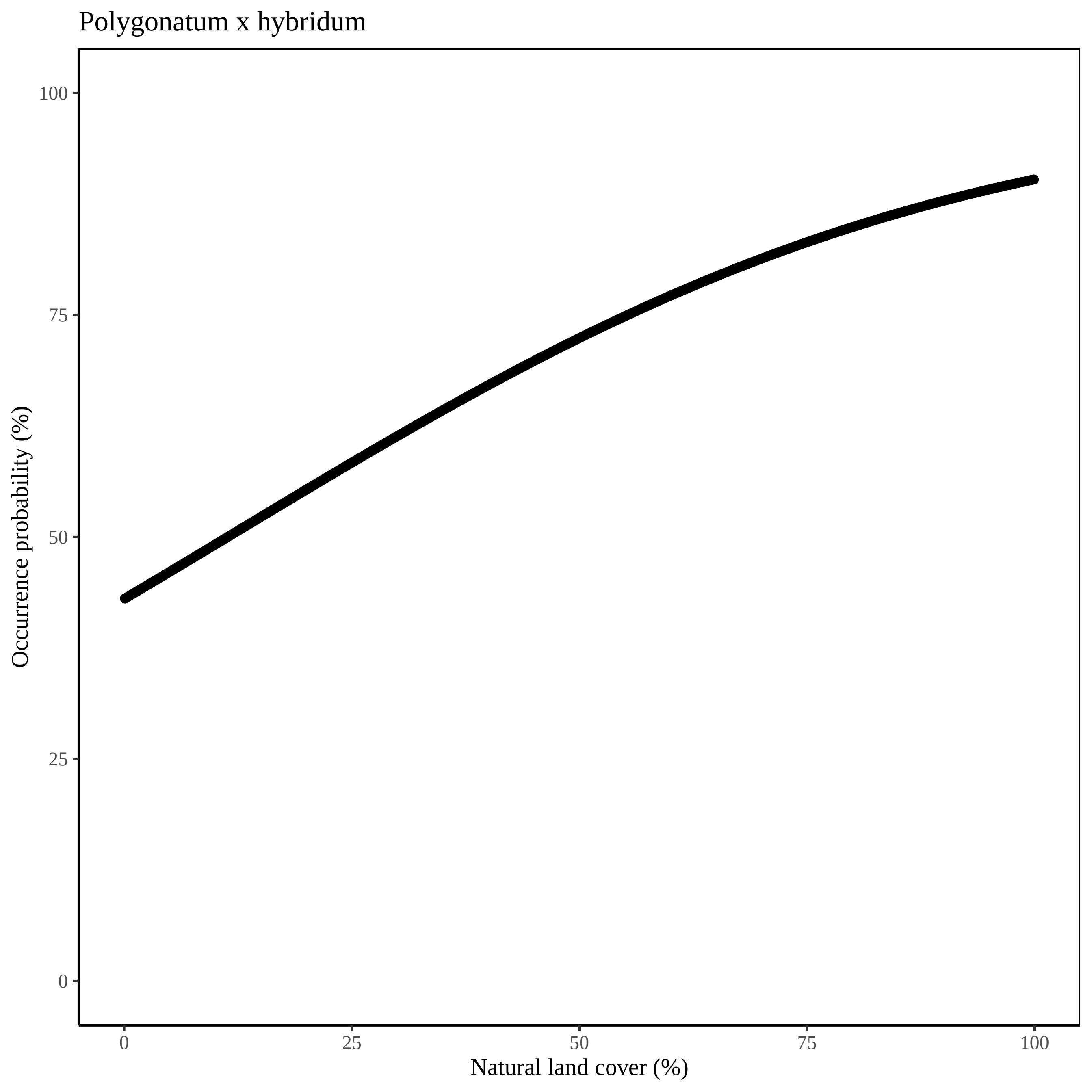
Polygonatum x hybridum
Occurrence probability (%)
100
75
50
25
0
0
25
50
75
100
Natural land cover (%)

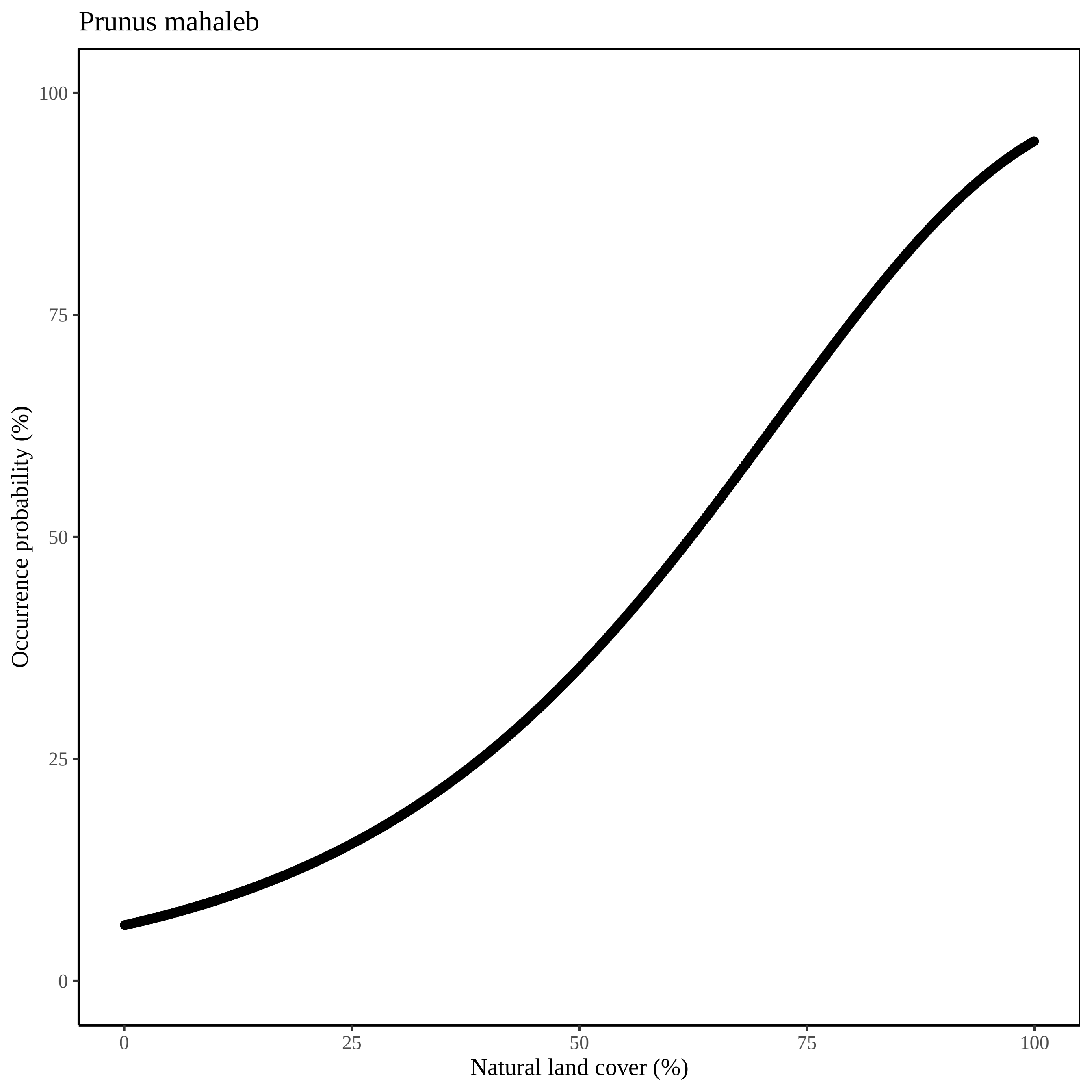
Prunus mahaleb
Occurrence probability (%)
100
75
50
25
0
0
25
50
75
100
Natural land cover (%)

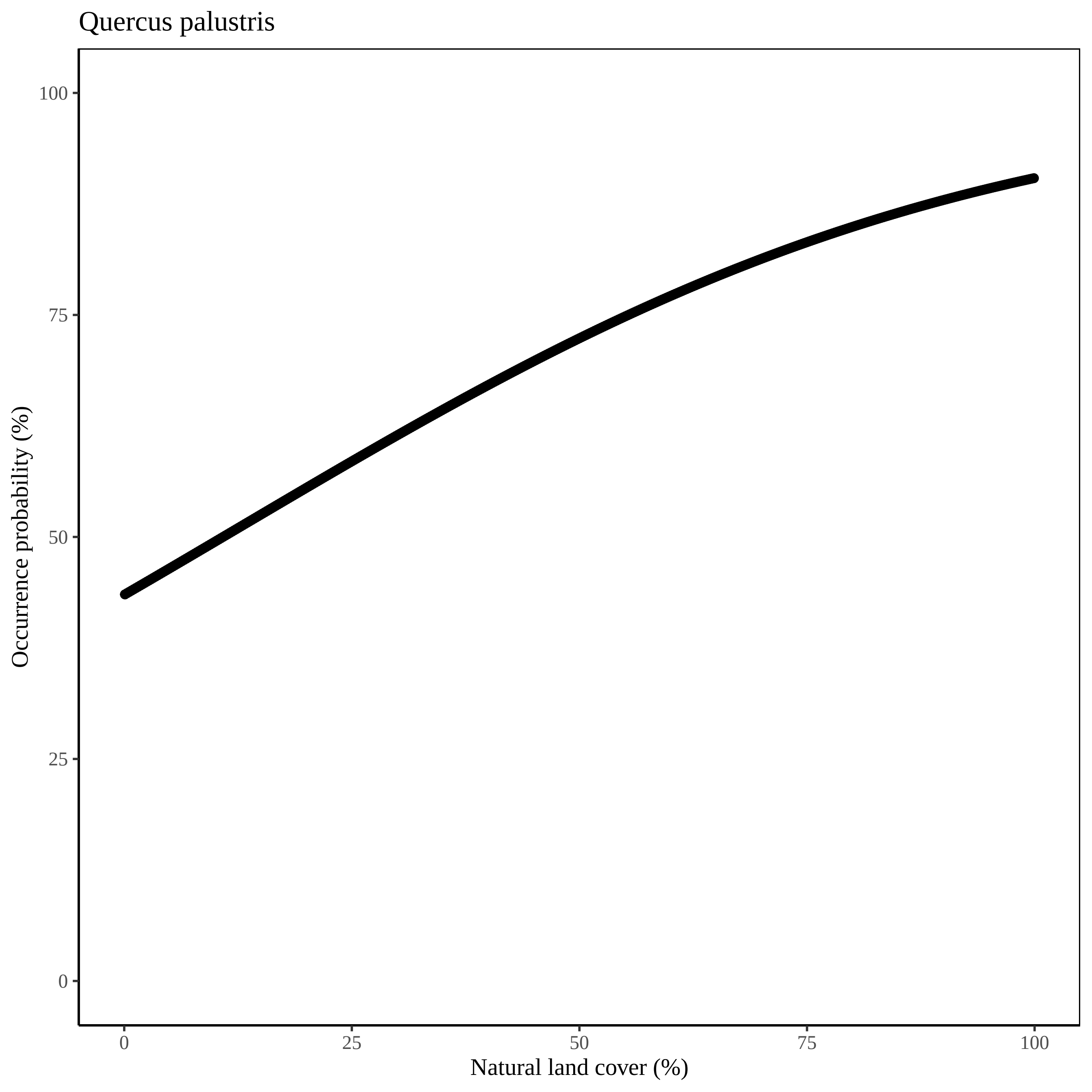
Quercus palustris
100
75
50
25
0
Occurrence probability (%)
0
25
50
75
100
Natural land cover (%)

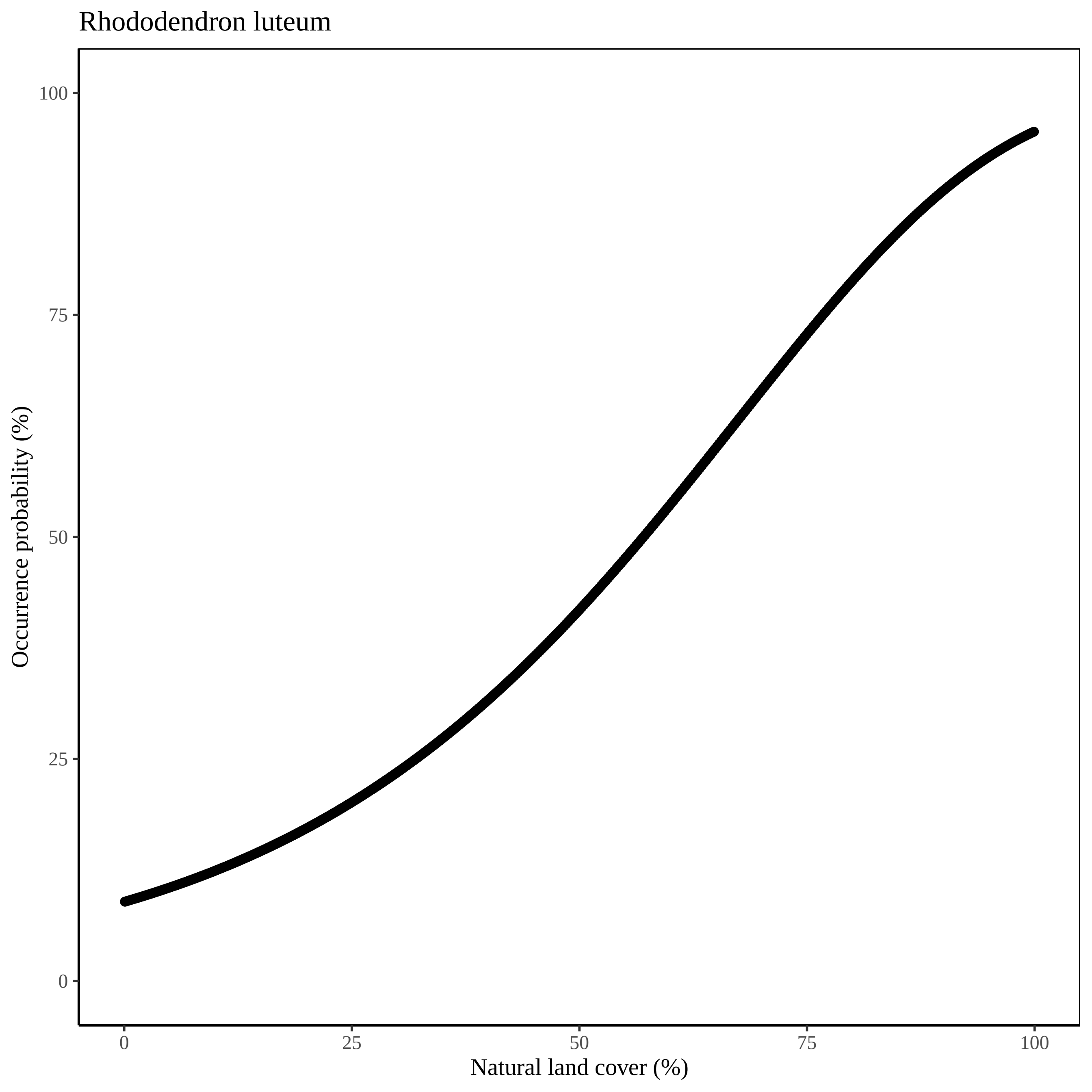

Rhododendron luteum
Occurrence probability (%)
100
75
50
25
0
0
25
50
75
100
Natural land cover (%)

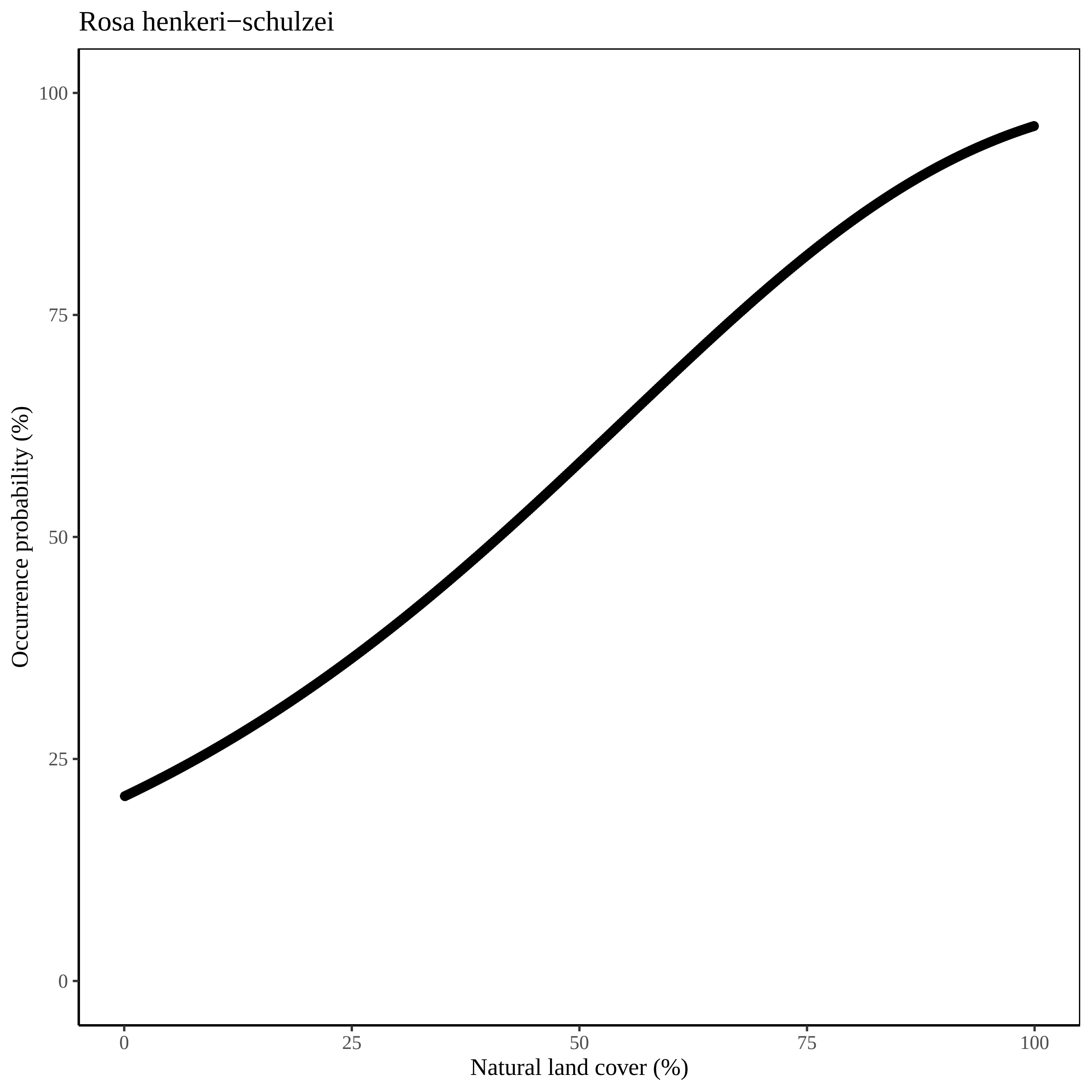

Rosa henkeri−schulzei
Occurrence probability (%)
100
75
50
25
0
0
25
50
75
100
Natural land cover (%)

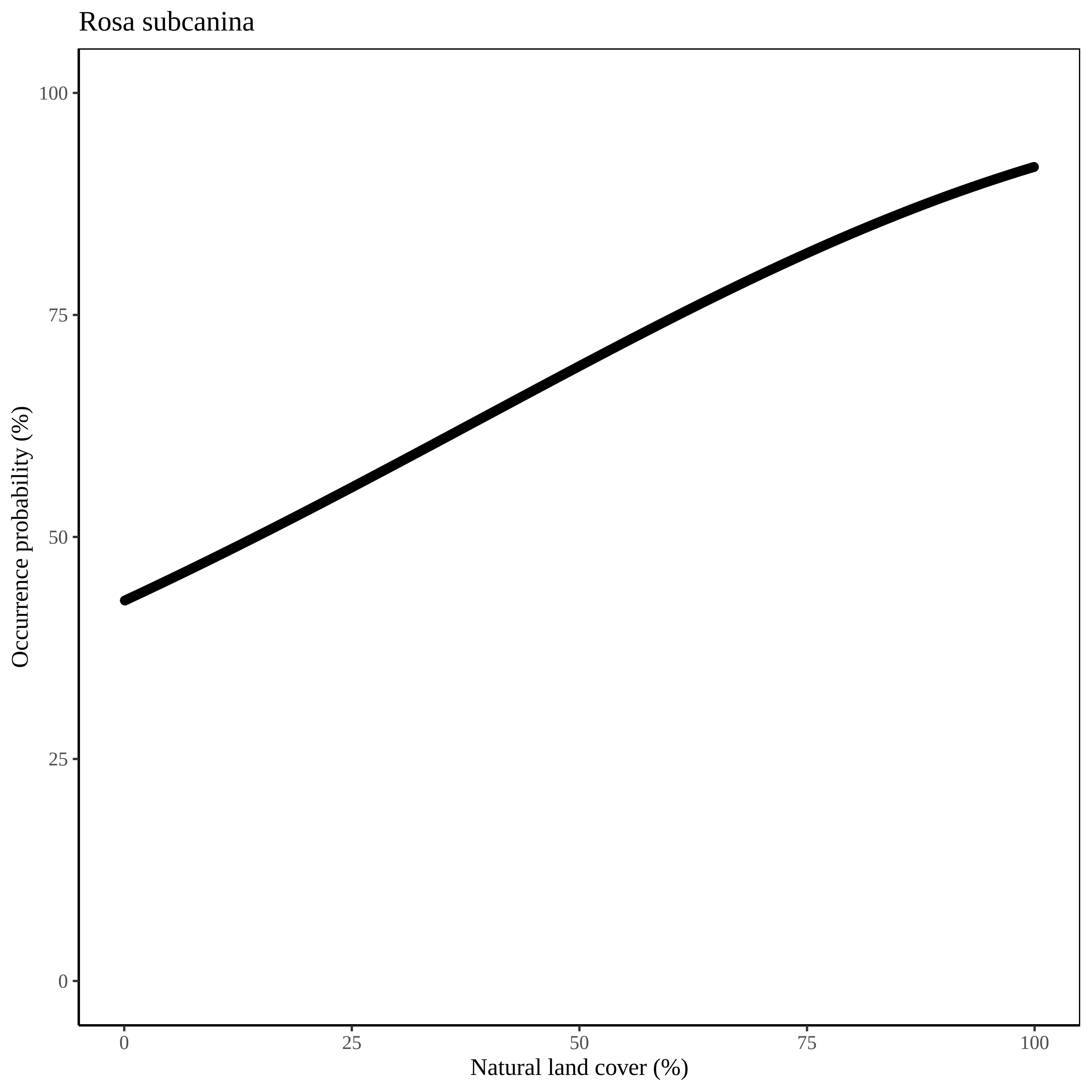
Rosa subcanina
Occurrence probability (%)
100
75
50
25
0
0
25
50
75
100
Natural land cover (%)

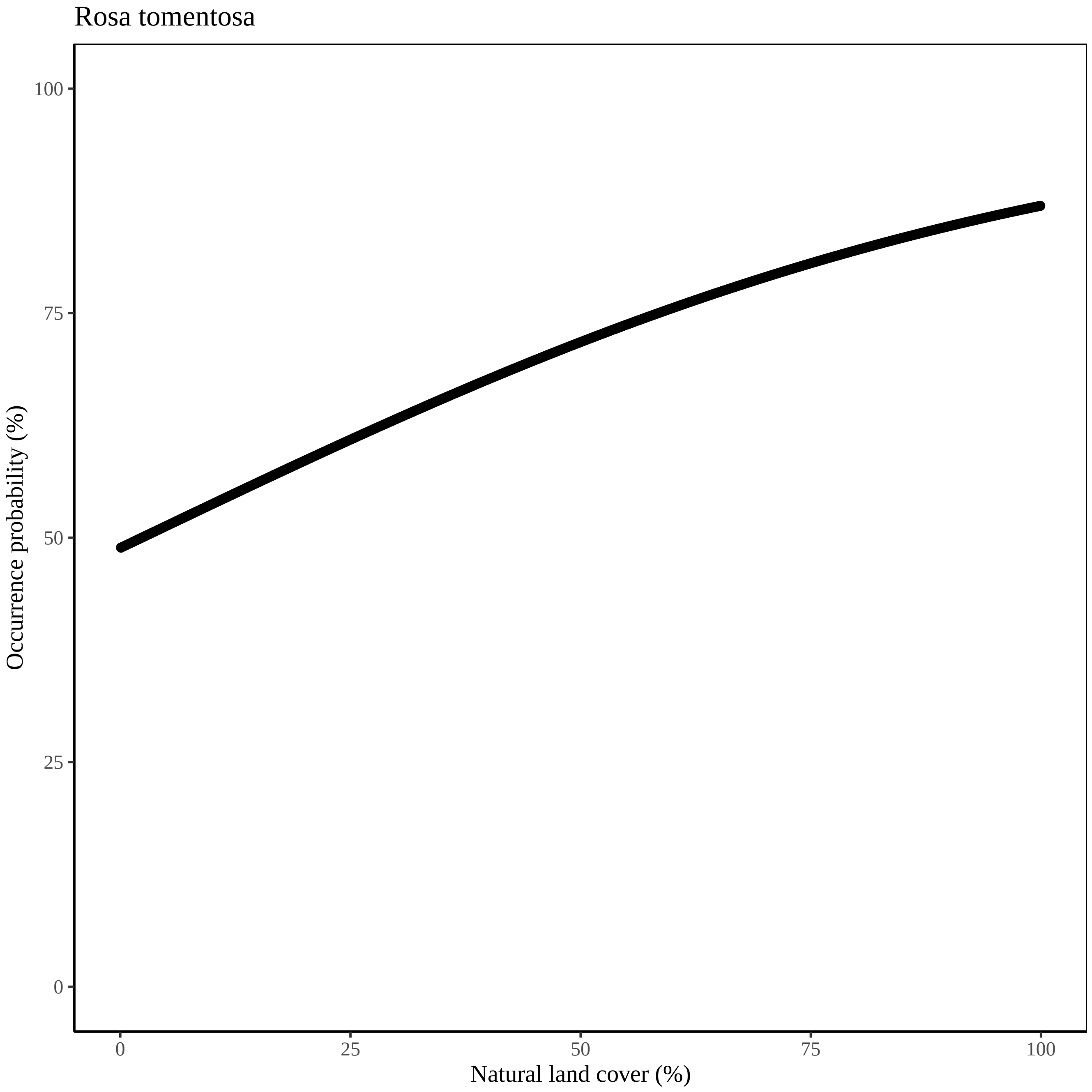

Rosa tomentosa
Occurrence probability (%)
100
75
50
25
0
0
25
50
75
100
Natural land cover (%)

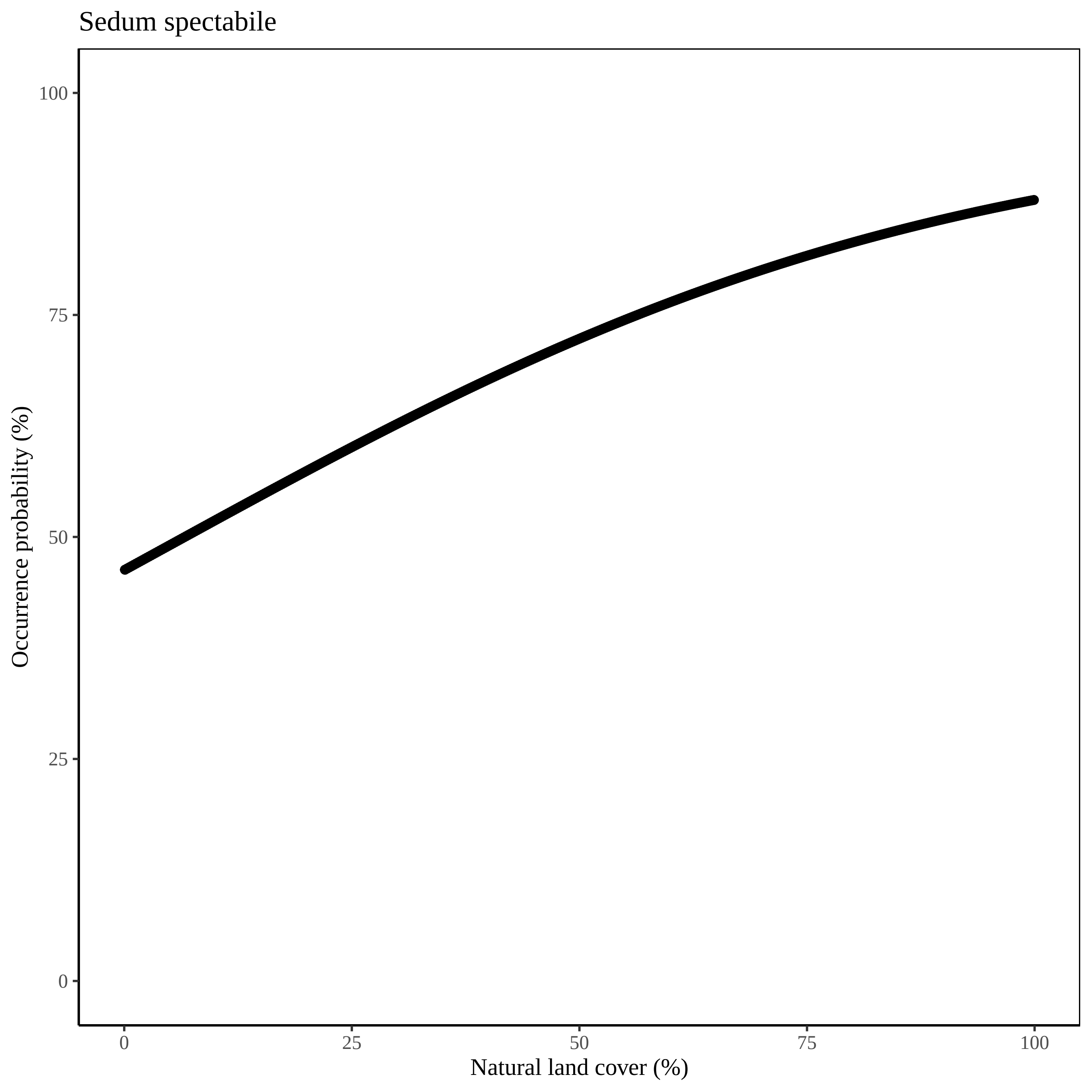

Sedum spectabile
Occurrence probability (%)
100
75
50
25
0
0
25
50
75
100
Natural land cover (%)

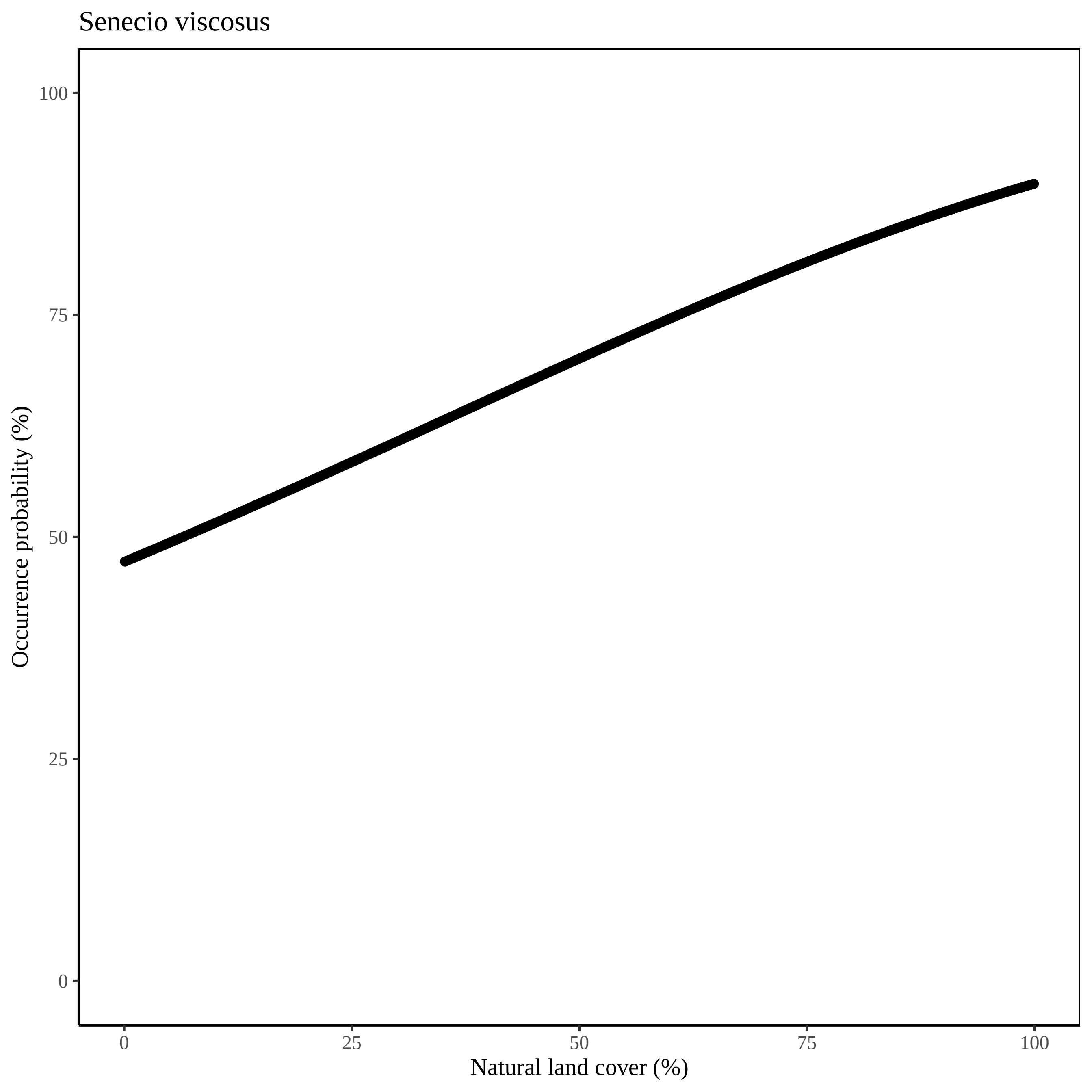
Senecio viscosus
Occurrence probability (%)
100
75
50
25
0
0
25
50
75
100
Natural land cover (%)

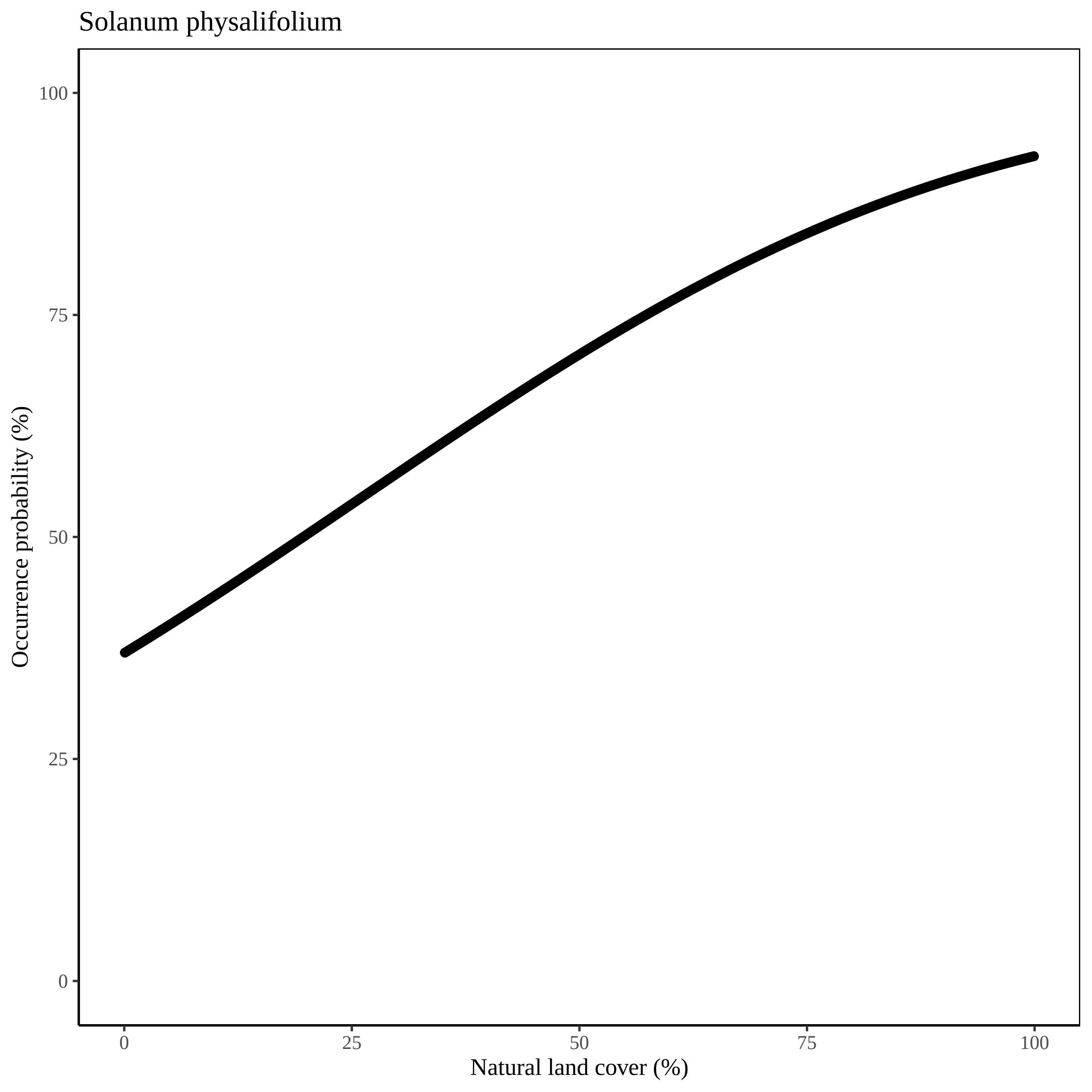

Solanum physalifolium
Occurrence probability (%)
100
75
50
25
0
0
25
50
75
100
Natural land cover (%)

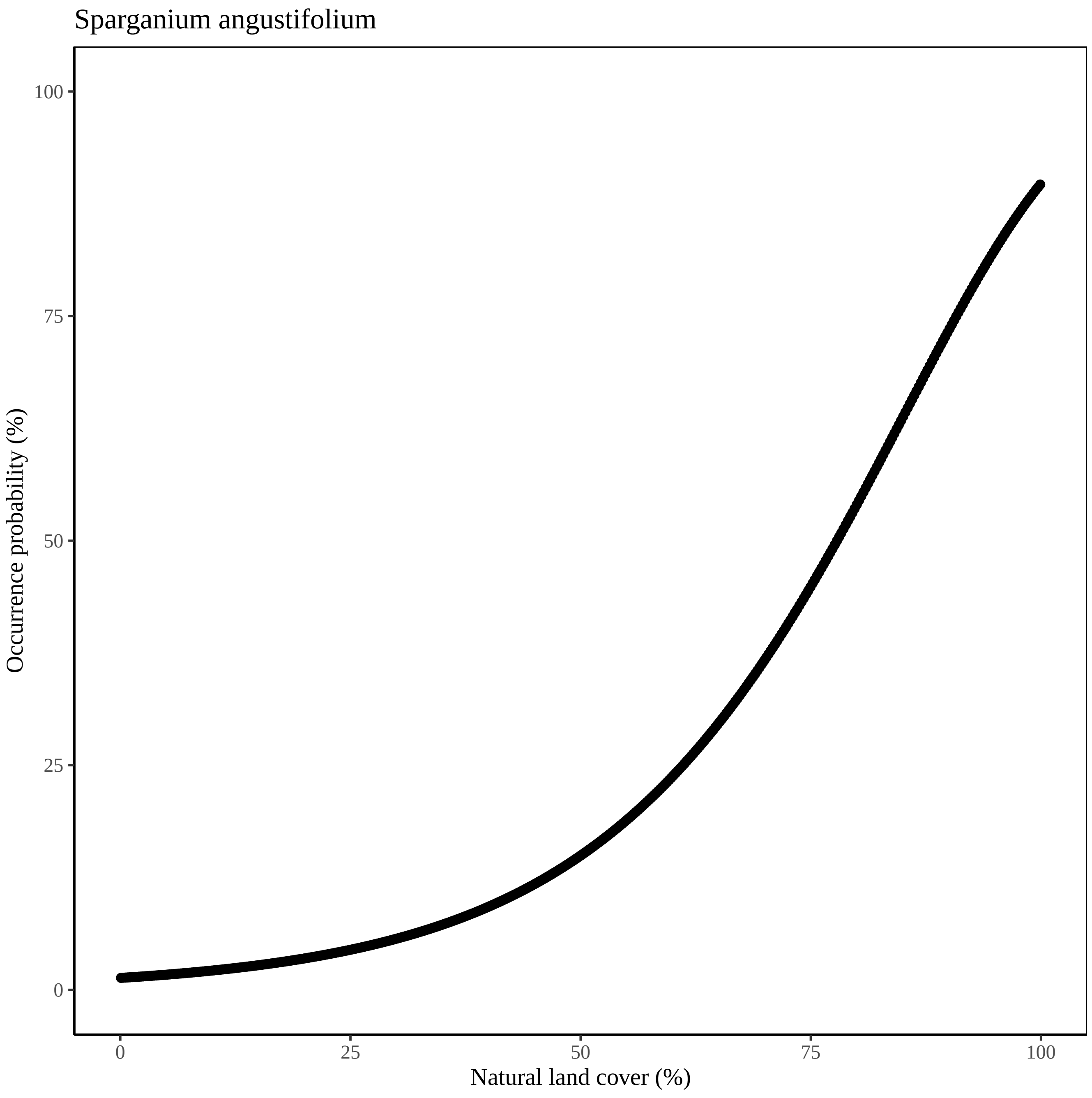
Sparganium angustifolium
Occurrence probability (%)
100
75
50
25
0
0
25
50
75
100
Natural land cover (%)

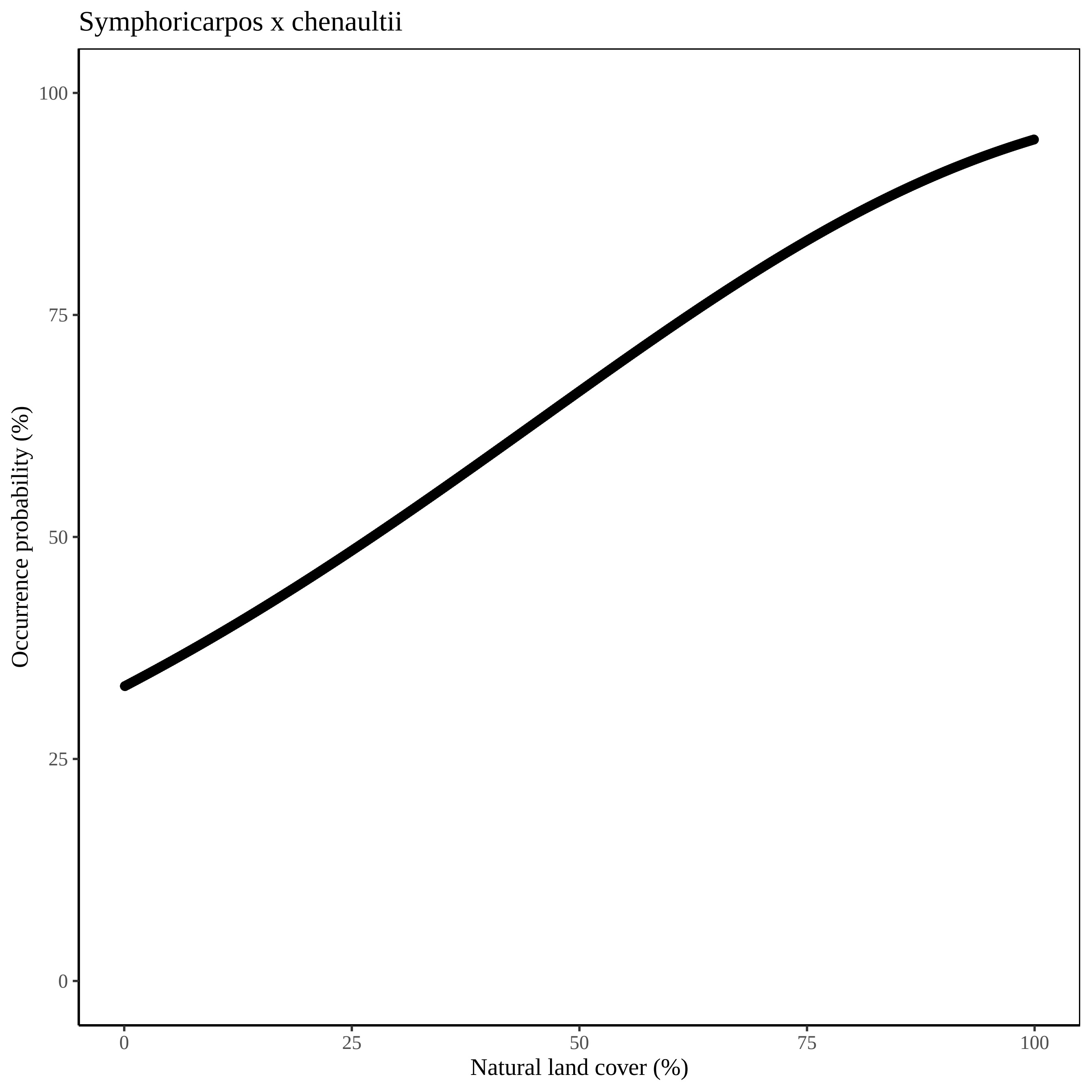

Symphoricarpos x chenaultii
Occurrence probability (%)
100
75
50
25
0
0
25
50
75
100
Natural land cover (%)

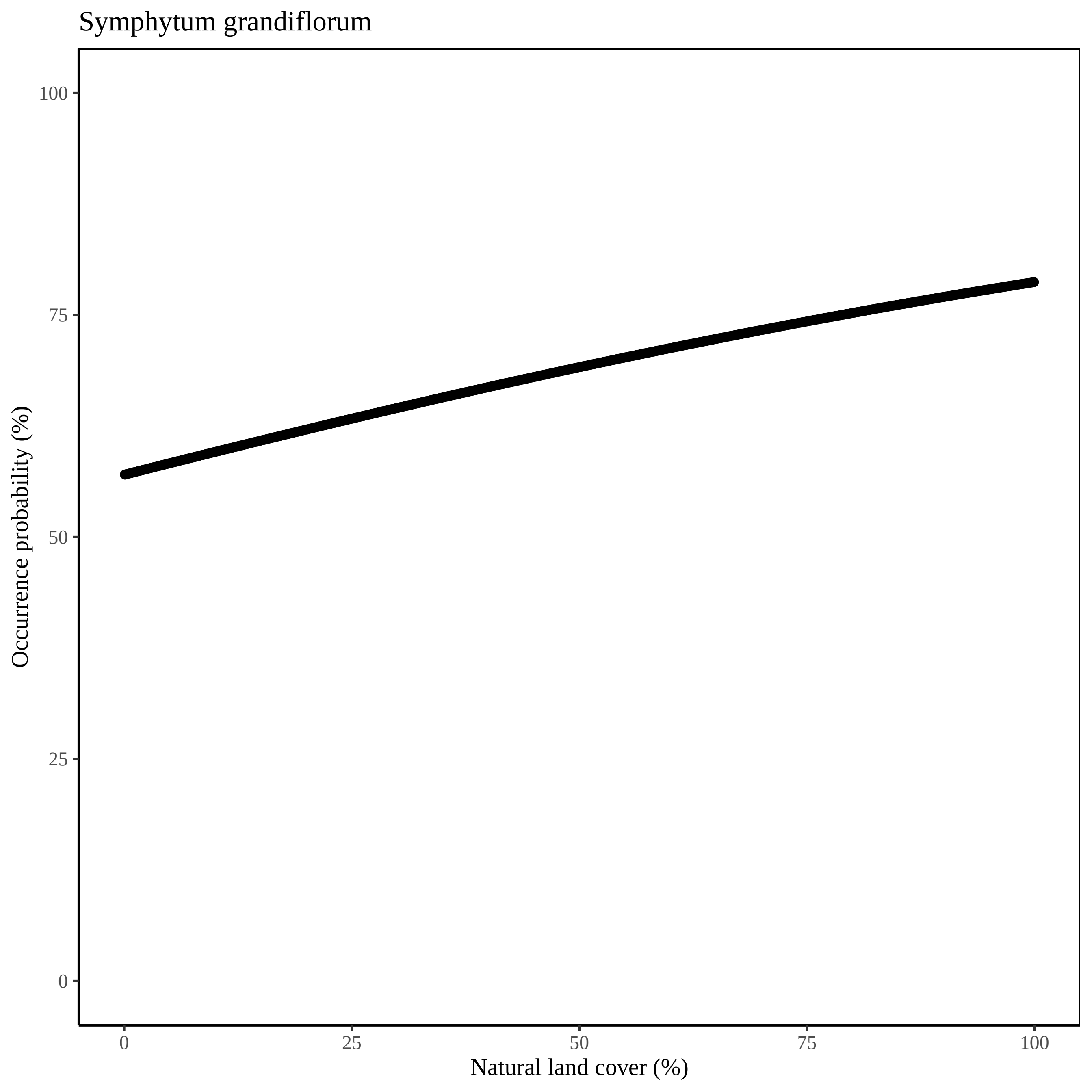

Symphytum grandiflorum
Occurrence probability (%)
100
75
50
25
0
0
25
50
75
100
Natural land cover (%)

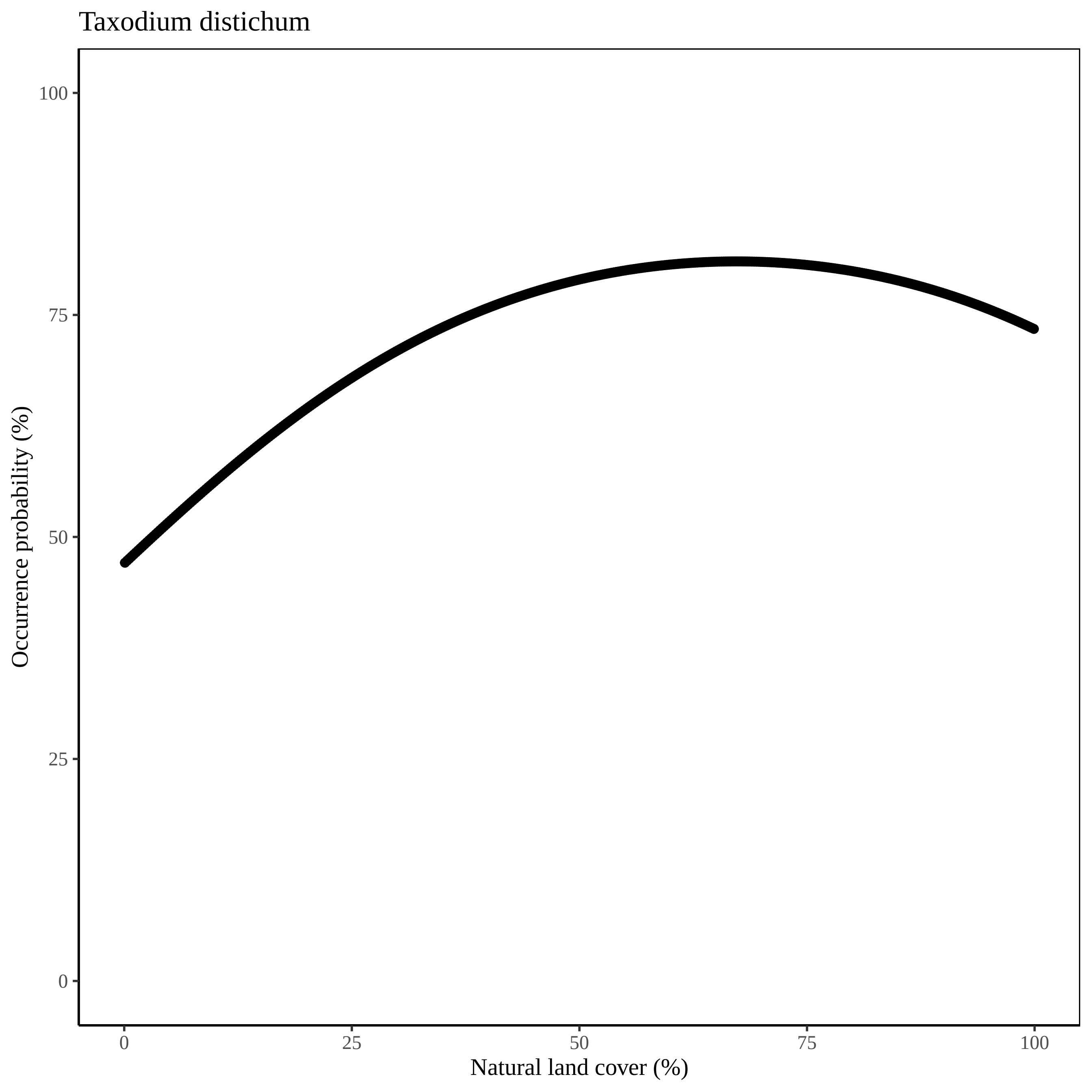
Taxodium distichum
Occurrence probability (%)
100
75
50
25
0
0
25
50
75
100
Natural land cover (%)

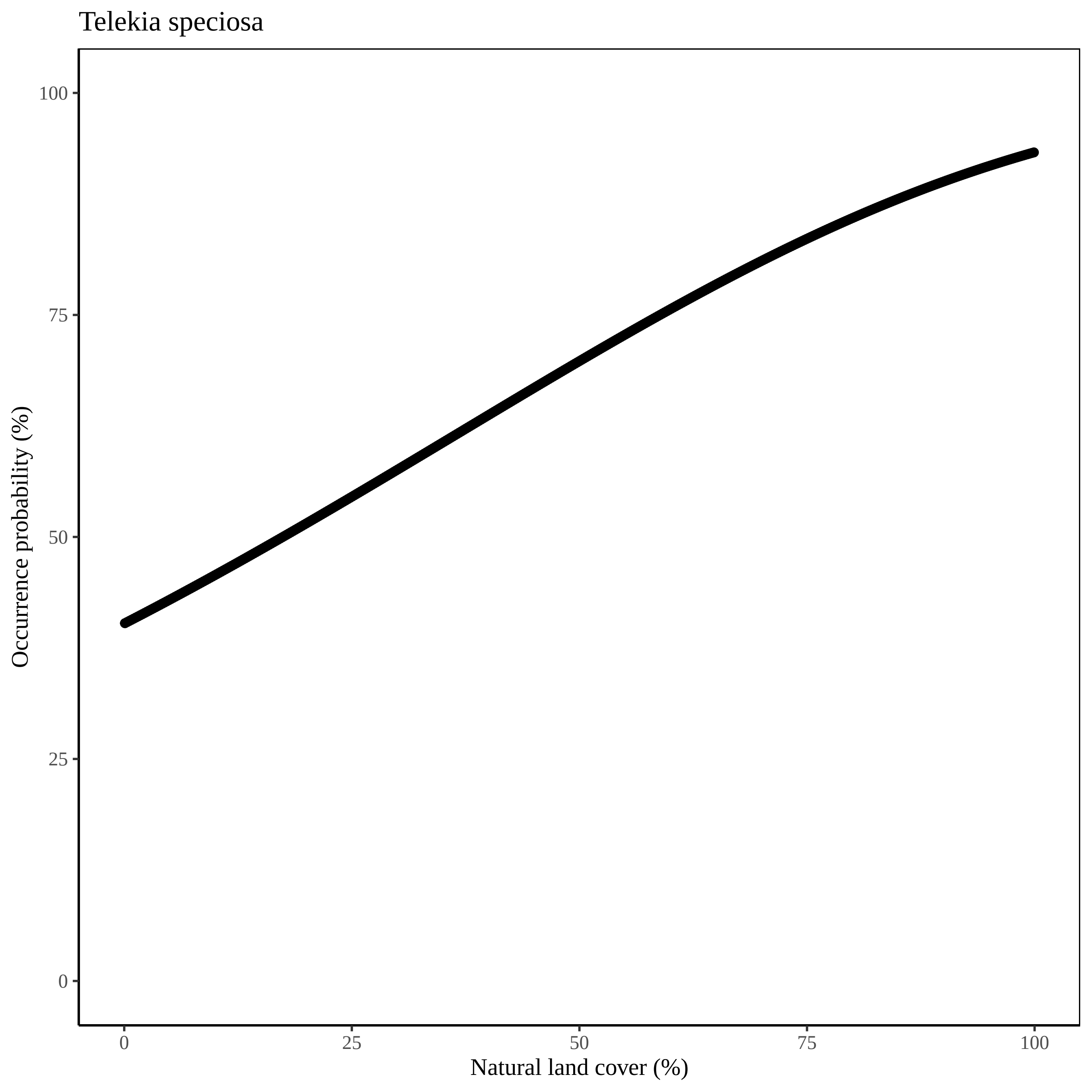

Telekia speciosa
Occurrence probability (%)
100
75
50
25
0
0
25
50
75
100
Natural land cover (%)

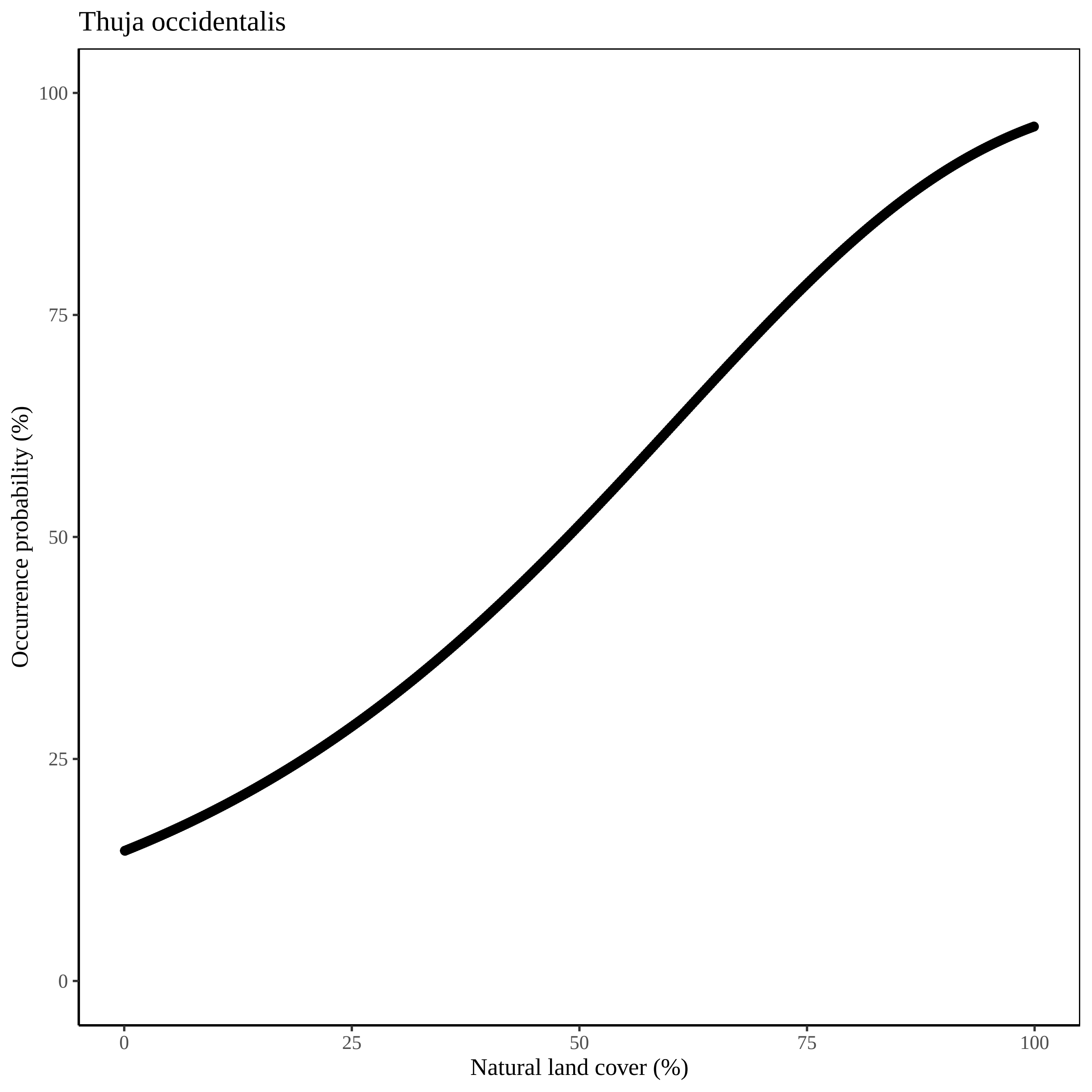

Thuja occidentalis
Occurrence probability (%)
100
75
50
25
0
0
25
50
75
100
Natural land cover (%)

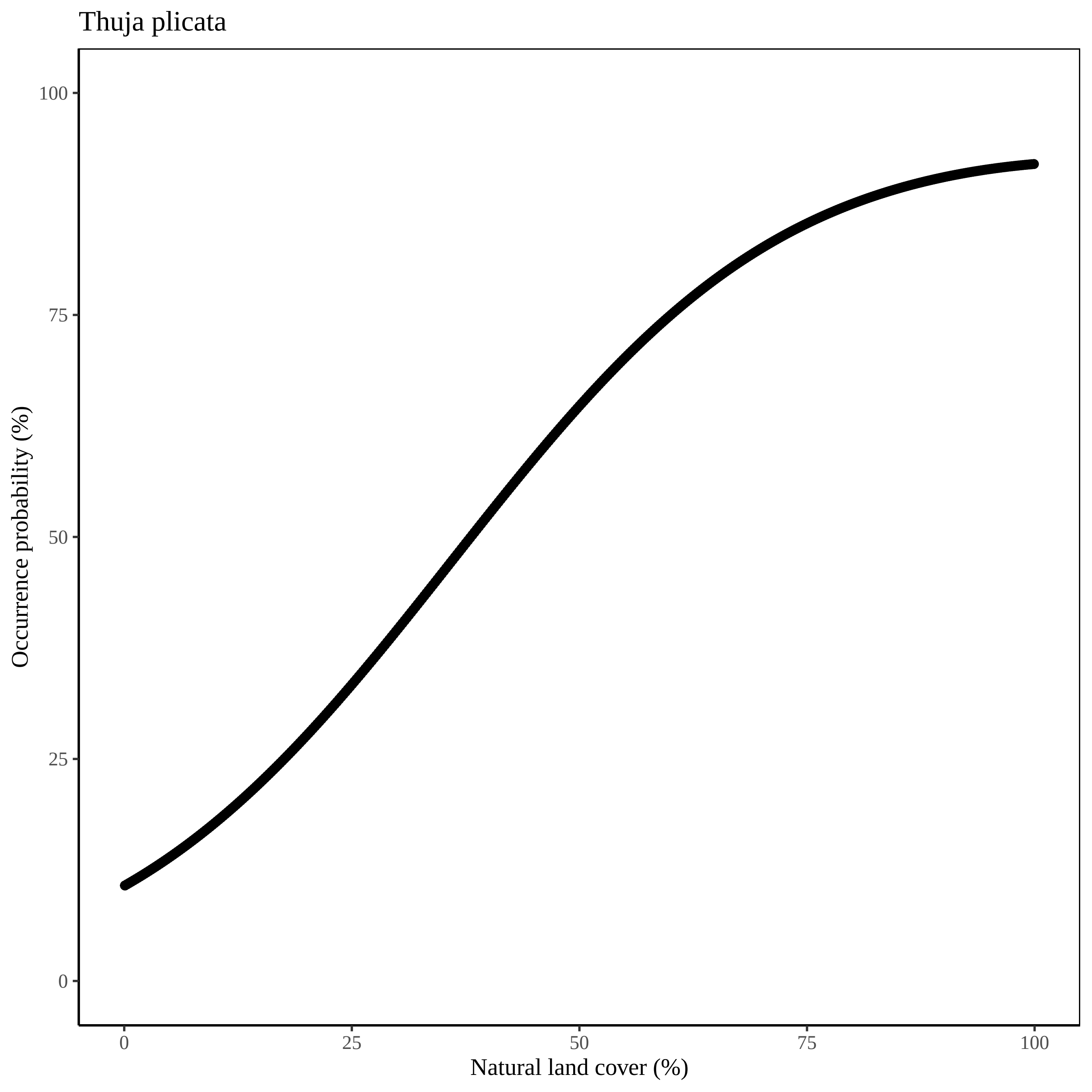
Thuja plicata
Occurrence probability (%)
100
75
50
25
0
0
25
50
75
100
Natural land cover (%)

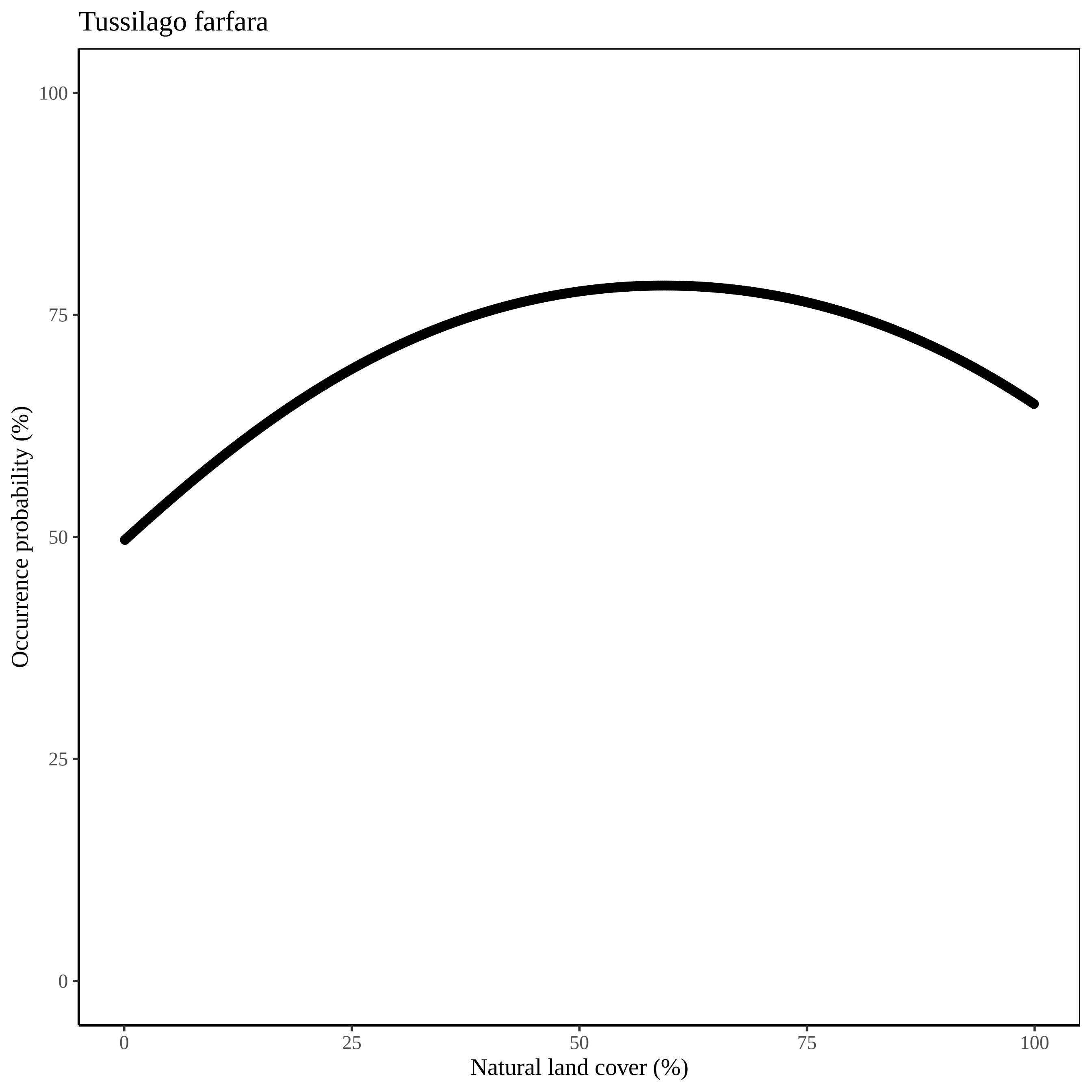

Tussilago farfara
Occurrence probability (%)
100
75
50
25
0
0
25
50
75
100
Natural land cover (%)

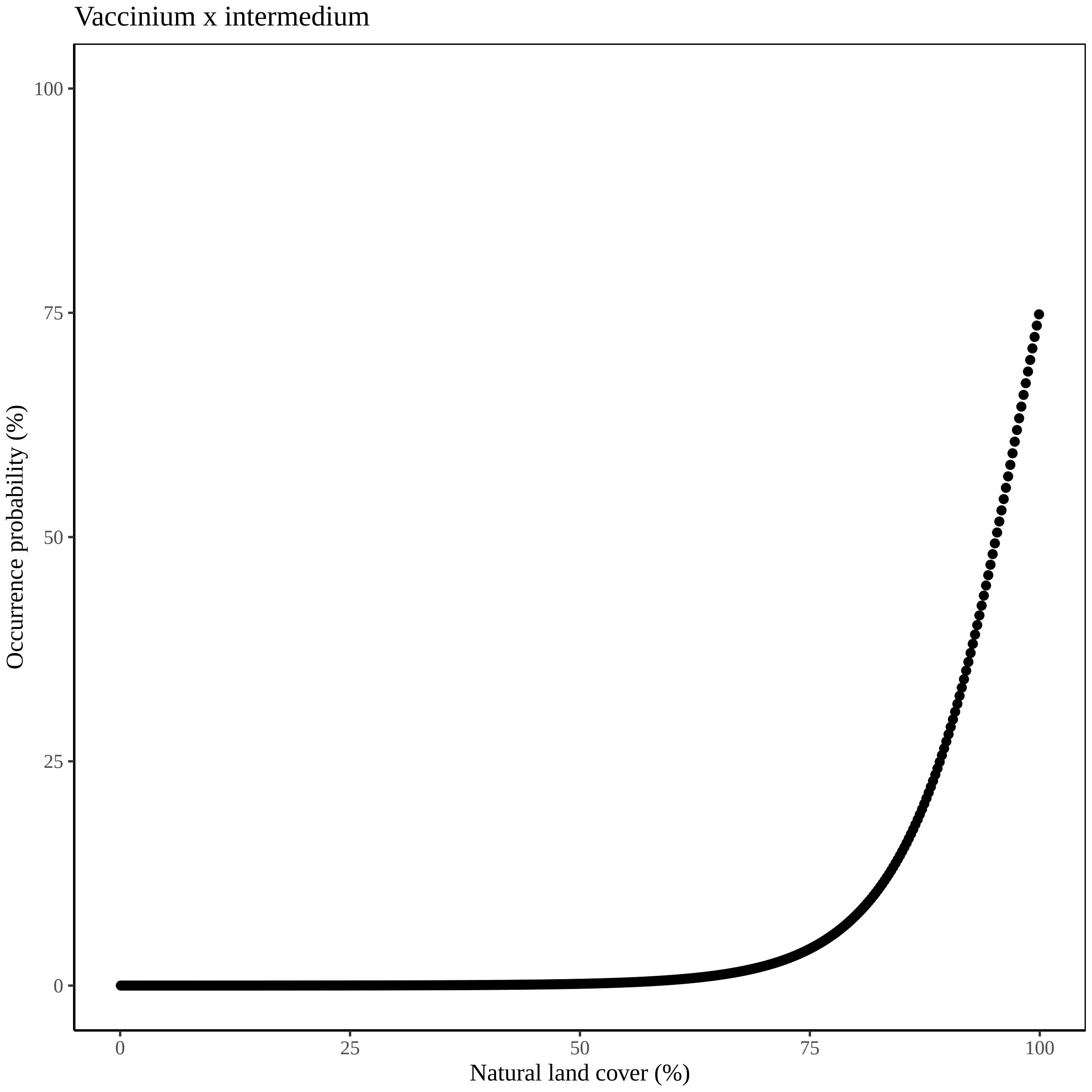

Vaccinium x intermedium
Occurrence probability (%)
100
75
50
25
0
0
25
50
75
100
Natural land cover (%)

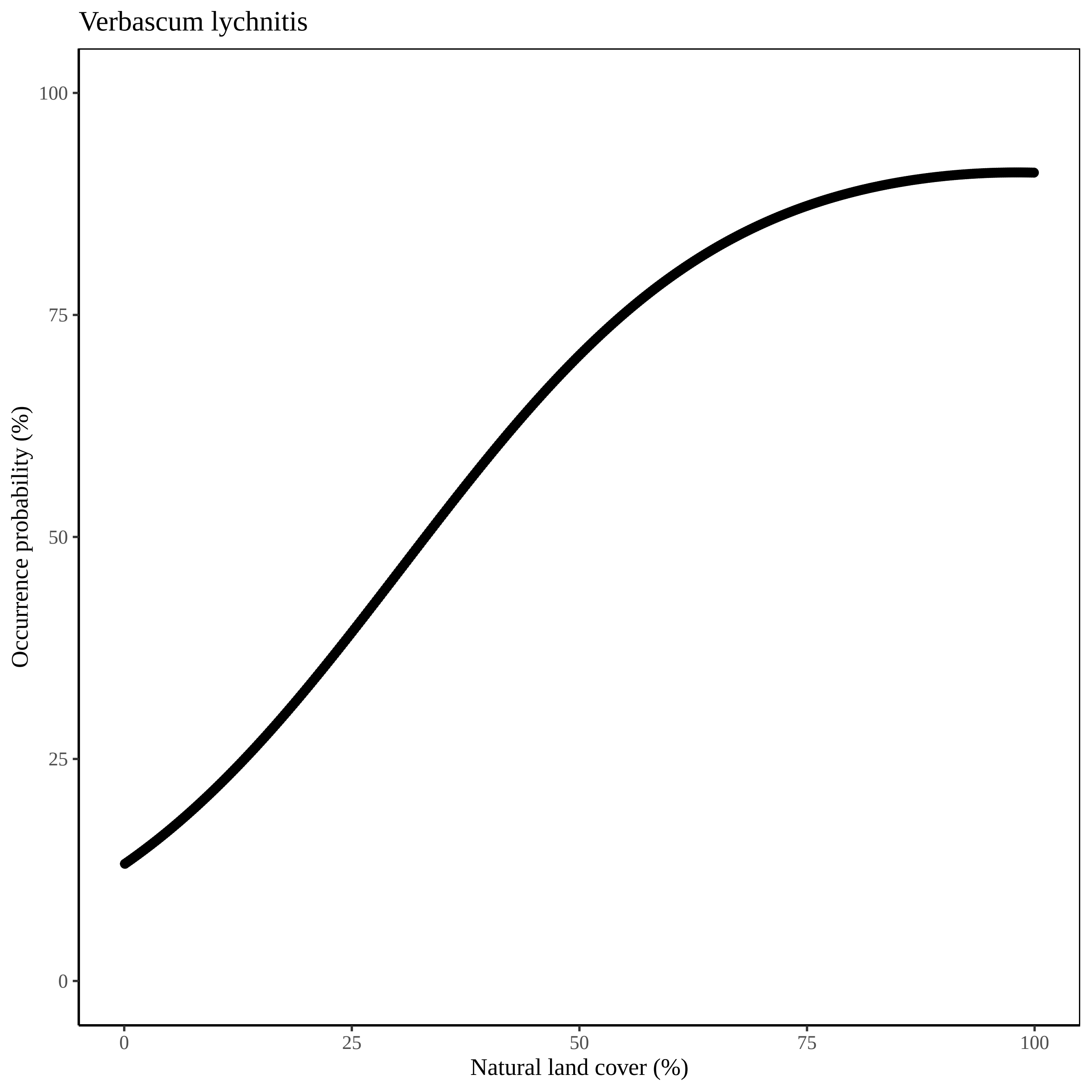

Verbascum lychnitis
Occurrence probability (%)
100
75
50
25
0
0
25
50
75
100
Natural land cover (%)

Verbena hastata

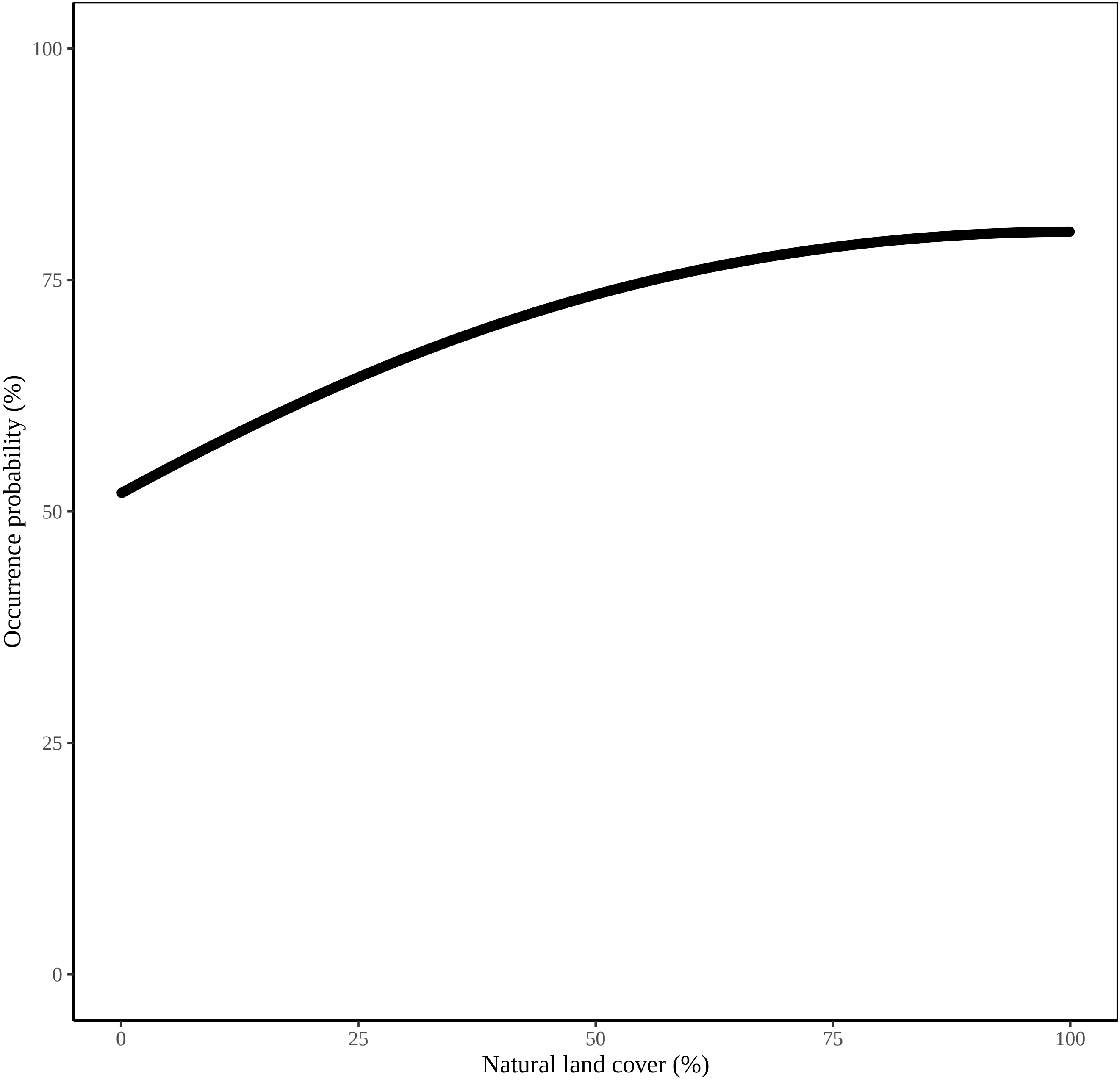

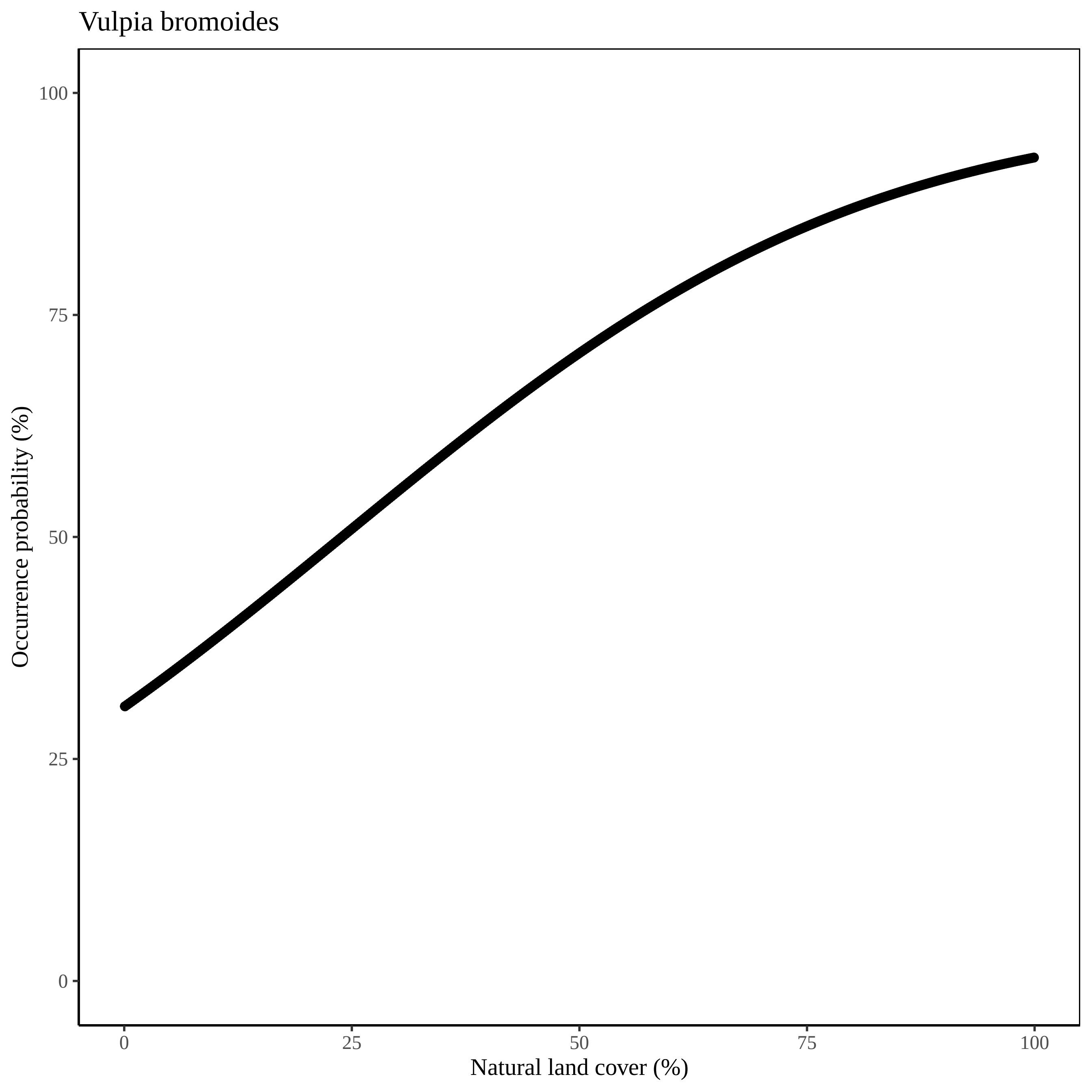
Vulpia bromoides
Occurrence probability (%)
100
75
50
25
0
0
25
50
75
100
Natural land cover (%)

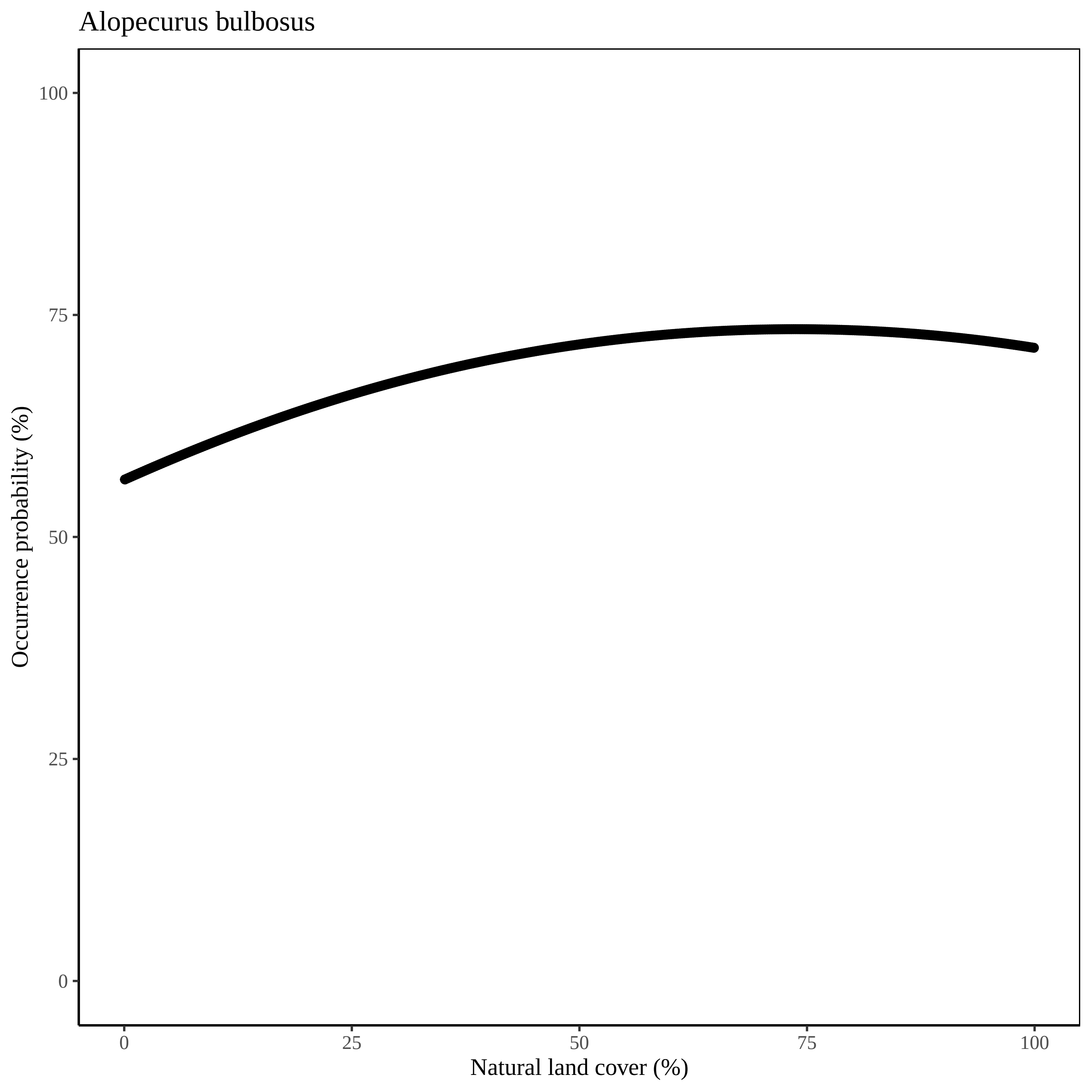

Alopecurus bulbosus
Occurrence probability (%)
100
75
50
25
0
0
25
50
75
100
Natural land cover (%)

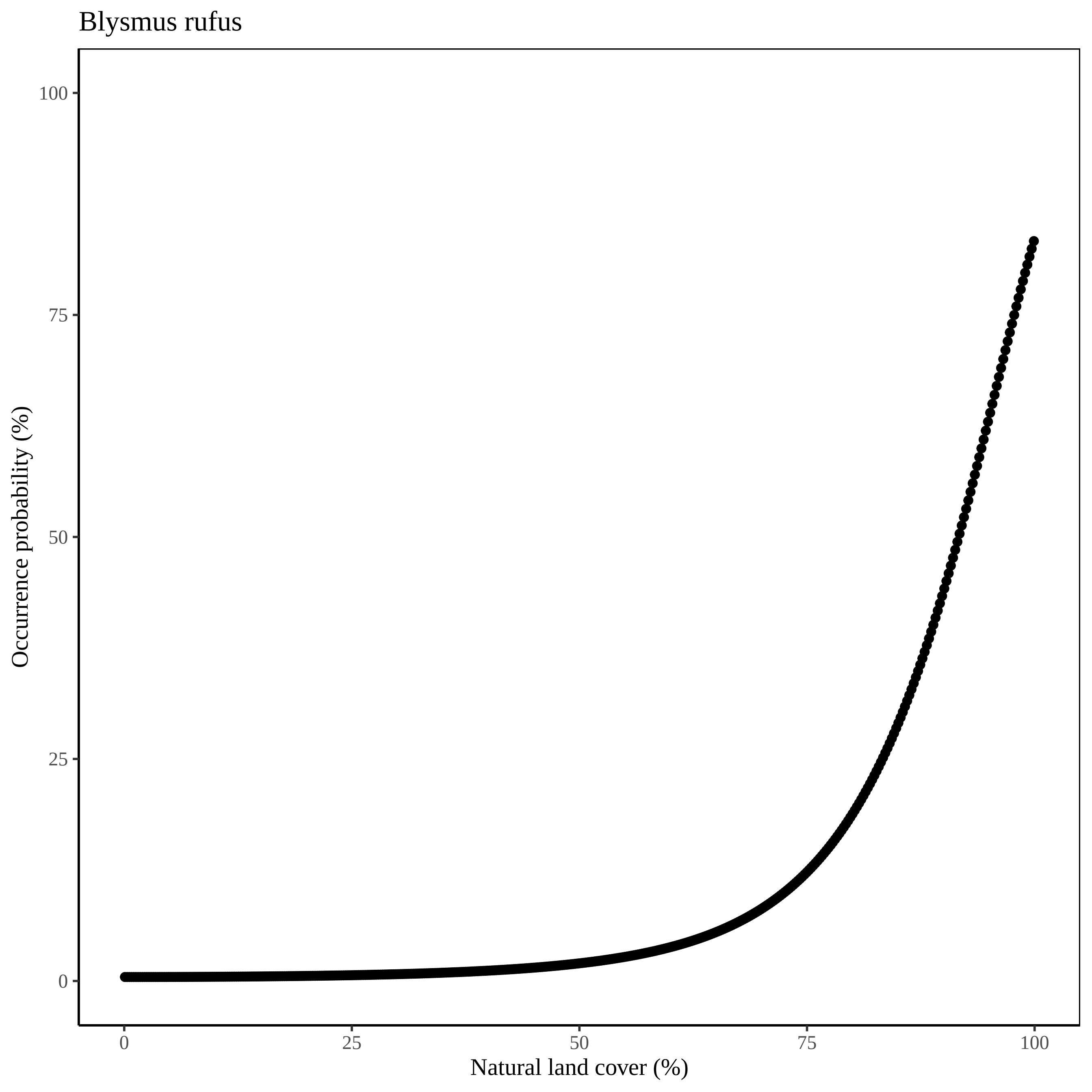
Blysmus rufus
Occurrence probability (%)
100
75
50
25
0
0
25
50
75
100
Natural land cover (%)

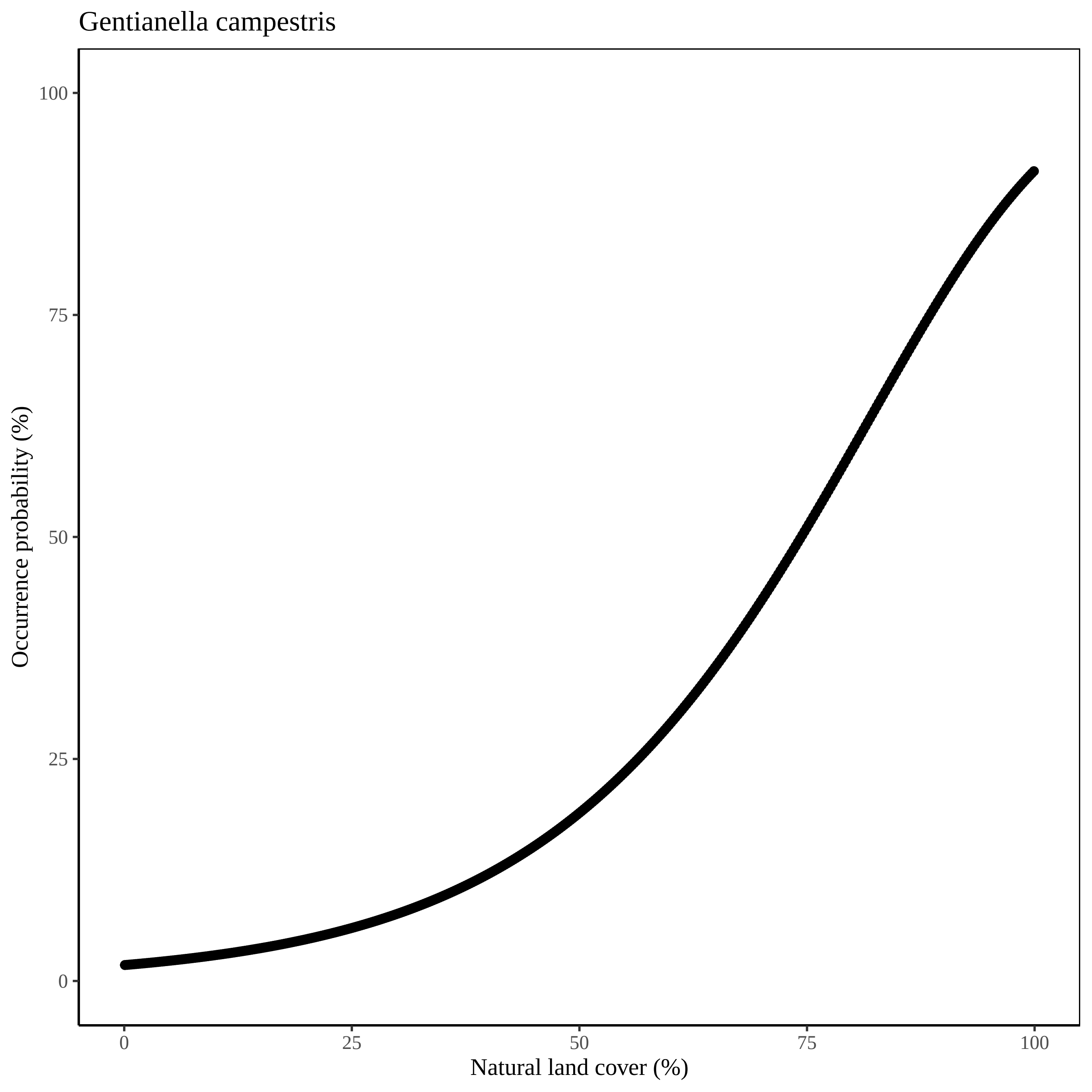

Gentianella campestris
Occurrence probability (%)
100
75
50
25
0
0
25
50
75
100
Natural land cover (%)

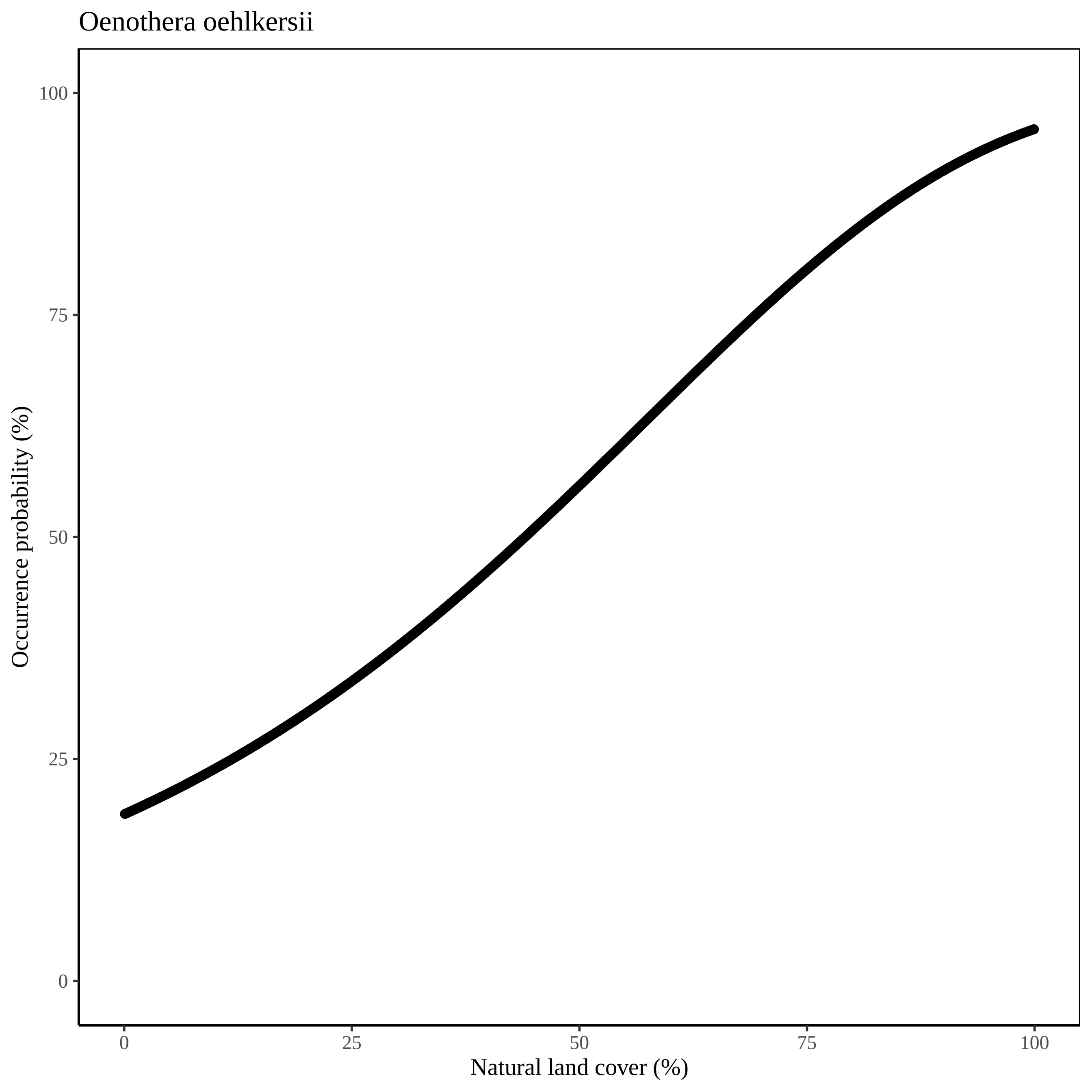

Oenothera oehlkersii
Occurrence probability (%)
100
75
50
25
0
0
25
50
75
100
Natural land cover (%)

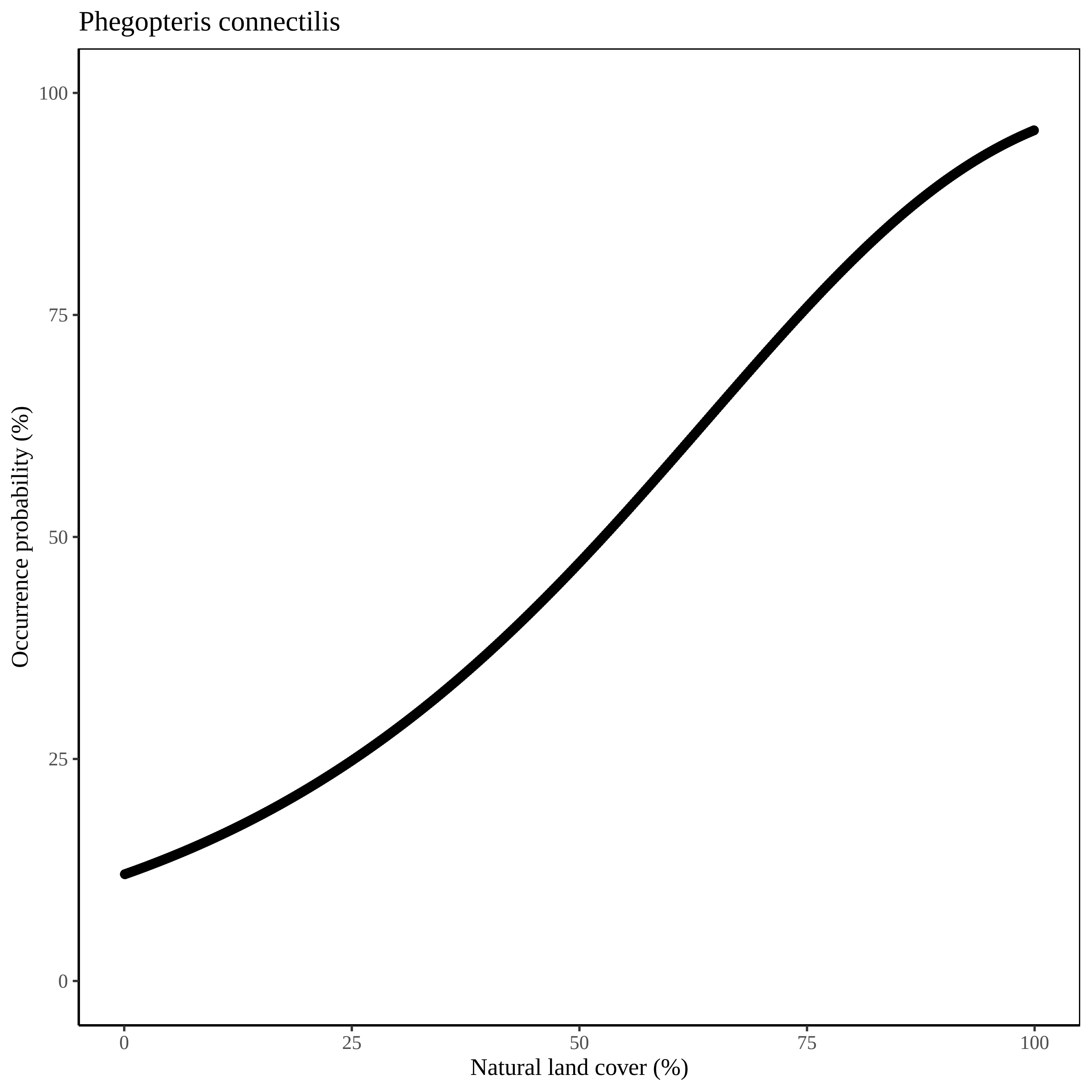

Phegopteris connectilis
Occurrence probability (%)
100
75
50
25
0
0
25
50
75
100
Natural land cover (%)

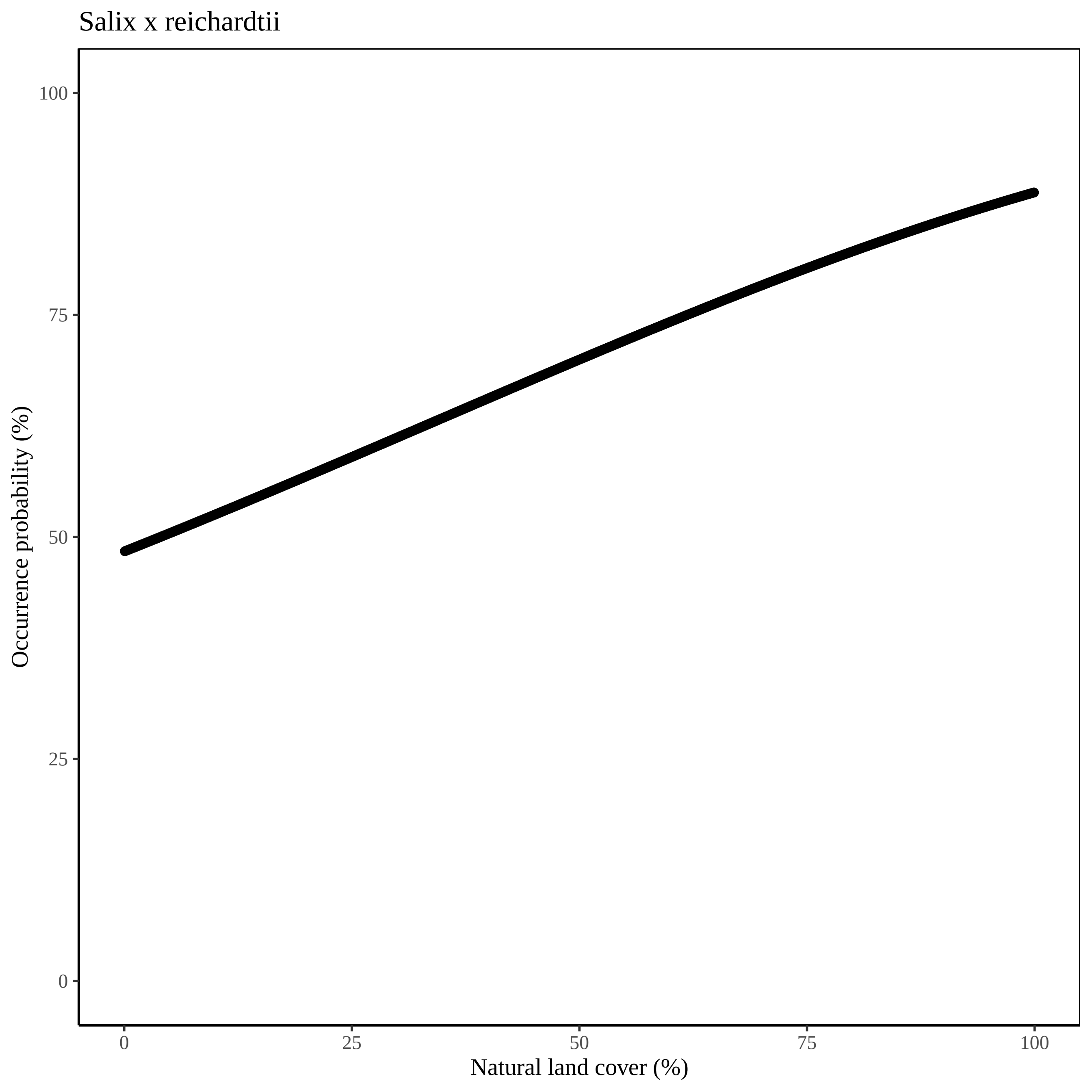

Salix x reichardtii
Occurrence probability (%)
100
75
50
25
0
0
25
50
75
100
Natural land cover (%)

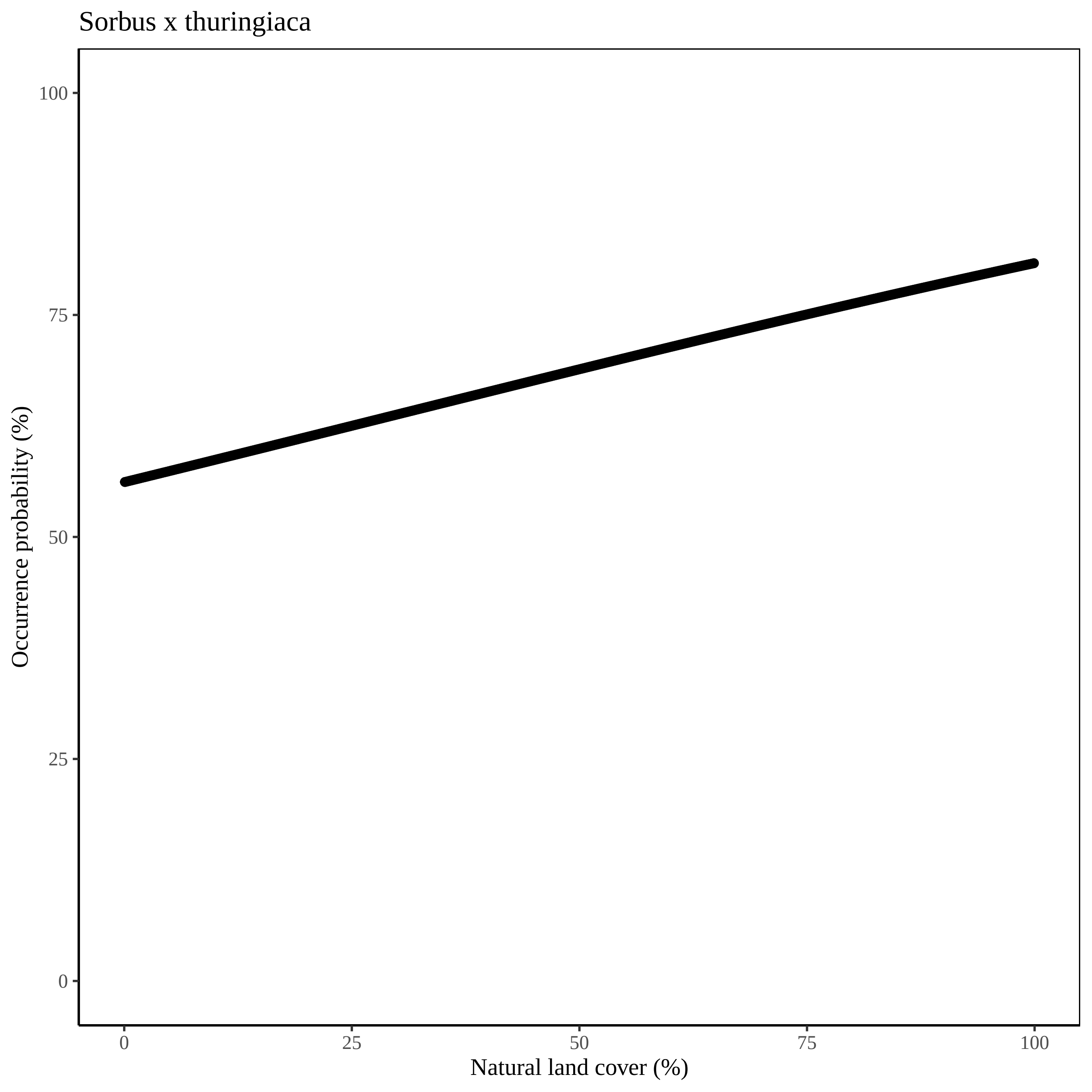

Sorbus x thuringiaca
Occurrence probability (%)
100
75
50
25
0
0
25
50
75
100
Natural land cover (%)

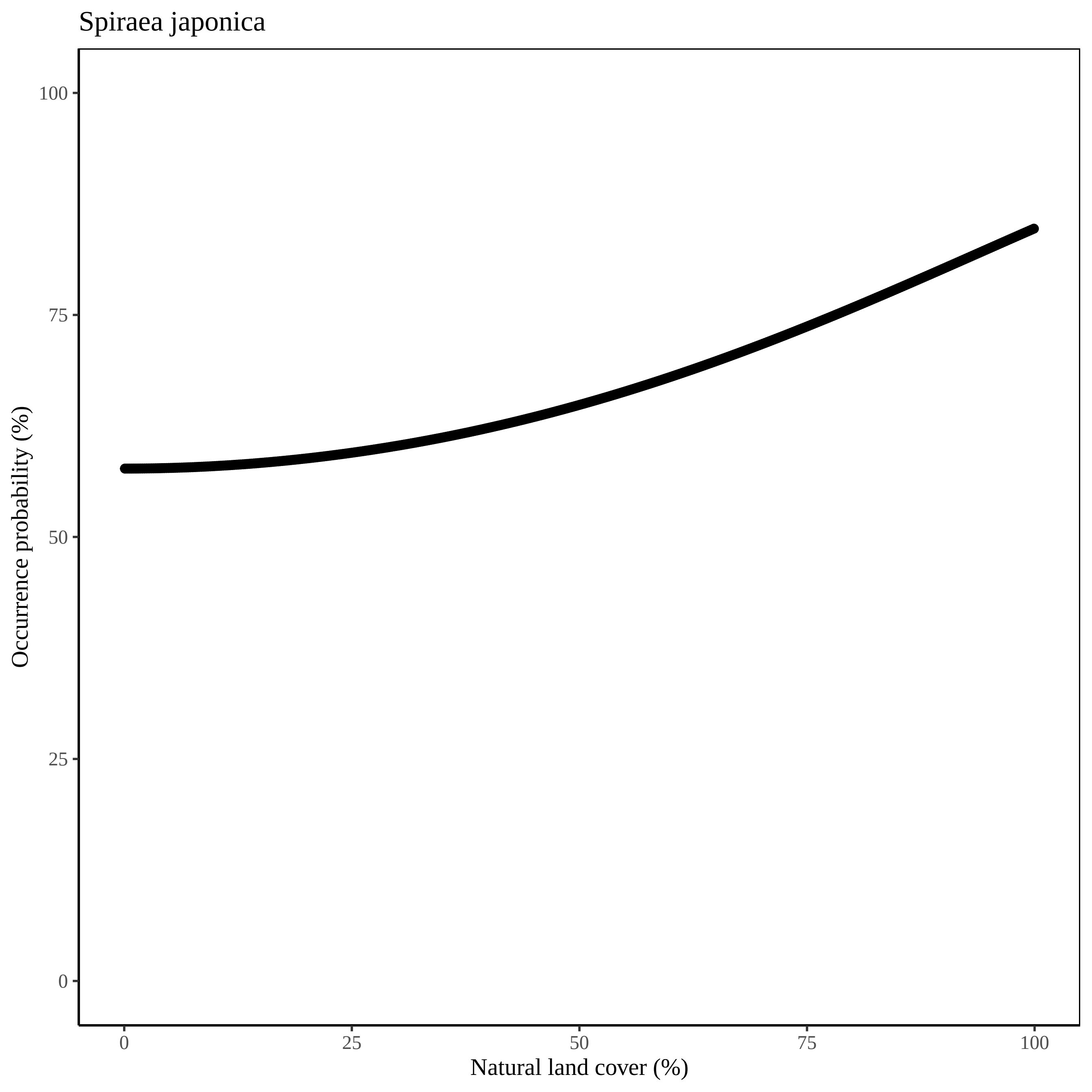
Spiraea japonica
Occurrence probability (%)
100
75
50
25
0
0
25
50
75
100
Natural land cover (%)

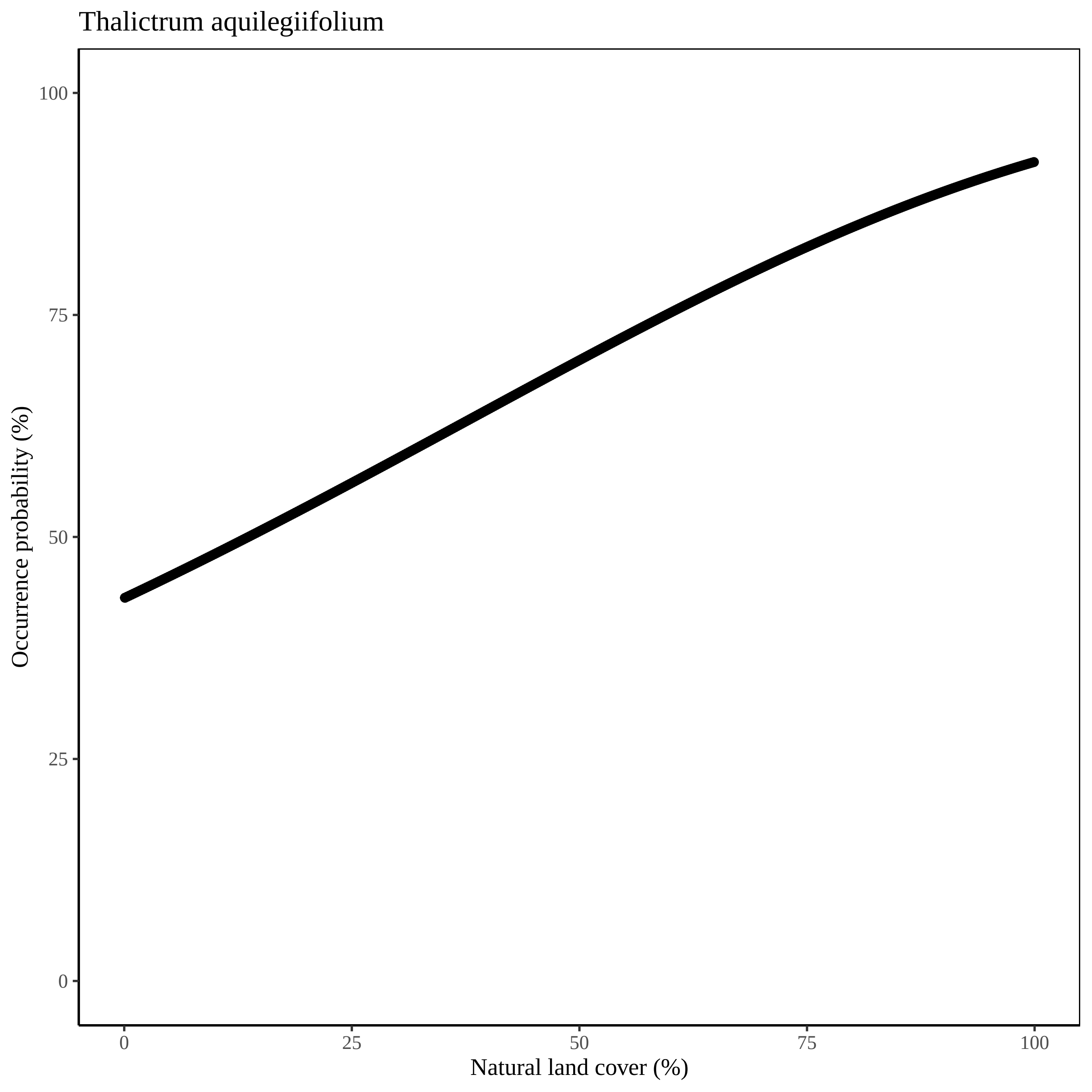
Thalictrum aquilegiifolium
Occurrence probability (%)
100
75
50
25
0
0
25
50
75
100
Natural land cover (%)

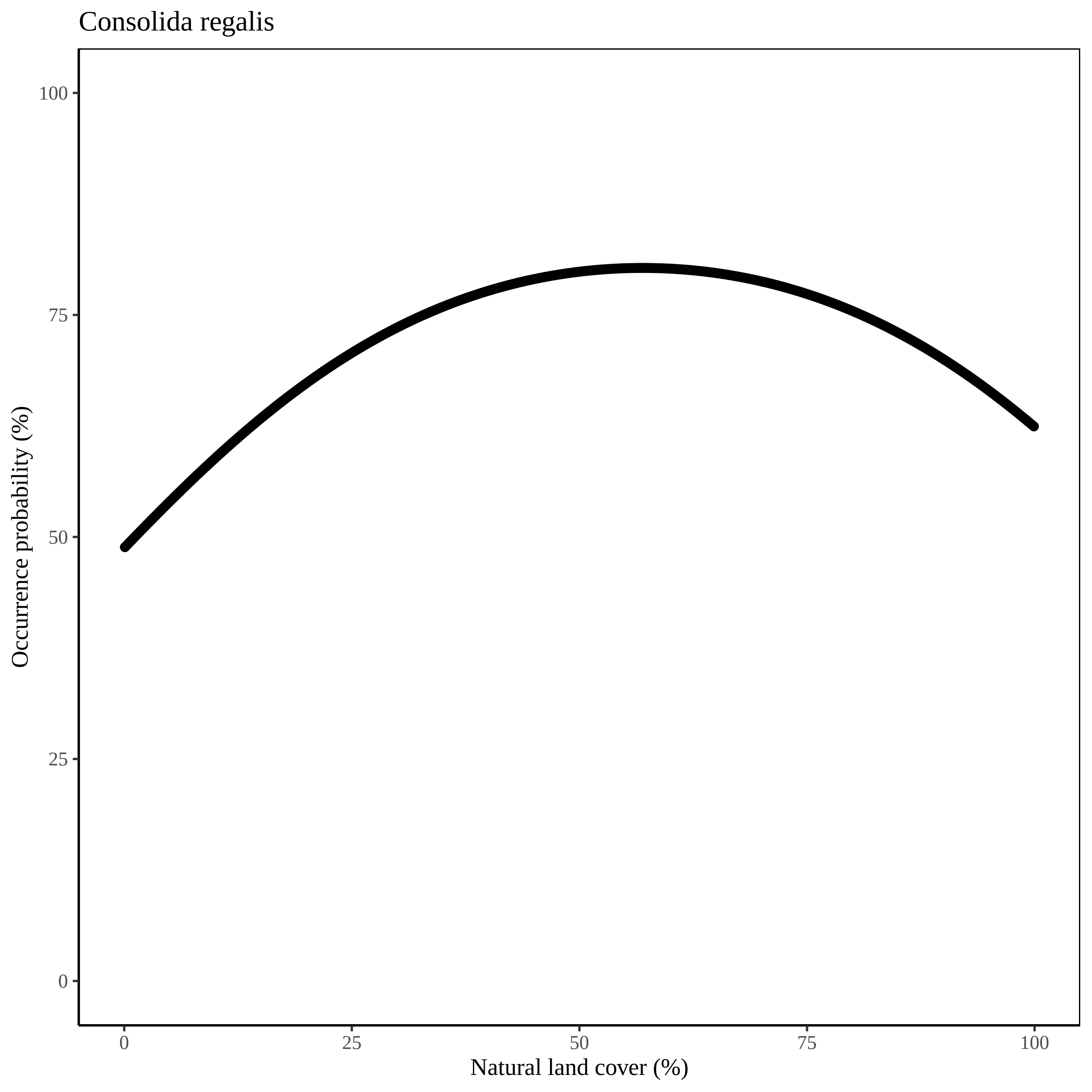

Consolida regalis
Occurrence probability (%)
100
75
50
25
0
0
25
50
75
100
Natural land cover (%)

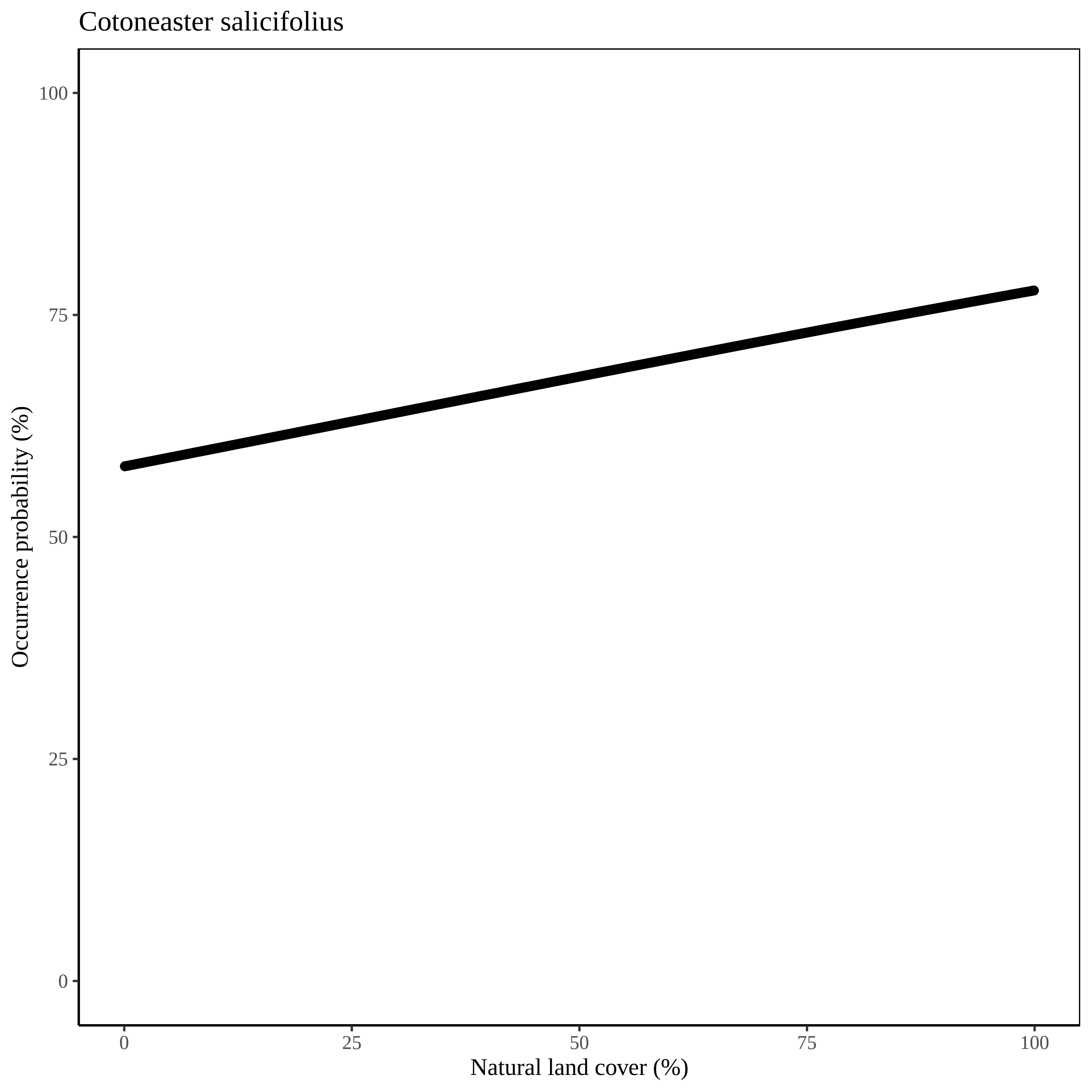
Cotoneaster salicifolius
Occurrence probability (%)
100
75
50
25
0
0
25
50
75
100
Natural land cover (%)

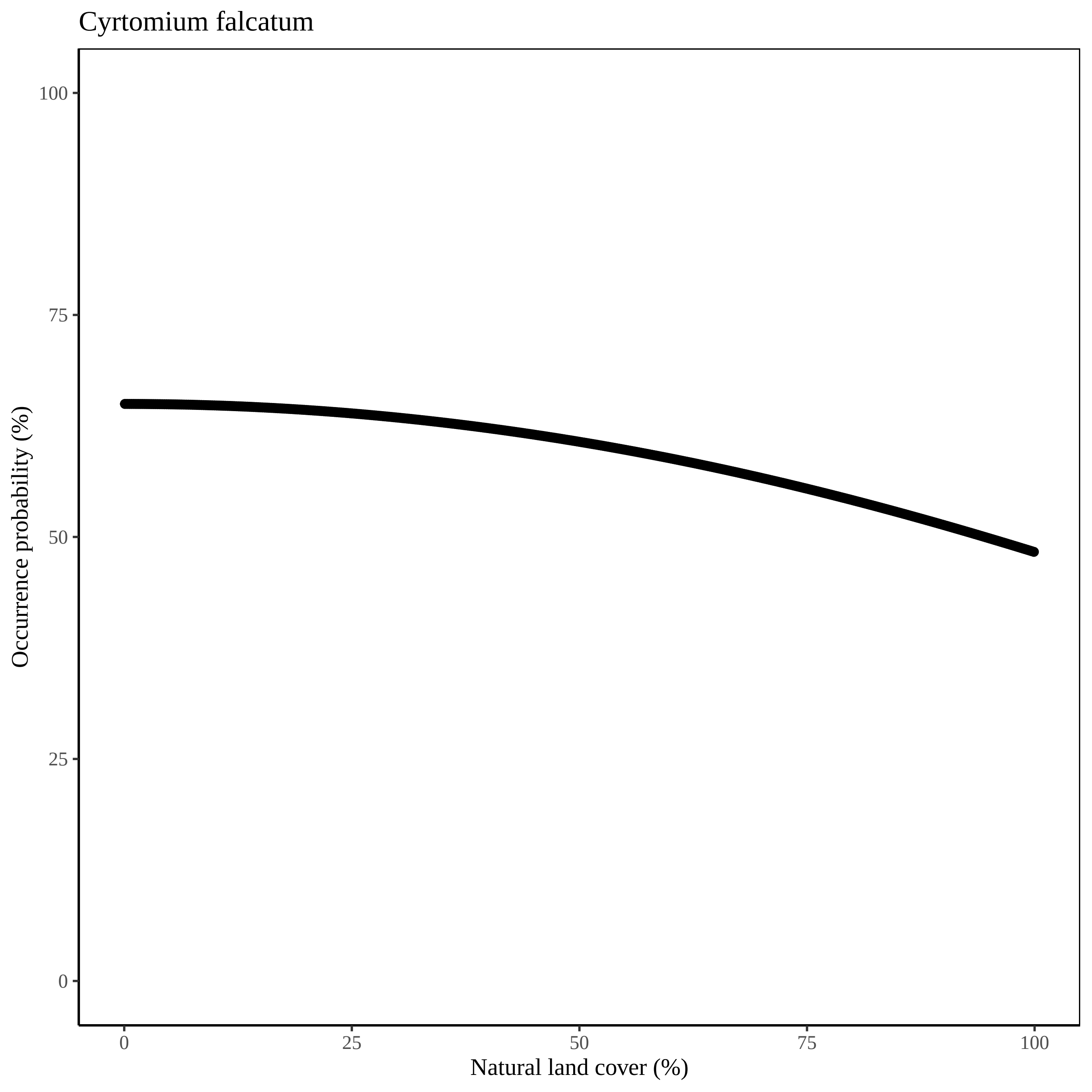
Cyrtomium falcatum
Occurrence probability (%)
100
75
50
25
0
0
25
50
75
100
Natural land cover (%)

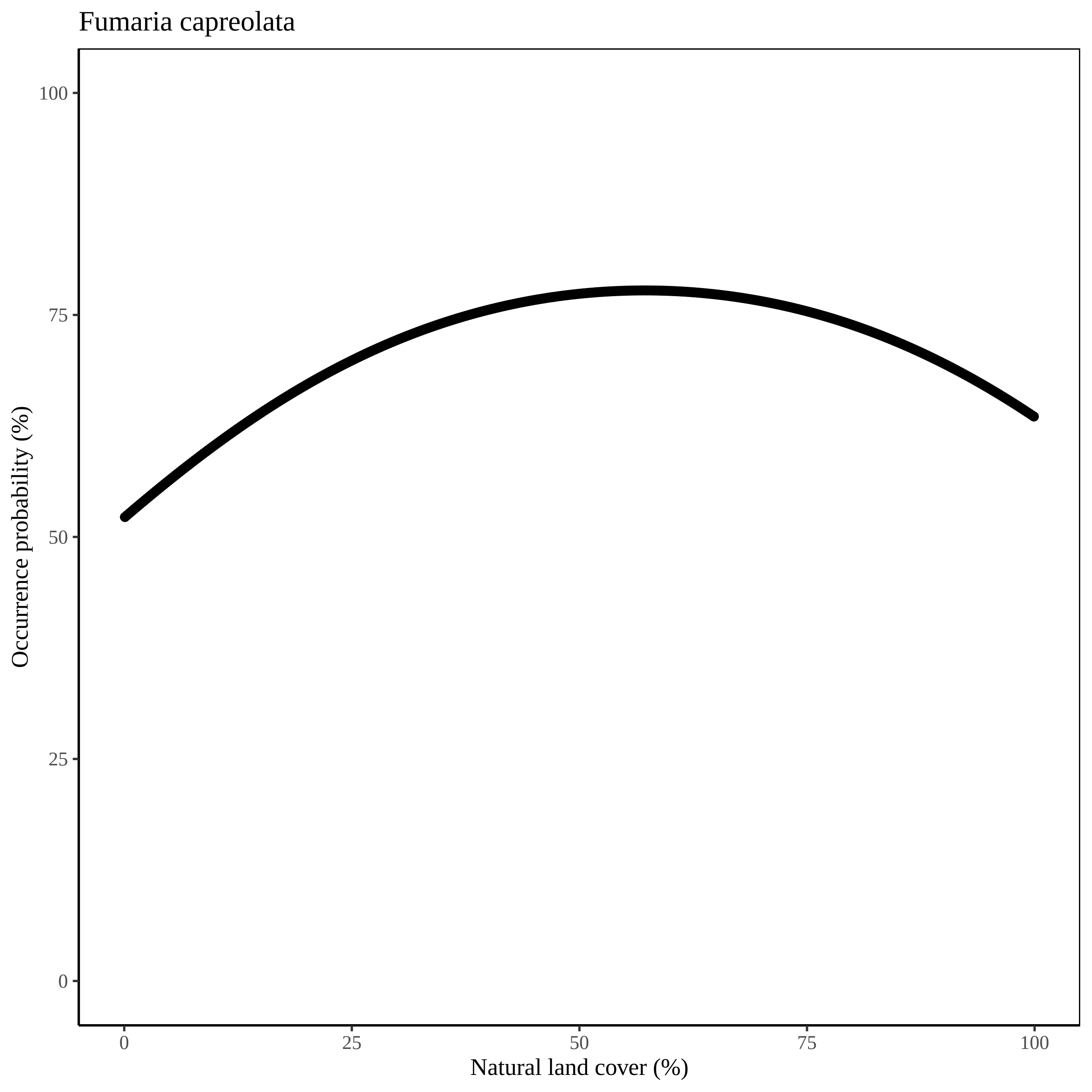

Fumaria capreolata
Occurrence probability (%)
100
75
50
25
0
0
25
50
75
100
Natural land cover (%)

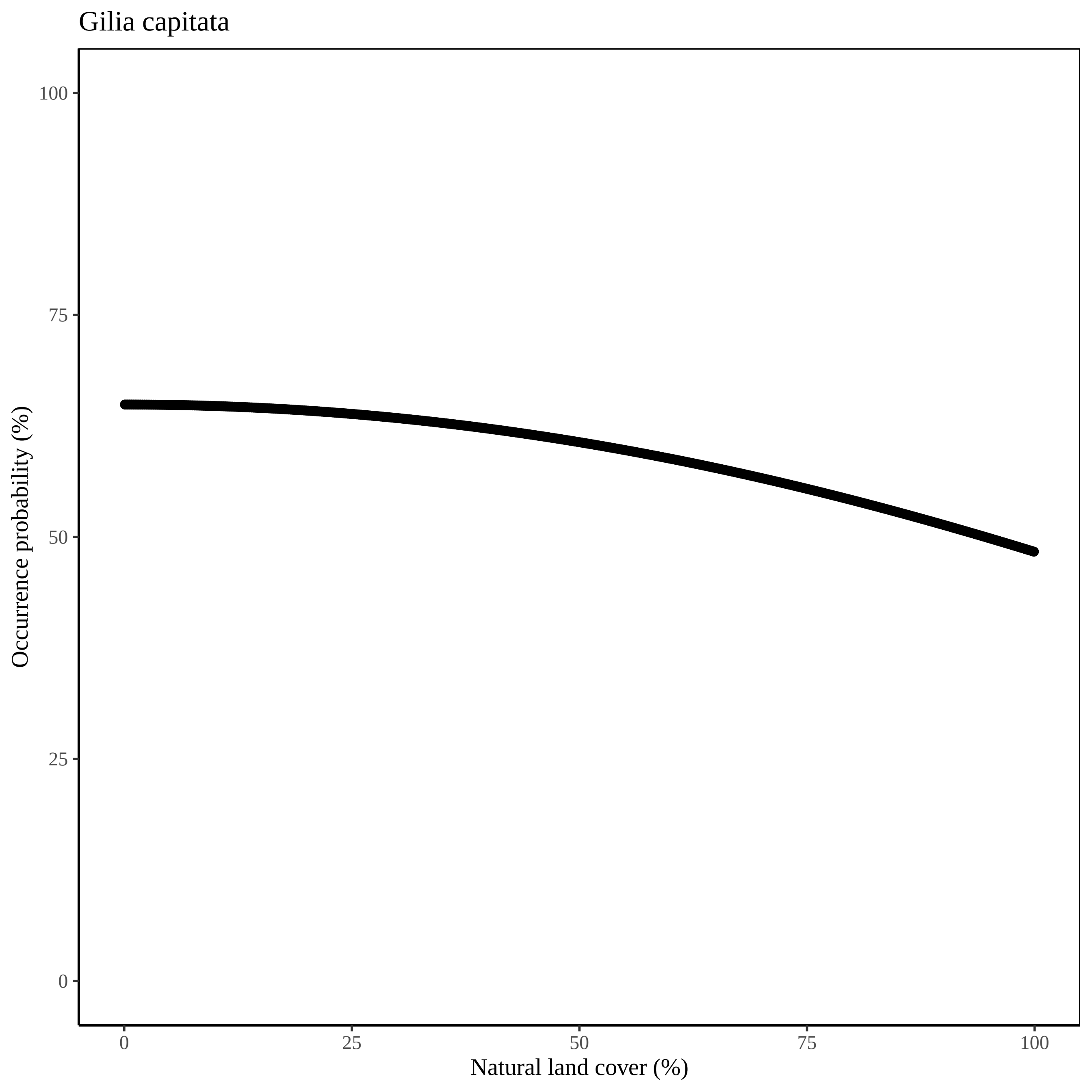
Gilia capitata
Occurrence probability (%)
100
75
50
25
0
0
25
50
75
100
Natural land cover (%)

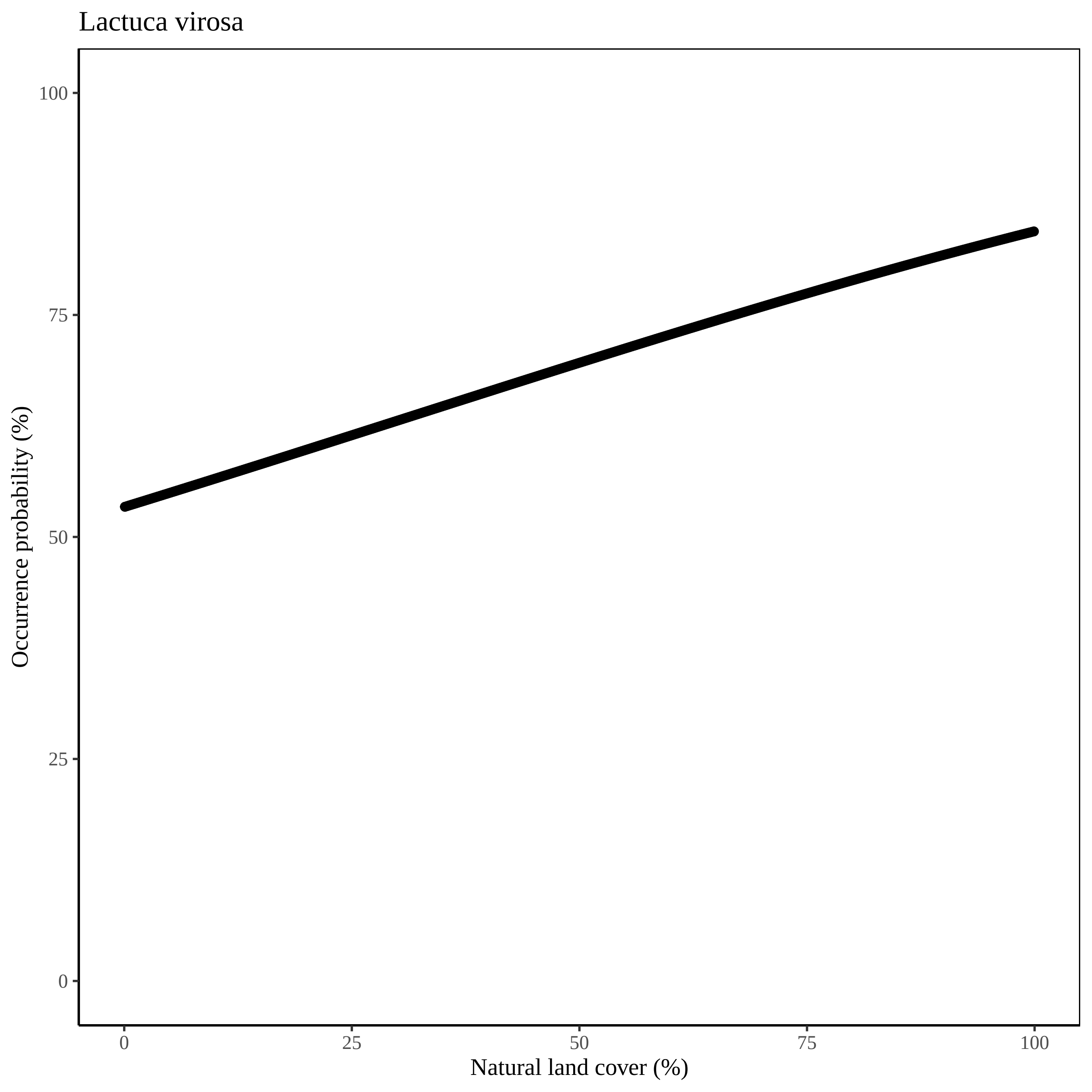
Lactuca virosa
Occurrence probability (%)
100
75
50
25
0
0
25
50
75
100
Natural land cover (%)

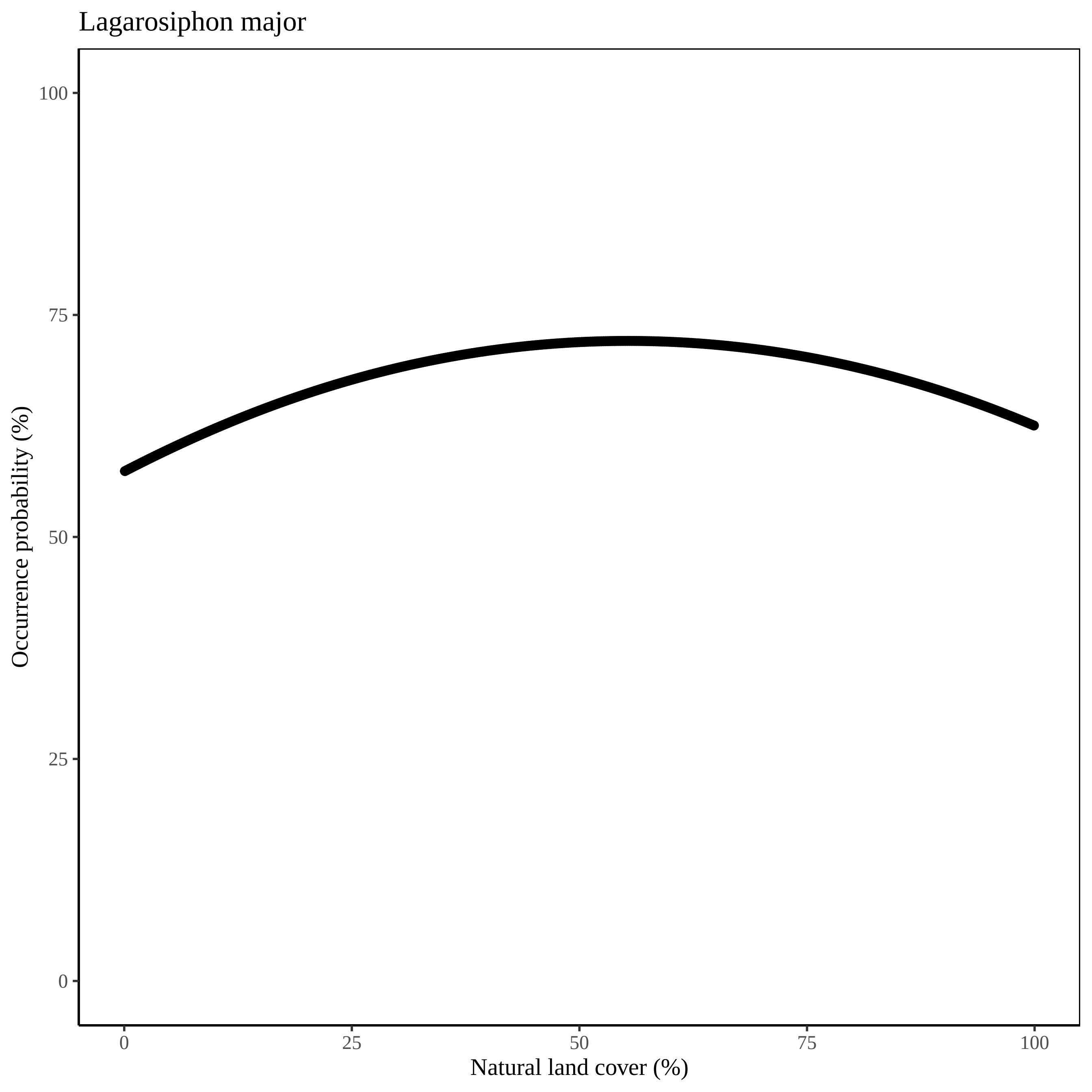
Lagarosiphon major
Occurrence probability (%)
100
75
50
25
0
0
25
50
75
100
Natural land cover (%)

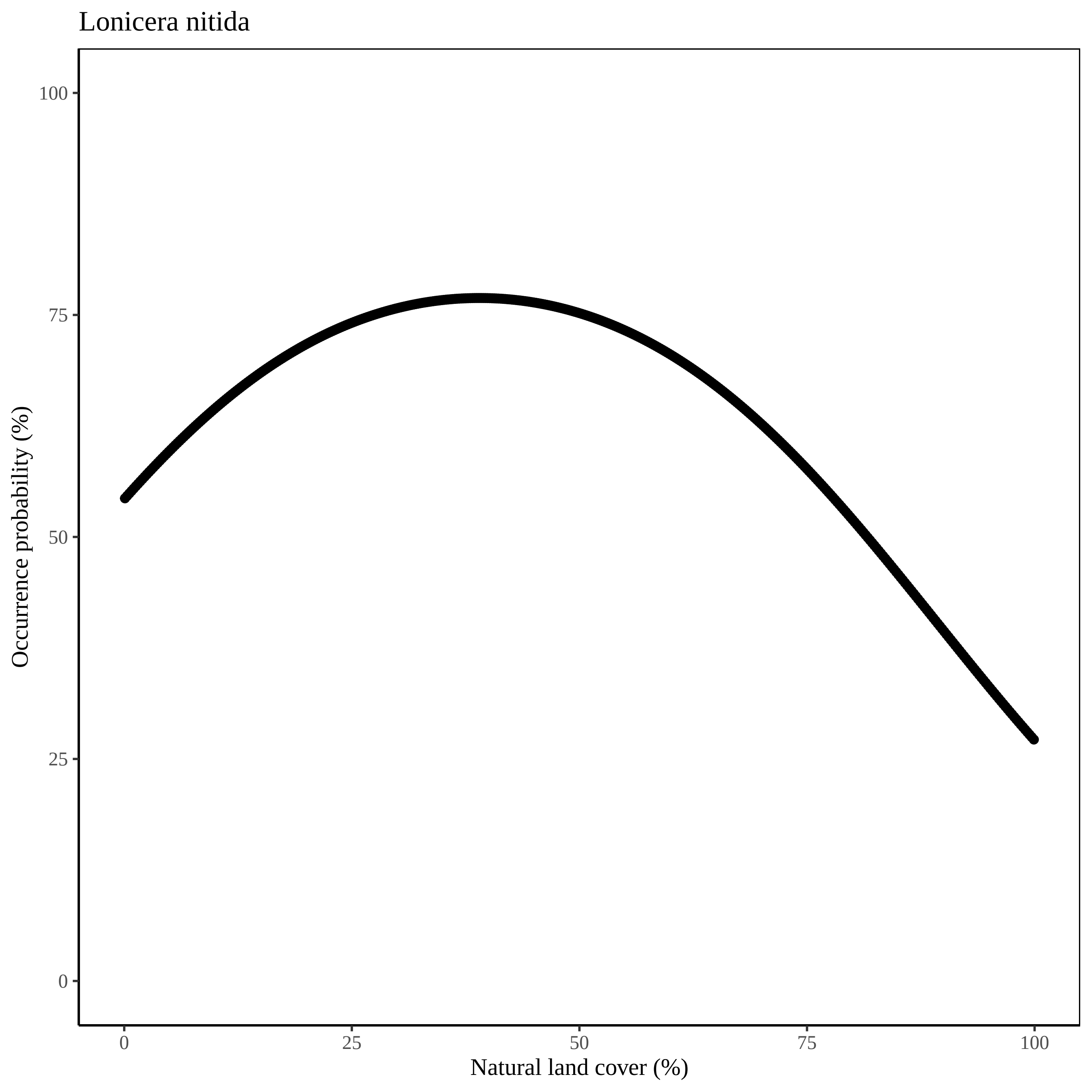

Lonicera nitida
Occurrence probability (%)
100
75
50
25
0
0
25
50
75
100
Natural land cover (%)

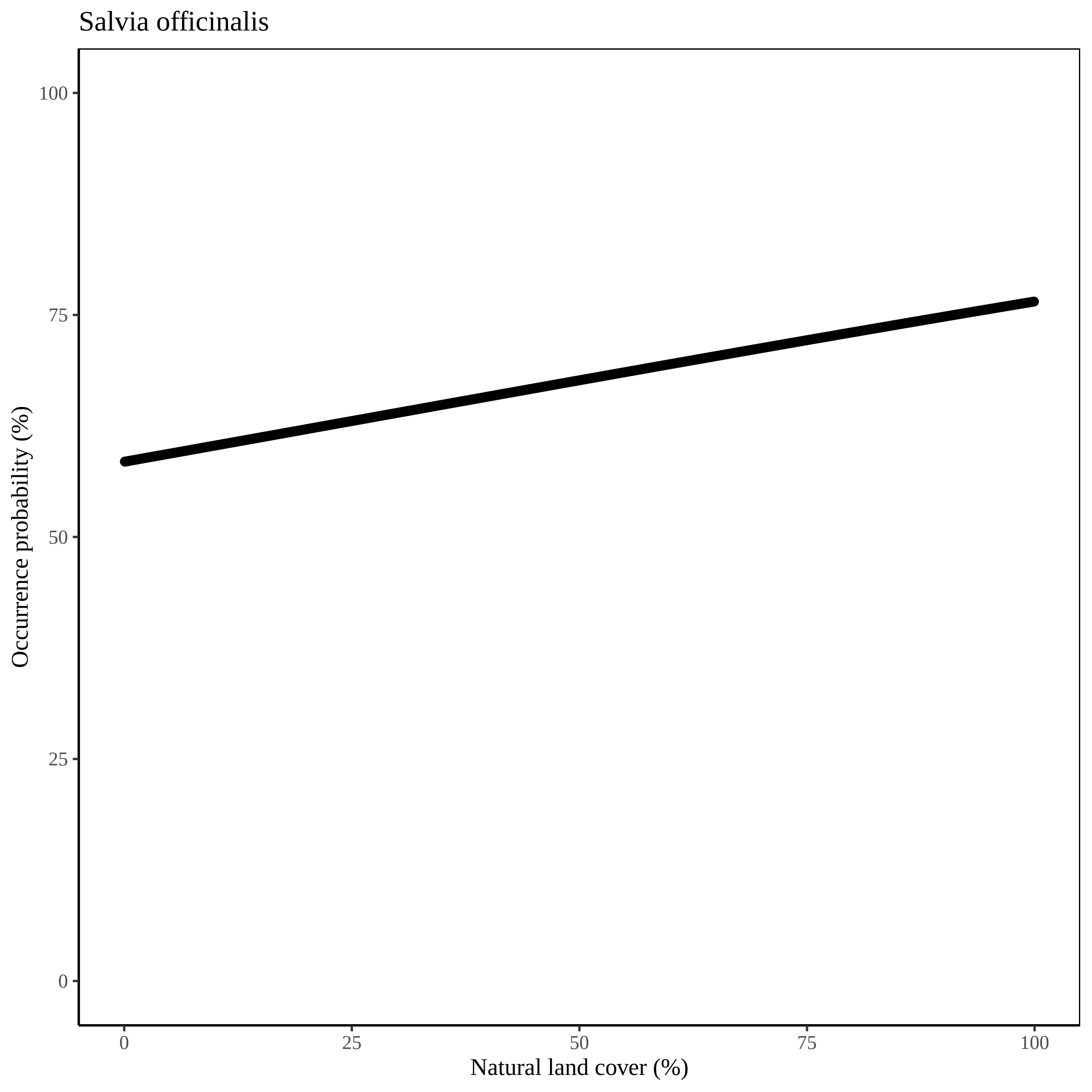

Salvia officinalis
Occurrence probability (%)
100
75
50
25
0
0
25
50
75
100
Natural land cover (%)

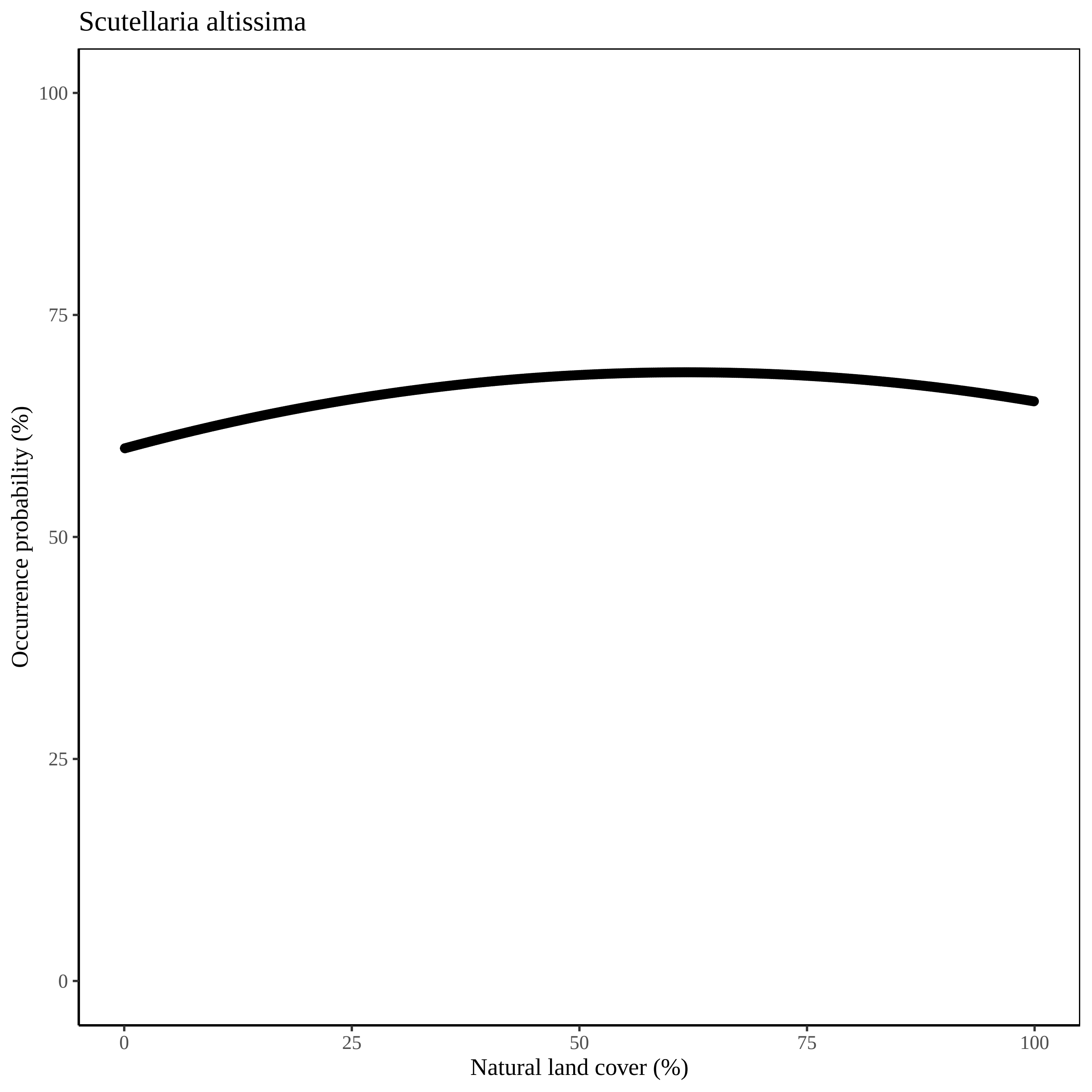

Scutellaria altissima
Occurrence probability (%)
100
75
50
25
0
0
25
50
75
100
Natural land cover (%)

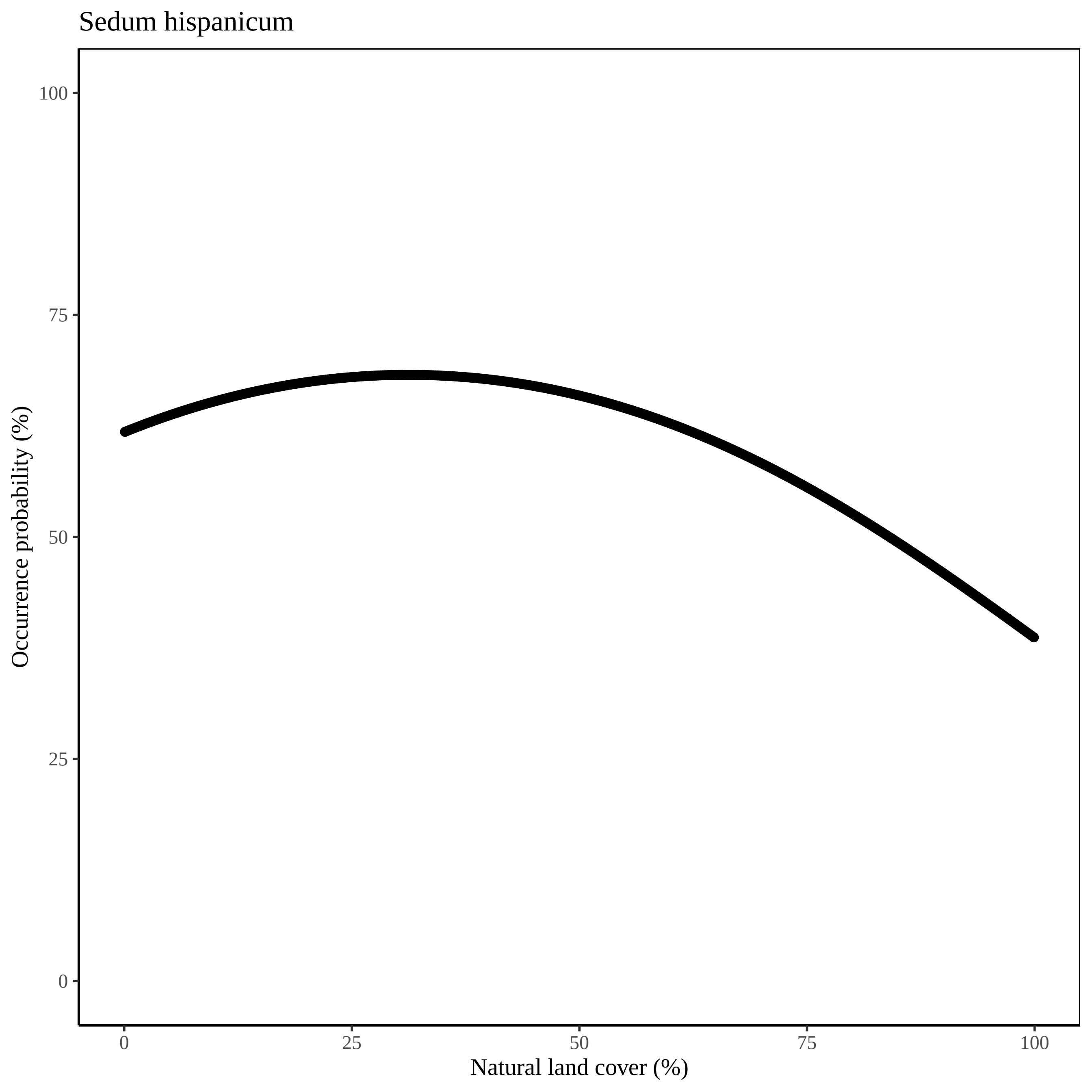
Sedum hispanicum
Occurrence probability (%)
100
75
50
25
0
0
25
50
75
100
Natural land cover (%)

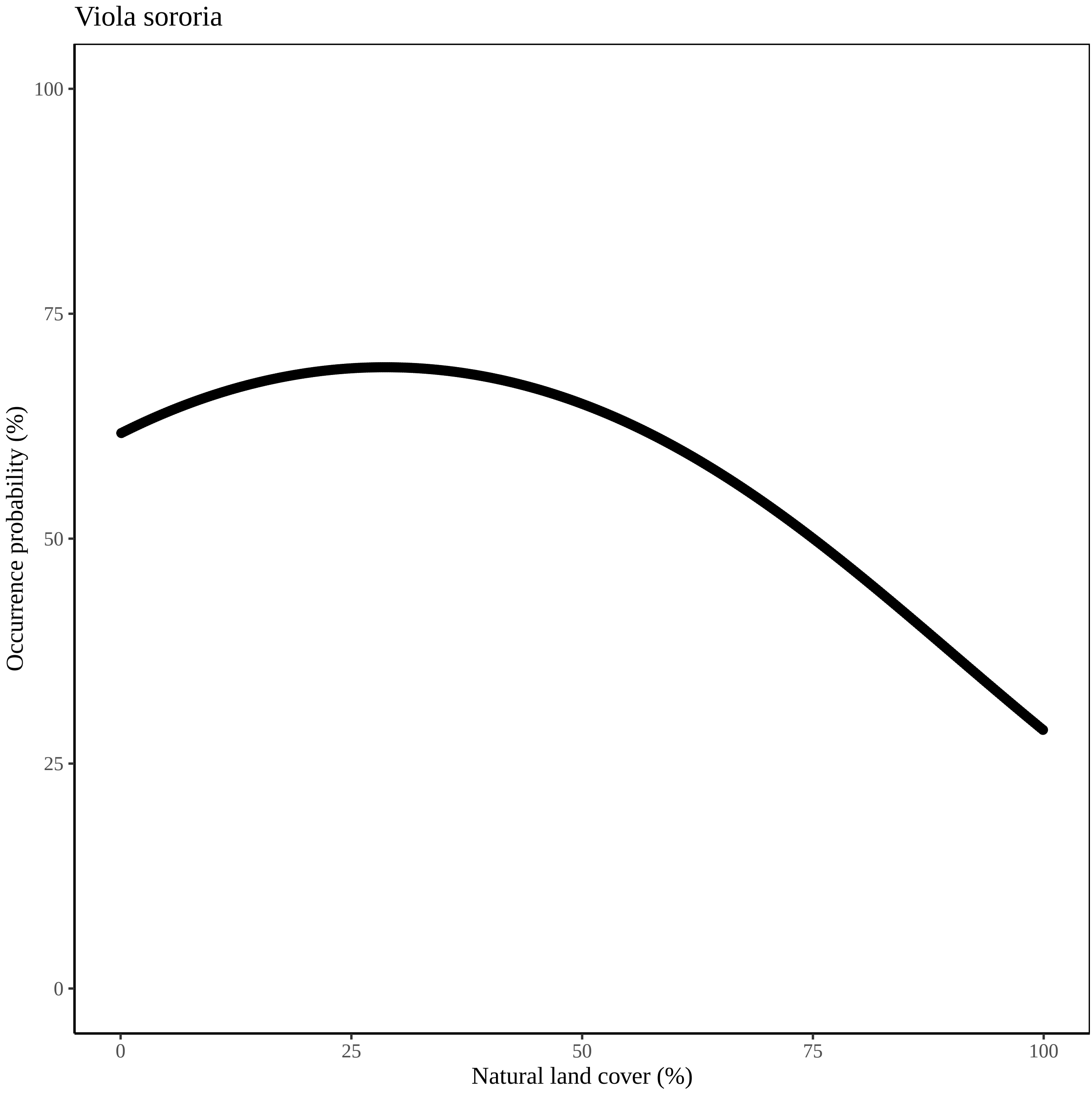
Viola sororia
Occurrence probability (%)
100
75
50
25
0
0
25
50
75
100
Natural land cover (%)

Abies alba

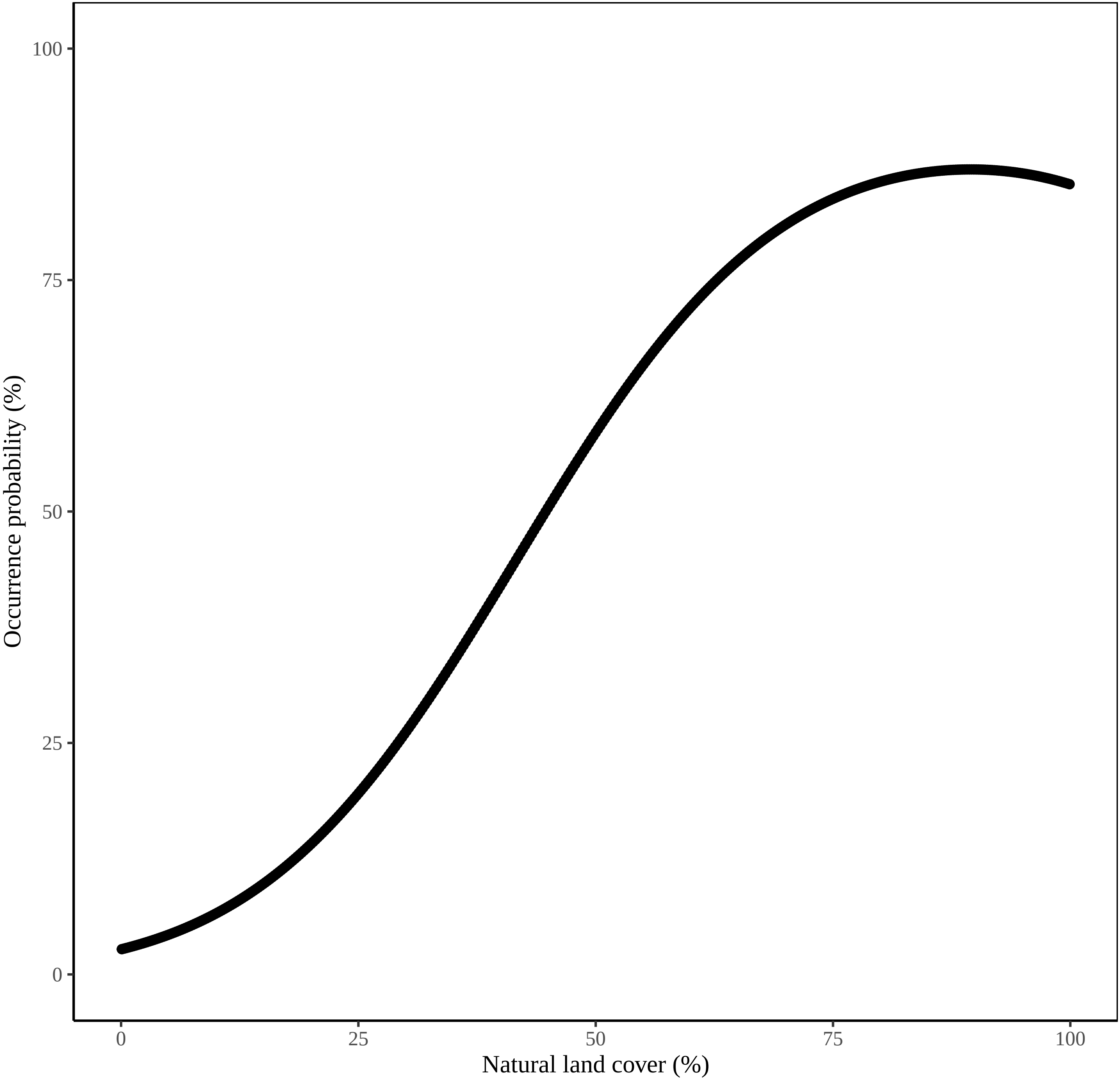

Occurrence probability (%)
100
75
50
25
0
0
25
50
75
100
Natural land cover (%)

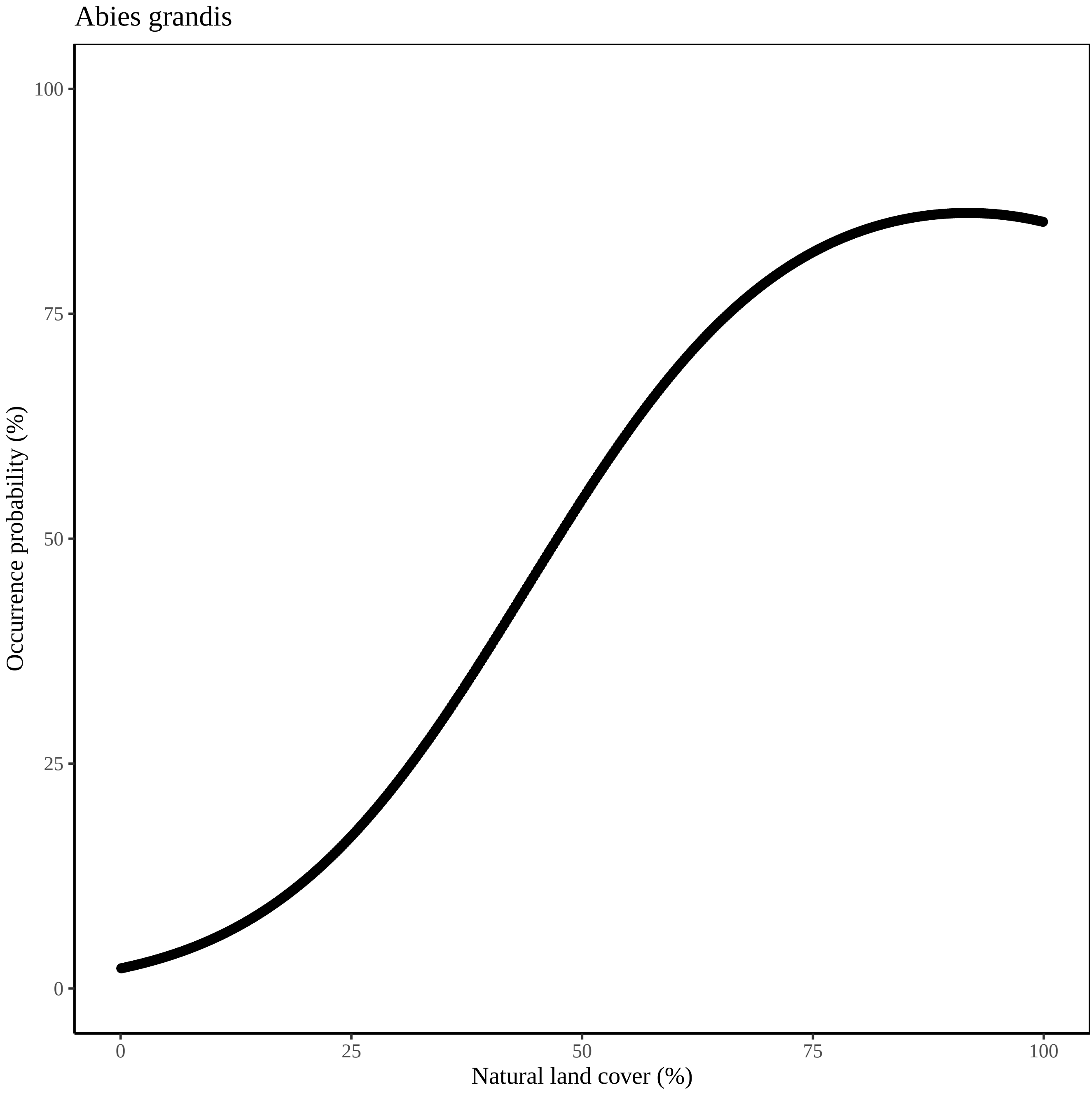
Abies grandis
Occurrence probability (%)
100
75
50
25
0
Natural land cover (%)
0
25
50
75
100

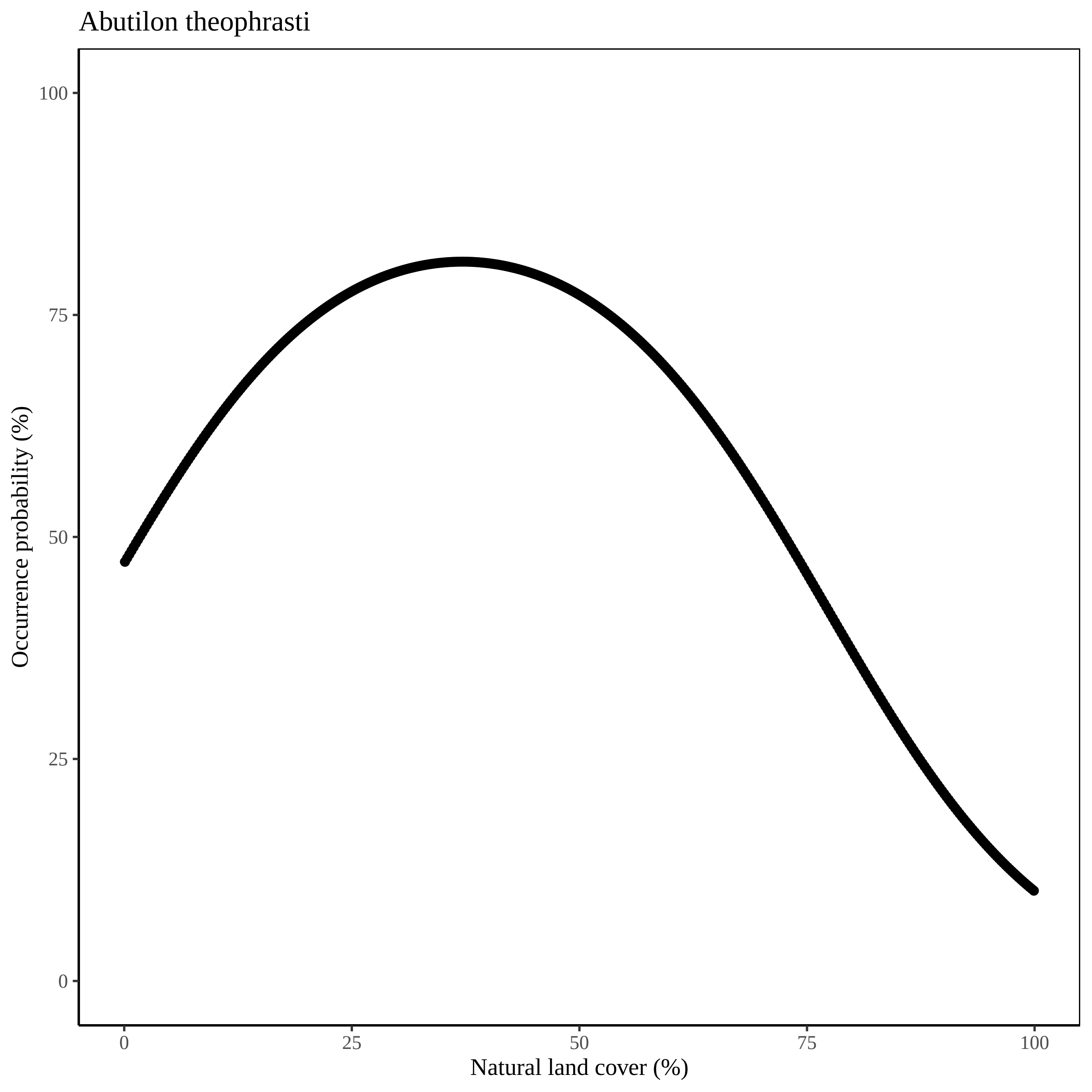

Abutilon theophrasti
Occurrence probability (%)
100
75
50
25
0
0
25
50
75
100
Natural land cover (%)

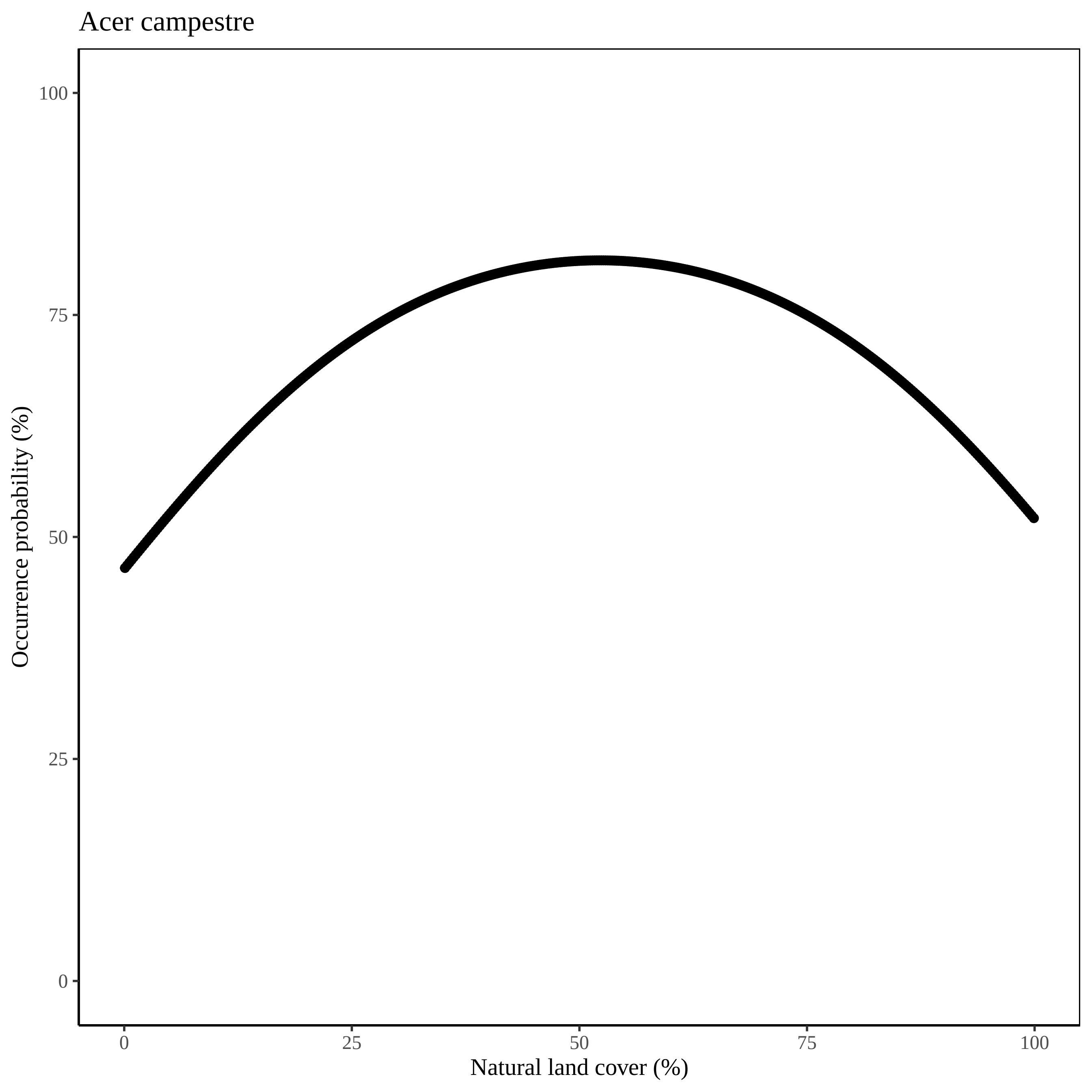

Acer campestre
Occurrence probability (%)
100
75
50
25
0
0
25
50
75
100
Natural land cover (%)

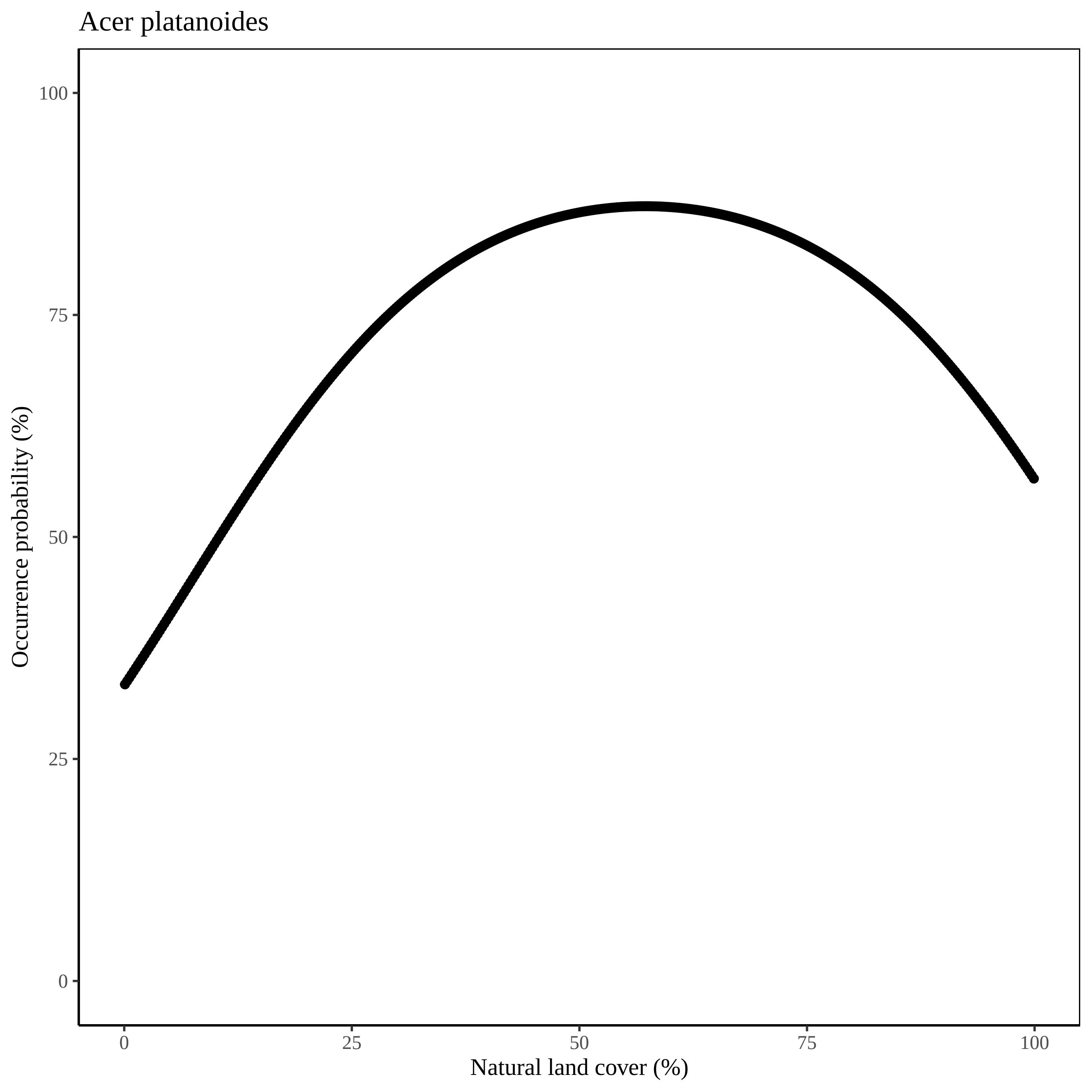

Acer platanoides
Occurrence probability (%)
100
75
50
25
0
0
25
50
75
100
Natural land cover (%)

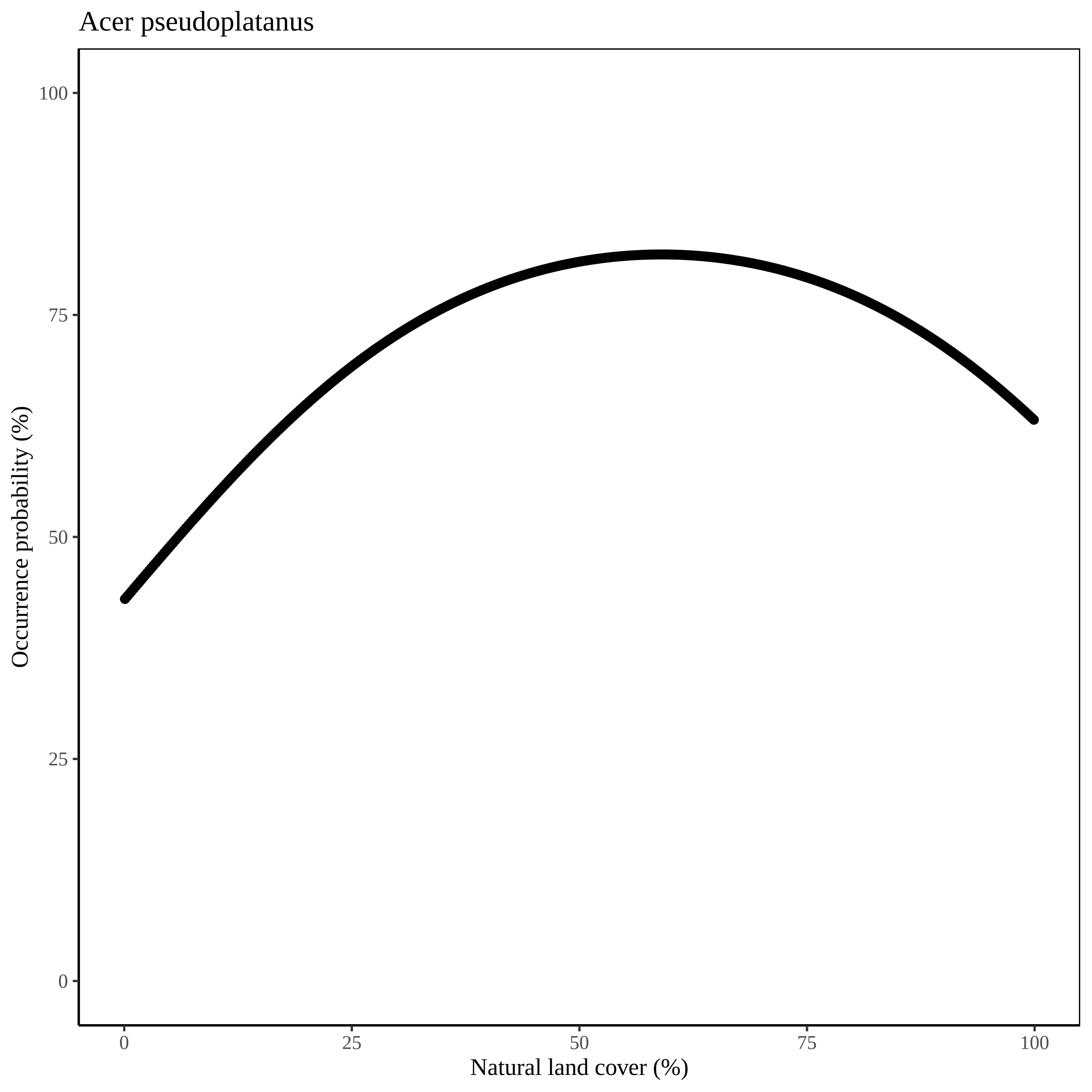
Acer pseudoplatanus
Occurrence probability (%)
Natural land cover (%)
0
25
50
75
100

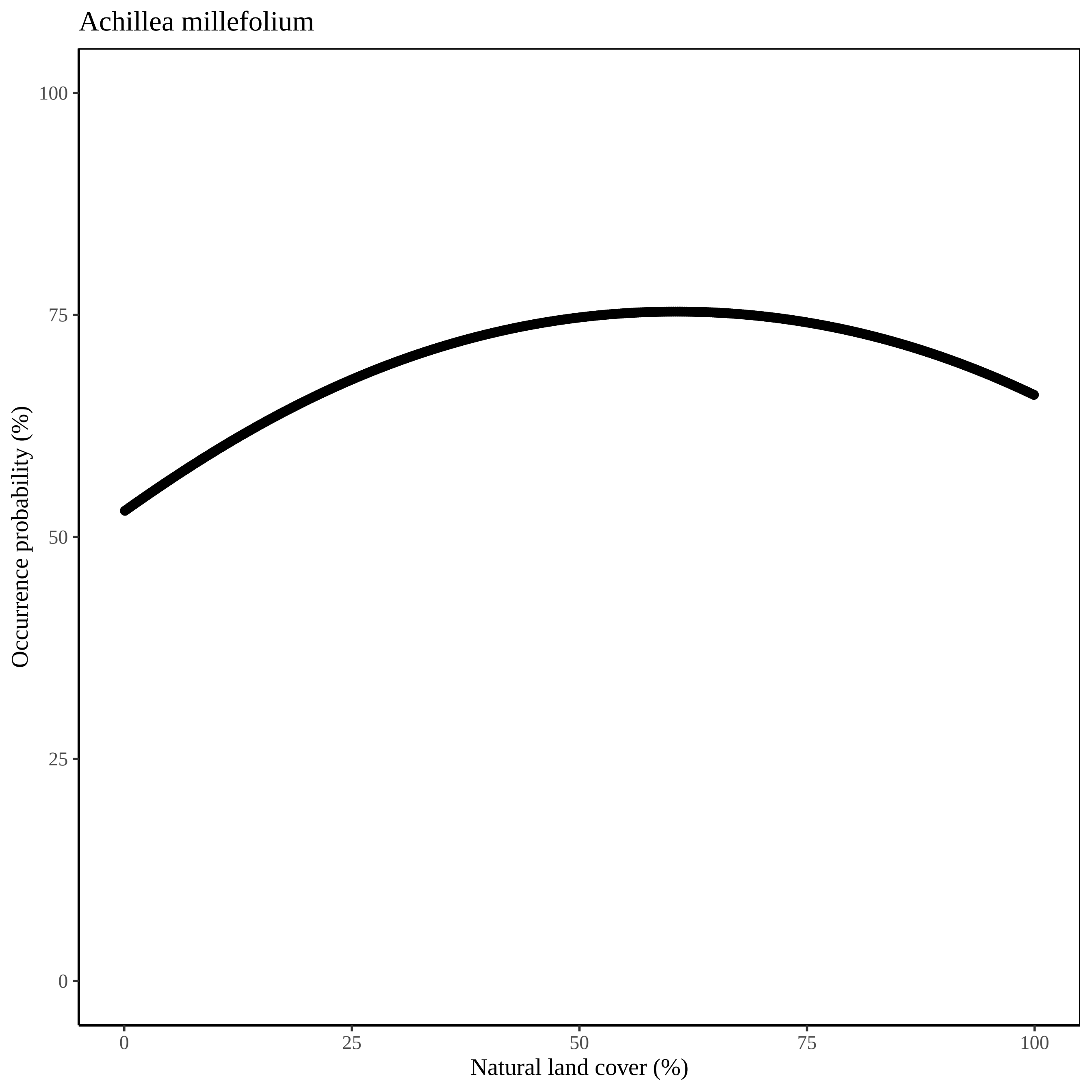

Achillea millefolium
Occurrence probability (%)
100
75
50
25
0
0
25
50
75
100
Natural land cover (%)

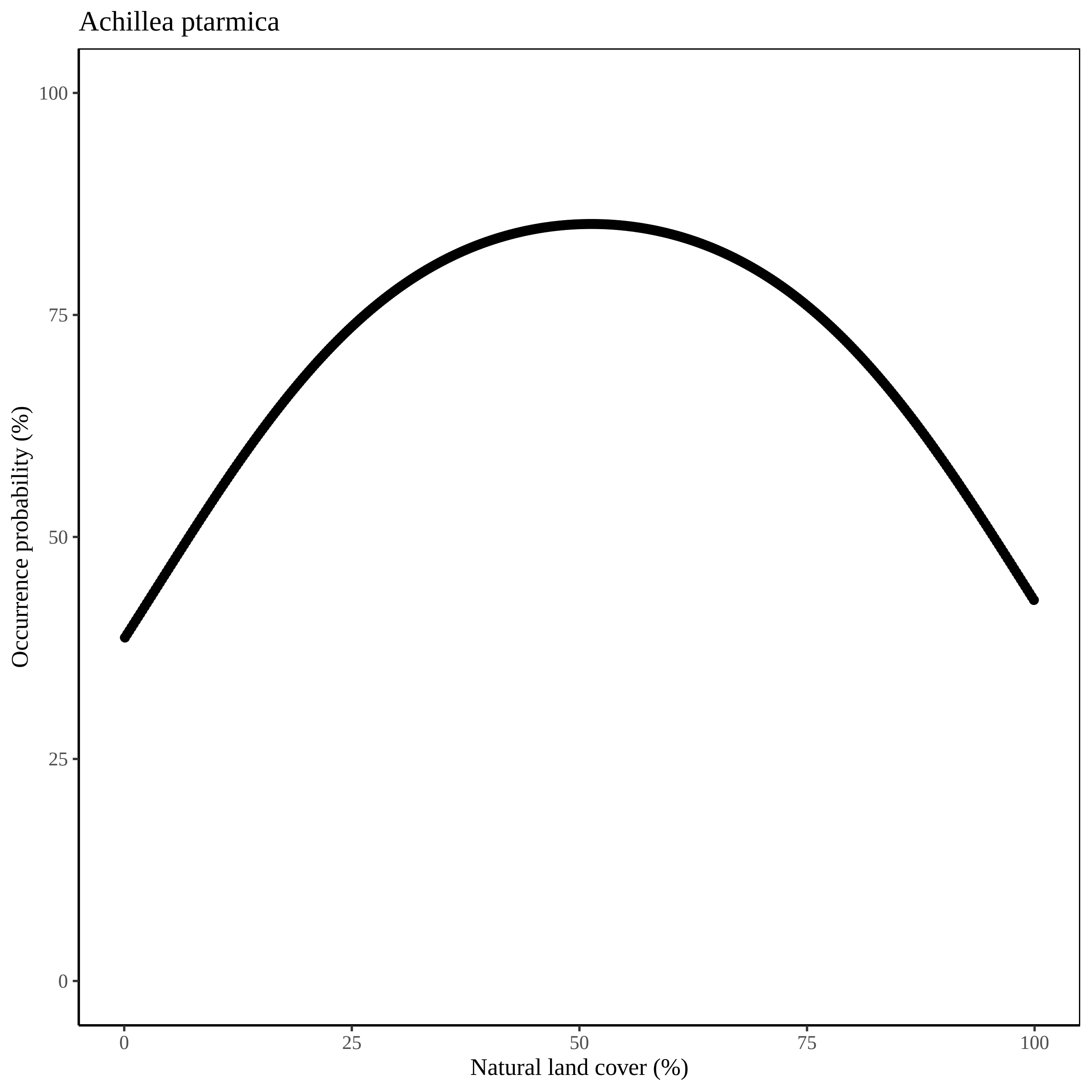

Achillea ptarmica
100
75
50
25
0
Occurrence probability (%)
0
25
50
75
100
Natural land cover (%)

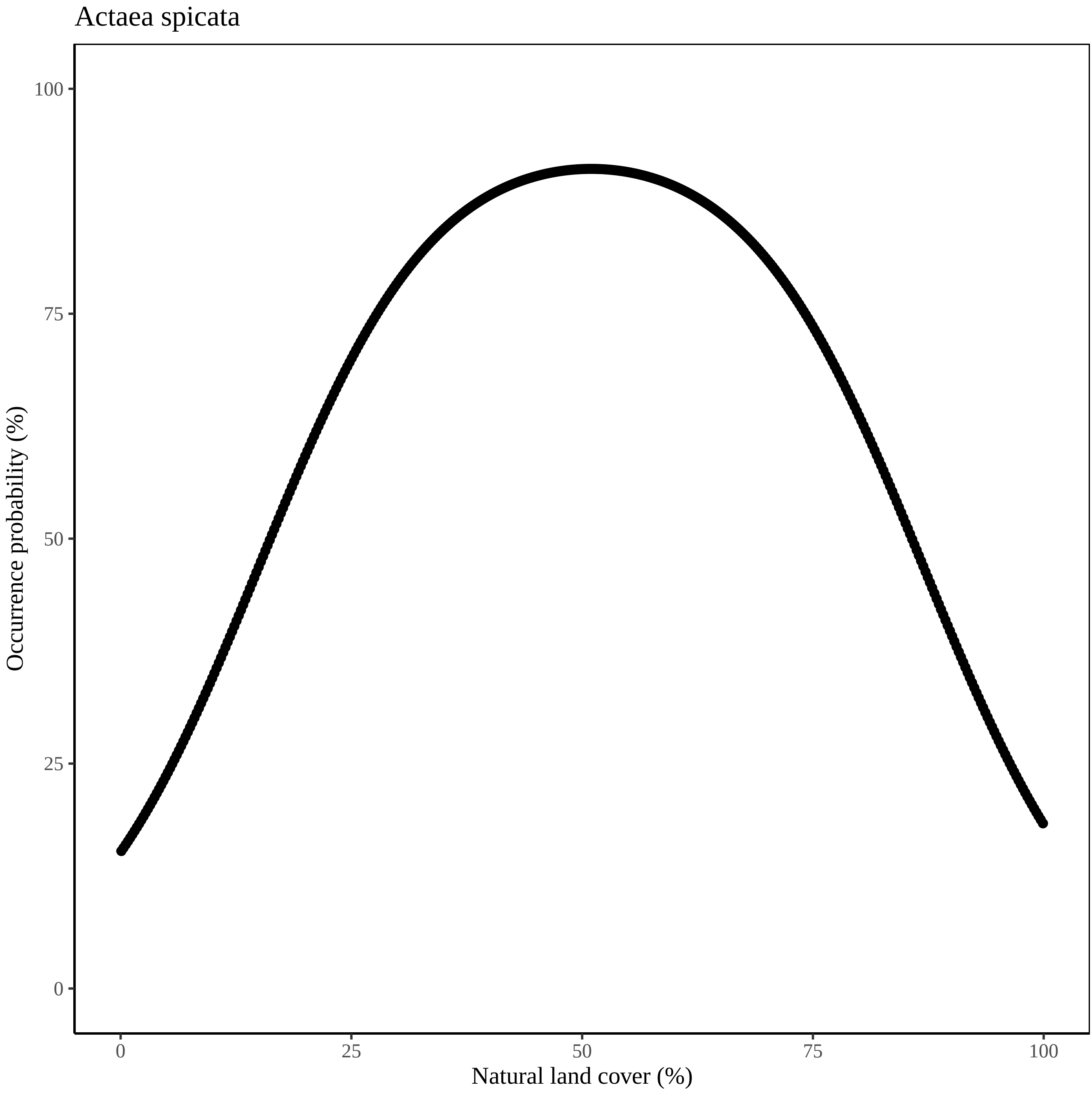

Actaea spicata
Occurrence probability (%)
100
75
50
25
0
0
25
50
75
100
Natural land cover (%)

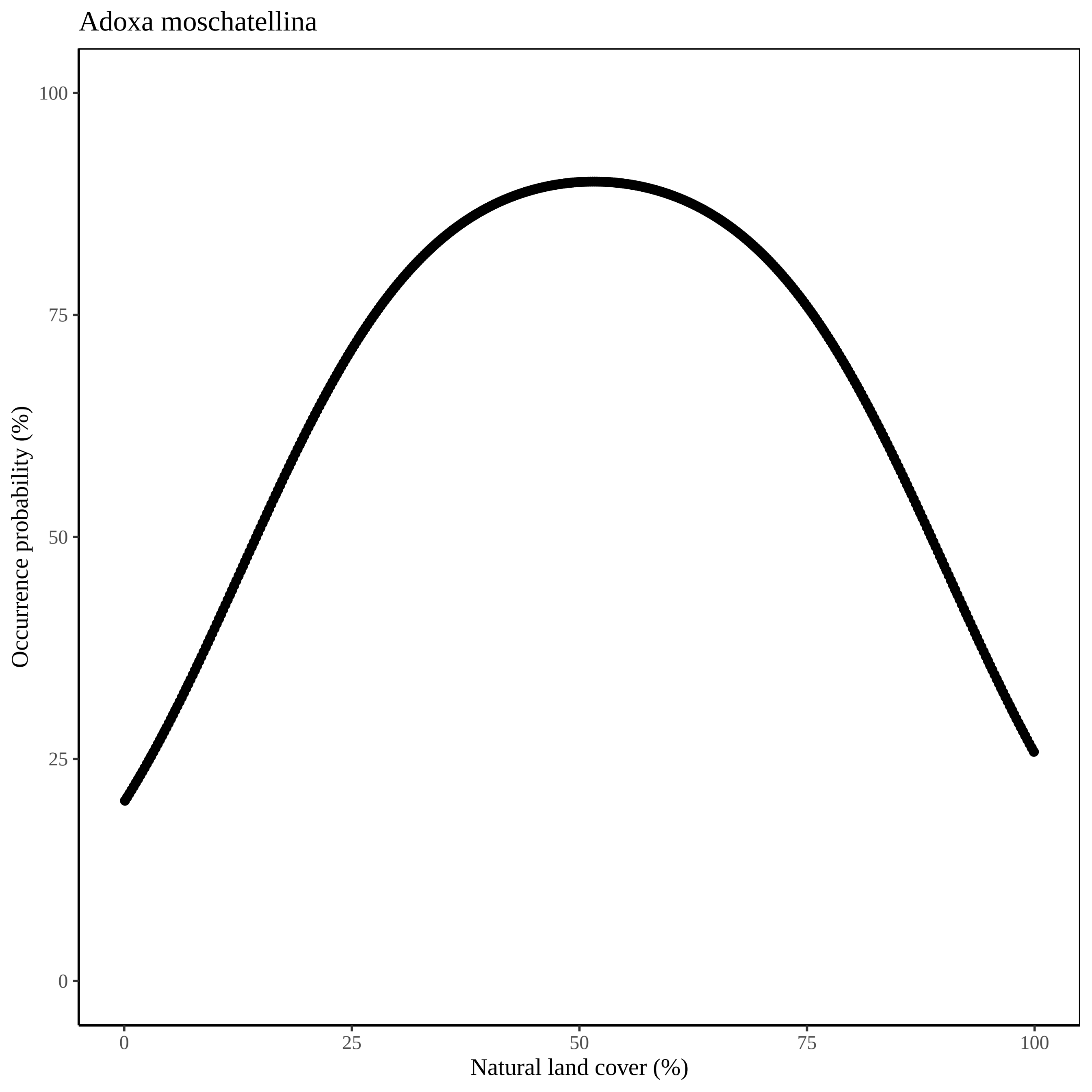
Adoxa moschatellina
100
75
50
25
0
Occurrence probability (%)
0
25
50
75
100
Natural land cover (%)

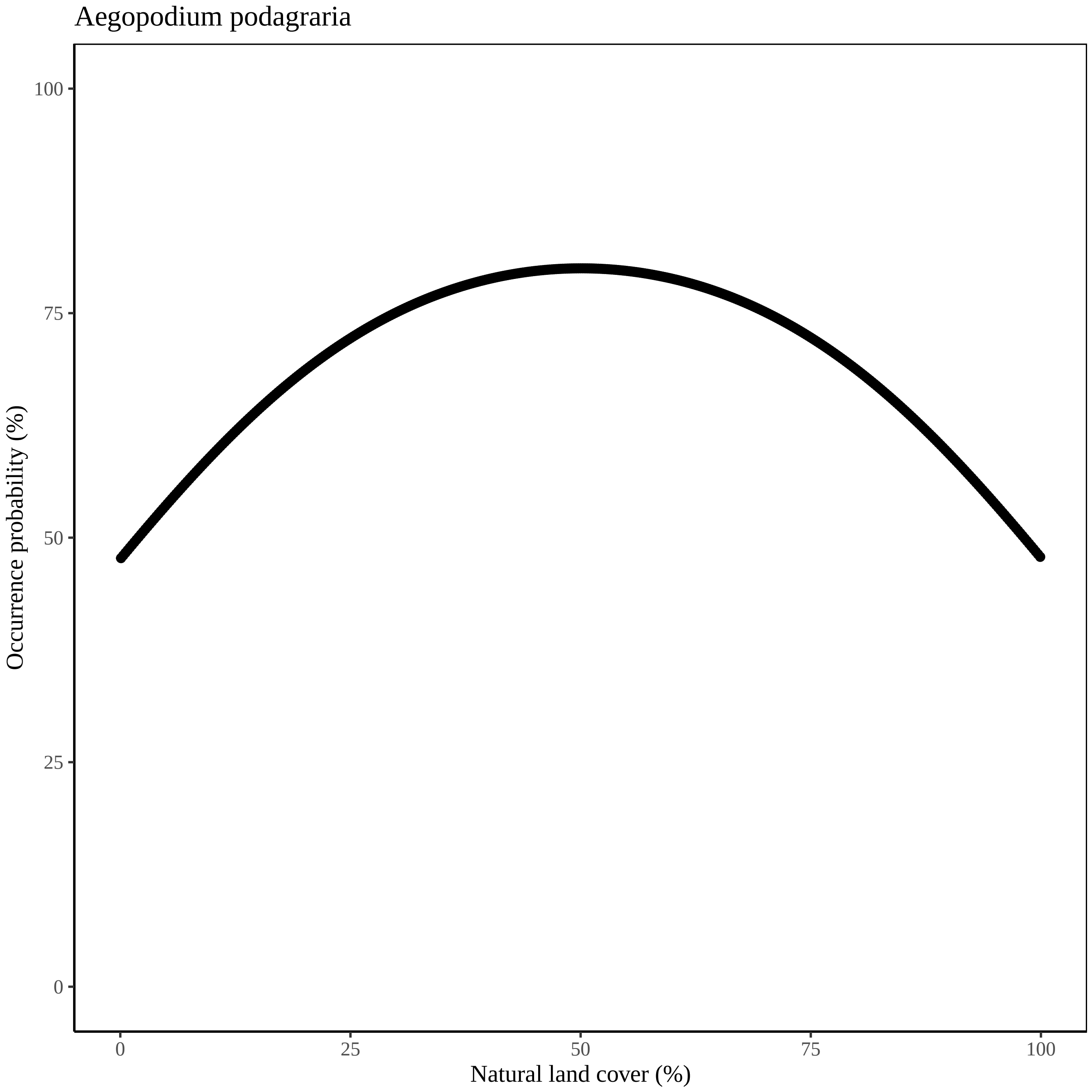

Aegopodium podagraria
Occurrence probability (%)
100
75
50
25
0
0
25
50
75
100
Natural land cover (%)

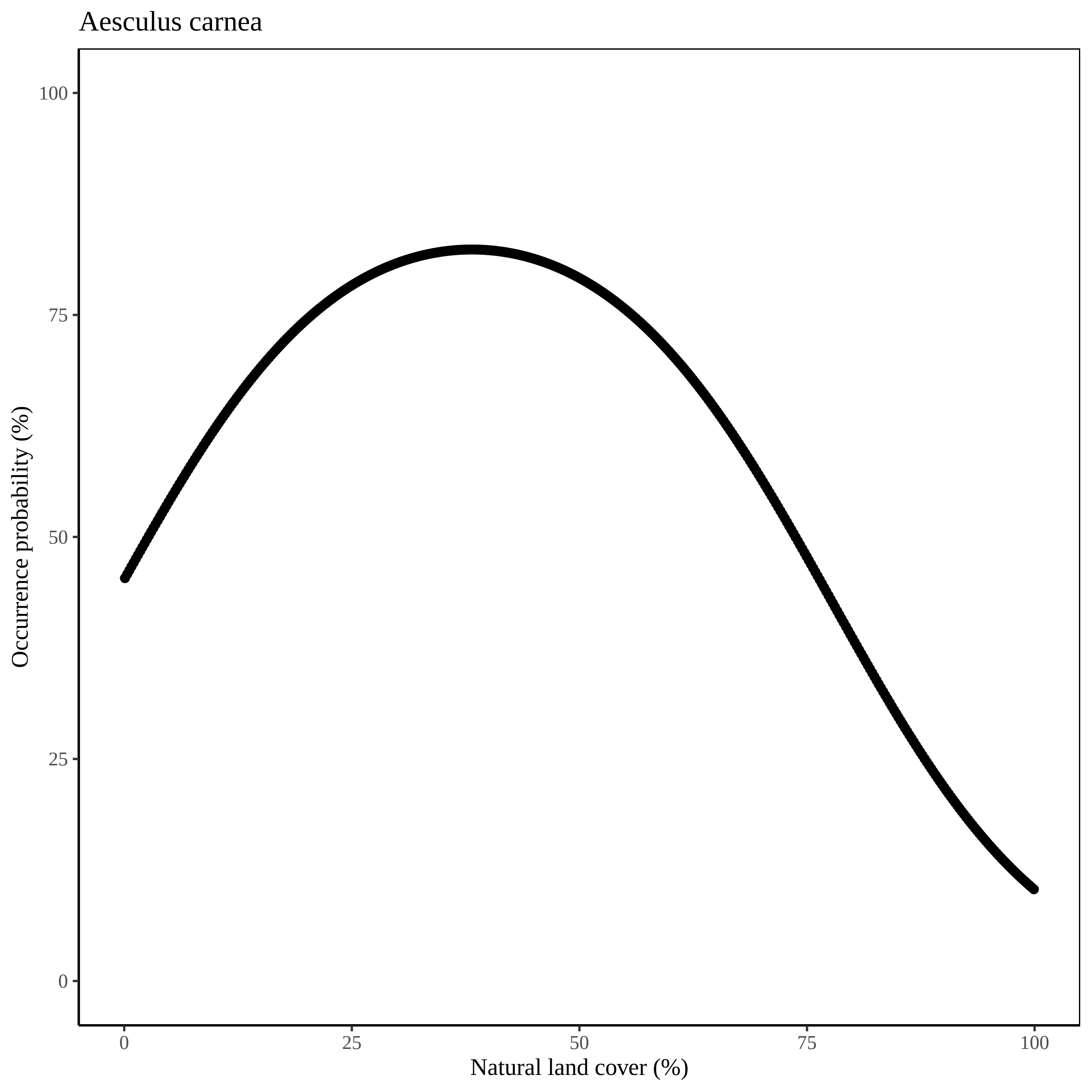
Aesculus carnea
Occurrence probability (%)
100
75
50
25
0
0
25
50
75
100
Natural land cover (%)

Aesculus hippocastanum

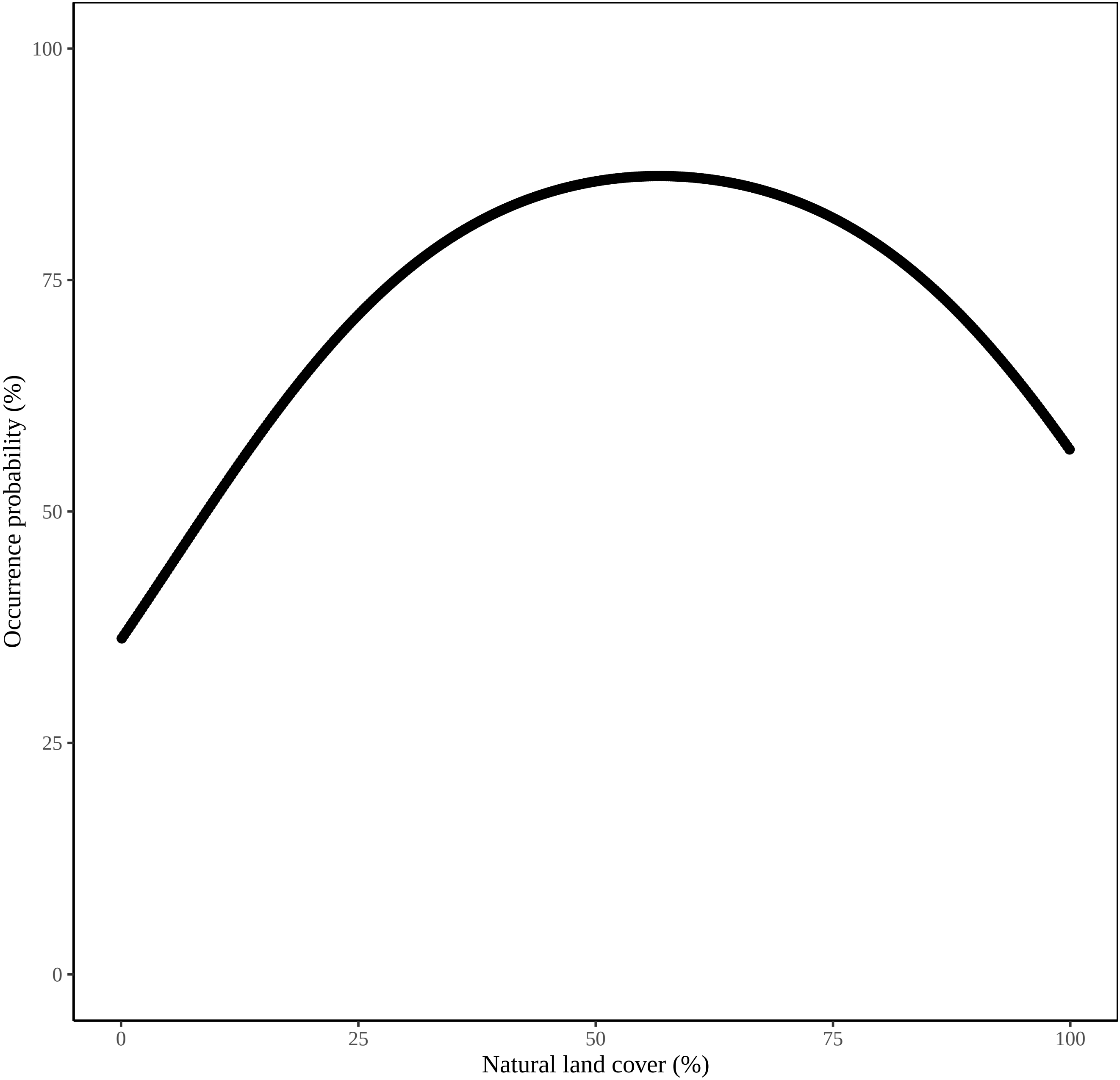

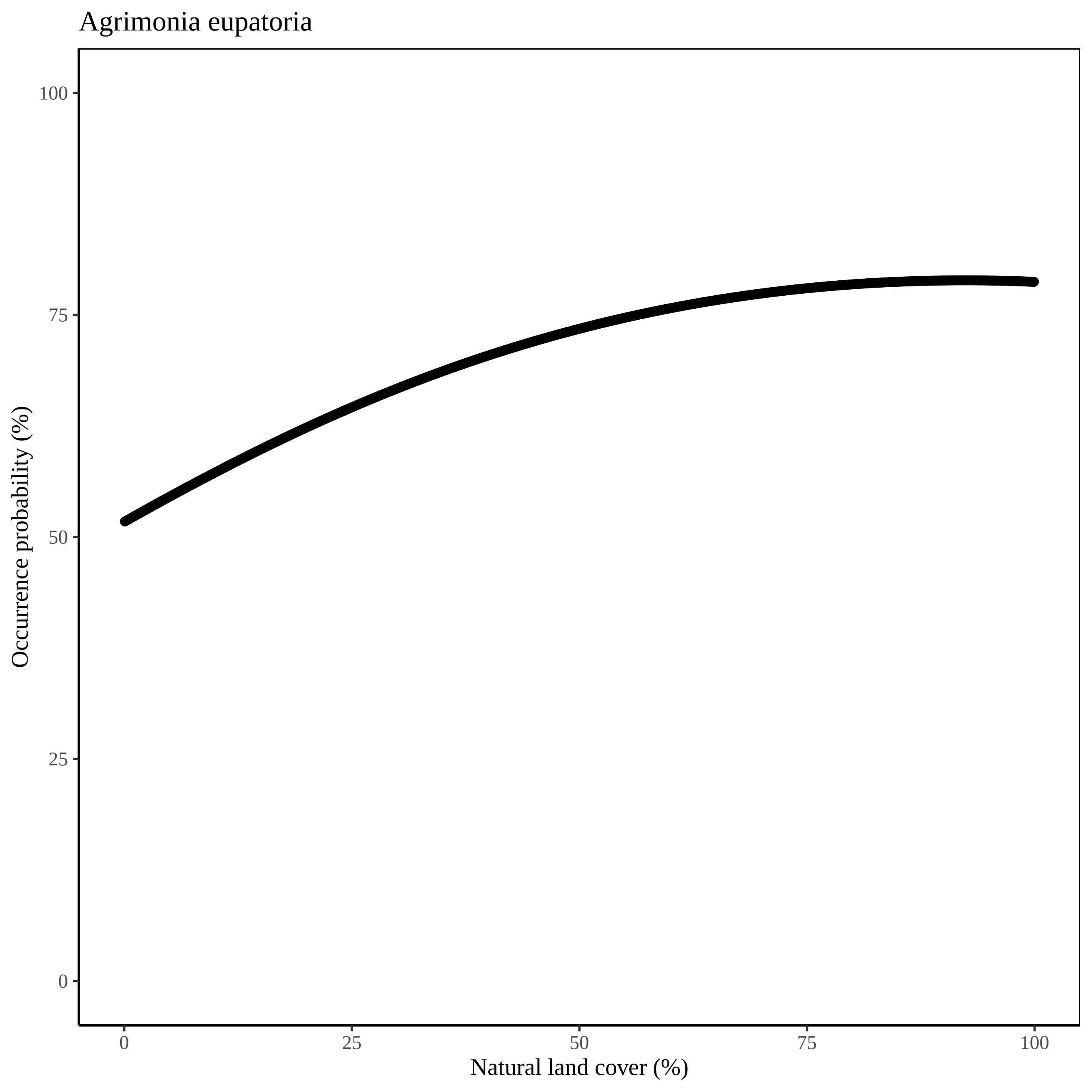
Agrimonia eupatoria
Occurrence probability (%)
100
75
50
25
0
0
25
50
75
100
Natural land cover (%)

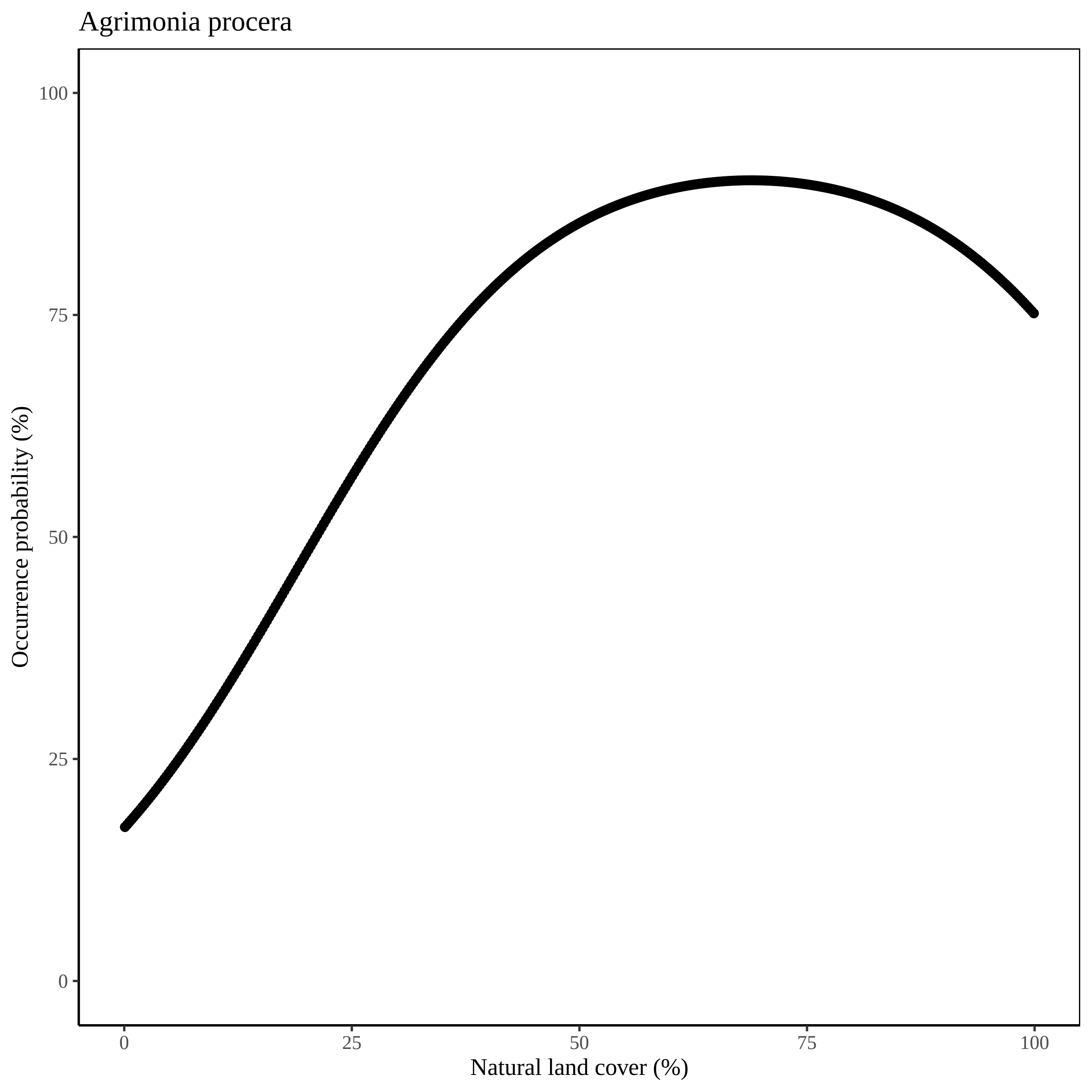

Agrimonia procera
Occurrence probability (%)
100
75
50
25
0
0
25
50
75
100
Natural land cover (%)

Agrostemma githago

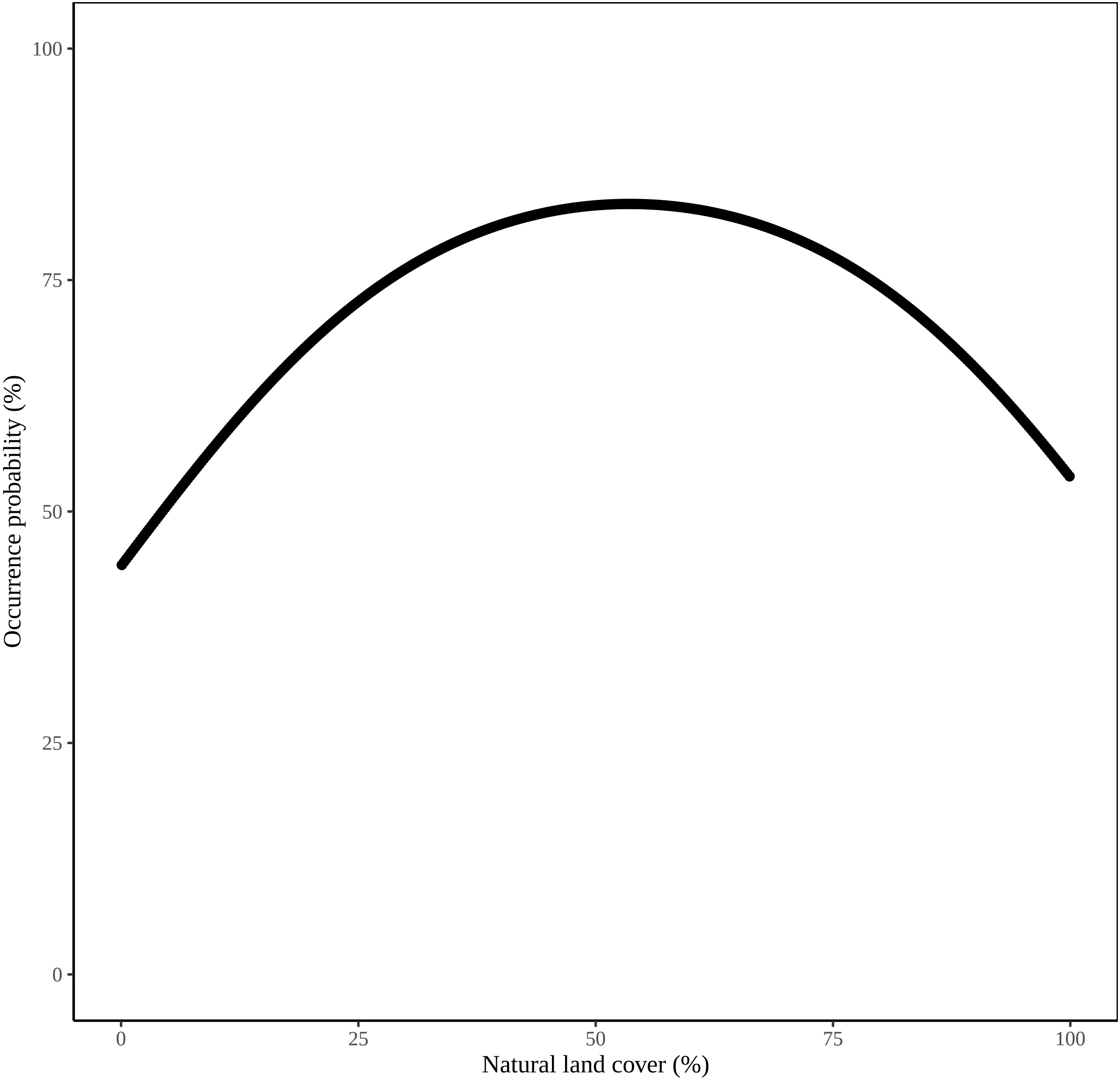

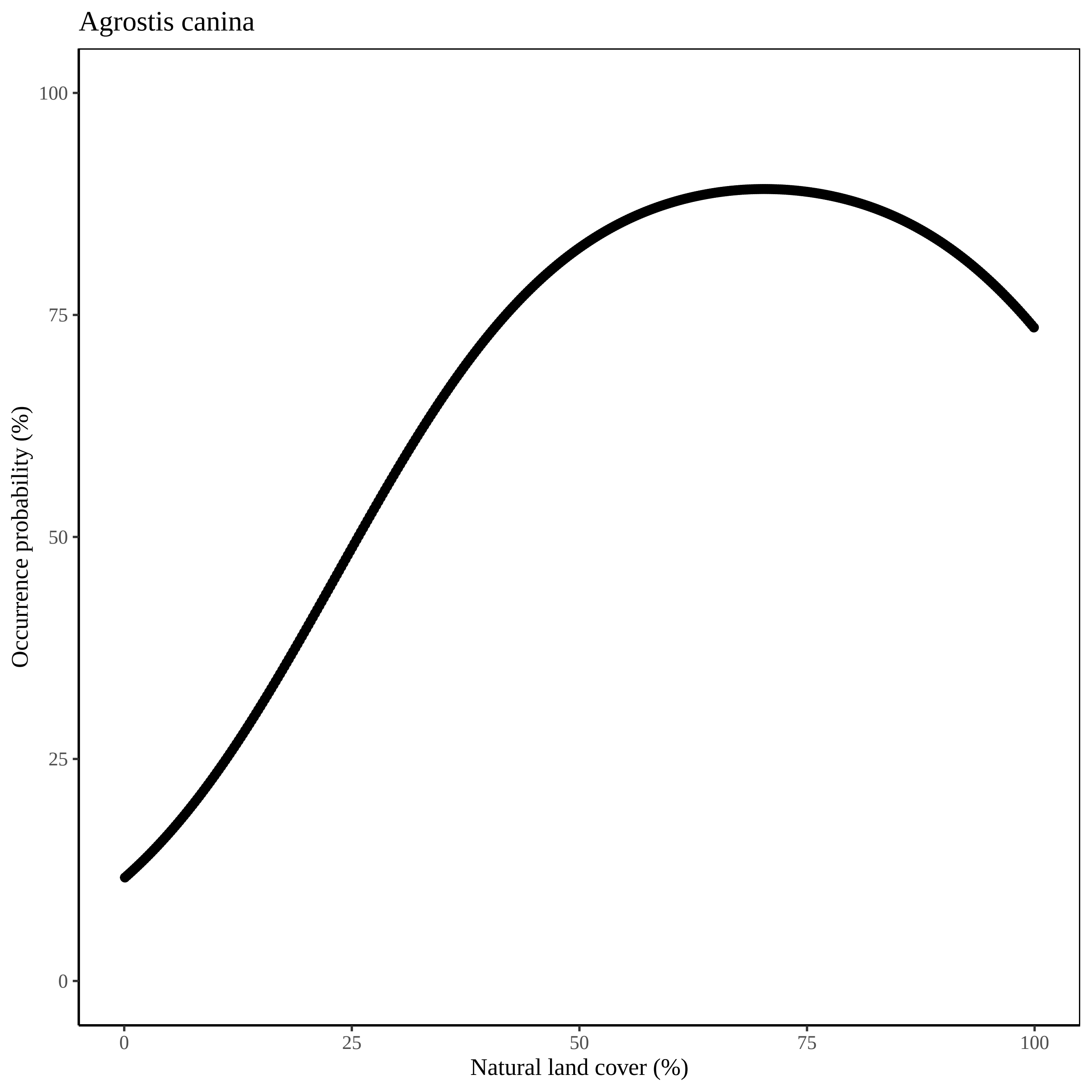

Agrostis canina
Occurrence probability (%)
100
75
50
25
0
0
25
50
75
100
Natural land cover (%)

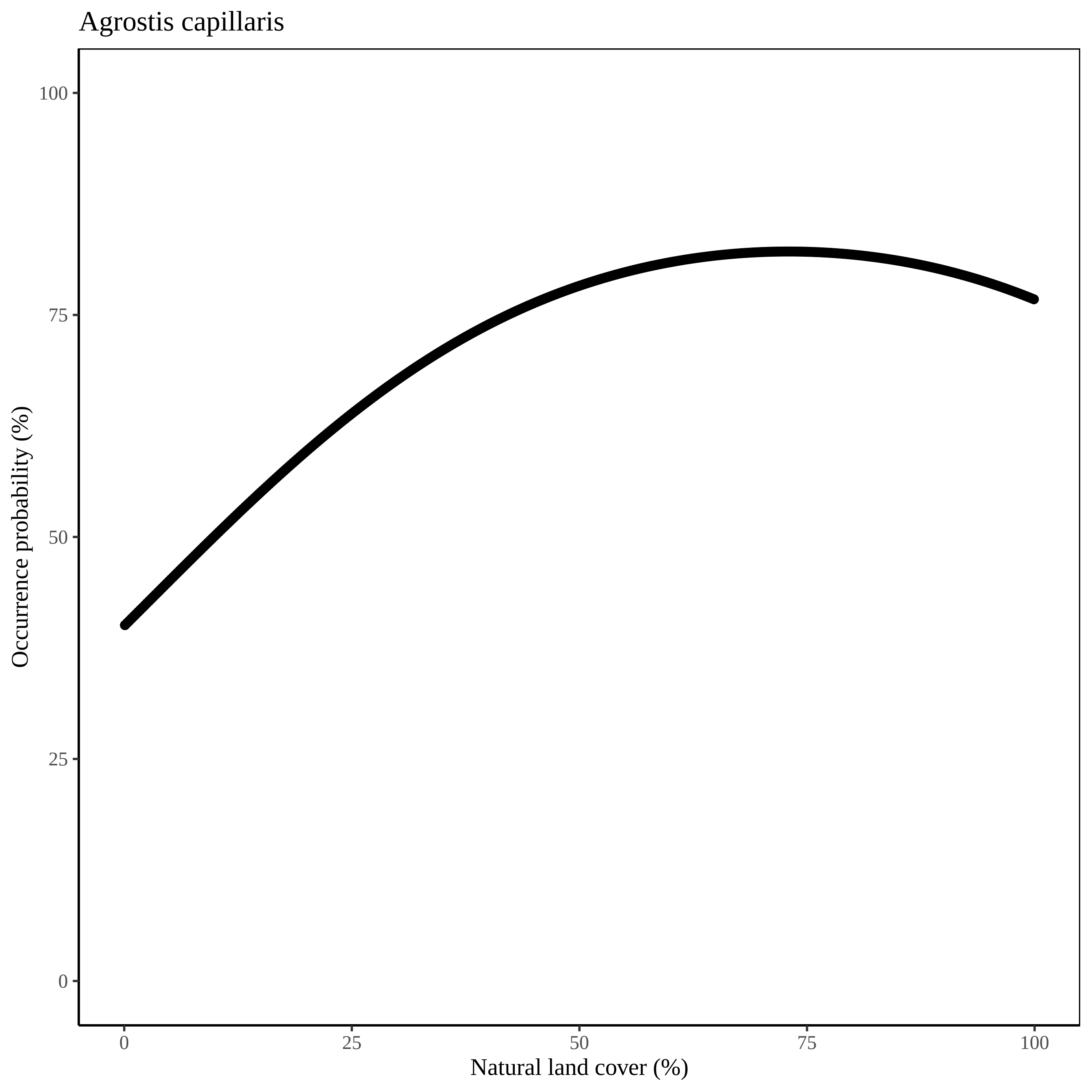
Agrostis capillaris
Occurrence probability (%)
100
75
50
25
0
0
25
50
75
100
Natural land cover (%)

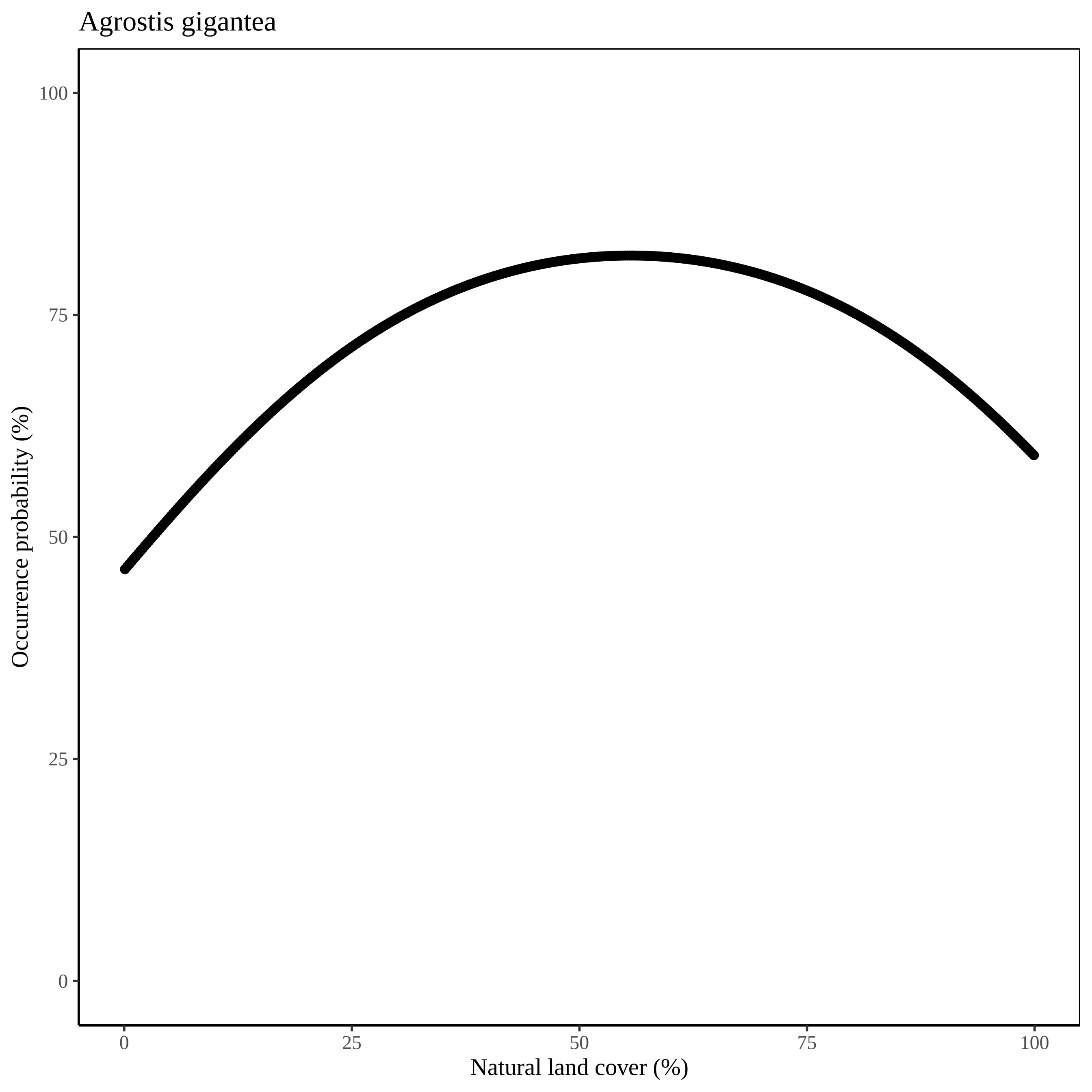

Agrostis gigantea
100
75
50
25
0
Occurrence probability (%)
0
25
50
75
100
Natural land cover (%)

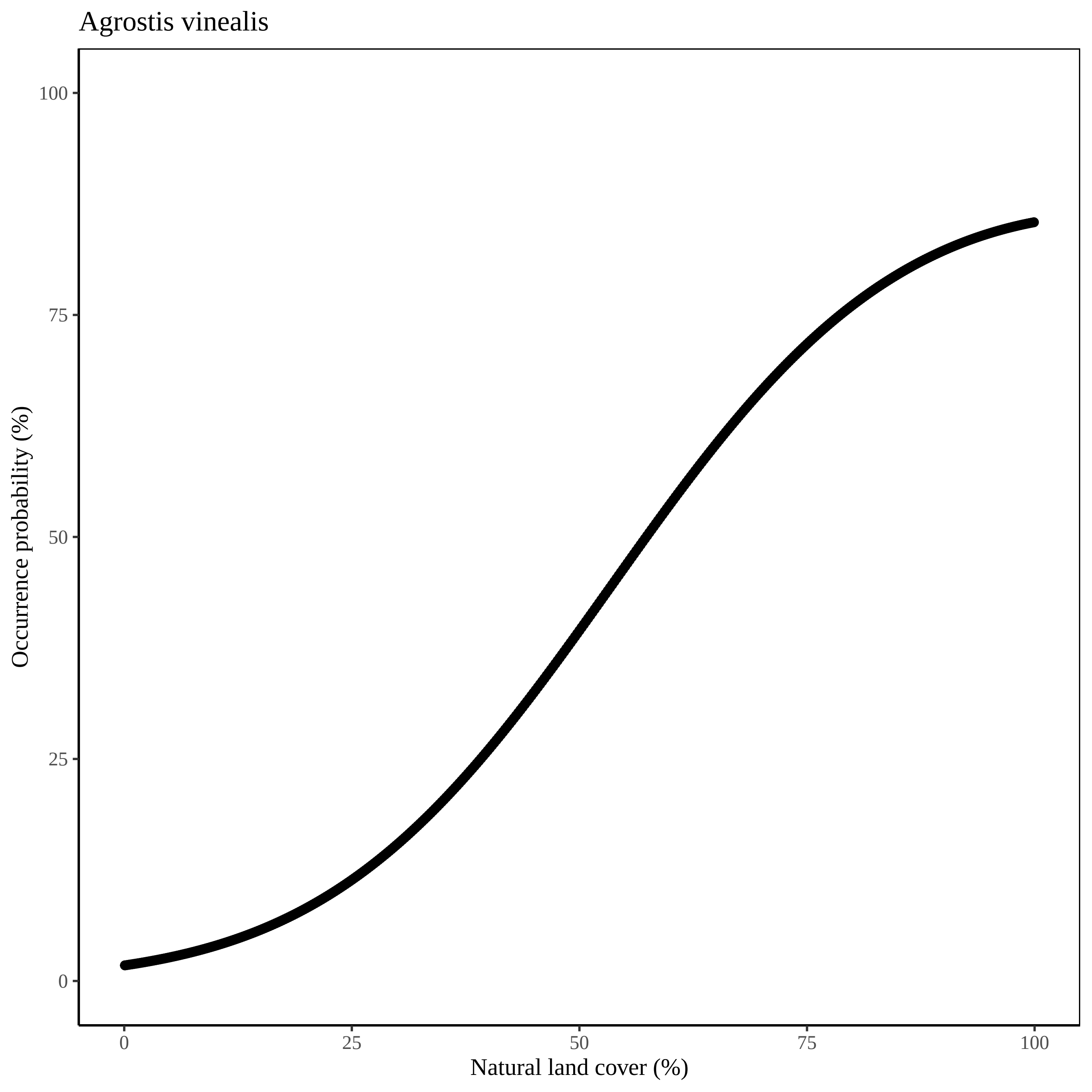
Agrostis vinealis
Occurrence probability (%)
100
75
50
25
0
0
25
50
75
100
Natural land cover (%)

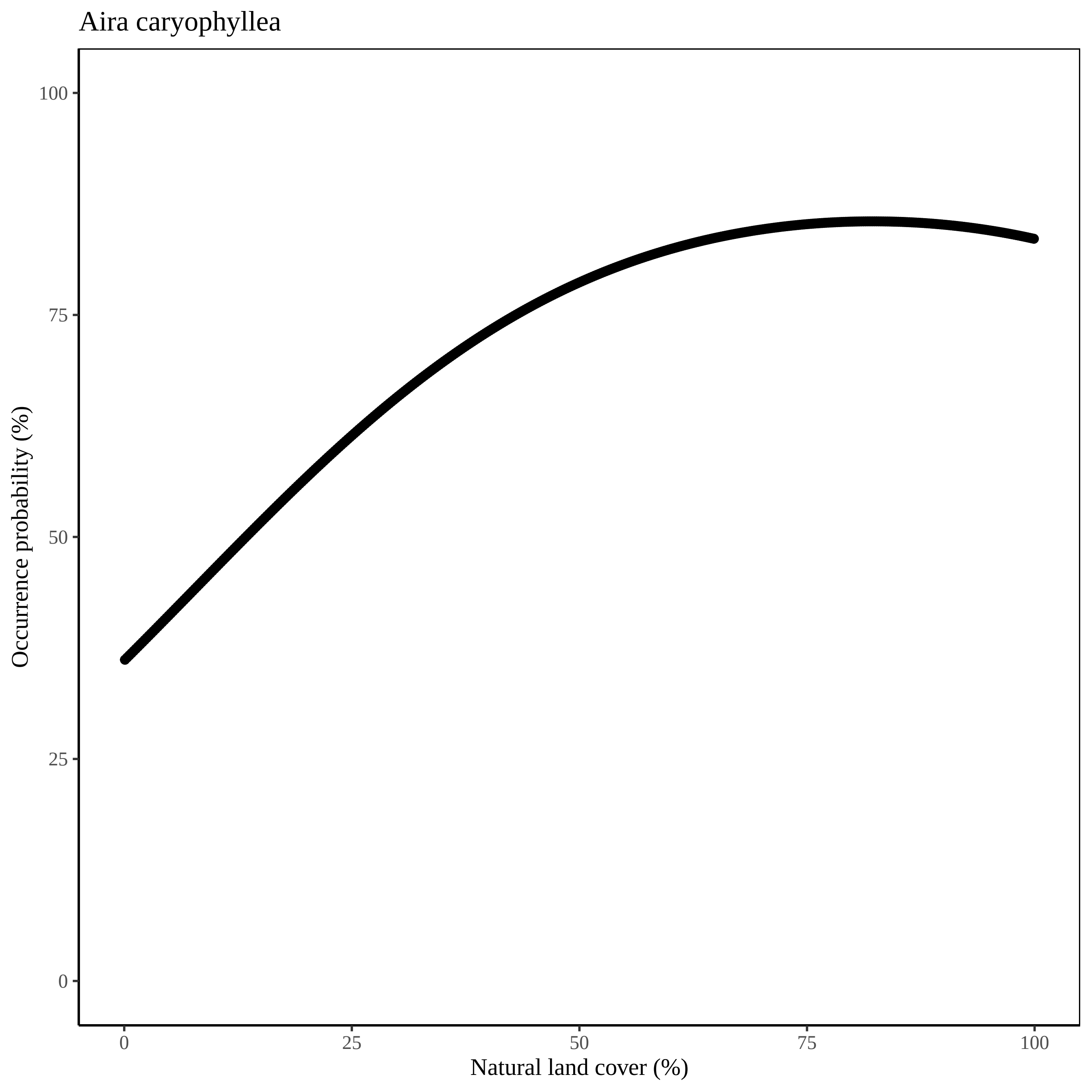
Aira caryophyllea
Occurrence probability (%)
100
75
50
25
0
0
25
50
75
100
Natural land cover (%)

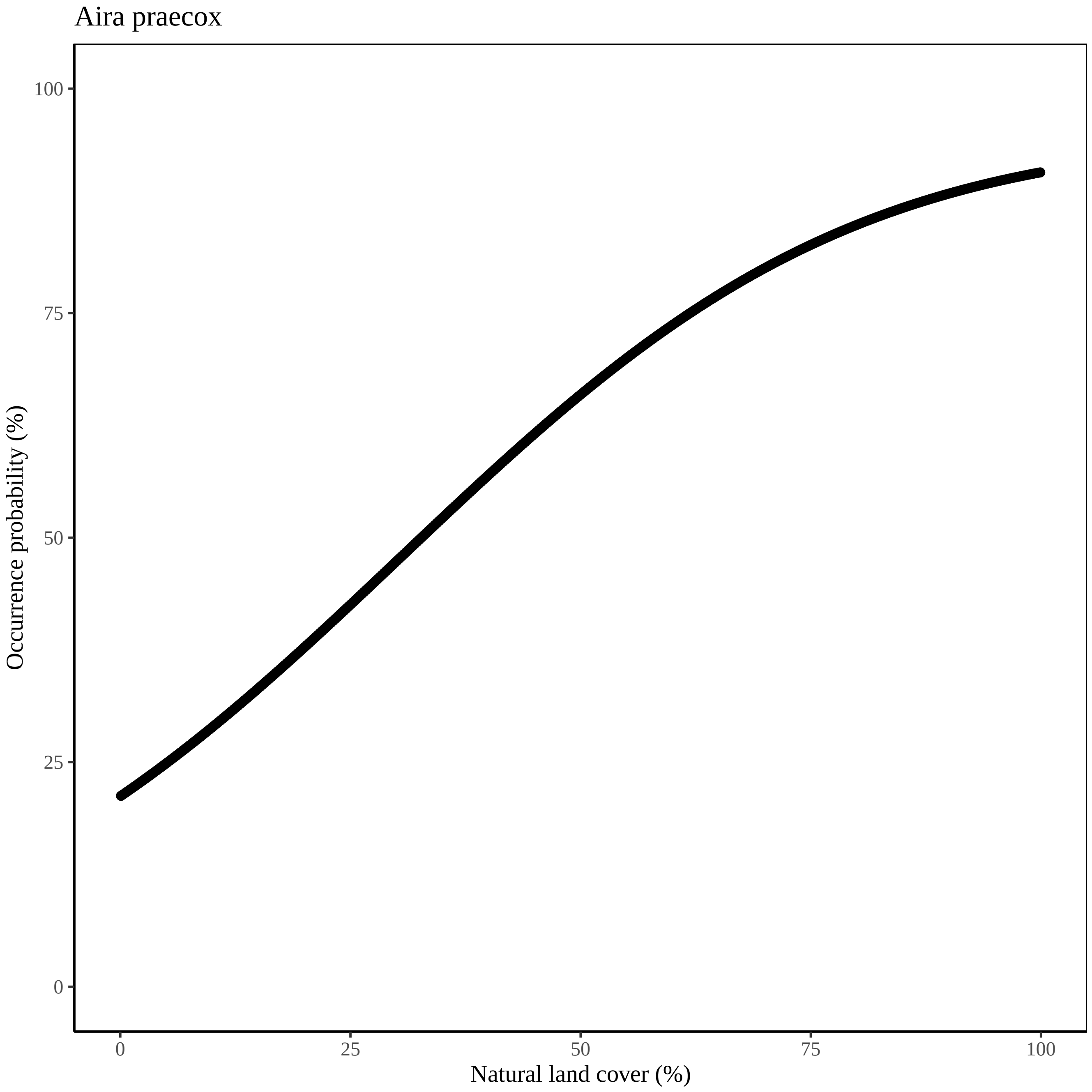
Aira praecox
Occurrence probability (%)
100
75
50
25
0
0
25
50
75
100
Natural land cover (%)

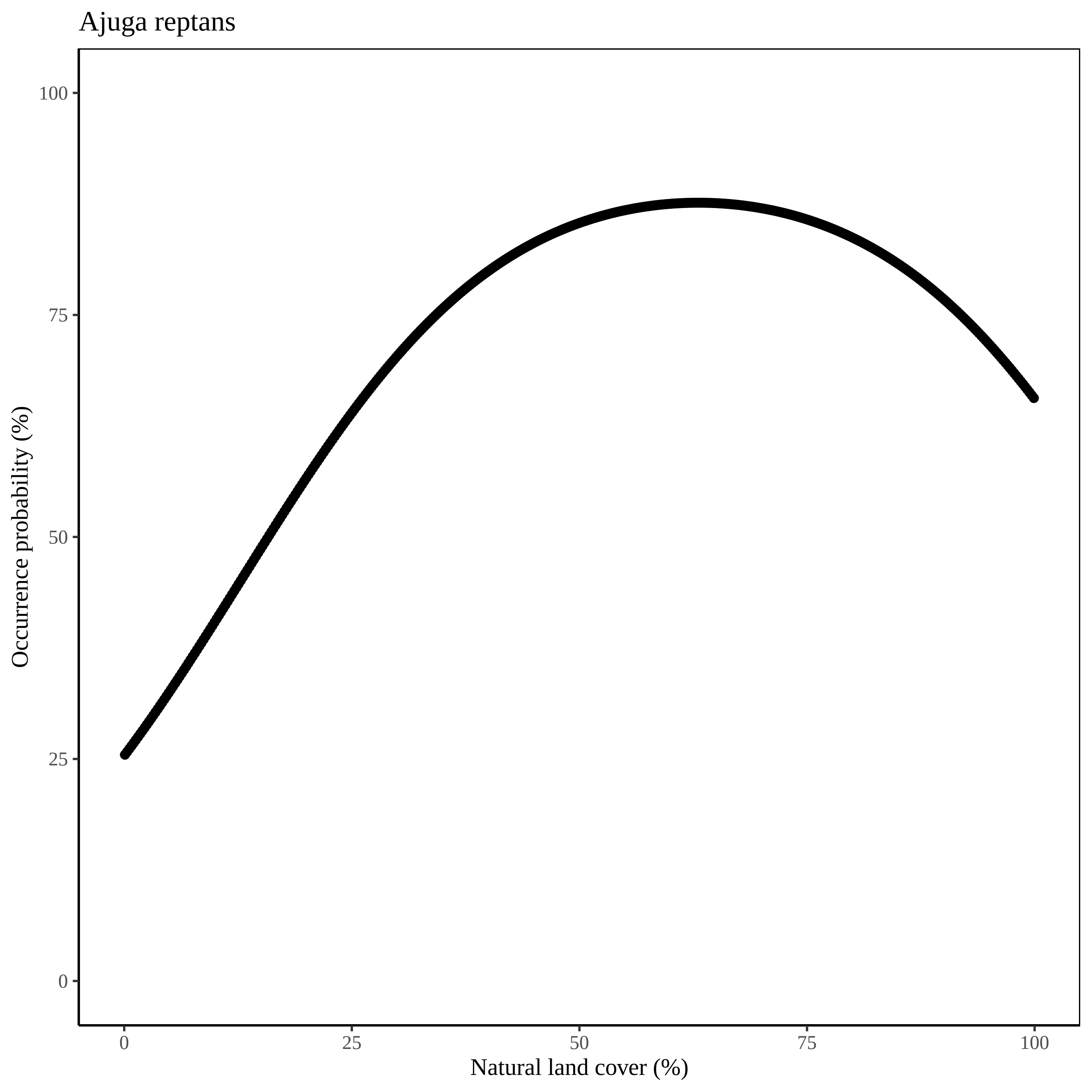

Ajuga reptans
Occurrence probability (%)
100
75
50
25
0
0
25
50
75
100
Natural land cover (%)

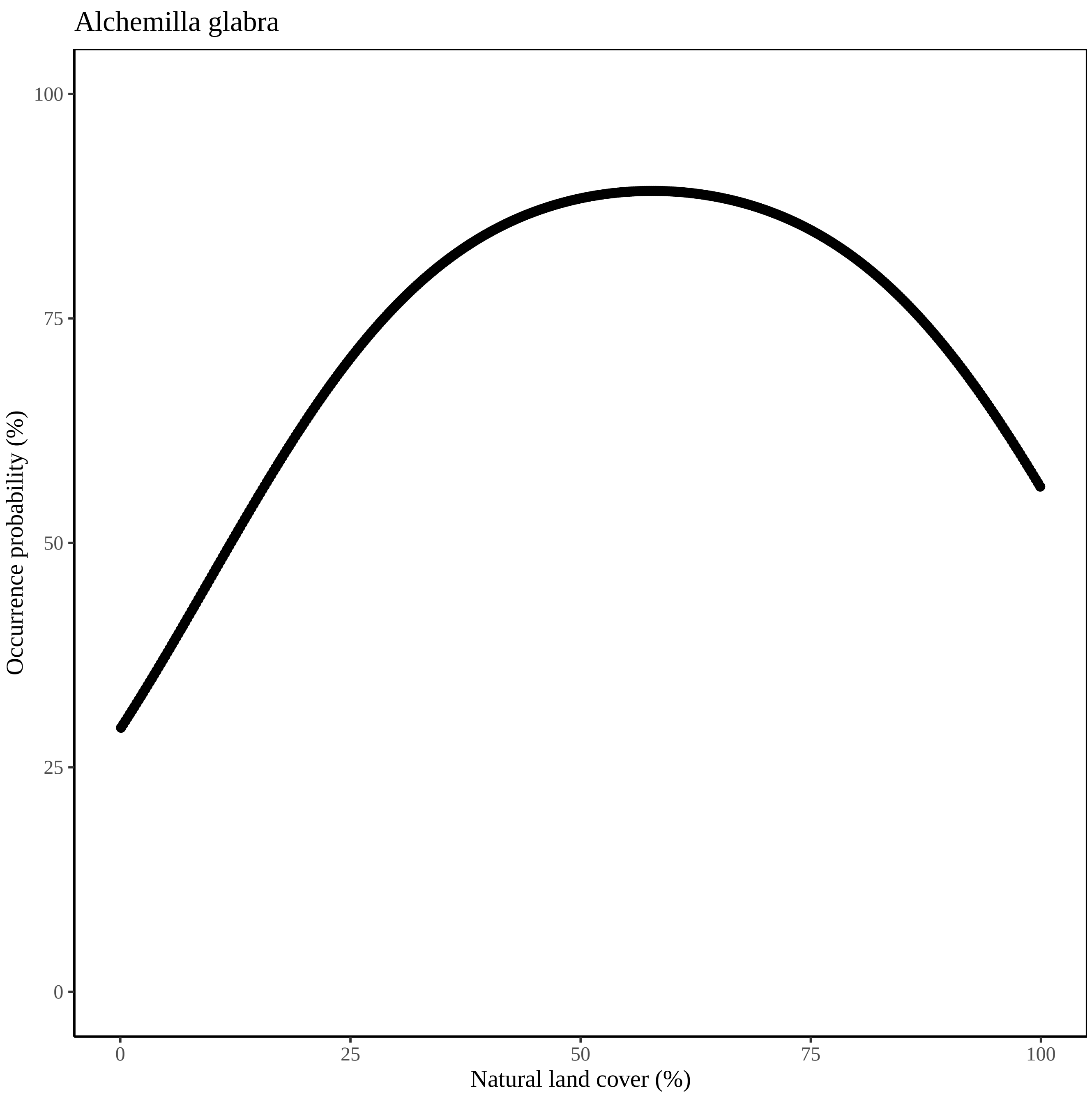

Alchemilla glabra
Occurrence probability (%)
100
75
50
25
0
0
25
50
75
100
Natural land cover (%)

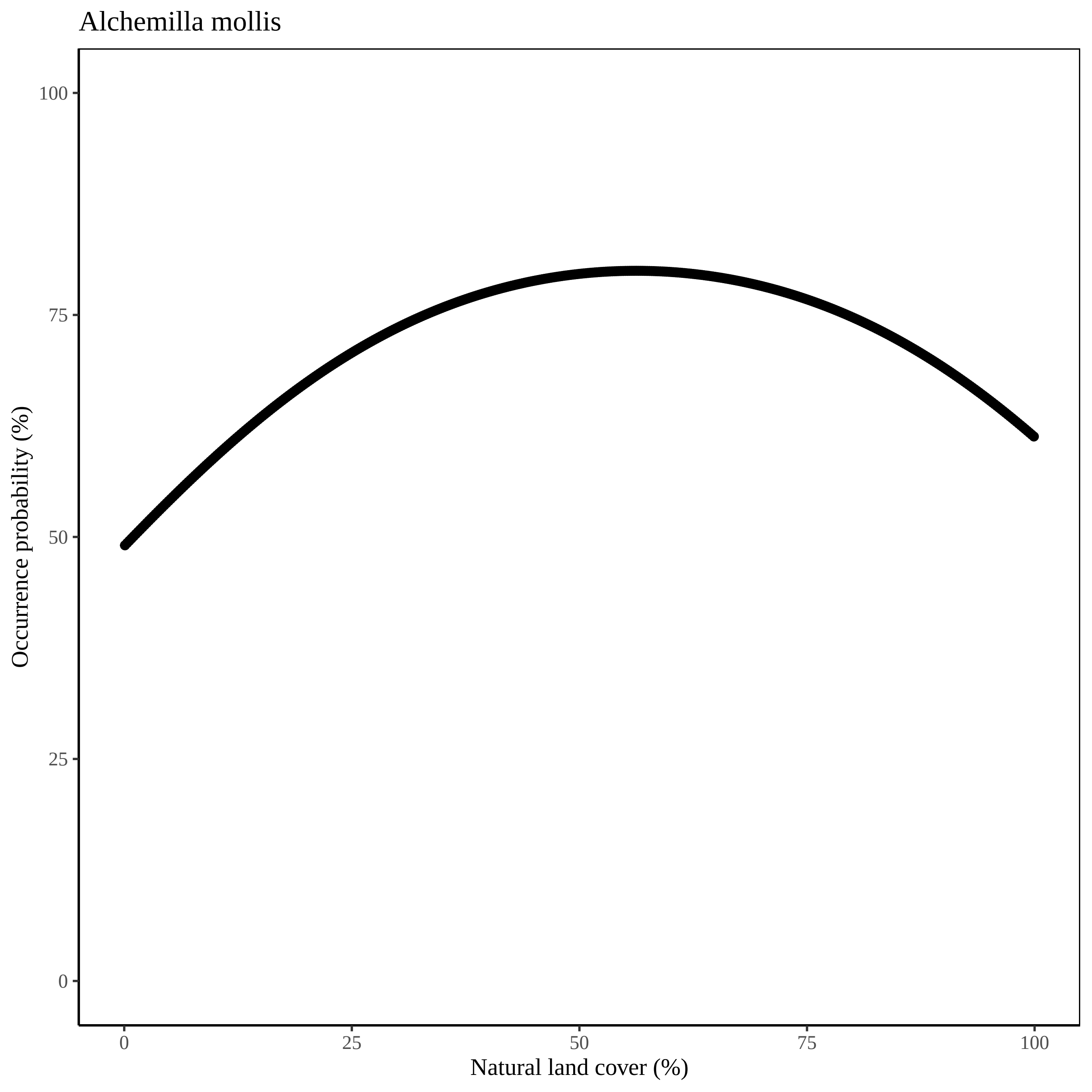

Alchemilla mollis
Occurrence probability (%)
100
75
50
25
0
0
25
50
75
100
Natural land cover (%)

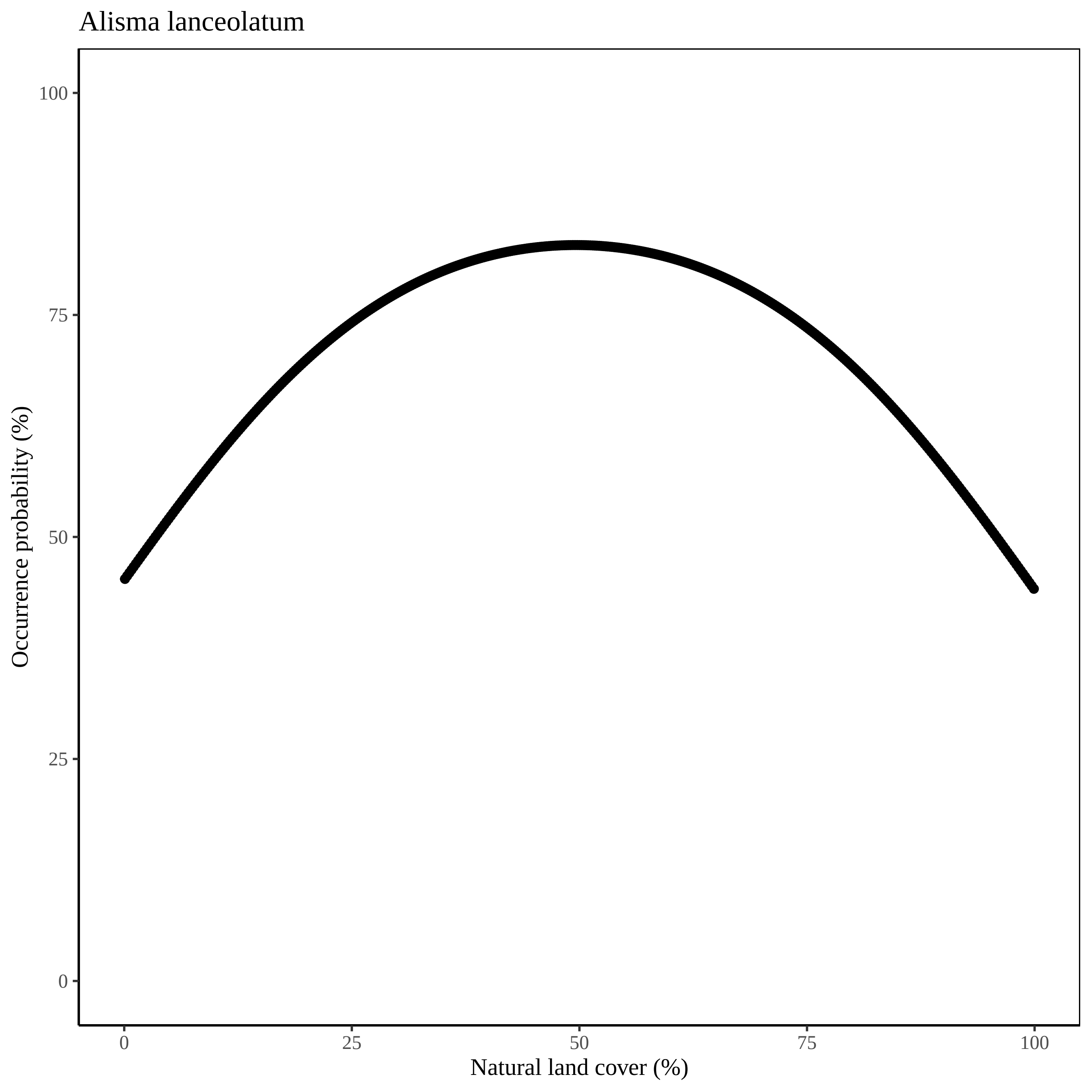
Alisma lanceolatum
100
75
50
25
0
Occurrence probability (%)
0
25
50
75
100
Natural land cover (%)

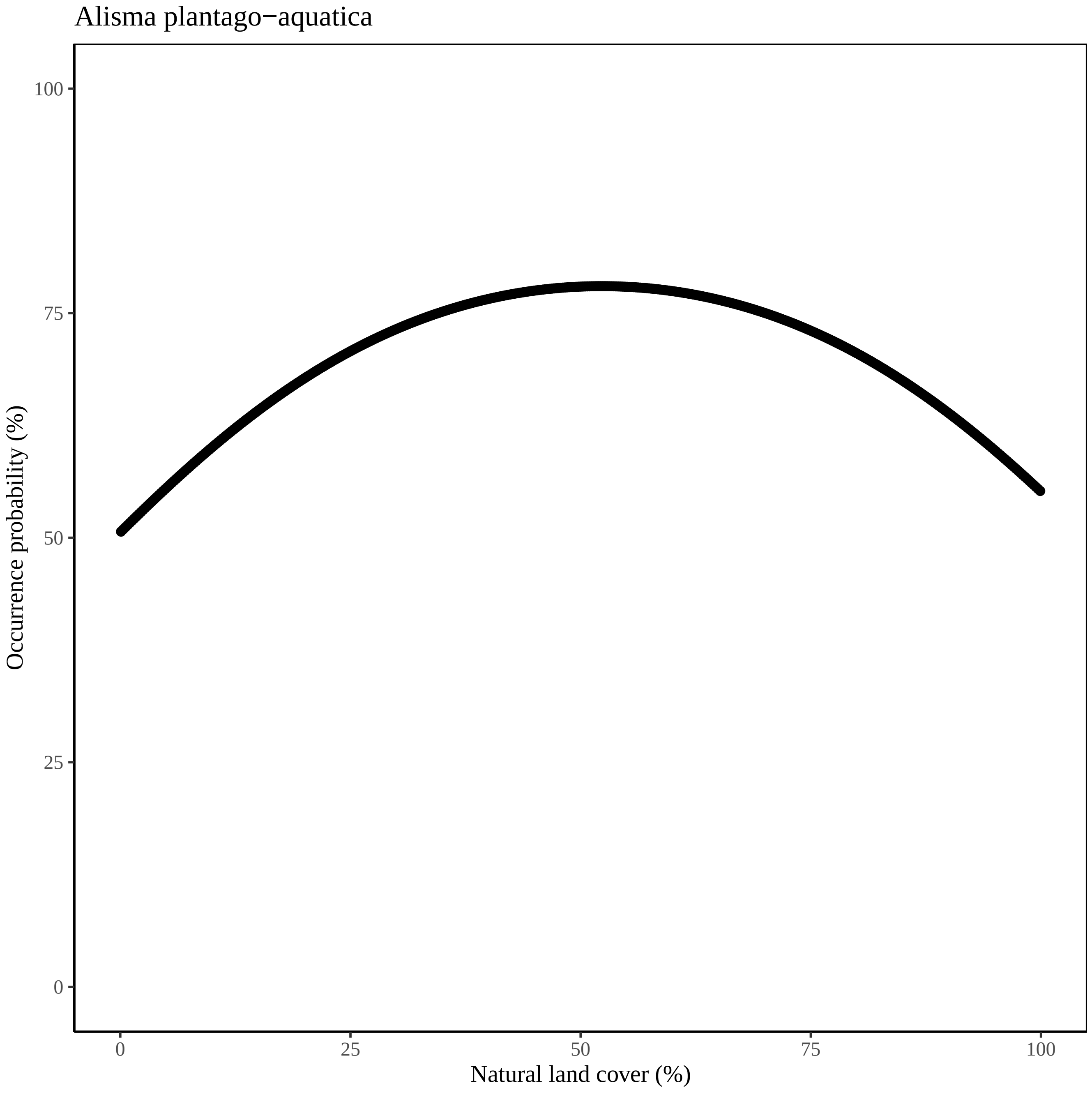
Alisma plantago−aquatica
Occurrence probability (%)
100
75
50
25
0
0
25
50
75
100
Natural land cover (%)

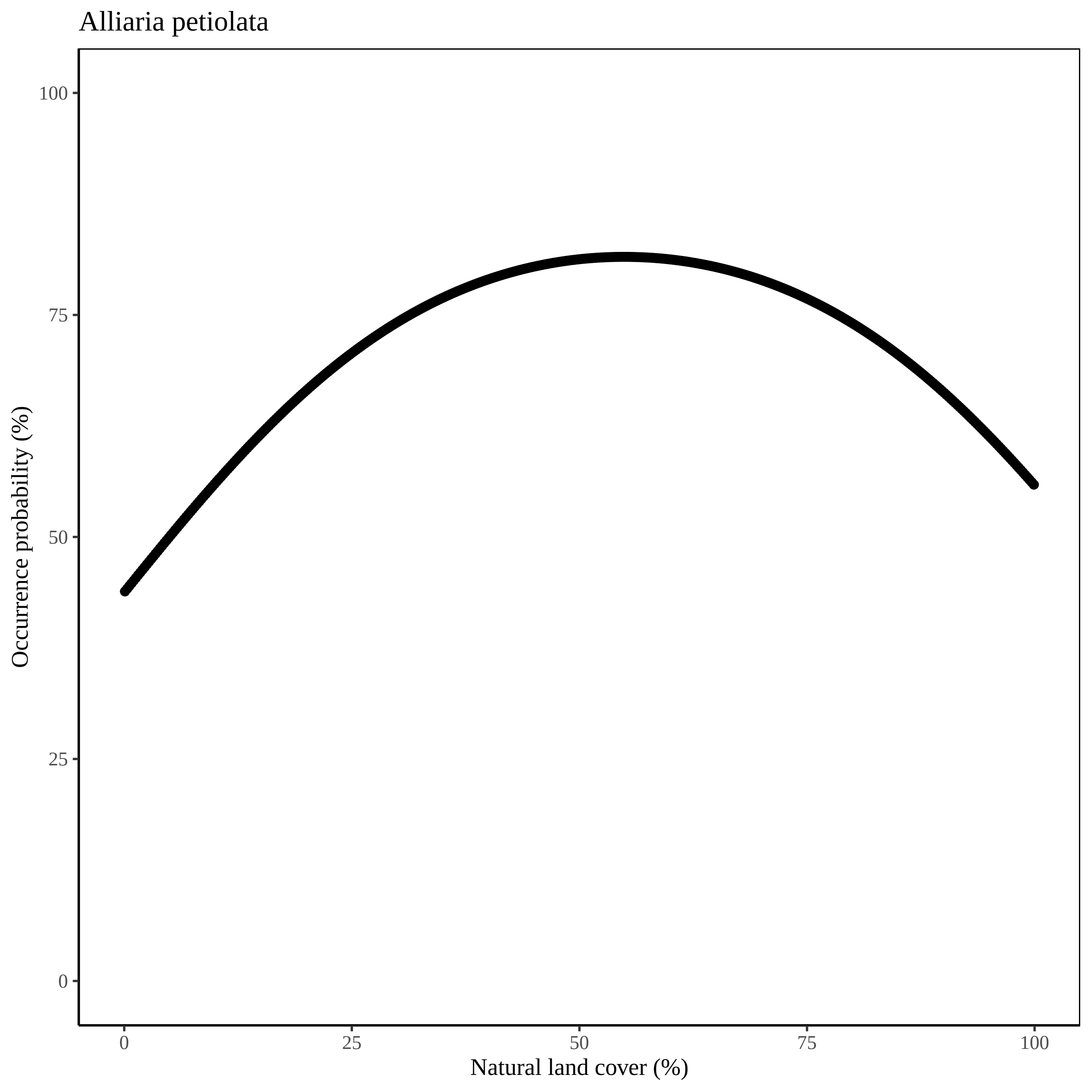
Alliaria petiolata
100
75
50
25
0
Occurrence probability (%)
0
25
50
75
100
Natural land cover (%)

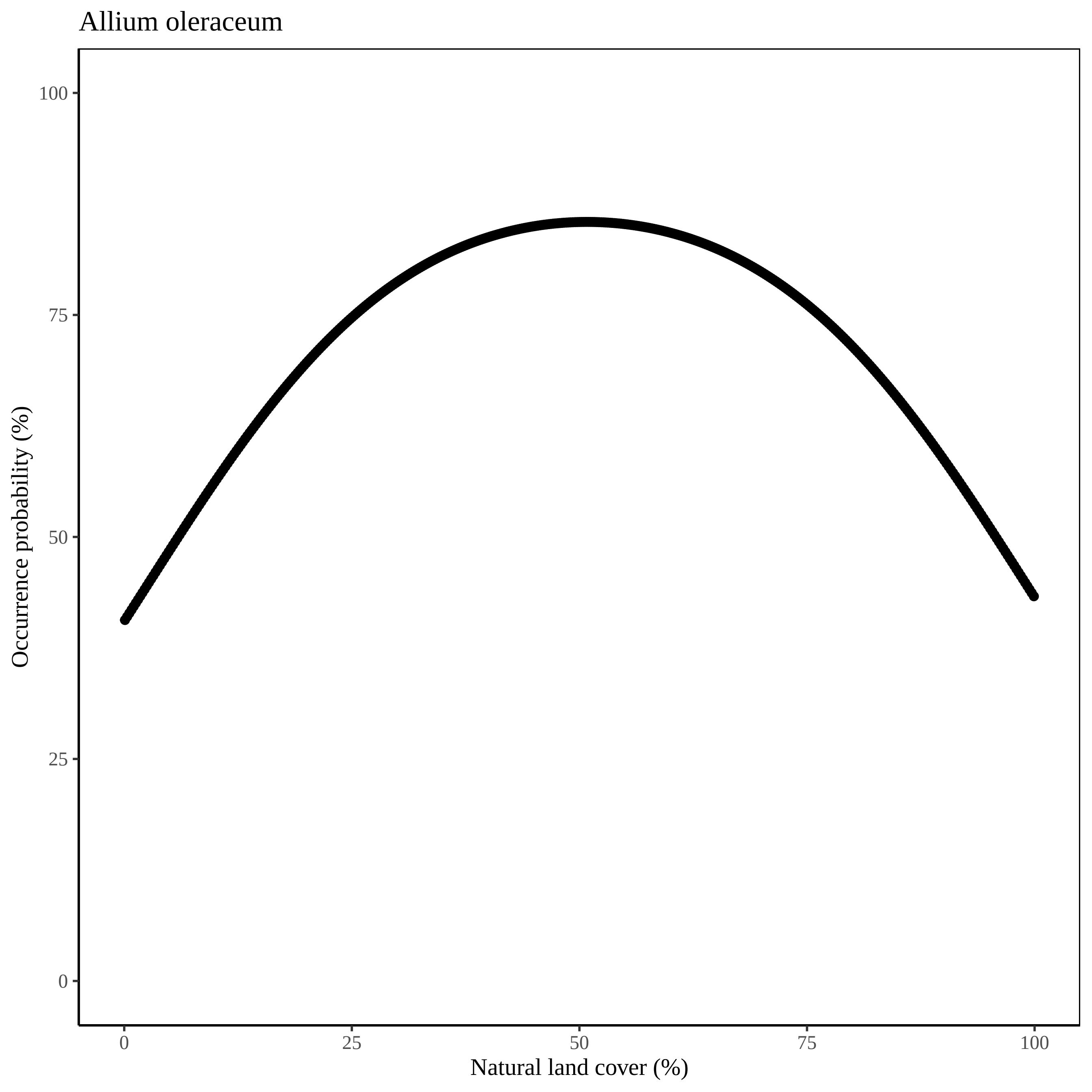

Allium oleraceum
100
75
50
25
0
Occurrence probability (%)
0
25
50
75
100
Natural land cover (%)

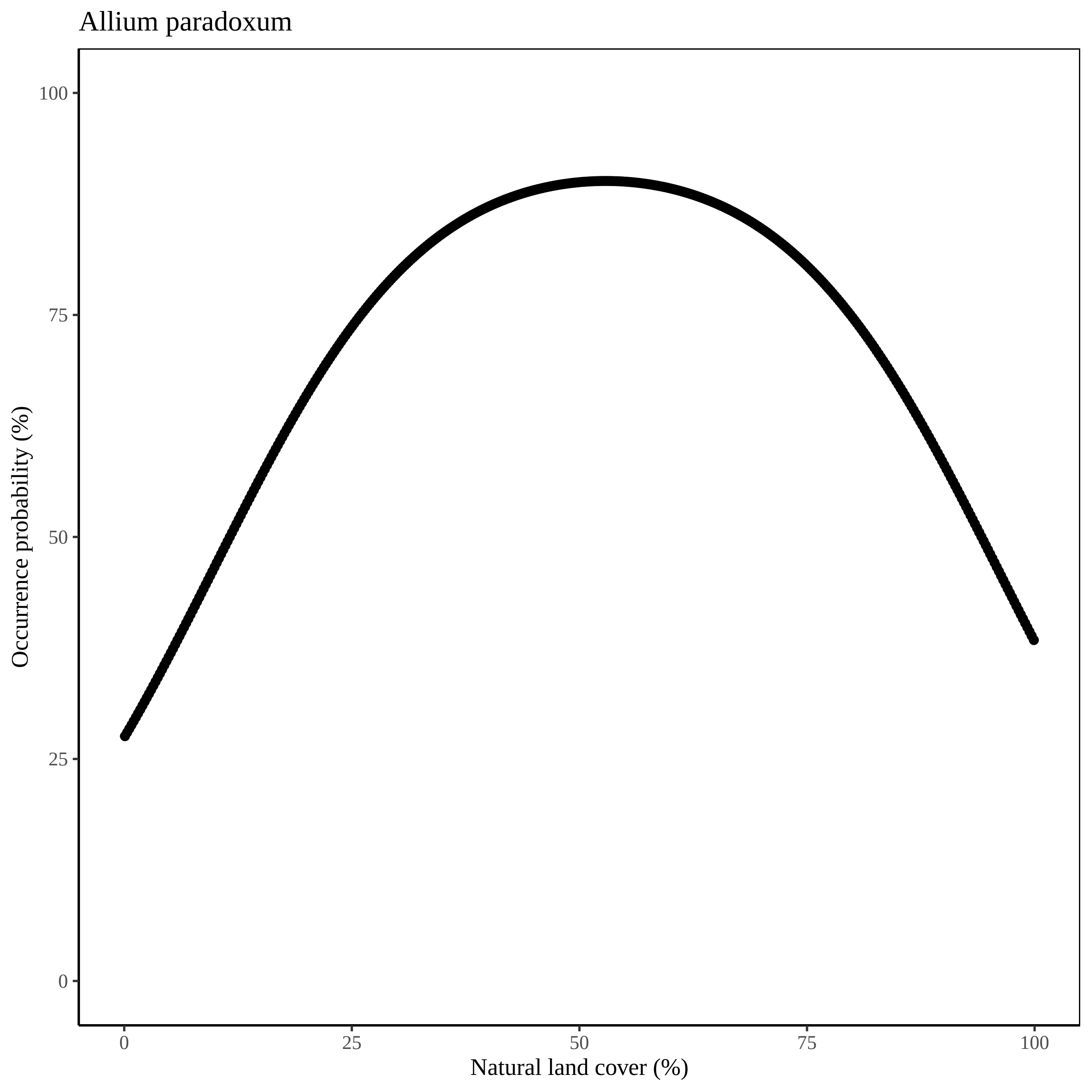
Allium paradoxum
Occurrence probability (%)
100
75
50
25
0
0
25
50
75
100
Natural land cover (%)

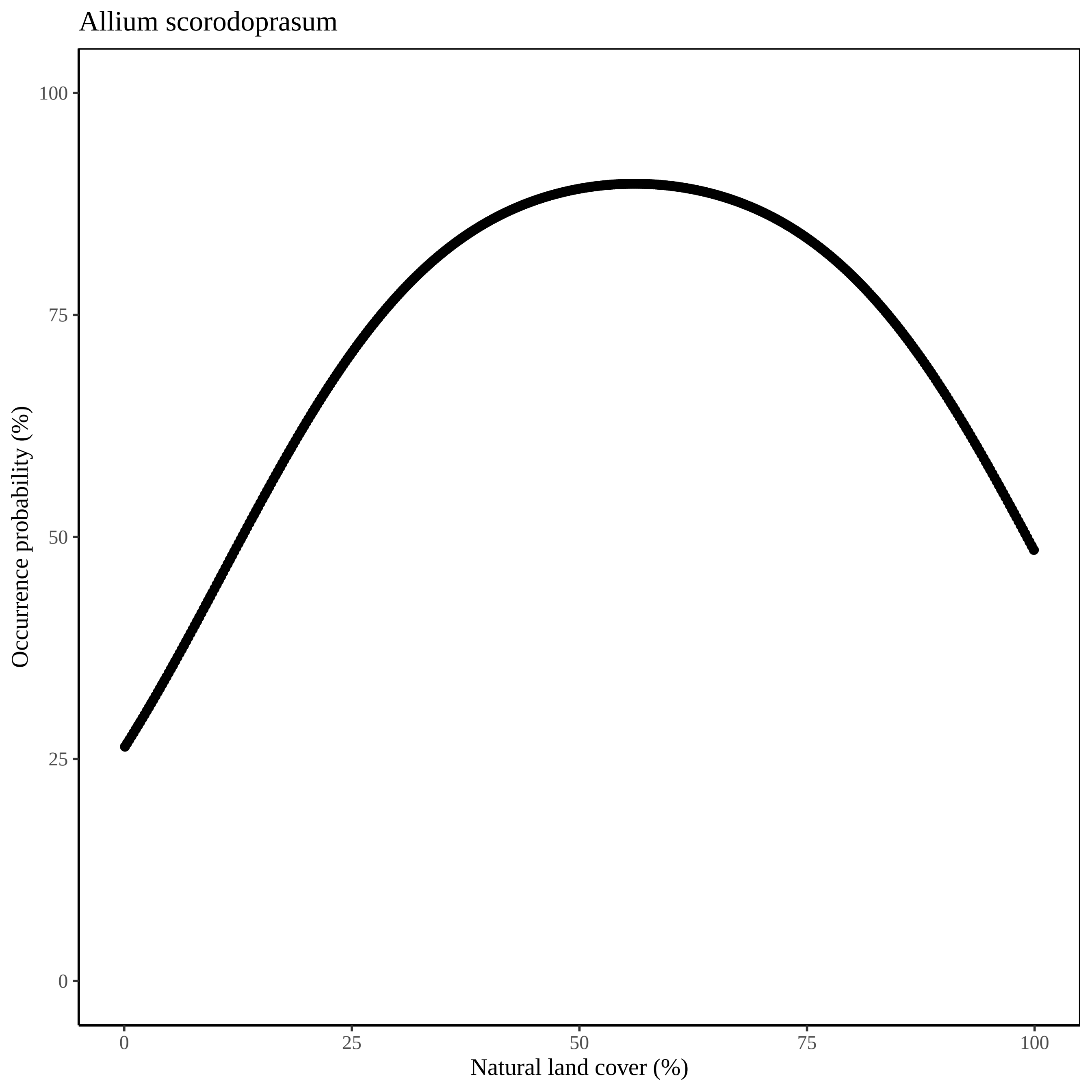

Allium scorodoprasum
Occurrence probability (%)
100
75
50
25
0
0
25
50
75
100
Natural land cover (%)

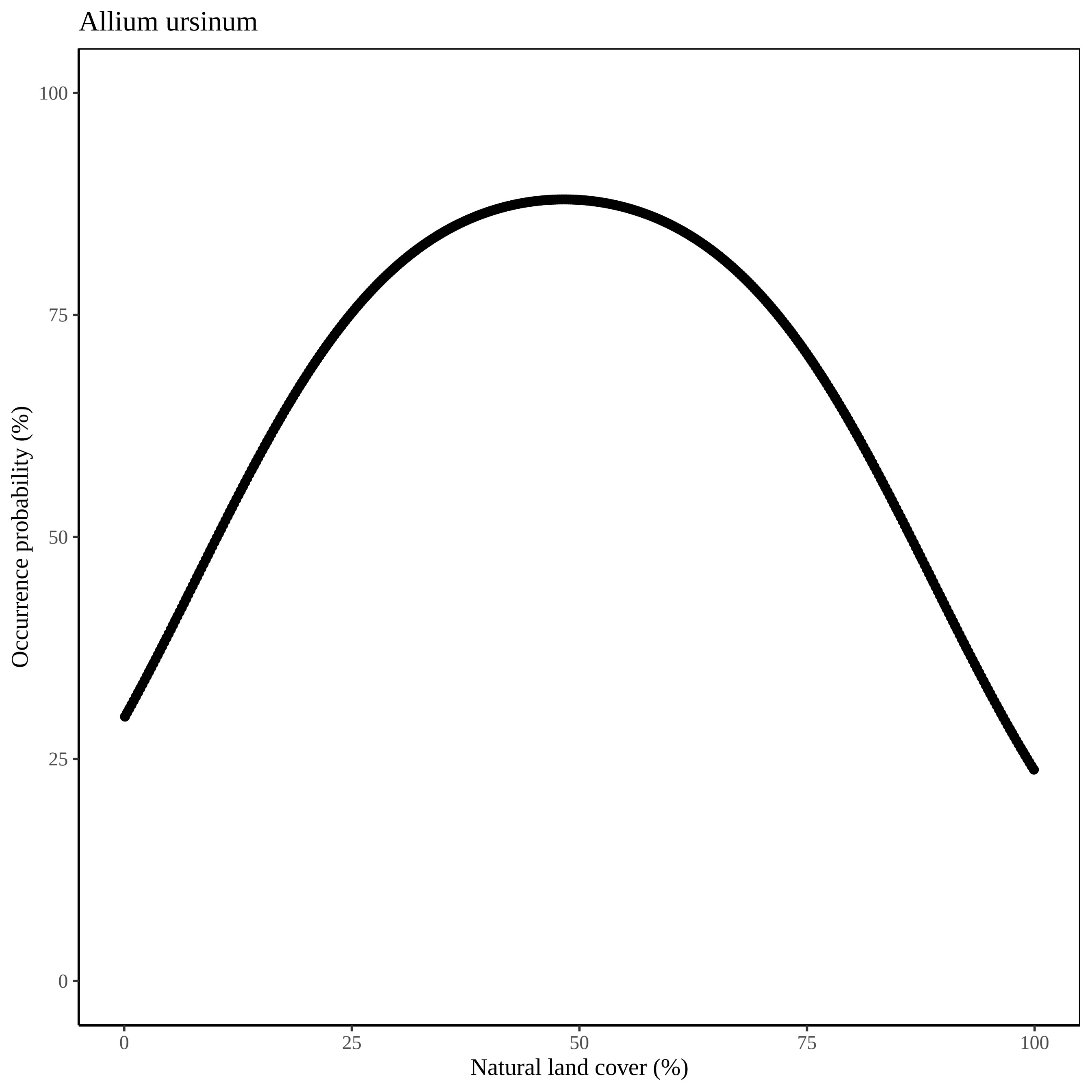
Allium ursinum
Occurrence probability (%)
100
75
50
25
0
0
25
50
75
100
Natural land cover (%)

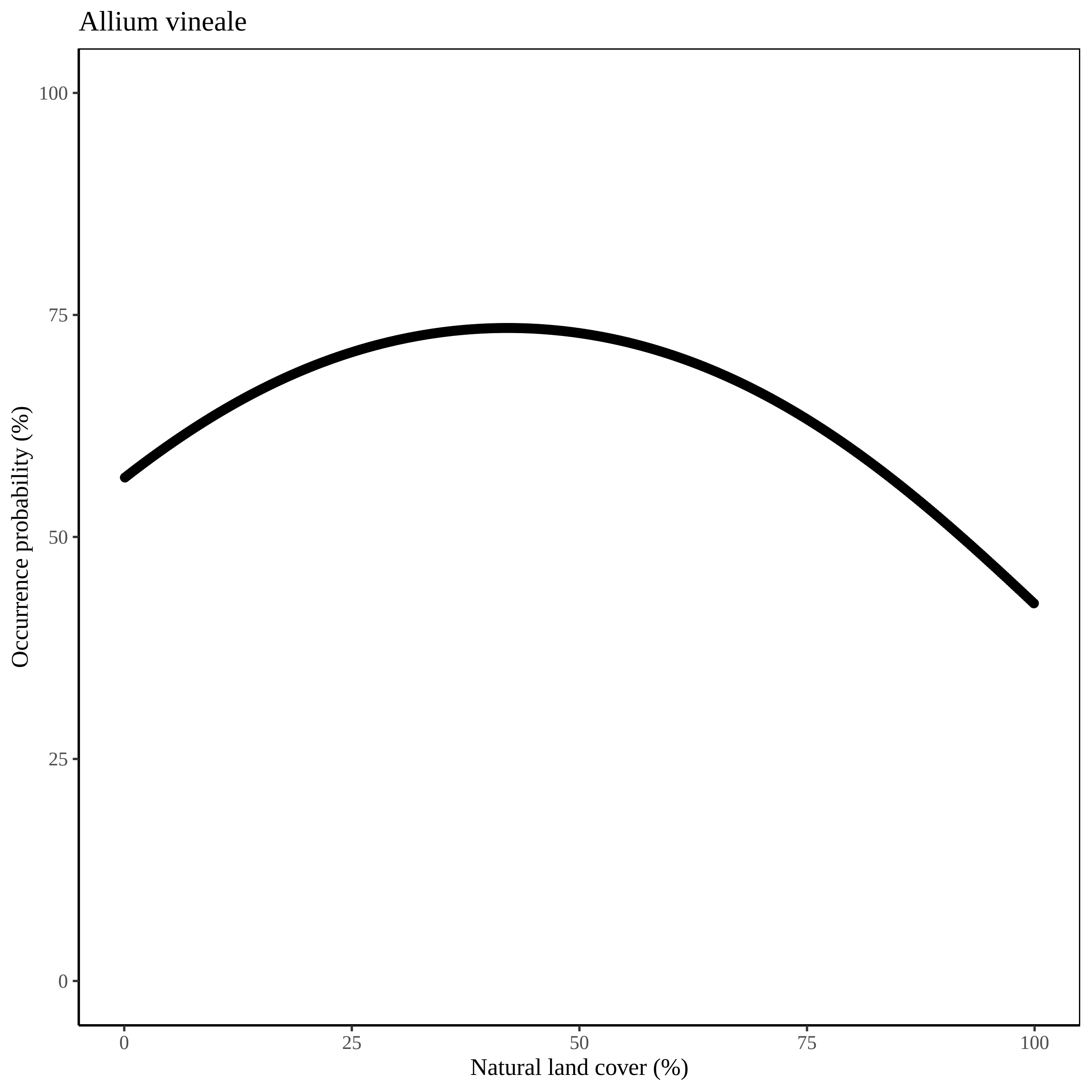
Allium vineale
100
75
50
25
0
Occurrence probability (%)
0
25
50
75
100
Natural land cover (%)

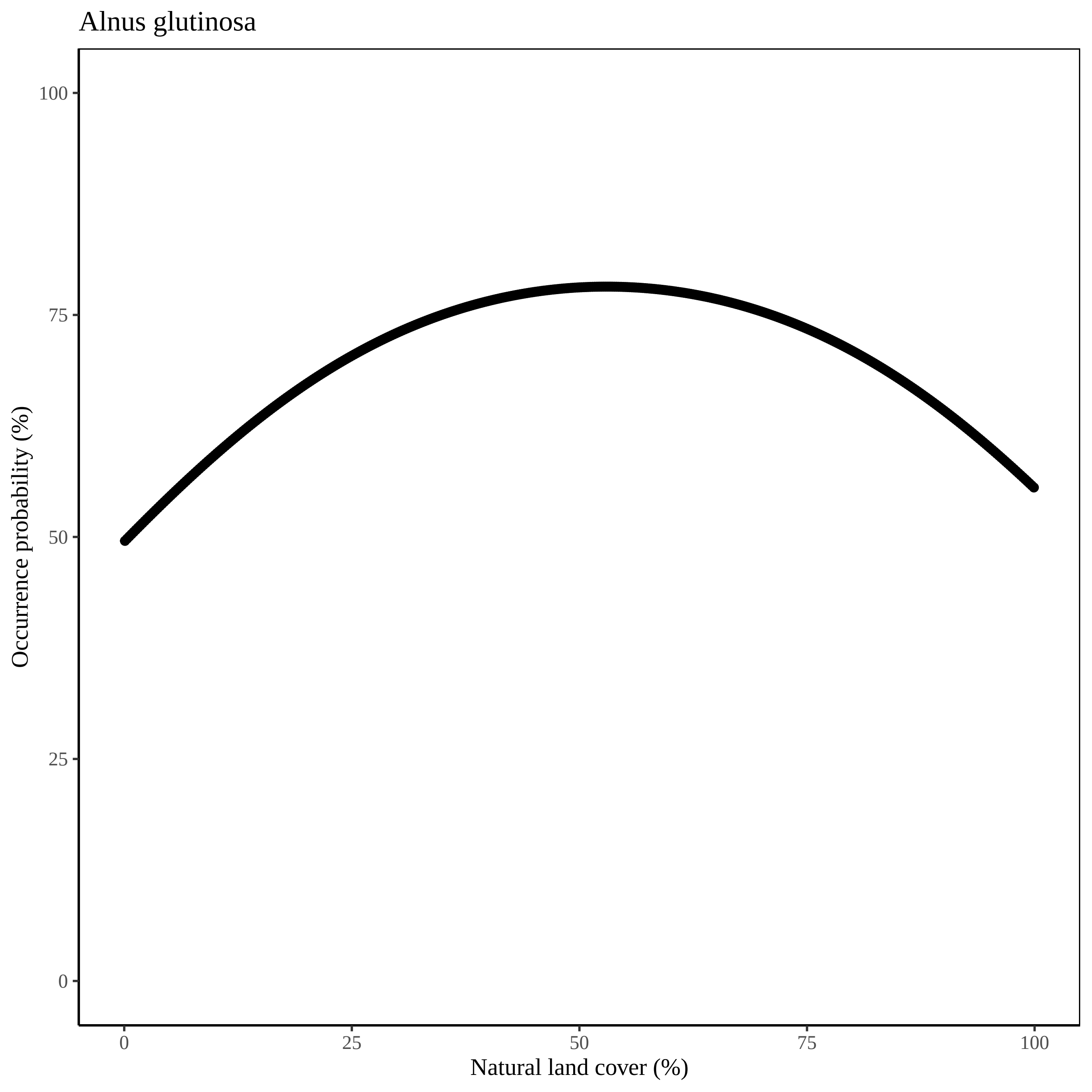
Alnus glutinosa
Occurrence probability (%)
100
75
50
25
0
0
25
50
75
100
Natural land cover (%)

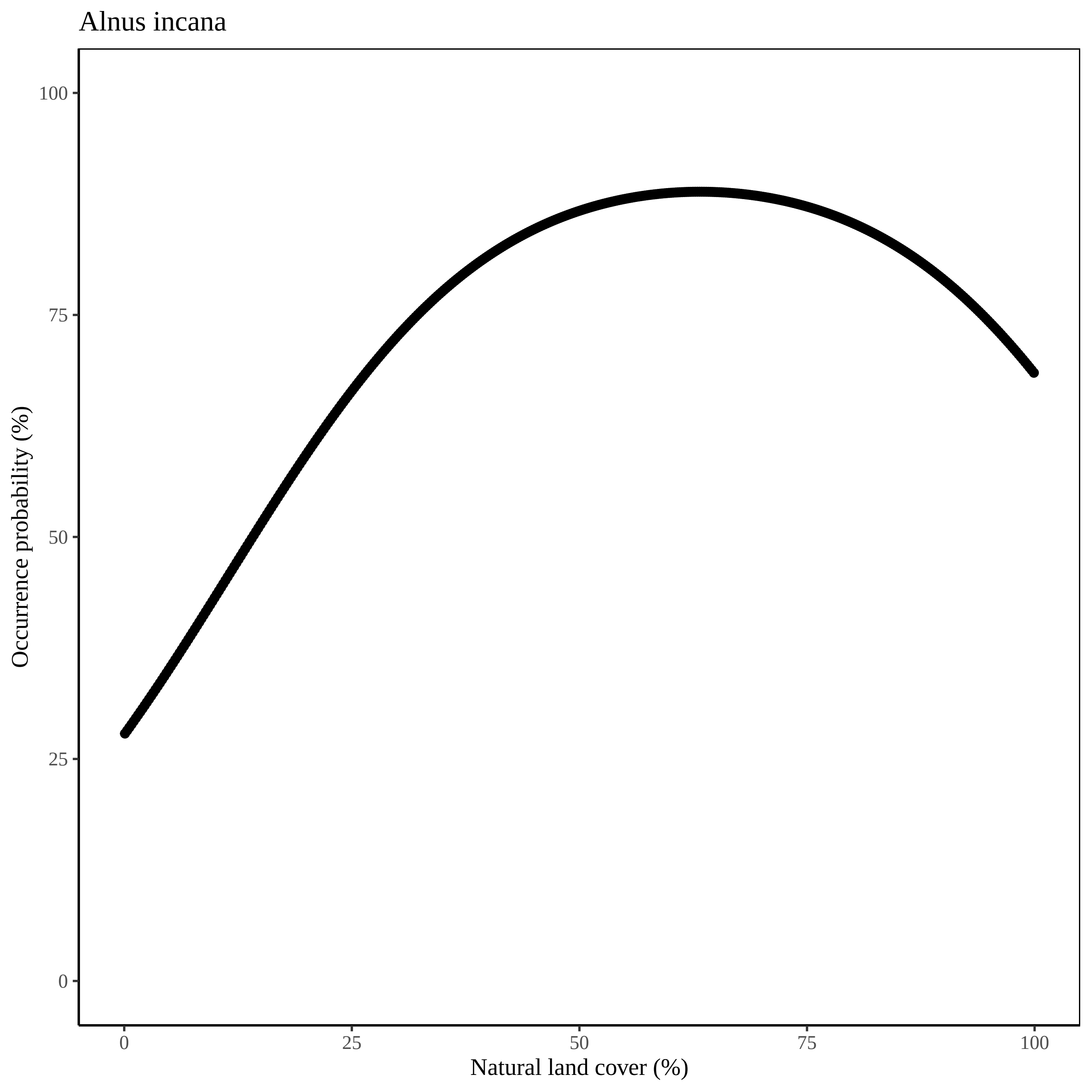
Alnus incana
Occurrence probability (%)
100
75
50
25
0
0
25
50
75
100
Natural land cover (%)

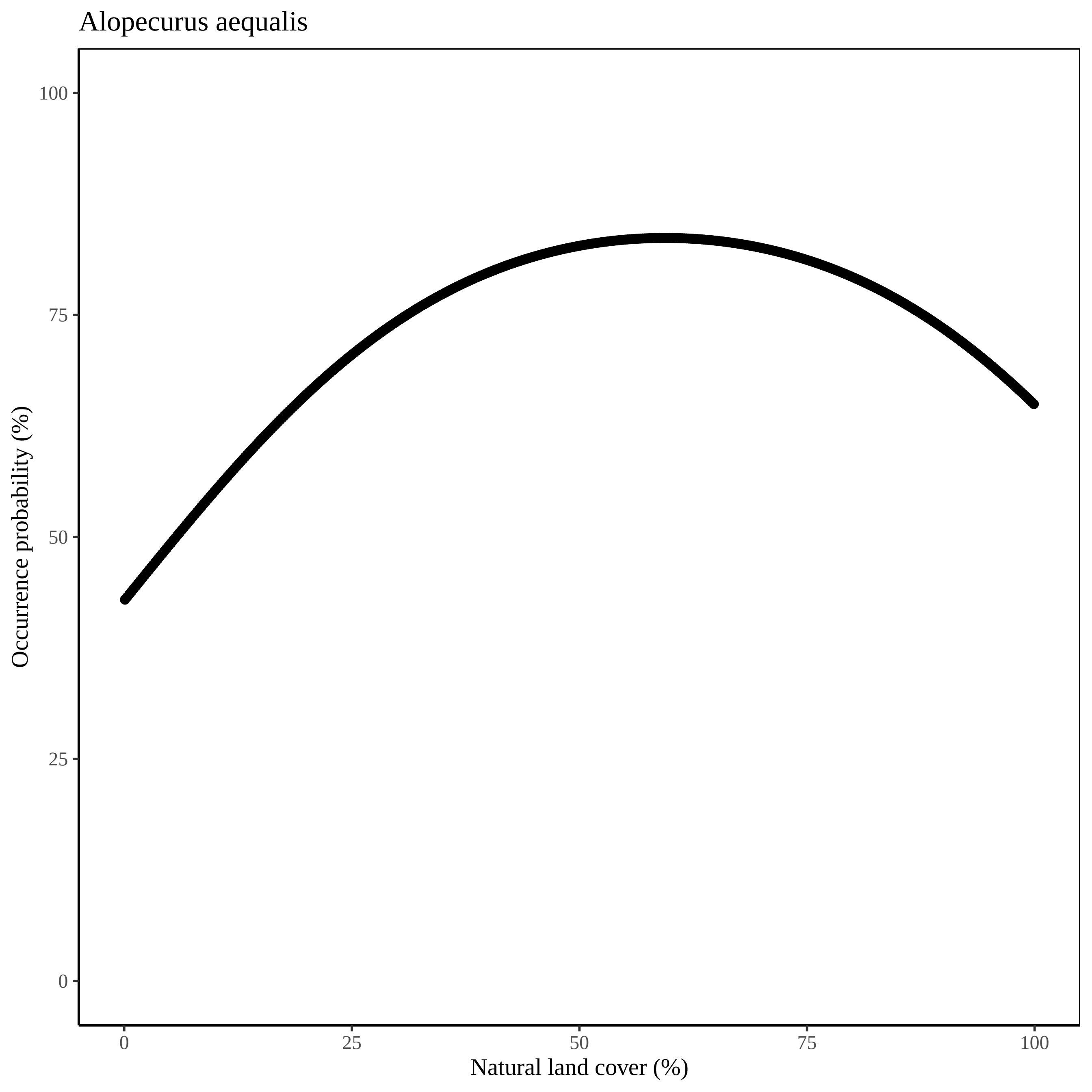

Alopecurus aequalis
100
75
50
25
0
Occurrence probability (%)
0
25
50
75
100
Natural land cover (%)

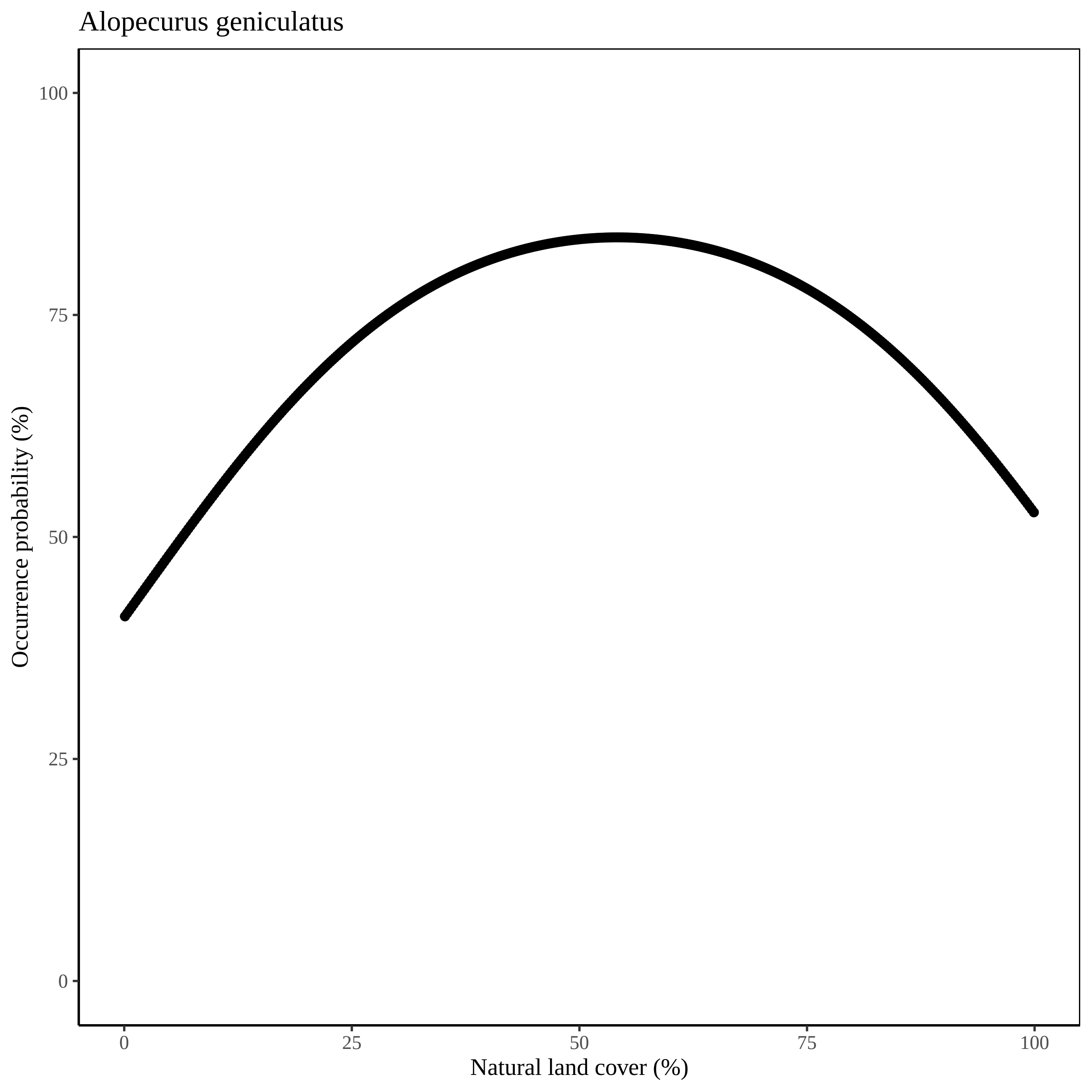

Alopecurus geniculatus
Occurrence probability (%)
100
75
50
25
0
0
25
50
75
100
Natural land cover (%)

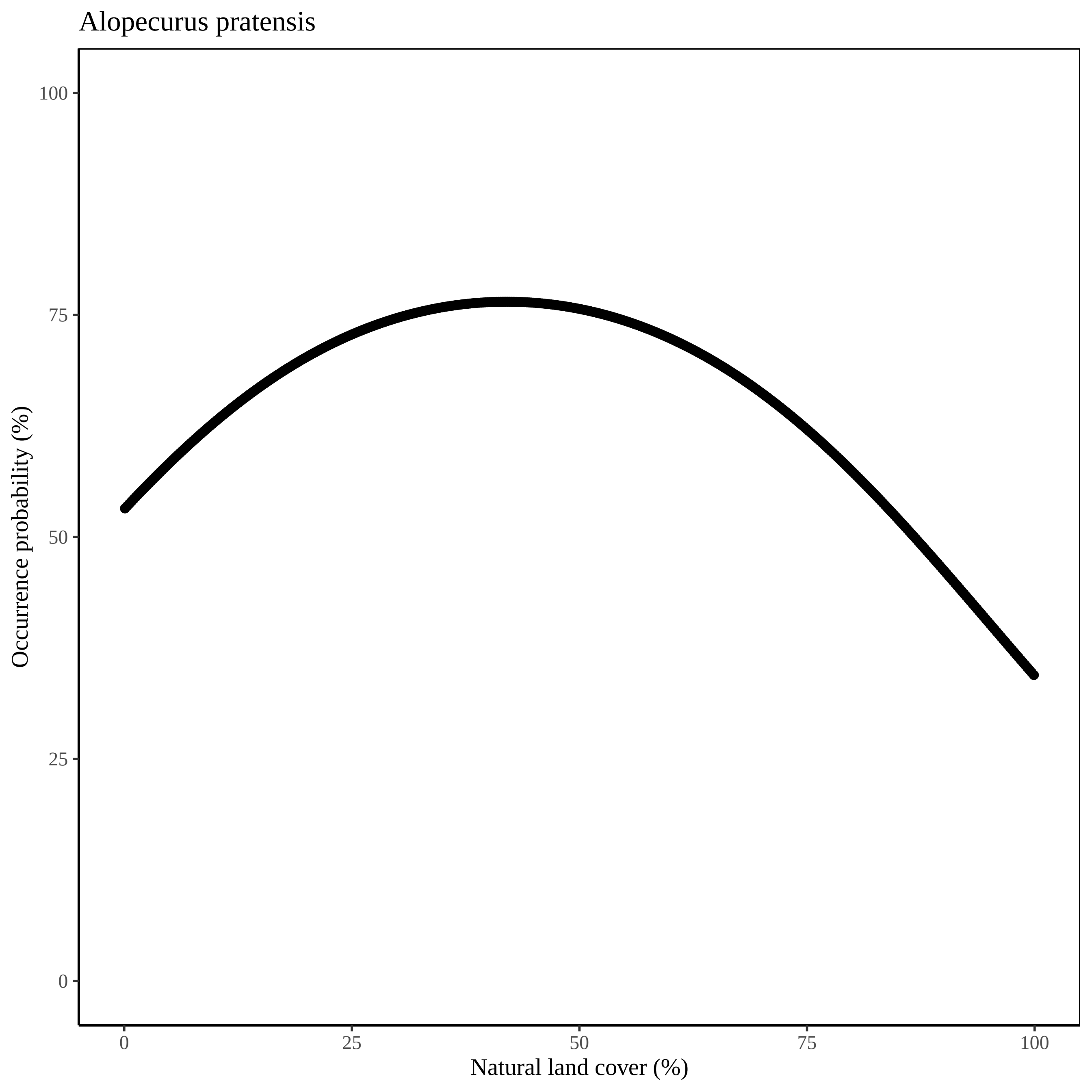
Alopecurus pratensis
Occurrence probability (%)
100
75
50
25
0
0
25
50
75
100
Natural land cover (%)

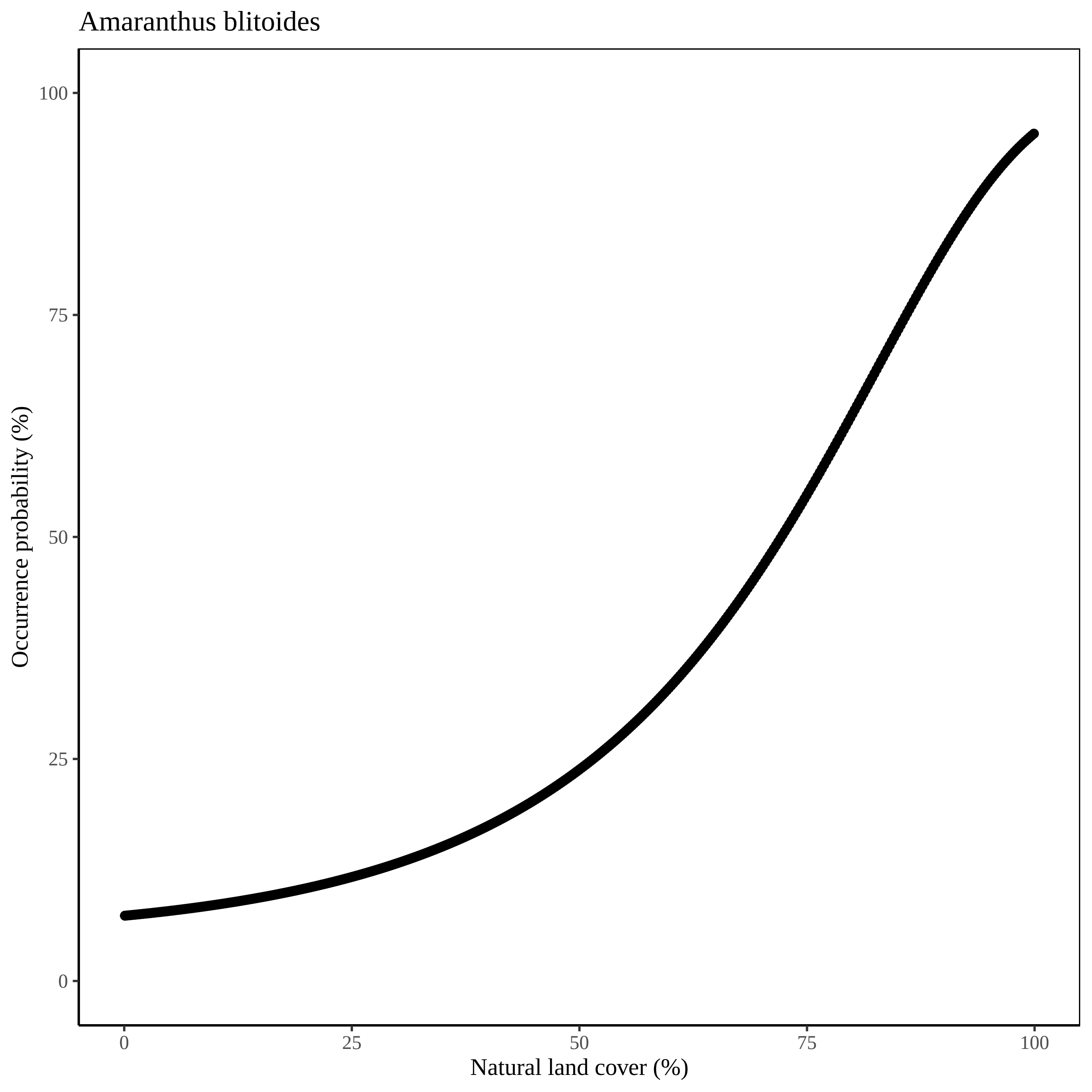

Amaranthus blitoides
Occurrence probability (%)
100
75
50
25
0
0
25
50
75
100
Natural land cover (%)

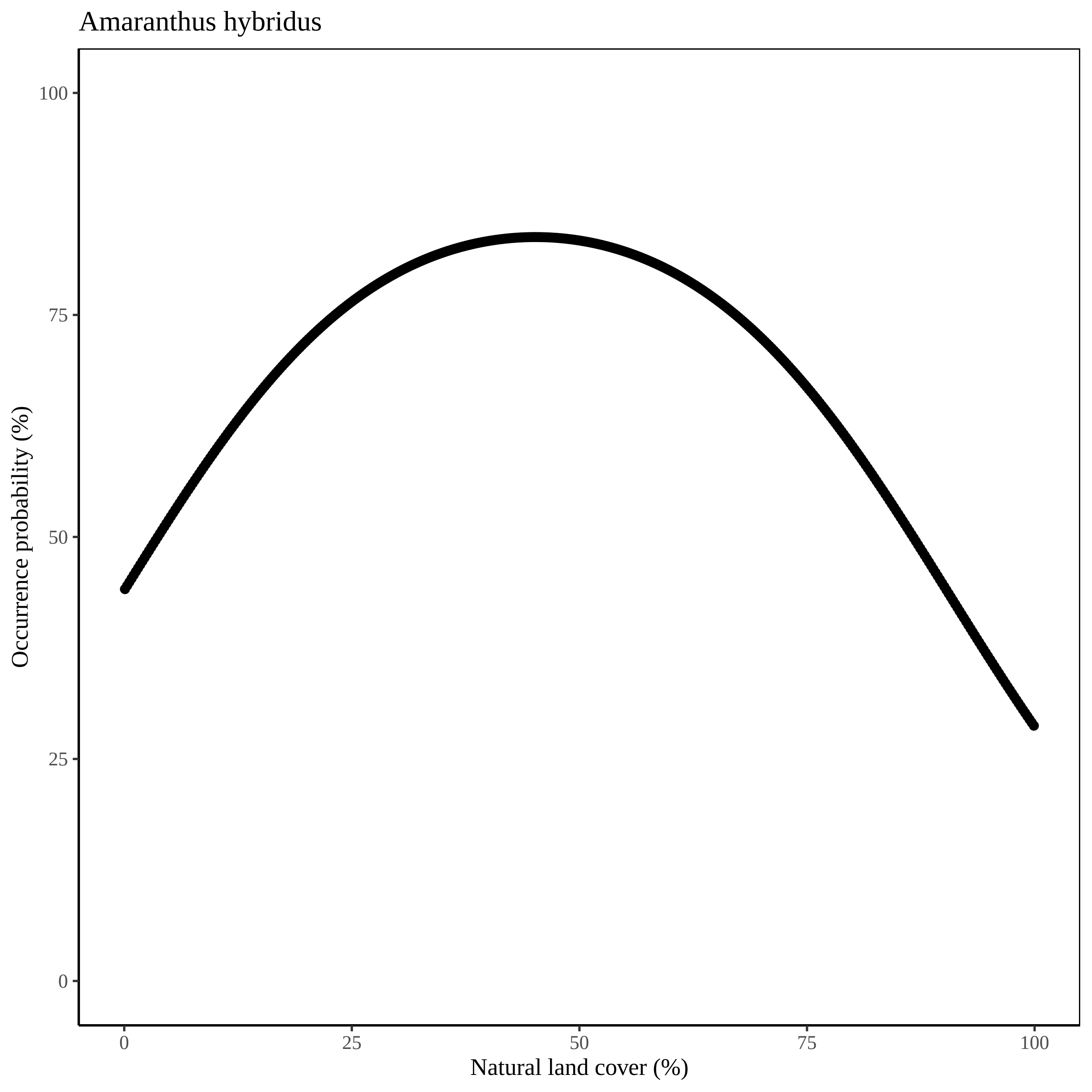

Amaranthus hybridus
100
75
50
25
0
Occurrence probability (%)
0
25
50
75
100
Natural land cover (%)

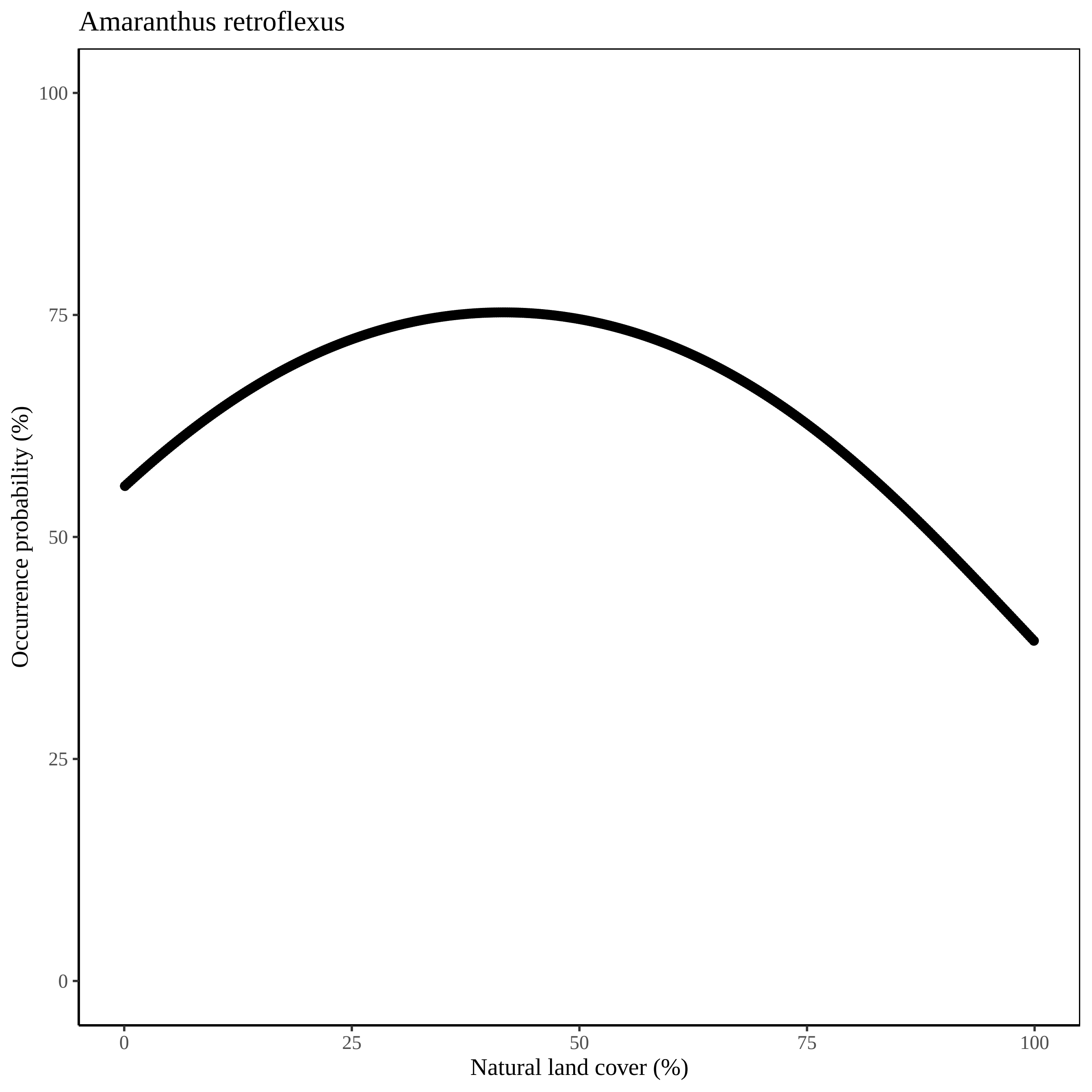

Amaranthus retroflexus
100
75
50
25
0
Occurrence probability (%)
0
25
50
75
100
Natural land cover (%)

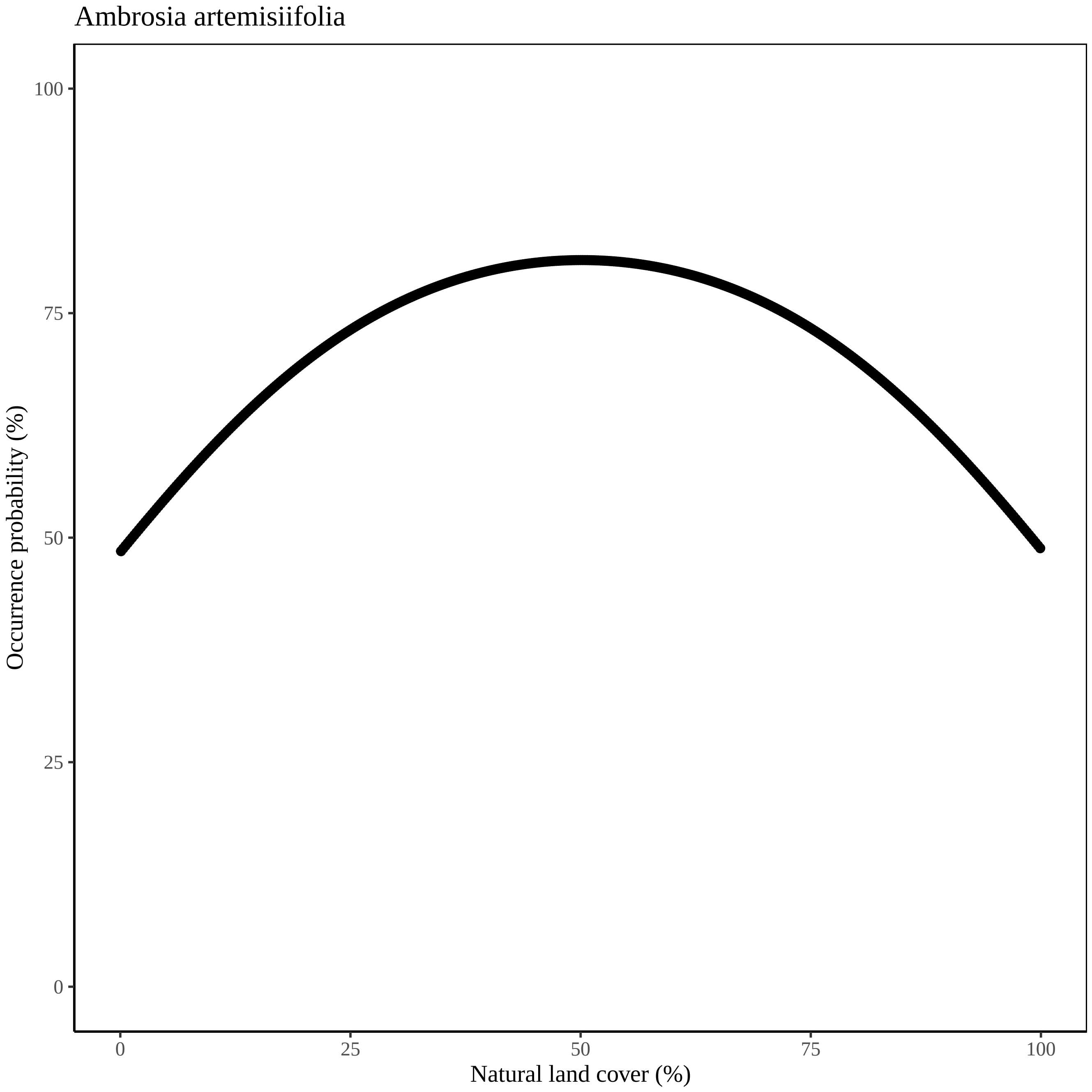

Ambrosia artemisiifolia
Occurrence probability (%)
0
25
50
75
100
Natural land cover (%)
0
25
50
75
100

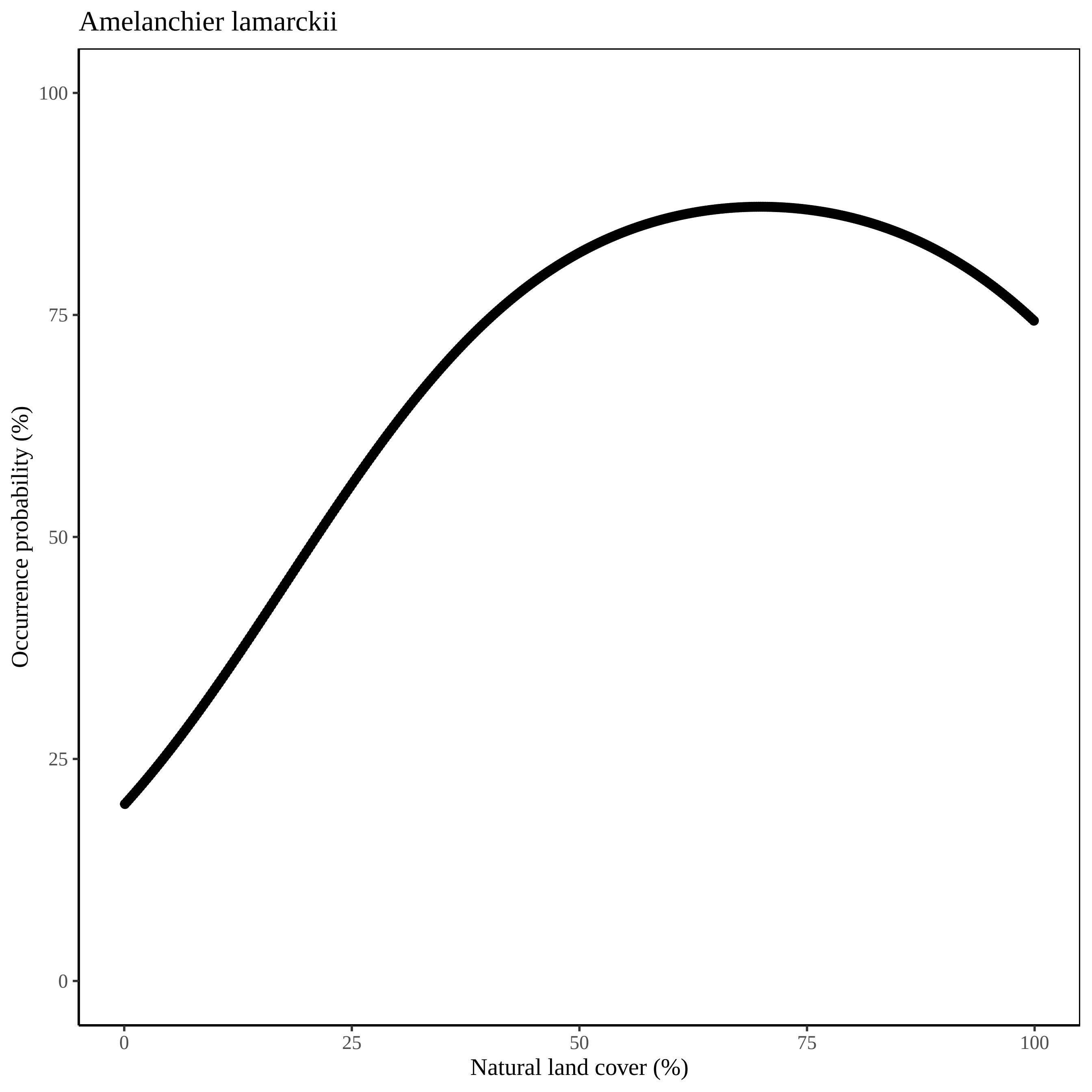

Amelanchier lamarckii
Occurrence probability (%)
100
75
50
25
0
0
25
50
75
100
Natural land cover (%)

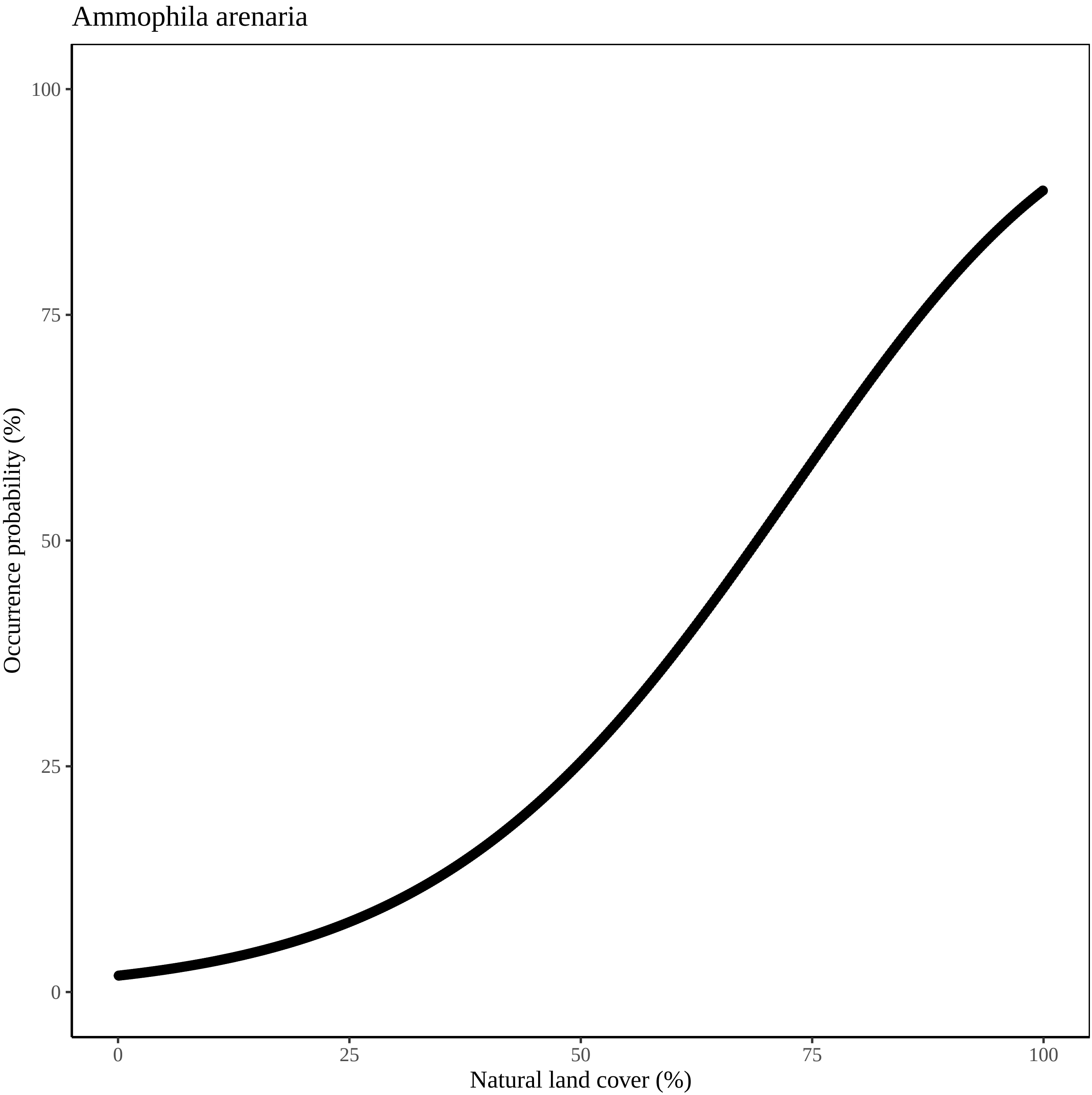
Ammophila arenaria
Occurrence probability (%)
100
75
50
25
0
0
25
50
75
100
Natural land cover (%)

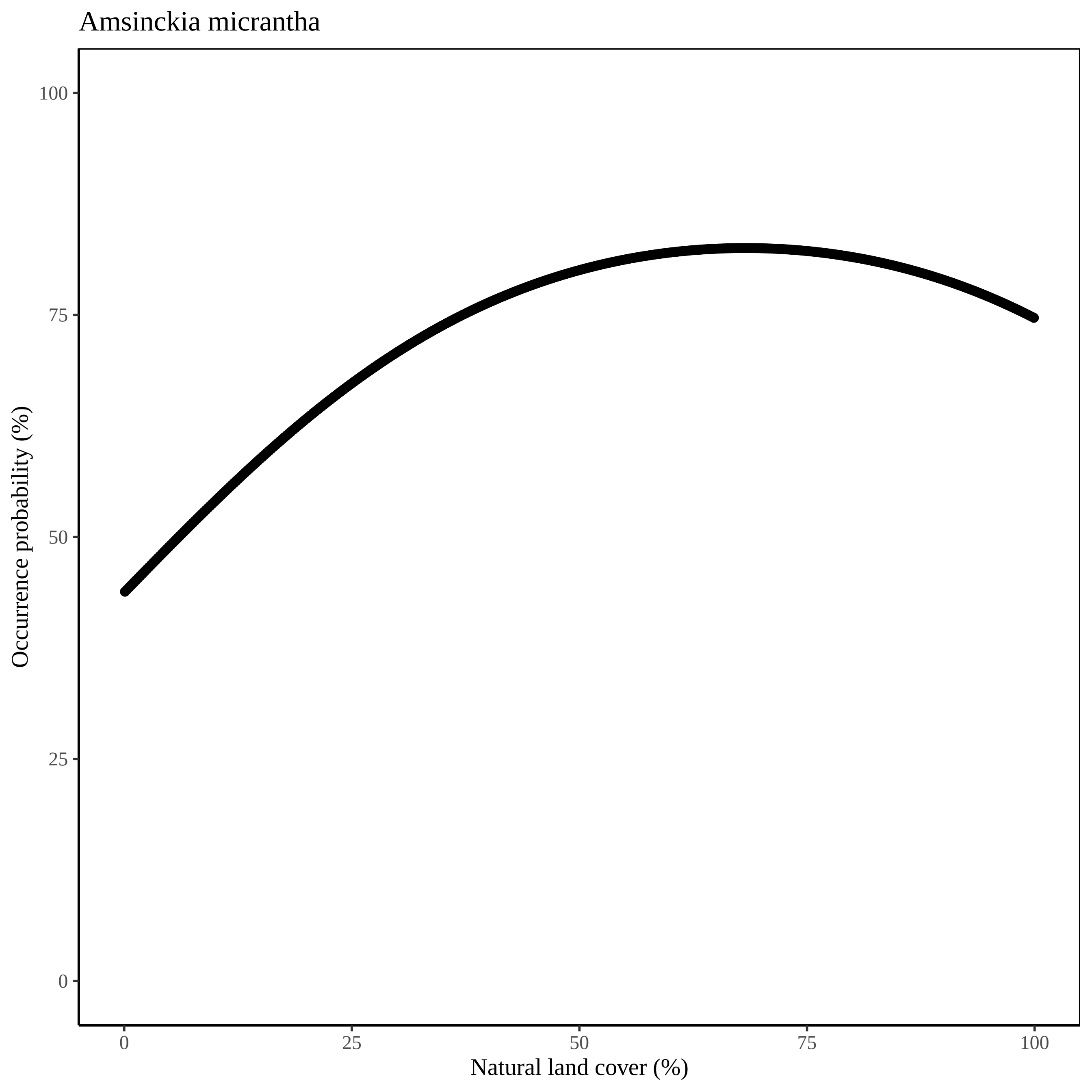

Amsinckia micrantha
100
75
50
25
0
Occurrence probability (%)
0
25
50
75
100
Natural land cover (%)

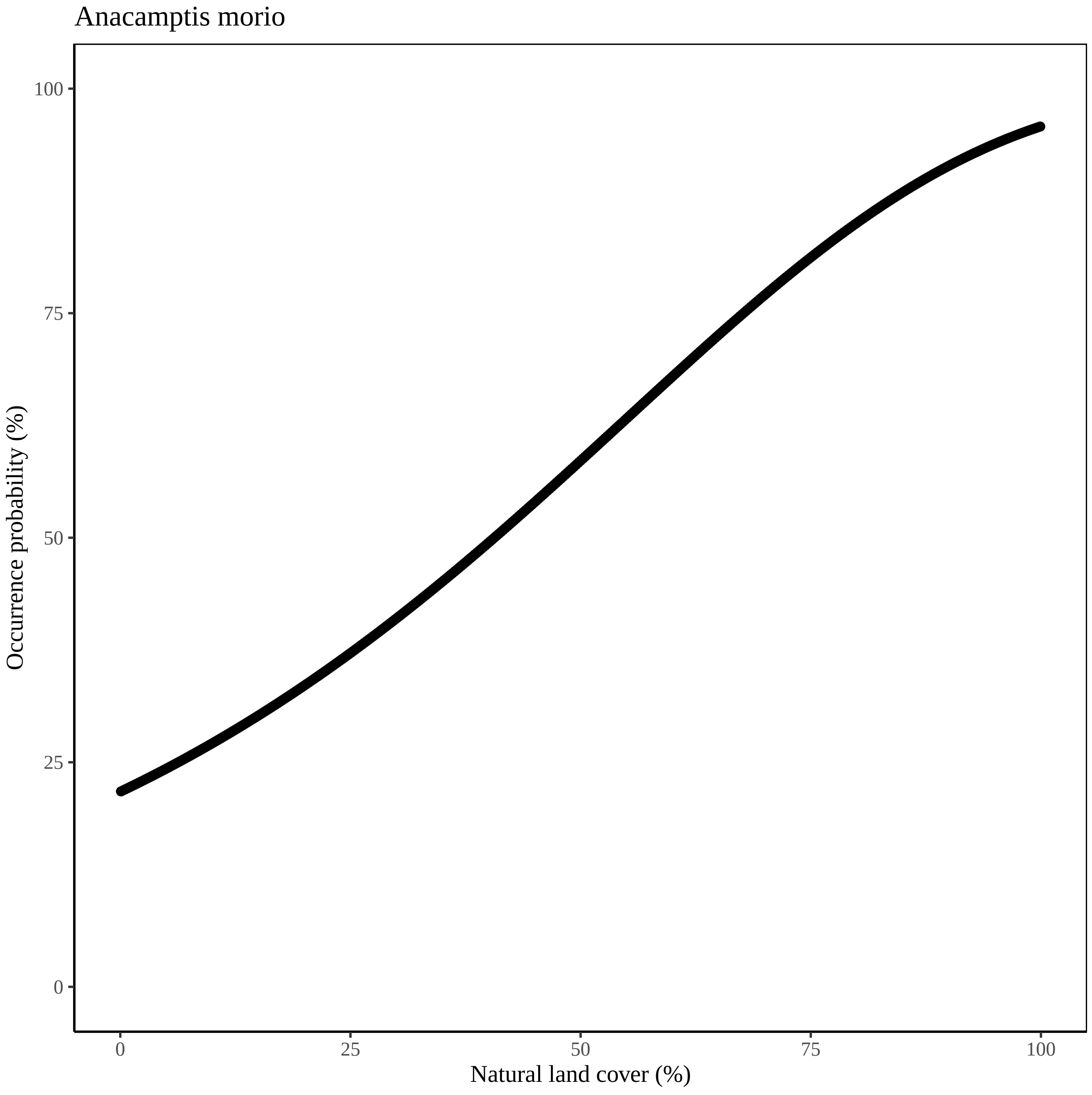

Anacamptis morio
Occurrence probability (%)
100
75
50
25
0
0
25
50
75
100
Natural land cover (%)

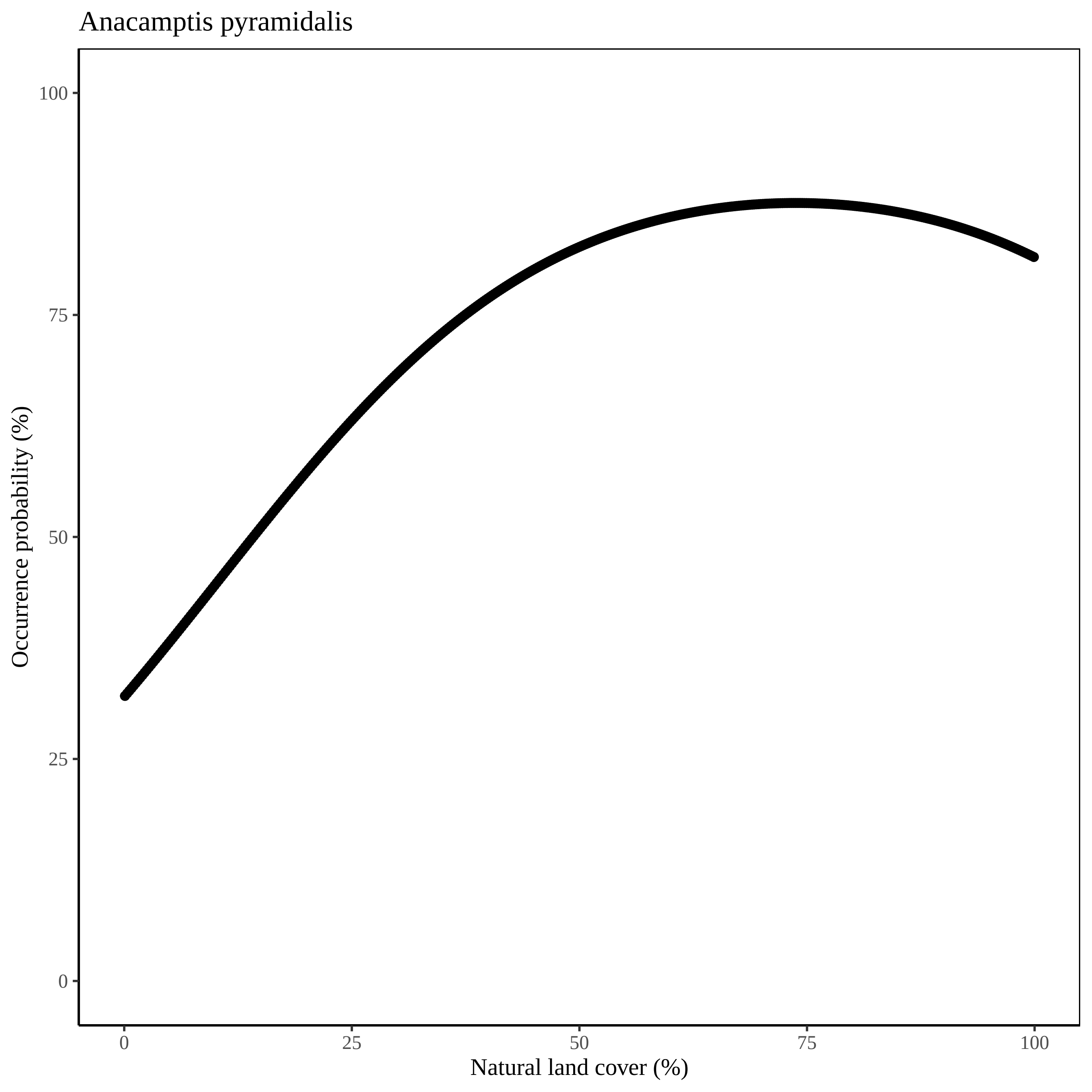
Anacamptis pyramidalis
Occurrence probability (%)
100
75
50
25
0
0
25
50
75
100
Natural land cover (%)

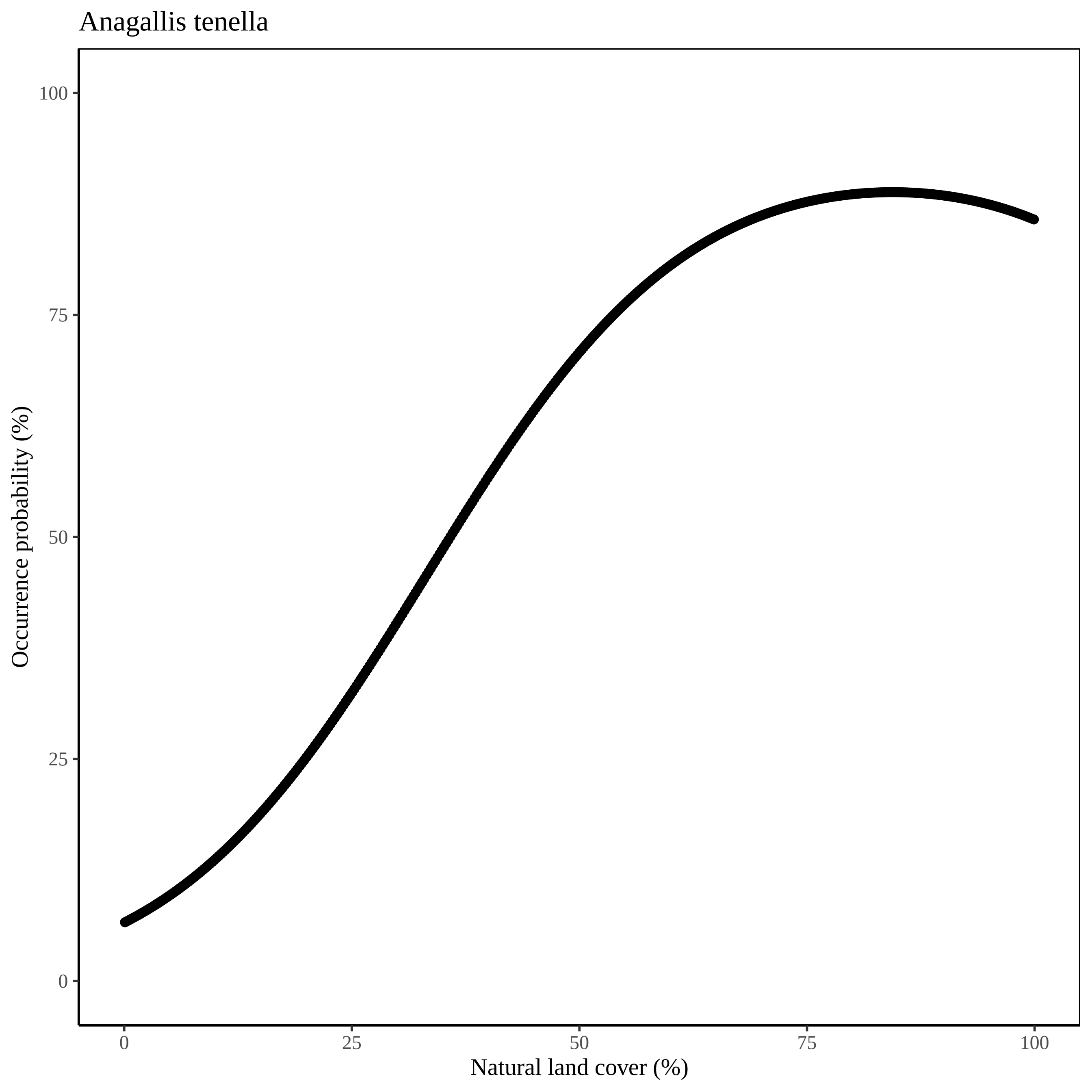
Anagallis tenella
Occurrence probability (%)
100
75
50
25
0
0
25
50
75
100
Natural land cover (%)

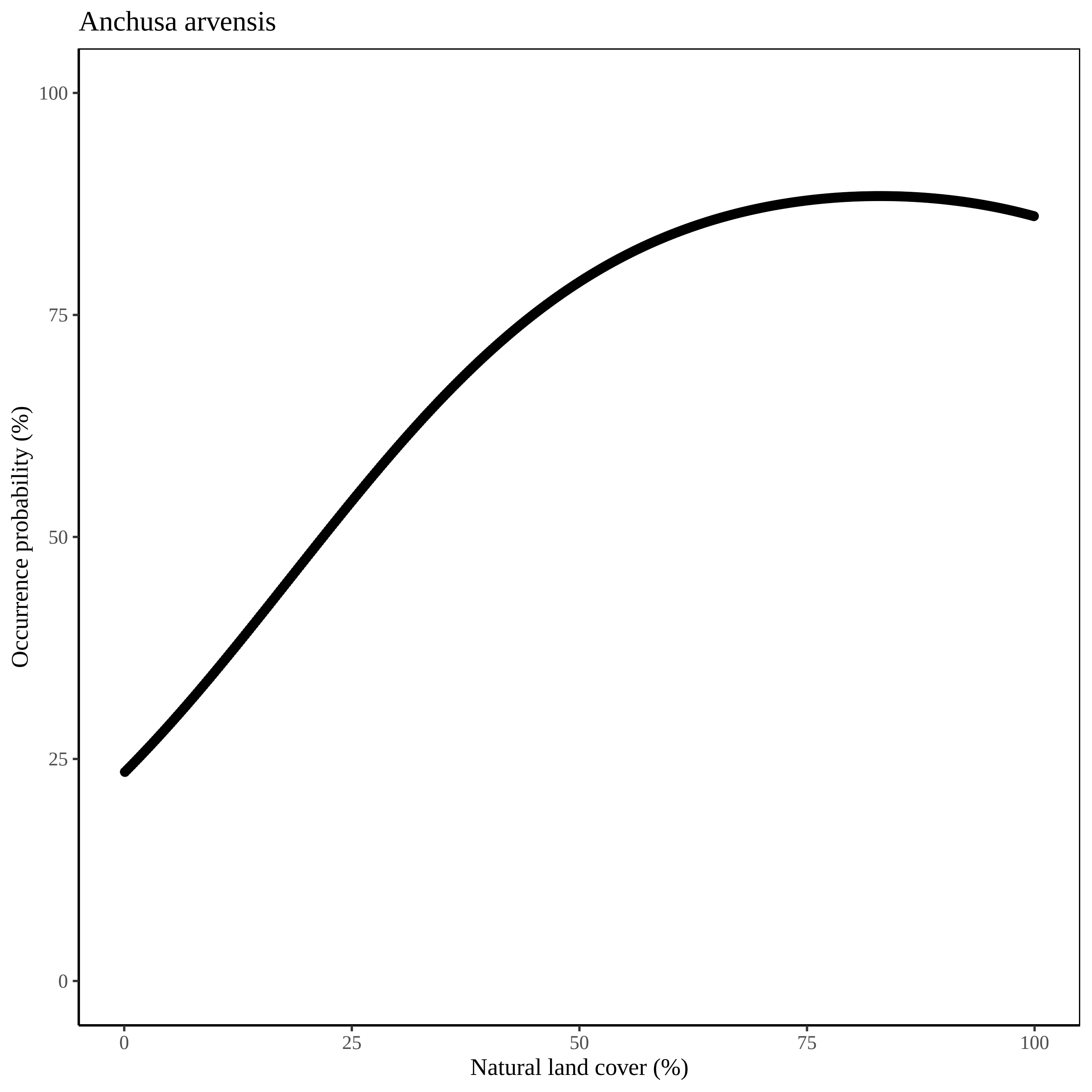
Anchusa arvensis
Occurrence probability (%)
100
75
50
25
0
0
25
50
75
100
Natural land cover (%)

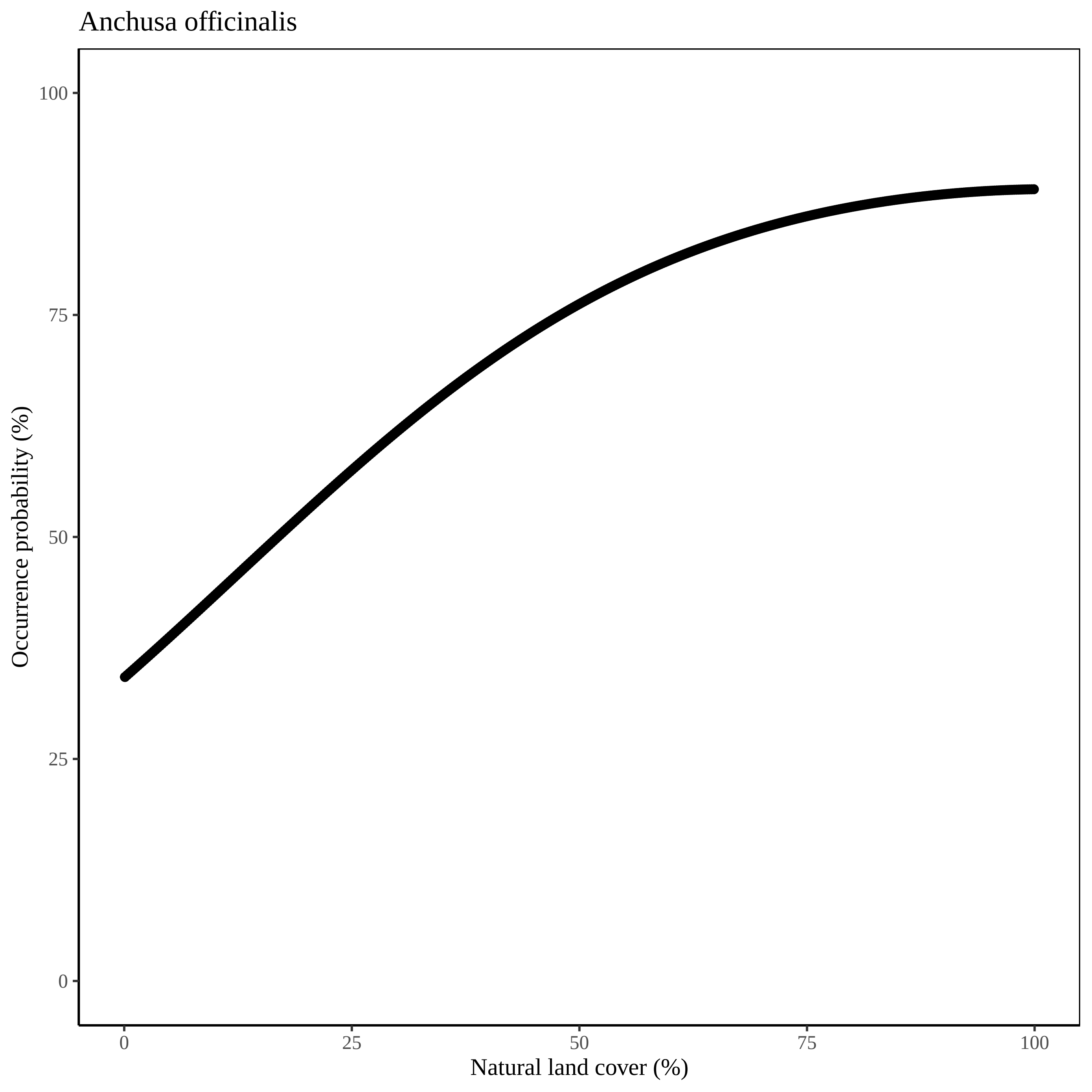
Anchusa officinalis
Occurrence probability (%)
100
75
50
25
0
0
25
50
75
100
Natural land cover (%)

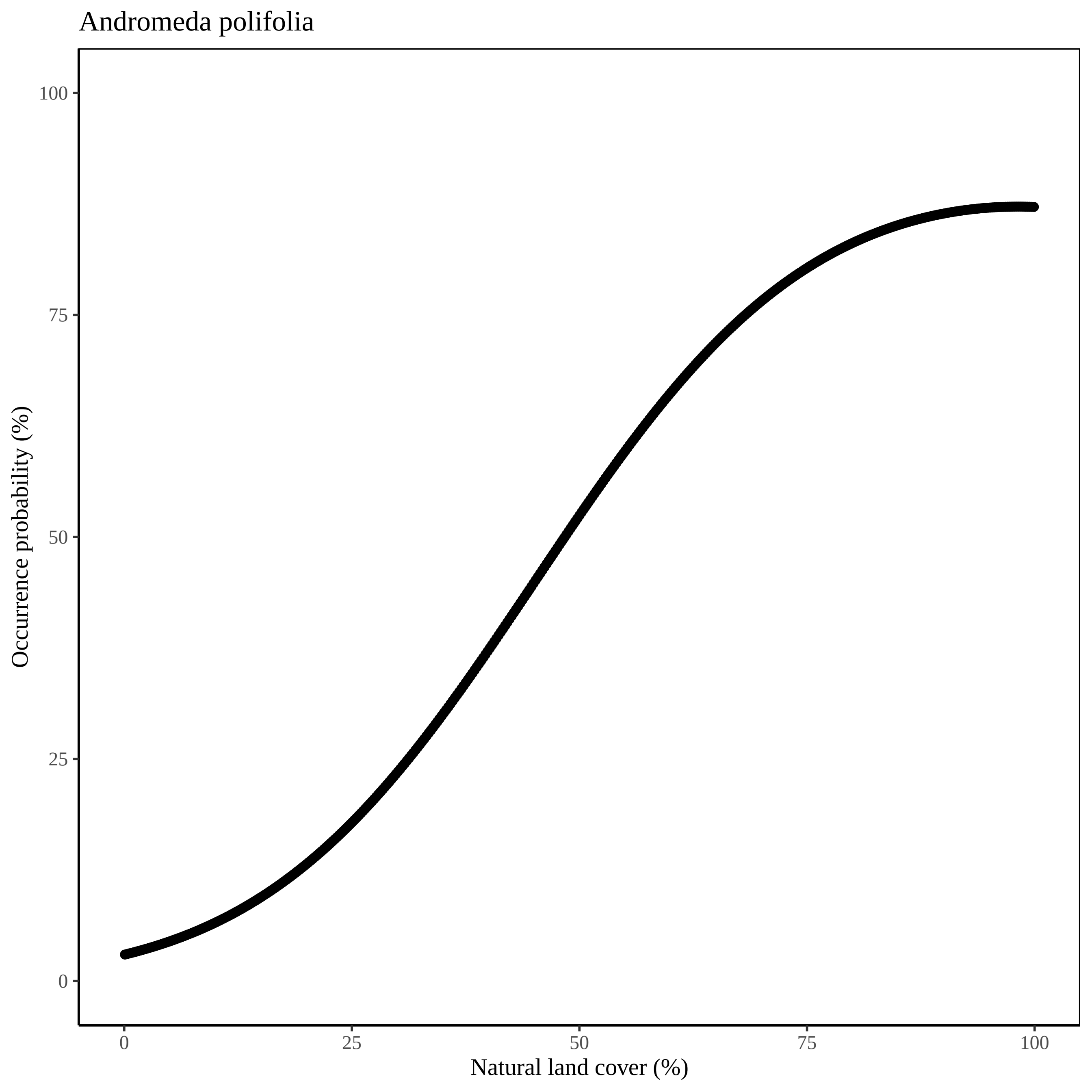

Andromeda polifolia
Occurrence probability (%)
0
25
50
75
100
Natural land cover (%)
0
25
50
75
100

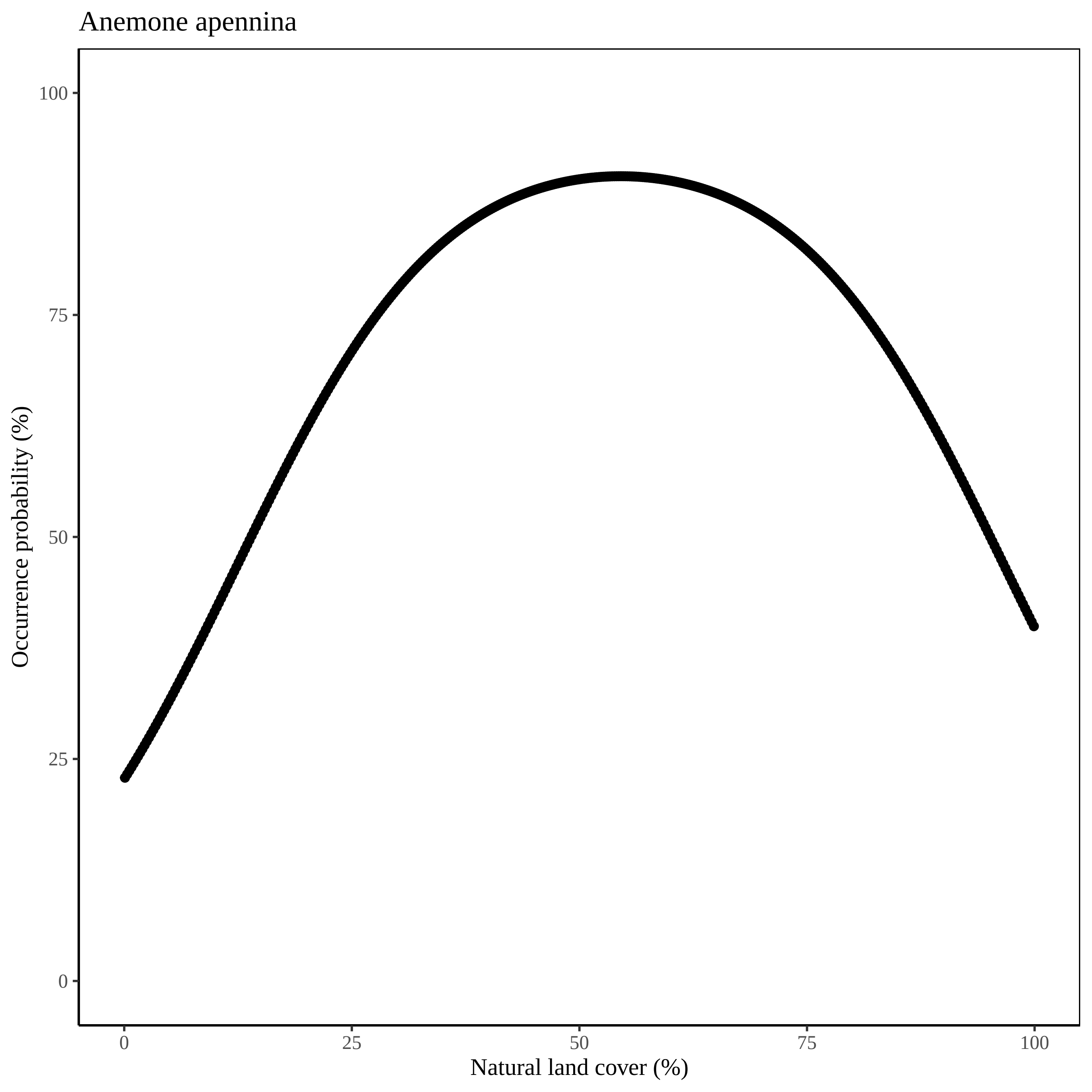

Anemone apennina
Occurrence probability (%)
100
75
50
25
0
0
25
50
75
100
Natural land cover (%)

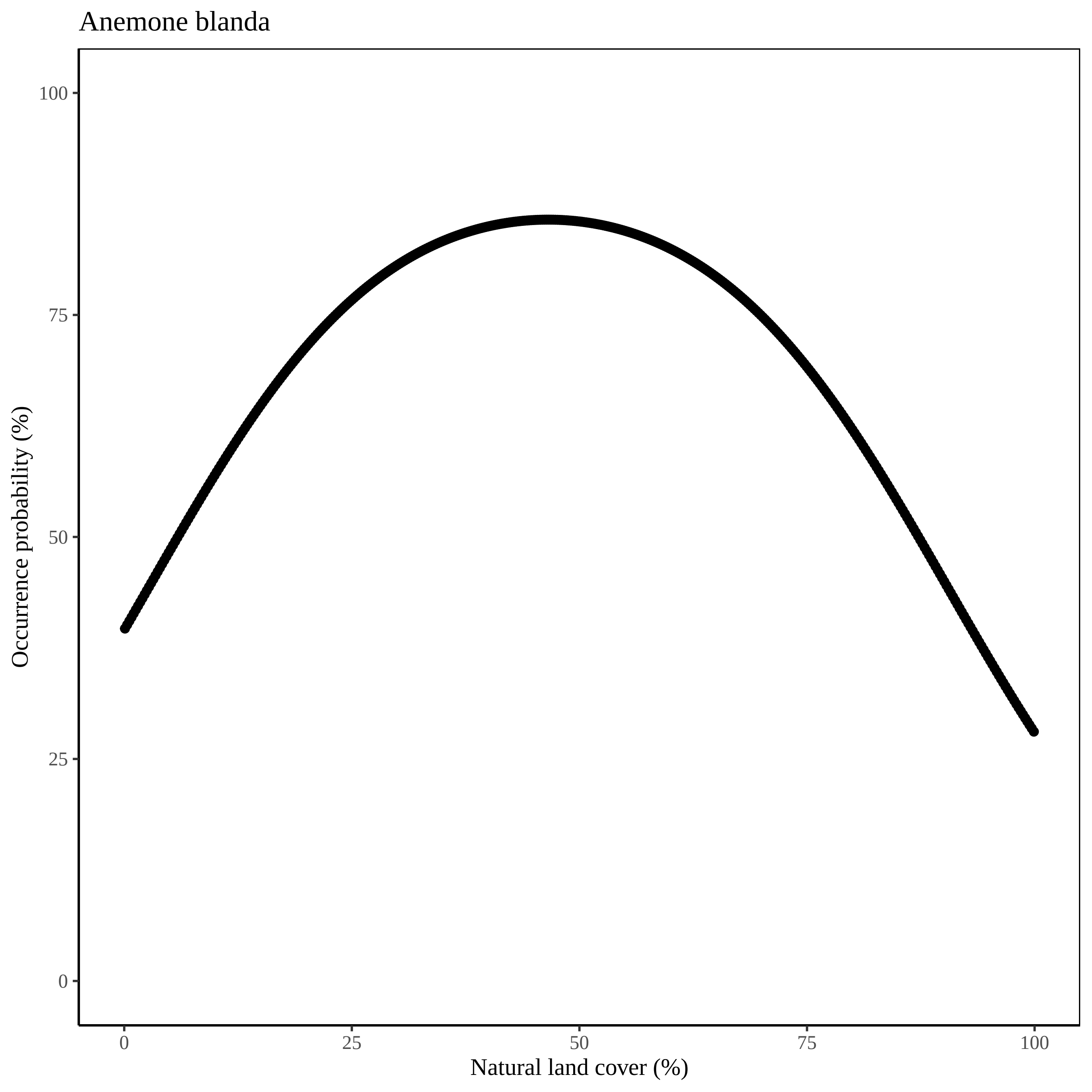

Anemone blanda
Occurrence probability (%)
100
75
50
25
0
0
25
50
75
100
Natural land cover (%)

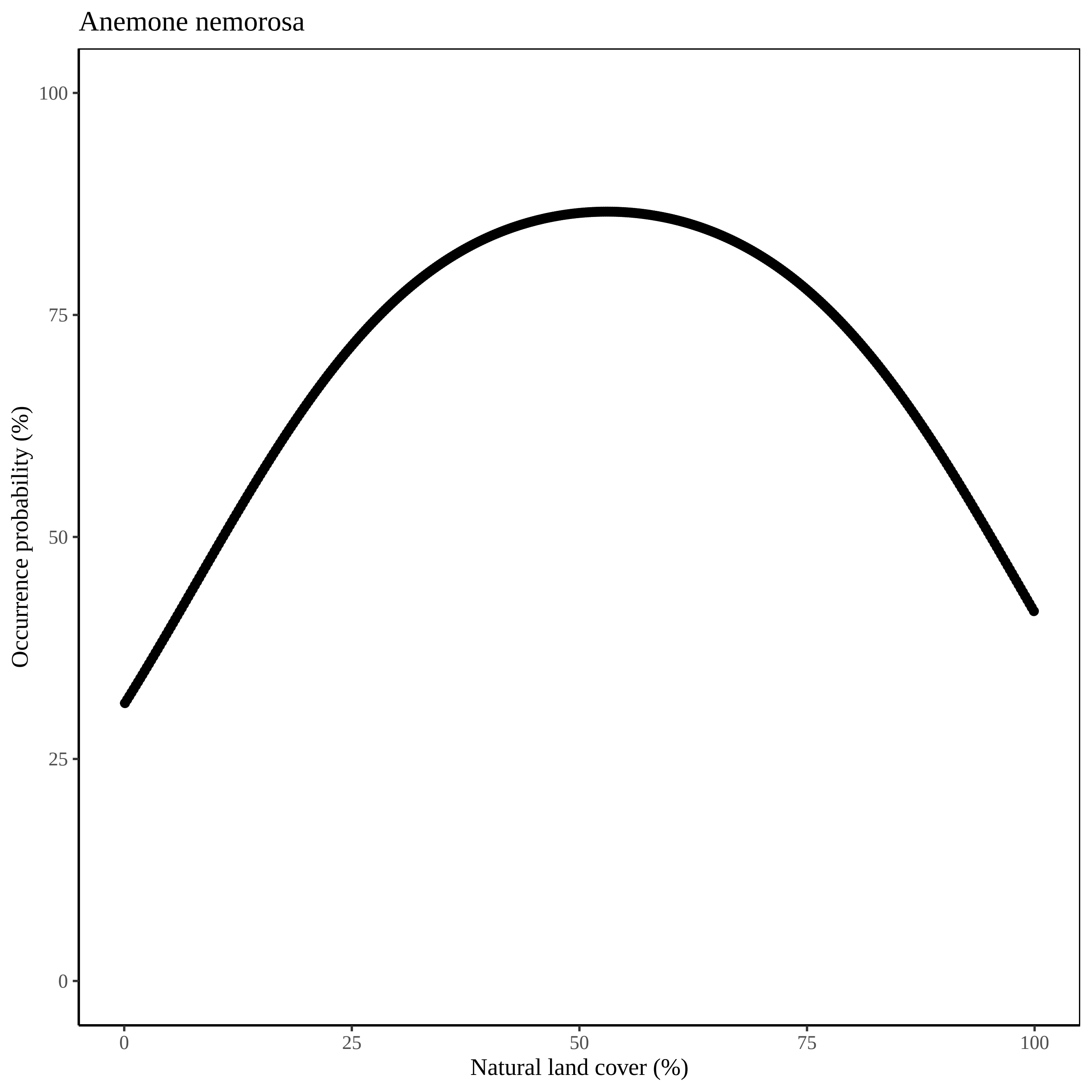

Anemone nemorosa
Occurrence probability (%)
100
75
50
25
0
0
25
50
75
100
Natural land cover (%)

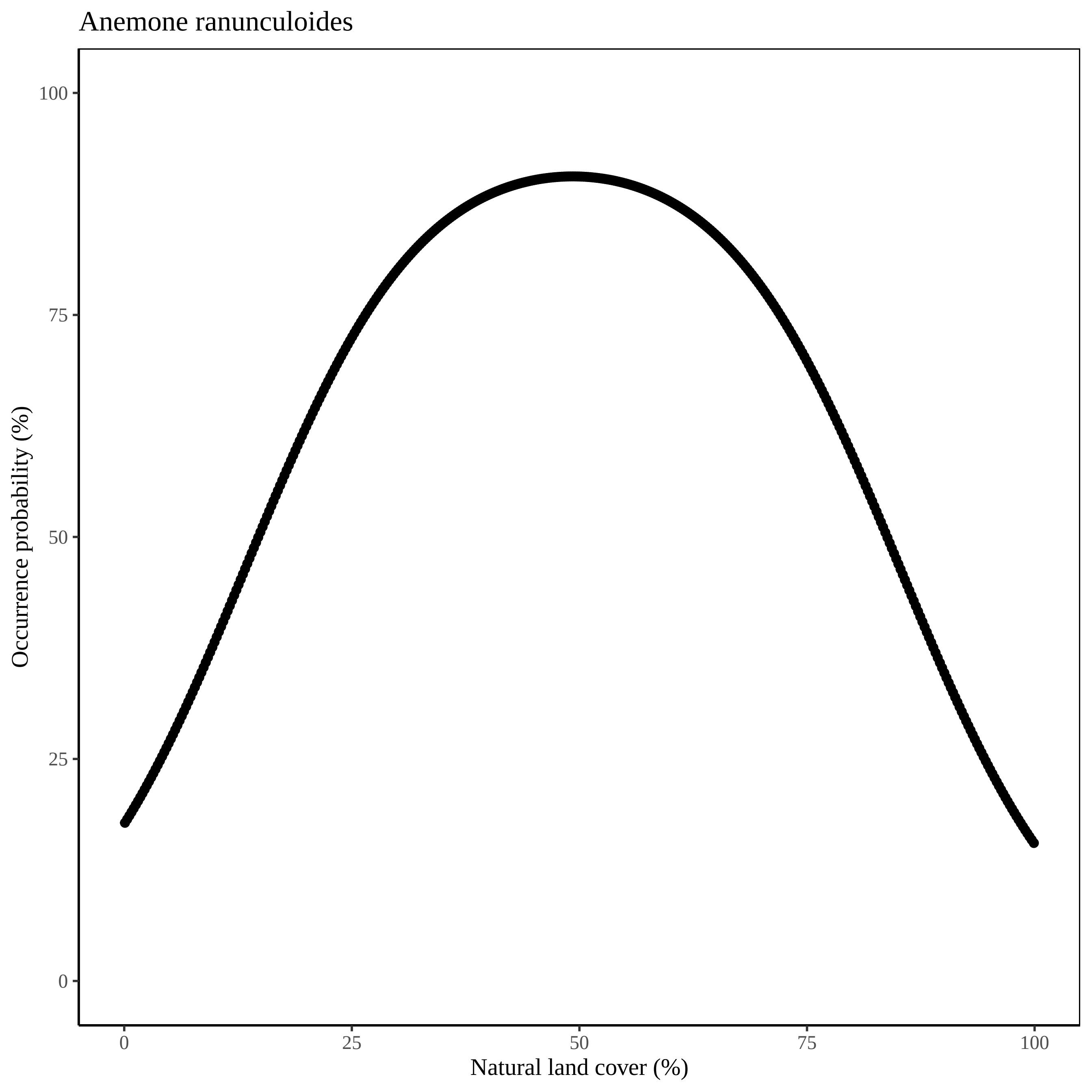

Anemone ranunculoides
Occurrence probability (%)
100
75
50
25
0
0
25
50
75
100
Natural land cover (%)

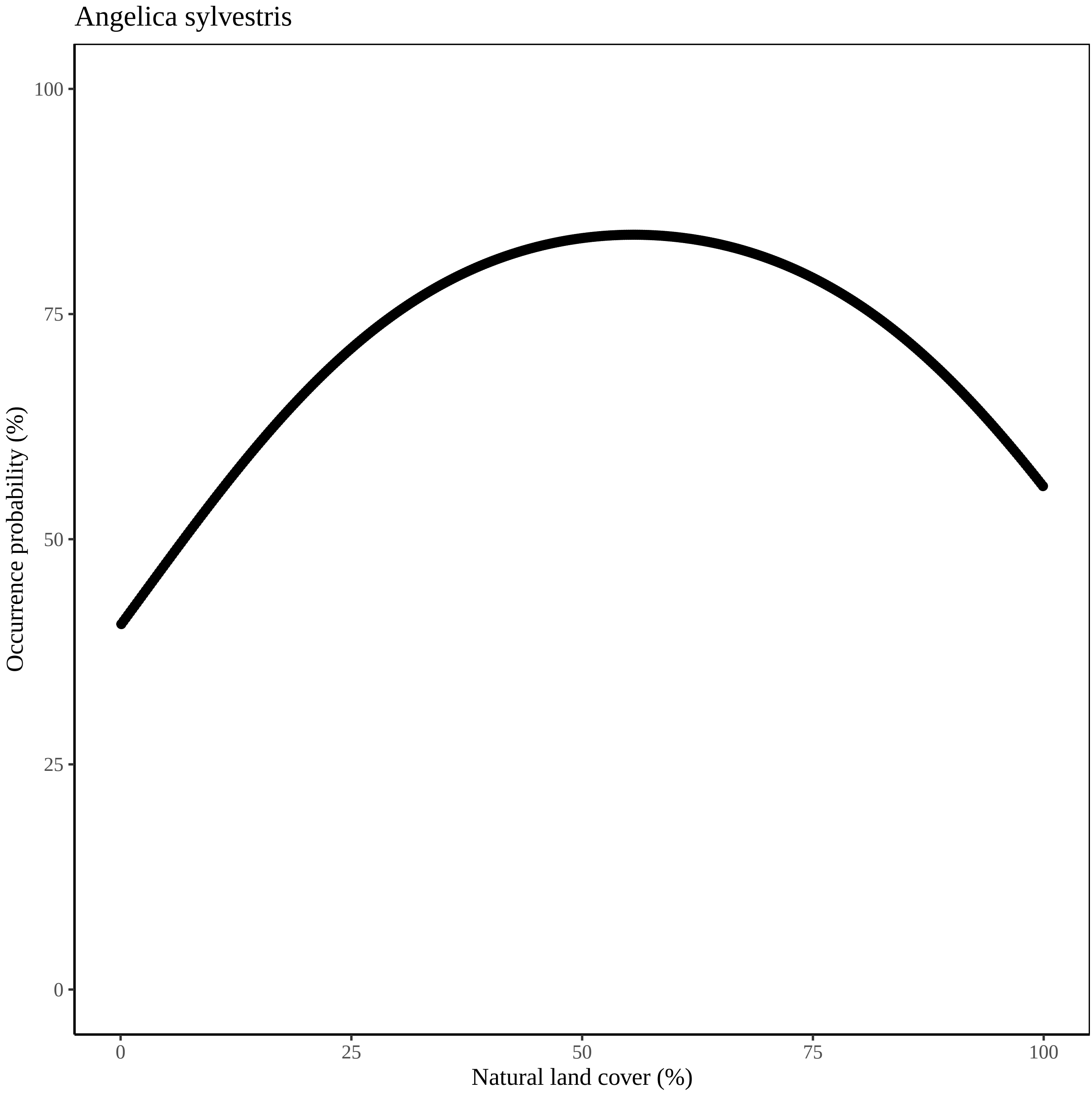
Angelica sylvestris
100
75
50
25
0
Occurrence probability (%)
0
25
50
75
100
Natural land cover (%)

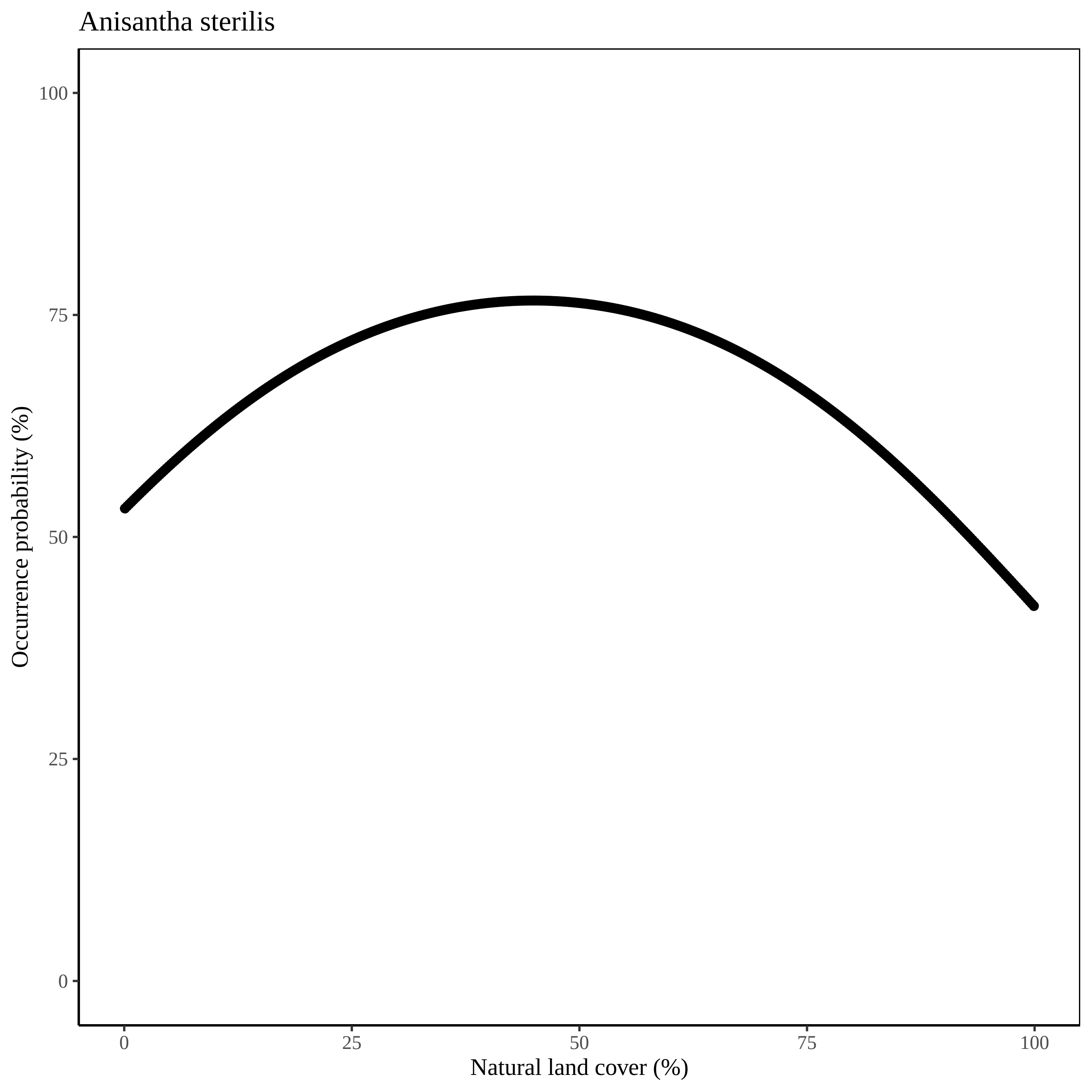

Anisantha sterilis
Occurrence probability (%)
100
75
50
25
0
0
25
50
75
100
Natural land cover (%)

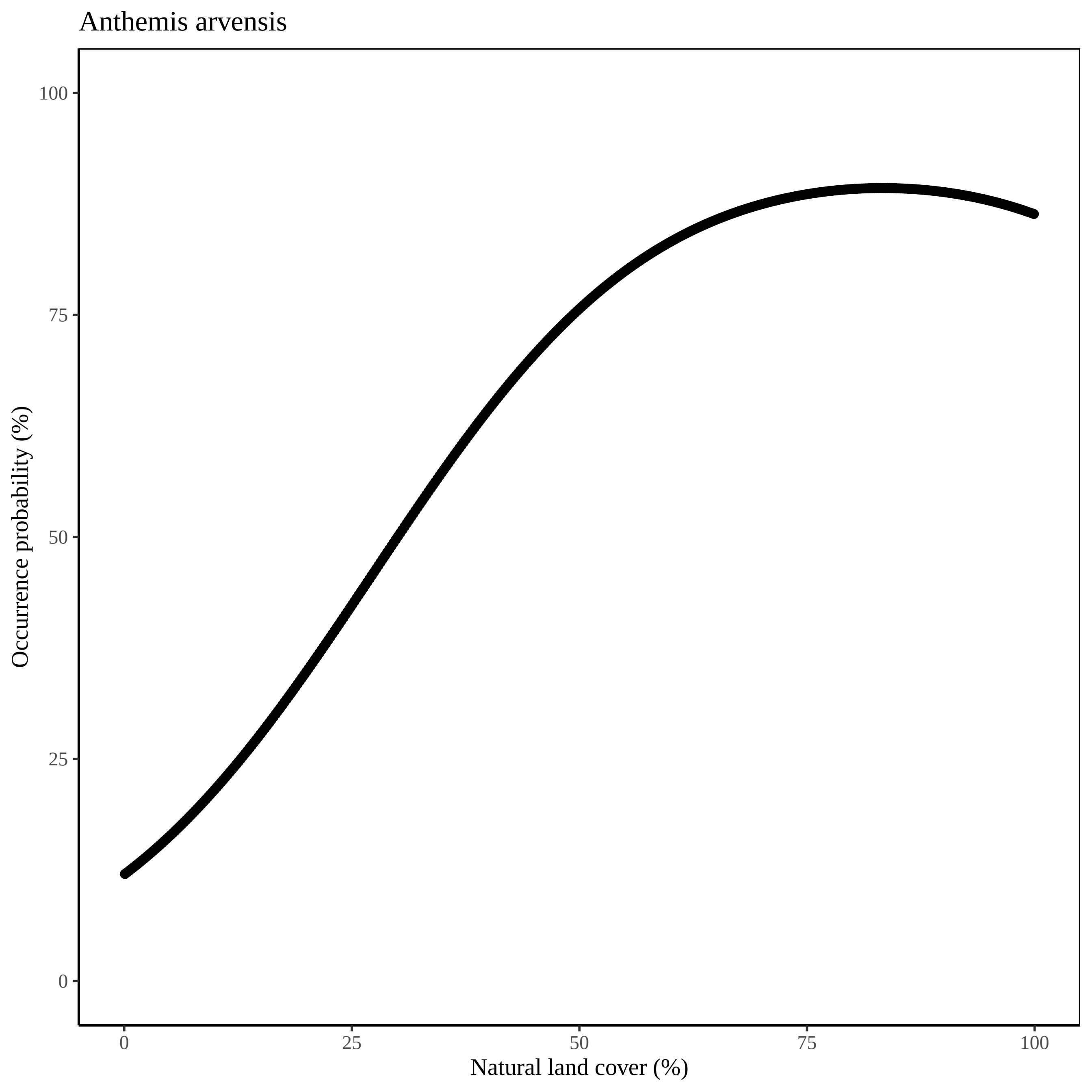

Anthemis arvensis
Occurrence probability (%)
100
75
50
25
0
0
25
50
75
100
Natural land cover (%)

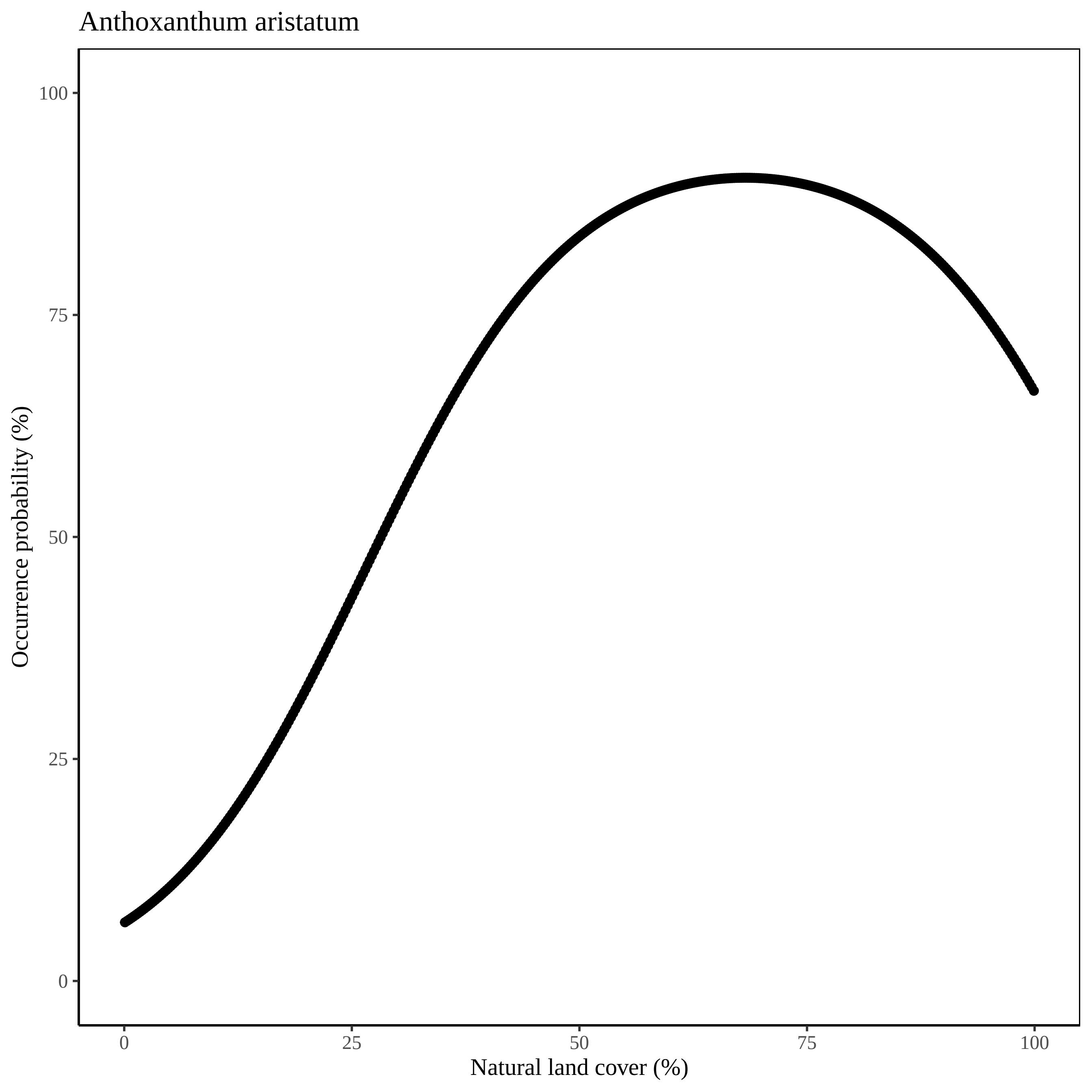

Anthoxanthum aristatum
Occurrence probability (%)
100
75
50
25
0
0
25
50
75
100
Natural land cover (%)

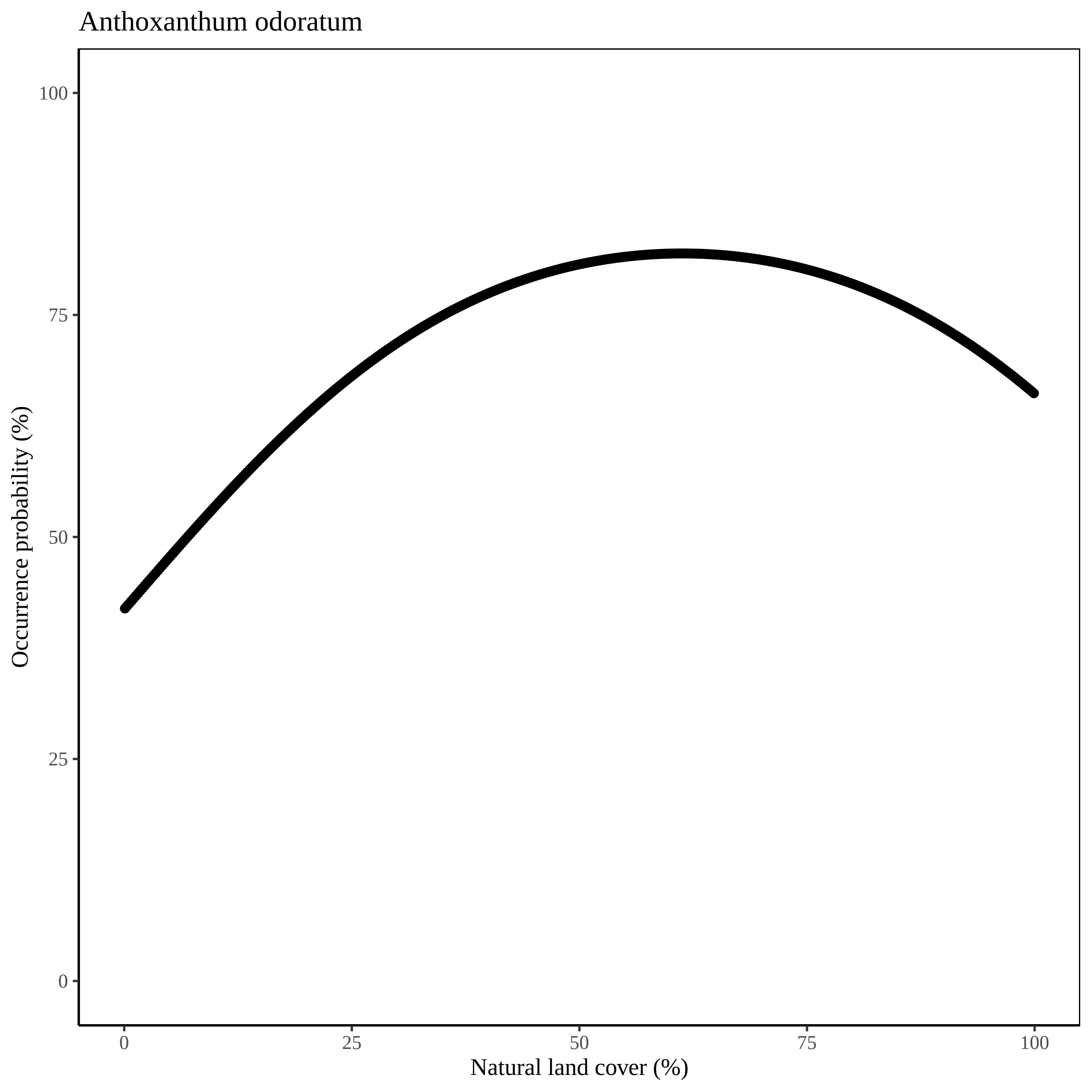

Anthoxanthum odoratum
Occurrence probability (%)
100
75
50
25
0
0
25
50
75
100
Natural land cover (%)

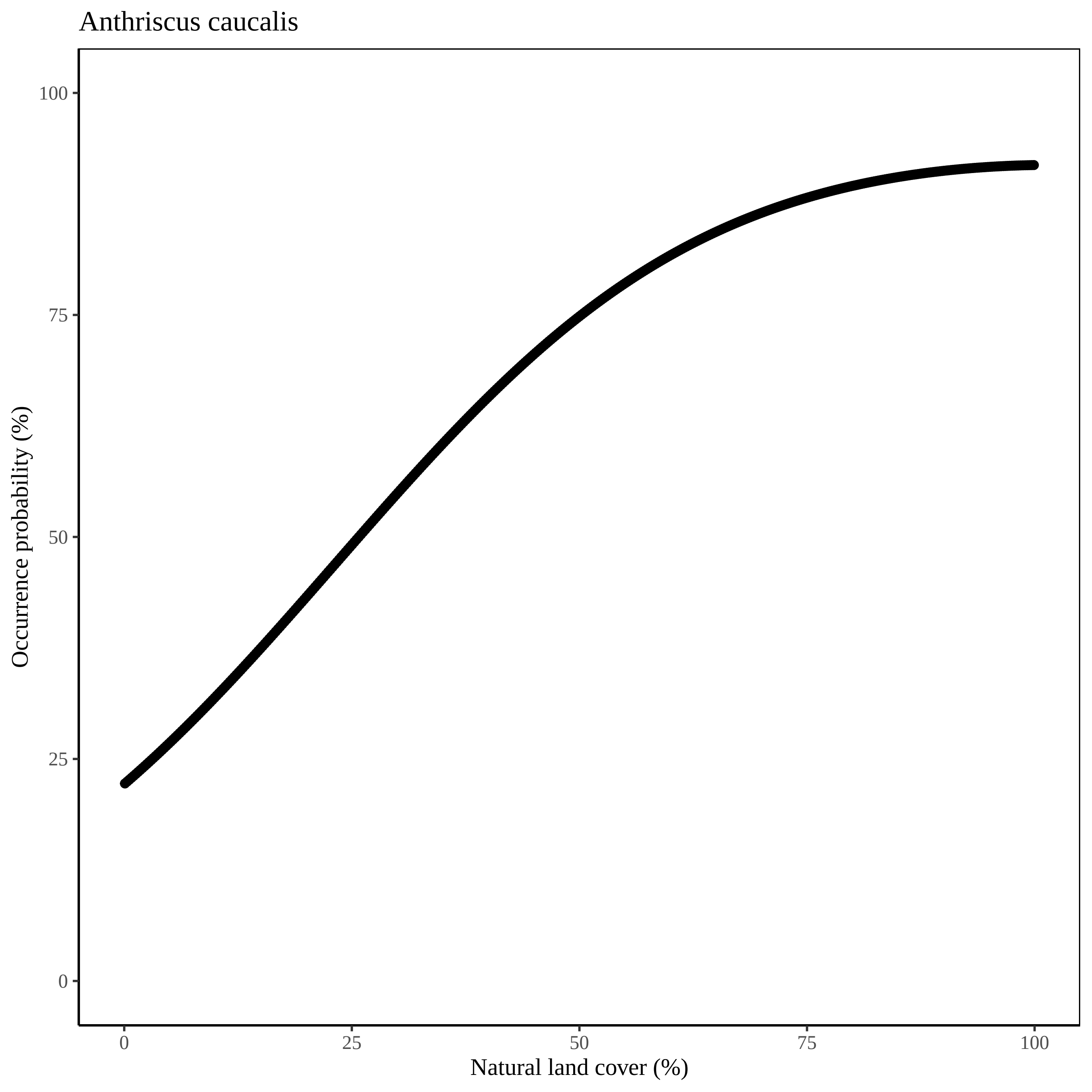

Anthriscus caucalis
Occurrence probability (%)
100
75
50
25
0
0
25
50
75
100
Natural land cover (%)

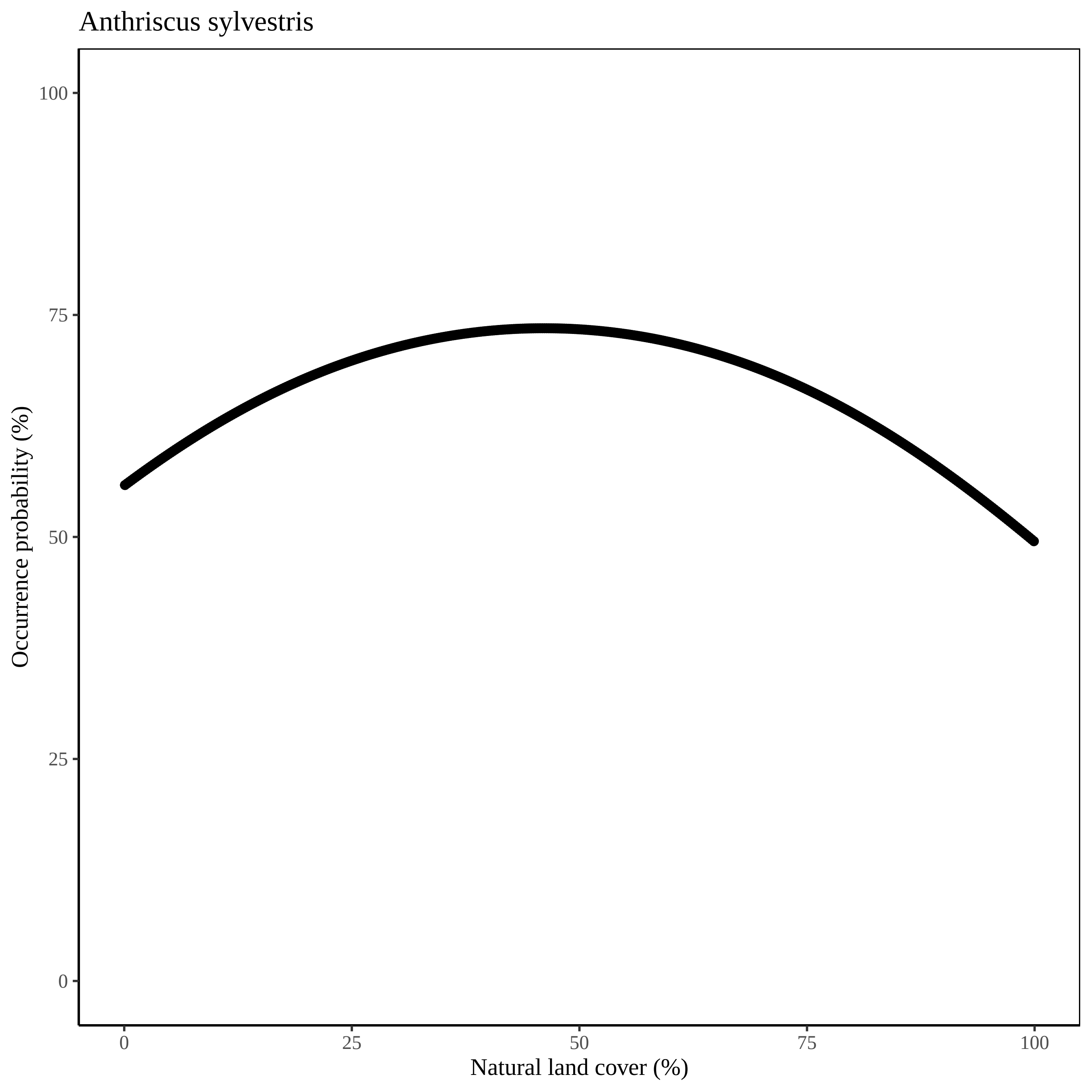

Anthriscus sylvestris
Occurrence probability (%)
100
75
50
25
0
0
25
50
75
100
Natural land cover (%)

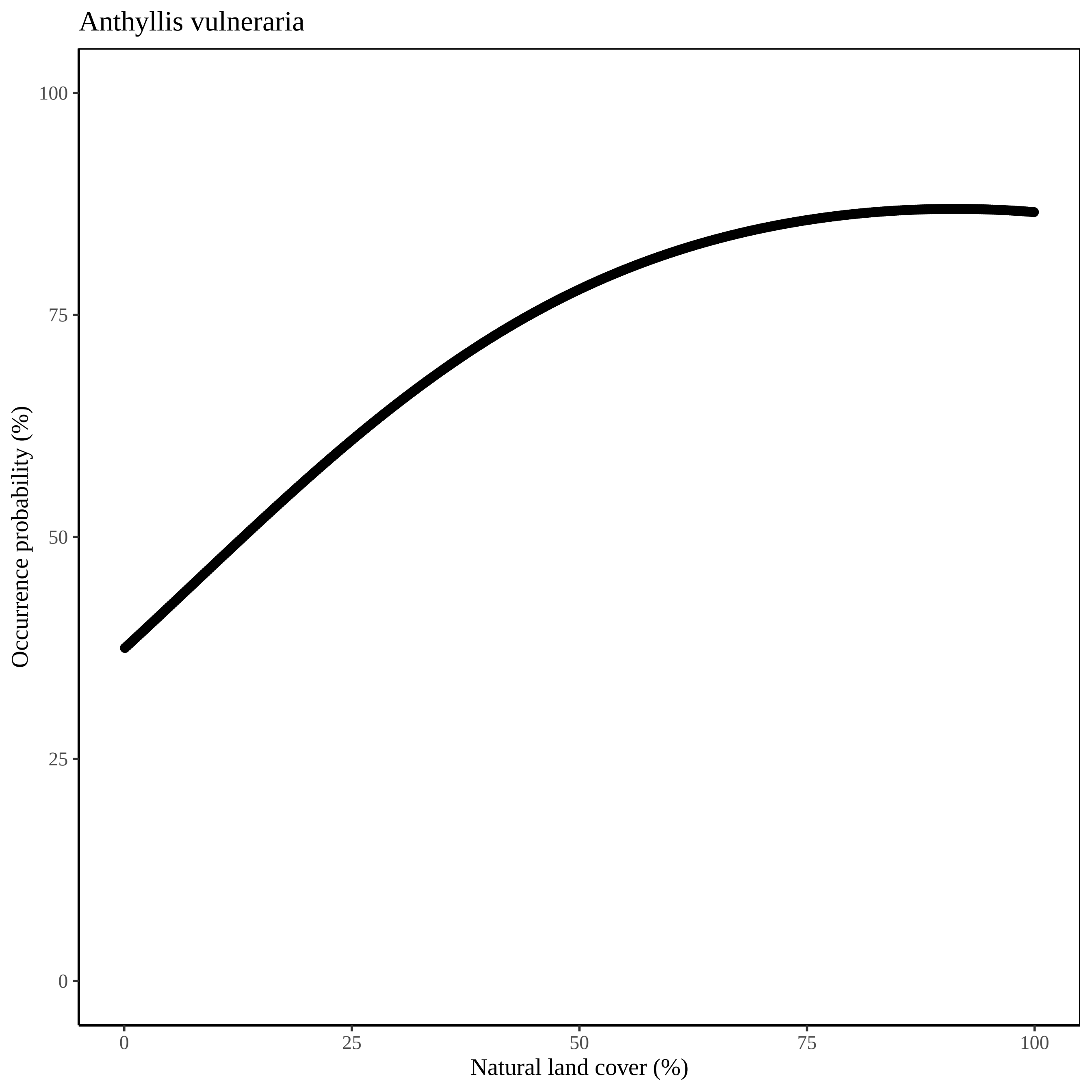

Anthyllis vulneraria
Occurrence probability (%)
100
75
50
25
0
0
25
50
75
100
Natural land cover (%)

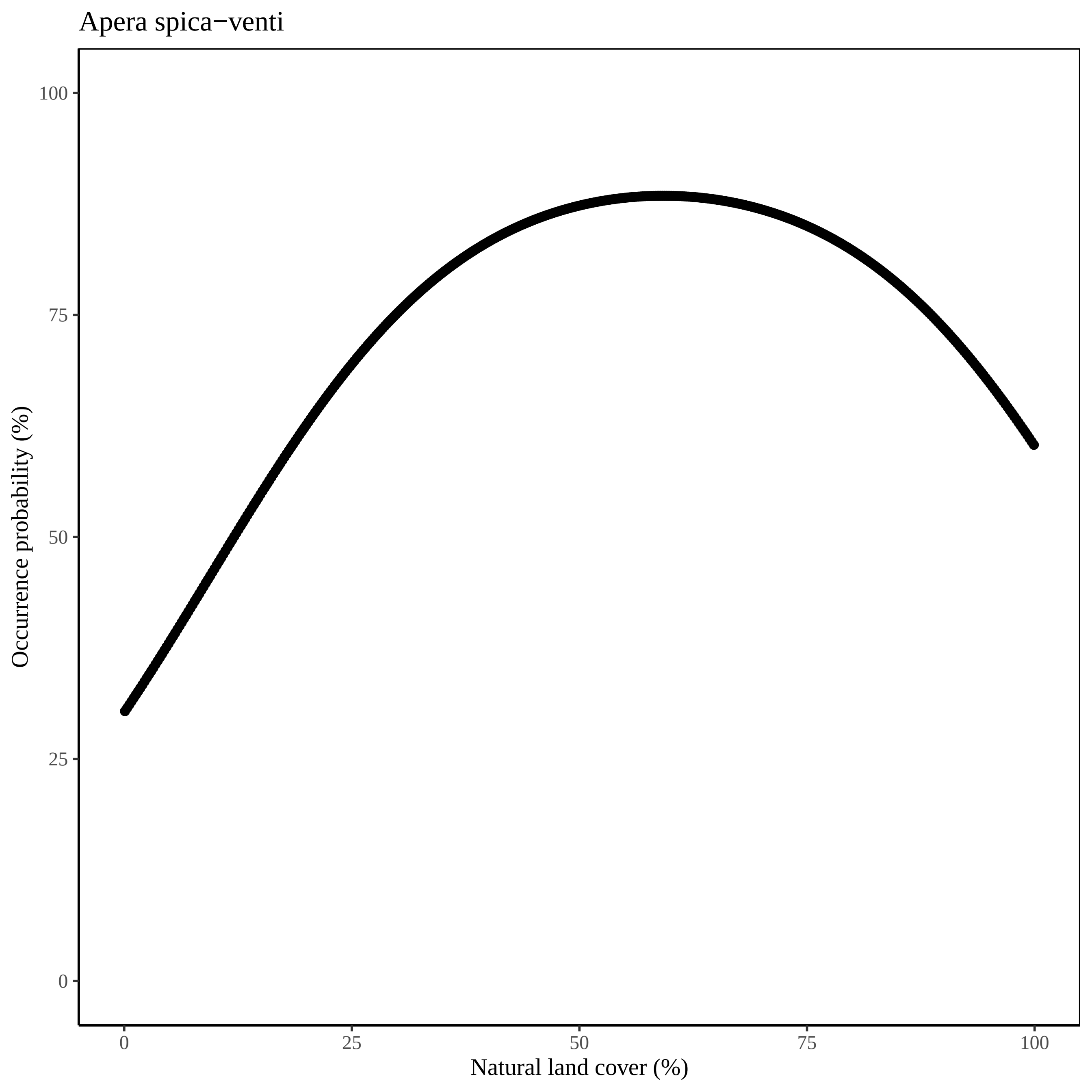

Apera spica−venti
Occurrence probability (%)
100
75
50
25
0
0
25
50
75
100
Natural land cover (%)

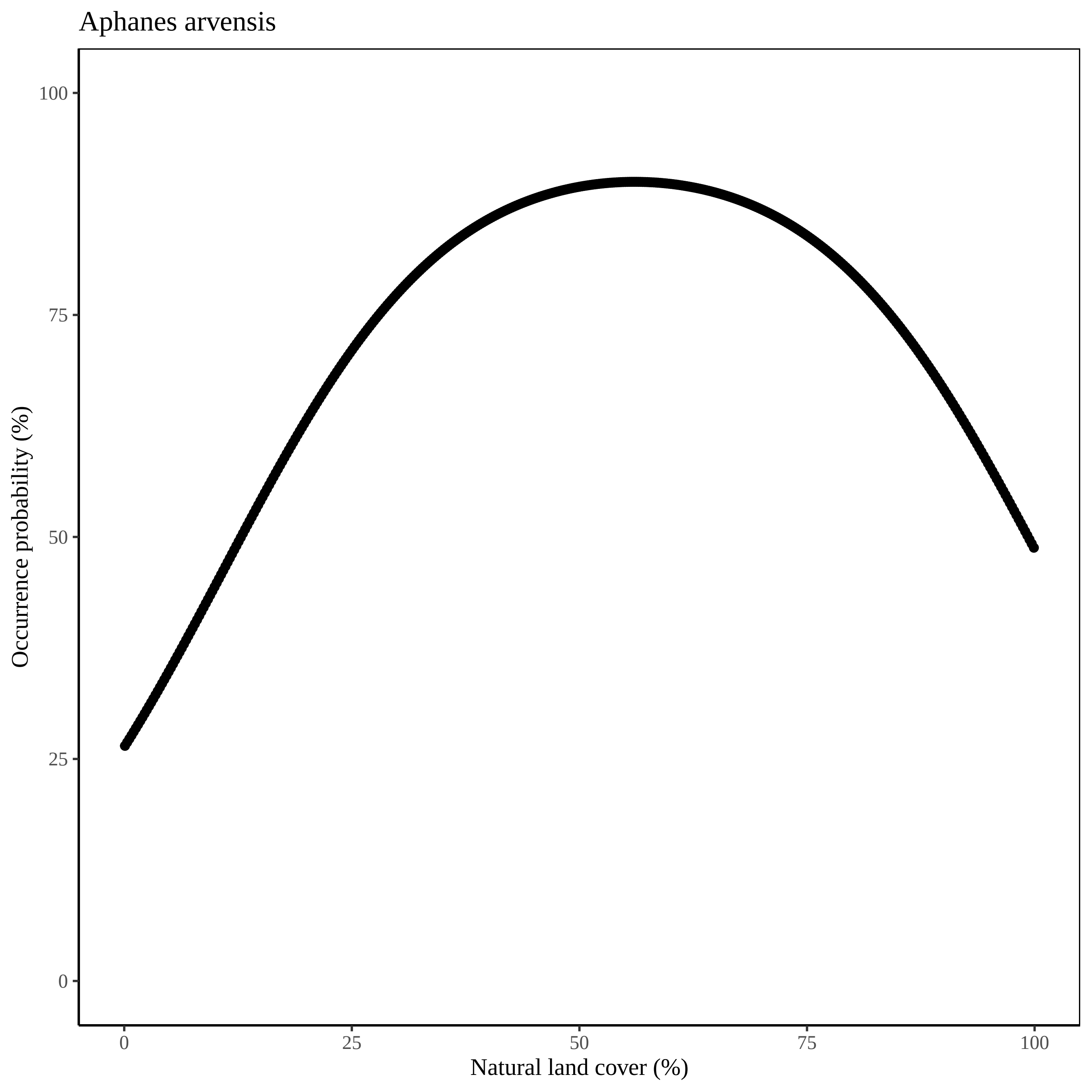

Aphanes arvensis
Occurrence probability (%)
100
75
50
25
0
0
25
50
75
100
Natural land cover (%)

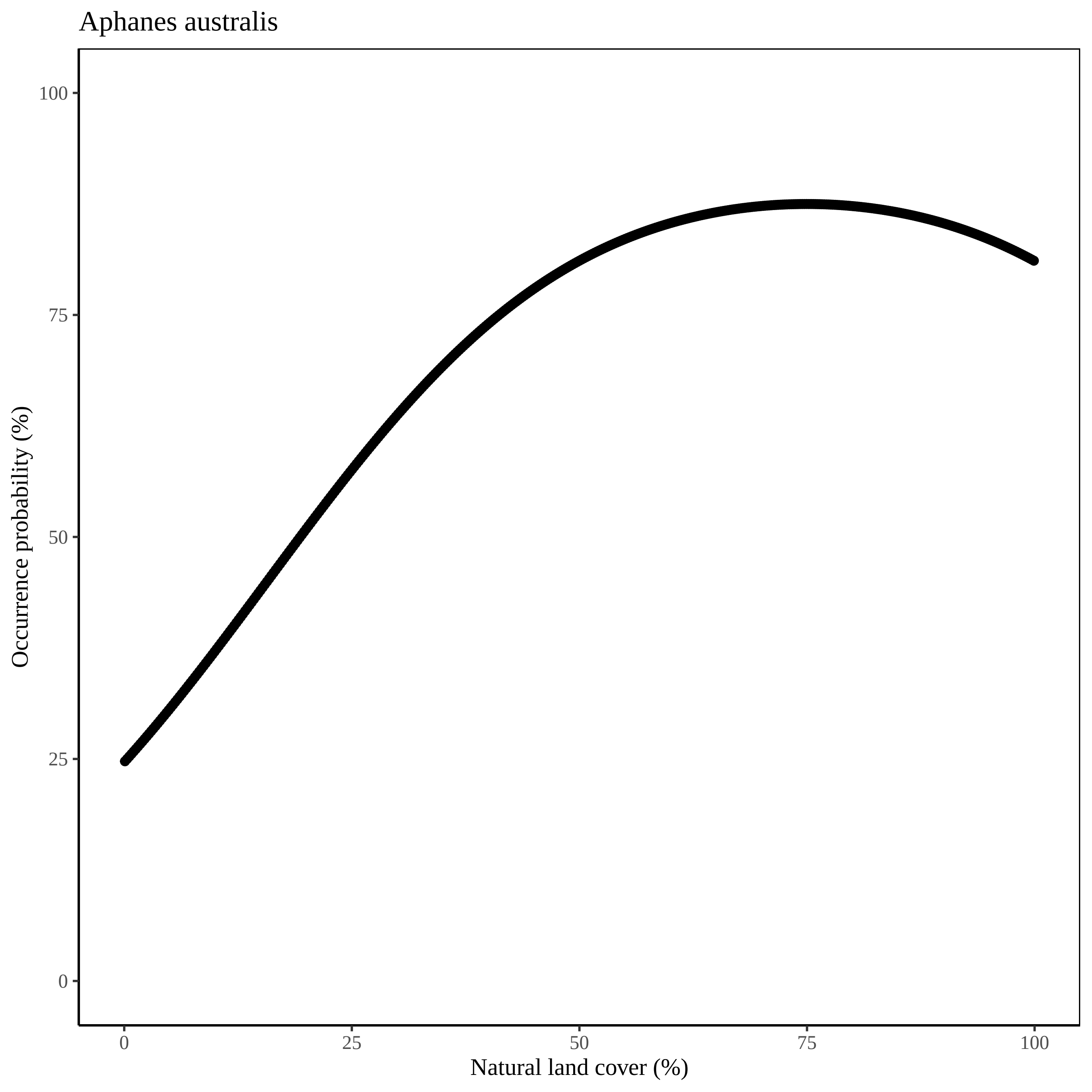
Aphanes australis
100
75
50
25
0
Occurrence probability (%)
0
25
50
75
100
Natural land cover (%)

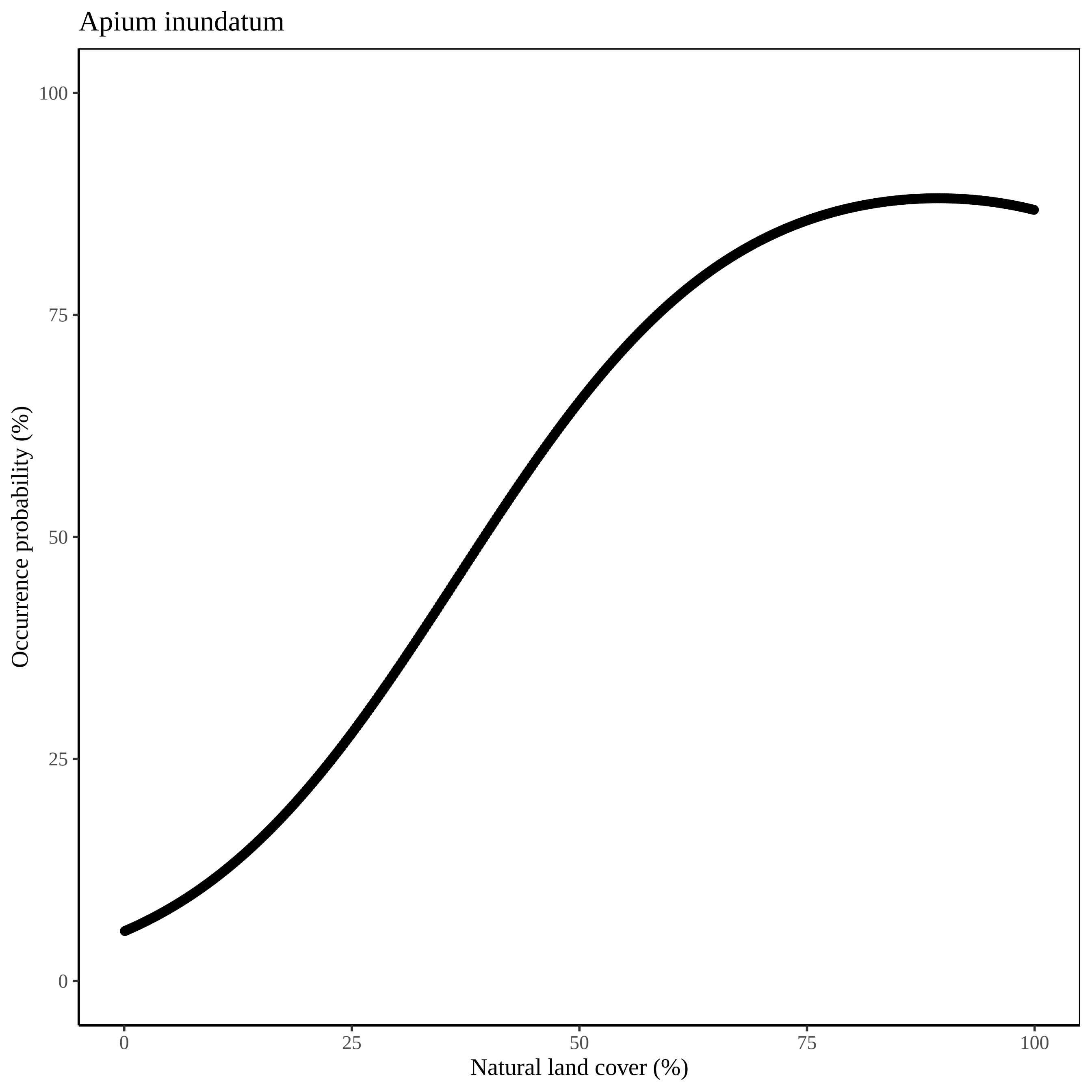
Apium inundatum
Occurrence probability (%)
100
75
50
25
0
0
25
50
75
100
Natural land cover (%)

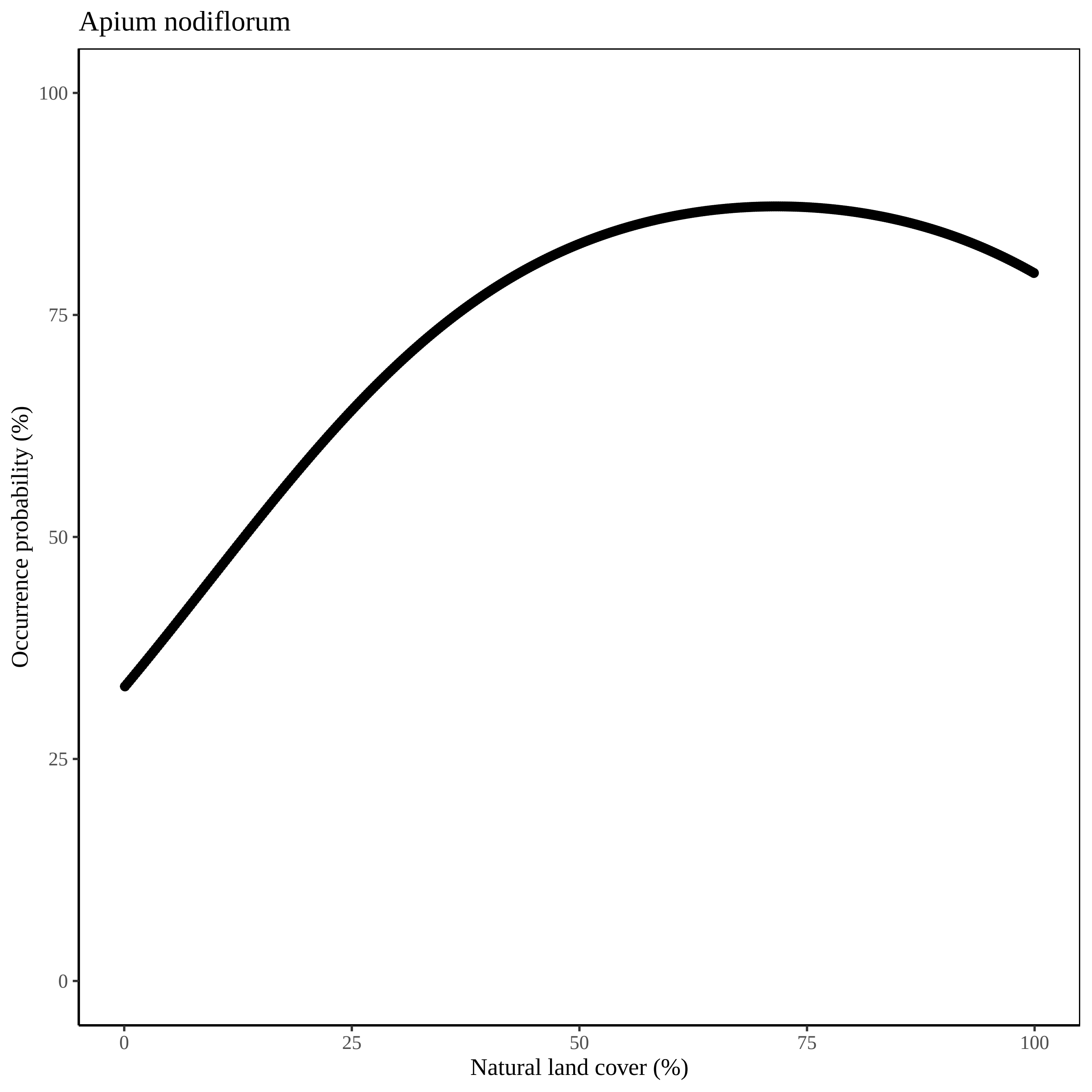

Apium nodiflorum
Occurrence probability (%)
0
25
50
75
100
0
25
50
75
100
Natural land cover (%)

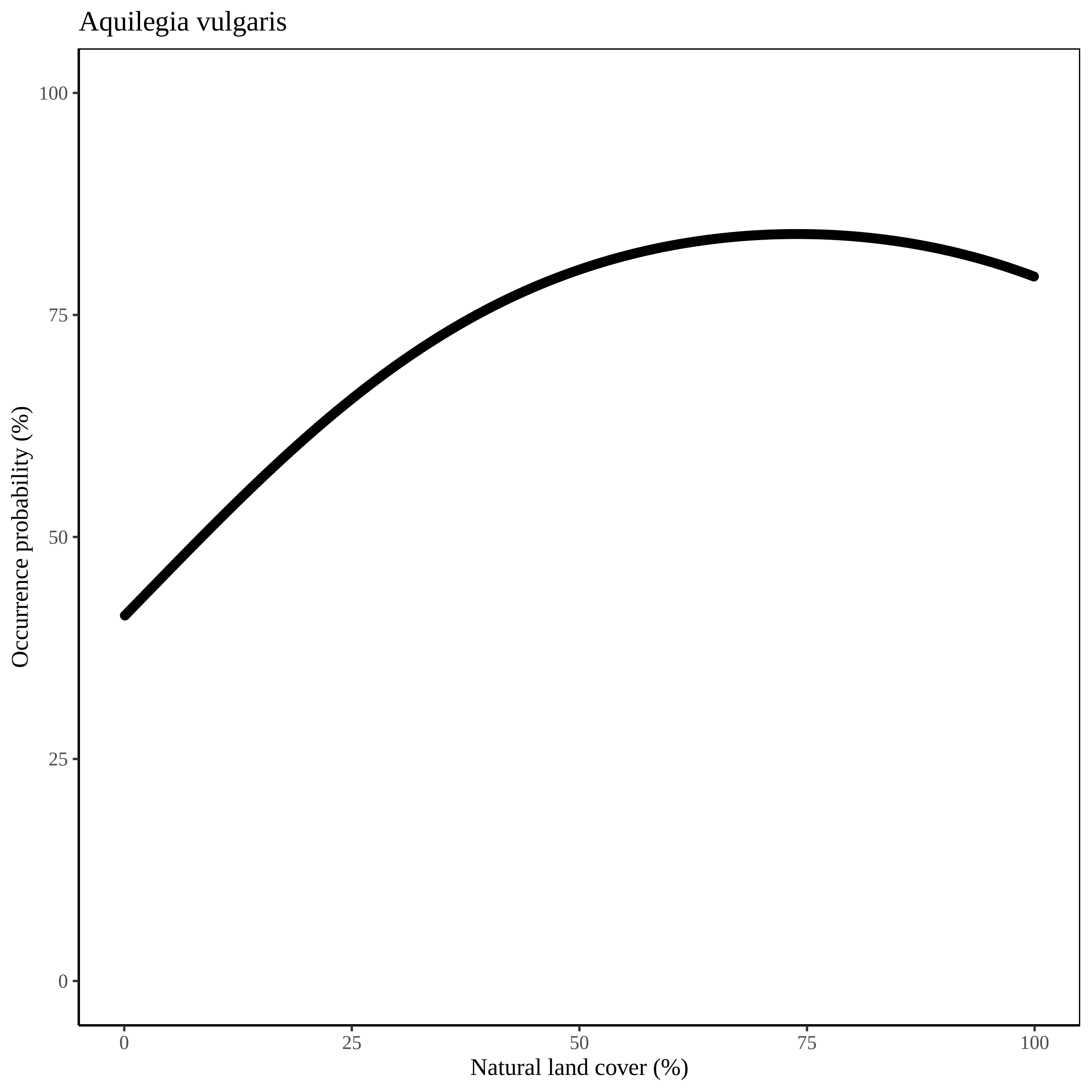

Aquilegia vulgaris
Occurrence probability (%)
Natural land cover (%)
0
25
50
75
100

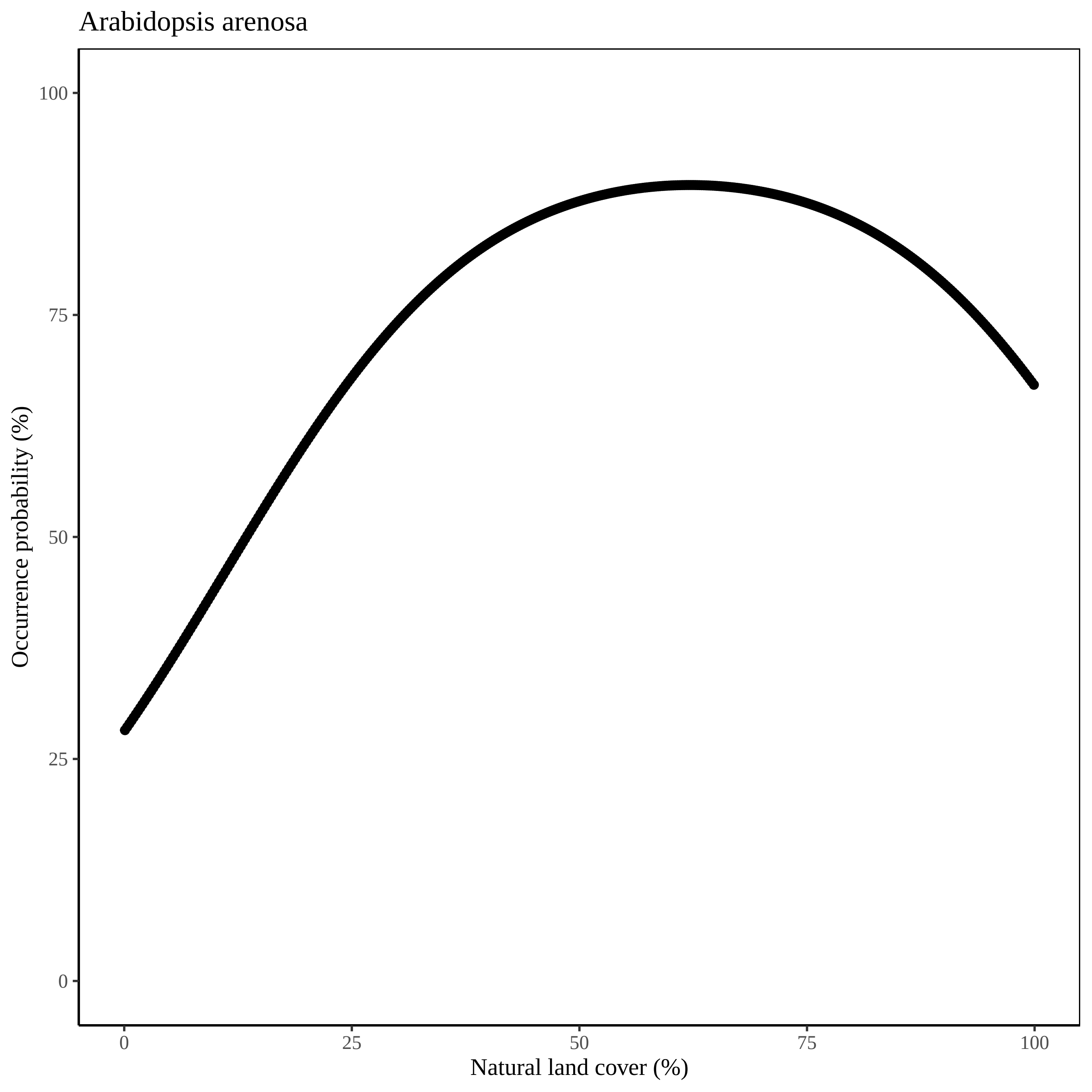

Arabidopsis arenosa
Occurrence probability (%)
100
75
50
25
0
0
25
50
75
100
Natural land cover (%)

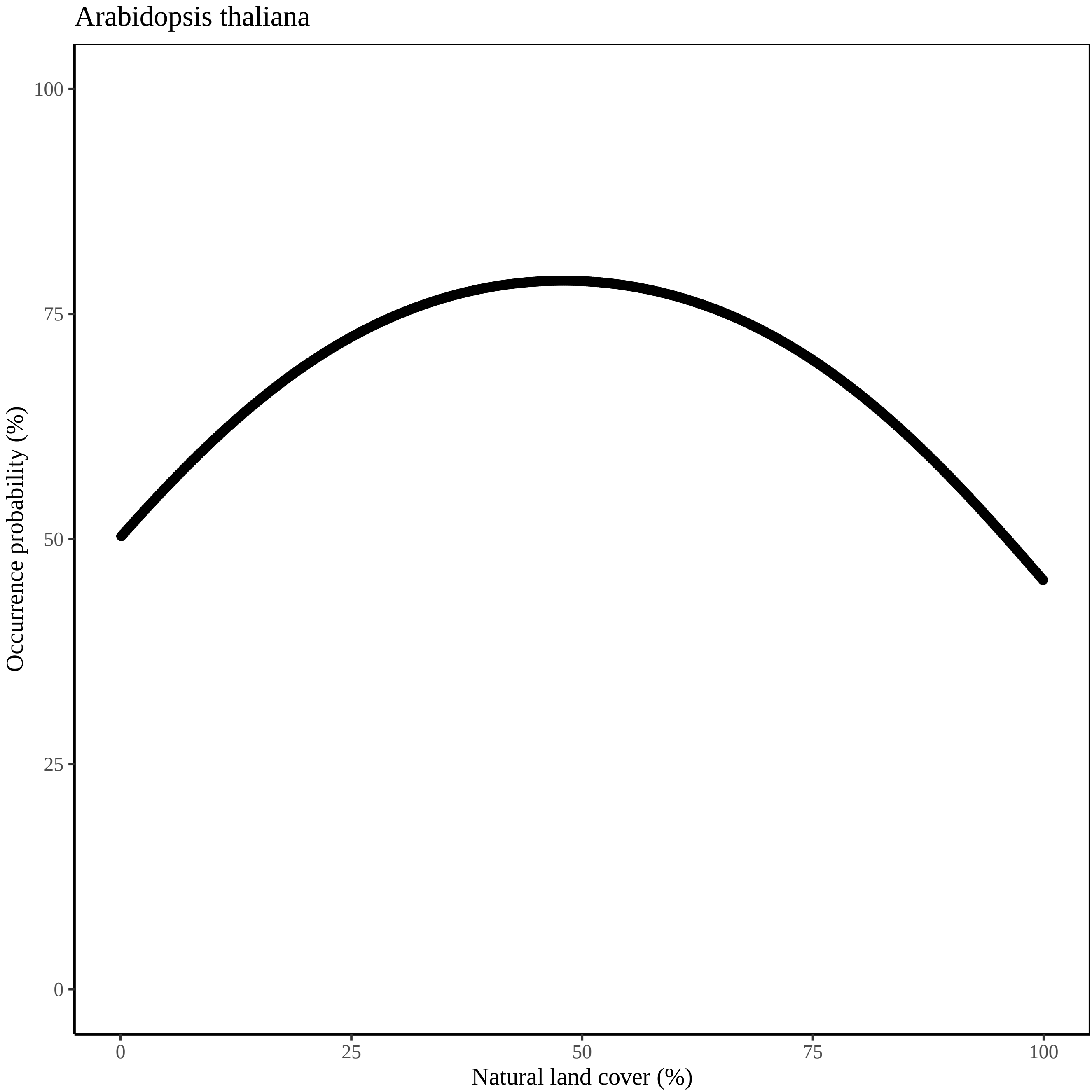
Arabidopsis thaliana
Occurrence probability (%)
100
75
50
25
0
0
25
50
75
100
Natural land cover (%)

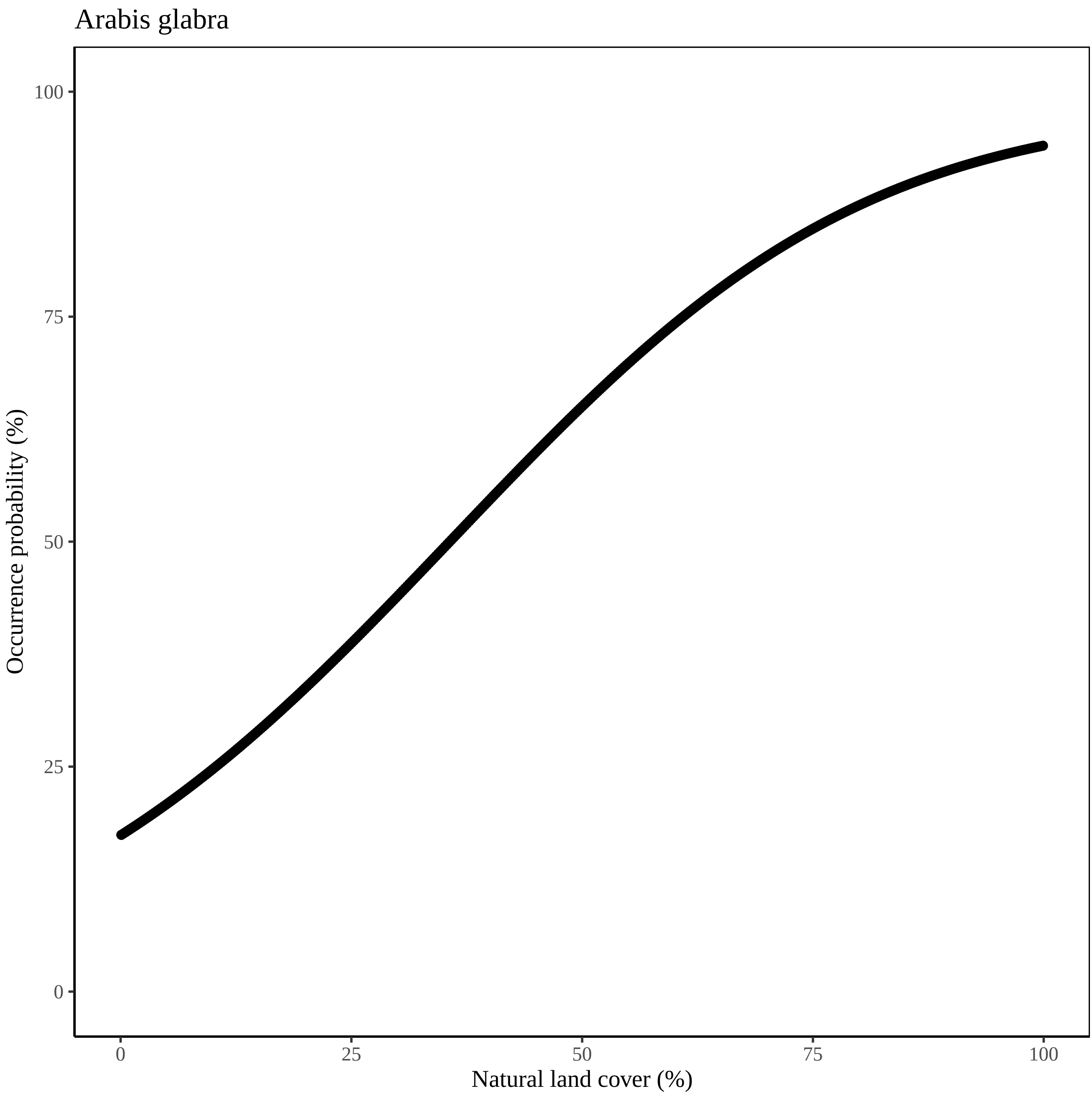
Arabis glabra
Occurrence probability (%)
100
75
50
25
0
0
25
50
75
100
Natural land cover (%)

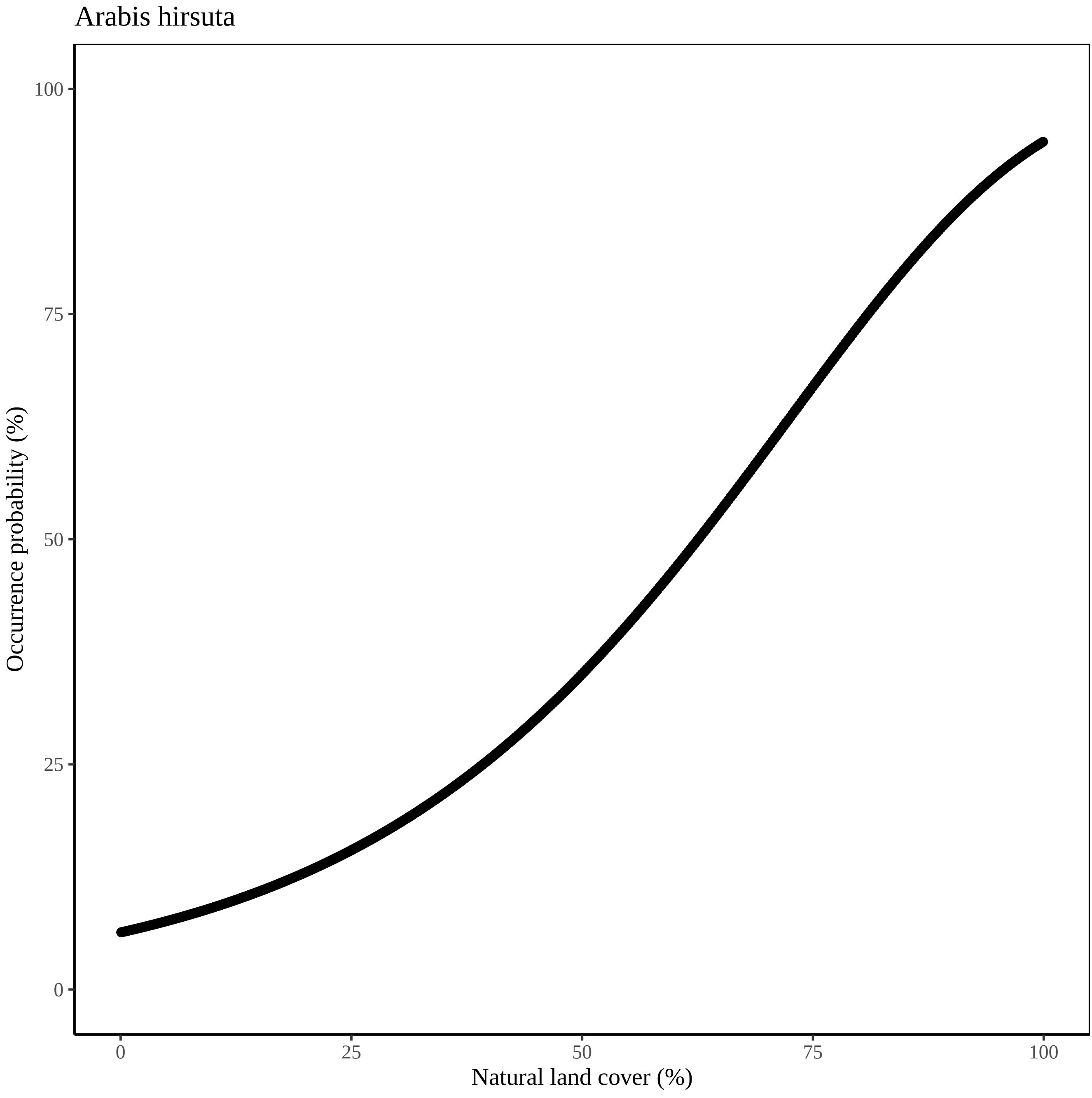

Arabis hirsuta
100
75
50
25
0
Occurrence probability (%)
0
25
50
75
100
Natural land cover (%)

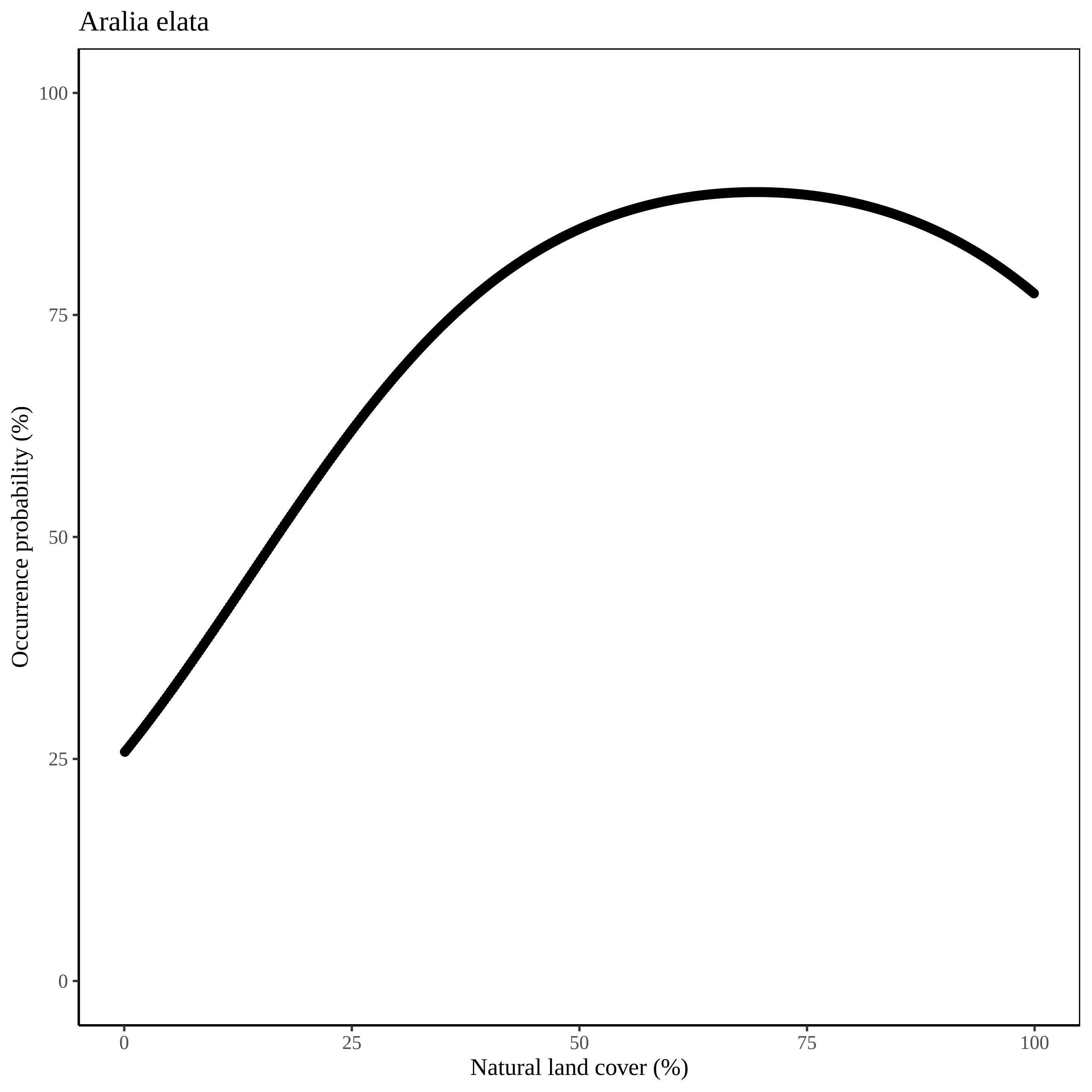

Aralia elata
Occurrence probability (%)
100
75
50
25
0
0
25
50
75
100
Natural land cover (%)

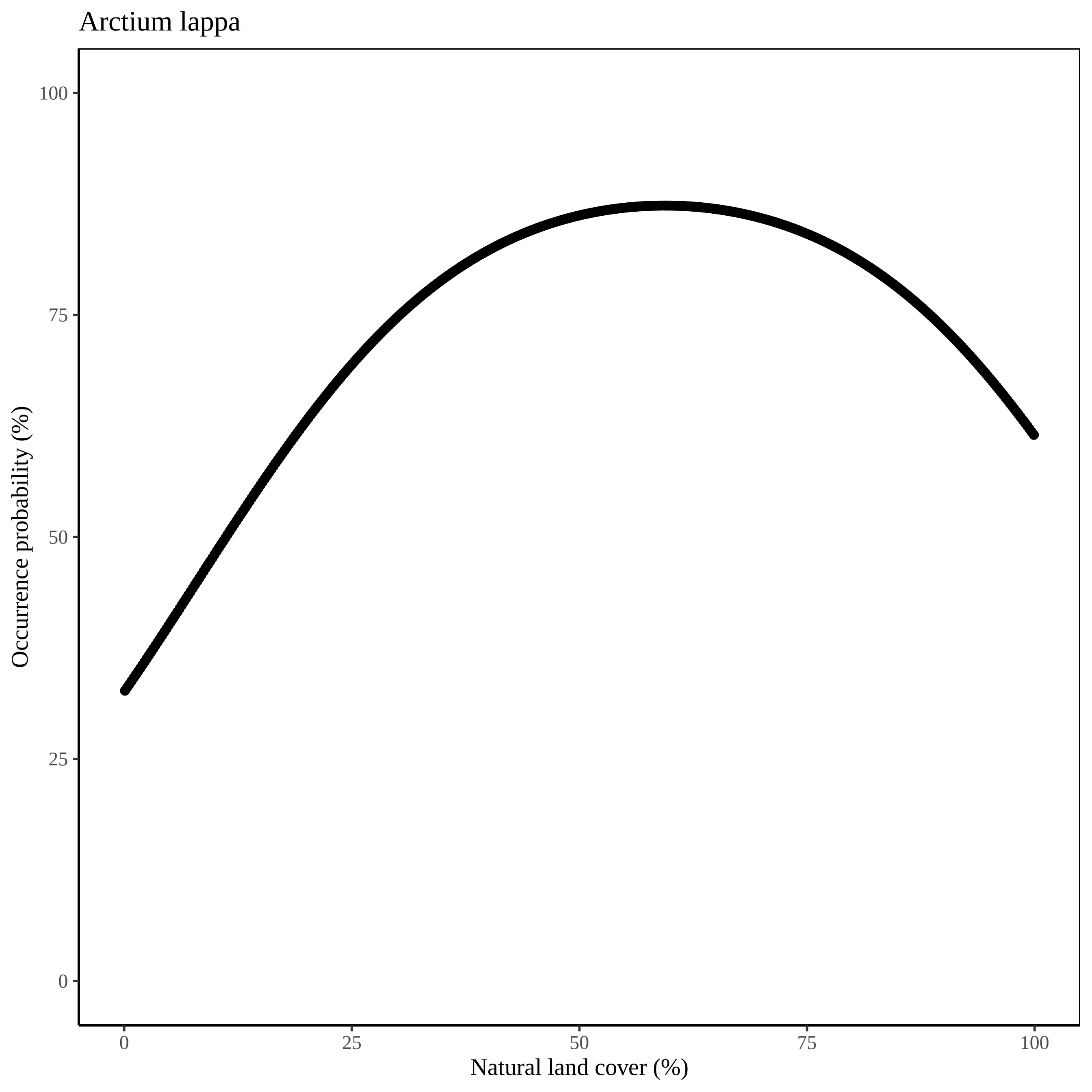
Arctium lappa
Occurrence probability (%)
100
75
50
25
0
0
25
50
75
100
Natural land cover (%)

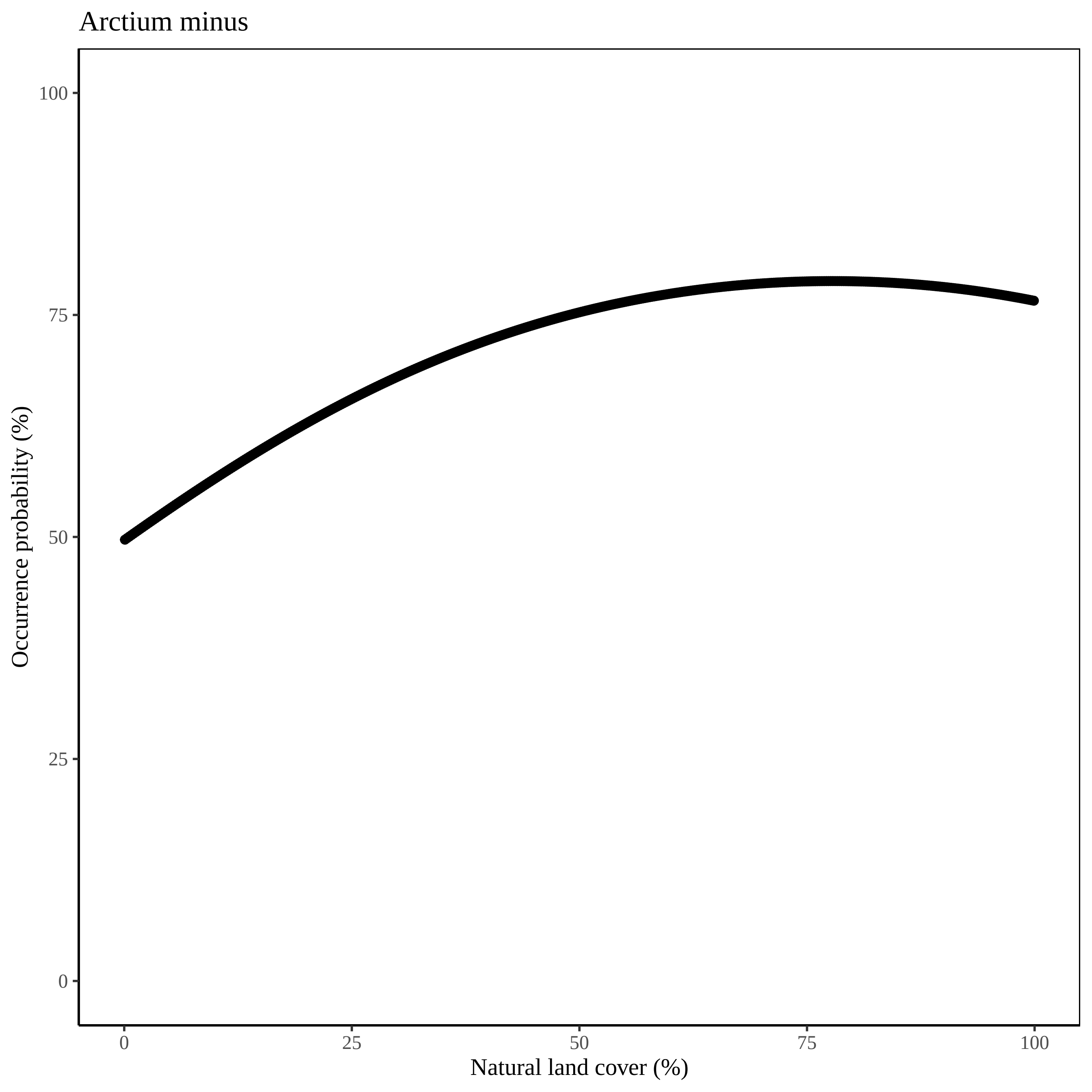
Arctium minus
Occurrence probability (%)
Natural land cover (%)
0
25
50
75
100
0
25
50
75
100

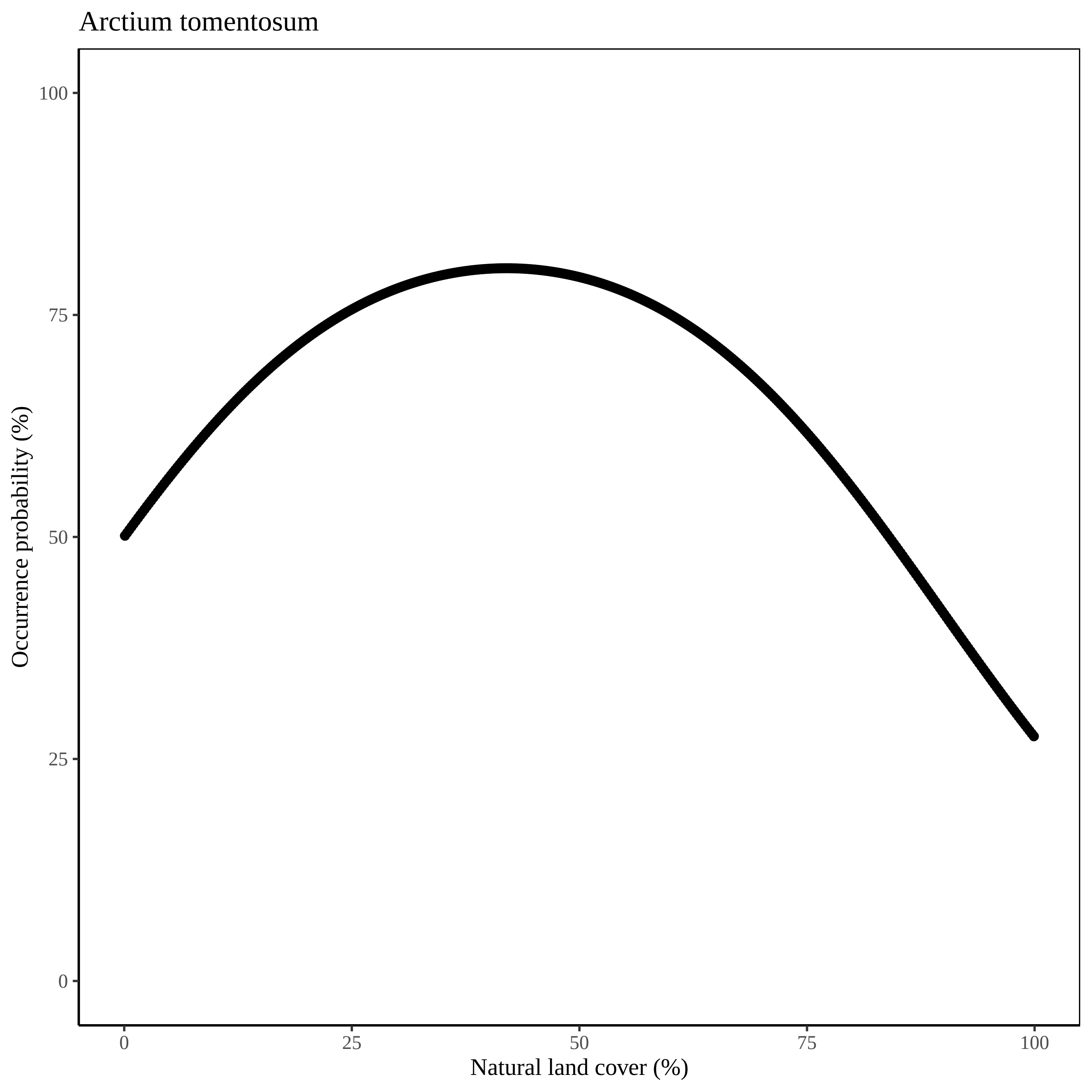

Arctium tomentosum
Occurrence probability (%)
100
75
50
25
0
0
25
50
75
100
Natural land cover (%)

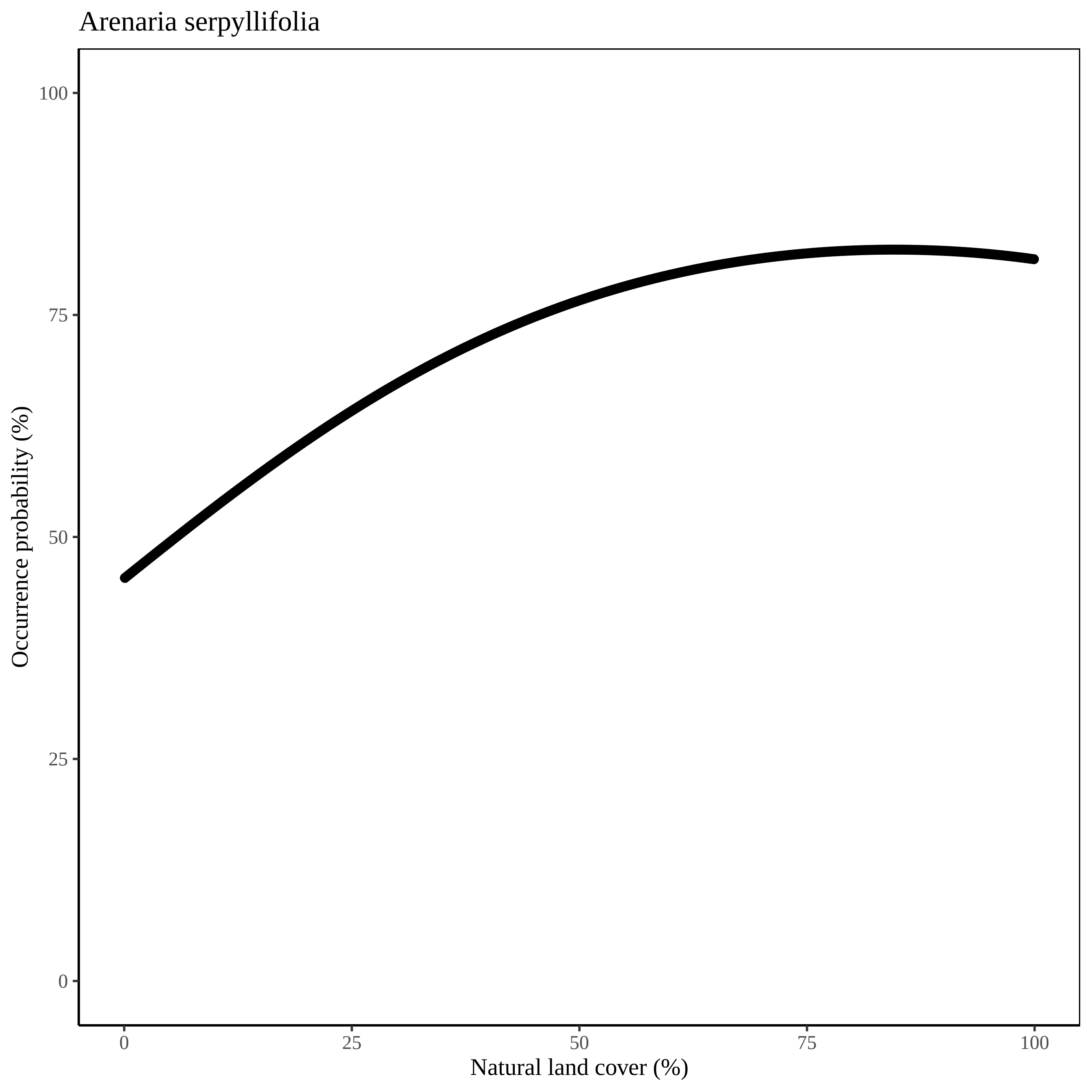
Arenaria serpyllifolia
Occurrence probability (%)
100
75
50
25
0
0
25
50
75
100
Natural land cover (%)

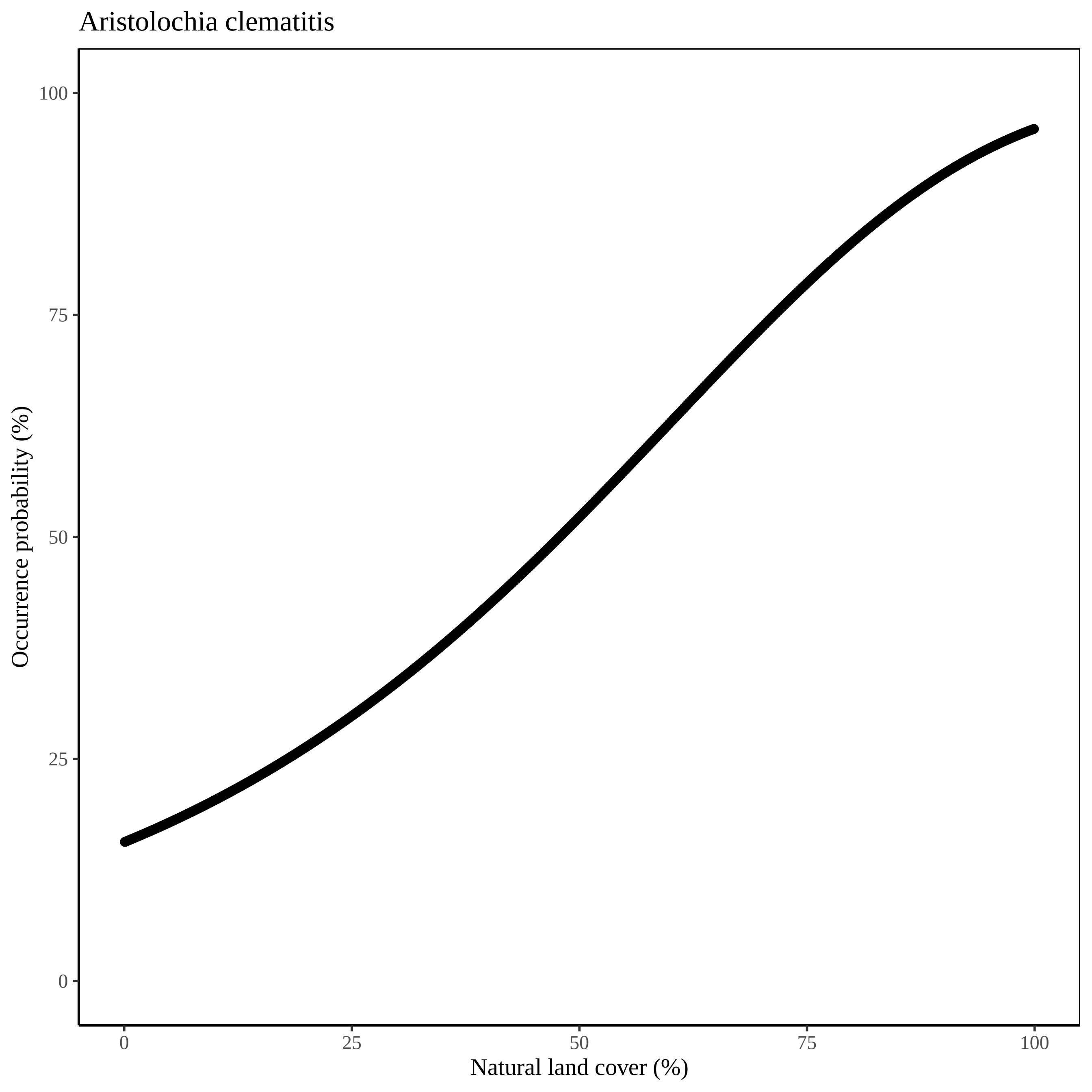

Aristolochia clematitis
Occurrence probability (%)
100
75
50
25
0
0
25
50
75
100
Natural land cover (%)

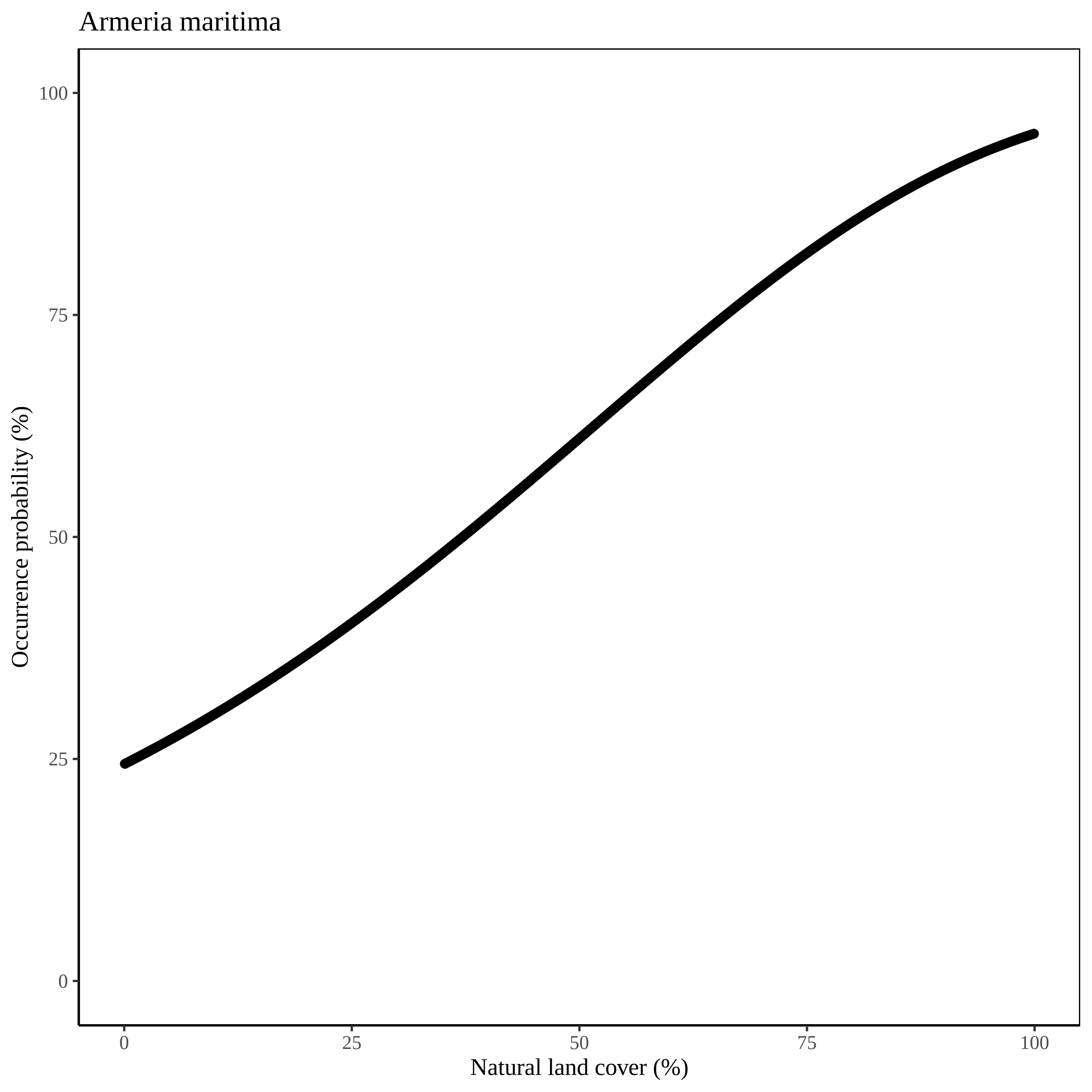

Armeria maritima
100
75
50
25
0
0
25
50
75
100
Occurrence probability (%)
Natural land cover (%)

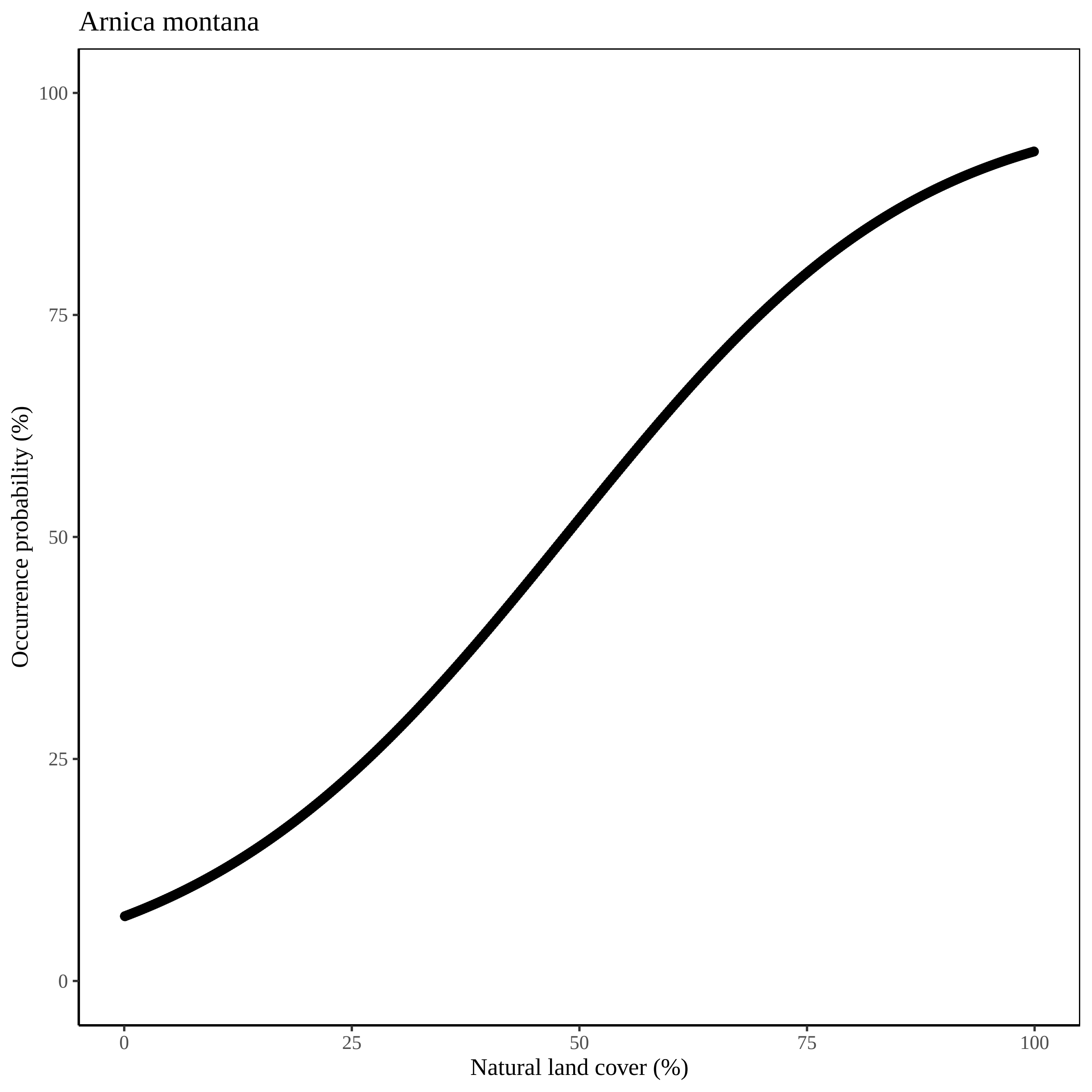

Arnica montana
Occurrence probability (%)
100
75
50
25
0
0
25
50
75
100
Natural land cover (%)

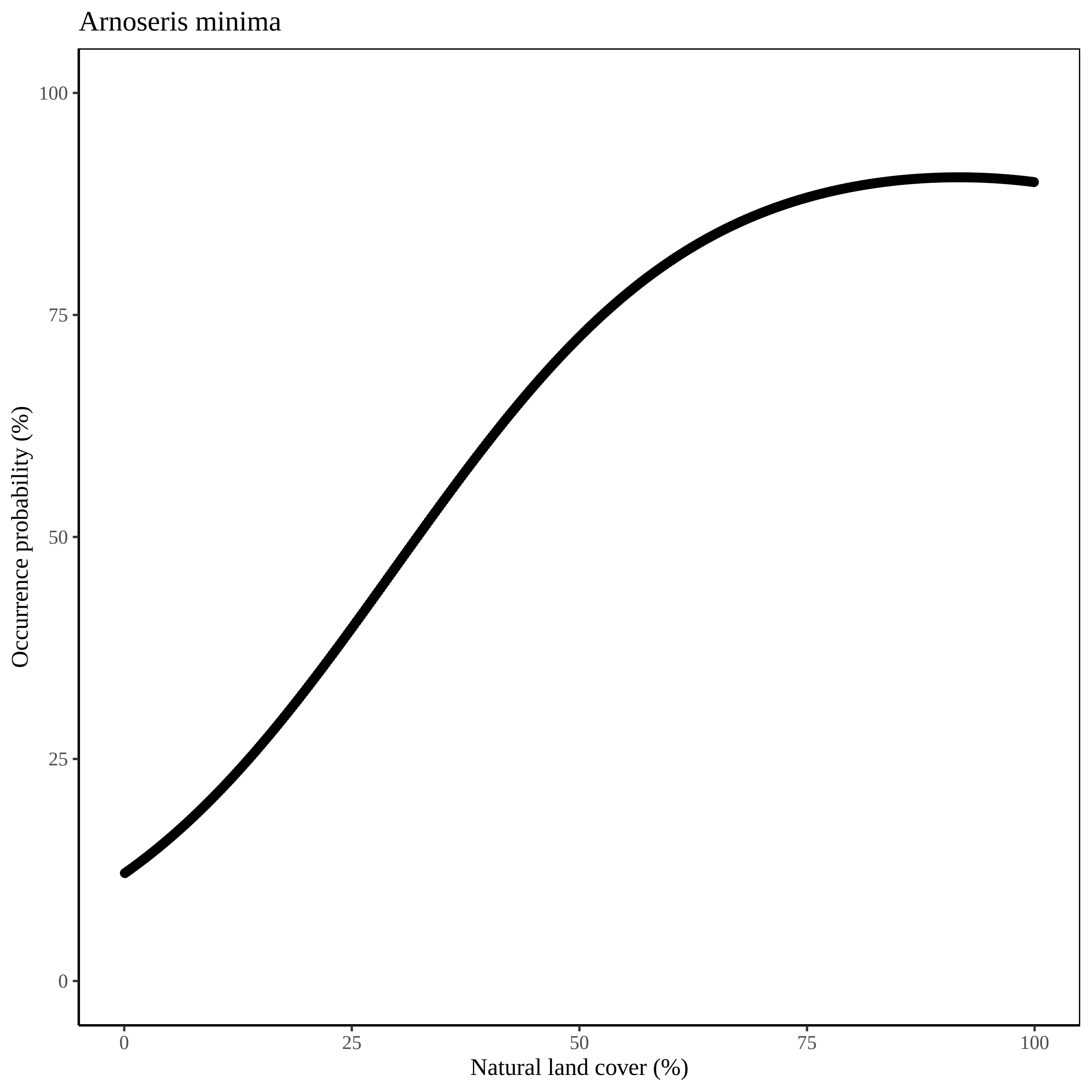

Arnoseris minima
Occurrence probability (%)
100
75
50
25
0
0
25
50
75
100
Natural land cover (%)

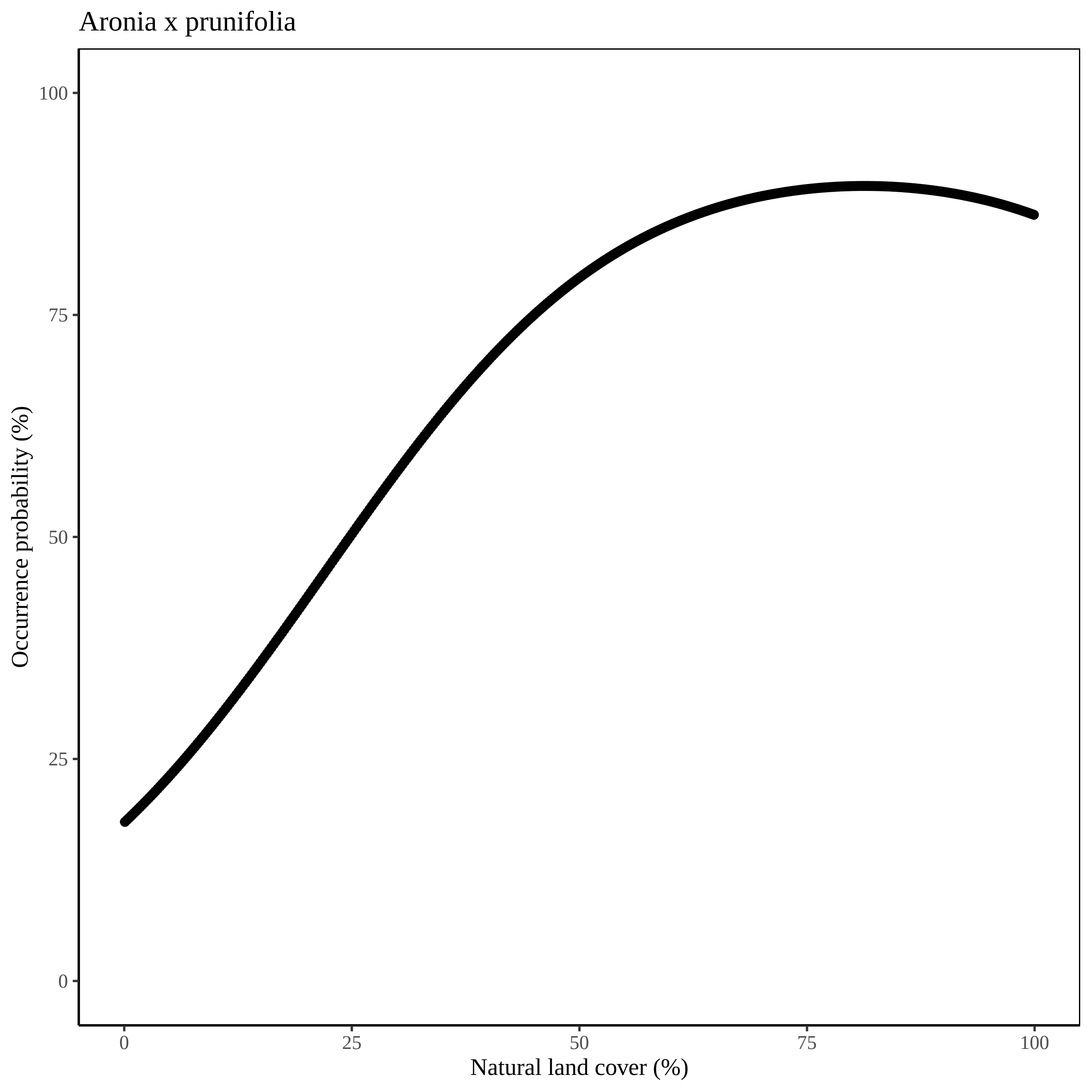
Aronia x prunifolia
100
75
50
25
0
Occurrence probability (%)
0
25
50
75
100
Natural land cover (%)

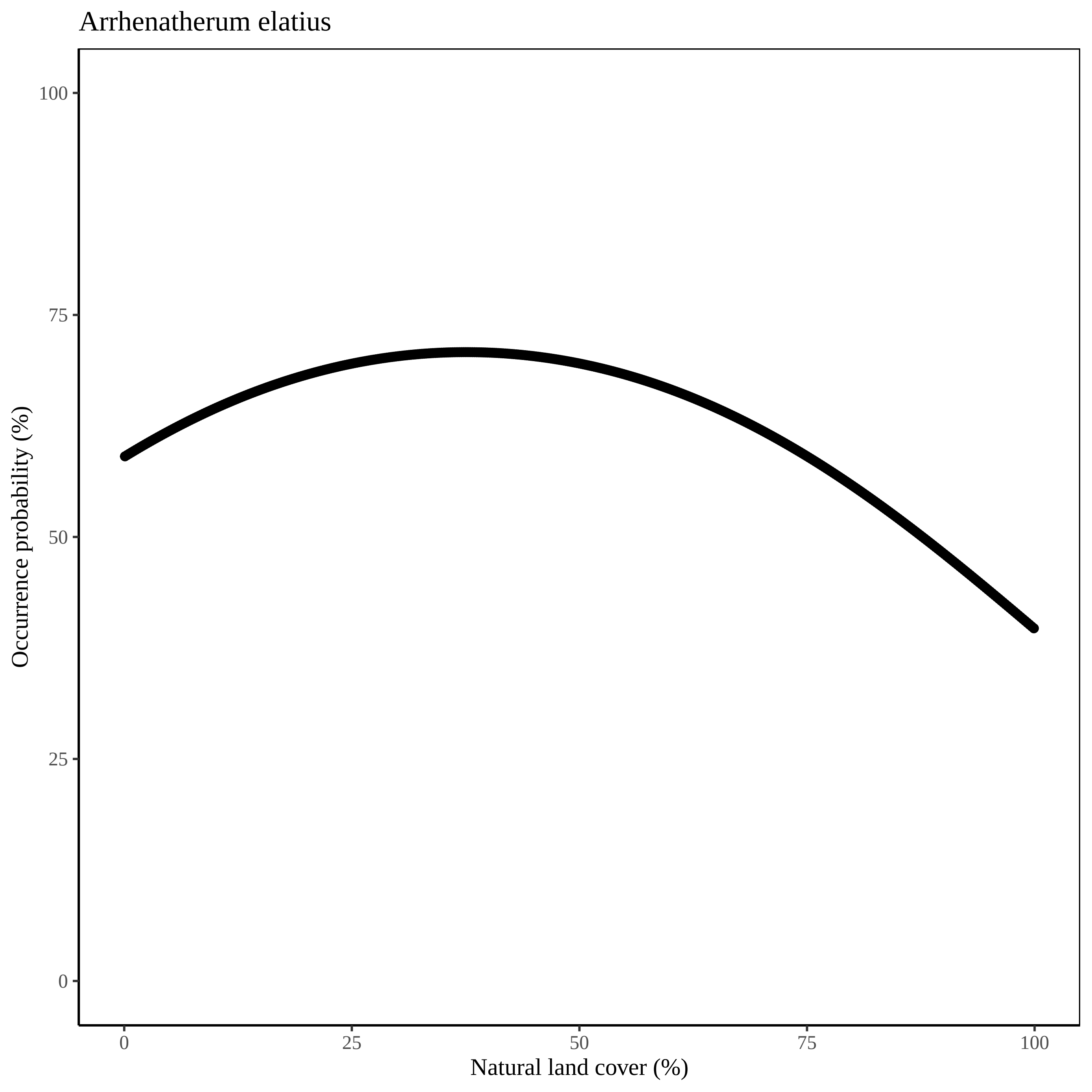
Arrhenatherum elatius
100
75
50
25
0
Occurrence probability (%)
0
25
50
75
100
Natural land cover (%)

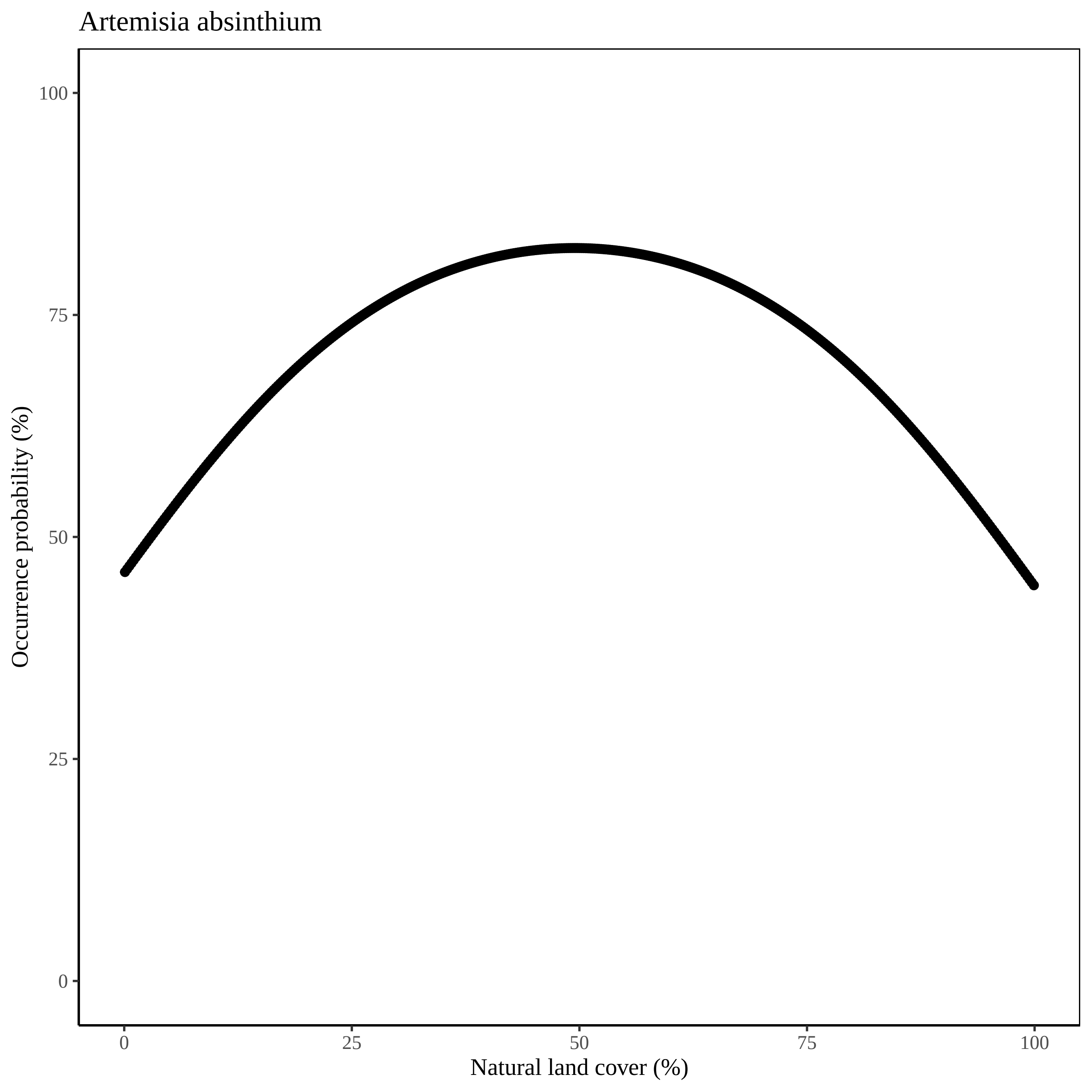

Artemisia absinthium
100
75
50
25
0
Occurrence probability (%)
0
25
50
75
100
Natural land cover (%)

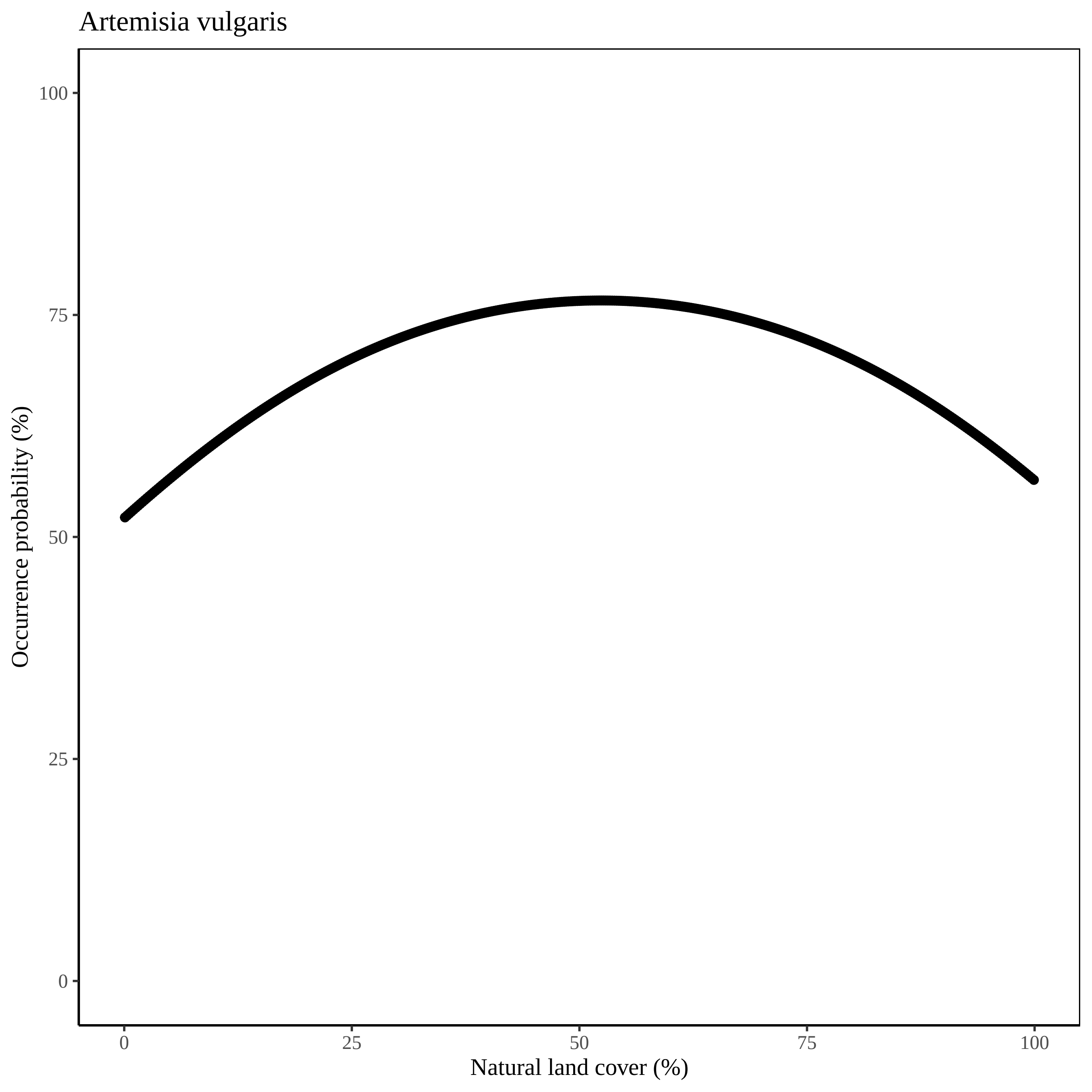

Artemisia vulgaris
100
75
50
25
0
Occurrence probability (%)
0
25
50
75
100
Natural land cover (%)

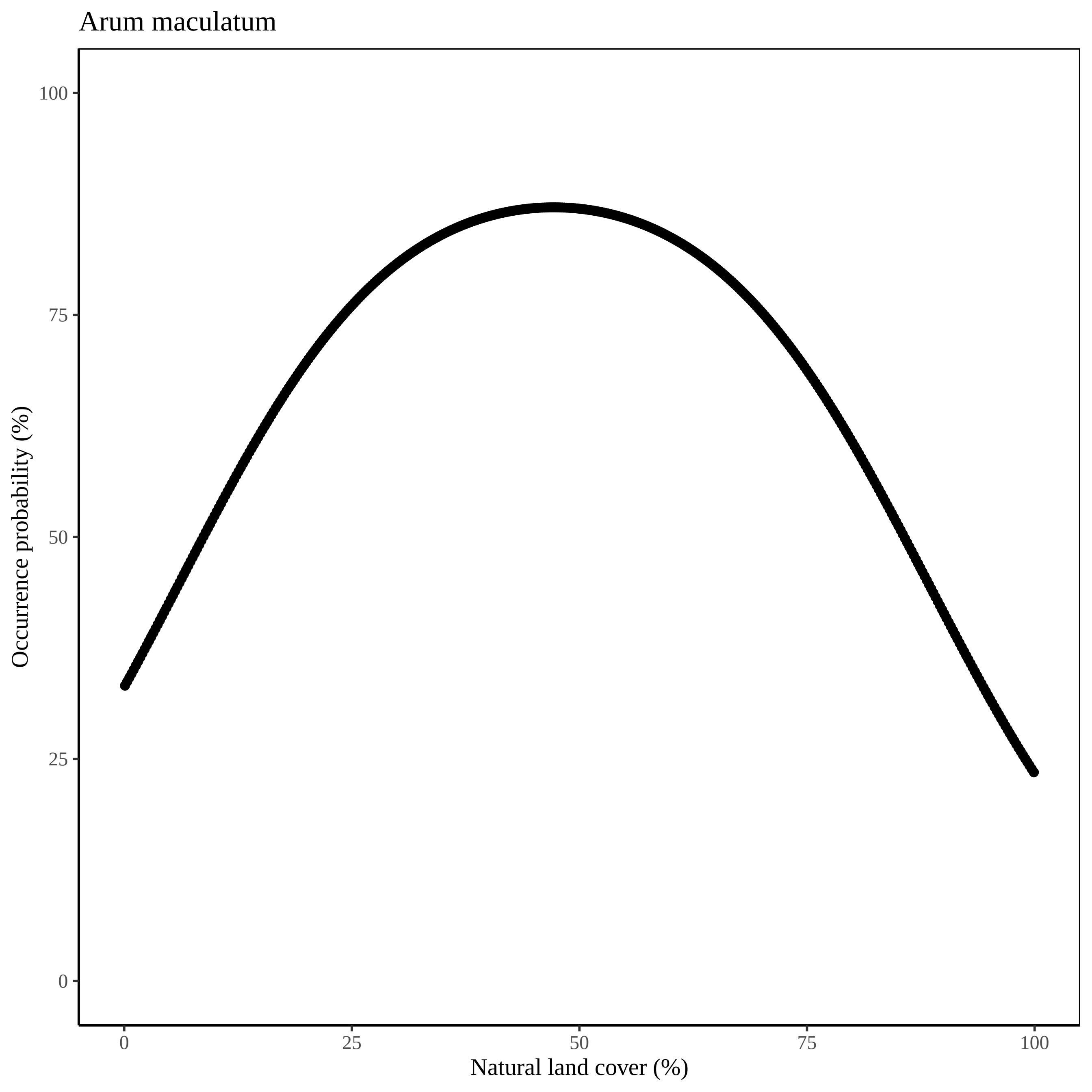

Arum maculatum
Occurrence probability (%)
100
75
50
25
0
0
25
50
75
100
Natural land cover (%)

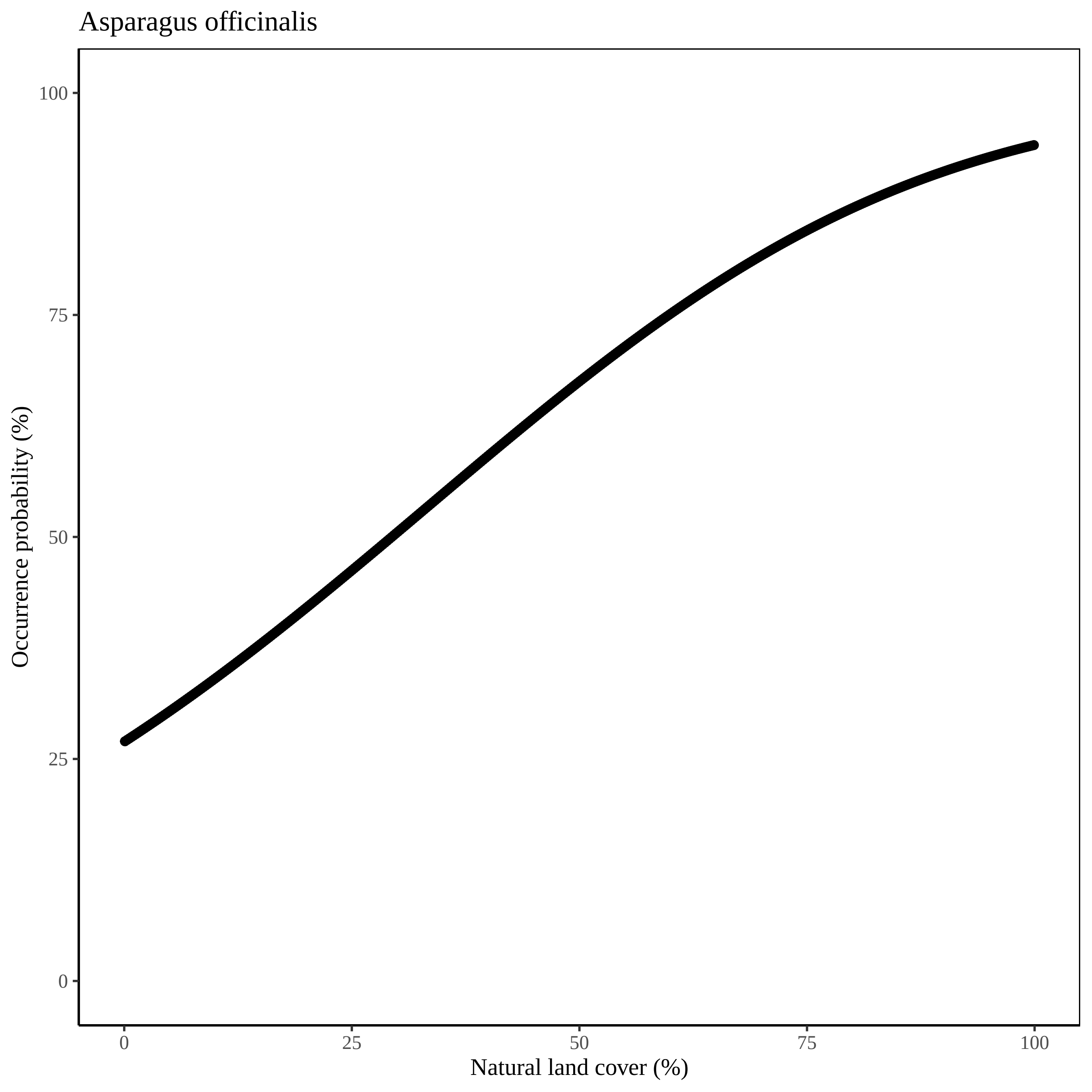

Asparagus officinalis
100
75
50
25
0
Occurrence probability (%)
0
25
50
75
100
Natural land cover (%)

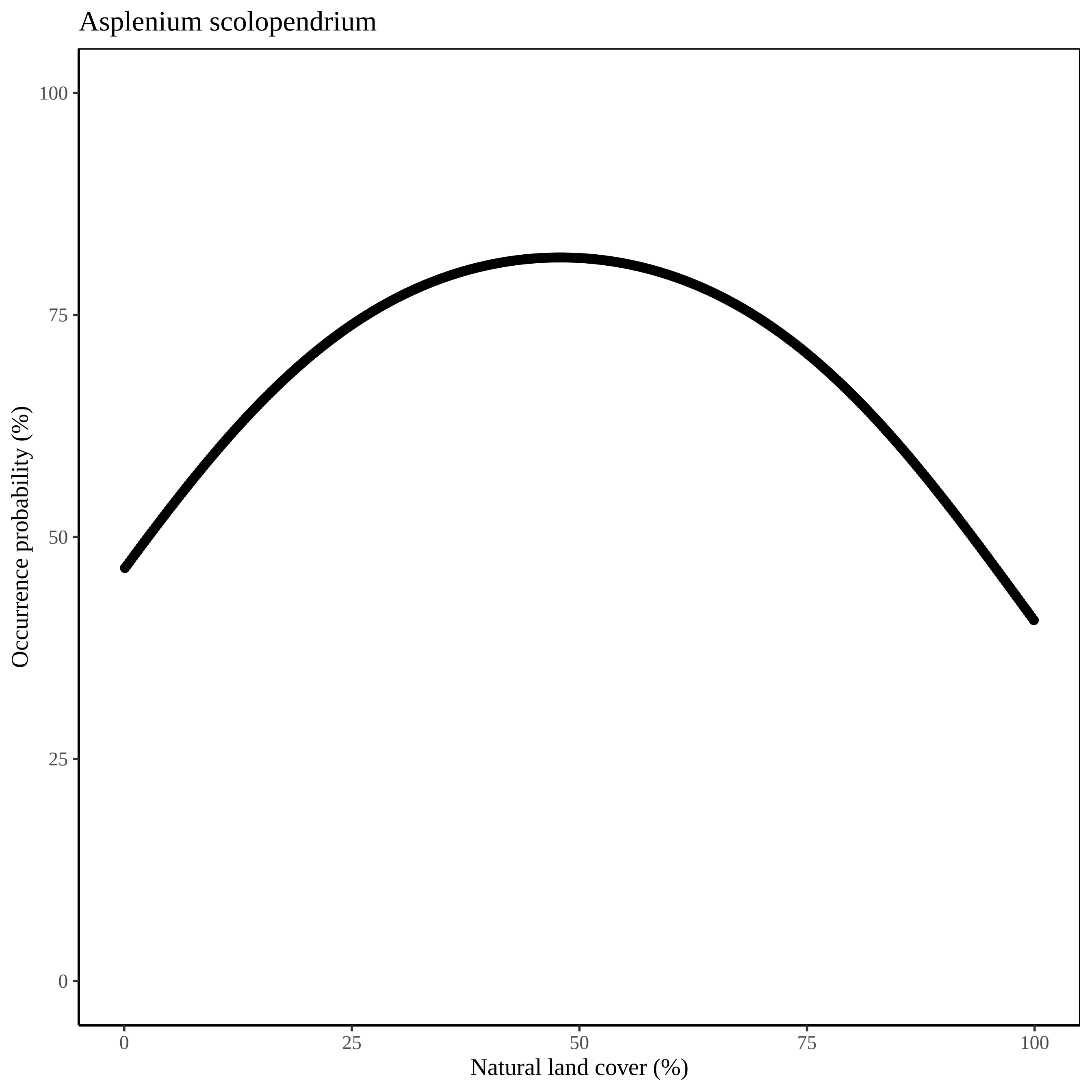
Asplenium scolopendrium
Occurrence probability (%)
100
75
50
25
0
0
25
50
75
100
Natural land cover (%)

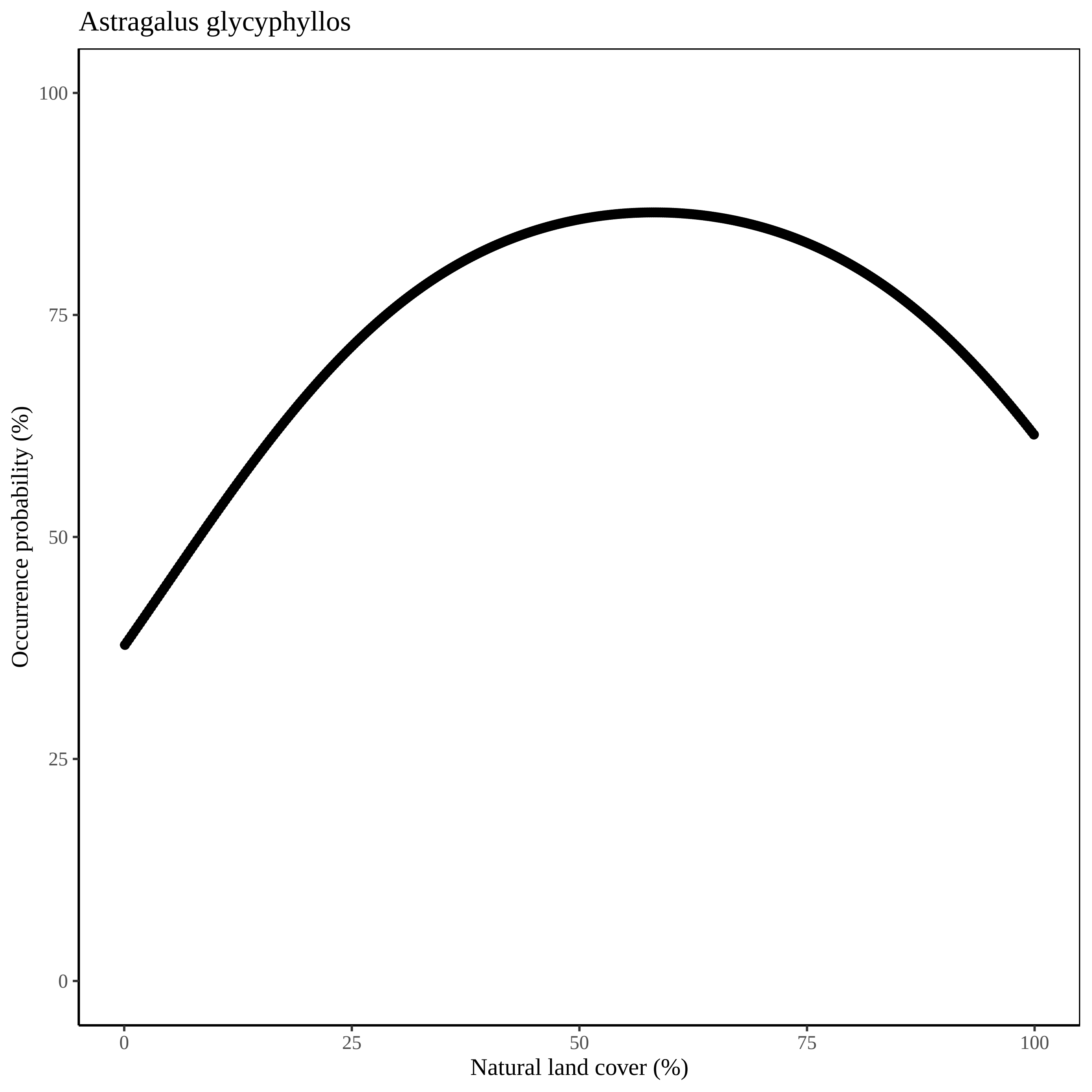

Astragalus glycyphyllos
Occurrence probability (%)
100
75
50
25
0
0
25
50
75
100
Natural land cover (%)

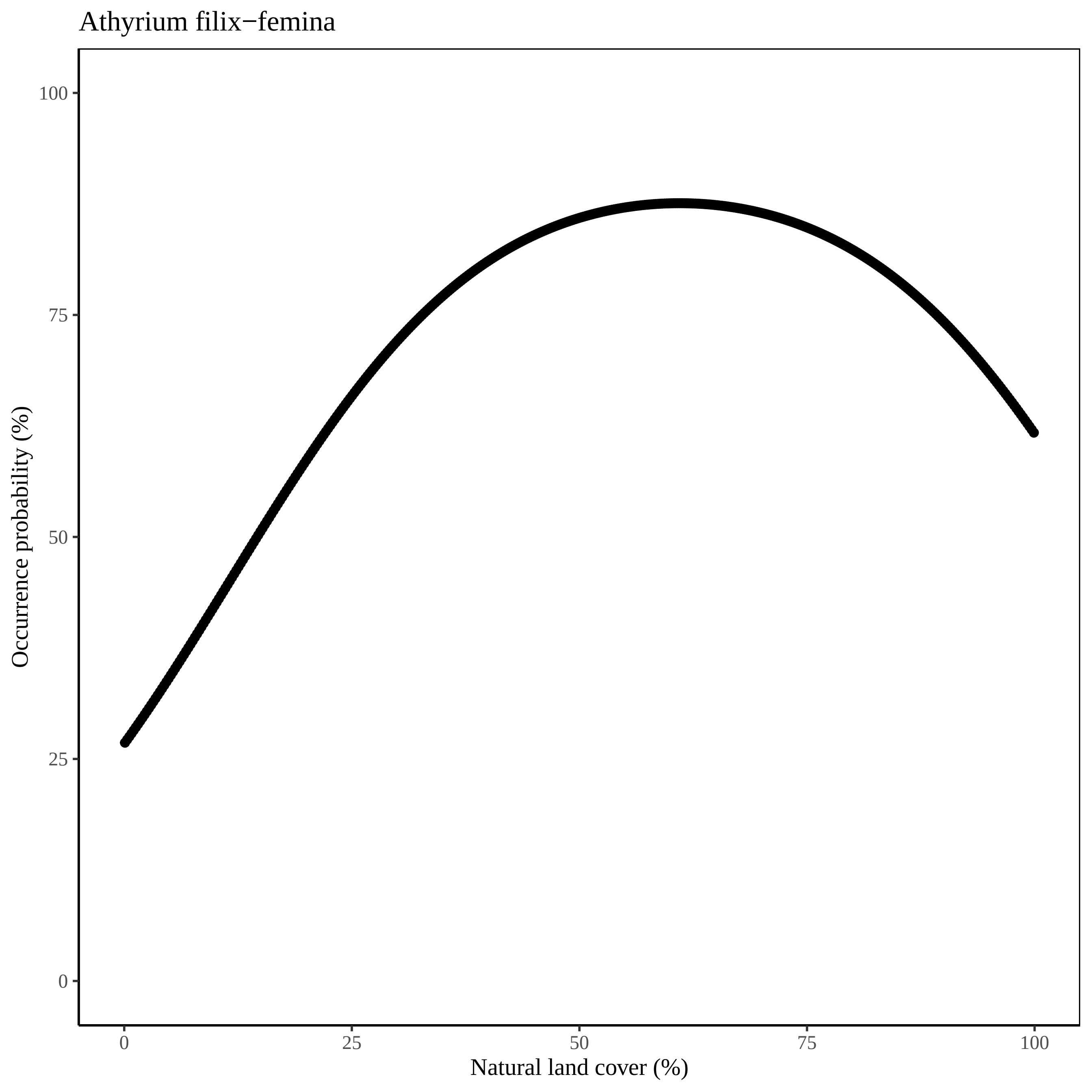
Athyrium filix−femina
Occurrence probability (%)
100
75
50
25
0
0
25
50
75
100
Natural land cover (%)

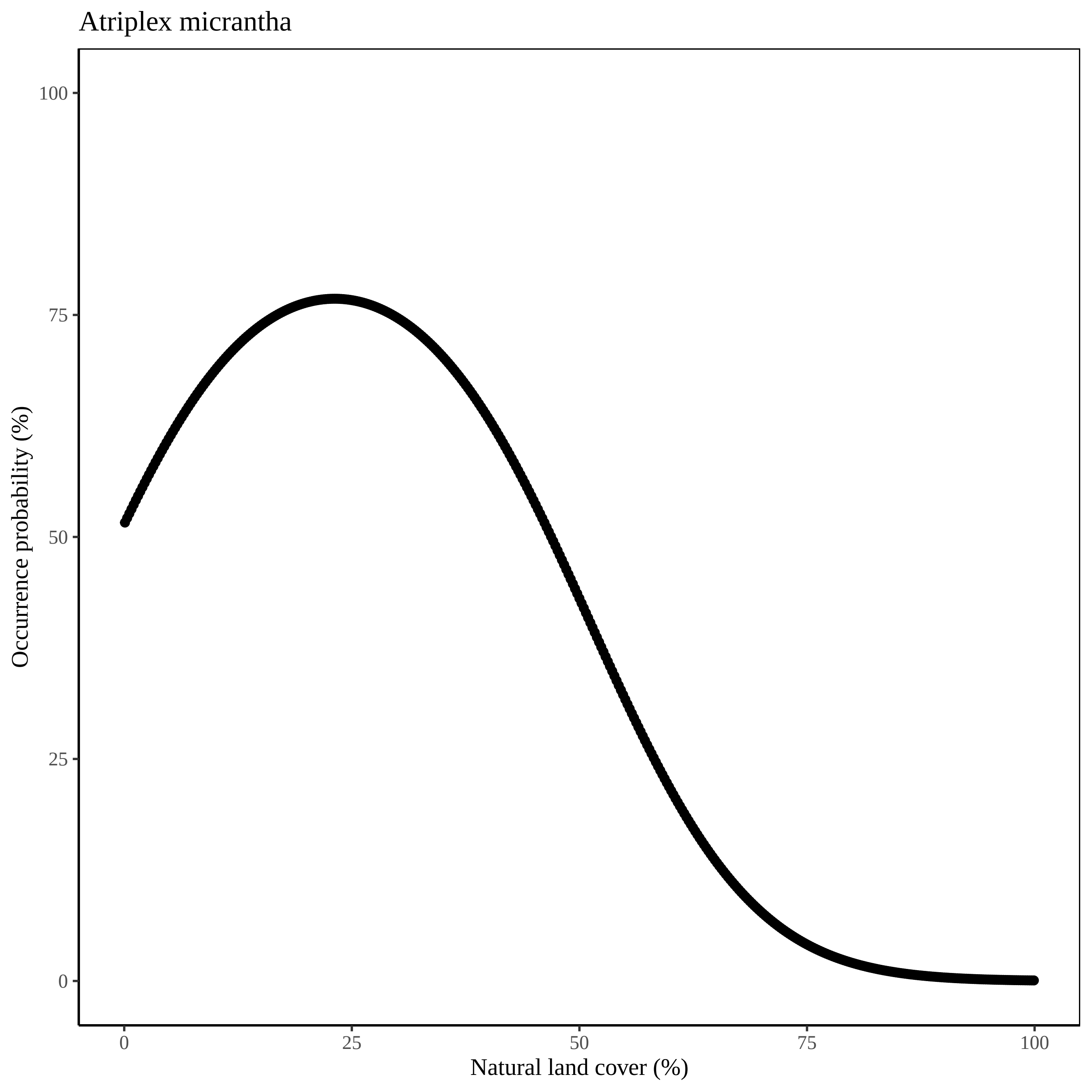

Atriplex micrantha
Occurrence probability (%)
100
75
50
25
0
0
25
50
75
100
Natural land cover (%)

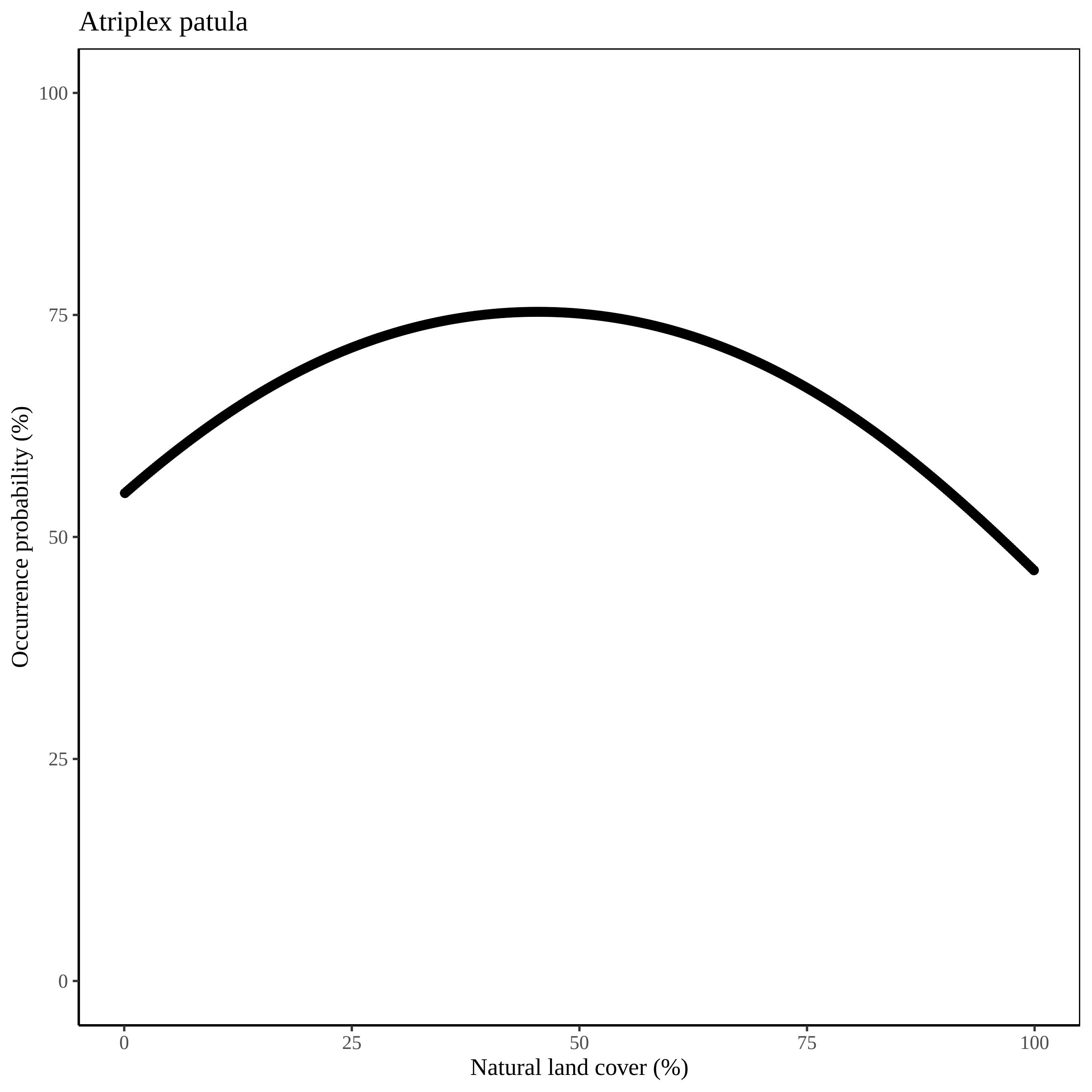

Atriplex patula
100
75
50
25
0
Occurrence probability (%)
0
25
50
75
100
Natural land cover (%)

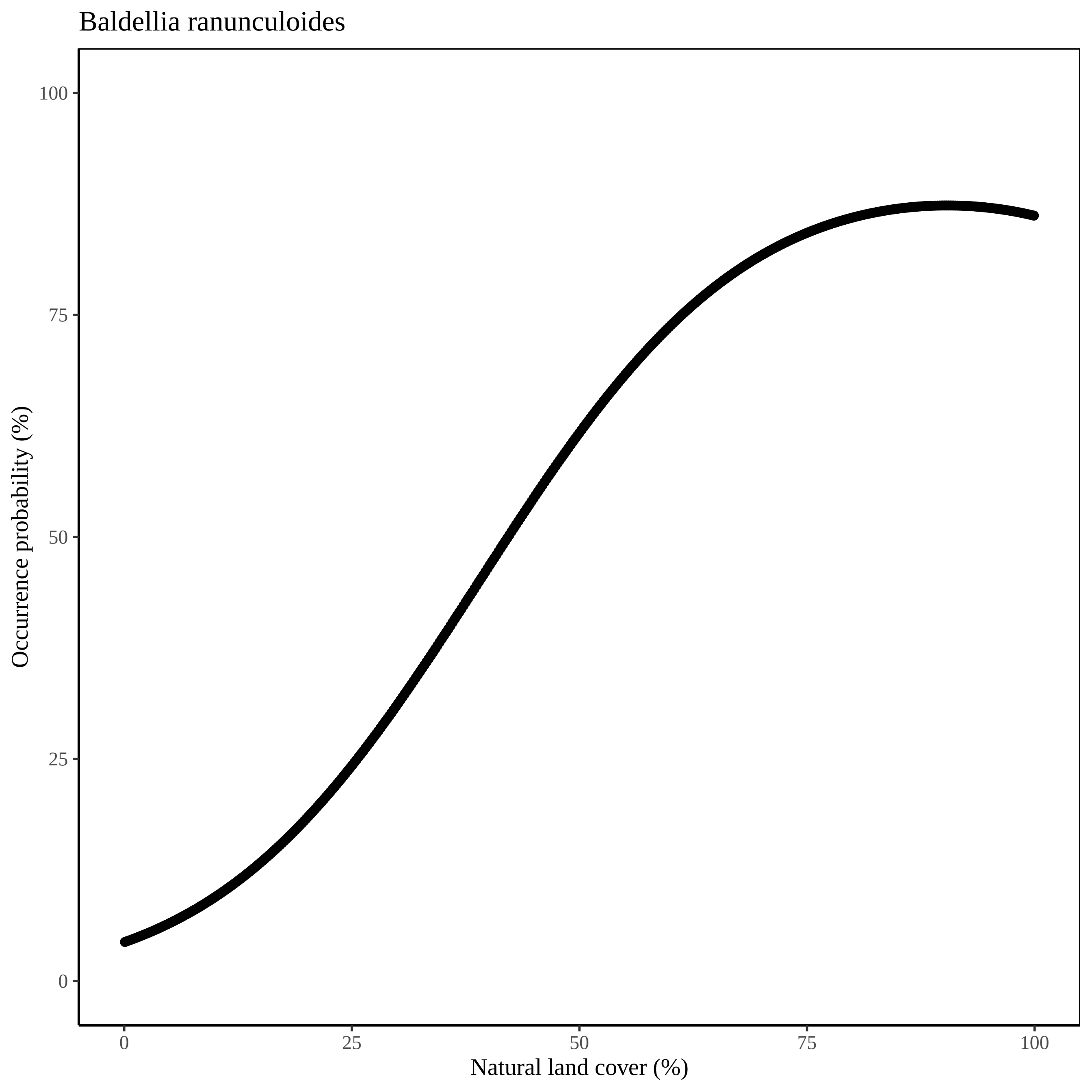
Baldellia ranunculoides
100
75
50
25
0
Occurrence probability (%)
0
25
50
75
100
Natural land cover (%)

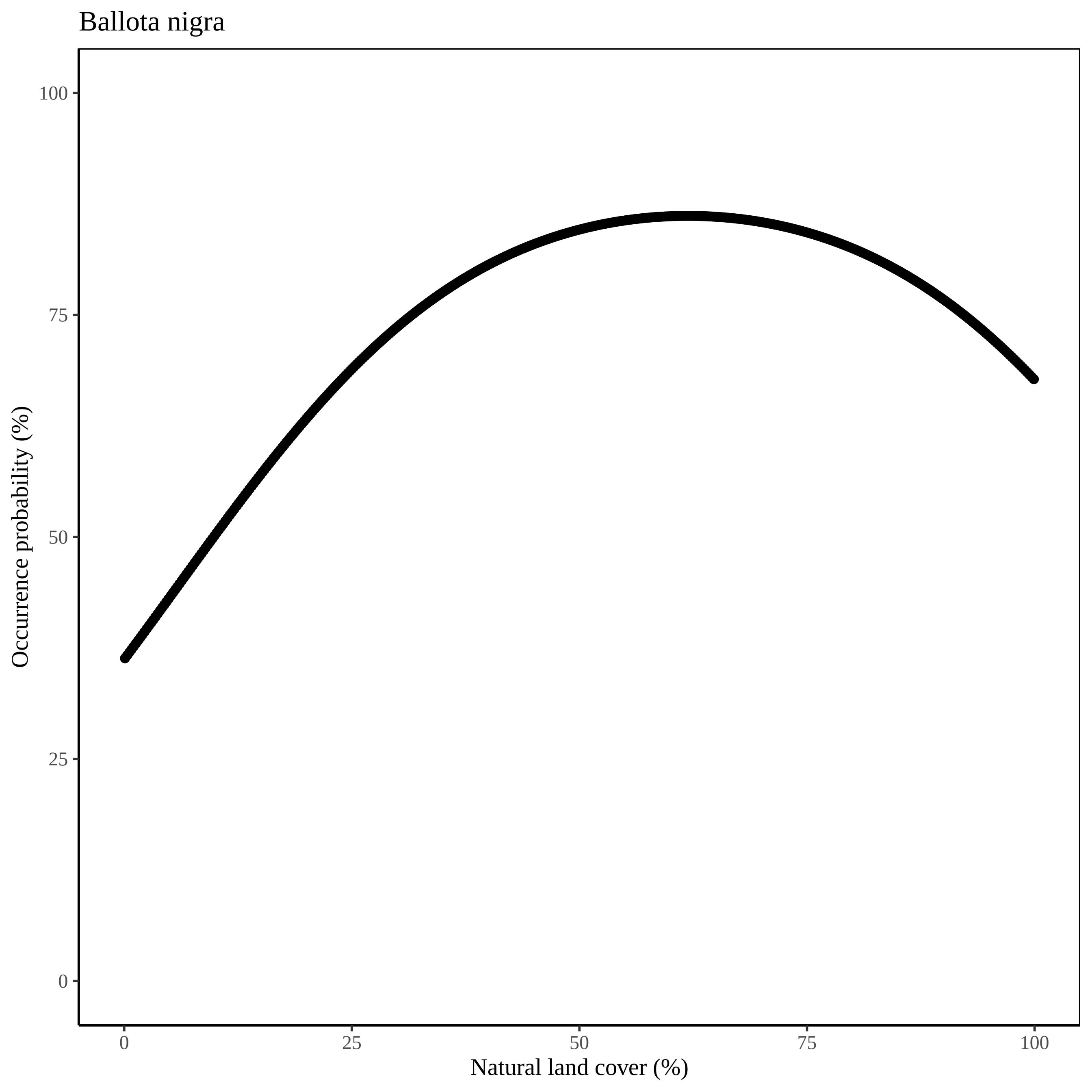
Ballota nigra
Occurrence probability (%)
100
75
50
25
0
0
25
50
75
100
Natural land cover (%)

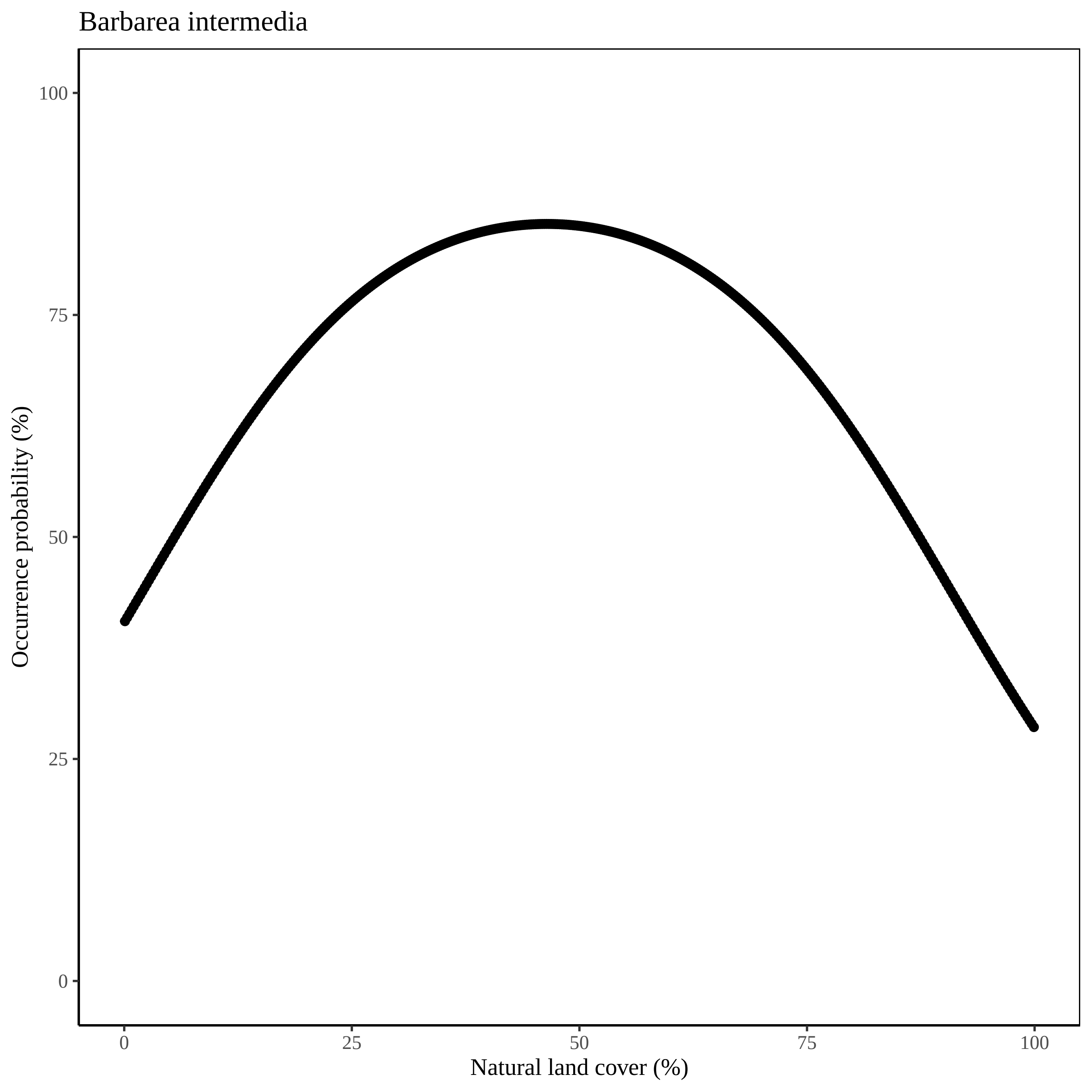
Barbarea intermedia
Occurrence probability (%)
100
75
50
25
0
0
25
50
75
100
Natural land cover (%)

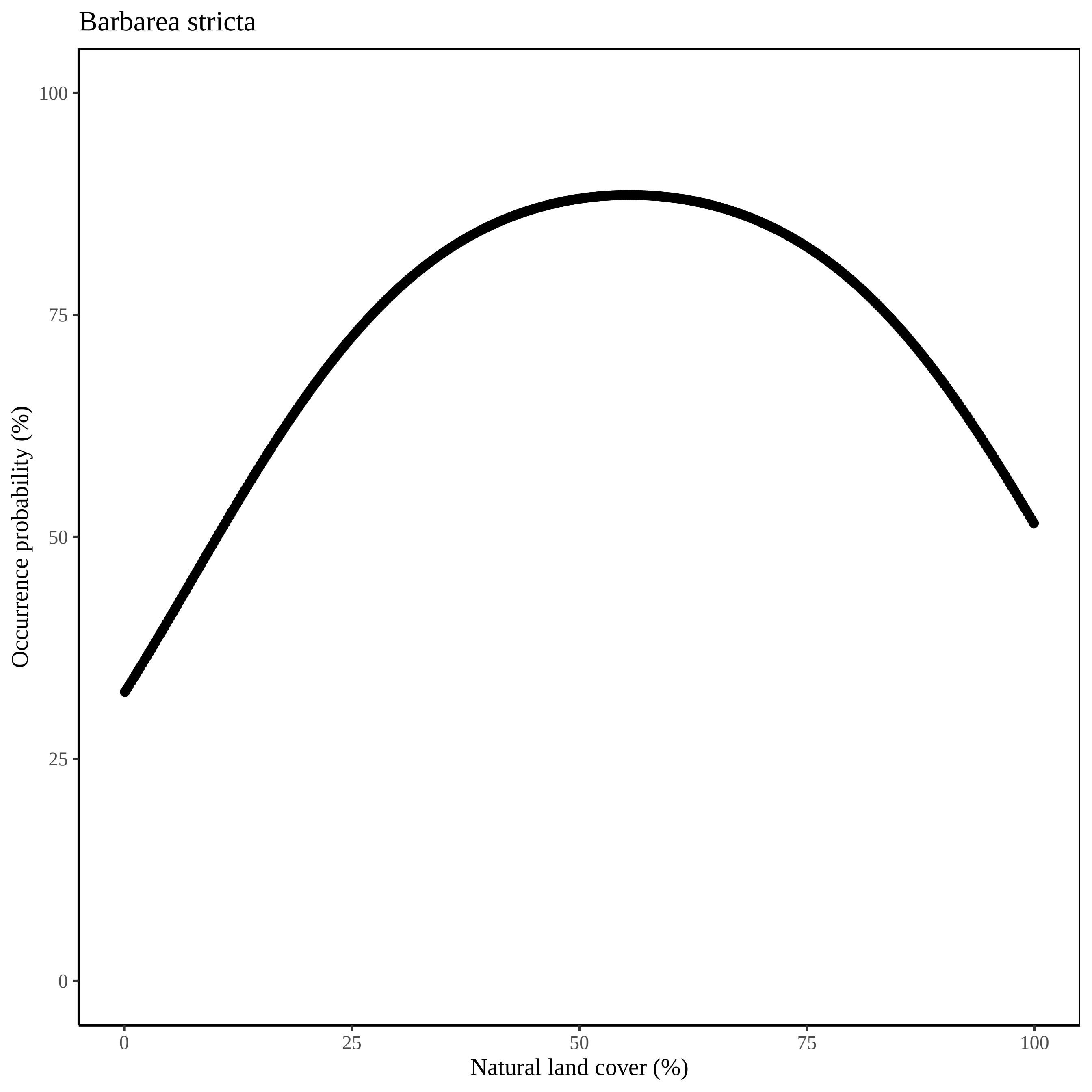

Barbarea stricta
100
75
50
25
0
Occurrence probability (%)
0
25
50
75
100
Natural land cover (%)

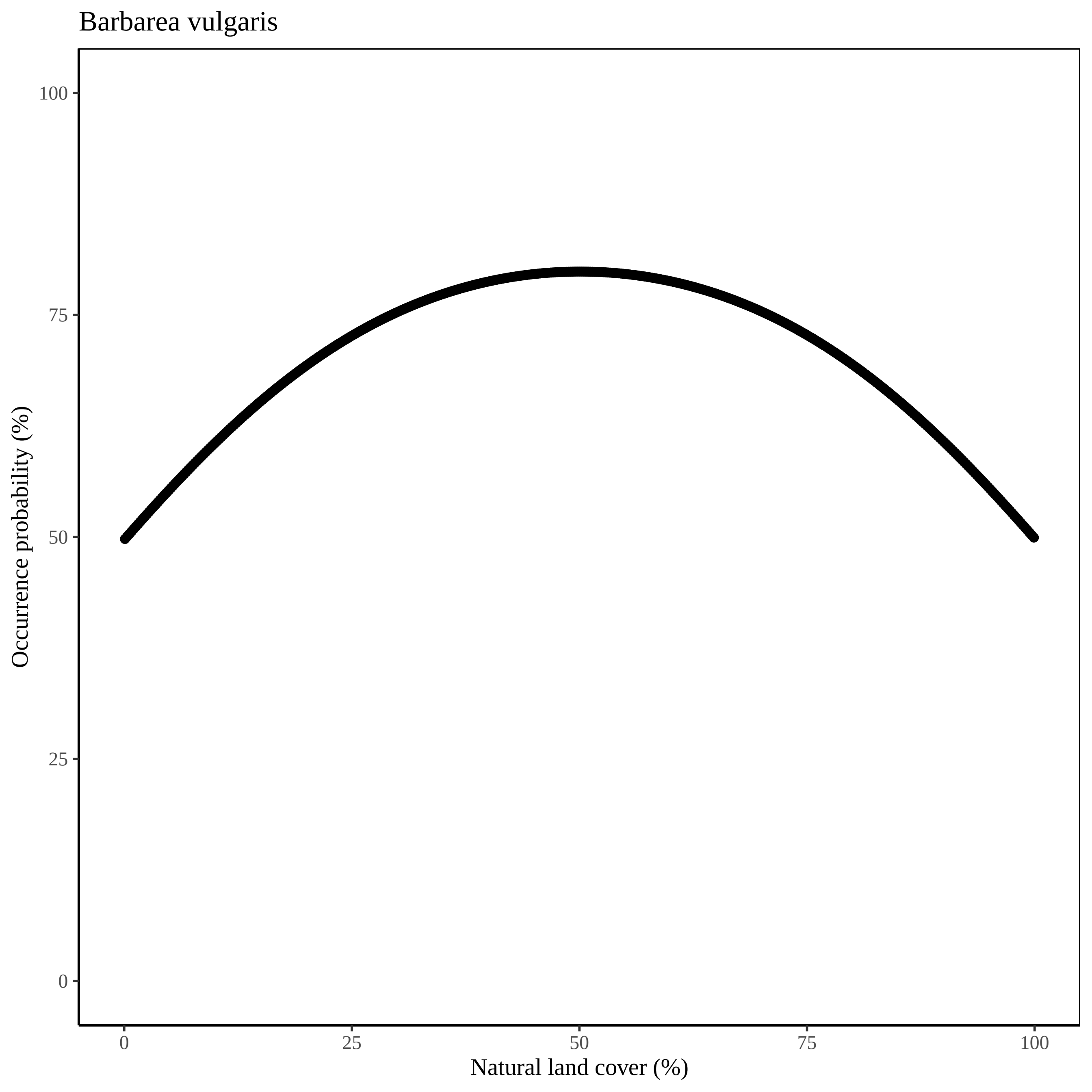

Barbarea vulgaris
Occurrence probability (%)
0
25
50
75
100
Natural land cover (%)
0
25
50
75
100

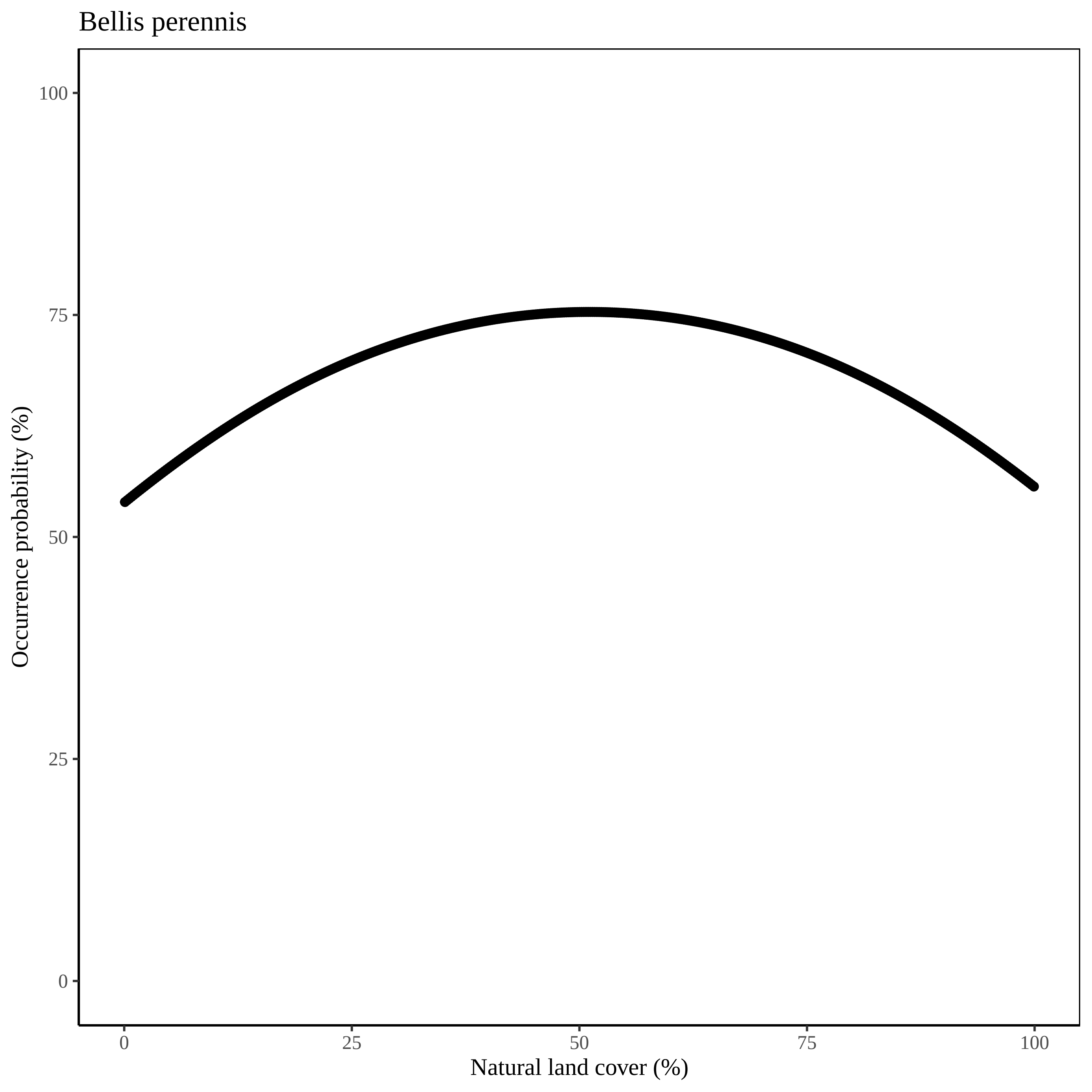

Bellis perennis
Occurrence probability (%)
100
75
50
25
0
0
25
50
75
100
Natural land cover (%)

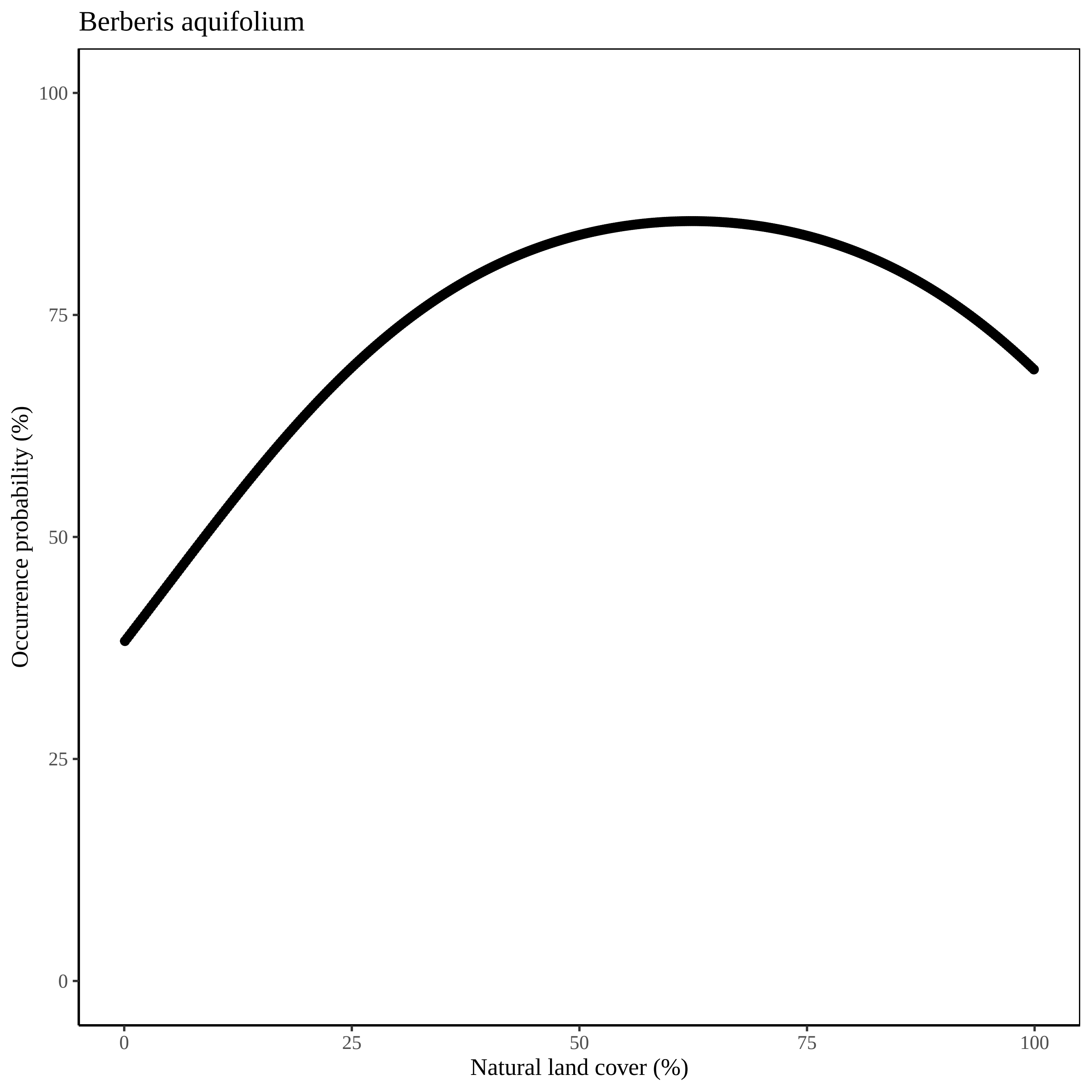
Berberis aquifolium
100
75
50
25
0
Occurrence probability (%)
0
25
50
75
100
Natural land cover (%)

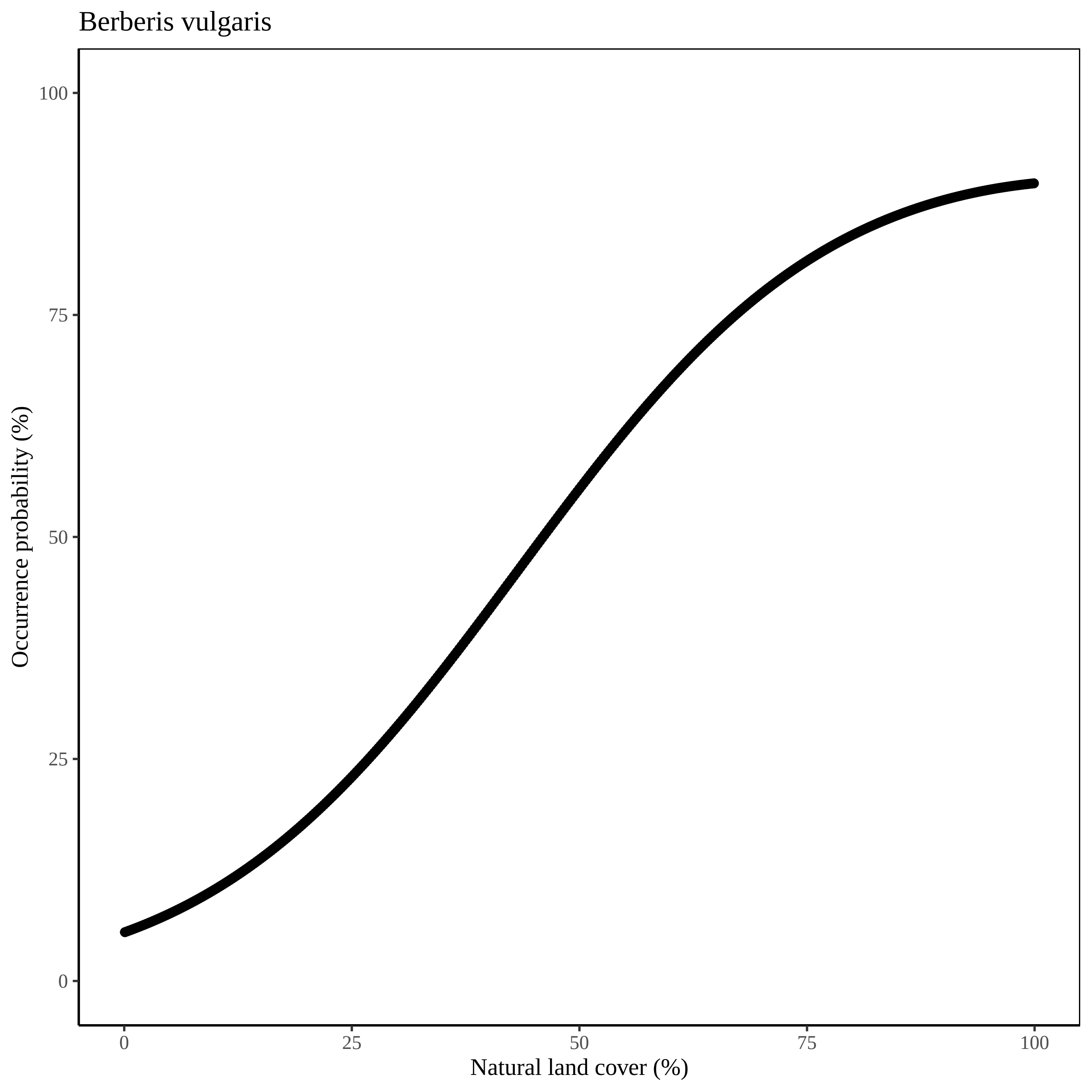

Berberis vulgaris
100
75
50
25
0
0
25
50
75
100
Natural land cover (%)
Occurrence probability (%)

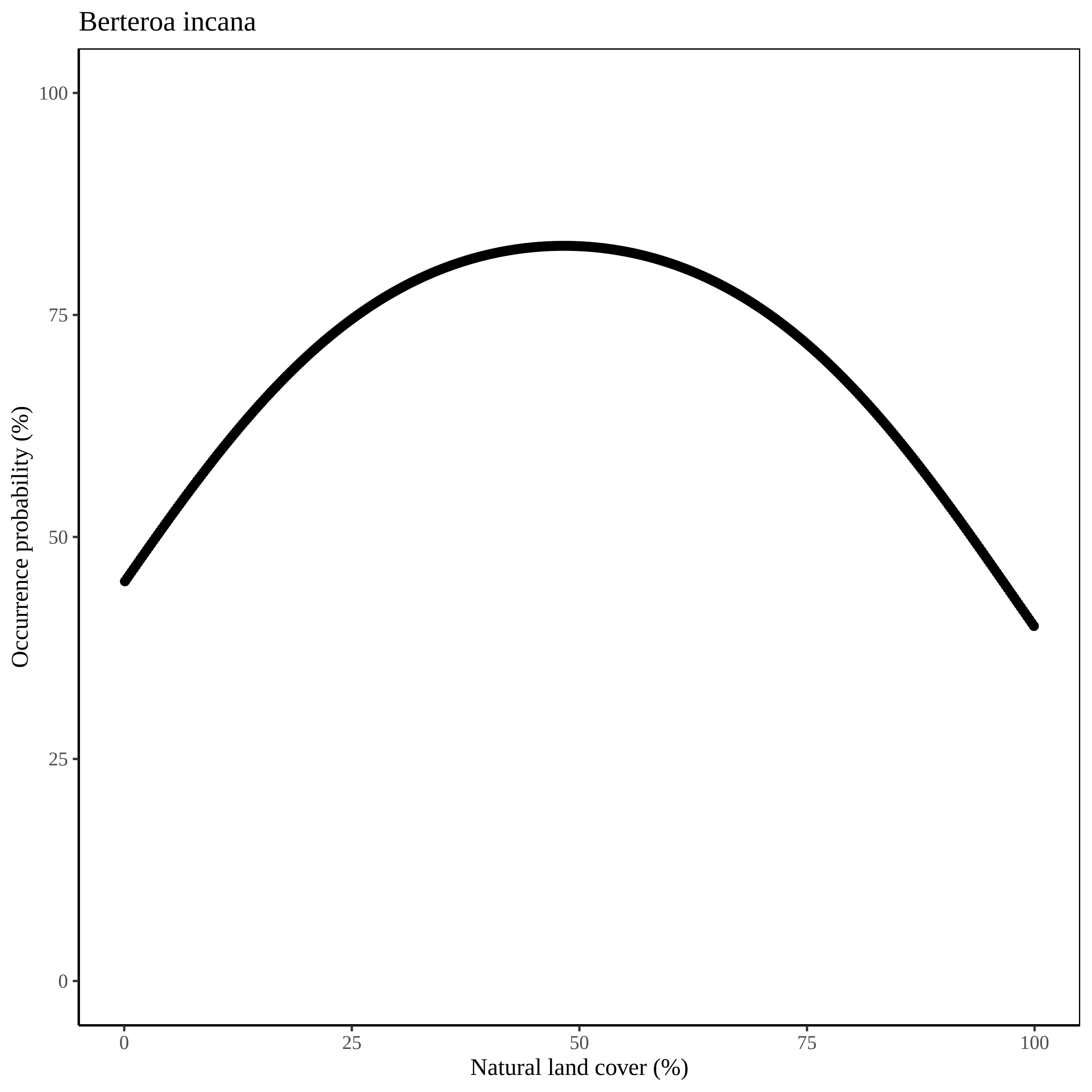
Berteroa incana
Occurrence probability (%)
100
75
50
25
0
0
25
50
75
100
Natural land cover (%)

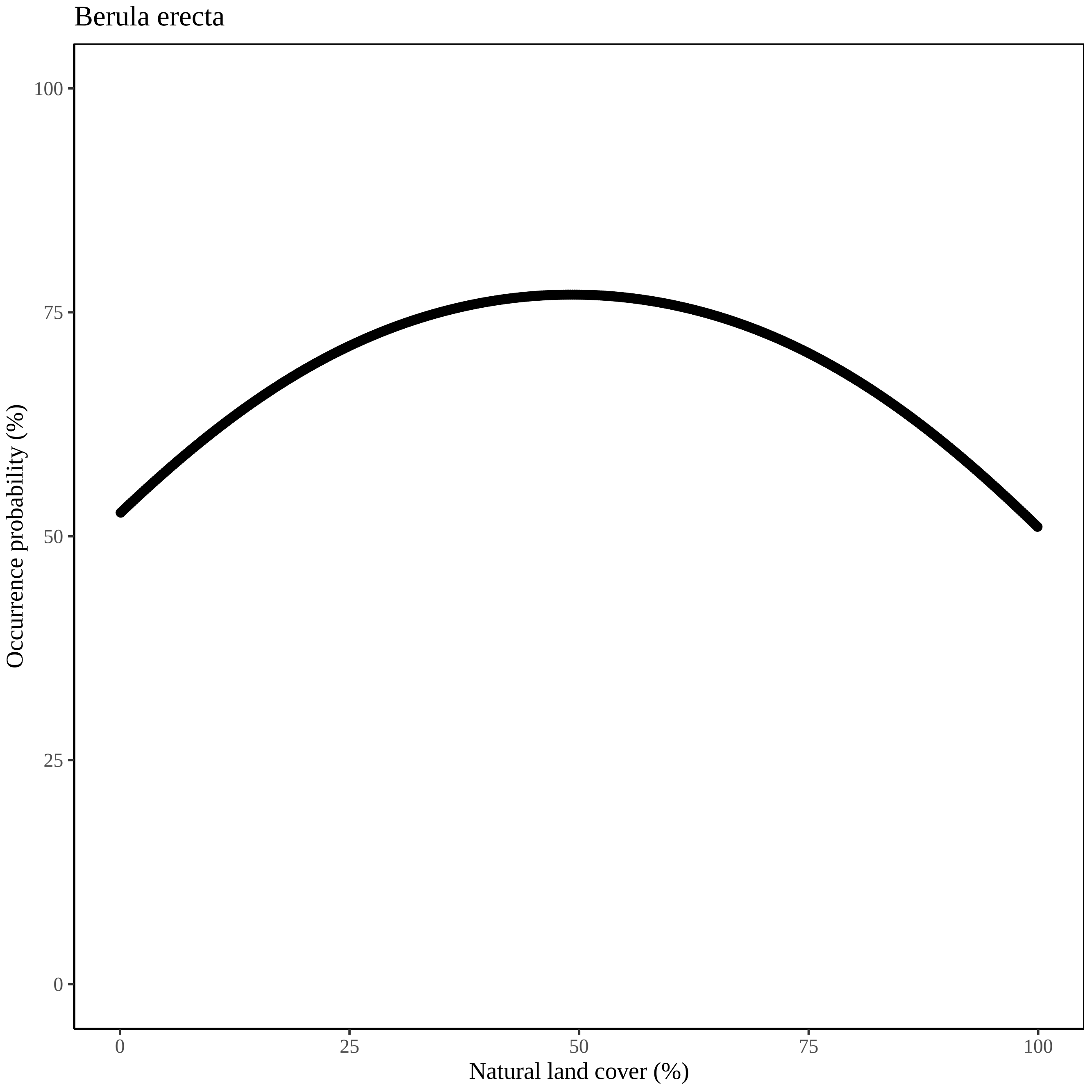

Berula erecta
Occurrence probability (%)
Natural land cover (%)
0
25
50
75
100
0
25
50
75
100

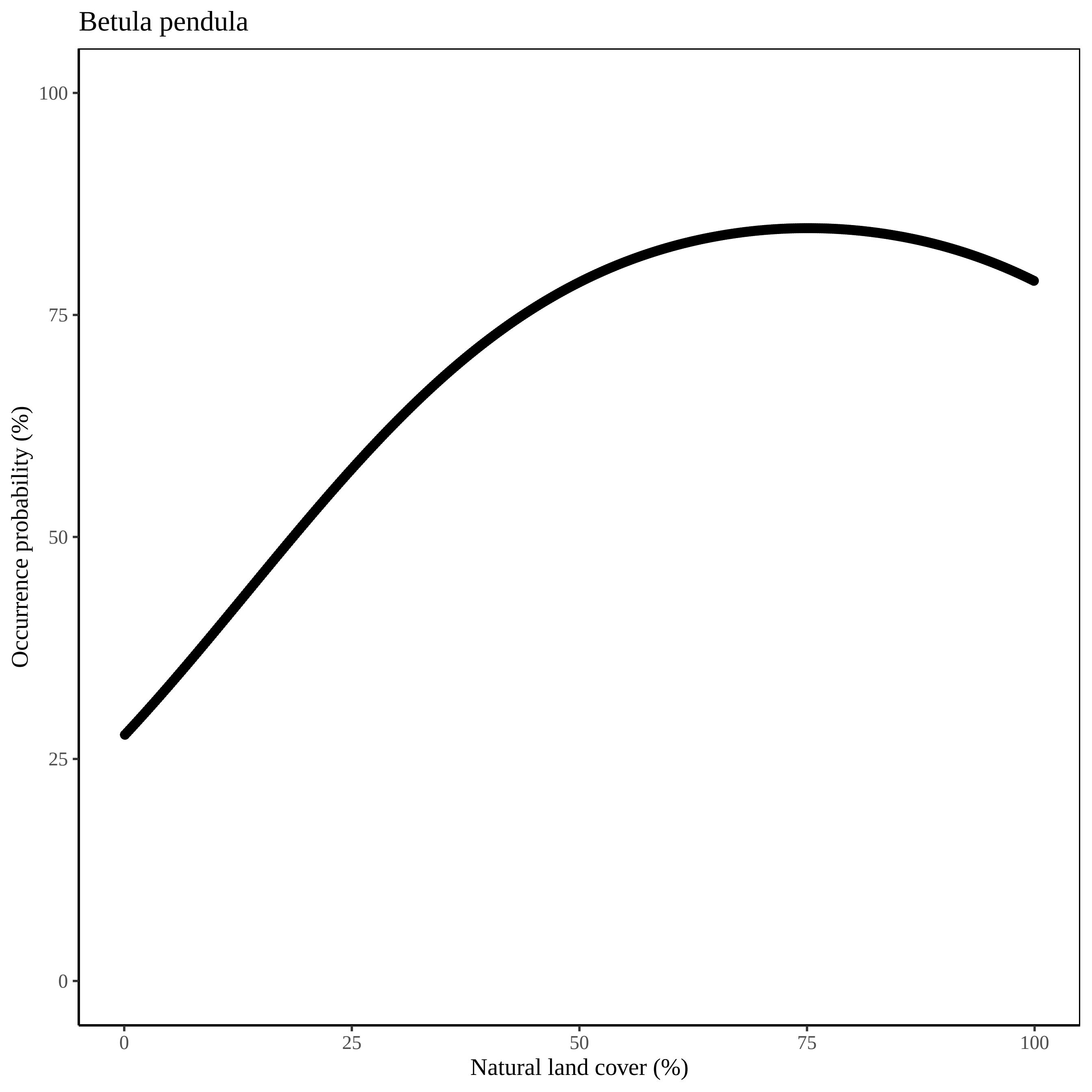
Betula pendula
Occurrence probability (%)
100
75
50
25
0
0
25
50
75
100
Natural land cover (%)

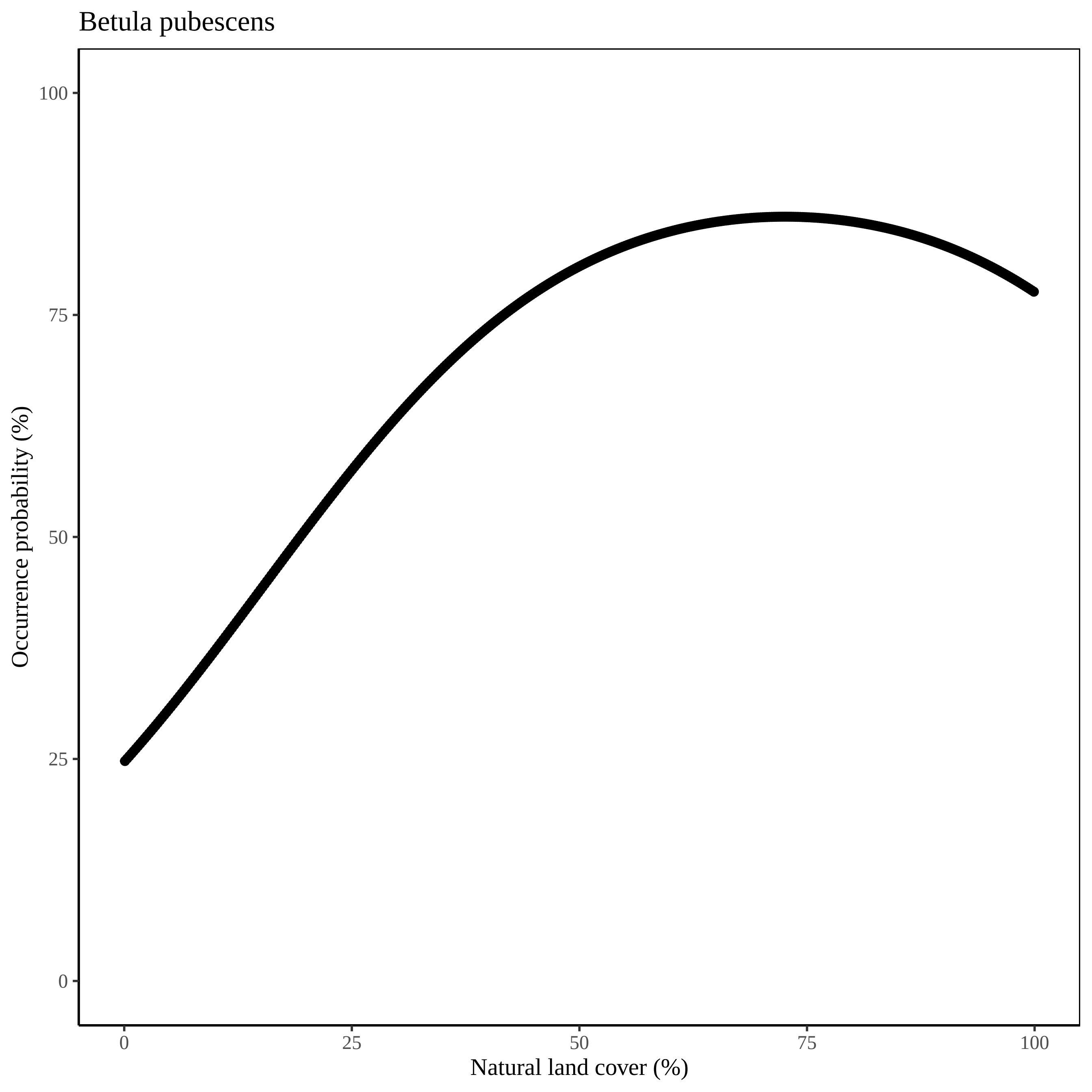
Betula pubescens
Occurrence probability (%)
100
75
50
25
0
0
25
50
75
100
Natural land cover (%)

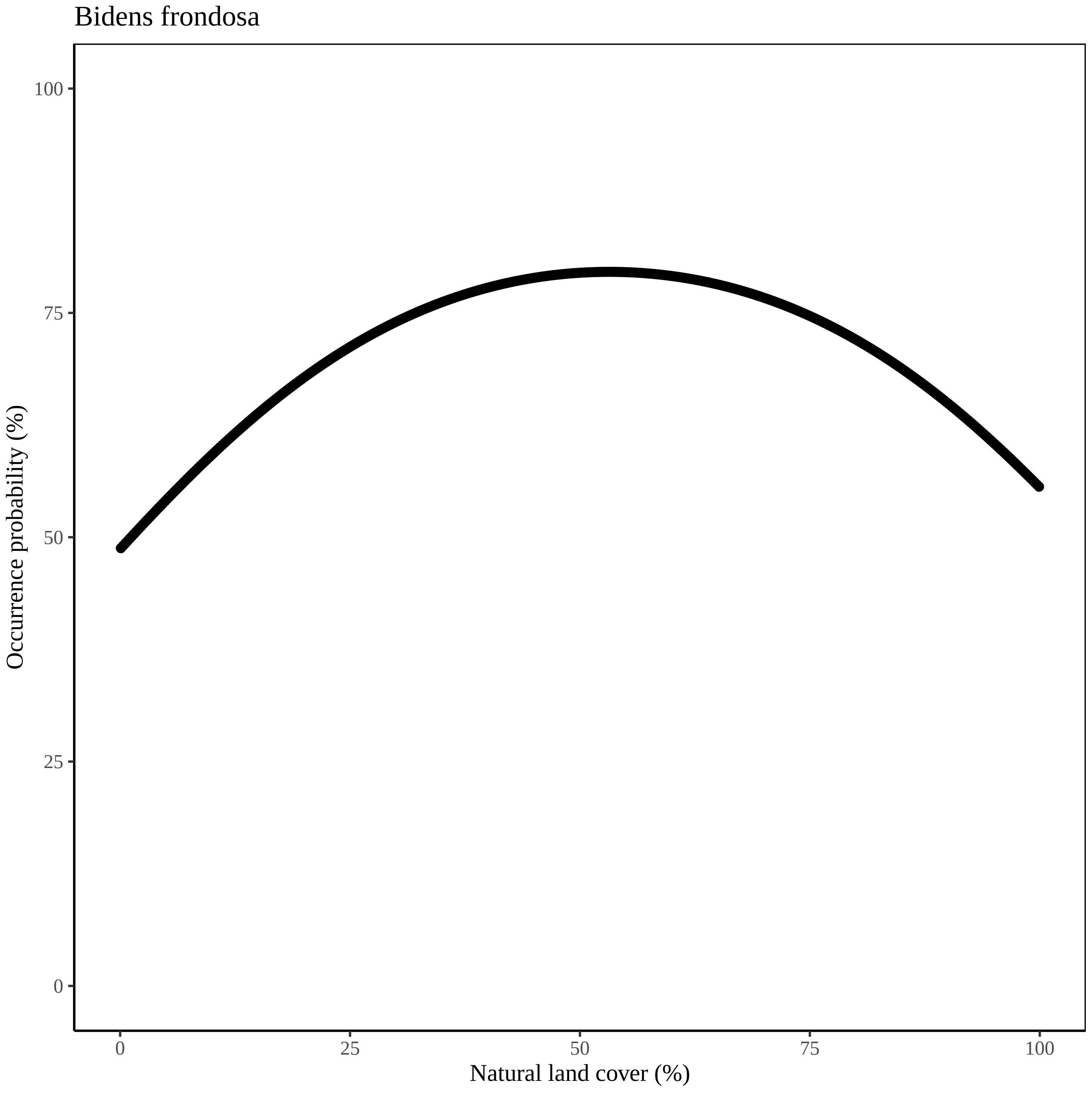
Bidens frondosa
Occurrence probability (%)
100
75
50
25
0
0
25
50
75
100
Natural land cover (%)

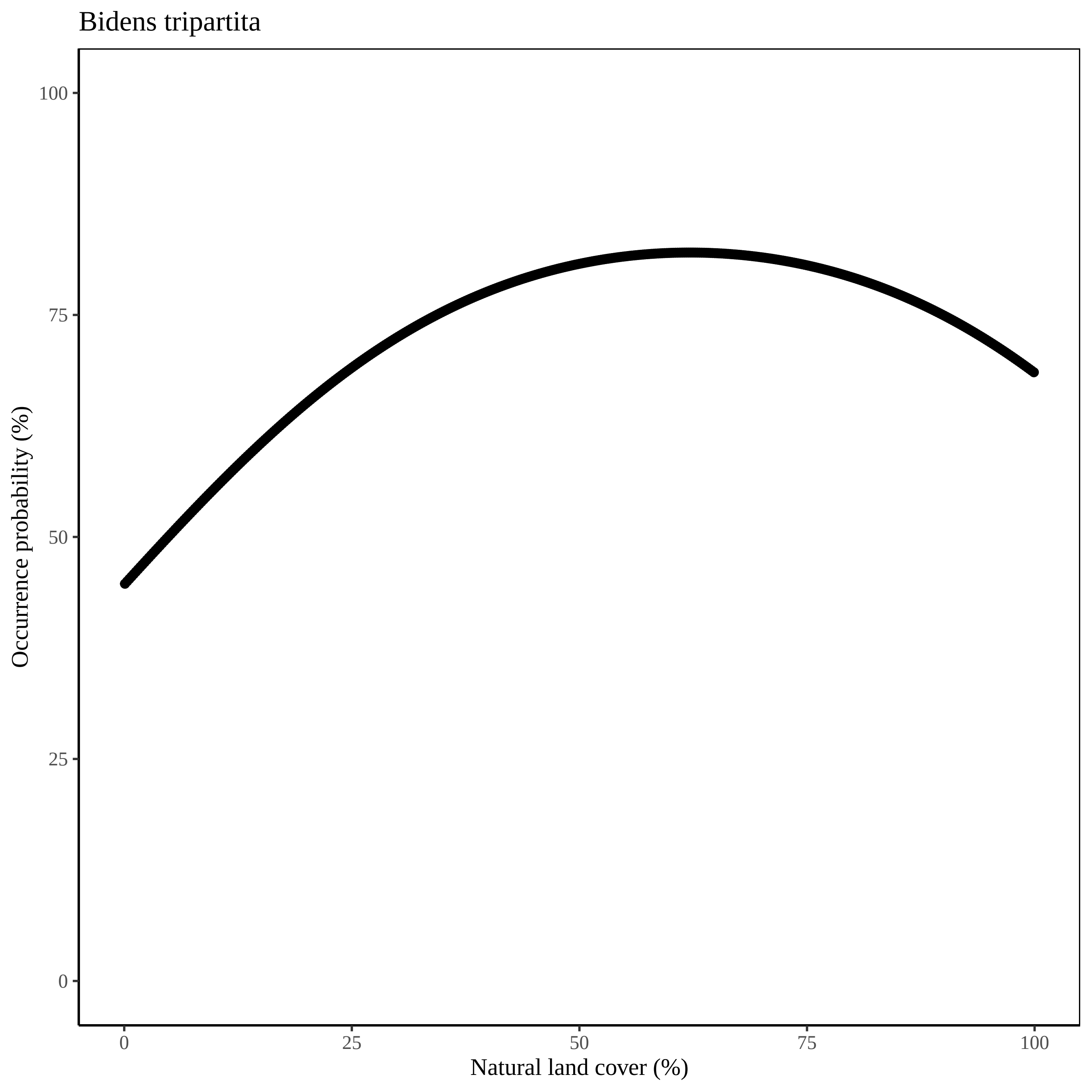
Bidens tripartita
Occurrence probability (%)
100
75
50
25
0
0
25
50
75
100
Natural land cover (%)

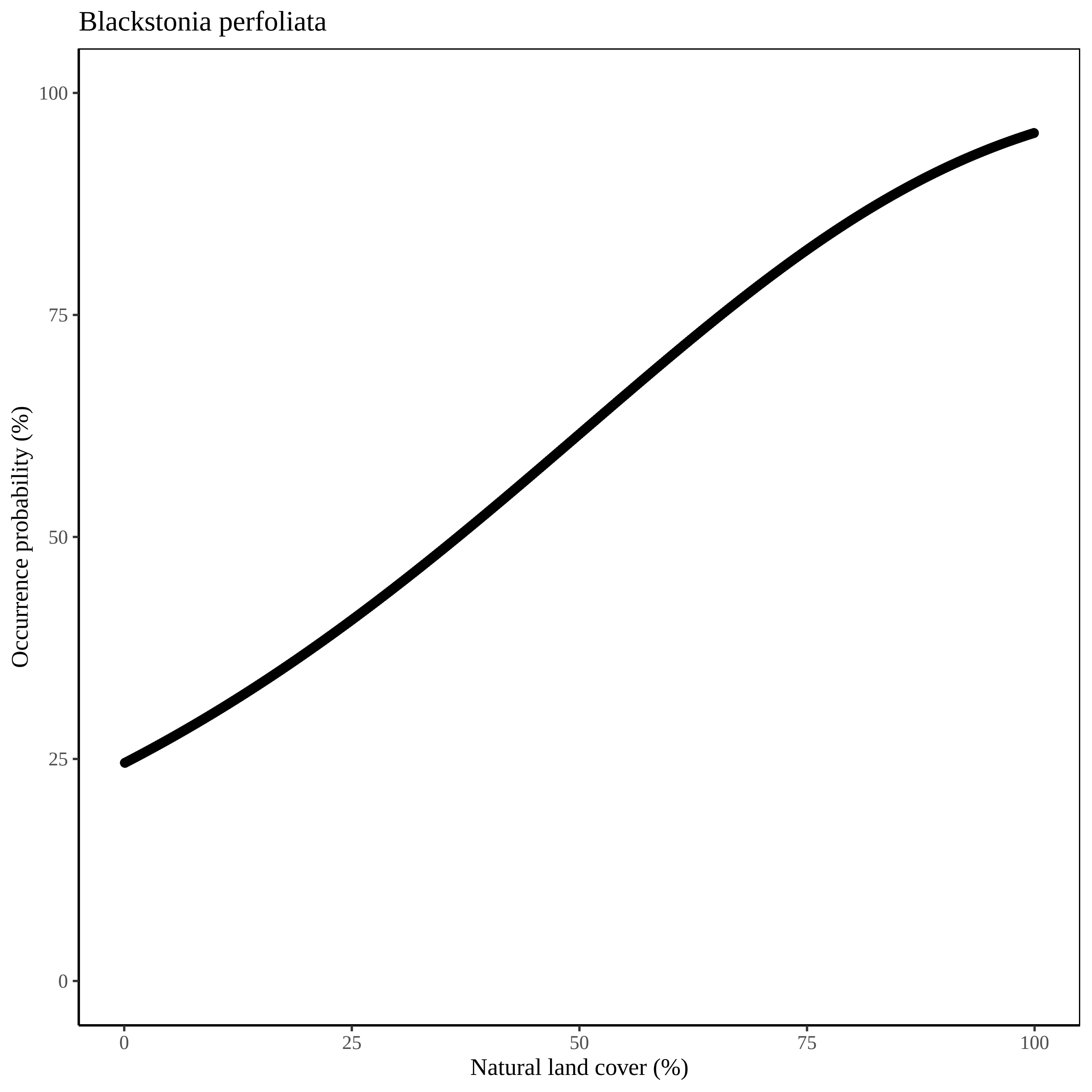
Blackstonia perfoliata
Occurrence probability (%)
100
75
50
25
0
0
25
50
75
100
Natural land cover (%)

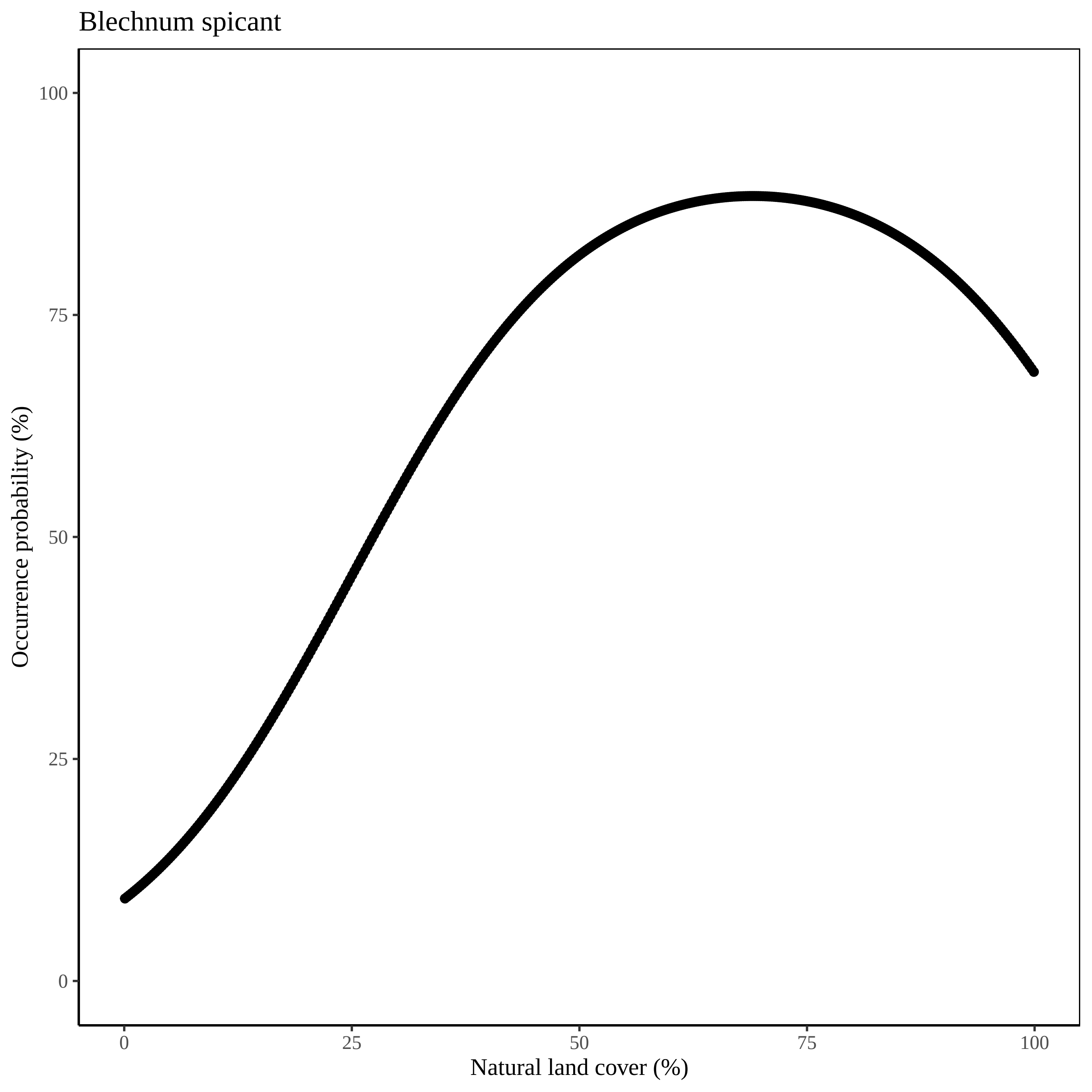

Blechnum spicant
Occurrence probability (%)
100
75
50
25
0
0
25
50
75
100
Natural land cover (%)

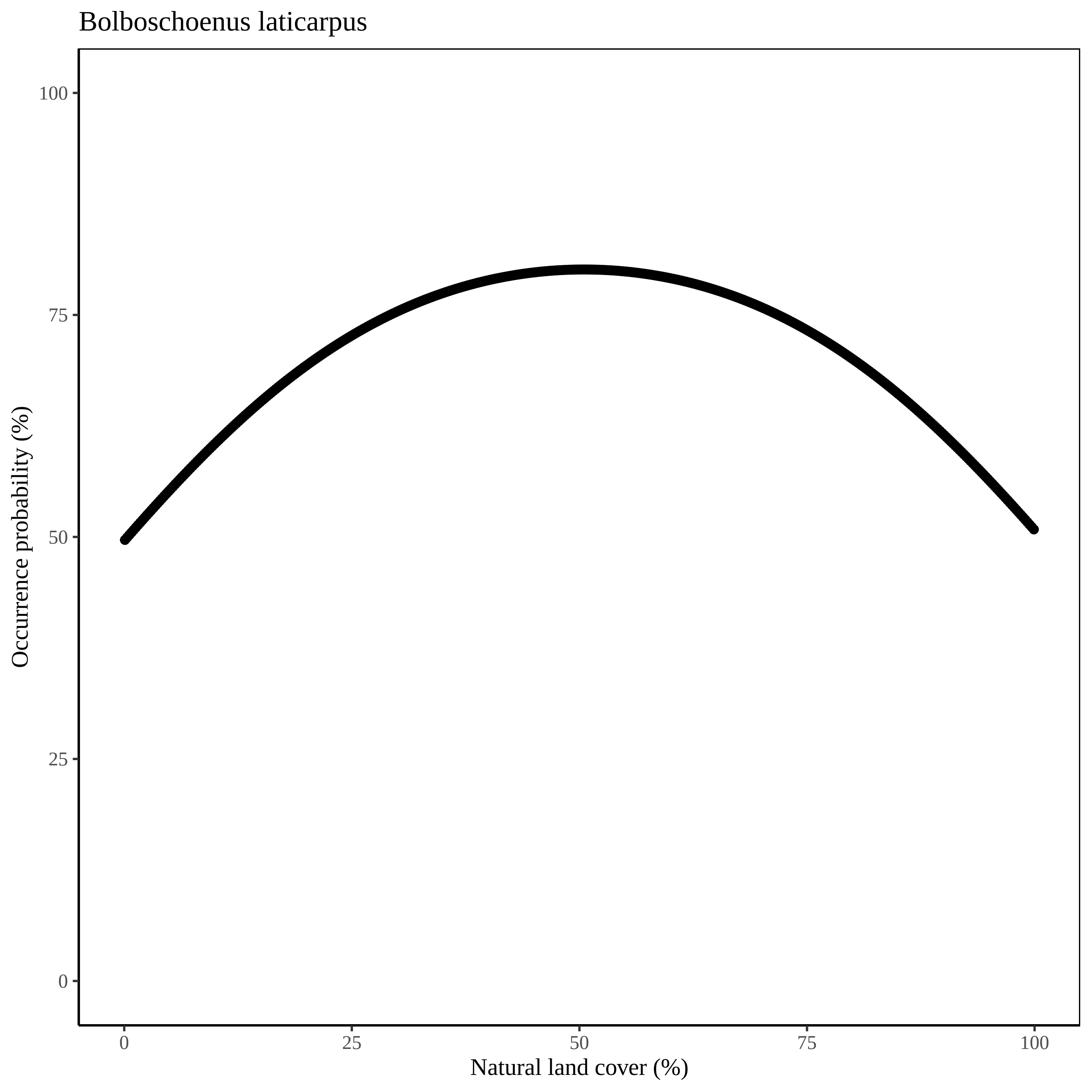

Bolboschoenus laticarpus
Occurrence probability (%)
100
75
50
25
0
0
25
50
75
100
Natural land cover (%)

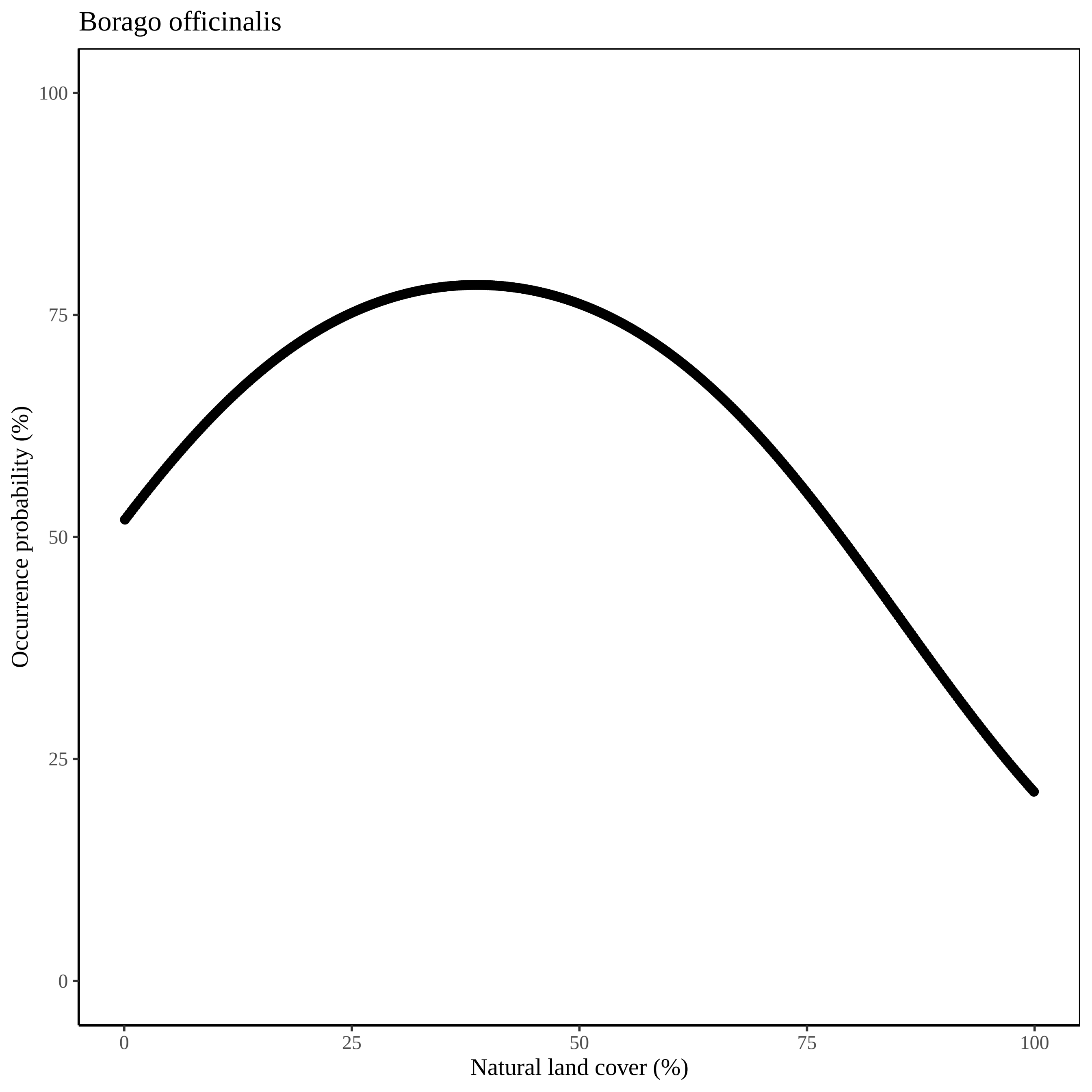

Borago officinalis
100
75
50
25
0
Occurrence probability (%)
0
25
50
75
100
Natural land cover (%)

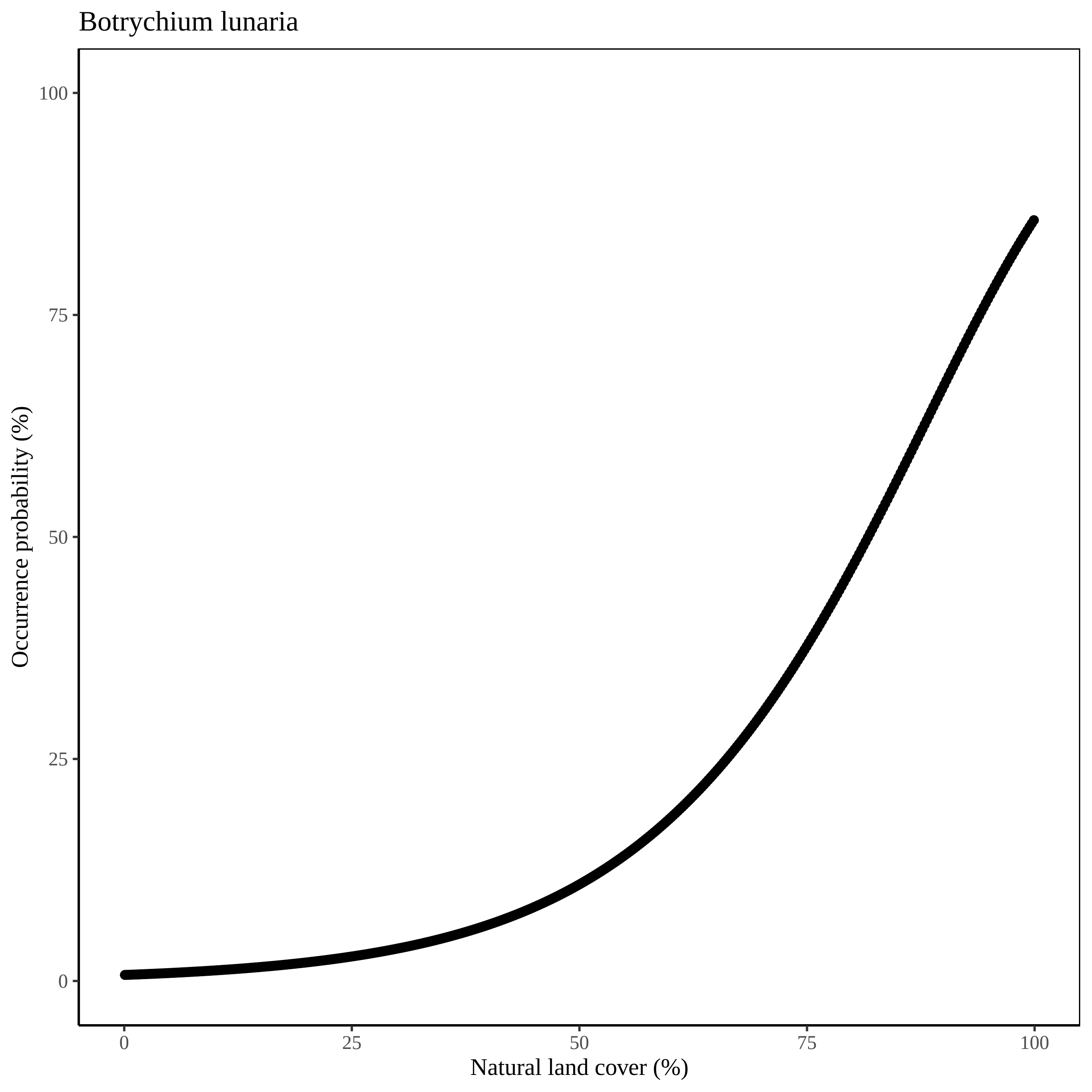
Botrychium lunaria
Occurrence probability (%)
100
75
50
25
0
0
25
50
75
100
Natural land cover (%)

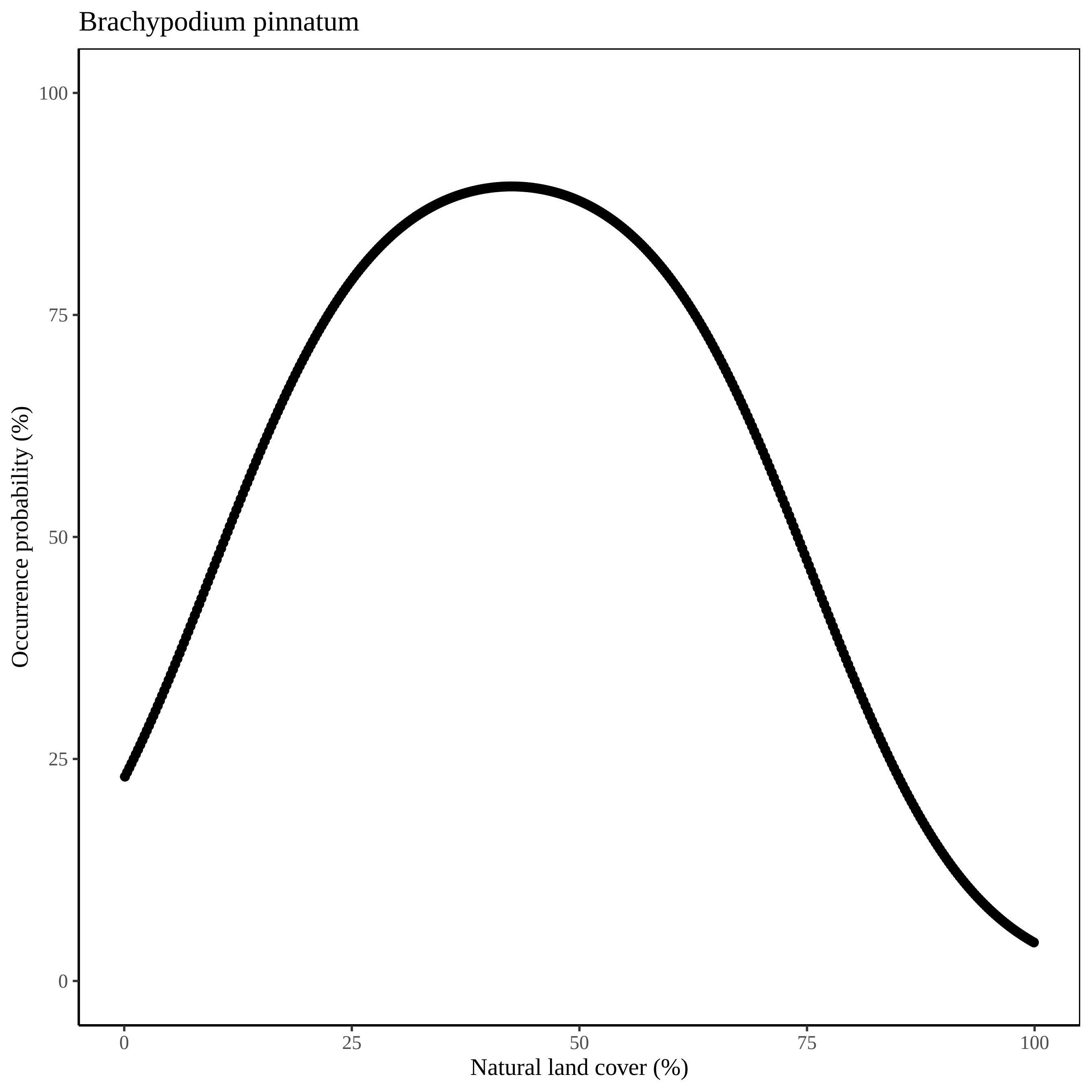
Brachypodium pinnatum
Occurrence probability (%)
100
75
50
25
0
0
25
50
75
100
Natural land cover (%)

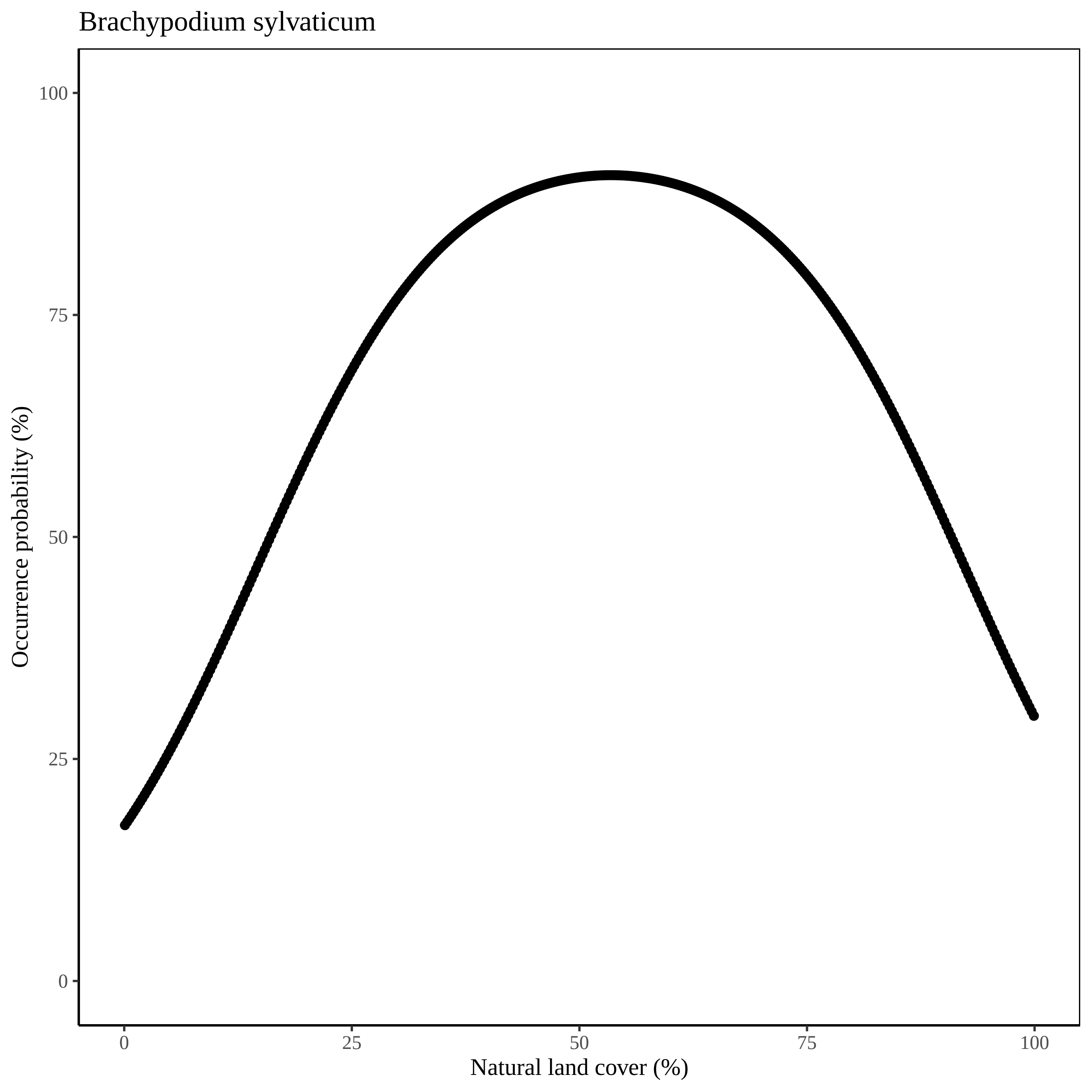

Brachypodium sylvaticum
100
75
50
25
0
Occurrence probability (%)
0
25
50
75
100
Natural land cover (%)

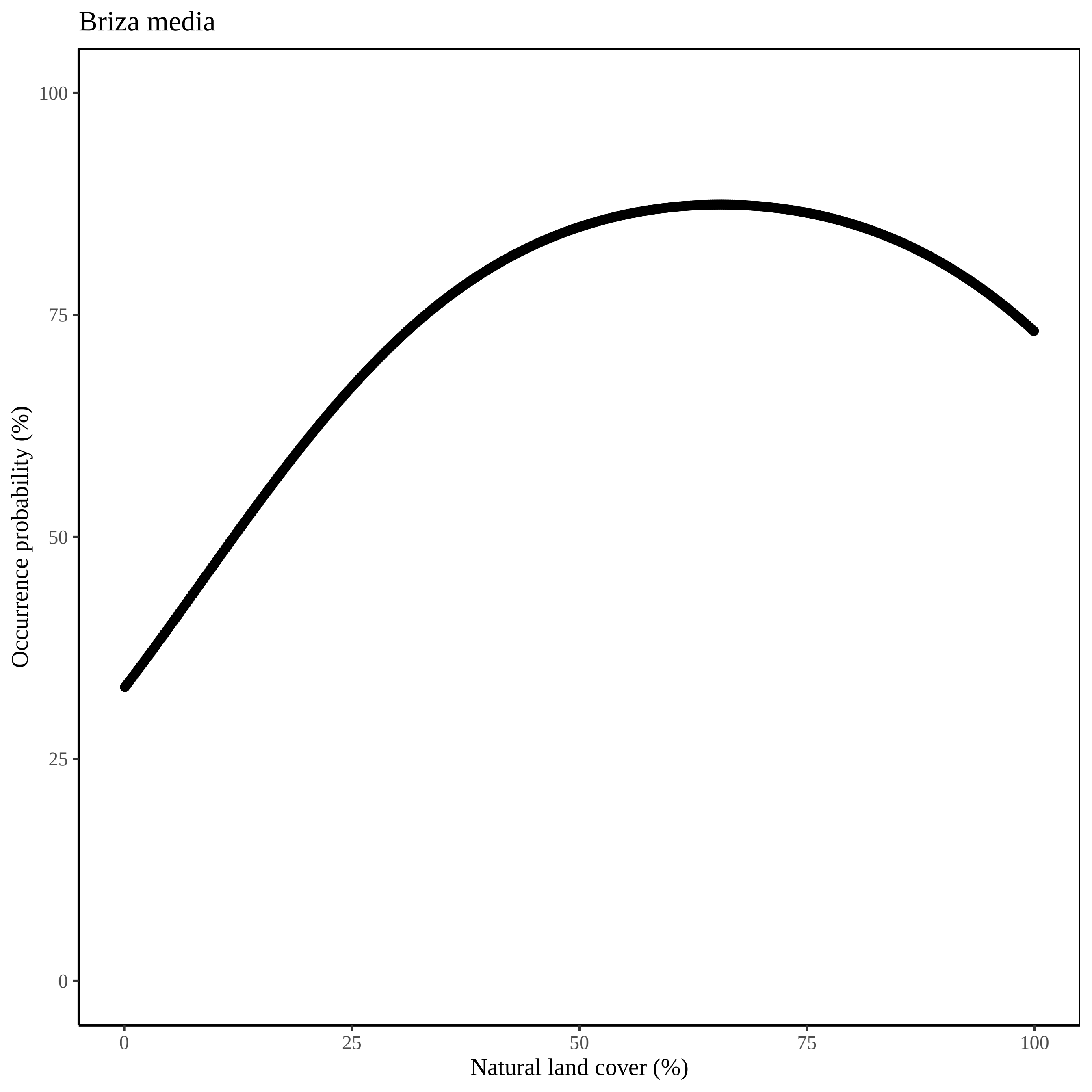

Briza media
Occurrence probability (%)
100
75
50
25
0
0
25
50
75
100
Natural land cover (%)

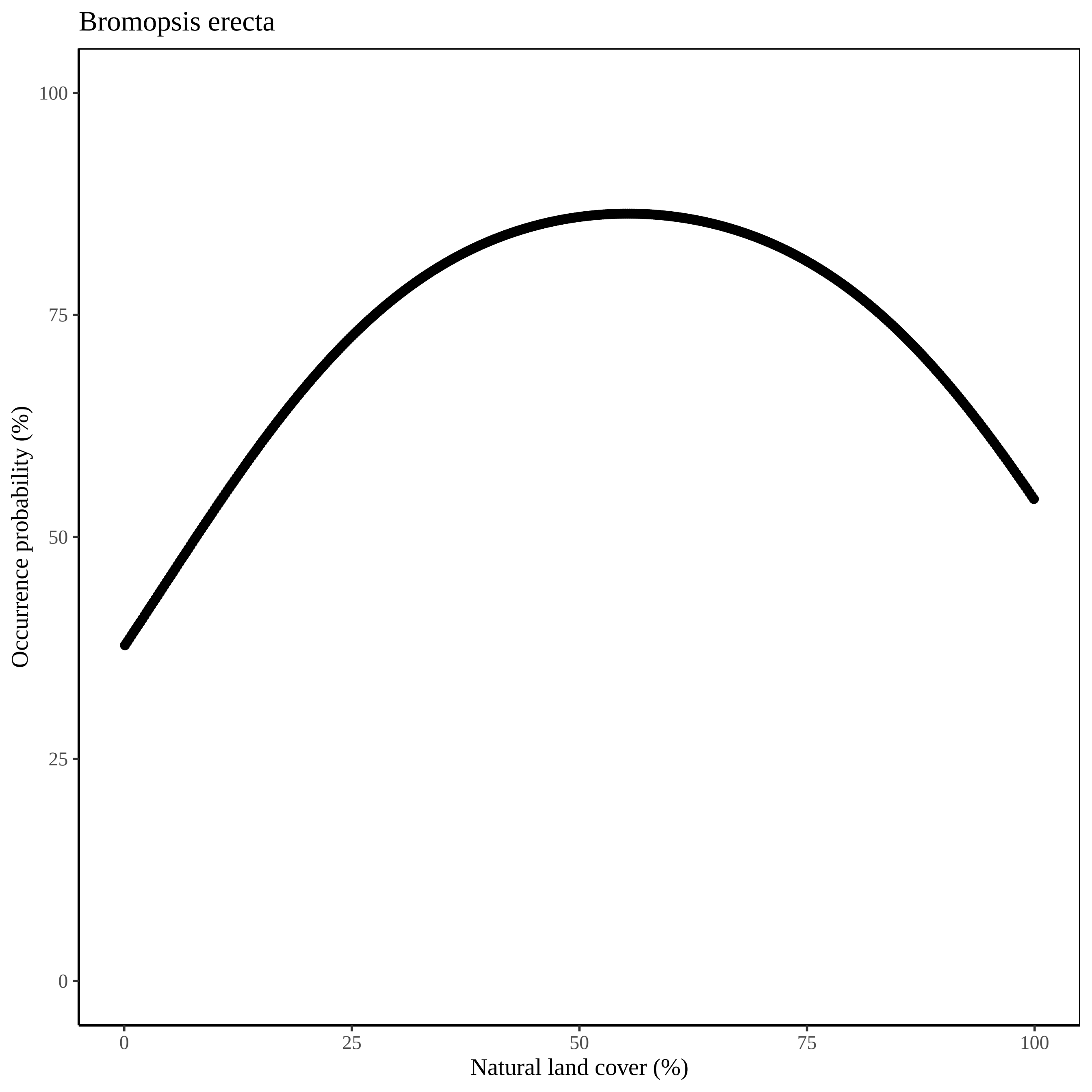

Bromopsis erecta
Occurrence probability (%)
100
75
50
25
0
0
25
50
75
100
Natural land cover (%)

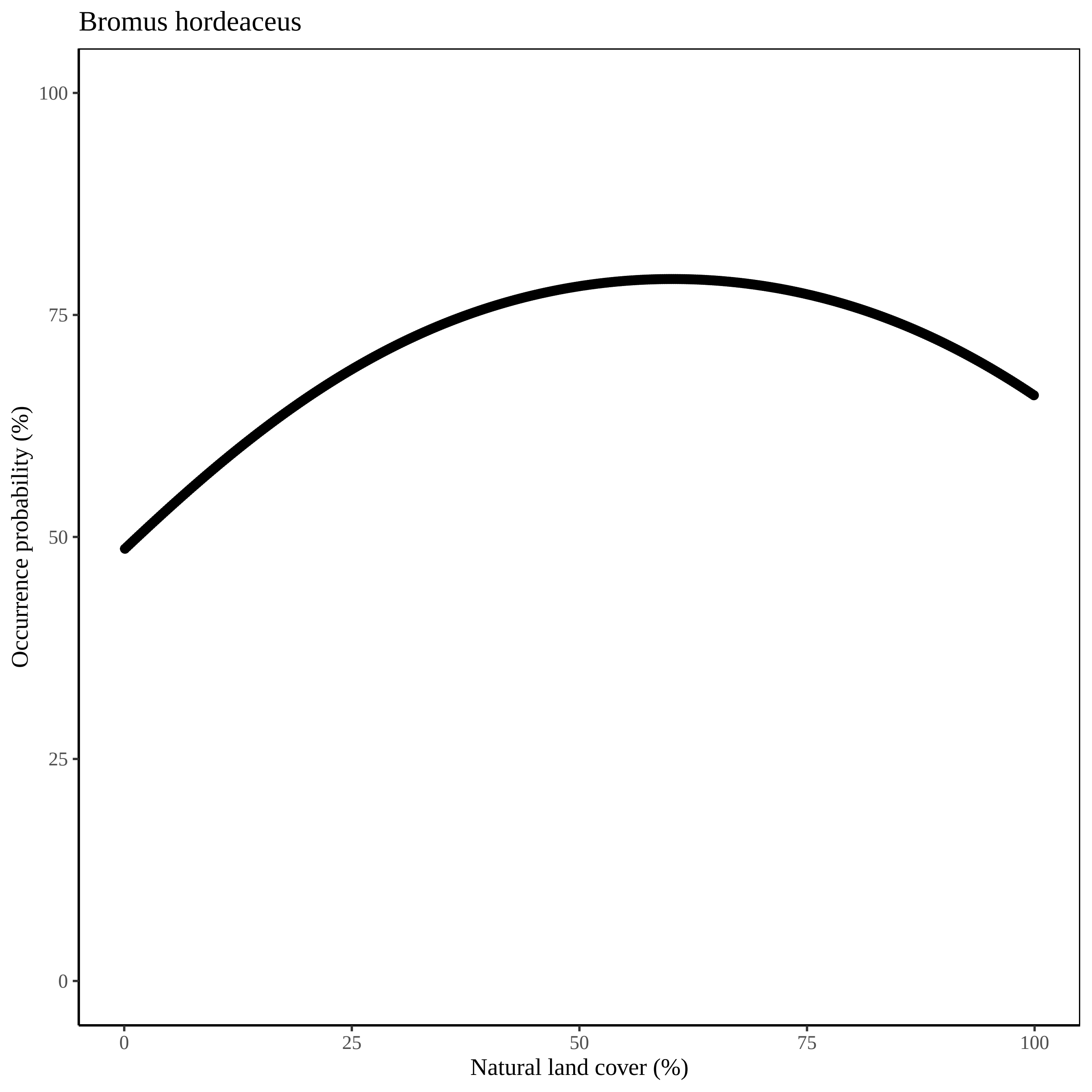

Bromus hordeaceus
Occurrence probability (%)
100
75
50
25
0
0
25
50
75
100
Natural land cover (%)

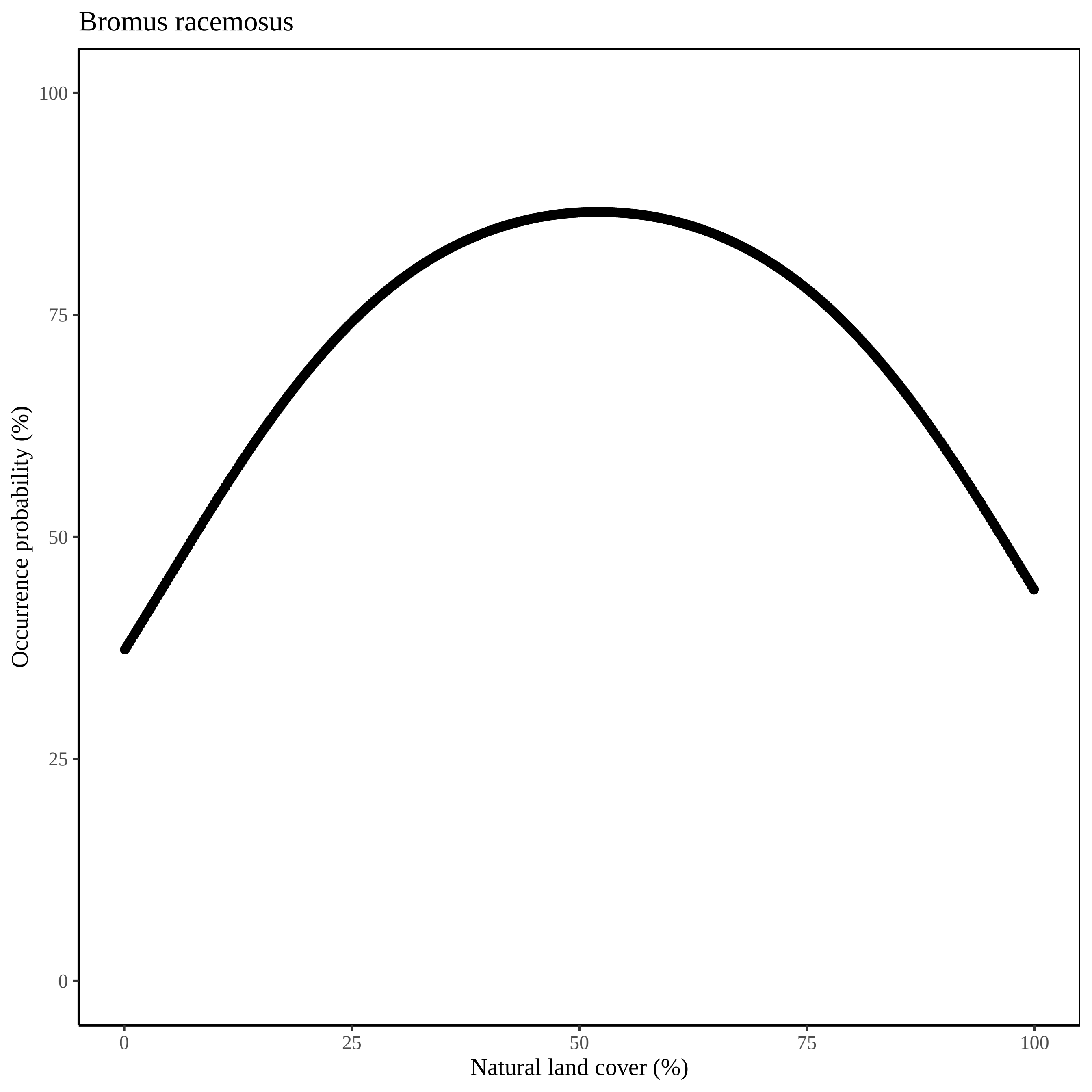
Bromus racemosus
Occurrence probability (%)
100
75
50
25
0
0
25
50
75
100
Natural land cover (%)

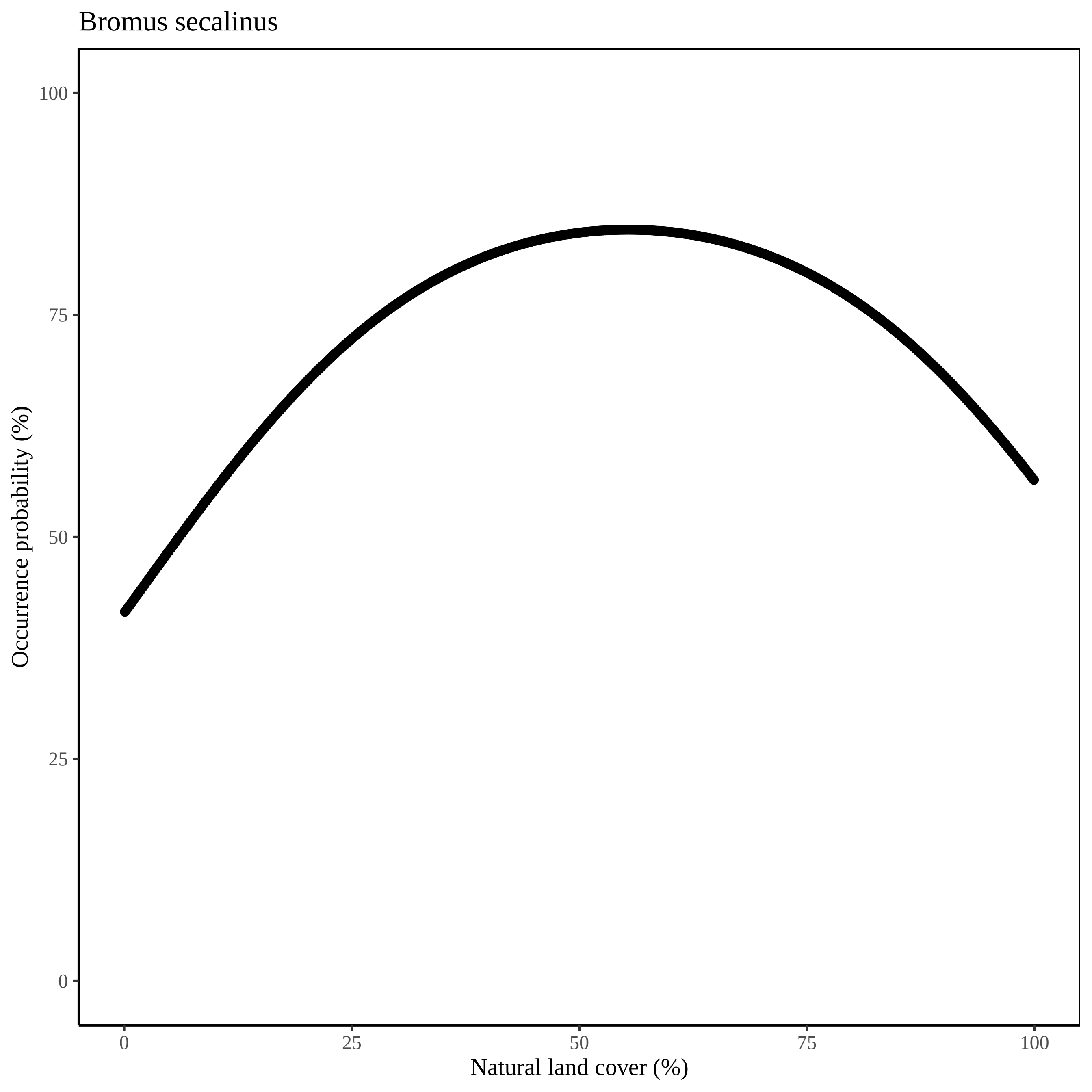
Bromus secalinus
Occurrence probability (%)
100
75
50
25
0
0
25
50
75
100
Natural land cover (%)

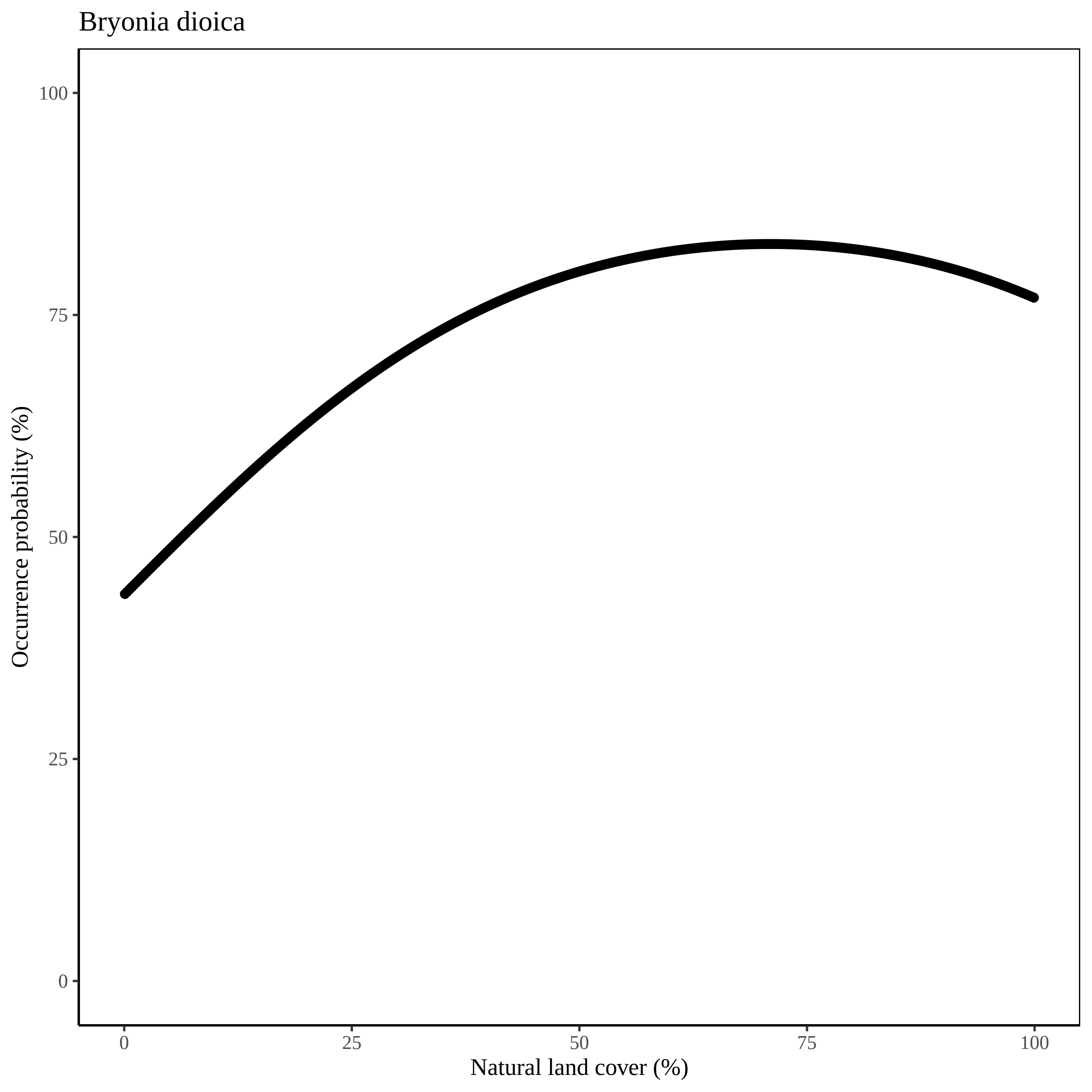
Bryonia dioica
Occurrence probability (%)
100
75
50
25
0
0
25
50
75
100
Natural land cover (%)

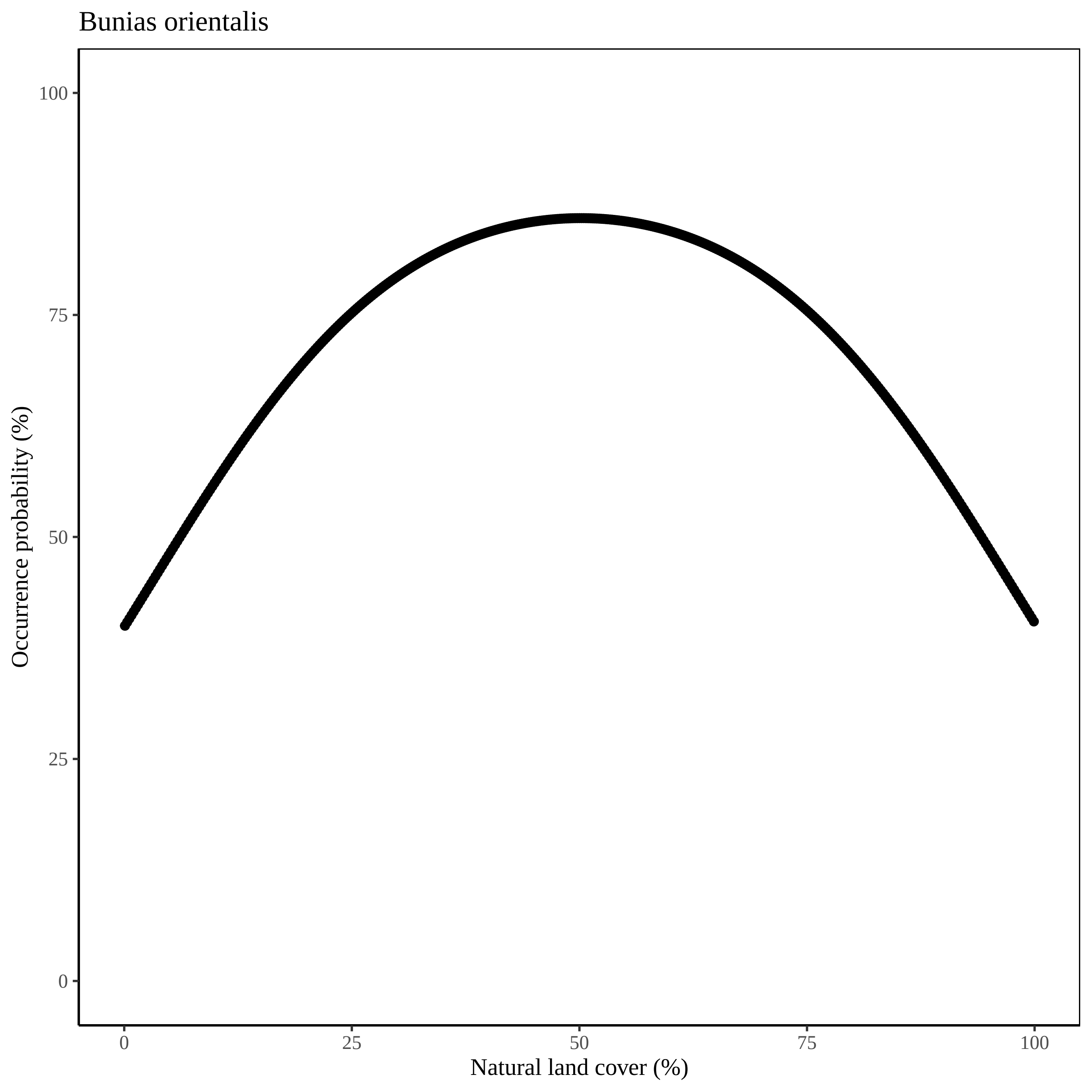
Bunias orientalis
Occurrence probability (%)
100
75
50
25
0
Natural land cover (%)
0
25
50
75
100

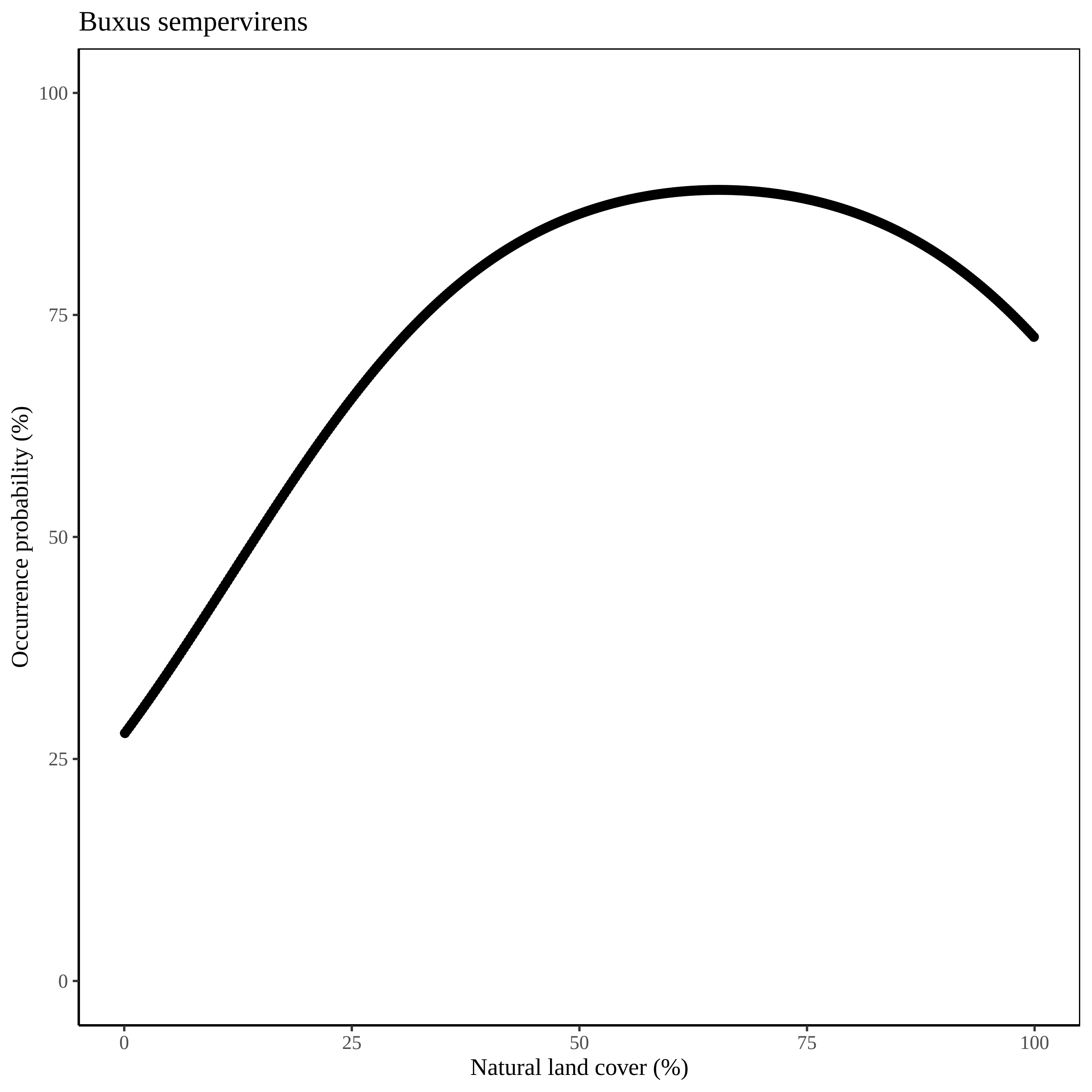

Buxus sempervirens
Occurrence probability (%)
100
75
50
25
0
0
25
50
75
100
Natural land cover (%)

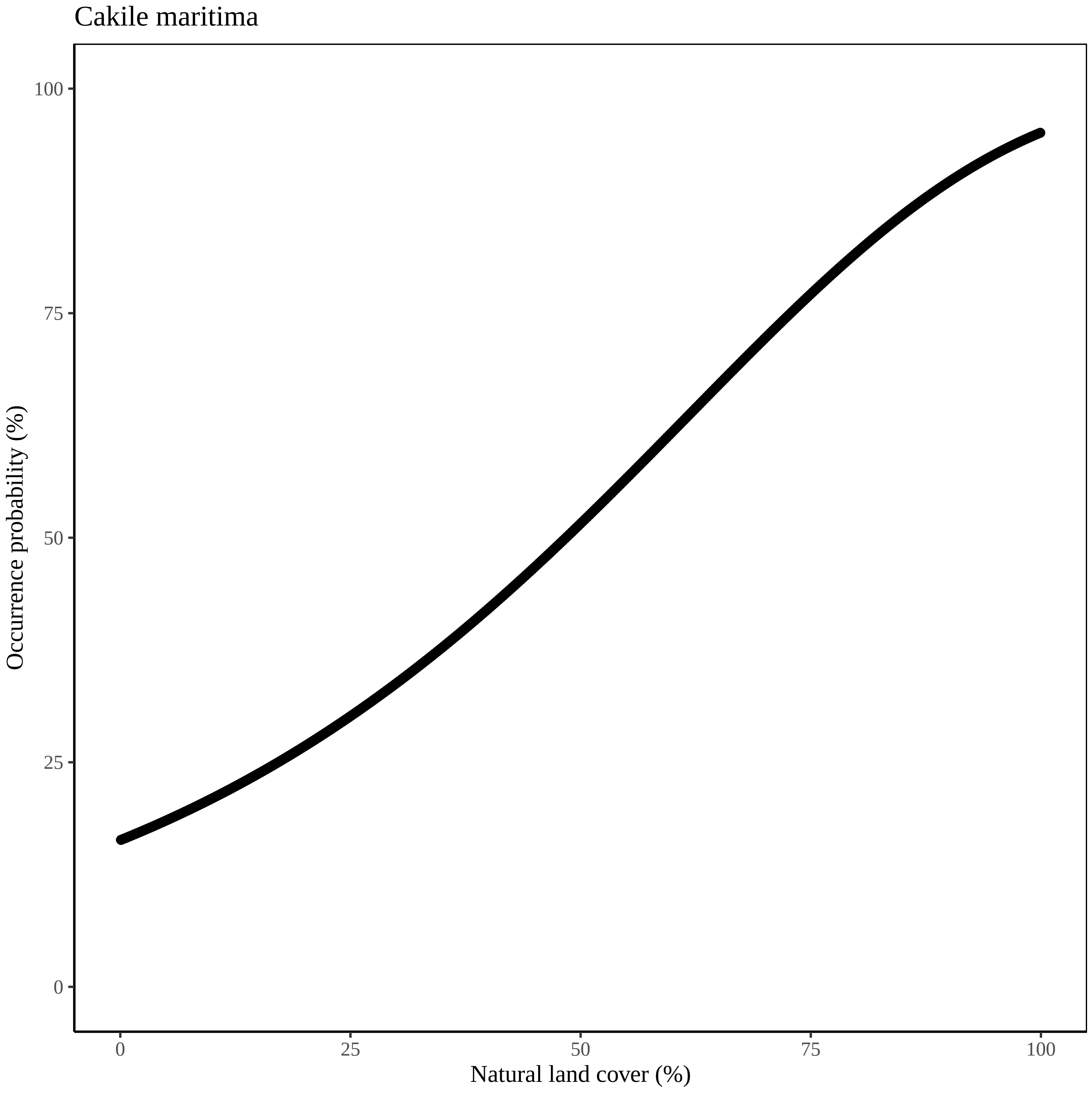

Cakile maritima
Occurrence probability (%)
100
75
50
25
0
0
25
50
75
100
Natural land cover (%)

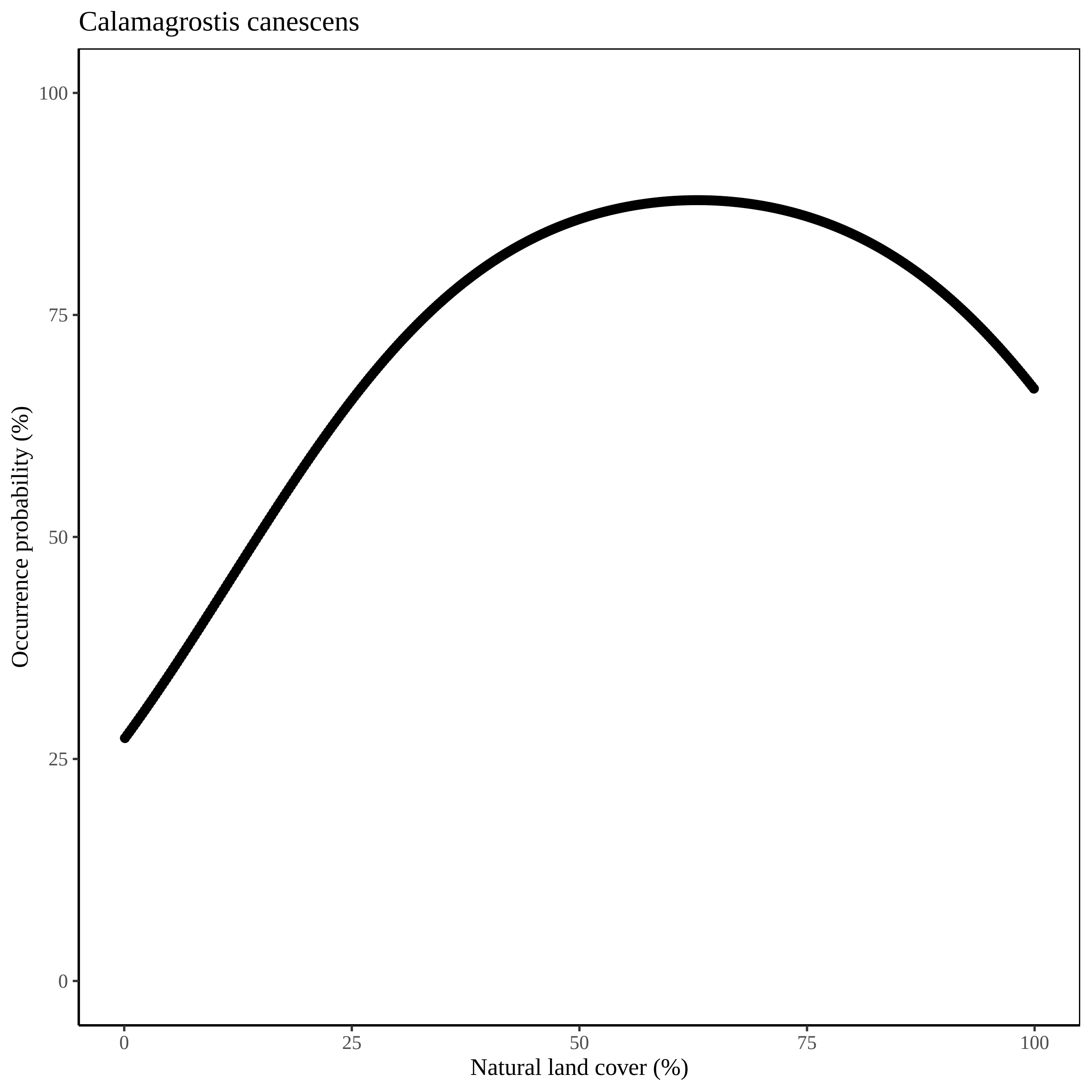
Calamagrostis canescens
Occurrence probability (%)
100
75
50
25
0
0
25
50
75
100
Natural land cover (%)

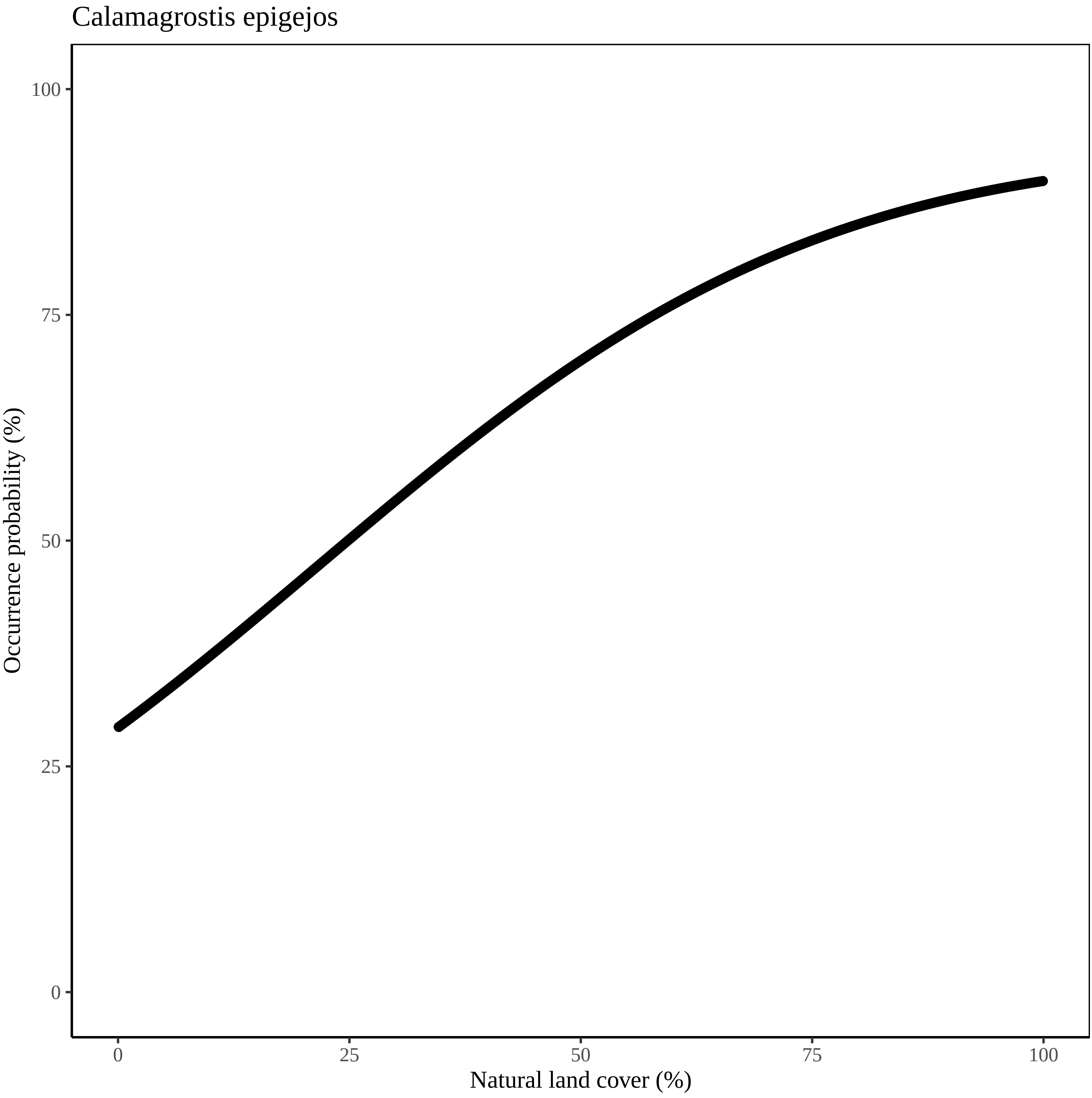

Calamagrostis epigejos
Occurrence probability (%)
100
75
50
25
0
0
25
50
75
100
Natural land cover (%)

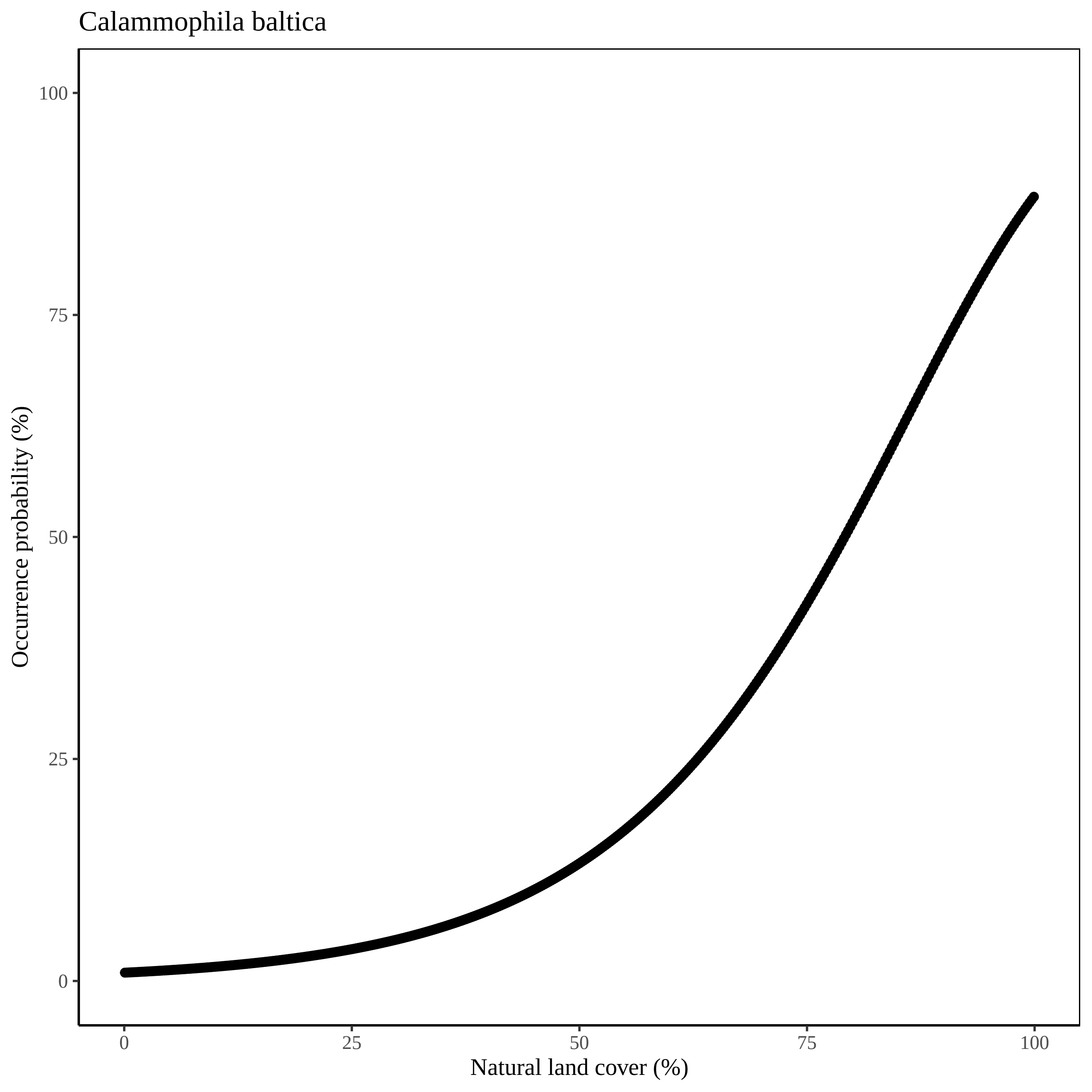

Calammophila baltica
Occurrence probability (%)
100
75
50
25
0
0
25
50
75
100
Natural land cover (%)

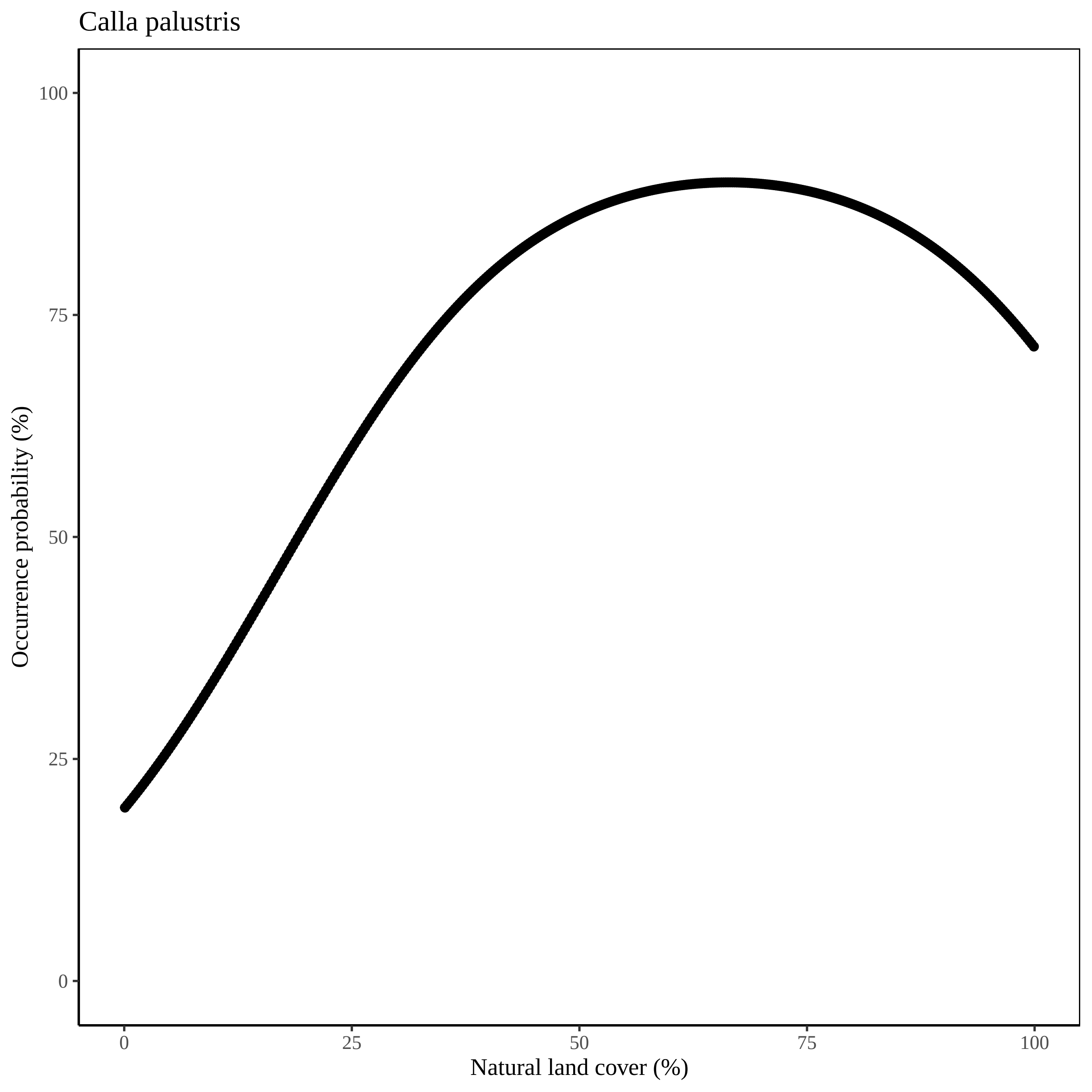

Calla palustris
Occurrence probability (%)
100
75
50
25
0
0
25
50
75
100
Natural land cover (%)

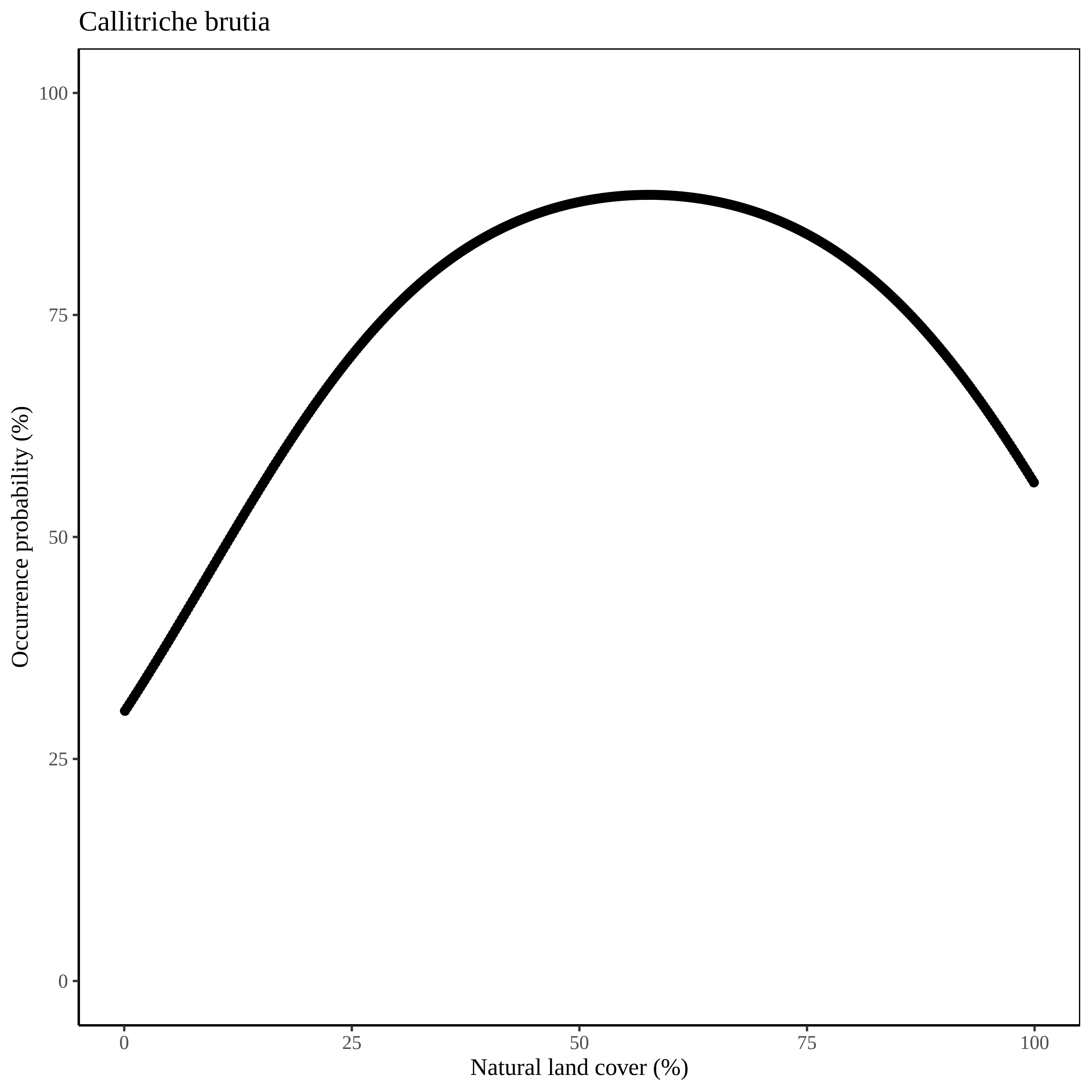
Callitriche brutia
Occurrence probability (%)
100
75
50
25
0
0
25
50
75
100
Natural land cover (%)

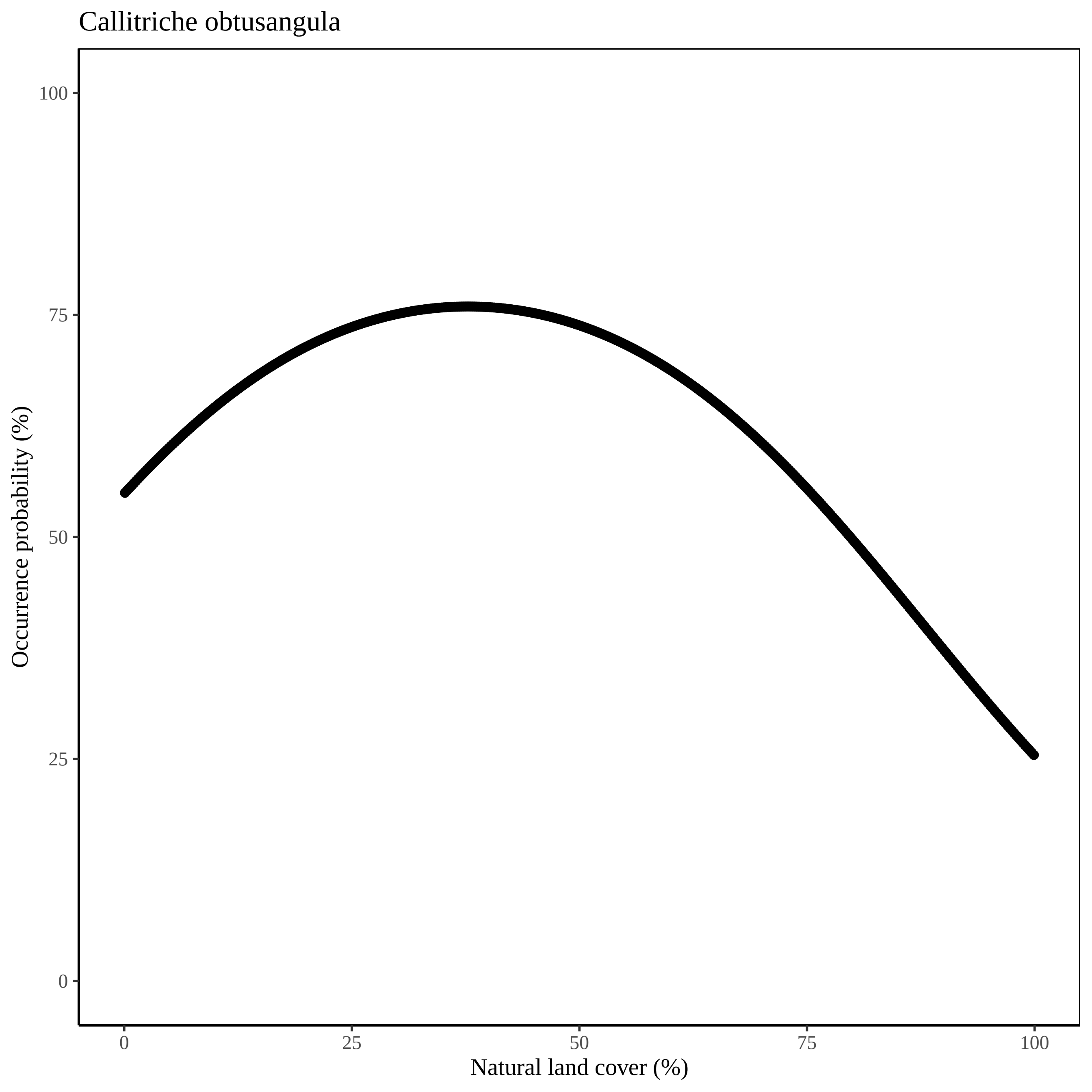

Callitriche obtusangula
Occurrence probability (%)
100
75
50
25
0
0
25
50
75
100
Natural land cover (%)

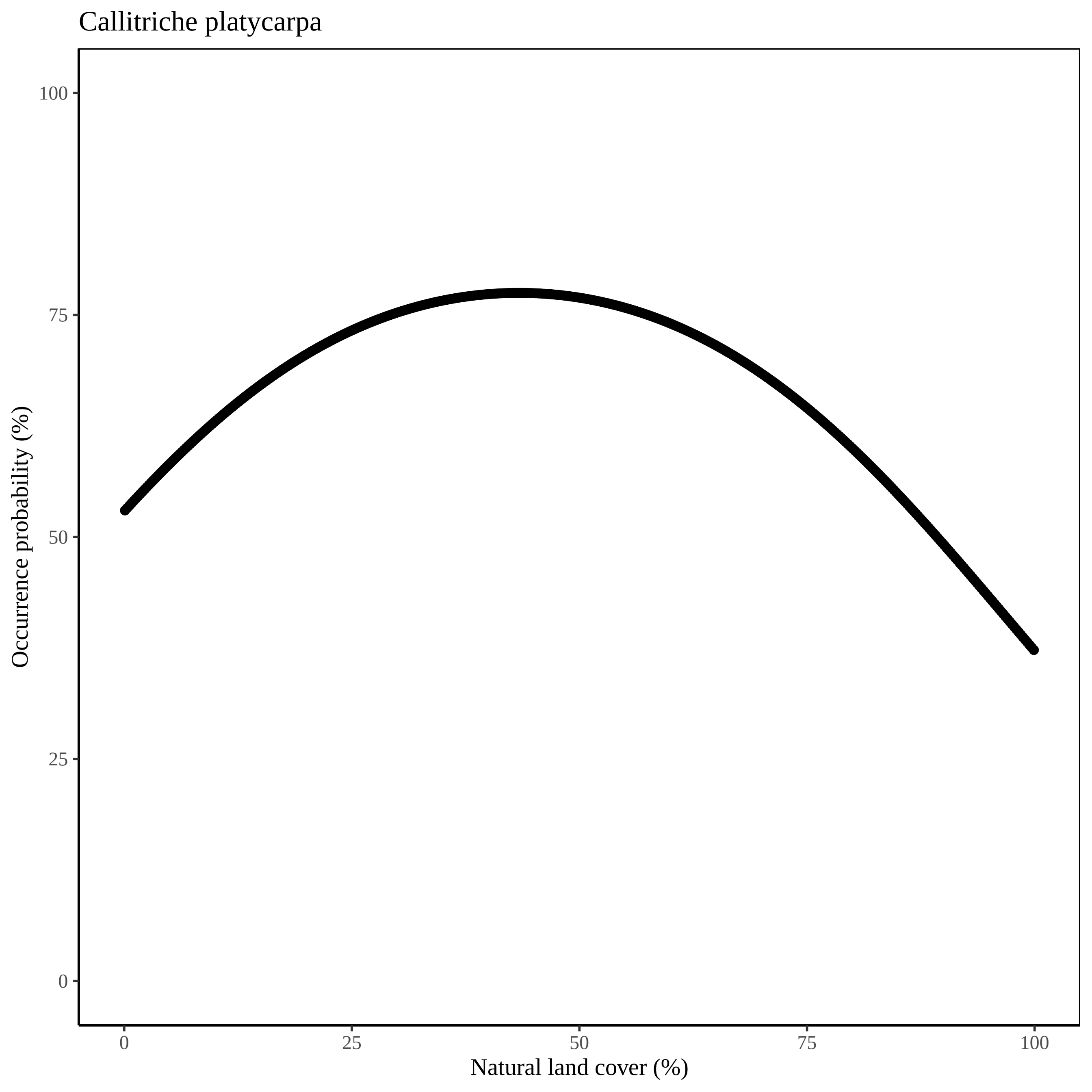

Callitriche platycarpa
Occurrence probability (%)
100
75
50
25
0
0
25
50
75
100
Natural land cover (%)

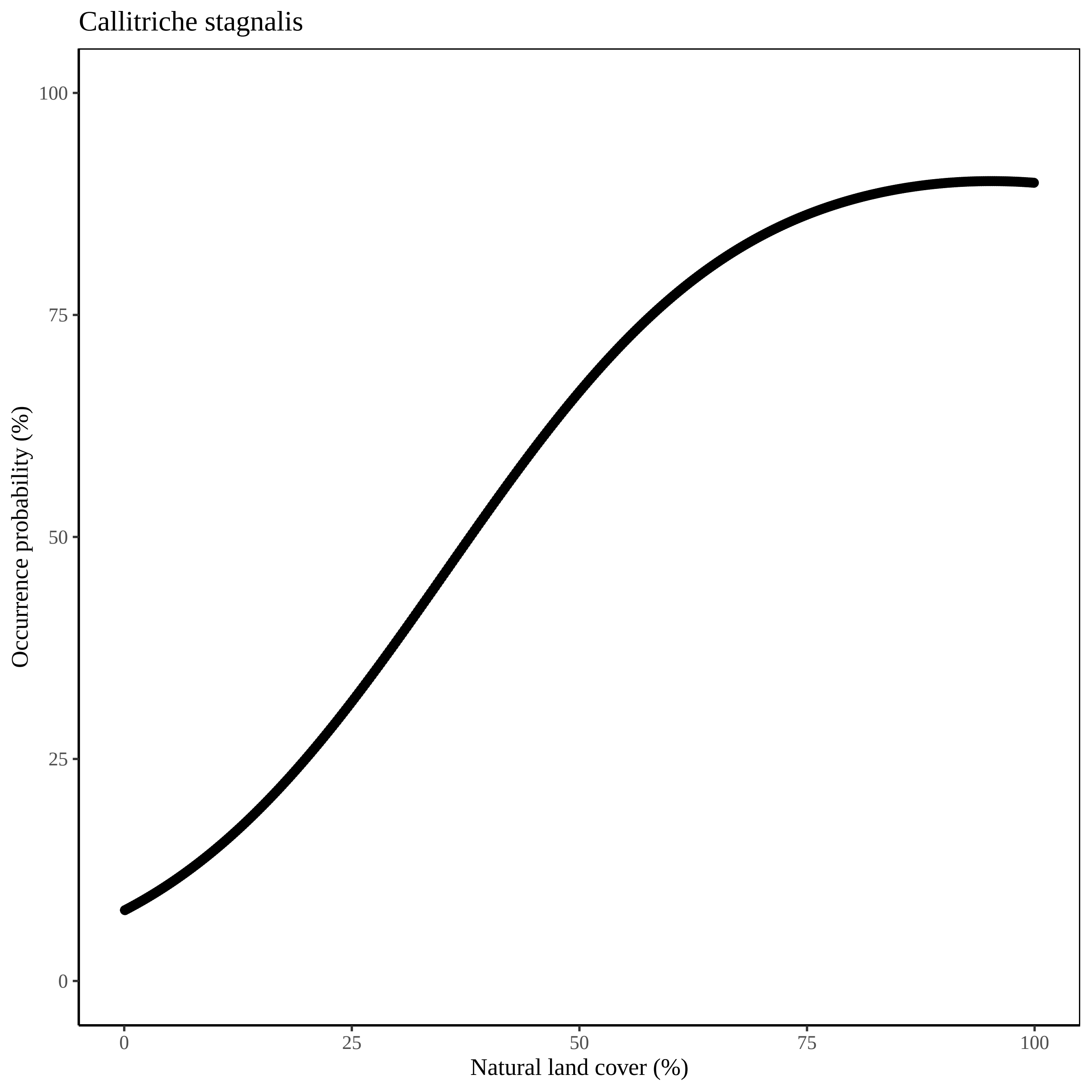
Callitriche stagnalis
Occurrence probability (%)
100
75
50
25
0
Natural land cover (%)
0
25
50
75
100

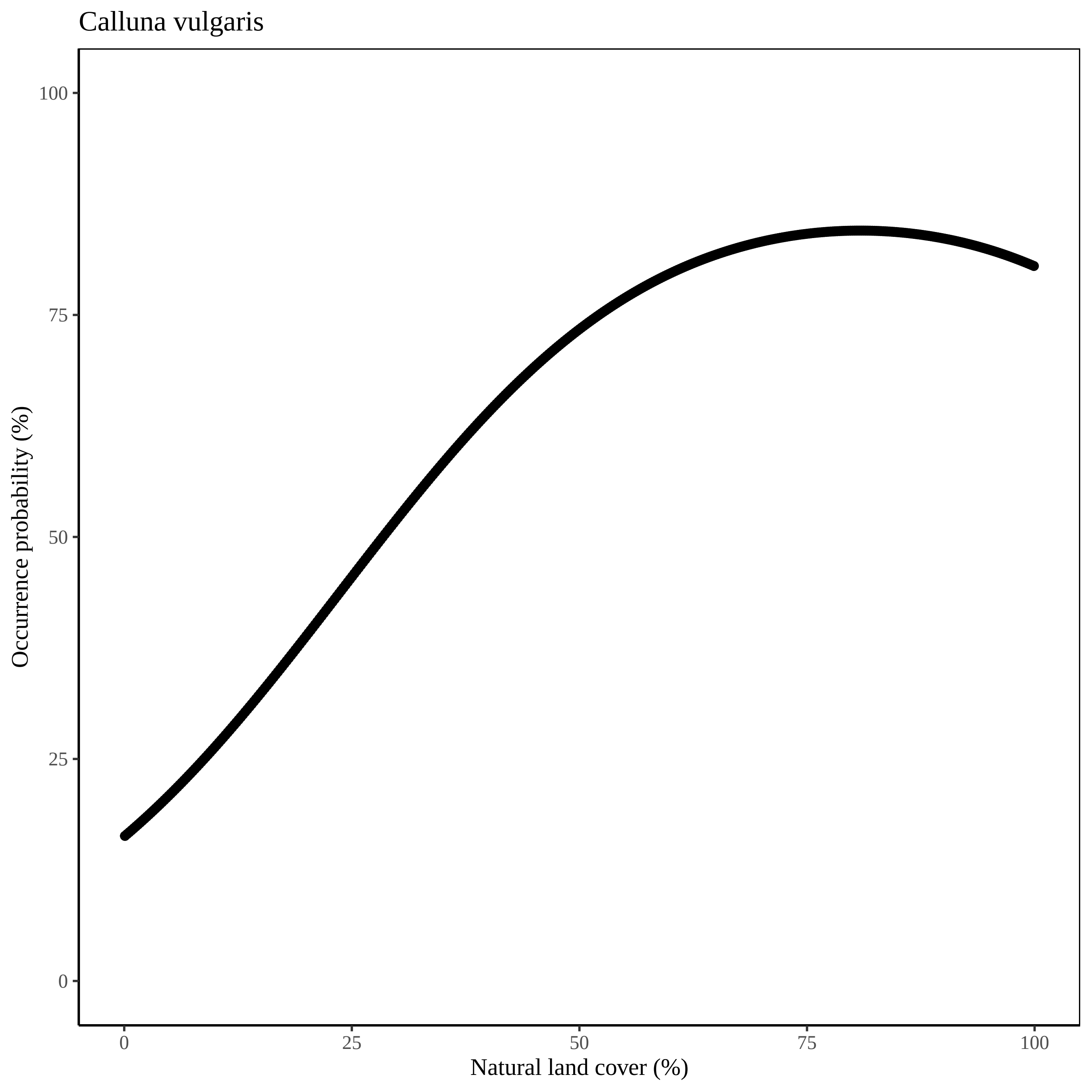
Calluna vulgaris
Occurrence probability (%)
100
75
50
25
0
0
25
50
75
100
Natural land cover (%)

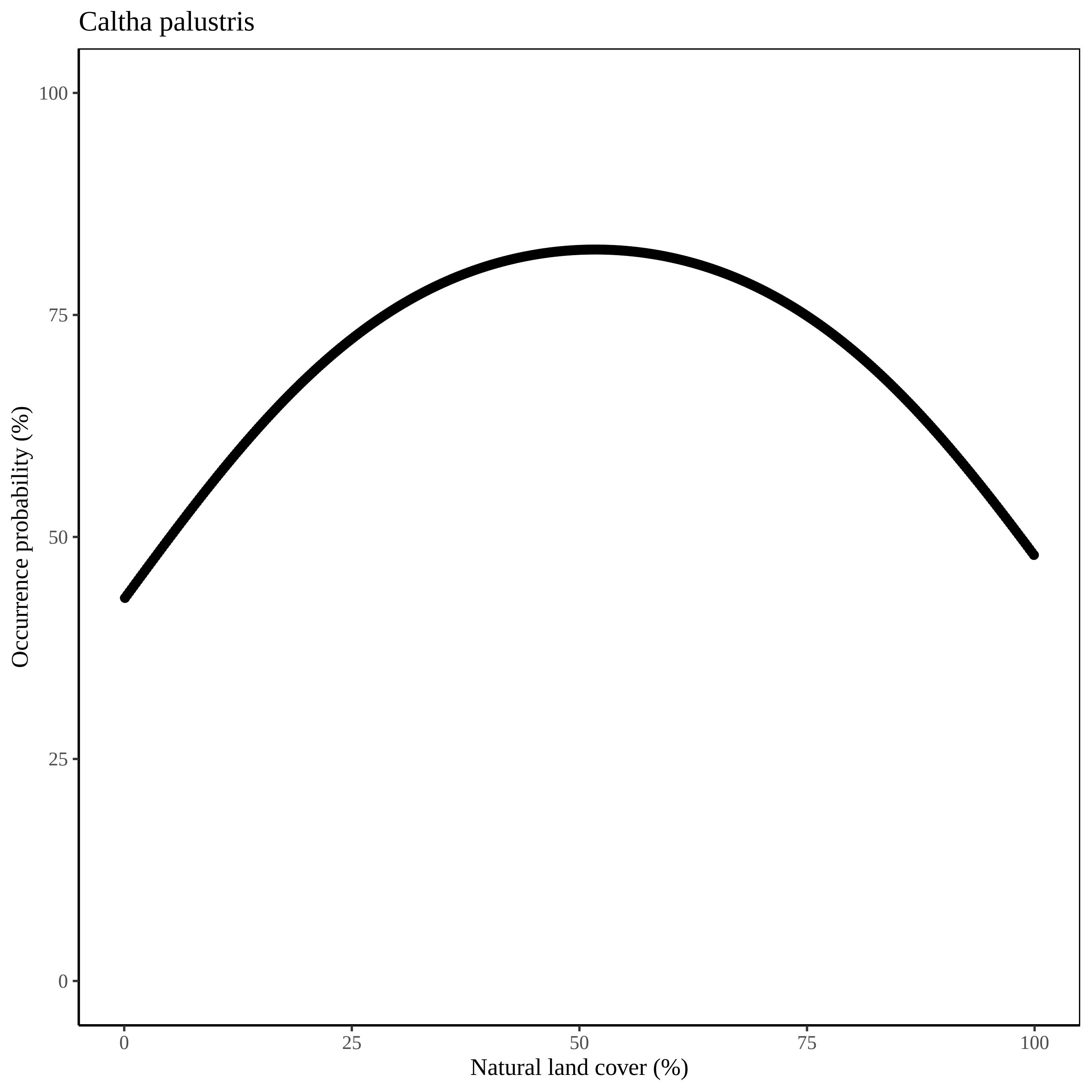

Caltha palustris
100
75
50
25
0
Occurrence probability (%)
0
25
50
75
100
Natural land cover (%)

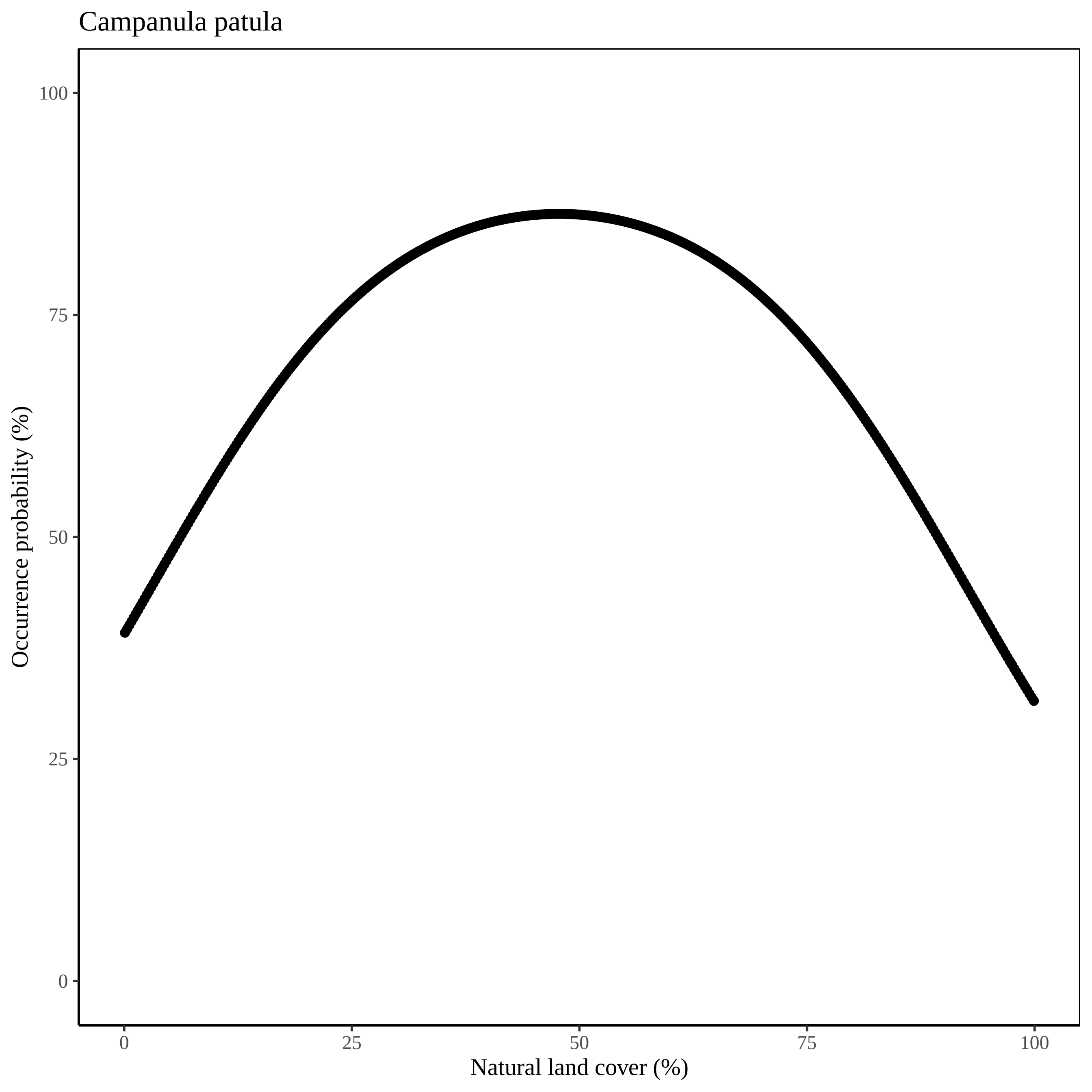

Campanula patula
Occurrence probability (%)
100
75
50
25
0
0
25
50
75
100
Natural land cover (%)

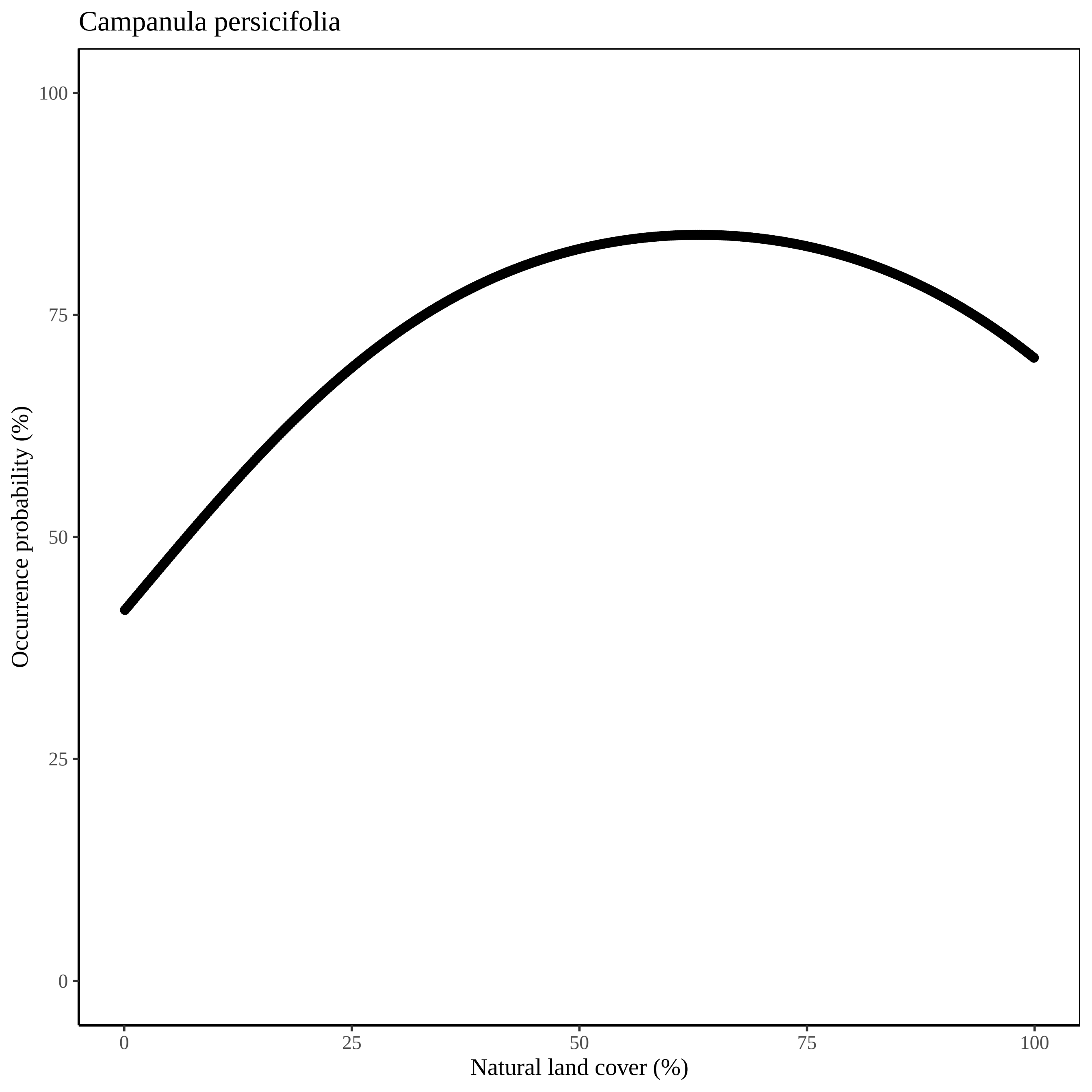

Campanula persicifolia
Occurrence probability (%)
100
75
50
25
0
0
25
50
75
100
Natural land cover (%)

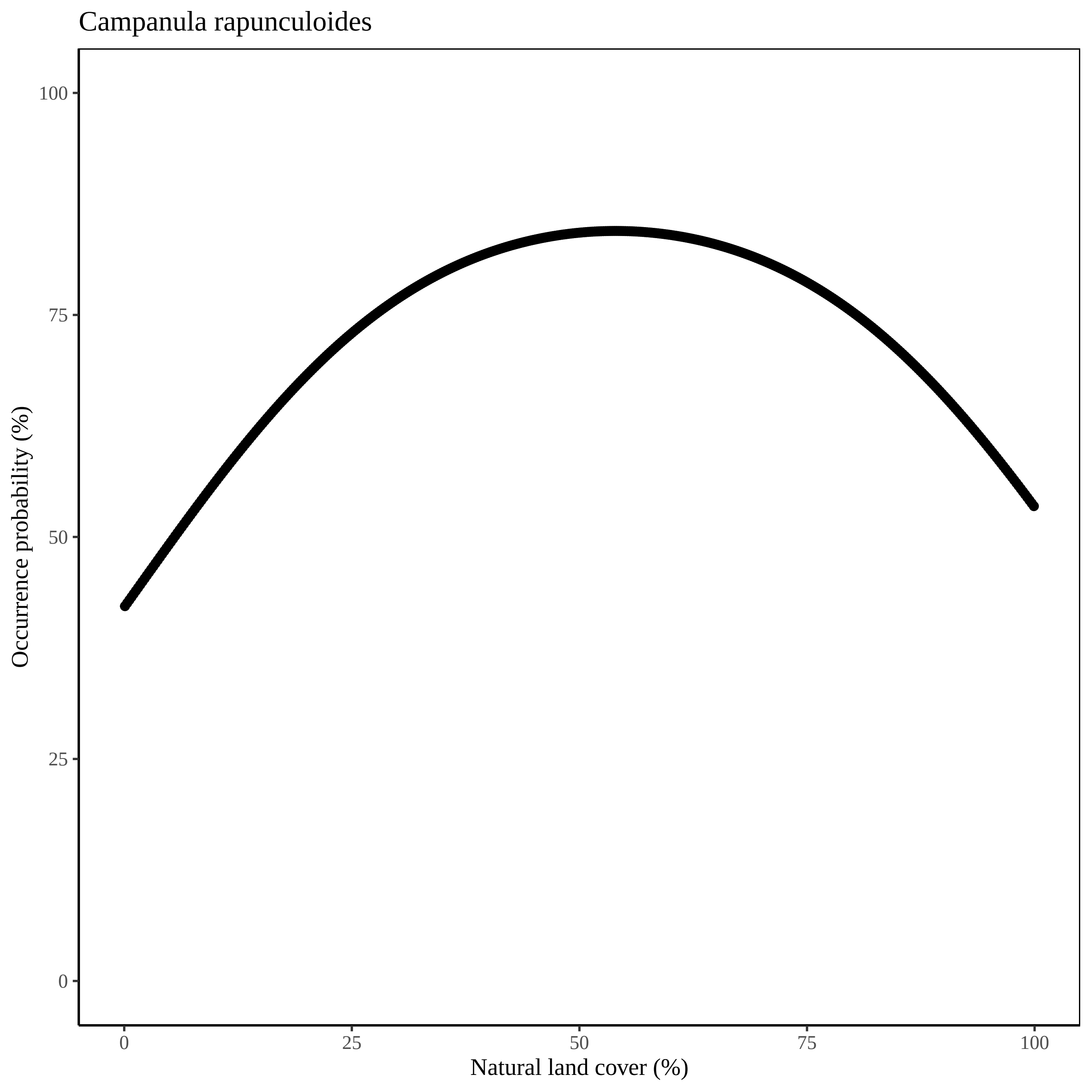

Campanula rapunculoides
Occurrence probability (%)
100
75
50
25
0
0
25
50
75
100
Natural land cover (%)

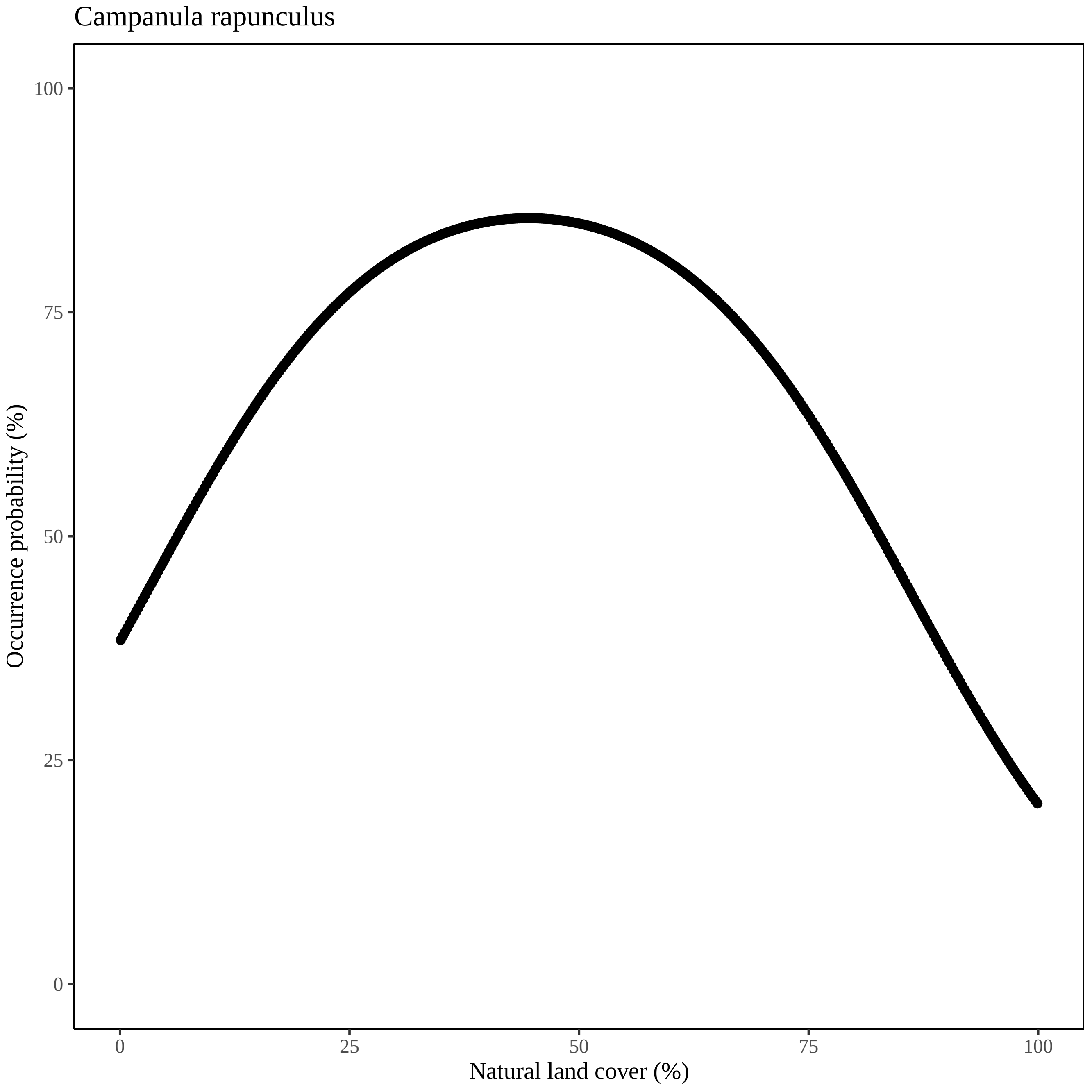

Campanula rapunculus
Occurrence probability (%)
100
75
50
25
0
0
25
50
75
100
Natural land cover (%)

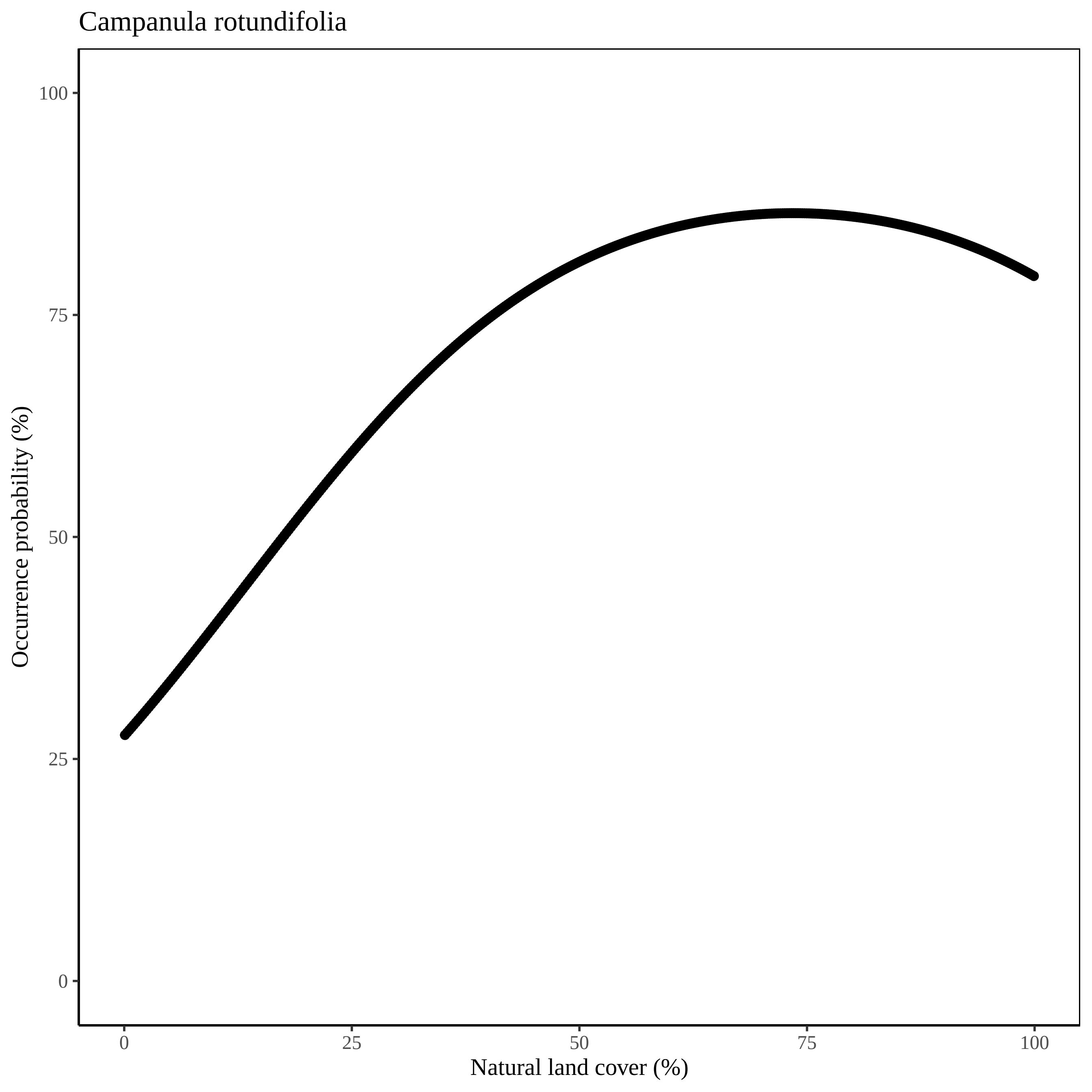

Campanula rotundifolia
Occurrence probability (%)
100
75
50
25
0
0
25
50
75
100
Natural land cover (%)

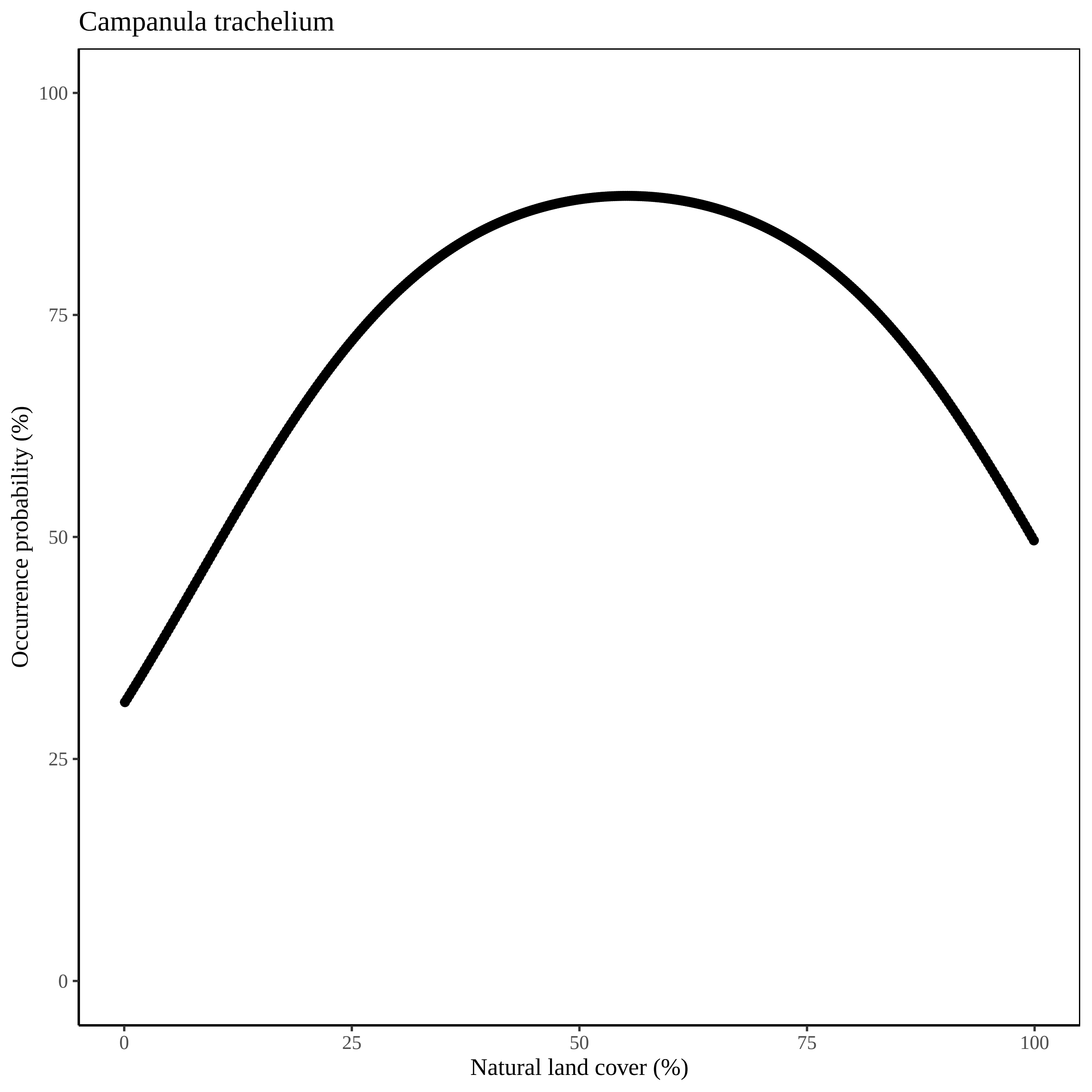

Campanula trachelium
Occurrence probability (%)
Natural land cover (%)
0
25
50
75
100

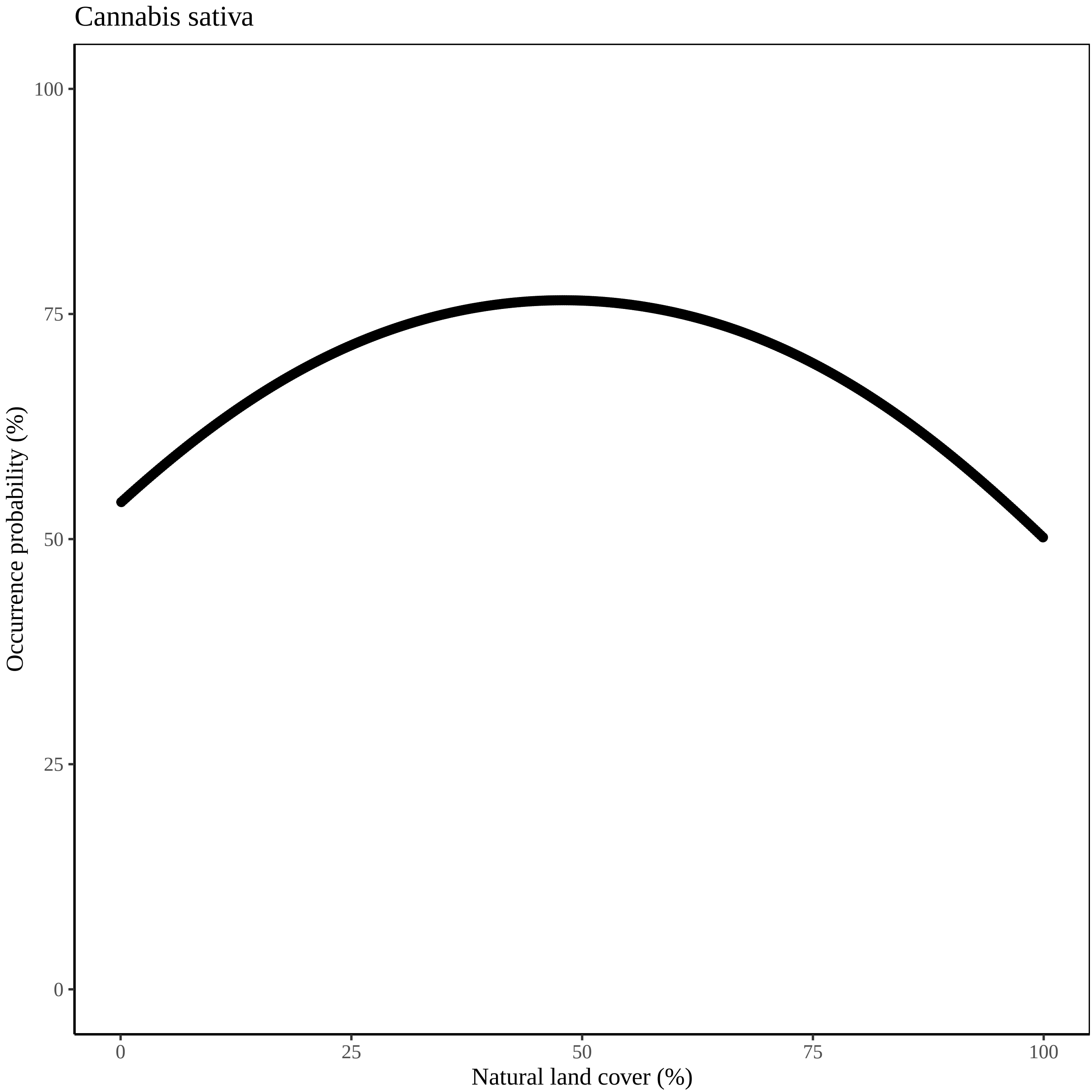

Cannabis sativa
100
75
50
25
0
Occurrence probability (%)
0
25
50
75
100
Natural land cover (%)

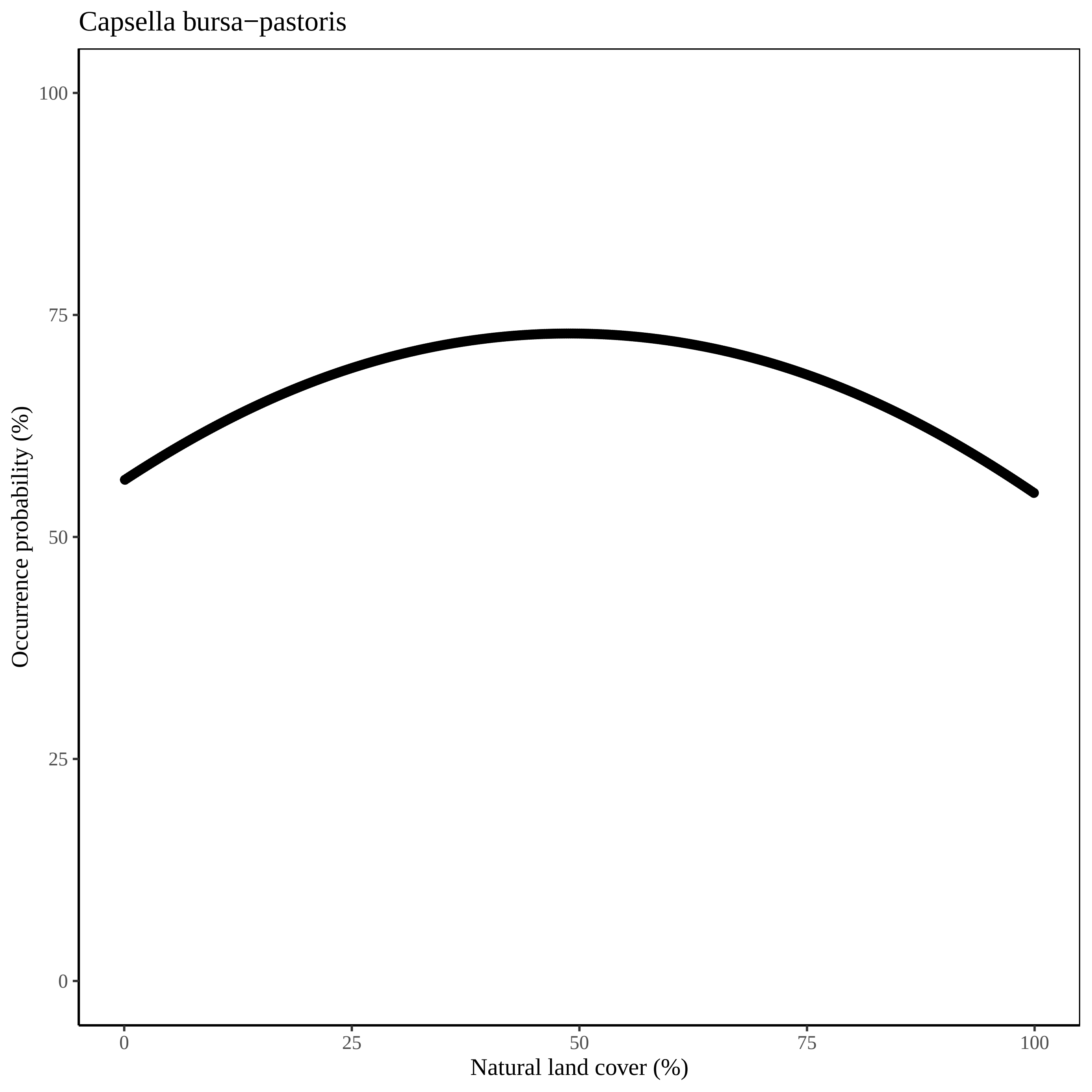
Capsella bursa−pastoris
Occurrence probability (%)
100
75
50
25
0
0
25
50
75
100
Natural land cover (%)

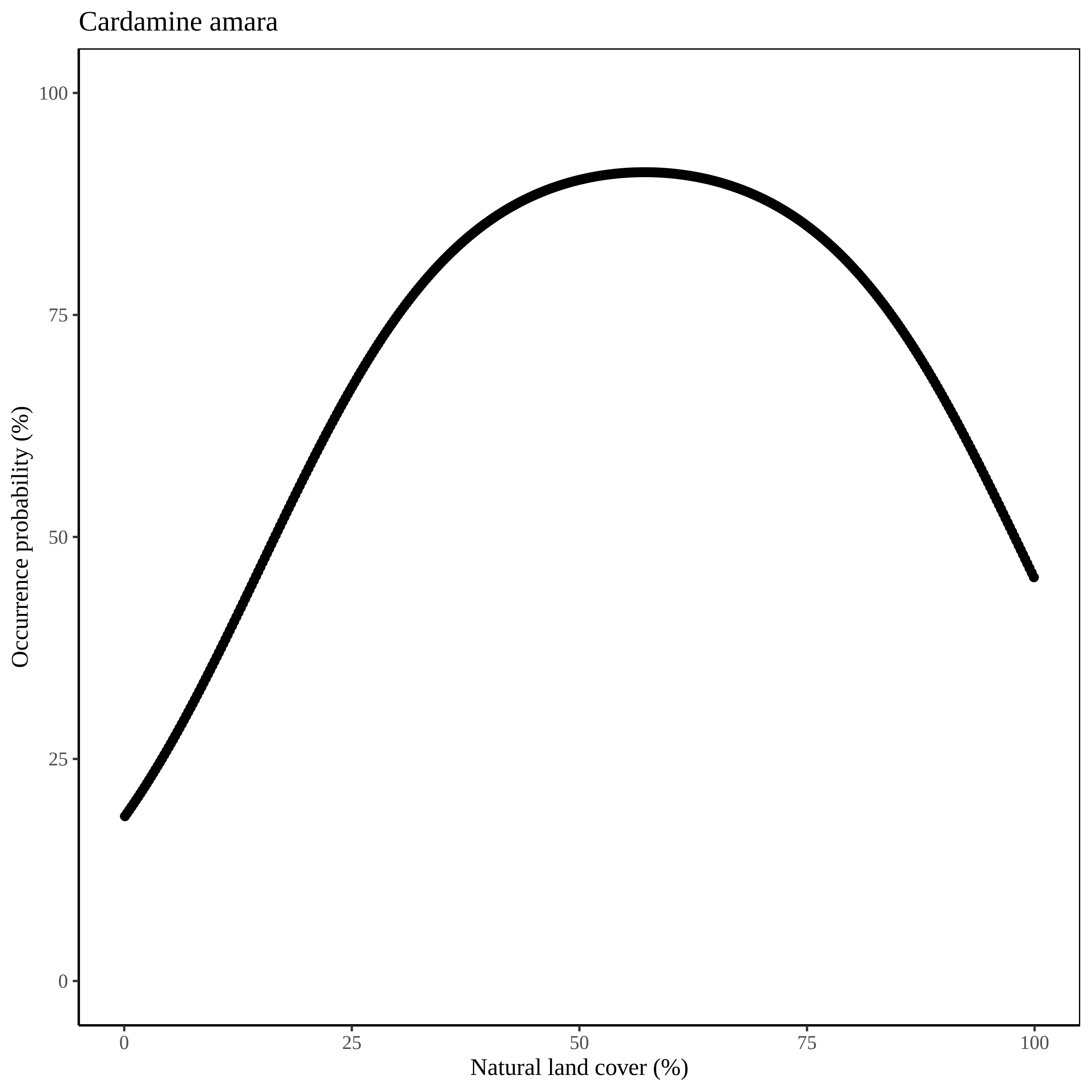
Cardamine amara
Occurrence probability (%)
100
75
50
25
0
0
25
50
75
100
Natural land cover (%)

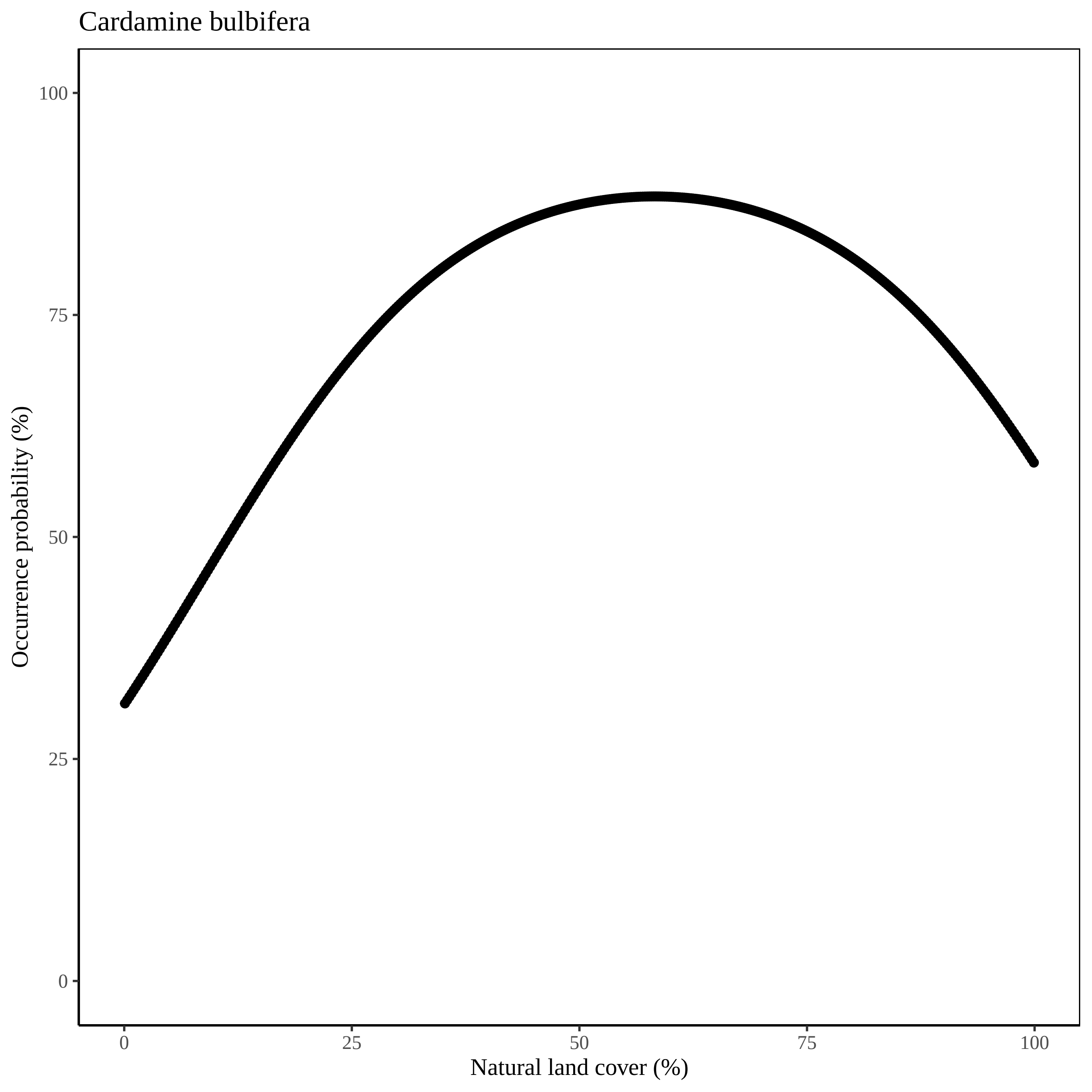

Cardamine bulbifera
100
75
50
25
0
Occurrence probability (%)
0
25
50
75
100
Natural land cover (%)

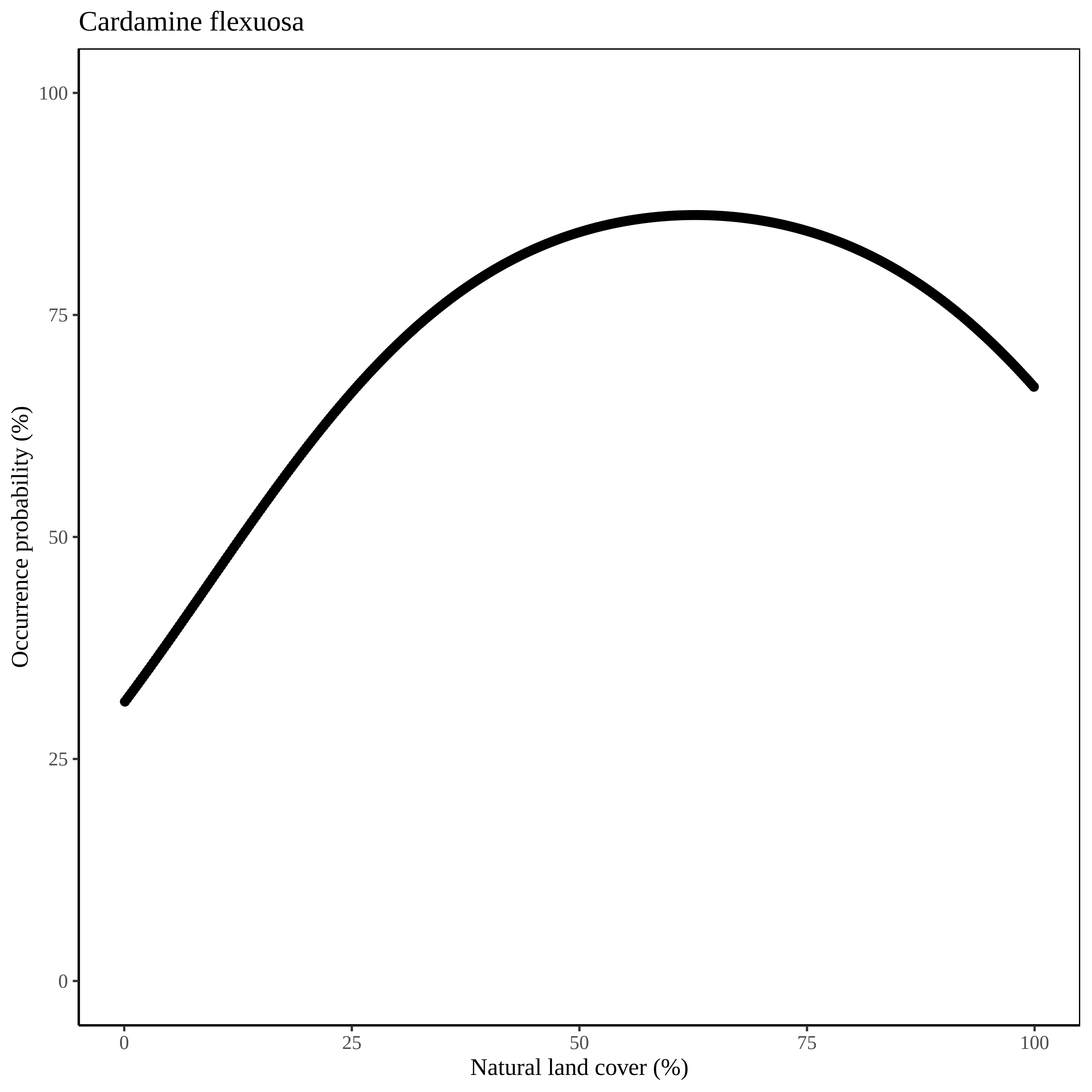

Cardamine flexuosa
Occurrence probability (%)
100
75
50
25
0
0
25
50
75
100
Natural land cover (%)

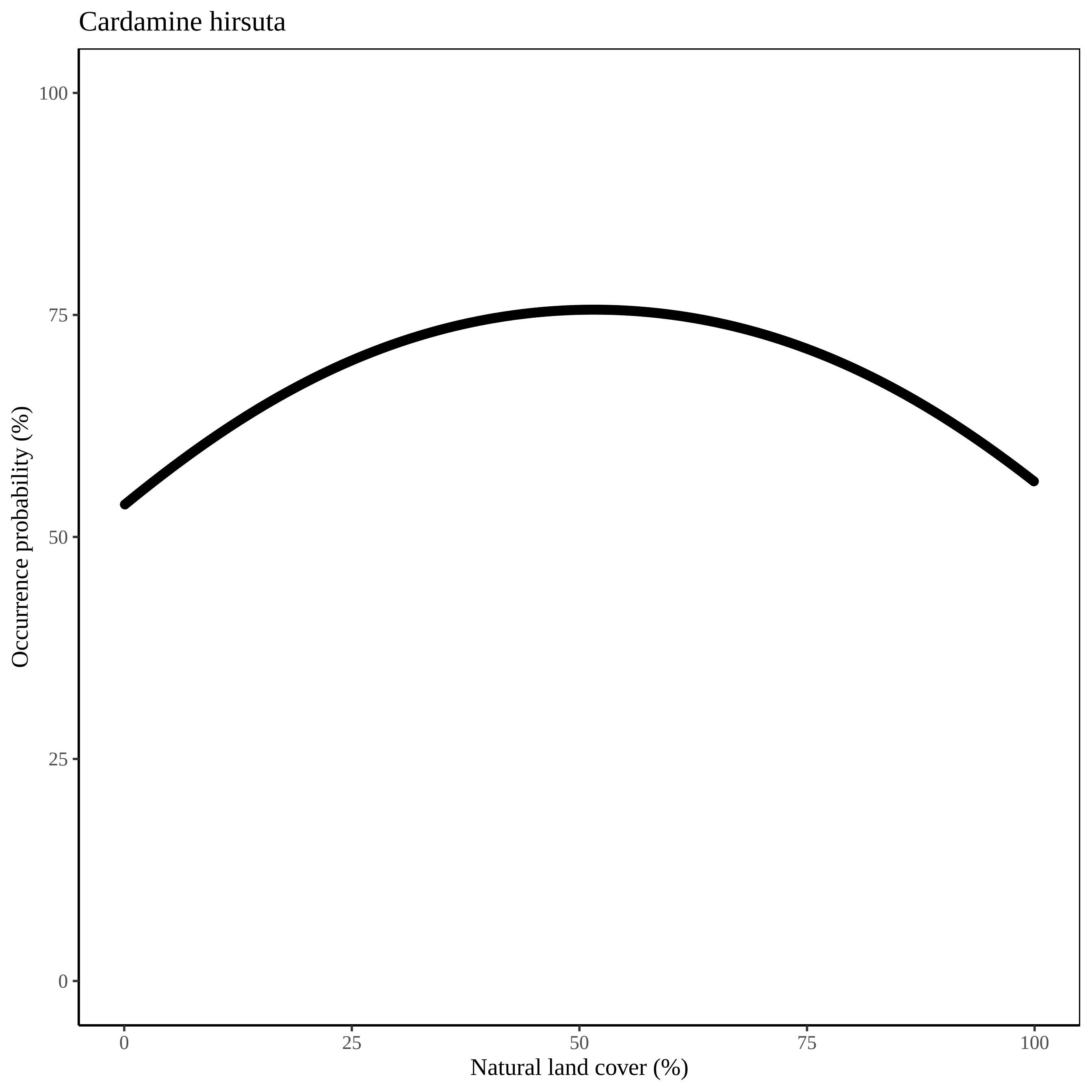

Cardamine hirsuta
Occurrence probability (%)
100
75
50
25
0
0
25
50
75
100
Natural land cover (%)

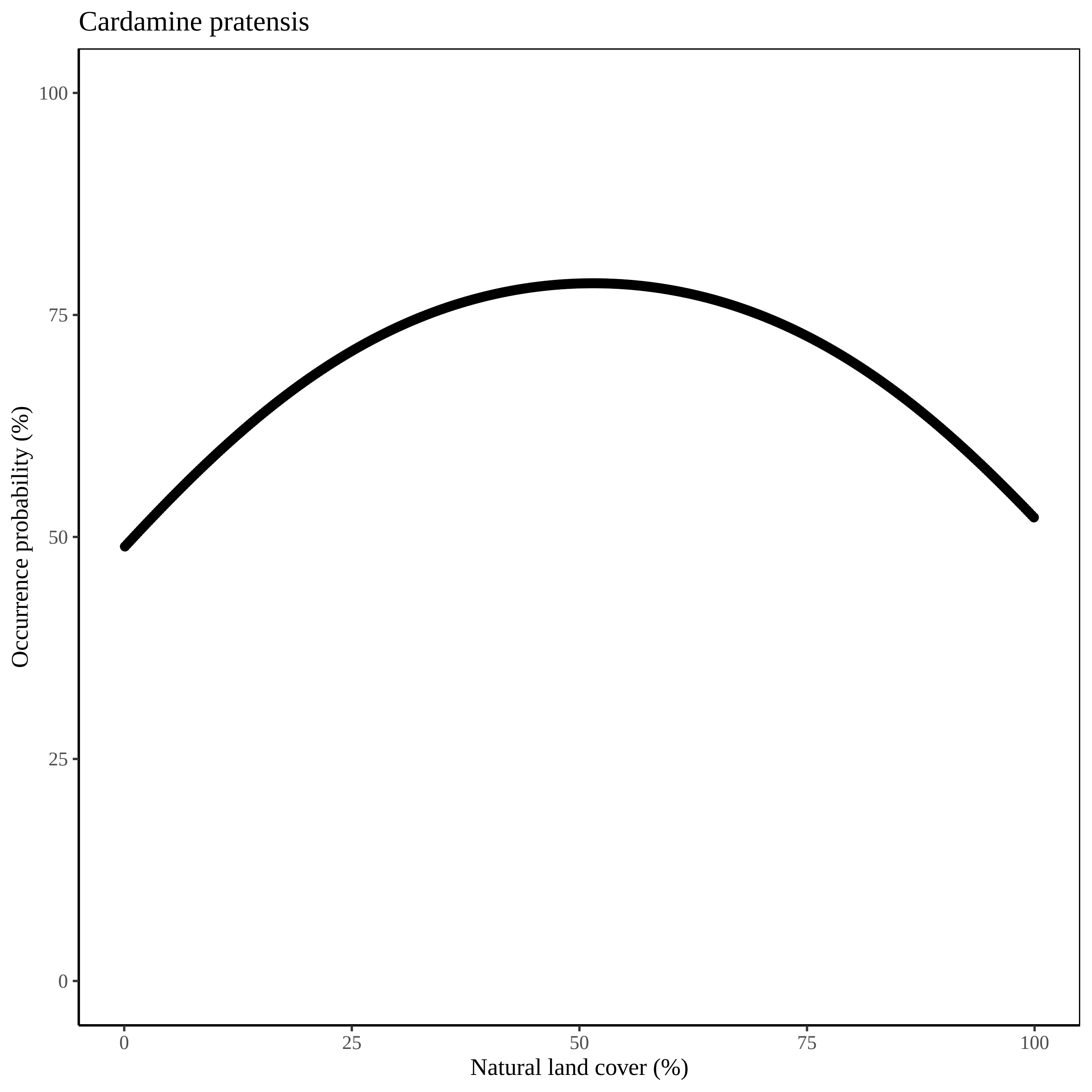

Cardamine pratensis
Occurrence probability (%)
100
75
50
25
0
0
25
50
75
100
Natural land cover (%)

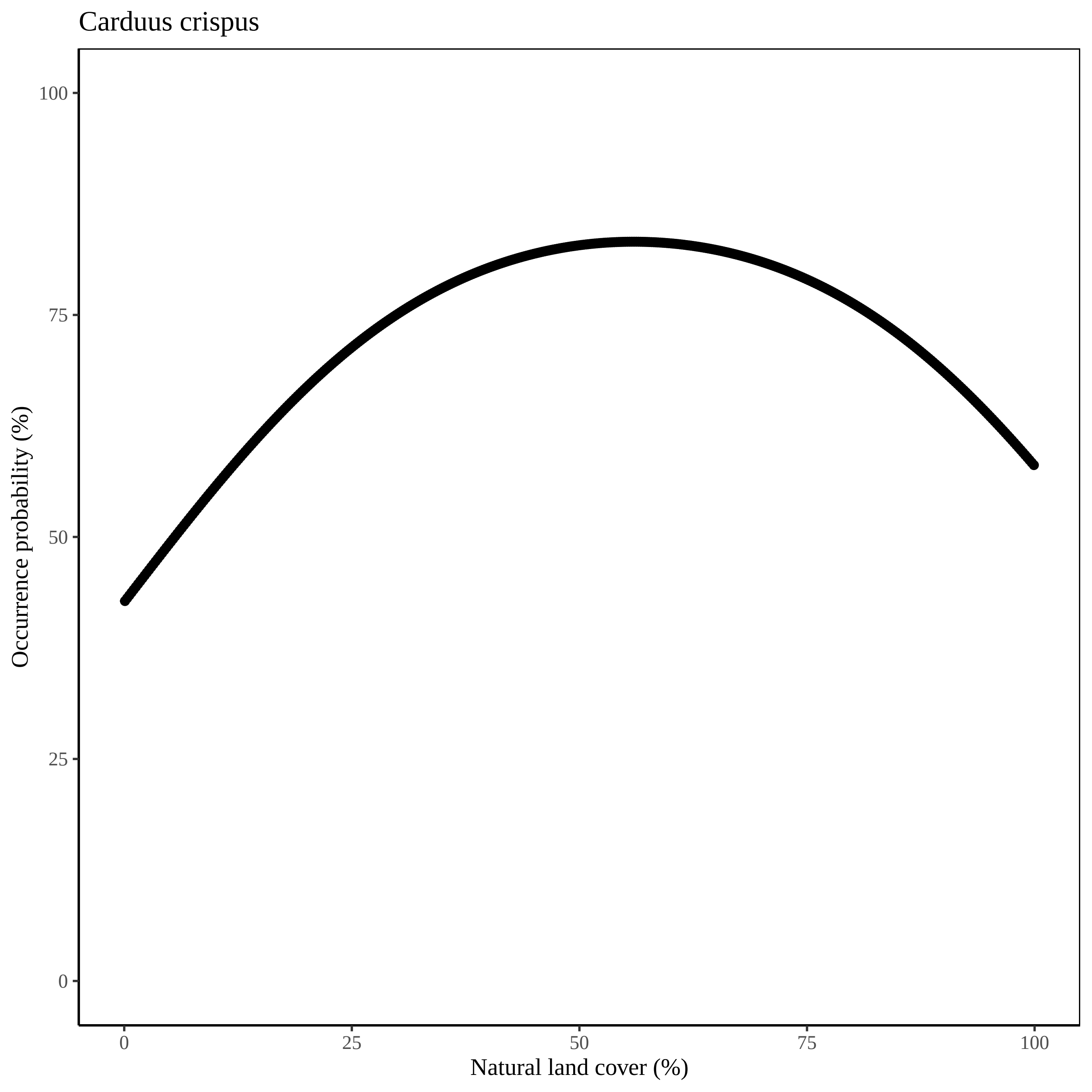
Carduus crispus
Occurrence probability (%)
100
75
50
25
0
0
25
50
75
100
Natural land cover (%)

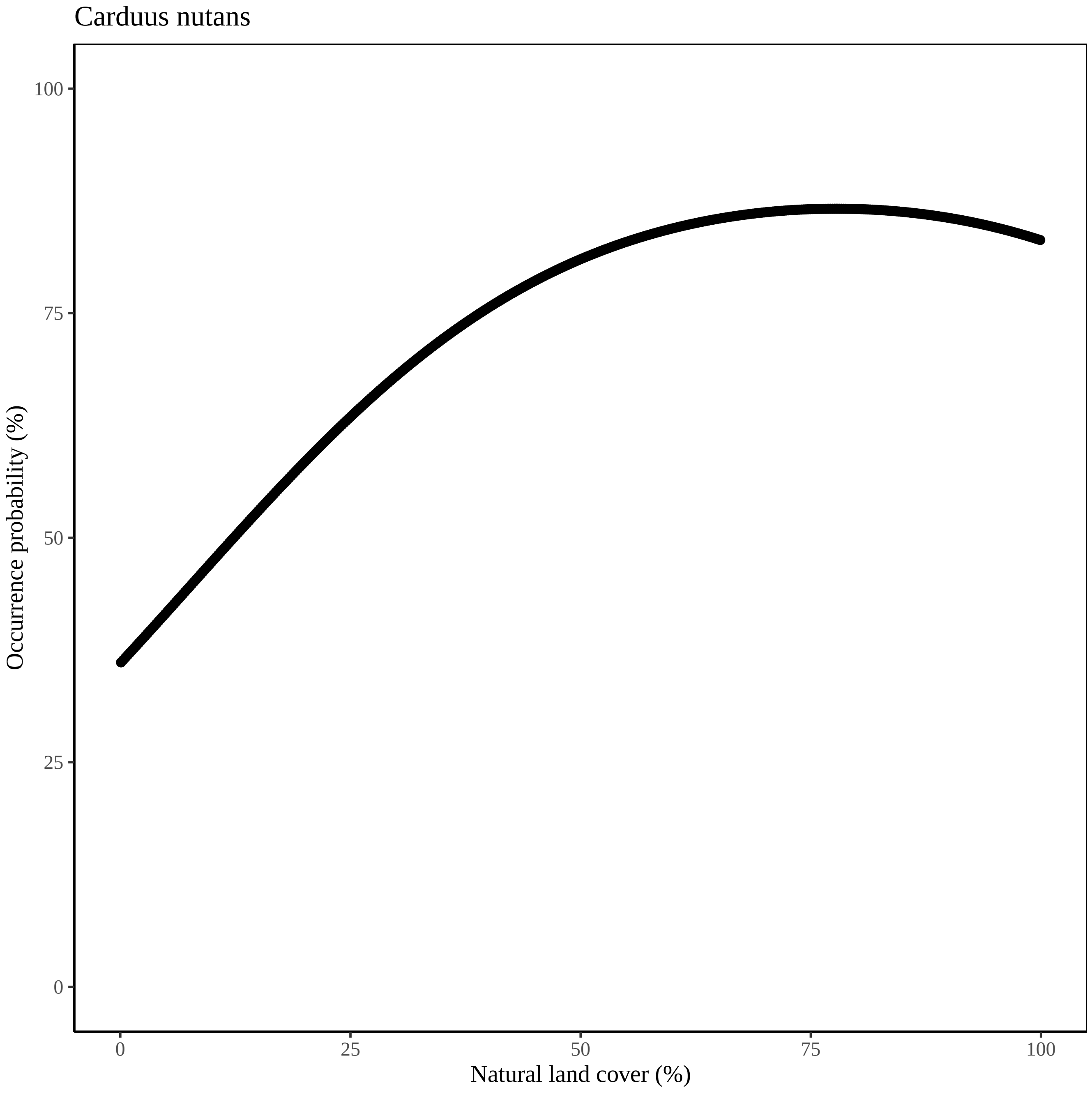

Carduus nutans
Occurrence probability (%)
100
75
50
25
0
0
25
50
75
100
Natural land cover (%)

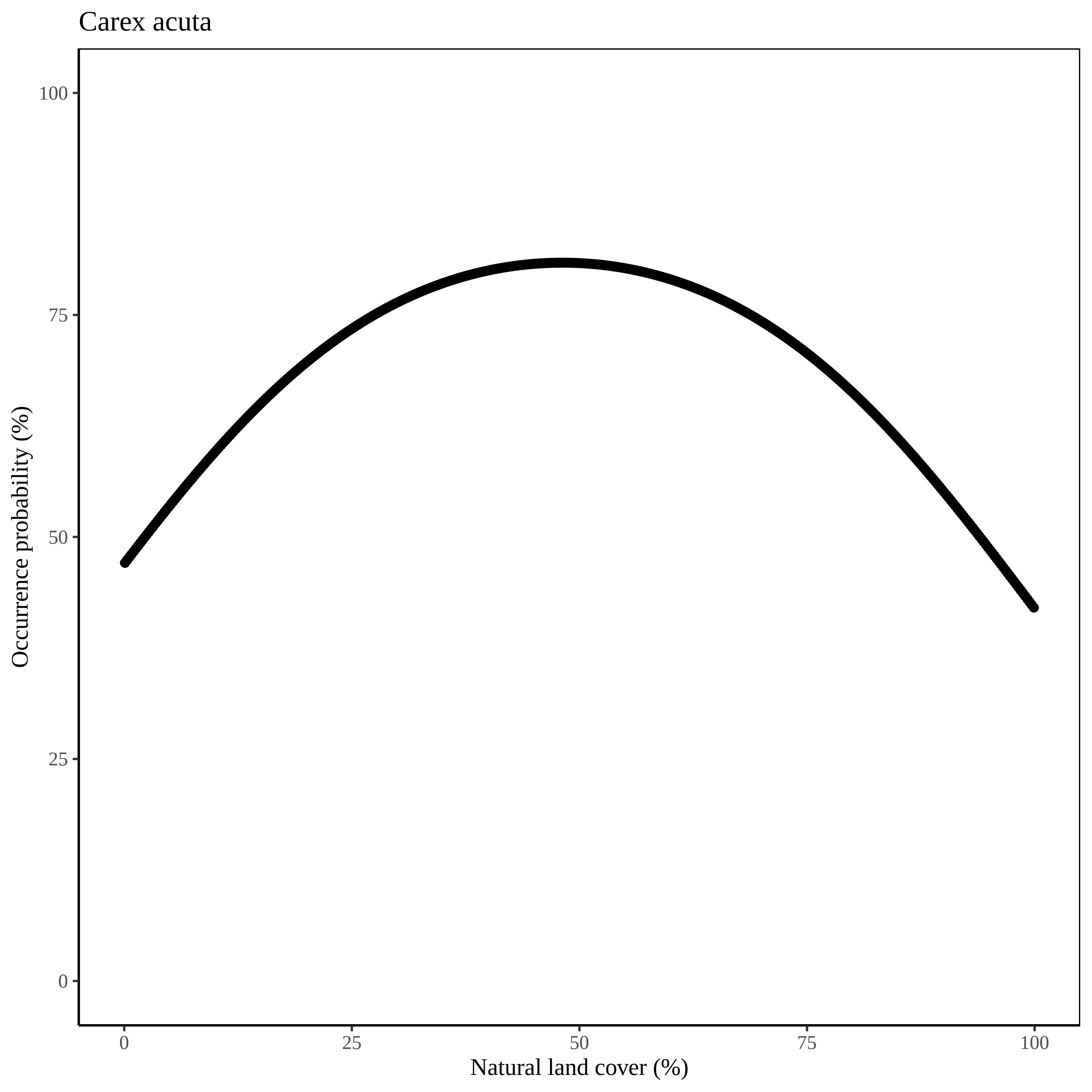
Carex acuta
Occurrence probability (%)
100
75
50
25
0
0
25
50
75
100
Natural land cover (%)

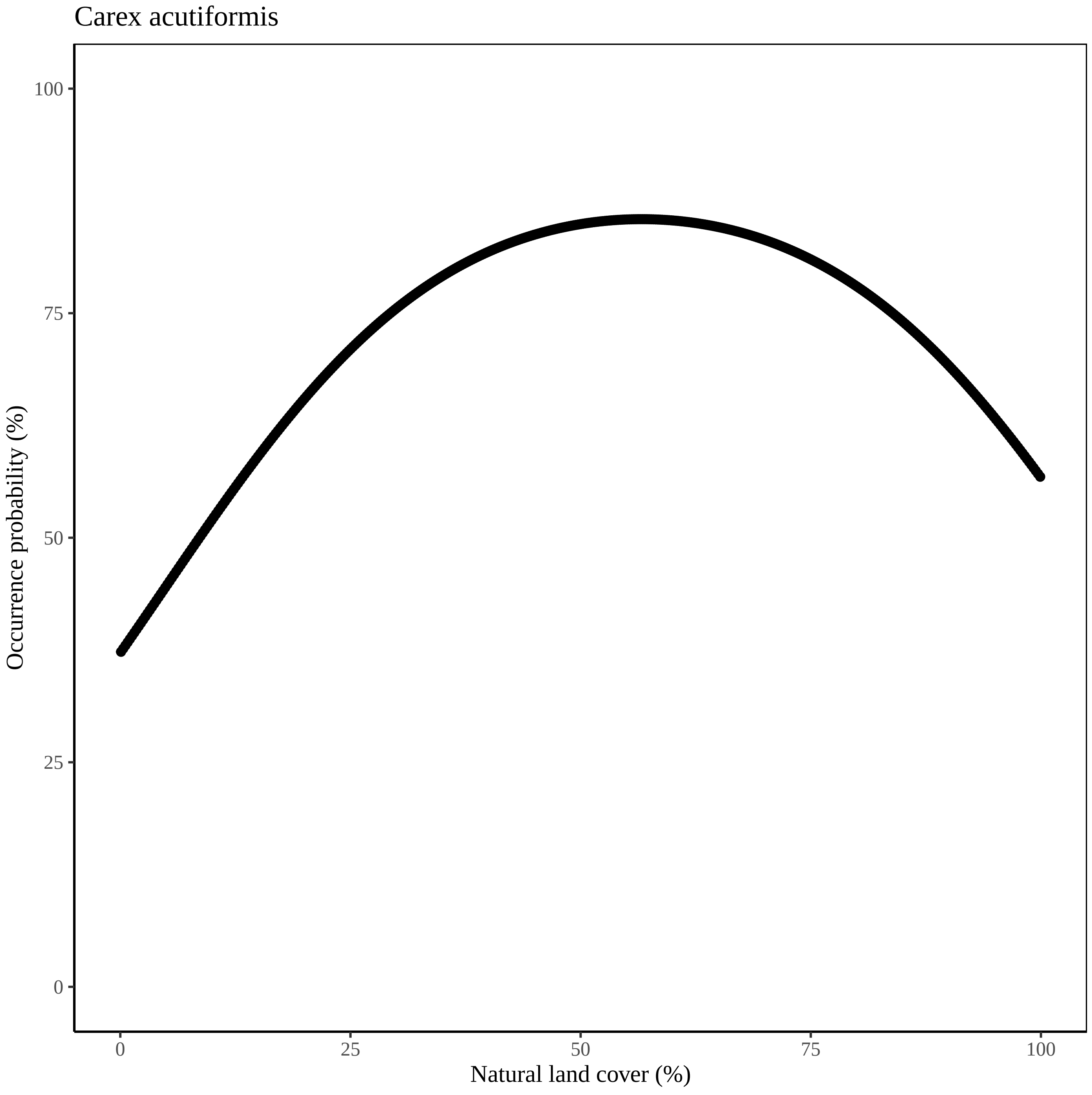
Carex acutiformis
Occurrence probability (%)
100
75
50
25
0
0
25
50
75
100
Natural land cover (%)

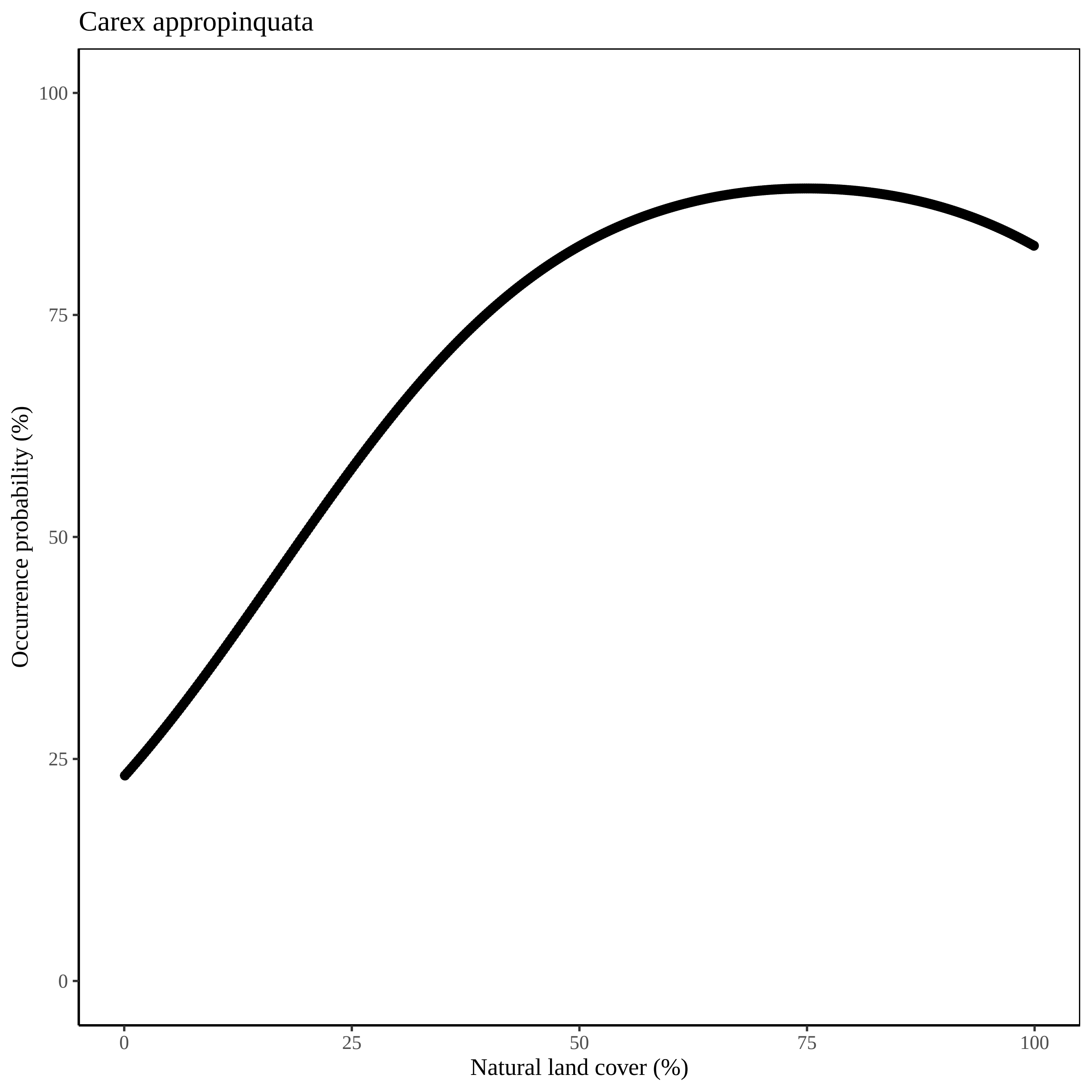

Carex appropinquata
100
75
50
25
0
Occurrence probability (%)
0
25
50
75
100
Natural land cover (%)

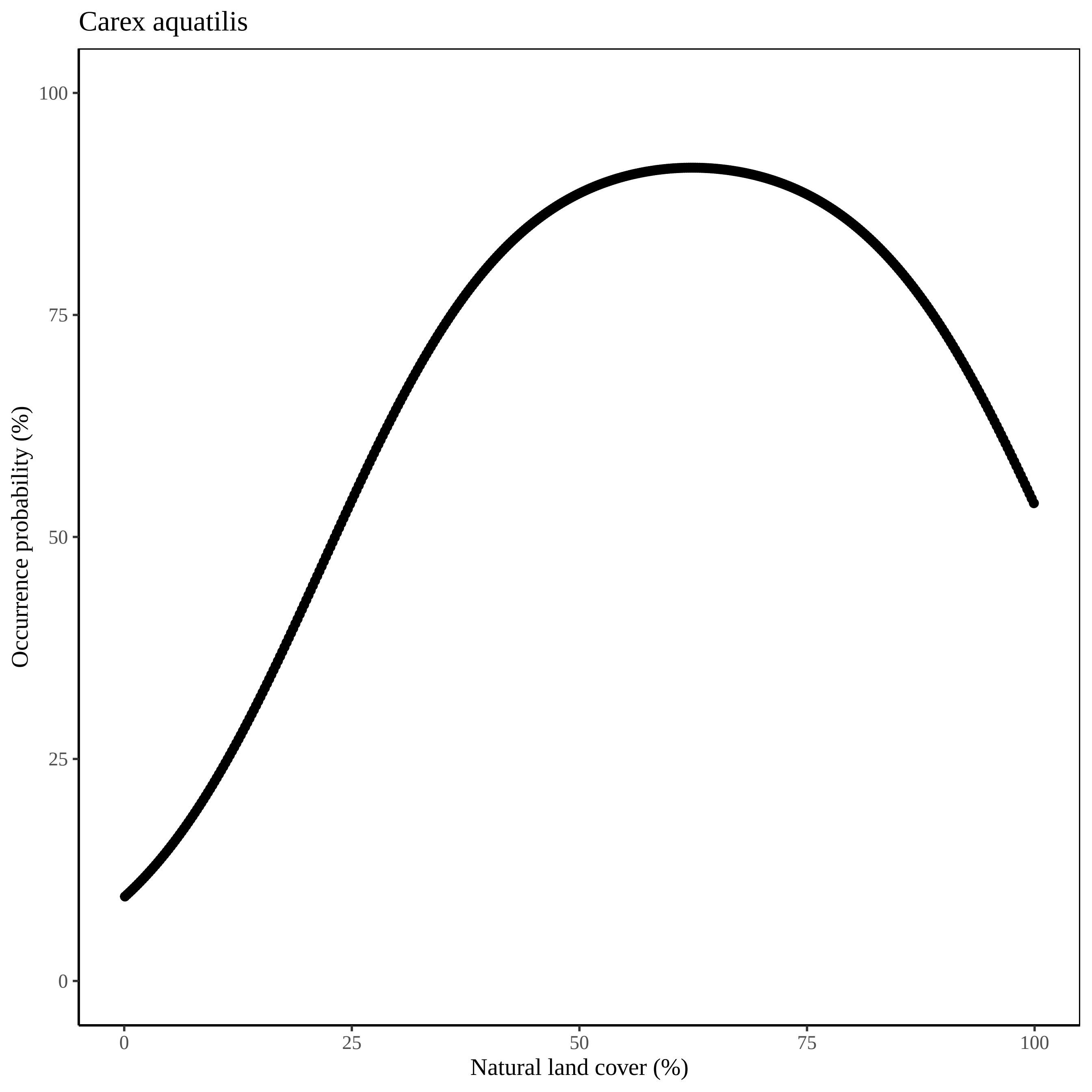

Carex aquatilis
100
75
50
25
0
Occurrence probability (%)
0
25
50
75
100
Natural land cover (%)

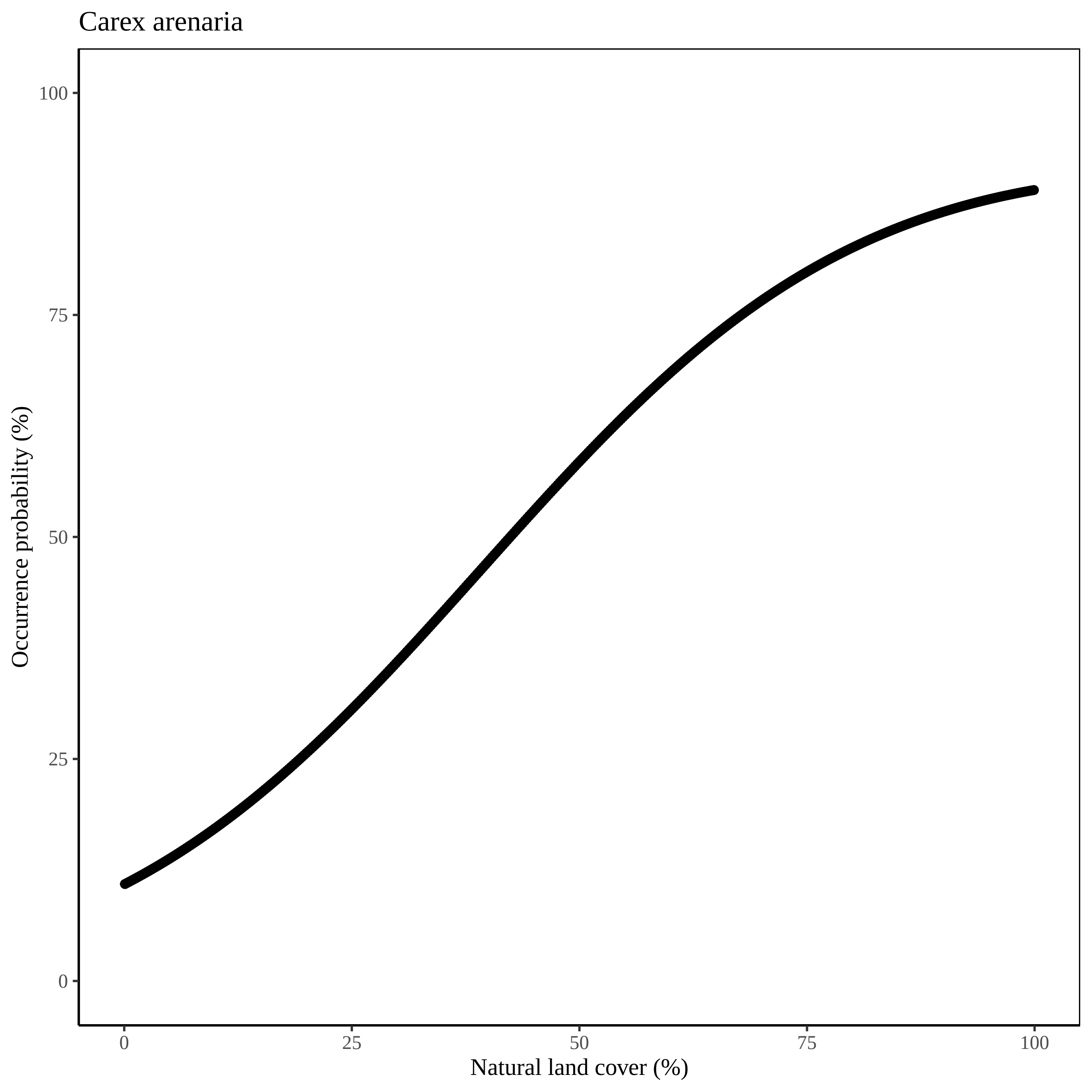

Carex arenaria
Occurrence probability (%)
100
75
50
25
0
0
25
50
75
100
Natural land cover (%)

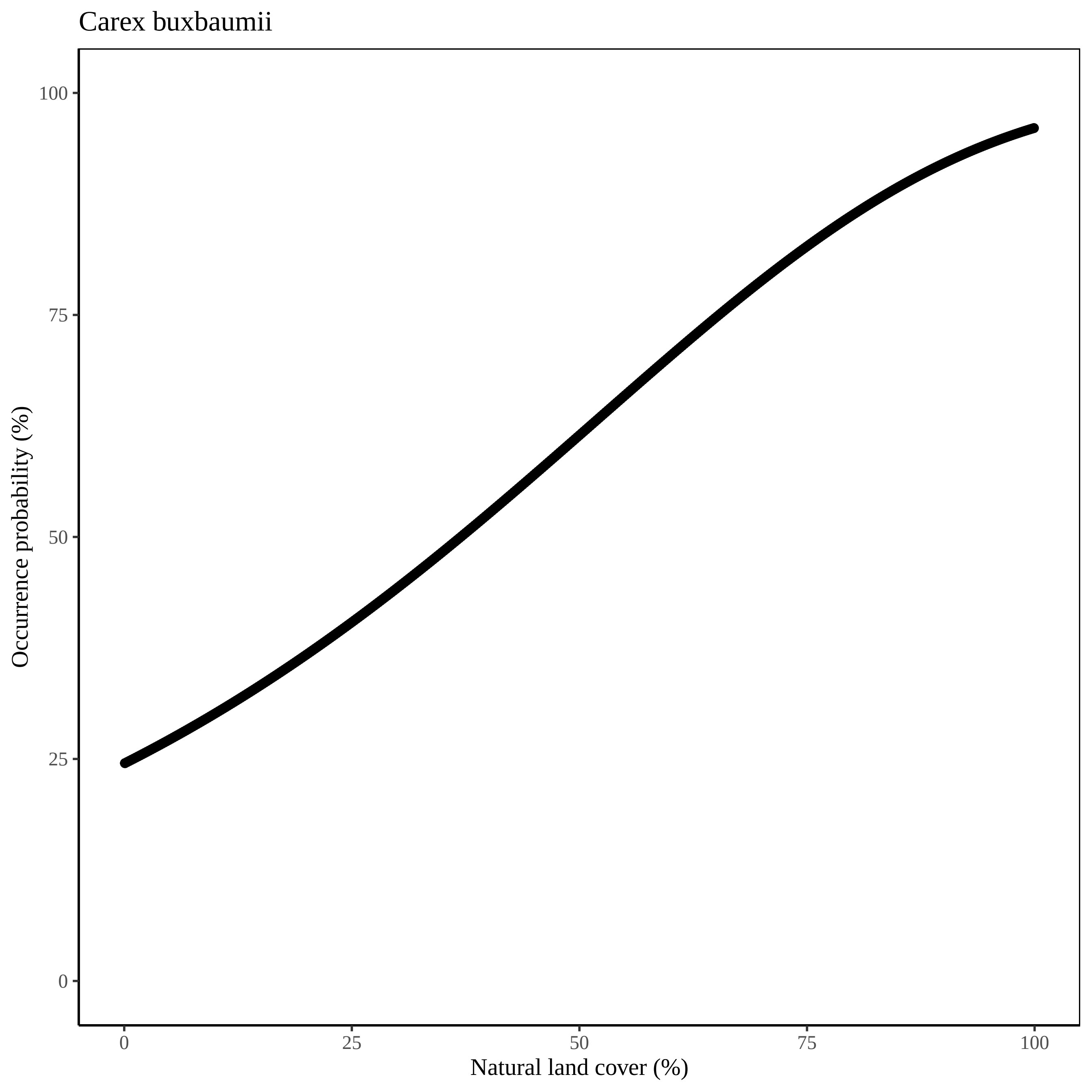

Carex buxbaumii
Occurrence probability (%)
100
75
50
25
0
0
25
50
75
100
Natural land cover (%)

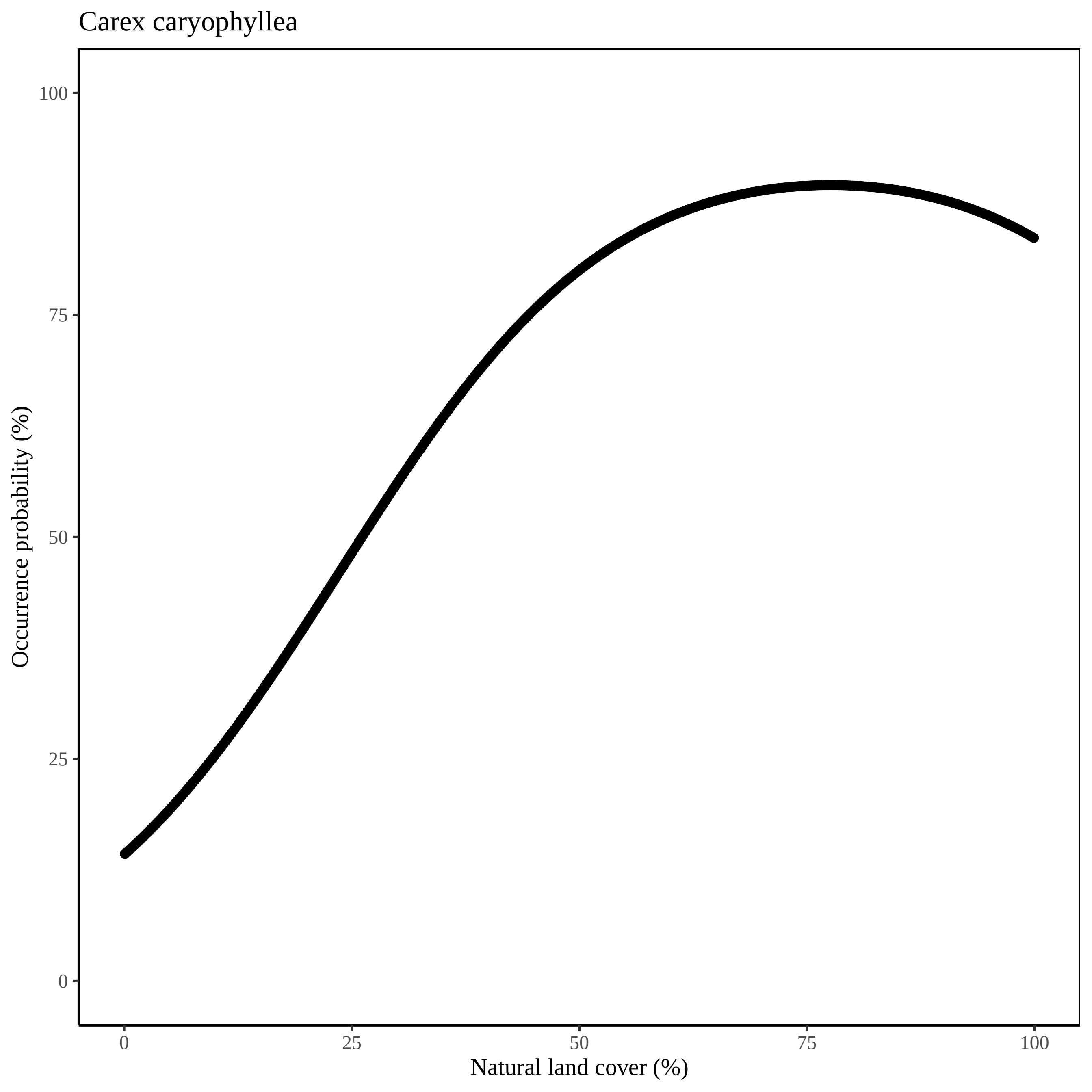
Carex caryophyllea
Occurrence probability (%)
100
75
50
25
0
0
25
50
75
100
Natural land cover (%)

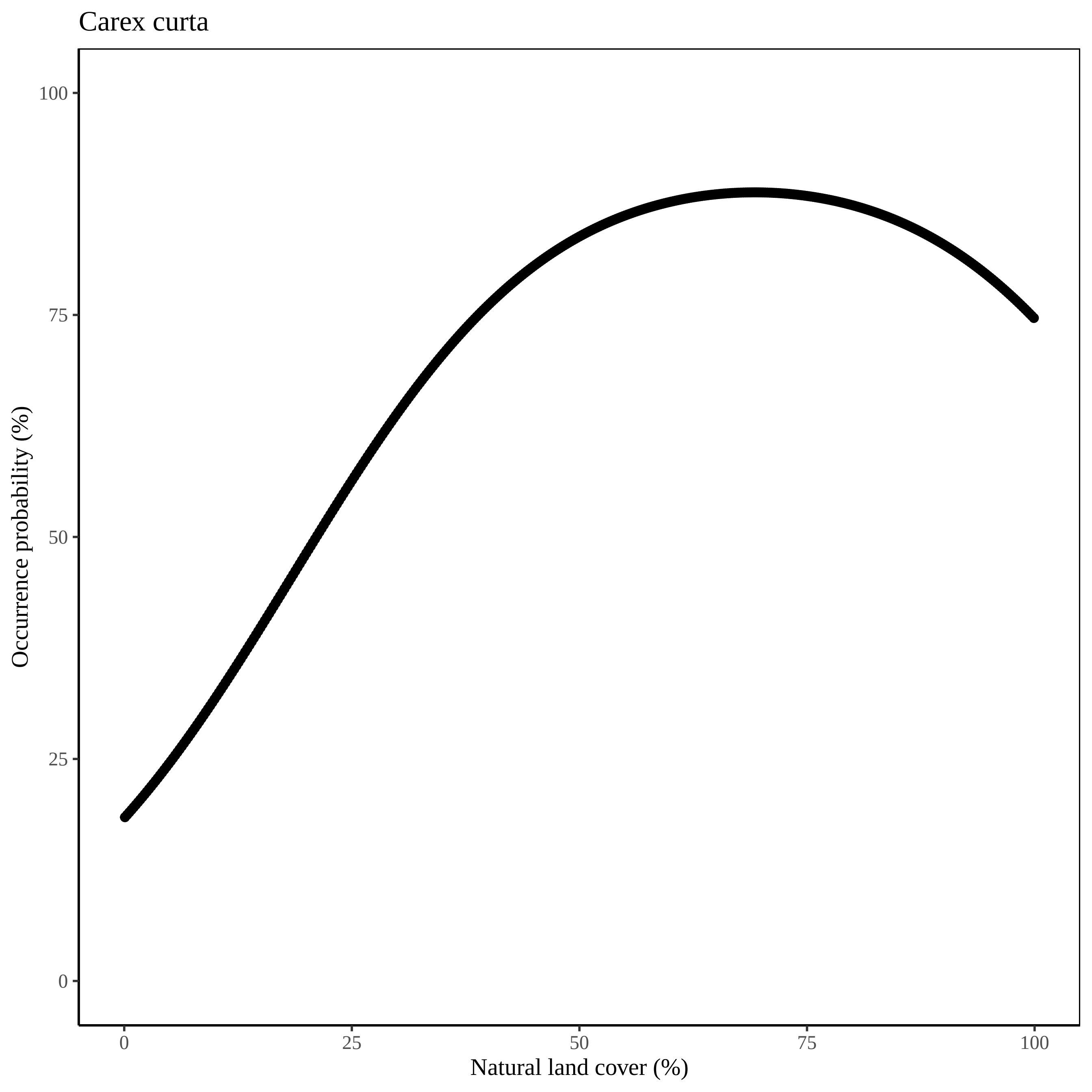
Carex curta
Occurrence probability (%)
100
75
50
25
0
0
25
50
75
100
Natural land cover (%)

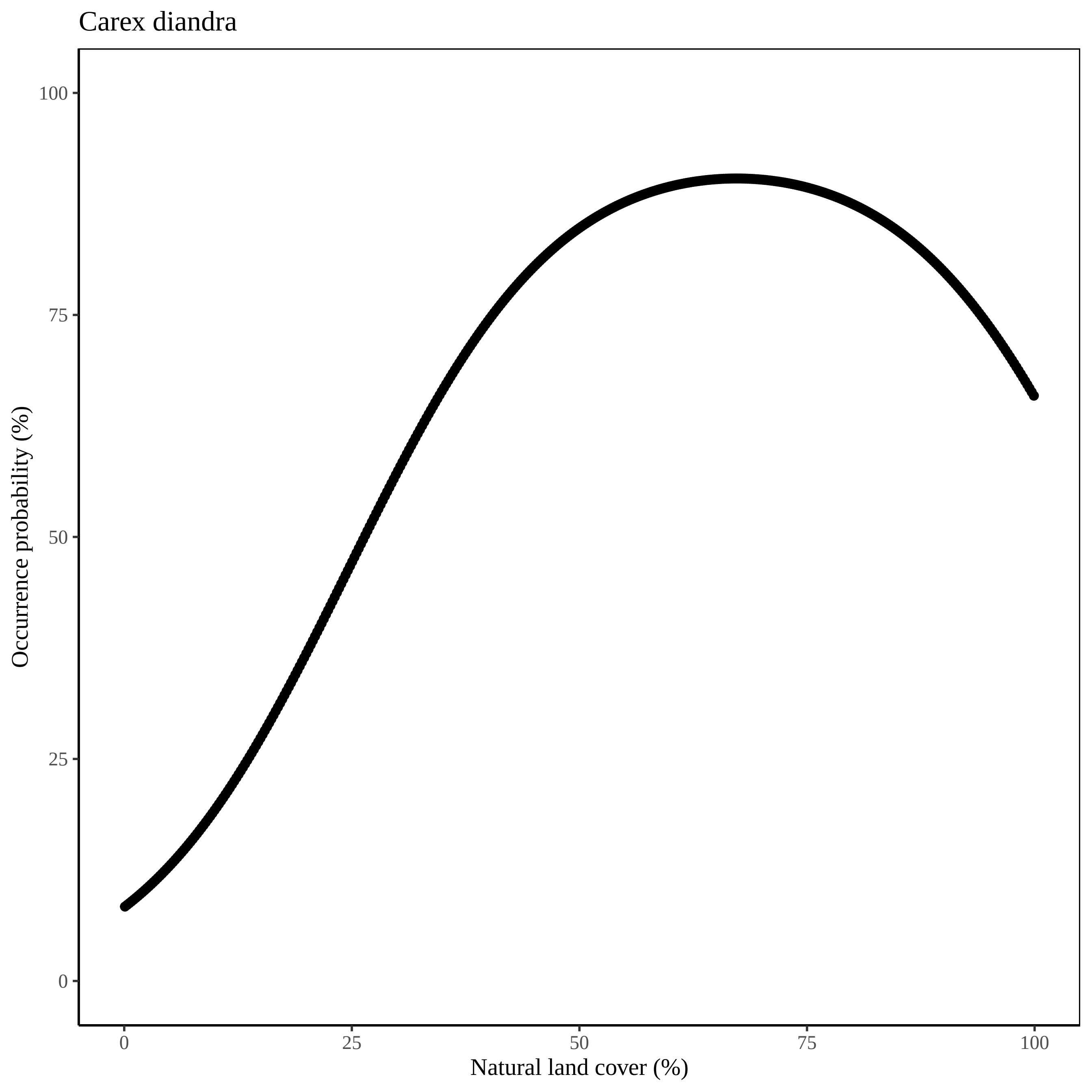
Carex diandra
100
75
50
25
0
Occurrence probability (%)
0
25
50
75
100
Natural land cover (%)

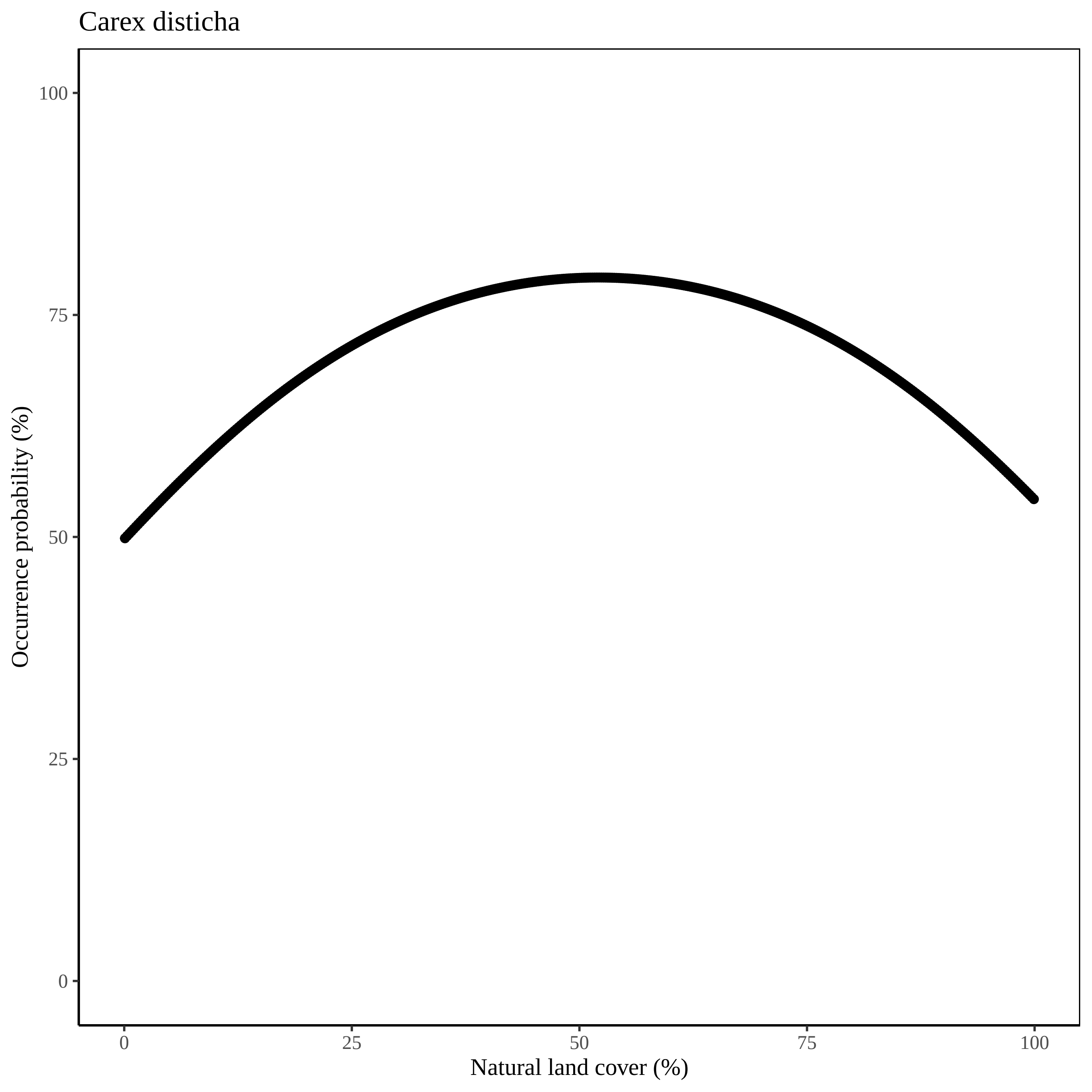
Carex disticha
Occurrence probability (%)
100
75
50
25
0
0
25
50
75
100
Natural land cover (%)

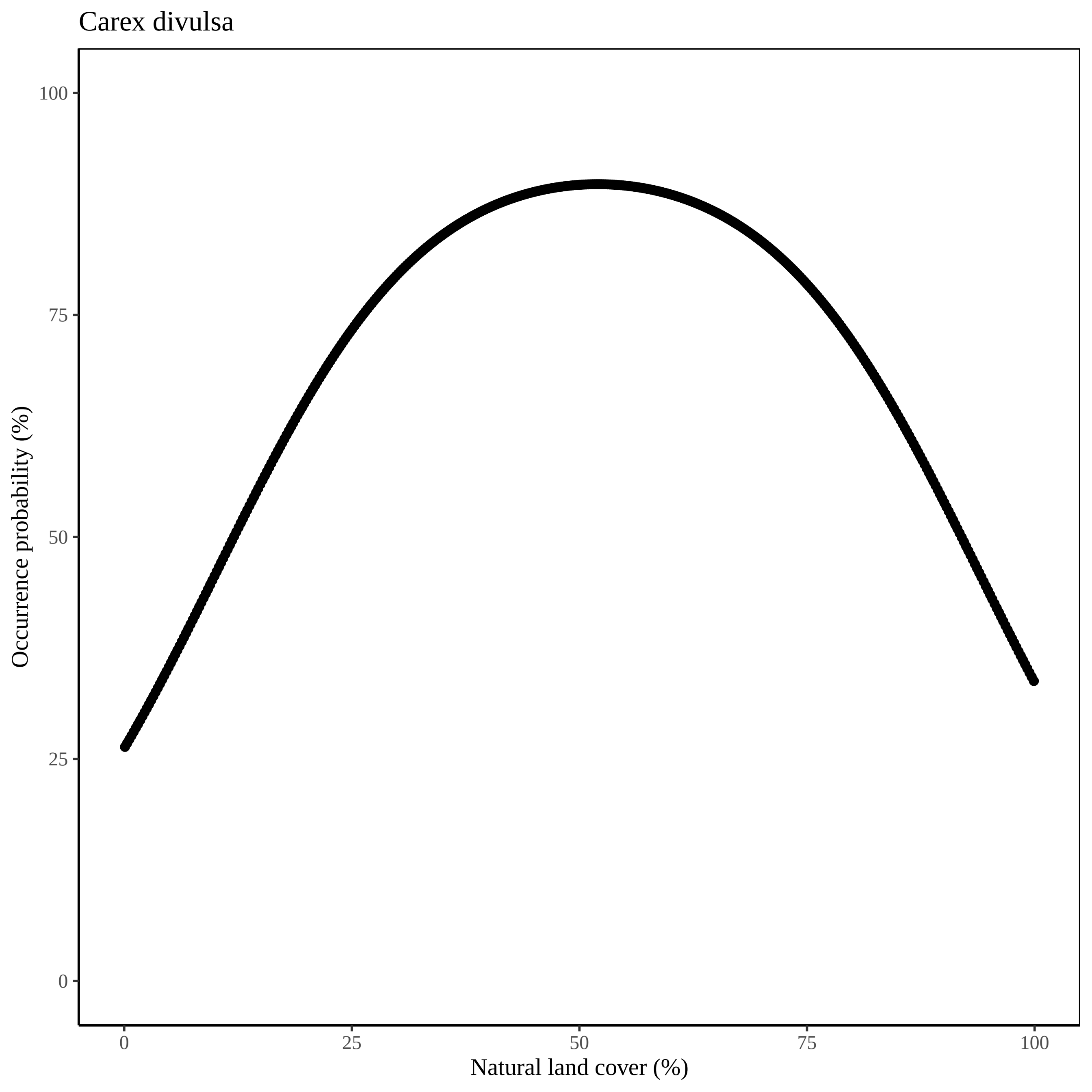

Carex divulsa
100
75
50
25
0
Occurrence probability (%)
0
25
50
75
100
Natural land cover (%)

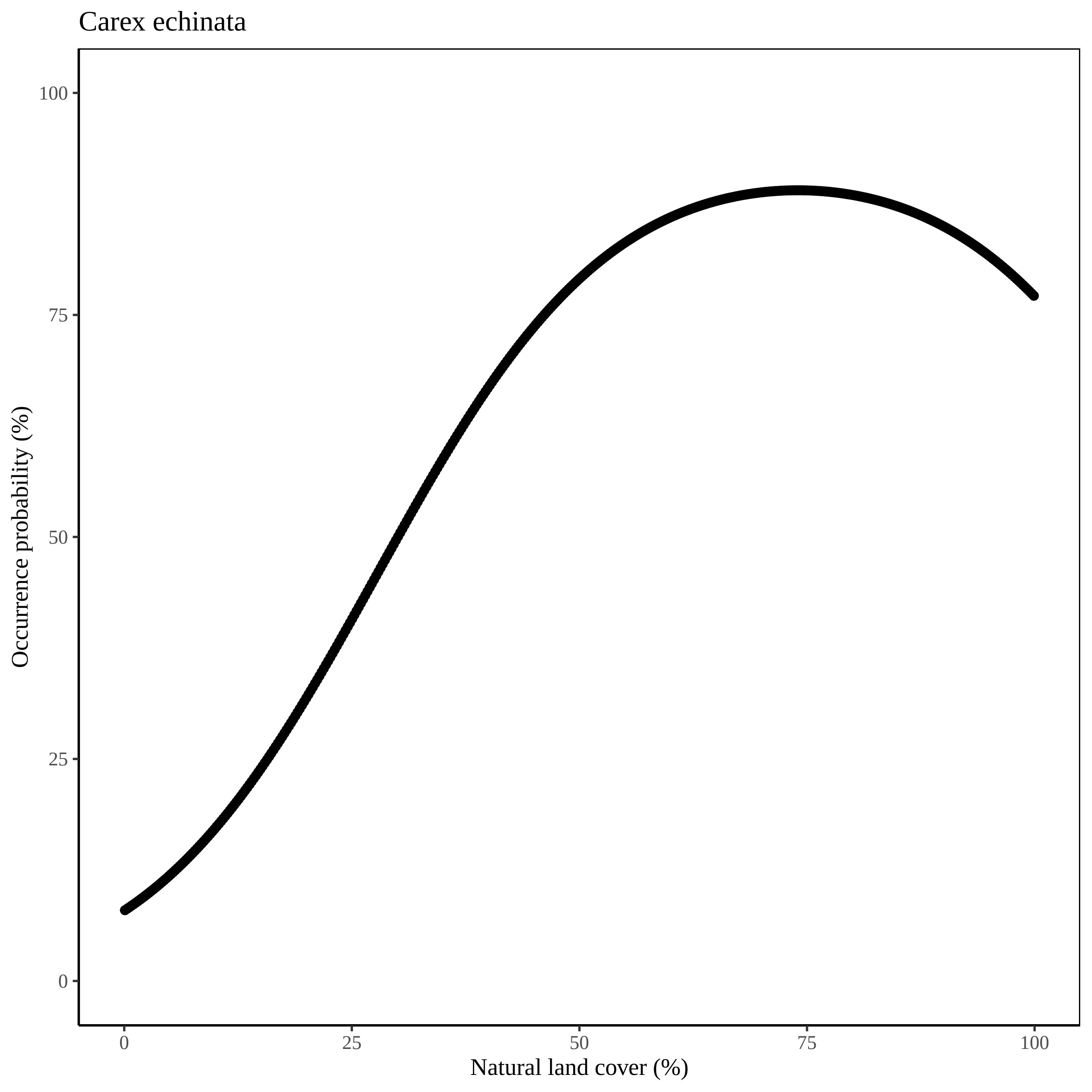
Carex echinata
Occurrence probability (%)
100
75
50
25
0
0
25
50
75
100
Natural land cover (%)

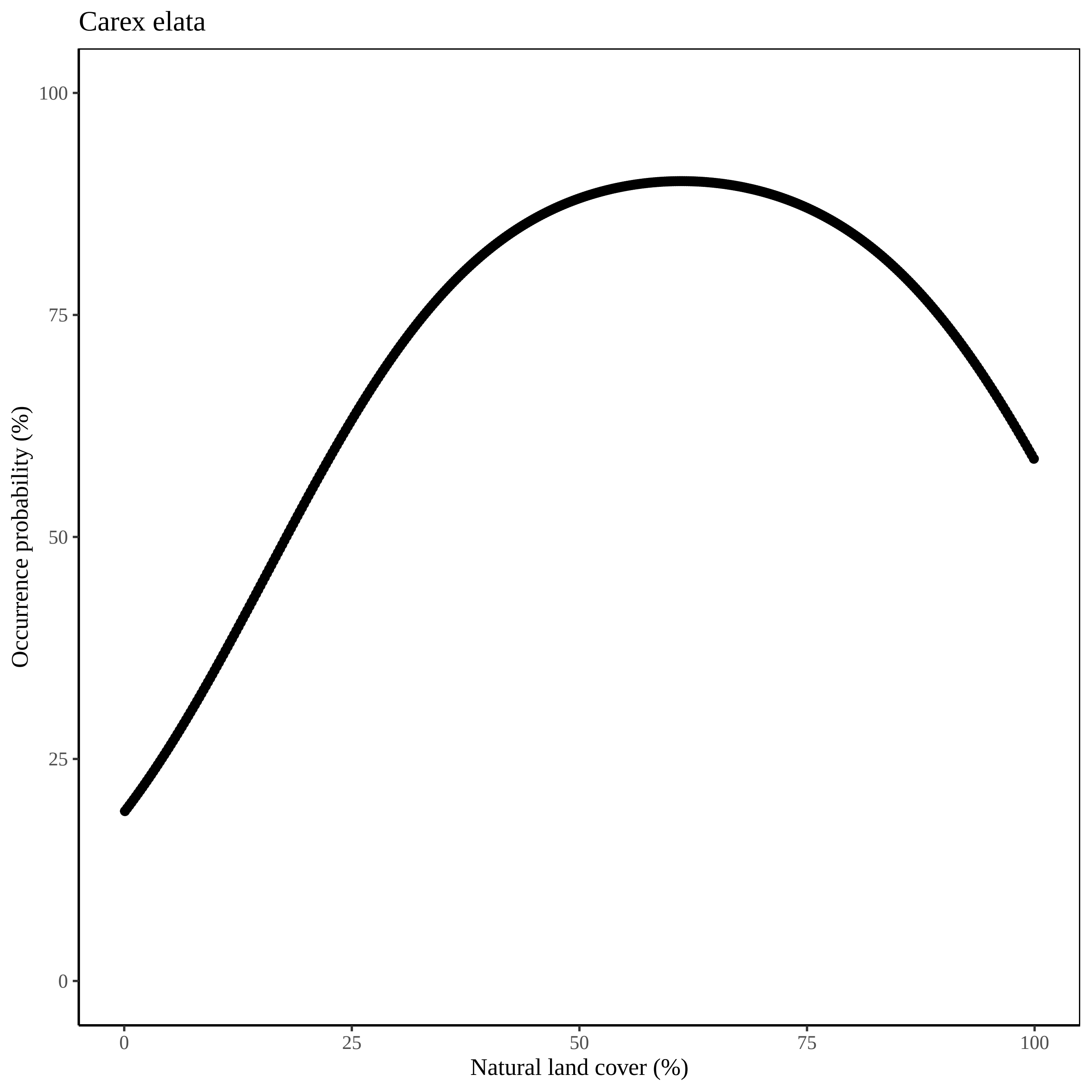
Carex elata
Occurrence probability (%)
100
75
50
25
0
0
25
50
75
100
Natural land cover (%)

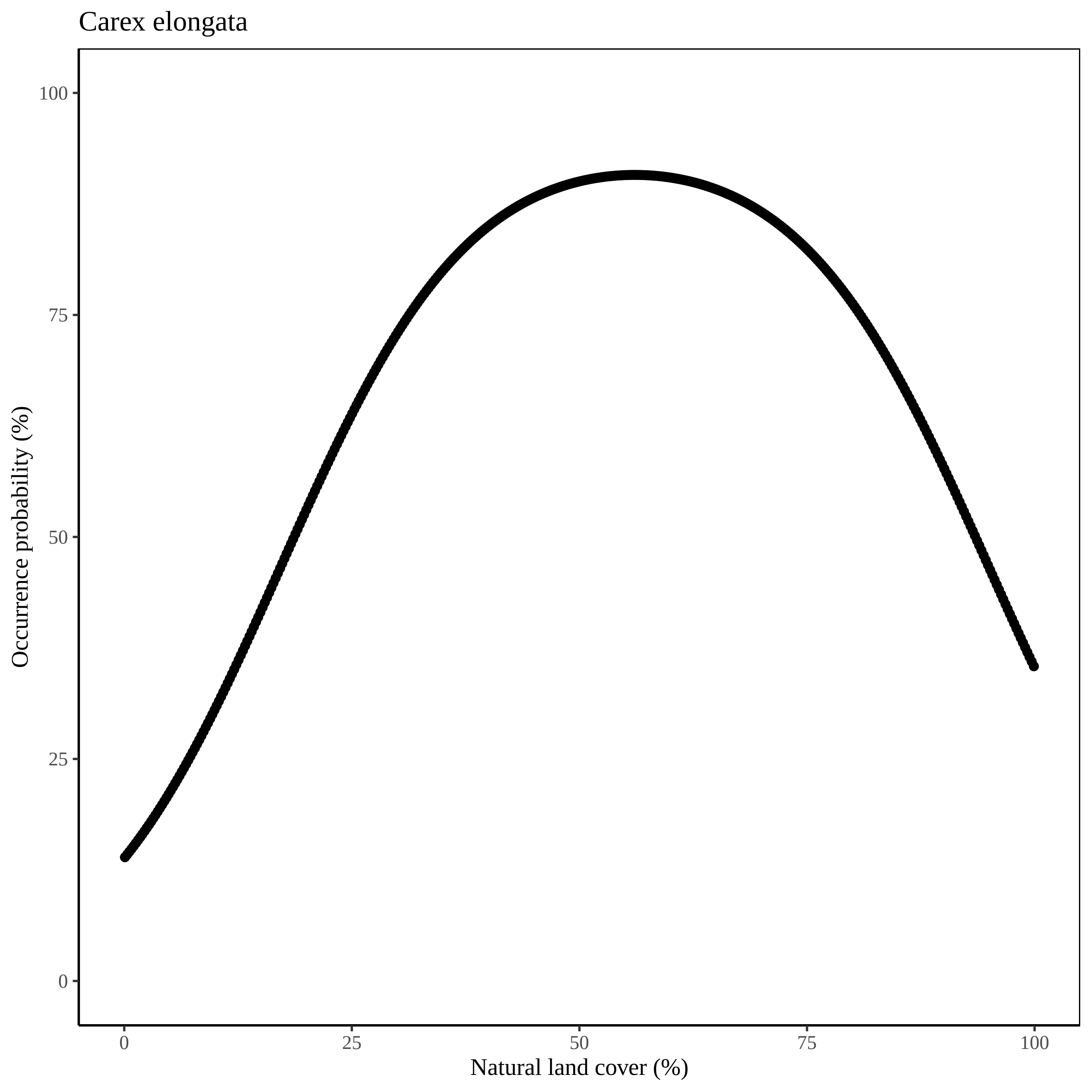
Carex elongata
100
75
50
25
0
Occurrence probability (%)
0
25
50
75
100
Natural land cover (%)

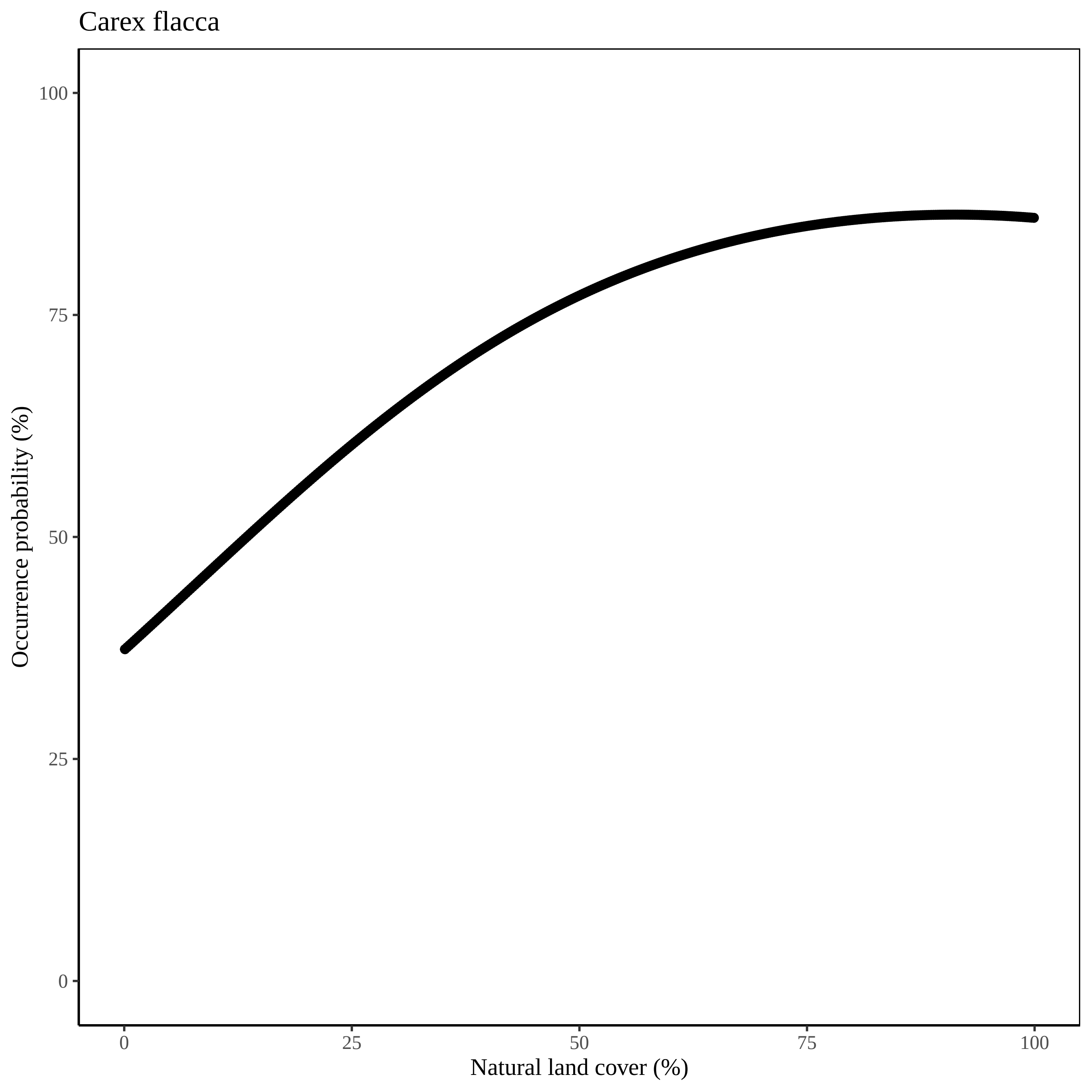
Carex flacca
Occurrence probability (%)
100
75
50
25
0
0
25
50
75
100
Natural land cover (%)

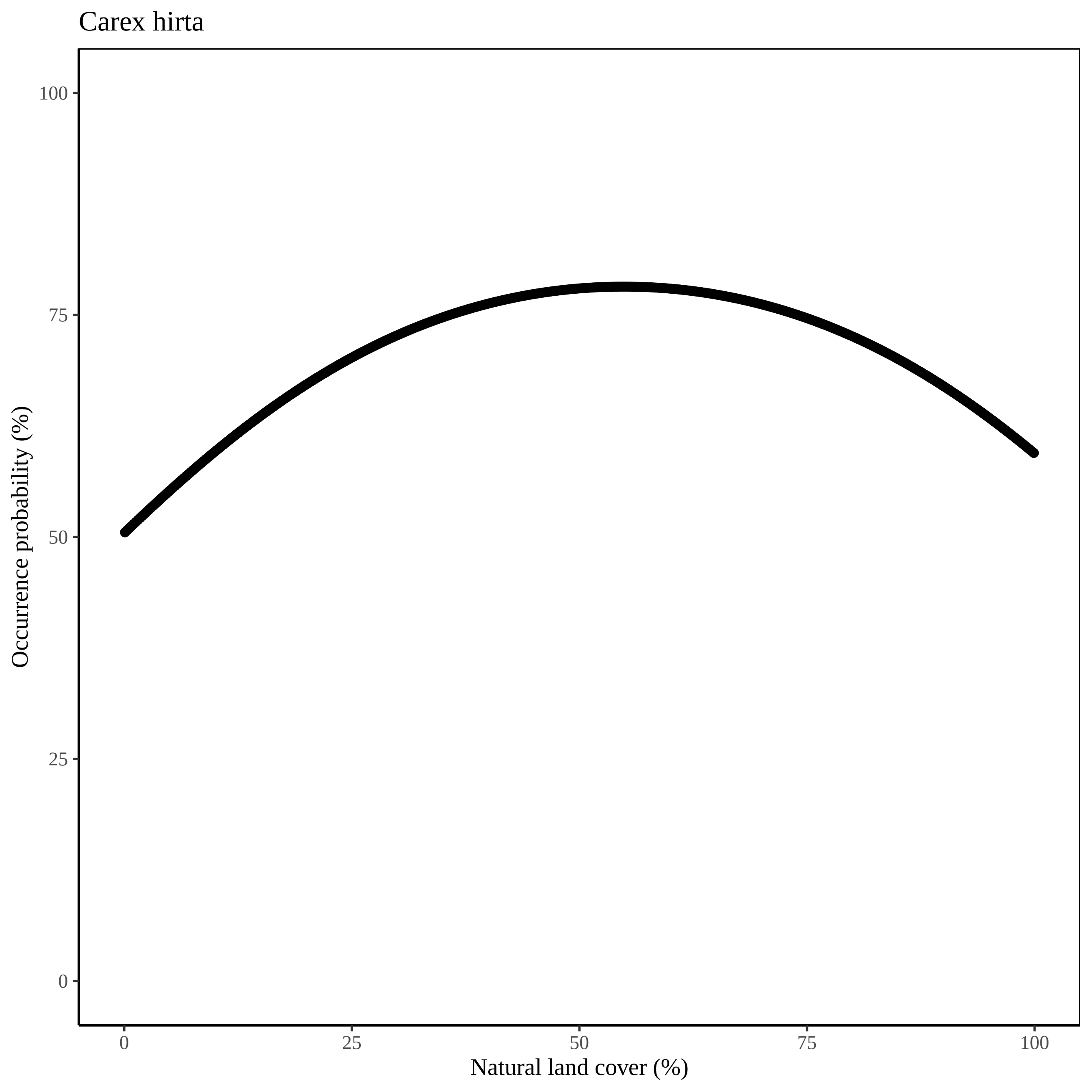
Carex hirta
Occurrence probability (%)
100
75
50
25
0
0
25
50
75
100
Natural land cover (%)

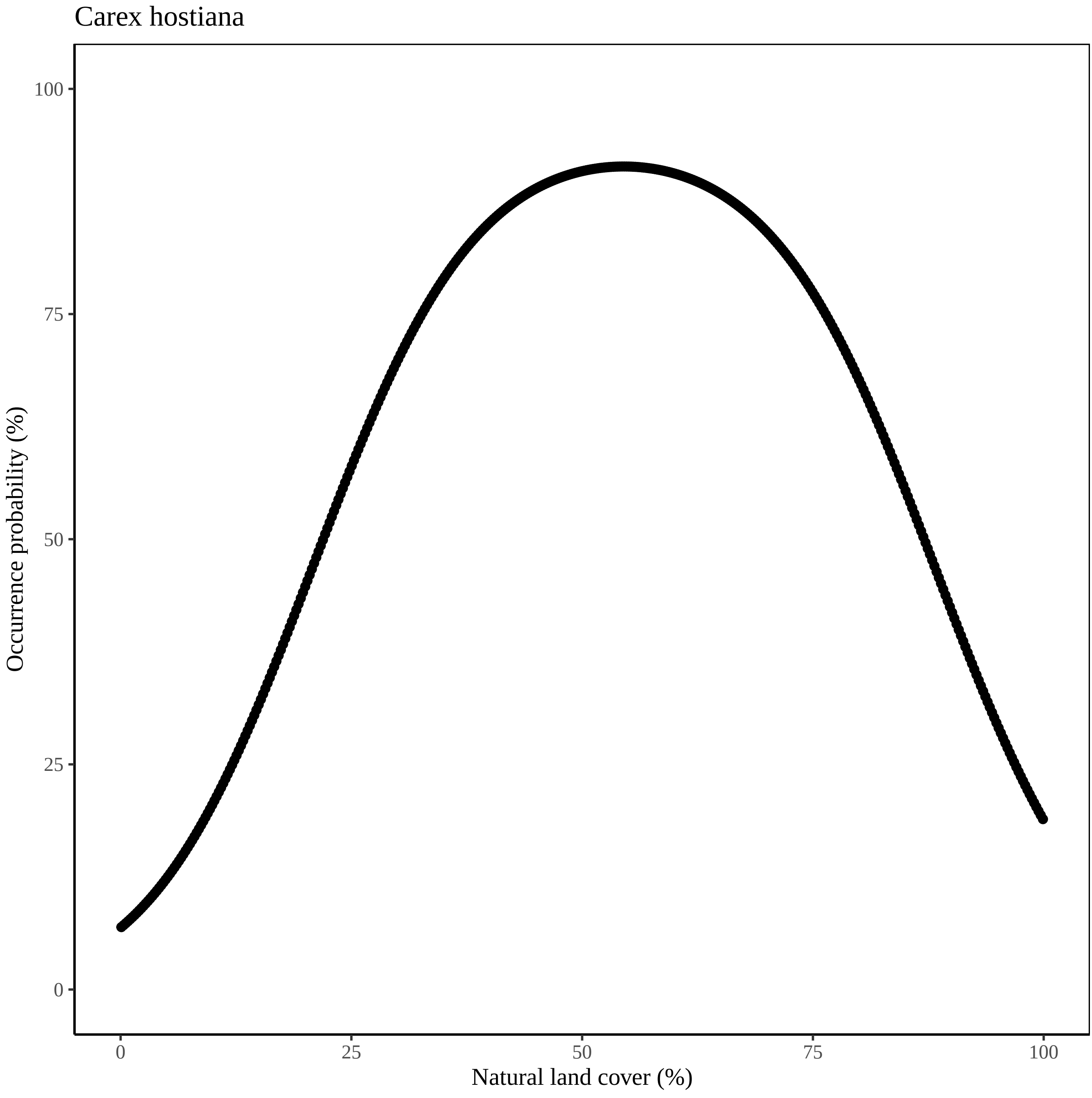

Carex hostiana
Occurrence probability (%)
100
75
50
25
0
0
25
50
75
100
Natural land cover (%)

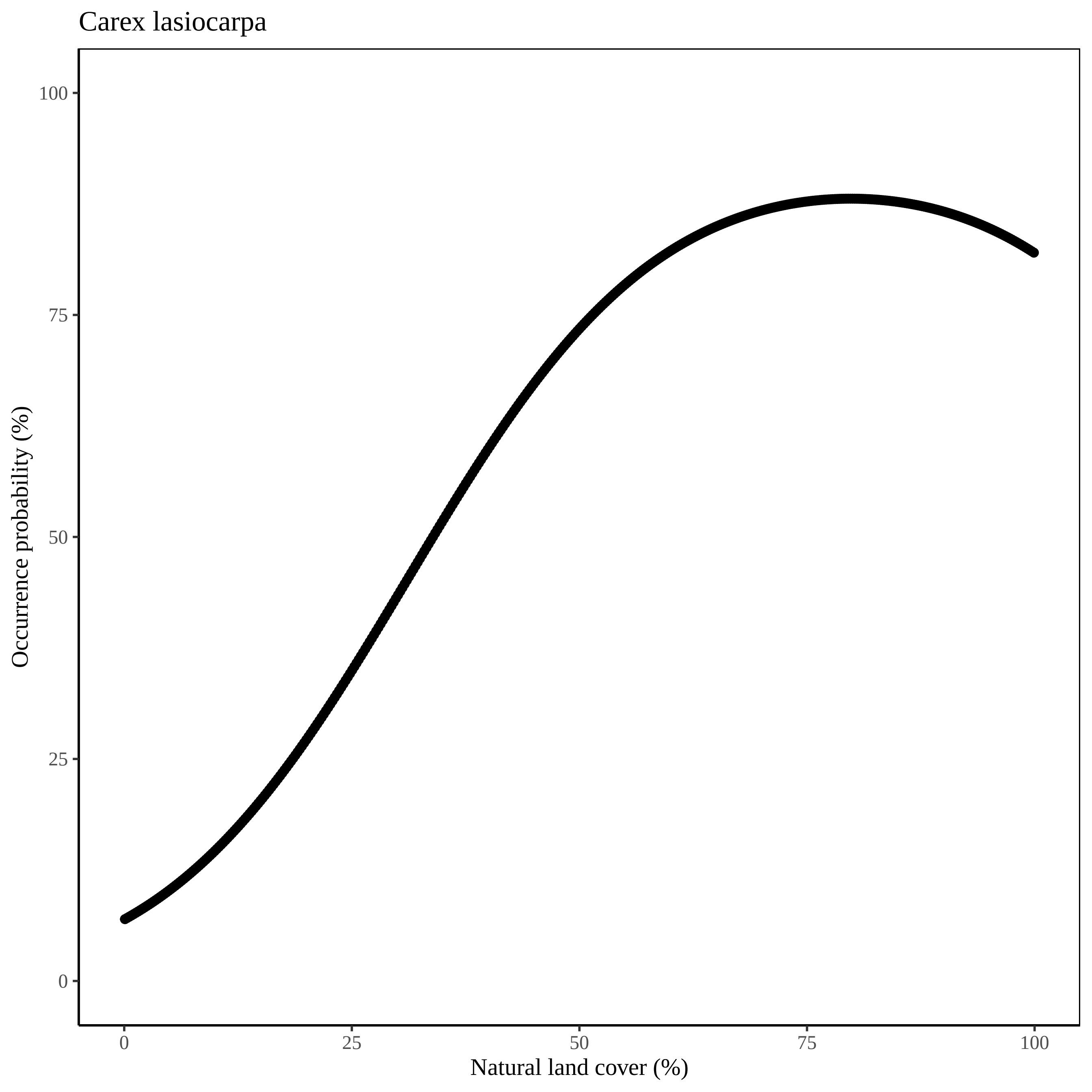
Carex lasiocarpa
Occurrence probability (%)
100
75
50
25
0
0
25
50
75
100
Natural land cover (%)

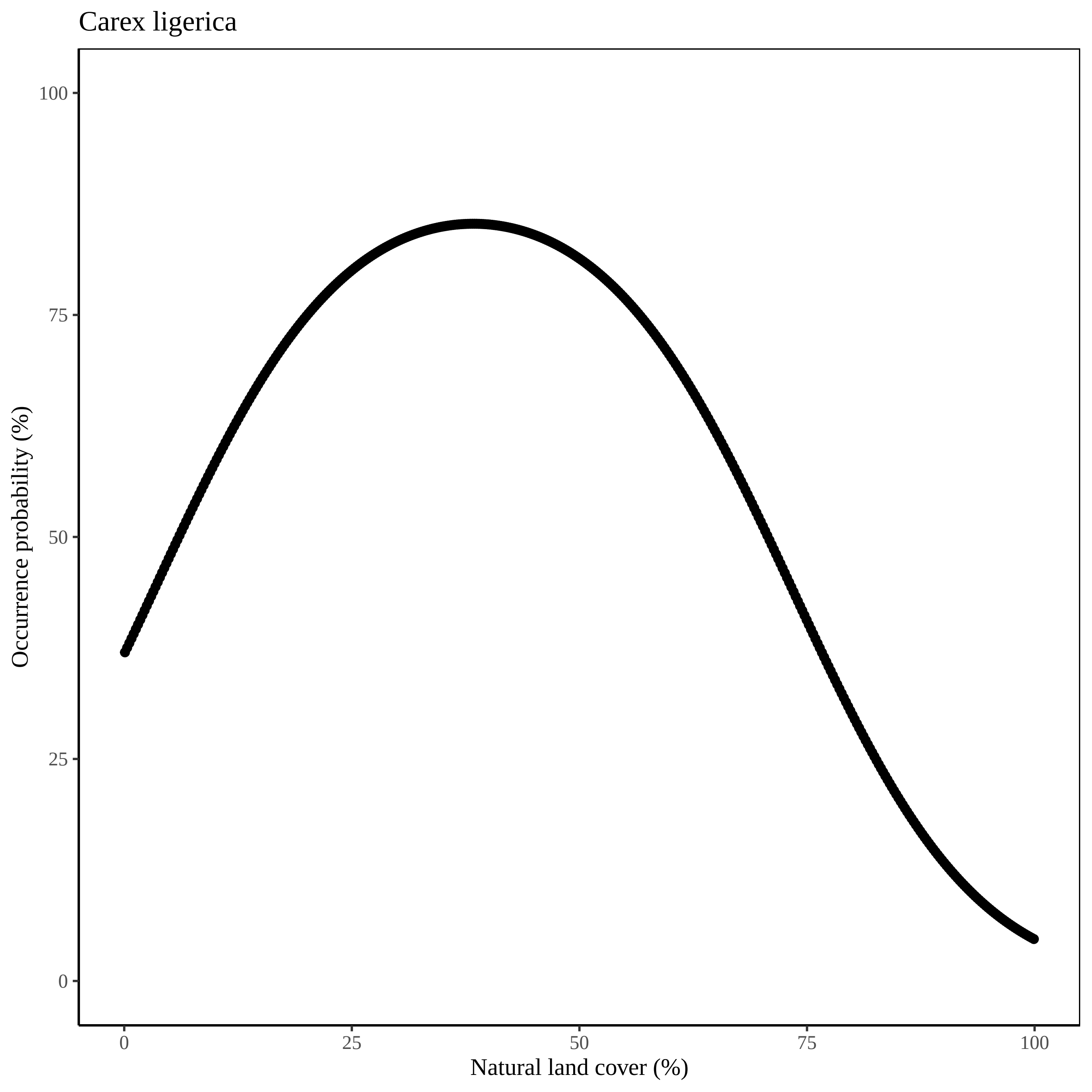
Carex ligerica
Occurrence probability (%)
Natural land cover (%)
0
25
50
75
100

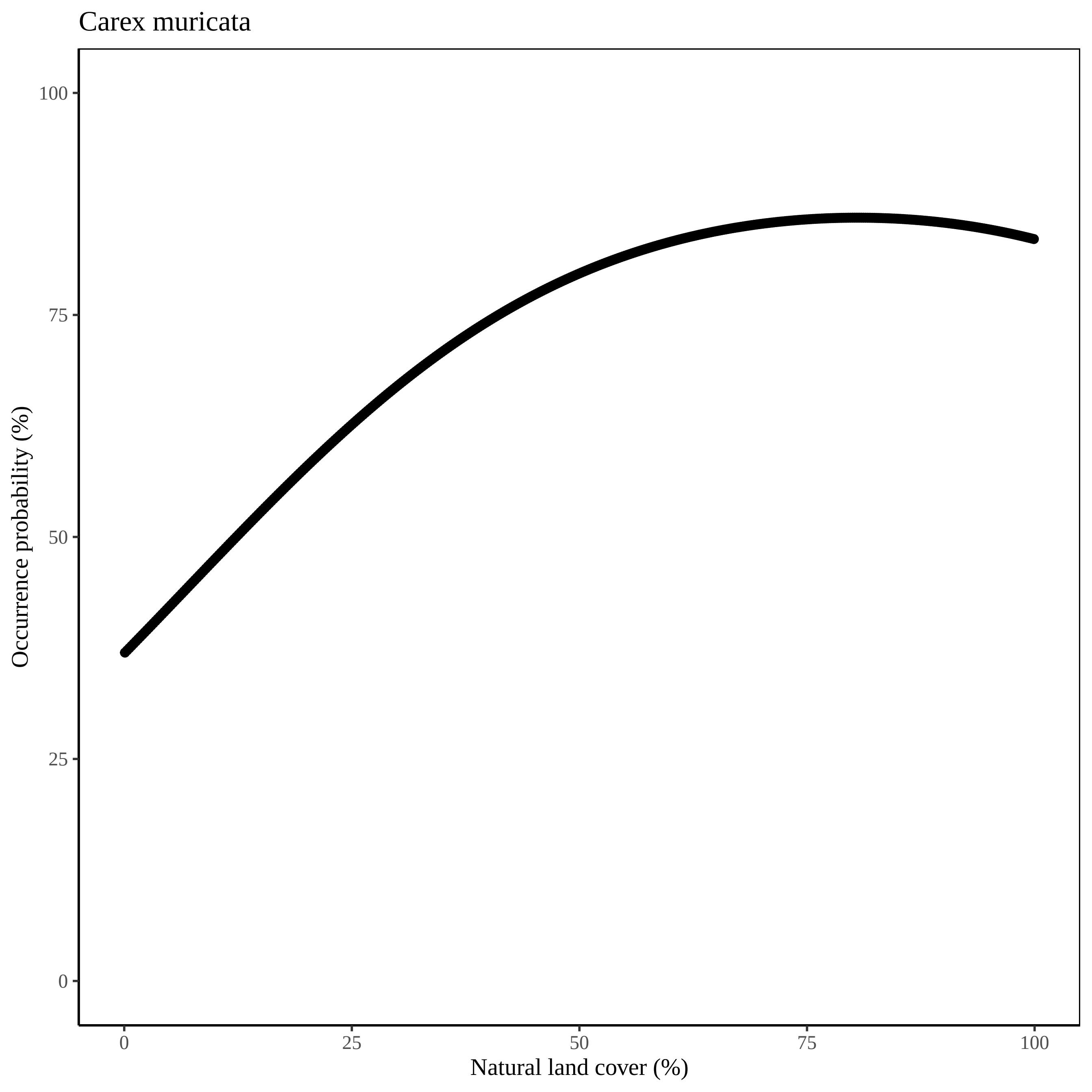
Carex muricata
Occurrence probability (%)
100
75
50
25
0
0
25
50
75
100
Natural land cover (%)

Carex nigra

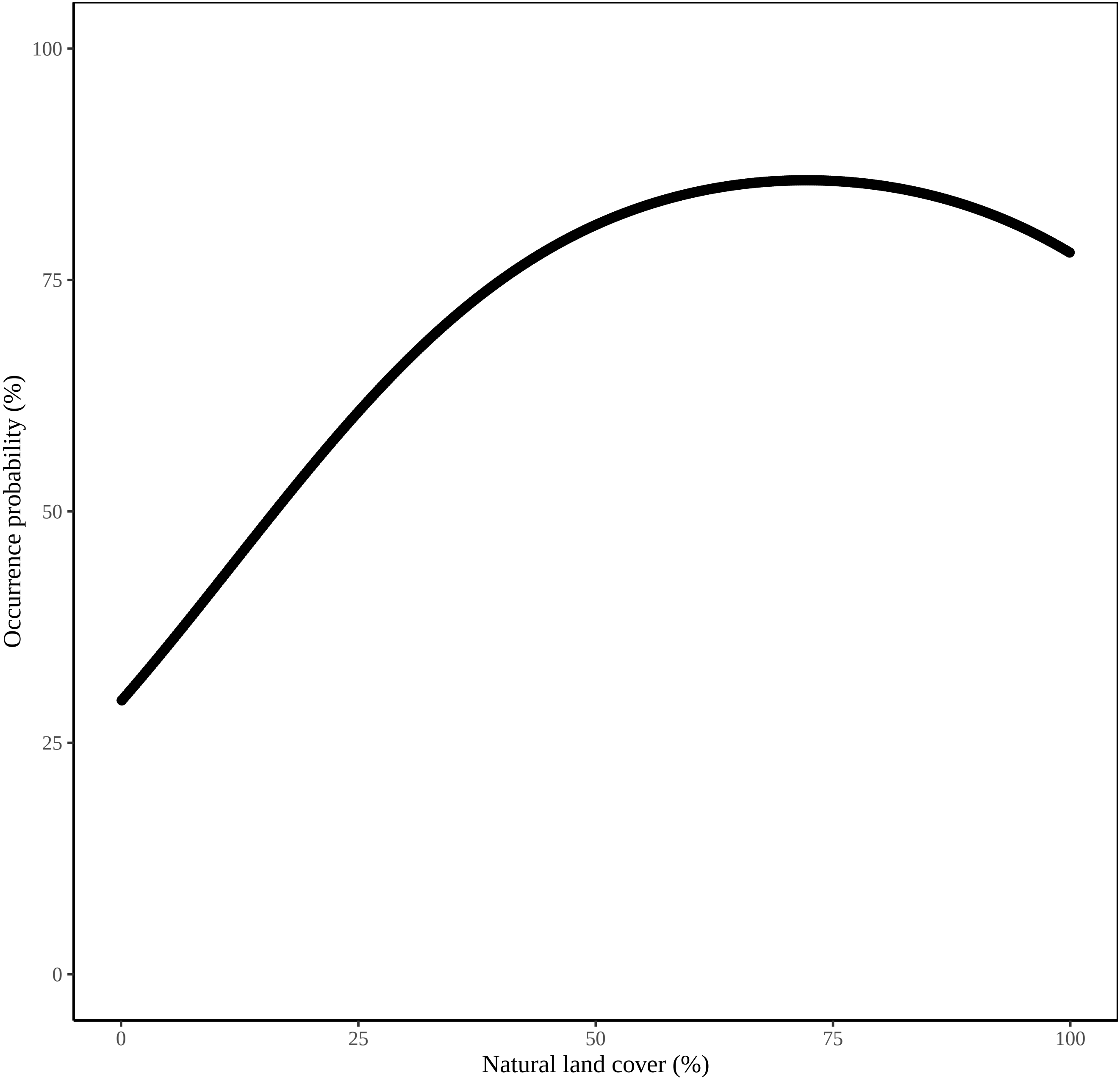
100
75
50
25
0
Occurrence probability (%)
0
25
50
75
100
Natural land cover (%)

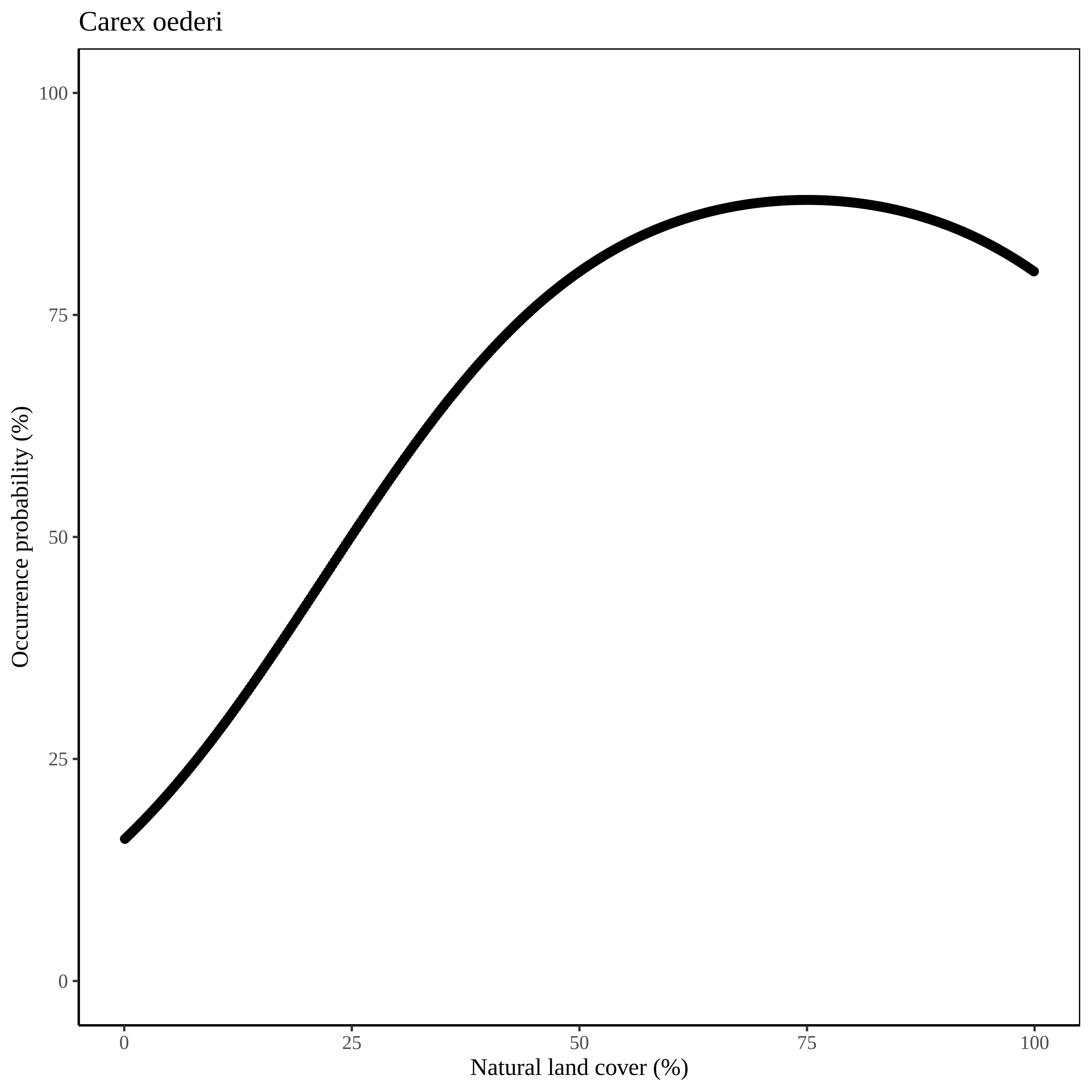
Carex oederi
100
75
50
25
0
0
25
50
75
100
Natural land cover (%)
Occurrence probability (%)

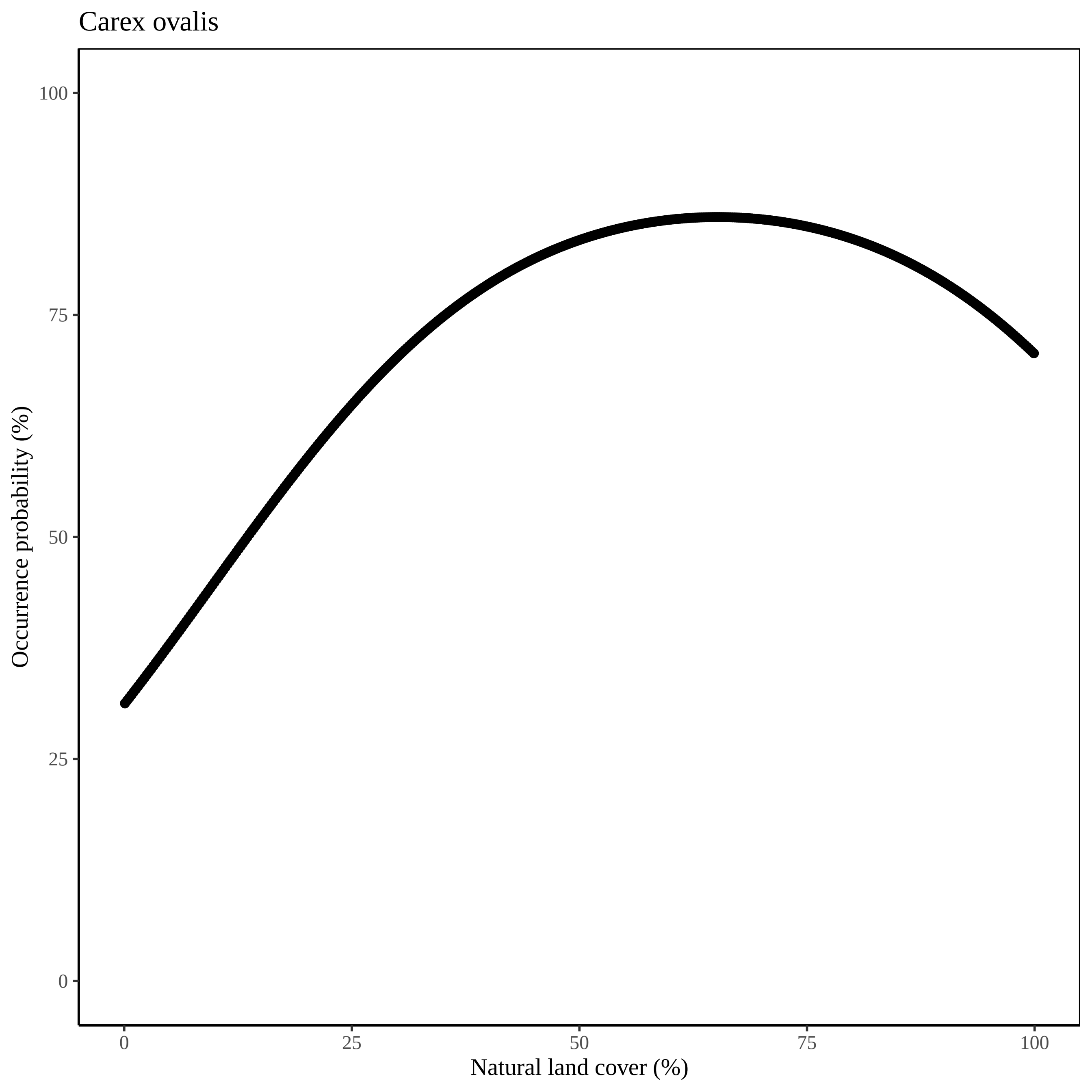

Carex ovalis
Occurrence probability (%)
100
75
50
25
0
0
25
50
75
100
Natural land cover (%)

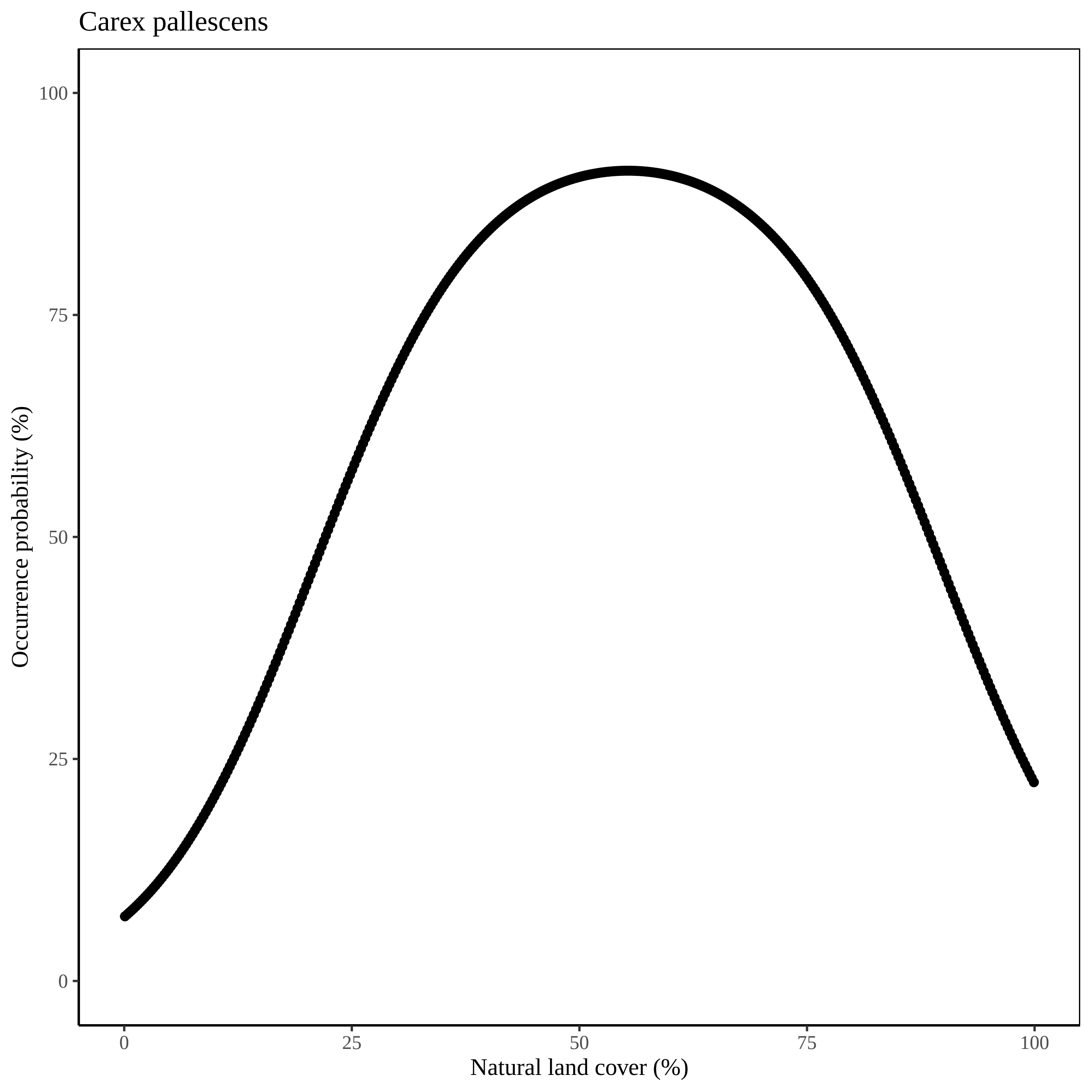
Carex pallescens
Occurrence probability (%)
100
75
50
25
0
0
25
50
75
100
Natural land cover (%)

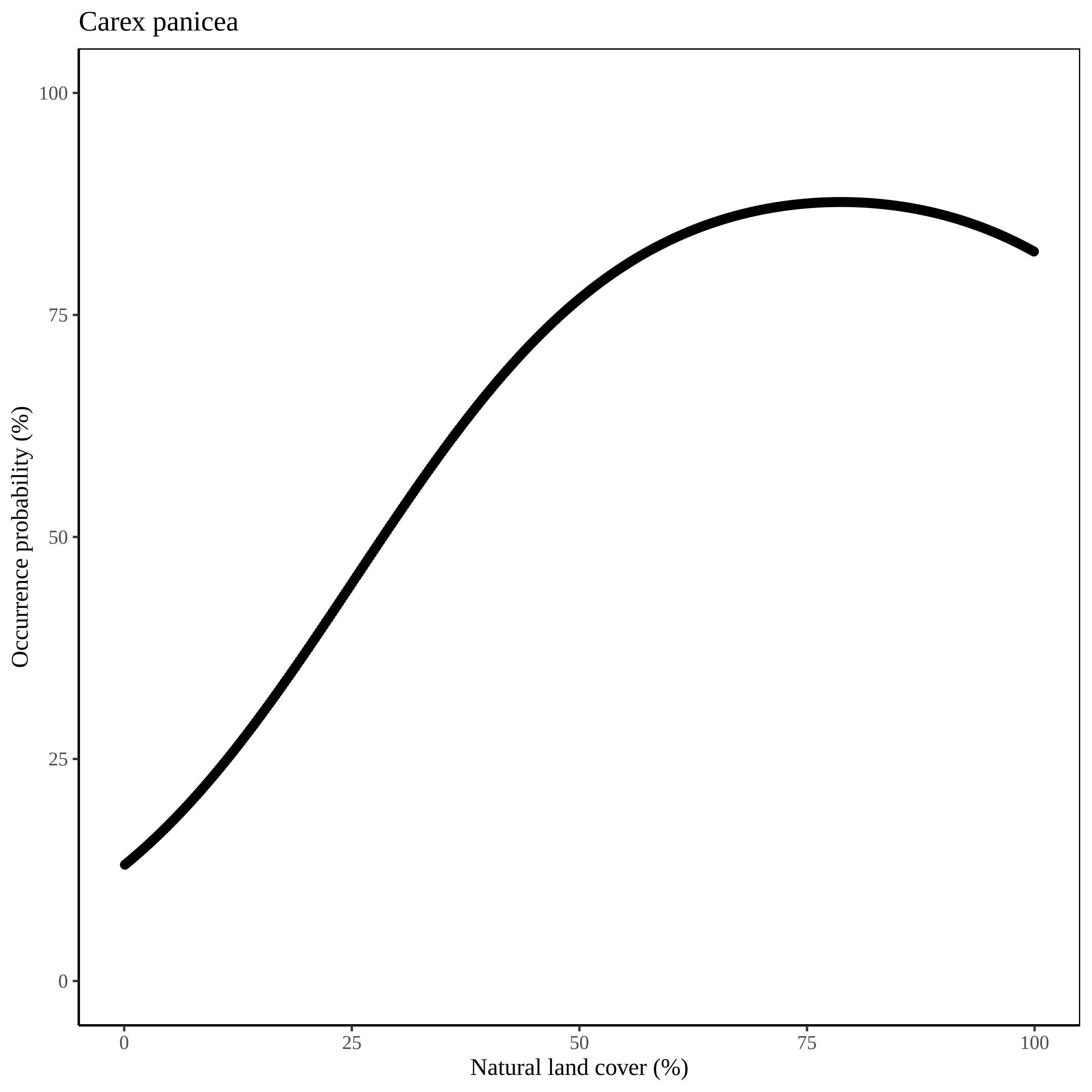
Carex panicea
Occurrence probability (%)
100
75
50
25
0
0
25
50
75
100
Natural land cover (%)

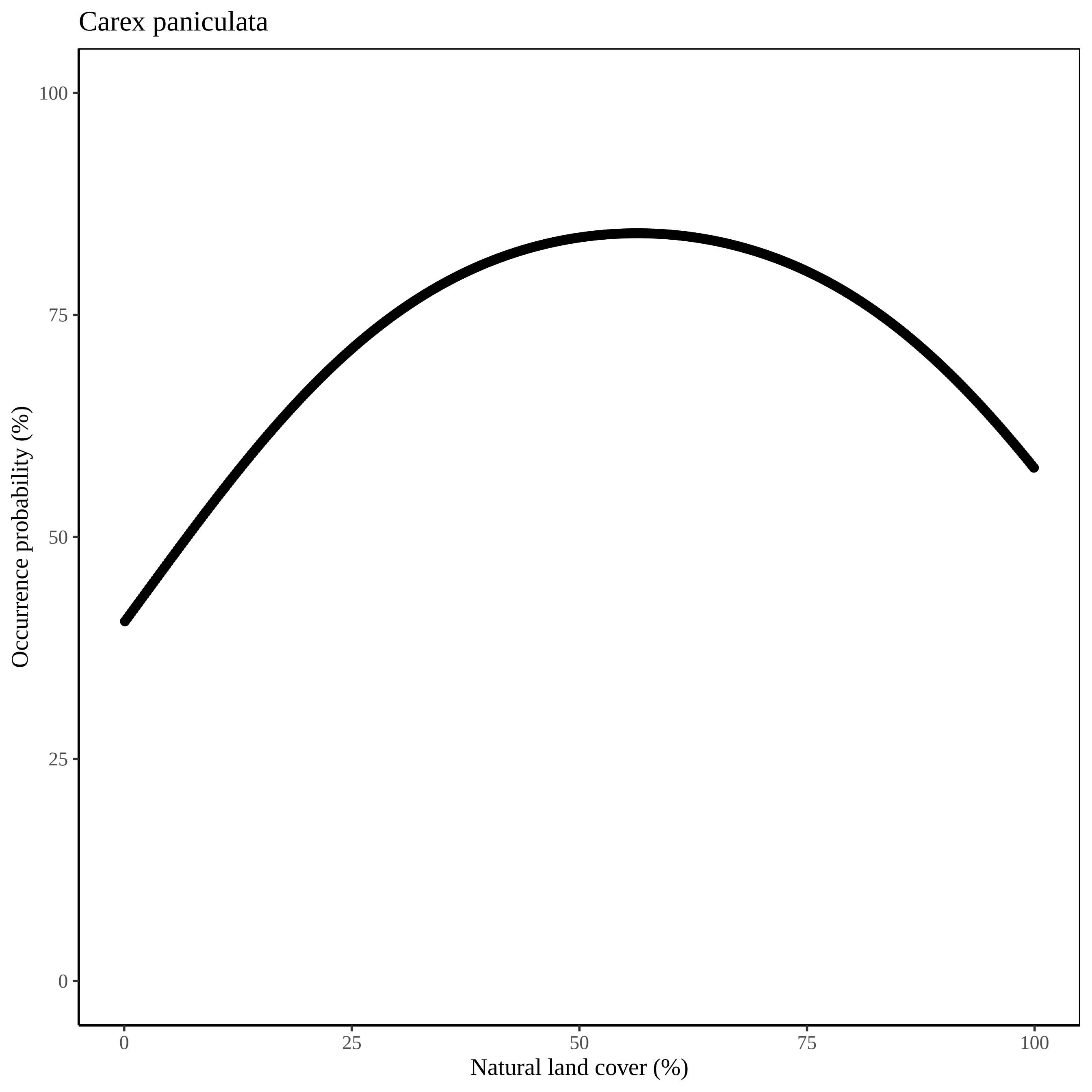

Carex paniculata
Occurrence probability (%)
100
75
50
25
0
0
25
50
75
100
Natural land cover (%)

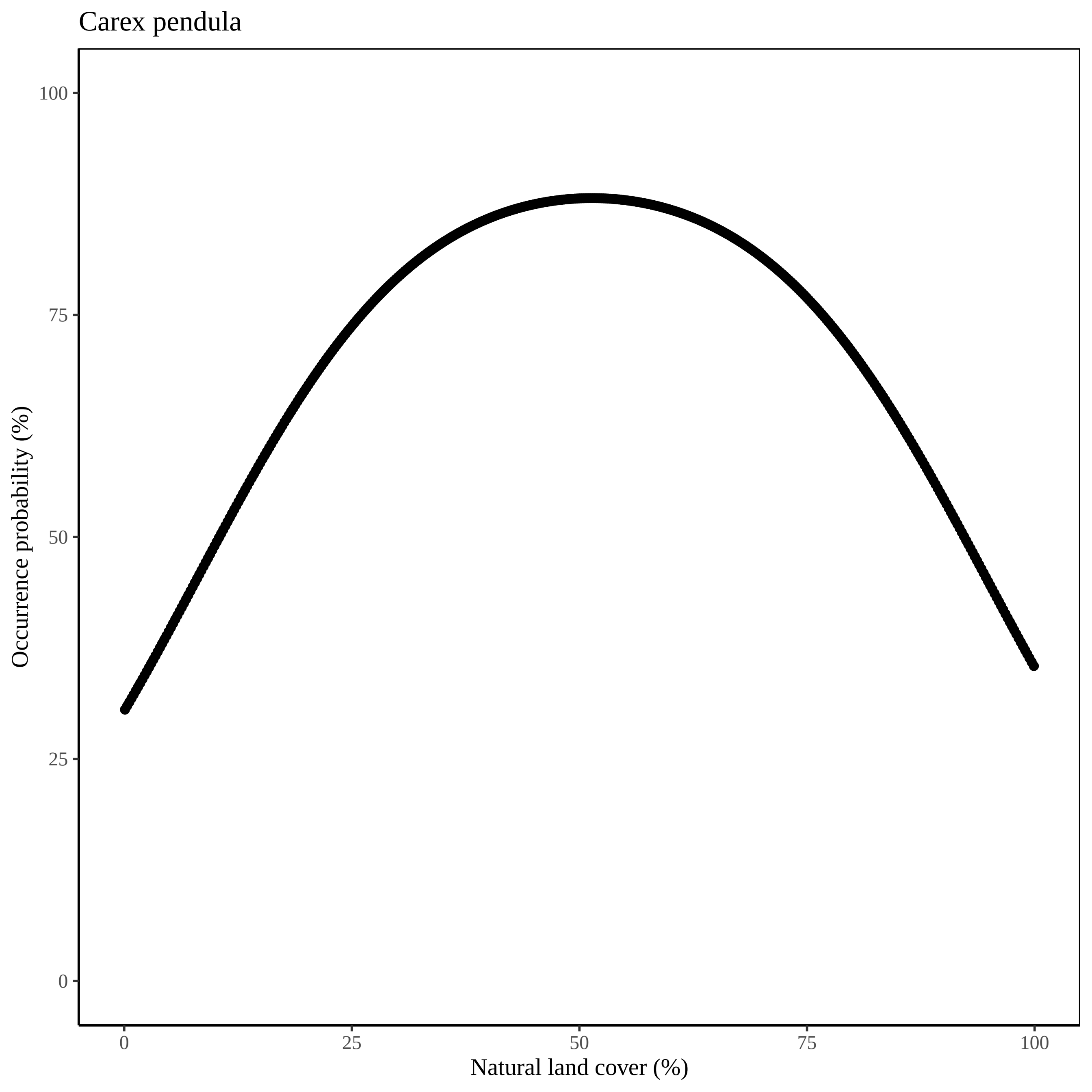

Carex pendula
100
75
50
25
0
Occurrence probability (%)
0
25
50
75
100
Natural land cover (%)

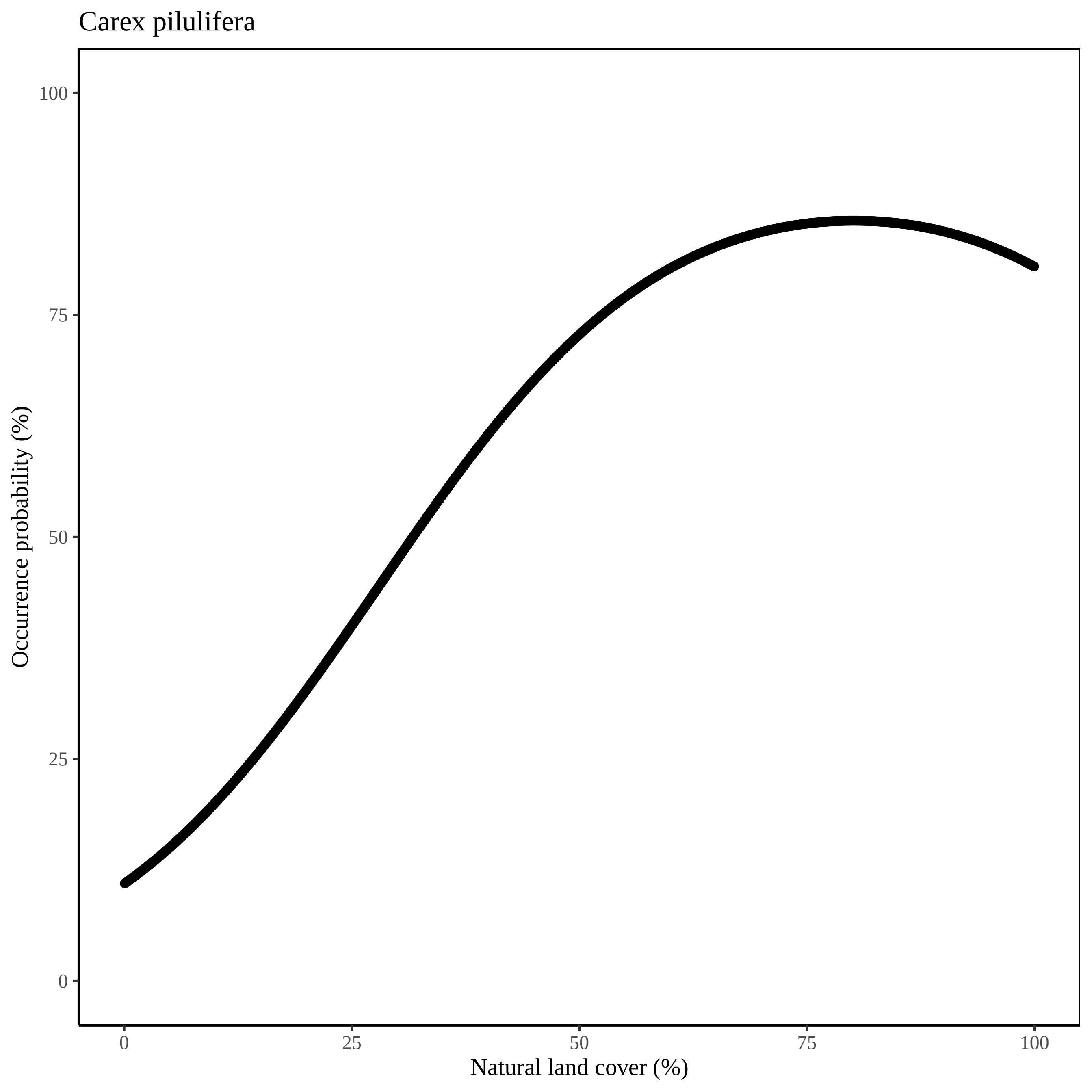
Carex pilulifera
Occurrence probability (%)
100
75
50
25
0
0
25
50
75
100
Natural land cover (%)

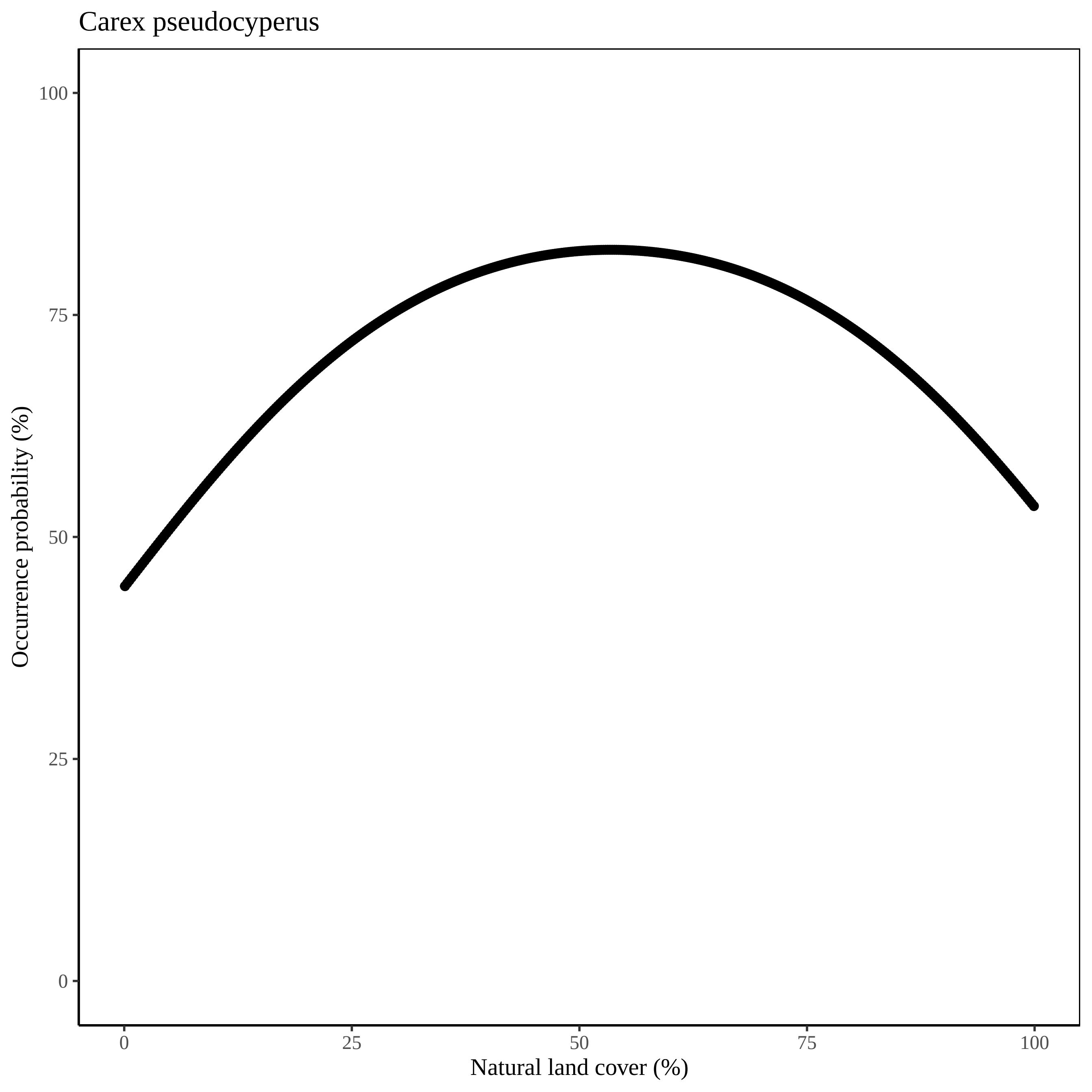

Carex pseudocyperus
100
75
50
25
0
Occurrence probability (%)
0
25
50
75
100
Natural land cover (%)

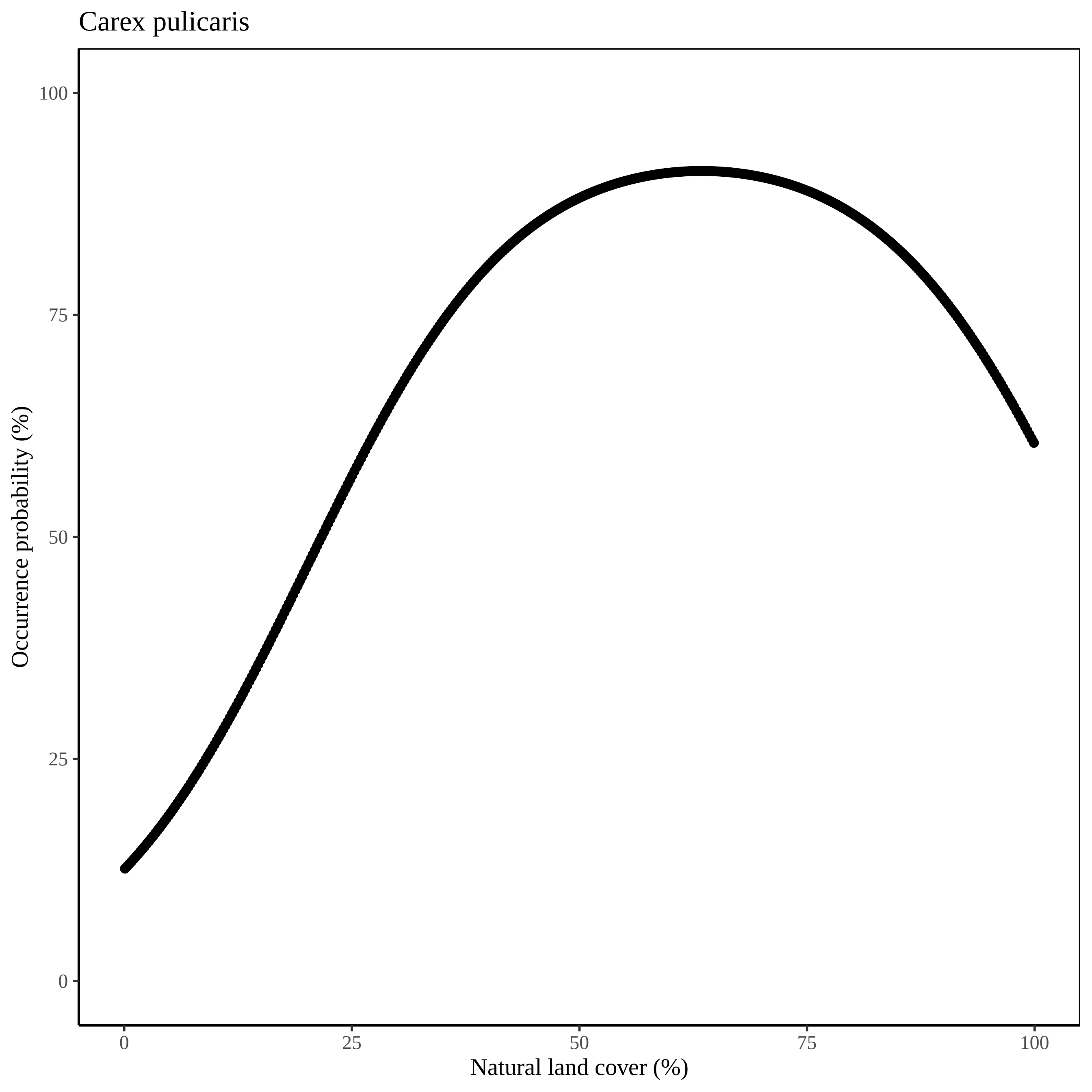

Carex pulicaris
100
75
50
25
0
Occurrence probability (%)
0
25
50
75
100
Natural land cover (%)

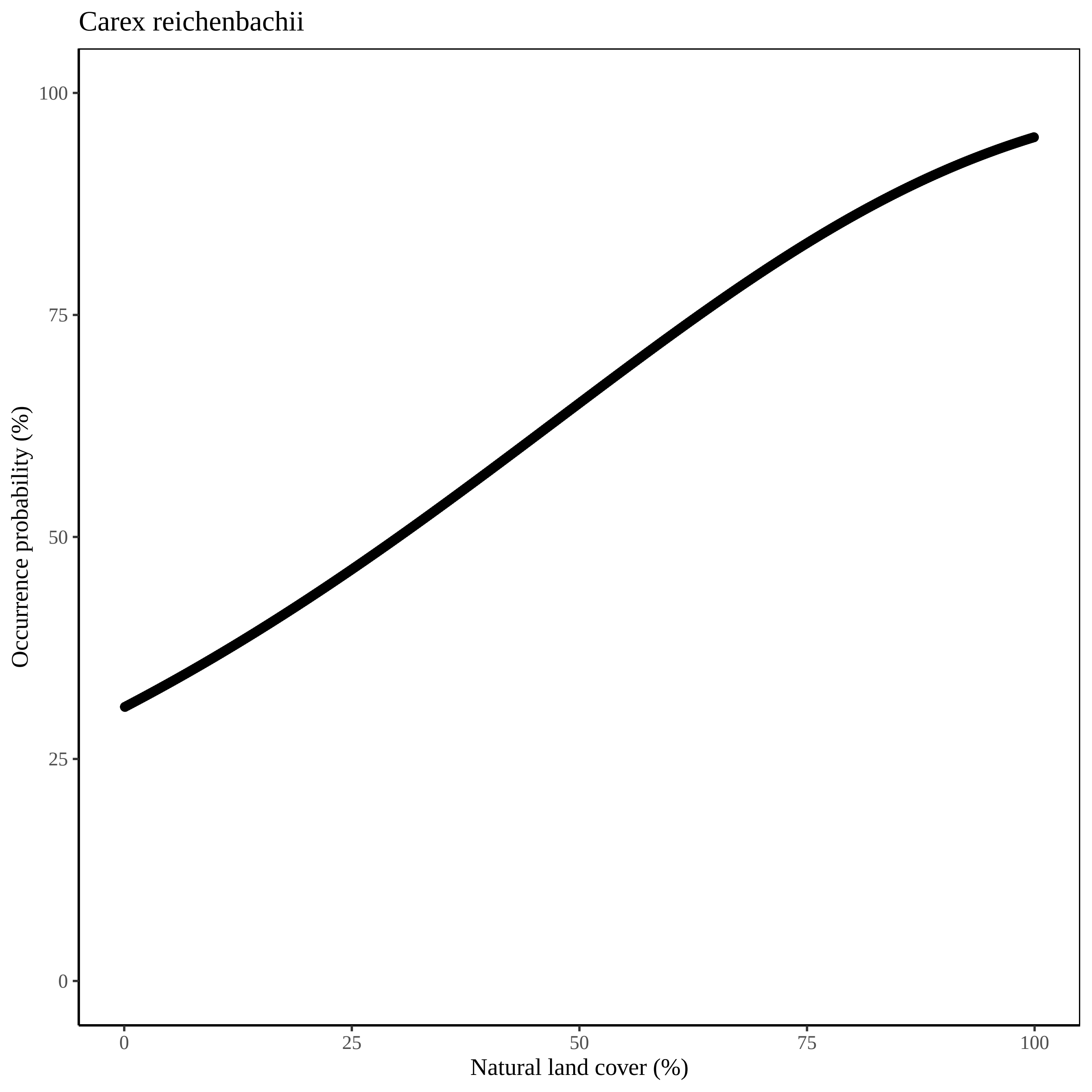

Carex reichenbachii
Occurrence probability (%)
100
75
50
25
0
0
25
50
75
100
Natural land cover (%)

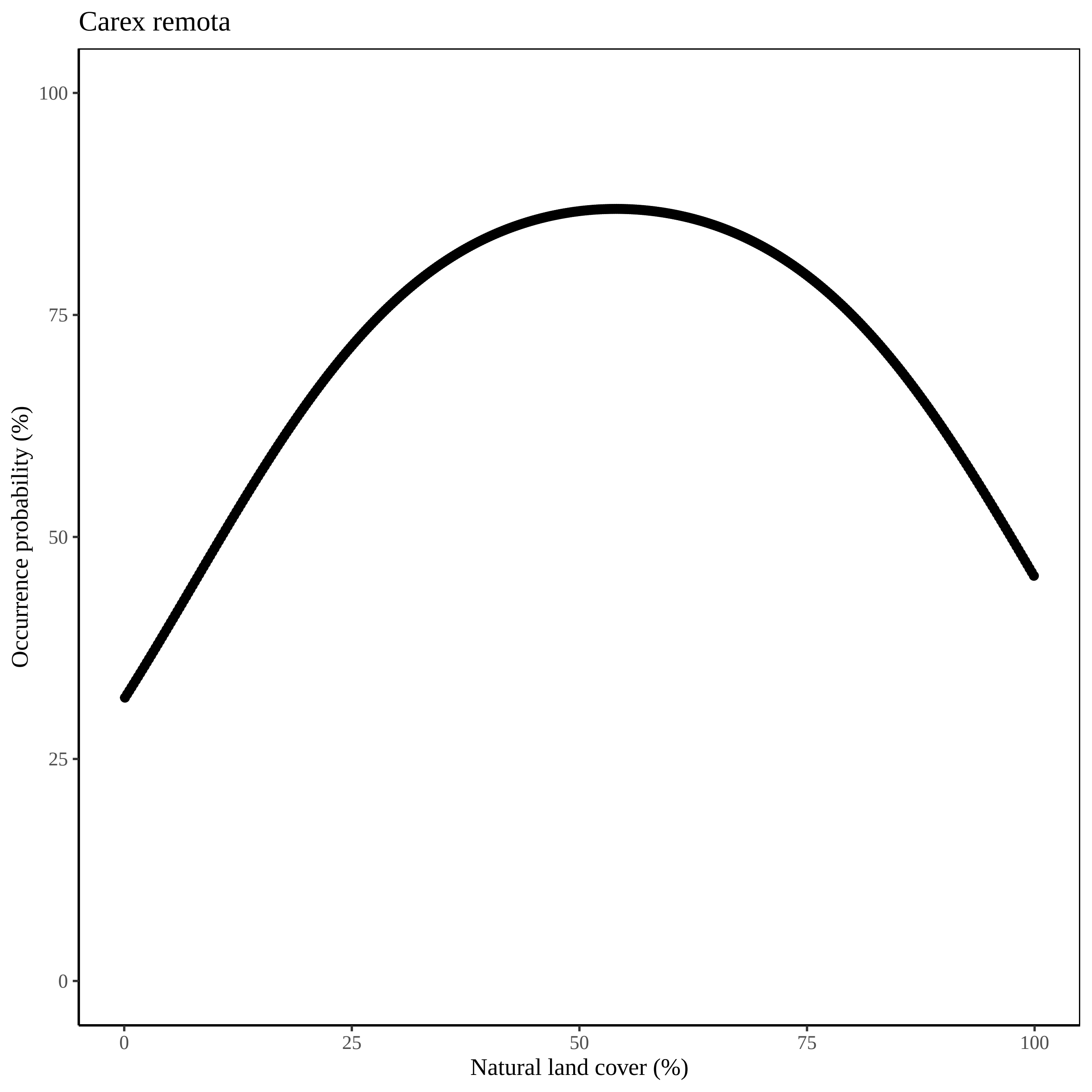

Carex remota
100
75
50
25
0
0
25
50
75
100
Occurrence probability (%)
Natural land cover (%)

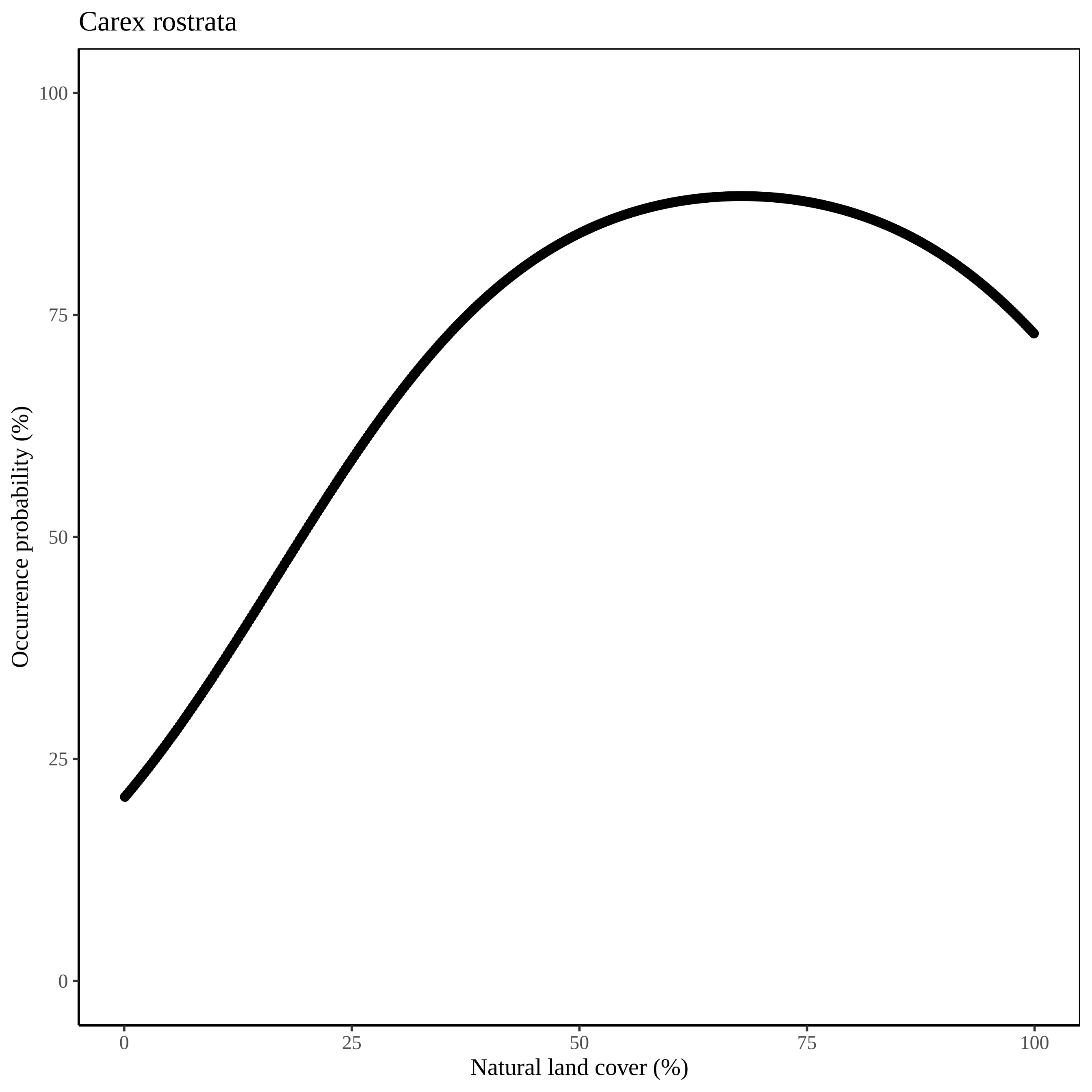
Carex rostrata
Occurrence probability (%)
100
75
50
25
0
0
25
50
75
100
Natural land cover (%)

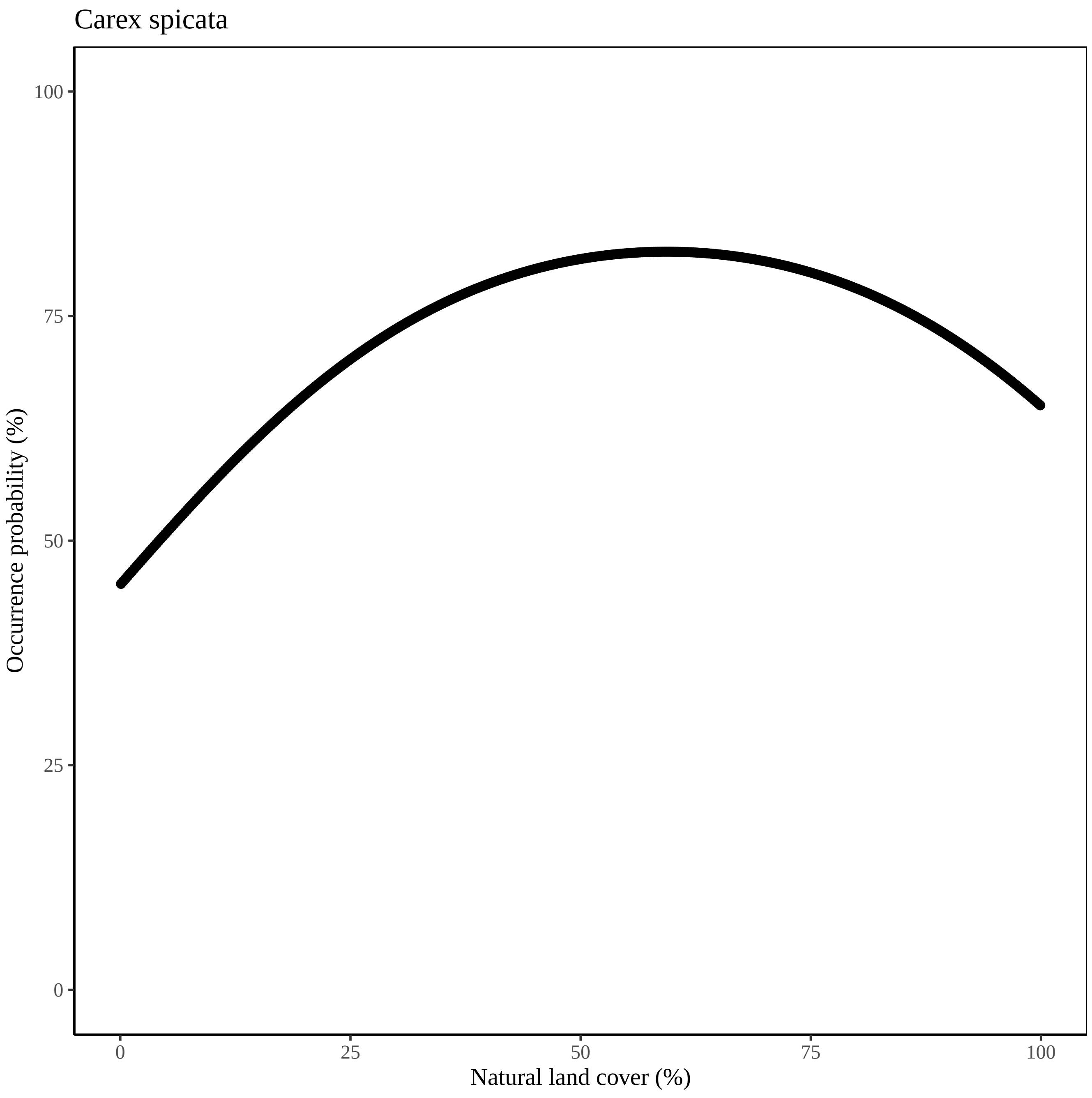
Carex spicata
Occurrence probability (%)
100
75
50
25
0
0
25
50
75
100
Natural land cover (%)

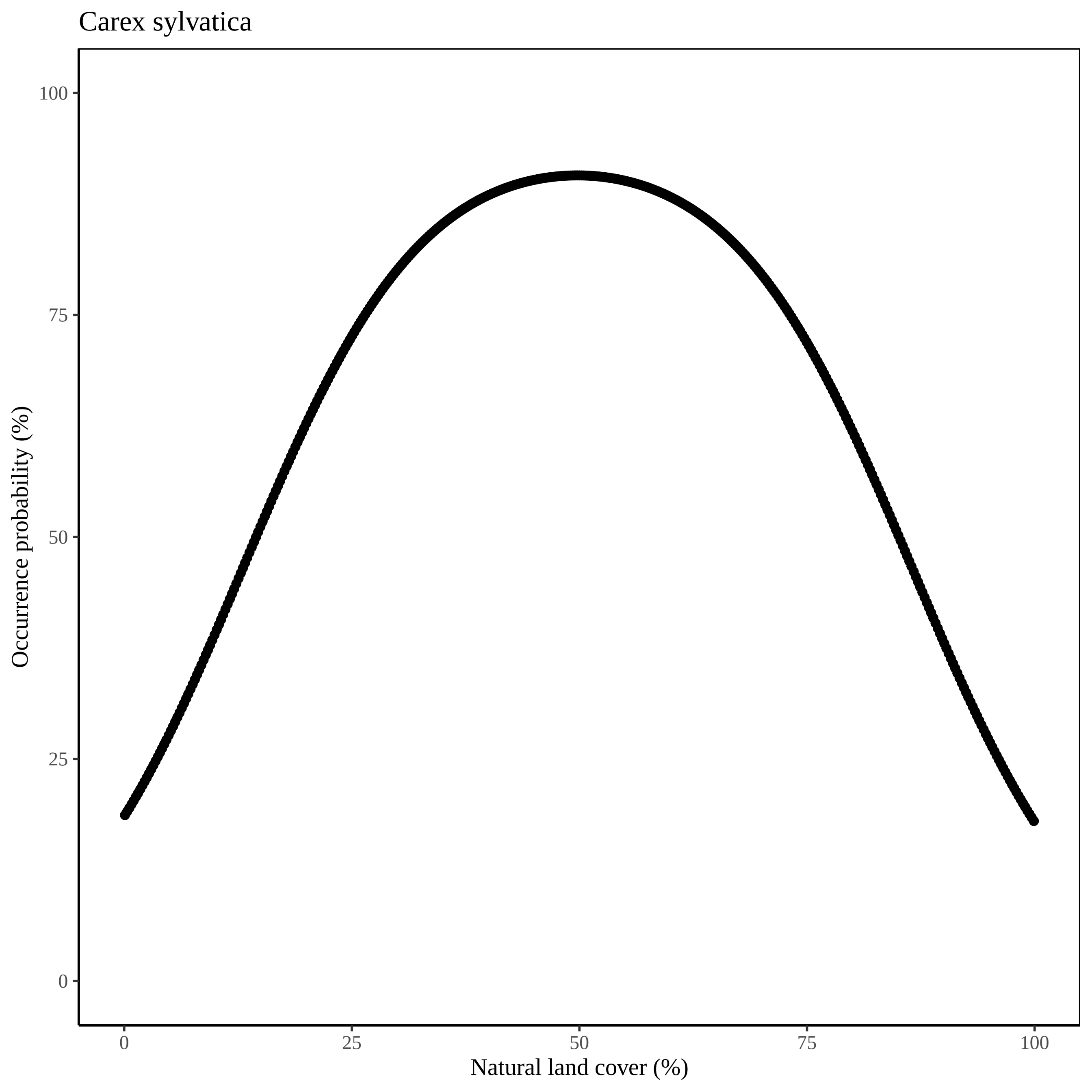
Carex sylvatica
Occurrence probability (%)
100
75
50
25
0
0
25
50
75
100
Natural land cover (%)

Carex trinervis

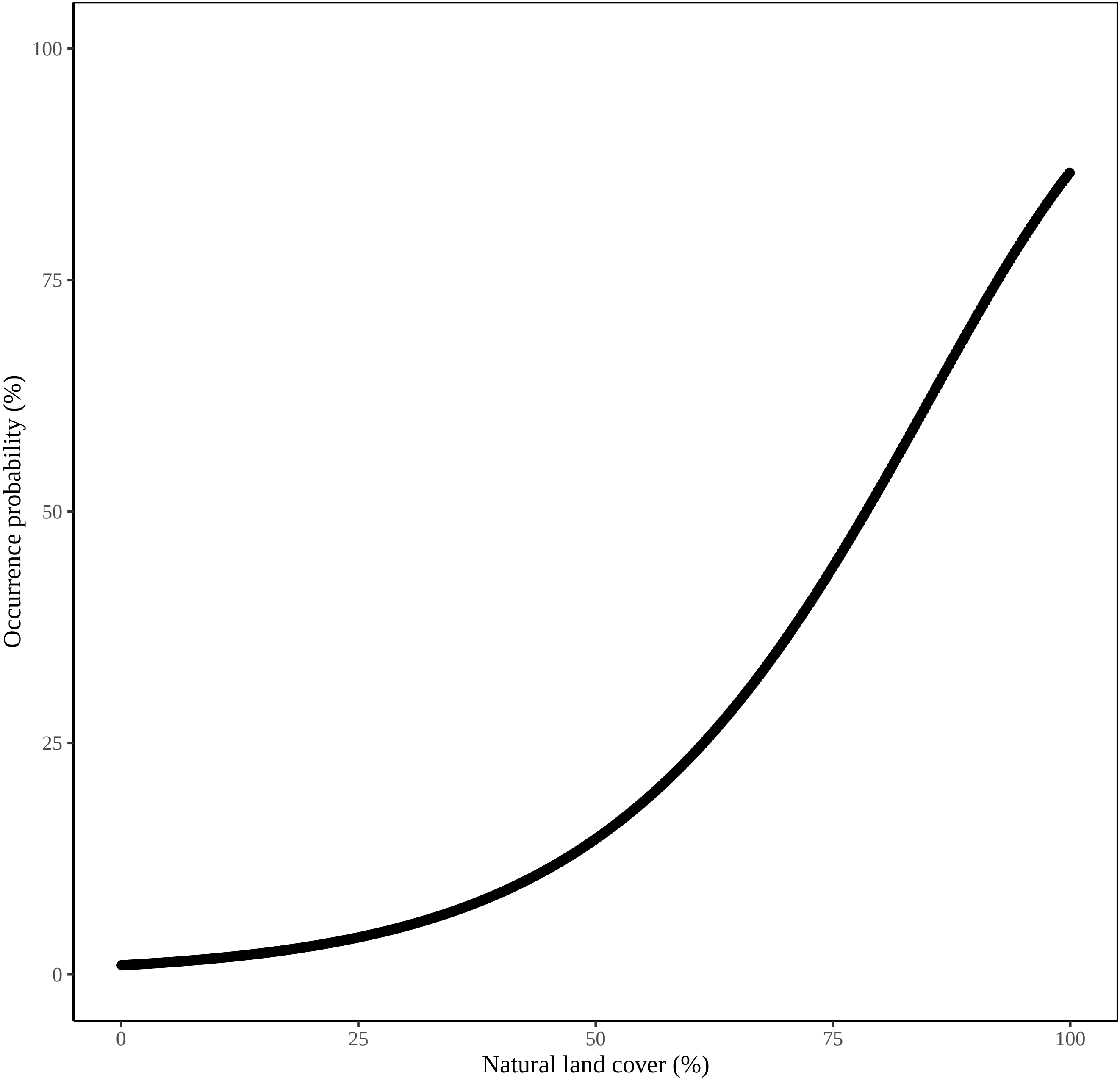

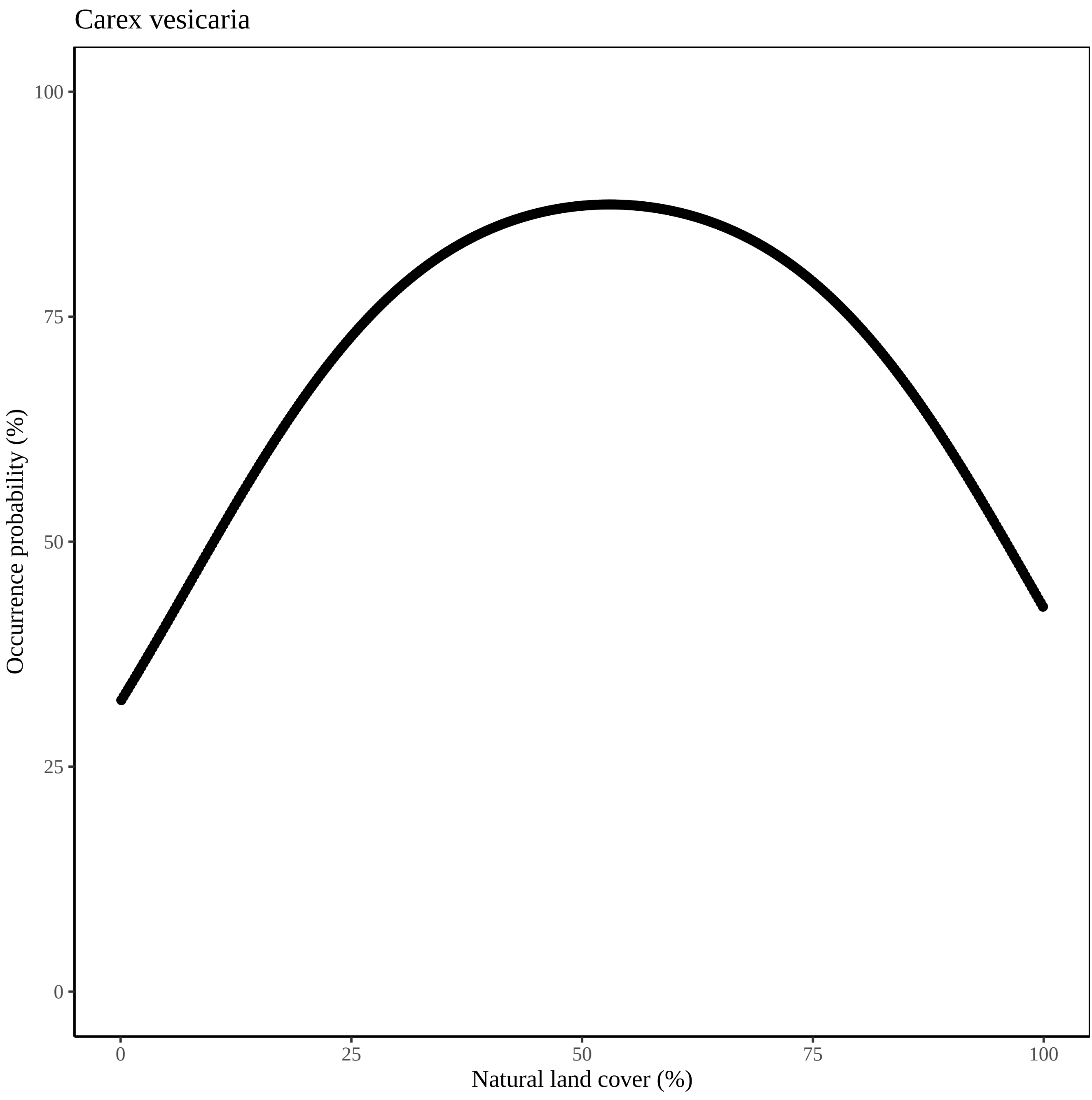
Carex vesicaria
Occurrence probability (%)
100
75
50
25
0
0
25
50
75
100
Natural land cover (%)

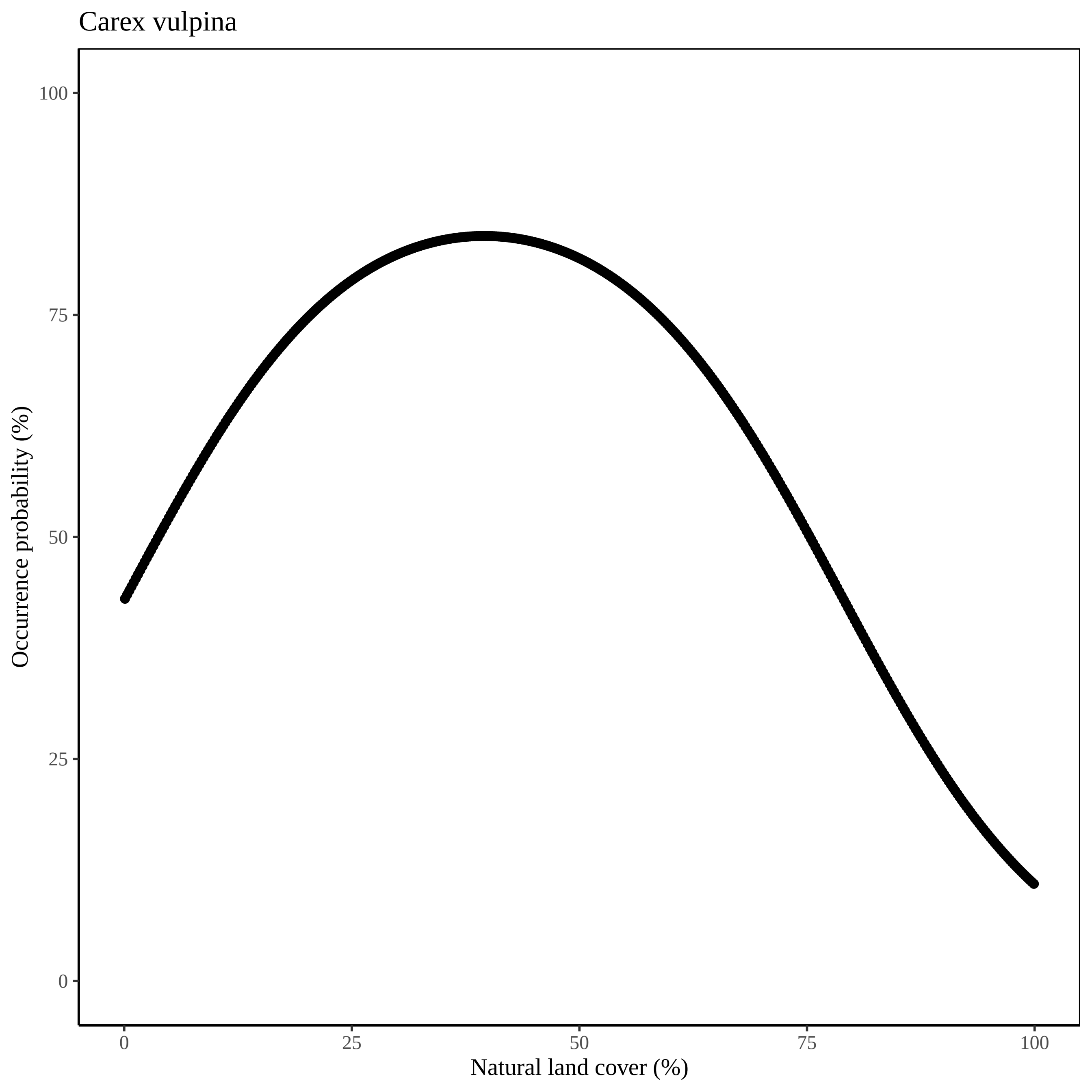
Carex vulpina
Occurrence probability (%)
100
75
50
25
0
0
25
50
75
100
Natural land cover (%)

Carex x elytroides

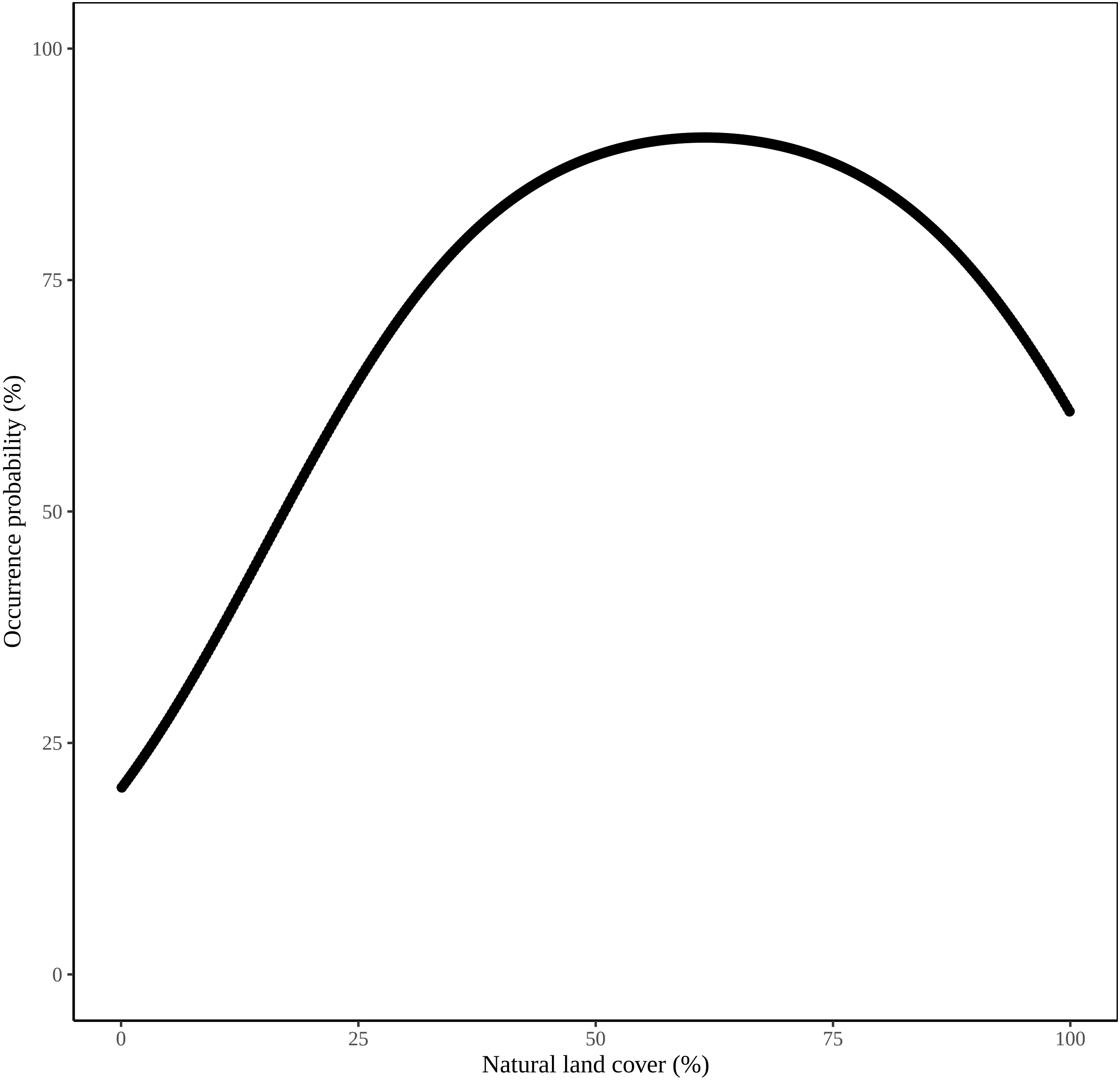

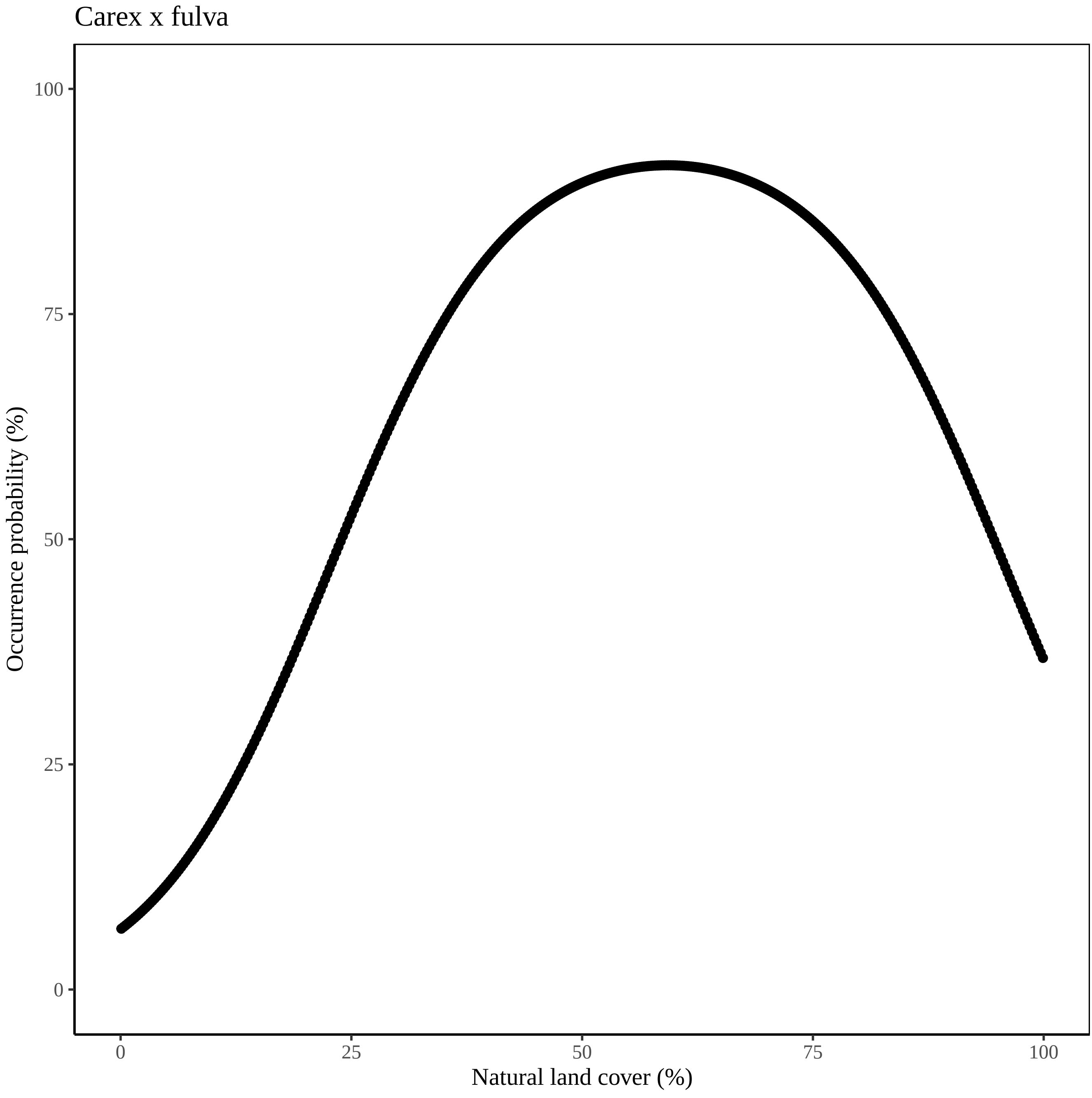

Carex x fulva
100
75
50
25
0
Occurrence probability (%)
0
25
50
75
100
Natural land cover (%)

Carex x timmiana

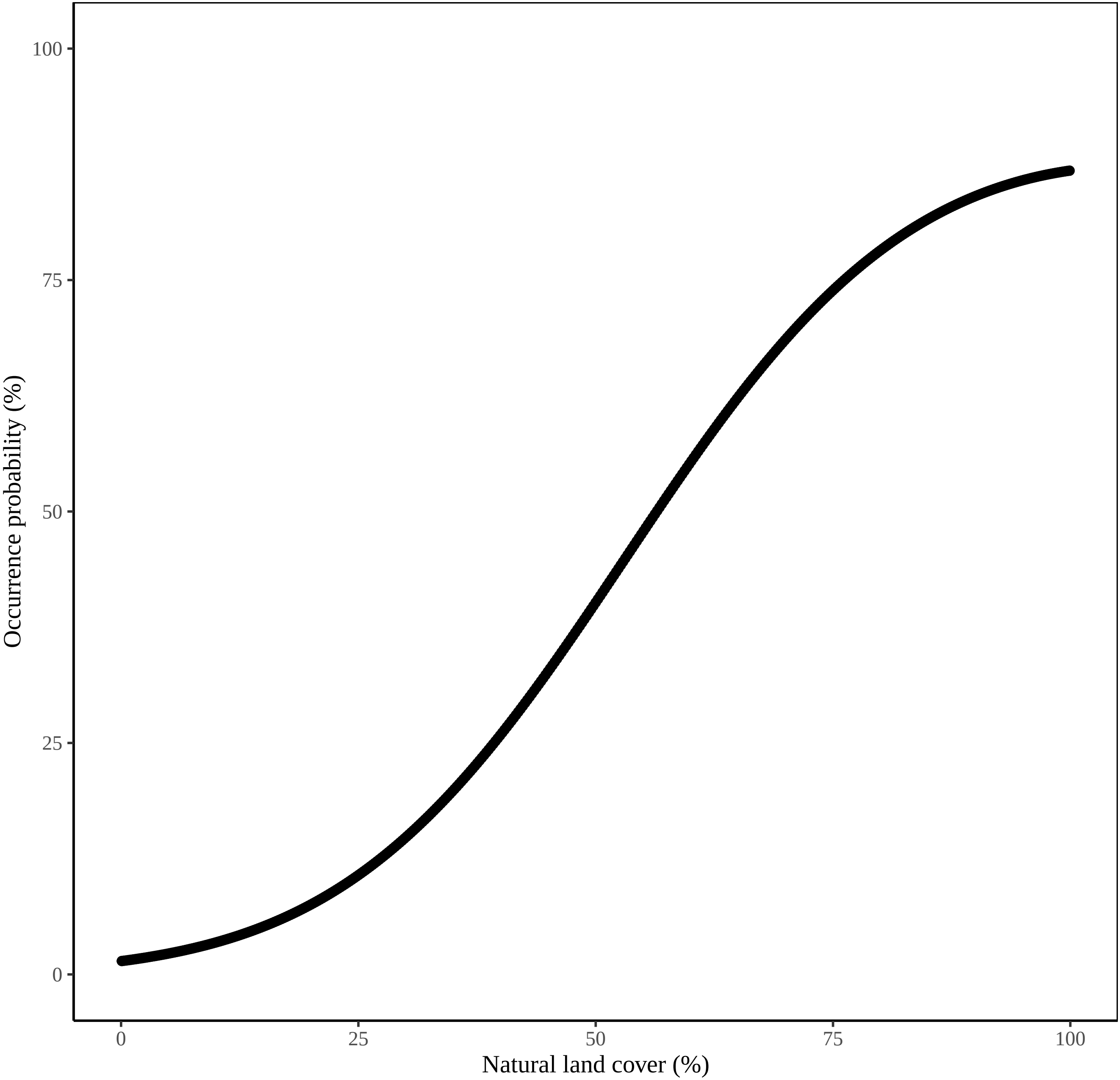

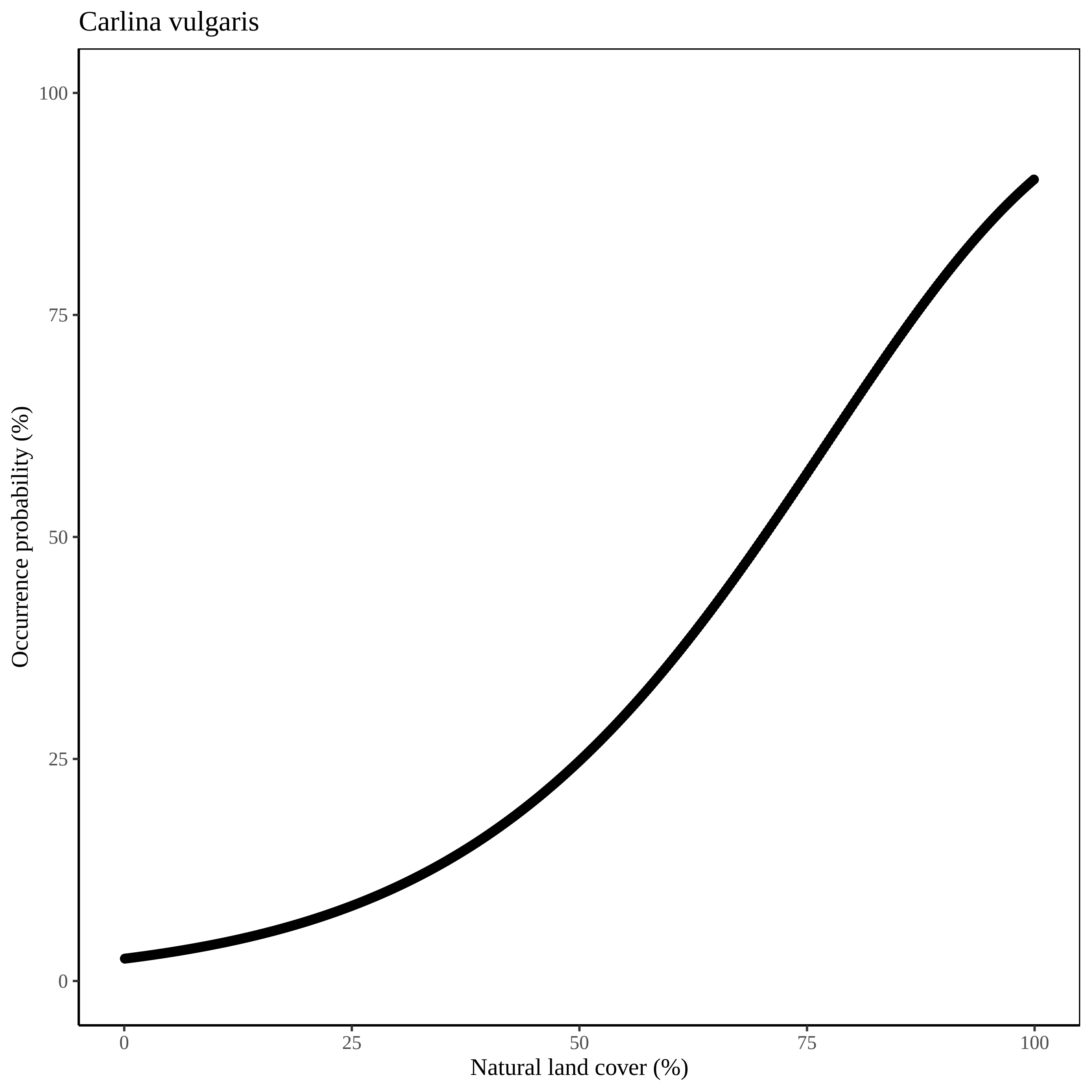
Carlina vulgaris
Occurrence probability (%)
100
75
50
25
0
0
25
50
75
100
Natural land cover (%)

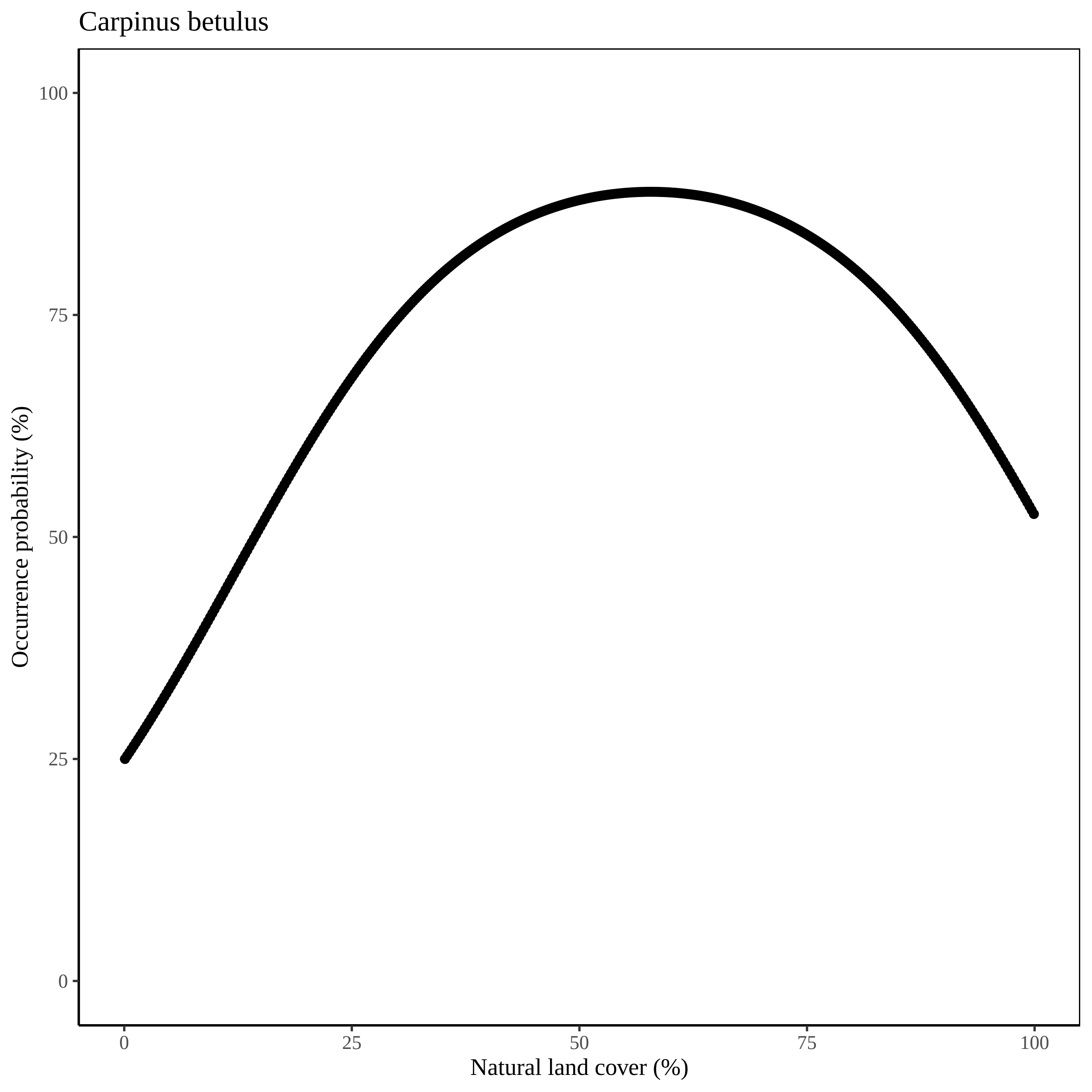
Carpinus betulus
Occurrence probability (%)
100
75
50
25
0
0
25
50
75
100
Natural land cover (%)

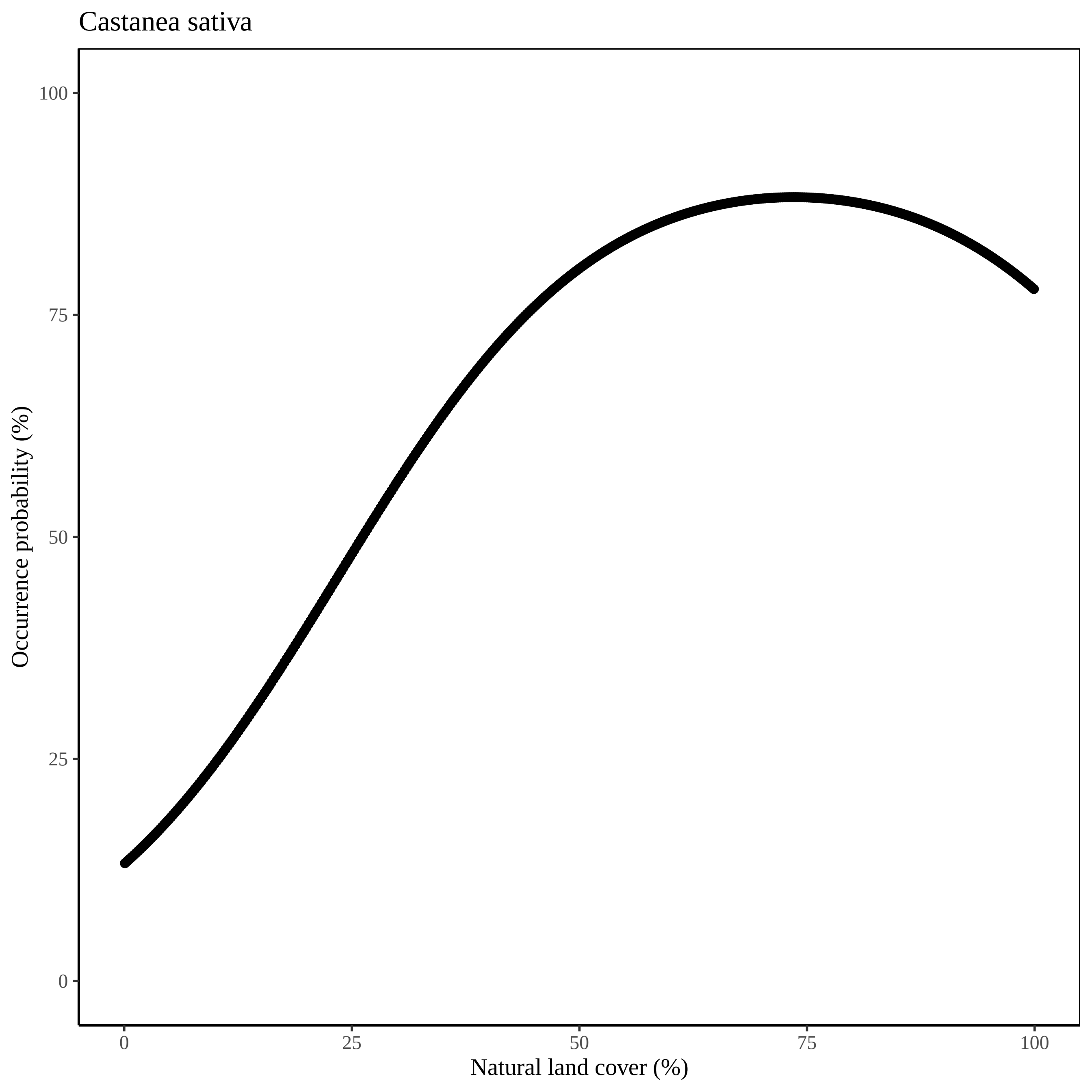
Castanea sativa
Occurrence probability (%)
100
75
50
25
0
0
25
50
75
100
Natural land cover (%)

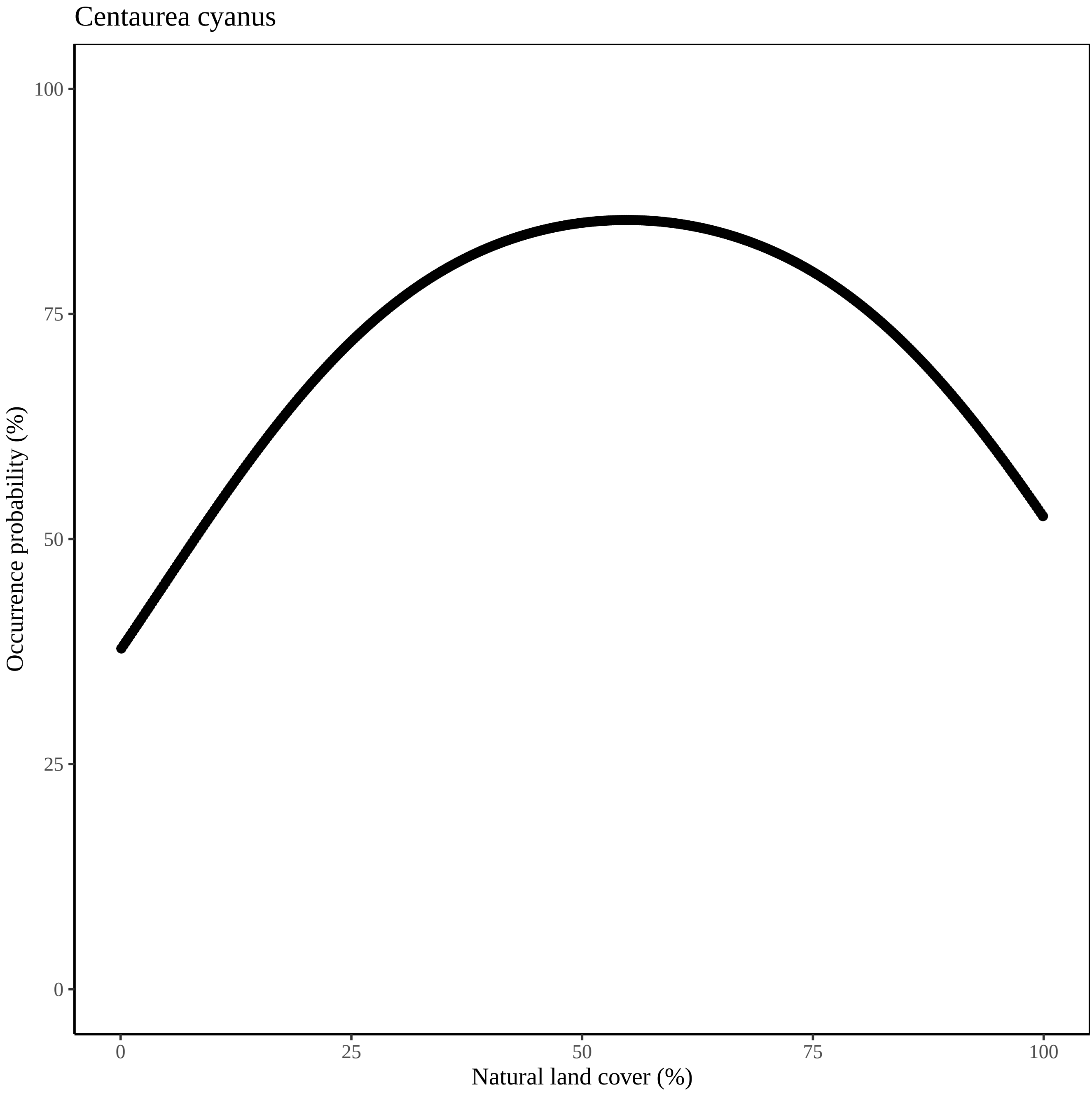
Centaurea cyanus
Occurrence probability (%)
100
75
50
25
0
0
25
50
75
100
Natural land cover (%)

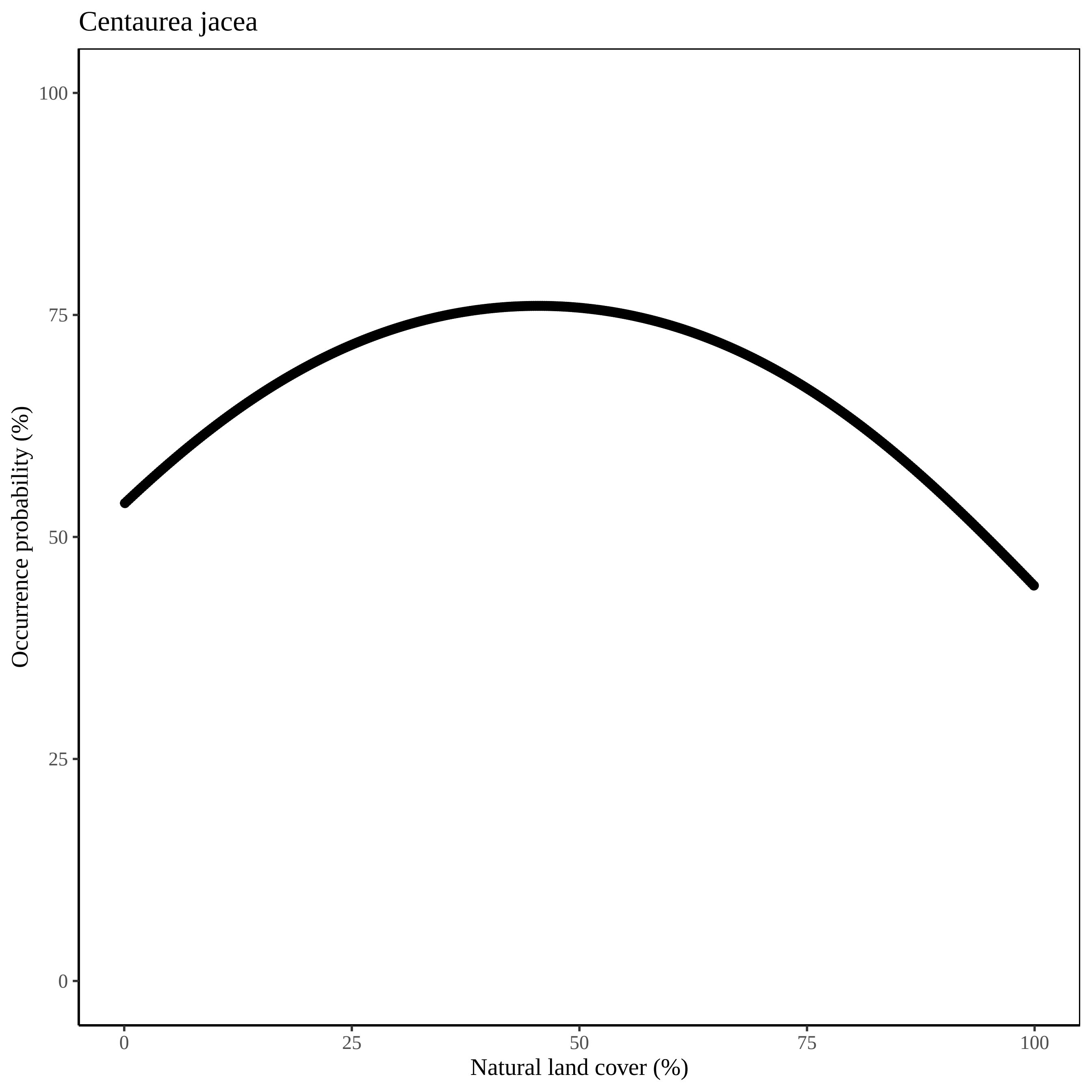

Centaurea jacea
Occurrence probability (%)
100
75
50
25
0
0
25
50
75
100
Natural land cover (%)

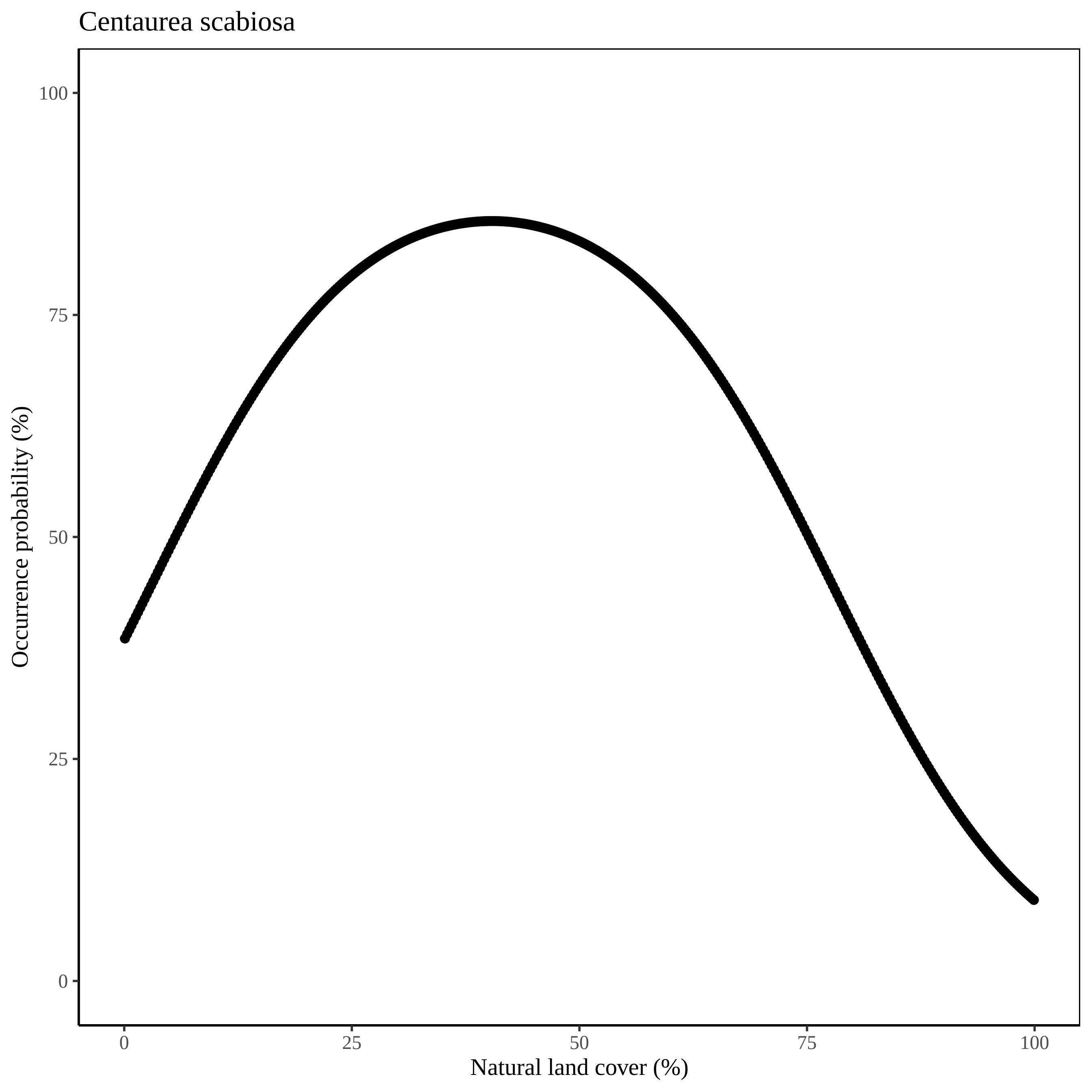

Centaurea scabiosa
100
75
50
25
0
Occurrence probability (%)
0
25
50
75
100
Natural land cover (%)

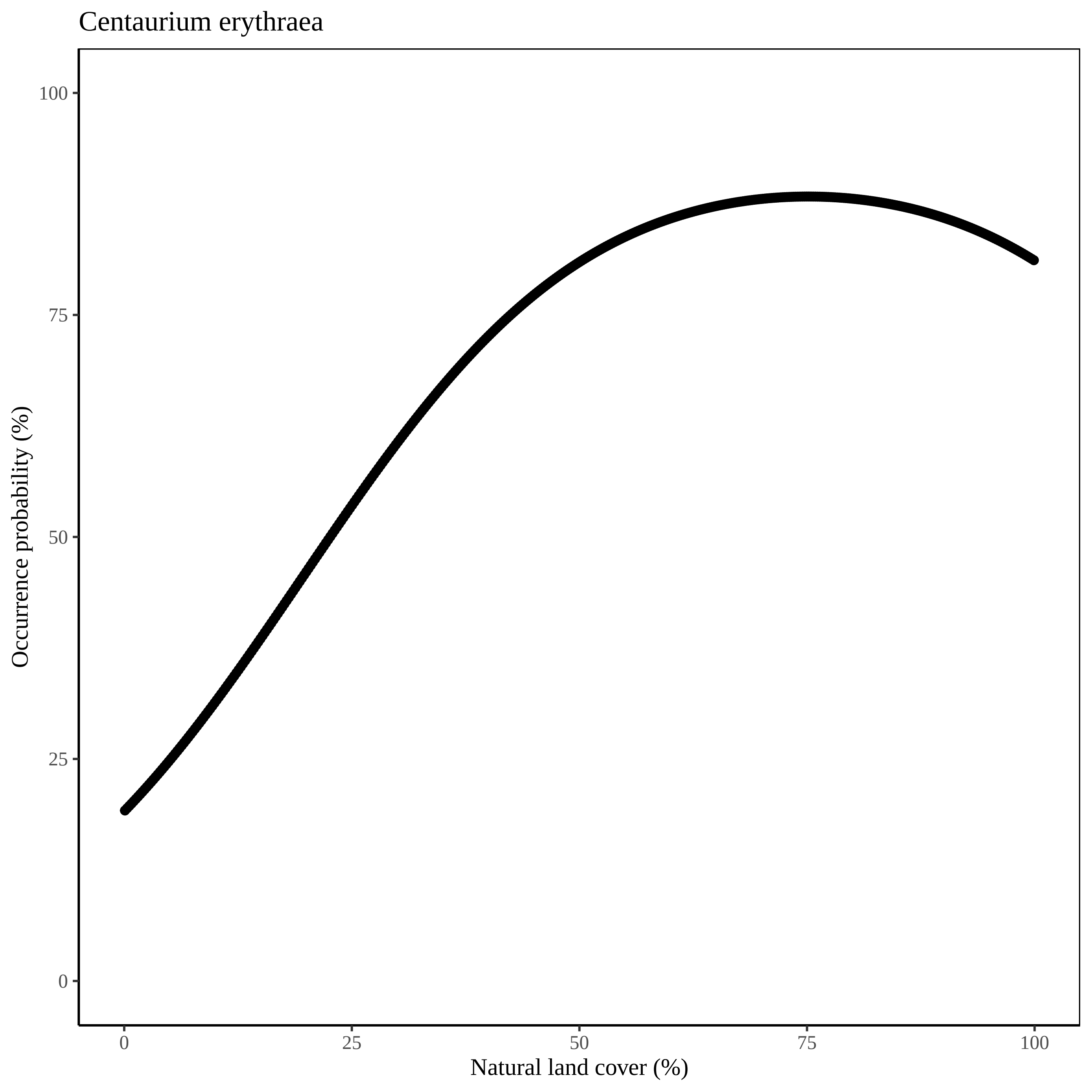
Centaurium erythraea
Occurrence probability (%)
100
75
50
25
0
0
25
50
75
100
Natural land cover (%)

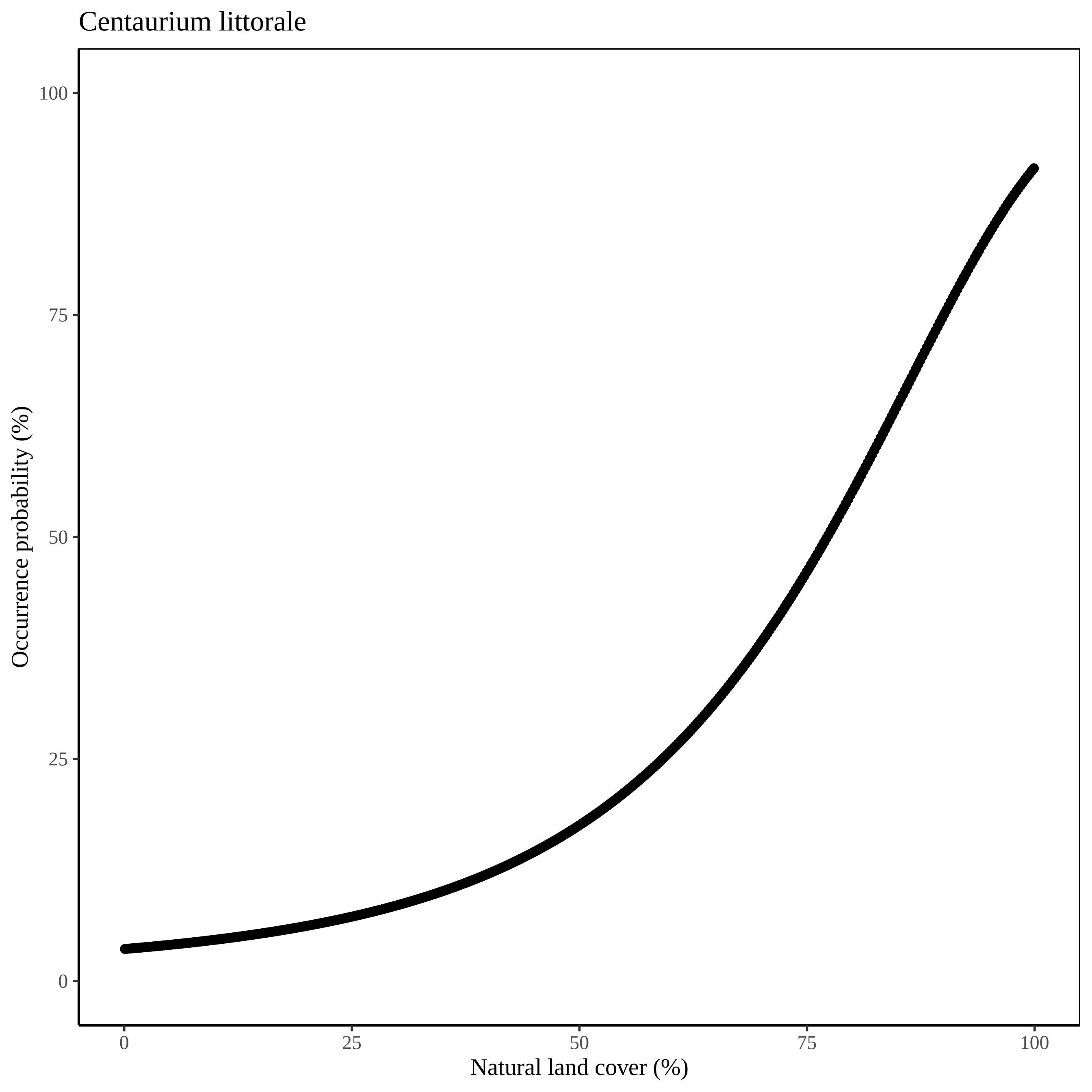
Centaurium littorale
Occurrence probability (%)
100
75
50
25
0
Natural land cover (%)
0
25
50
75
100

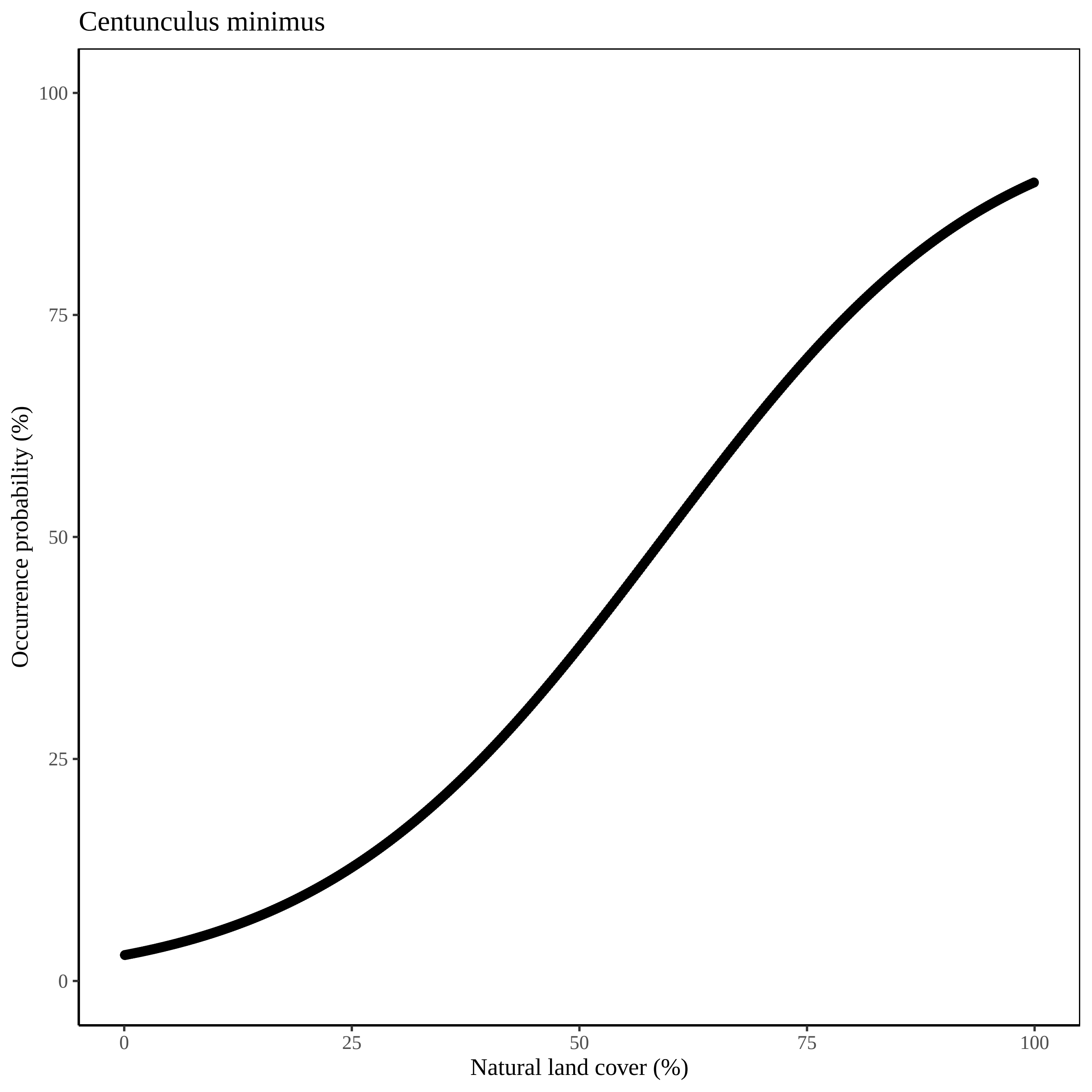
Centunculus minimus
Occurrence probability (%)
100
75
50
25
0
0
25
50
75
100
Natural land cover (%)

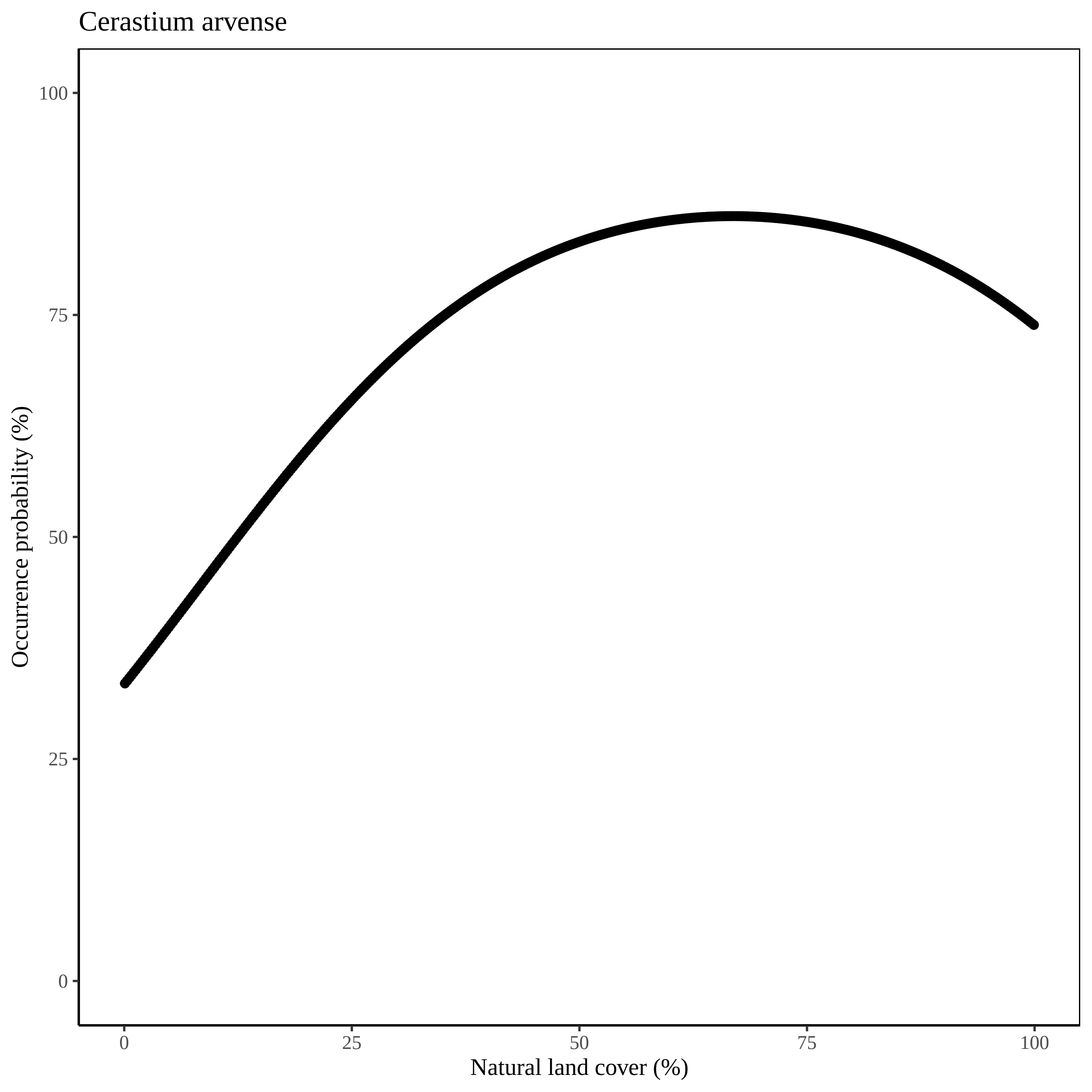

Cerastium arvense
Occurrence probability (%)
100
75
50
25
0
0
25
50
75
100
Natural land cover (%)

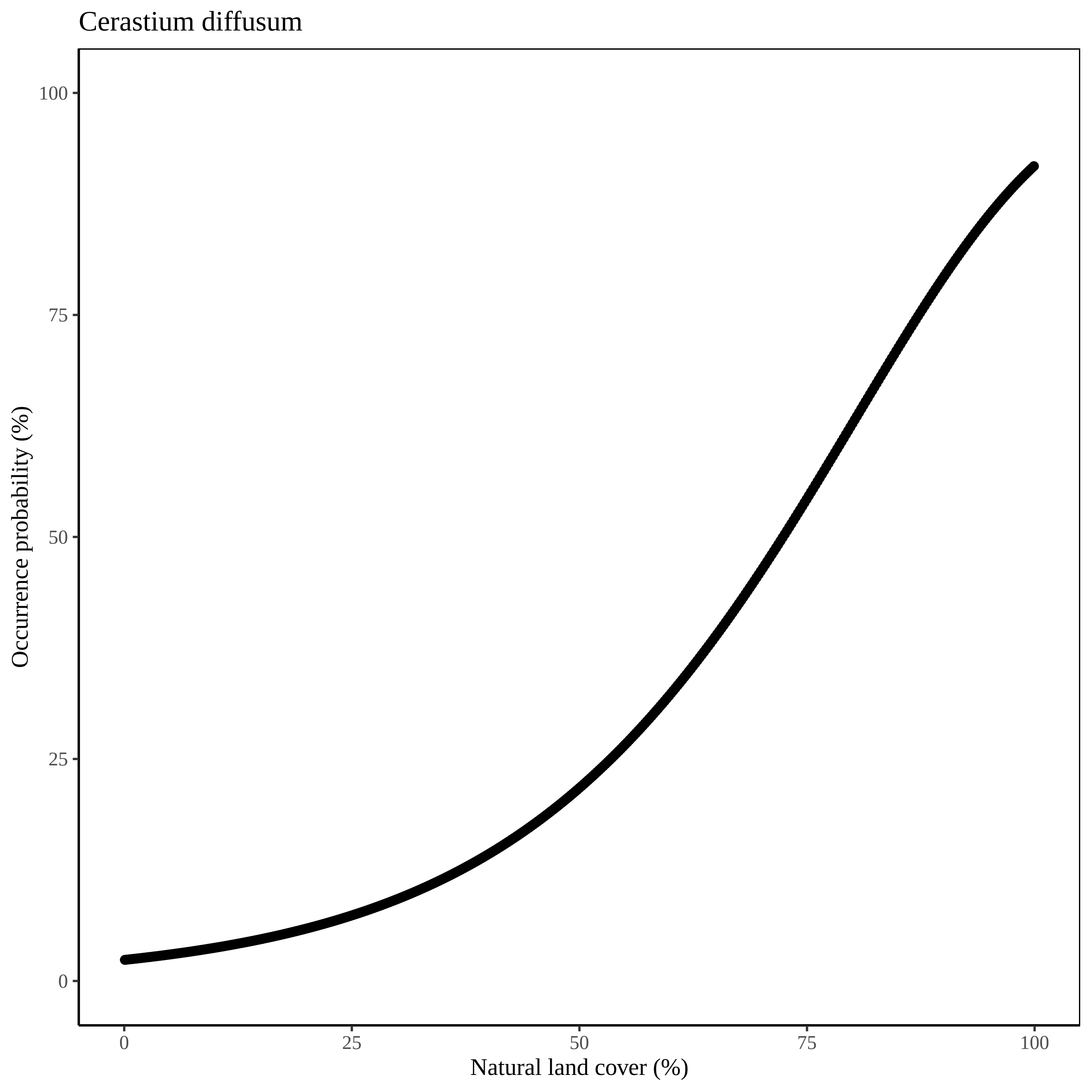

Cerastium diffusum
Occurrence probability (%)
100
75
50
25
0
0
25
50
75
100
Natural land cover (%)

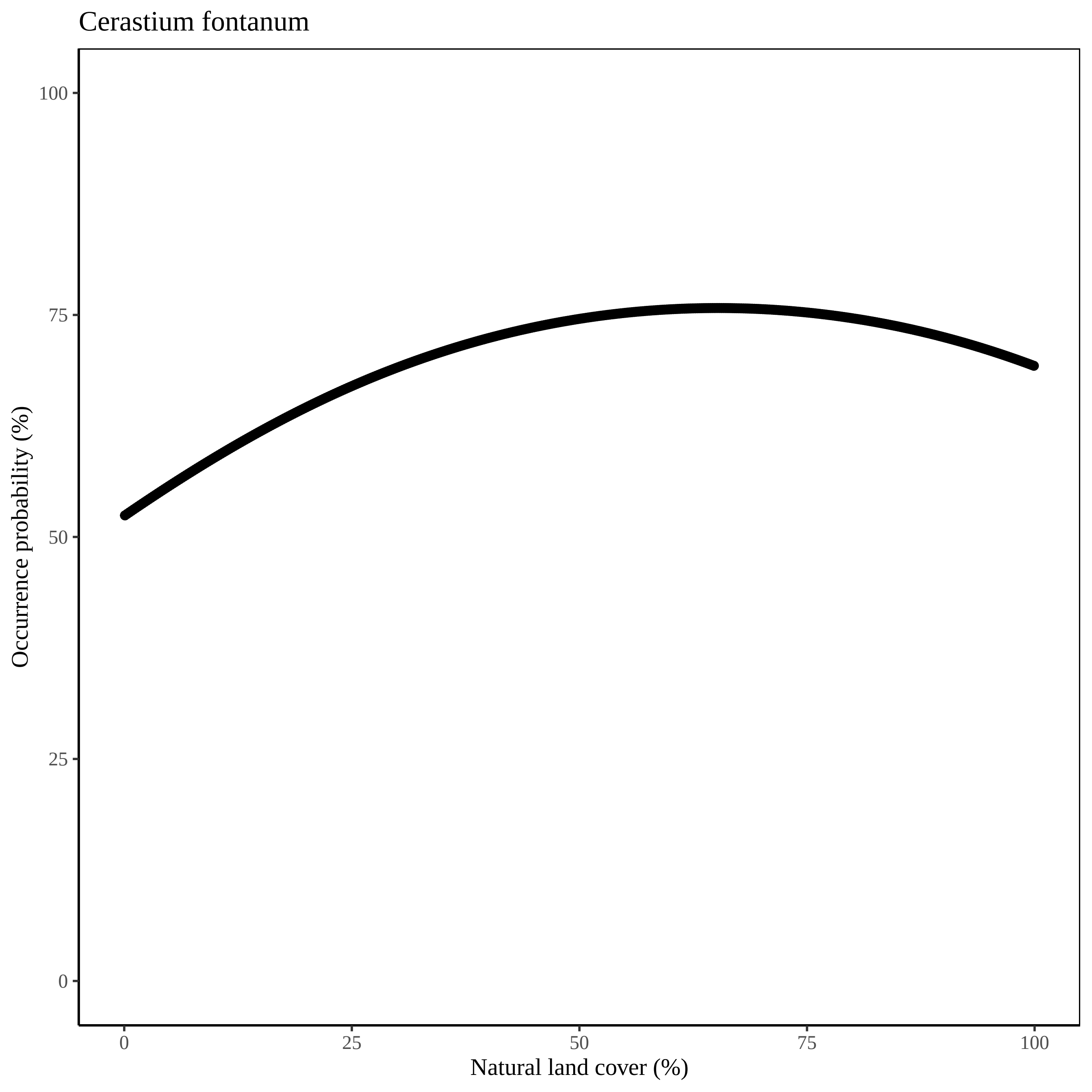
Cerastium fontanum
Occurrence probability (%)
100
75
50
25
0
0
25
50
75
100
Natural land cover (%)

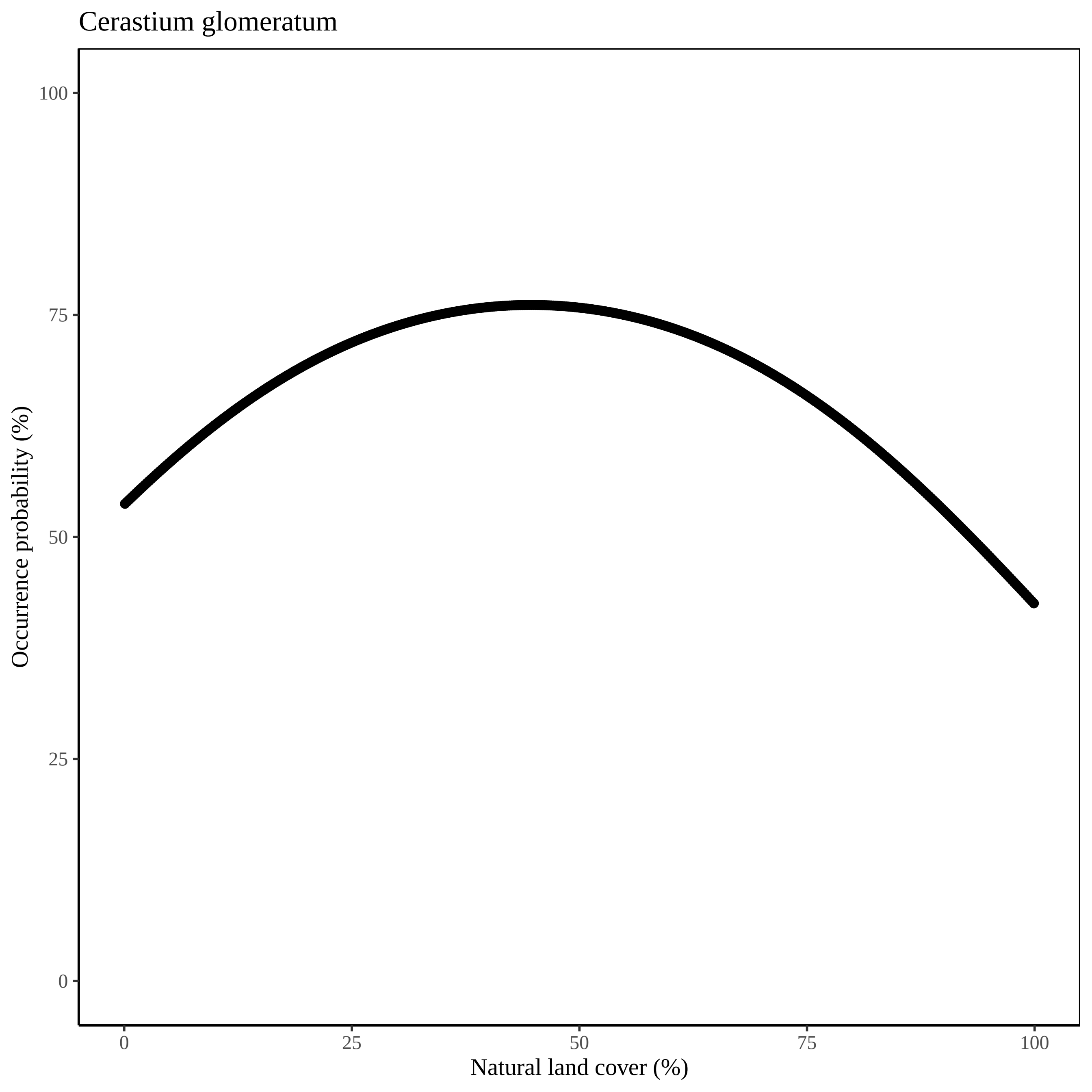
Cerastium glomeratum
100
75
50
25
0
Occurrence probability (%)
0
25
50
75
100
Natural land cover (%)

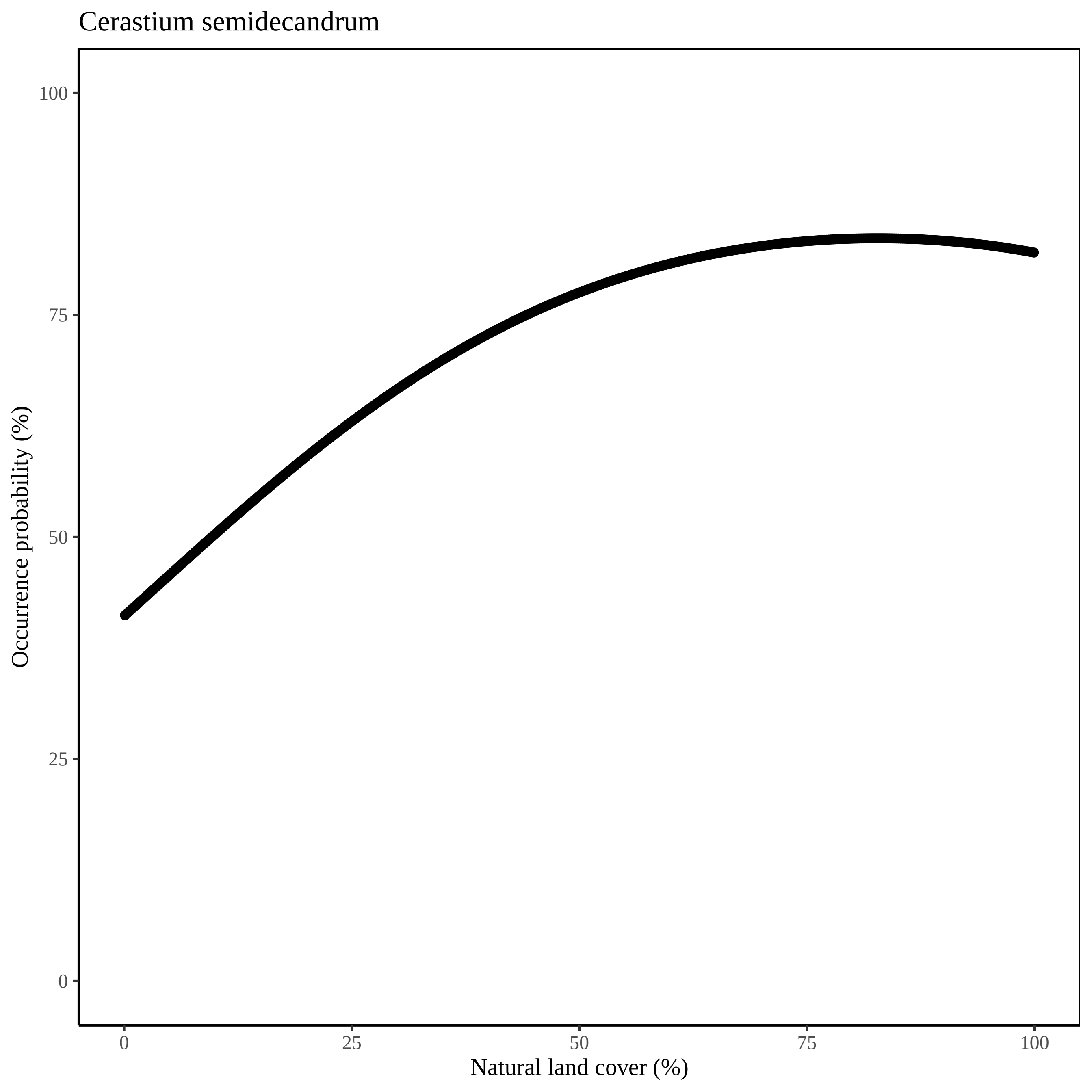
Cerastium semidecandrum
Occurrence probability (%)
100
75
50
25
0
0
25
50
75
100
Natural land cover (%)

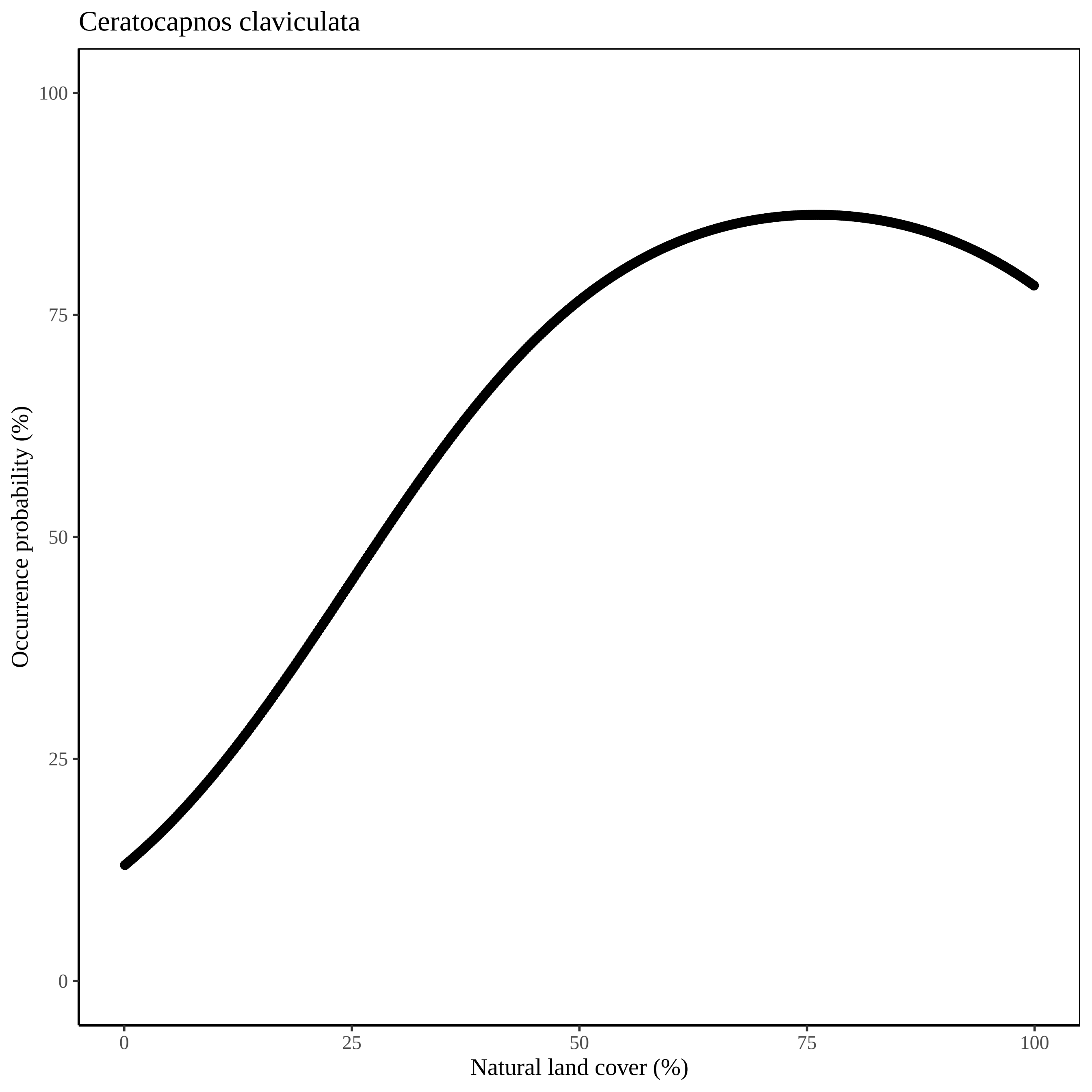

Ceratocapnos claviculata
Occurrence probability (%)
100
75
50
25
0
0
25
50
75
100
Natural land cover (%)

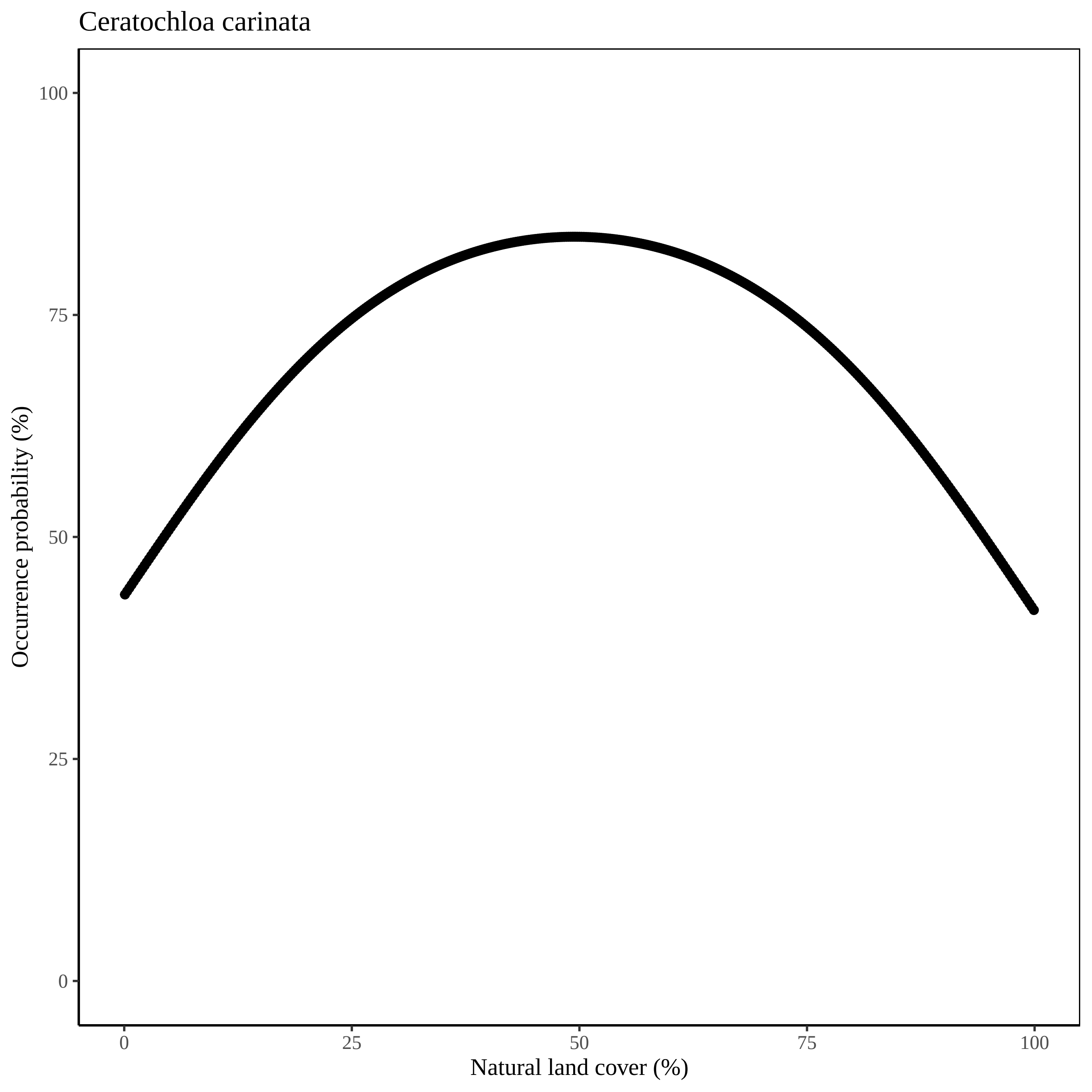
Ceratochloa carinata
Occurrence probability (%)
100
75
50
25
0
0
25
50
75
100
Natural land cover (%)

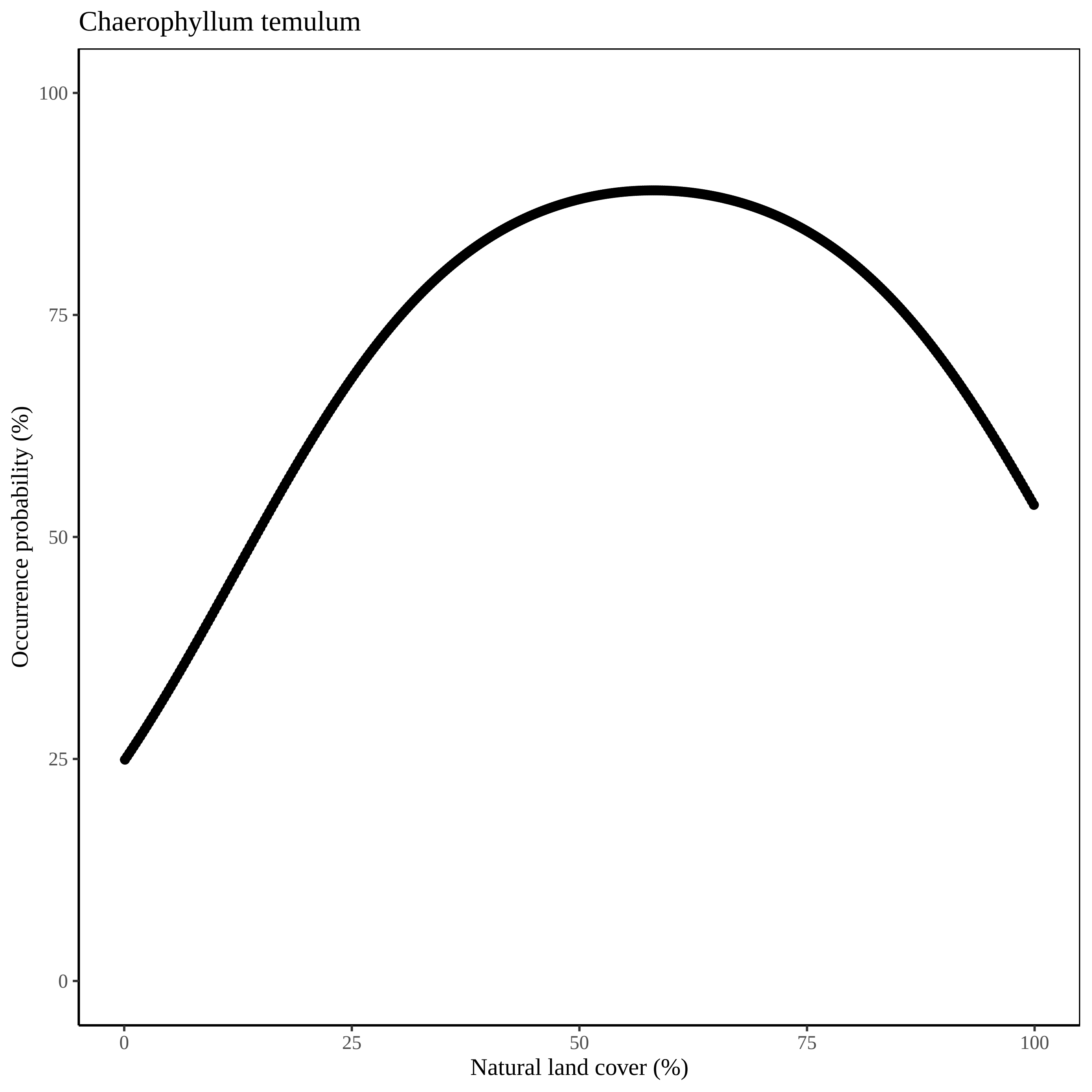

Chaerophyllum temulum
Occurrence probability (%)
100
75
50
25
0
0
25
50
75
100
Natural land cover (%)

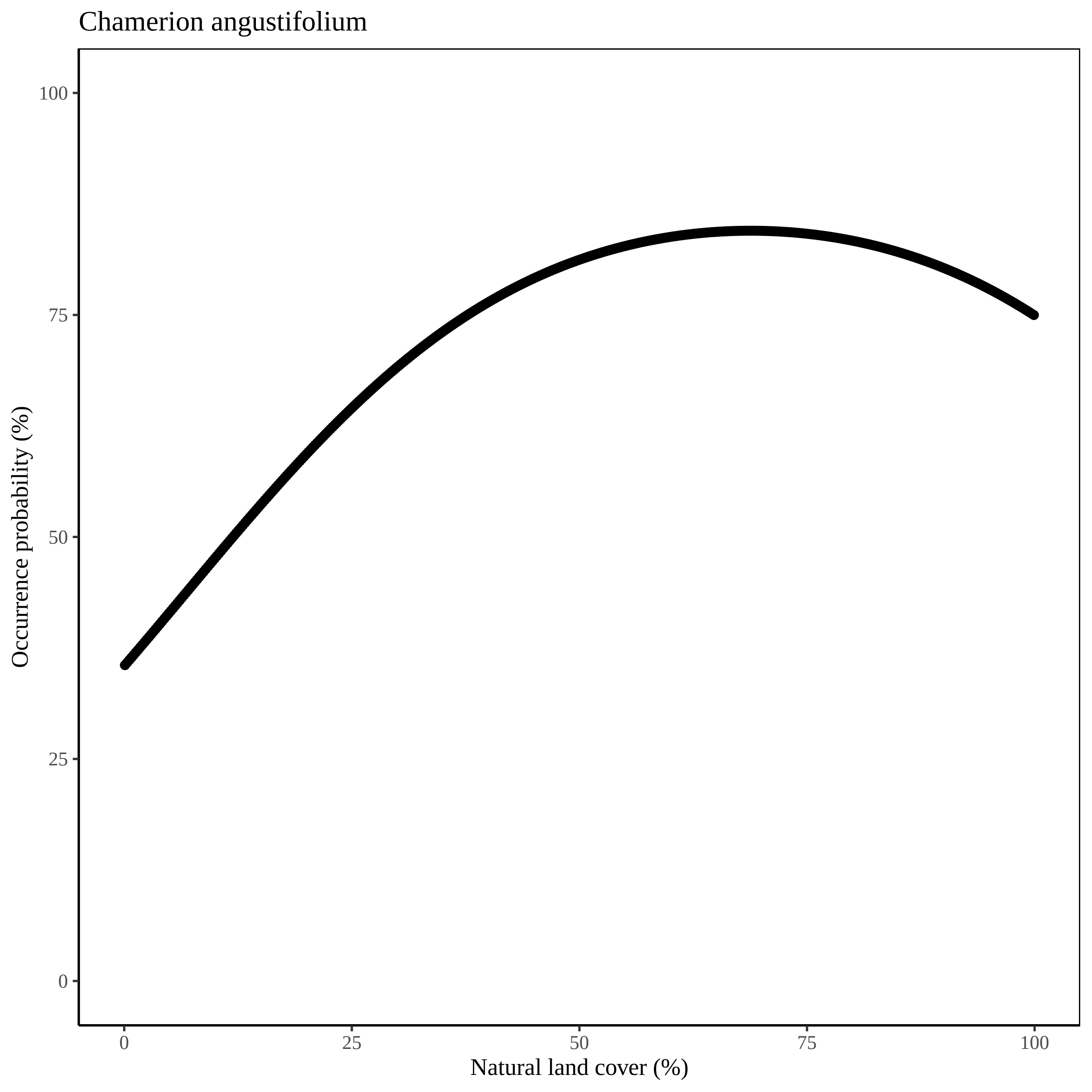

Chamerion angustifolium
100
75
50
25
0
0
25
50
75
100
Occurrence probability (%)
Natural land cover (%)

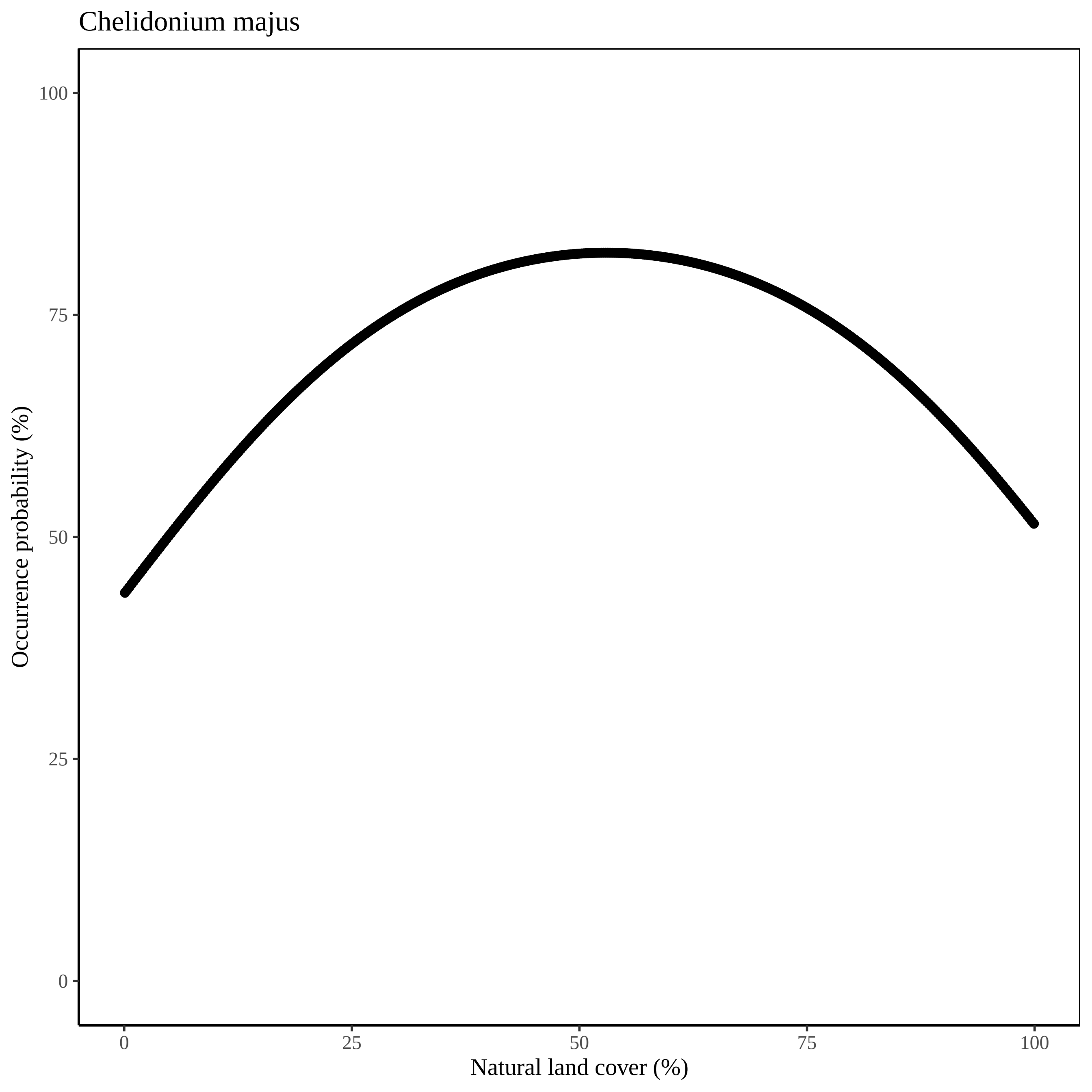
Chelidonium majus
Occurrence probability (%)
100
75
50
25
0
0
25
50
75
100
Natural land cover (%)

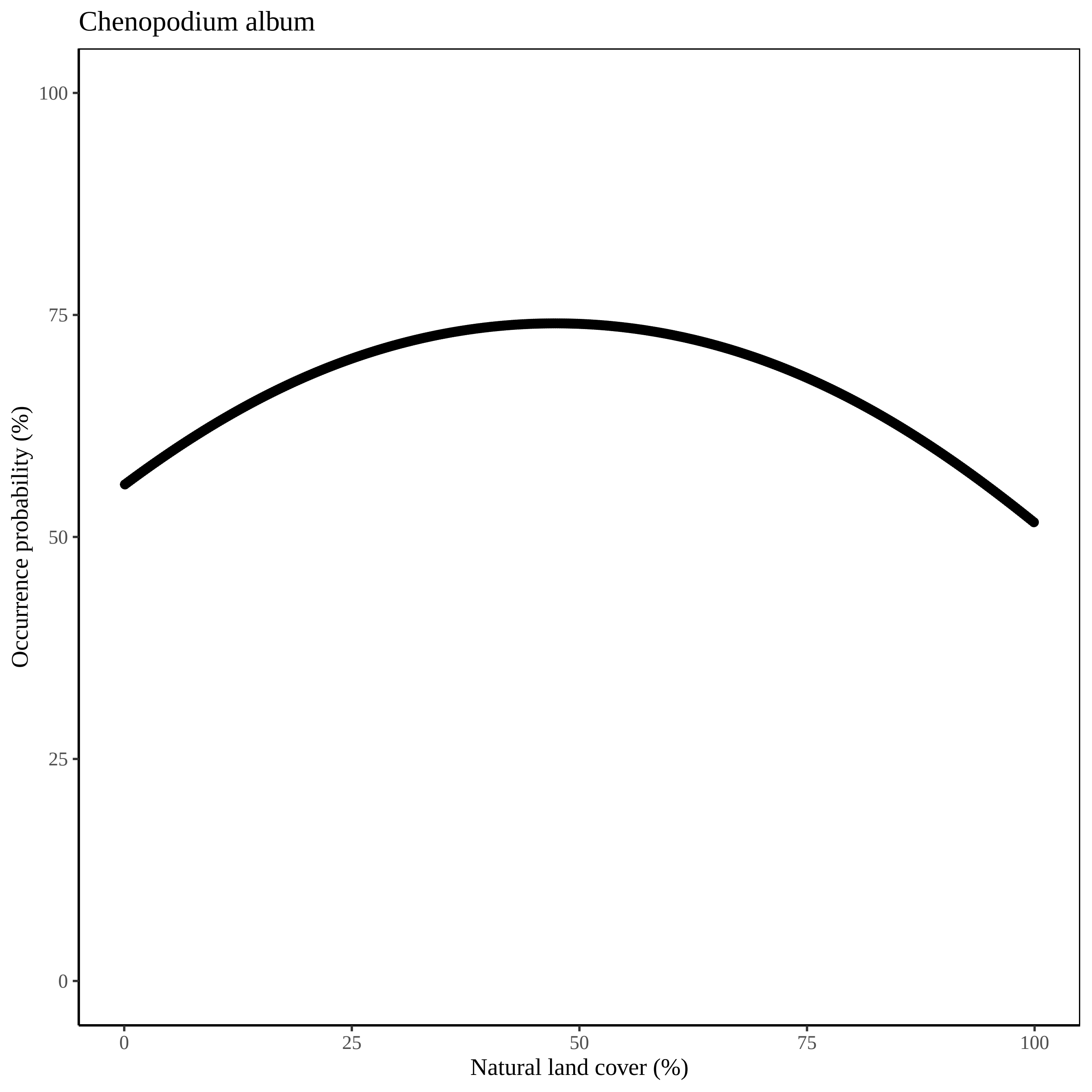

Chenopodium album
Occurrence probability (%)
100
75
50
25
0
0
25
50
75
100
Natural land cover (%)

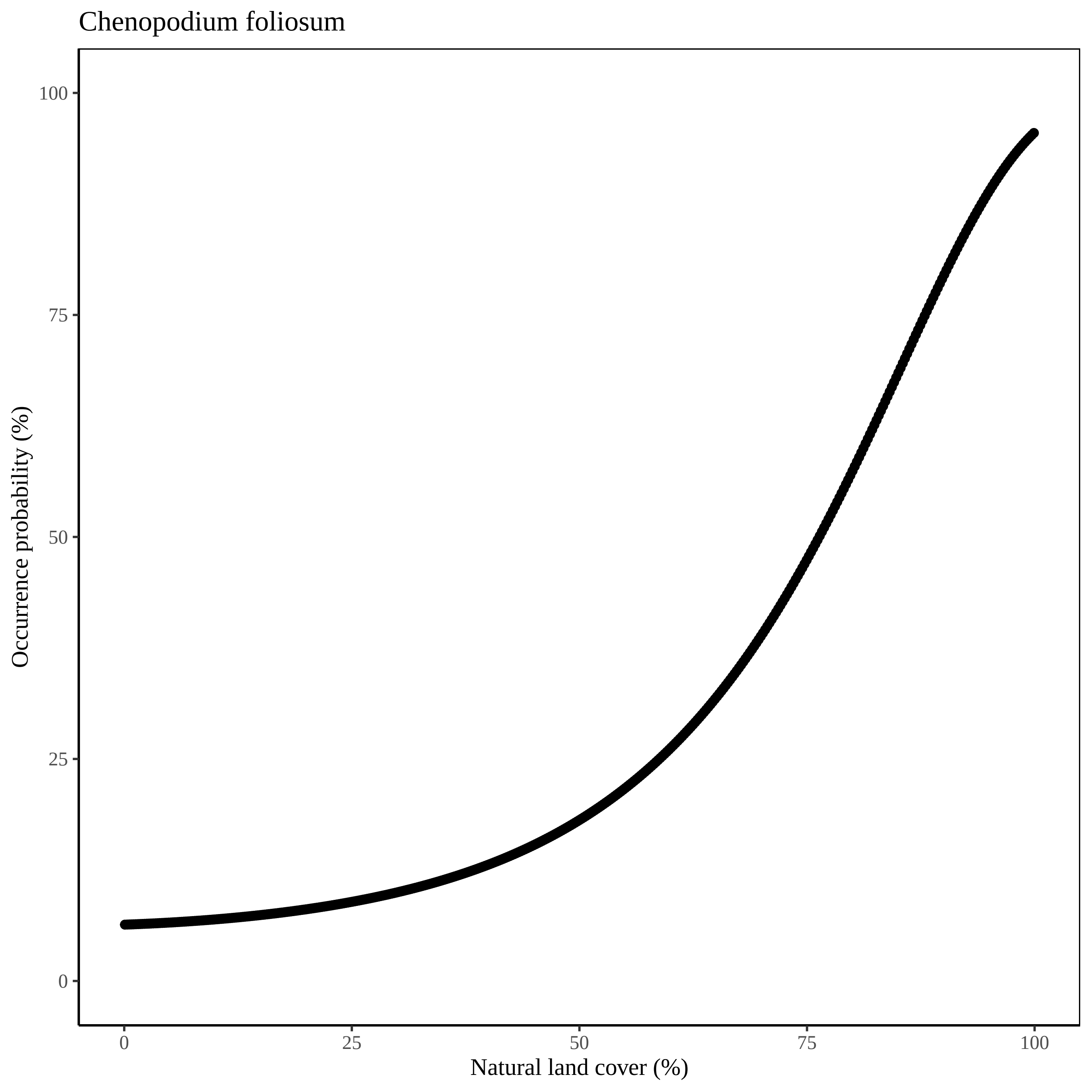
Chenopodium foliosum
Occurrence probability (%)
100
75
50
25
0
0
25
50
75
100
Natural land cover (%)

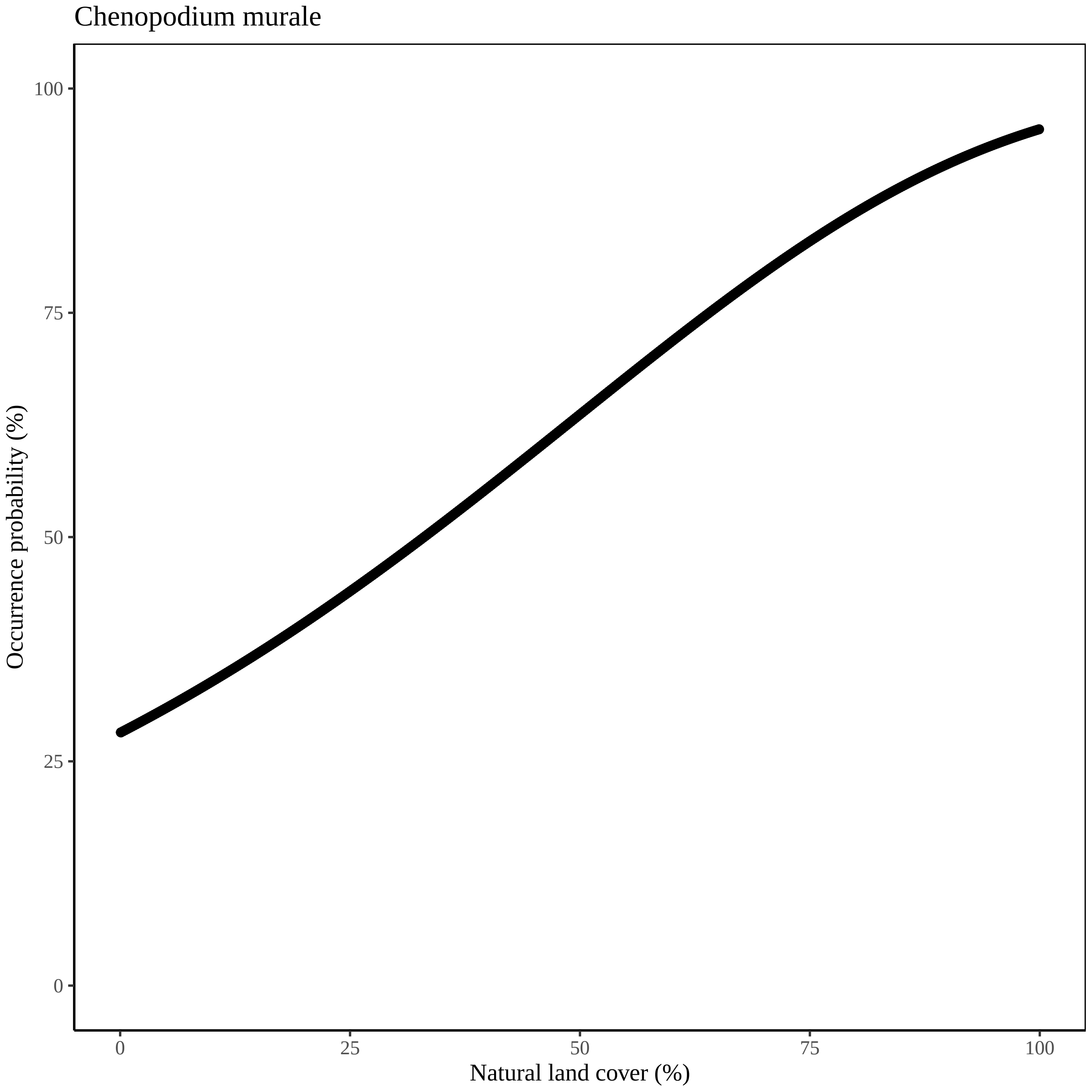

Chenopodium murale
Occurrence probability (%)
100
75
50
25
0
0
25
50
75
100
Natural land cover (%)

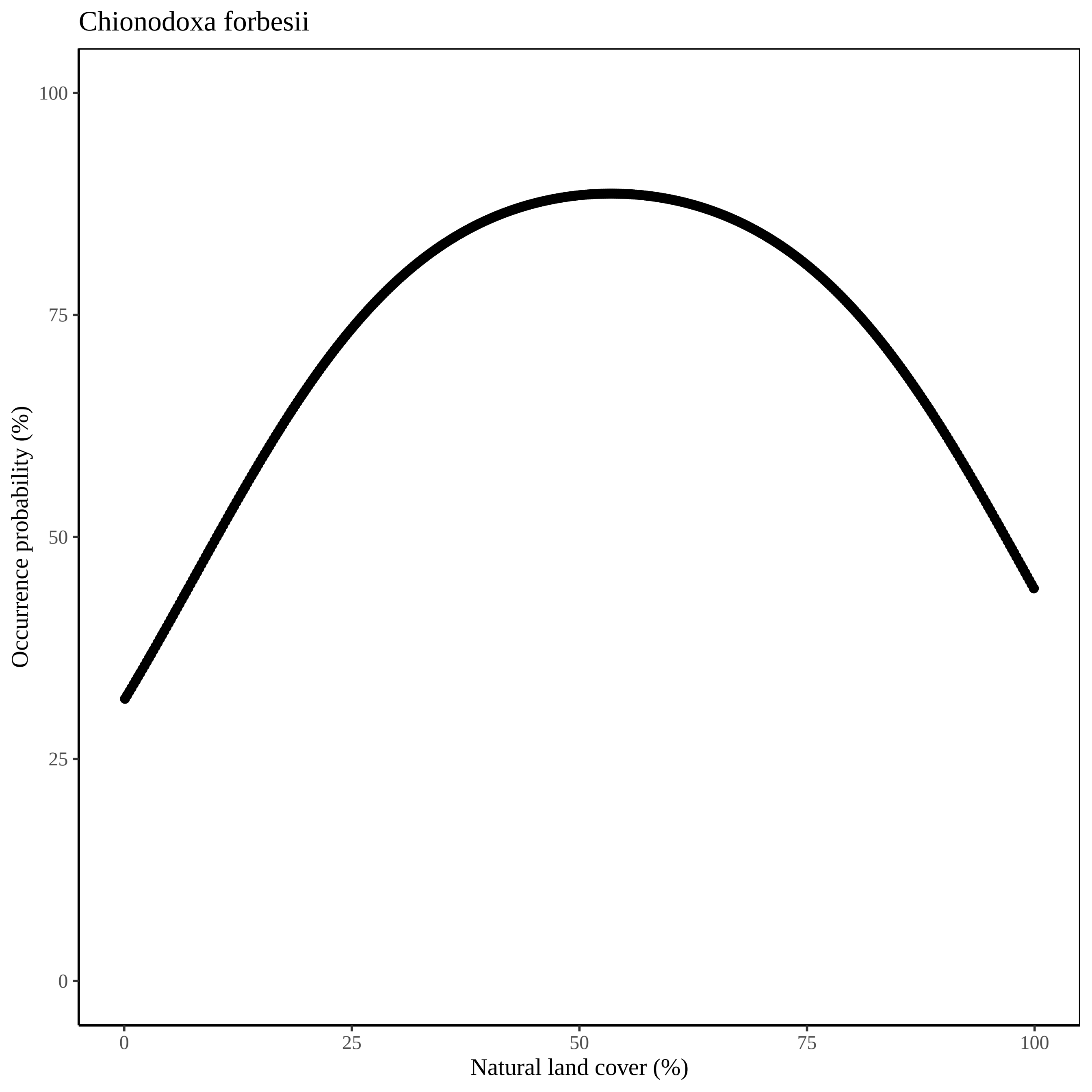

Chionodoxa forbesii
Occurrence probability (%)
100
75
50
25
0
0
25
50
75
100
Natural land cover (%)

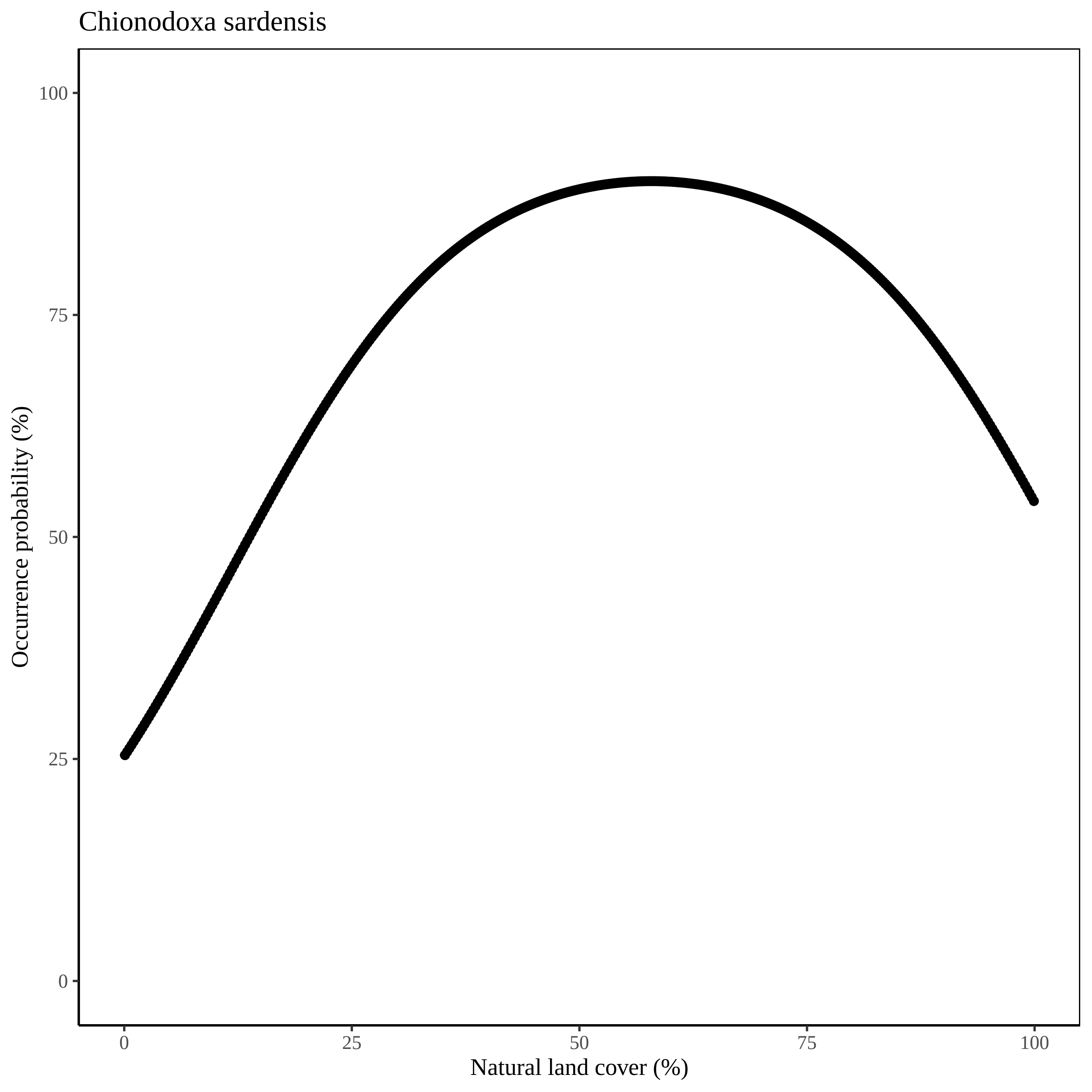

Chionodoxa sardensis
Occurrence probability (%)
100
75
50
25
0
0
25
50
75
100
Natural land cover (%)

Chrysosplenium alternifolium

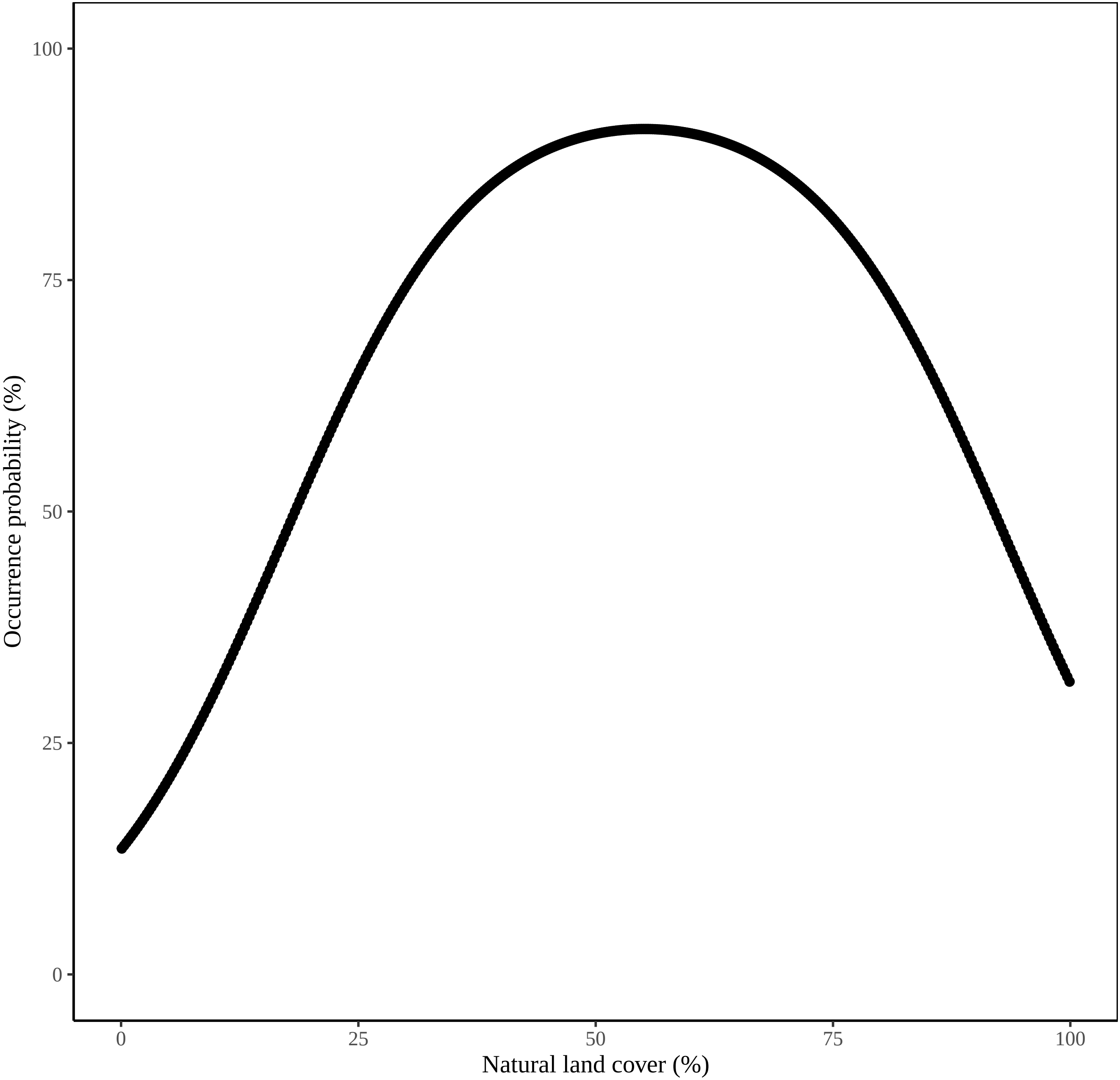

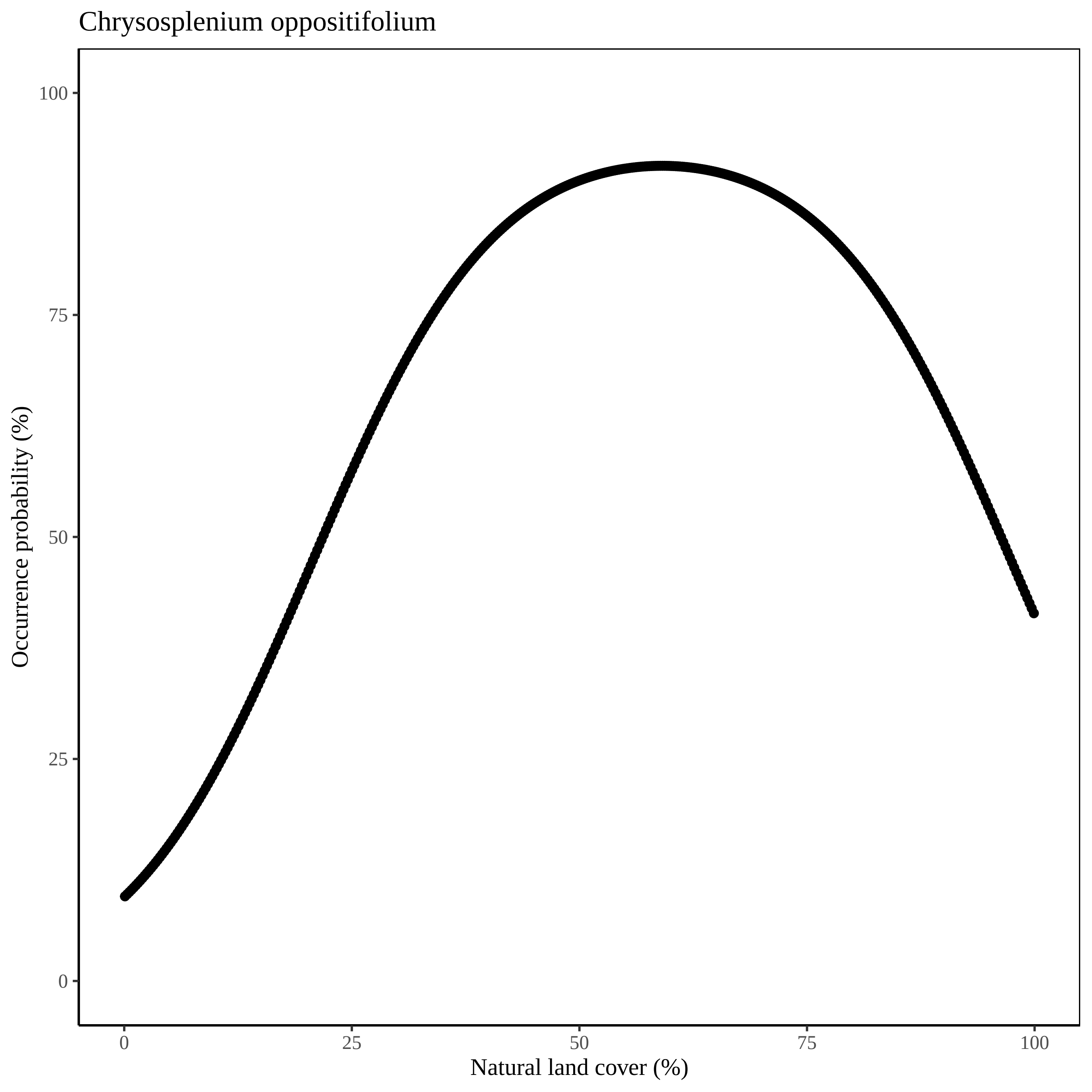
Chrysosplenium oppositifolium
Occurrence probability (%)
100
75
50
25
0
0
25
50
75
100
Natural land cover (%)

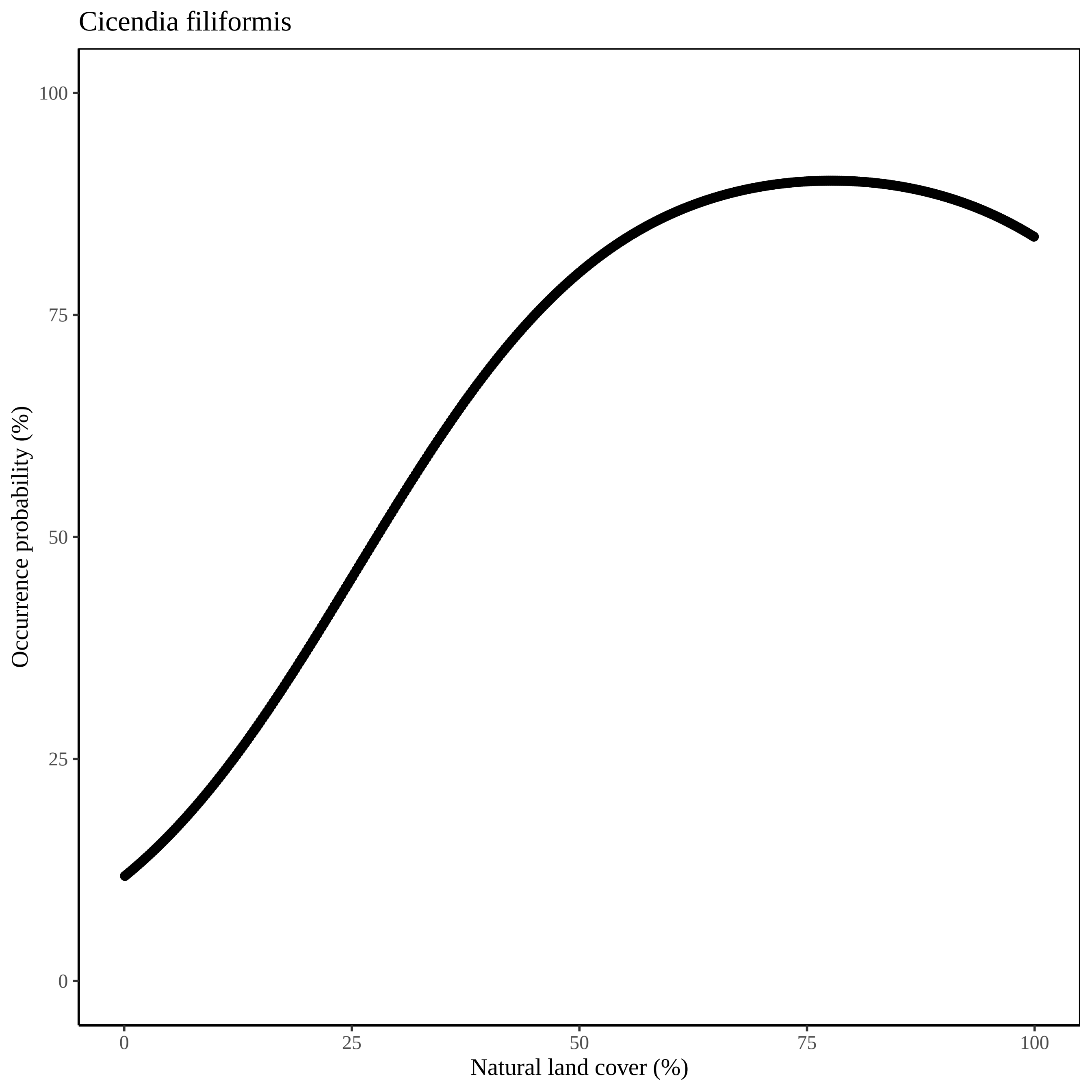
Cicendia filiformis
Occurrence probability (%)
100
75
50
25
0
0
25
50
75
100
Natural land cover (%)

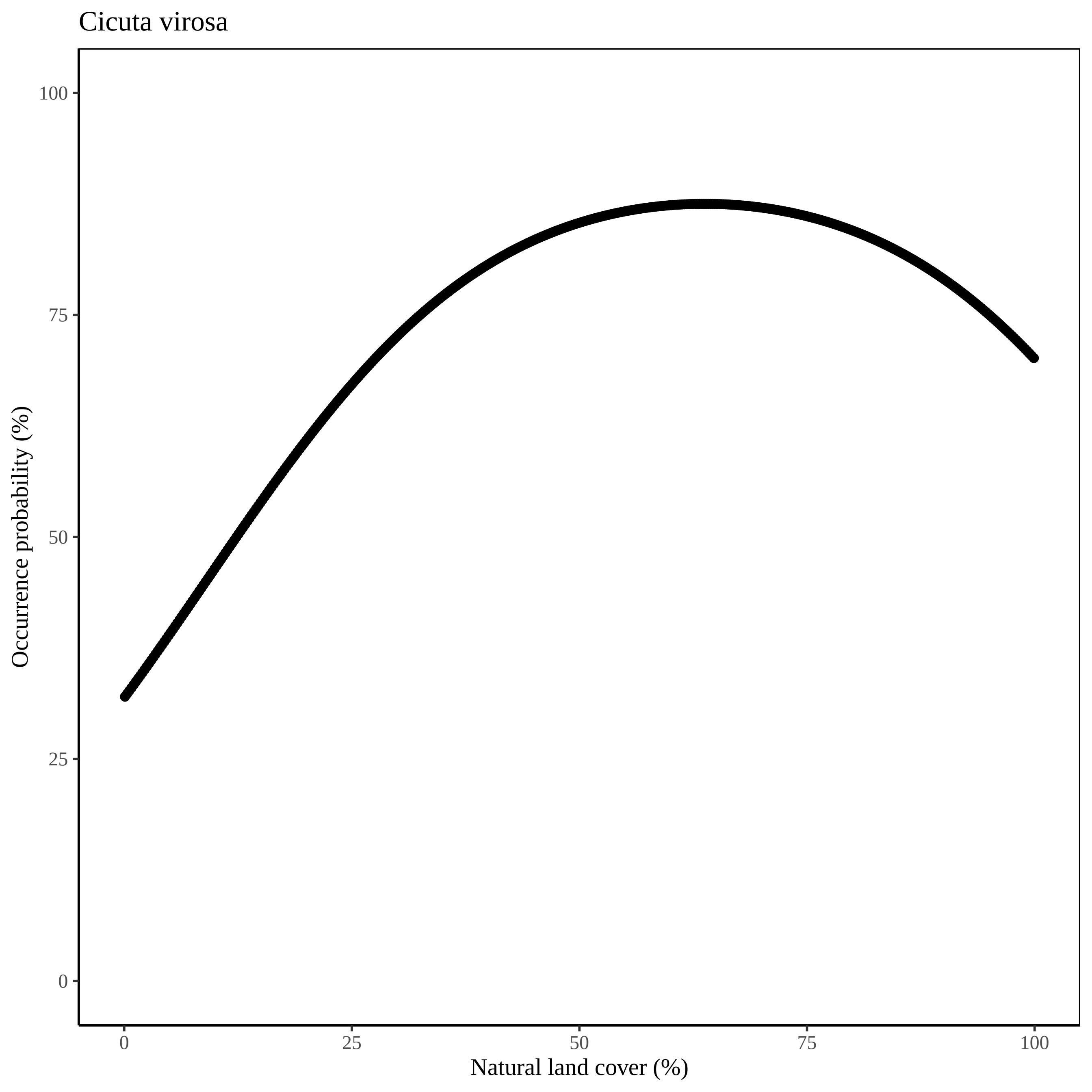
Cicuta virosa
100
75
50
25
0
Occurrence probability (%)
0
25
50
75
100
Natural land cover (%)

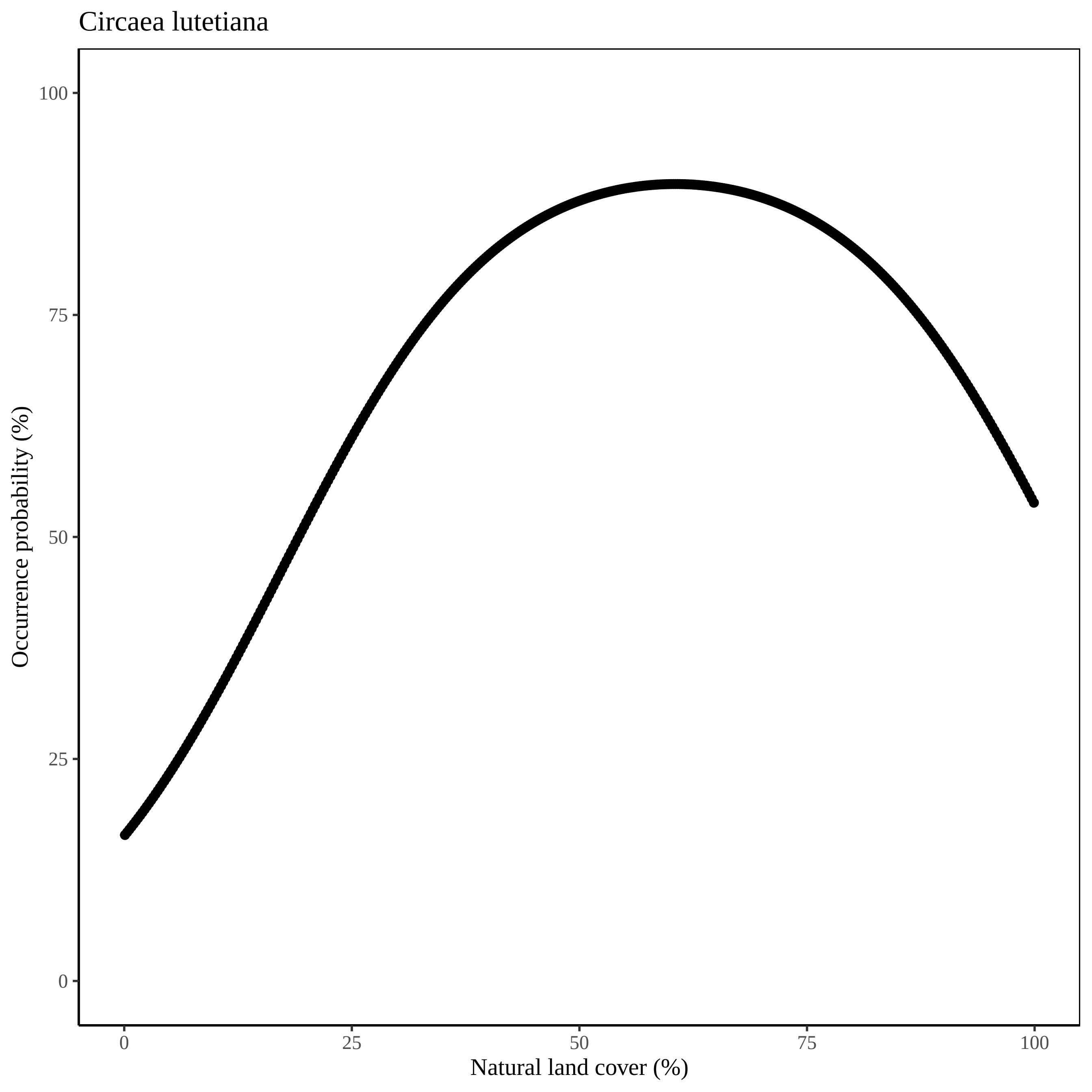
Circaea lutetiana
Occurrence probability (%)
100
75
50
25
0
0
25
50
75
100
Natural land cover (%)

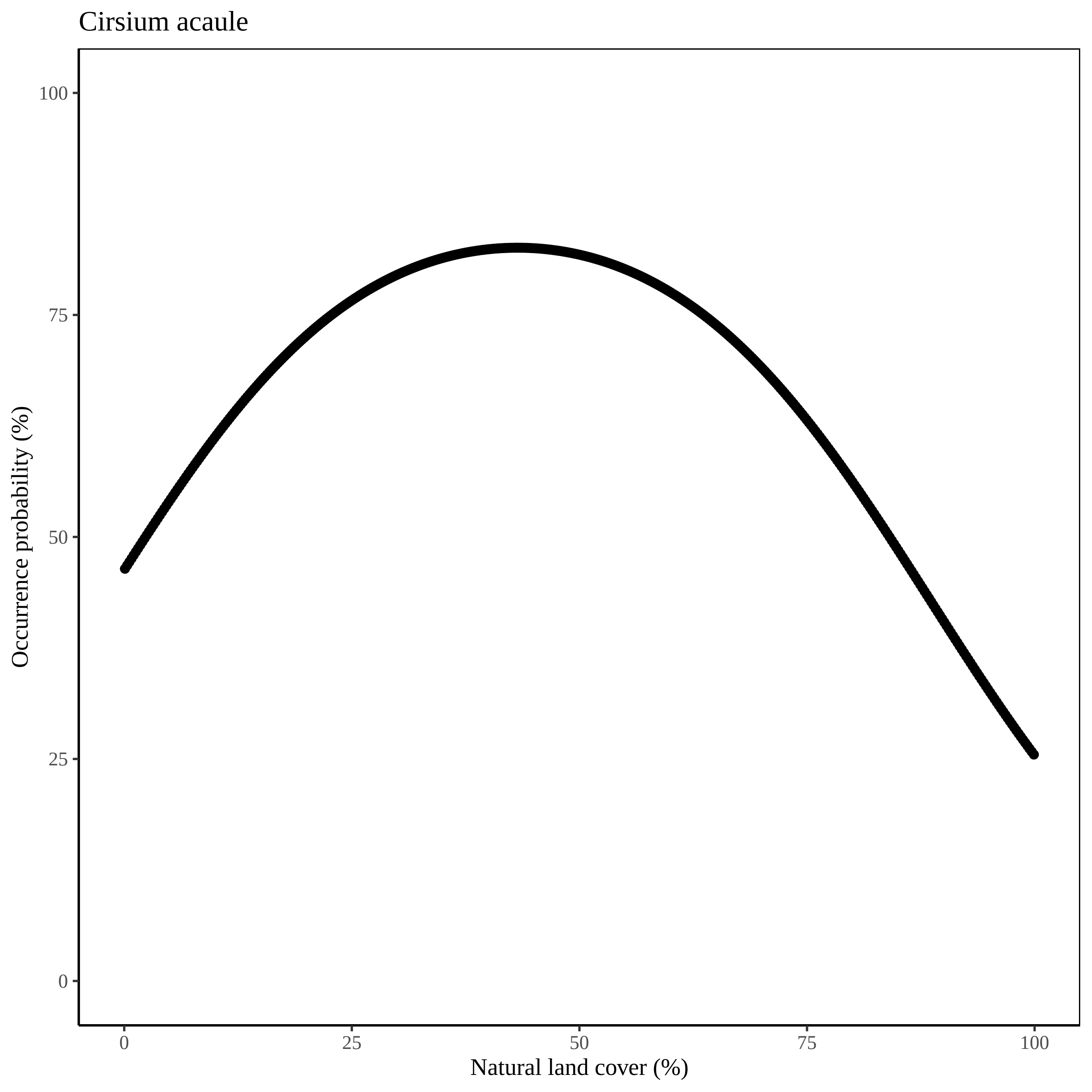

Cirsium acaule
Occurrence probability (%)
100
75
50
25
0
0
25
50
75
100
Natural land cover (%)

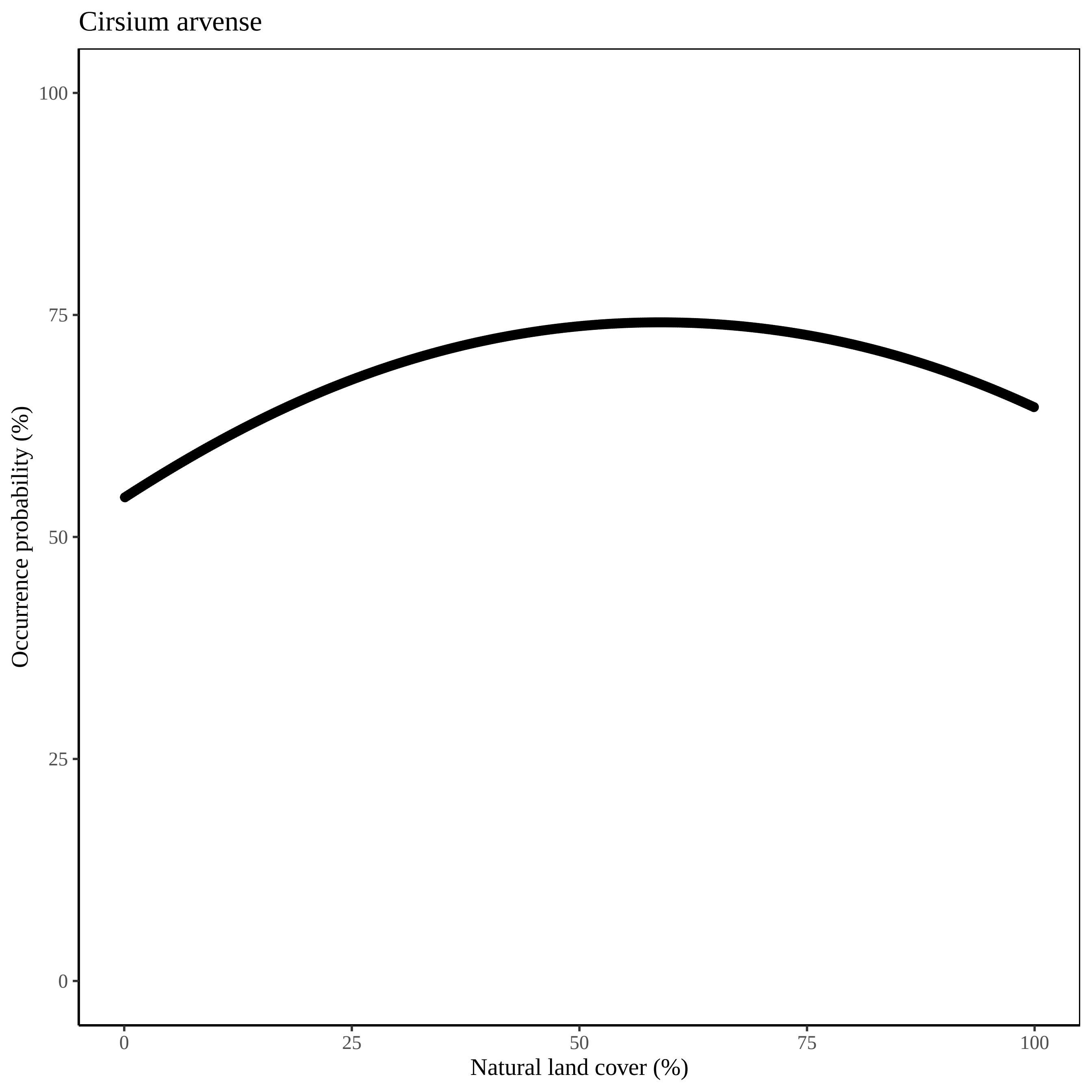

Cirsium arvense
Occurrence probability (%)
100
75
50
25
0
0
25
50
75
100
Natural land cover (%)

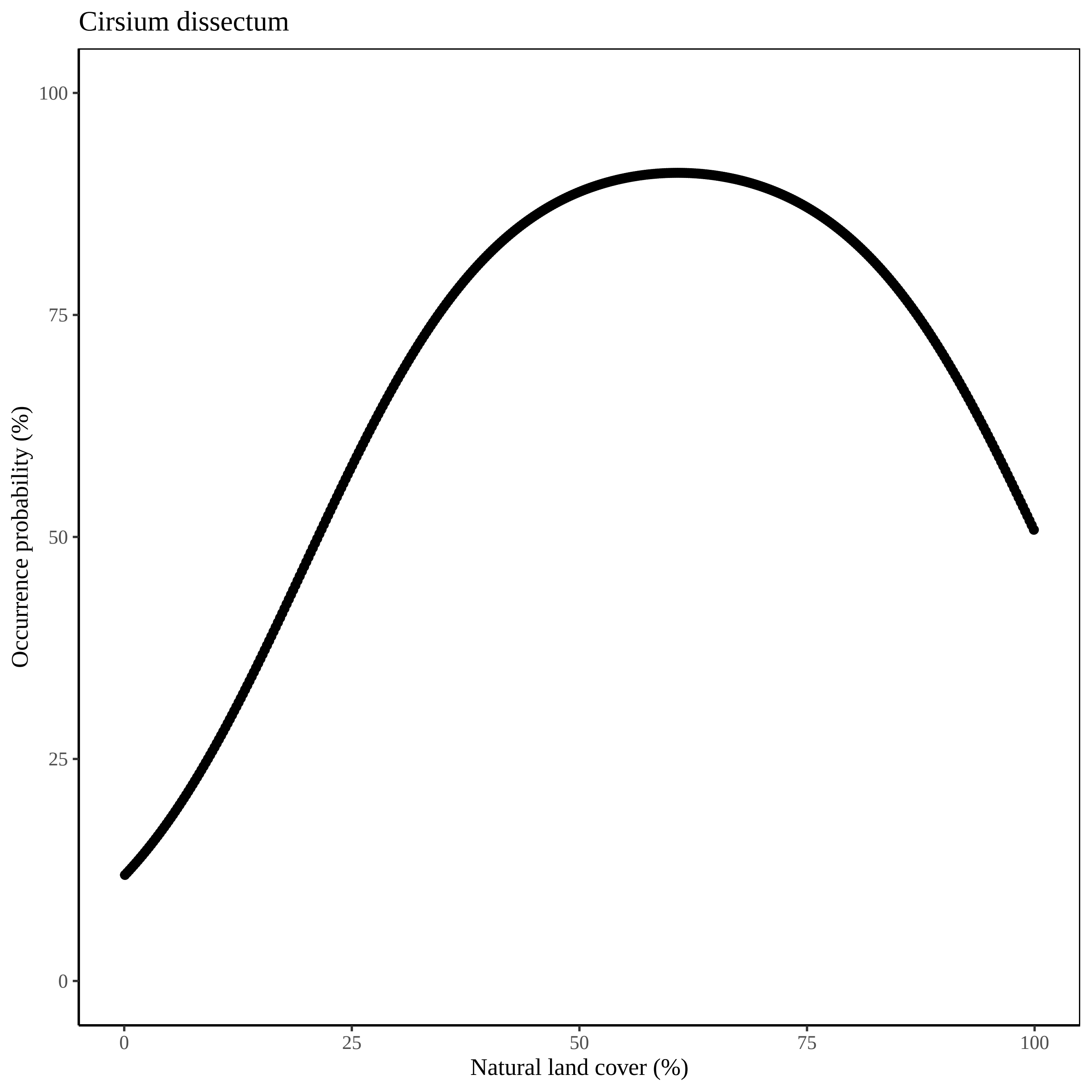

Cirsium dissectum
Occurrence probability (%)
100
75
50
25
0
0
25
50
75
100
Natural land cover (%)

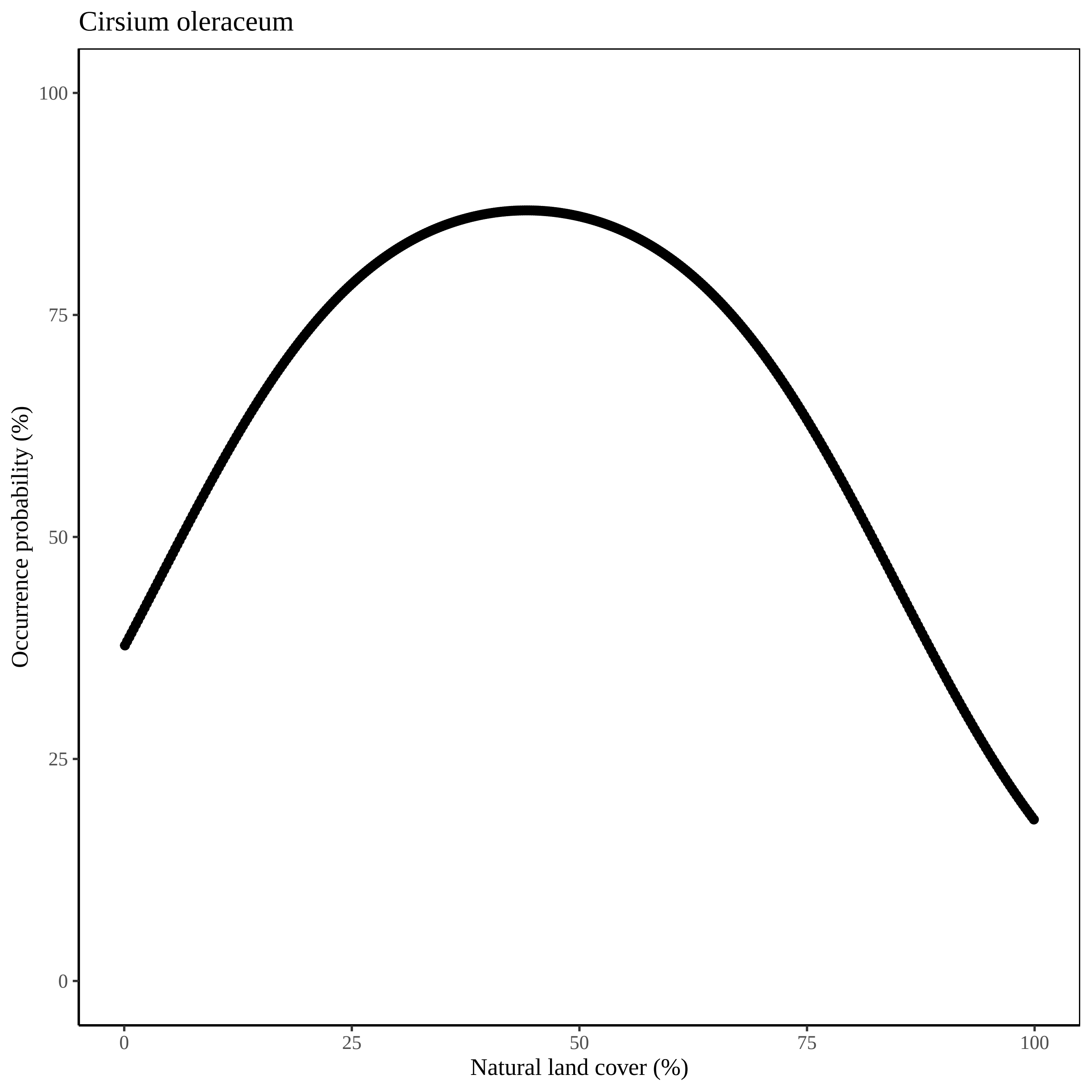
Cirsium oleraceum
Occurrence probability (%)
100
75
50
25
0
0
25
50
75
100
Natural land cover (%)

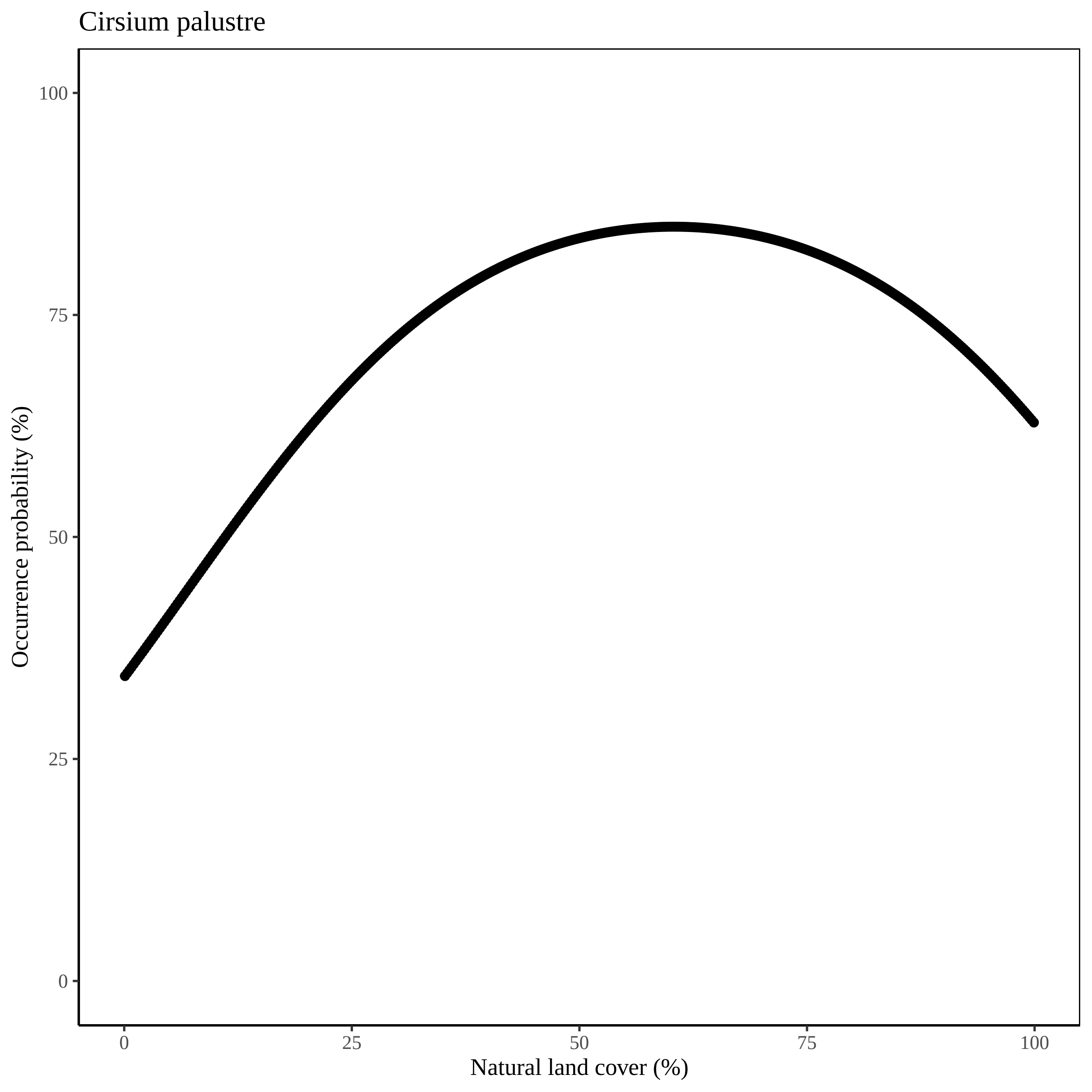

Cirsium palustre
Occurrence probability (%)
100
75
50
25
0
0
25
50
75
100
Natural land cover (%)

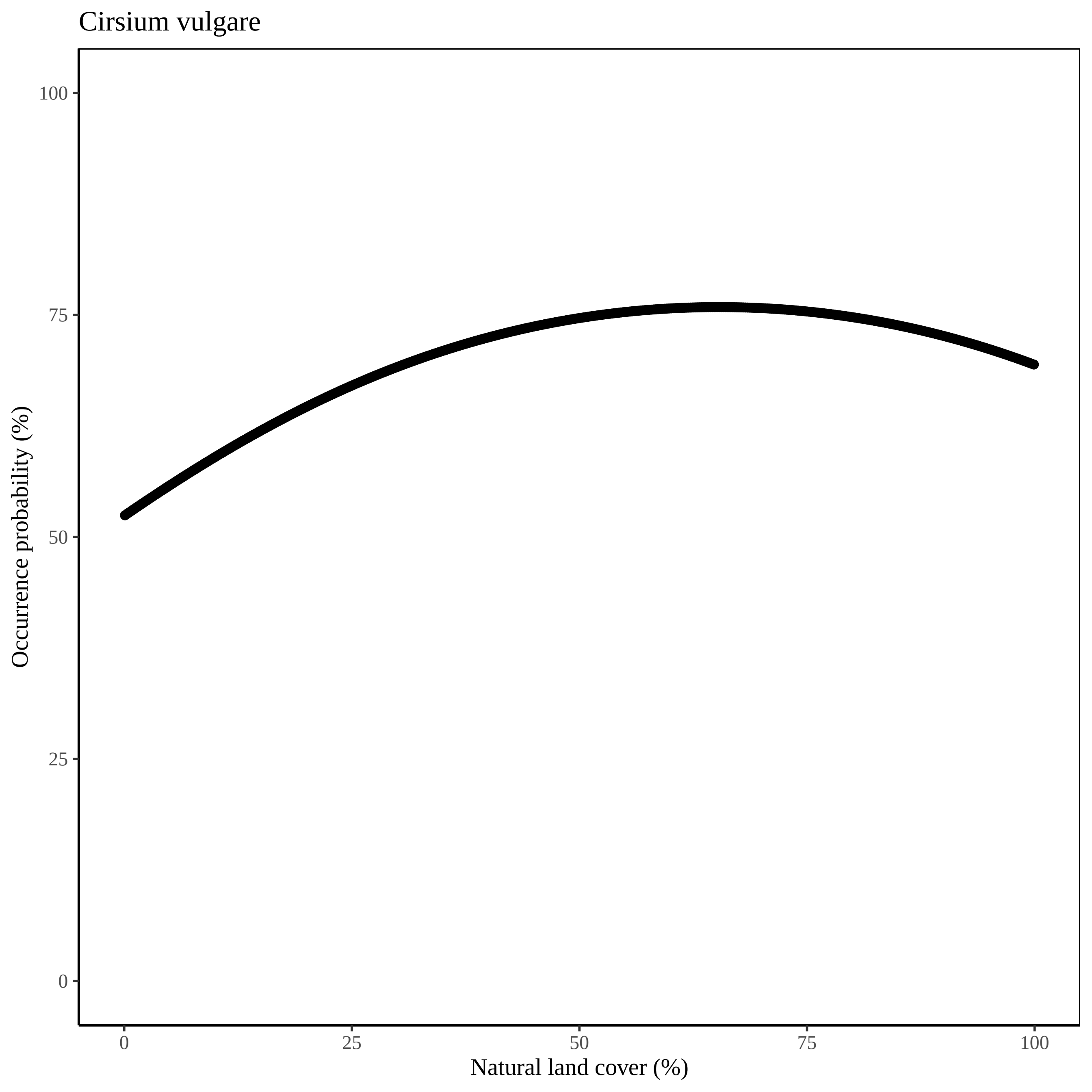

Cirsium vulgare
Occurrence probability (%)
100
75
50
25
0
0
25
50
75
100
Natural land cover (%)

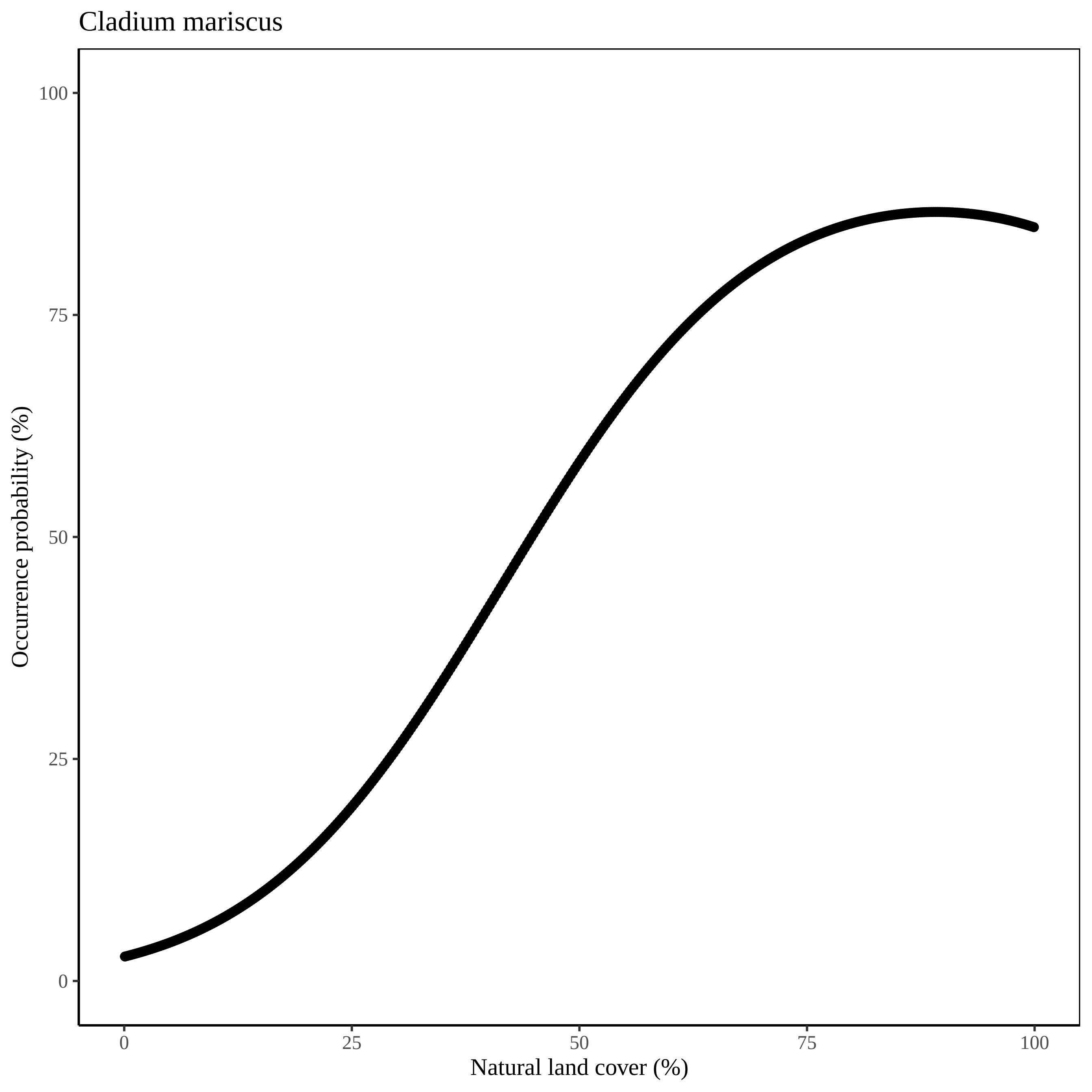
Cladium mariscus
Occurrence probability (%)
100
75
50
25
0
0
25
50
75
100
Natural land cover (%)

Claytonia perfoliata

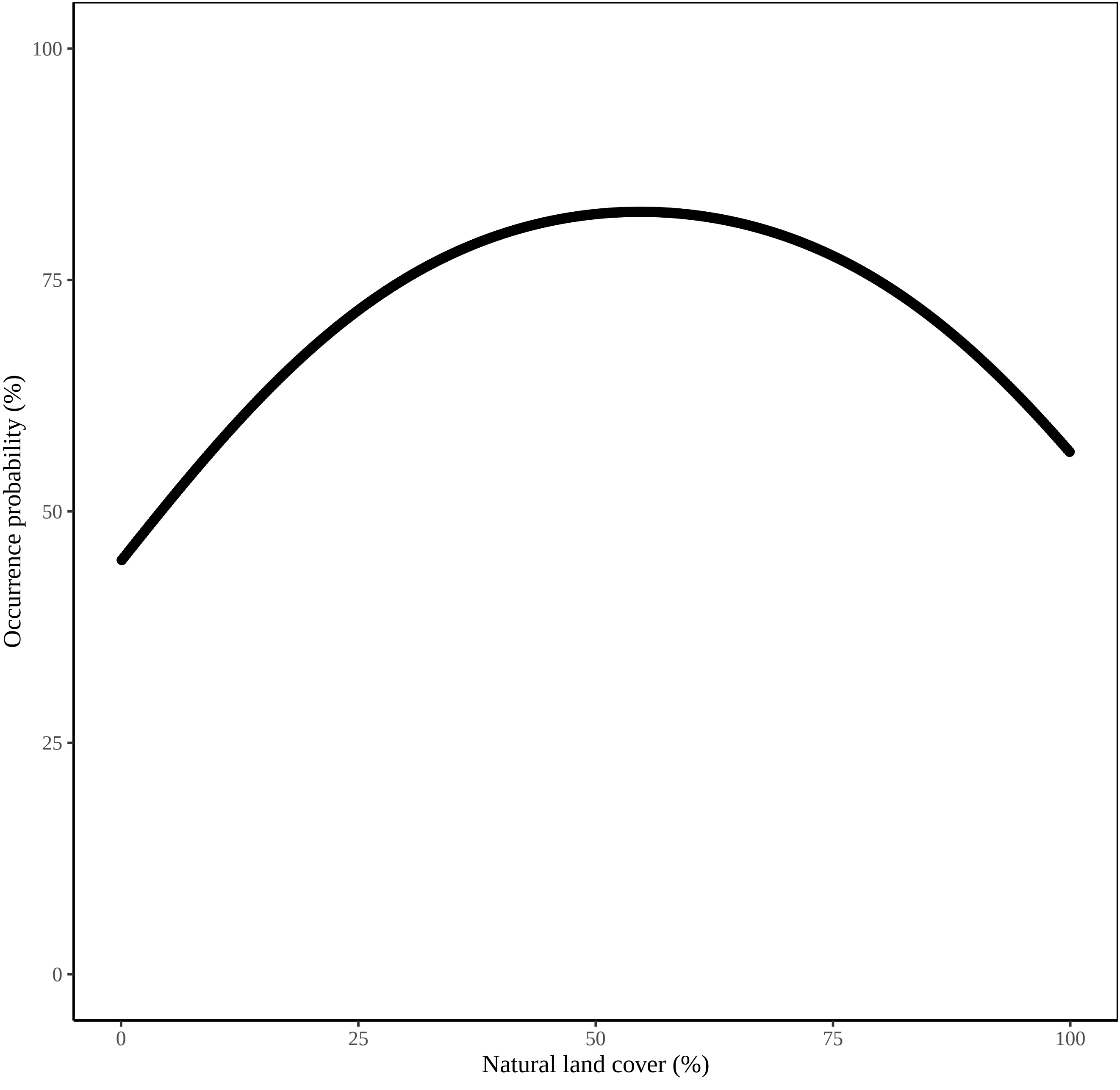

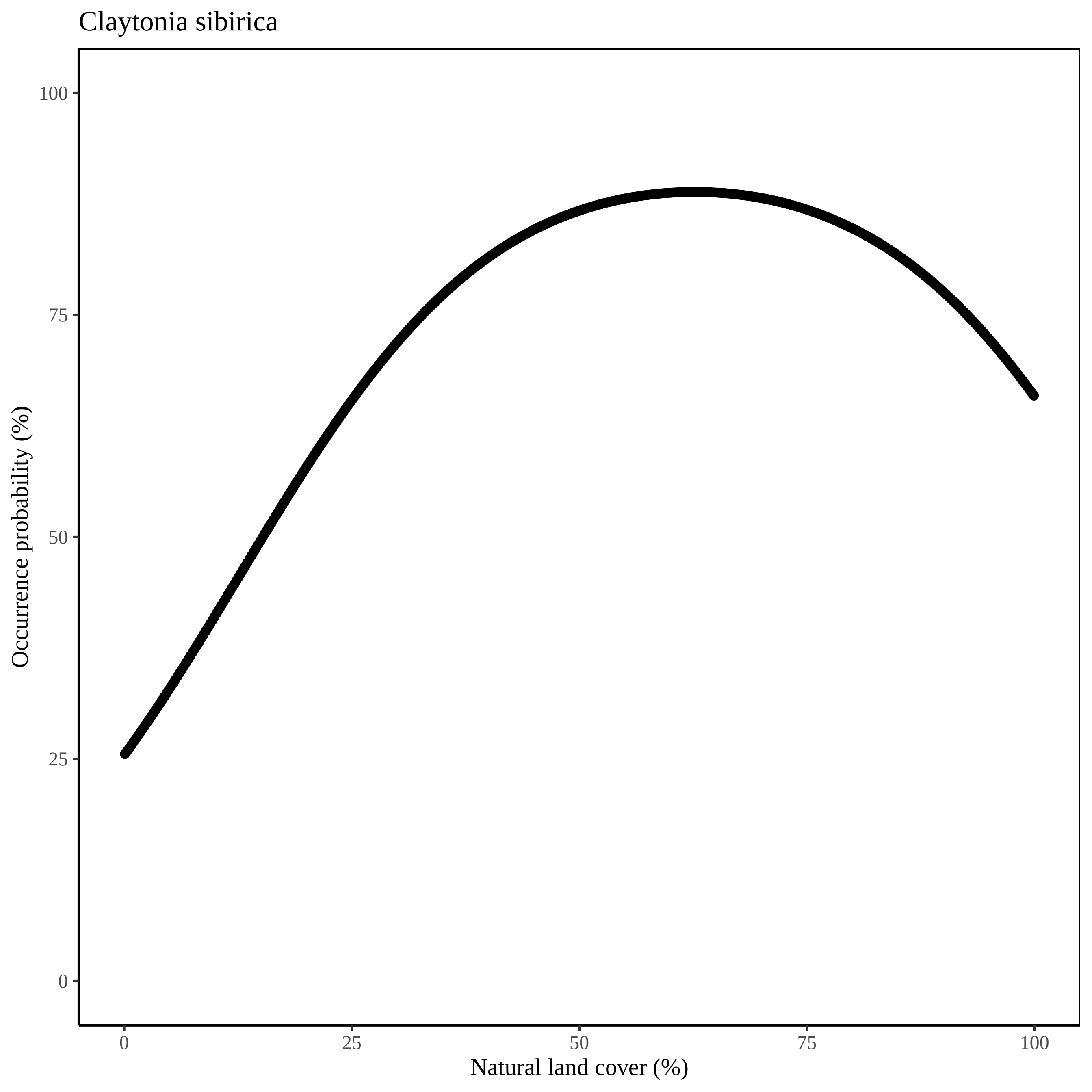
Claytonia sibirica
100
75
50
25
0
Occurrence probability (%)
0
25
50
75
100
Natural land cover (%)

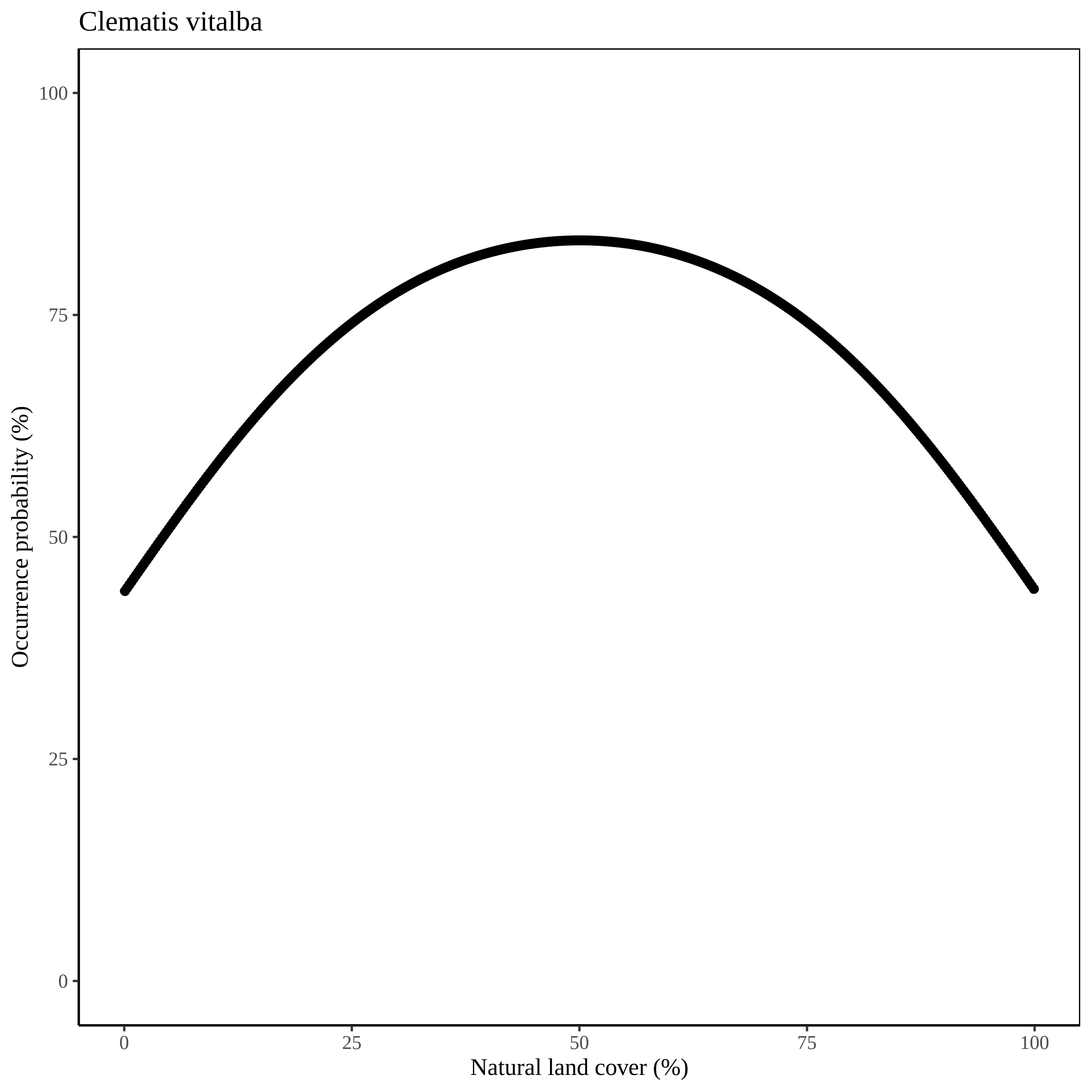
Clematis vitalba
Occurrence probability (%)
100
75
50
25
0
0
25
50
75
100
Natural land cover (%)

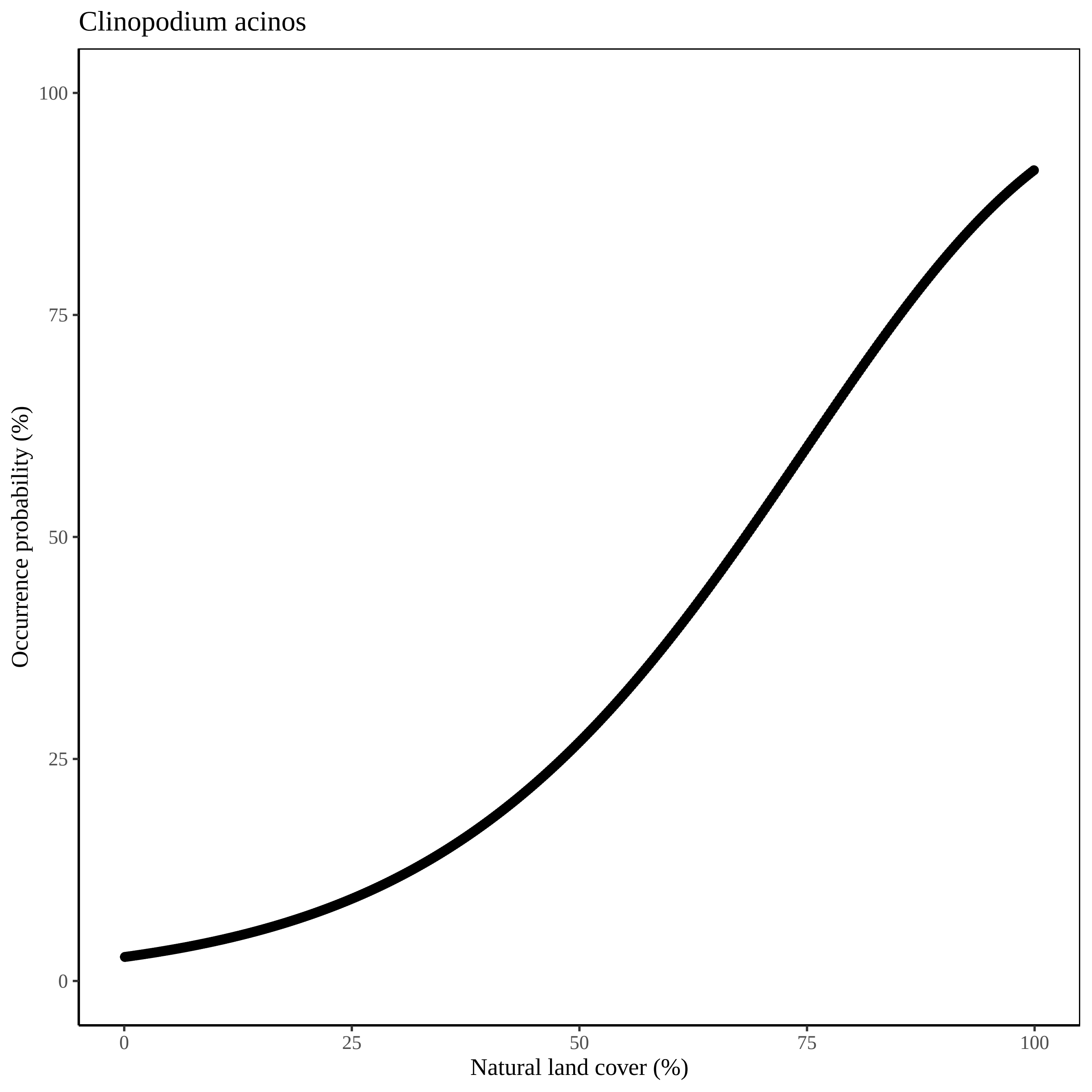

Clinopodium acinos
Occurrence probability (%)
100
75
50
25
0
0
25
50
75
100
Natural land cover (%)

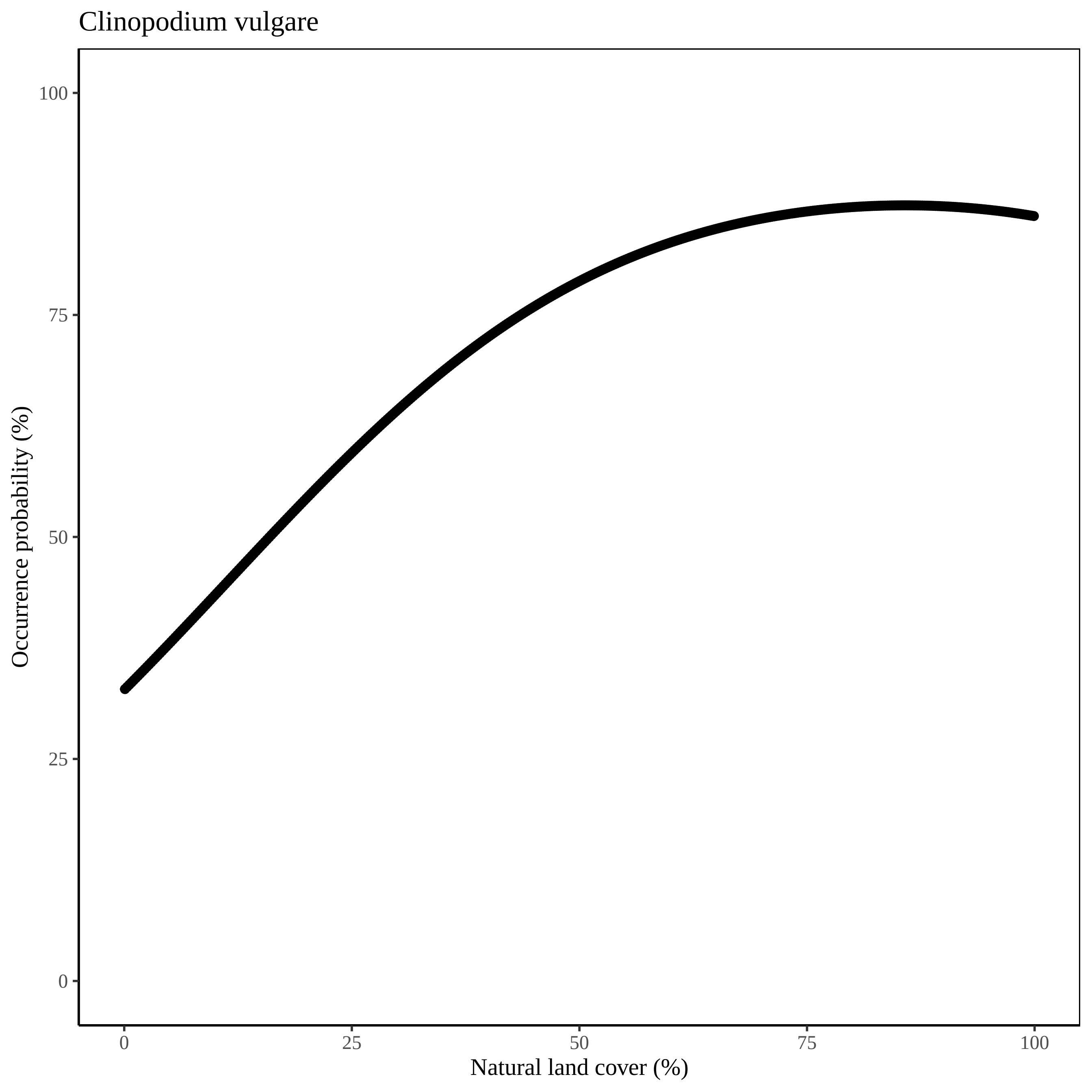

Clinopodium vulgare
Occurrence probability (%)
100
75
50
25
0
0
25
50
75
100
Natural land cover (%)

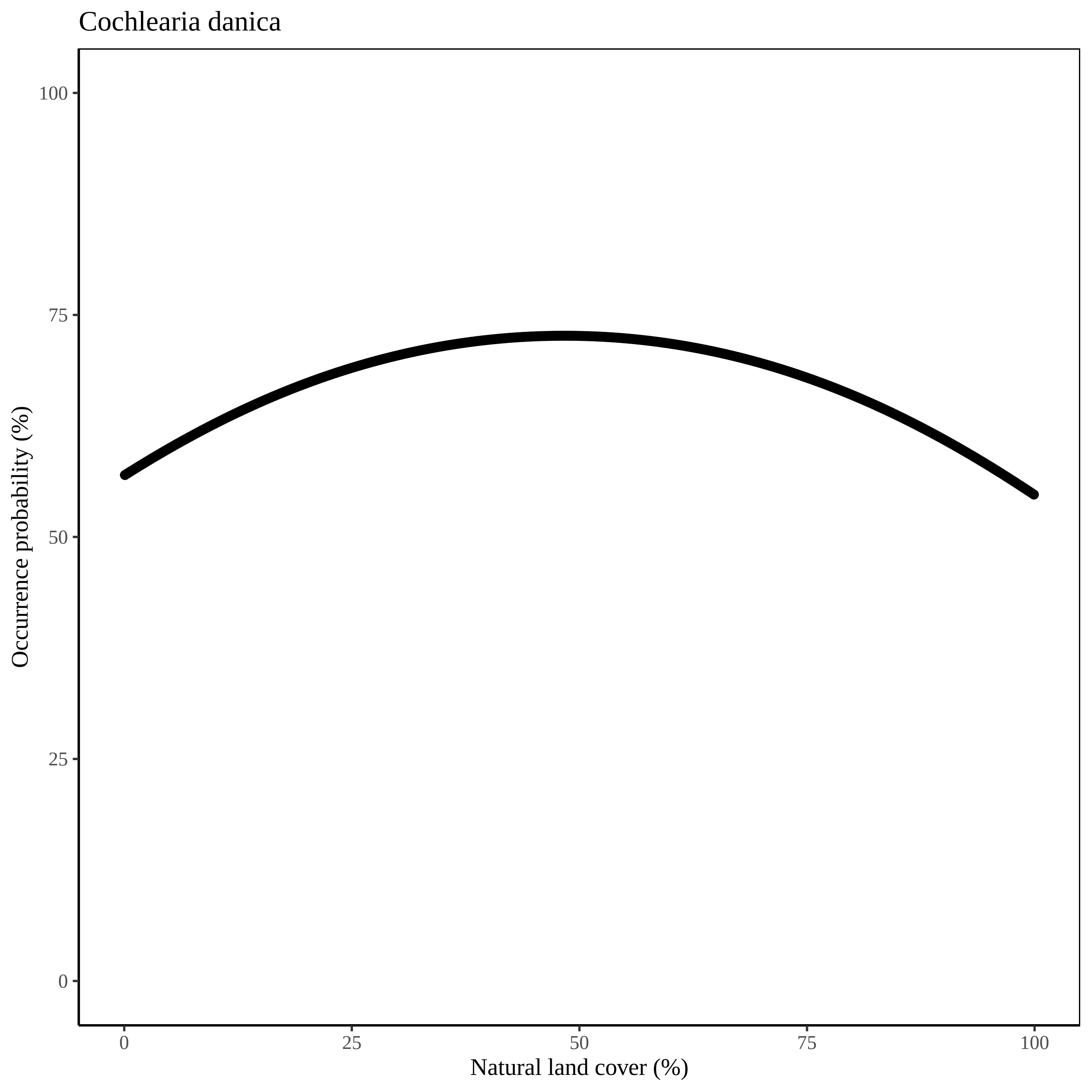
Cochlearia danica
Occurrence probability (%)
100
75
50
25
0
0
25
50
75
100
Natural land cover (%)

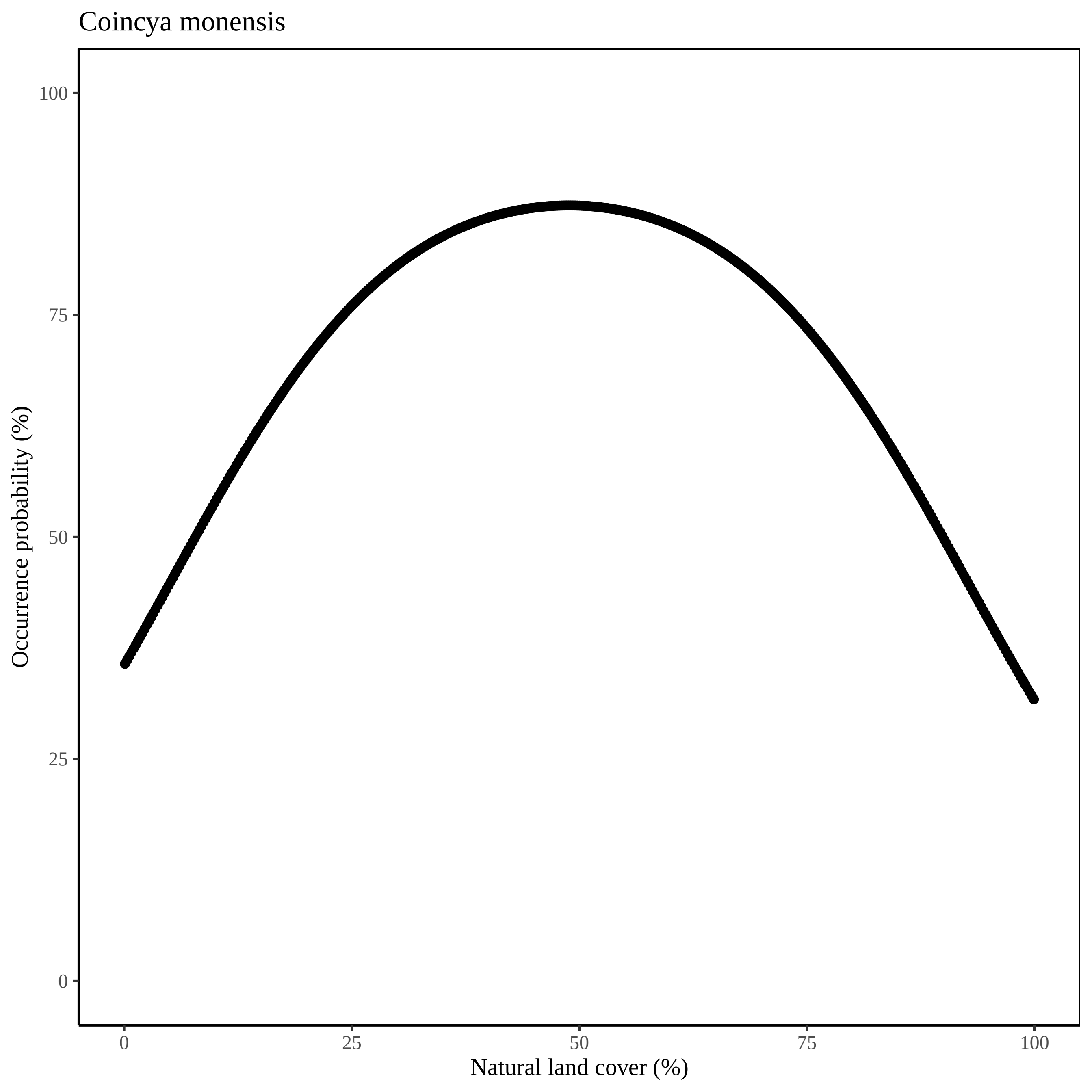
Coincya monensis
Occurrence probability (%)
0
25
50
75
100
Natural land cover (%)
0
25
50
75
100

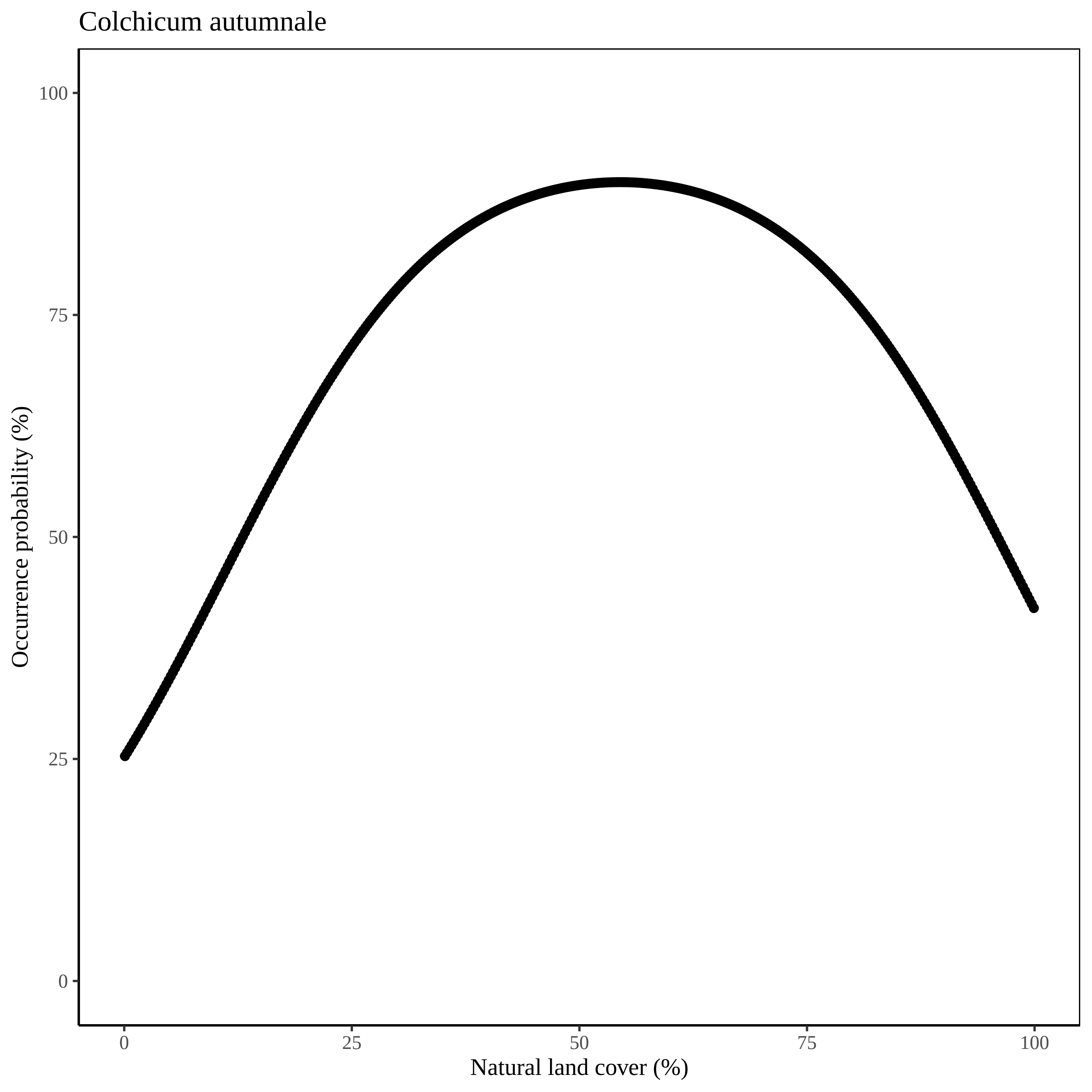

Colchicum autumnale
Occurrence probability (%)
100
75
50
25
0
0
25
50
75
100
Natural land cover (%)

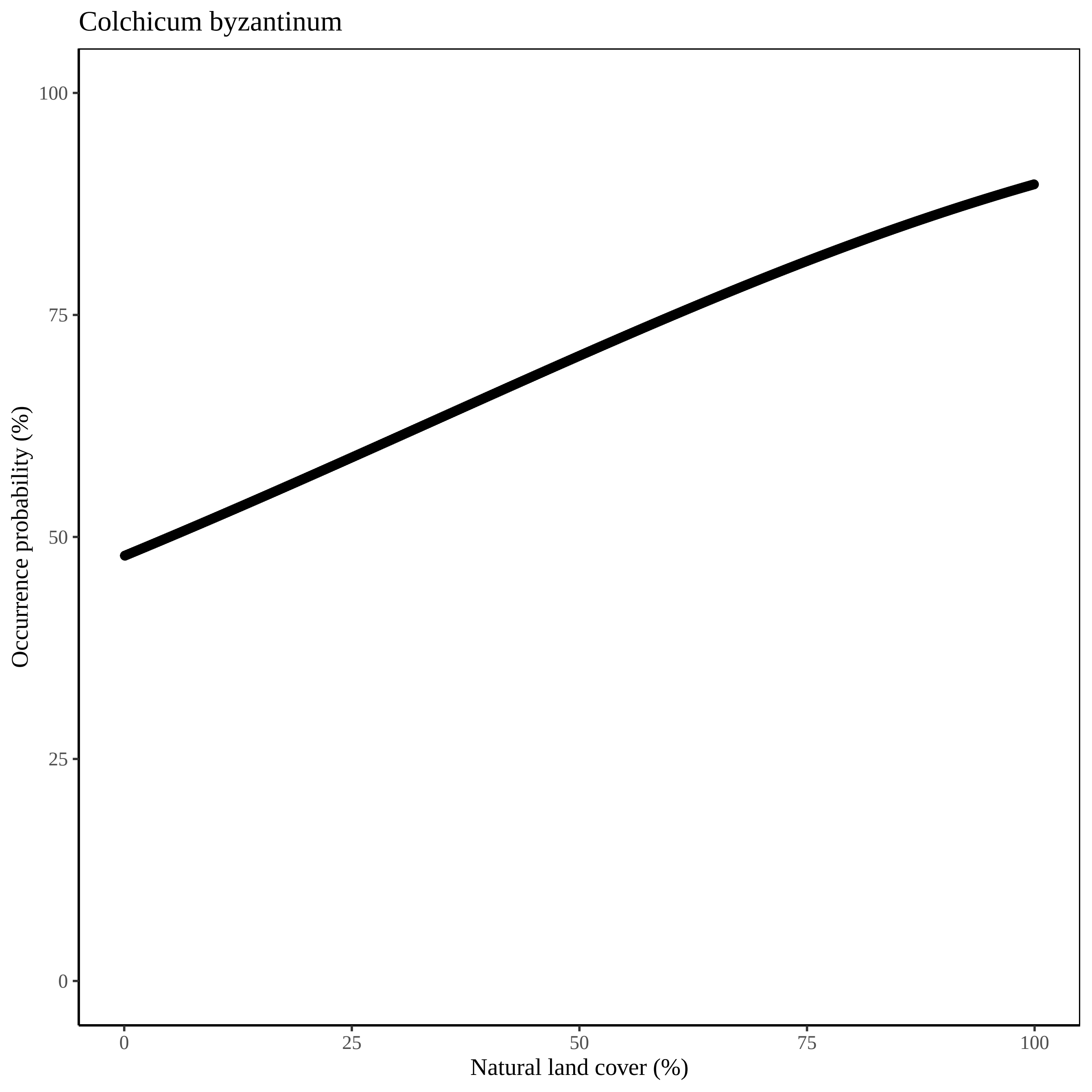
Colchicum byzantinum
Occurrence probability (%)
100
75
50
25
0
0
25
50
75
100
Natural land cover (%)

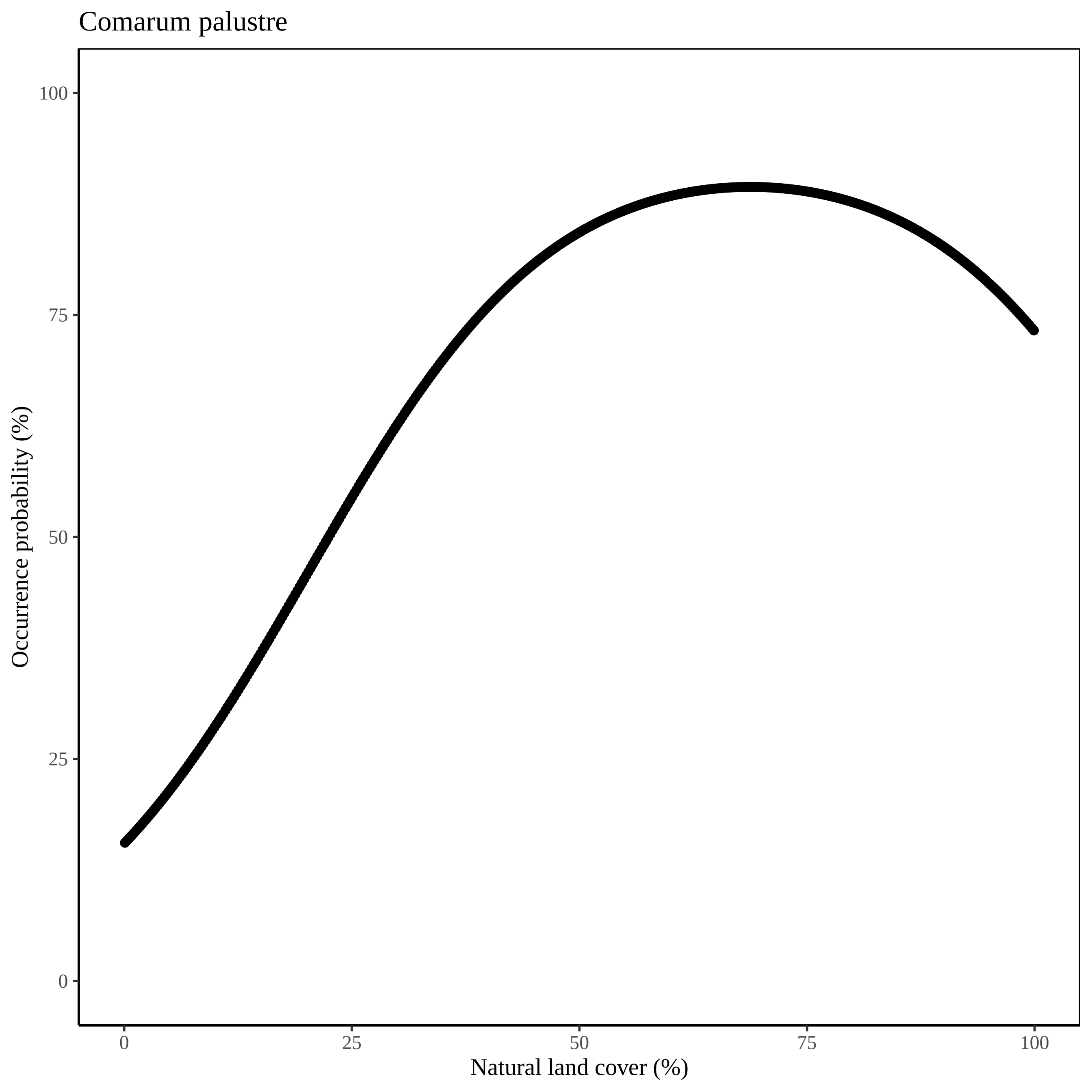

Comarum palustre
Occurrence probability (%)
100
75
50
25
0
0
25
50
75
100
Natural land cover (%)

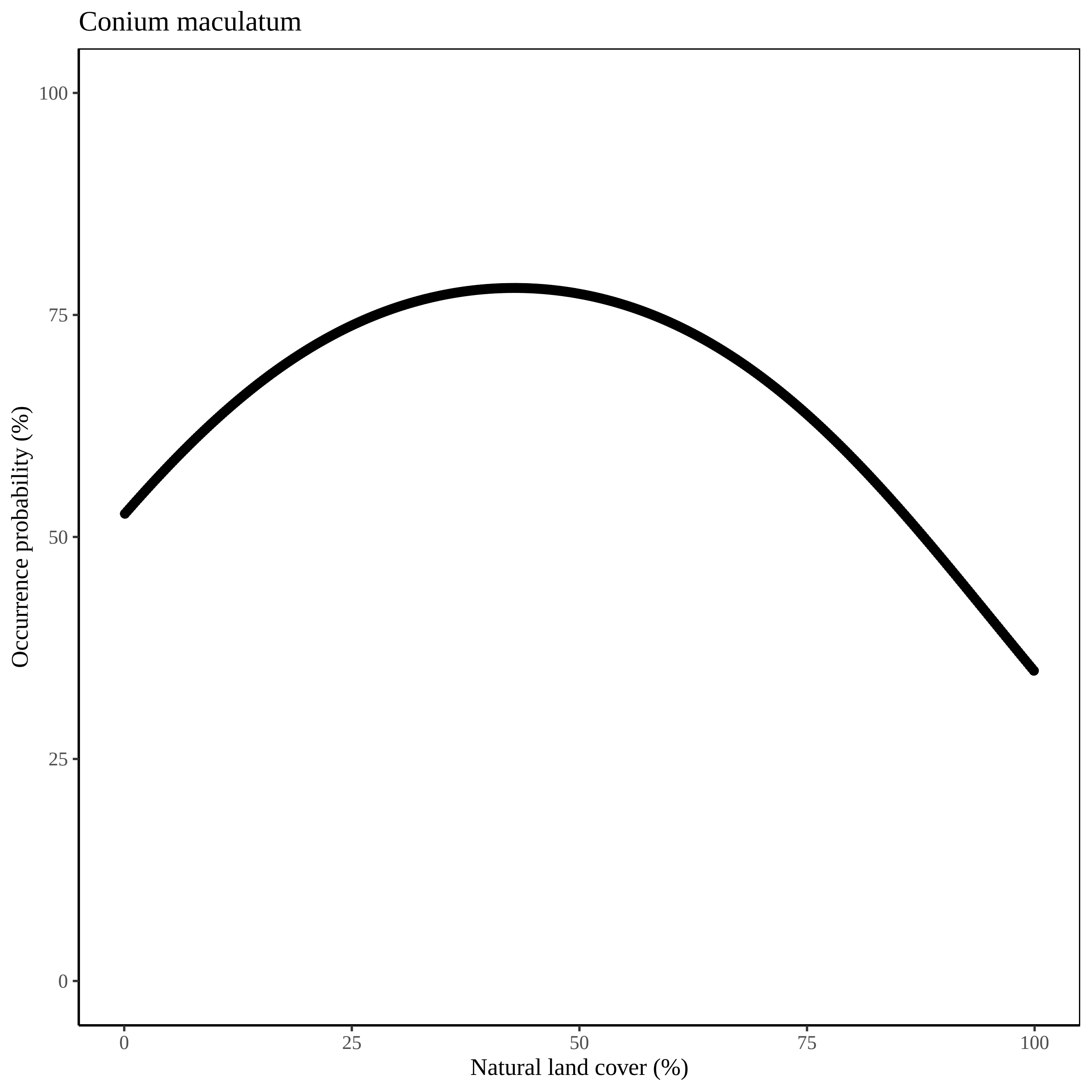

Conium maculatum
Occurrence probability (%)
100
75
50
25
0
0
25
50
75
100
Natural land cover (%)

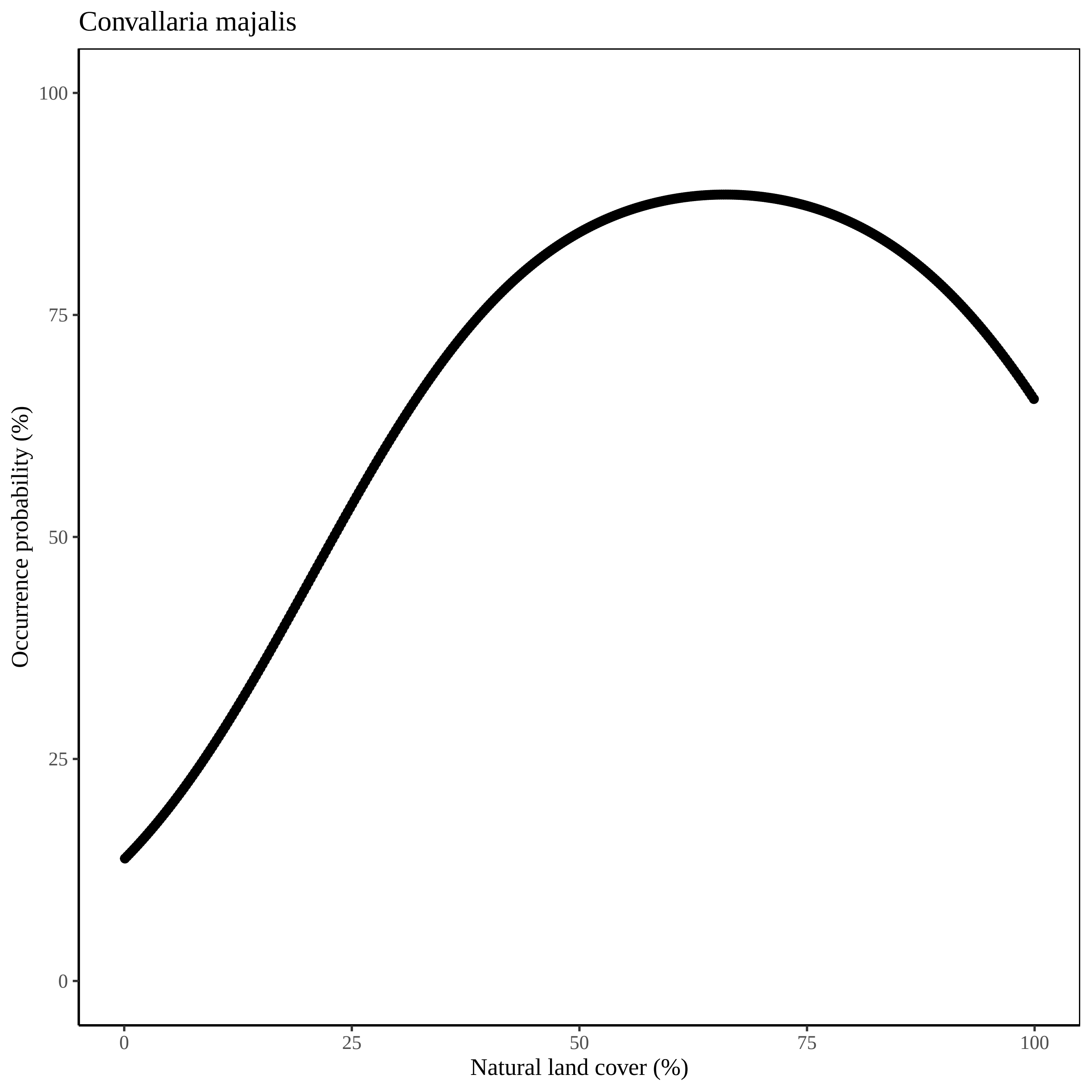

Convallaria majalis
Occurrence probability (%)
100
75
50
25
0
0
25
50
75
100
Natural land cover (%)

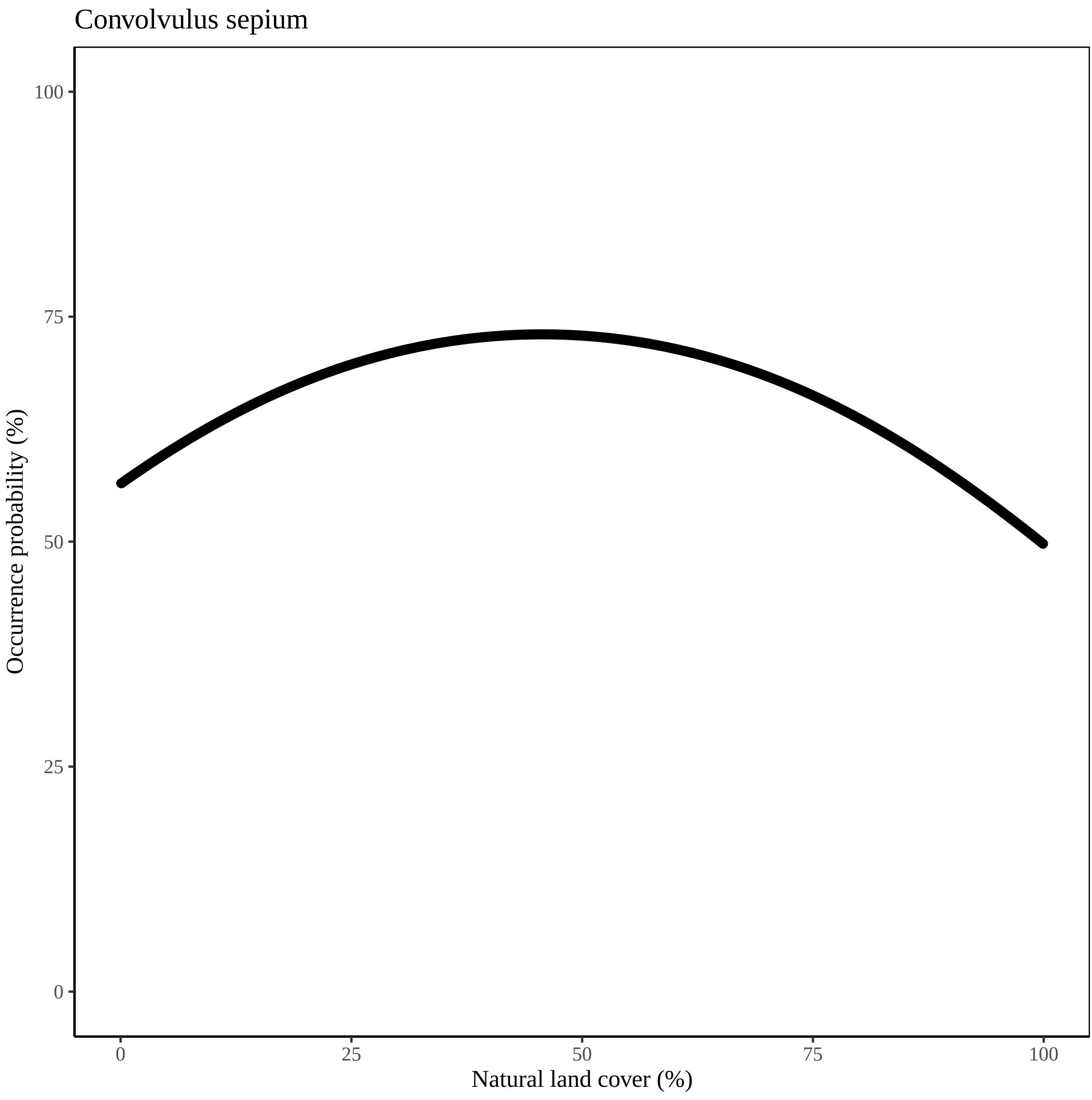

Convolvulus sepium
Occurrence probability (%)
100
75
50
25
0
0
25
50
75
100
Natural land cover (%)

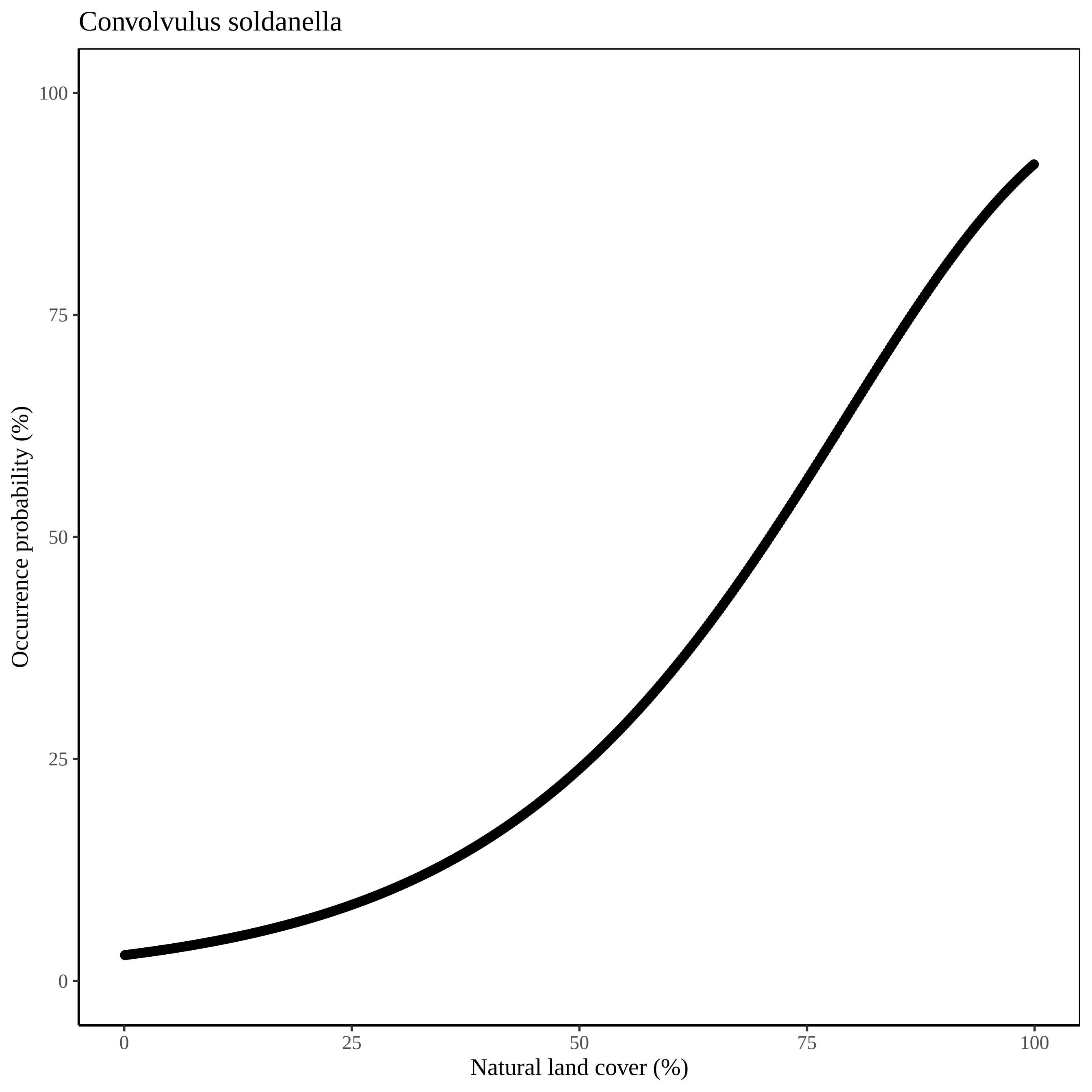
Convolvulus soldanella
Occurrence probability (%)
100
75
50
25
0
0
25
50
75
100
Natural land cover (%)

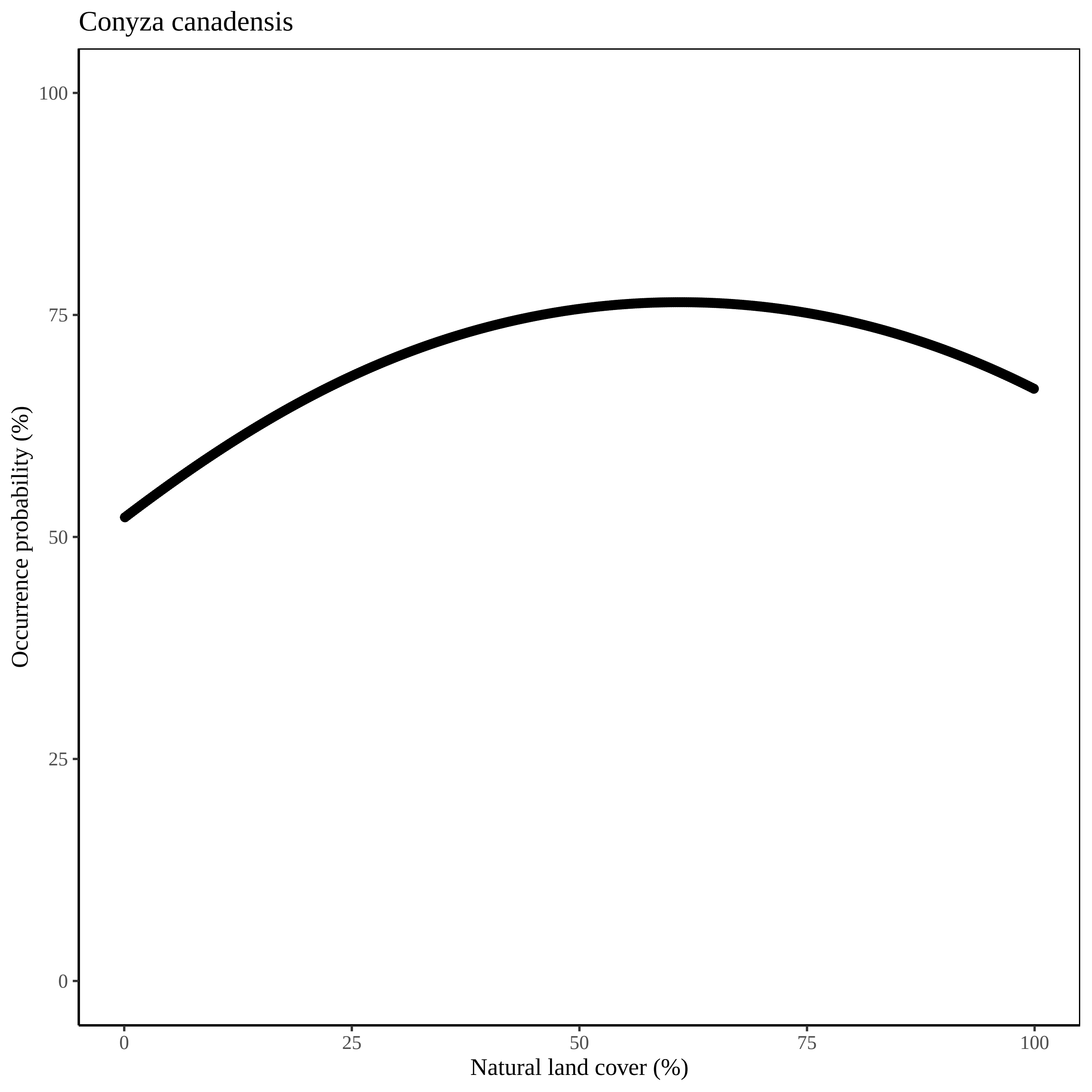
Conyza canadensis
Occurrence probability (%)
100
75
50
25
0
0
25
50
75
100
Natural land cover (%)

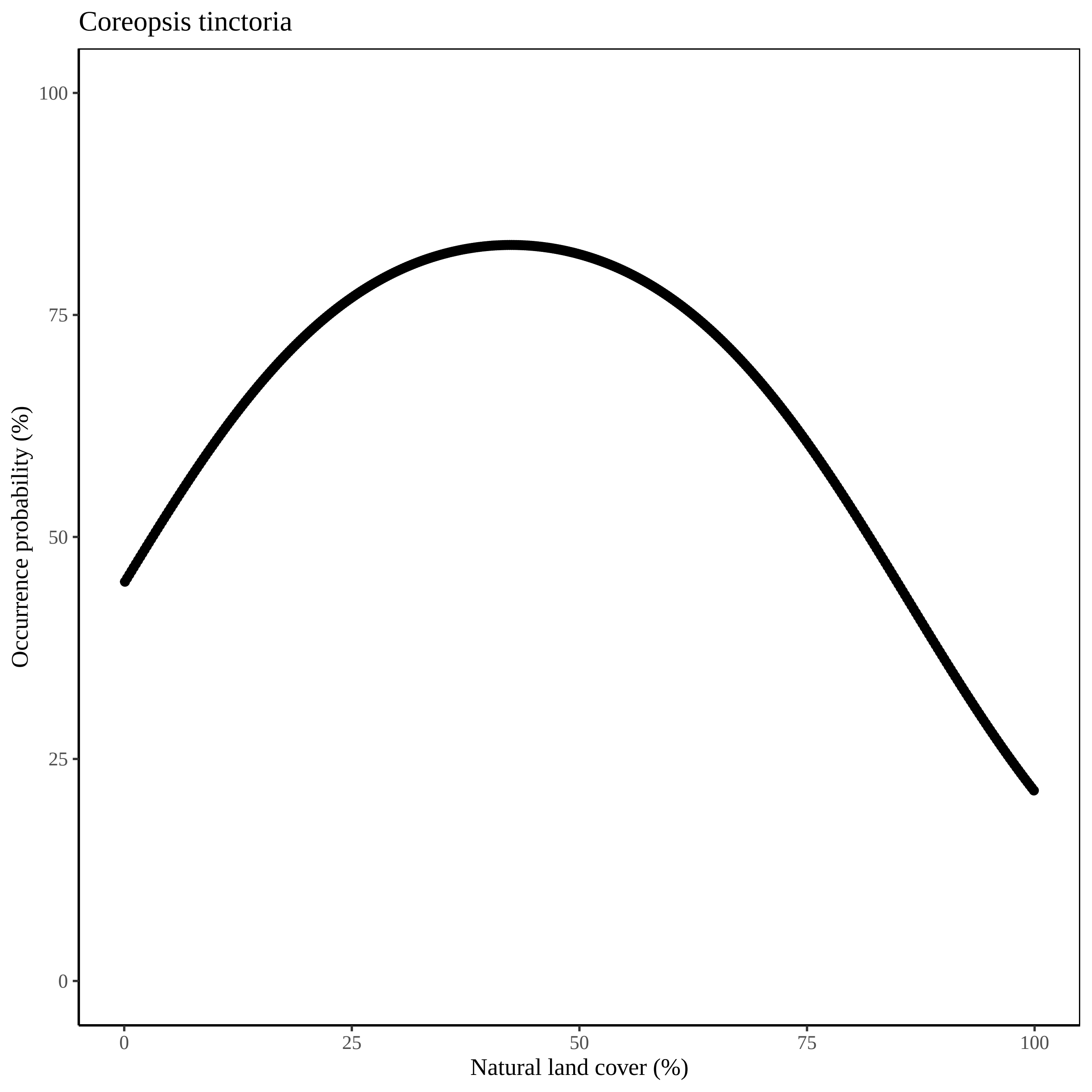

Coreopsis tinctoria
100
75
50
25
0
Occurrence probability (%)
0
25
50
75
100
Natural land cover (%)

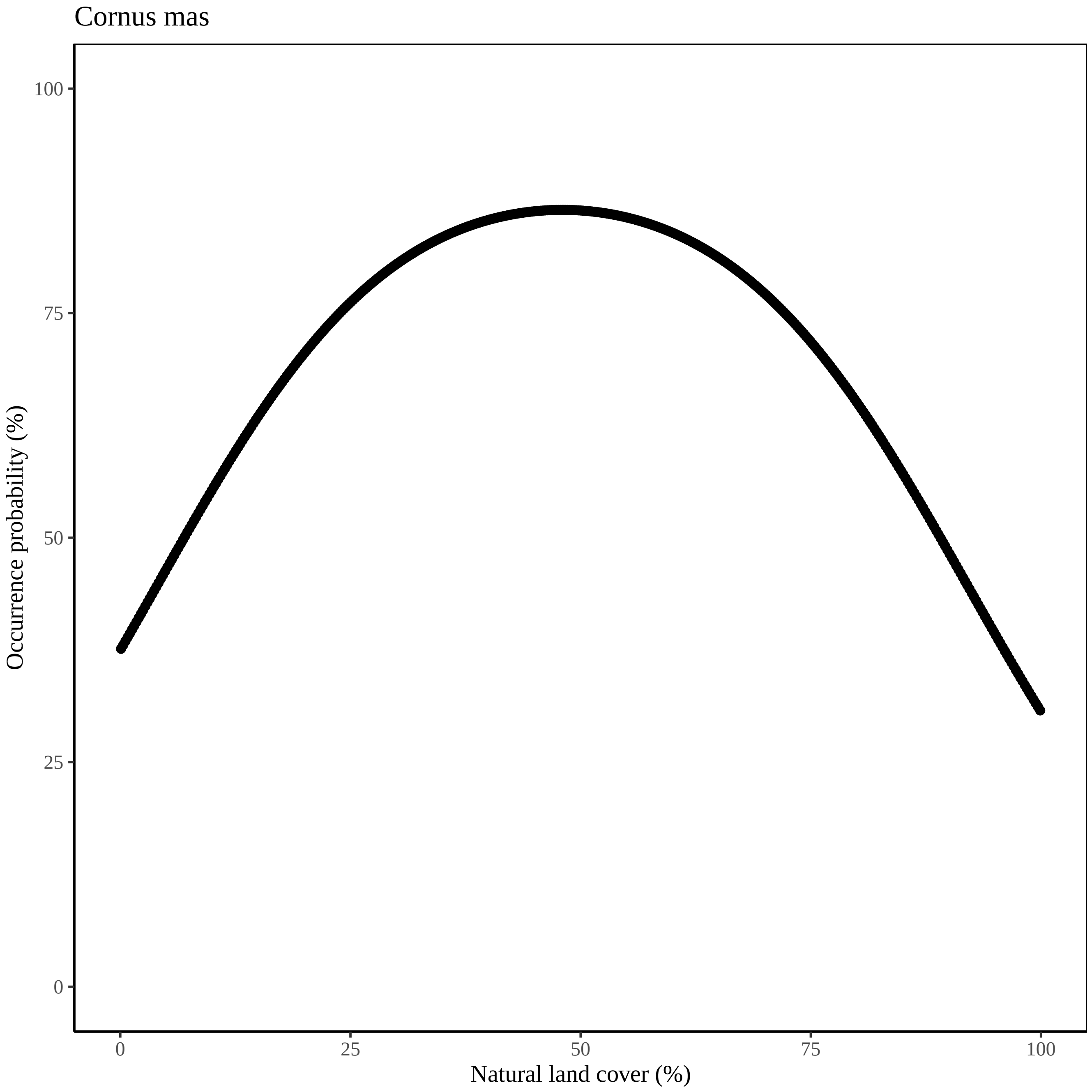

Cornus mas
100
75
50
25
0
Occurrence probability (%)
0
25
50
75
100
Natural land cover (%)

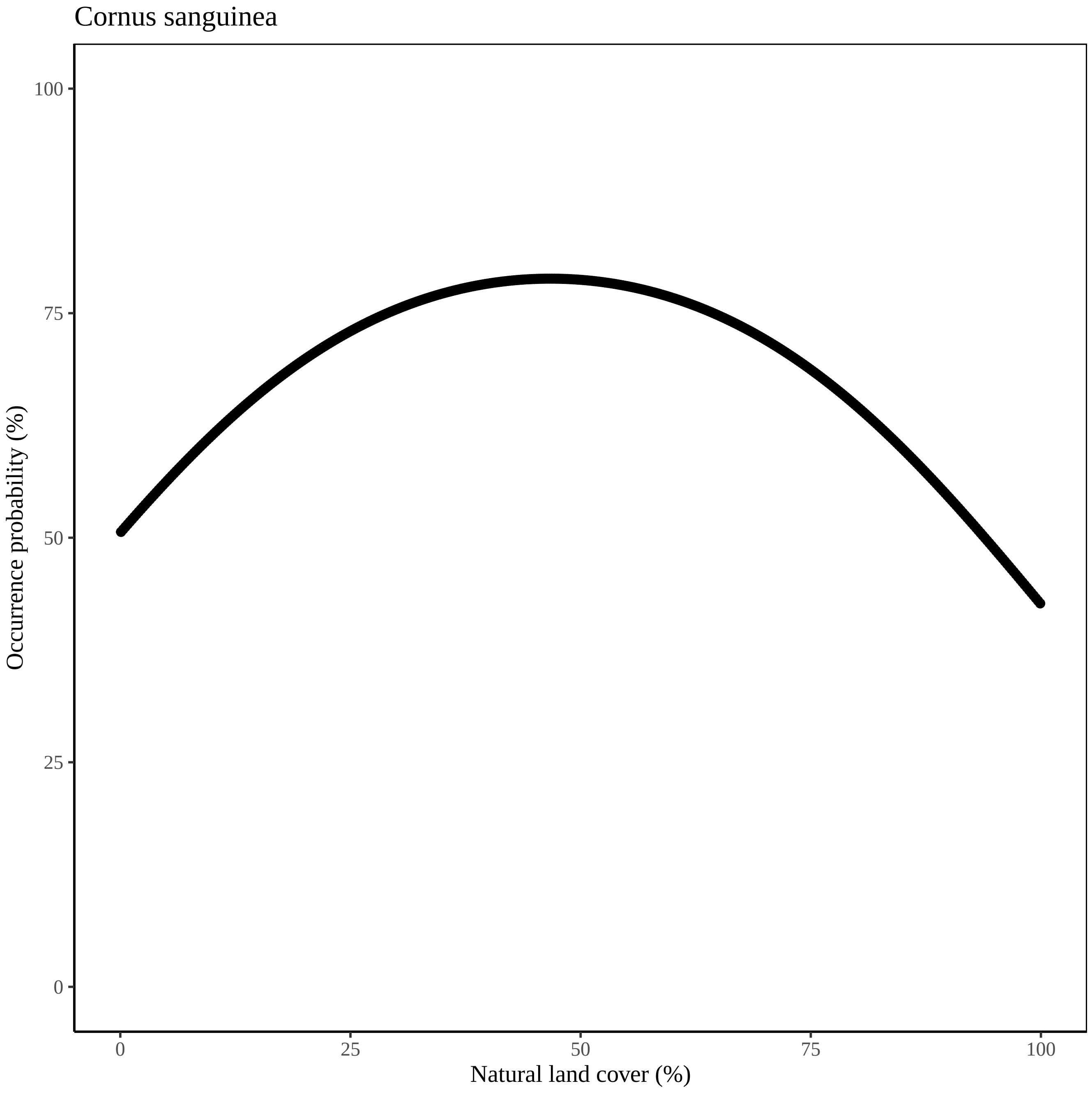
Cornus sanguinea
Occurrence probability (%)
100
75
50
25
0
0
25
50
75
100
Natural land cover (%)

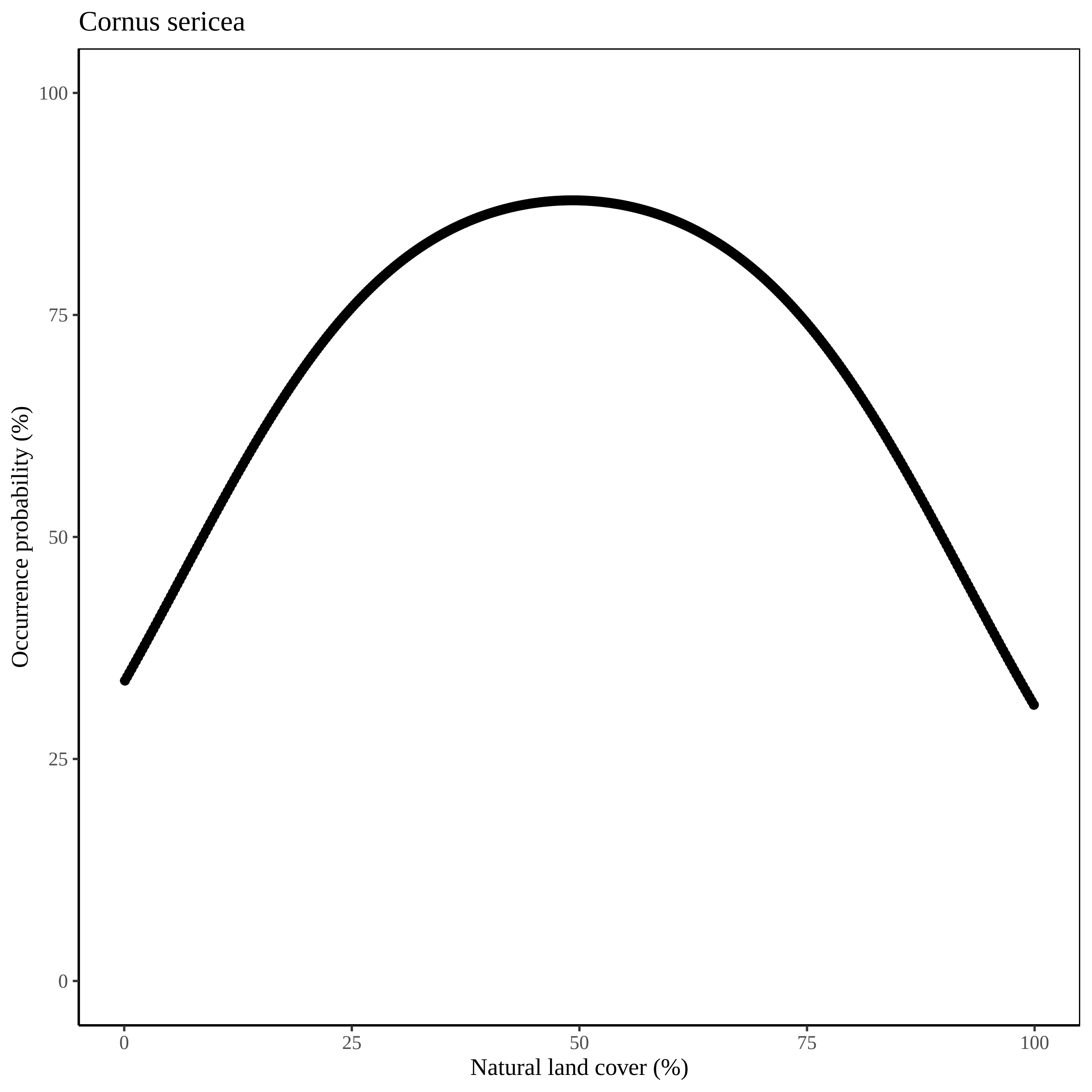
Cornus sericea
Occurrence probability (%)
100
75
50
25
0
0
25
50
75
100
Natural land cover (%)

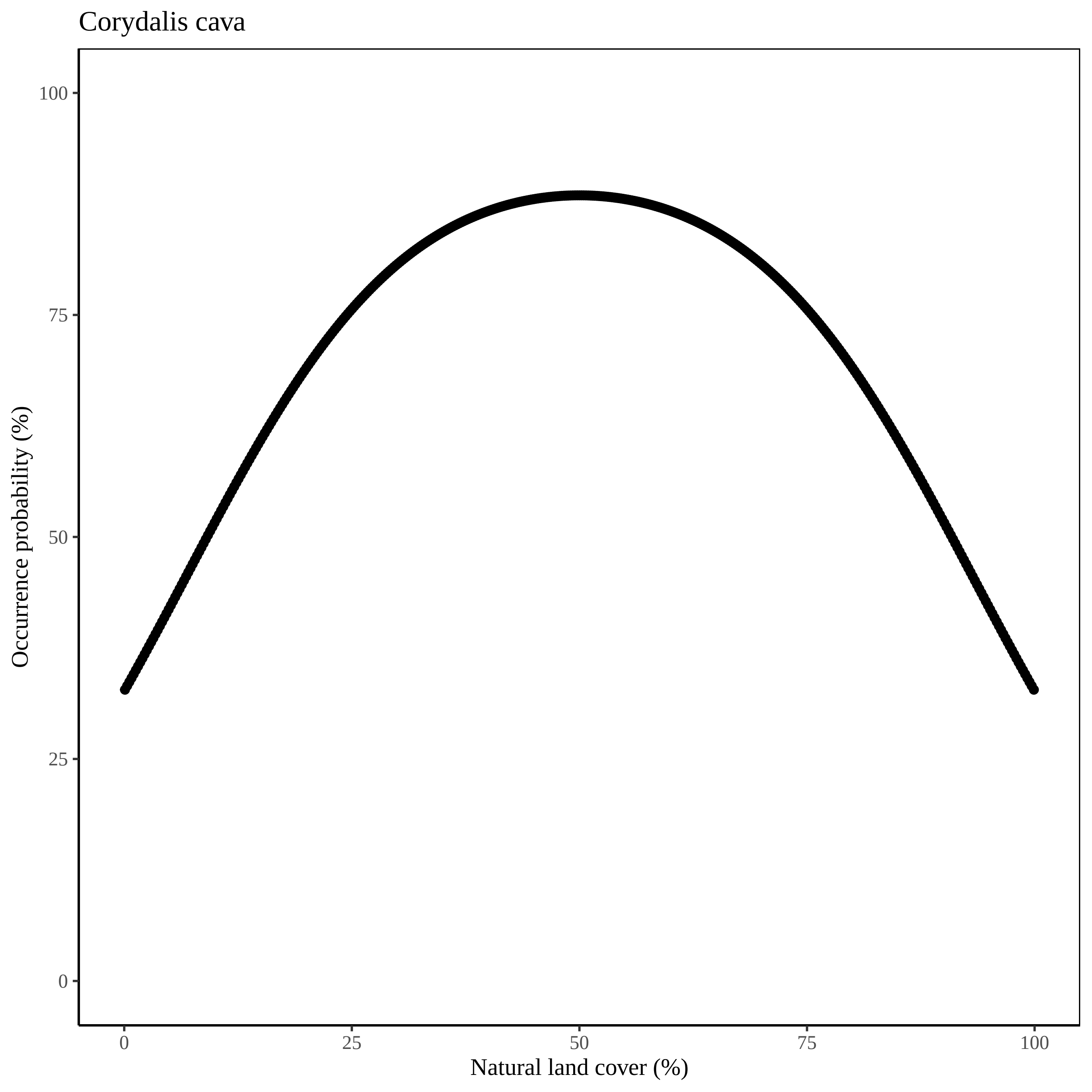
Corydalis cava
Occurrence probability (%)
100
75
50
25
0
0
25
50
75
100
Natural land cover (%)

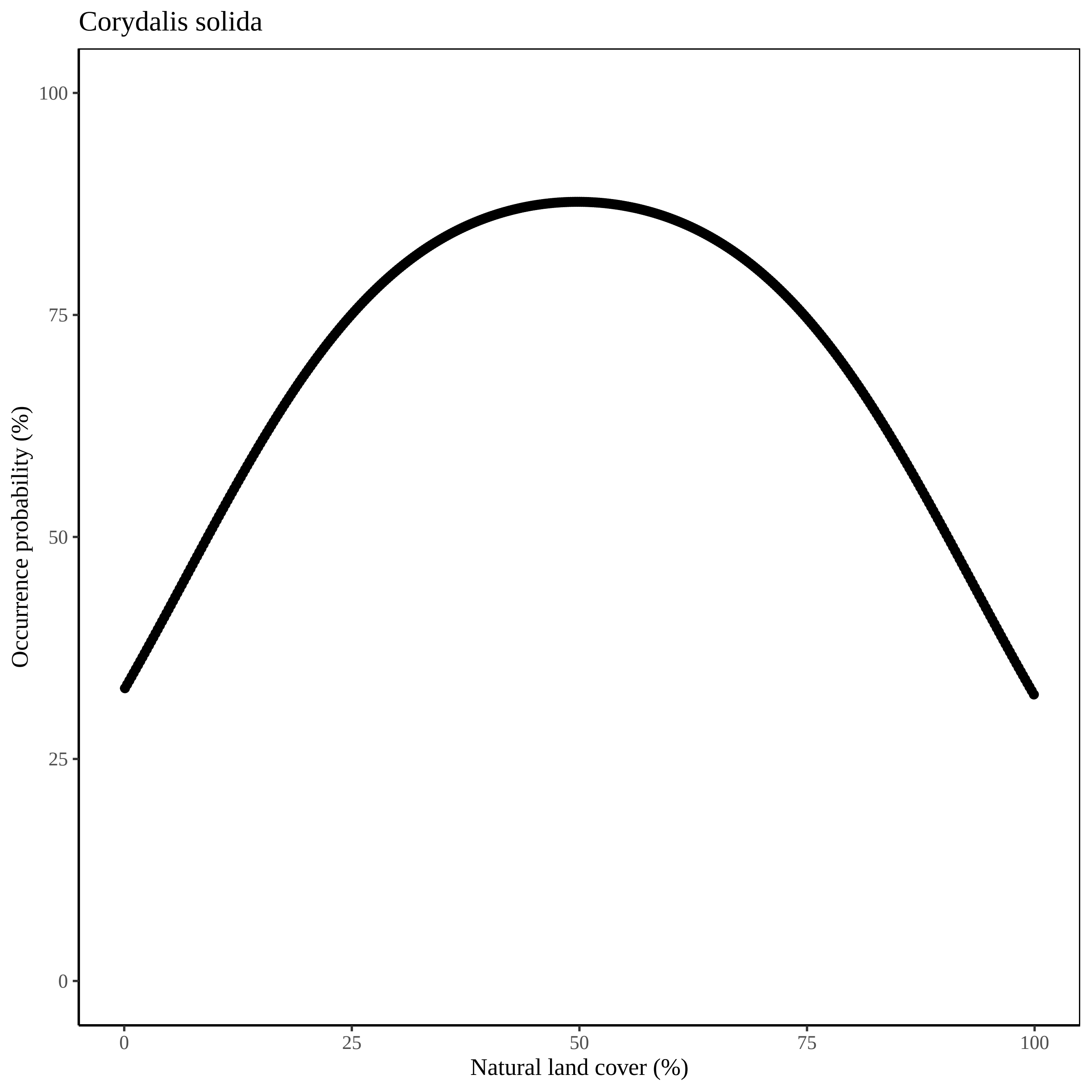
Corydalis solida
Occurrence probability (%)
100
75
50
25
0
0
25
50
75
100
Natural land cover (%)

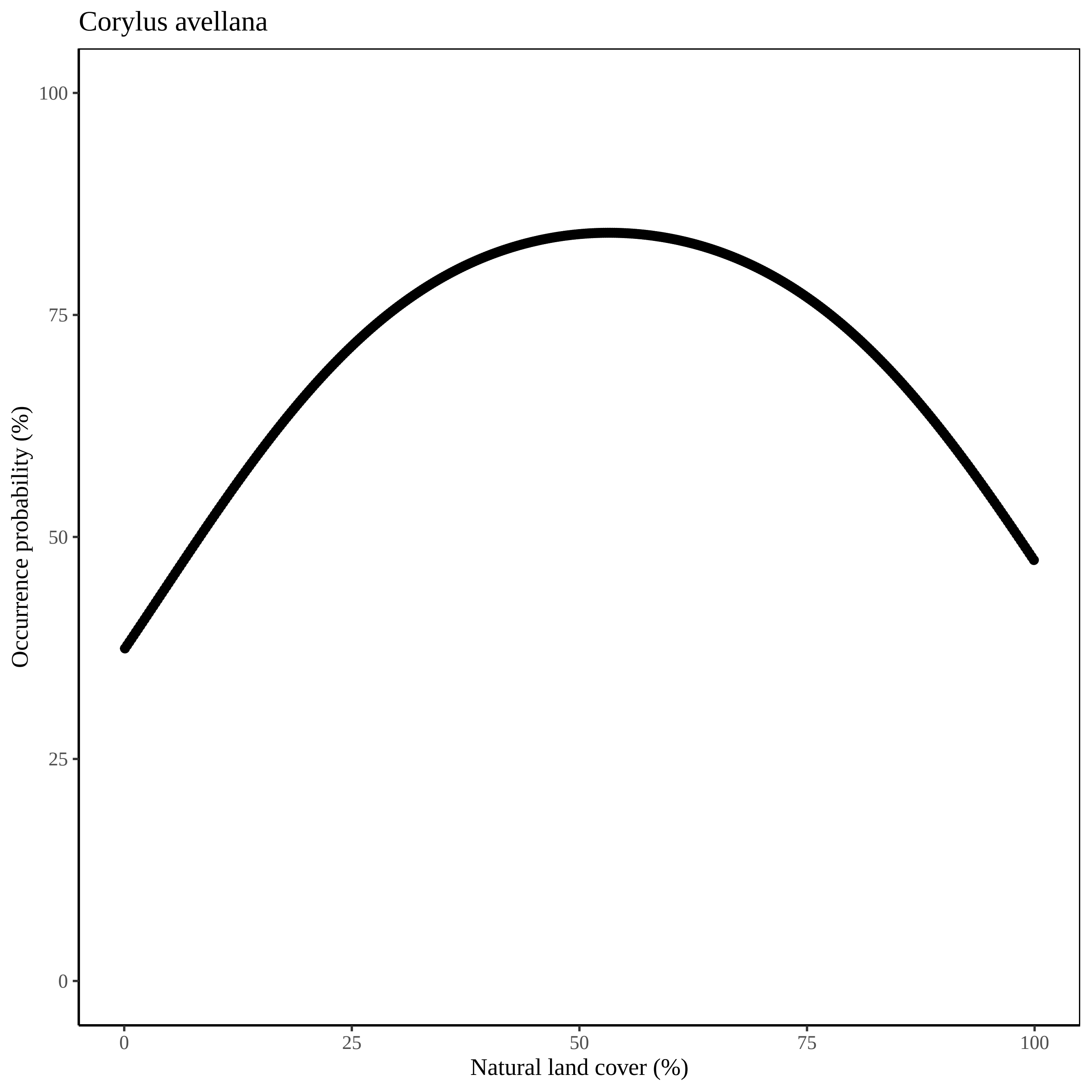
Corylus avellana
Occurrence probability (%)
100
75
50
25
0
0
25
50
75
100
Natural land cover (%)

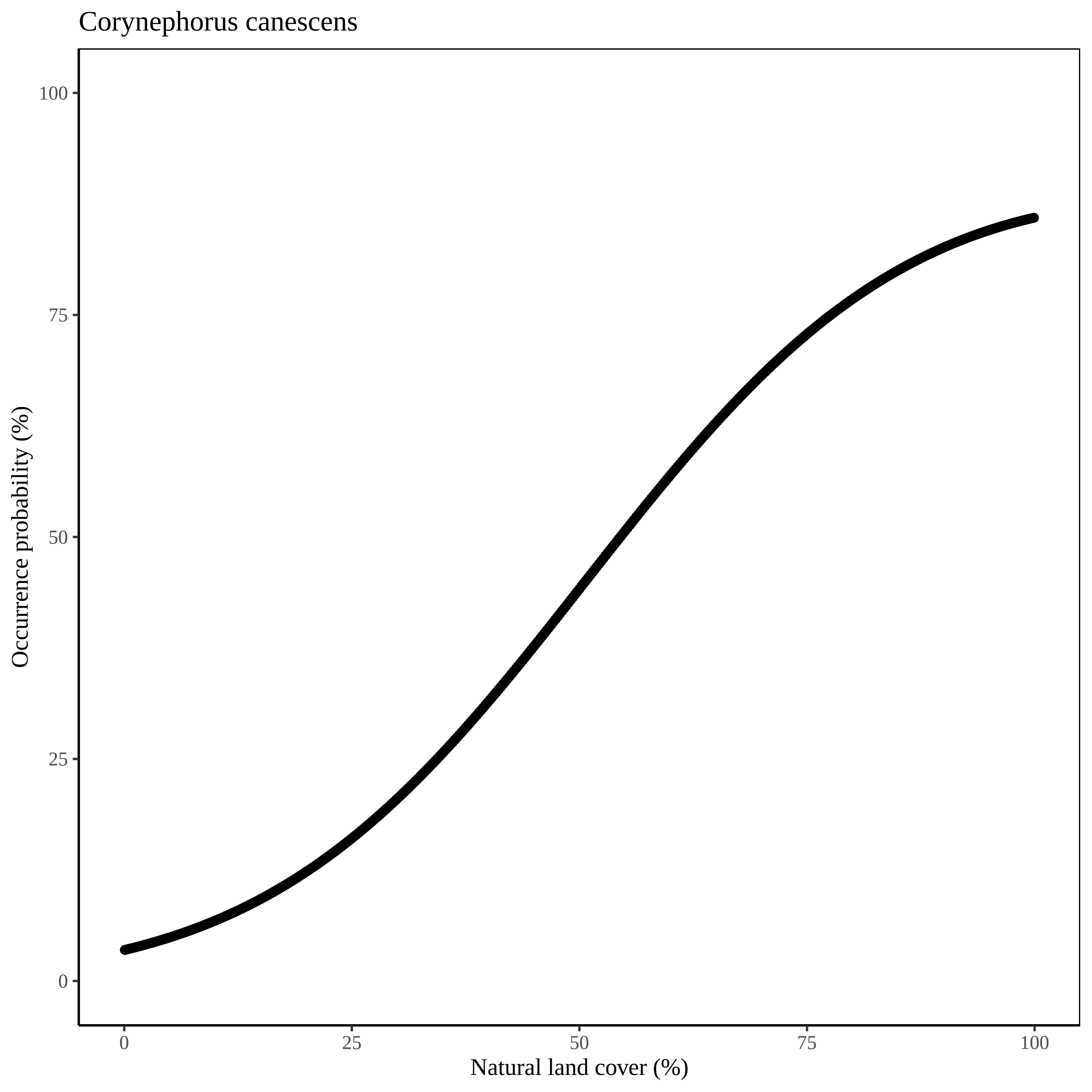
Corynephorus canescens
Occurrence probability (%)
100
75
50
25
0
Natural land cover (%)
0
25
50
75
100

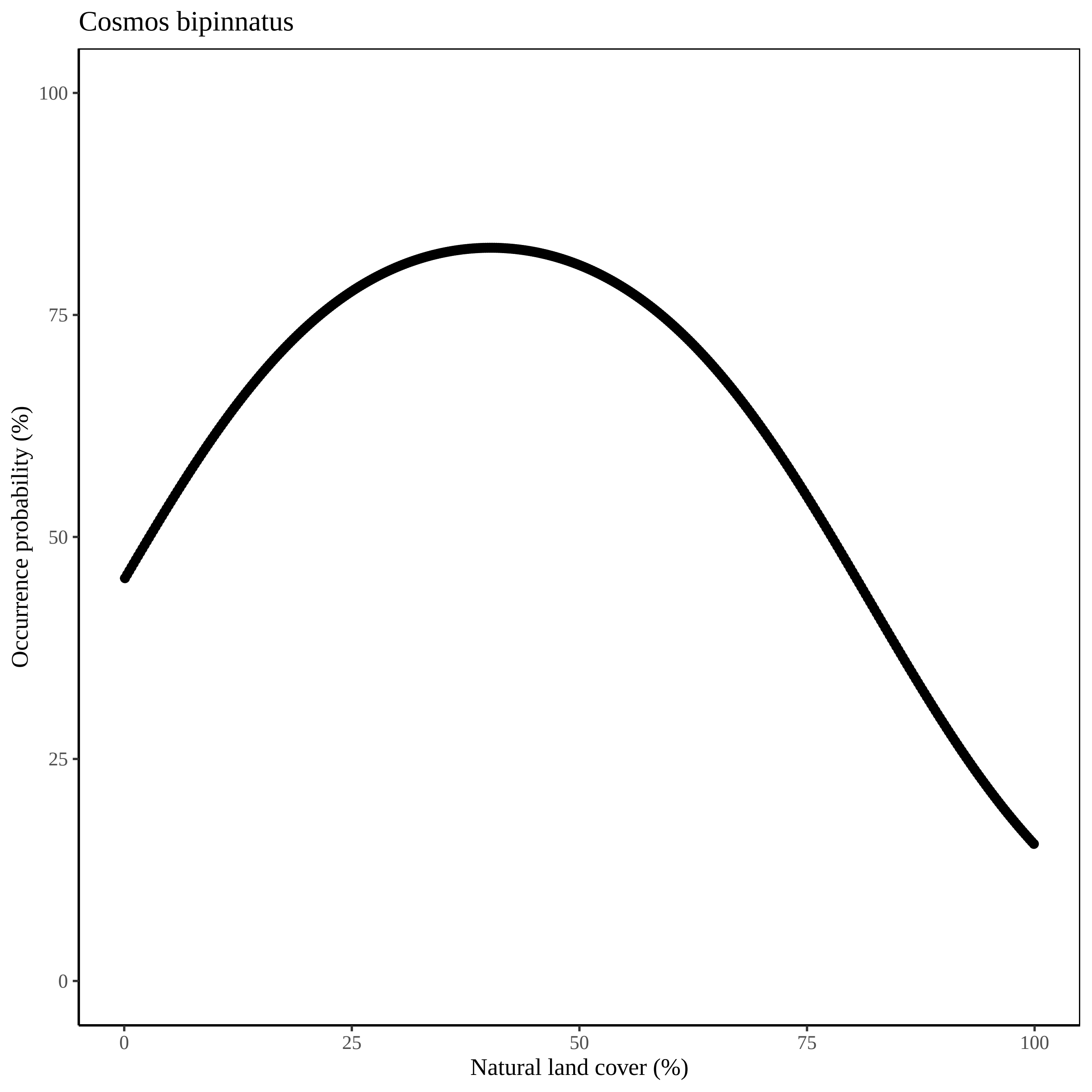

Cosmos bipinnatus
100
75
50
25
0
Occurrence probability (%)
0
25
50
75
100
Natural land cover (%)

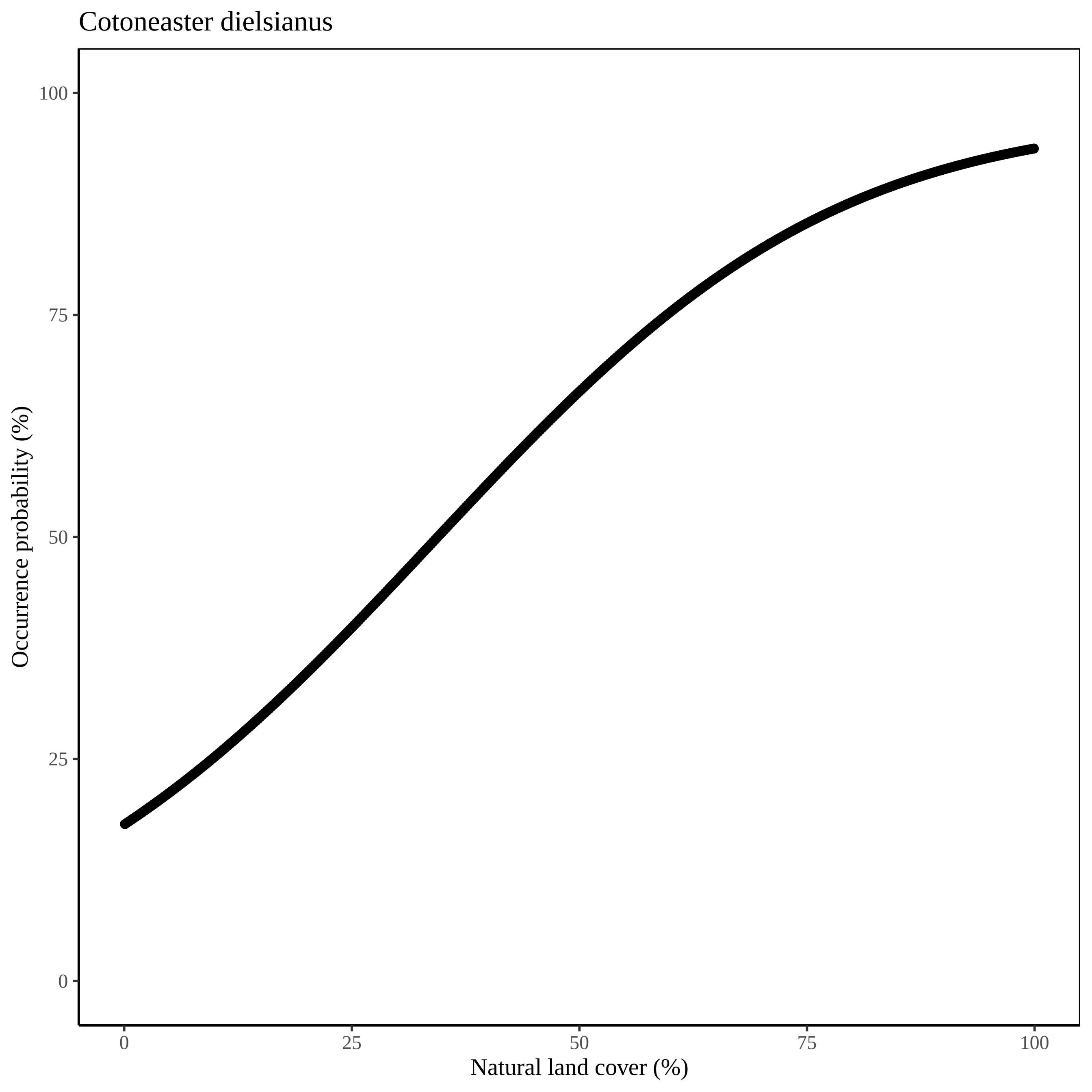
Cotoneaster dielsianus
Occurrence probability (%)
100
75
50
25
0
0
25
50
75
100
Natural land cover (%)

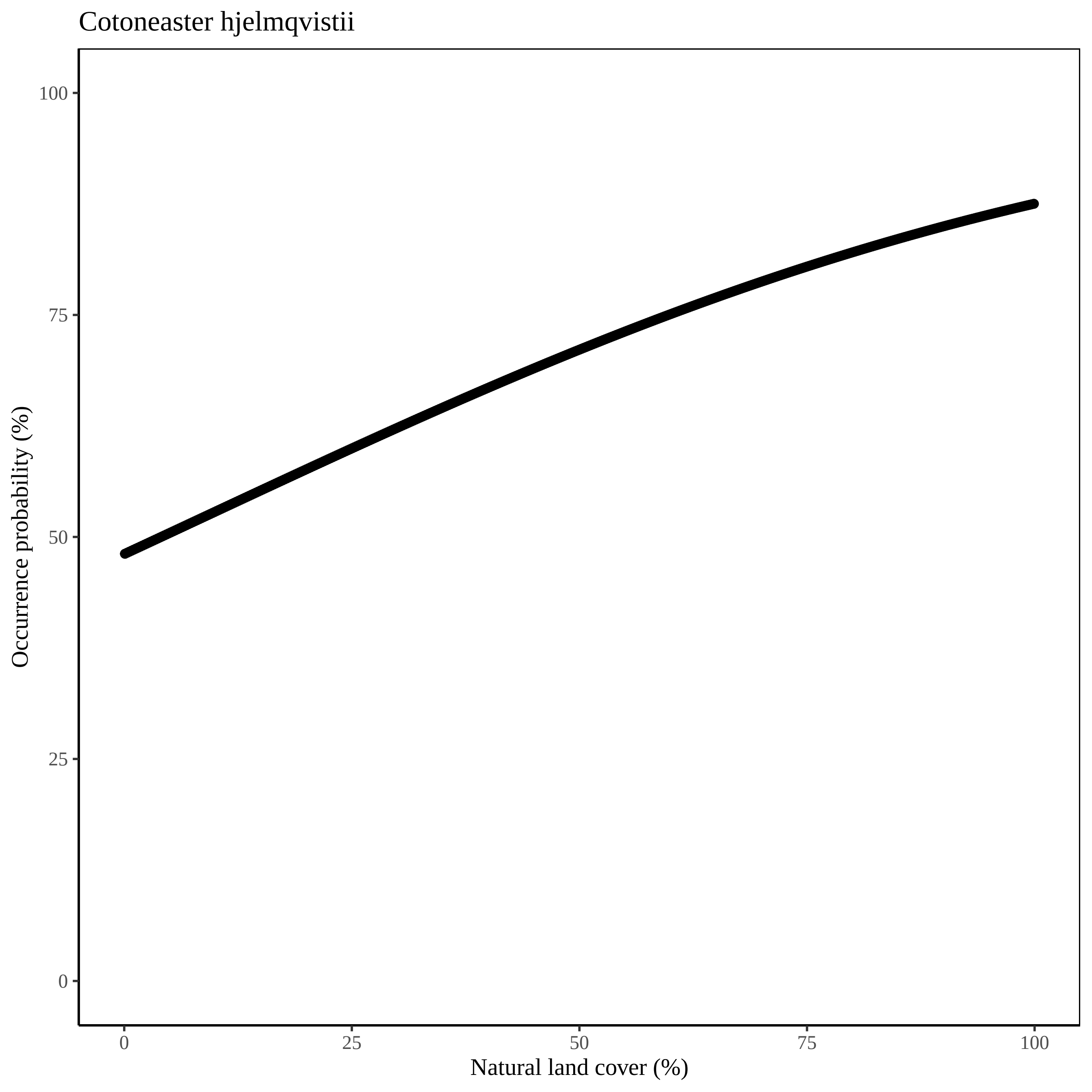

Cotoneaster hjelmqvistii
Occurrence probability (%)
100
75
50
25
0
0
25
50
75
100
Natural land cover (%)

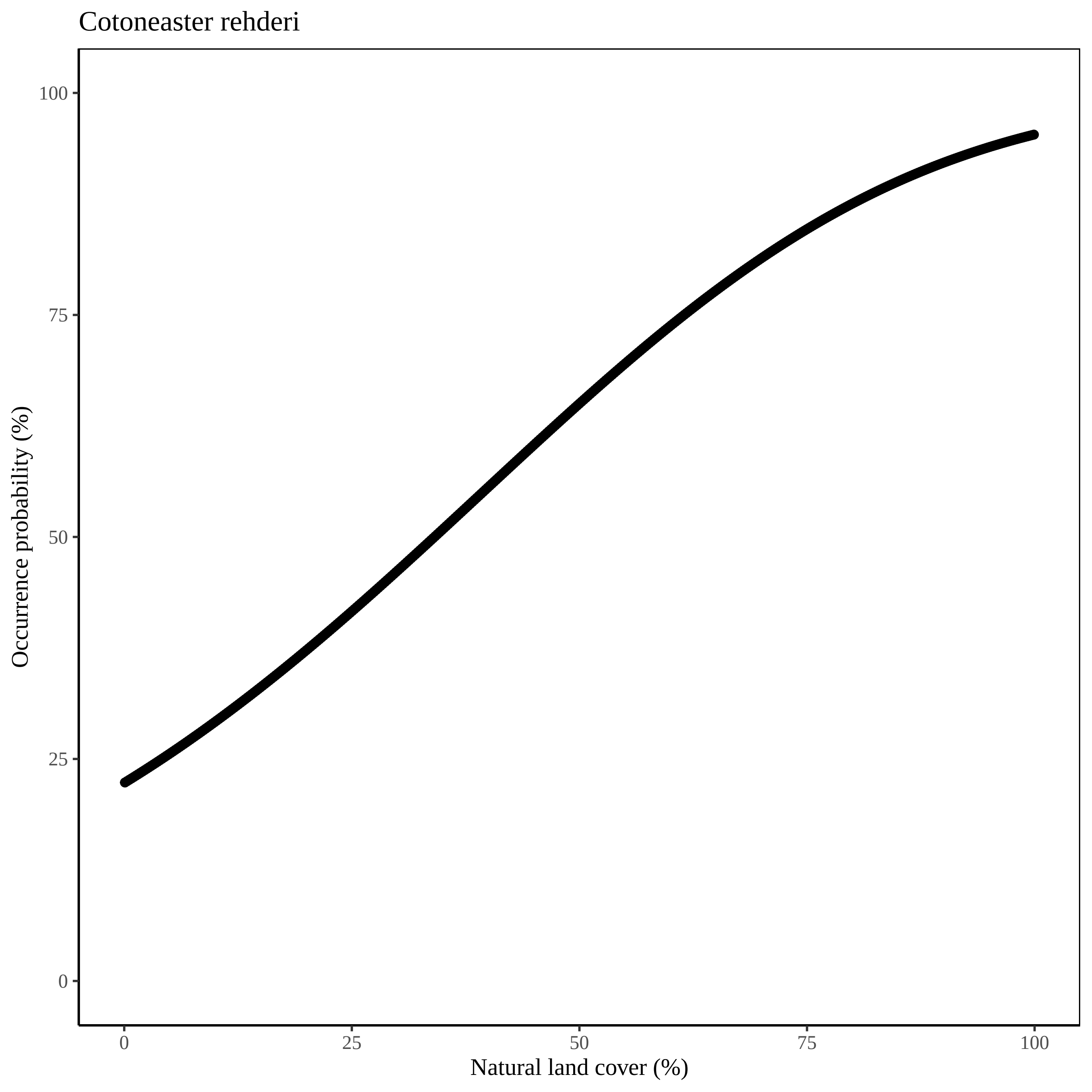
Cotoneaster rehderi
Occurrence probability (%)
100
75
50
25
0
0
25
50
75
100
Natural land cover (%)

Crassula helmsii

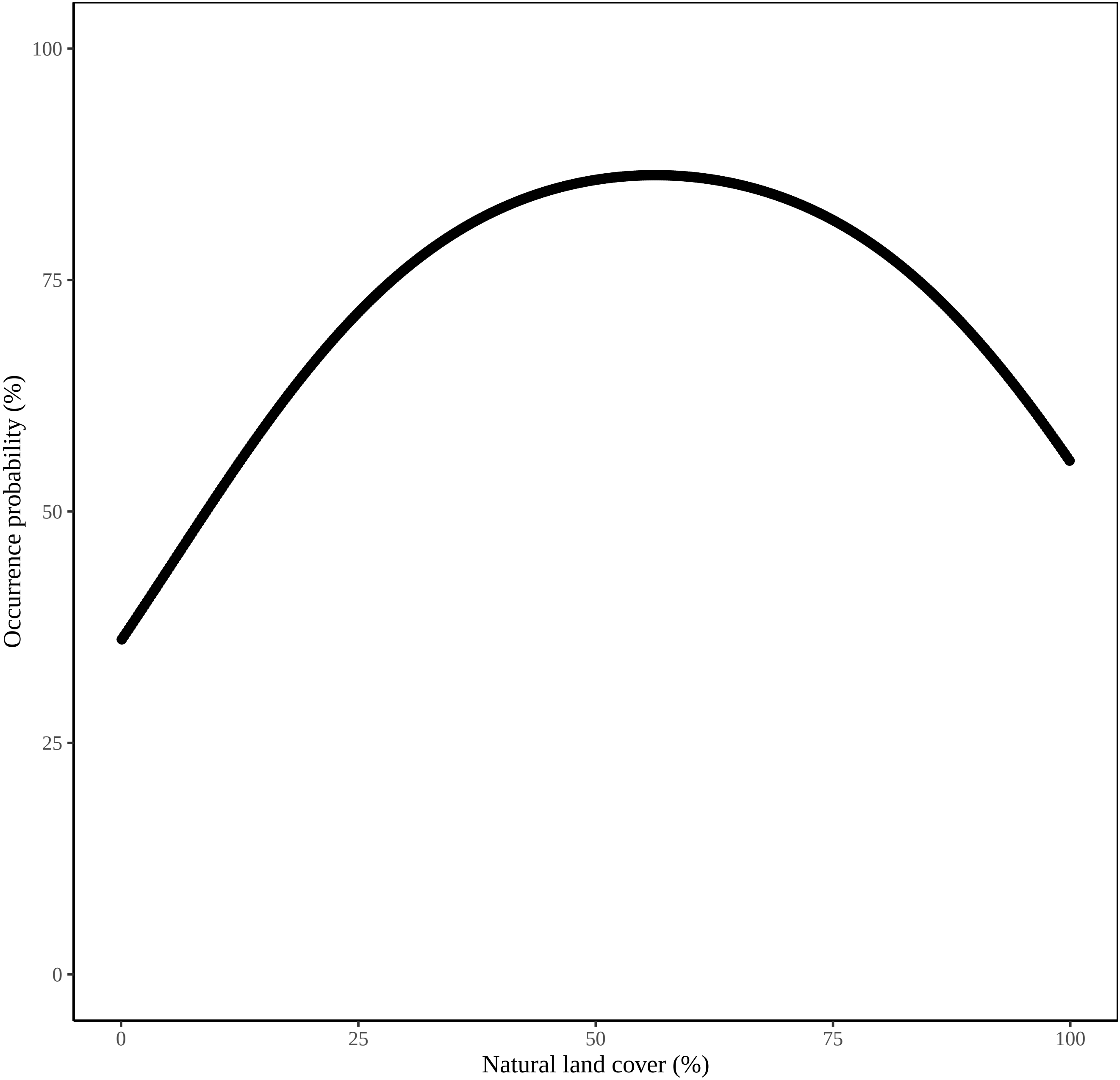

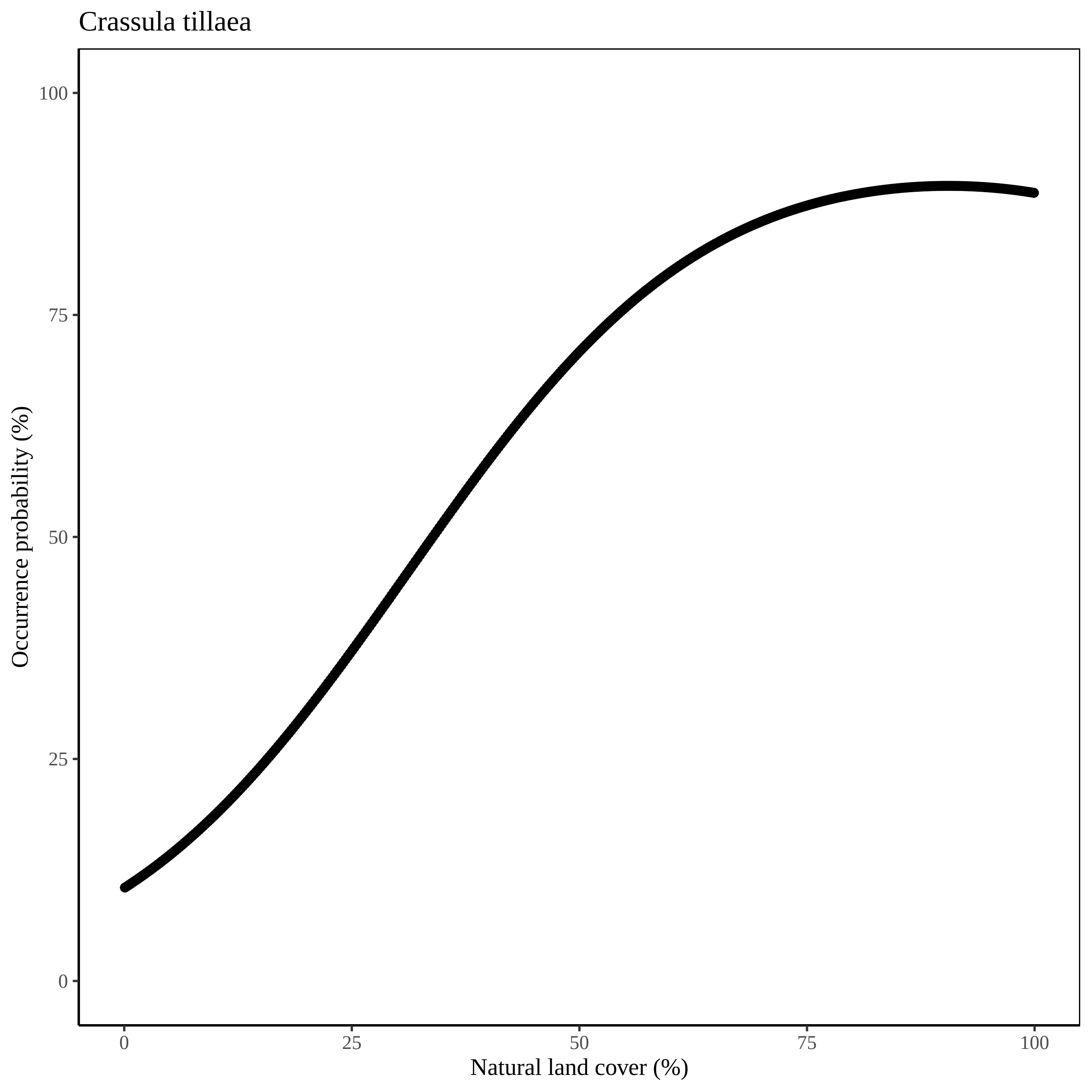

Crassula tillaea
Occurrence probability (%)
100
75
50
25
0
0
25
50
75
100
Natural land cover (%)

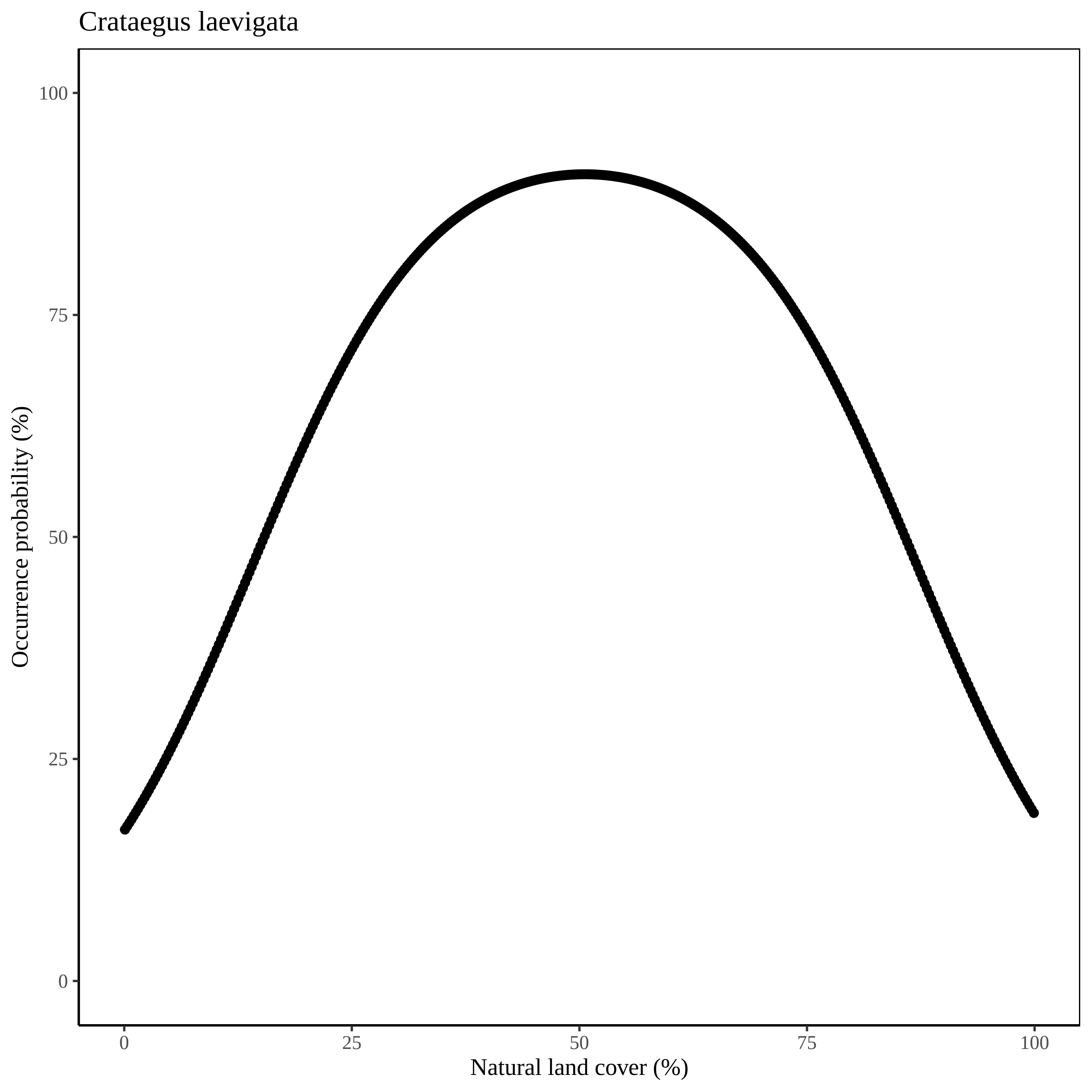

Crataegus laevigata
Occurrence probability (%)
100
75
50
25
0
0
25
50
75
100
Natural land cover (%)

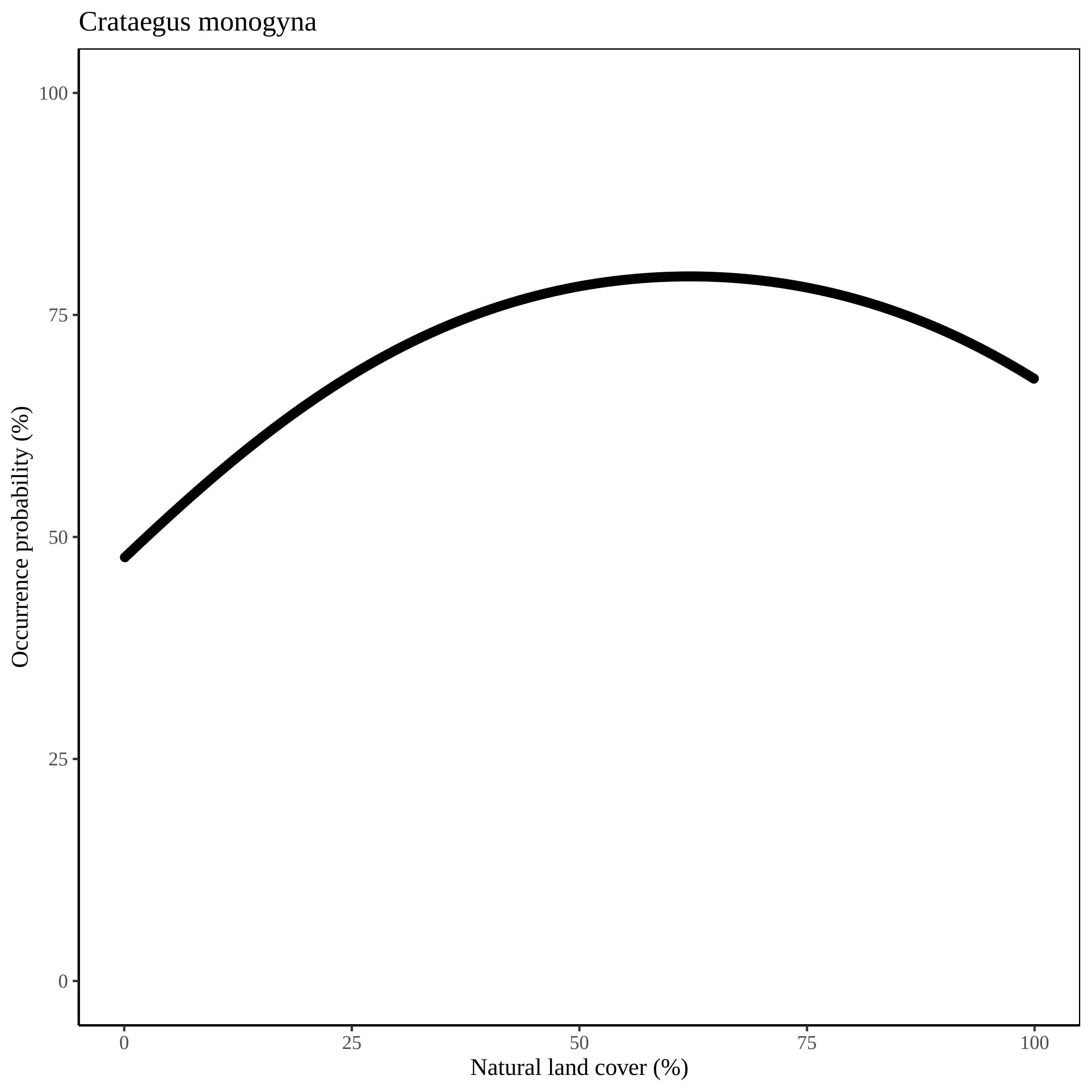
Crataegus monogyna
Occurrence probability (%)
100
75
50
25
0
0
25
50
75
100
Natural land cover (%)

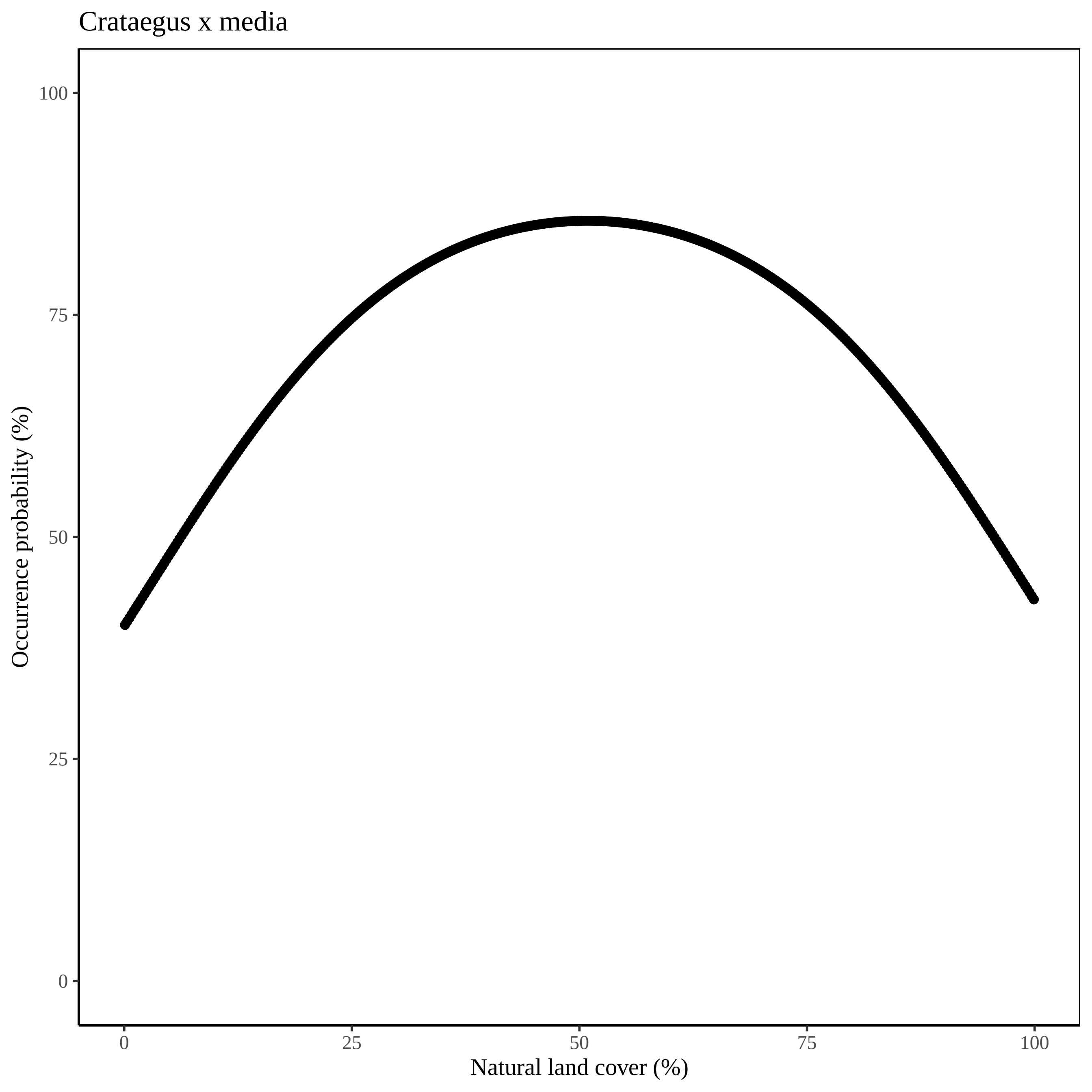
Crataegus x media
Occurrence probability (%)
100
75
50
25
0
0
25
50
75
100
Natural land cover (%)

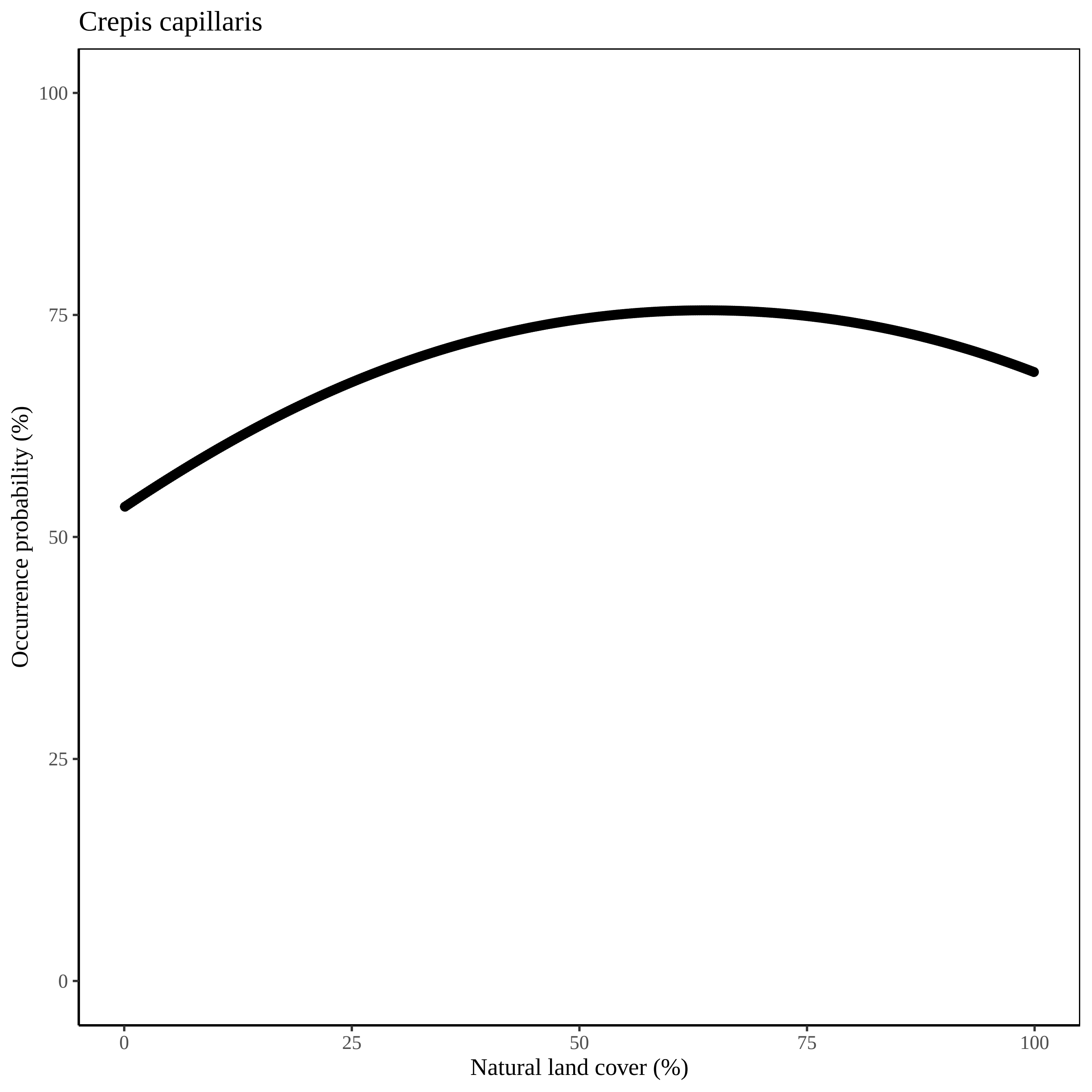
Crepis capillaris
Occurrence probability (%)
100
75
50
25
0
0
25
50
75
100
Natural land cover (%)

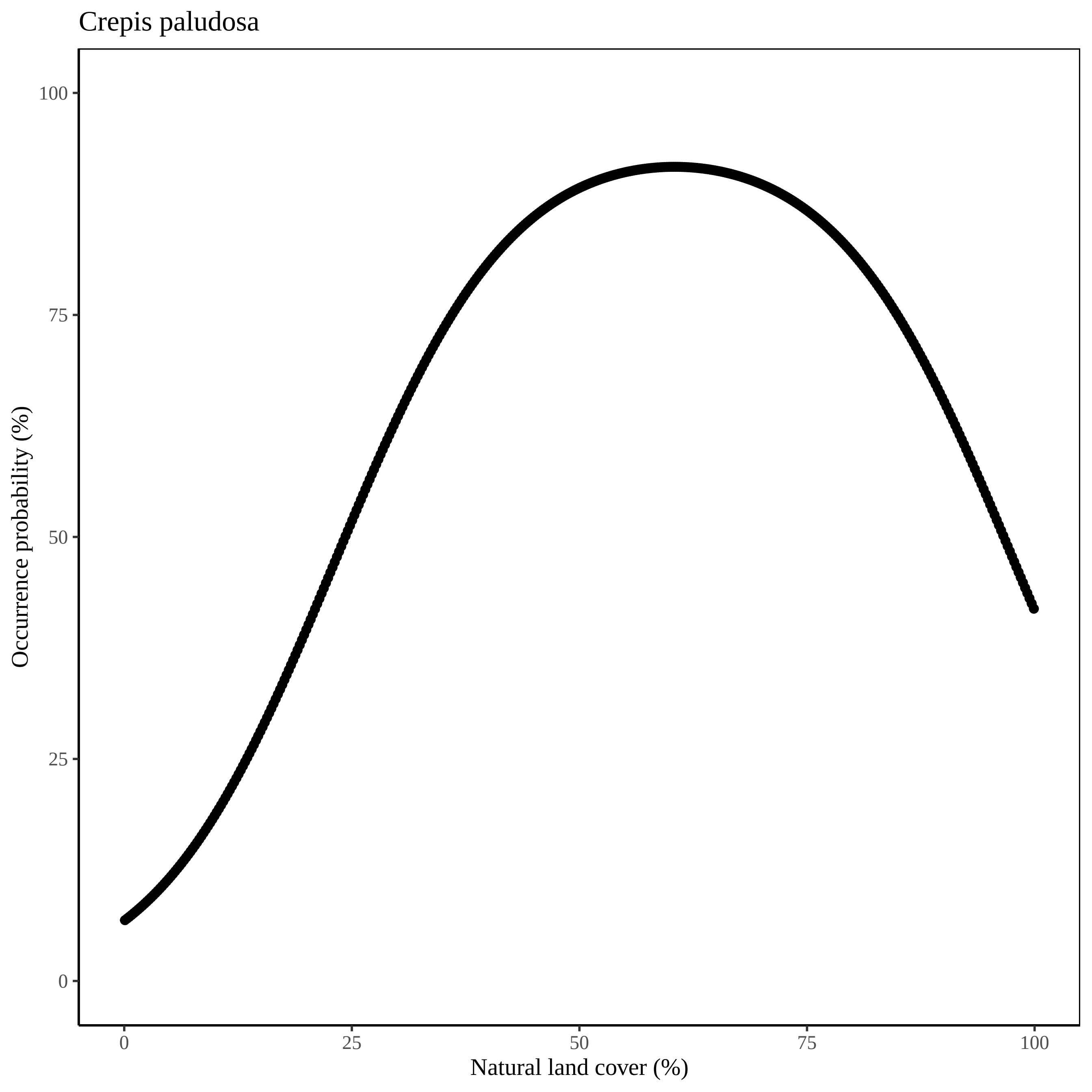
Crepis paludosa
Occurrence probability (%)
100
75
50
25
0
0
25
50
75
100
Natural land cover (%)

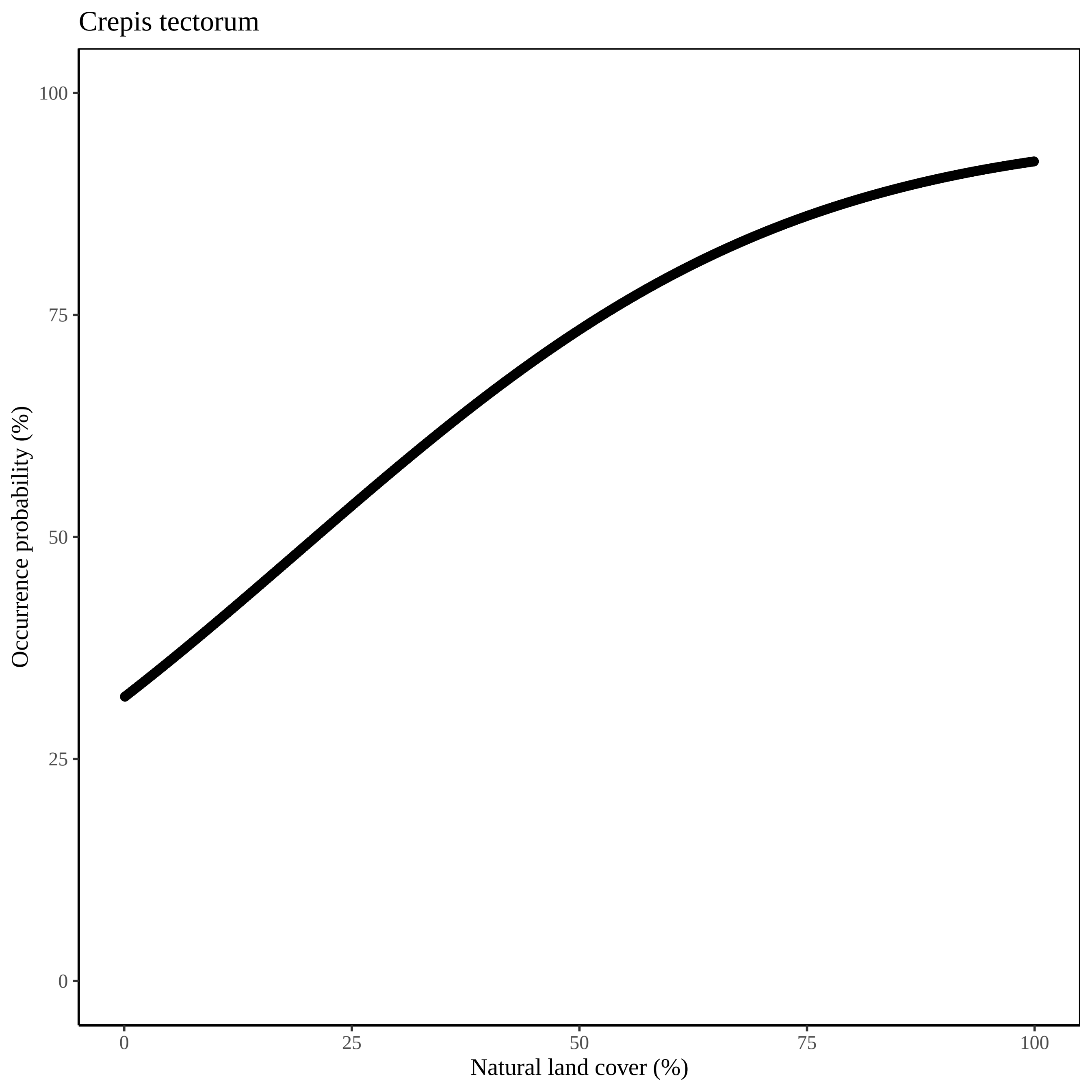

Crepis tectorum
Occurrence probability (%)
100
75
50
25
0
0
25
50
75
100
Natural land cover (%)

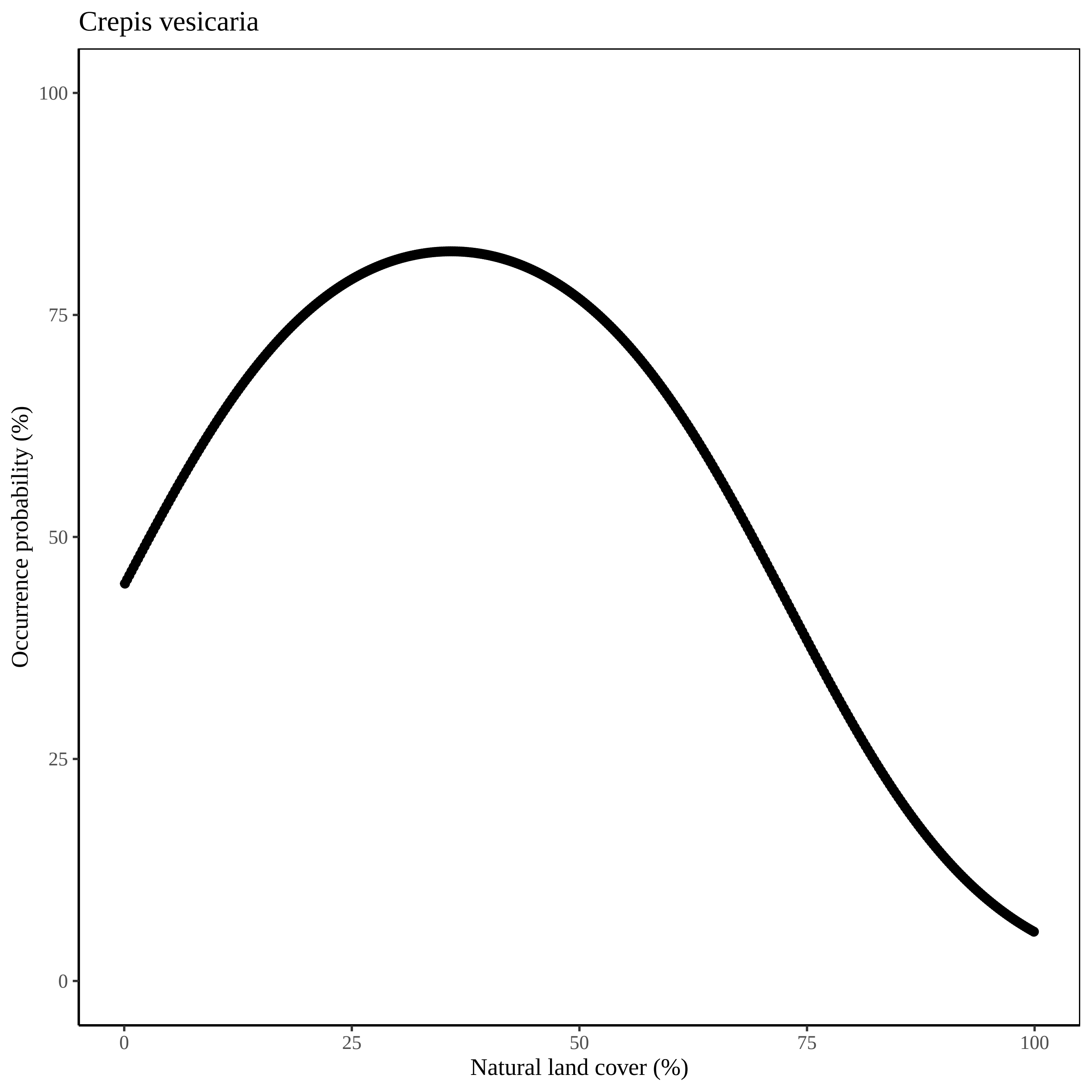
Crepis vesicaria
Occurrence probability (%)
100
75
50
25
0
0
25
50
75
100
Natural land cover (%)

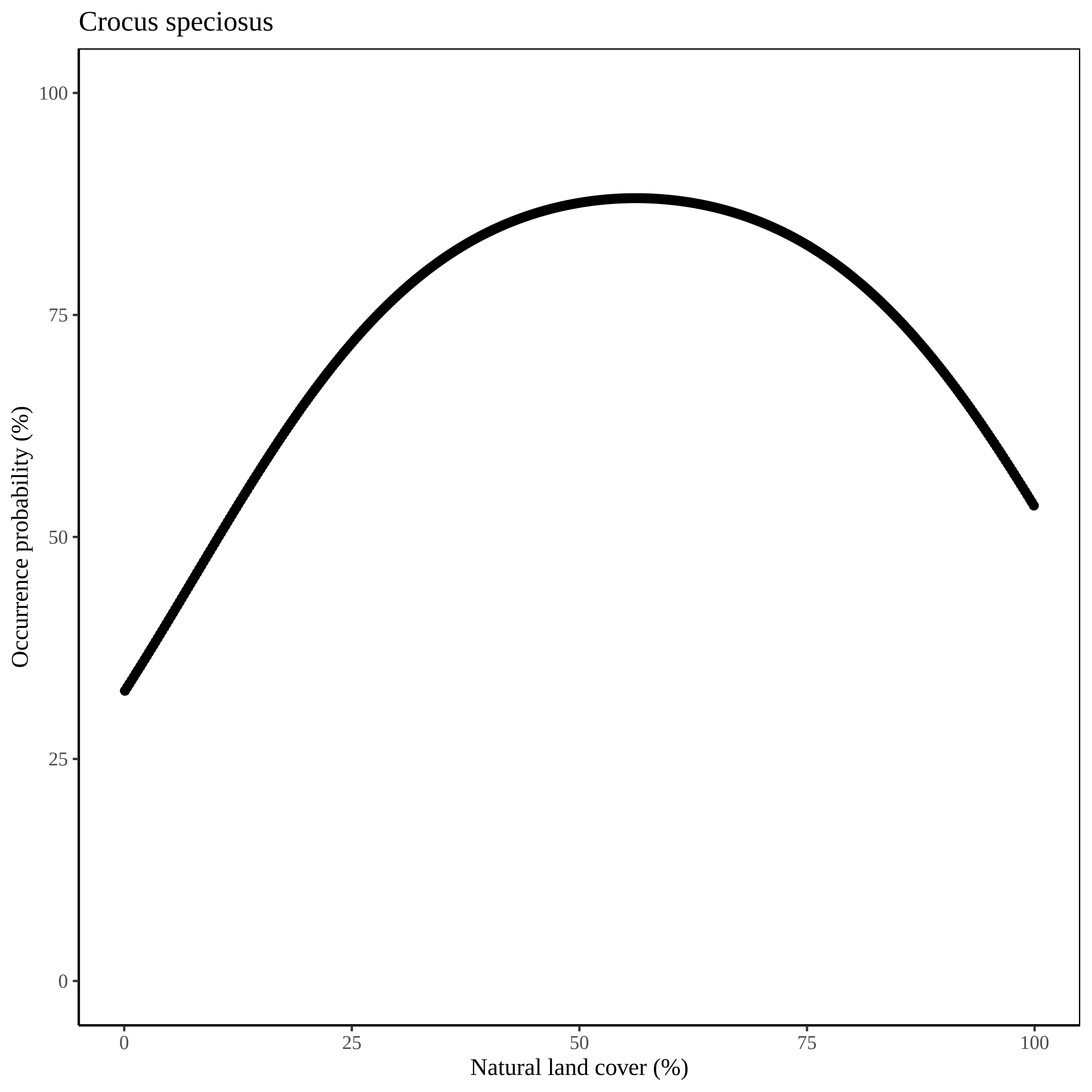
Crocus speciosus
Occurrence probability (%)
100
75
50
25
0
0
25
50
75
100
Natural land cover (%)

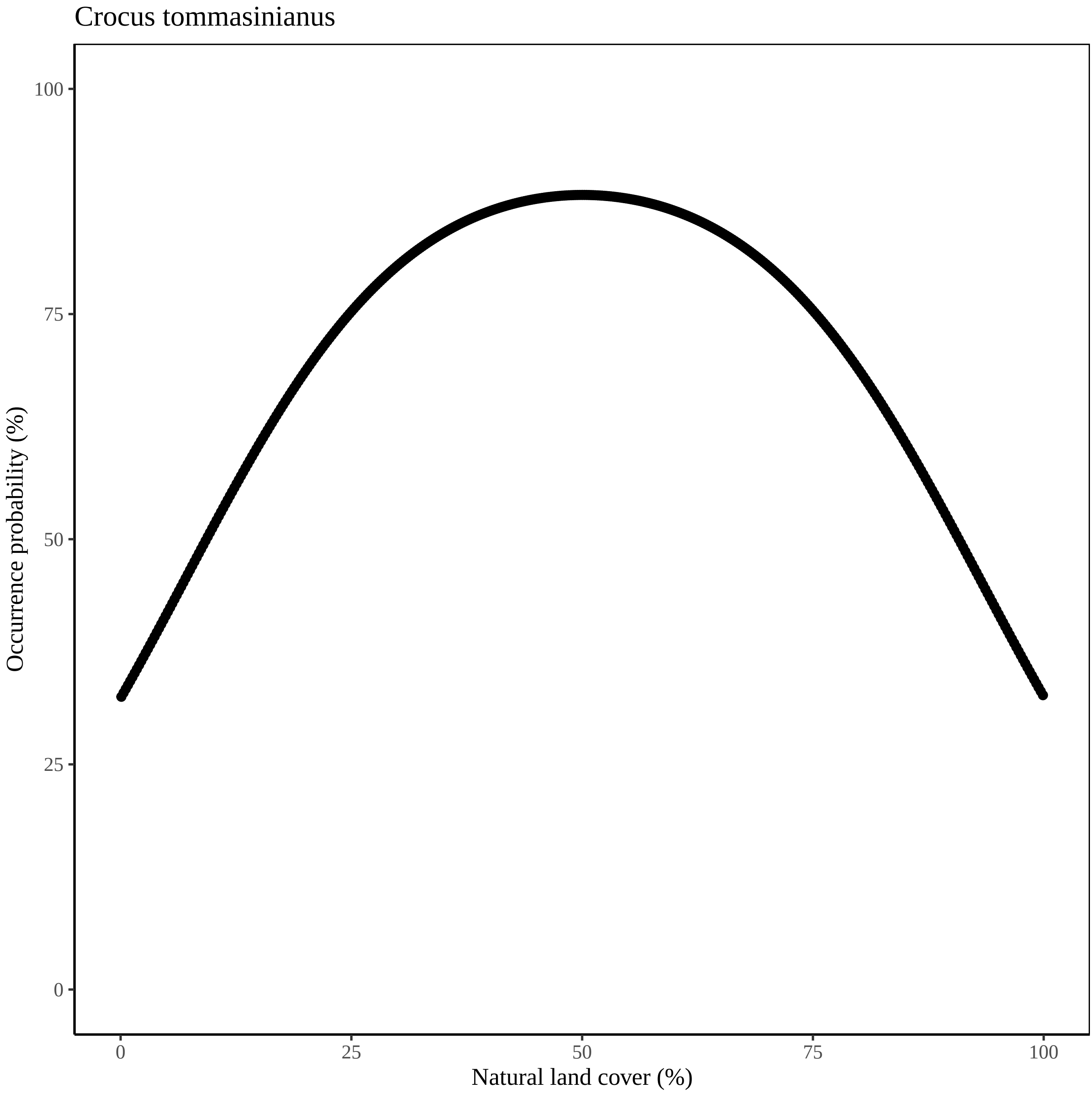
Crocus tommasinianus
Occurrence probability (%)
100
75
50
25
0
0
25
50
75
100
Natural land cover (%)

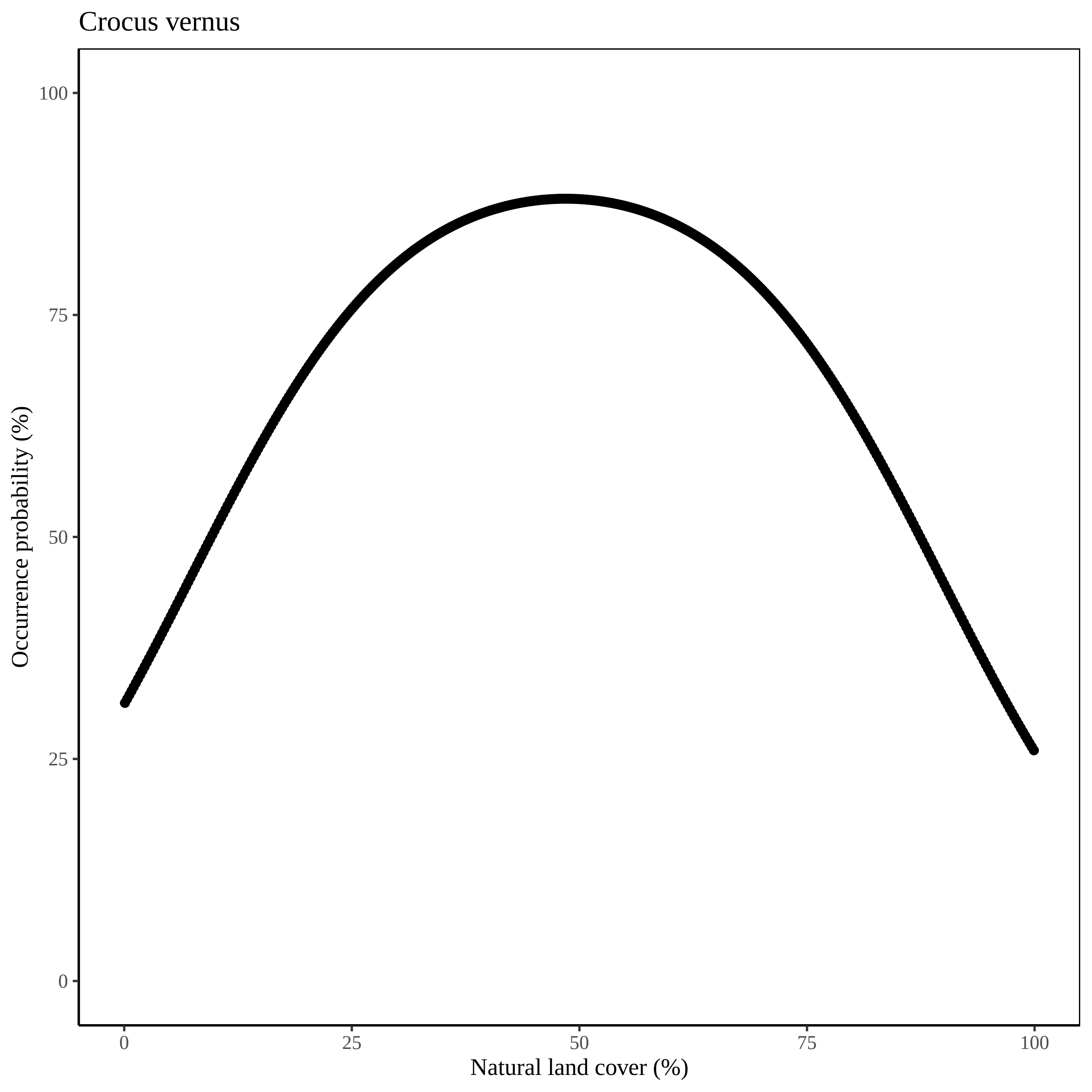
Crocus vernus
Occurrence probability (%)
100
75
50
25
0
0
25
50
75
100
Natural land cover (%)

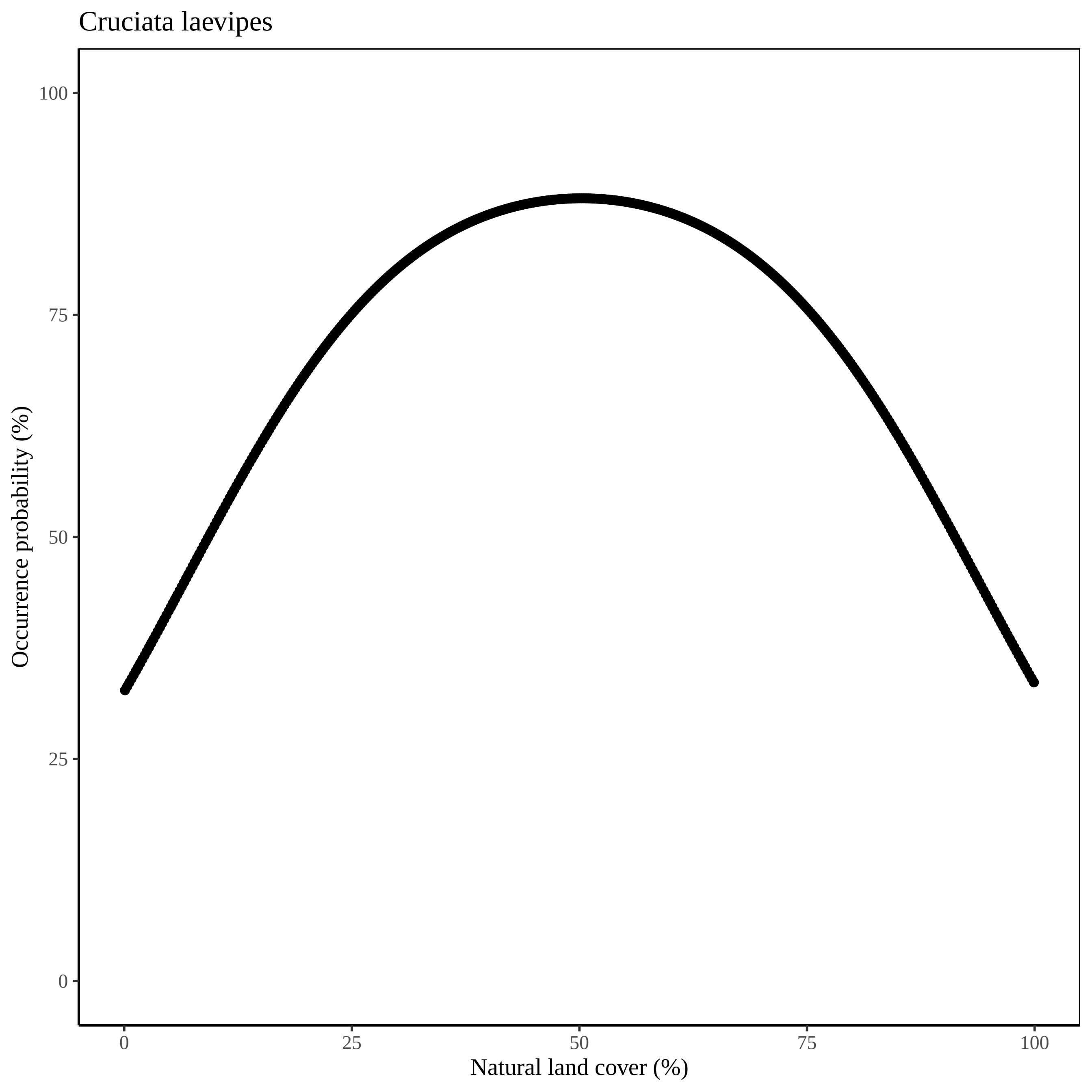

Cruciata laevipes
Occurrence probability (%)
0
25
50
75
100
Natural land cover (%)
0
25
50
75
100

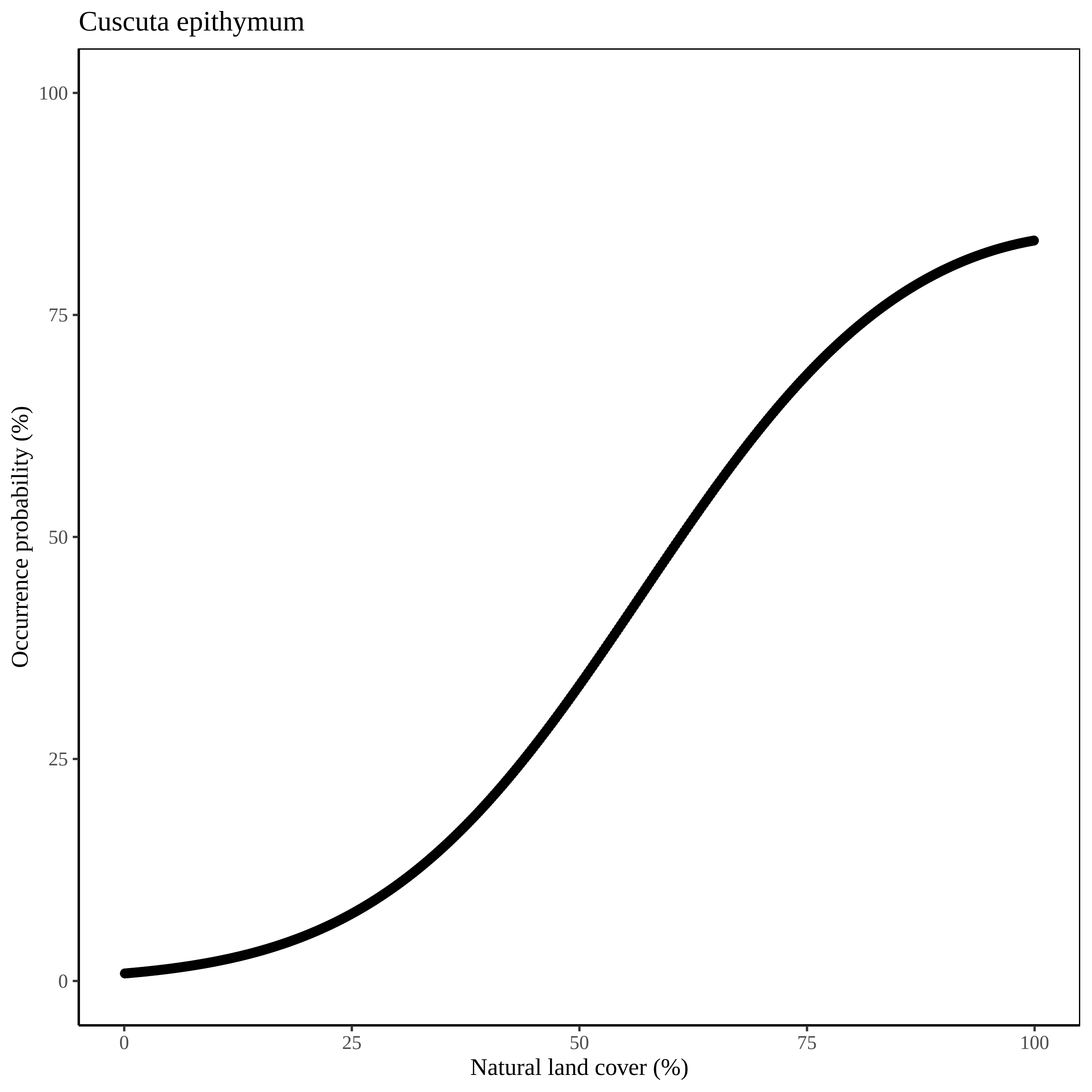
Cuscuta epithymum
Occurrence probability (%)
100
75
50
25
0
0
25
50
75
100
Natural land cover (%)

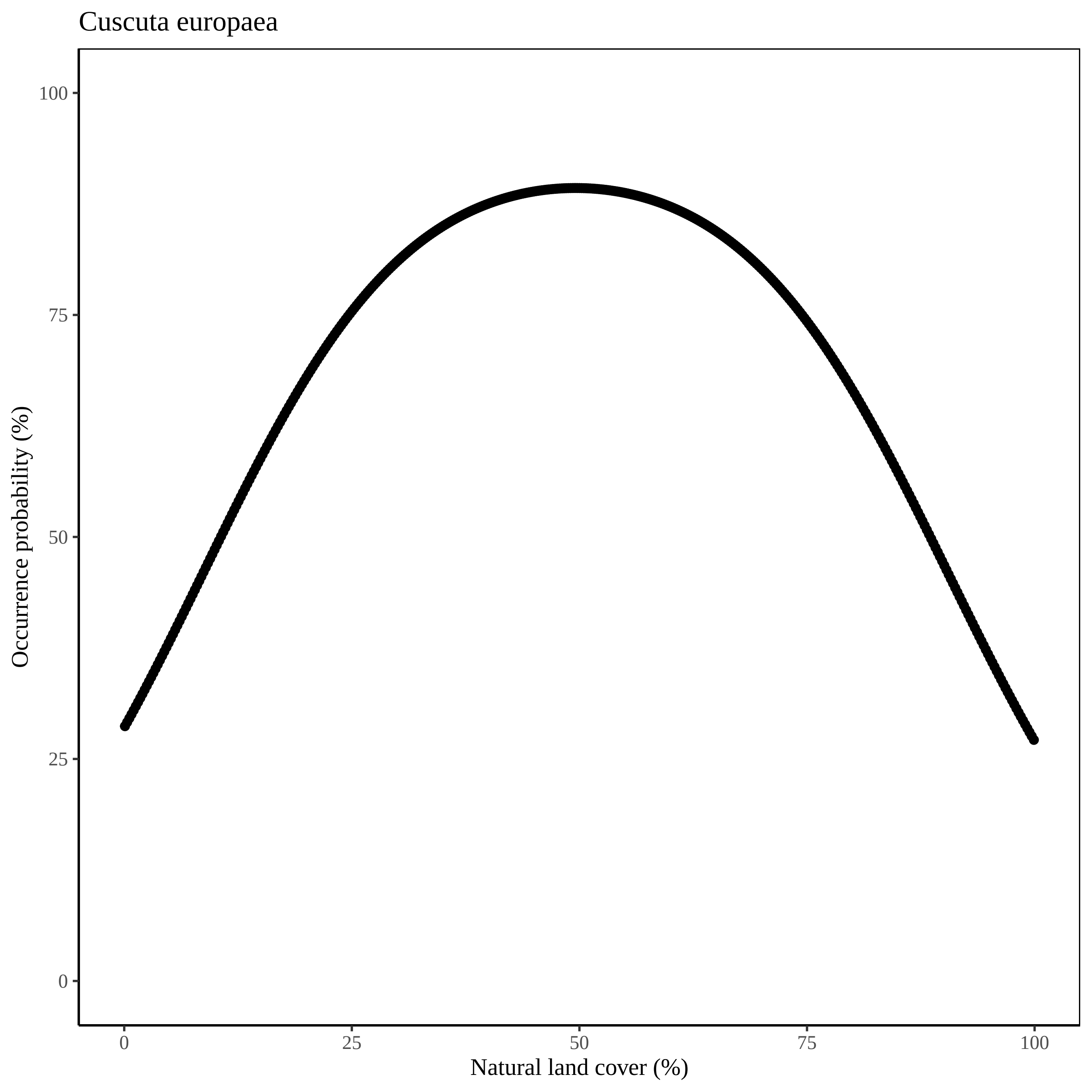

Cuscuta europaea
Occurrence probability (%)
100
75
50
25
0
0
25
50
75
100
Natural land cover (%)

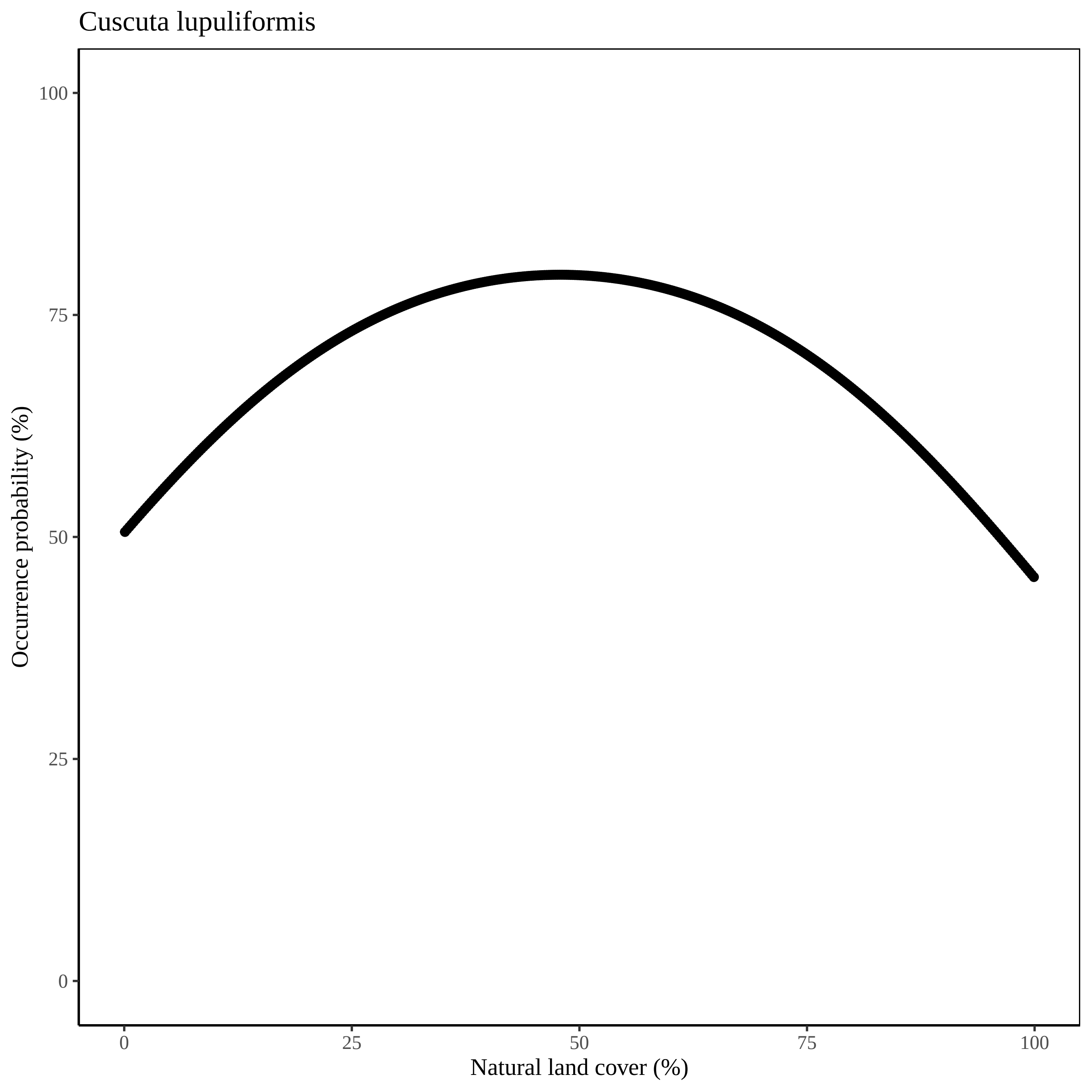
Cuscuta lupuliformis
100
75
50
25
0
Occurrence probability (%)
0
25
50
75
100
Natural land cover (%)

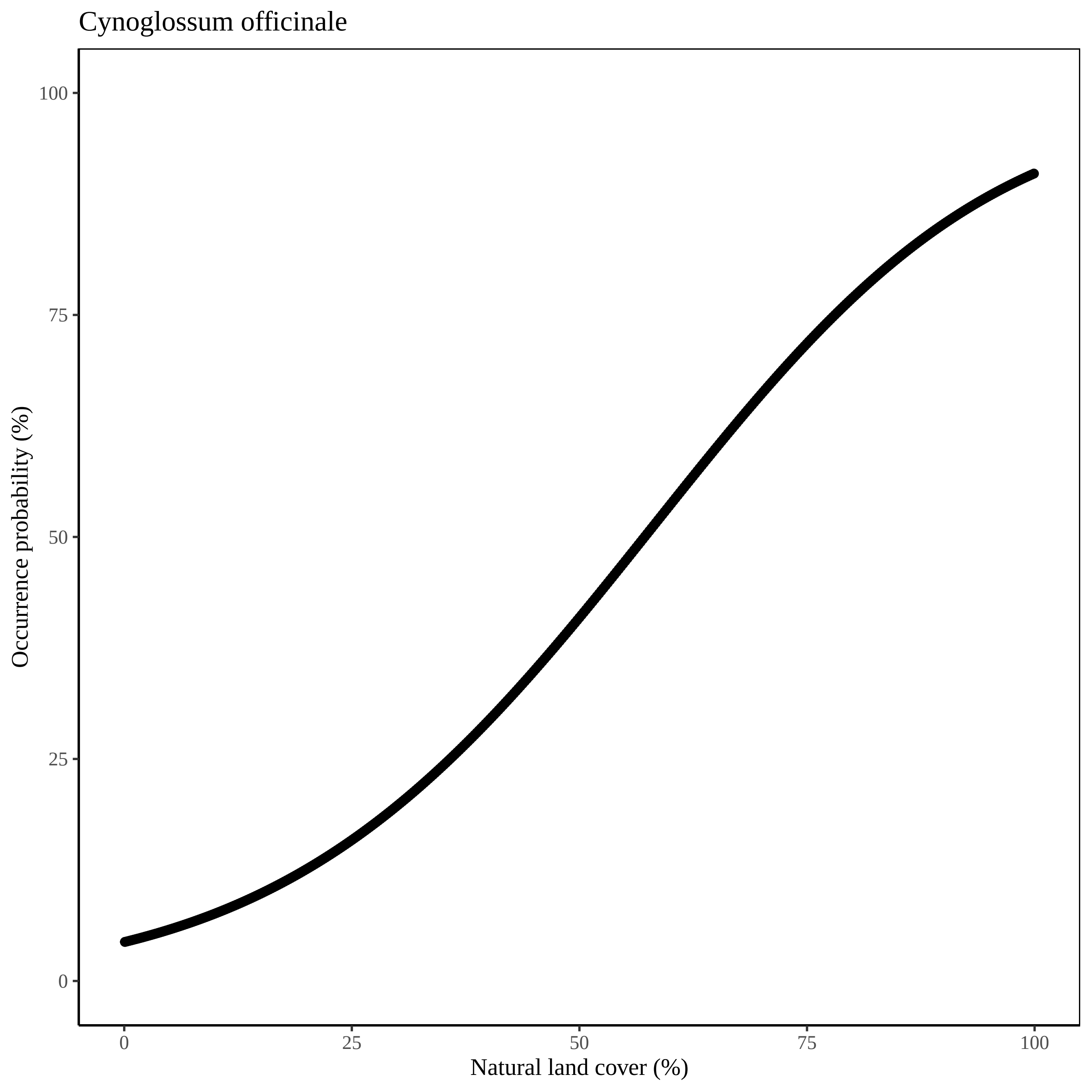

Cynoglossum officinale
Occurrence probability (%)
100
75
50
25
0
0
25
50
75
100
Natural land cover (%)

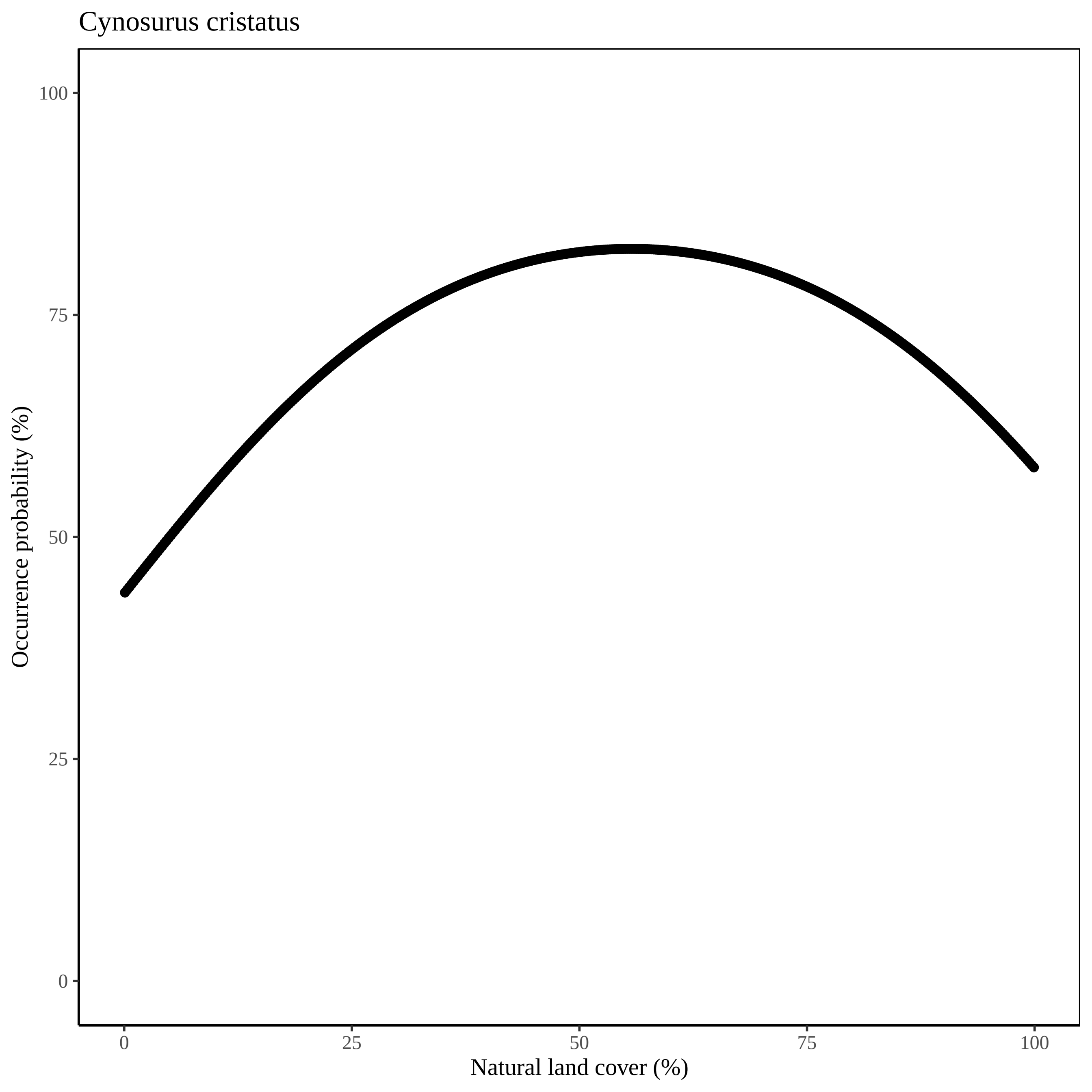
Cynosurus cristatus
Occurrence probability (%)
100
75
50
25
0
Natural land cover (%)
0
25
50
75
100

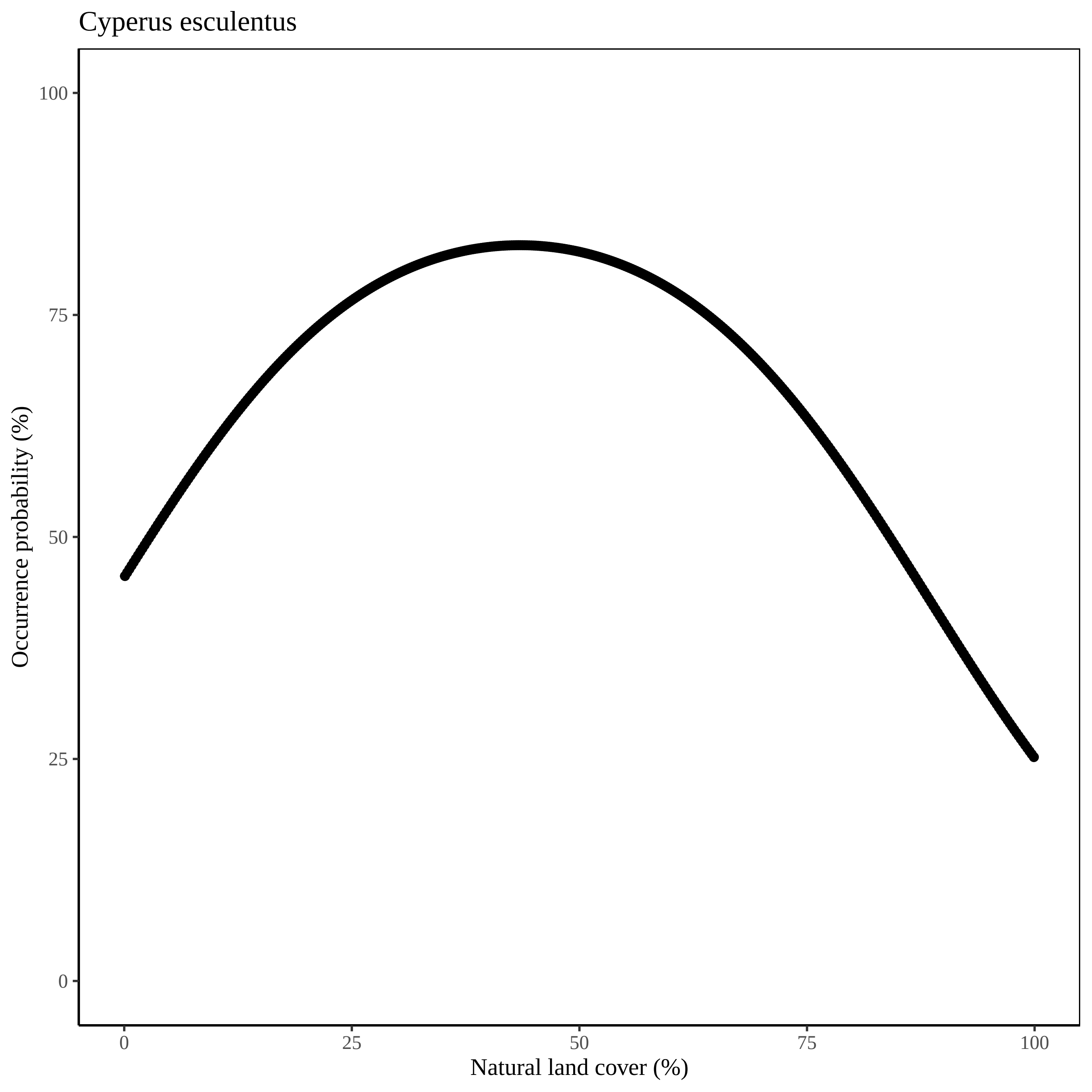
Cyperus esculentus
Occurrence probability (%)
100
75
50
25
0
0
25
50
75
100
Natural land cover (%)

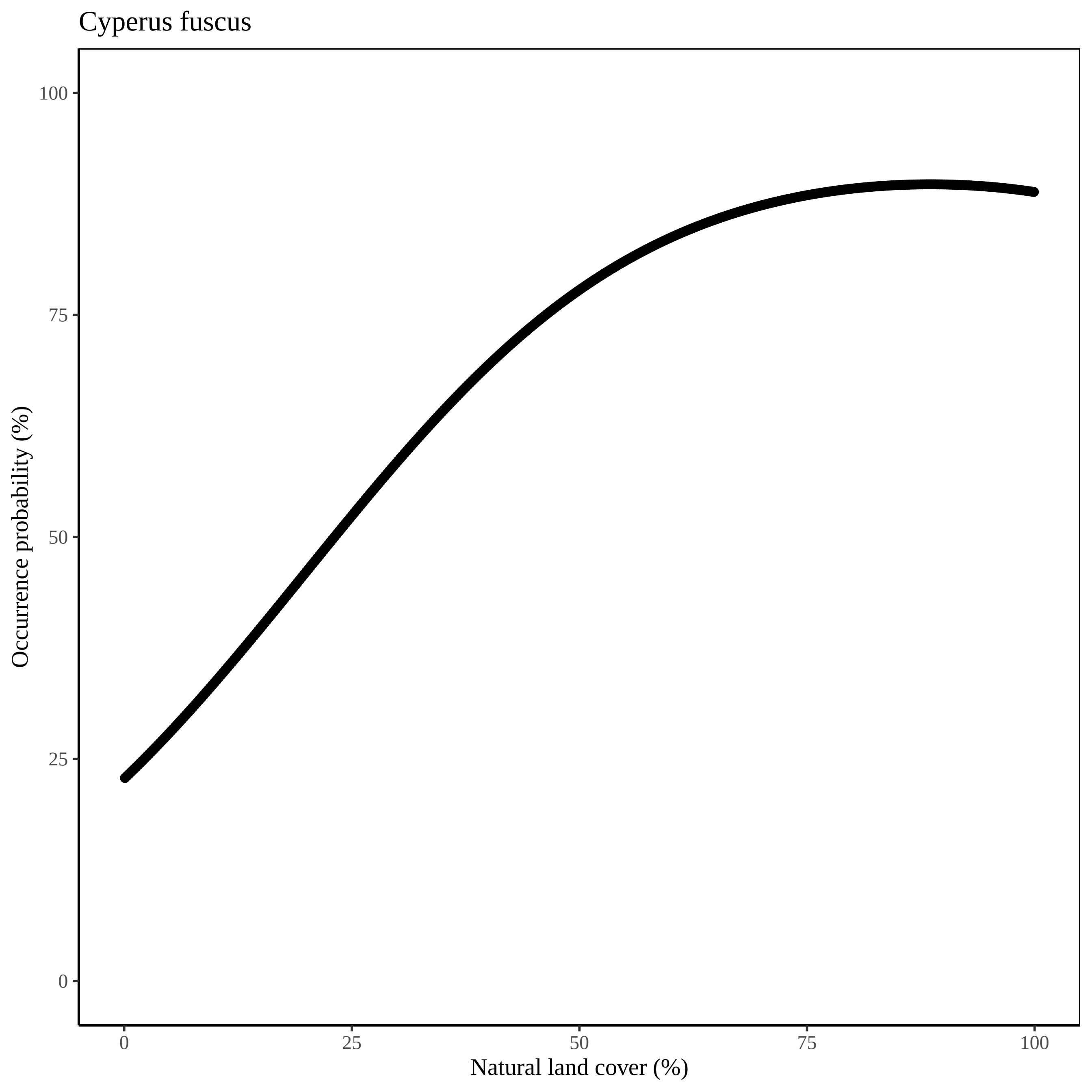

Cyperus fuscus
Occurrence probability (%)
100
75
50
25
0
0
25
50
75
100
Natural land cover (%)

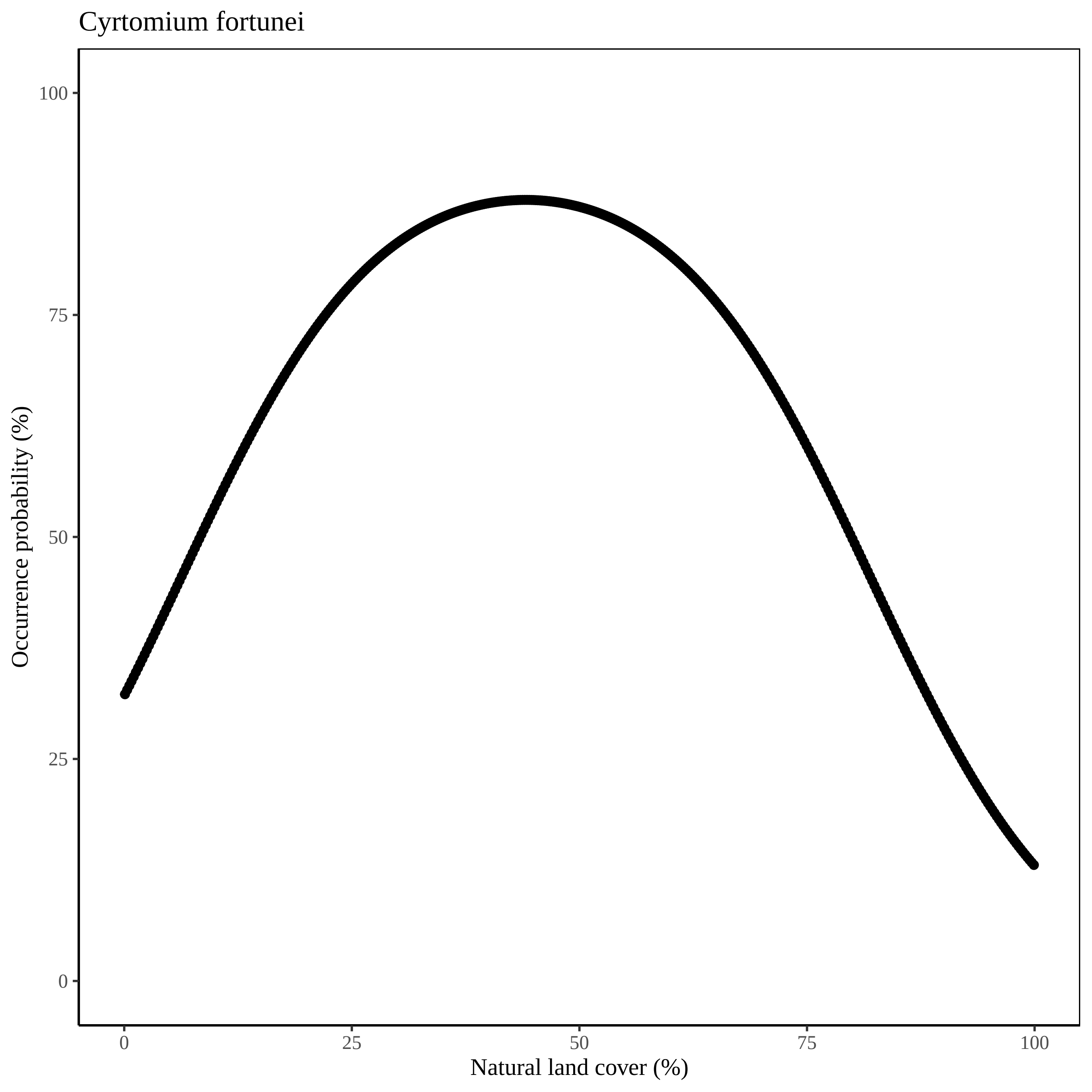

Cyrtomium fortunei
Occurrence probability (%)
100
75
50
25
0
0
25
50
75
100
Natural land cover (%)

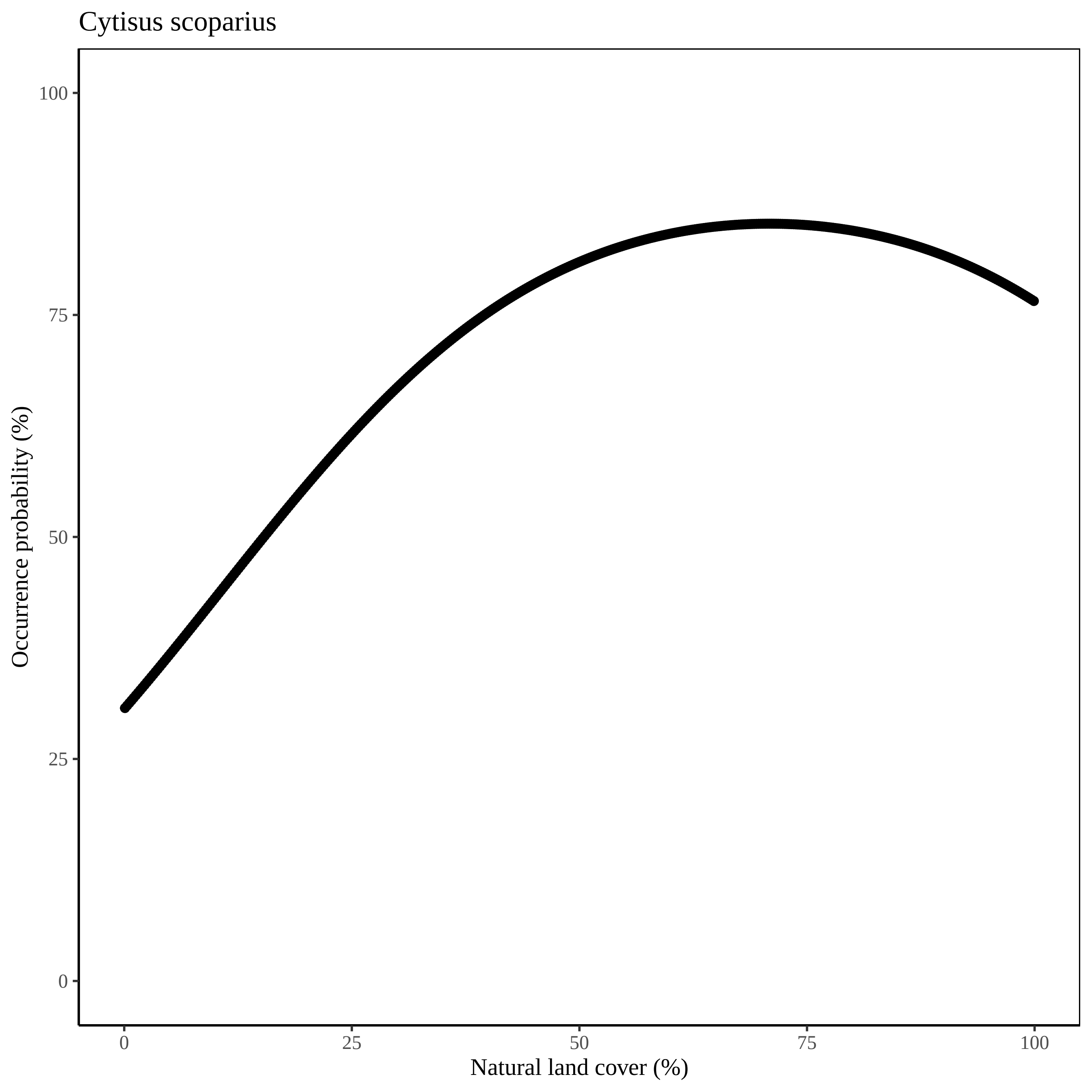
Cytisus scoparius
Occurrence probability (%)
100
75
50
25
0
0
25
50
75
100
Natural land cover (%)

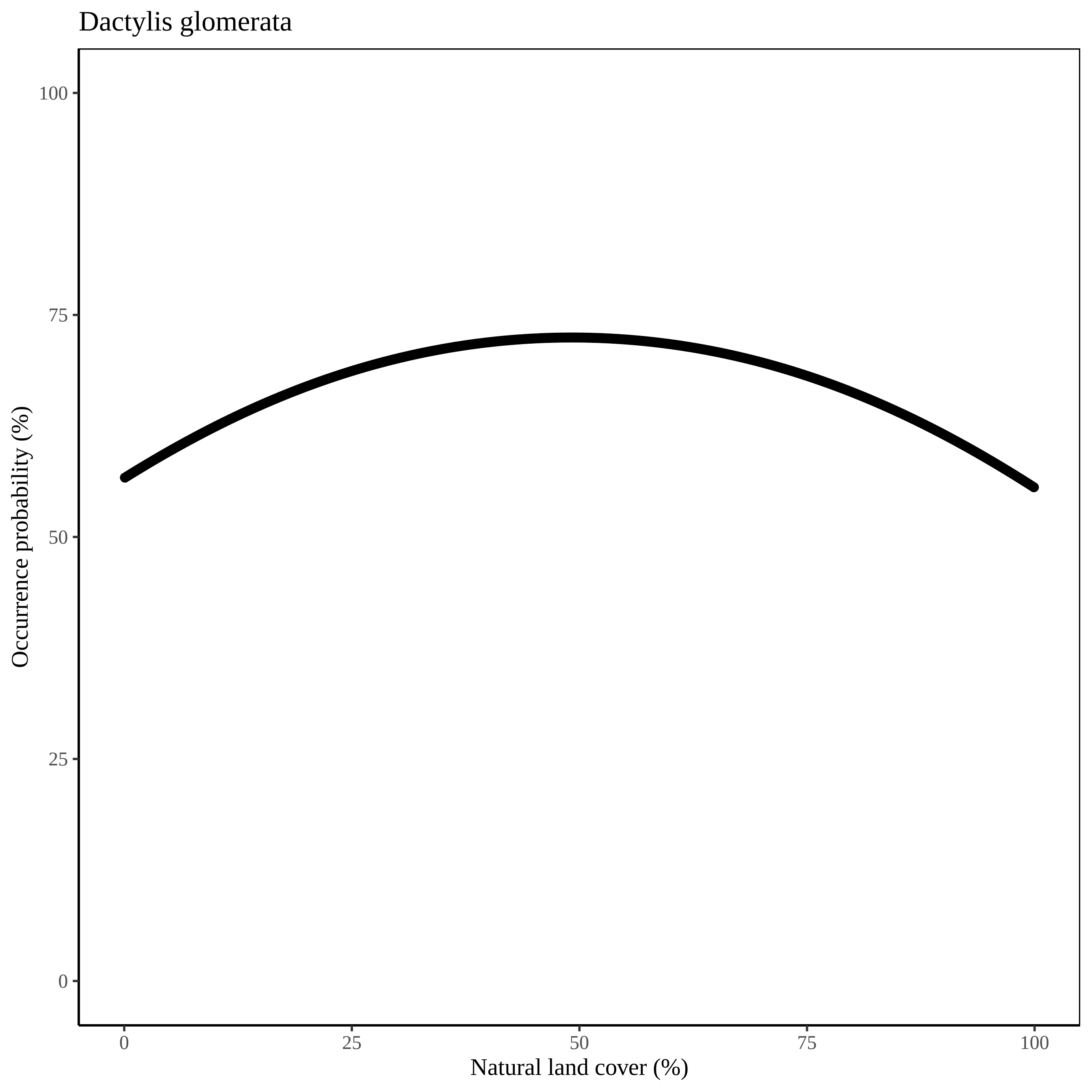
Dactylis glomerata
Occurrence probability (%)
100
75
50
25
0
0
25
50
75
100
Natural land cover (%)

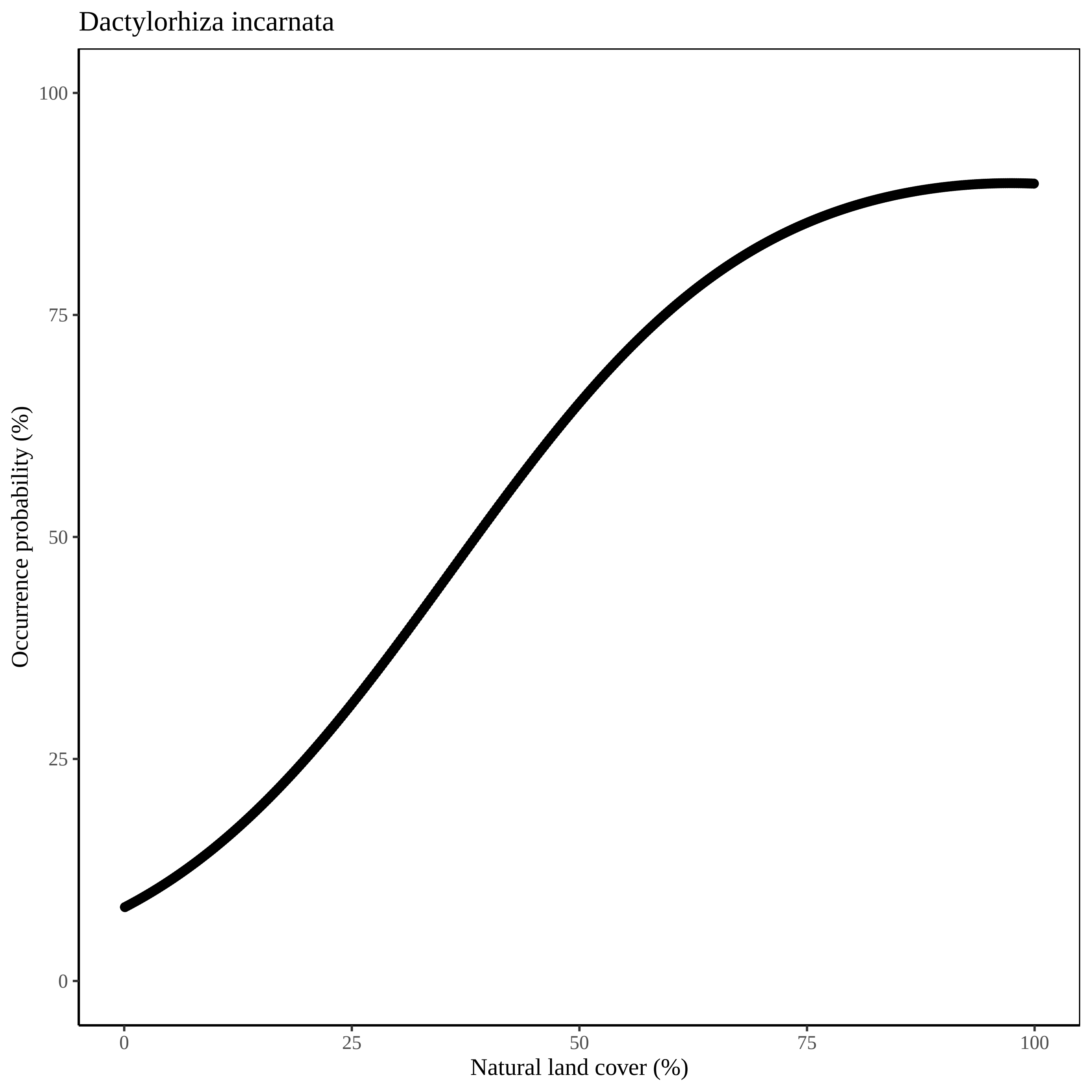

Dactylorhiza incarnata
Occurrence probability (%)
100
75
50
25
0
0
25
50
75
100
Natural land cover (%)

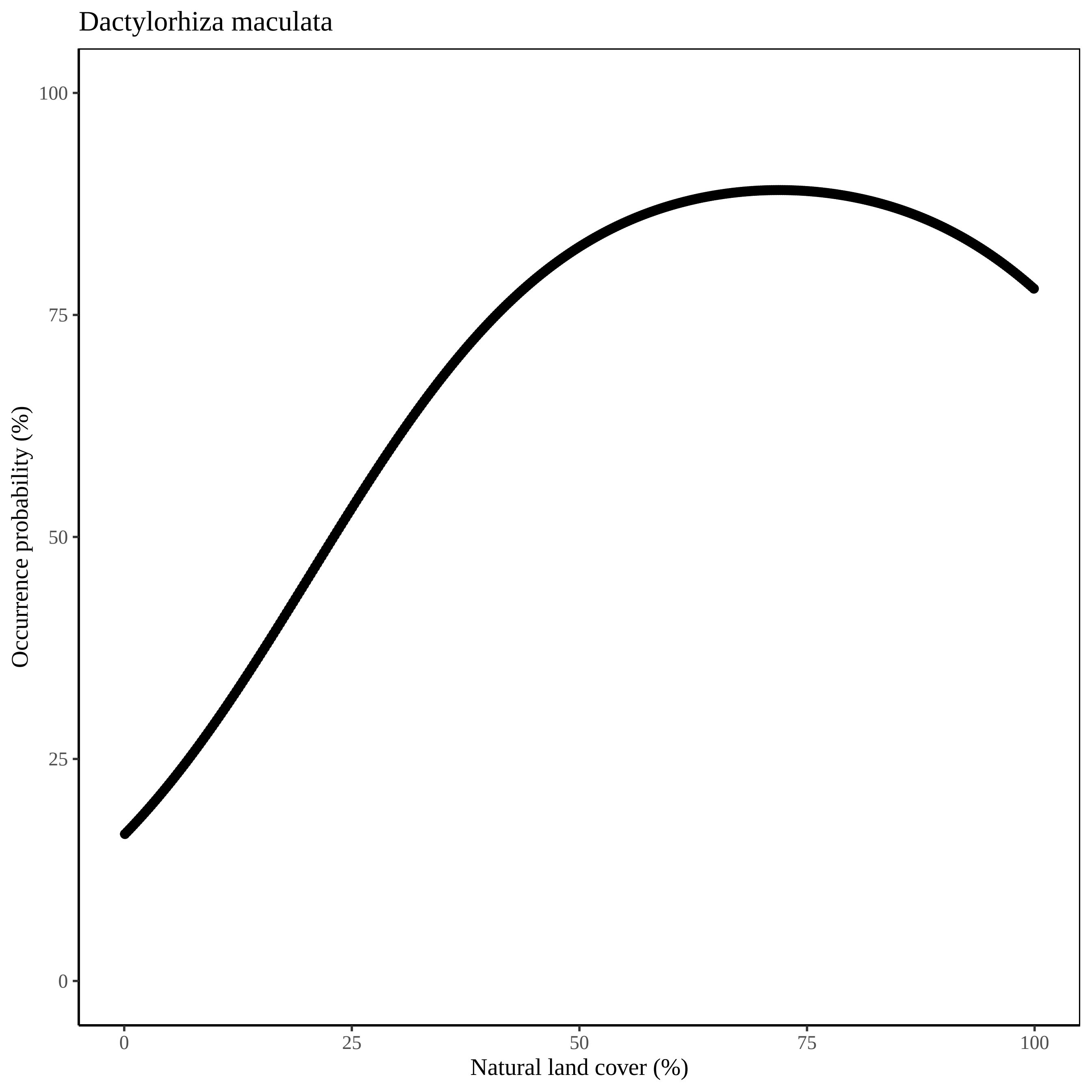
Dactylorhiza maculata
Occurrence probability (%)
100
75
50
25
0
0
25
50
75
100
Natural land cover (%)

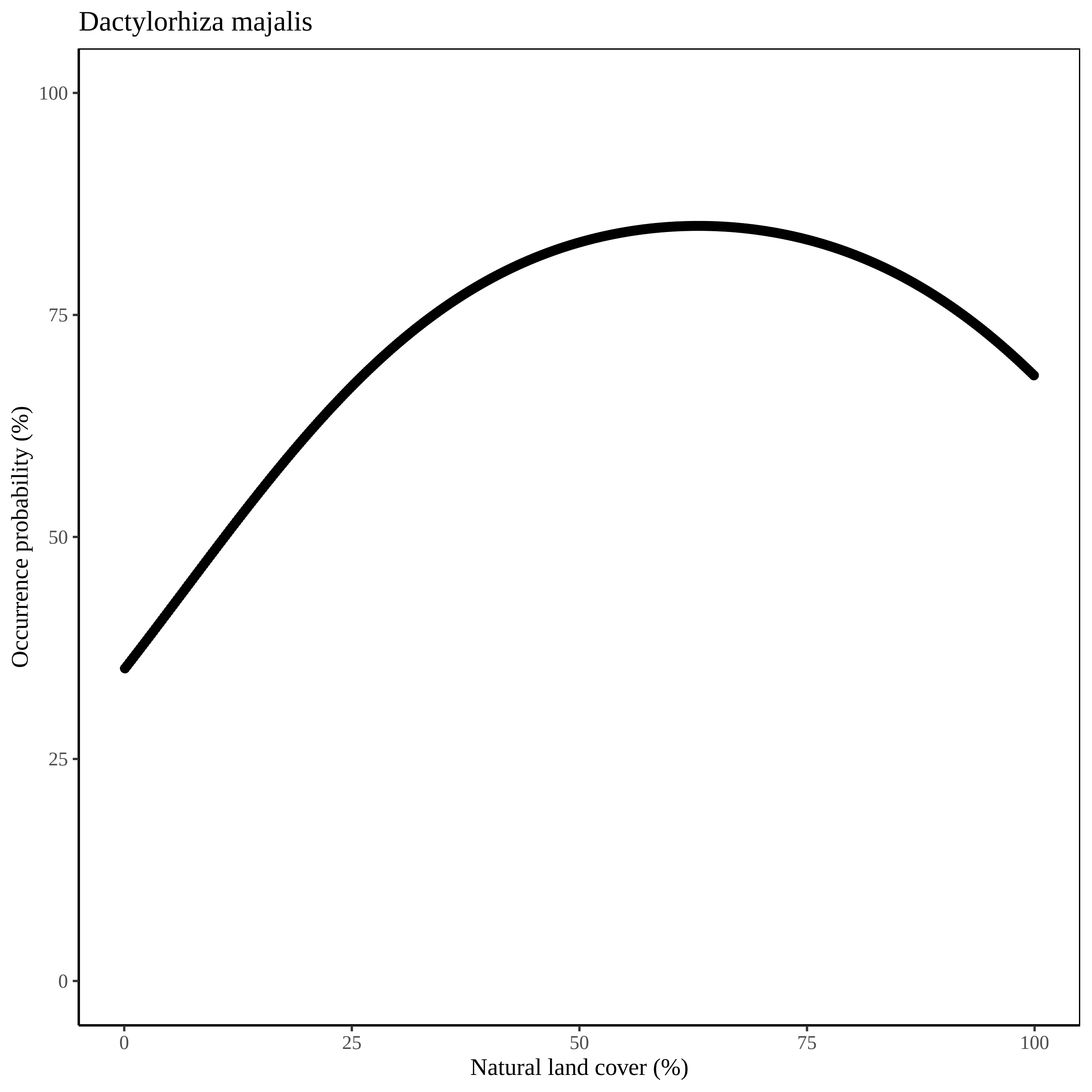
Dactylorhiza majalis
Occurrence probability (%)
100
75
50
25
0
0
25
50
75
100
Natural land cover (%)

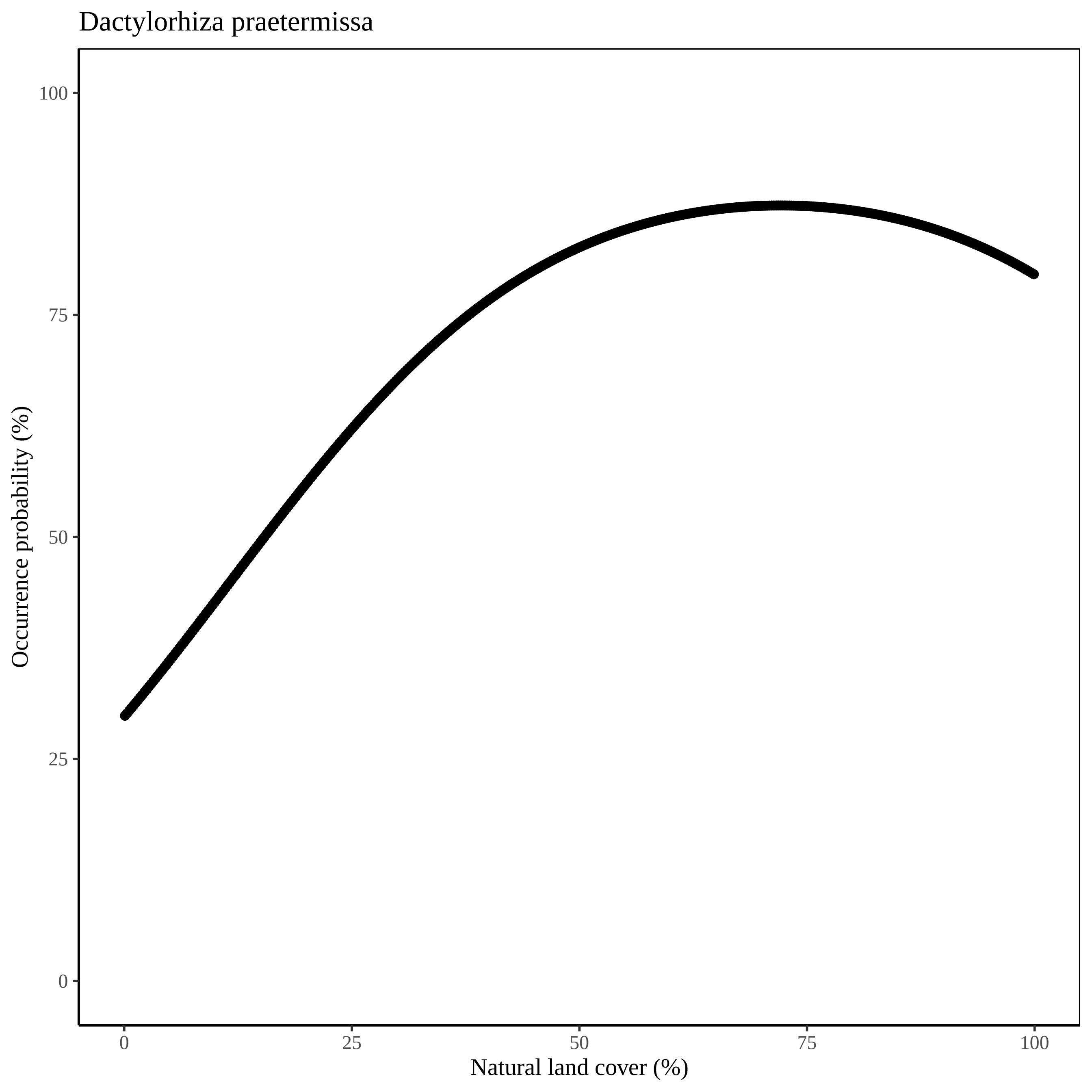

Dactylorhiza praetermissa
Occurrence probability (%)
0
25
50
75
100
Natural land cover (%)
0
25
50
75
100

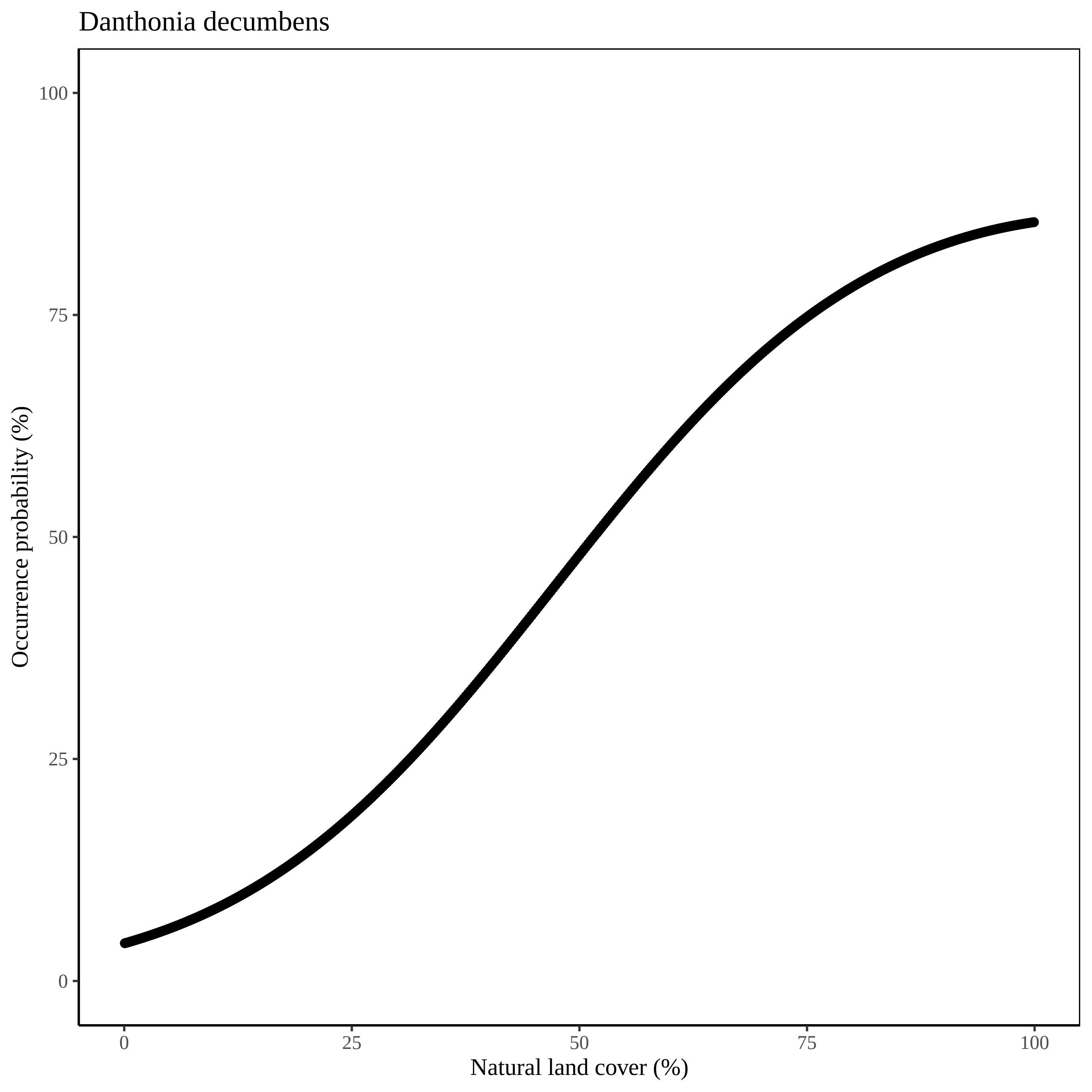

Danthonia decumbens
Occurrence probability (%)
Natural land cover (%)
0
25
50
75
100

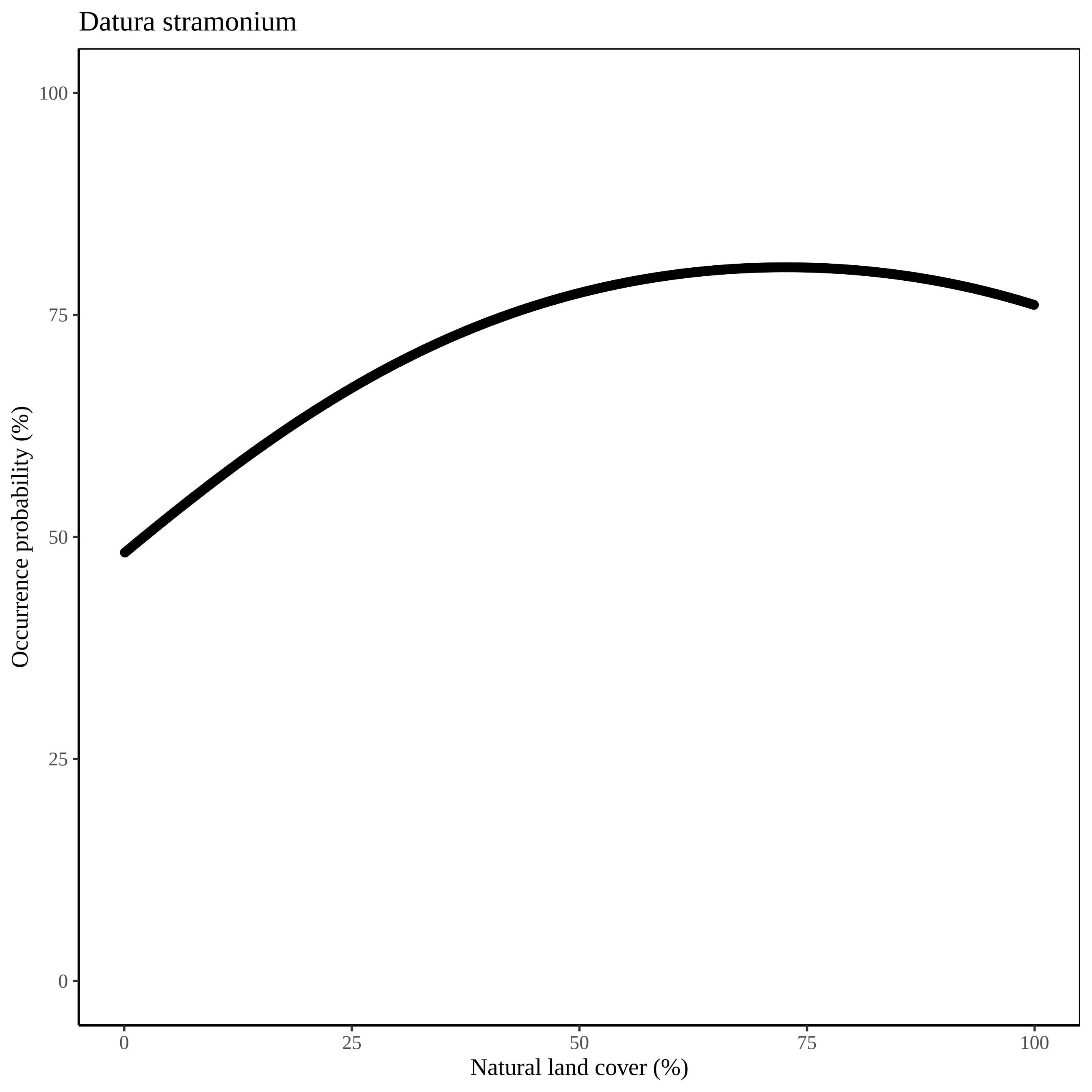
Datura stramonium
Occurrence probability (%)
100
75
50
25
0
0
25
50
75
100
Natural land cover (%)

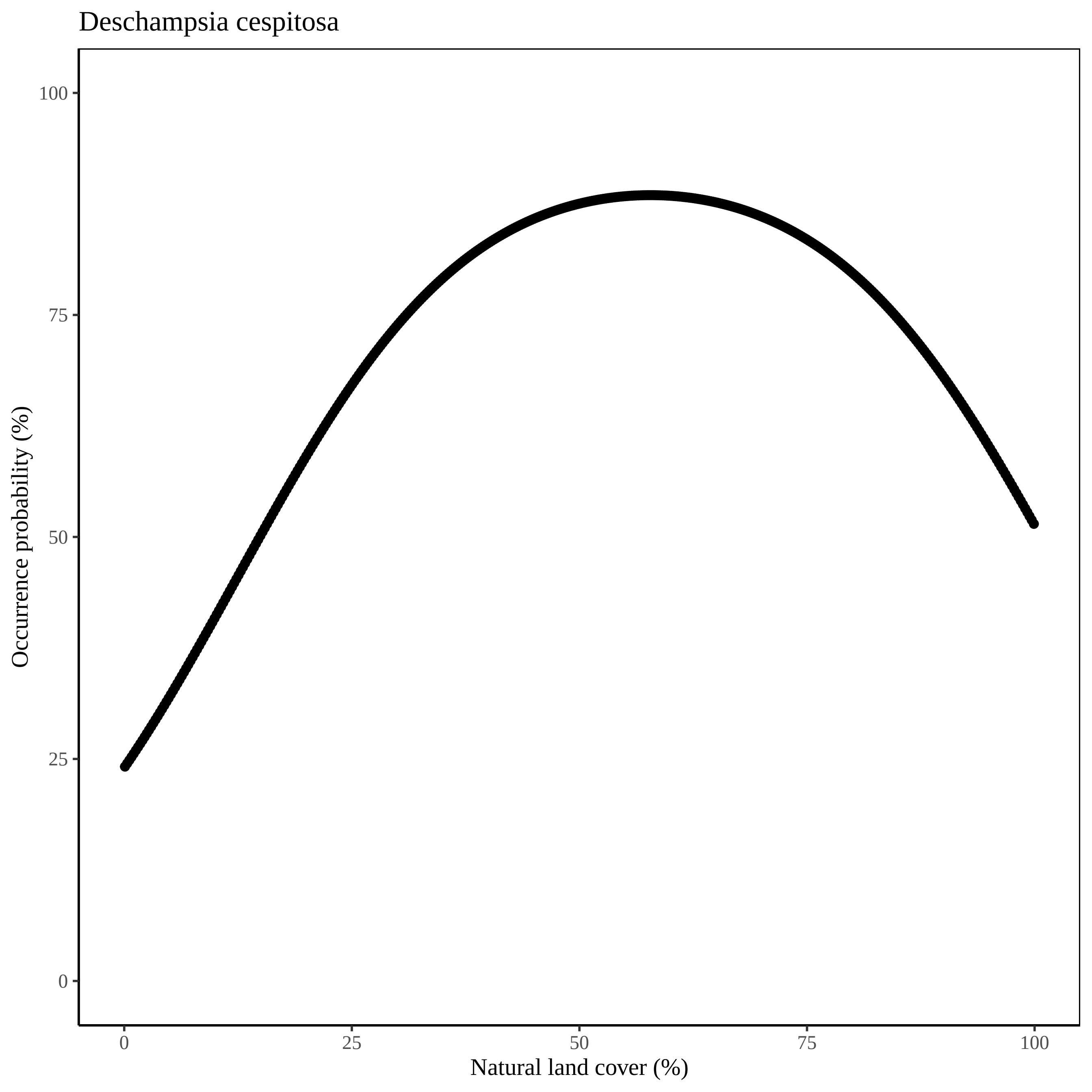
Deschampsia cespitosa
100
75
50
25
0
Occurrence probability (%)
0
25
50
75
100
Natural land cover (%)

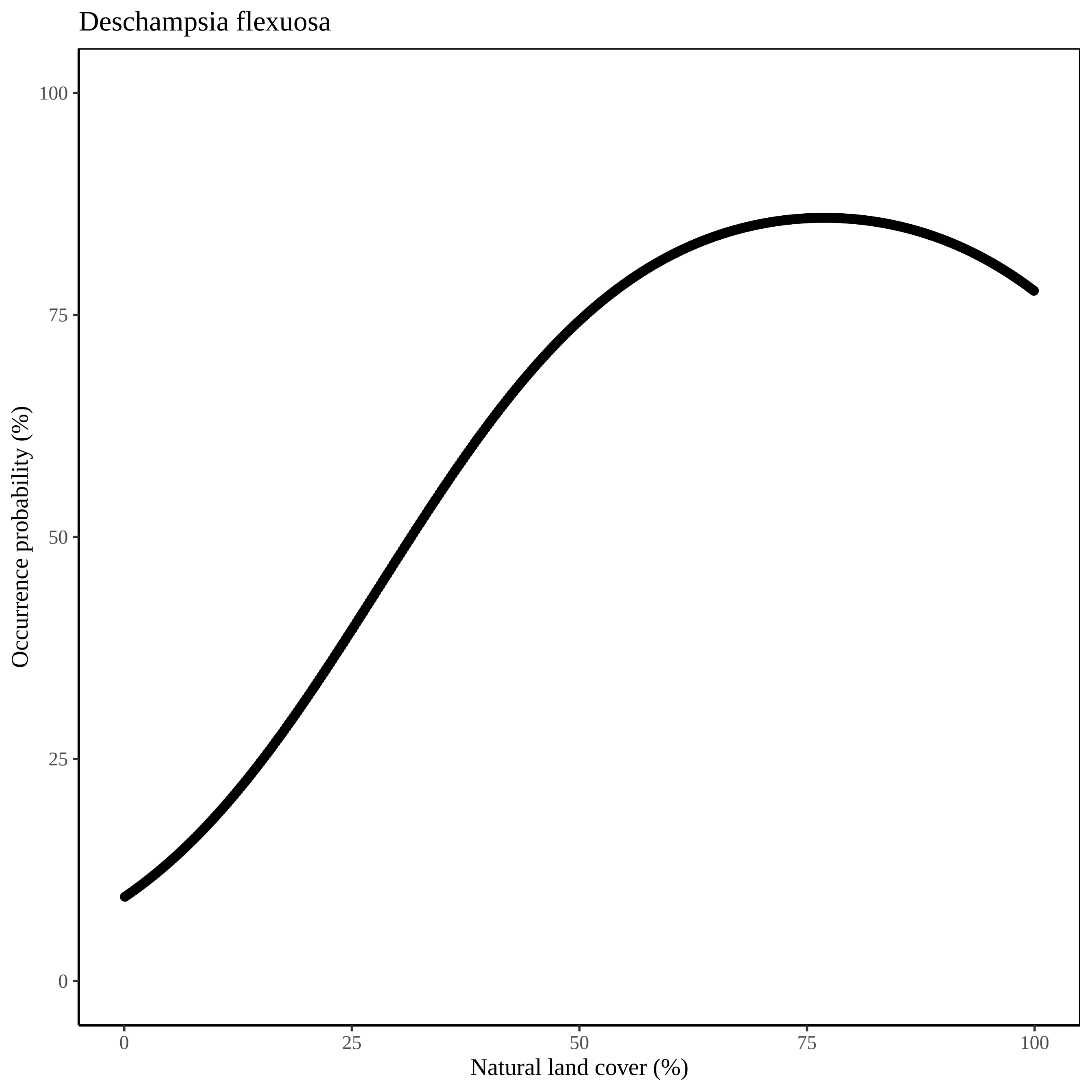

Deschampsia flexuosa
Occurrence probability (%)
100
75
50
25
0
0
25
50
75
100
Natural land cover (%)

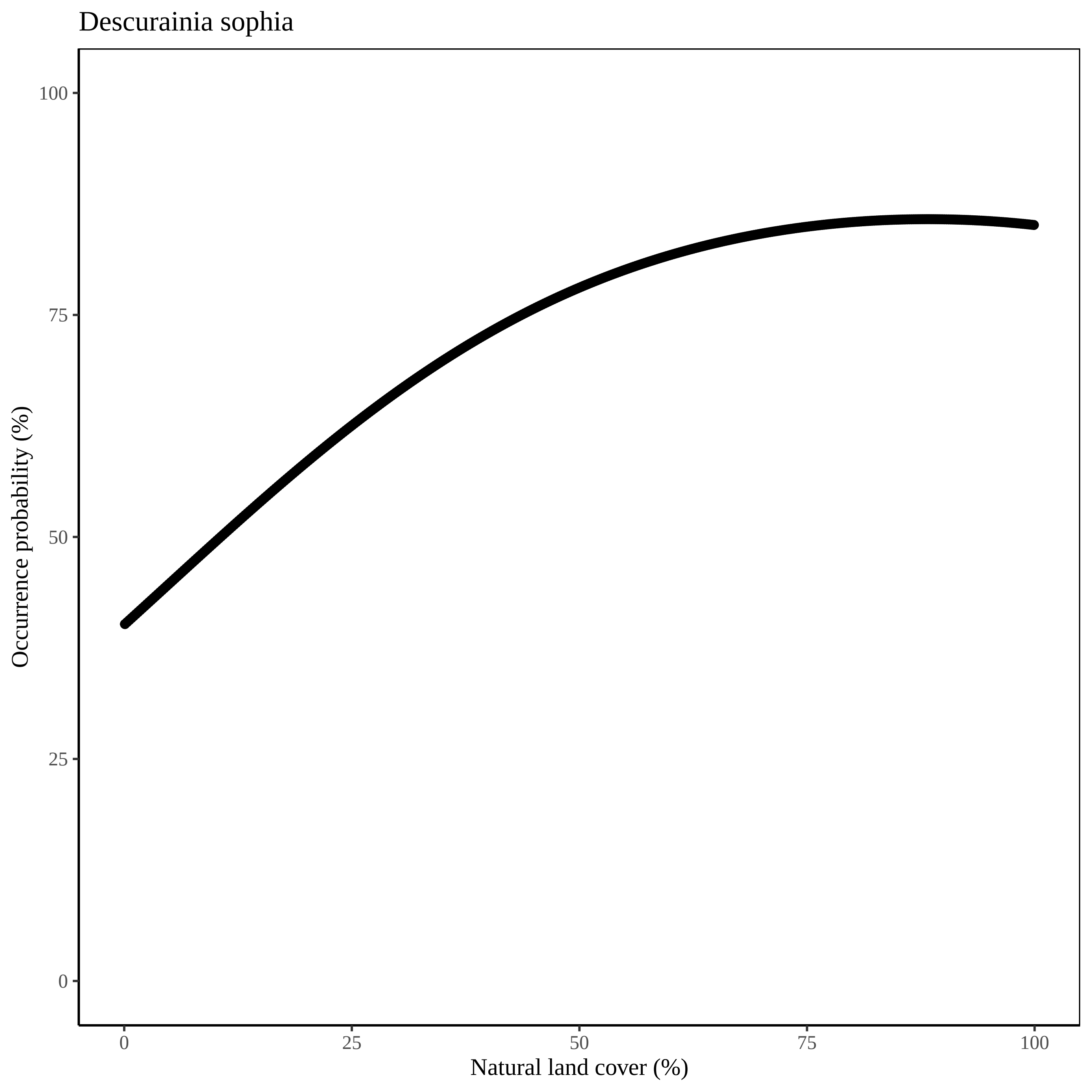
Descurainia sophia
Occurrence probability (%)
100
75
50
25
0
0
25
50
75
100
Natural land cover (%)

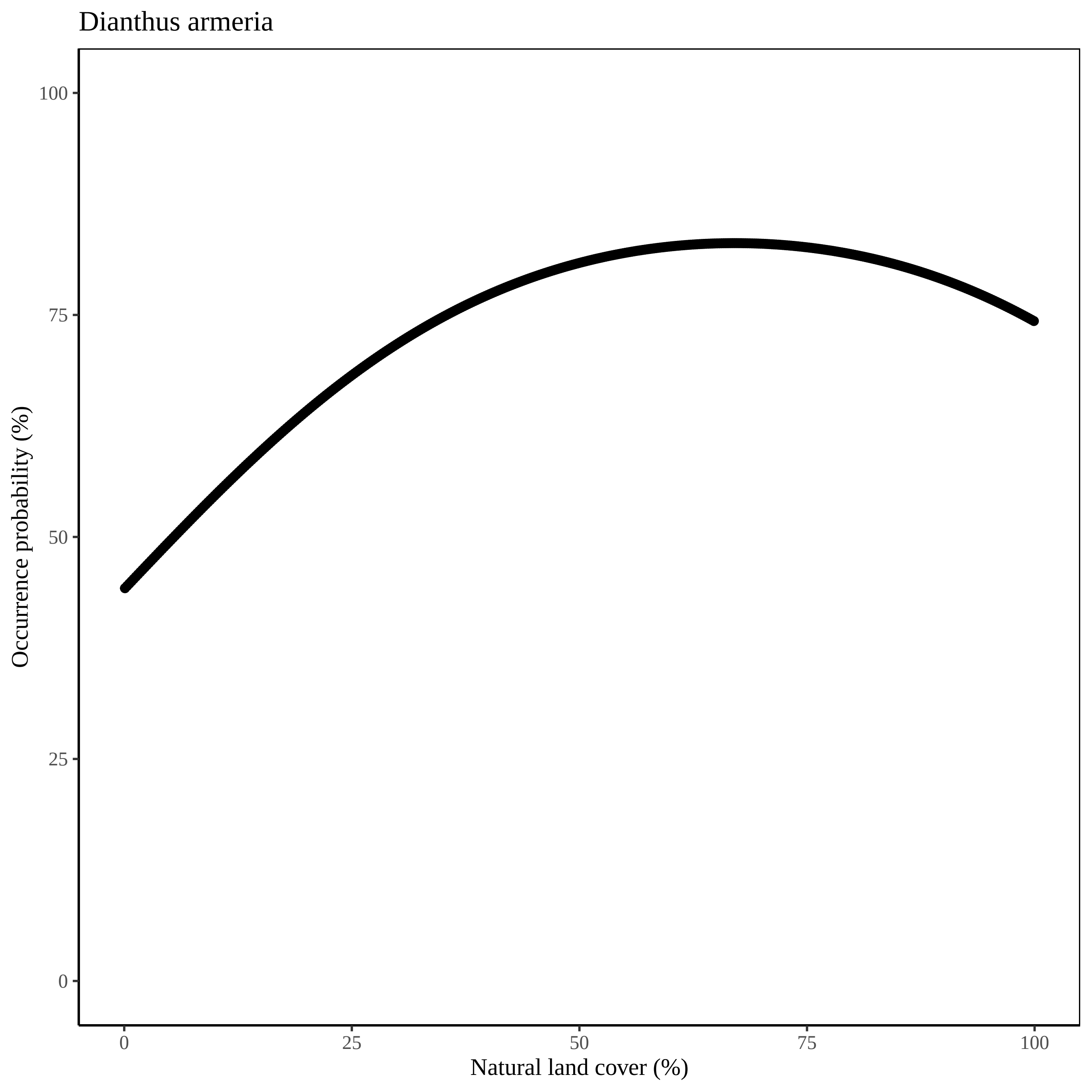

Dianthus armeria
Occurrence probability (%)
100
75
50
25
0
0
25
50
75
100
Natural land cover (%)

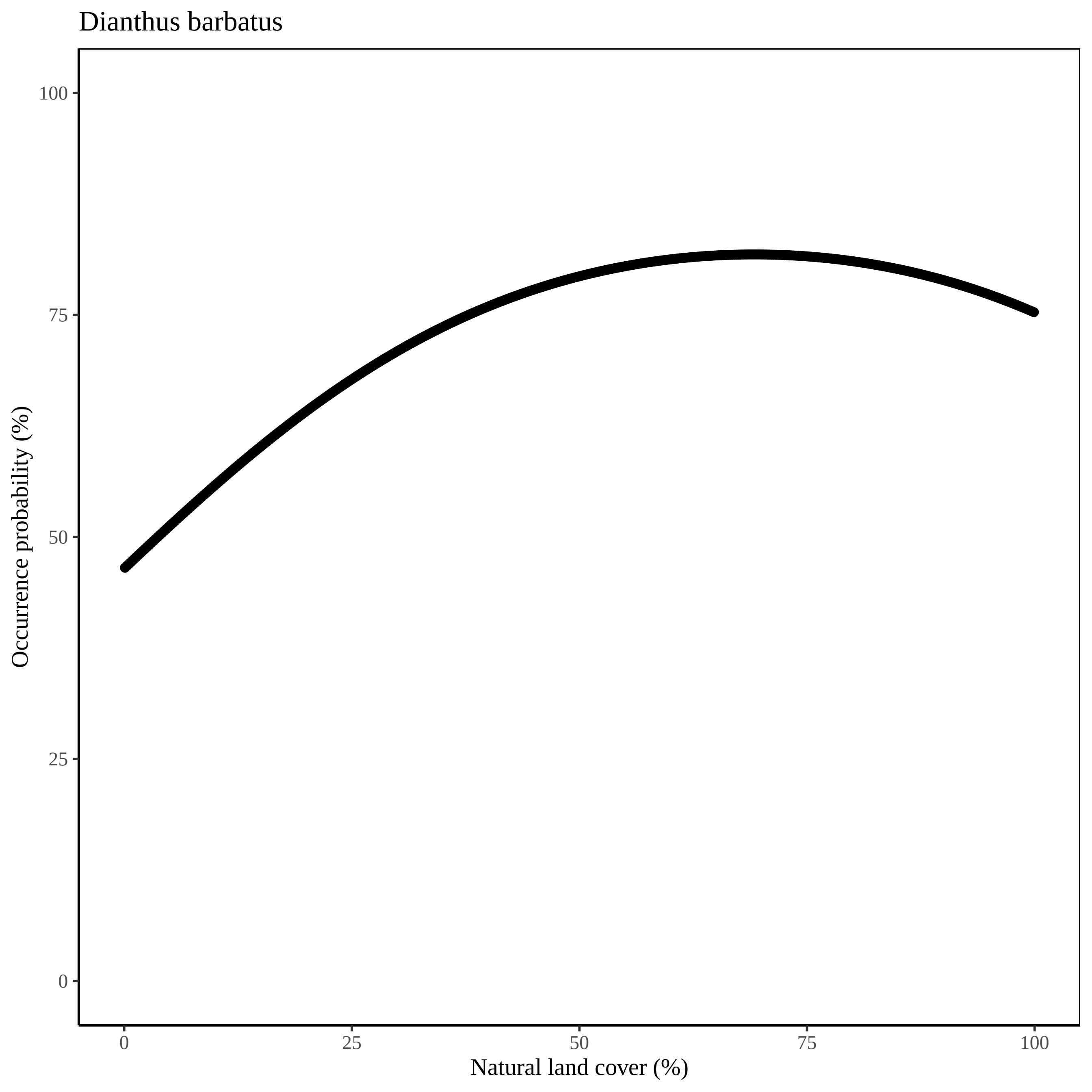

Dianthus barbatus
Occurrence probability (%)
100
75
50
25
0
0
25
50
75
100
Natural land cover (%)

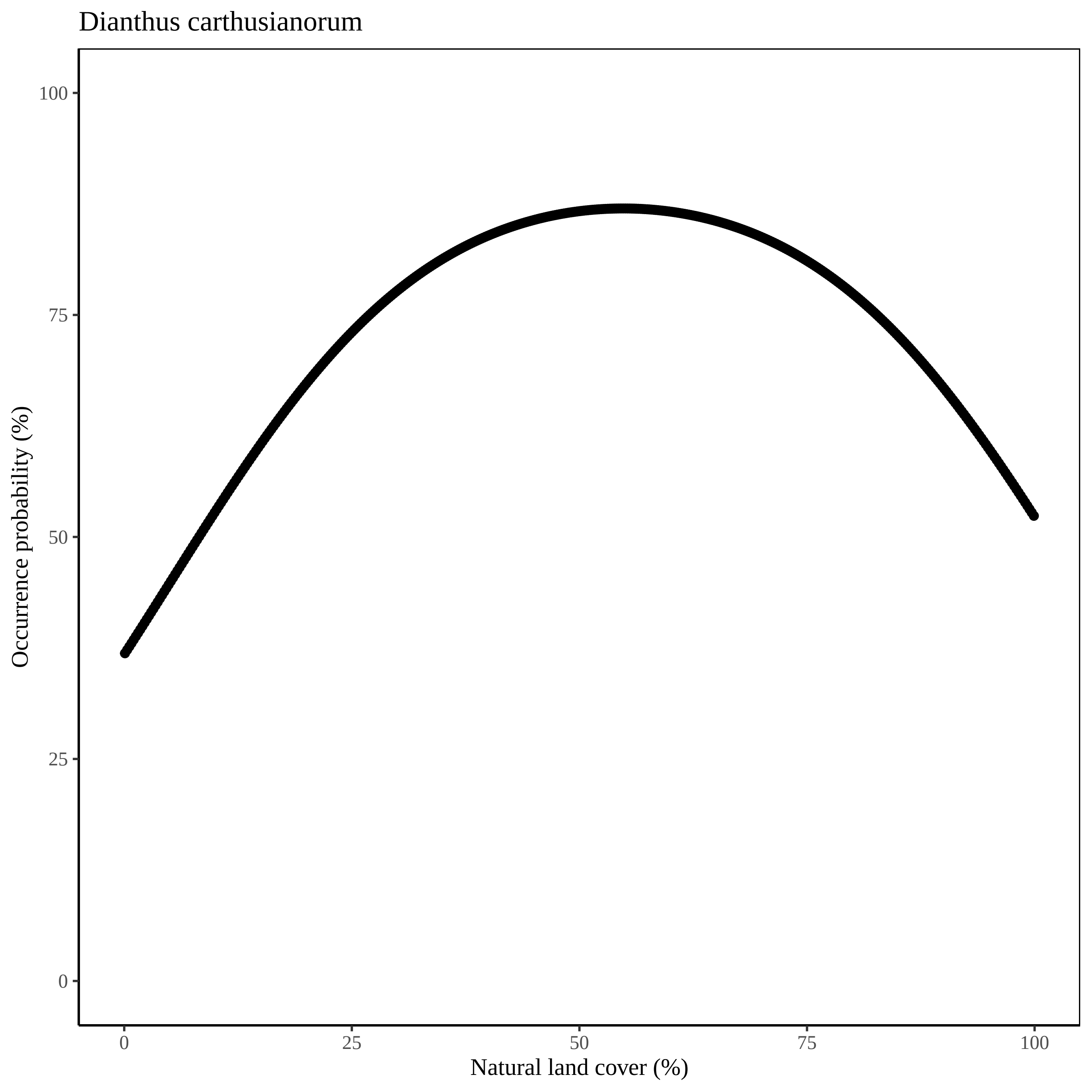
Dianthus carthusianorum
Occurrence probability (%)
100
75
50
25
0
0
25
50
75
100
Natural land cover (%)

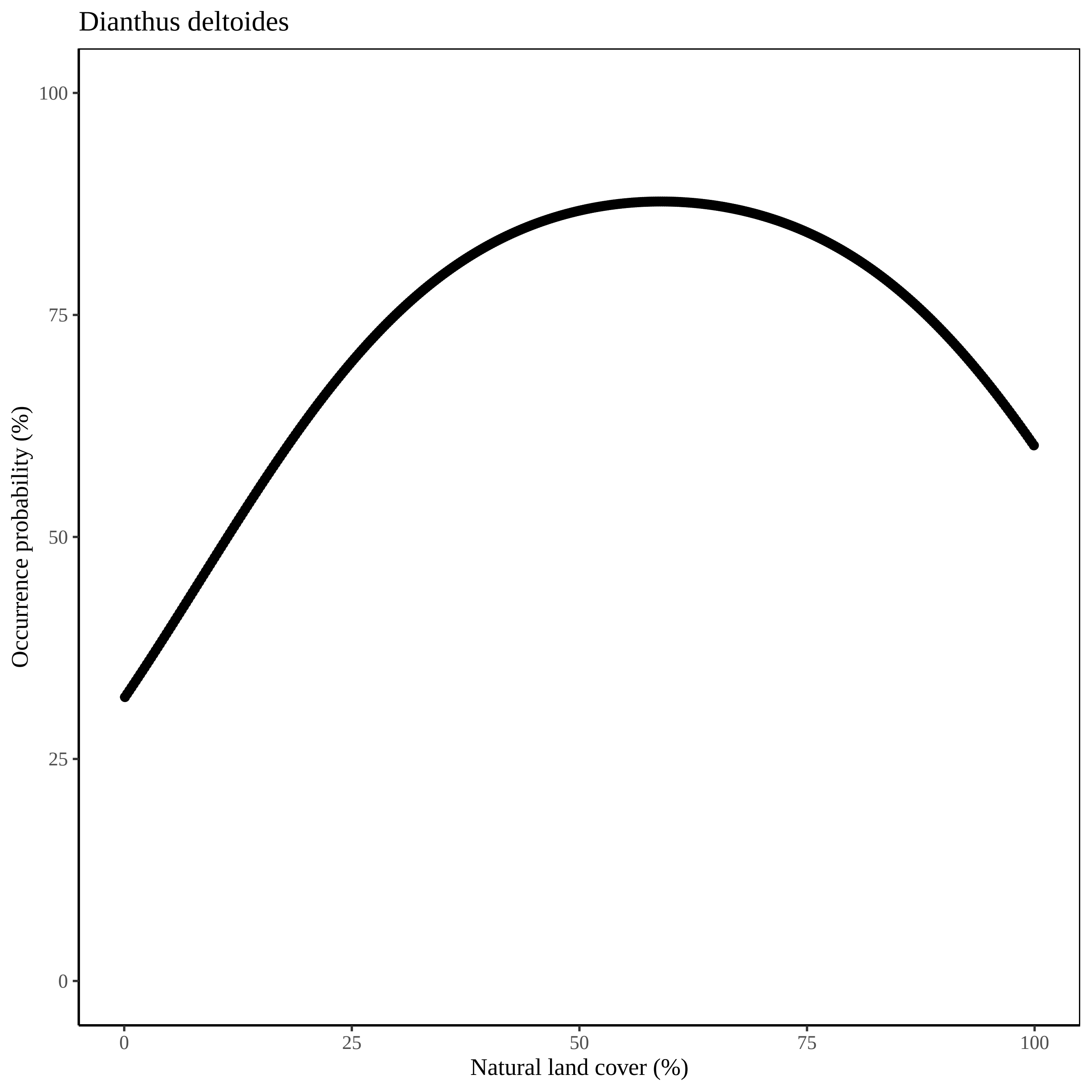

Dianthus deltoides
100
75
50
25
0
Occurrence probability (%)
0
25
50
75
100
Natural land cover (%)

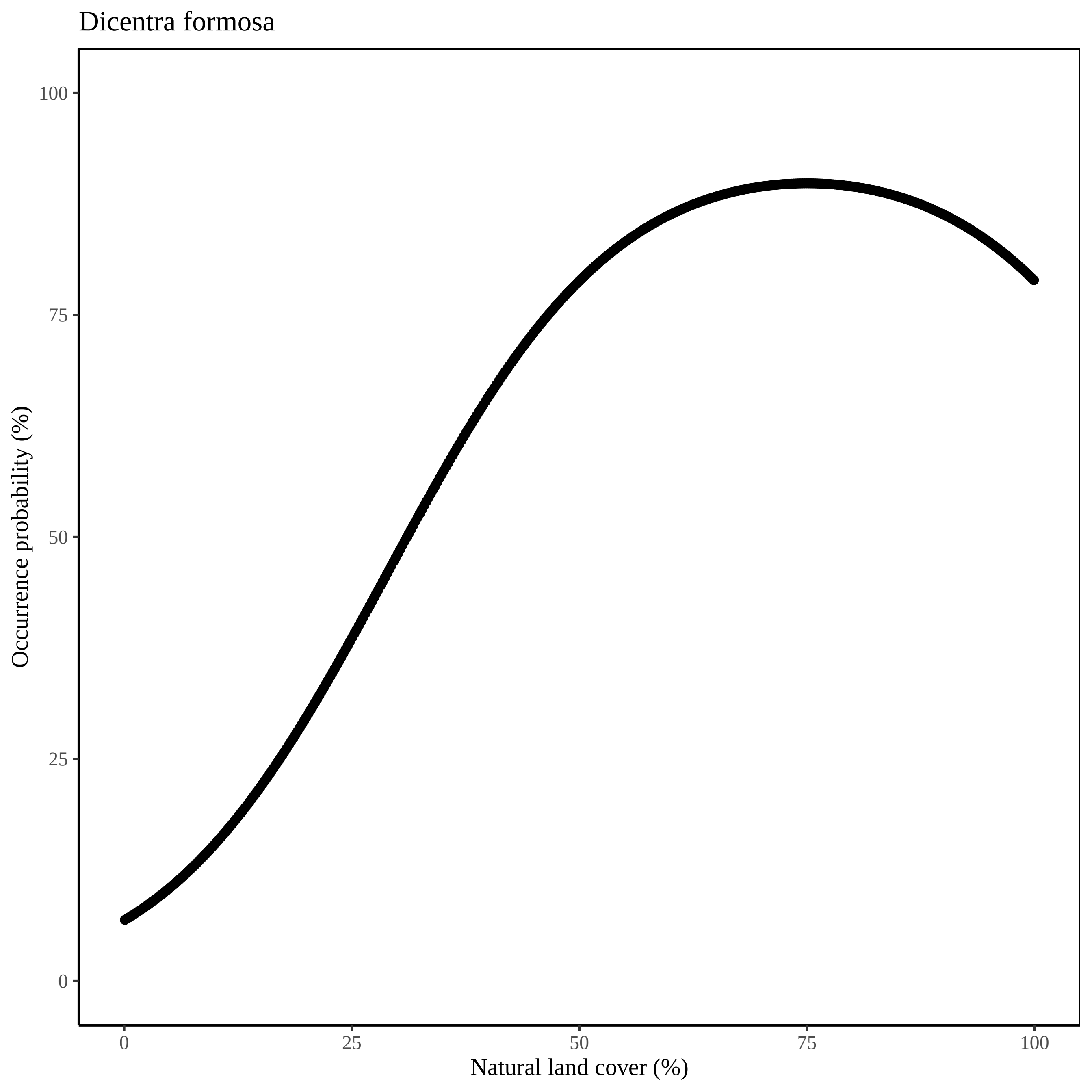

Dicentra formosa
Occurrence probability (%)
100
75
50
25
0
Natural land cover (%)
0
25
50
75
100

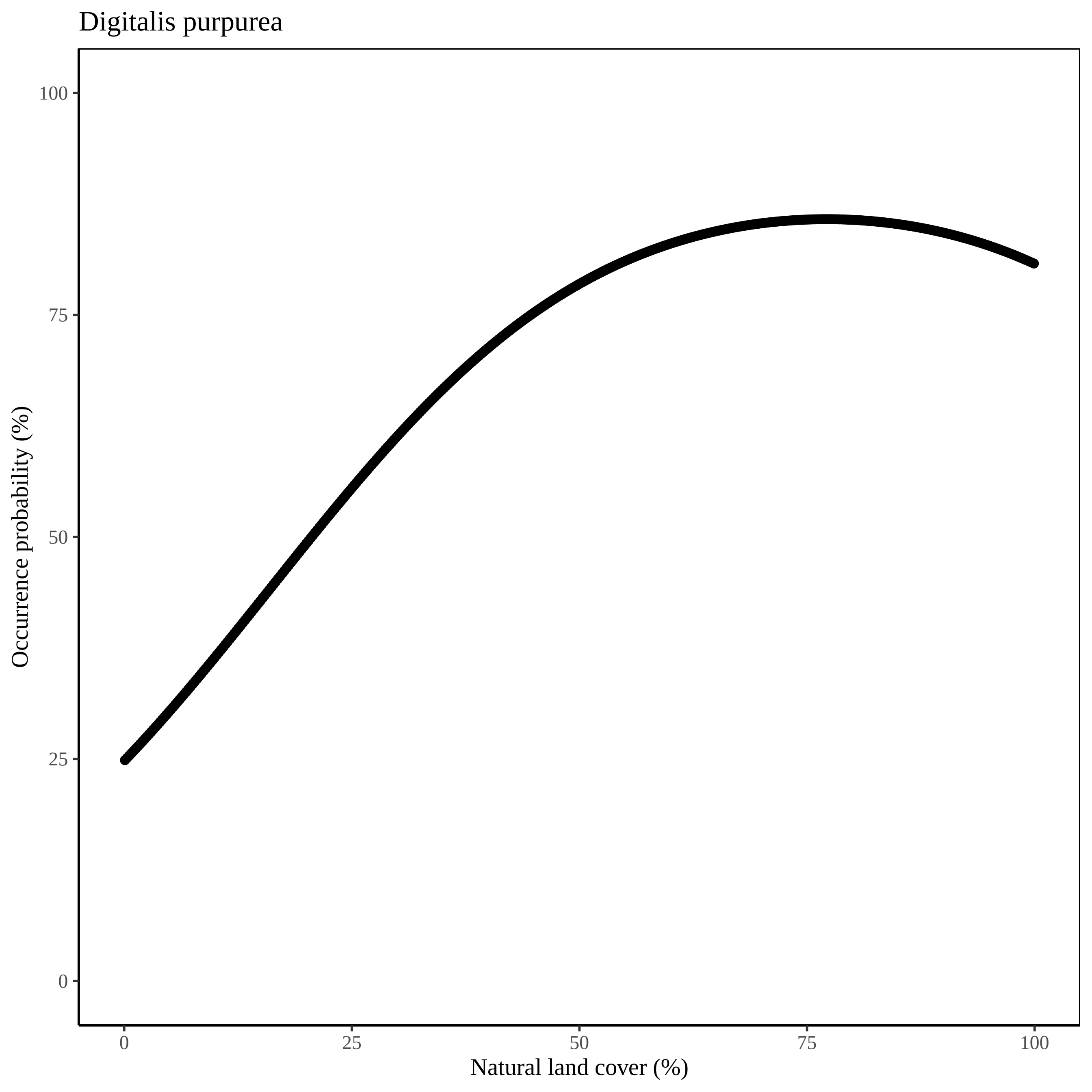

Digitalis purpurea
100
75
50
25
0
Occurrence probability (%)
0
25
50
75
100
Natural land cover (%)

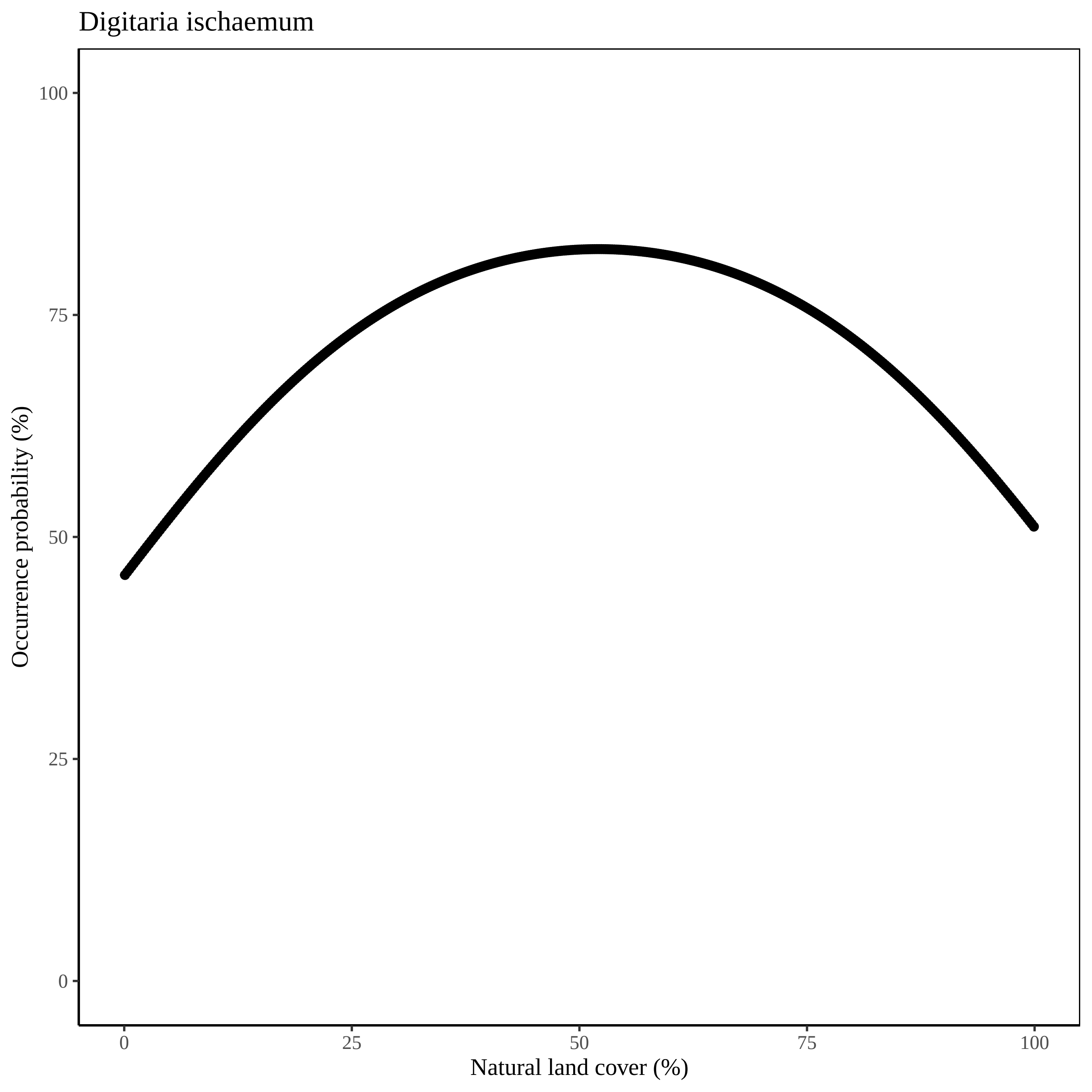
Digitaria ischaemum
Occurrence probability (%)
Natural land cover (%)
0
25
50
75
100
0
25
50
75
100

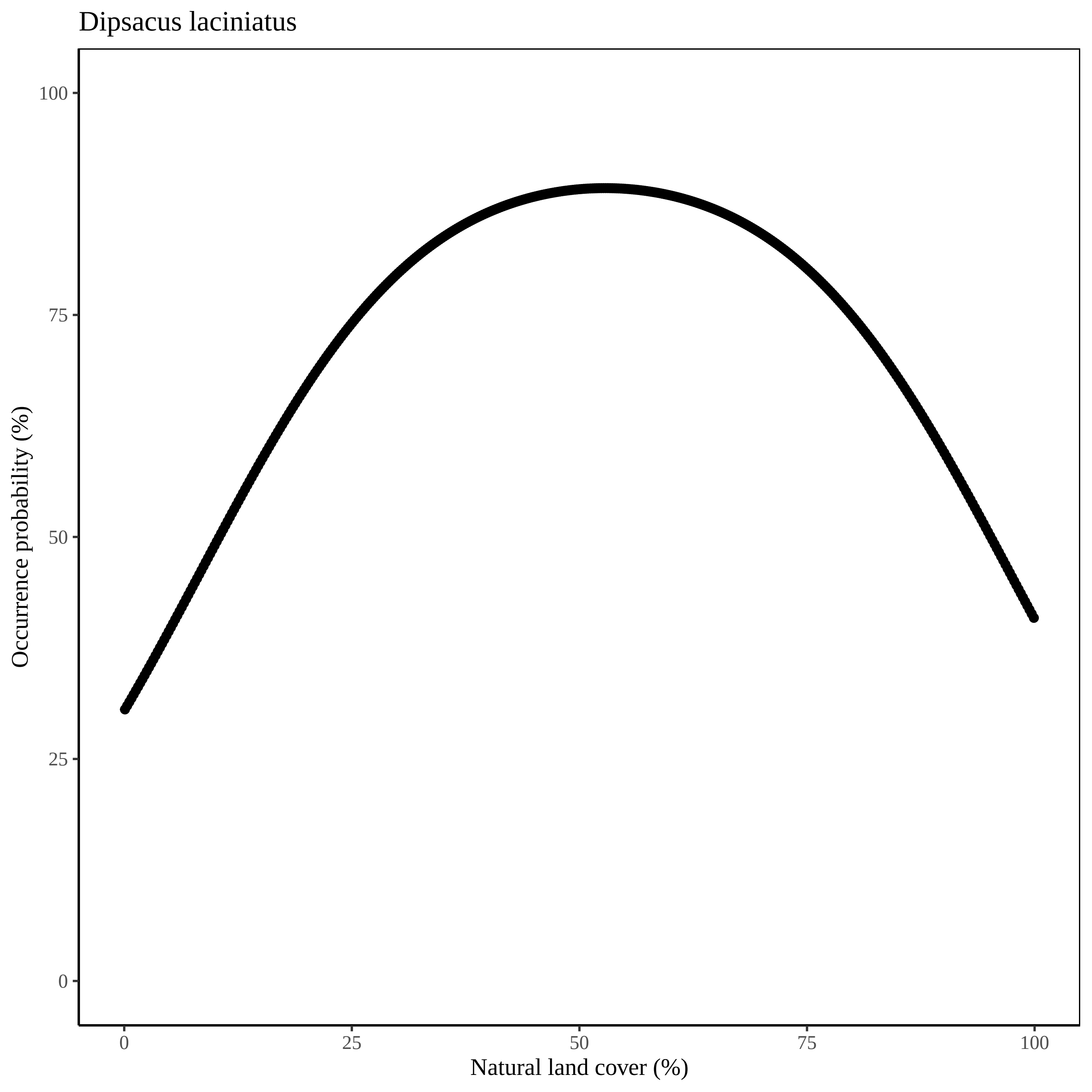
Dipsacus laciniatus
Occurrence probability (%)
100
75
50
25
0
0
25
50
75
100
Natural land cover (%)

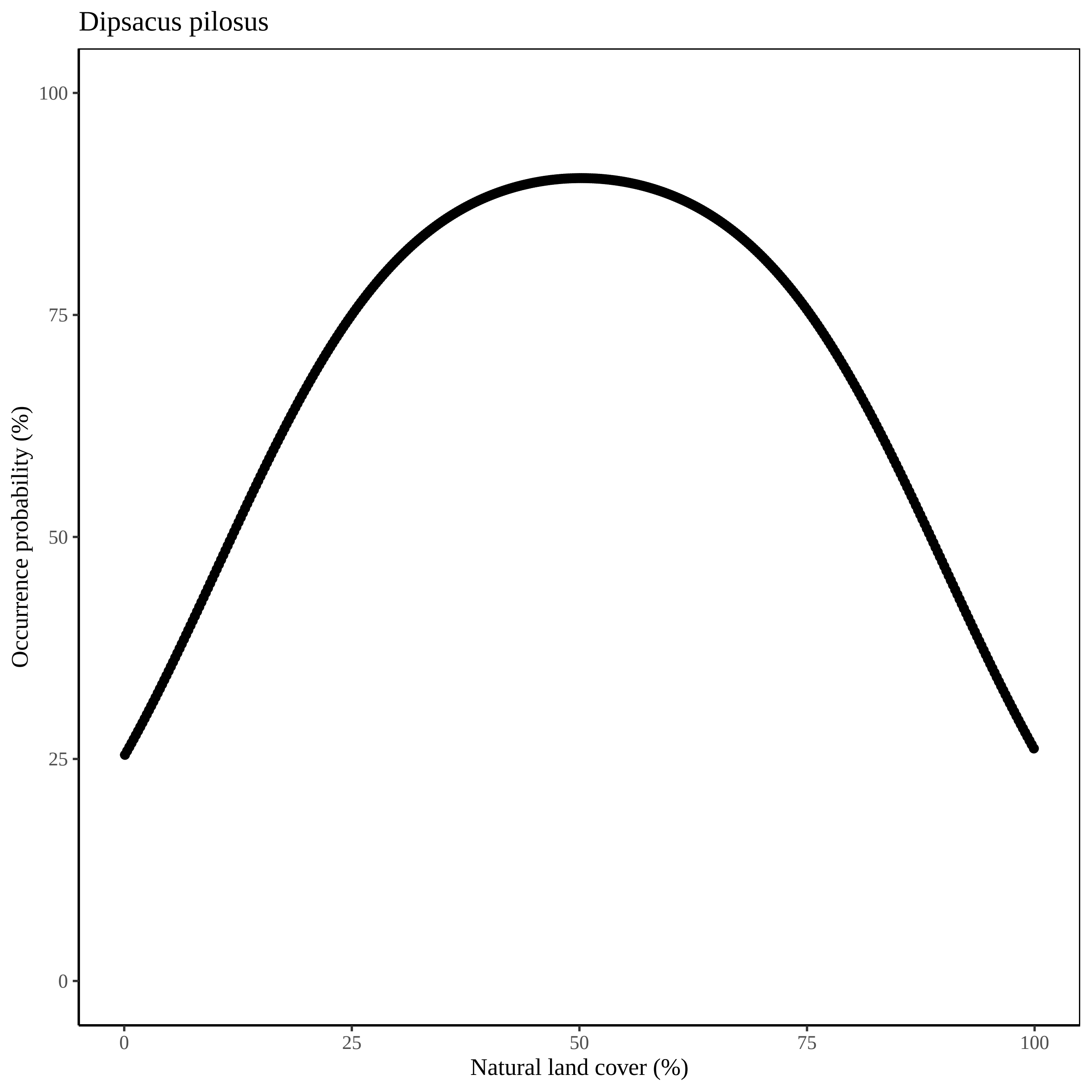
Dipsacus pilosus
100
75
50
25
0
Occurrence probability (%)
0
25
50
75
100
Natural land cover (%)

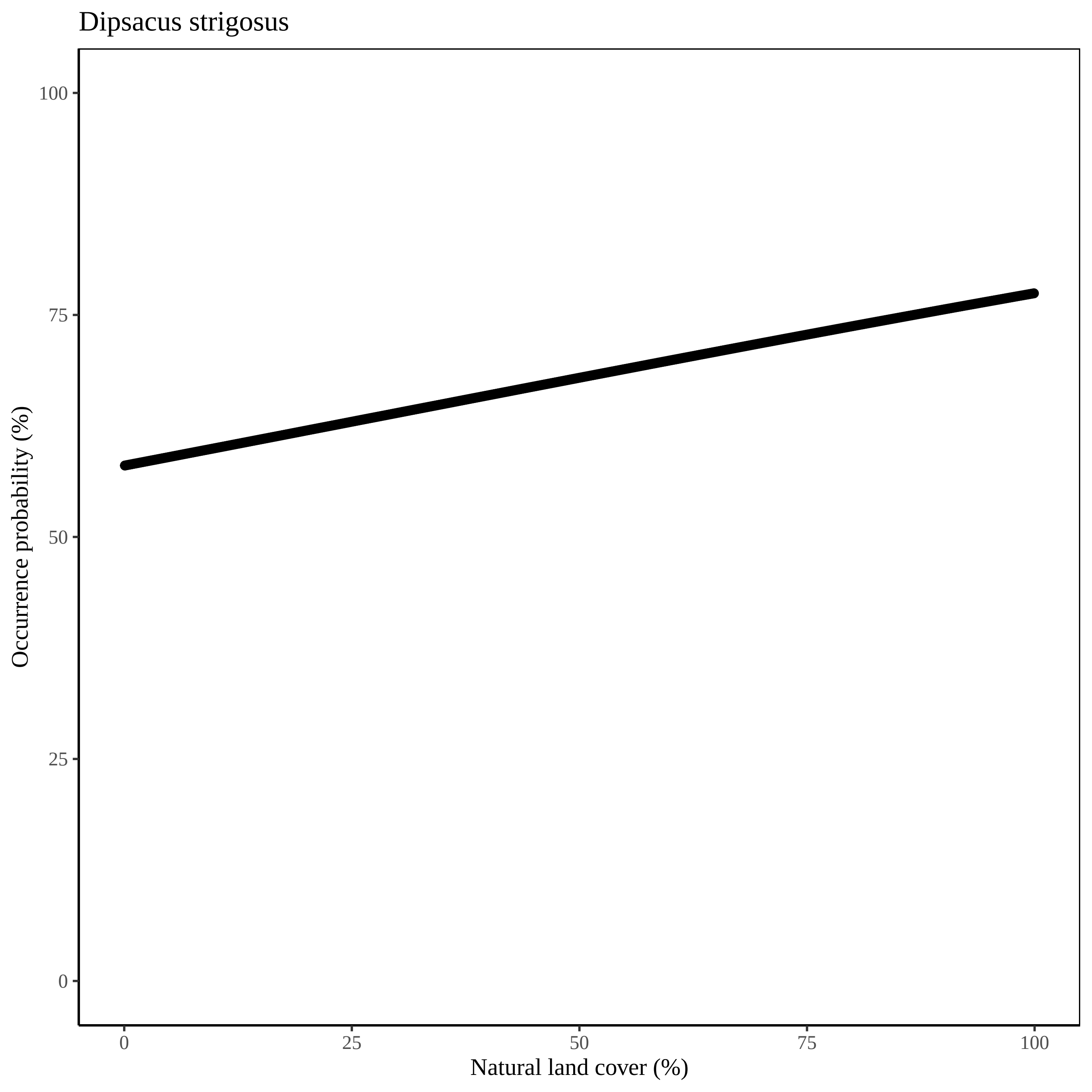
Dipsacus strigosus
Occurrence probability (%)
100
75
50
25
0
0
25
50
75
100
Natural land cover (%)

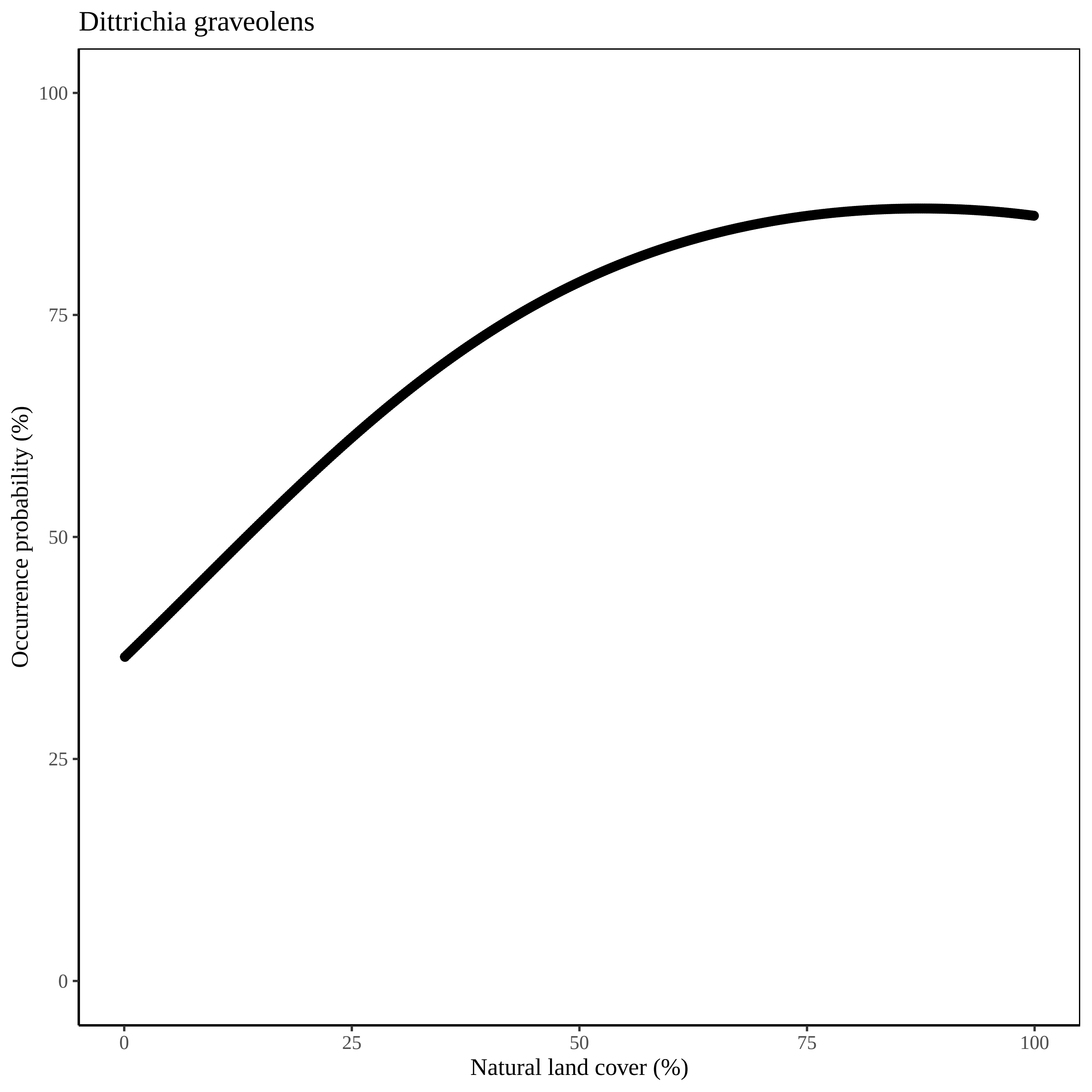
Dittrichia graveolens
Occurrence probability (%)
100
75
50
25
0
0
25
50
75
100
Natural land cover (%)

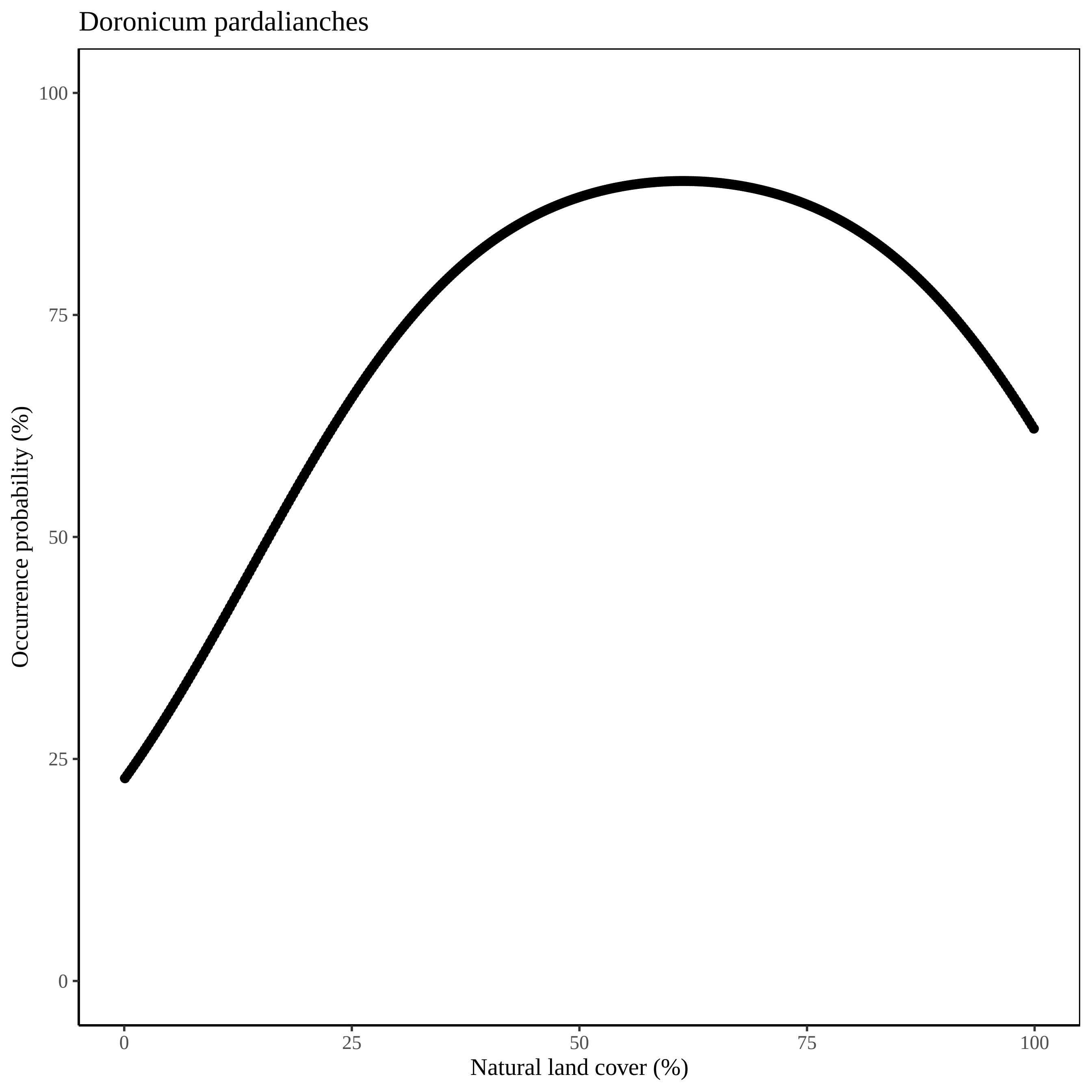
Doronicum pardalianches
Occurrence probability (%)
0
25
50
75
100
Natural land cover (%)
0
25
50
75
100

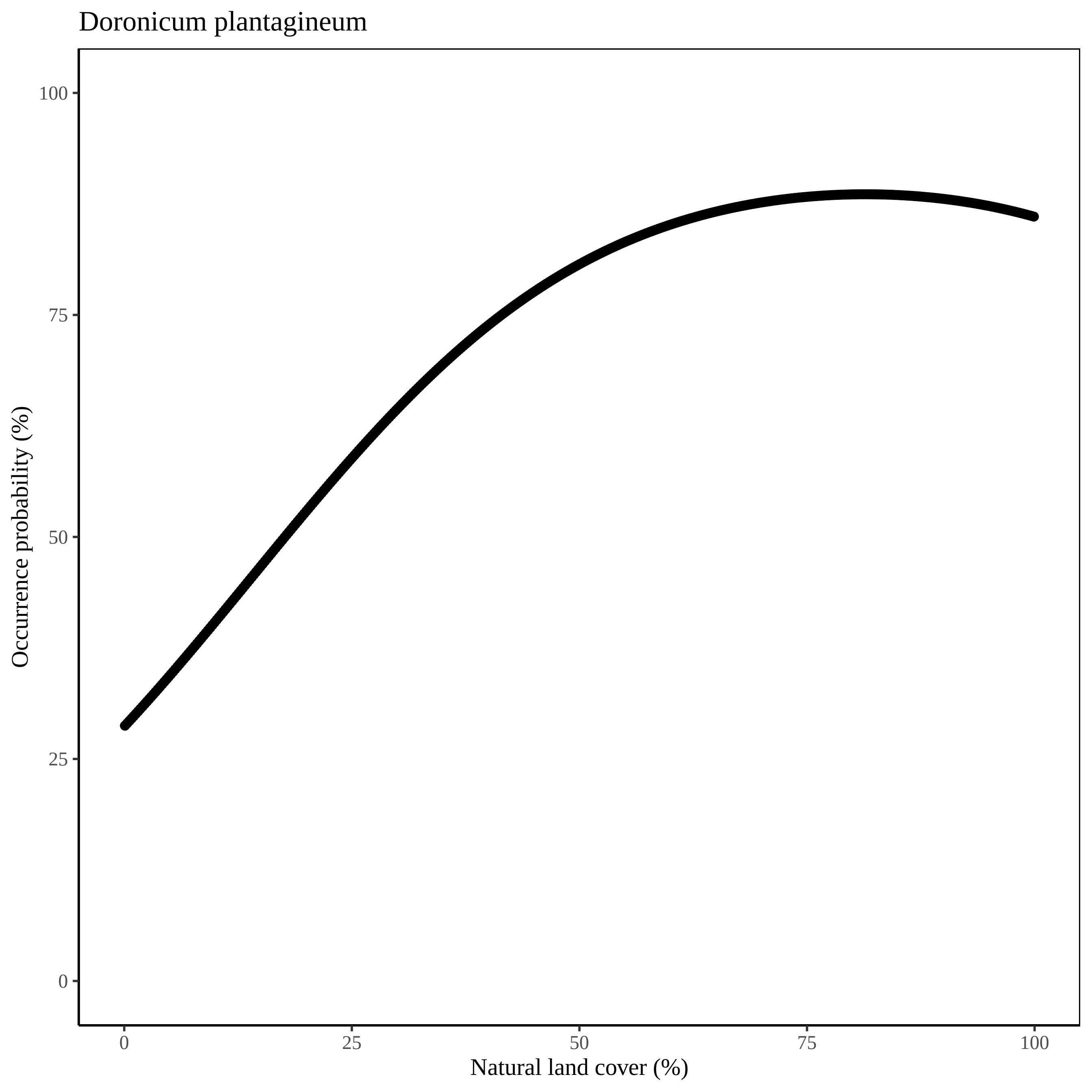

Doronicum plantagineum
Occurrence probability (%)
100
75
50
25
0
0
25
50
75
100
Natural land cover (%)

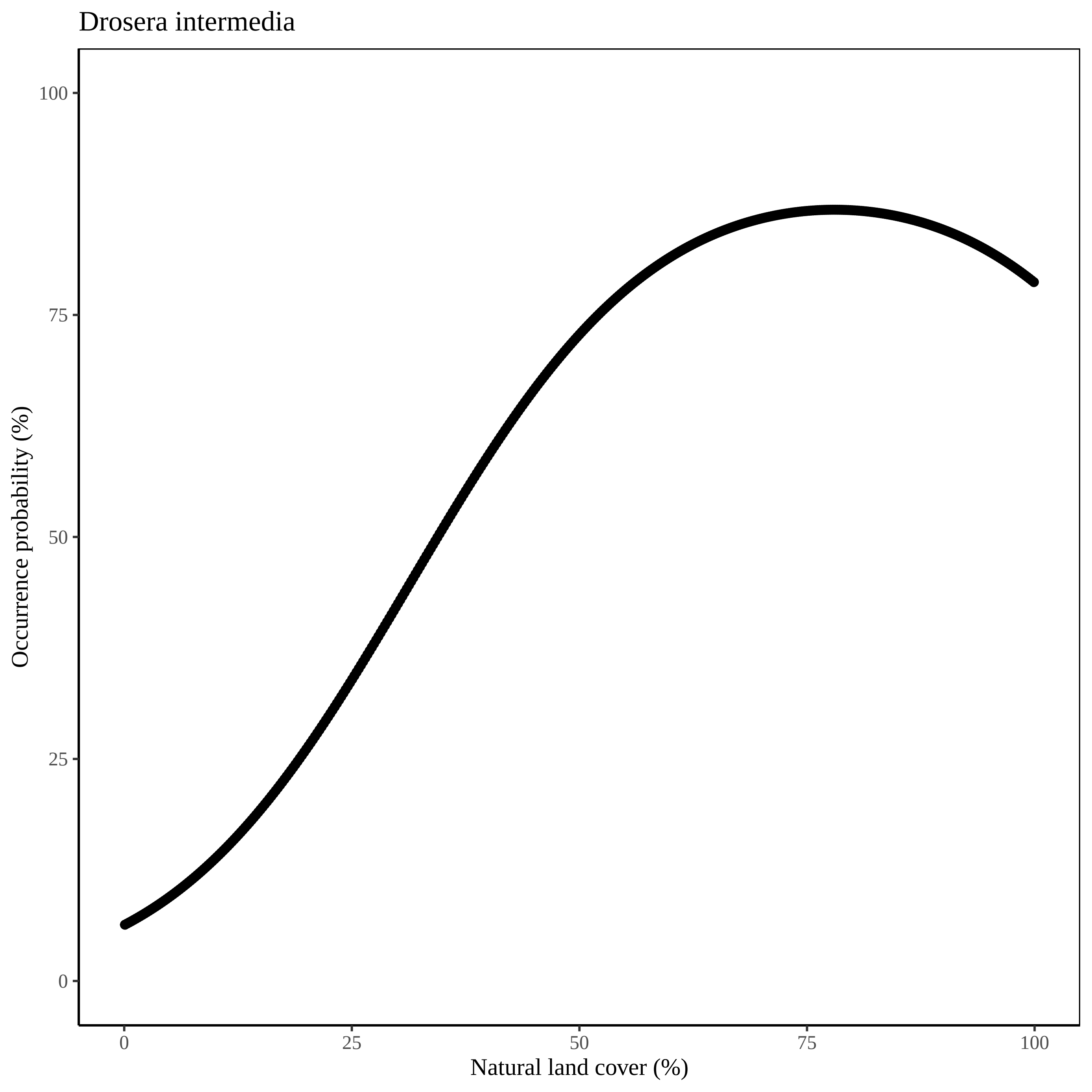

Drosera intermedia
Occurrence probability (%)
100
75
50
25
0
0
25
50
75
100
Natural land cover (%)

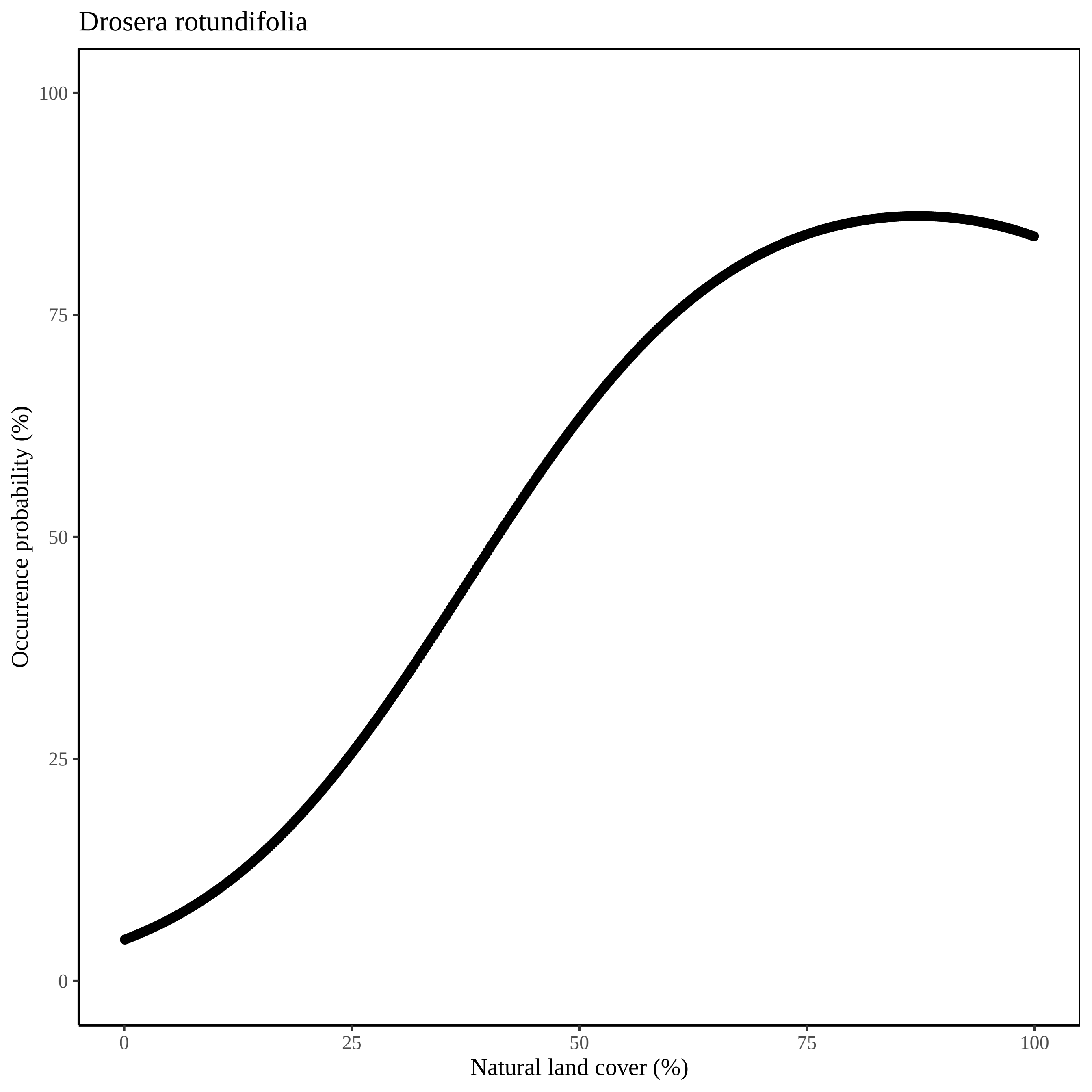
Drosera rotundifolia
Occurrence probability (%)
100
75
50
25
0
0
25
50
75
100
Natural land cover (%)

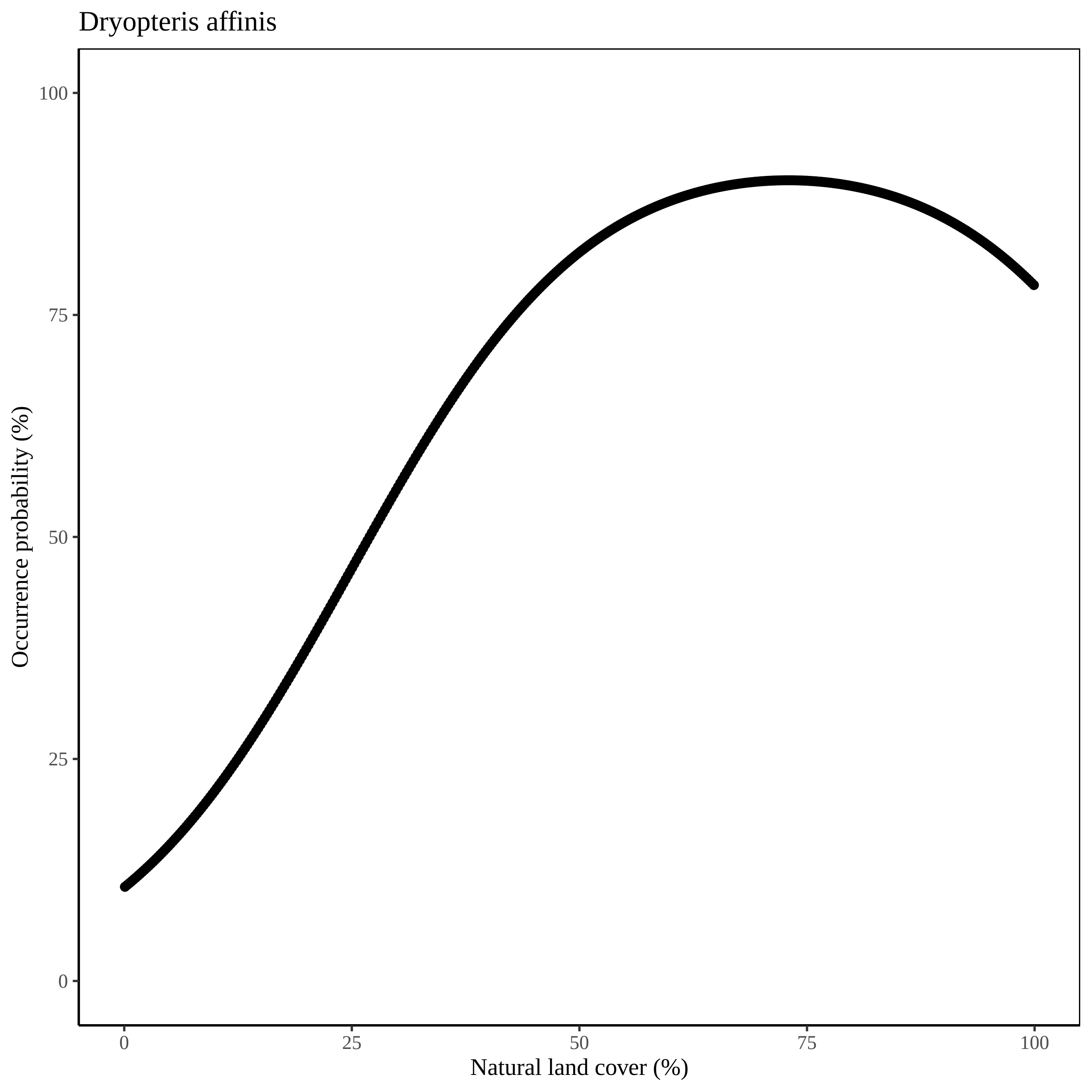

Dryopteris affinis
Occurrence probability (%)
100
75
50
25
0
Natural land cover (%)
0
25
50
75
100

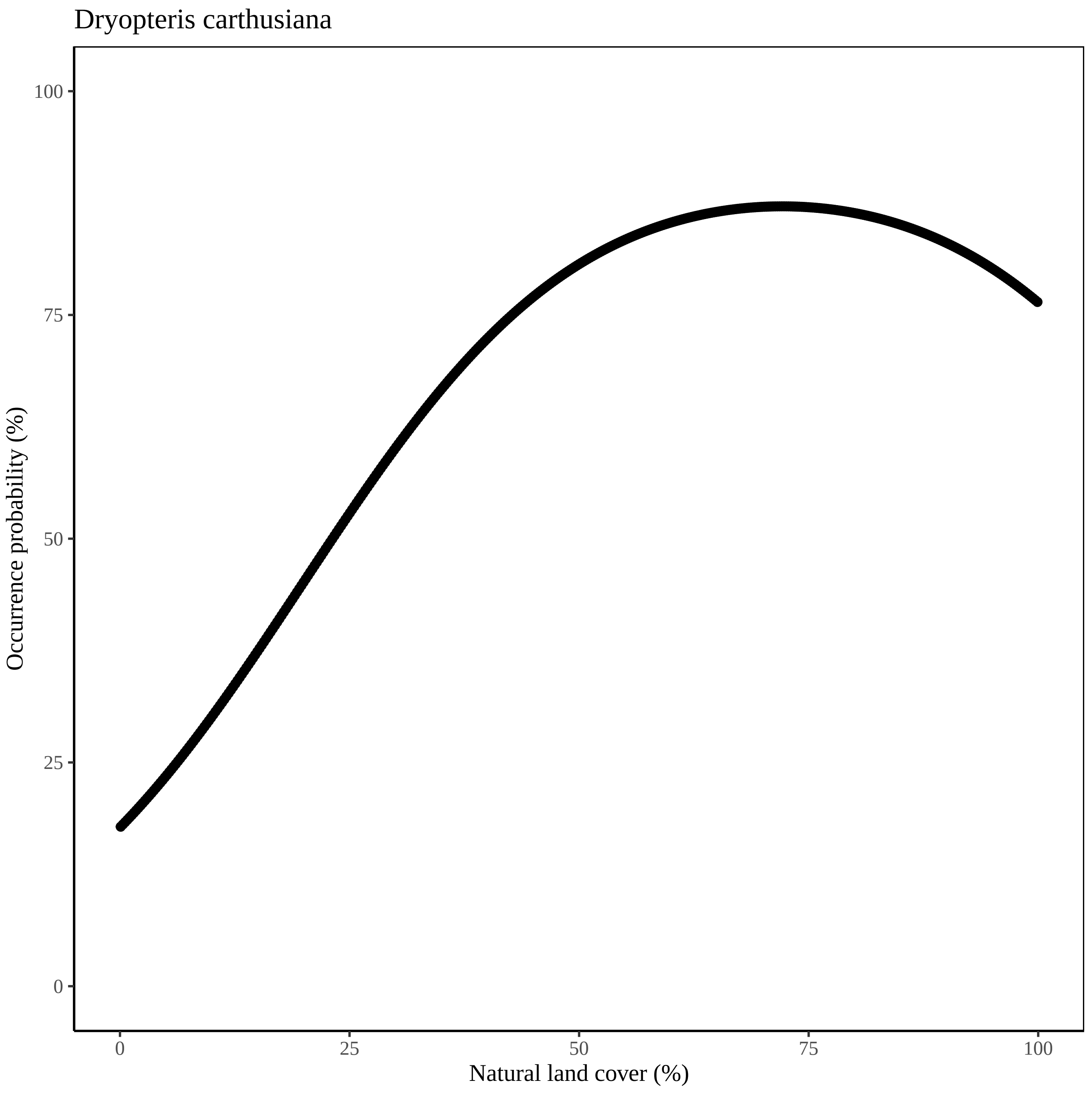

Dryopteris carthusiana
Occurrence probability (%)
100
75
50
25
0
0
25
50
75
100
Natural land cover (%)

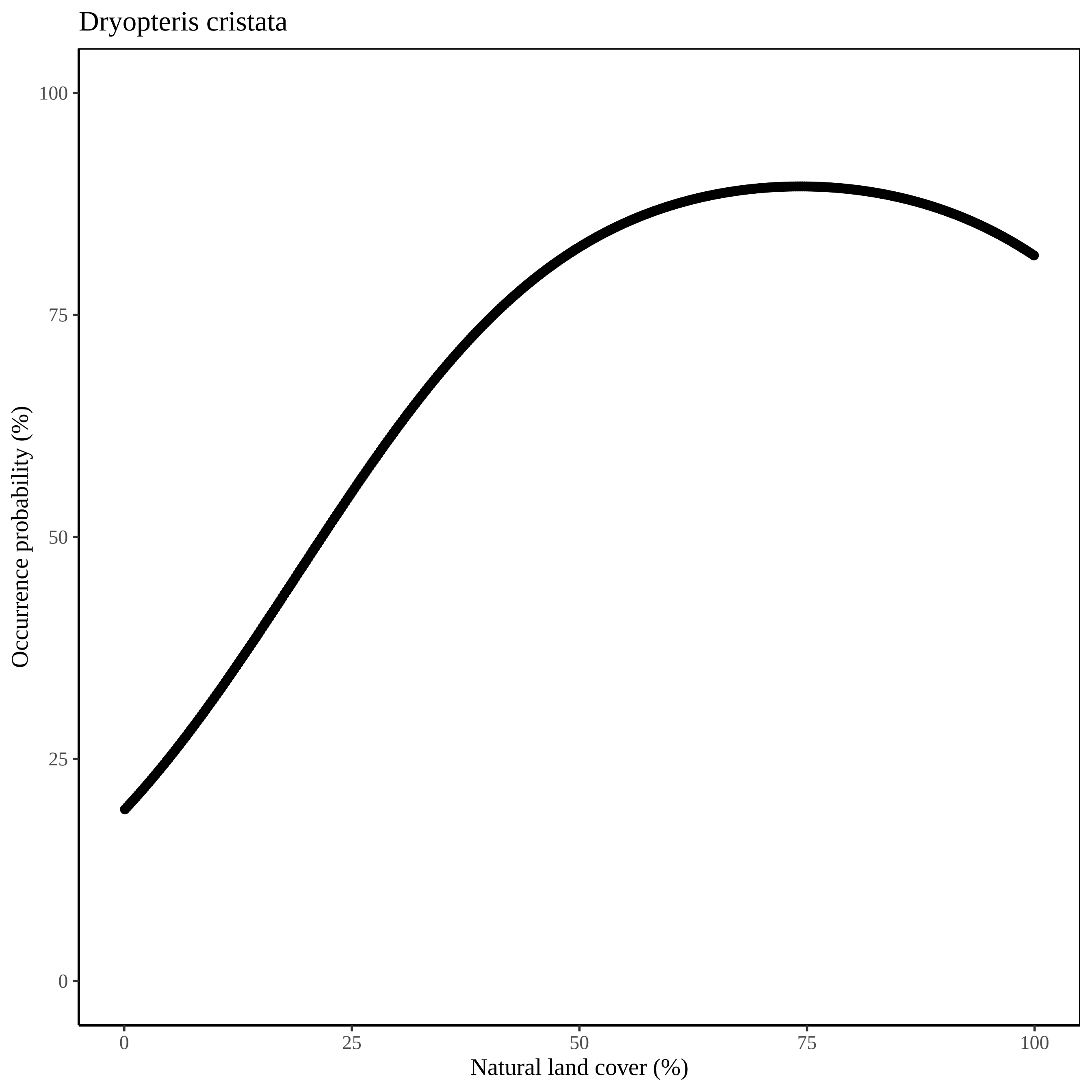

Dryopteris cristata
Occurrence probability (%)
100
75
50
25
0
0
25
50
75
100
Natural land cover (%)

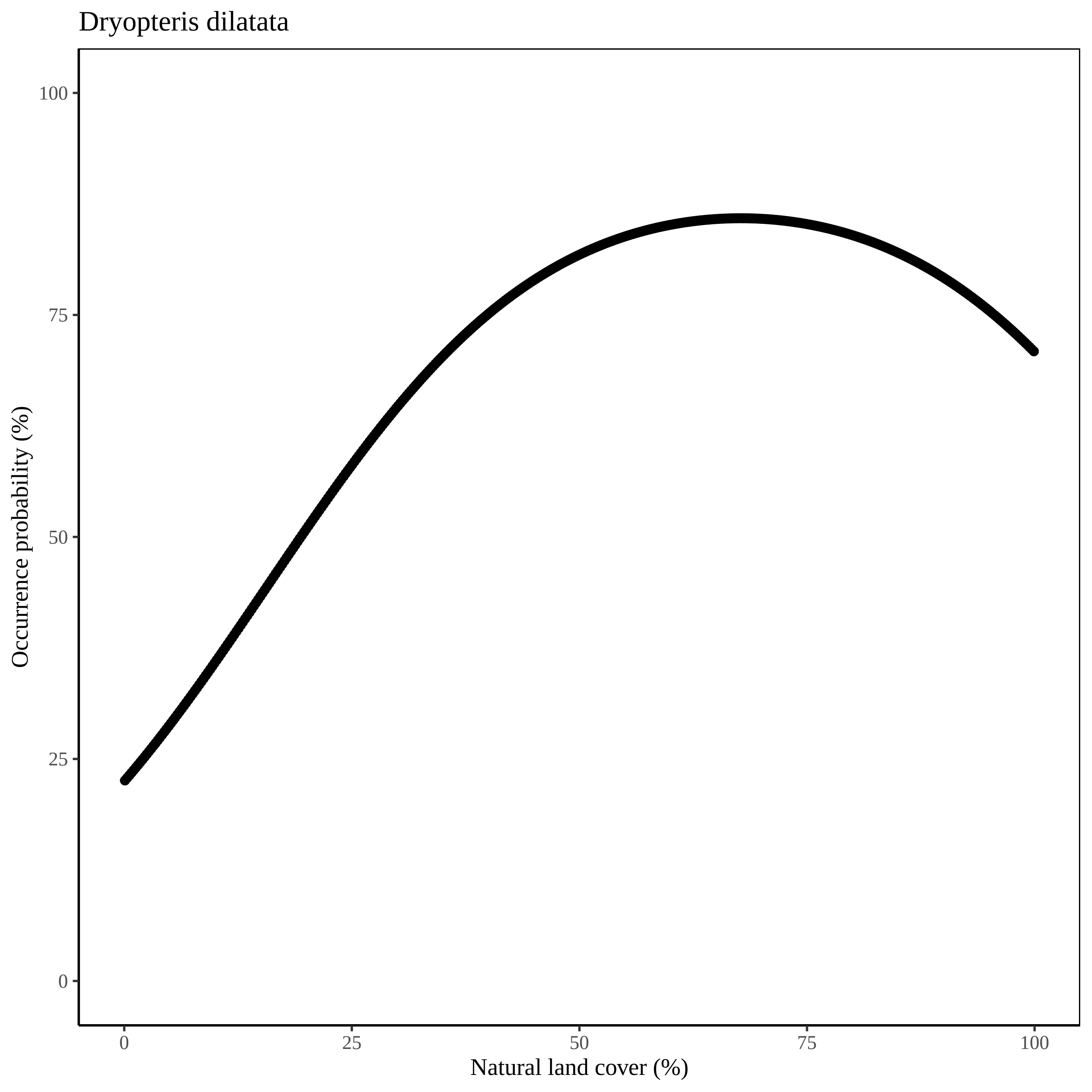

Dryopteris dilatata
Occurrence probability (%)
100
75
50
25
0
0
25
50
75
100
Natural land cover (%)

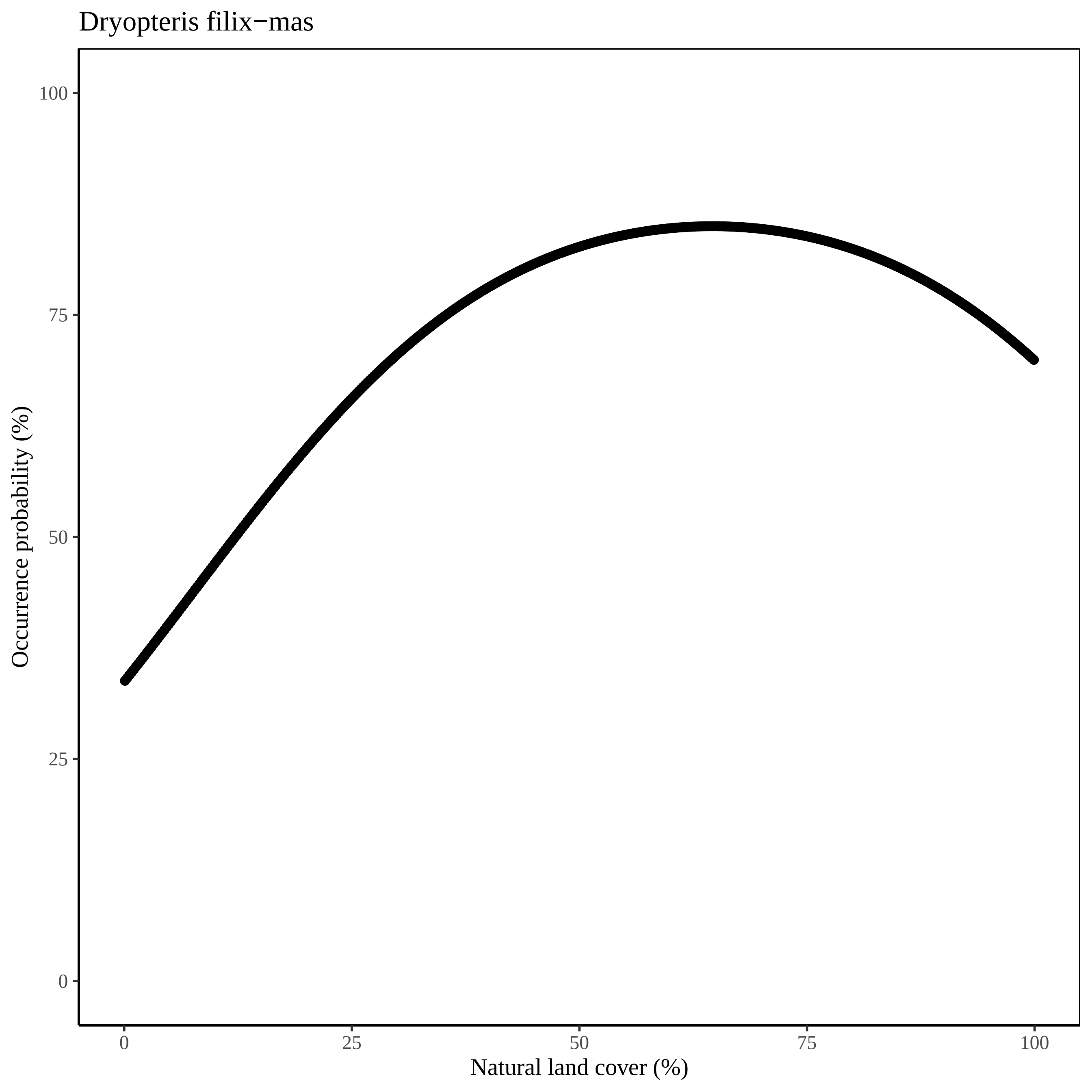
Dryopteris filix−mas
Occurrence probability (%)
100
75
50
25
0
0
25
50
75
100
Natural land cover (%)

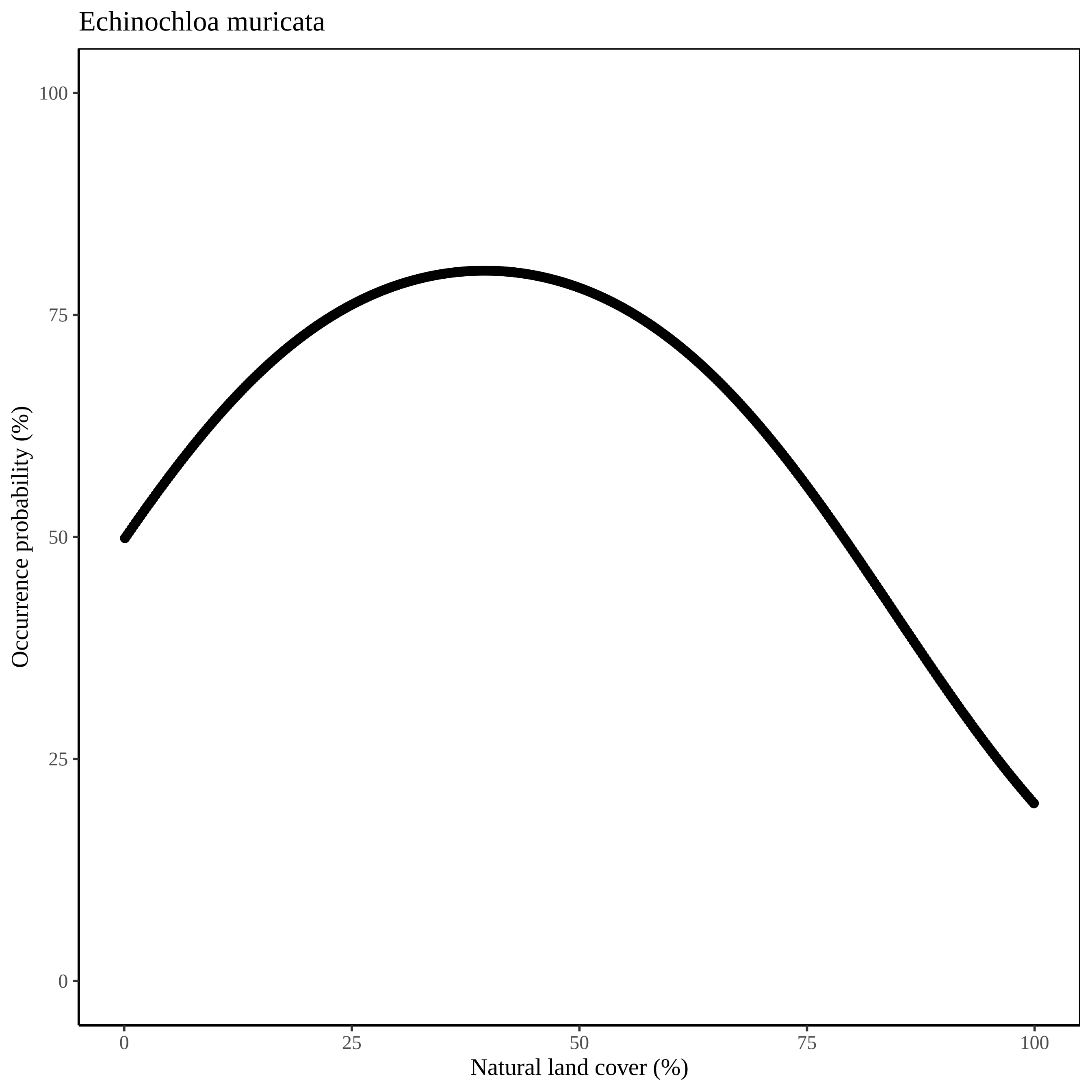
Echinochloa muricata
Occurrence probability (%)
100
75
50
25
0
0
25
50
75
100
Natural land cover (%)

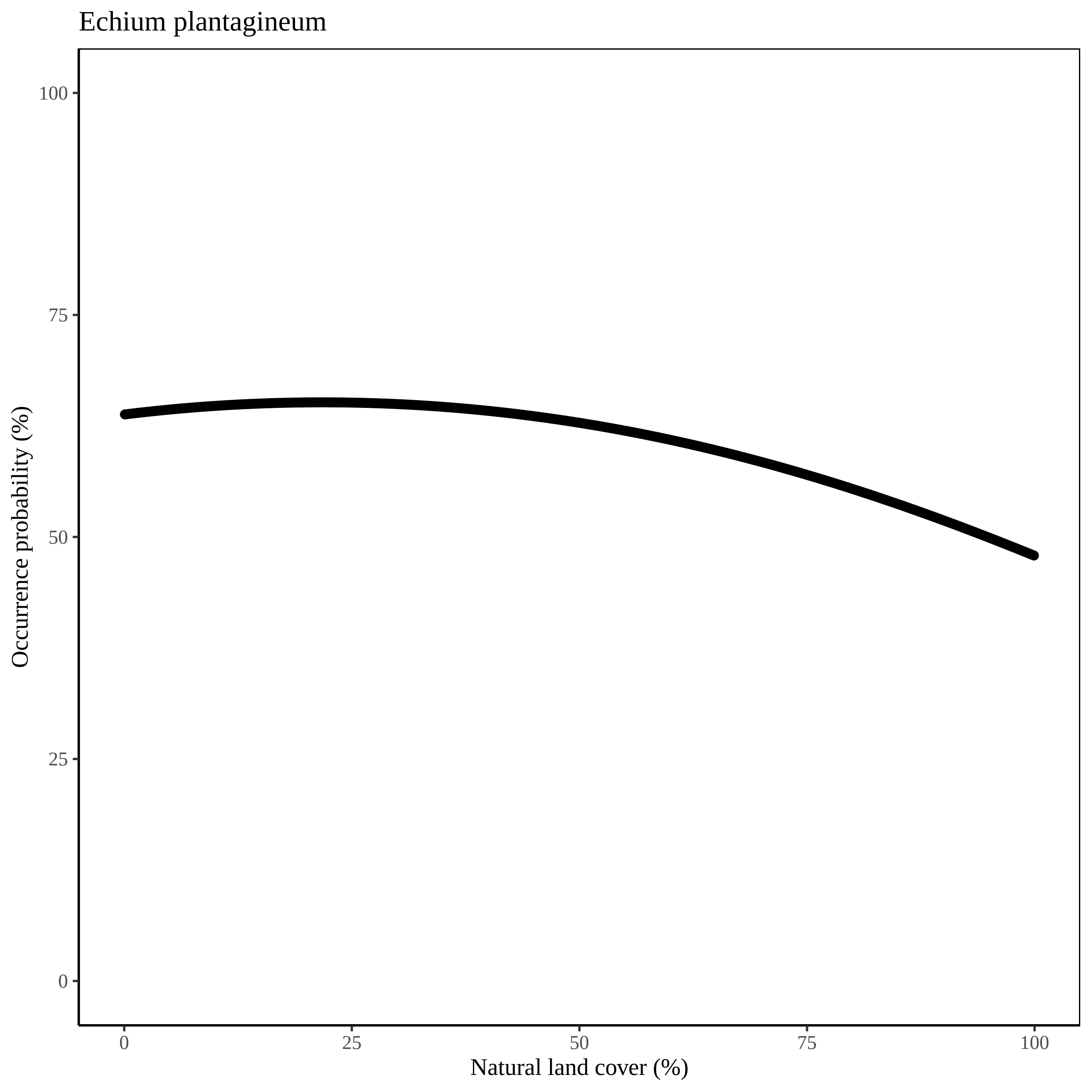

Echium plantagineum
100
75
50
25
0
Occurrence probability (%)
0
25
50
75
100
Natural land cover (%)

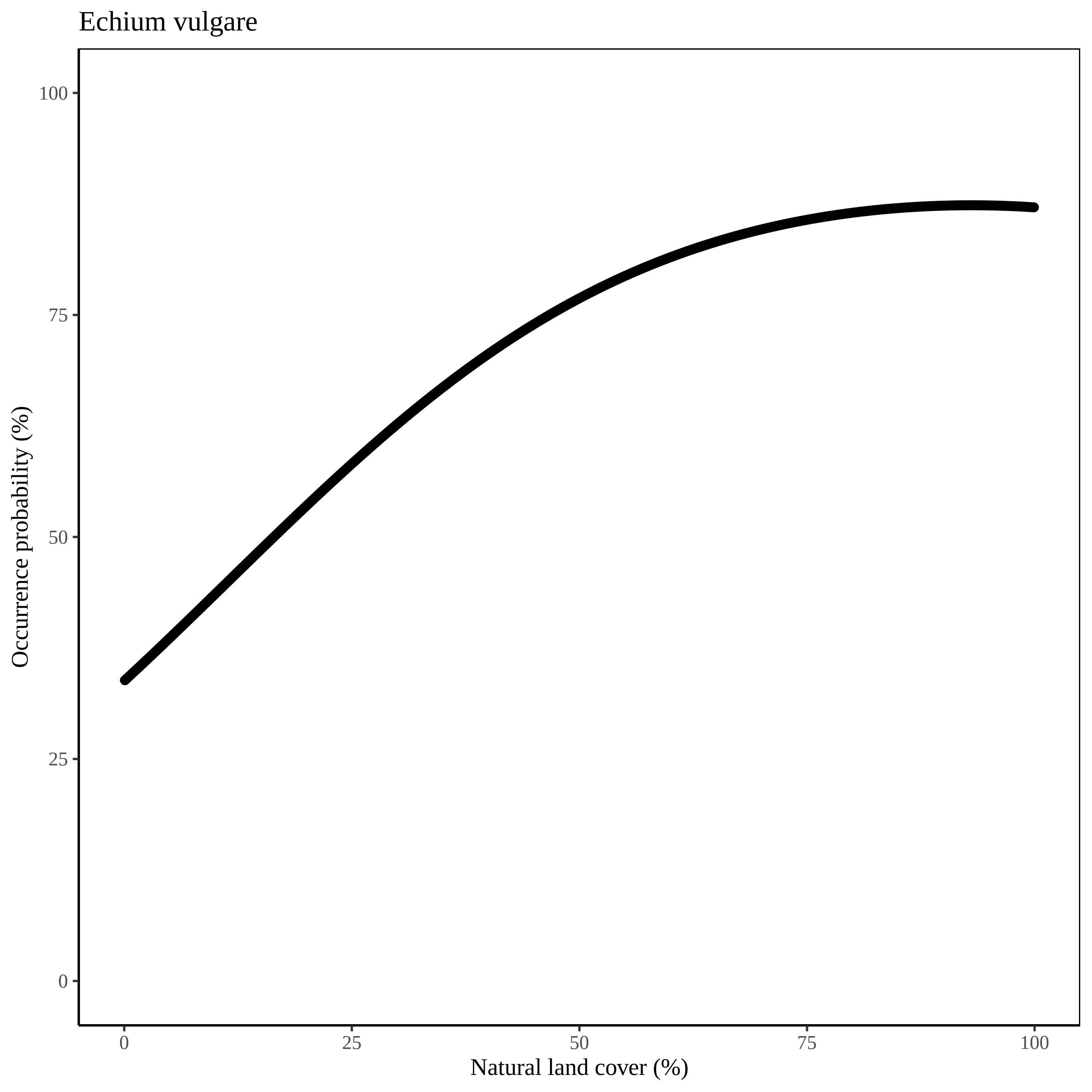

Echium vulgare
Occurrence probability (%)
100
75
50
25
0
0
25
50
75
100
Natural land cover (%)

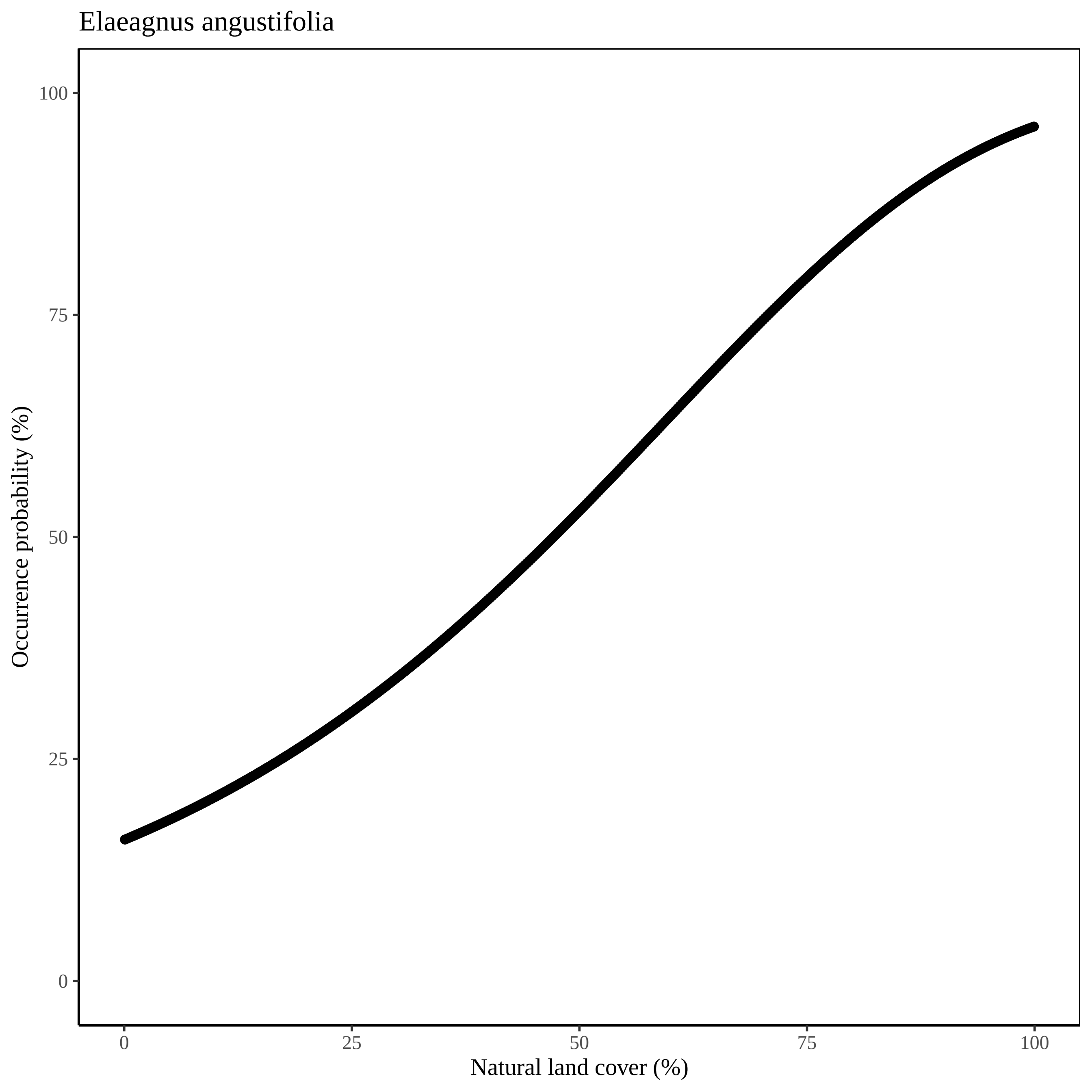
Elaeagnus angustifolia
Occurrence probability (%)
100
75
50
25
0
0
25
50
75
100
Natural land cover (%)

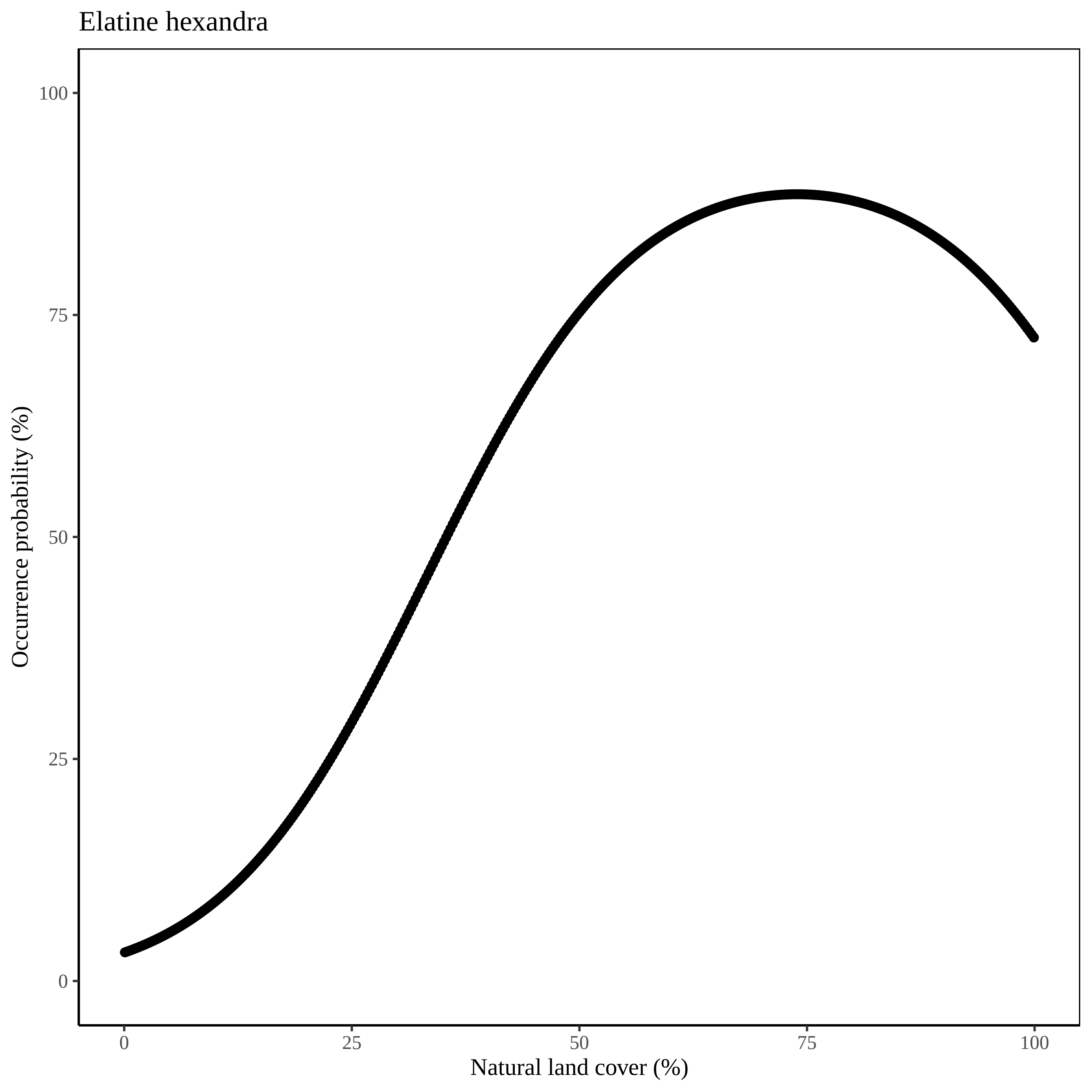

Elatine hexandra
Occurrence probability (%)
100
75
50
25
0
0
25
50
75
100
Natural land cover (%)

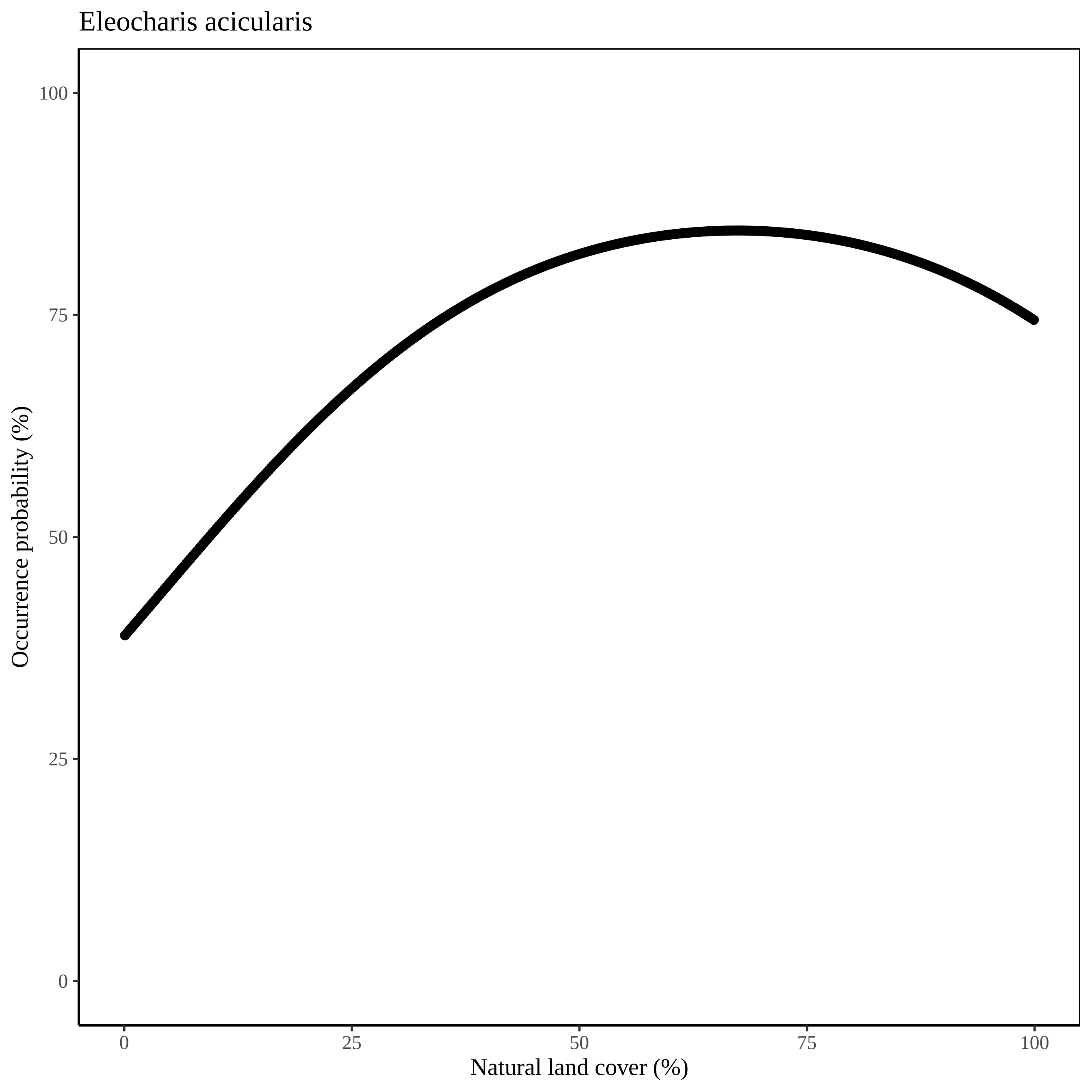
Eleocharis acicularis
Occurrence probability (%)
100
75
50
25
0
0
25
50
75
100
Natural land cover (%)

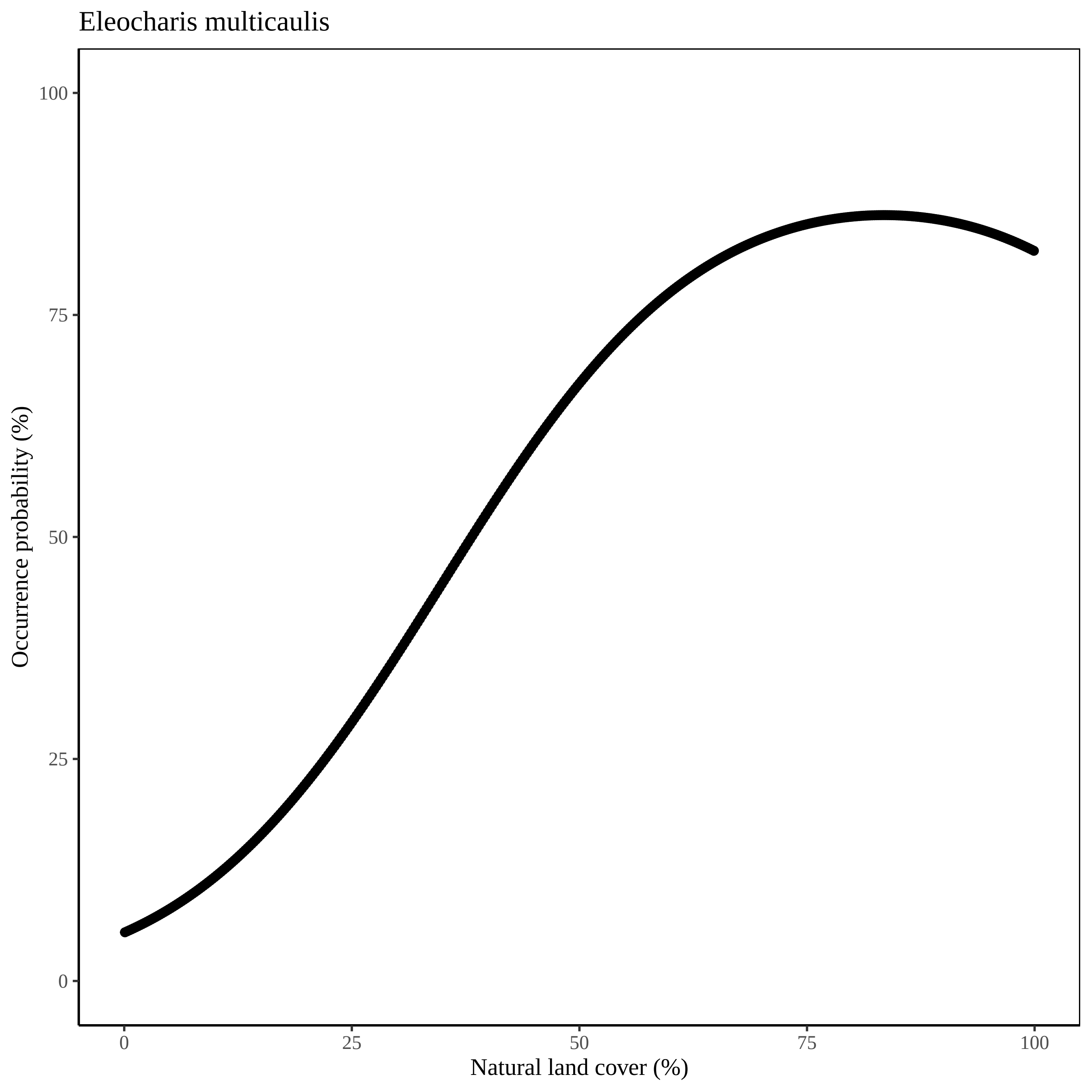

Eleocharis multicaulis
Occurrence probability (%)
100
75
50
25
0
0
25
50
75
100
Natural land cover (%)

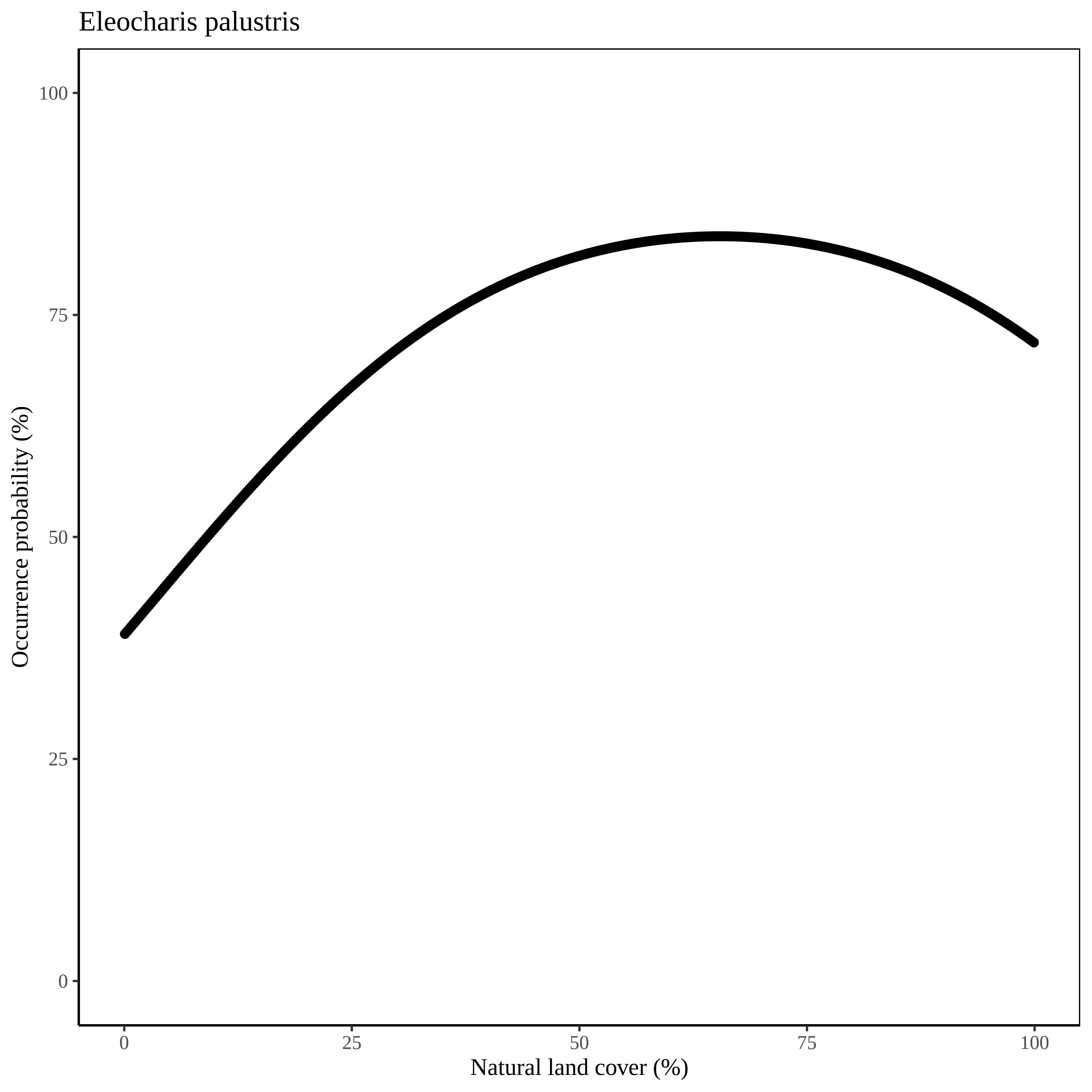
Eleocharis palustris
Occurrence probability (%)
100
75
50
25
0
0
25
50
75
100
Natural land cover (%)

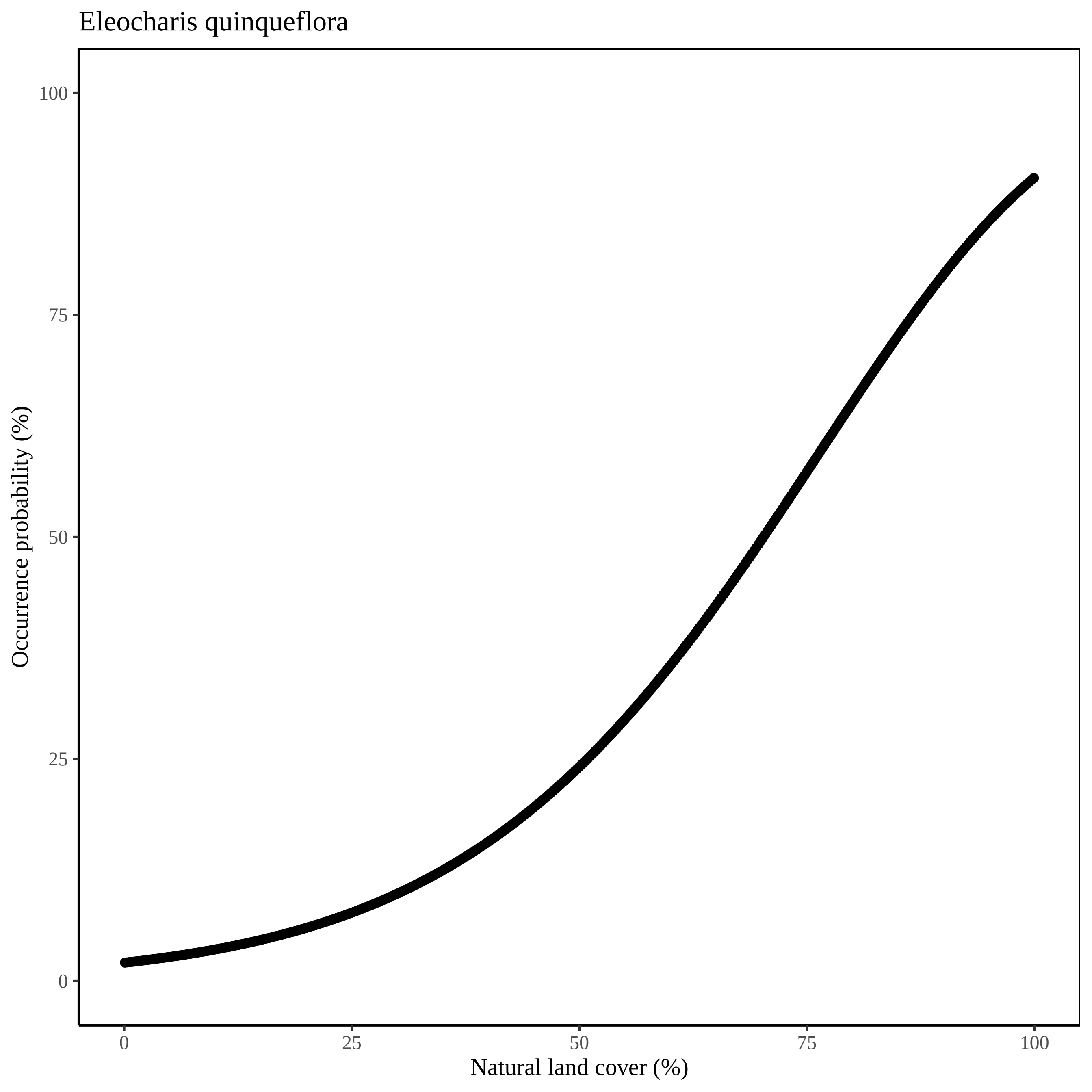

Eleocharis quinqueflora
Occurrence probability (%)
100
75
50
25
0
0
25
50
75
100
Natural land cover (%)

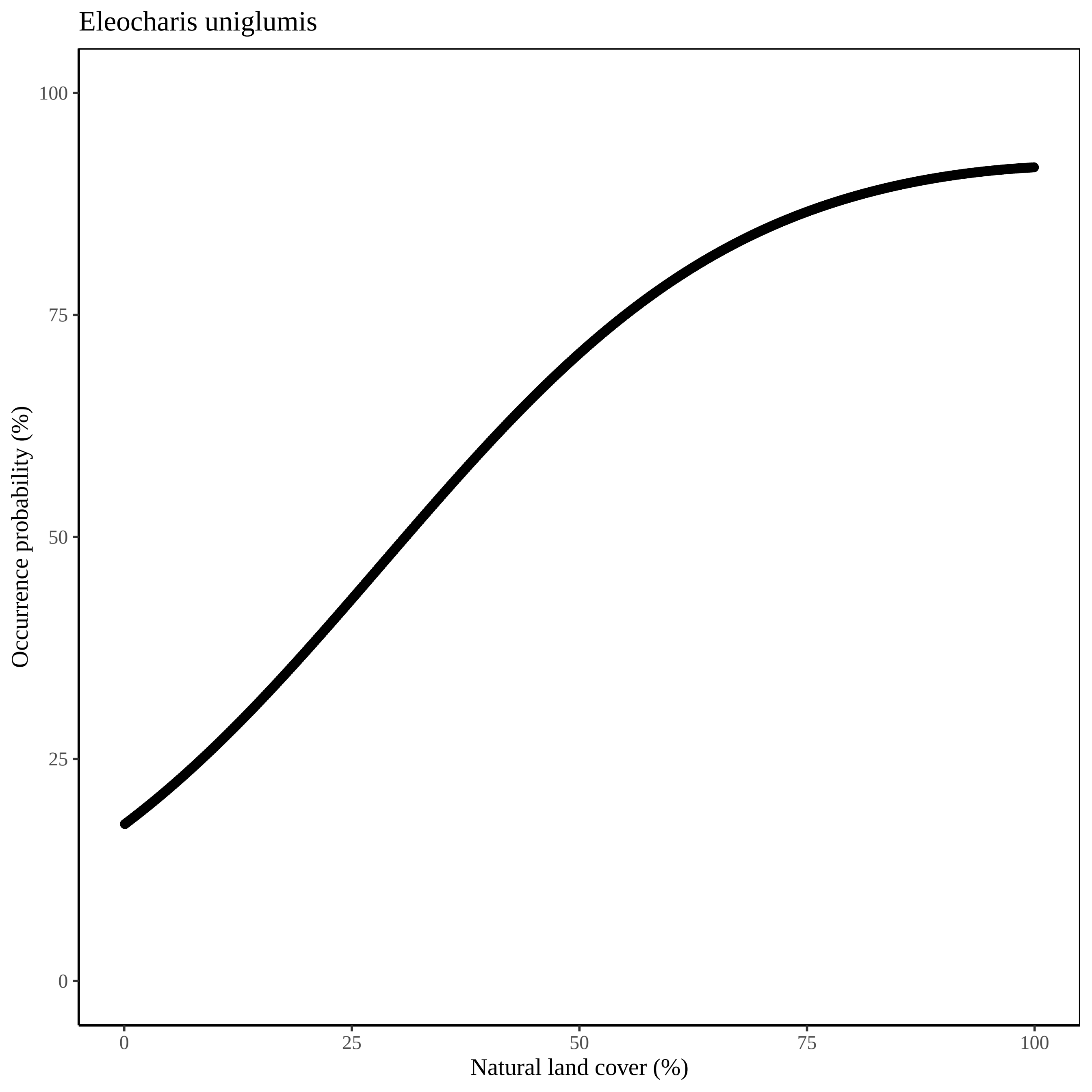
Eleocharis uniglumis
Occurrence probability (%)
0
25
50
75
100
Natural land cover (%)
0
25
50
75
100

Eleogiton fluitans

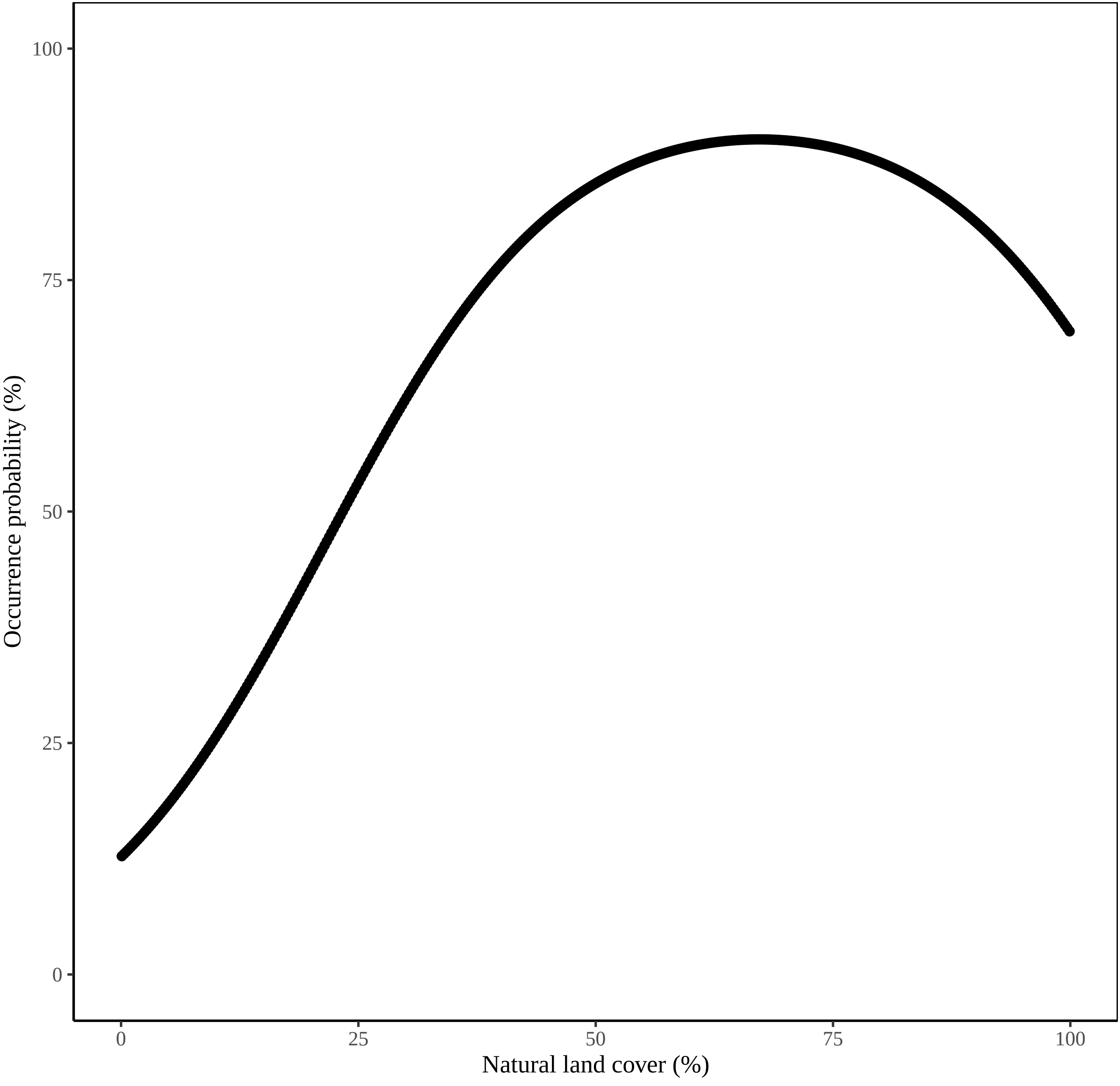

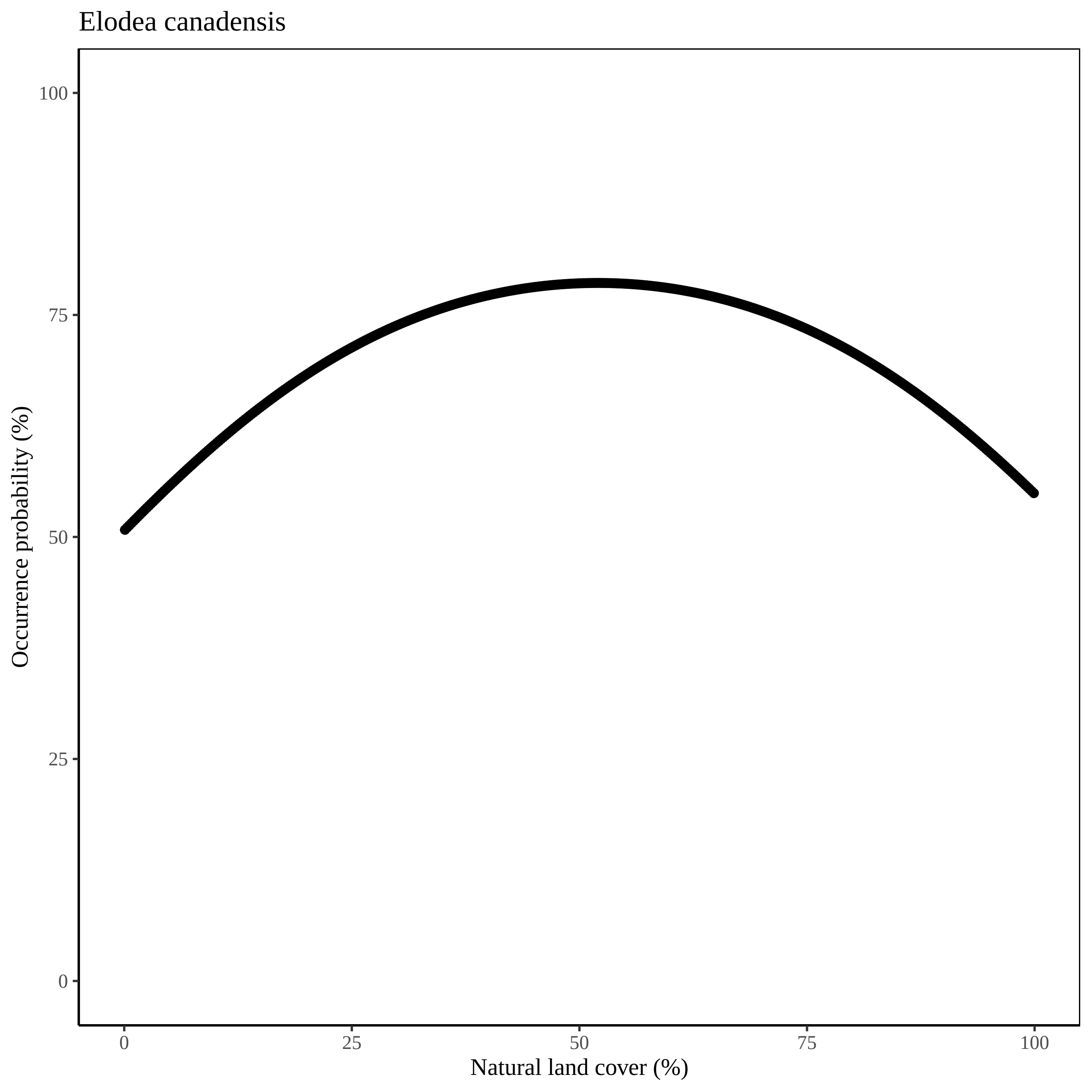
Elodea canadensis
Occurrence probability (%)
100
75
50
25
0
0
25
50
75
100
Natural land cover (%)

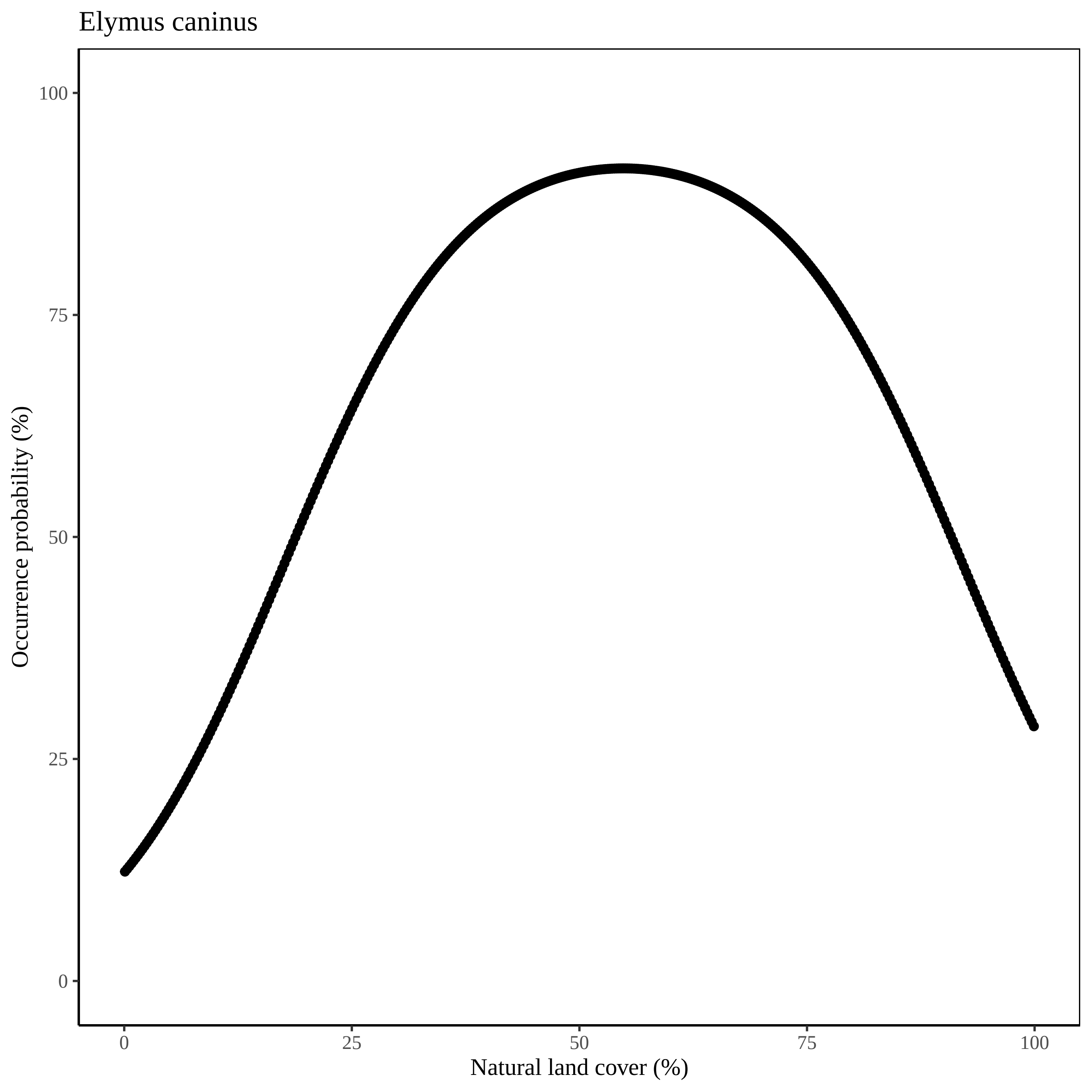

Elymus caninus
100
75
50
25
0
Occurrence probability (%)
0
25
50
75
100
Natural land cover (%)

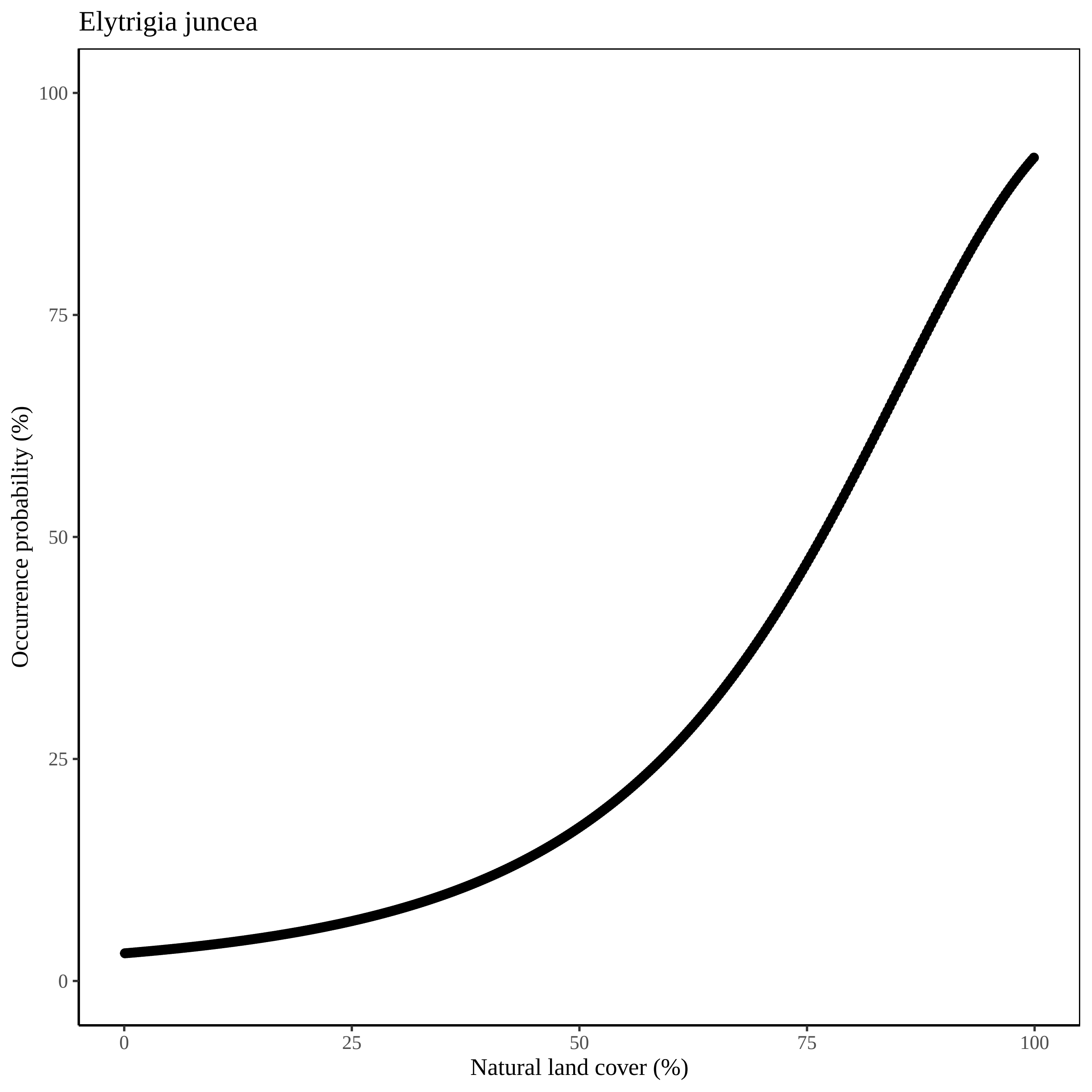

Elytrigia juncea
Occurrence probability (%)
100
75
50
25
0
0
25
50
75
100
Natural land cover (%)

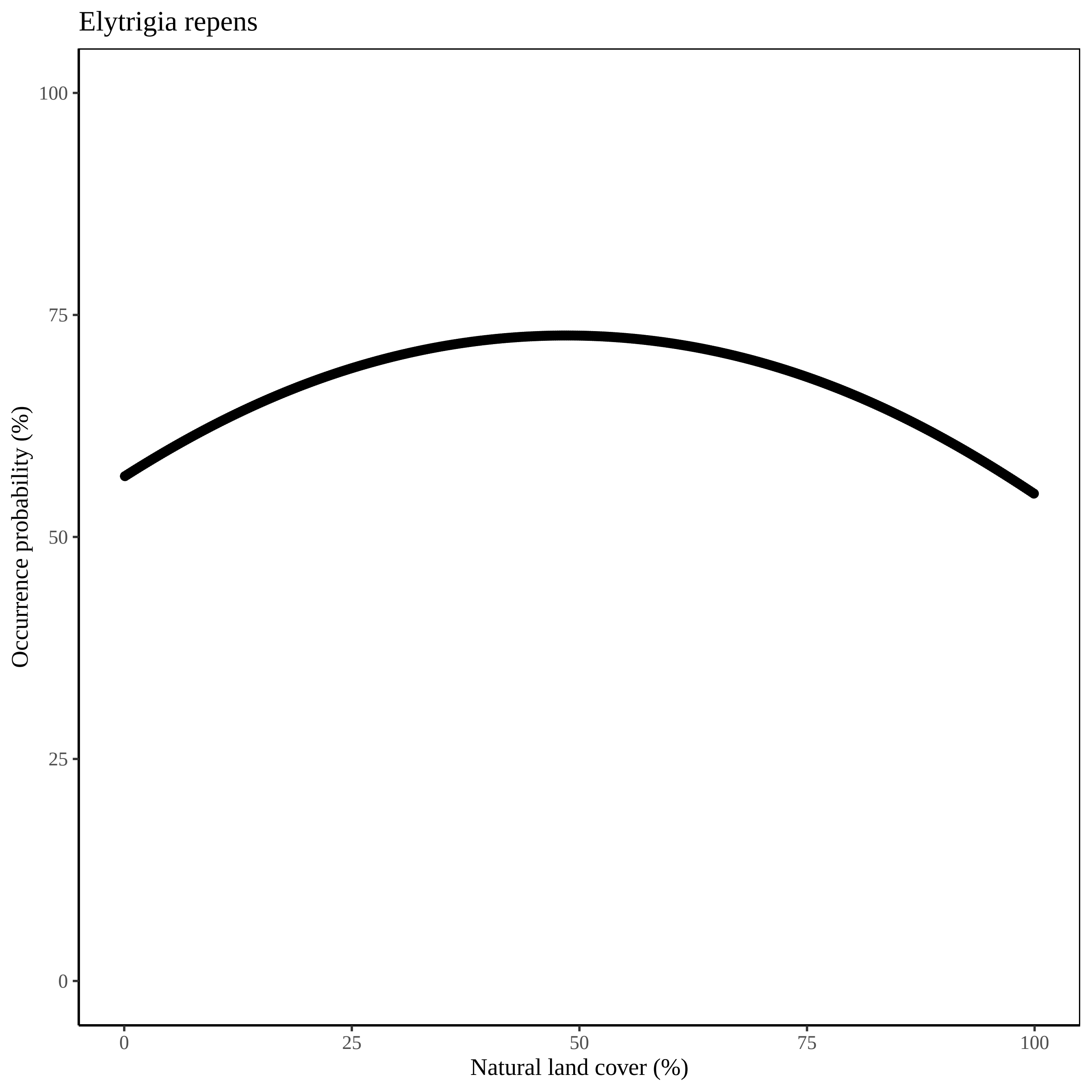
Elytrigia repens
Occurrence probability (%)
100
75
50
25
0
0
25
50
75
100
Natural land cover (%)

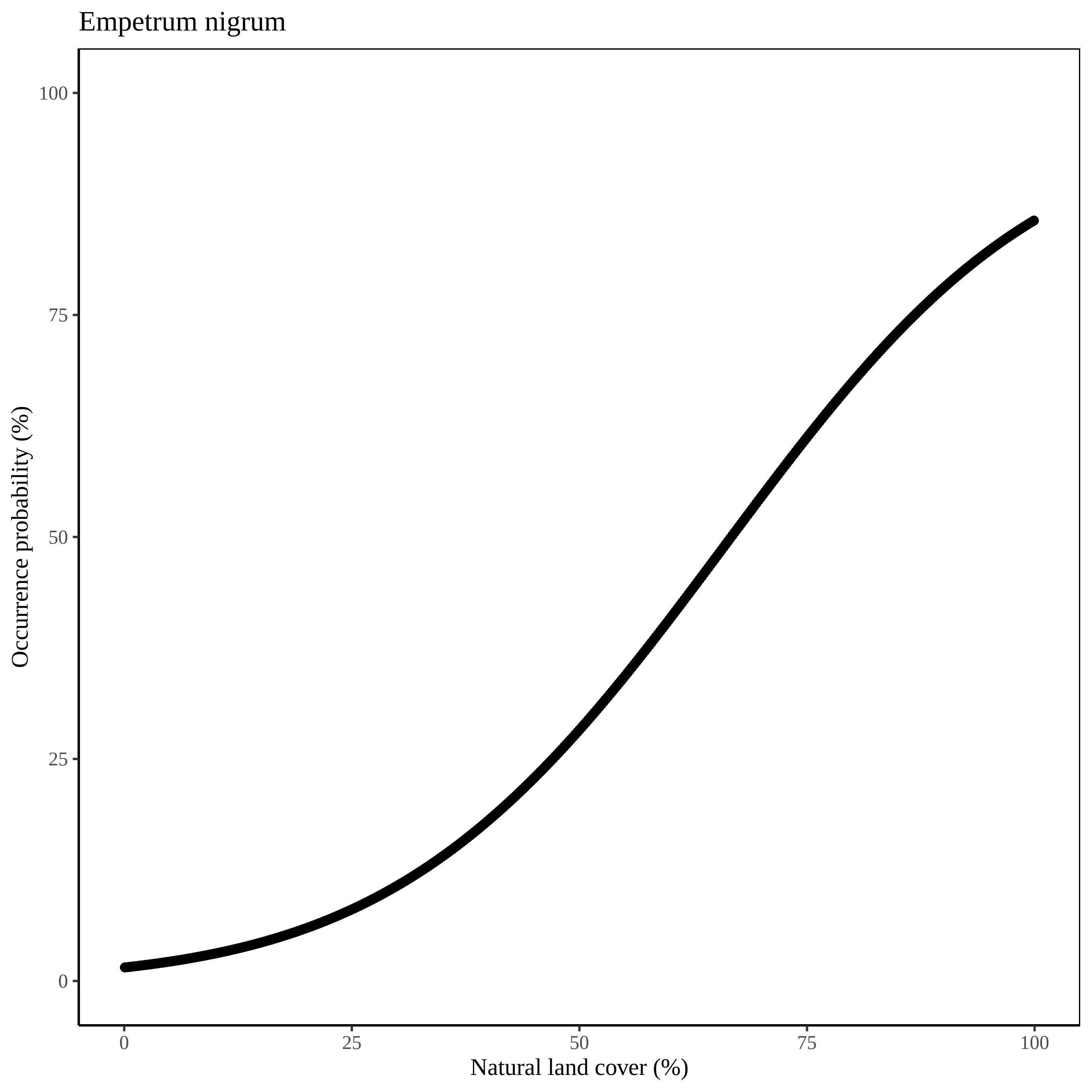

Empetrum nigrum
Occurrence probability (%)
100
75
50
25
0
0
25
50
75
100
Natural land cover (%)

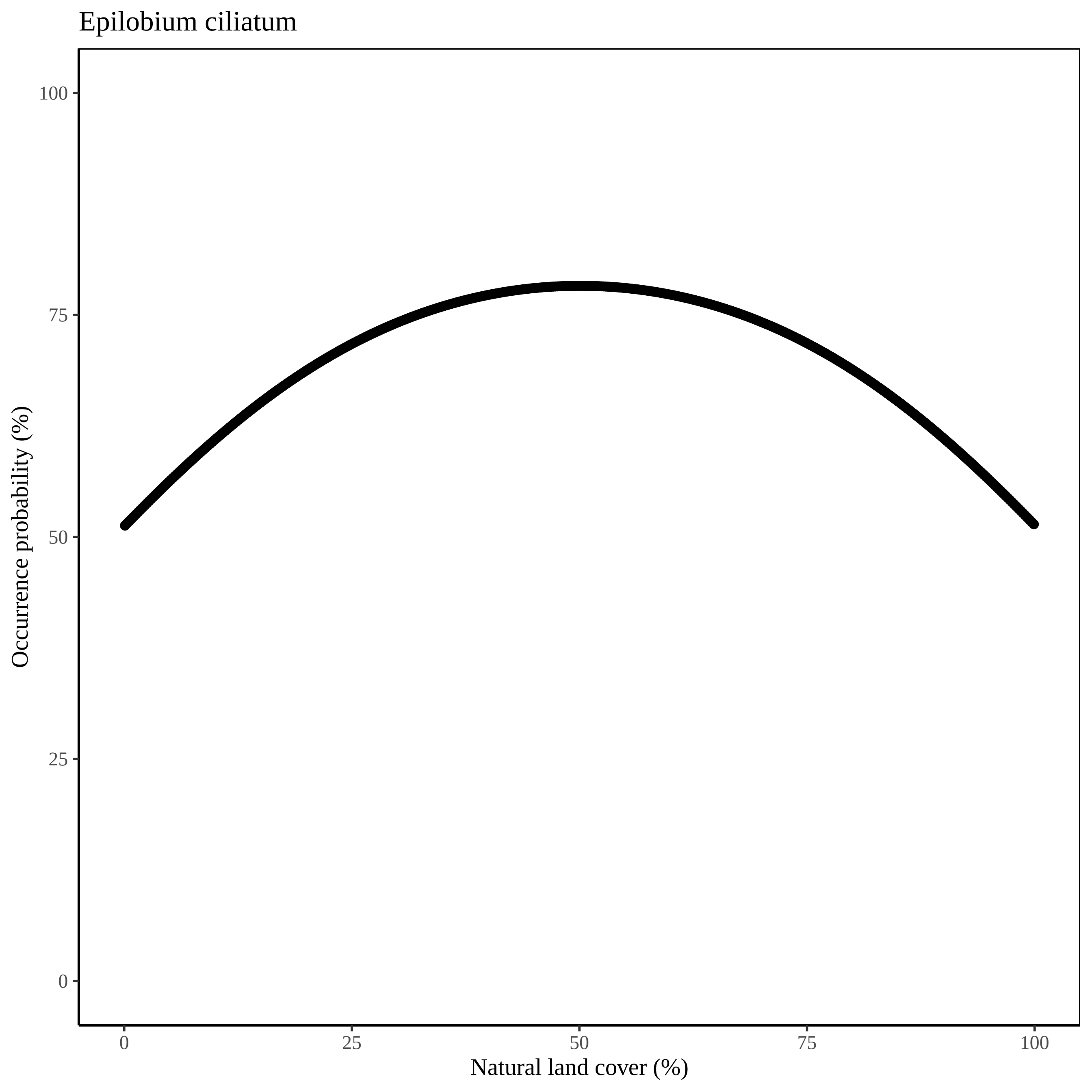
Epilobium ciliatum
Occurrence probability (%)
100
75
50
25
0
0
25
50
75
100
Natural land cover (%)

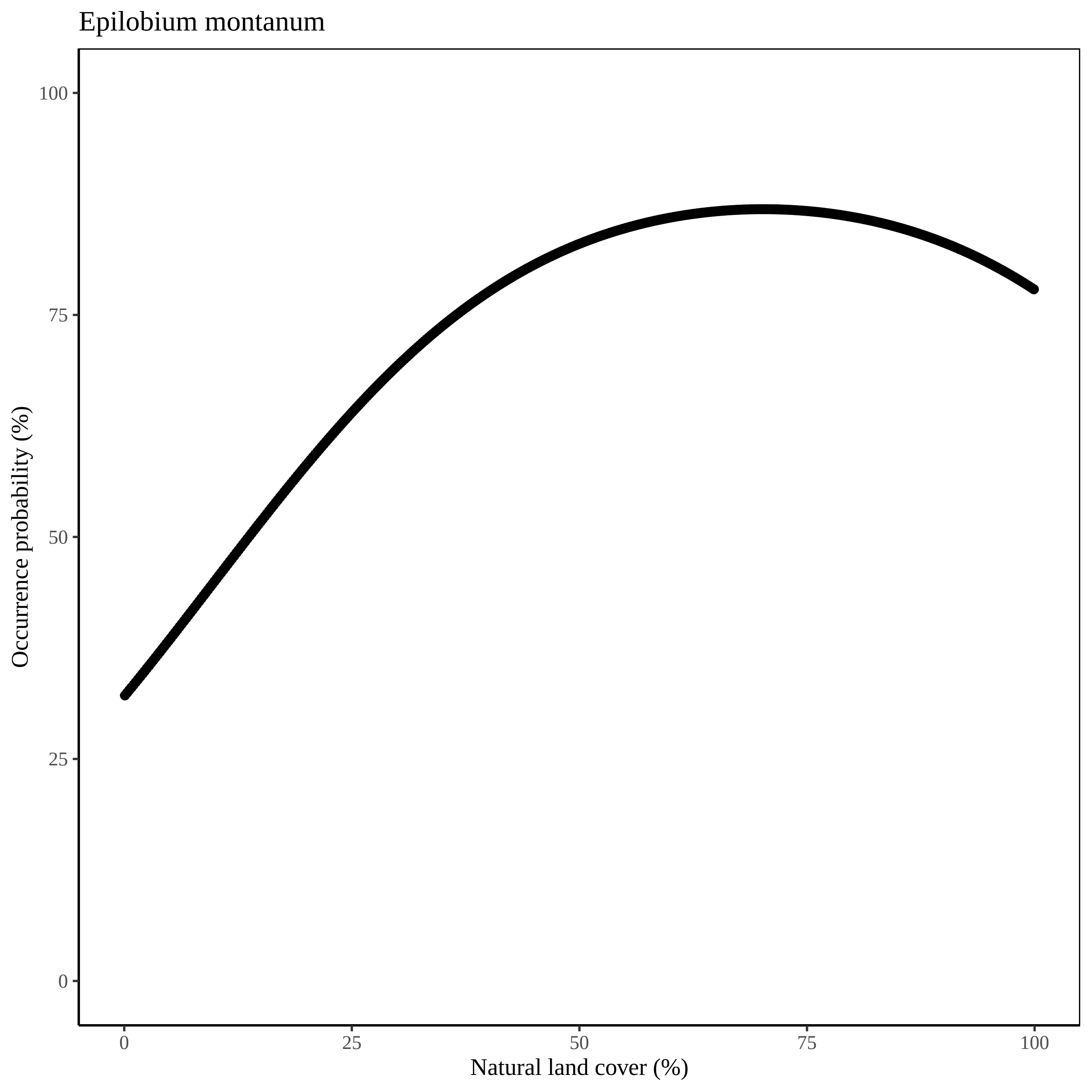
Epilobium montanum
Occurrence probability (%)
100
75
50
25
0
0
25
50
75
100
Natural land cover (%)

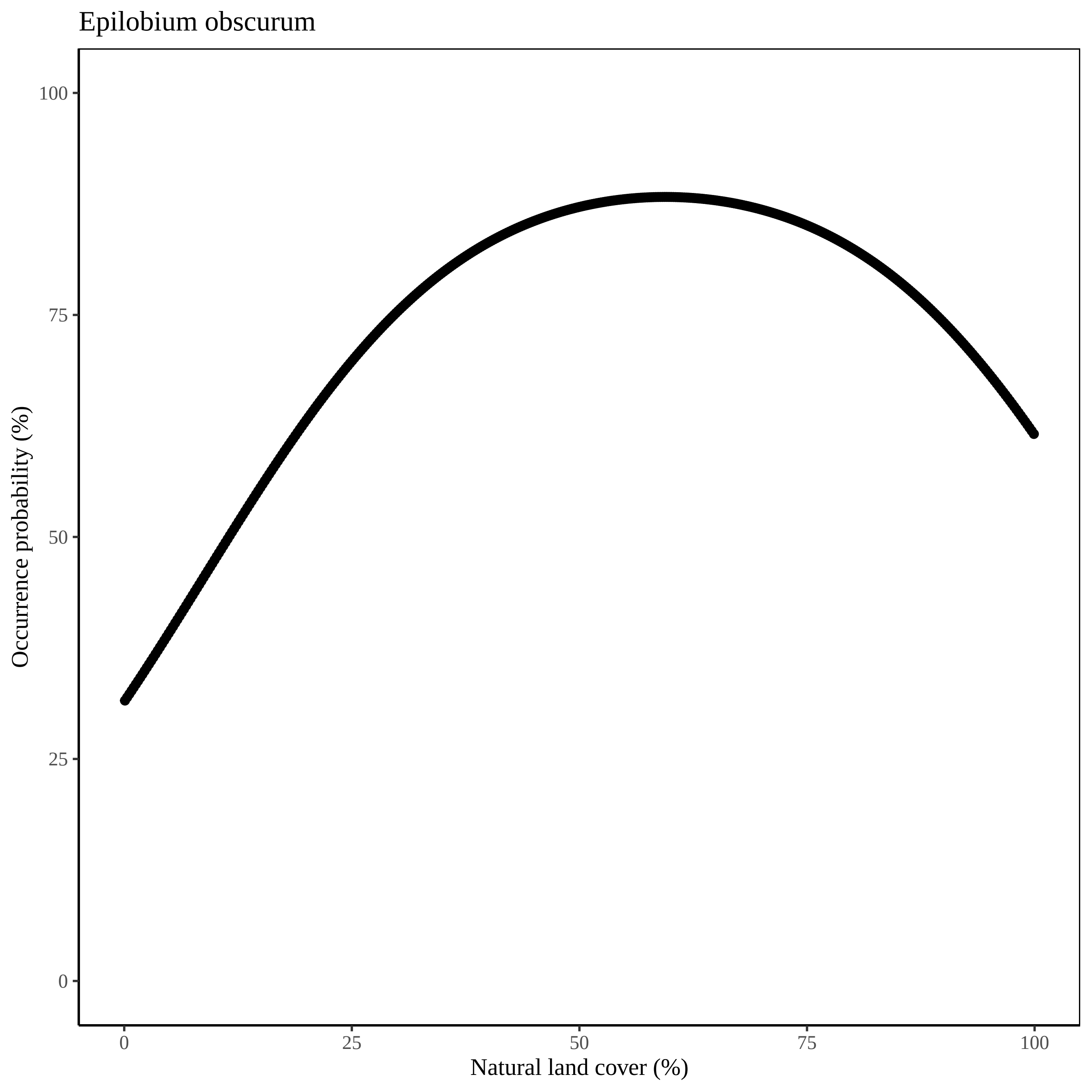
Epilobium obscurum
Occurrence probability (%)
100
75
50
25
0
0
25
50
75
100
Natural land cover (%)

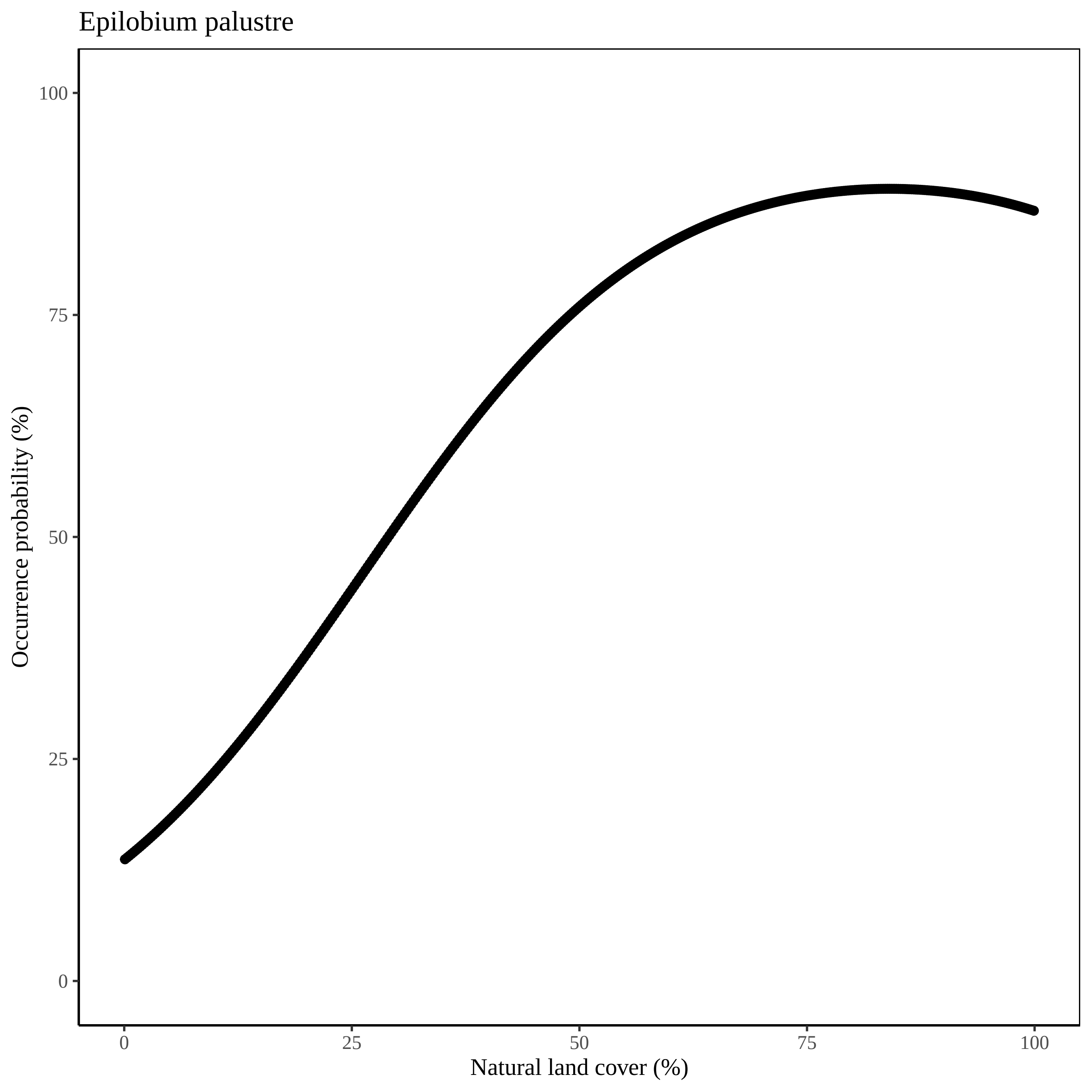

Epilobium palustre
100
75
50
25
0
Occurrence probability (%)
0
25
50
75
100
Natural land cover (%)

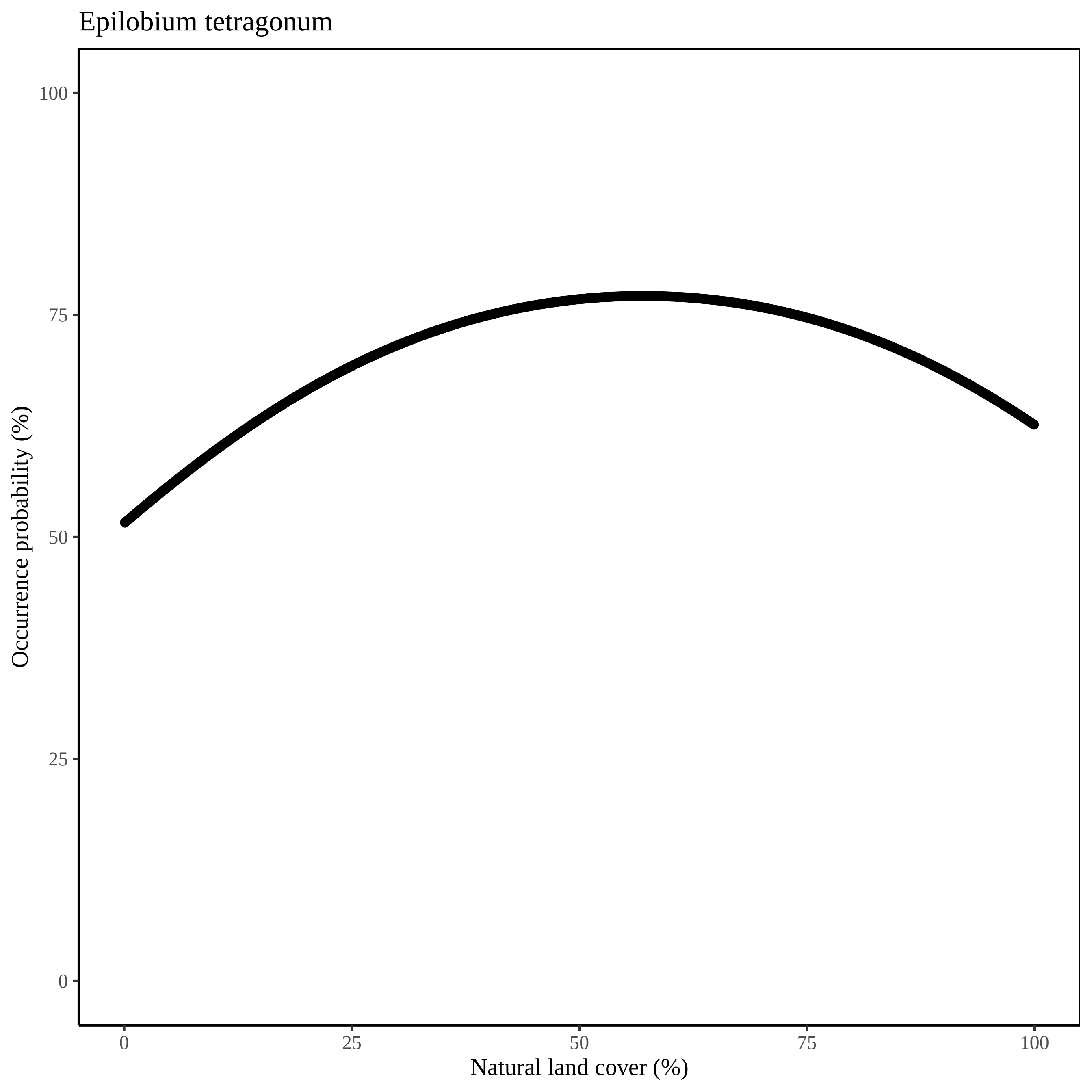

Epilobium tetragonum
Occurrence probability (%)
100
75
50
25
0
0
25
50
75
100
Natural land cover (%)

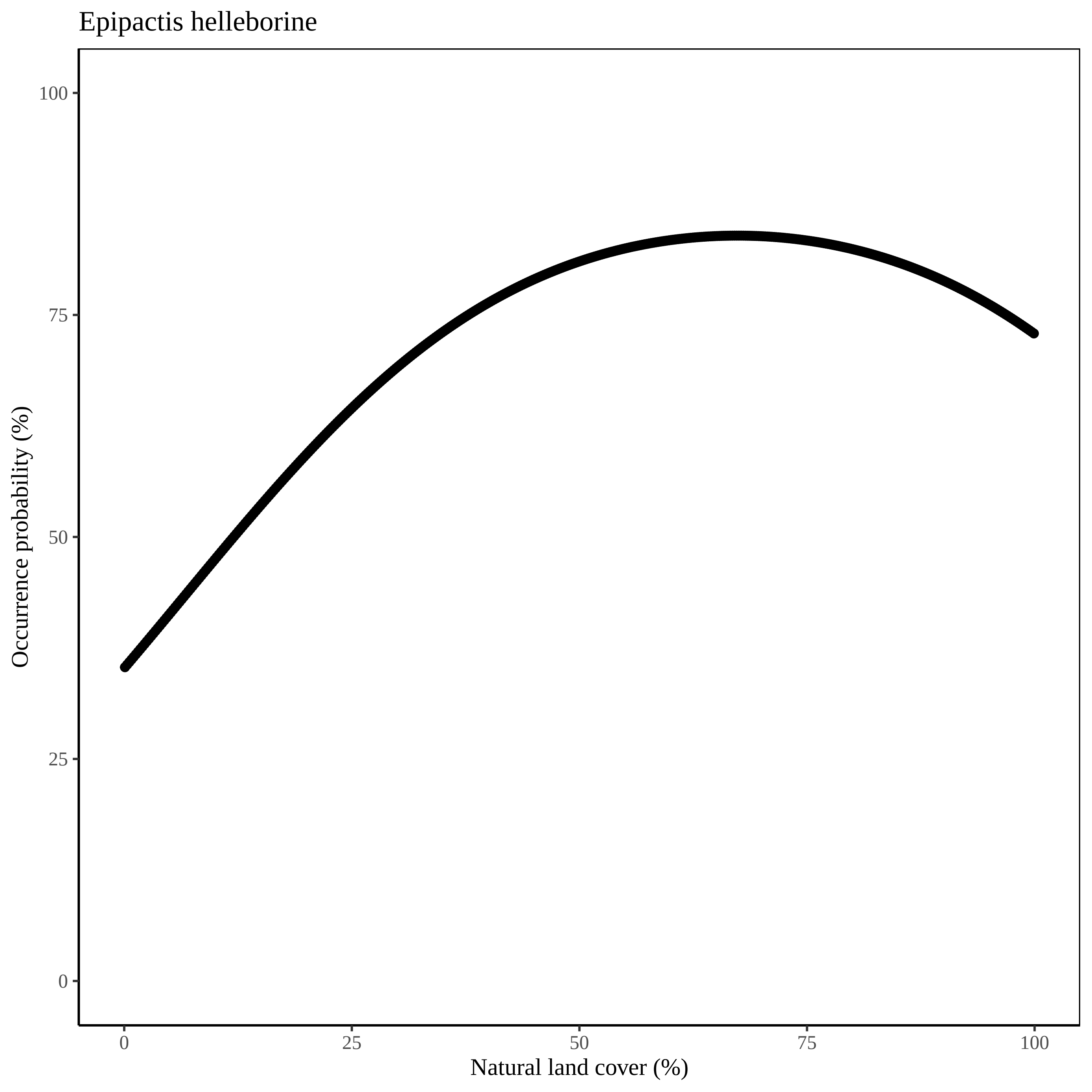
Epipactis helleborine
Occurrence probability (%)
100
75
50
25
0
0
25
50
75
100
Natural land cover (%)

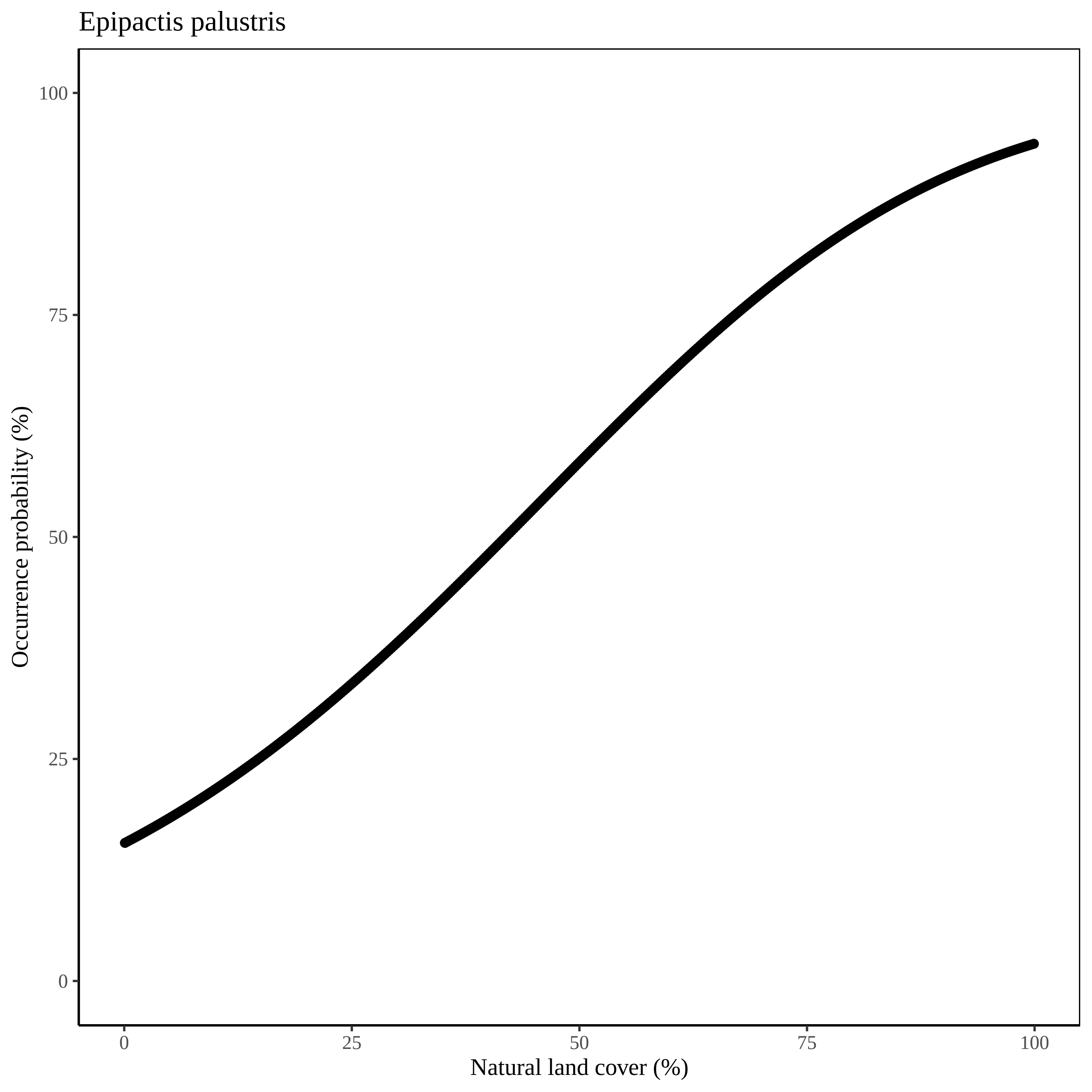
Epipactis palustris
Occurrence probability (%)
100
75
50
25
0
0
25
50
75
100
Natural land cover (%)

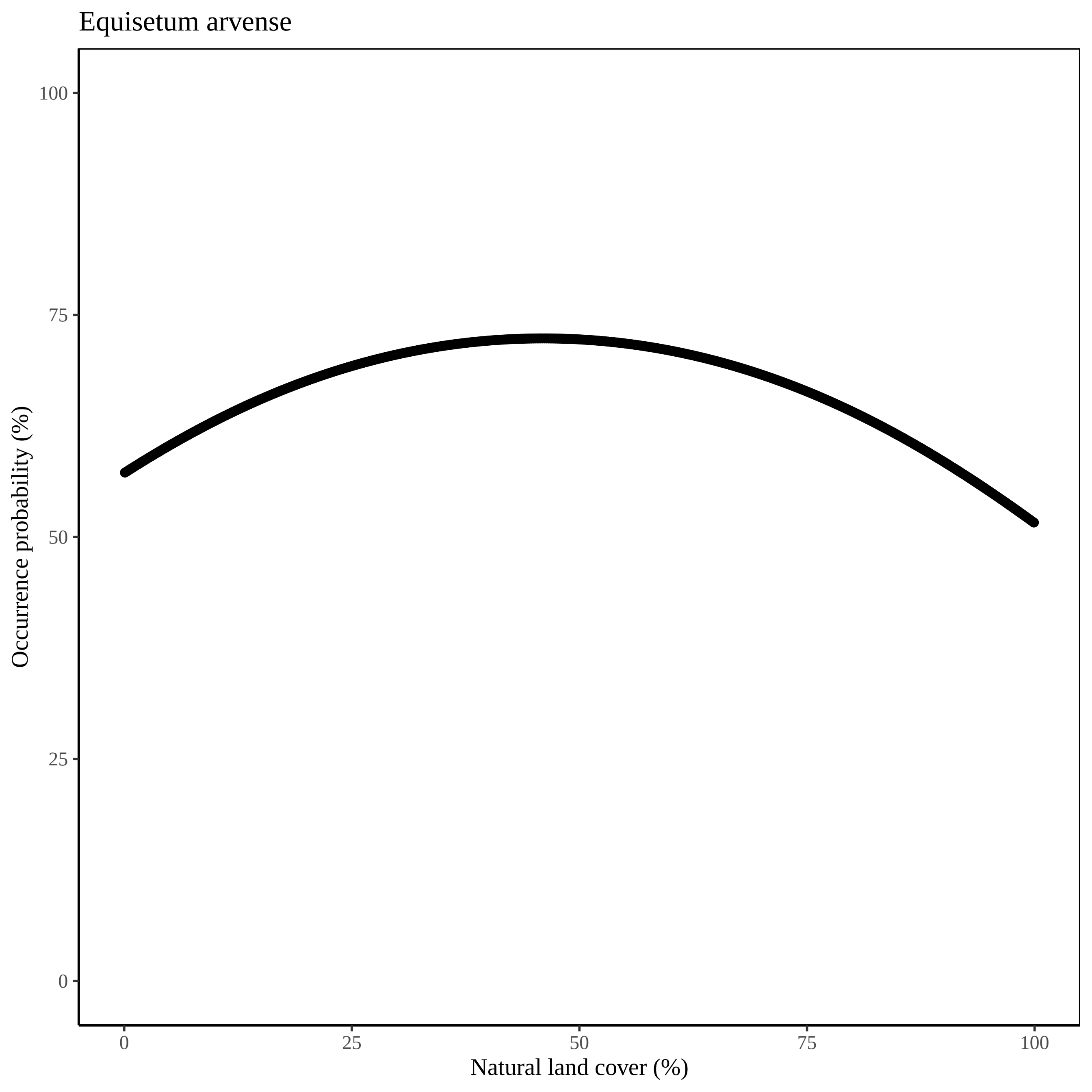
Equisetum arvense
Occurrence probability (%)
100
75
50
25
0
0
25
50
75
100
Natural land cover (%)

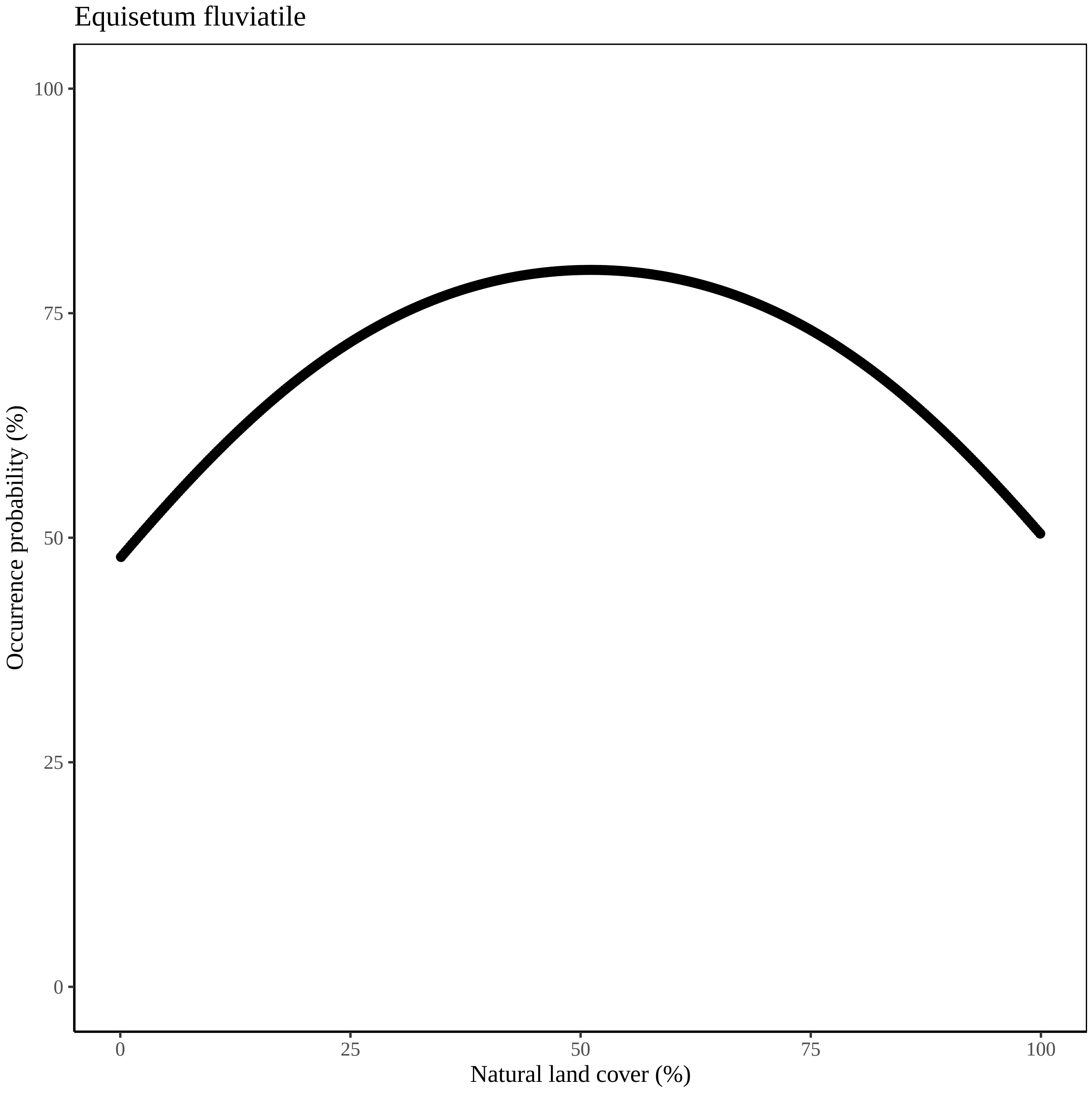

Equisetum fluviatile
100
75
50
25
0
Occurrence probability (%)
0
25
50
75
100
Natural land cover (%)

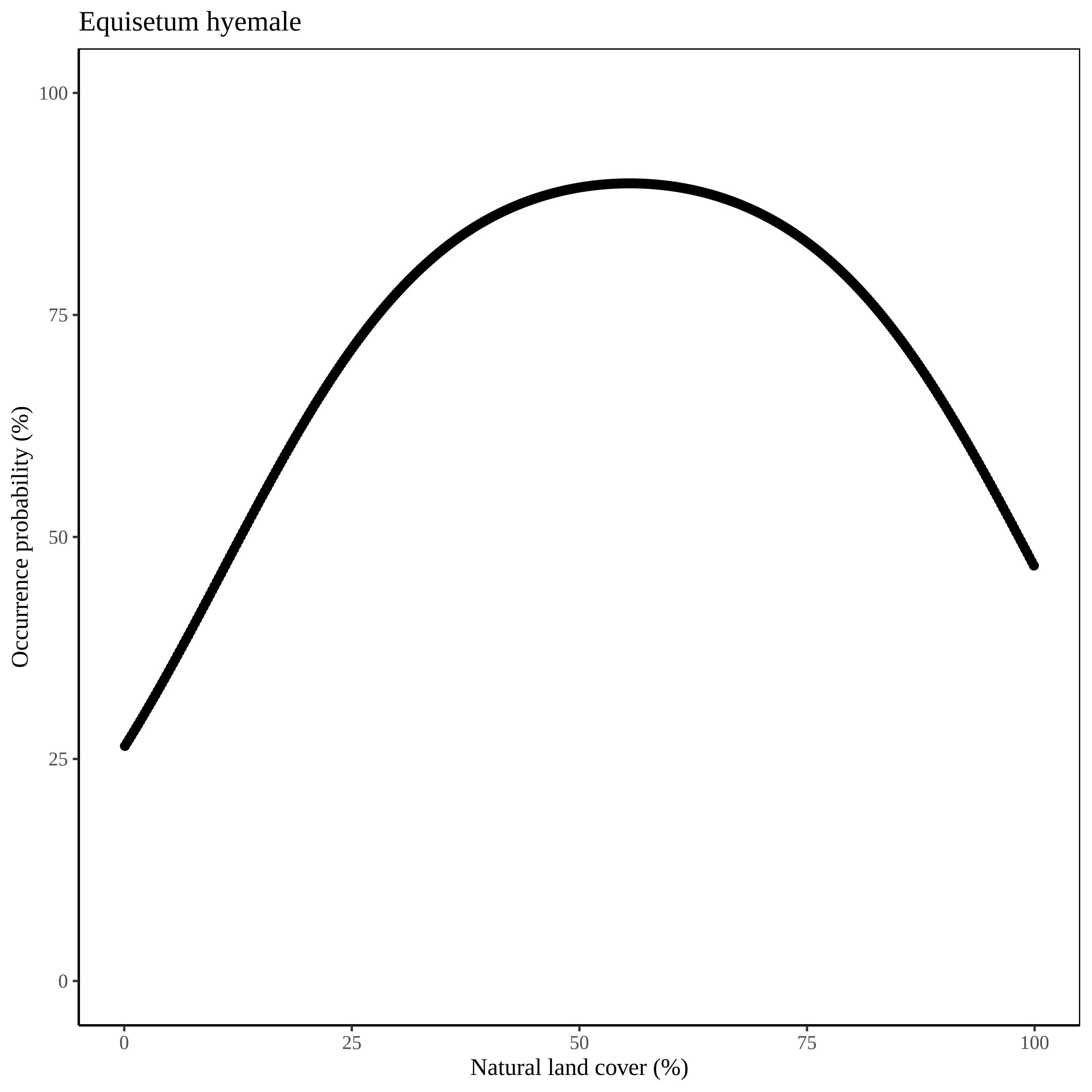
Equisetum hyemale
Occurrence probability (%)
100
75
50
25
0
0
25
50
75
100
Natural land cover (%)

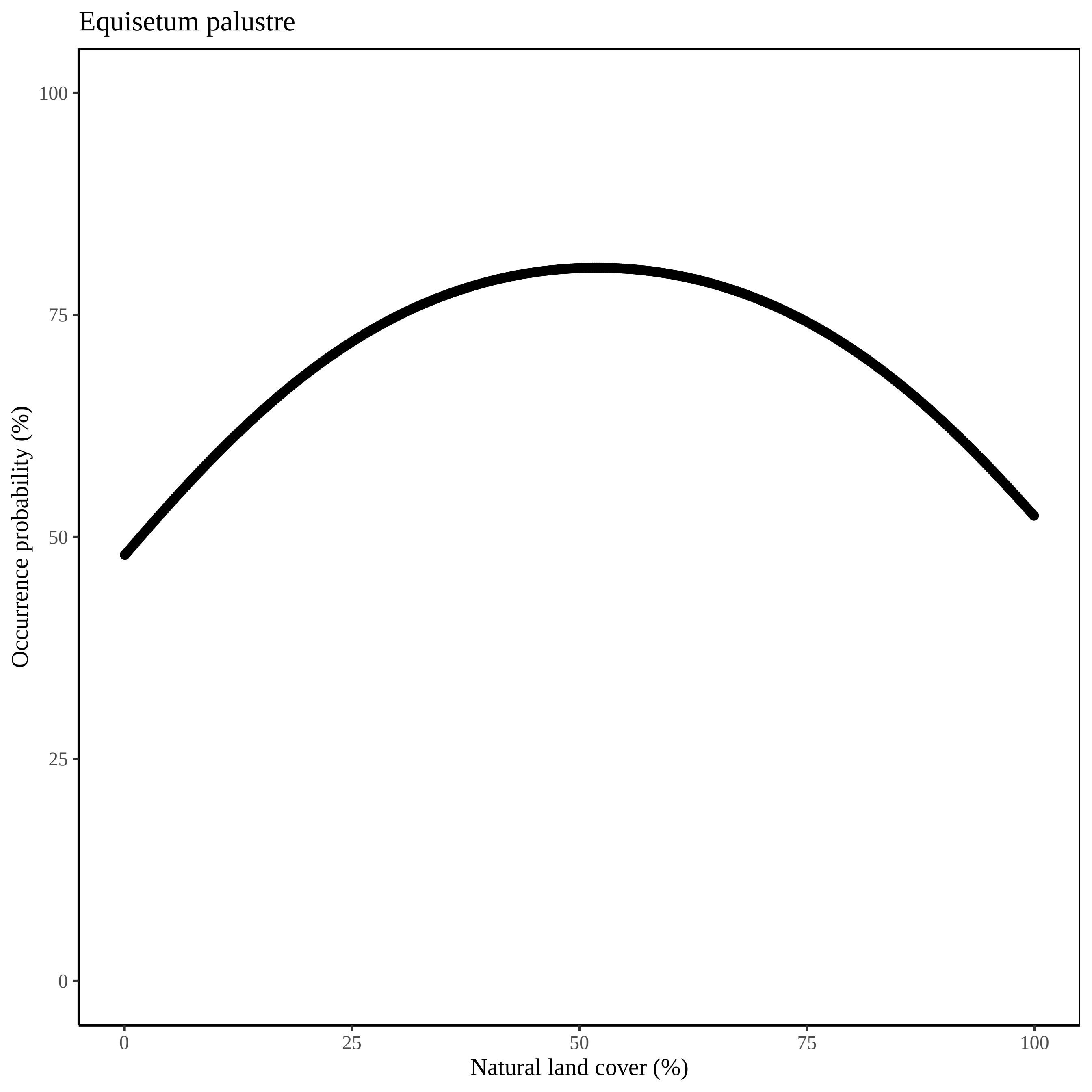
Equisetum palustre
Occurrence probability (%)
100
75
50
25
0
0
25
50
75
100
Natural land cover (%)

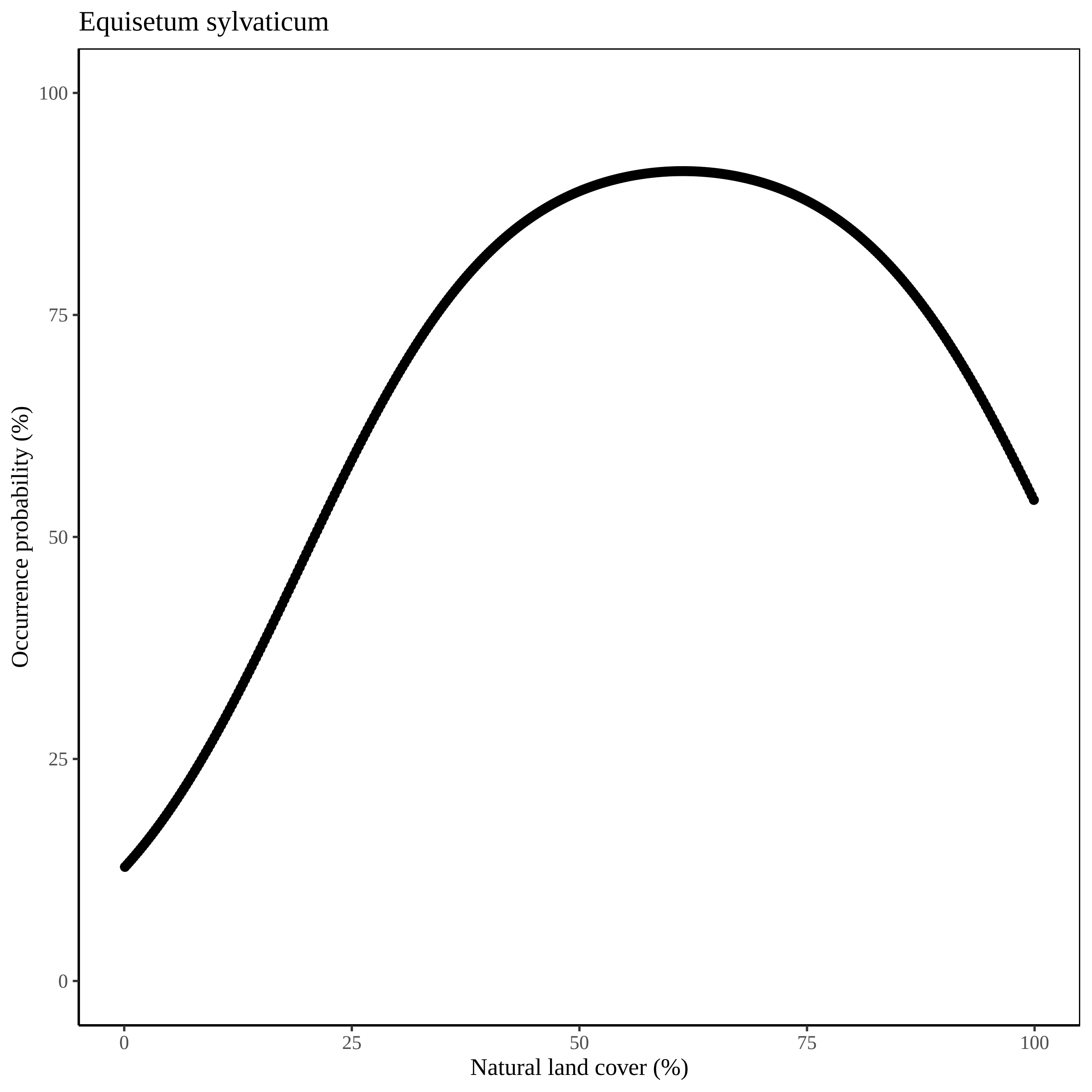
Equisetum sylvaticum
Occurrence probability (%)
100
75
50
25
0
0
25
50
75
100
Natural land cover (%)

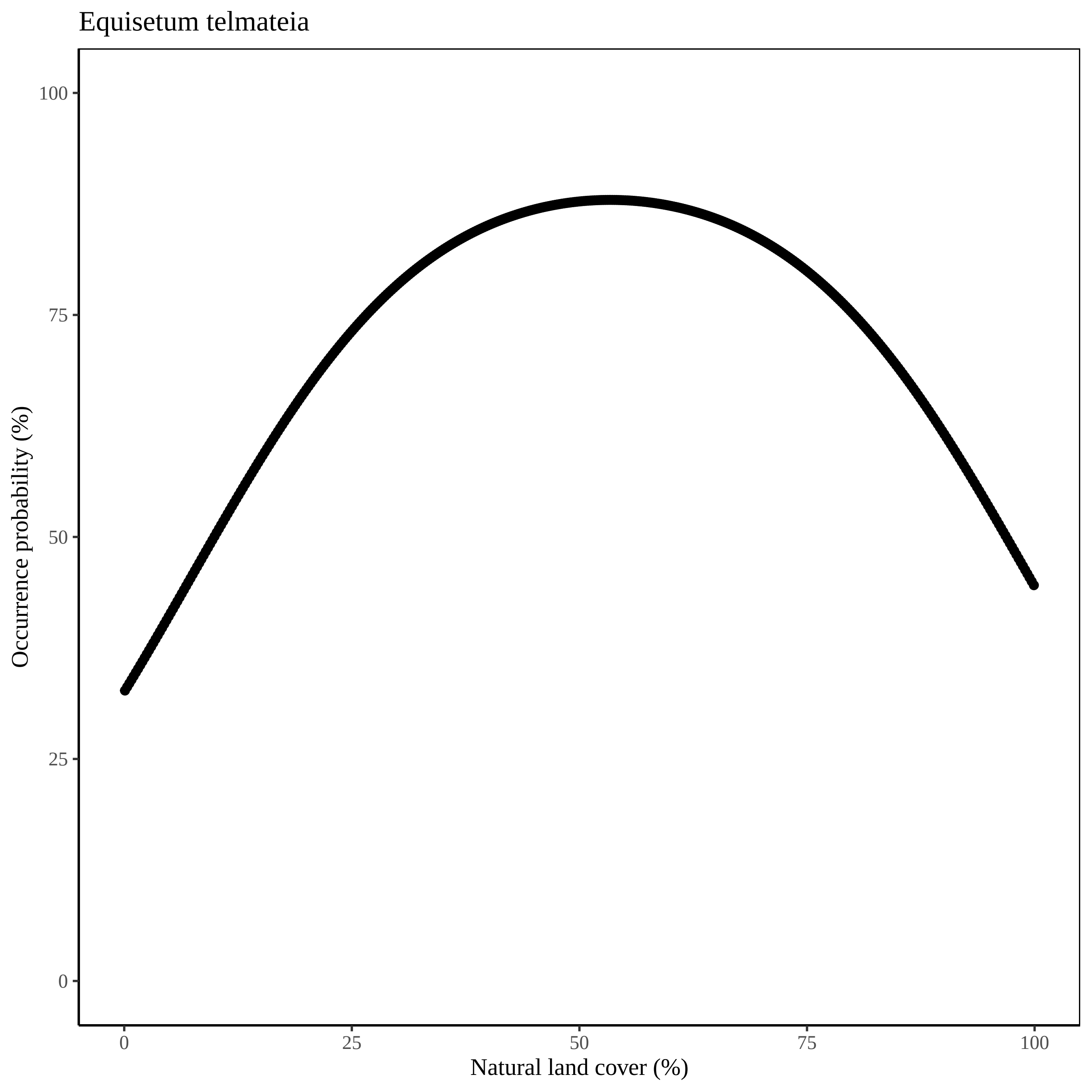
Equisetum telmateia
Occurrence probability (%)
100
75
50
25
0
0
25
50
75
100
Natural land cover (%)

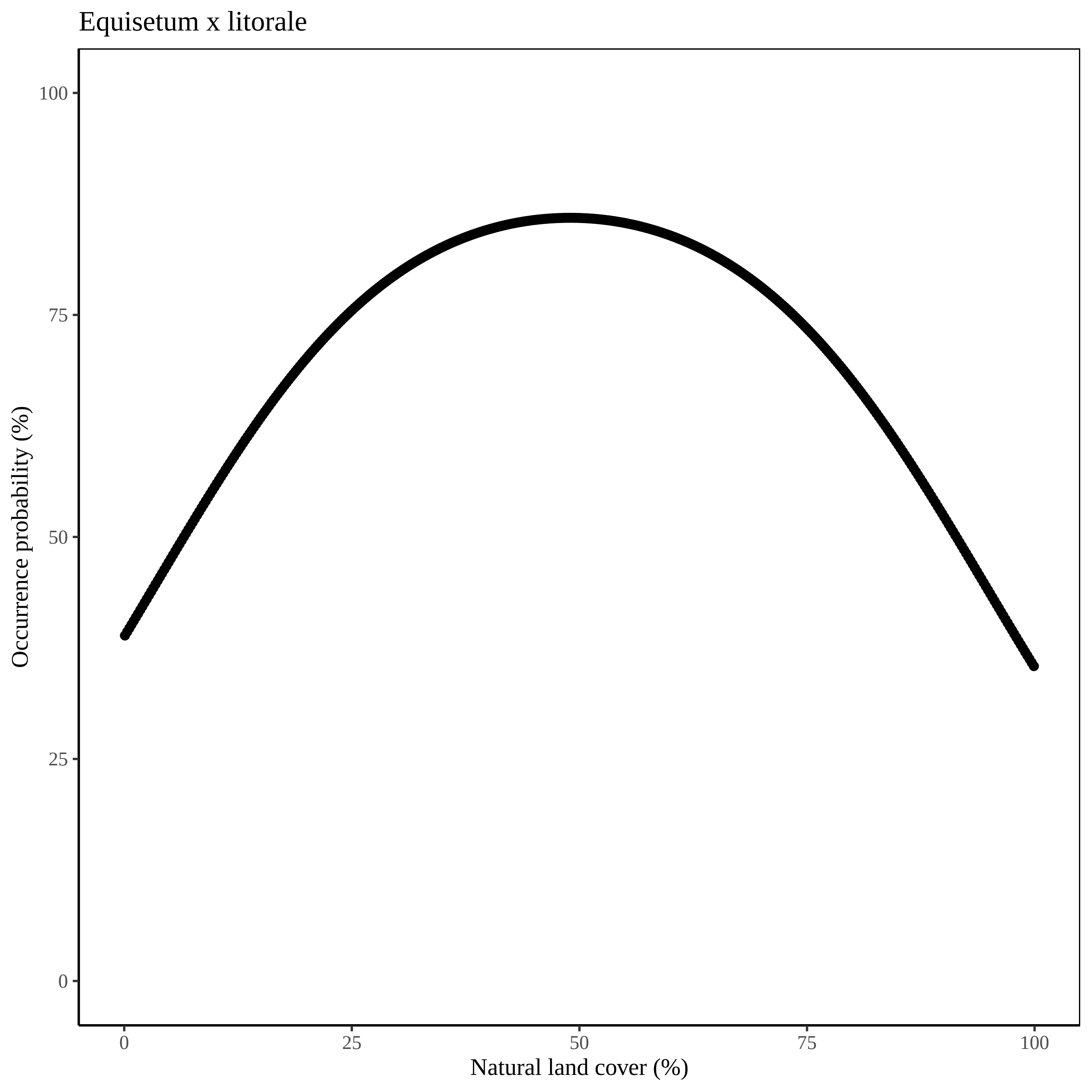
Equisetum x litorale
Occurrence probability (%)
100
75
50
25
0
0
25
50
75
100
Natural land cover (%)

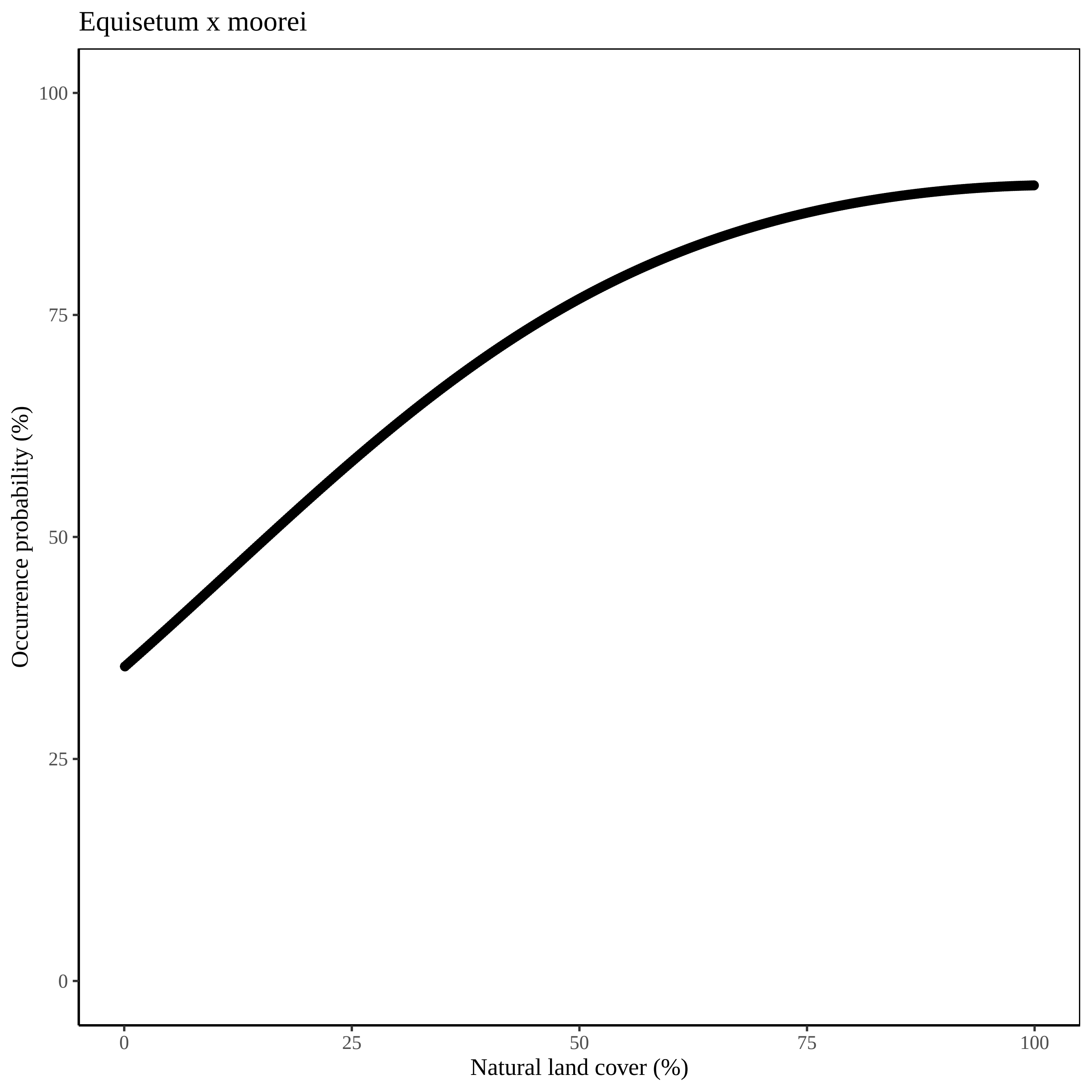
Equisetum x moorei
Occurrence probability (%)
100
75
50
25
0
0
25
50
75
100
Natural land cover (%)

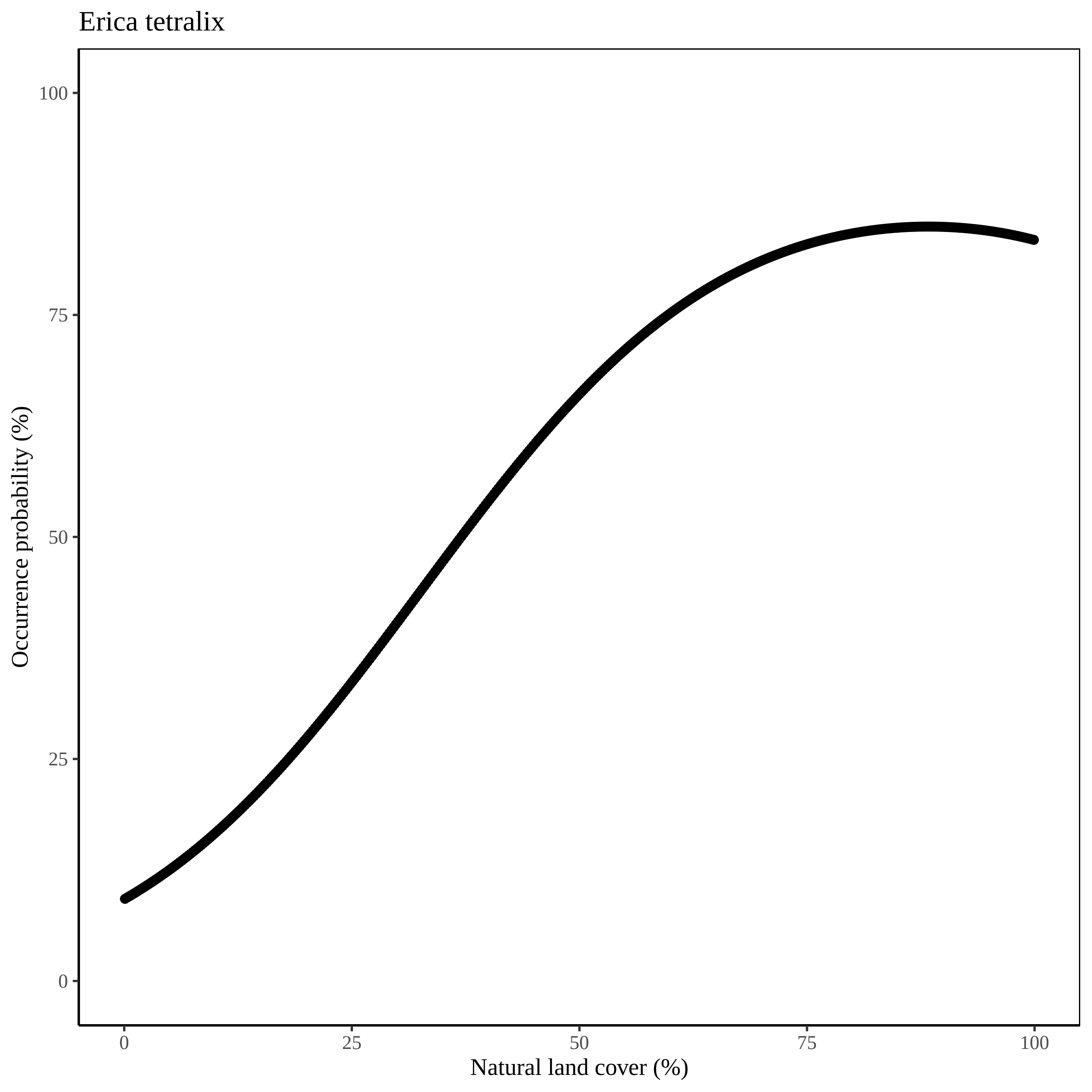

Erica tetralix
Occurrence probability (%)
100
75
50
25
0
0
25
50
75
100
Natural land cover (%)

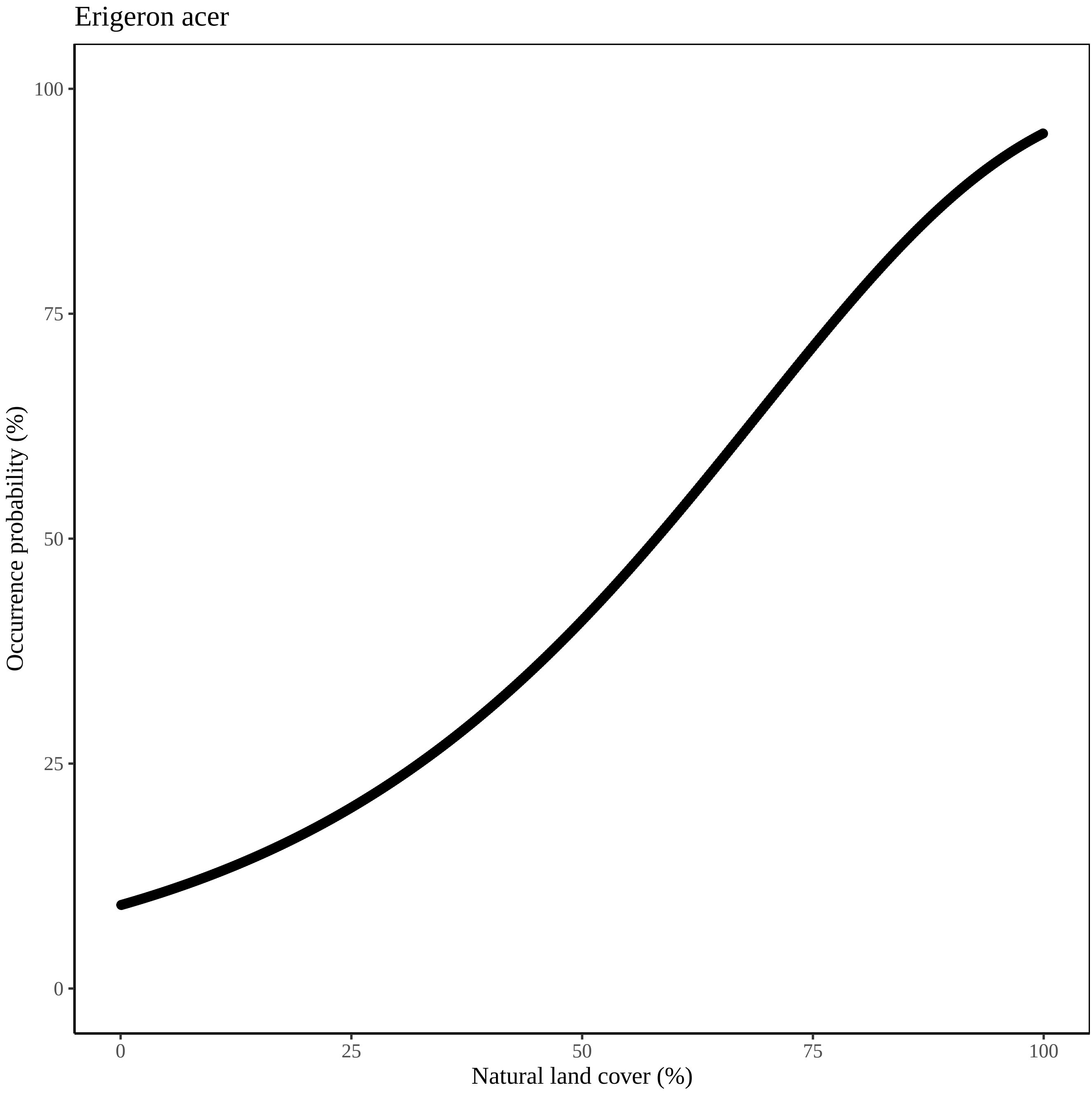

Erigeron acer
100
75
50
25
0
Occurrence probability (%)
0
25
50
75
100
Natural land cover (%)

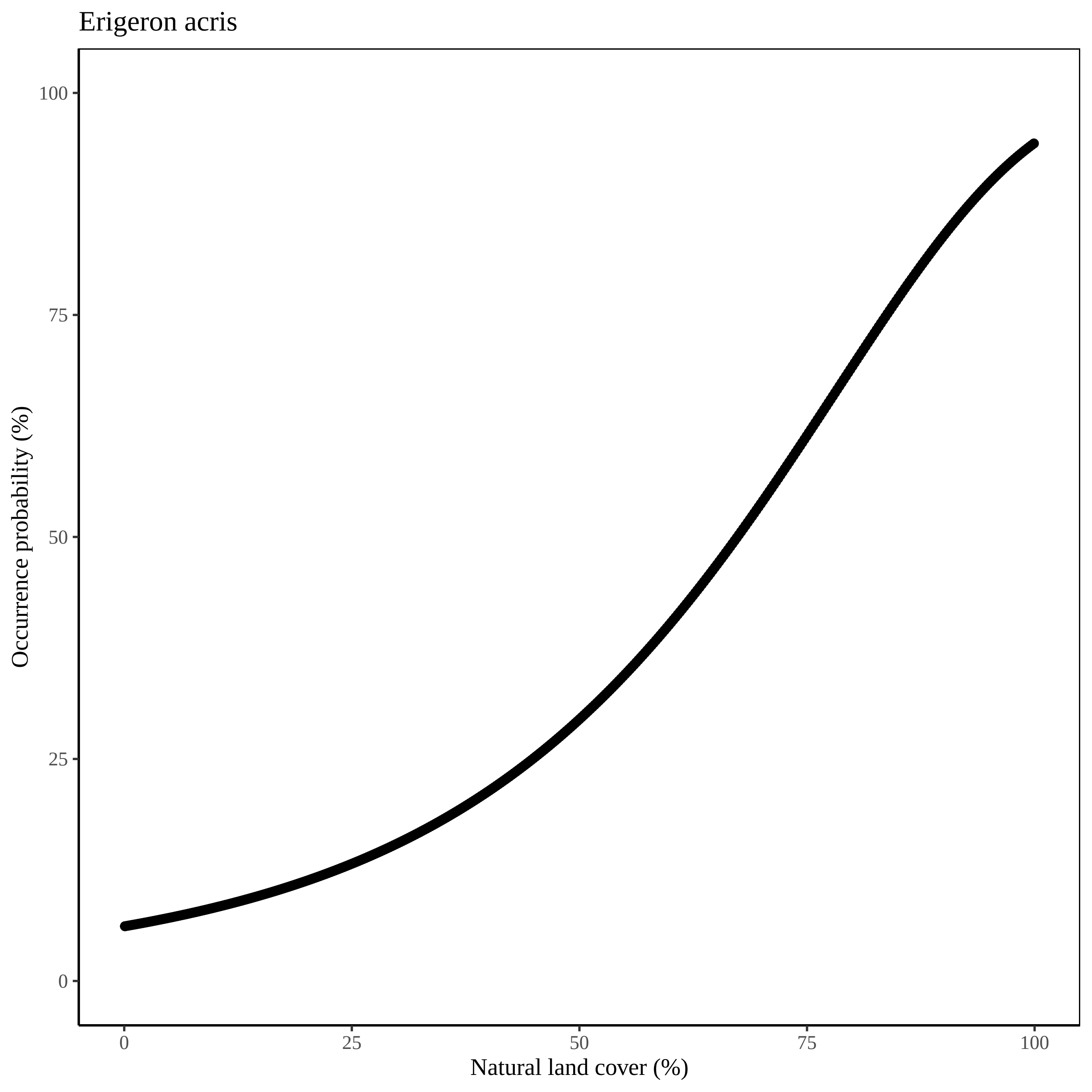
Erigeron acris
Occurrence probability (%)
100
75
50
25
0
0
25
50
75
100
Natural land cover (%)

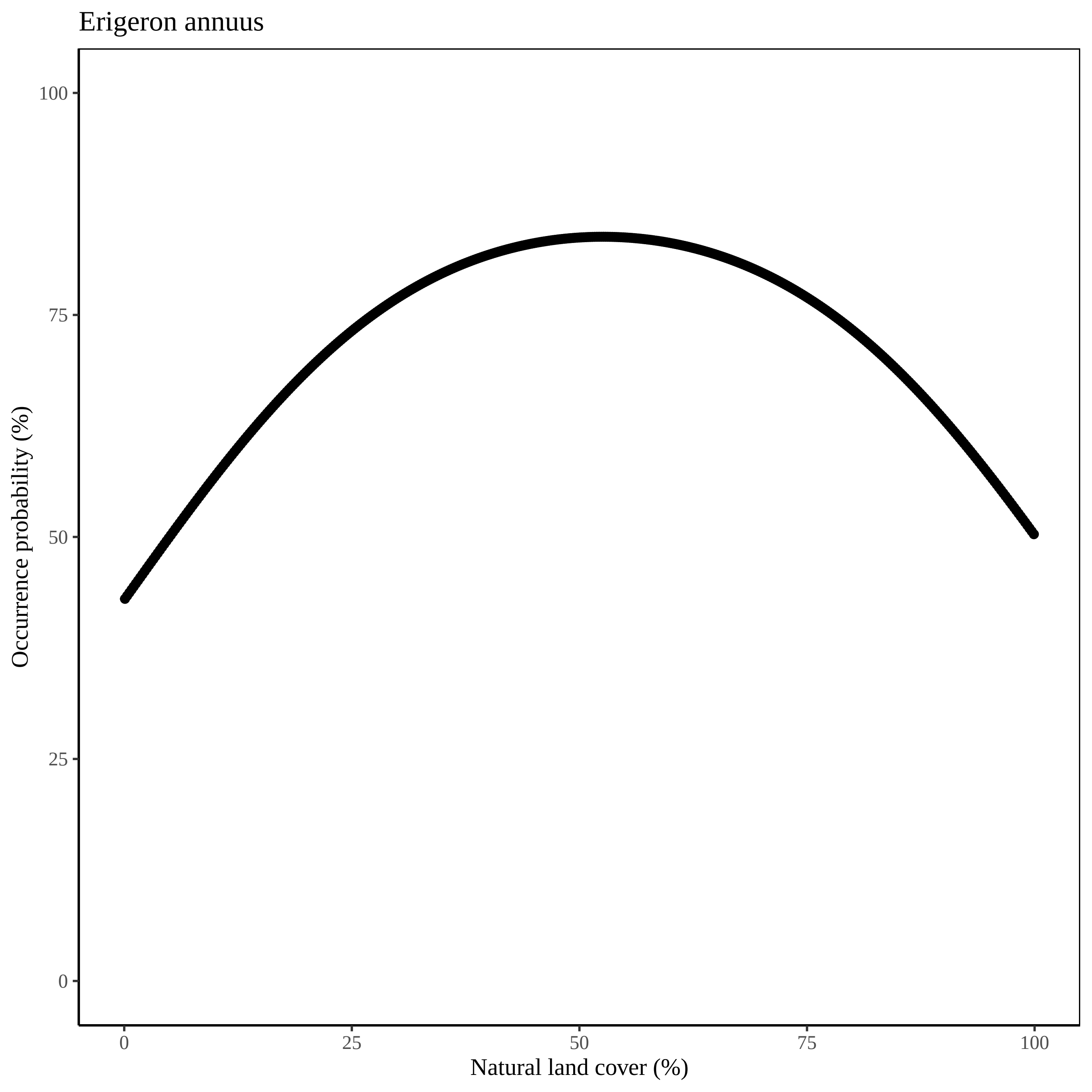
Erigeron annuus
Occurrence probability (%)
100
75
50
25
0
0
25
50
75
100
Natural land cover (%)

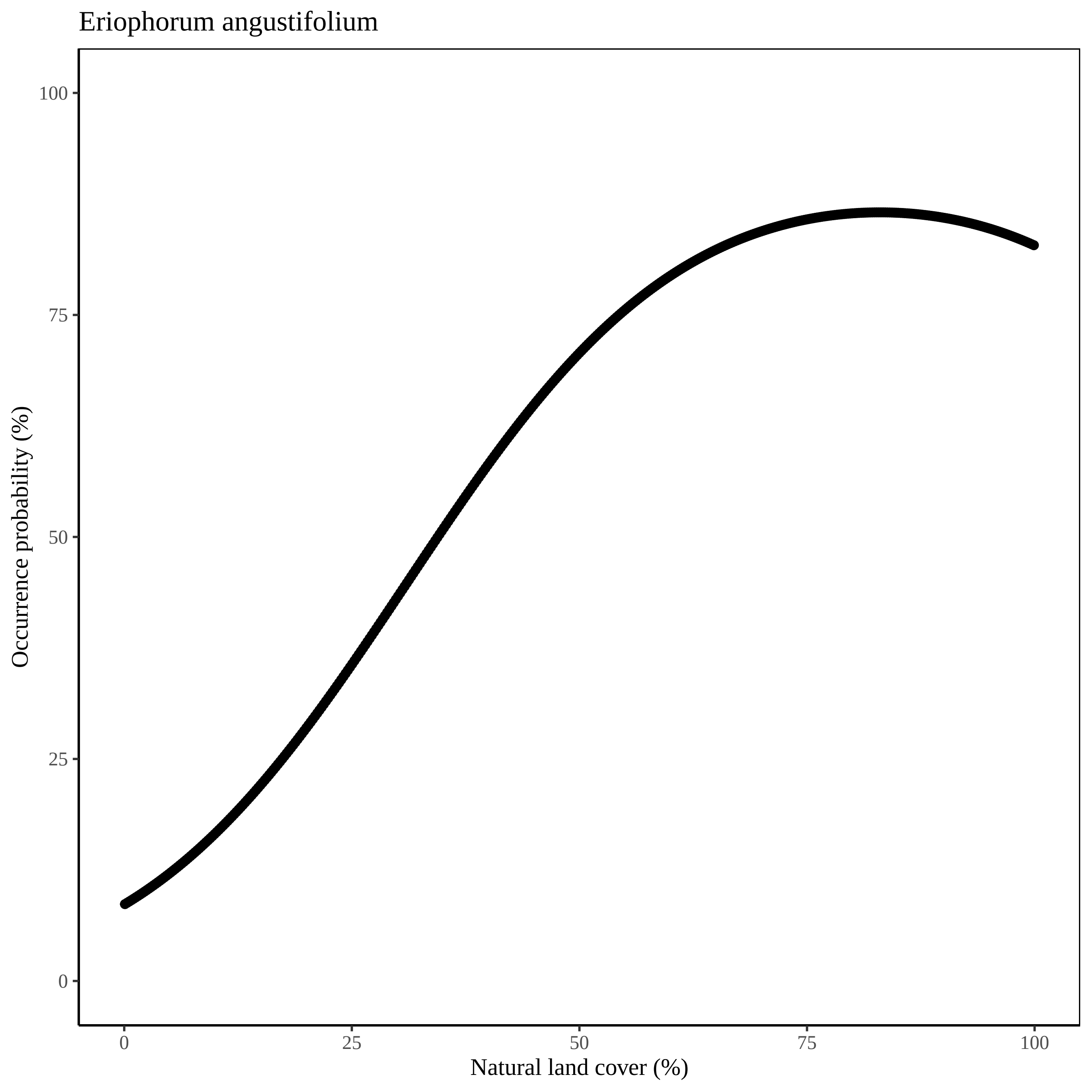

Eriophorum angustifolium
100
75
50
25
0
Occurrence probability (%)
0
25
50
75
100
Natural land cover (%)

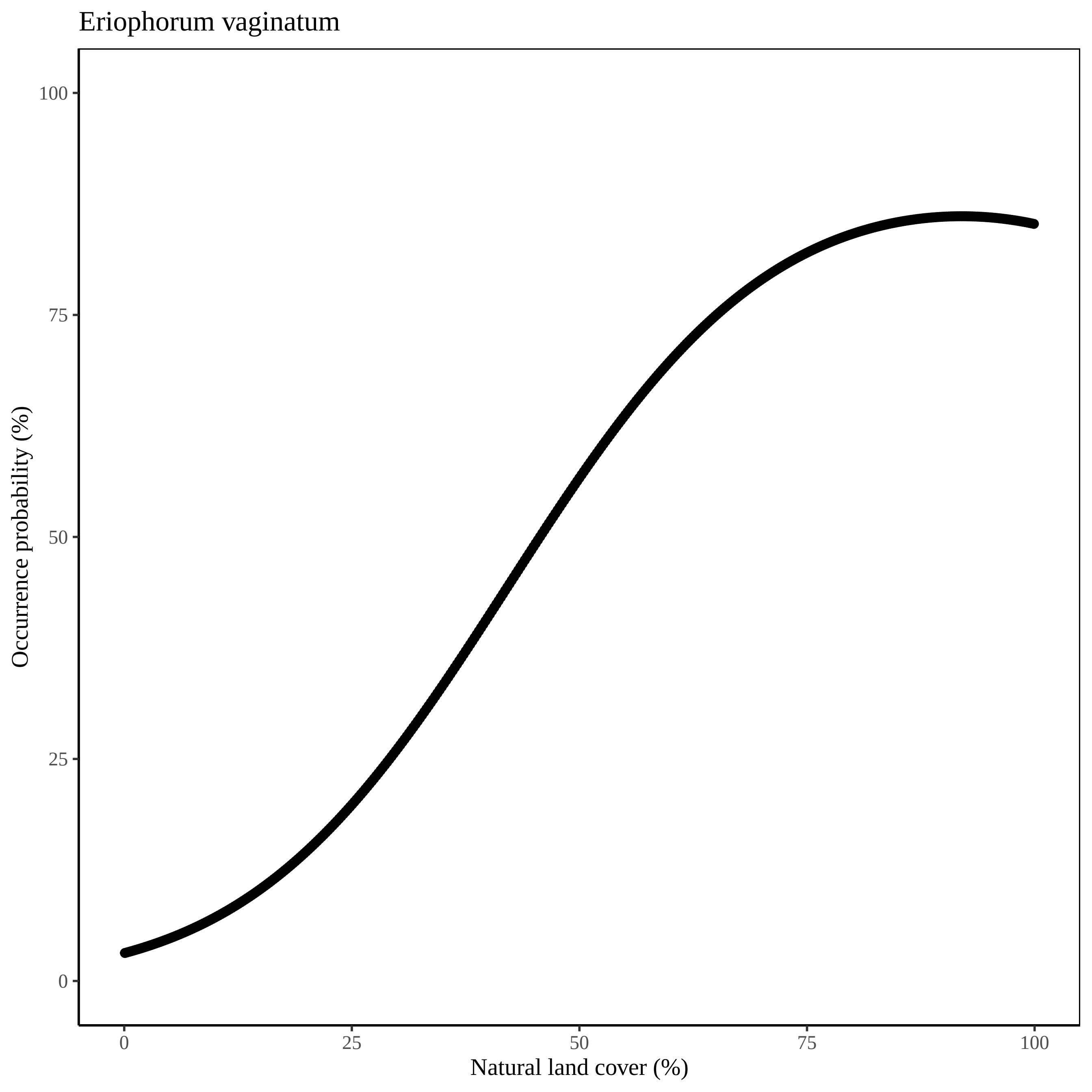

Eriophorum vaginatum
Occurrence probability (%)
100
75
50
25
0
0
25
50
75
100
Natural land cover (%)

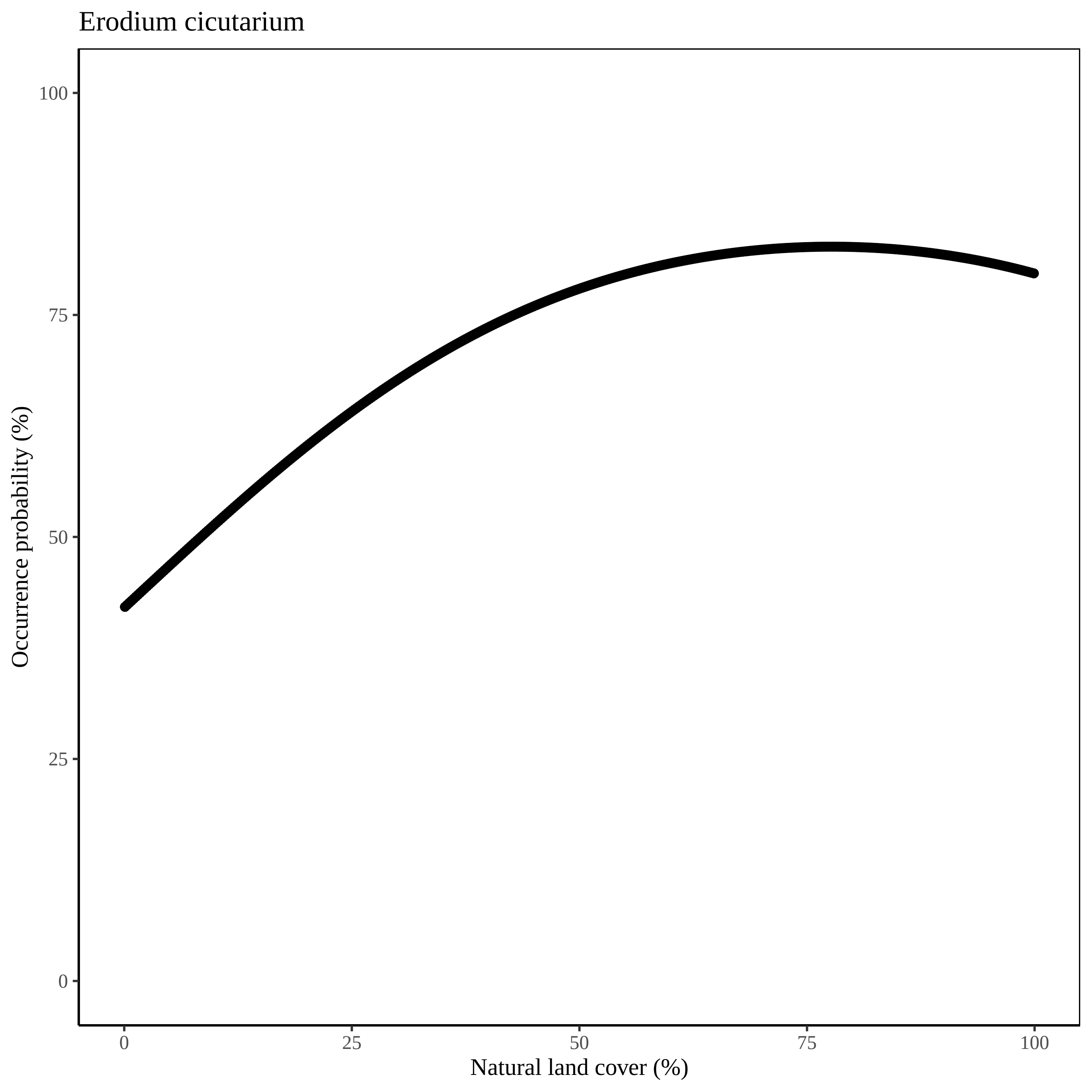

Erodium cicutarium
100
75
50
25
0
Occurrence probability (%)
0
25
50
75
100
Natural land cover (%)

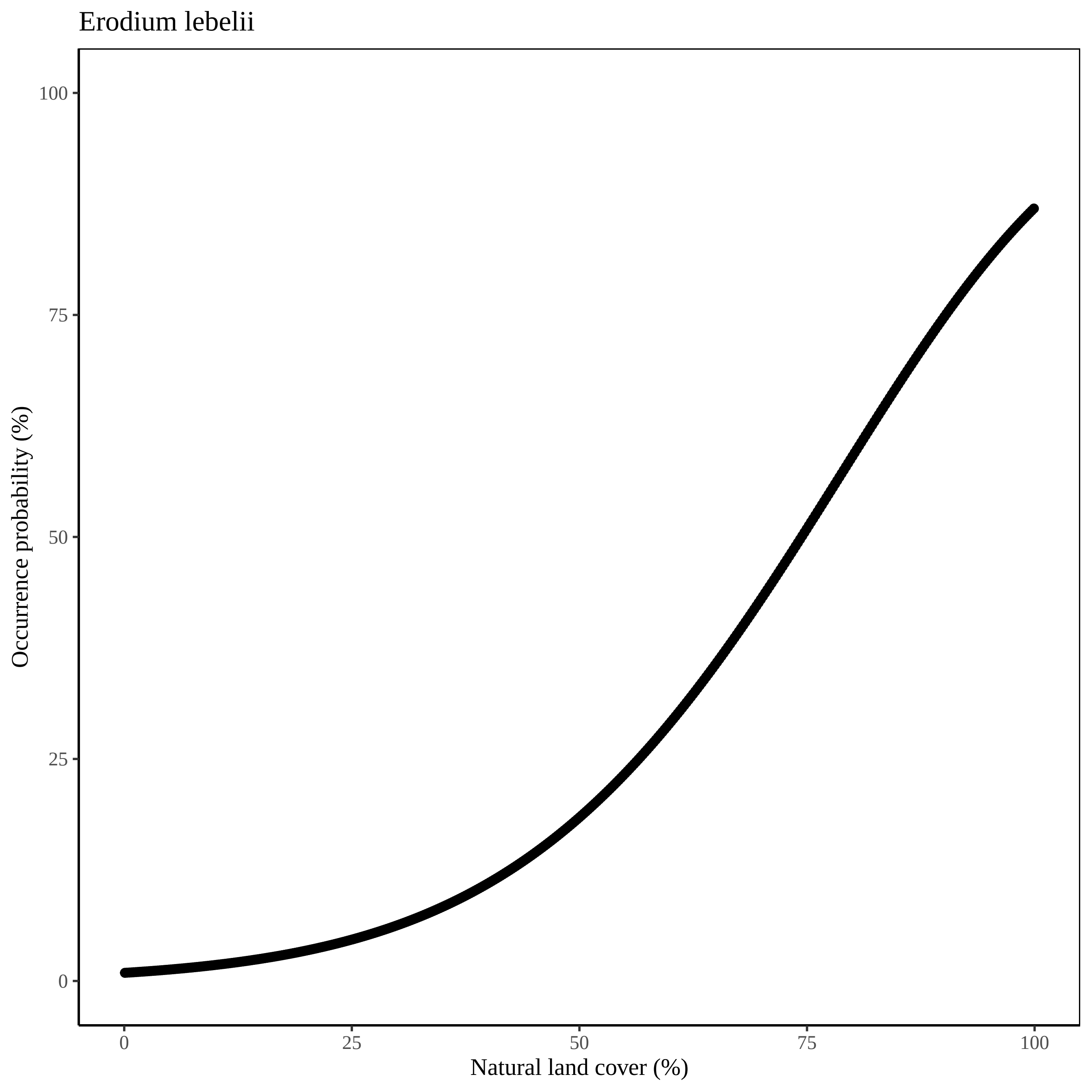

Erodium lebelii
Occurrence probability (%)
100
75
50
25
0
0
25
50
75
100
Natural land cover (%)

Erophila verna

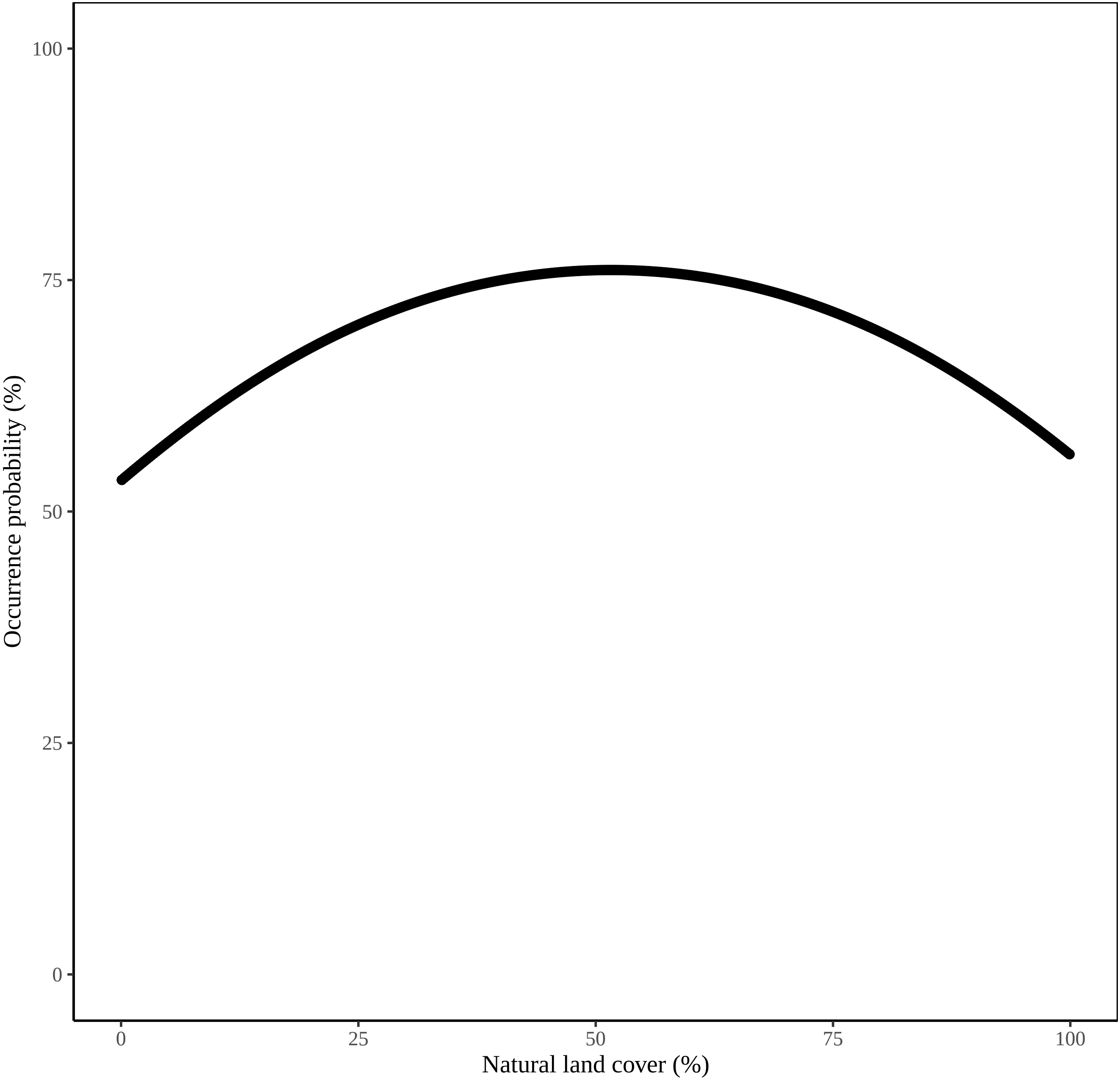

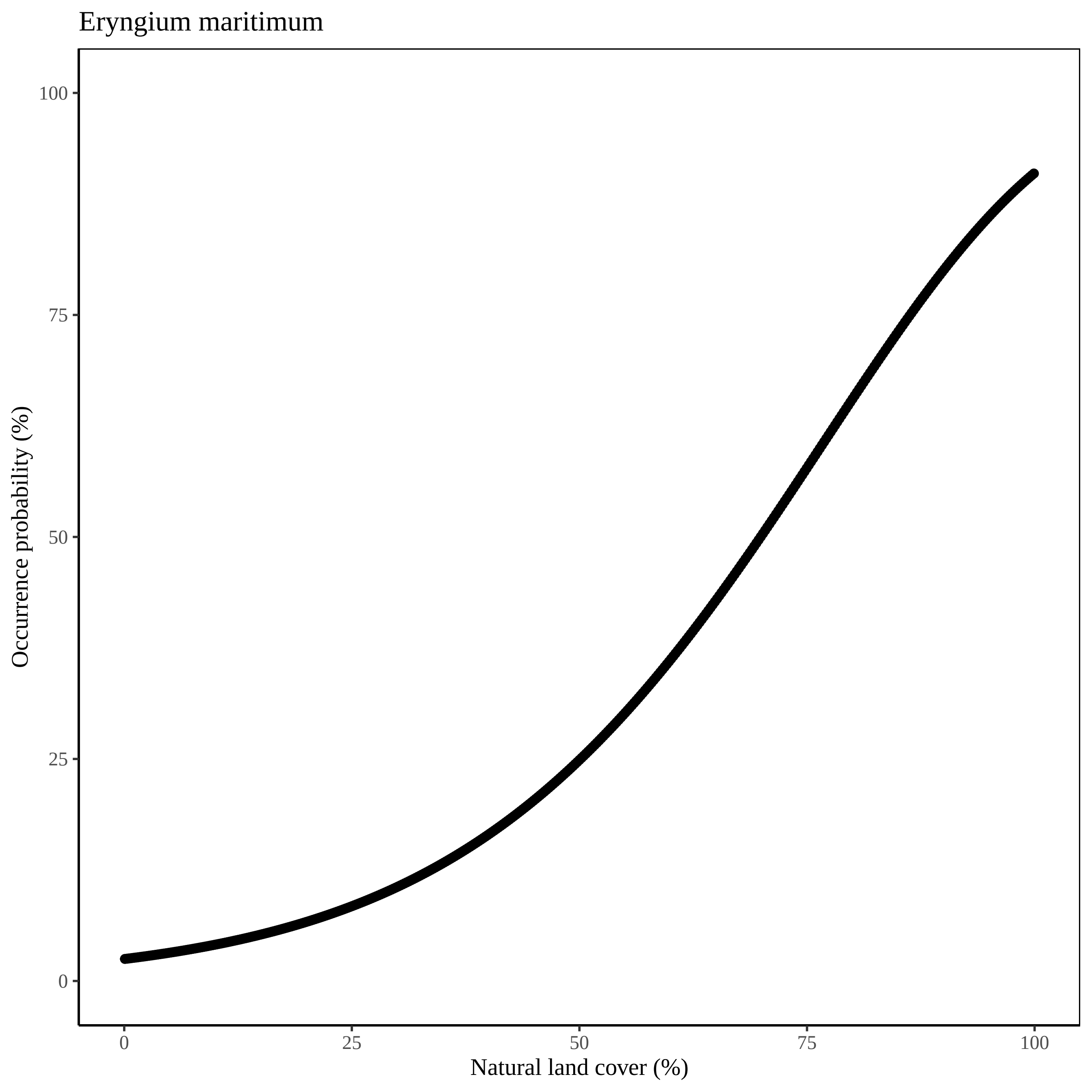

Eryngium maritimum
Occurrence probability (%)
100
75
50
25
0
0
25
50
75
100
Natural land cover (%)

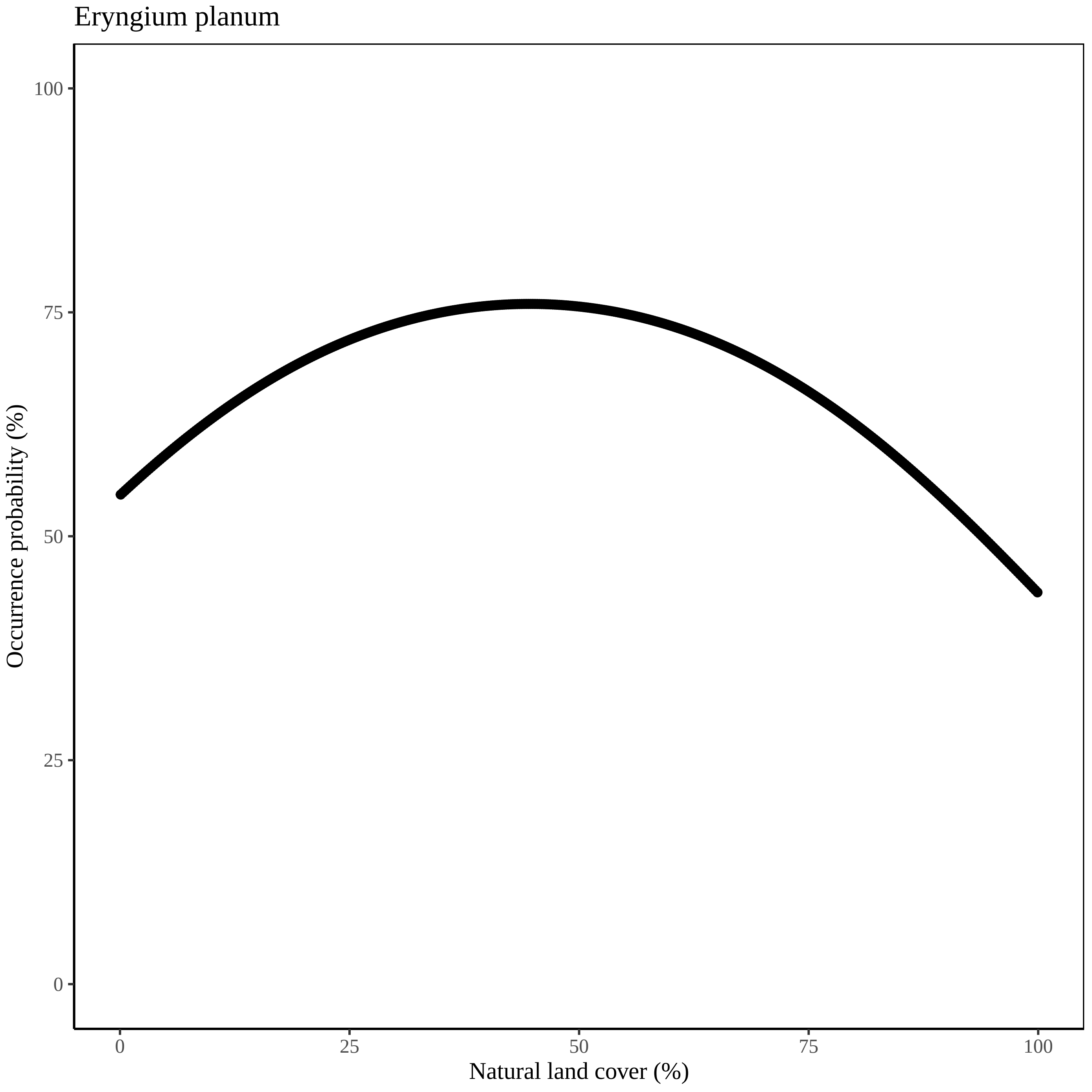

Eryngium planum
Occurrence probability (%)
100
75
50
25
0
0
25
50
75
100
Natural land cover (%)

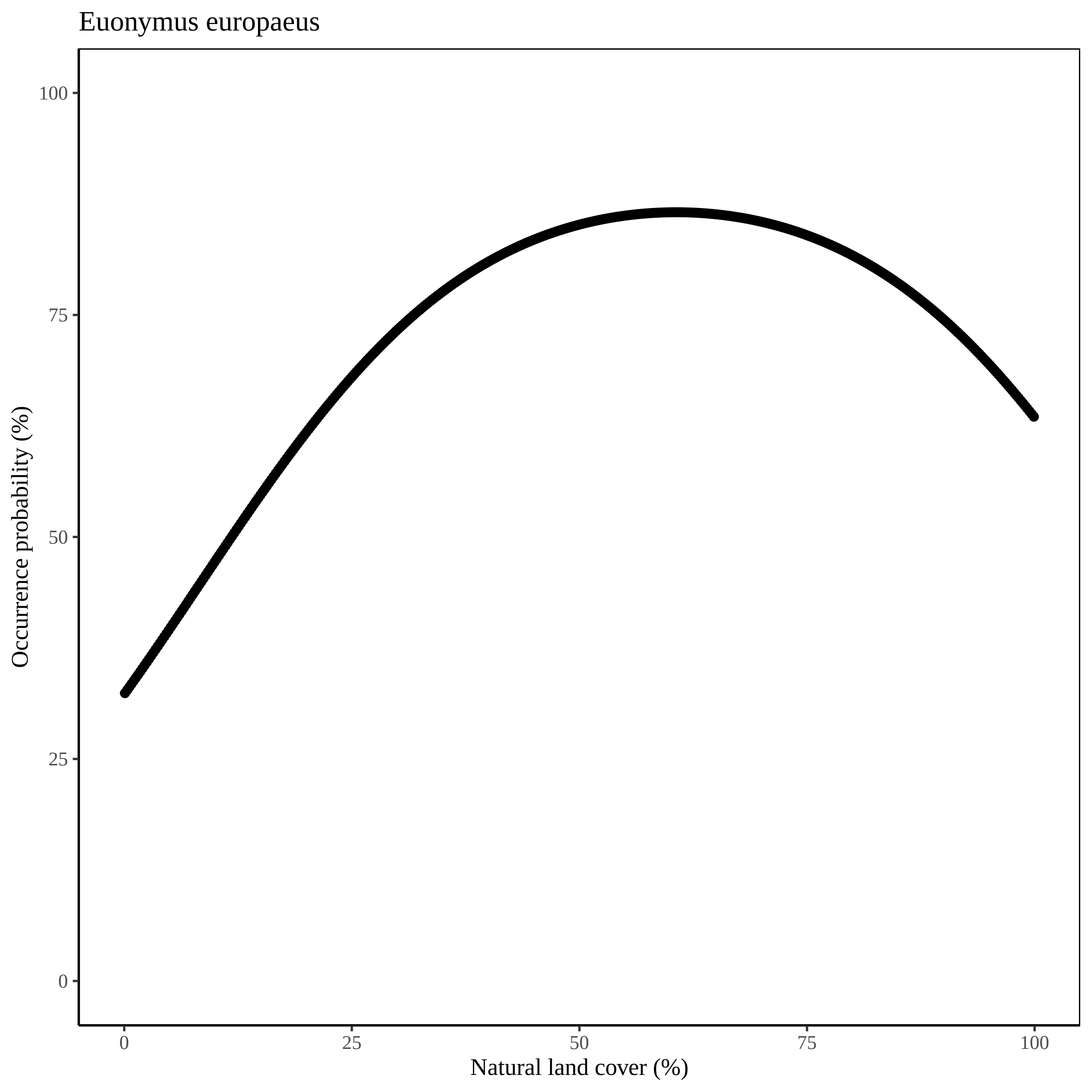

Euonymus europaeus
Occurrence probability (%)
100
75
50
25
0
0
25
50
75
100
Natural land cover (%)

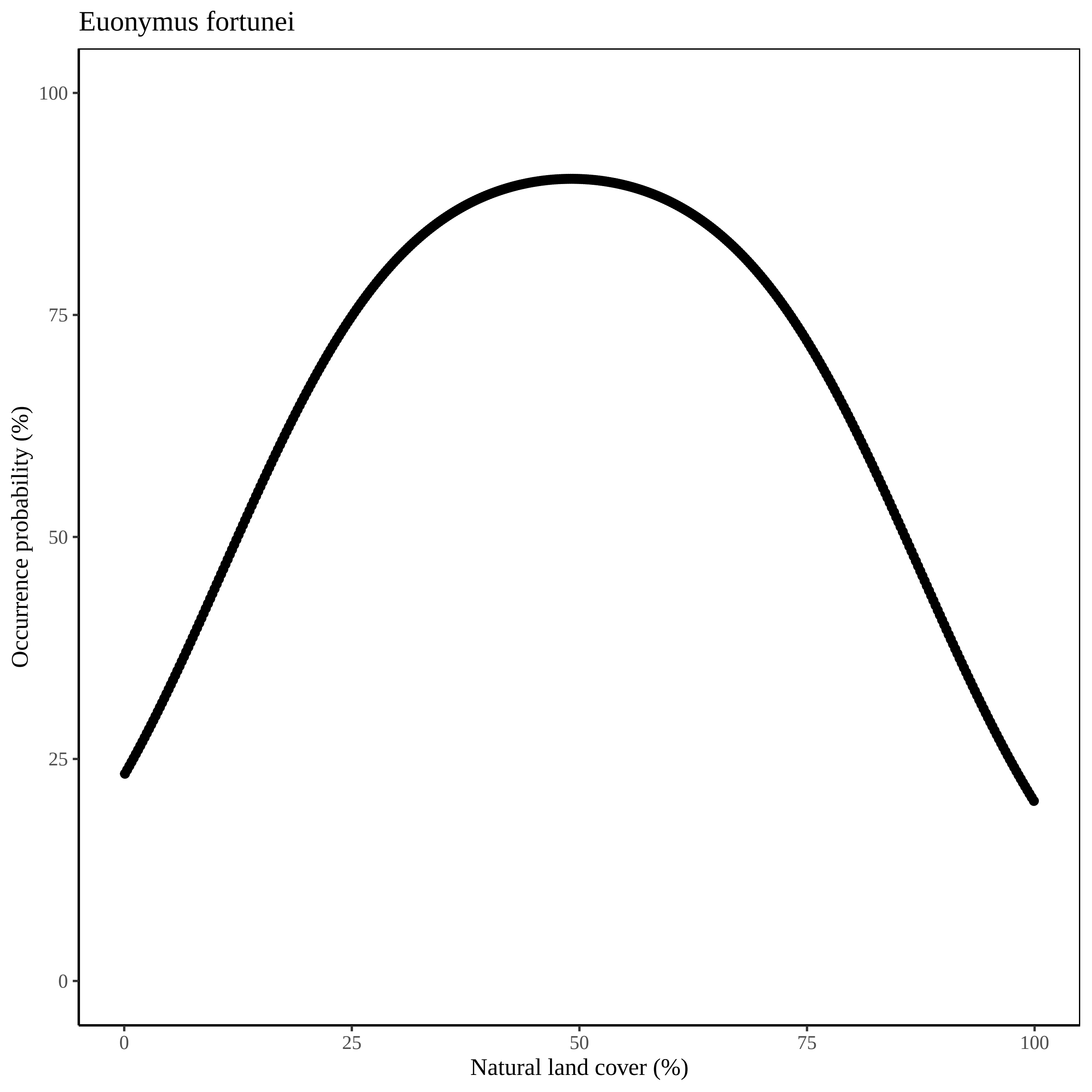

Euonymus fortunei
Occurrence probability (%)
100
75
50
25
0
0
25
50
75
100
Natural land cover (%)

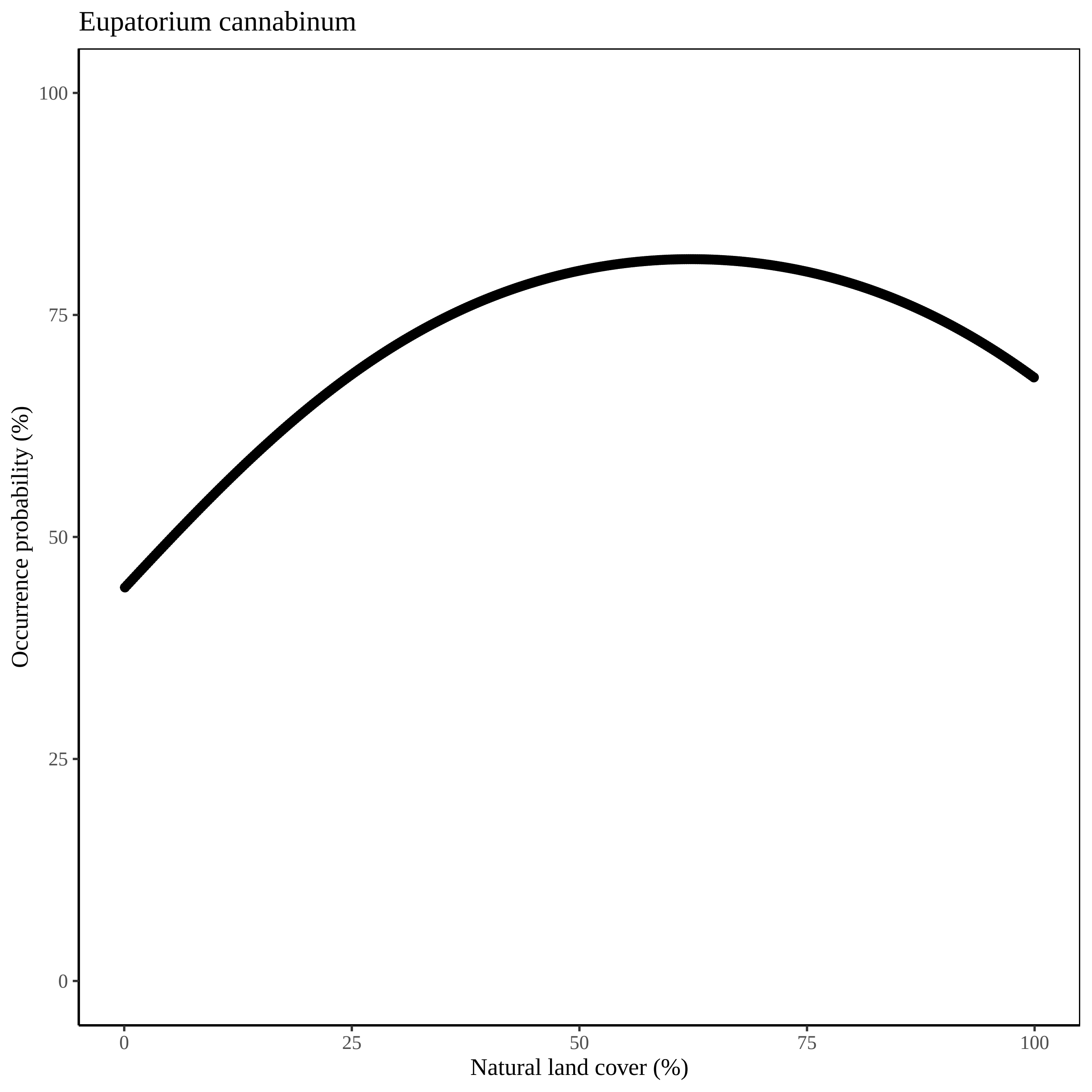
Eupatorium cannabinum
Occurrence probability (%)
100
75
50
25
0
0
25
50
75
100
Natural land cover (%)

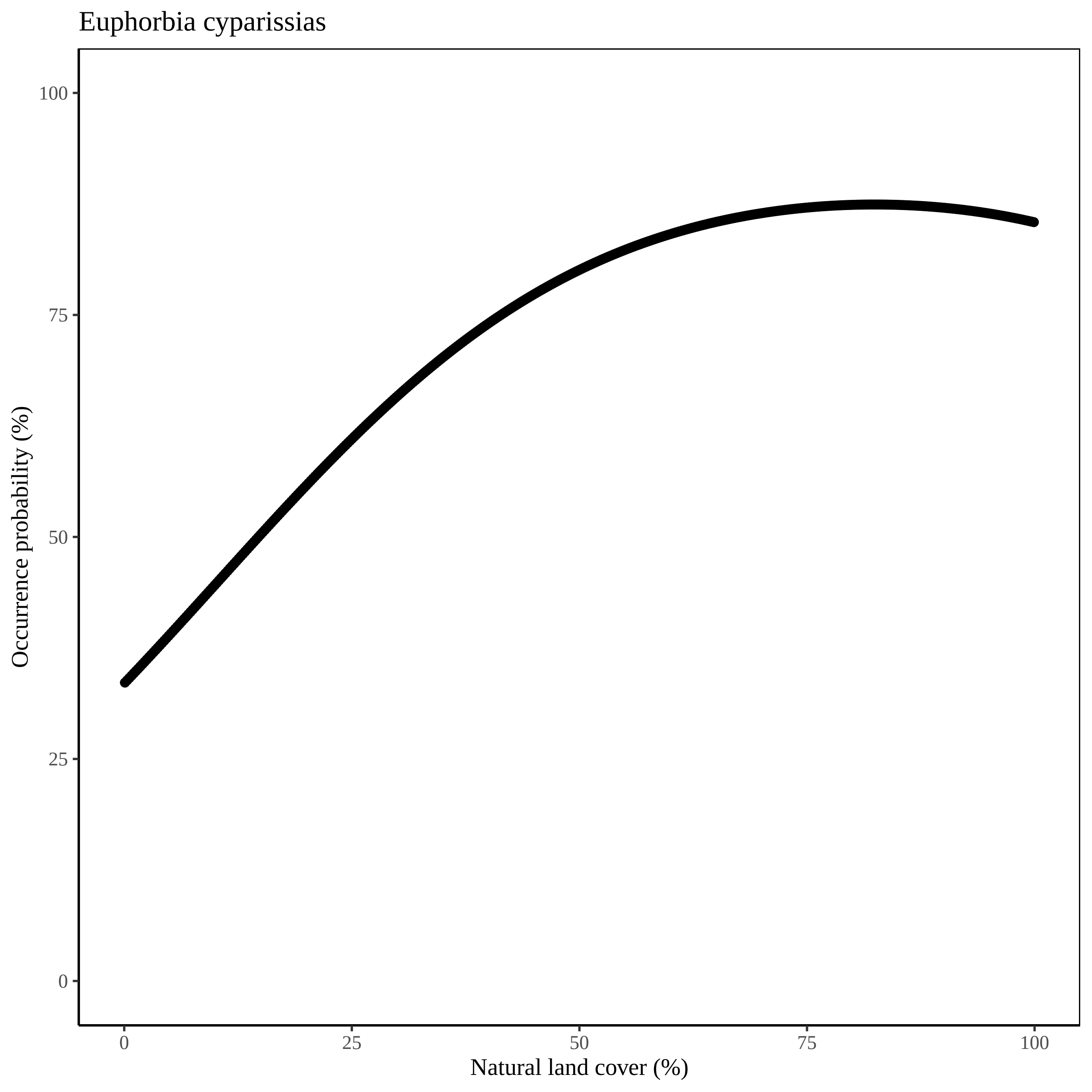
Euphorbia cyparissias
Occurrence probability (%)
100
75
50
25
0
0
25
50
75
100
Natural land cover (%)

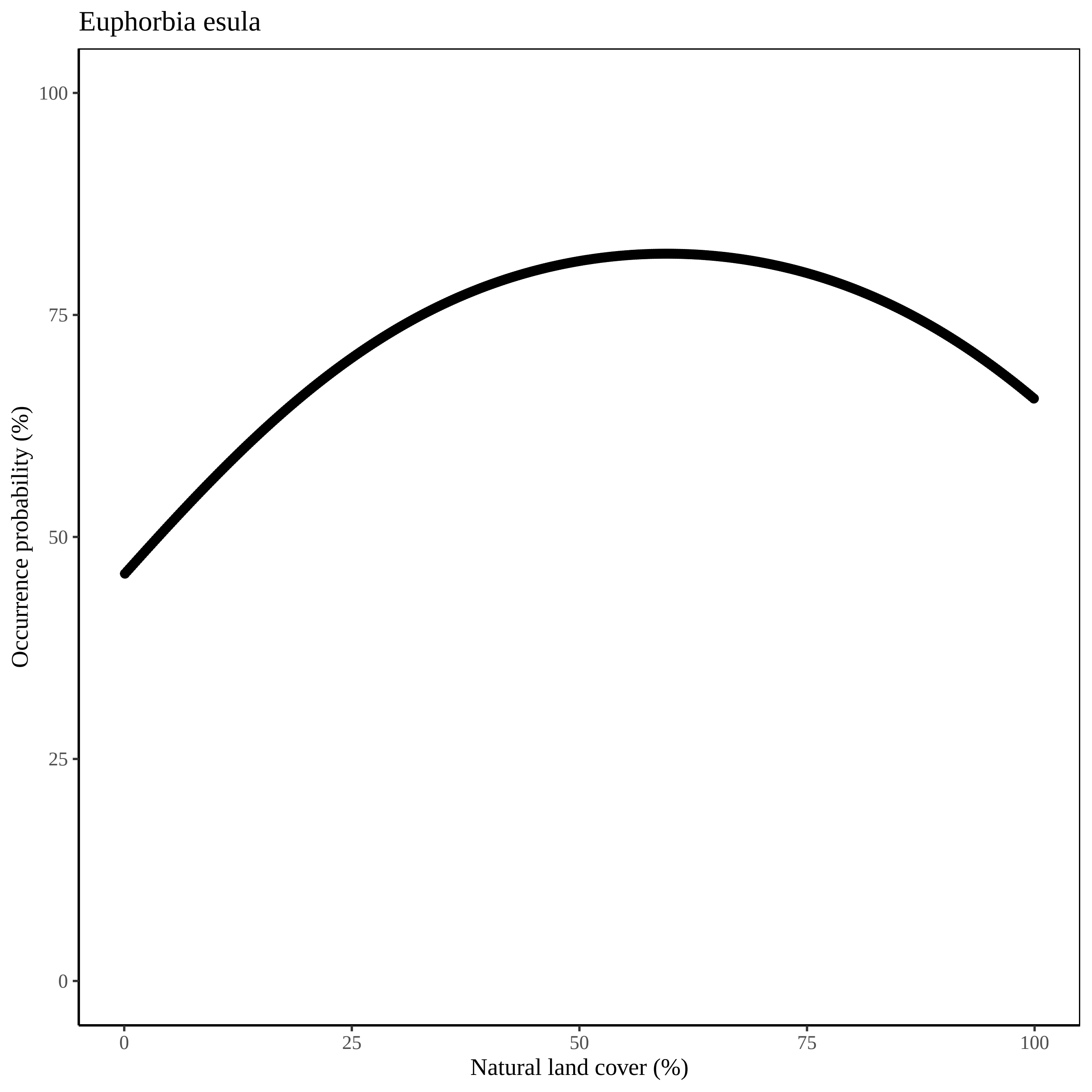
Euphorbia esula
Occurrence probability (%)
100
75
50
25
0
0
25
50
75
100
Natural land cover (%)

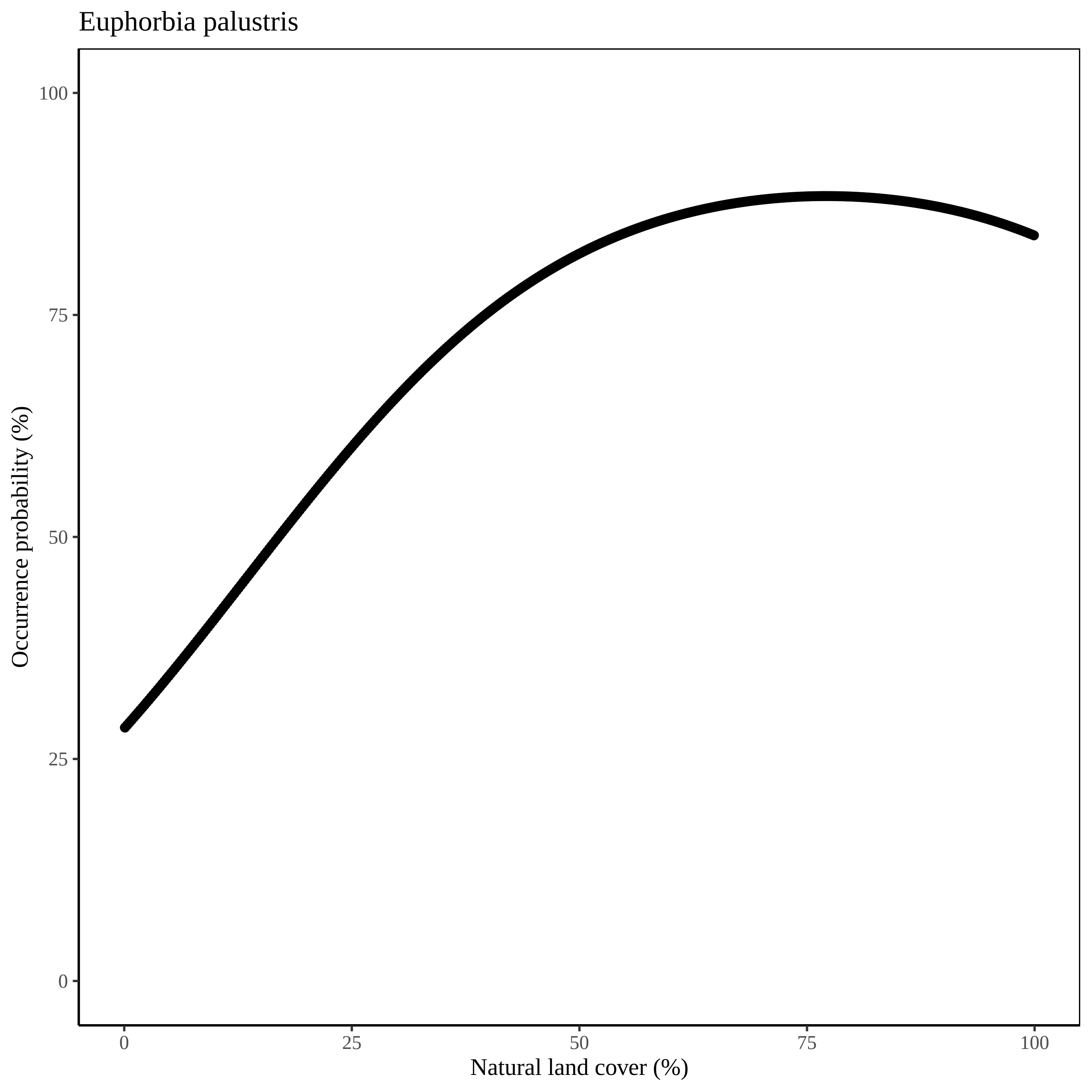
Euphorbia palustris
Occurrence probability (%)
100
75
50
25
0
0
25
50
75
100
Natural land cover (%)

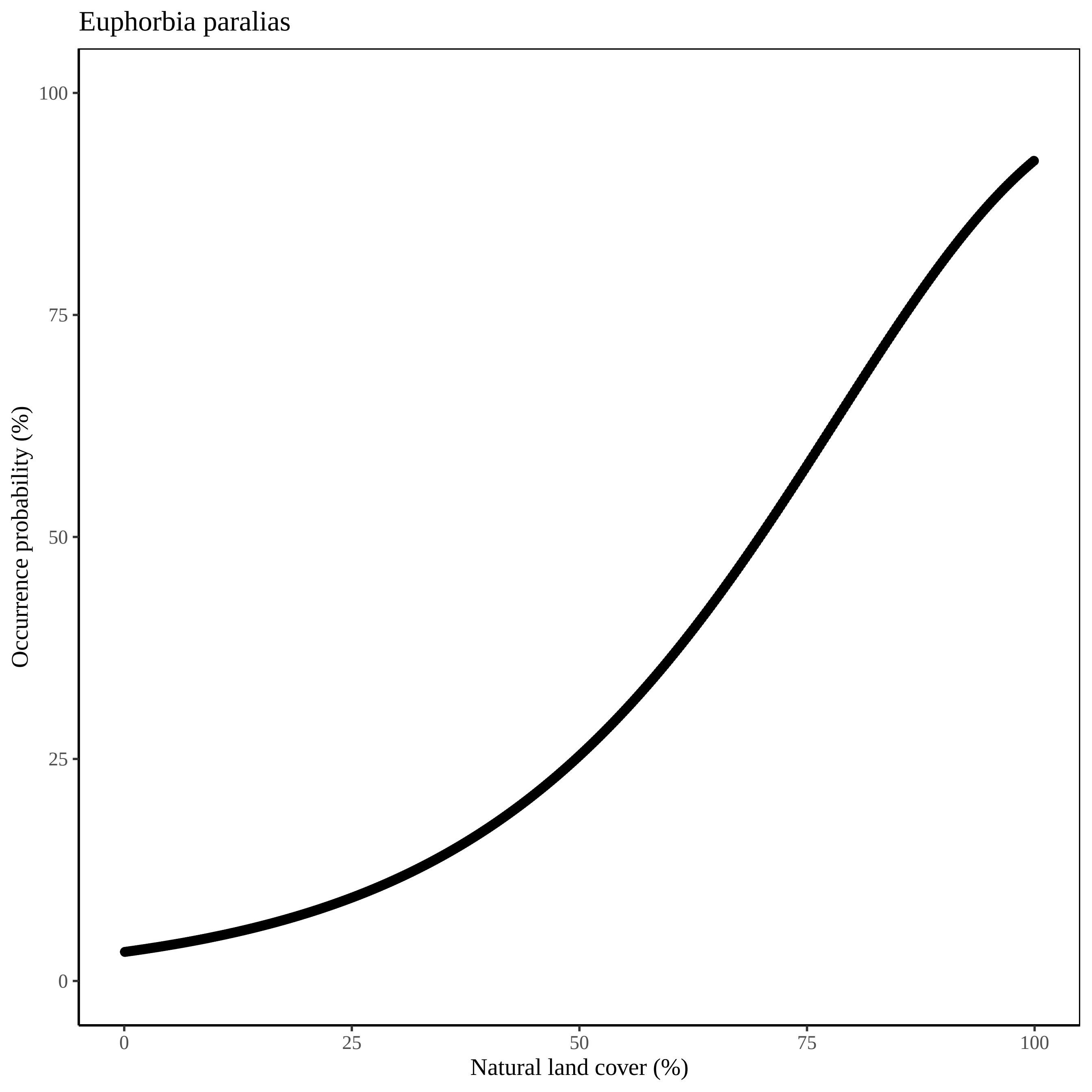

Euphorbia paralias
Occurrence probability (%)
100
75
50
25
0
0
25
50
75
100
Natural land cover (%)

Euphrasia stricta

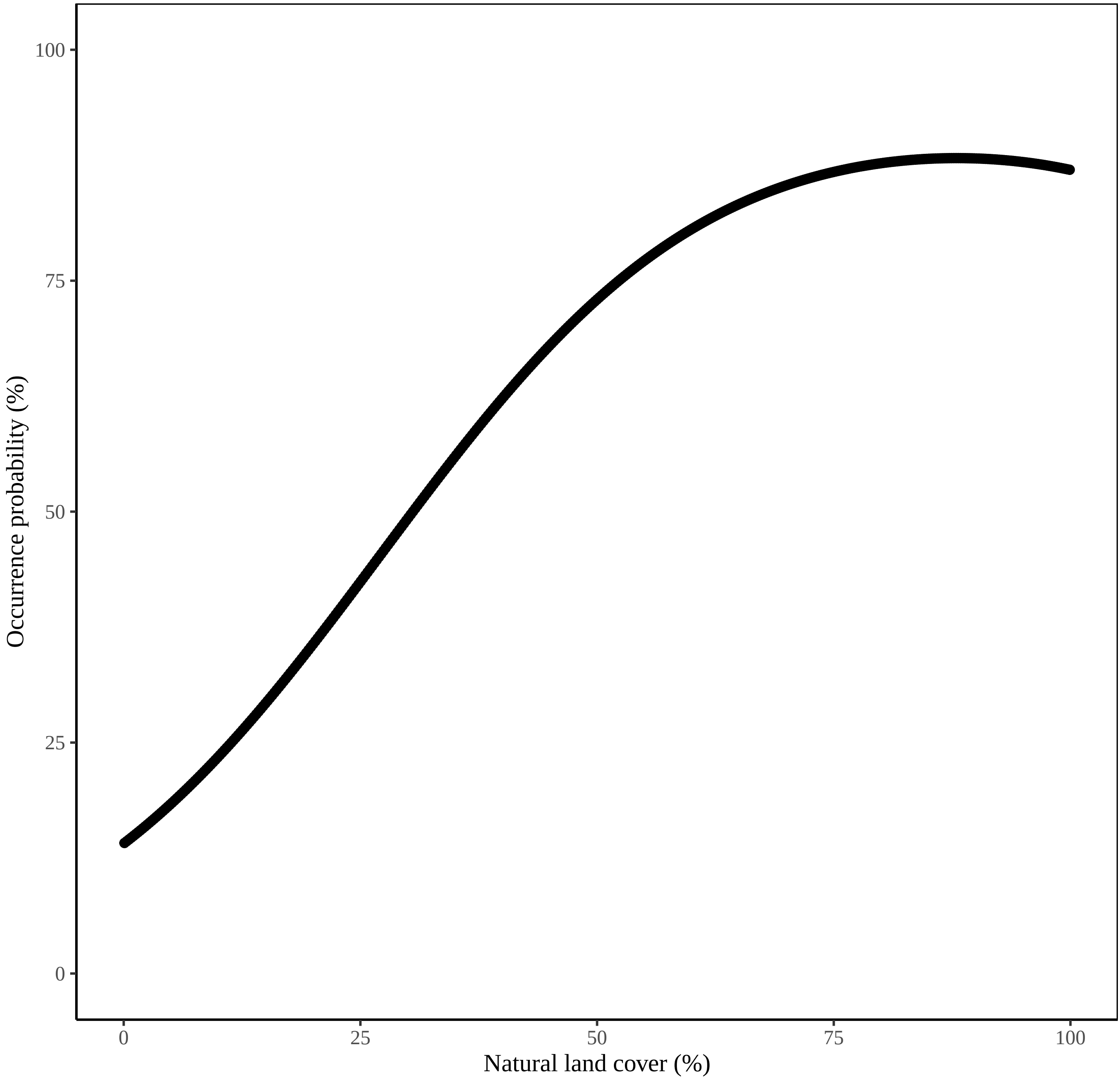

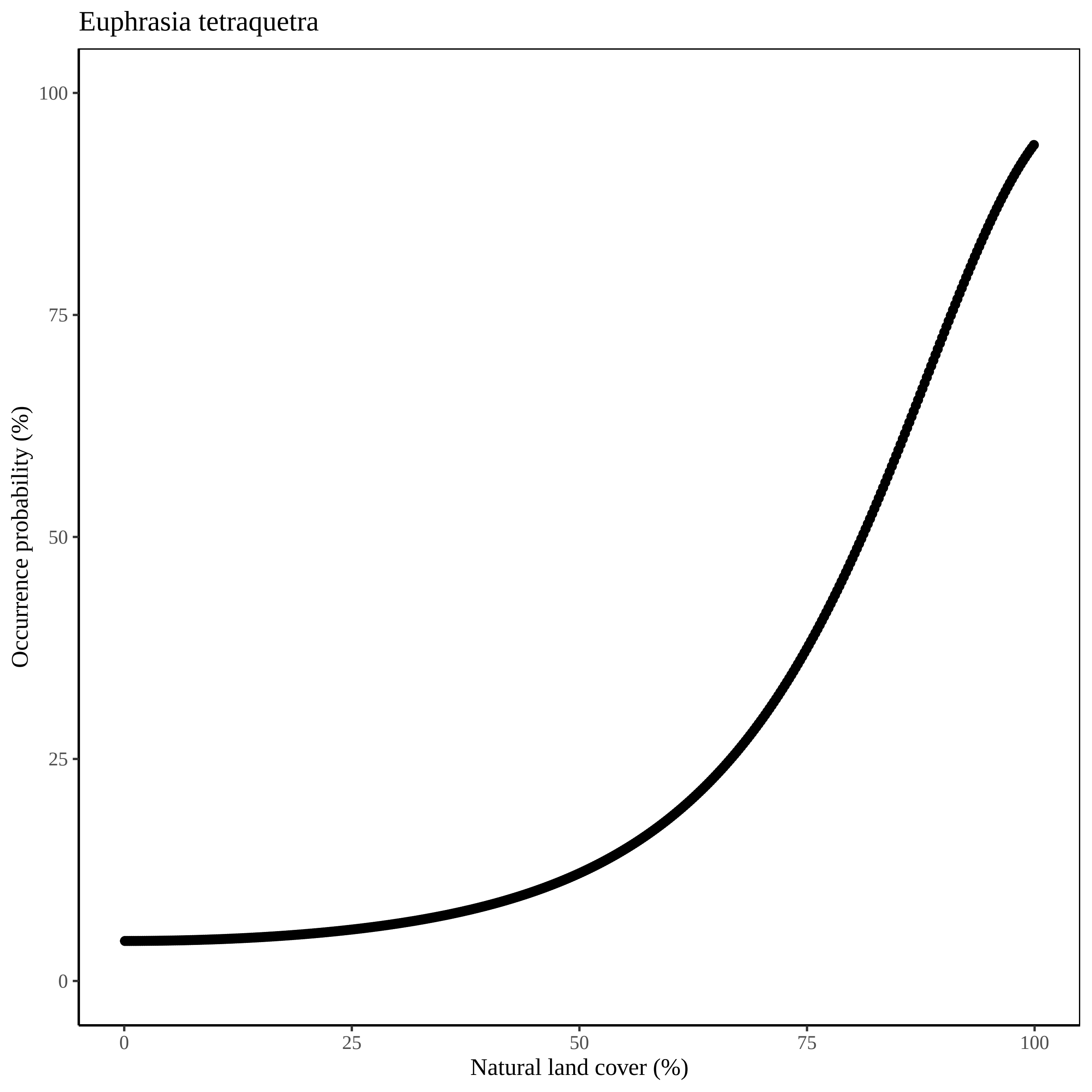

Euphrasia tetraquetra
Occurrence probability (%)
100
75
50
25
0
Natural land cover (%)
0
25
50
75
100

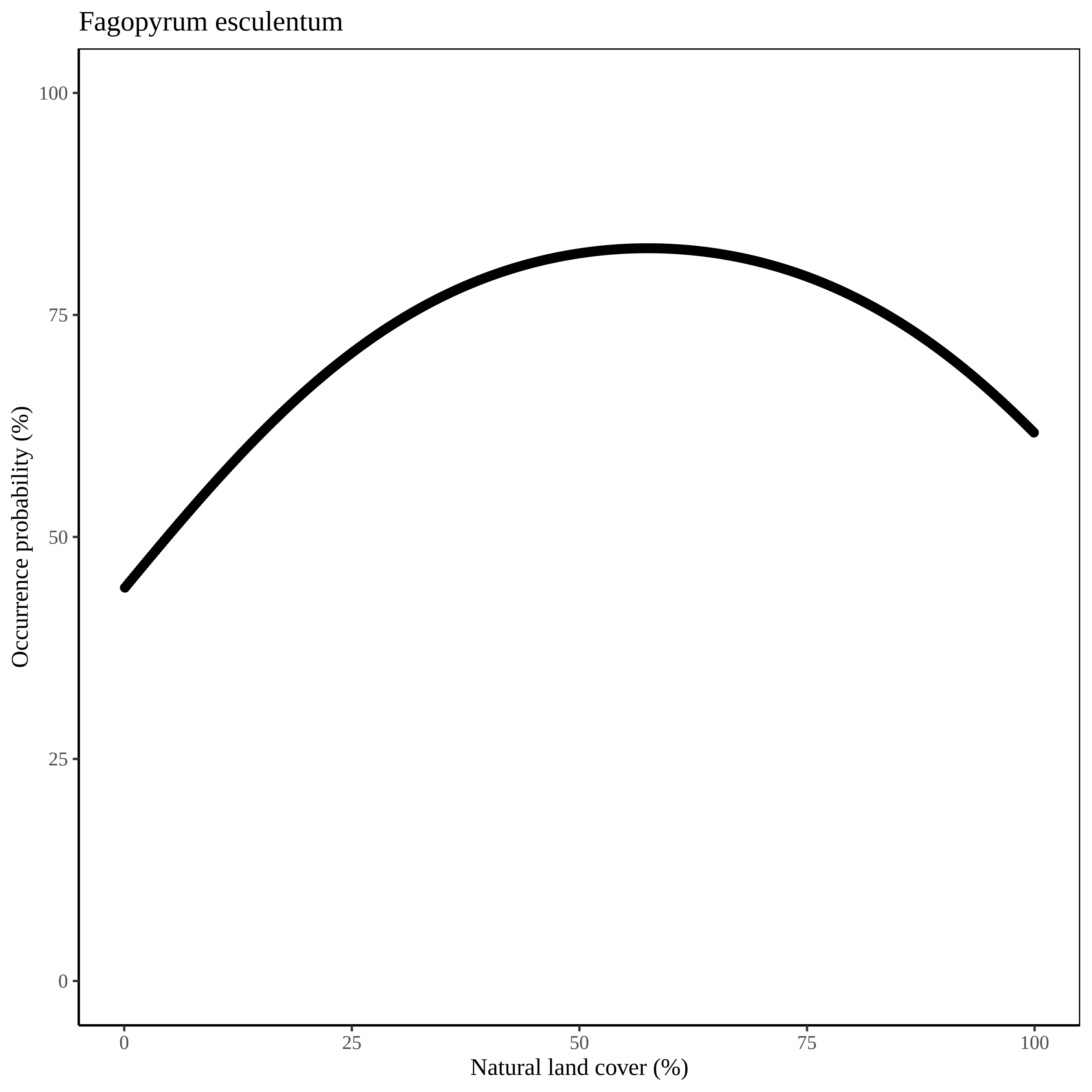

Fagopyrum esculentum
Occurrence probability (%)
100
75
50
25
0
0
25
50
75
100
Natural land cover (%)

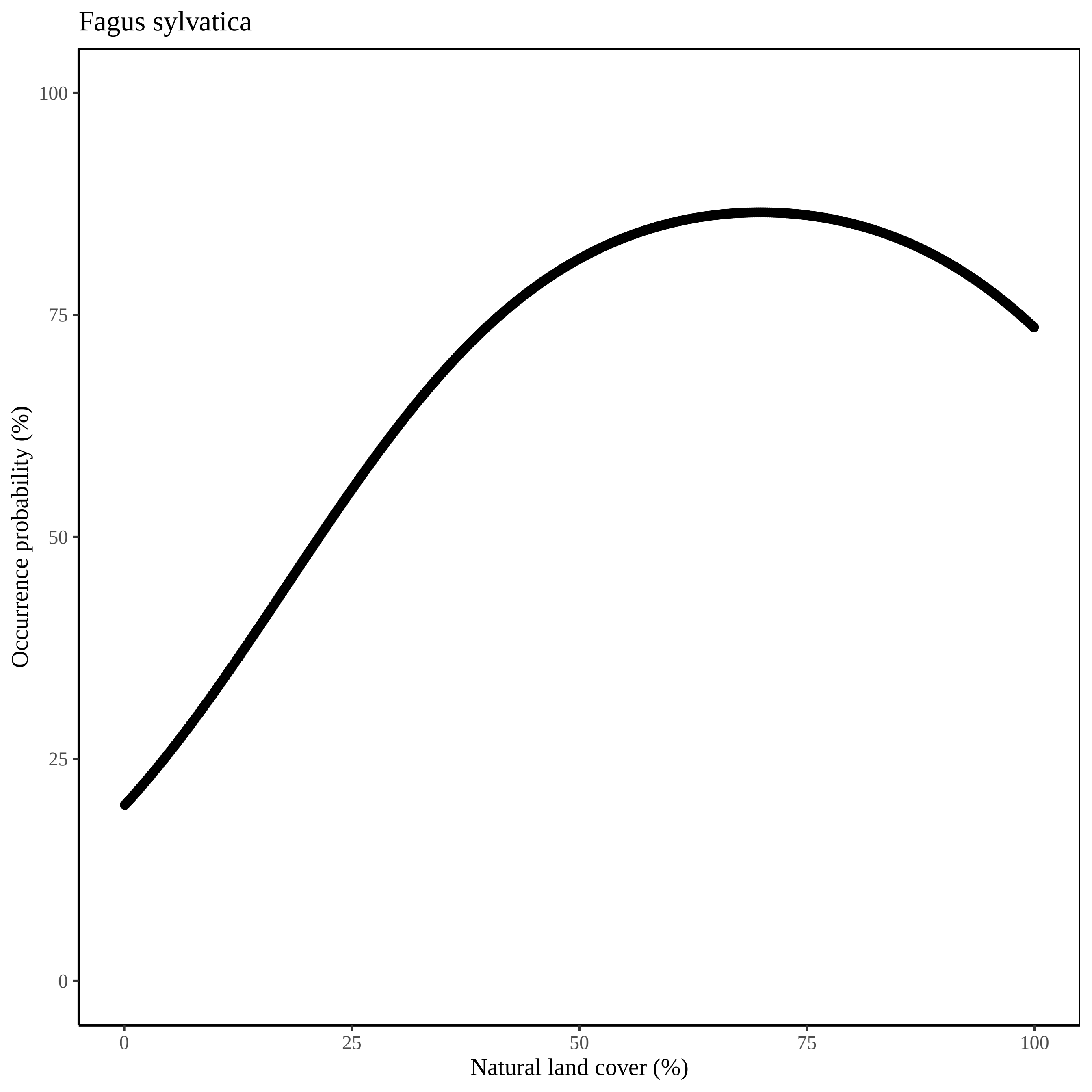

Fagus sylvatica
Occurrence probability (%)
100
75
50
25
0
0
25
50
75
100
Natural land cover (%)

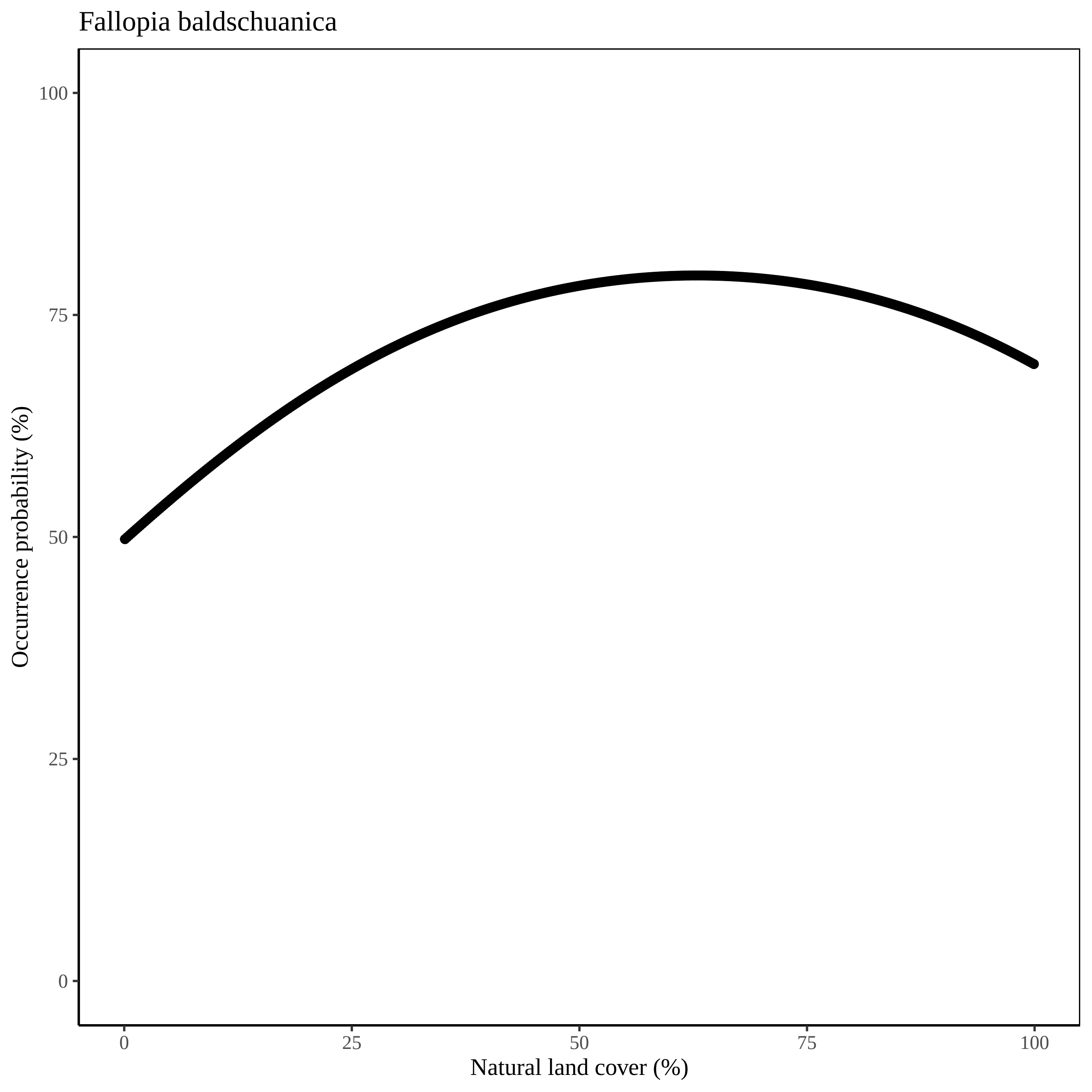

Fallopia baldschuanica
100
75
50
25
0
Occurrence probability (%)
0
25
50
75
100
Natural land cover (%)

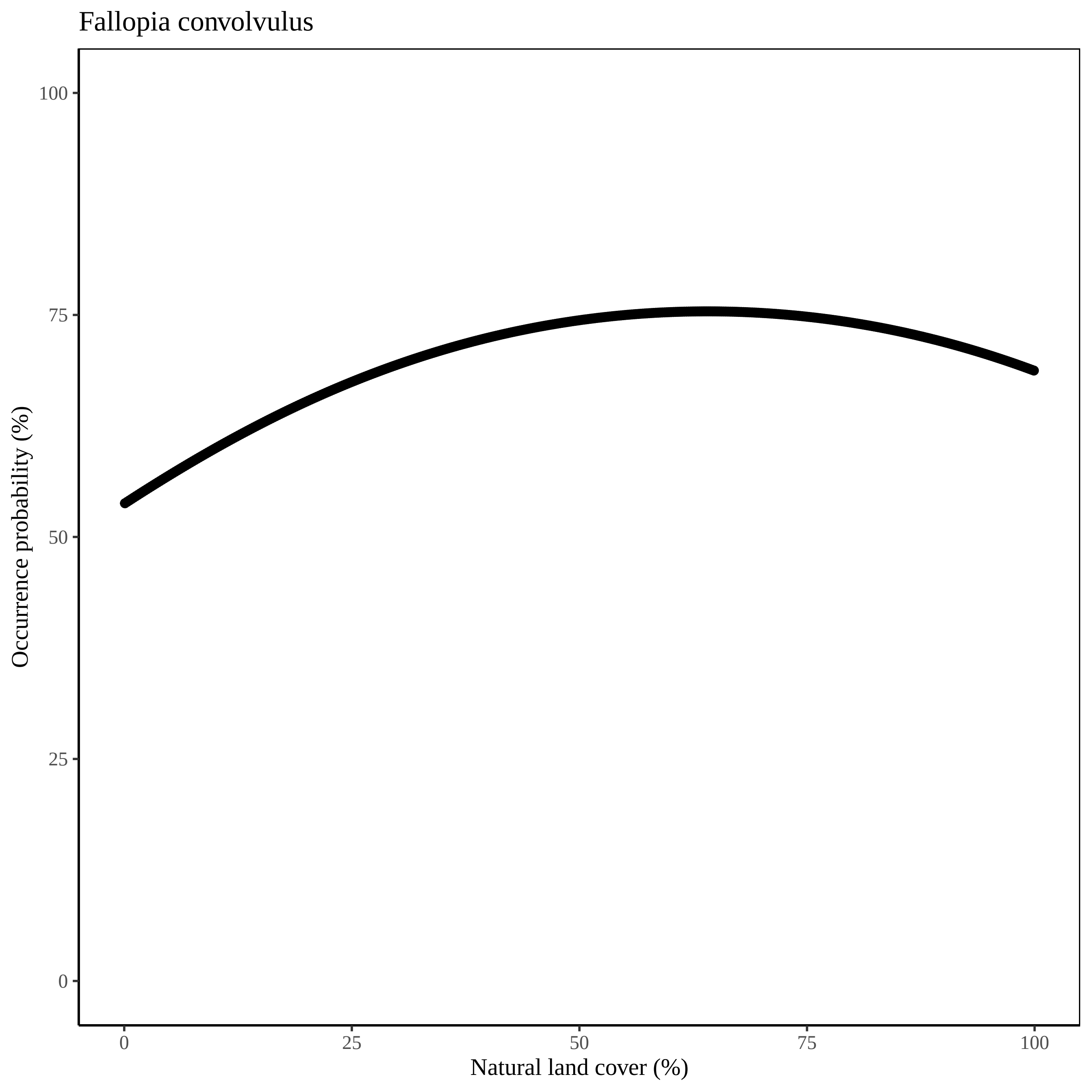

Fallopia convolvulus
100
75
50
25
0
Occurrence probability (%)
0
25
50
75
100
Natural land cover (%)

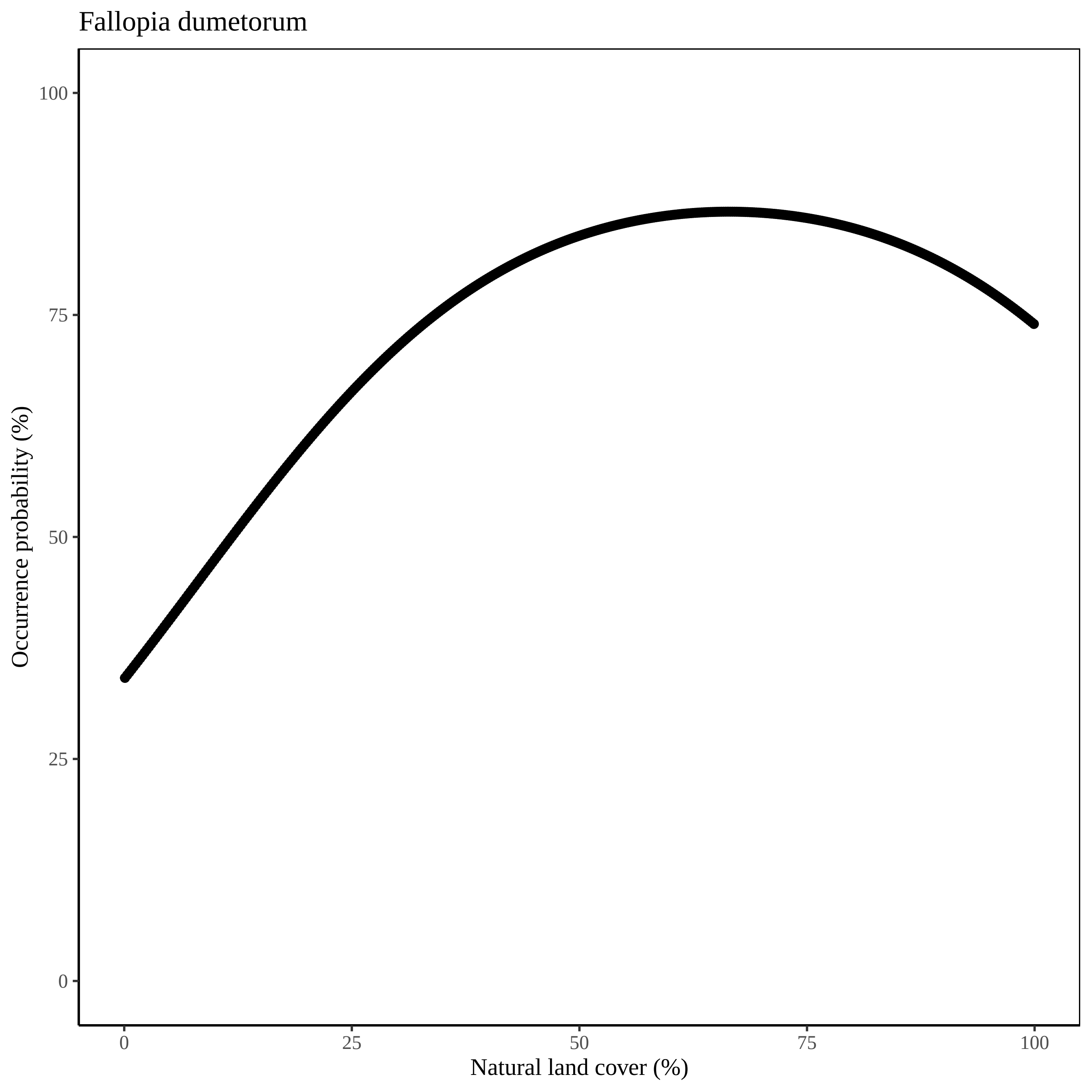

Fallopia dumetorum
Occurrence probability (%)
100
75
50
25
0
0
25
50
75
100
Natural land cover (%)

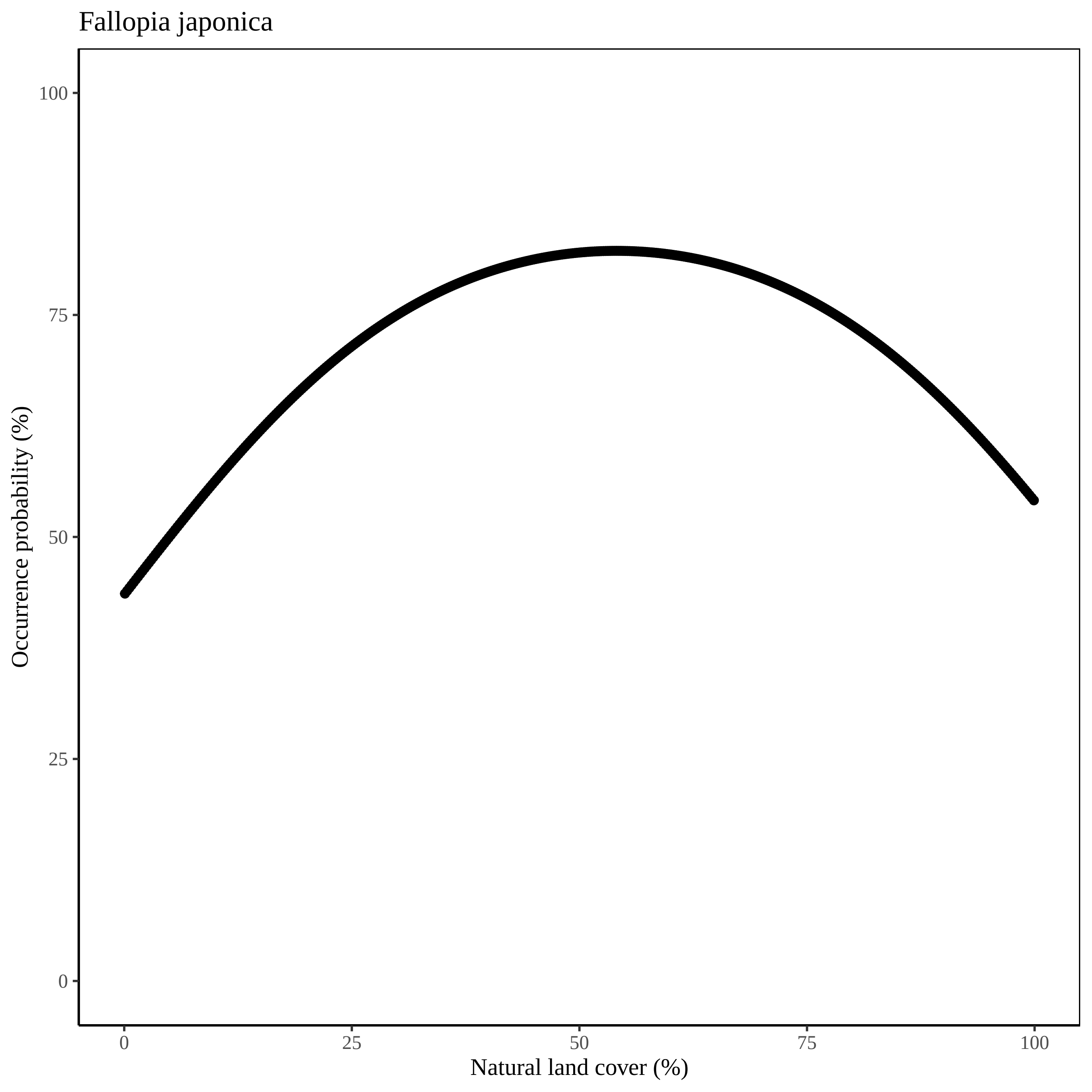
Fallopia japonica
Occurrence probability (%)
100
75
50
25
0
0
25
50
75
100
Natural land cover (%)

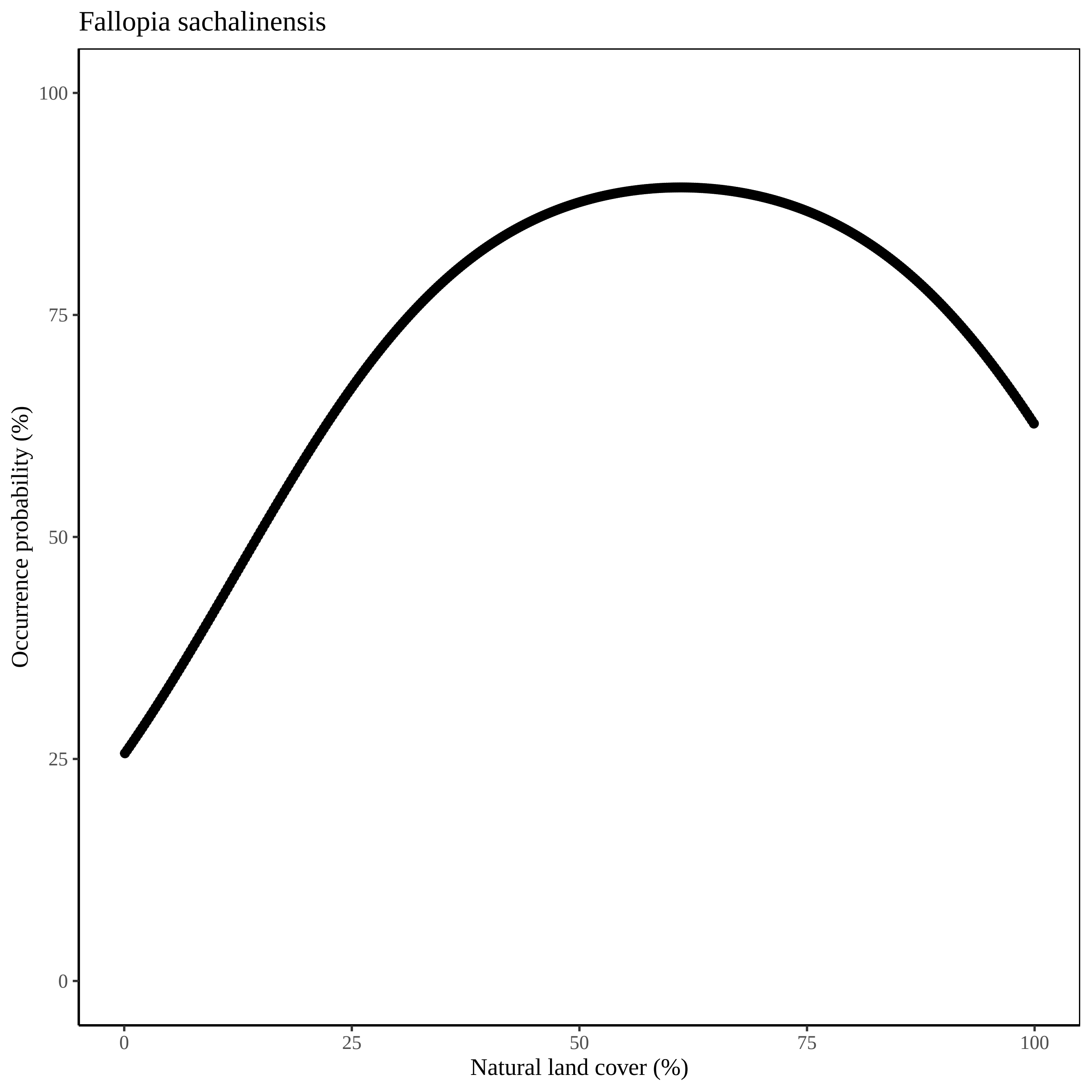
Fallopia sachalinensis
Occurrence probability (%)
100
75
50
25
0
0
25
50
75
100
Natural land cover (%)

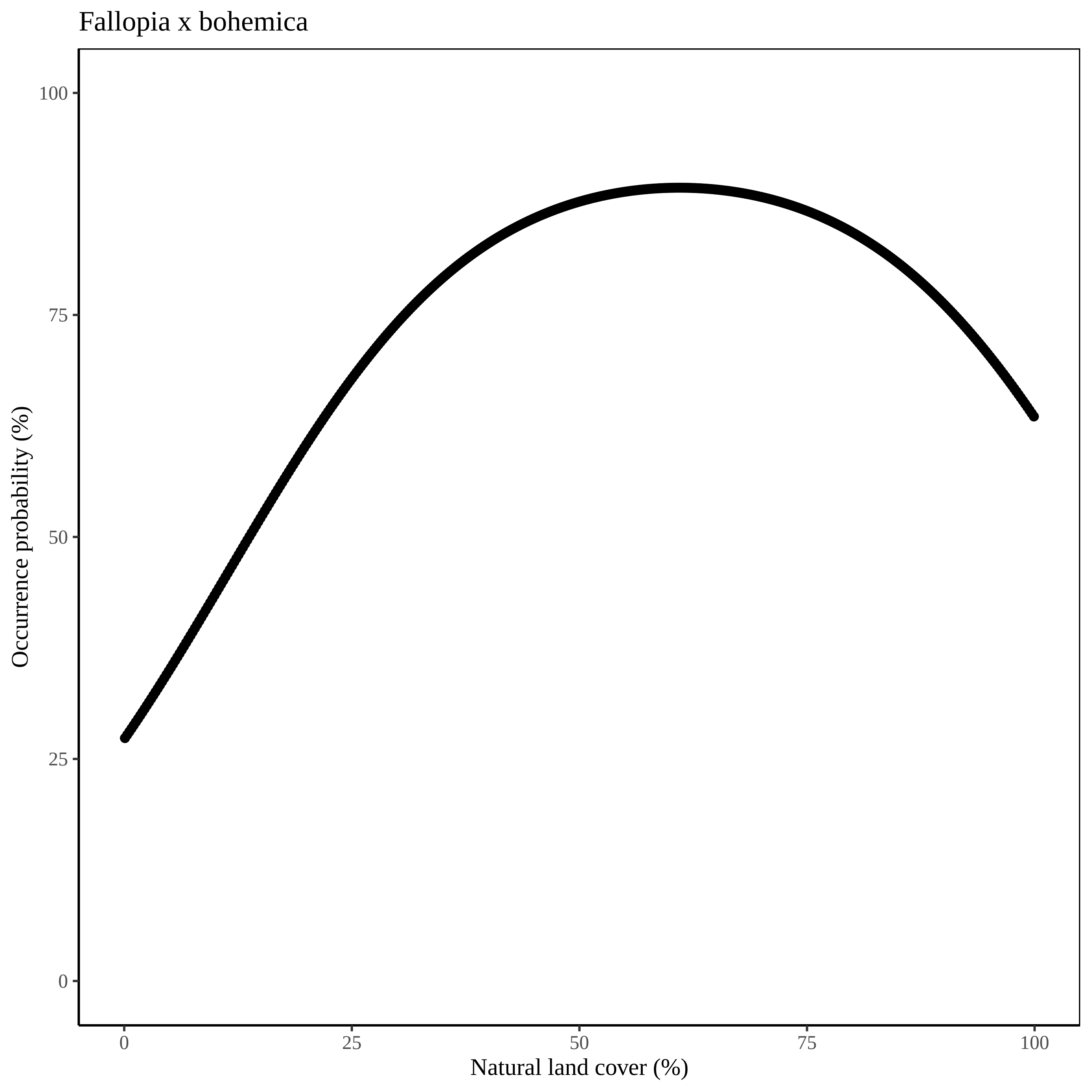
Fallopia x bohemica
Occurrence probability (%)
100
75
50
25
0
0
25
50
75
100
Natural land cover (%)

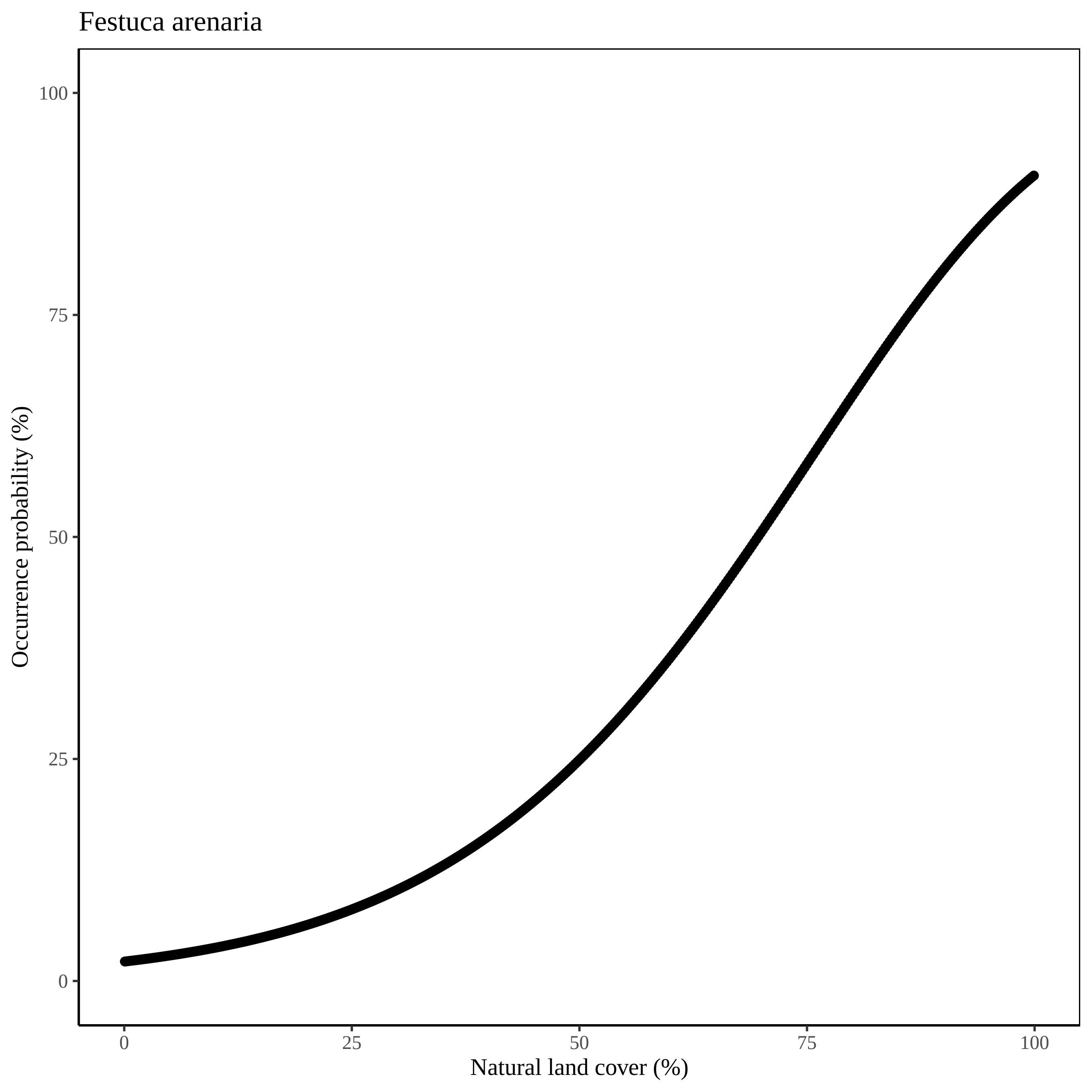
Festuca arenaria
100
75
50
25
0
0
25
50
75
100
Occurrence probability (%)
Natural land cover (%)

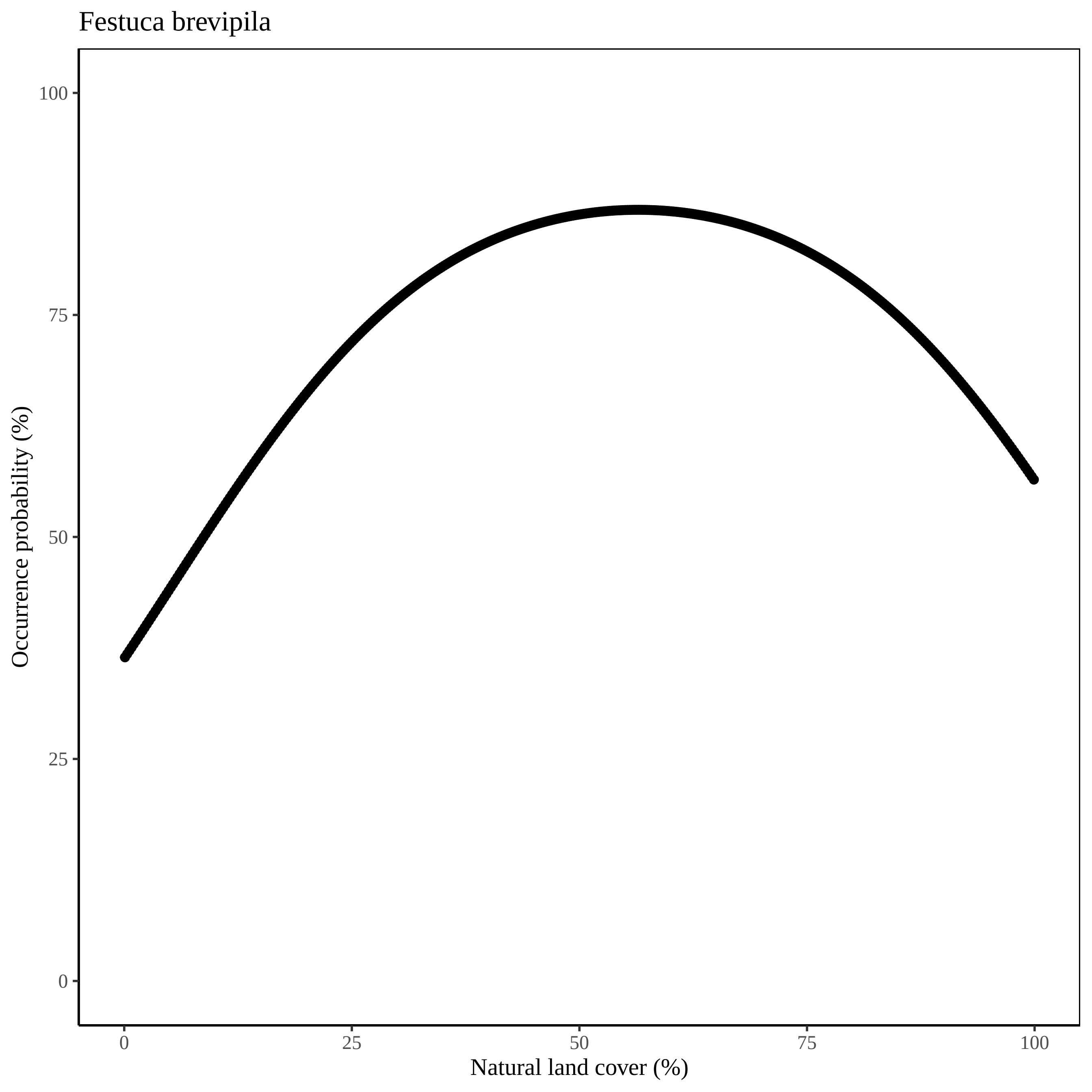
Festuca brevipila
100
75
50
25
0
Occurrence probability (%)
0
25
50
75
100
Natural land cover (%)

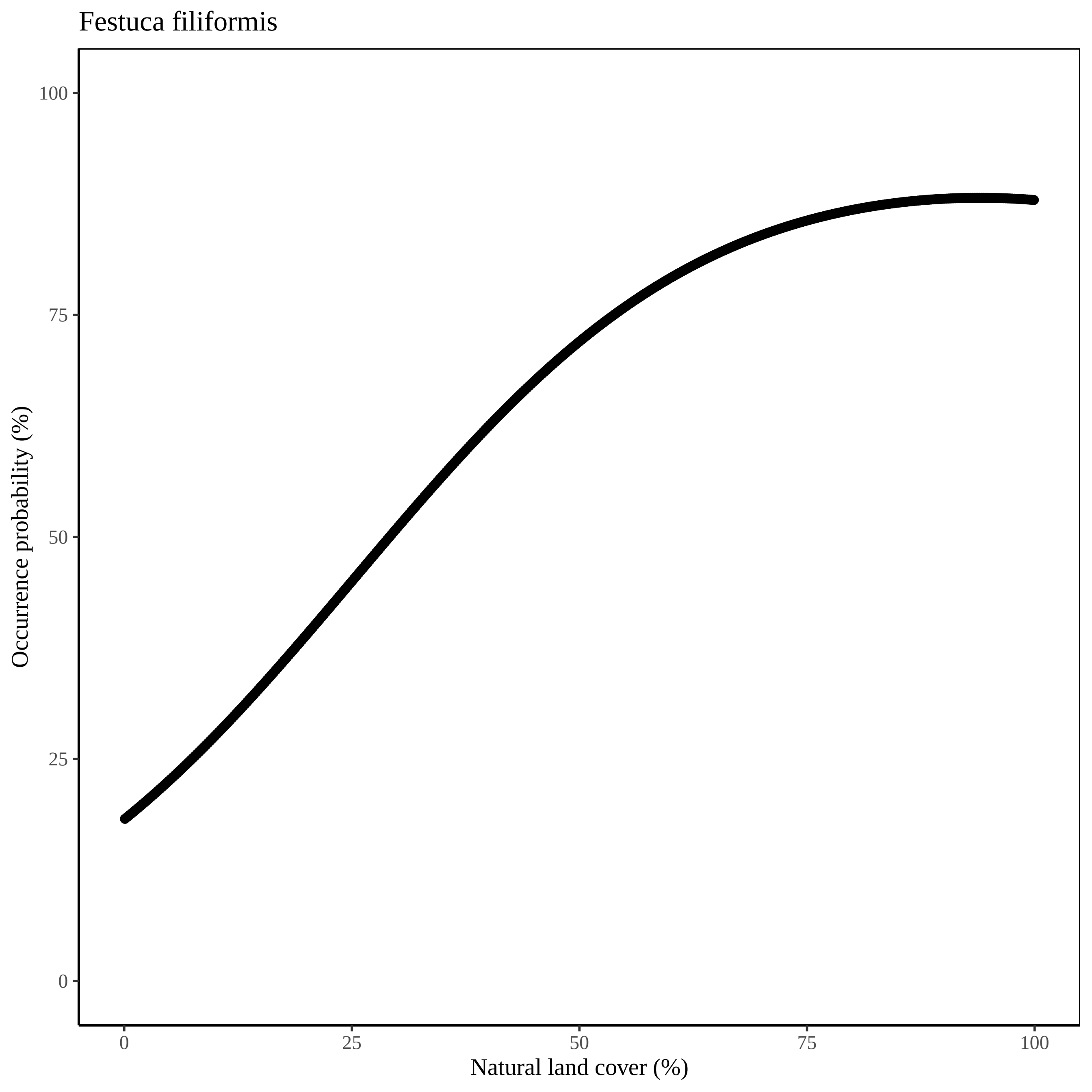
Festuca filiformis
Occurrence probability (%)
100
75
50
25
0
0
25
50
75
100
Natural land cover (%)

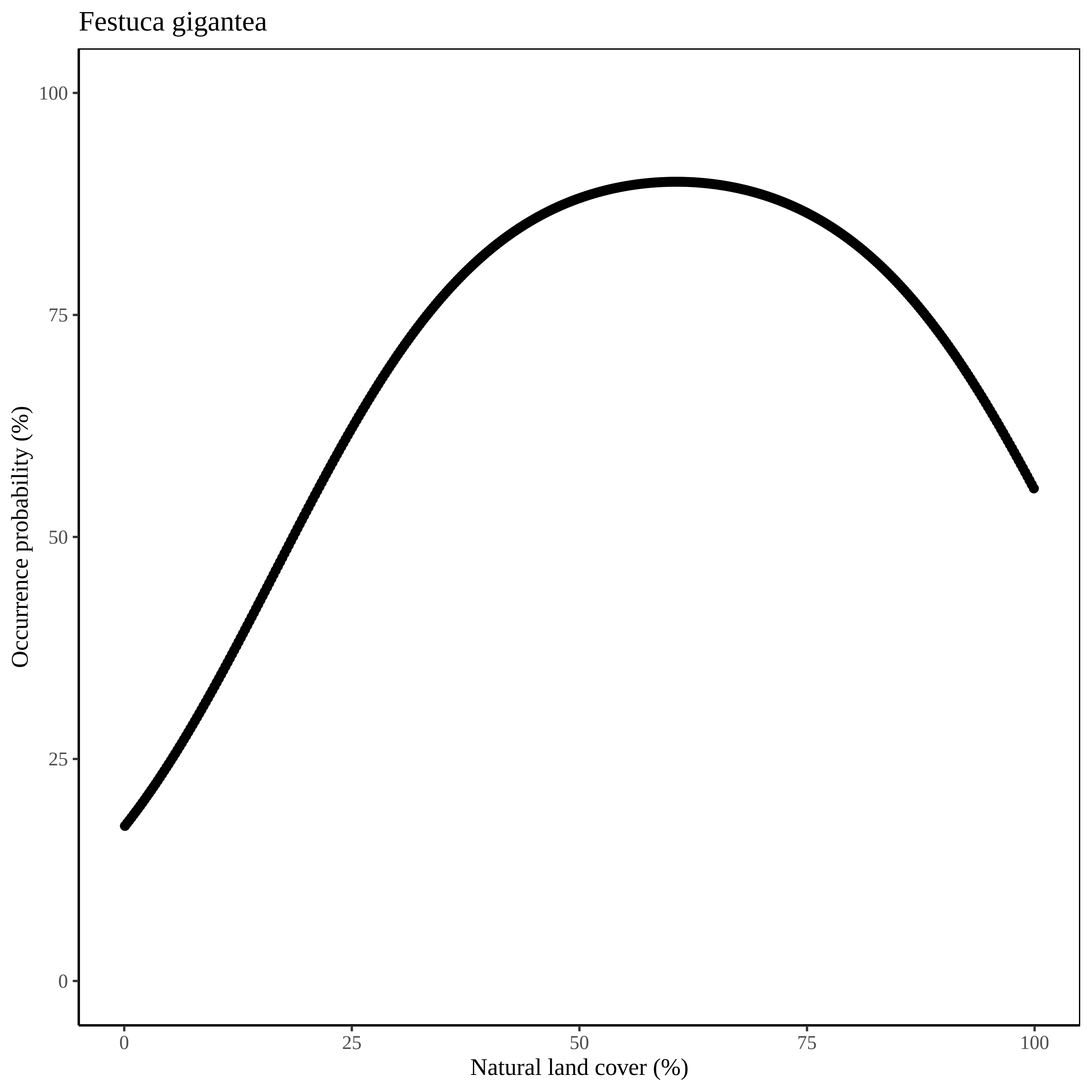
Festuca gigantea
Occurrence probability (%)
100
75
50
25
0
0
25
50
75
100
Natural land cover (%)

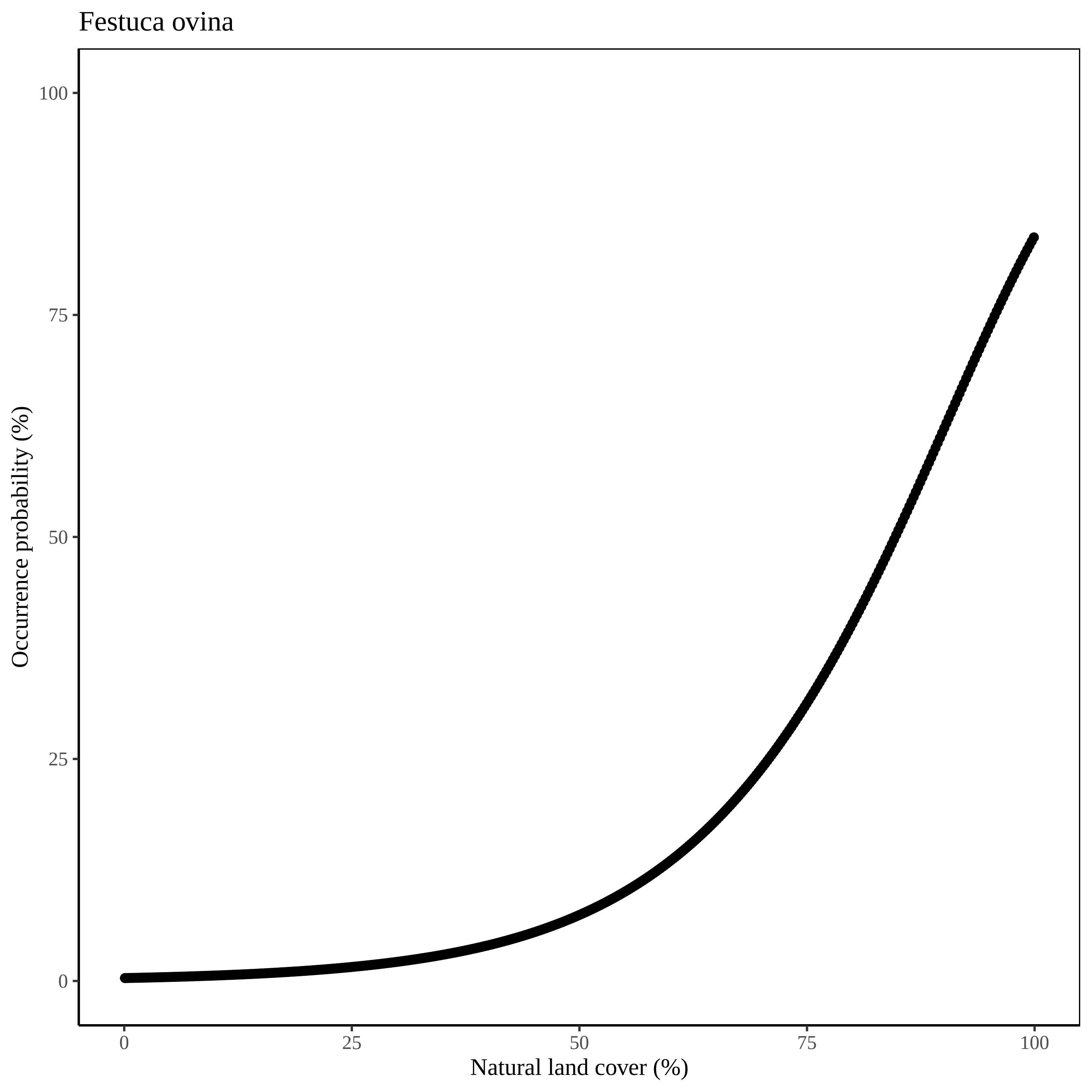

Festuca ovina
Occurrence probability (%)
100
75
50
25
0
0
25
50
75
100
Natural land cover (%)

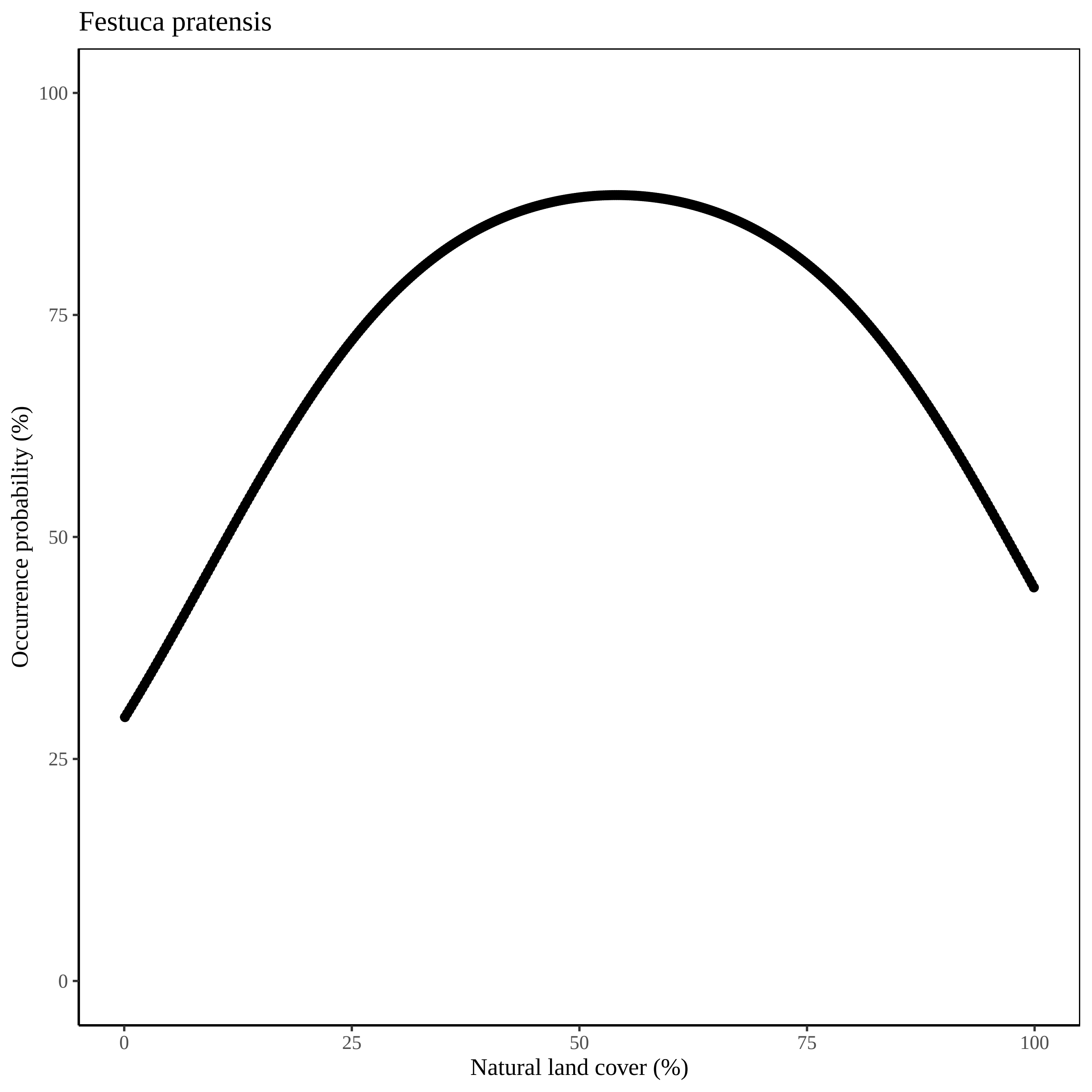
Festuca pratensis
Occurrence probability (%)
100
75
50
25
0
0
25
50
75
100
Natural land cover (%)

Festuca rubra

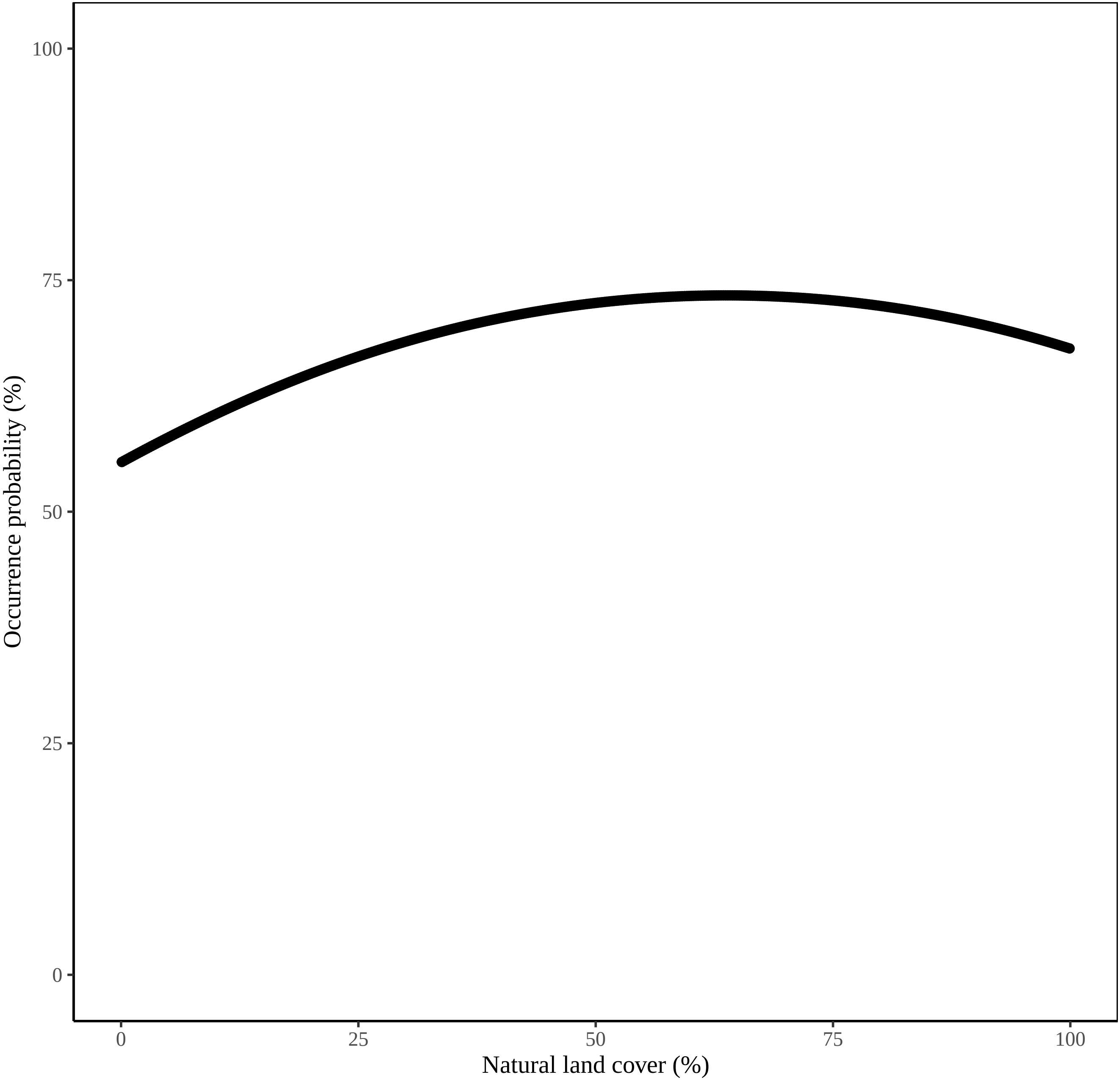

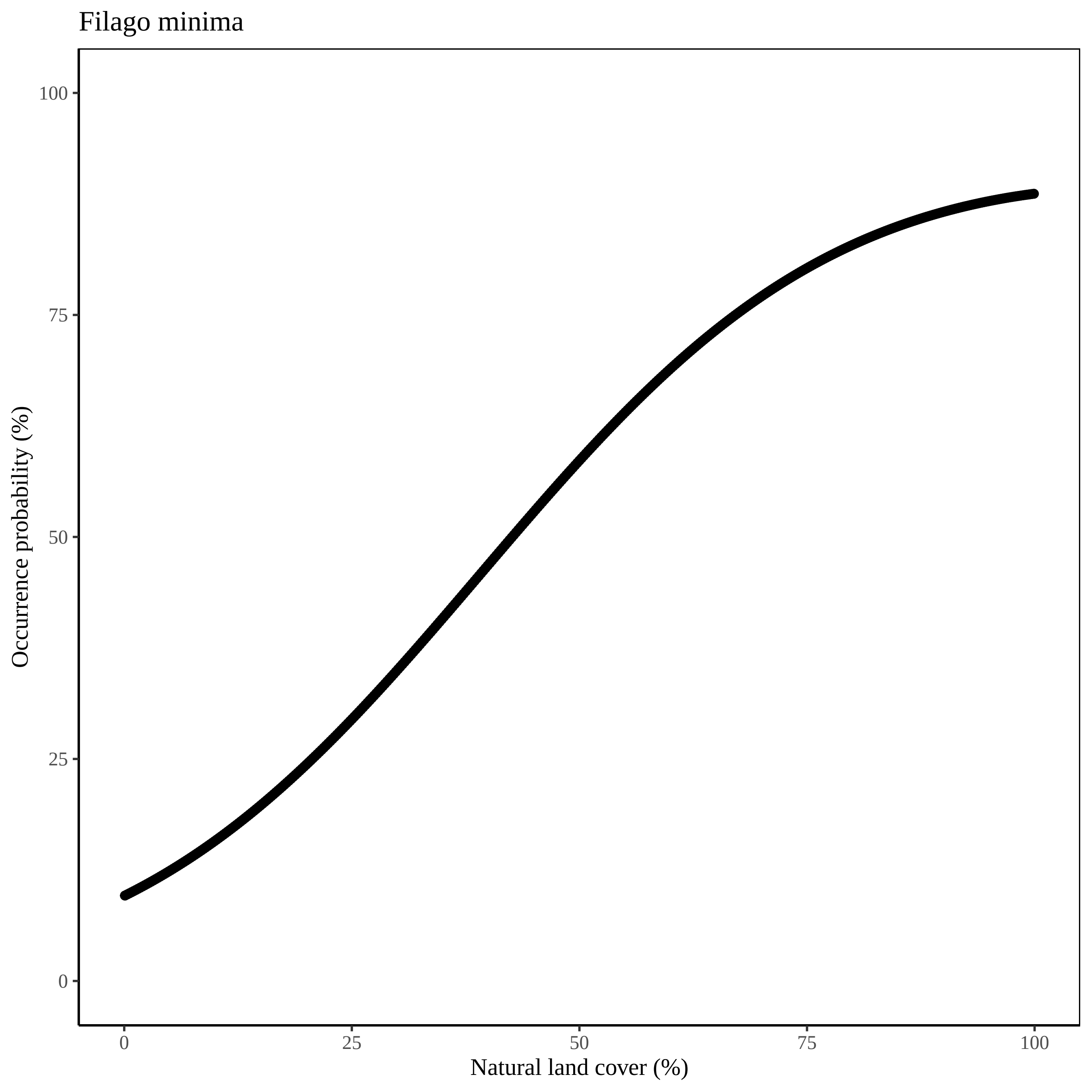

Filago minima
100
75
50
25
0
Occurrence probability (%)
0
25
50
75
100
Natural land cover (%)

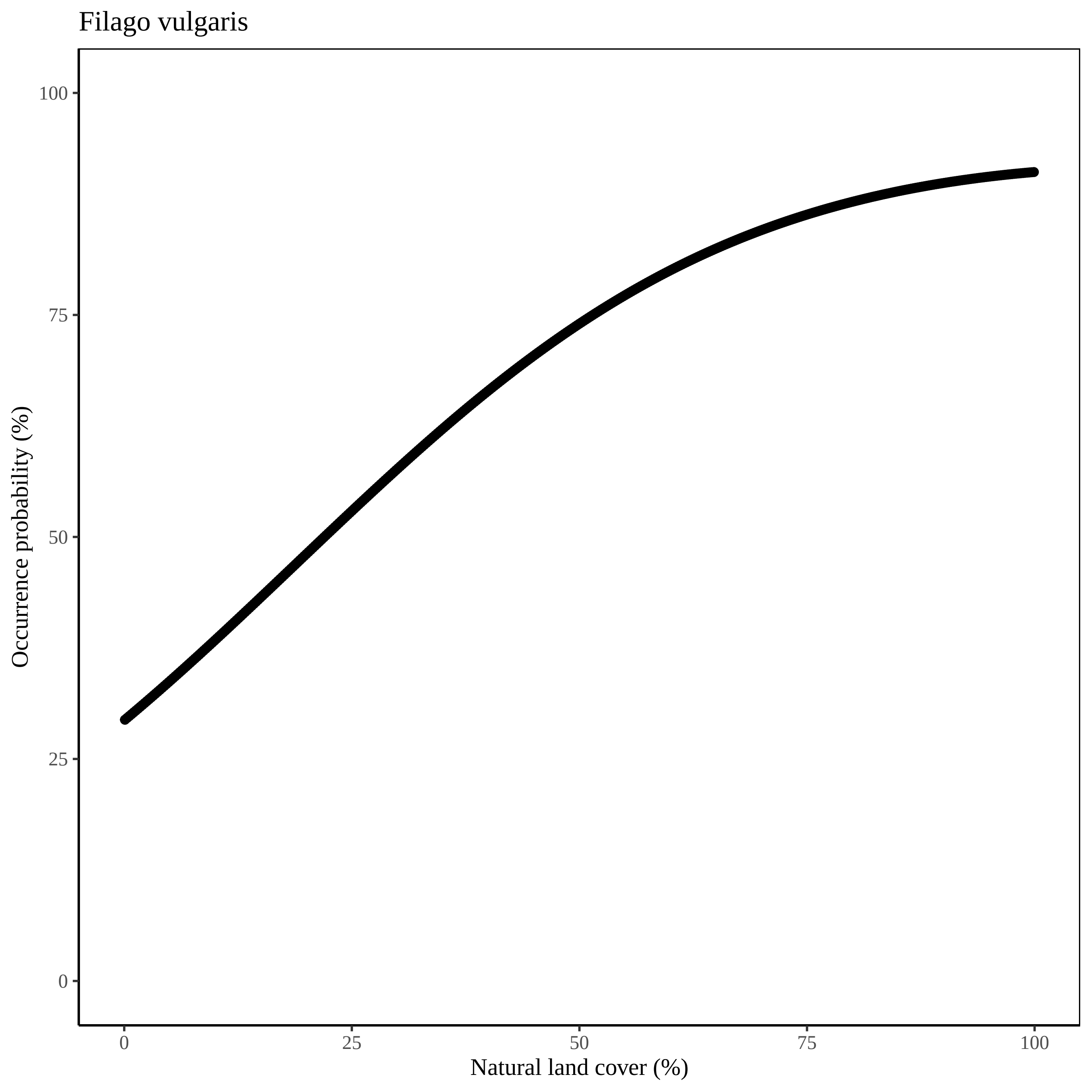

Filago vulgaris
100
75
50
25
0
Occurrence probability (%)
0
25
50
75
100
Natural land cover (%)

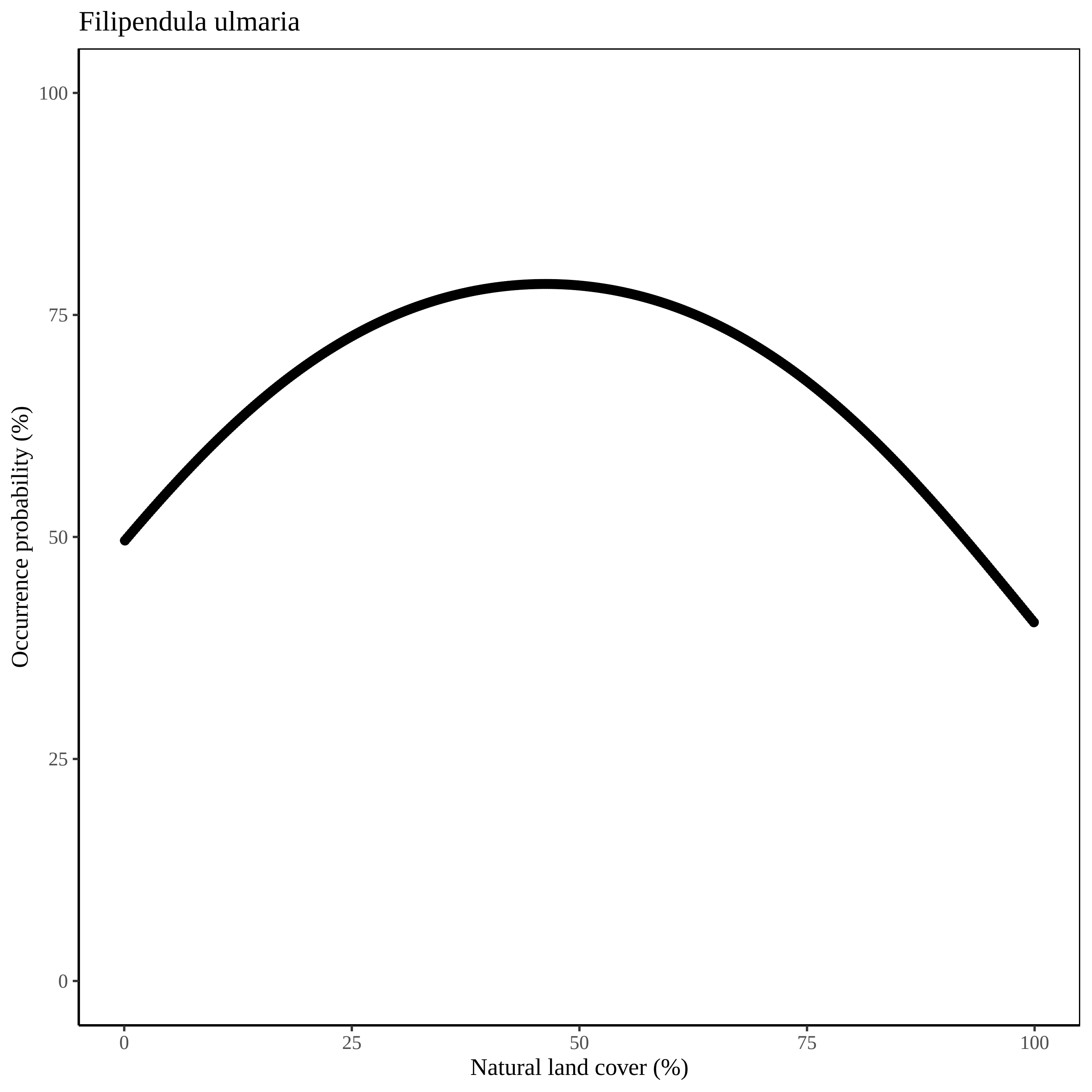

Filipendula ulmaria
Occurrence probability (%)
100
75
50
25
0
0
25
50
75
100
Natural land cover (%)

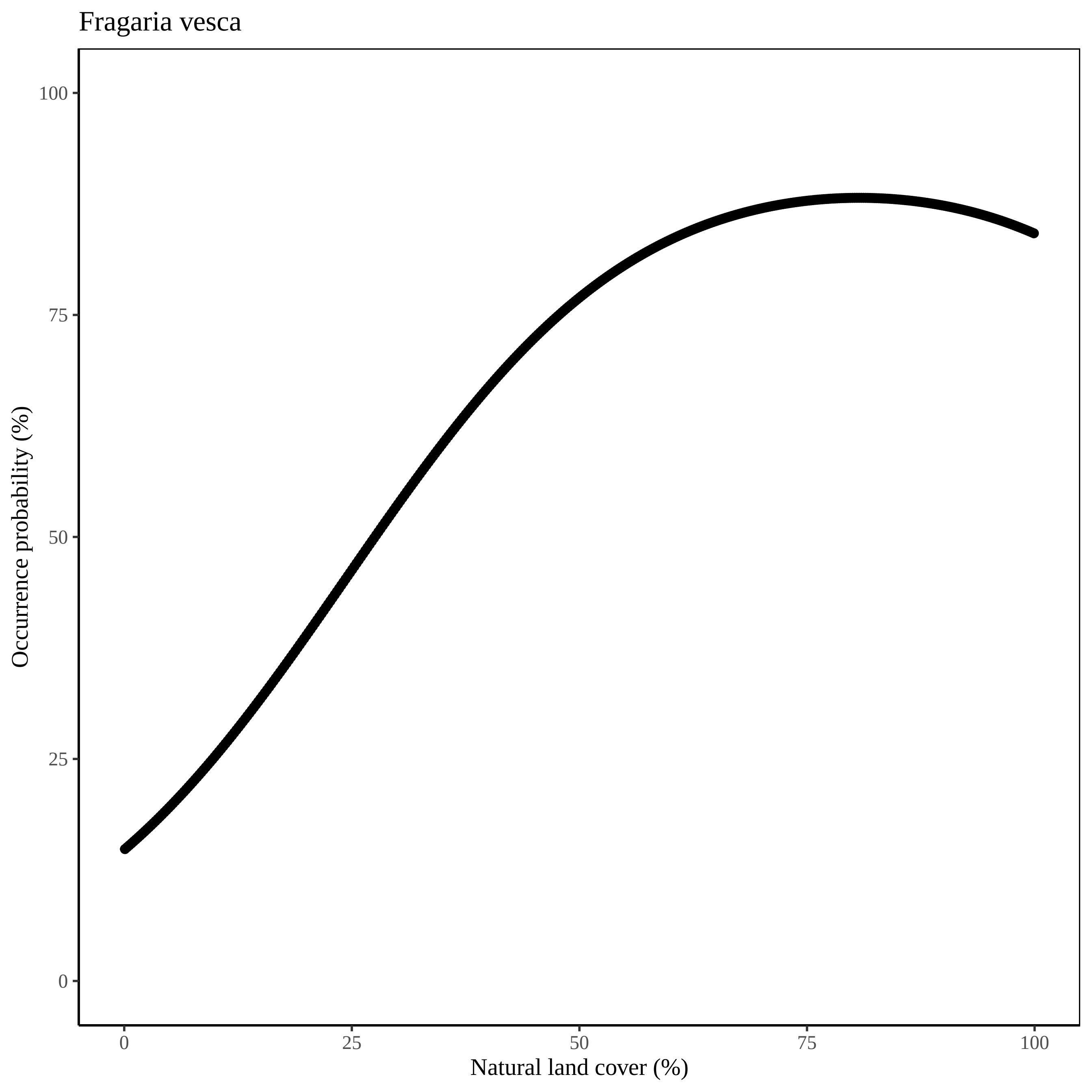

Fragaria vesca
100
75
50
25
0
Occurrence probability (%)
0
25
50
75
100
Natural land cover (%)

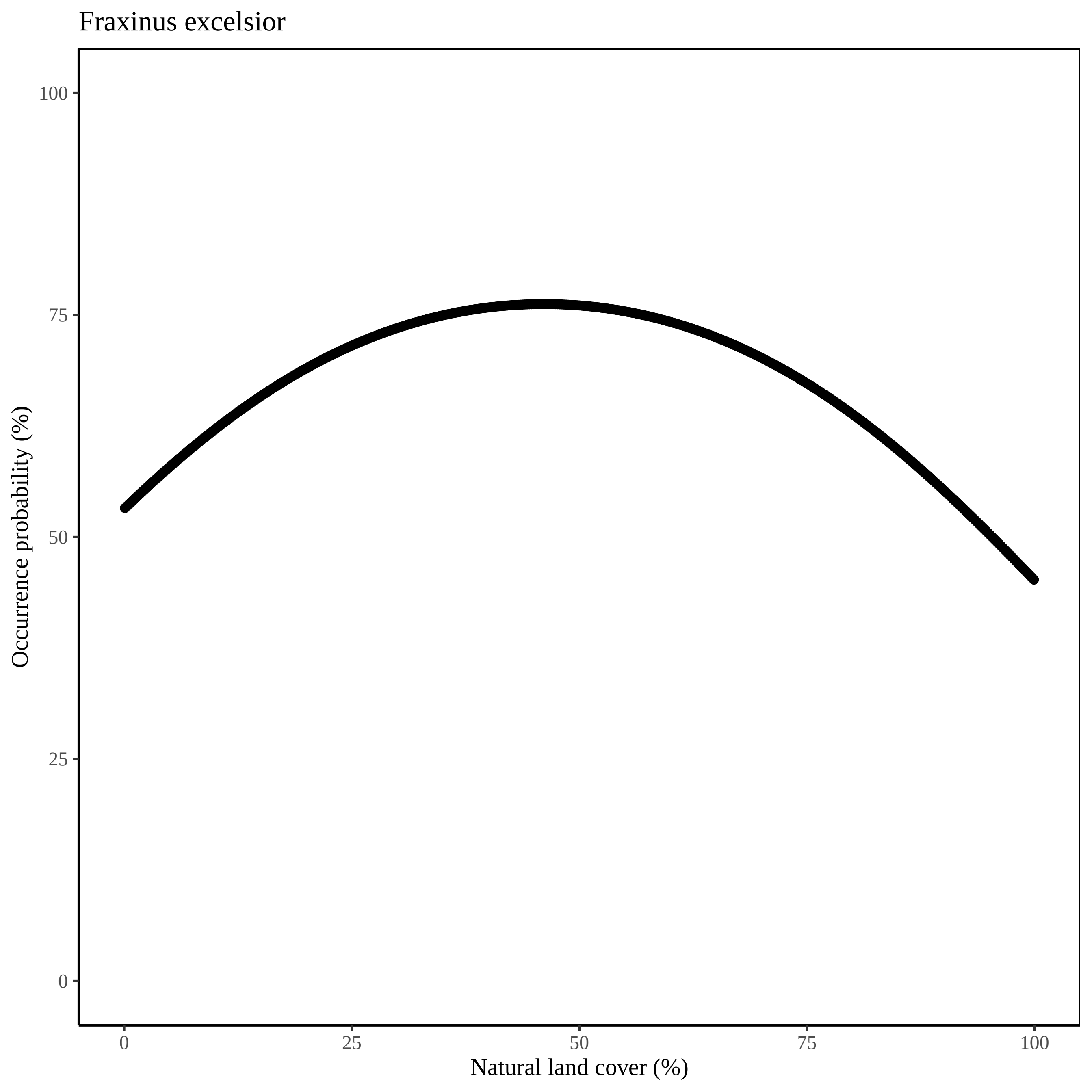
Fraxinus excelsior
Occurrence probability (%)
100
75
50
25
0
0
25
50
75
100
Natural land cover (%)

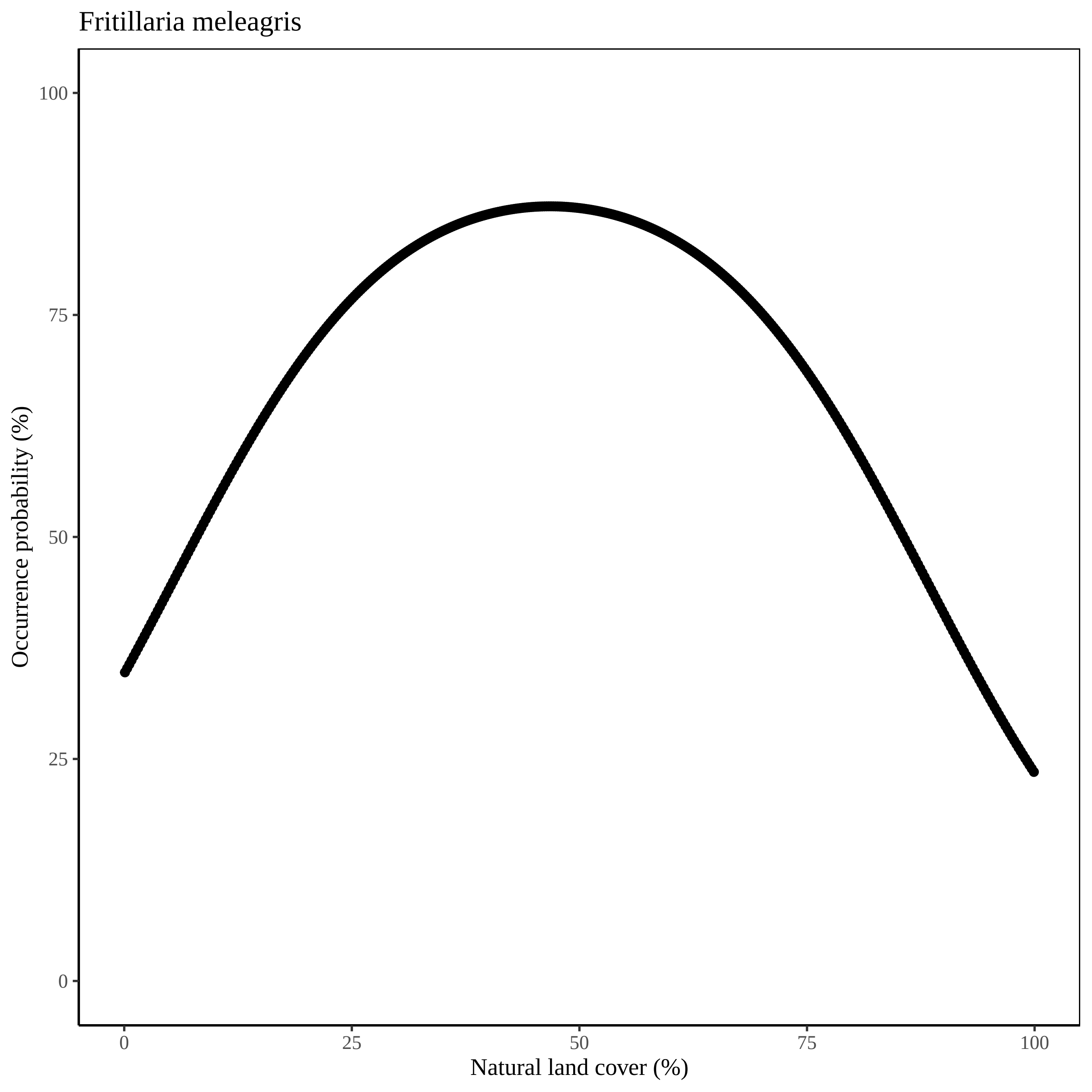
Fritillaria meleagris
Occurrence probability (%)
100
75
50
25
0
0
25
50
75
100
Natural land cover (%)

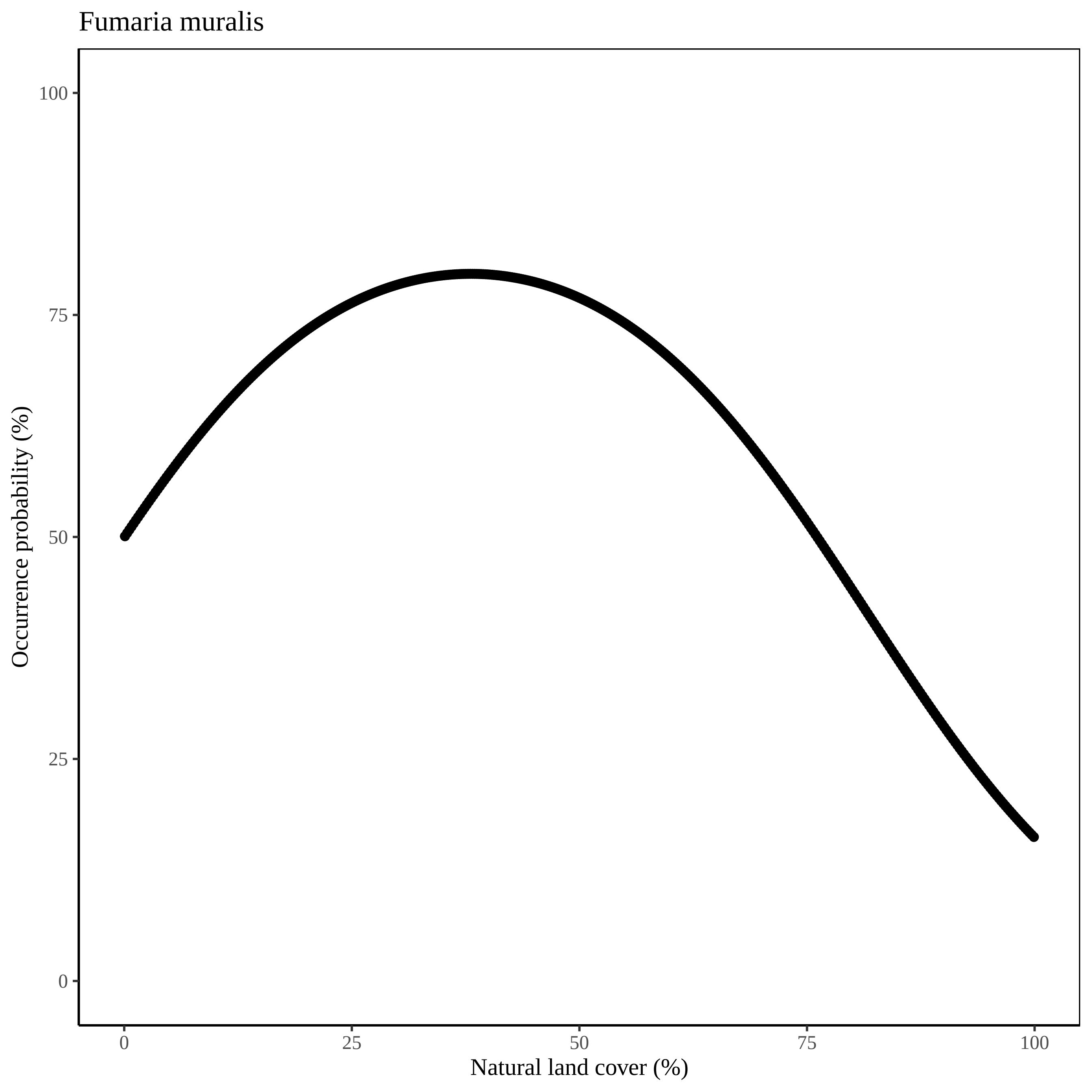
Fumaria muralis
Occurrence probability (%)
Natural land cover (%)
0
25
50
75
100
0
25
50
75
100

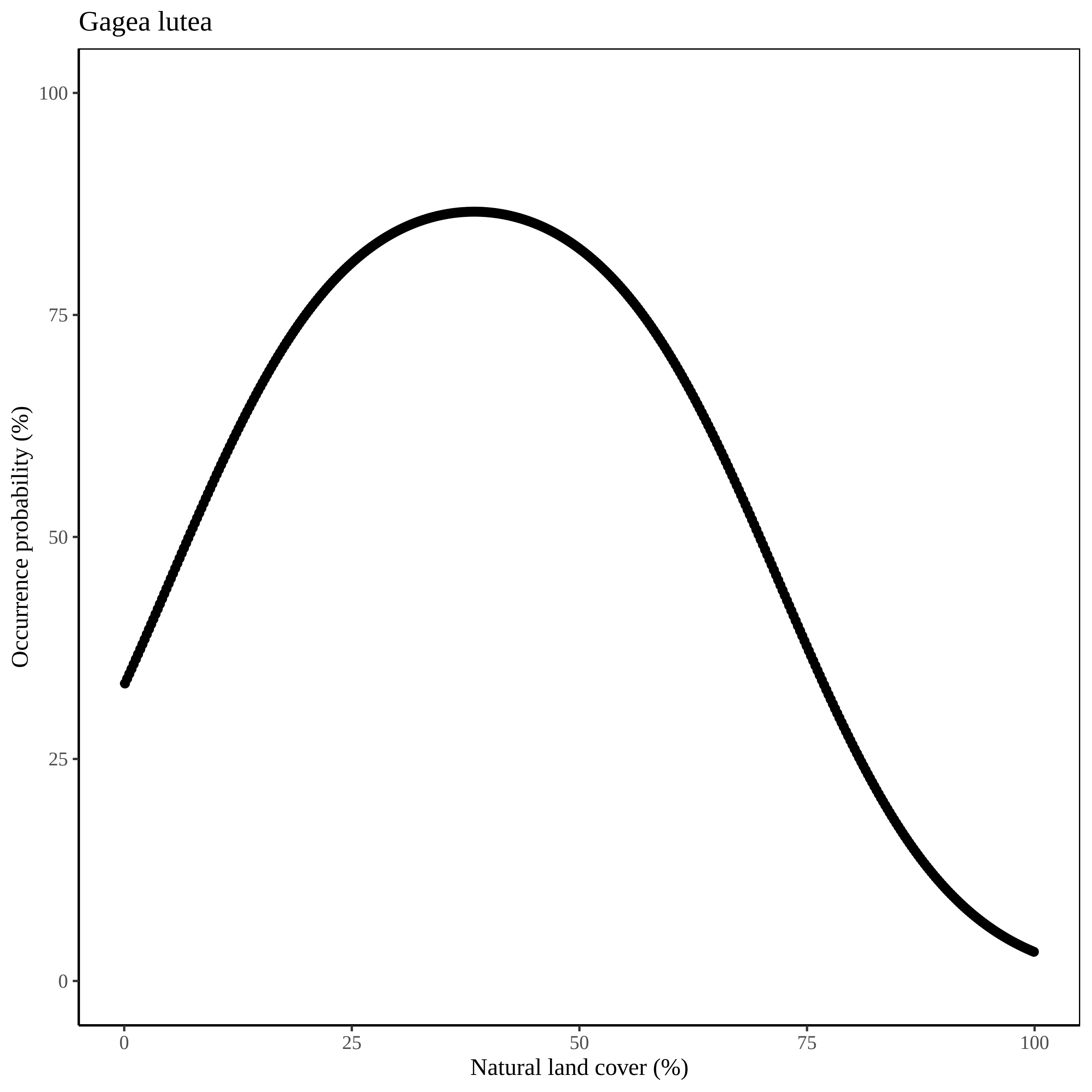
Gagea lutea
Occurrence probability (%)
100
75
50
25
0
0
25
50
75
100
Natural land cover (%)

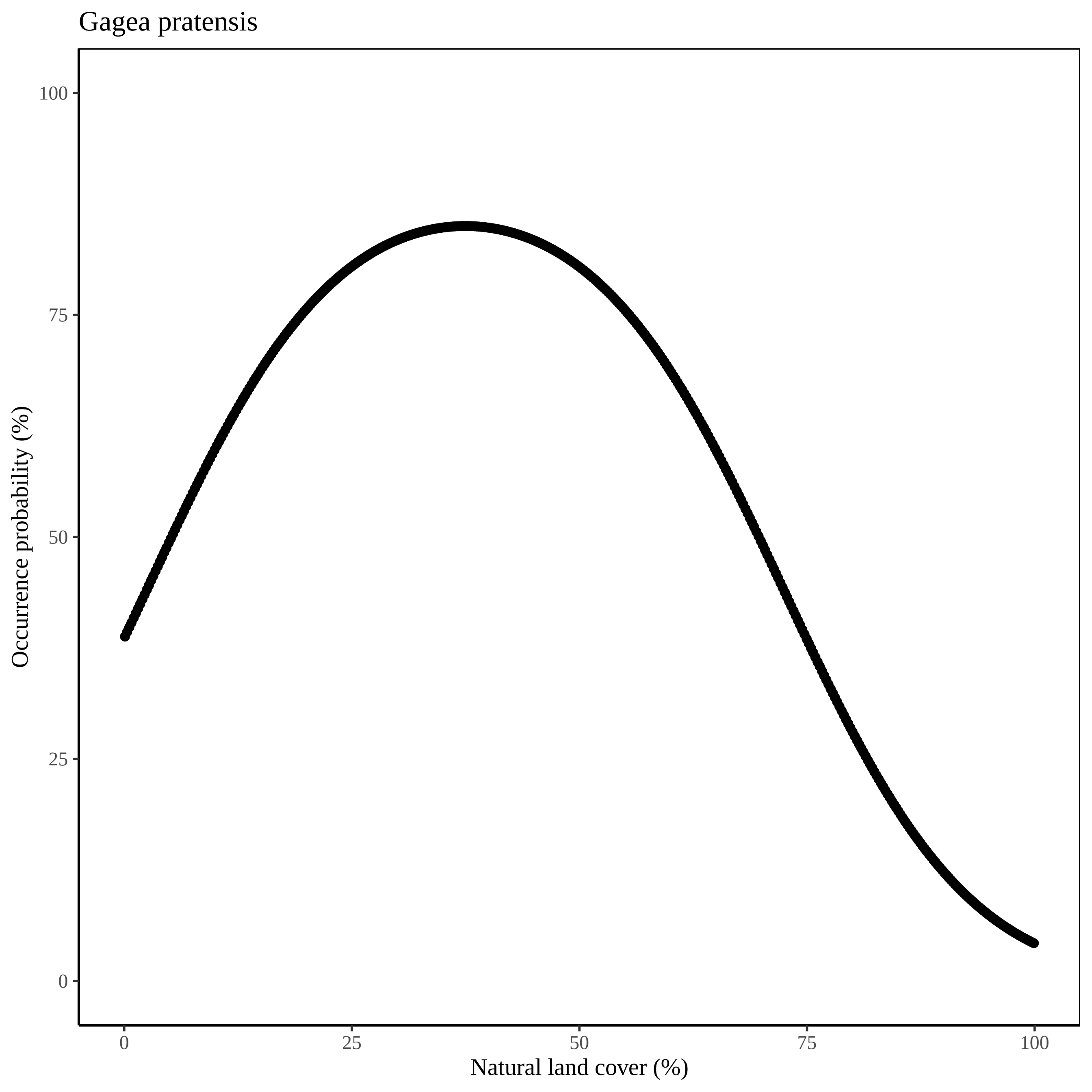
Gagea pratensis
Occurrence probability (%)
100
75
50
25
0
0
25
50
75
100
Natural land cover (%)

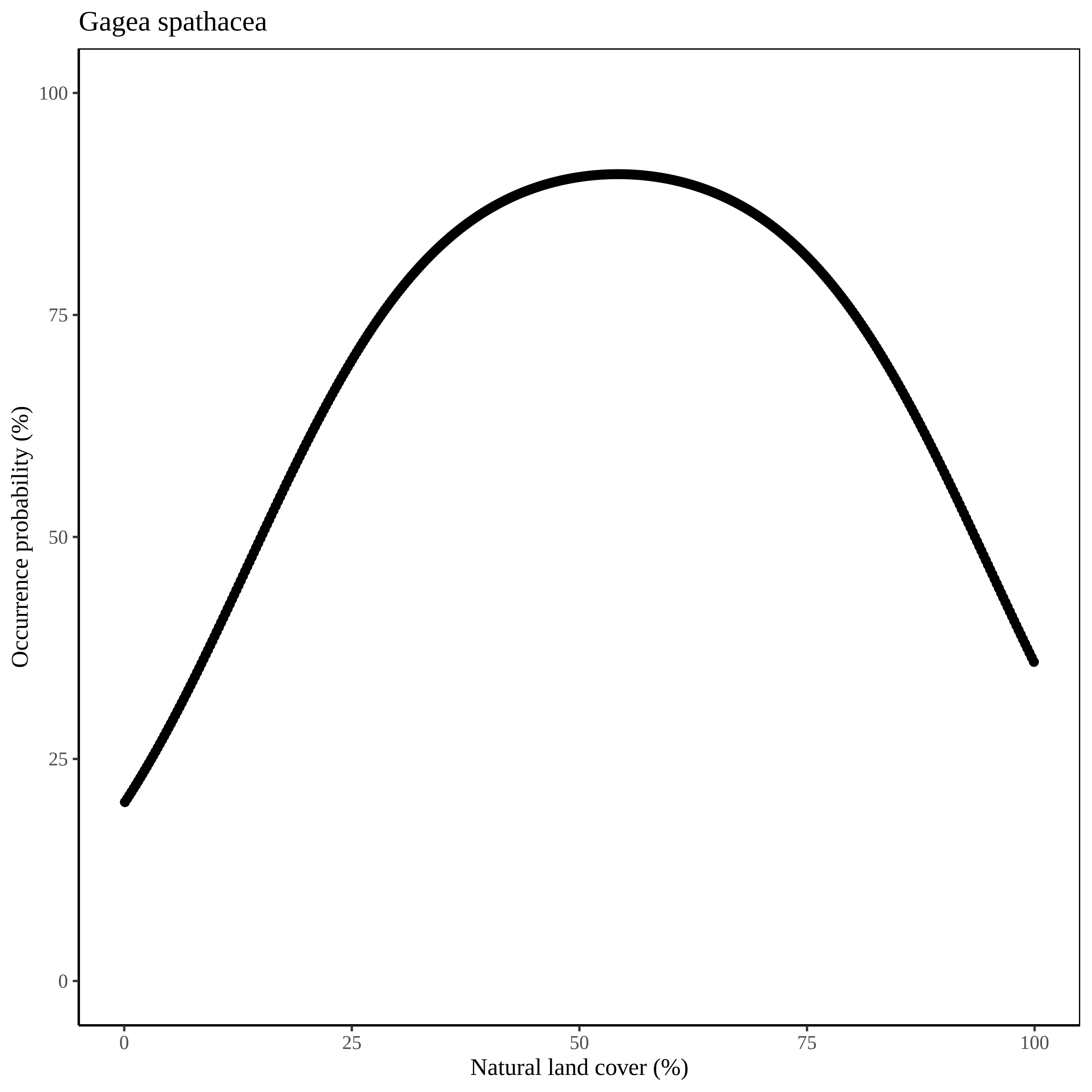
Gagea spathacea
Occurrence probability (%)
Natural land cover (%)
0
25
50
75
100

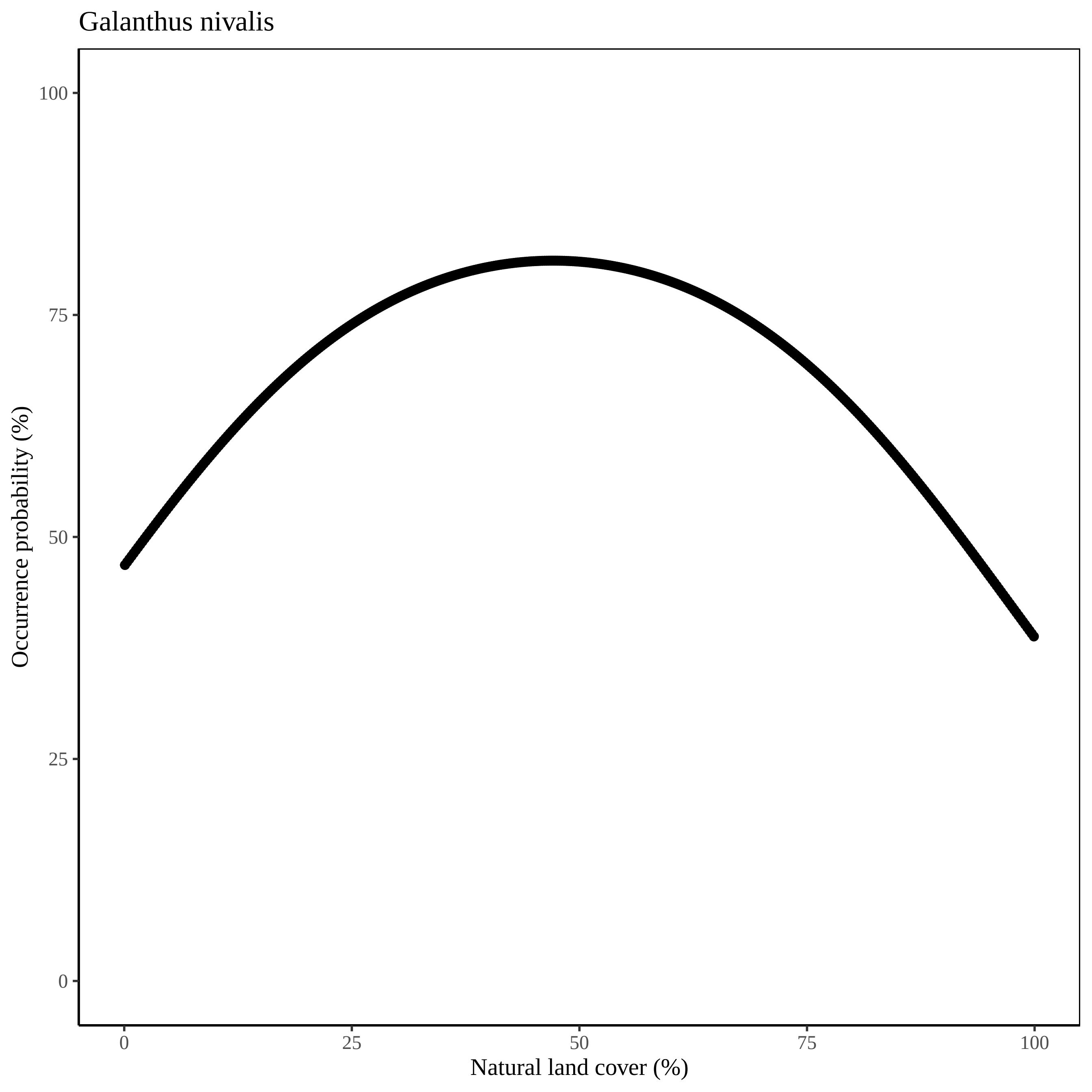

Galanthus nivalis
Occurrence probability (%)
100
75
50
25
0
0
25
50
75
100
Natural land cover (%)

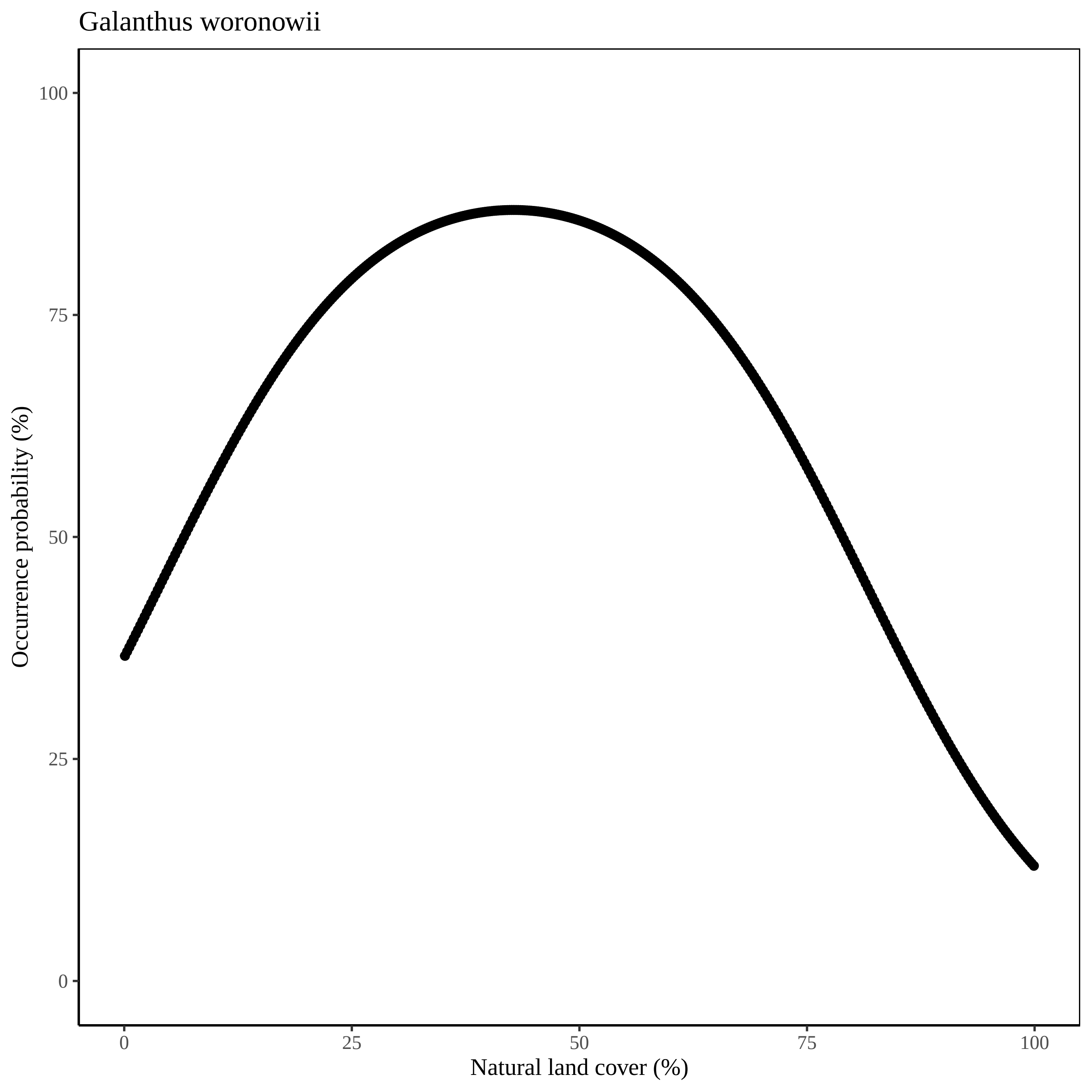
Galanthus woronowii
Occurrence probability (%)
100
75
50
25
0
0
25
50
75
100
Natural land cover (%)

Galeopsis bifida

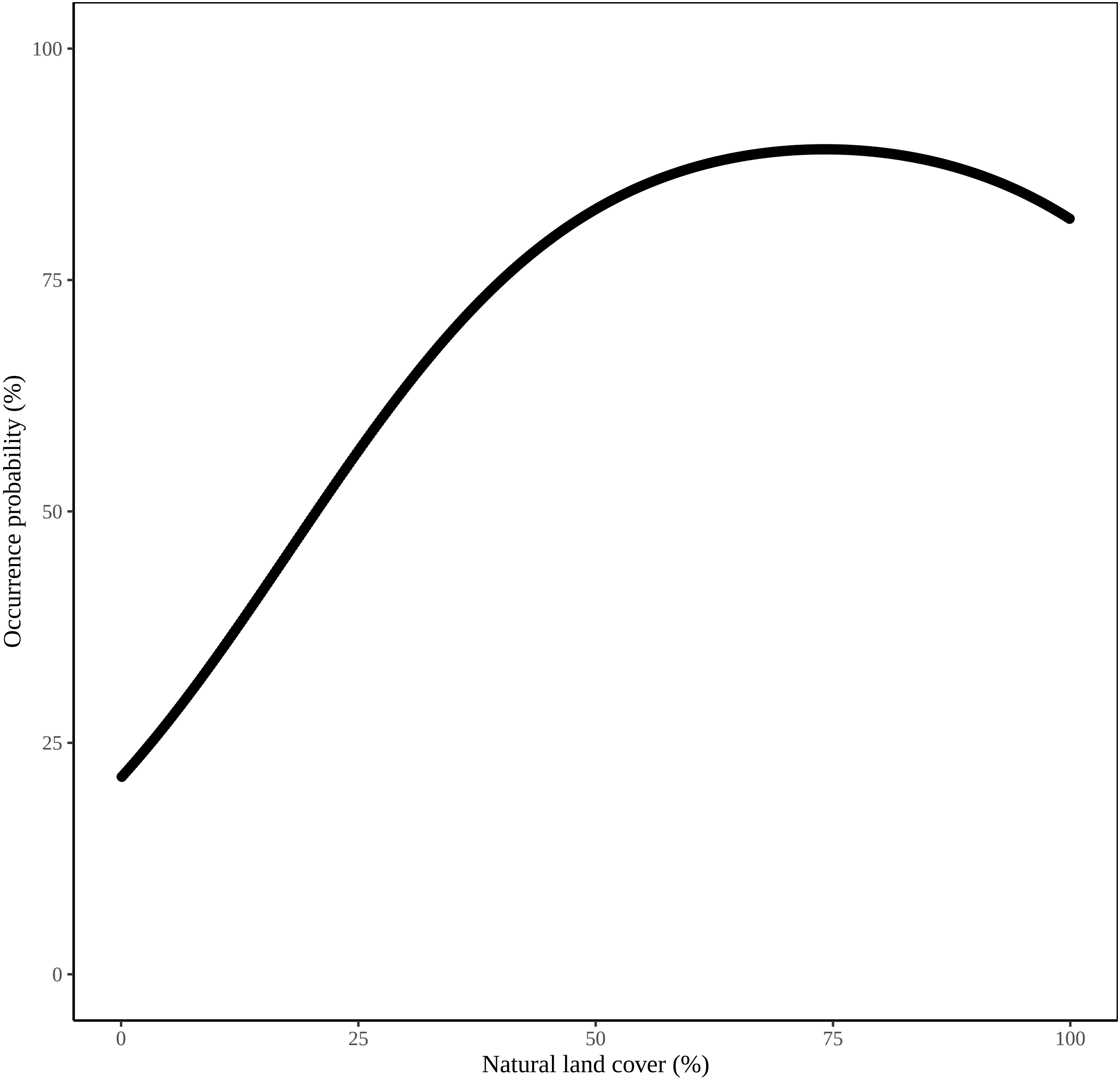

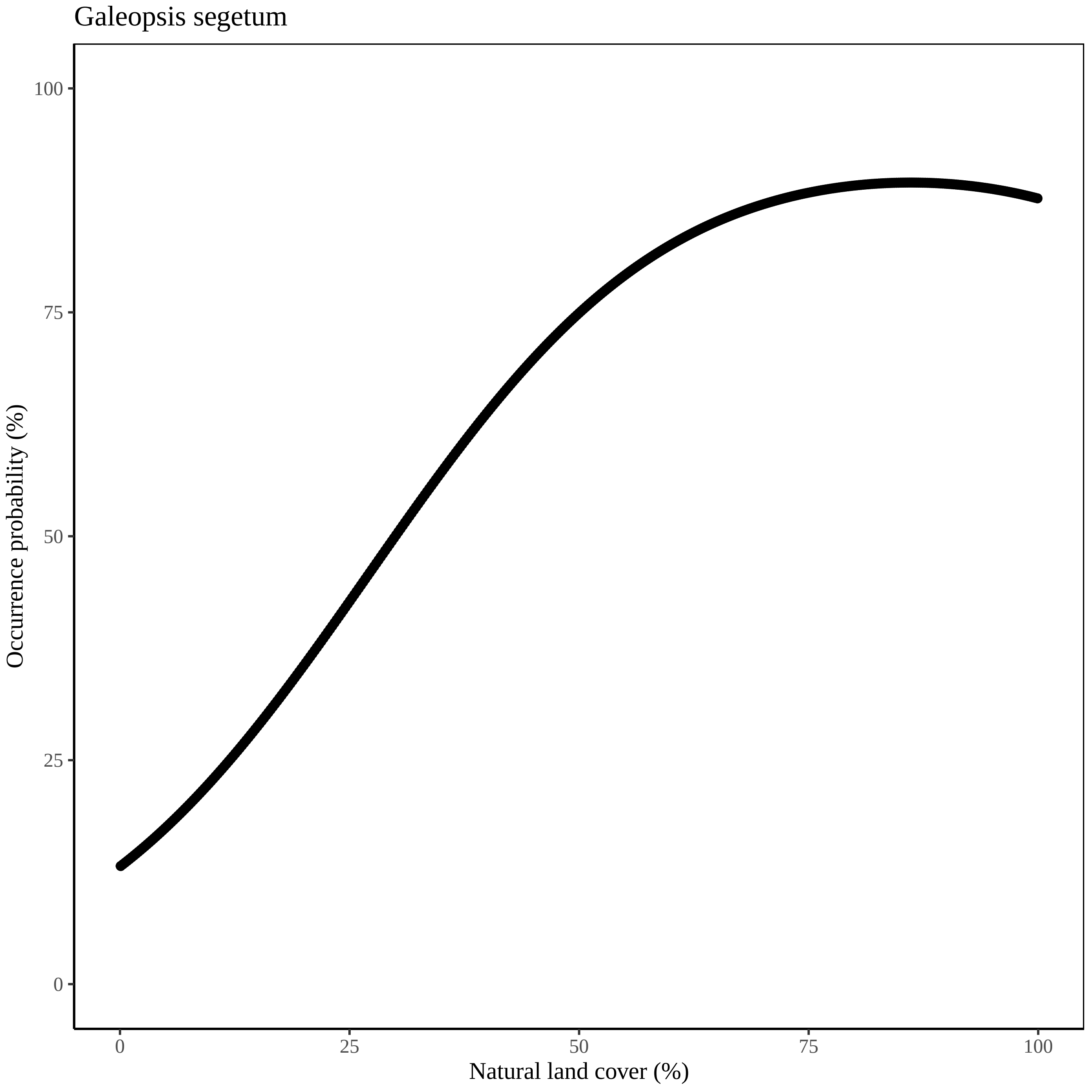
Galeopsis segetum
100
75
50
25
0
Occurrence probability (%)
0
25
50
75
100
Natural land cover (%)

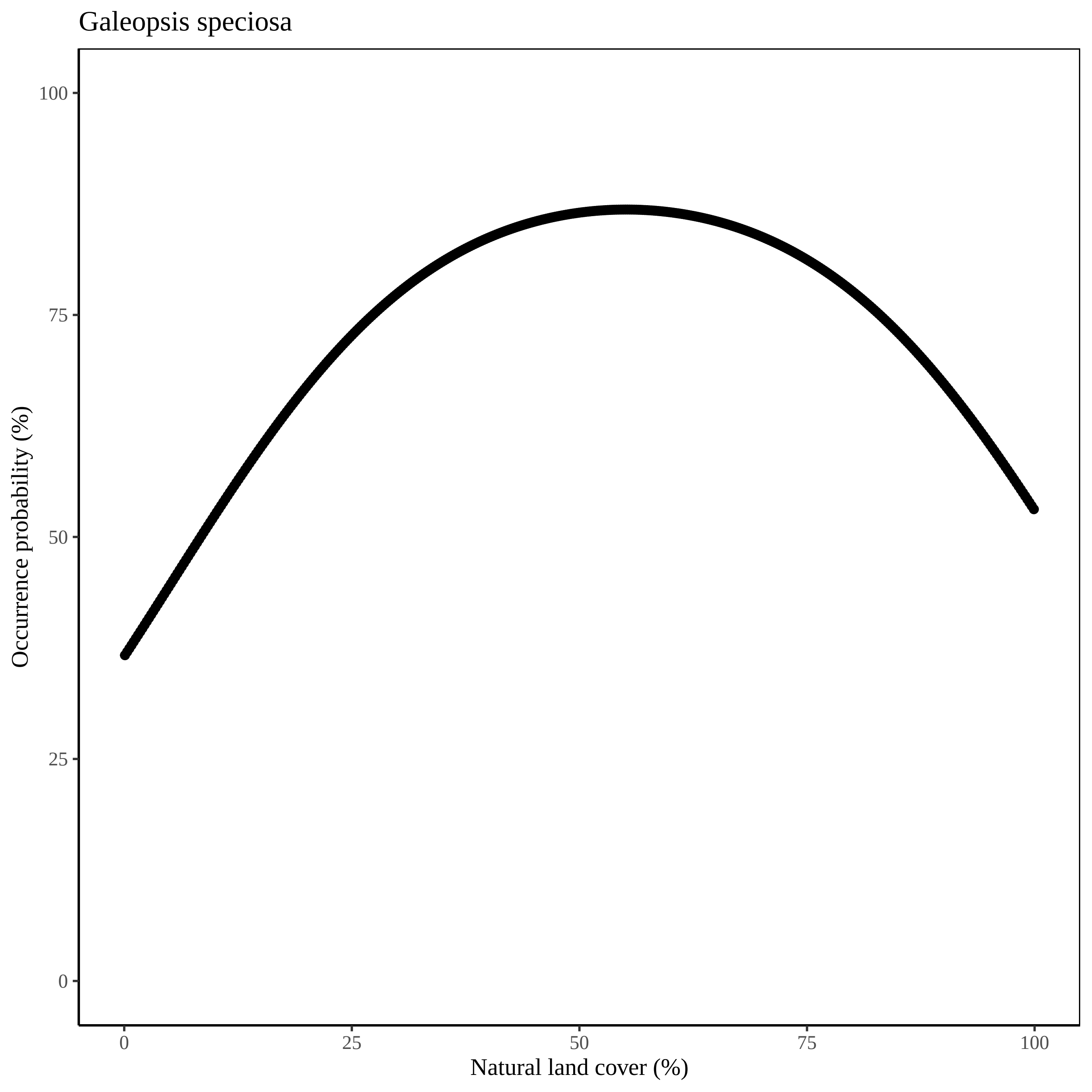
Galeopsis speciosa
Occurrence probability (%)
100
75
50
25
0
0
25
50
75
100
Natural land cover (%)

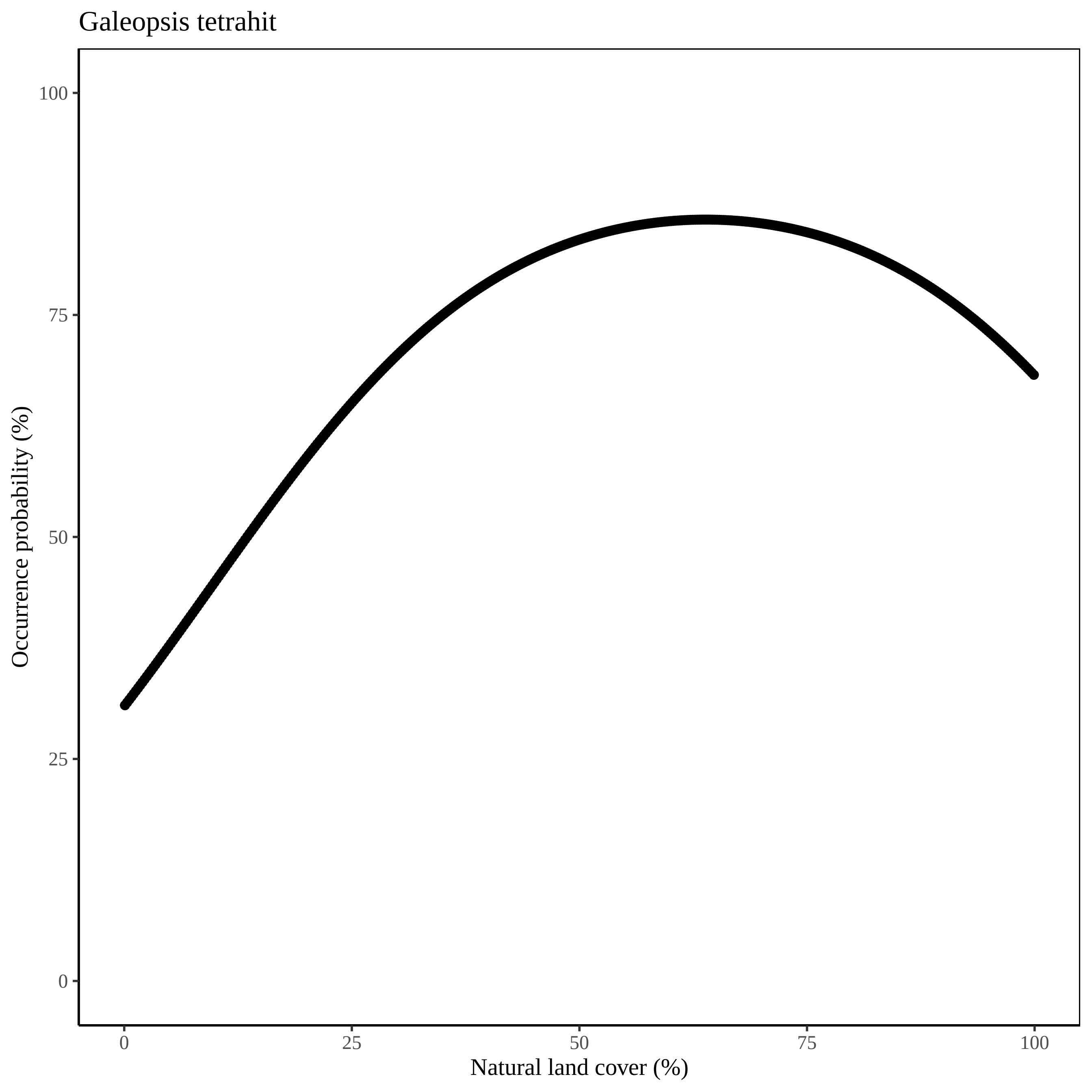

Galeopsis tetrahit
Occurrence probability (%)
100
75
50
25
0
0
25
50
75
100
Natural land cover (%)

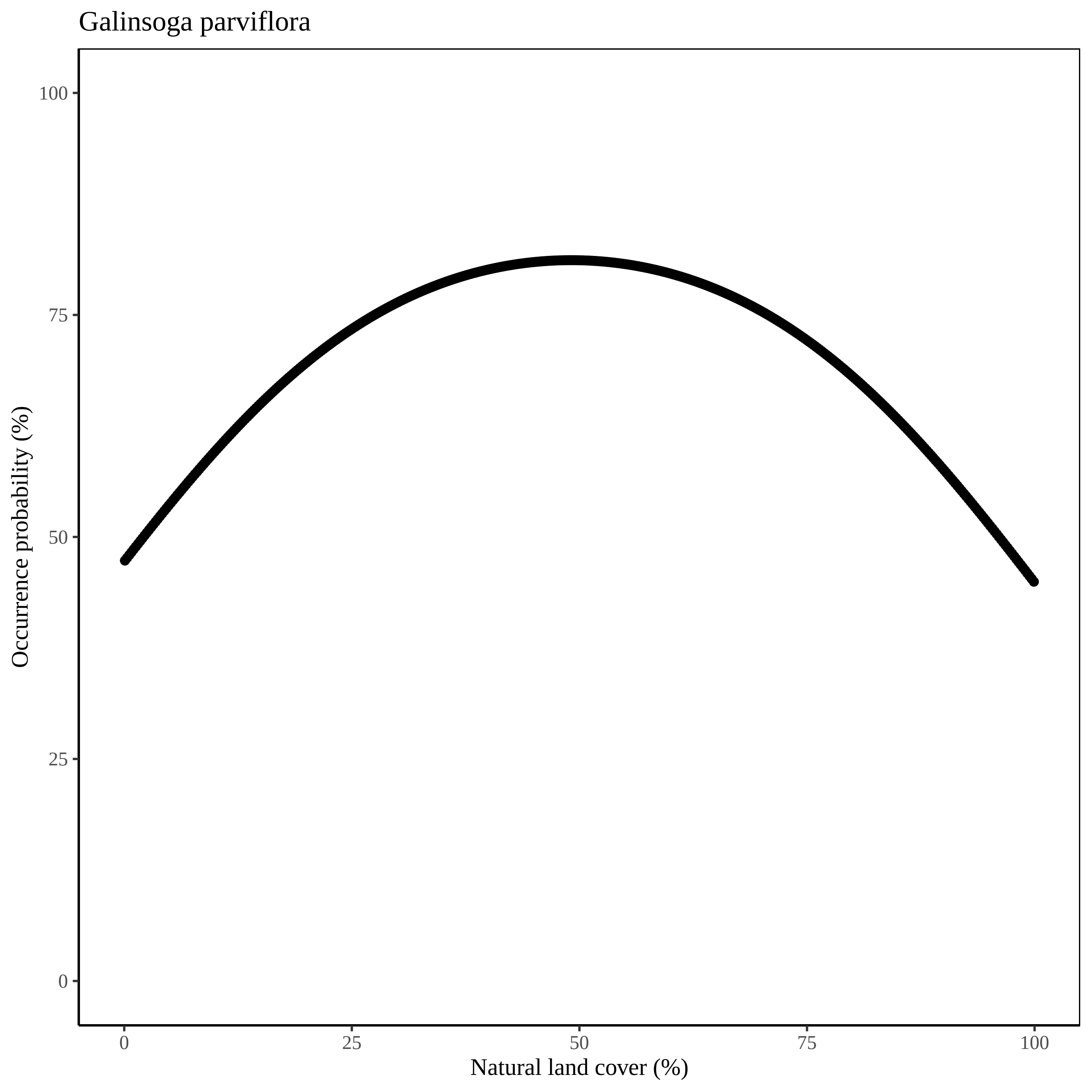
Galinsoga parviflora
Occurrence probability (%)
100
75
50
25
0
0
25
50
75
100
Natural land cover (%)

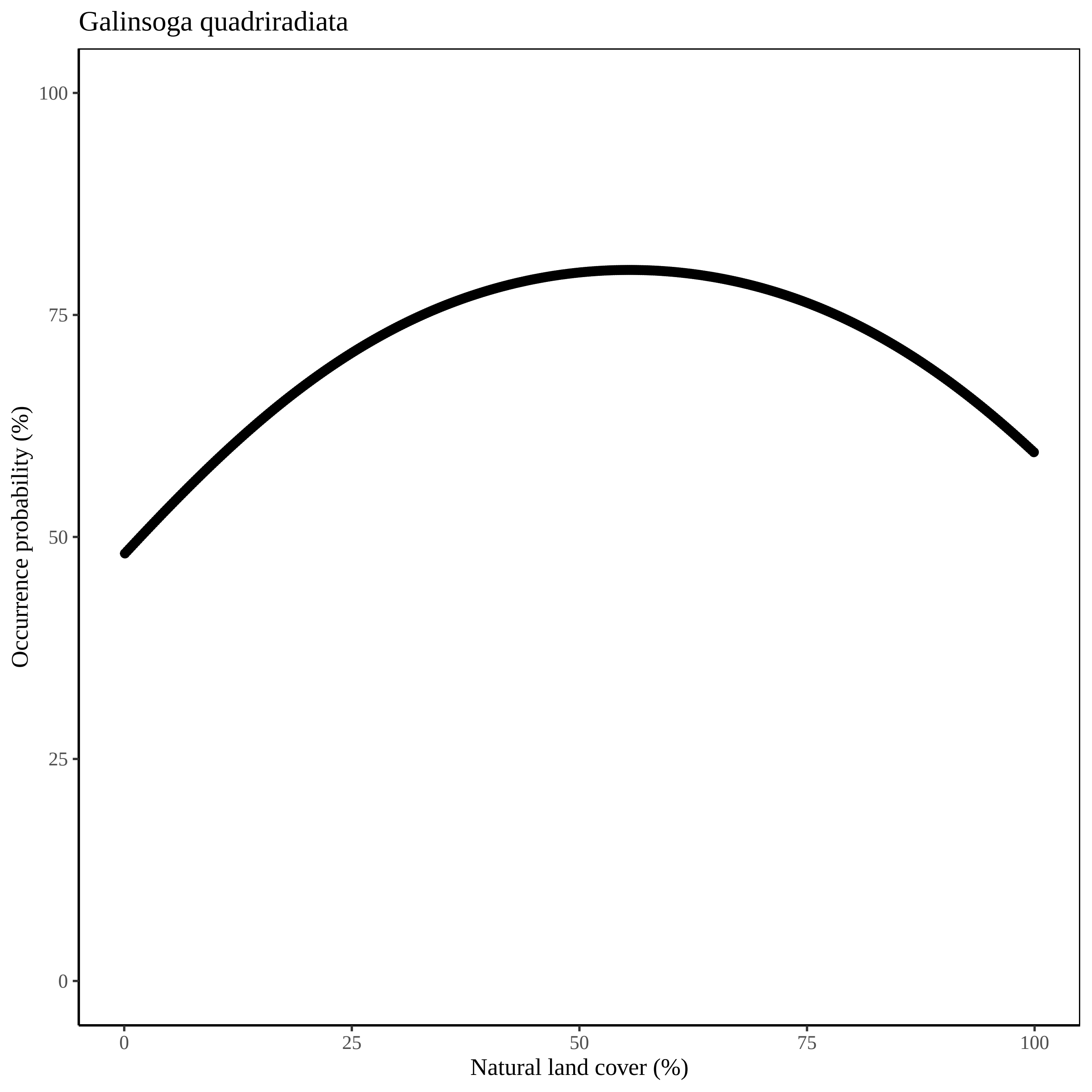

Galinsoga quadriradiata
Occurrence probability (%)
100
75
50
25
0
0
25
50
75
100
Natural land cover (%)

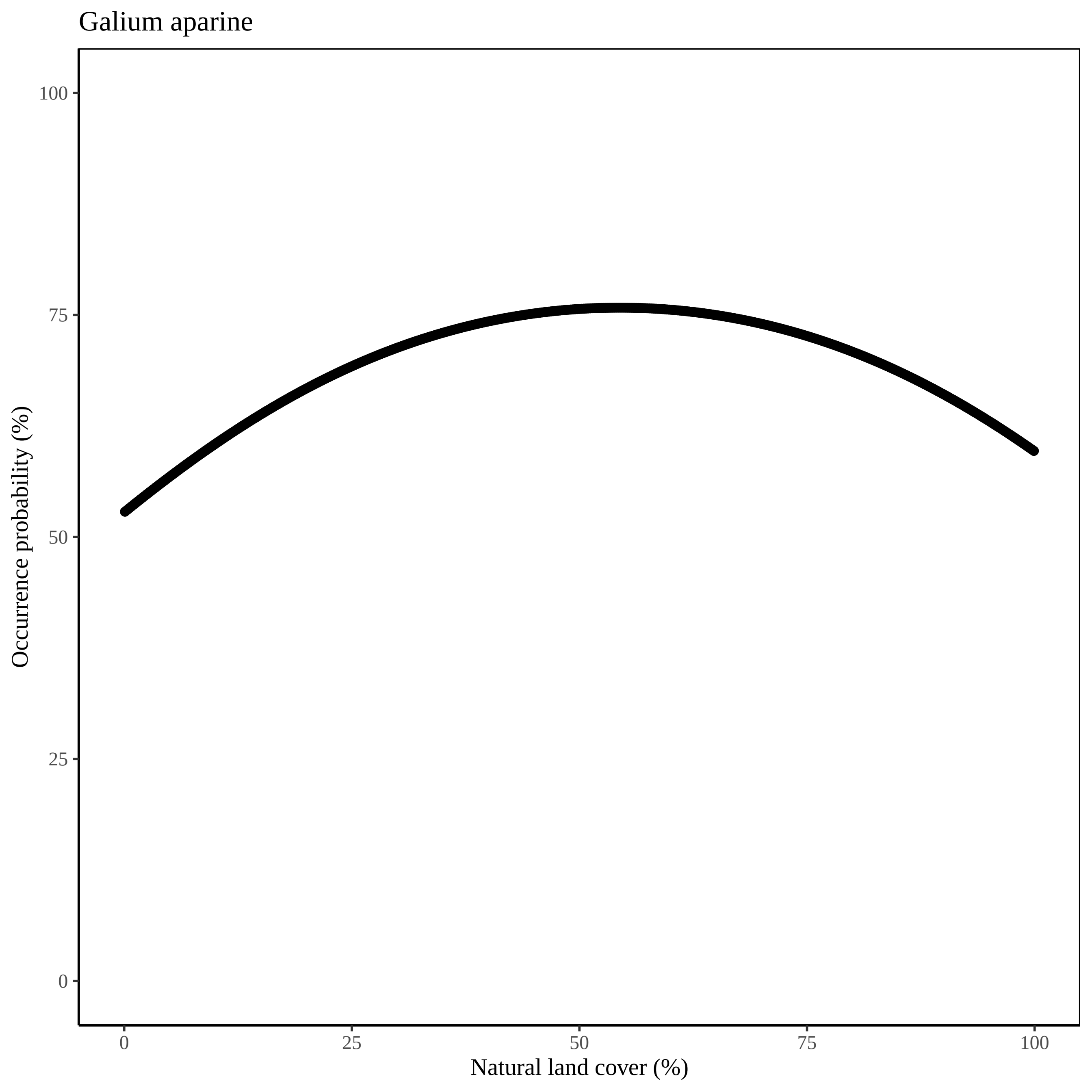
Galium aparine
Occurrence probability (%)
100
75
50
25
0
0
25
50
75
100
Natural land cover (%)

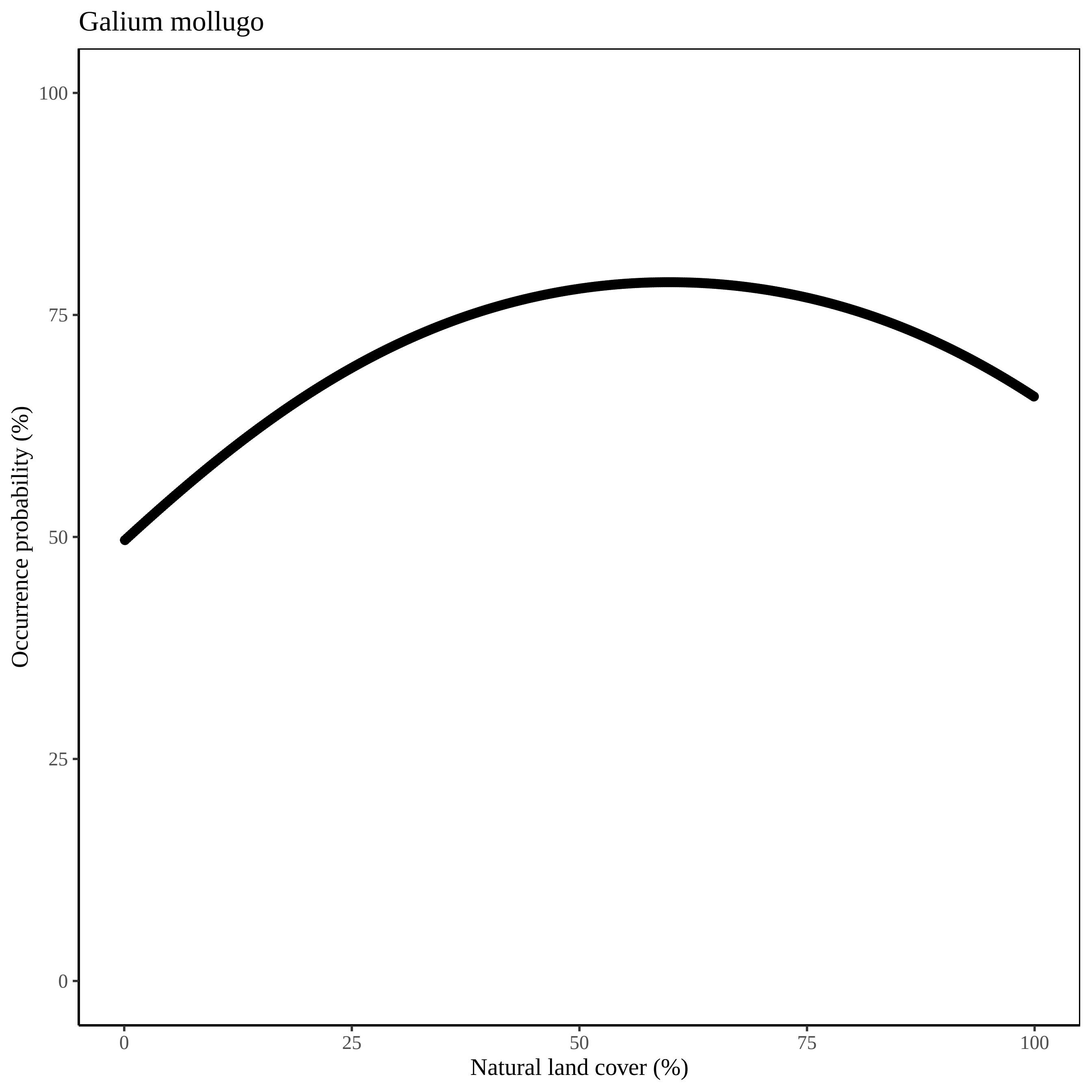

Galium mollugo
Occurrence probability (%)
100
75
50
25
0
0
25
50
75
100
Natural land cover (%)

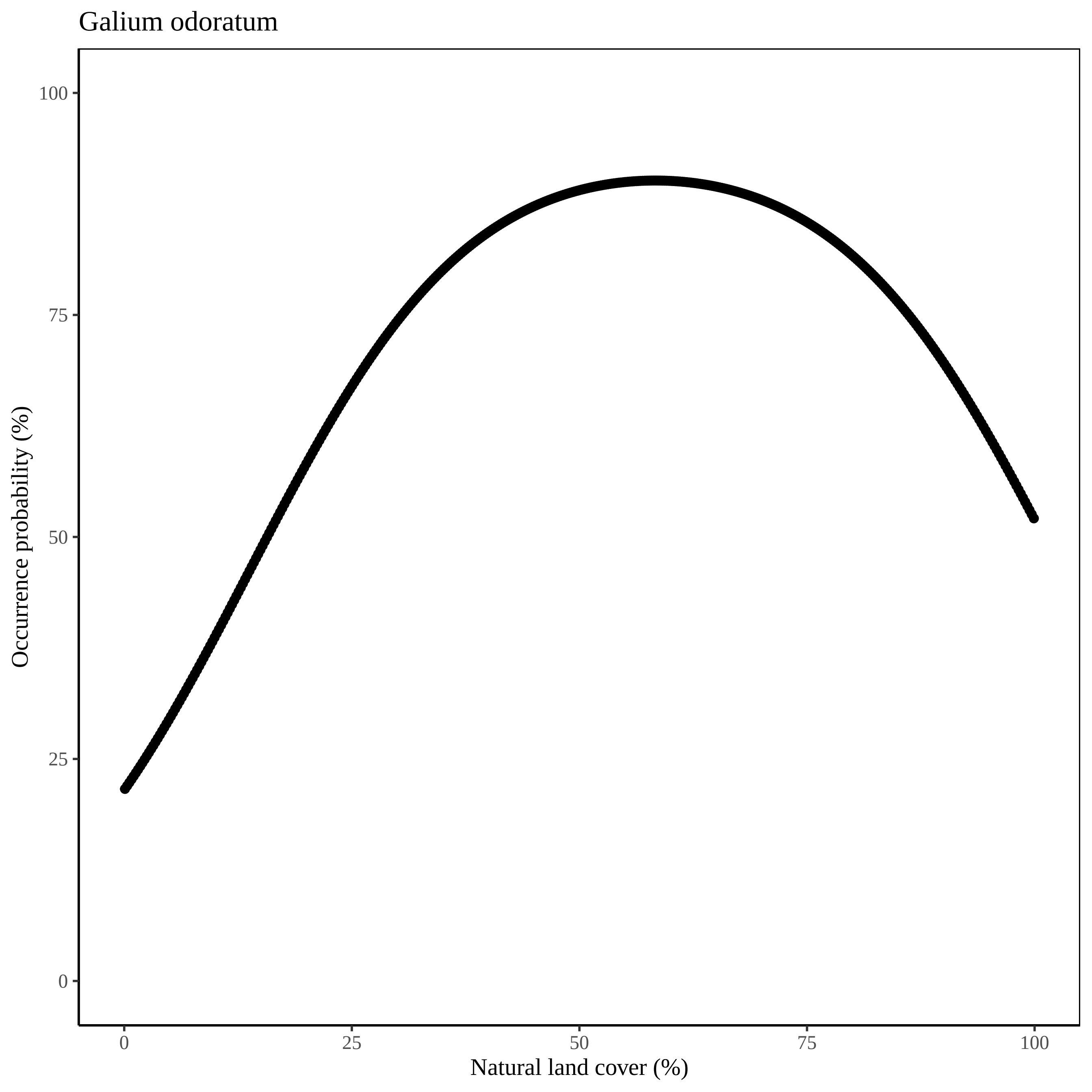
Galium odoratum
Occurrence probability (%)
100
75
50
25
0
0
25
50
75
100
Natural land cover (%)

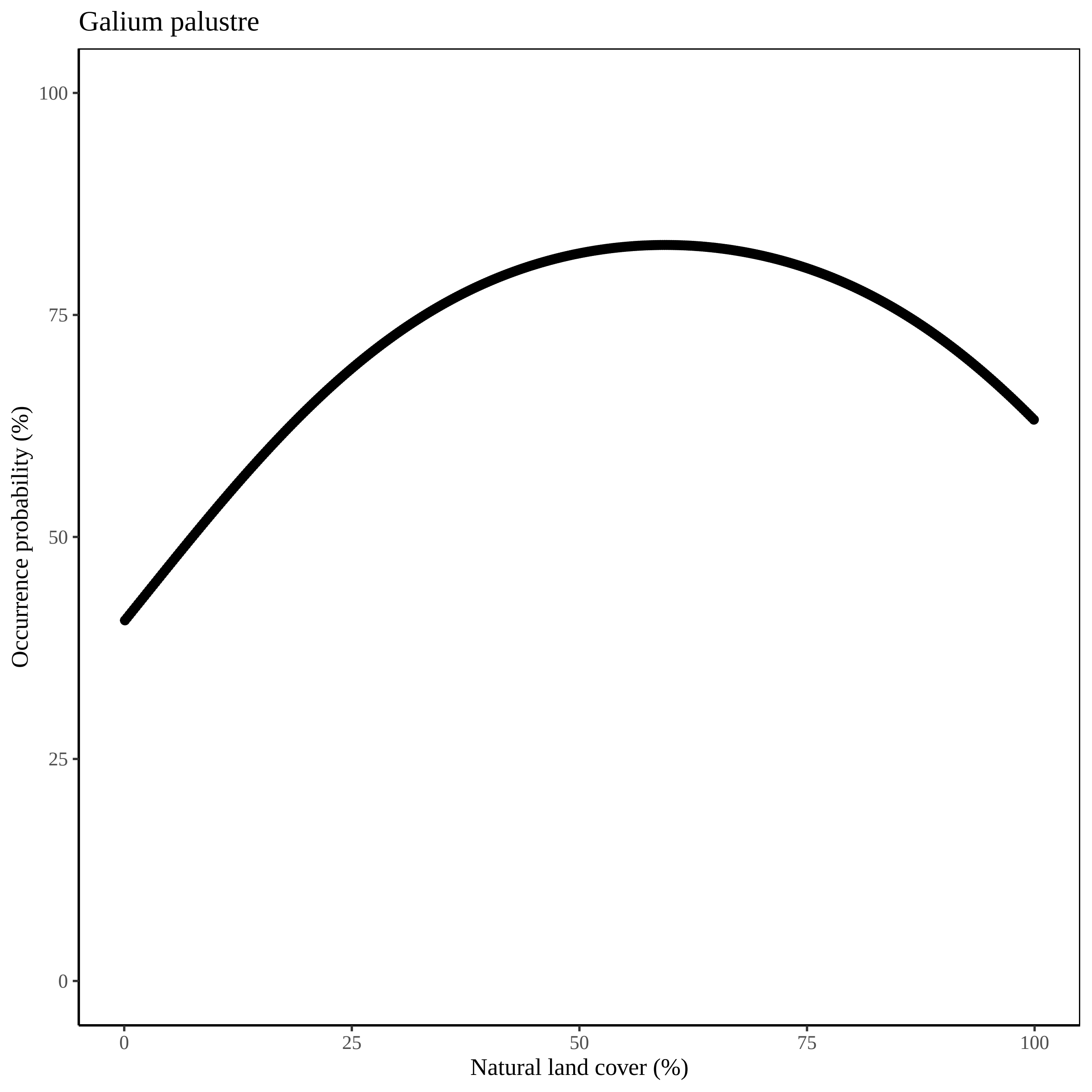
Galium palustre
Occurrence probability (%)
100
75
50
25
0
0
25
50
75
100
Natural land cover (%)

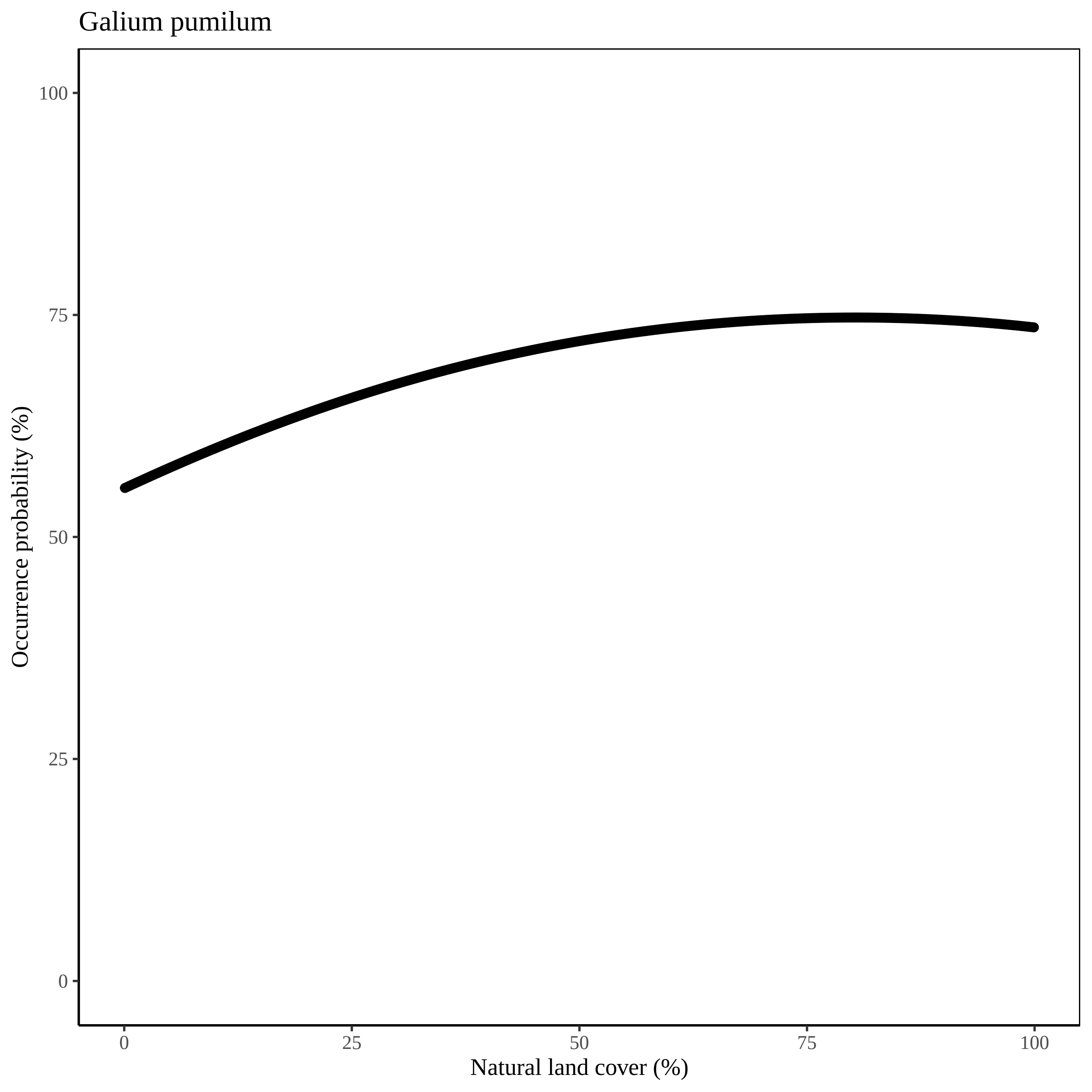

Galium pumilum
100
75
50
25
0
Occurrence probability (%)
0
25
50
75
100
Natural land cover (%)

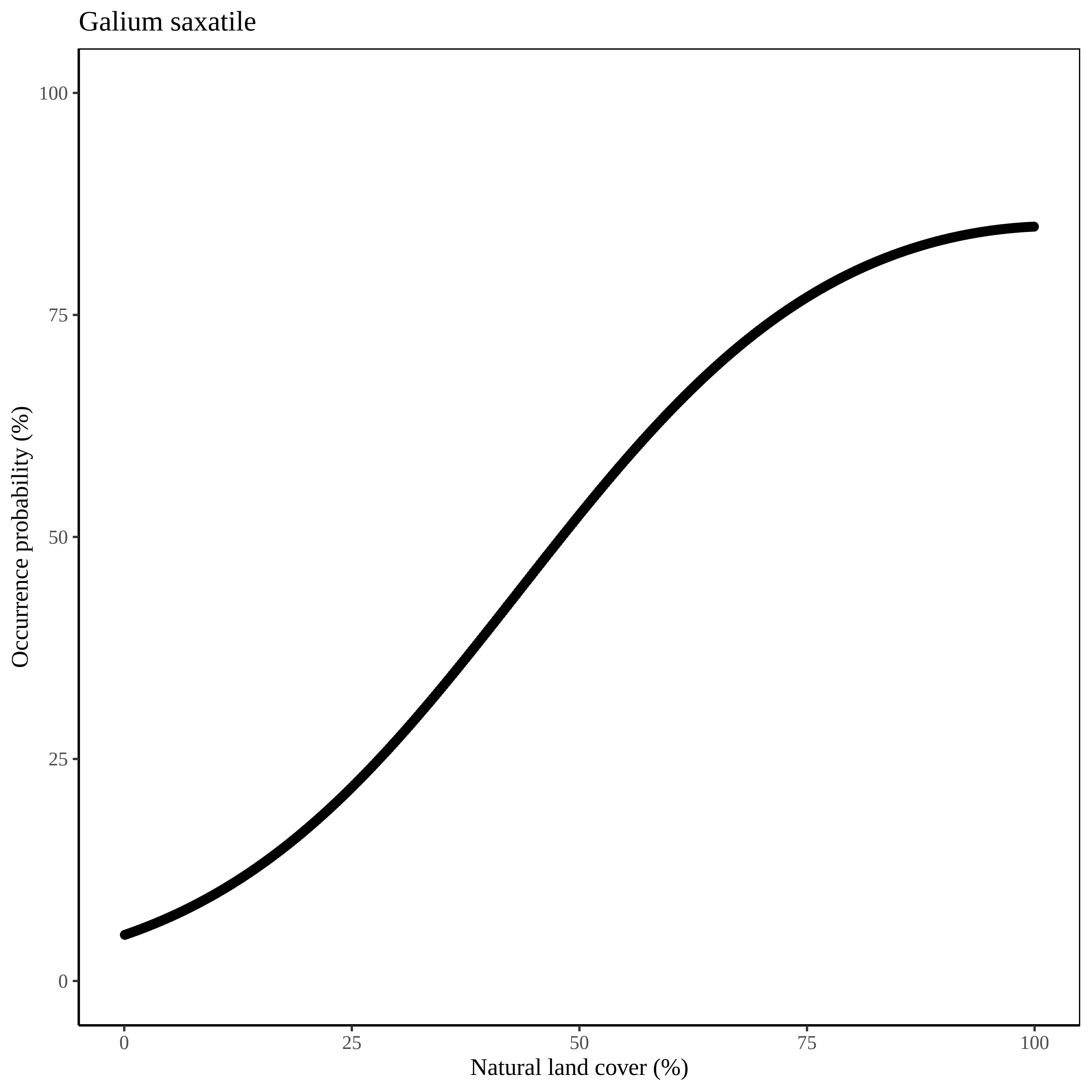
Galium saxatile
Occurrence probability (%)
100
75
50
25
0
0
25
50
75
100
Natural land cover (%)

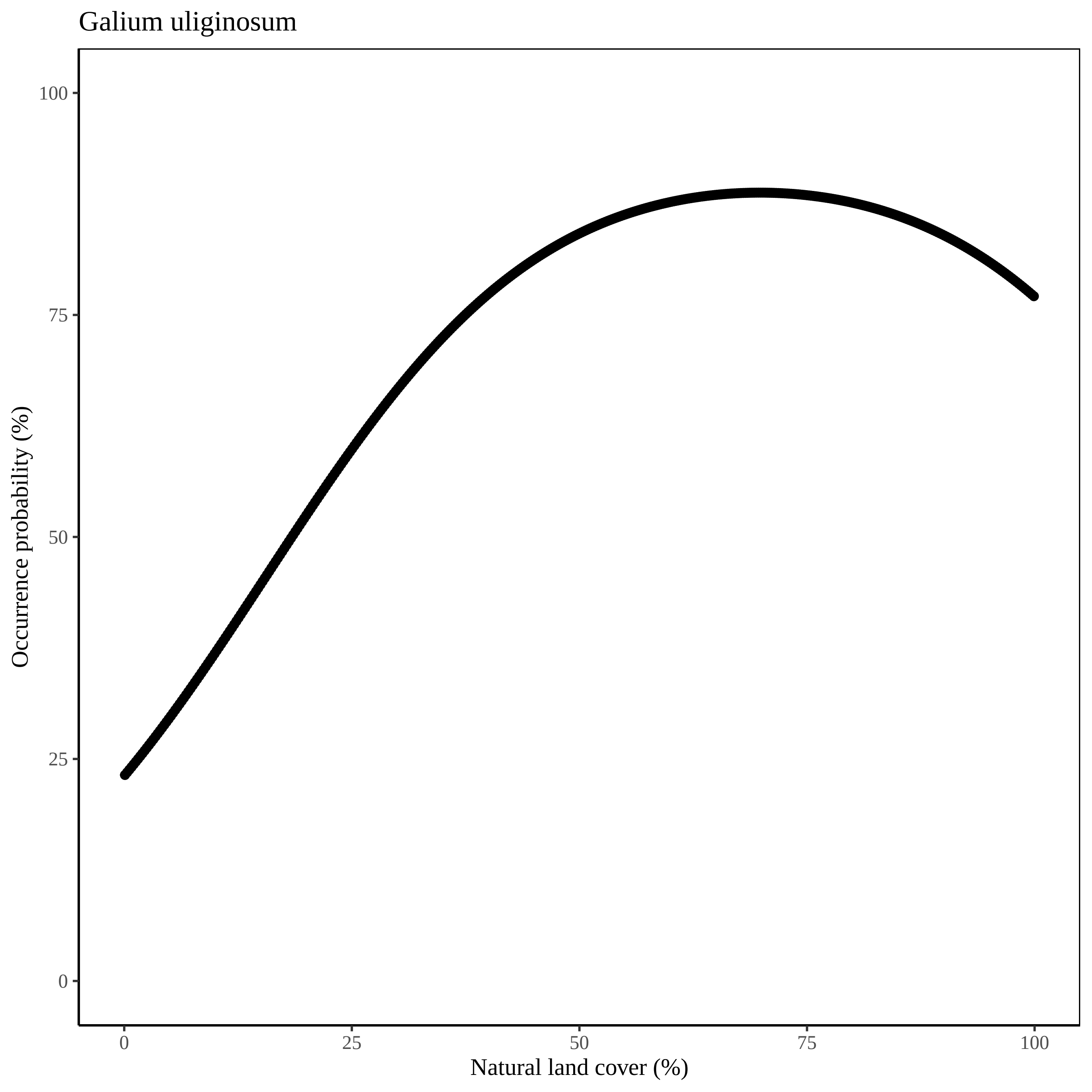
Galium uliginosum
100
75
50
25
0
Occurrence probability (%)
0
25
50
75
100
Natural land cover (%)

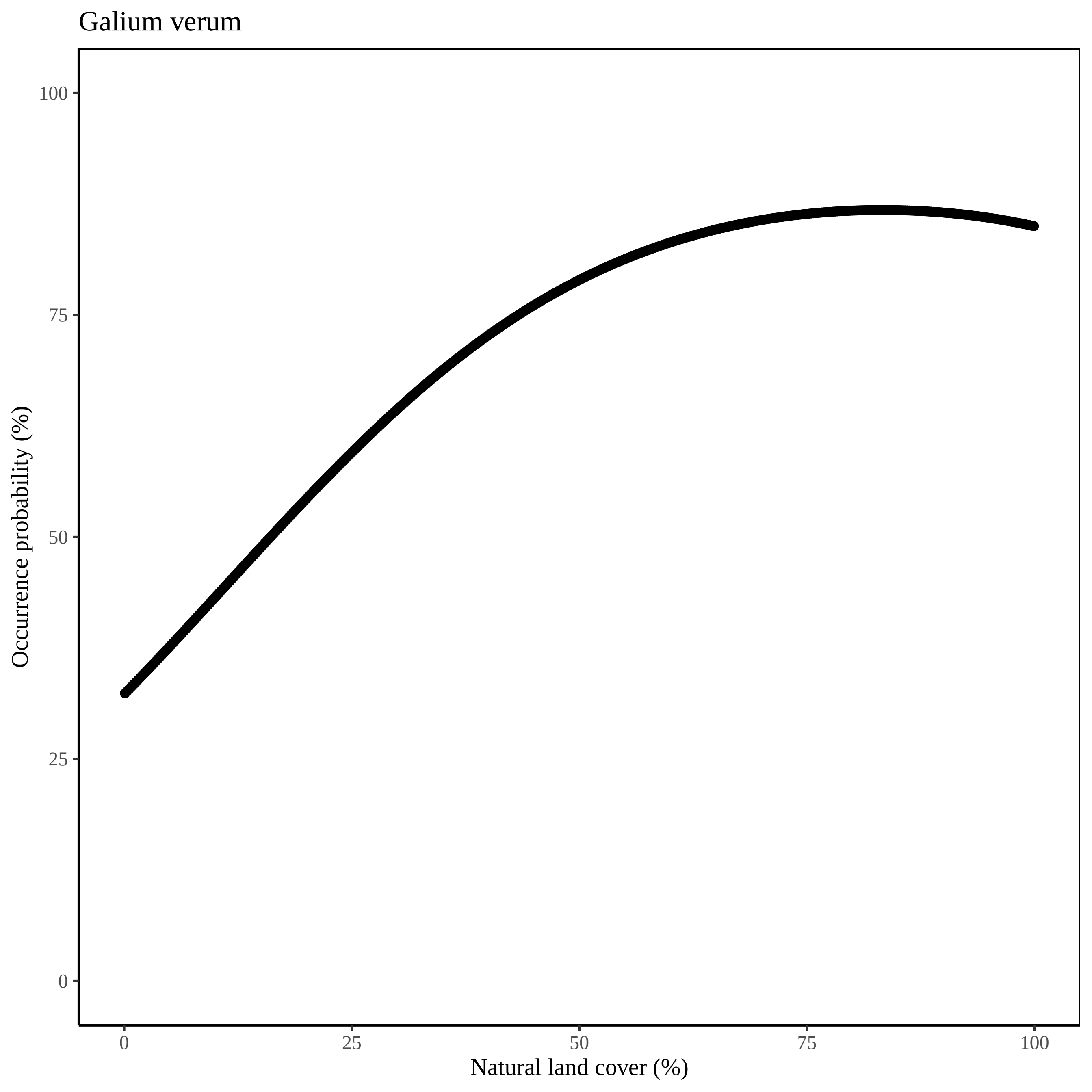
Galium verum
Occurrence probability (%)
100
75
50
25
0
0
25
50
75
100
Natural land cover (%)

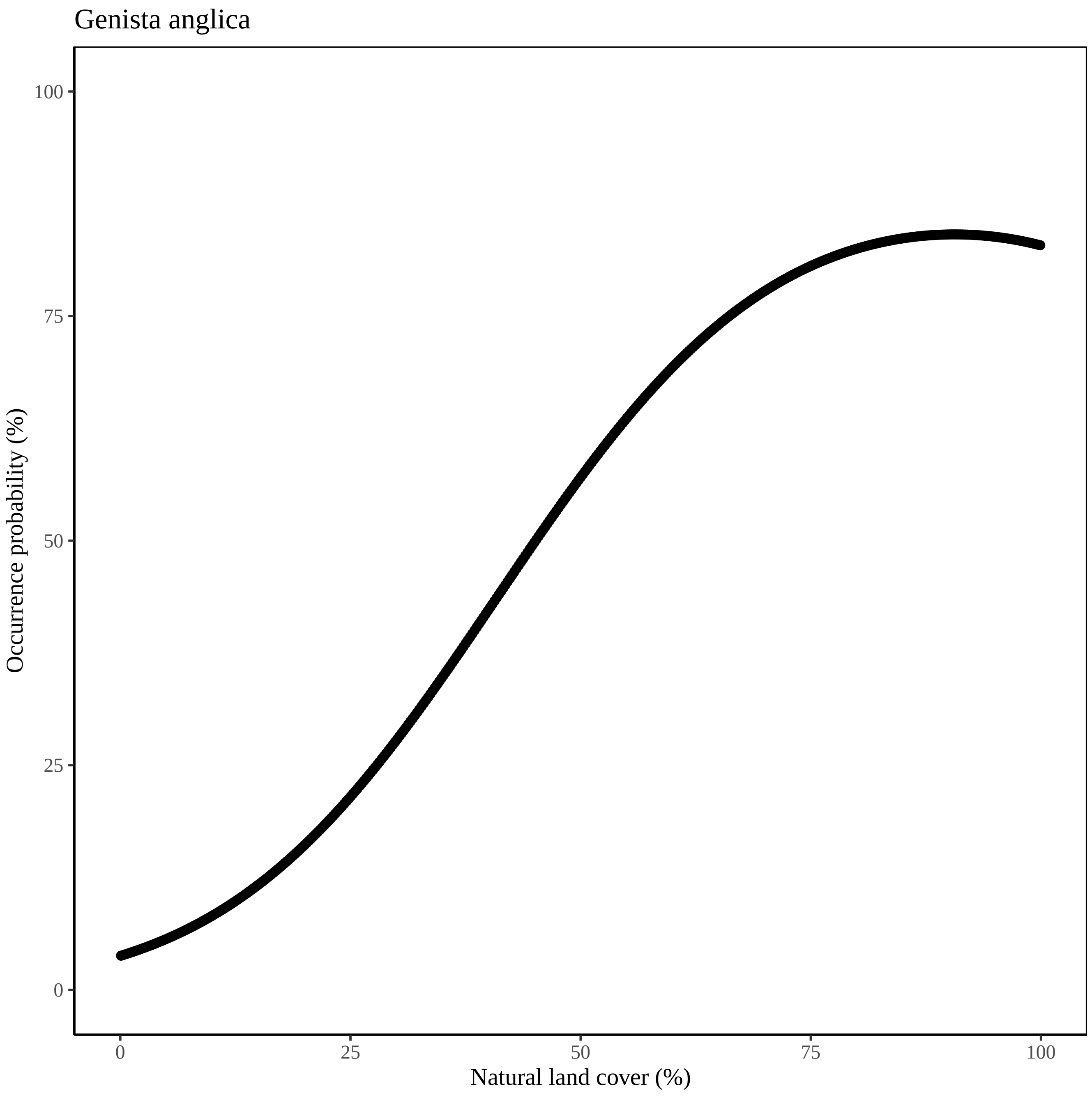
Genista anglica
Occurrence probability (%)
100
75
50
25
0
0
25
50
75
100
Natural land cover (%)

Genista pilosa

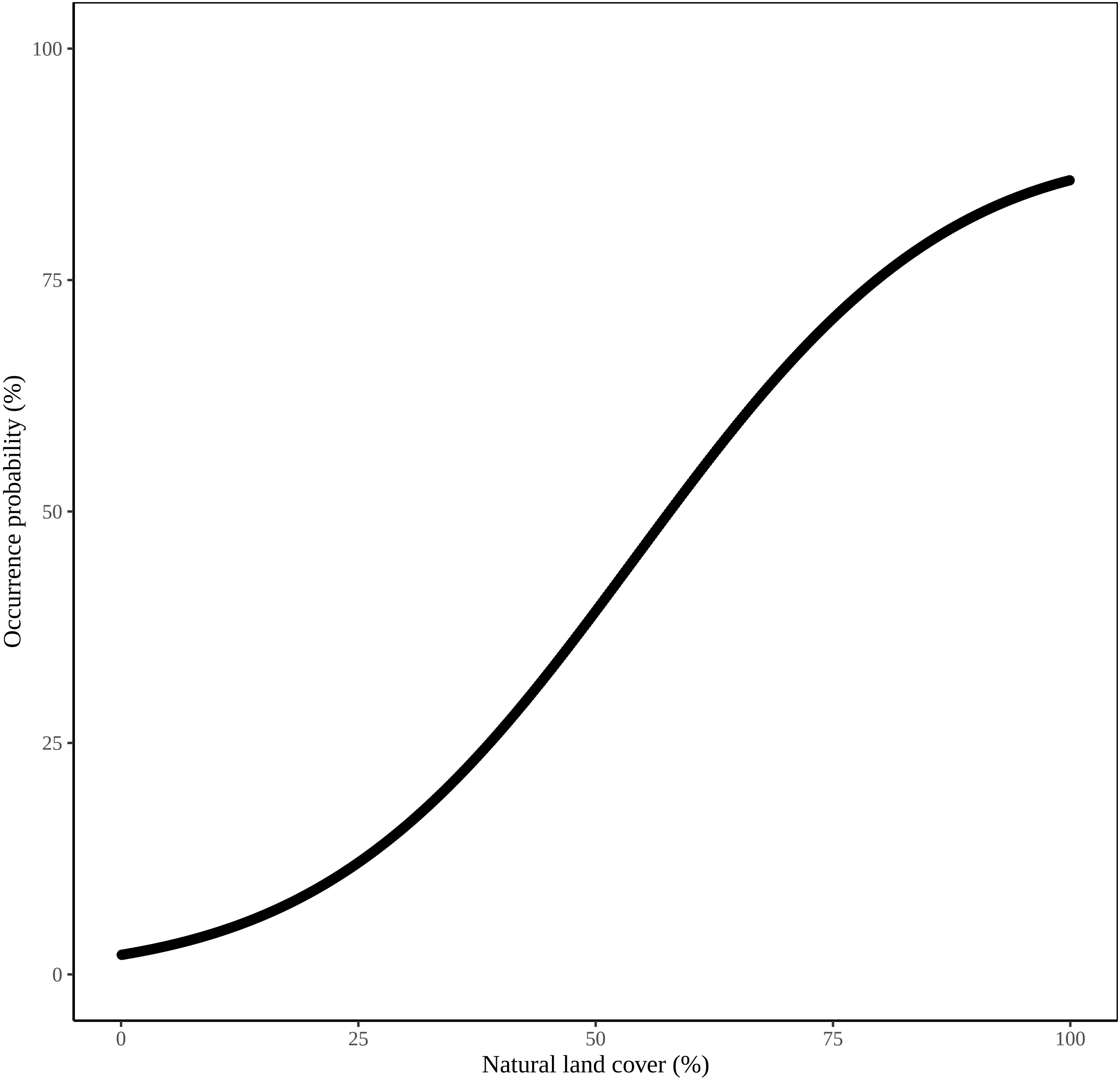

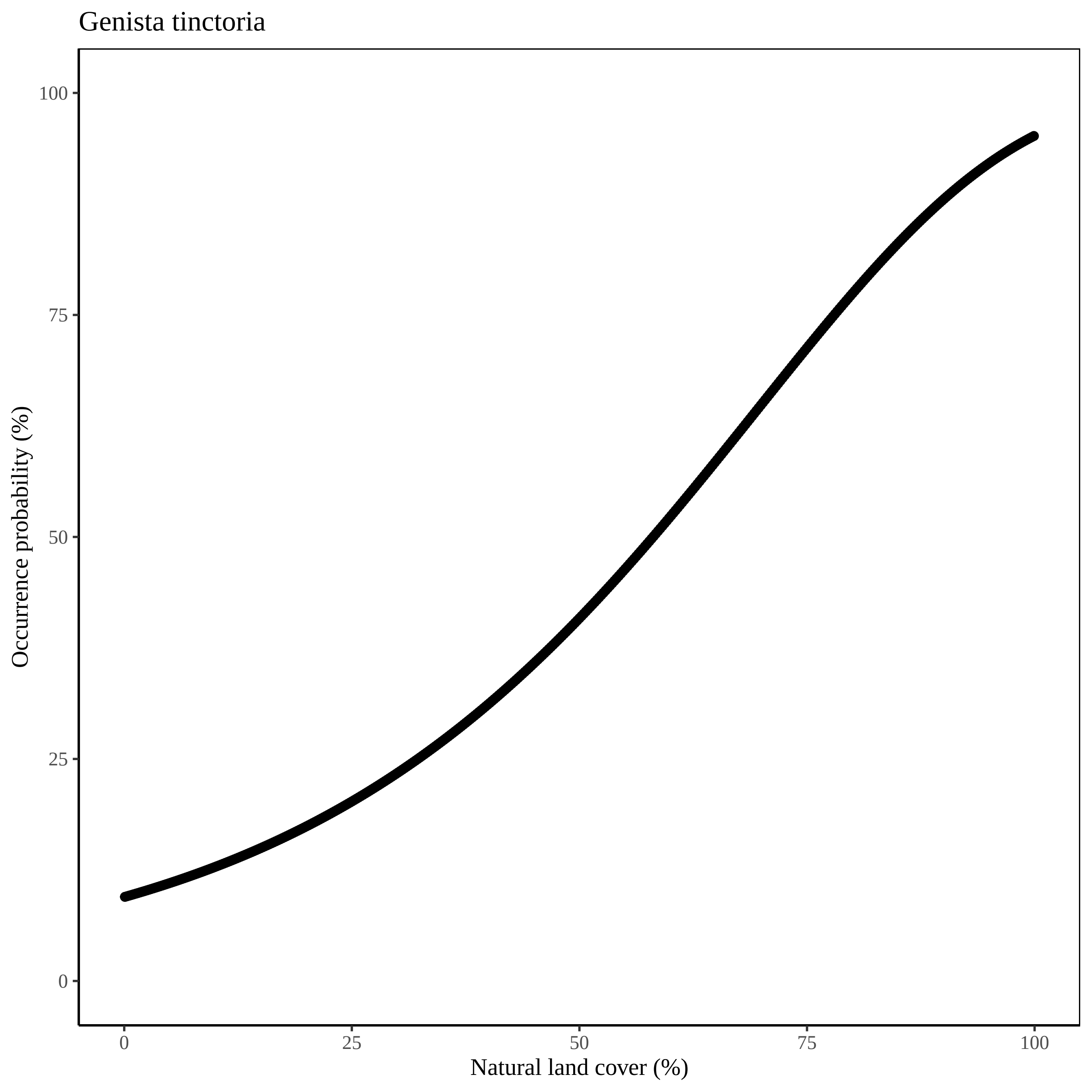
Genista tinctoria
Occurrence probability (%)
100
75
50
25
0
0
25
50
75
100
Natural land cover (%)

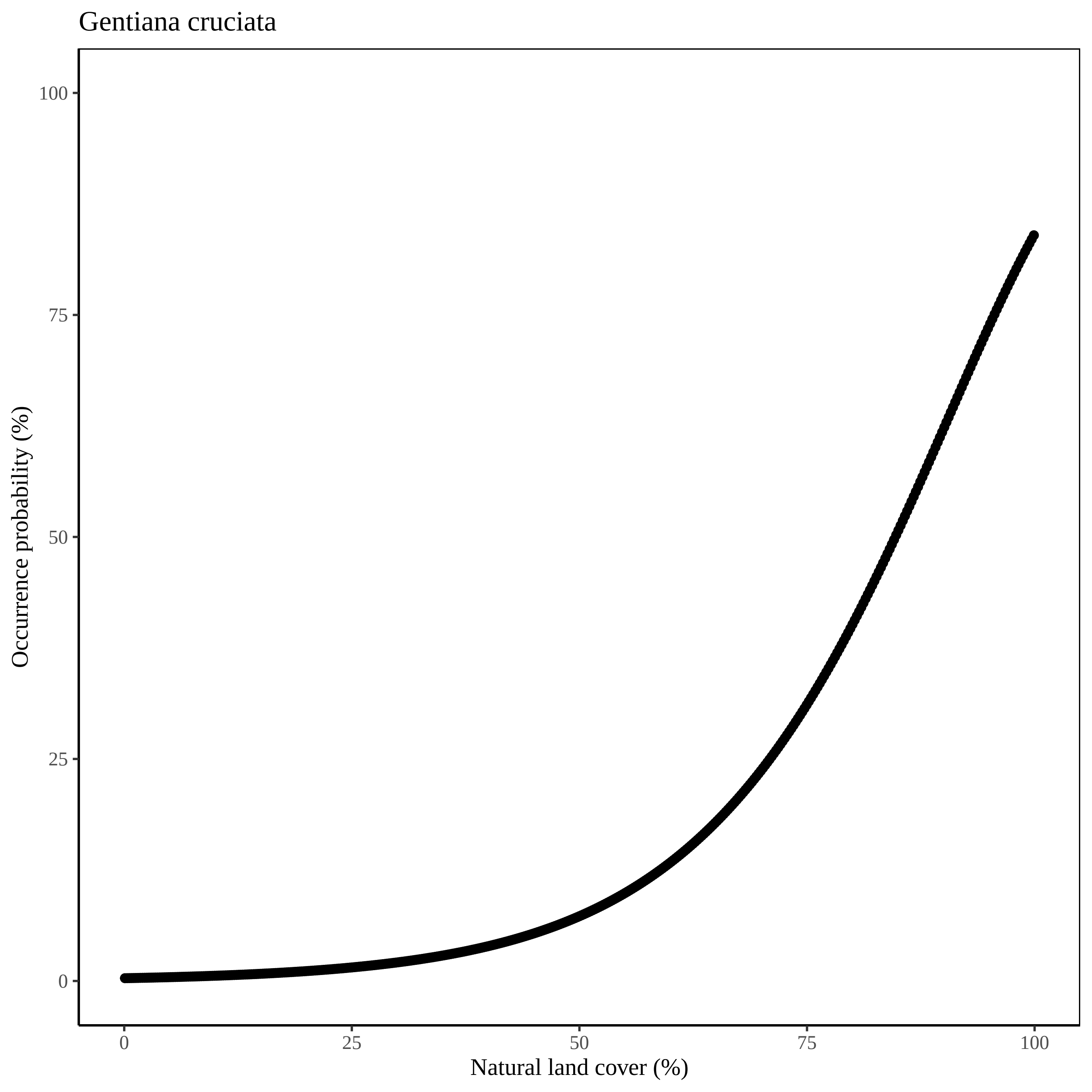
Gentiana cruciata
Occurrence probability (%)
100
75
50
25
0
0
25
50
75
100
Natural land cover (%)

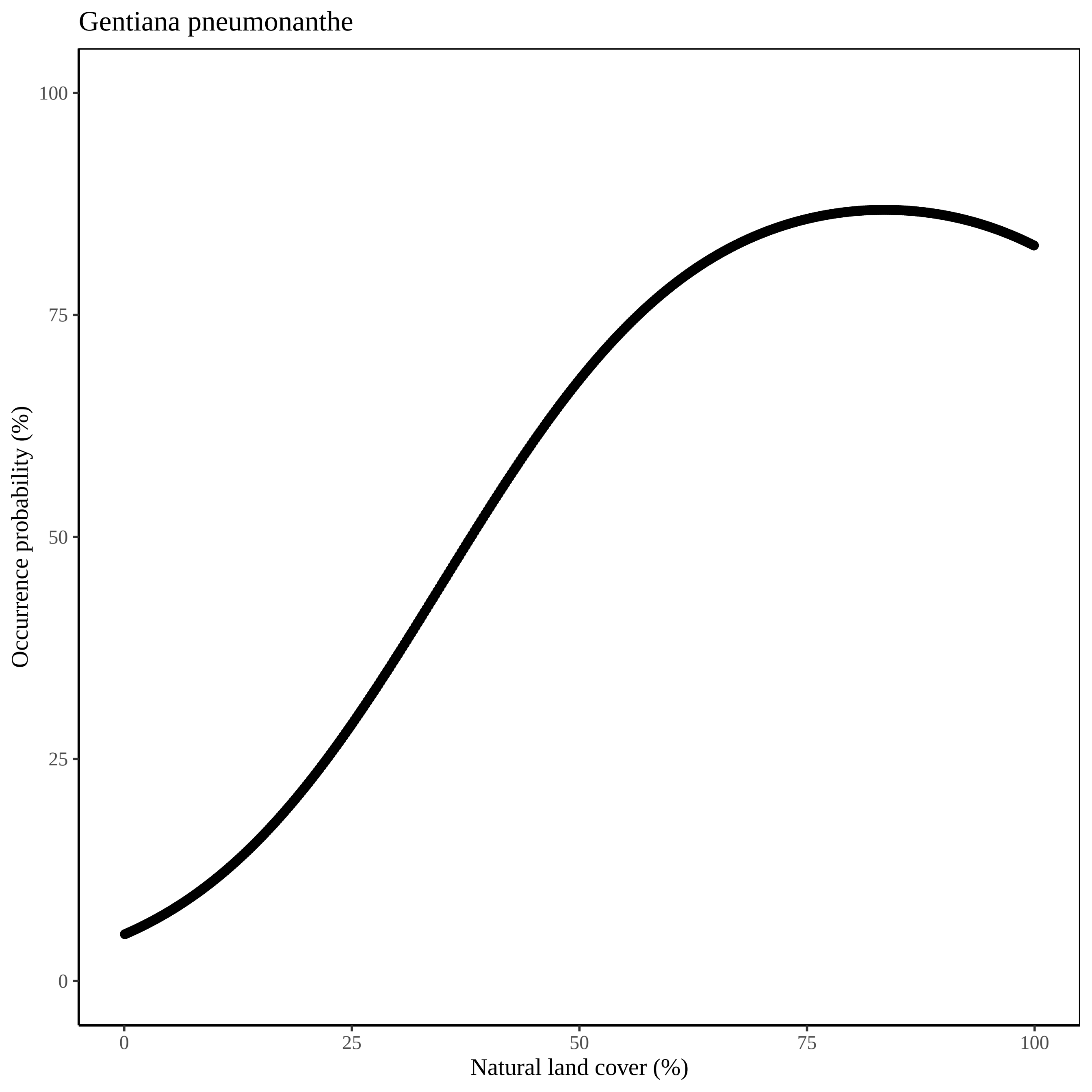

Gentiana pneumonanthe
Occurrence probability (%)
Natural land cover (%)
0
25
50
75
100

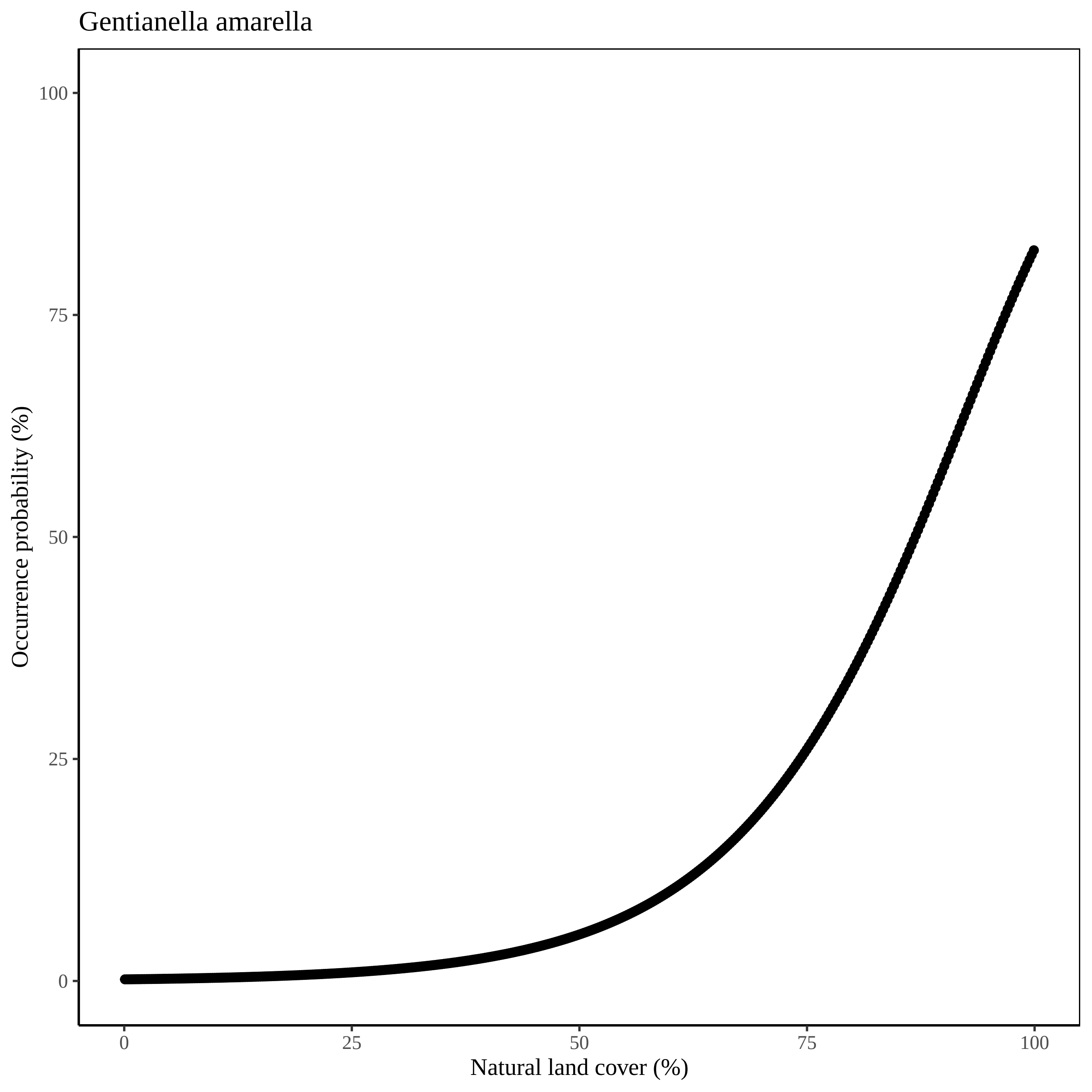
Gentianella amarella
Occurrence probability (%)
100
75
50
25
0
0
25
50
75
100
Natural land cover (%)

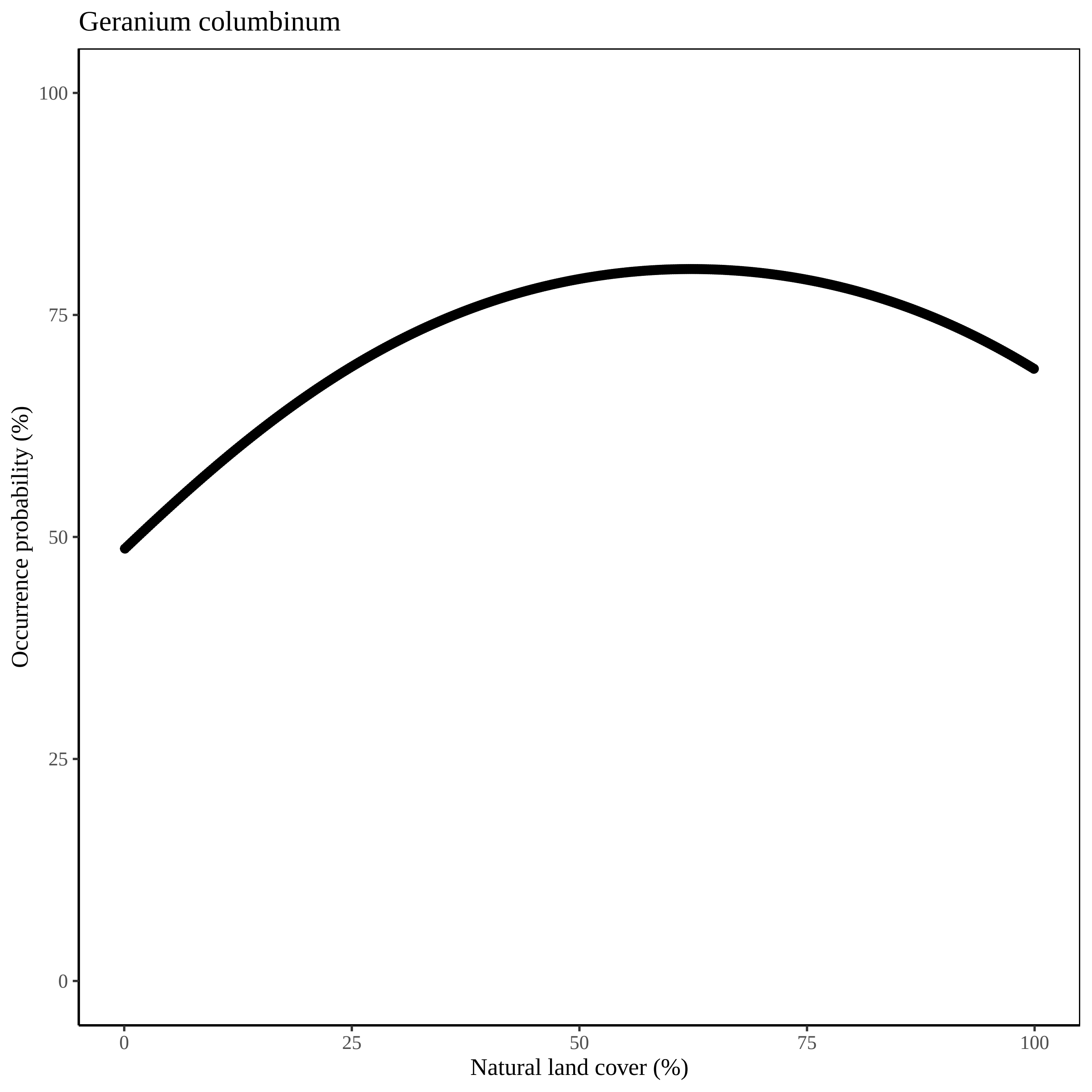

Geranium columbinum
Occurrence probability (%)
100
75
50
25
0
0
25
50
75
100
Natural land cover (%)

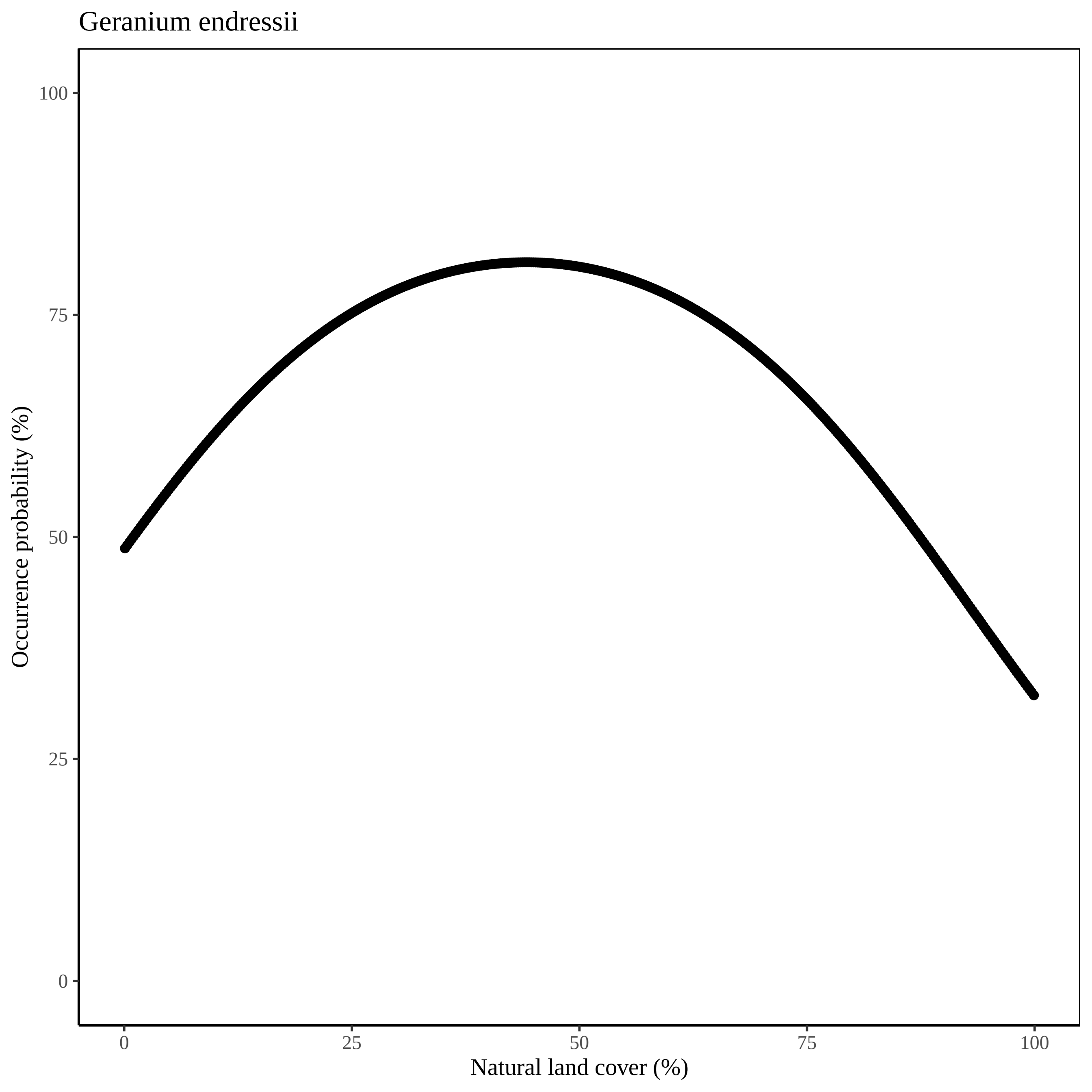

Geranium endressii
Occurrence probability (%)
100
75
50
25
0
0
25
50
75
100
Natural land cover (%)

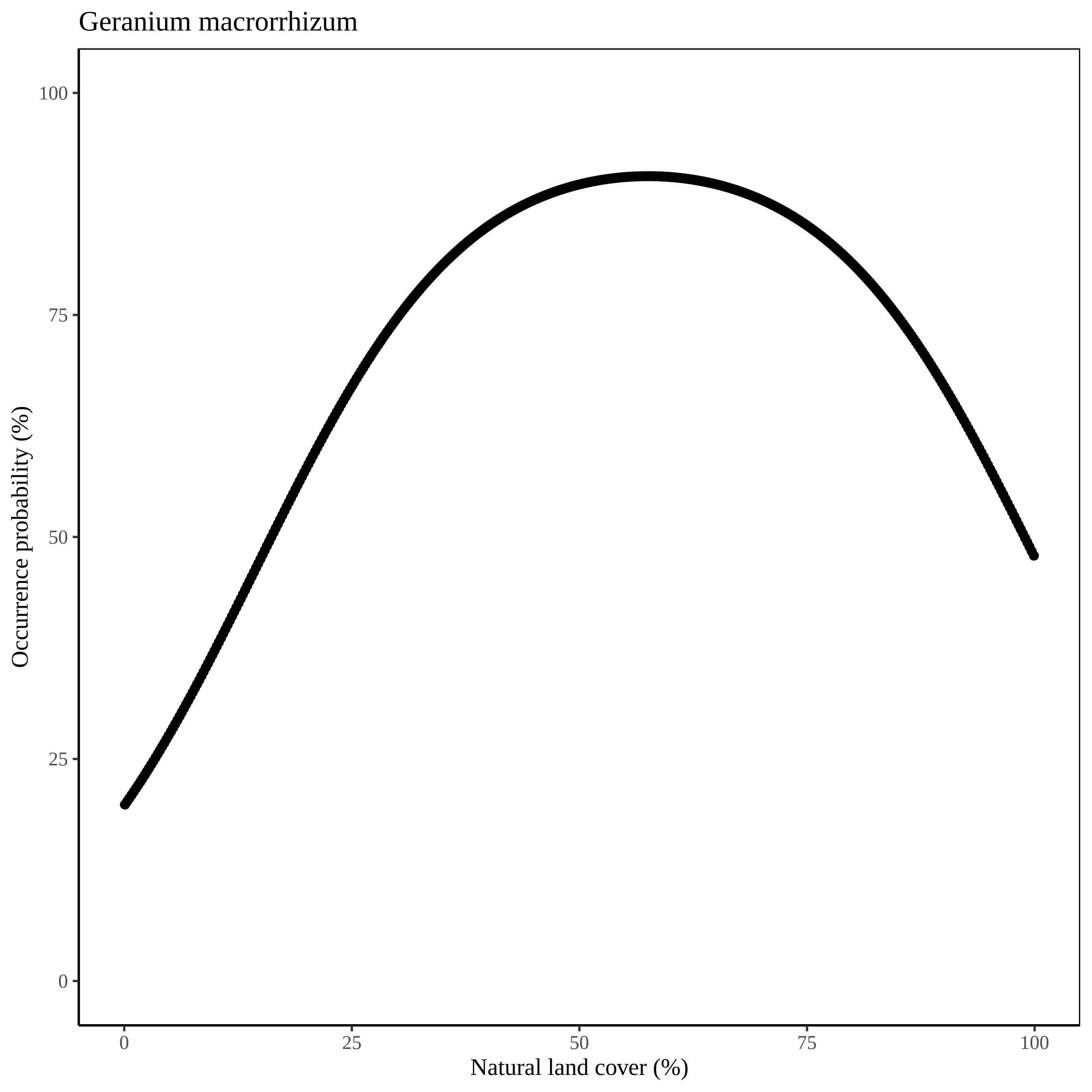

Geranium macrorrhizum
Occurrence probability (%)
Natural land cover (%)
0
25
50
75
100

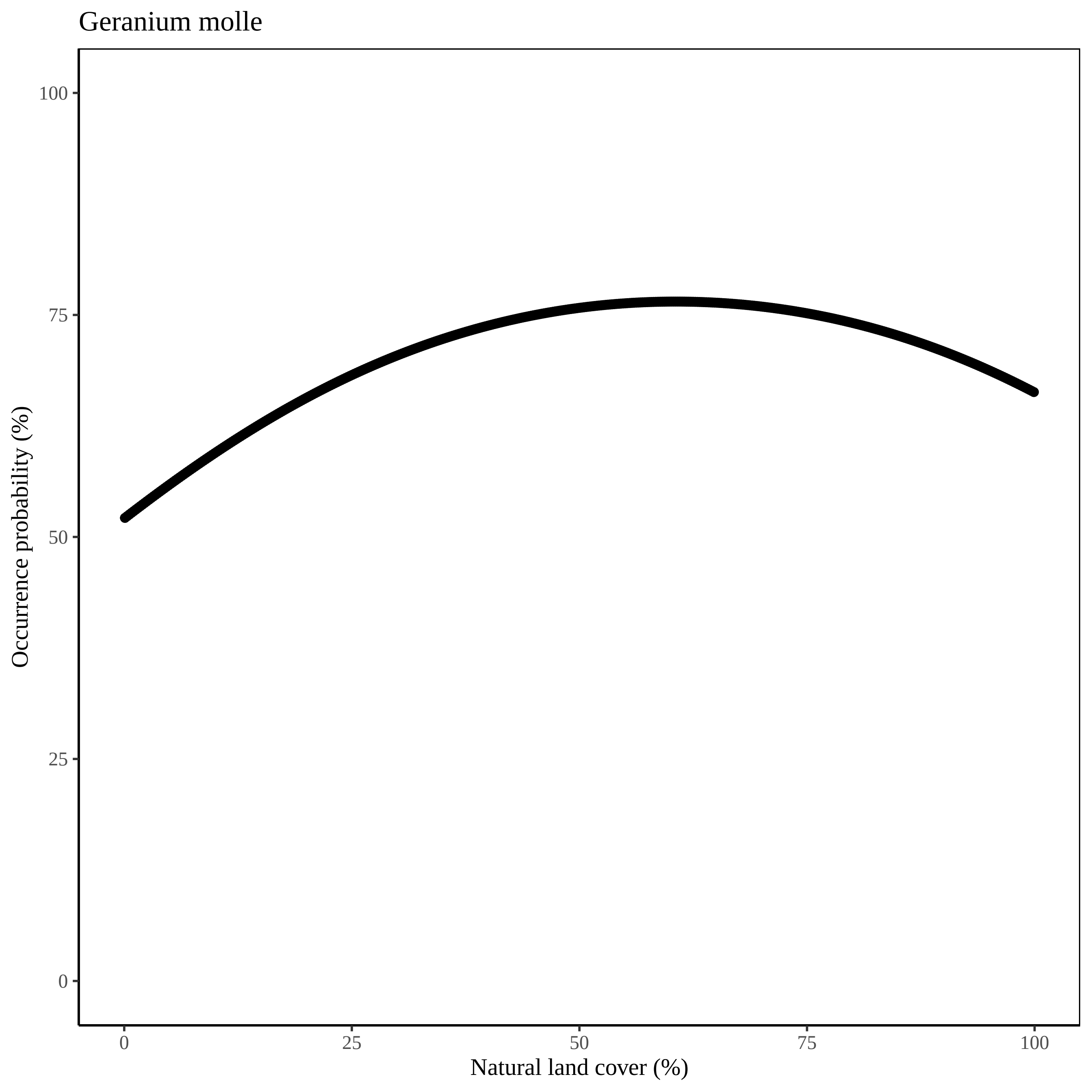

Geranium molle
100
75
50
25
0
0
25
50
75
100
Occurrence probability (%)
Natural land cover (%)

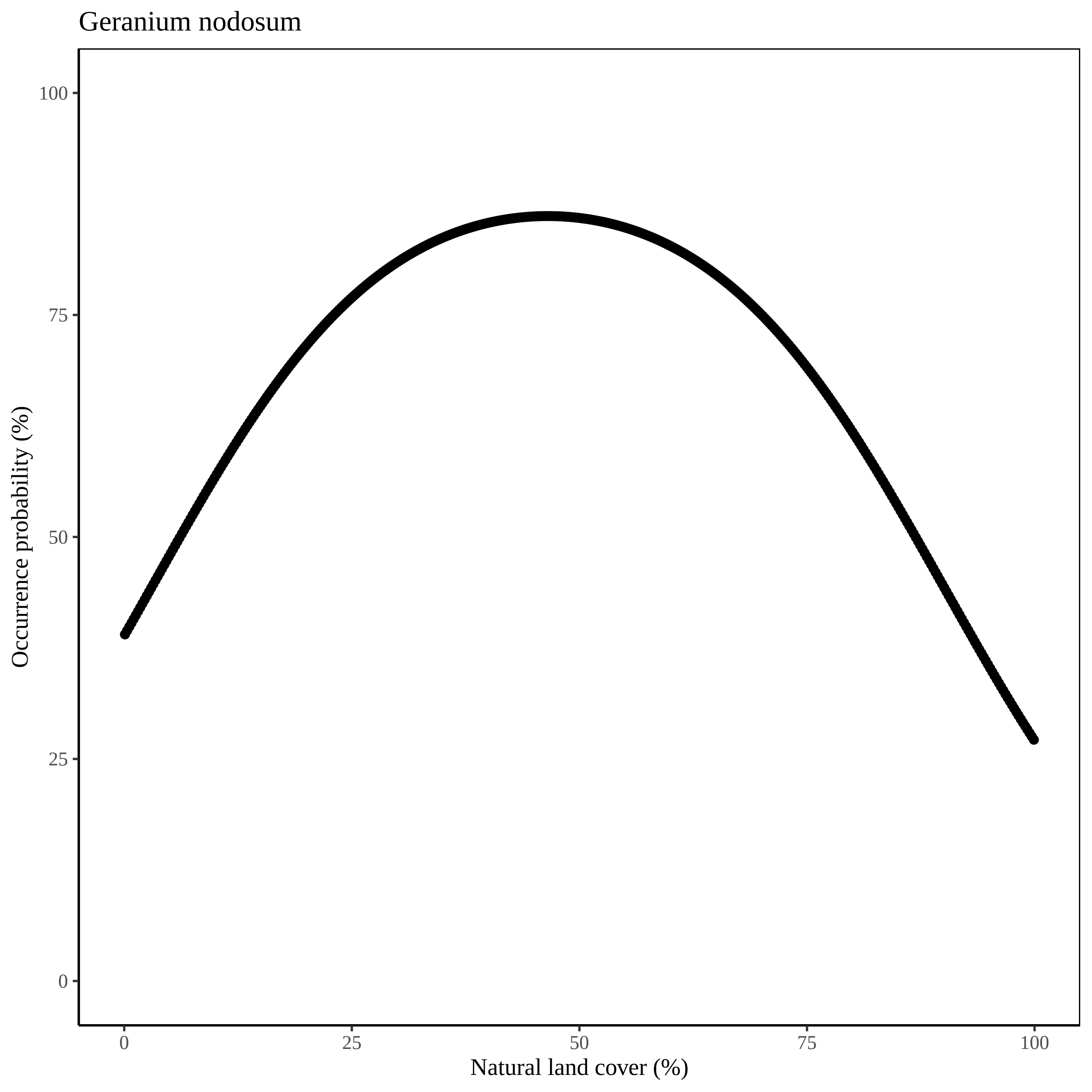
Geranium nodosum
Occurrence probability (%)
100
75
50
25
0
0
25
50
75
100
Natural land cover (%)

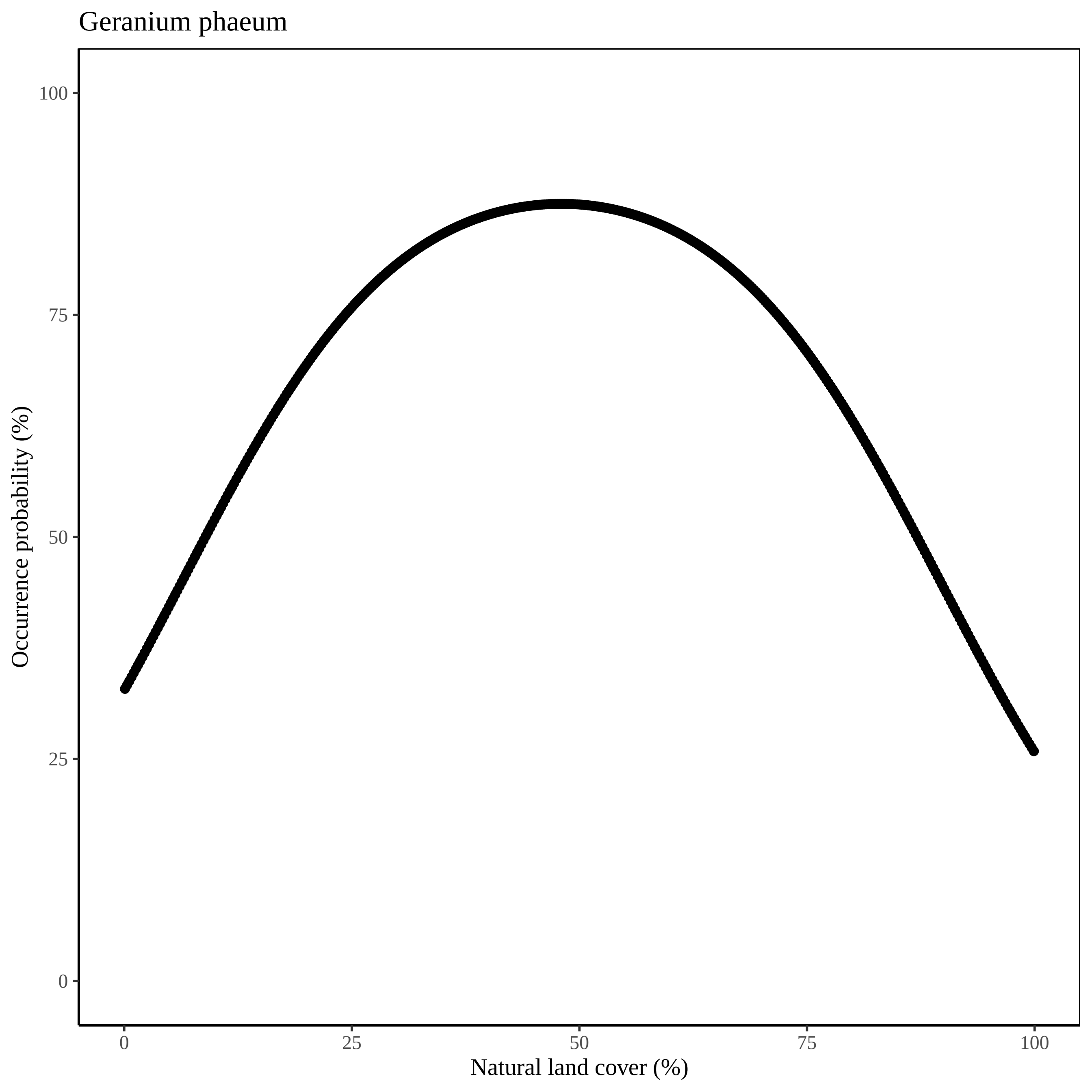

Geranium phaeum
Occurrence probability (%)
100
75
50
25
0
0
25
50
75
100
Natural land cover (%)

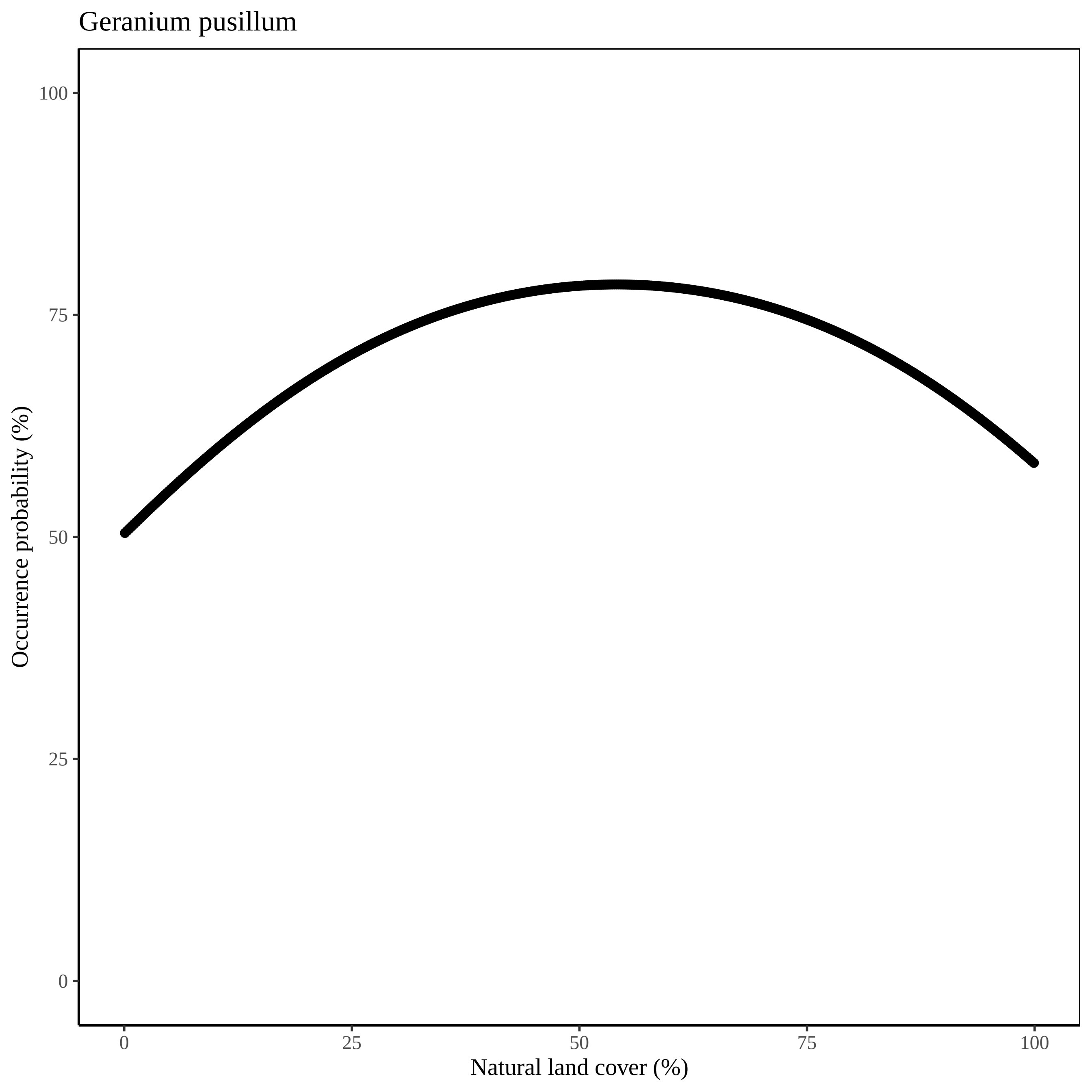

Geranium pusillum
Occurrence probability (%)
100
75
50
25
0
0
25
50
75
100
Natural land cover (%)

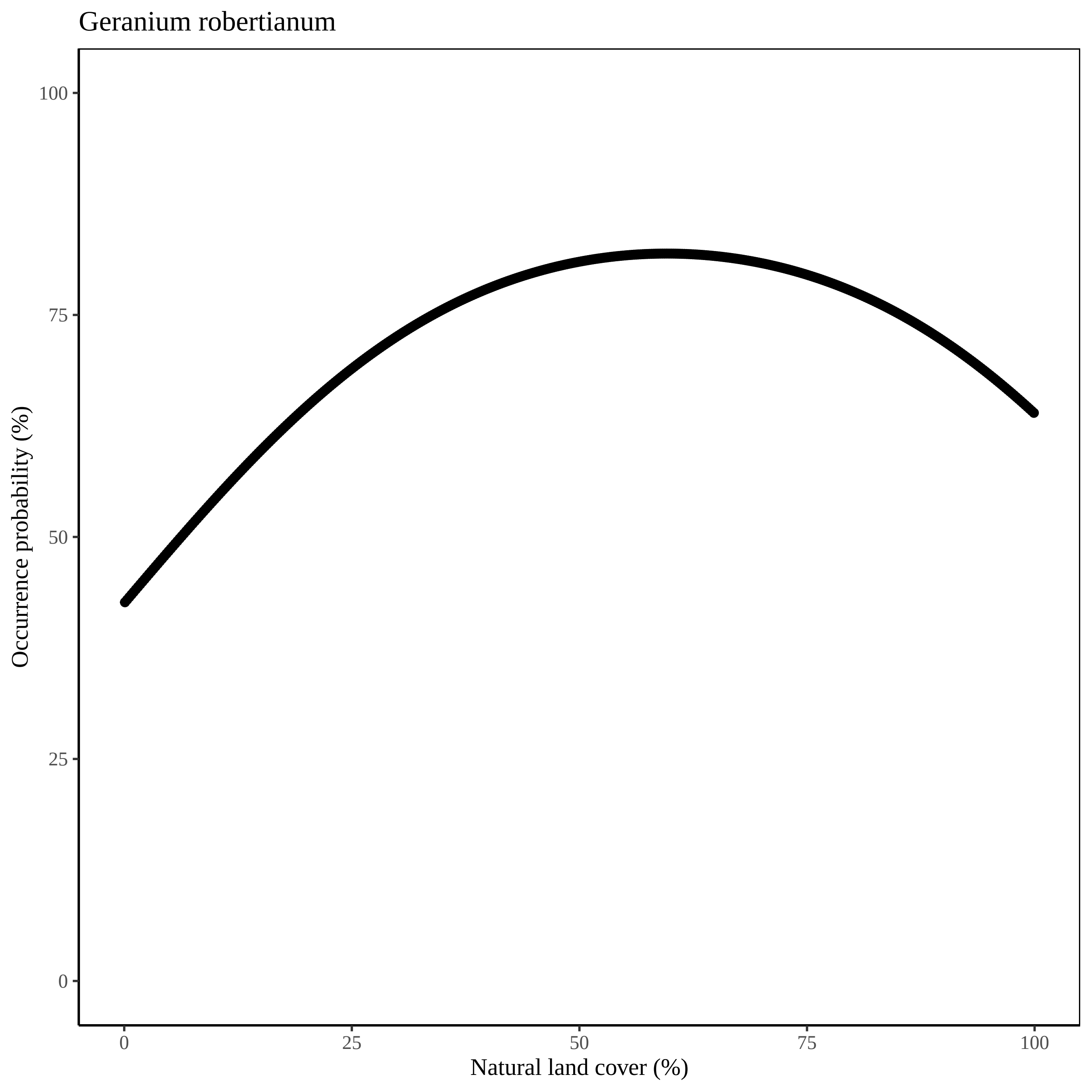

Geranium robertianum
Occurrence probability (%)
100
75
50
25
0
0
25
50
75
100
Natural land cover (%)

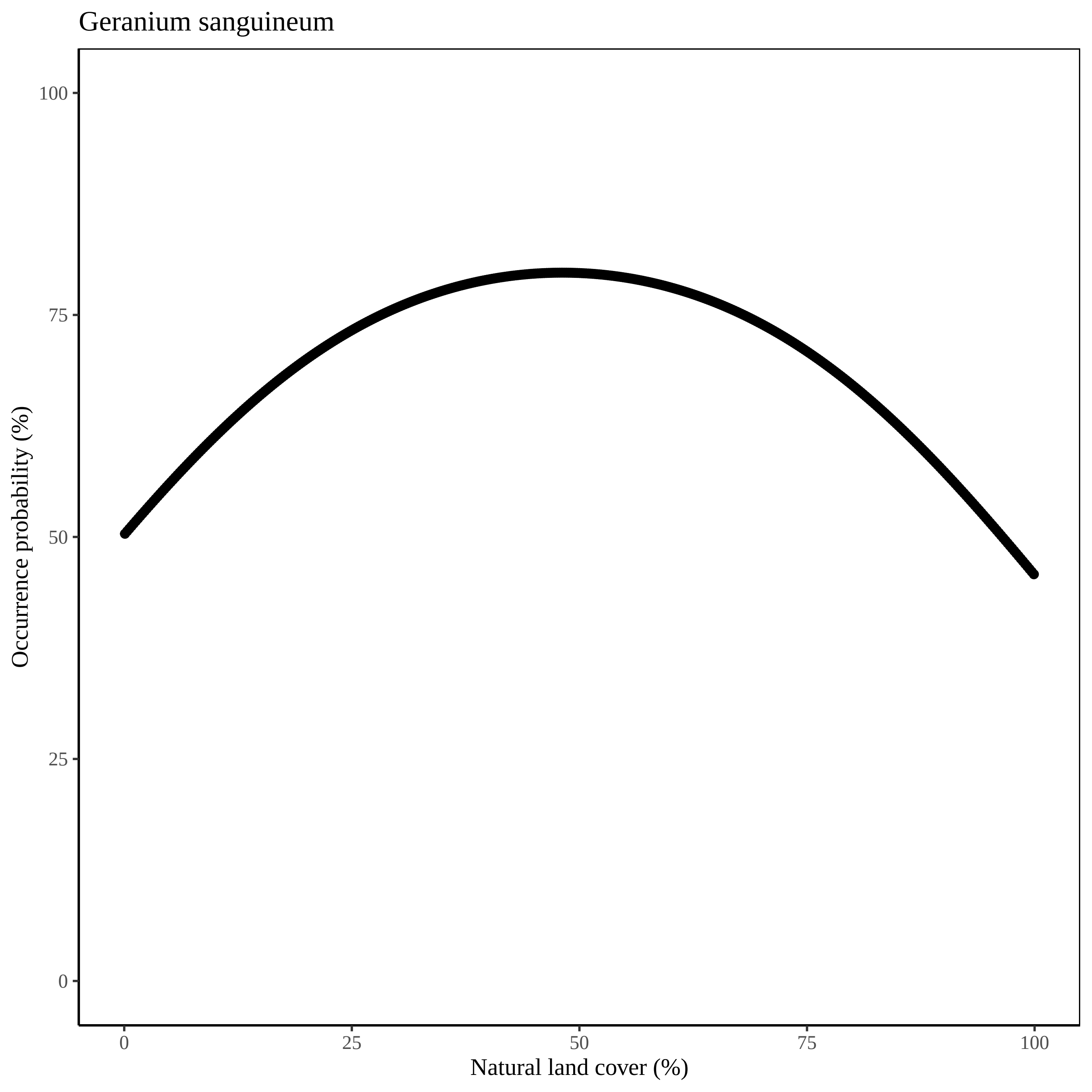
Geranium sanguineum
Occurrence probability (%)
100
75
50
25
0
0
25
50
75
100
Natural land cover (%)

Geum macrophyllum

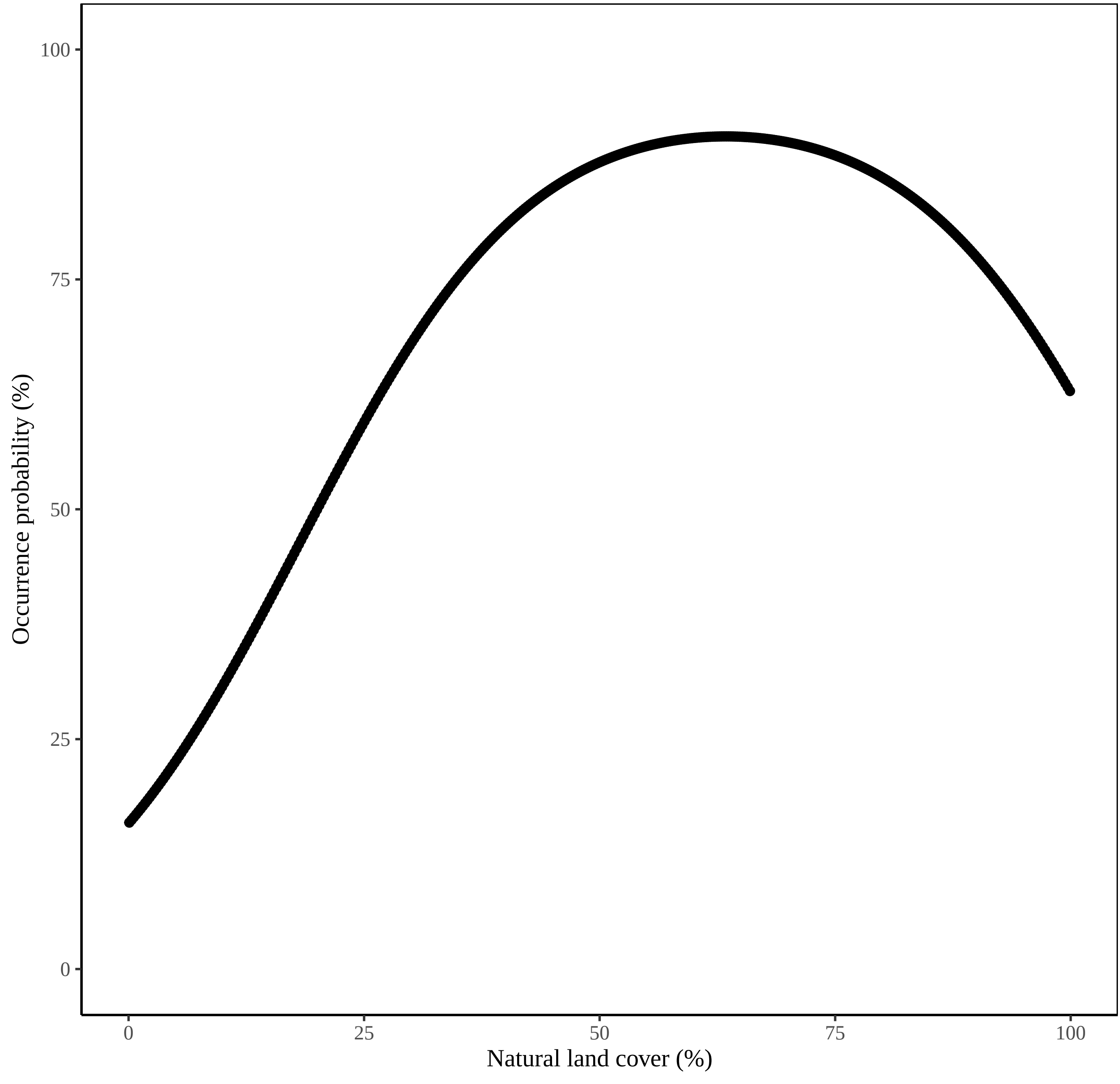

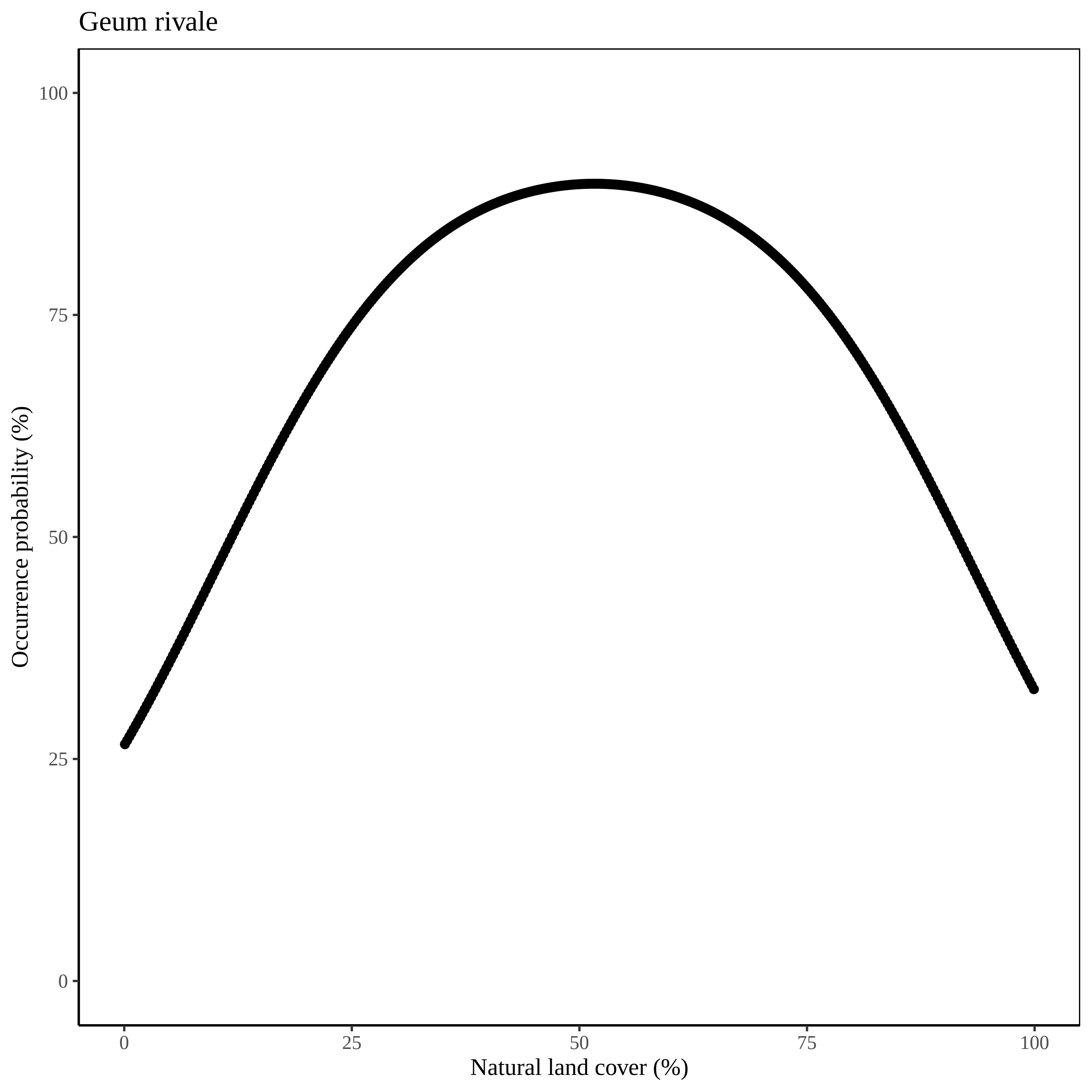

Geum rivale
100
75
50
25
0
0
25
50
75
100
Occurrence probability (%)
Natural land cover (%)

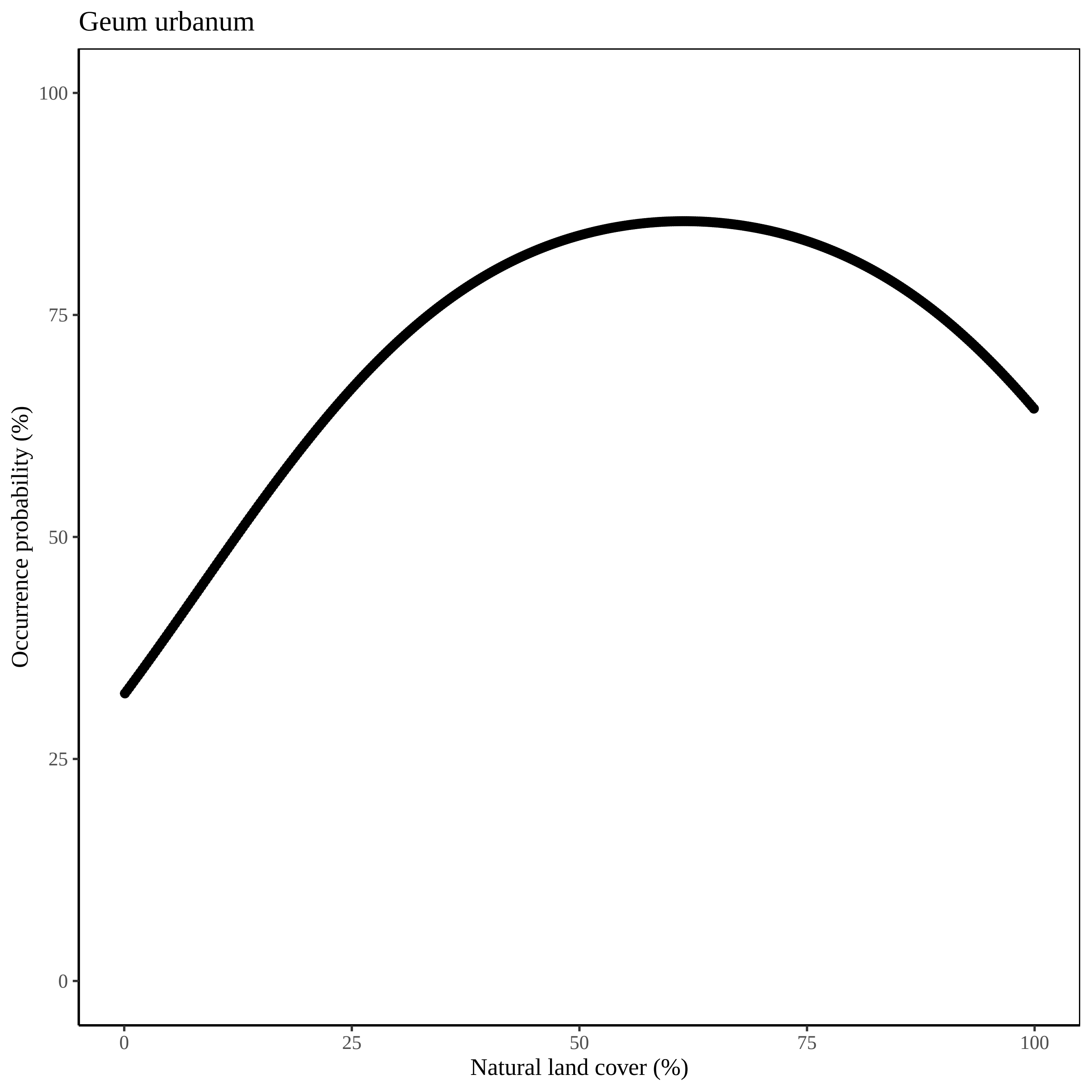
Geum urbanum
100
75
50
25
0
0
25
50
75
100
Occurrence probability (%)
Natural land cover (%)

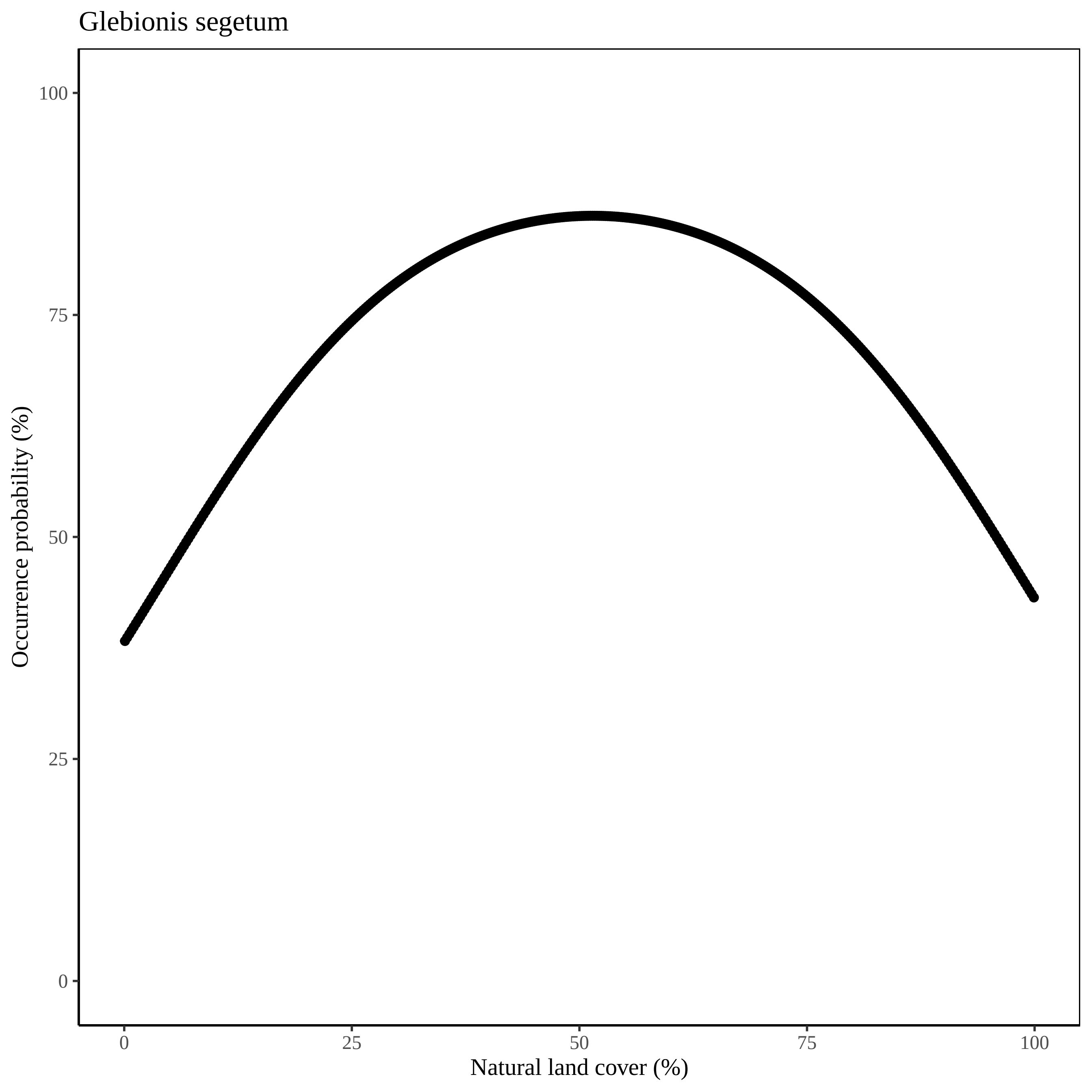
Glebionis segetum
Occurrence probability (%)
100
75
50
25
0
0
25
50
75
100
Natural land cover (%)

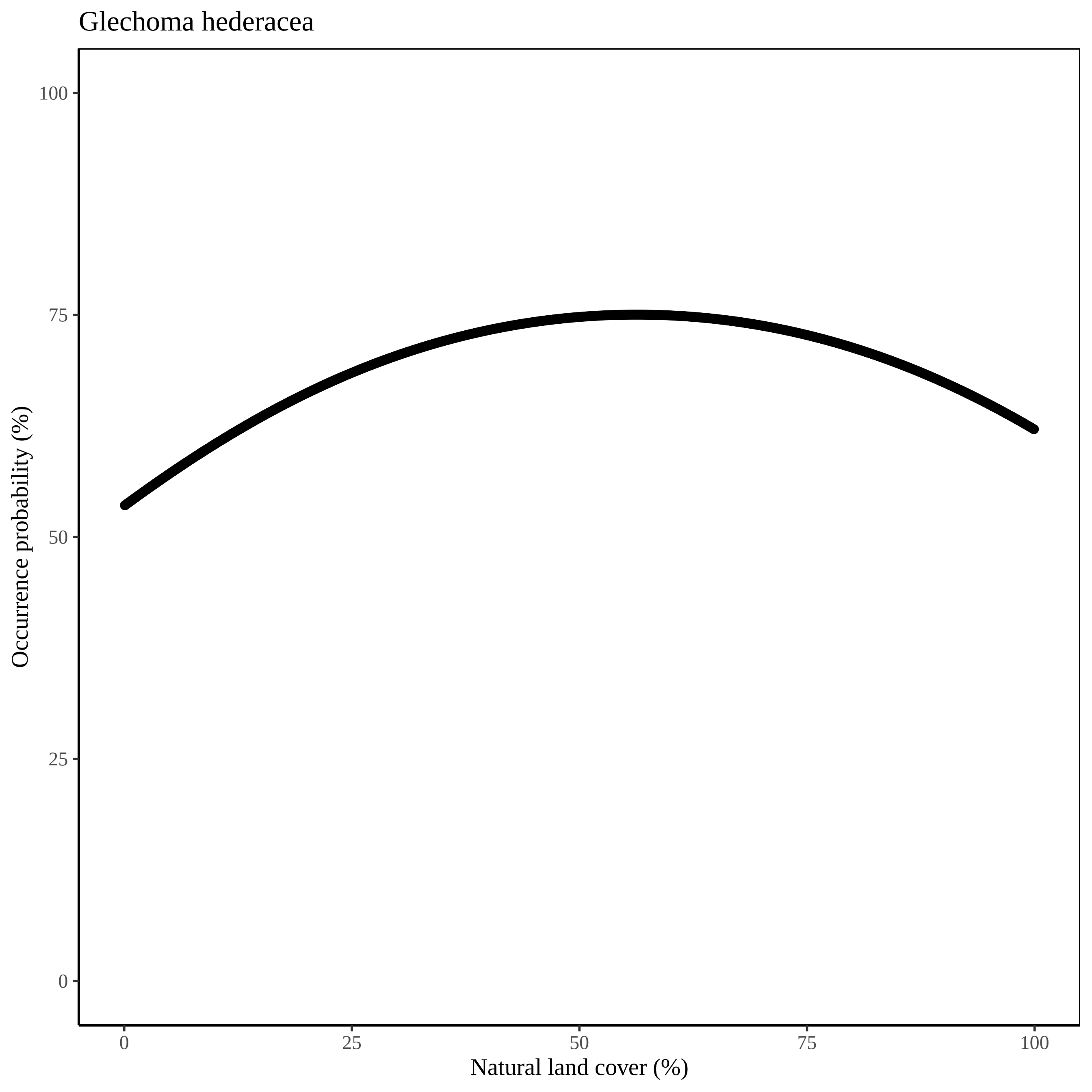

Glechoma hederacea
Occurrence probability (%)
100
75
50
25
0
0
25
50
75
100
Natural land cover (%)

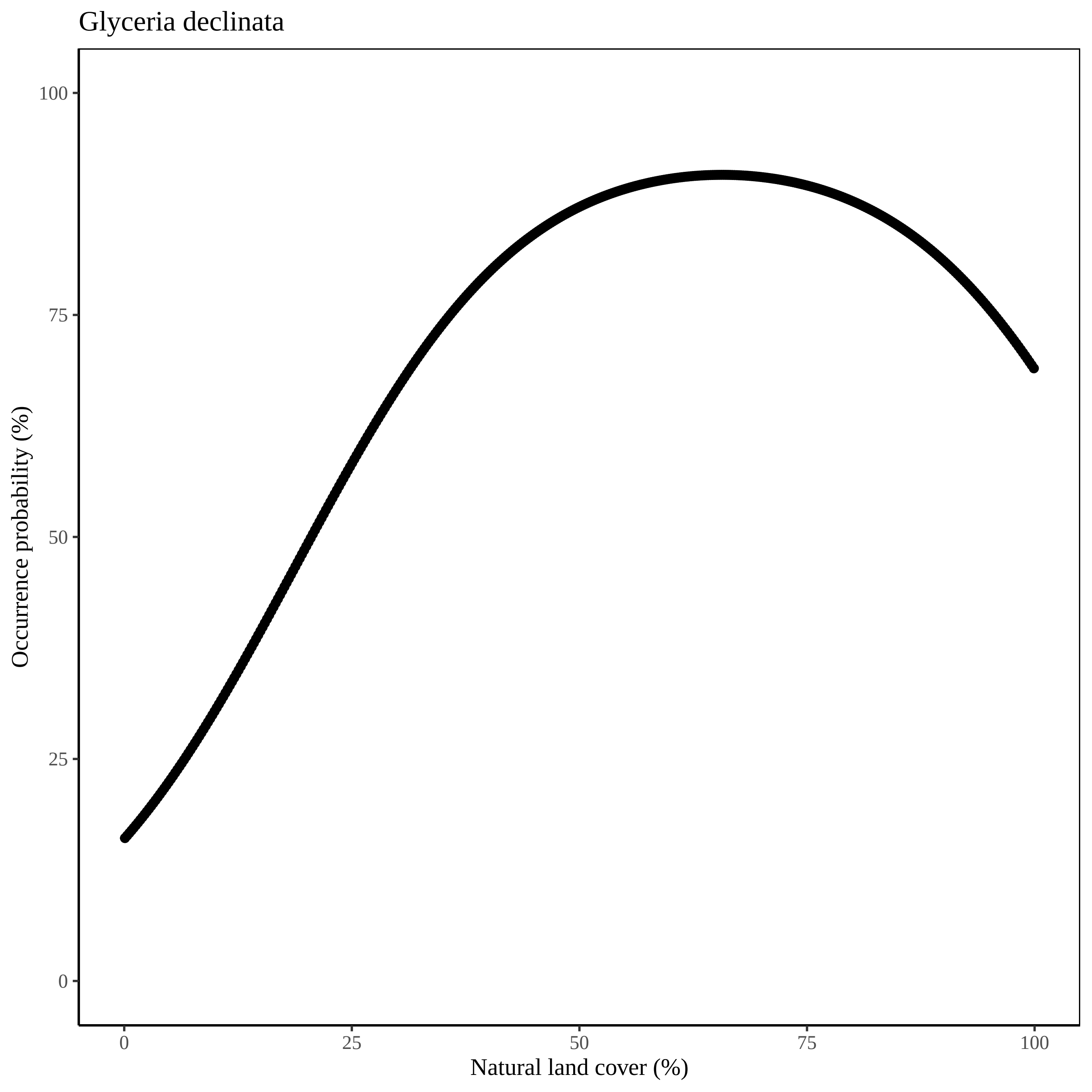
Glyceria declinata
Occurrence probability (%)
100
75
50
25
0
0
25
50
75
100
Natural land cover (%)

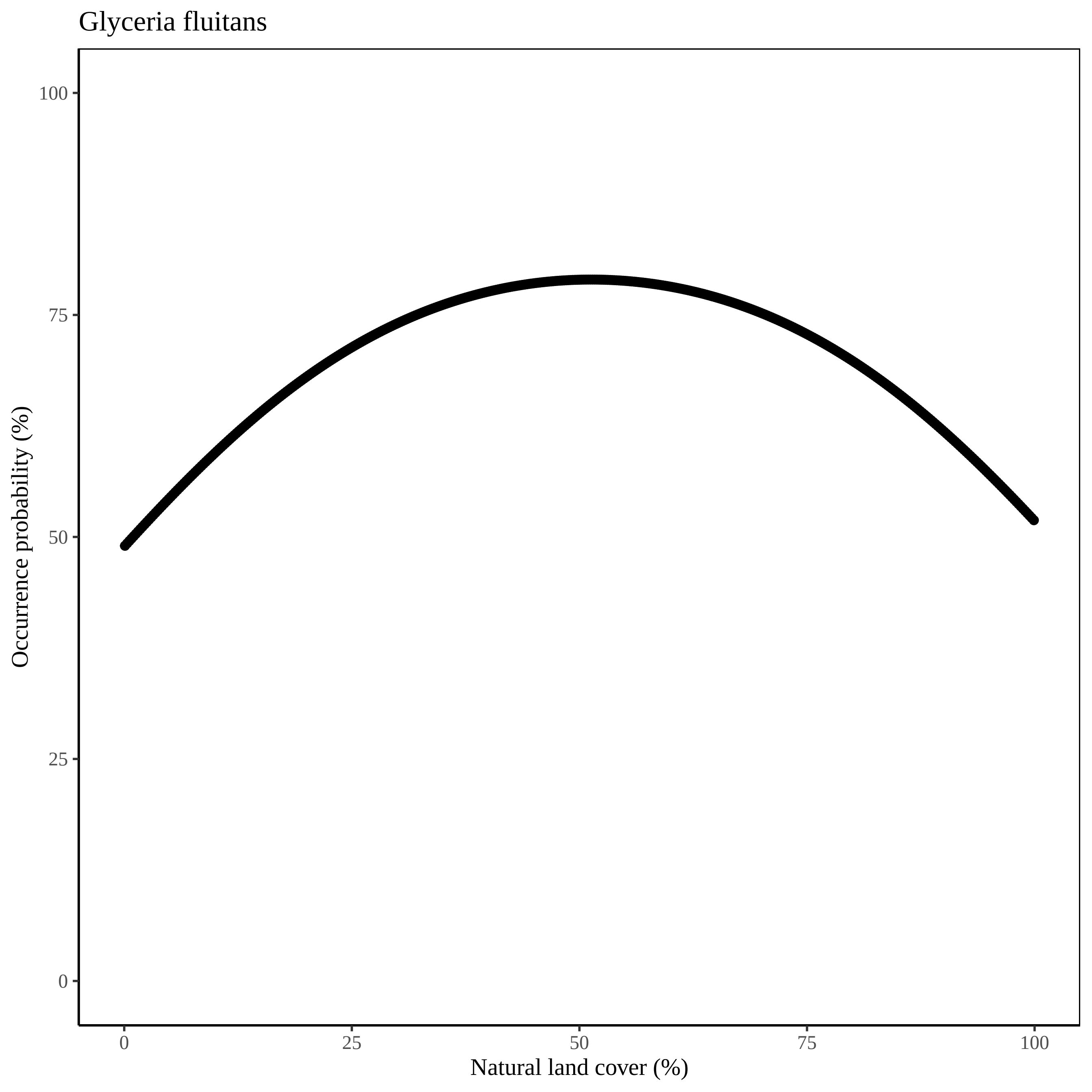

Glyceria fluitans
100
75
50
25
0
Occurrence probability (%)
0
25
50
75
100
Natural land cover (%)

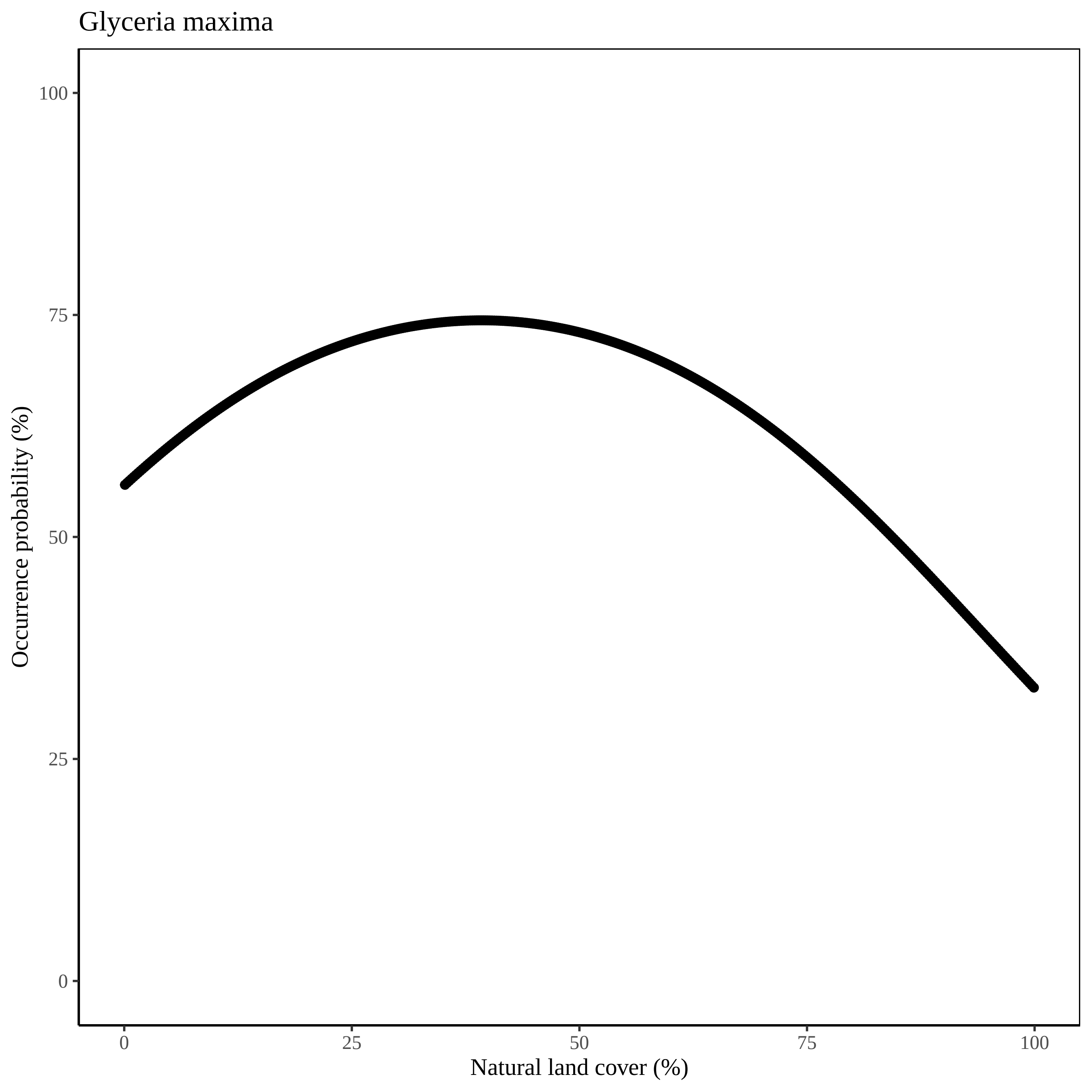

Glyceria maxima
Occurrence probability (%)
100
75
50
25
0
0
25
50
75
100
Natural land cover (%)

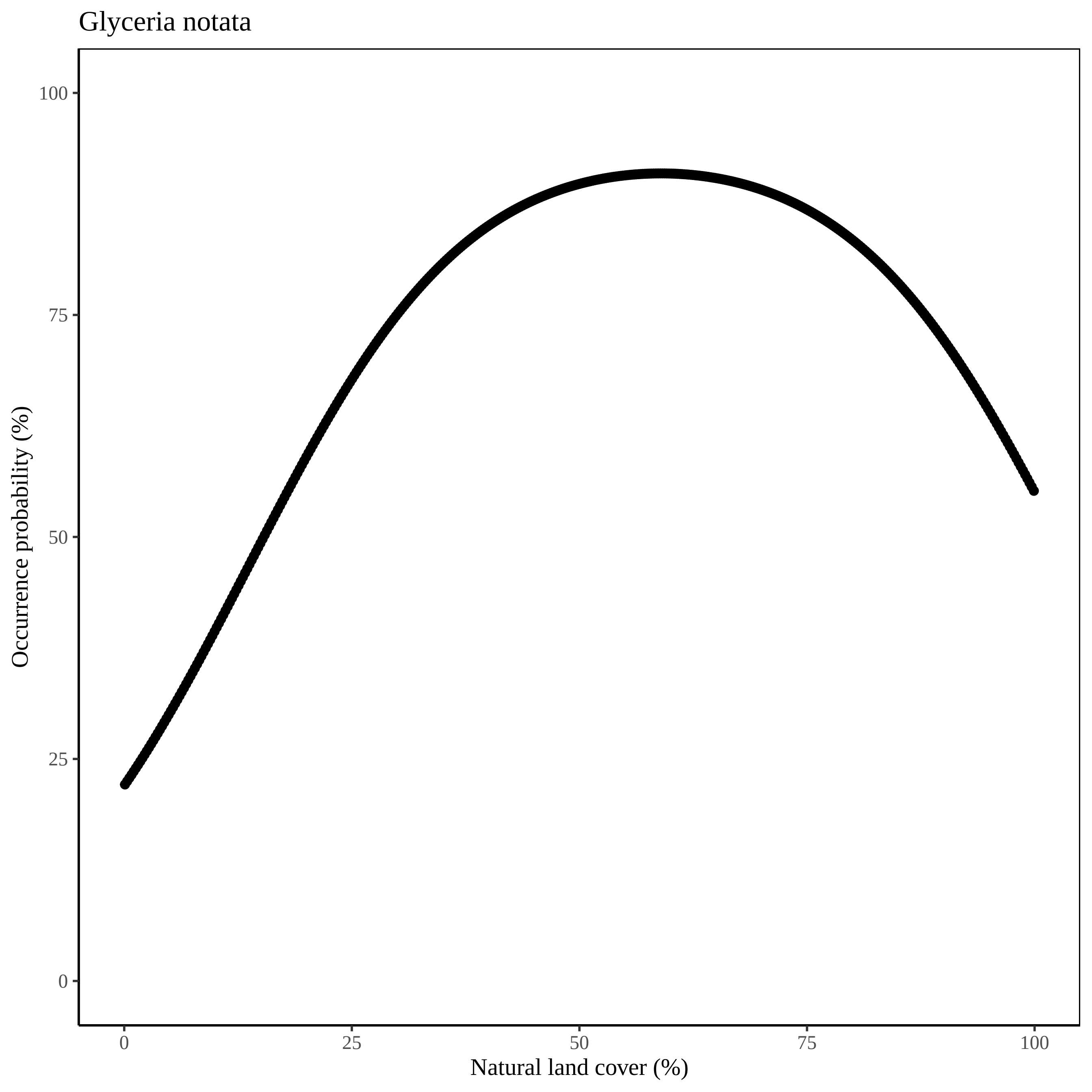

Glyceria notata
Occurrence probability (%)
100
75
50
25
0
0
25
50
75
100
Natural land cover (%)

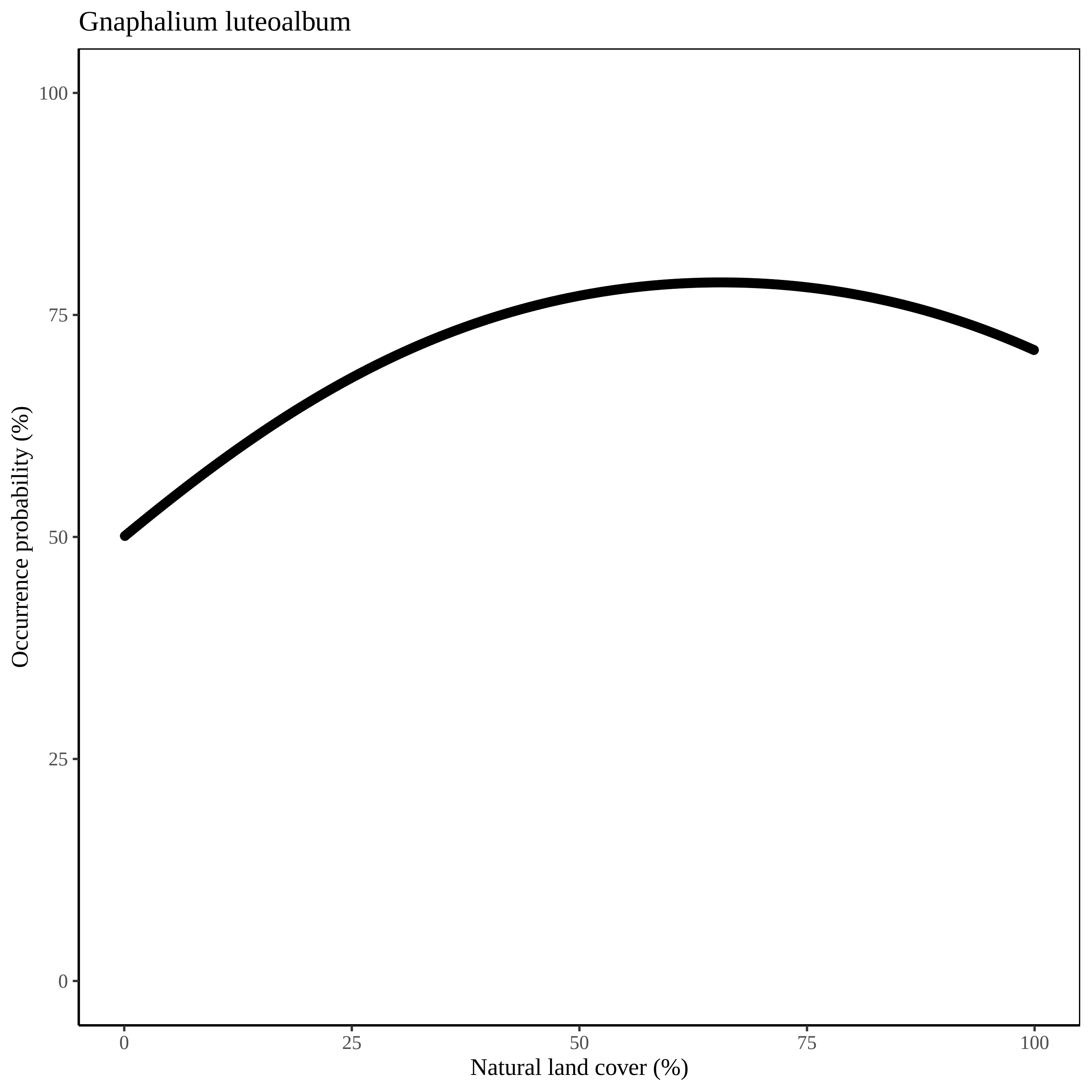

Gnaphalium luteoalbum
Occurrence probability (%)
100
75
50
25
0
0
25
50
75
100
Natural land cover (%)

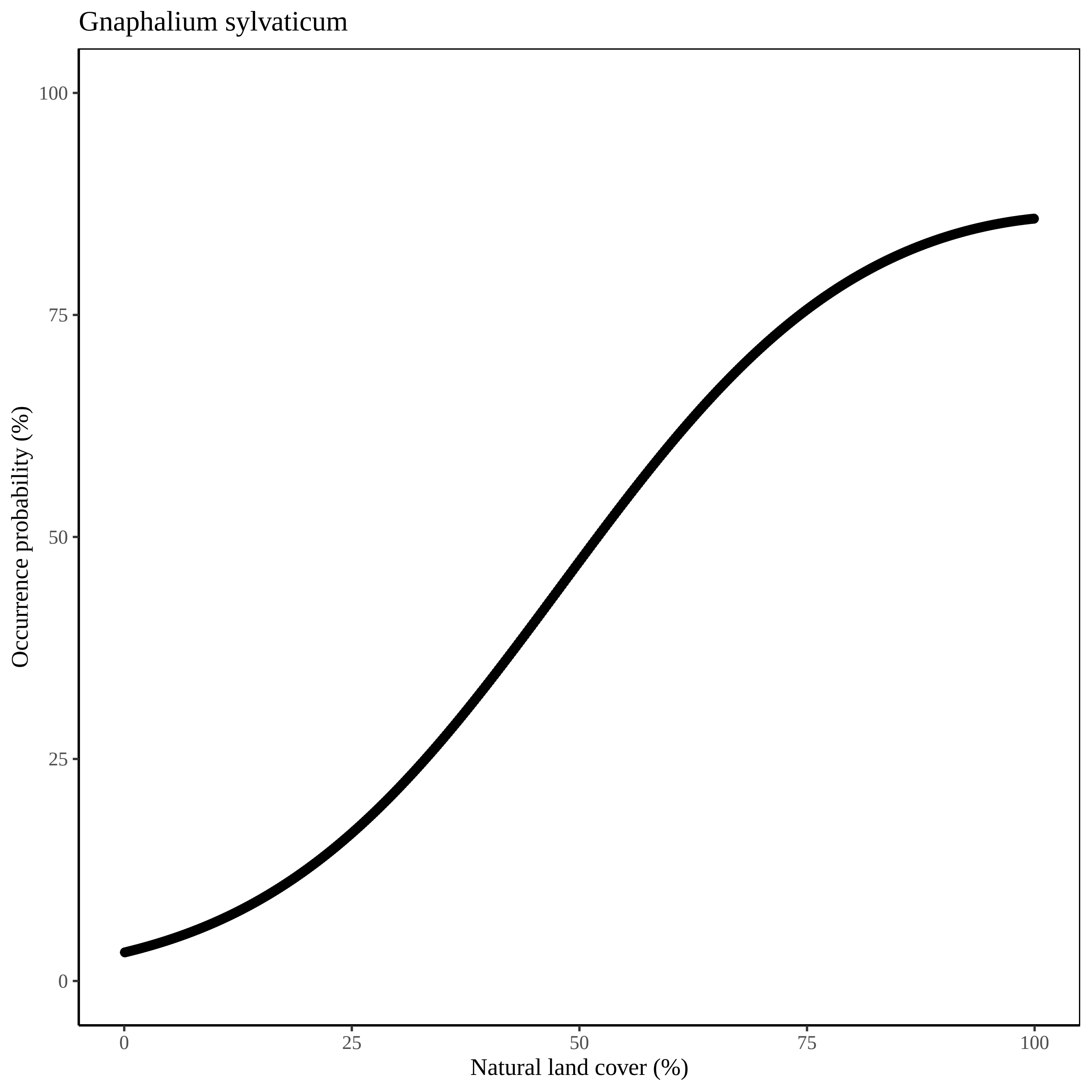
Gnaphalium sylvaticum
Occurrence probability (%)
100
75
50
25
0
0
25
50
75
100
Natural land cover (%)

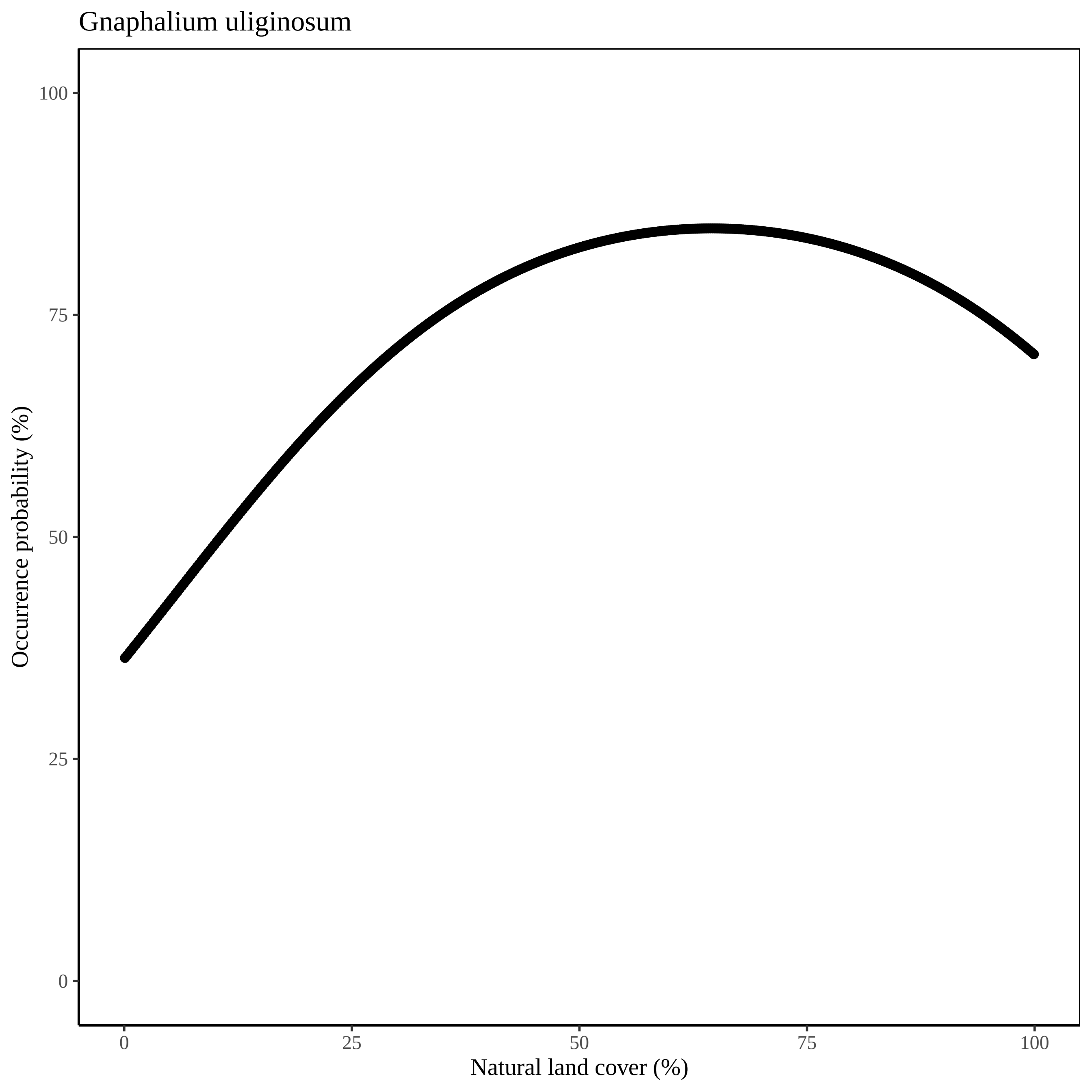
Gnaphalium uliginosum
Occurrence probability (%)
100
75
50
25
0
0
25
50
75
100
Natural land cover (%)

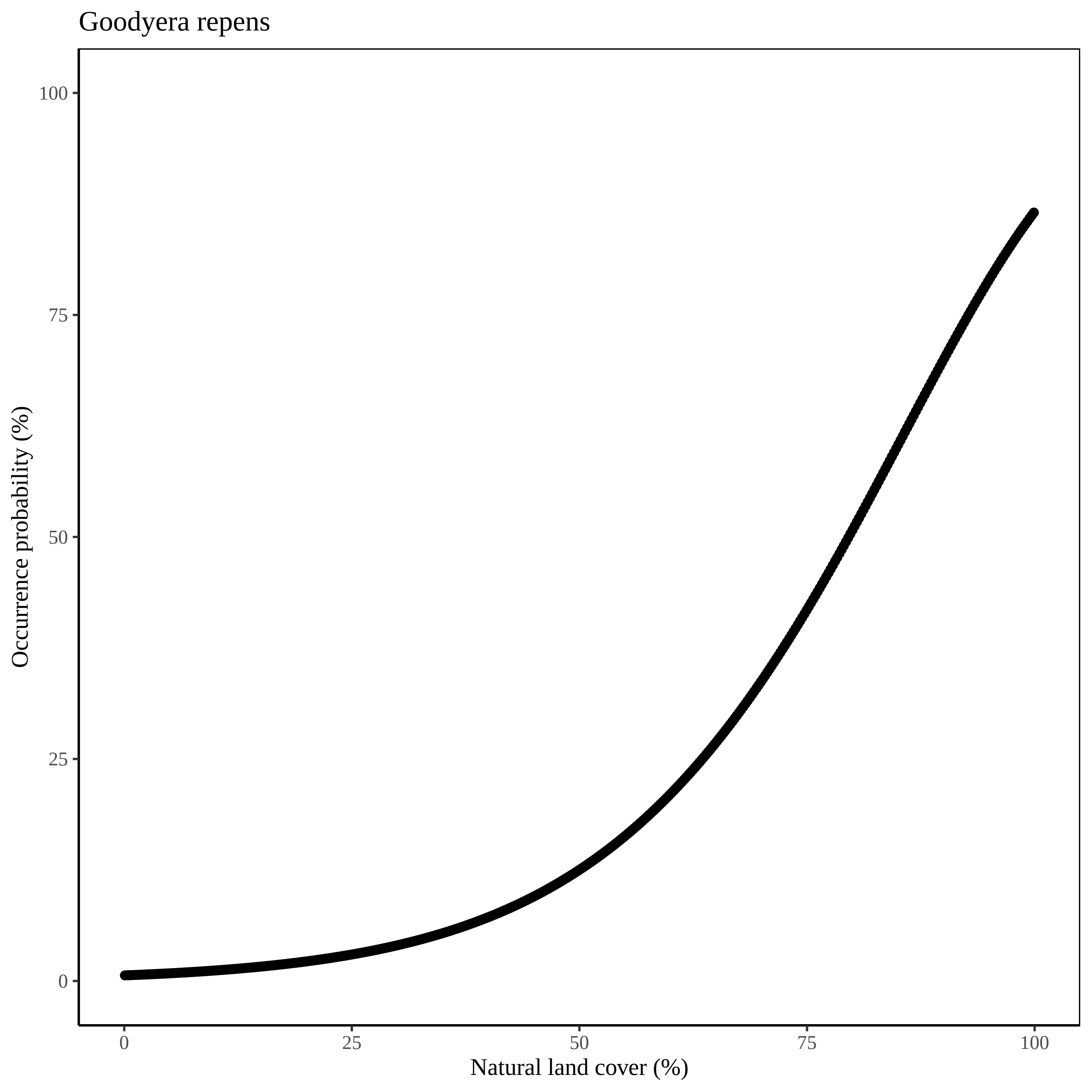
Goodyera repens
Occurrence probability (%)
100
75
50
25
0
0
25
50
75
100
Natural land cover (%)

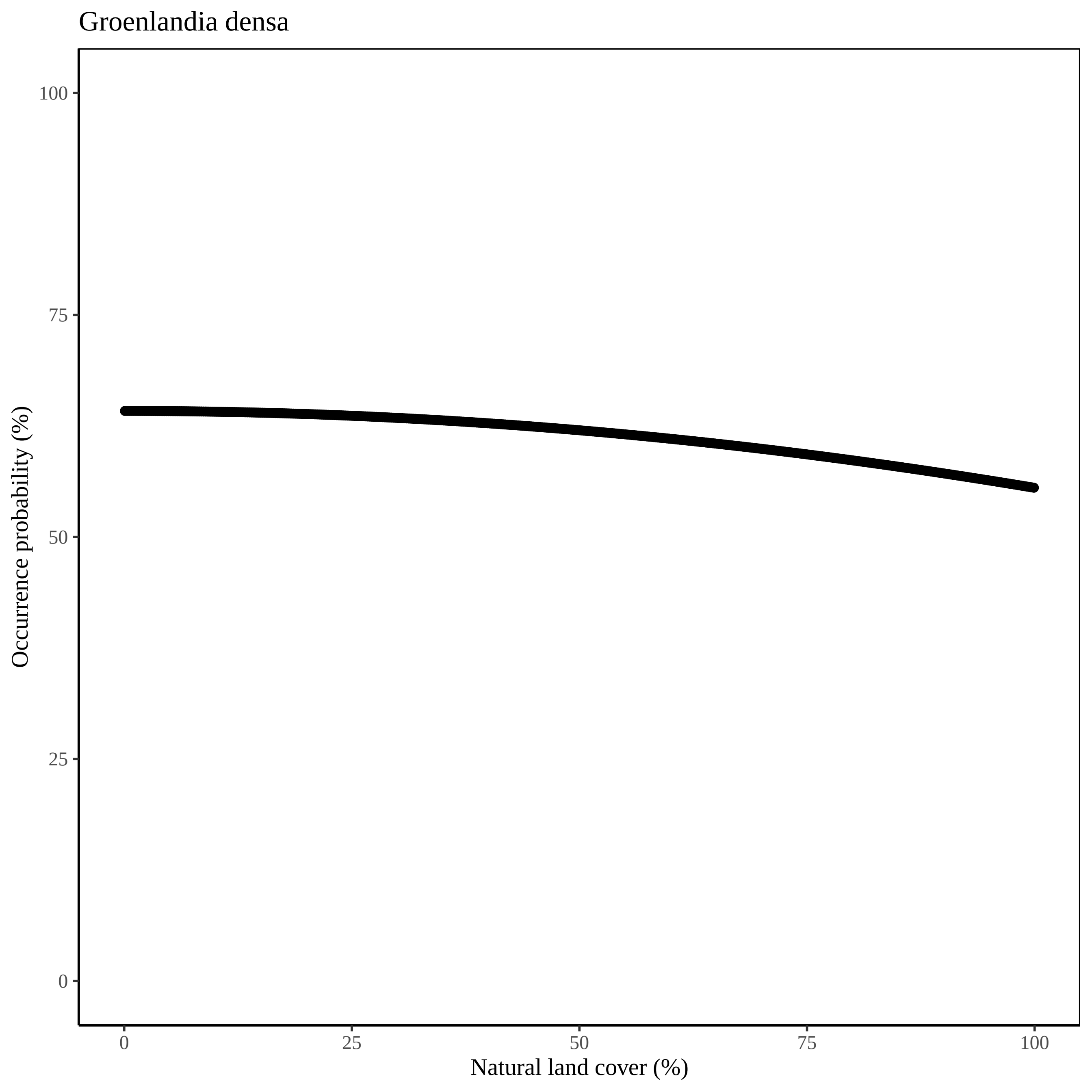
Groenlandia densa
Occurrence probability (%)
100
75
50
25
0
Natural land cover (%)
0
25
50
75
100

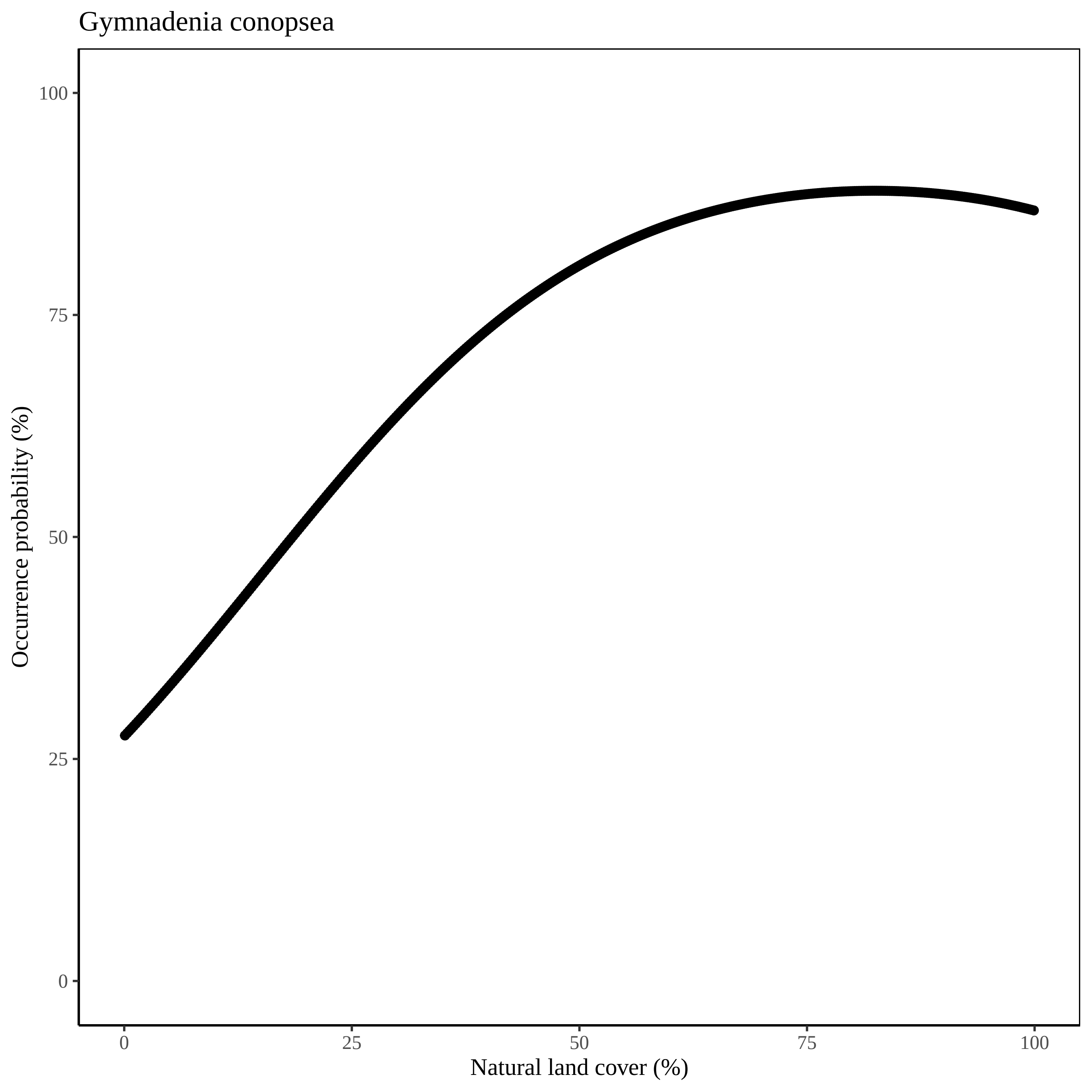

Gymnadenia conopsea
Occurrence probability (%)
100
75
50
25
0
0
25
50
75
100
Natural land cover (%)

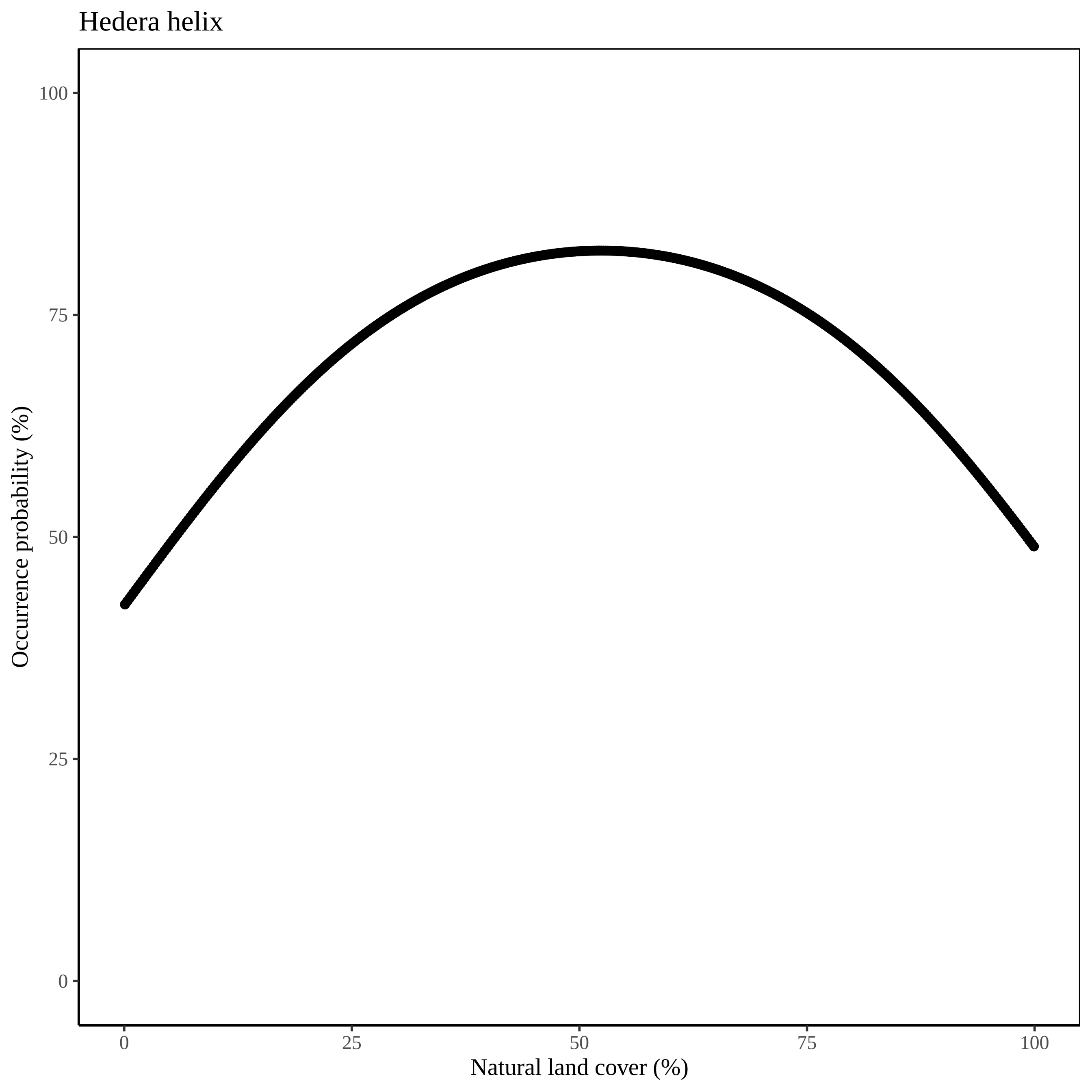

Hedera helix
Occurrence probability (%)
100
75
50
25
0
0
25
50
75
100
Natural land cover (%)

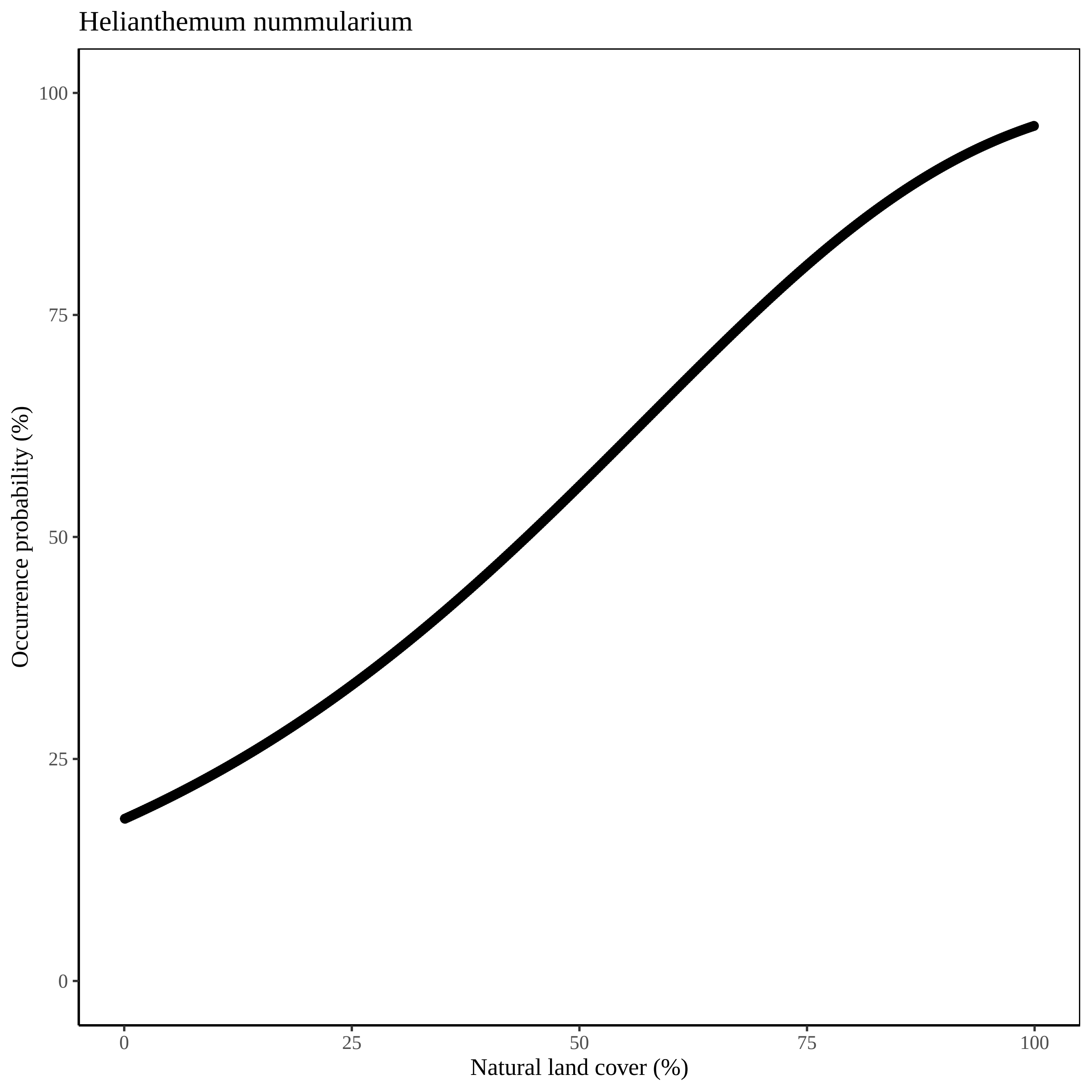
Helianthemum nummularium
Occurrence probability (%)
100
75
50
25
0
0
25
50
75
100
Natural land cover (%)

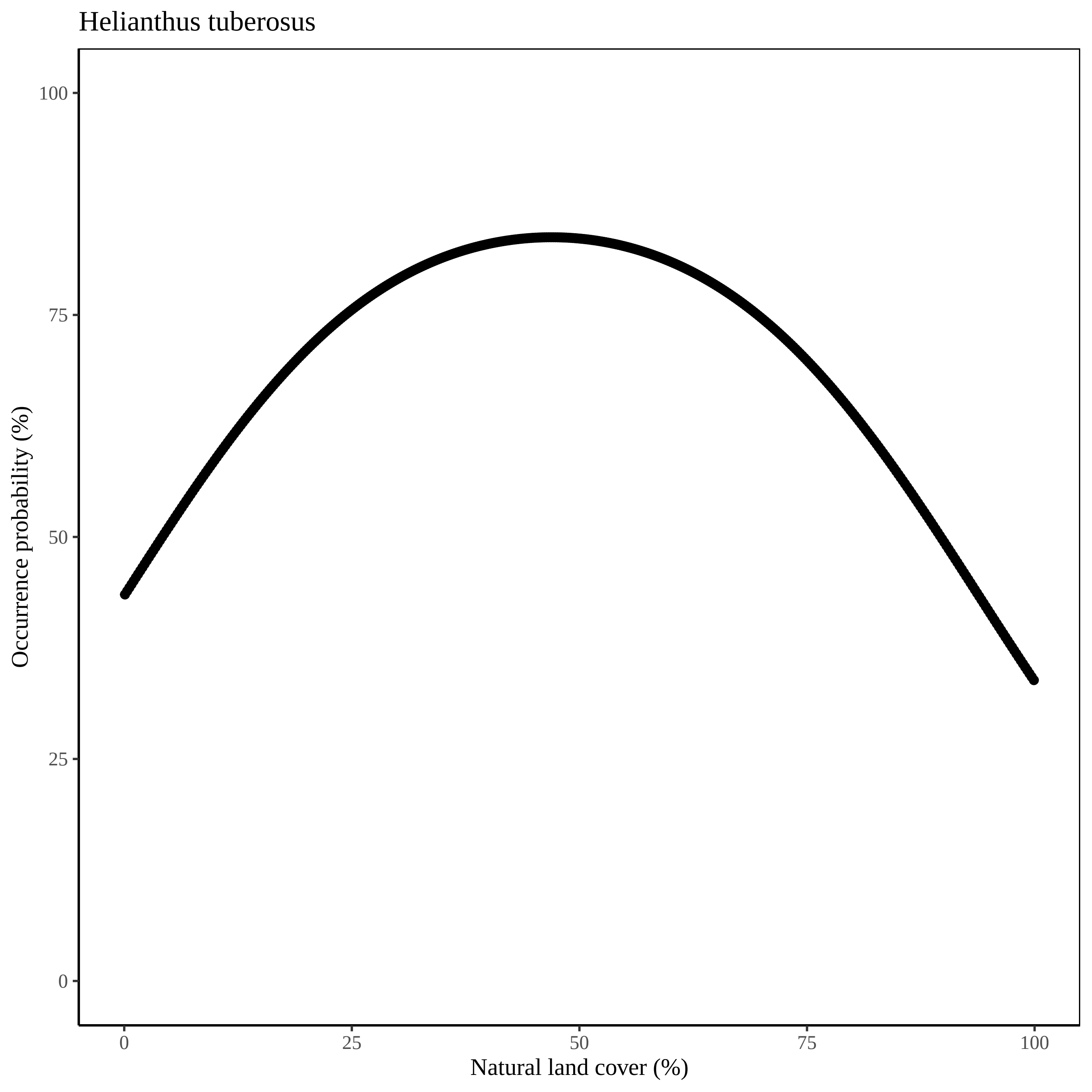

Helianthus tuberosus
Occurrence probability (%)
100
75
50
25
0
0
25
50
75
100
Natural land cover (%)

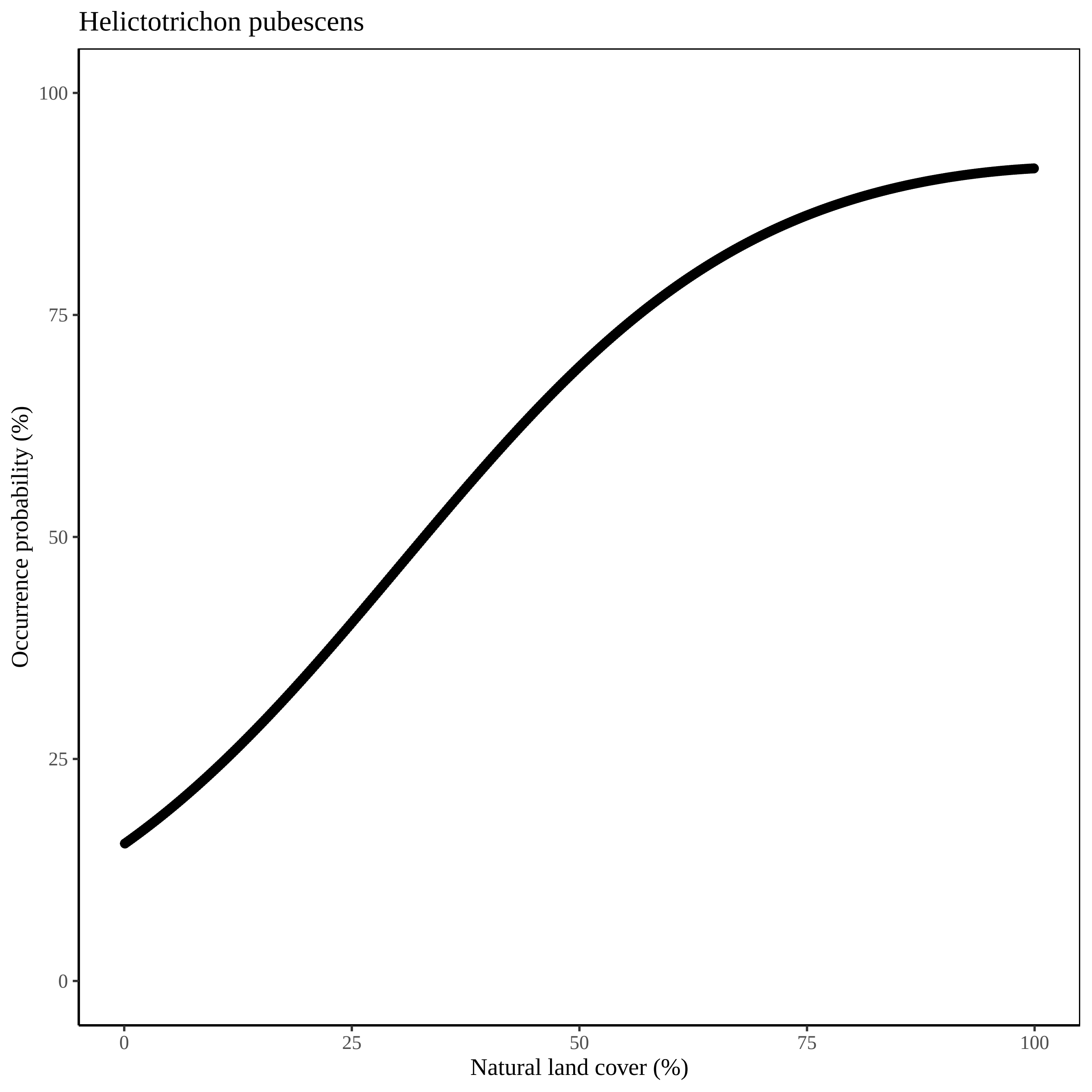
Helictotrichon pubescens
100
75
50
25
0
Occurrence probability (%)
0
25
50
75
100
Natural land cover (%)

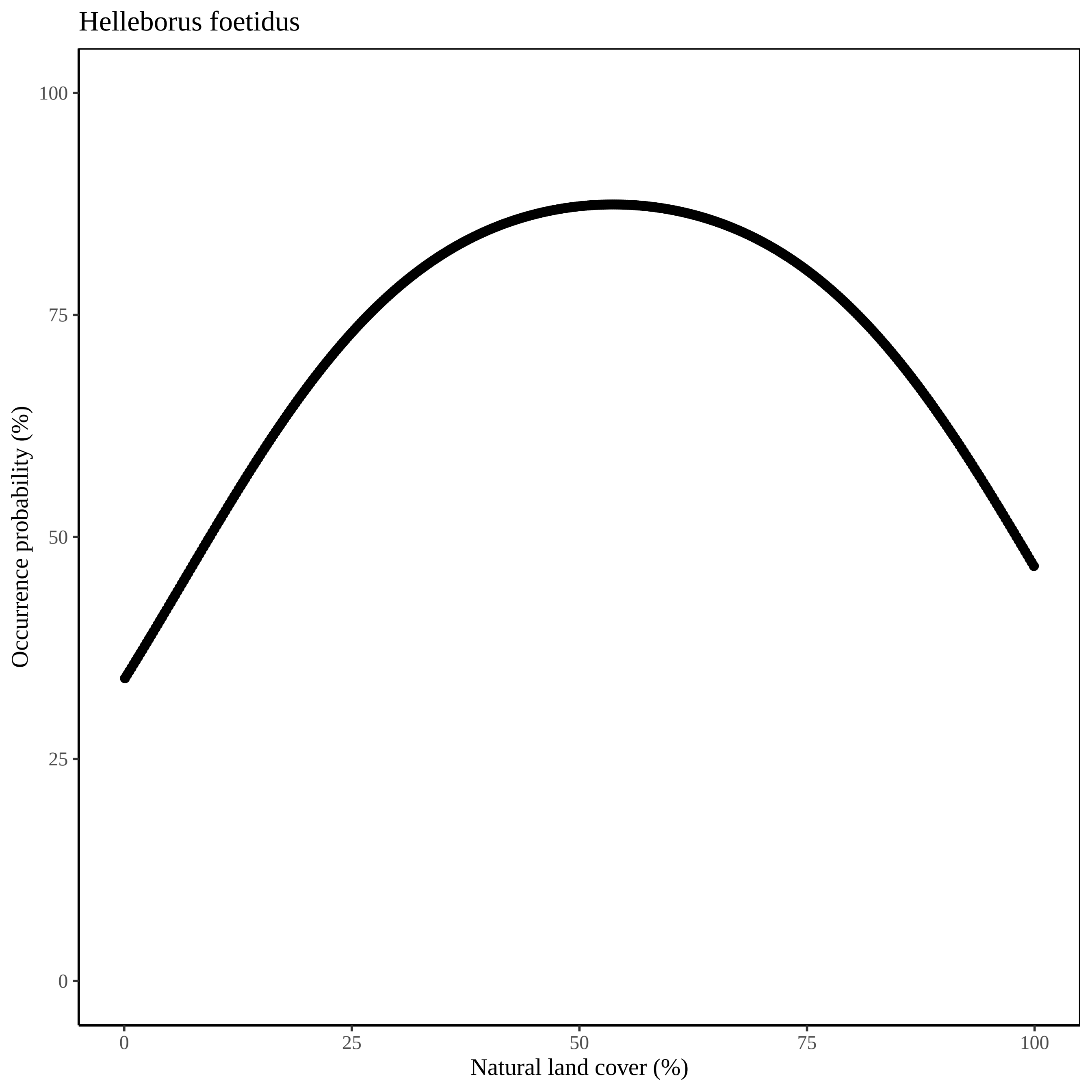

Helleborus foetidus
Occurrence probability (%)
100
75
50
25
0
0
25
50
75
100
Natural land cover (%)

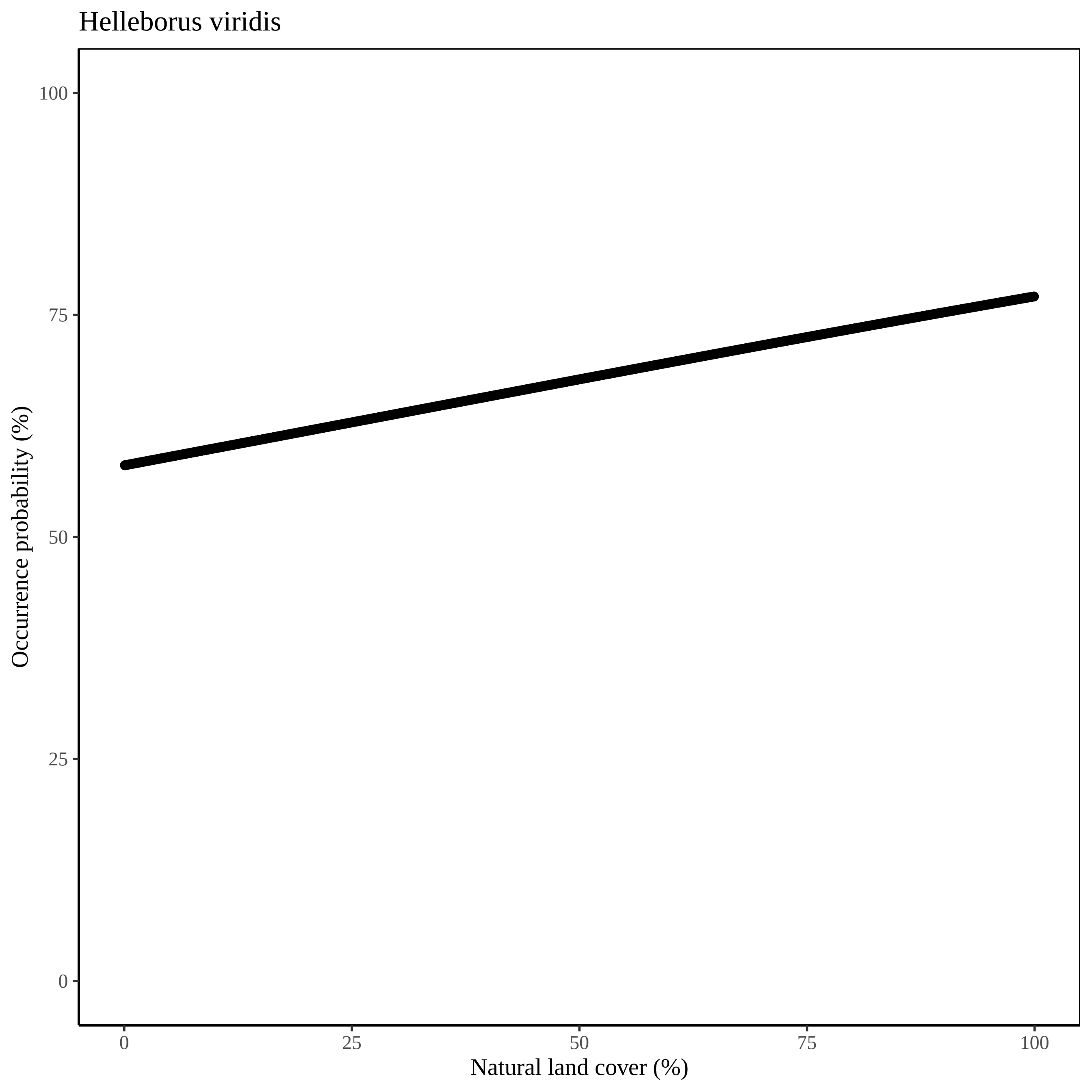
Helleborus viridis
Occurrence probability (%)
100
75
50
25
0
0
25
50
75
100
Natural land cover (%)

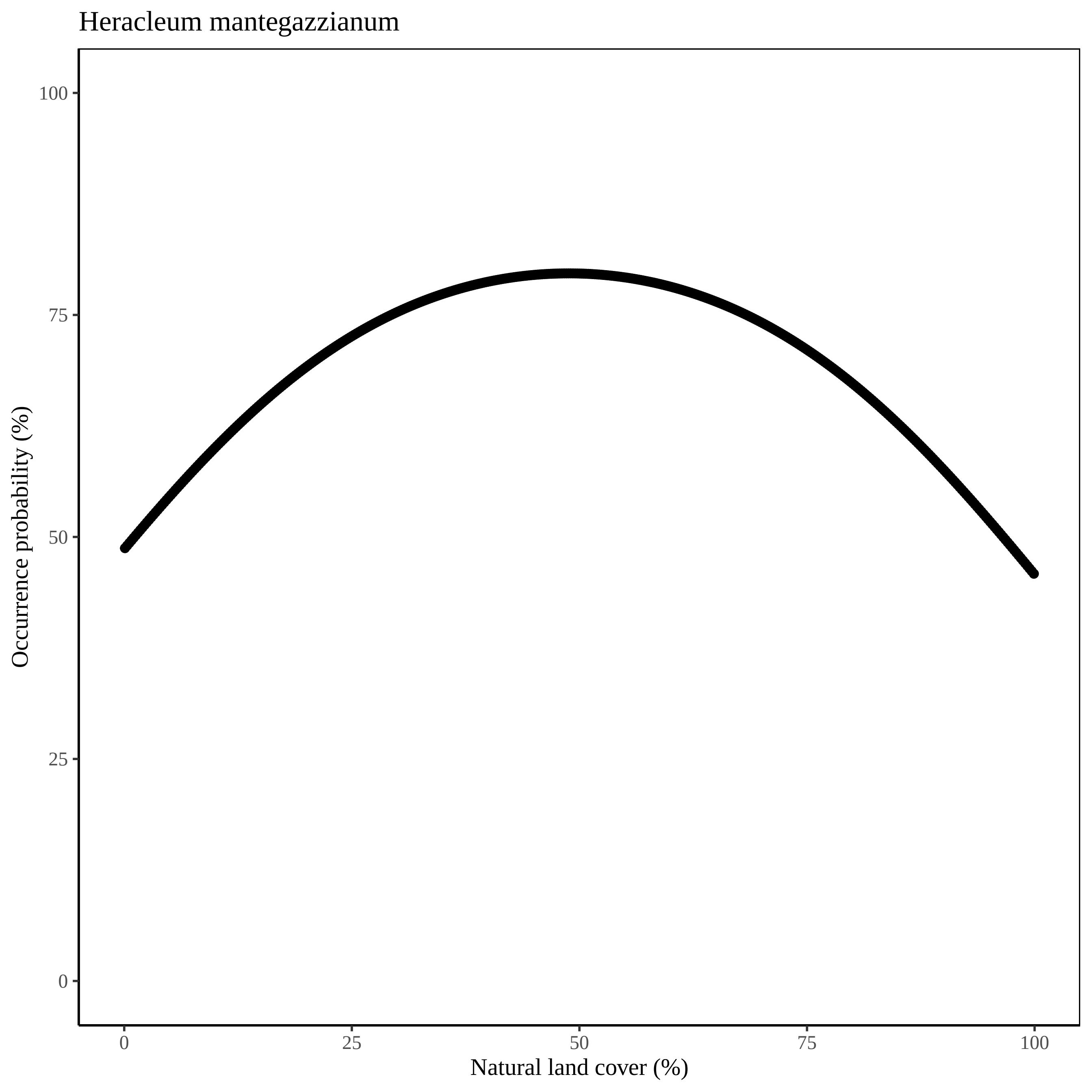
Heracleum mantegazzianum
Occurrence probability (%)
100
75
50
25
0
0
25
50
75
100
Natural land cover (%)

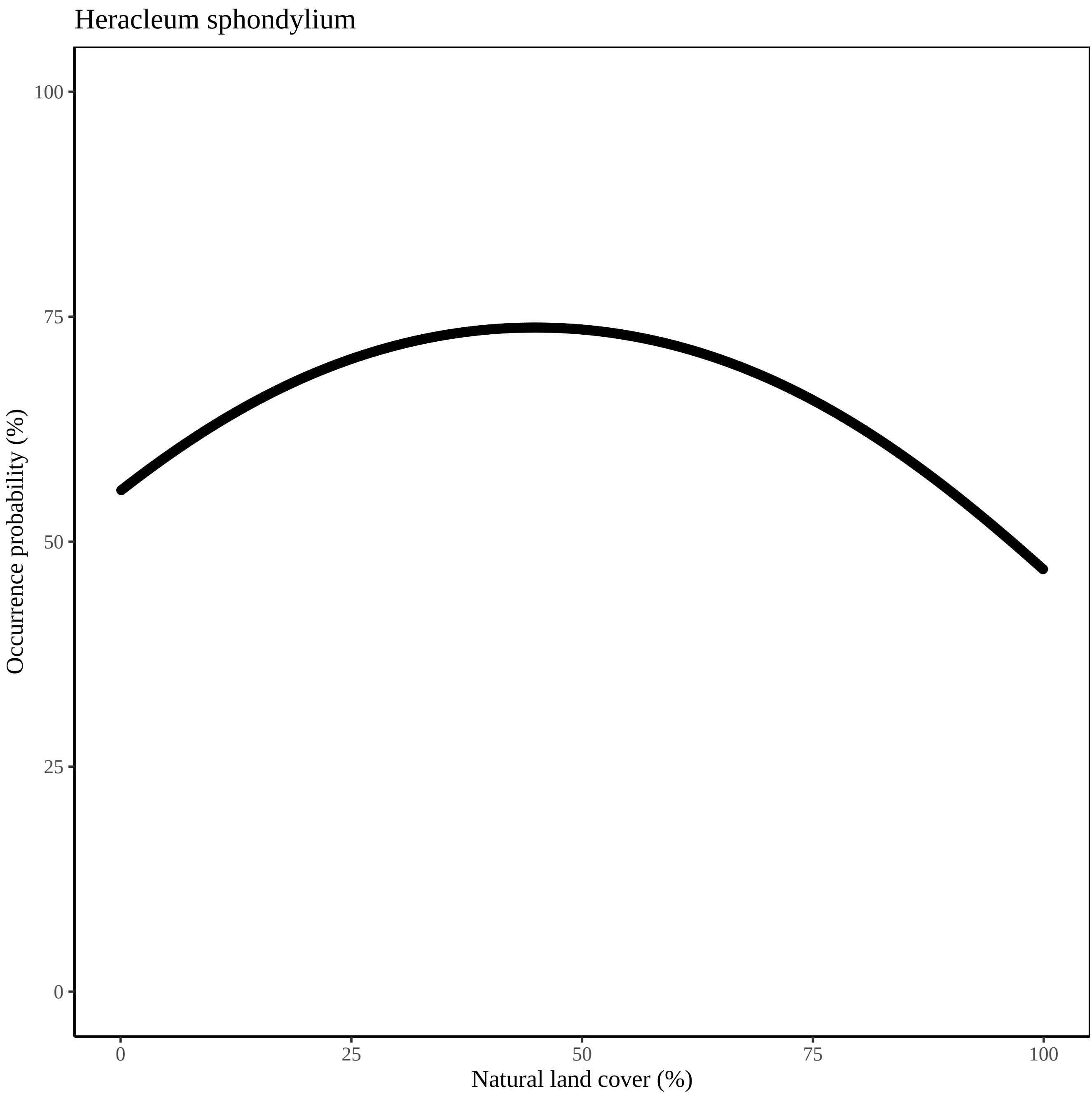
Heracleum sphondylium
Occurrence probability (%)
100
75
50
25
0
0
25
50
75
100
Natural land cover (%)

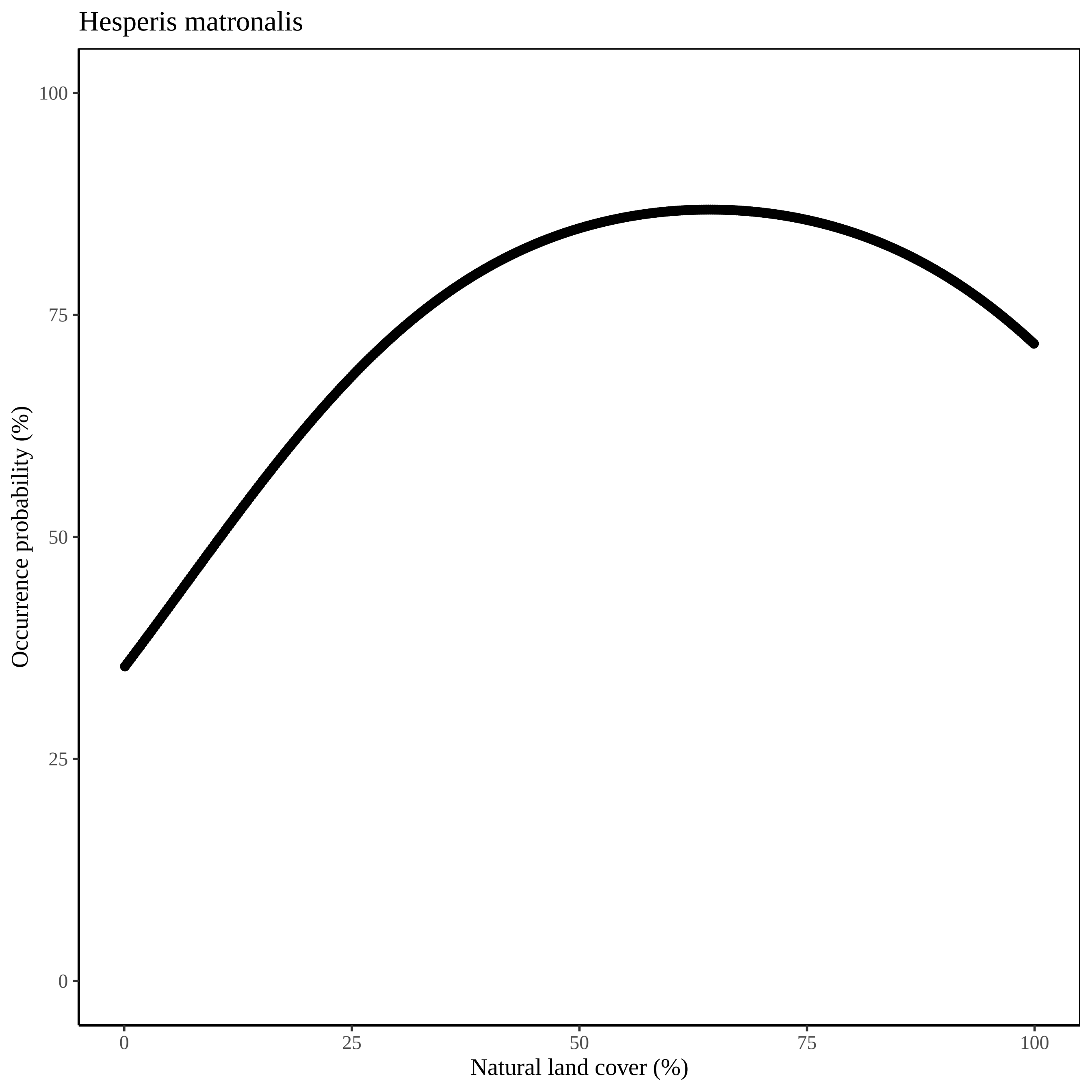
Hesperis matronalis
Occurrence probability (%)
100
75
50
25
0
0
25
50
75
100
Natural land cover (%)

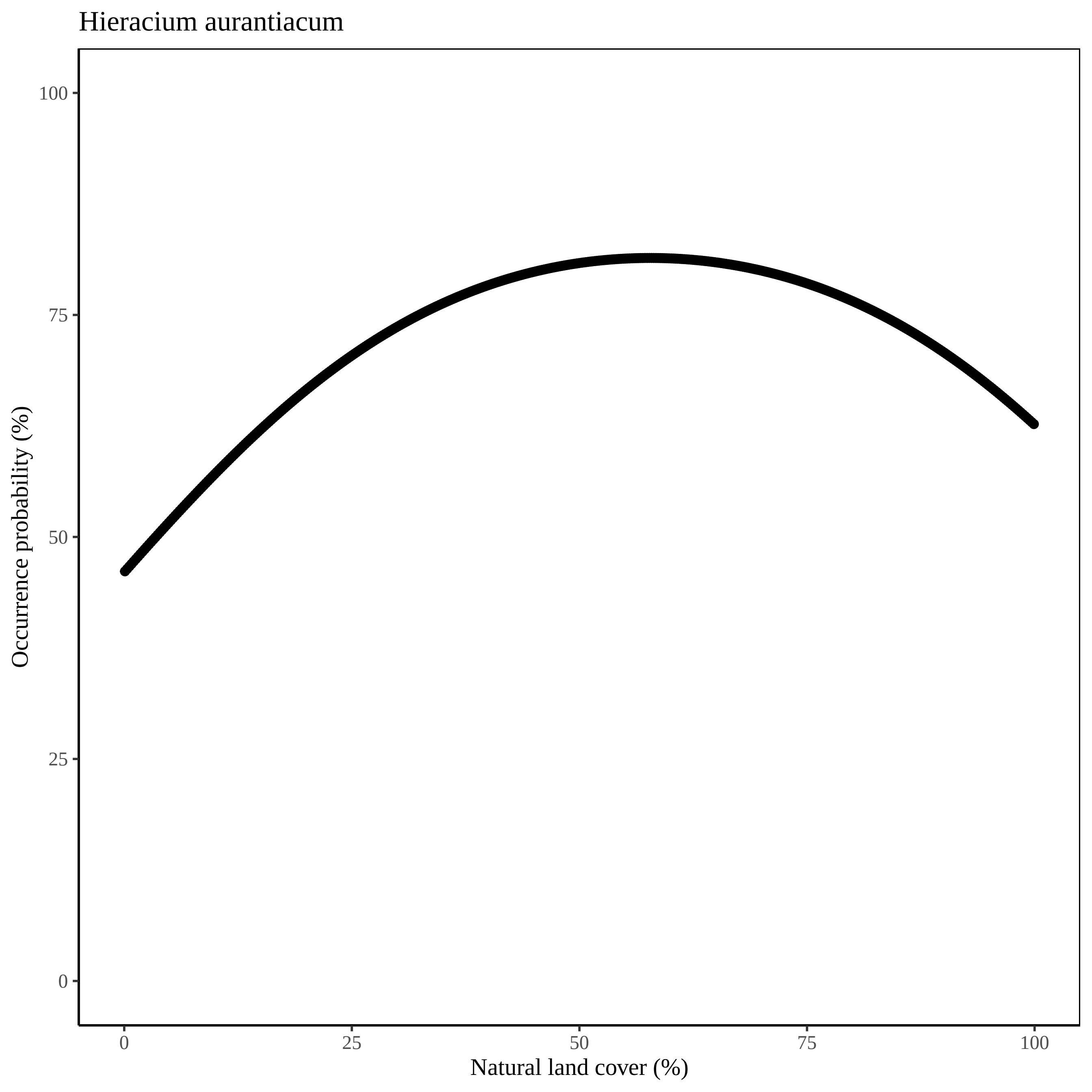
Hieracium aurantiacum
Occurrence probability (%)
100
75
50
25
0
0
25
50
75
100
Natural land cover (%)

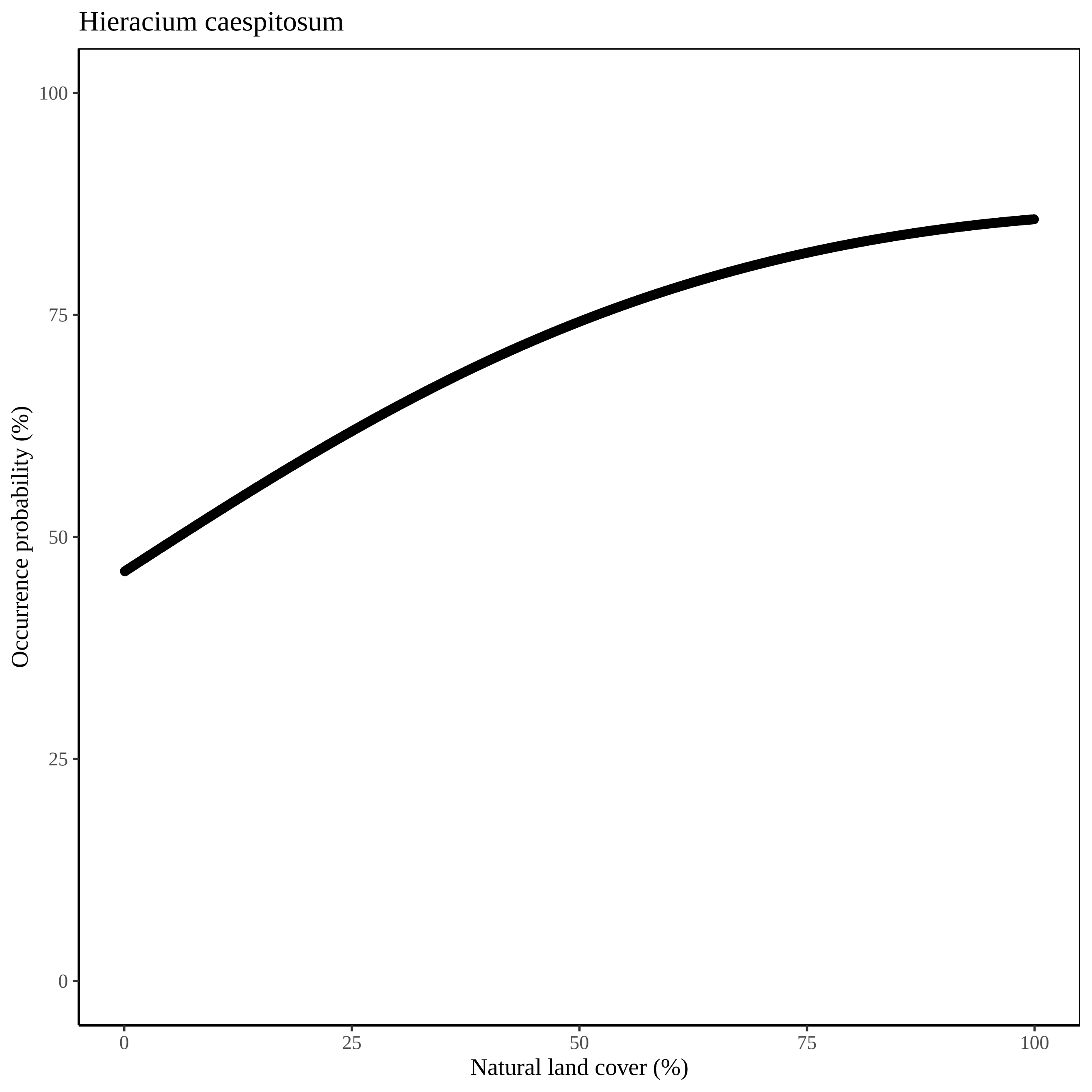

Hieracium caespitosum
100
75
50
25
0
Occurrence probability (%)
0
25
50
75
100
Natural land cover (%)

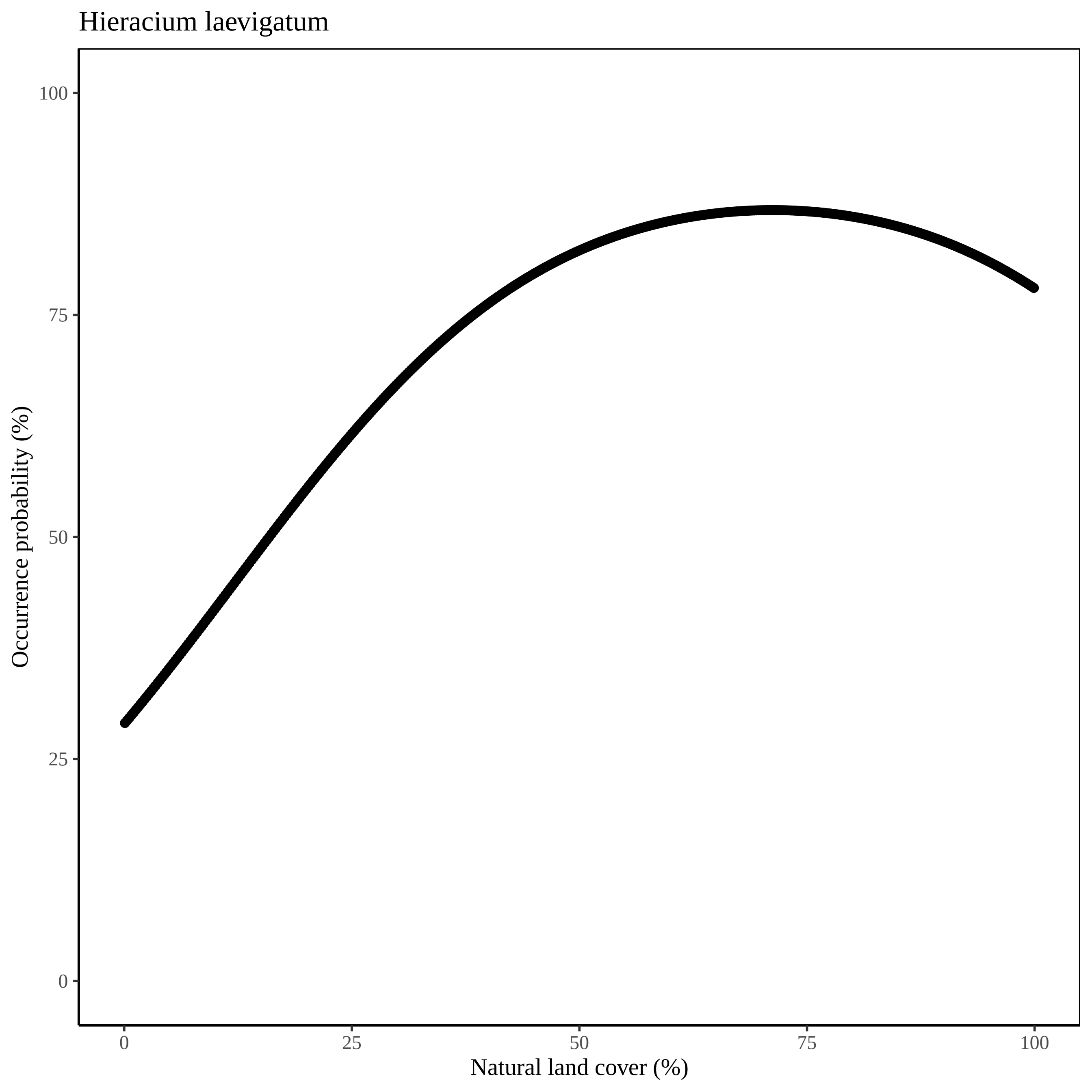

Hieracium laevigatum
Occurrence probability (%)
100
75
50
25
0
0
25
50
75
100
Natural land cover (%)

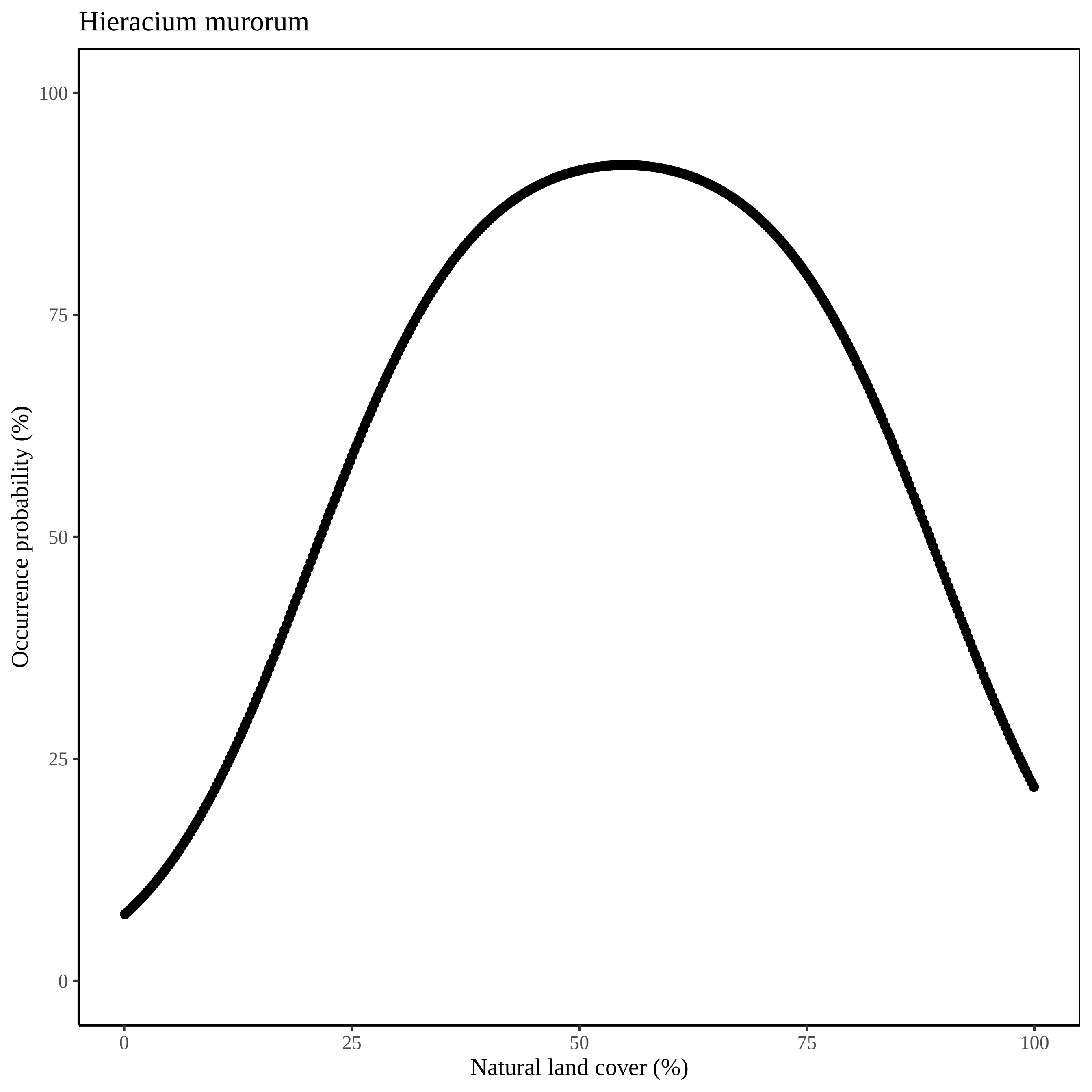

Hieracium murorum
Occurrence probability (%)
100
75
50
25
0
0
25
50
75
100
Natural land cover (%)

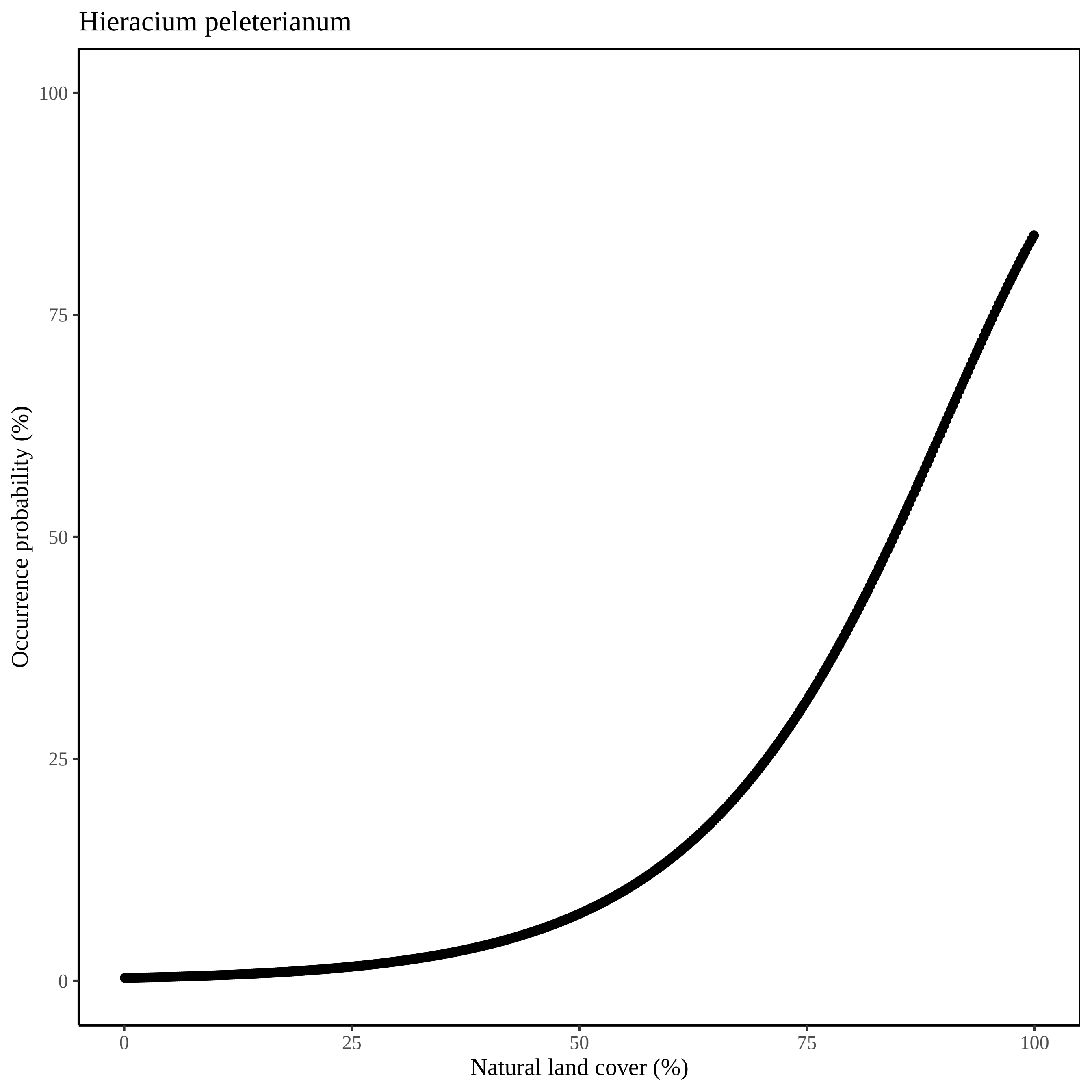
Hieracium peleterianum
Occurrence probability (%)
100
75
50
25
0
0
25
50
75
100
Natural land cover (%)

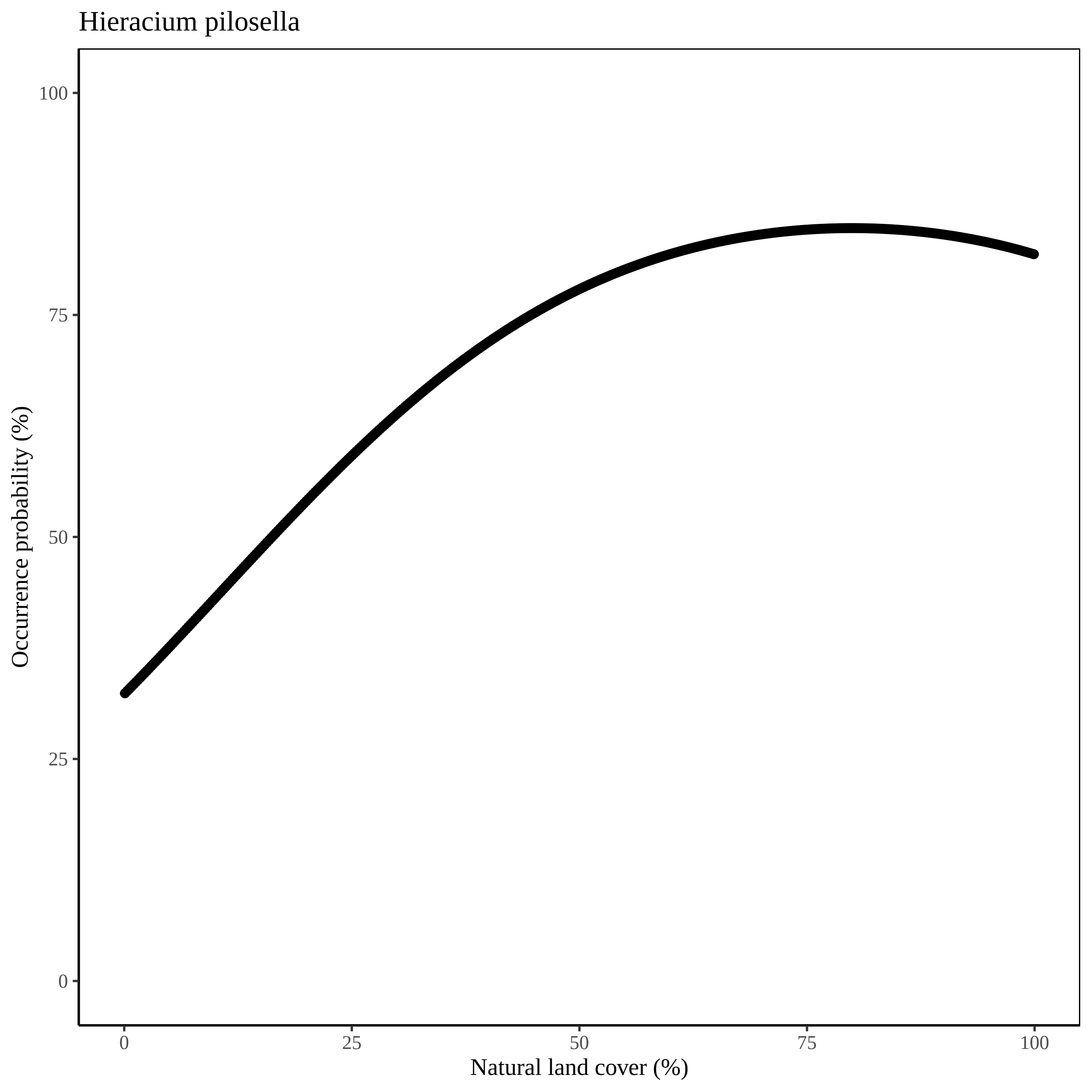

Hieracium pilosella
Occurrence probability (%)
100
75
50
25
0
0
25
50
75
100
Natural land cover (%)

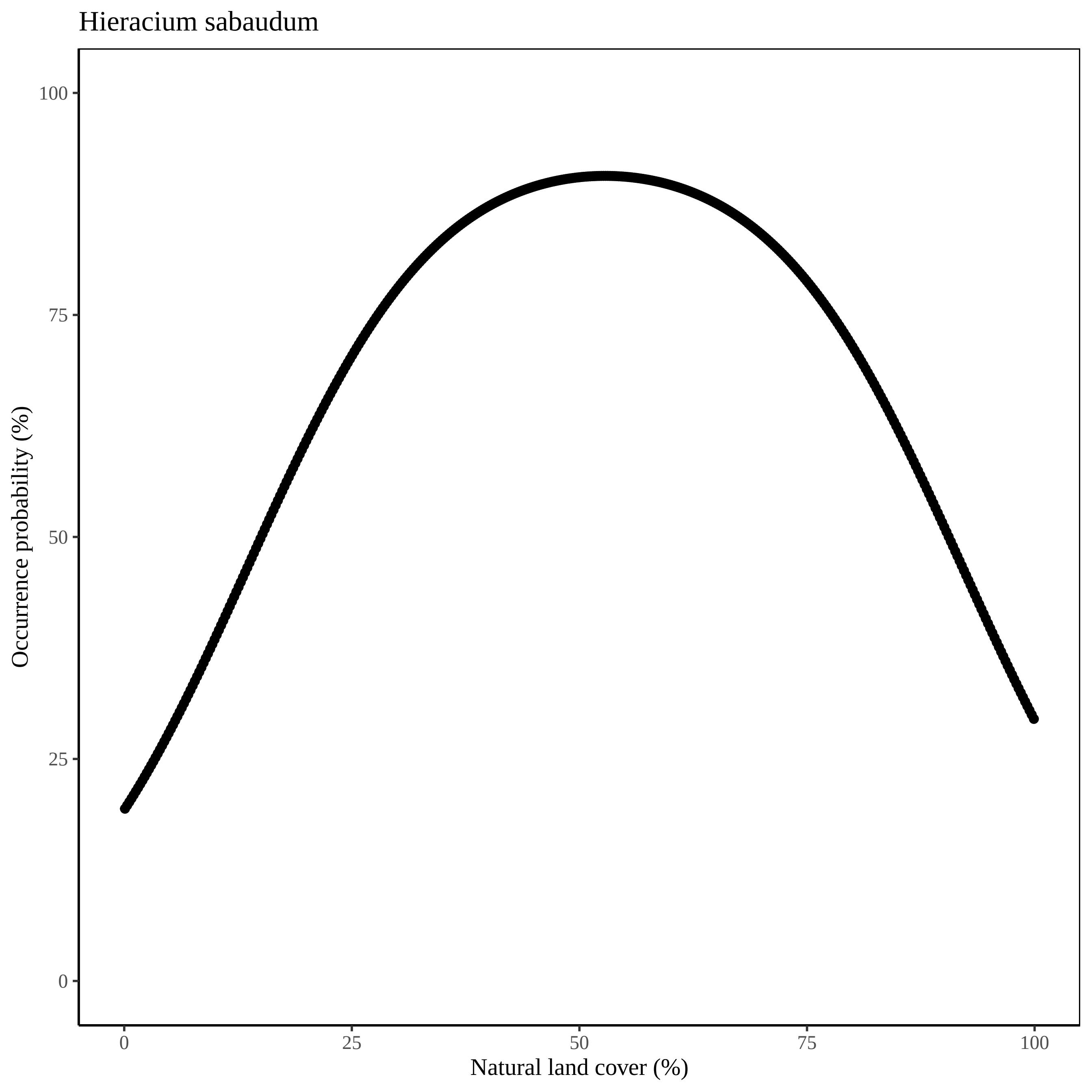
Hieracium sabaudum
Occurrence probability (%)
100
75
50
25
0
0
25
50
75
100
Natural land cover (%)

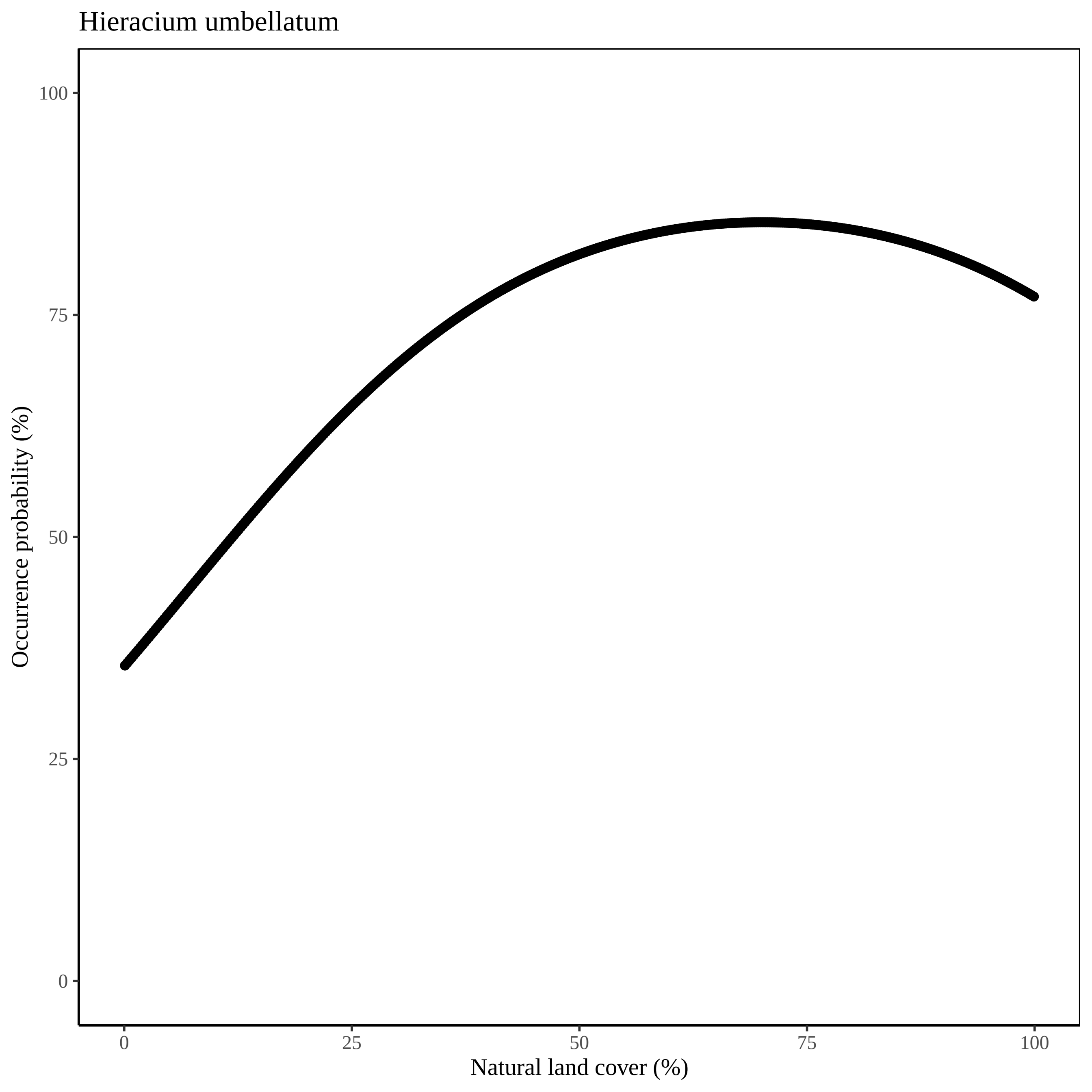
Hieracium umbellatum
Occurrence probability (%)
100
75
50
25
0
0
25
50
75
100
Natural land cover (%)

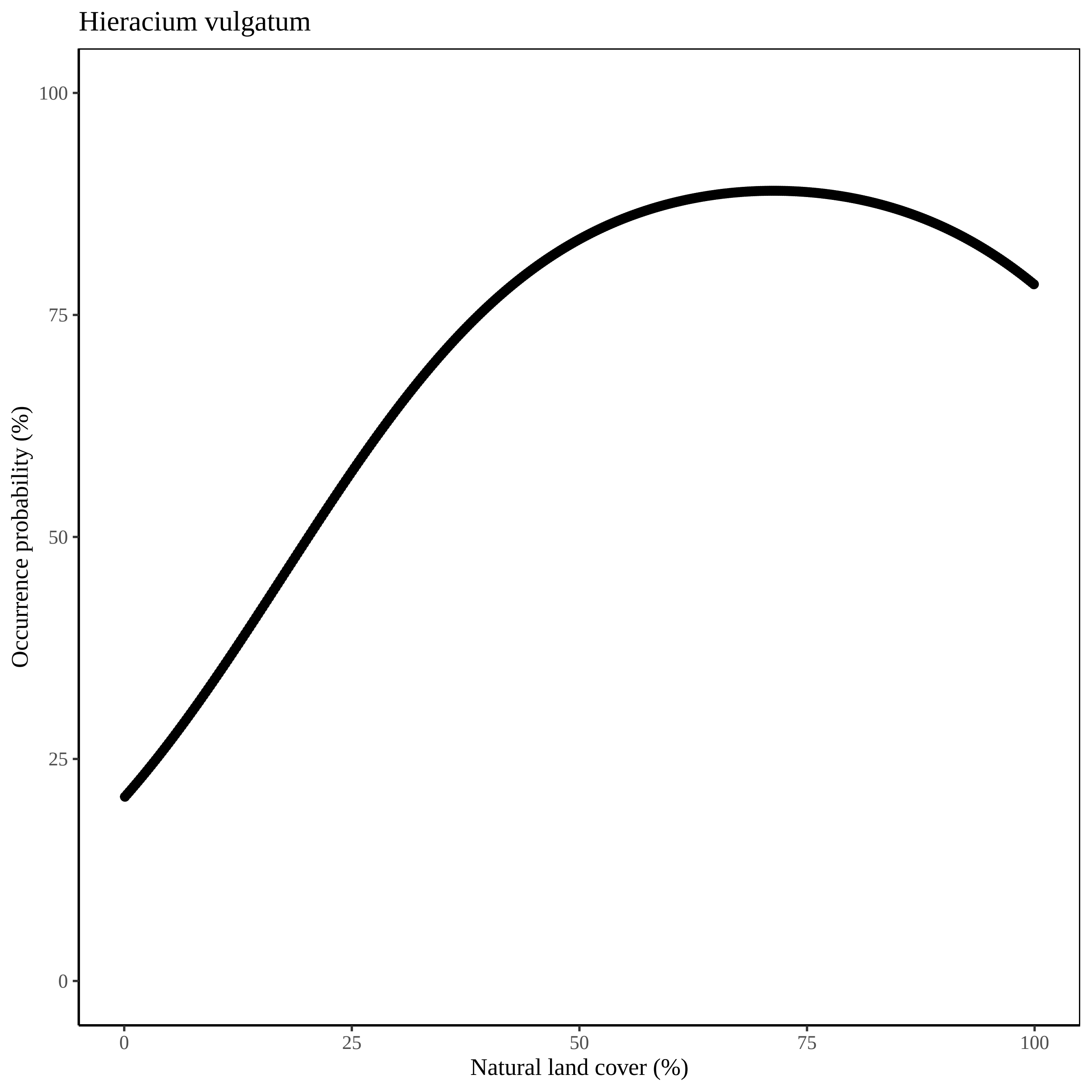
Hieracium vulgatum
Occurrence probability (%)
100
75
50
25
0
0
25
50
75
100
Natural land cover (%)

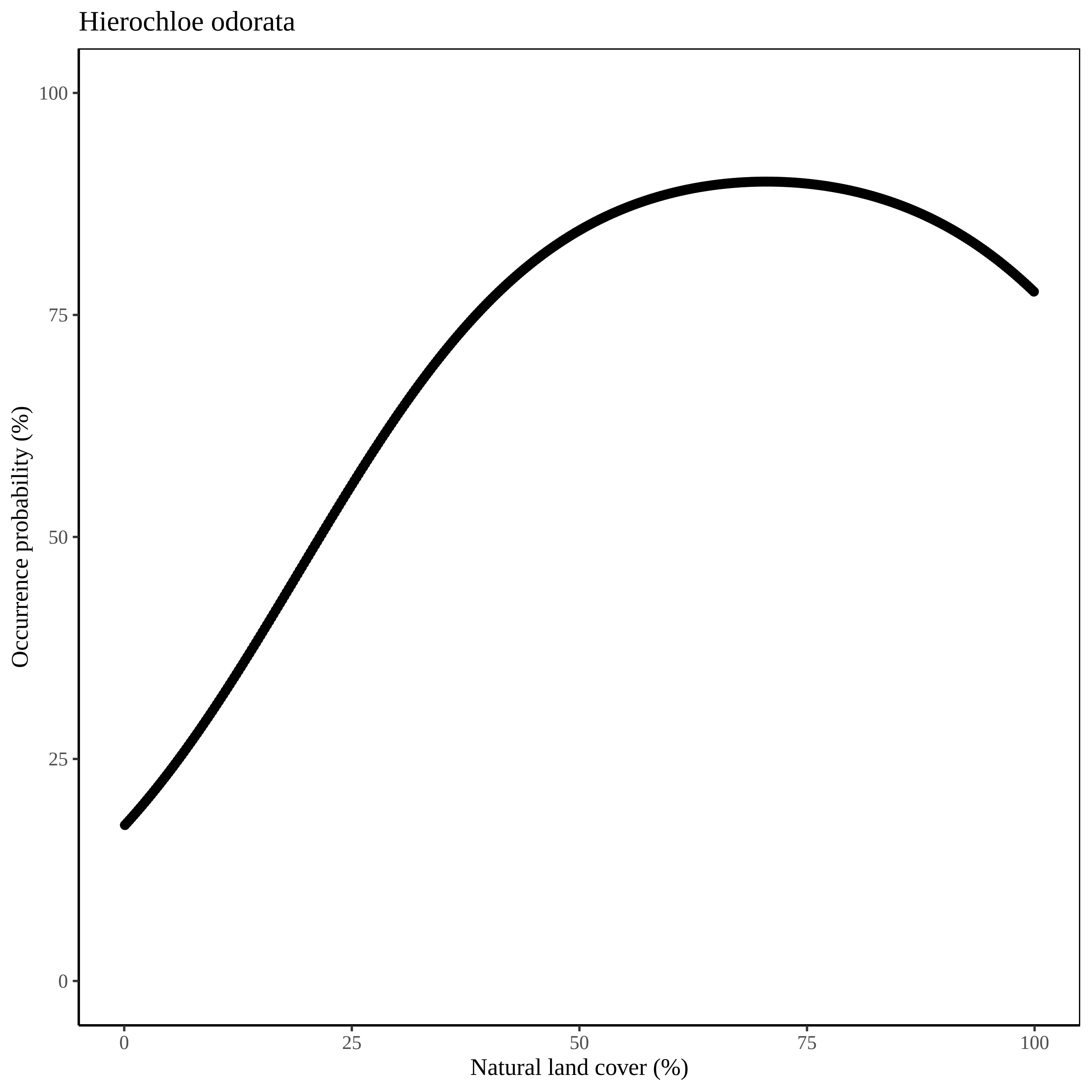

Hierochloe odorata
Occurrence probability (%)
100
75
50
25
0
0
25
50
75
100
Natural land cover (%)

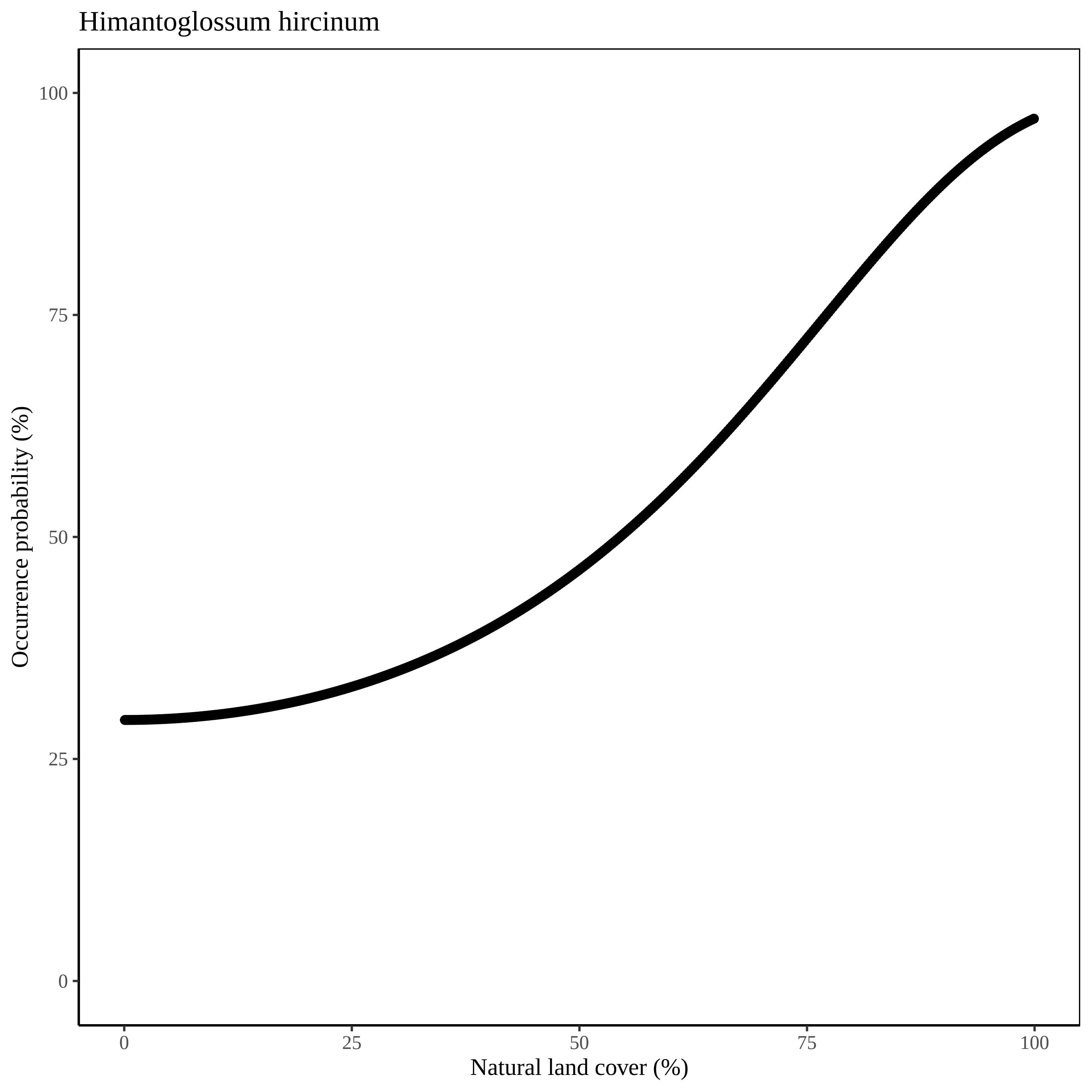
Himantoglossum hircinum
Occurrence probability (%)
100
75
50
25
0
0
25
50
75
100
Natural land cover (%)

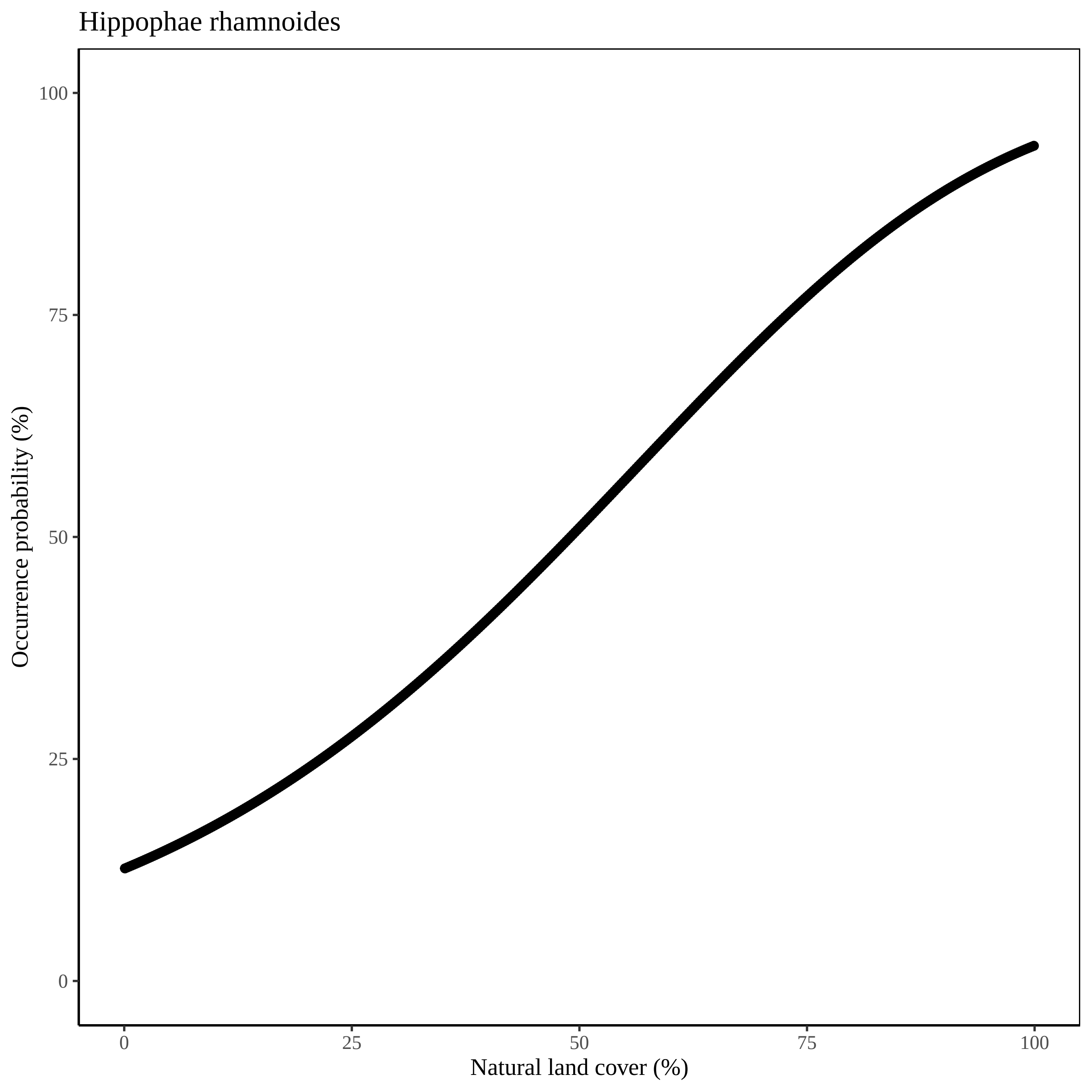
Hippophae rhamnoides
Occurrence probability (%)
100
75
50
25
0
0
25
50
75
100
Natural land cover (%)

Hippuris vulgaris

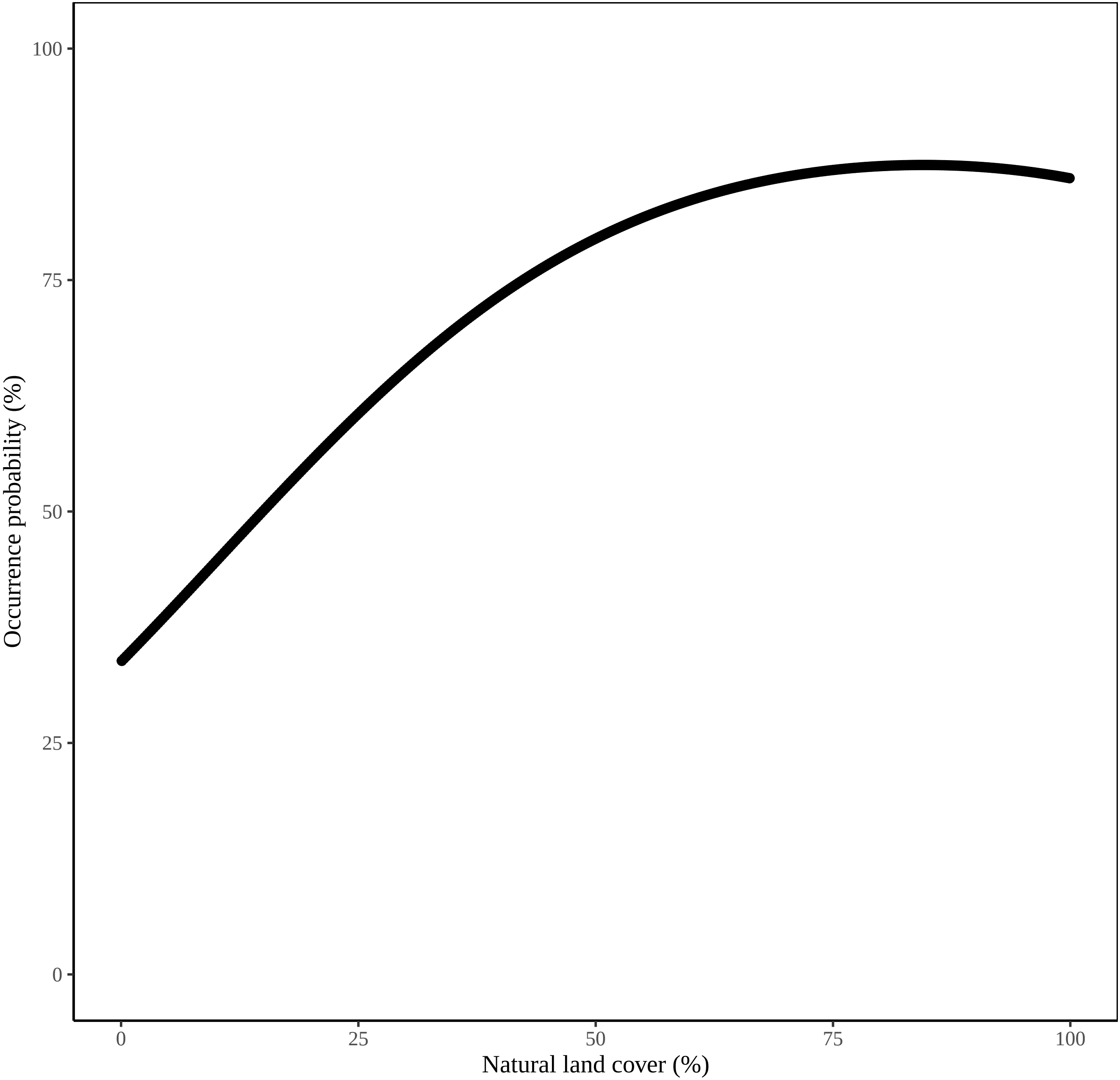

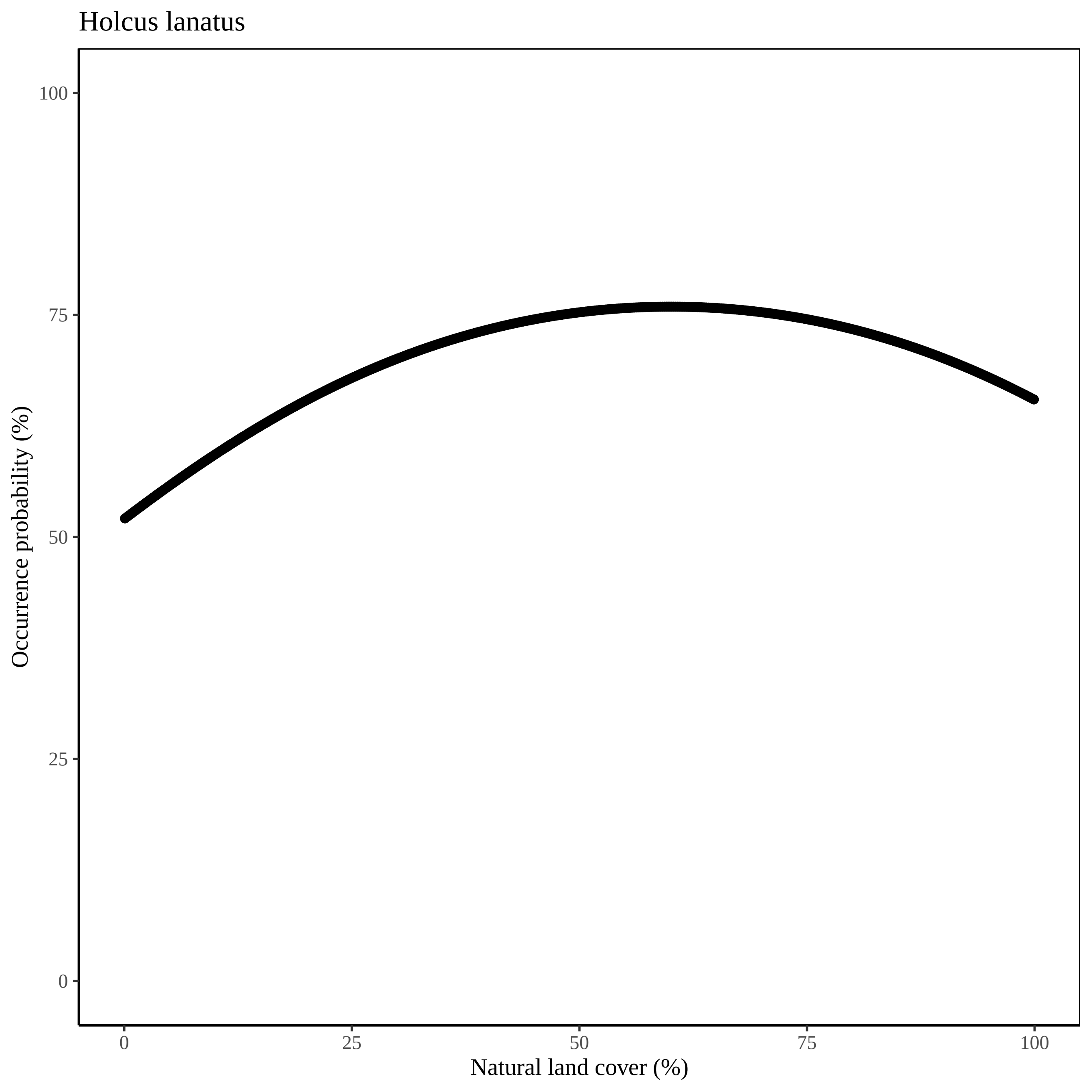
Holcus lanatus
Occurrence probability (%)
100
75
50
25
0
0
25
50
75
100
Natural land cover (%)

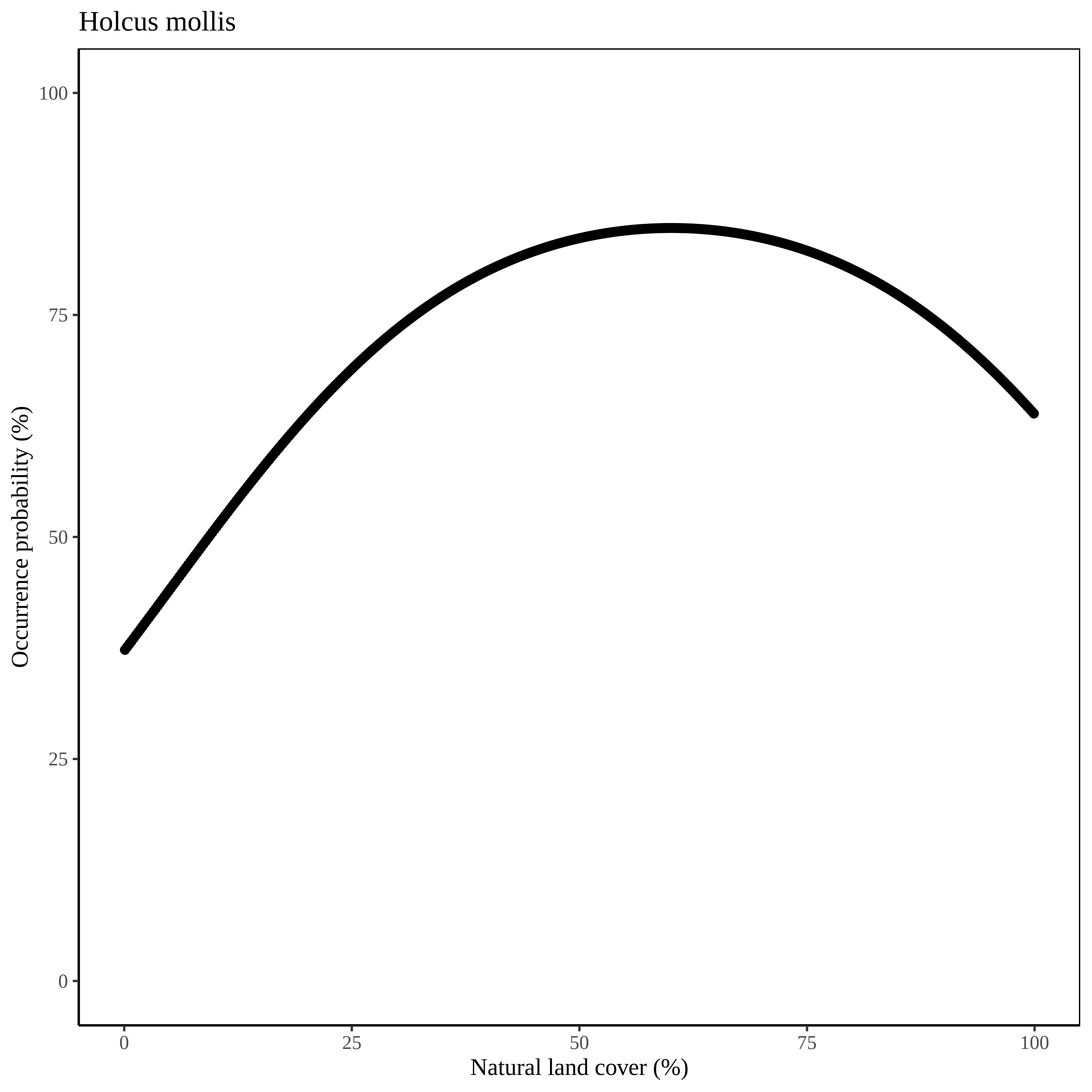

Holcus mollis
100
75
50
25
0
Occurrence probability (%)
0
25
50
75
100
Natural land cover (%)

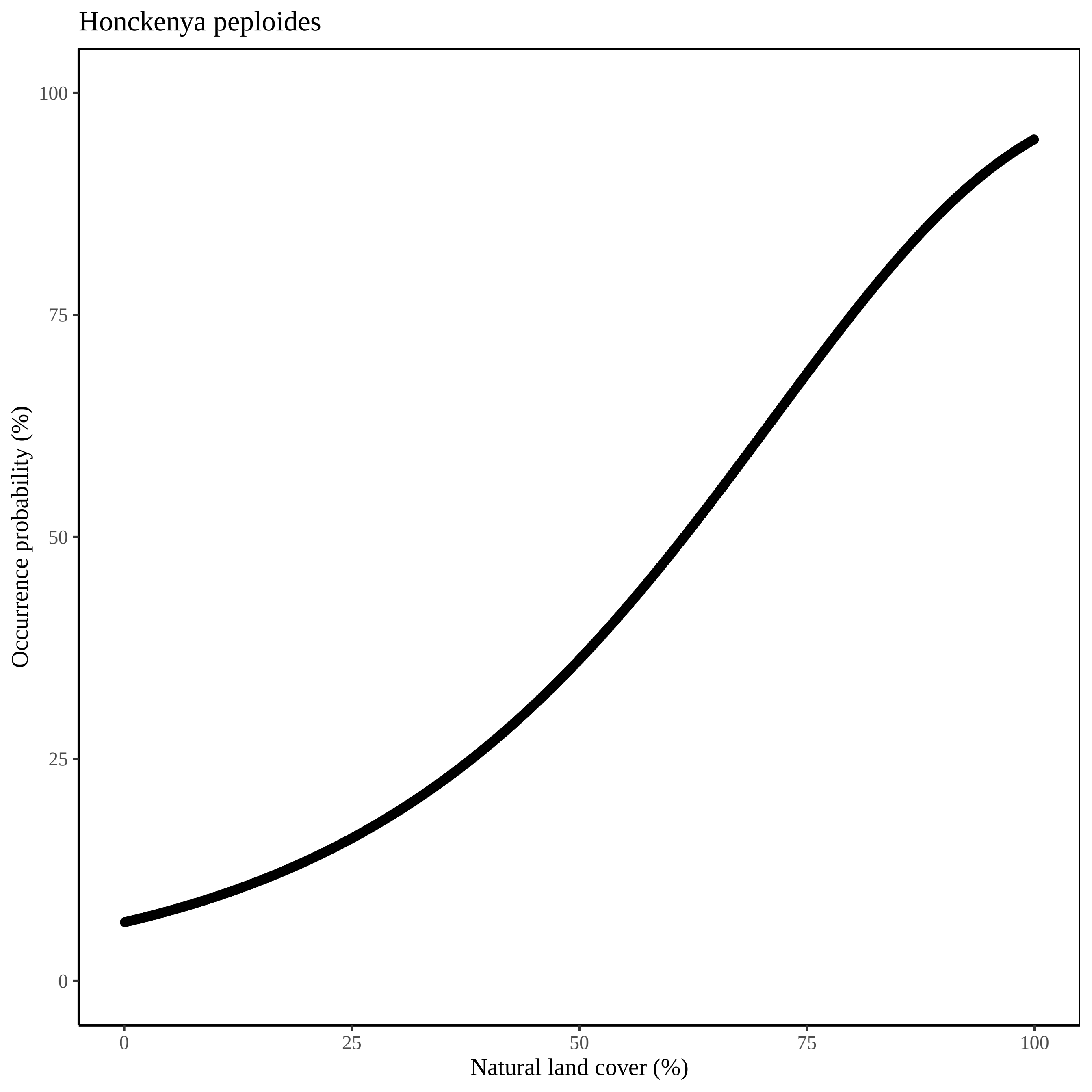
Honckenya peploides
Occurrence probability (%)
100
75
50
25
0
0
25
50
75
100
Natural land cover (%)

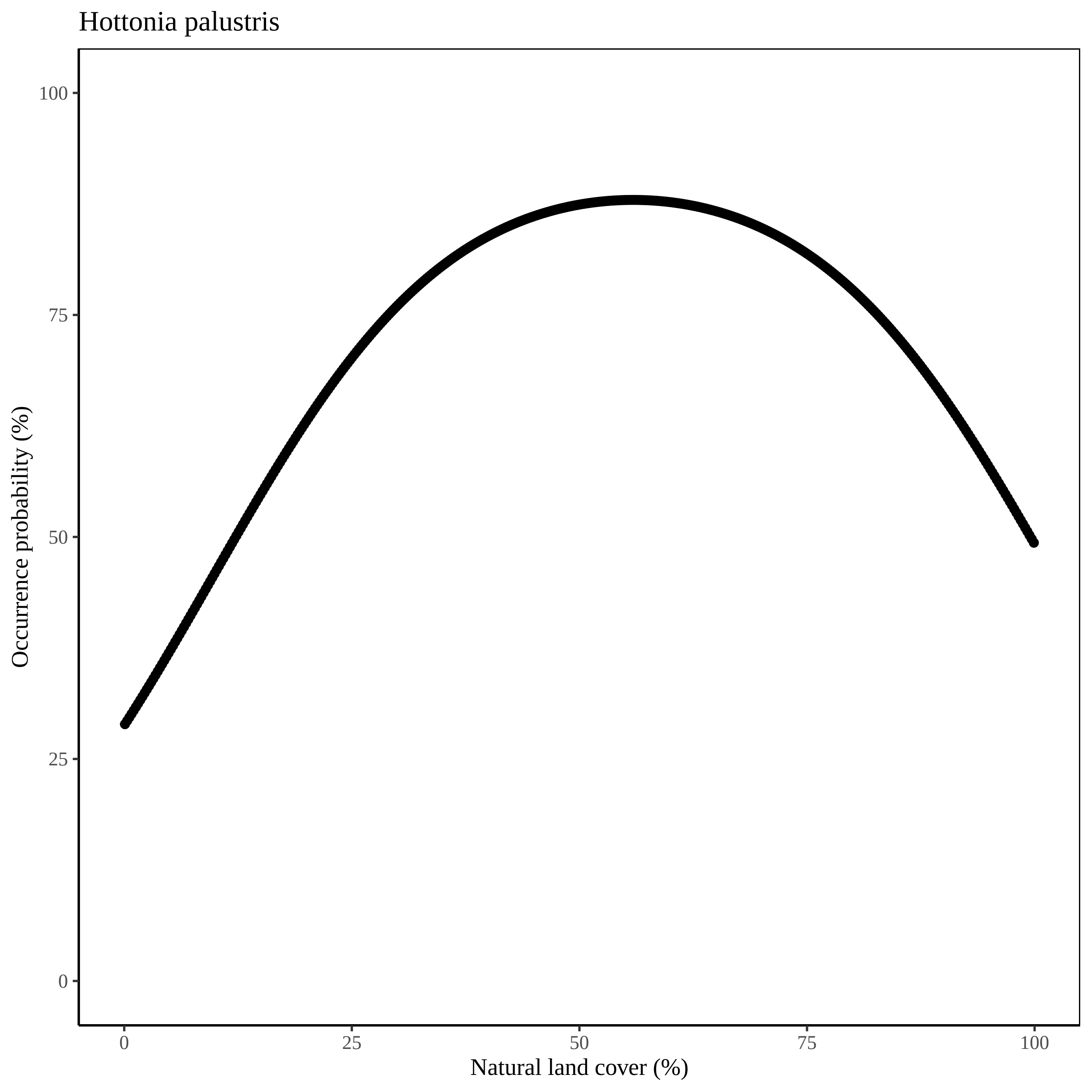
Hottonia palustris
Occurrence probability (%)
Natural land cover (%)
0
25
50
75
100
0
25
50
75
100

Humulus lupulus

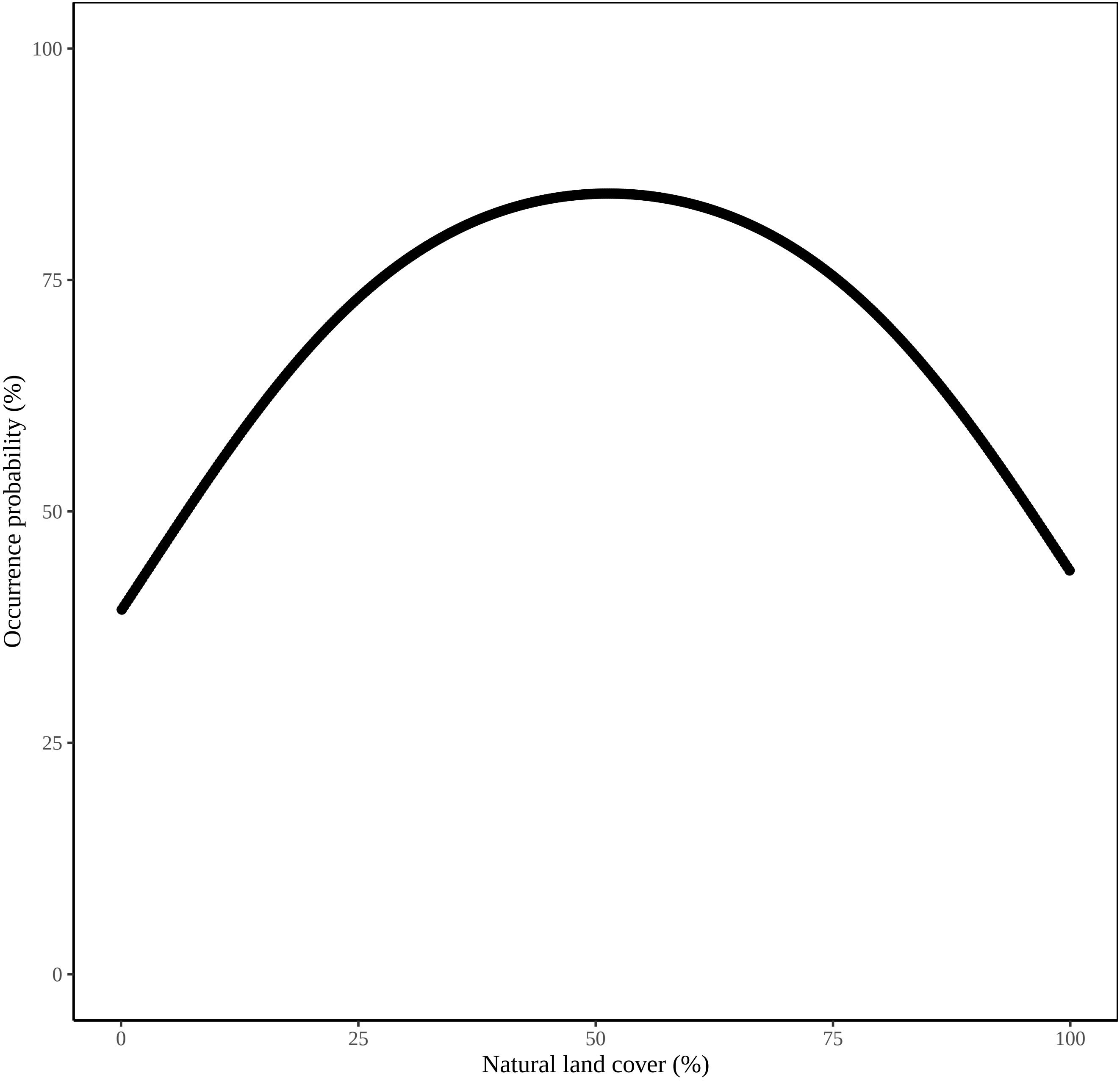

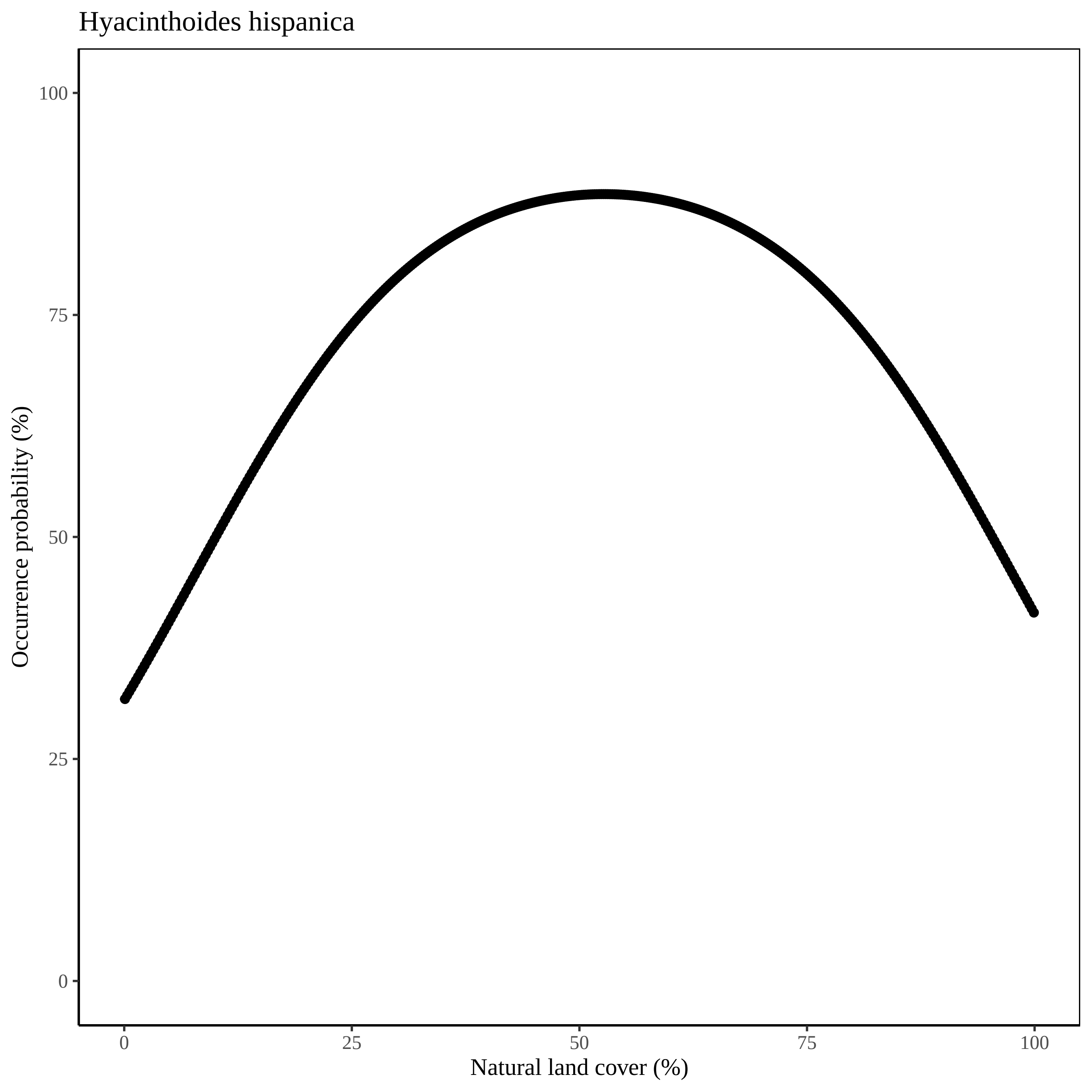
Hyacinthoides hispanica
Occurrence probability (%)
100
75
50
25
0
0
25
50
75
100
Natural land cover (%)

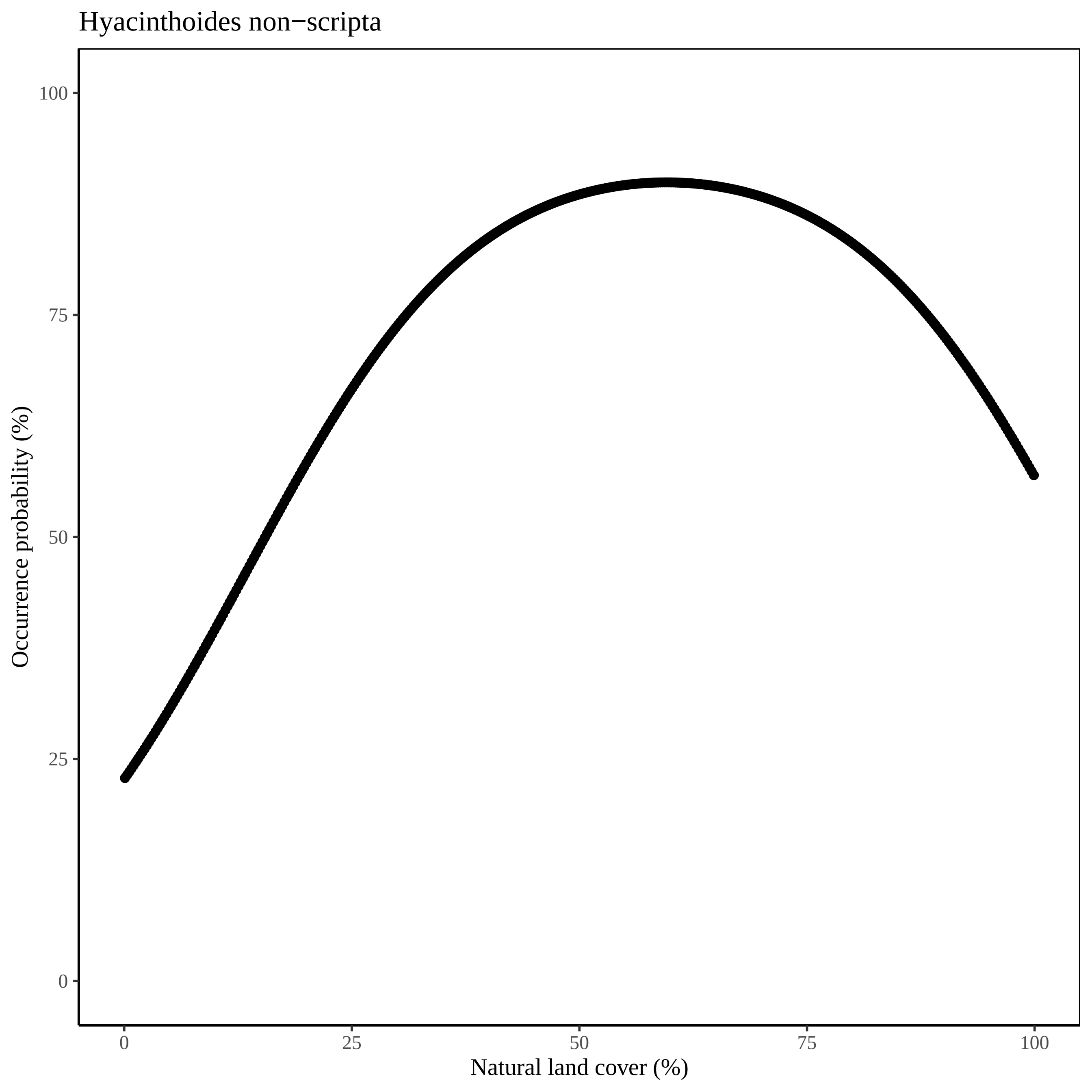

Hyacinthoides non−scripta
Occurrence probability (%)
100
75
50
25
0
0
25
50
75
100
Natural land cover (%)

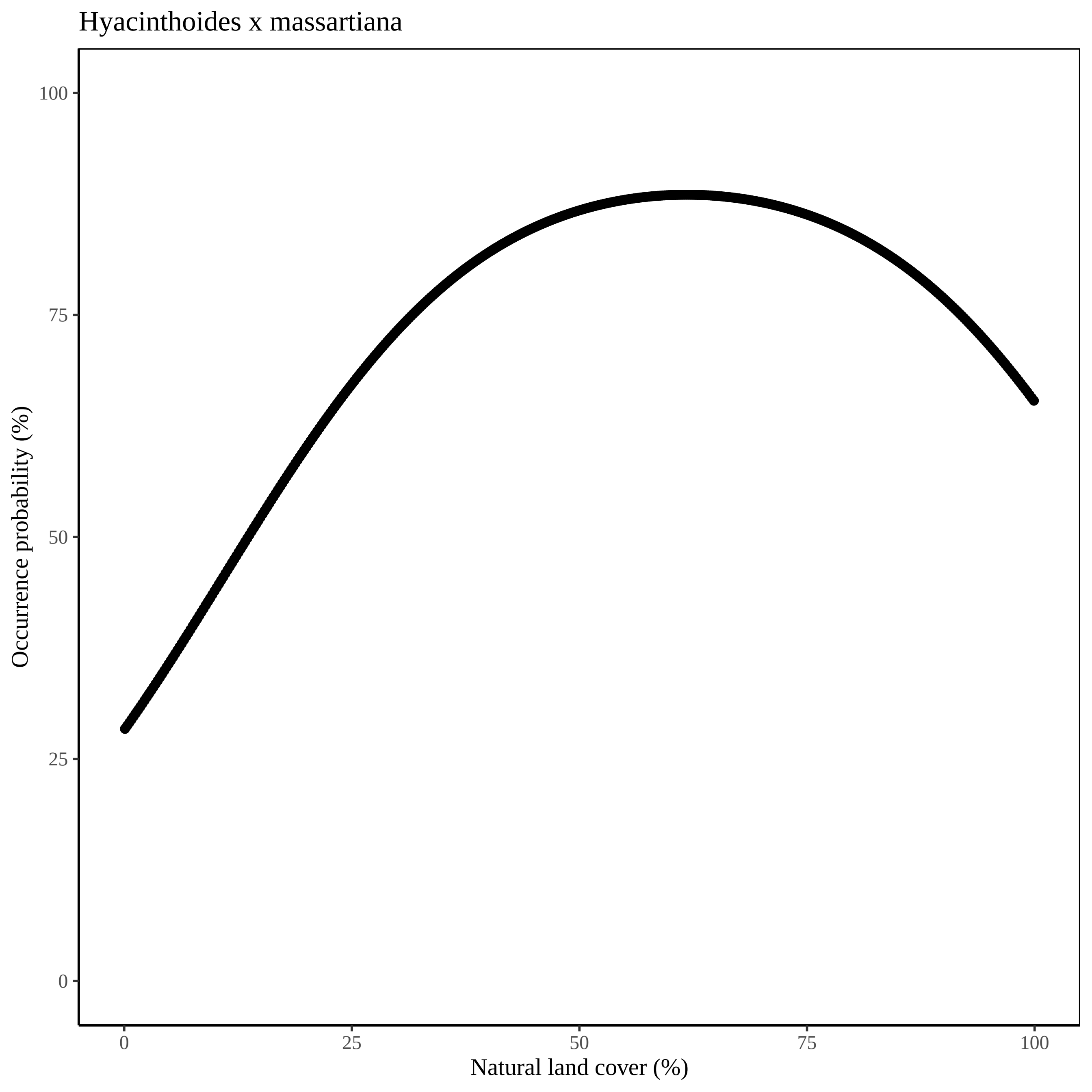

Hyacinthoides x massartiana
Occurrence probability (%)
100
75
50
25
0
0
25
50
75
100
Natural land cover (%)

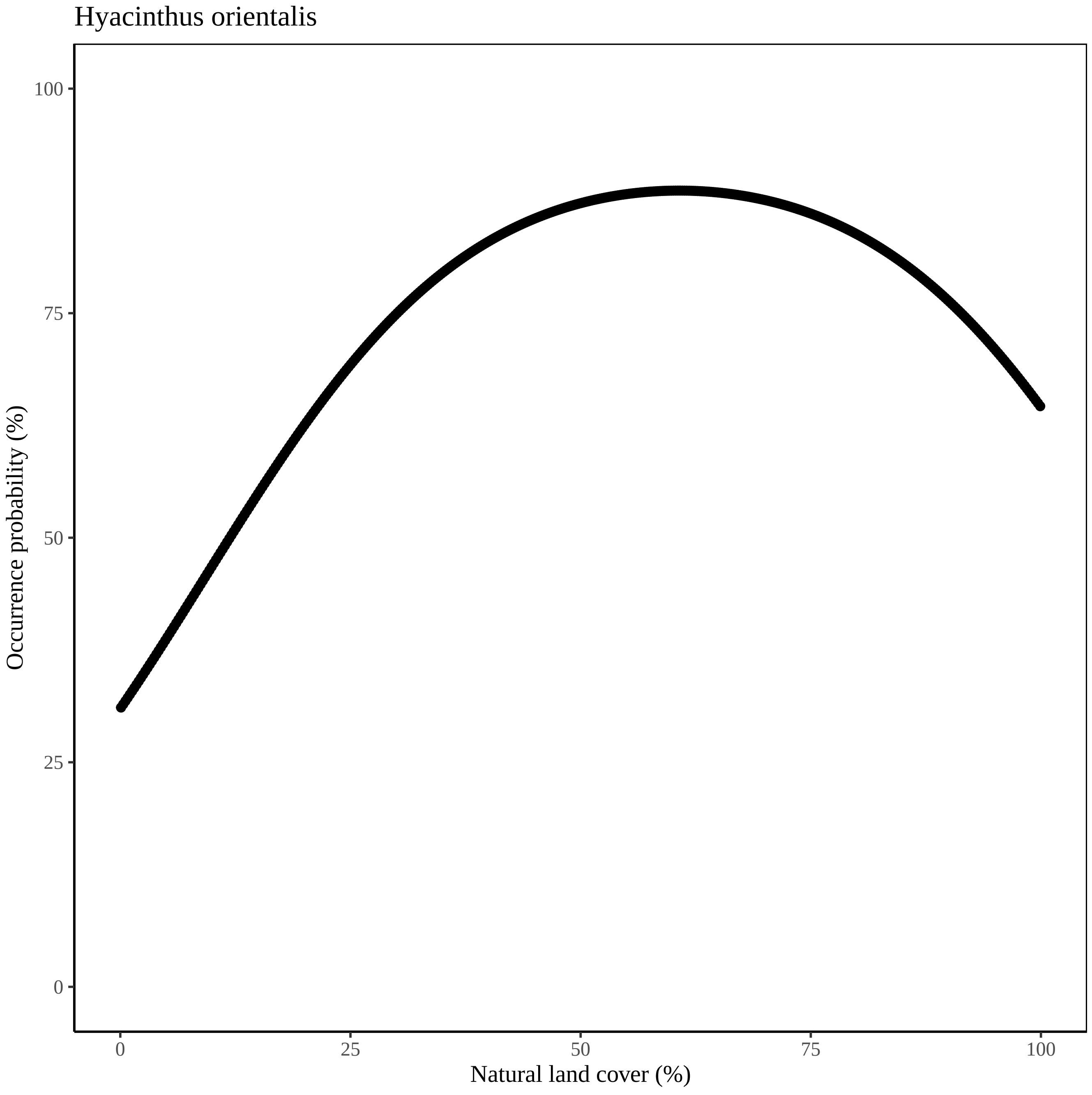

Hyacinthus orientalis
Occurrence probability (%)
100
75
50
25
0
0
25
50
75
100
Natural land cover (%)

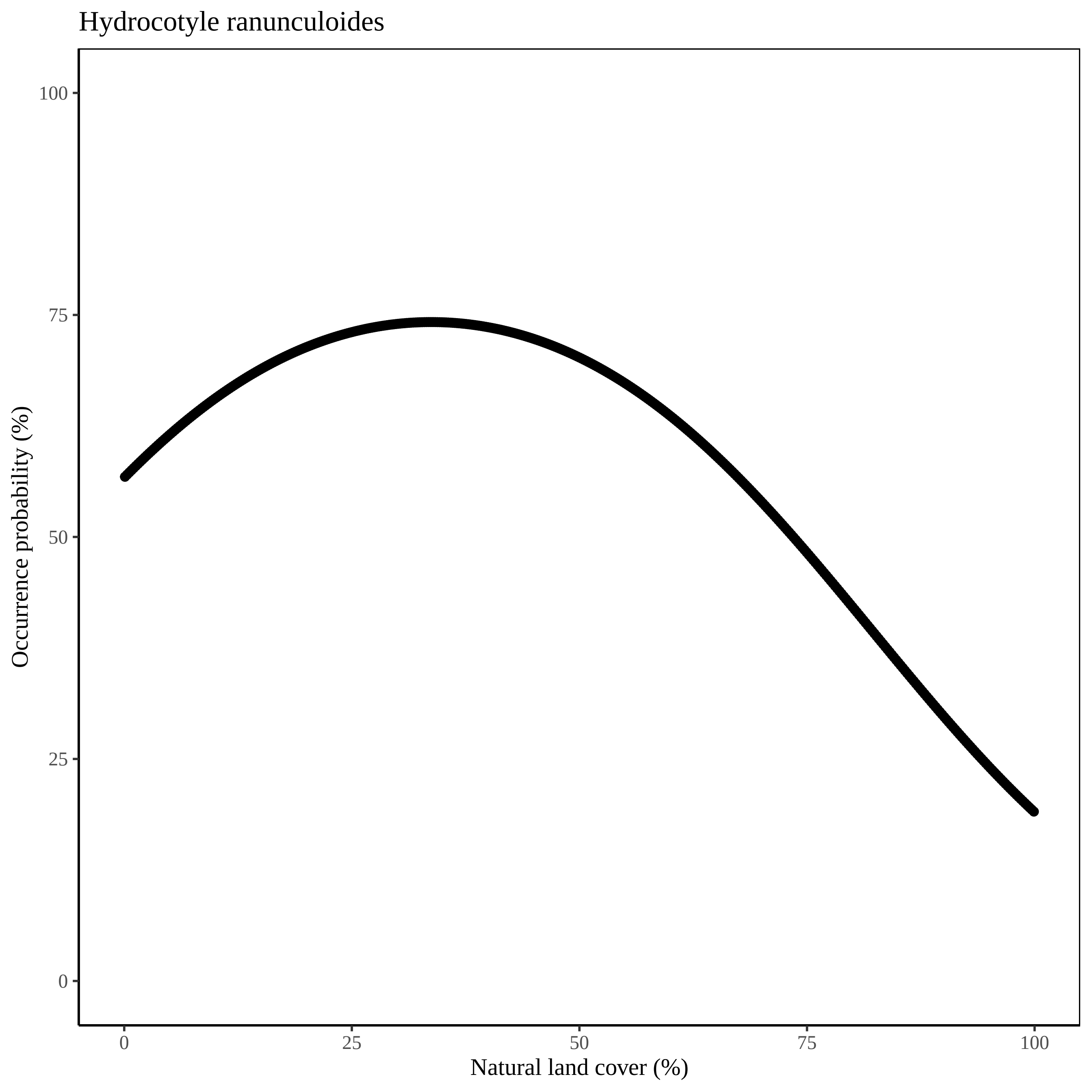

Hydrocotyle ranunculoides
Occurrence probability (%)
100
75
50
25
0
0
25
50
75
100
Natural land cover (%)

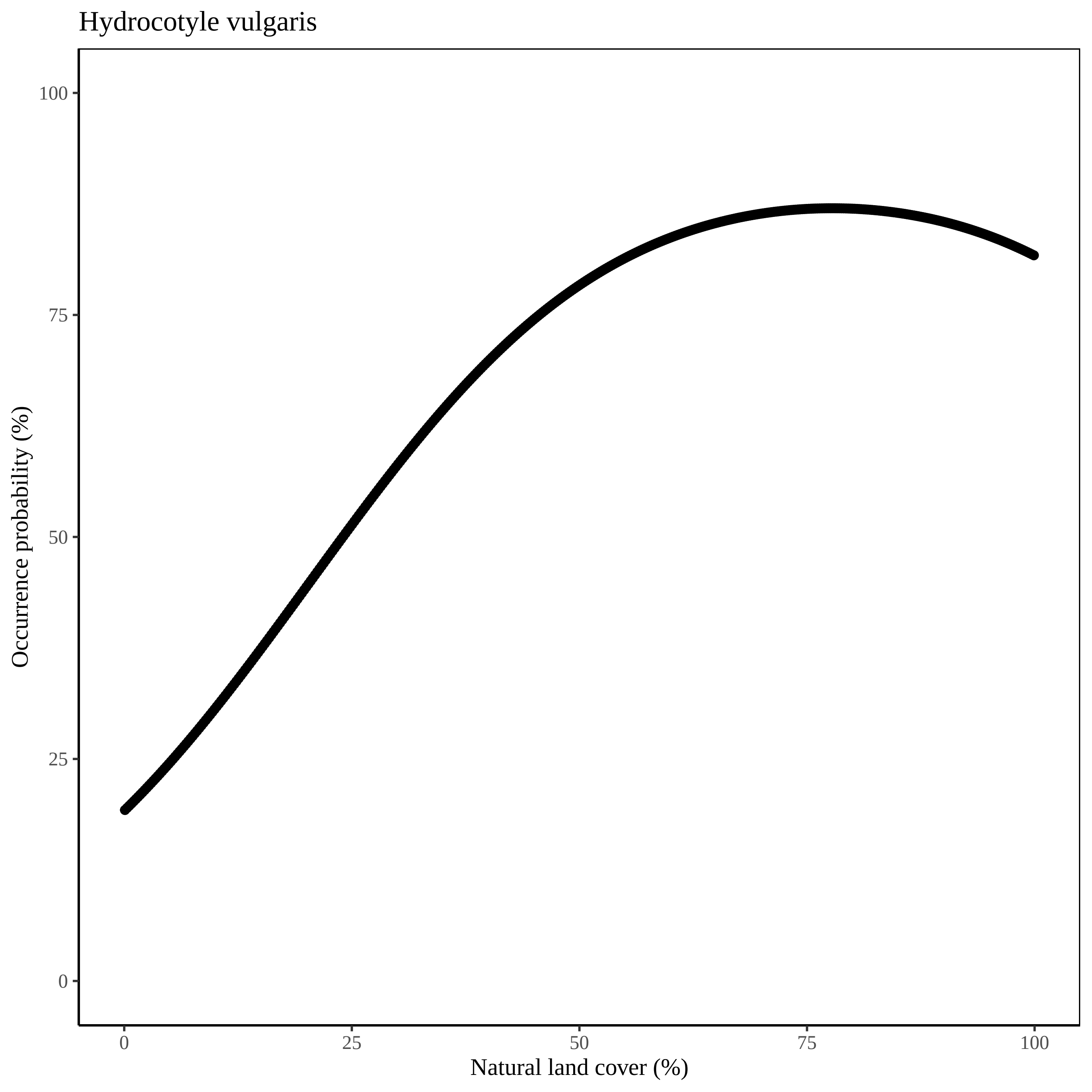
Hydrocotyle vulgaris
Occurrence probability (%)
100
75
50
25
0
0
25
50
75
100
Natural land cover (%)

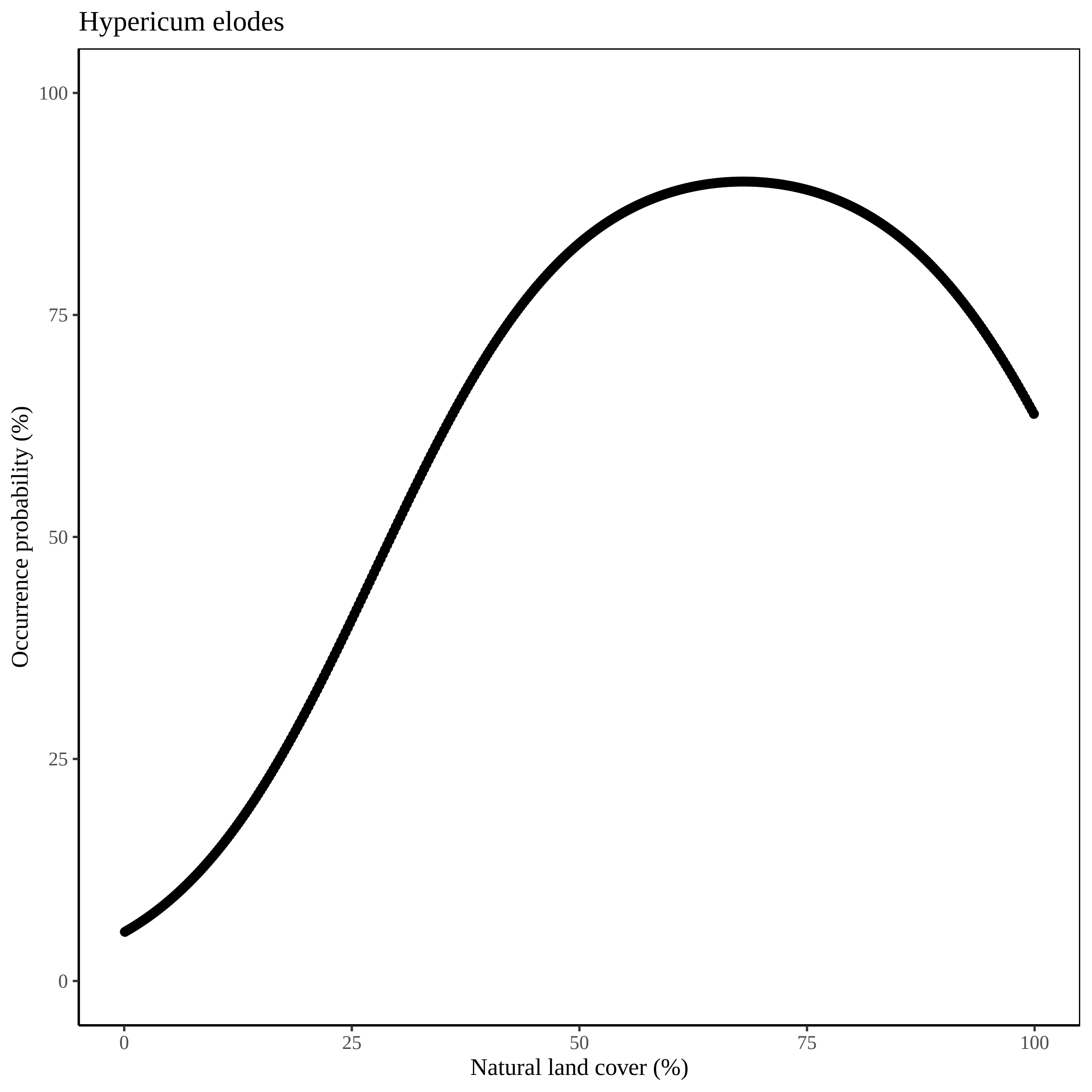

Hypericum elodes
Occurrence probability (%)
100
75
50
25
0
0
25
50
75
100
Natural land cover (%)

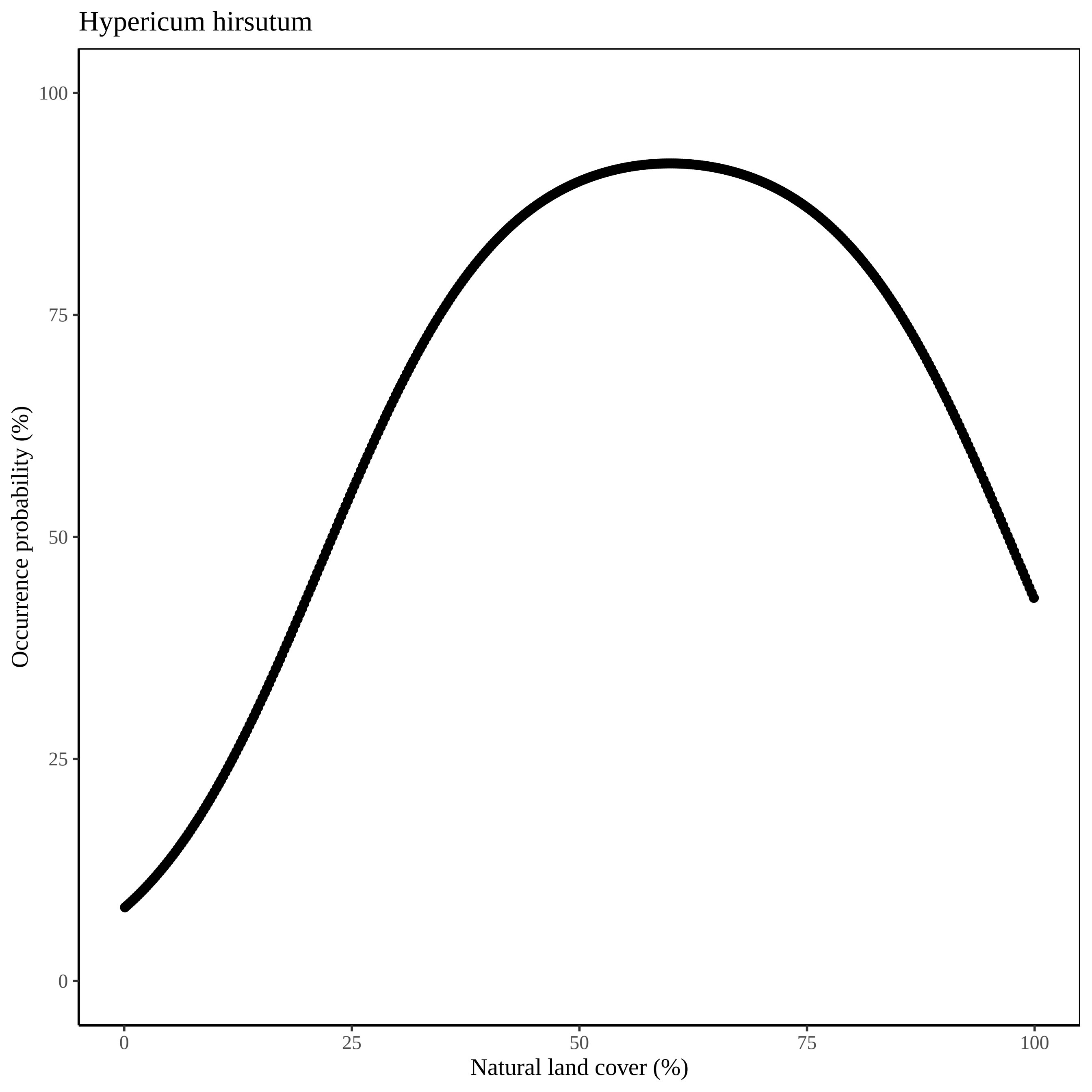
Hypericum hirsutum
Occurrence probability (%)
100
75
50
25
0
0
25
50
75
100
Natural land cover (%)

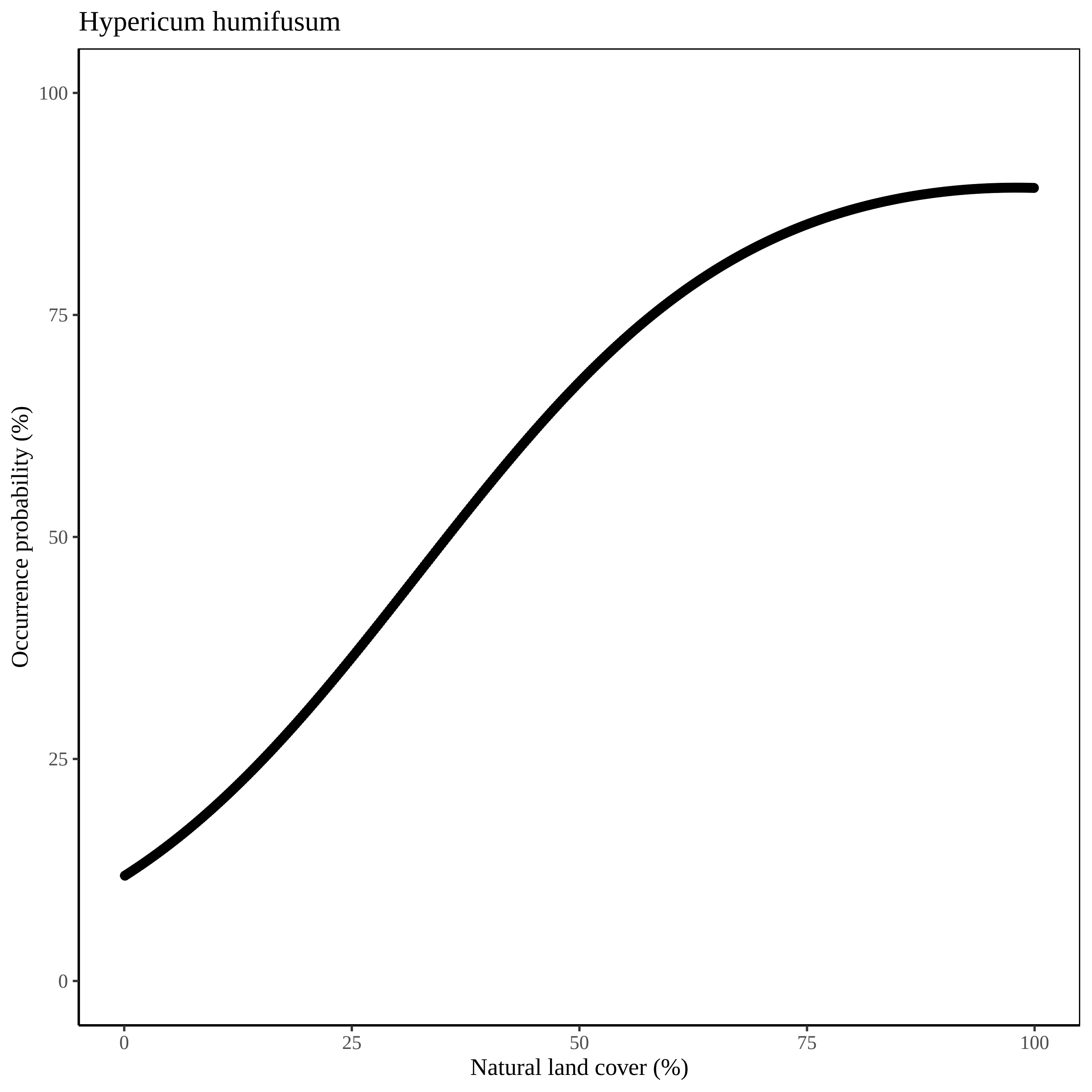

Hypericum humifusum
Occurrence probability (%)
100
75
50
25
0
0
25
50
75
100
Natural land cover (%)

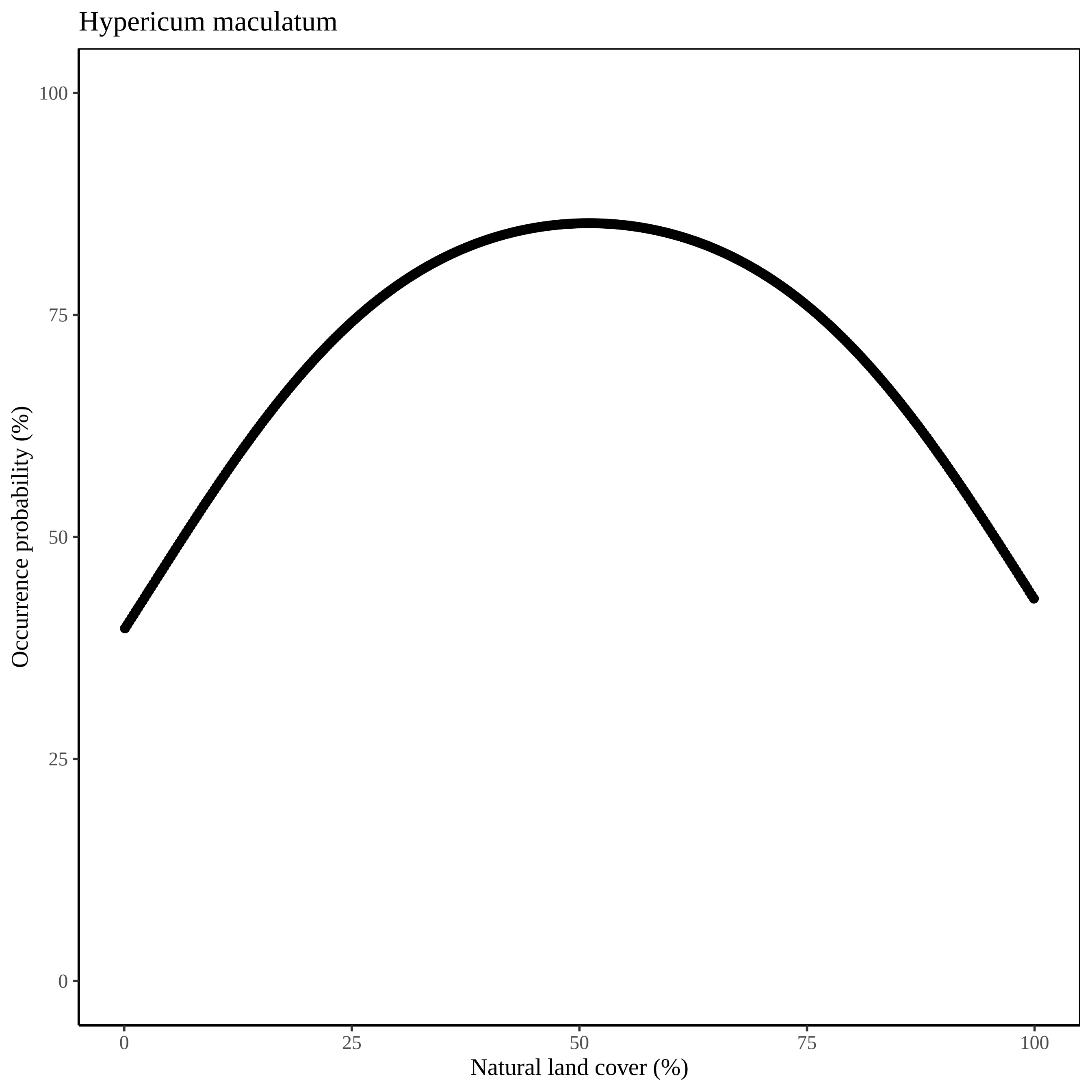
Hypericum maculatum
Occurrence probability (%)
100
75
50
25
0
0
25
50
75
100
Natural land cover (%)

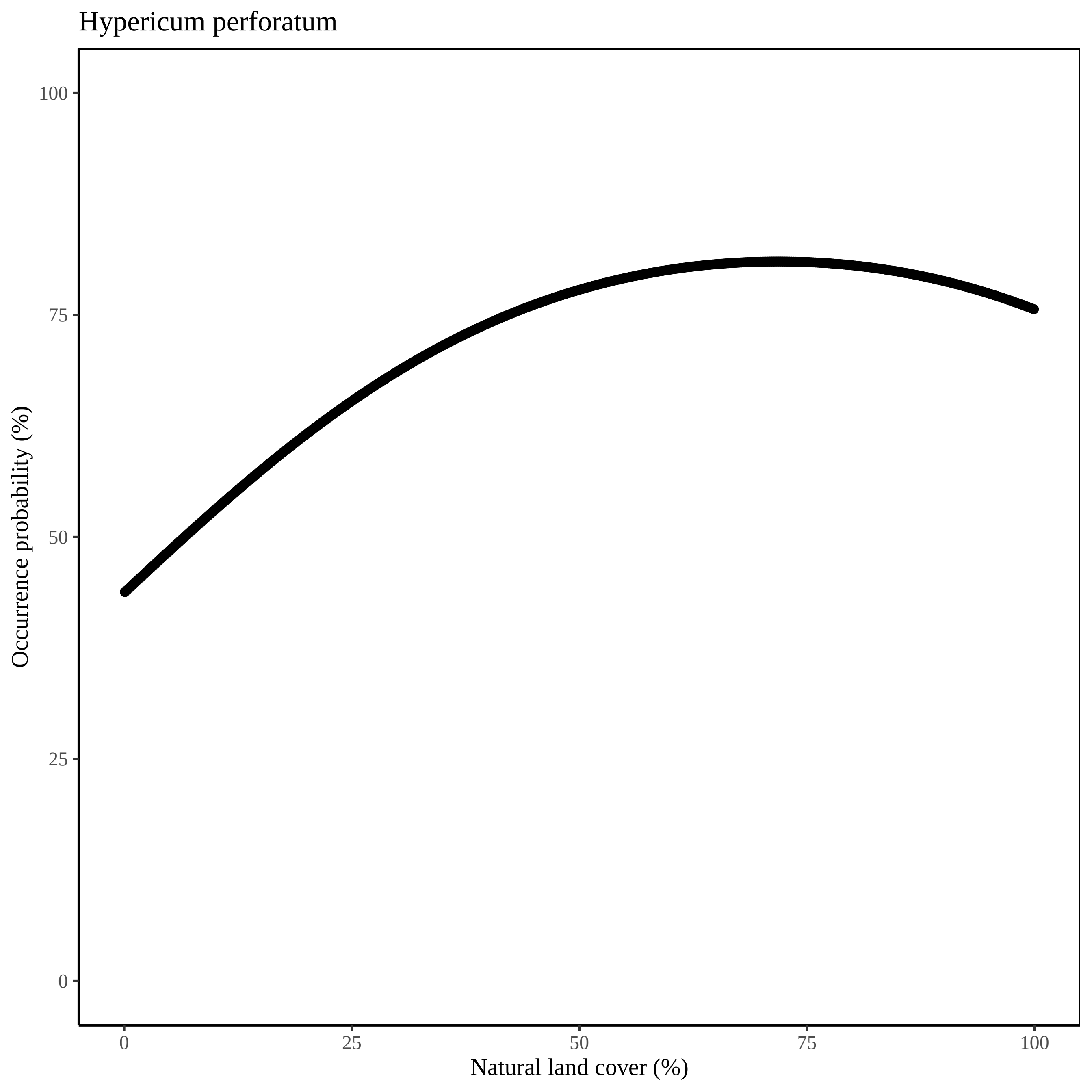
Hypericum perforatum
Occurrence probability (%)
100
75
50
25
0
0
25
50
75
100
Natural land cover (%)

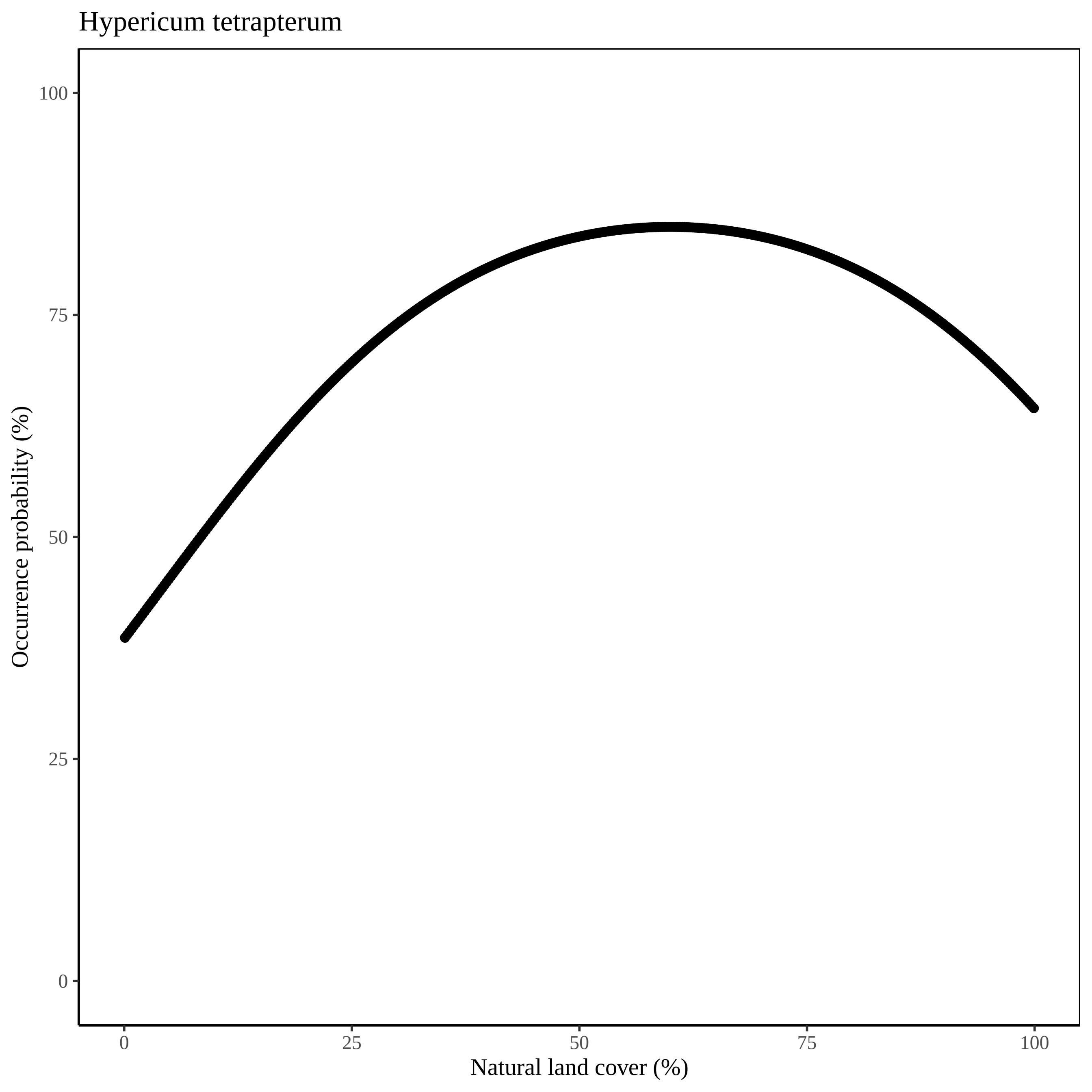
Hypericum tetrapterum
Occurrence probability (%)
100
75
50
25
0
0
25
50
75
100
Natural land cover (%)

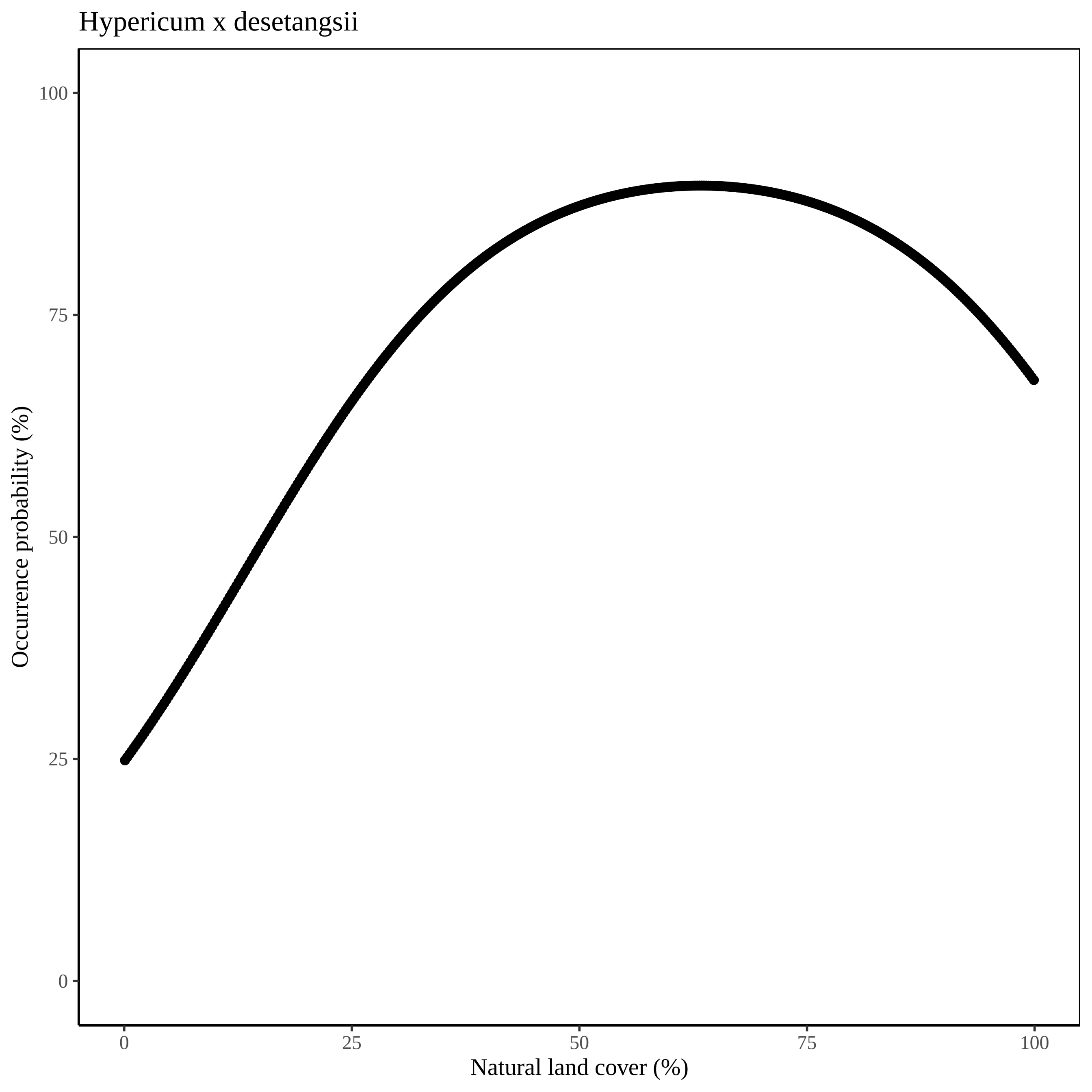

Hypericum x desetangsii
Occurrence probability (%)
100
75
50
25
0
0
25
50
75
100
Natural land cover (%)

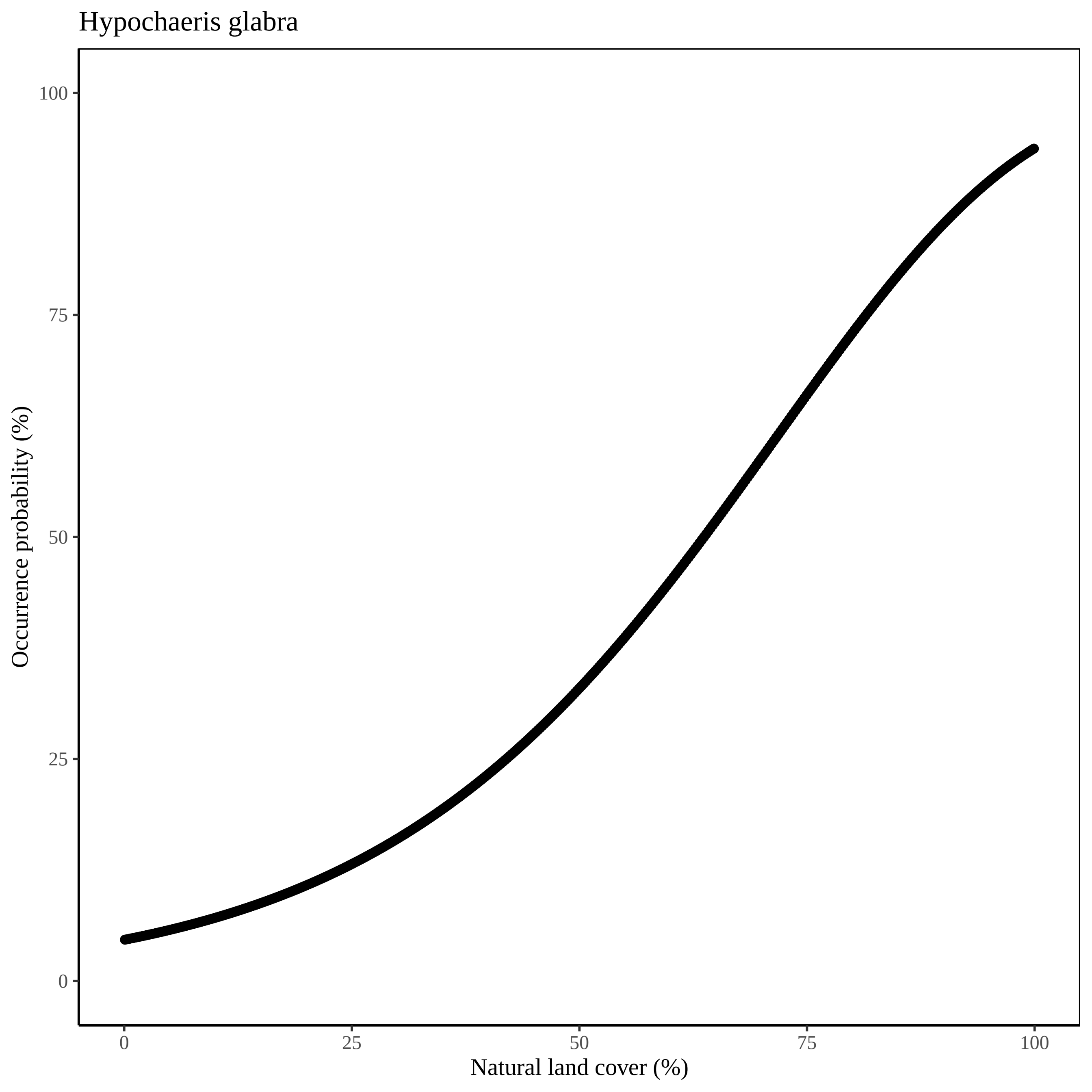
Hypochaeris glabra
Occurrence probability (%)
100
75
50
25
0
0
25
50
75
100
Natural land cover (%)

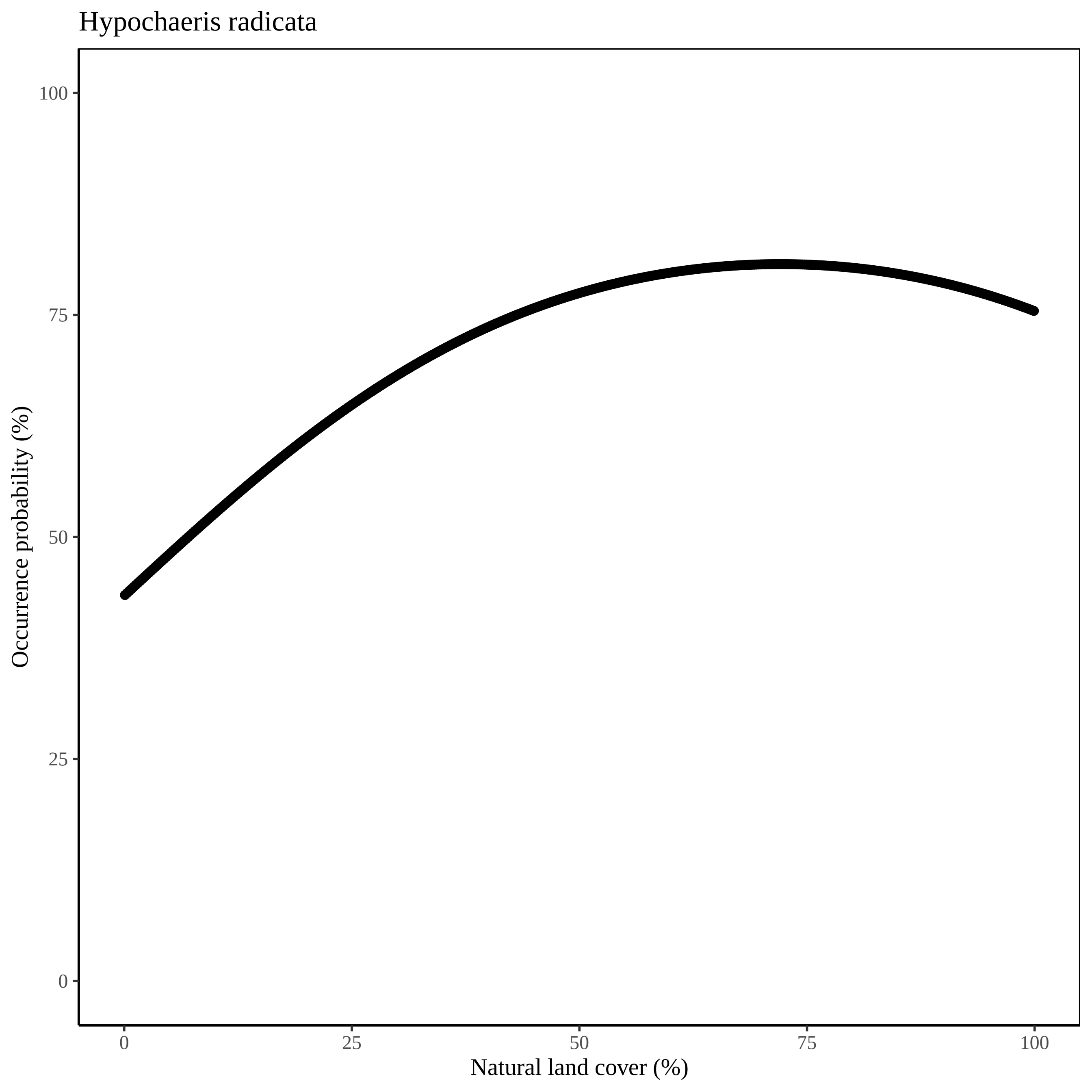
Hypochaeris radicata
Occurrence probability (%)
100
75
50
25
0
0
25
50
75
100
Natural land cover (%)

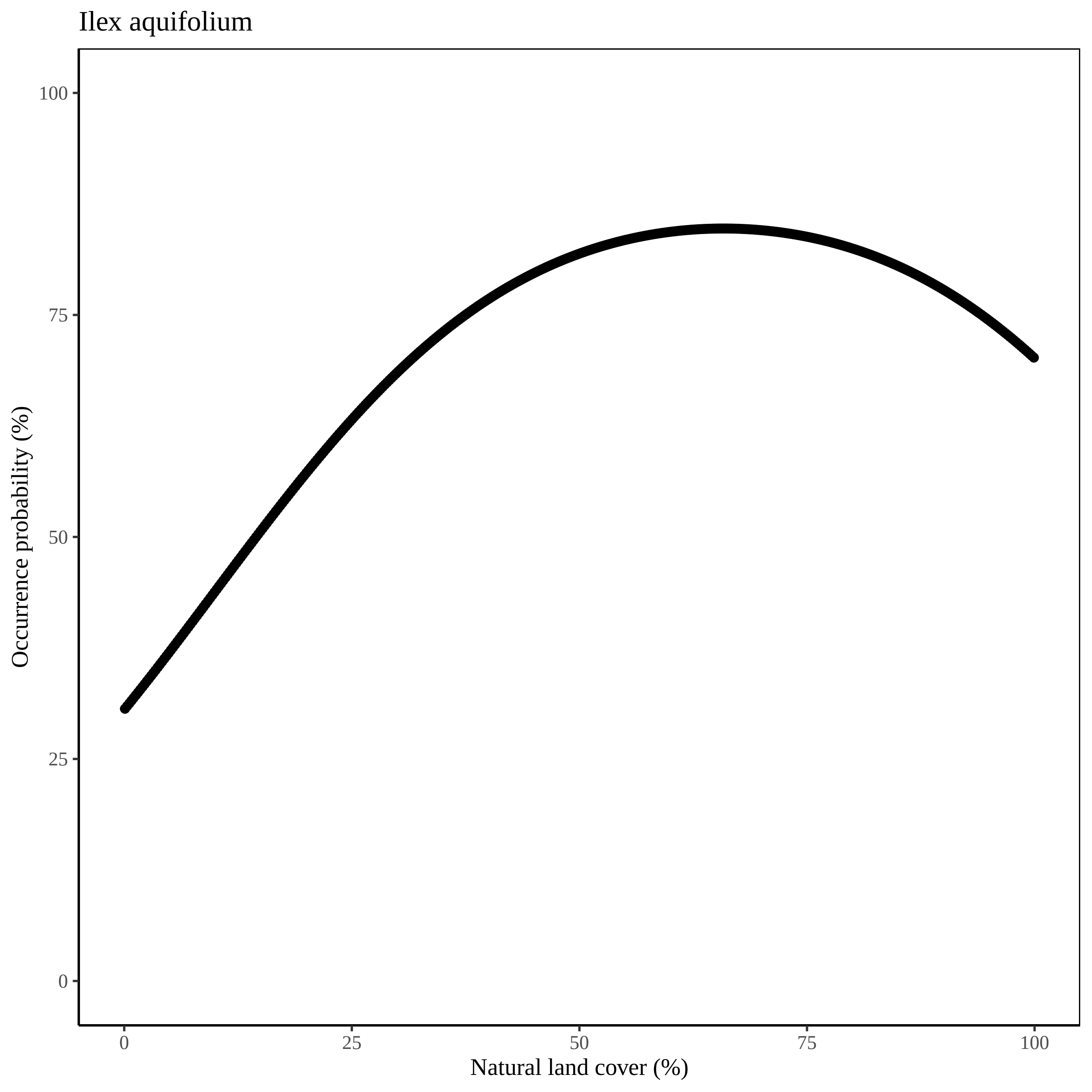
Ilex aquifolium
100
75
50
25
0
Occurrence probability (%)
0
25
50
75
100
Natural land cover (%)

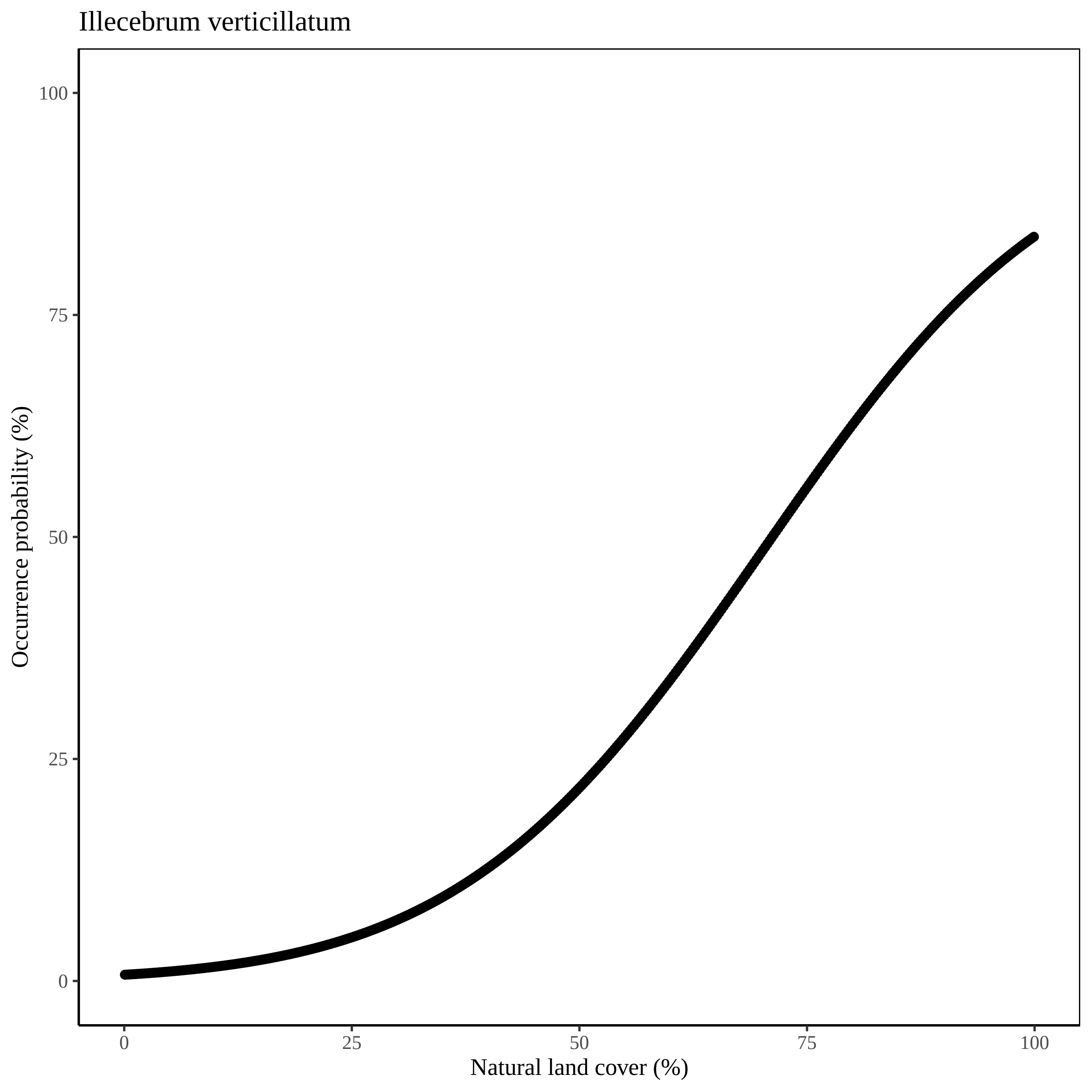

Illecebrum verticillatum
Occurrence probability (%)
100
75
50
25
0
0
25
50
75
100
Natural land cover (%)

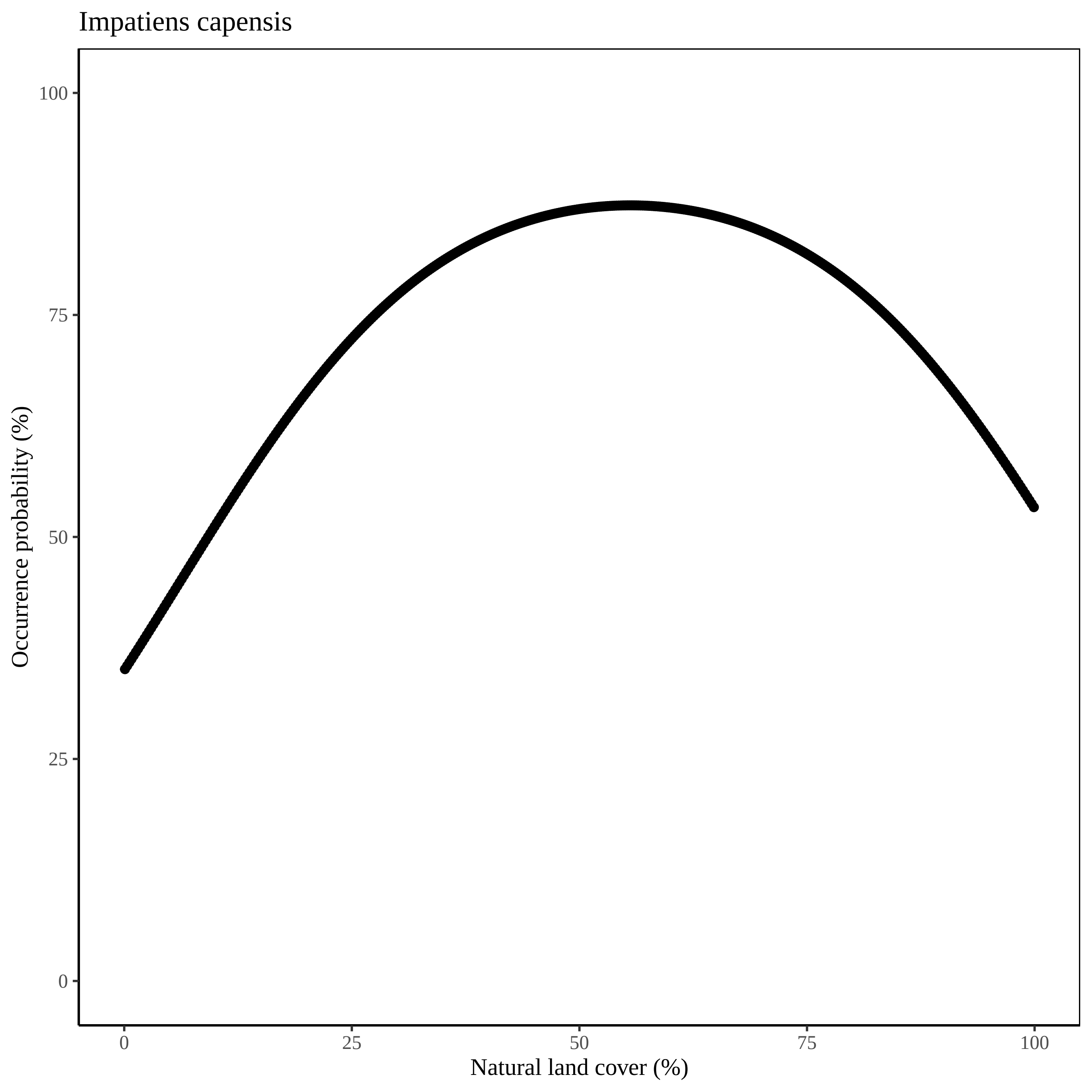
Impatiens capensis
Occurrence probability (%)
100
75
50
25
0
0
25
50
75
100
Natural land cover (%)

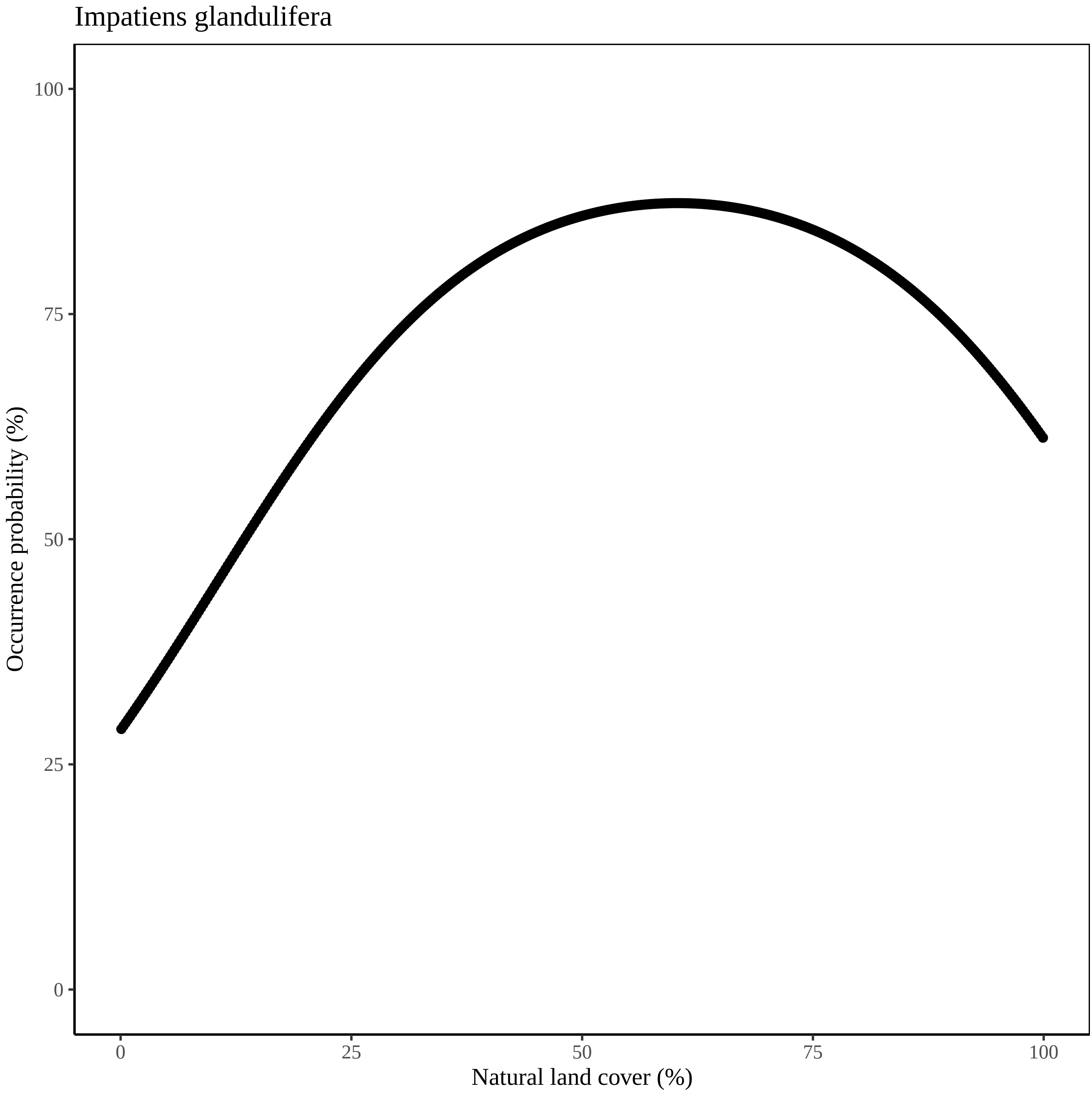

Impatiens glandulifera
100
75
50
25
0
Occurrence probability (%)
0
25
50
75
100
Natural land cover (%)

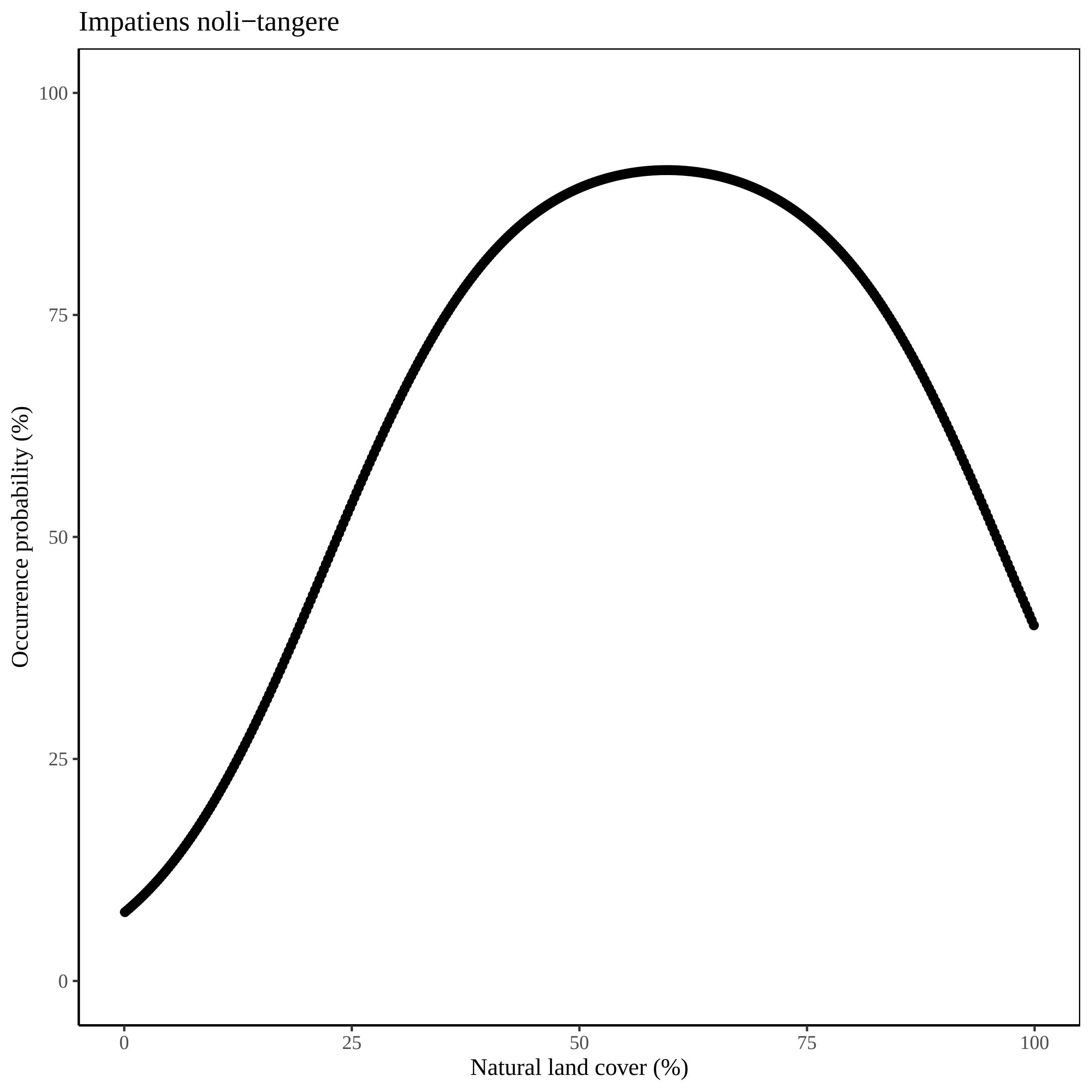

Impatiens noli−tangere
Occurrence probability (%)
100
75
50
25
0
0
25
50
75
100
Natural land cover (%)

Impatiens parviflora

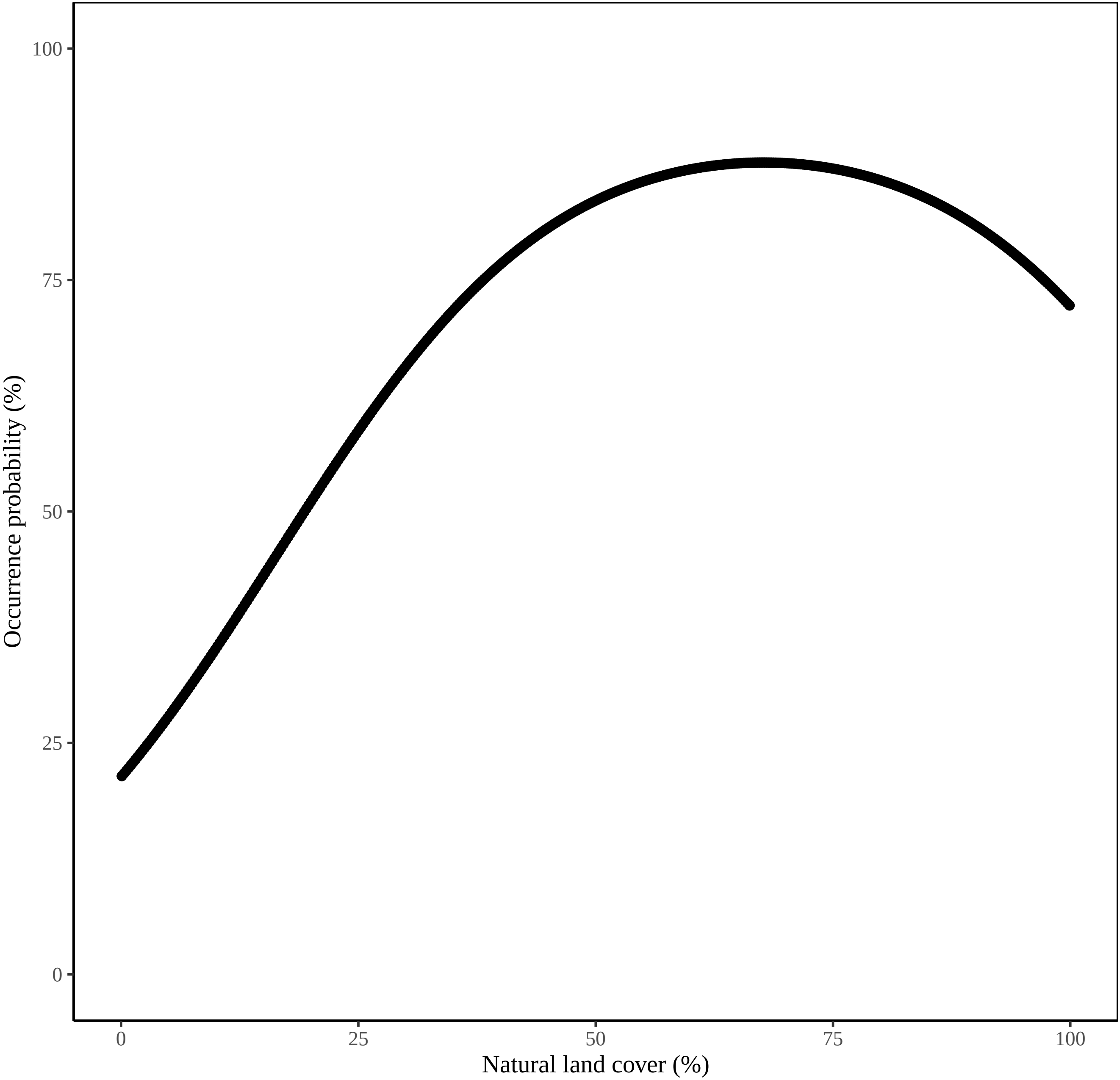

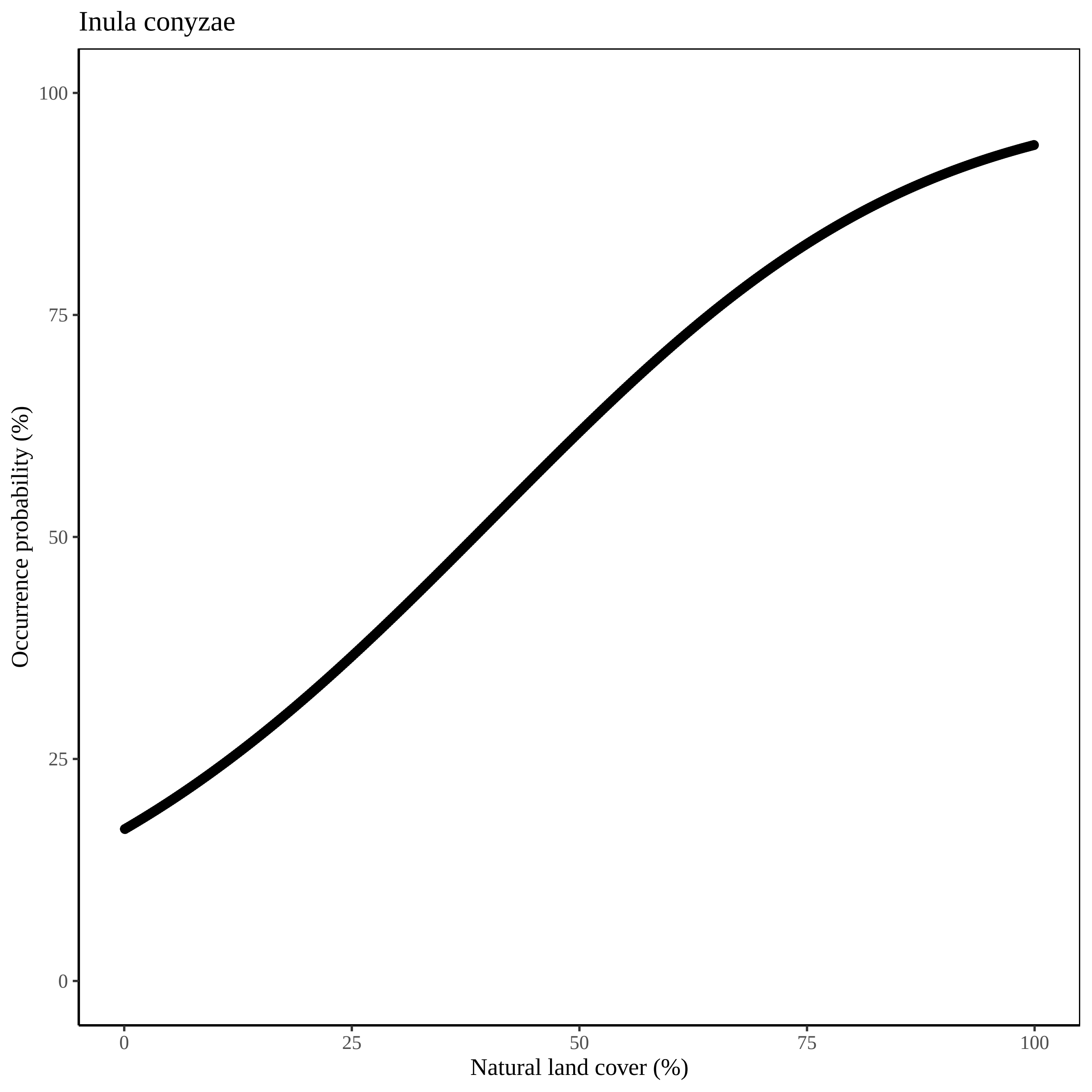

Inula conyzae
Occurrence probability (%)
100
75
50
25
0
0
25
50
75
100
Natural land cover (%)

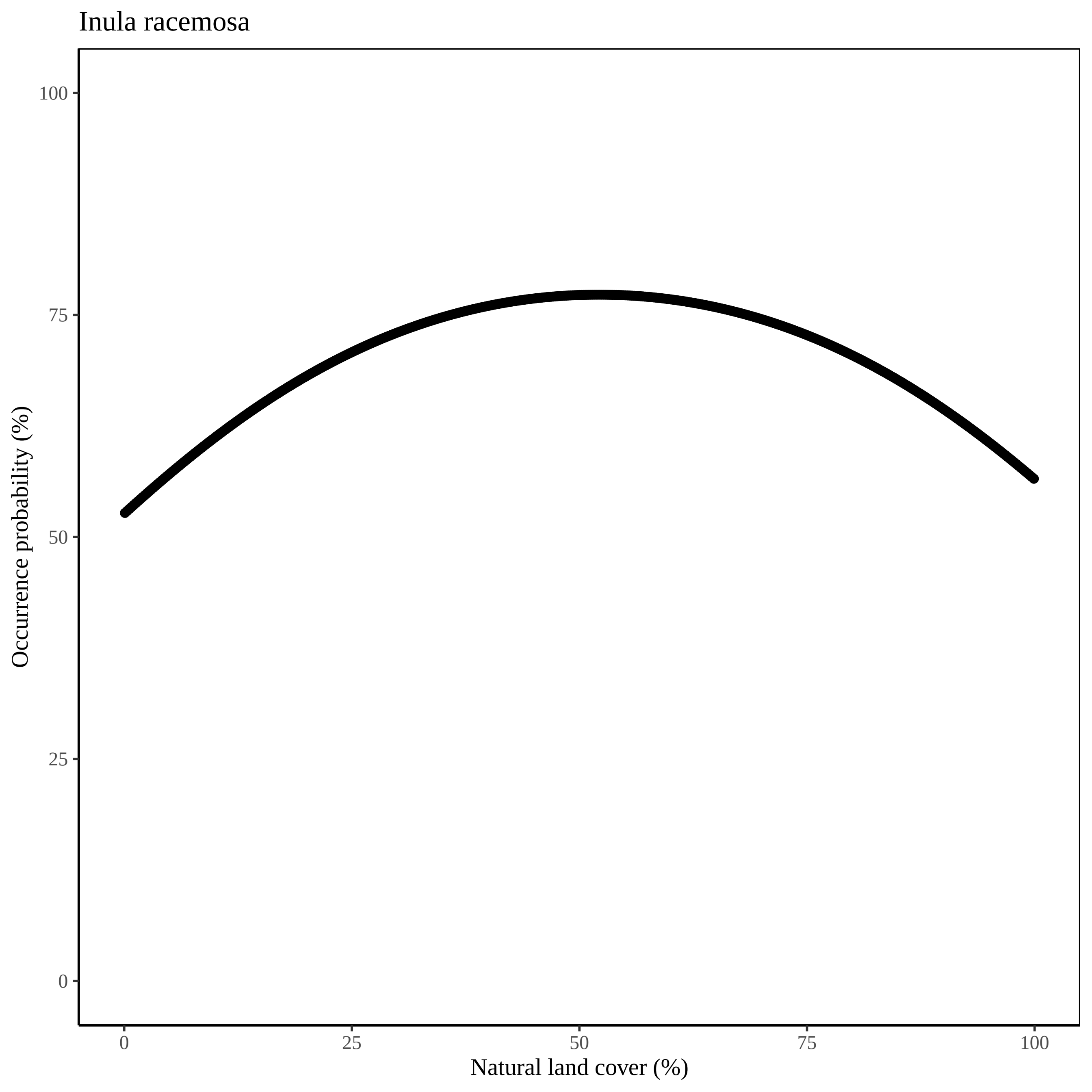

Inula racemosa
Occurrence probability (%)
100
75
50
25
0
0
25
50
75
100
Natural land cover (%)

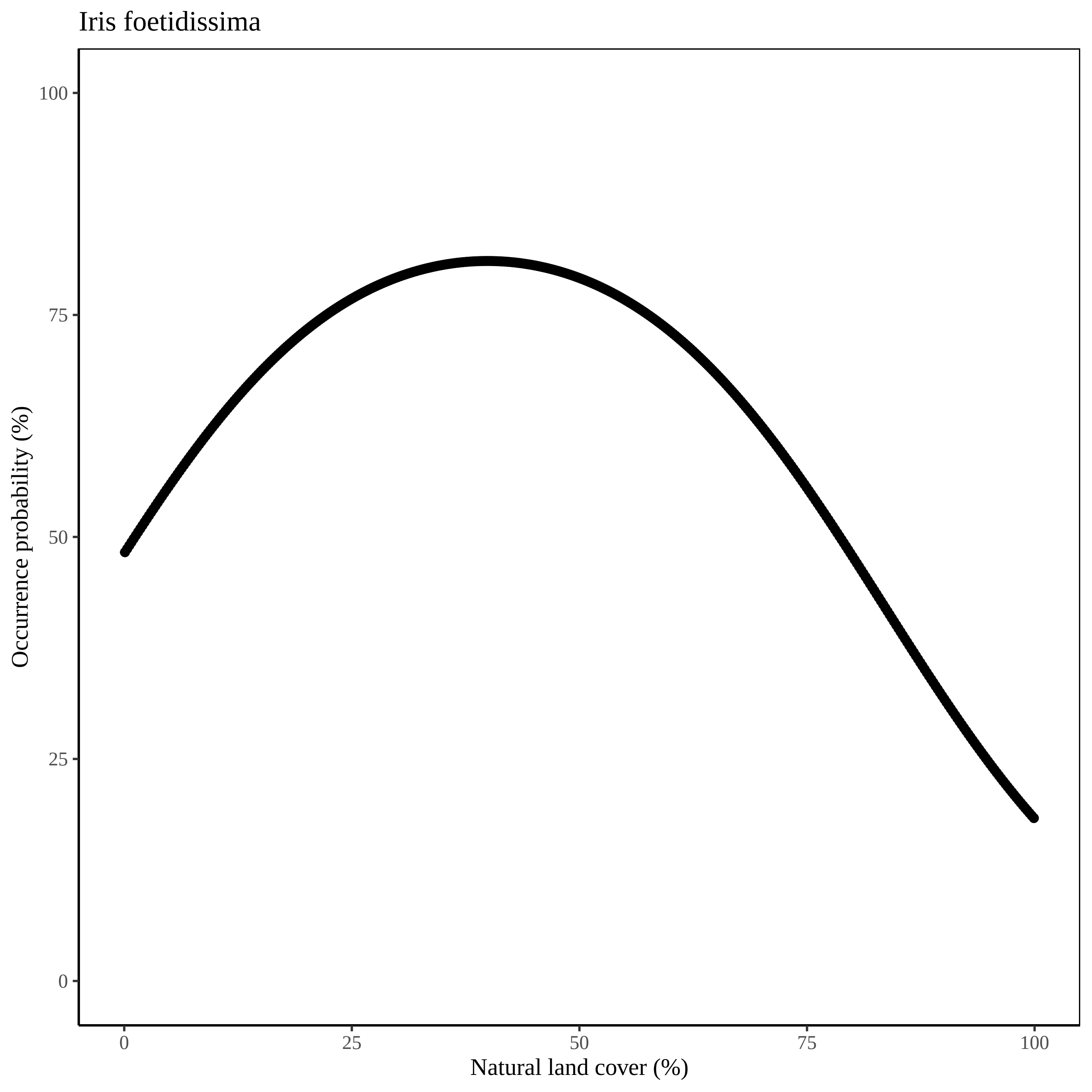
Iris foetidissima
Occurrence probability (%)
100
75
50
25
0
0
25
50
75
100
Natural land cover (%)

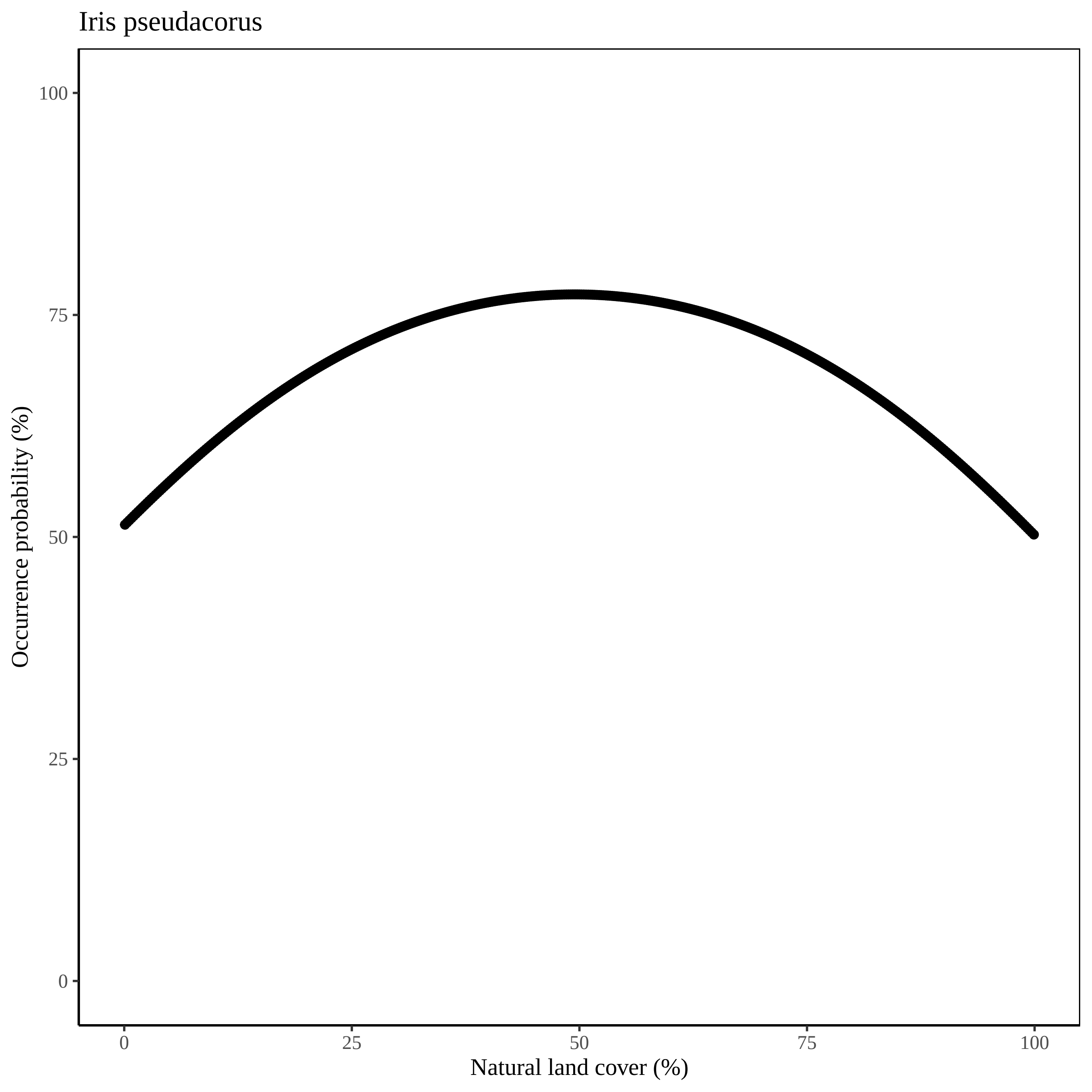
Iris pseudacorus
Occurrence probability (%)
100
75
50
25
0
0
25
50
75
100
Natural land cover (%)

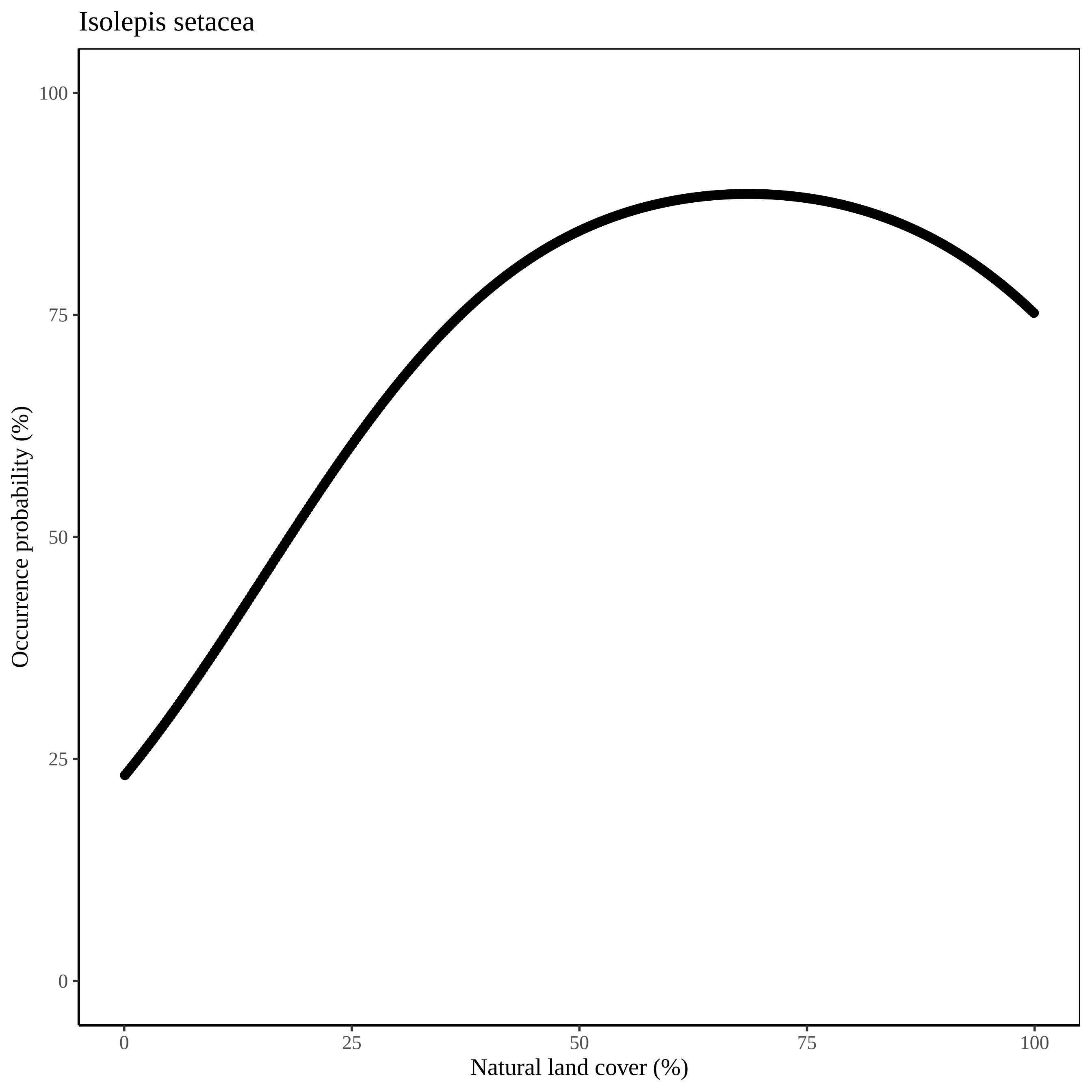
Isolepis setacea
Occurrence probability (%)
100
75
50
25
0
0
25
50
75
100
Natural land cover (%)

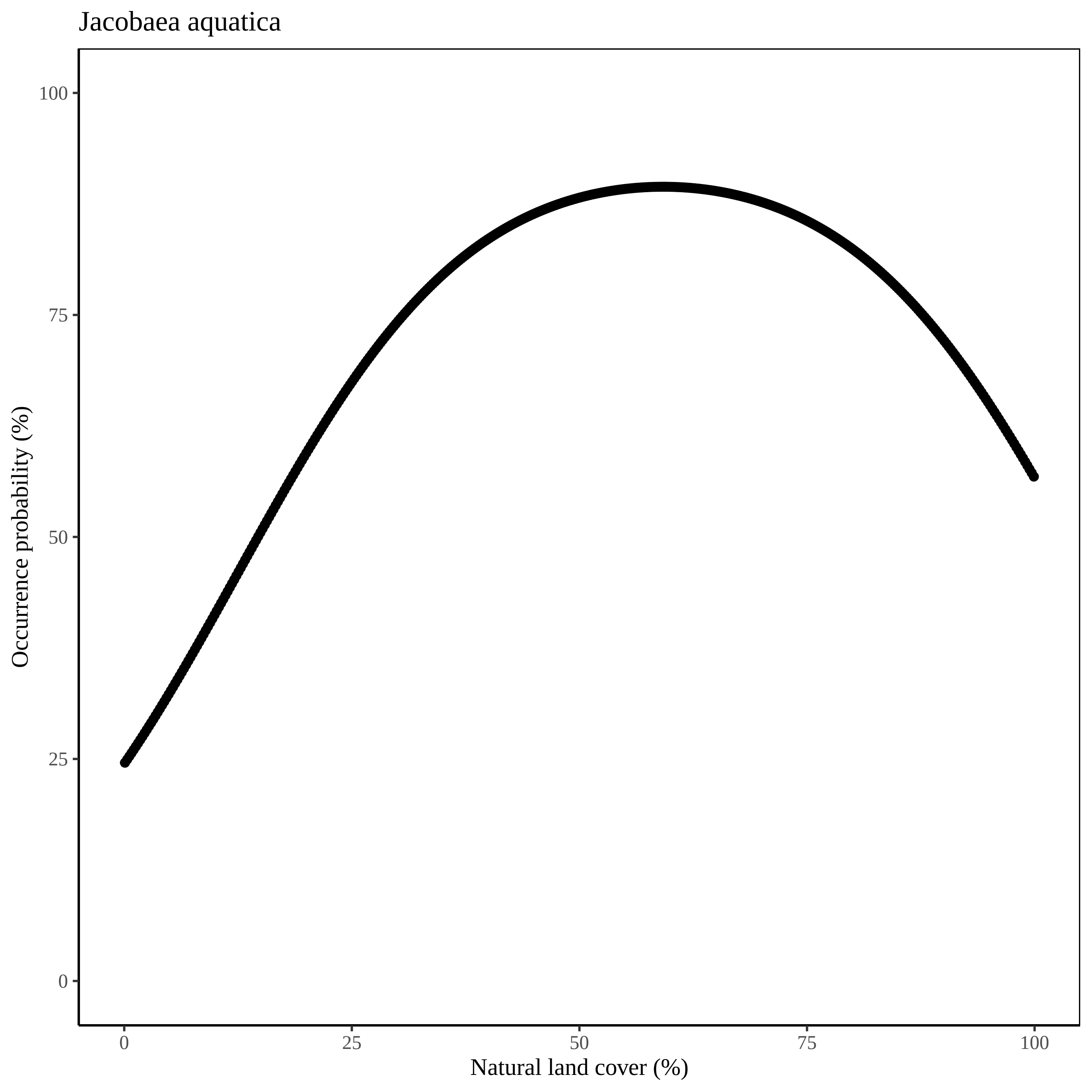

Jacobaea aquatica
100
75
50
25
0
Occurrence probability (%)
0
25
50
75
100
Natural land cover (%)

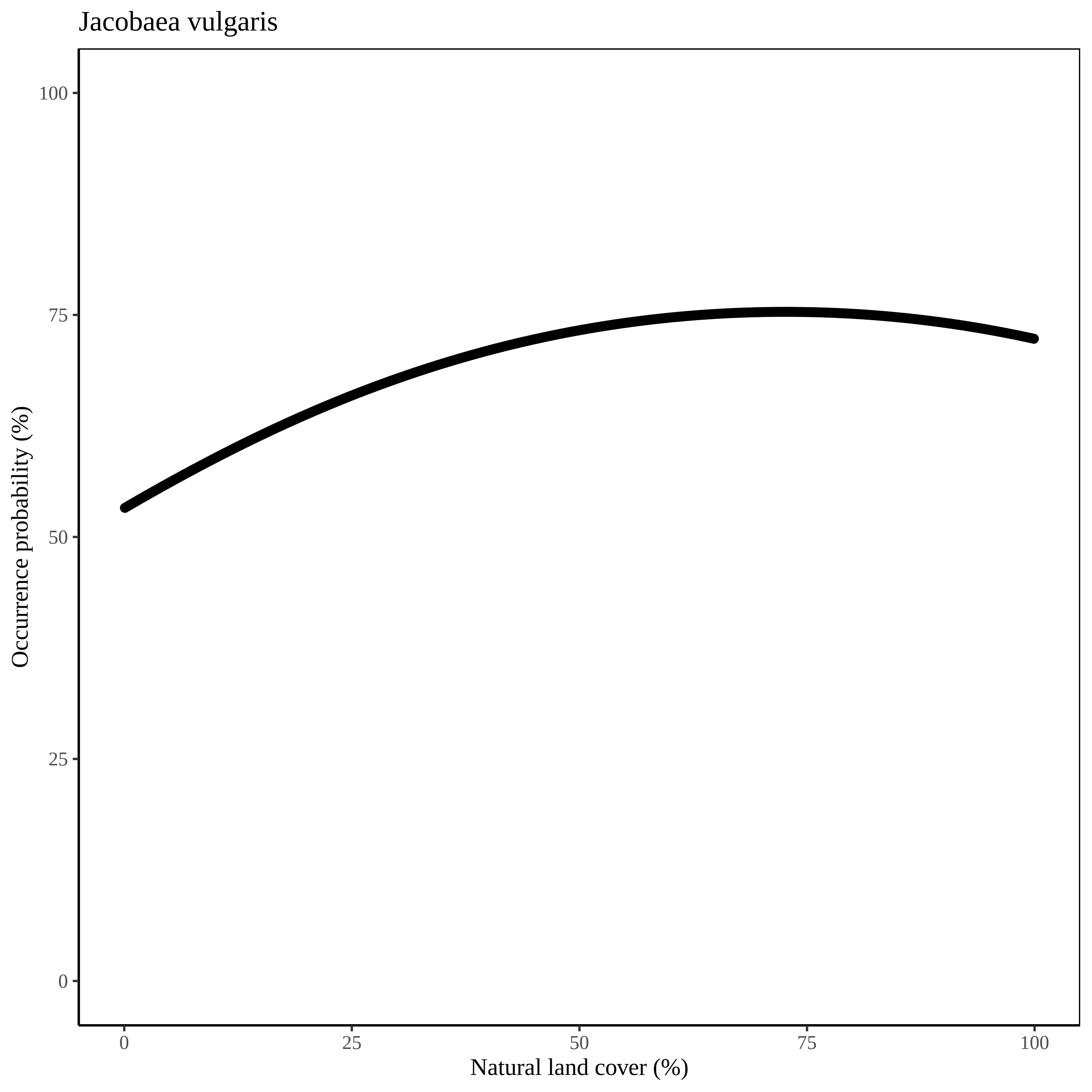

Jacobaea vulgaris
Occurrence probability (%)
100
75
50
25
0
0
25
50
75
100
Natural land cover (%)

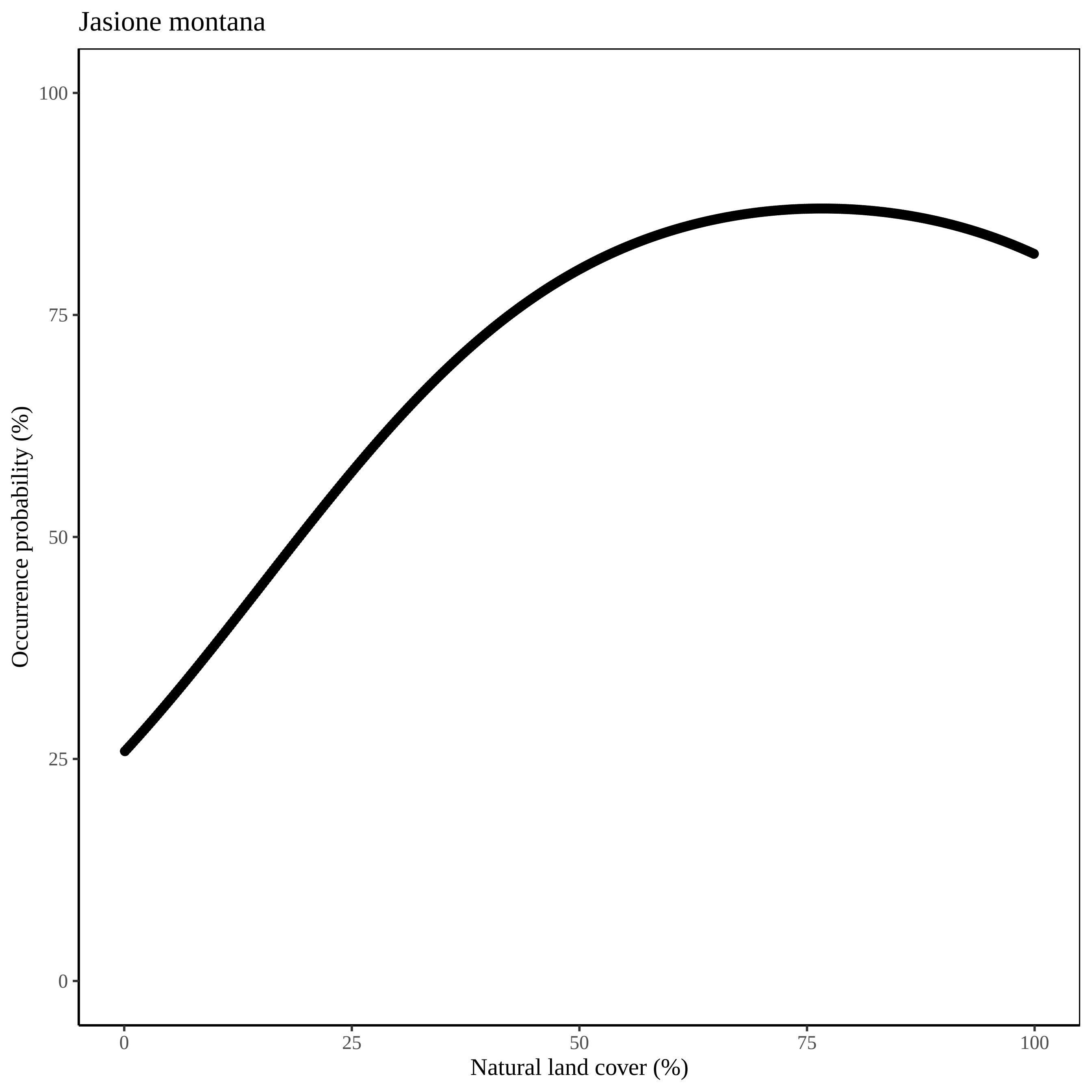

Jasione montana
Occurrence probability (%)
100
75
50
25
0
0
25
50
75
100
Natural land cover (%)

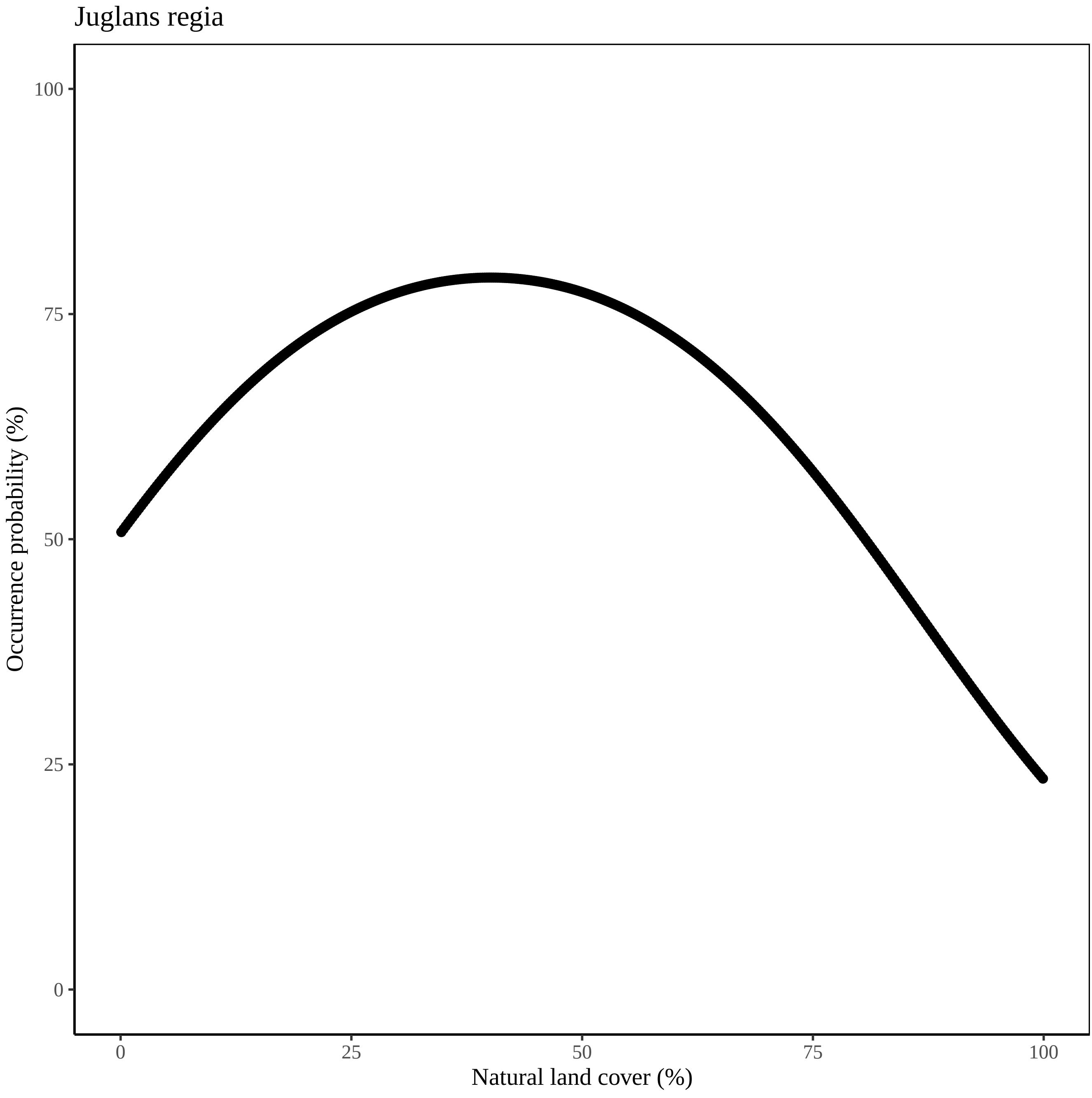

Juglans regia
Occurrence probability (%)
100
75
50
25
0
0
25
50
75
100
Natural land cover (%)

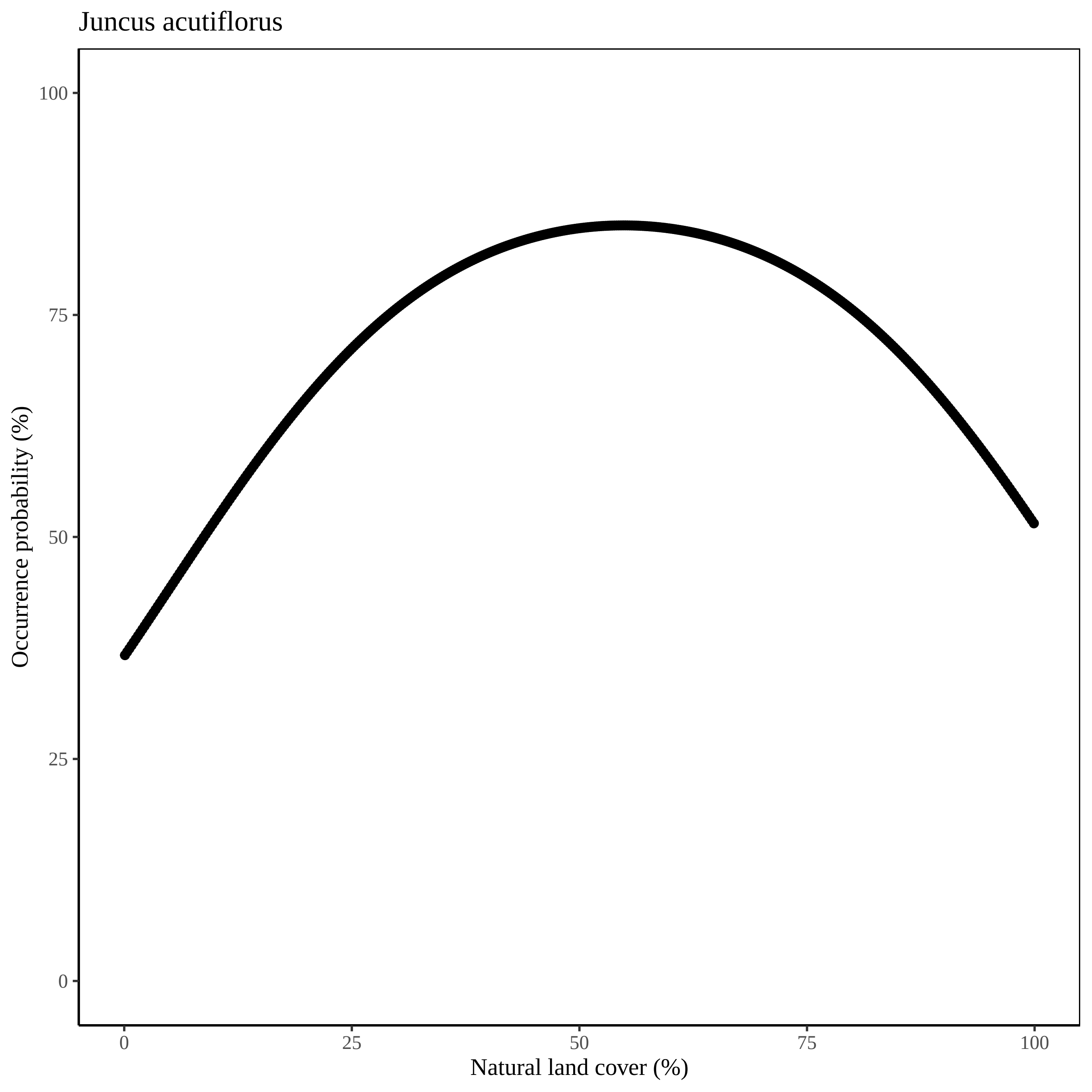

Juncus acutiflorus
Occurrence probability (%)
100
75
50
25
0
0
25
50
75
100
Natural land cover (%)

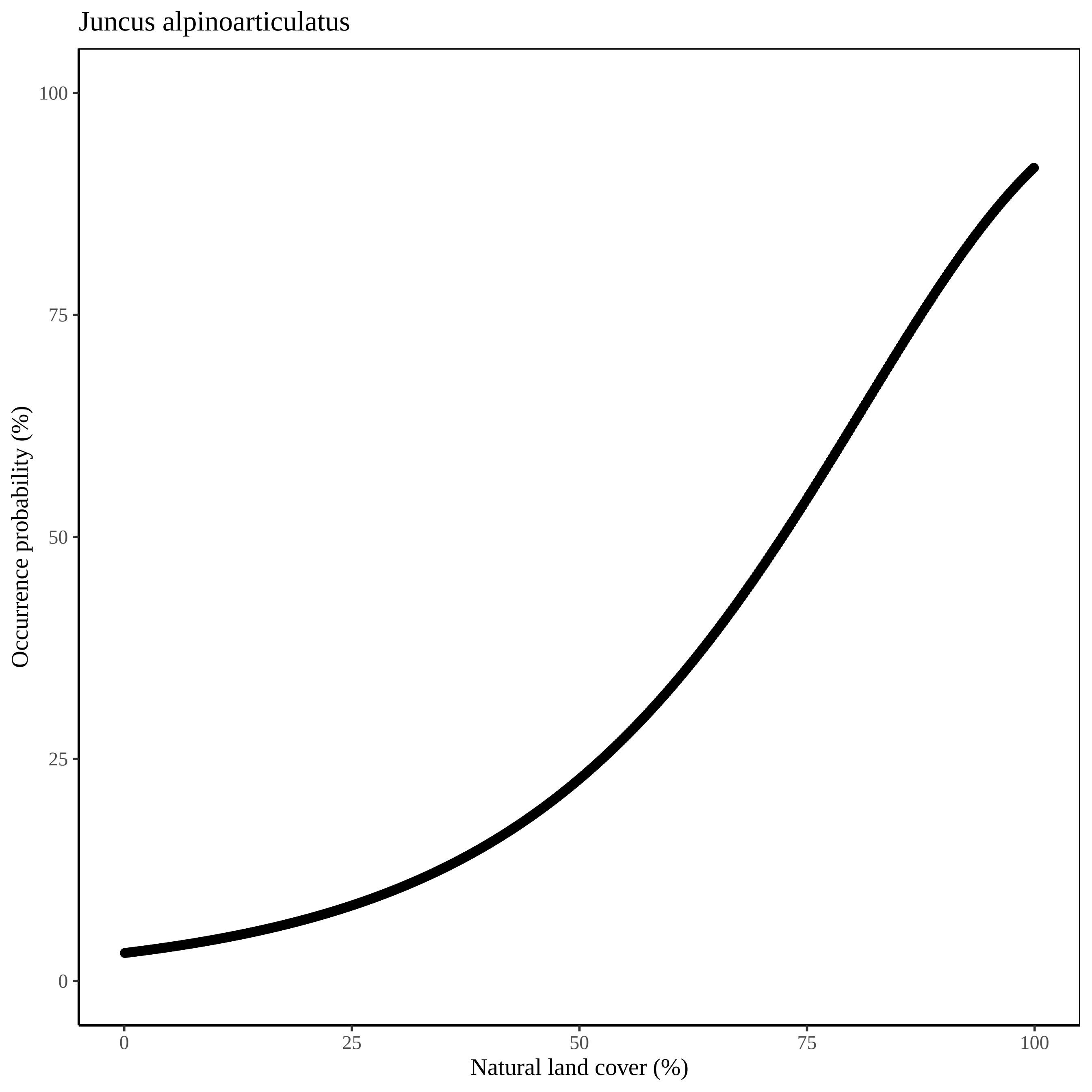
Juncus alpinoarticulatus
Occurrence probability (%)
100
75
50
25
0
0
25
50
75
100
Natural land cover (%)

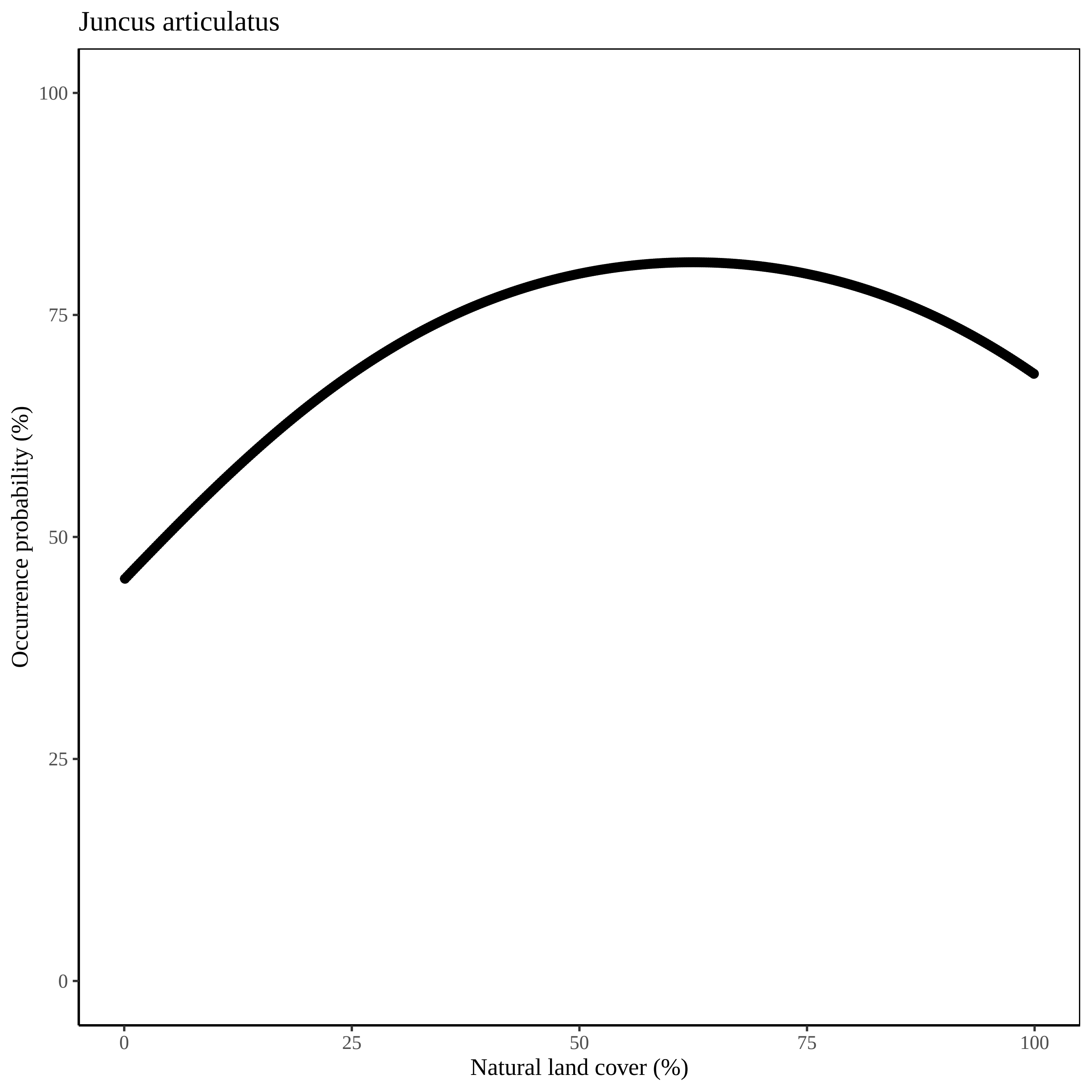

Juncus articulatus
100
75
50
25
0
Occurrence probability (%)
0
25
50
75
100
Natural land cover (%)

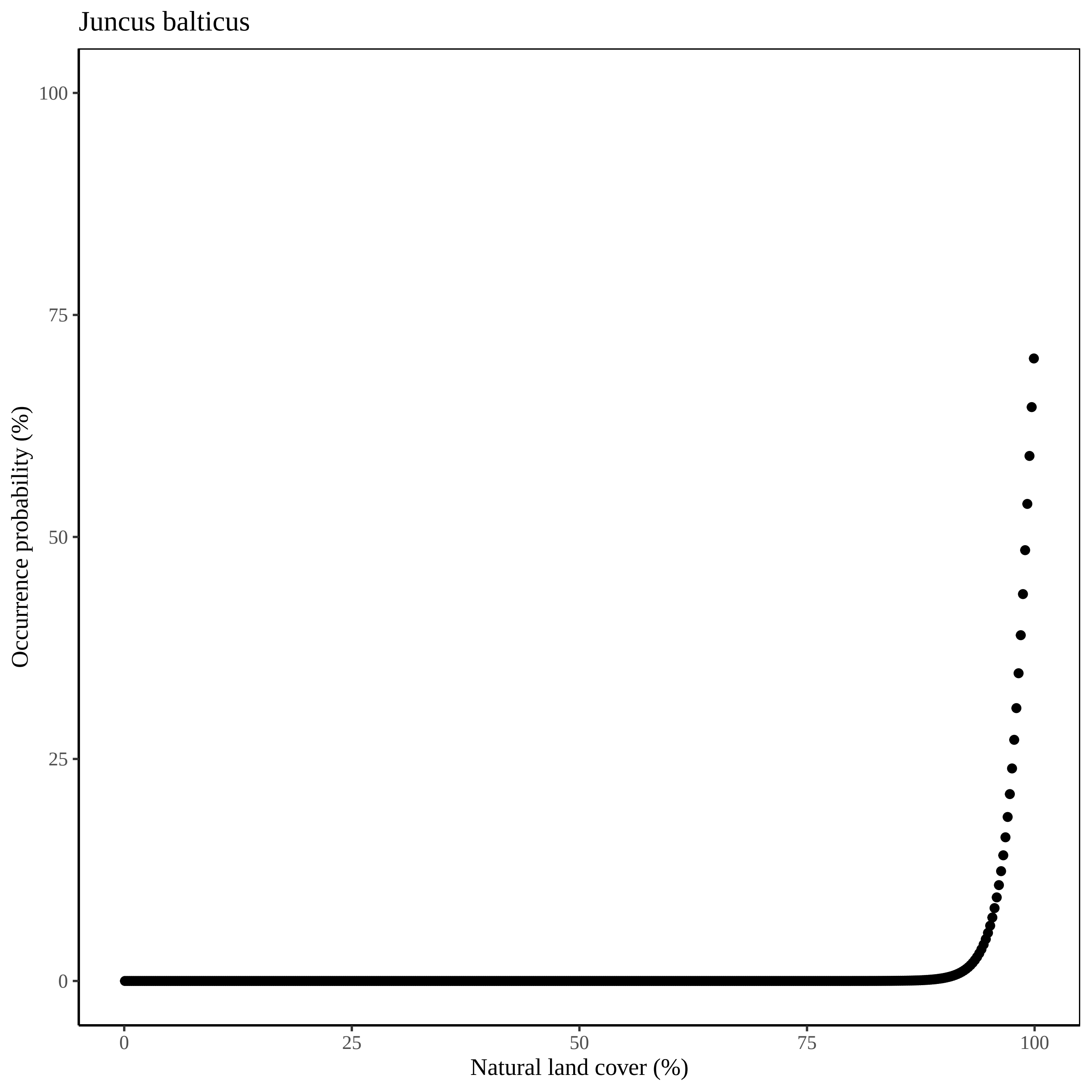

Juncus balticus
Occurrence probability (%)
100
75
50
25
0
Natural land cover (%)
0
25
50
75
100

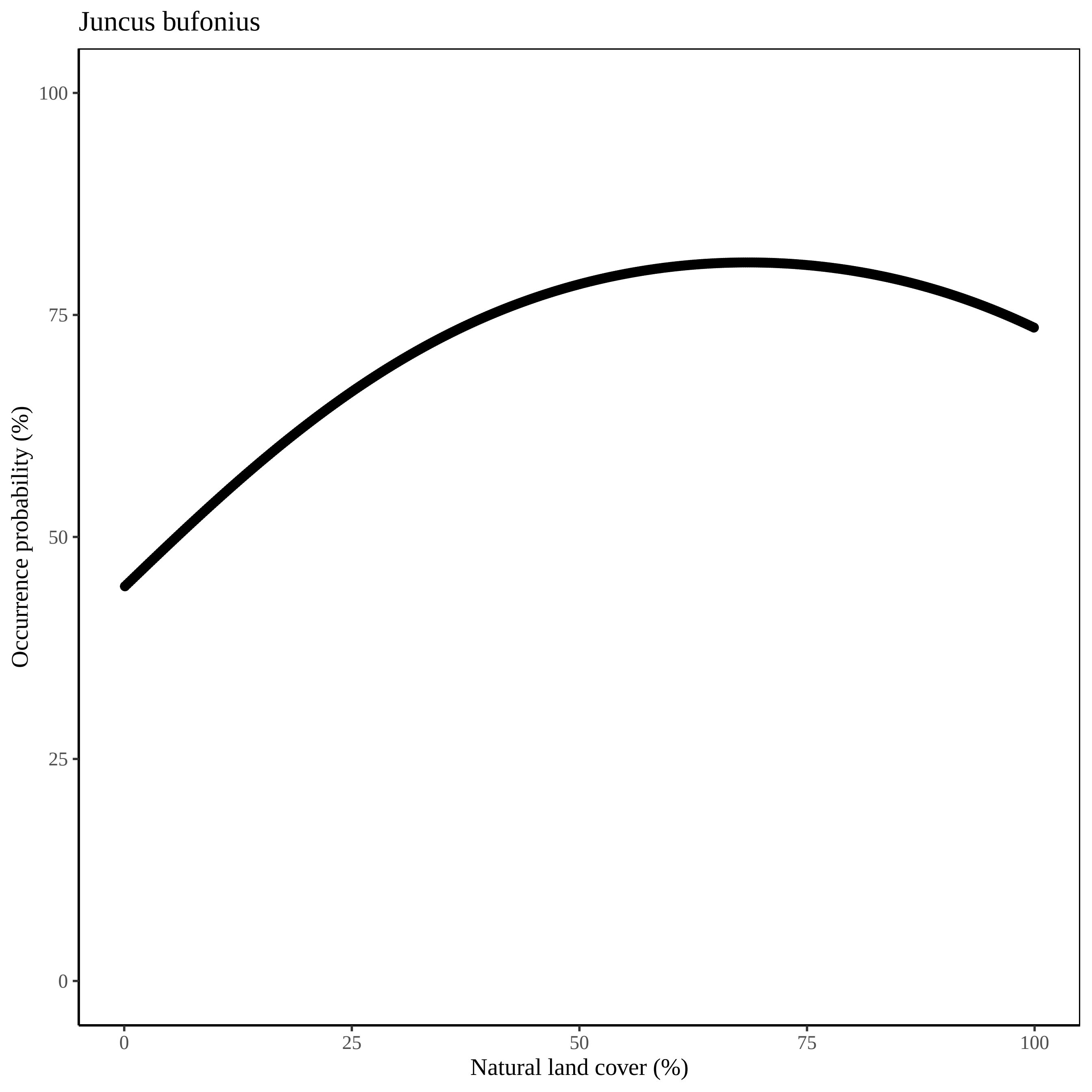

Juncus bufonius
Occurrence probability (%)
100
75
50
25
0
0
25
50
75
100
Natural land cover (%)

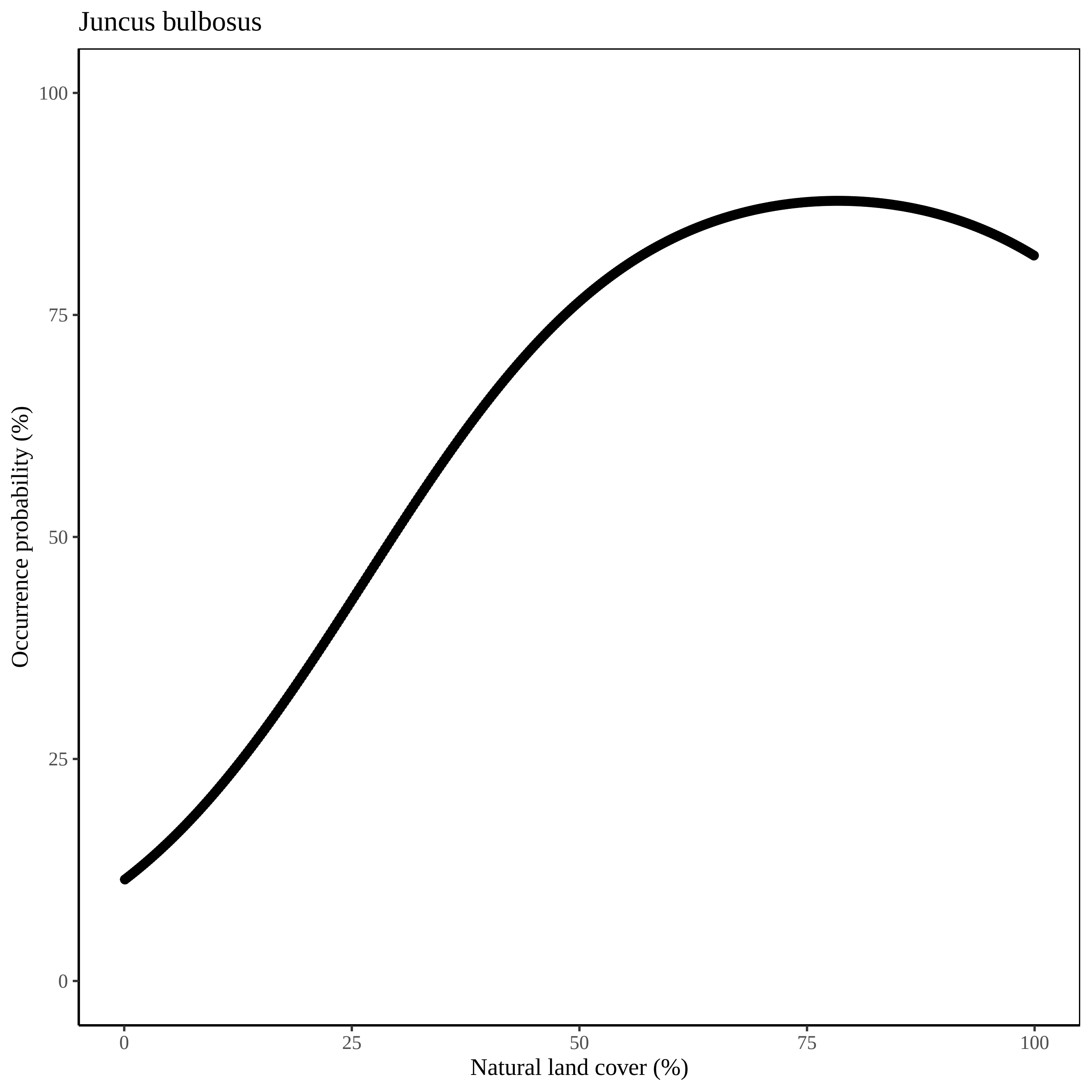
Juncus bulbosus
Occurrence probability (%)
100
75
50
25
0
0
25
50
75
100
Natural land cover (%)

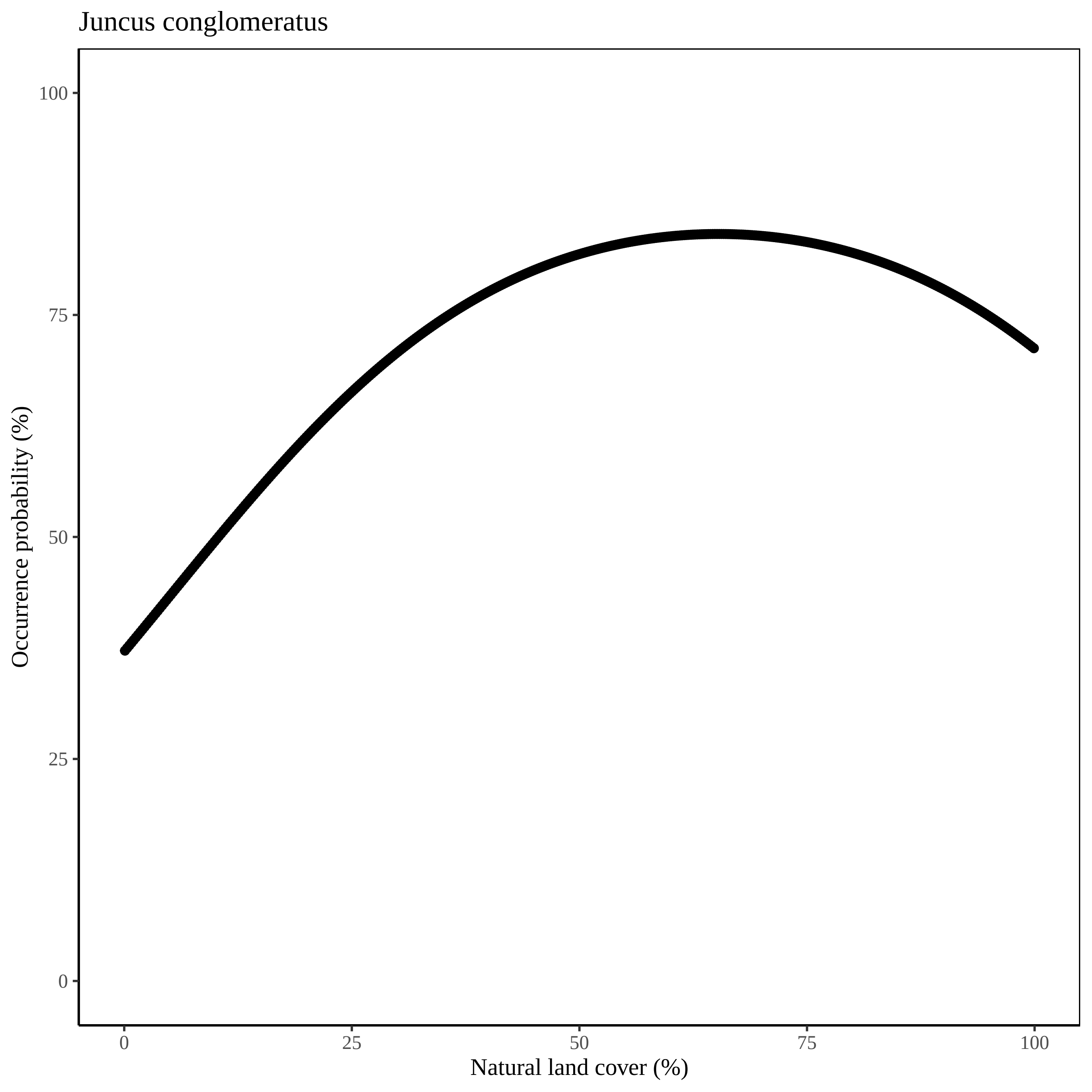

Juncus conglomeratus
Occurrence probability (%)
100
75
50
25
0
0
25
50
75
100
Natural land cover (%)

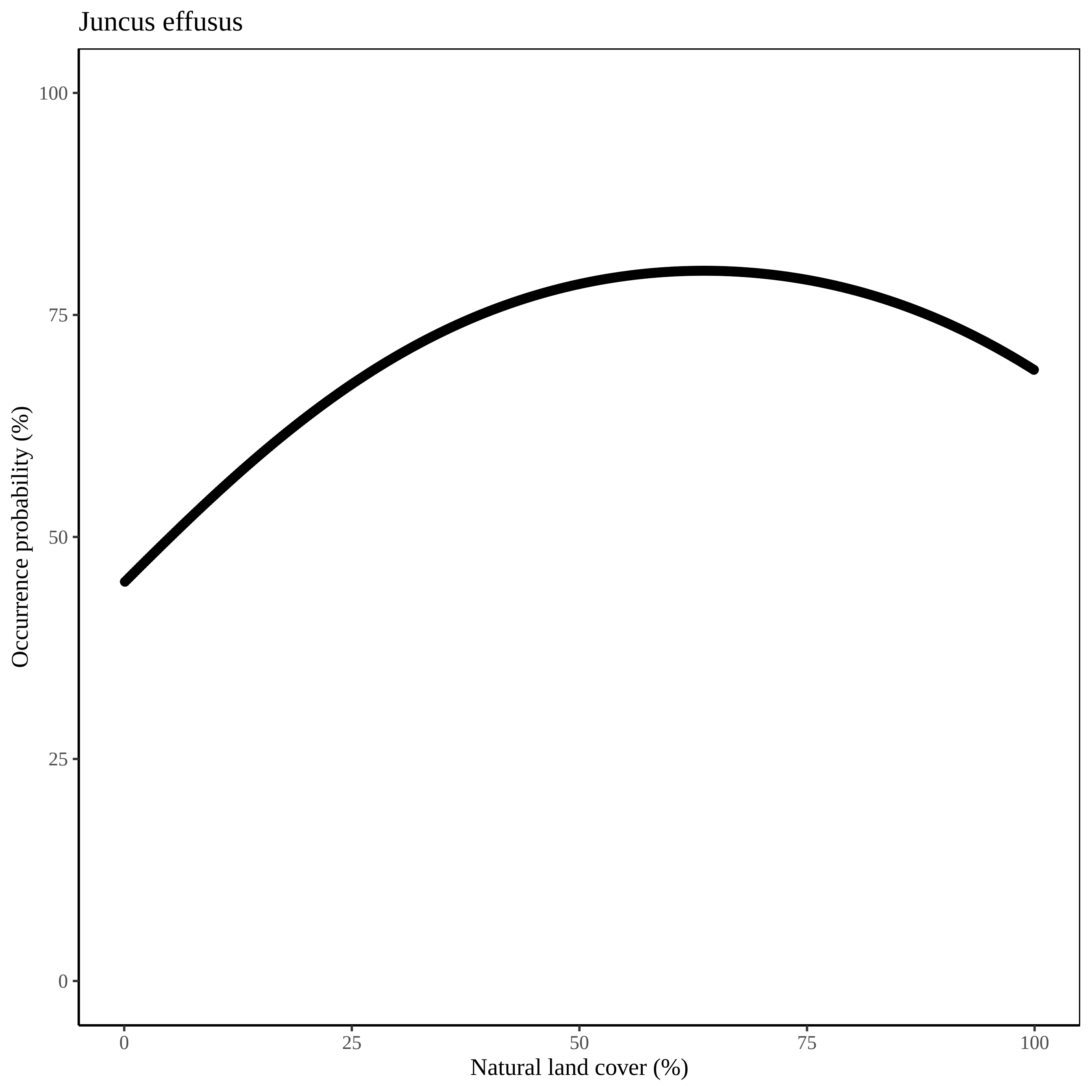
Juncus effusus
100
75
50
25
0
Occurrence probability (%)
0
25
50
75
100
Natural land cover (%)

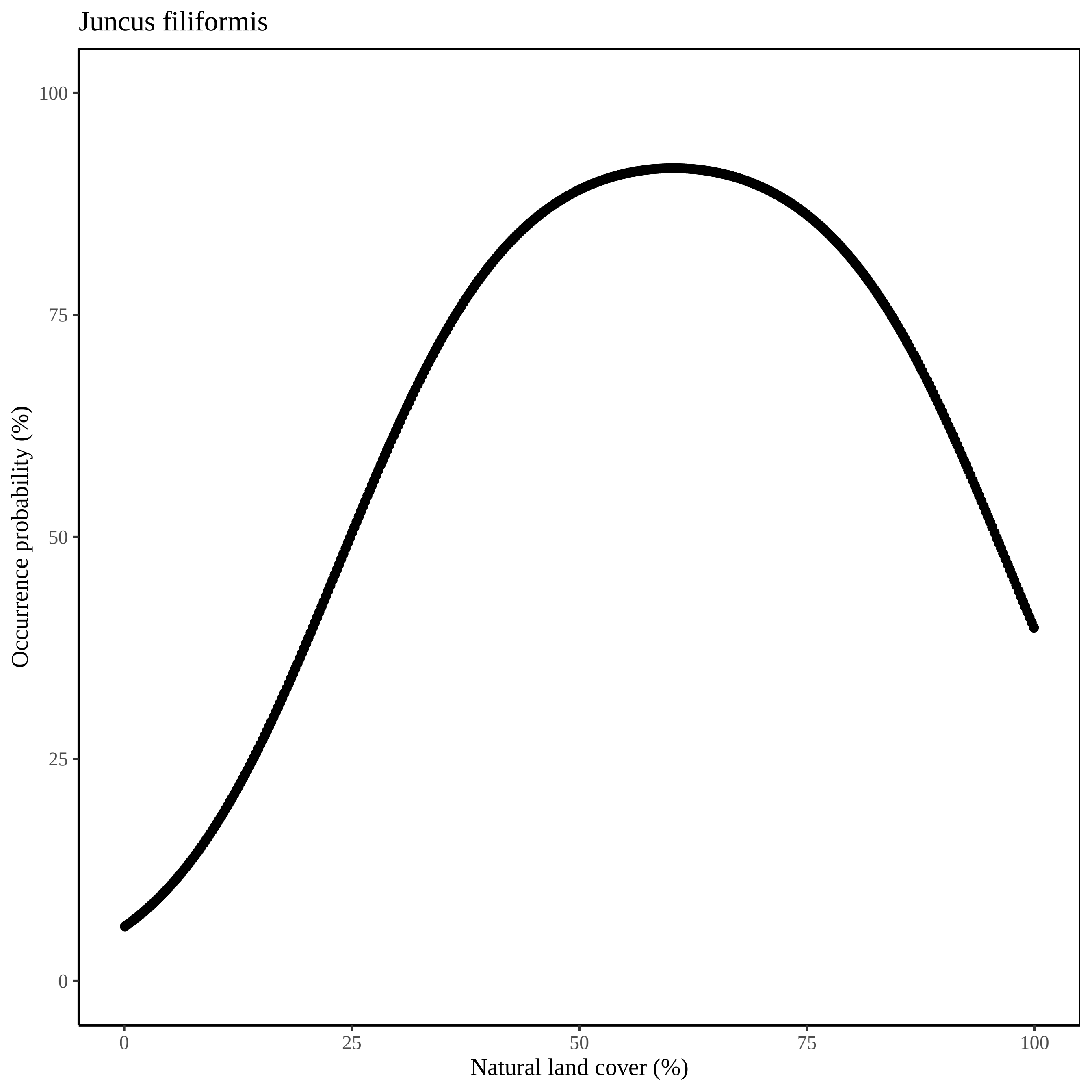
Juncus filiformis
Occurrence probability (%)
100
75
50
25
0
Natural land cover (%)
0
25
50
75
100

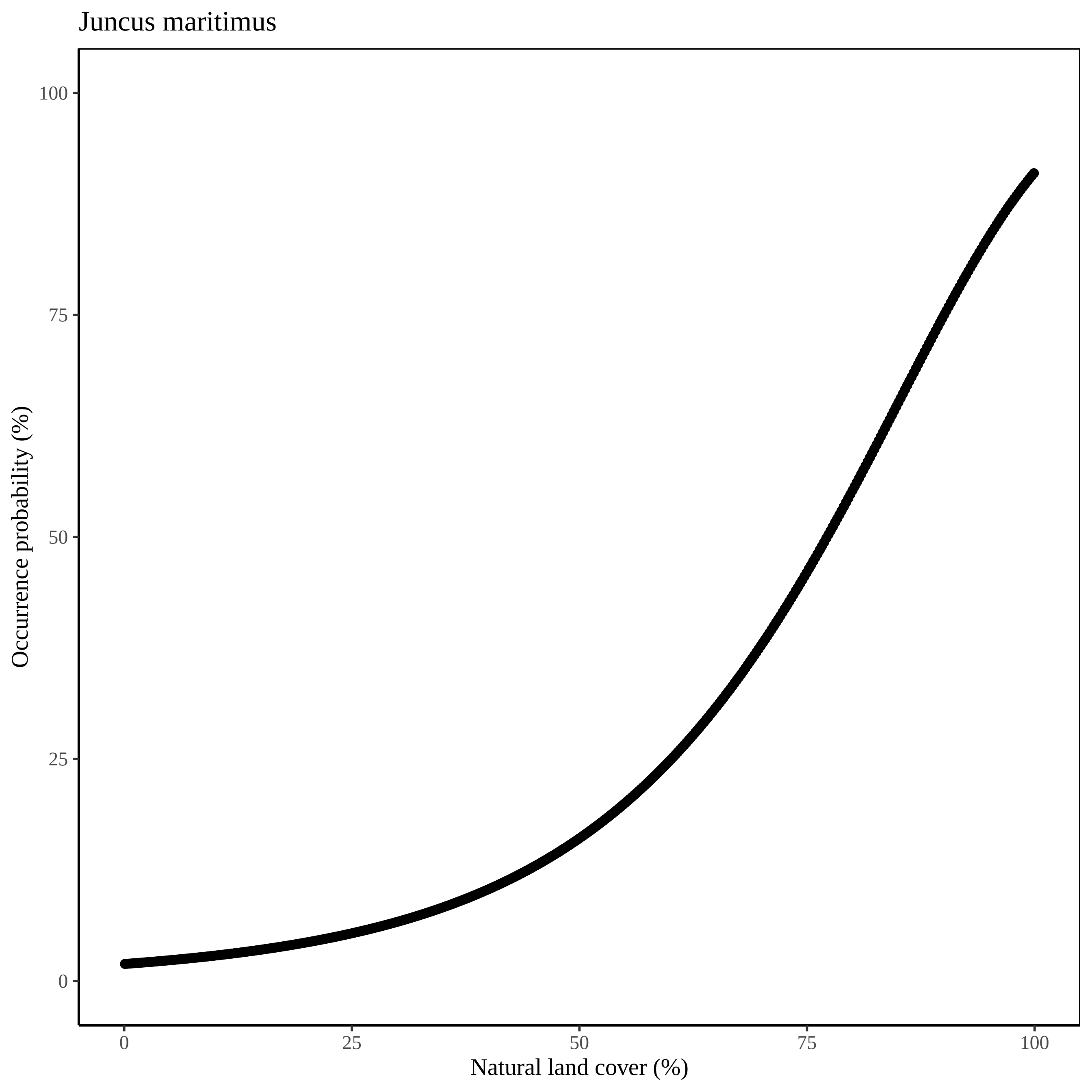

Juncus maritimus
Occurrence probability (%)
100
75
50
25
0
0
25
50
75
100
Natural land cover (%)

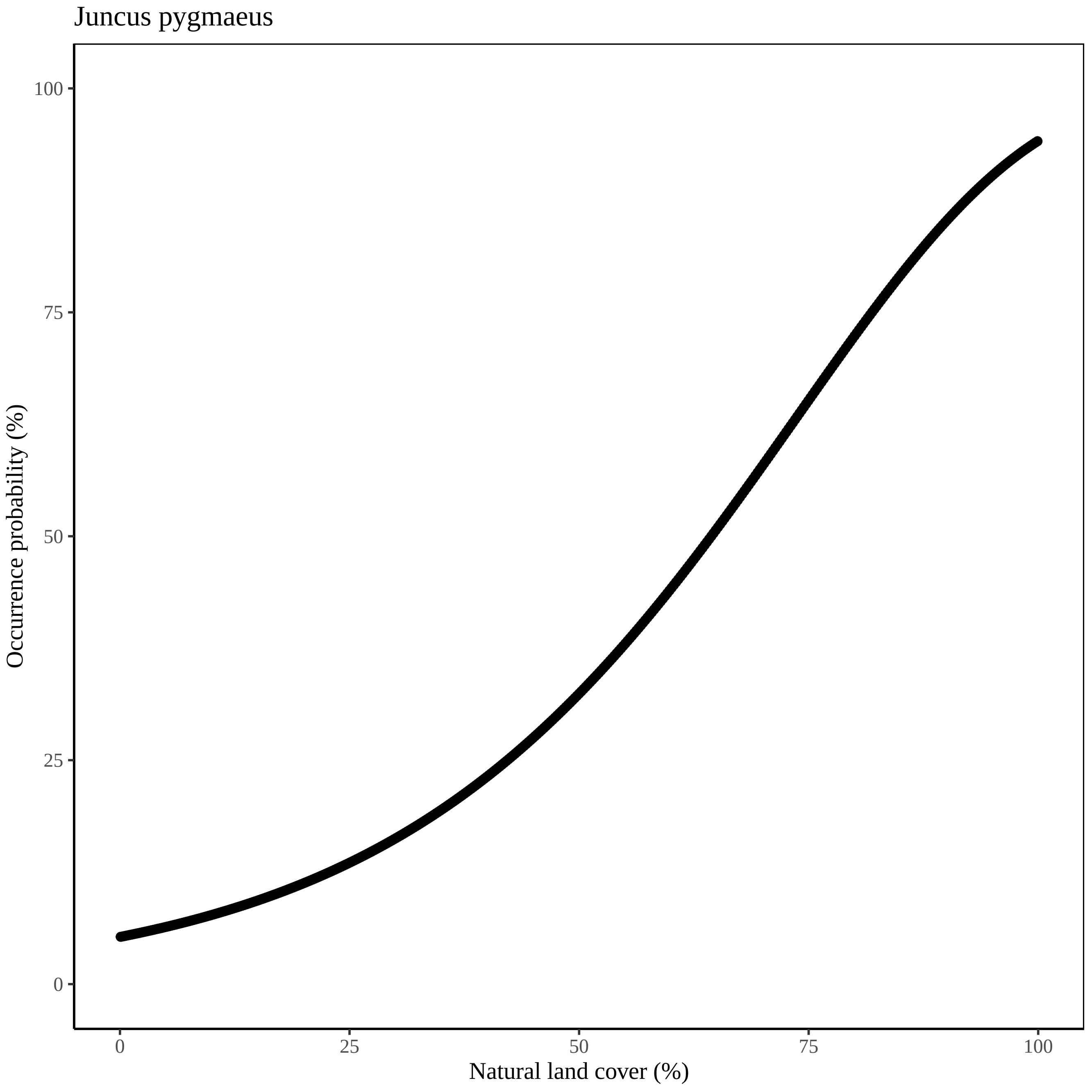
Juncus pygmaeus
Occurrence probability (%)
100
75
50
25
0
0
25
50
75
100
Natural land cover (%)

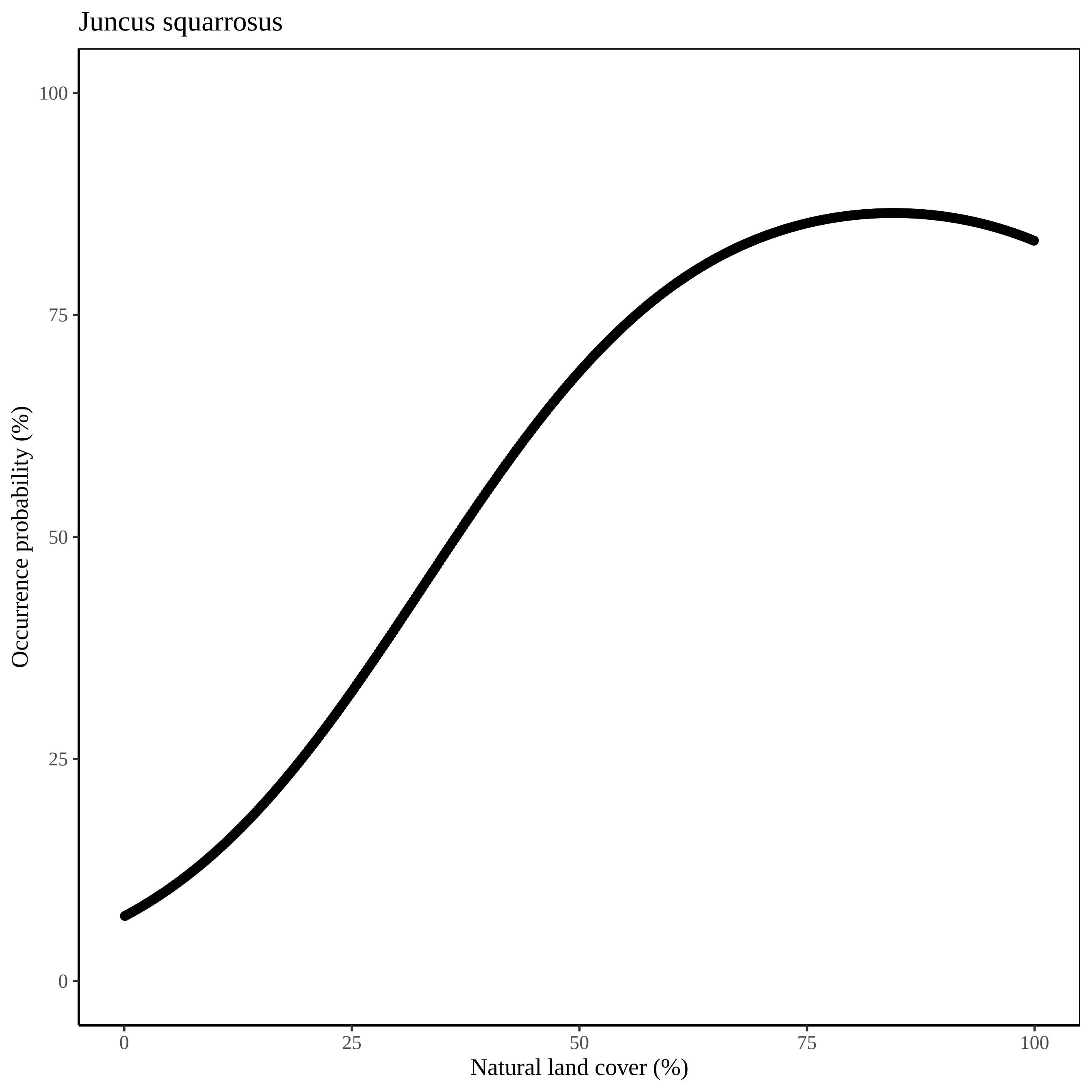
Juncus squarrosus
Occurrence probability (%)
100
75
50
25
0
0
25
50
75
100
Natural land cover (%)

Juncus subnodulosus

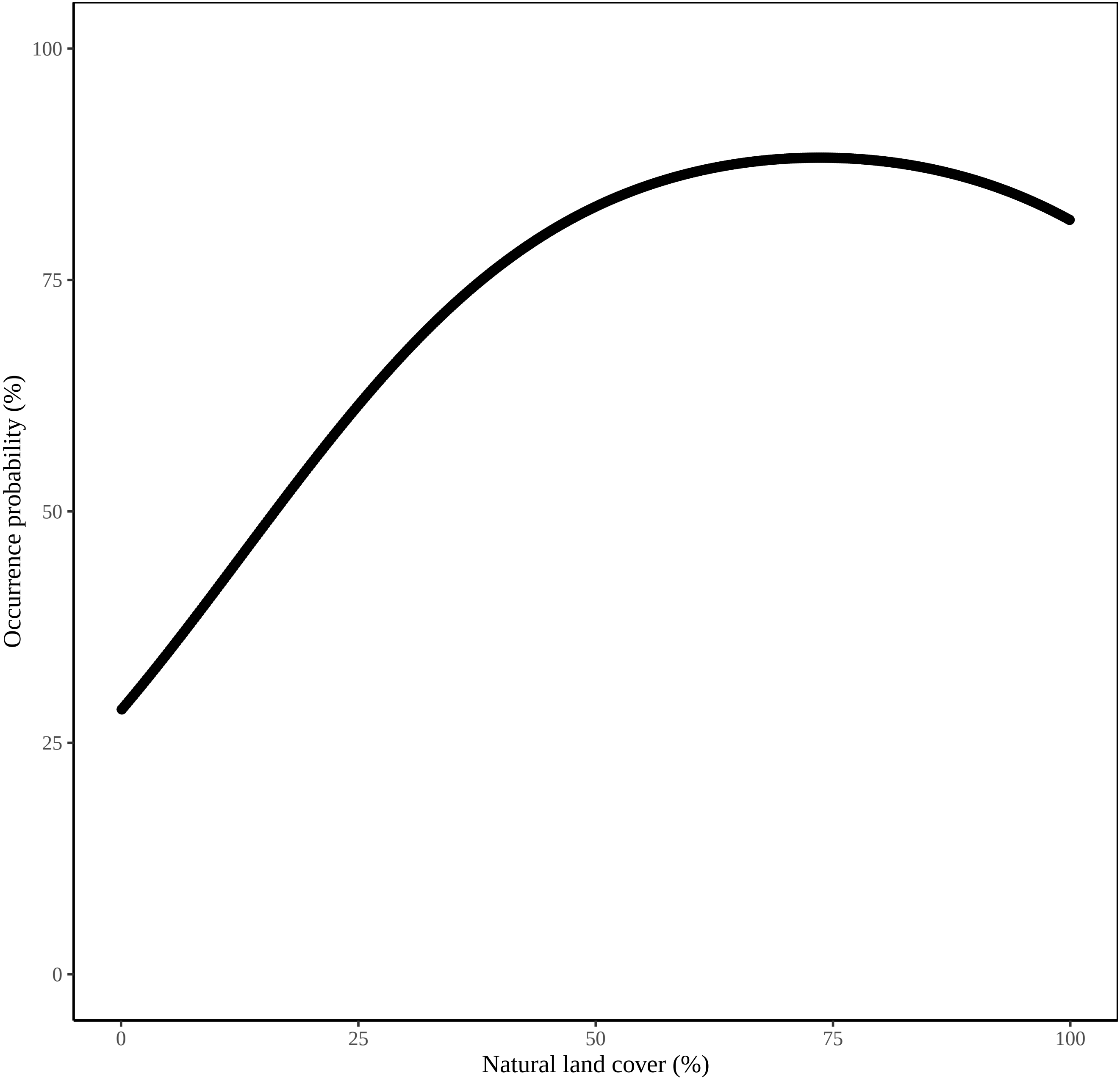

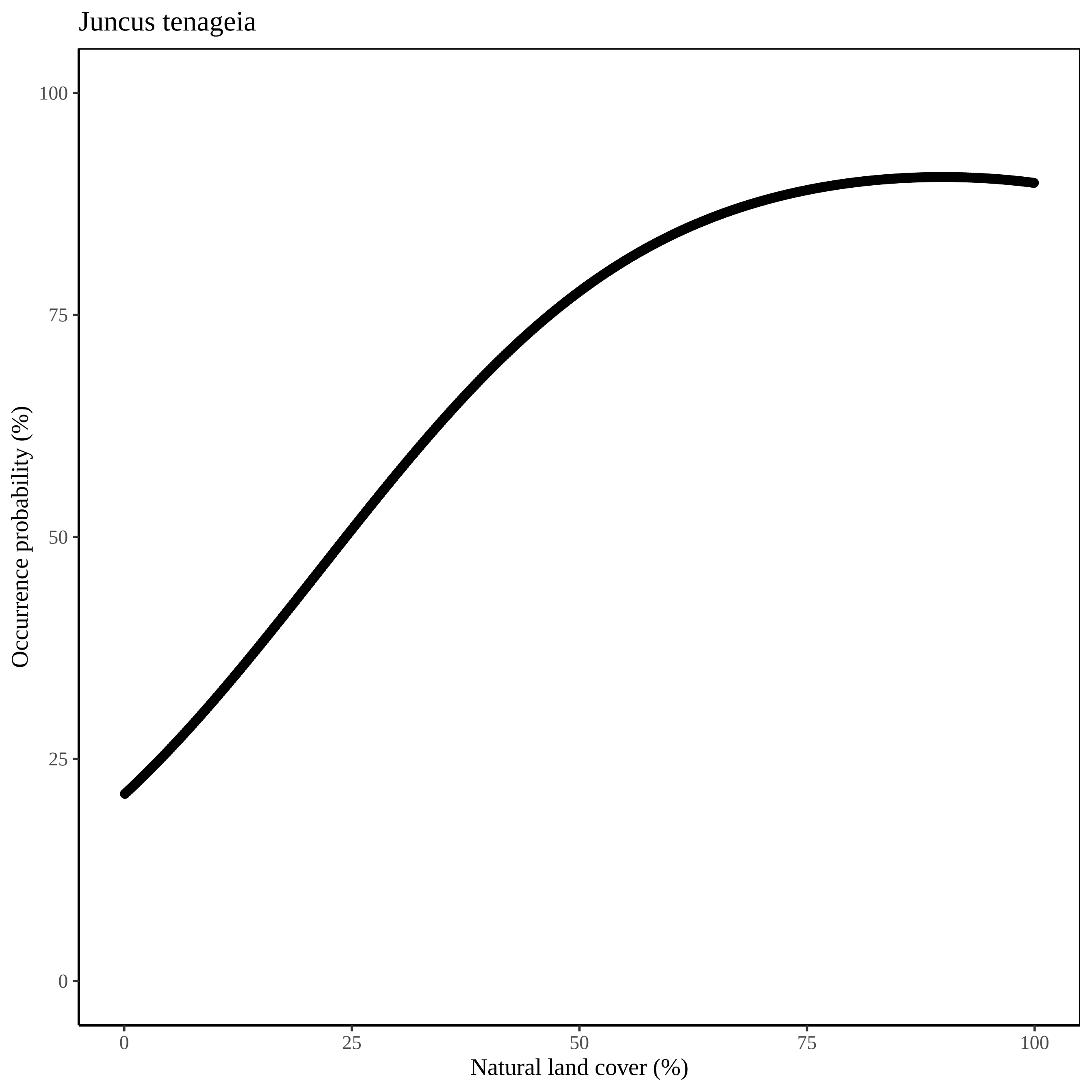

Juncus tenageia
Occurrence probability (%)
100
75
50
25
0
0
25
50
75
100
Natural land cover (%)

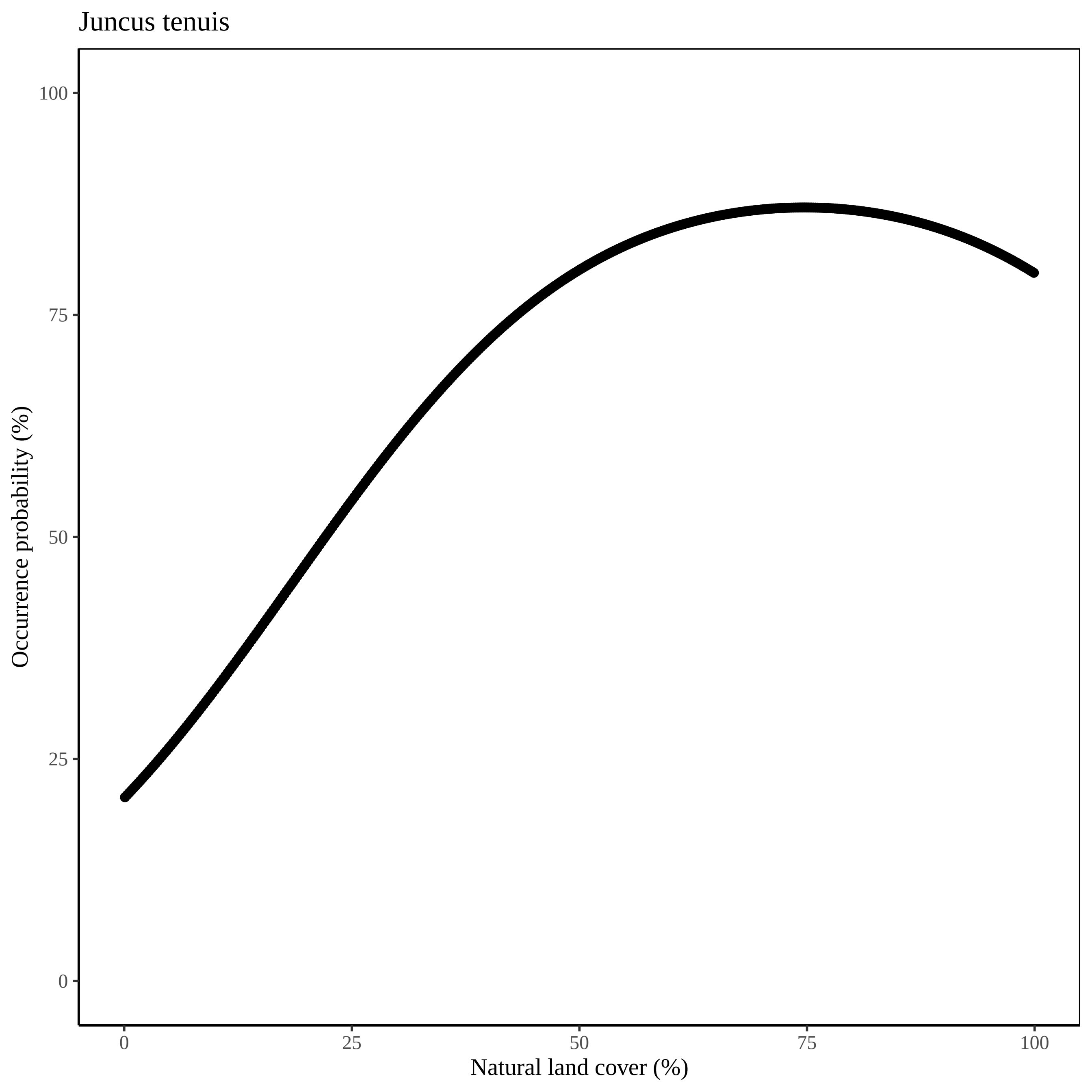
Juncus tenuis
Occurrence probability (%)
100
75
50
25
0
0
25
50
75
100
Natural land cover (%)

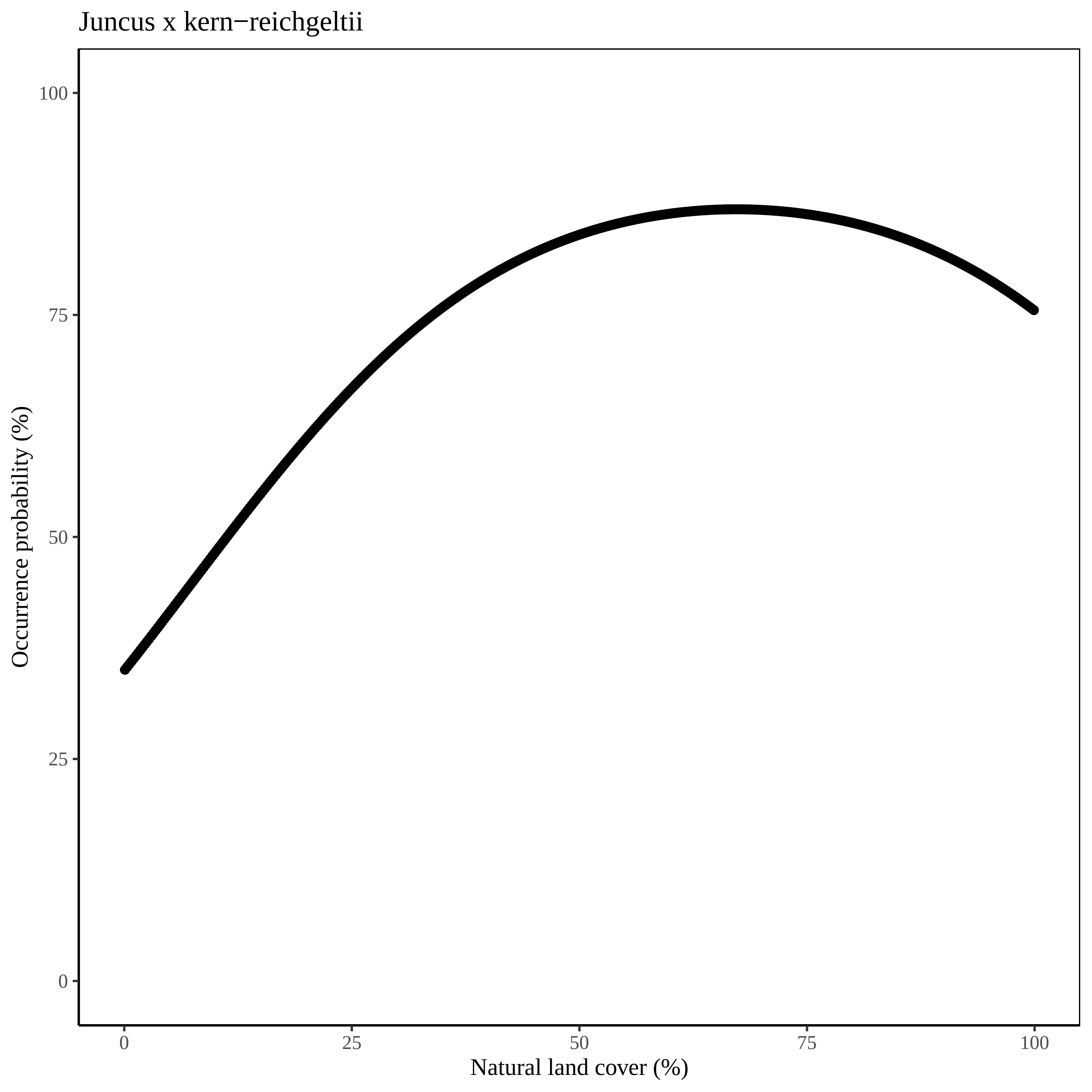
Juncus x kern−reichgeltii
Occurrence probability (%)
100
75
50
25
0
0
25
50
75
100
Natural land cover (%)

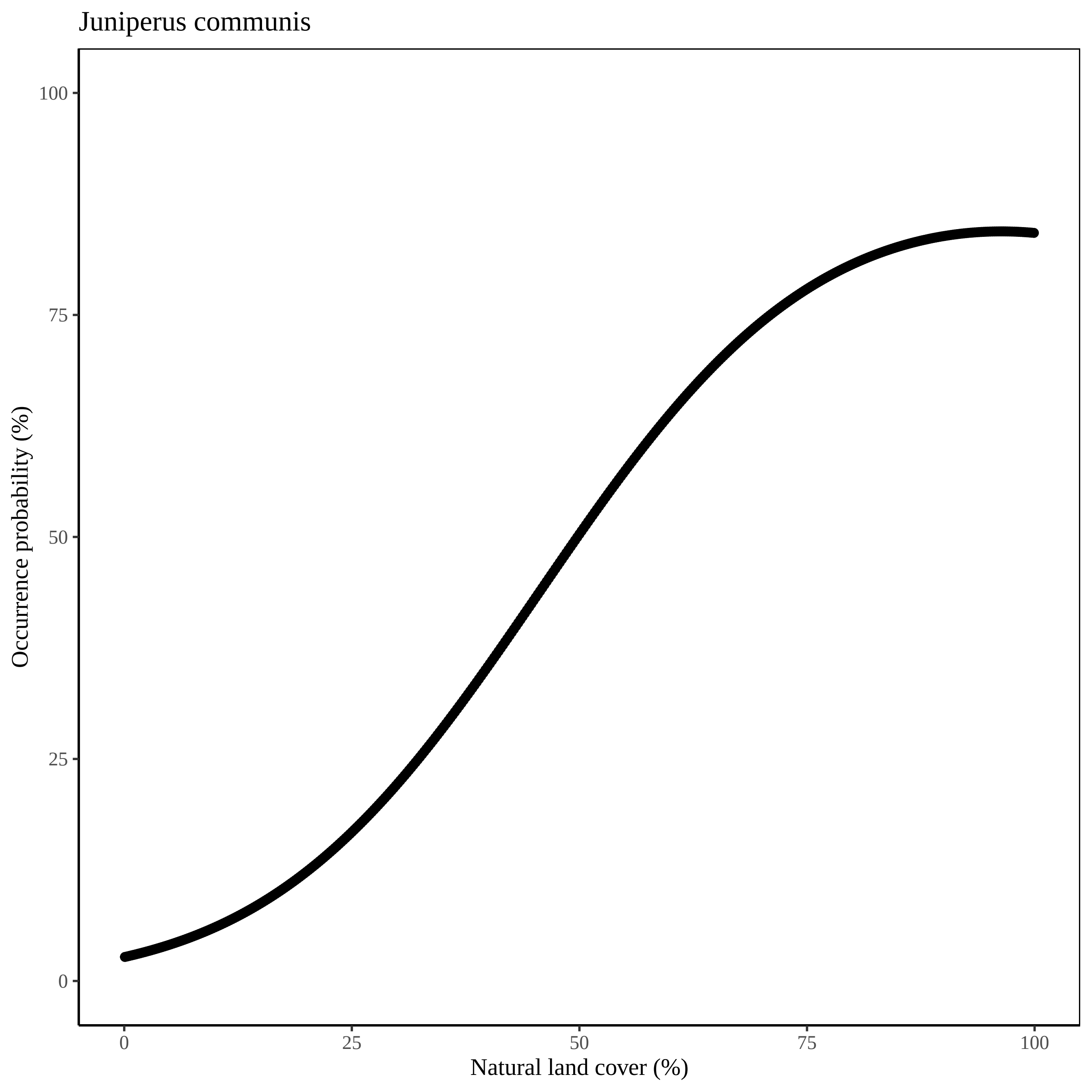

Juniperus communis
Occurrence probability (%)
100
75
50
25
0
0
25
50
75
100
Natural land cover (%)

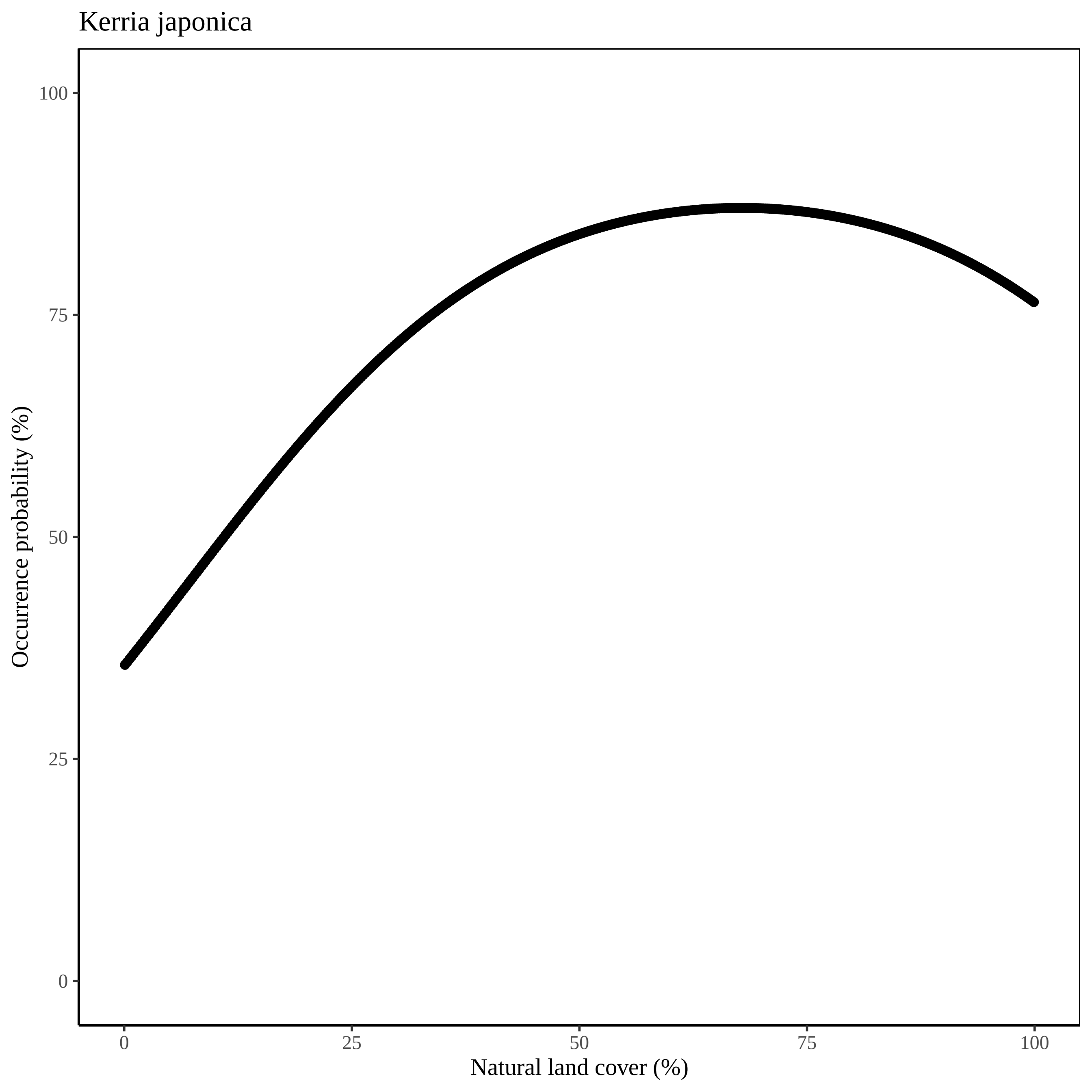
Kerria japonica
Occurrence probability (%)
100
75
50
25
0
0
25
50
75
100
Natural land cover (%)

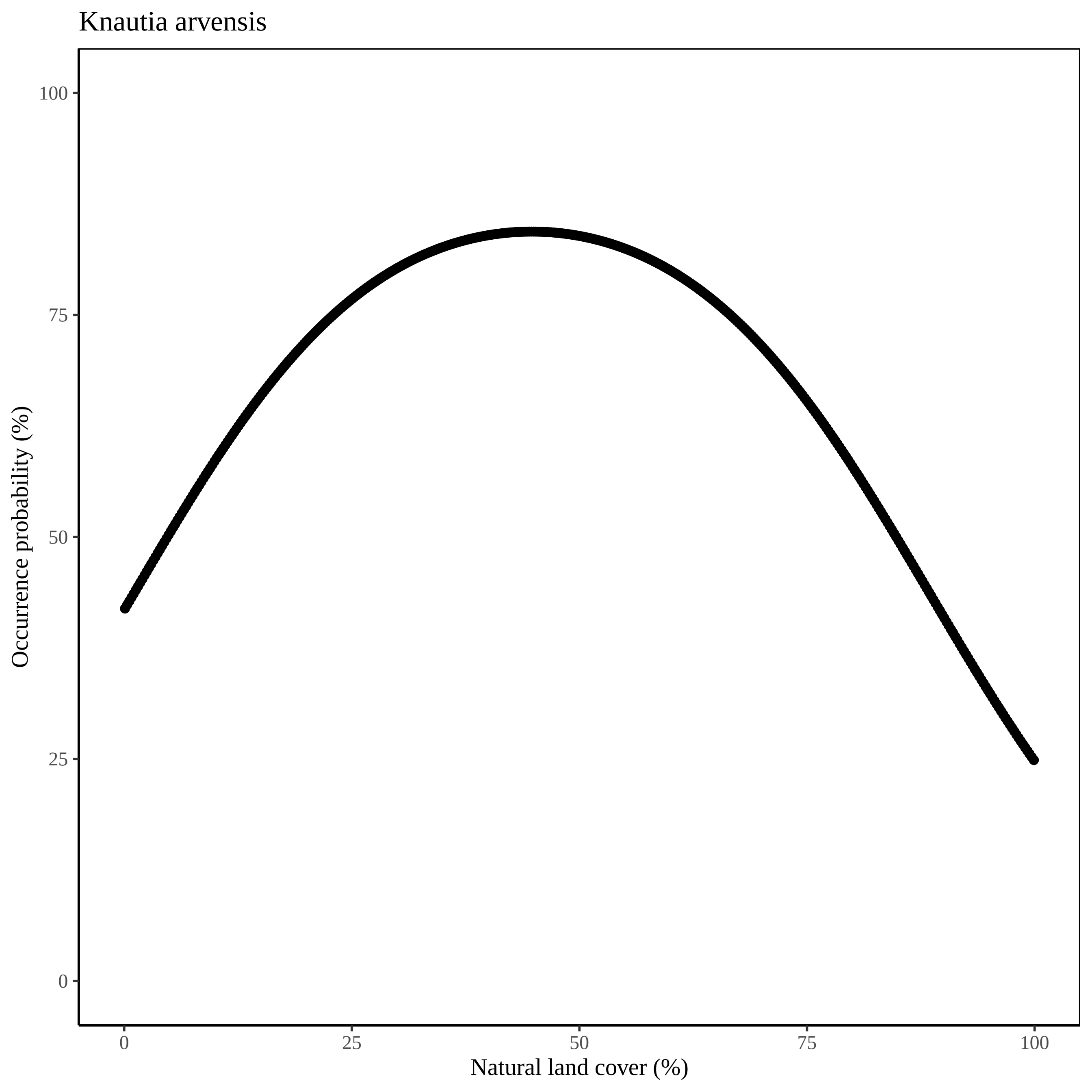

Knautia arvensis
100
75
50
25
0
Occurrence probability (%)
0
25
50
75
100
Natural land cover (%)

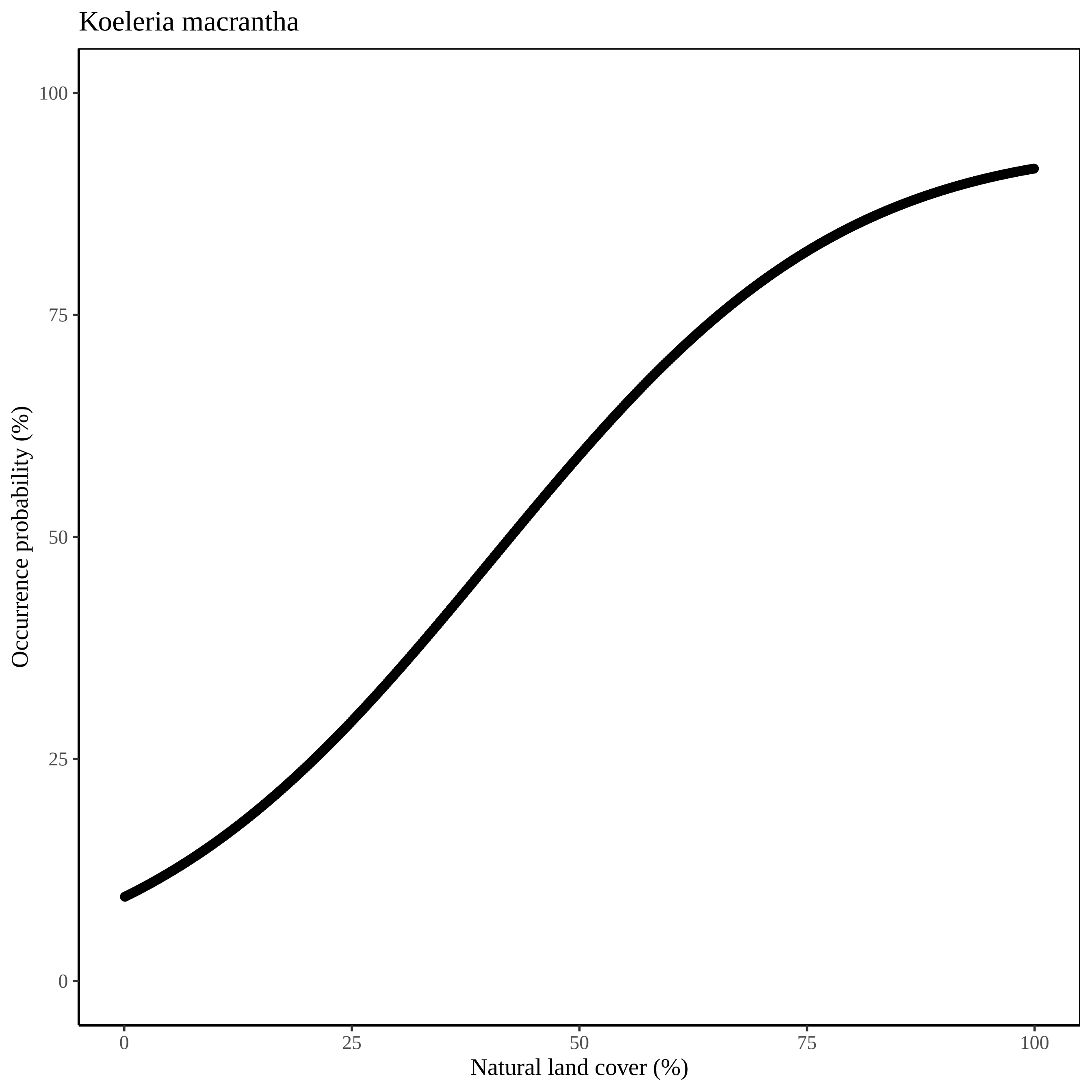

Koeleria macrantha
Occurrence probability (%)
100
75
50
25
0
0
25
50
75
100
Natural land cover (%)

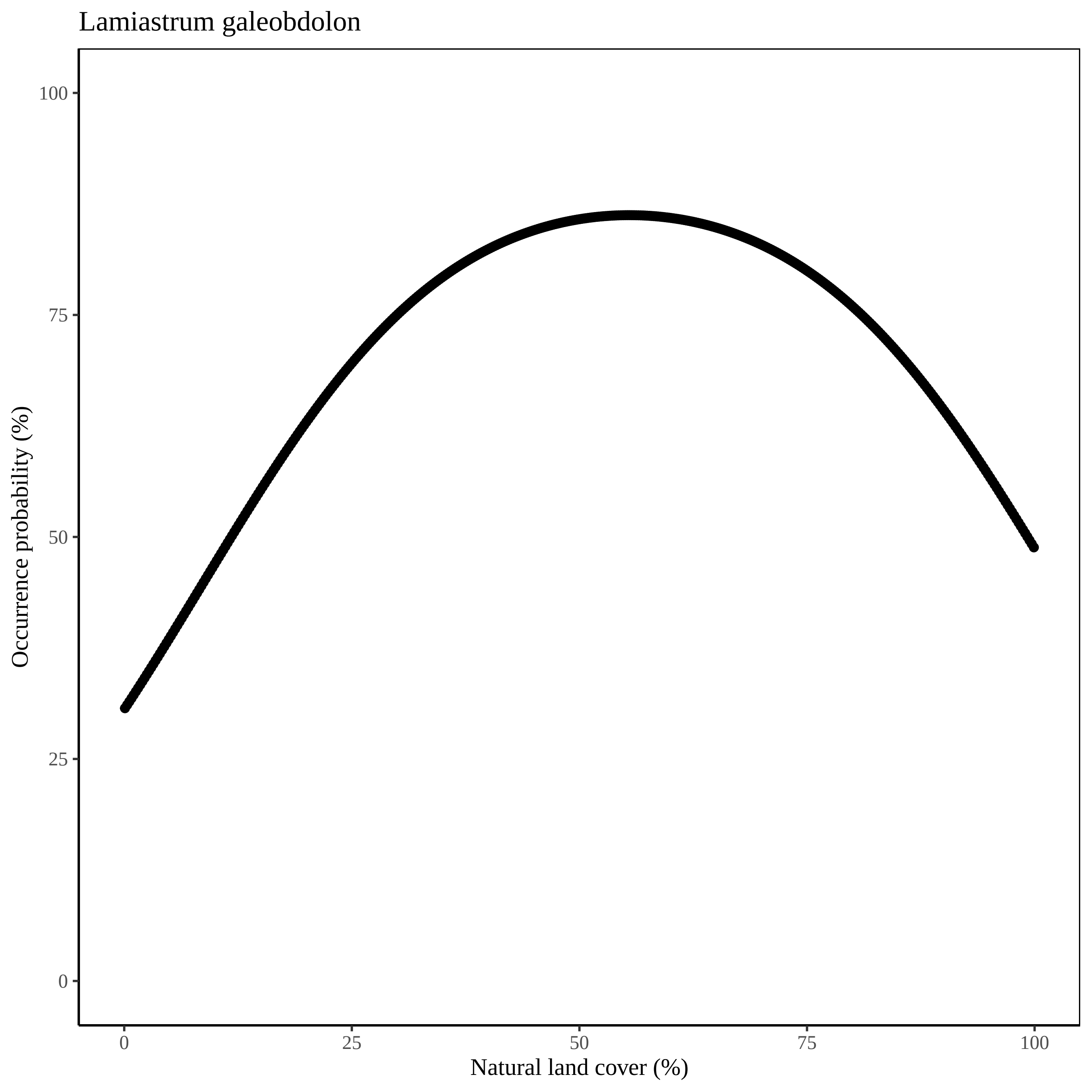
Lamiastrum galeobdolon
Occurrence probability (%)
100
75
50
25
0
0
25
50
75
100
Natural land cover (%)

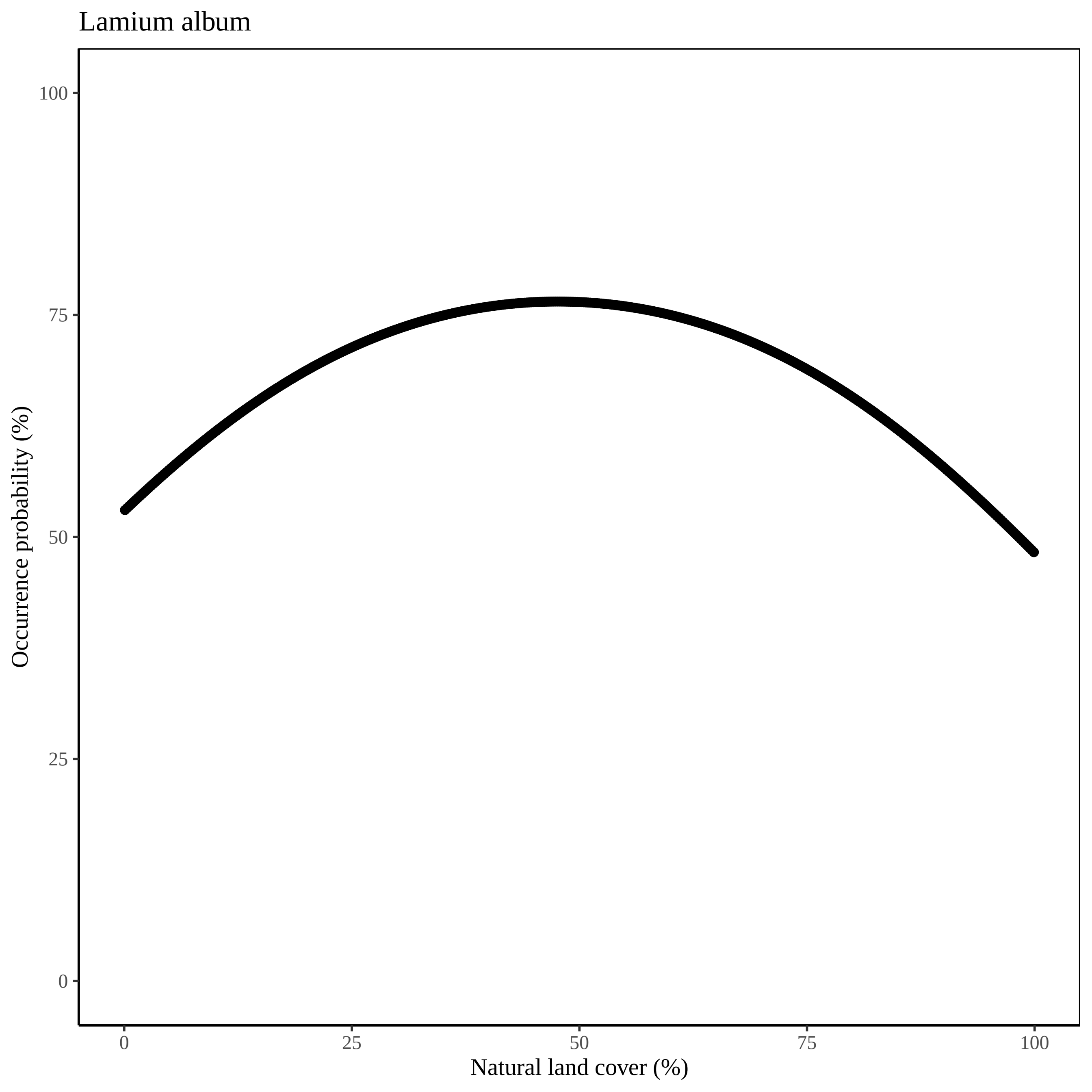
Lamium album
Occurrence probability (%)
100
75
50
25
0
0
25
50
75
100
Natural land cover (%)

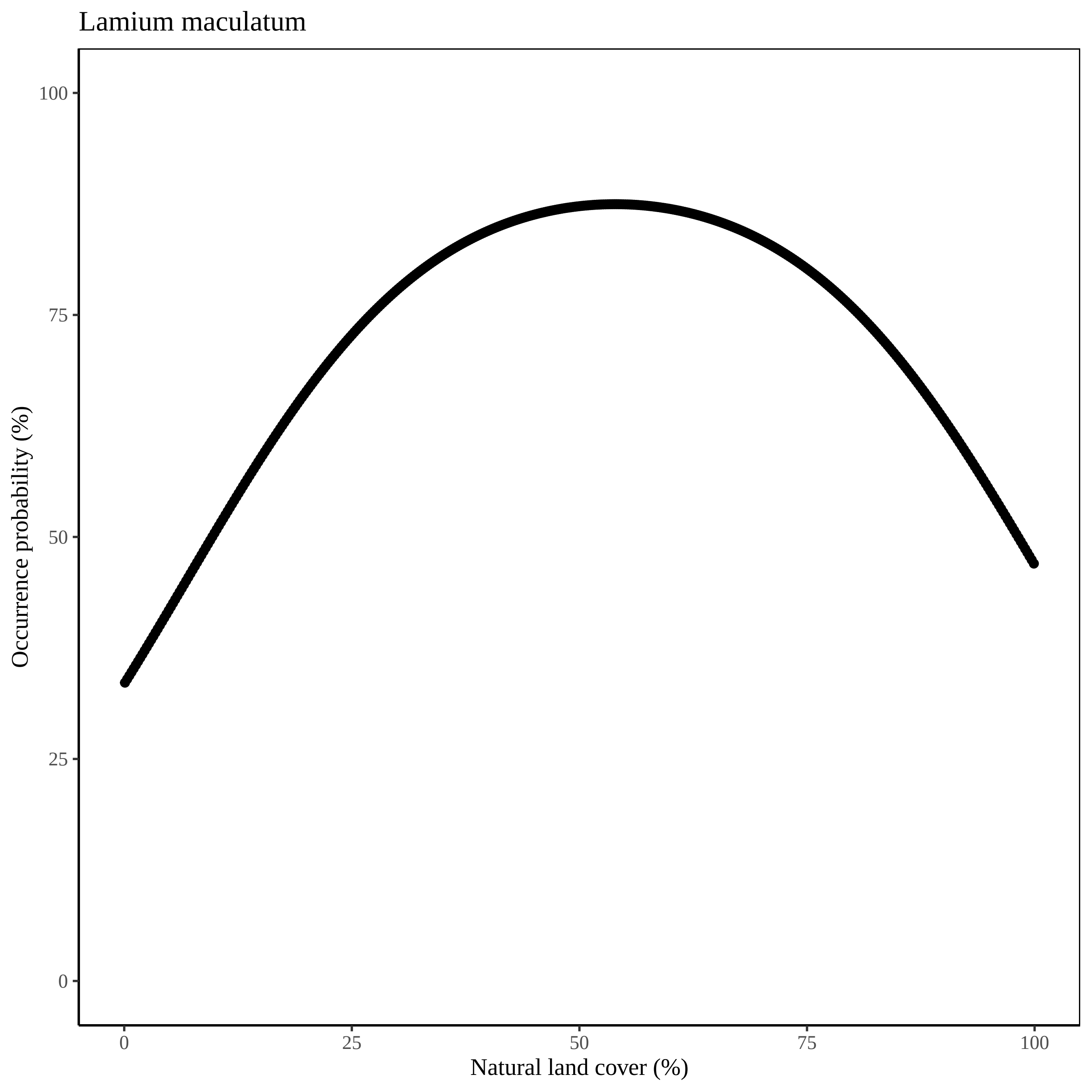
Lamium maculatum
Occurrence probability (%)
100
75
50
25
0
0
25
50
75
100
Natural land cover (%)

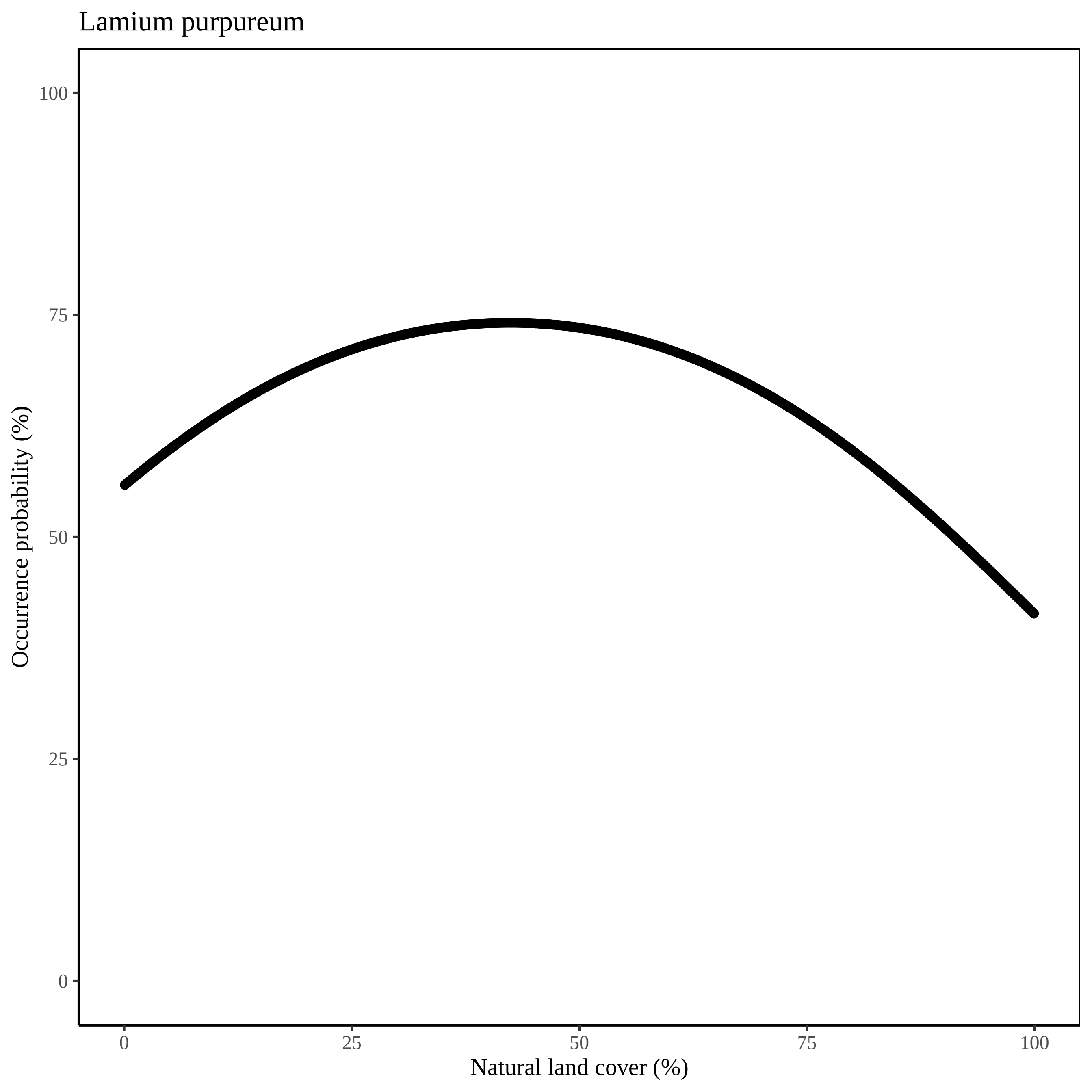

Lamium purpureum
100
75
50
25
0
Occurrence probability (%)
0
25
50
75
100
Natural land cover (%)

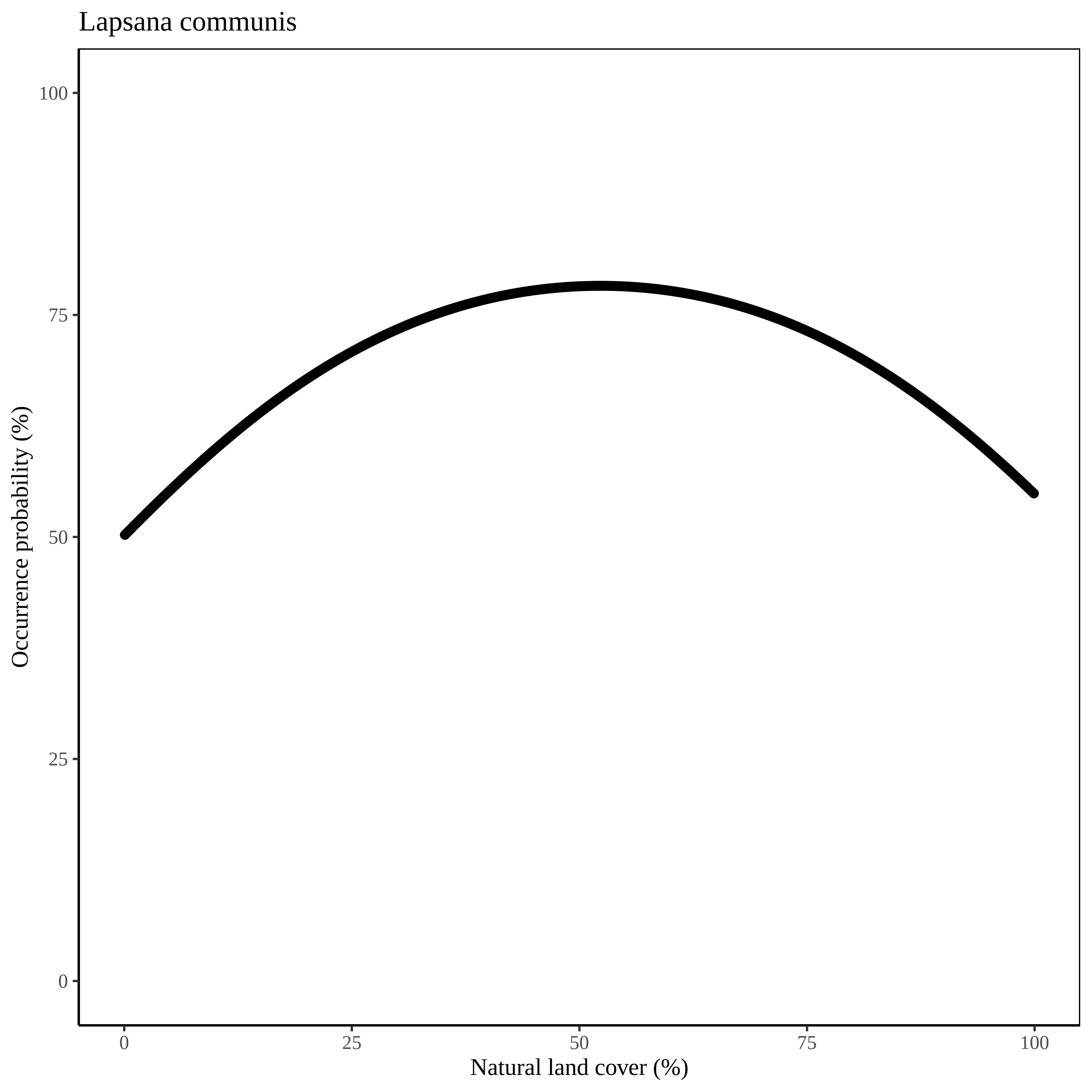
Lapsana communis
Occurrence probability (%)
100
75
50
25
0
0
25
50
75
100
Natural land cover (%)

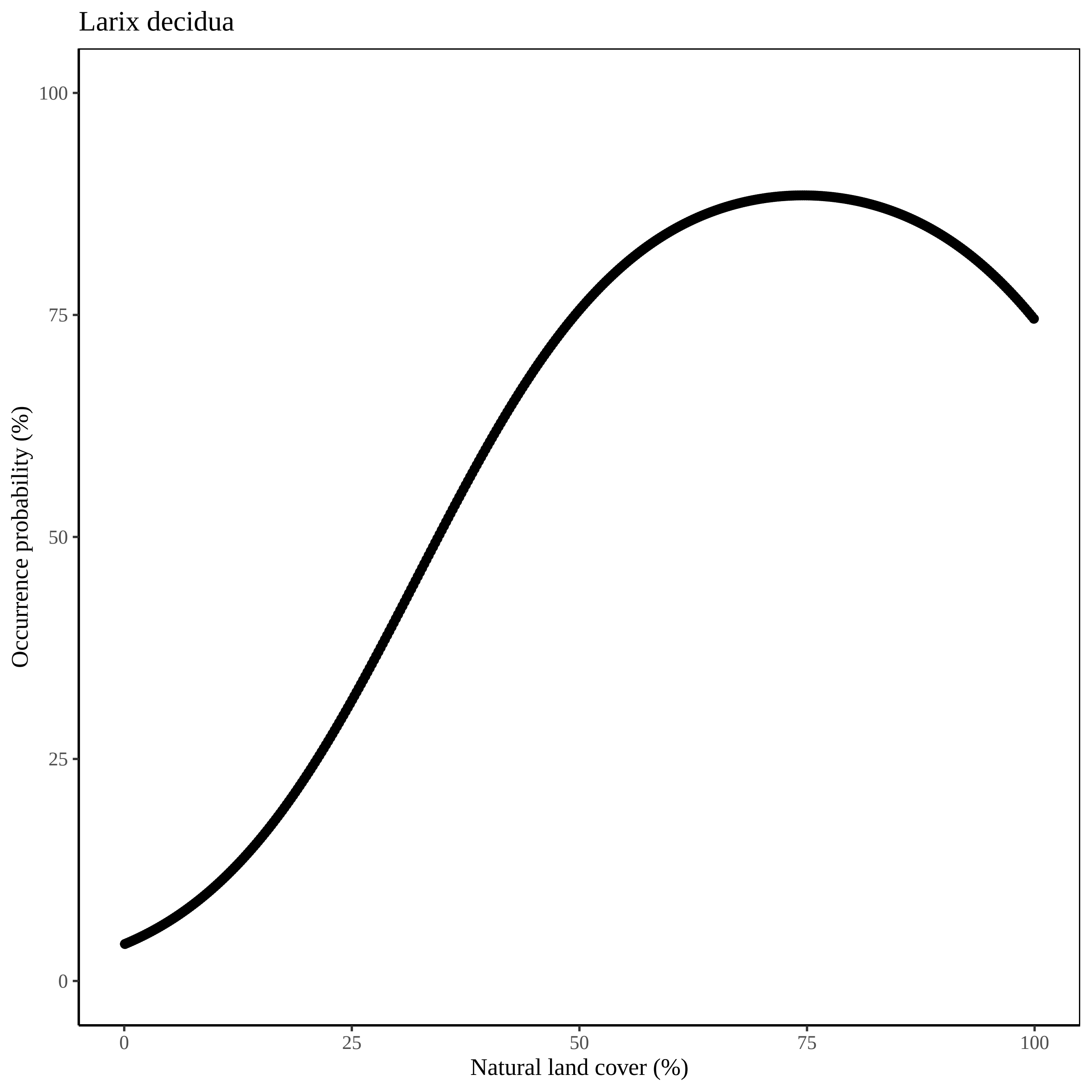

Larix decidua
Occurrence probability (%)
100
75
50
25
0
0
25
50
75
100
Natural land cover (%)

Larix kaempferi

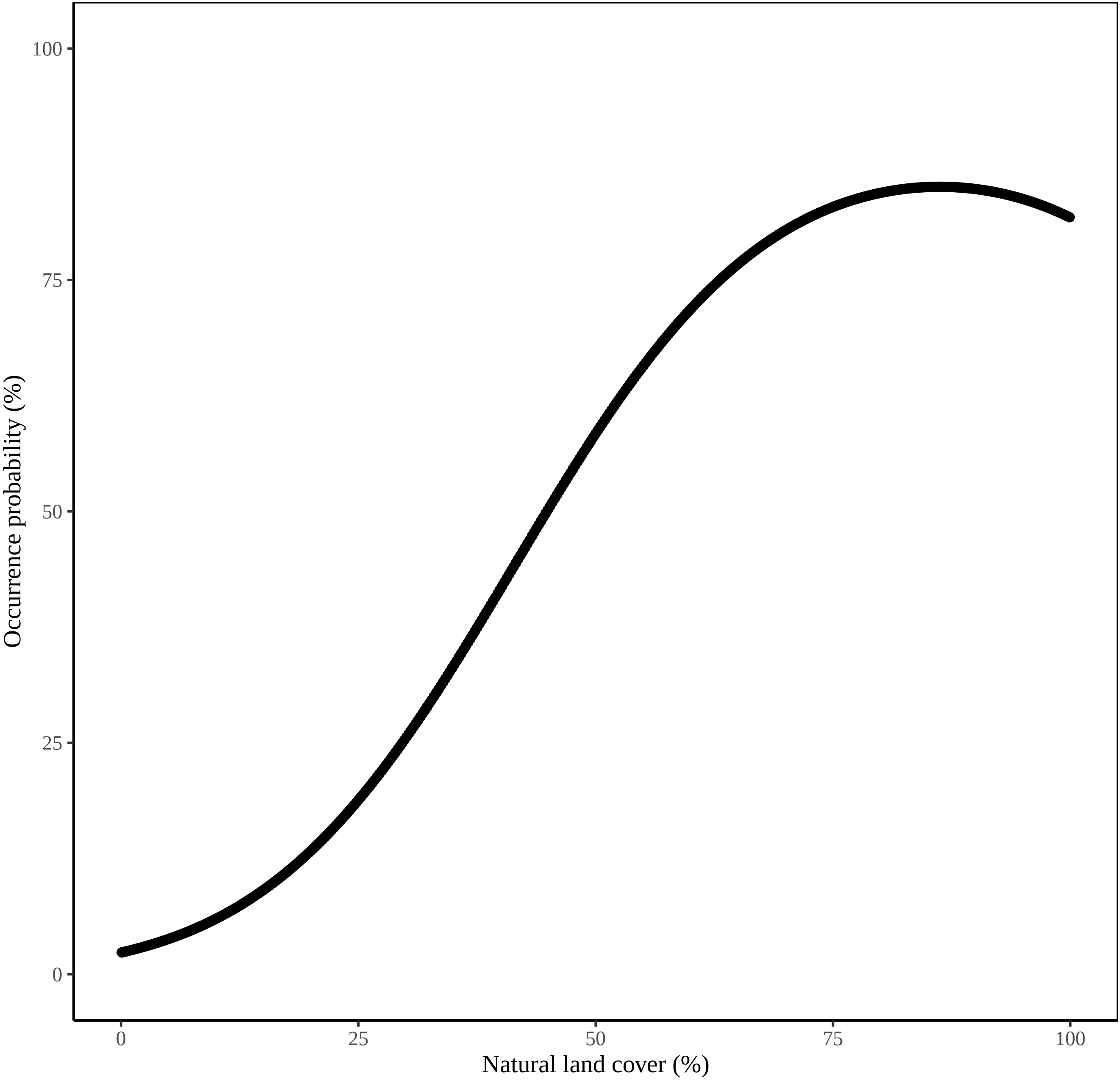

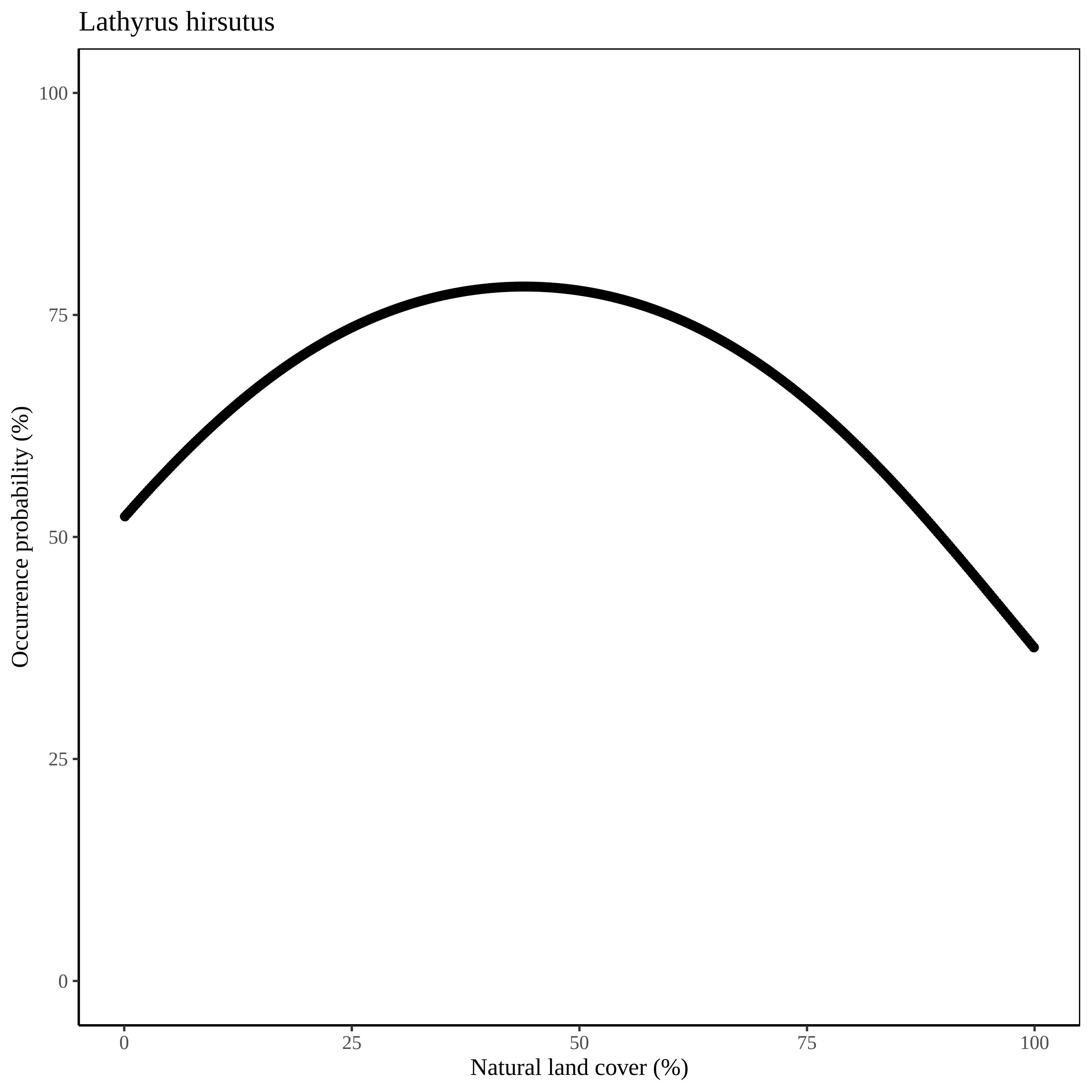
Lathyrus hirsutus
Occurrence probability (%)
100
75
50
25
0
0
25
50
75
100
Natural land cover (%)

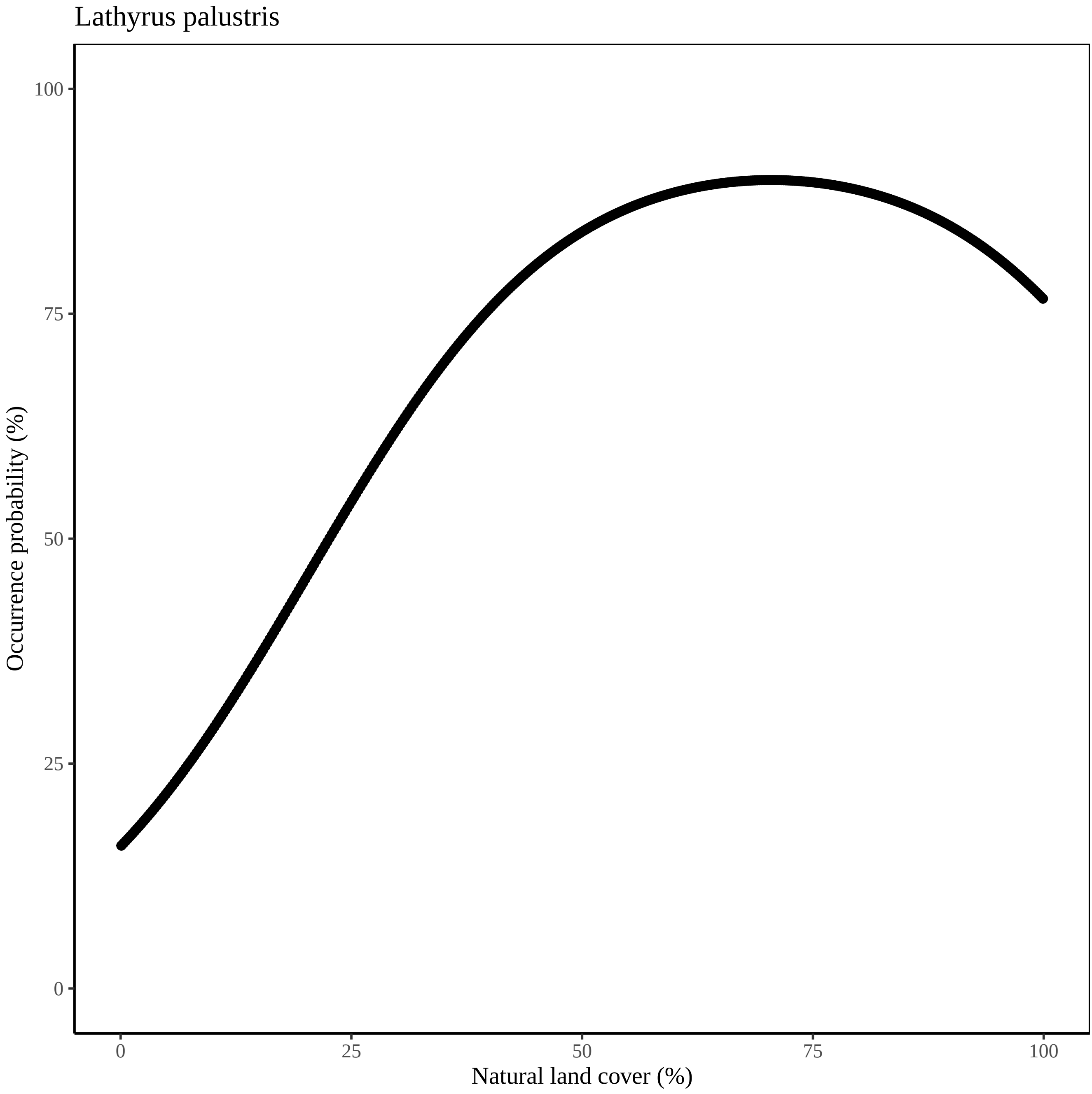

Lathyrus palustris
100
75
50
25
0
Occurrence probability (%)
0
25
50
75
100
Natural land cover (%)

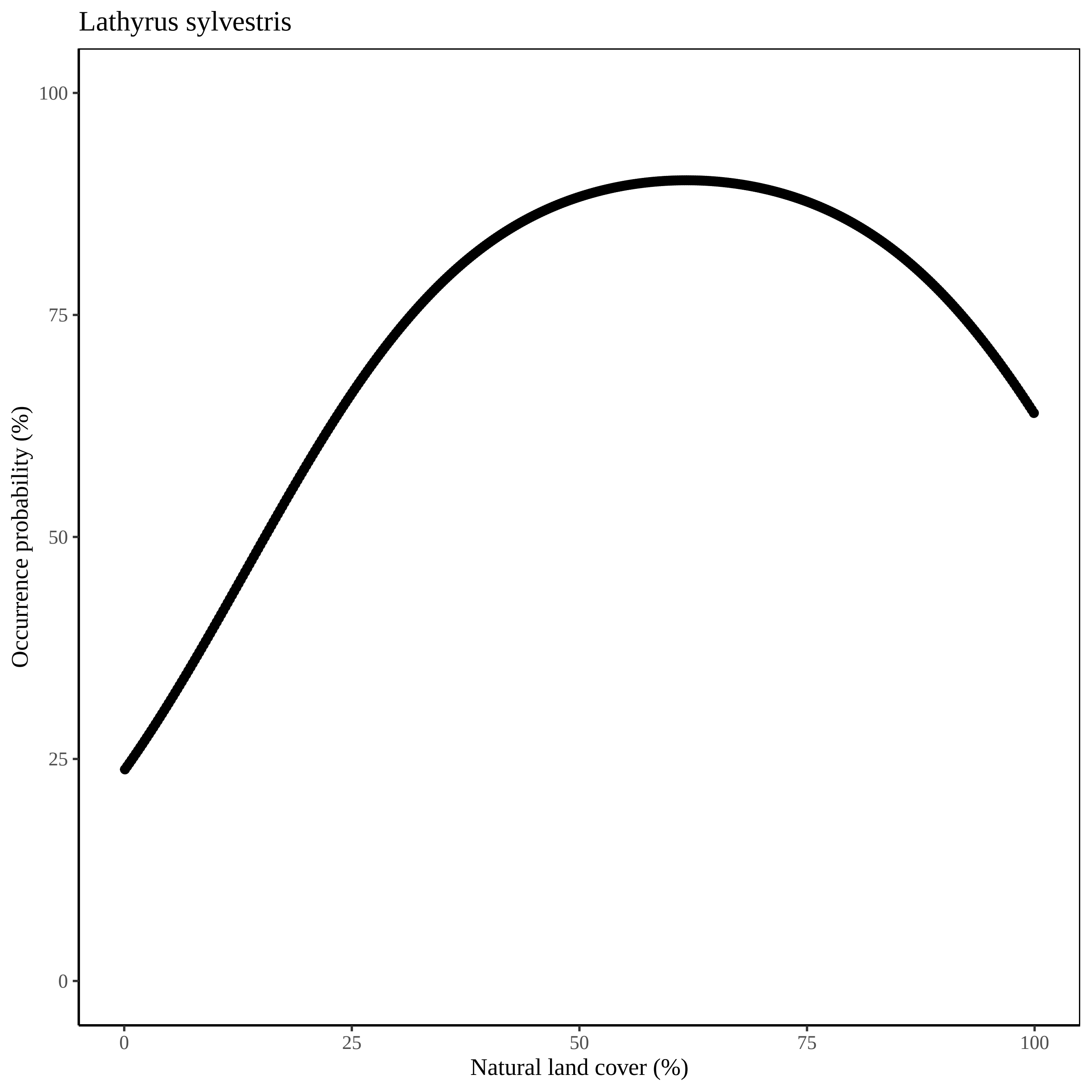
Lathyrus sylvestris
Occurrence probability (%)
100
75
50
25
0
0
25
50
75
100
Natural land cover (%)

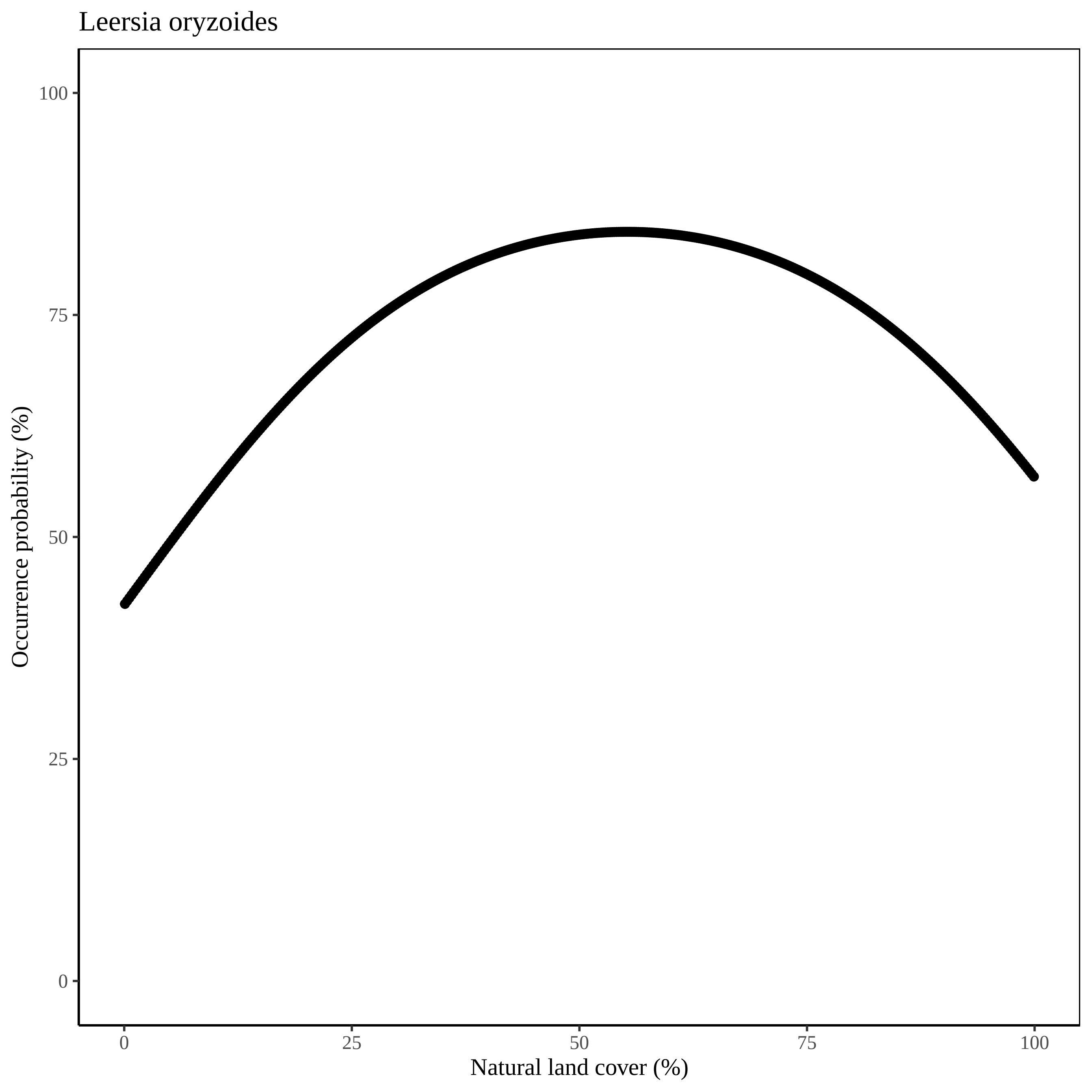

Leersia oryzoides
Occurrence probability (%)
100
75
50
25
0
0
25
50
75
100
Natural land cover (%)

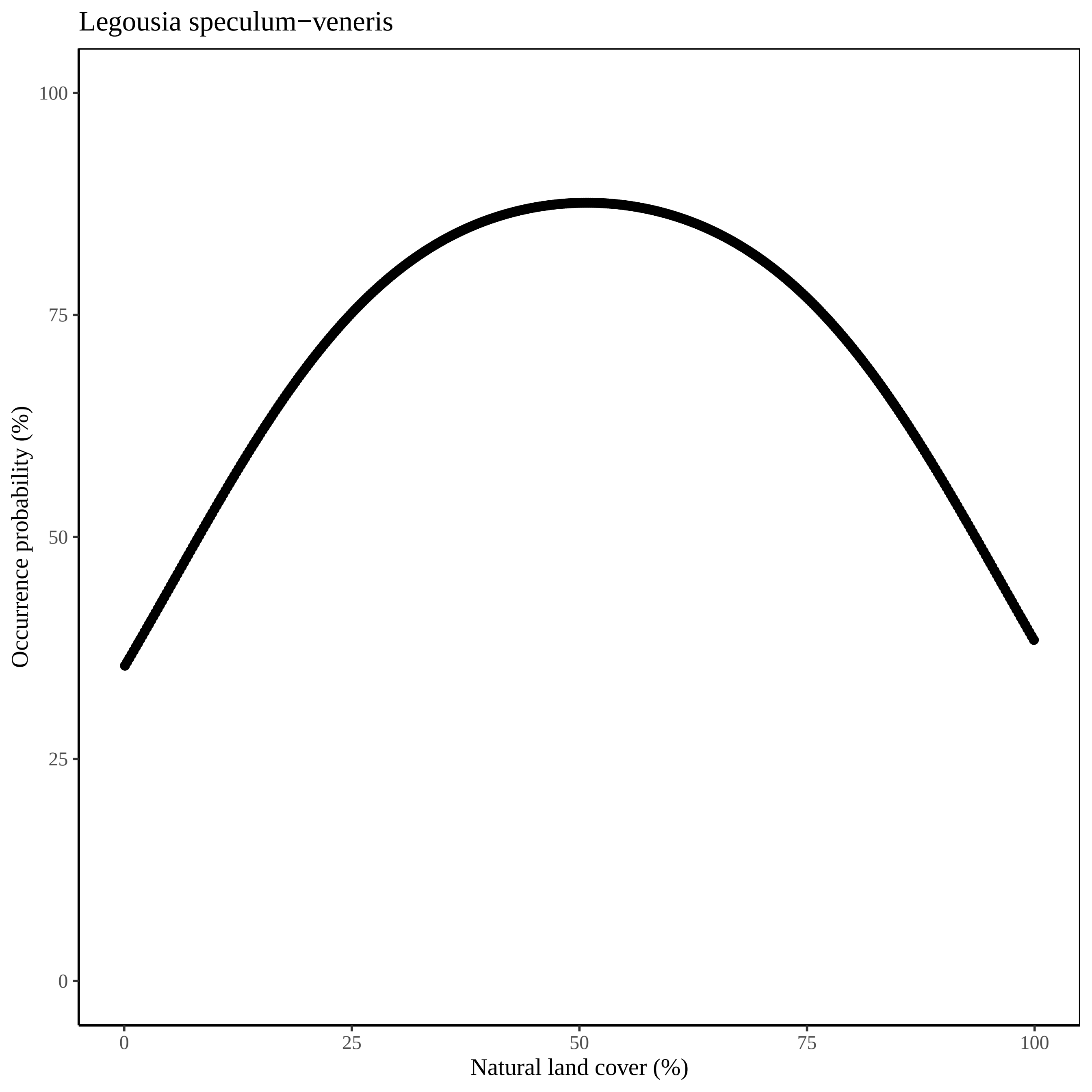
Legousia speculum−veneris
100
75
50
25
0
Occurrence probability (%)
0
25
50
75
100
Natural land cover (%)

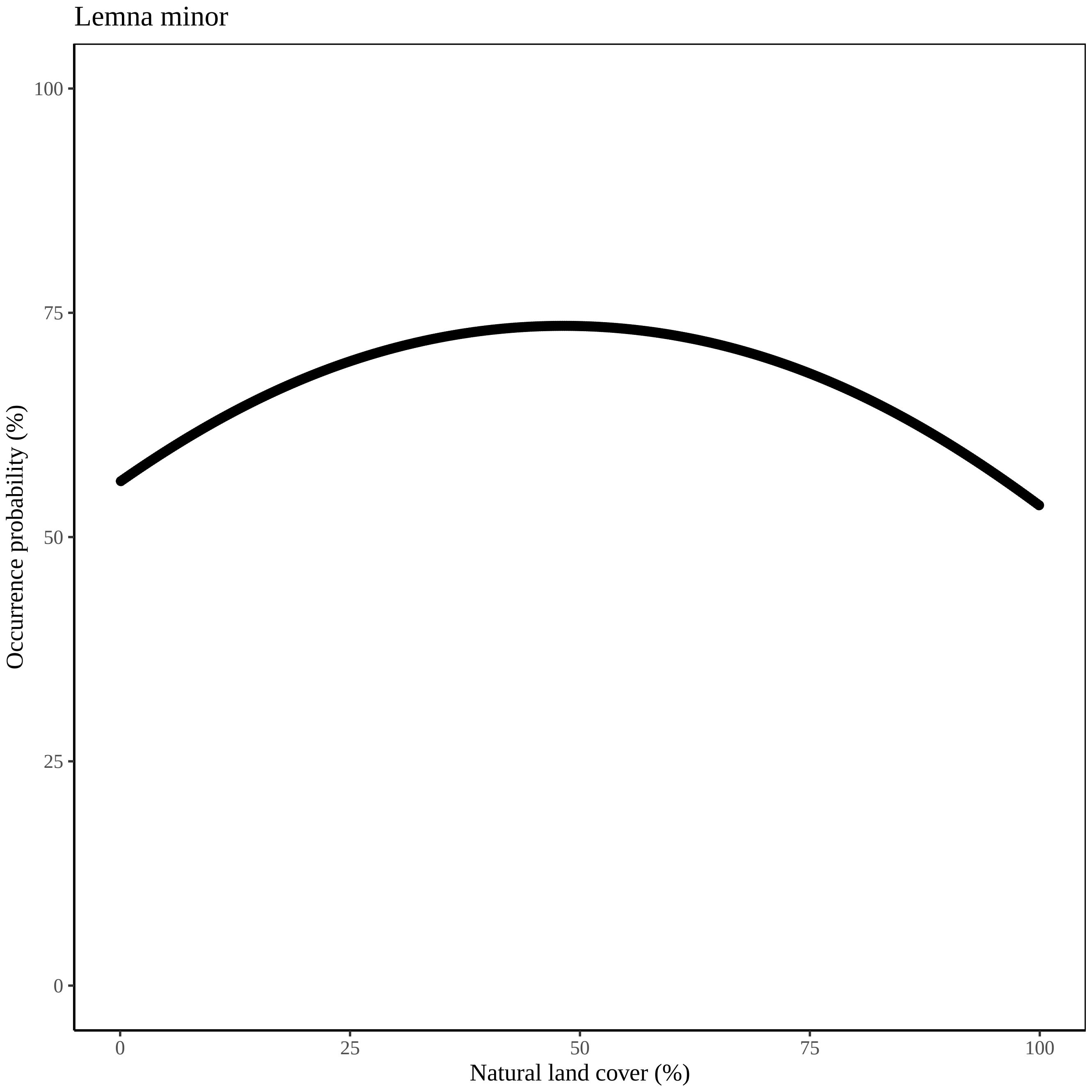

Lemna minor
Occurrence probability (%)
100
75
50
25
0
0
25
50
75
100
Natural land cover (%)

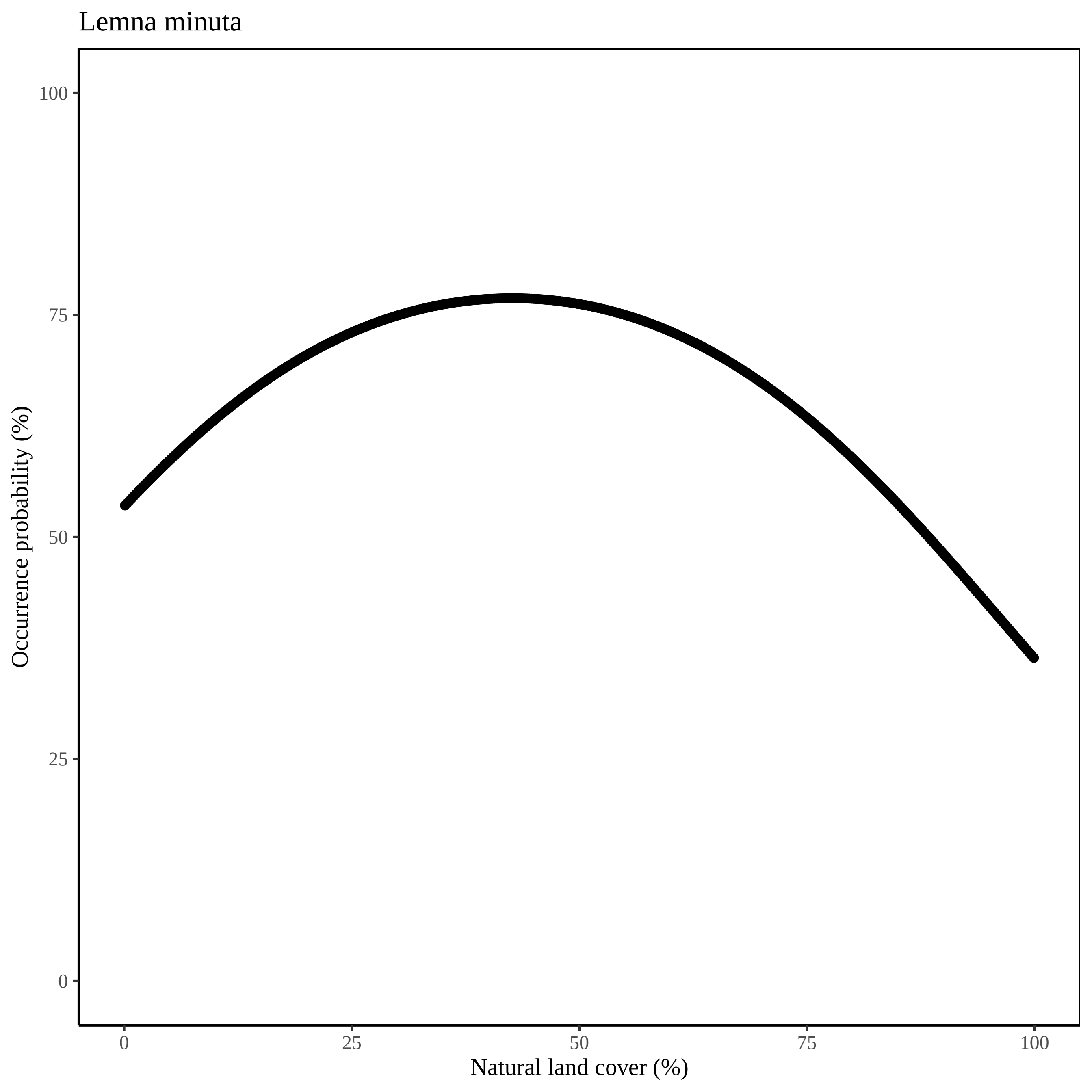

Lemna minuta
100
75
50
25
0
Occurrence probability (%)
0
25
50
75
100
Natural land cover (%)

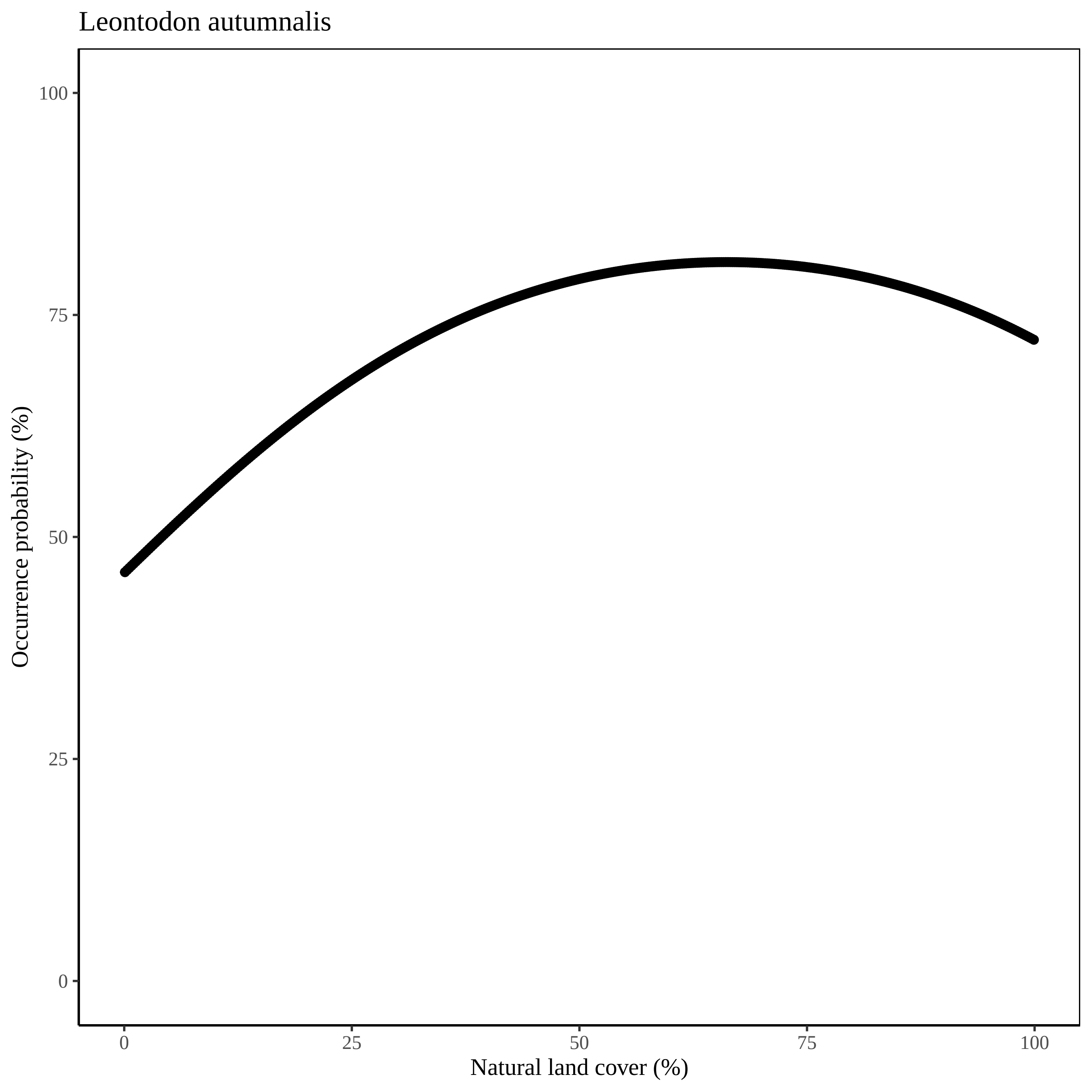

Leontodon autumnalis
Occurrence probability (%)
100
75
50
25
0
0
25
50
75
100
Natural land cover (%)

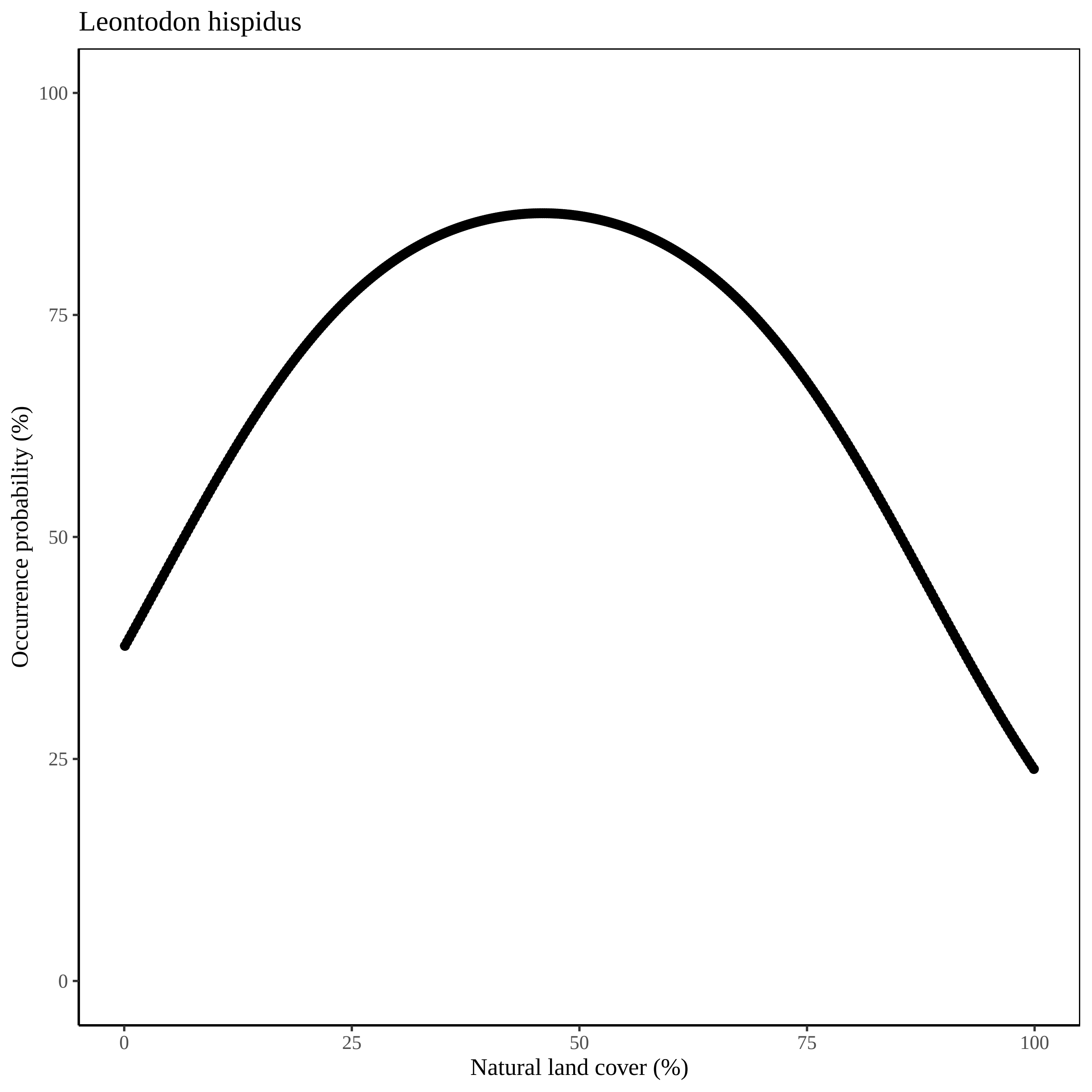
Leontodon hispidus
100
75
50
25
0
Occurrence probability (%)
0
25
50
75
100
Natural land cover (%)

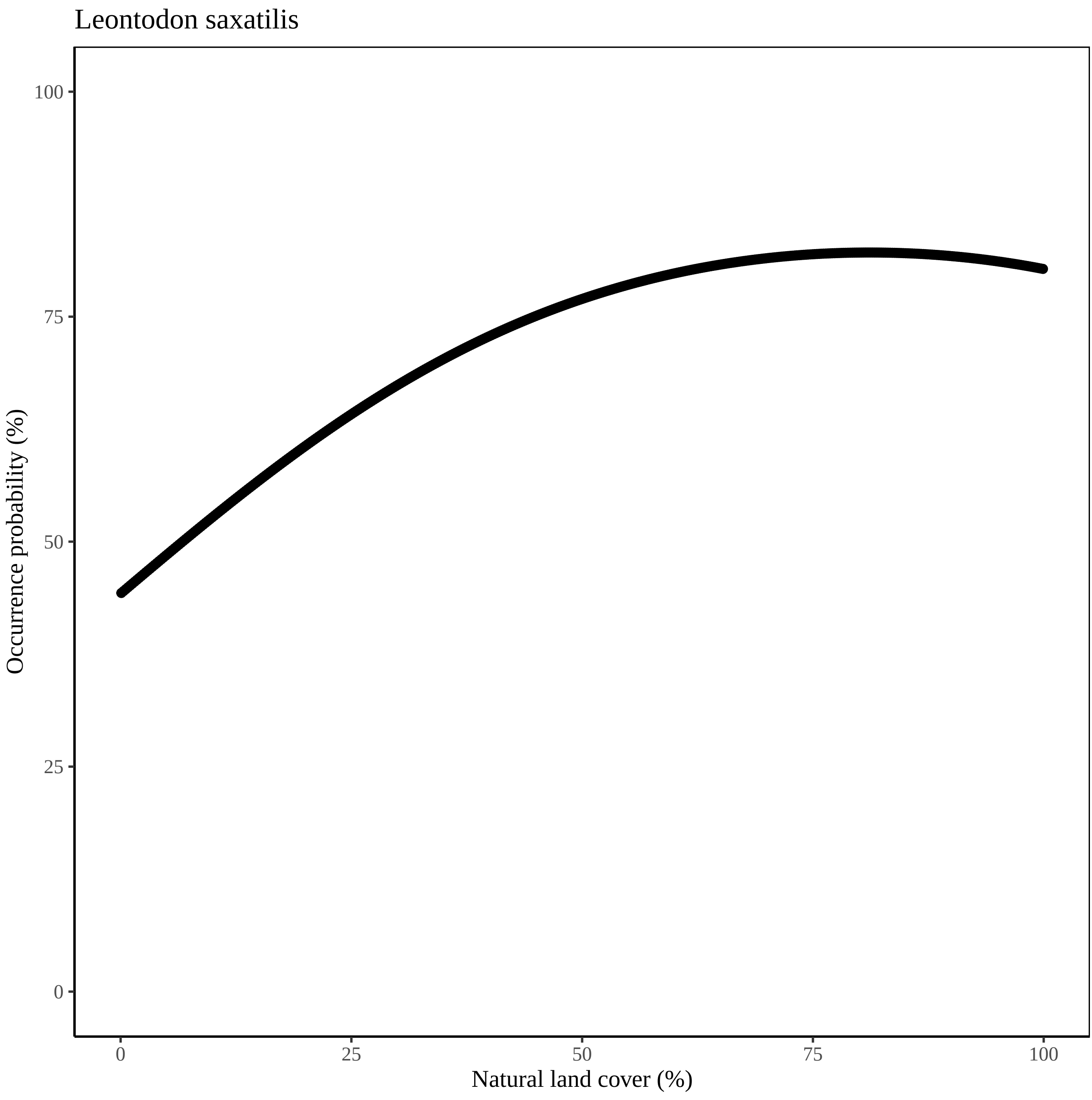

Leontodon saxatilis
100
75
50
25
0
Occurrence probability (%)
0
25
50
75
100
Natural land cover (%)

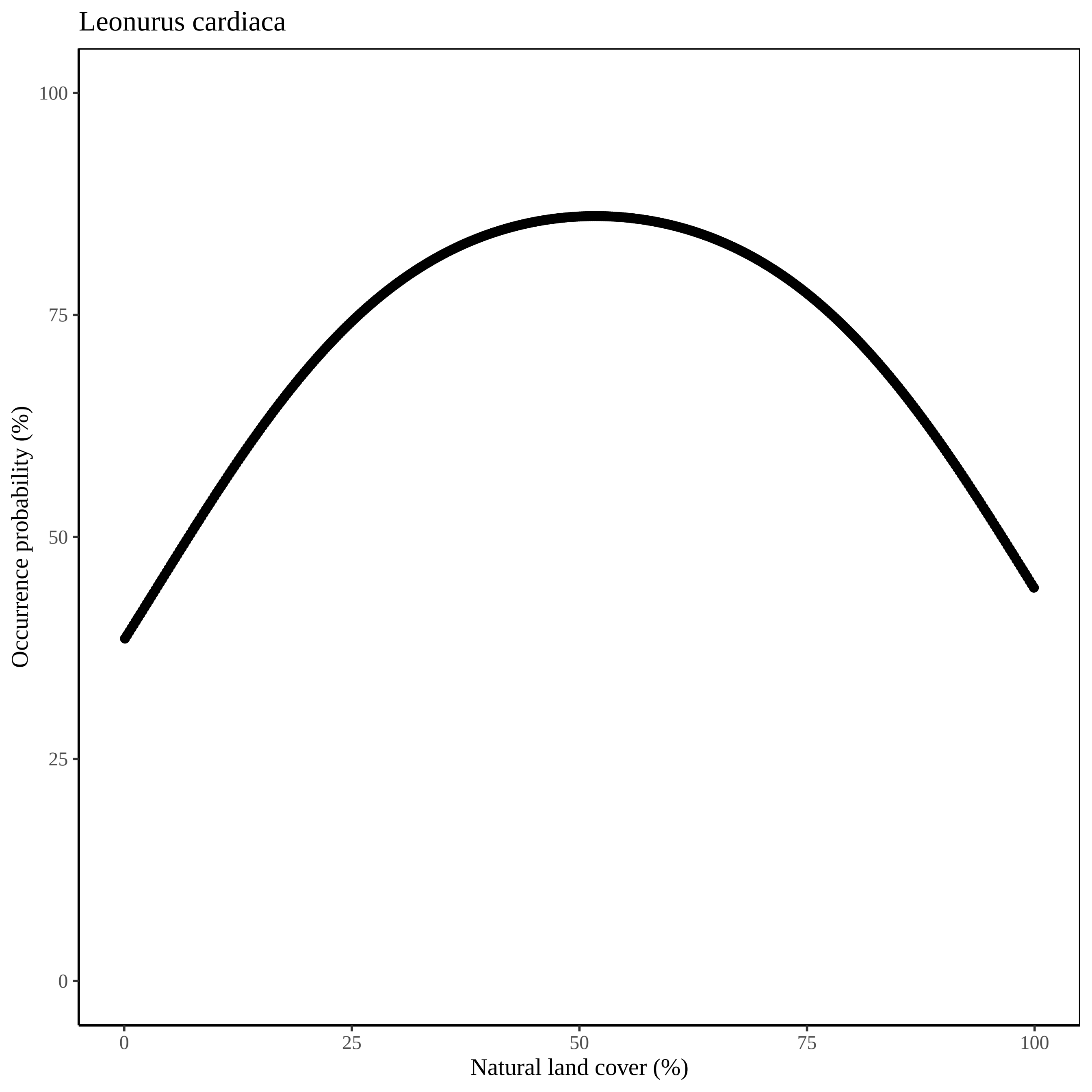
Leonurus cardiaca
Occurrence probability (%)
100
75
50
25
0
Natural land cover (%)
0
25
50
75
100

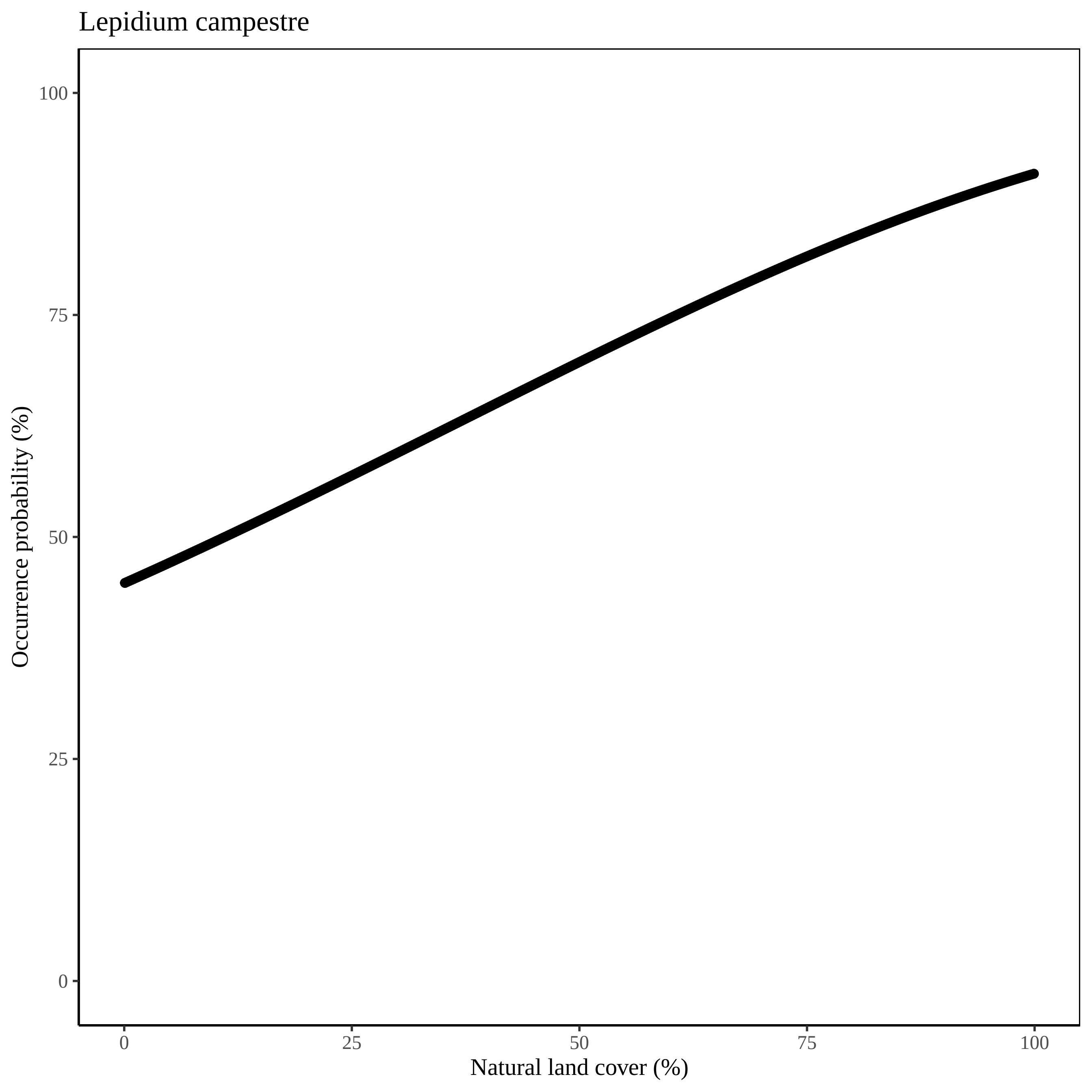

Lepidium campestre
Occurrence probability (%)
100
75
50
25
0
0
25
50
75
100
Natural land cover (%)

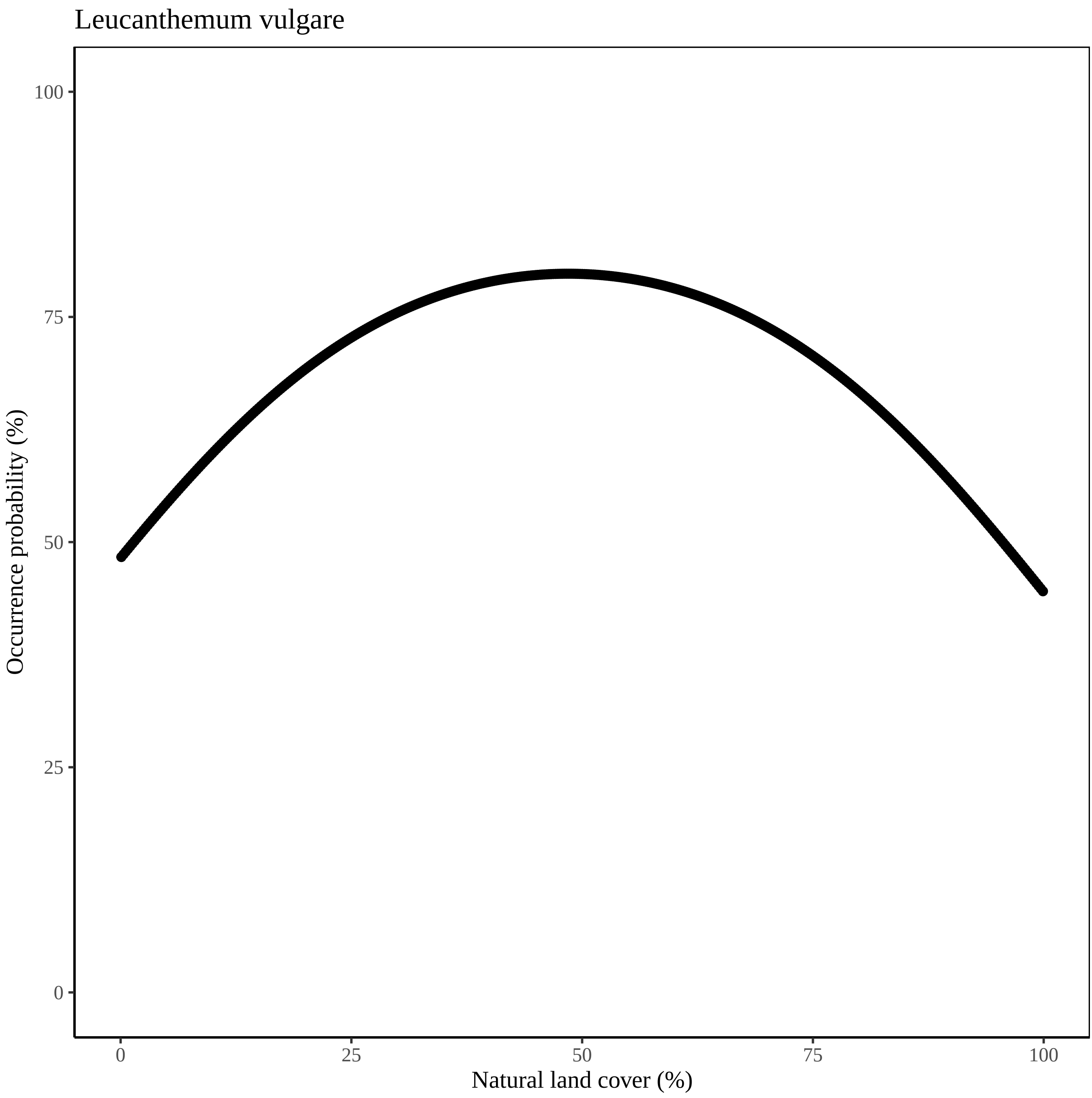

Leucanthemum vulgare
100
75
50
25
0
Occurrence probability (%)
0
25
50
75
100
Natural land cover (%)

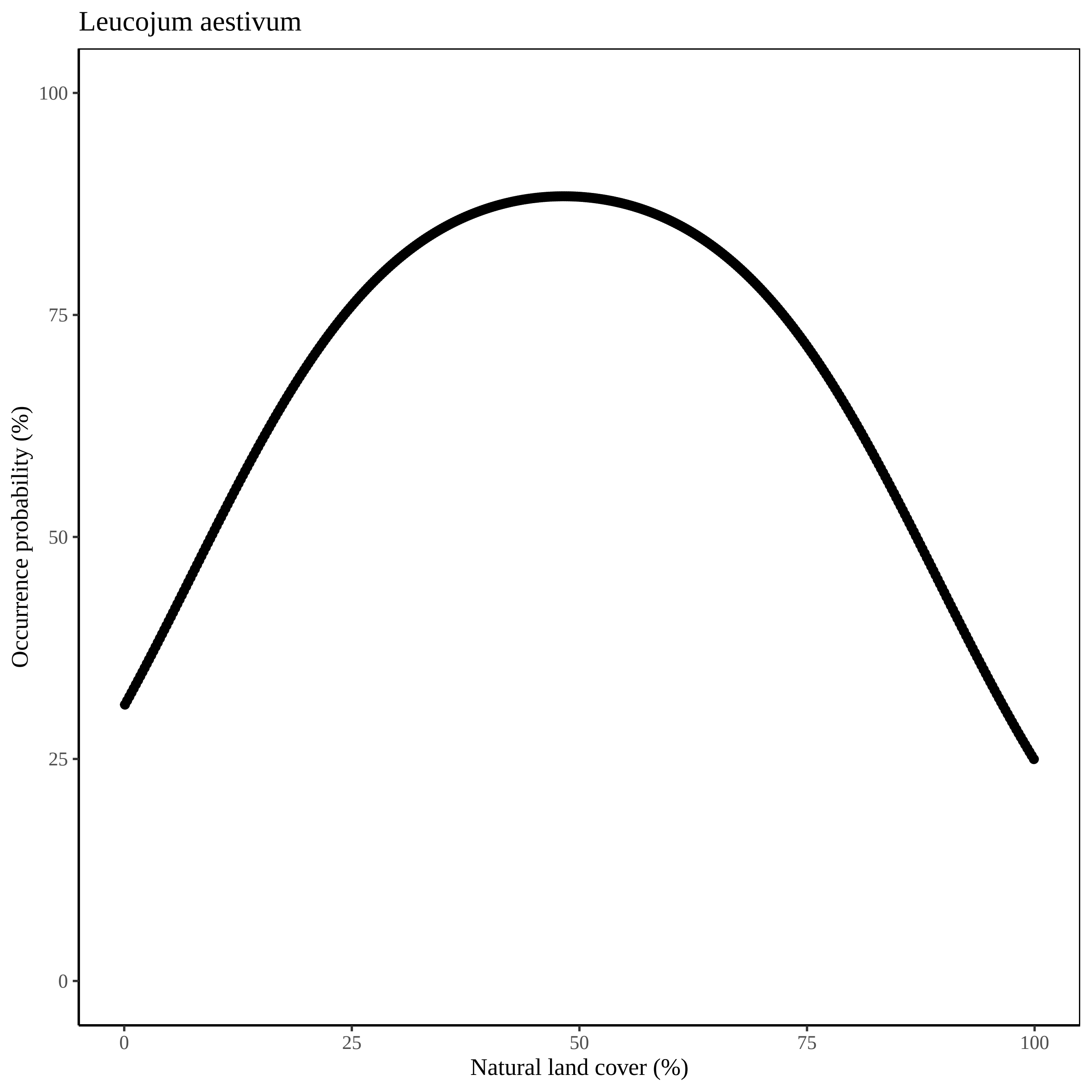
Leucojum aestivum
Occurrence probability (%)
100
75
50
25
0
0
25
50
75
100
Natural land cover (%)

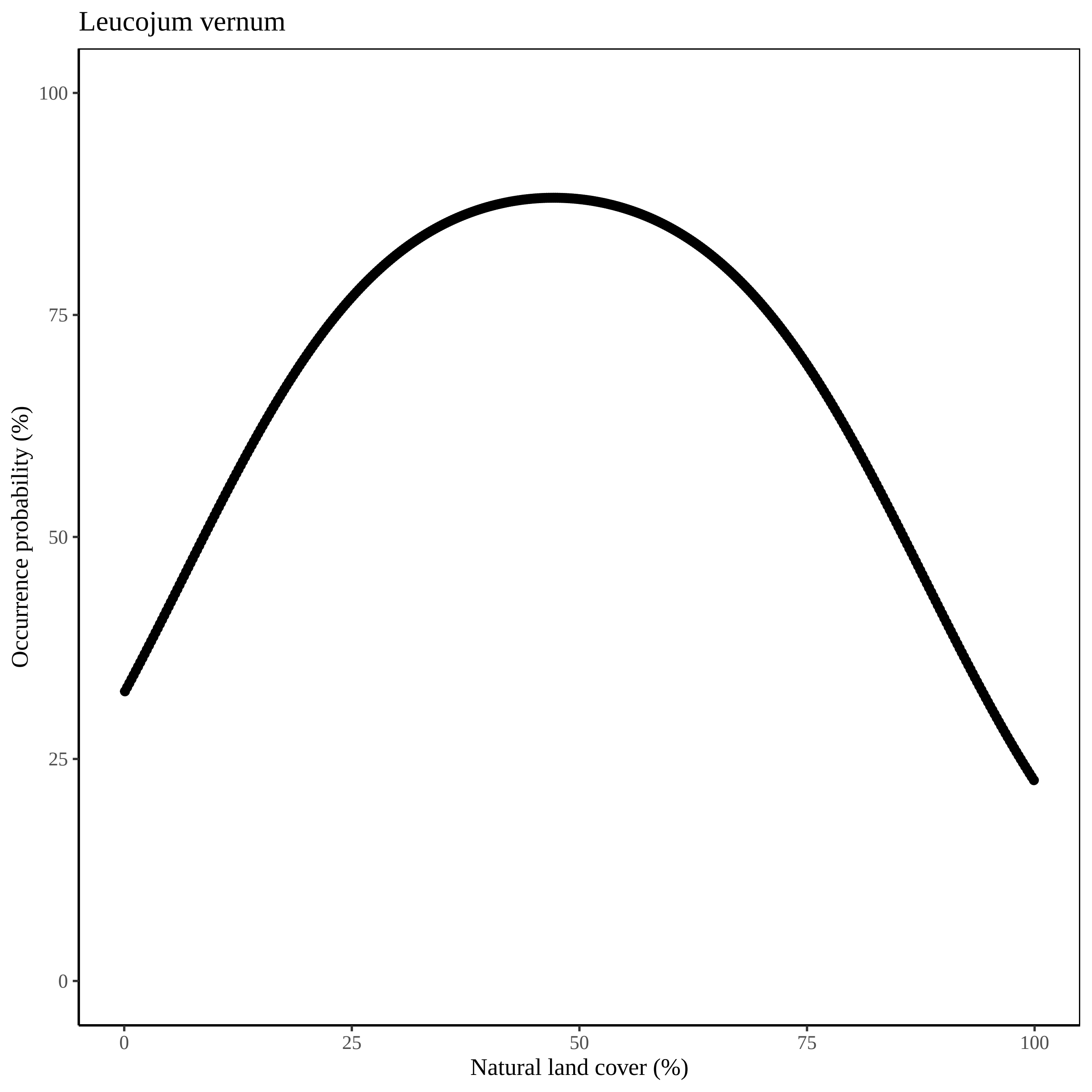
Leucojum vernum
Occurrence probability (%)
100
75
50
25
0
0
25
50
75
100
Natural land cover (%)

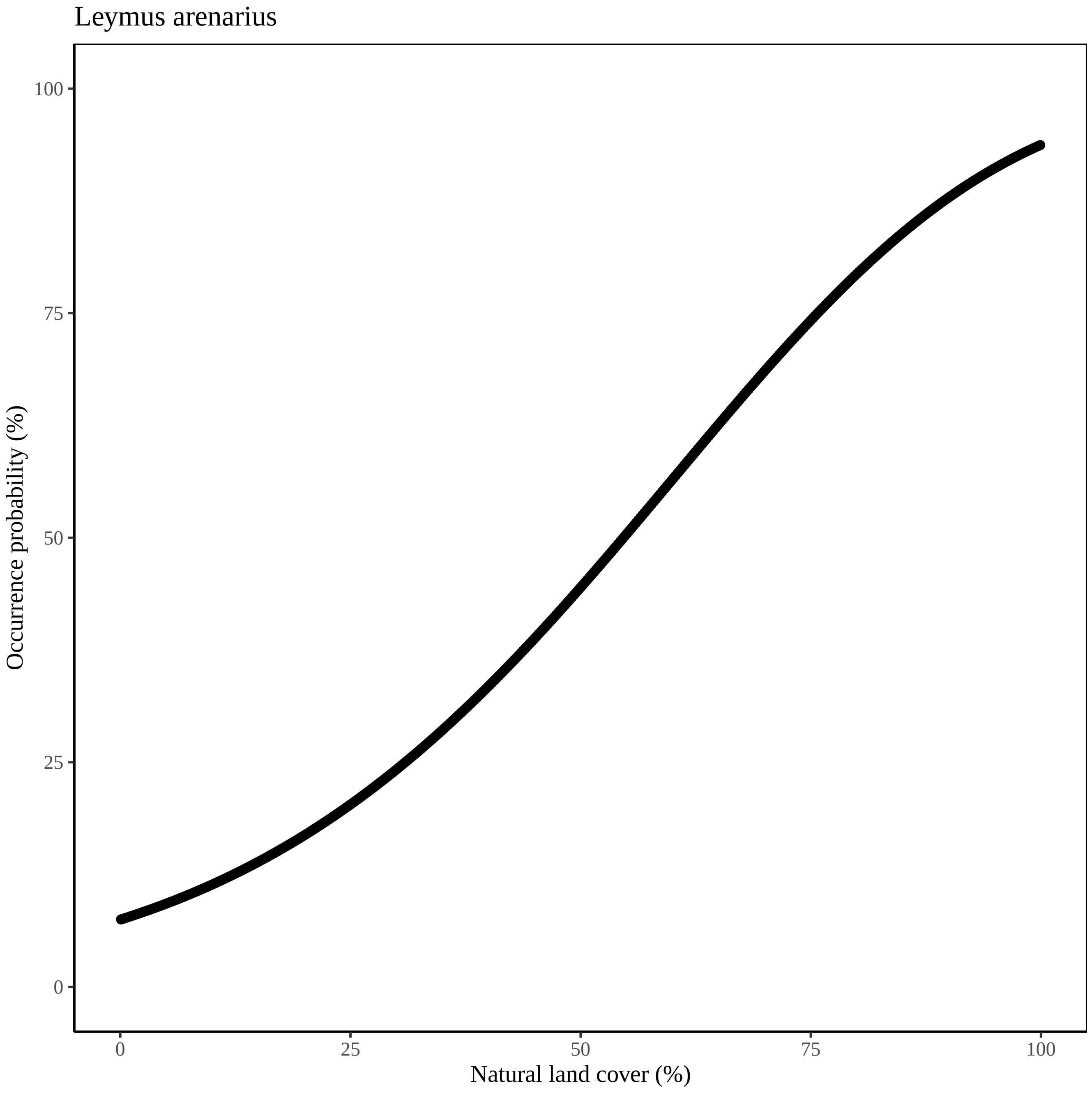

Leymus arenarius
Occurrence probability (%)
100
75
50
25
0
0
25
50
75
100
Natural land cover (%)

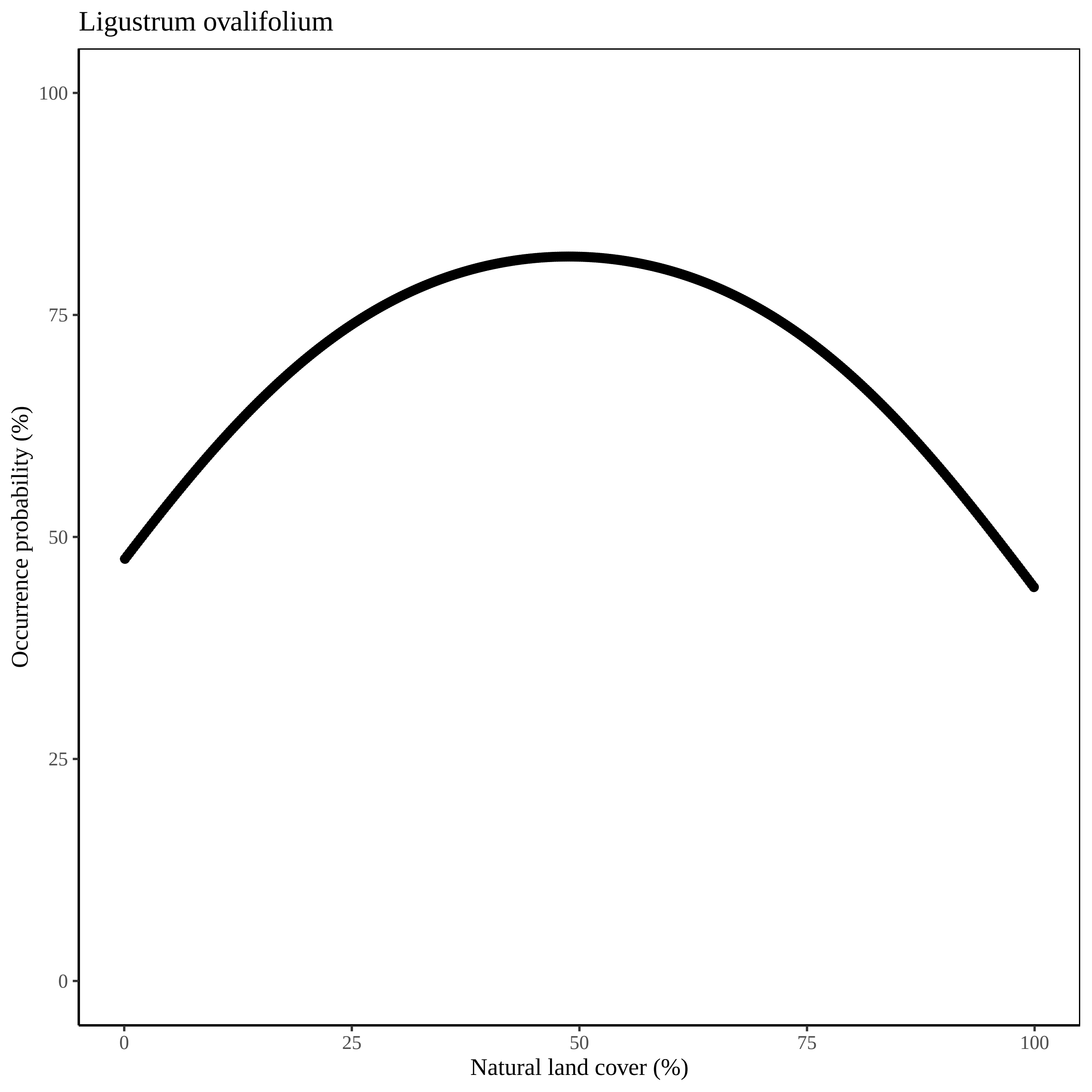
Ligustrum ovalifolium
Occurrence probability (%)
100
75
50
25
0
0
25
50
75
100
Natural land cover (%)

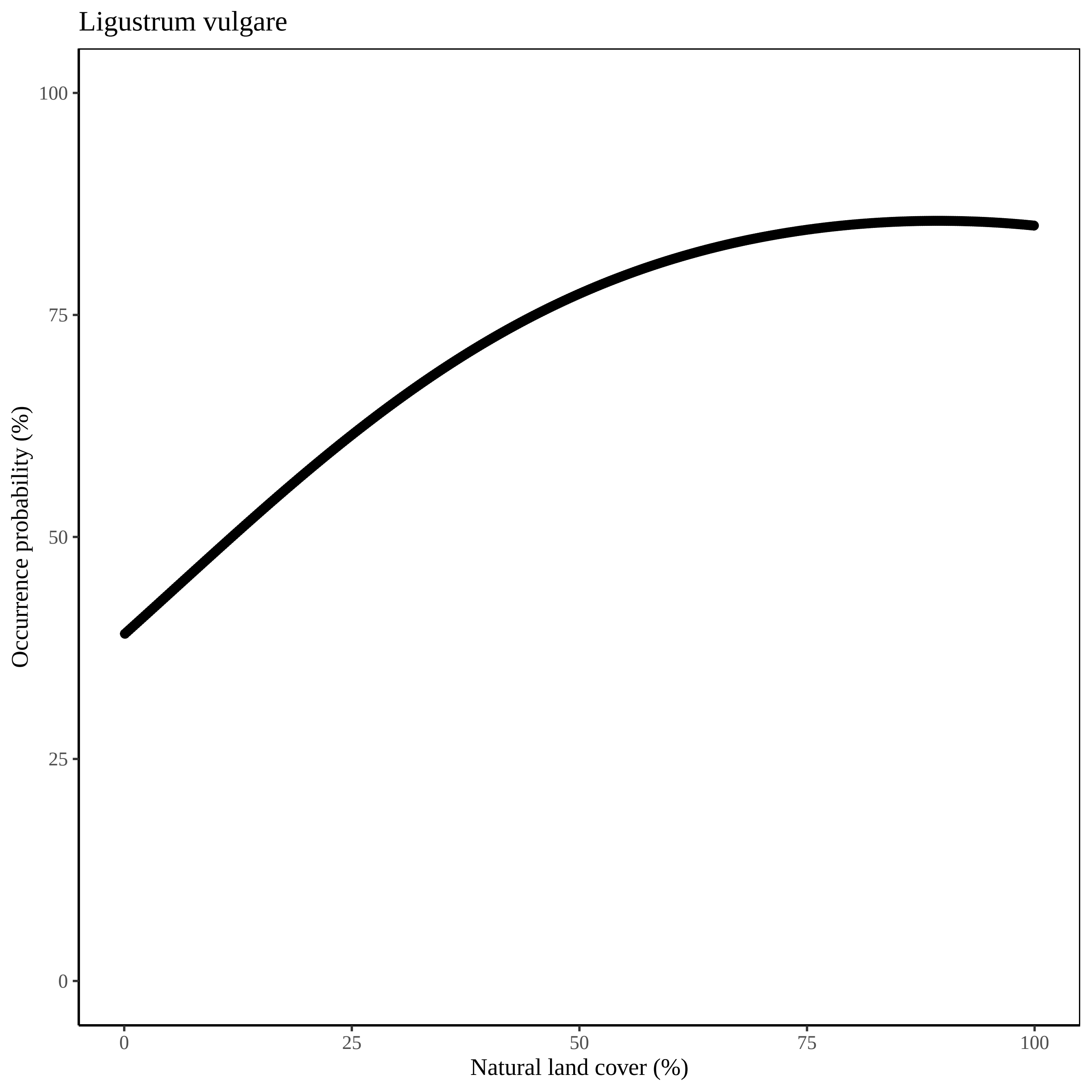

Ligustrum vulgare
Occurrence probability (%)
100
75
50
25
0
0
25
50
75
100
Natural land cover (%)

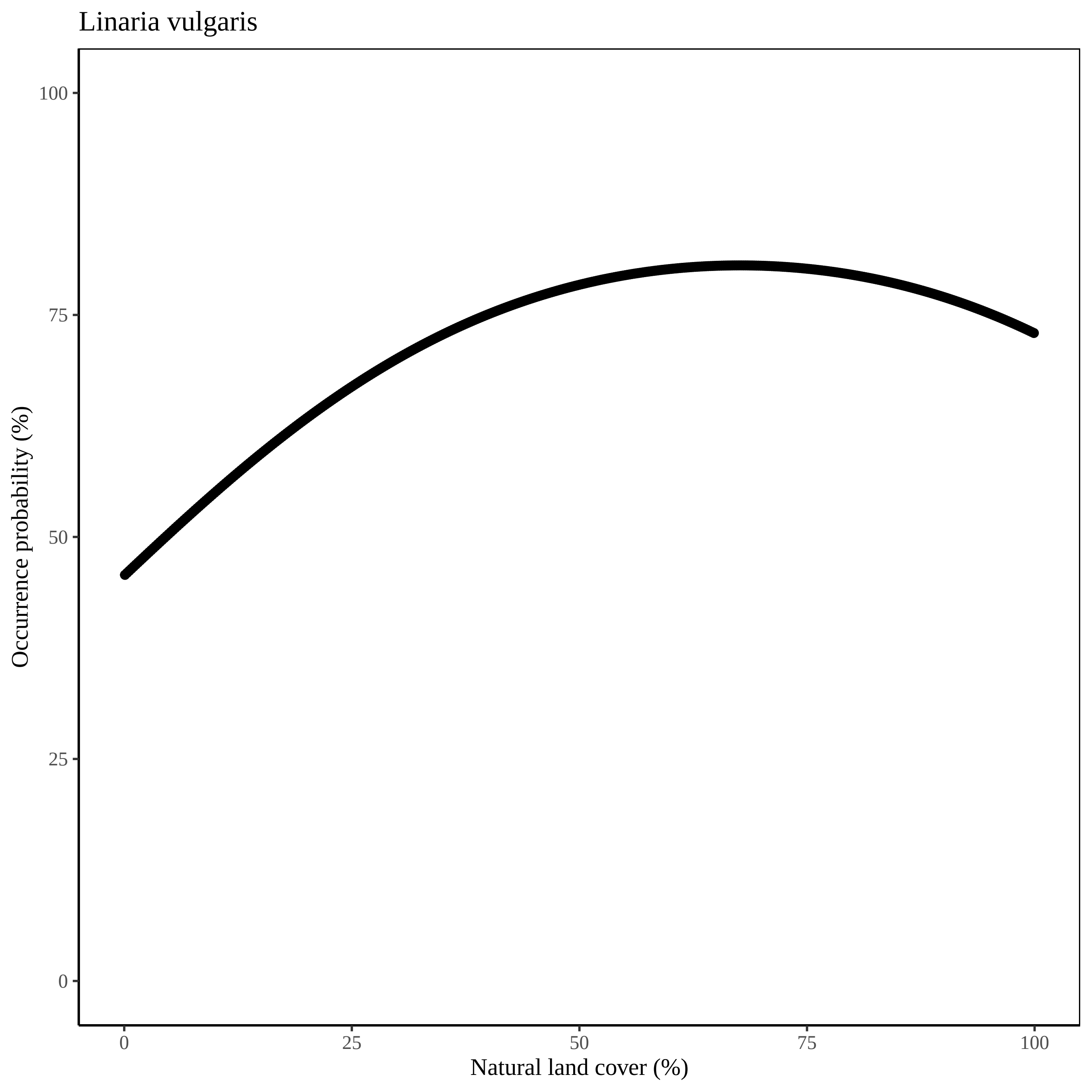
Linaria vulgaris
Occurrence probability (%)
100
75
50
25
0
0
25
50
75
100
Natural land cover (%)

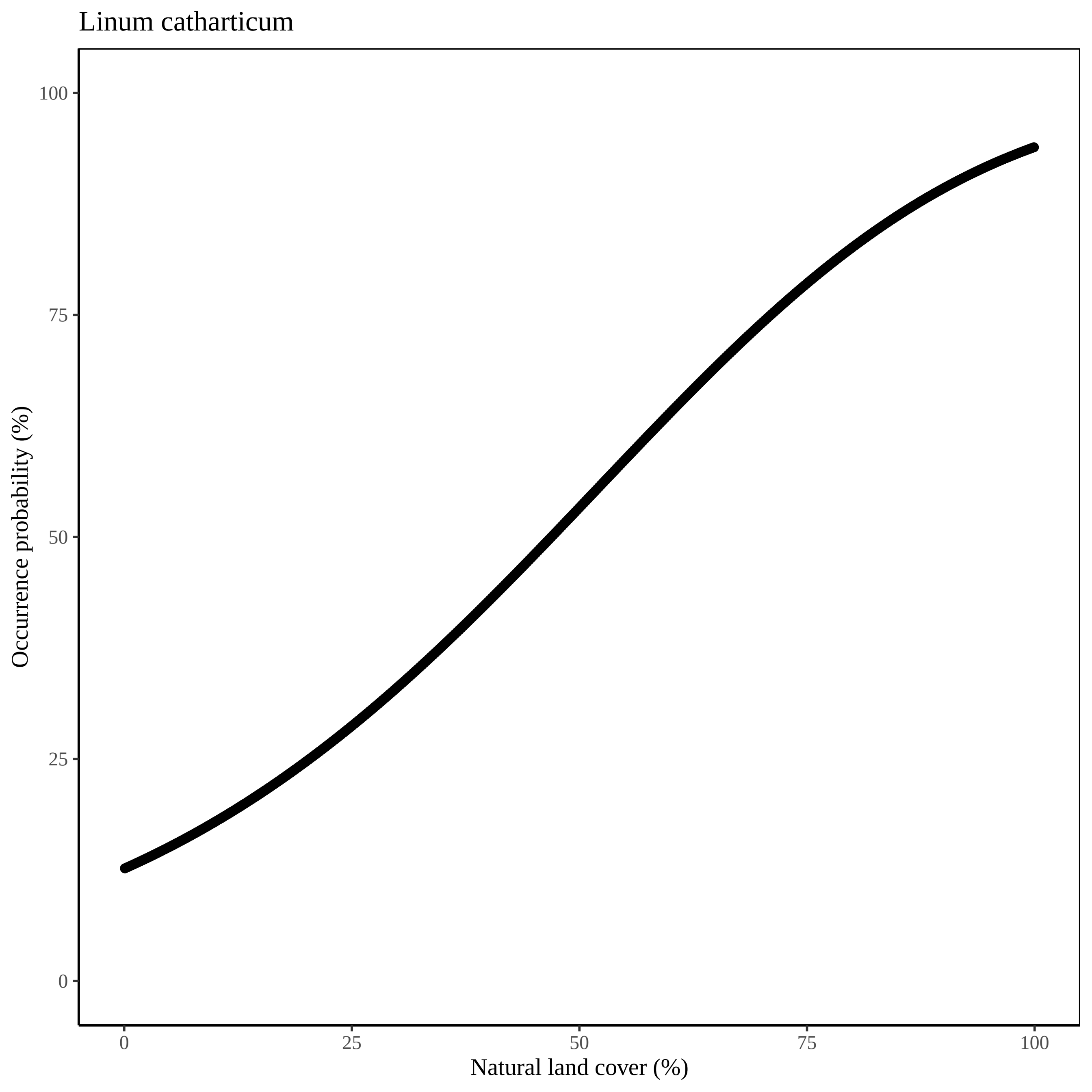
Linum catharticum
Occurrence probability (%)
100
75
50
25
0
0
25
50
75
100
Natural land cover (%)

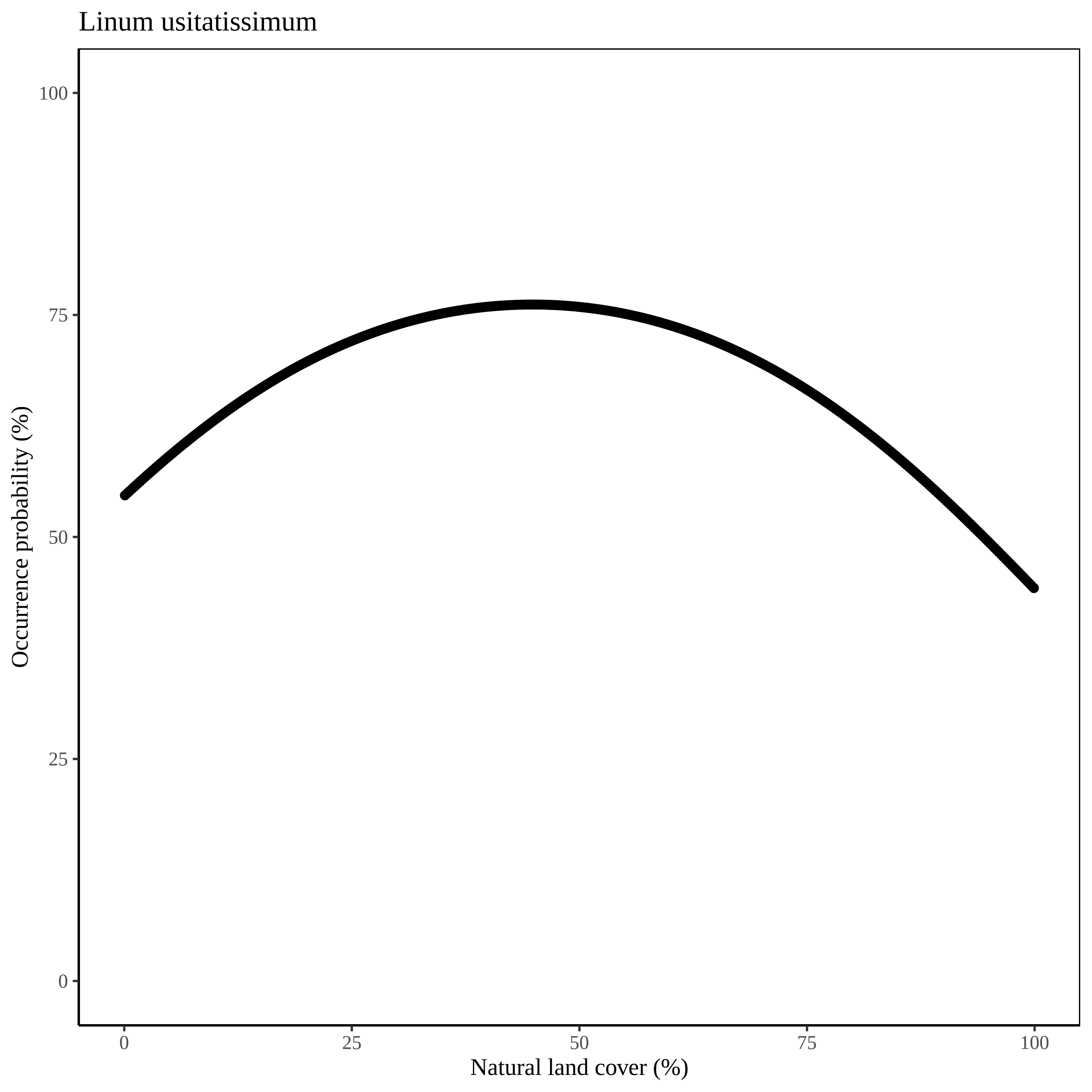
Linum usitatissimum
Occurrence probability (%)
100
75
50
25
0
0
25
50
75
100
Natural land cover (%)

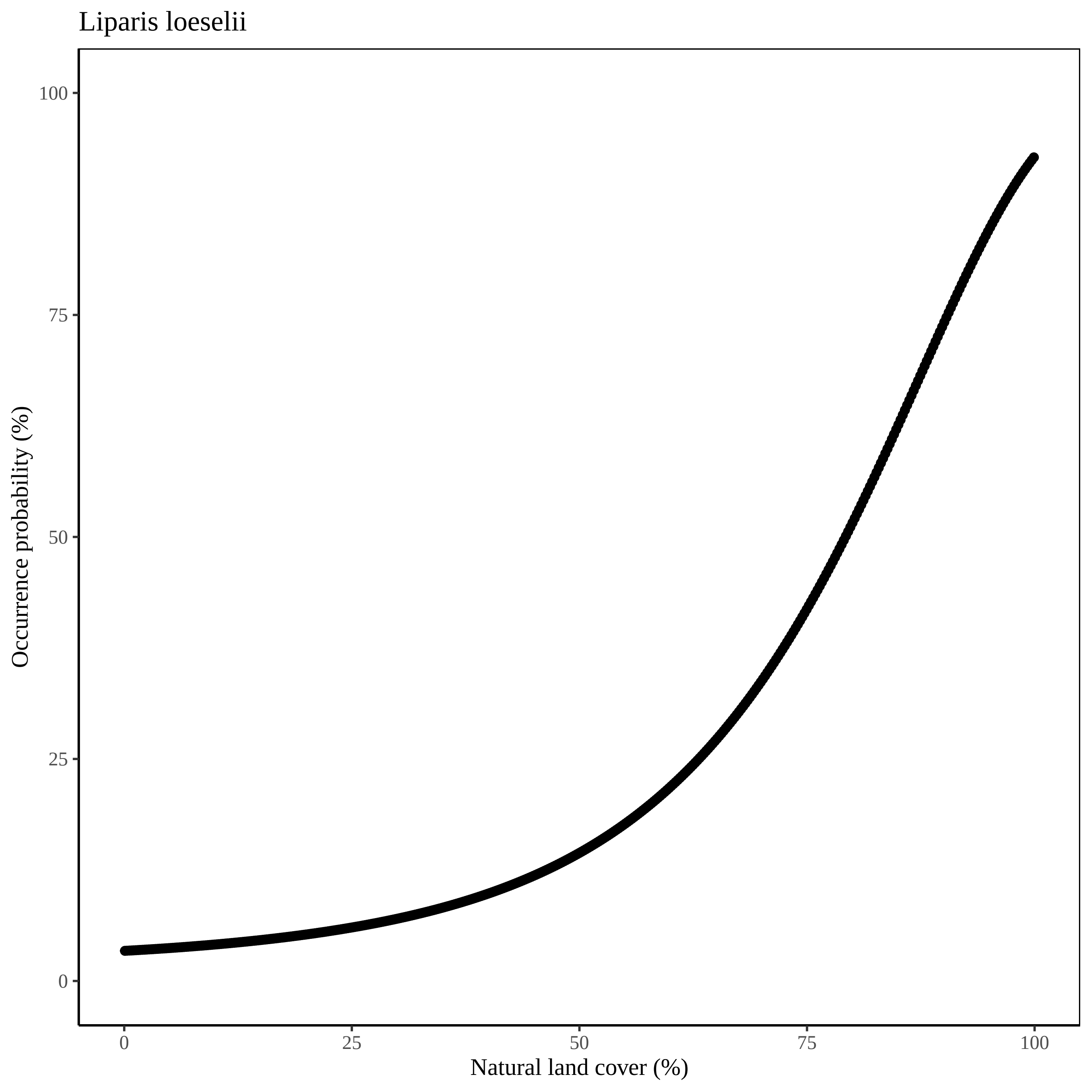
Liparis loeselii
Occurrence probability (%)
100
75
50
25
0
0
25
50
75
100
Natural land cover (%)

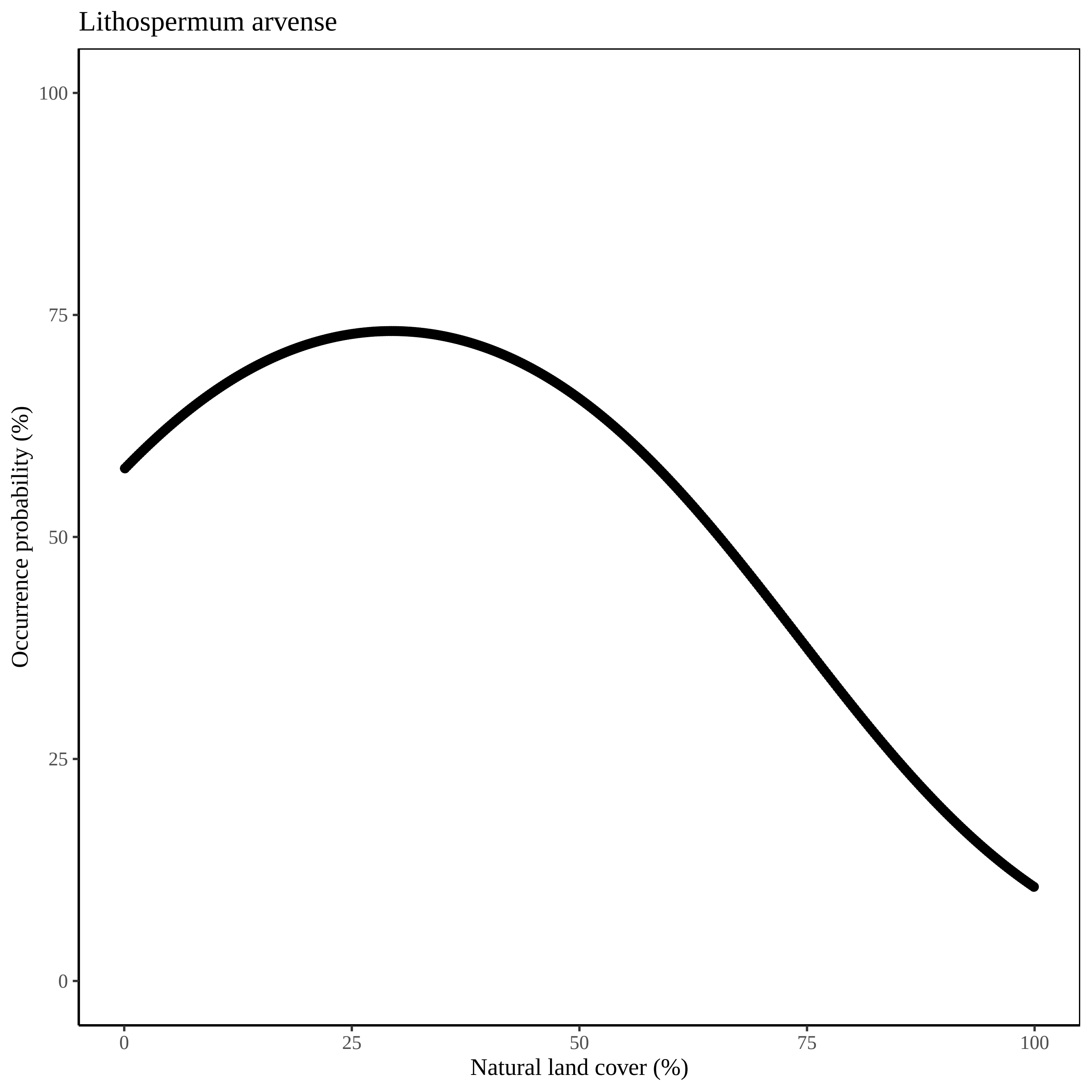
Lithospermum arvense
Occurrence probability (%)
100
75
50
25
0
0
25
50
75
100
Natural land cover (%)

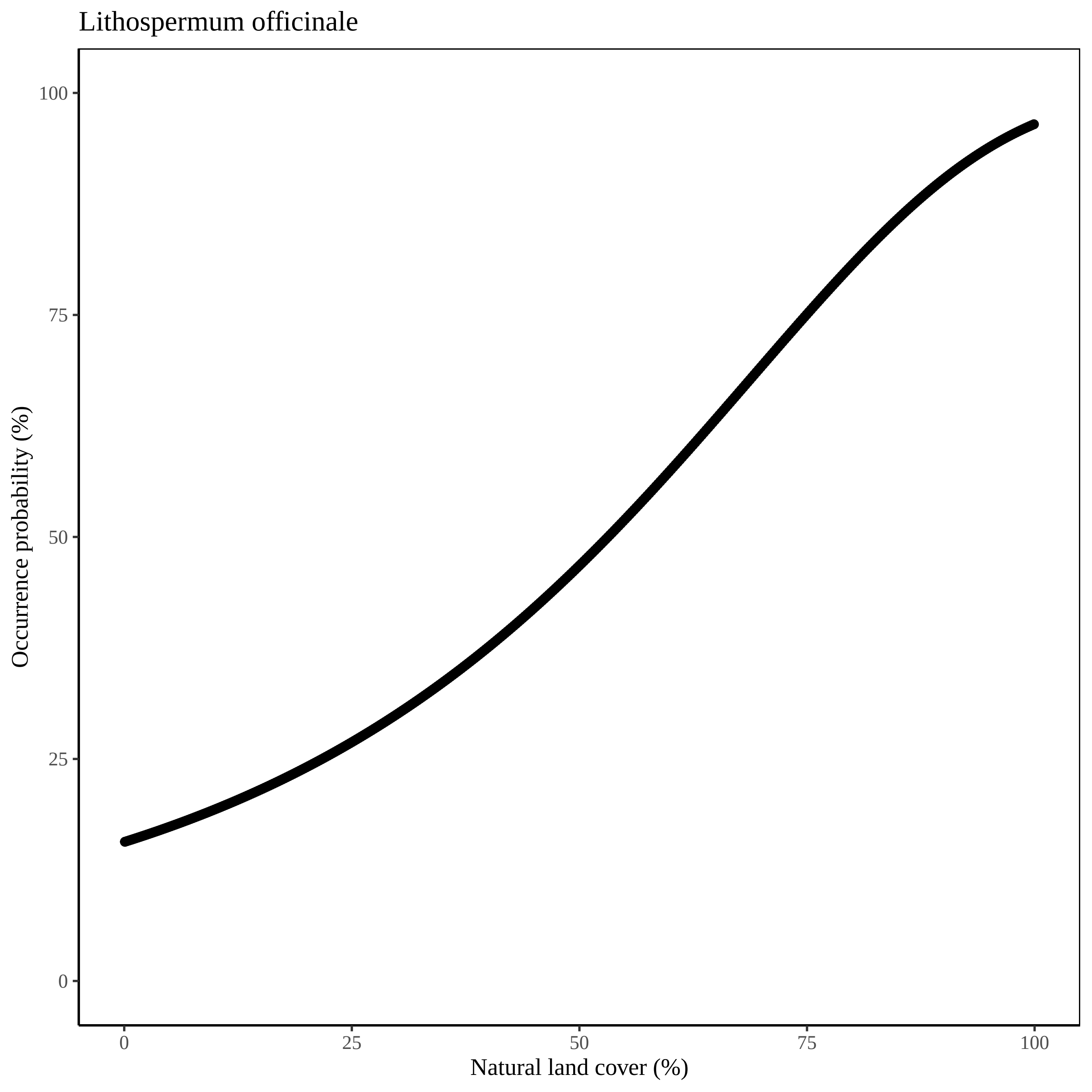
Lithospermum officinale
Occurrence probability (%)
100
75
50
25
0
0
25
50
75
100
Natural land cover (%)

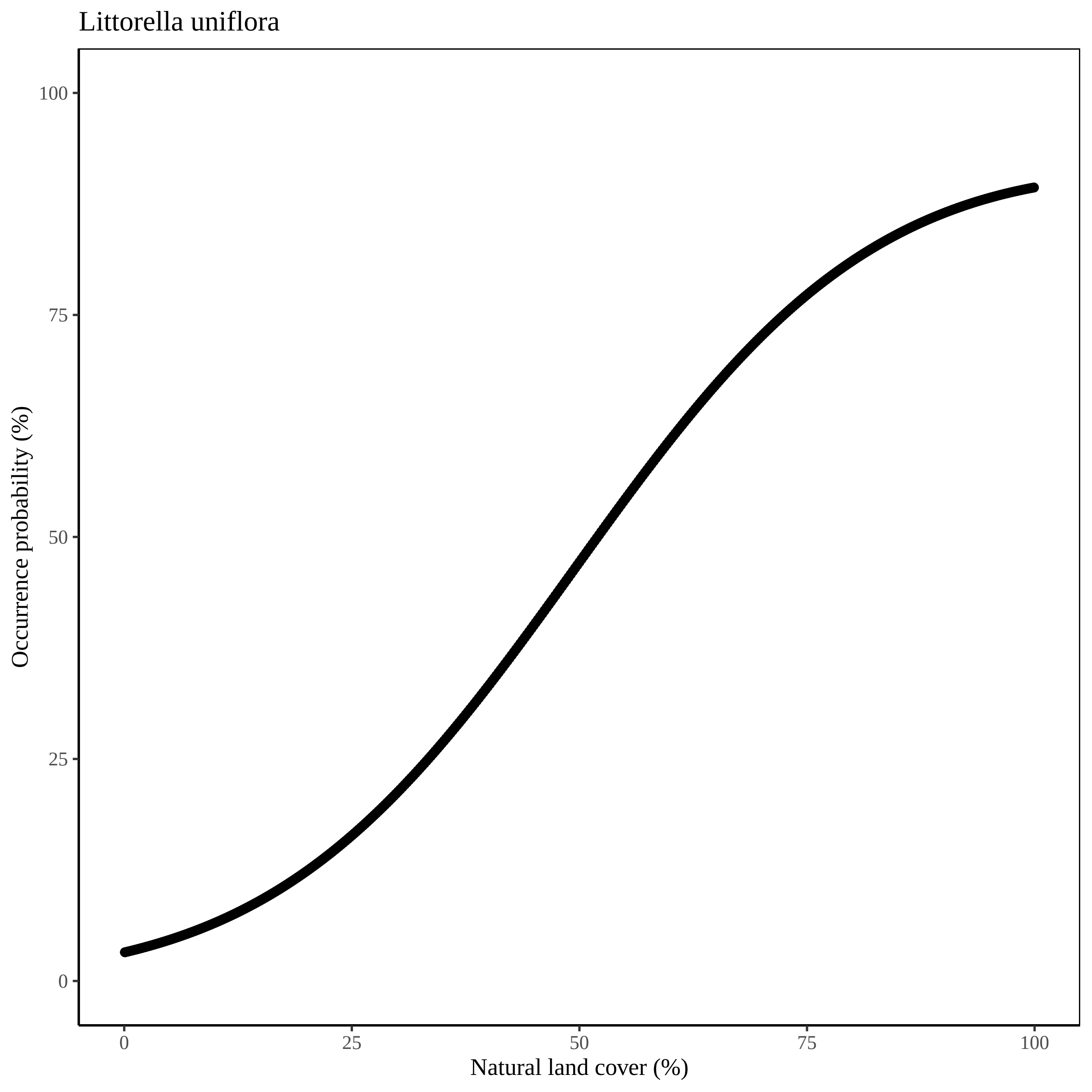
Littorella uniflora
Occurrence probability (%)
100
75
50
25
0
0
25
50
75
100
Natural land cover (%)

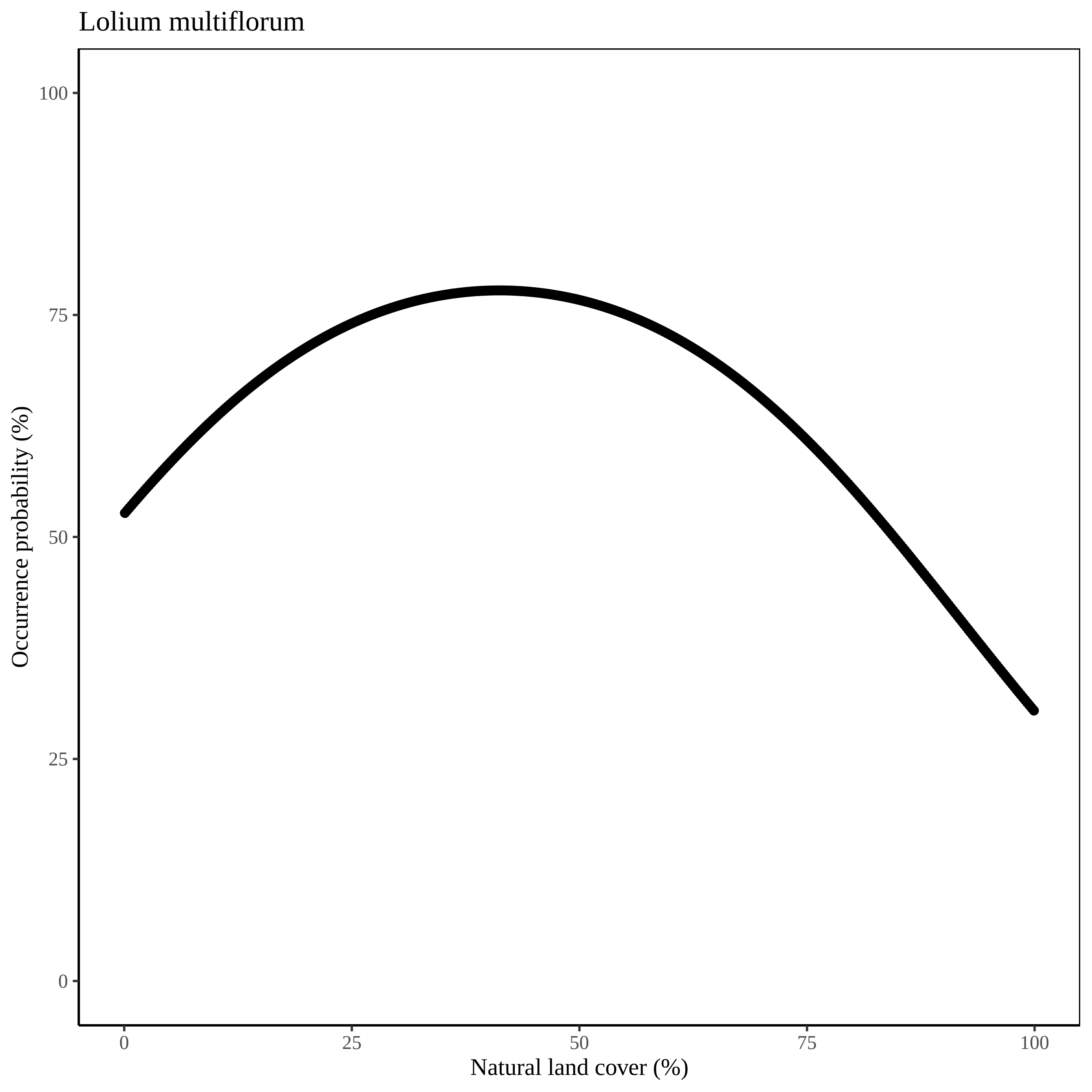

Lolium multiflorum
Occurrence probability (%)
100
75
50
25
0
0
25
50
75
100
Natural land cover (%)

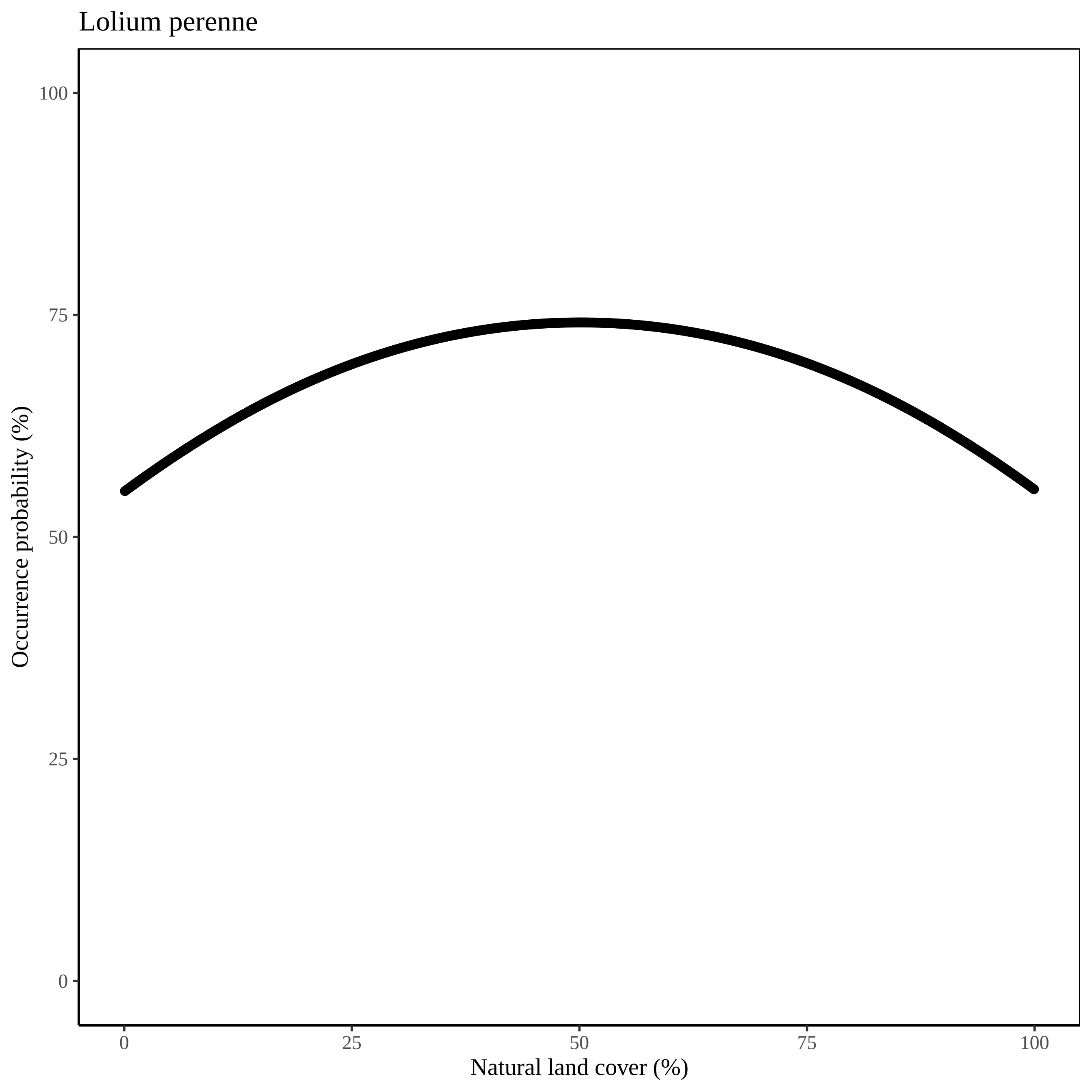
Lolium perenne
100
75
50
25
0
Occurrence probability (%)
0
25
50
75
100
Natural land cover (%)

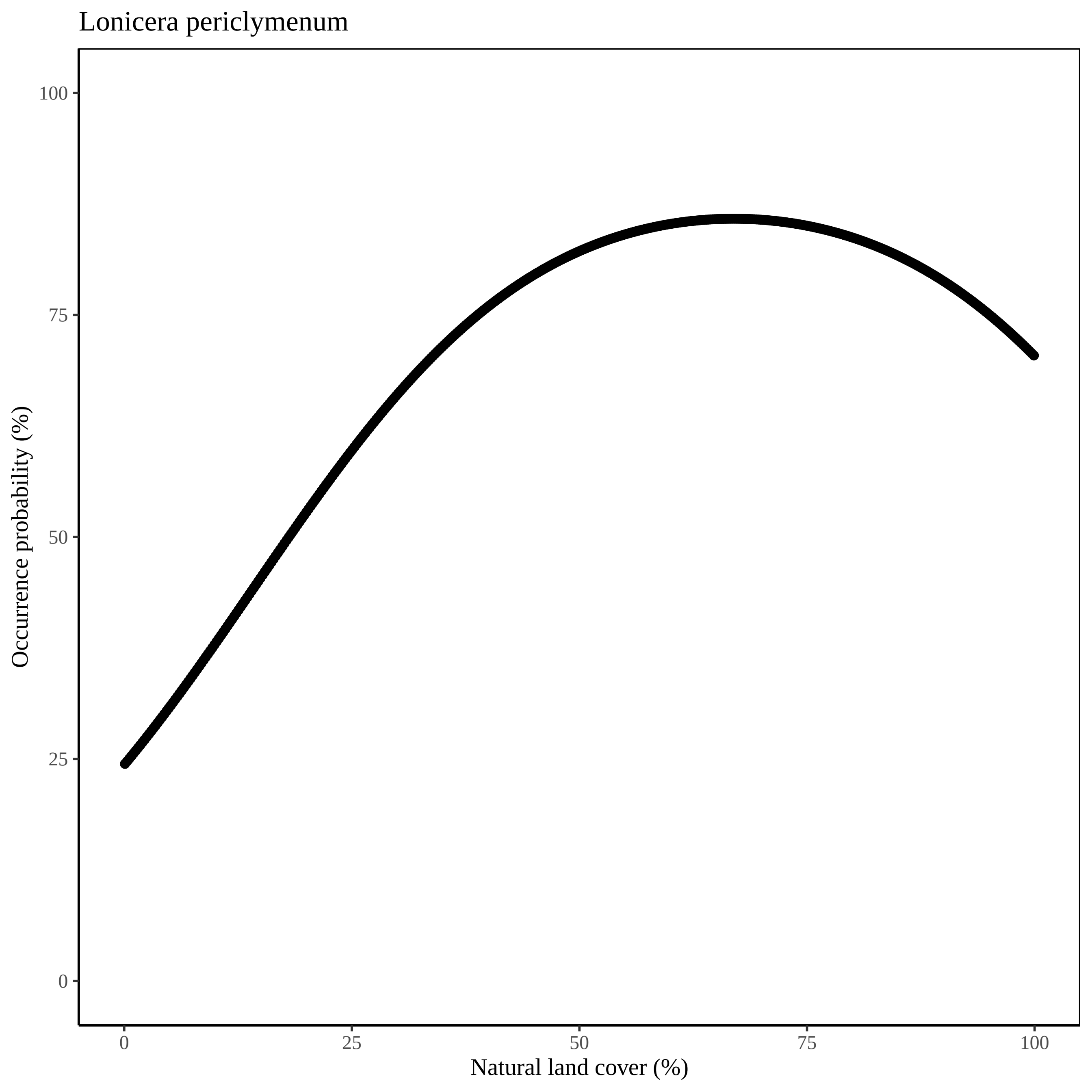
Lonicera periclymenum
Occurrence probability (%)
100
75
50
25
0
0
25
50
75
100
Natural land cover (%)

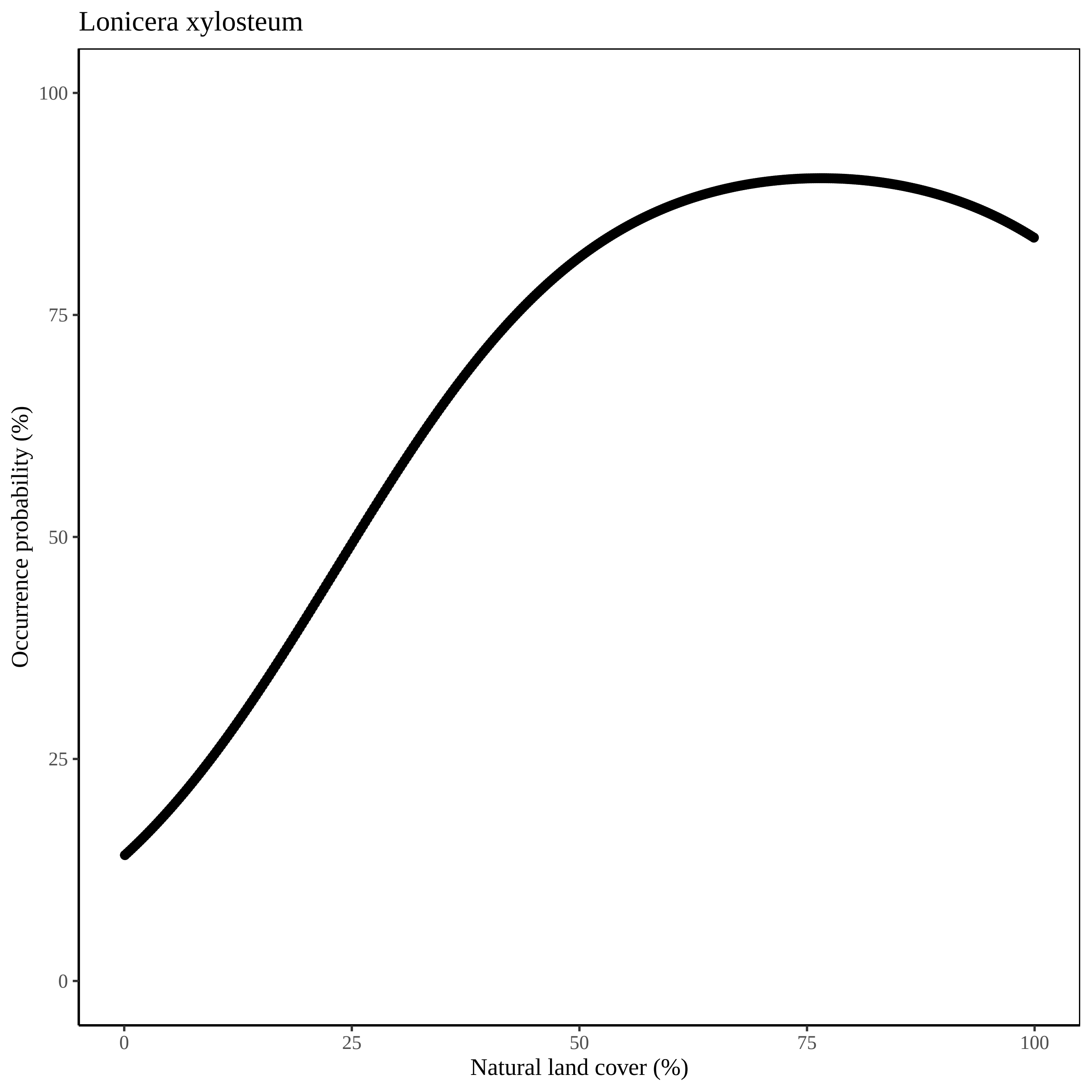
Lonicera xylosteum
Occurrence probability (%)
100
75
50
25
0
0
25
50
75
100
Natural land cover (%)

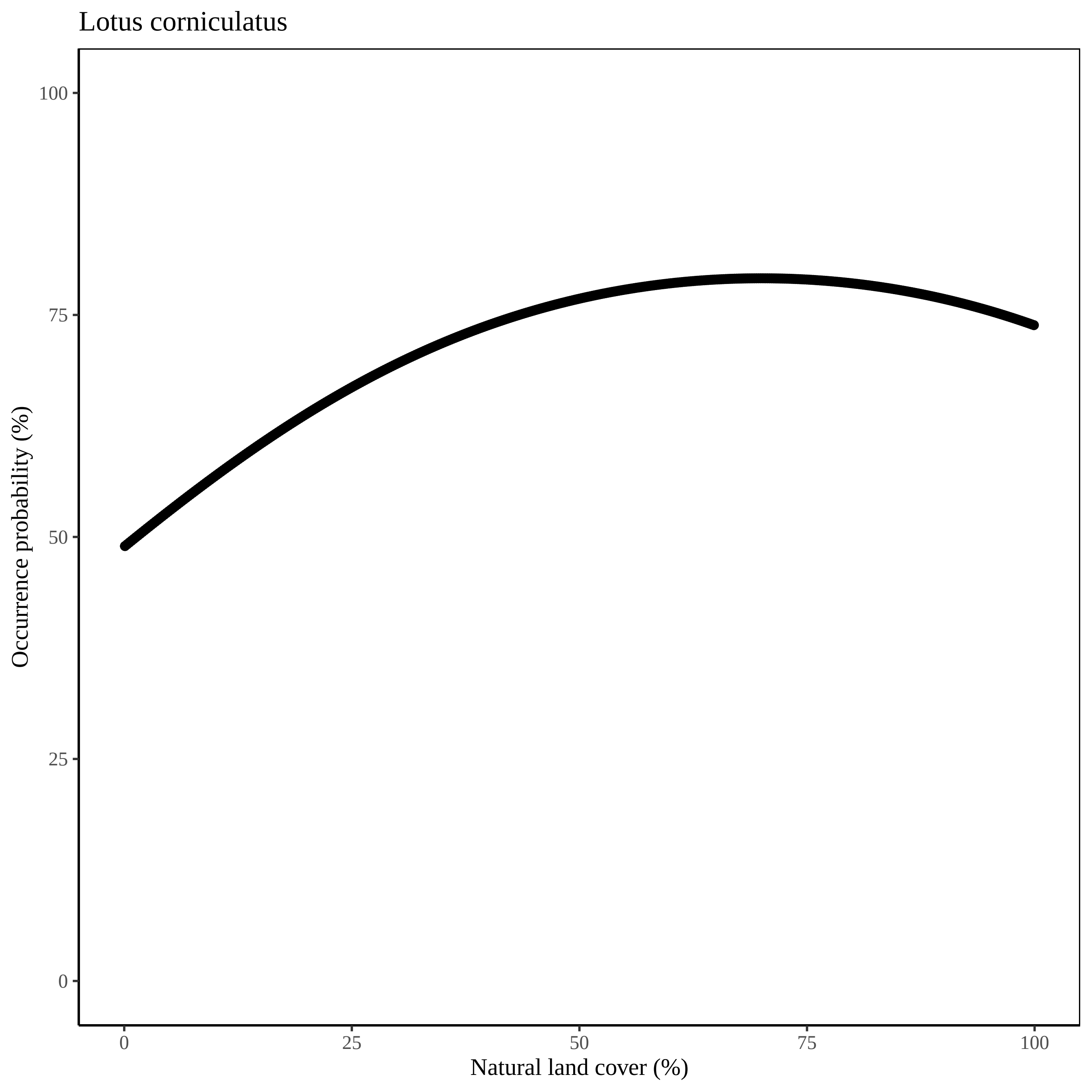
Lotus corniculatus
100
75
50
25
0
Occurrence probability (%)
0
25
50
75
100
Natural land cover (%)

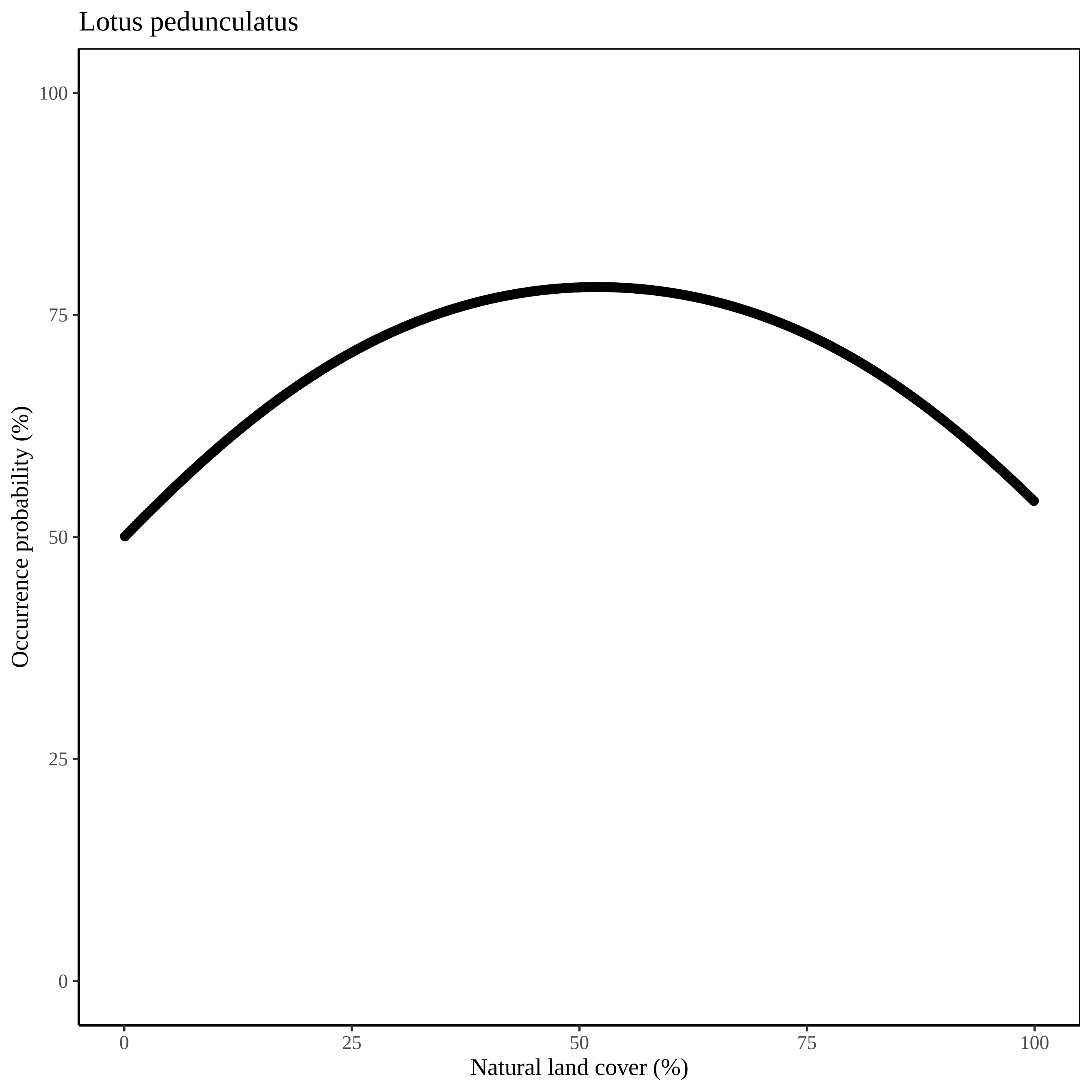
Lotus pedunculatus
Occurrence probability (%)
100
75
50
25
0
0
25
50
75
100
Natural land cover (%)

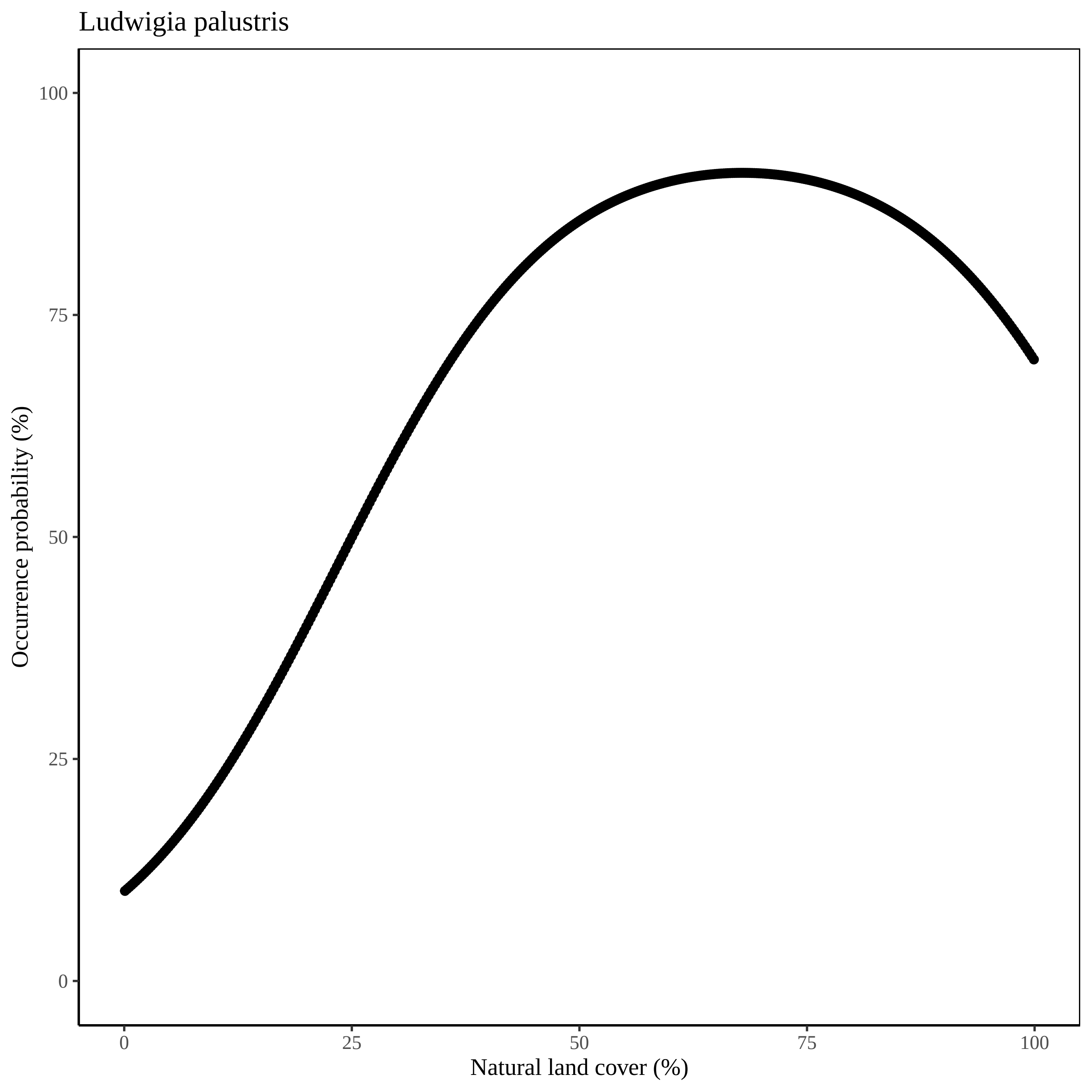

Ludwigia palustris
100
75
50
25
0
Occurrence probability (%)
0
25
50
75
100
Natural land cover (%)

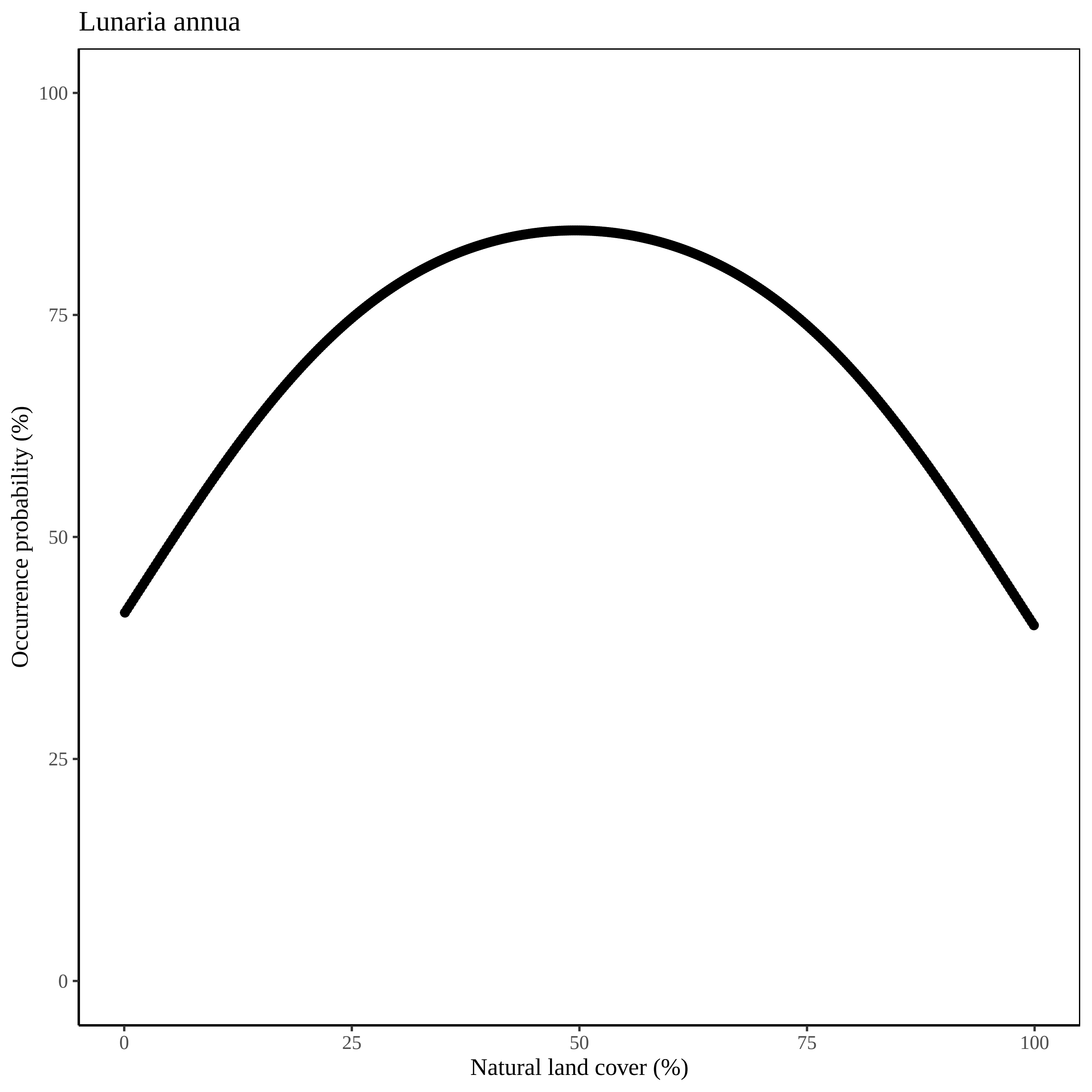
Lunaria annua
Occurrence probability (%)
100
75
50
25
0
0
25
50
75
100
Natural land cover (%)

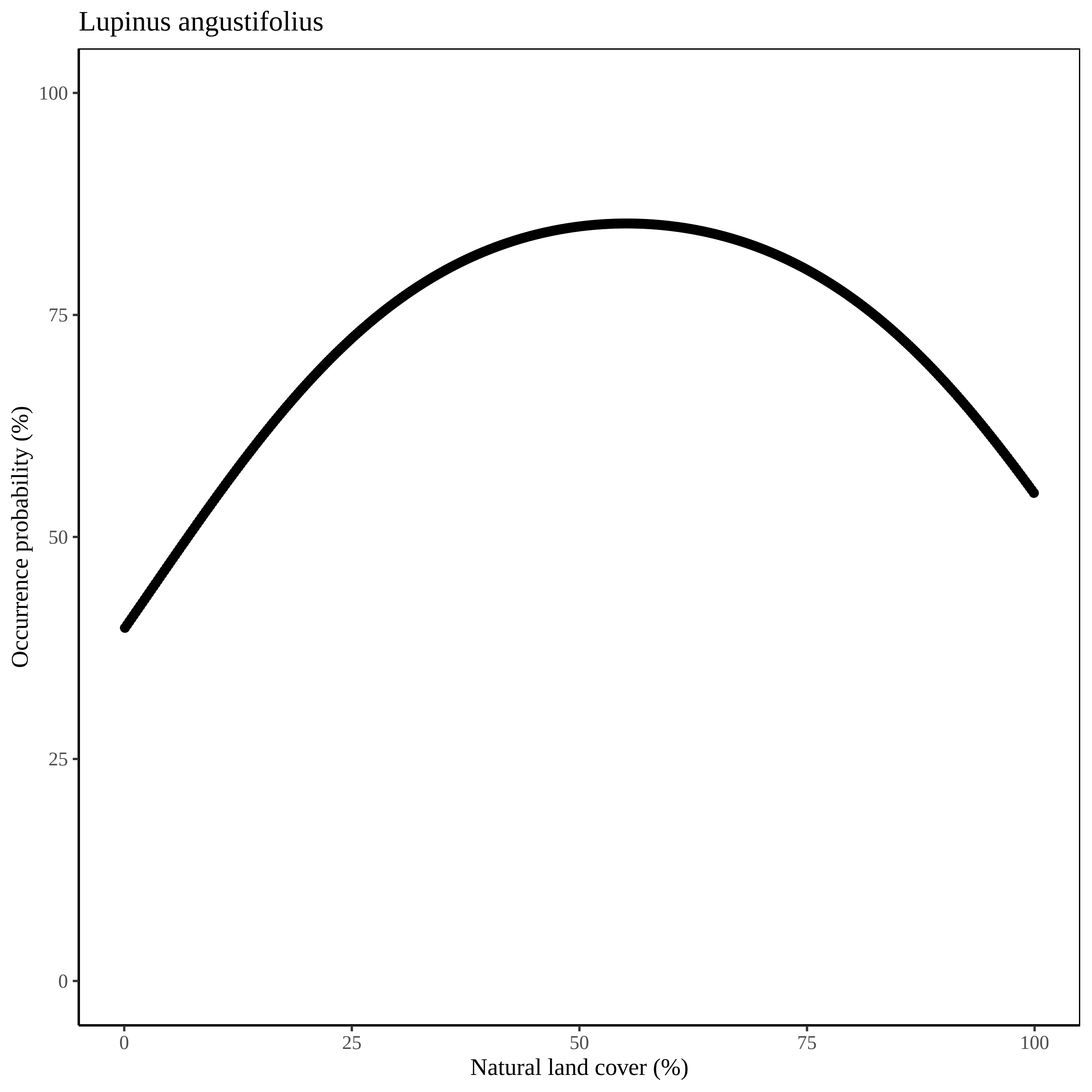
Lupinus angustifolius
Occurrence probability (%)
100
75
50
25
0
0
25
50
75
100
Natural land cover (%)

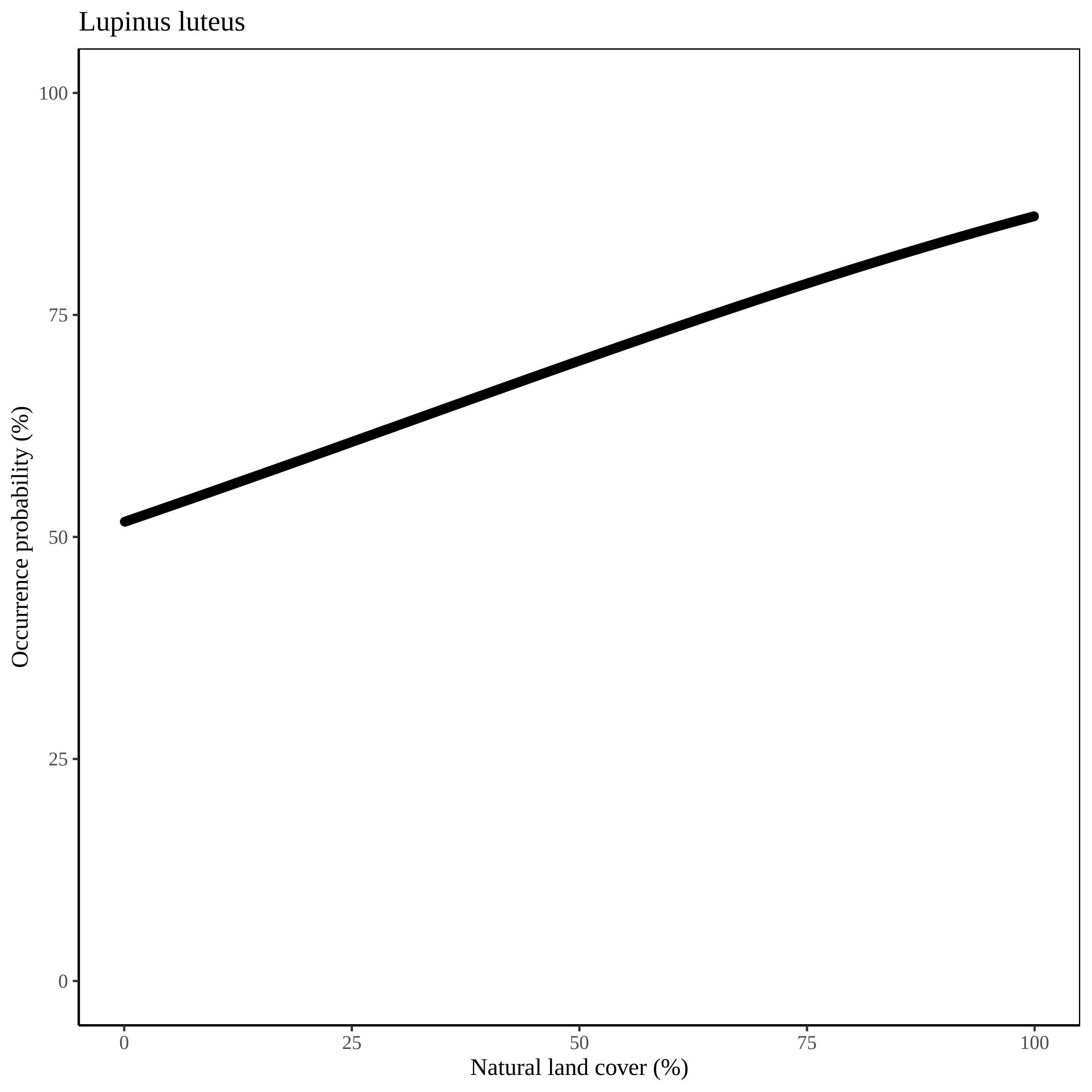
Lupinus luteus
Occurrence probability (%)
100
75
50
25
0
0
25
50
75
100
Natural land cover (%)

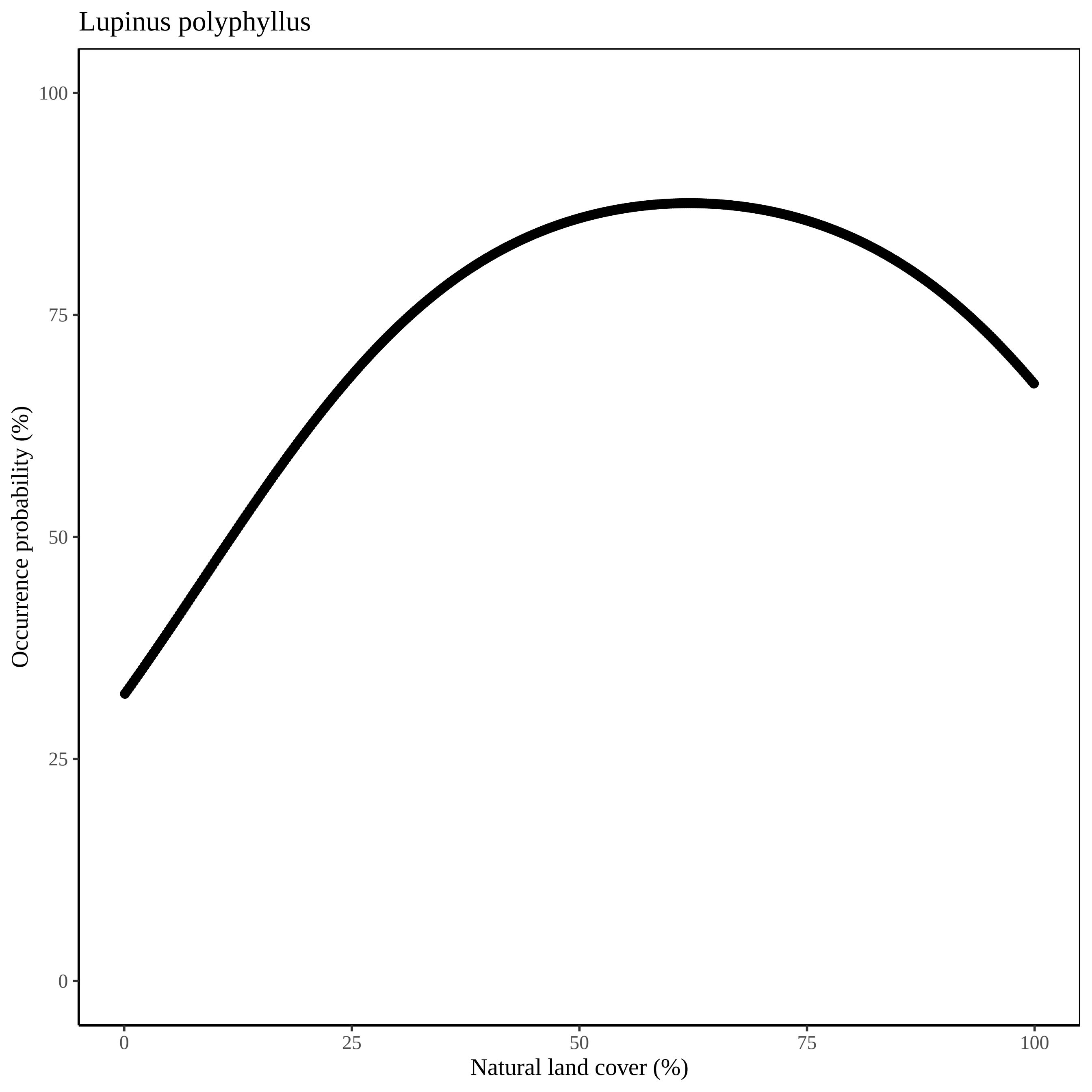
Lupinus polyphyllus
100
75
50
25
0
Occurrence probability (%)
0
25
50
75
100
Natural land cover (%)

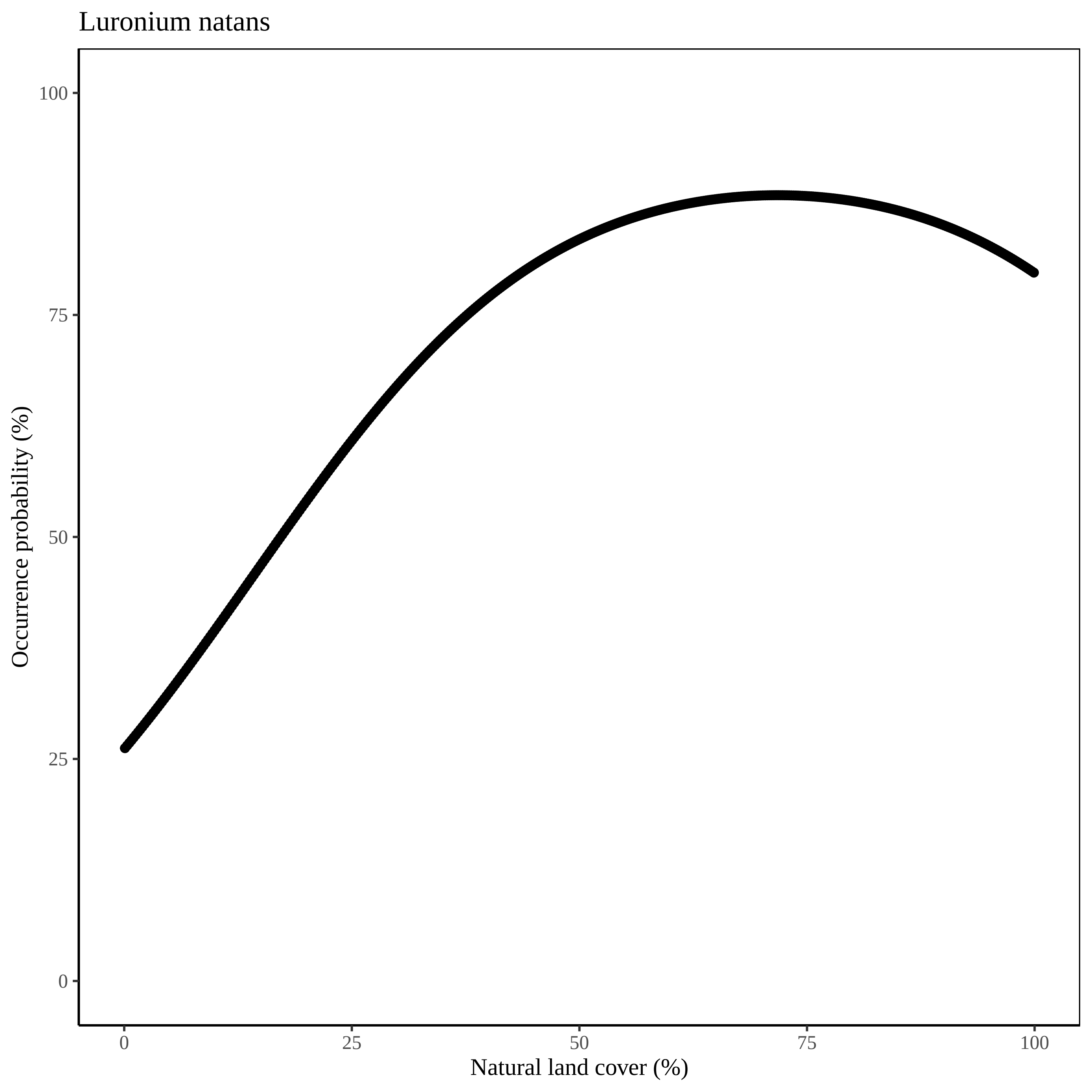
Luronium natans
Occurrence probability (%)
Natural land cover (%)
0
25
50
75
100

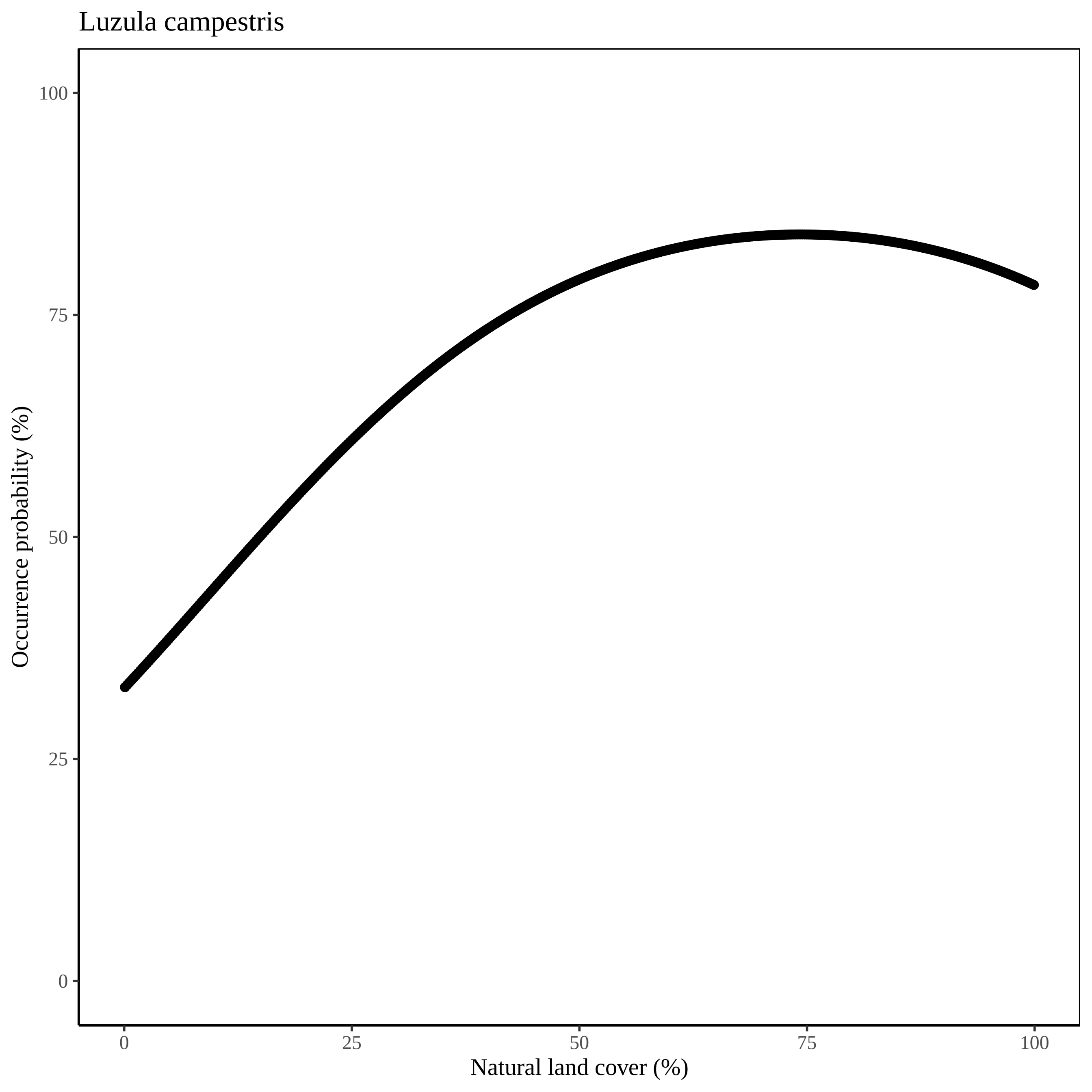
Luzula campestris
Occurrence probability (%)
100
75
50
25
0
0
25
50
75
100
Natural land cover (%)

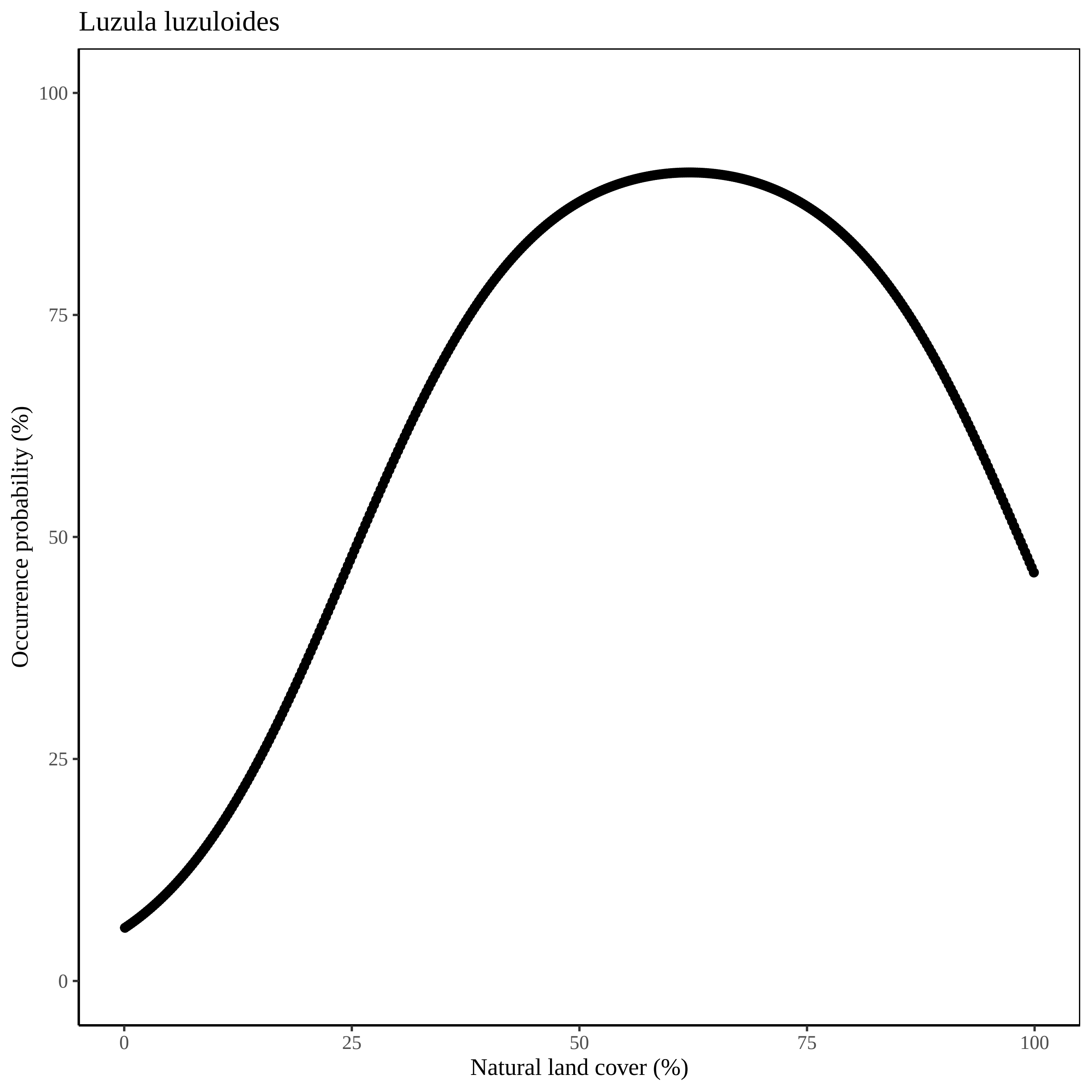
Luzula luzuloides
Occurrence probability (%)
100
75
50
25
0
0
25
50
75
100
Natural land cover (%)

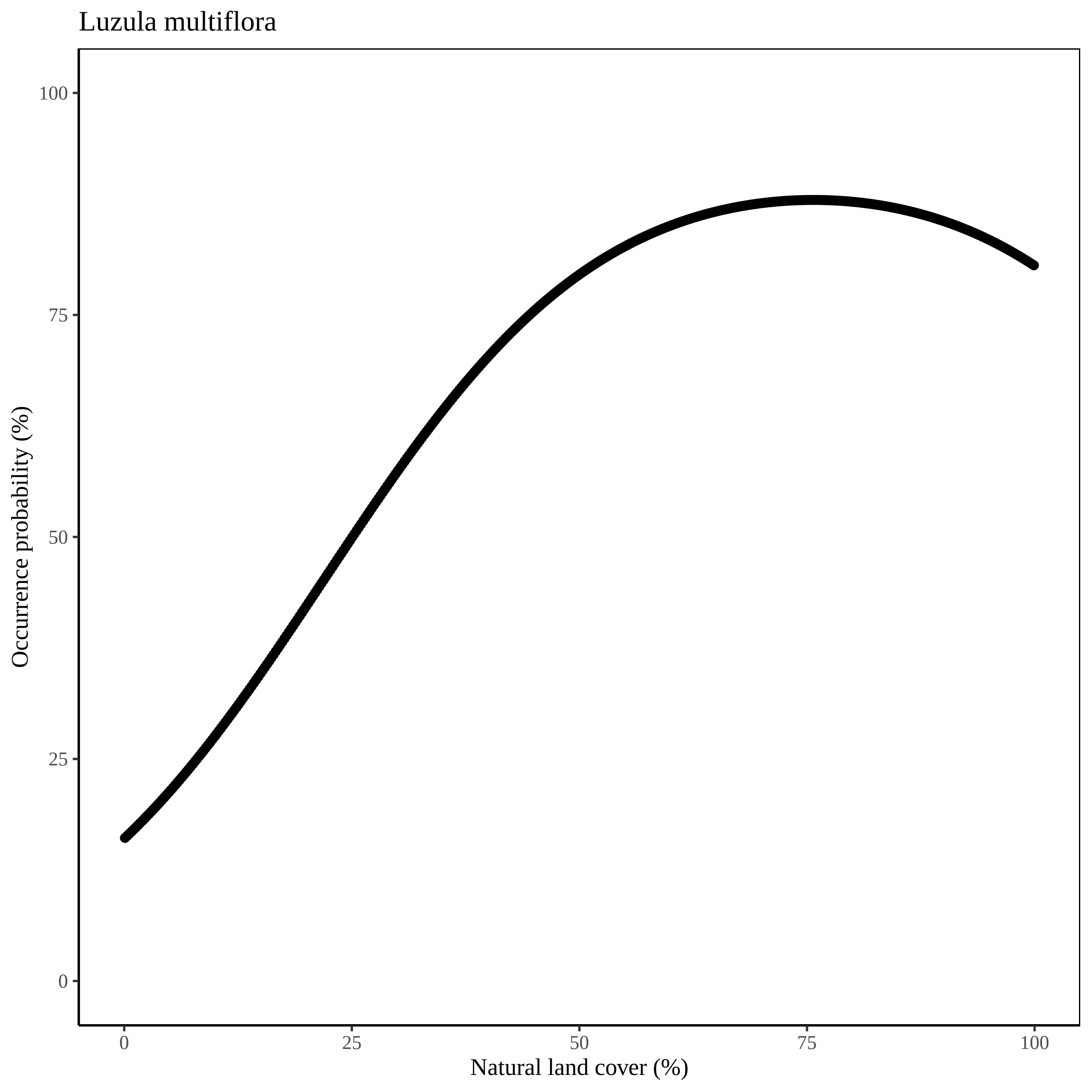
Luzula multiflora
Occurrence probability (%)
100
75
50
25
0
0
25
50
75
100
Natural land cover (%)

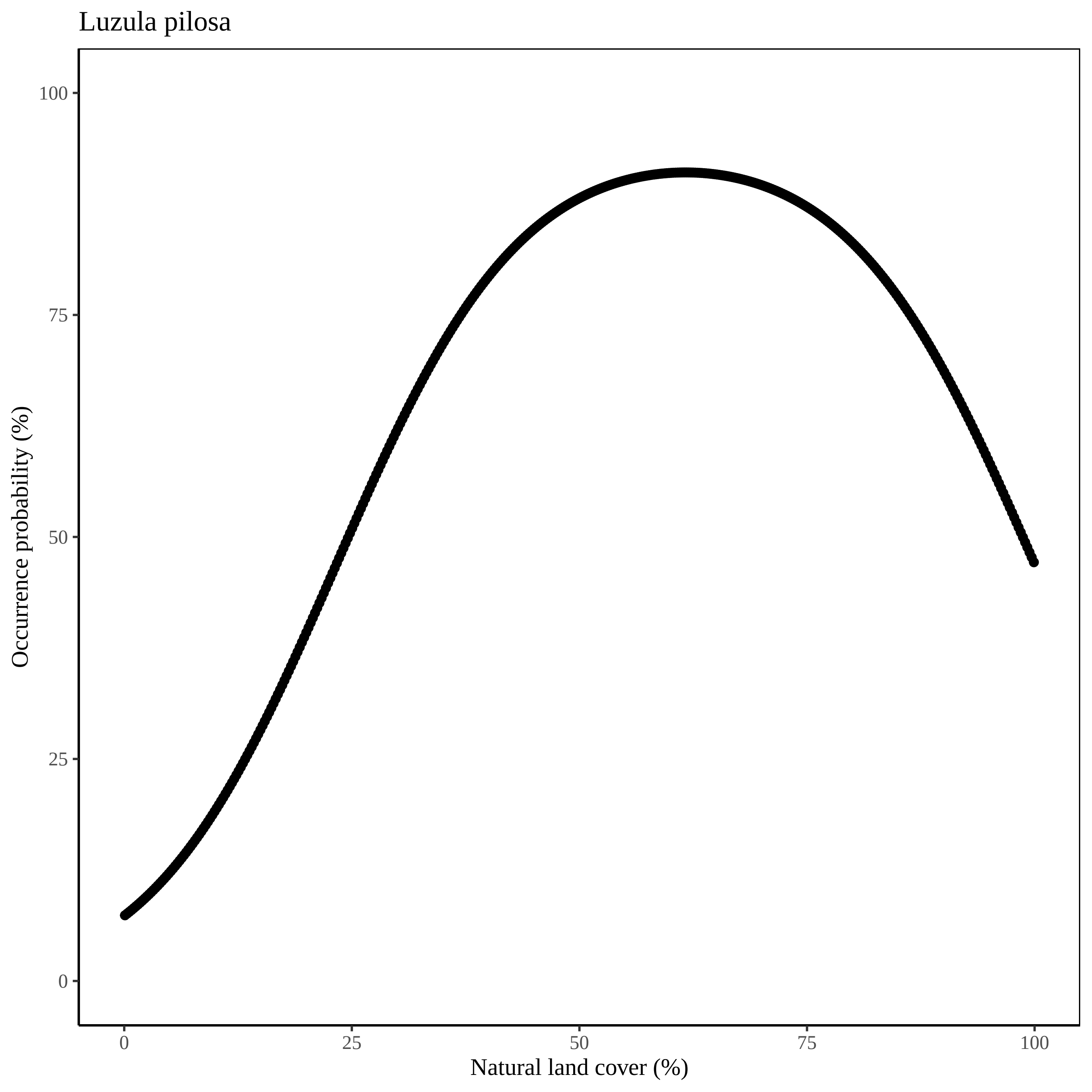
Luzula pilosa
Occurrence probability (%)
100
75
50
25
0
0
25
50
75
100
Natural land cover (%)

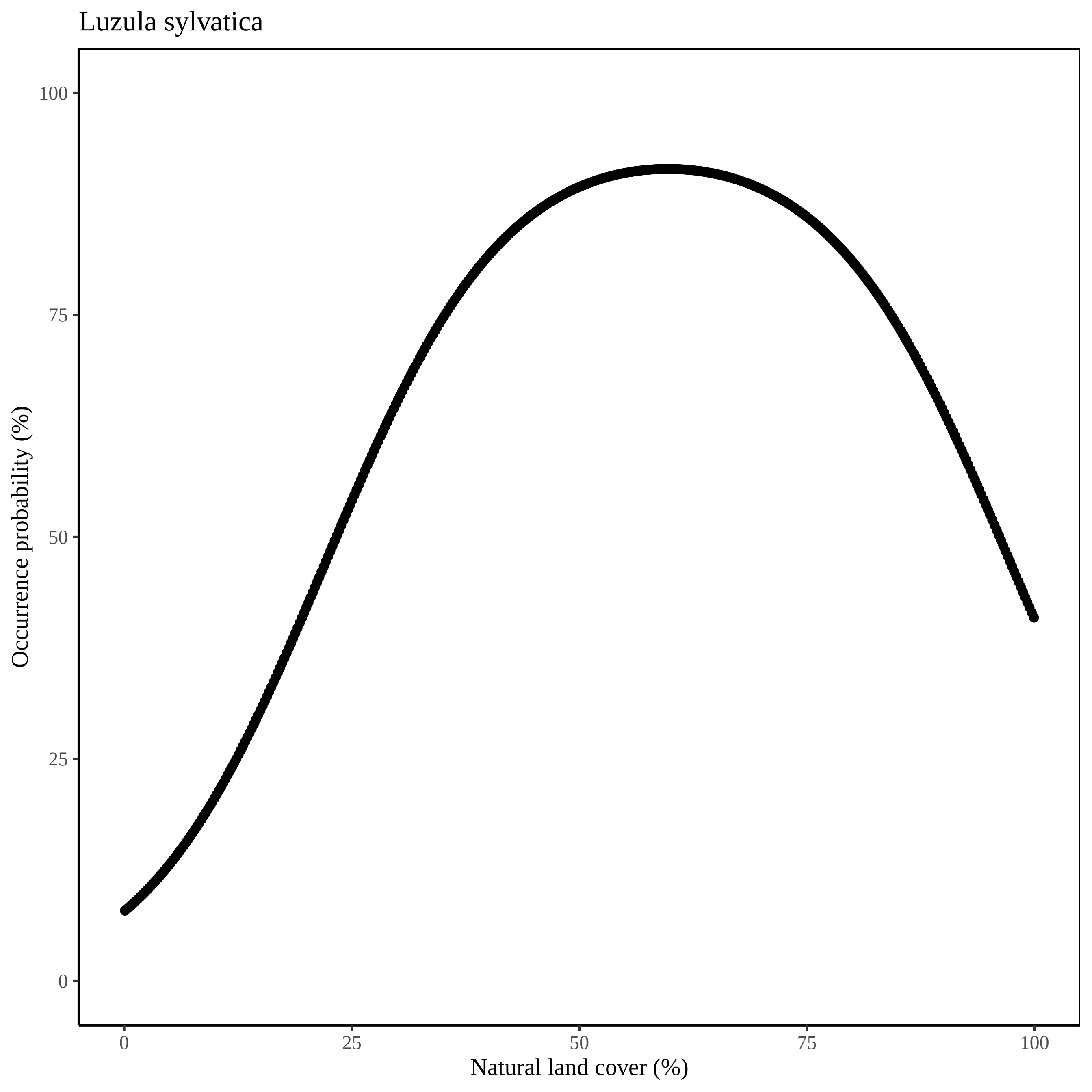

Luzula sylvatica
100
75
50
25
0
Occurrence probability (%)
0
25
50
75
100
Natural land cover (%)

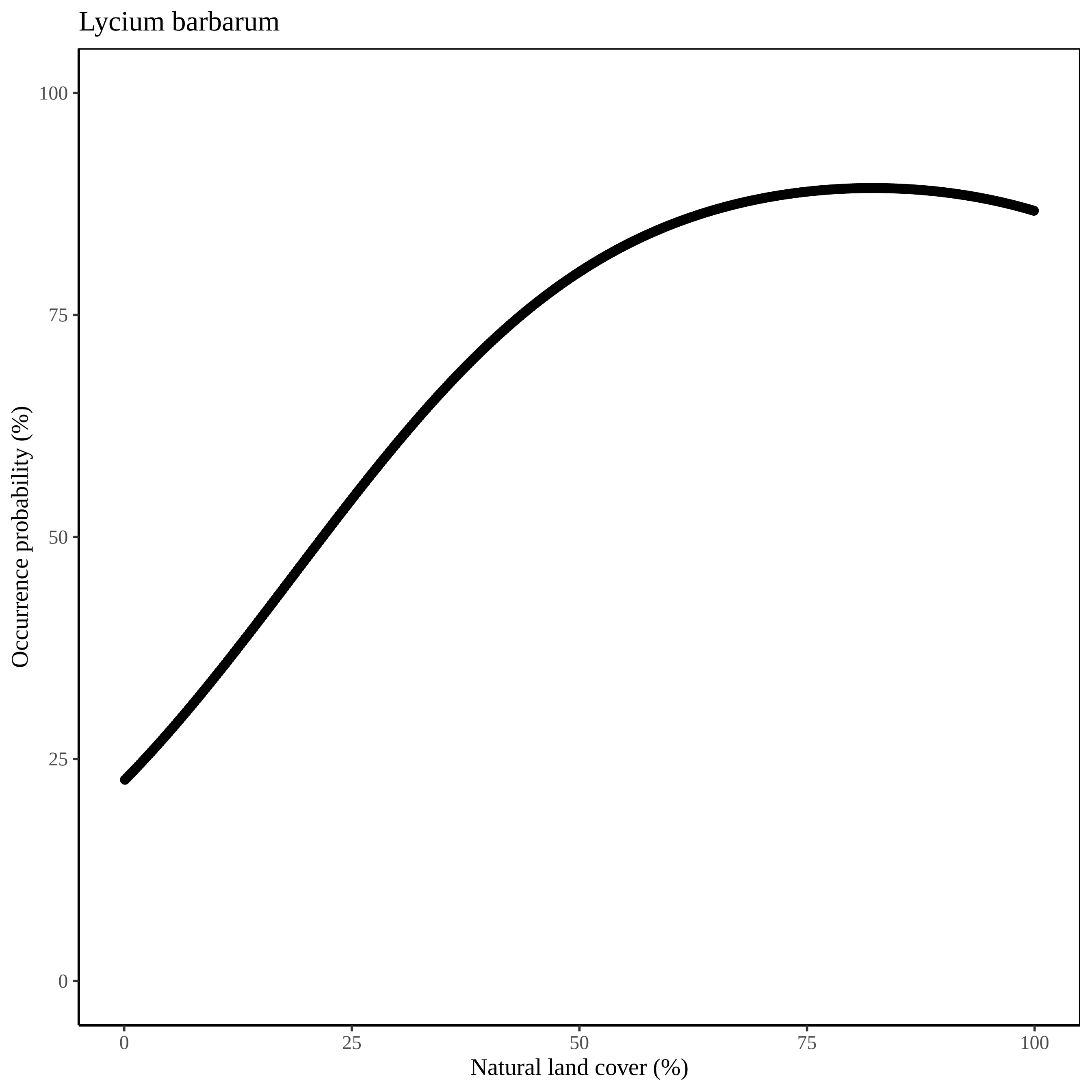
Lycium barbarum
Occurrence probability (%)
100
75
50
25
0
0
25
50
75
100
Natural land cover (%)

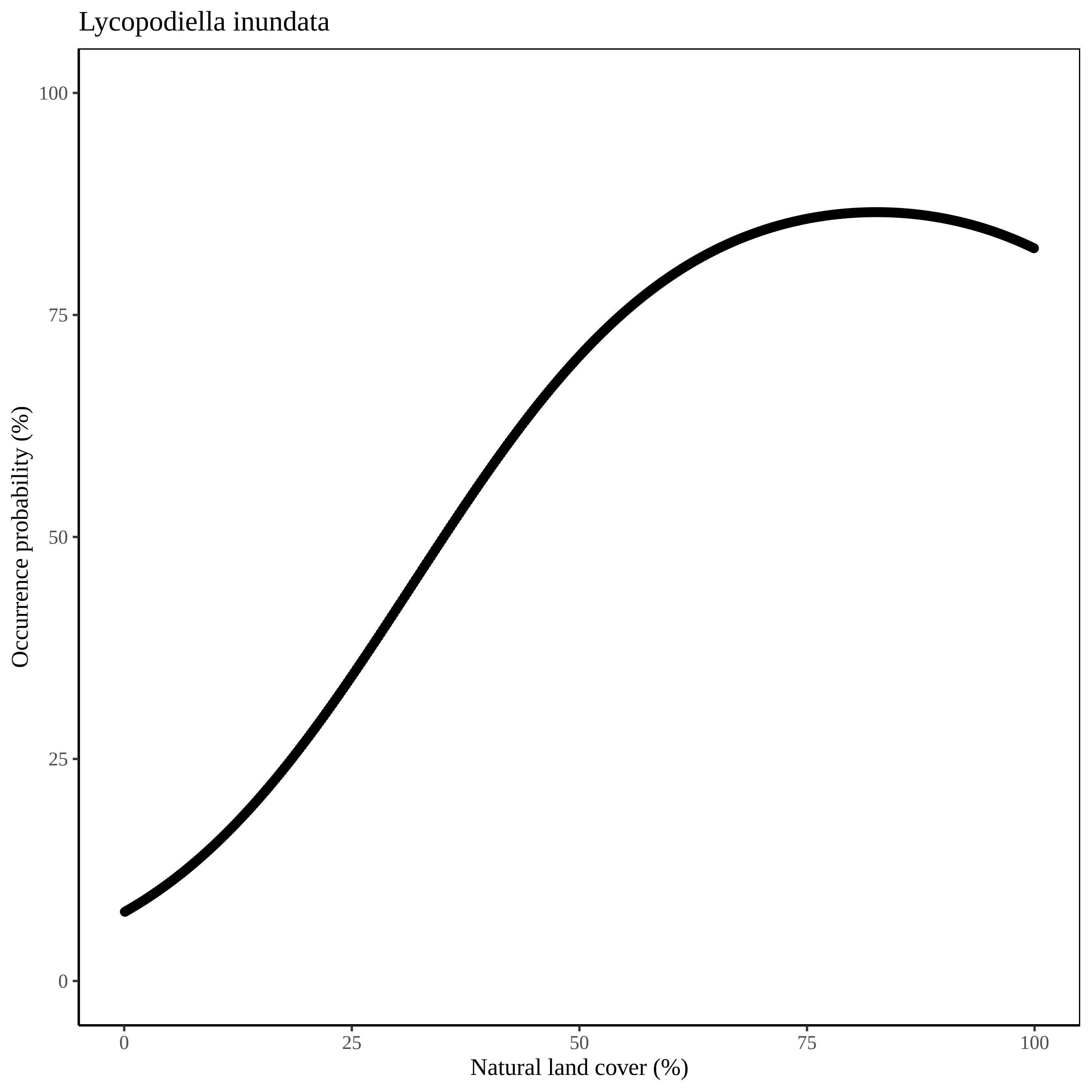
Lycopodiella inundata
Occurrence probability (%)
100
75
50
25
0
0
25
50
75
100
Natural land cover (%)

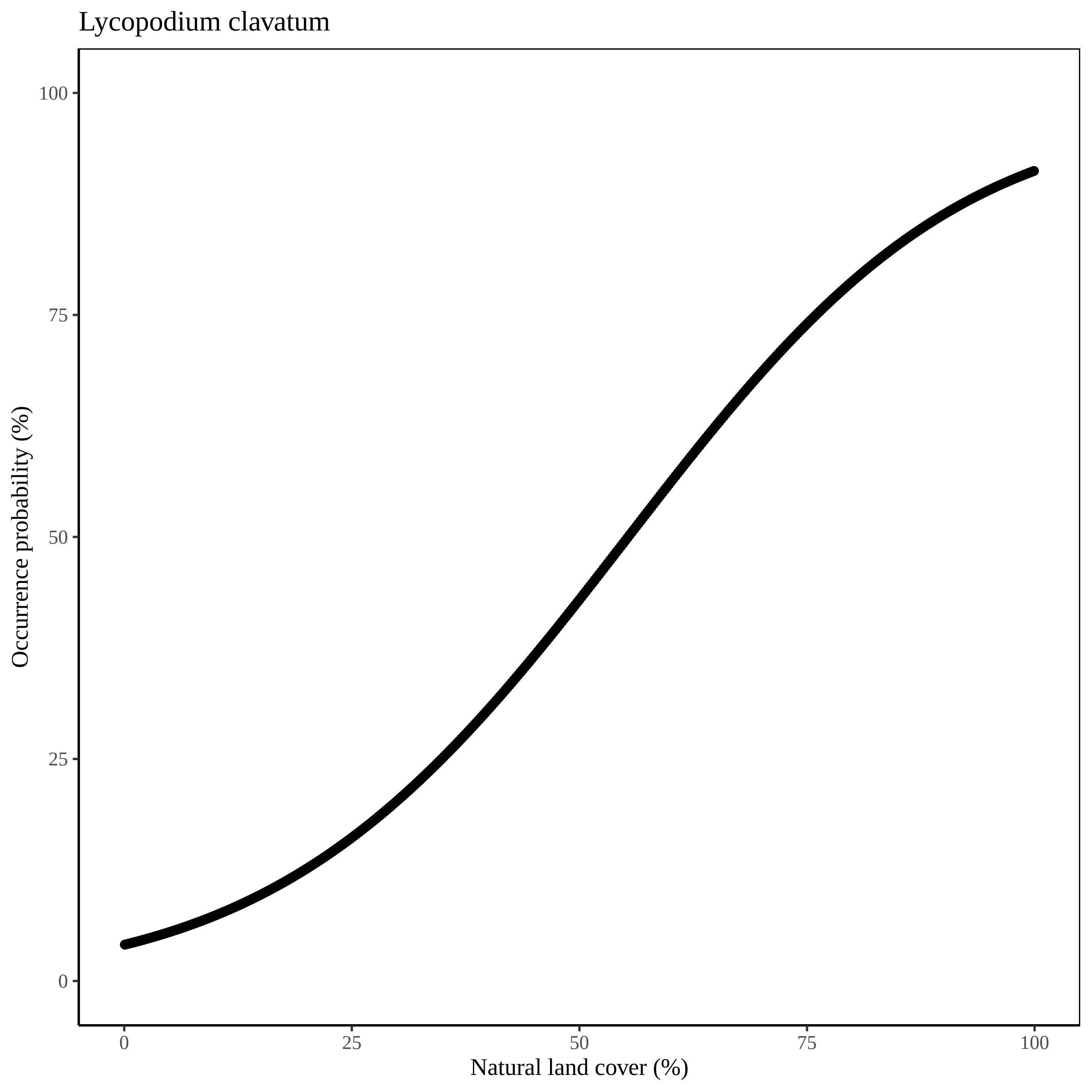
Lycopodium clavatum
Occurrence probability (%)
100
75
50
25
0
0
25
50
75
100
Natural land cover (%)

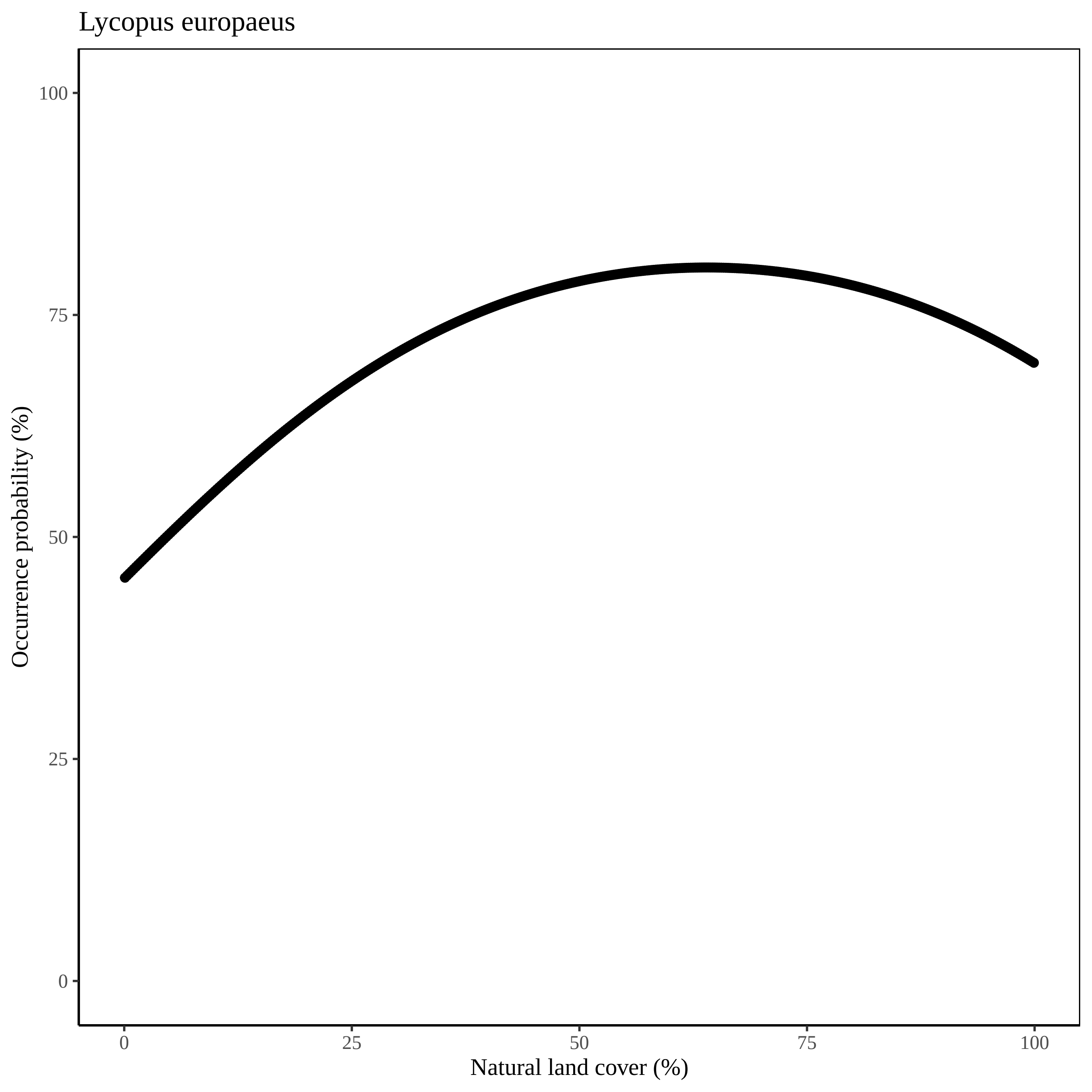

Lycopus europaeus
Occurrence probability (%)
100
75
50
25
0
0
25
50
75
100
Natural land cover (%)

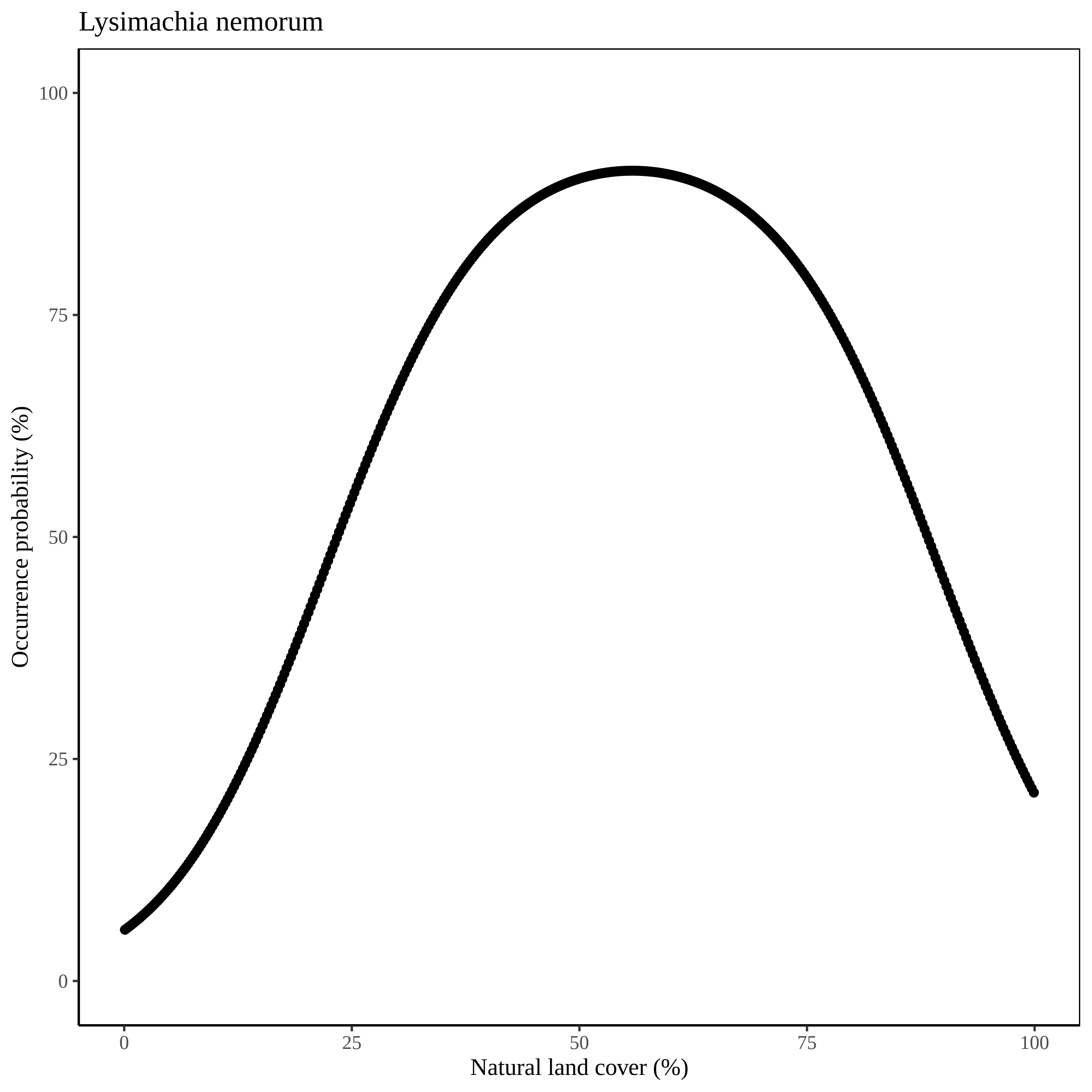

Lysimachia nemorum
Occurrence probability (%)
100
75
50
25
0
Natural land cover (%)
0
25
50
75
100

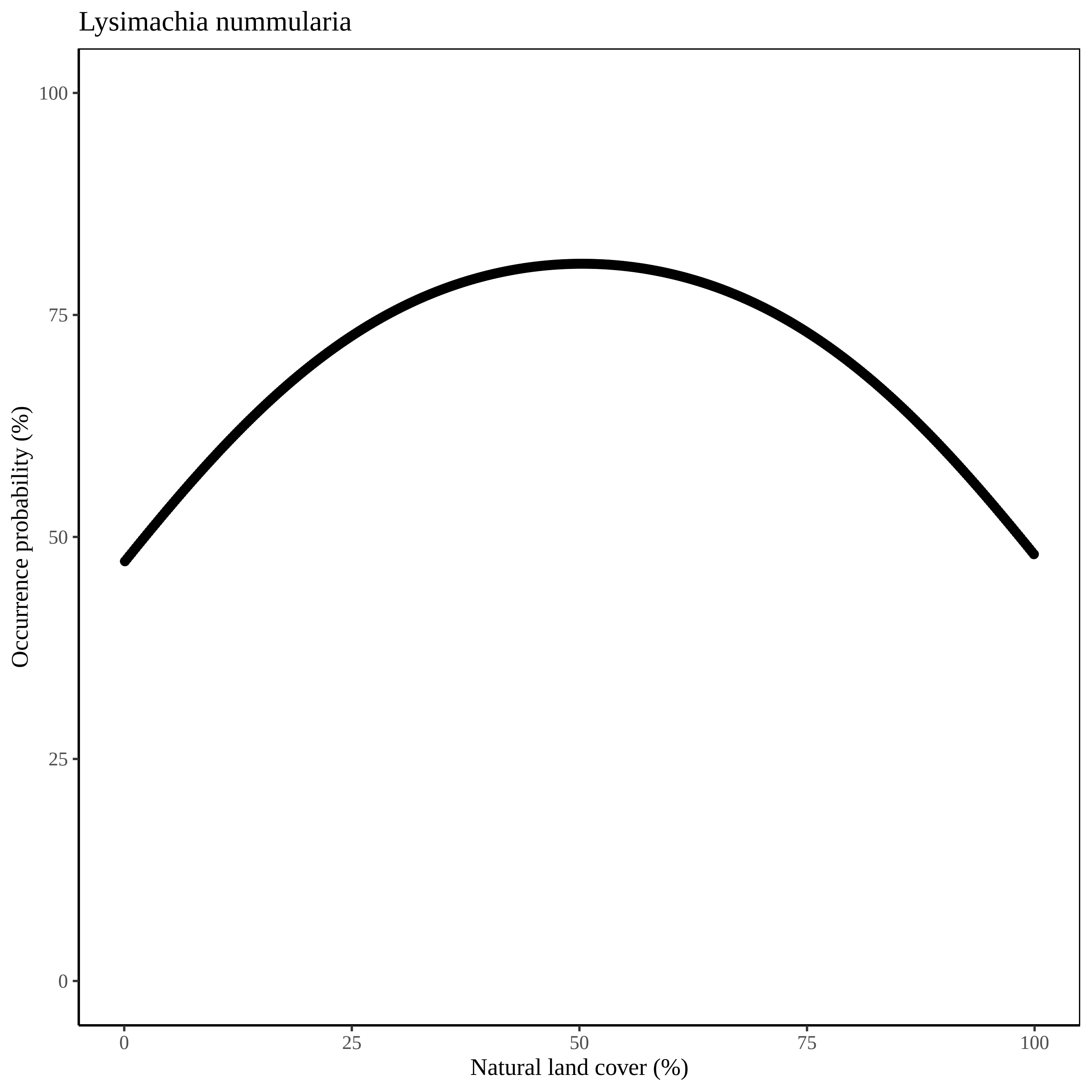
Lysimachia nummularia
Occurrence probability (%)
100
75
50
25
0
0
25
50
75
100
Natural land cover (%)

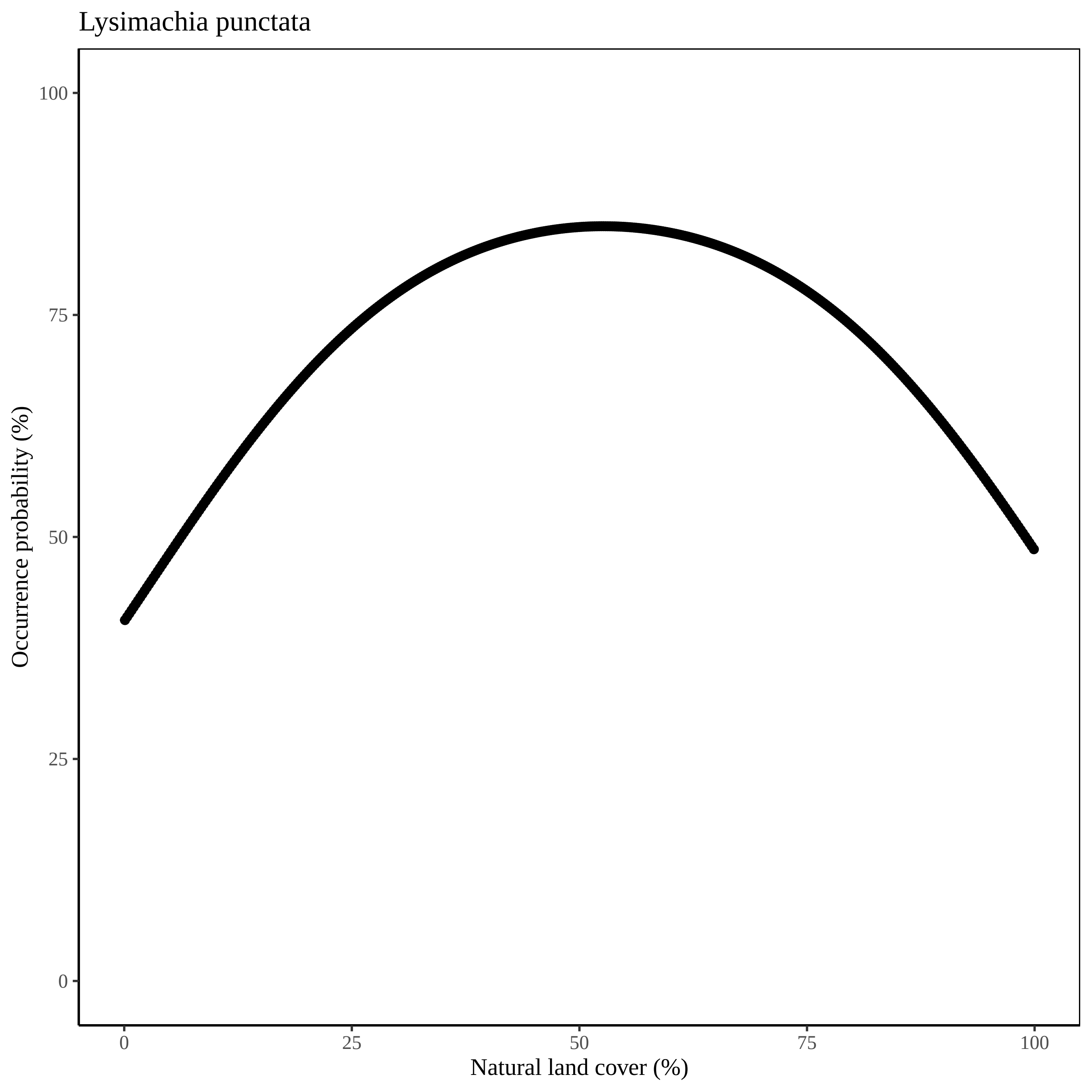
Lysimachia punctata
Occurrence probability (%)
100
75
50
25
0
0
25
50
75
100
Natural land cover (%)

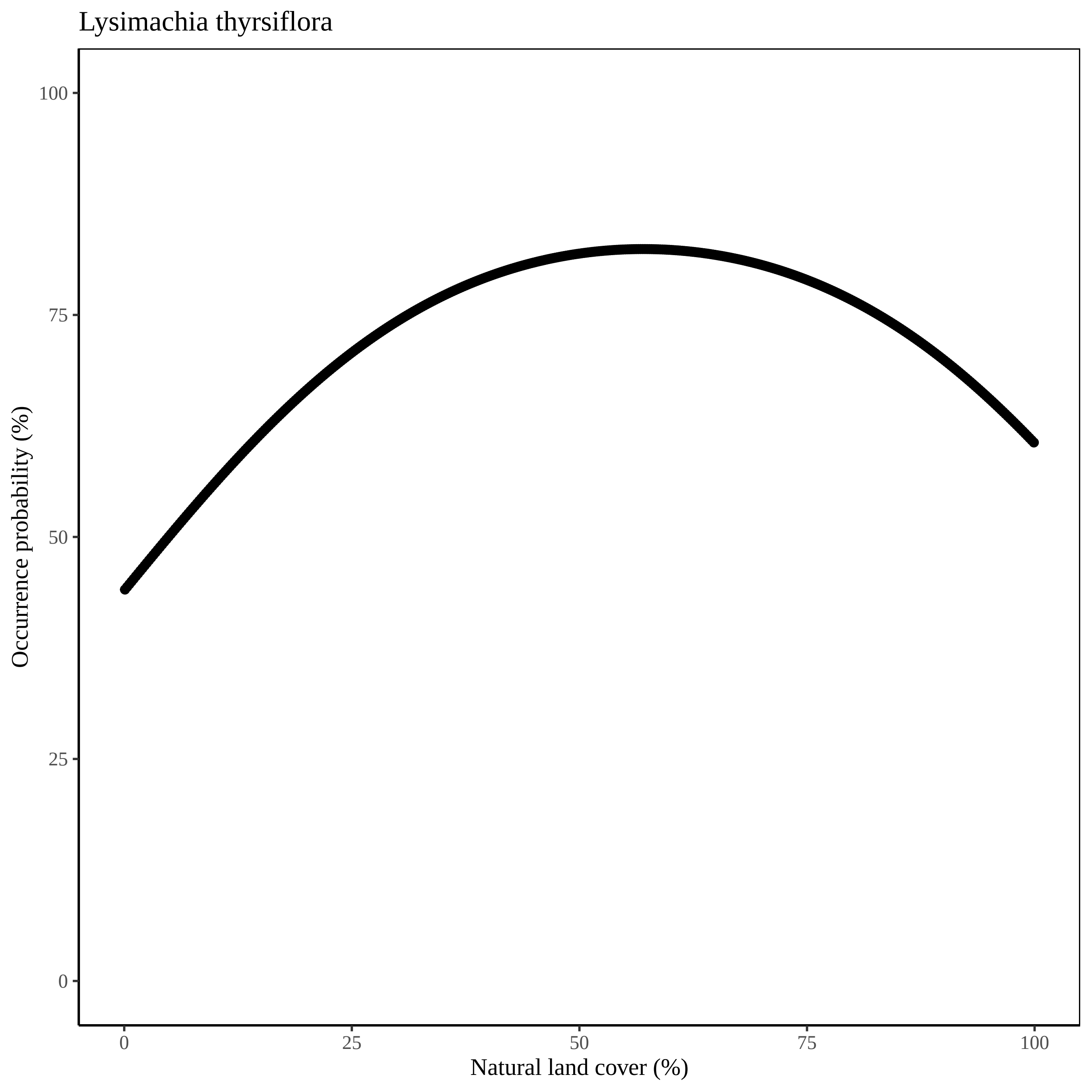
Lysimachia thyrsiflora
Occurrence probability (%)
100
75
50
25
0
0
25
50
75
100
Natural land cover (%)

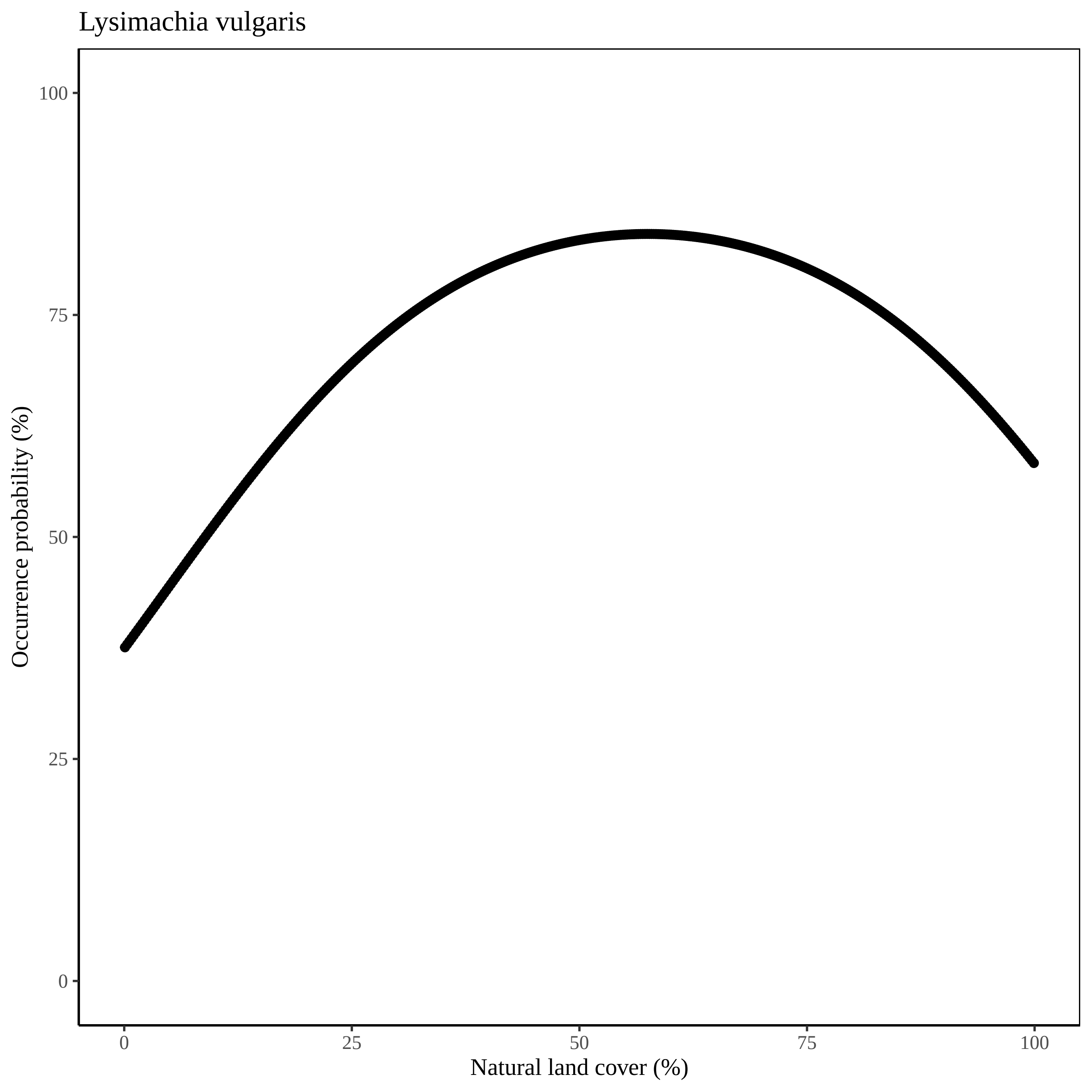

Lysimachia vulgaris
100
75
50
25
0
Occurrence probability (%)
0
25
50
75
100
Natural land cover (%)

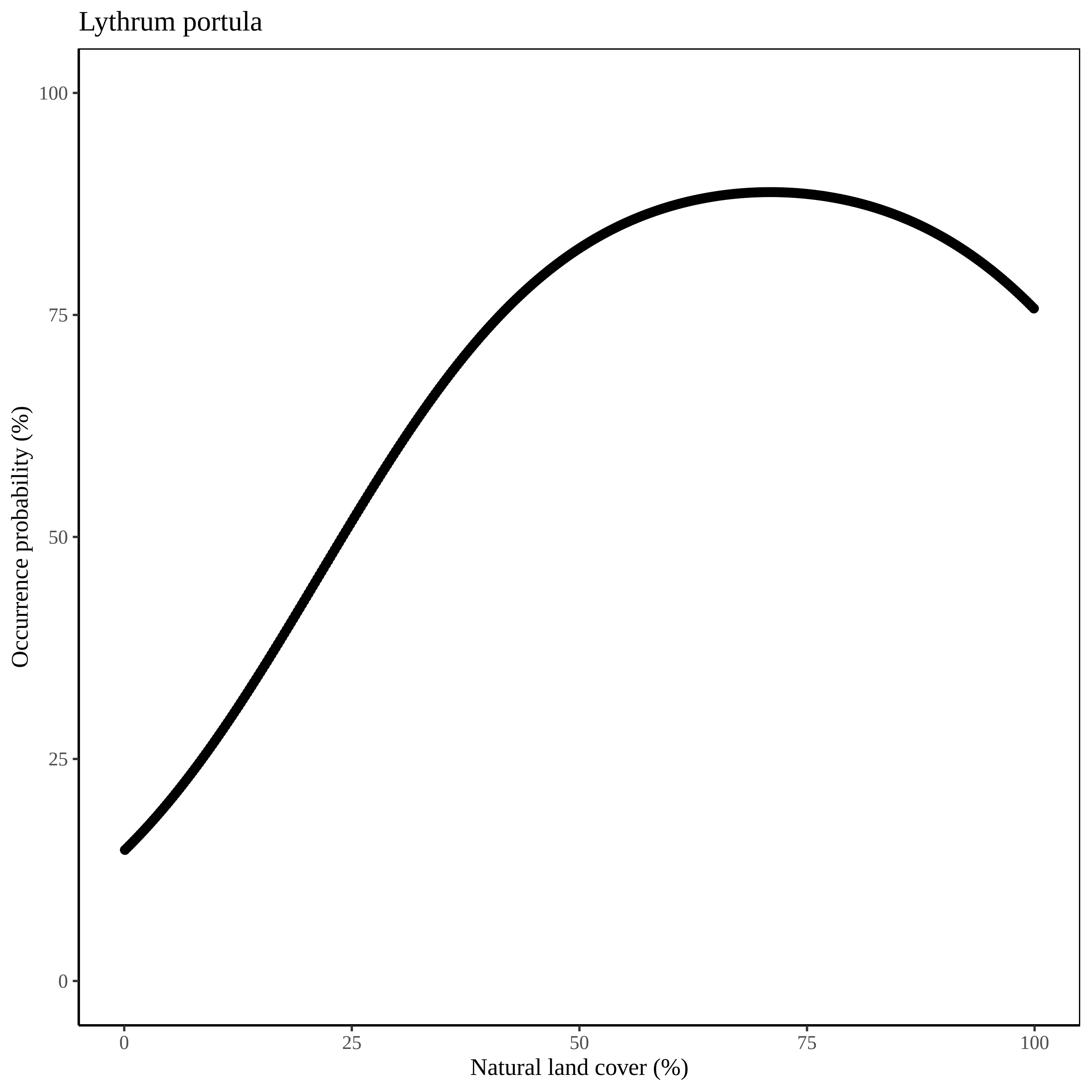
Lythrum portula
Occurrence probability (%)
100
75
50
25
0
0
25
50
75
100
Natural land cover (%)

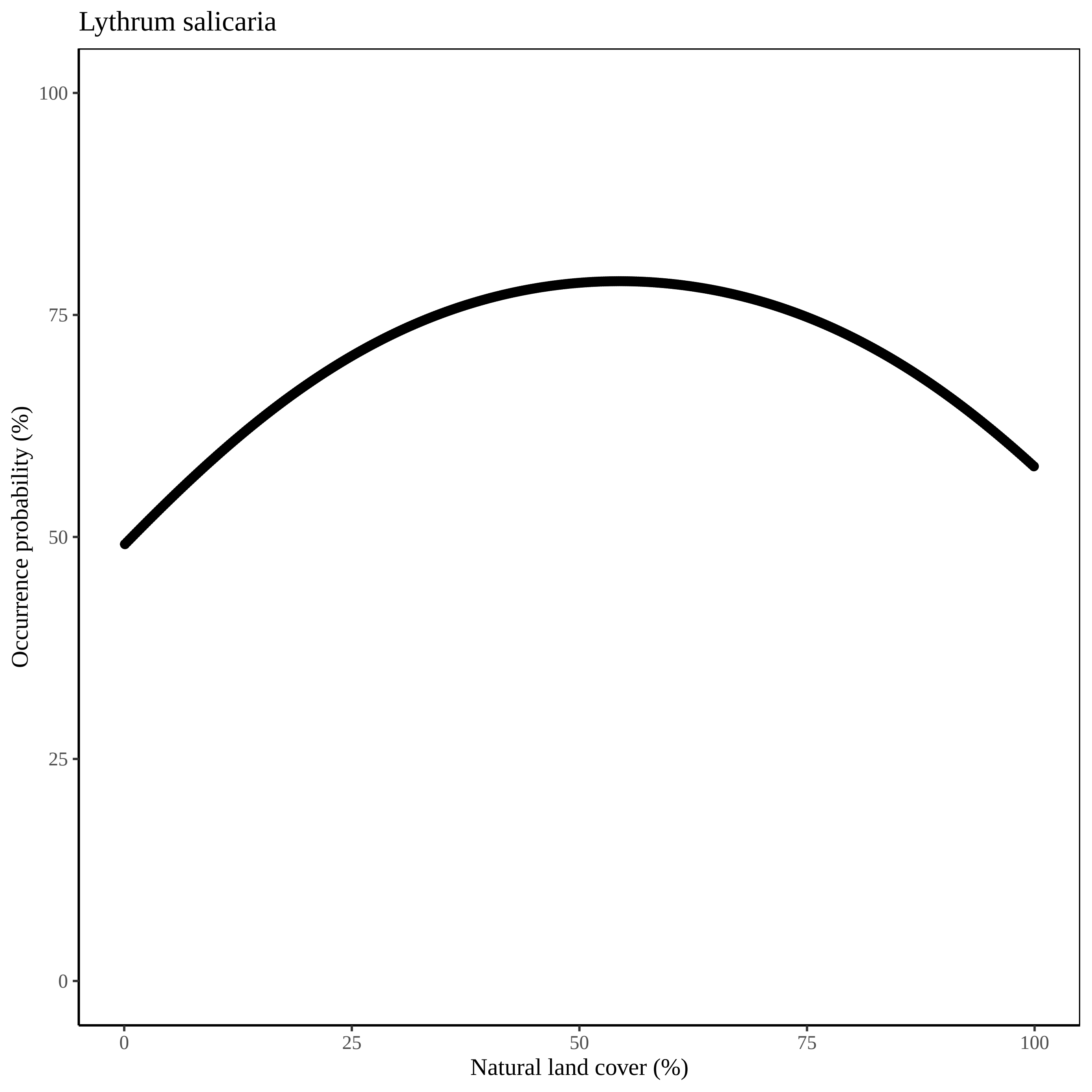
Lythrum salicaria
Occurrence probability (%)
100
75
50
25
0
0
25
50
75
100
Natural land cover (%)

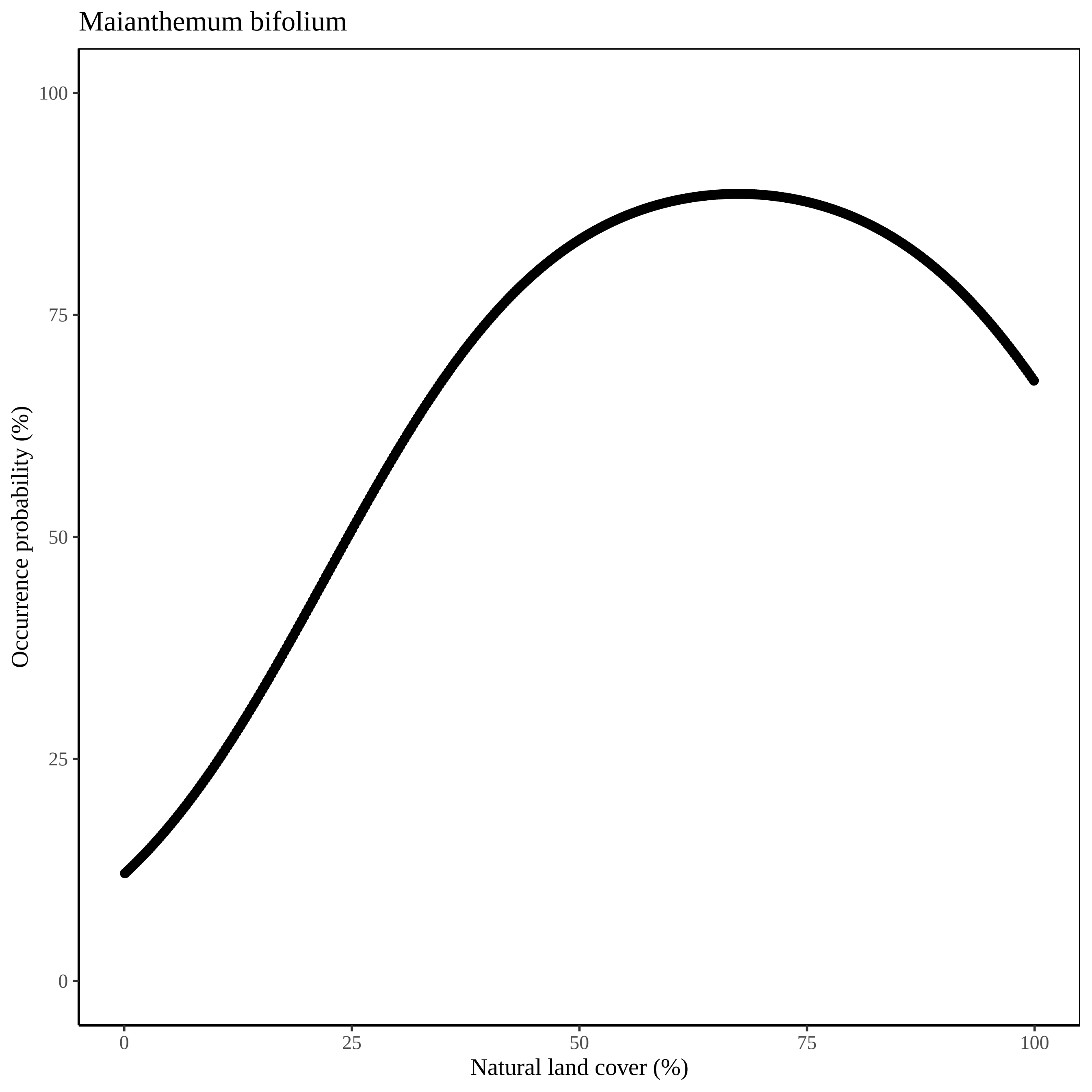
Maianthemum bifolium
Occurrence probability (%)
100
75
50
25
0
0
25
50
75
100
Natural land cover (%)

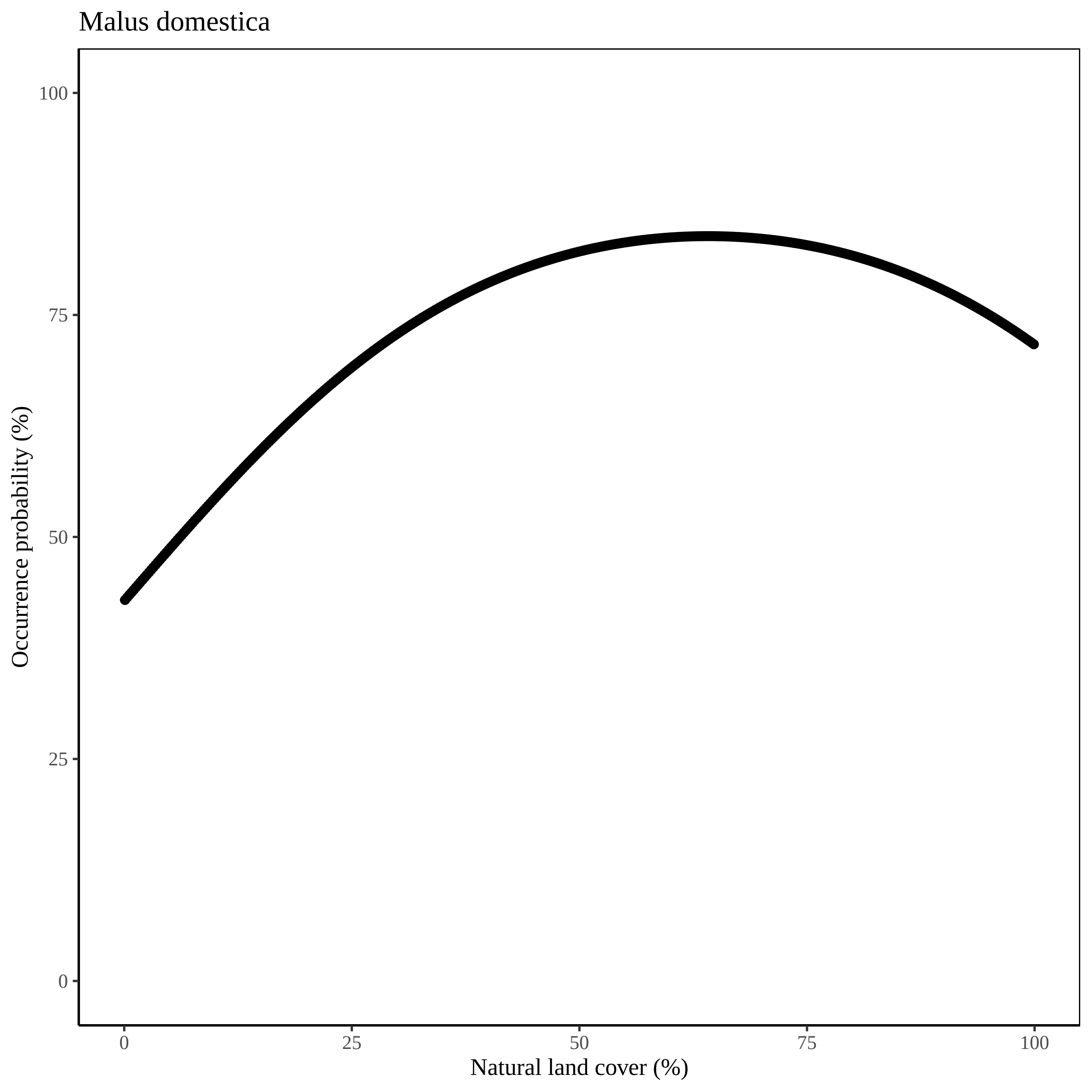
Malus domestica
Occurrence probability (%)
100
75
50
25
0
0
25
50
75
100
Natural land cover (%)

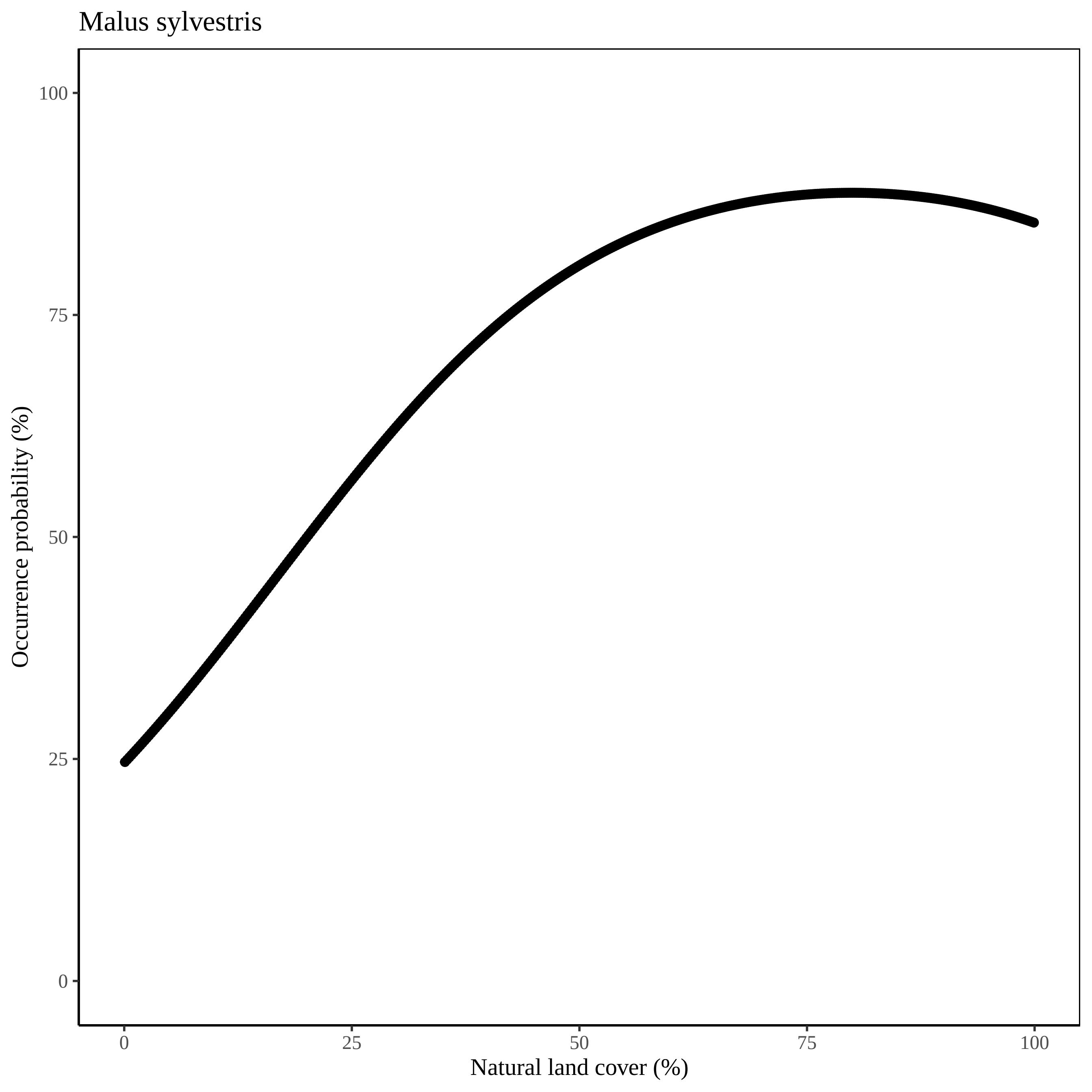
Malus sylvestris
Occurrence probability (%)
100
75
50
25
0
0
25
50
75
100
Natural land cover (%)

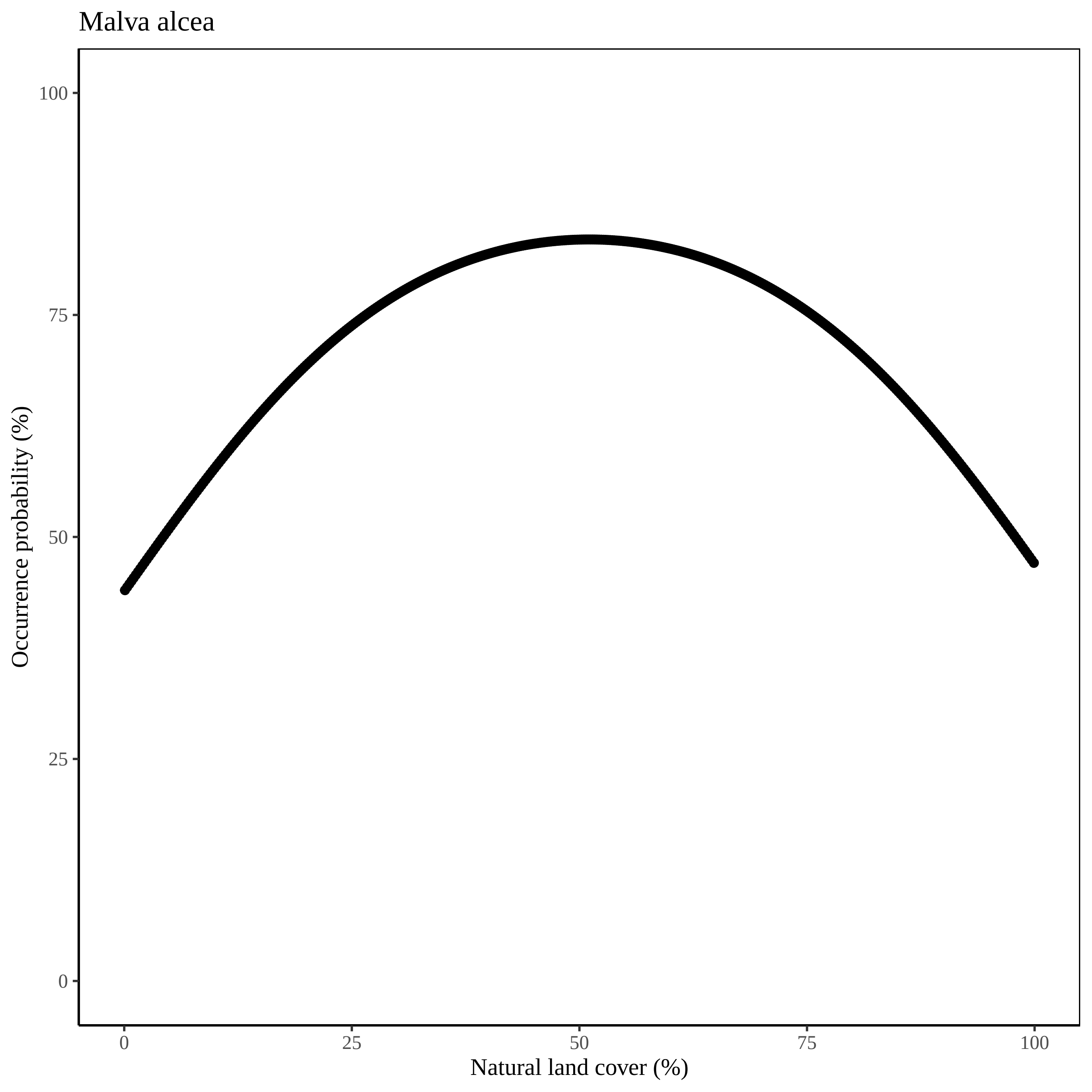

Malva alcea
100
75
50
25
0
Occurrence probability (%)
0
25
50
75
100
Natural land cover (%)

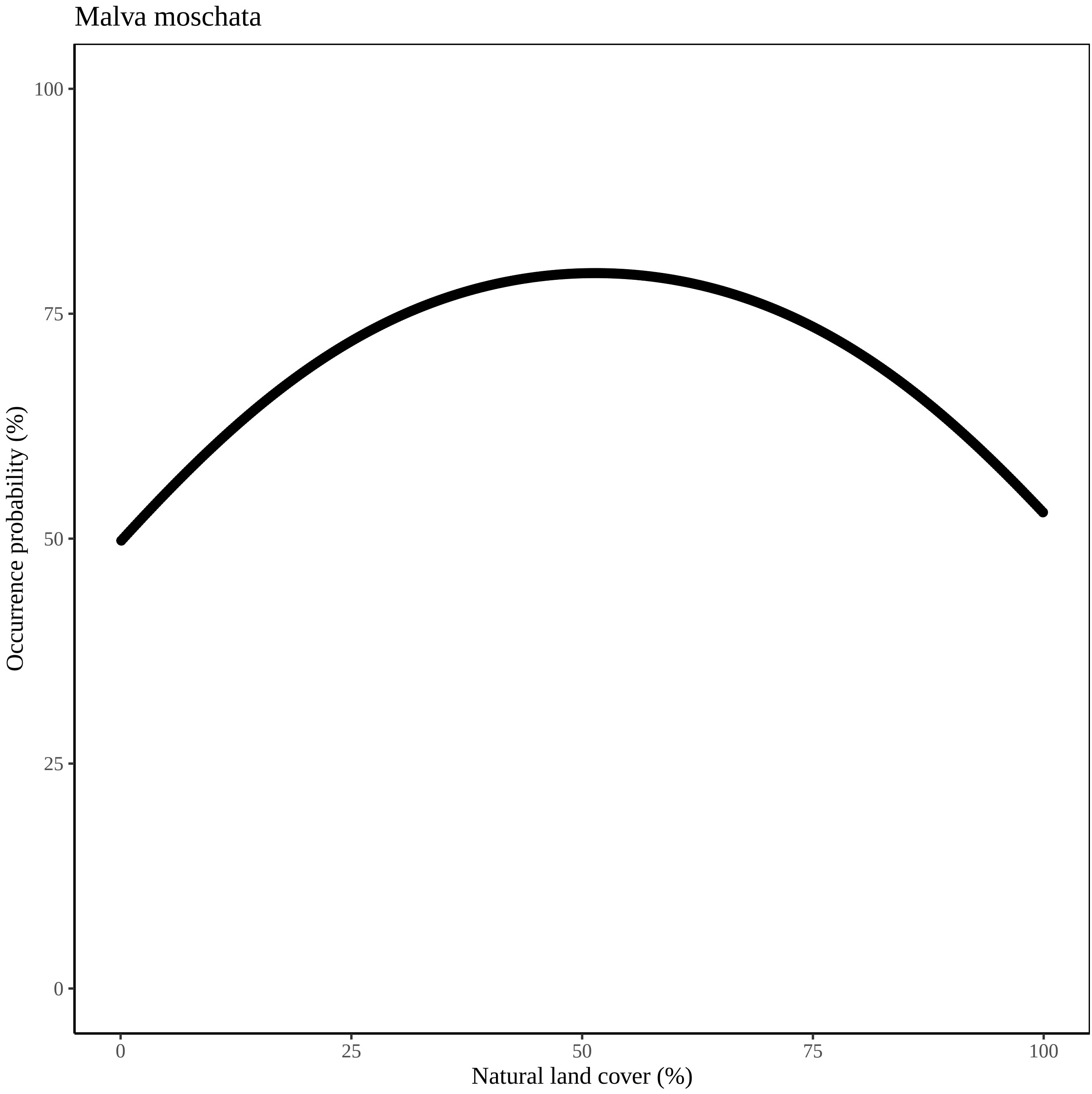
Malva moschata
Occurrence probability (%)
100
75
50
25
0
0
25
50
75
100
Natural land cover (%)

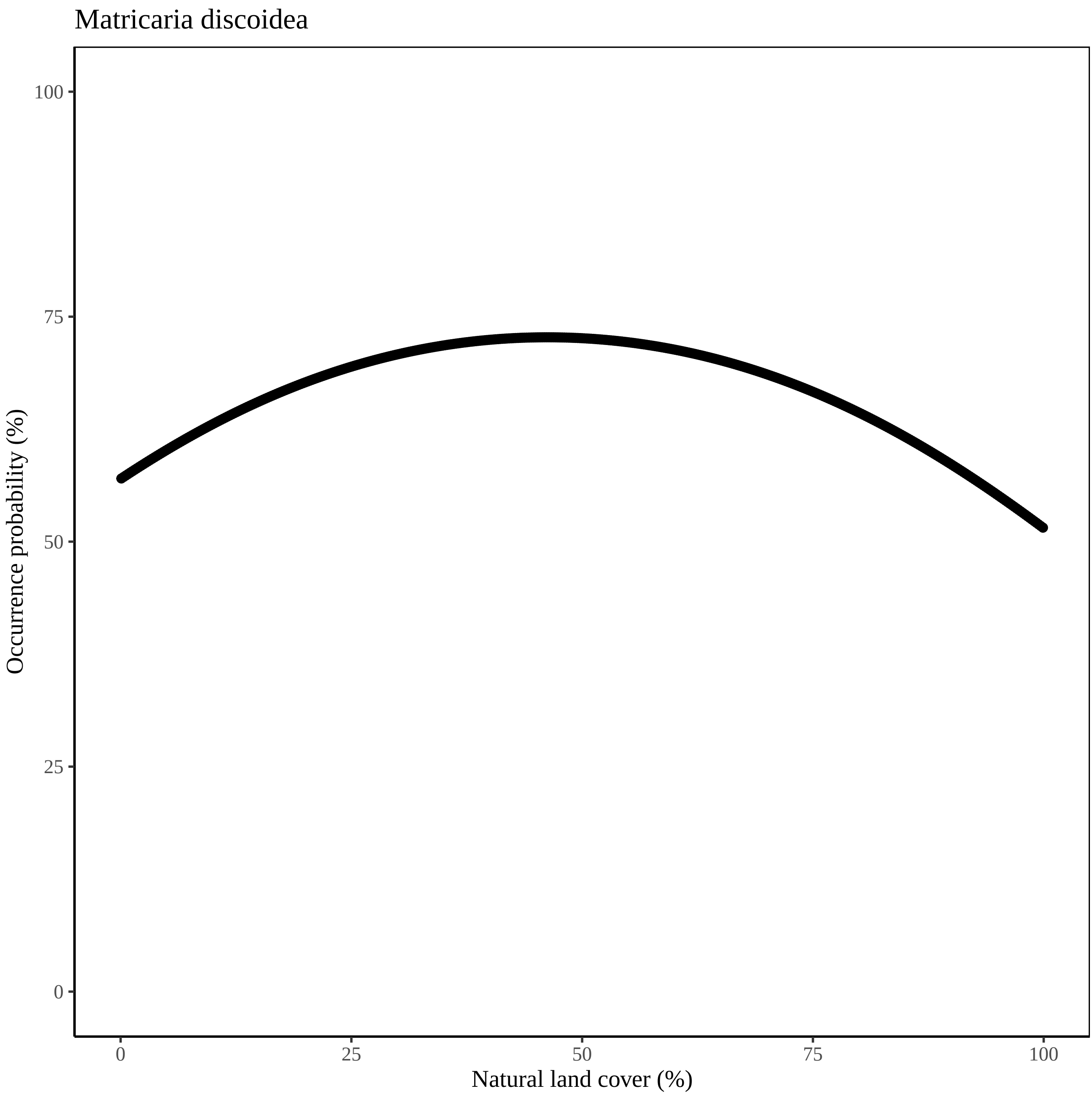
Matricaria discoidea
Occurrence probability (%)
100
75
50
25
0
0
25
50
75
100
Natural land cover (%)

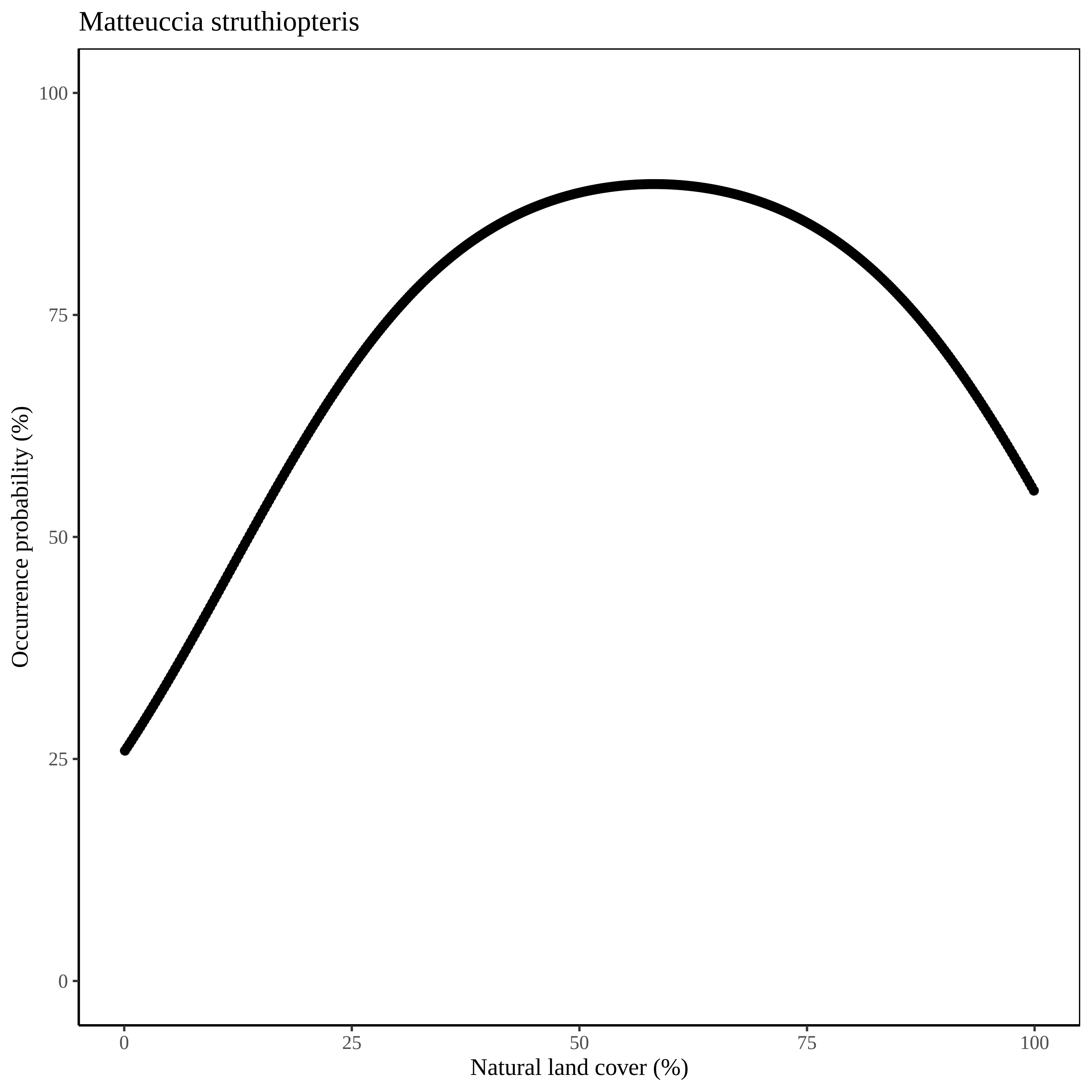
Matteuccia struthiopteris
Occurrence probability (%)
100
75
50
25
0
0
25
50
75
100
Natural land cover (%)

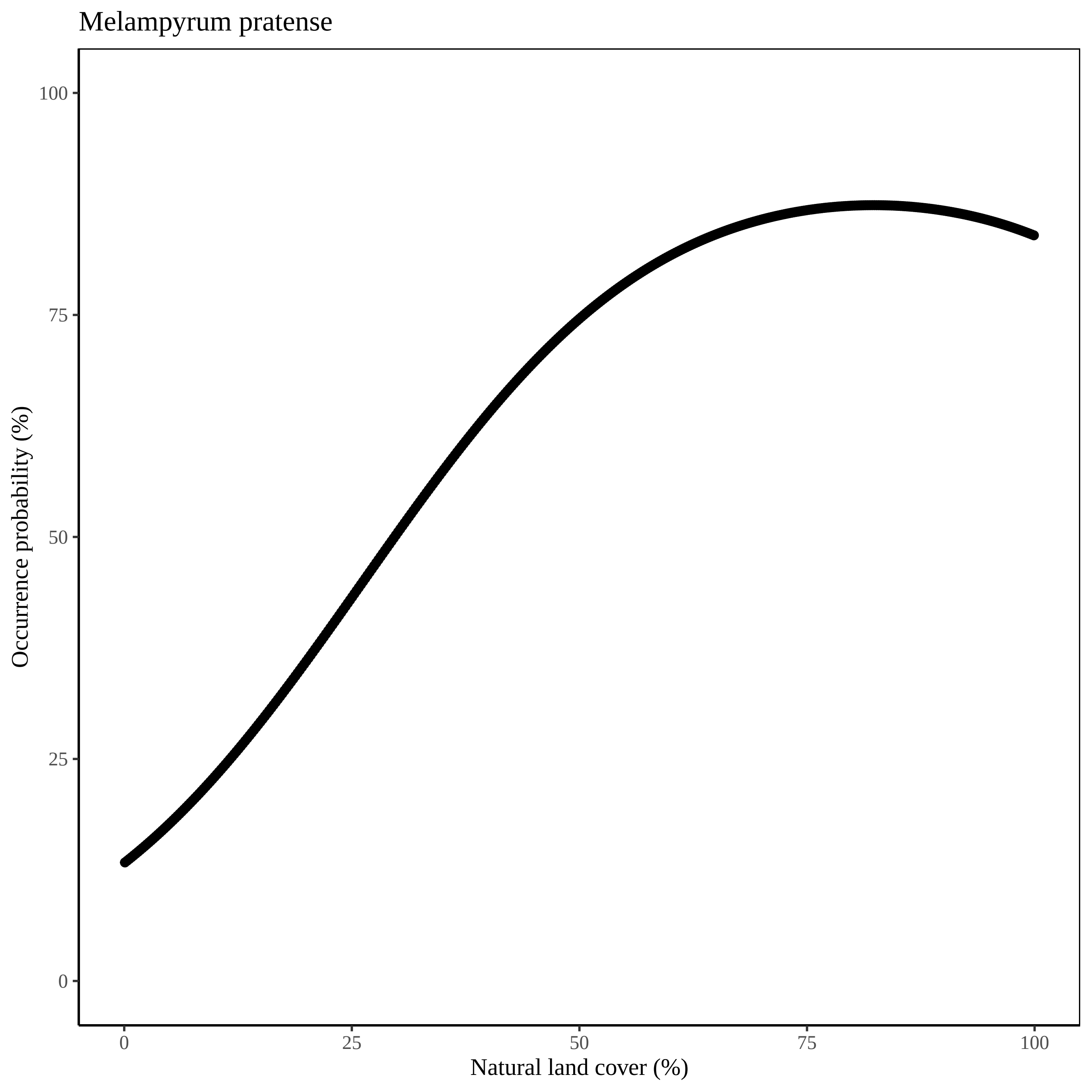
Melampyrum pratense
Occurrence probability (%)
0
25
50
75
100
Natural land cover (%)
0
25
50
75
100

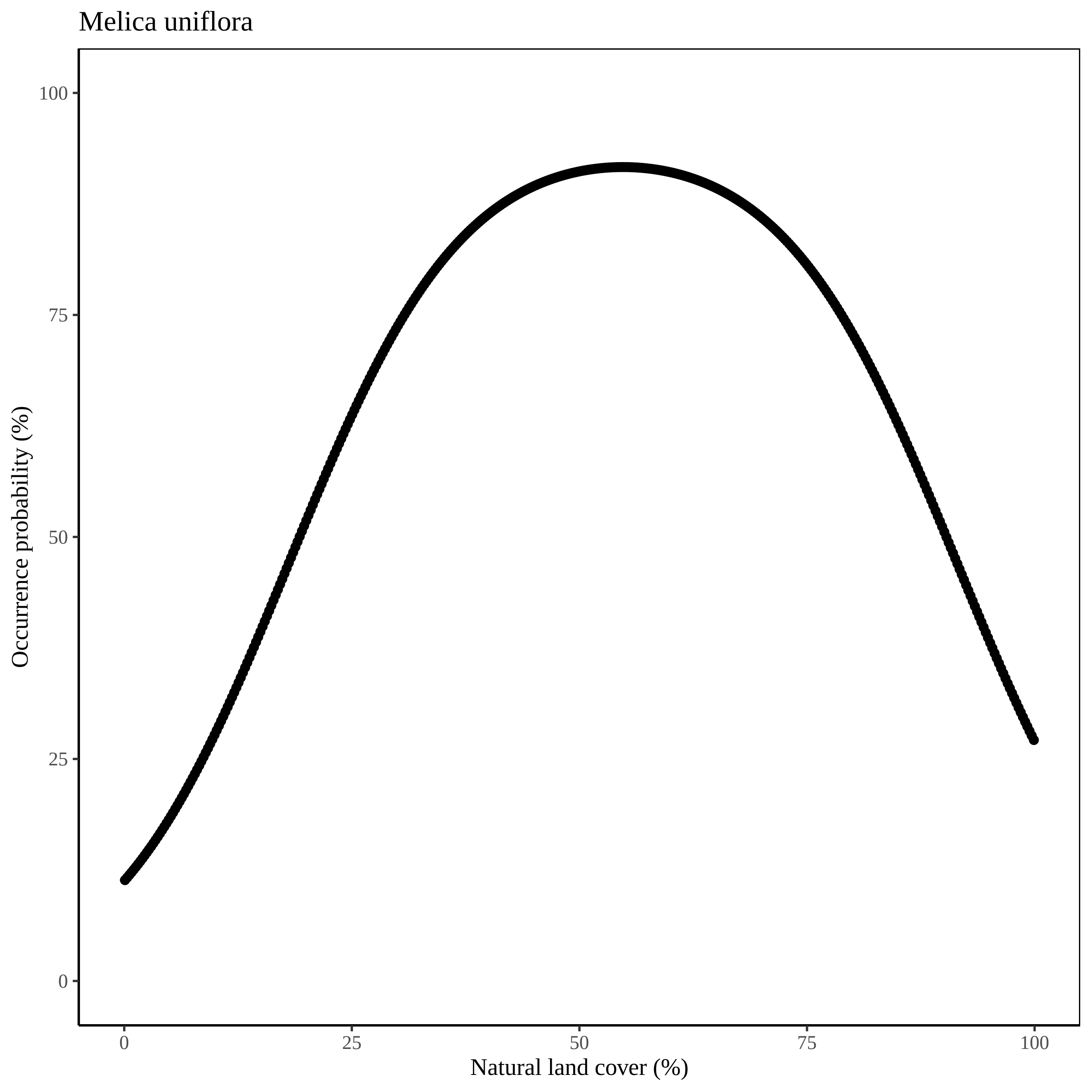
Melica uniflora
Occurrence probability (%)
100
75
50
25
0
0
25
50
75
100
Natural land cover (%)

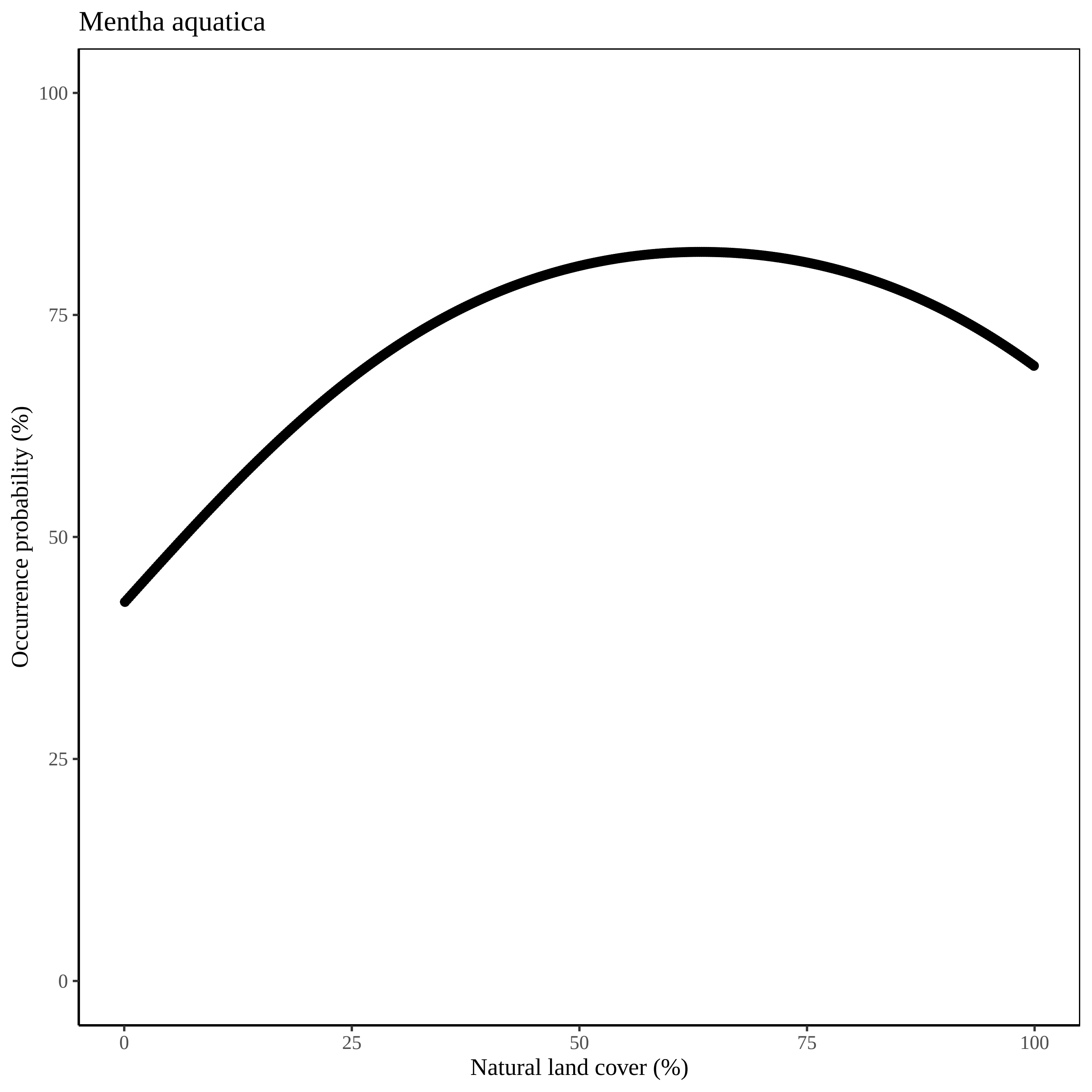
Mentha aquatica
Occurrence probability (%)
100
75
50
25
0
0
25
50
75
100
Natural land cover (%)

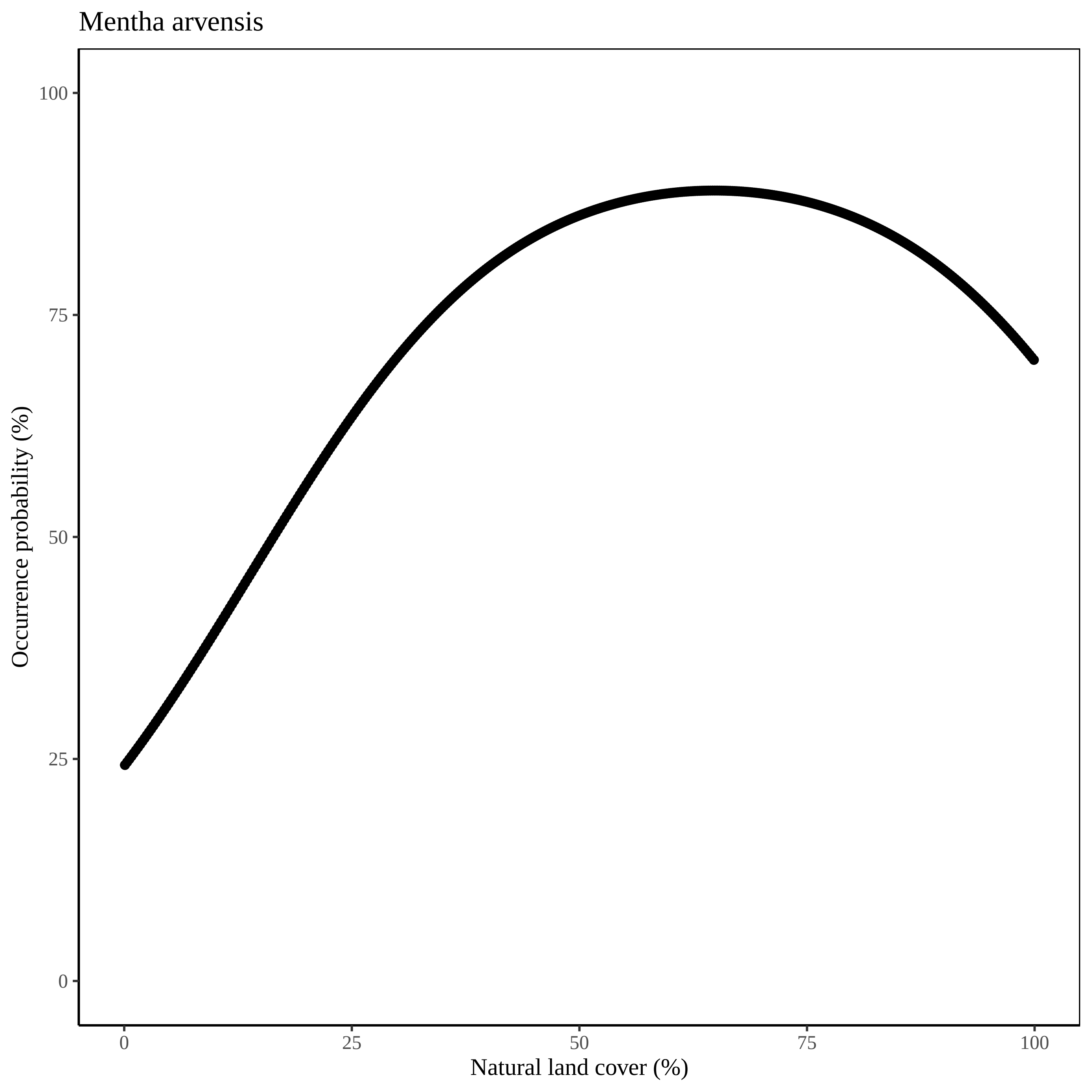
Mentha arvensis
Occurrence probability (%)
100
75
50
25
0
0
25
50
75
100
Natural land cover (%)

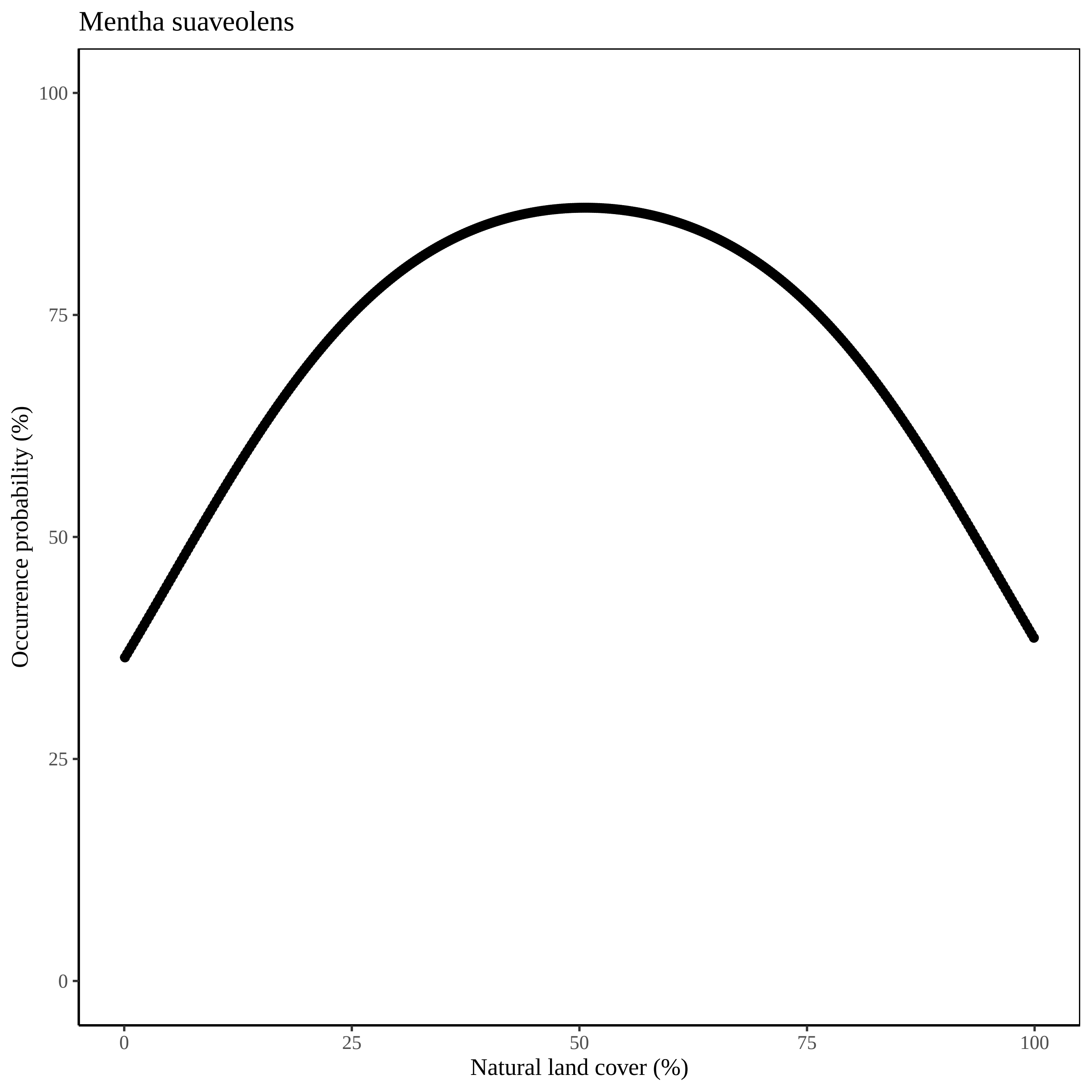
Mentha suaveolens
Occurrence probability (%)
100
75
50
25
0
0
25
50
75
100
Natural land cover (%)

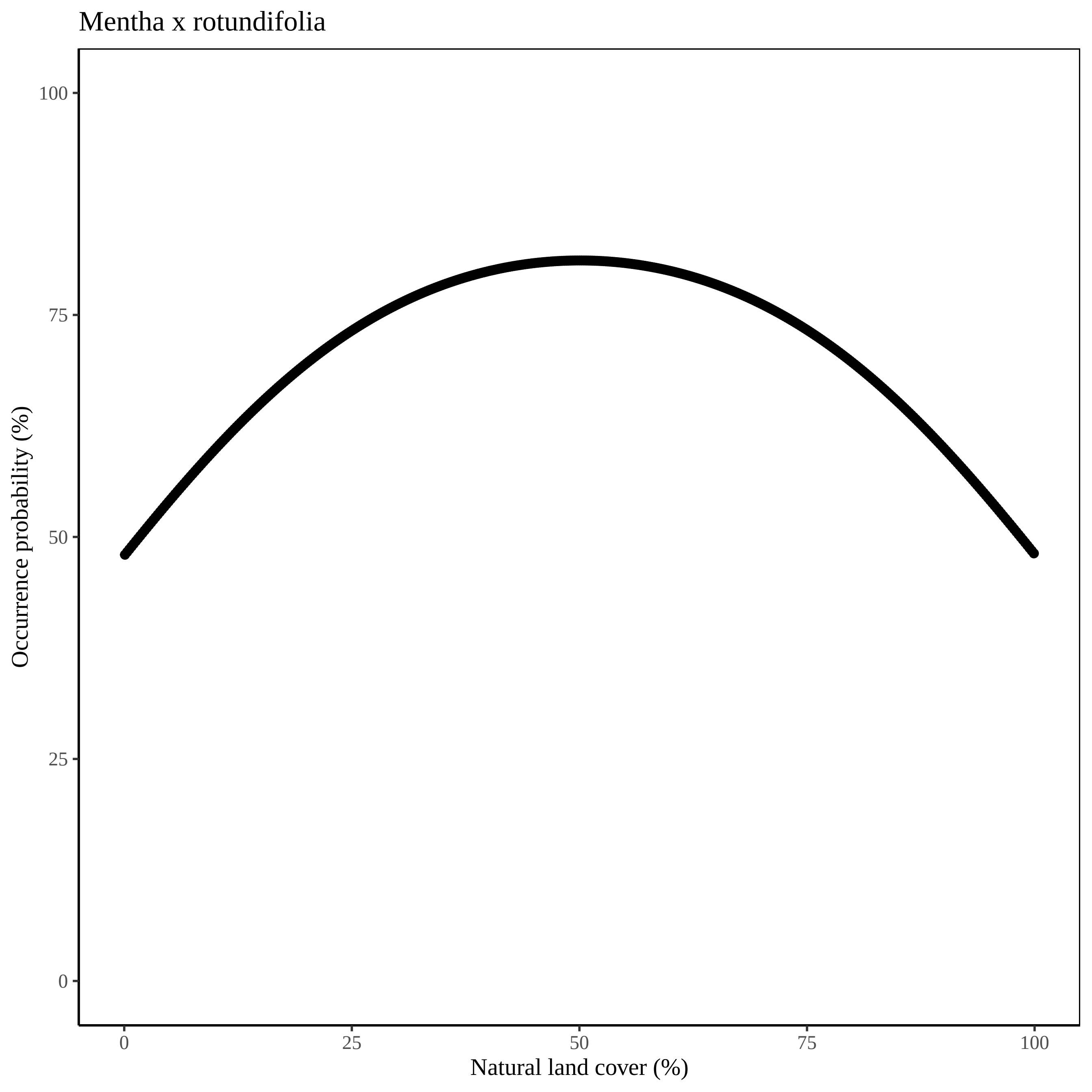
Mentha x rotundifolia
Occurrence probability (%)
100
75
50
25
0
0
25
50
75
100
Natural land cover (%)

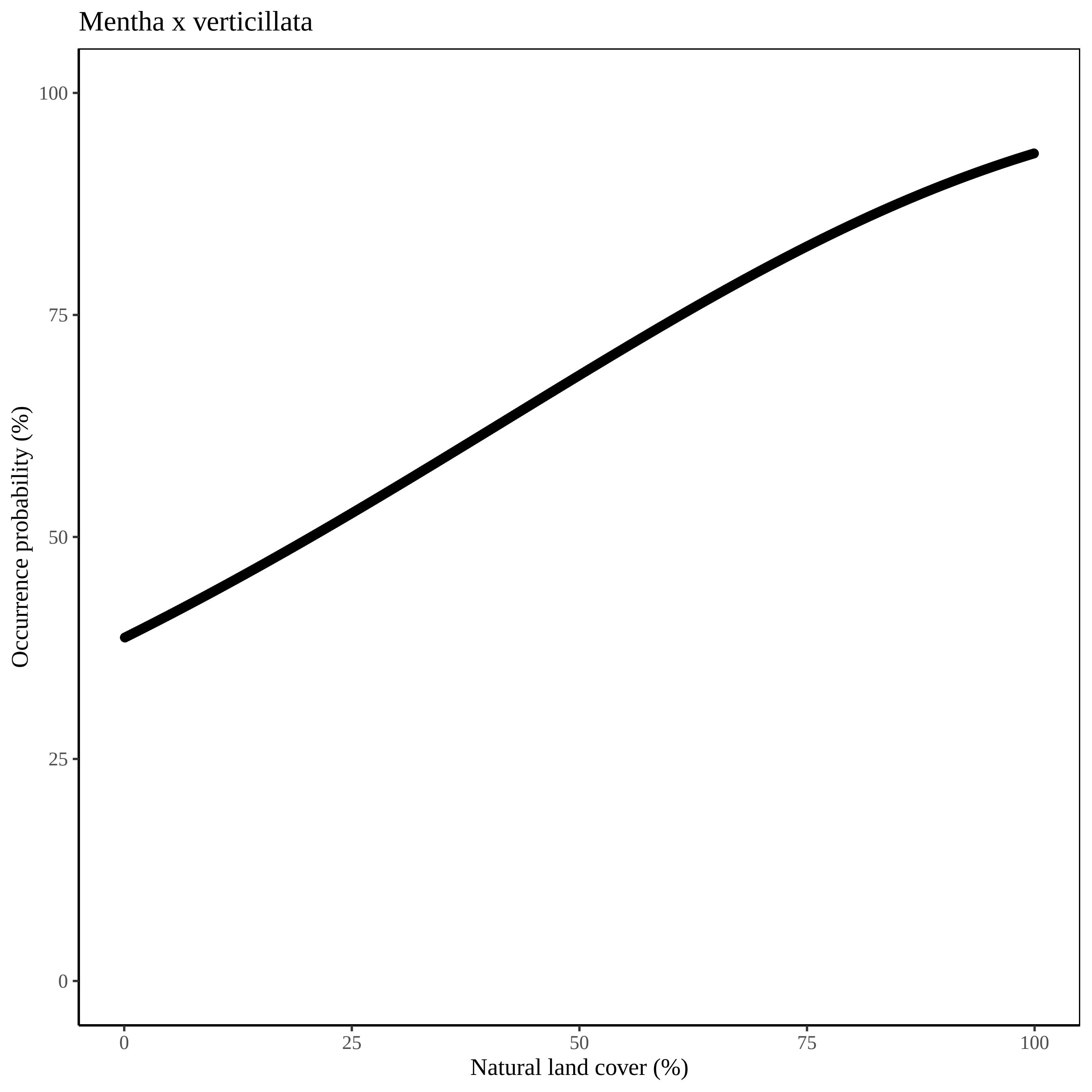

Mentha x verticillata
Occurrence probability (%)
100
75
50
25
0
0
25
50
75
100
Natural land cover (%)

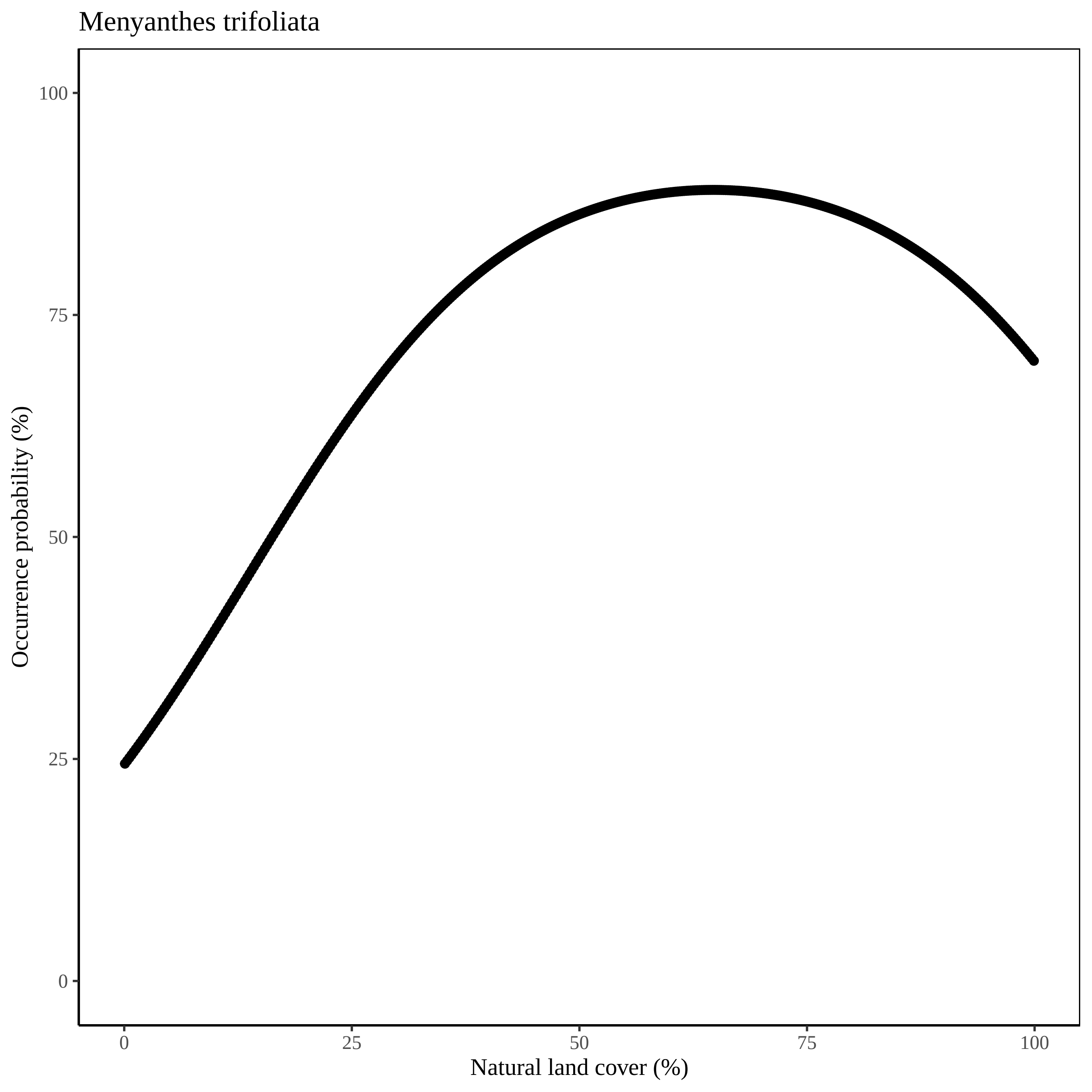
Menyanthes trifoliata
100
75
50
25
0
Occurrence probability (%)
0
25
50
75
100
Natural land cover (%)

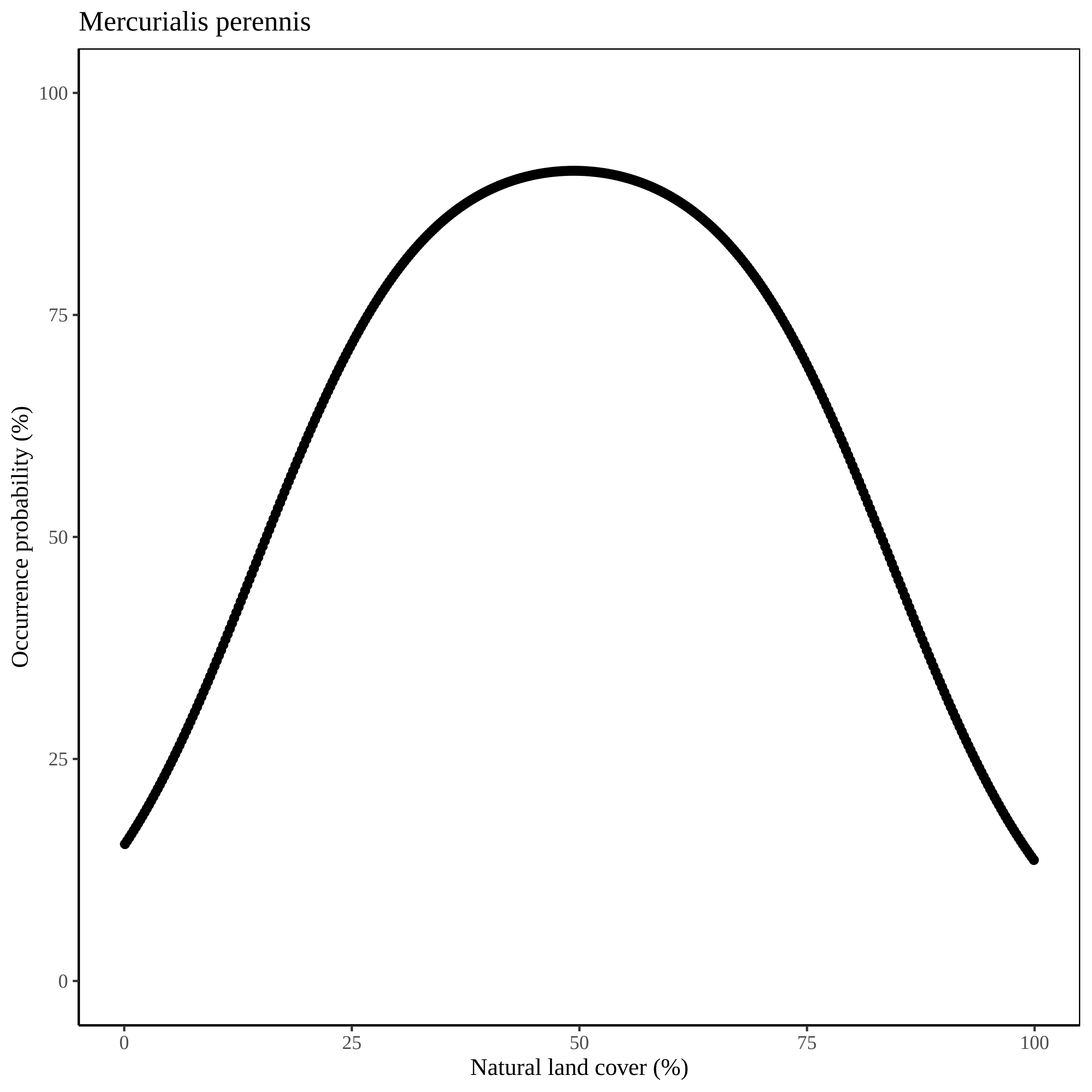
Mercurialis perennis
Occurrence probability (%)
100
75
50
25
0
0
25
50
75
100
Natural land cover (%)

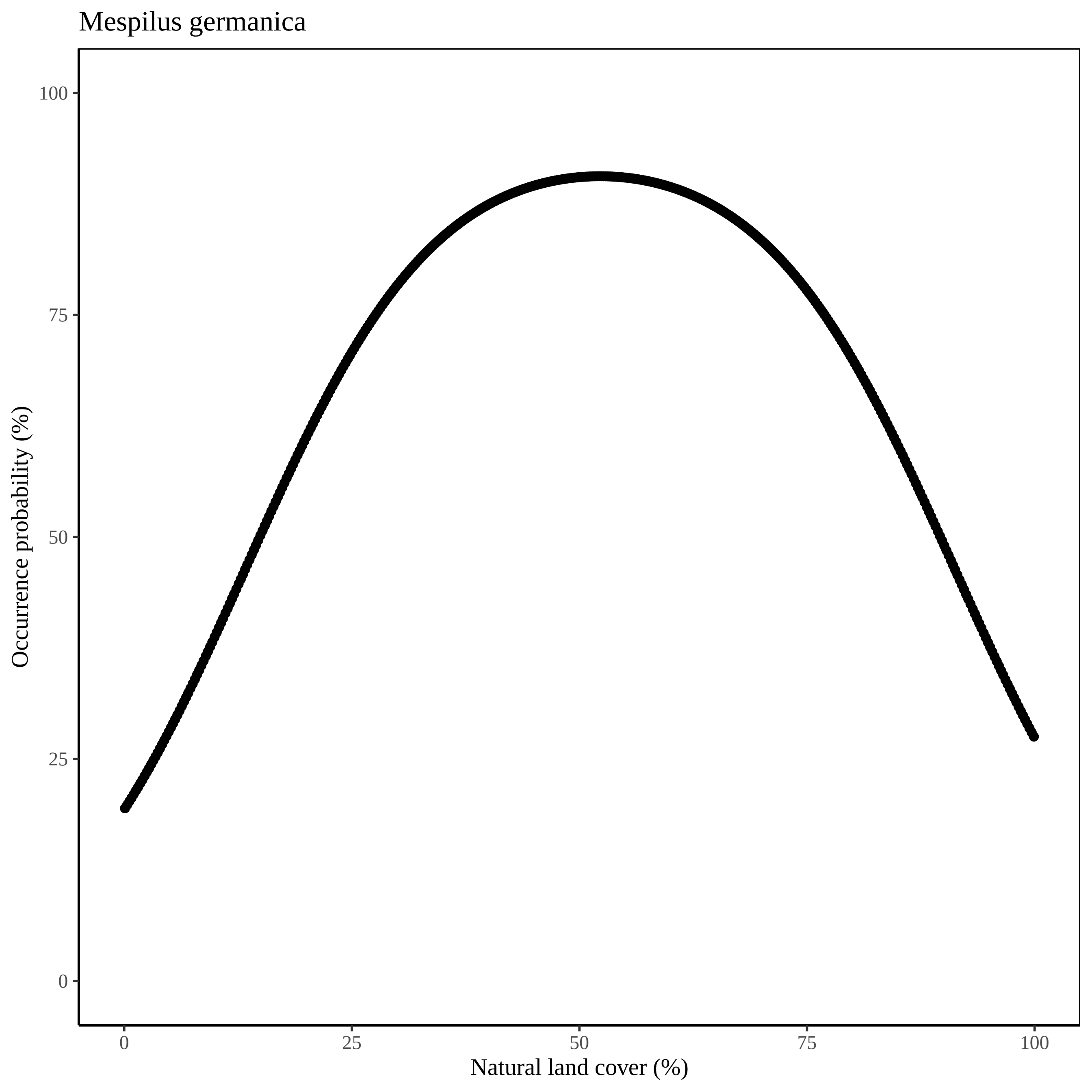
Mespilus germanica
Occurrence probability (%)
100
75
50
25
0
0
25
50
75
100
Natural land cover (%)

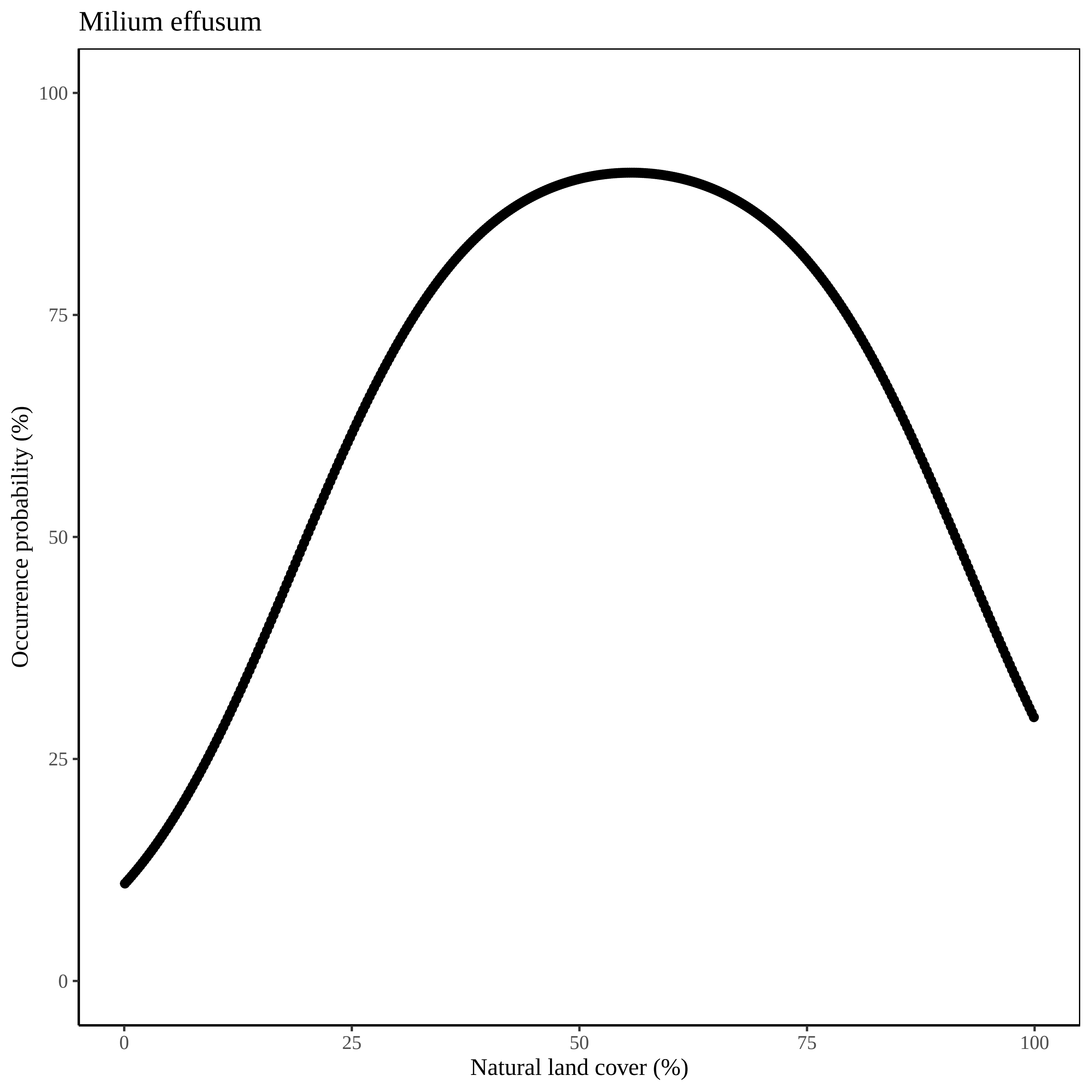
Milium effusum
100
75
50
25
0
Occurrence probability (%)
0
25
50
75
100
Natural land cover (%)

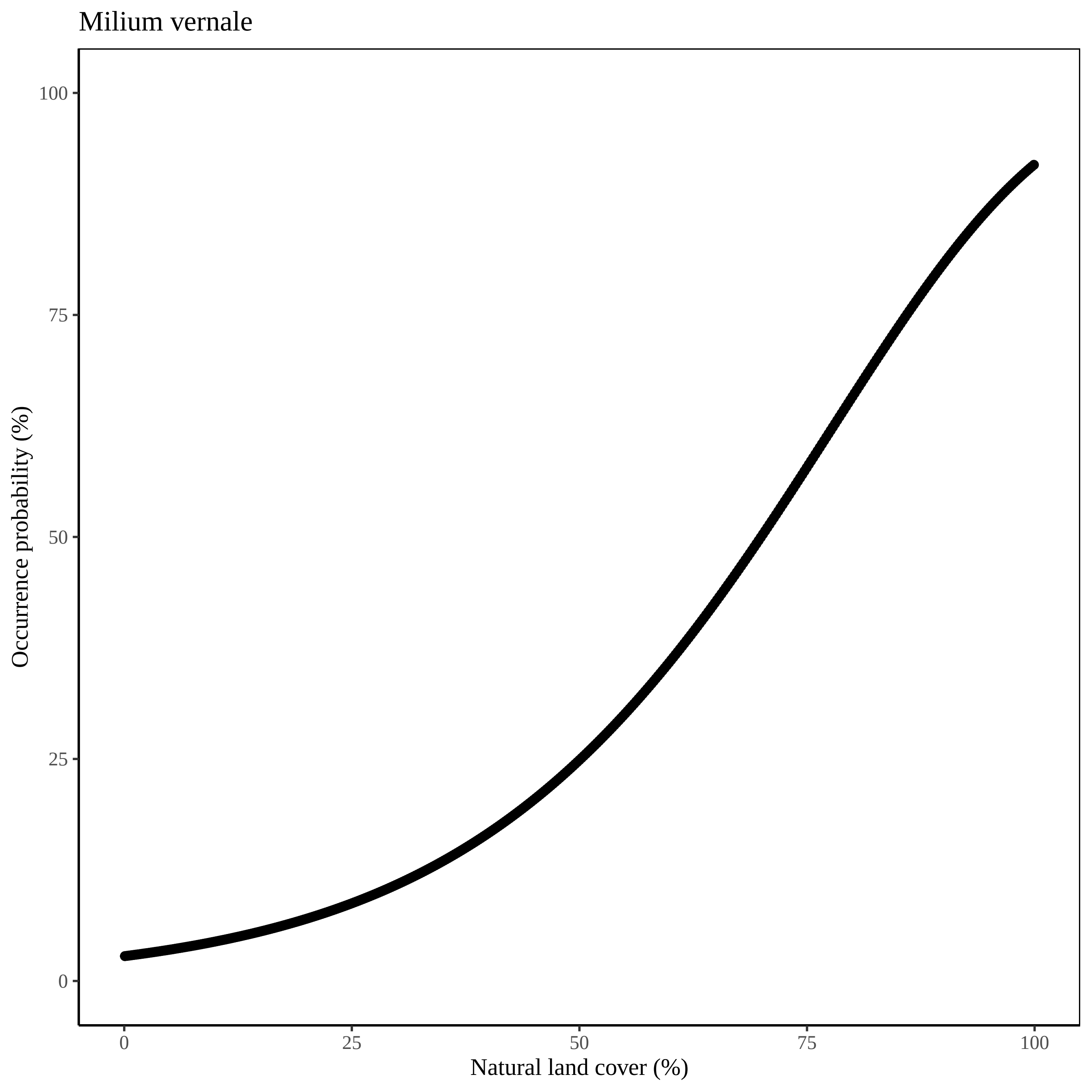
Milium vernale
Occurrence probability (%)
100
75
50
25
0
0
25
50
75
100
Natural land cover (%)

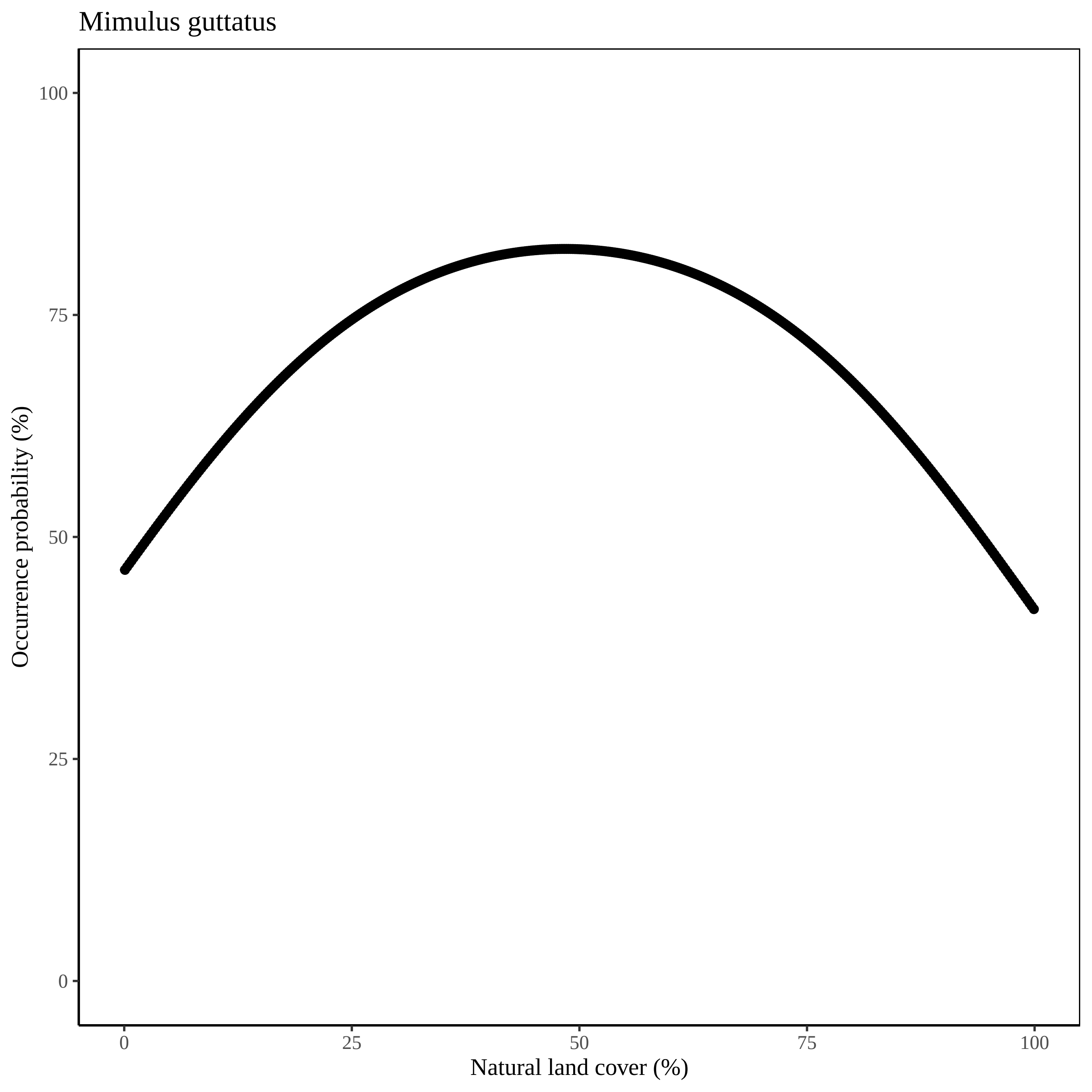
Mimulus guttatus
100
75
50
25
0
Occurrence probability (%)
0
25
50
75
100
Natural land cover (%)

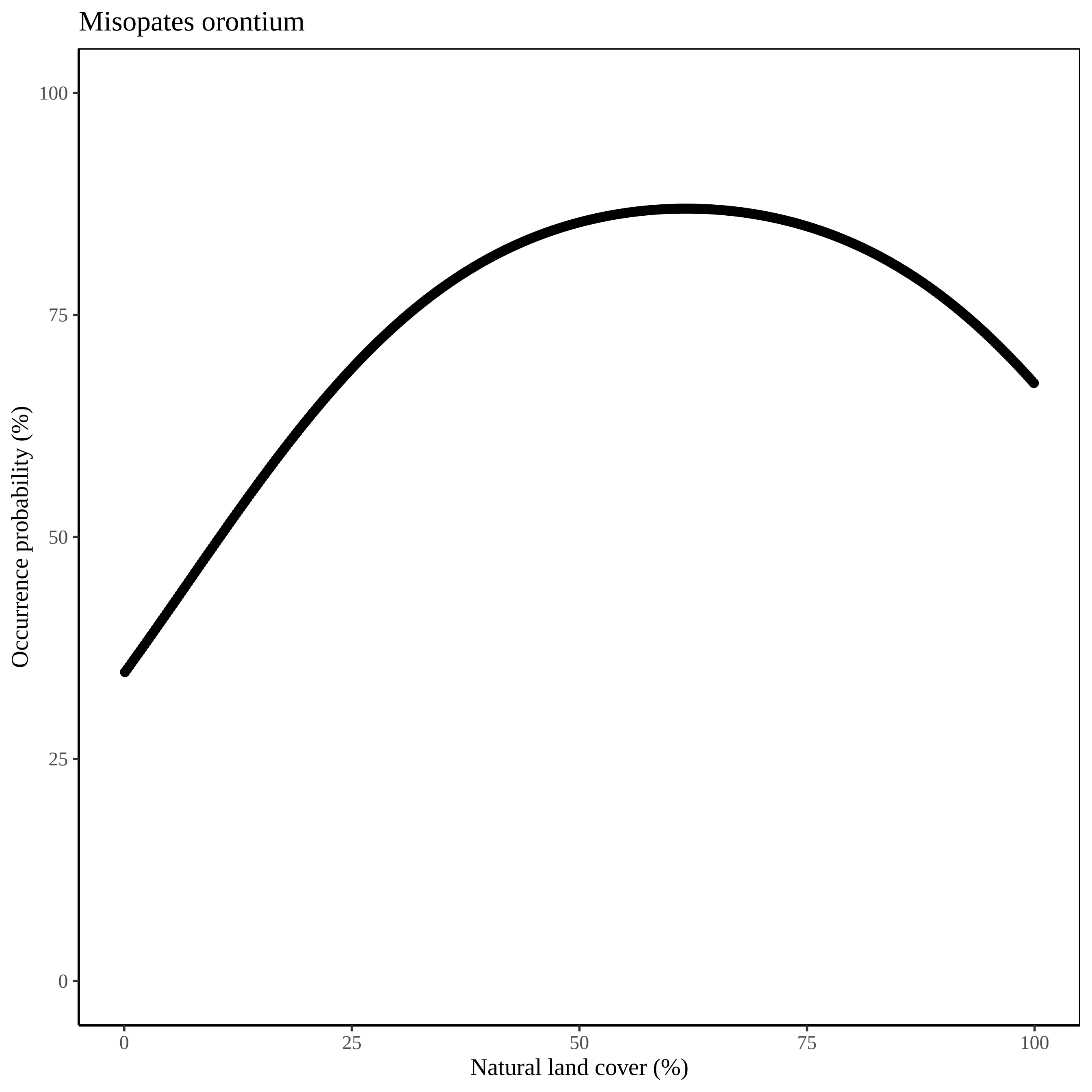
Misopates orontium
100
75
50
25
0
0
25
50
75
100
Occurrence probability (%)
Natural land cover (%)

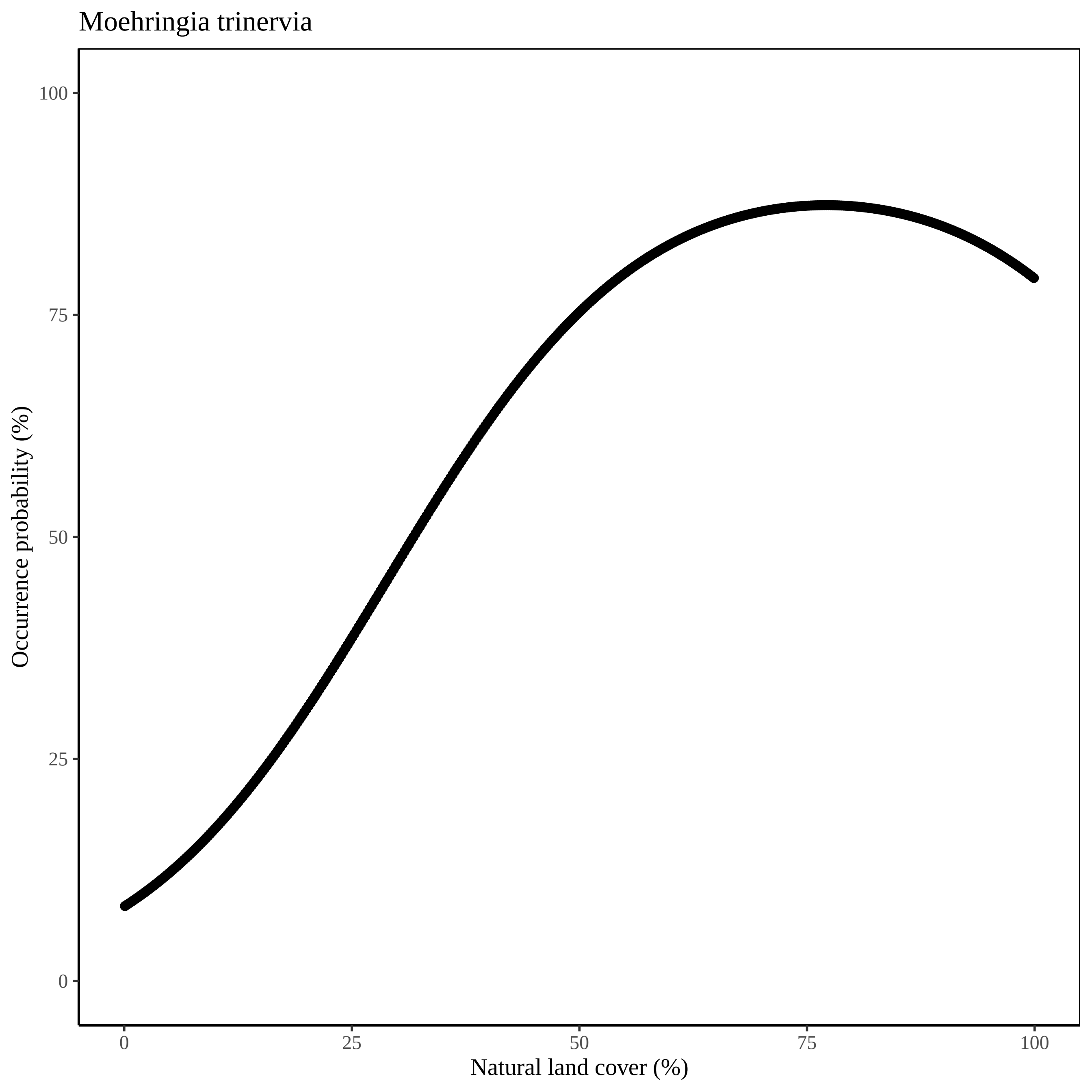
Moehringia trinervia
Occurrence probability (%)
100
75
50
25
0
0
25
50
75
100
Natural land cover (%)

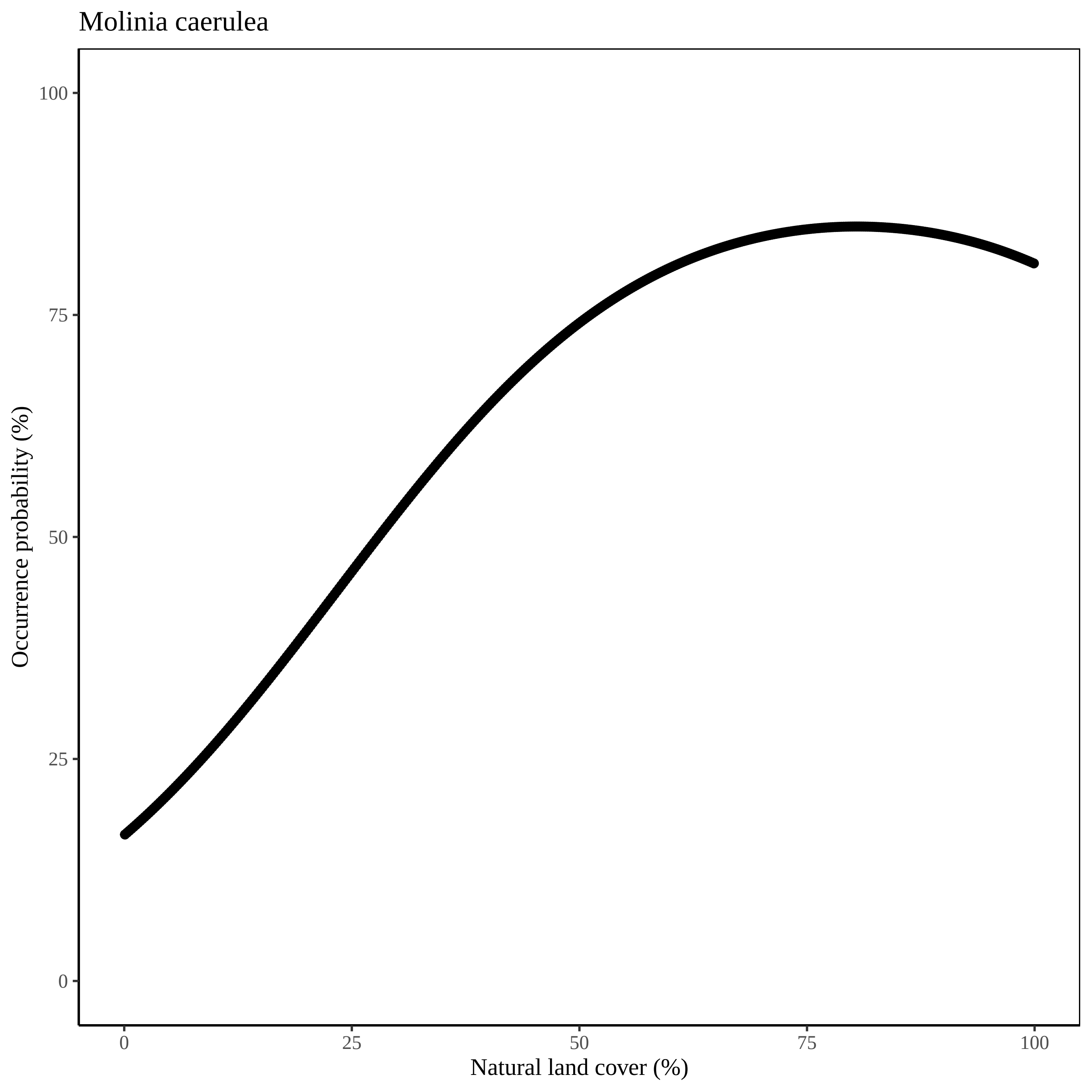
Molinia caerulea
100
75
50
25
0
Occurrence probability (%)
0
25
50
75
100
Natural land cover (%)

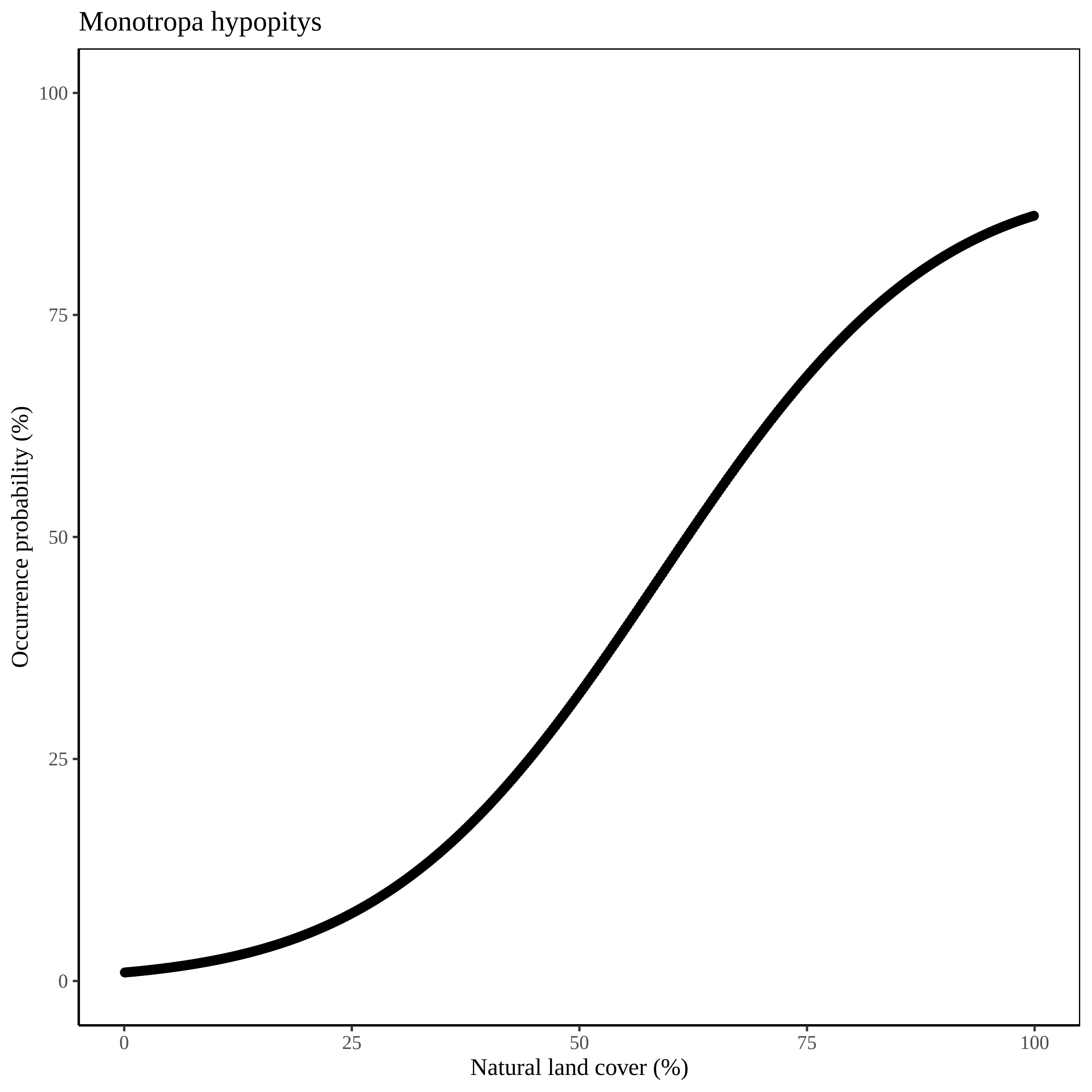
Monotropa hypopitys
Occurrence probability (%)
0
25
50
75
100
Natural land cover (%)
0
25
50
75
100

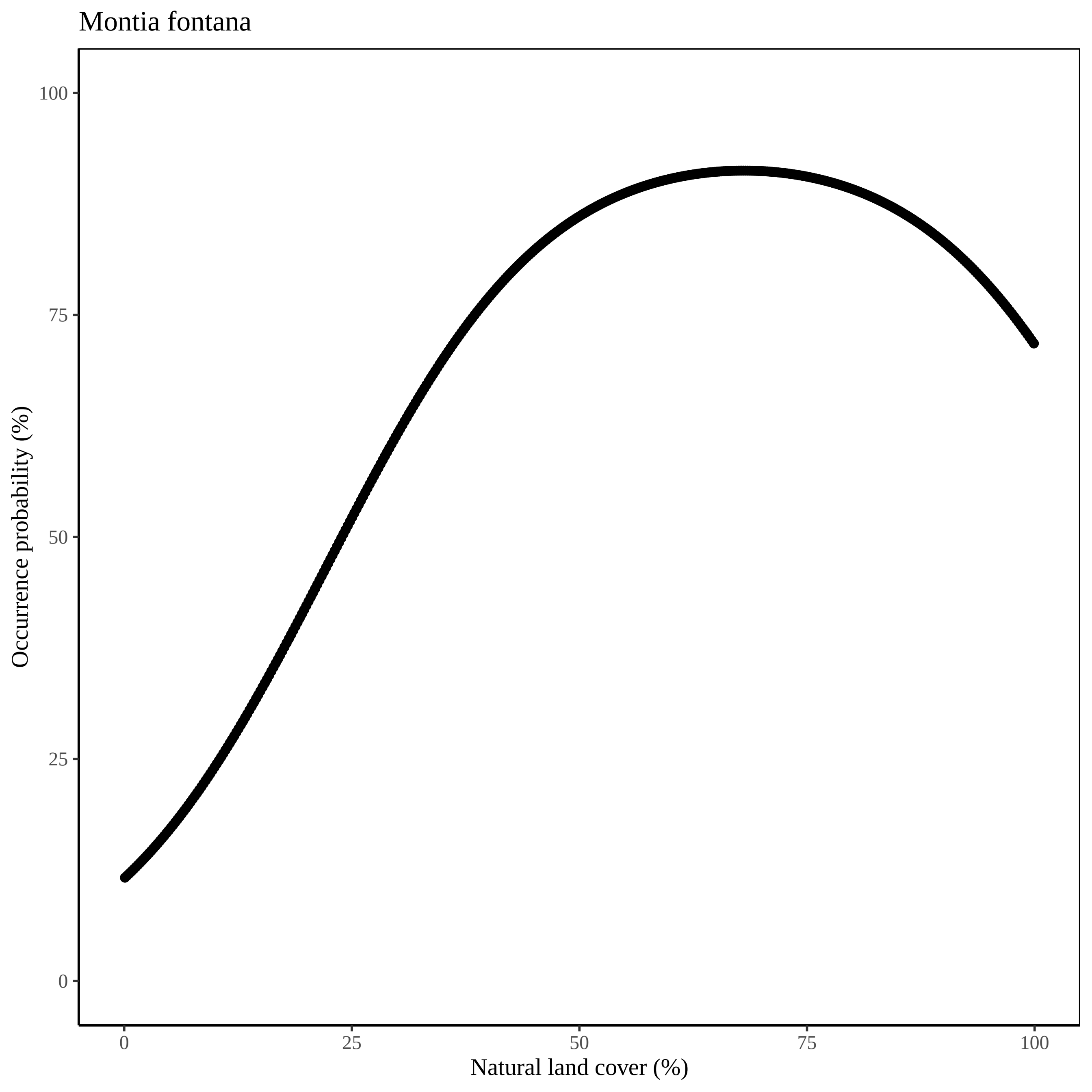
Montia fontana
Occurrence probability (%)
100
75
50
25
0
0
25
50
75
100
Natural land cover (%)

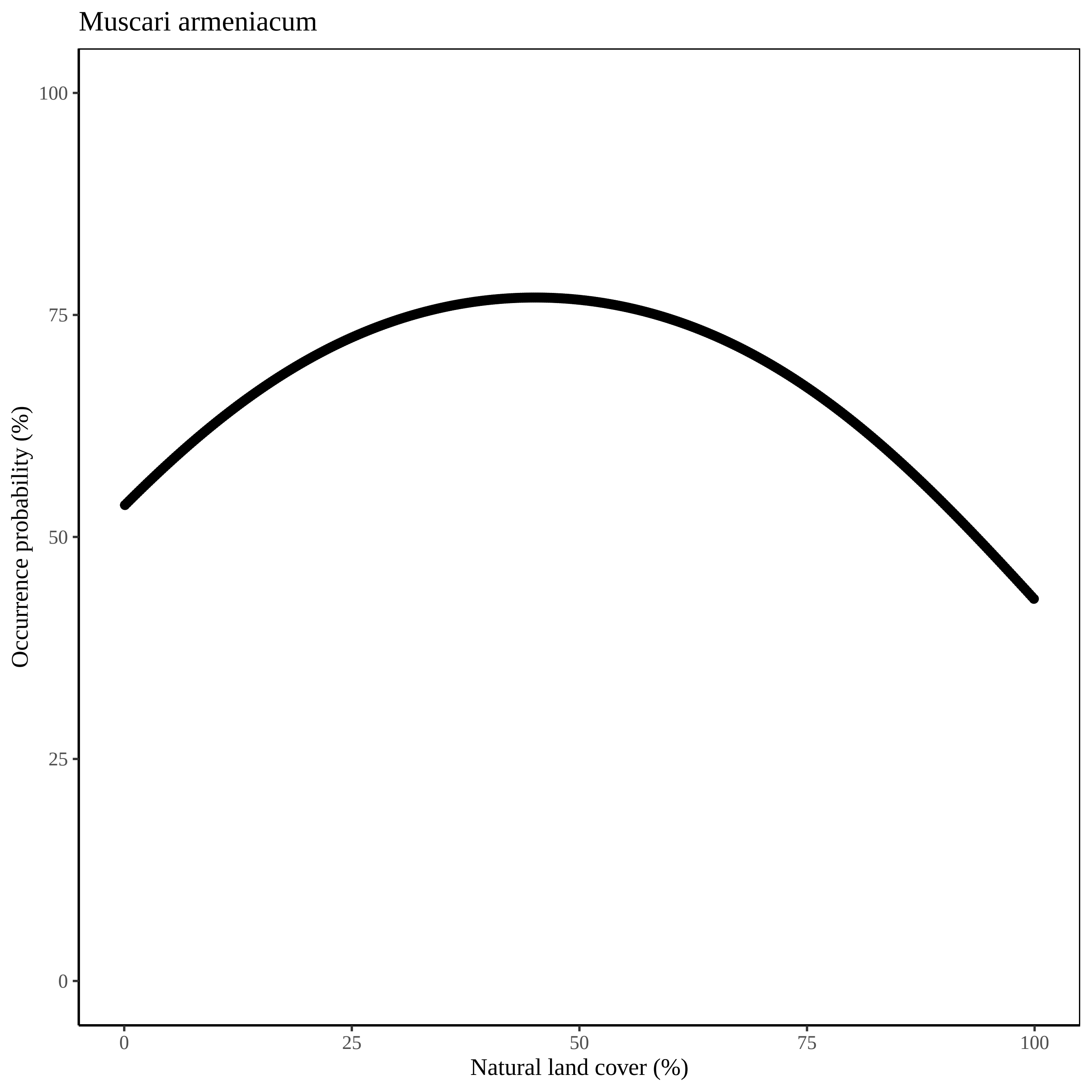

Muscari armeniacum
100
75
50
25
0
Occurrence probability (%)
0
25
50
75
100
Natural land cover (%)

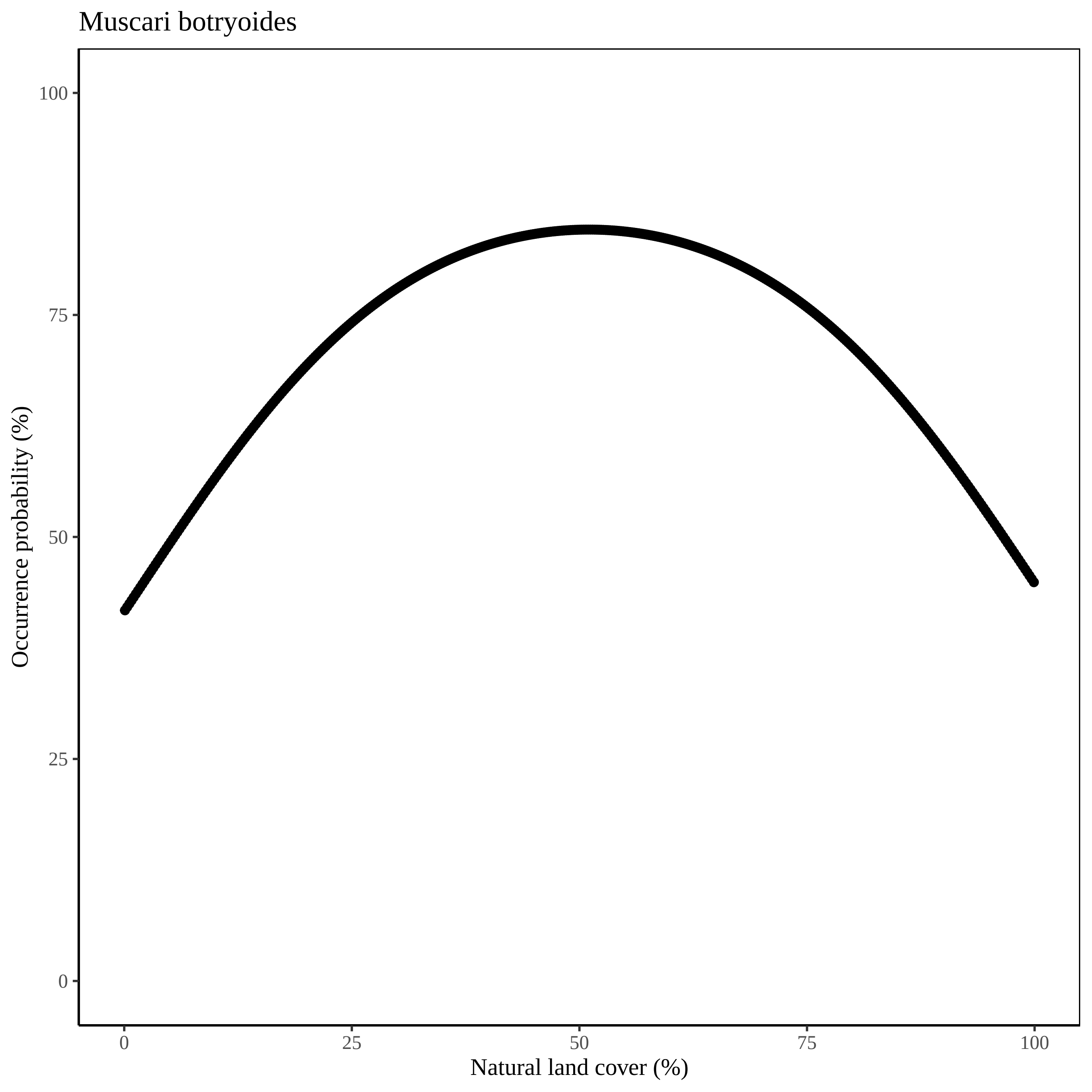
Muscari botryoides
Occurrence probability (%)
100
75
50
25
0
0
25
50
75
100
Natural land cover (%)

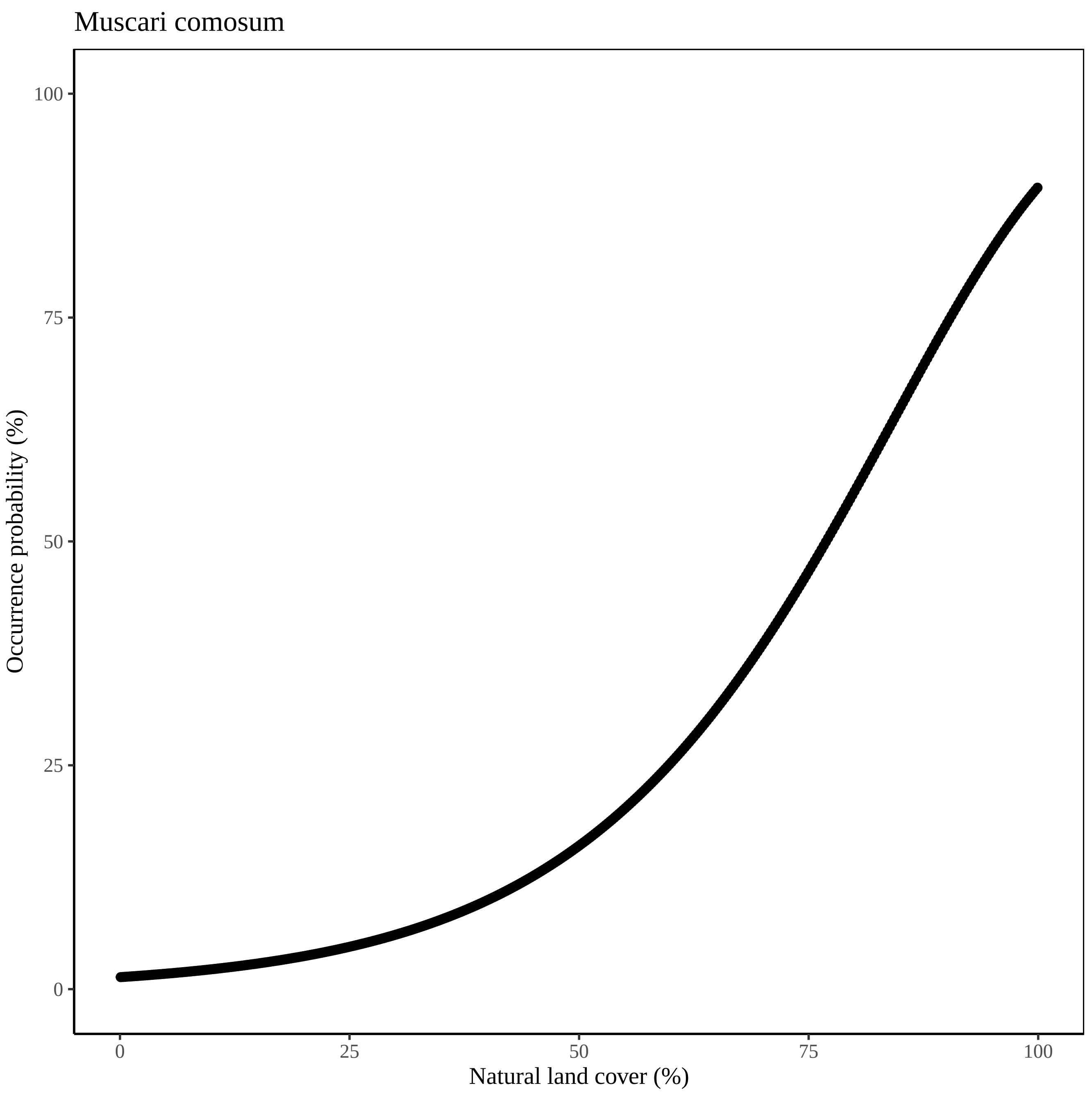

Muscari comosum
Occurrence probability (%)
100
75
50
25
0
0
25
50
75
100
Natural land cover (%)

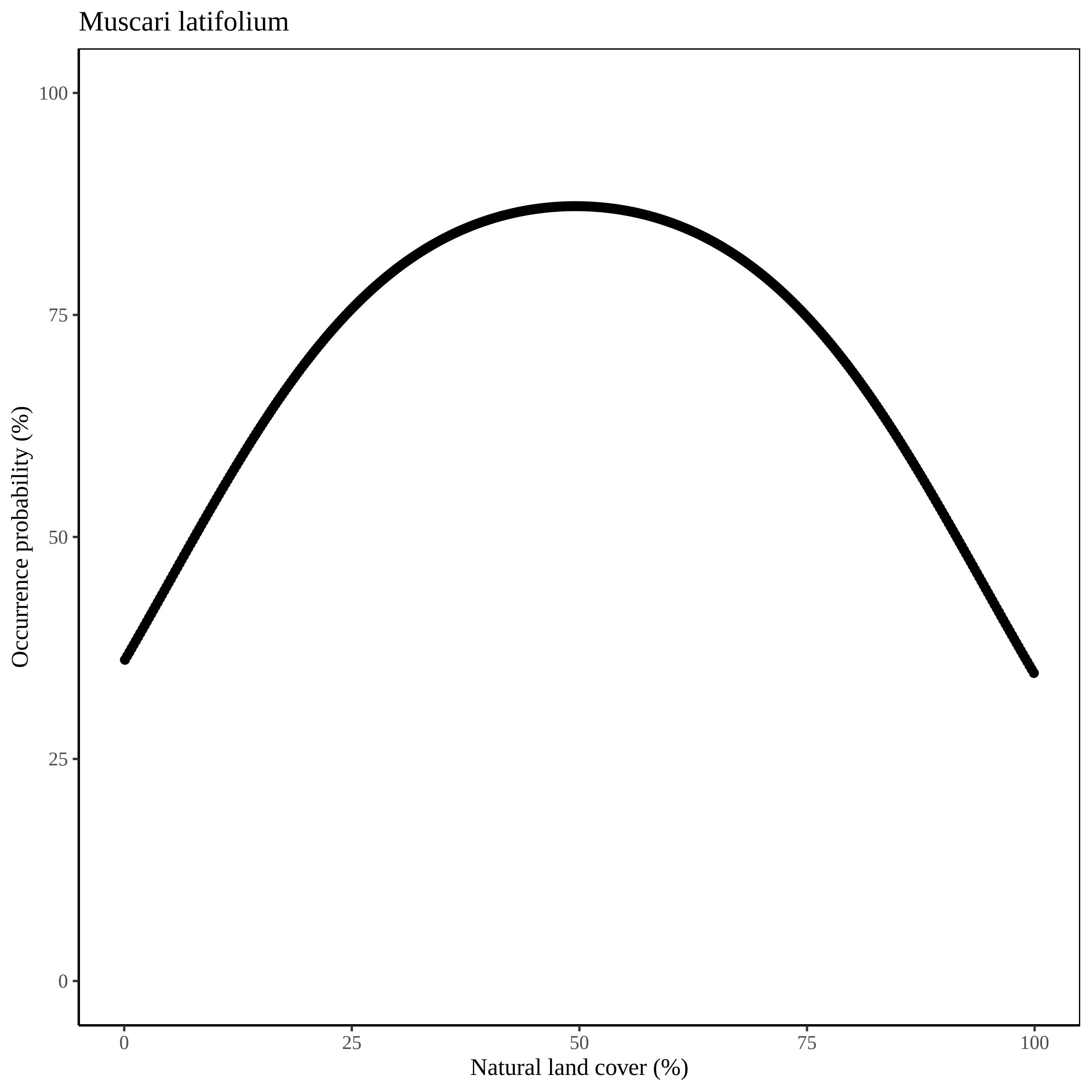
Muscari latifolium
Occurrence probability (%)
100
75
50
25
0
0
25
50
75
100
Natural land cover (%)

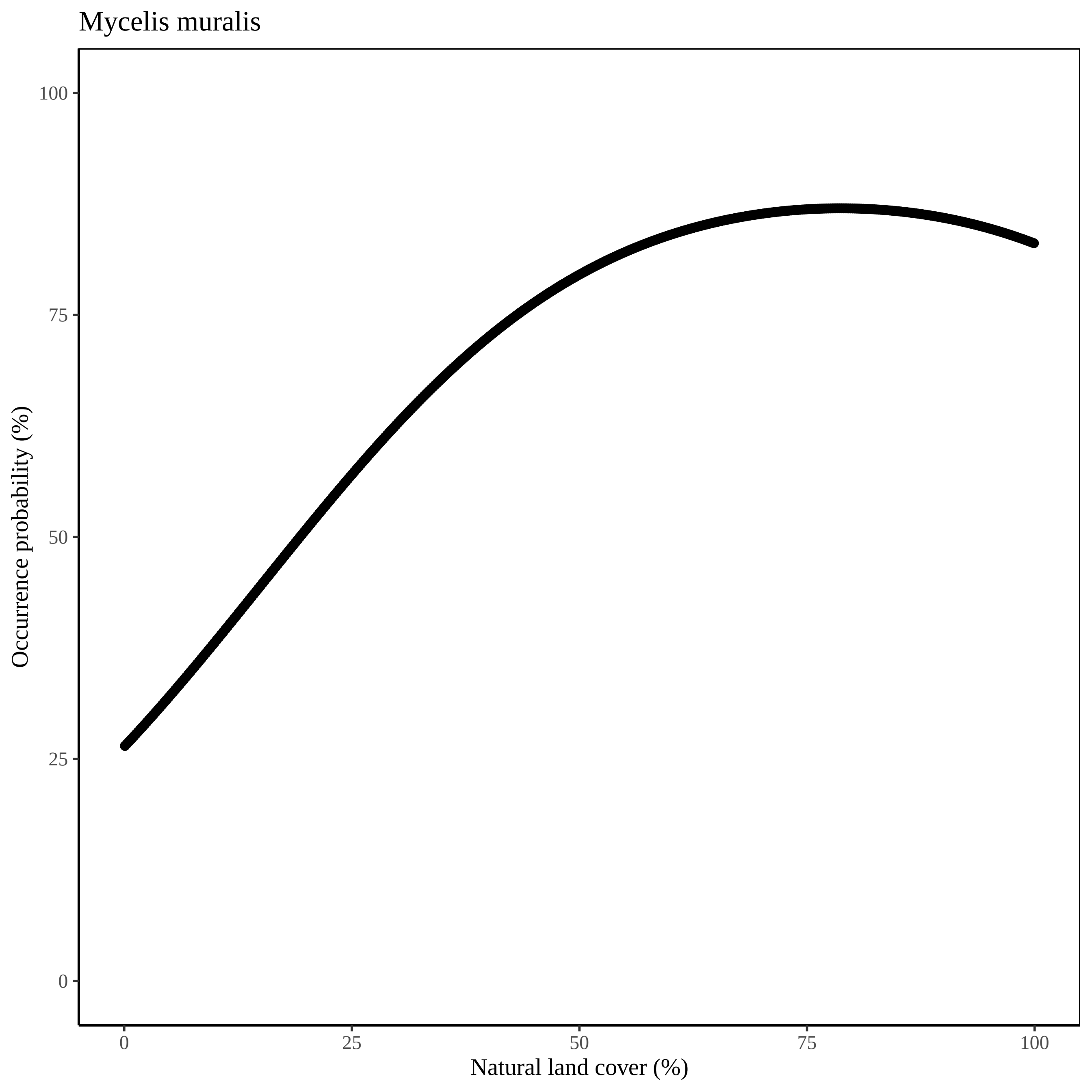
Mycelis muralis
100
75
50
25
0
Occurrence probability (%)
0
25
50
75
100
Natural land cover (%)

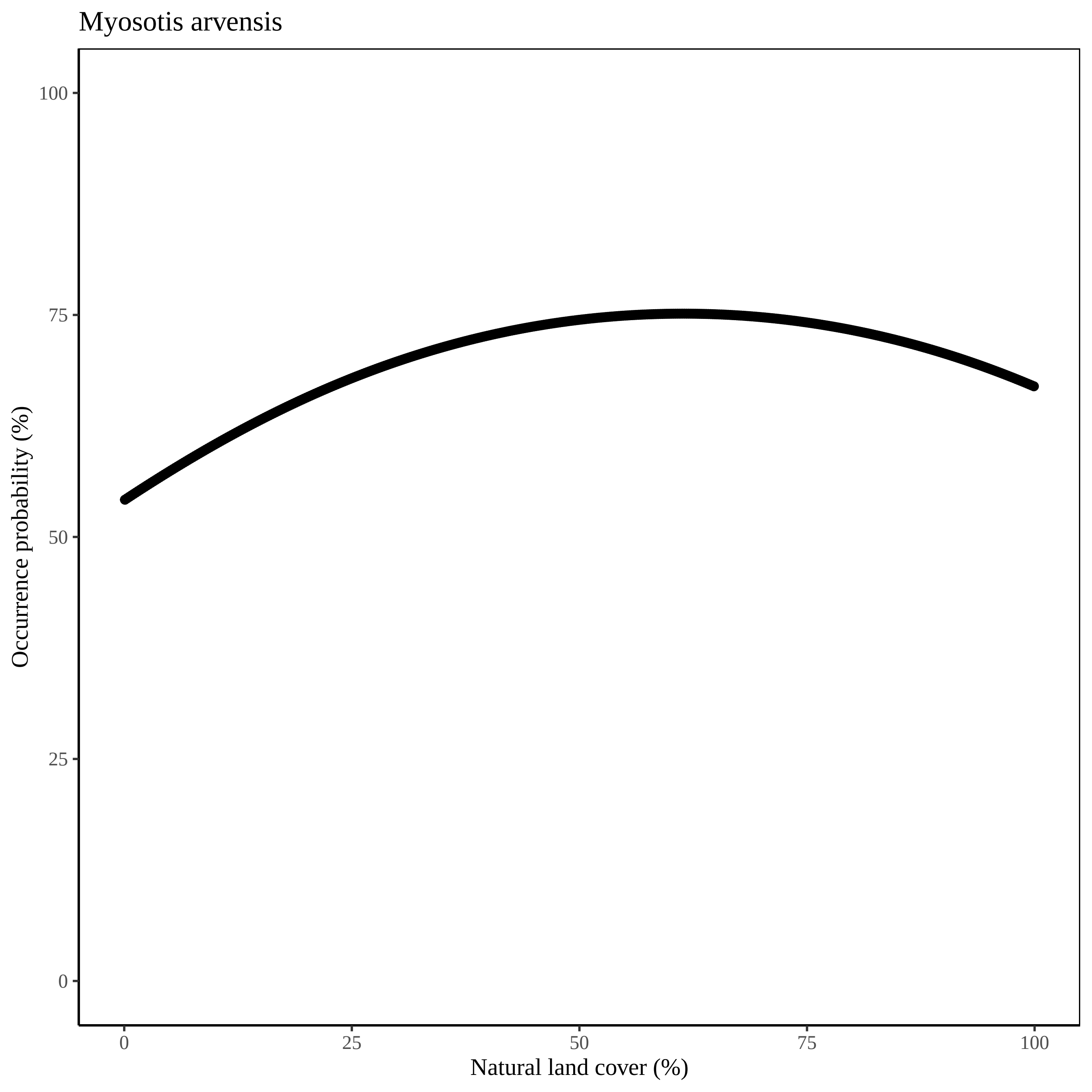

Myosotis arvensis
100
75
50
25
0
Occurrence probability (%)
0
25
50
75
100
Natural land cover (%)

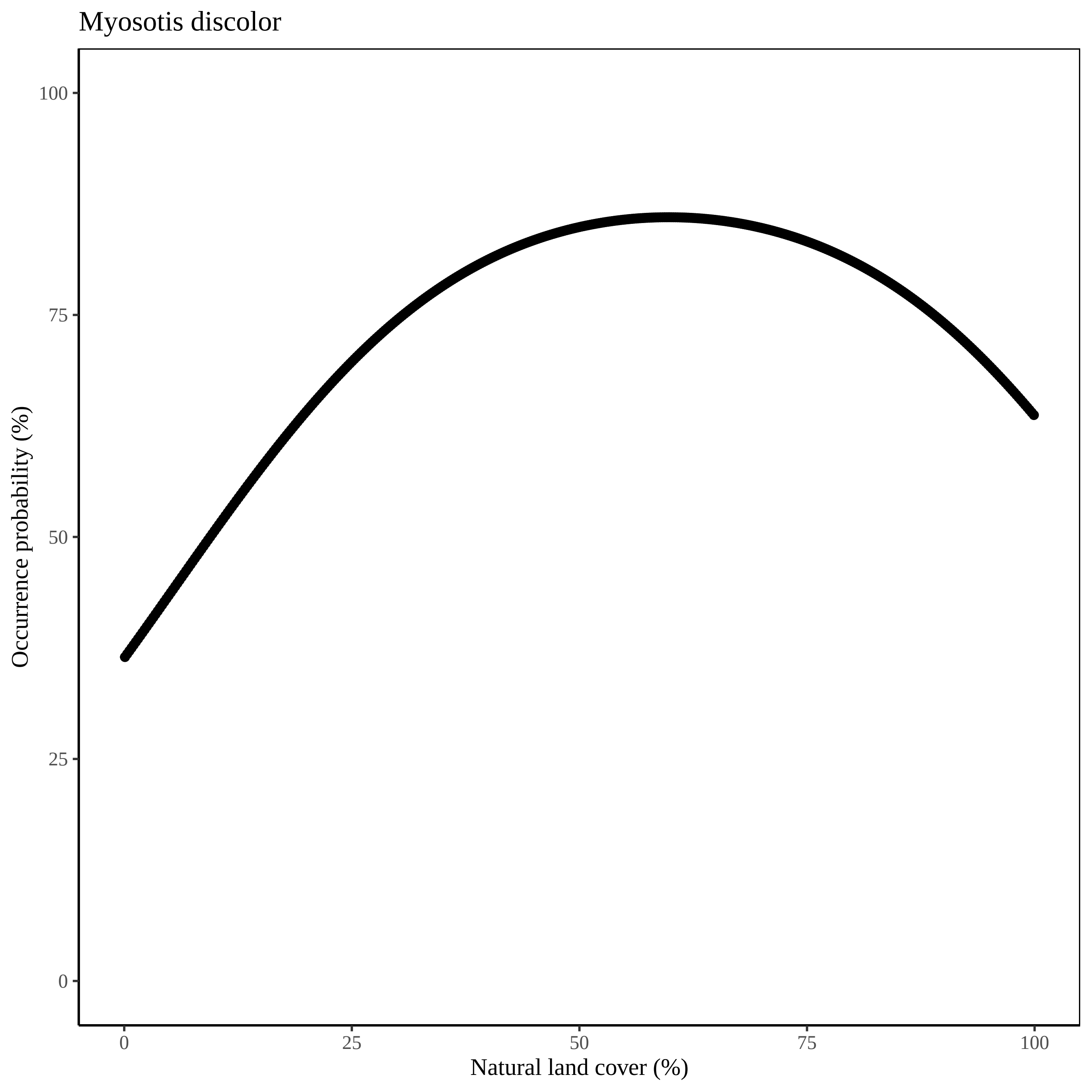
Myosotis discolor
Occurrence probability (%)
100
75
50
25
0
0
25
50
75
100
Natural land cover (%)

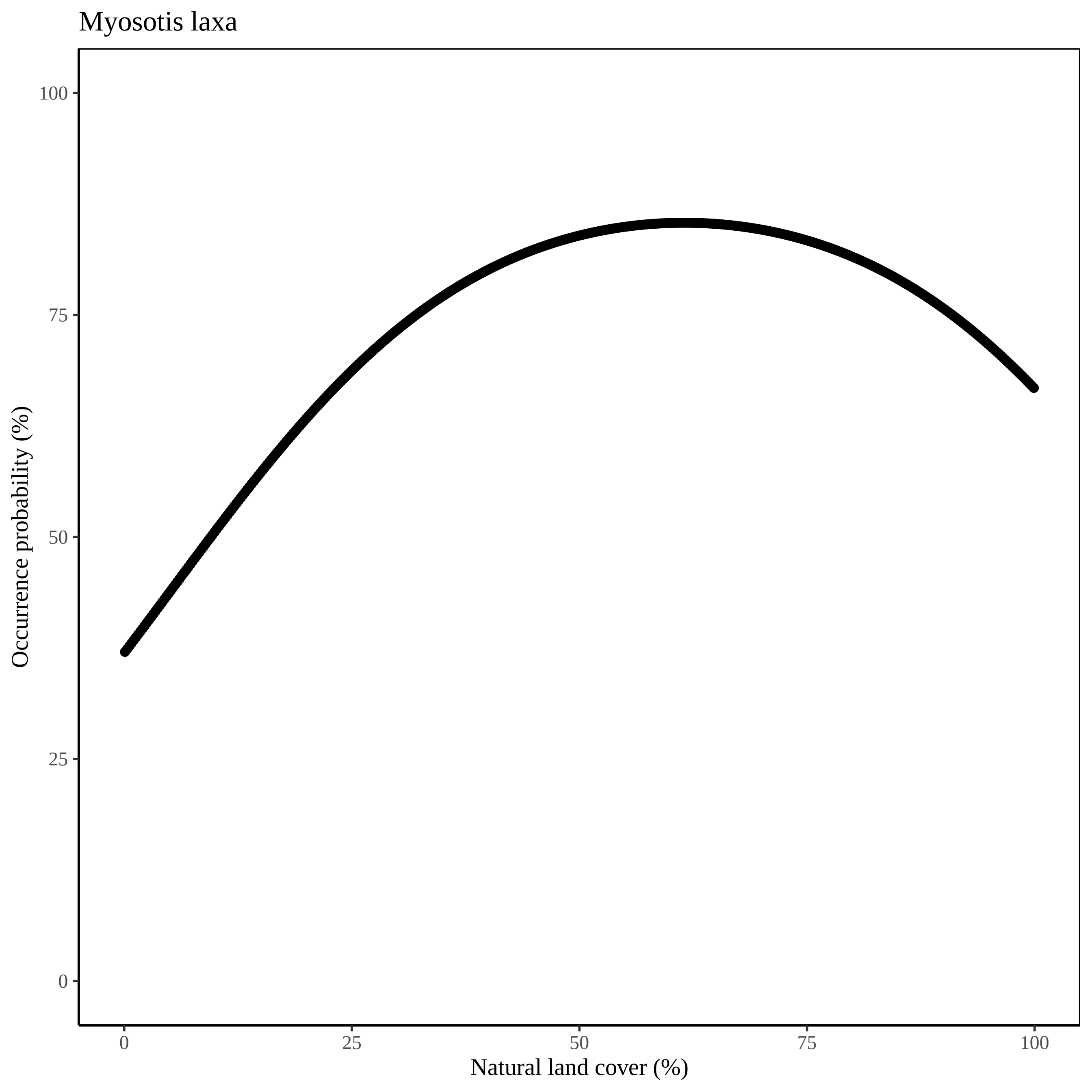
Myosotis laxa
Occurrence probability (%)
100
75
50
25
0
0
25
50
75
100
Natural land cover (%)

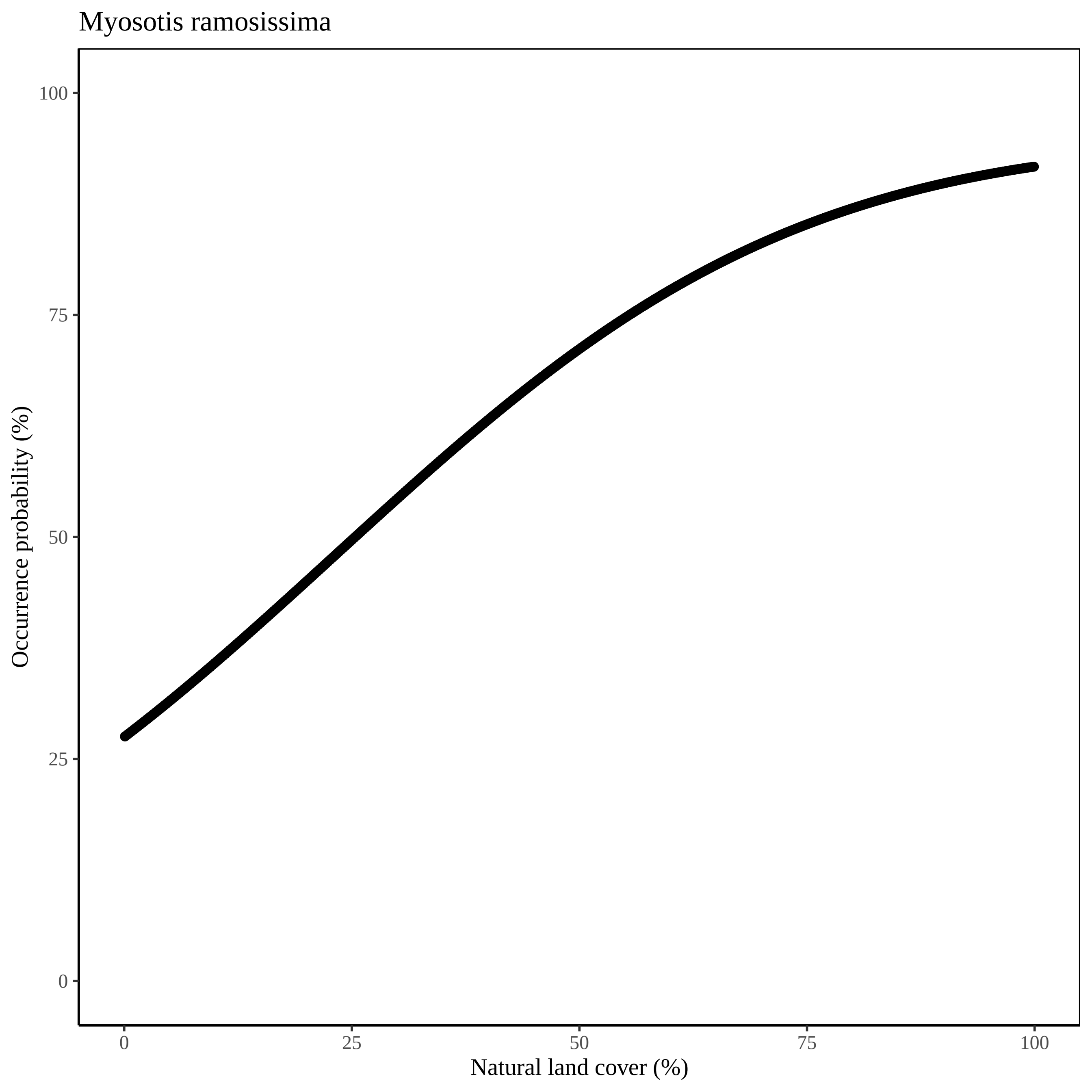
Myosotis ramosissima
Occurrence probability (%)
100
75
50
25
0
0
25
50
75
100
Natural land cover (%)

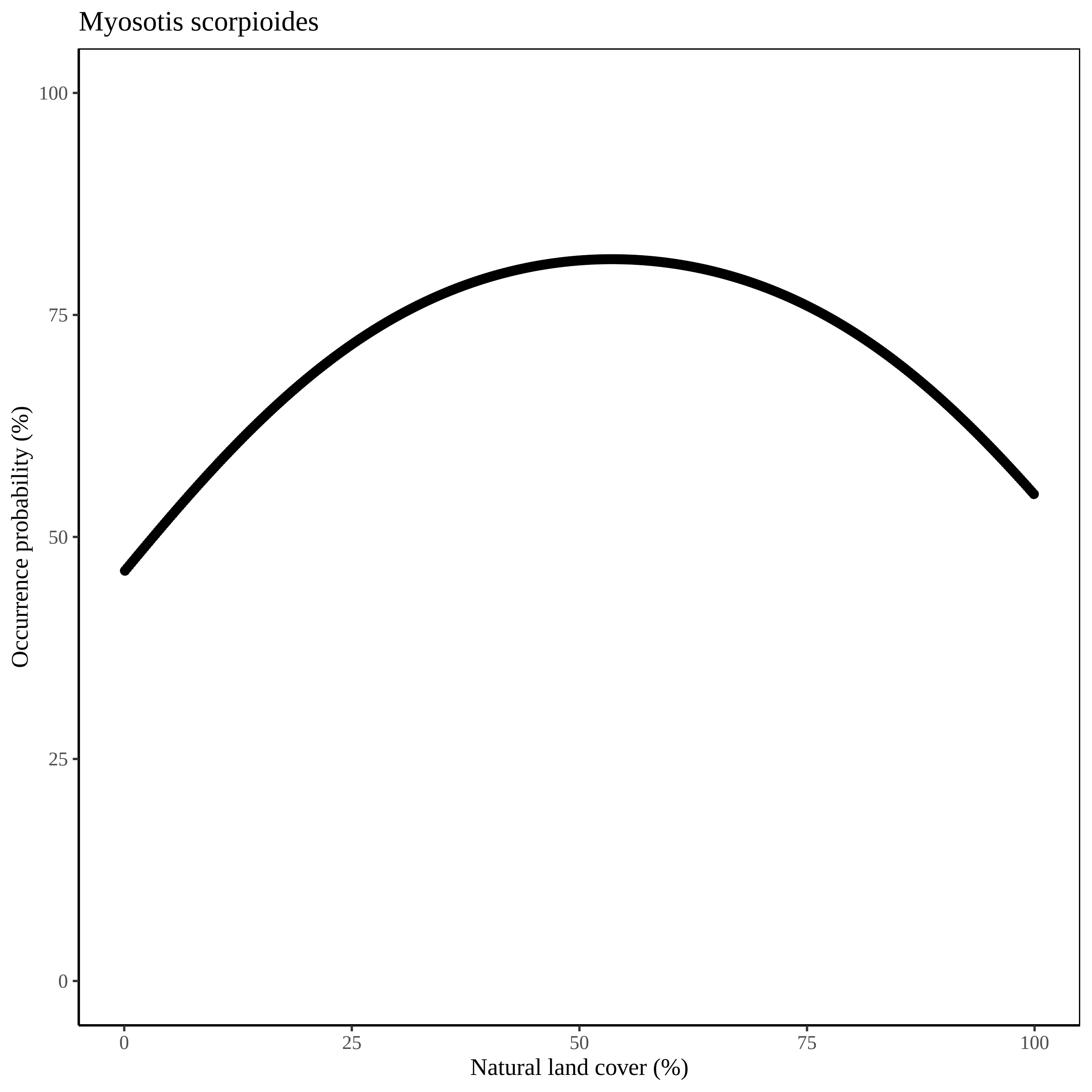
Myosotis scorpioides
Occurrence probability (%)
100
75
50
25
0
0
25
50
75
100
Natural land cover (%)

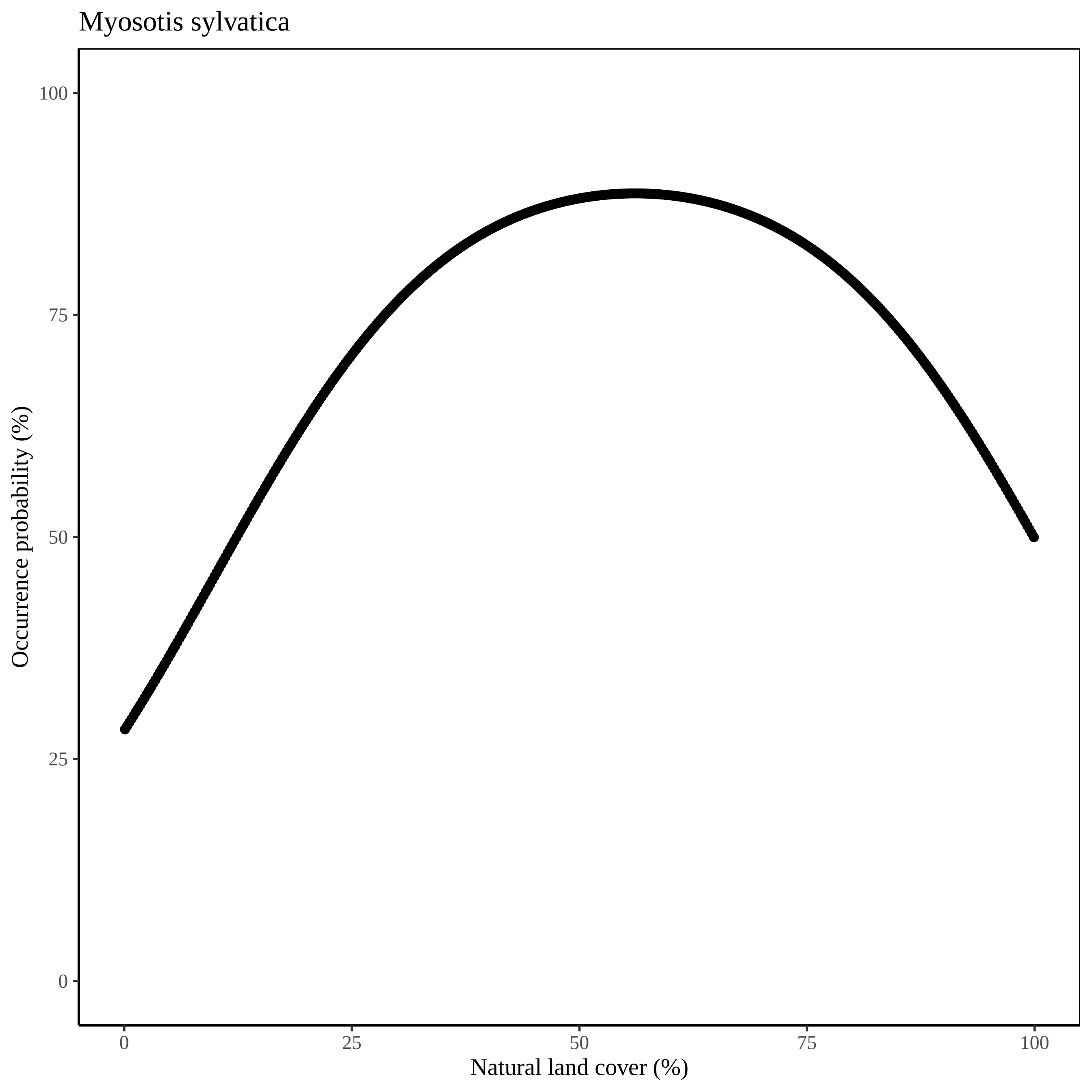
Myosotis sylvatica
Occurrence probability (%)
100
75
50
25
0
0
25
50
75
100
Natural land cover (%)

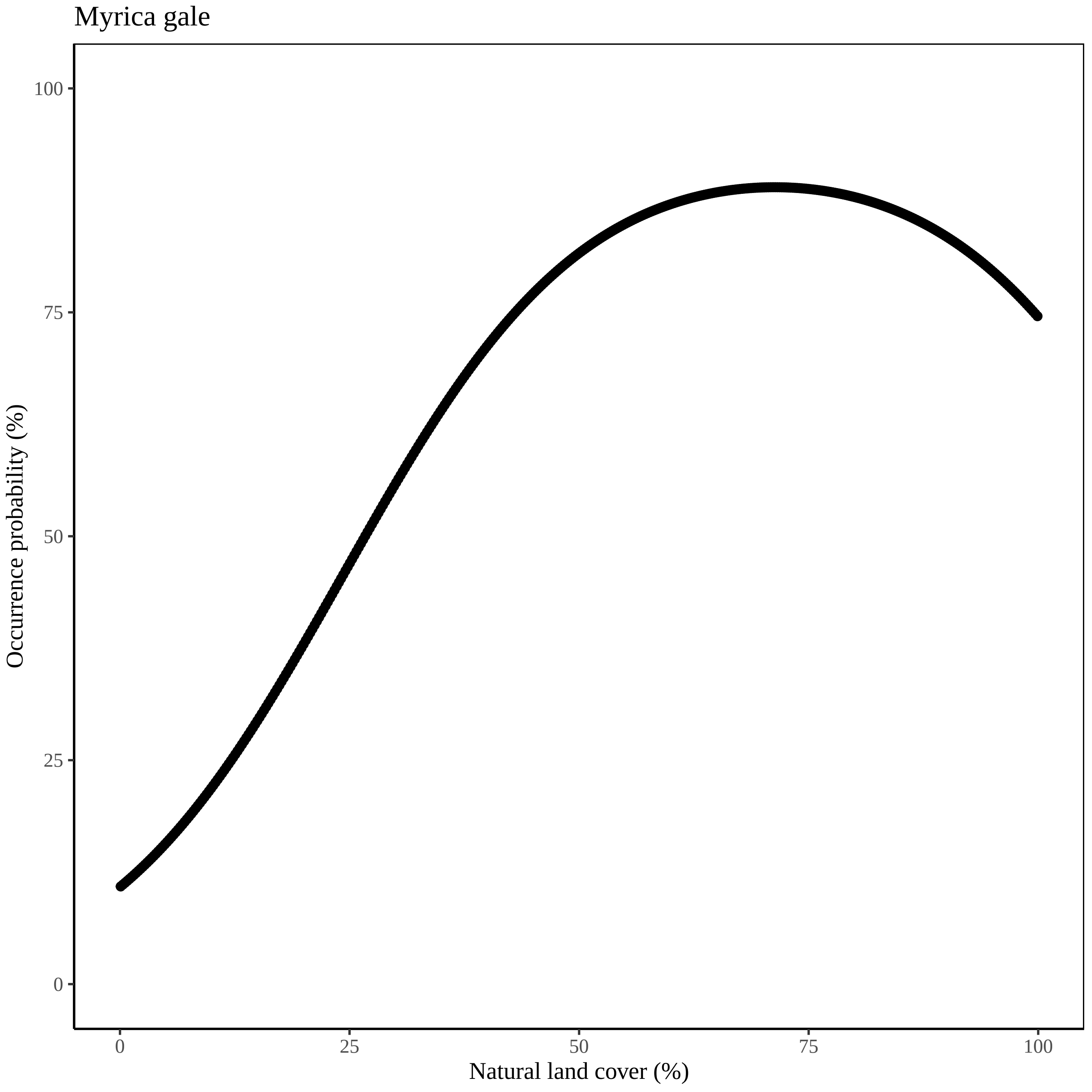
Myrica gale
Occurrence probability (%)
100
75
50
25
0
0
25
50
75
100
Natural land cover (%)

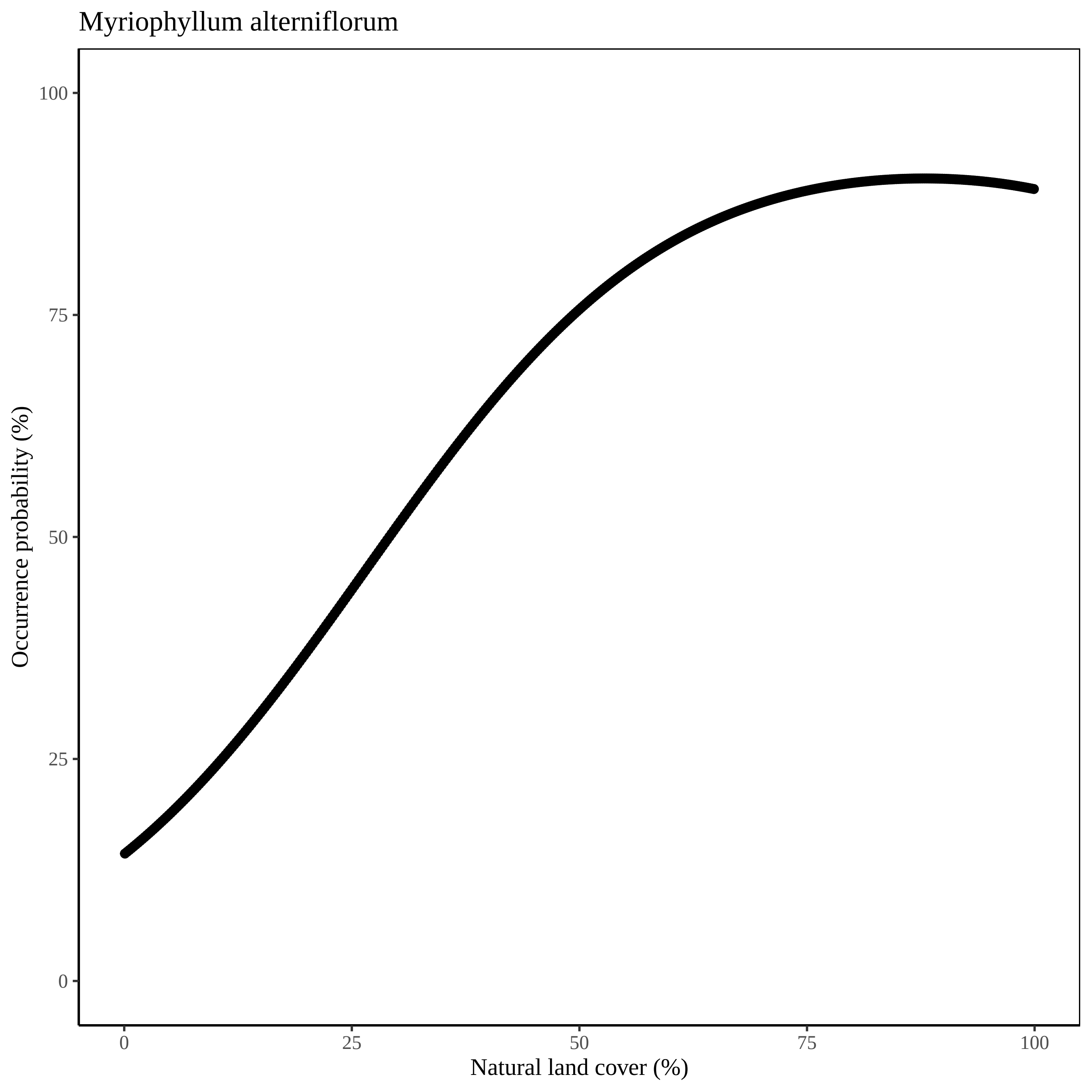
Myriophyllum alterniflorum
Occurrence probability (%)
100
75
50
25
0
0
25
50
75
100
Natural land cover (%)

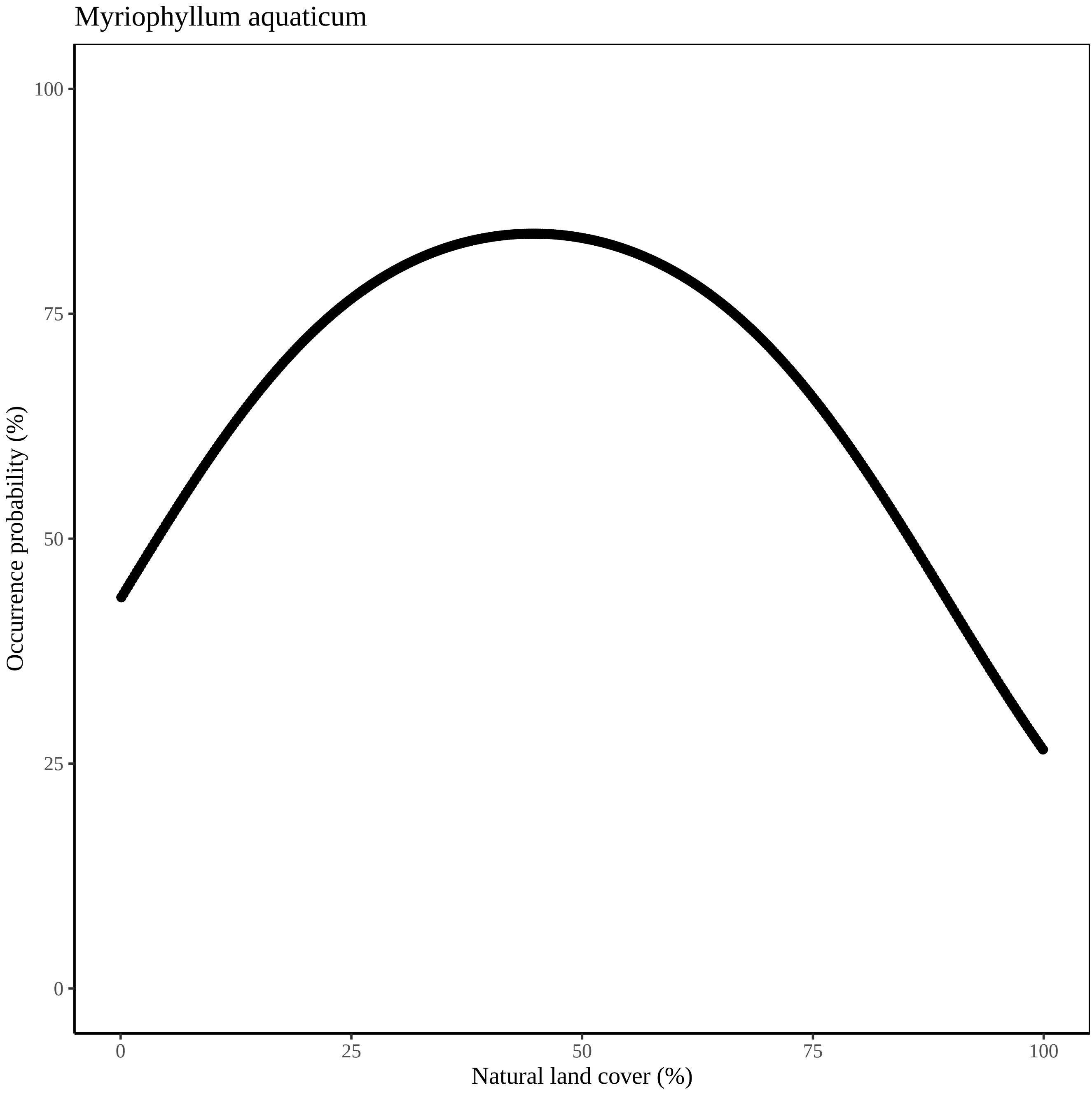
Myriophyllum aquaticum
Occurrence probability (%)
100
75
50
25
0
0
25
50
75
100
Natural land cover (%)

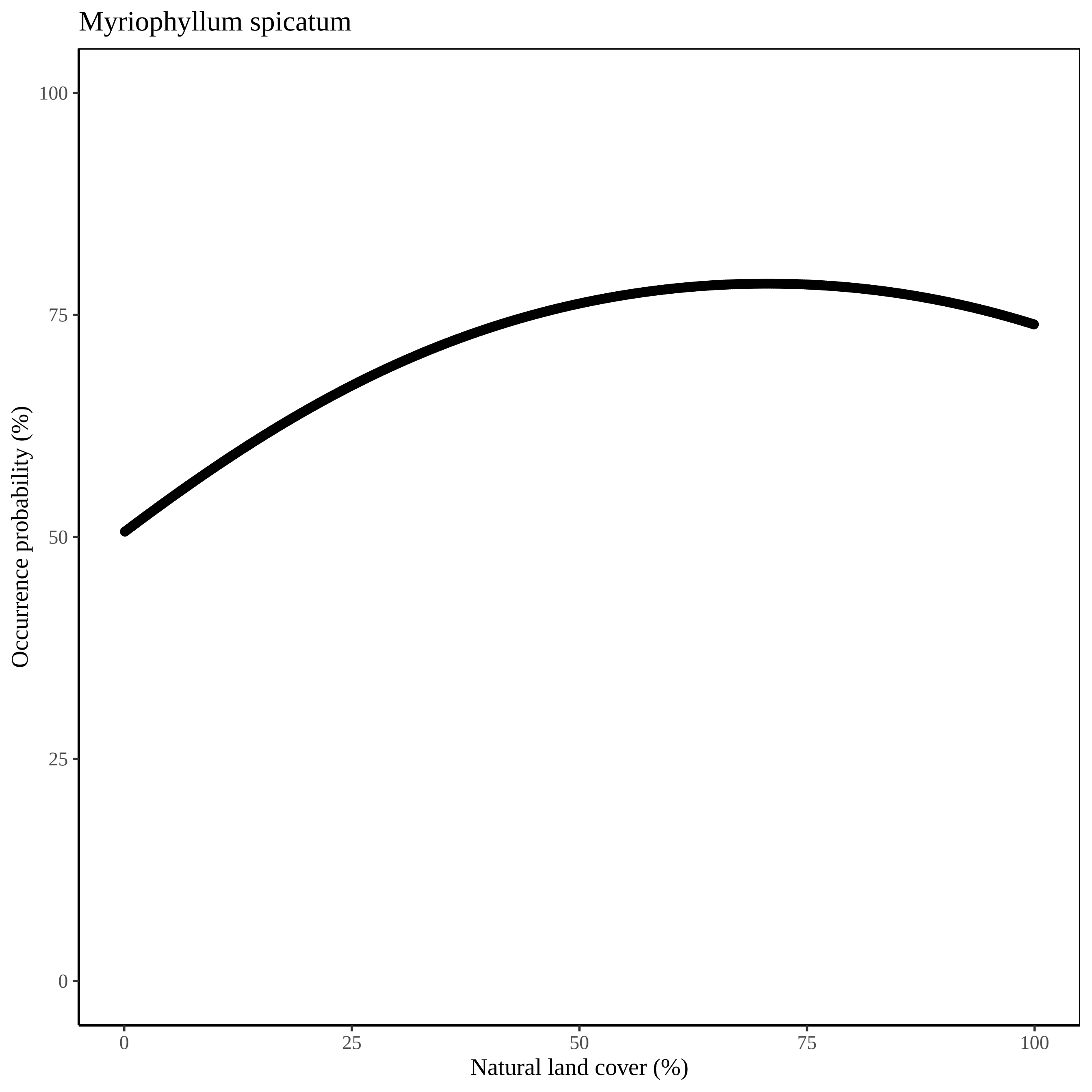
Myriophyllum spicatum
100
75
50
25
0
Occurrence probability (%)
0
25
50
75
100
Natural land cover (%)

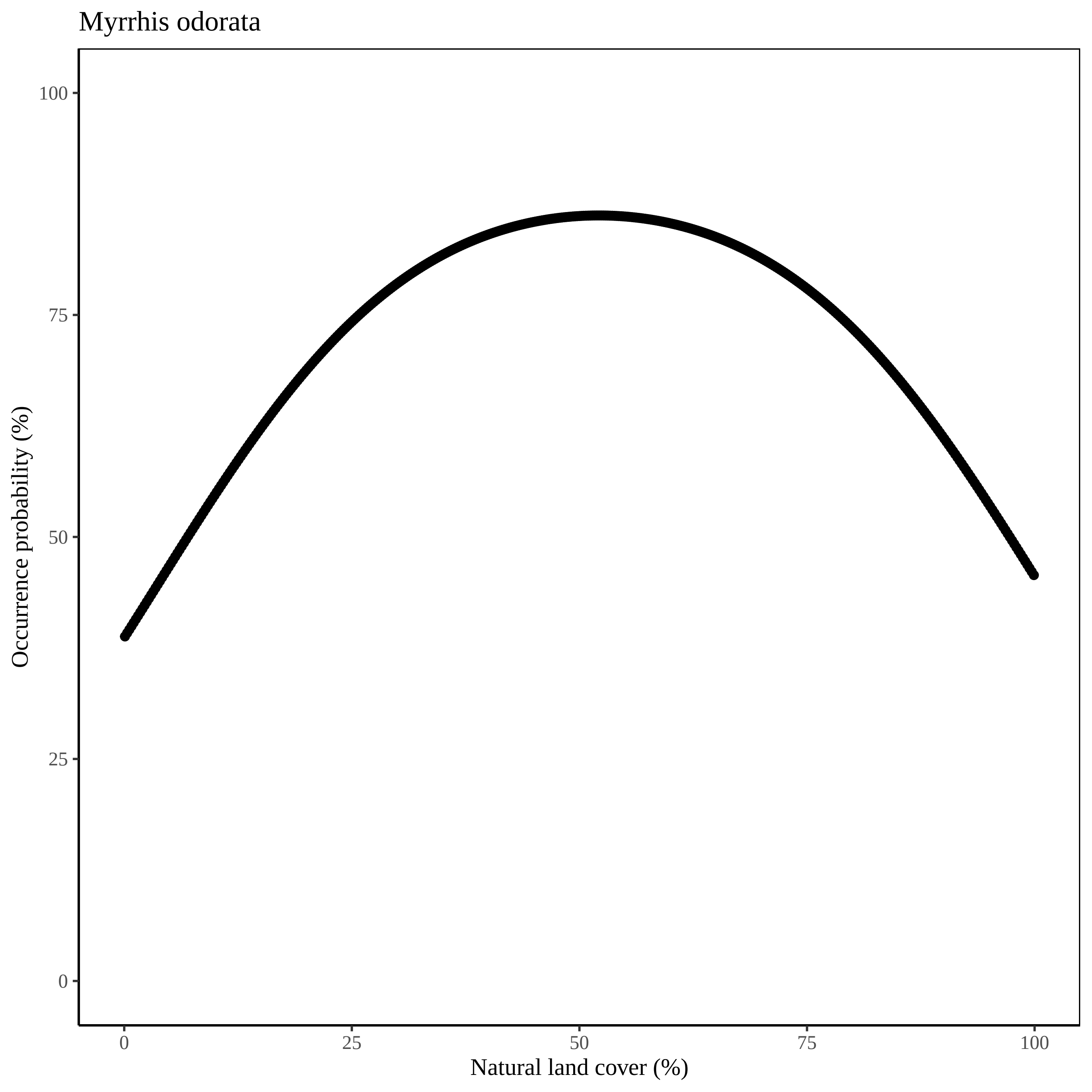
Myrrhis odorata
Occurrence probability (%)
100
75
50
25
0
0
25
50
75
100
Natural land cover (%)

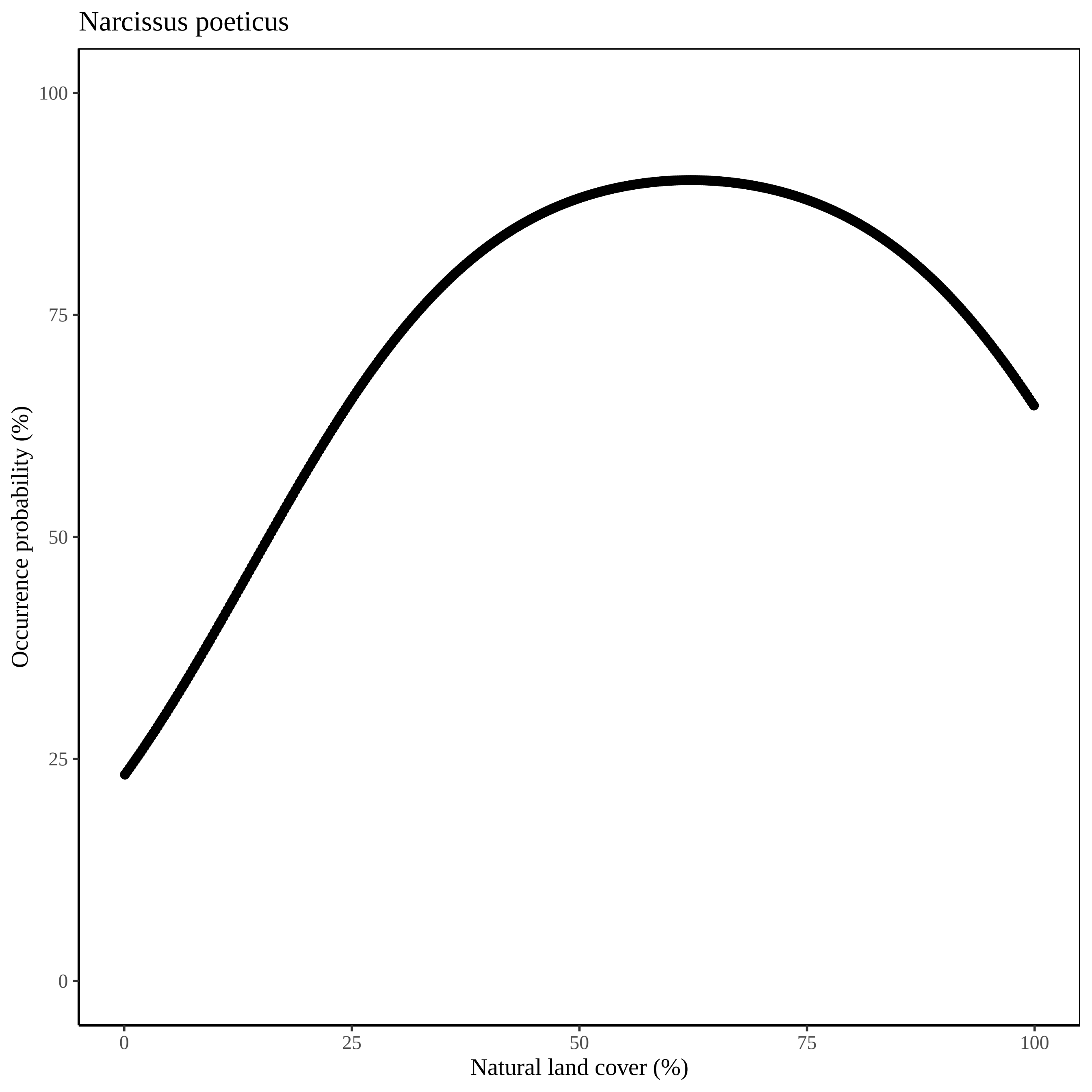

Narcissus poeticus
Occurrence probability (%)
100
75
50
25
0
0
25
50
75
100
Natural land cover (%)

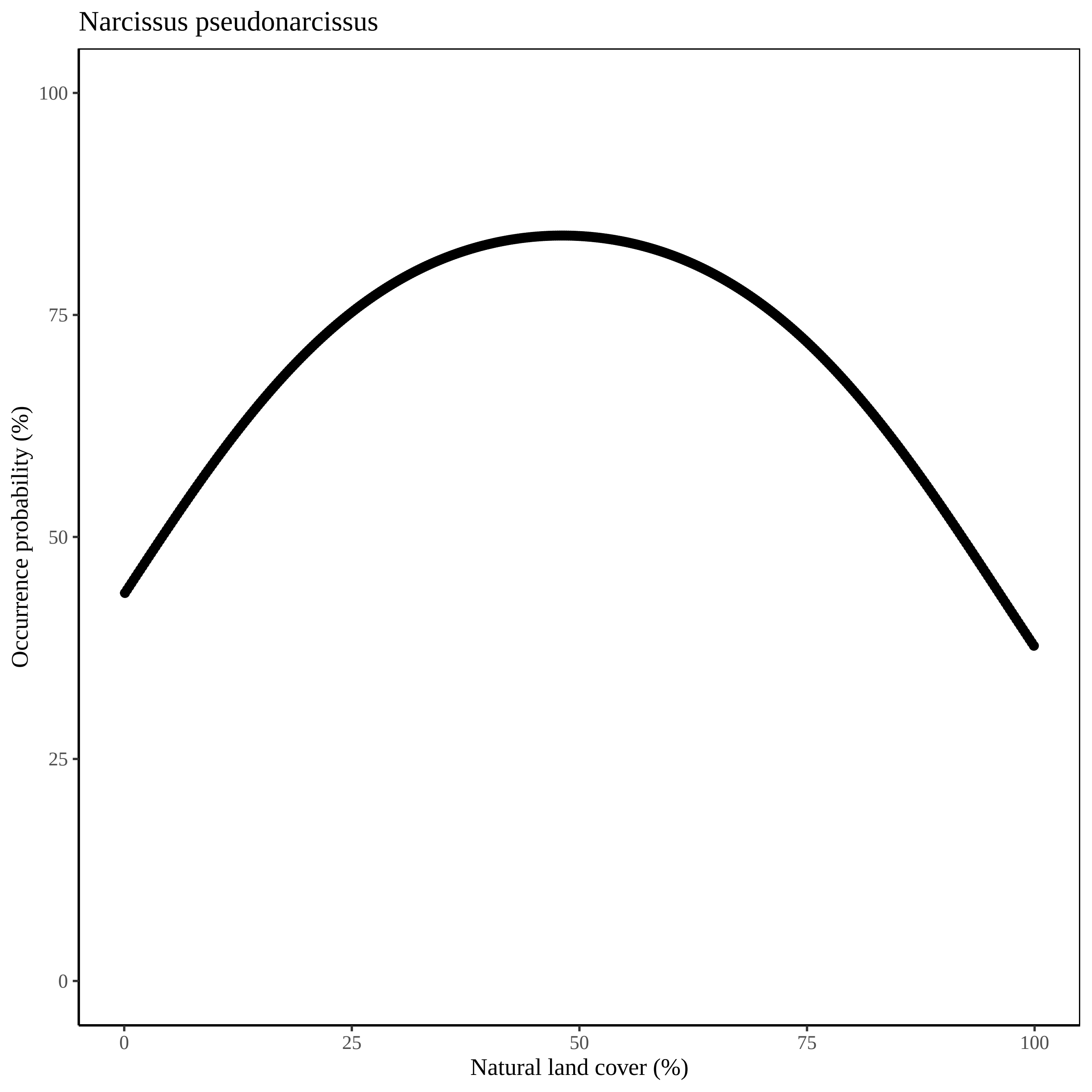
Narcissus pseudonarcissus
Occurrence probability (%)
100
75
50
25
0
0
25
50
75
100
Natural land cover (%)

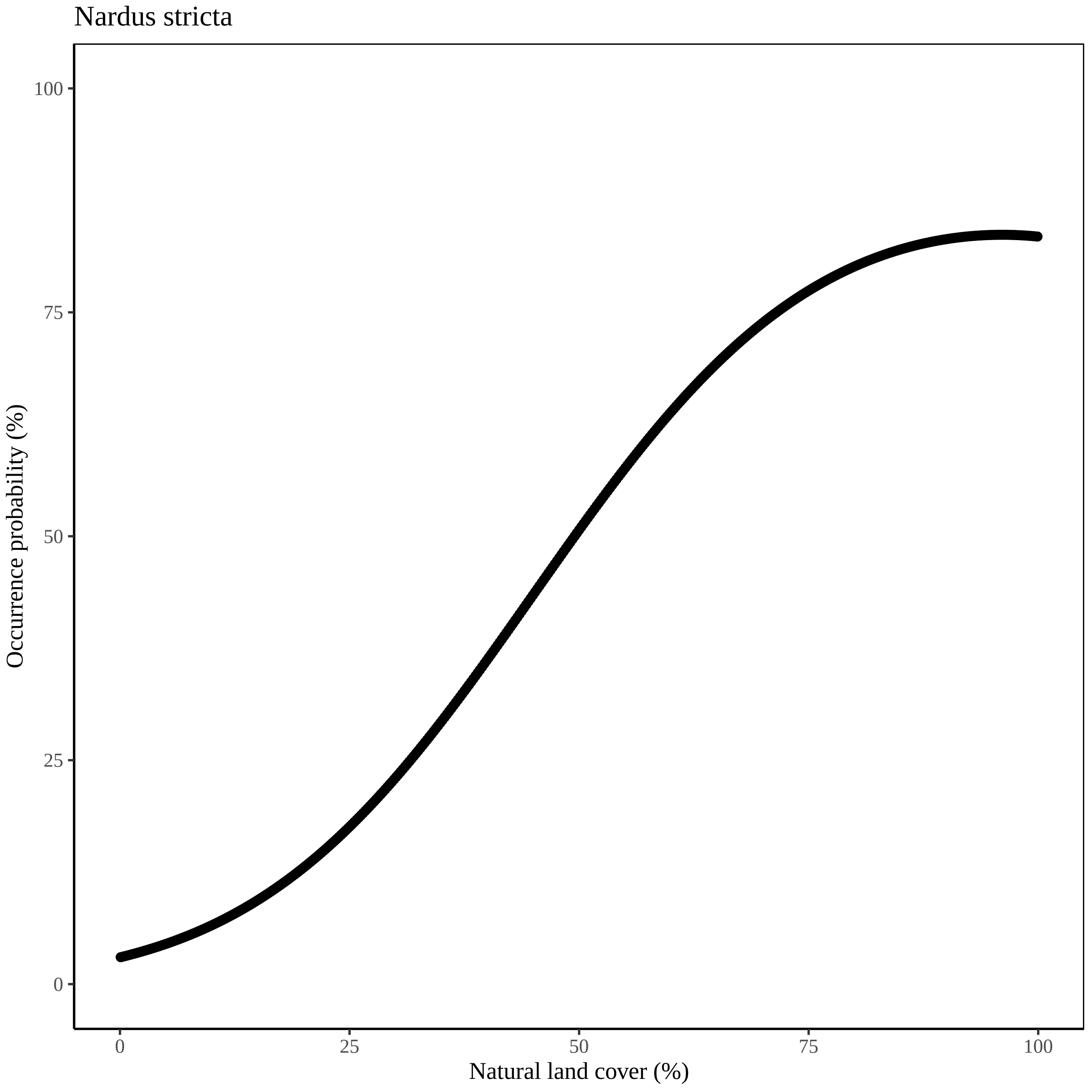
Nardus stricta
Occurrence probability (%)
100
75
50
25
0
Natural land cover (%)
0
25
50
75
100

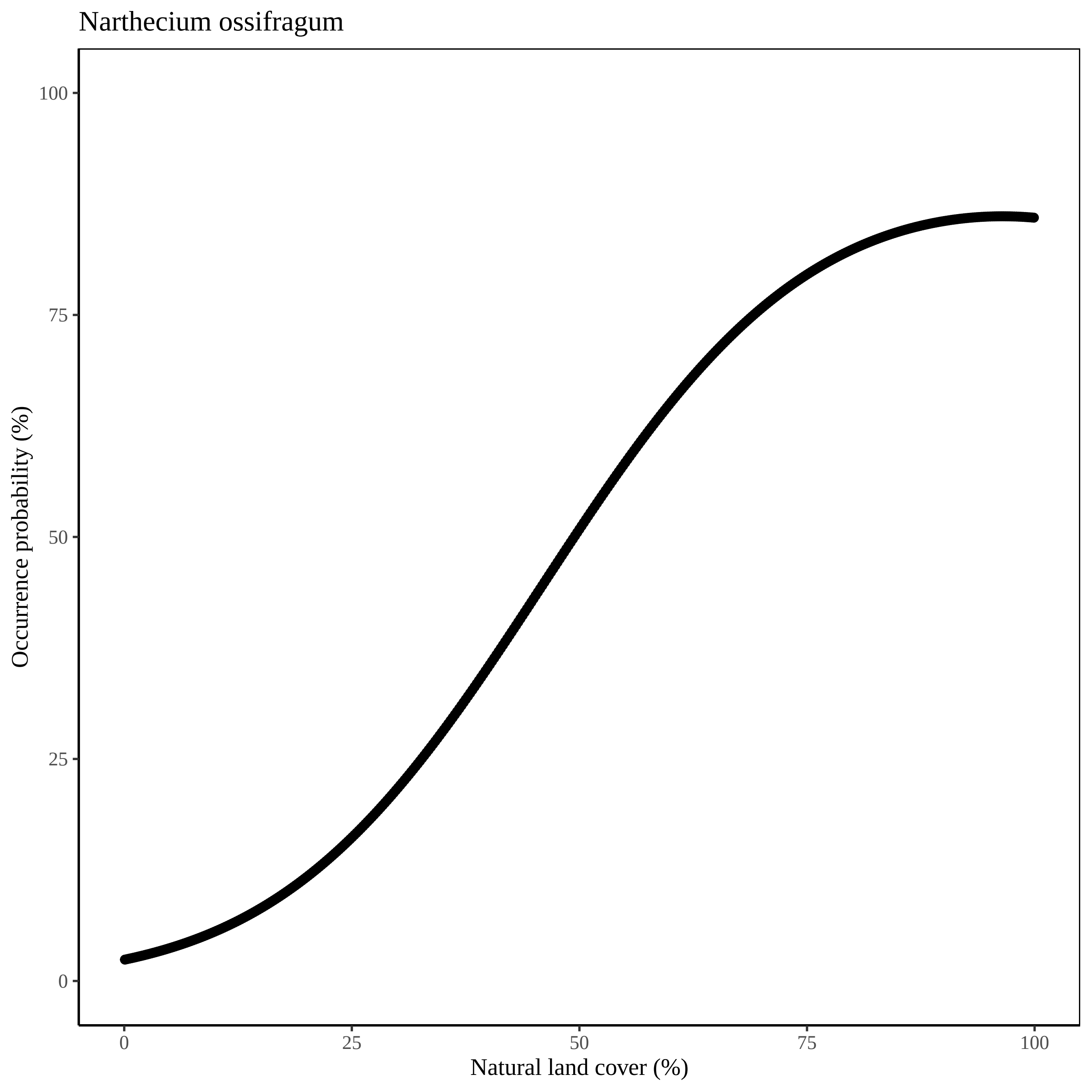

Narthecium ossifragum
Occurrence probability (%)
100
75
50
25
0
0
25
50
75
100
Natural land cover (%)

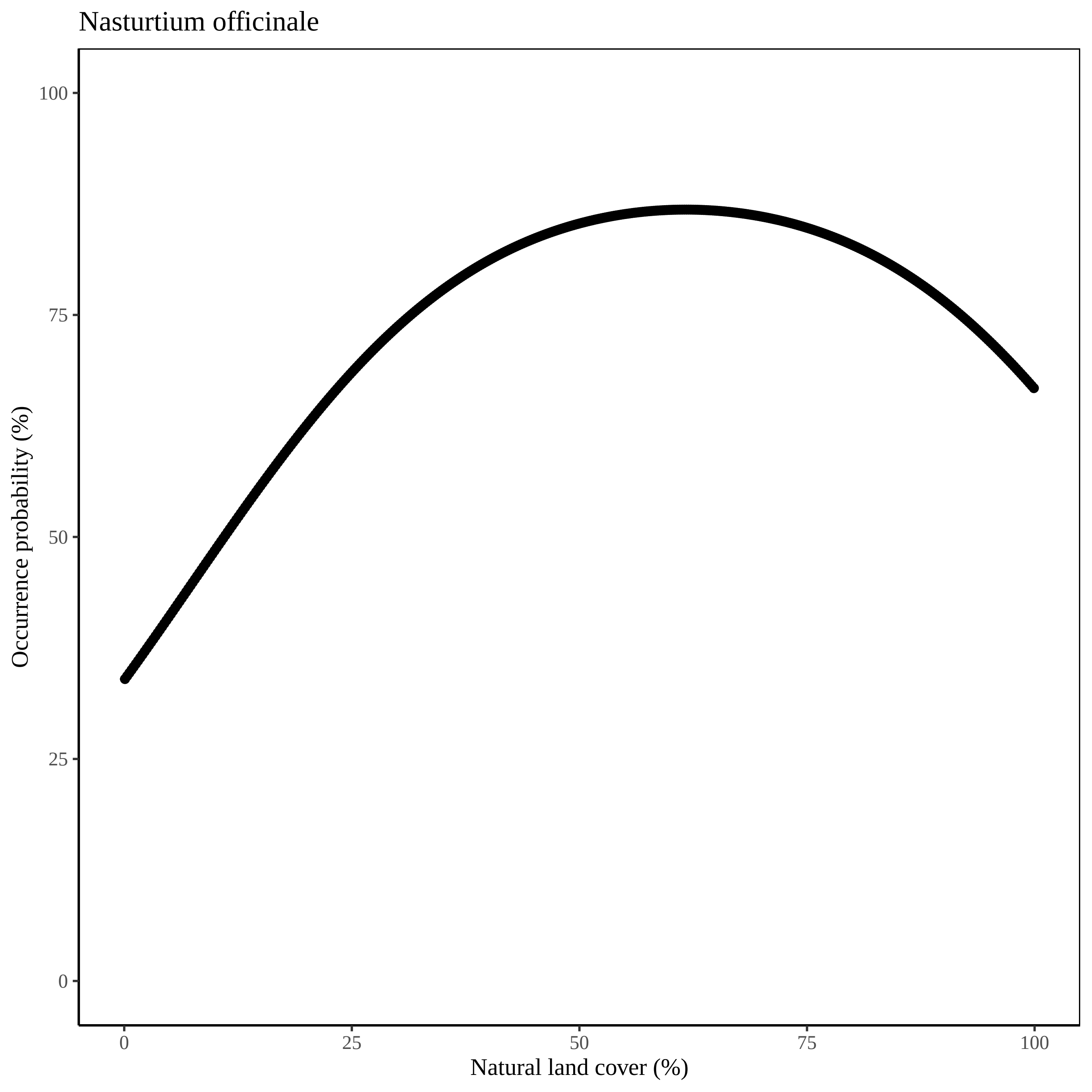
Nasturtium officinale
Occurrence probability (%)
100
75
50
25
0
0
25
50
75
100
Natural land cover (%)

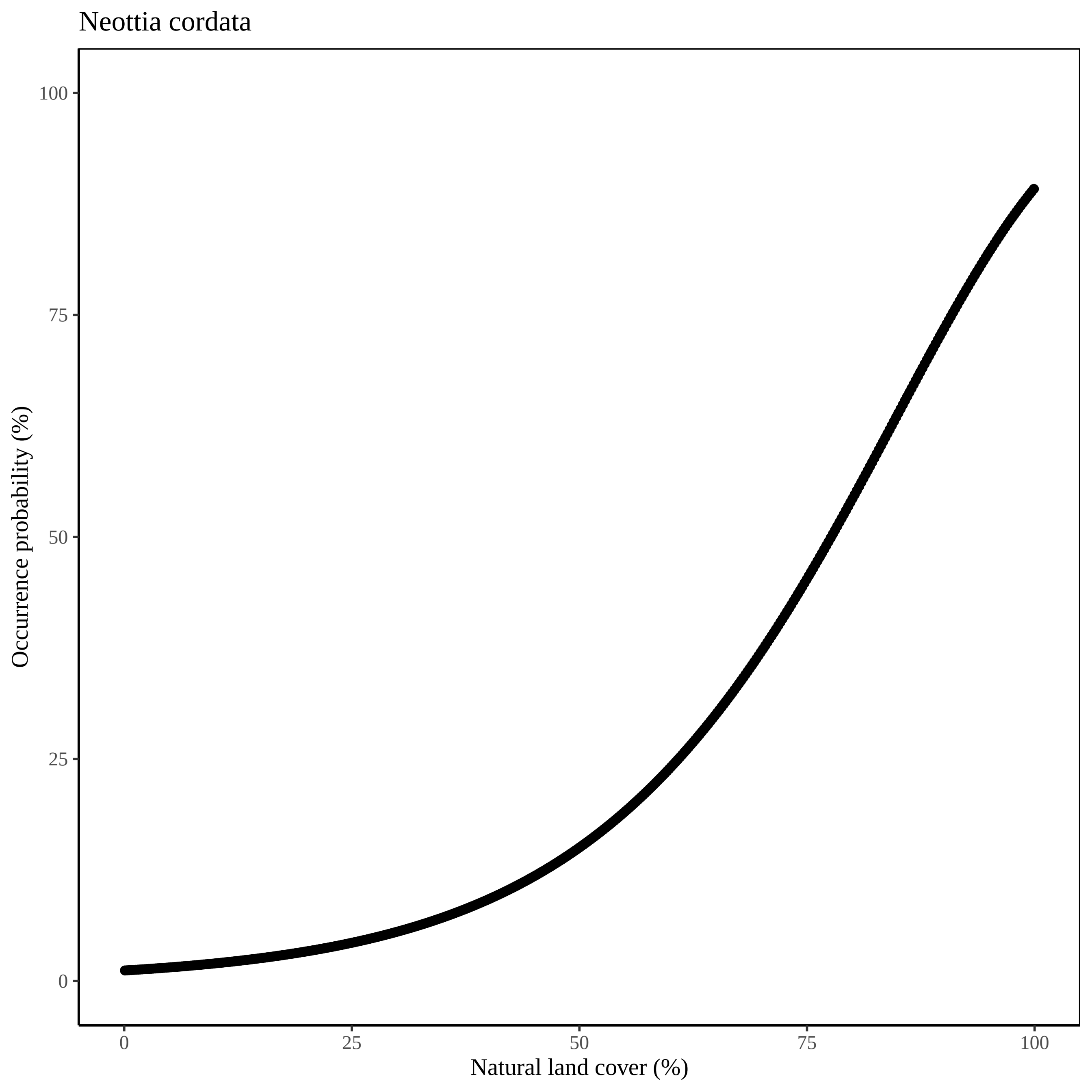
Neottia cordata
Occurrence probability (%)
100
75
50
25
0
0
25
50
75
100
Natural land cover (%)

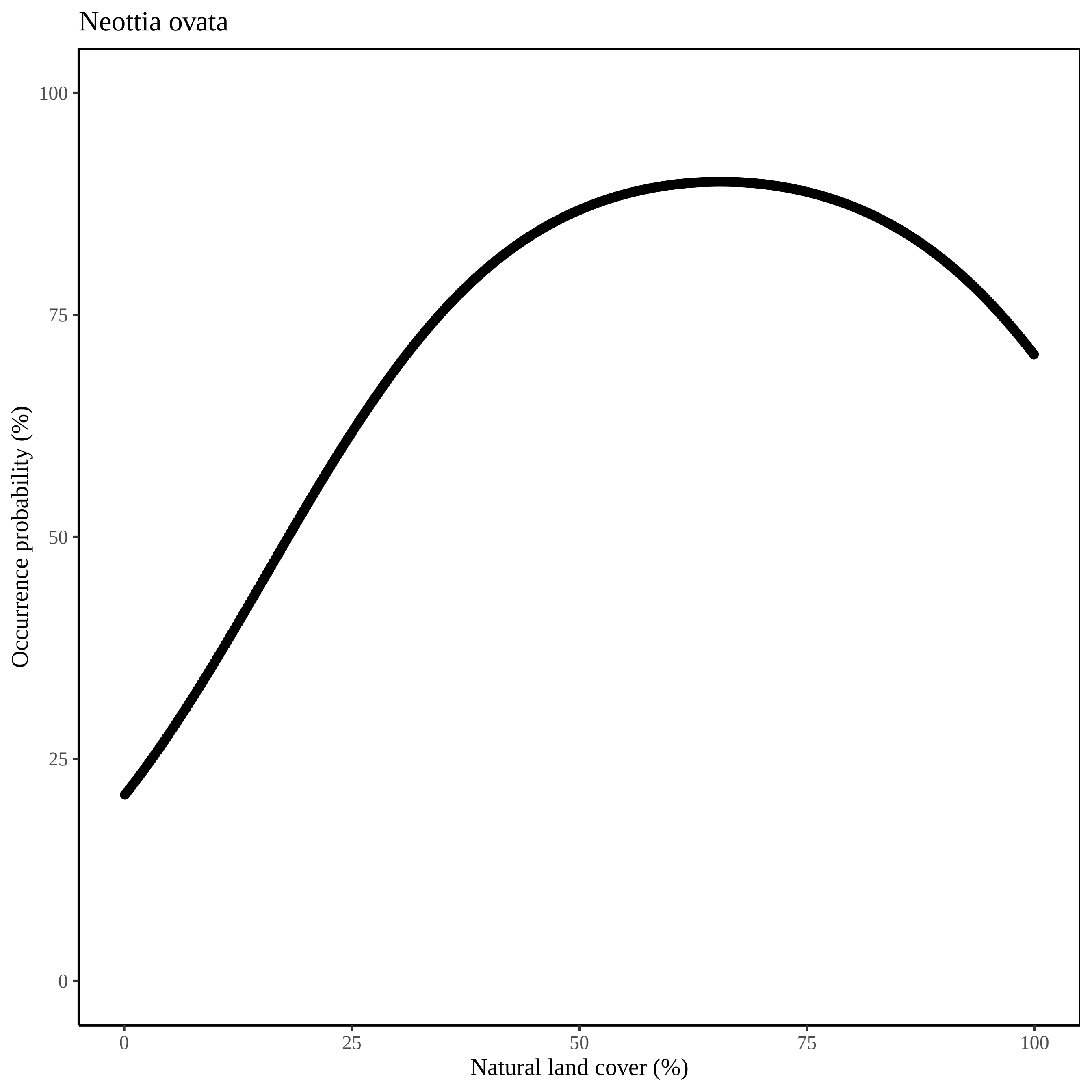
Neottia ovata
Occurrence probability (%)
0
25
50
75
100
Natural land cover (%)
0
25
50
75
100

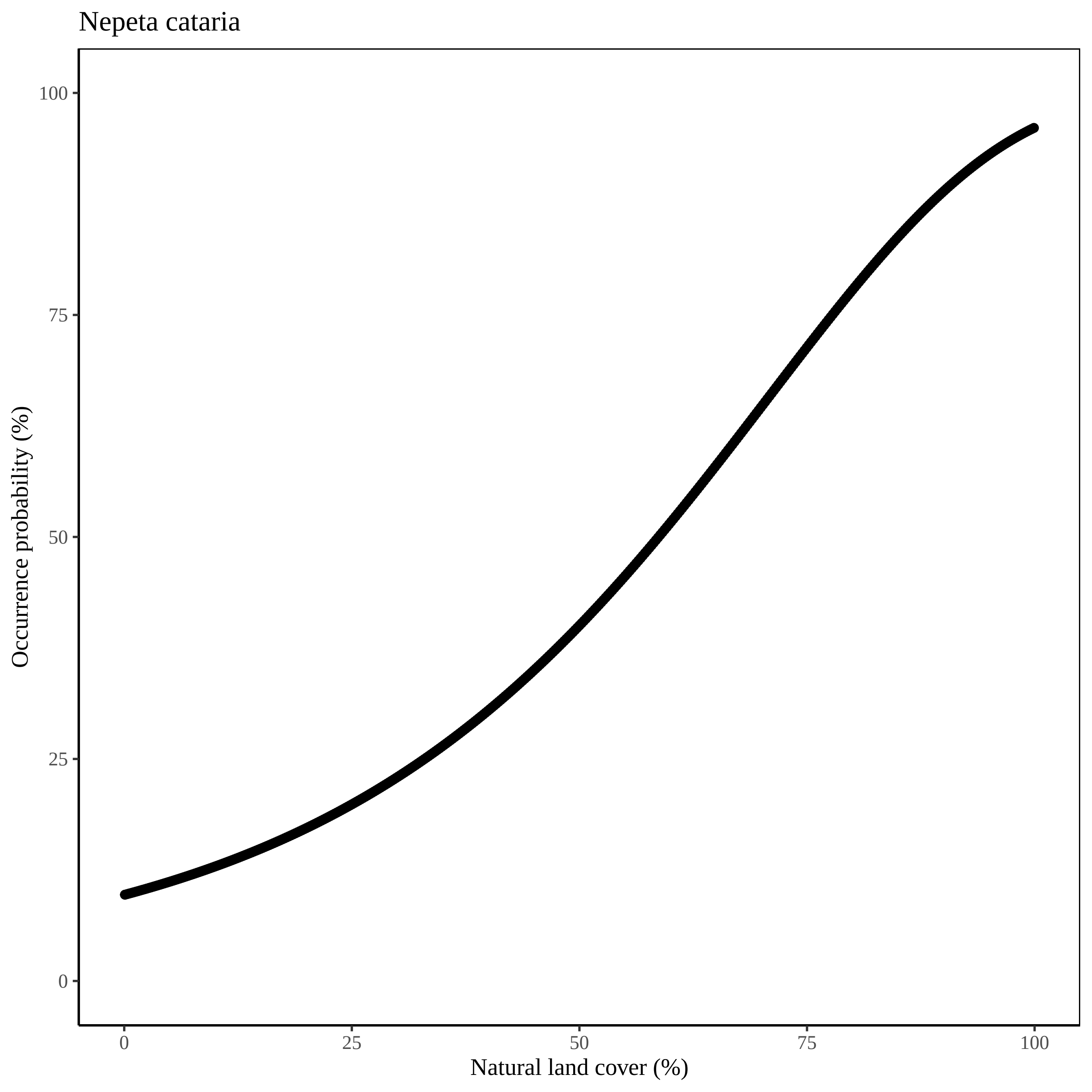
Nepeta cataria
Occurrence probability (%)
0
25
50
75
100
0
25
50
75
100
Natural land cover (%)

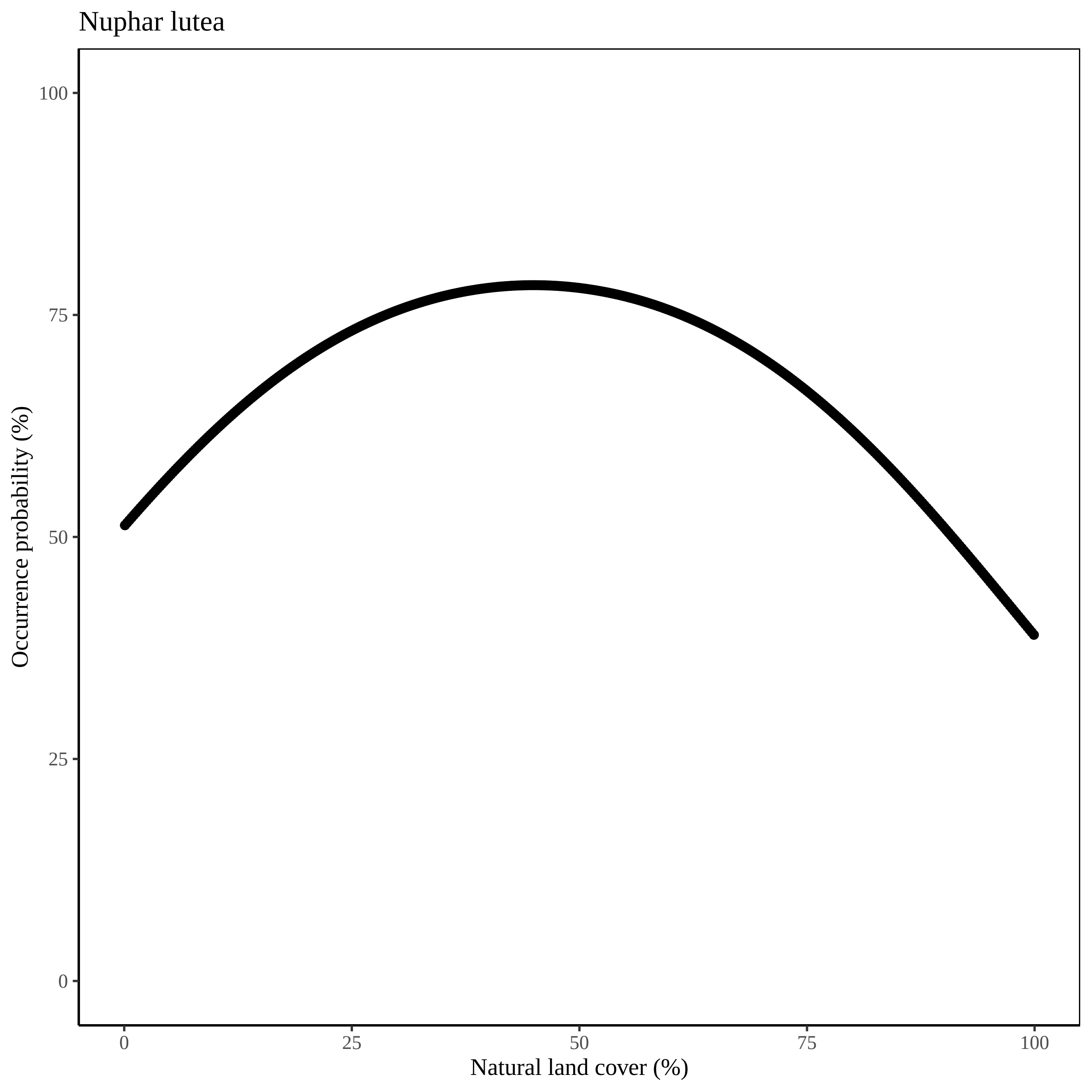
Nuphar lutea
Occurrence probability (%)
0
25
50
75
100
Natural land cover (%)
0
25
50
75
100

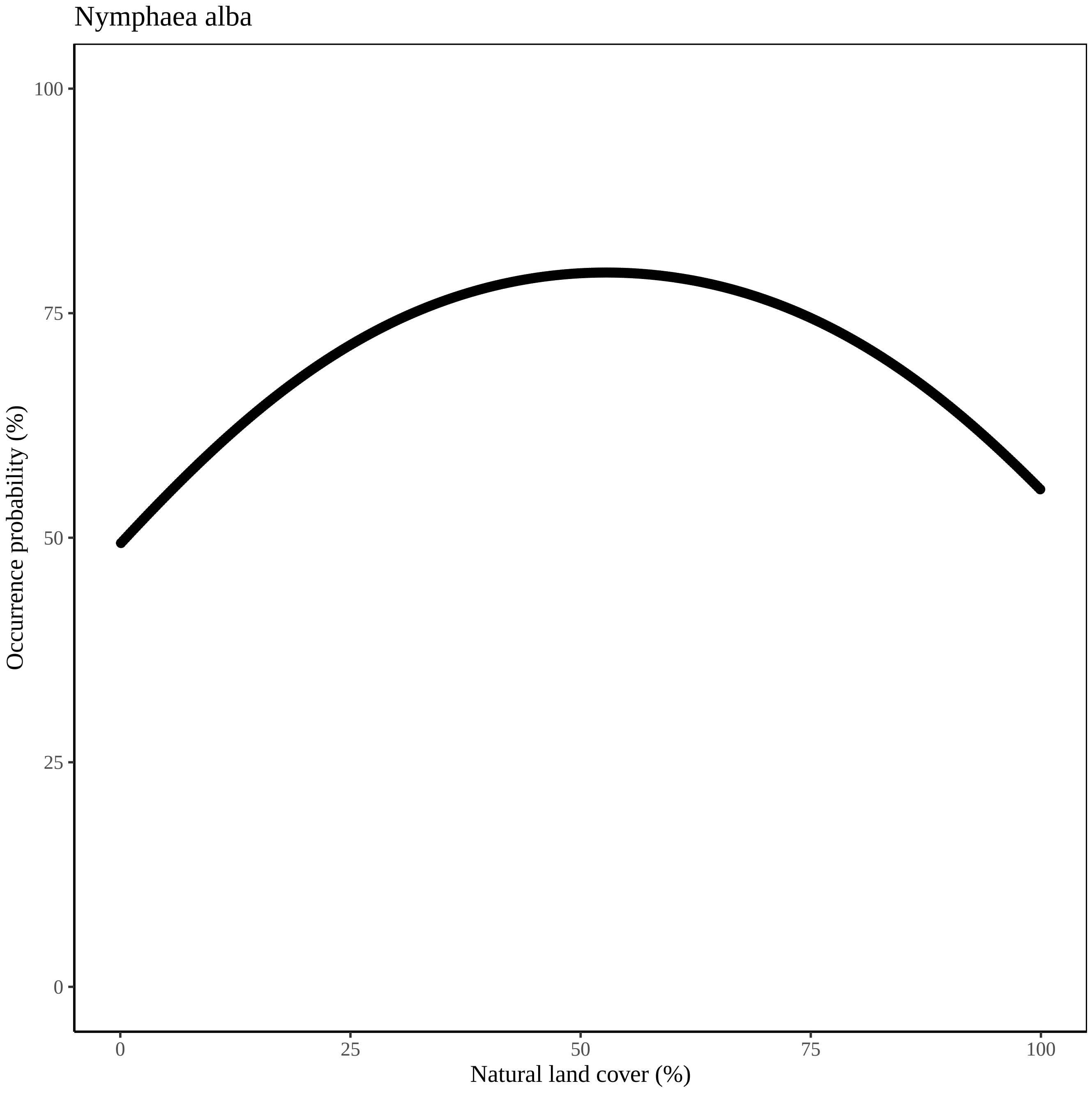
Nymphaea alba
Occurrence probability (%)
100
75
50
25
0
0
25
50
75
100
Natural land cover (%)

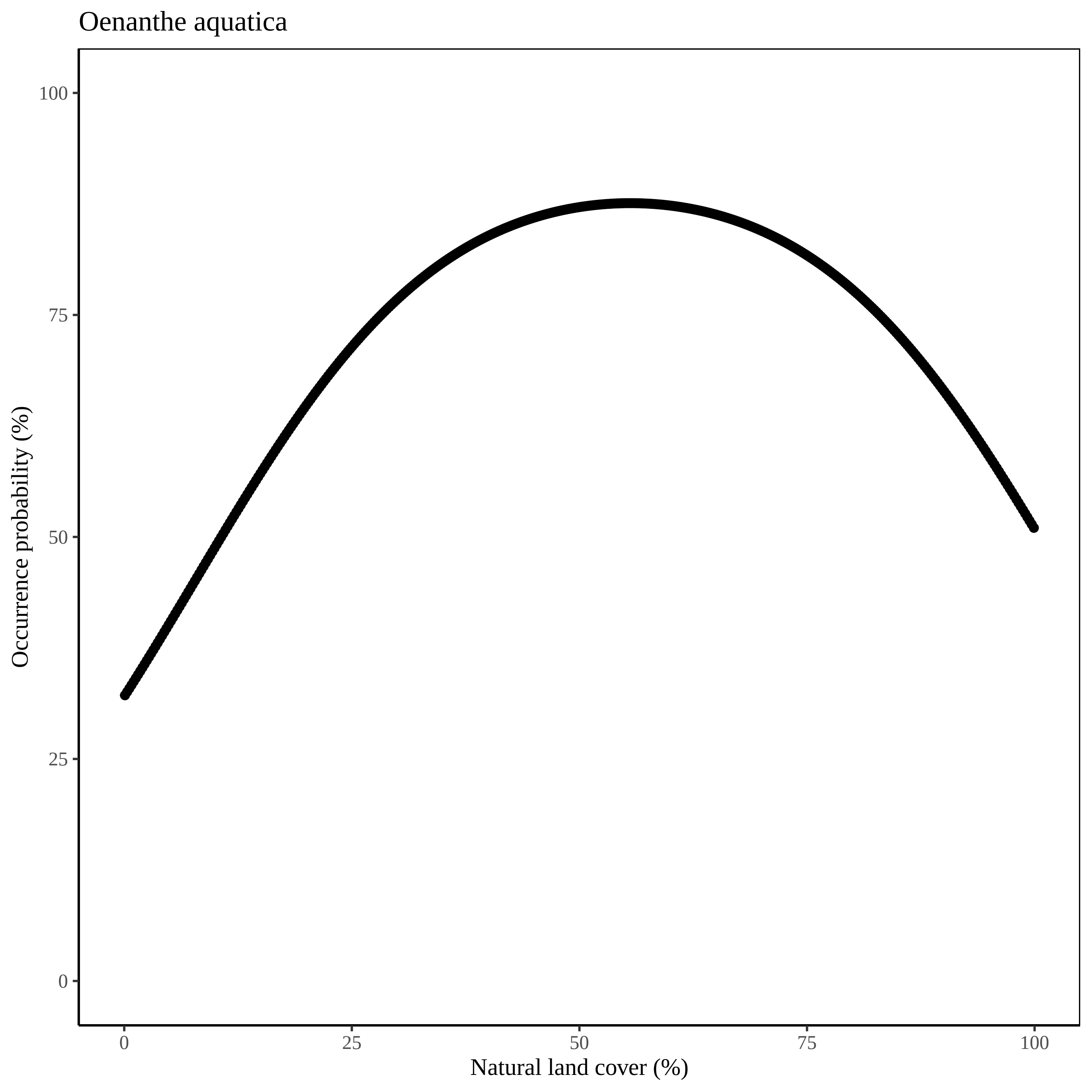
Oenanthe aquatica
Occurrence probability (%)
100
75
50
25
0
0
25
50
75
100
Natural land cover (%)

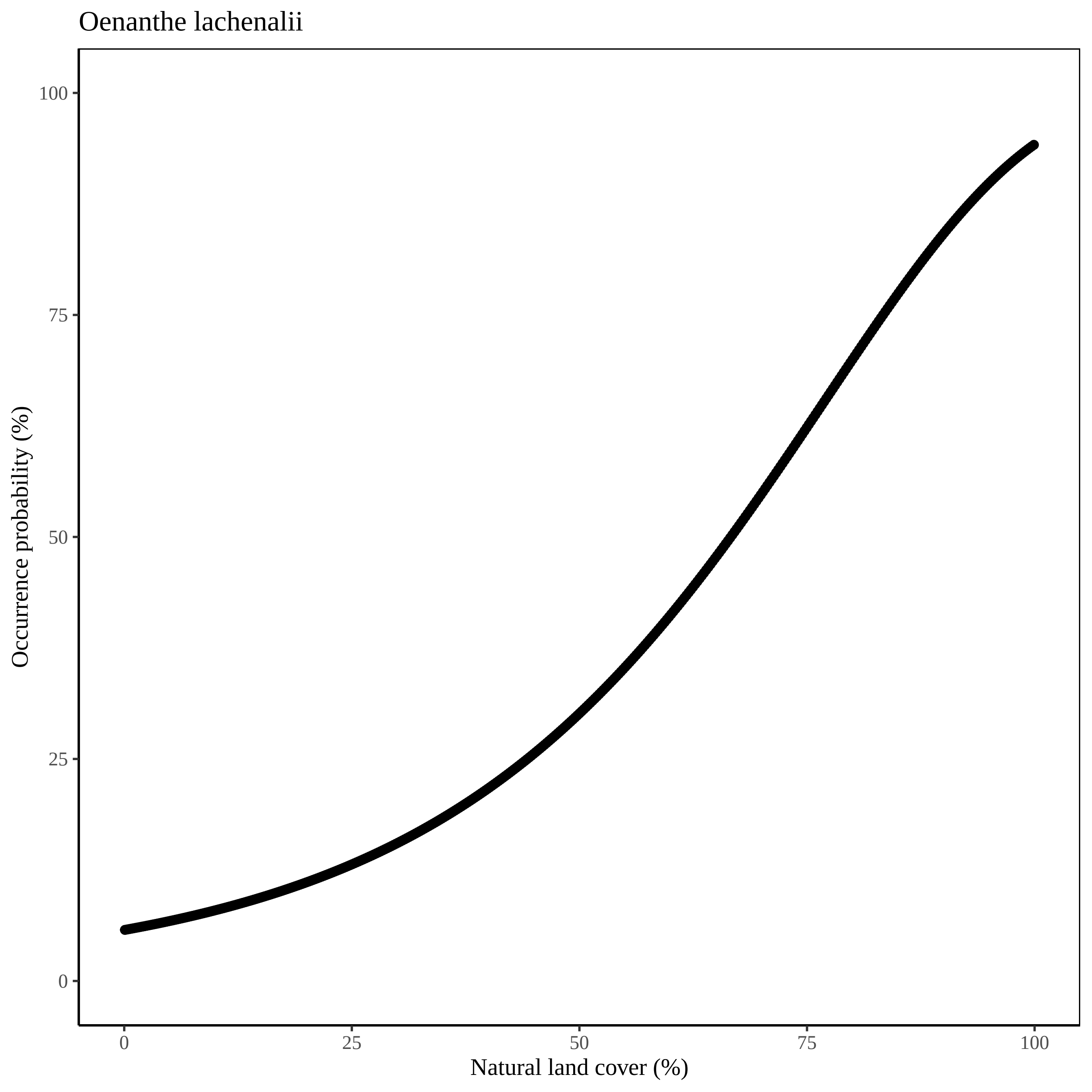
Oenanthe lachenalii
Occurrence probability (%)
100
75
50
25
0
0
25
50
75
100
Natural land cover (%)

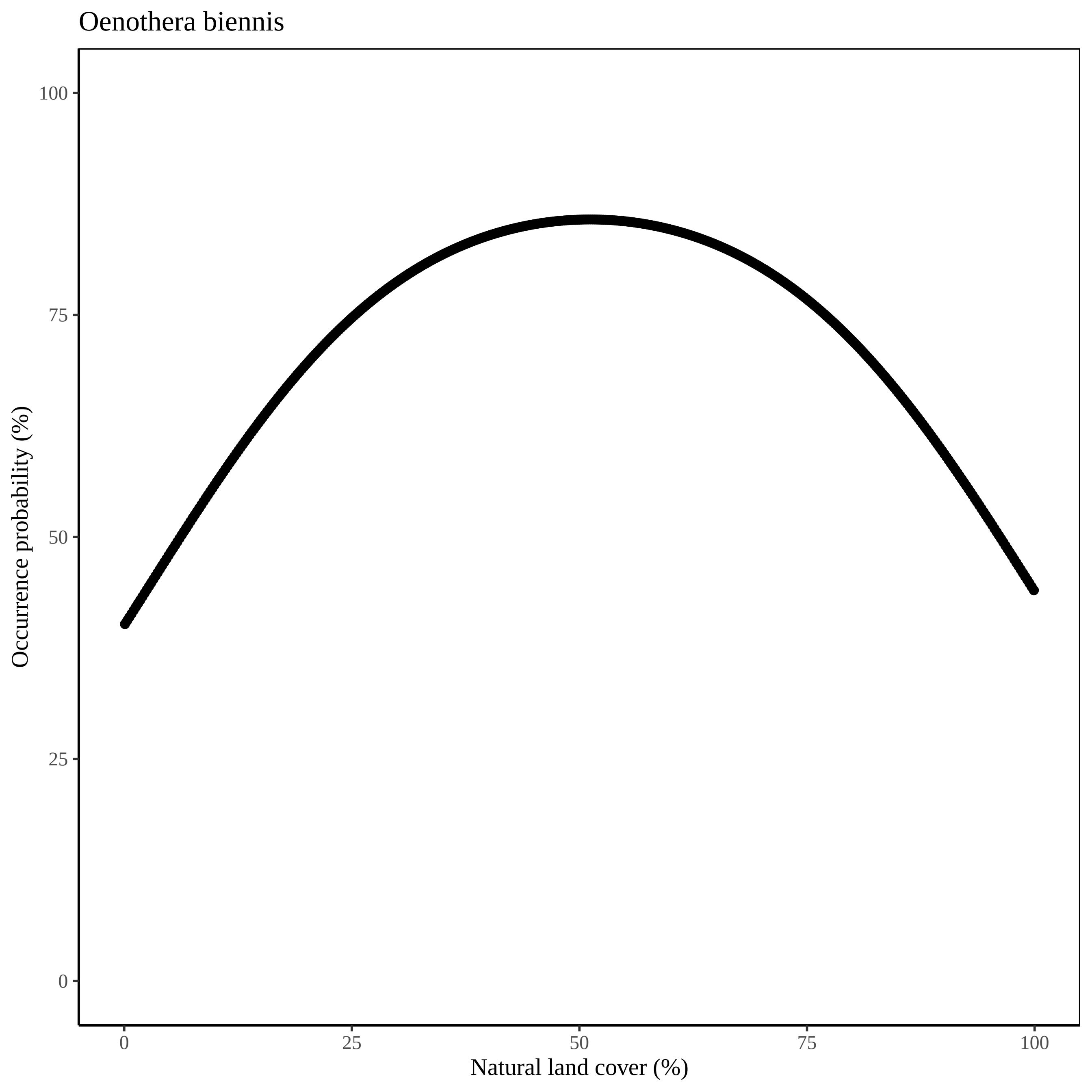

Oenothera biennis
100
75
50
25
0
Occurrence probability (%)
0
25
50
75
100
Natural land cover (%)

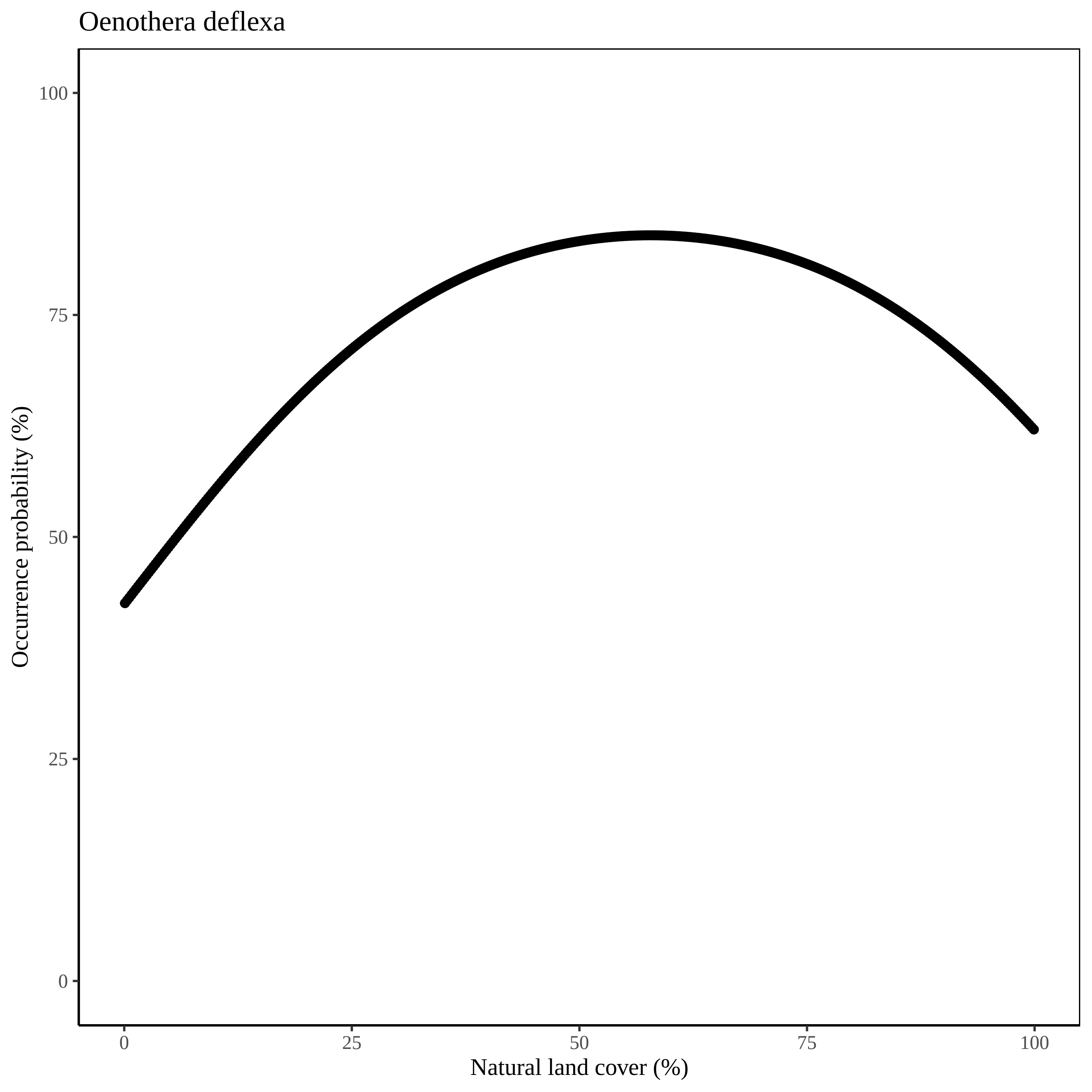

Oenothera deflexa
Occurrence probability (%)
100
75
50
25
0
0
25
50
75
100
Natural land cover (%)

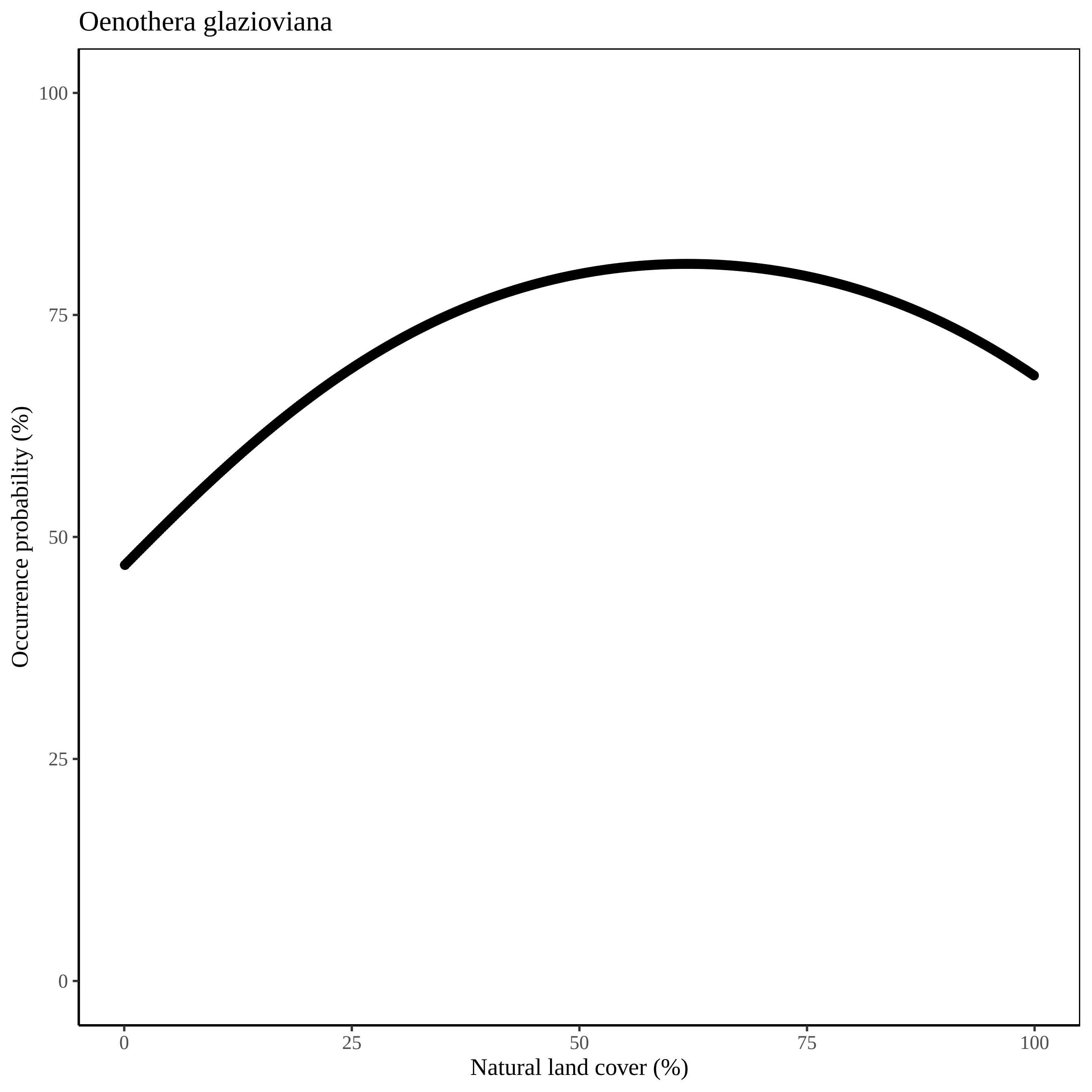
Oenothera glazioviana
Occurrence probability (%)
100
75
50
25
0
0
25
50
75
100
Natural land cover (%)

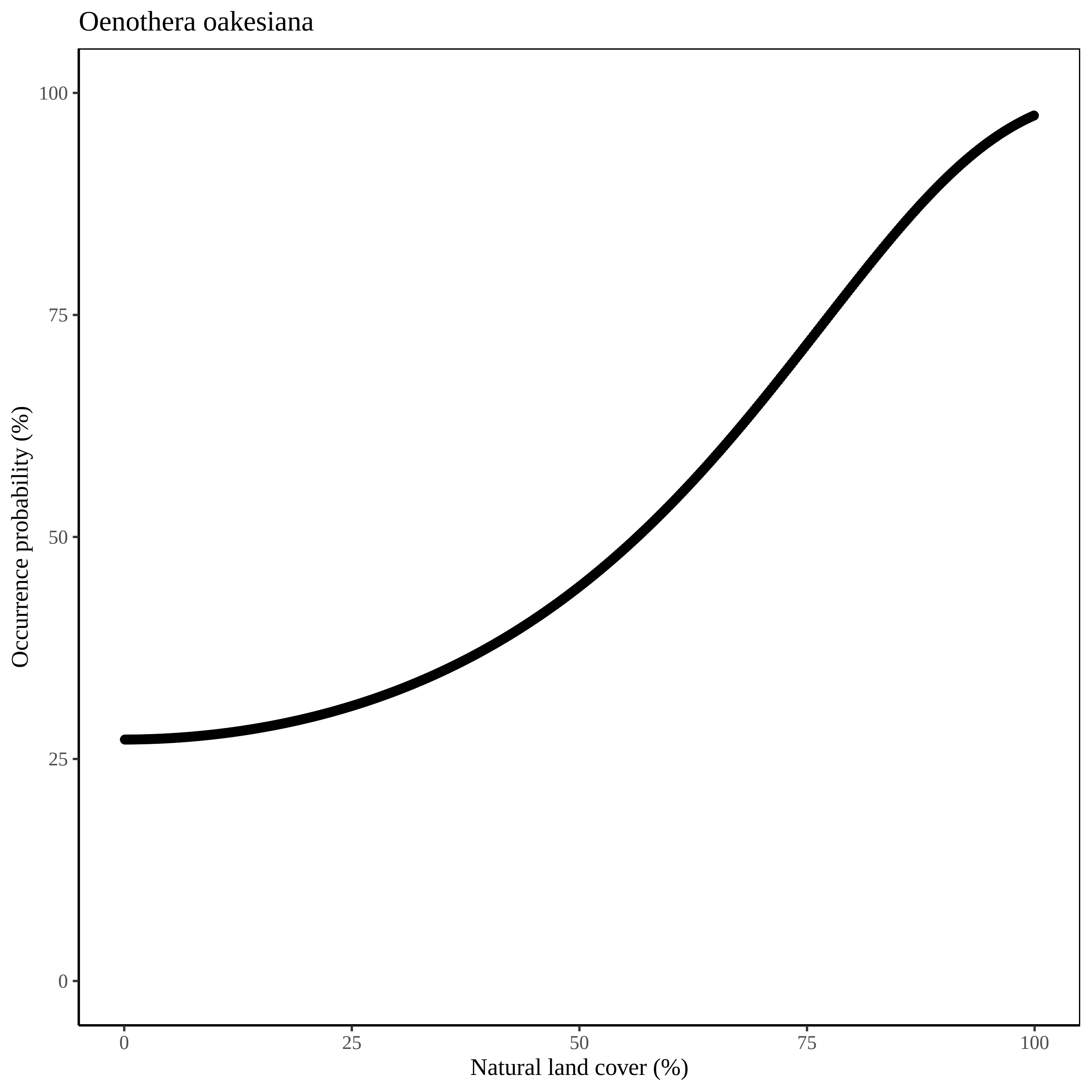
Oenothera oakesiana
Occurrence probability (%)
100
75
50
25
0
0
25
50
75
100
Natural land cover (%)

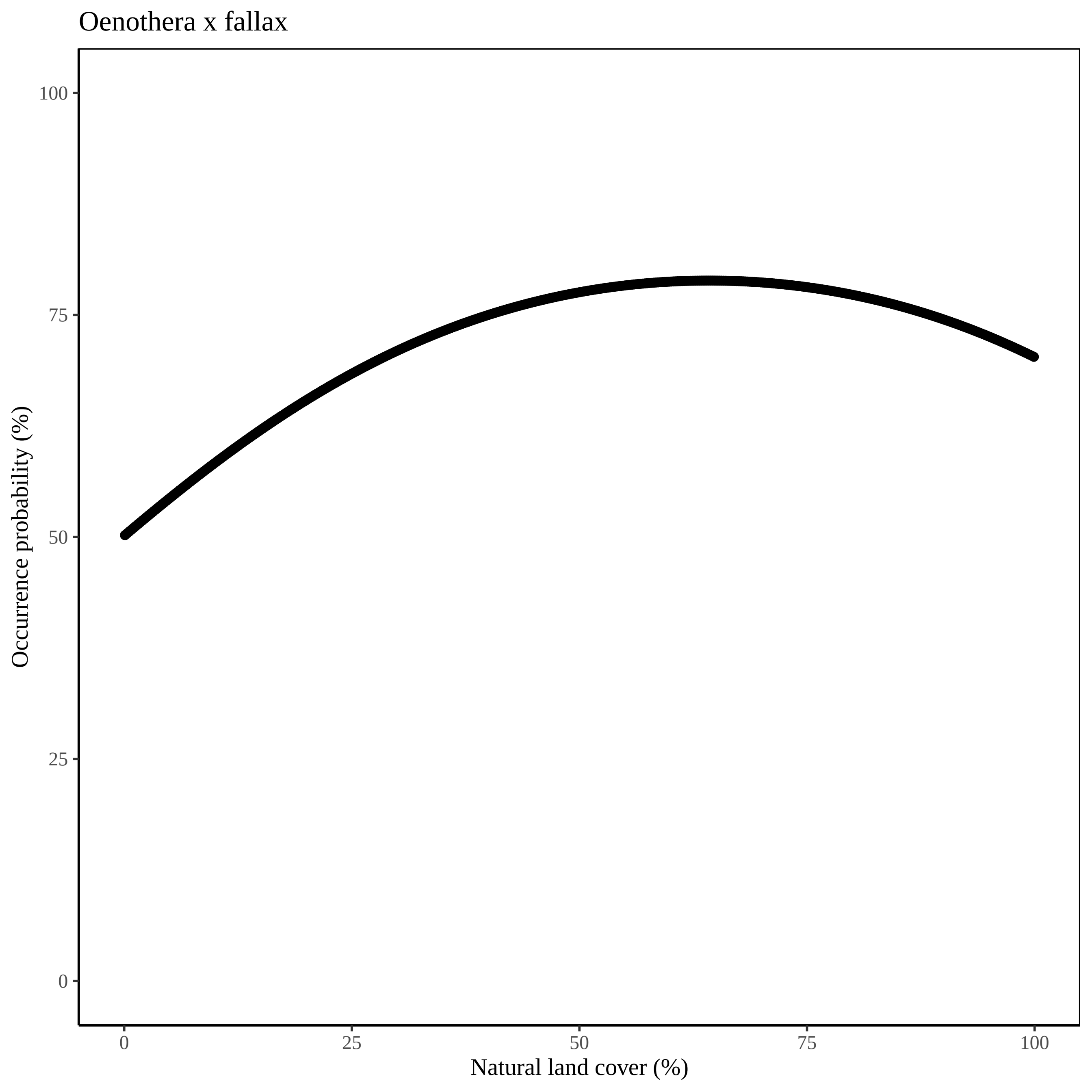
Oenothera x fallax
100
75
50
25
0
Occurrence probability (%)
0
25
50
75
100
Natural land cover (%)

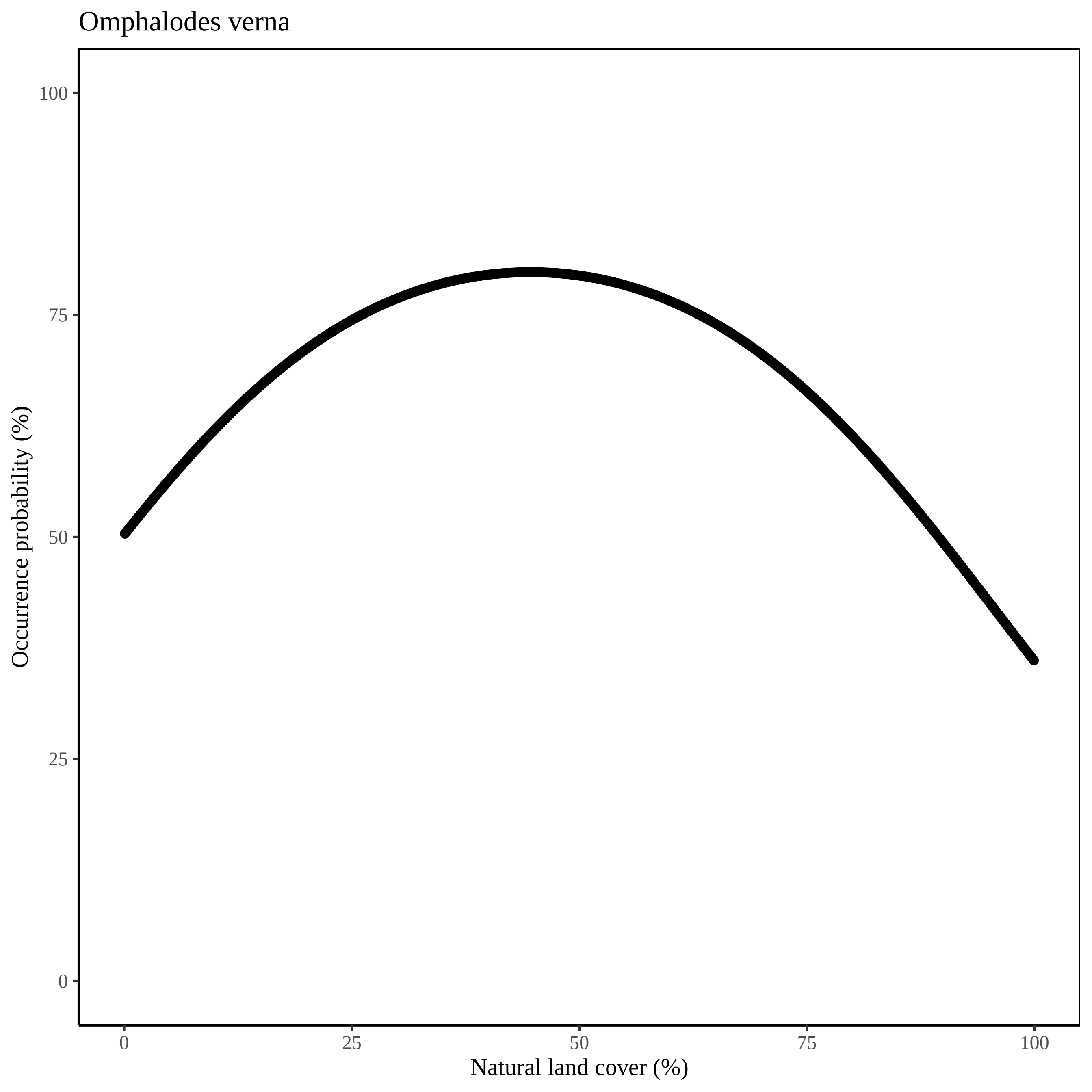

Omphalodes verna
100
75
50
25
0
Occurrence probability (%)
0
25
50
75
100
Natural land cover (%)

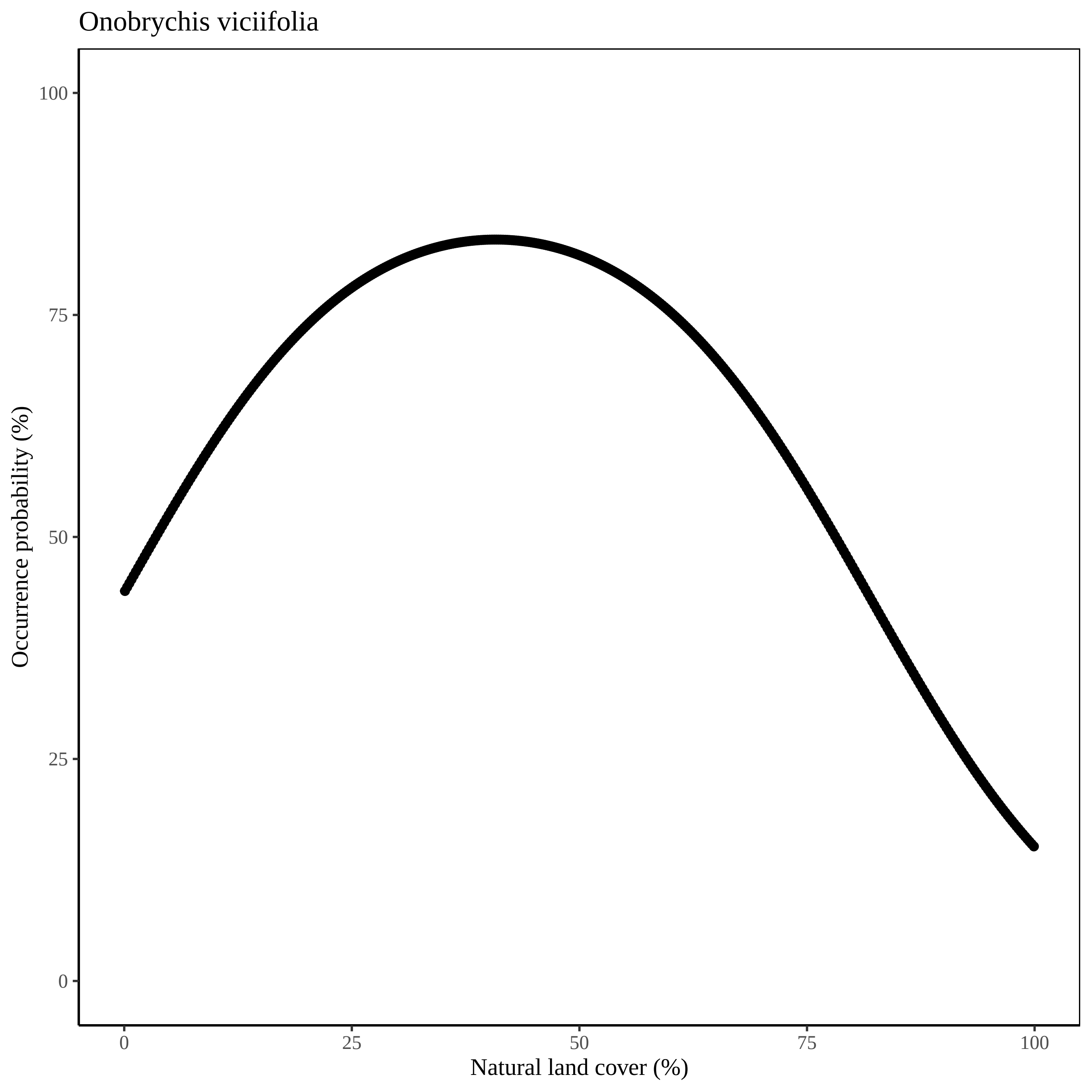
Onobrychis viciifolia
Occurrence probability (%)
100
75
50
25
0
0
25
50
75
100
Natural land cover (%)

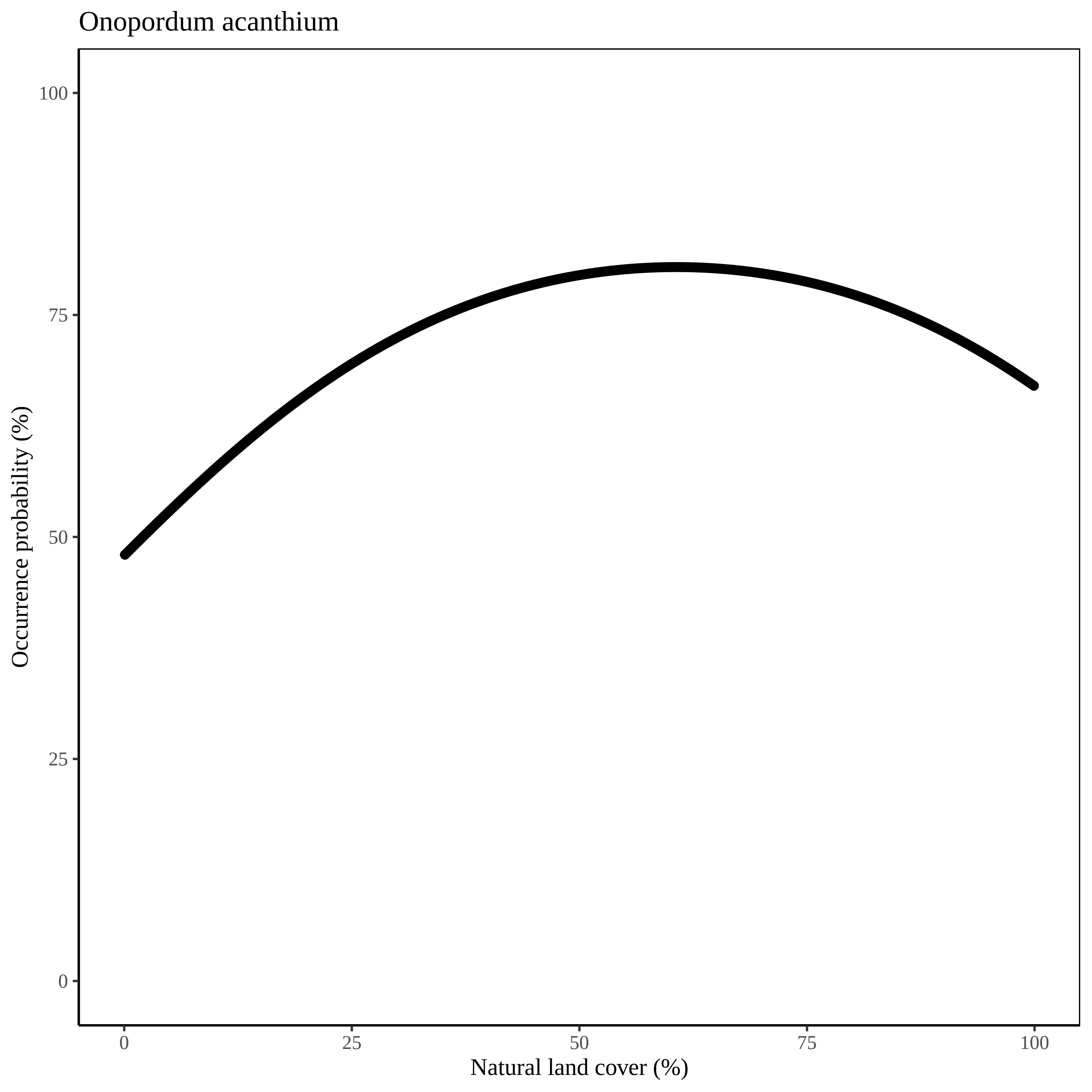
Onopordum acanthium
Occurrence probability (%)
100
75
50
25
0
0
25
50
75
100
Natural land cover (%)

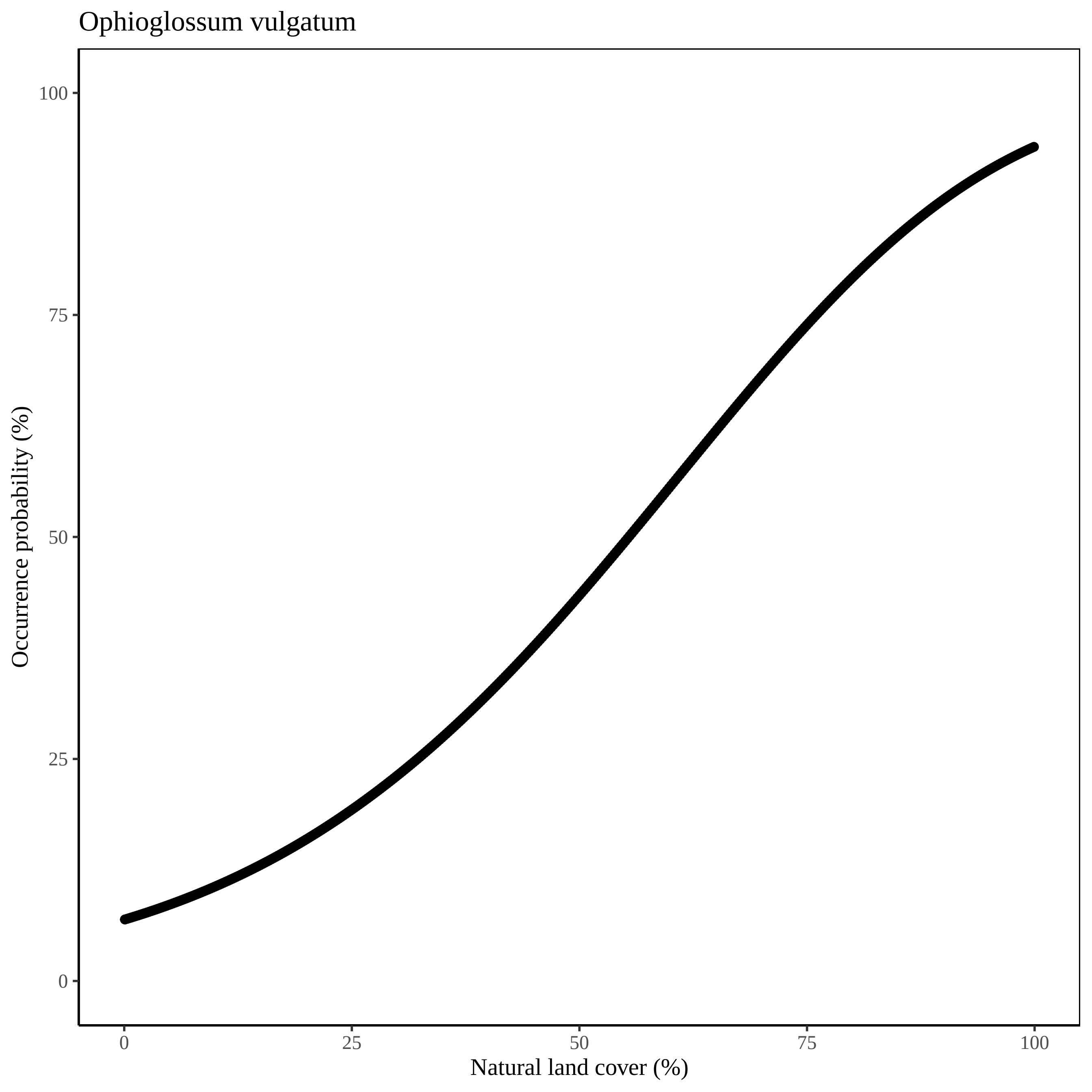
Ophioglossum vulgatum
100
75
50
25
0
Occurrence probability (%)
0
25
50
75
100
Natural land cover (%)

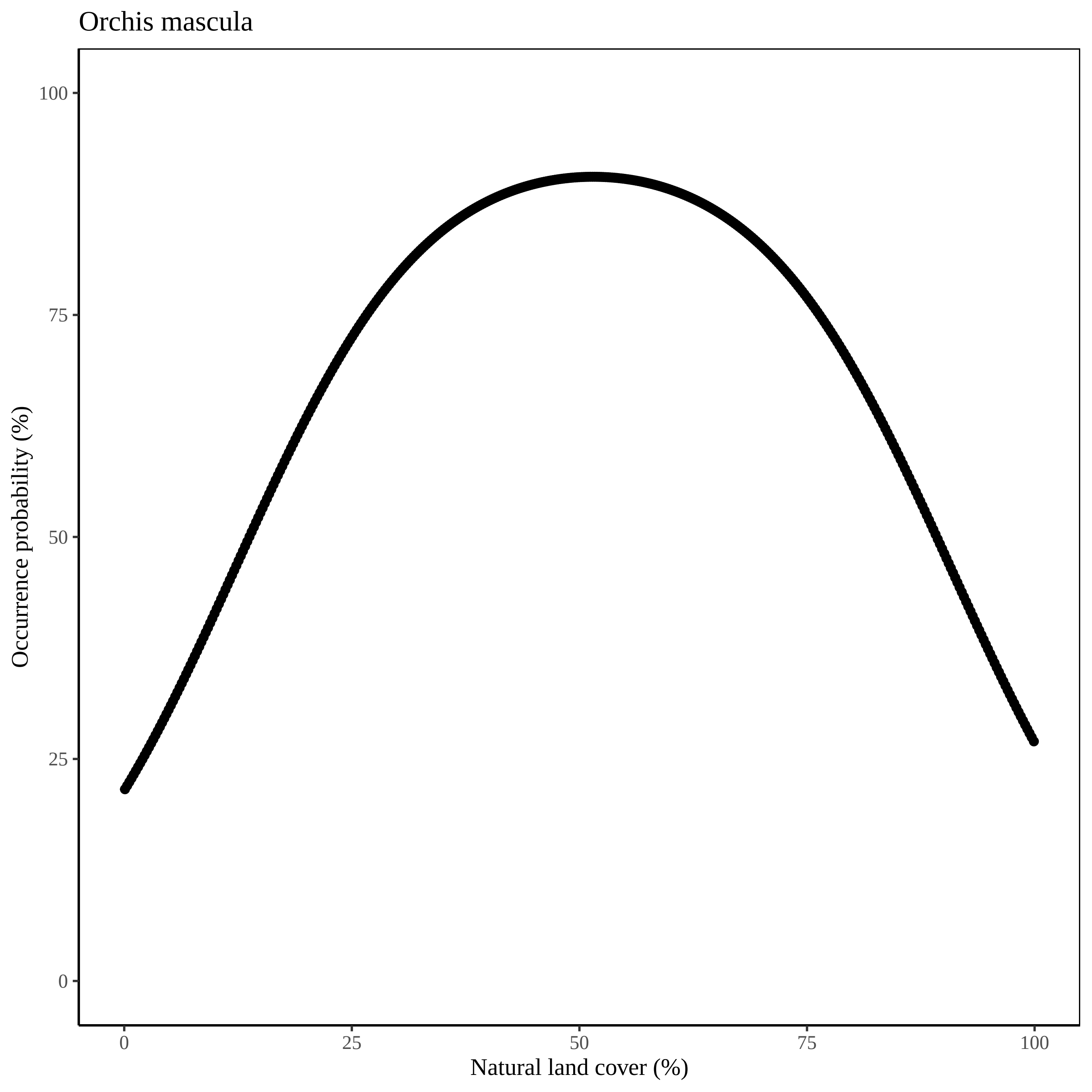
Orchis mascula
Occurrence probability (%)
100
75
50
25
0
0
25
50
75
100
Natural land cover (%)

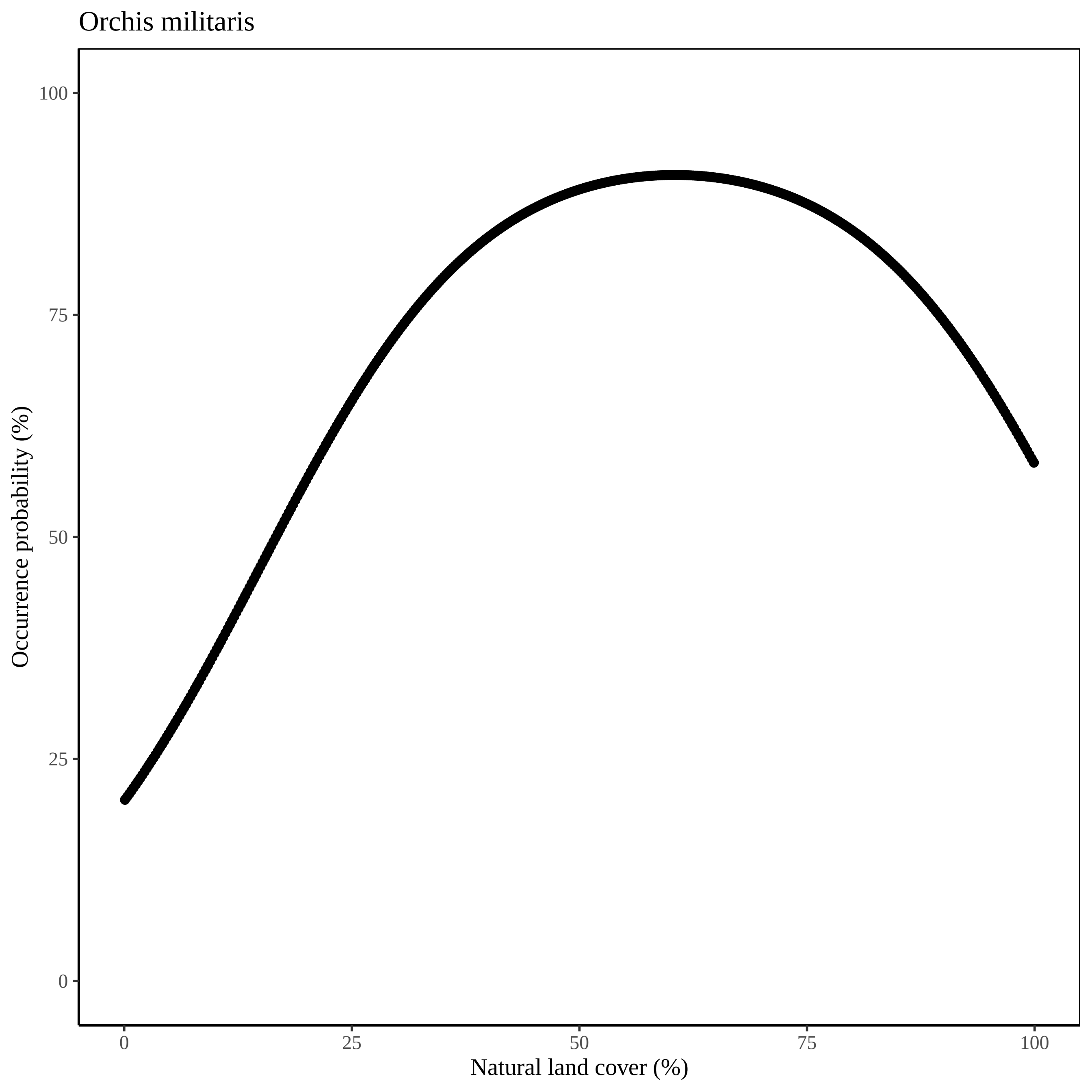
Orchis militaris
Occurrence probability (%)
100
75
50
25
0
0
25
50
75
100
Natural land cover (%)

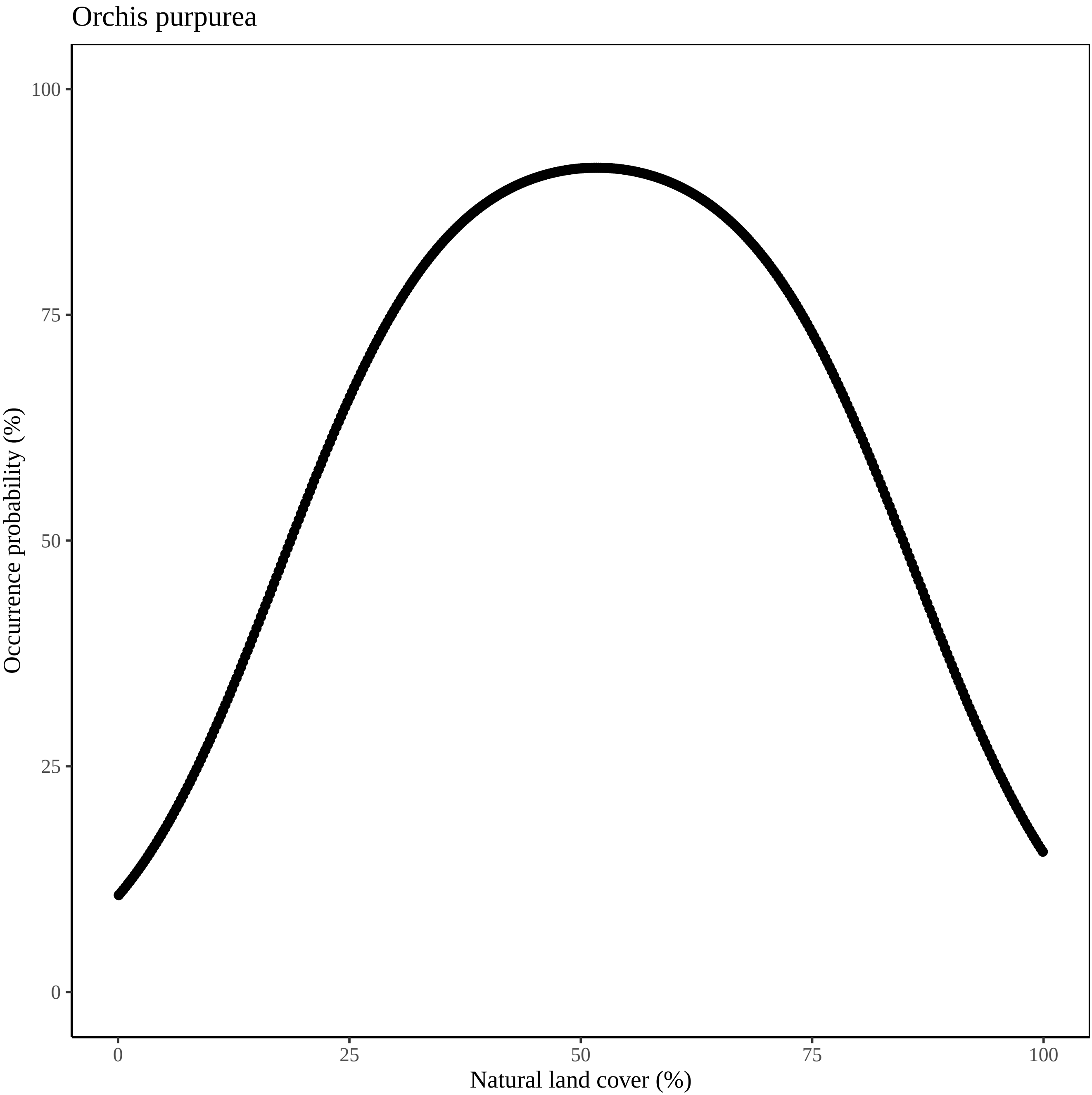
Orchis purpurea
Occurrence probability (%)
100
75
50
25
0
0
25
50
75
100
Natural land cover (%)

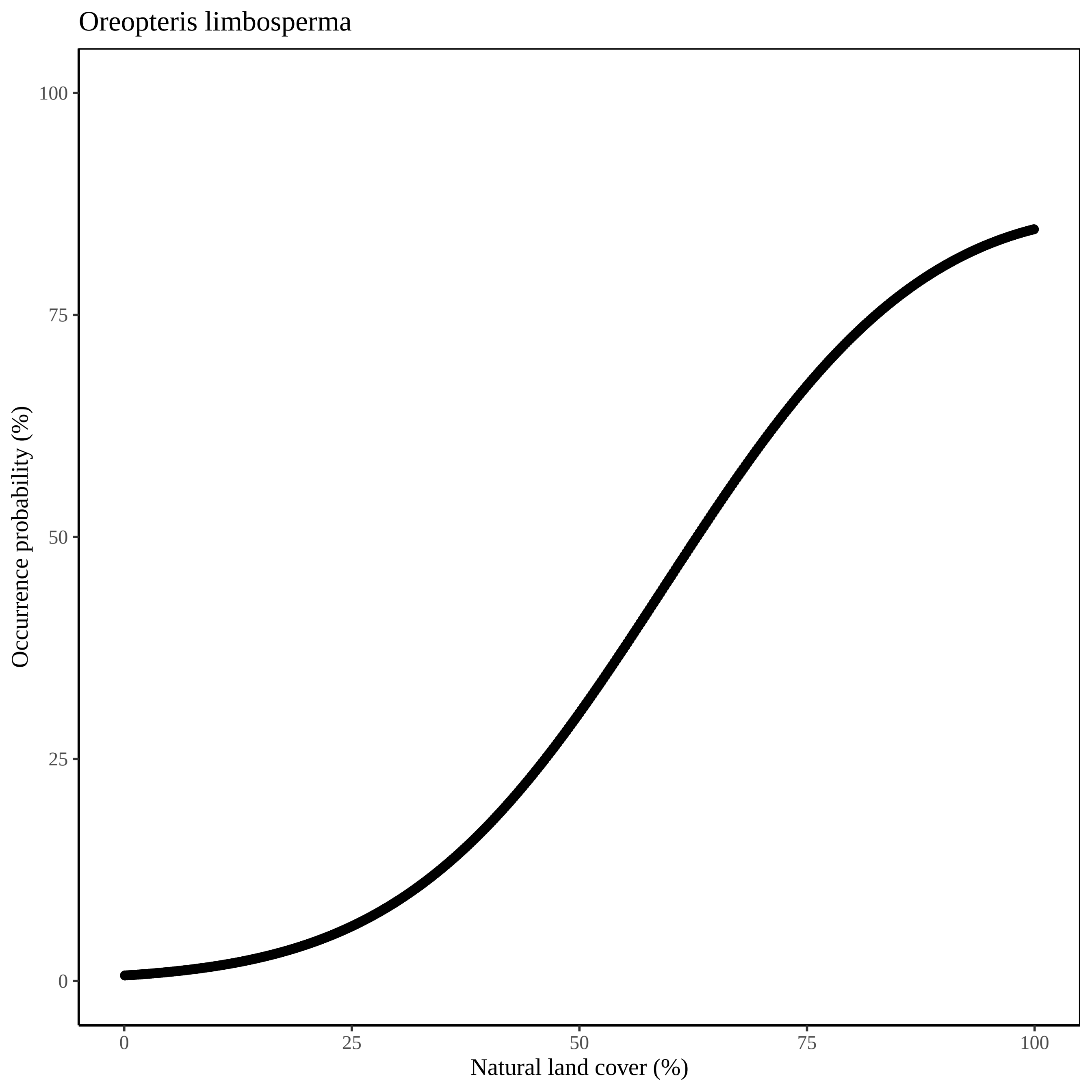
Oreopteris limbosperma
Occurrence probability (%)
100
75
50
25
0
0
25
50
75
100
Natural land cover (%)

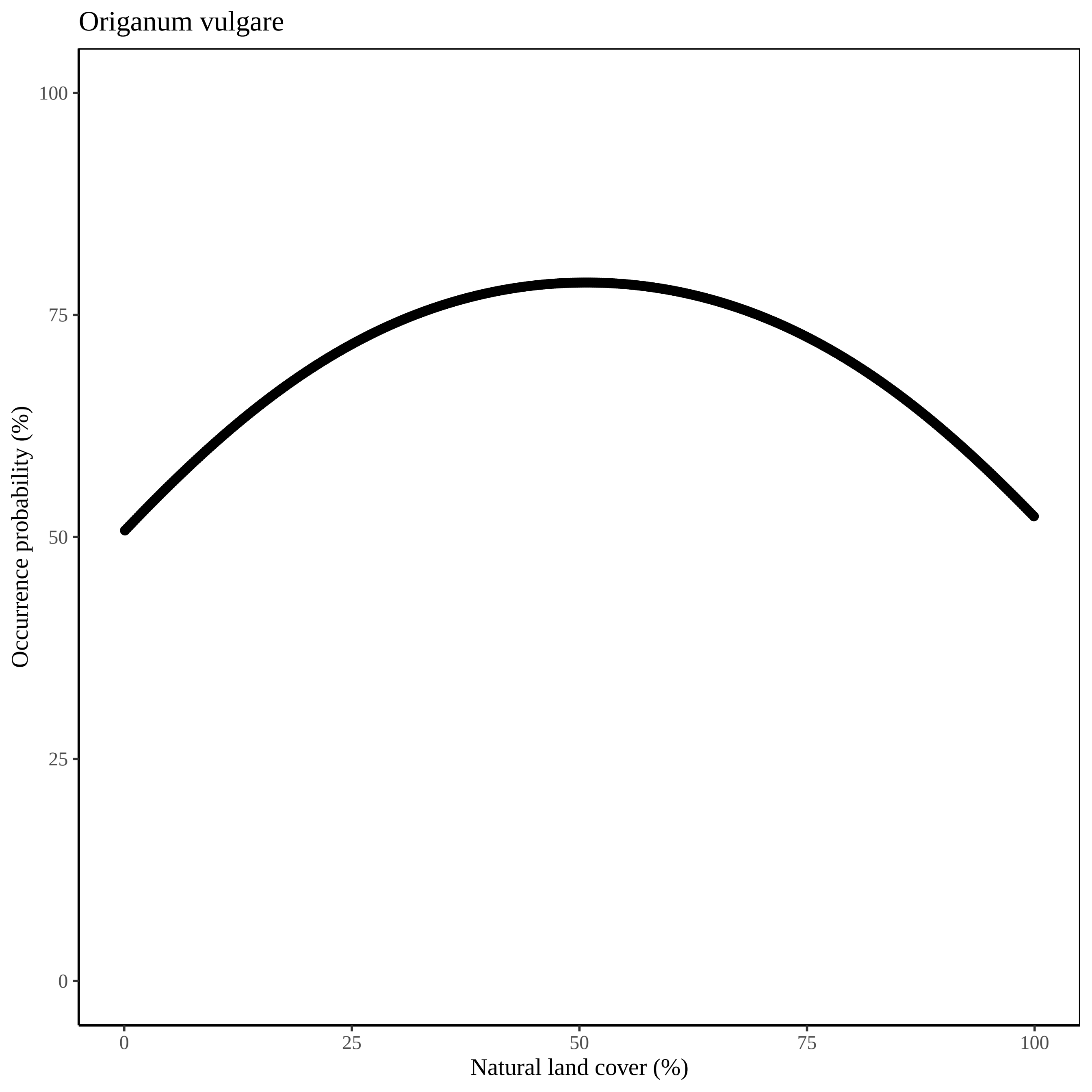
Origanum vulgare
Occurrence probability (%)
100
75
50
25
0
0
25
50
75
100
Natural land cover (%)

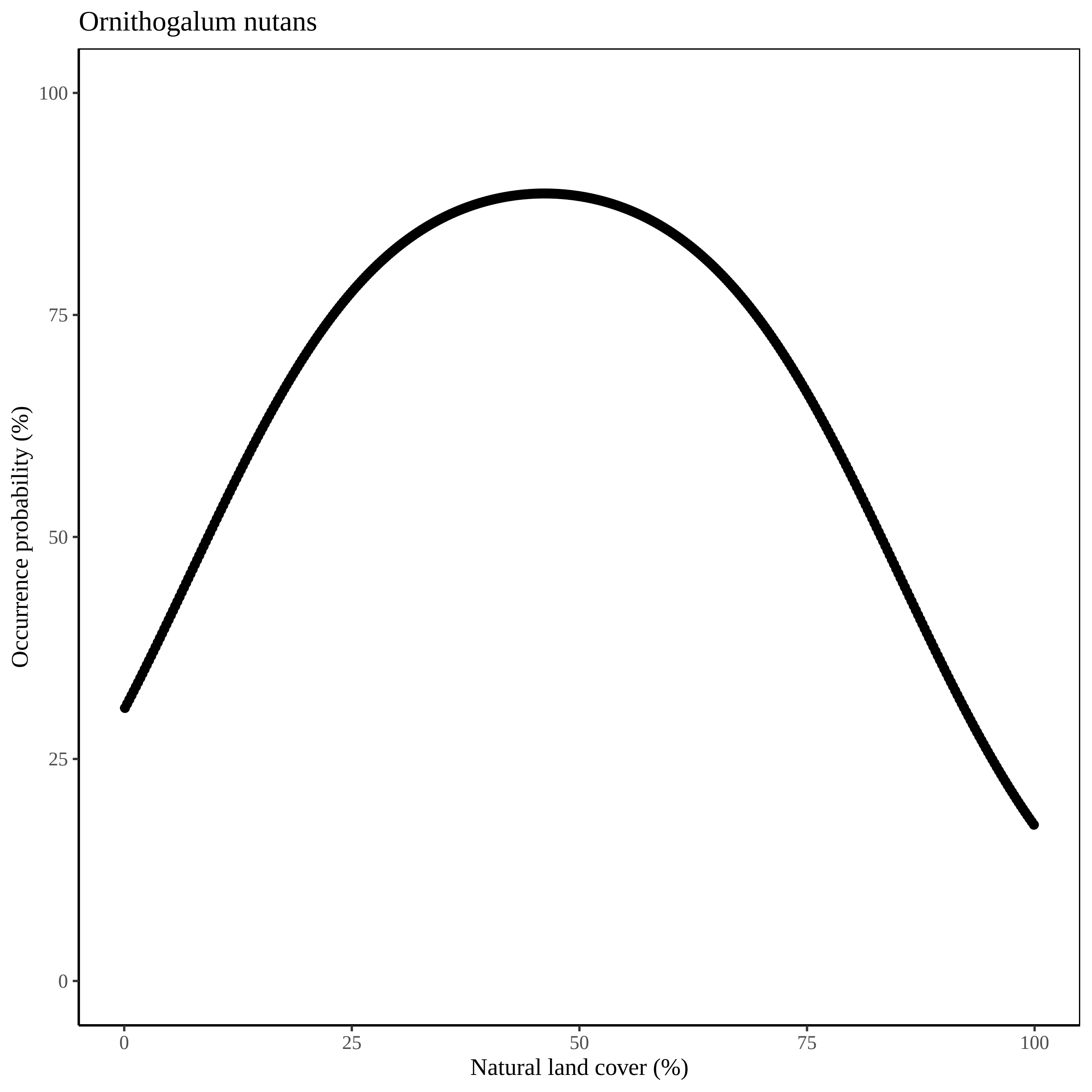
Ornithogalum nutans
Occurrence probability (%)
100
75
50
25
0
0
25
50
75
100
Natural land cover (%)

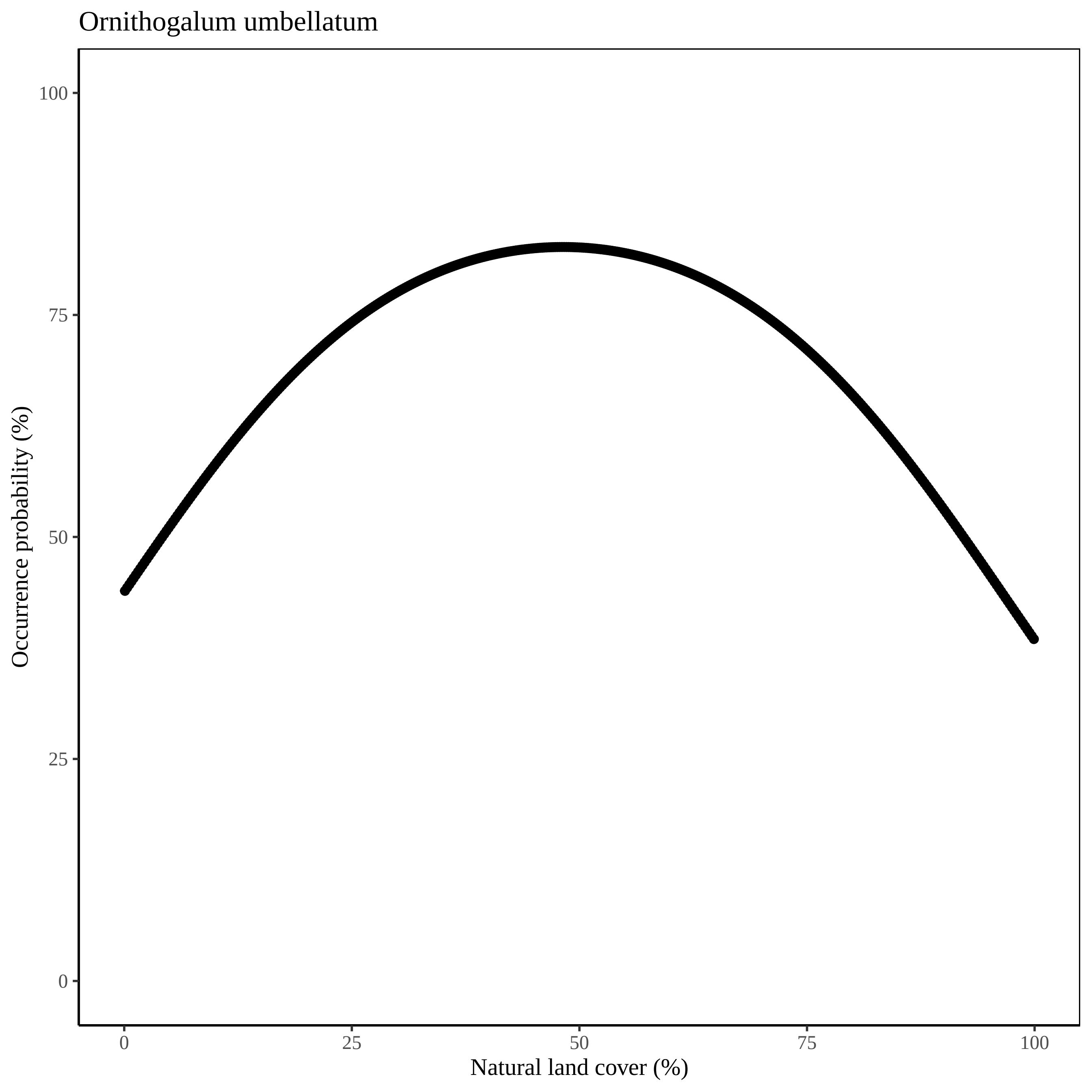

Ornithogalum umbellatum
100
75
50
25
0
Occurrence probability (%)
0
25
50
75
100
Natural land cover (%)

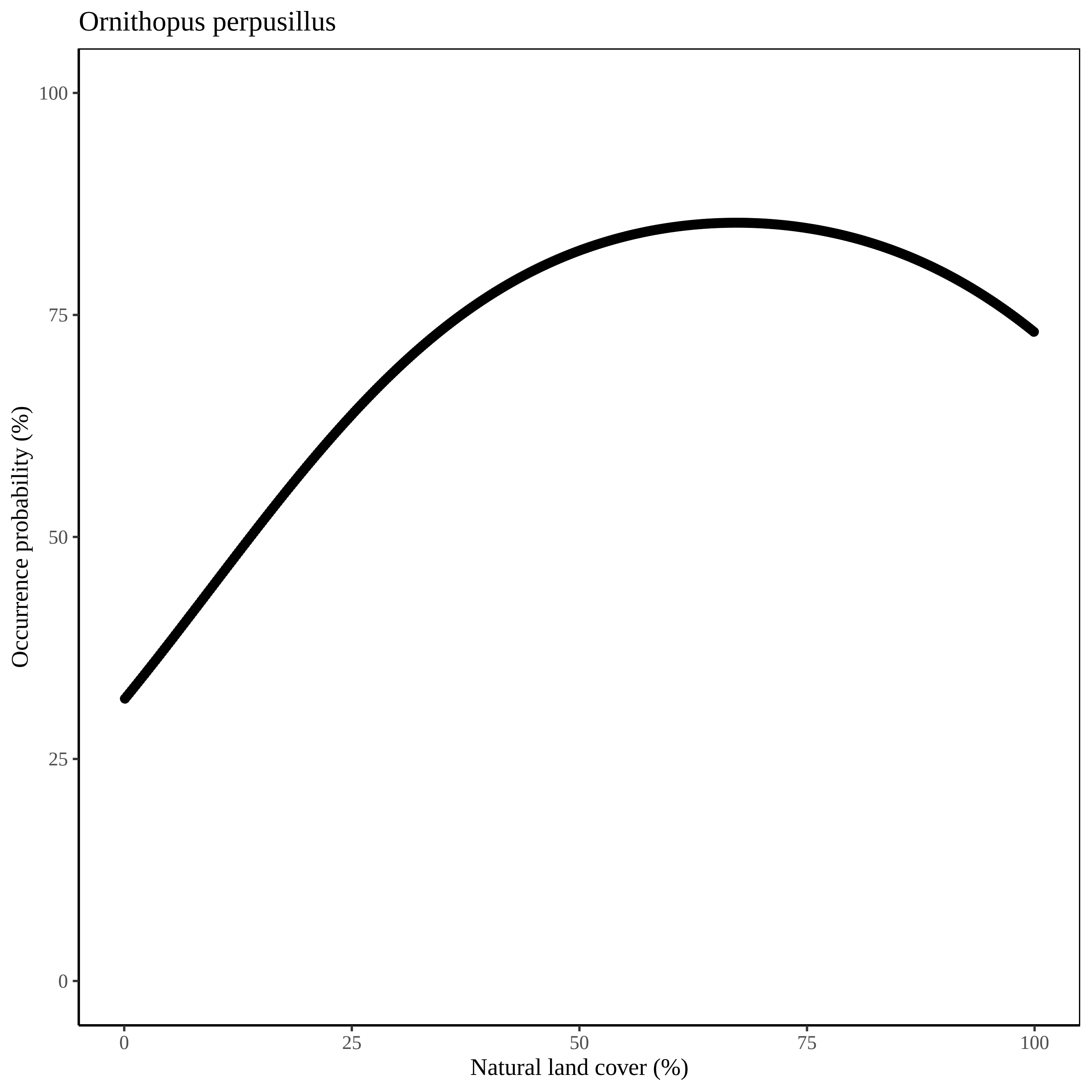
Ornithopus perpusillus
Occurrence probability (%)
0
25
50
75
100
Natural land cover (%)
0
25
50
75
100

Ornithopus sativus

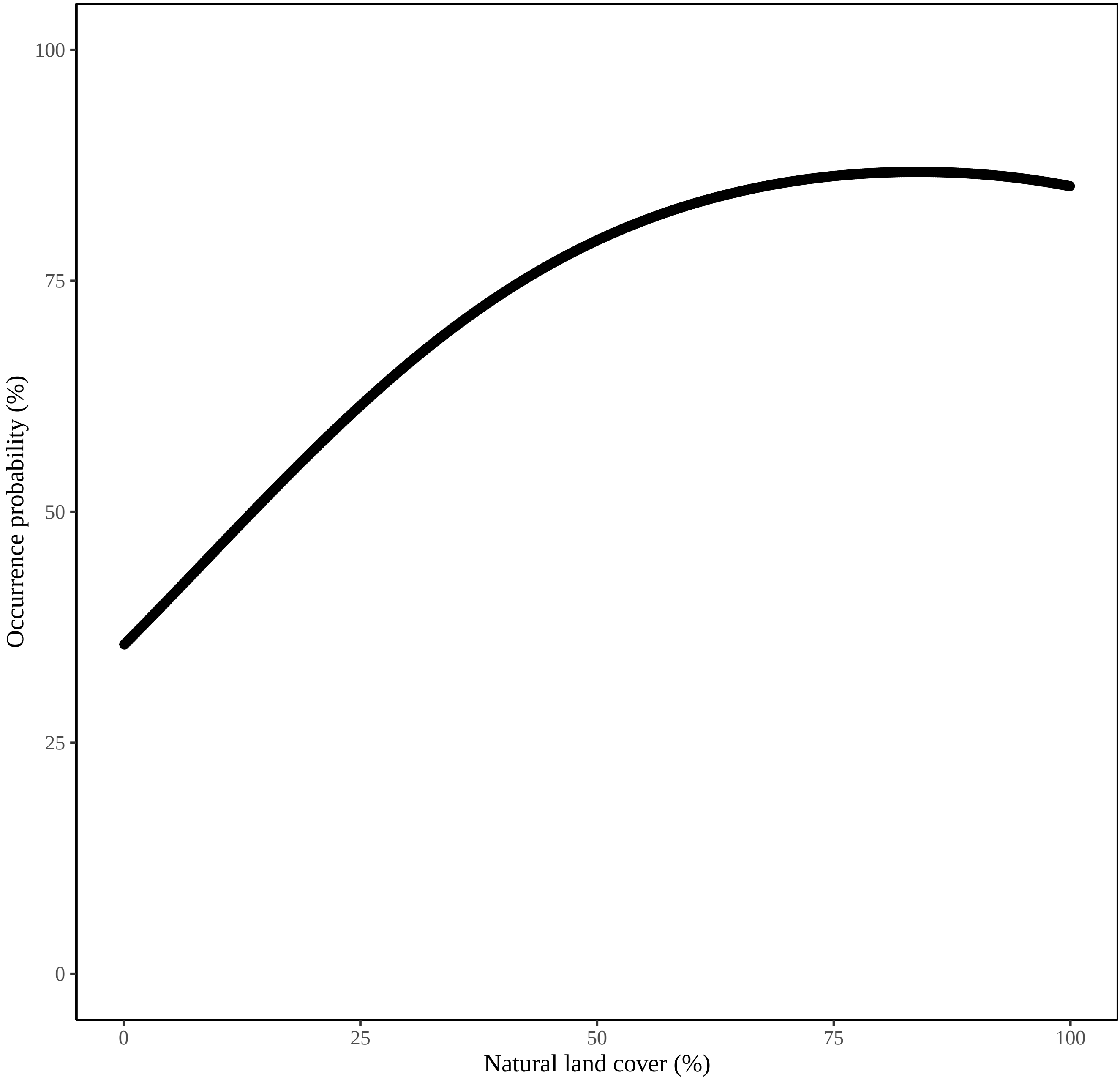

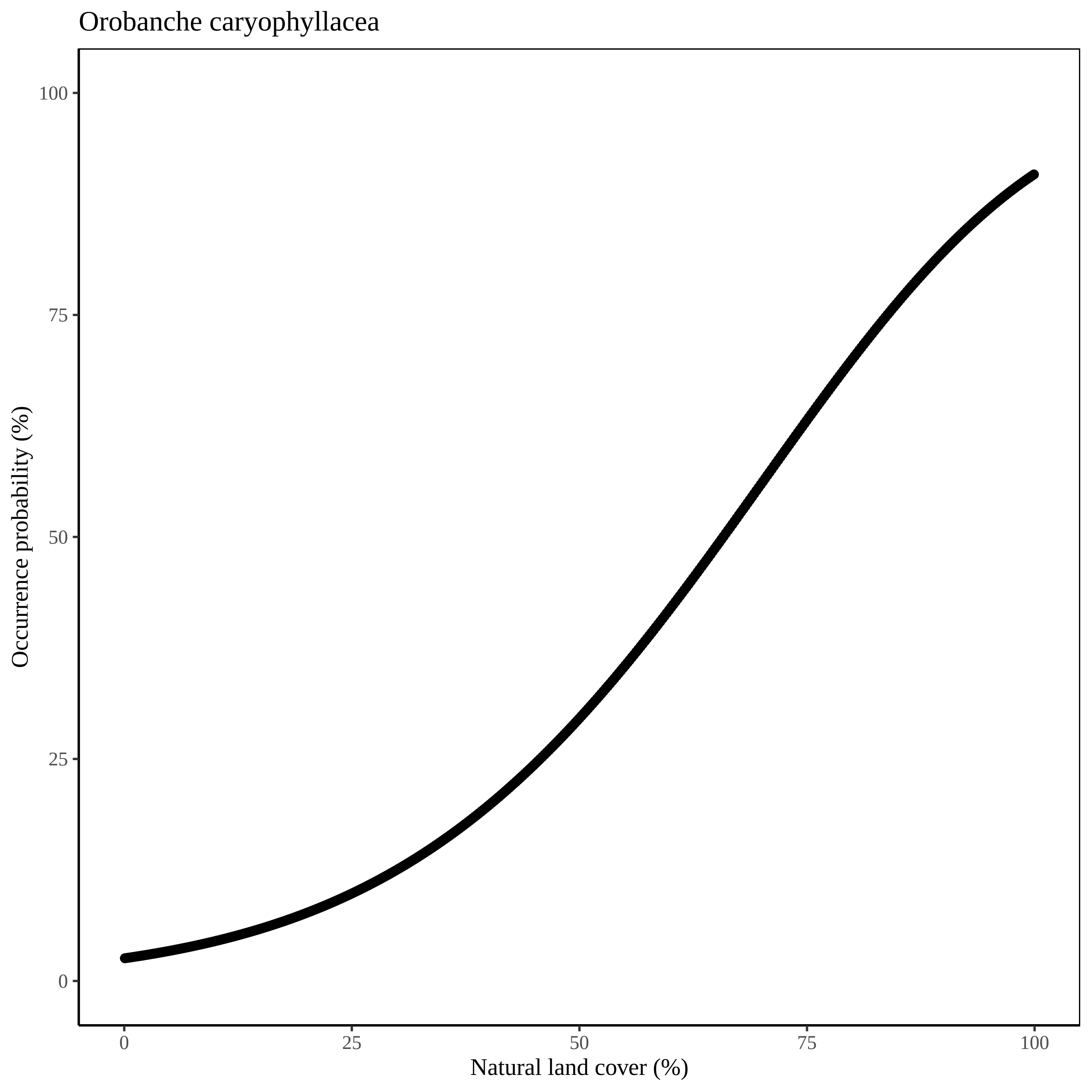
Orobanche caryophyllacea
Occurrence probability (%)
100
75
50
25
0
0
25
50
75
100
Natural land cover (%)

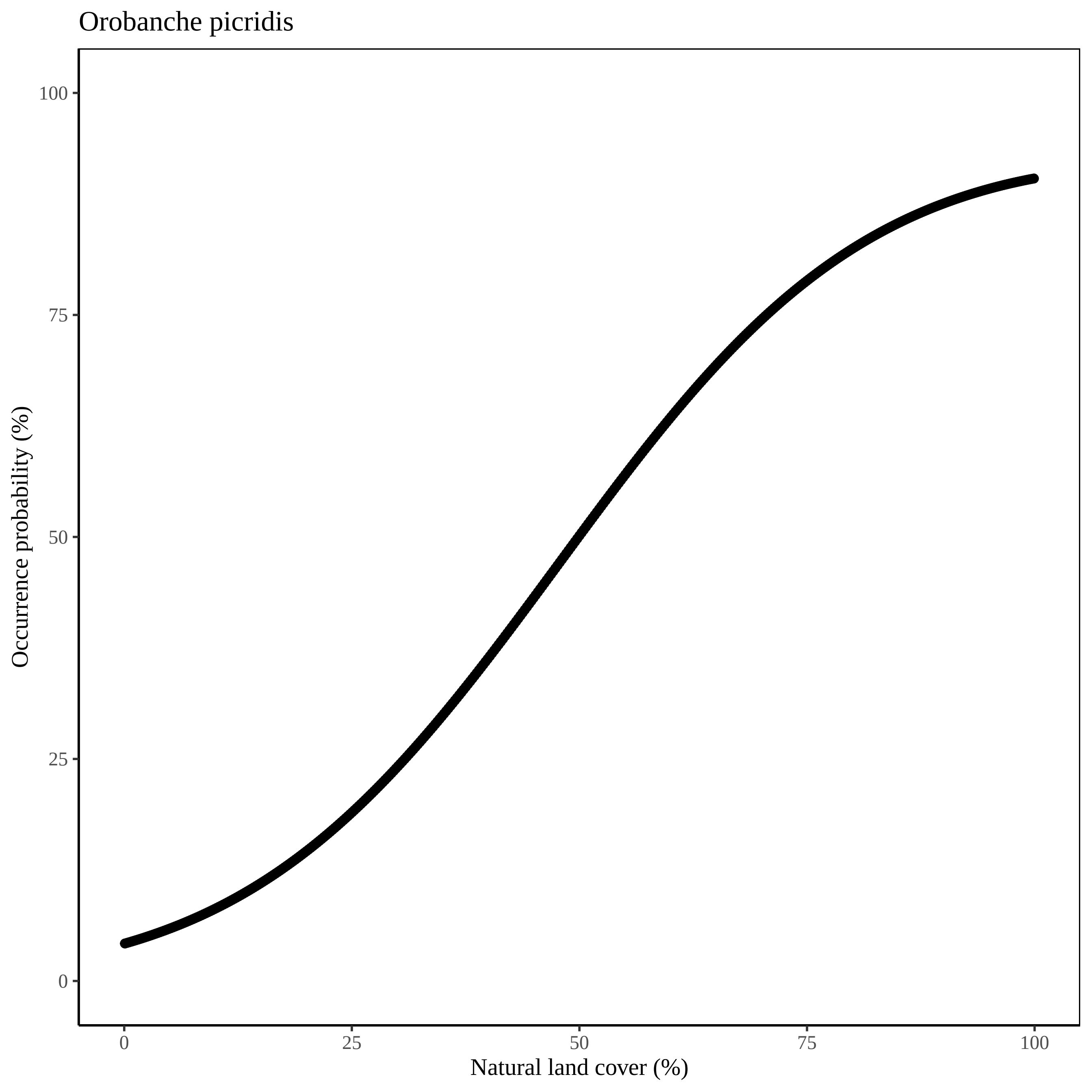

Orobanche picridis
Occurrence probability (%)
100
75
50
25
0
0
25
50
75
100
Natural land cover (%)

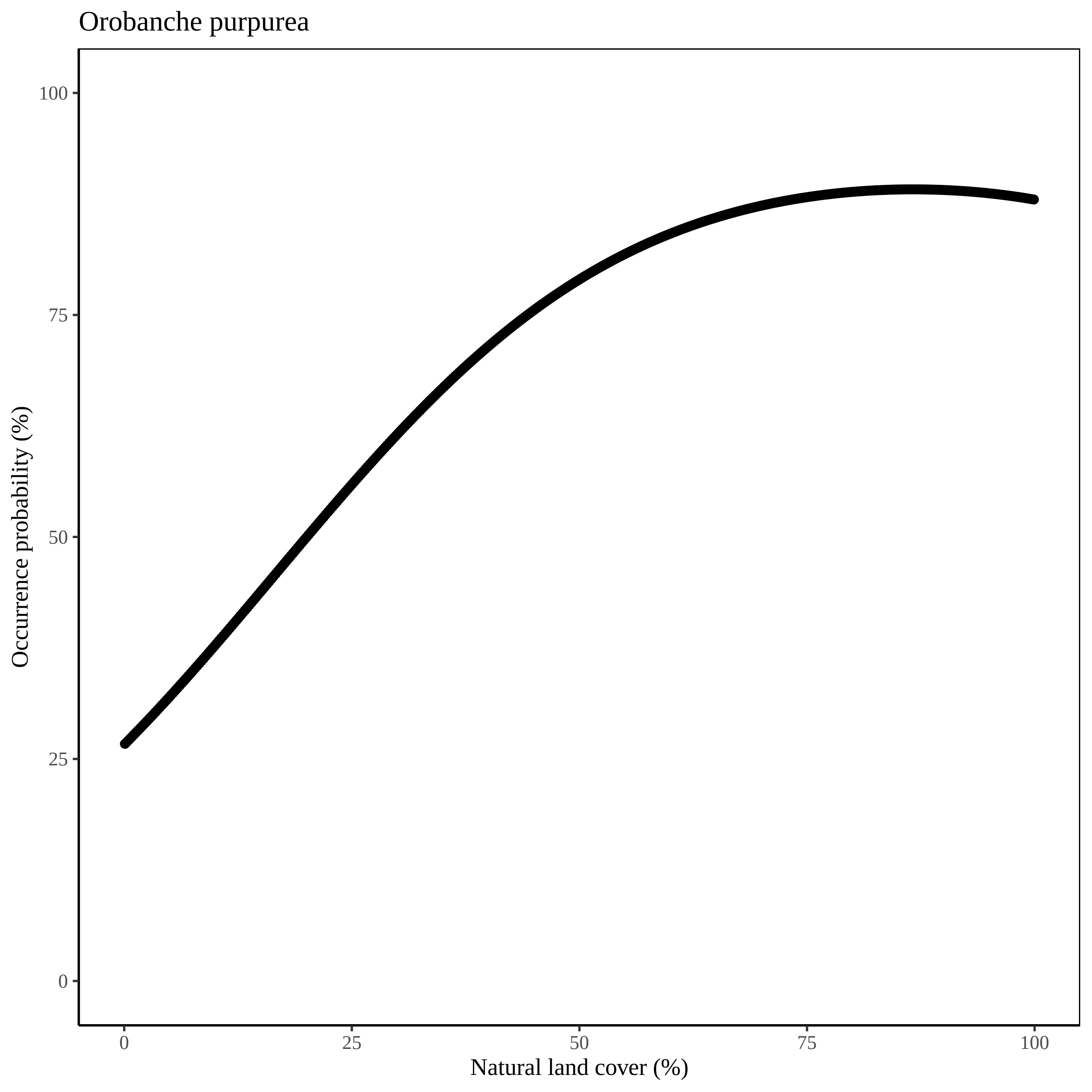
Orobanche purpurea
Occurrence probability (%)
100
75
50
25
0
0
25
50
75
100
Natural land cover (%)

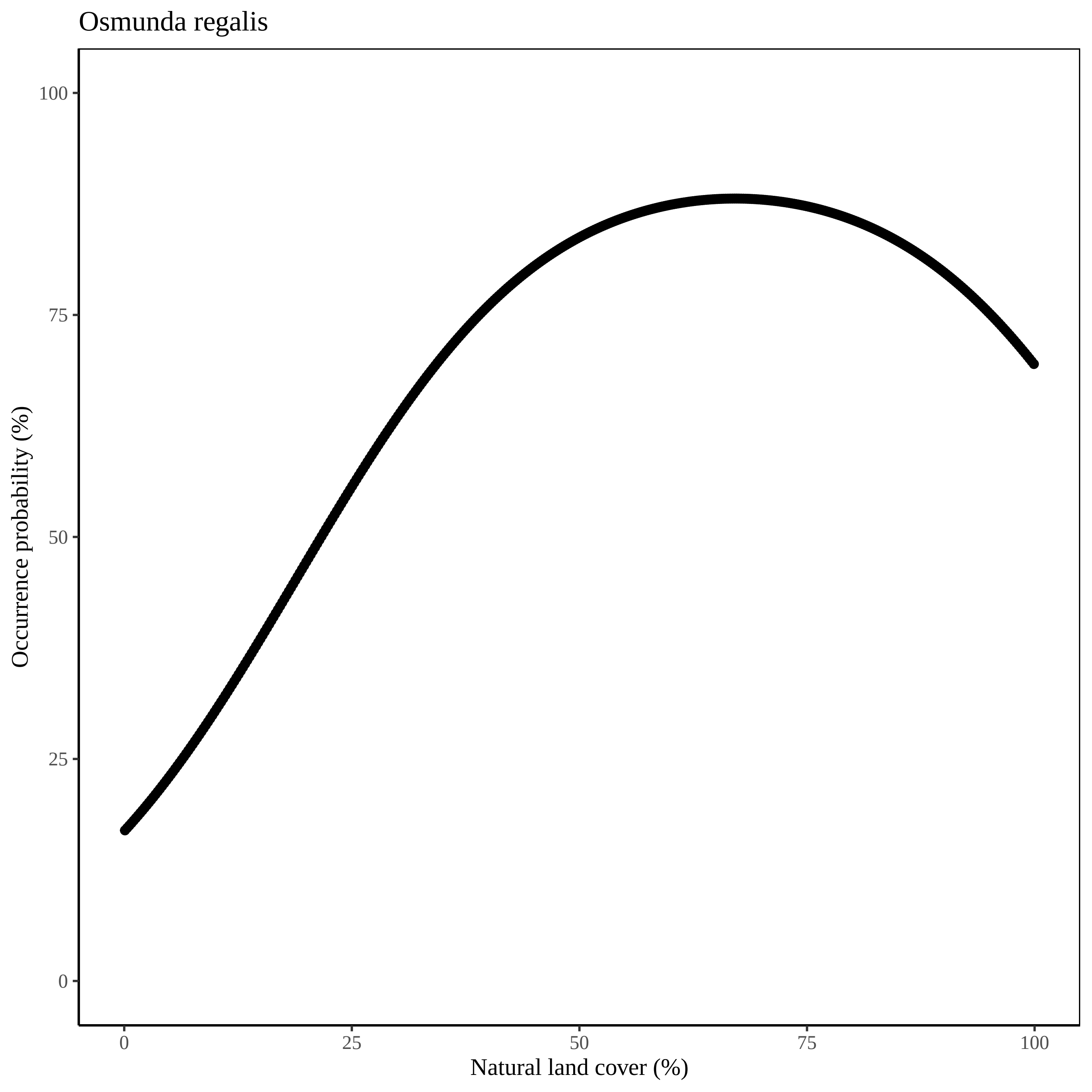
Osmunda regalis
Occurrence probability (%)
Natural land cover (%)
0
25
50
75
100

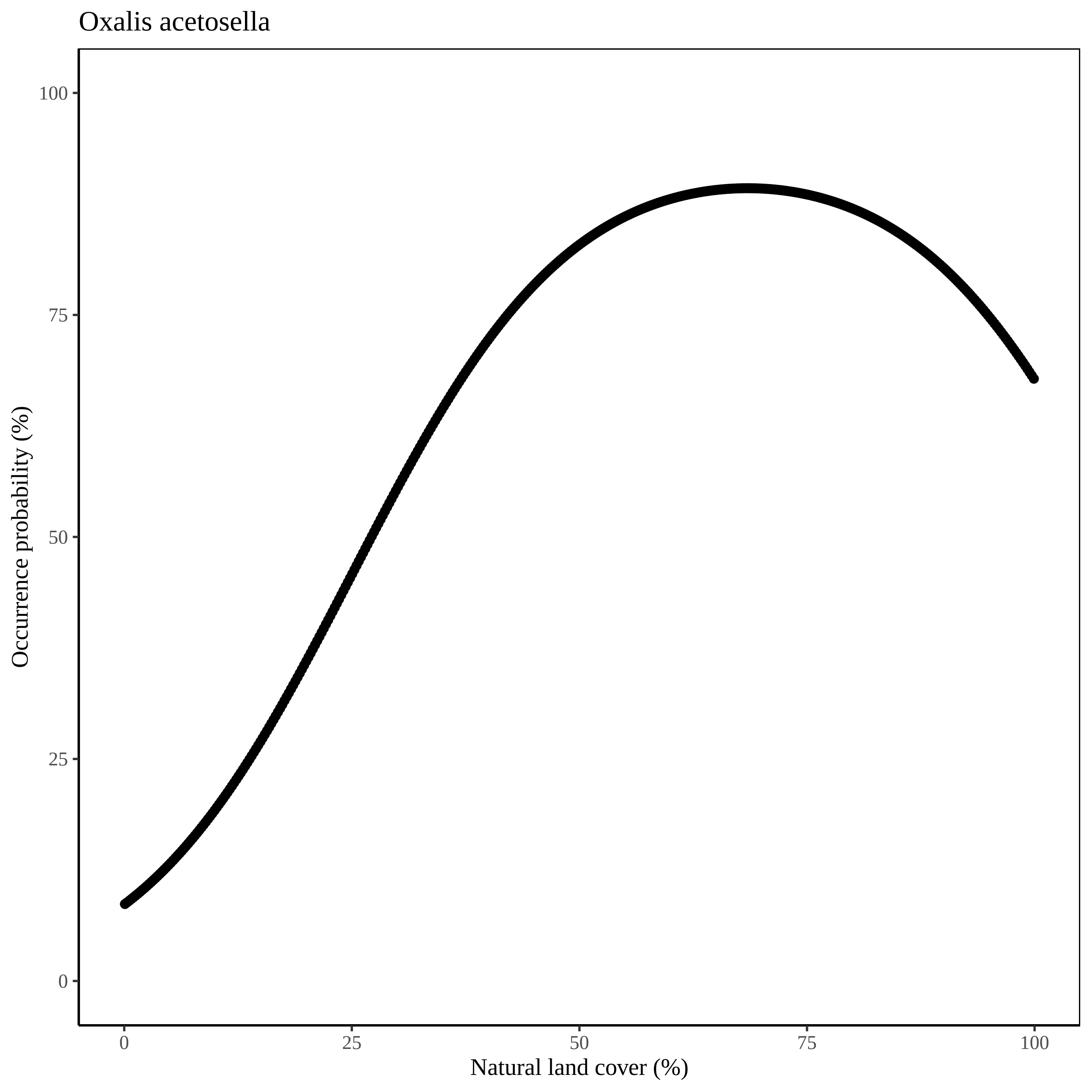

Oxalis acetosella
Occurrence probability (%)
100
75
50
25
0
0
25
50
75
100
Natural land cover (%)

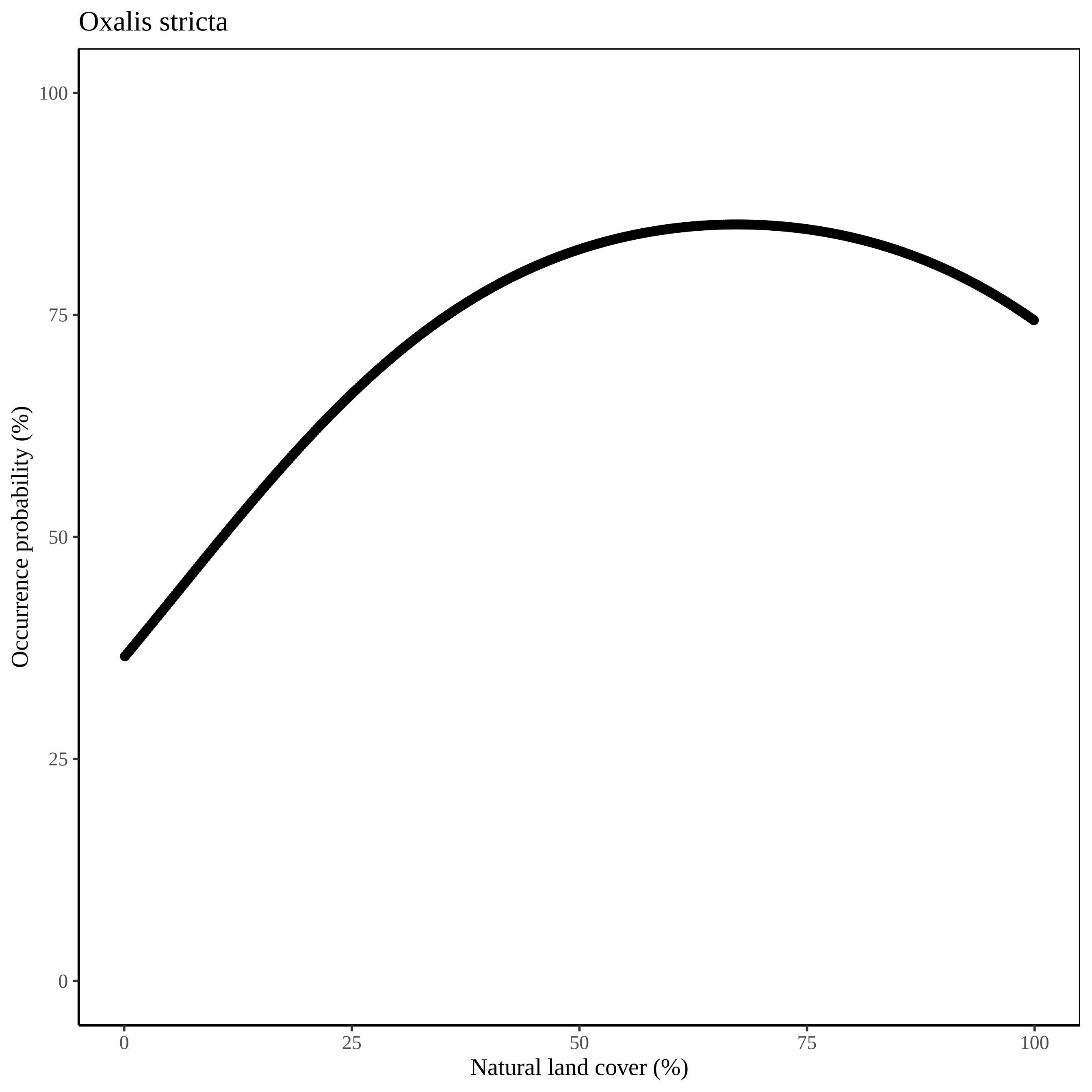

Oxalis stricta
Occurrence probability (%)
Natural land cover (%)
0
25
50
75
100

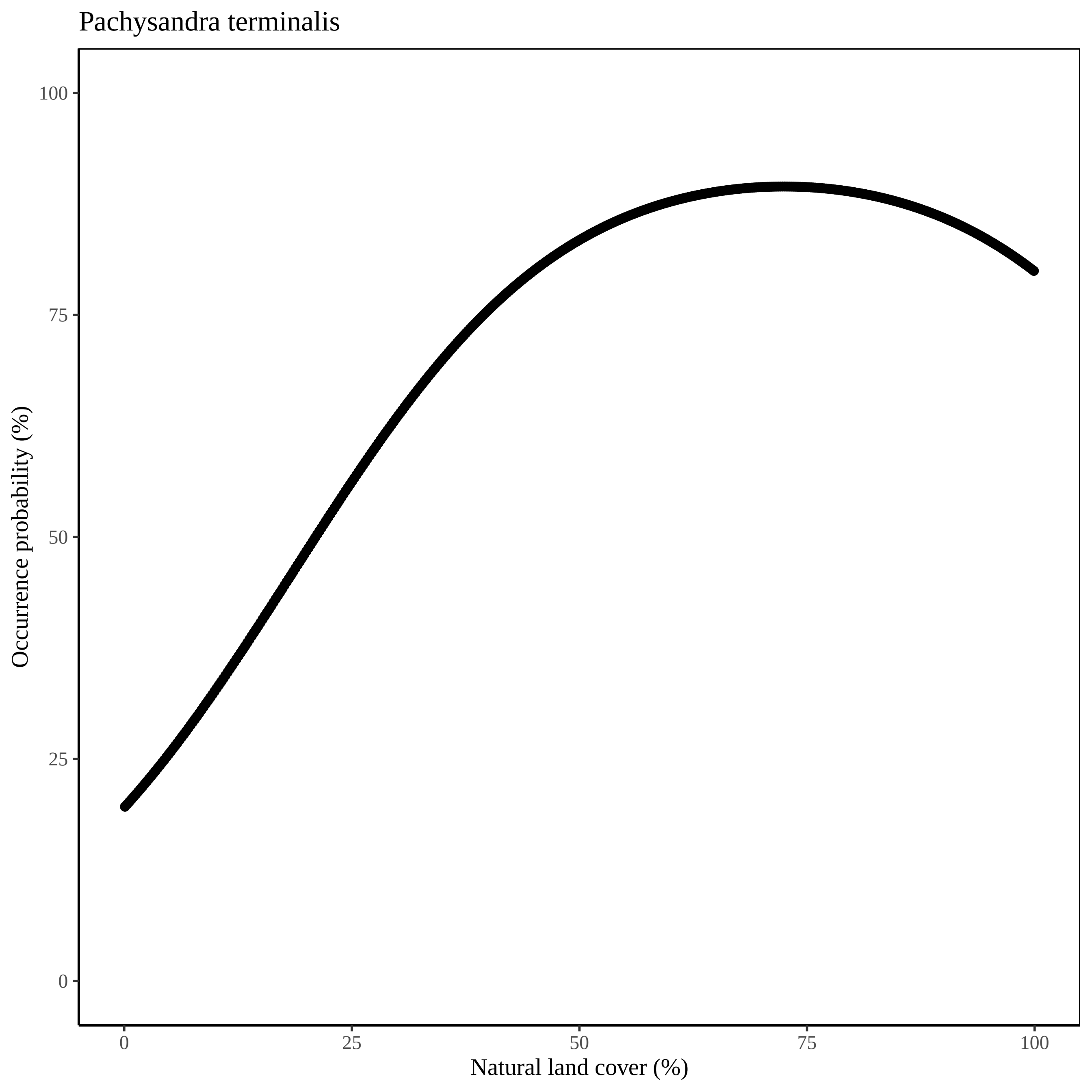

Pachysandra terminalis
Occurrence probability (%)
100
75
50
25
0
0
25
50
75
100
Natural land cover (%)

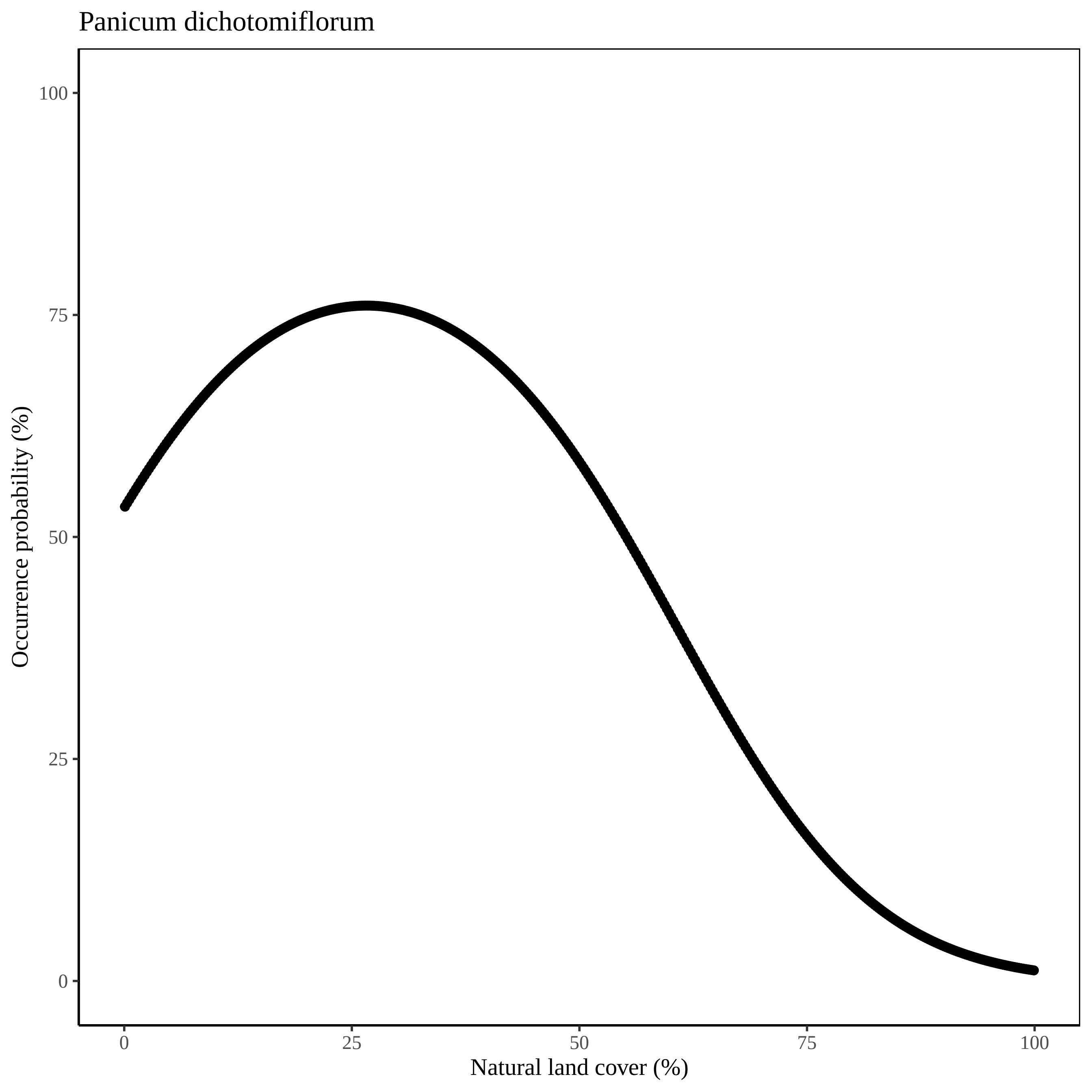

Panicum dichotomiflorum
Occurrence probability (%)
100
75
50
25
0
0
25
50
75
100
Natural land cover (%)

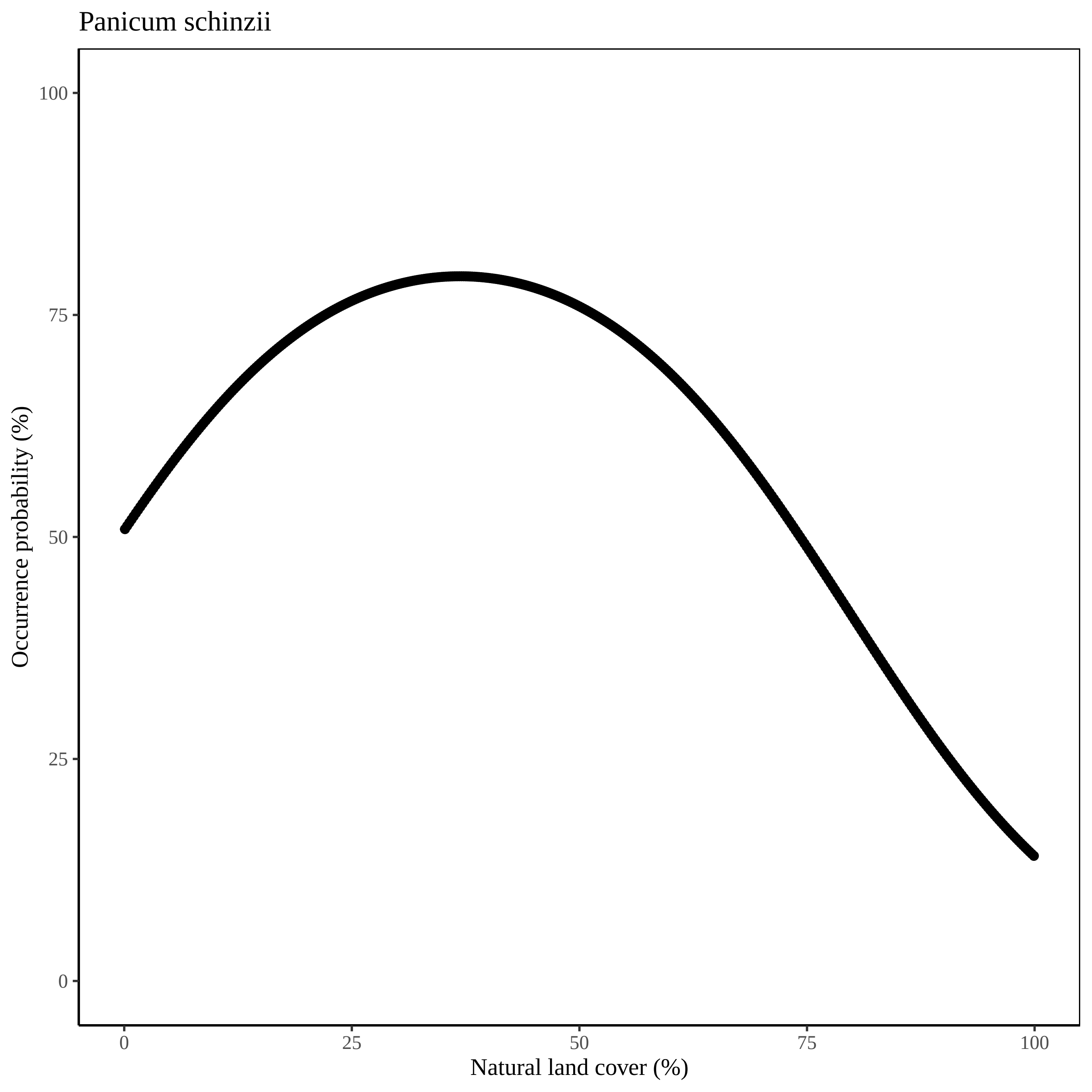
Panicum schinzii
100
75
50
25
0
Occurrence probability (%)
0
25
50
75
100
Natural land cover (%)

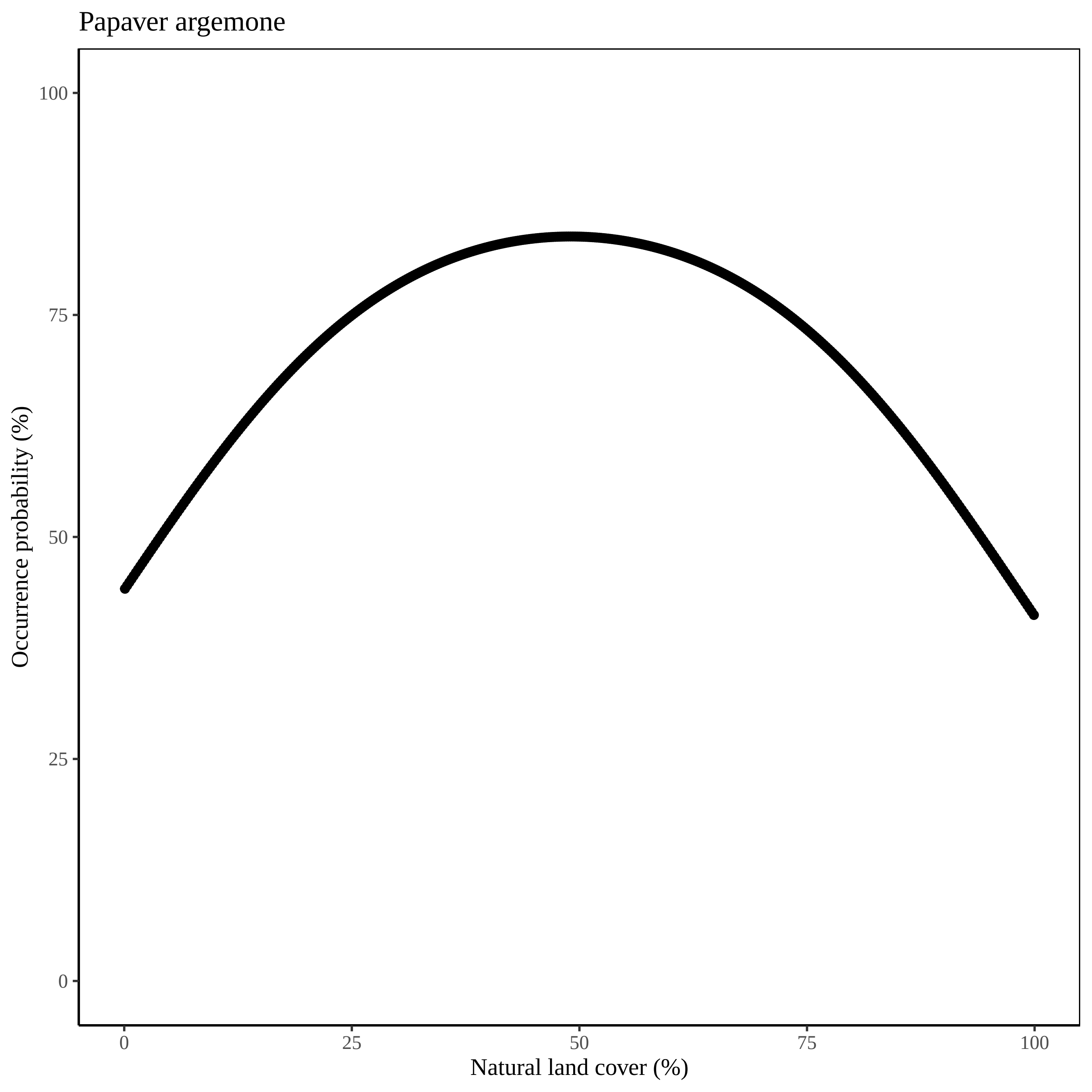
Papaver argemone
Occurrence probability (%)
100
75
50
25
0
0
25
50
75
100
Natural land cover (%)

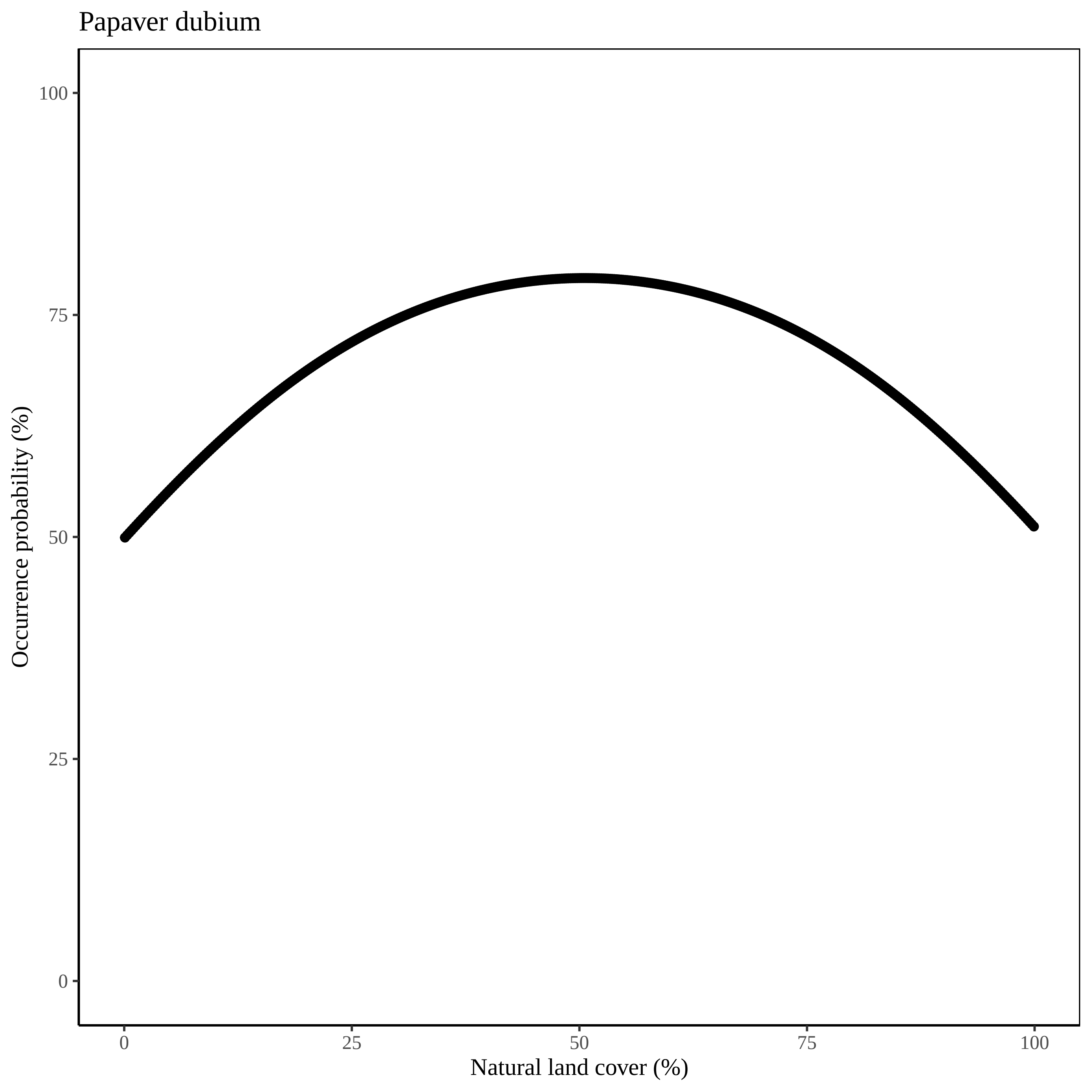
Papaver dubium
Occurrence probability (%)
100
75
50
25
0
0
25
50
75
100
Natural land cover (%)

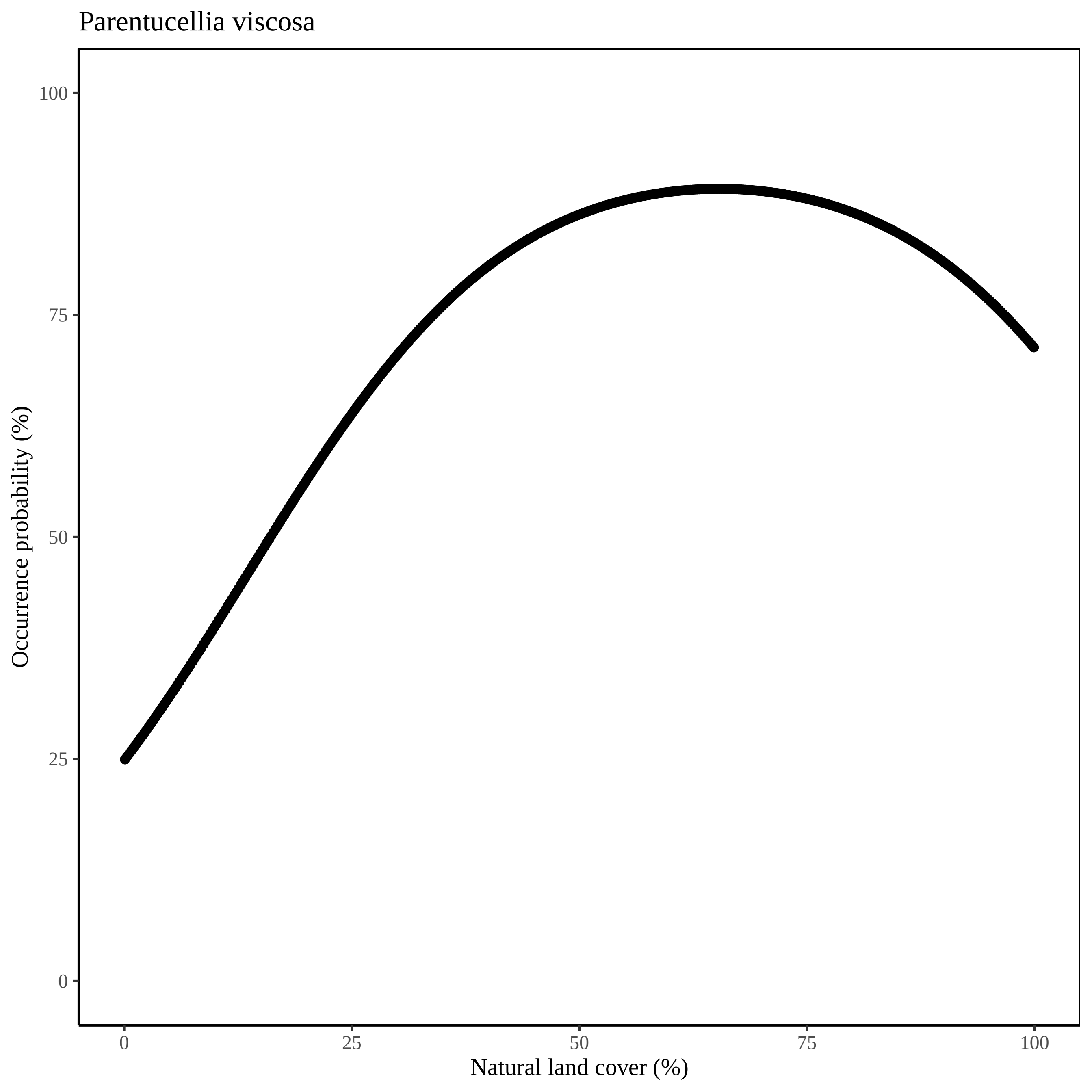
Parentucellia viscosa
Occurrence probability (%)
100
75
50
25
0
0
25
50
75
100
Natural land cover (%)

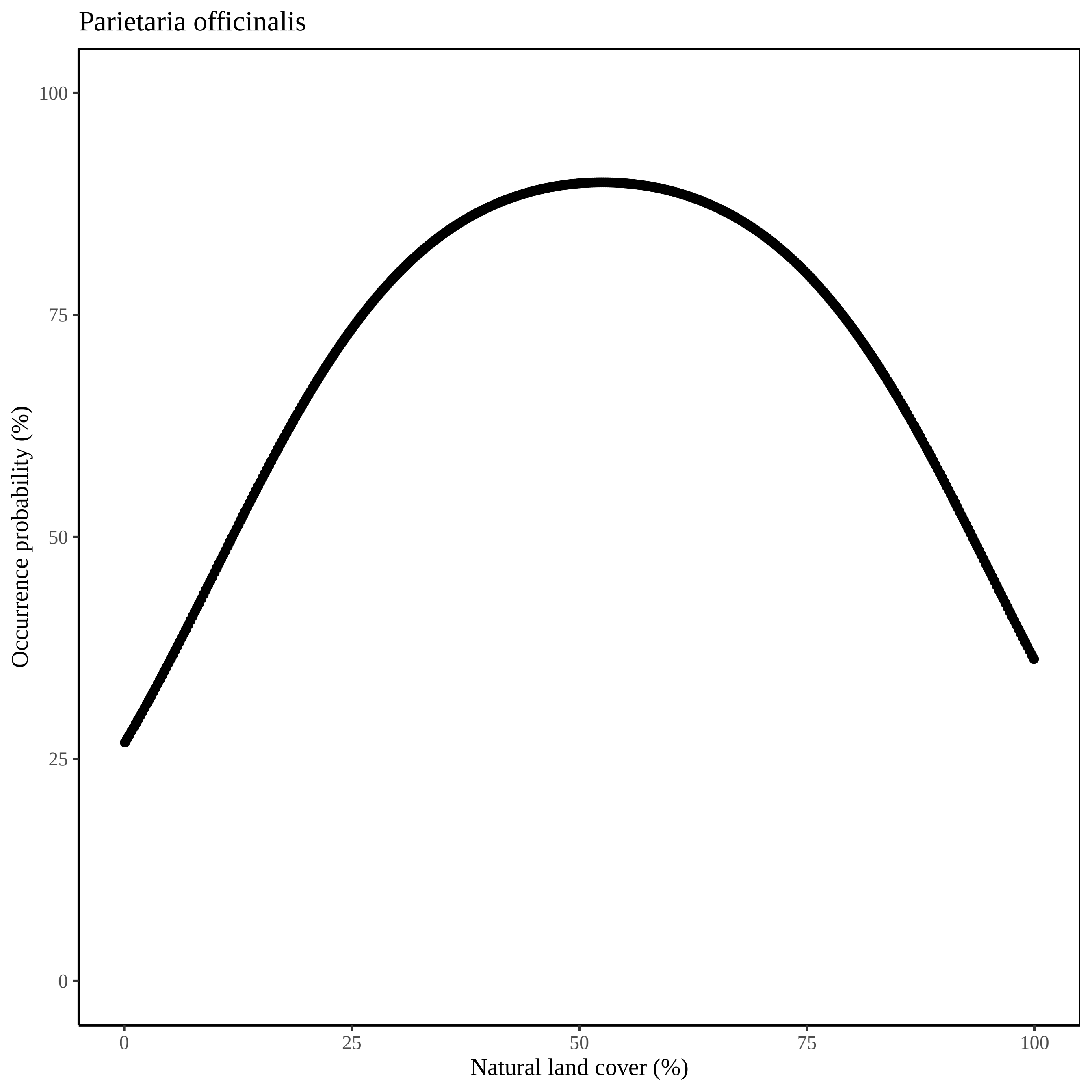
Parietaria officinalis
100
75
50
25
0
Occurrence probability (%)
0
25
50
75
100
Natural land cover (%)

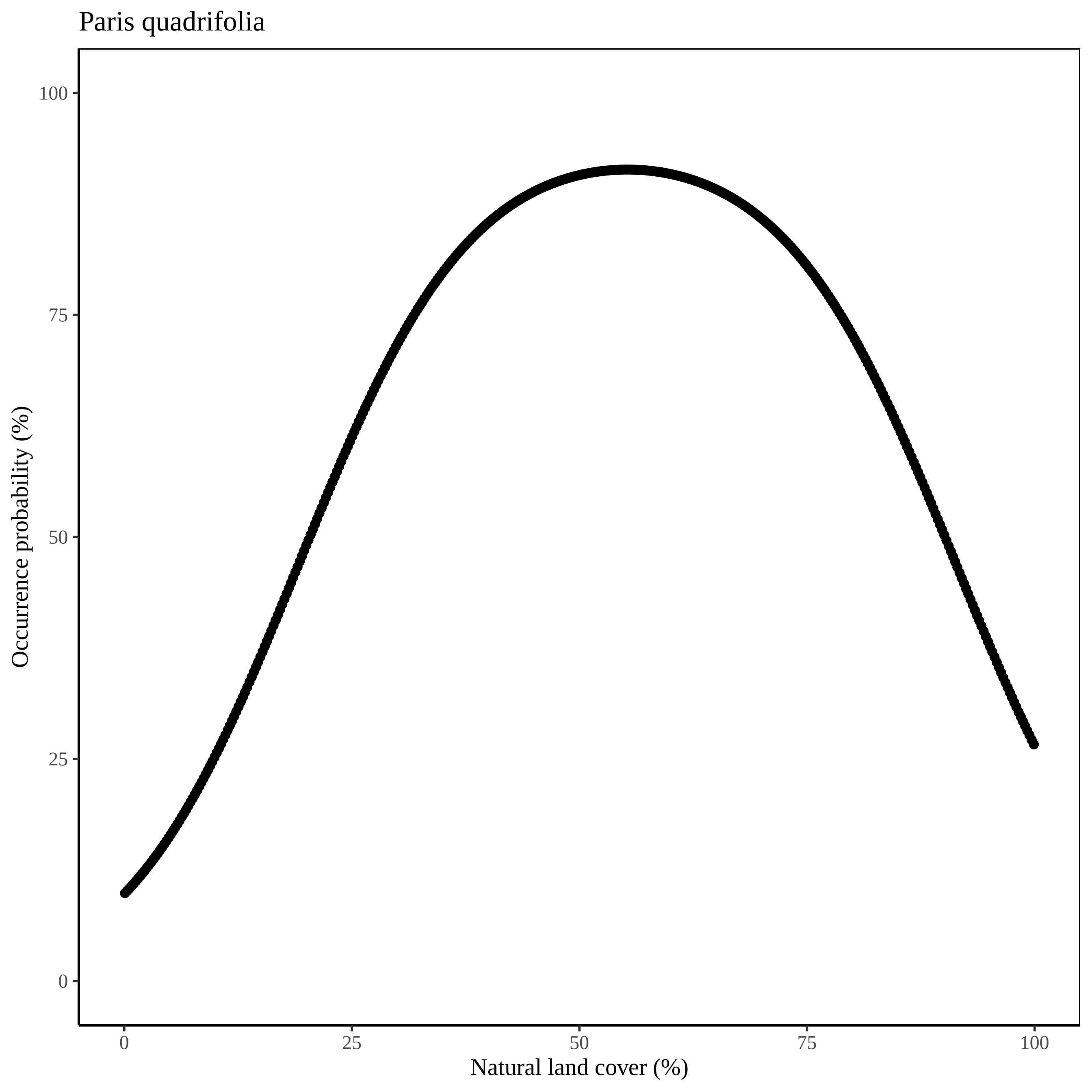
Paris quadrifolia
Occurrence probability (%)
100
75
50
25
0
0
25
50
75
100
Natural land cover (%)

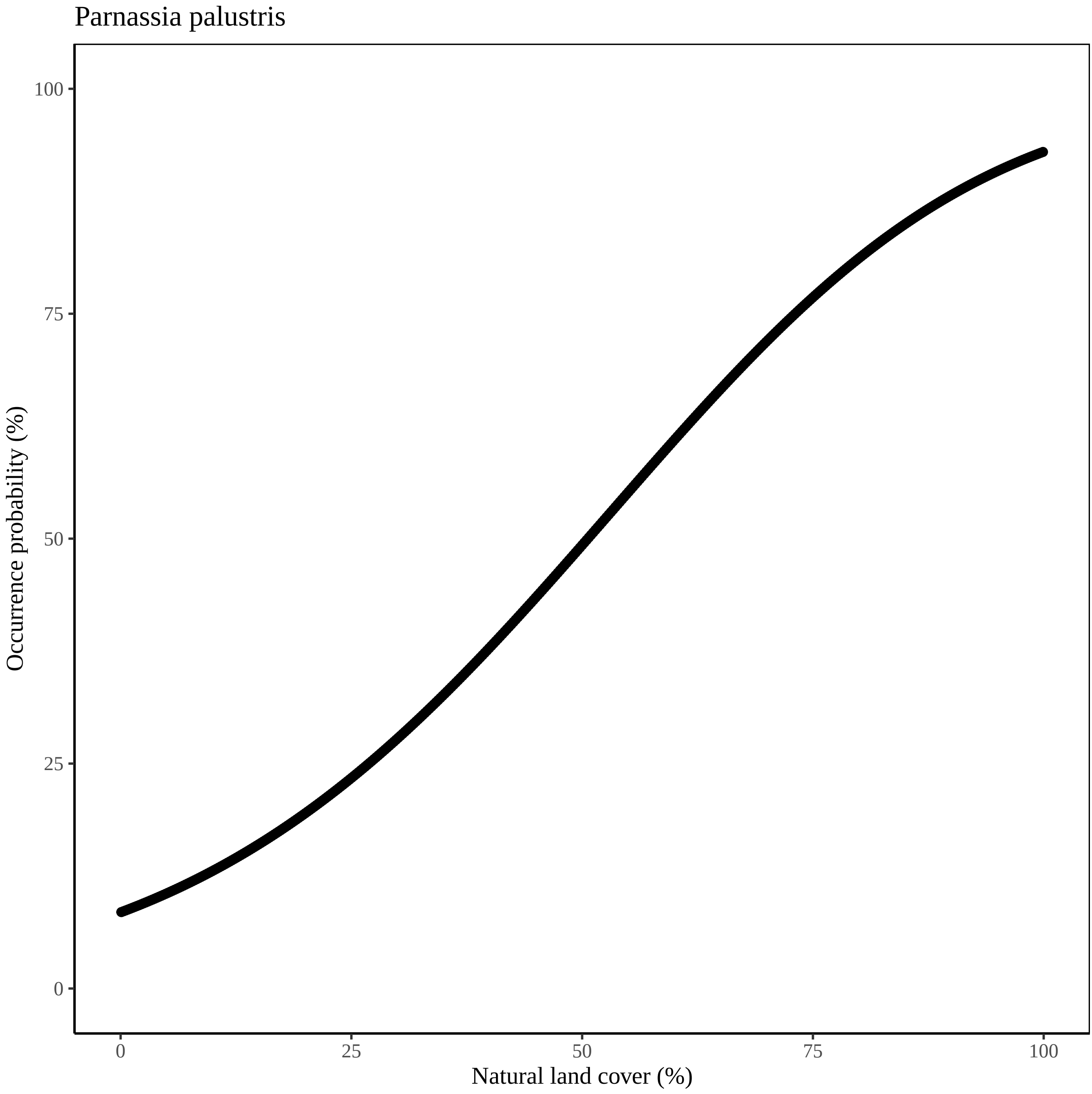
Parnassia palustris
Occurrence probability (%)
100
75
50
25
0
Natural land cover (%)
0
25
50
75
100

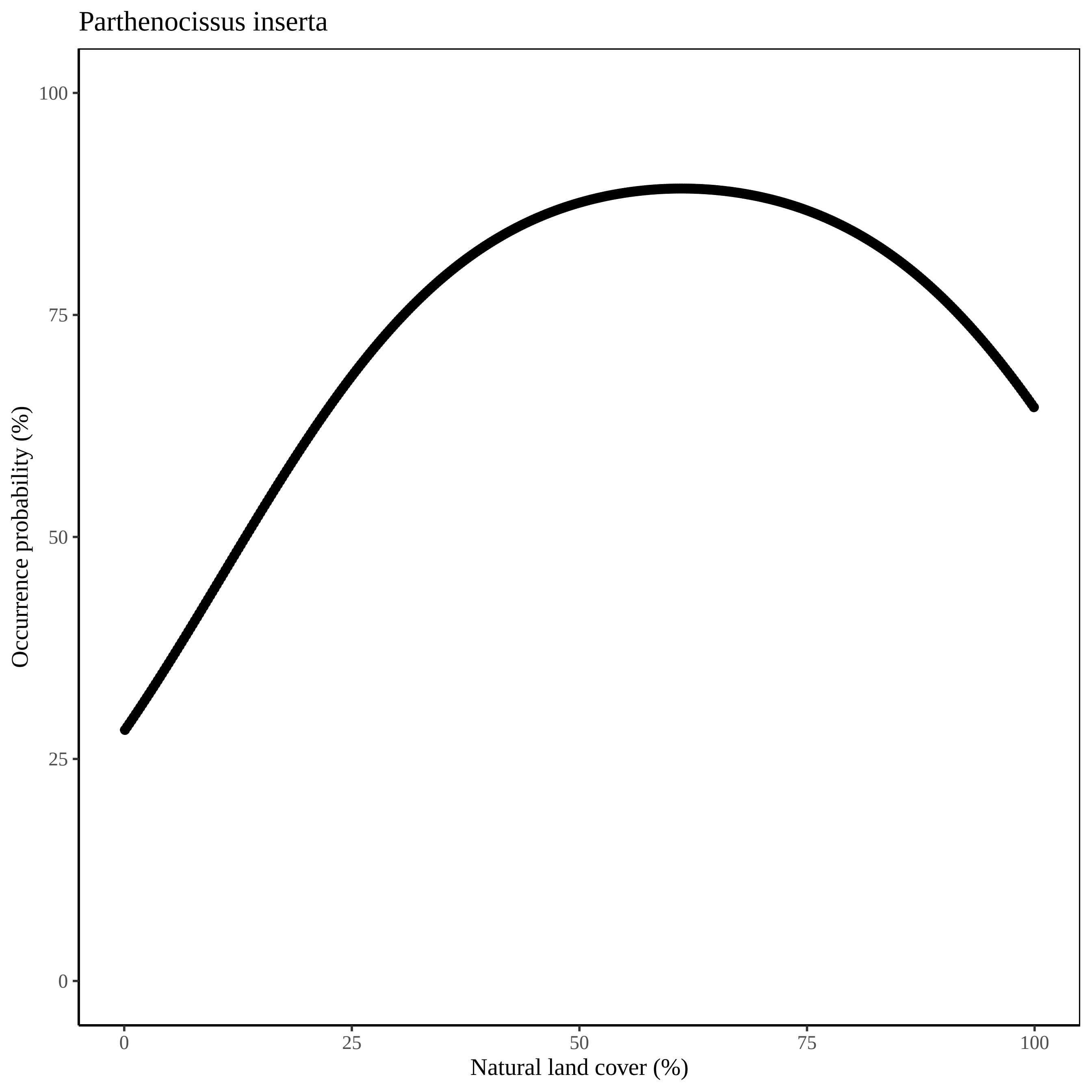
Parthenocissus inserta
Occurrence probability (%)
100
75
50
25
0
0
25
50
75
100
Natural land cover (%)

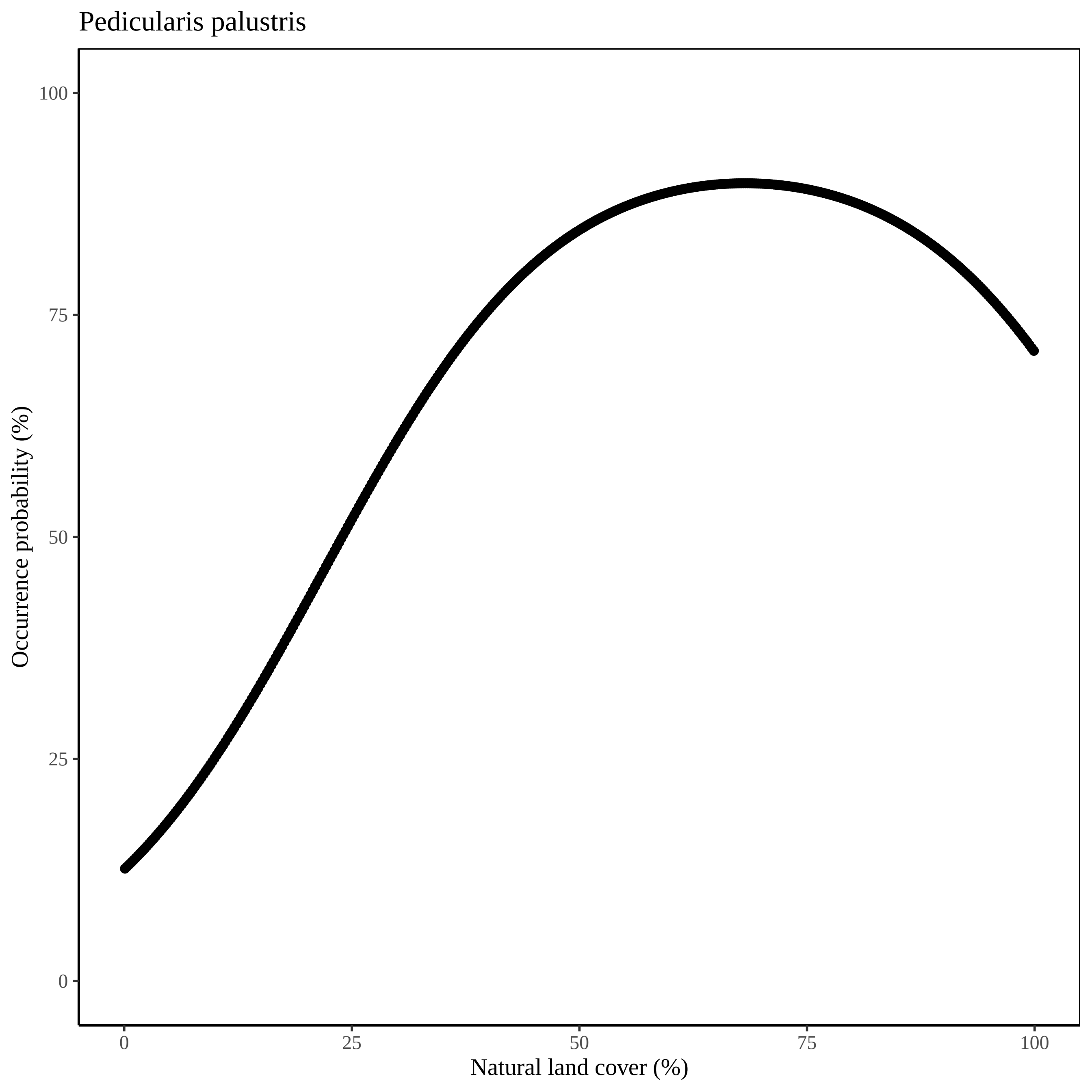
Pedicularis palustris
Occurrence probability (%)
Natural land cover (%)
0
25
50
75
100
0
25
50
75
100

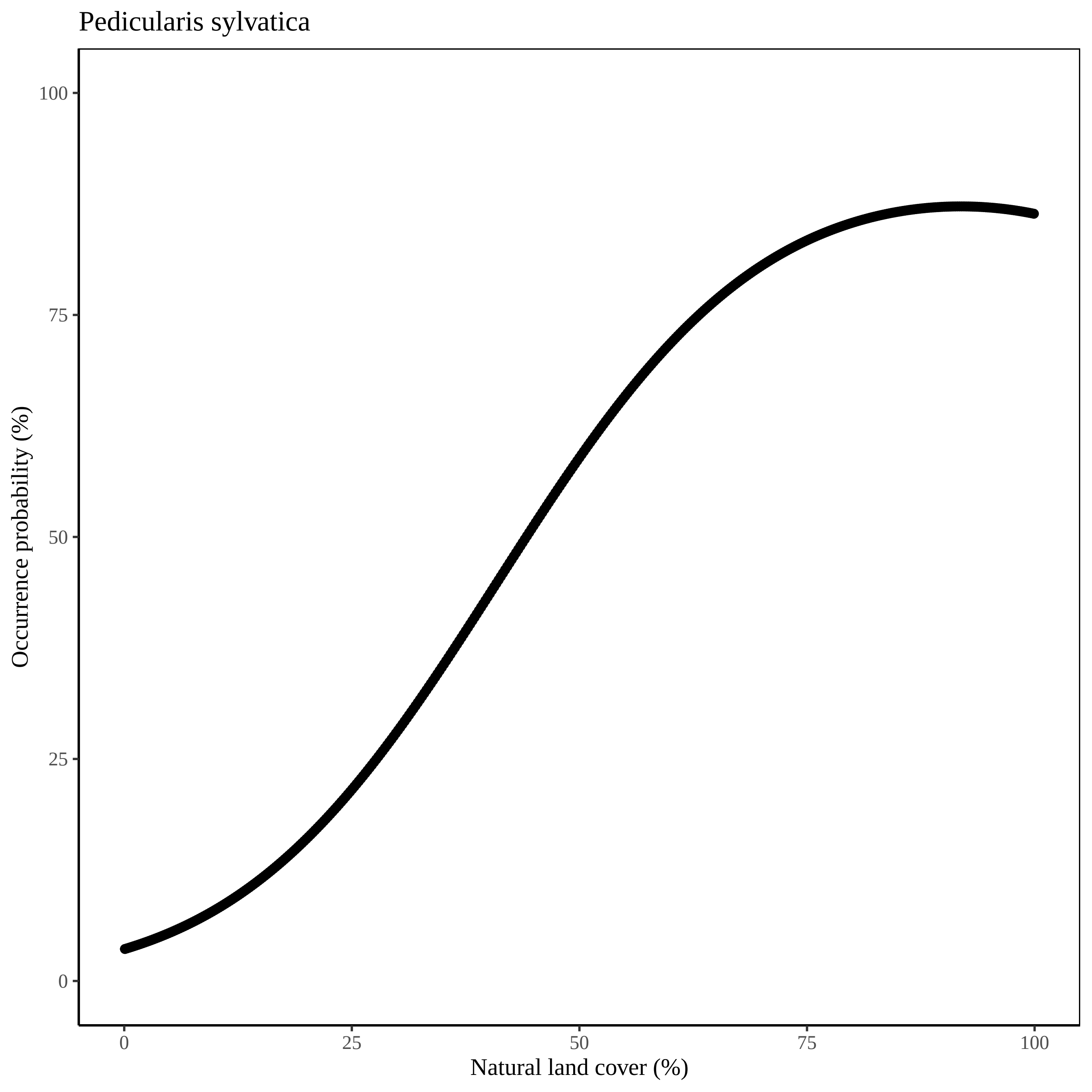
Pedicularis sylvatica
Occurrence probability (%)
100
75
50
25
0
0
25
50
75
100
Natural land cover (%)

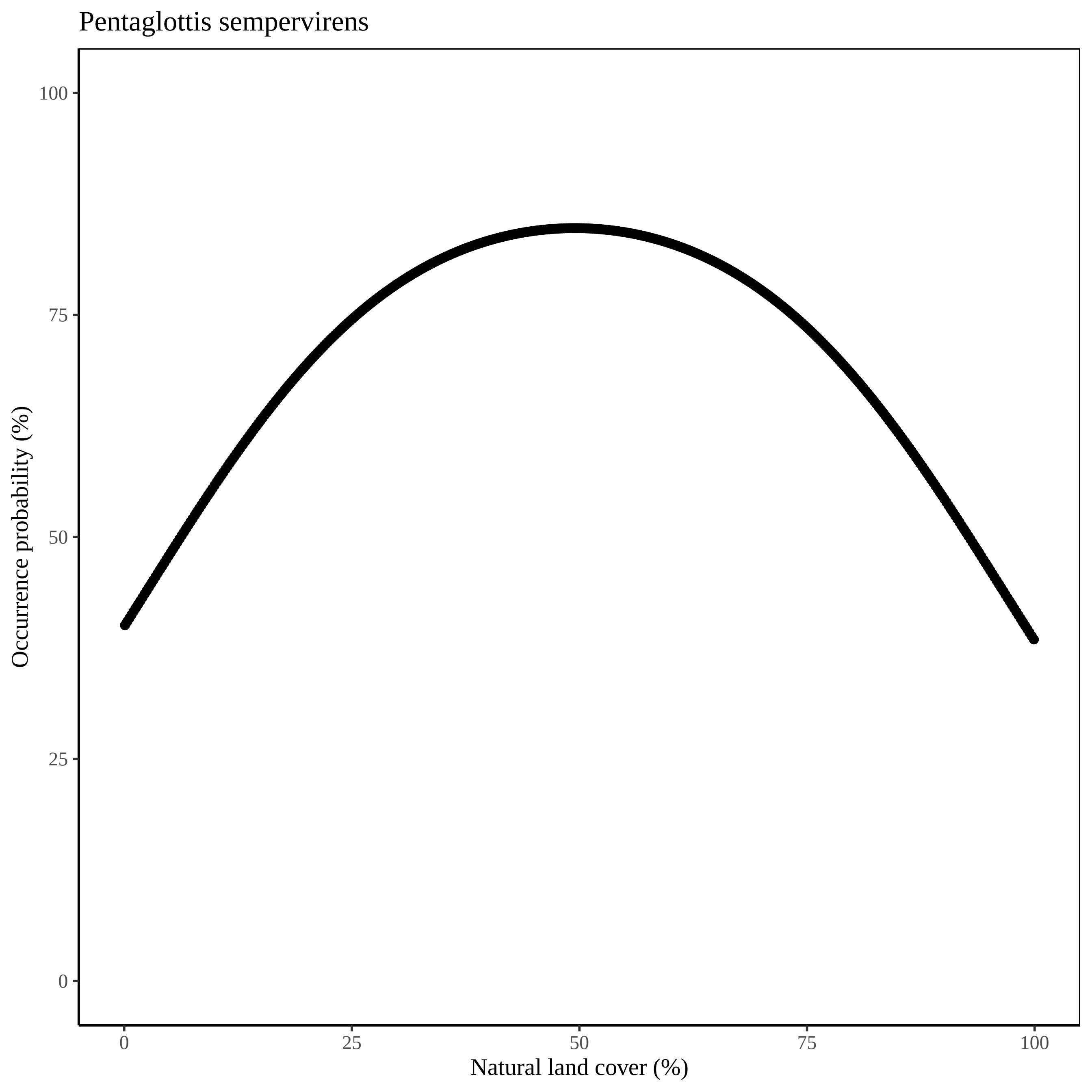
Pentaglottis sempervirens
100
75
50
25
0
Occurrence probability (%)
0
25
50
75
100
Natural land cover (%)

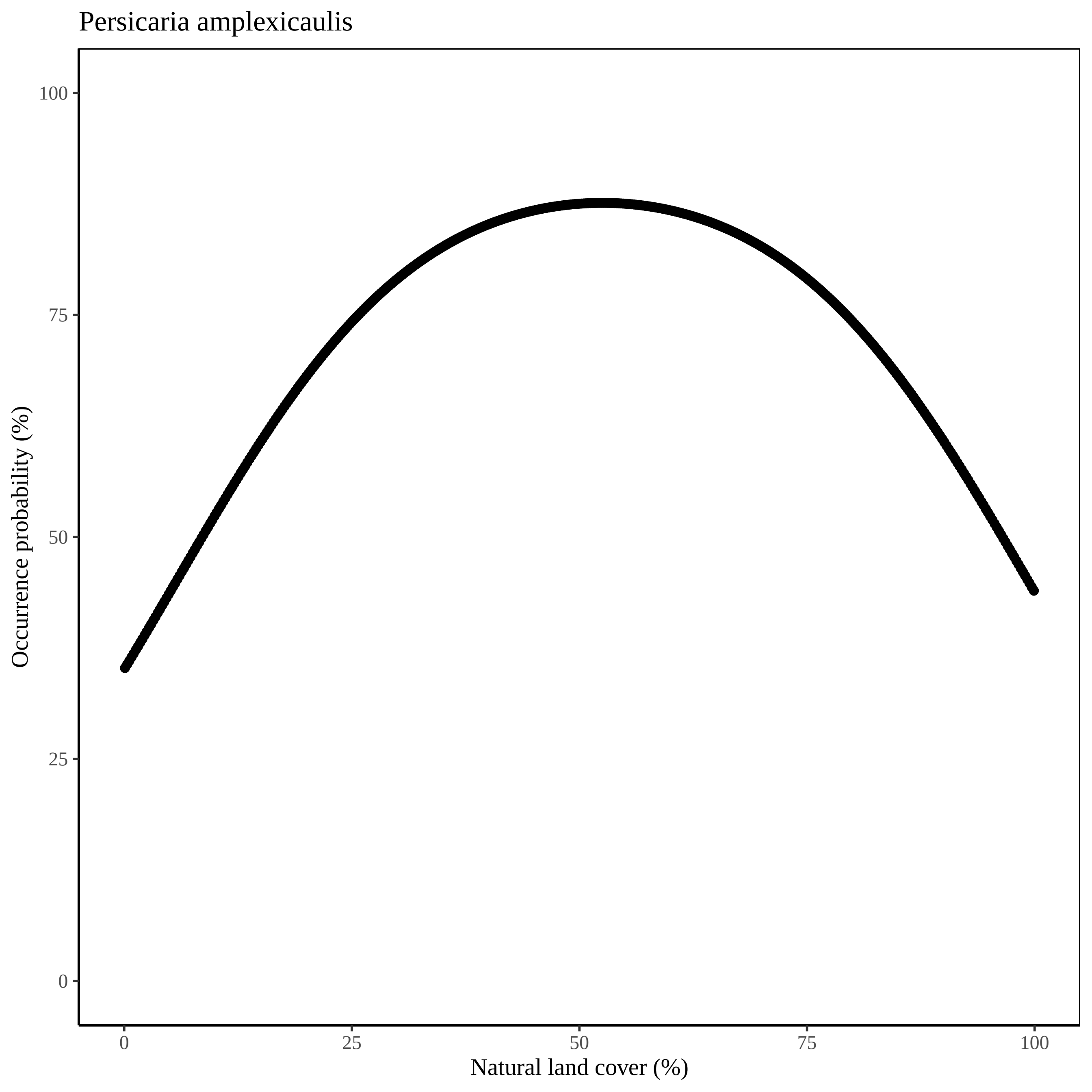
Persicaria amplexicaulis
Occurrence probability (%)
100
75
50
25
0
0
25
50
75
100
Natural land cover (%)

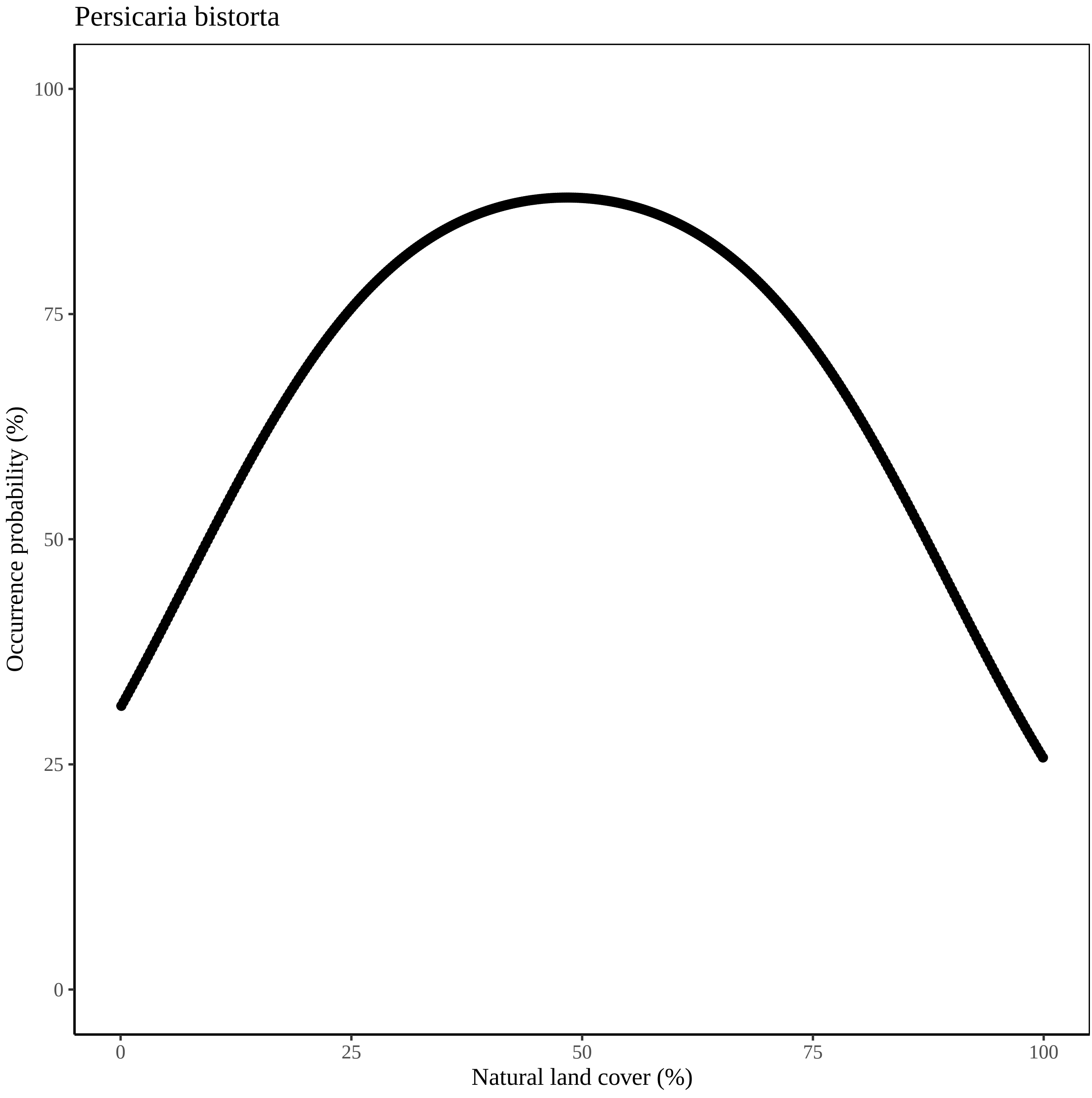
Persicaria bistorta
Occurrence probability (%)
100
75
50
25
0
0
25
50
75
100
Natural land cover (%)

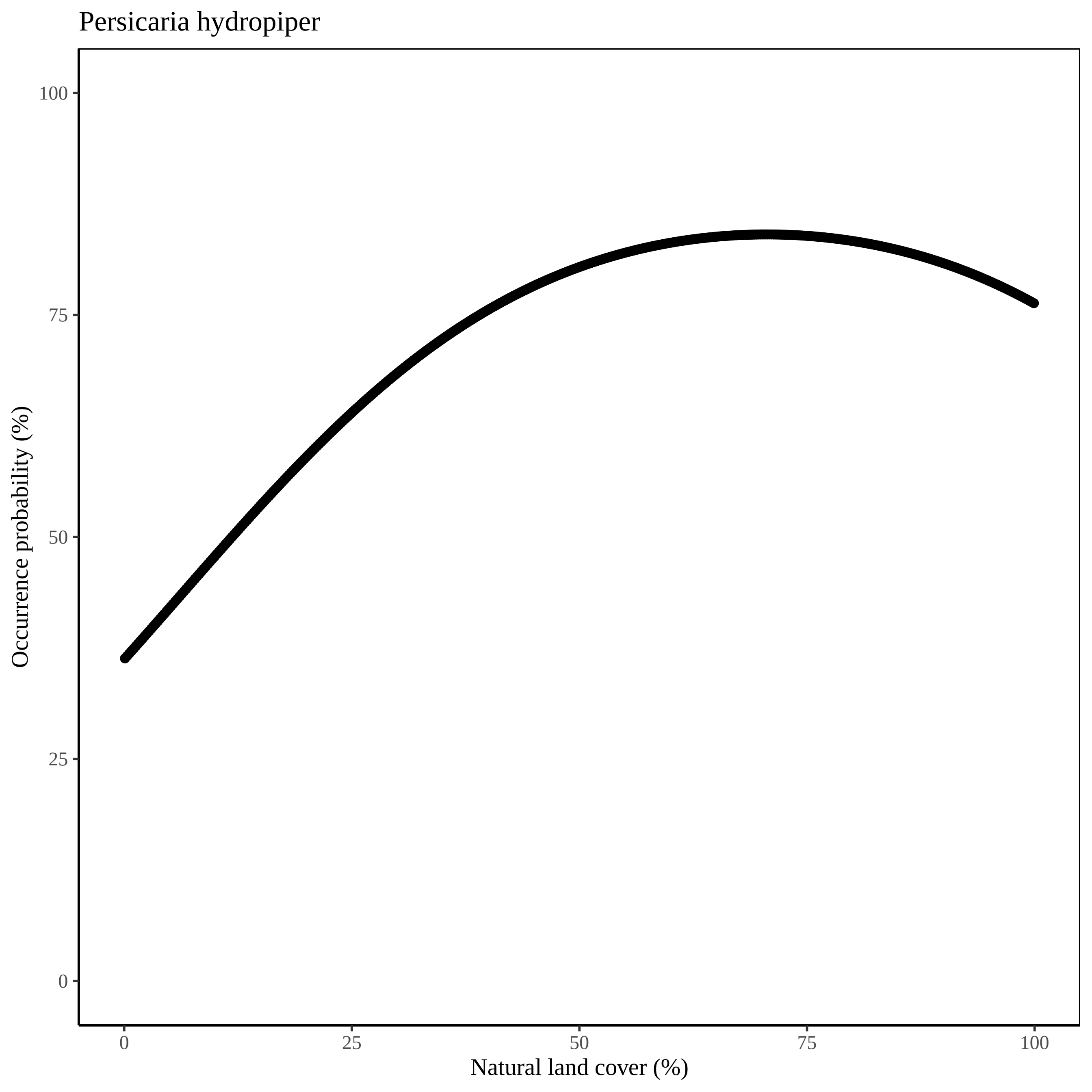
Persicaria hydropiper
Occurrence probability (%)
100
75
50
25
0
0
25
50
75
100
Natural land cover (%)

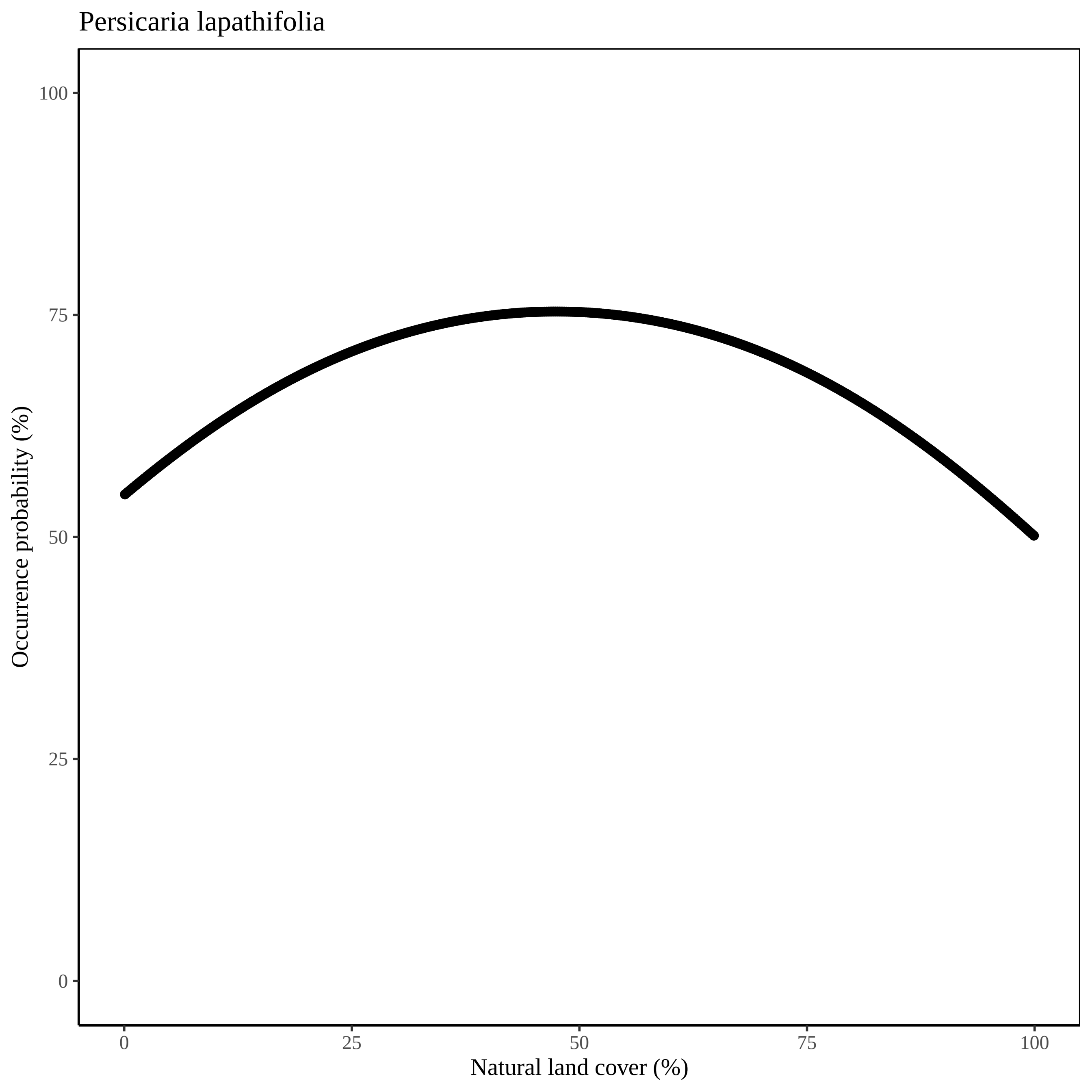
Persicaria lapathifolia
Occurrence probability (%)
100
75
50
25
0
0
25
50
75
100
Natural land cover (%)

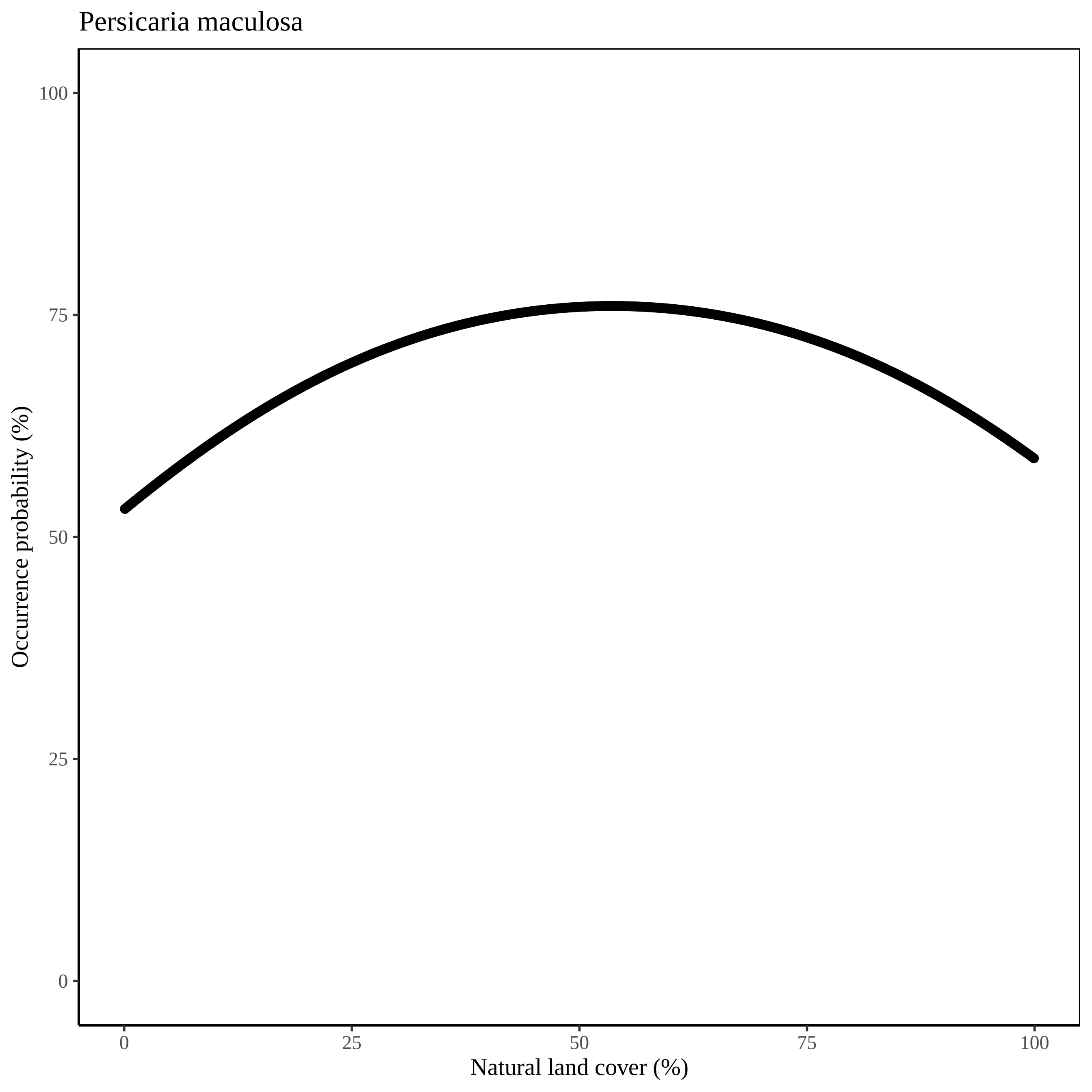
Persicaria maculosa
Occurrence probability (%)
100
75
50
25
0
0
25
50
75
100
Natural land cover (%)

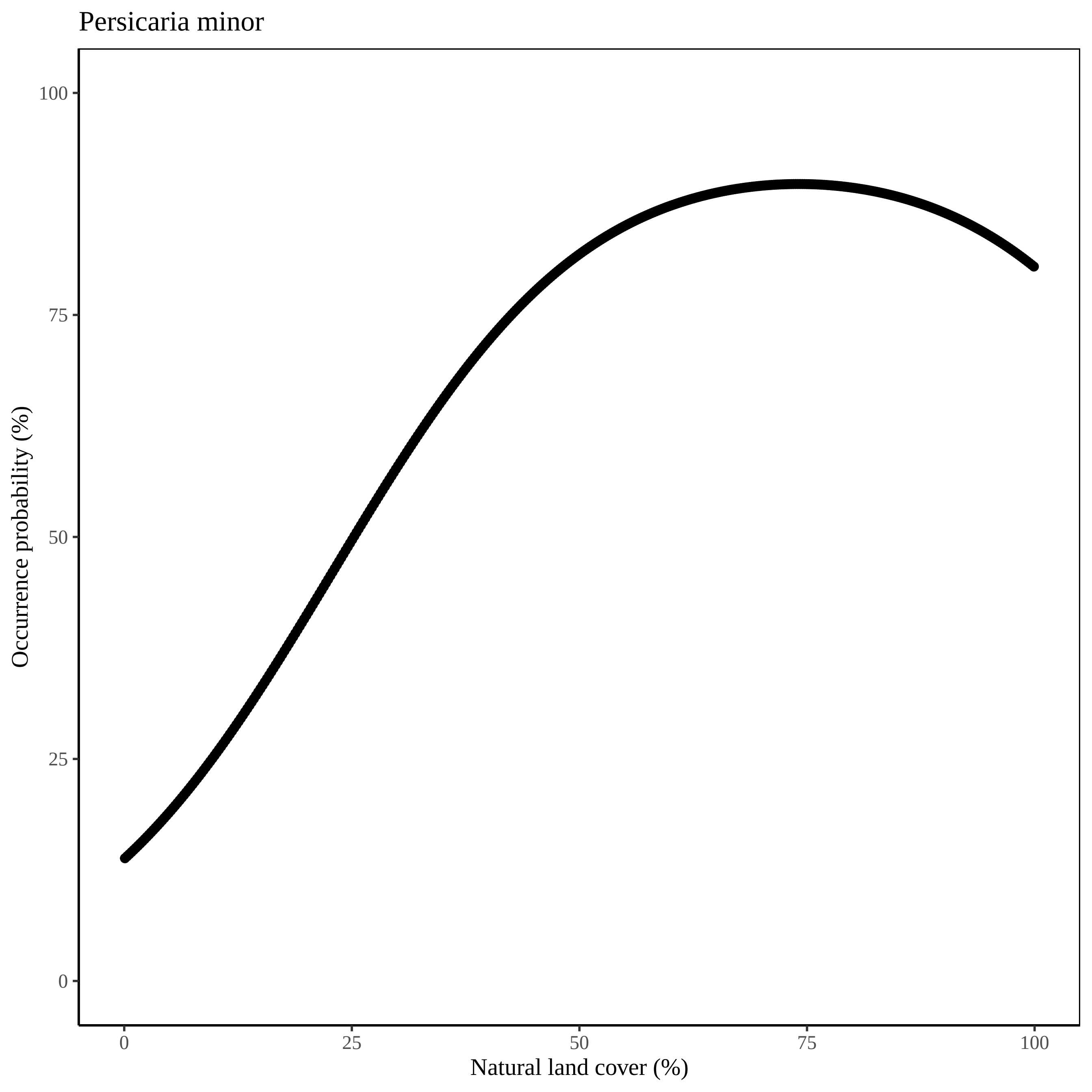
Persicaria minor
Occurrence probability (%)
100
75
50
25
0
0
25
50
75
100
Natural land cover (%)

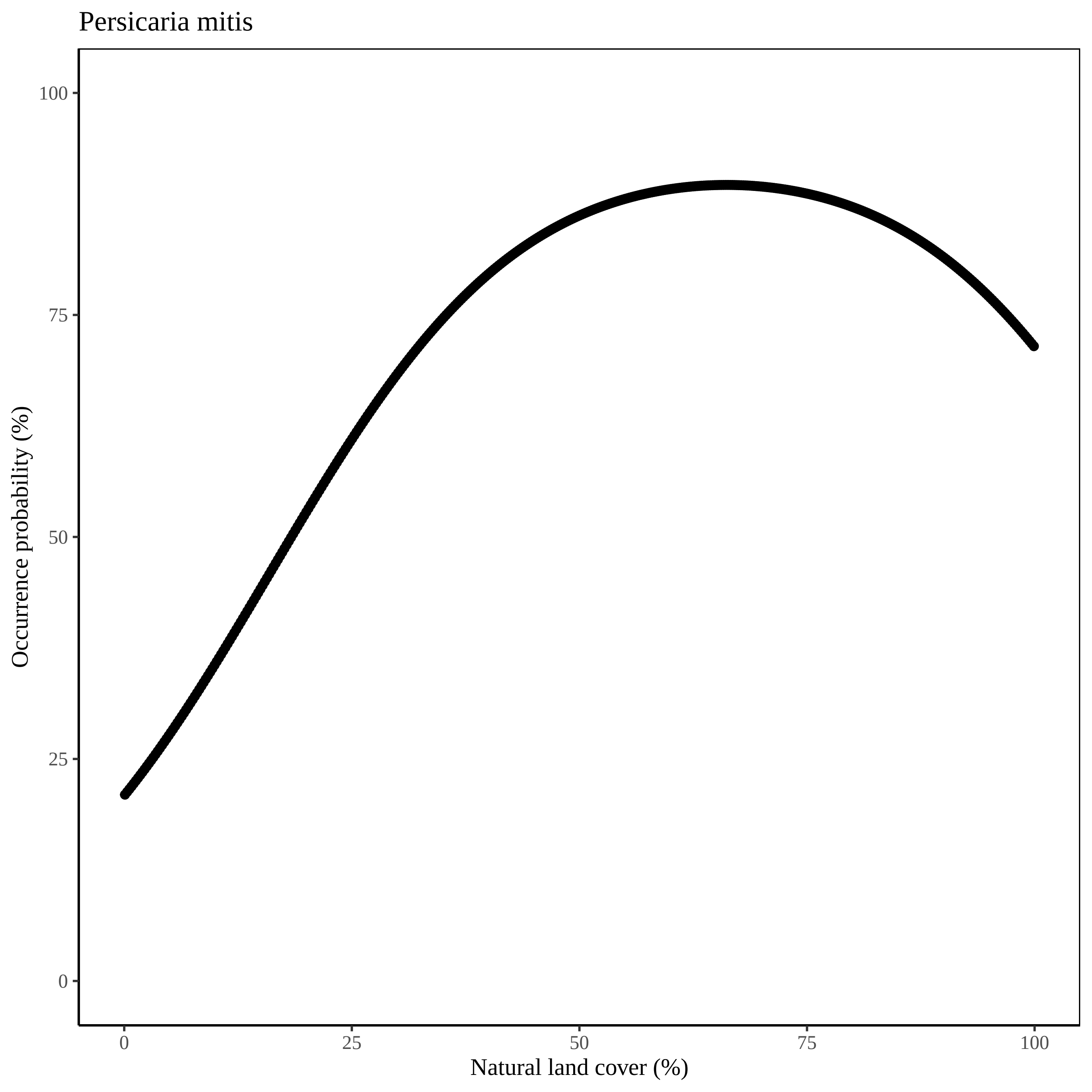
Persicaria mitis
100
75
50
25
0
0
25
50
75
100
Occurrence probability (%)
Natural land cover (%)

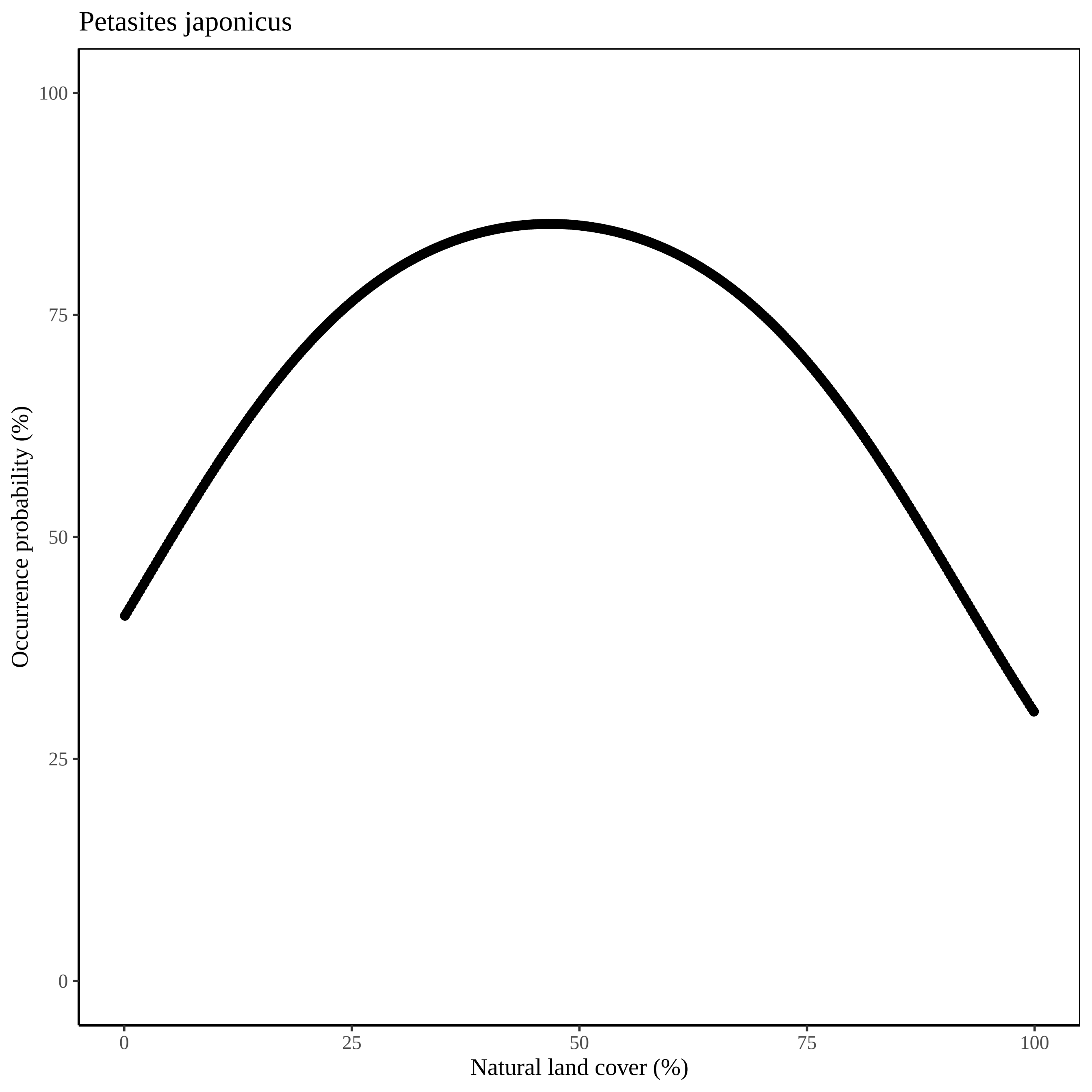
Petasites japonicus
Occurrence probability (%)
100
75
50
25
0
0
25
50
75
100
Natural land cover (%)

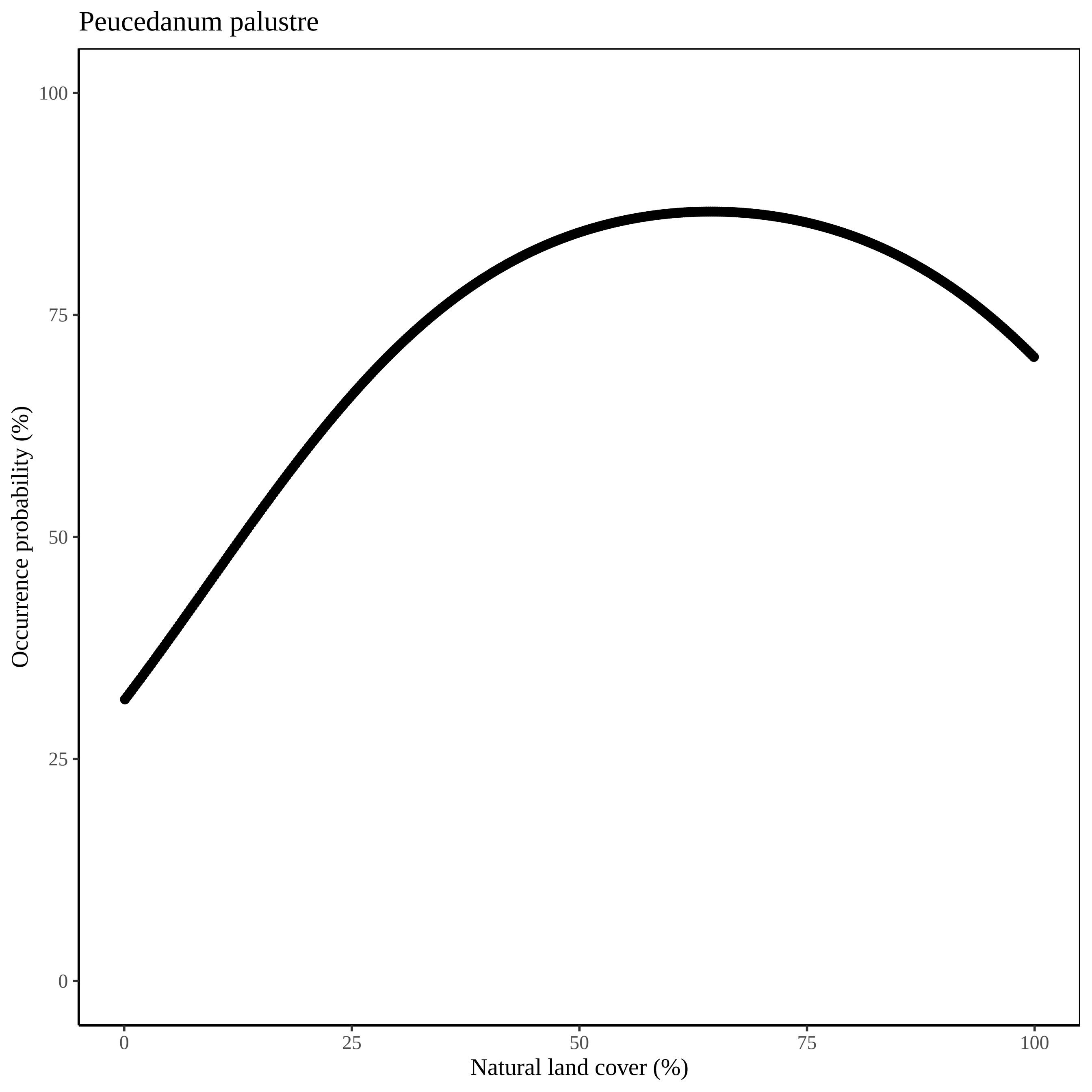
Peucedanum palustre
Occurrence probability (%)
100
75
50
25
0
0
25
50
75
100
Natural land cover (%)

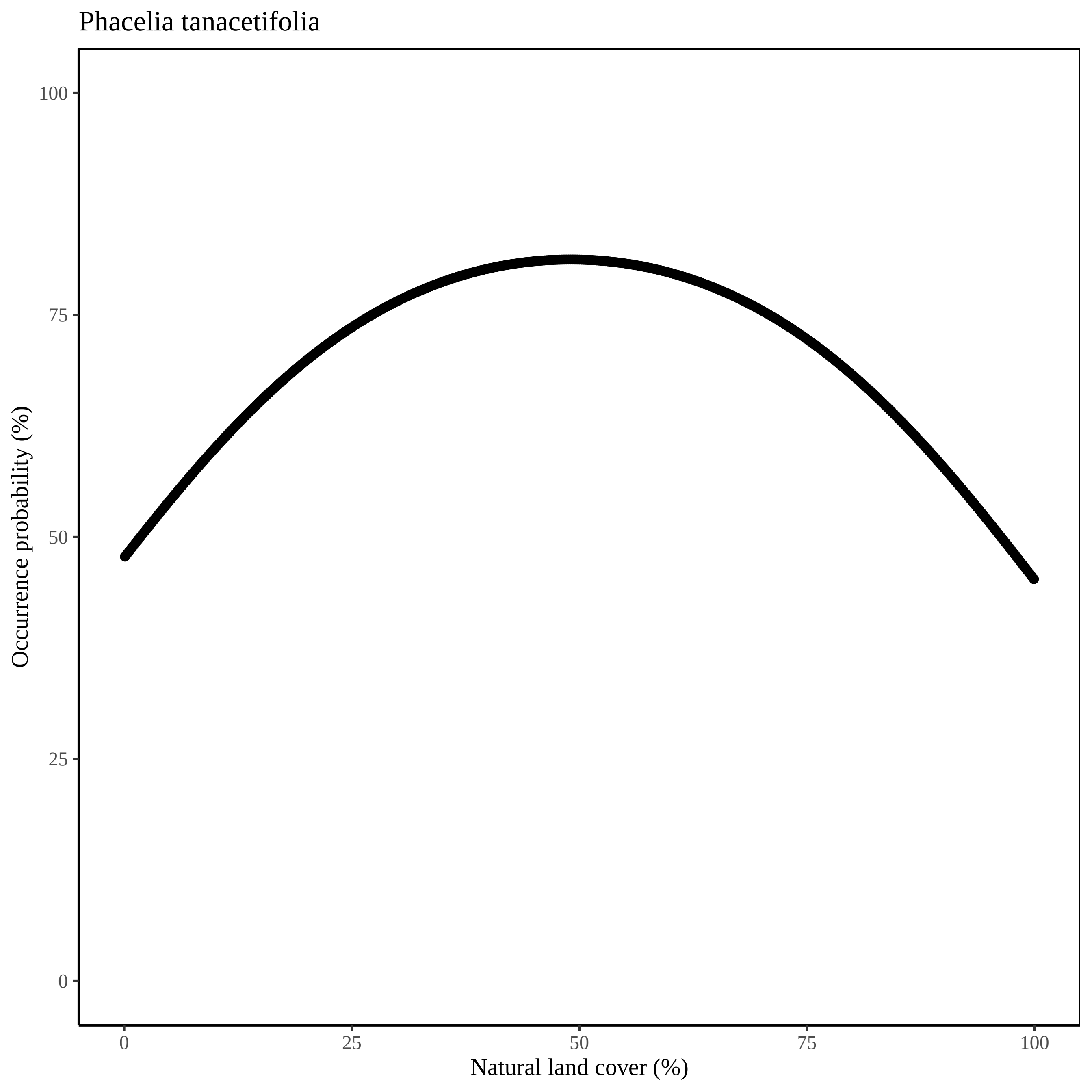
Phacelia tanacetifolia
100
75
50
25
0
Occurrence probability (%)
0
25
50
75
100
Natural land cover (%)

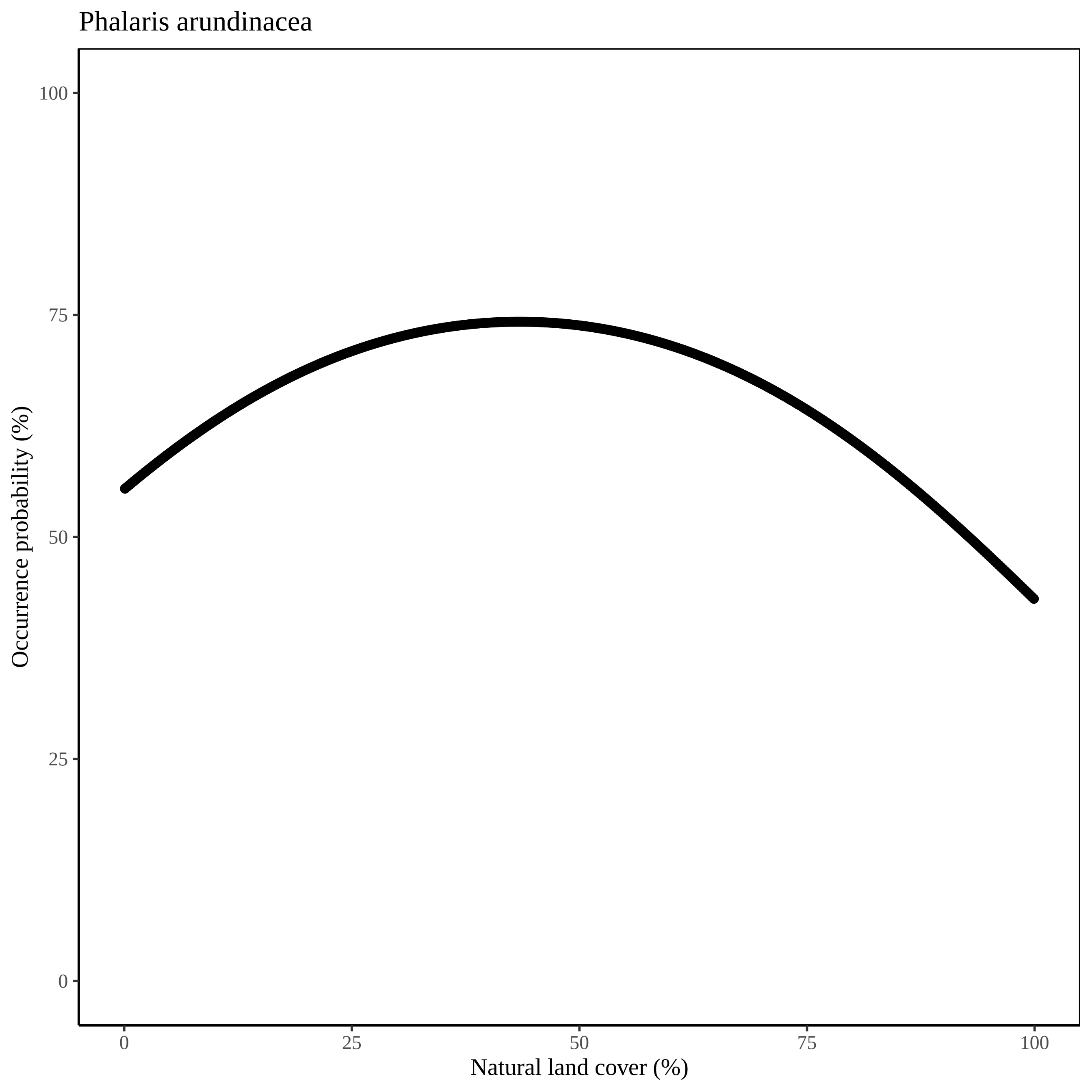

Phalaris arundinacea
100
75
50
25
0
Occurrence probability (%)
0
25
50
75
100
Natural land cover (%)

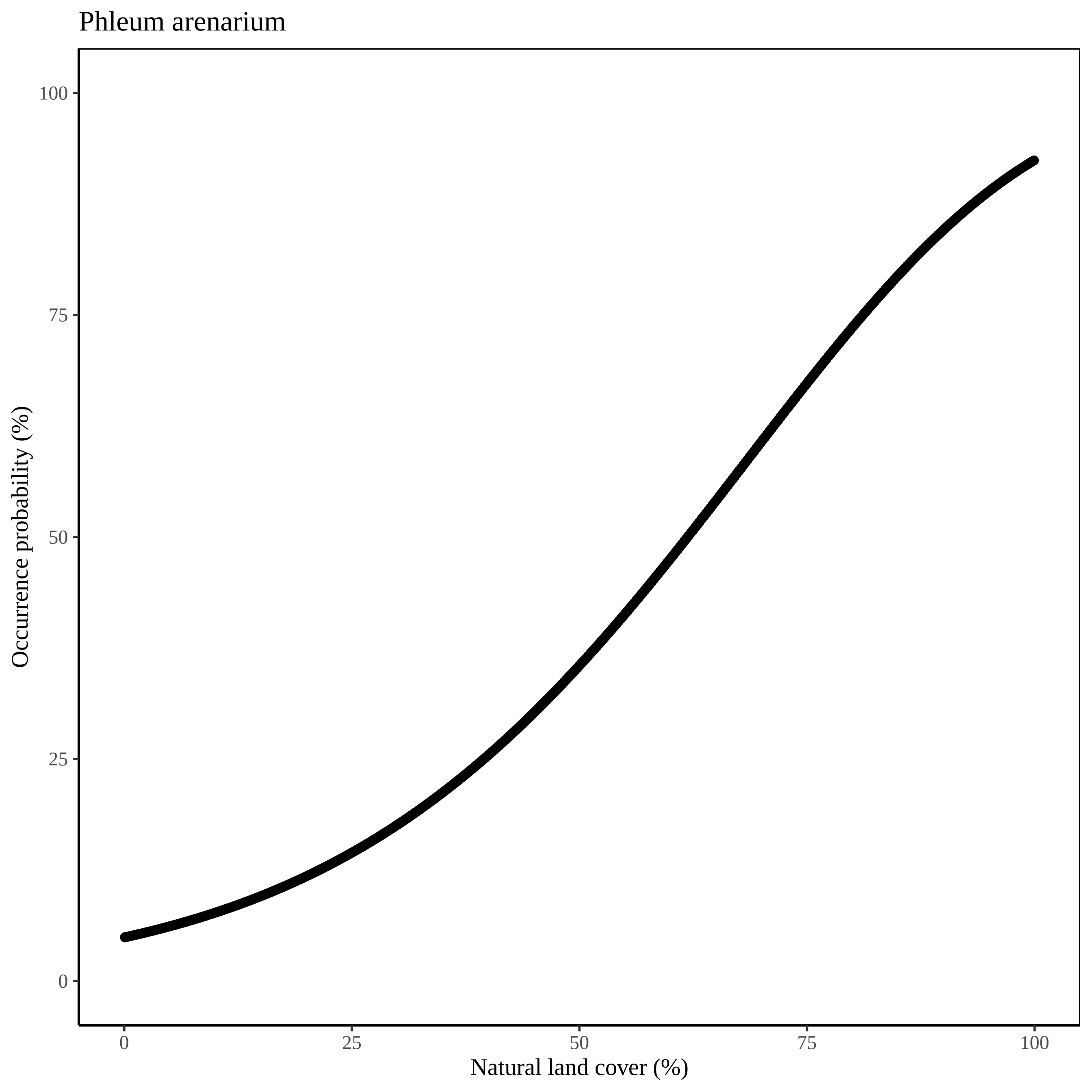
Phleum arenarium
Occurrence probability (%)
100
75
50
25
0
0
25
50
75
100
Natural land cover (%)

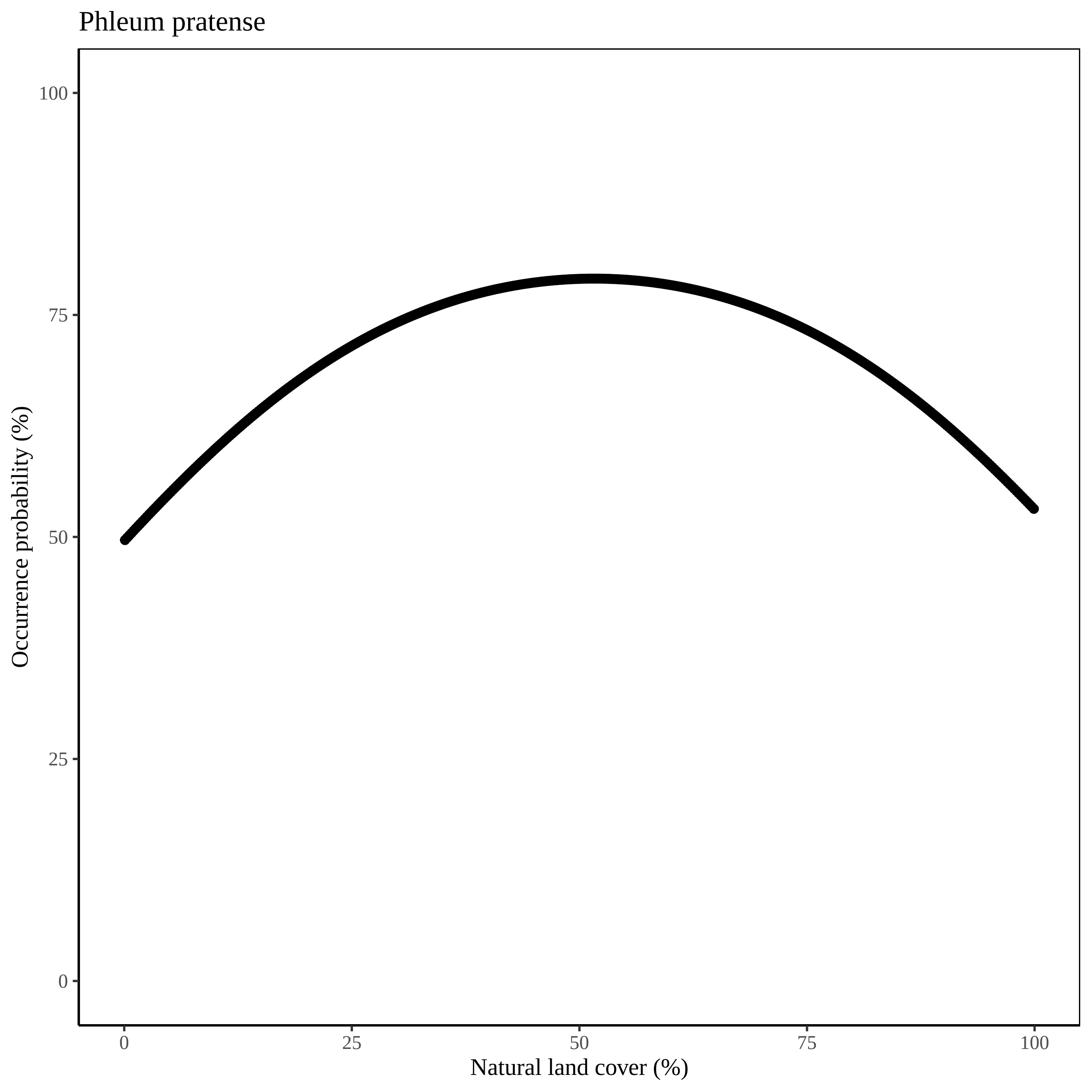
Phleum pratense
Occurrence probability (%)
100
75
50
25
0
0
25
50
75
100
Natural land cover (%)

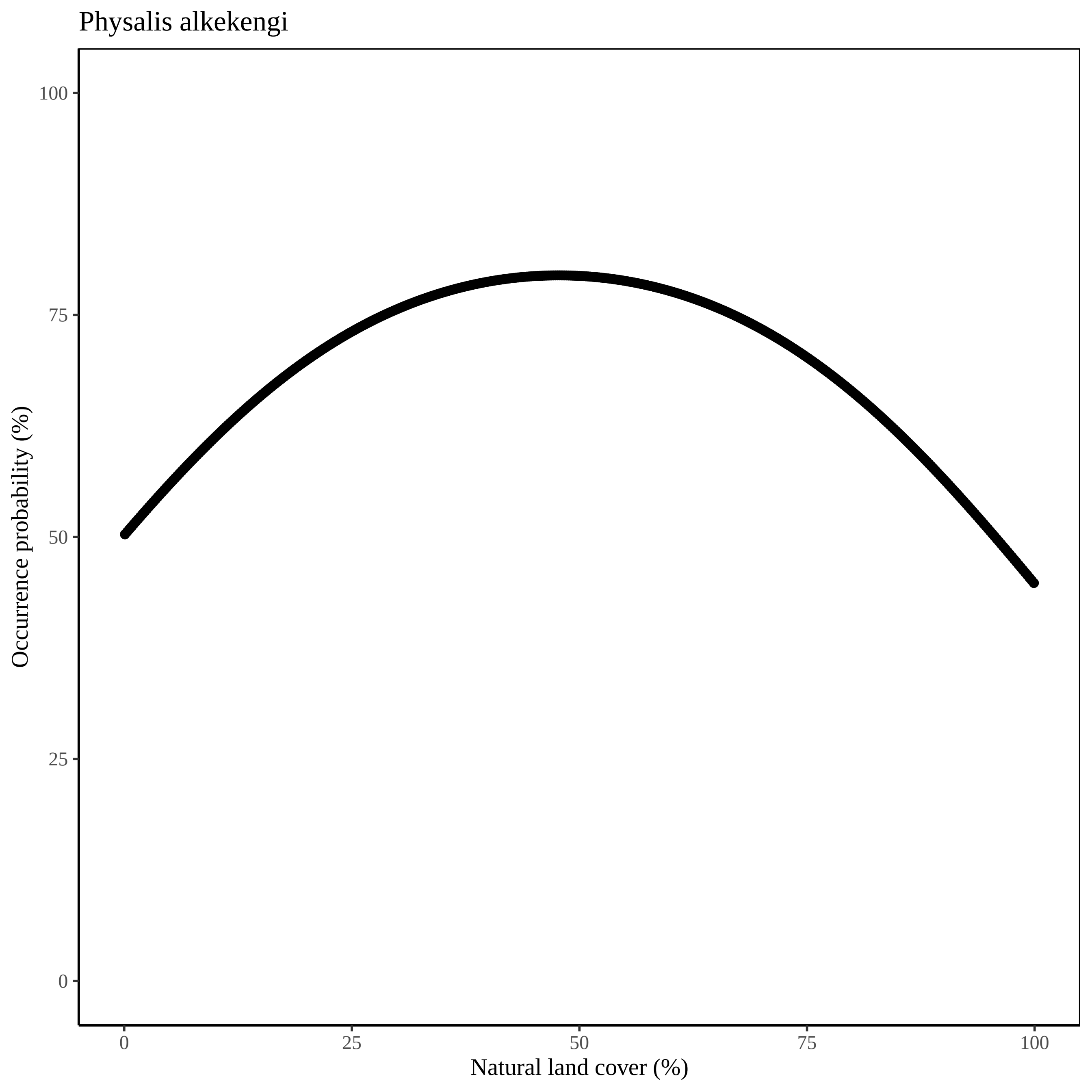
Physalis alkekengi
Occurrence probability (%)
100
75
50
25
0
0
25
50
75
100
Natural land cover (%)

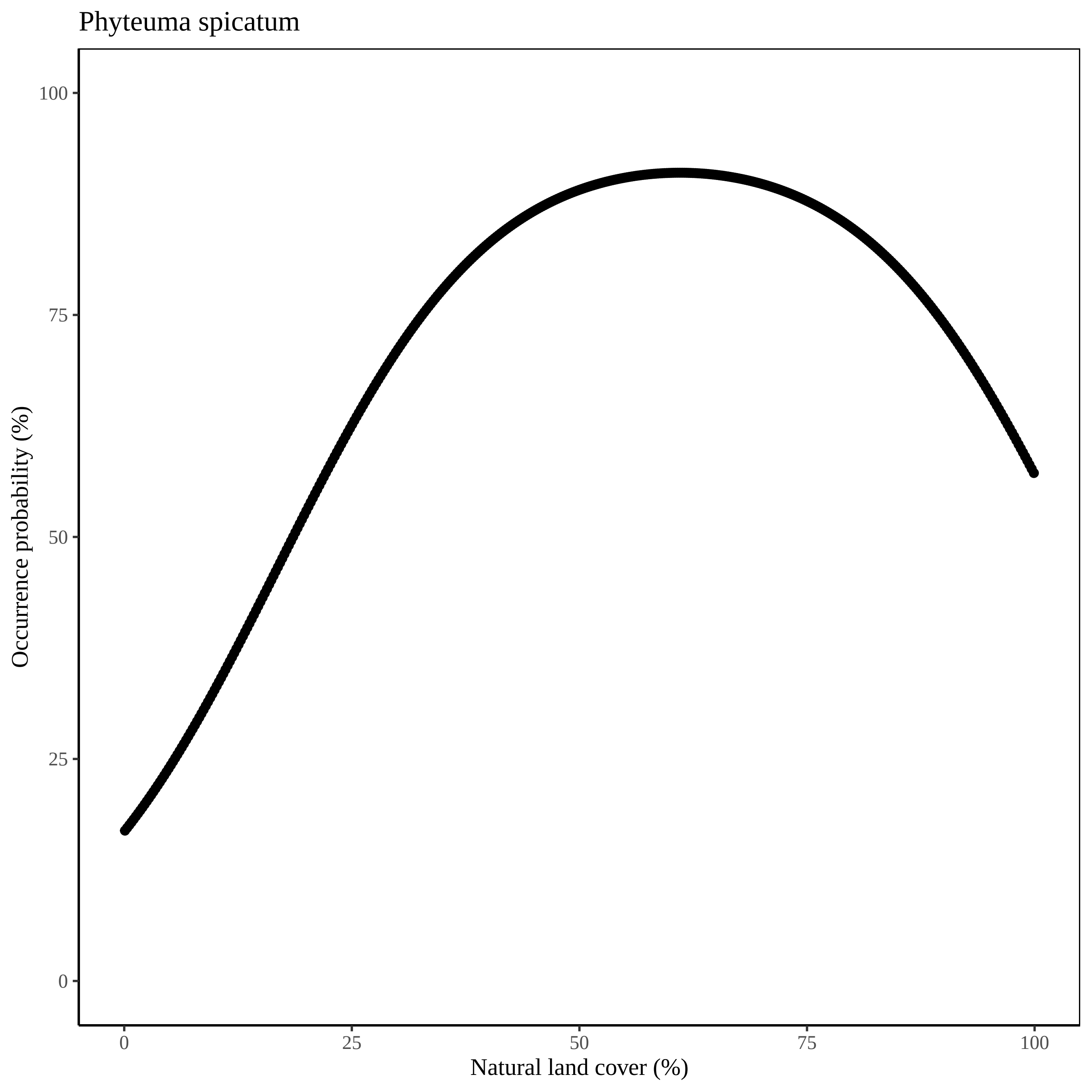
Phyteuma spicatum
Occurrence probability (%)
Natural land cover (%)
0
25
50
75
100

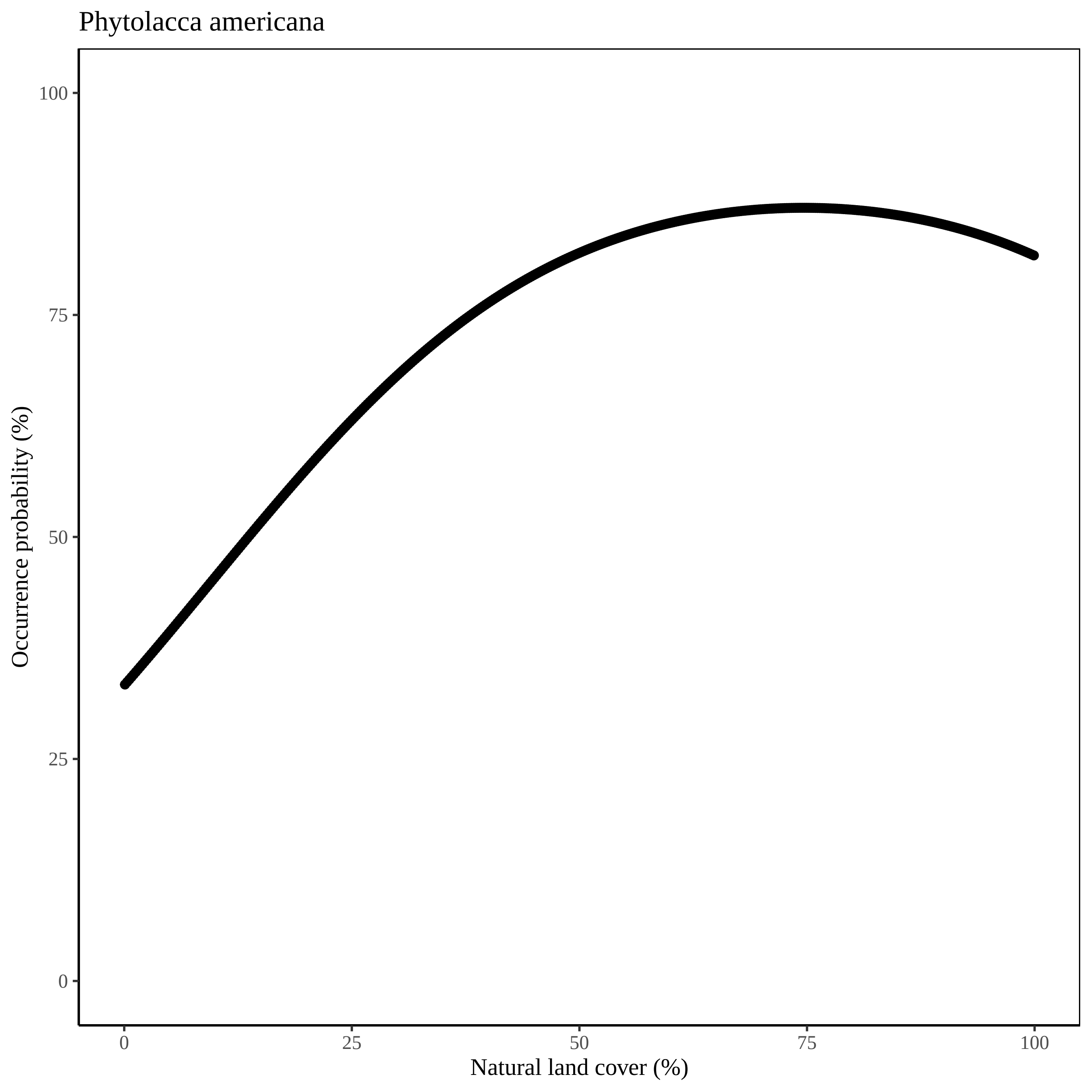

Phytolacca americana
Occurrence probability (%)
100
75
50
25
0
0
25
50
75
100
Natural land cover (%)

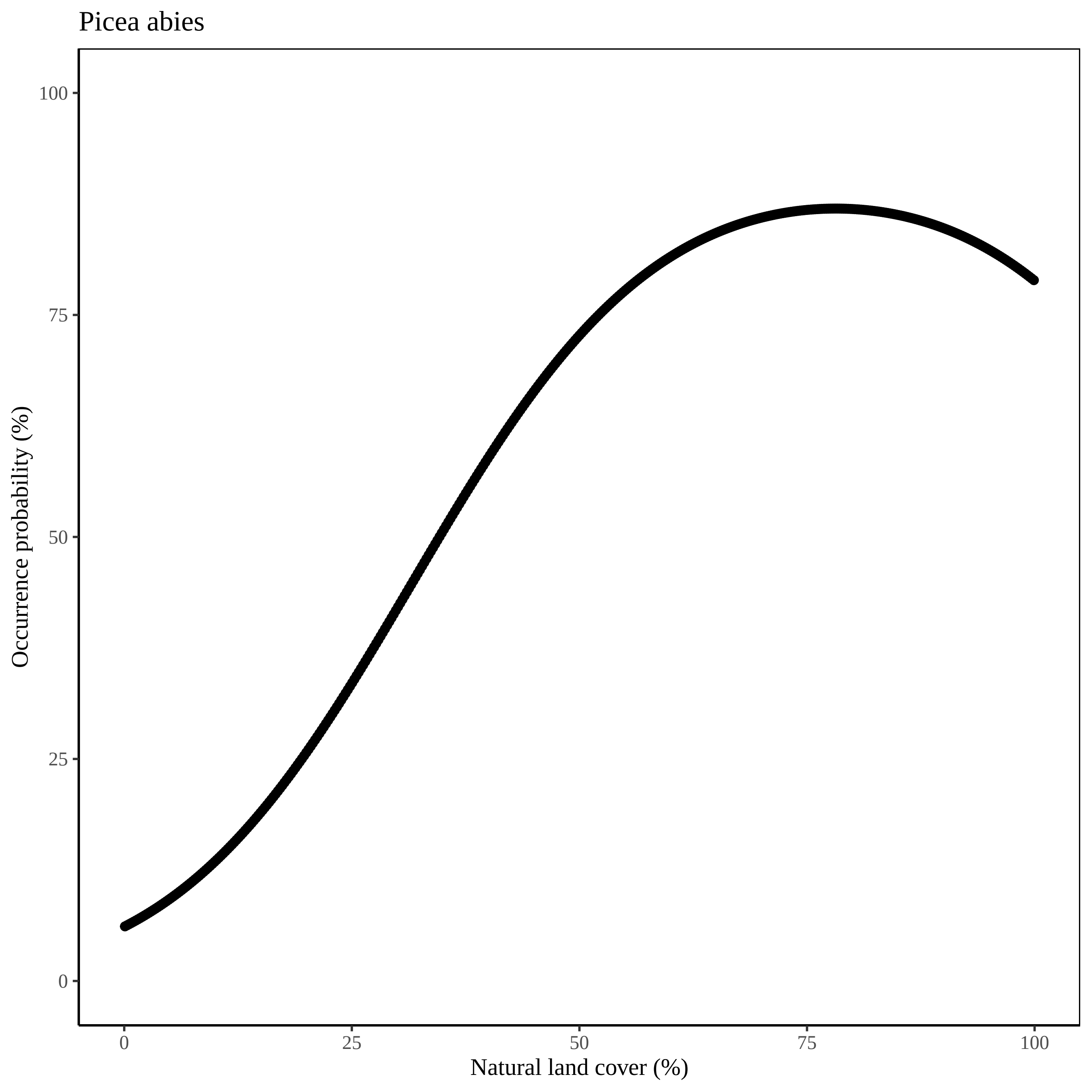
Picea abies
Occurrence probability (%)
100
75
50
25
0
0
25
50
75
100
Natural land cover (%)

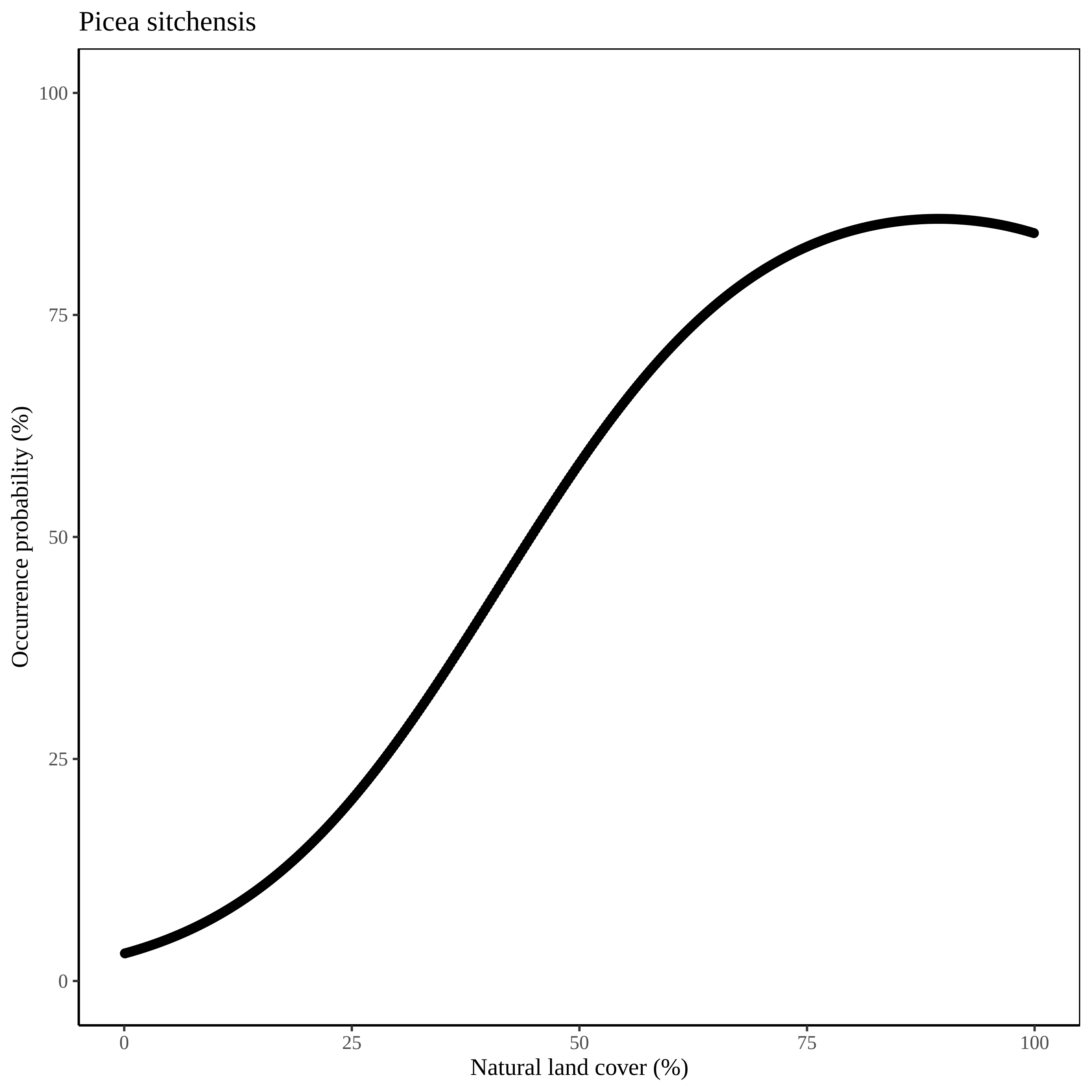
Picea sitchensis
Occurrence probability (%)
100
75
50
25
0
0
25
50
75
100
Natural land cover (%)

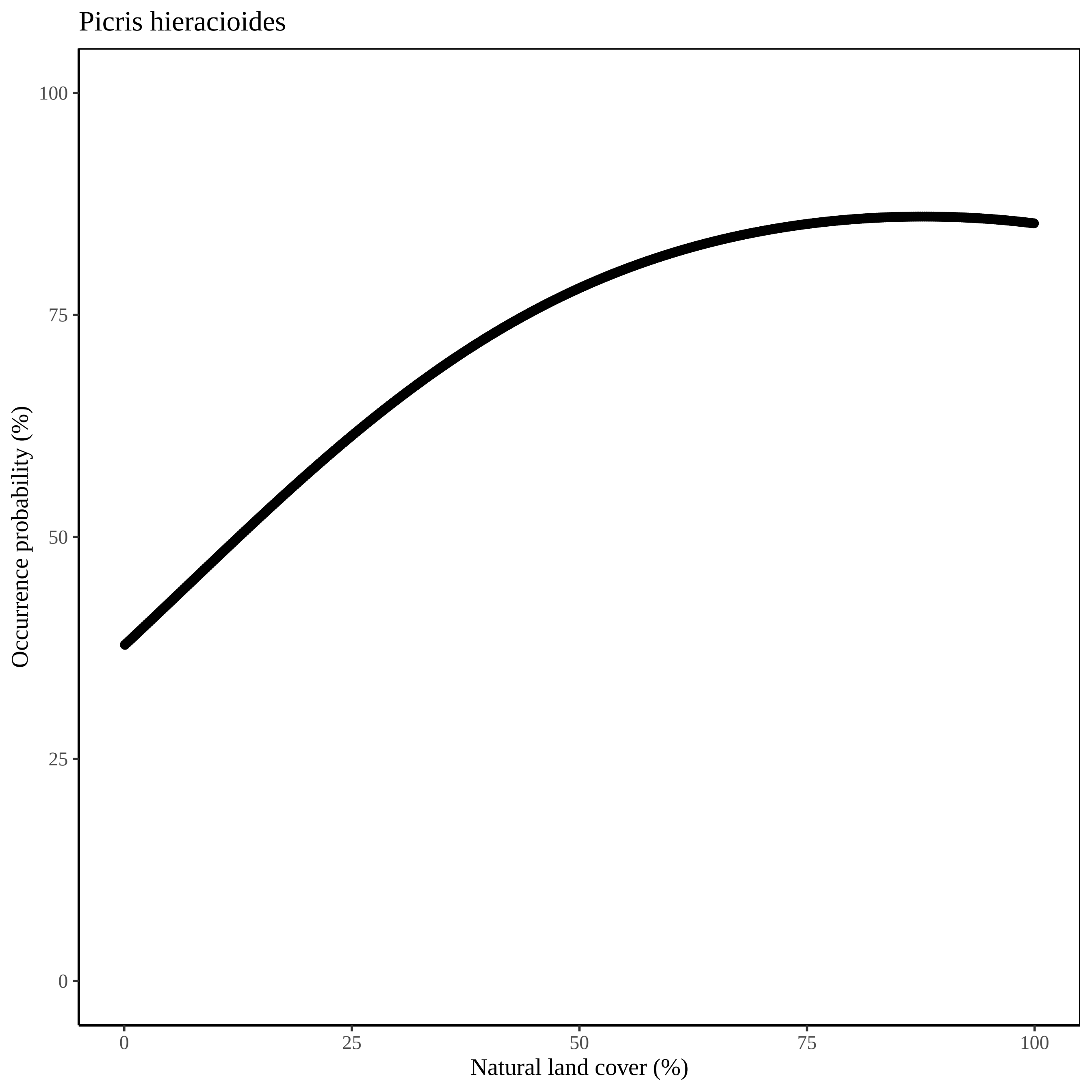
Picris hieracioides
Occurrence probability (%)
100
75
50
25
0
0
25
50
75
100
Natural land cover (%)

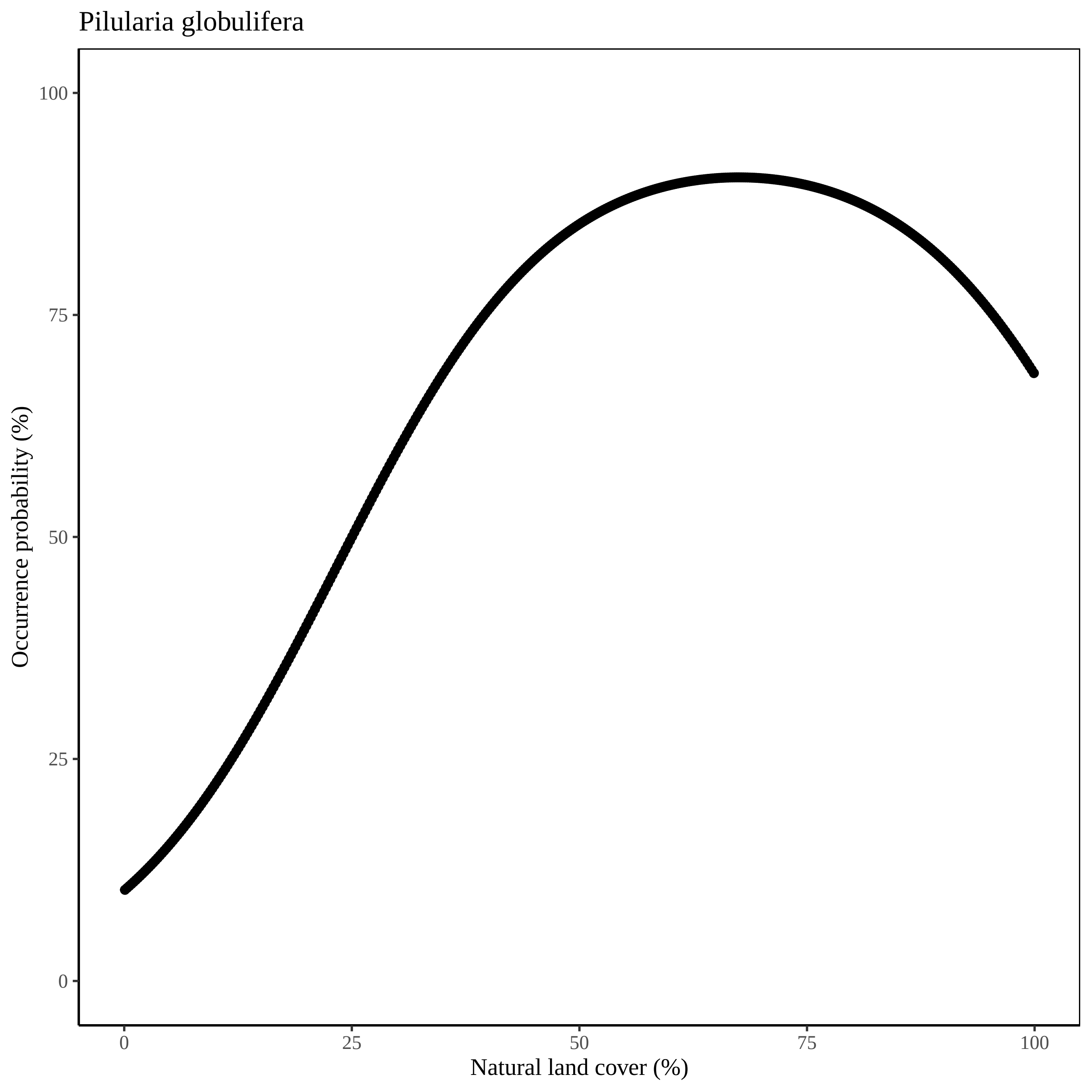
Pilularia globulifera
Occurrence probability (%)
0
25
50
75
100
Natural land cover (%)
0
25
50
75
100

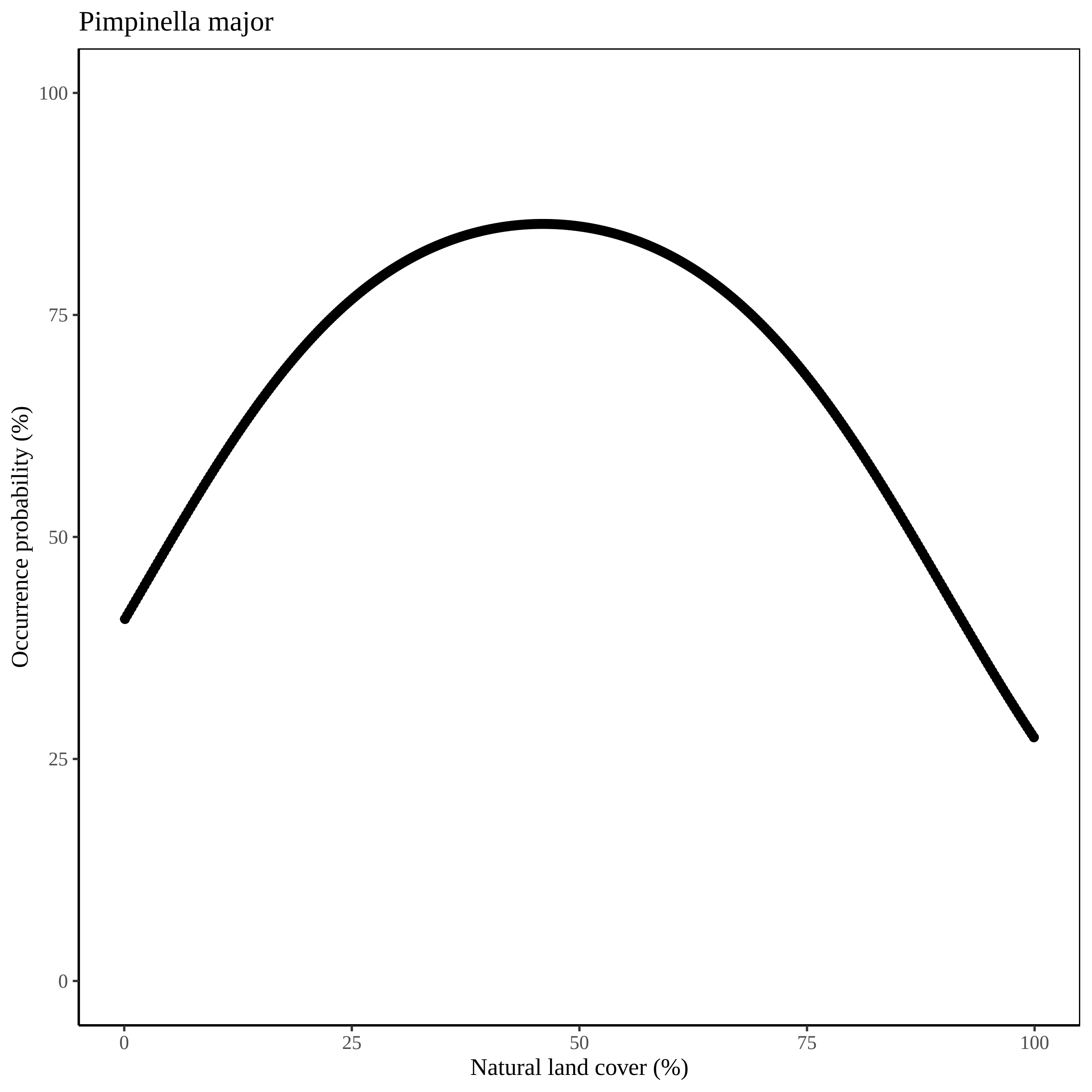
Pimpinella major
100
75
50
25
0
Occurrence probability (%)
0
25
50
75
100
Natural land cover (%)

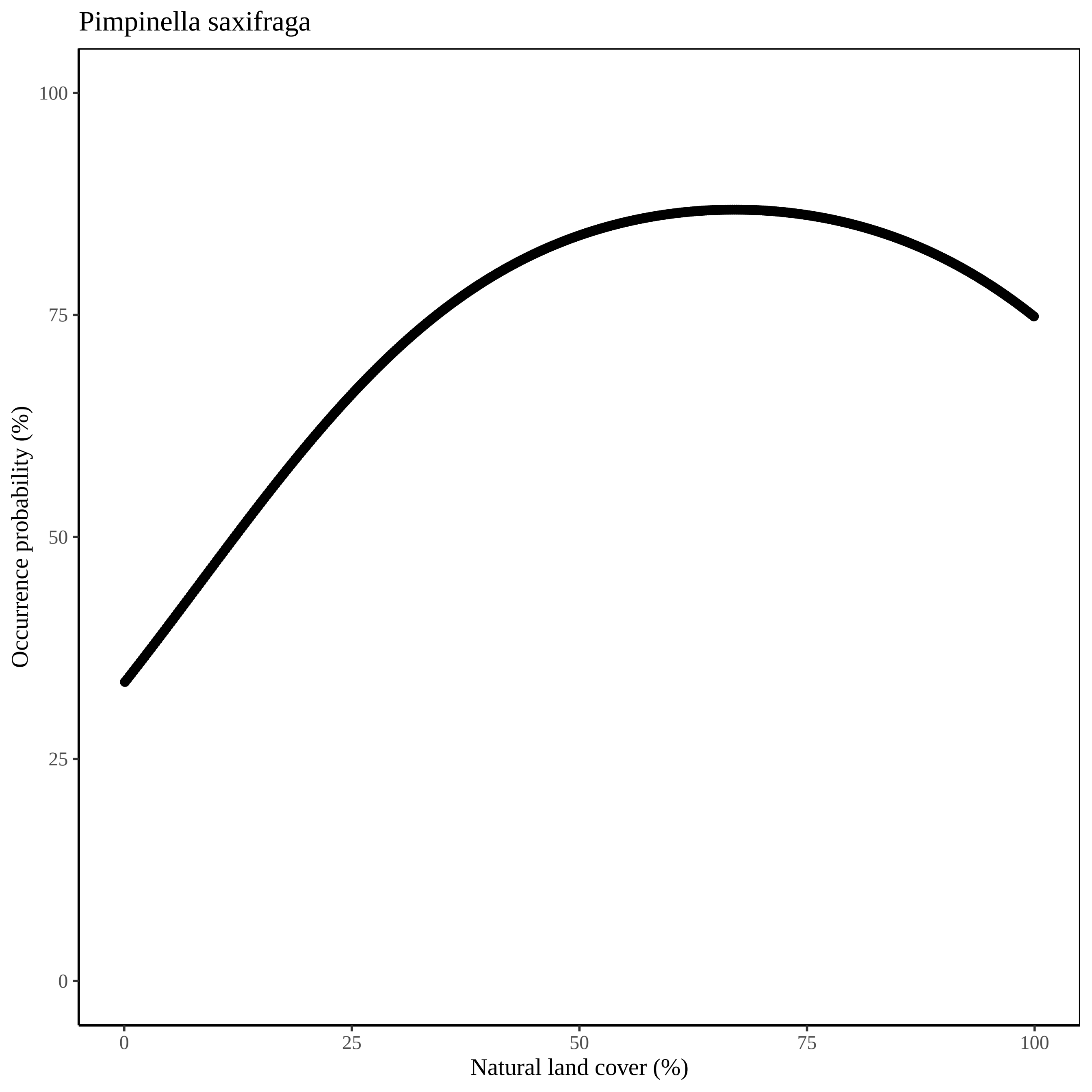
Pimpinella saxifraga
Occurrence probability (%)
100
75
50
25
0
0
25
50
75
100
Natural land cover (%)

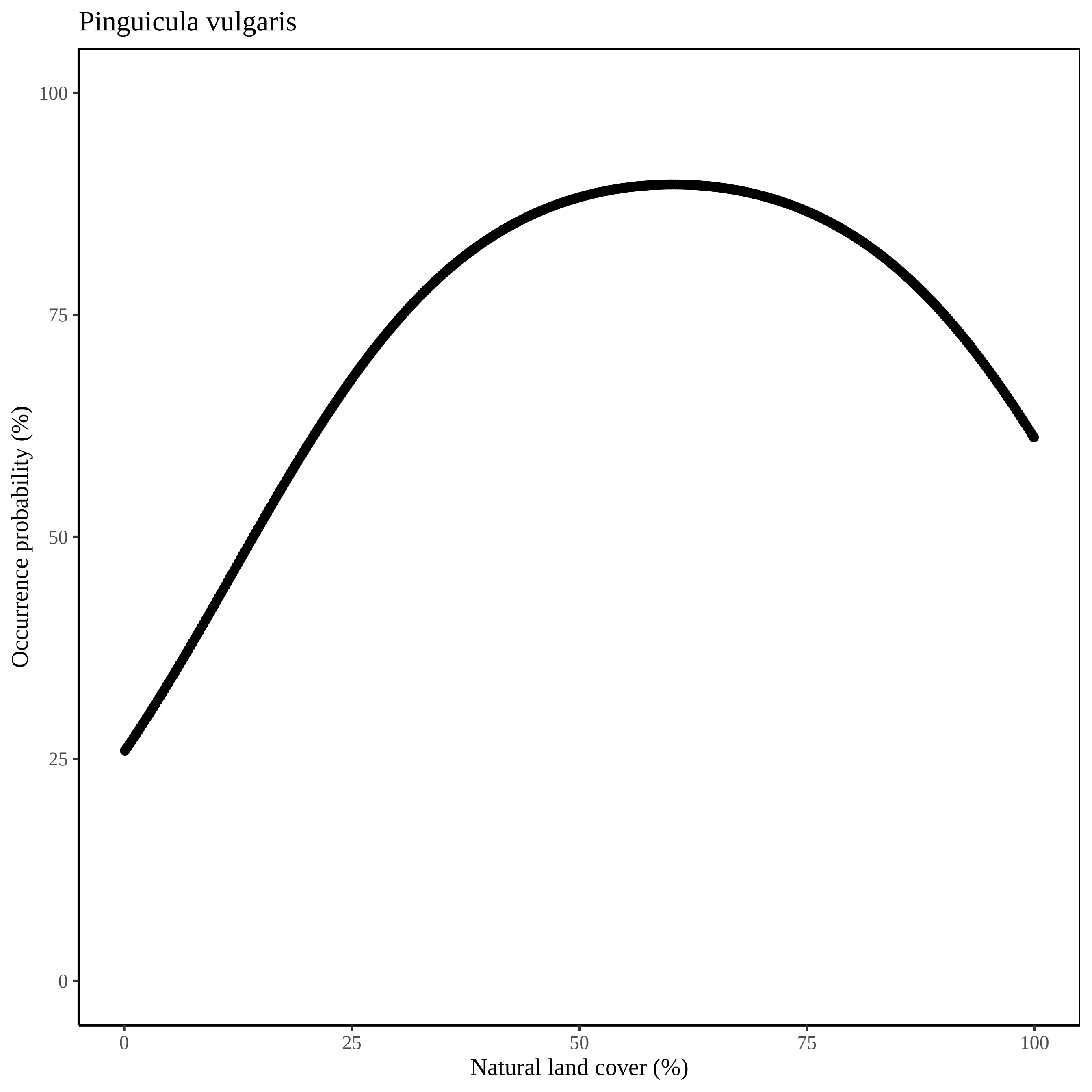
Pinguicula vulgaris
Occurrence probability (%)
100
75
50
25
0
0
25
50
75
100
Natural land cover (%)

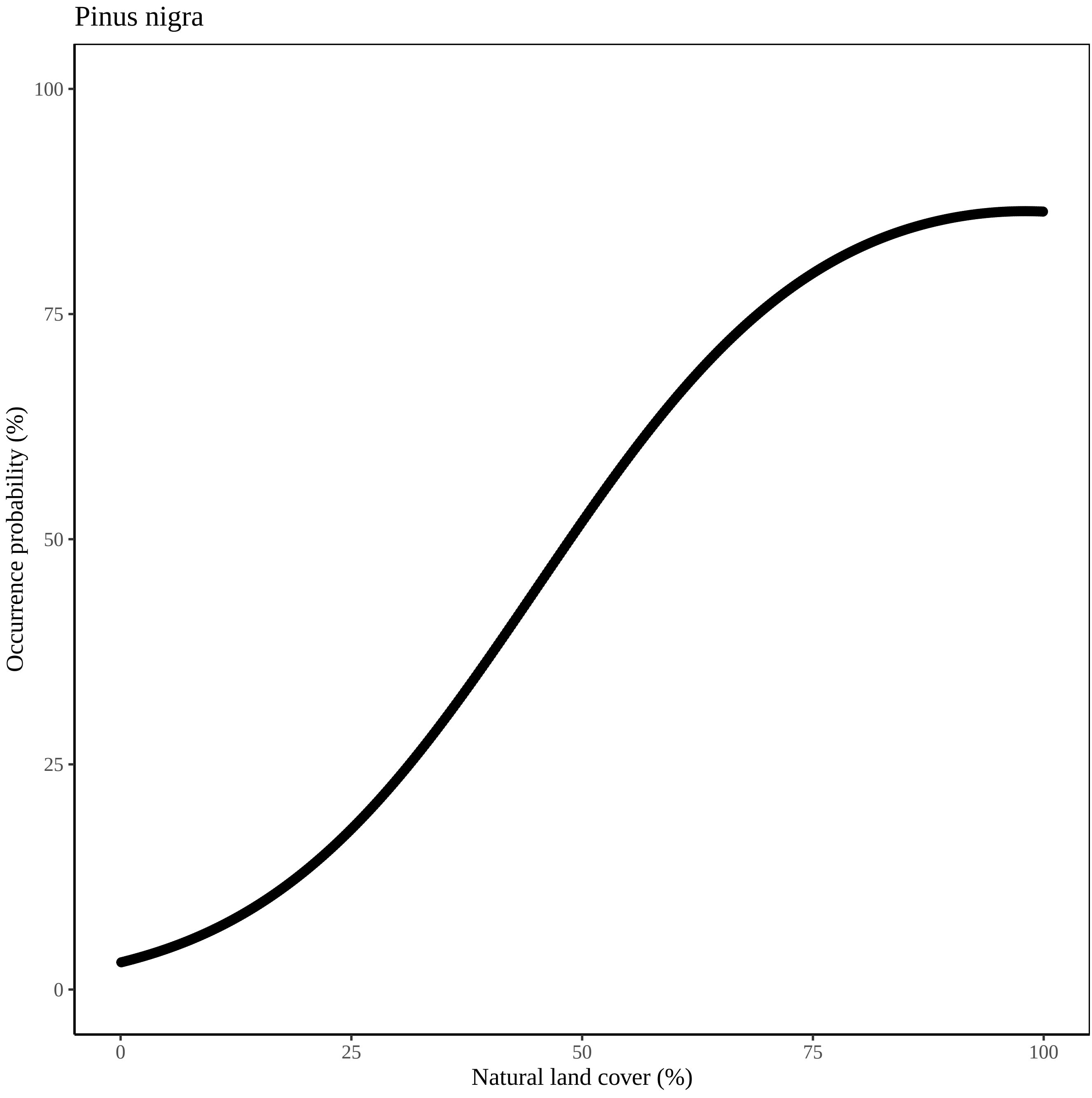

Pinus nigra
100
75
50
25
0
Occurrence probability (%)
0
25
50
75
100
Natural land cover (%)

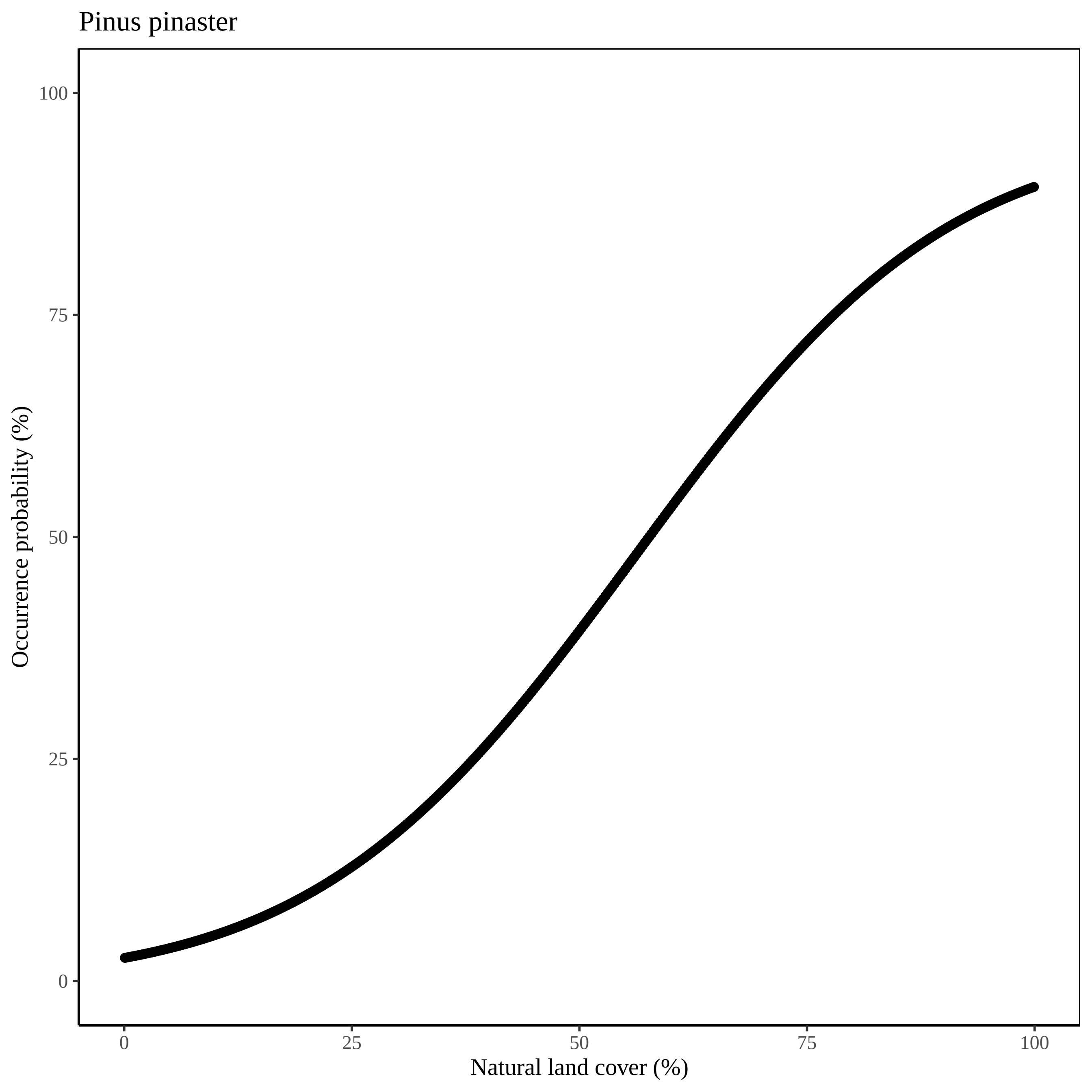
Pinus pinaster
Occurrence probability (%)
100
75
50
25
0
0
25
50
75
100
Natural land cover (%)

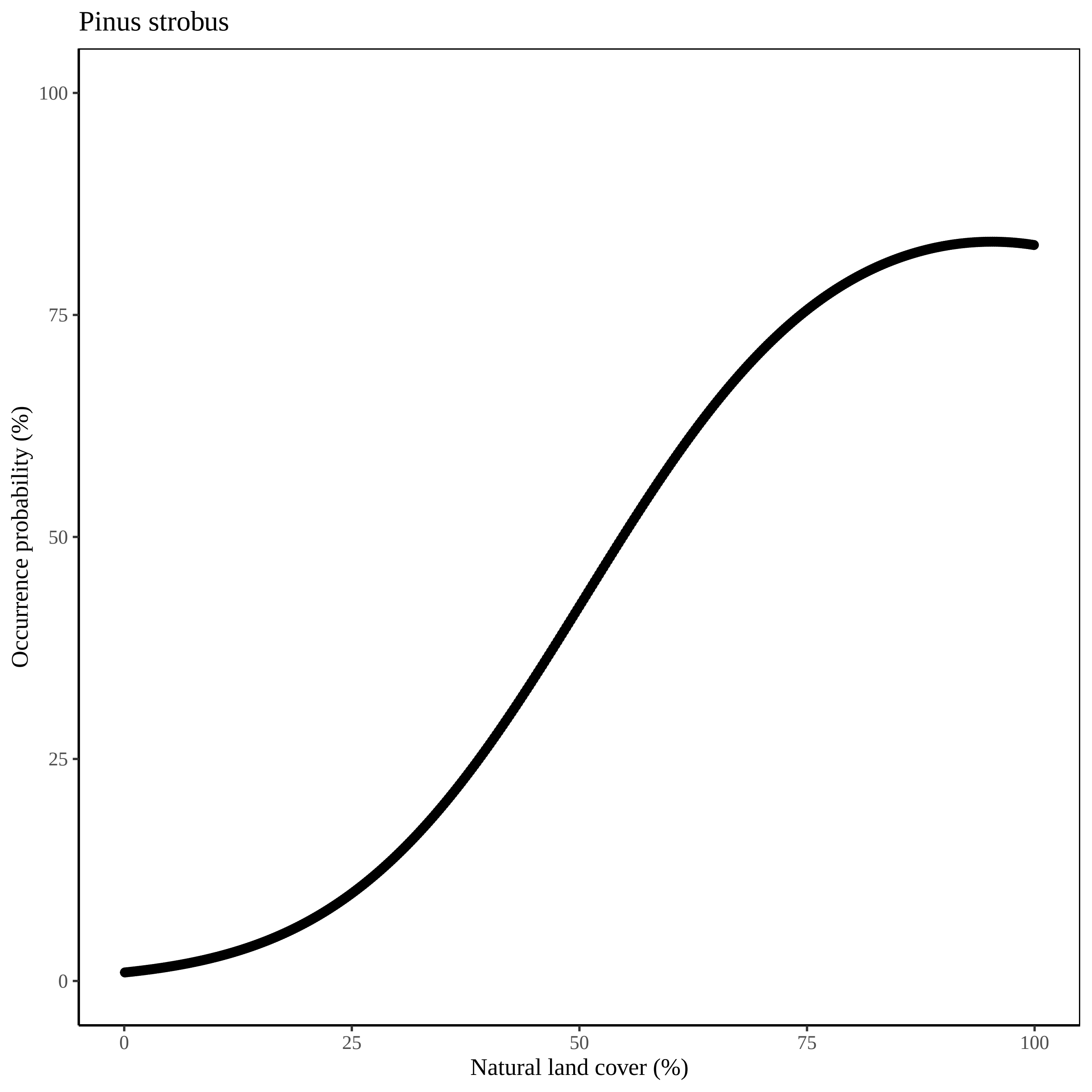
Pinus strobus
Occurrence probability (%)
100
75
50
25
0
0
25
50
75
100
Natural land cover (%)

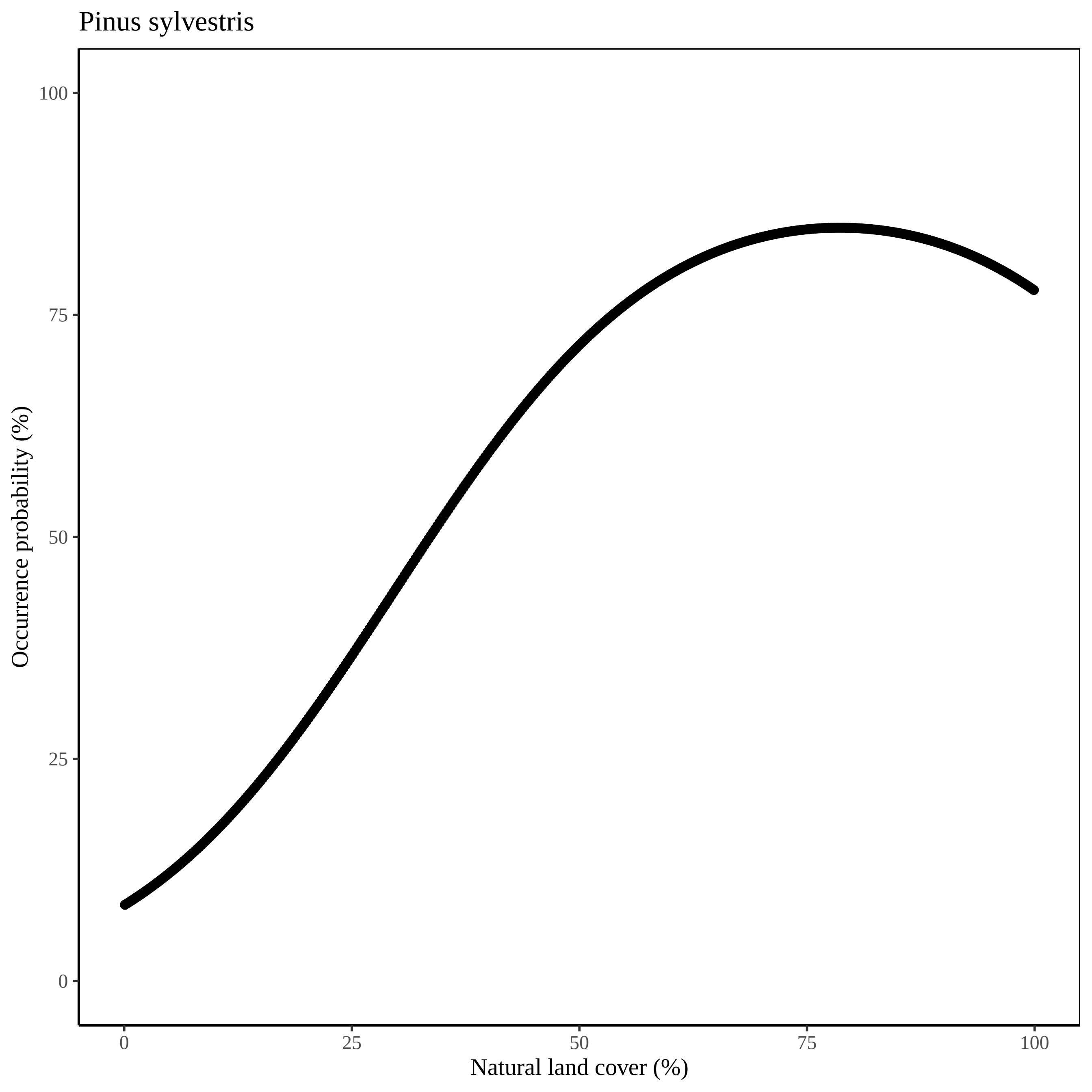

Pinus sylvestris
100
75
50
25
0
Occurrence probability (%)
0
25
50
75
100
Natural land cover (%)

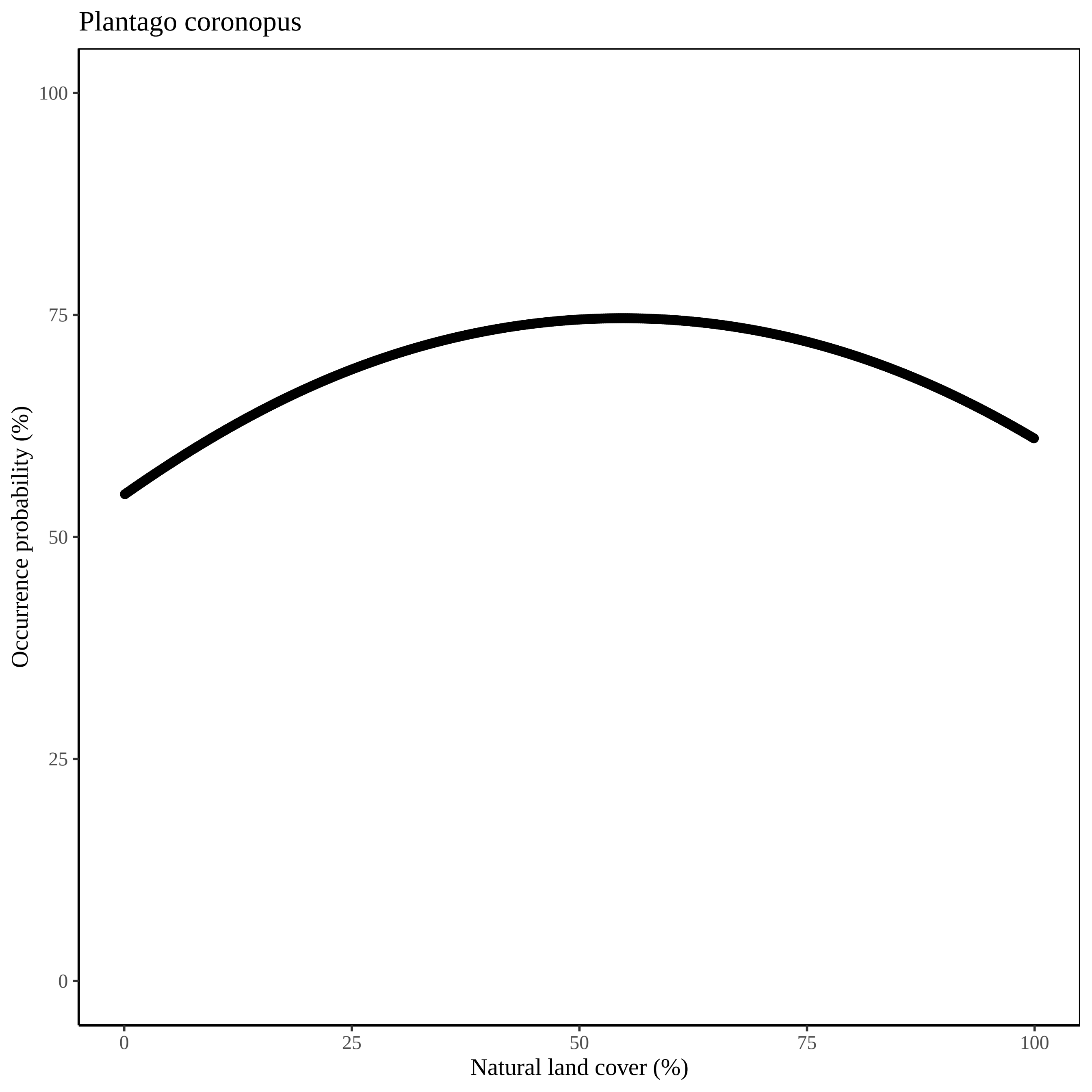

Plantago coronopus
Occurrence probability (%)
100
75
50
25
0
0
25
50
75
100
Natural land cover (%)

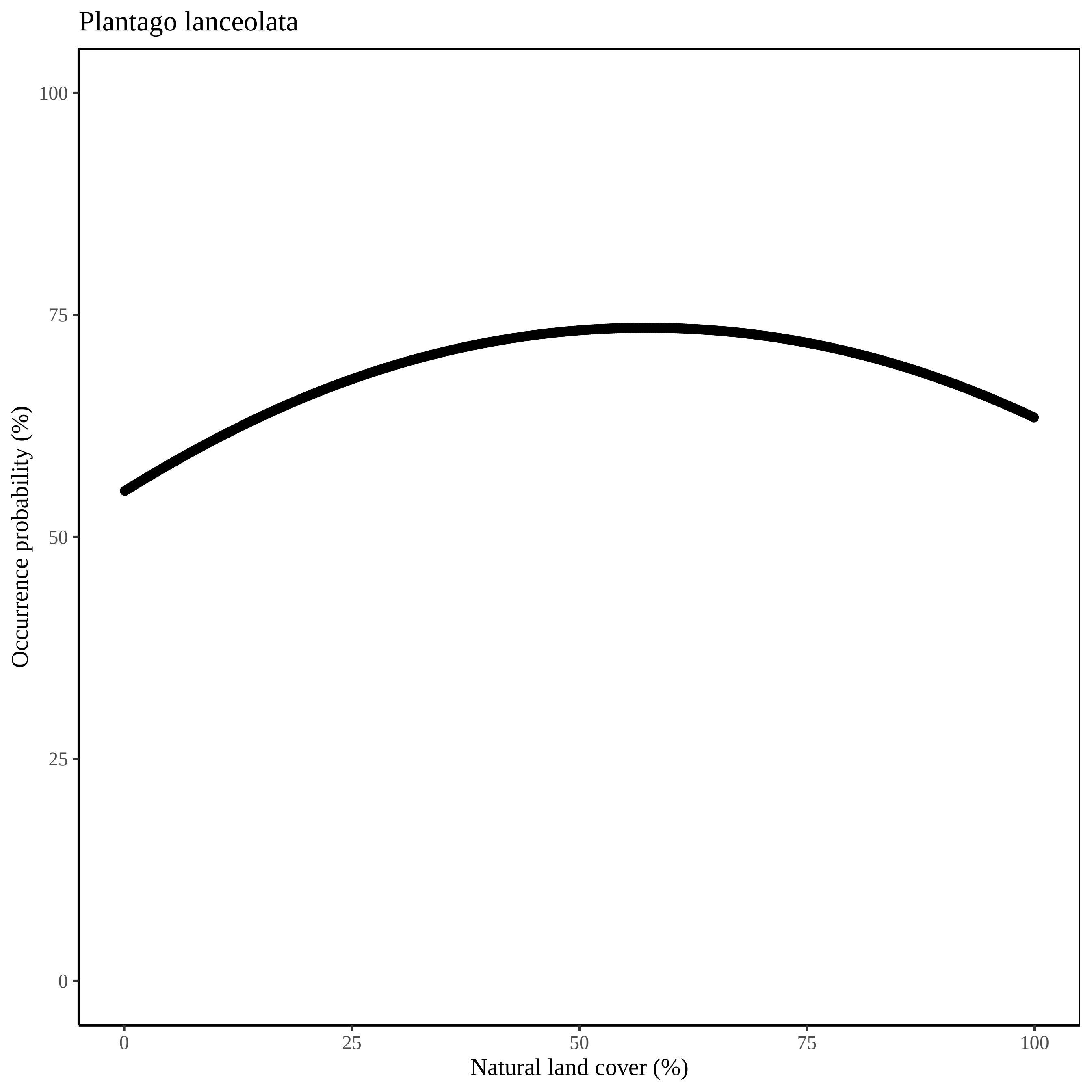
Plantago lanceolata
Occurrence probability (%)
100
75
50
25
0
0
25
50
75
100
Natural land cover (%)

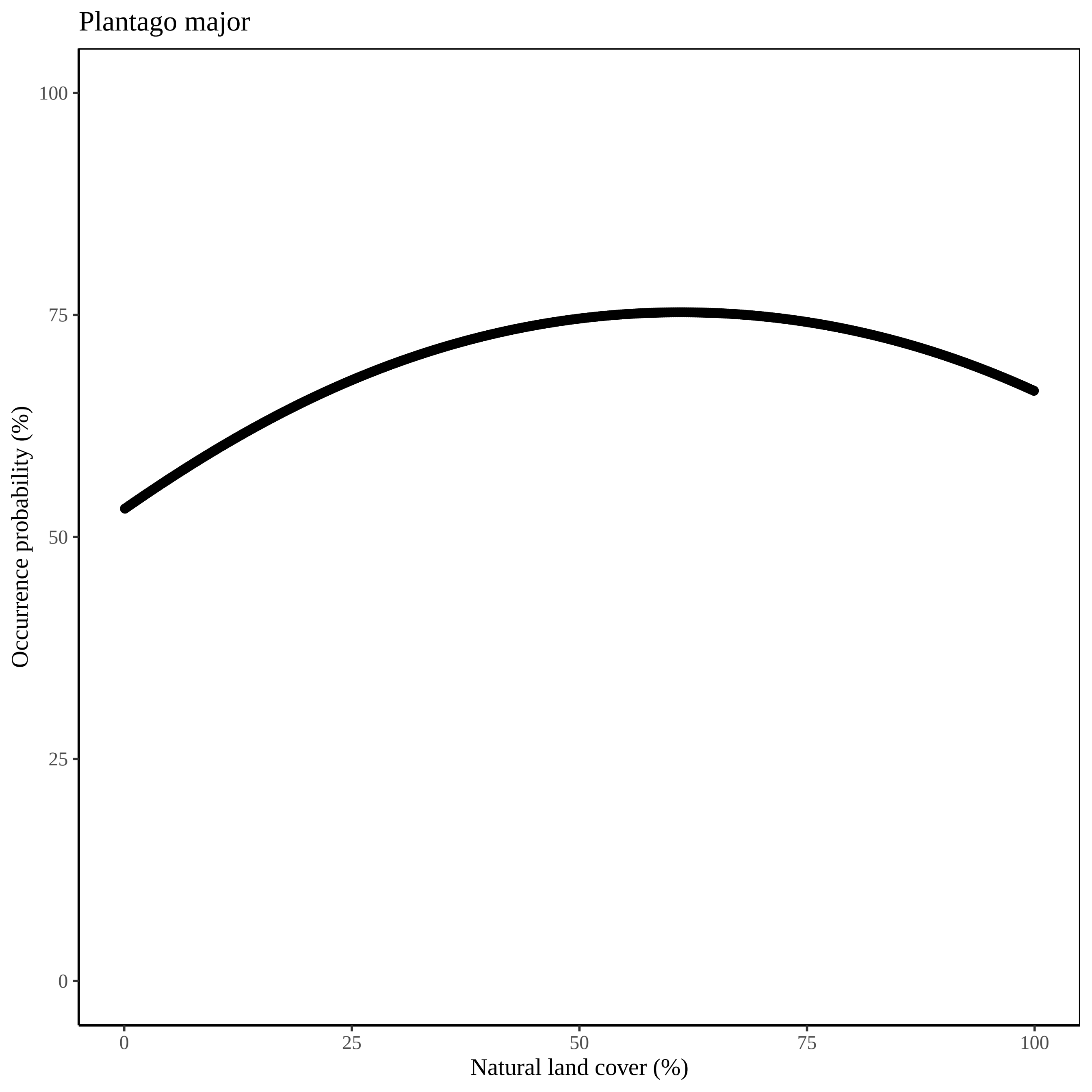
Plantago major
Occurrence probability (%)
100
75
50
25
0
0
25
50
75
100
Natural land cover (%)

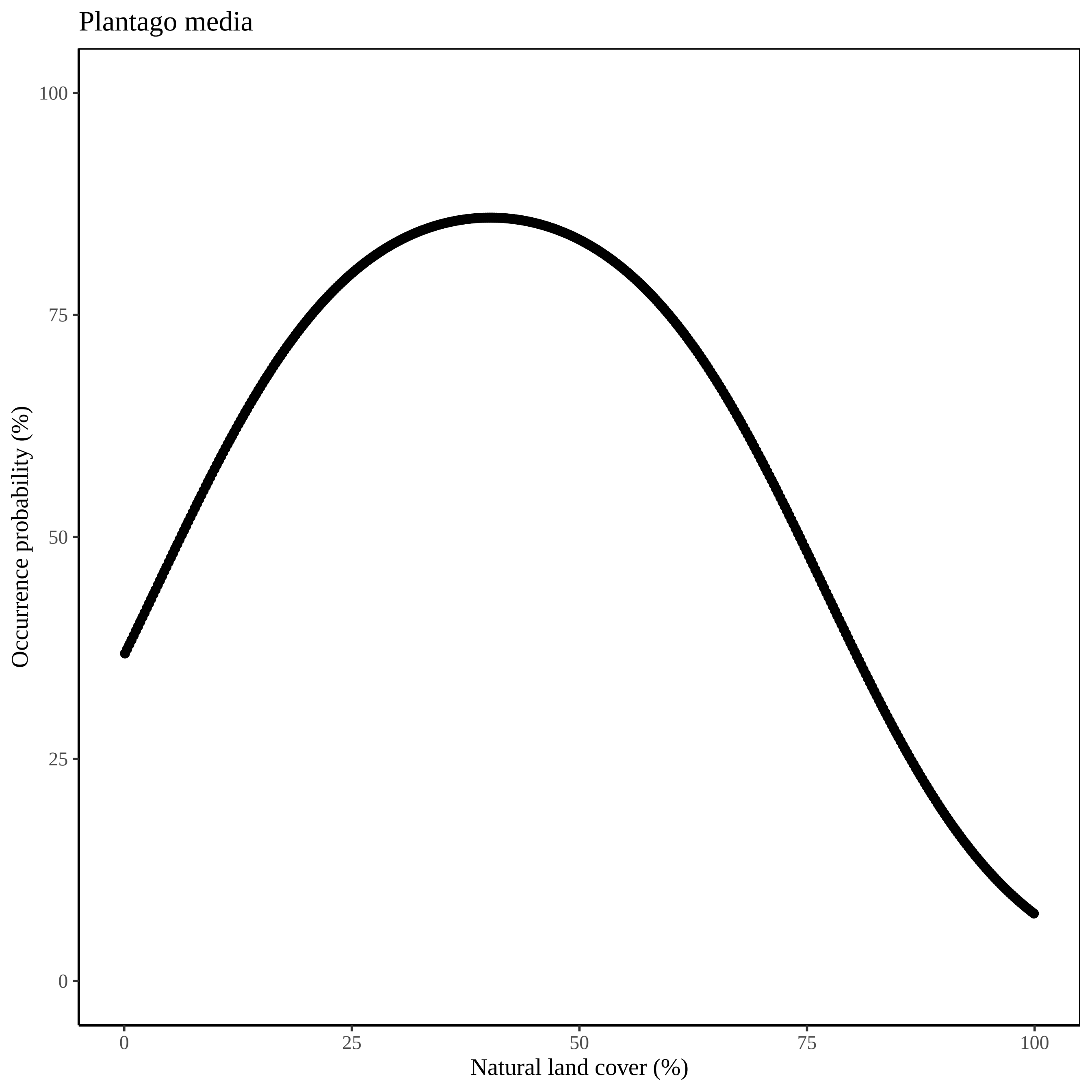
Plantago media
Occurrence probability (%)
100
75
50
25
0
0
25
50
75
100
Natural land cover (%)

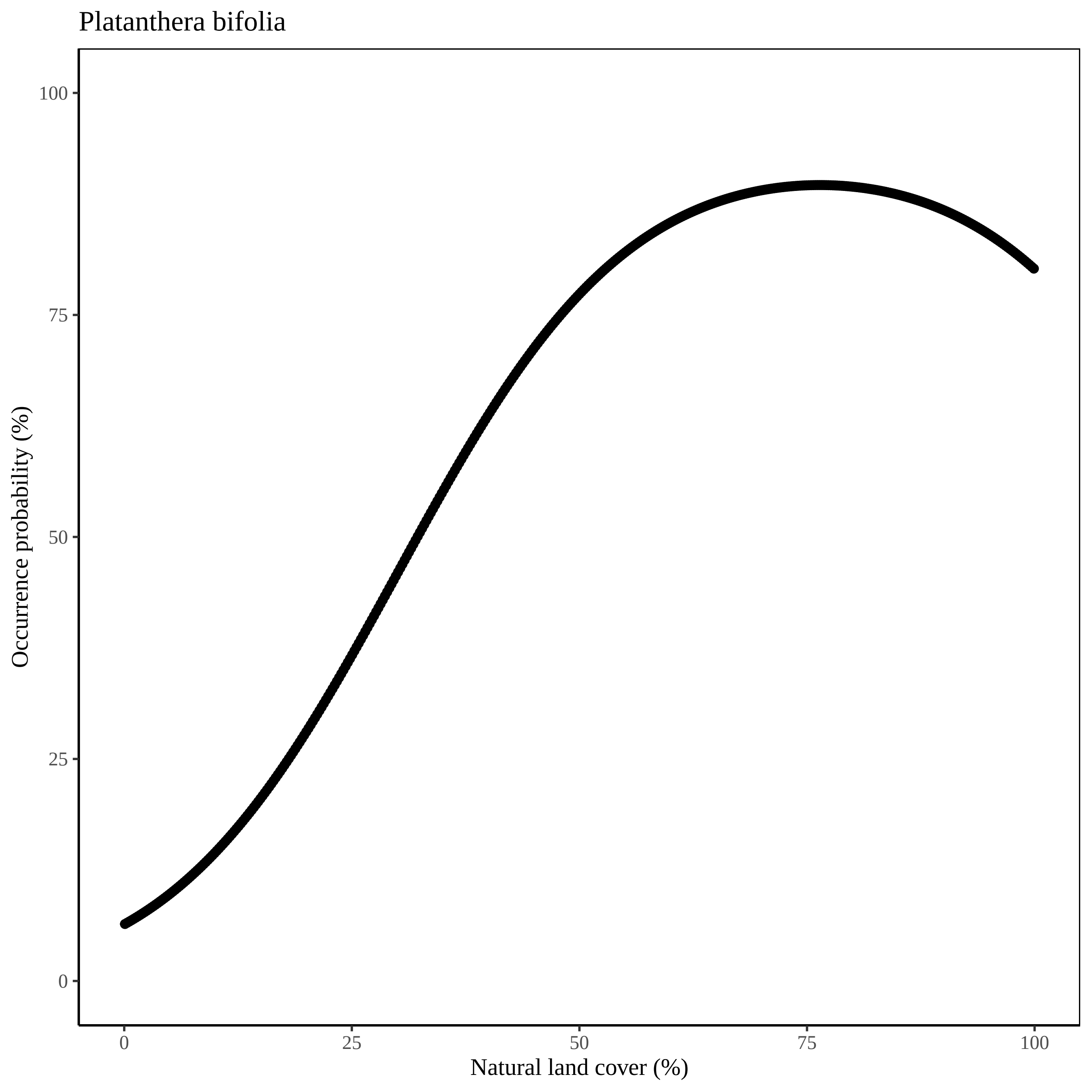
Platanthera bifolia
Occurrence probability (%)
100
75
50
25
0
0
25
50
75
100
Natural land cover (%)

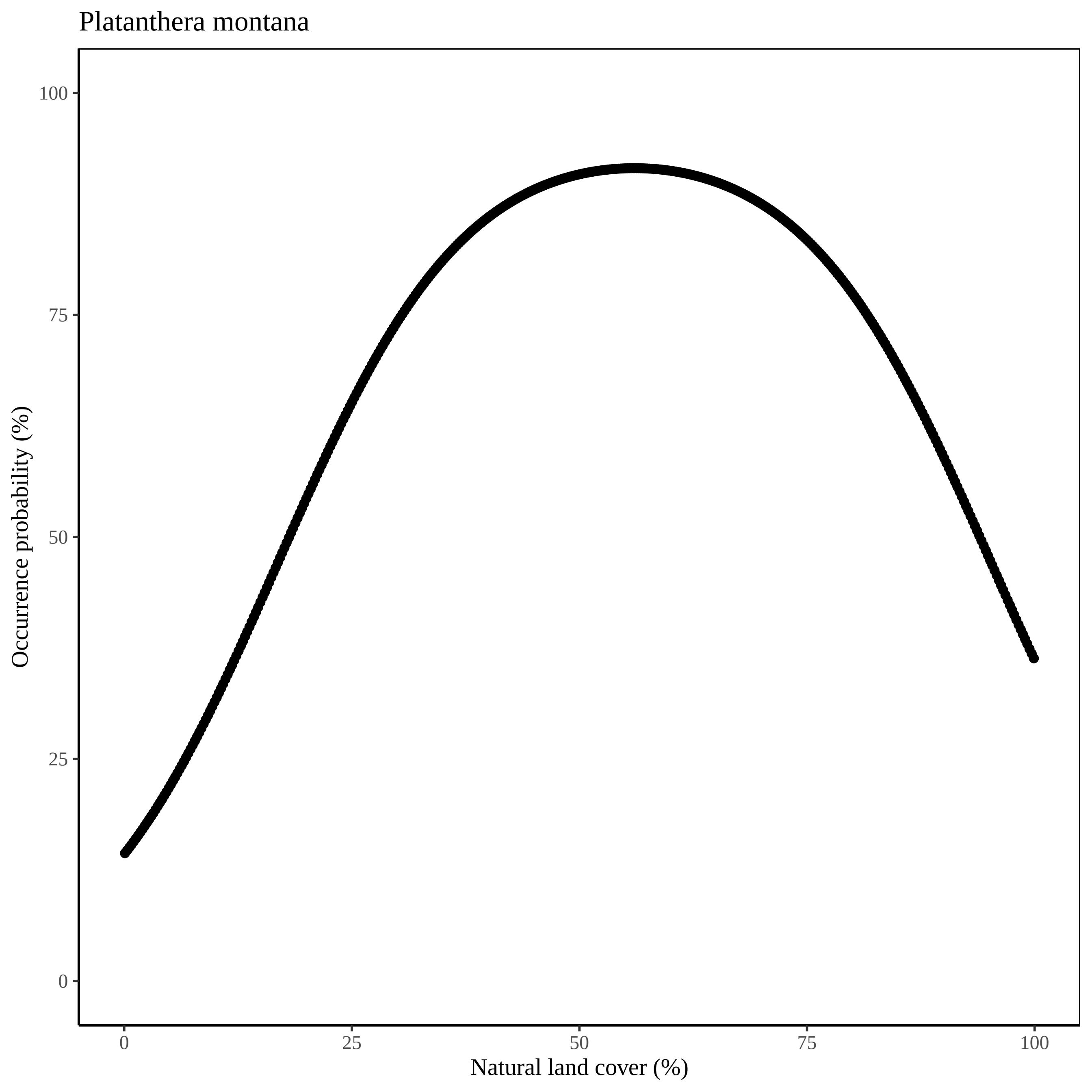
Platanthera montana
Occurrence probability (%)
100
75
50
25
0
0
25
50
75
100
Natural land cover (%)

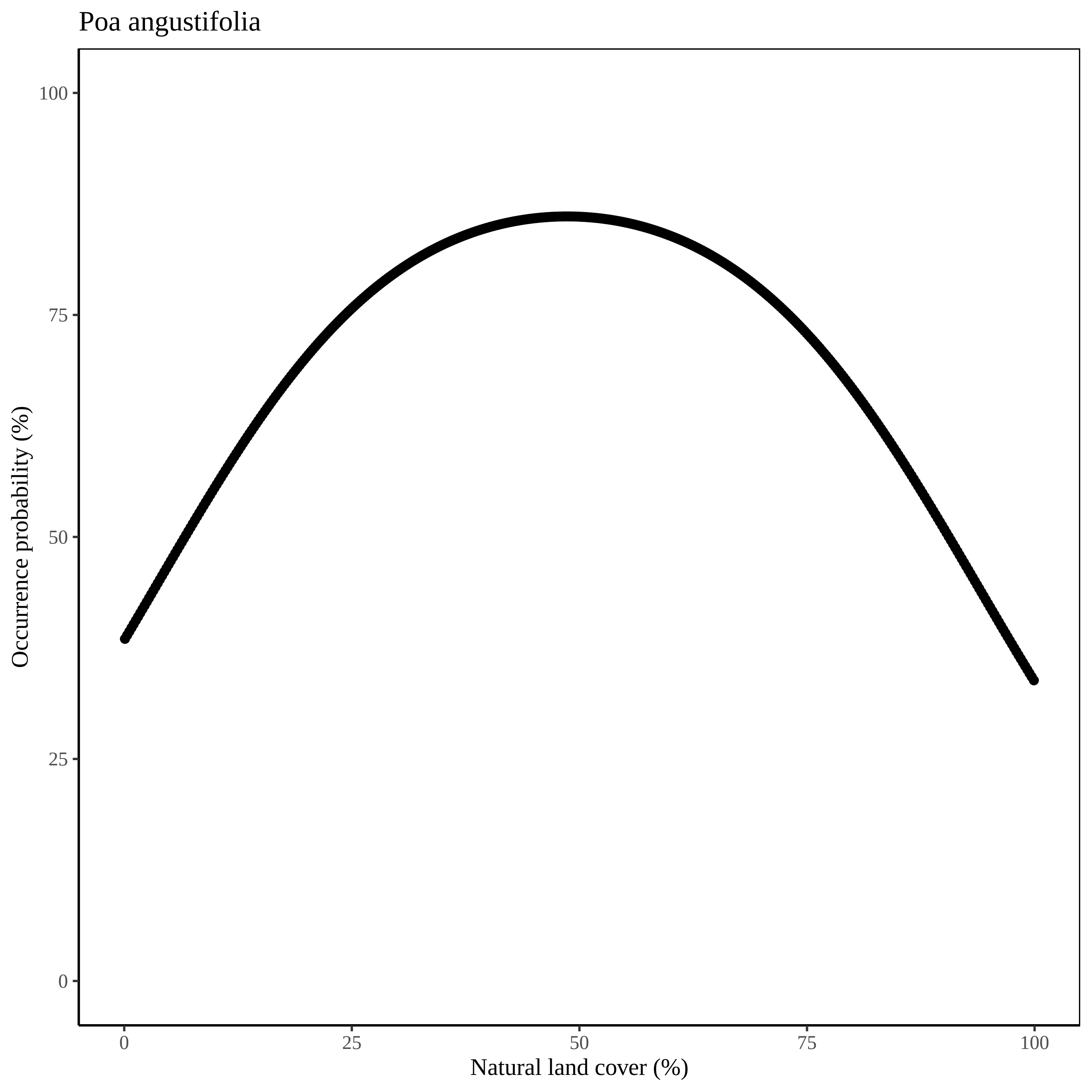
Poa angustifolia
Occurrence probability (%)
100
75
50
25
0
0
25
50
75
100
Natural land cover (%)

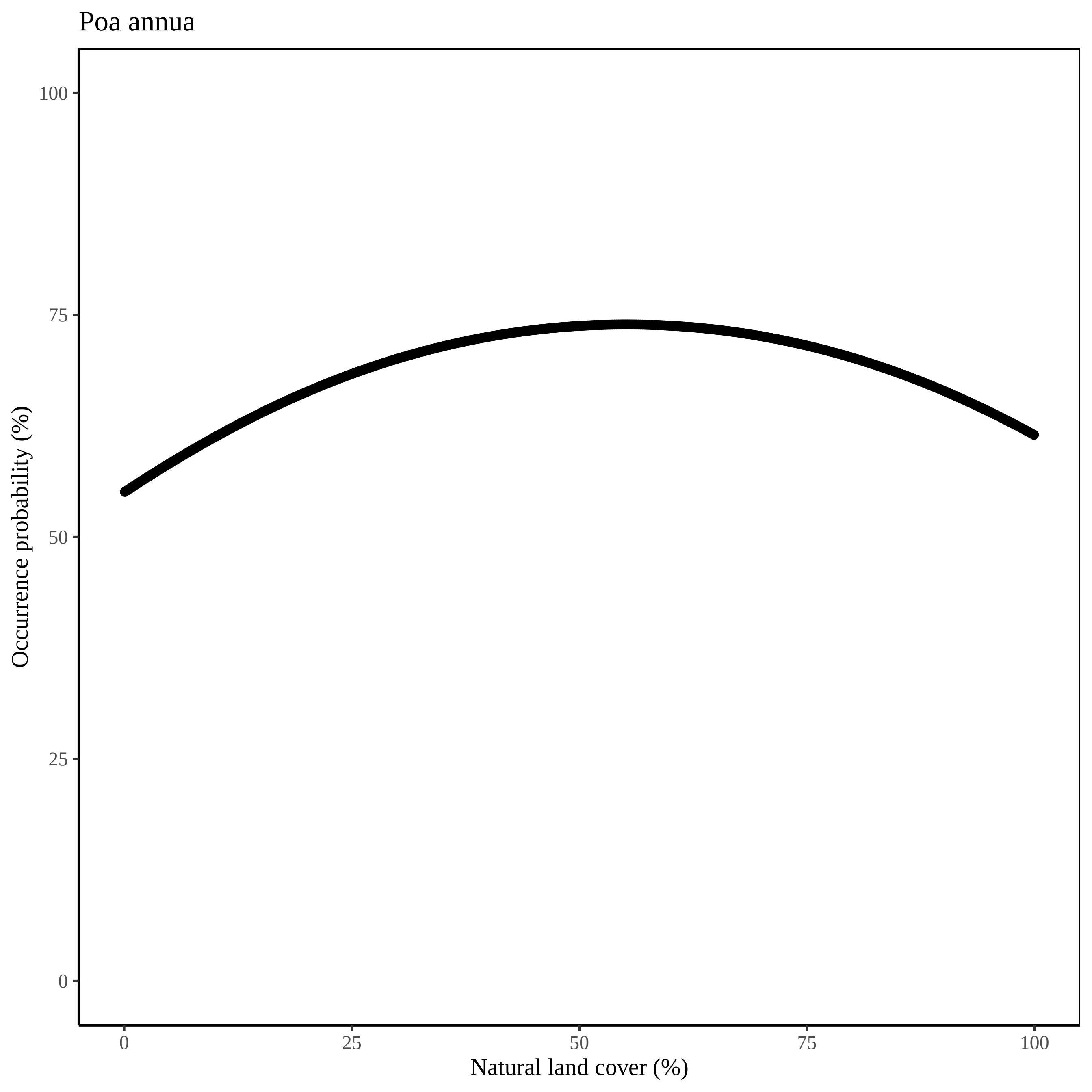

Poa annua
Occurrence probability (%)
100
75
50
25
0
0
25
50
75
100
Natural land cover (%)

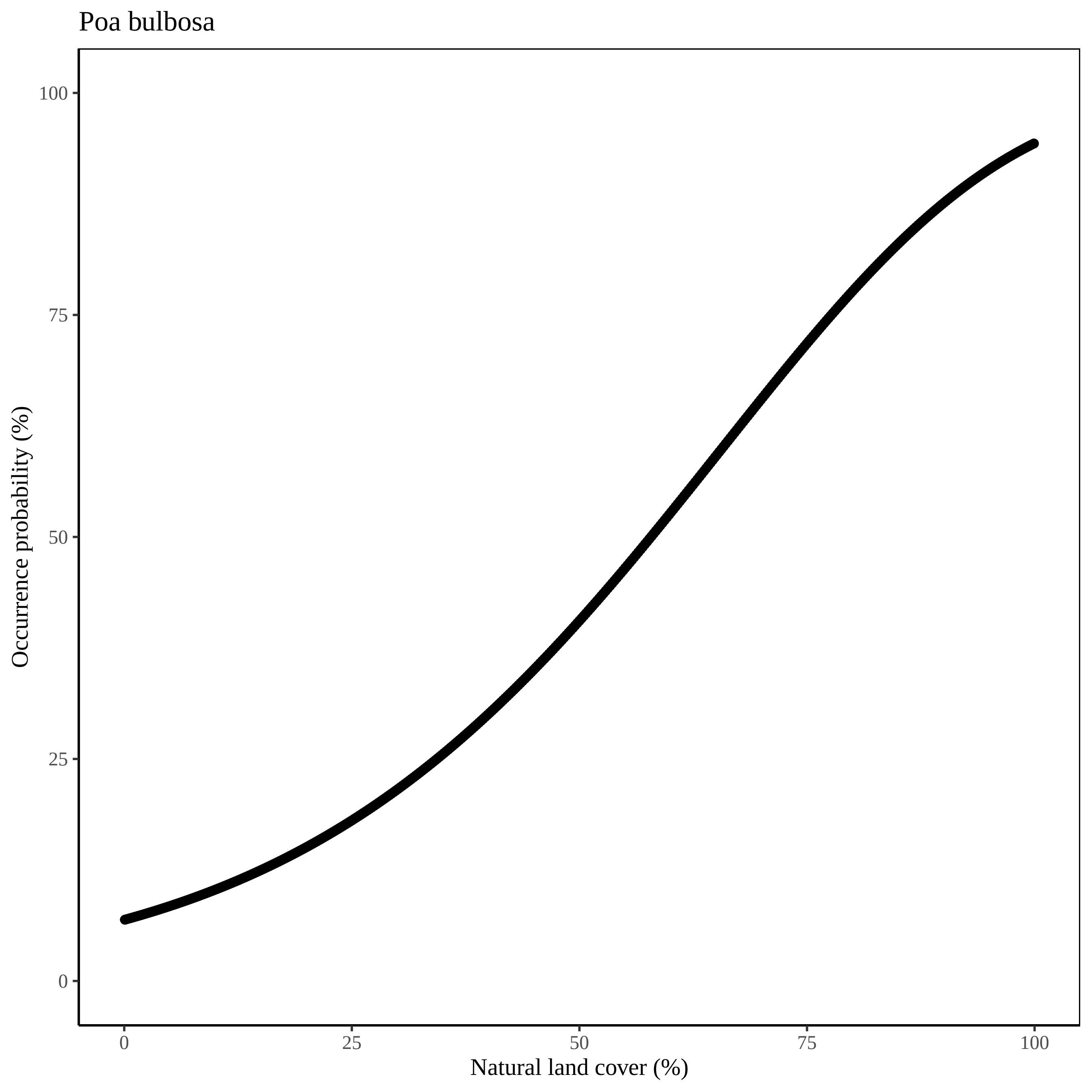
Poa bulbosa
Occurrence probability (%)
100
75
50
25
0
0
25
50
75
100
Natural land cover (%)

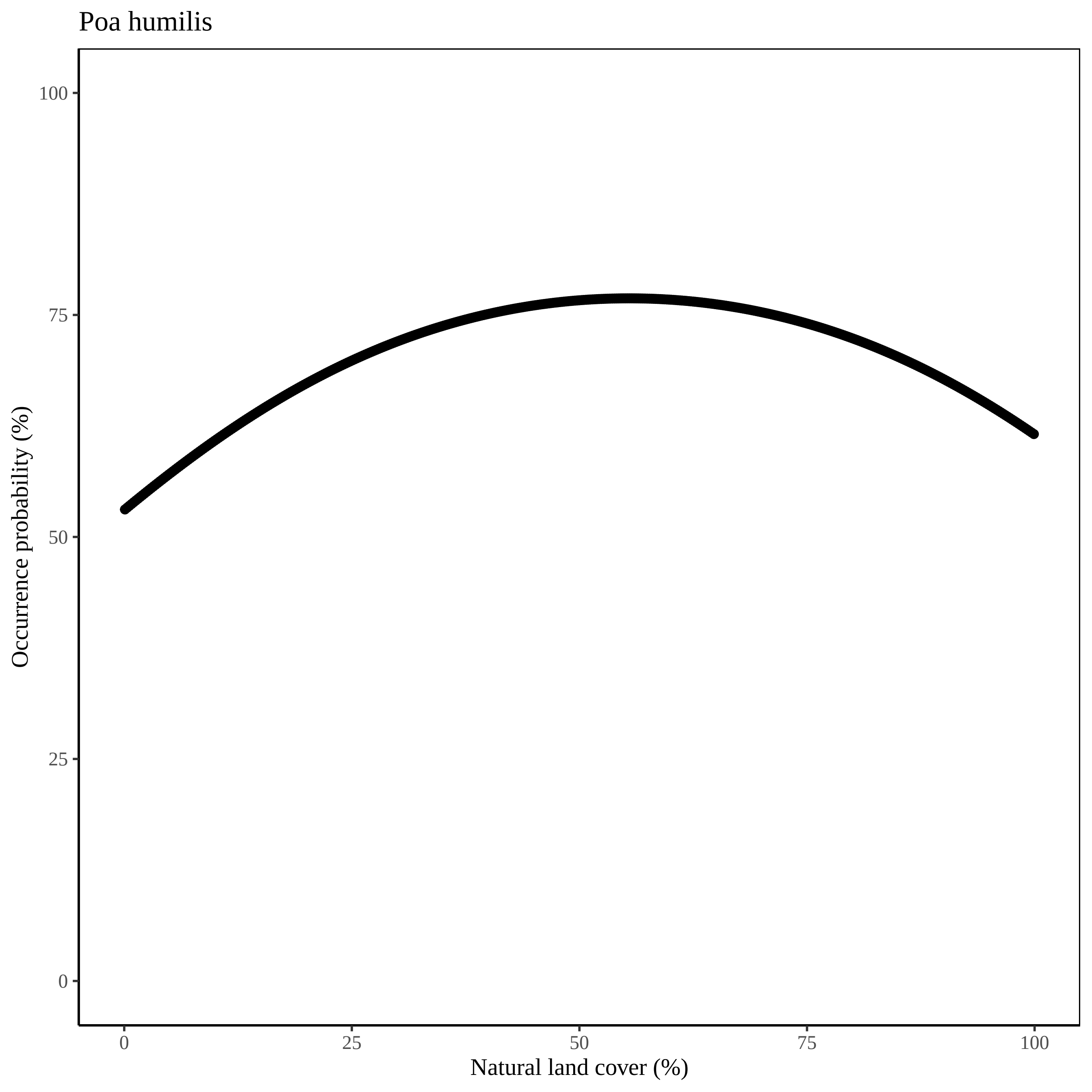
Poa humilis
100
75
50
25
0
Occurrence probability (%)
0
25
50
75
100
Natural land cover (%)

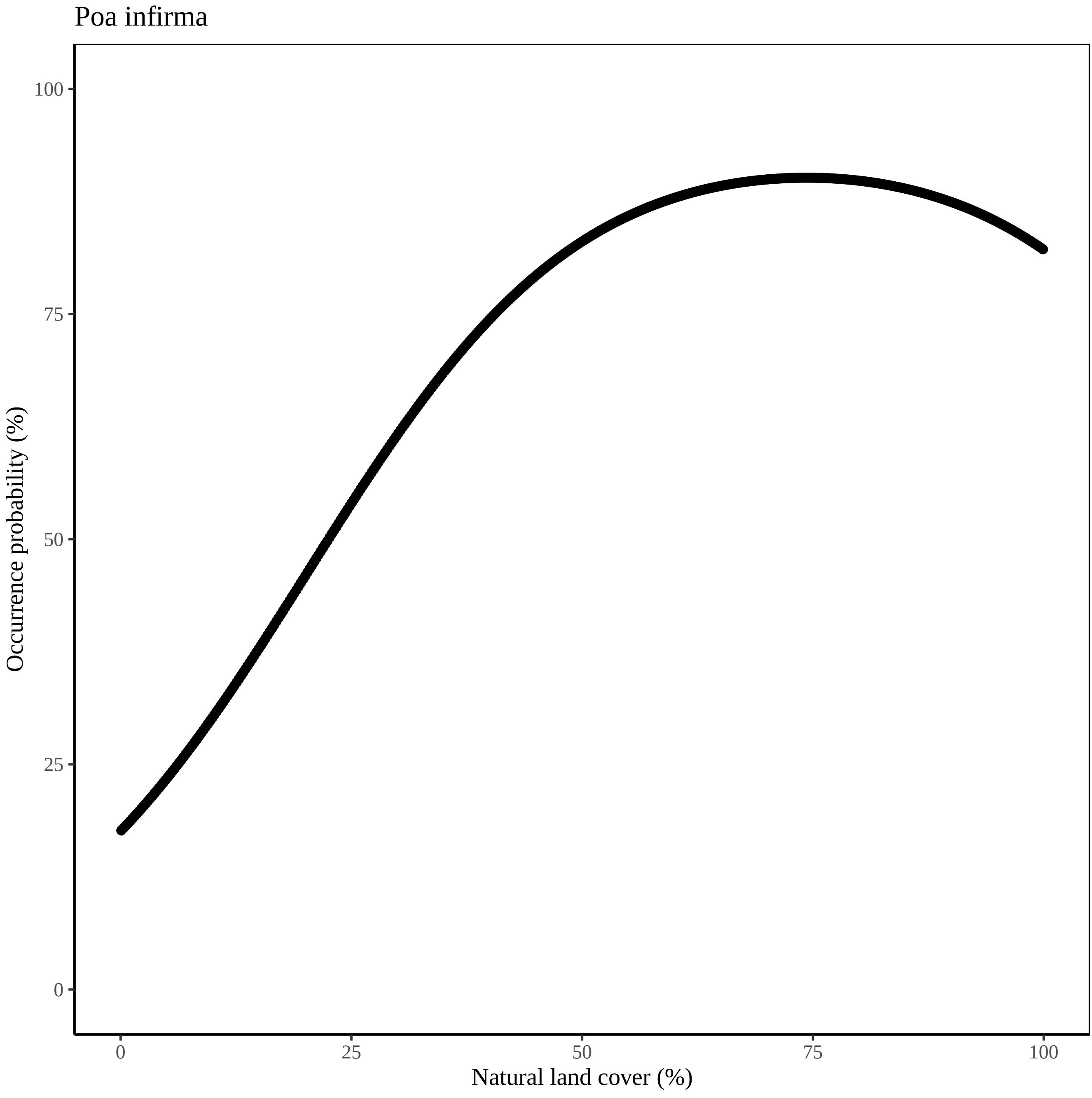
Poa infirma
100
75
50
25
0
Occurrence probability (%)
0
25
50
75
100
Natural land cover (%)

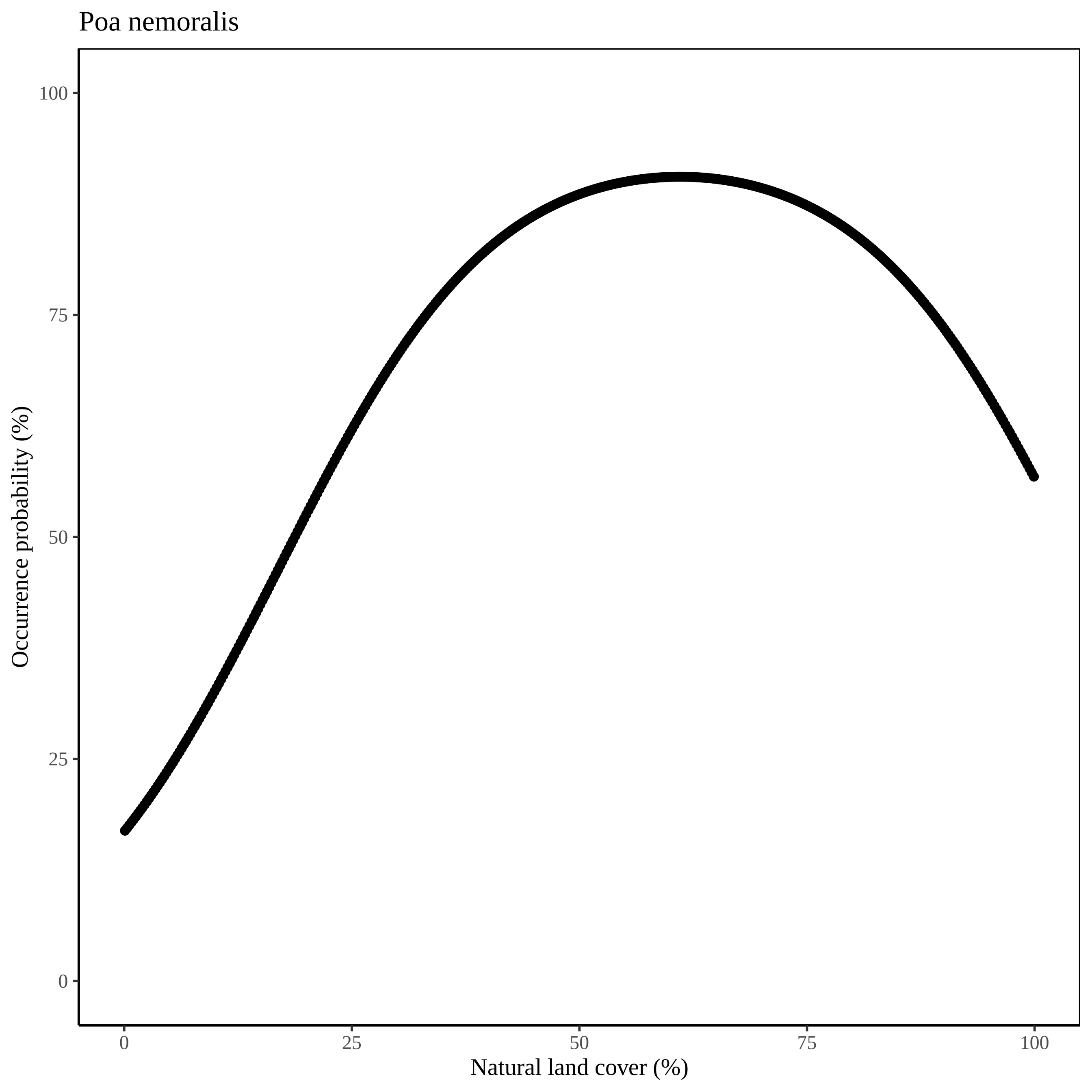
Poa nemoralis
Occurrence probability (%)
100
75
50
25
0
0
25
50
75
100
Natural land cover (%)

Poa palustris

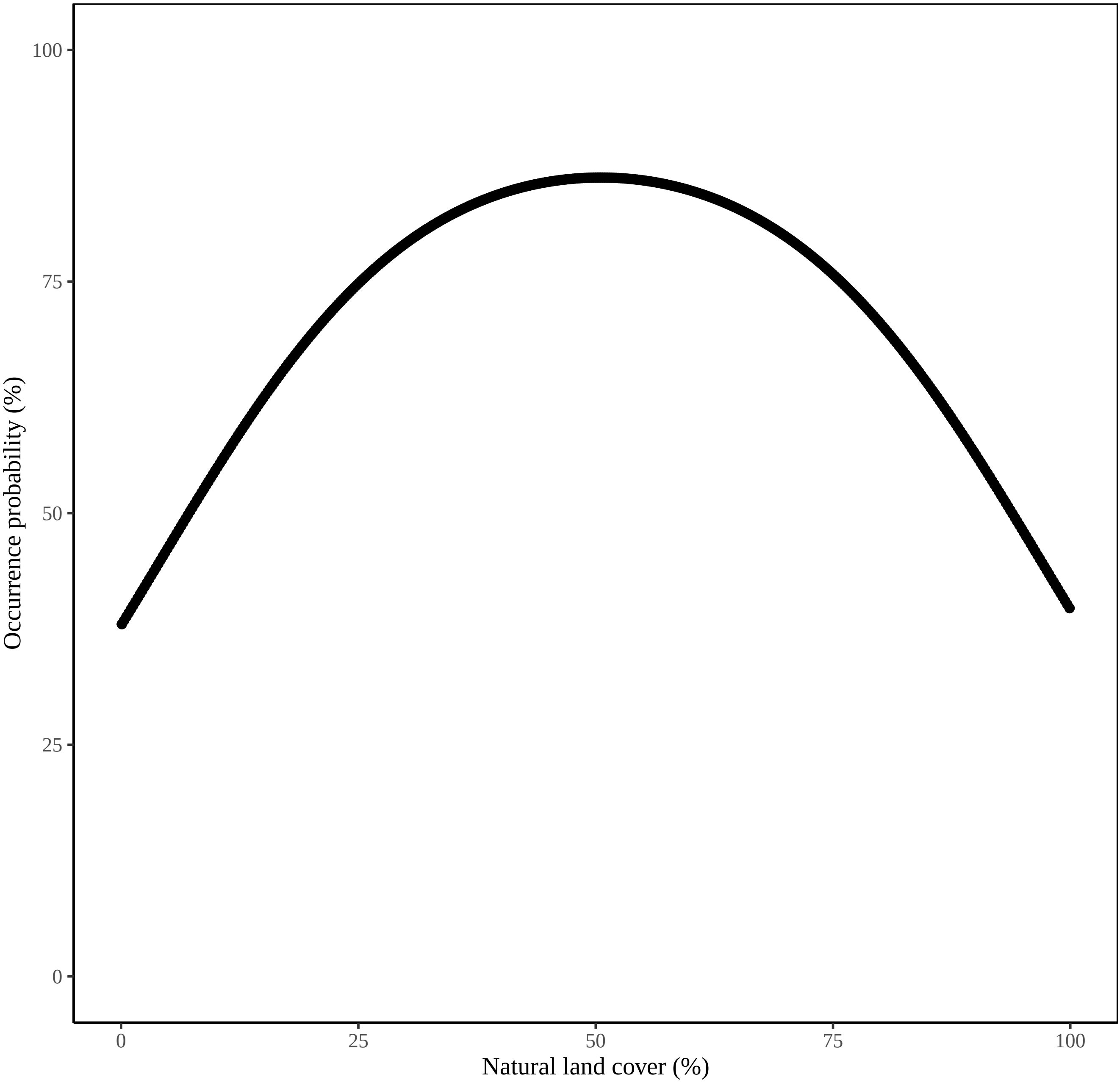

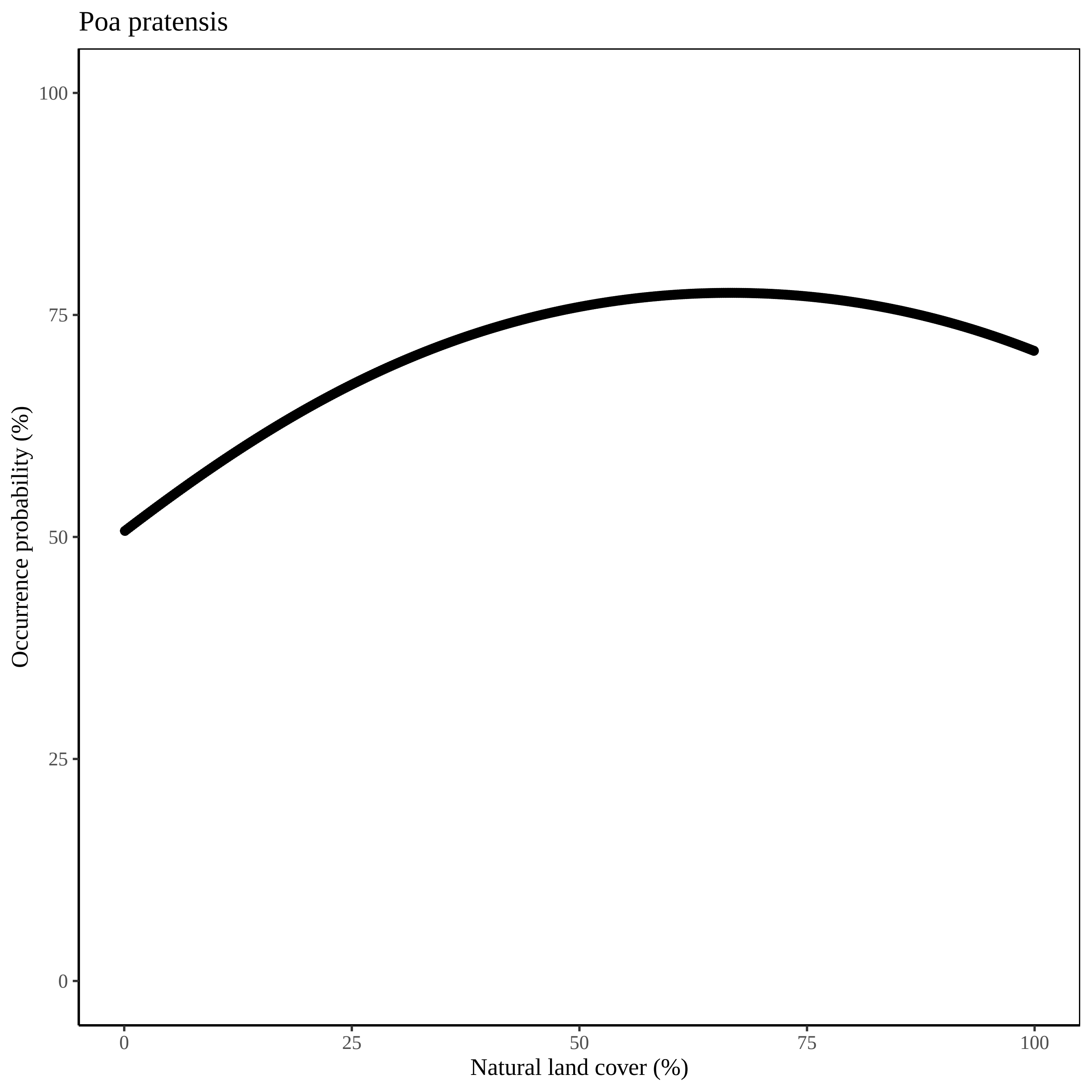

Poa pratensis
Occurrence probability (%)
100
75
50
25
0
0
25
50
75
100
Natural land cover (%)

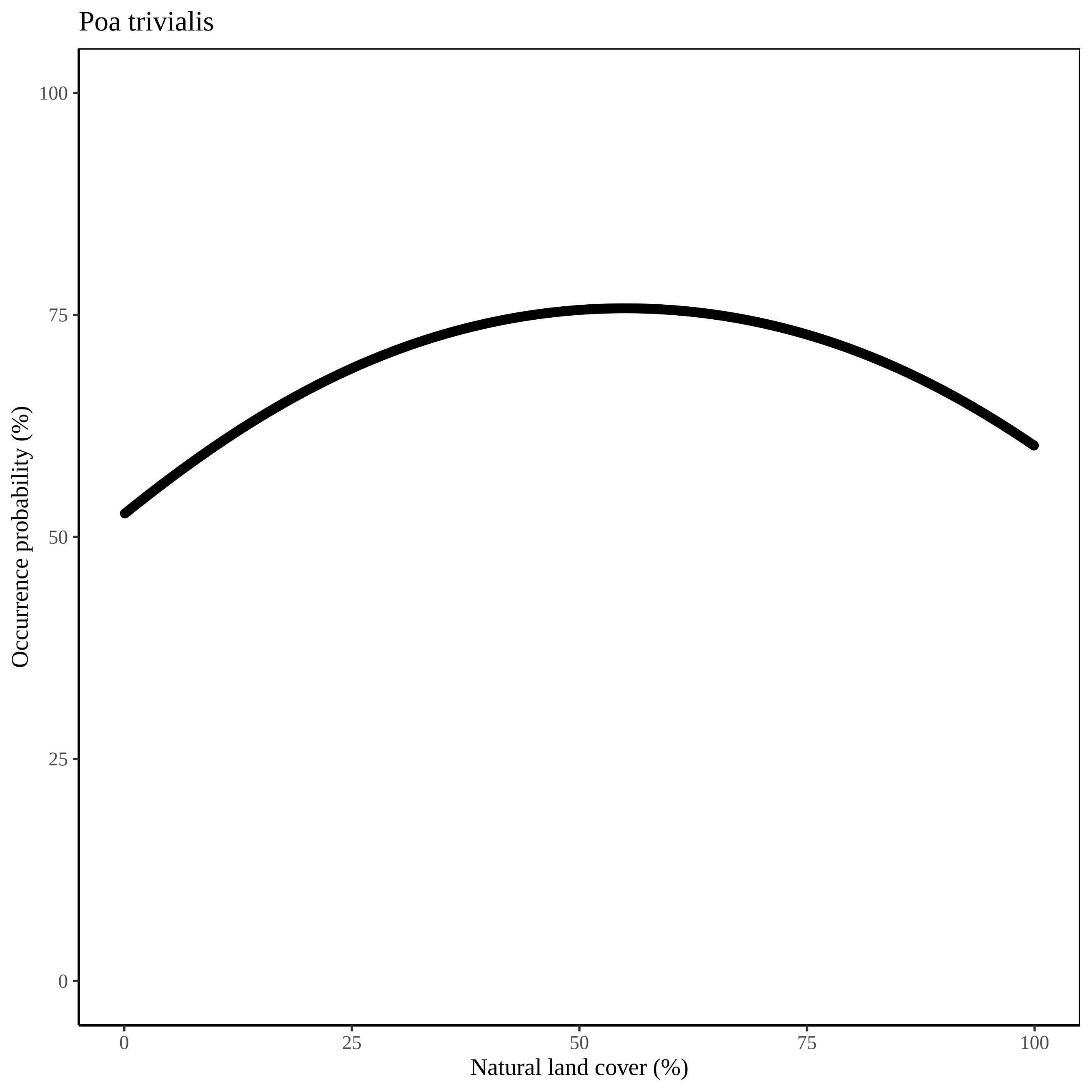
Poa trivialis
Occurrence probability (%)
100
75
50
25
0
0
25
50
75
100
Natural land cover (%)

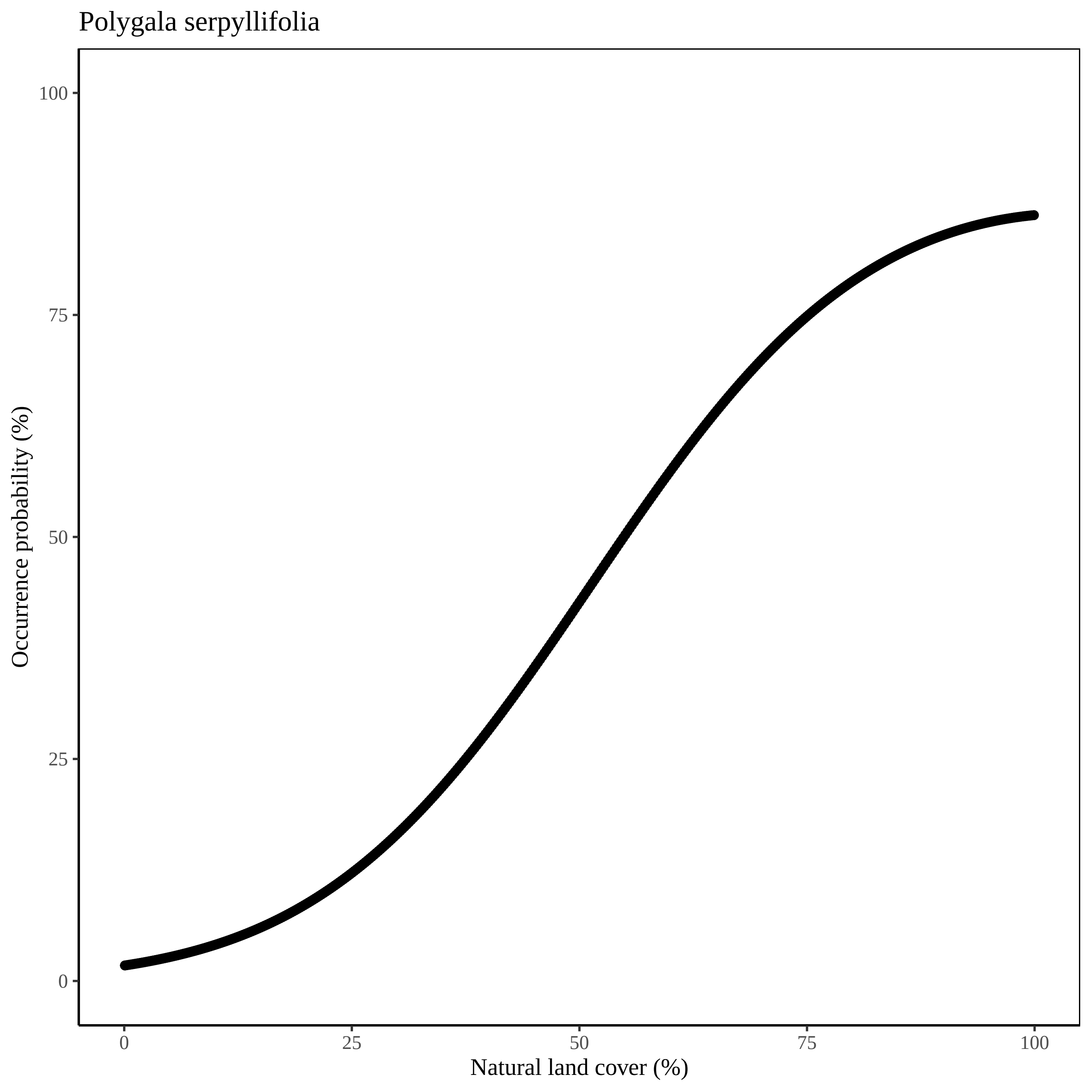
Polygala serpyllifolia
100
75
50
25
0
Occurrence probability (%)
0
25
50
75
100
Natural land cover (%)

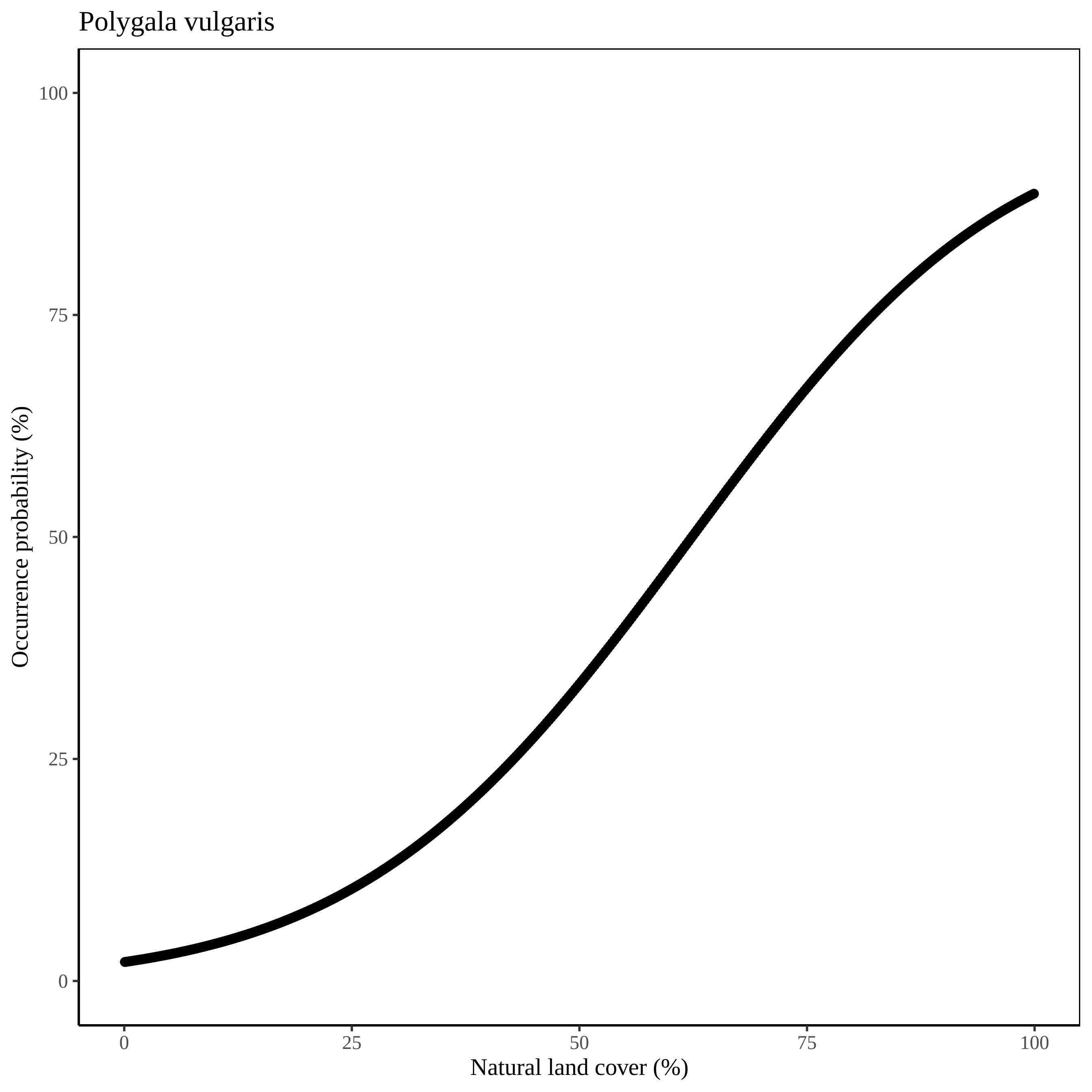
Polygala vulgaris
Occurrence probability (%)
100
75
50
25
0
0
25
50
75
100
Natural land cover (%)

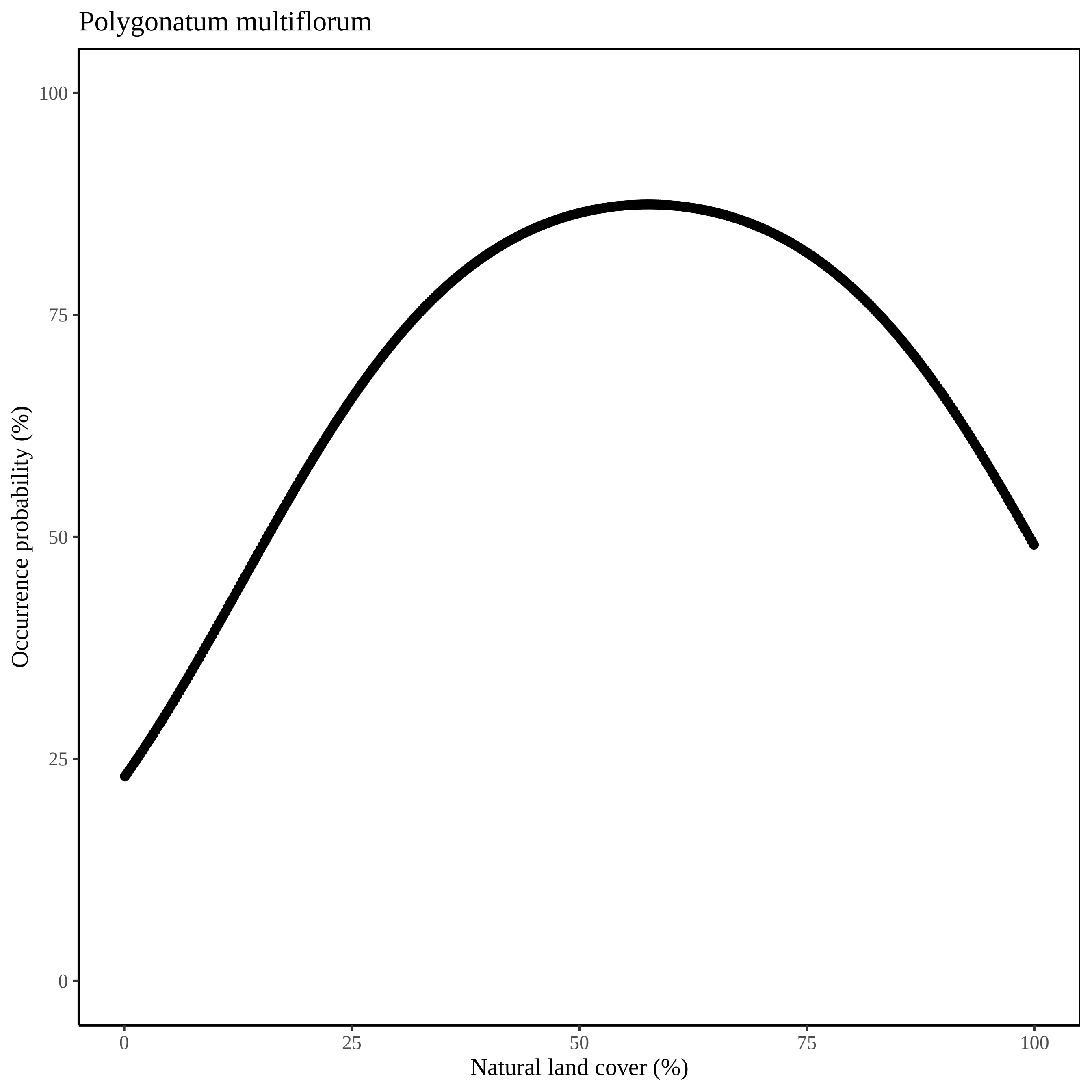
Polygonatum multiflorum
Occurrence probability (%)
100
75
50
25
0
0
25
50
75
100
Natural land cover (%)

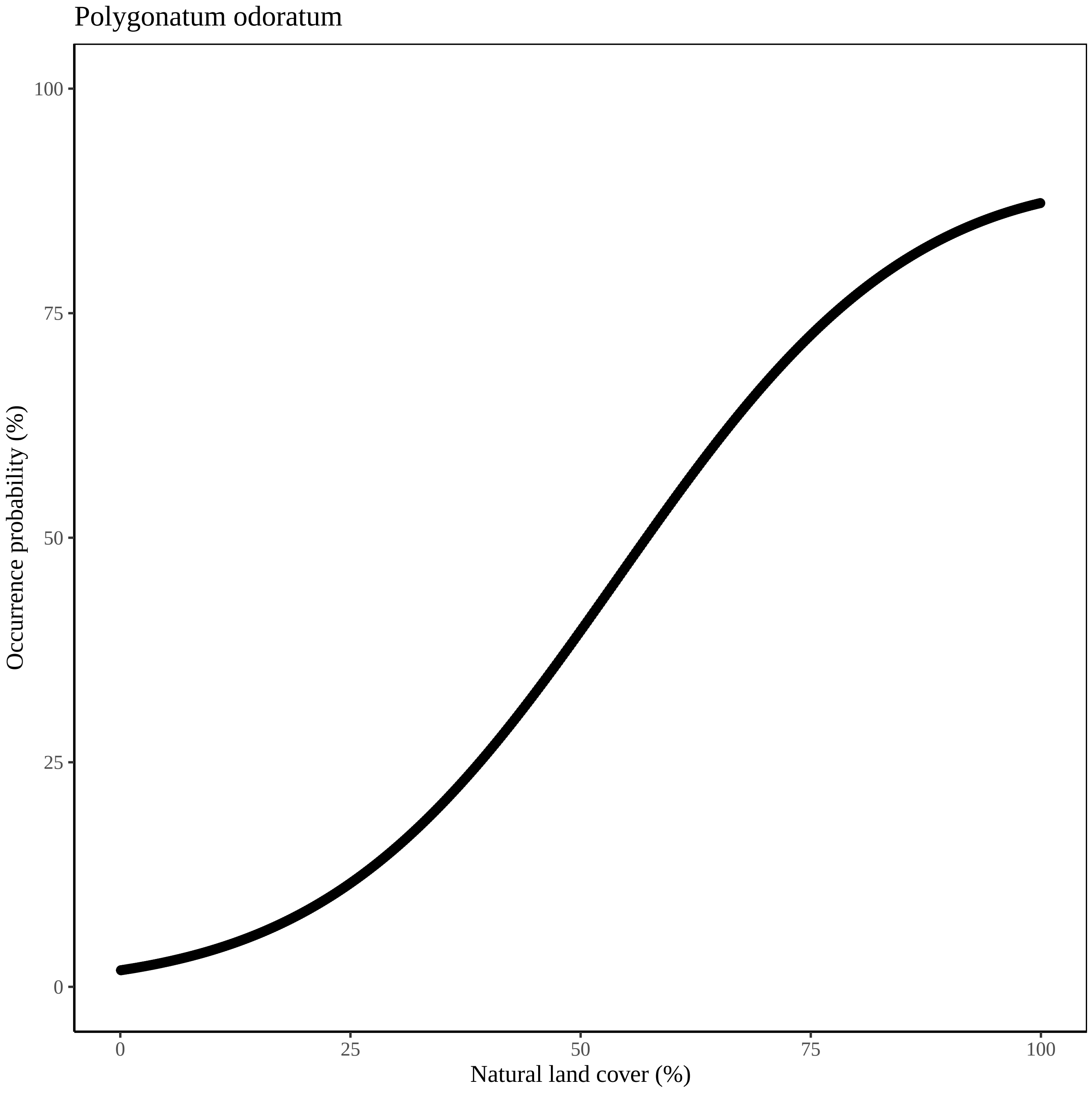
Polygonatum odoratum
Occurrence probability (%)
100
75
50
25
0
0
25
50
75
100
Natural land cover (%)

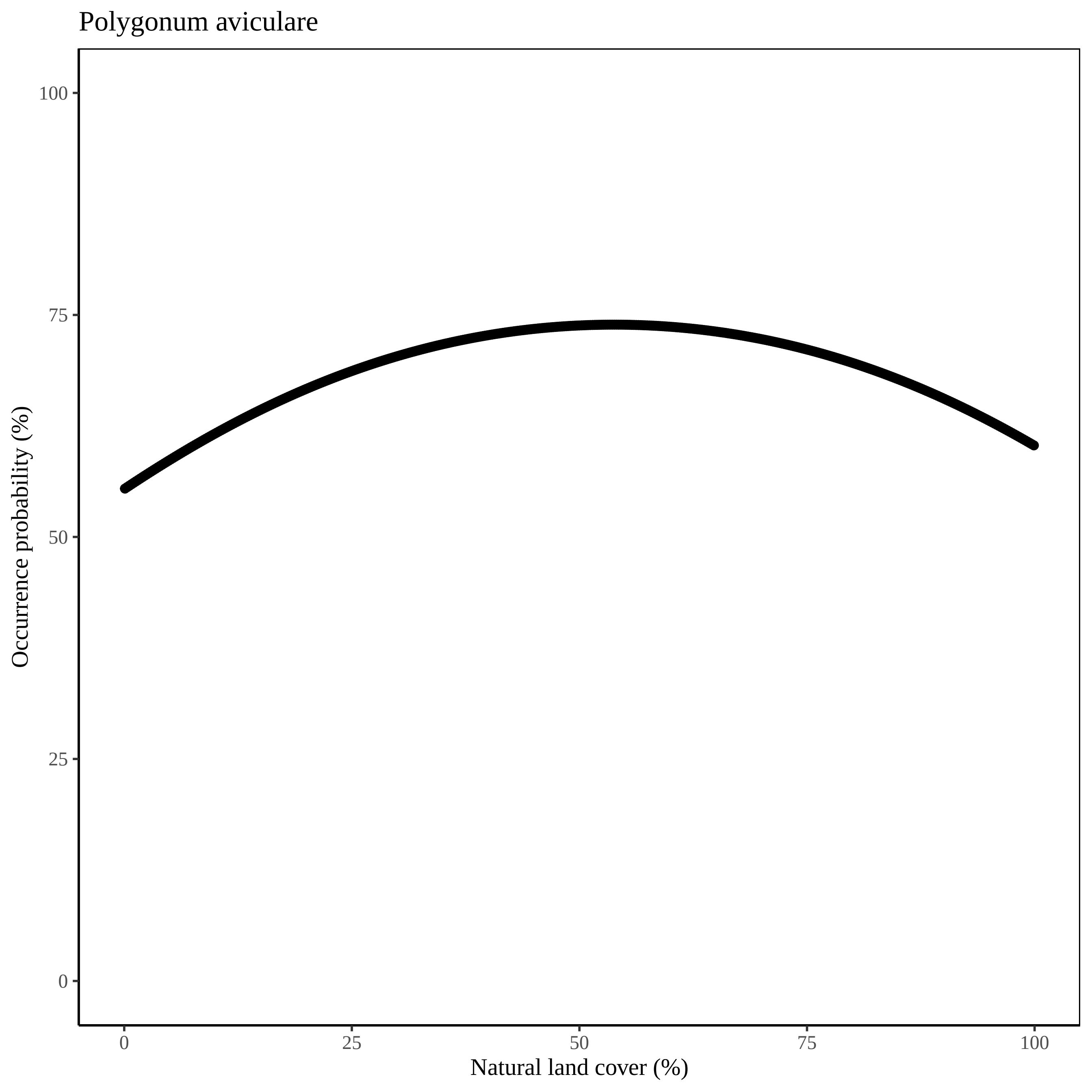

Polygonum aviculare
100
75
50
25
0
Occurrence probability (%)
0
25
50
75
100
Natural land cover (%)

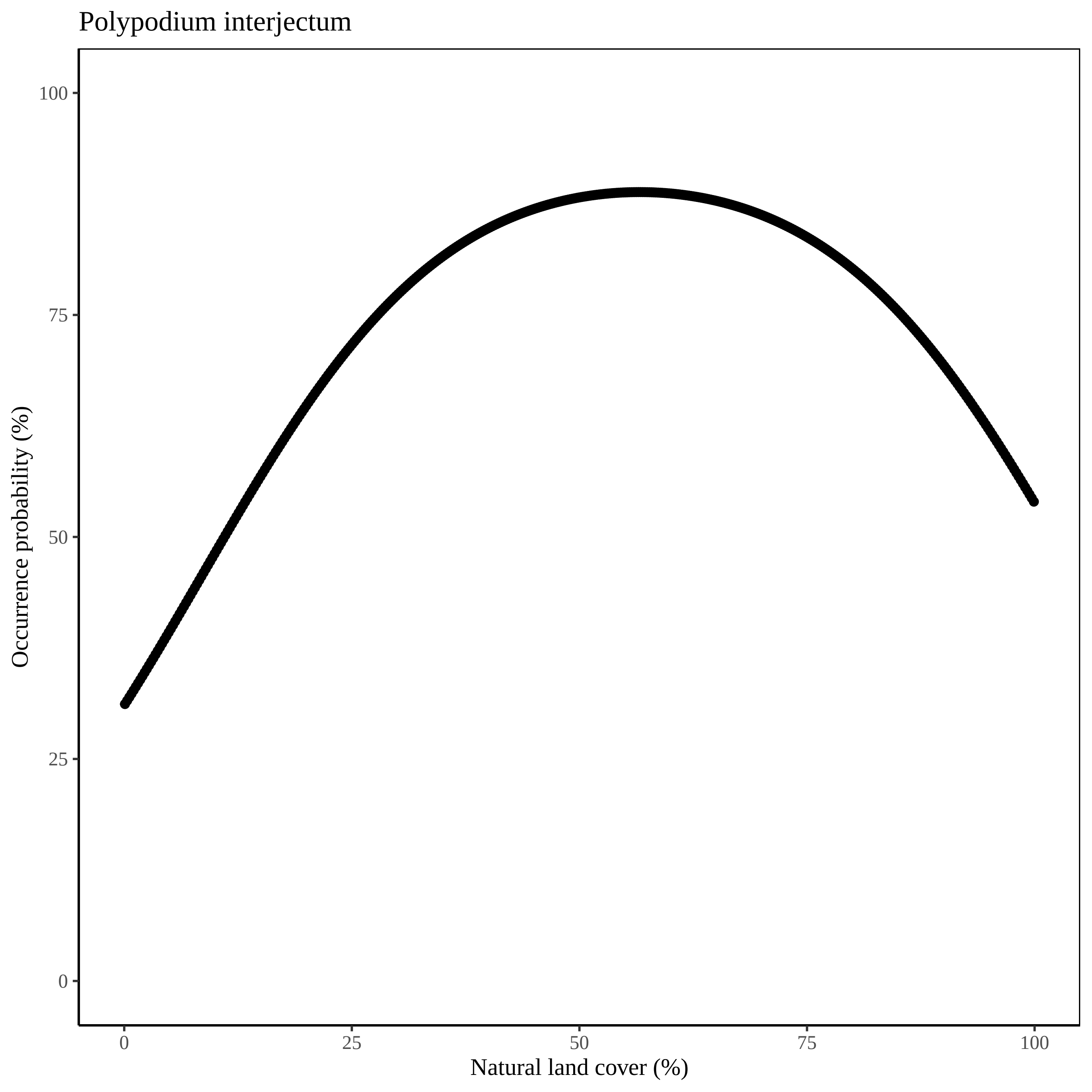

Polypodium interjectum
Occurrence probability (%)
100
75
50
25
0
0
25
50
75
100
Natural land cover (%)

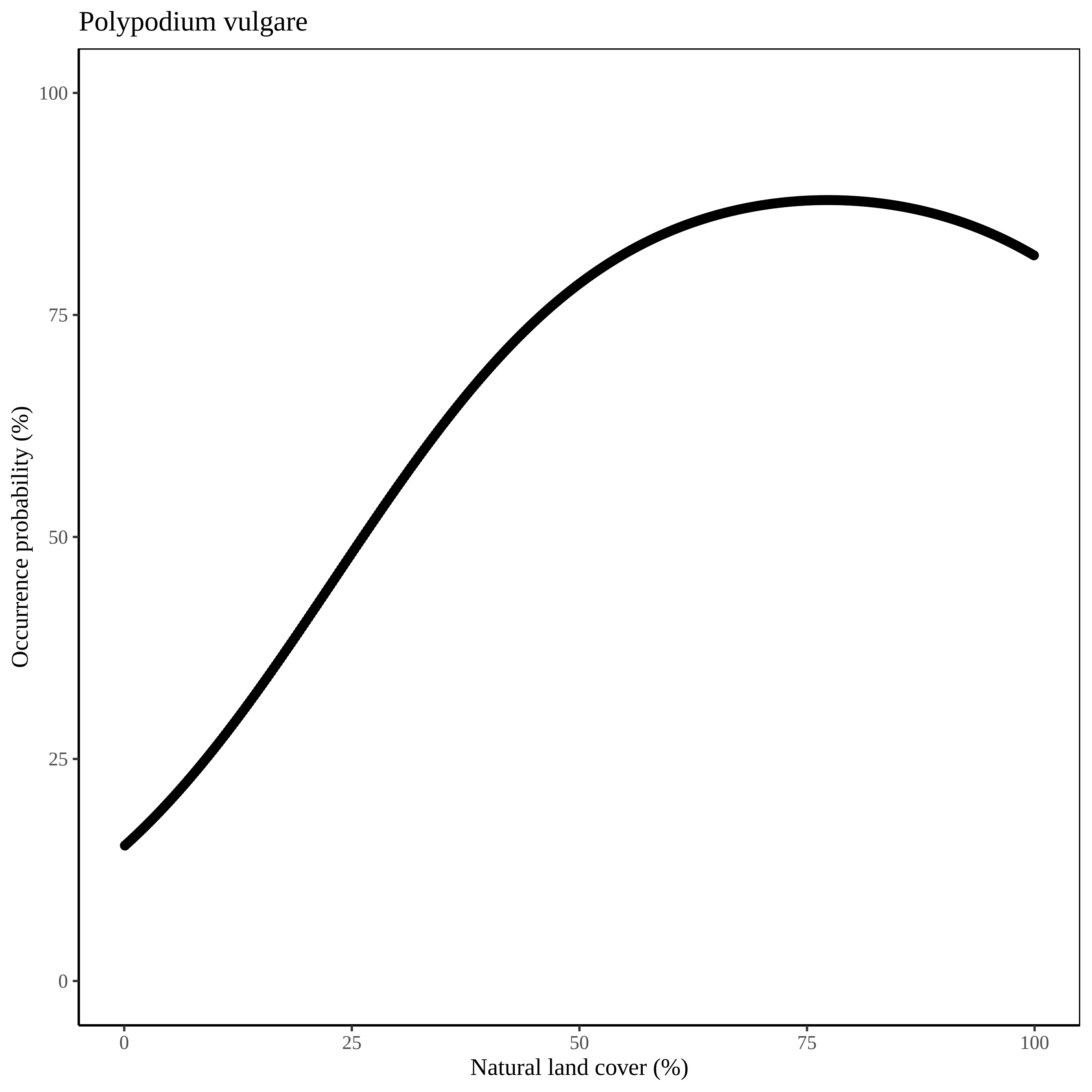
Polypodium vulgare
Occurrence probability (%)
100
75
50
25
0
0
25
50
75
100
Natural land cover (%)

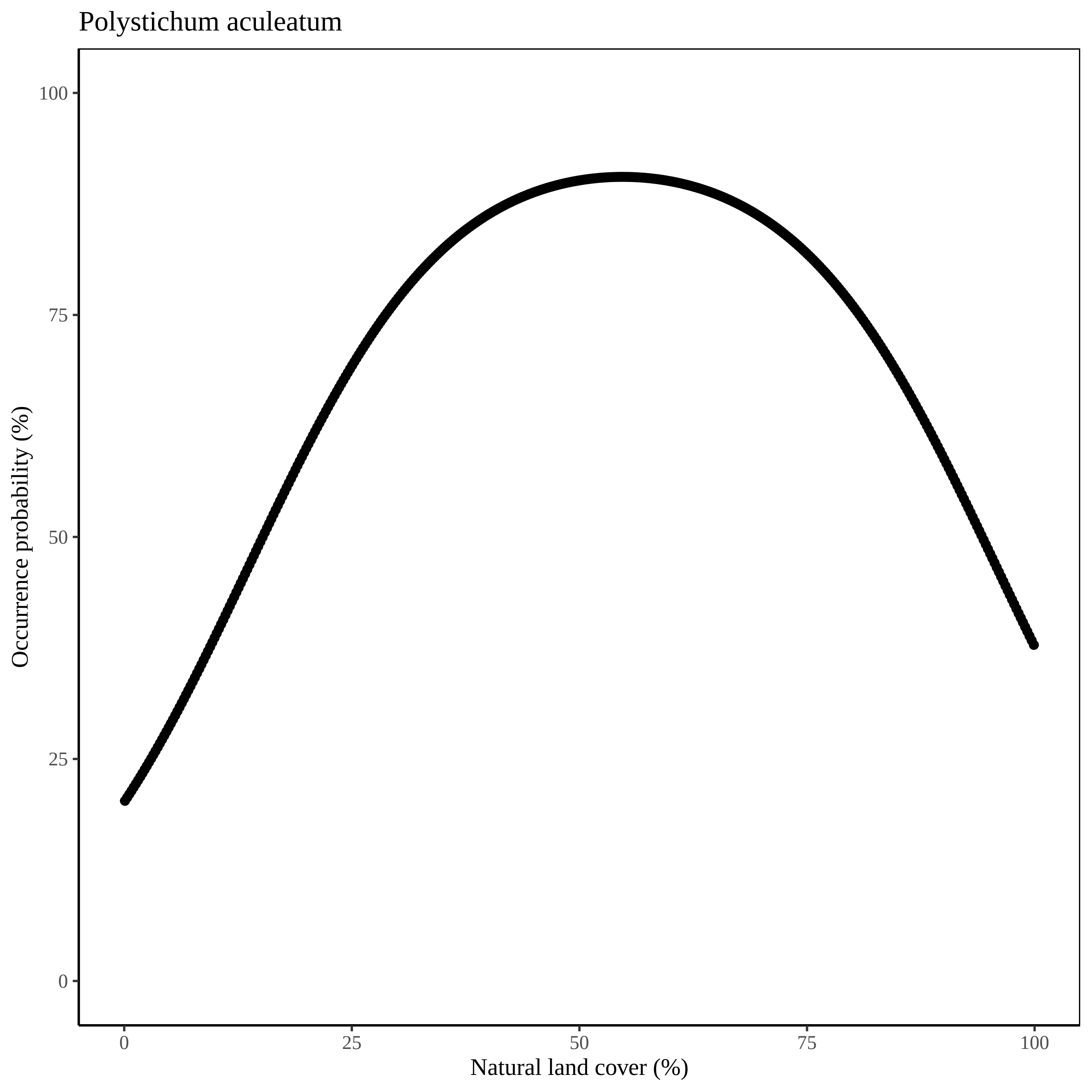
Polystichum aculeatum
Occurrence probability (%)
100
75
50
25
0
0
25
50
75
100
Natural land cover (%)

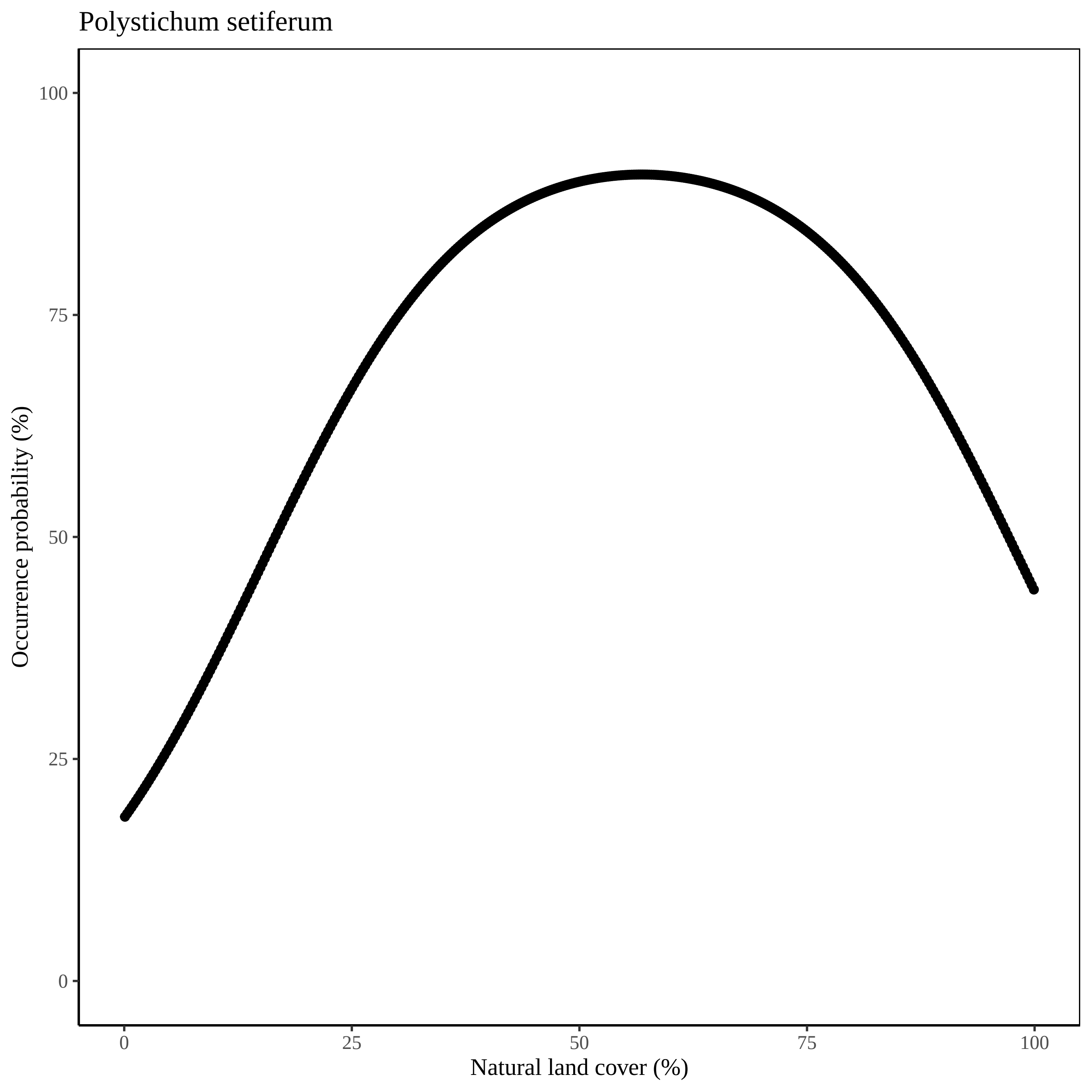
Polystichum setiferum
Occurrence probability (%)
100
75
50
25
0
0
25
50
75
100
Natural land cover (%)

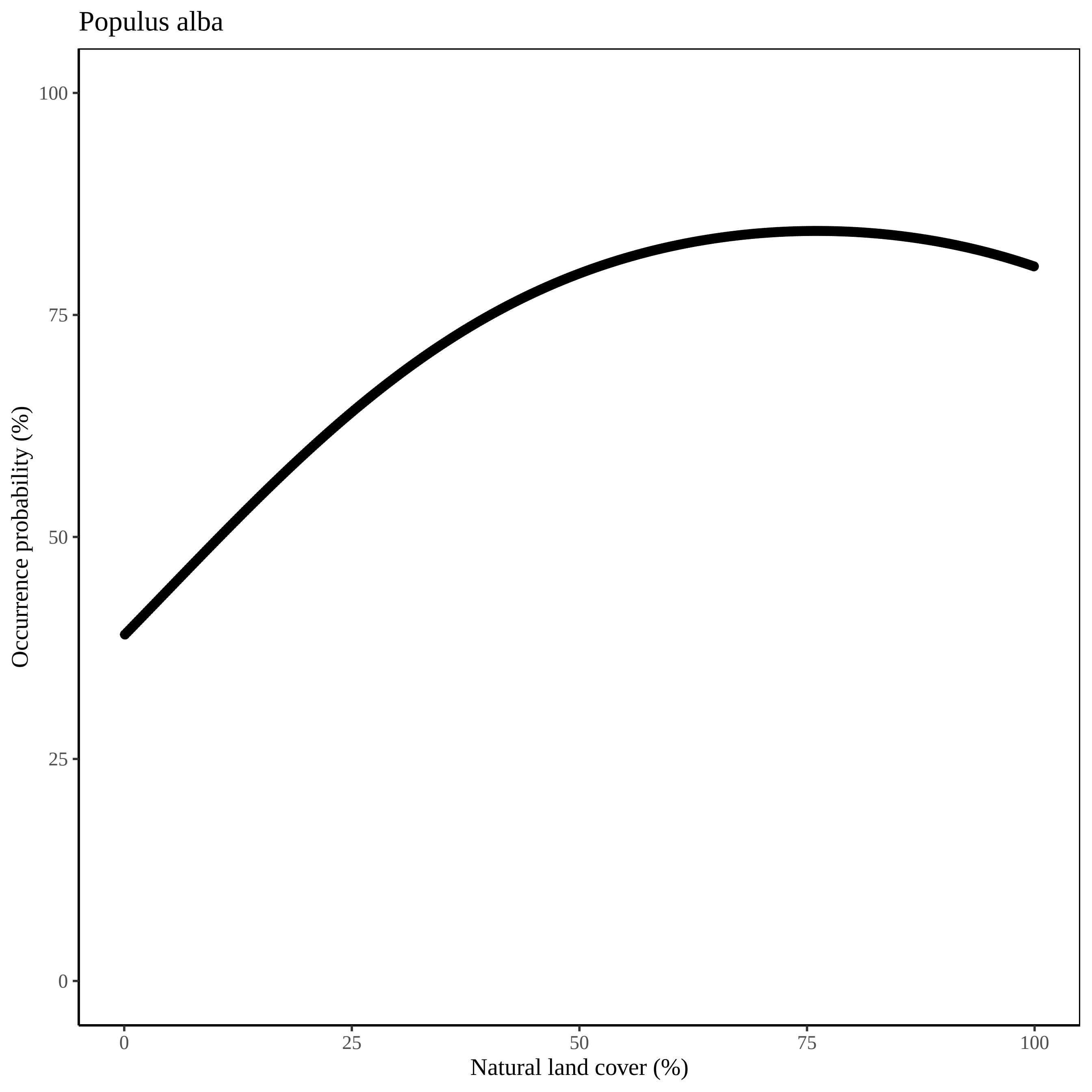

Populus alba
Occurrence probability (%)
100
75
50
25
0
0
25
50
75
100
Natural land cover (%)

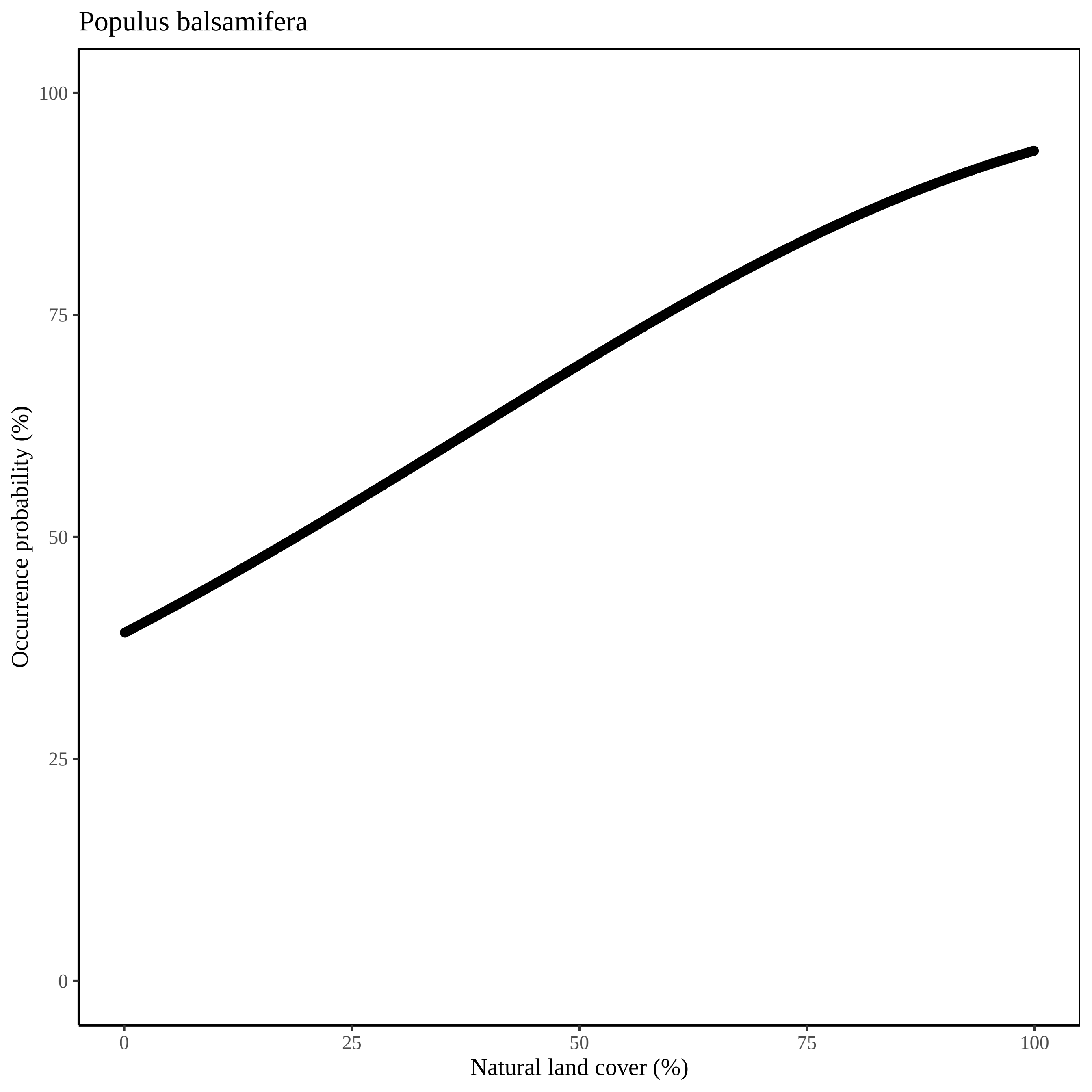

Populus balsamifera
Occurrence probability (%)
100
75
50
25
0
0
25
50
75
100
Natural land cover (%)

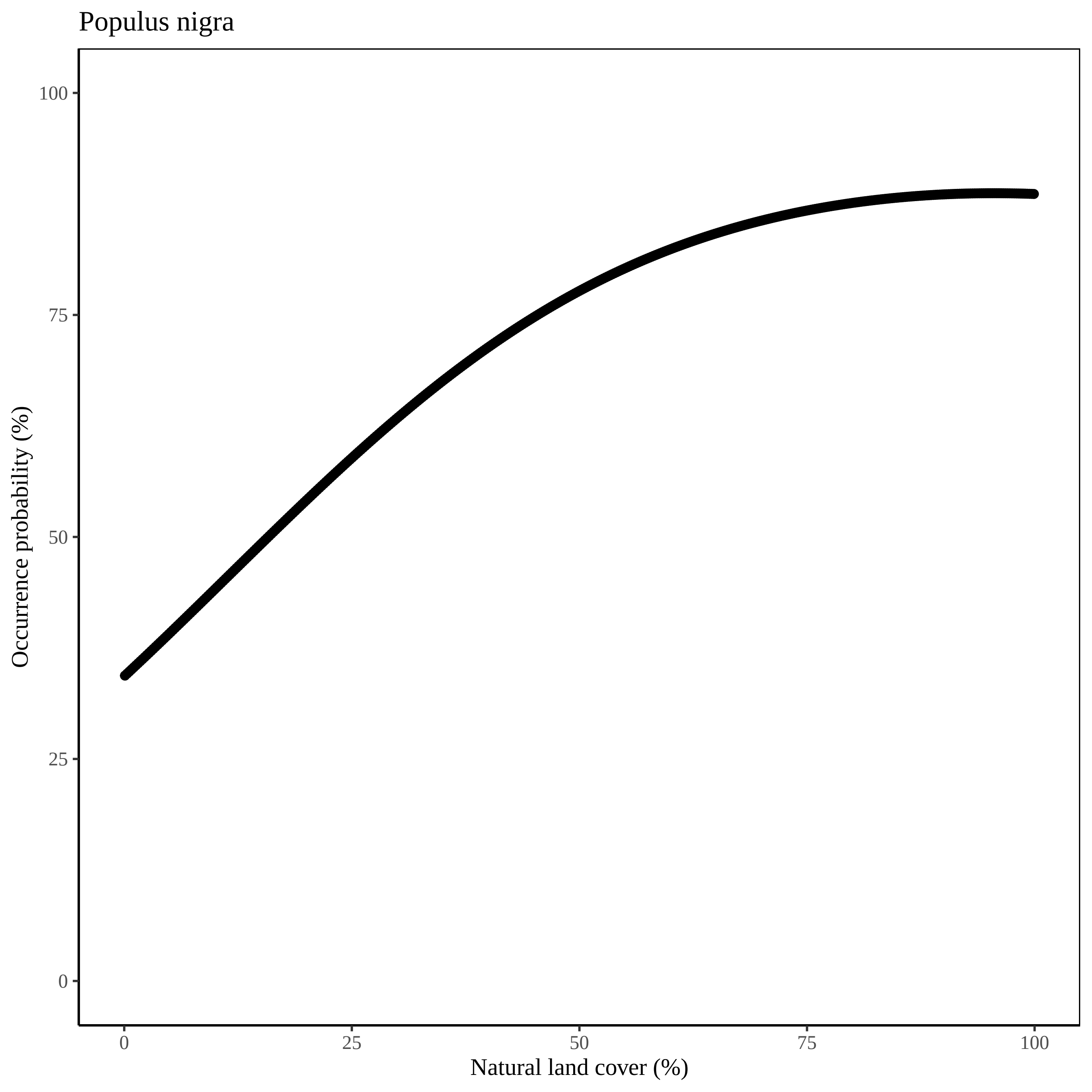
Populus nigra
Occurrence probability (%)
100
75
50
25
0
0
25
50
75
100
Natural land cover (%)

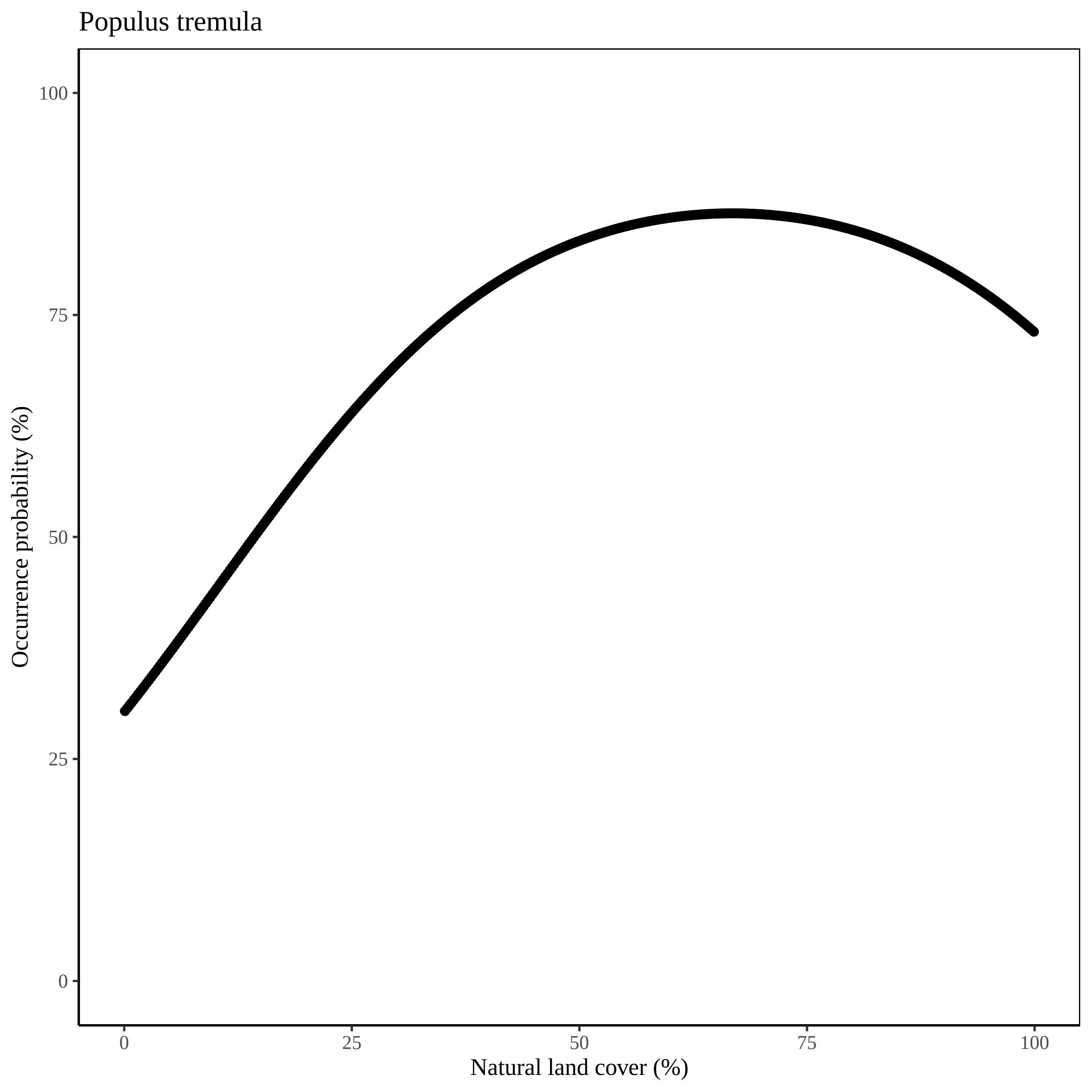
Populus tremula
Occurrence probability (%)
100
75
50
25
0
0
25
50
75
100
Natural land cover (%)

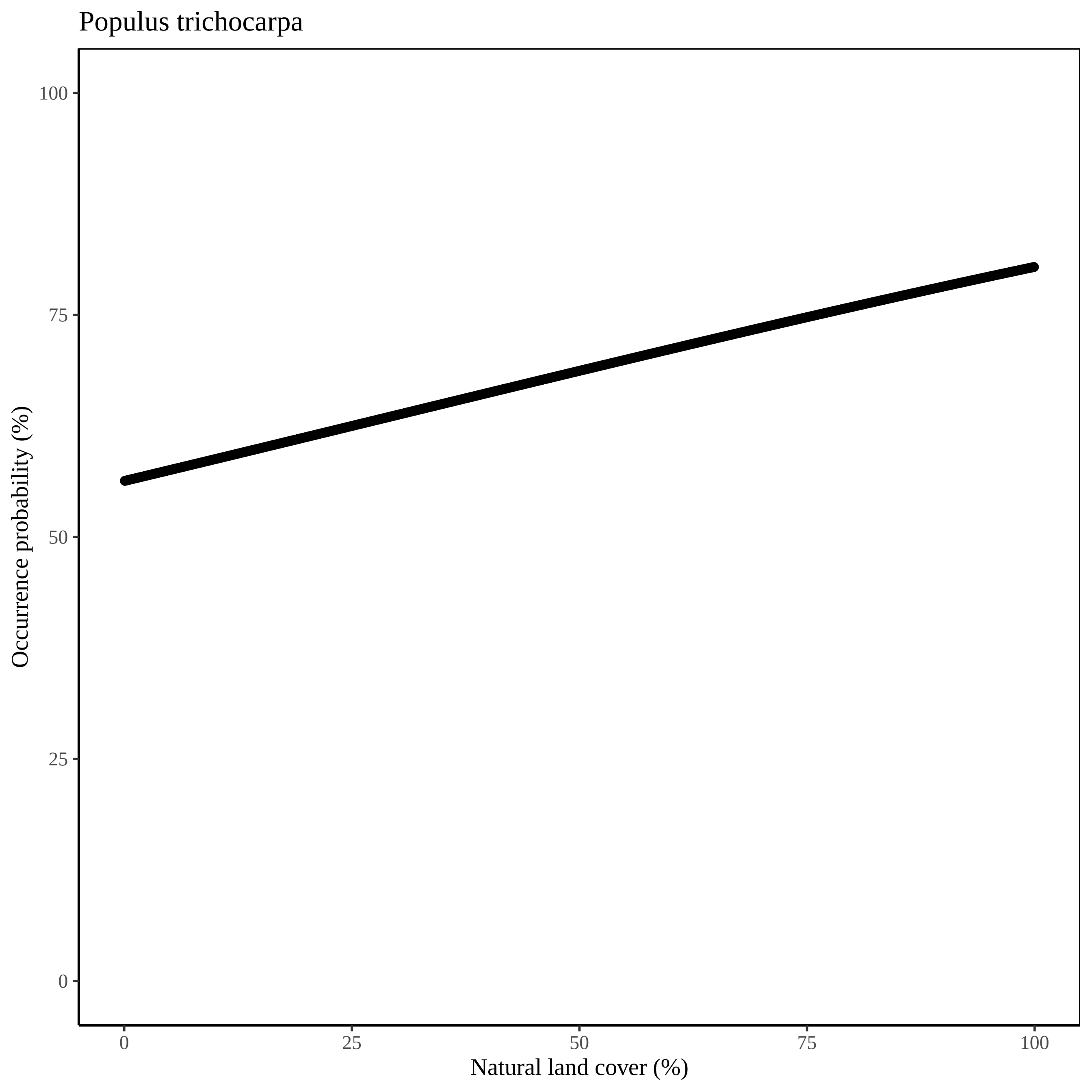

Populus trichocarpa
Occurrence probability (%)
100
75
50
25
0
0
25
50
75
100
Natural land cover (%)

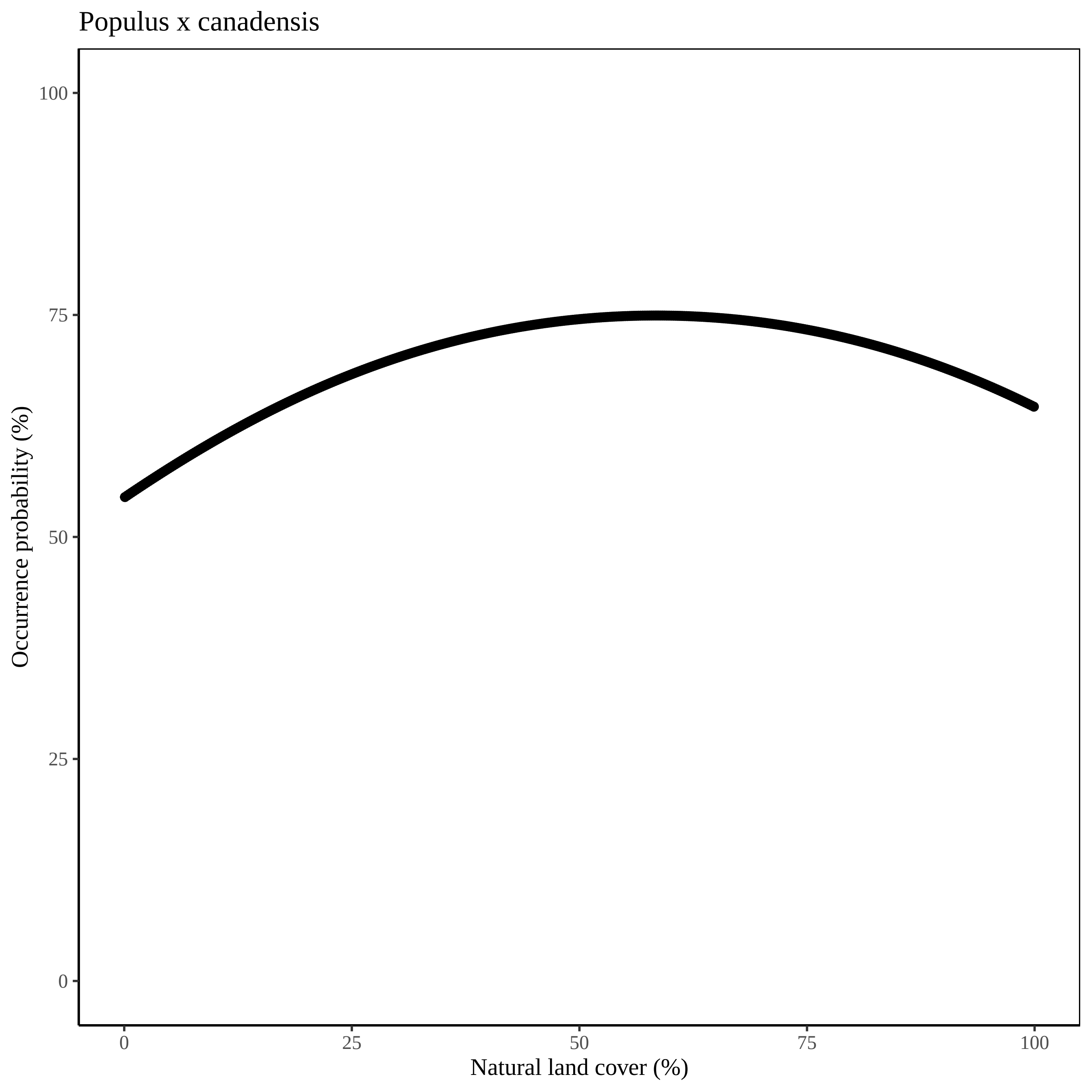
Populus x canadensis
Occurrence probability (%)
100
75
50
25
0
Natural land cover (%)
0
25
50
75
100

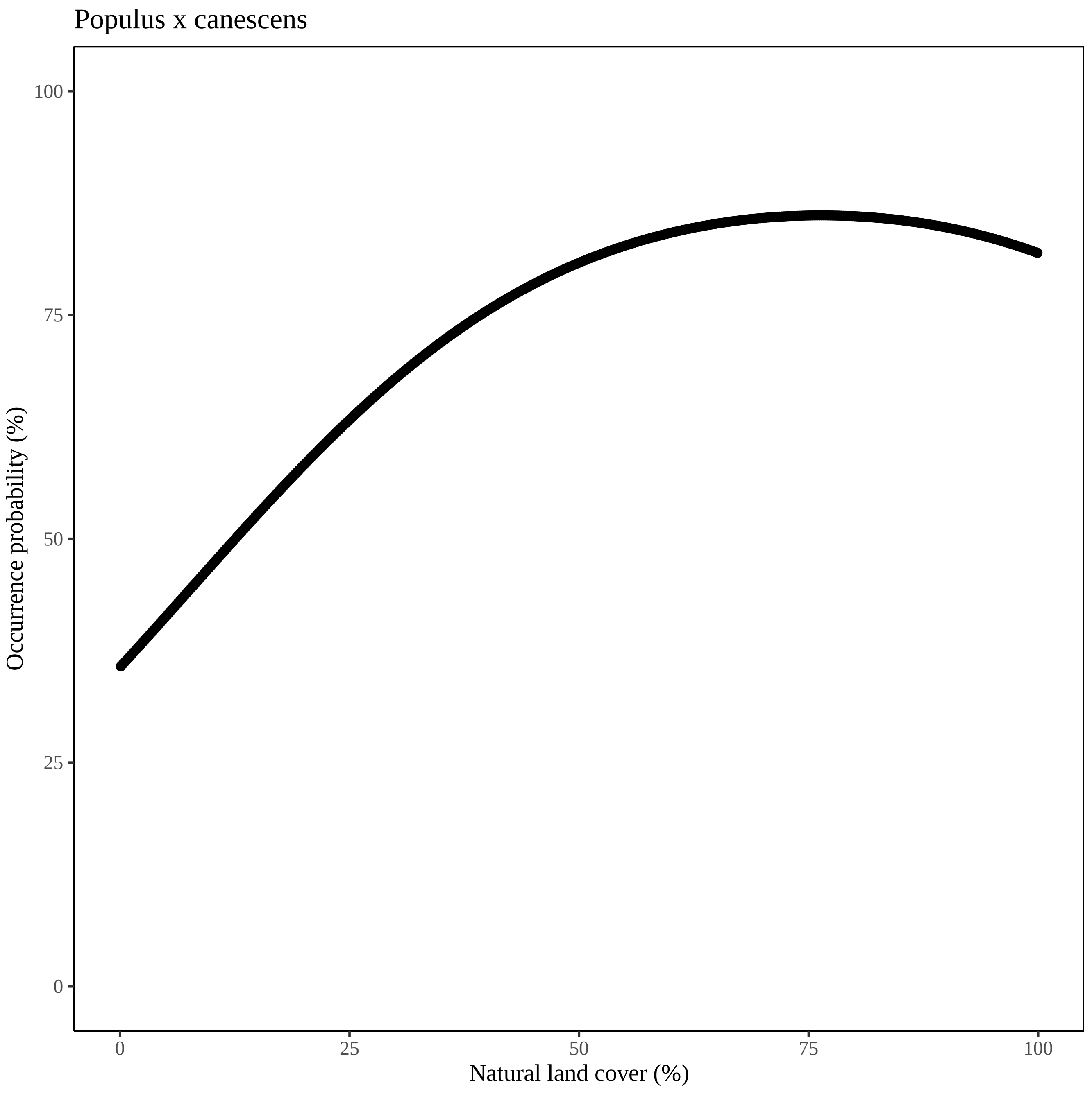

Populus x canescens
Occurrence probability (%)
100
75
50
25
0
0
25
50
75
100
Natural land cover (%)

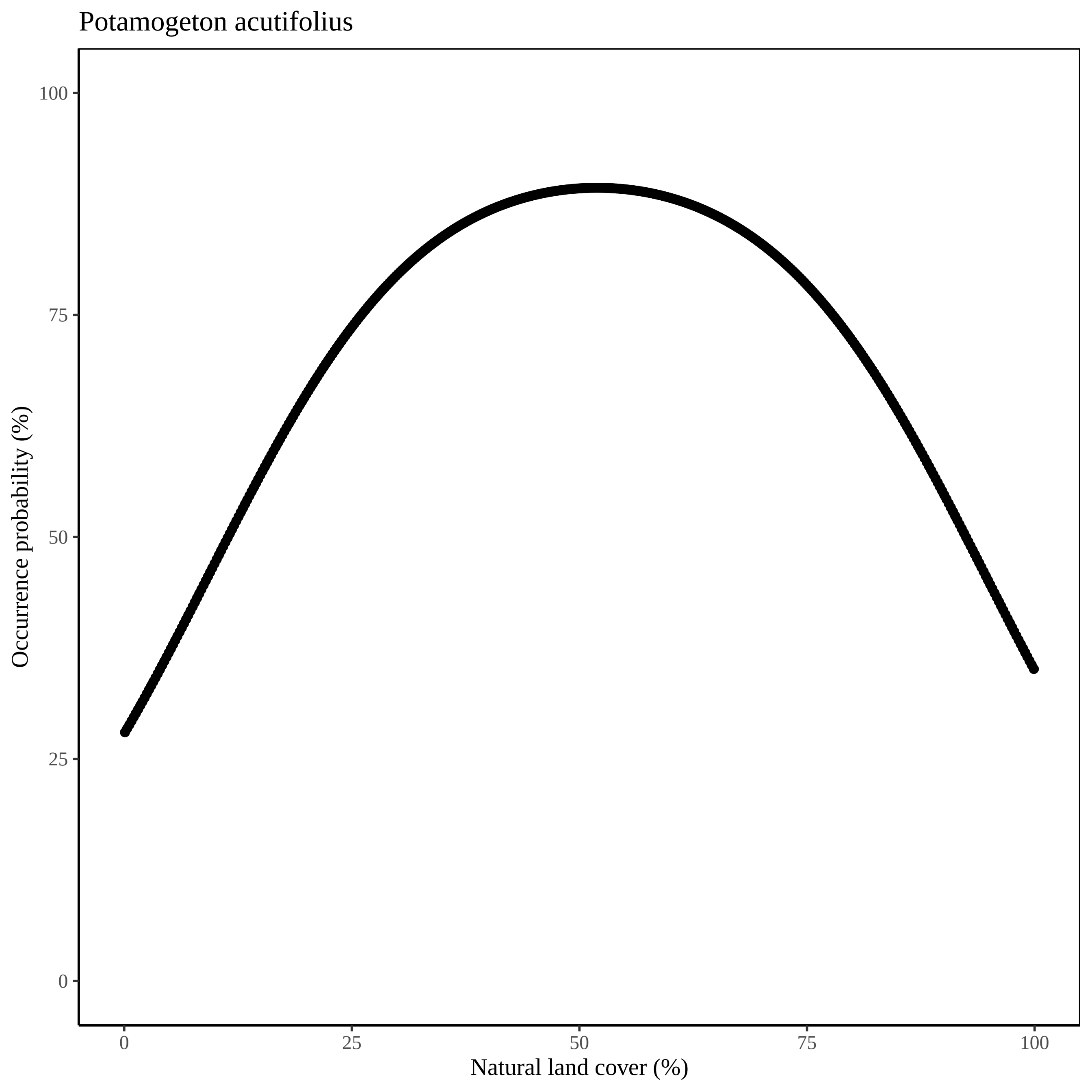
Potamogeton acutifolius
Occurrence probability (%)
100
75
50
25
0
0
25
50
75
100
Natural land cover (%)

Potamogeton alpinus

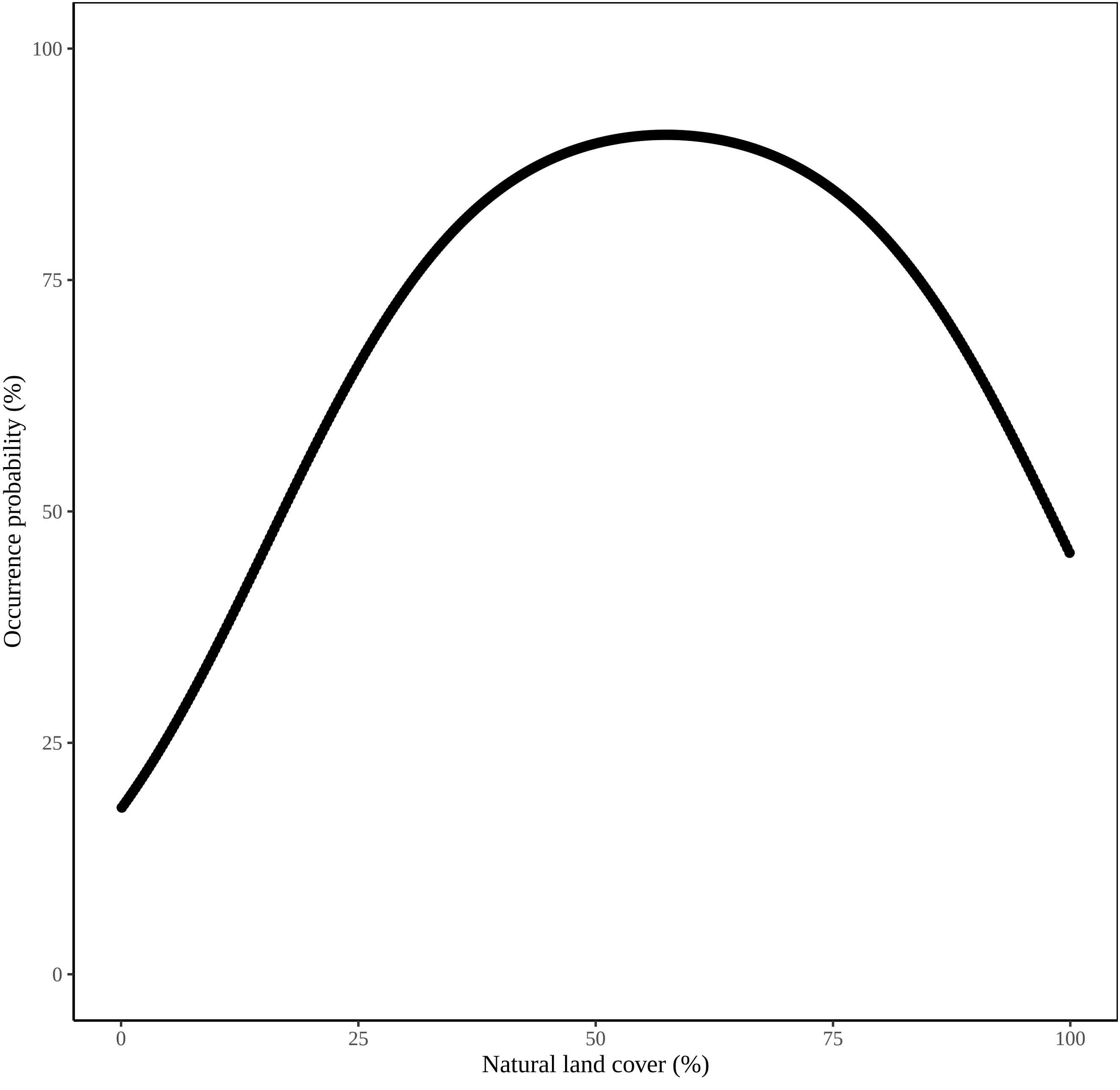

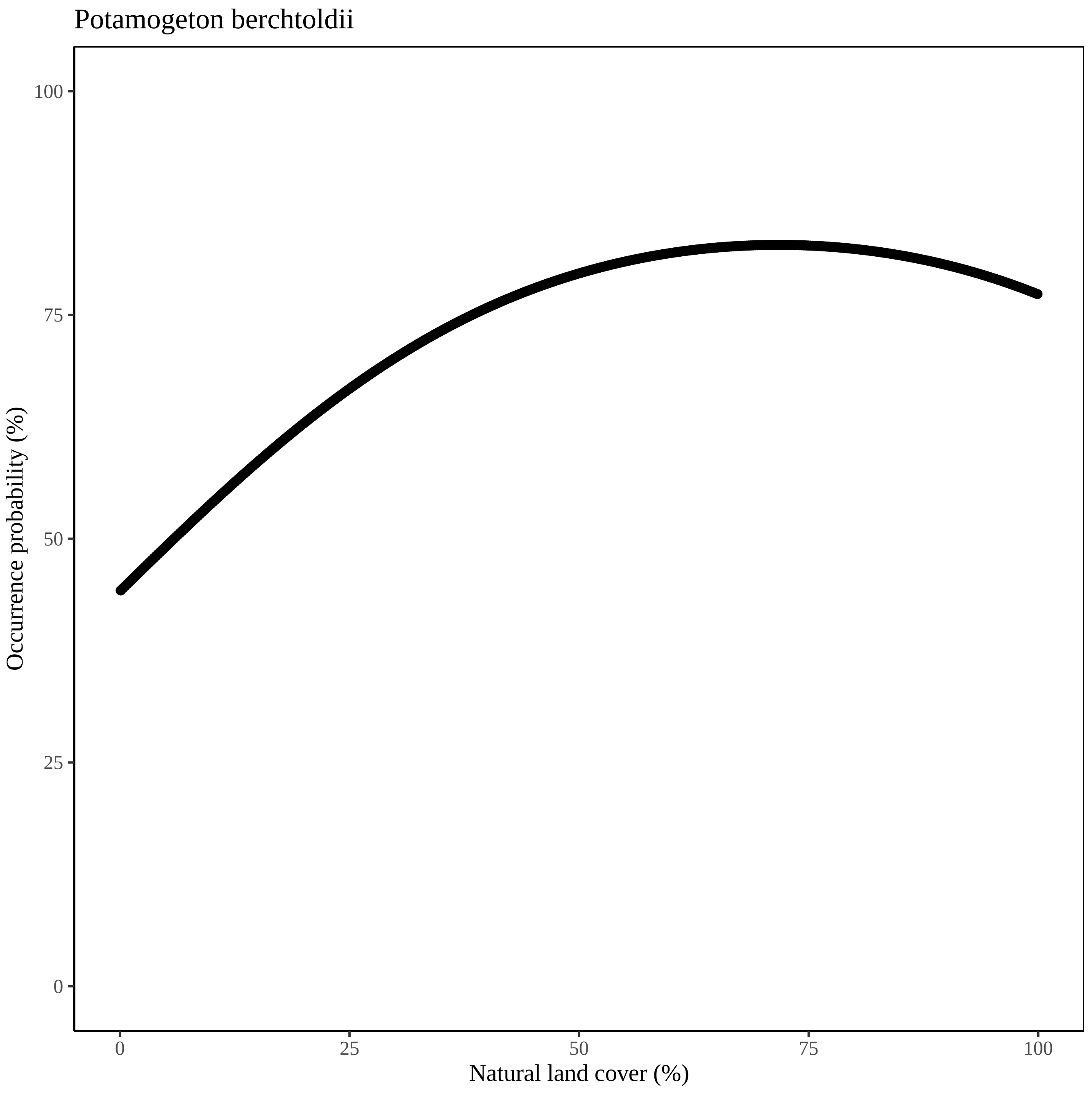

Potamogeton berchtoldii
Occurrence probability (%)
100
75
50
25
0
0
25
50
75
100
Natural land cover (%)

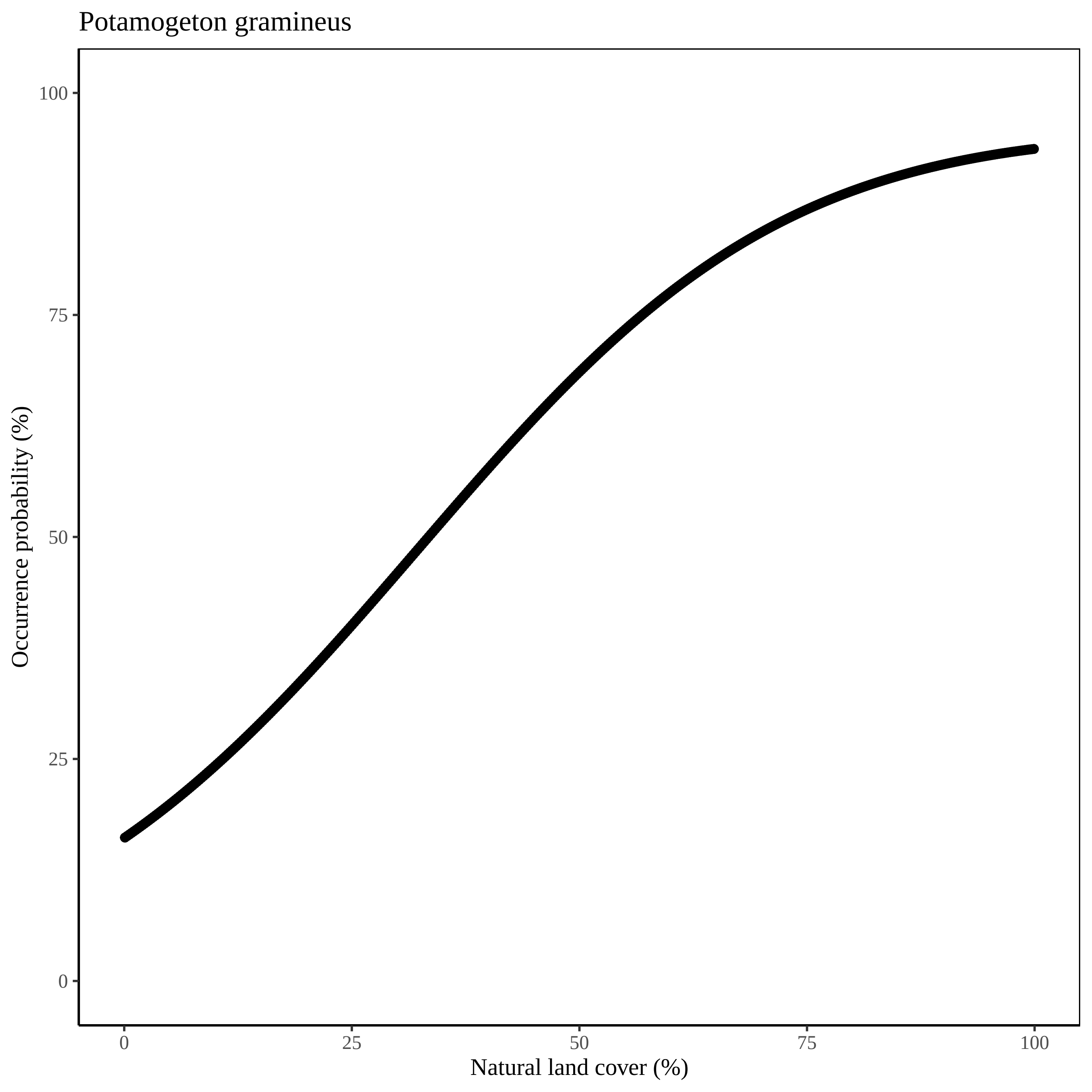

Potamogeton gramineus
Occurrence probability (%)
100
75
50
25
0
0
25
50
75
100
Natural land cover (%)

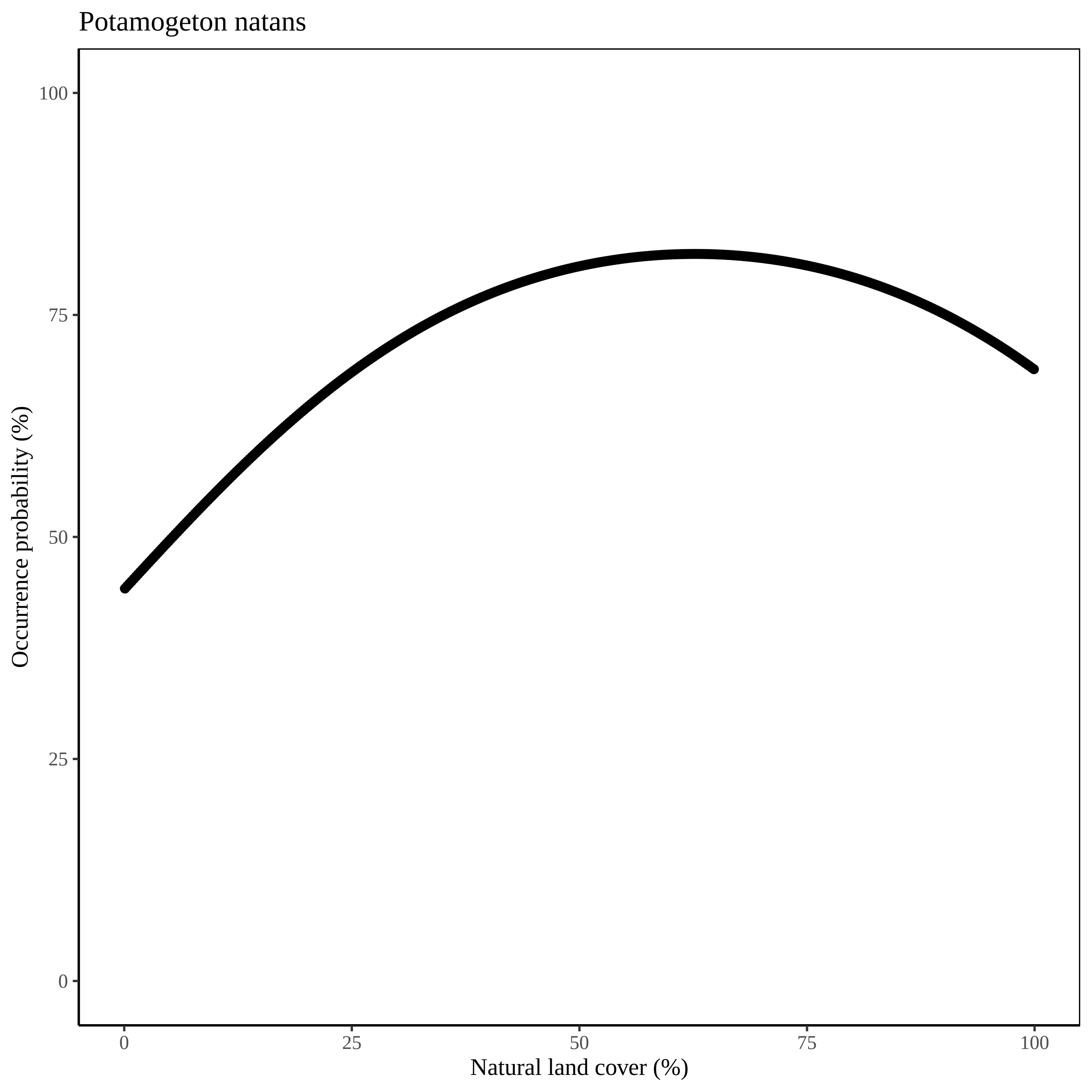
Potamogeton natans
Occurrence probability (%)
100
75
50
25
0
0
25
50
75
100
Natural land cover (%)

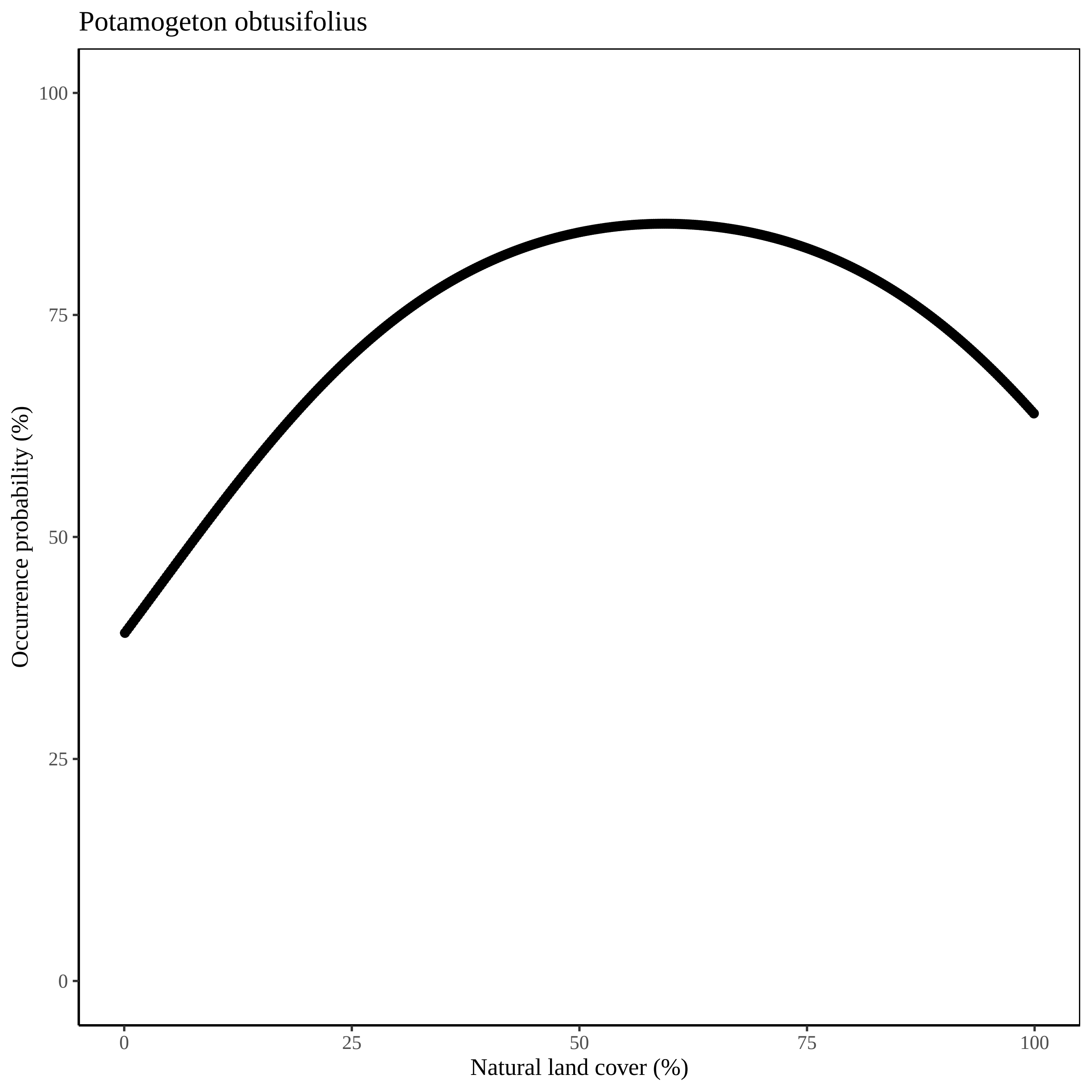
Potamogeton obtusifolius
Occurrence probability (%)
Natural land cover (%)
0
25
50
75
100
0
25
50
75
100

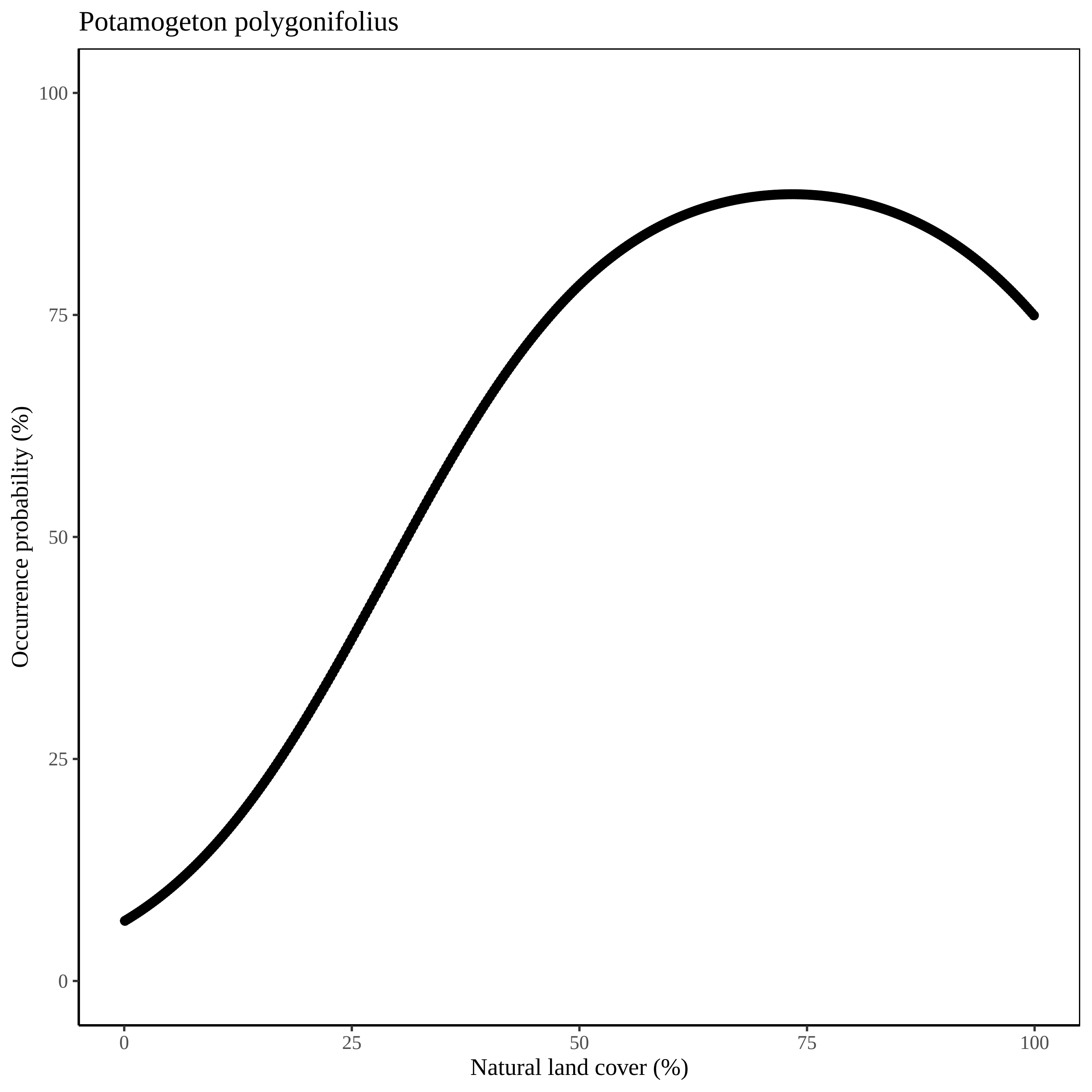
Potamogeton polygonifolius
Occurrence probability (%)
100
75
50
25
0
0
25
50
75
100
Natural land cover (%)

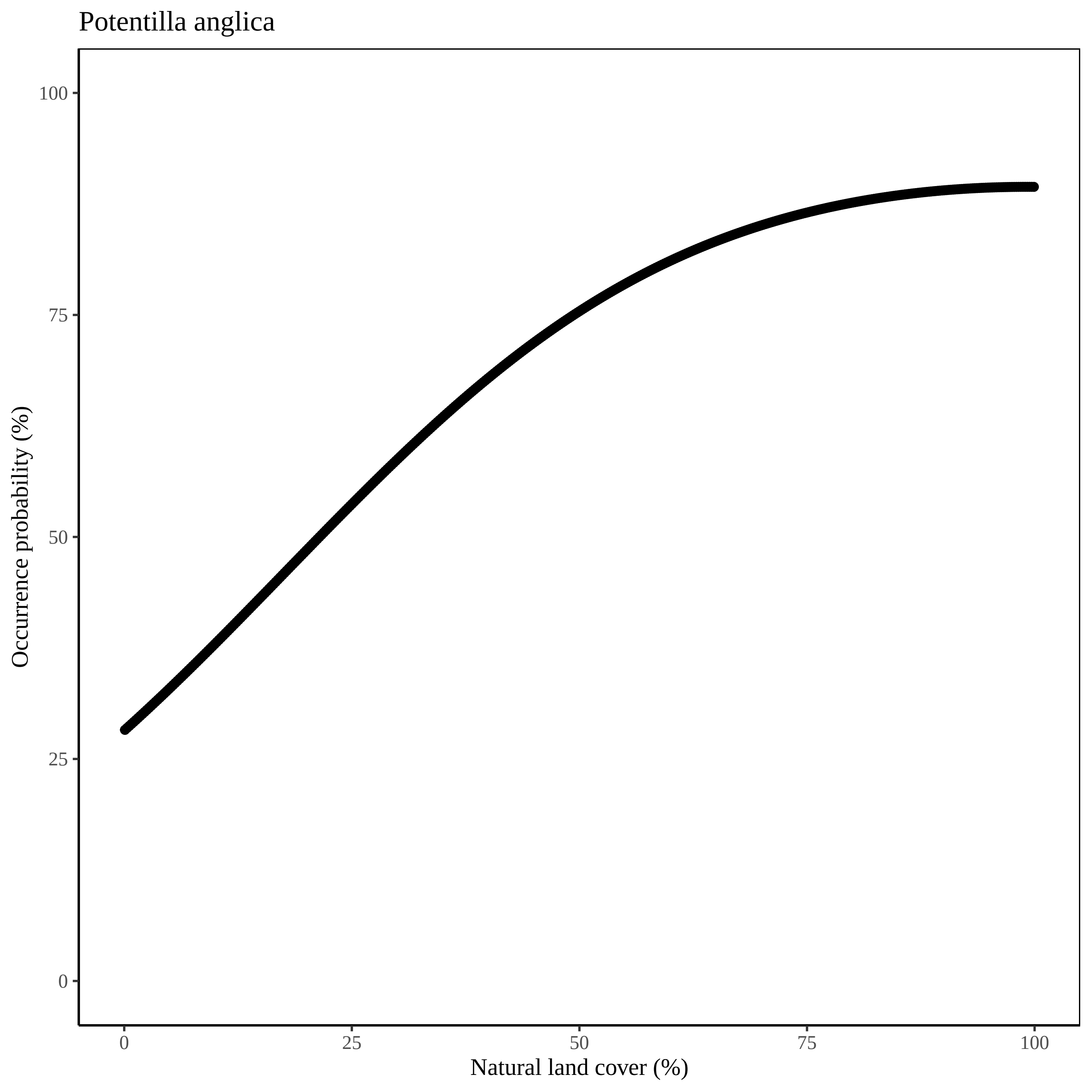

Potentilla anglica
100
75
50
25
0
Occurrence probability (%)
0
25
50
75
100
Natural land cover (%)

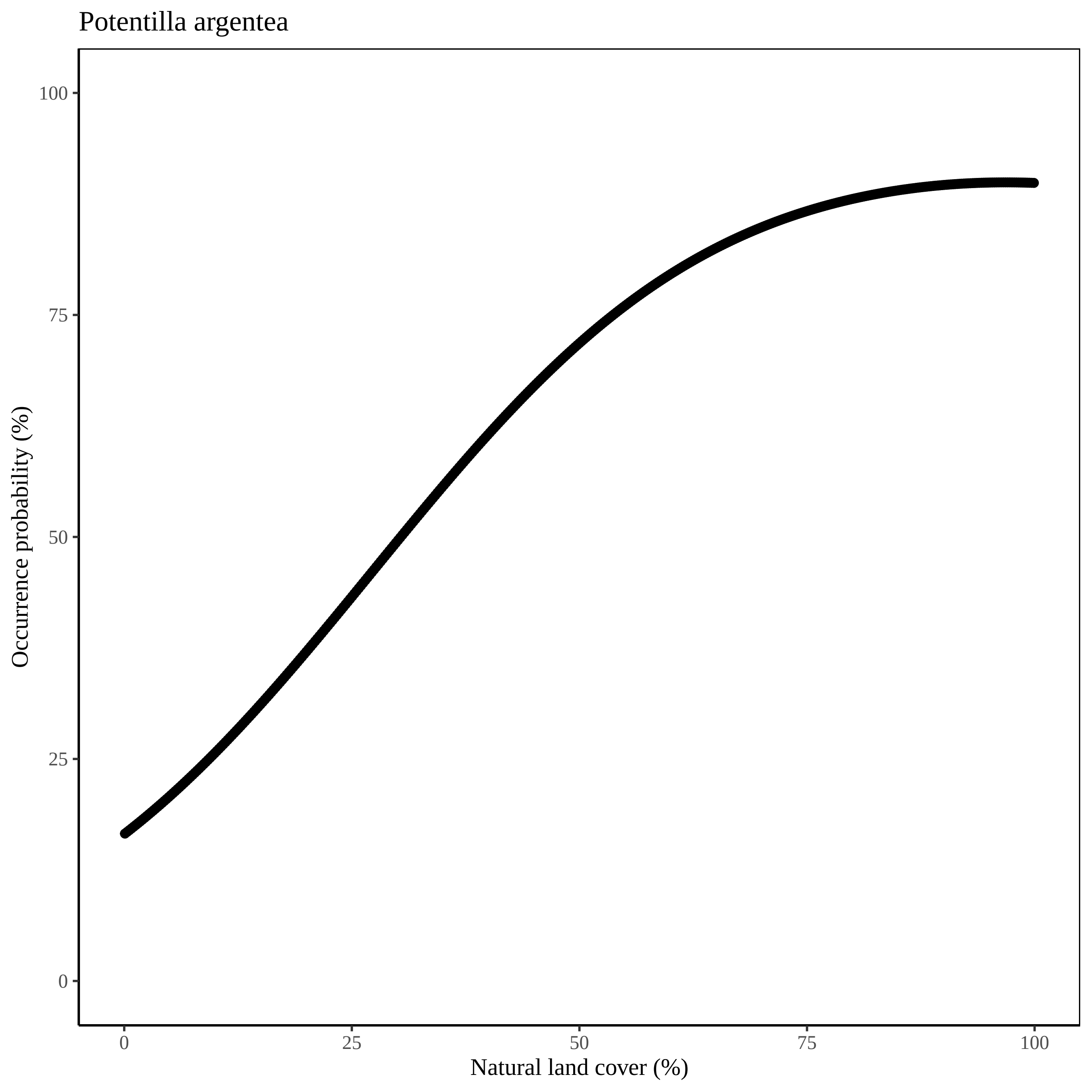
Potentilla argentea
Occurrence probability (%)
100
75
50
25
0
0
25
50
75
100
Natural land cover (%)

Potentilla erecta

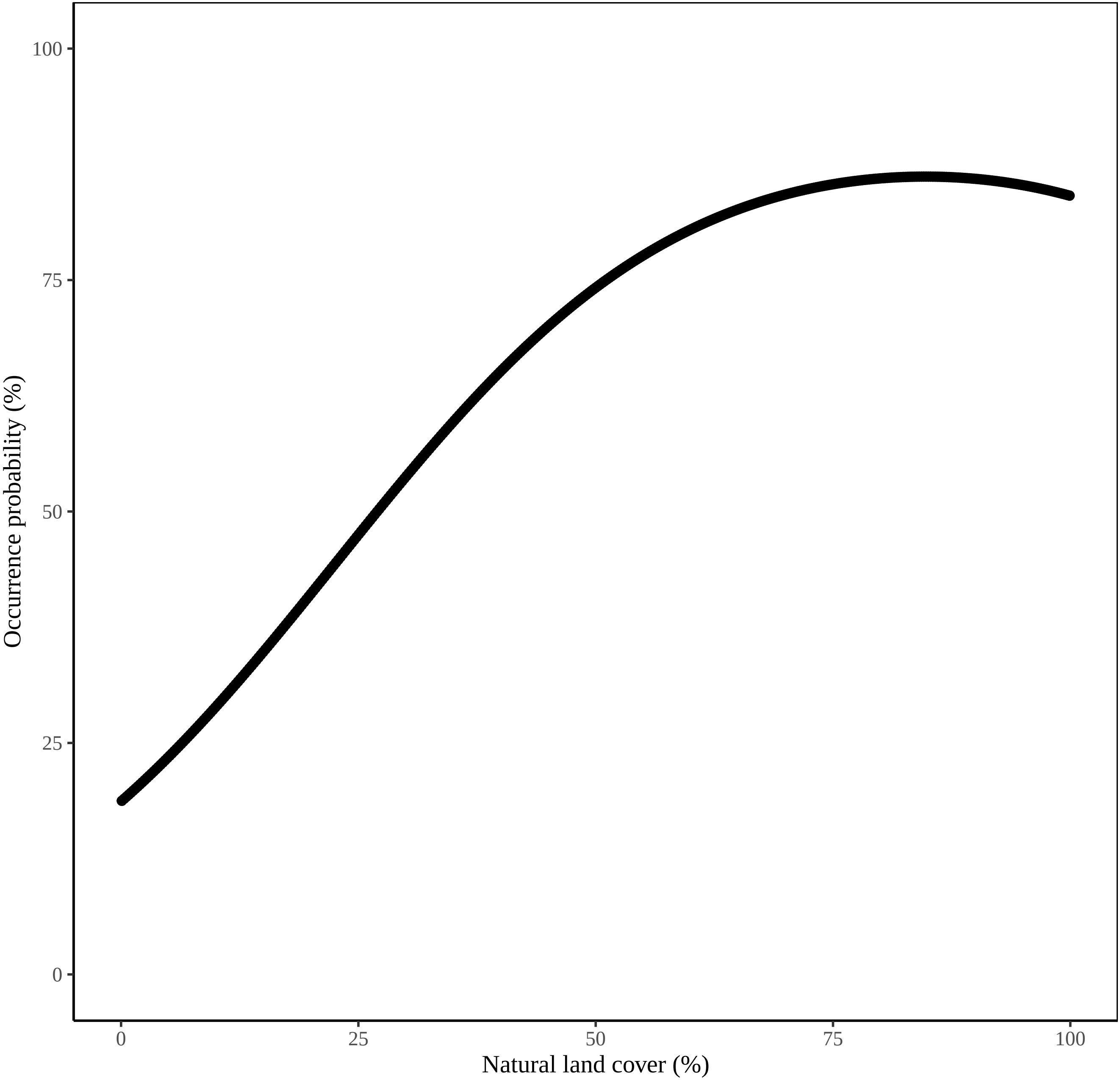

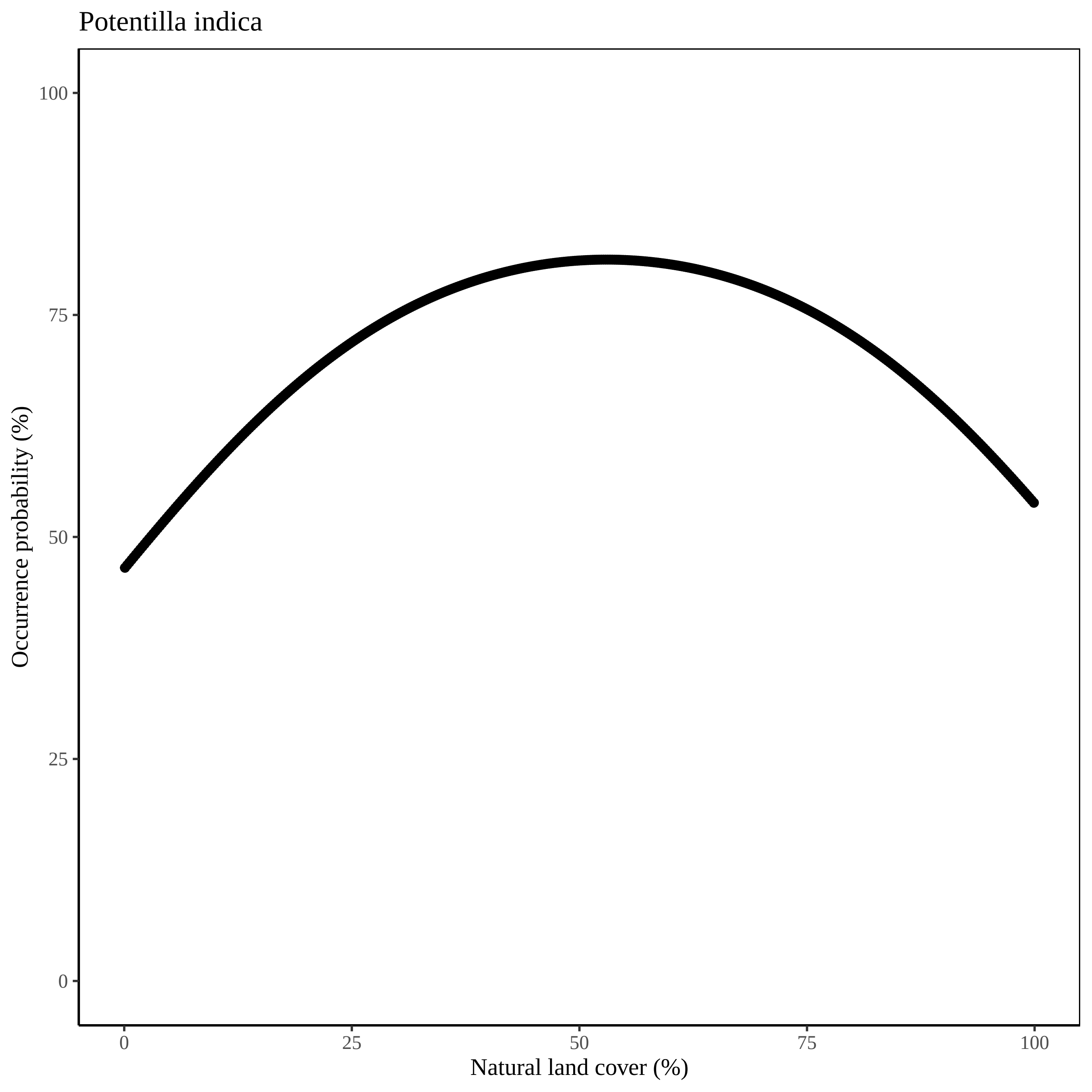
Potentilla indica
Occurrence probability (%)
100
75
50
25
0
0
25
50
75
100
Natural land cover (%)

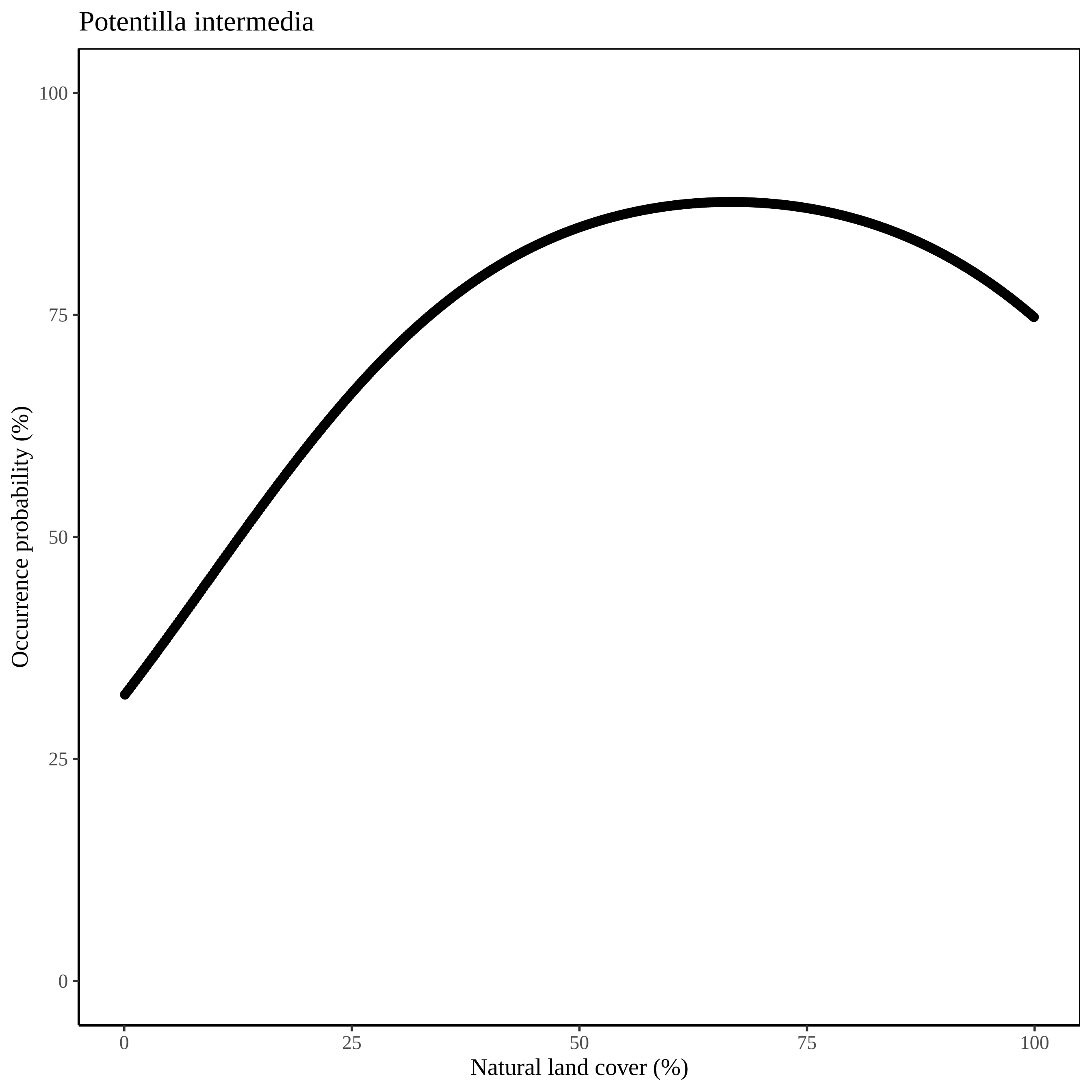
Potentilla intermedia
100
75
50
25
0
Occurrence probability (%)
0
25
50
75
100
Natural land cover (%)

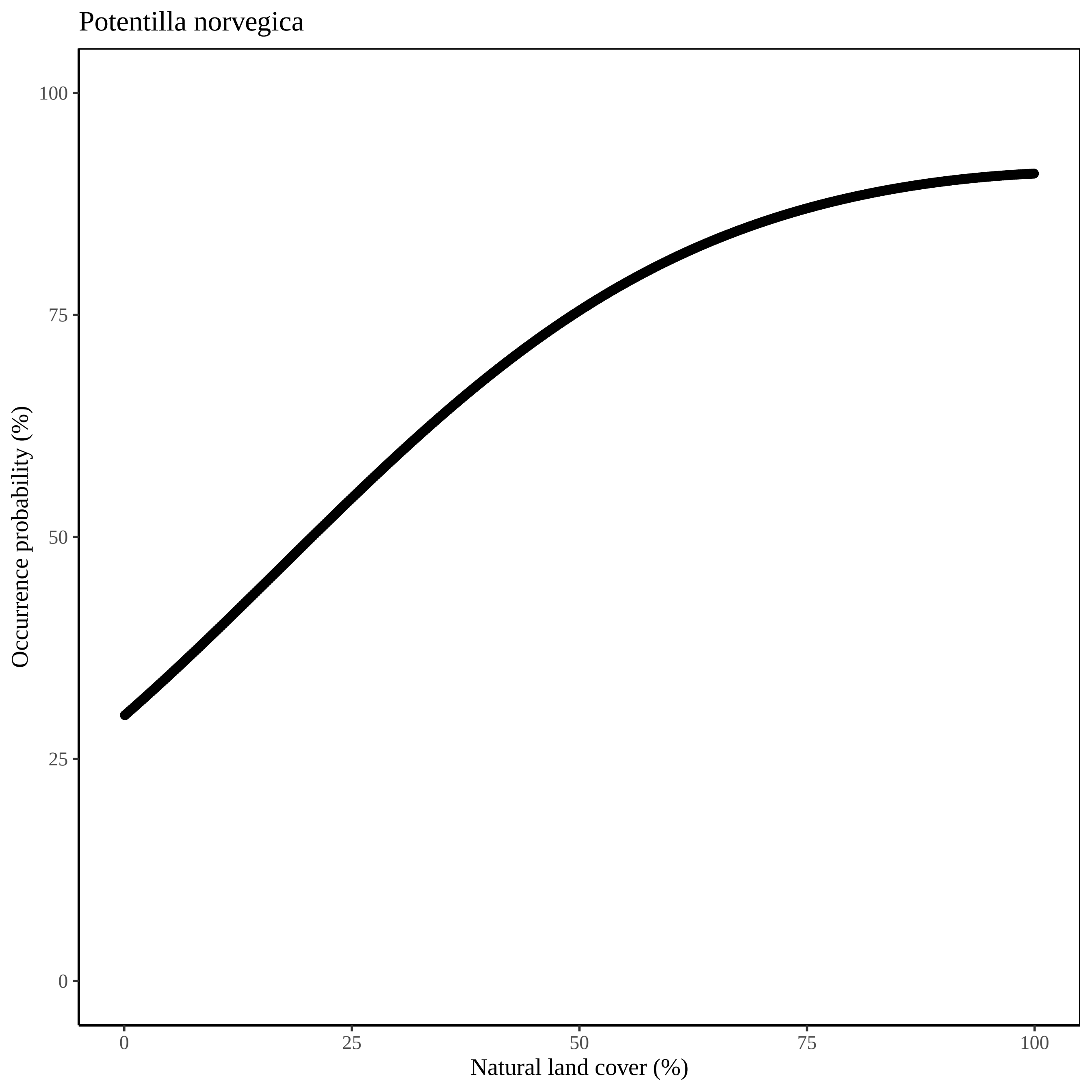
Potentilla norvegica
Occurrence probability (%)
100
75
50
25
0
0
25
50
75
100
Natural land cover (%)

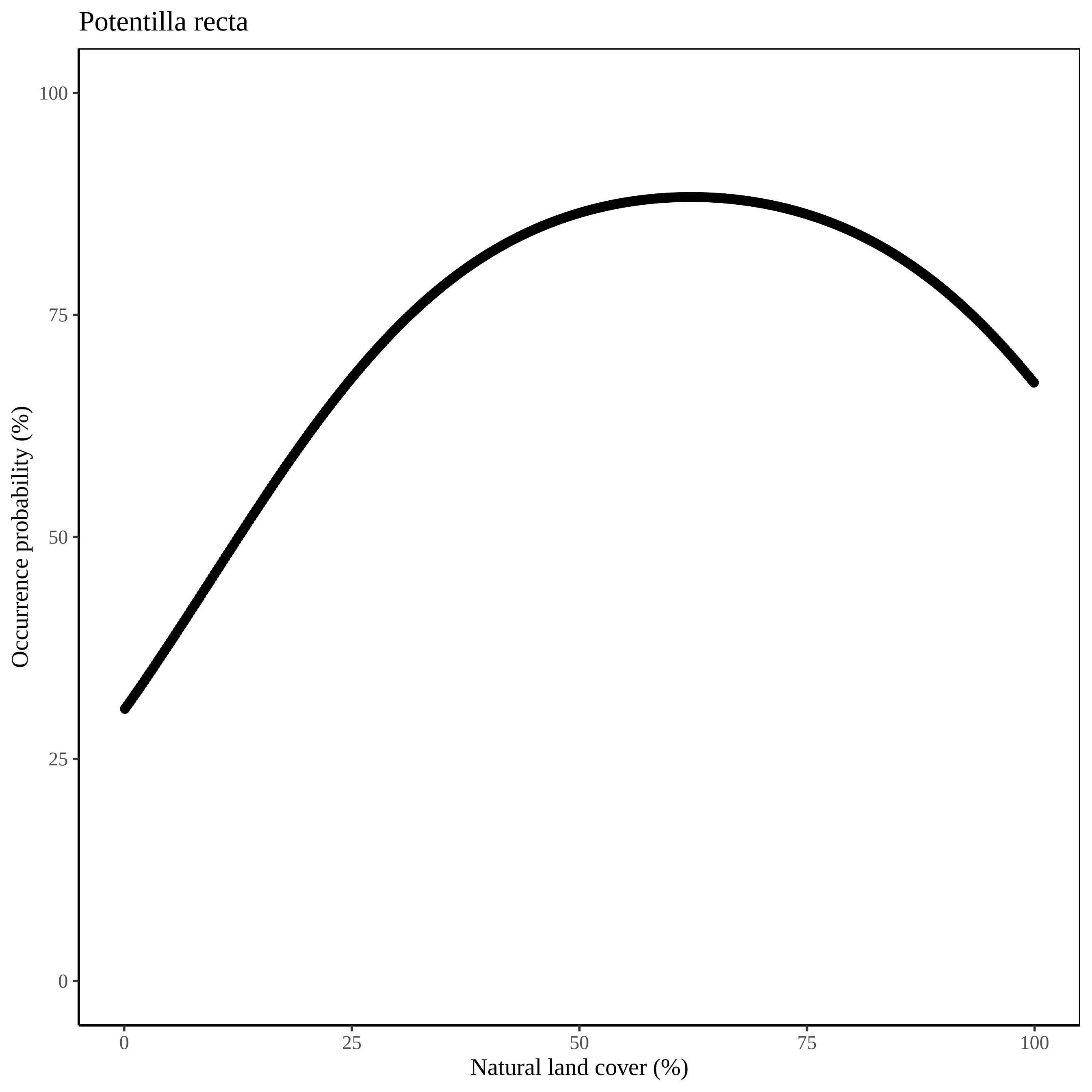

Potentilla recta
Occurrence probability (%)
100
75
50
25
0
0
25
50
75
100
Natural land cover (%)

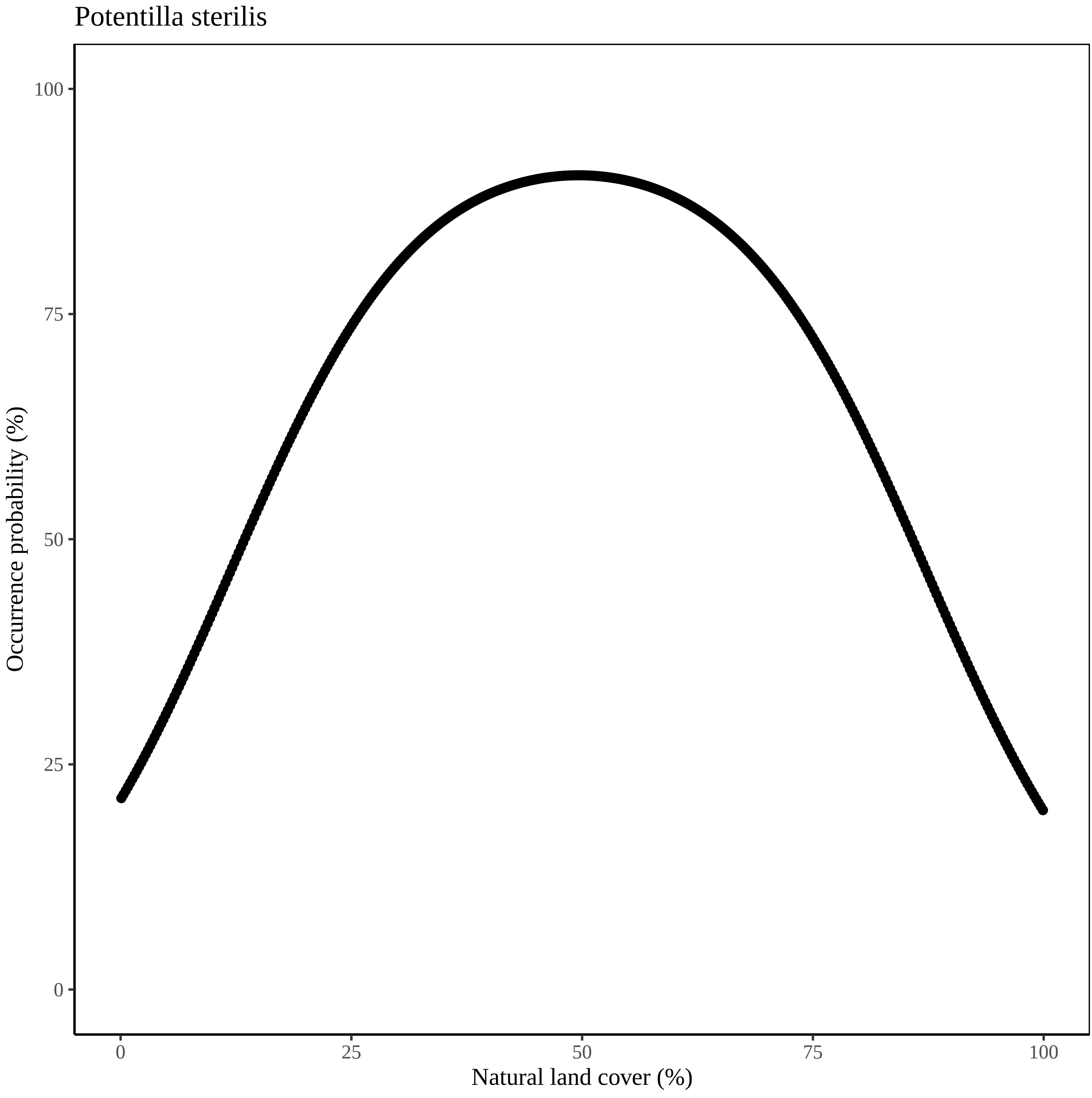

Potentilla sterilis
100
75
50
25
0
Occurrence probability (%)
0
25
50
75
100
Natural land cover (%)

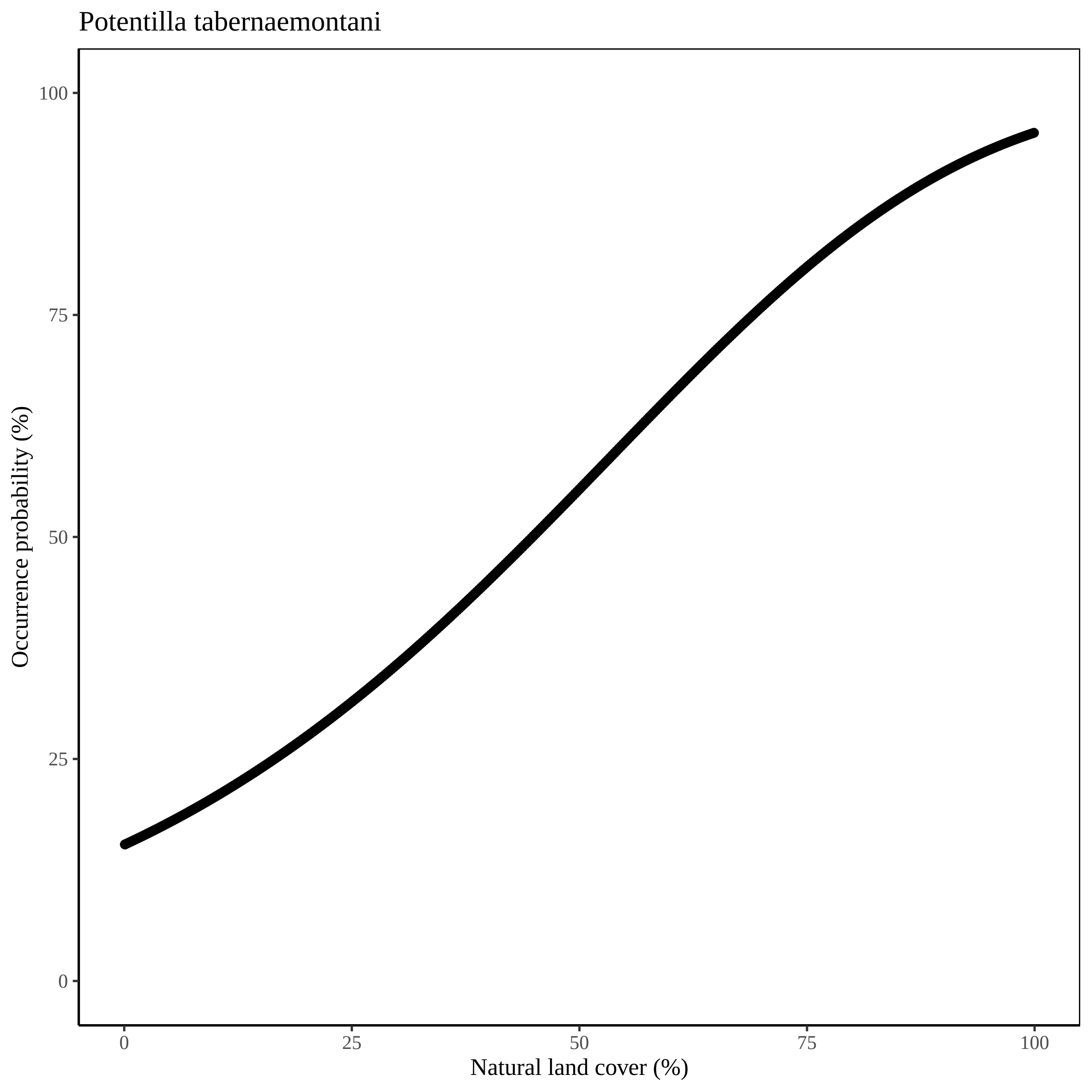
Potentilla tabernaemontani
Occurrence probability (%)
100
75
50
25
0
0
25
50
75
100
Natural land cover (%)

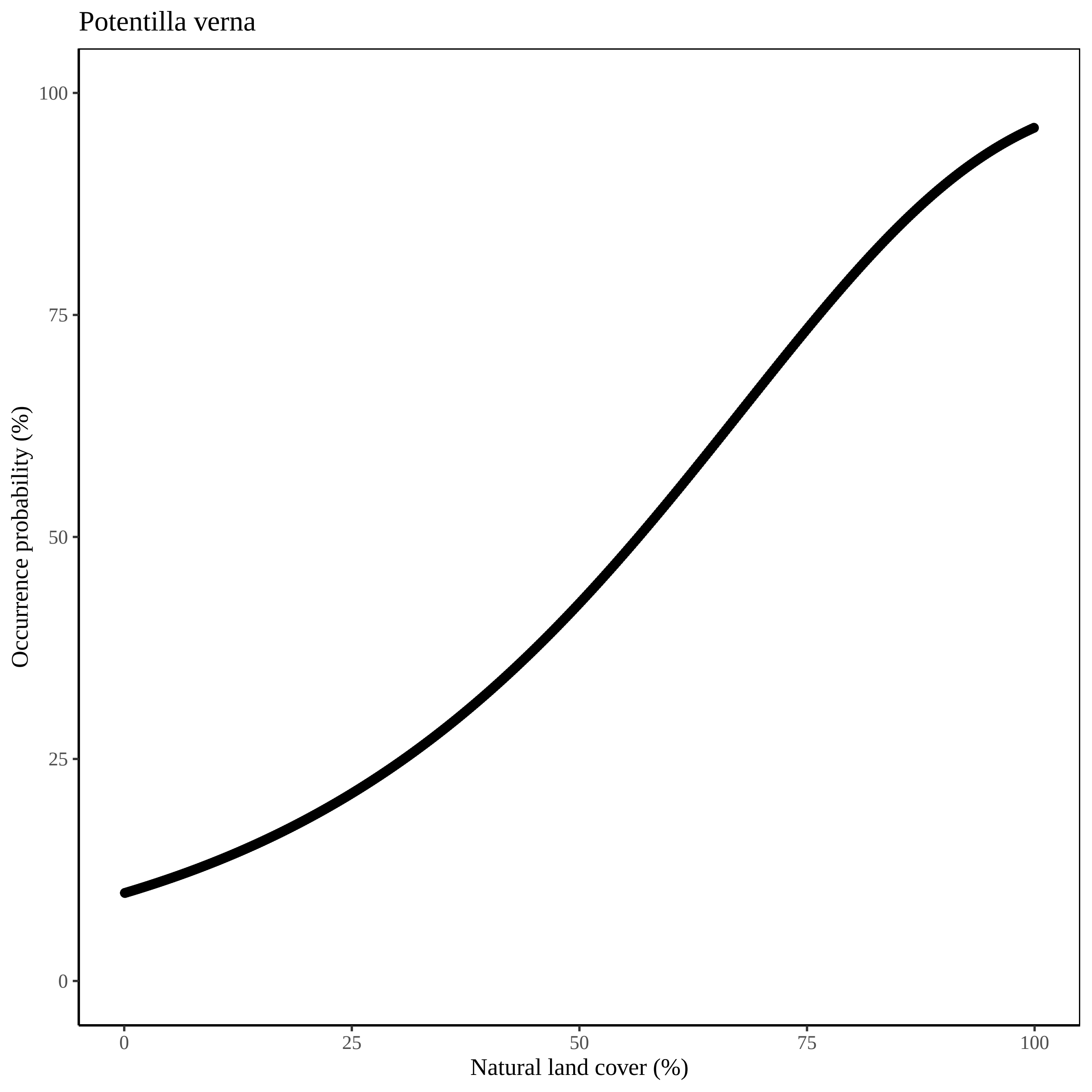
Potentilla verna
Occurrence probability (%)
100
75
50
25
0
0
25
50
75
100
Natural land cover (%)

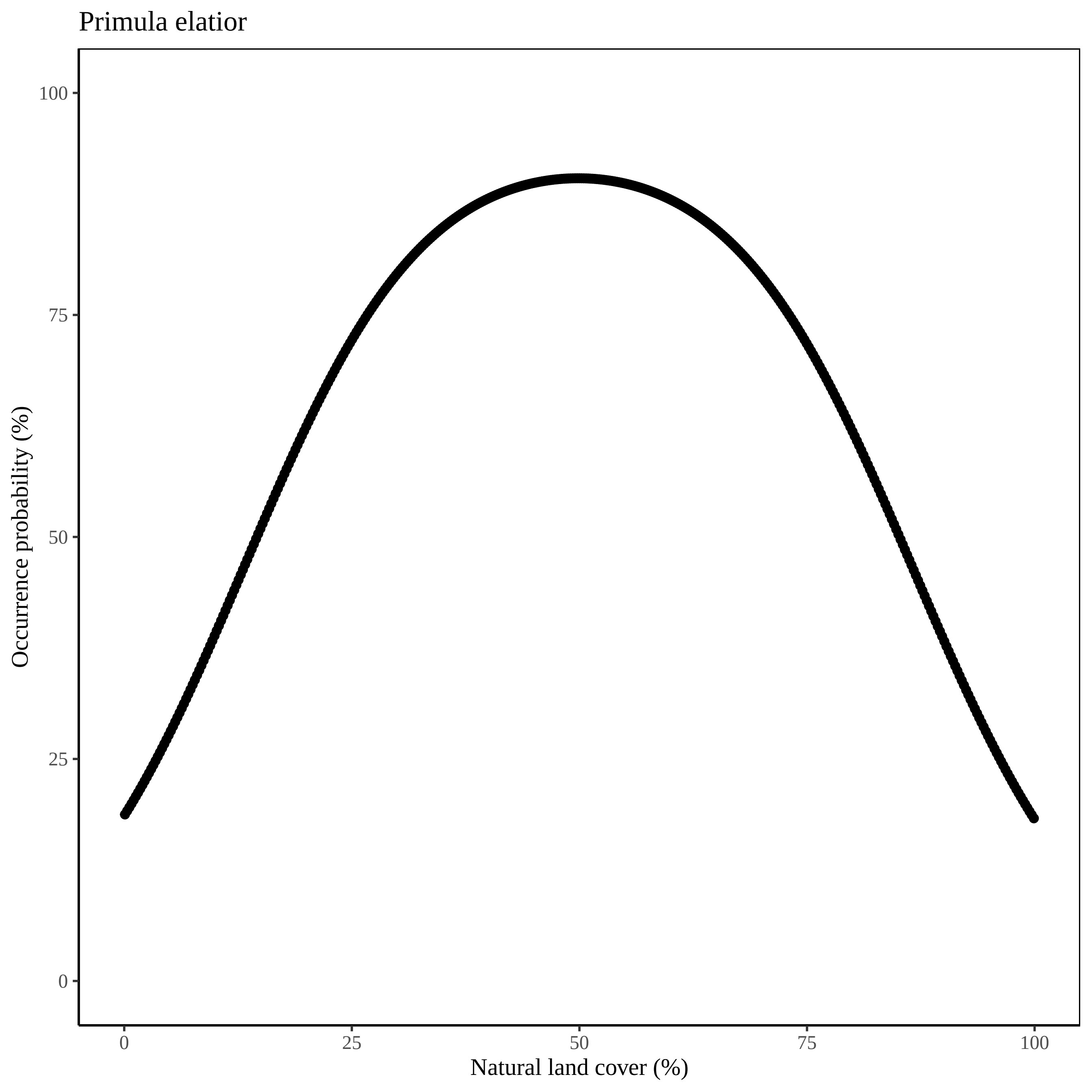
Primula elatior
Occurrence probability (%)
100
75
50
25
0
0
25
50
75
100
Natural land cover (%)

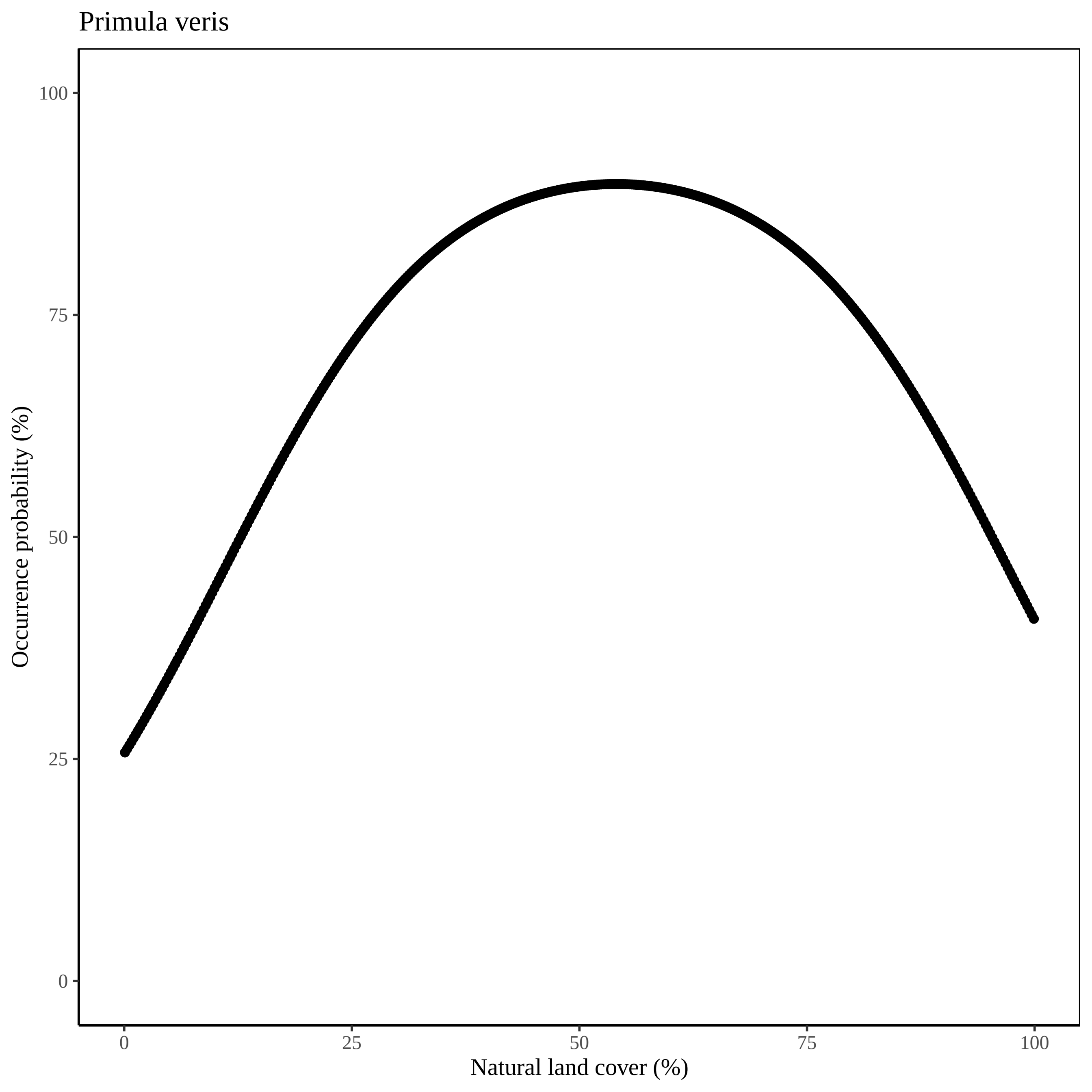

Primula veris
Occurrence probability (%)
100
75
50
25
0
0
25
50
75
100
Natural land cover (%)

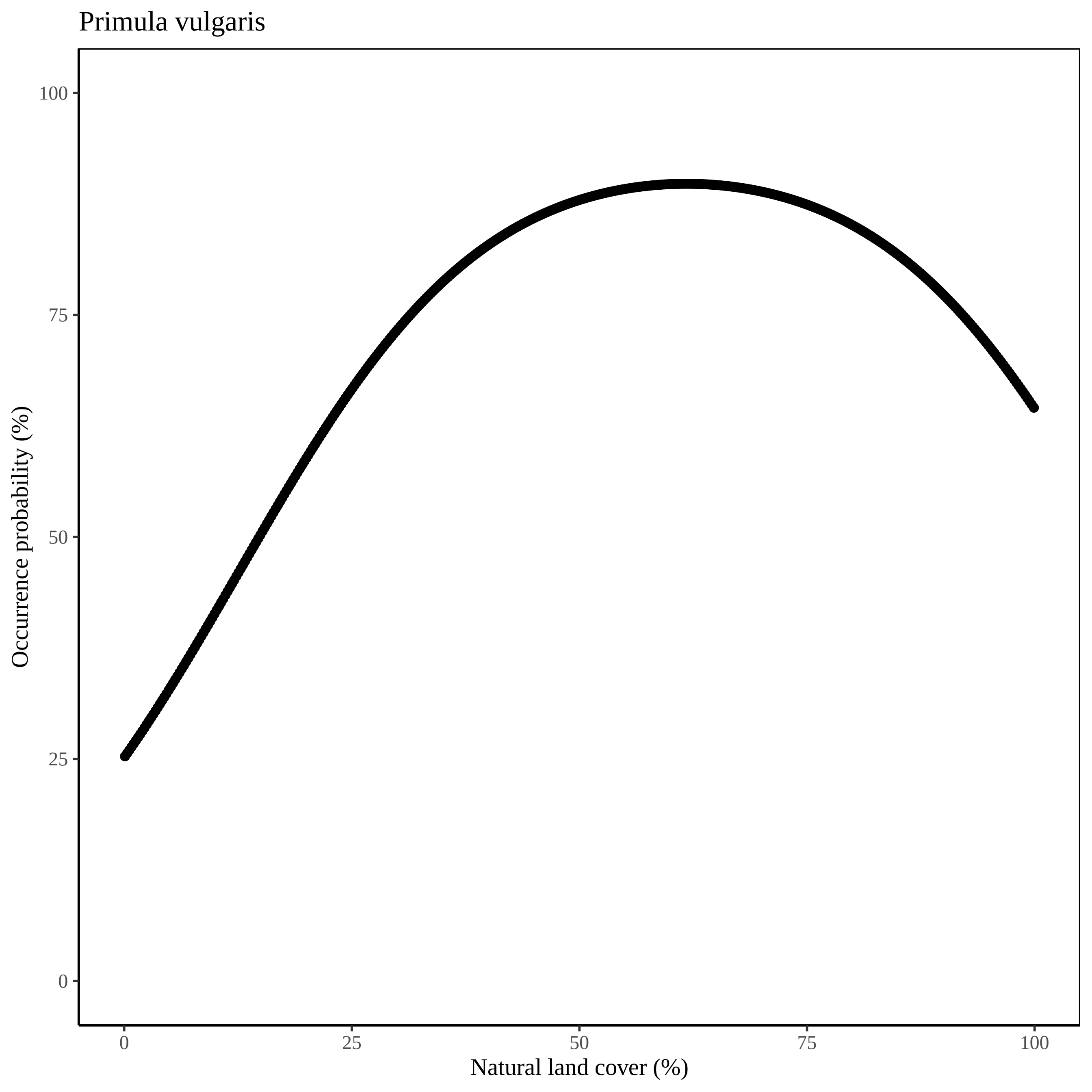
Primula vulgaris
Occurrence probability (%)
100
75
50
25
0
Natural land cover (%)
0
25
50
75
100

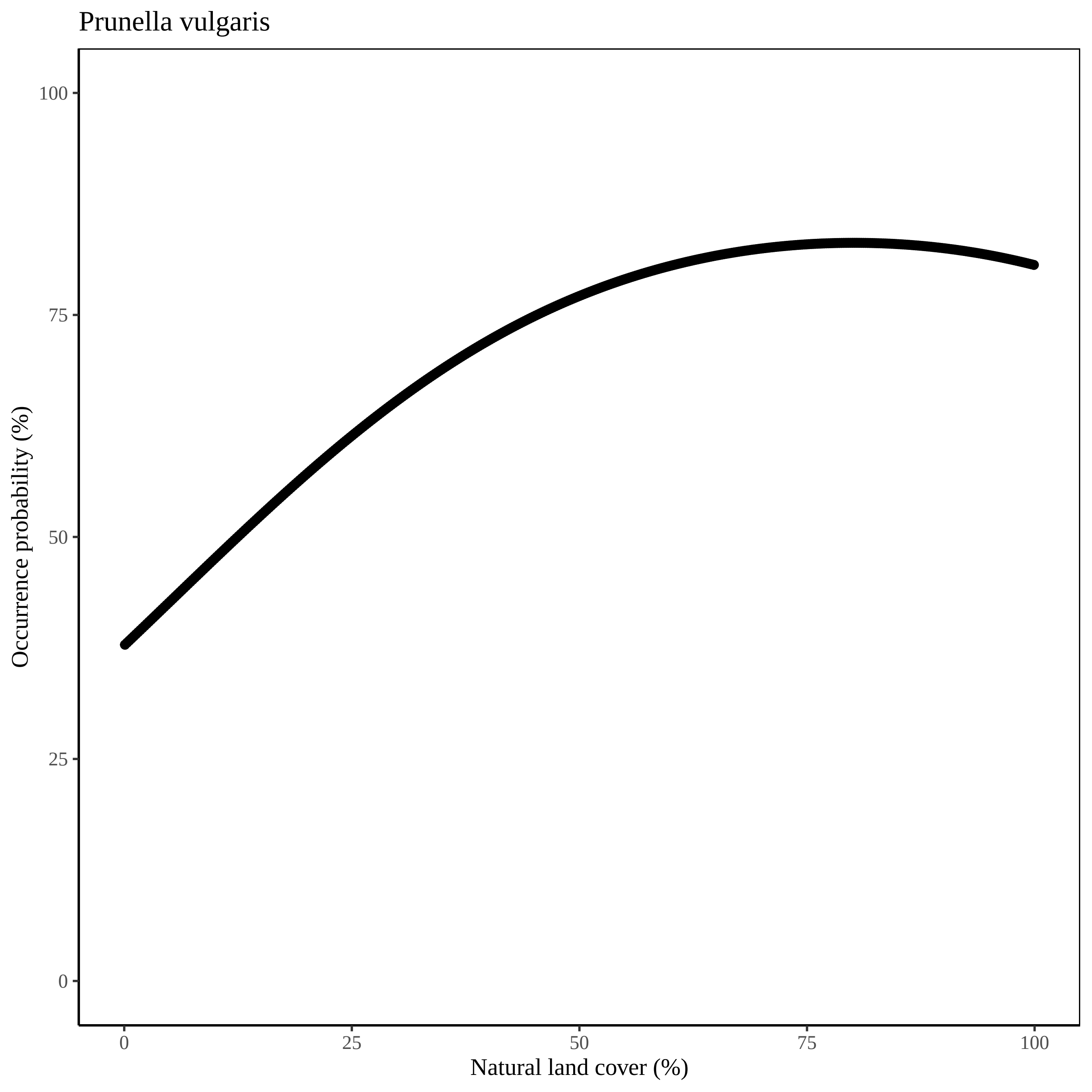
Prunella vulgaris
Occurrence probability (%)
100
75
50
25
0
0
25
50
75
100
Natural land cover (%)

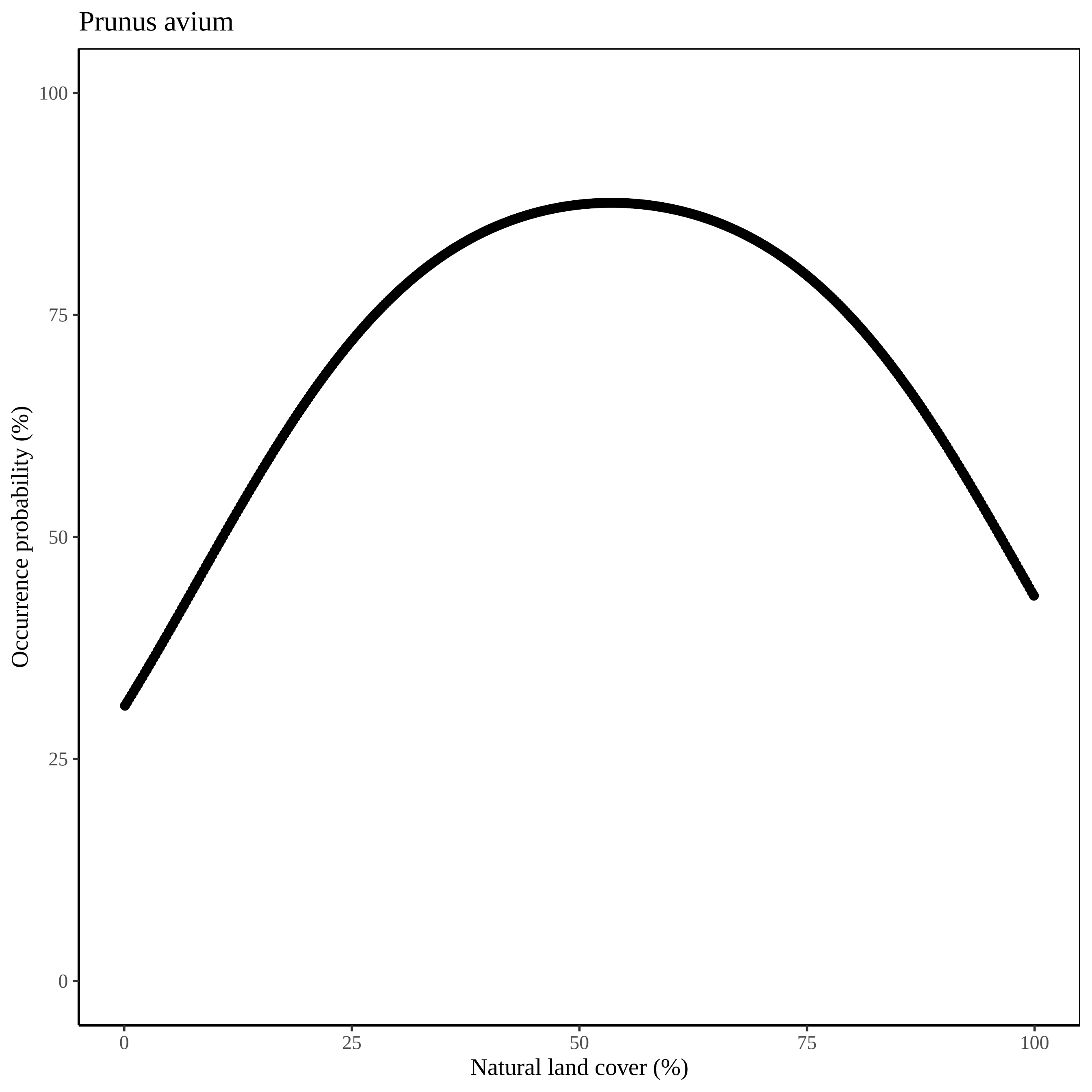
Prunus avium
Occurrence probability (%)
100
75
50
25
0
0
25
50
75
100
Natural land cover (%)

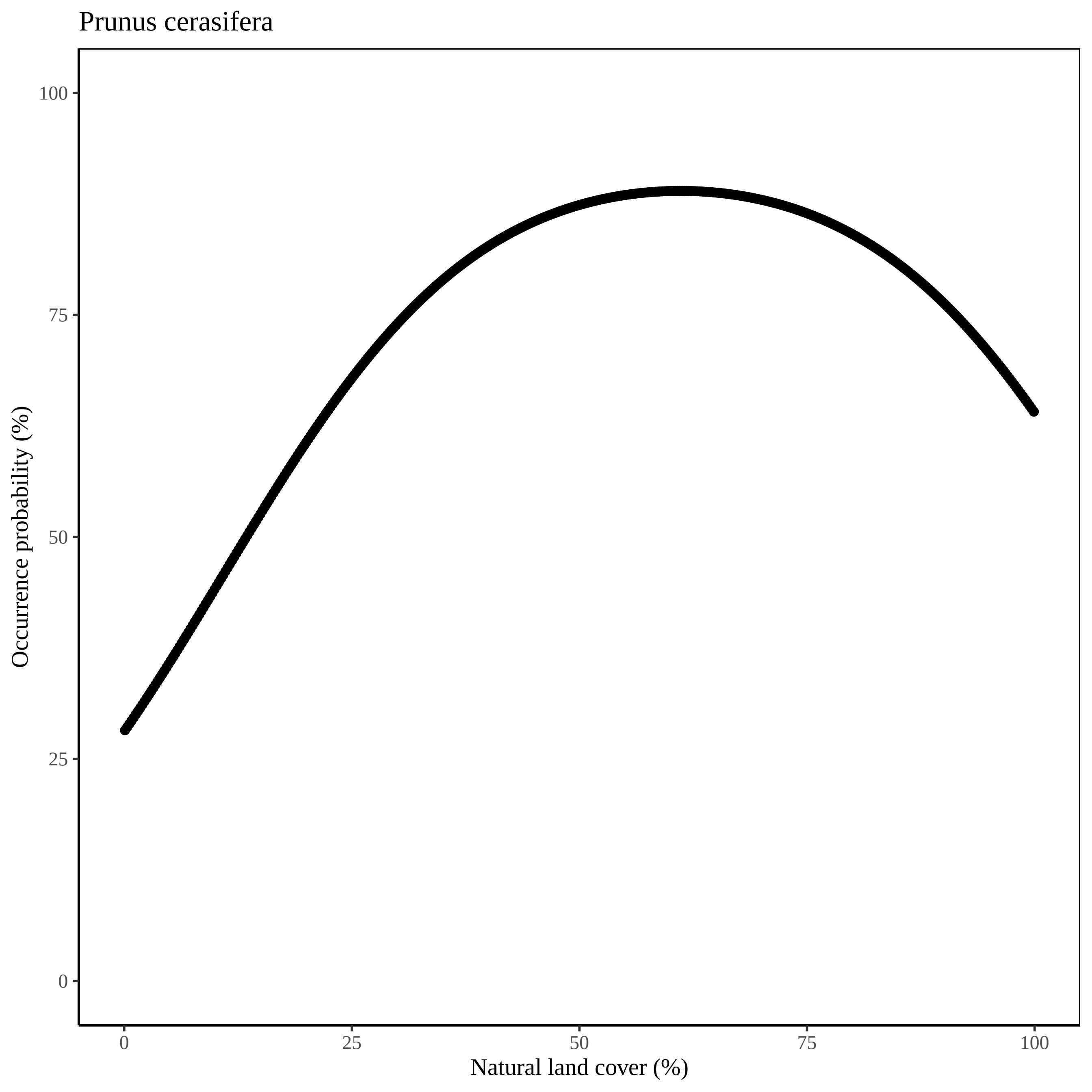
Prunus cerasifera
Occurrence probability (%)
100
75
50
25
0
0
25
50
75
100
Natural land cover (%)

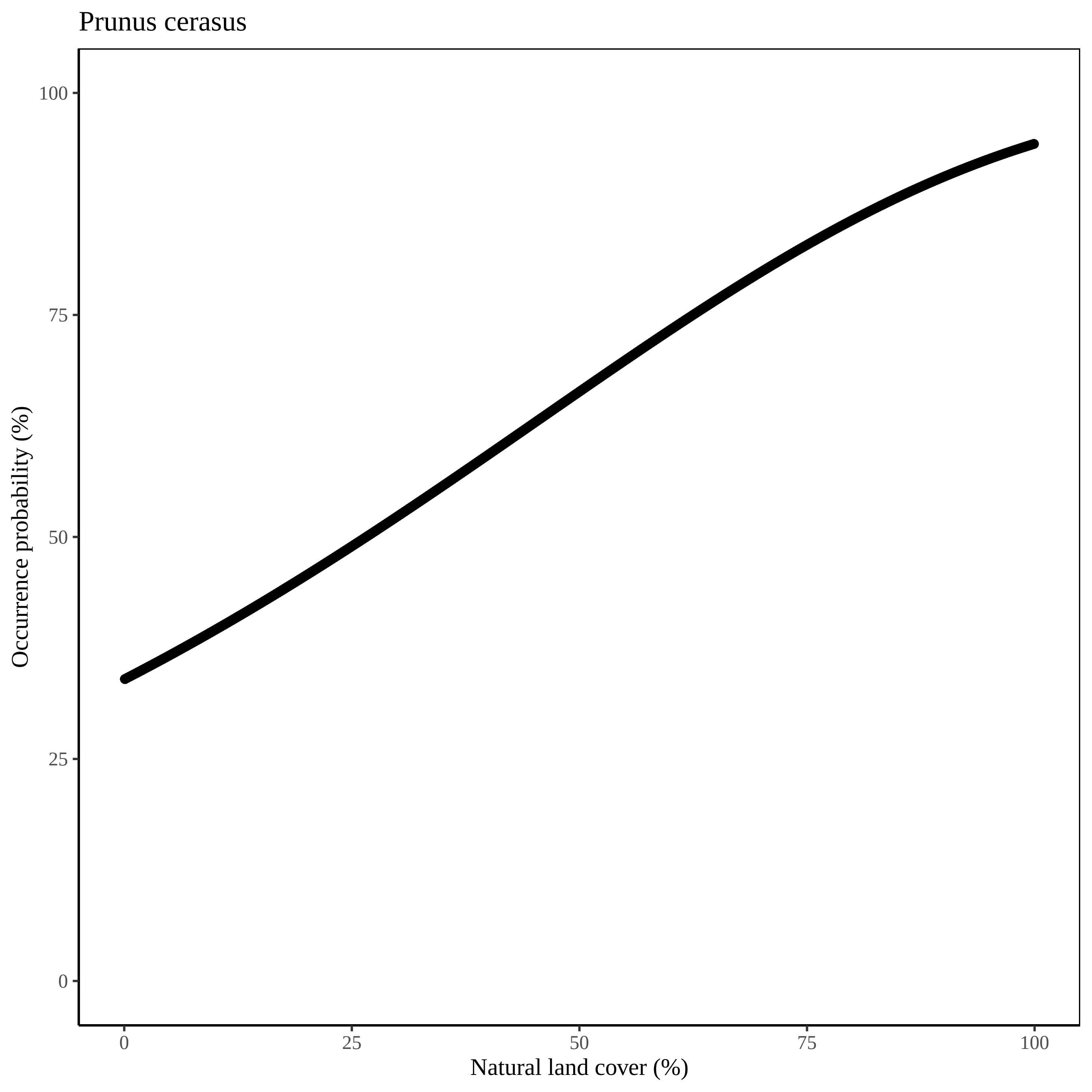
Prunus cerasus
100
75
50
25
0
Occurrence probability (%)
0
25
50
75
100
Natural land cover (%)

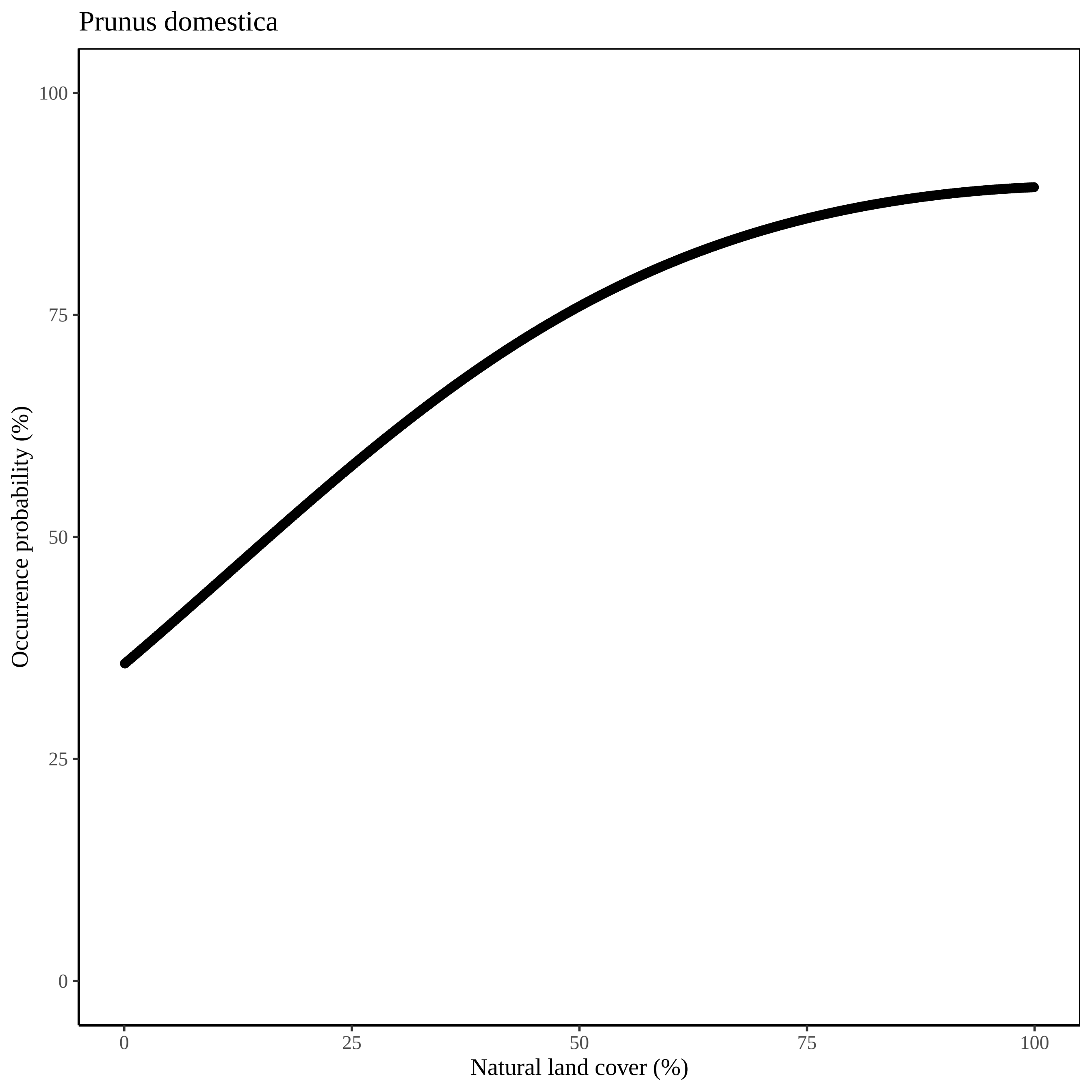

Prunus domestica
100
75
50
25
0
Occurrence probability (%)
0
25
50
75
100
Natural land cover (%)

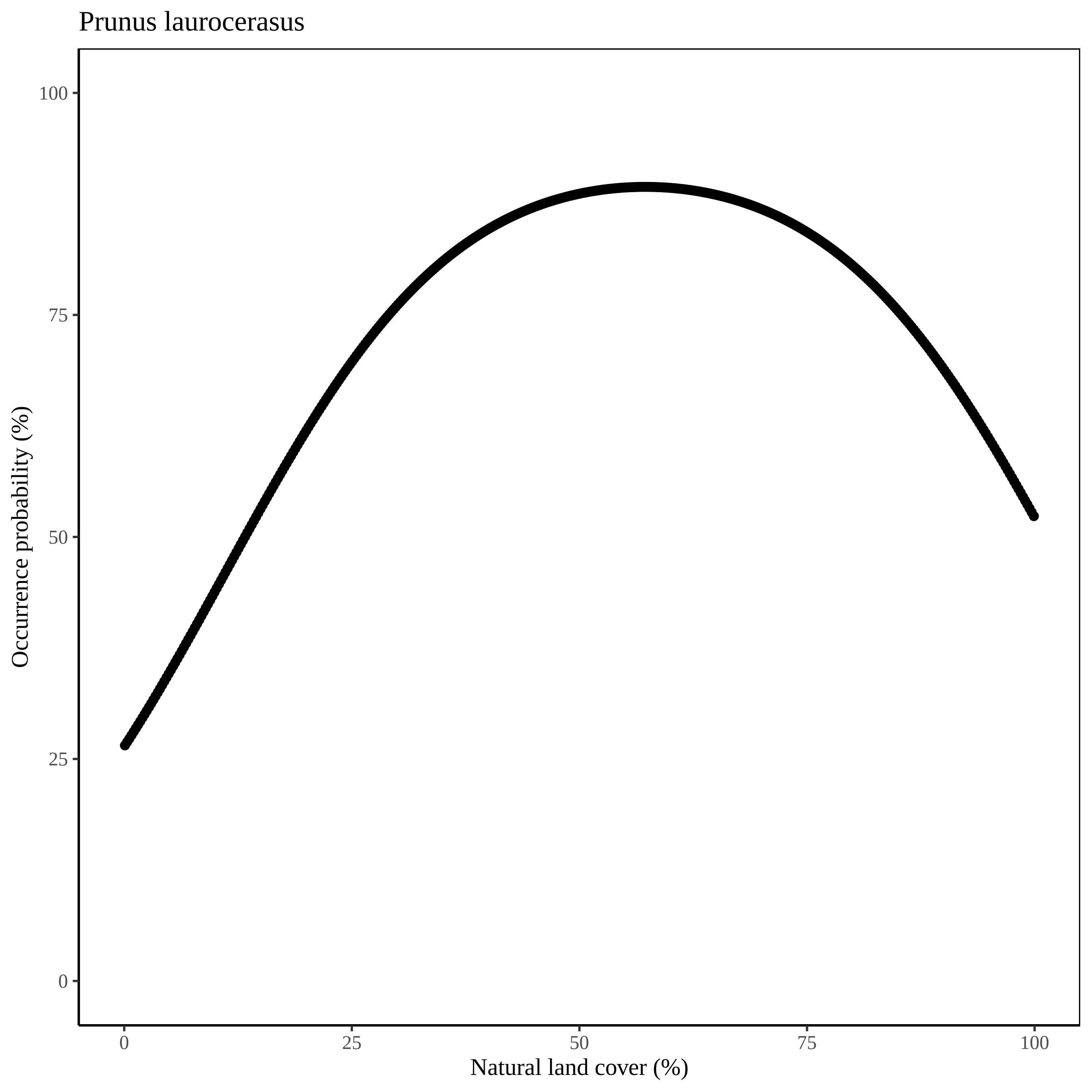
Prunus laurocerasus
100
75
50
25
0
Occurrence probability (%)
0
25
50
75
100
Natural land cover (%)

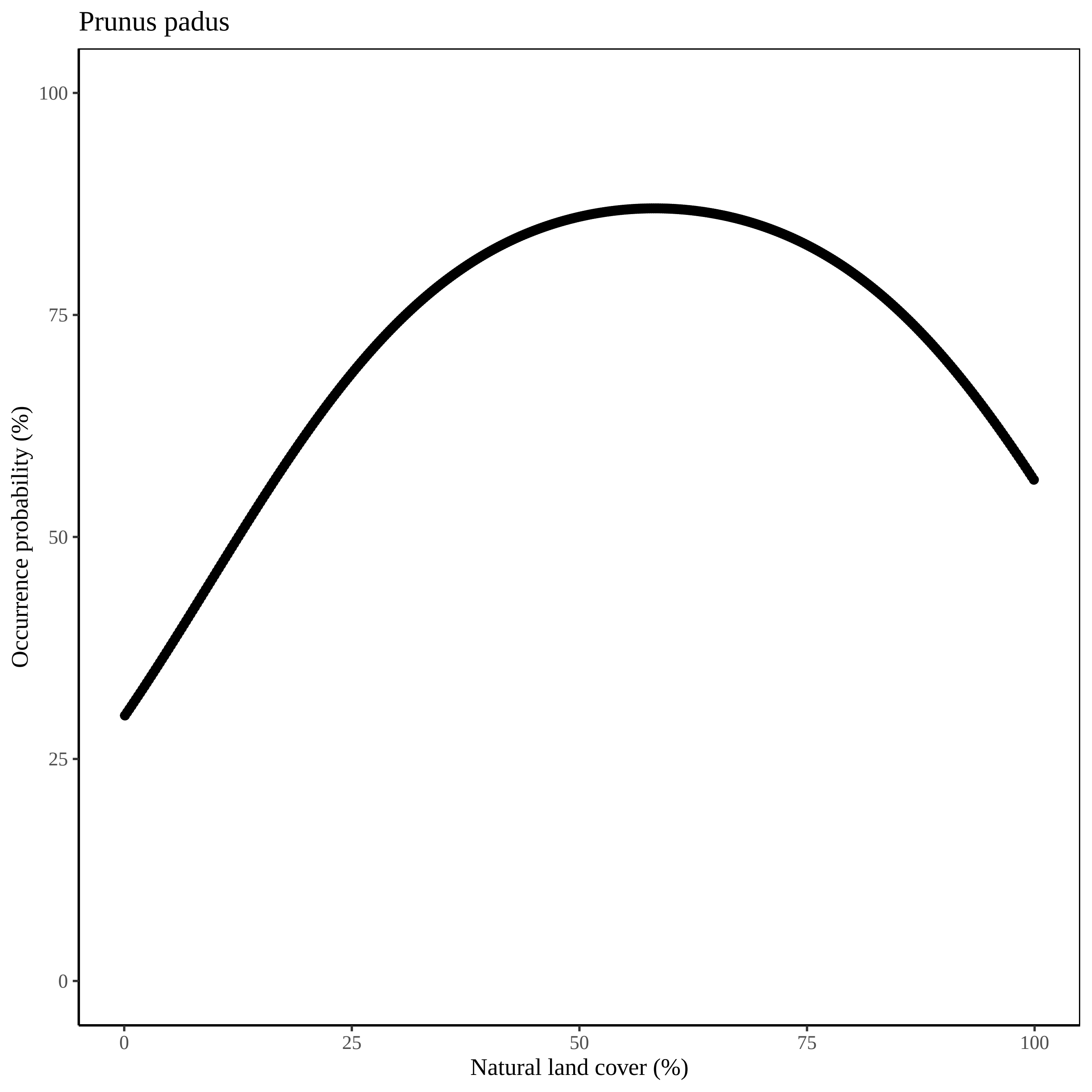
Prunus padus
Occurrence probability (%)
100
75
50
25
0
0
25
50
75
100
Natural land cover (%)

Prunus serotina

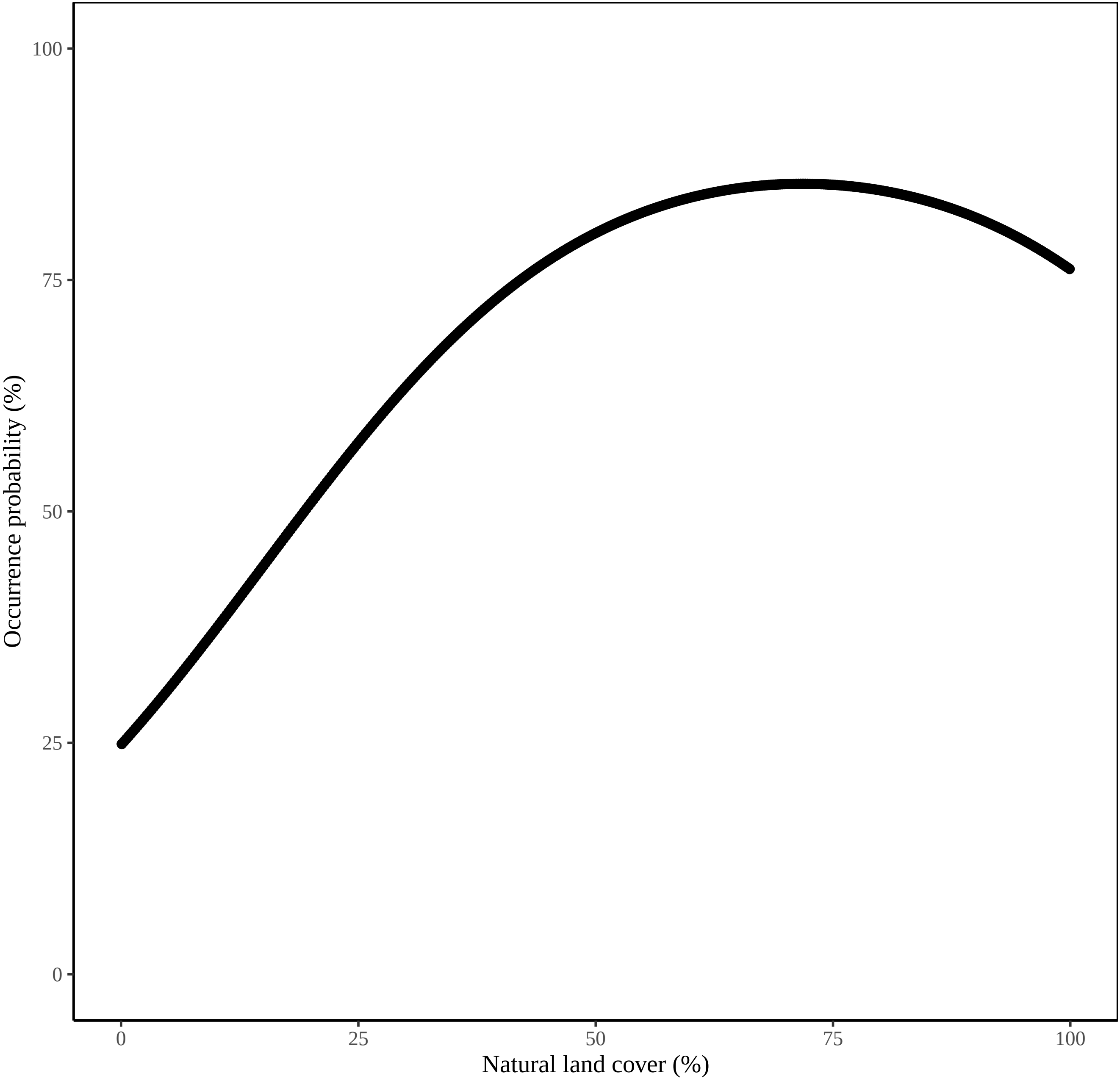

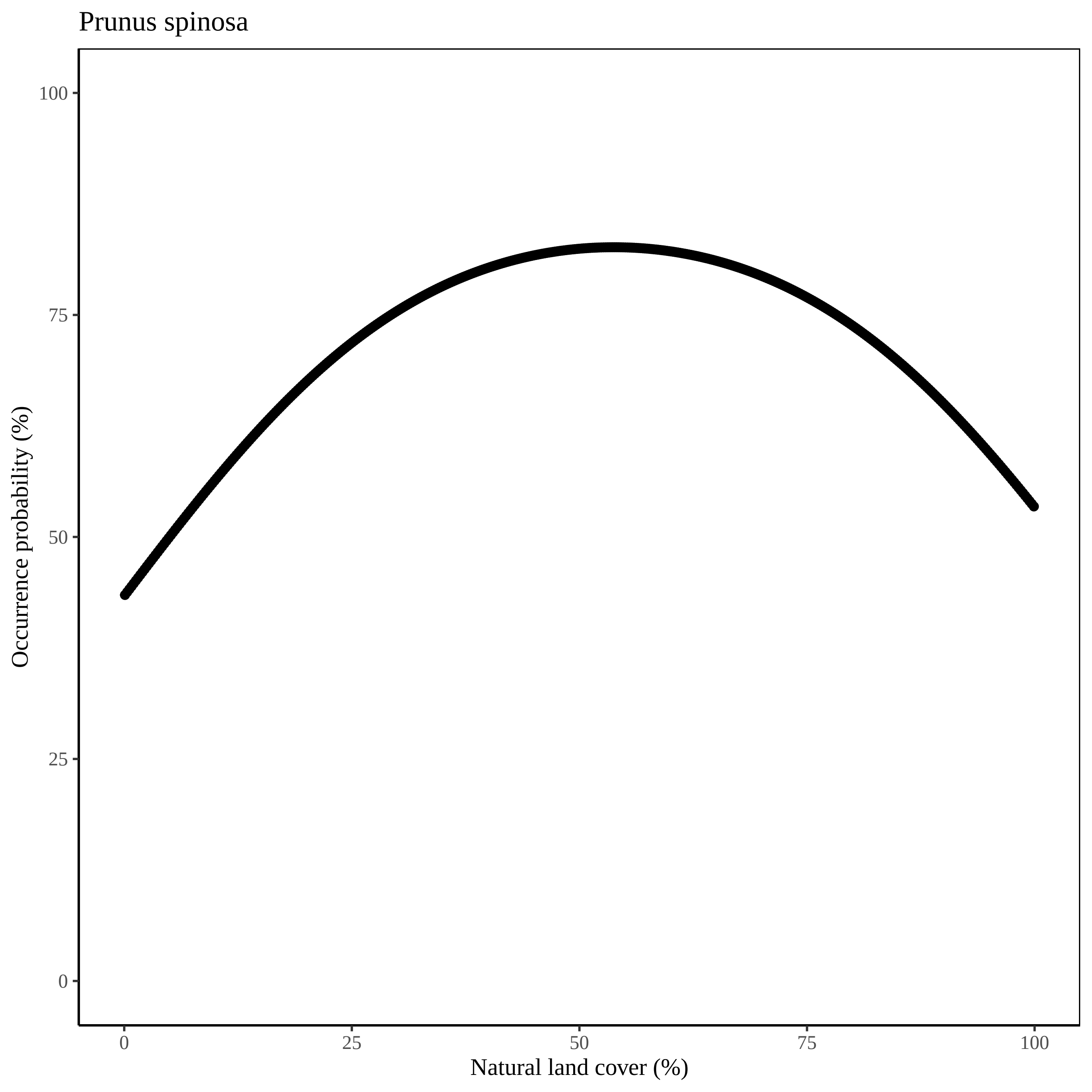

Prunus spinosa
Occurrence probability (%)
100
75
50
25
0
0
25
50
75
100
Natural land cover (%)

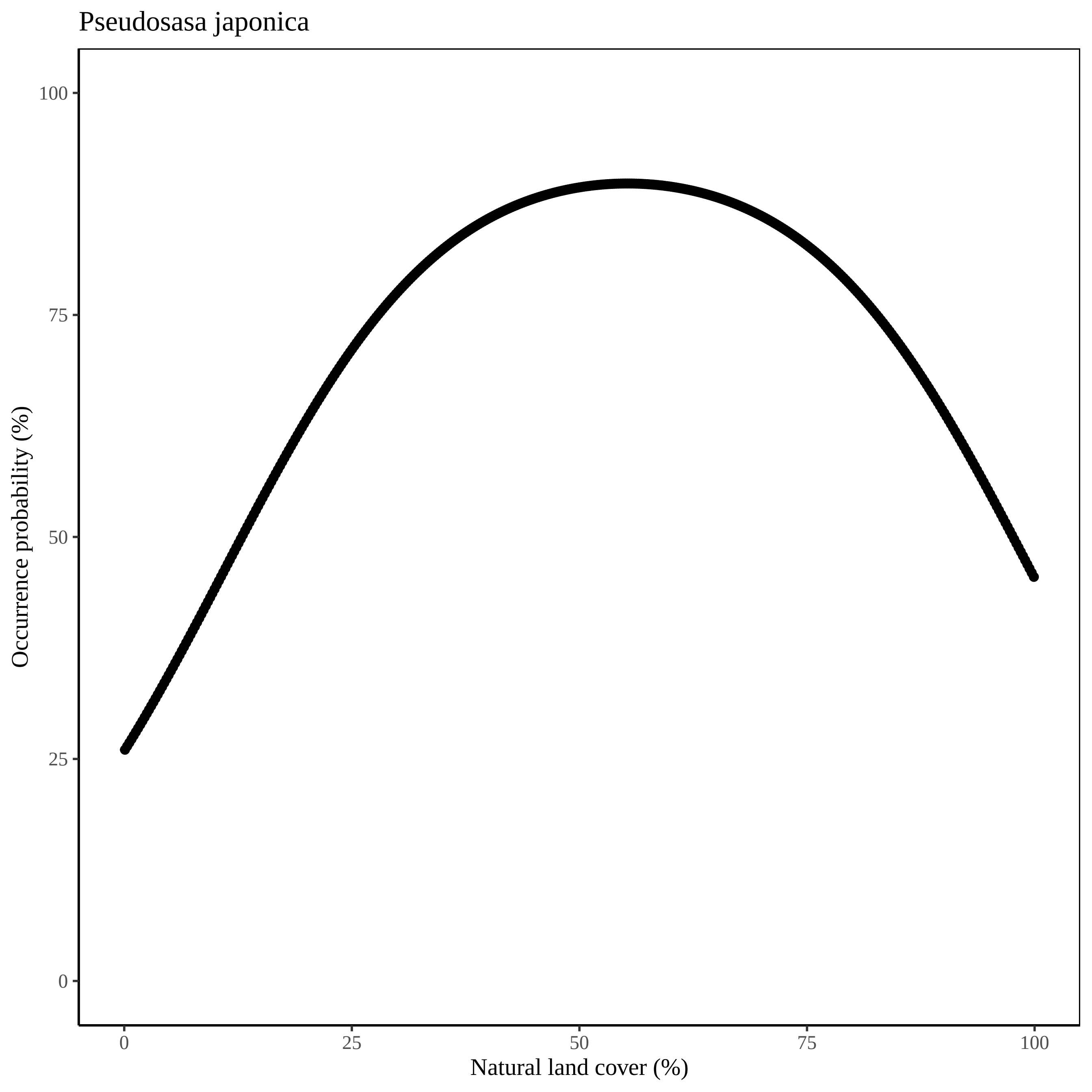
Pseudosasa japonica
Occurrence probability (%)
100
75
50
25
0
0
25
50
75
100
Natural land cover (%)

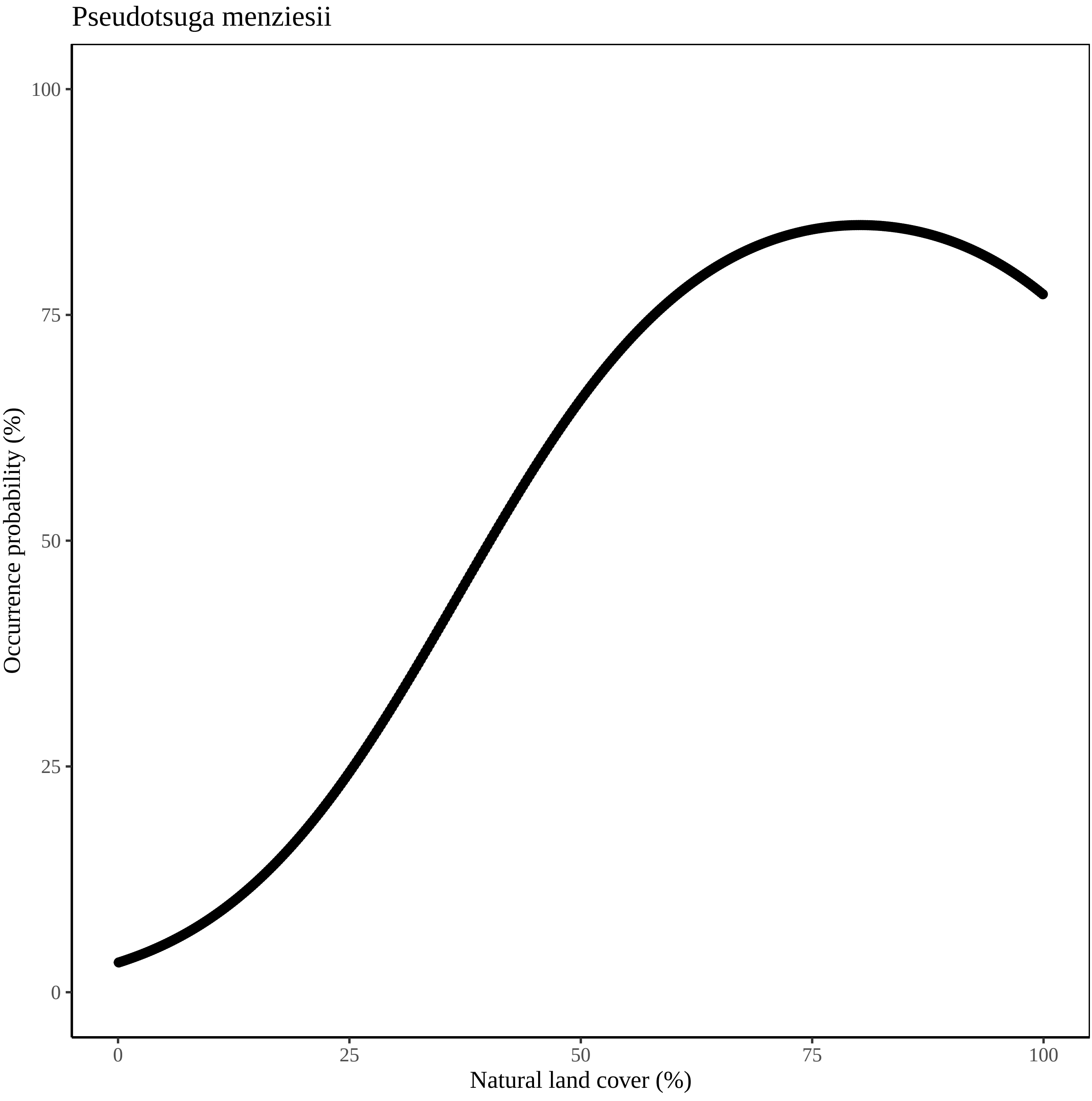
Pseudotsuga menziesii
Occurrence probability (%)
100
75
50
25
0
0
25
50
75
100
Natural land cover (%)

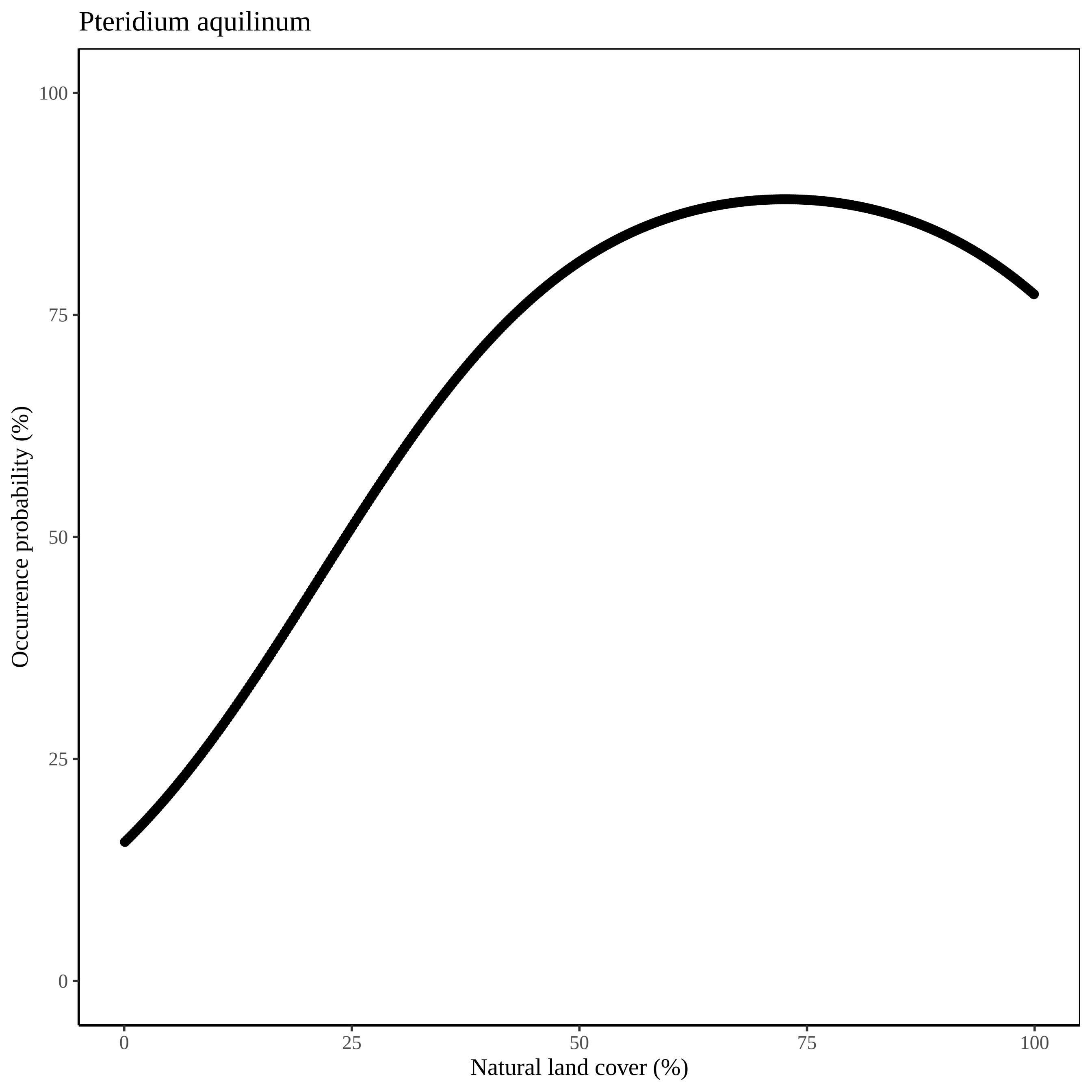

Pteridium aquilinum
Occurrence probability (%)
100
75
50
25
0
0
25
50
75
100
Natural land cover (%)

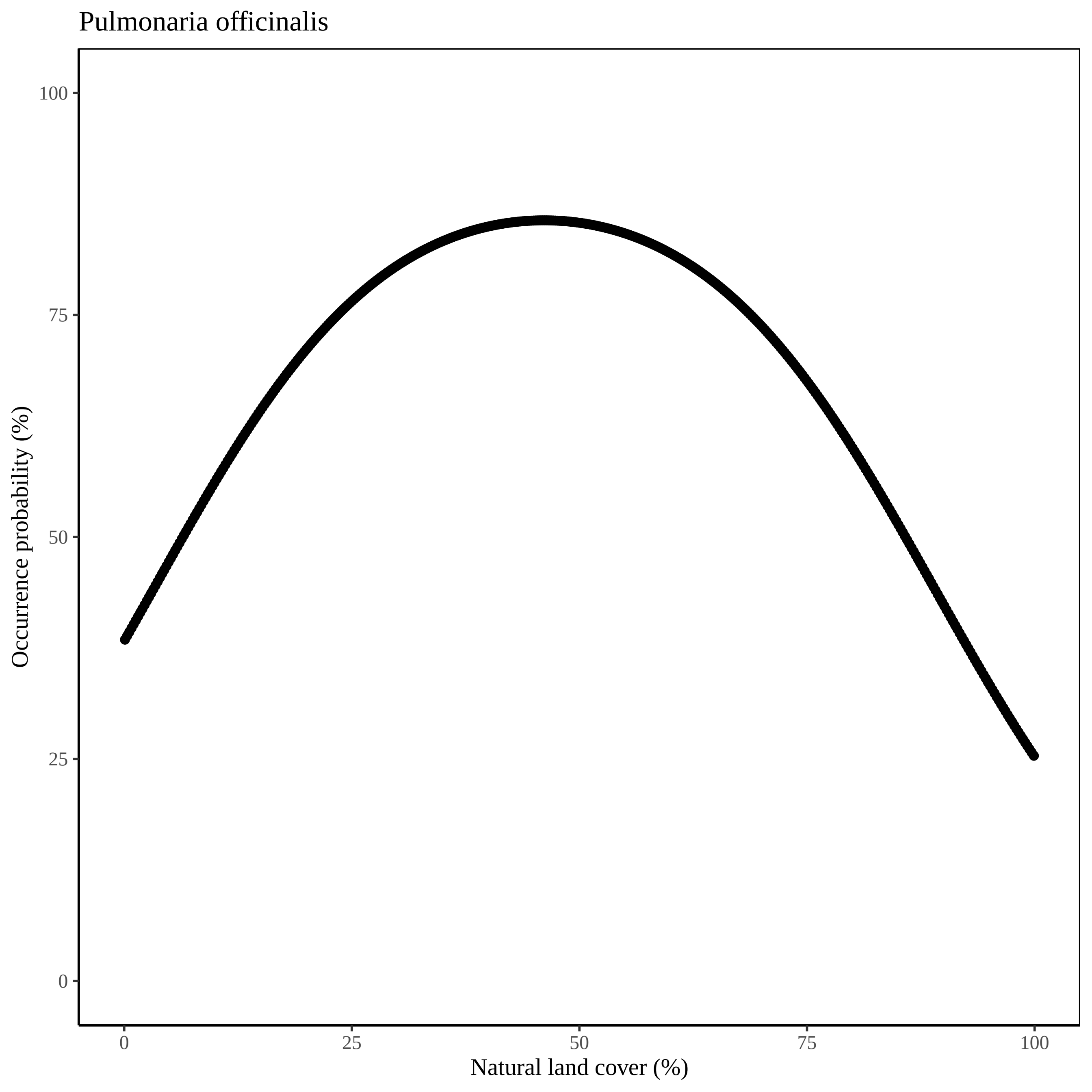
Pulmonaria officinalis
Occurrence probability (%)
0
25
50
75
100
Natural land cover (%)
0
25
50
75
100

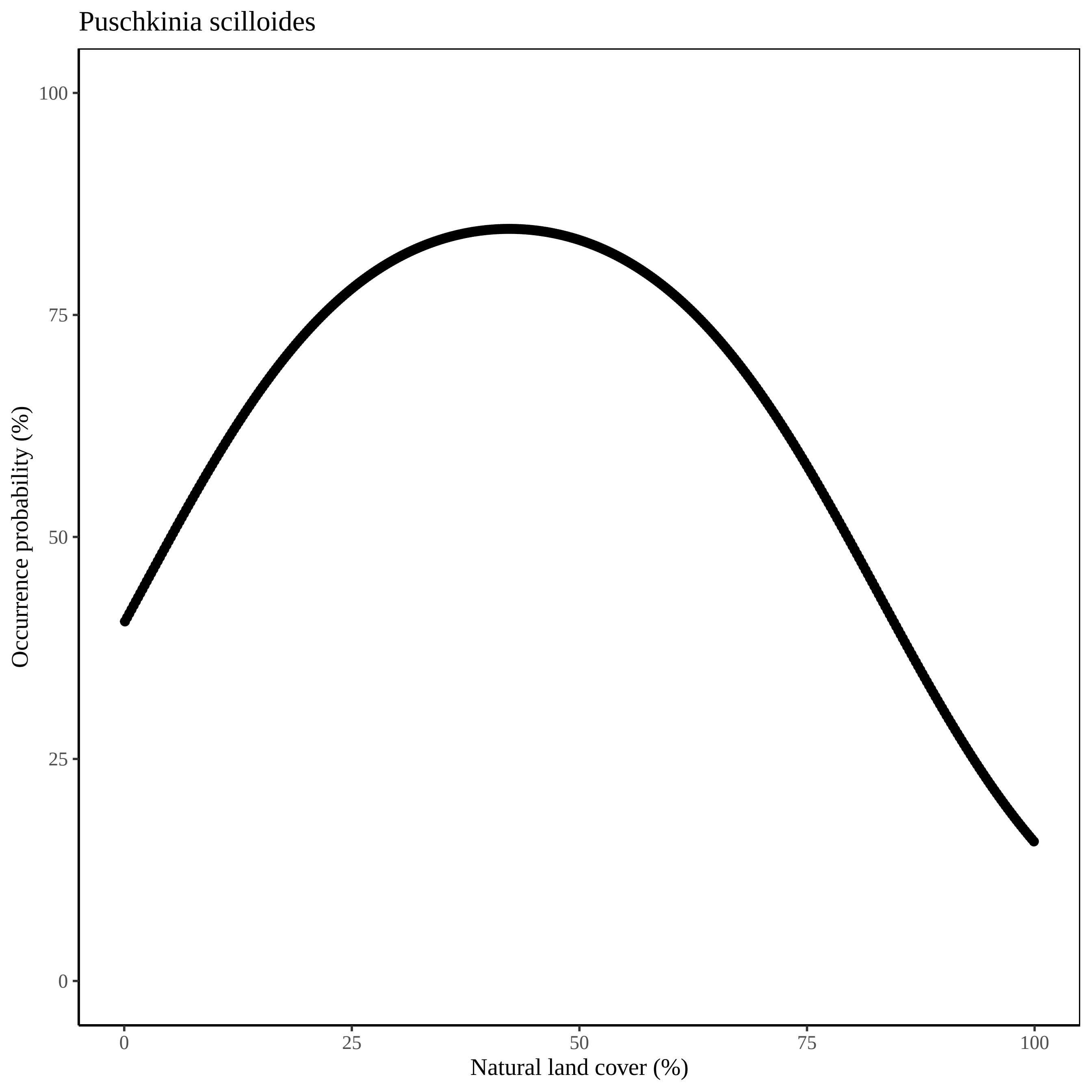
Puschkinia scilloides
100
75
50
25
0
Occurrence probability (%)
0
25
50
75
100
Natural land cover (%)

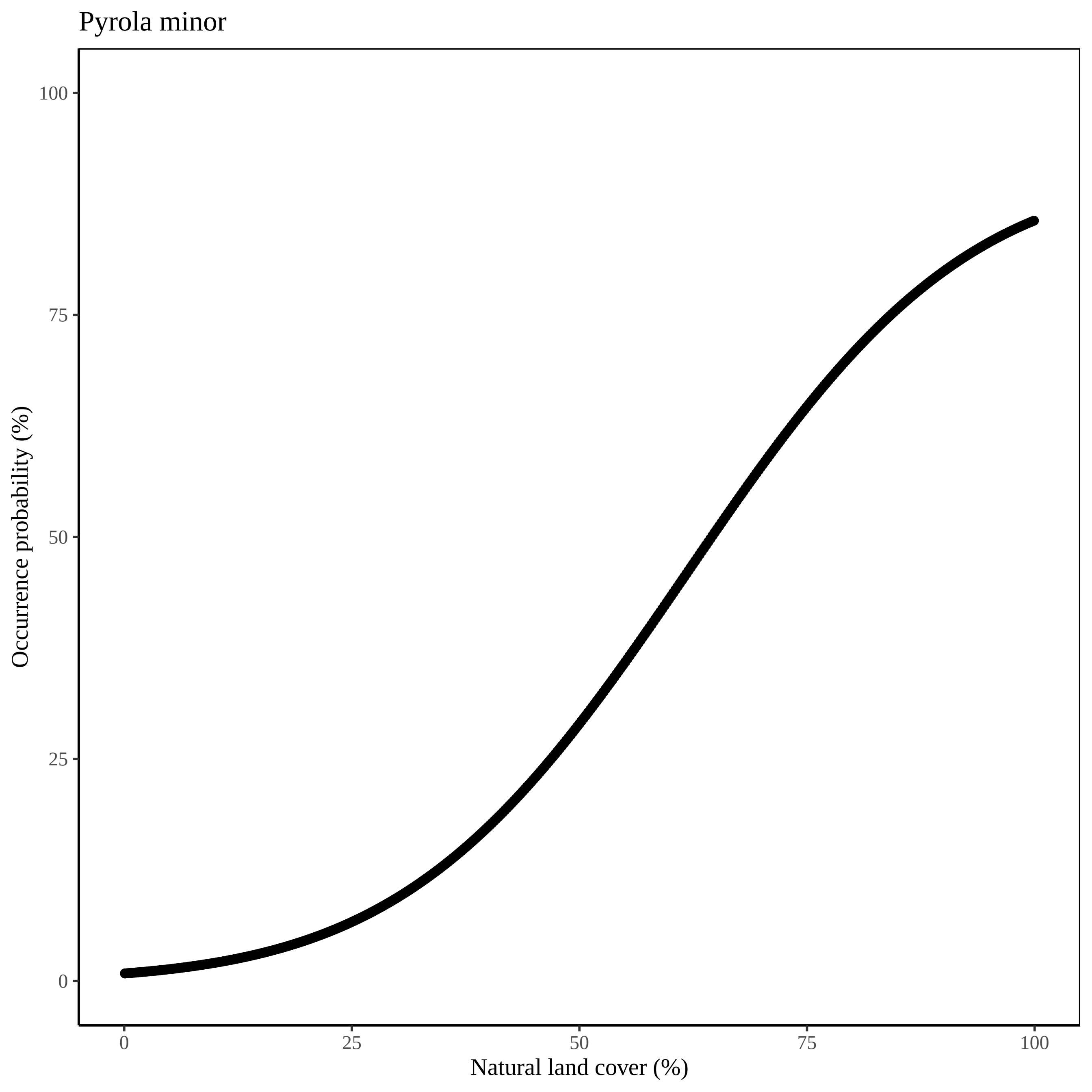

Pyrola minor
Occurrence probability (%)
Natural land cover (%)
0
25
50
75
100
0
25
50
75
100

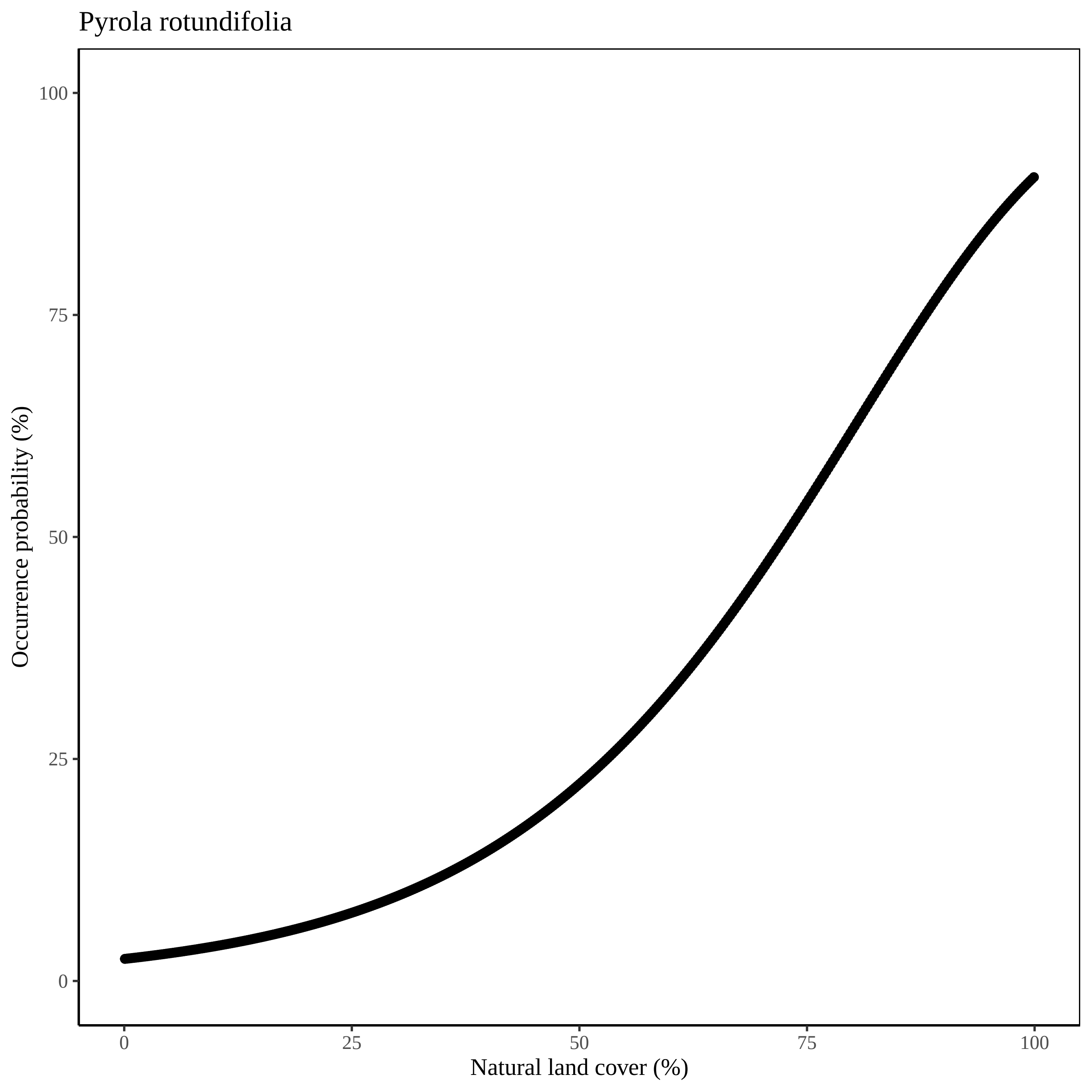
Pyrola rotundifolia
Occurrence probability (%)
100
75
50
25
0
0
25
50
75
100
Natural land cover (%)

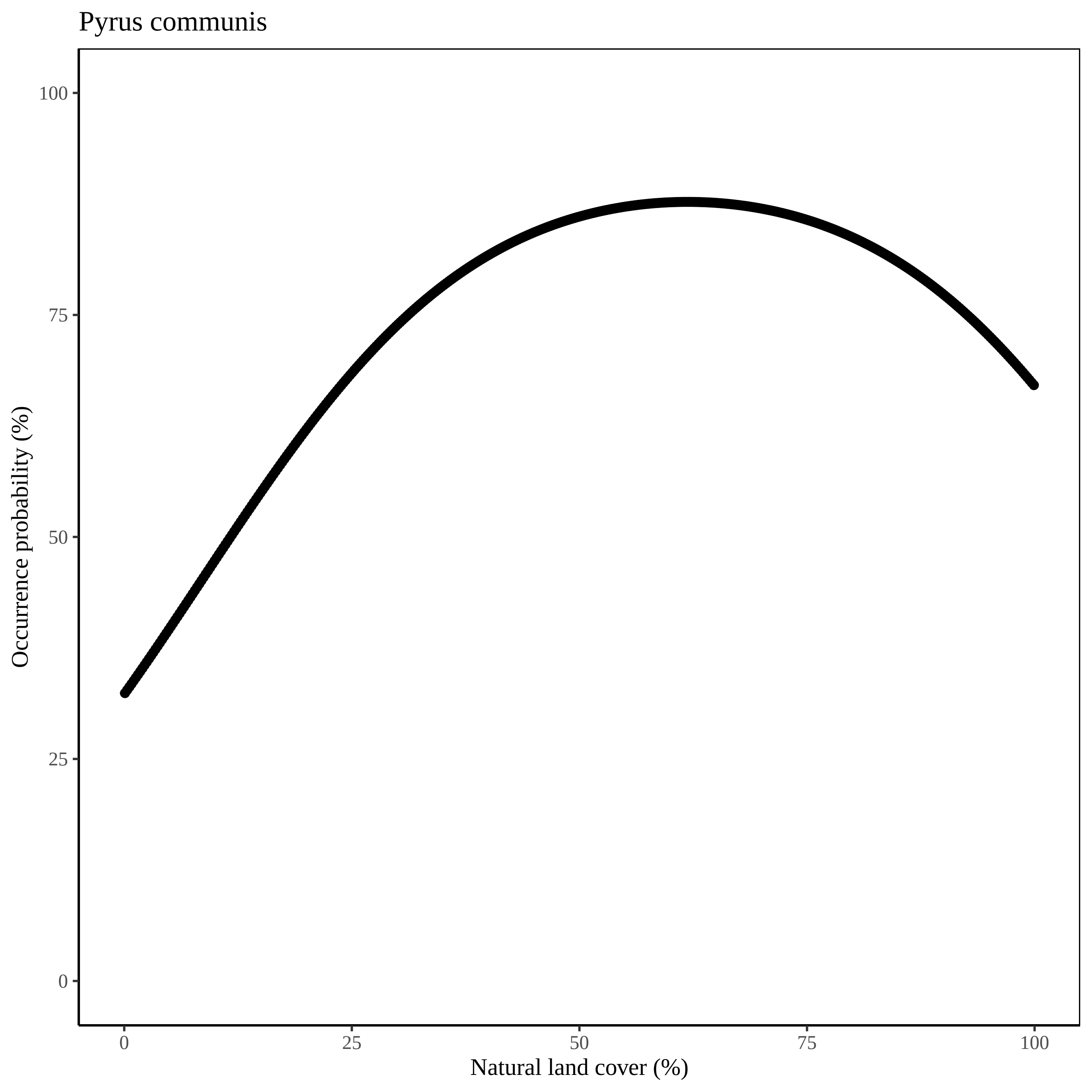

Pyrus communis
Occurrence probability (%)
100
75
50
25
0
0
25
50
75
100
Natural land cover (%)

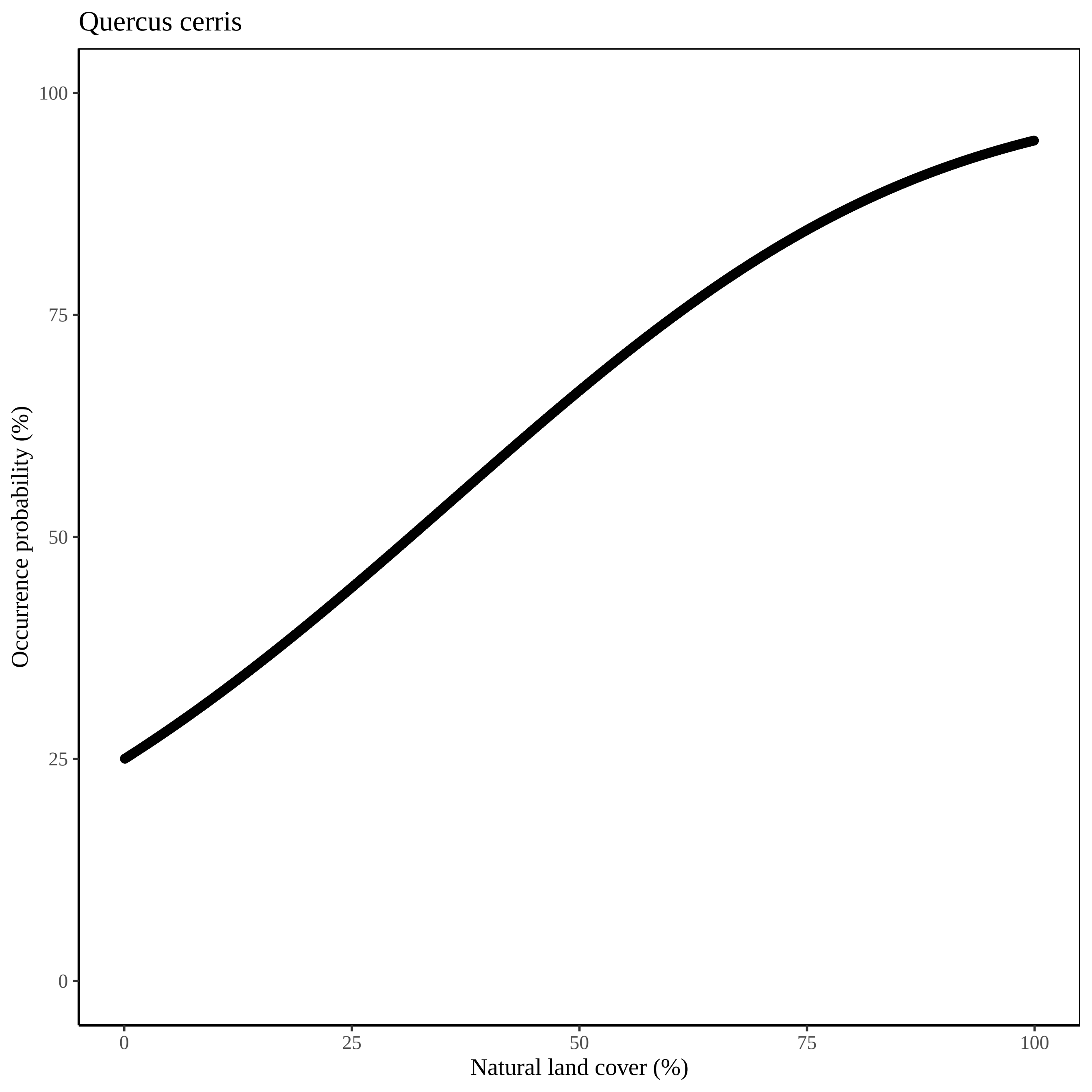
Quercus cerris
100
75
50
25
0
0
25
50
75
100
Occurrence probability (%)
Natural land cover (%)

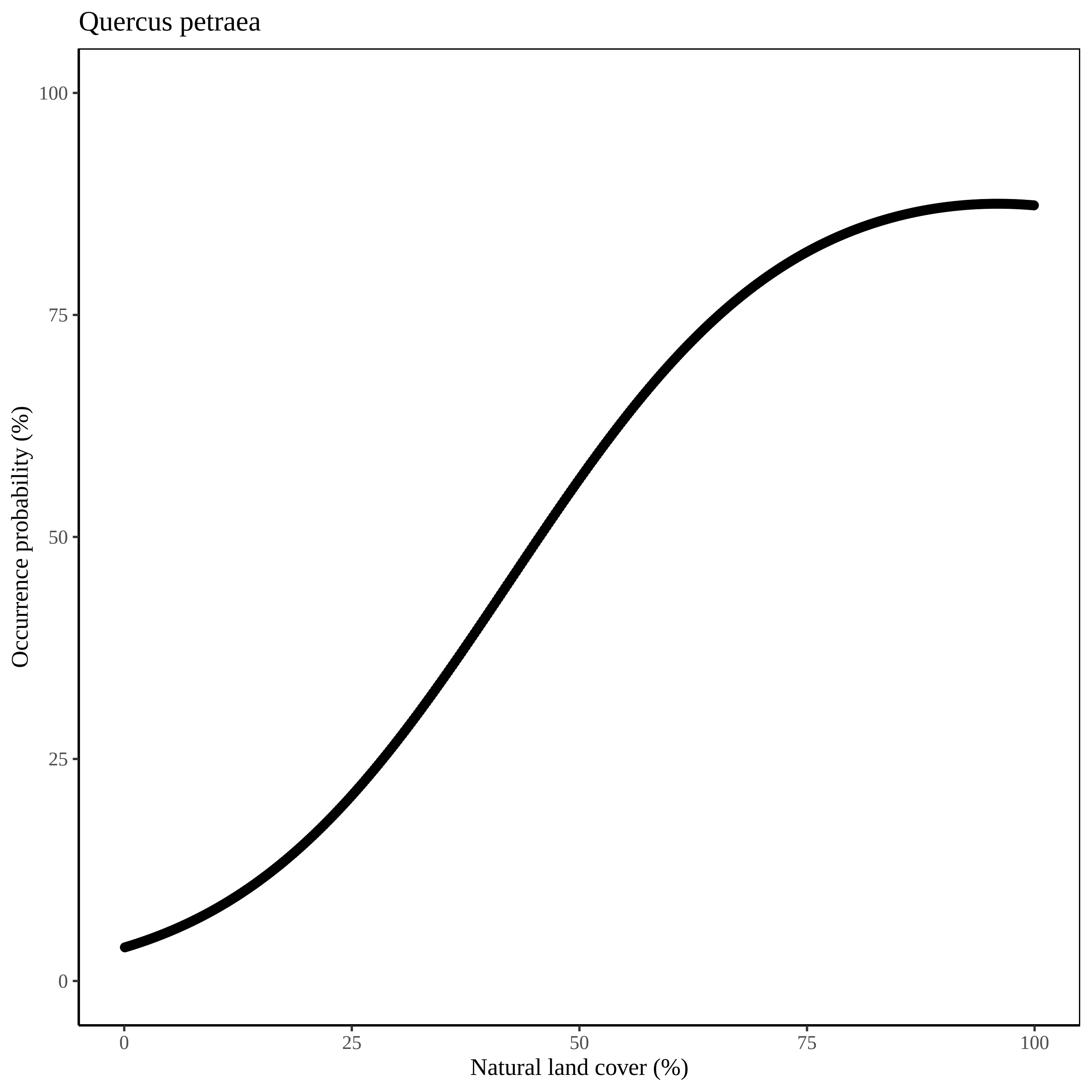
Quercus petraea
Occurrence probability (%)
100
75
50
25
0
0
25
50
75
100
Natural land cover (%)

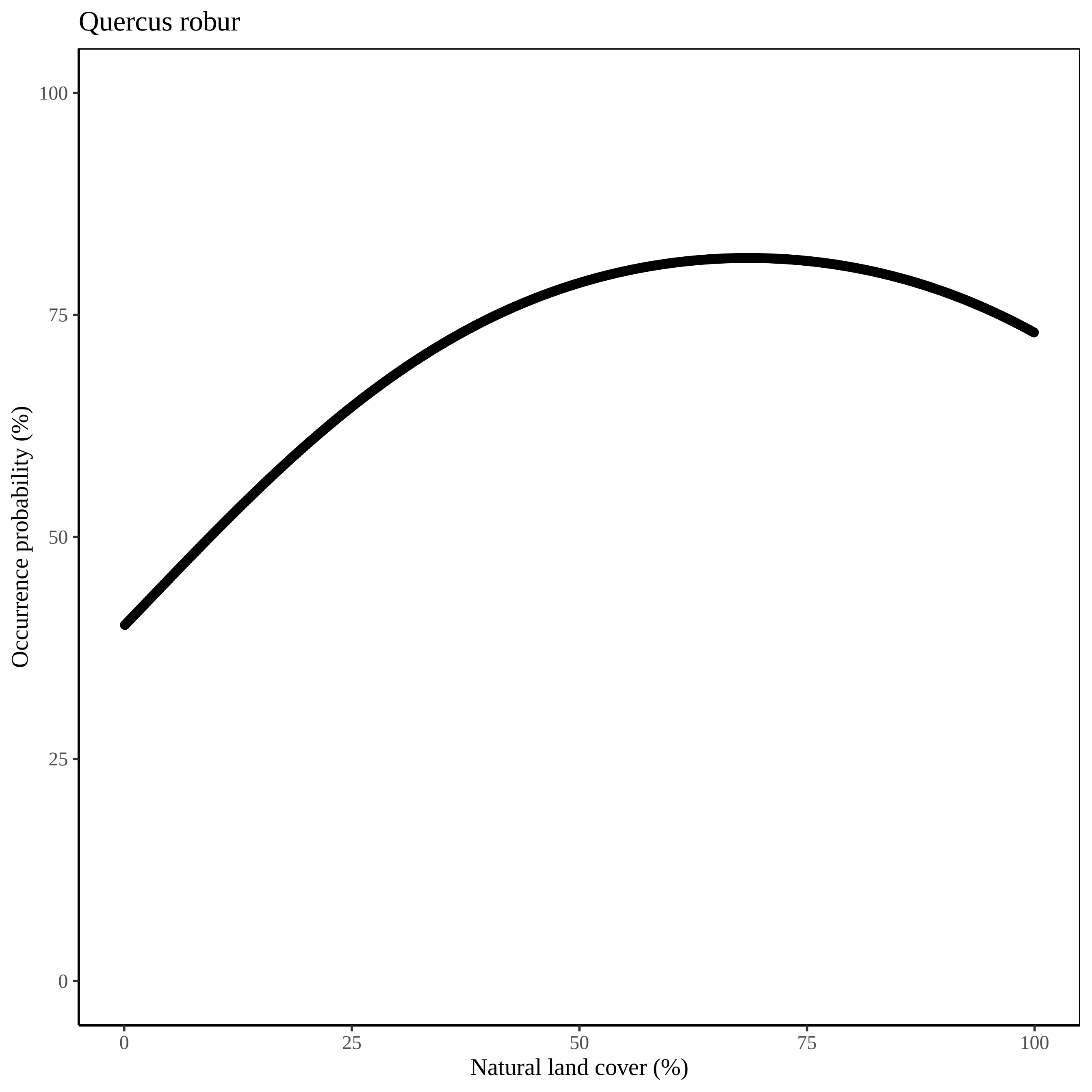
Quercus robur
Occurrence probability (%)
100
75
50
25
0
0
25
50
75
100
Natural land cover (%)

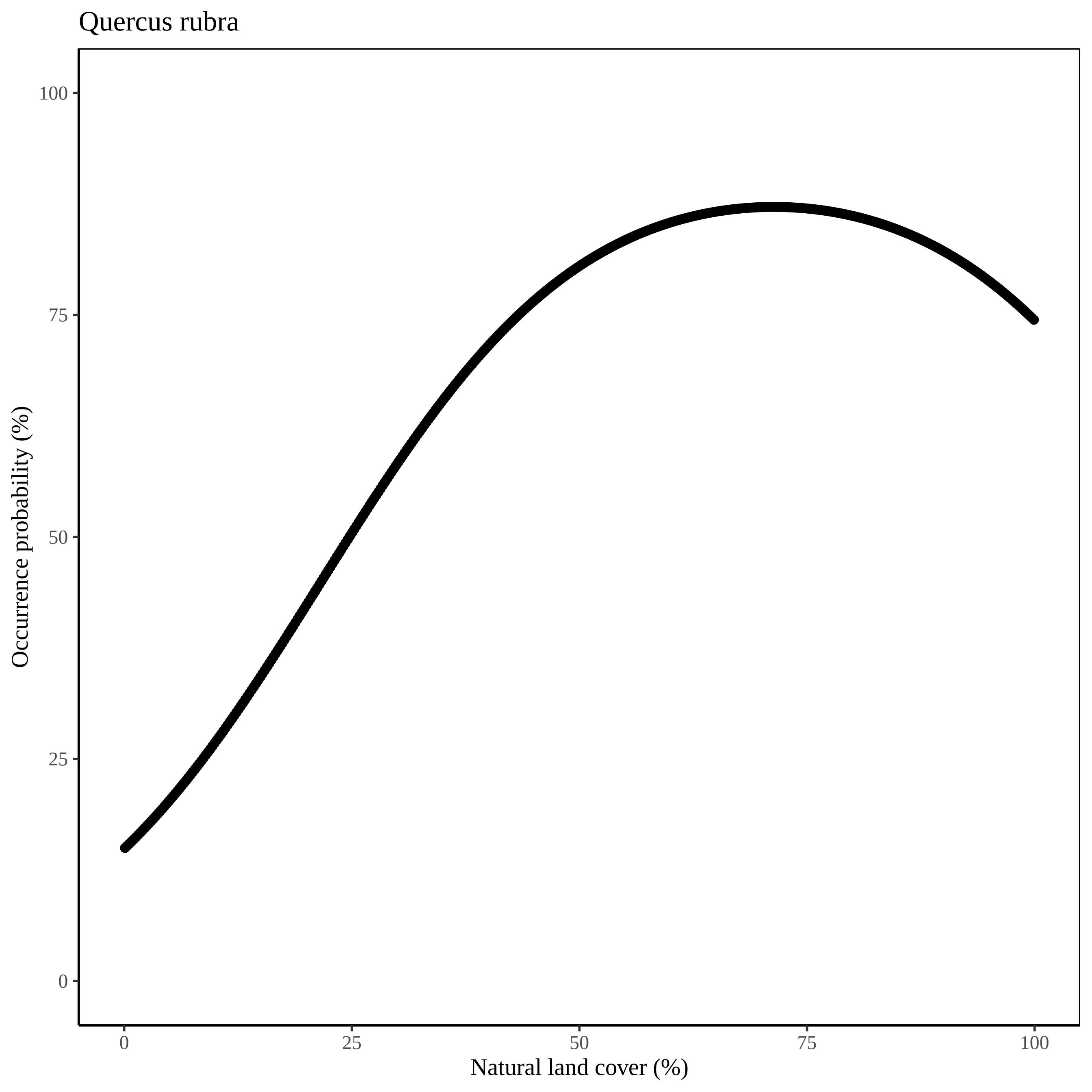
Quercus rubra
Occurrence probability (%)
100
75
50
25
0
0
25
50
75
100
Natural land cover (%)

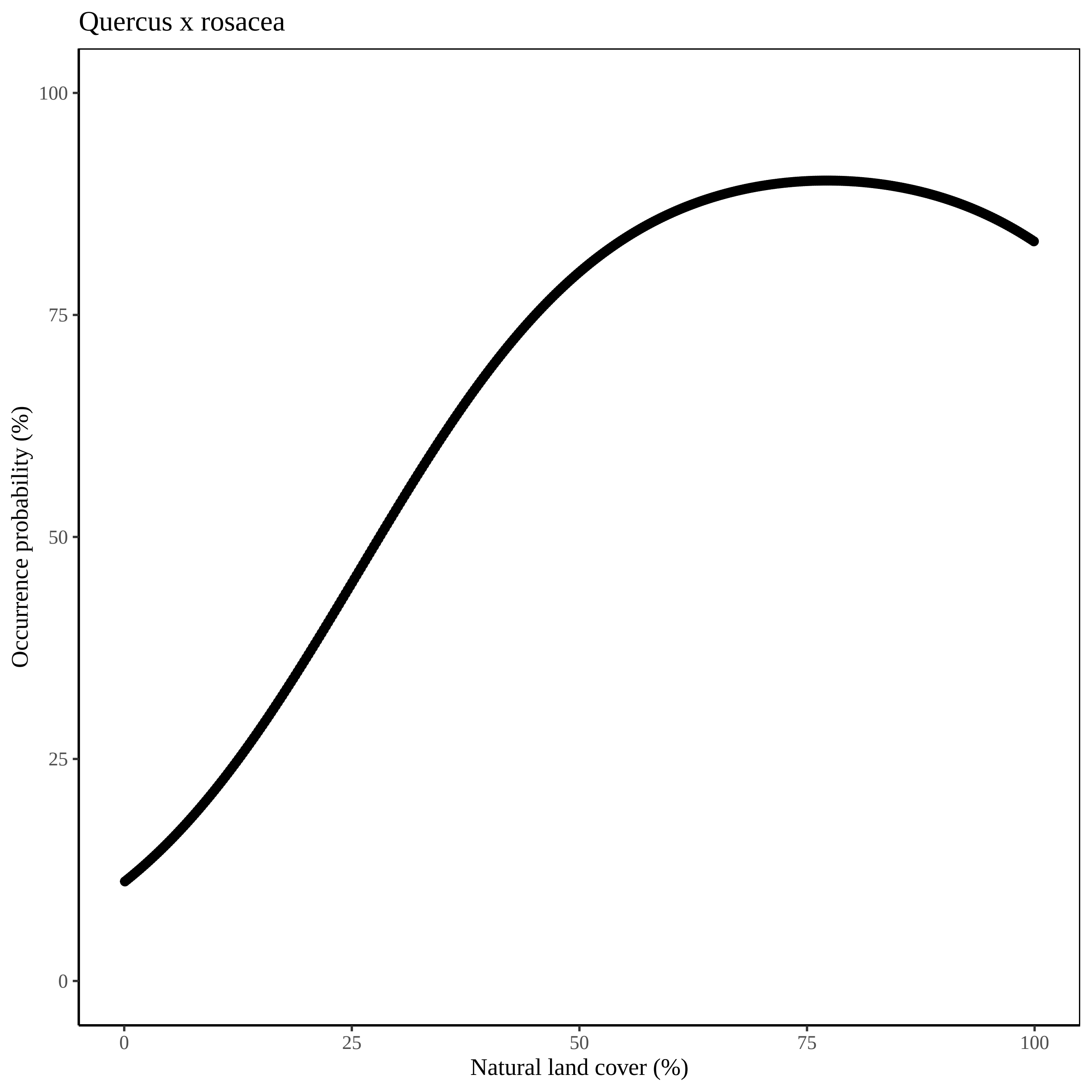
Quercus x rosacea
Occurrence probability (%)
100
75
50
25
0
0
25
50
75
100
Natural land cover (%)

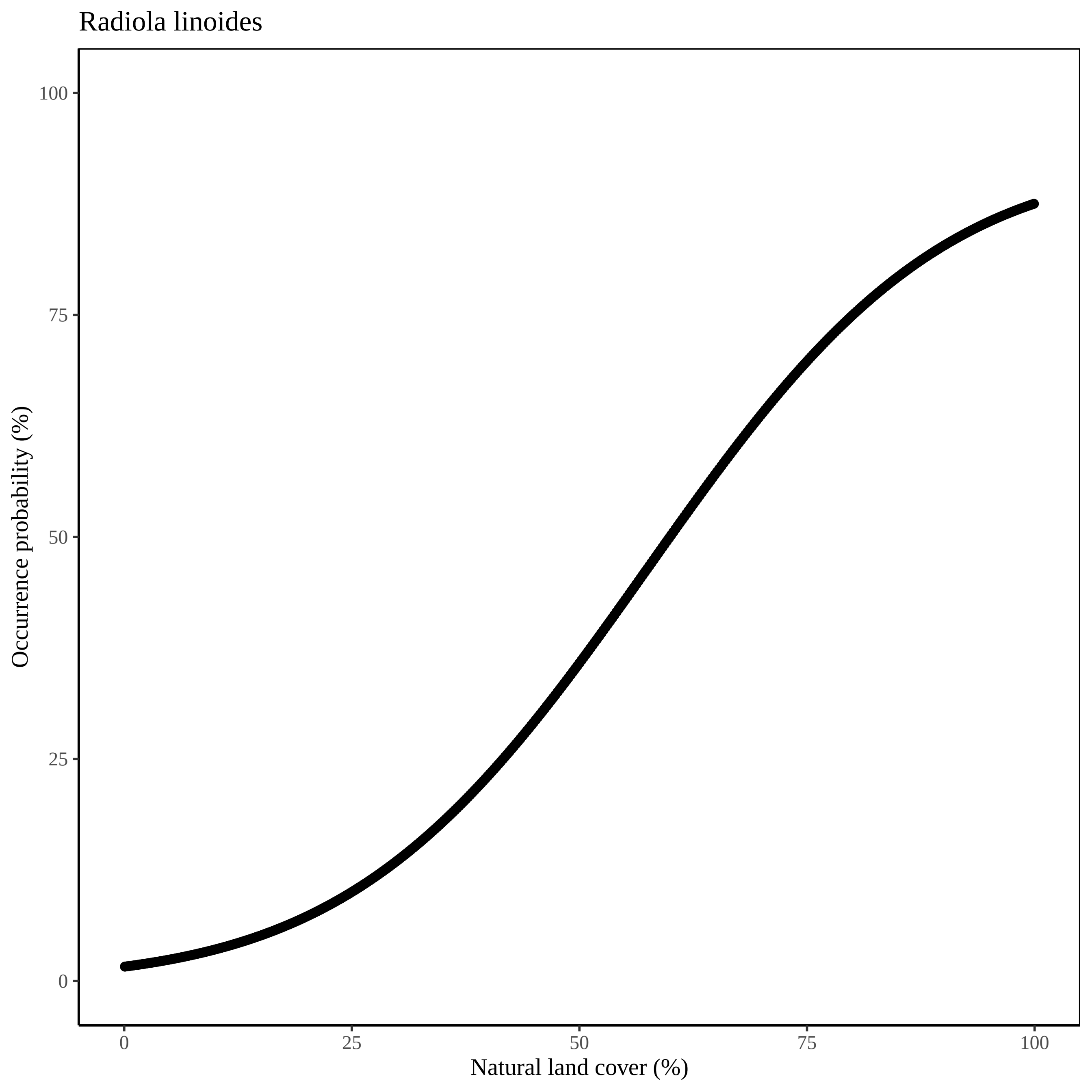

Radiola linoides
Occurrence probability (%)
100
75
50
25
0
0
25
50
75
100
Natural land cover (%)

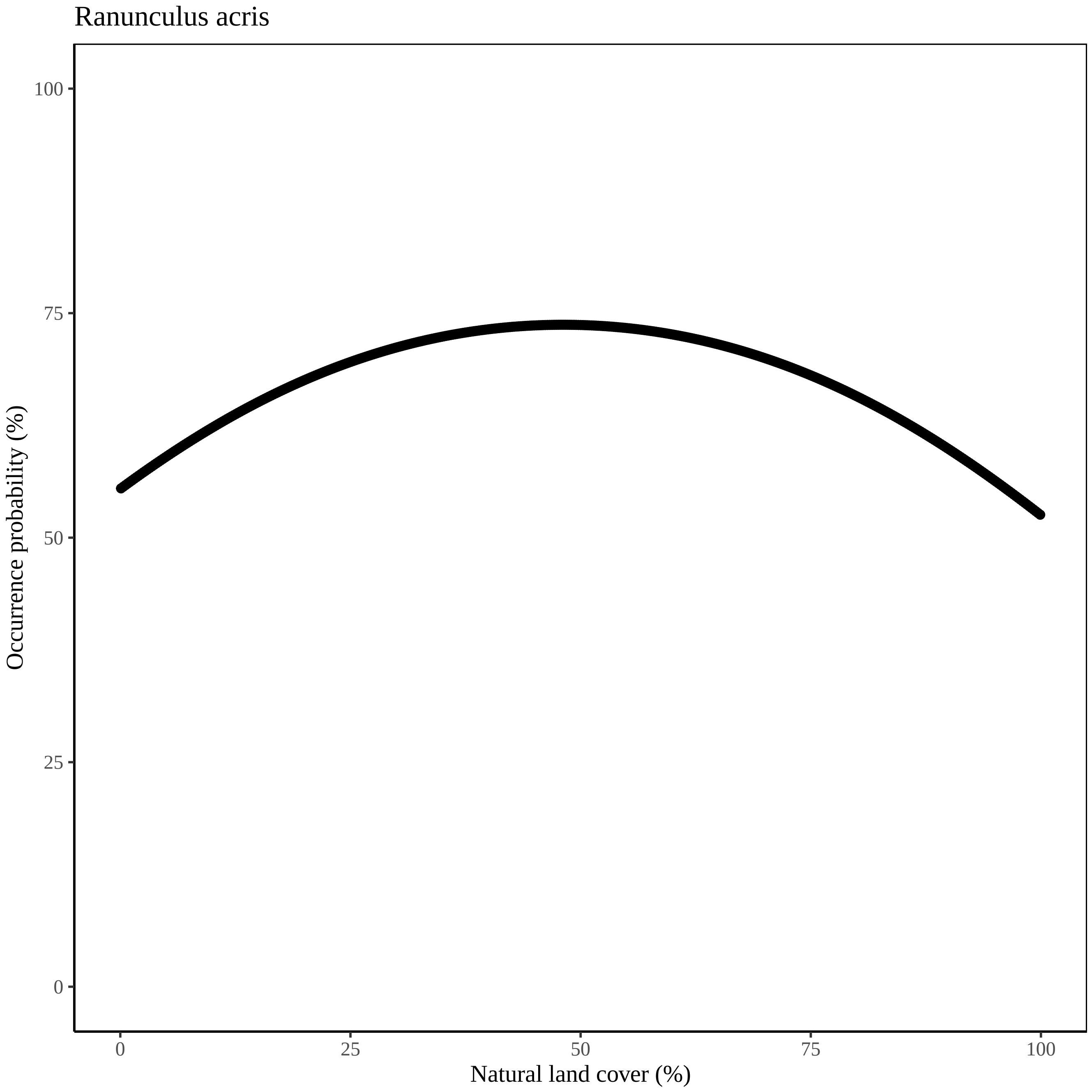
Ranunculus acris
Occurrence probability (%)
100
75
50
25
0
0
25
50
75
100
Natural land cover (%)

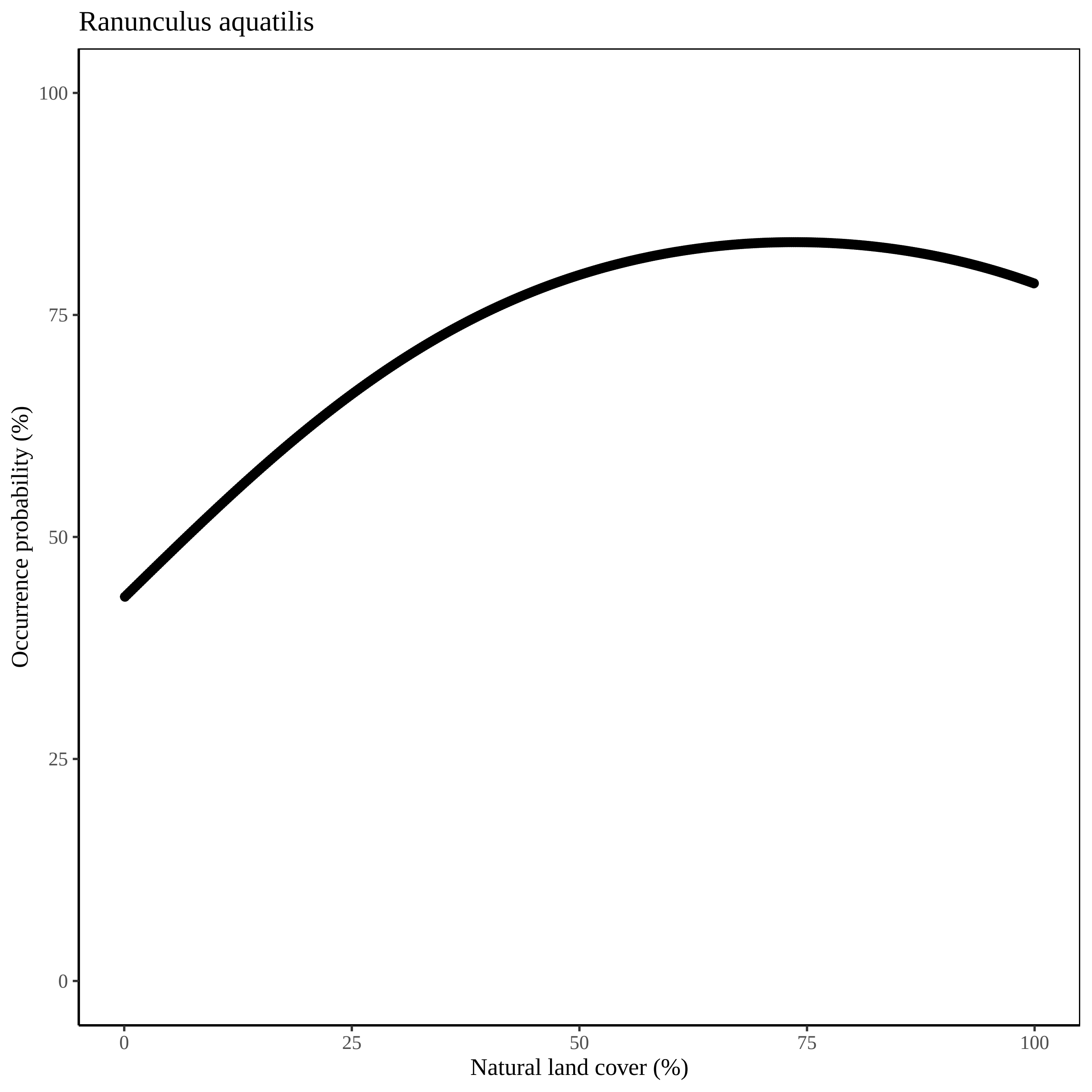
Ranunculus aquatilis
Occurrence probability (%)
100
75
50
25
0
0
25
50
75
100
Natural land cover (%)

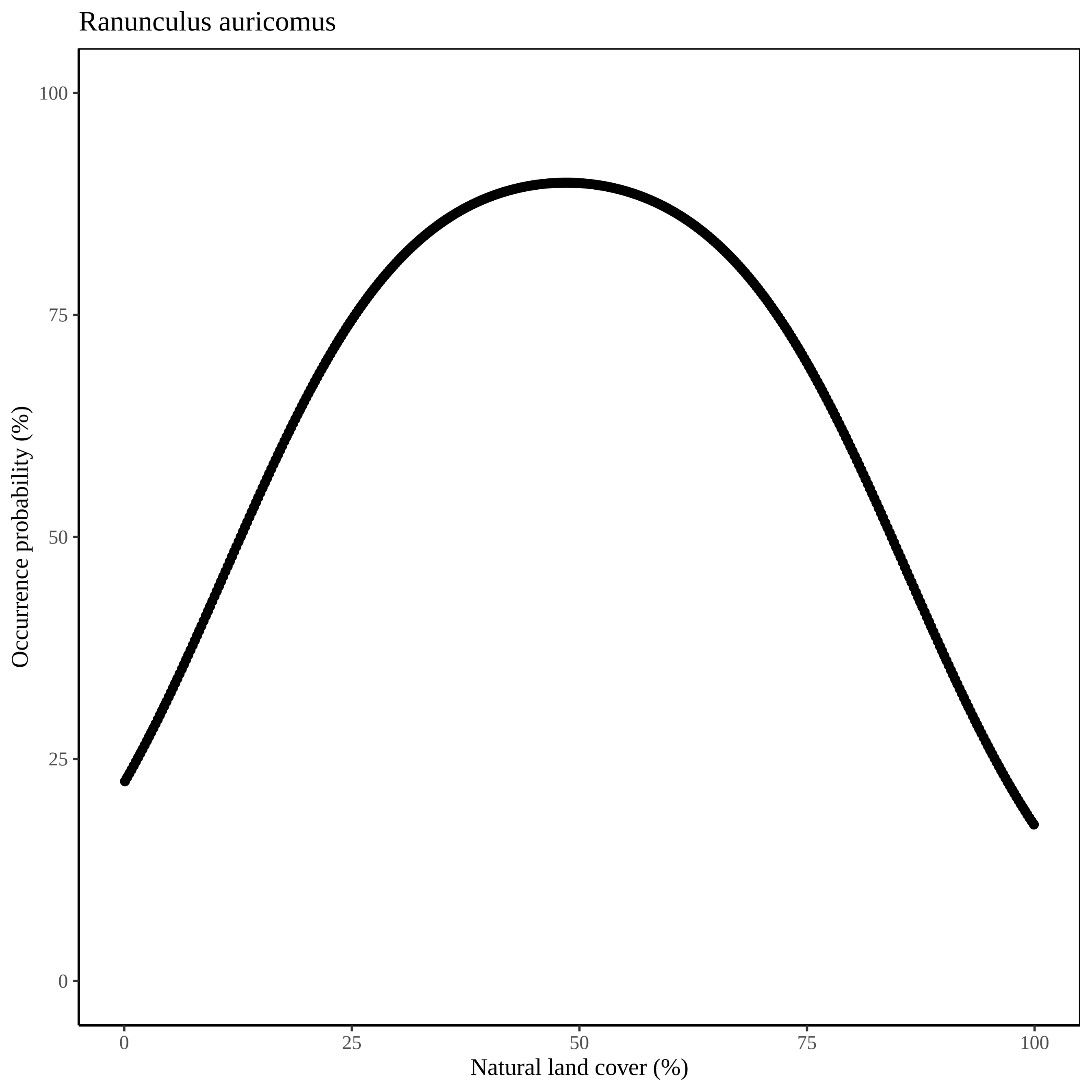
Ranunculus auricomus
Occurrence probability (%)
100
75
50
25
0
0
25
50
75
100
Natural land cover (%)

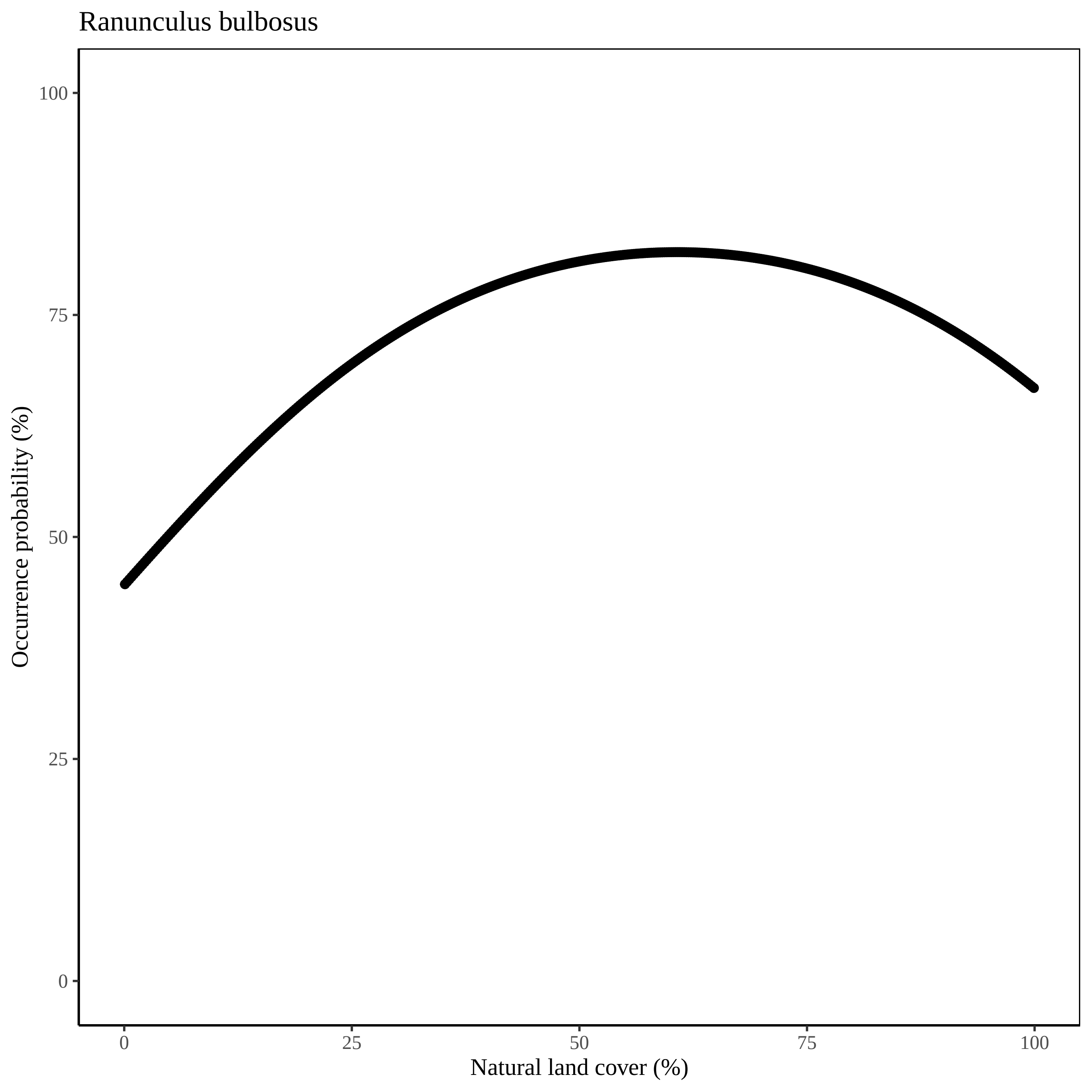
Ranunculus bulbosus
Occurrence probability (%)
100
75
50
25
0
0
25
50
75
100
Natural land cover (%)

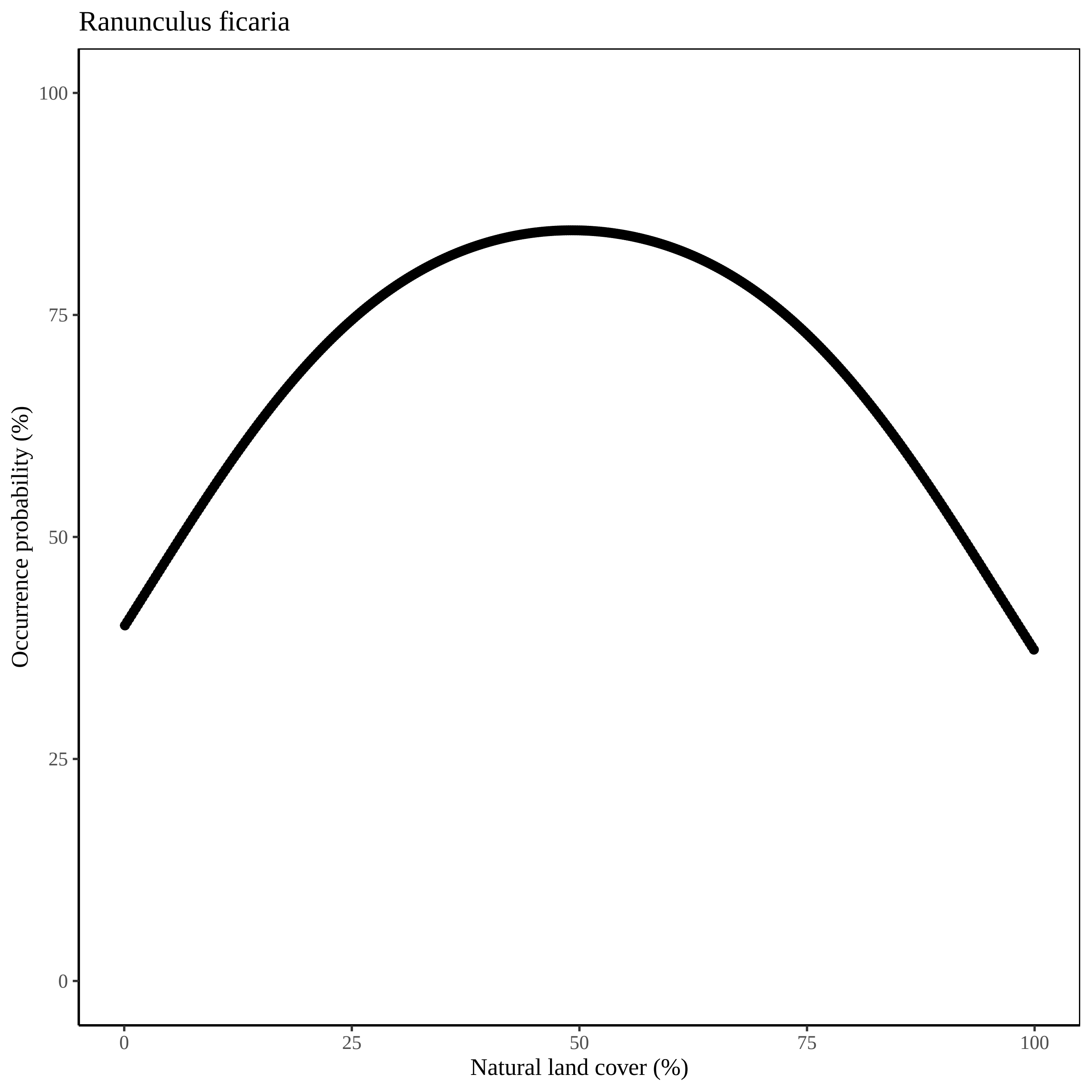
Ranunculus ficaria
Occurrence probability (%)
100
75
50
25
0
0
25
50
75
100
Natural land cover (%)

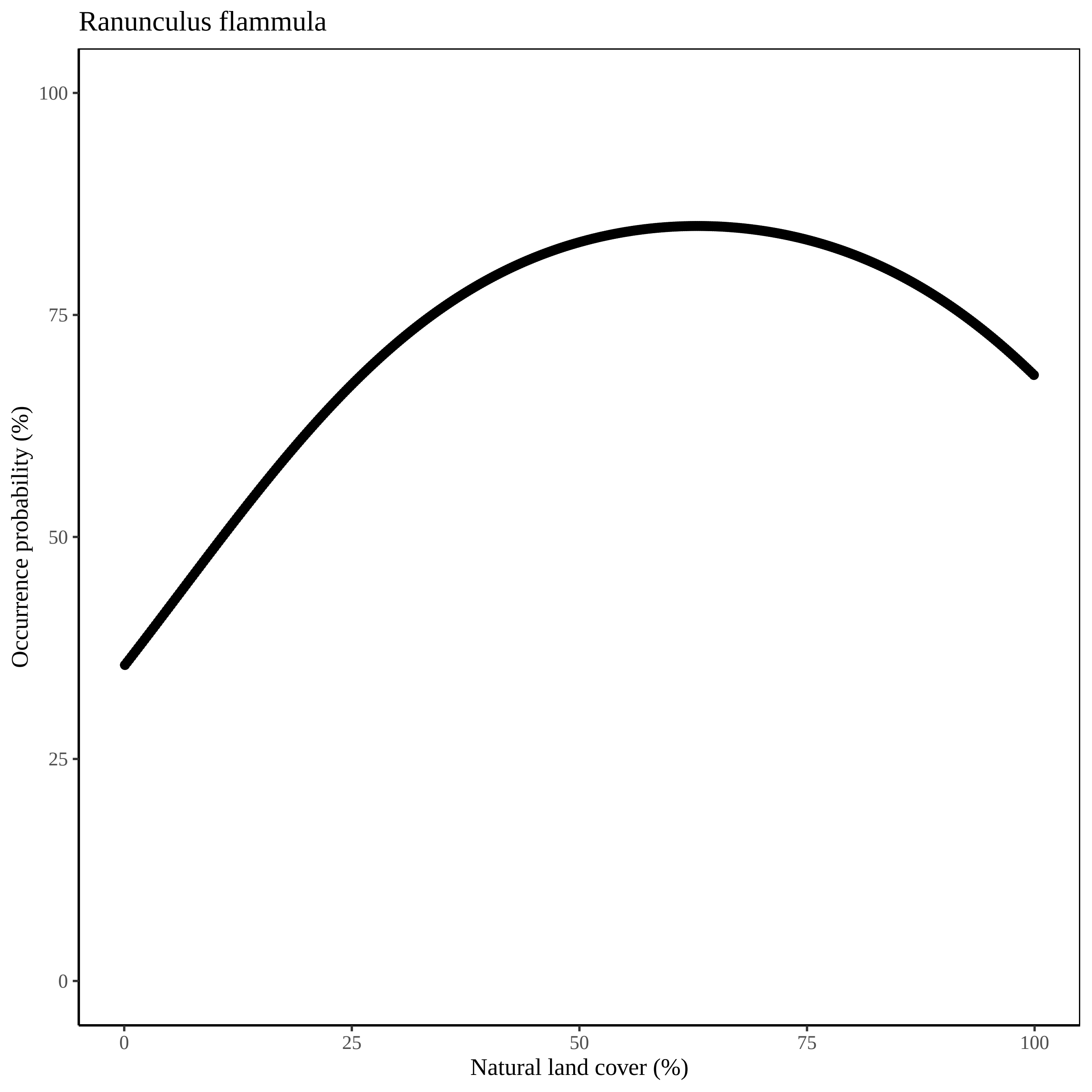
Ranunculus flammula
100
75
50
25
0
Occurrence probability (%)
0
25
50
75
100
Natural land cover (%)

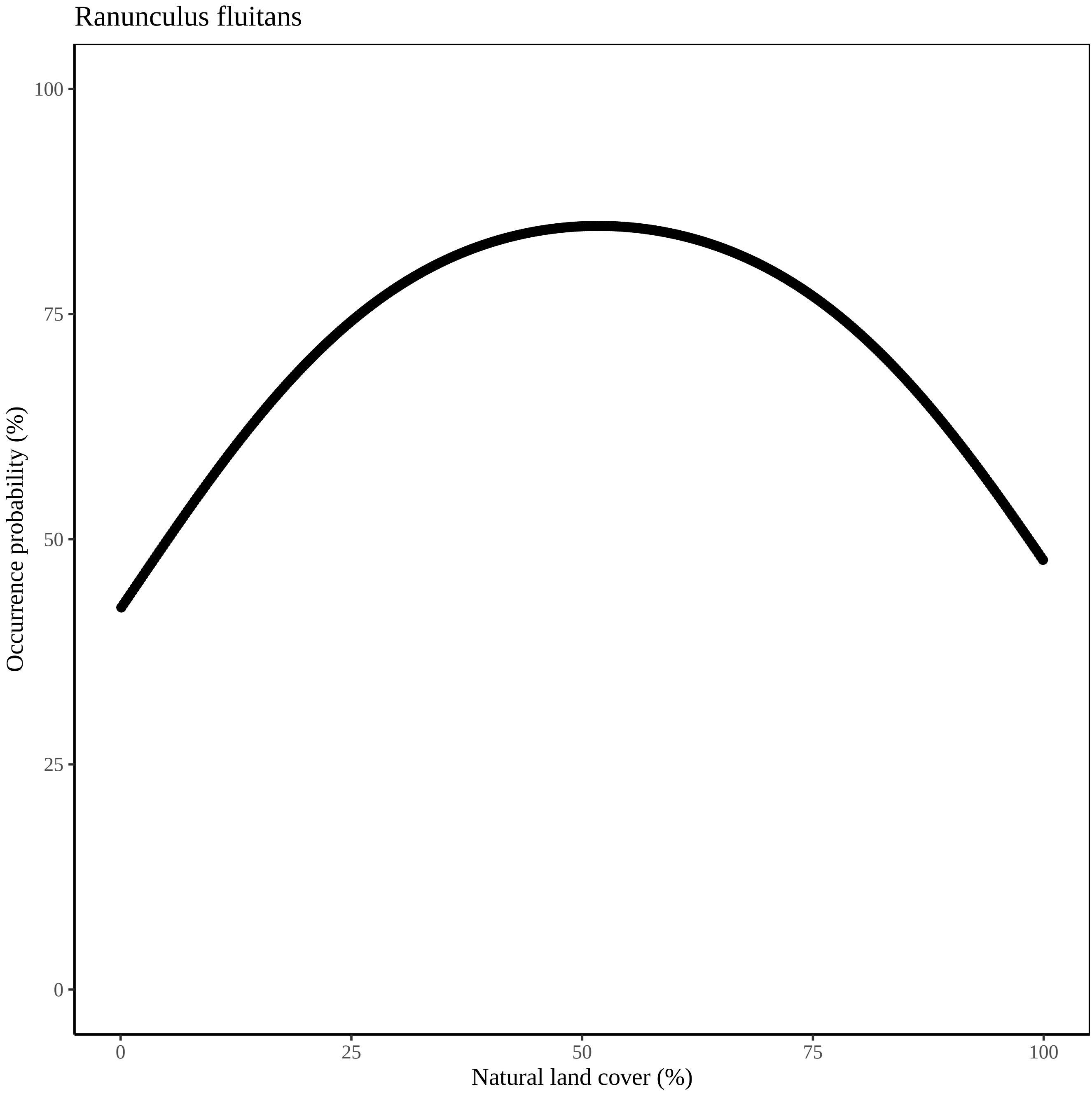
Ranunculus fluitans
Occurrence probability (%)
100
75
50
25
0
0
25
50
75
100
Natural land cover (%)

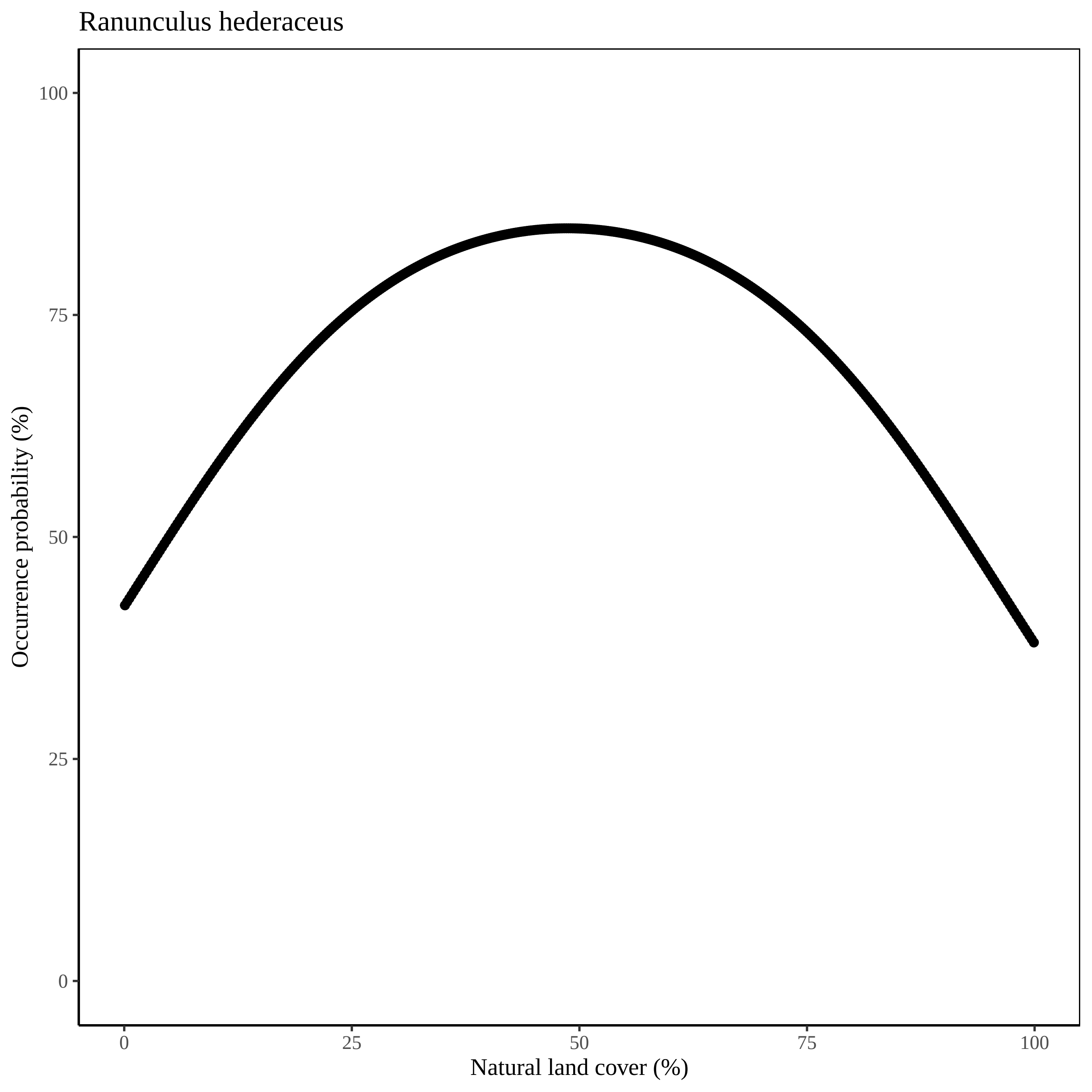
Ranunculus hederaceus
100
75
50
25
0
Occurrence probability (%)
0
25
50
75
100
Natural land cover (%)

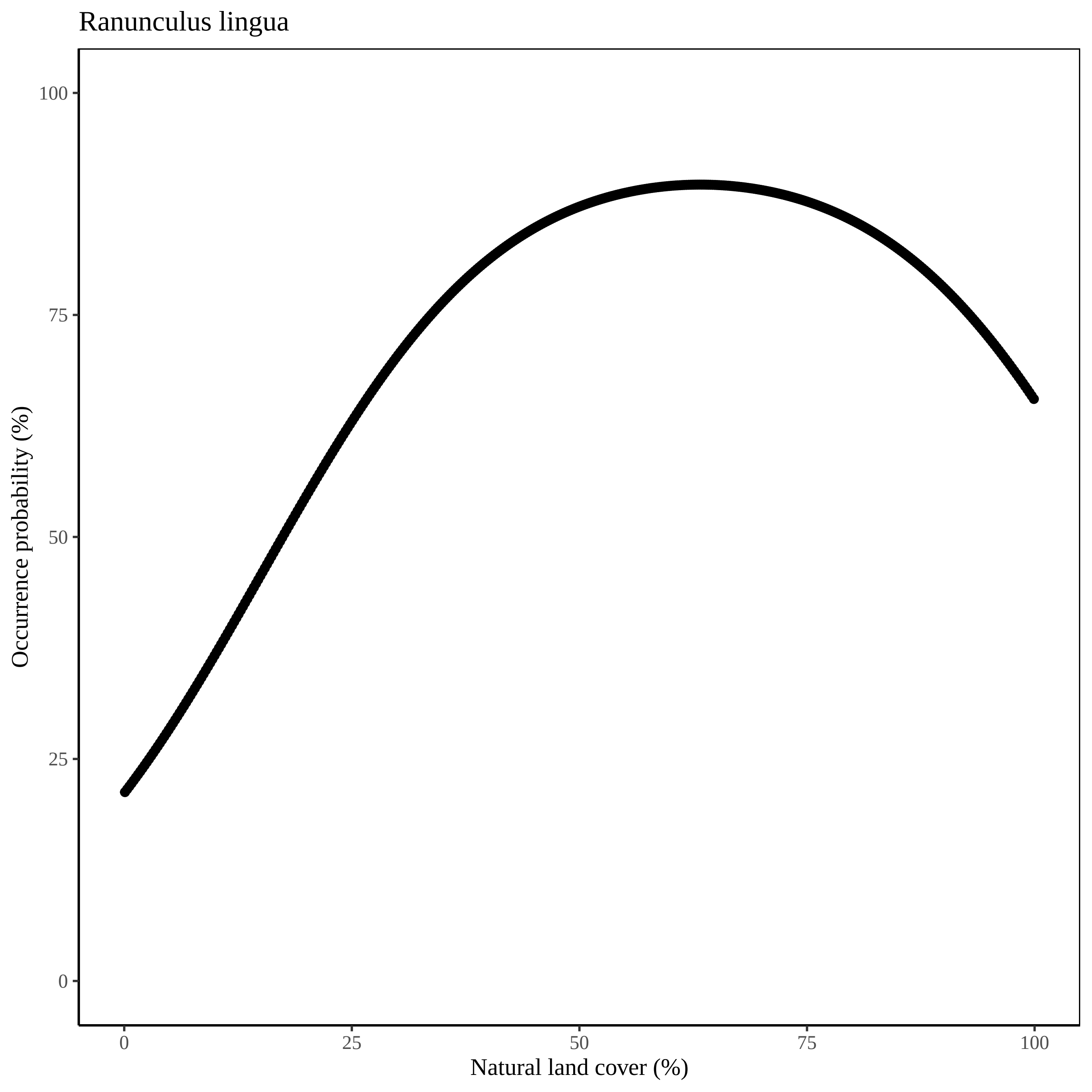
Ranunculus lingua
Occurrence probability (%)
100
75
50
25
0
Natural land cover (%)
0
25
50
75
100

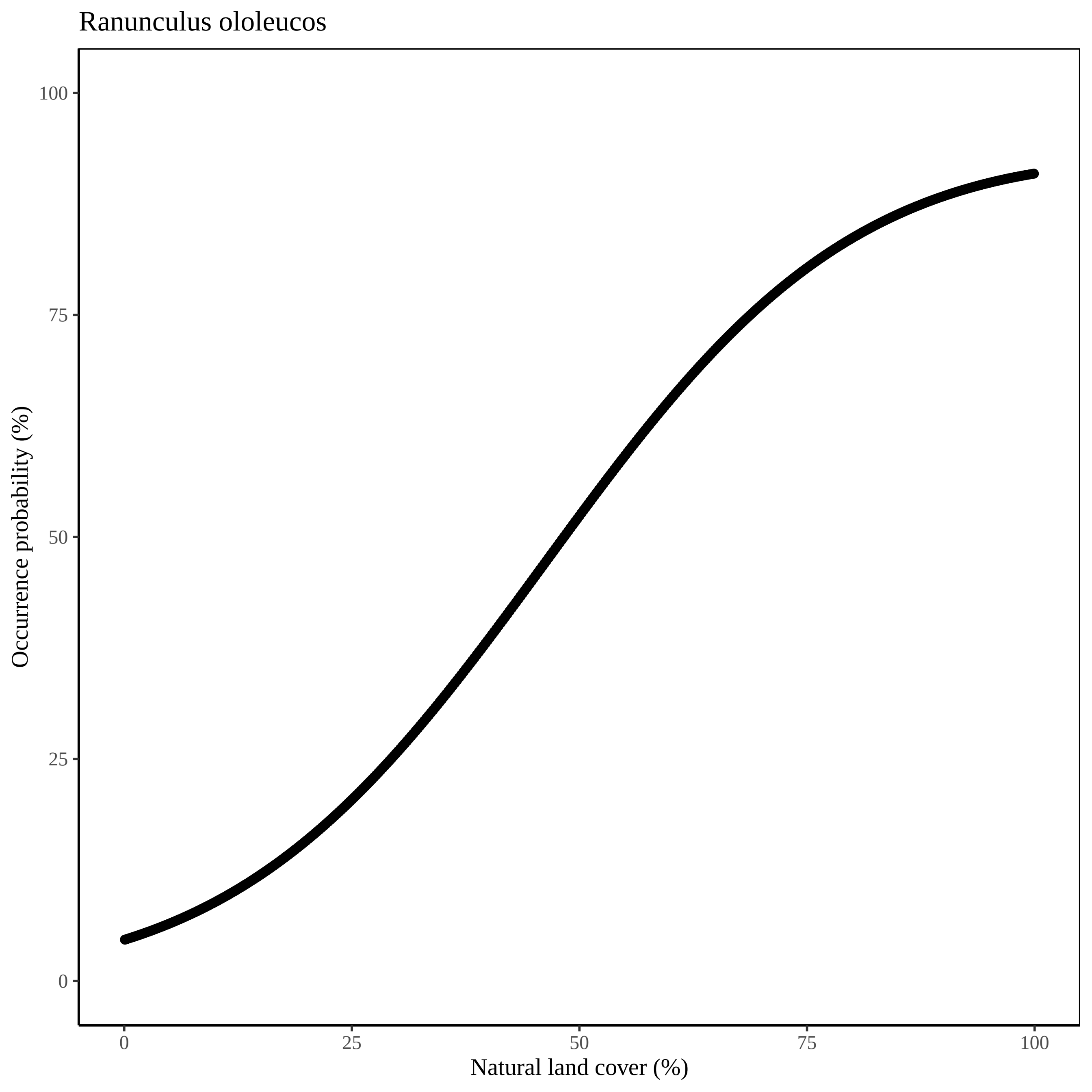
Ranunculus ololeucos
Occurrence probability (%)
100
75
50
25
0
0
25
50
75
100
Natural land cover (%)

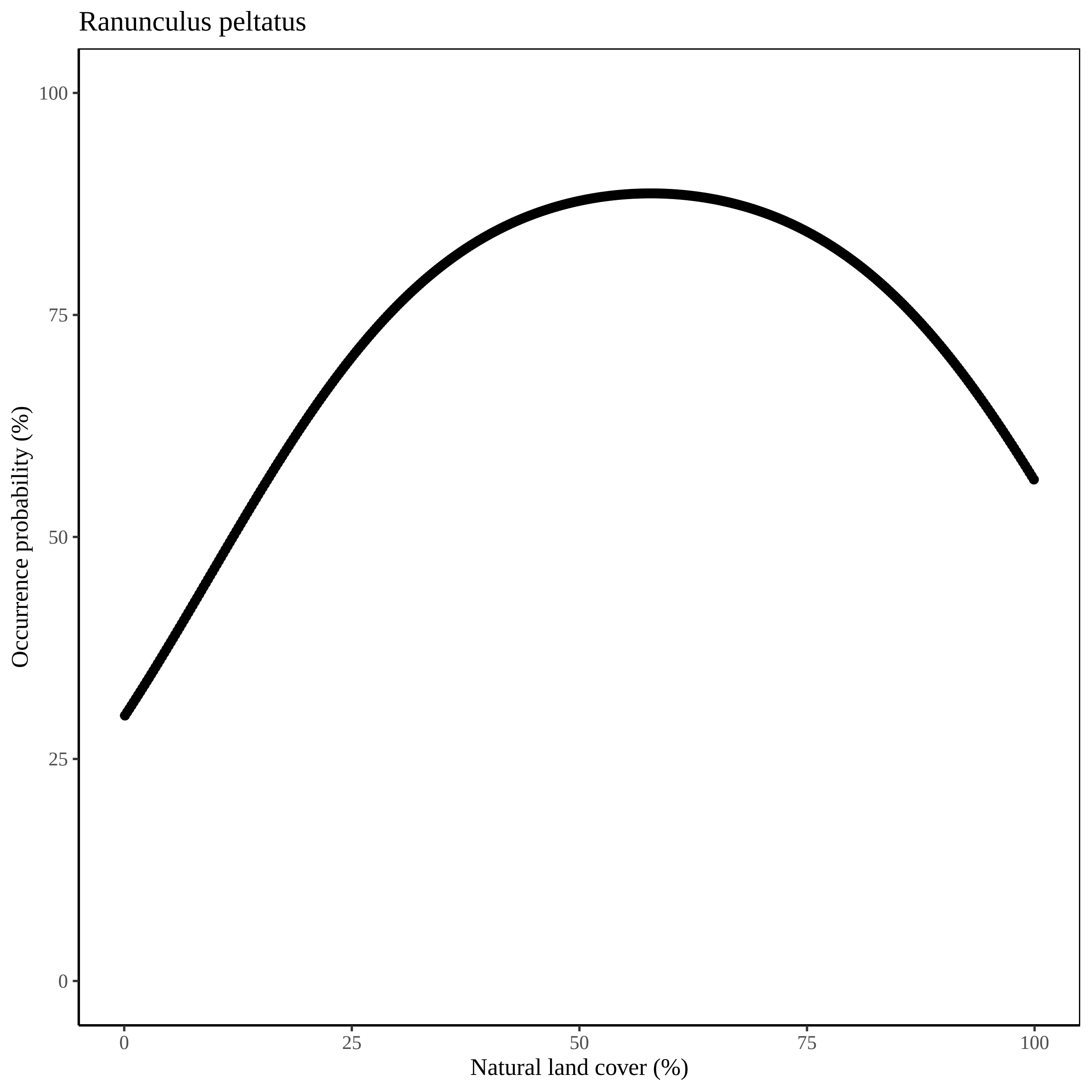

Ranunculus peltatus
100
75
50
25
0
Occurrence probability (%)
0
25
50
75
100
Natural land cover (%)

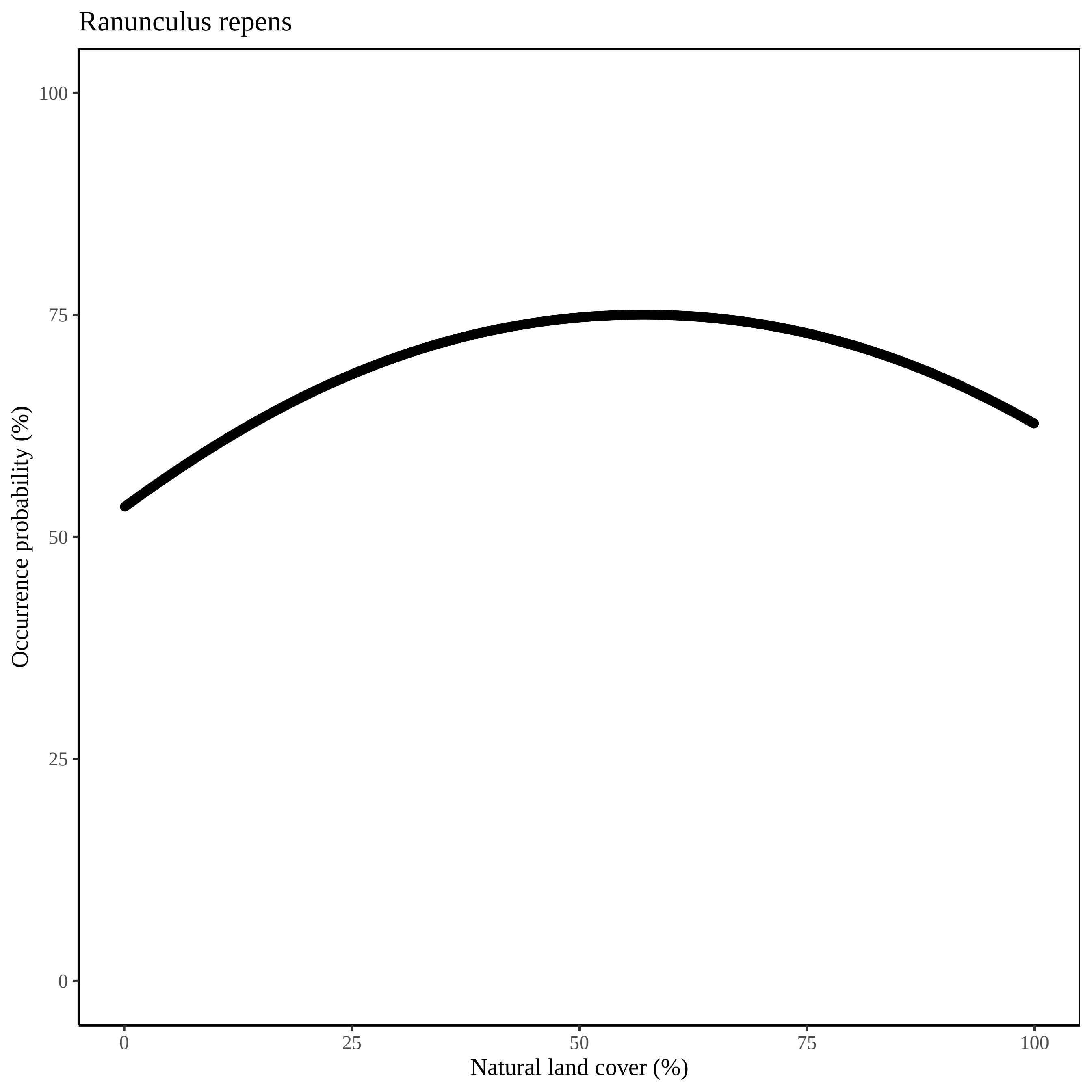
Ranunculus repens
100
75
50
25
0
Occurrence probability (%)
0
25
50
75
100
Natural land cover (%)

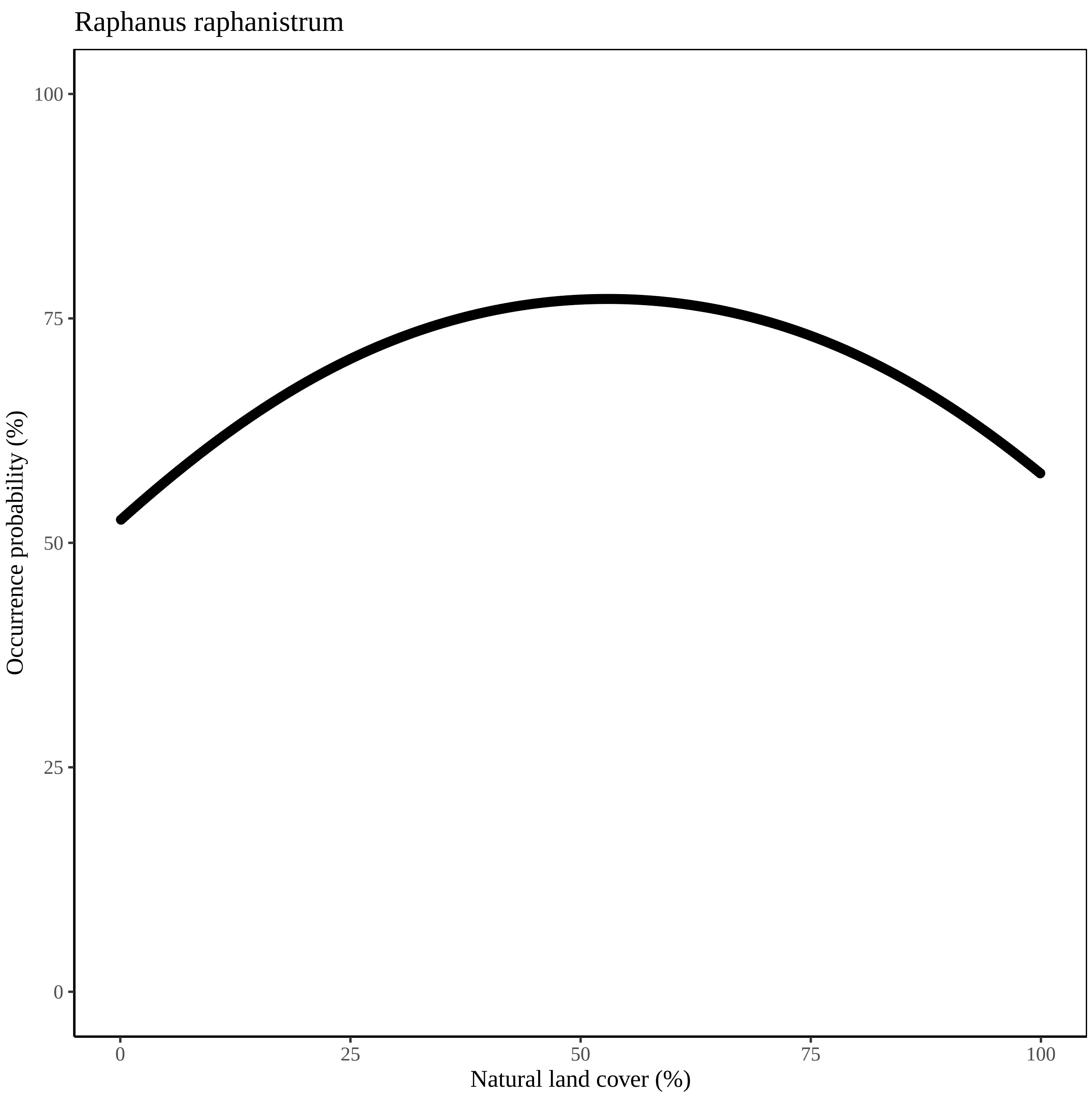
Raphanus raphanistrum
Occurrence probability (%)
100
75
50
25
0
0
25
50
75
100
Natural land cover (%)

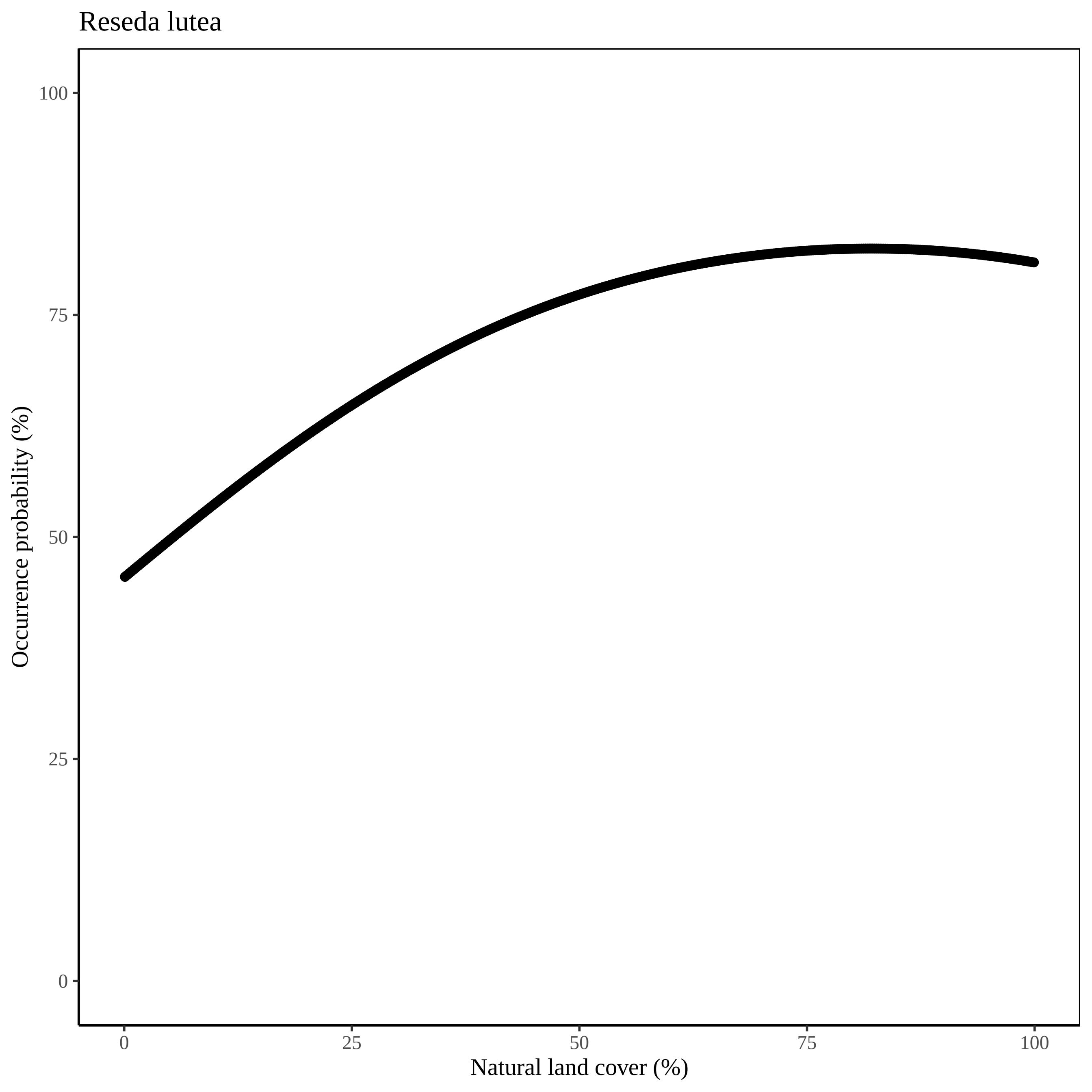
Reseda lutea
Occurrence probability (%)
100
75
50
25
0
0
25
50
75
100
Natural land cover (%)

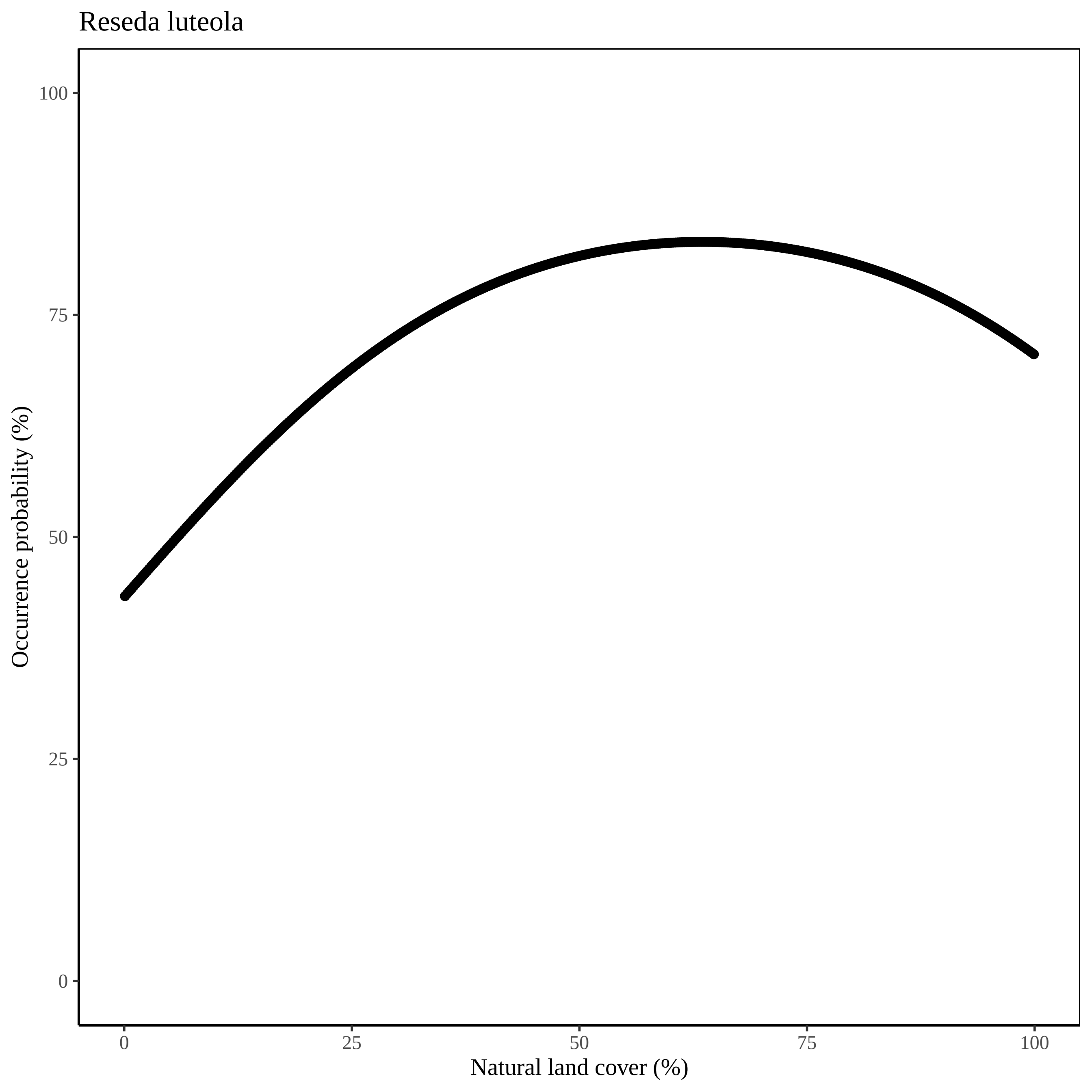
Reseda luteola
Occurrence probability (%)
100
75
50
25
0
0
25
50
75
100
Natural land cover (%)

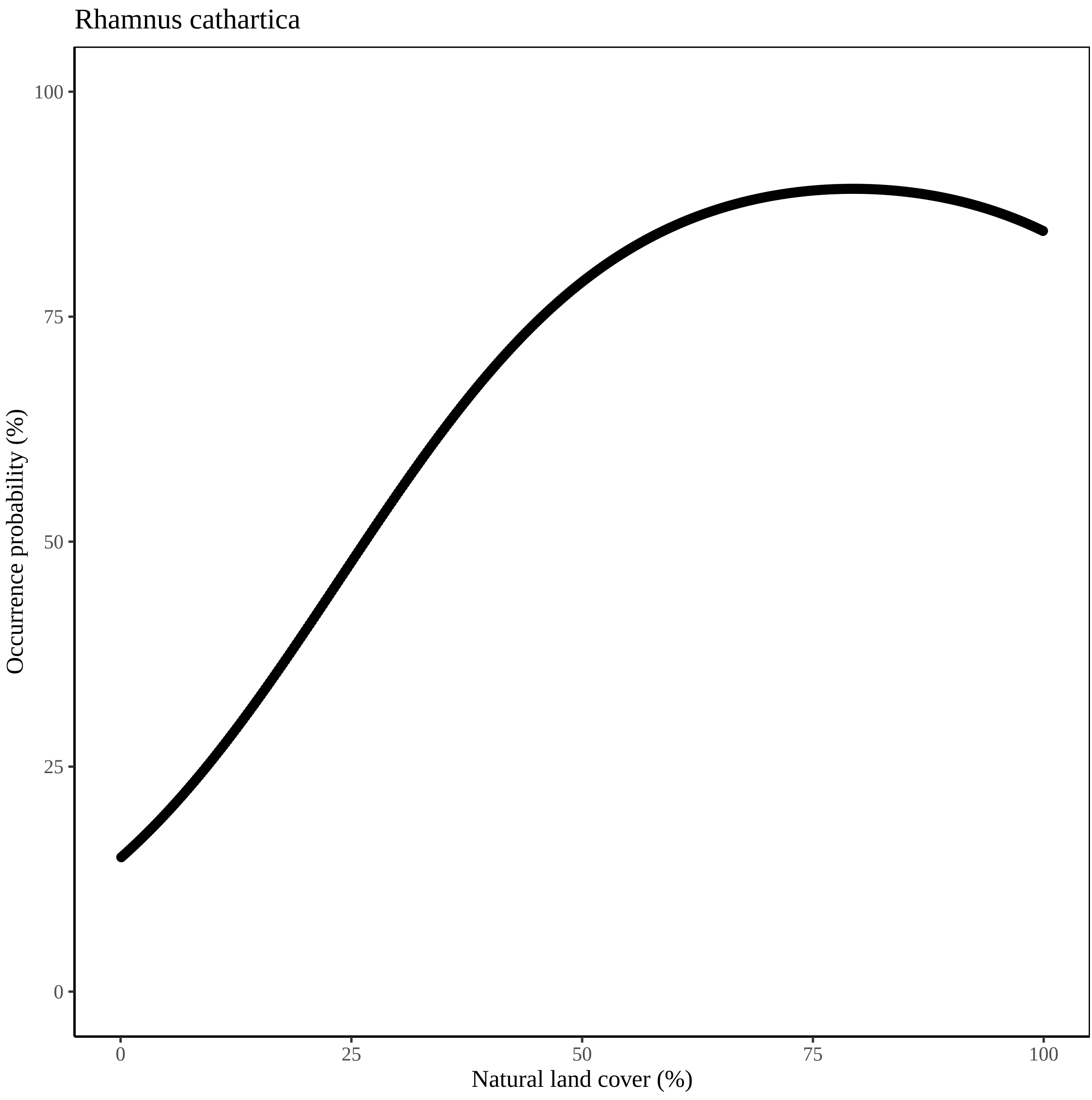
Rhamnus cathartica
Occurrence probability (%)
100
75
50
25
0
0
25
50
75
100
Natural land cover (%)

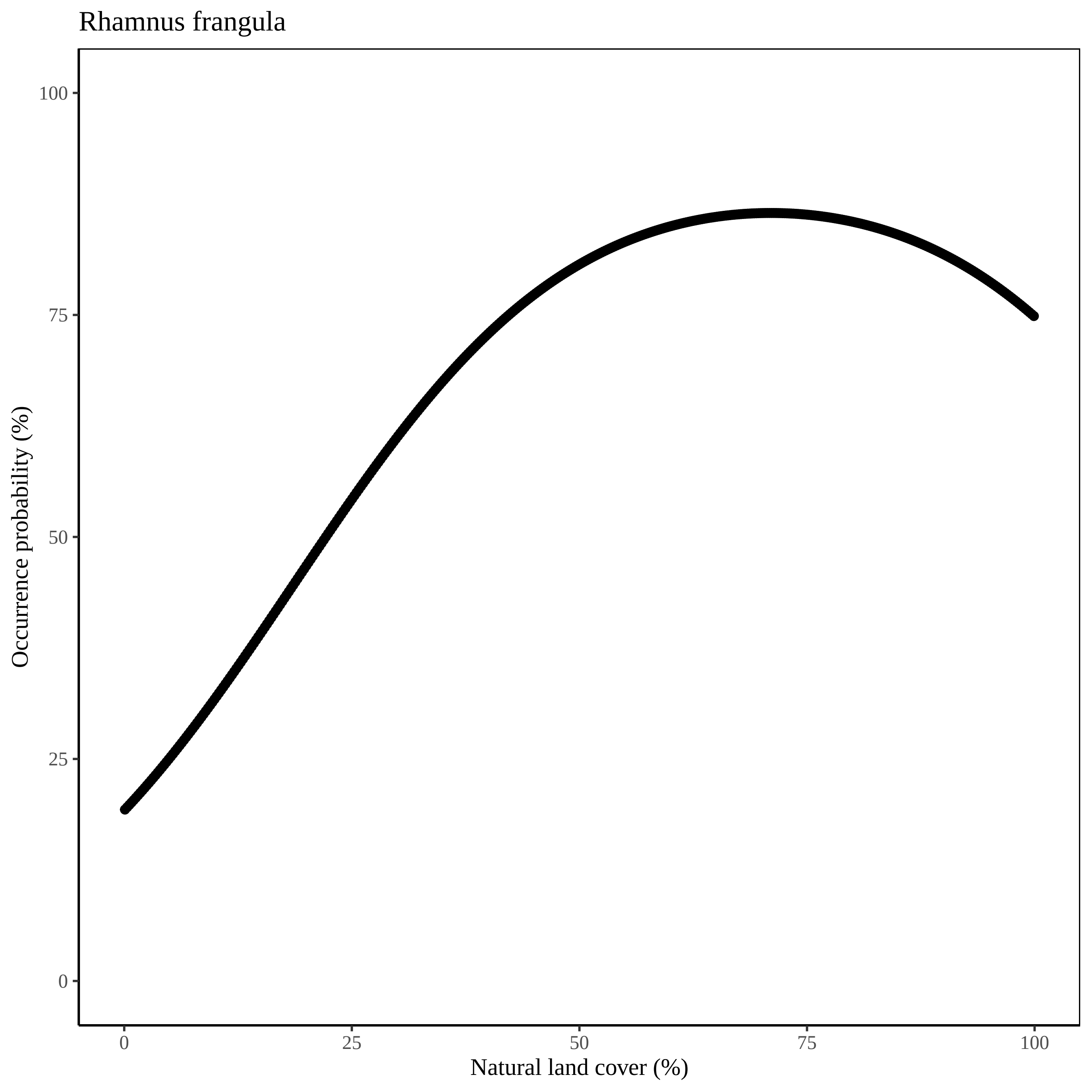
Rhamnus frangula
Occurrence probability (%)
100
75
50
25
0
0
25
50
75
100
Natural land cover (%)

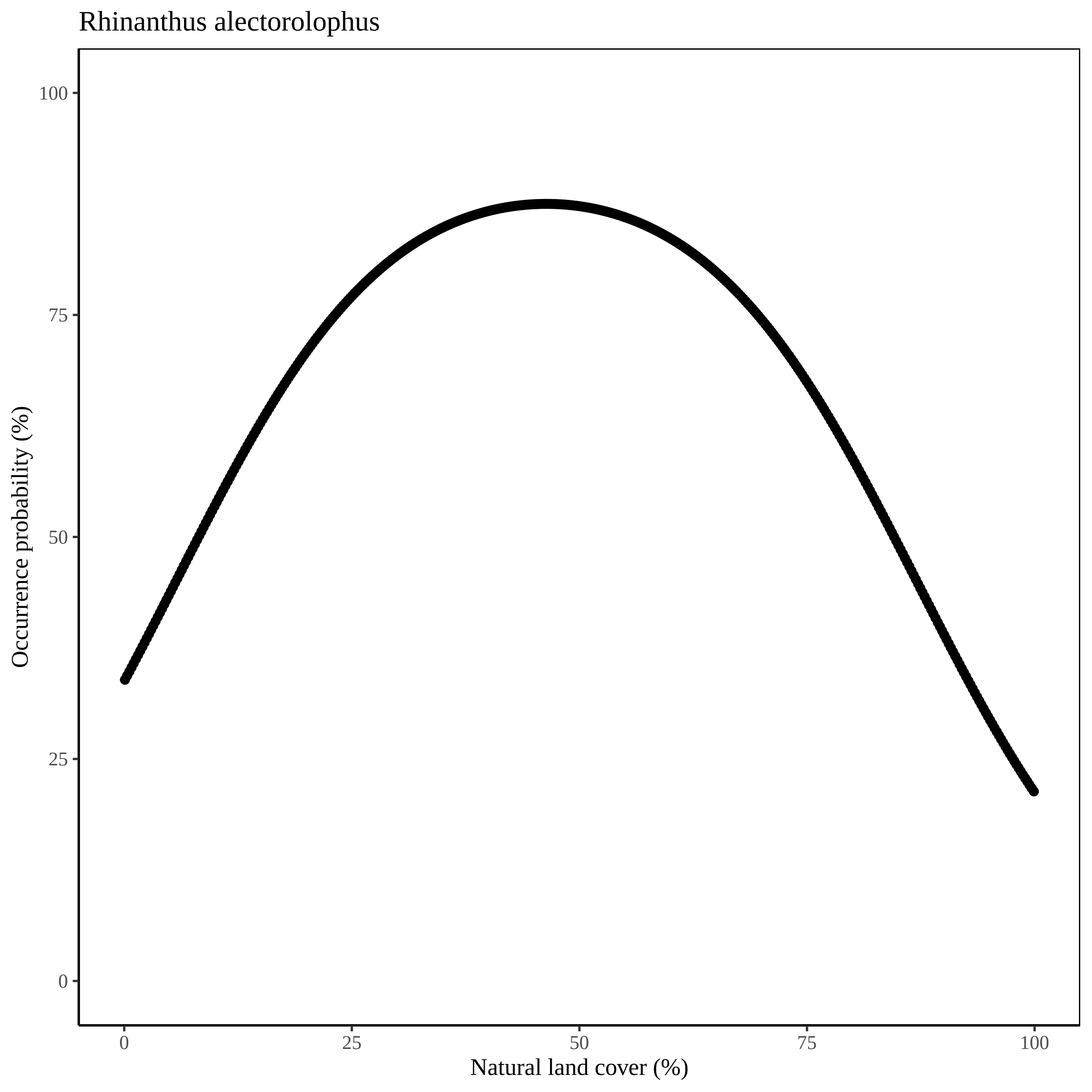

Rhinanthus alectorolophus
100
75
50
25
0
0
25
50
75
100
Occurrence probability (%)
Natural land cover (%)

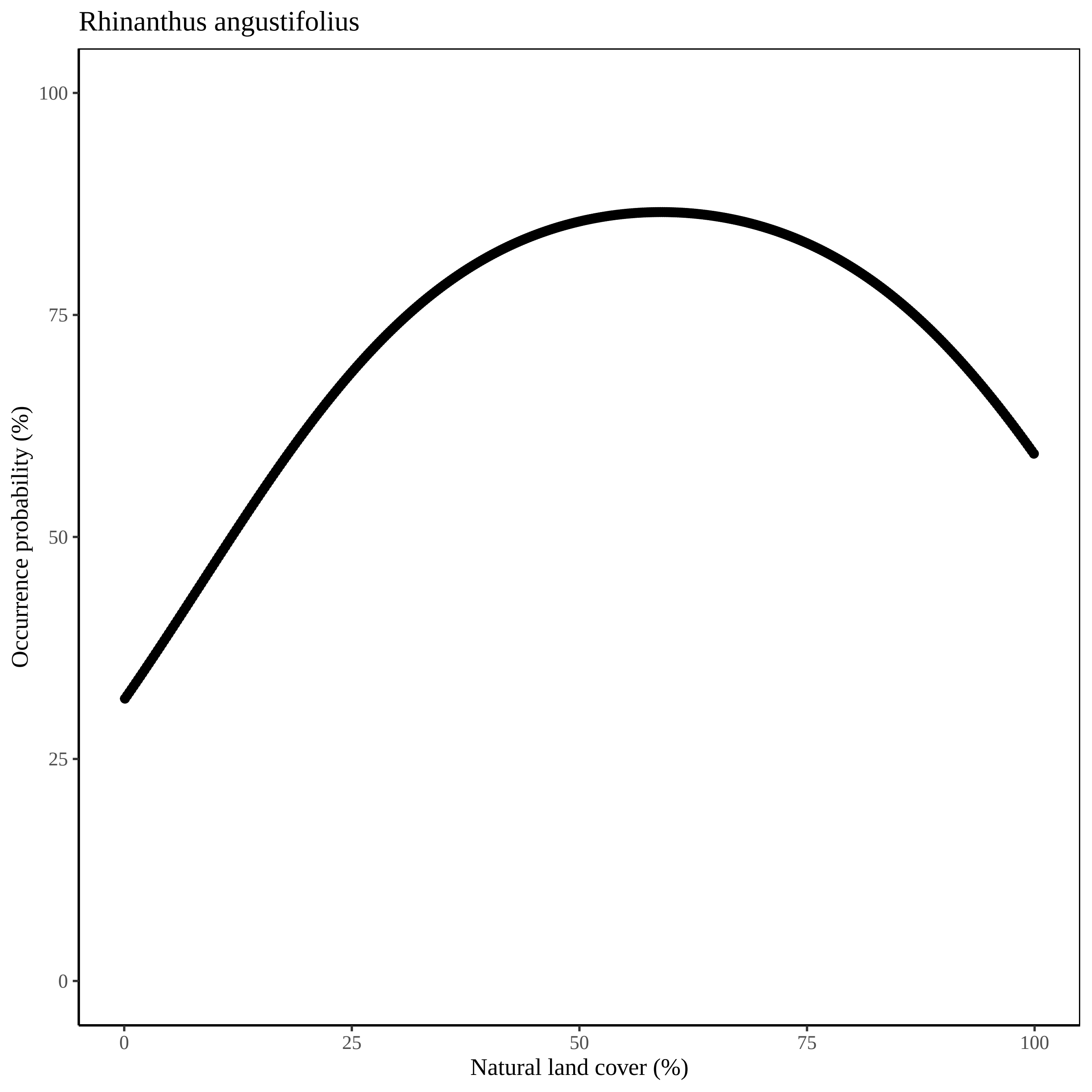
Rhinanthus angustifolius
100
75
50
25
0
Occurrence probability (%)
0
25
50
75
100
Natural land cover (%)

Rhinanthus minor

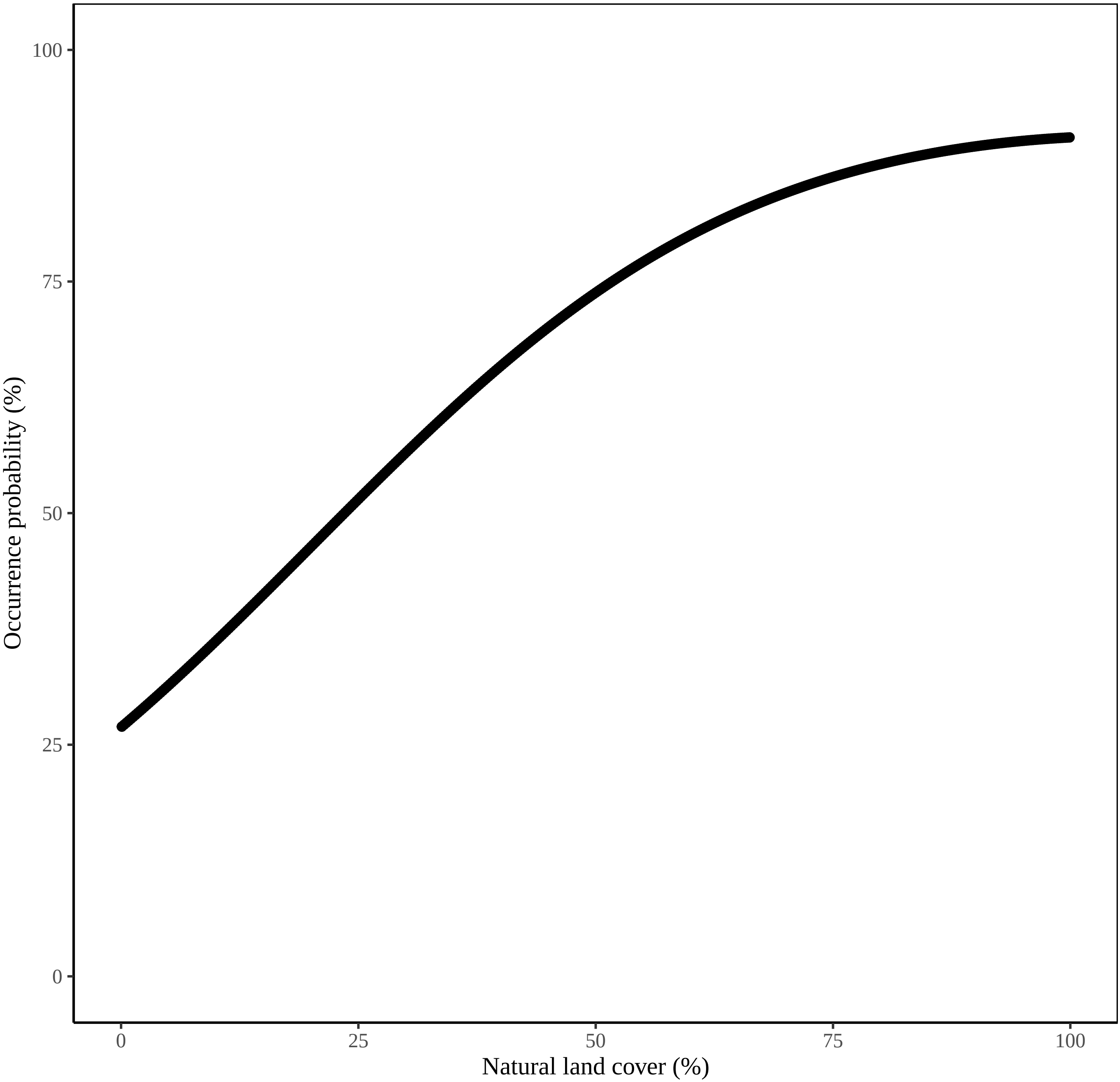

Rhododendron ponticum

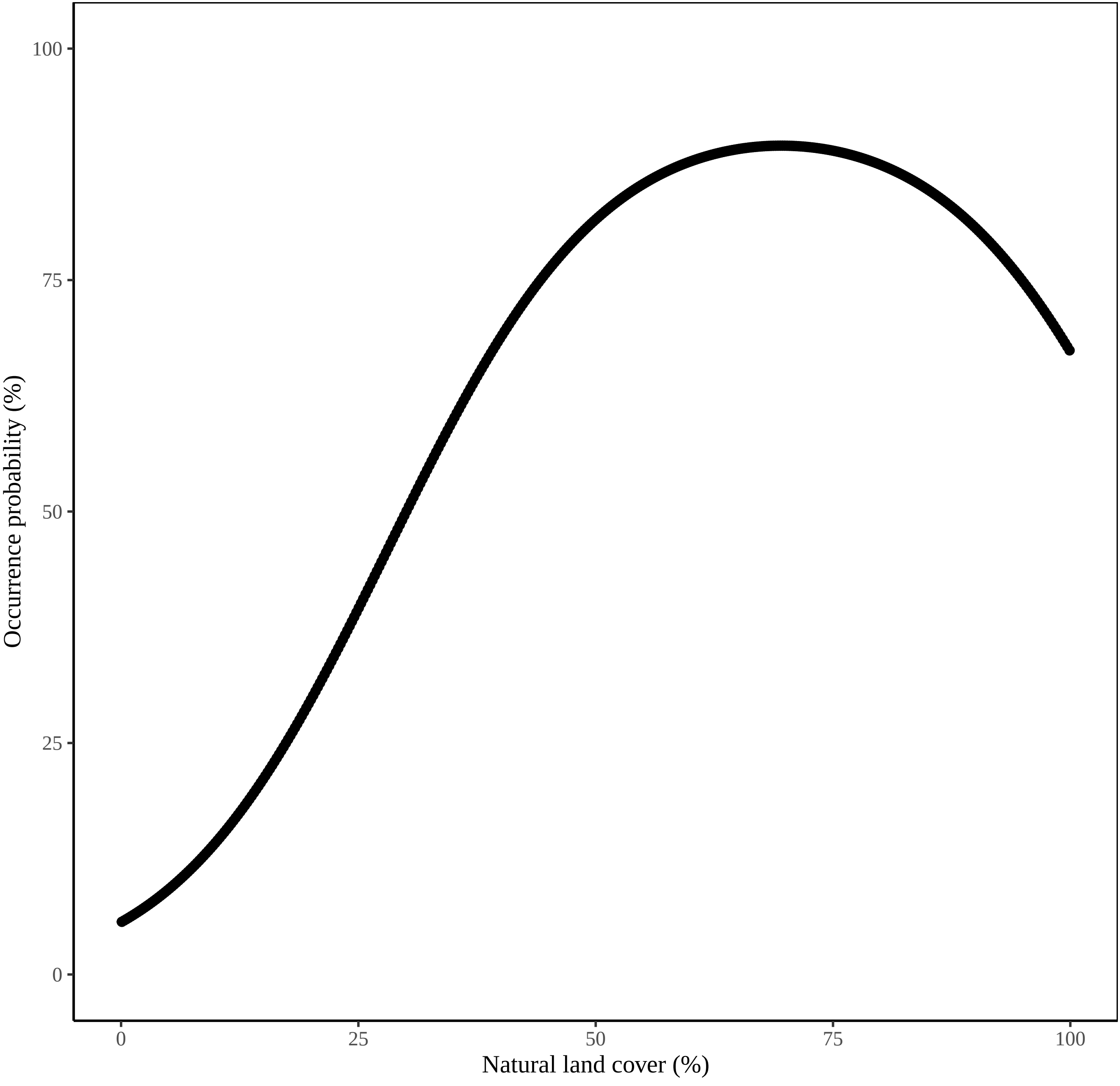

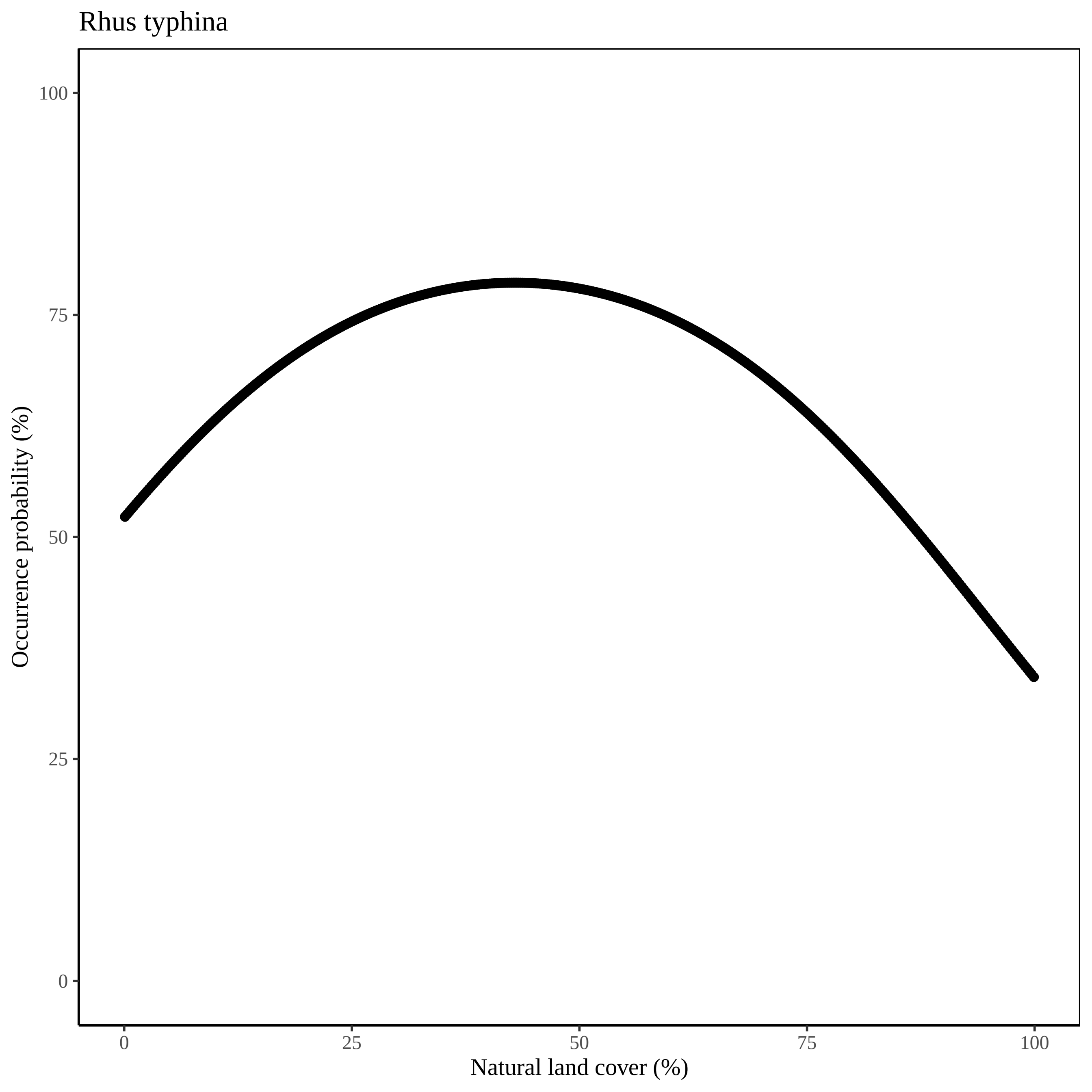

Rhus typhina
100
75
50
25
0
Occurrence probability (%)
0
25
50
75
100
Natural land cover (%)

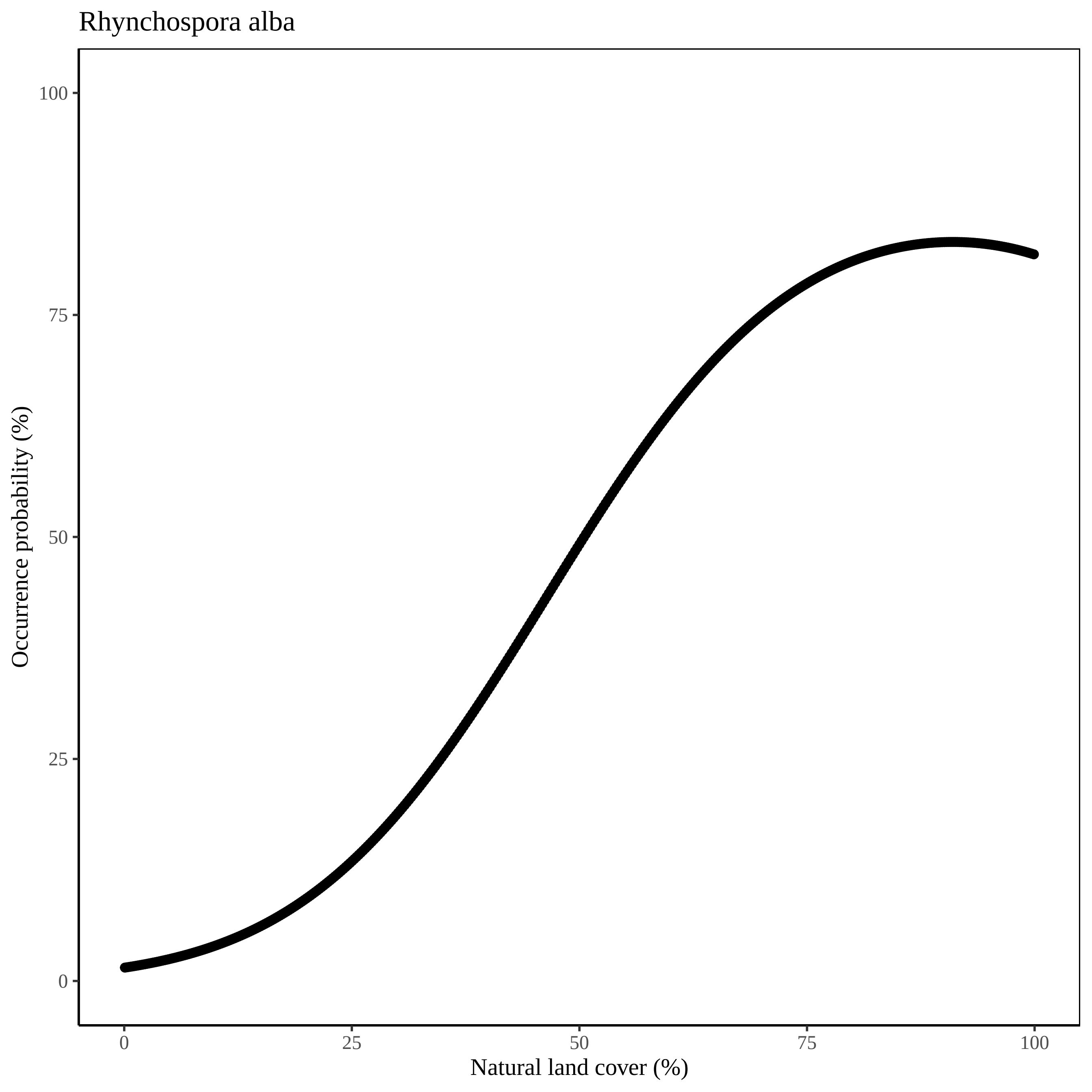
Rhynchospora alba
100
75
50
25
0
Occurrence probability (%)
0
25
50
75
100
Natural land cover (%)

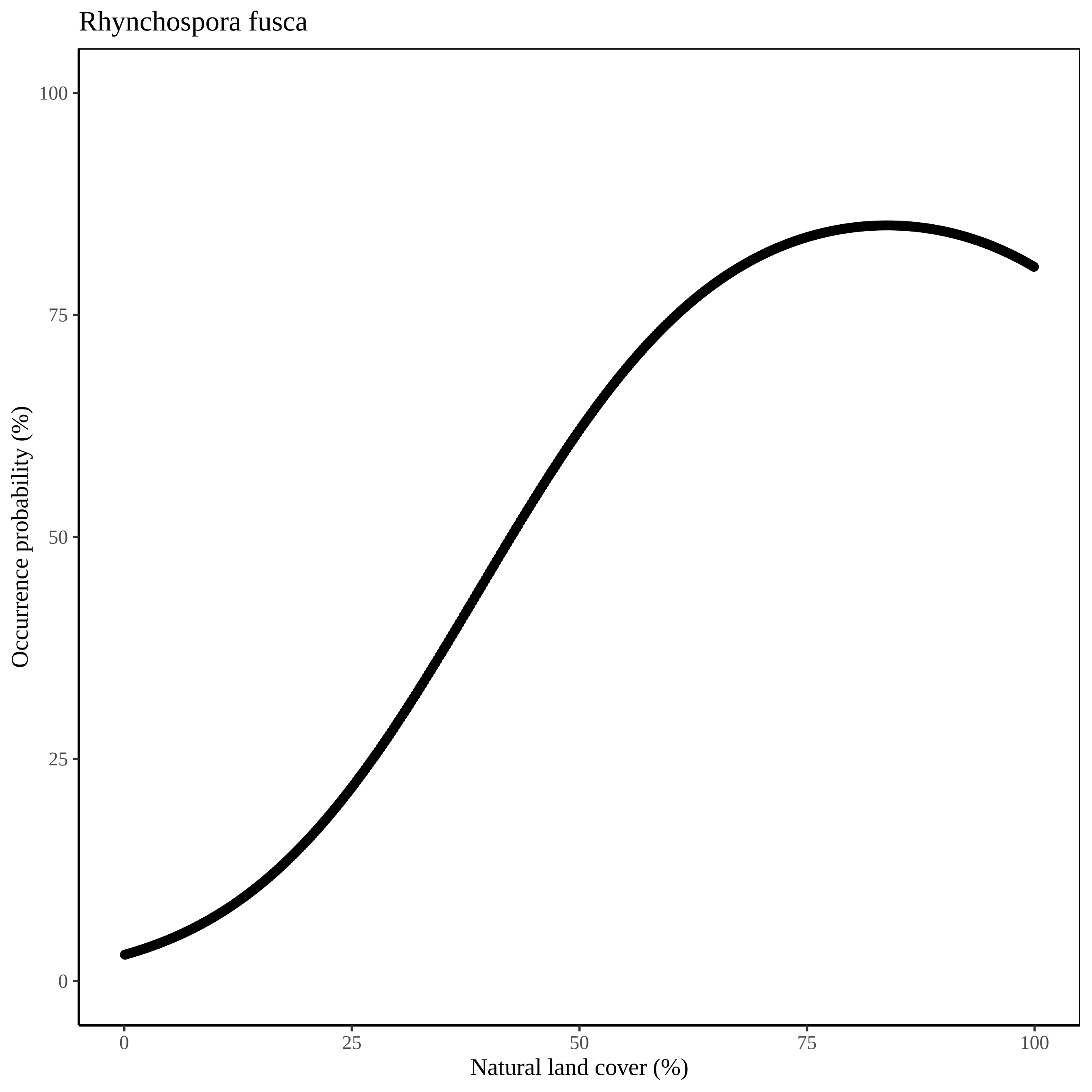
Rhynchospora fusca
Occurrence probability (%)
100
75
50
25
0
0
25
50
75
100
Natural land cover (%)

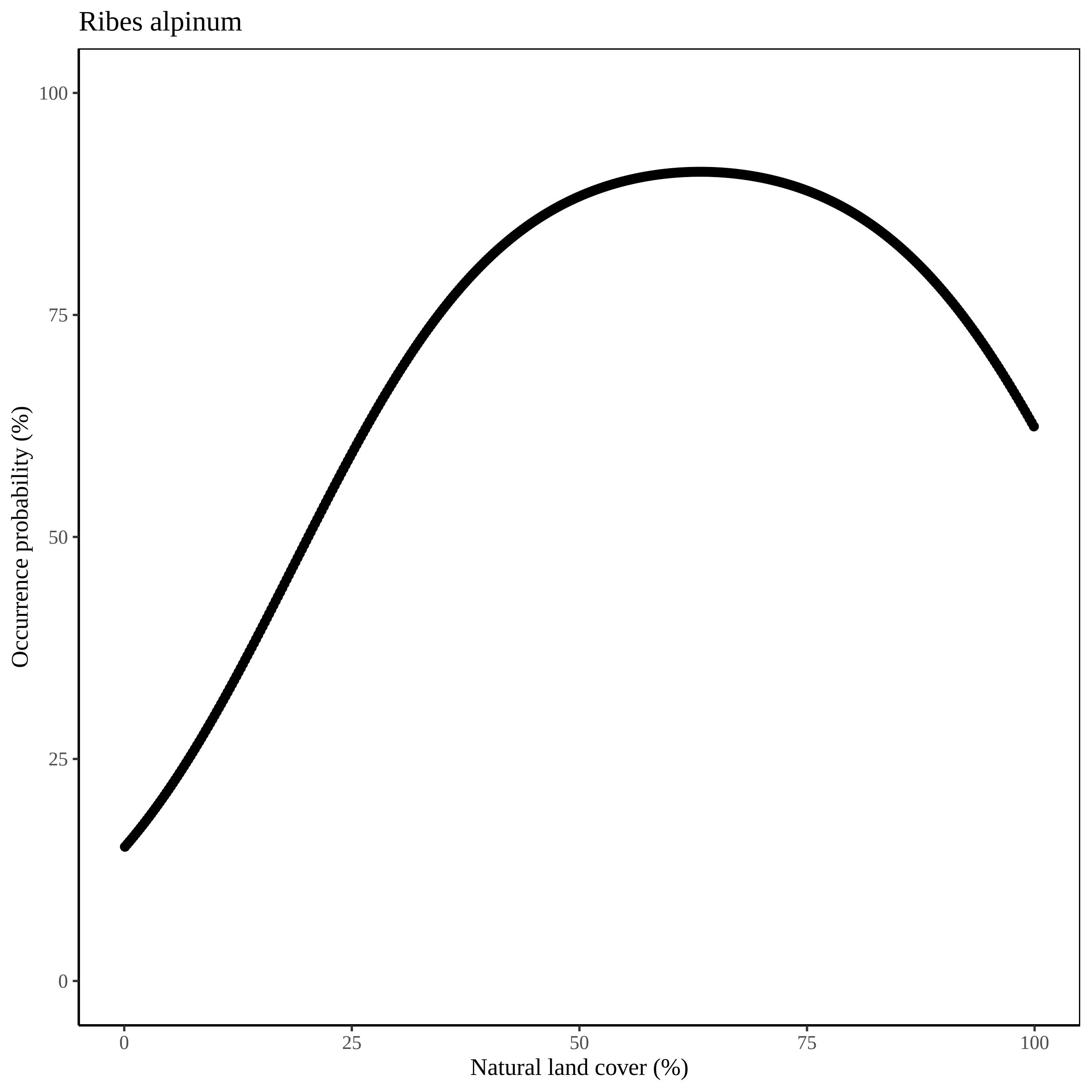
Ribes alpinum
Occurrence probability (%)
100
75
50
25
0
0
25
50
75
100
Natural land cover (%)

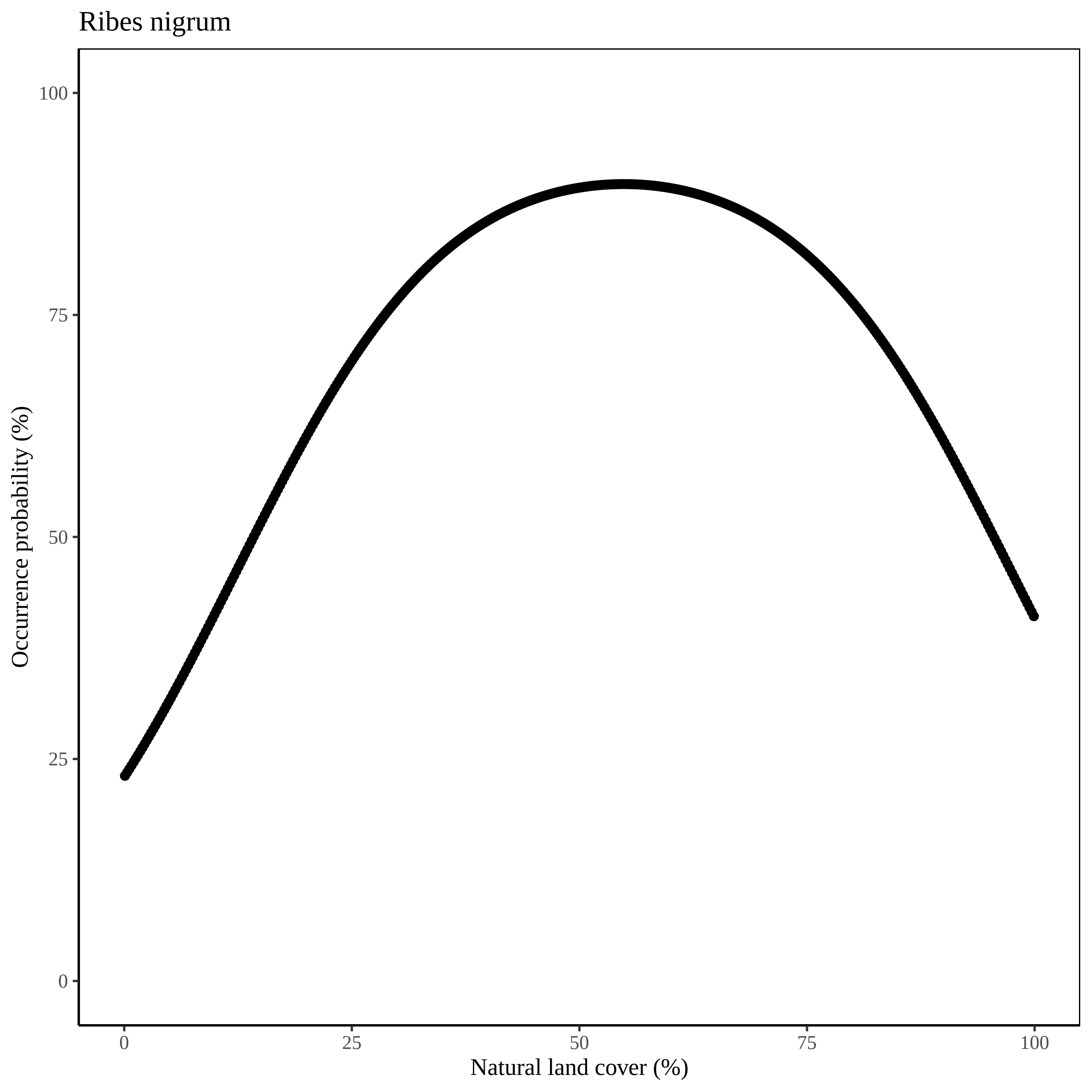

Ribes nigrum
Occurrence probability (%)
100
75
50
25
0
0
25
50
75
100
Natural land cover (%)

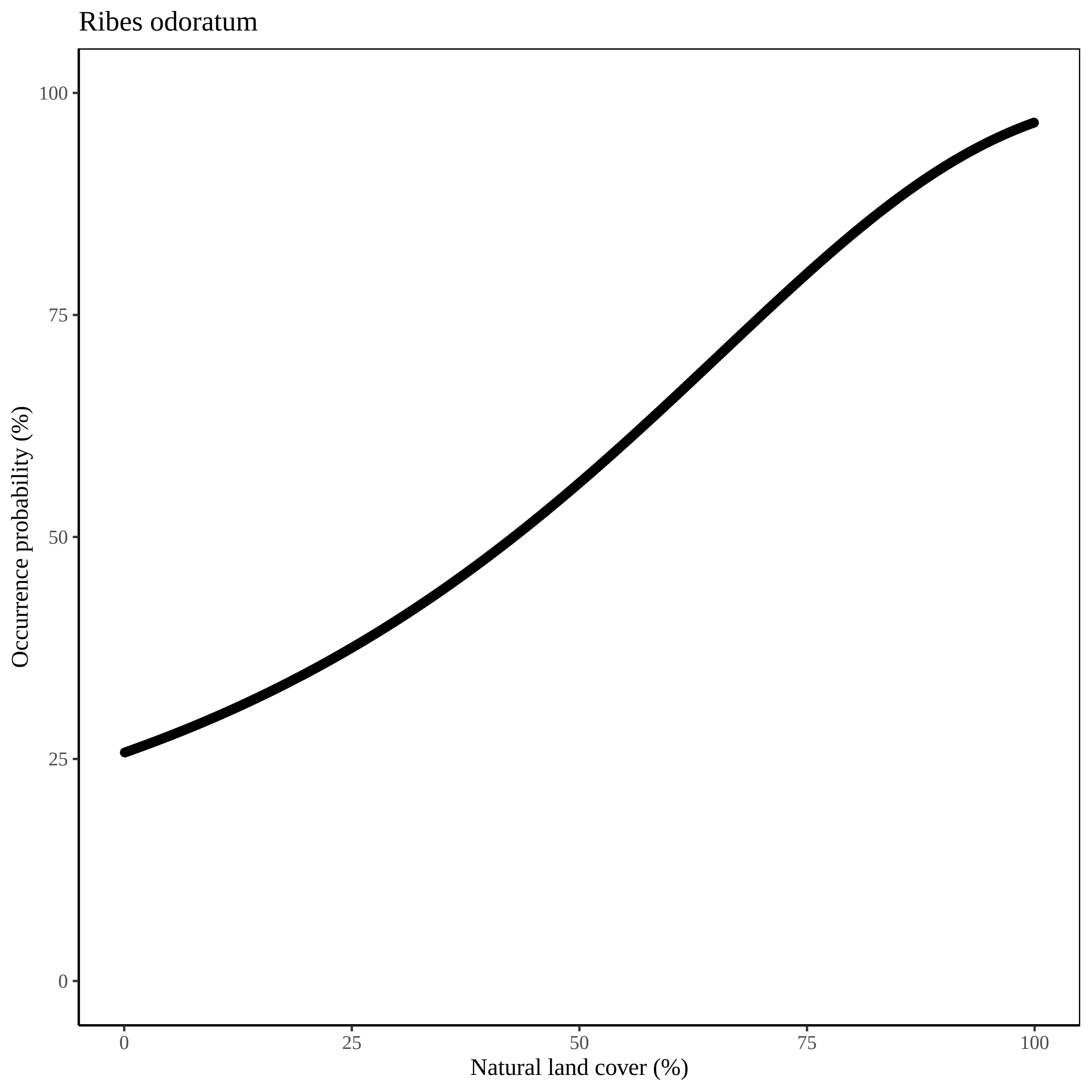
Ribes odoratum
Occurrence probability (%)
100
75
50
25
0
0
25
50
75
100
Natural land cover (%)

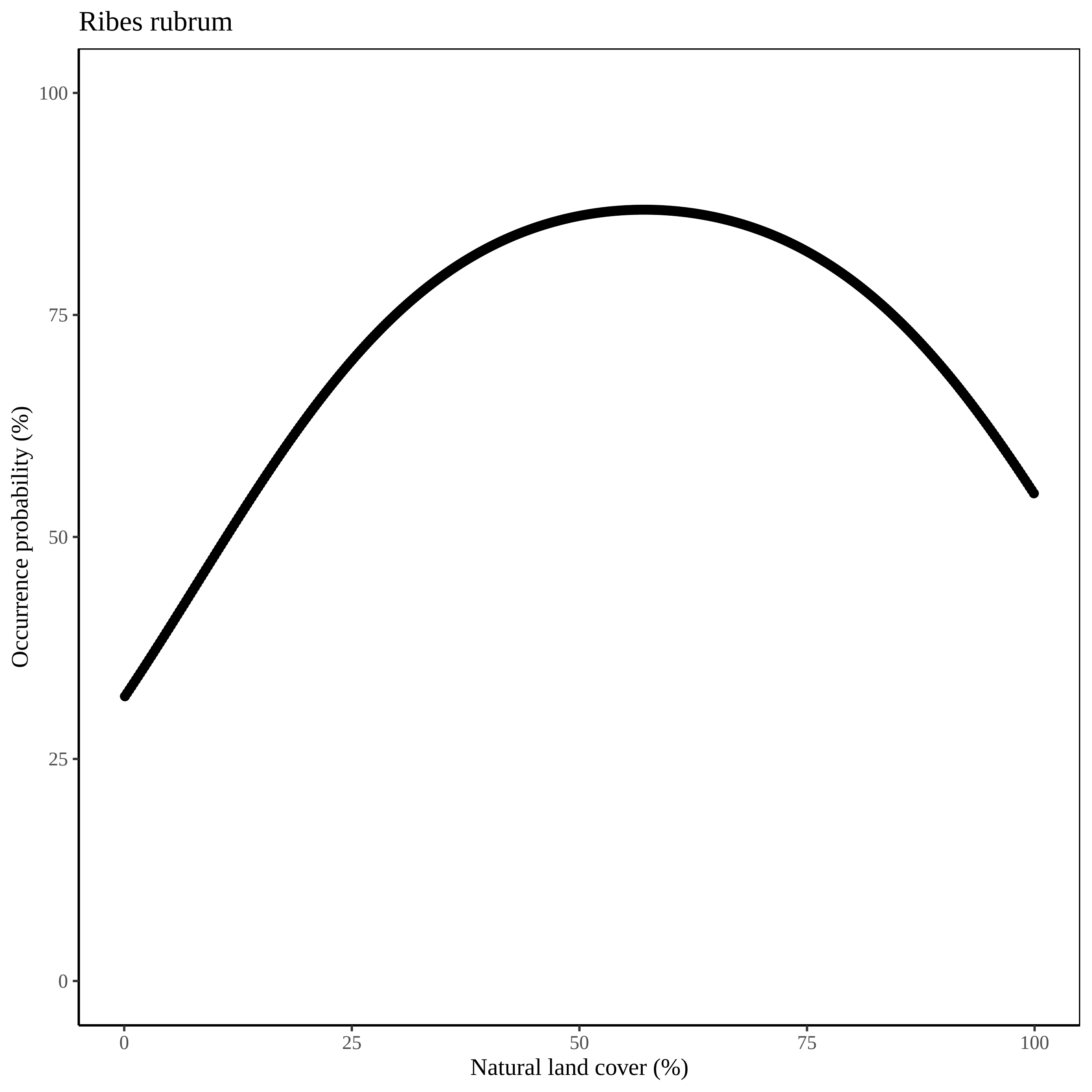

Ribes rubrum
100
75
50
25
0
Occurrence probability (%)
0
25
50
75
100
Natural land cover (%)

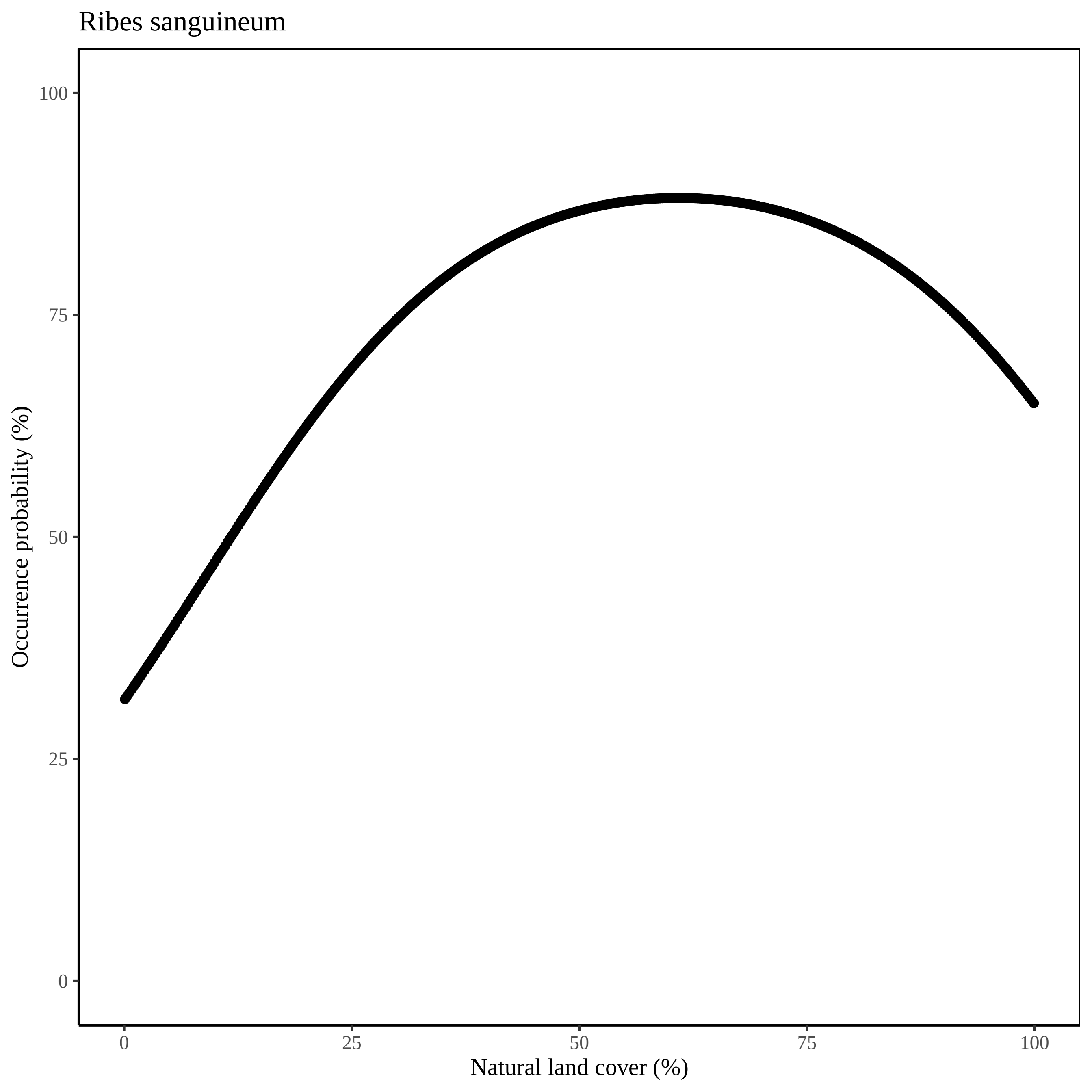
Ribes sanguineum
Occurrence probability (%)
100
75
50
25
0
0
25
50
75
100
Natural land cover (%)

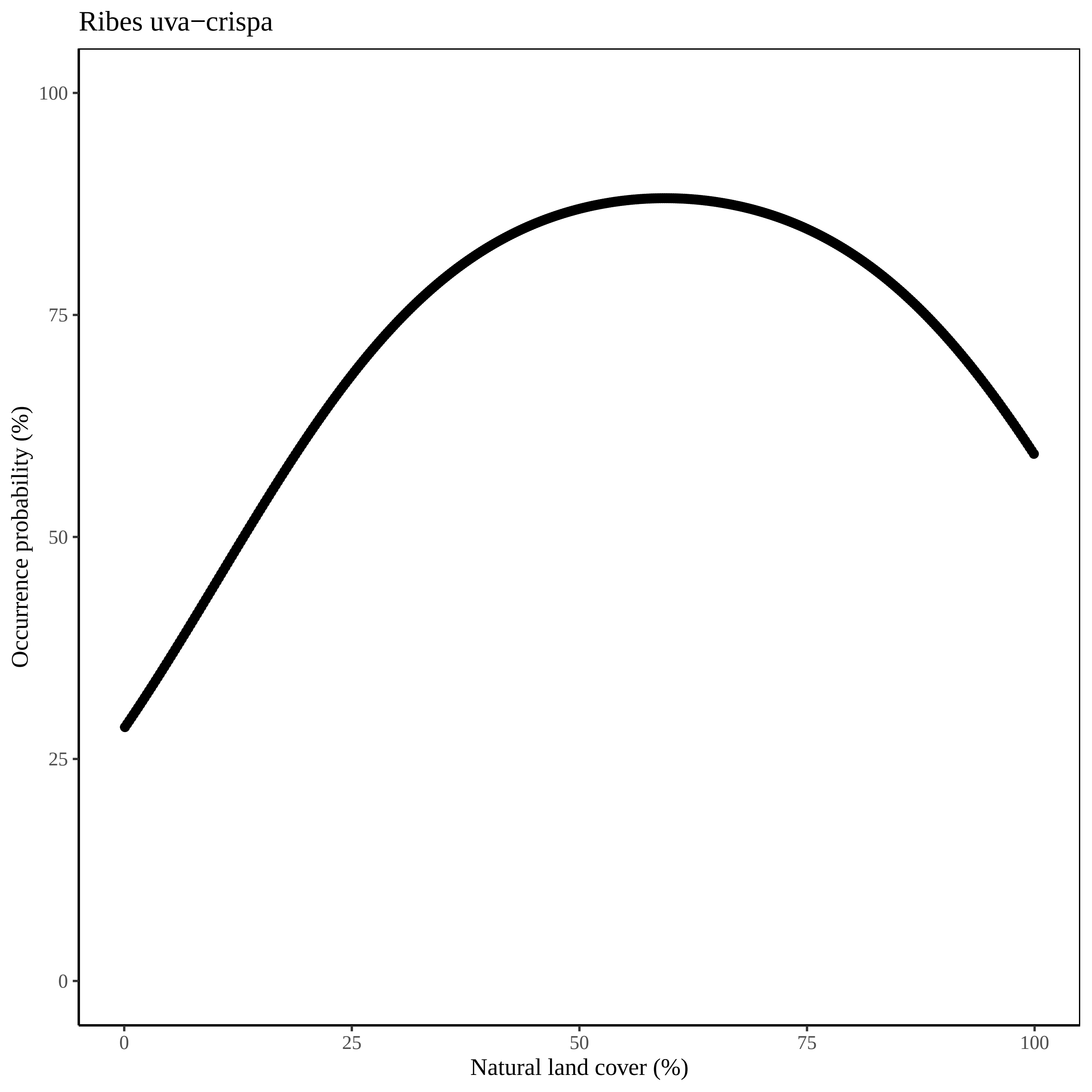
Ribes uva−crispa
Occurrence probability (%)
100
75
50
25
0
0
25
50
75
100
Natural land cover (%)

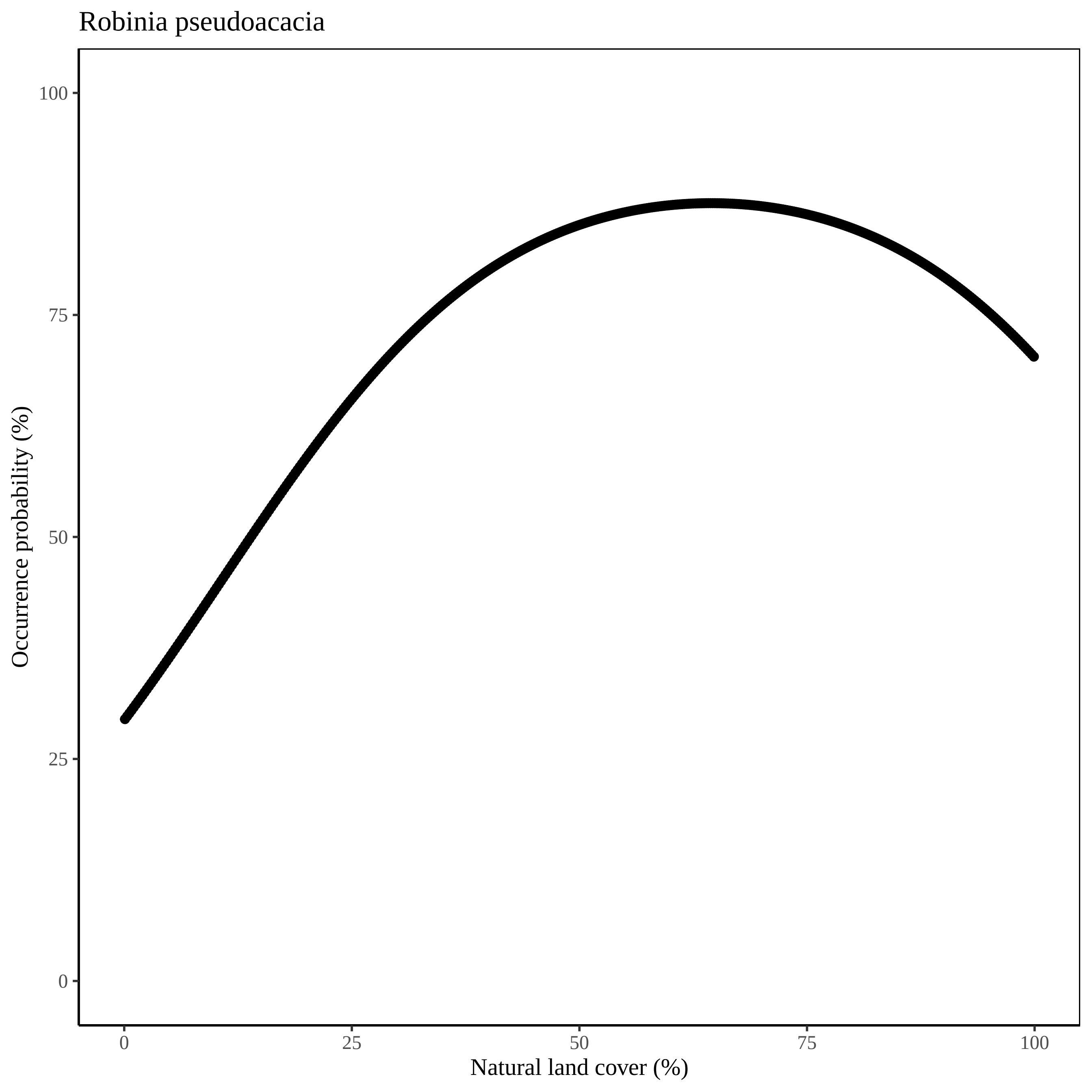
Robinia pseudoacacia
Occurrence probability (%)
100
75
50
25
0
0
25
50
75
100
Natural land cover (%)

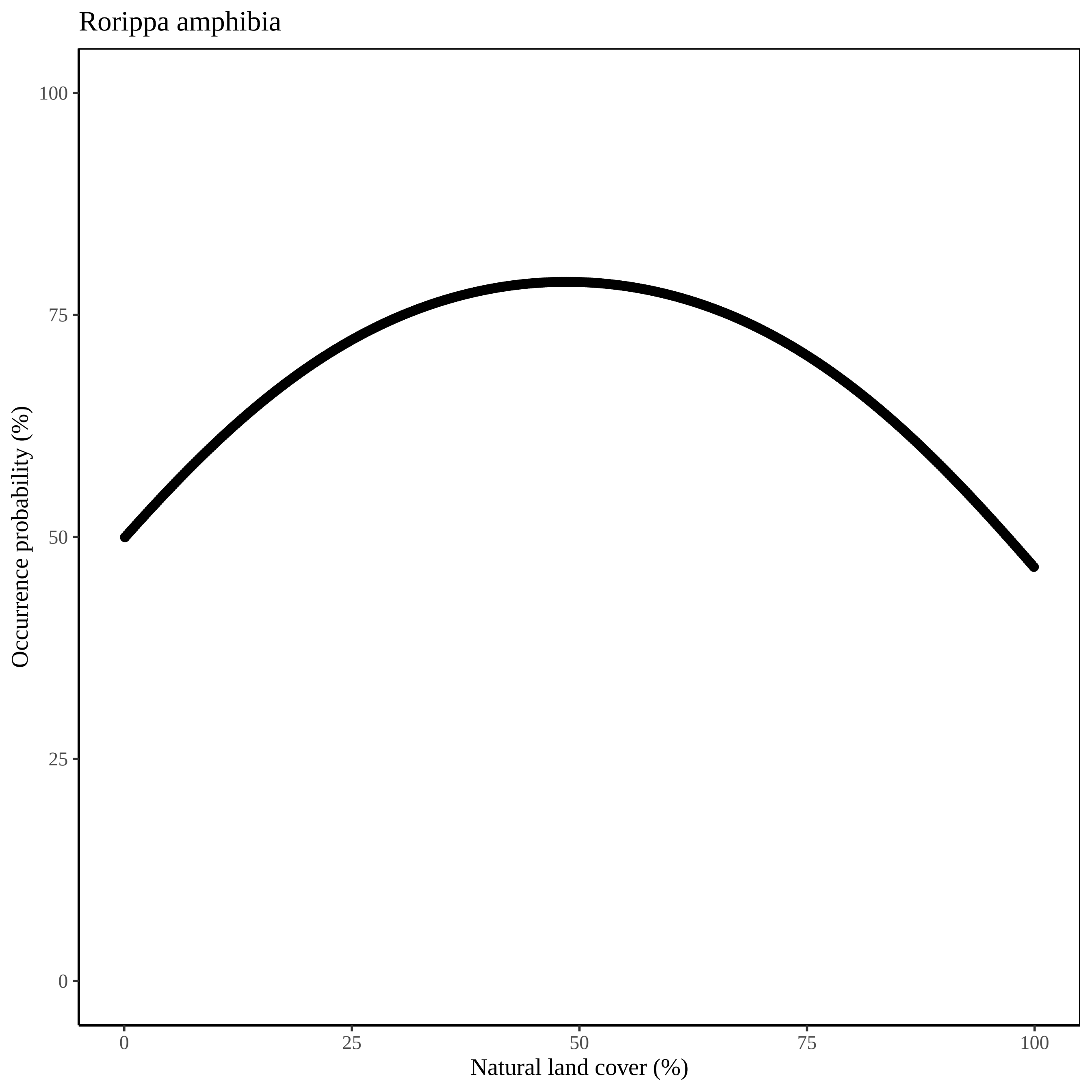
Rorippa amphibia
Occurrence probability (%)
100
75
50
25
0
0
25
50
75
100
Natural land cover (%)

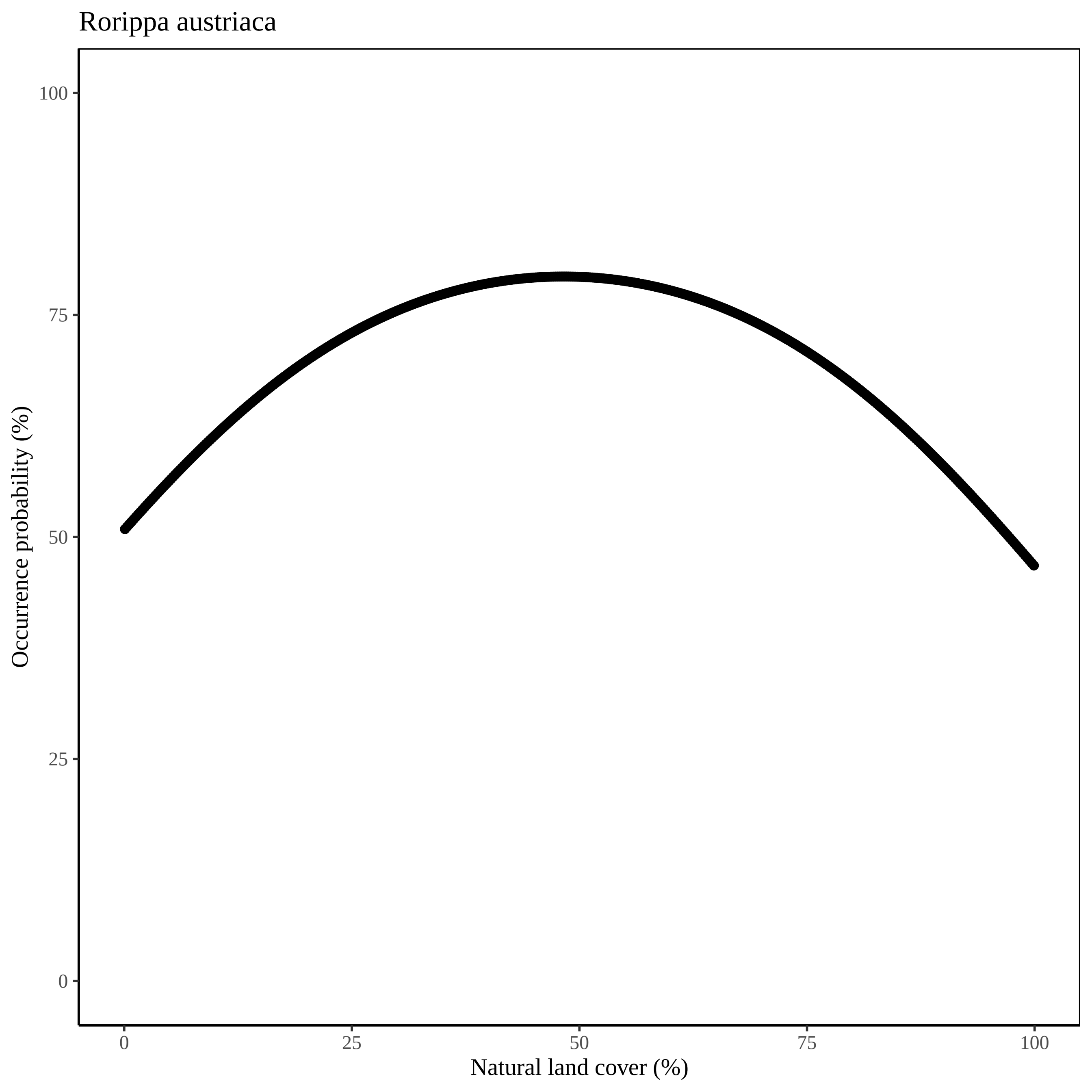
Rorippa austriaca
Occurrence probability (%)
100
75
50
25
0
0
25
50
75
100
Natural land cover (%)

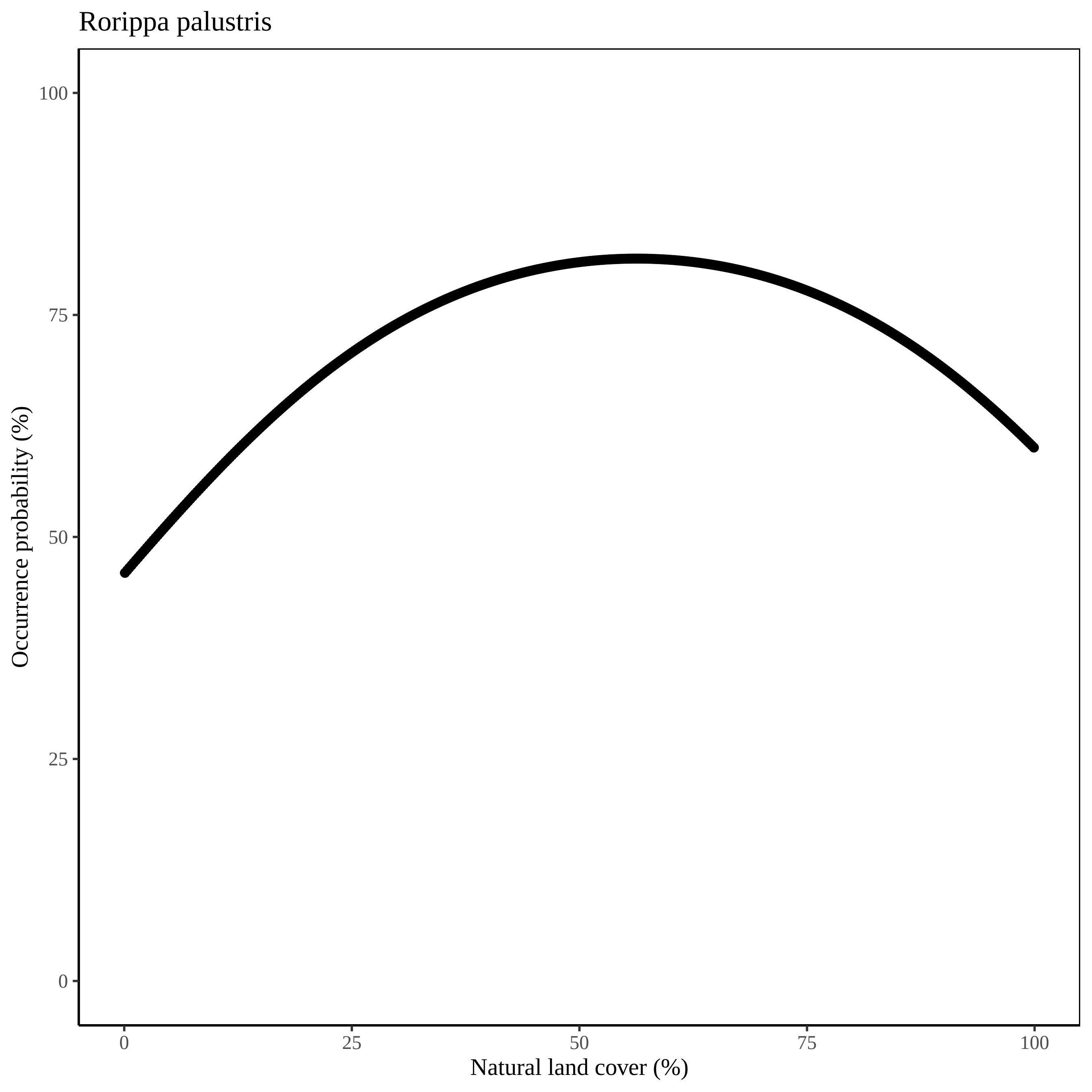

Rorippa palustris
Occurrence probability (%)
100
75
50
25
0
0
25
50
75
100
Natural land cover (%)

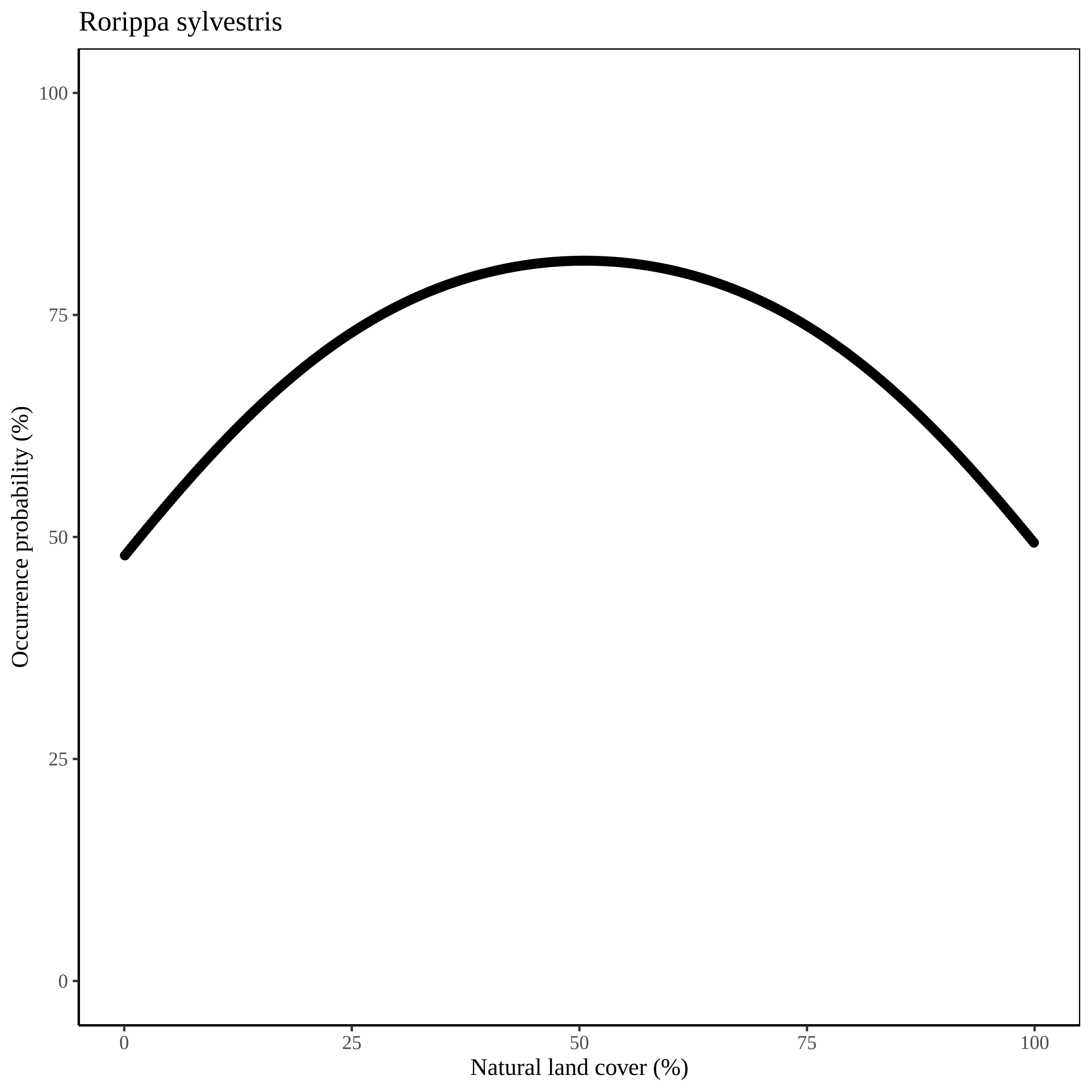
Rorippa sylvestris
Occurrence probability (%)
100
75
50
25
0
0
25
50
75
100
Natural land cover (%)

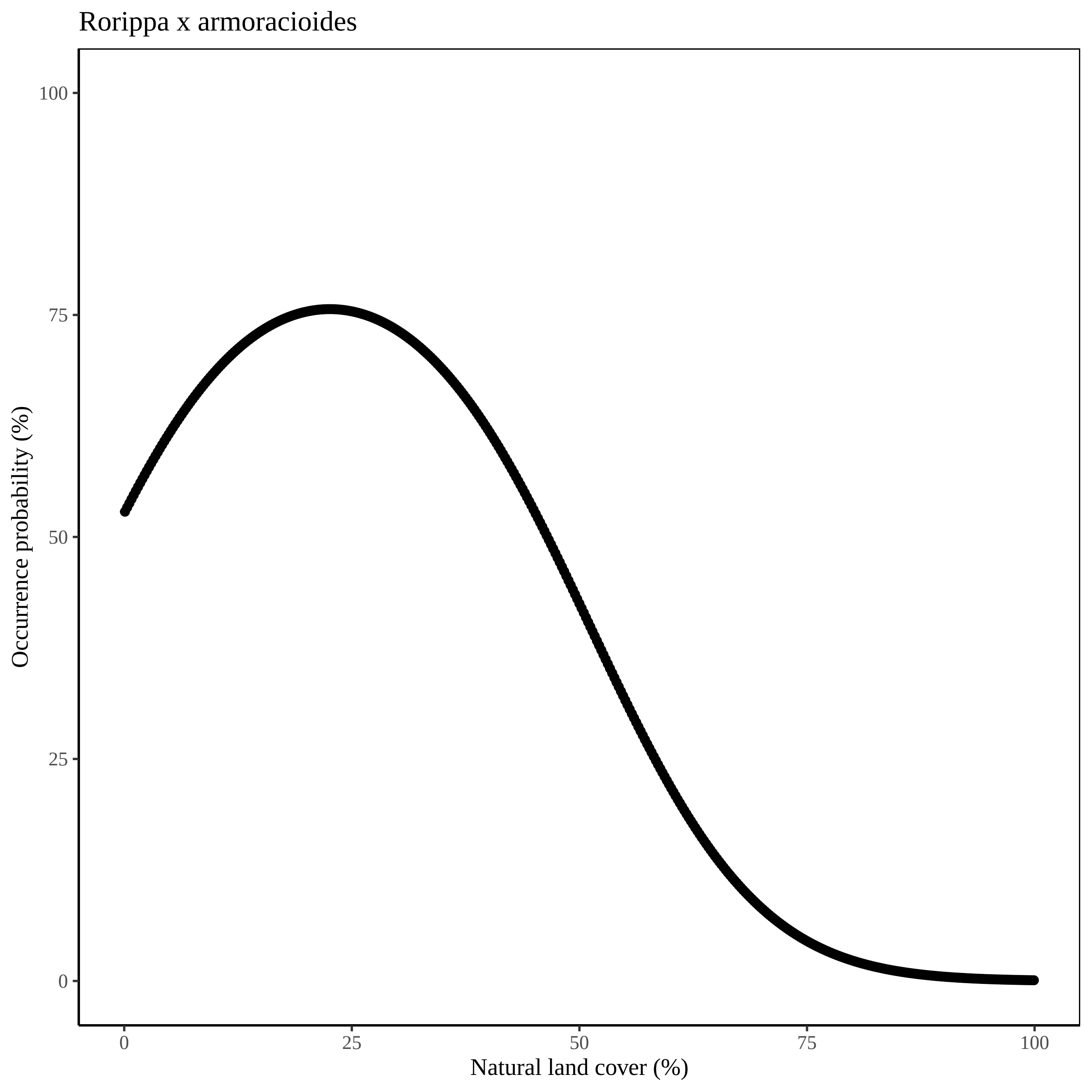
Rorippa x armoracioides
Occurrence probability (%)
100
75
50
25
0
0
25
50
75
100
Natural land cover (%)

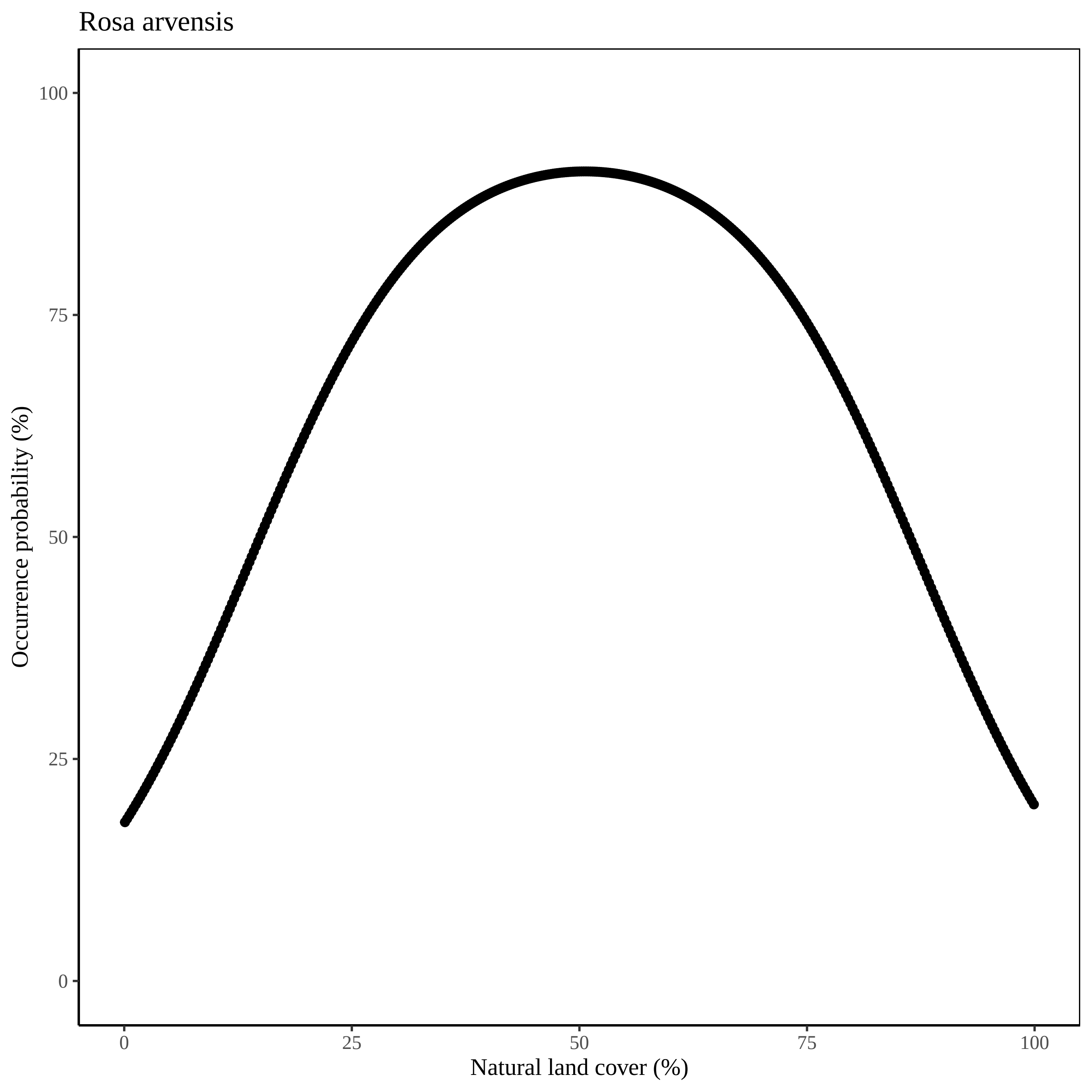
Rosa arvensis
Occurrence probability (%)
100
75
50
25
0
0
25
50
75
100
Natural land cover (%)

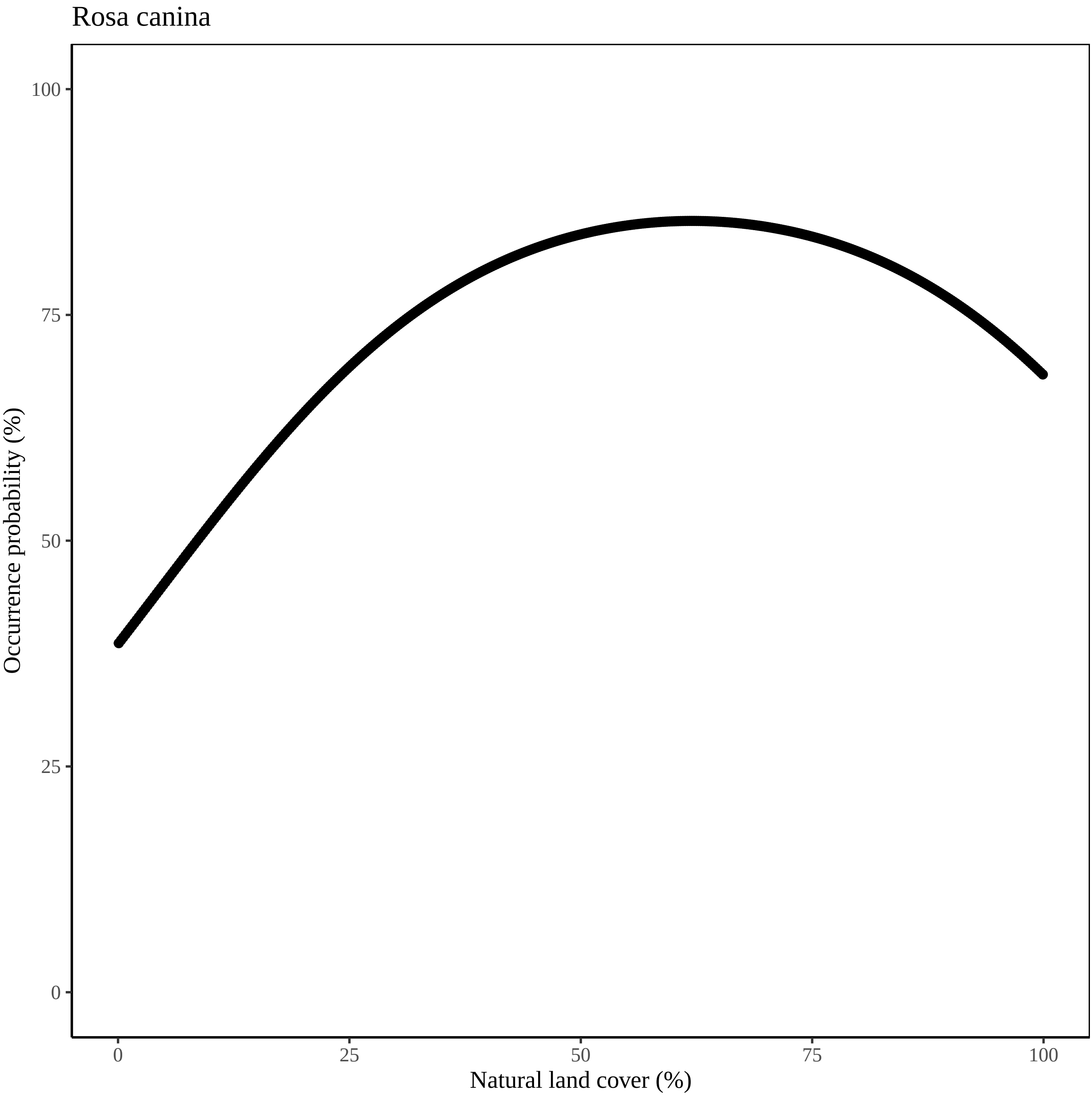
Rosa canina
Occurrence probability (%)
100
75
50
25
0
0
25
50
75
100
Natural land cover (%)

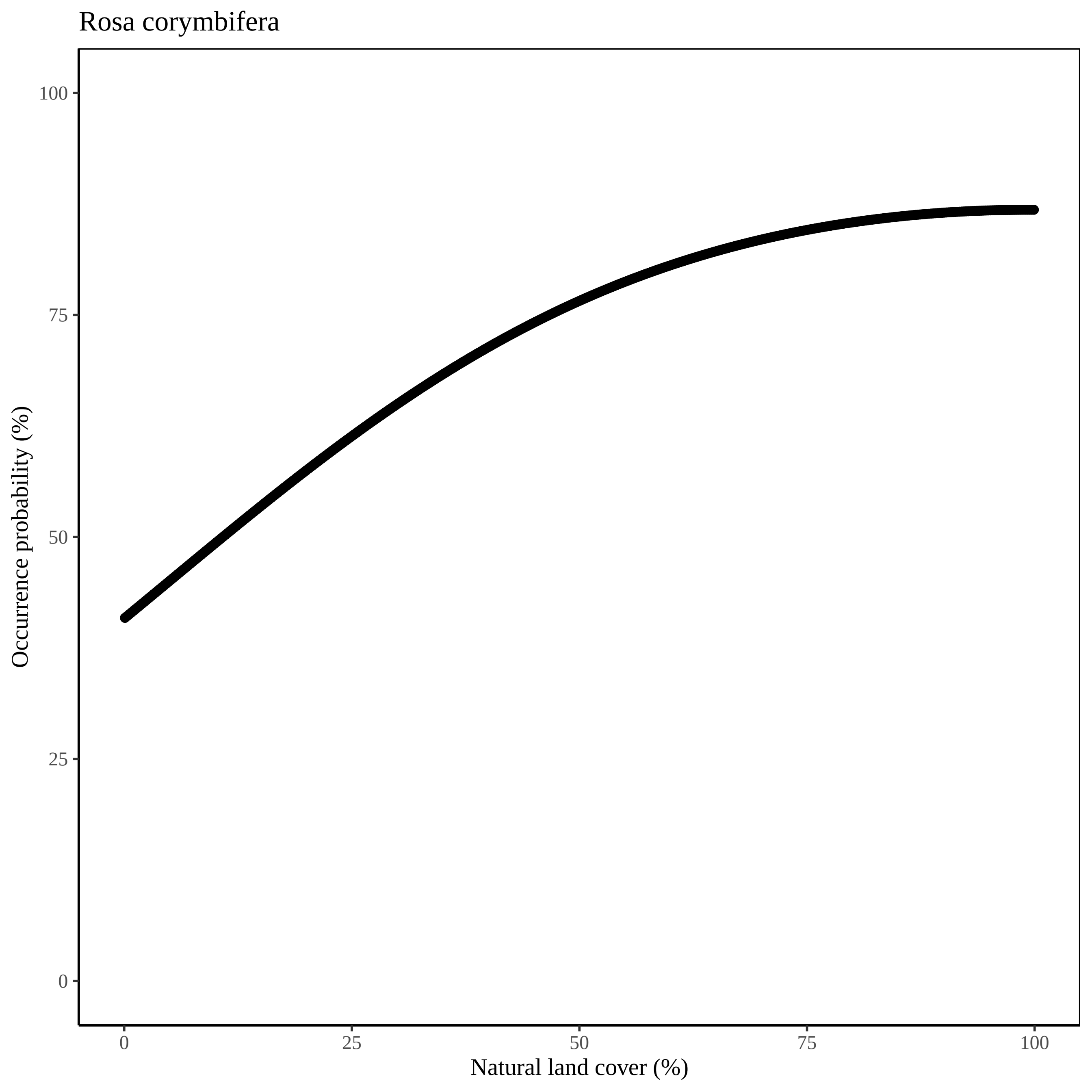
Rosa corymbifera
Occurrence probability (%)
100
75
50
25
0
0
25
50
75
100
Natural land cover (%)

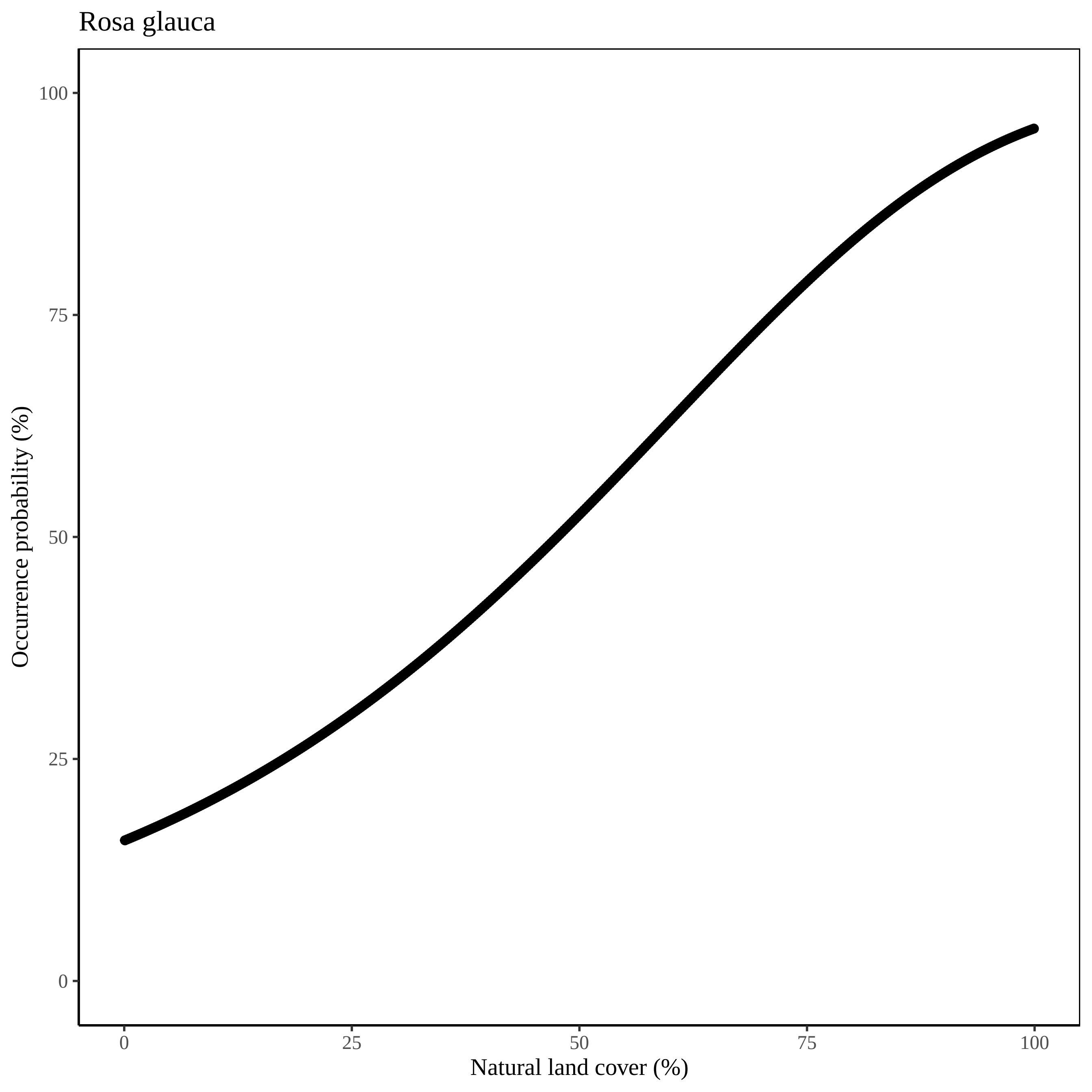
Rosa glauca
Occurrence probability (%)
100
75
50
25
0
0
25
50
75
100
Natural land cover (%)

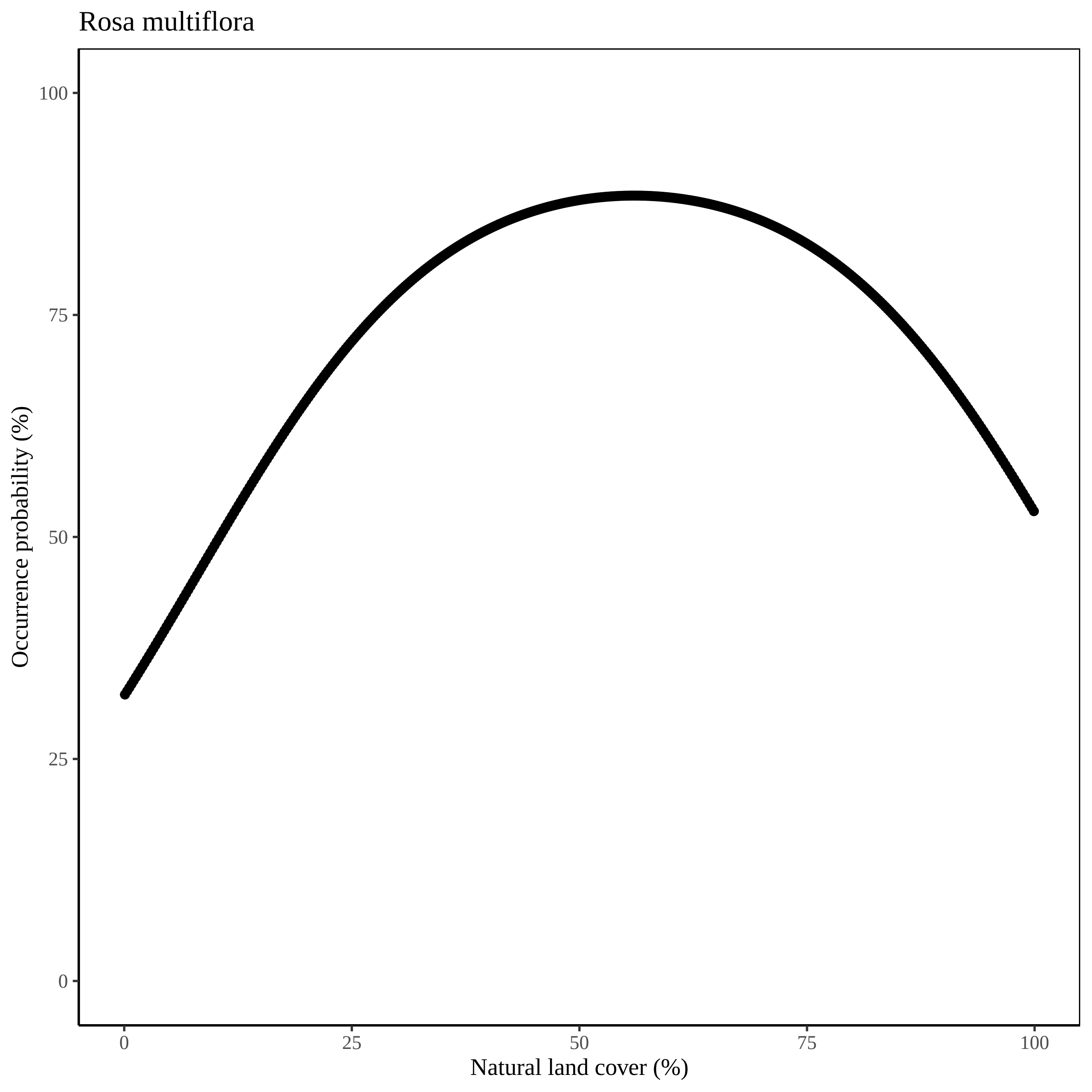
Rosa multiflora
Occurrence probability (%)
100
75
50
25
0
0
25
50
75
100
Natural land cover (%)

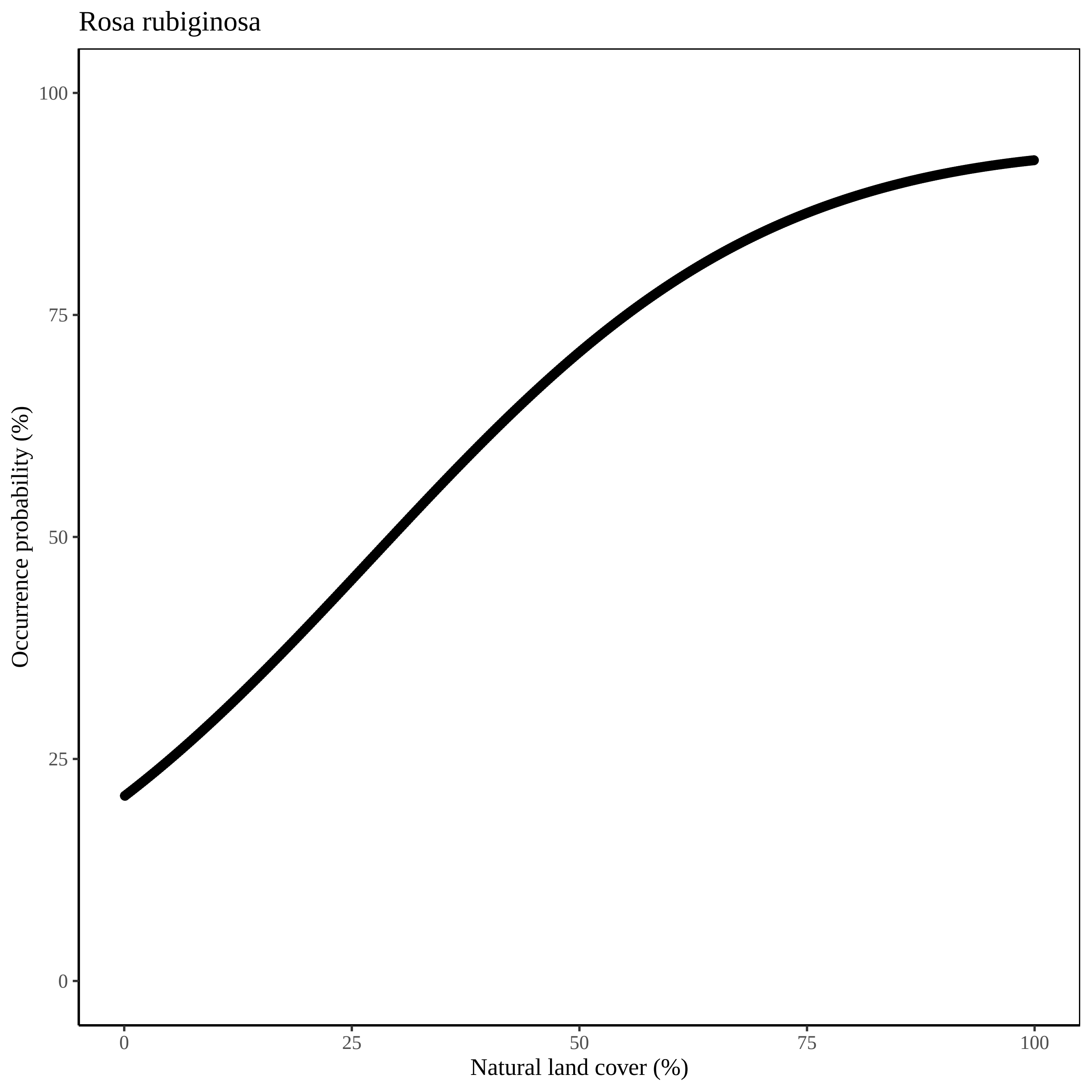
Rosa rubiginosa
Occurrence probability (%)
100
75
50
25
0
0
25
50
75
100
Natural land cover (%)

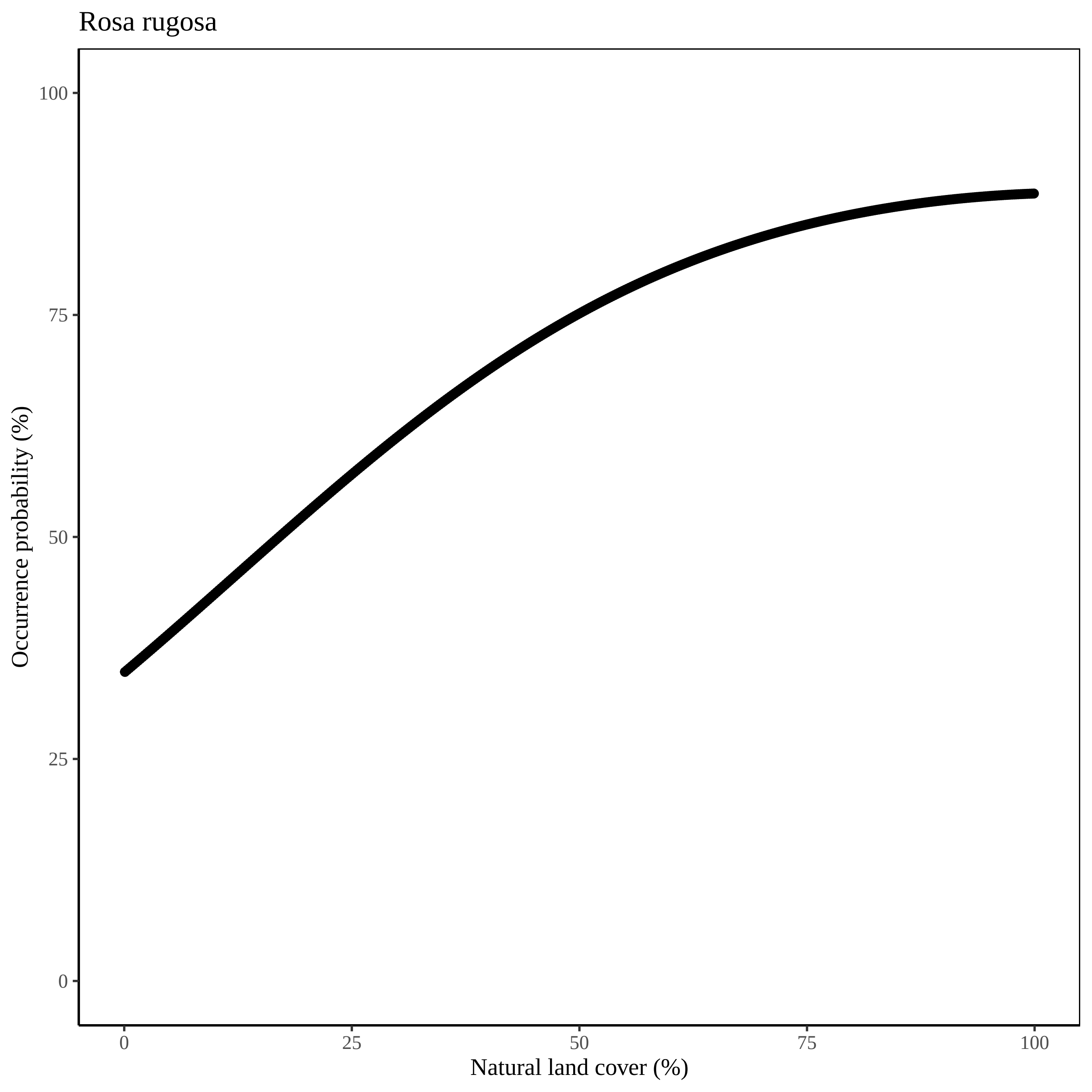

Rosa rugosa
Occurrence probability (%)
100
75
50
25
0
0
25
50
75
100
Natural land cover (%)

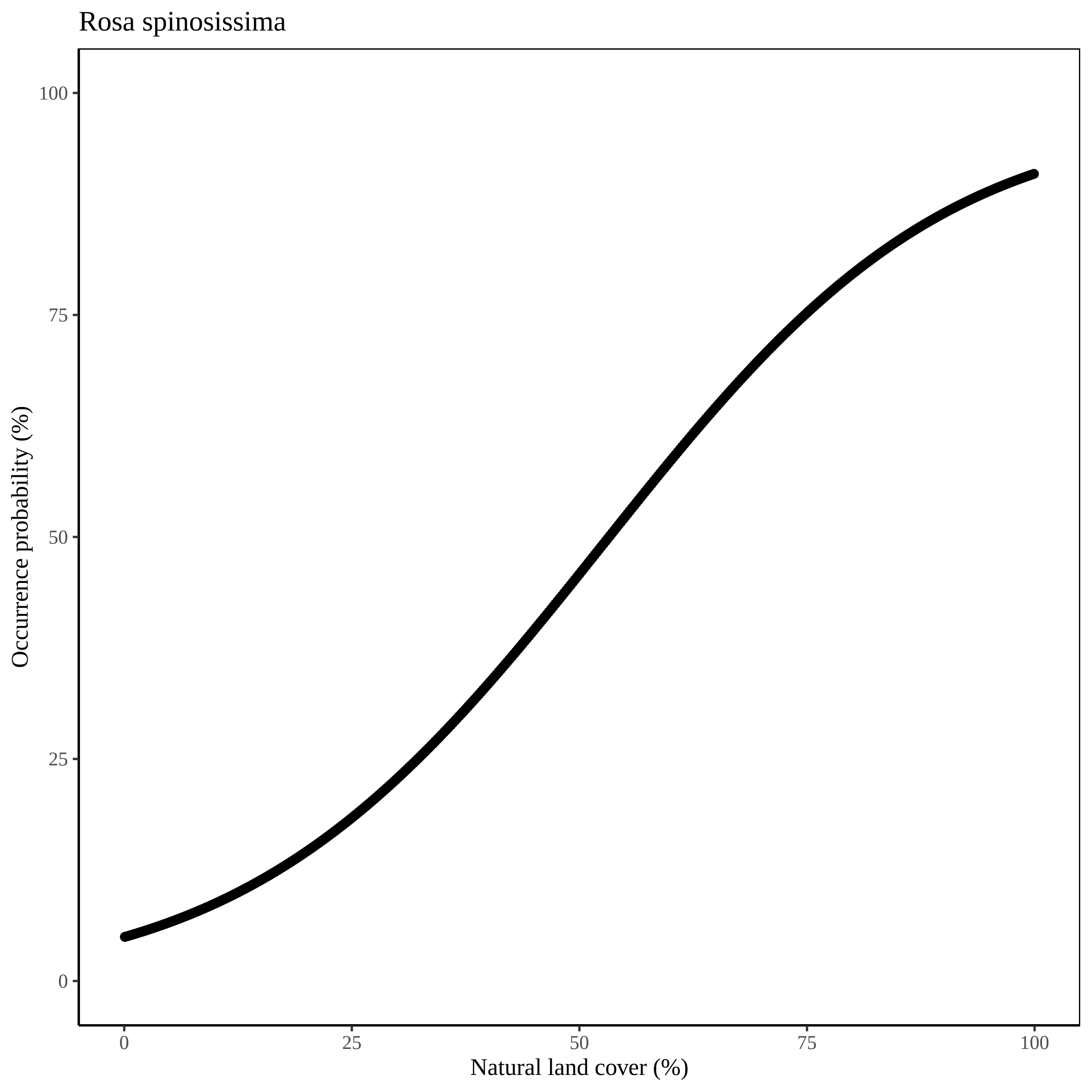
Rosa spinosissima
Occurrence probability (%)
100
75
50
25
0
0
25
50
75
100
Natural land cover (%)

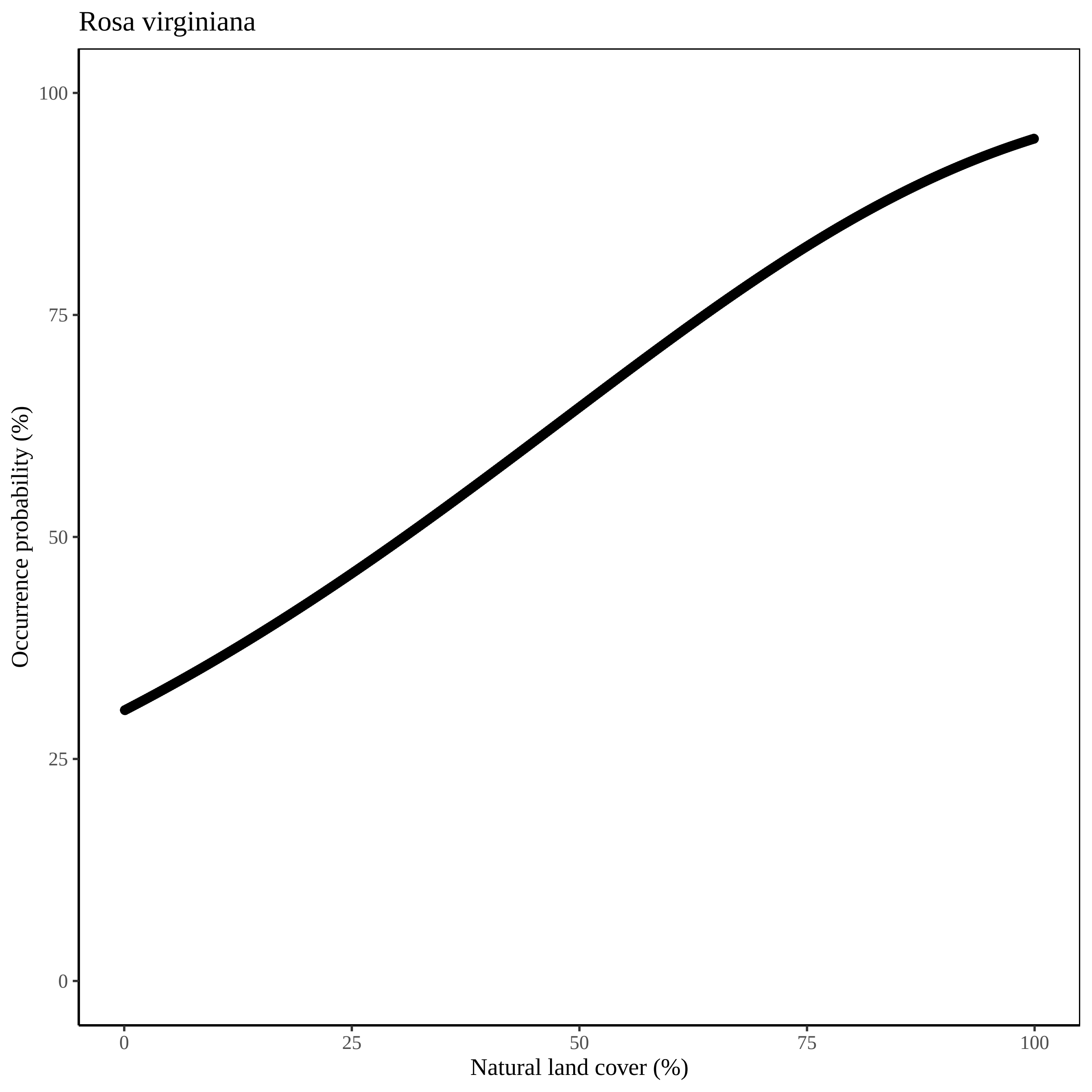
Rosa virginiana
Occurrence probability (%)
100
75
50
25
0
0
25
50
75
100
Natural land cover (%)

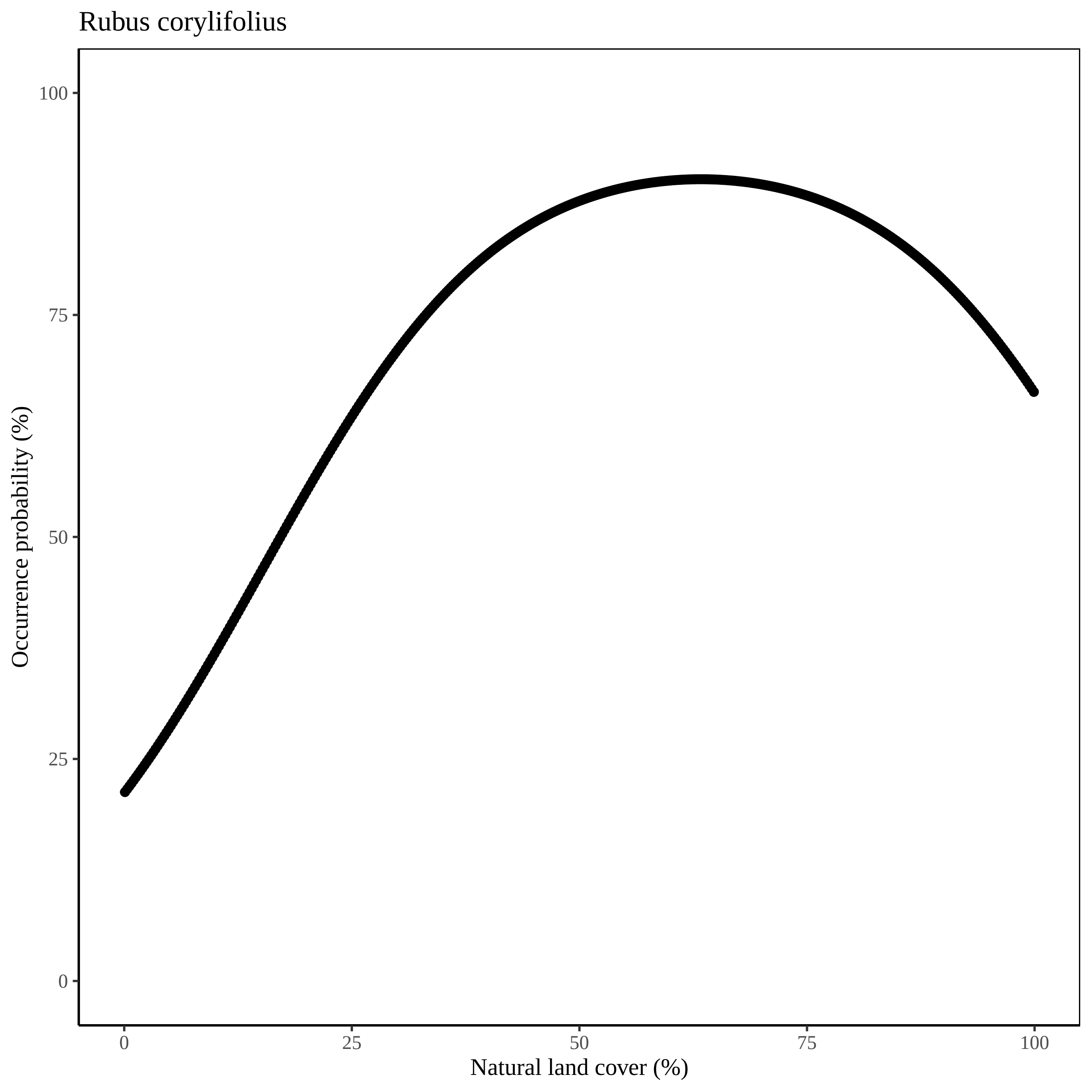
Rubus corylifolius
Occurrence probability (%)
100
75
50
25
0
0
25
50
75
100
Natural land cover (%)

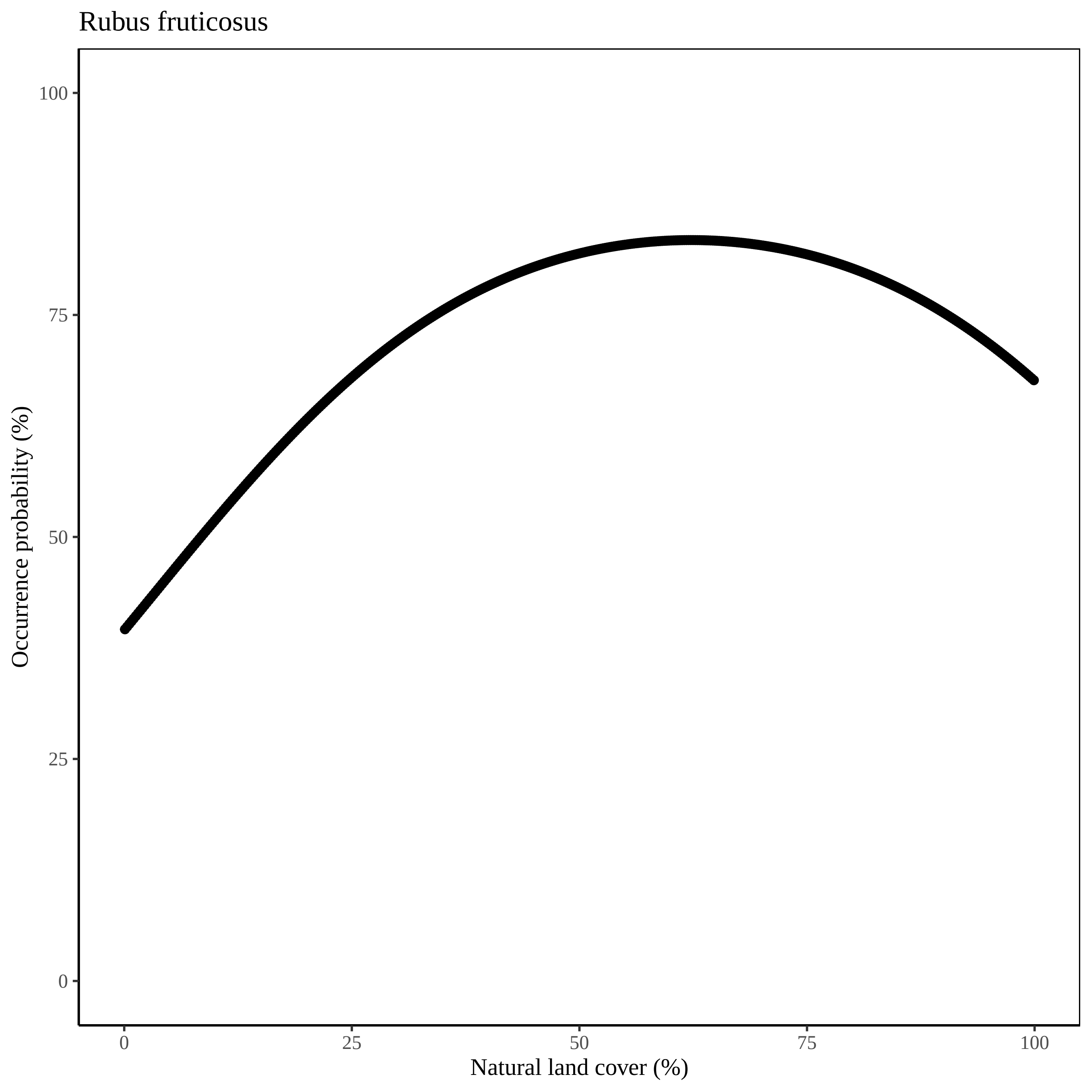

Rubus fruticosus
Occurrence probability (%)
100
75
50
25
0
0
25
50
75
100
Natural land cover (%)

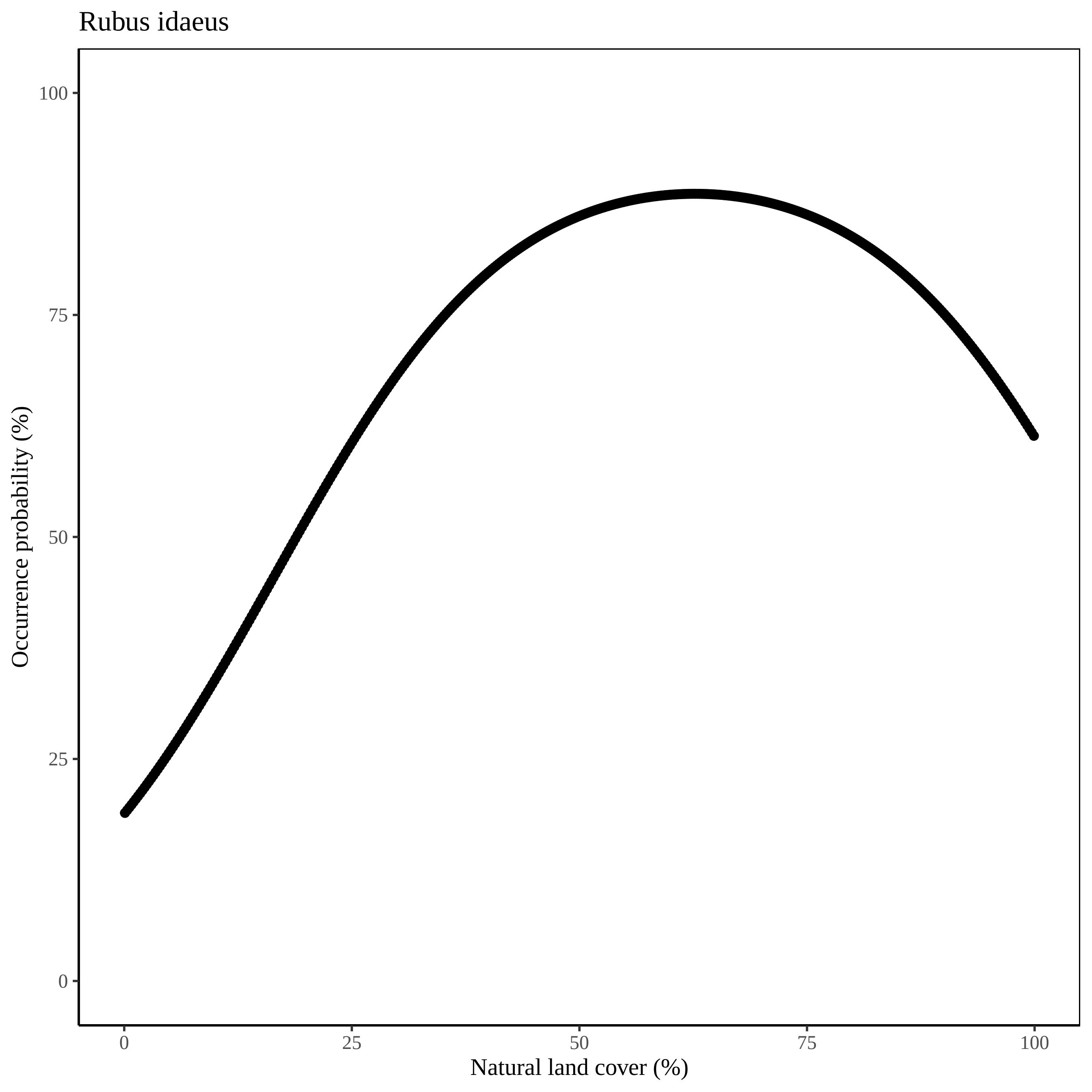
Rubus idaeus
Occurrence probability (%)
100
75
50
25
0
0
25
50
75
100
Natural land cover (%)

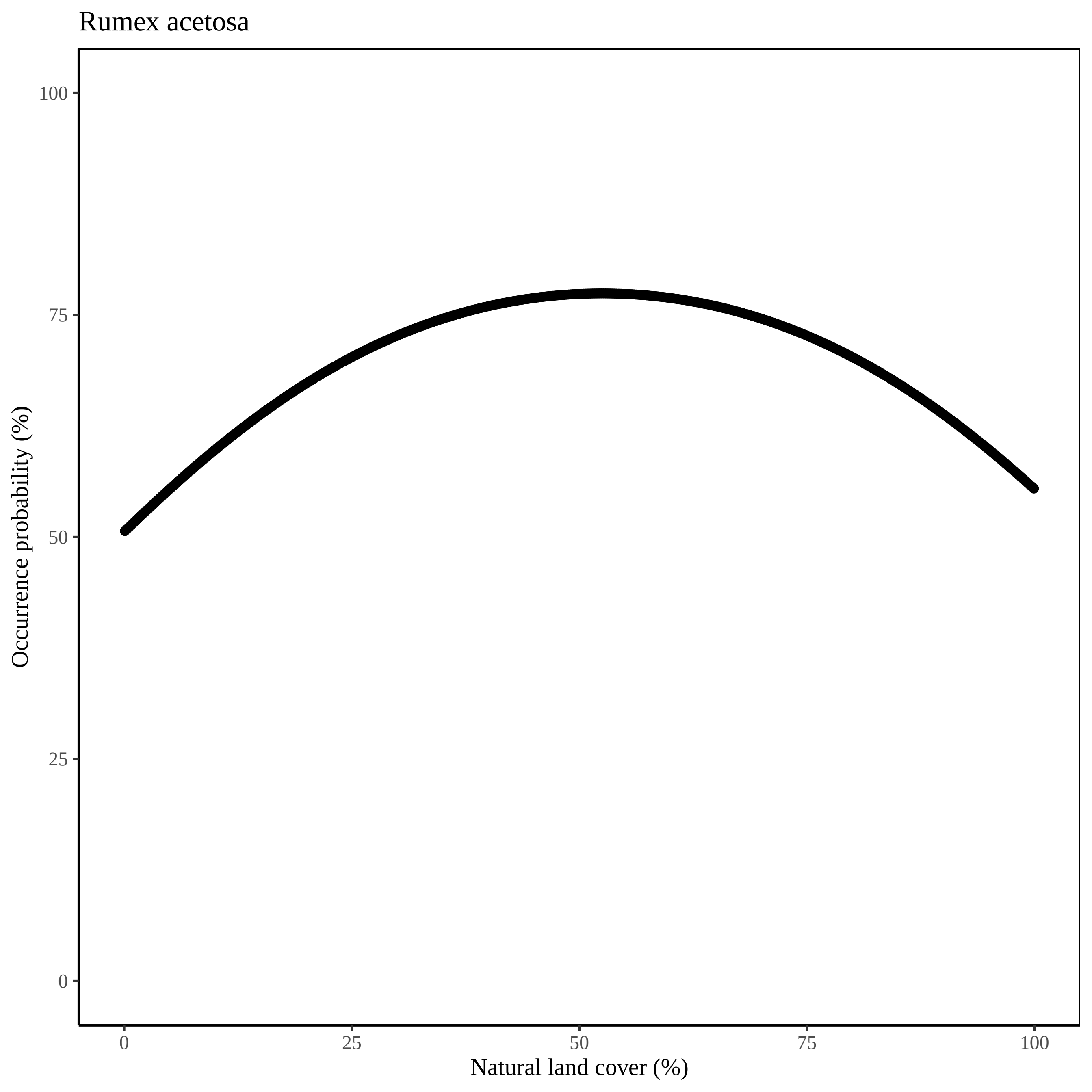

Rumex acetosa
Occurrence probability (%)
100
75
50
25
0
0
25
50
75
100
Natural land cover (%)

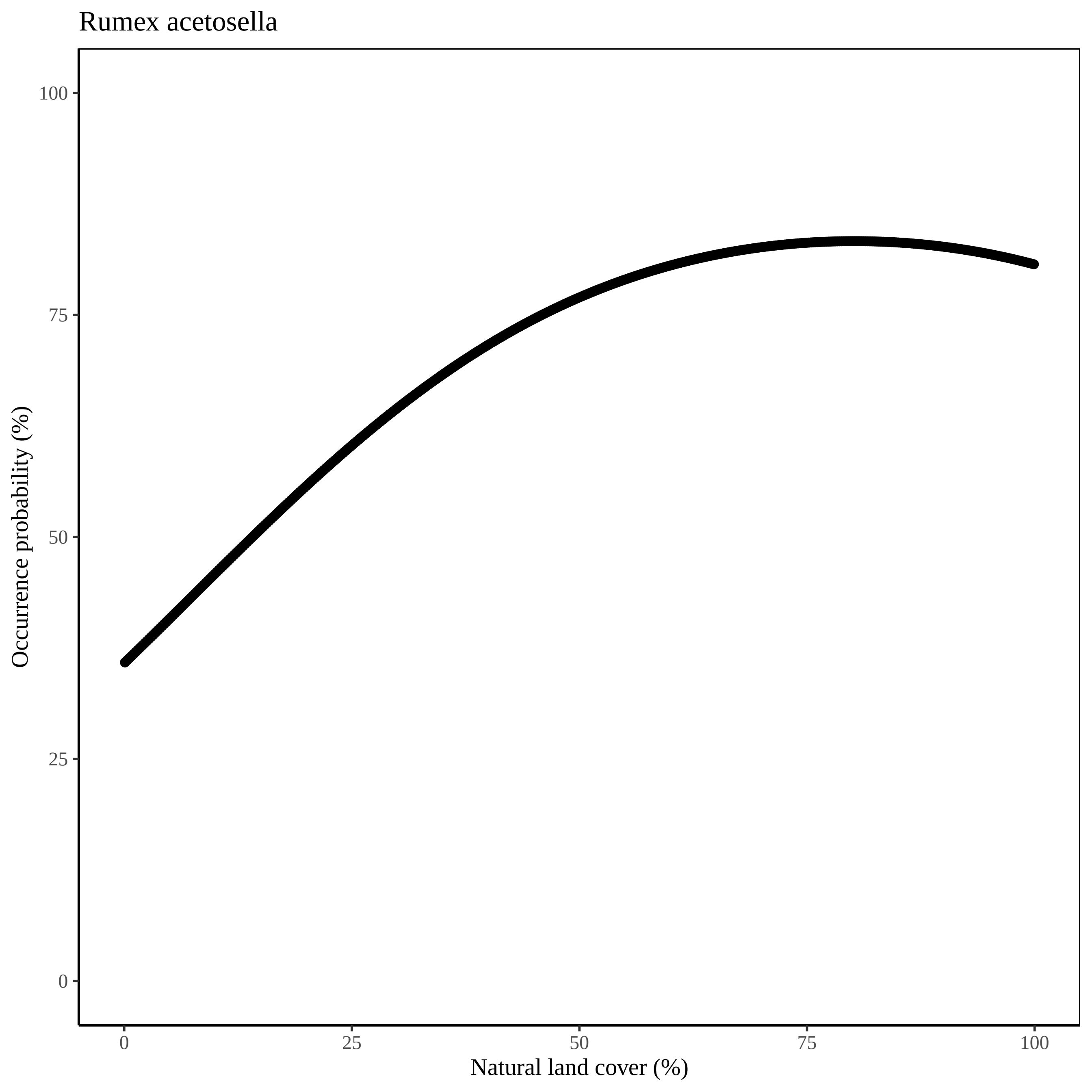
Rumex acetosella
Occurrence probability (%)
100
75
50
25
0
0
25
50
75
100
Natural land cover (%)

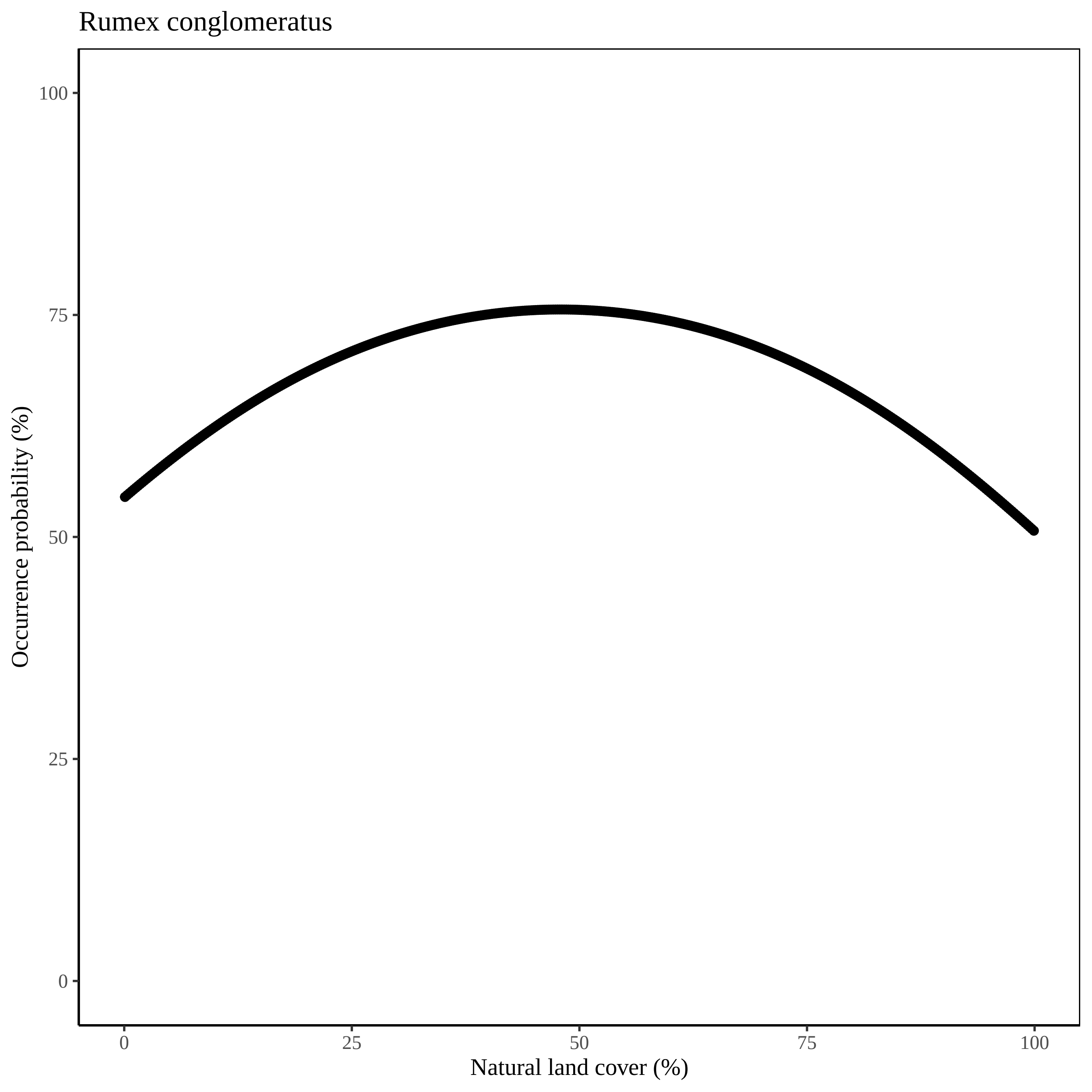

Rumex conglomeratus
100
75
50
25
0
Occurrence probability (%)
0
25
50
75
100
Natural land cover (%)

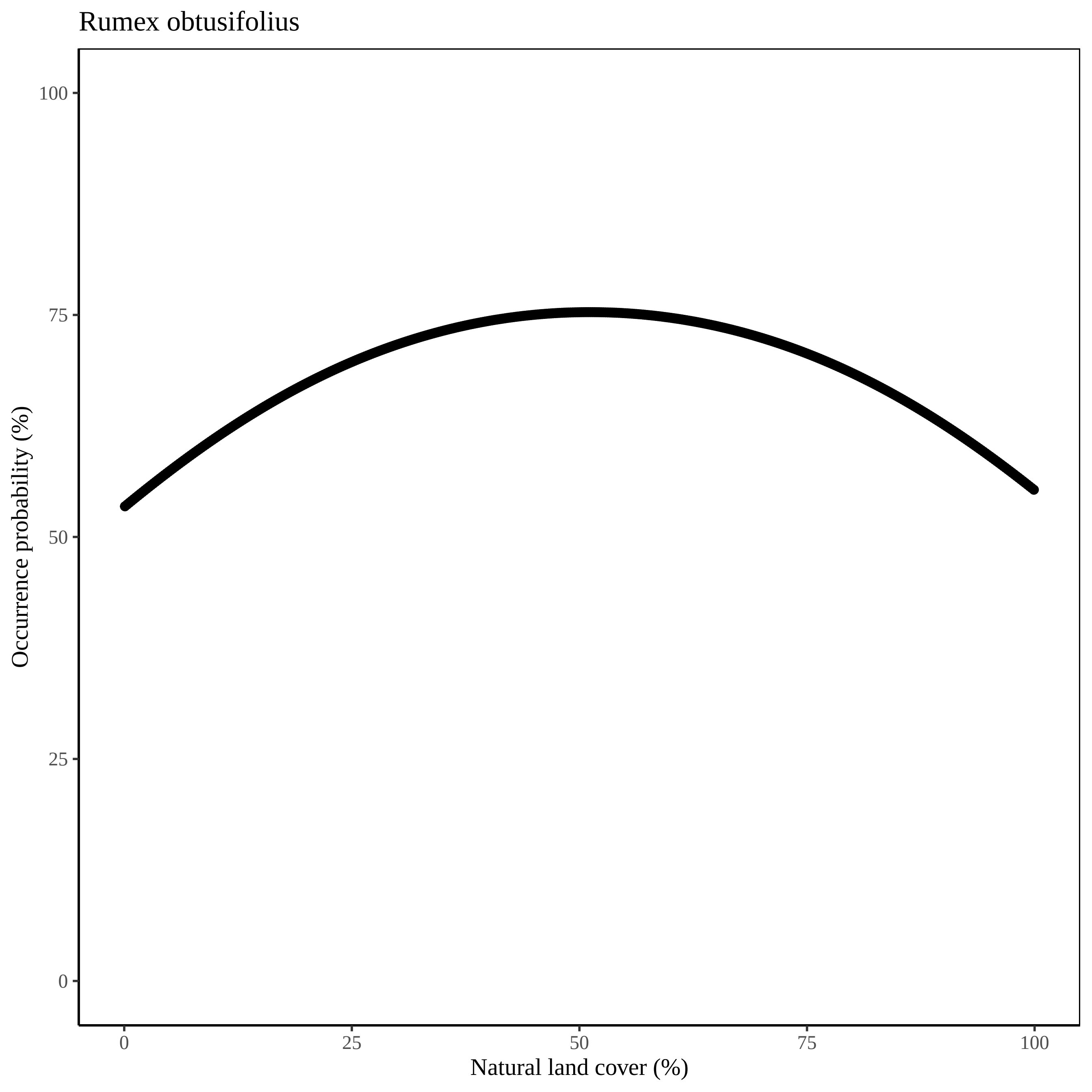

Rumex obtusifolius
100
75
50
25
0
Occurrence probability (%)
0
25
50
75
100
Natural land cover (%)

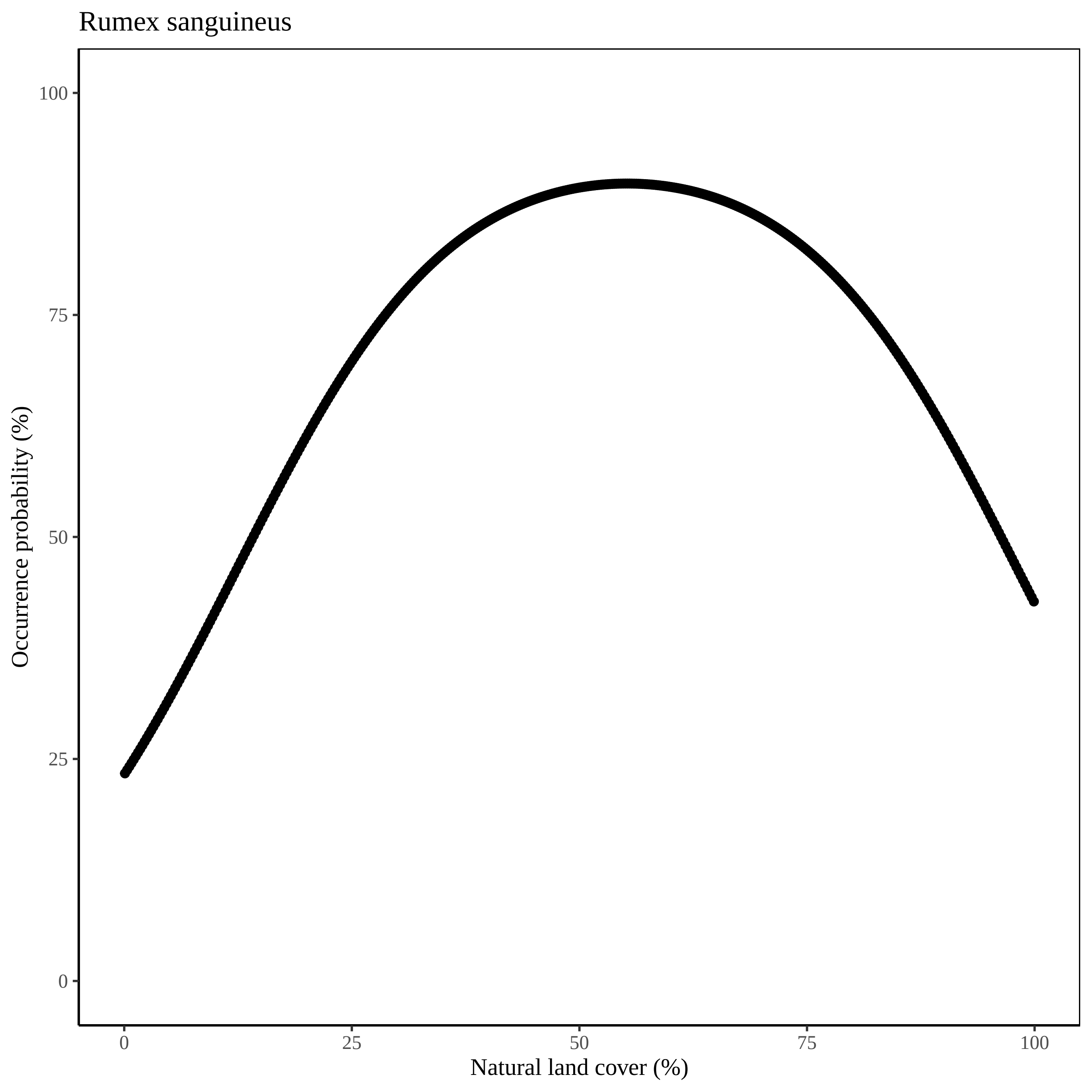
Rumex sanguineus
Occurrence probability (%)
100
75
50
25
0
0
25
50
75
100
Natural land cover (%)

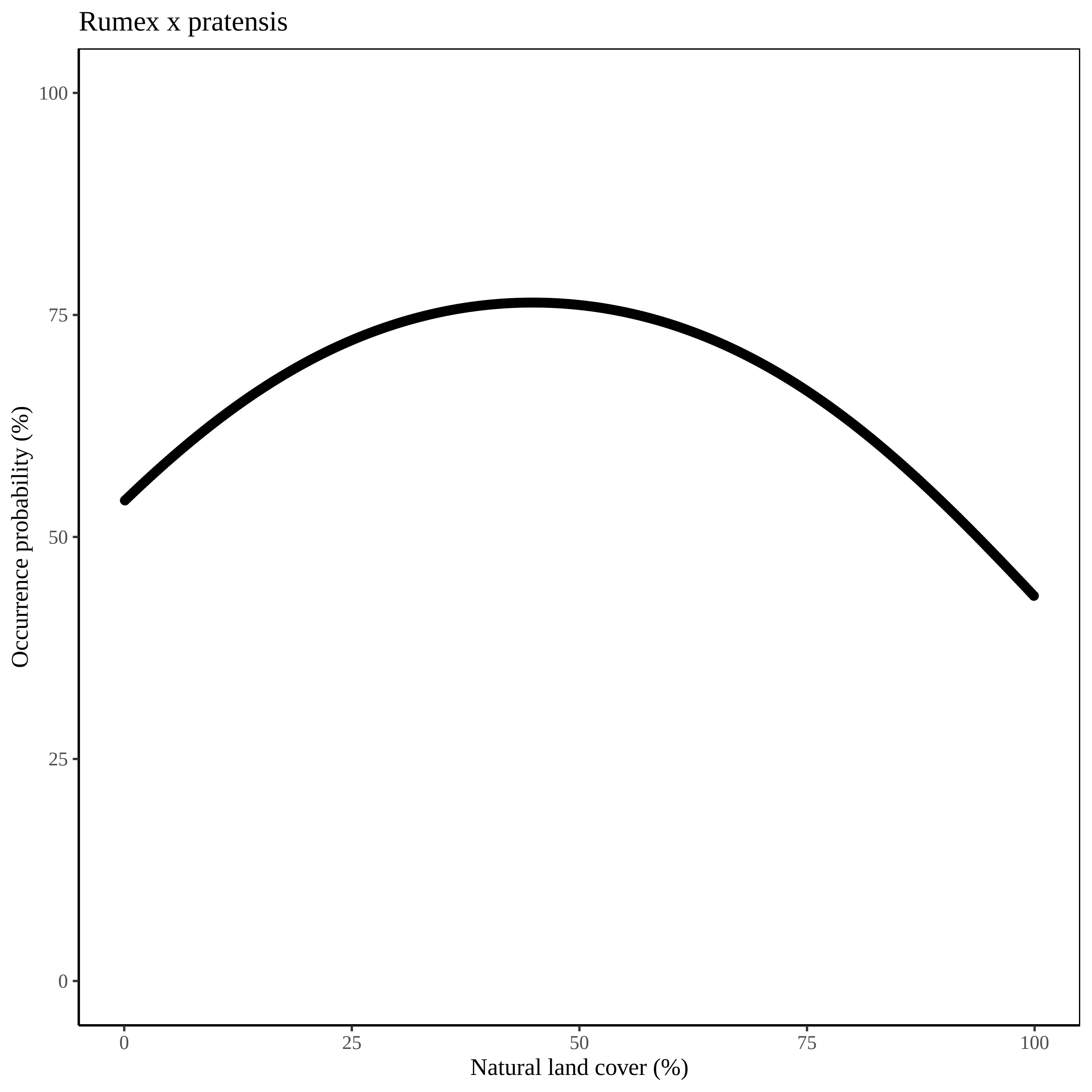
Rumex x pratensis
Occurrence probability (%)
100
75
50
25
0
0
25
50
75
100
Natural land cover (%)

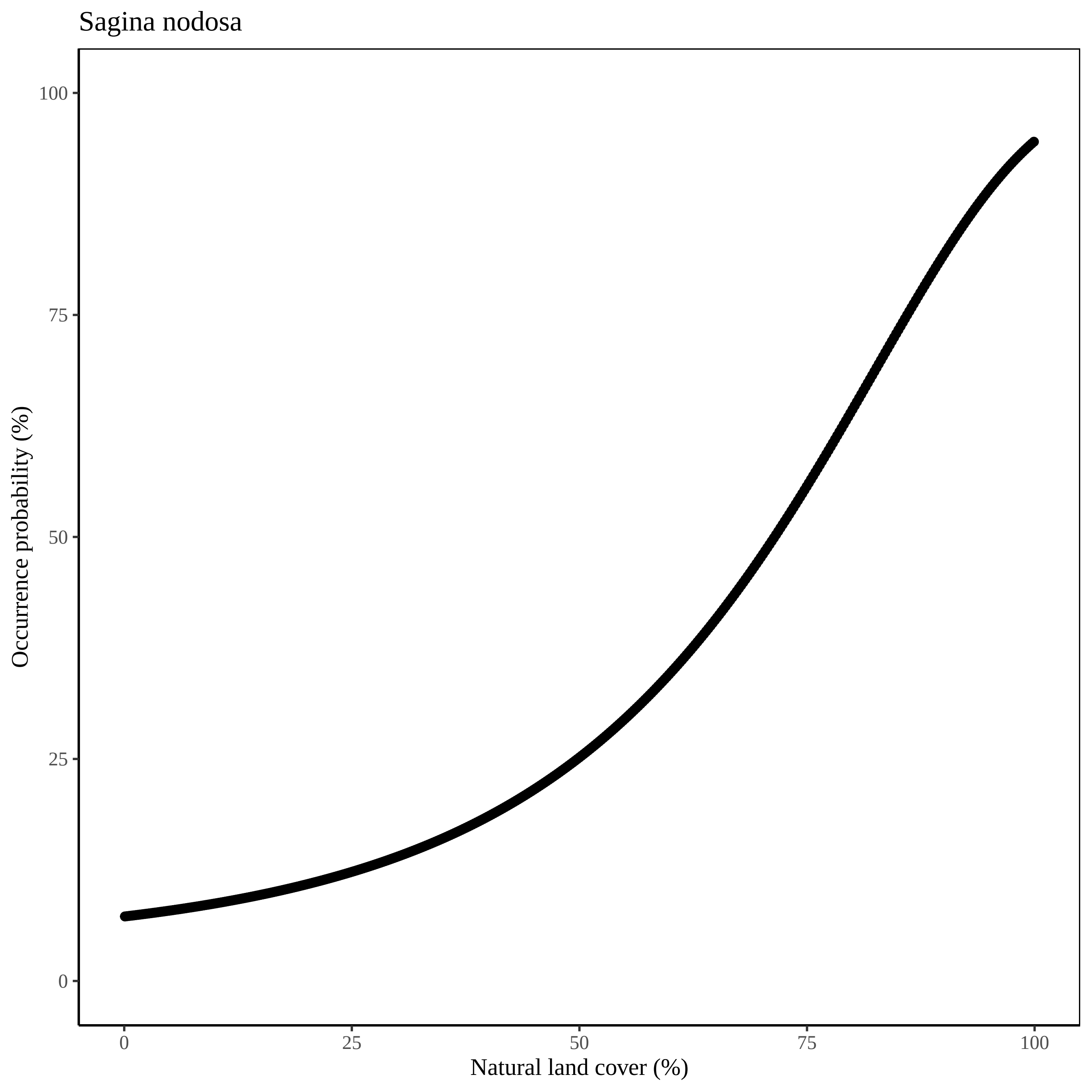
Sagina nodosa
100
75
50
25
0
Occurrence probability (%)
0
25
50
75
100
Natural land cover (%)

Sagina procumbens

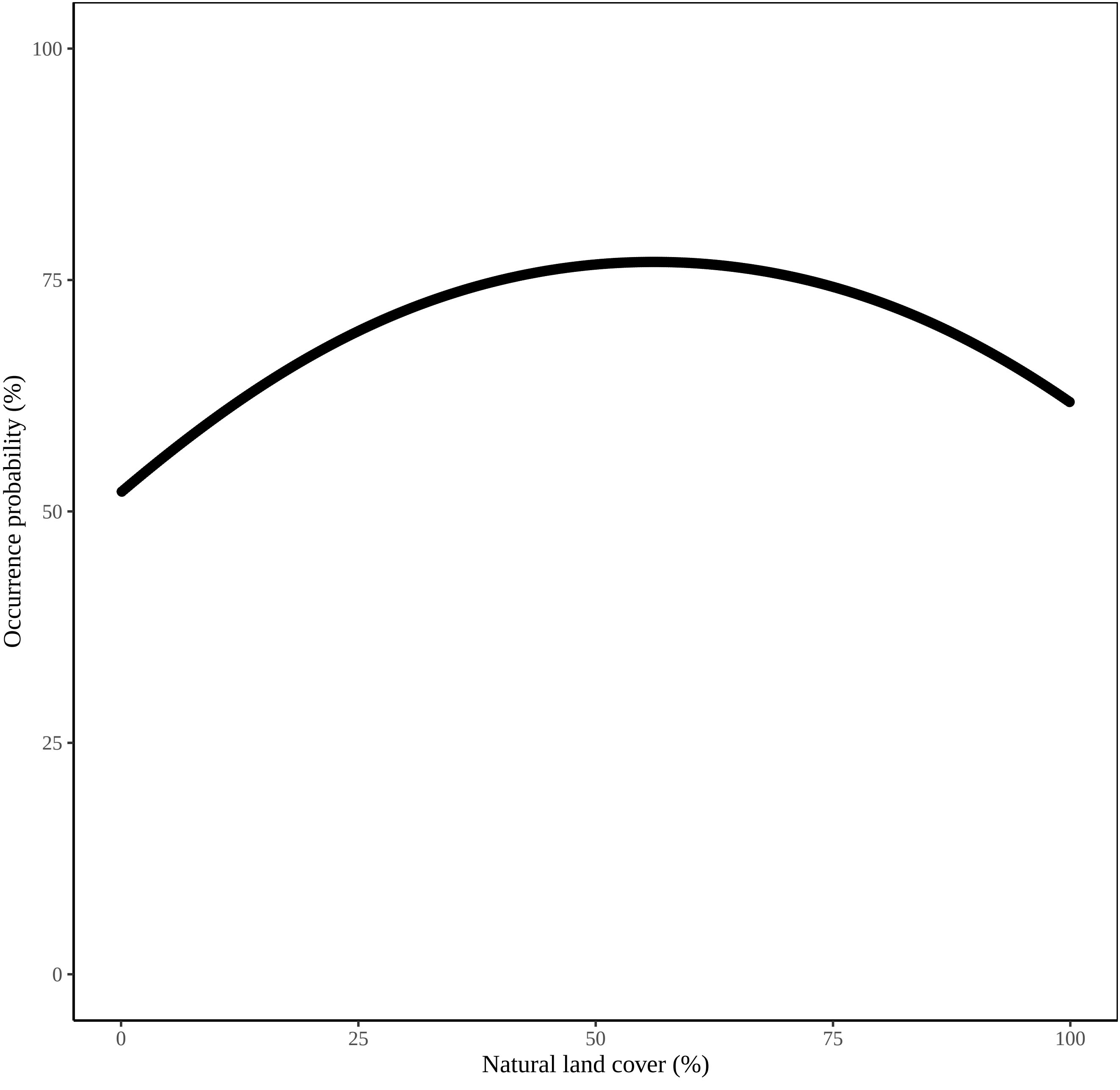

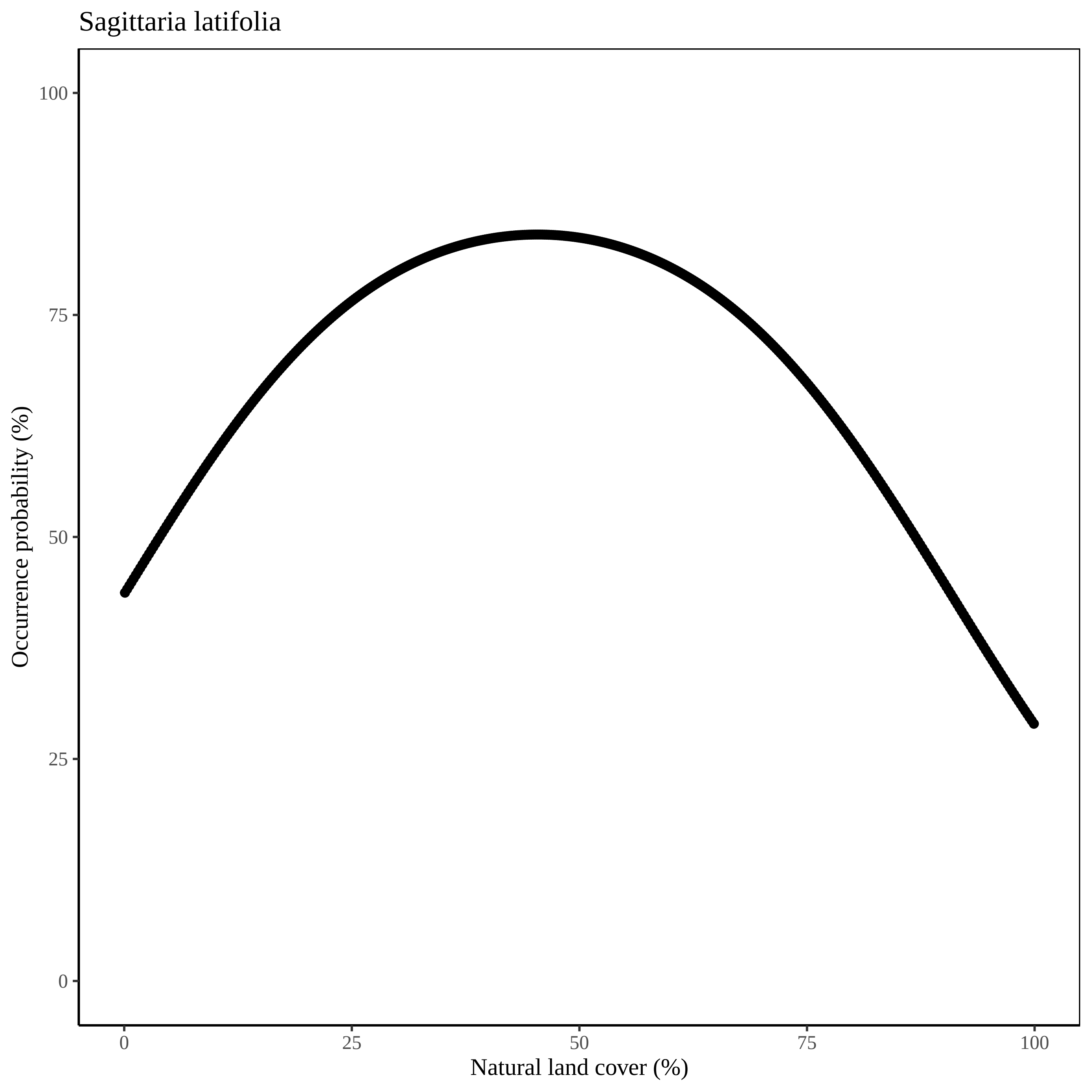
Sagittaria latifolia
Occurrence probability (%)
100
75
50
25
0
0
25
50
75
100
Natural land cover (%)

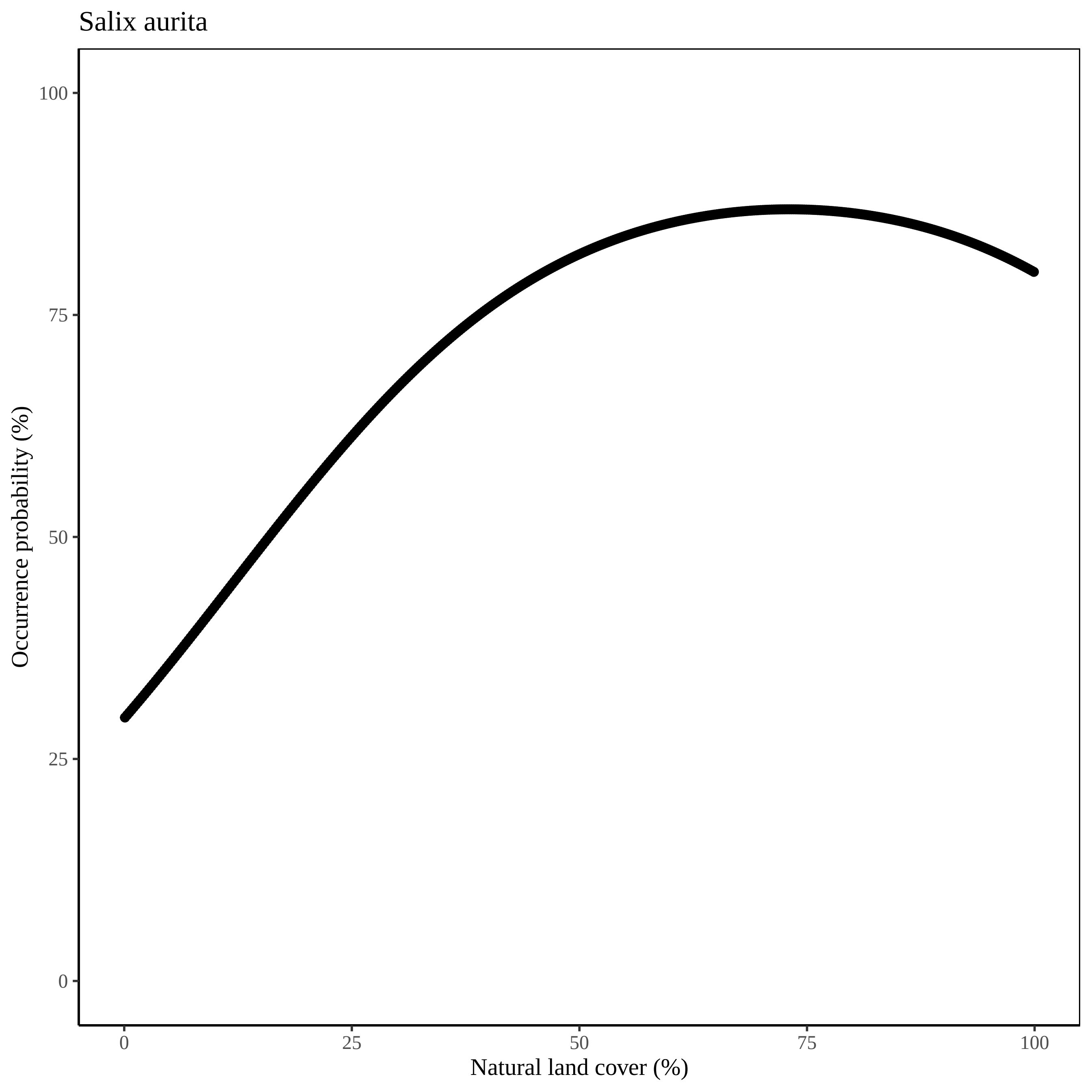
Salix aurita
Occurrence probability (%)
100
75
50
25
0
0
25
50
75
100
Natural land cover (%)

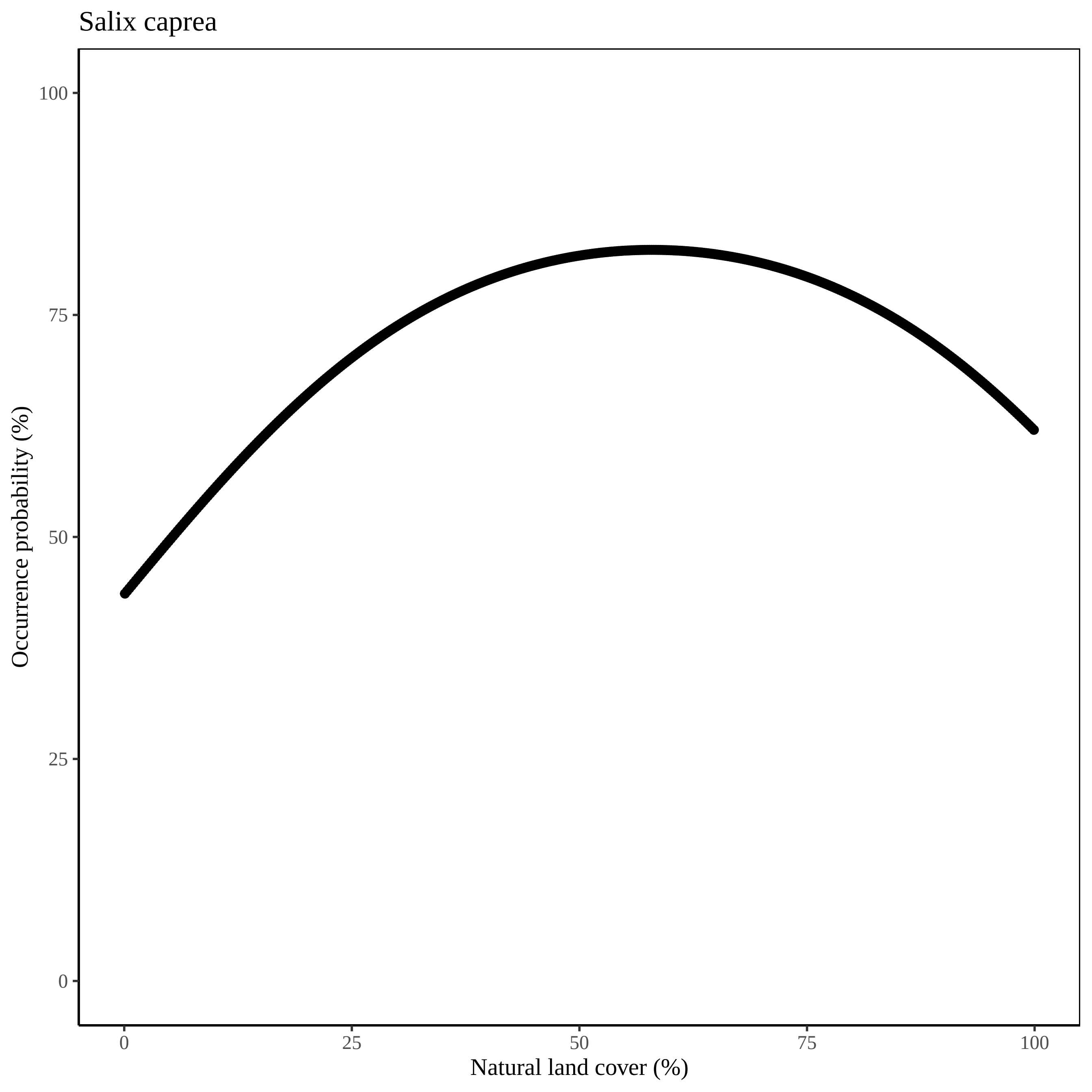
Salix caprea
Occurrence probability (%)
100
75
50
25
0
0
25
50
75
100
Natural land cover (%)

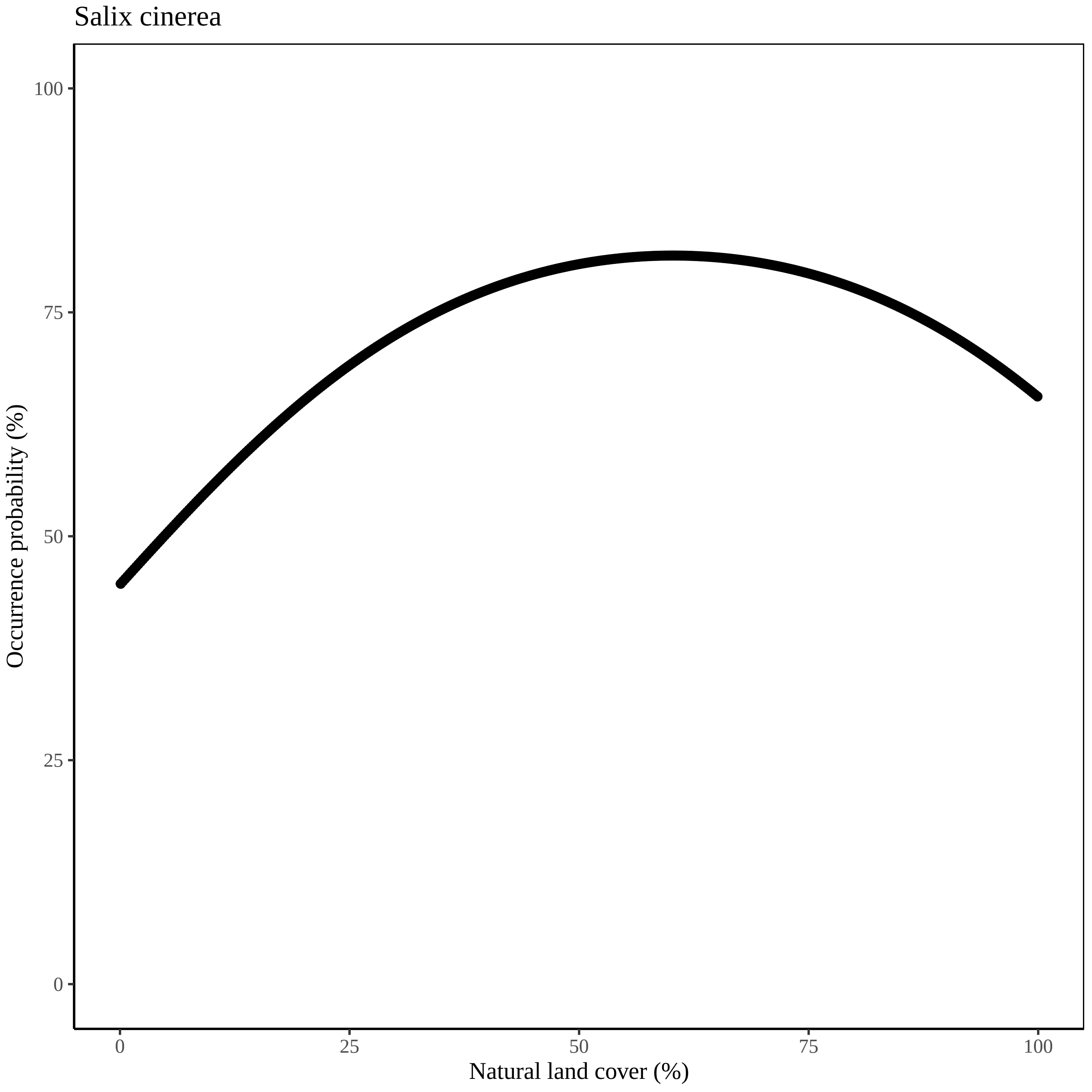

Salix cinerea
100
75
50
25
0
Occurrence probability (%)
0
25
50
75
100
Natural land cover (%)

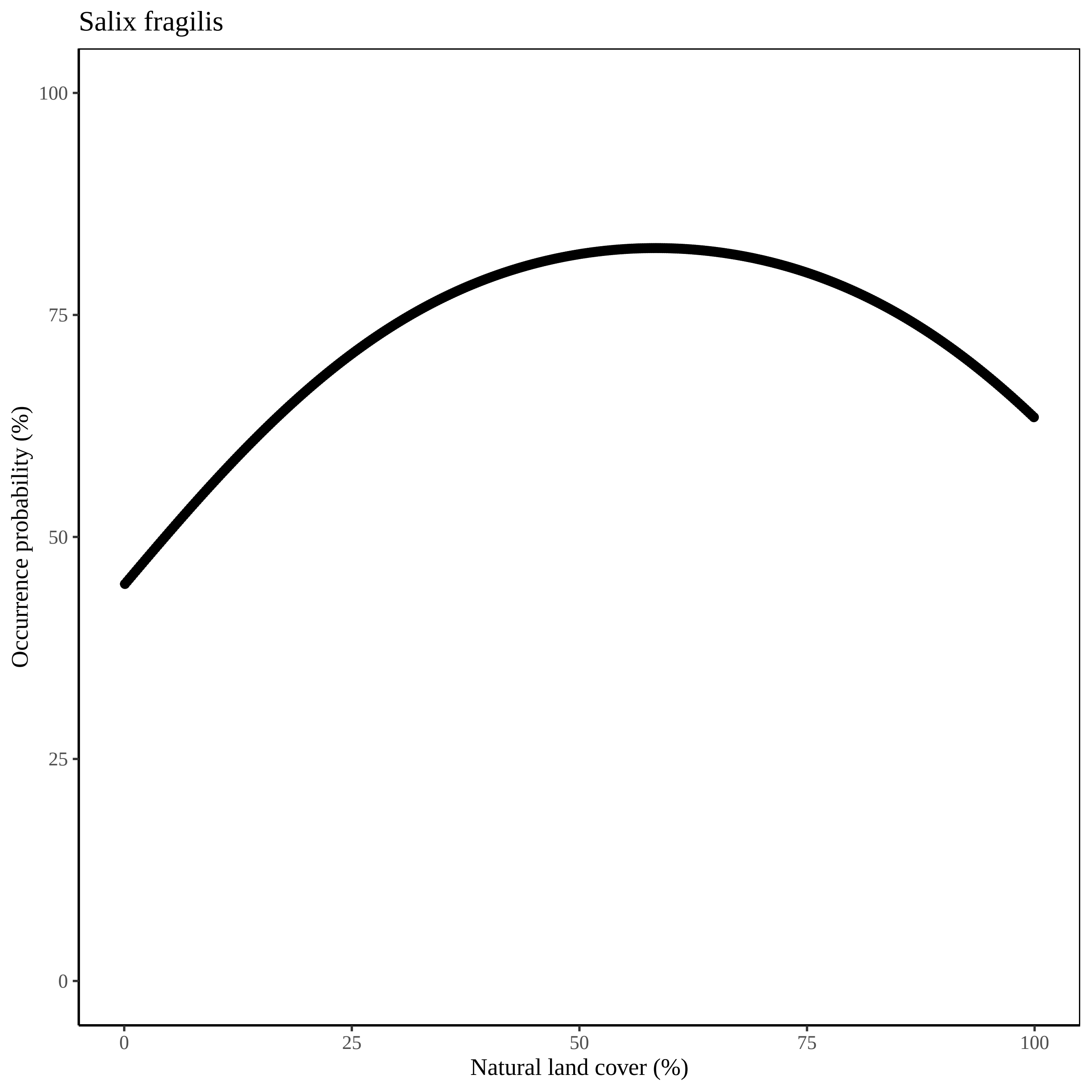
Salix fragilis
Occurrence probability (%)
100
75
50
25
0
0
25
50
75
100
Natural land cover (%)

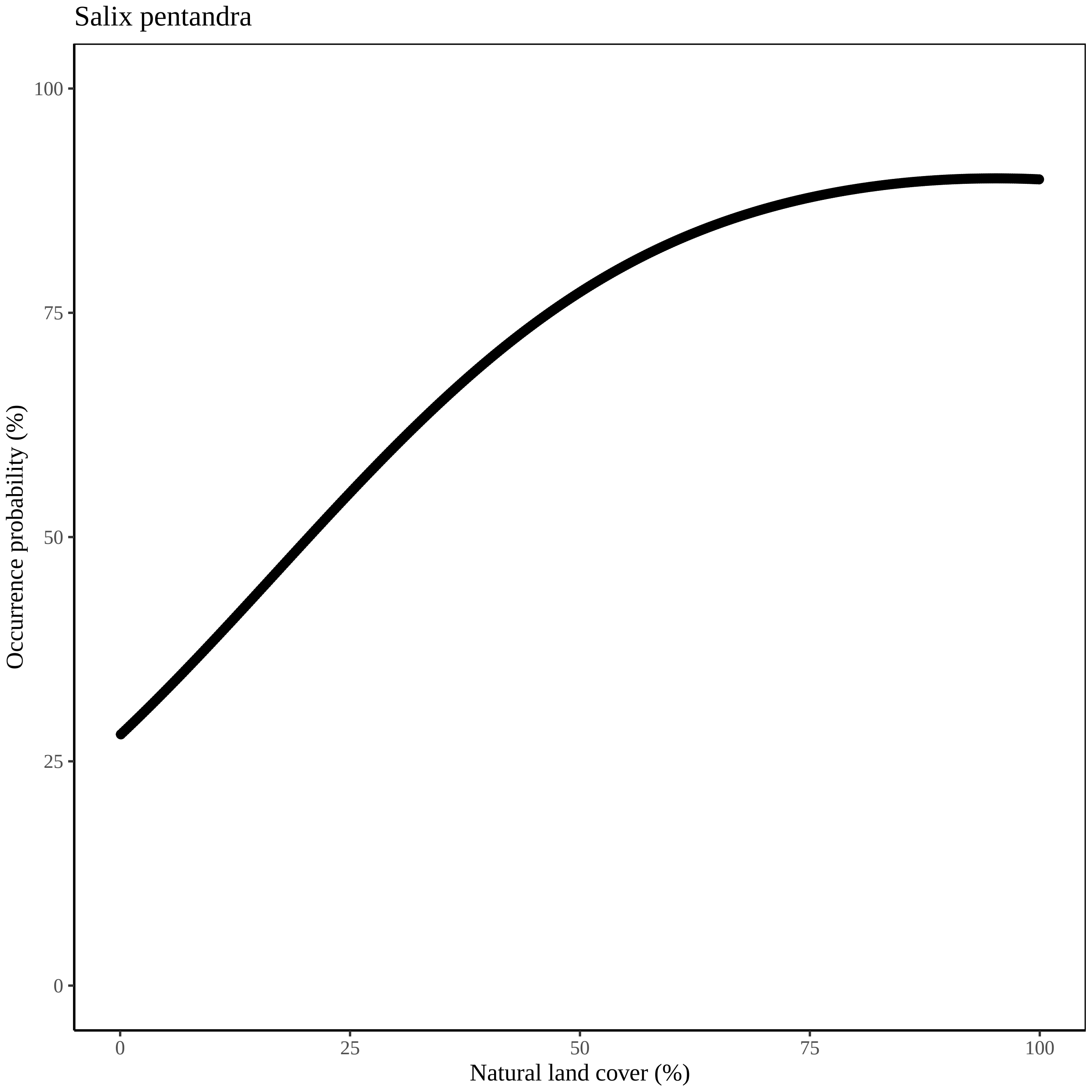
Salix pentandra
Occurrence probability (%)
100
75
50
25
0
0
25
50
75
100
Natural land cover (%)

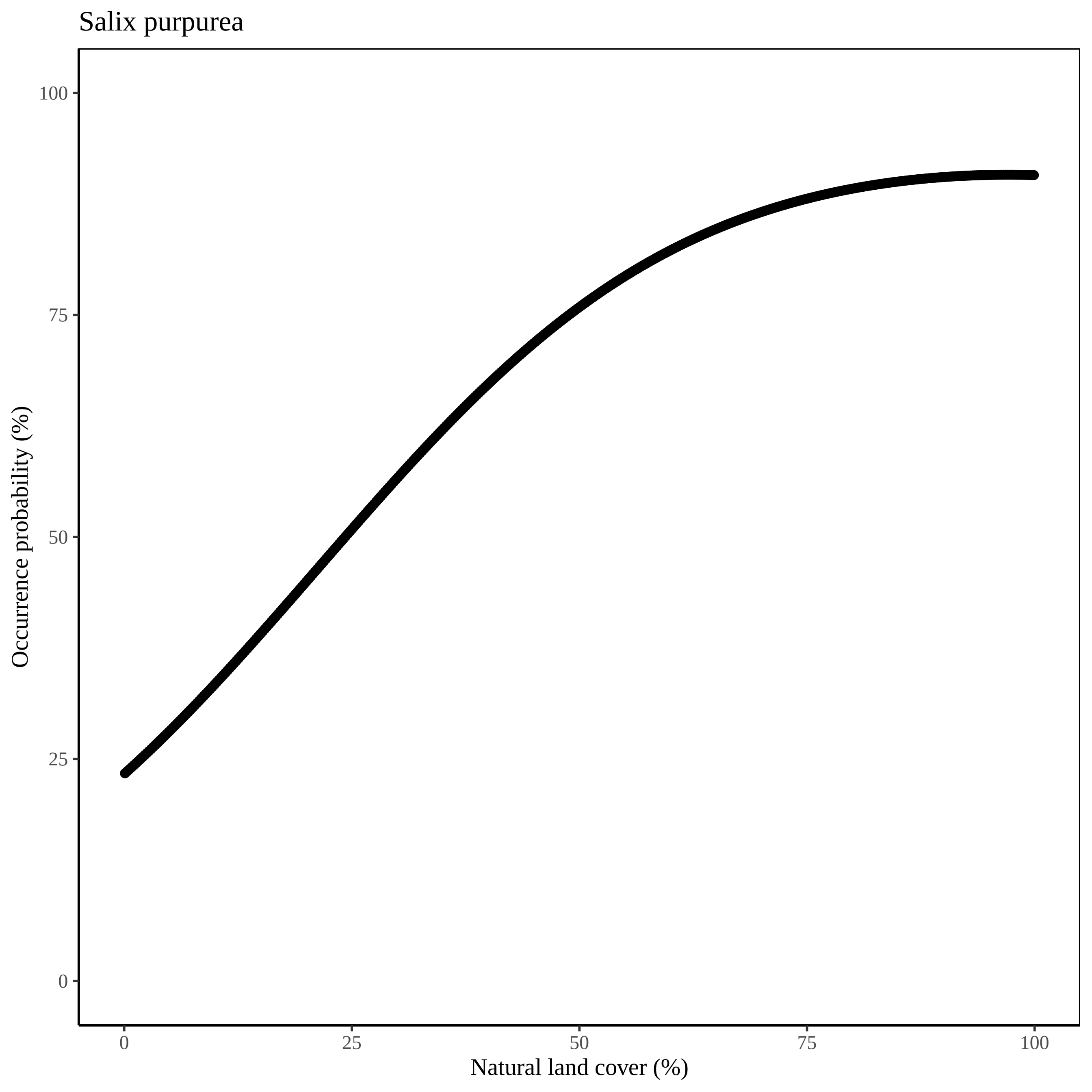
Salix purpurea
Occurrence probability (%)
100
75
50
25
0
0
25
50
75
100
Natural land cover (%)

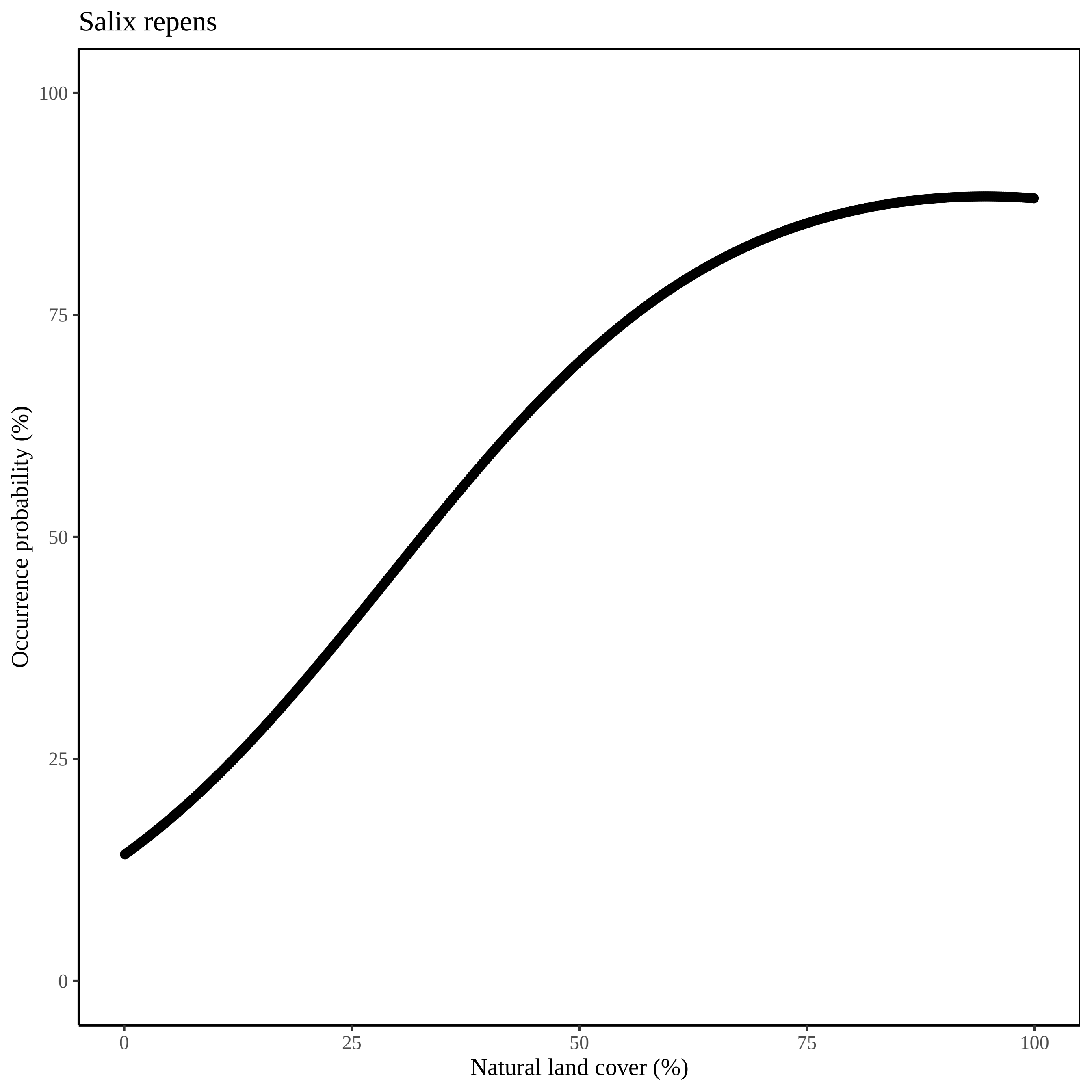
Salix repens
Occurrence probability (%)
100
75
50
25
0
0
25
50
75
100
Natural land cover (%)

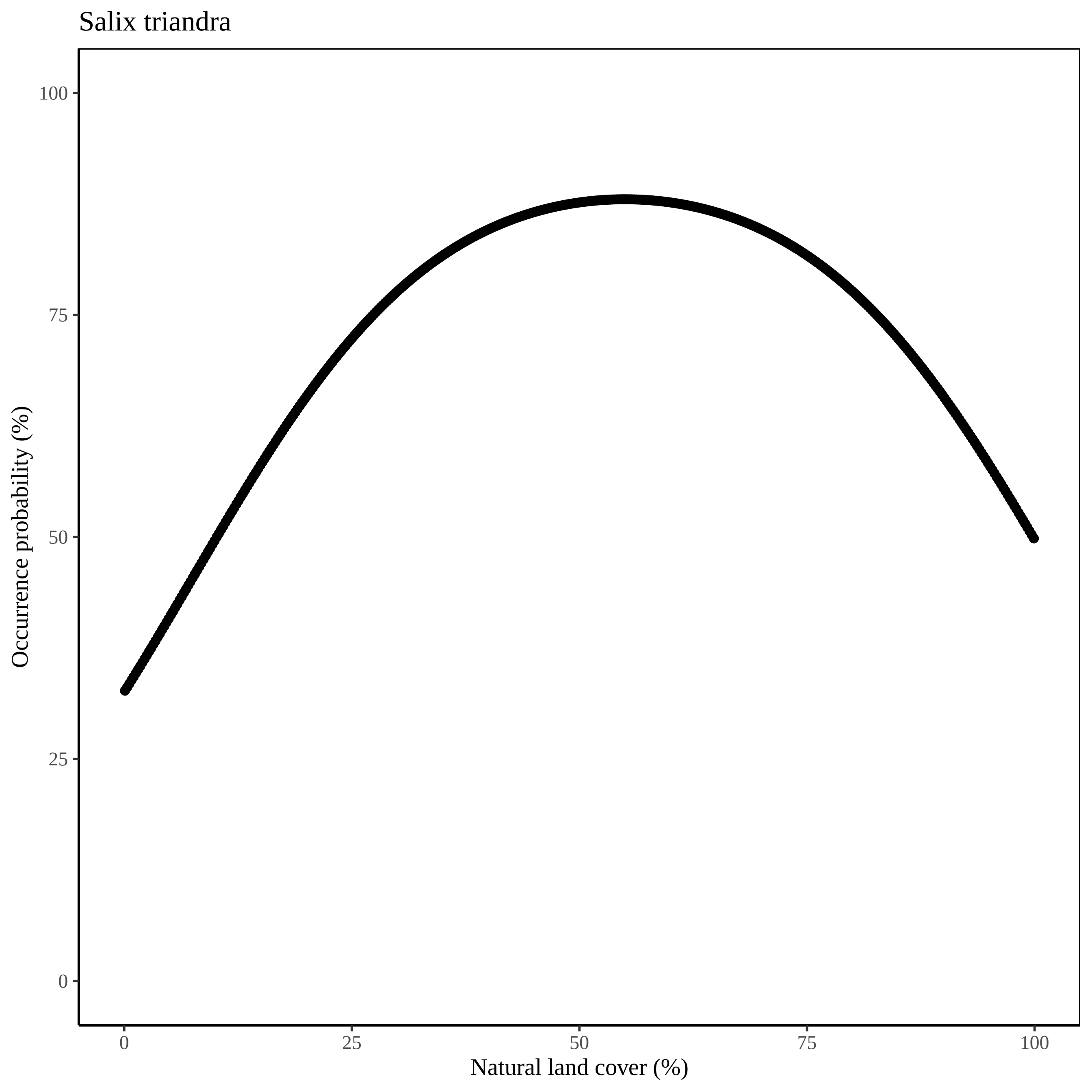

Salix triandra
Occurrence probability (%)
100
75
50
25
0
0
25
50
75
100
Natural land cover (%)

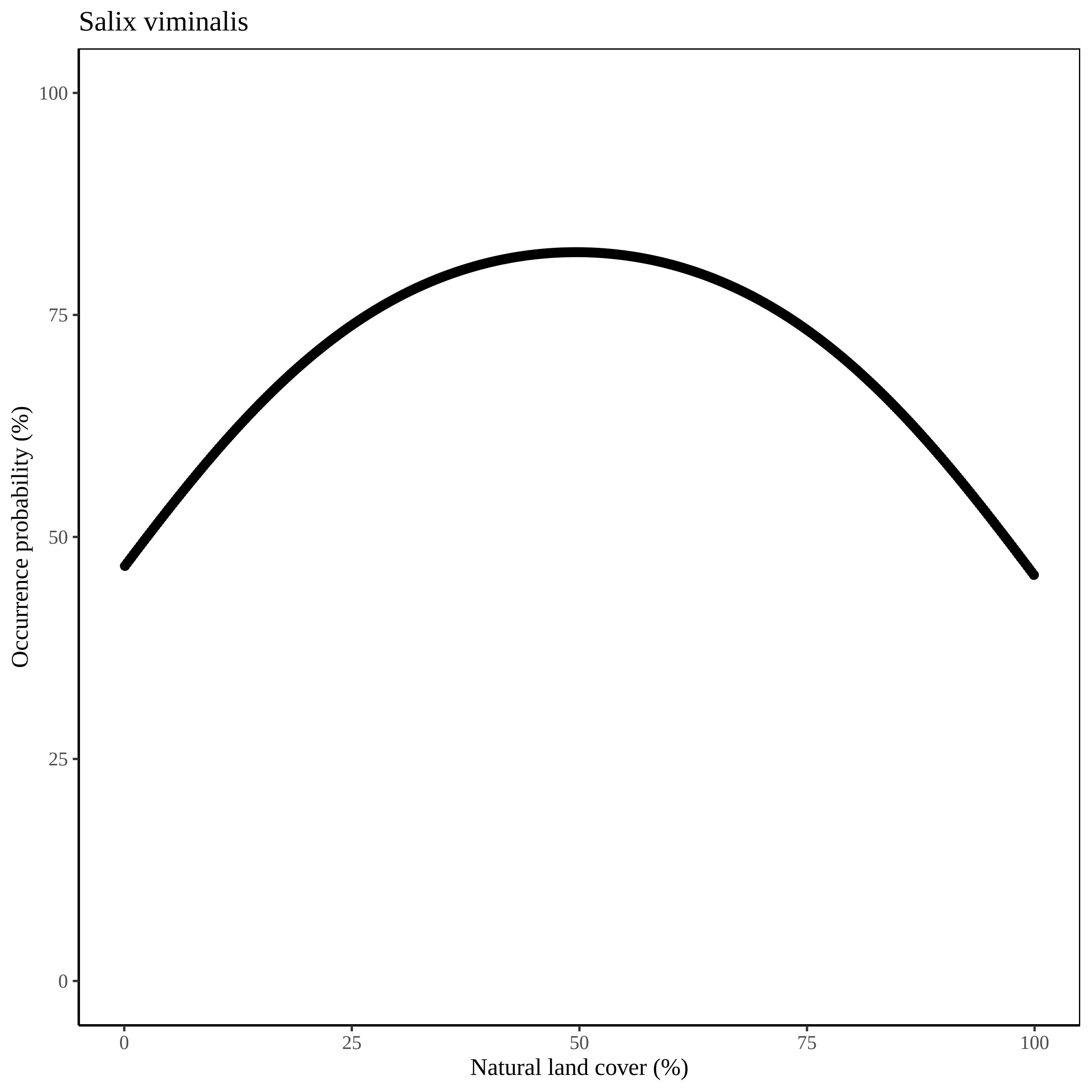
Salix viminalis
100
75
50
25
0
Occurrence probability (%)
0
25
50
75
100
Natural land cover (%)

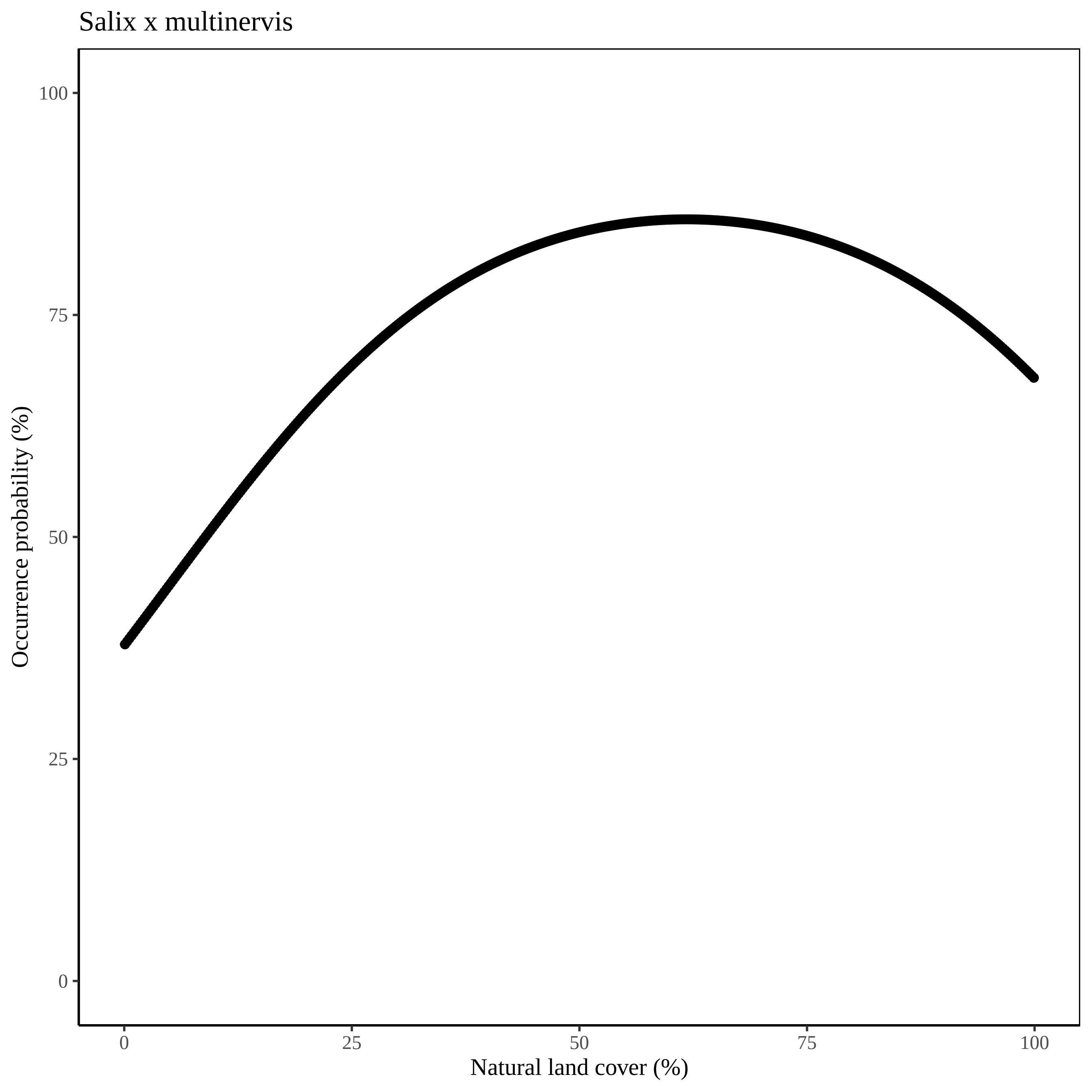
Salix x multinervis
100
75
50
25
0
Occurrence probability (%)
0
25
50
75
100
Natural land cover (%)

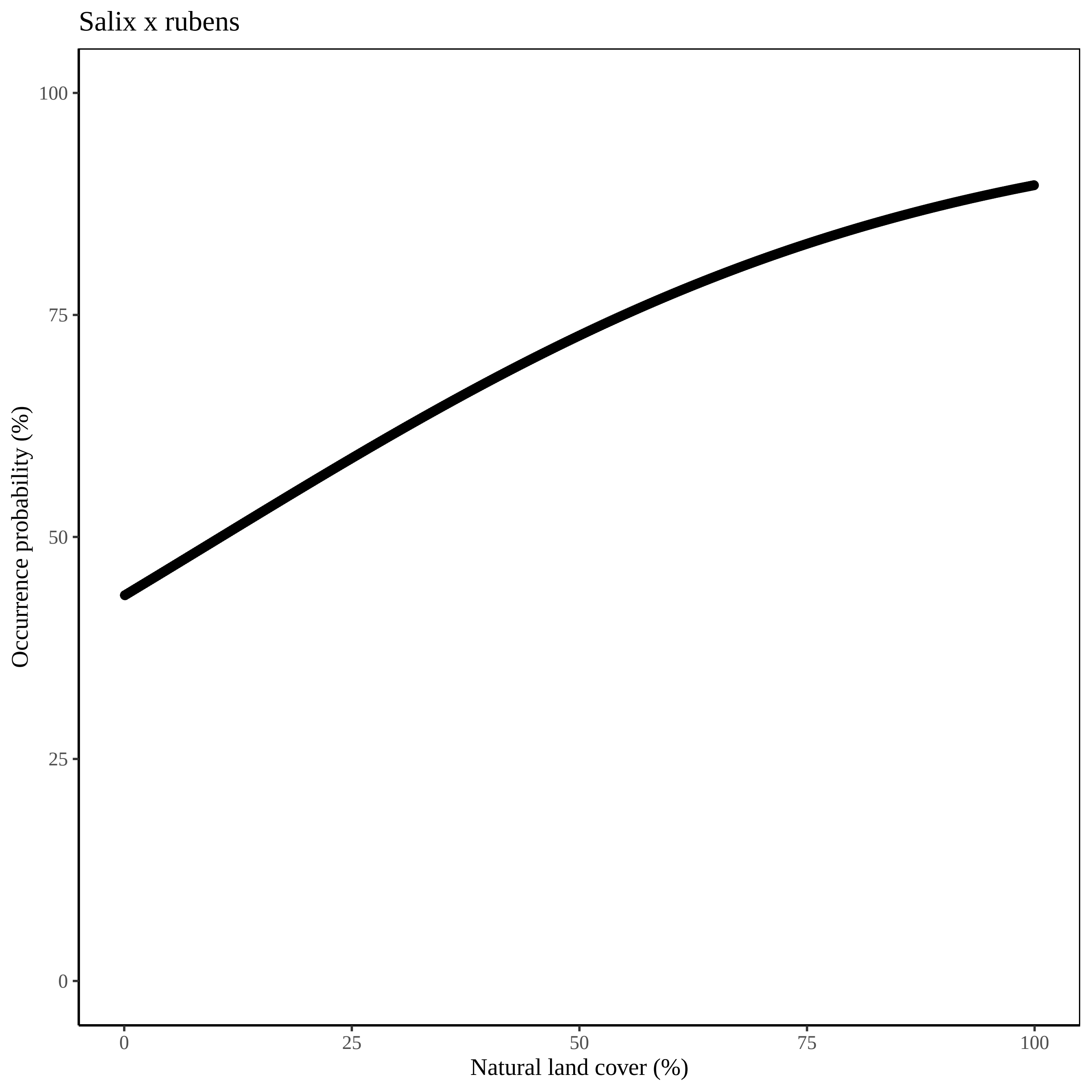
Salix x rubens
Occurrence probability (%)
100
75
50
25
0
0
25
50
75
100
Natural land cover (%)

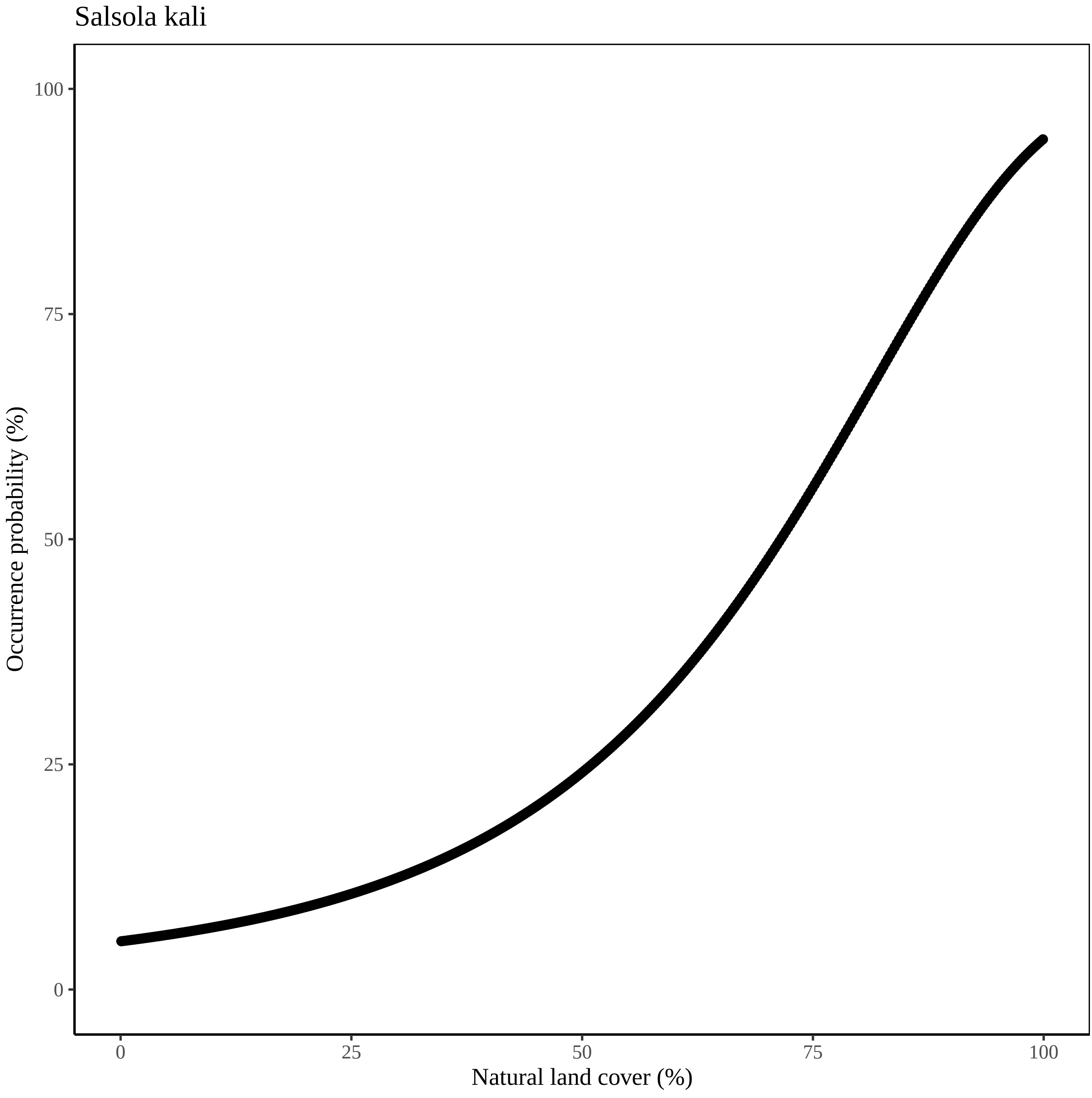
Salsola kali
Occurrence probability (%)
100
75
50
25
0
0
25
50
75
100
Natural land cover (%)

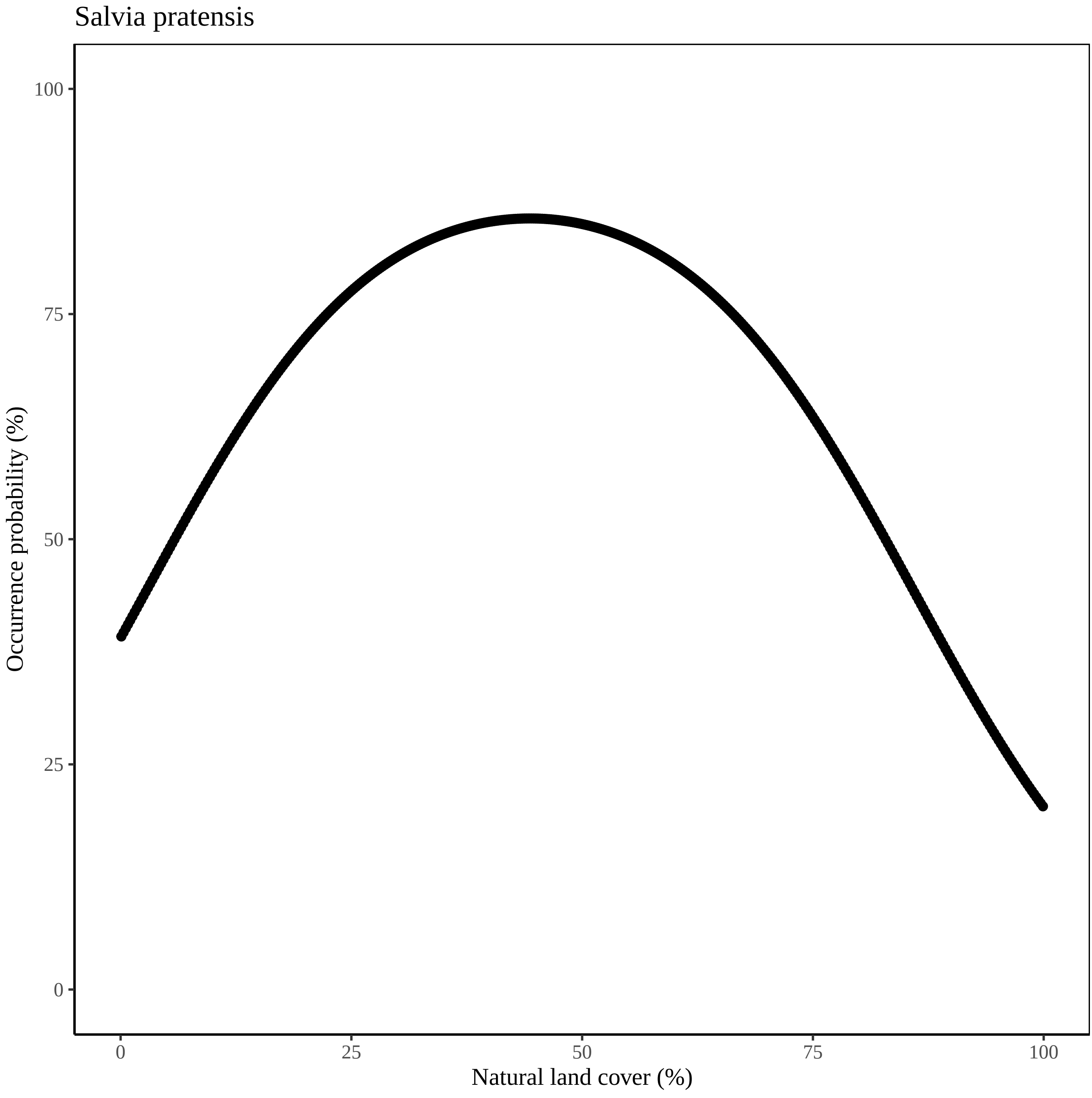

Salvia pratensis
Occurrence probability (%)
100
75
50
25
0
0
25
50
75
100
Natural land cover (%)

Sambucus ebulus

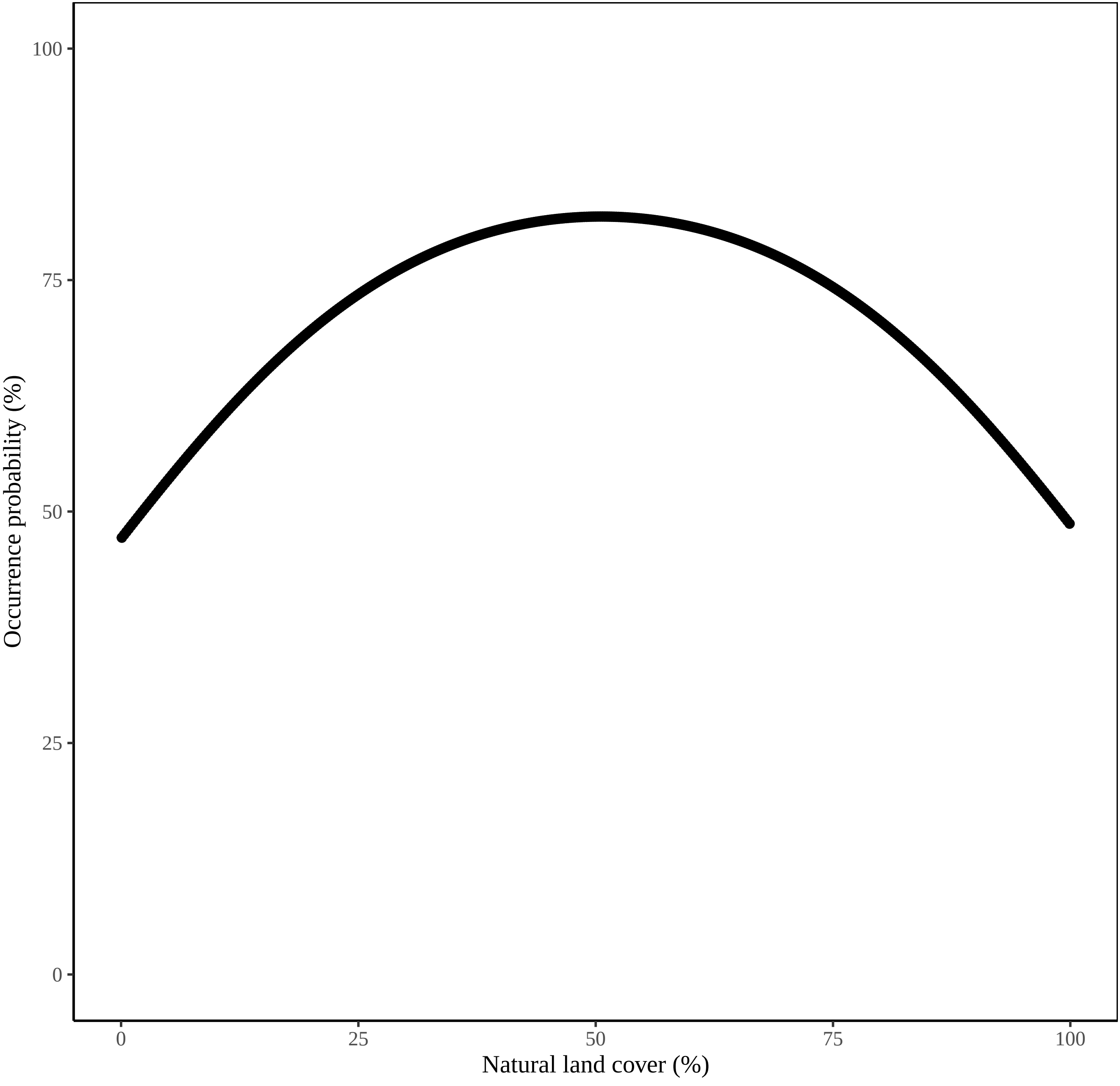

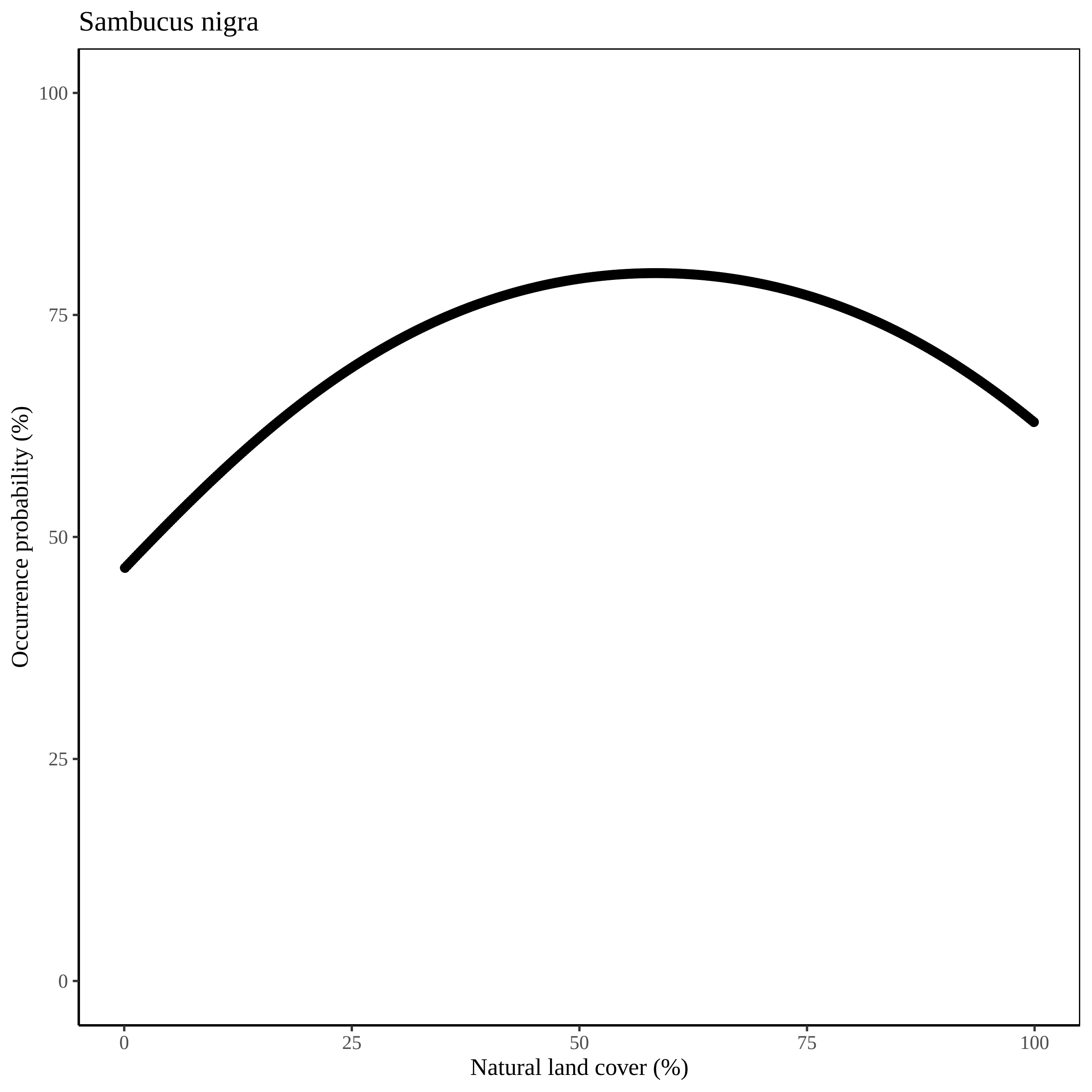
Sambucus nigra
100
75
50
25
0
Occurrence probability (%)
0
25
50
75
100
Natural land cover (%)

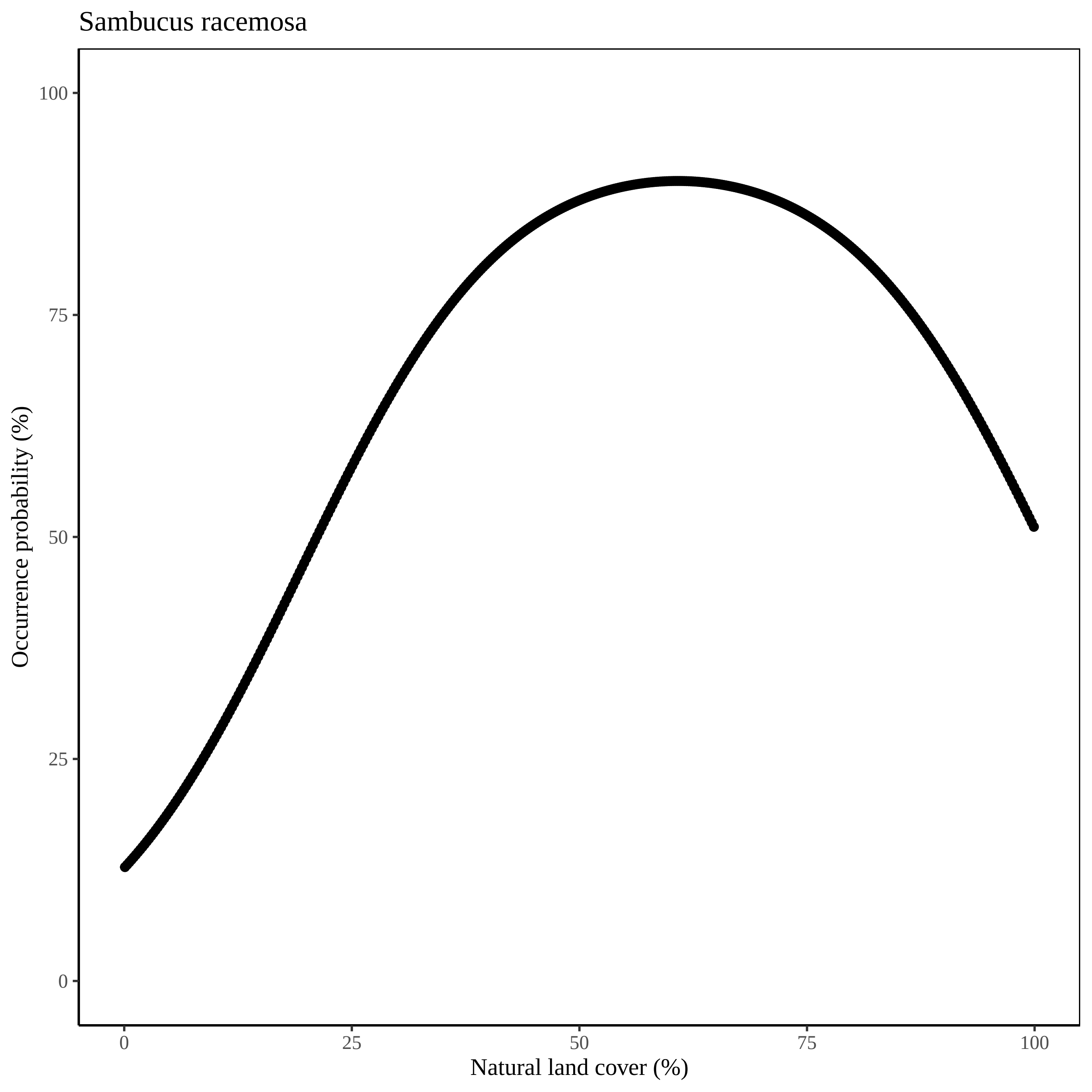
Sambucus racemosa
Occurrence probability (%)
100
75
50
25
0
0
25
50
75
100
Natural land cover (%)

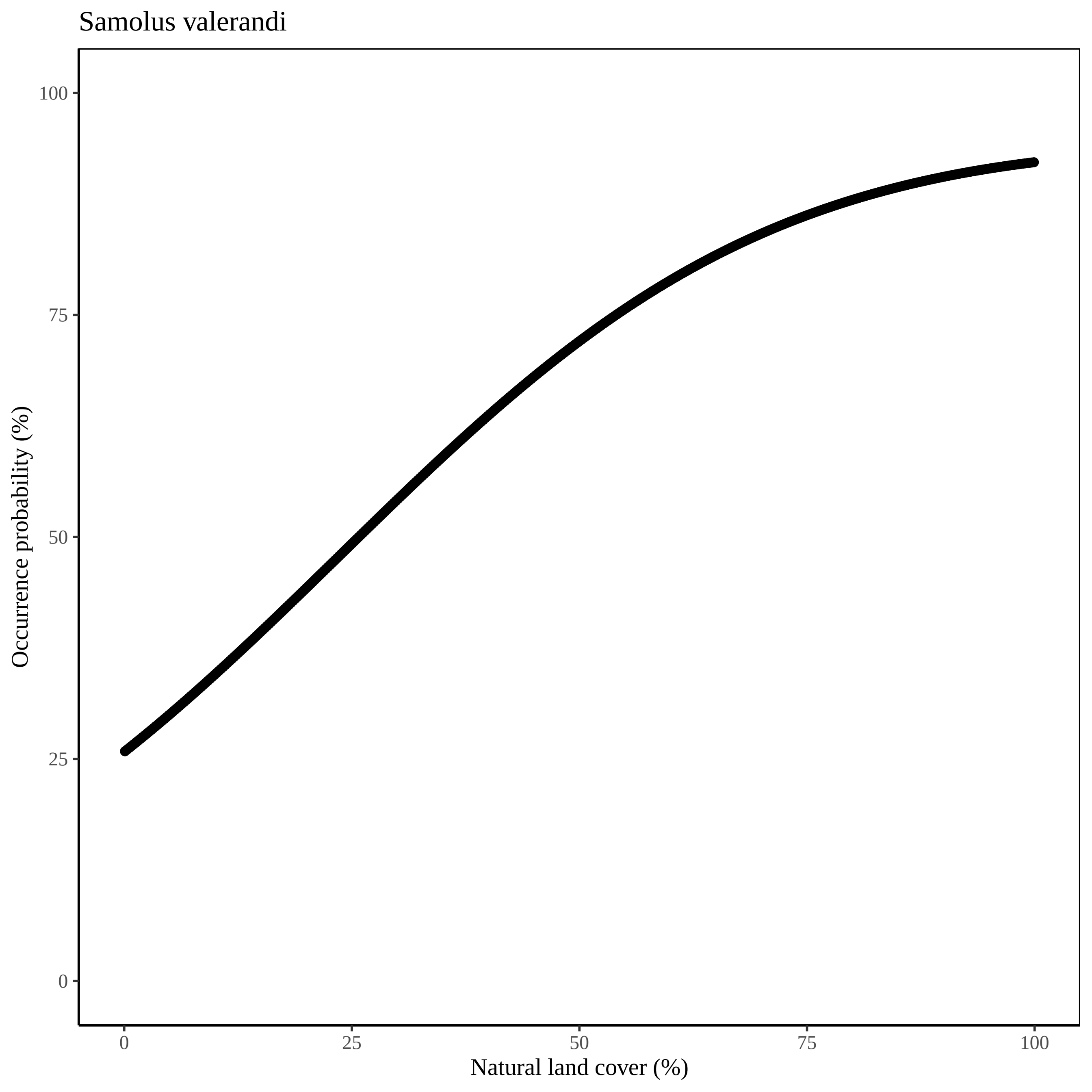
Samolus valerandi
100
75
50
25
0
Occurrence probability (%)
0
25
50
75
100
Natural land cover (%)

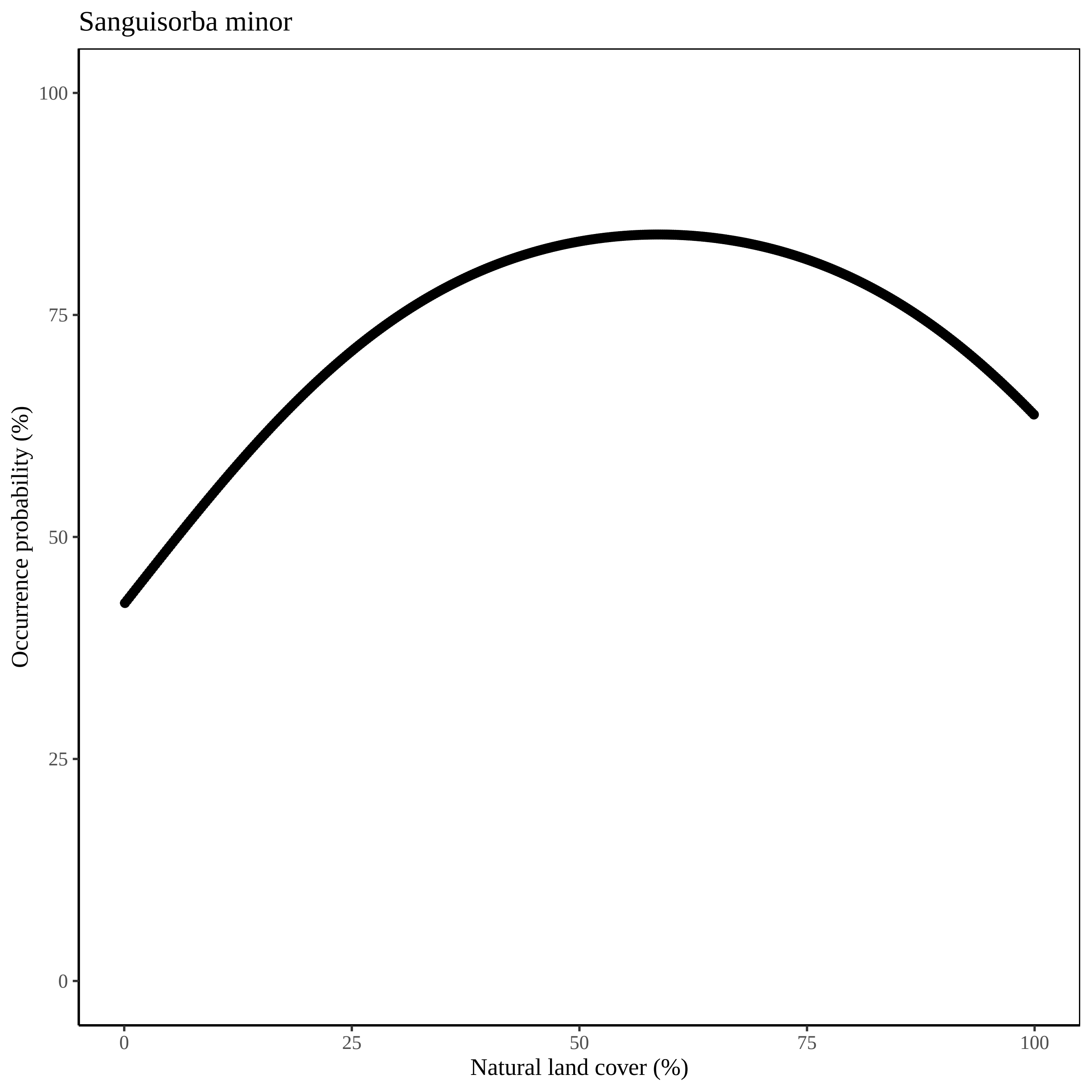
Sanguisorba minor
Occurrence probability (%)
100
75
50
25
0
0
25
50
75
100
Natural land cover (%)

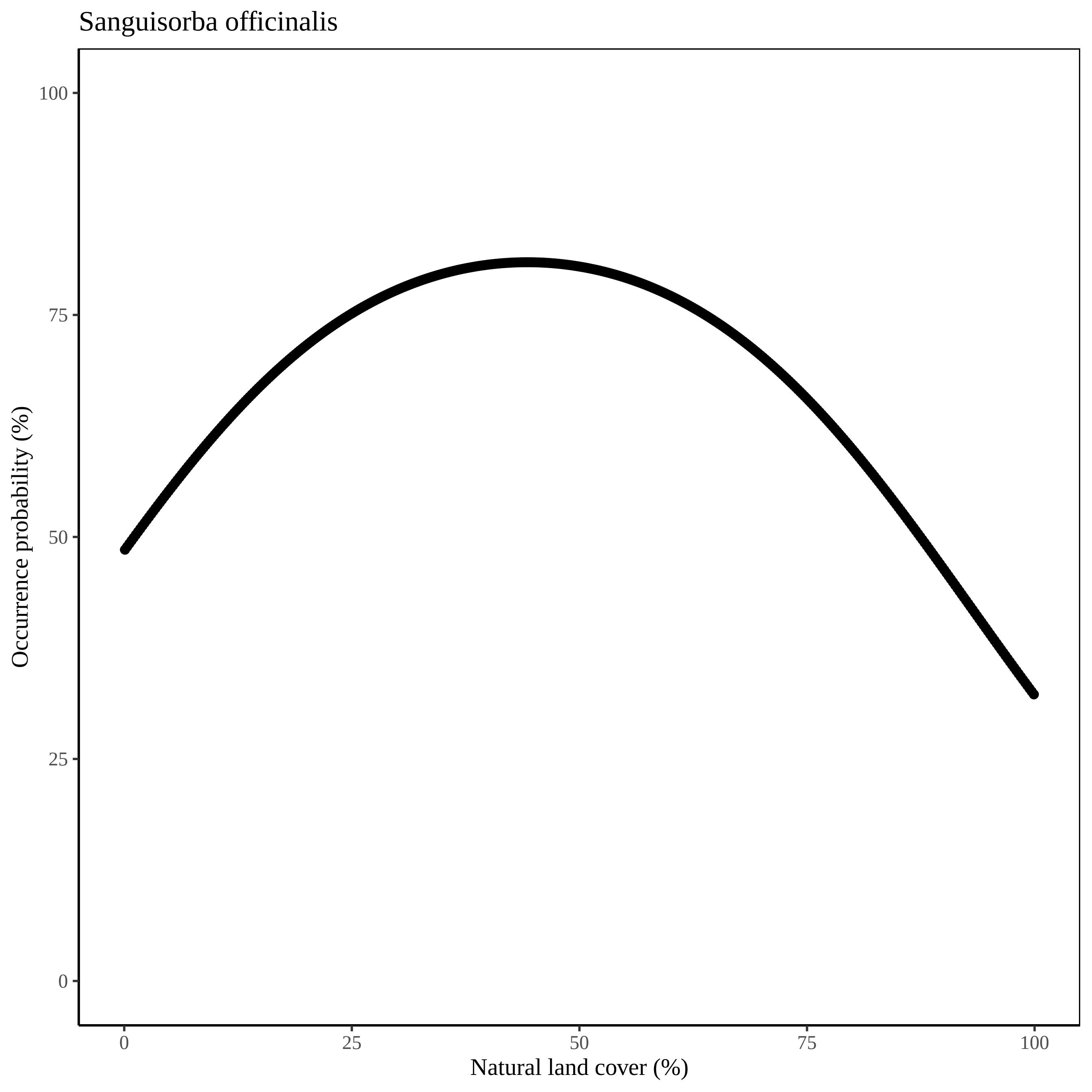
Sanguisorba officinalis
100
75
50
25
0
Occurrence probability (%)
0
25
50
75
100
Natural land cover (%)

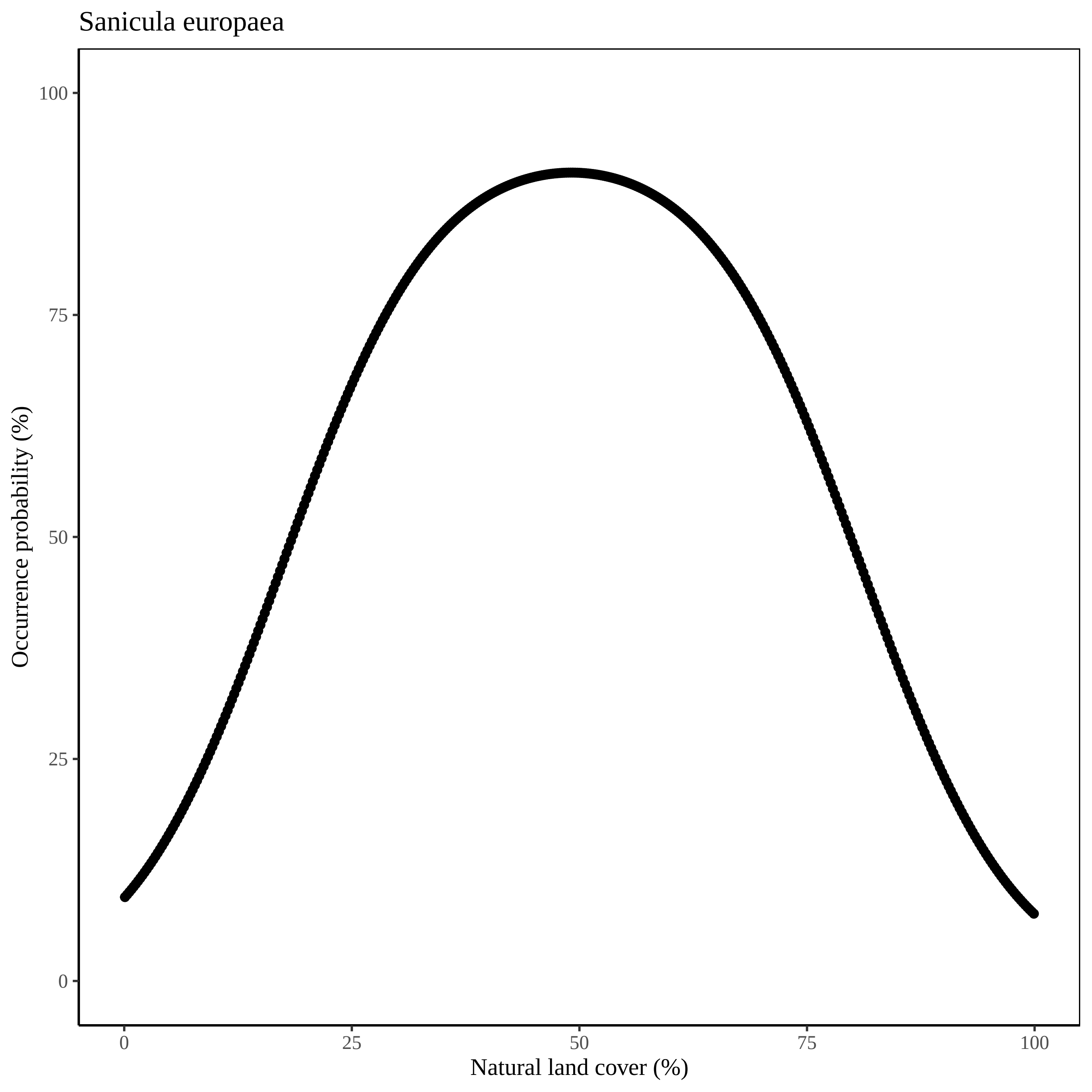

Sanicula europaea
Occurrence probability (%)
100
75
50
25
0
0
25
50
75
100
Natural land cover (%)

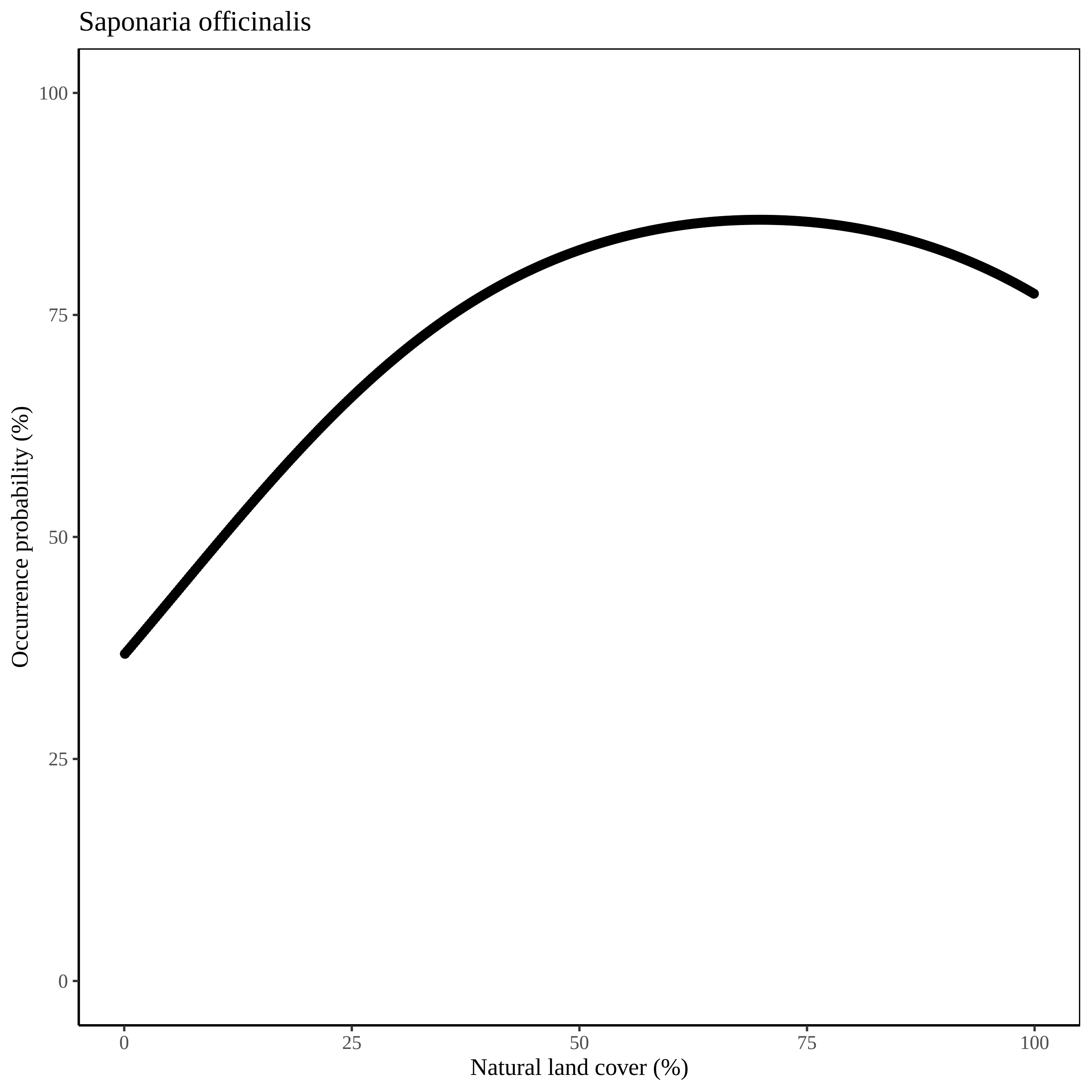

Saponaria officinalis
Occurrence probability (%)
100
75
50
25
0
0
25
50
75
100
Natural land cover (%)

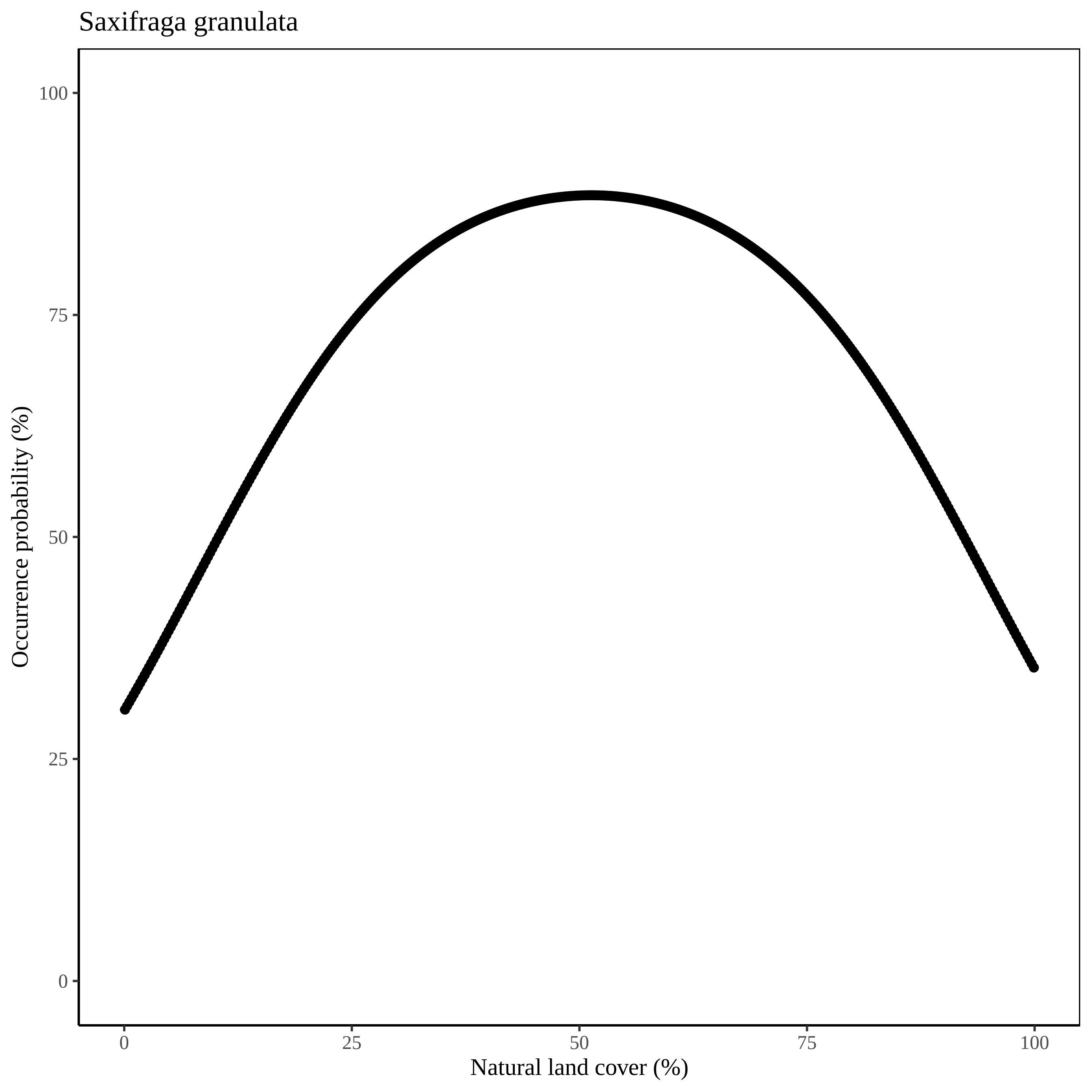

Saxifraga granulata
Occurrence probability (%)
100
75
50
25
0
0
25
50
75
100
Natural land cover (%)

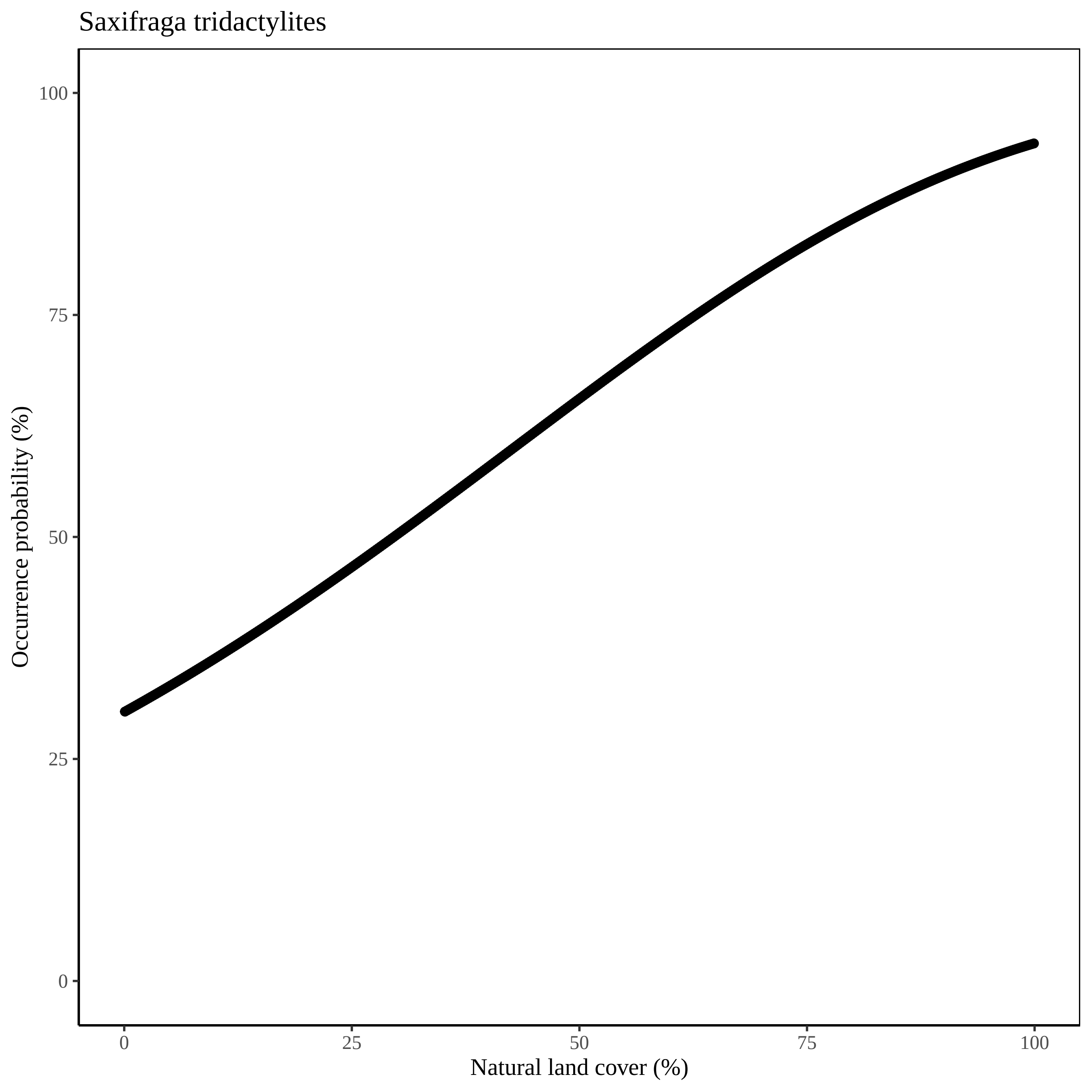

Saxifraga tridactylites
Occurrence probability (%)
100
75
50
25
0
0
25
50
75
100
Natural land cover (%)

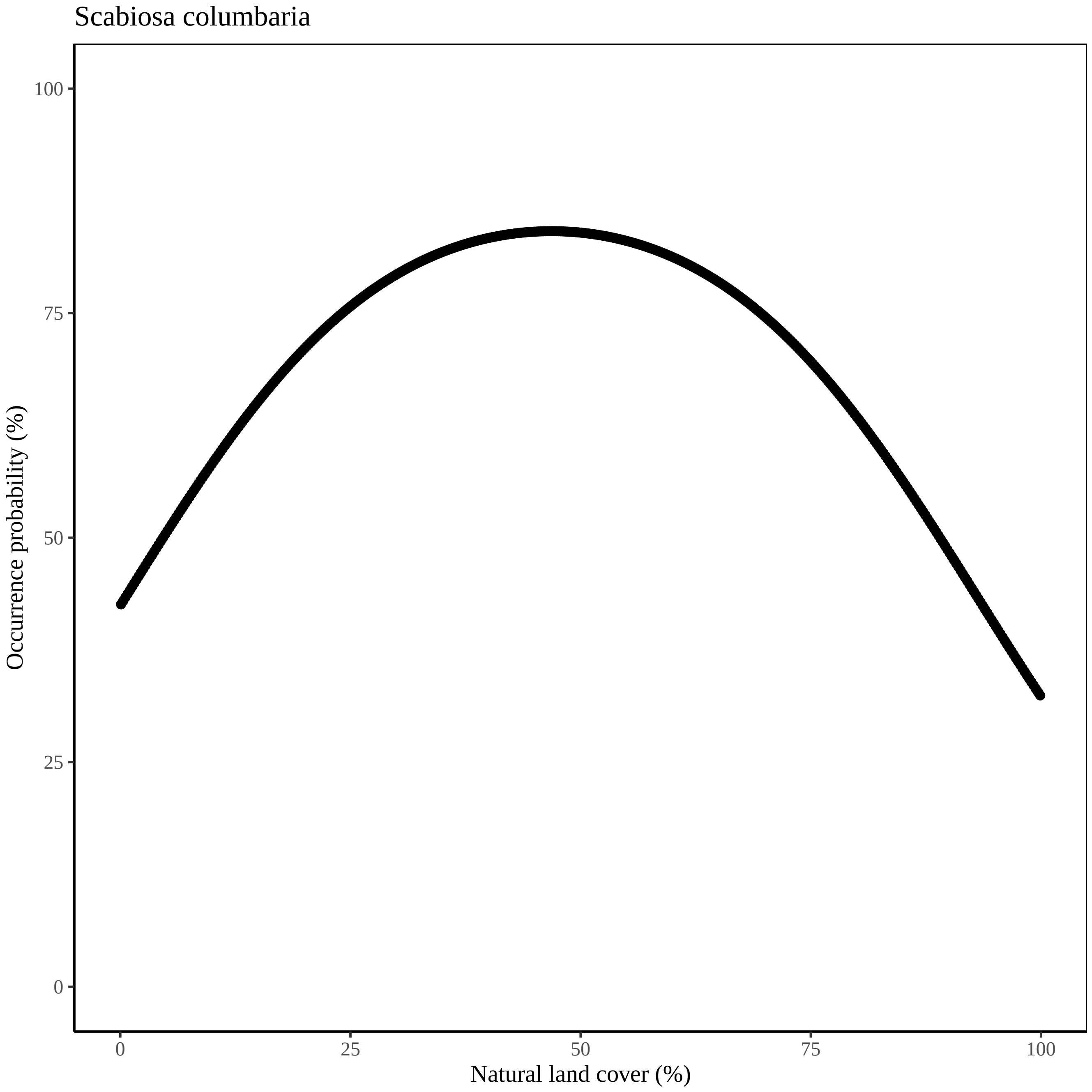
Scabiosa columbaria
100
75
50
25
0
Occurrence probability (%)
0
25
50
75
100
Natural land cover (%)

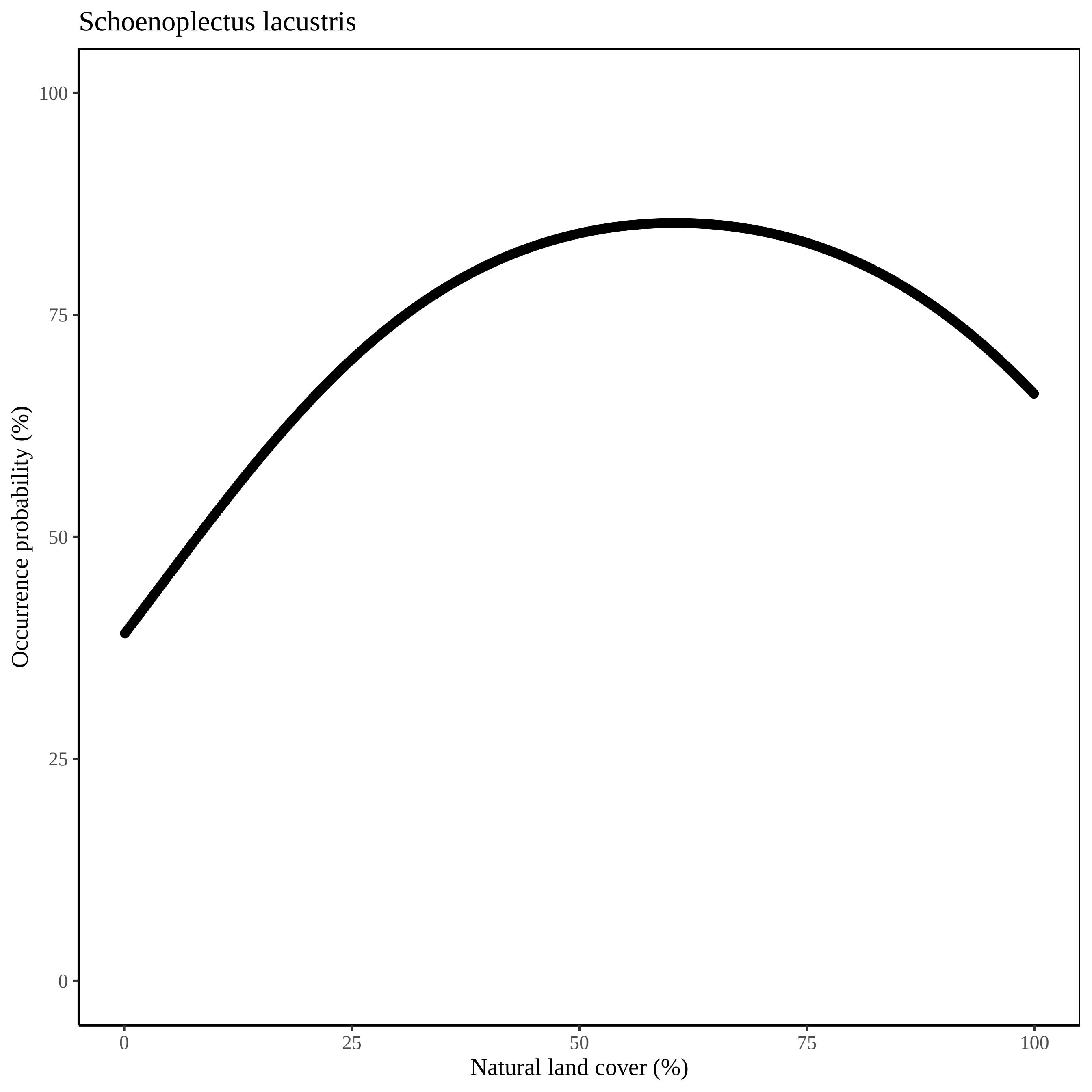
Schoenoplectus lacustris
Occurrence probability (%)
100
75
50
25
0
0
25
50
75
100
Natural land cover (%)

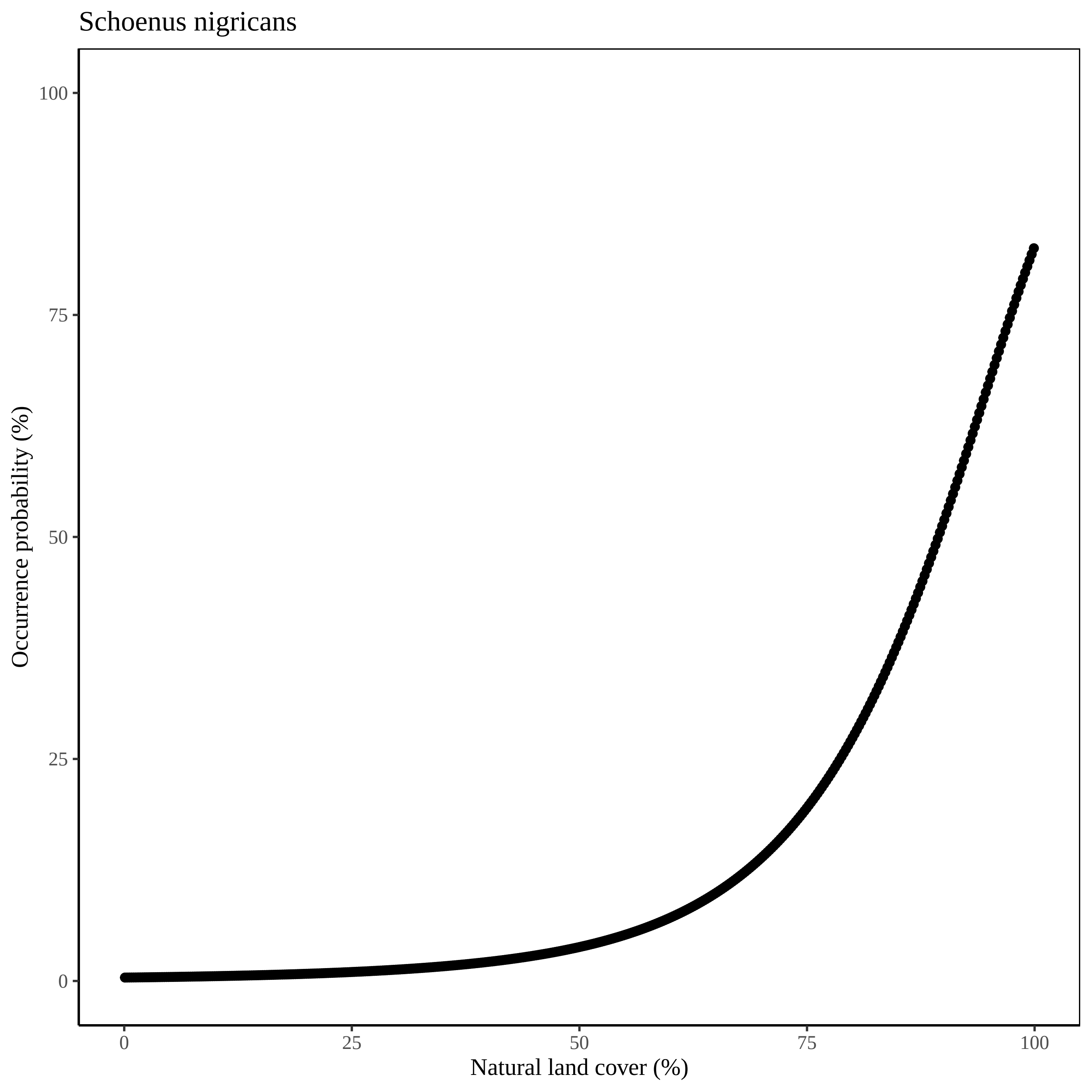

Schoenus nigricans
Occurrence probability (%)
100
75
50
25
0
0
25
50
75
100
Natural land cover (%)

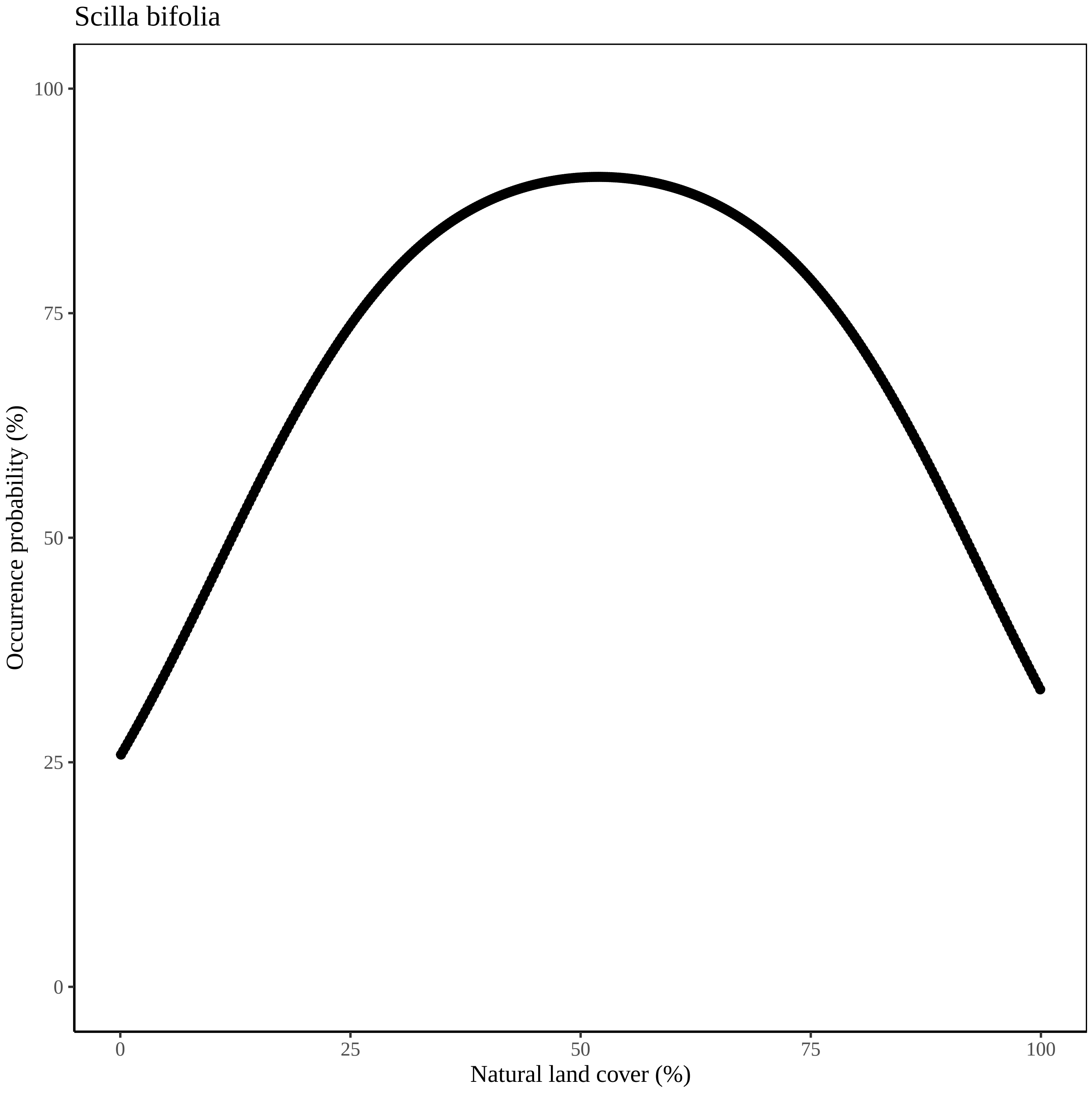

Scilla bifolia
Occurrence probability (%)
100
75
50
25
0
0
25
50
75
100
Natural land cover (%)

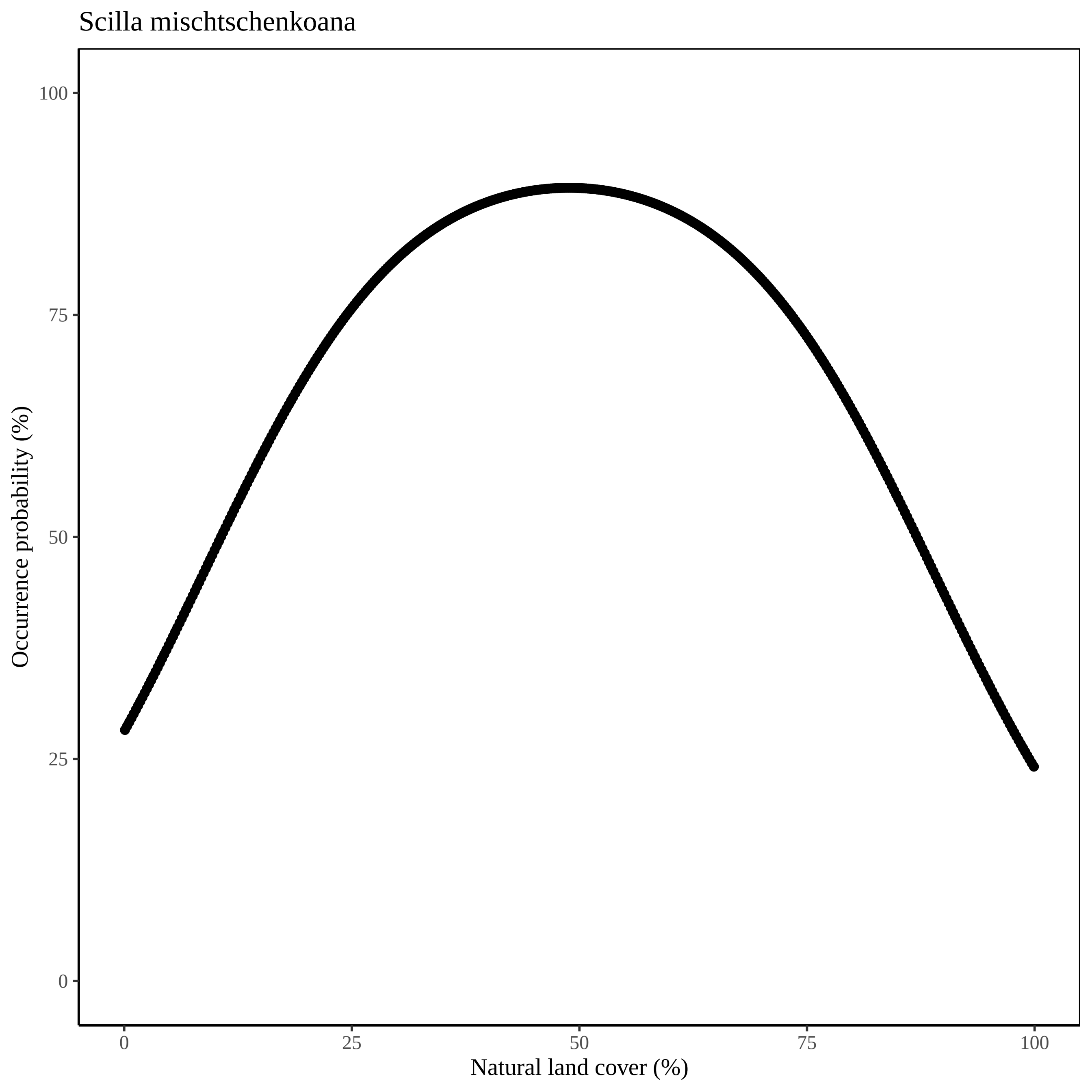
Scilla mischtschenkoana
Occurrence probability (%)
100
75
50
25
0
0
25
50
75
100
Natural land cover (%)

Scilla siberica

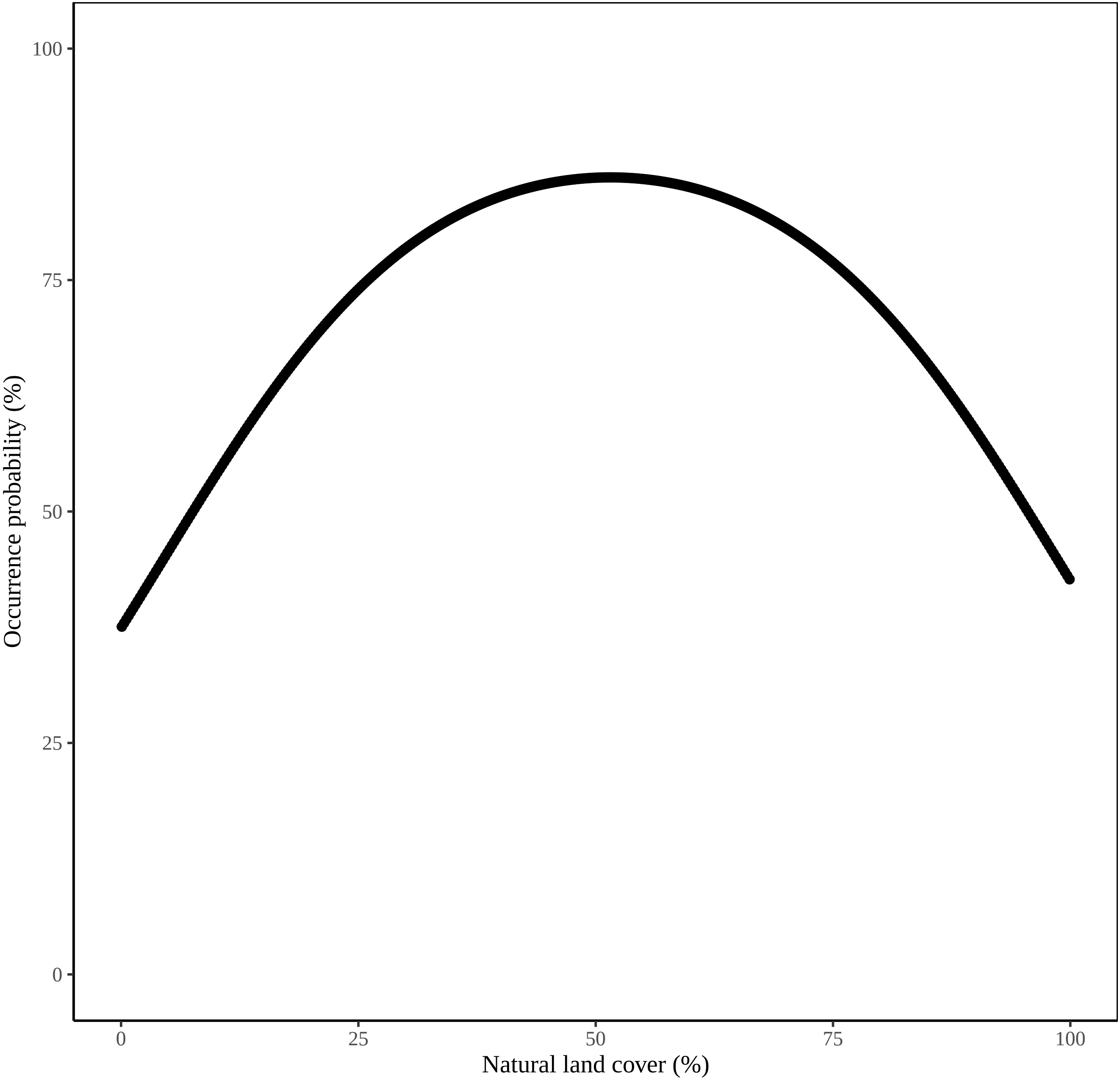

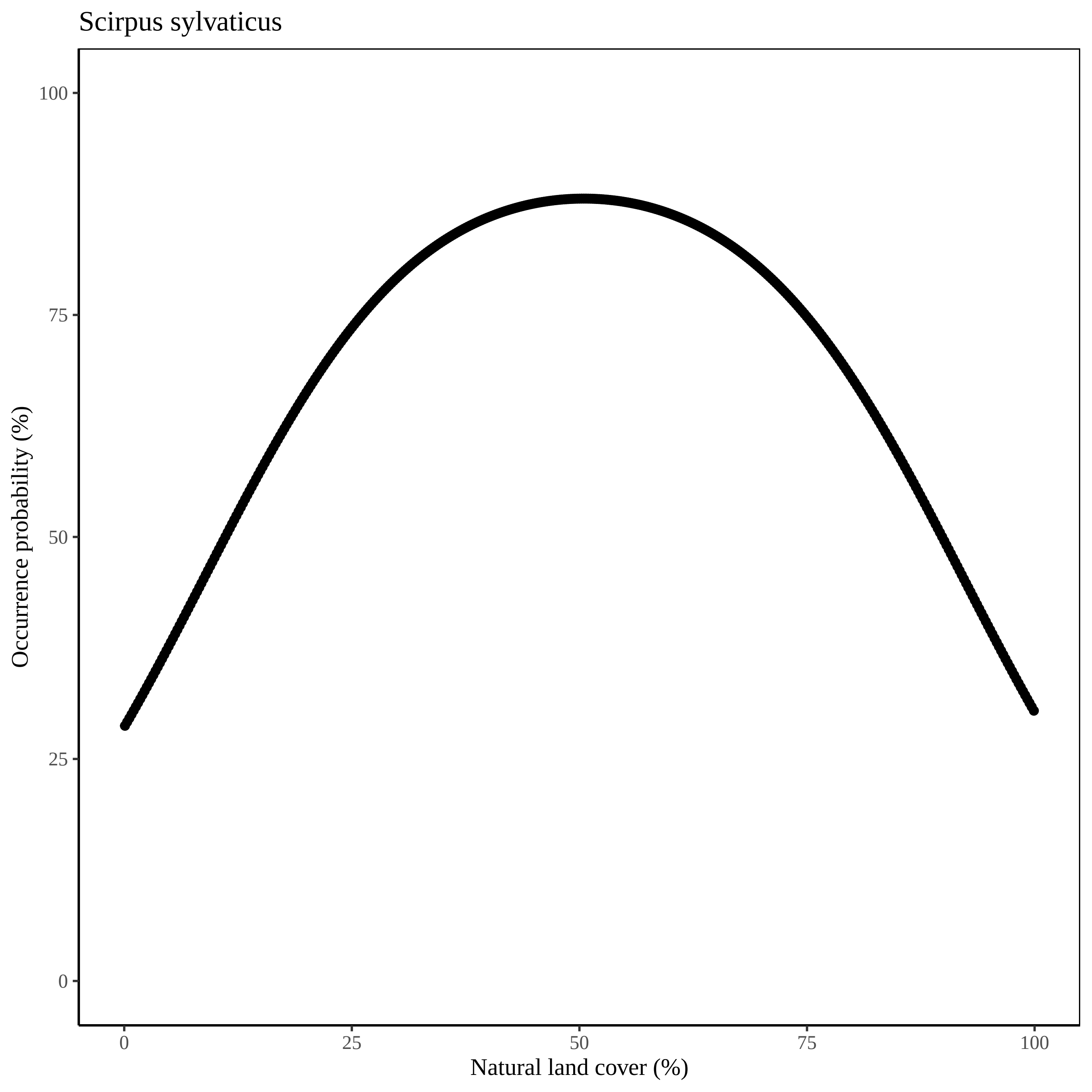
Scirpus sylvaticus
Occurrence probability (%)
100
75
50
25
0
0
25
50
75
100
Natural land cover (%)

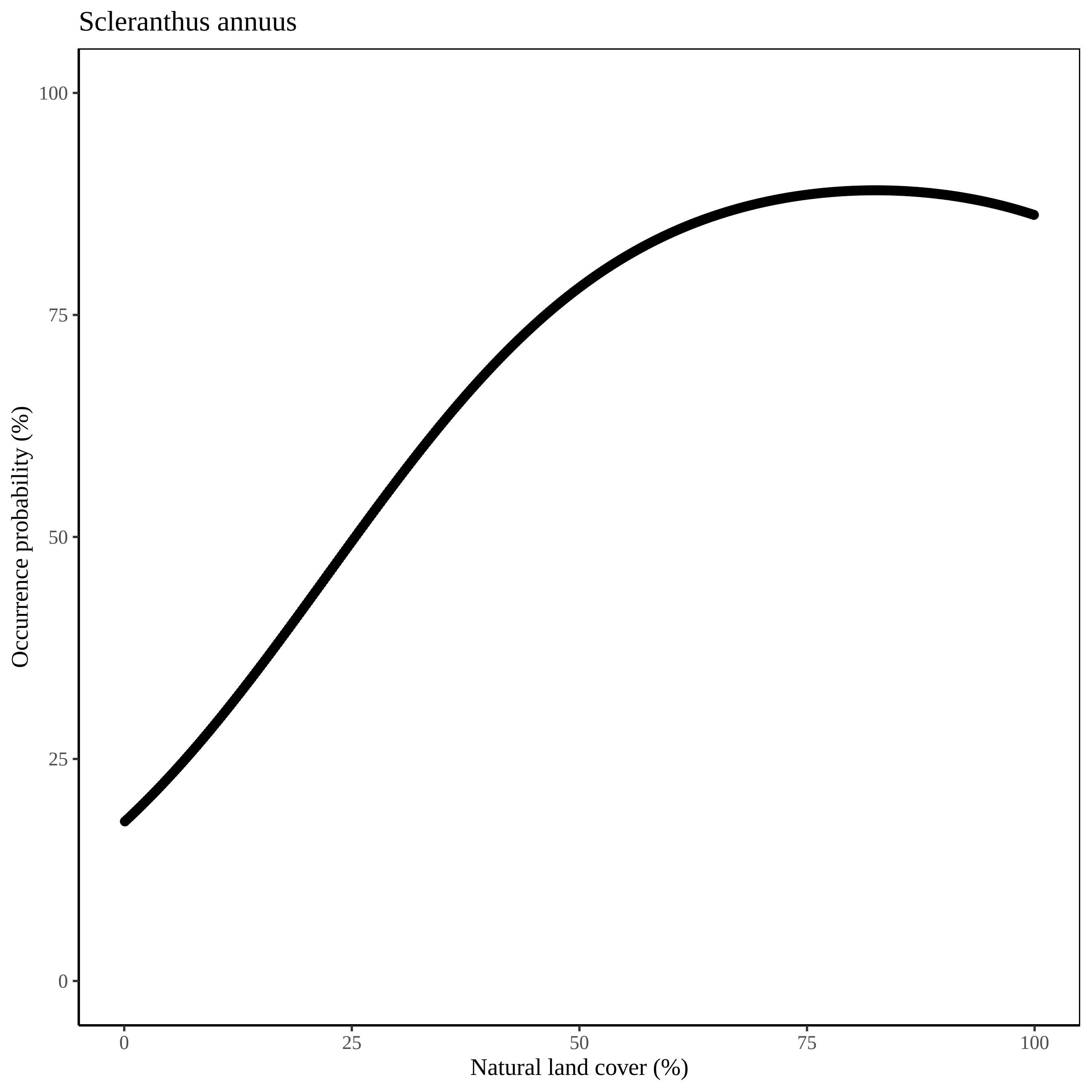
Scleranthus annuus
100
75
50
25
0
Occurrence probability (%)
0
25
50
75
100
Natural land cover (%)

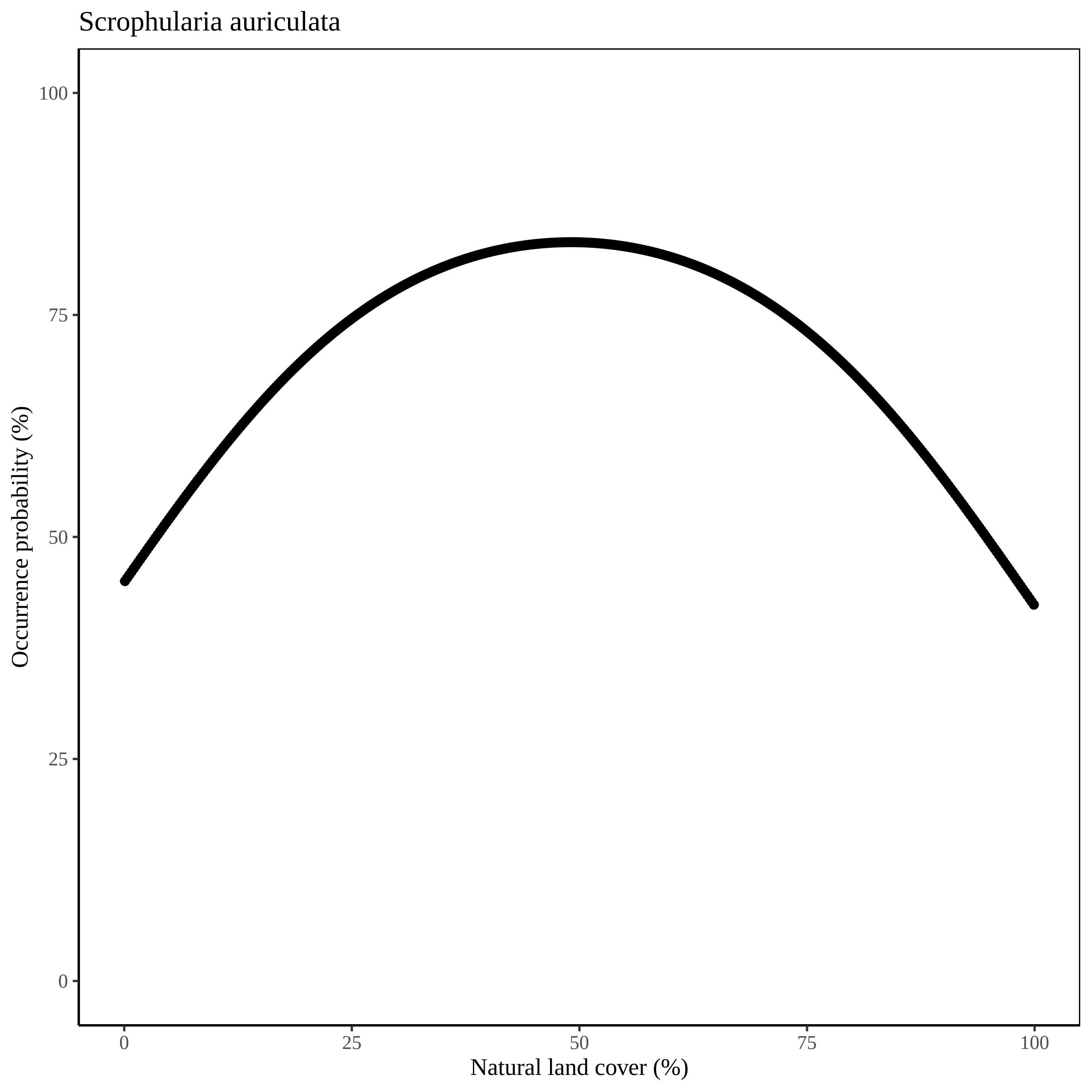

Scrophularia auriculata
100
75
50
25
0
Occurrence probability (%)
0
25
50
75
100
Natural land cover (%)

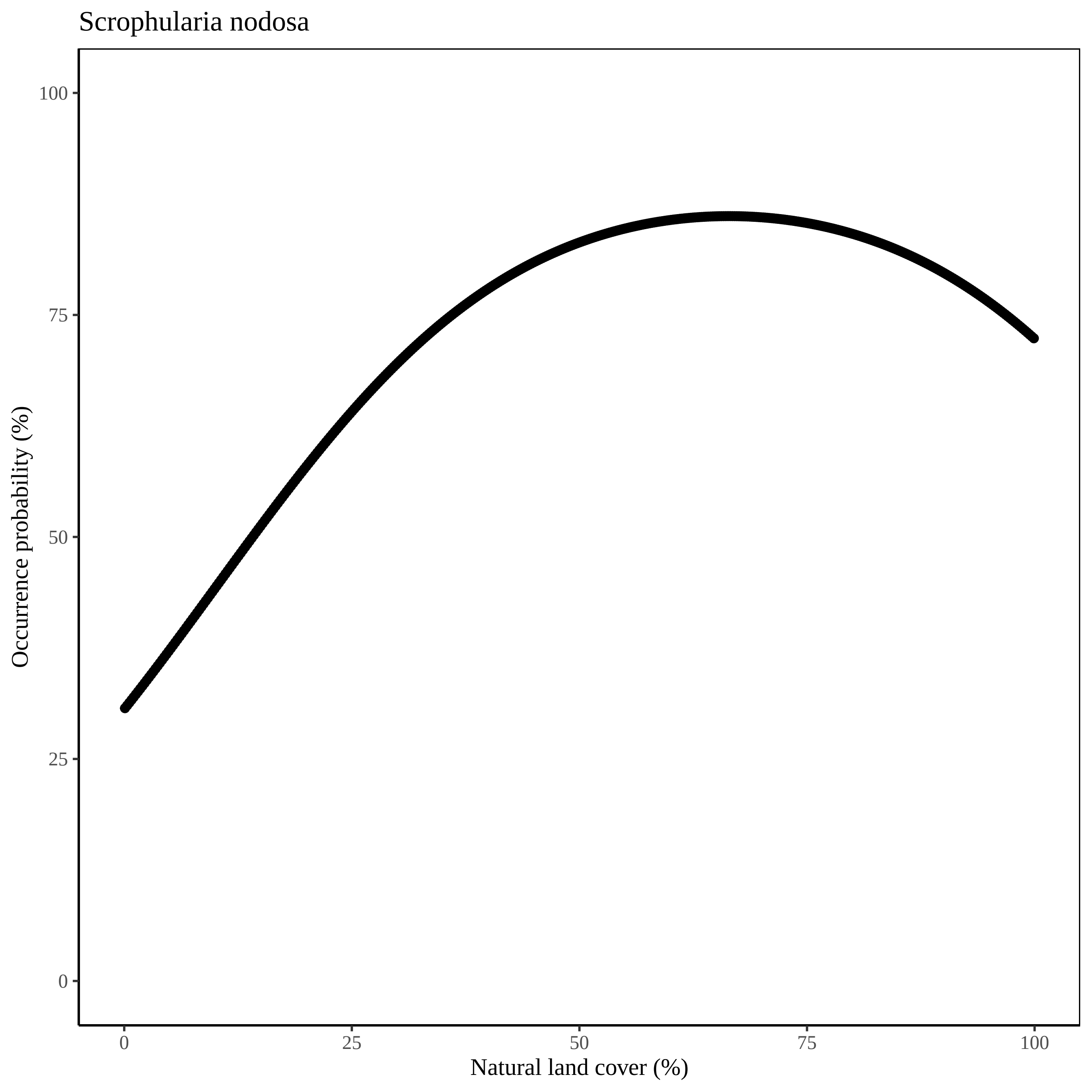
Scrophularia nodosa
Occurrence probability (%)
100
75
50
25
0
0
25
50
75
100
Natural land cover (%)

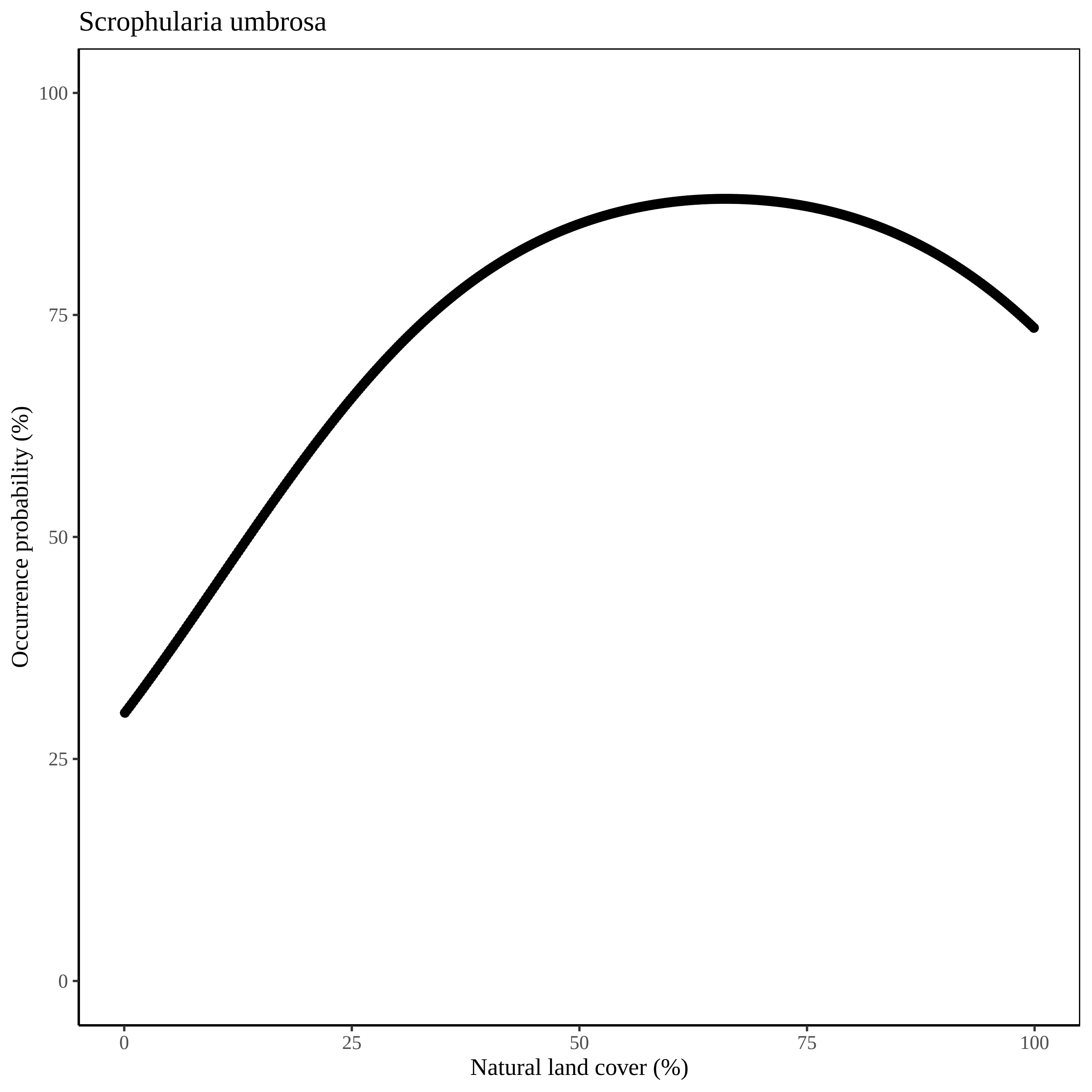

Scrophularia umbrosa
Occurrence probability (%)
100
75
50
25
0
0
25
50
75
100
Natural land cover (%)

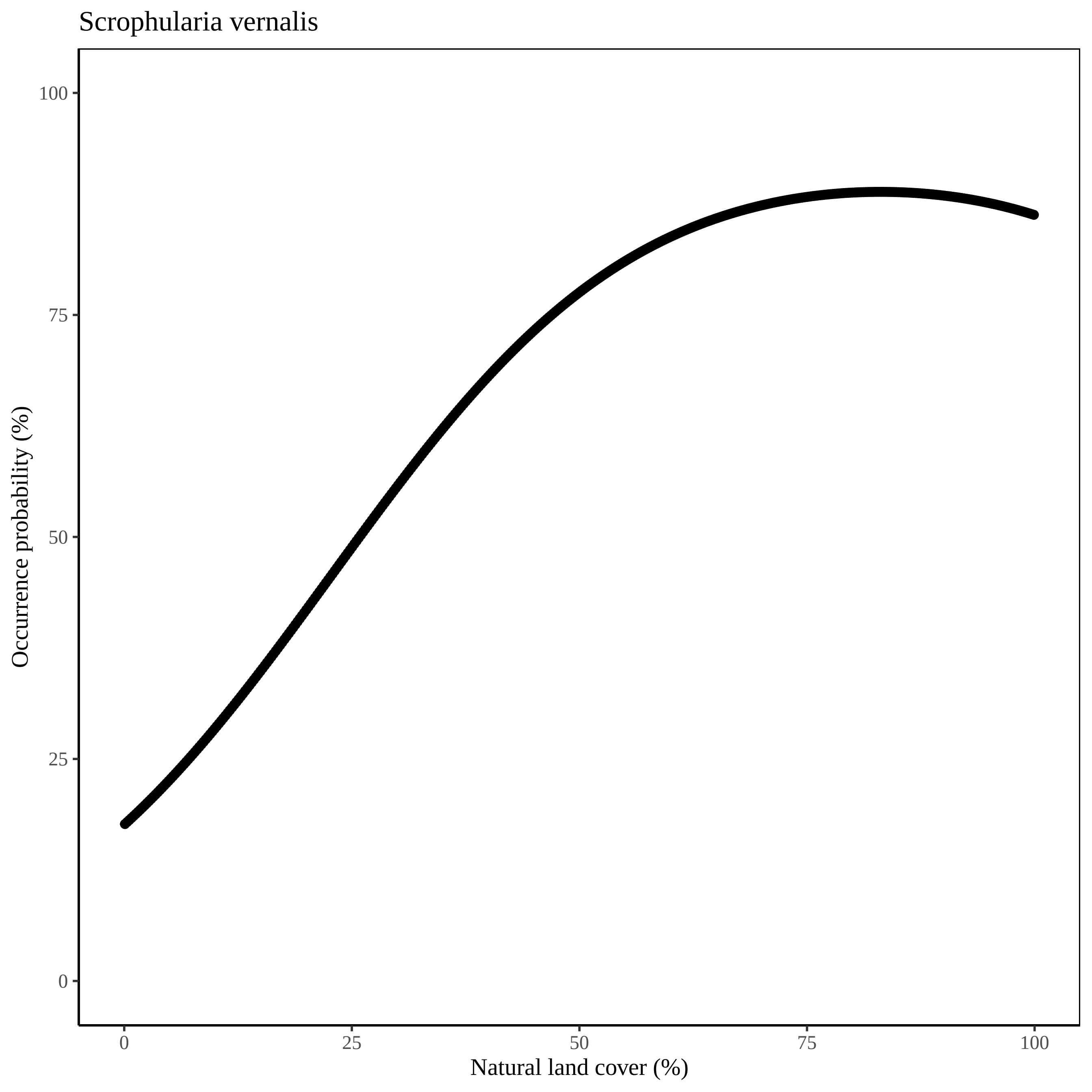
Scrophularia vernalis
Occurrence probability (%)
100
75
50
25
0
0
25
50
75
100
Natural land cover (%)

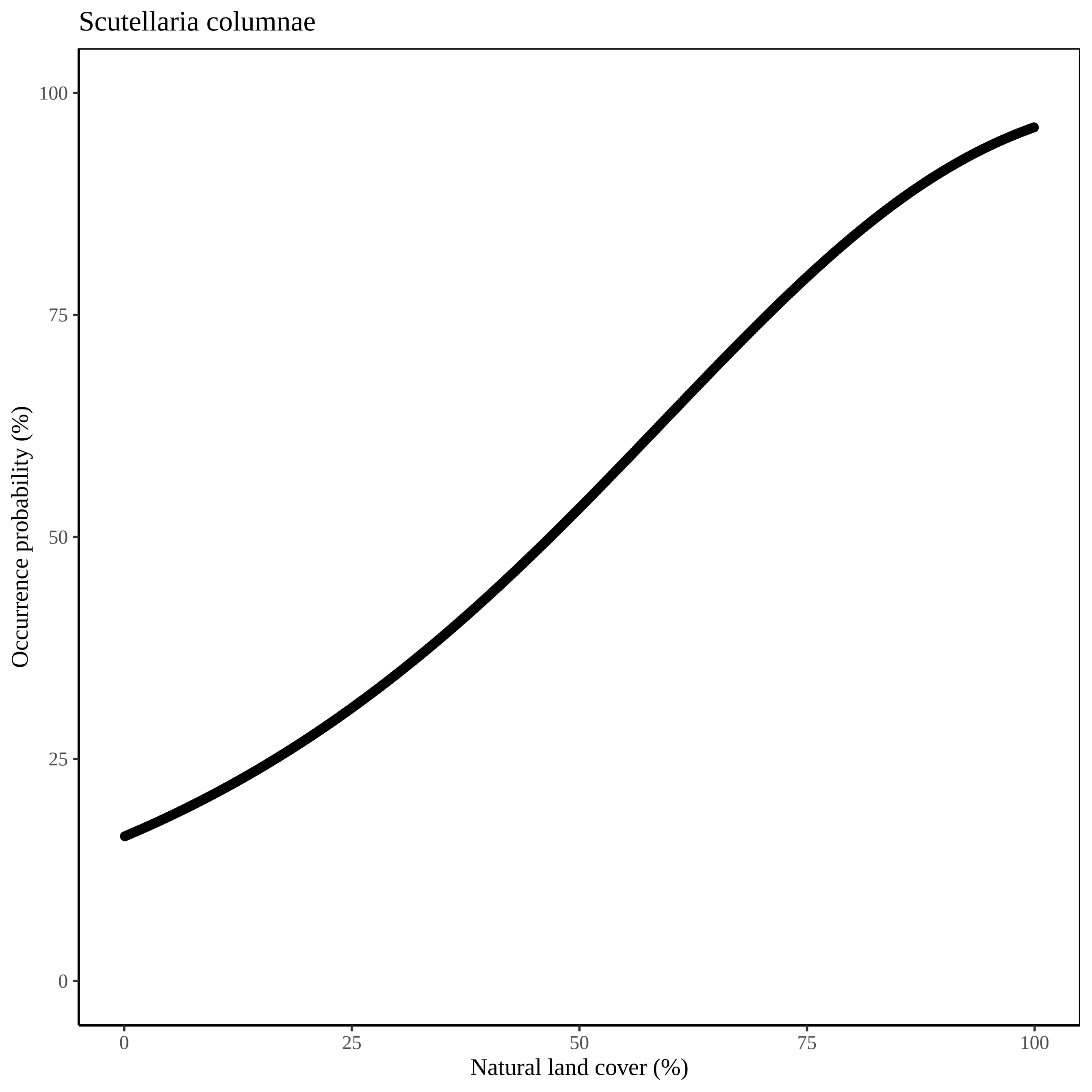

Scutellaria columnae
100
75
50
25
0
0
25
50
75
100
Occurrence probability (%)
Natural land cover (%)

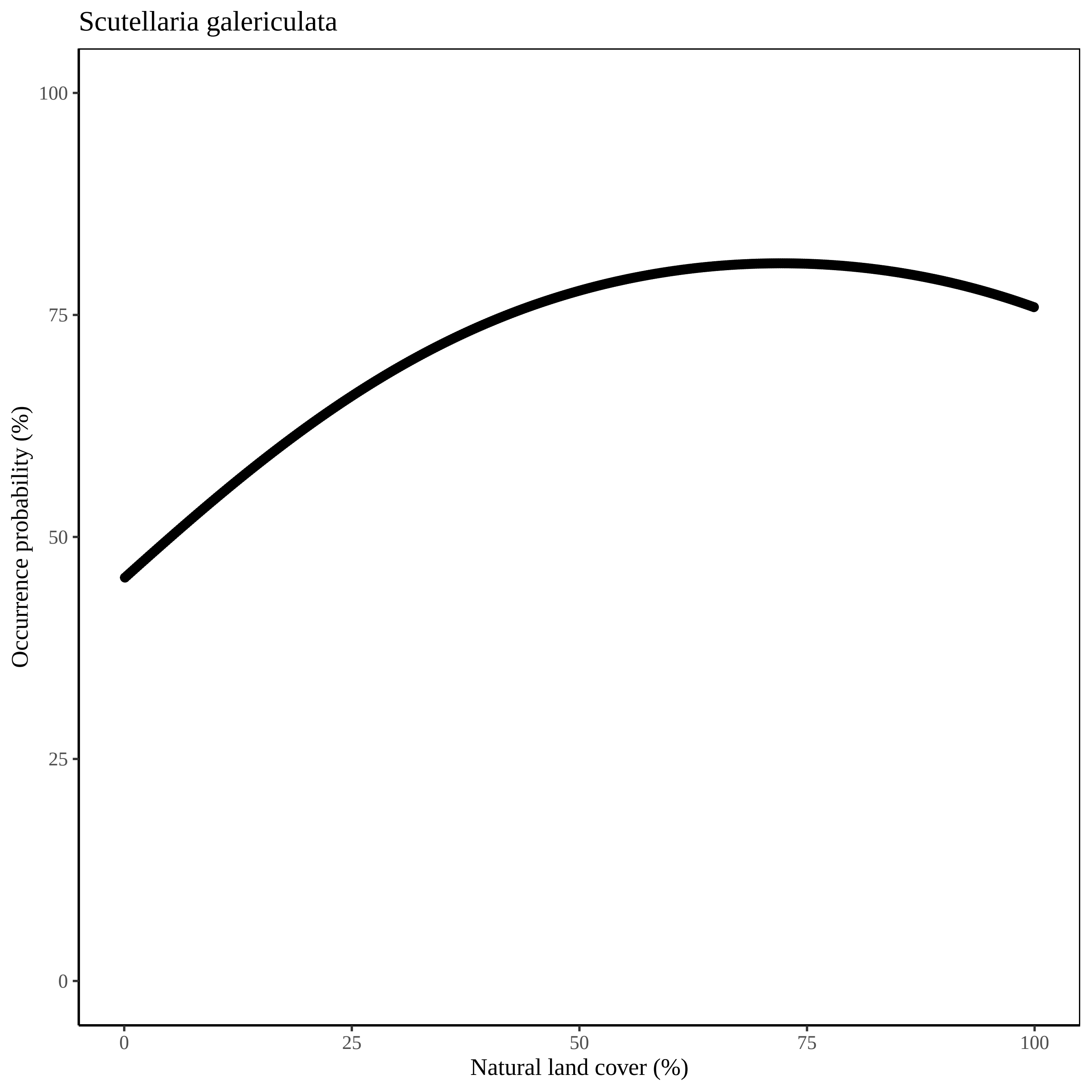
Scutellaria galericulata
Occurrence probability (%)
100
75
50
25
0
0
25
50
75
100
Natural land cover (%)

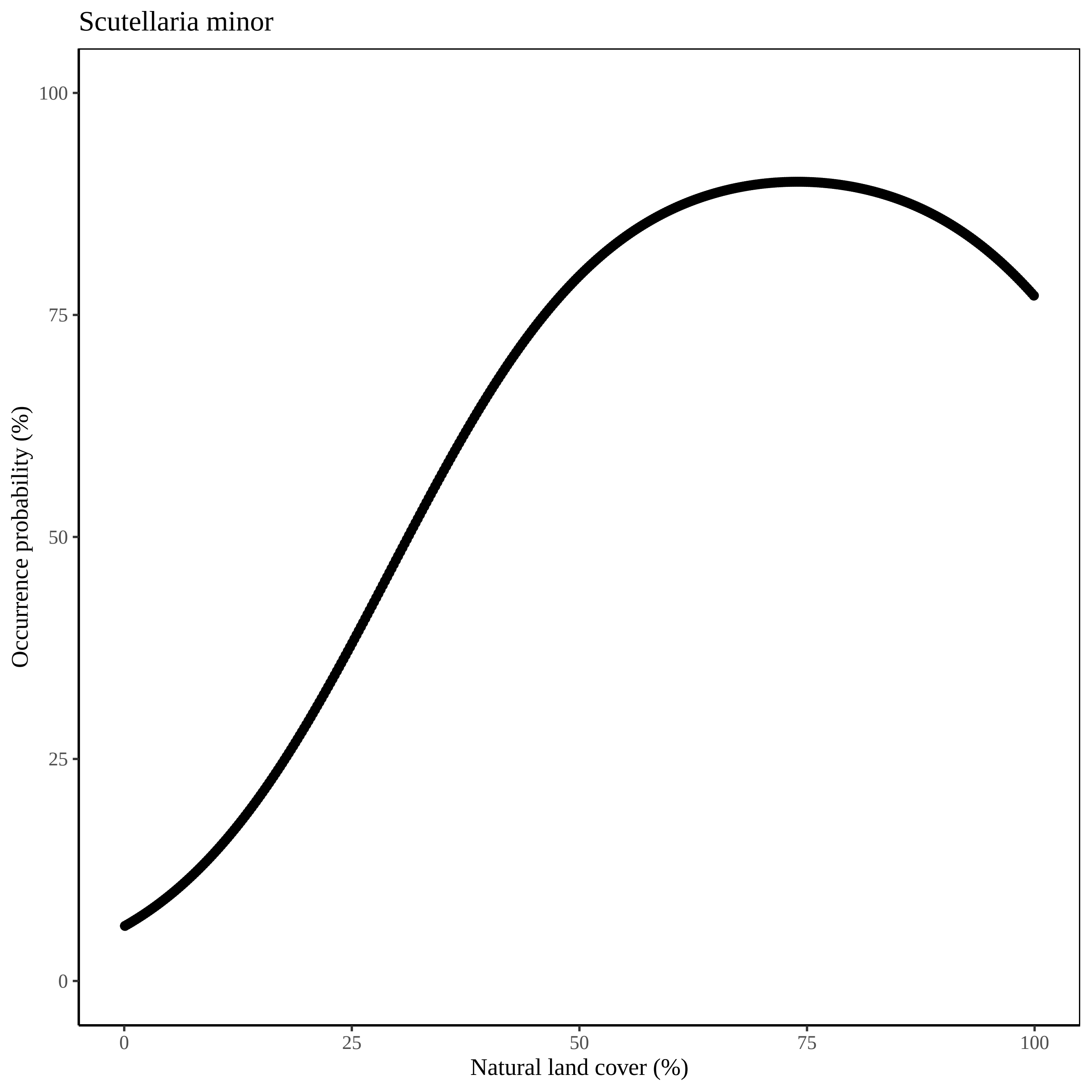
Scutellaria minor
100
75
50
25
0
0
25
50
75
100
Occurrence probability (%)
Natural land cover (%)

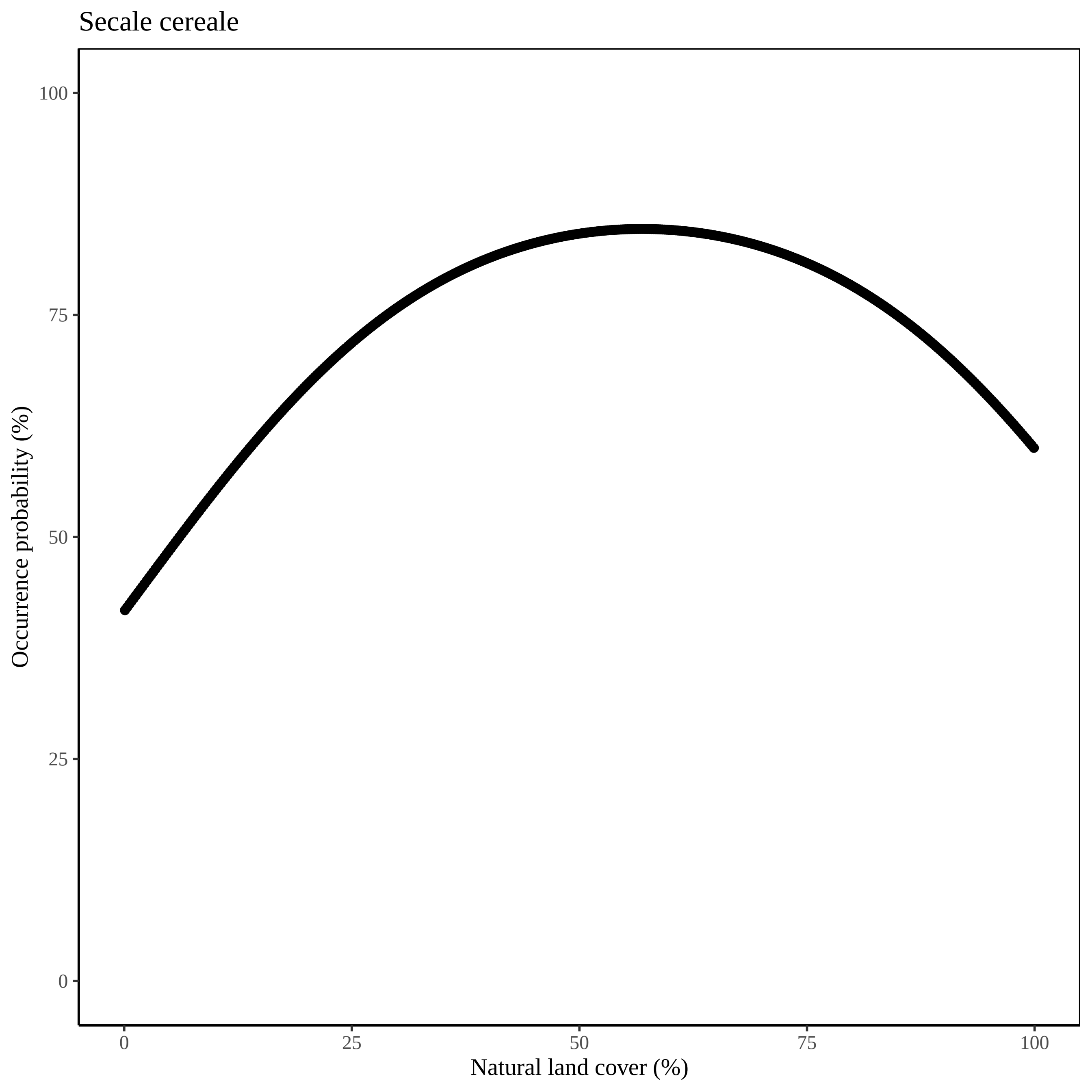
Secale cereale
100
75
50
25
0
Occurrence probability (%)
0
25
50
75
100
Natural land cover (%)

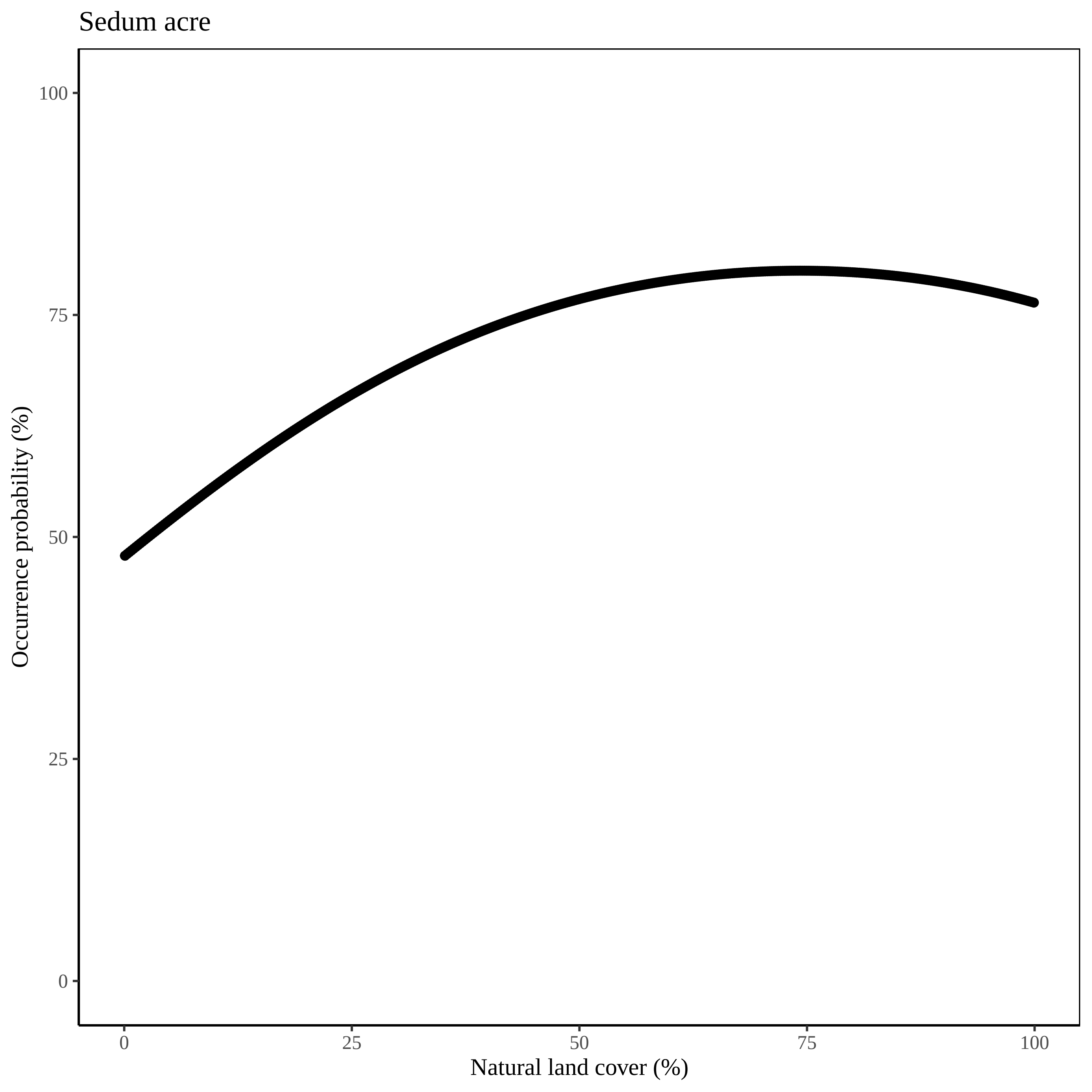
Sedum acre
Occurrence probability (%)
100
75
50
25
0
0
25
50
75
100
Natural land cover (%)

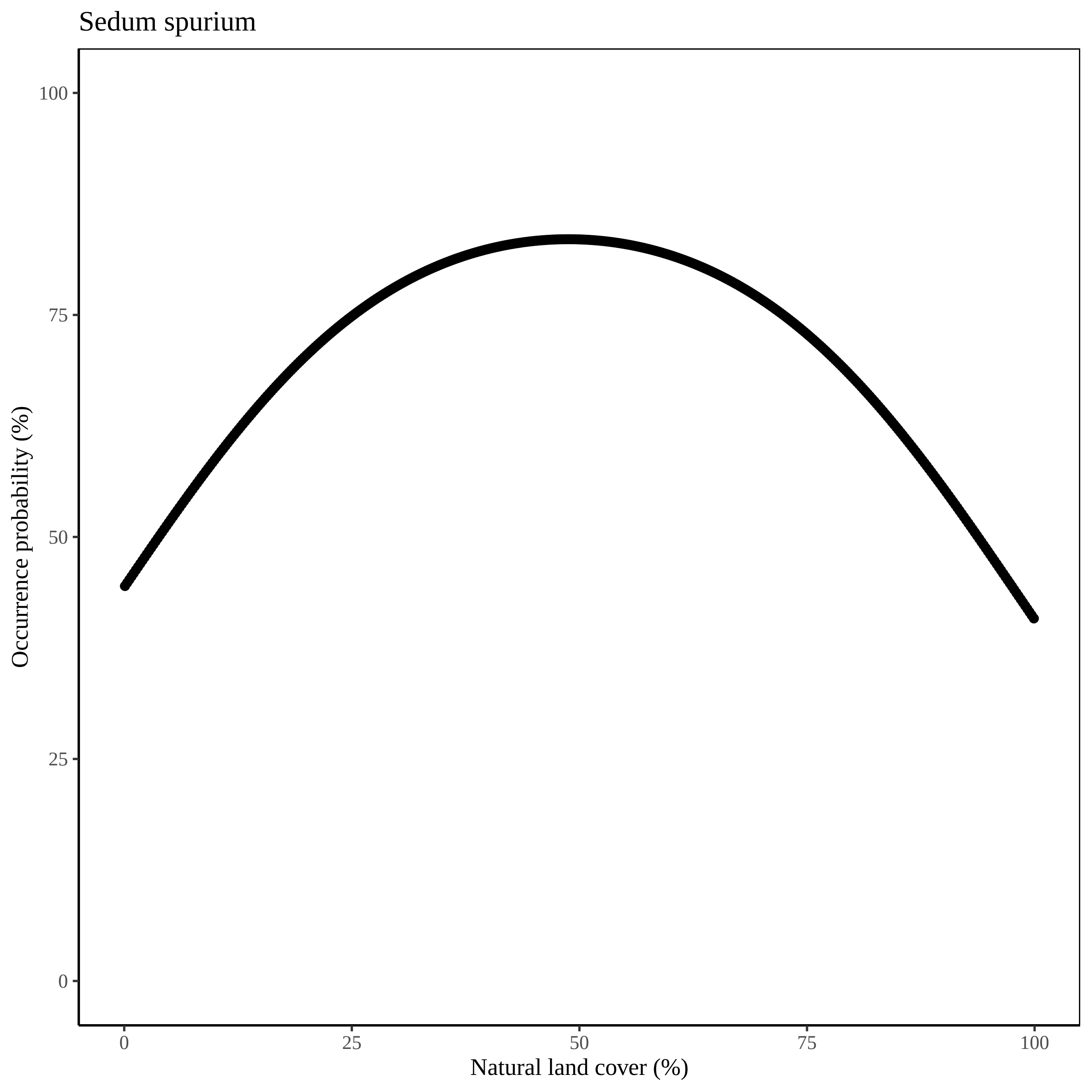

Sedum spurium
Occurrence probability (%)
100
75
50
25
0
0
25
50
75
100
Natural land cover (%)

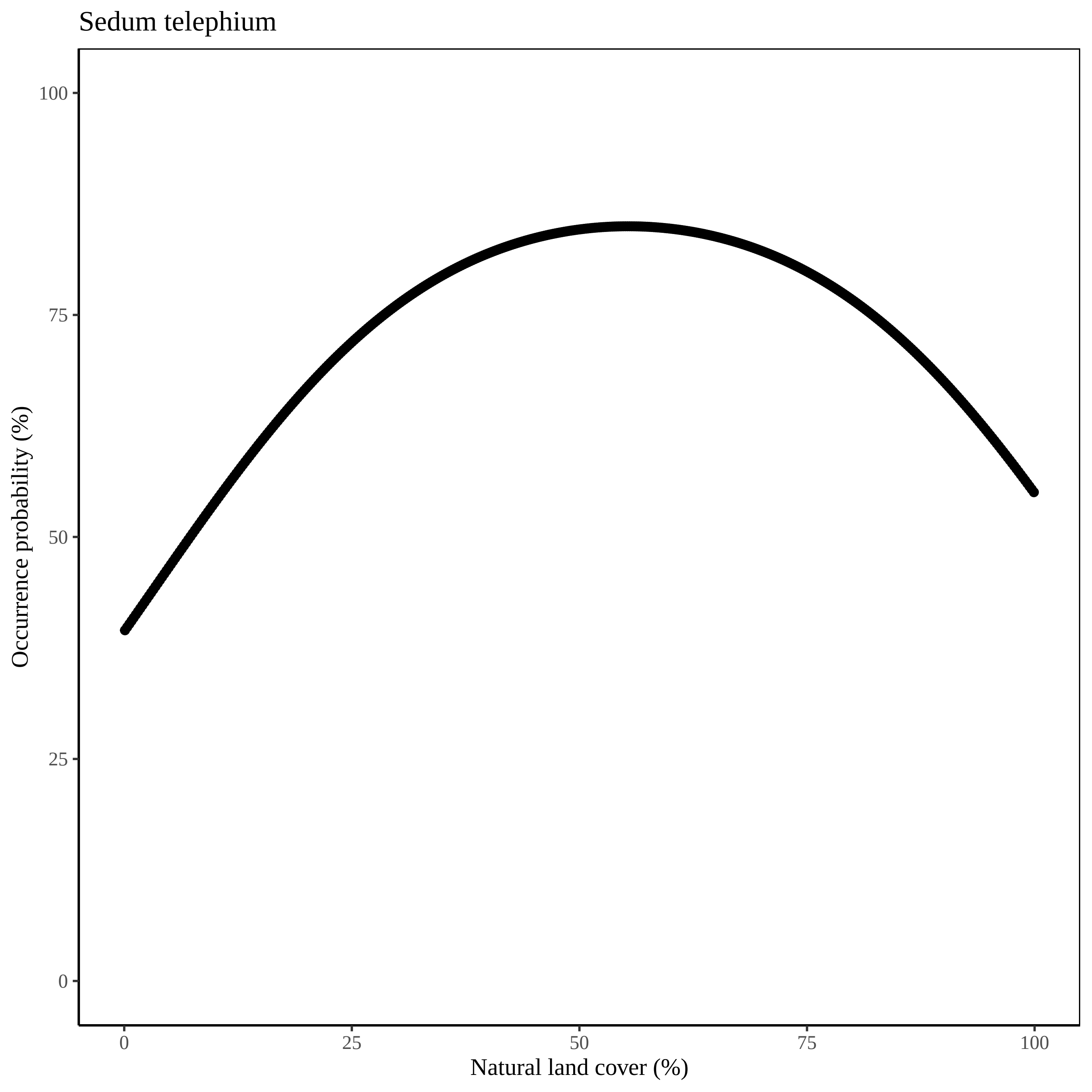

Sedum telephium
Occurrence probability (%)
100
75
50
25
0
0
25
50
75
100
Natural land cover (%)

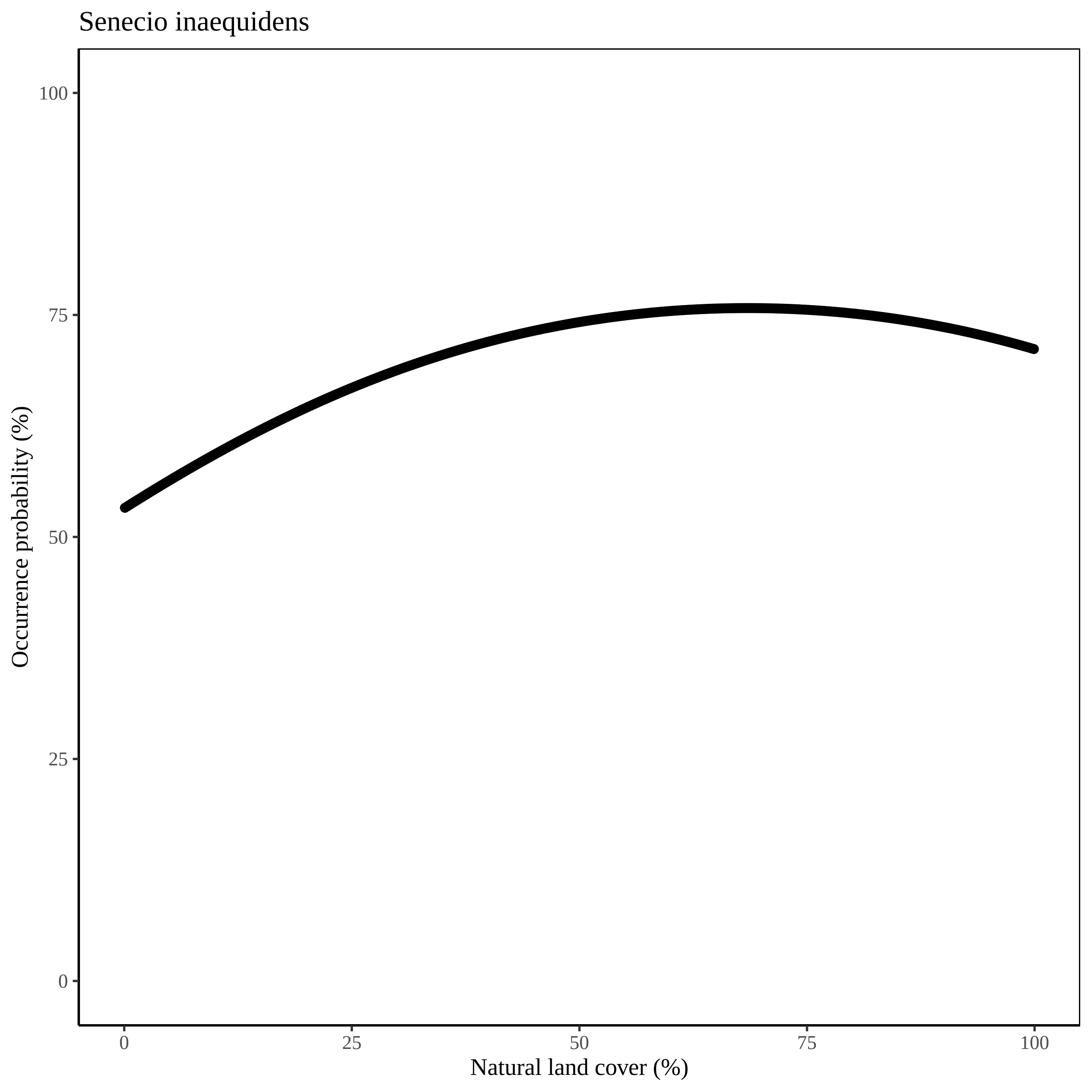

Senecio inaequidens
Occurrence probability (%)
100
75
50
25
0
0
25
50
75
100
Natural land cover (%)

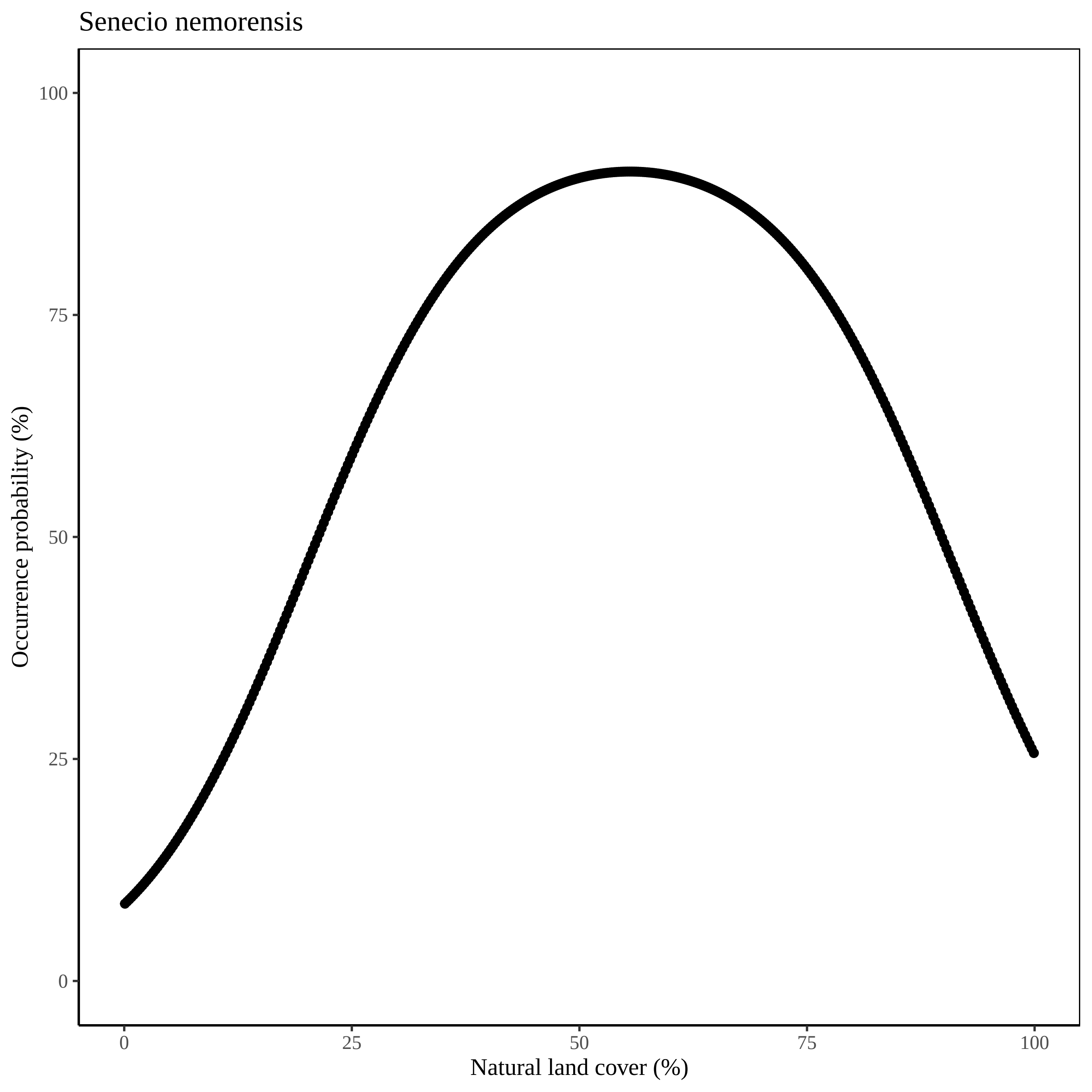
Senecio nemorensis
Occurrence probability (%)
100
75
50
25
0
0
25
50
75
100
Natural land cover (%)

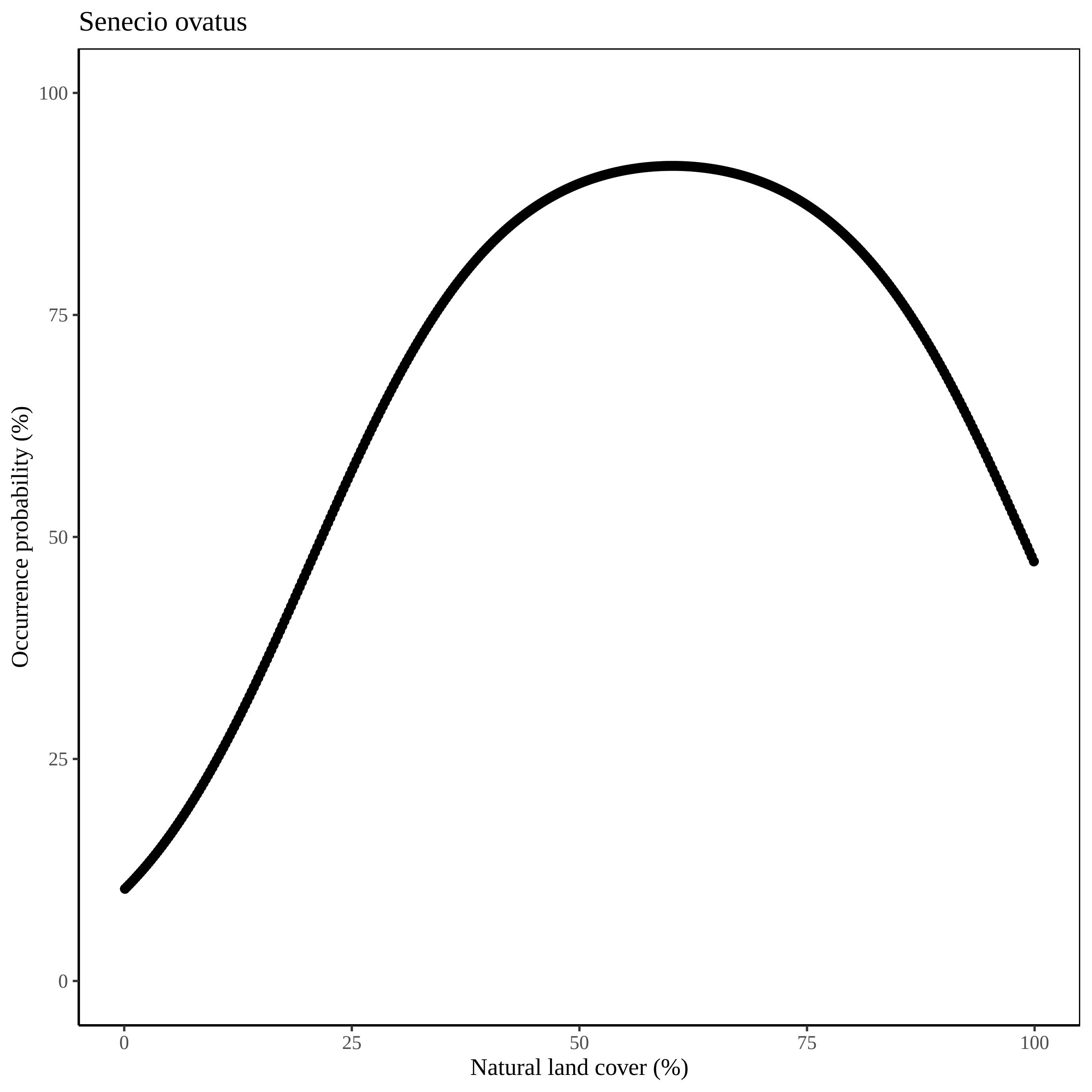

Senecio ovatus
Occurrence probability (%)
100
75
50
25
0
0
25
50
75
100
Natural land cover (%)

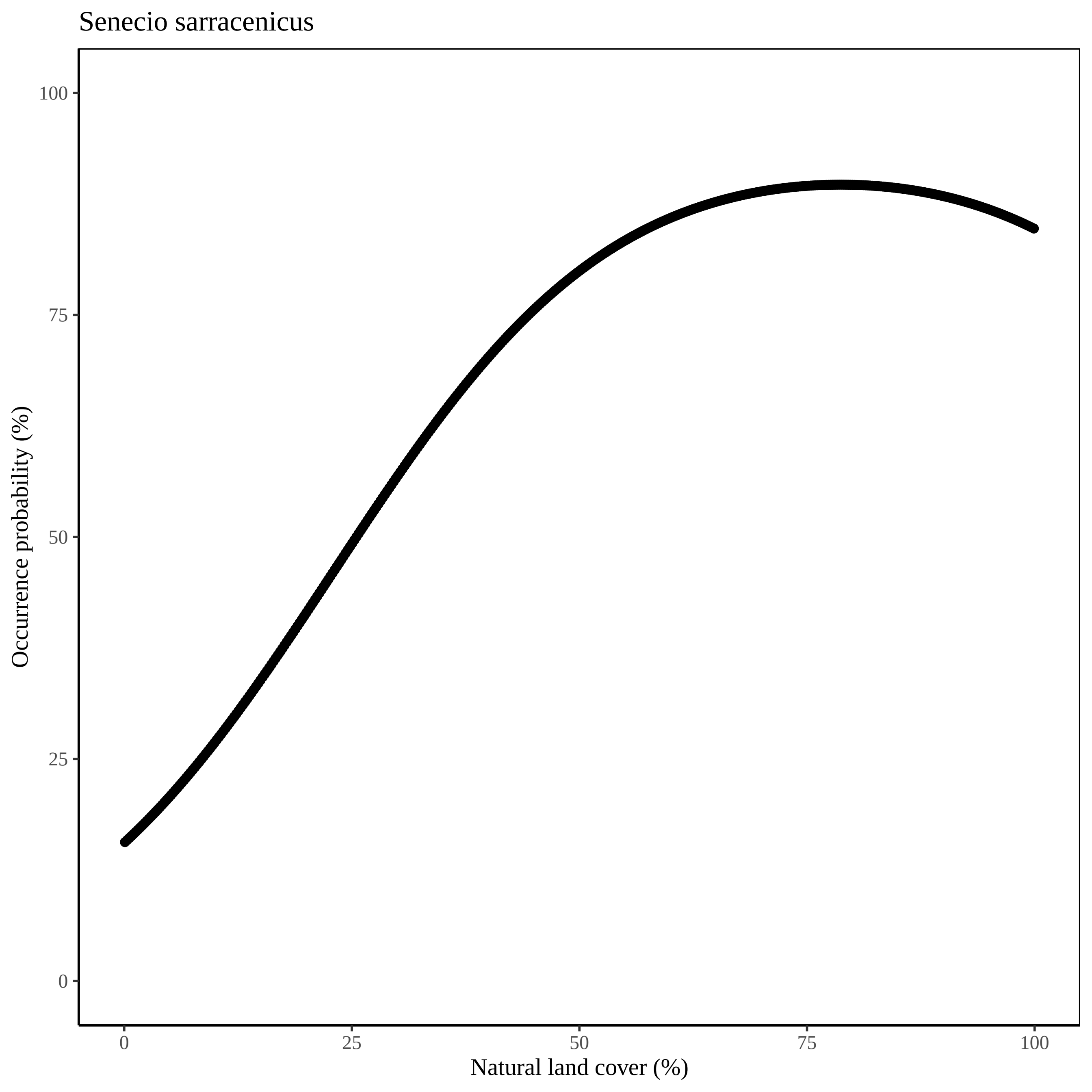
Senecio sarracenicus
Occurrence probability (%)
100
75
50
25
0
0
25
50
75
100
Natural land cover (%)

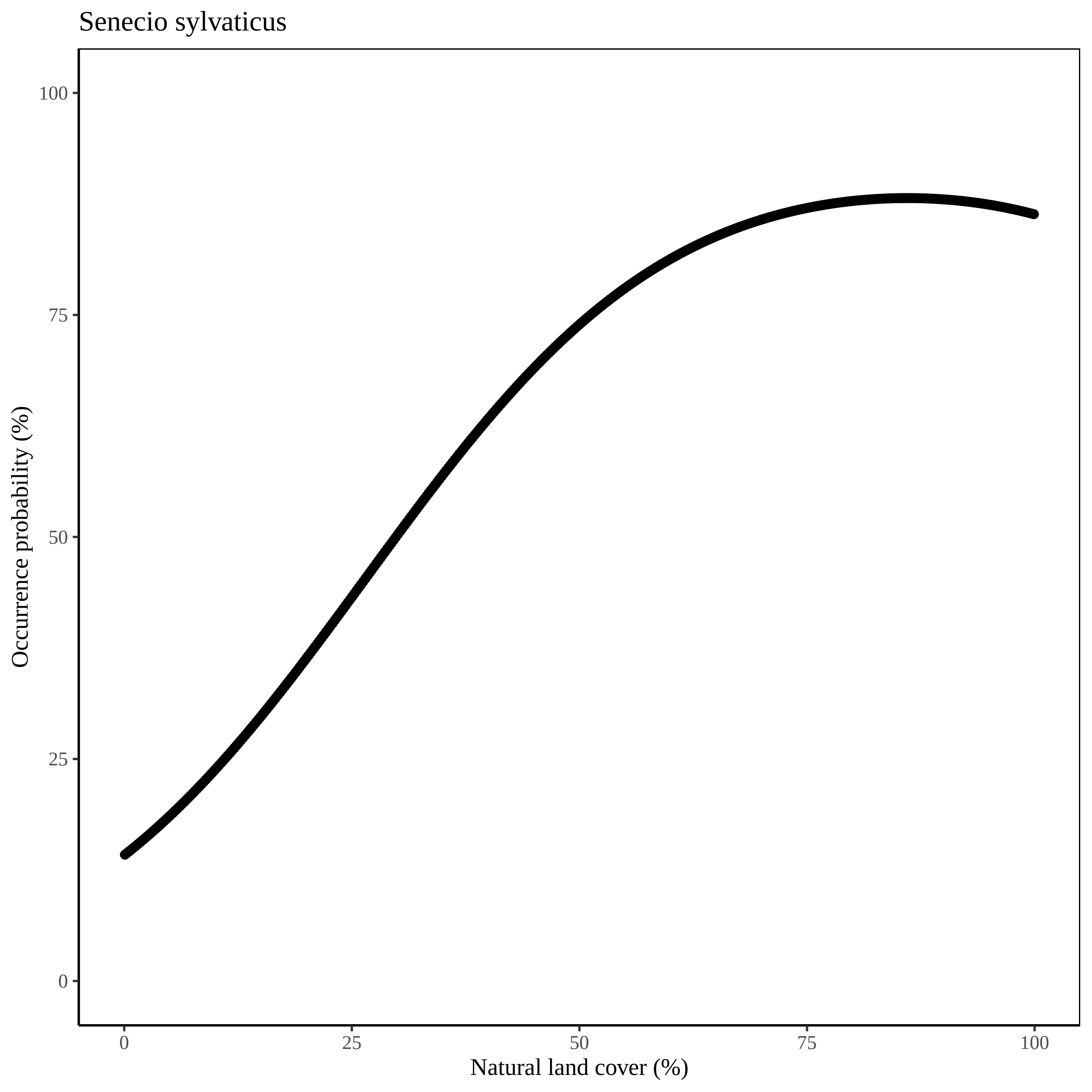
Senecio sylvaticus
Occurrence probability (%)
100
75
50
25
0
0
25
50
75
100
Natural land cover (%)

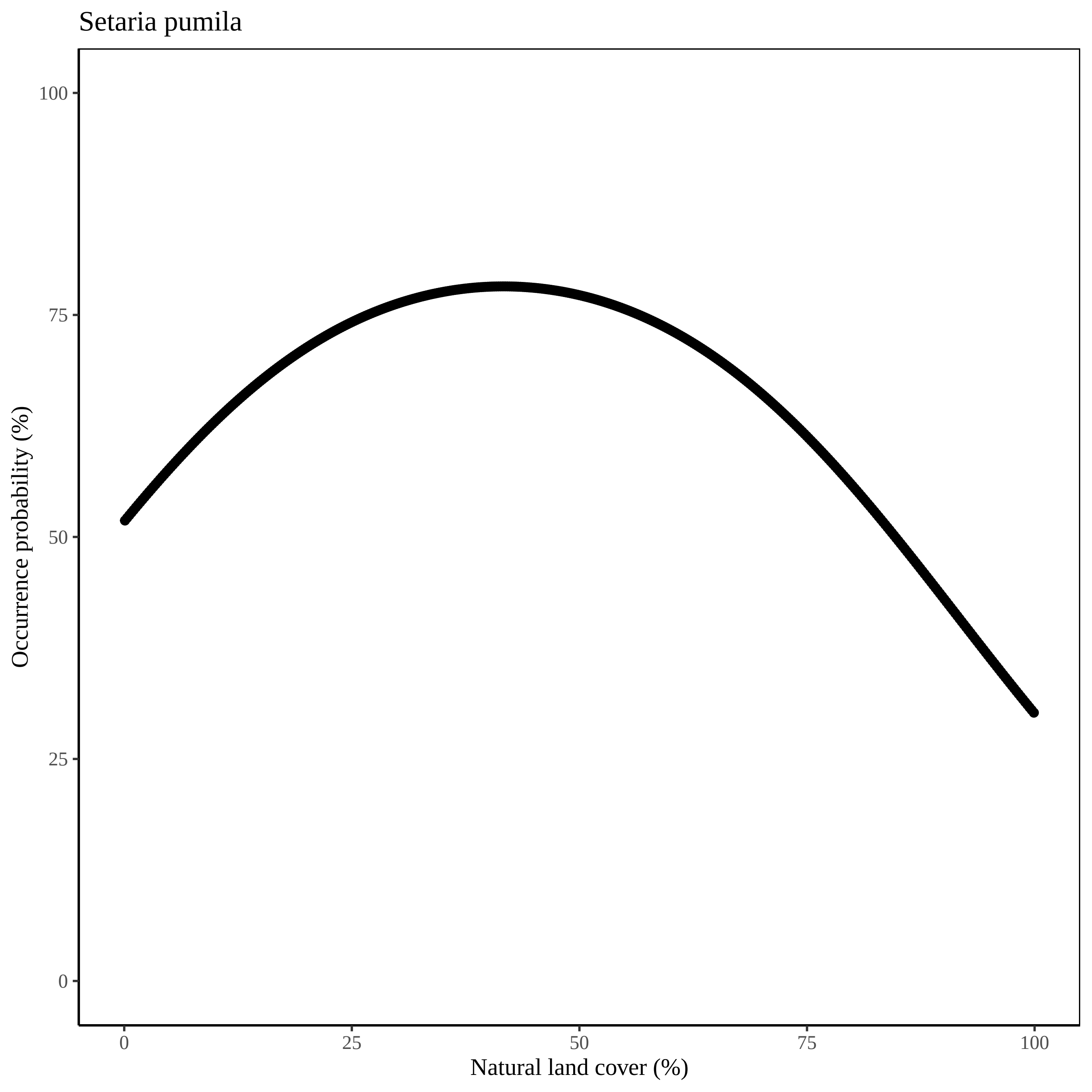
Setaria pumila
Occurrence probability (%)
Natural land cover (%)
0
25
50
75
100

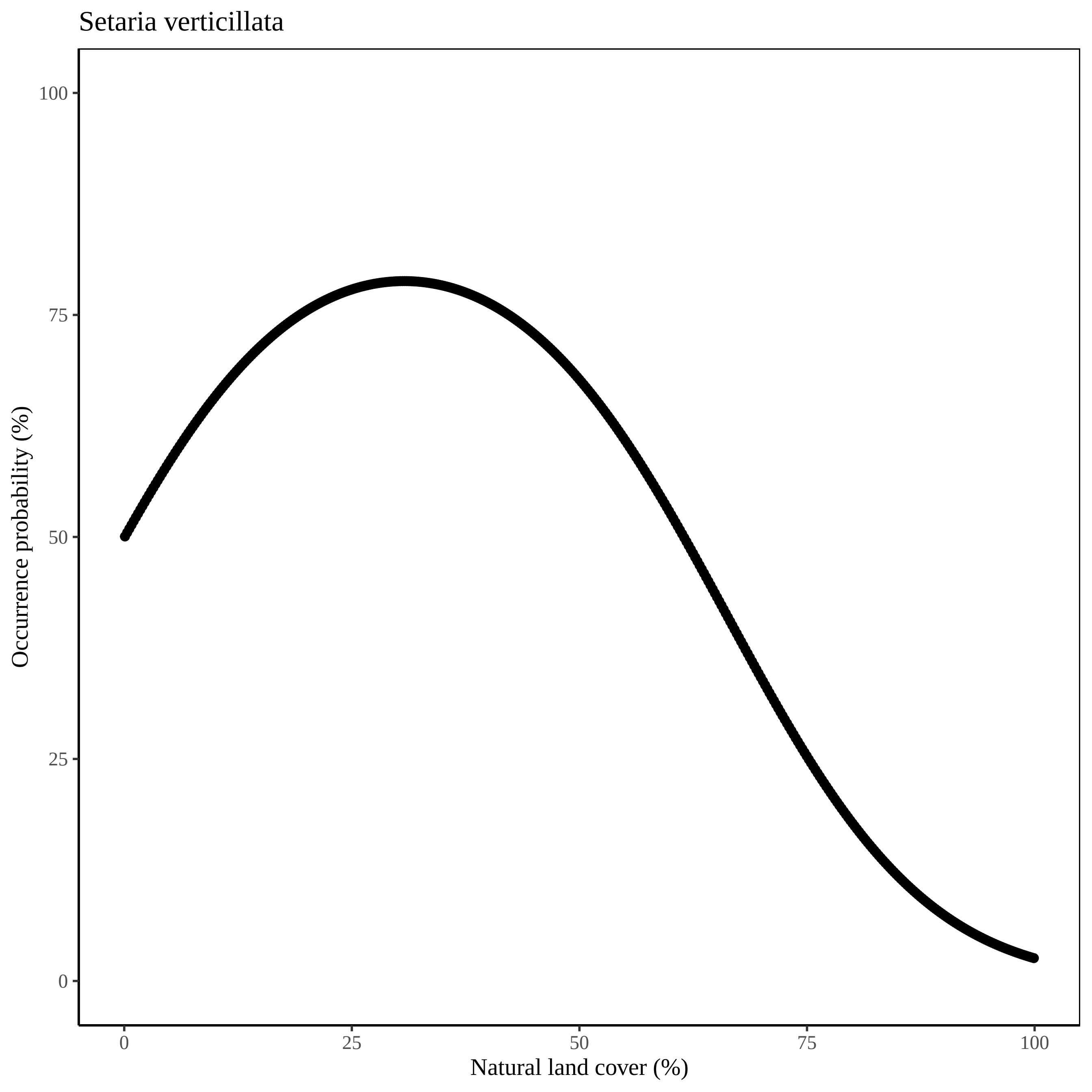
Setaria verticillata
Occurrence probability (%)
100
75
50
25
0
0
25
50
75
100
Natural land cover (%)

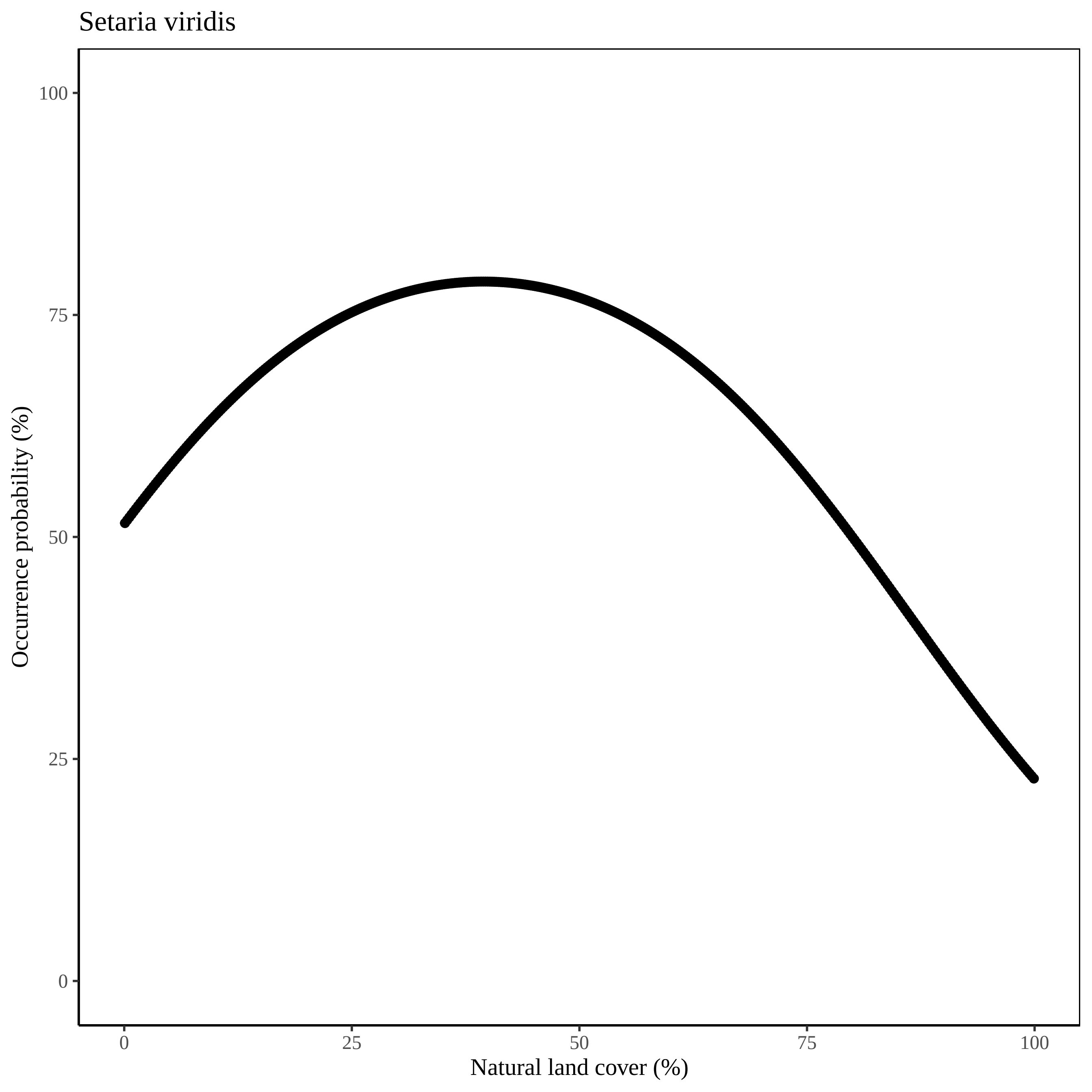
Setaria viridis
Occurrence probability (%)
100
75
50
25
0
0
25
50
75
100
Natural land cover (%)

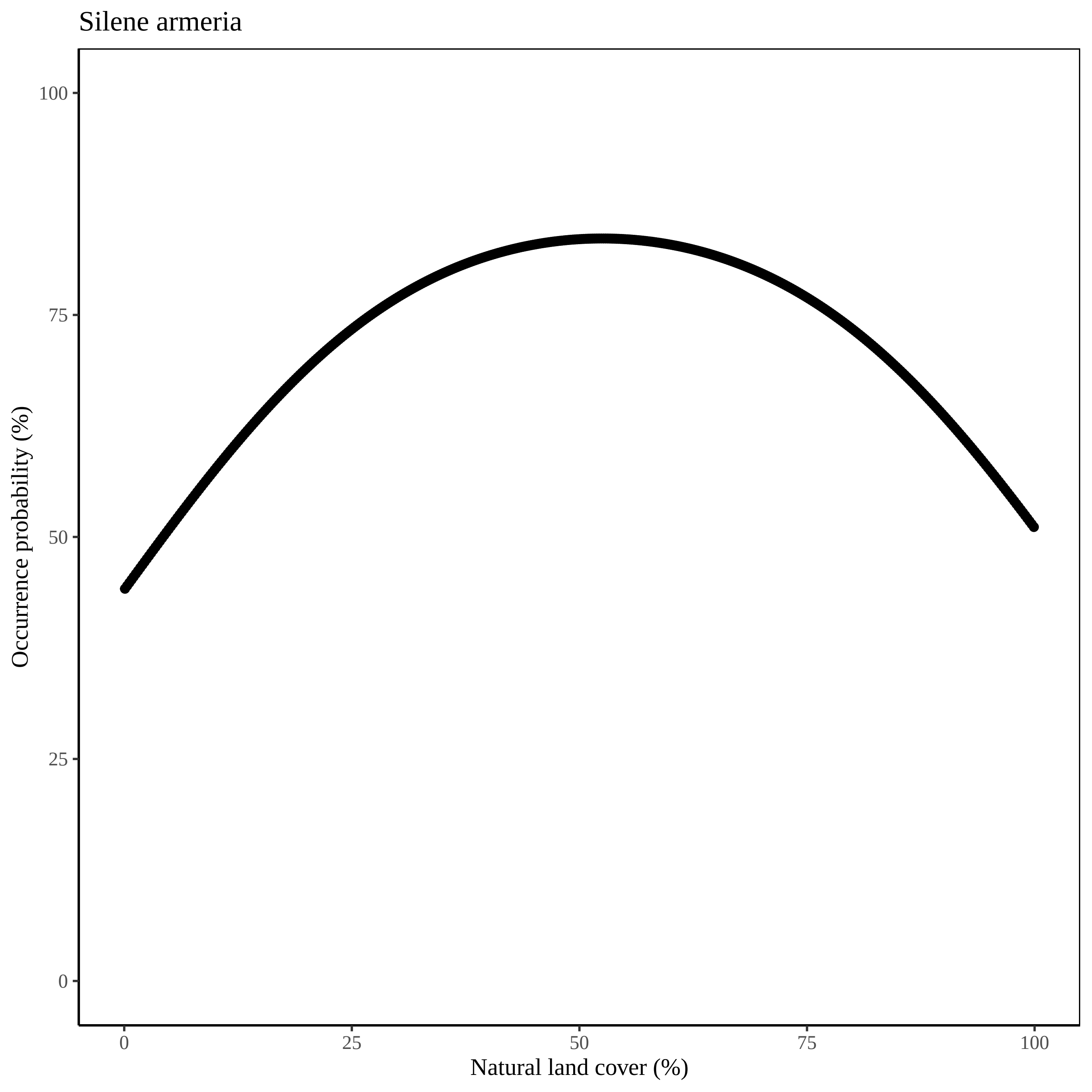
Silene armeria
Occurrence probability (%)
100
75
50
25
0
0
25
50
75
100
Natural land cover (%)

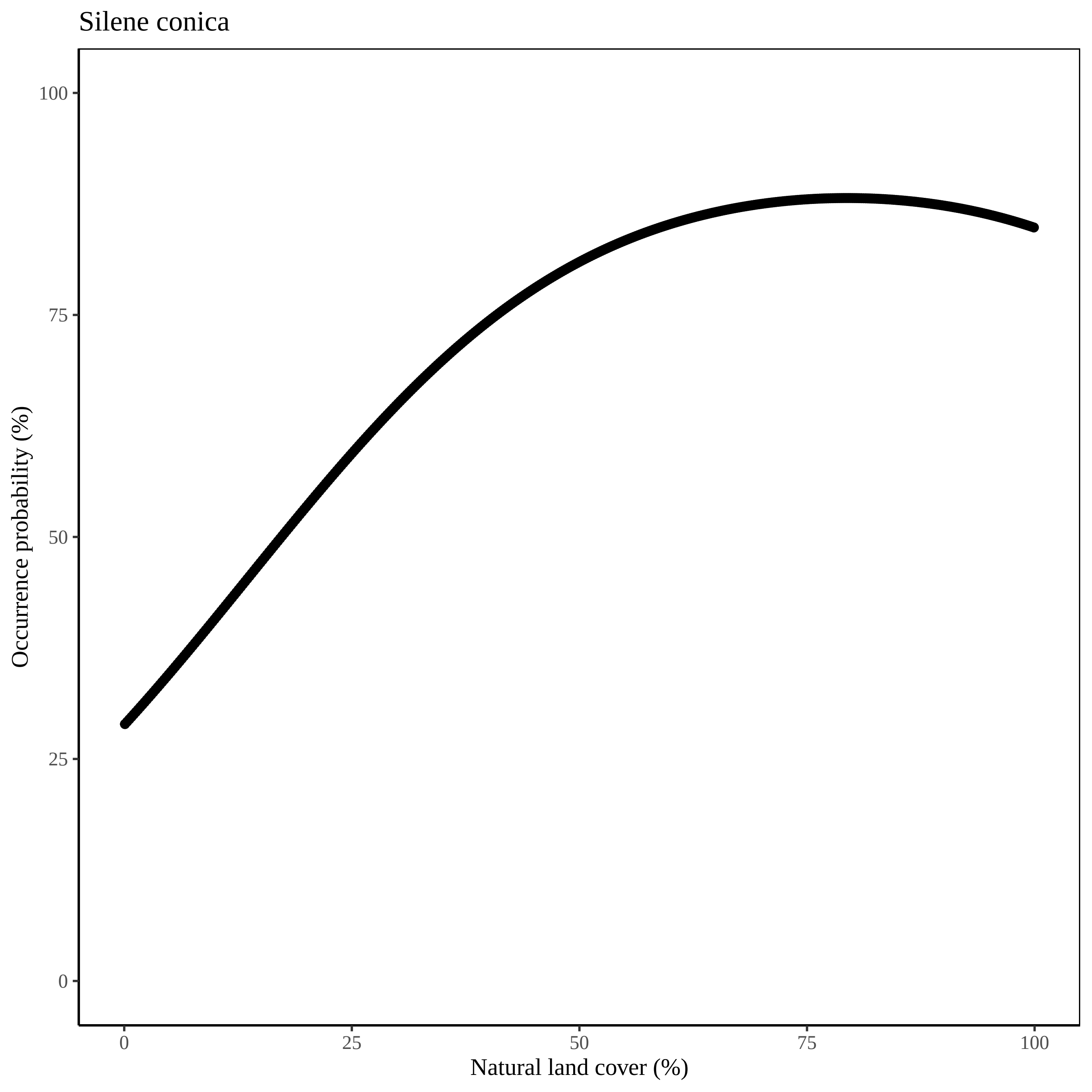
Silene conica
Occurrence probability (%)
100
75
50
25
0
0
25
50
75
100
Natural land cover (%)

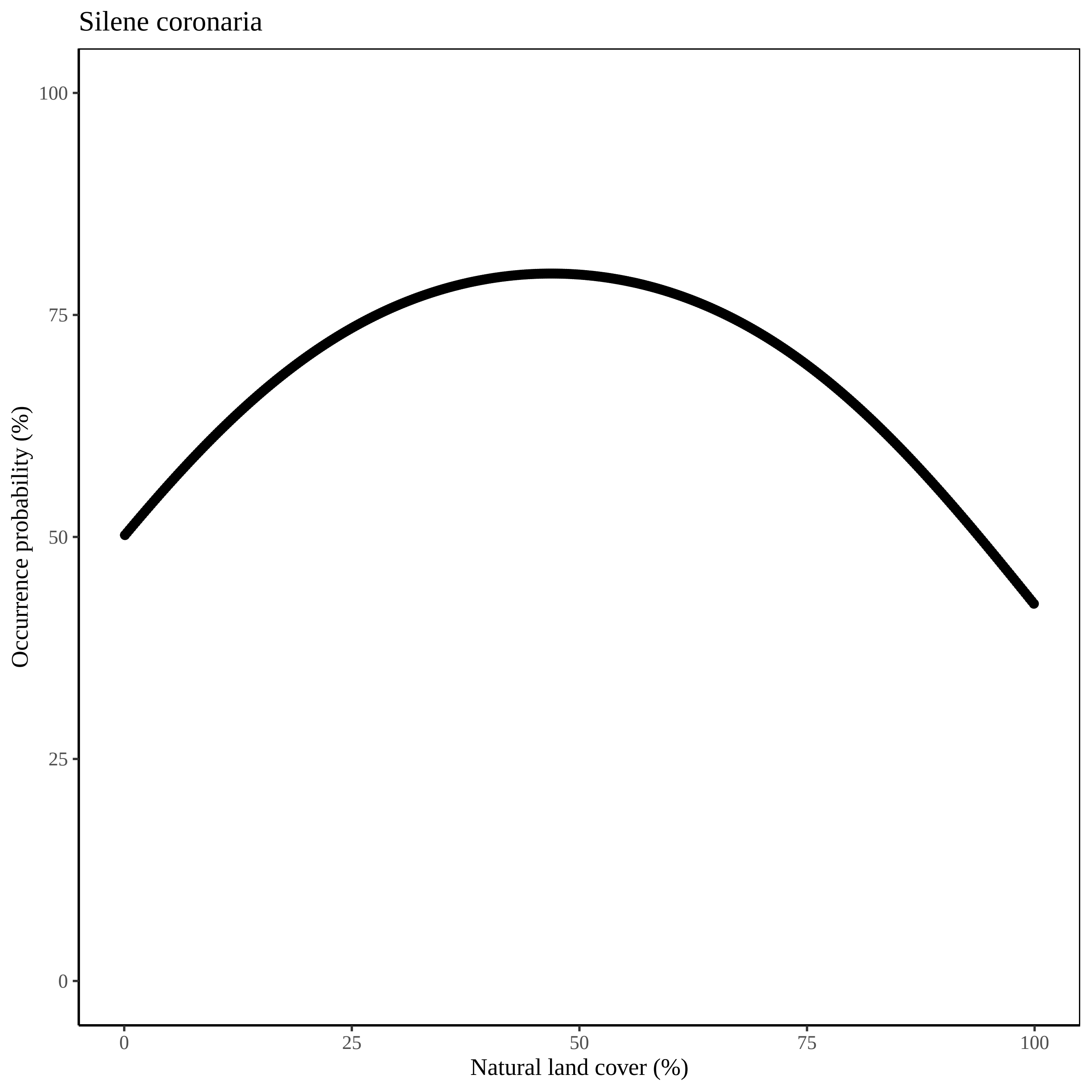

Silene coronaria
Occurrence probability (%)
100
75
50
25
0
0
25
50
75
100
Natural land cover (%)

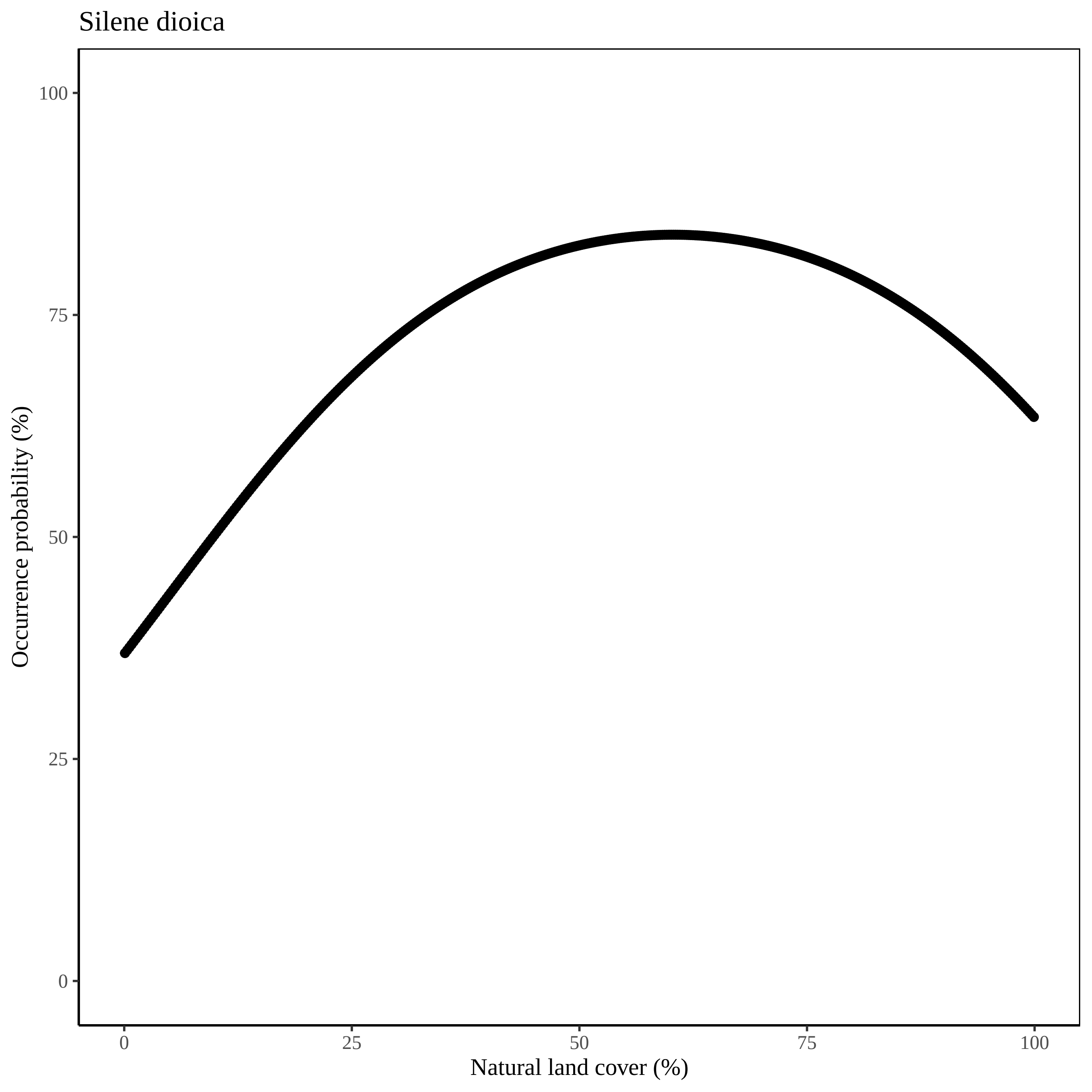
Silene dioica
Occurrence probability (%)
100
75
50
25
0
0
25
50
75
100
Natural land cover (%)

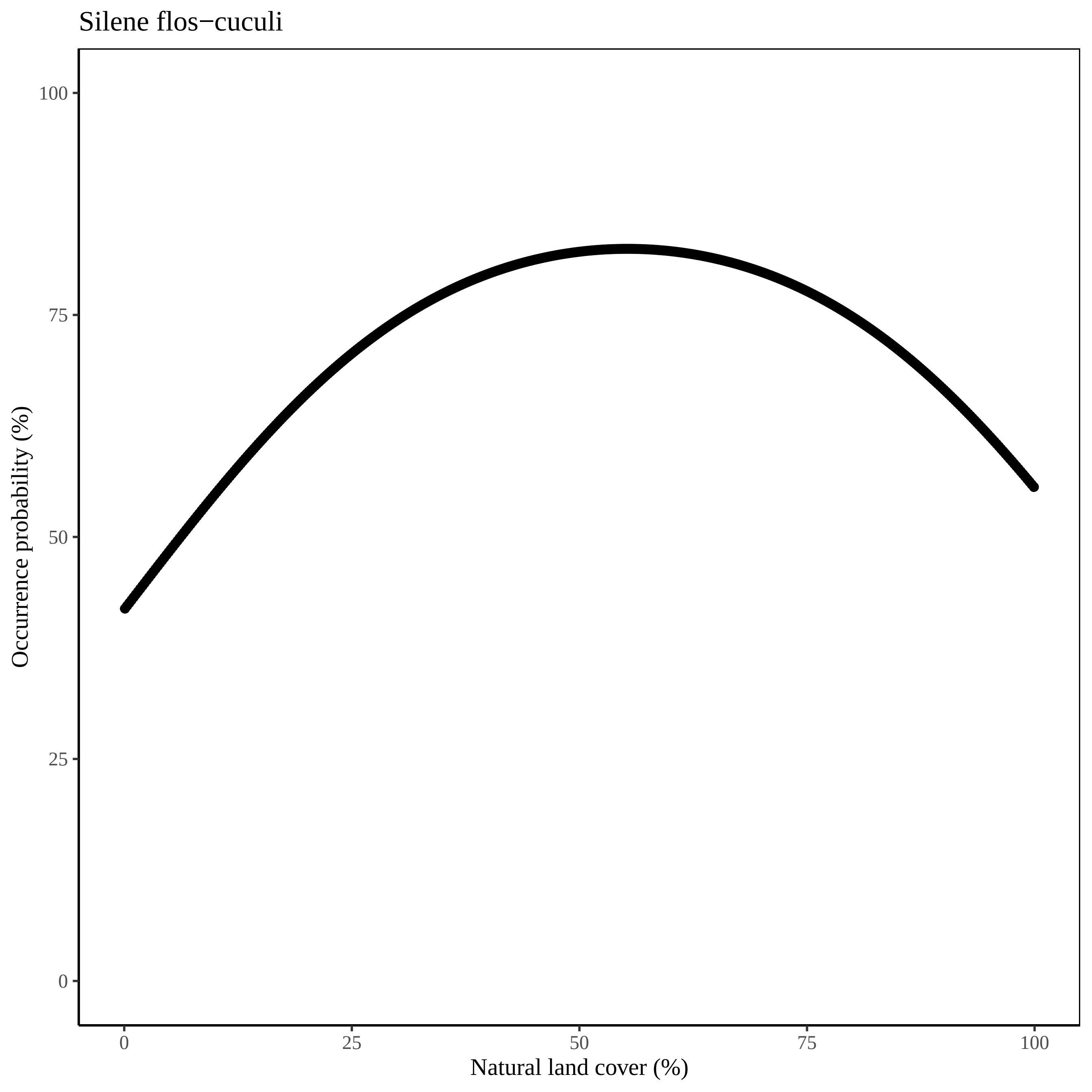
Silene flos−cuculi
Occurrence probability (%)
100
75
50
25
0
0
25
50
75
100
Natural land cover (%)

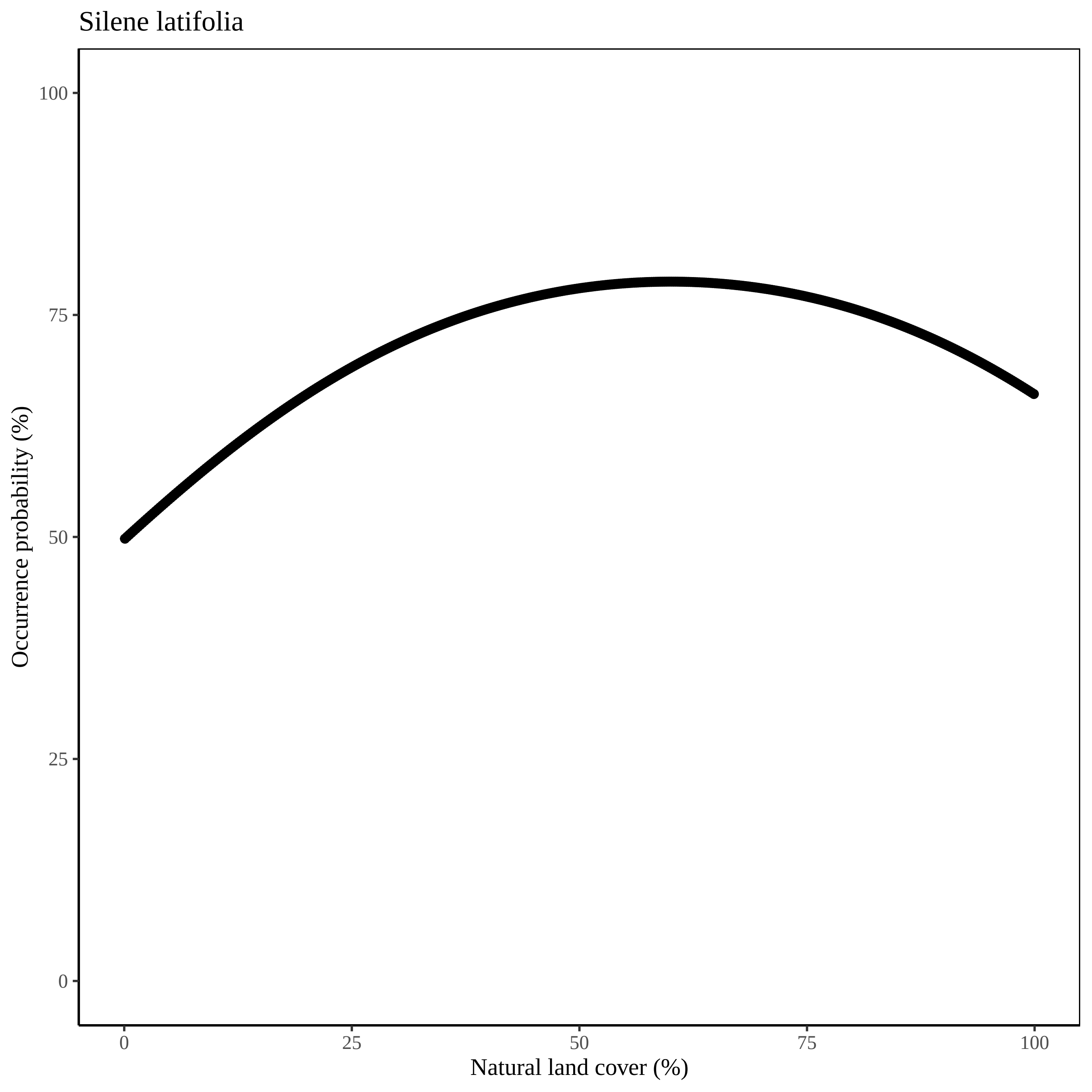
Silene latifolia
Occurrence probability (%)
100
75
50
25
0
0
25
50
75
100
Natural land cover (%)

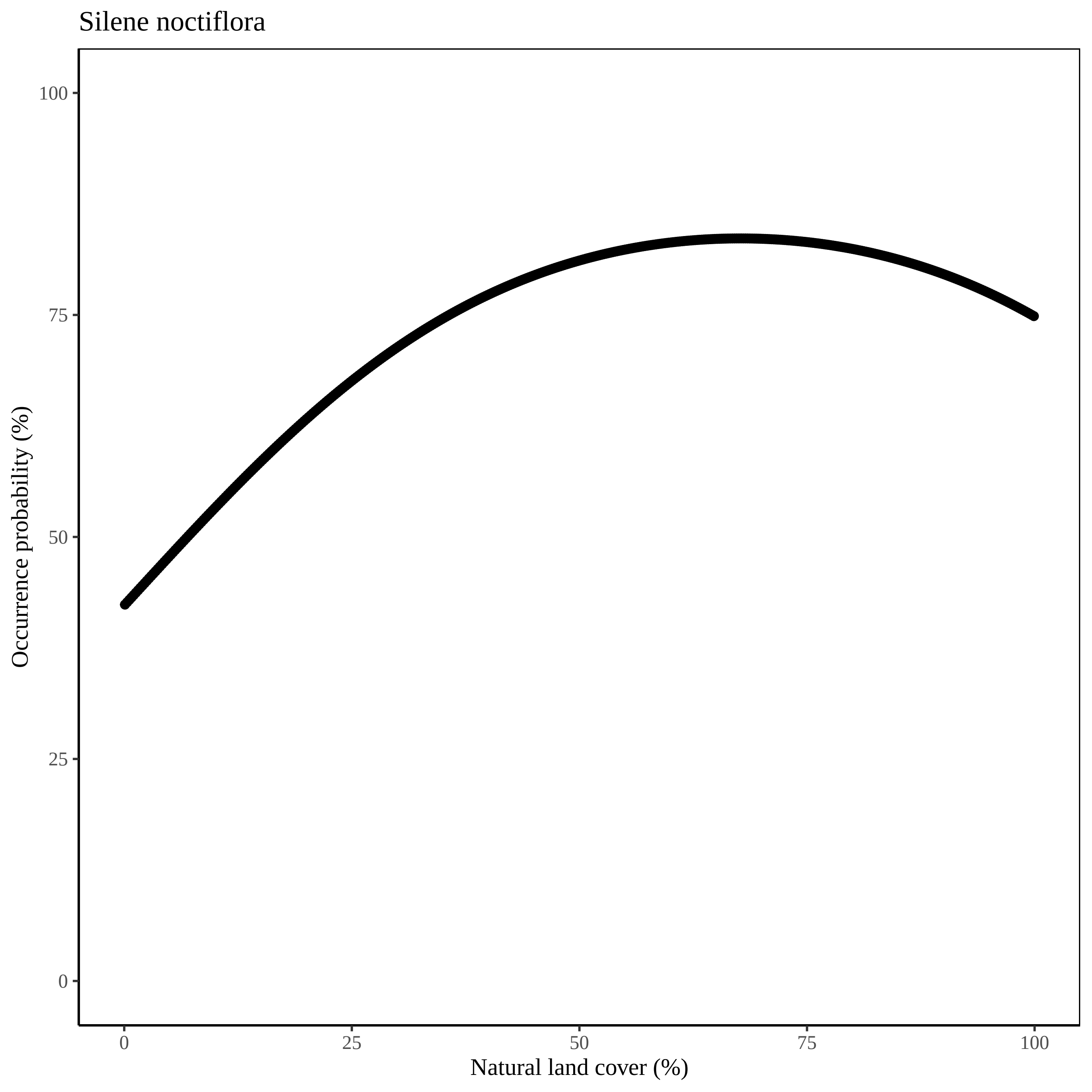
Silene noctiflora
Occurrence probability (%)
100
75
50
25
0
0
25
50
75
100
Natural land cover (%)

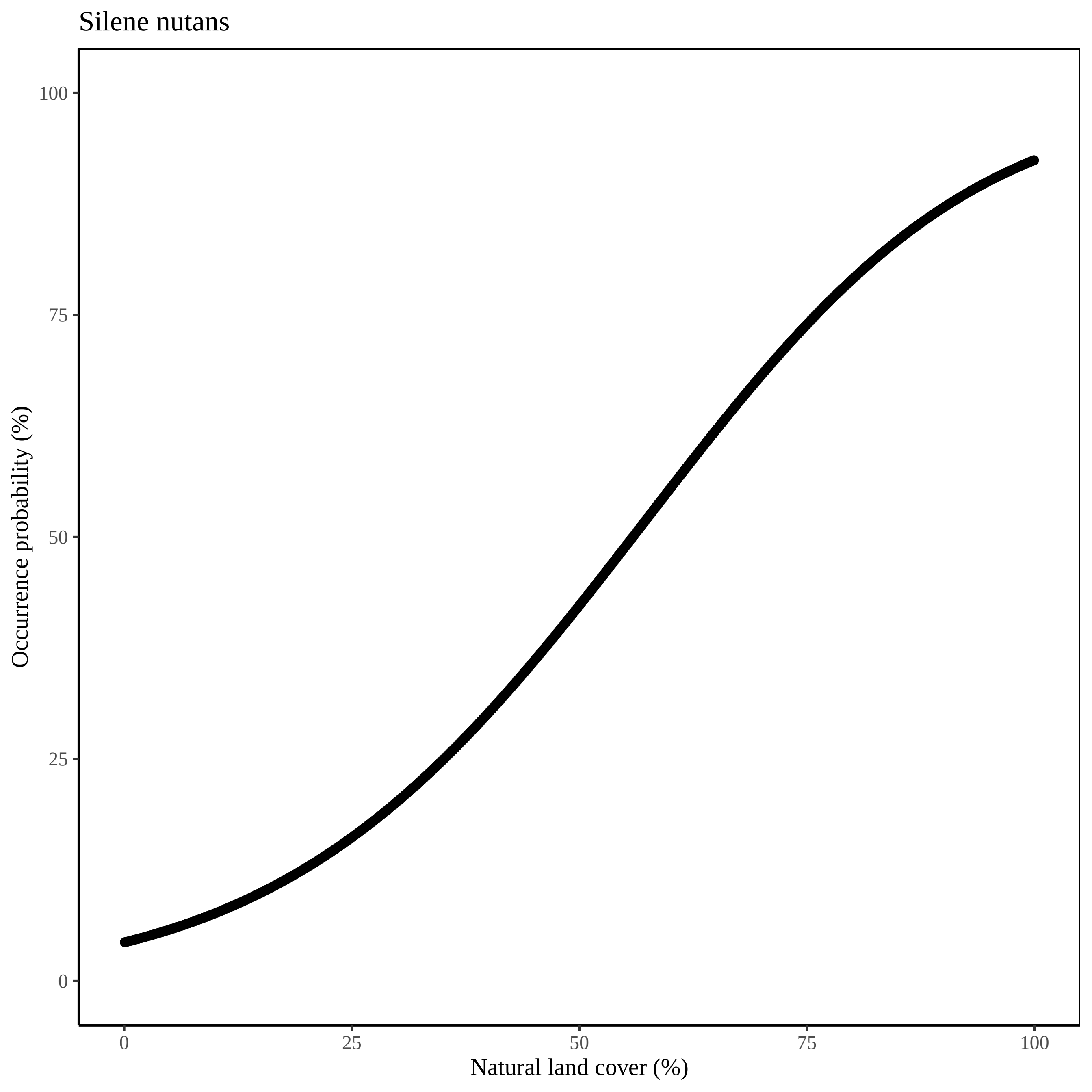
Silene nutans
Occurrence probability (%)
100
75
50
25
0
0
25
50
75
100
Natural land cover (%)

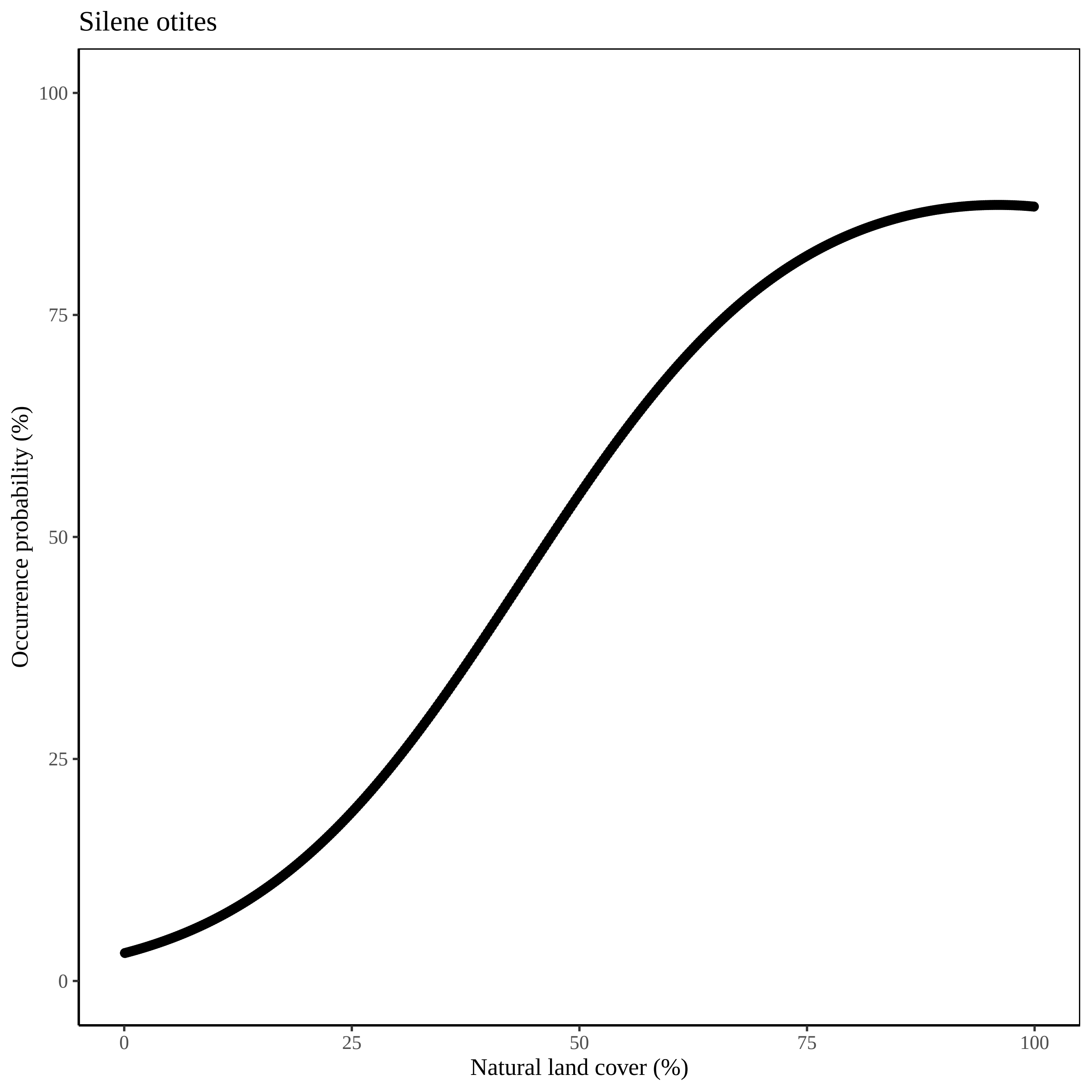
Silene otites
Occurrence probability (%)
100
75
50
25
0
0
25
50
75
100
Natural land cover (%)

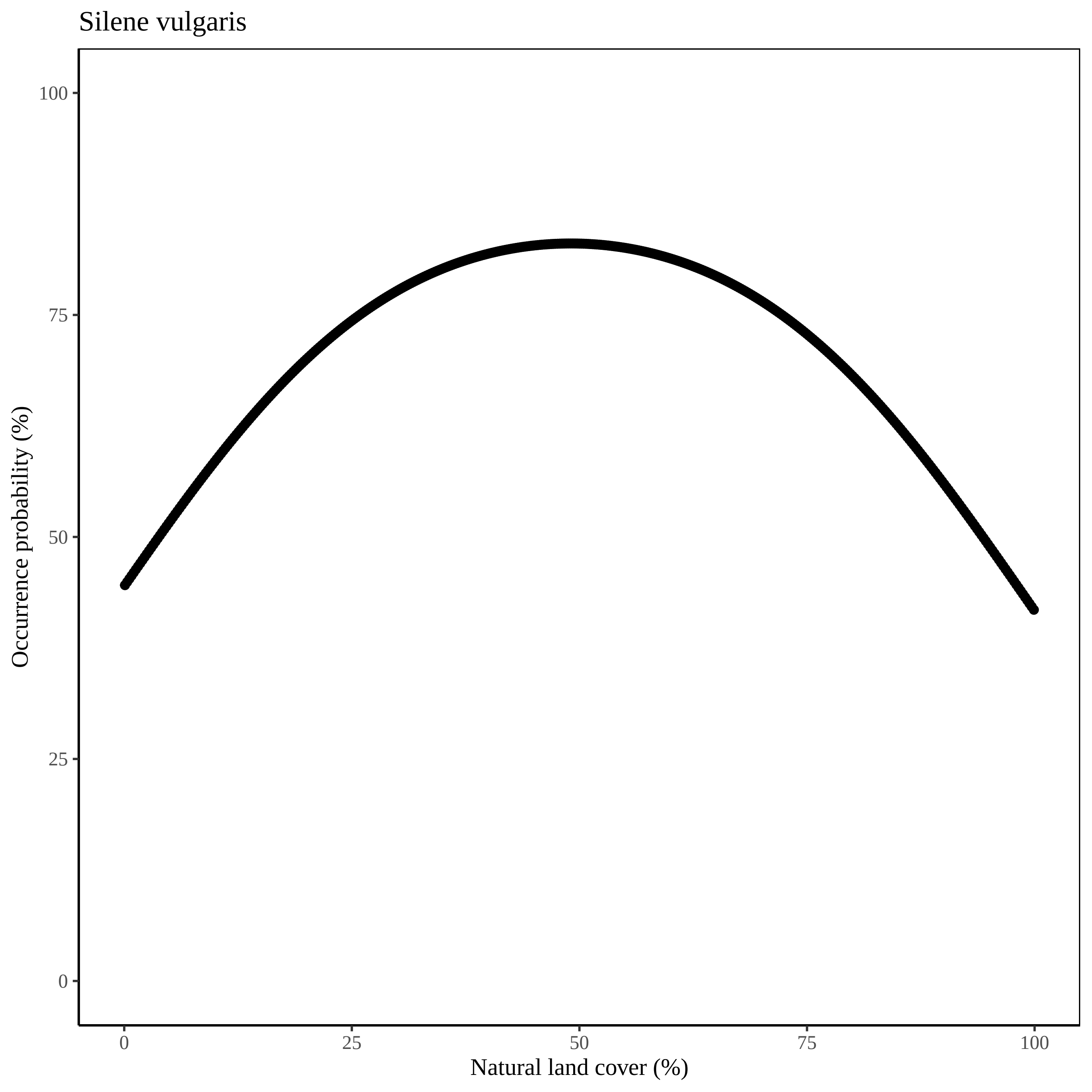
Silene vulgaris
Occurrence probability (%)
100
75
50
25
0
0
25
50
75
100
Natural land cover (%)

Silene x hampeana

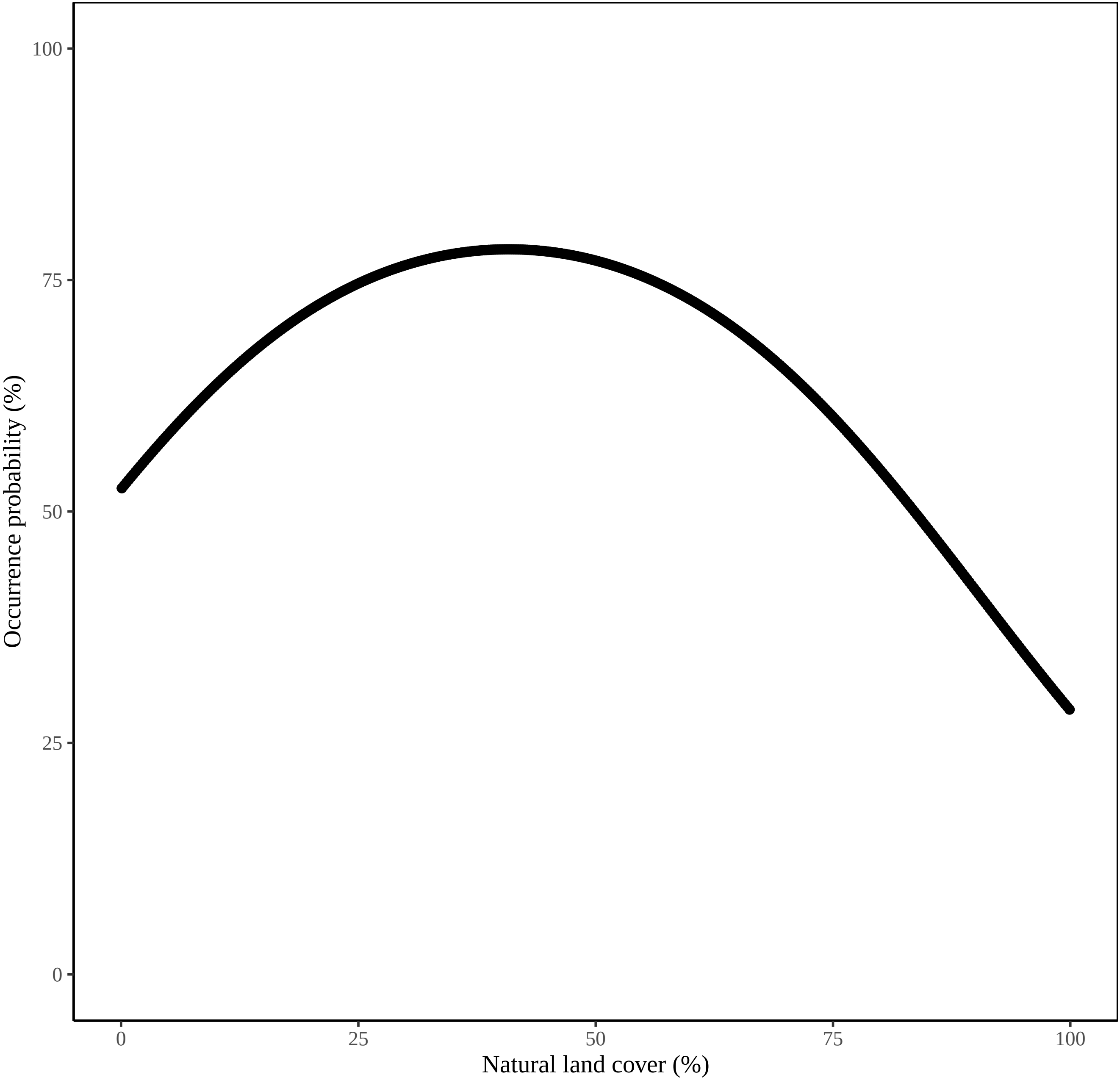

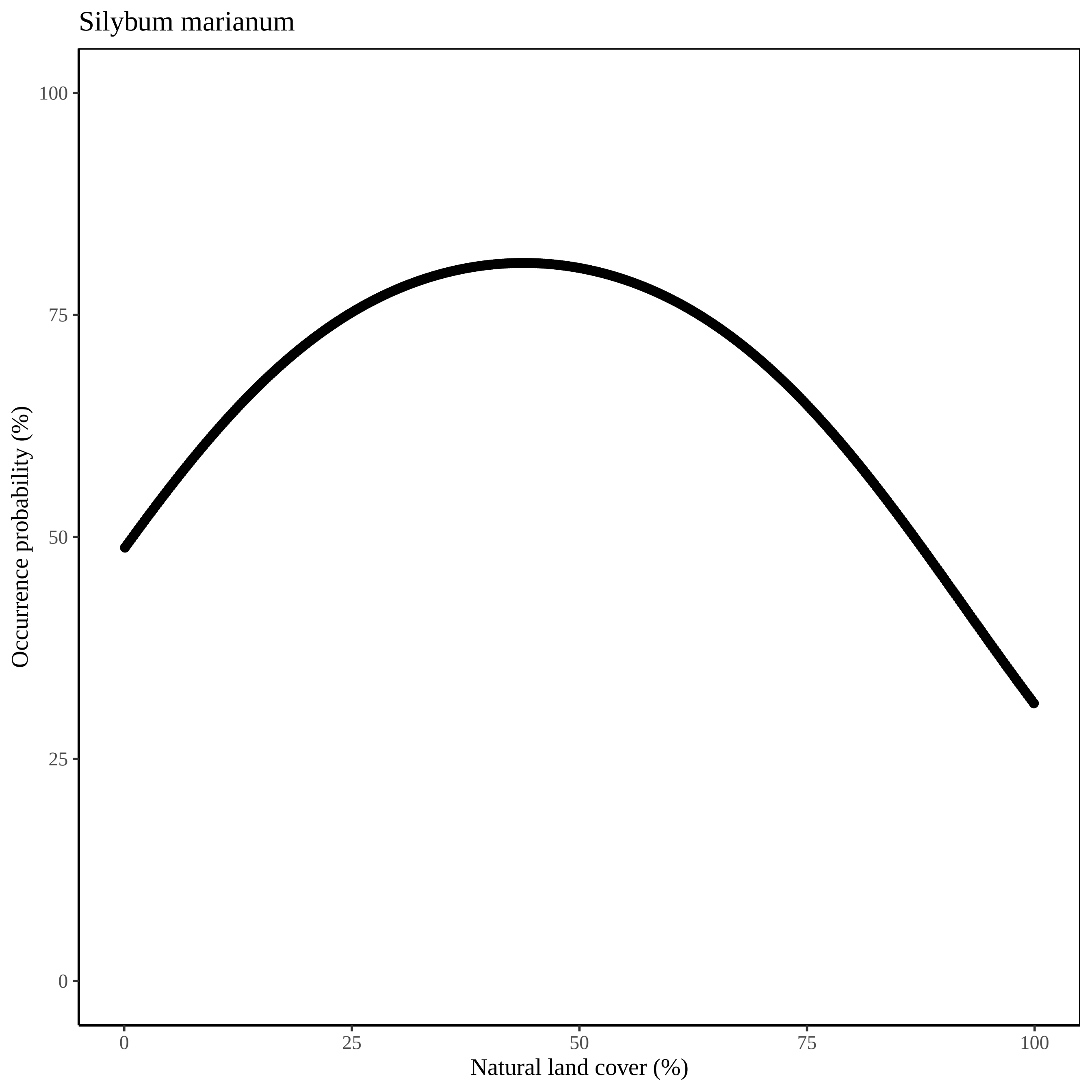
Silybum marianum
Occurrence probability (%)
100
75
50
25
0
0
25
50
75
100
Natural land cover (%)

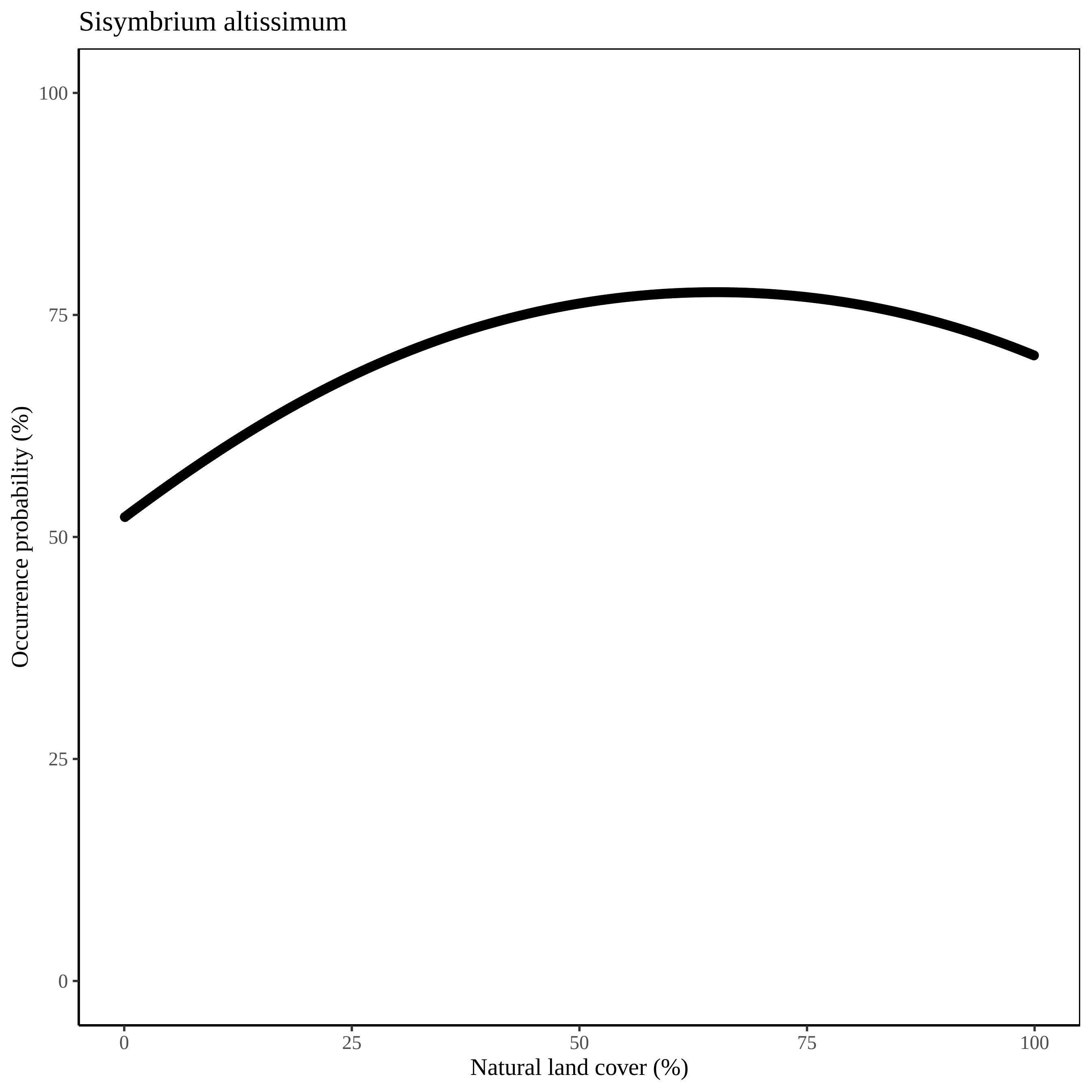
Sisymbrium altissimum
Occurrence probability (%)
100
75
50
25
0
0
25
50
75
100
Natural land cover (%)

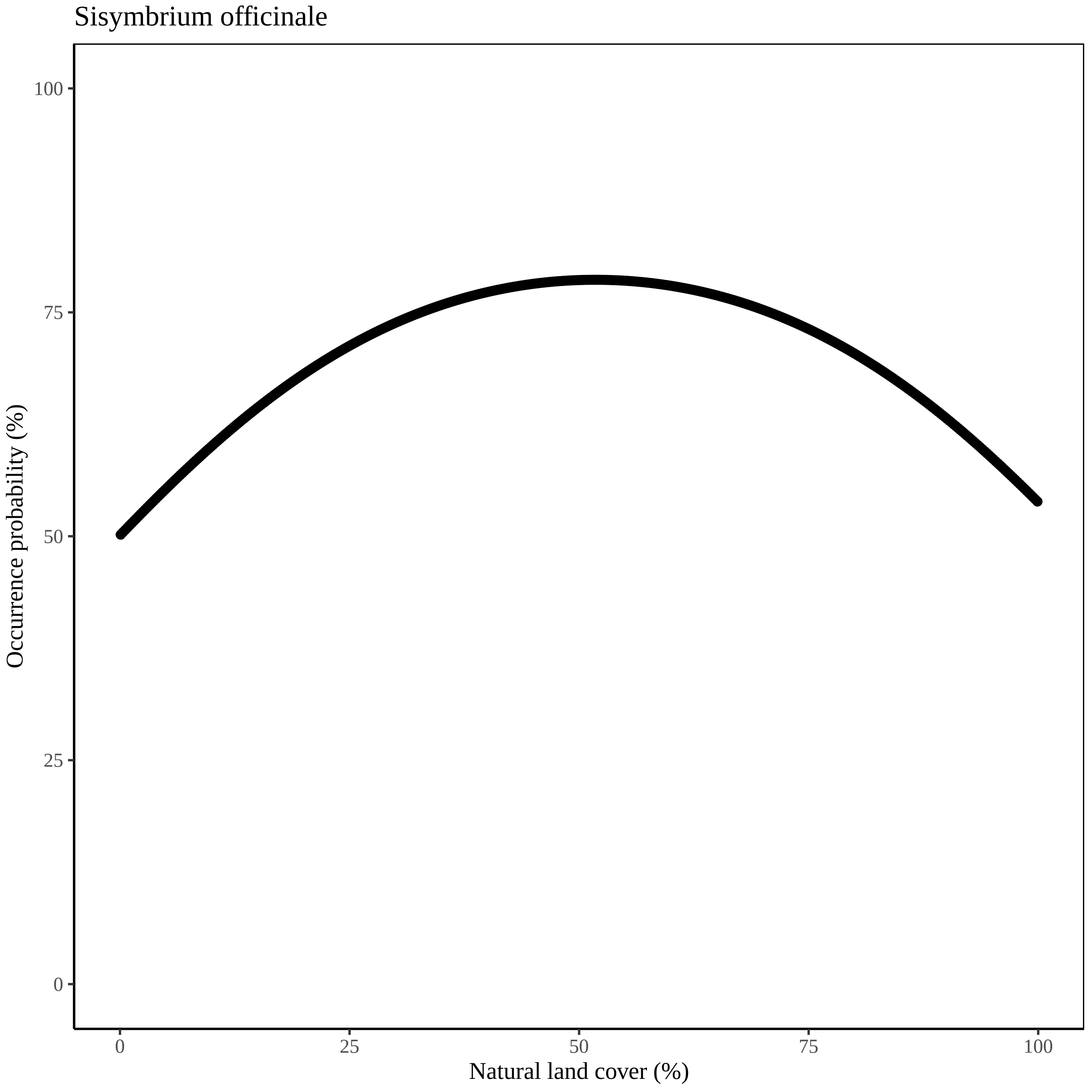
Sisymbrium officinale
100
75
50
25
0
Occurrence probability (%)
0
25
50
75
100
Natural land cover (%)

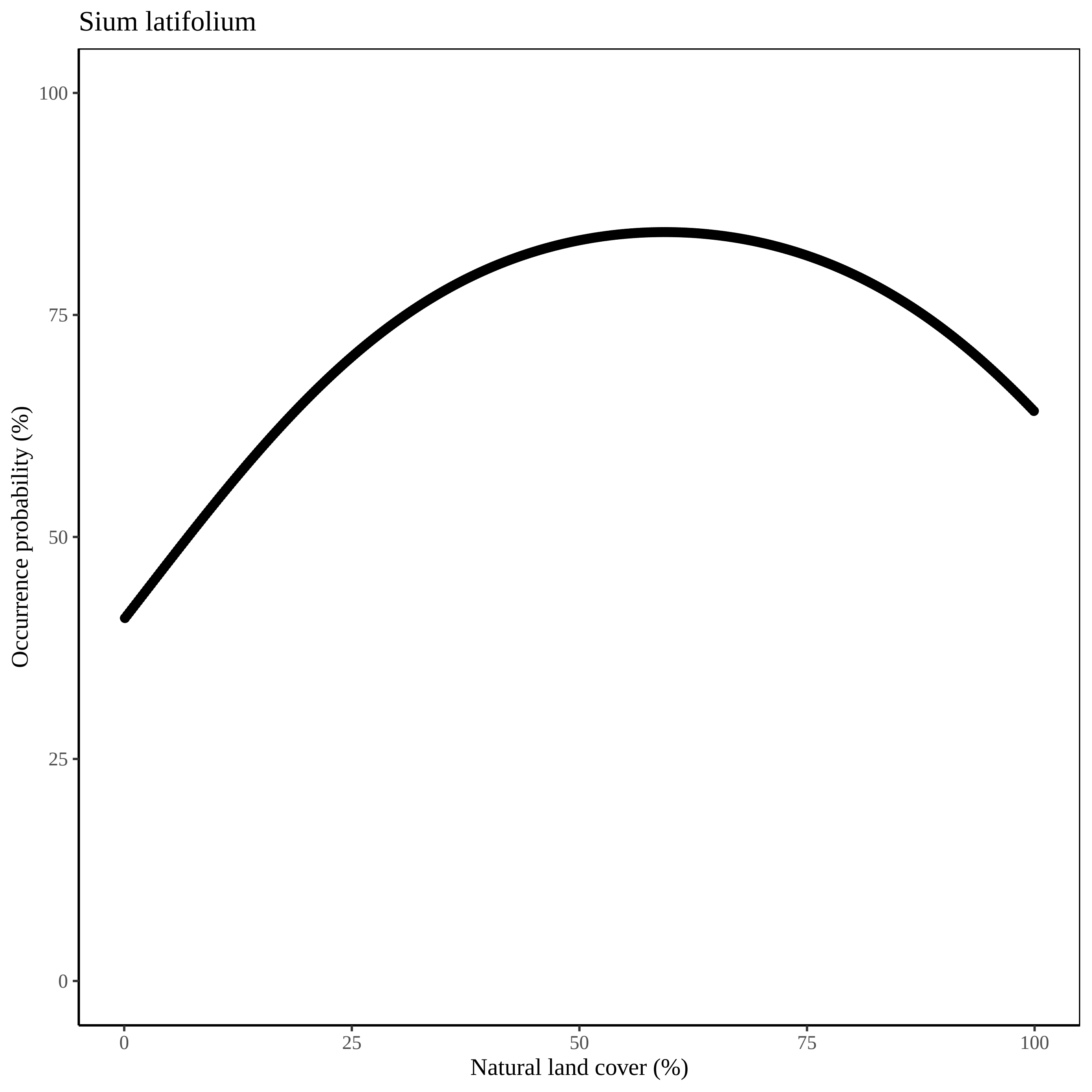
Sium latifolium
Occurrence probability (%)
100
75
50
25
0
0
25
50
75
100
Natural land cover (%)

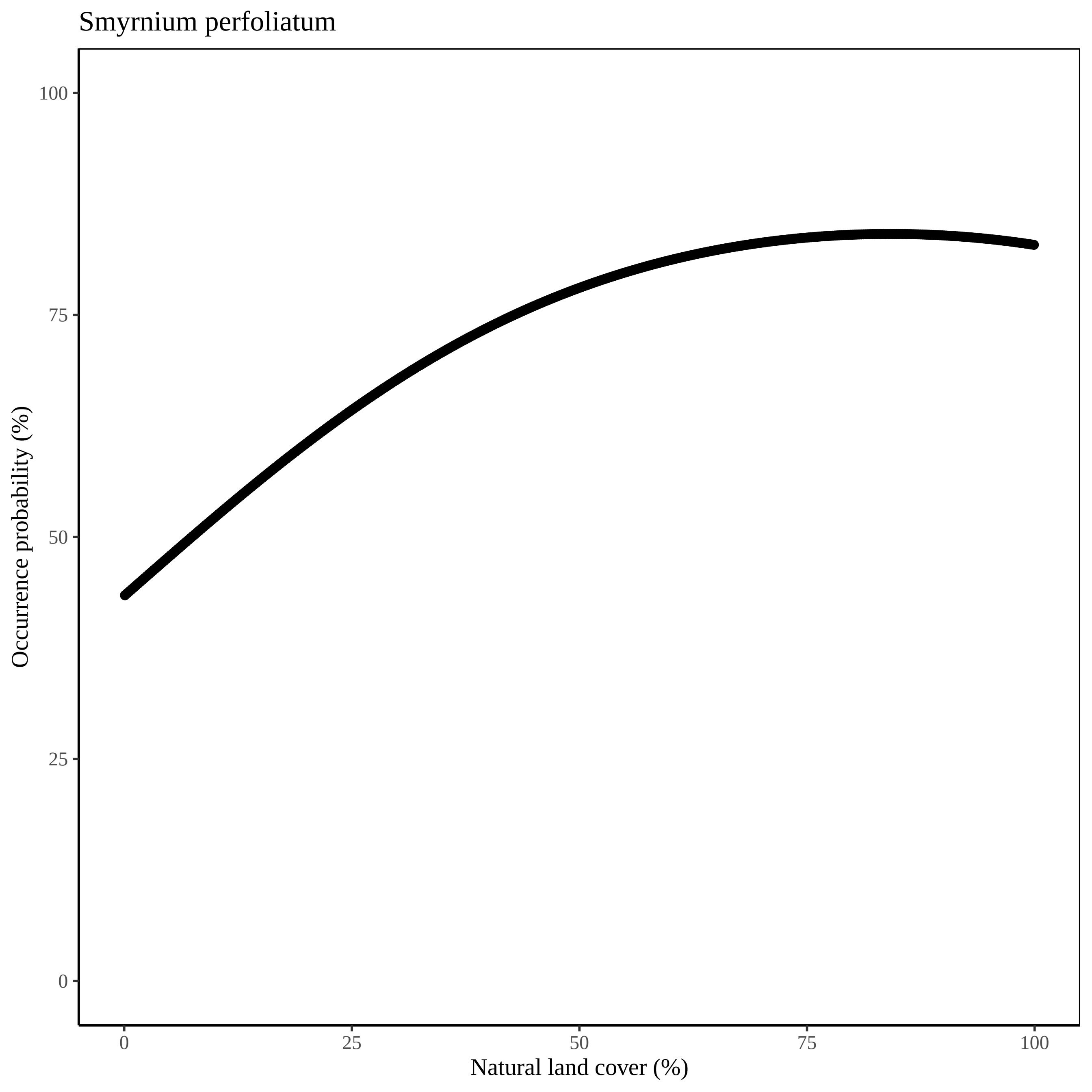
Smyrnium perfoliatum
Occurrence probability (%)
100
75
50
25
0
0
25
50
75
100
Natural land cover (%)

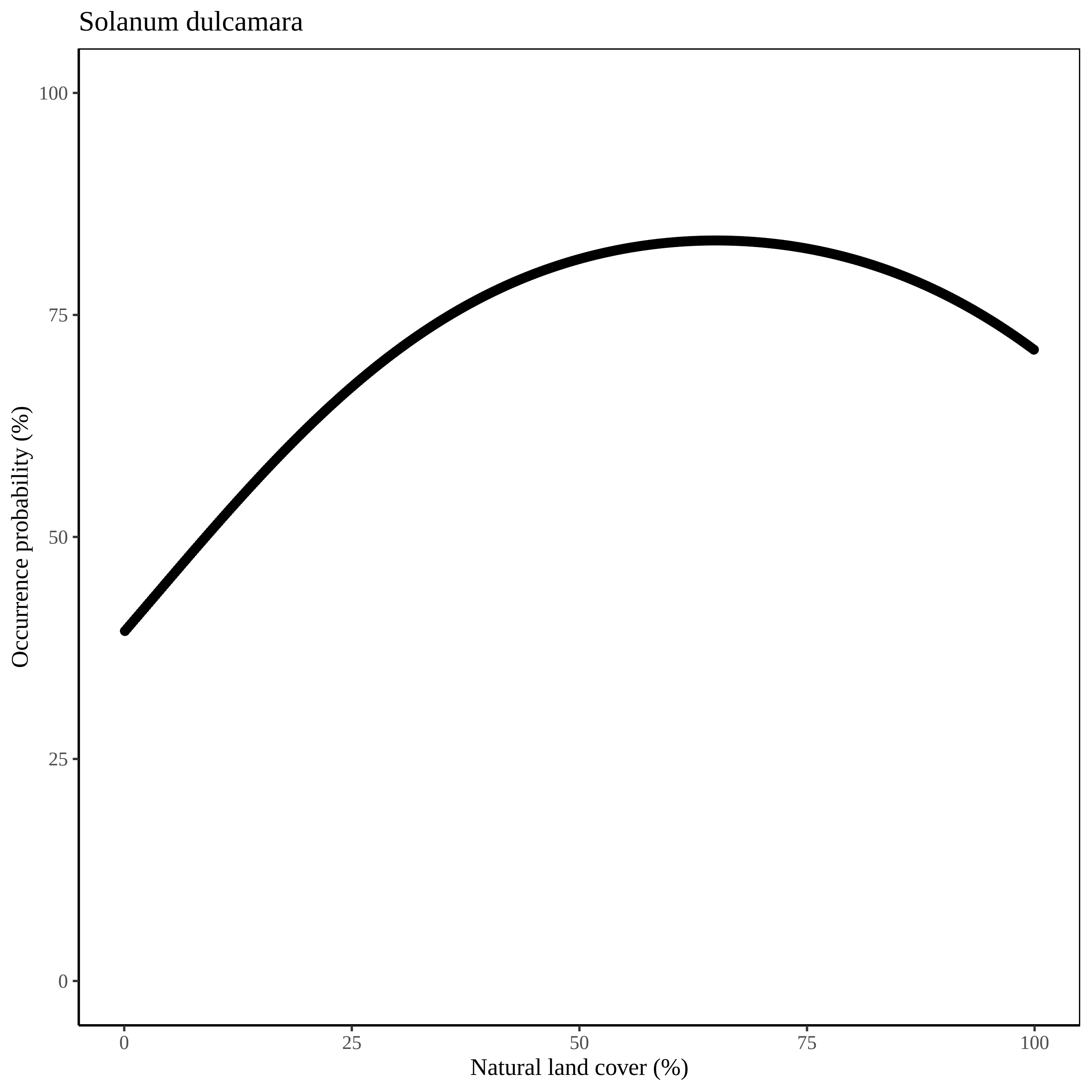
Solanum dulcamara
Occurrence probability (%)
100
75
50
25
0
0
25
50
75
100
Natural land cover (%)

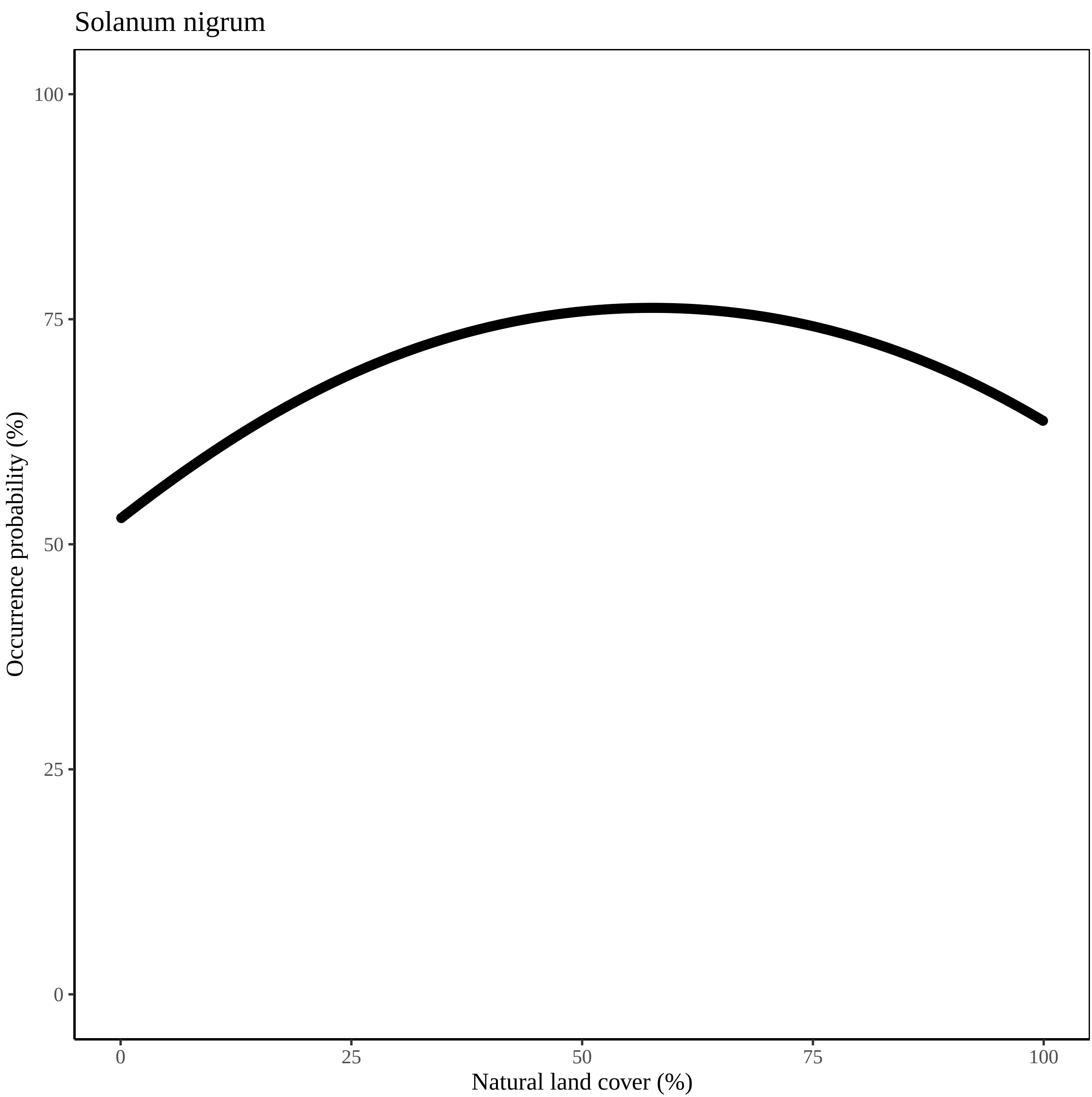
Solanum nigrum
Occurrence probability (%)
100
75
50
25
0
0
25
50
75
100
Natural land cover (%)

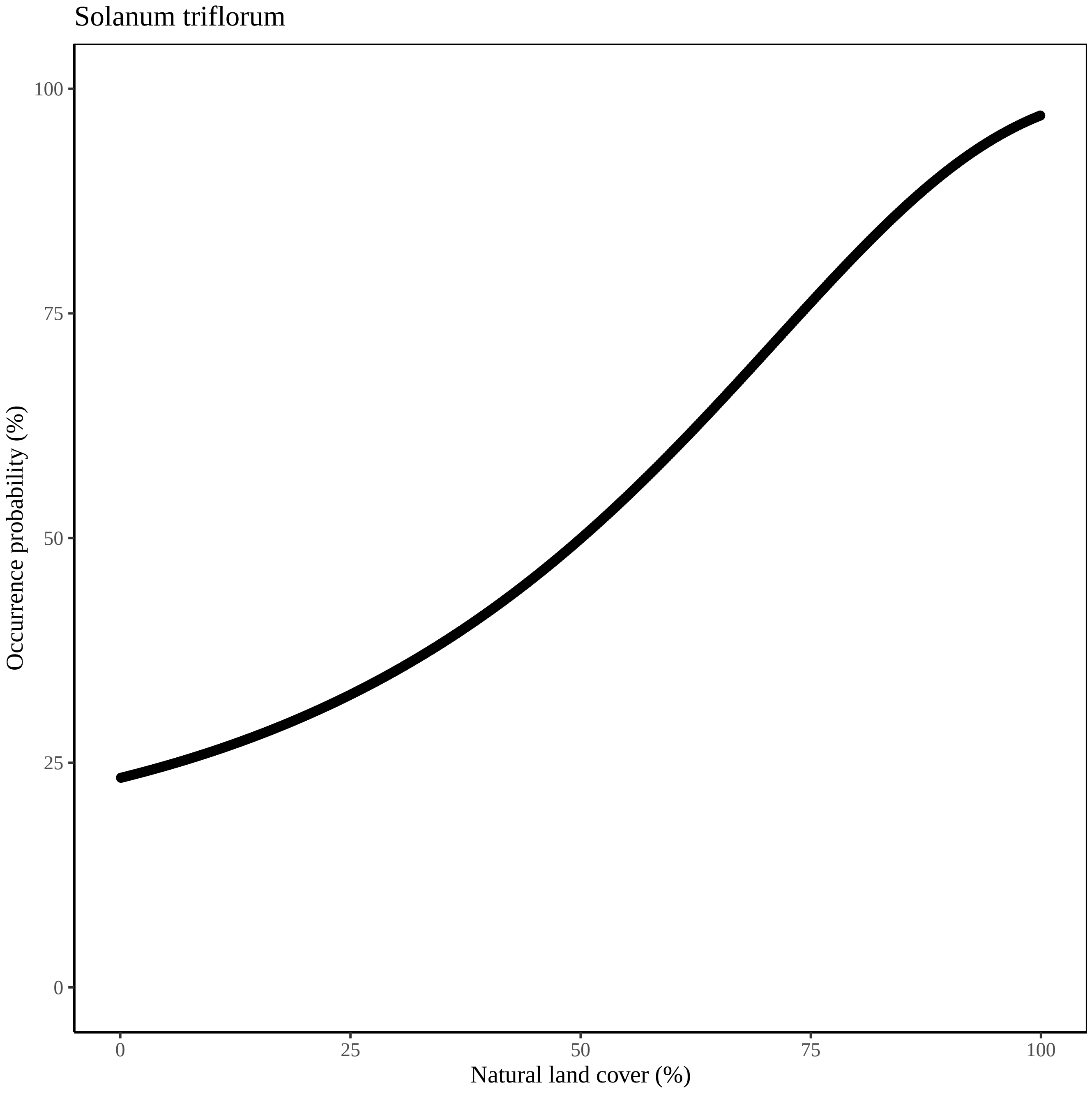

Solanum triflorum
Occurrence probability (%)
100
75
50
25
0
0
25
50
75
100
Natural land cover (%)

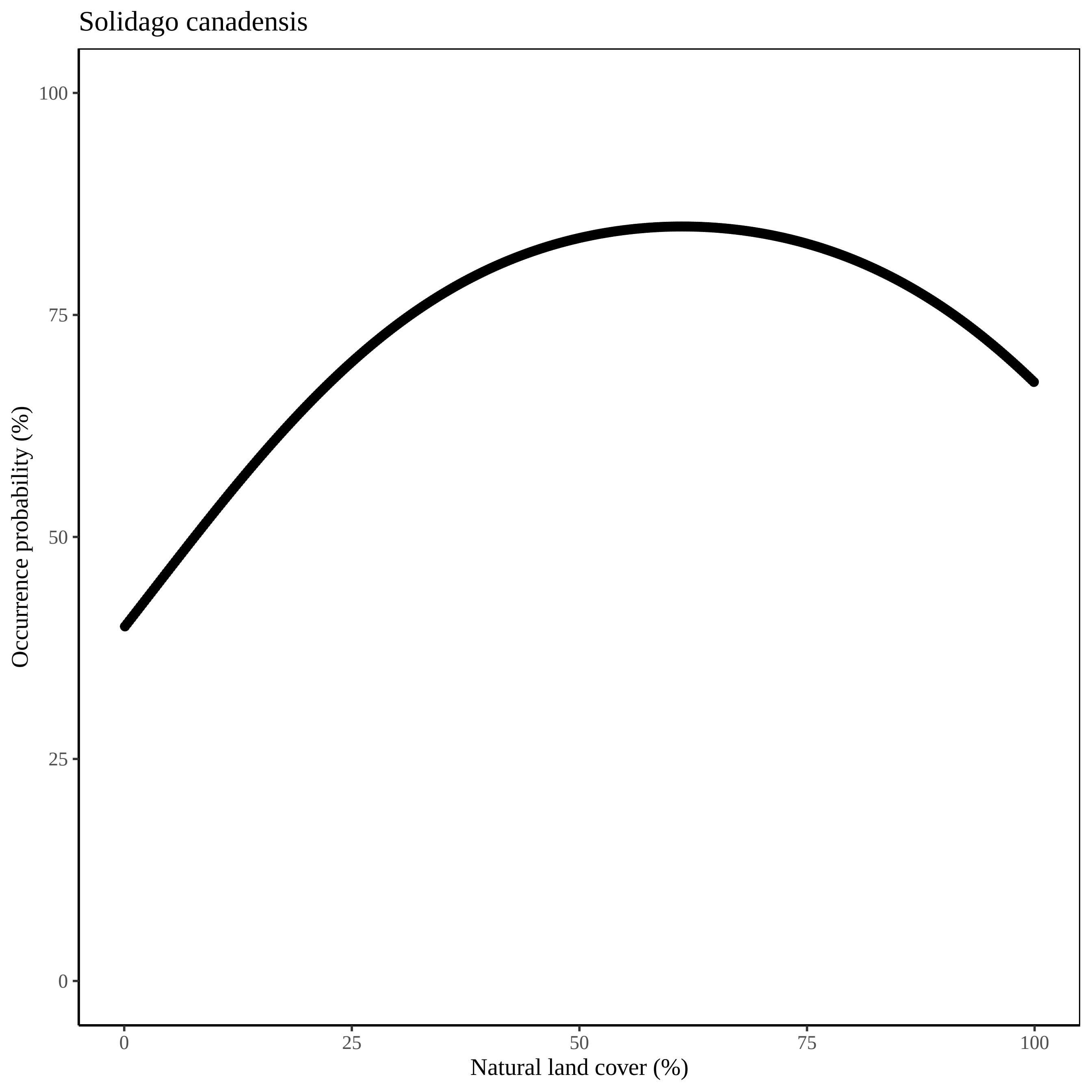
Solidago canadensis
Occurrence probability (%)
100
75
50
25
0
0
25
50
75
100
Natural land cover (%)

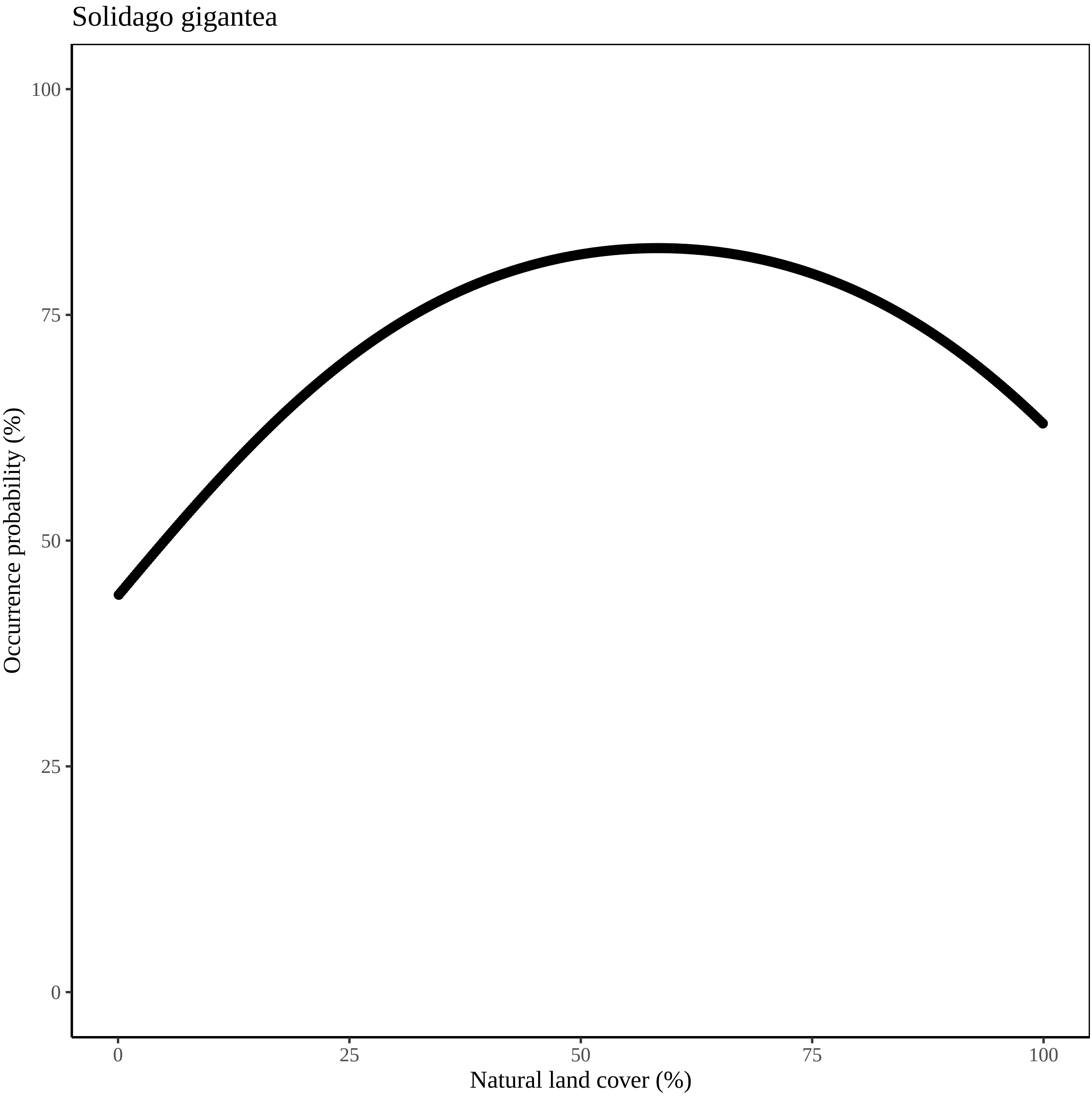
Solidago gigantea
Occurrence probability (%)
100
75
50
25
0
0
25
50
75
100
Natural land cover (%)

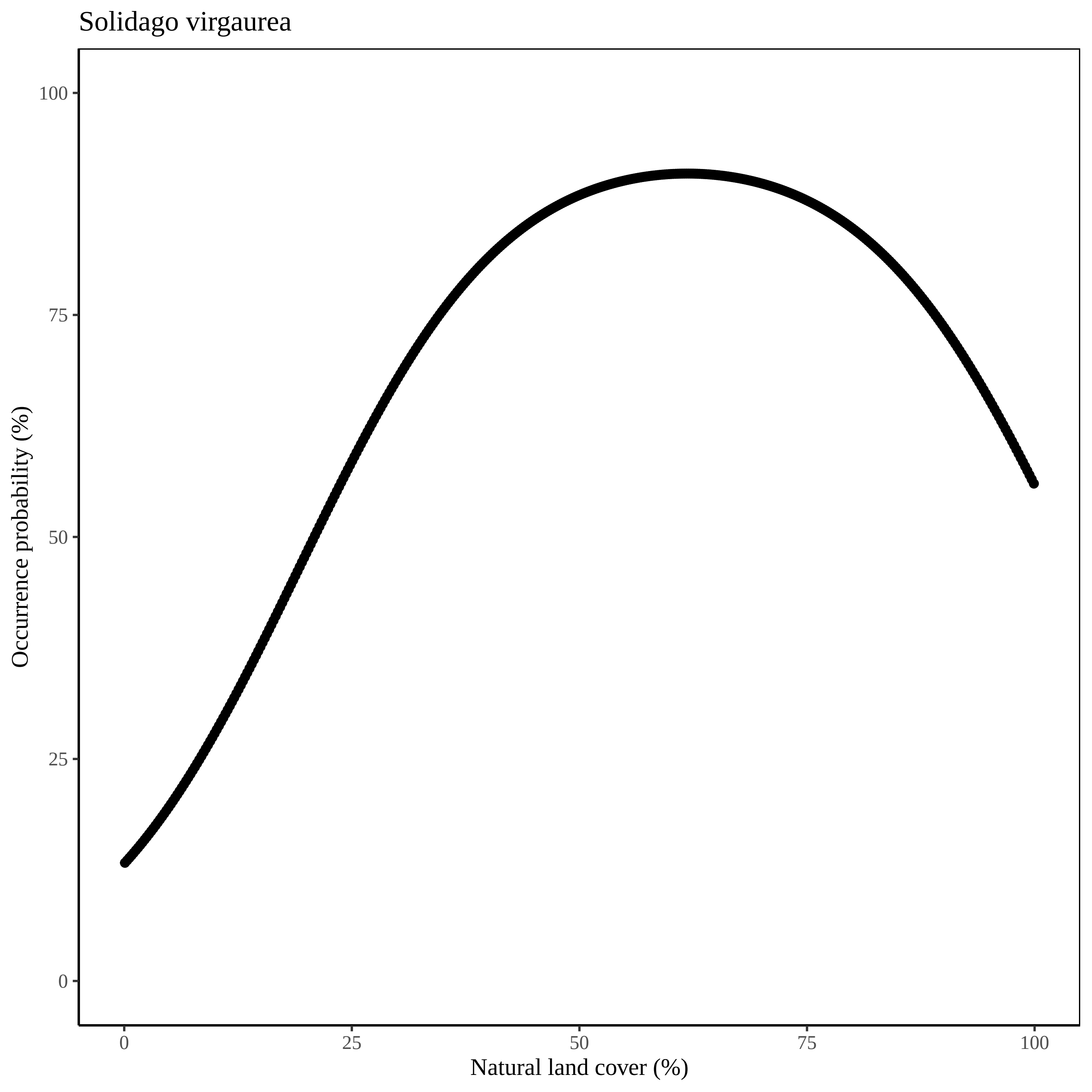
Solidago virgaurea
Occurrence probability (%)
100
75
50
25
0
0
25
50
75
100
Natural land cover (%)

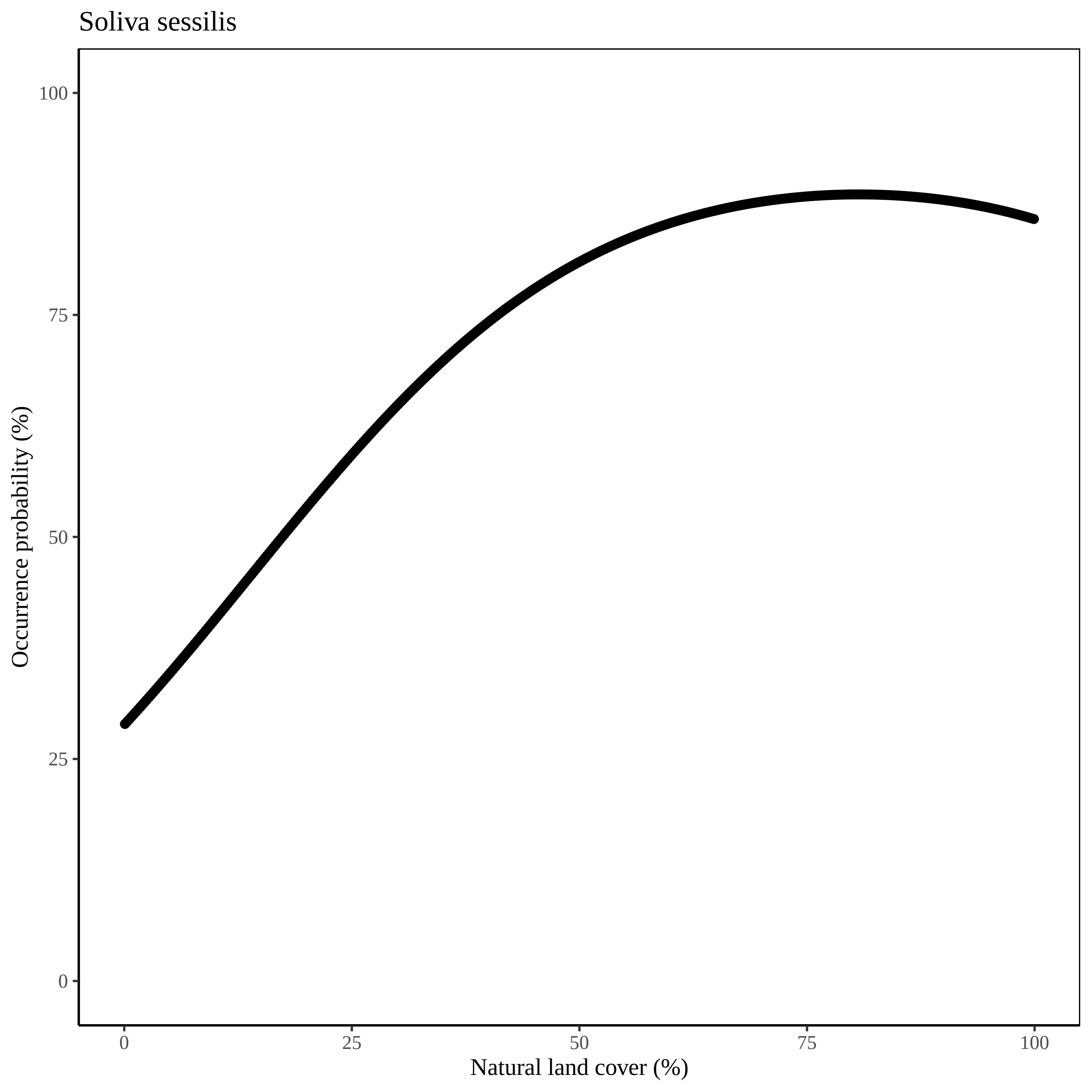
Soliva sessilis
Occurrence probability (%)
100
75
50
25
0
0
25
50
75
100
Natural land cover (%)

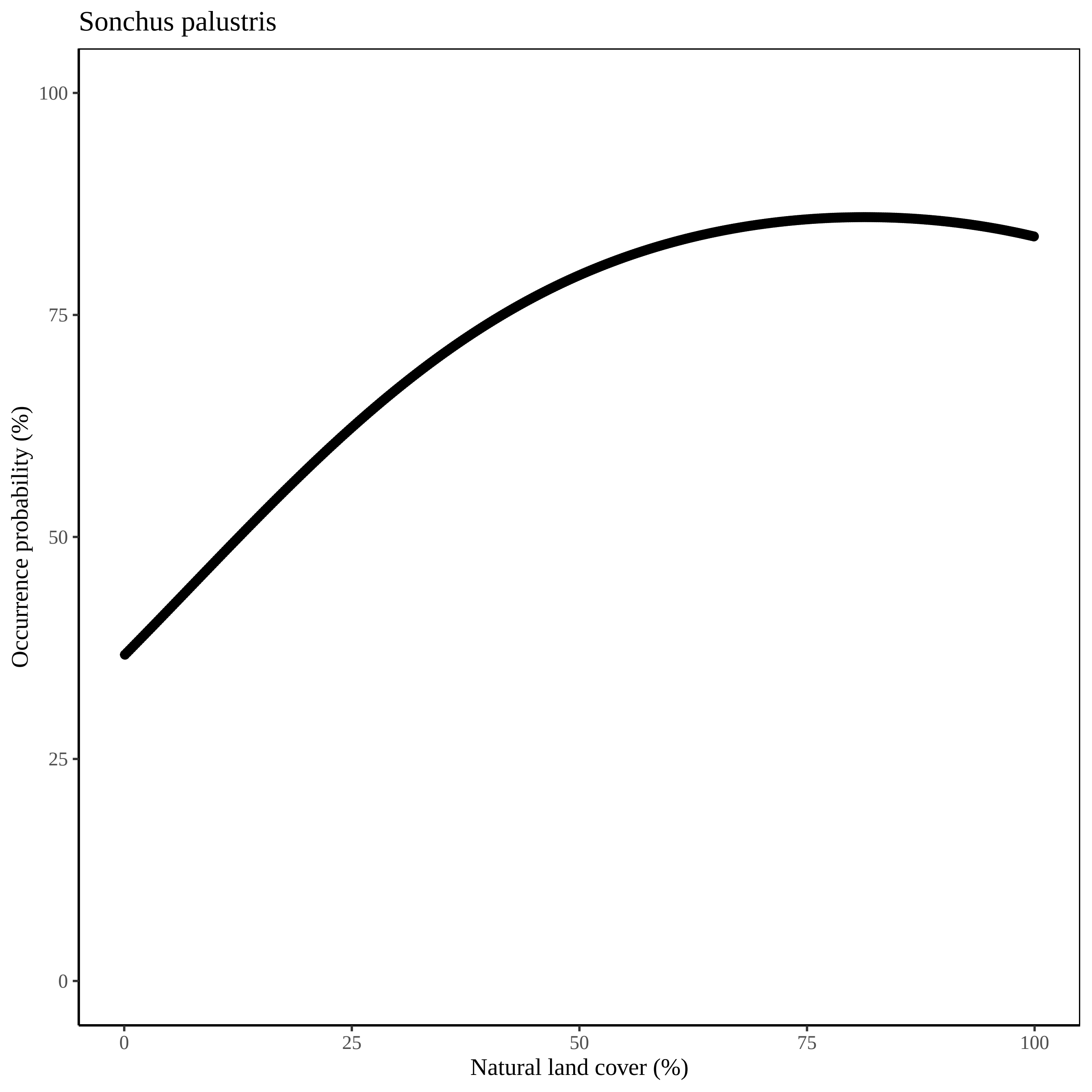
Sonchus palustris
Occurrence probability (%)
100
75
50
25
0
0
25
50
75
100
Natural land cover (%)

Sorbaria sorbifolia

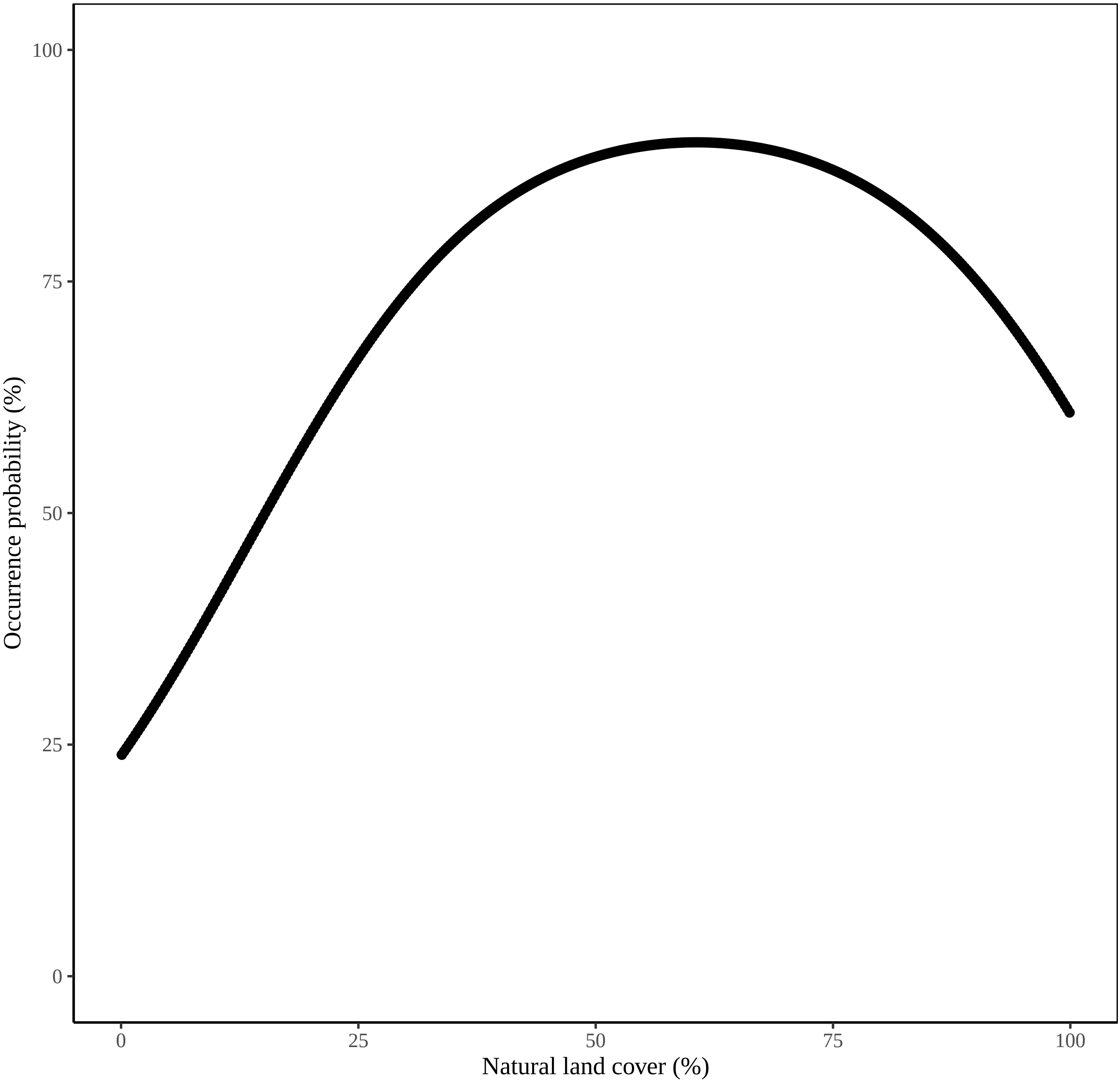

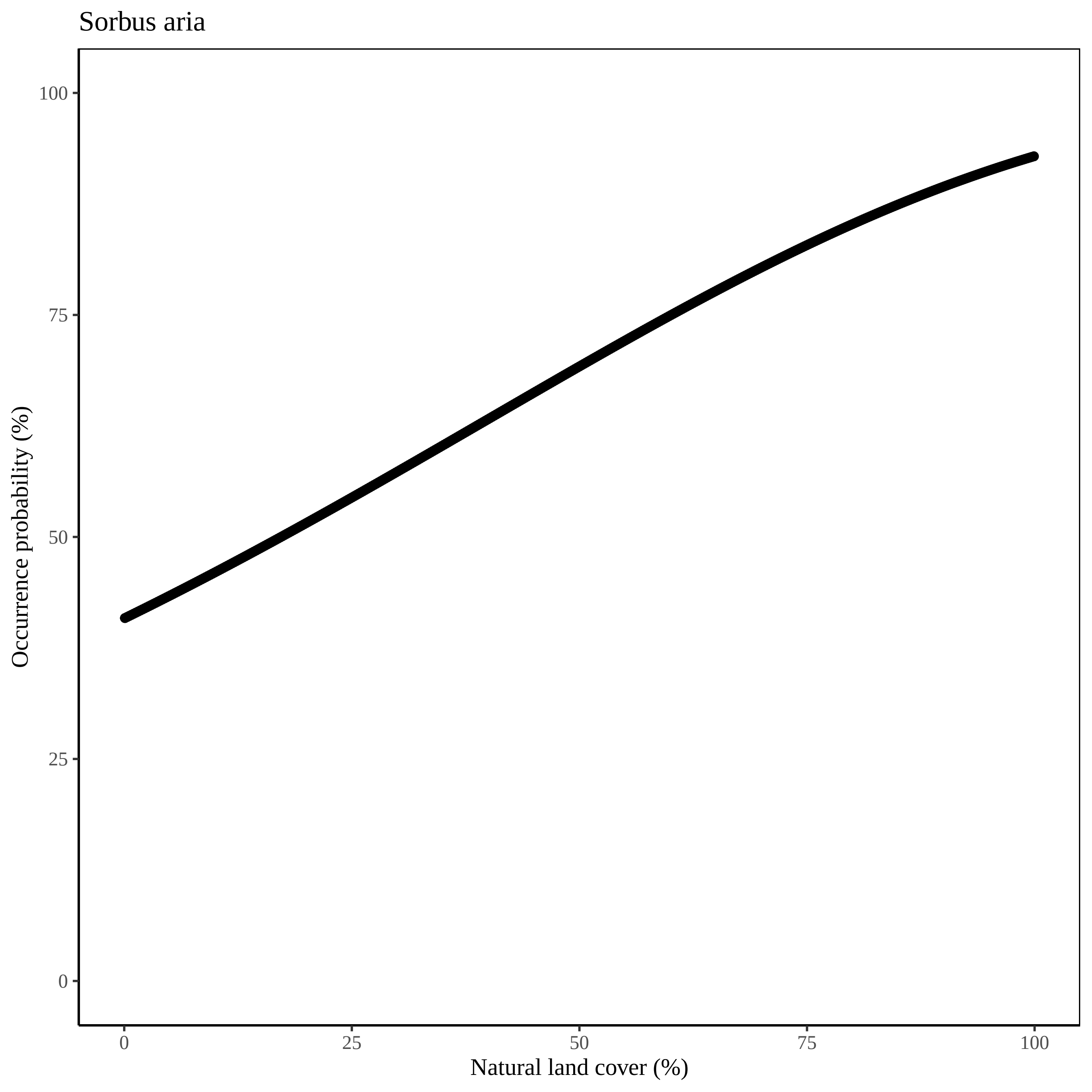
Sorbus aria
Occurrence probability (%)
100
75
50
25
0
0
25
50
75
100
Natural land cover (%)

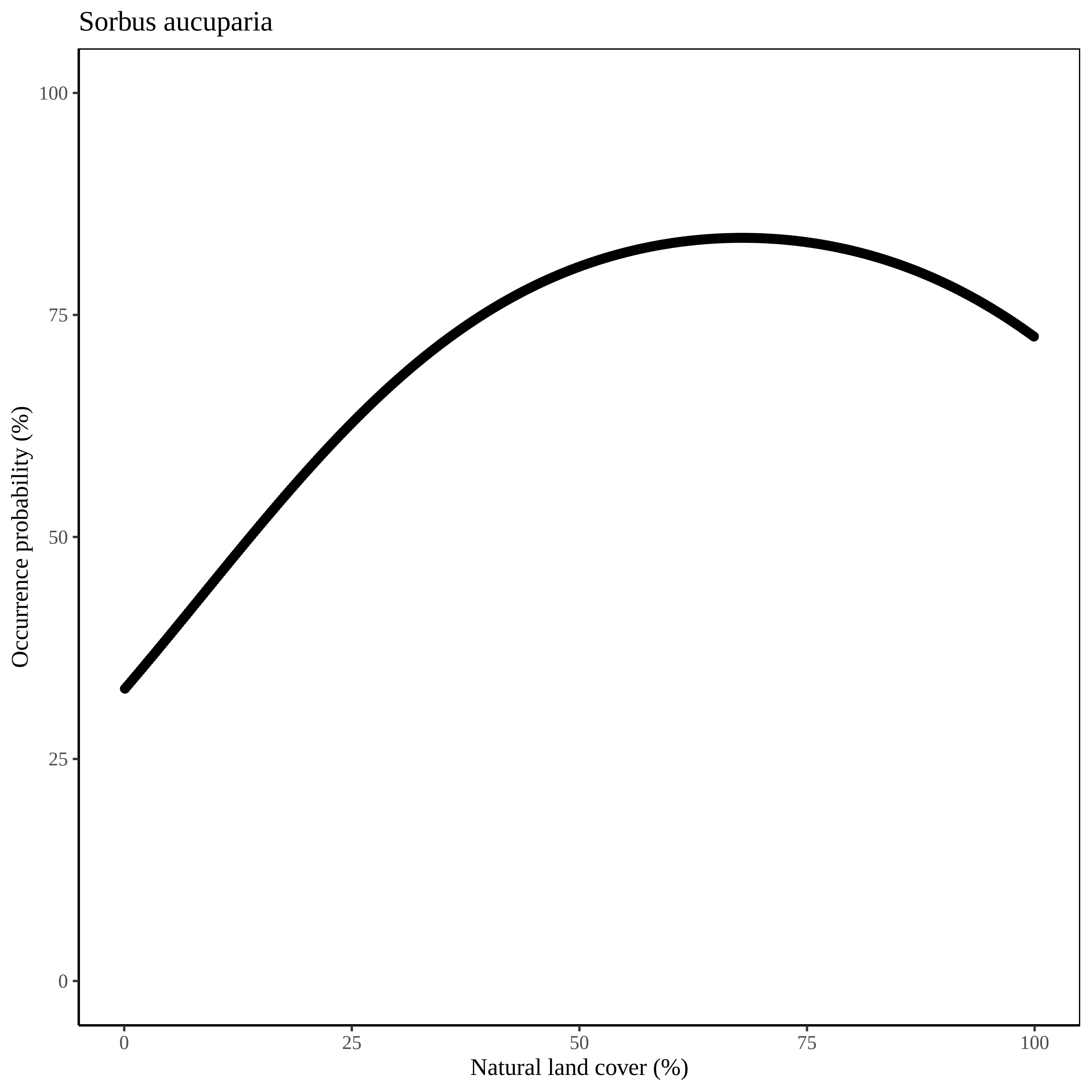
Sorbus aucuparia
100
75
50
25
0
Occurrence probability (%)
0
25
50
75
100
Natural land cover (%)

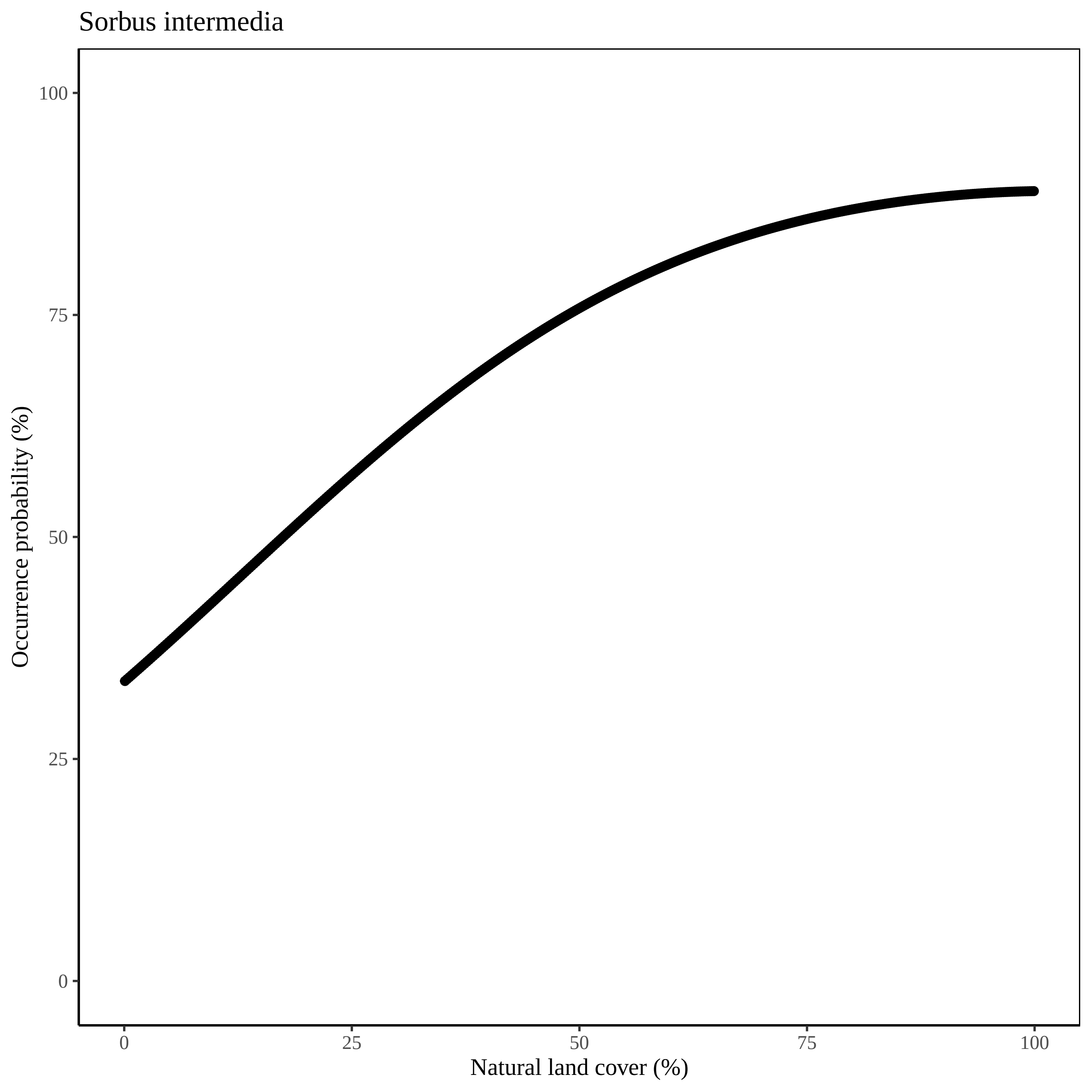

Sorbus intermedia
Occurrence probability (%)
100
75
50
25
0
0
25
50
75
100
Natural land cover (%)

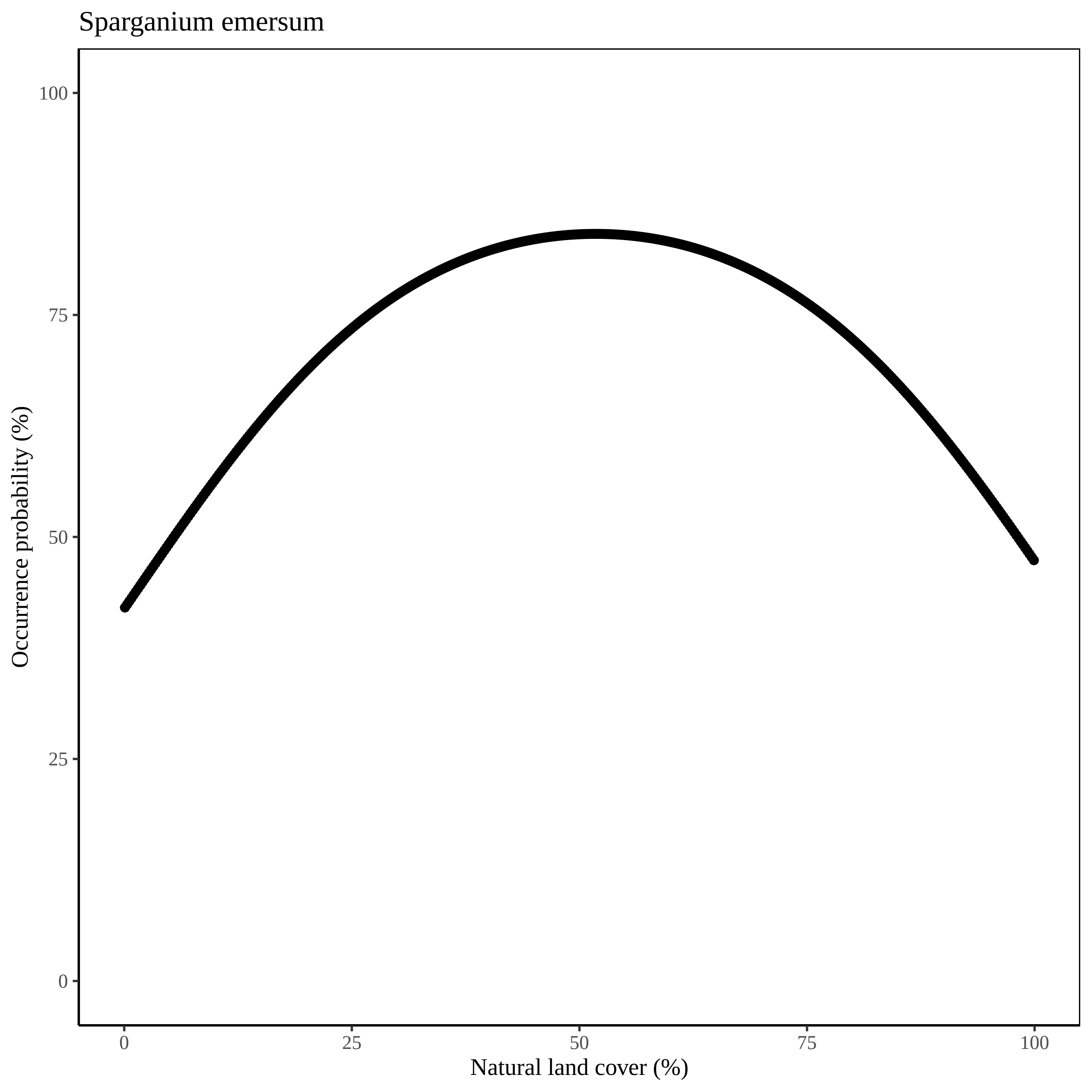
Sparganium emersum
Occurrence probability (%)
100
75
50
25
0
0
25
50
75
100
Natural land cover (%)

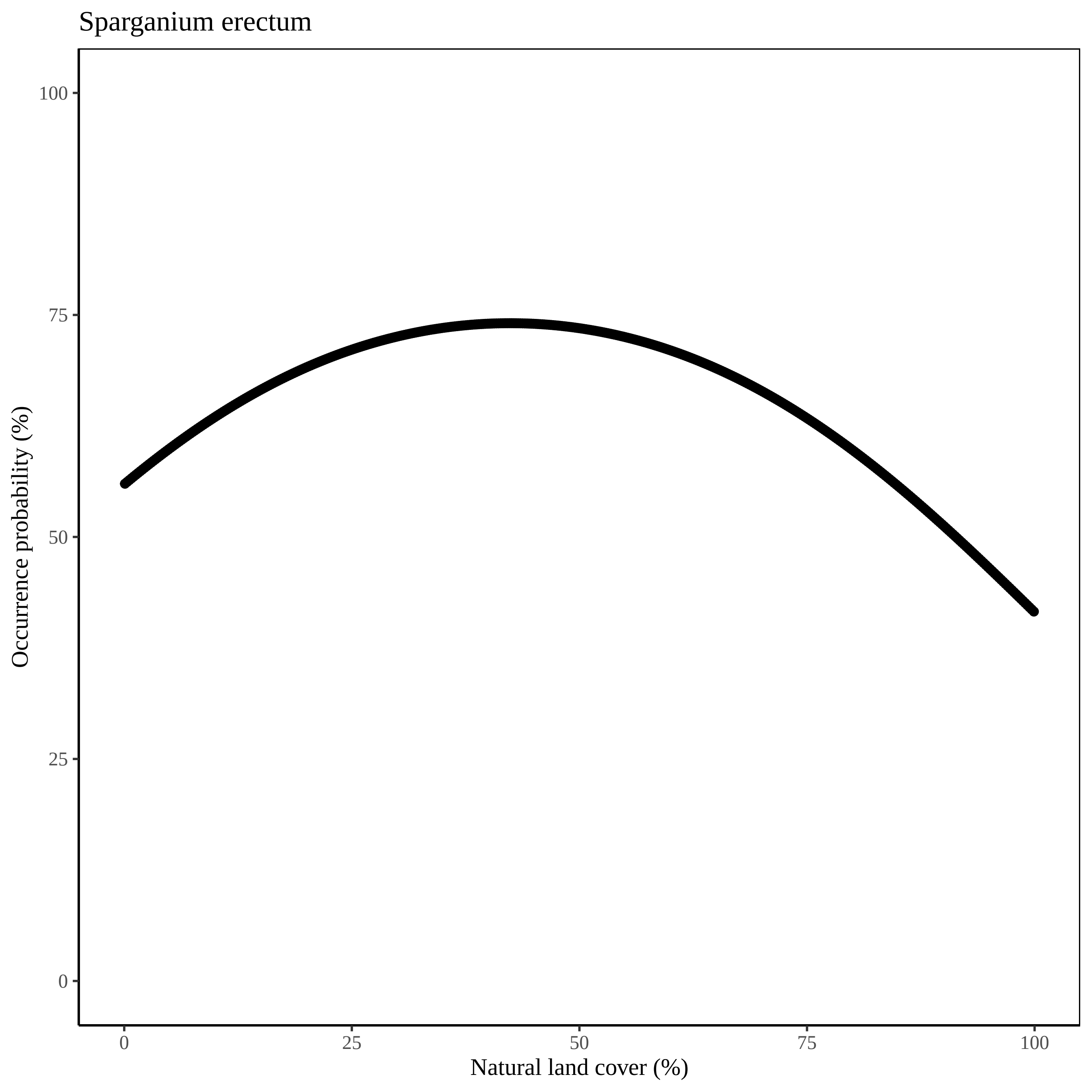
Sparganium erectum
Occurrence probability (%)
100
75
50
25
0
0
25
50
75
100
Natural land cover (%)

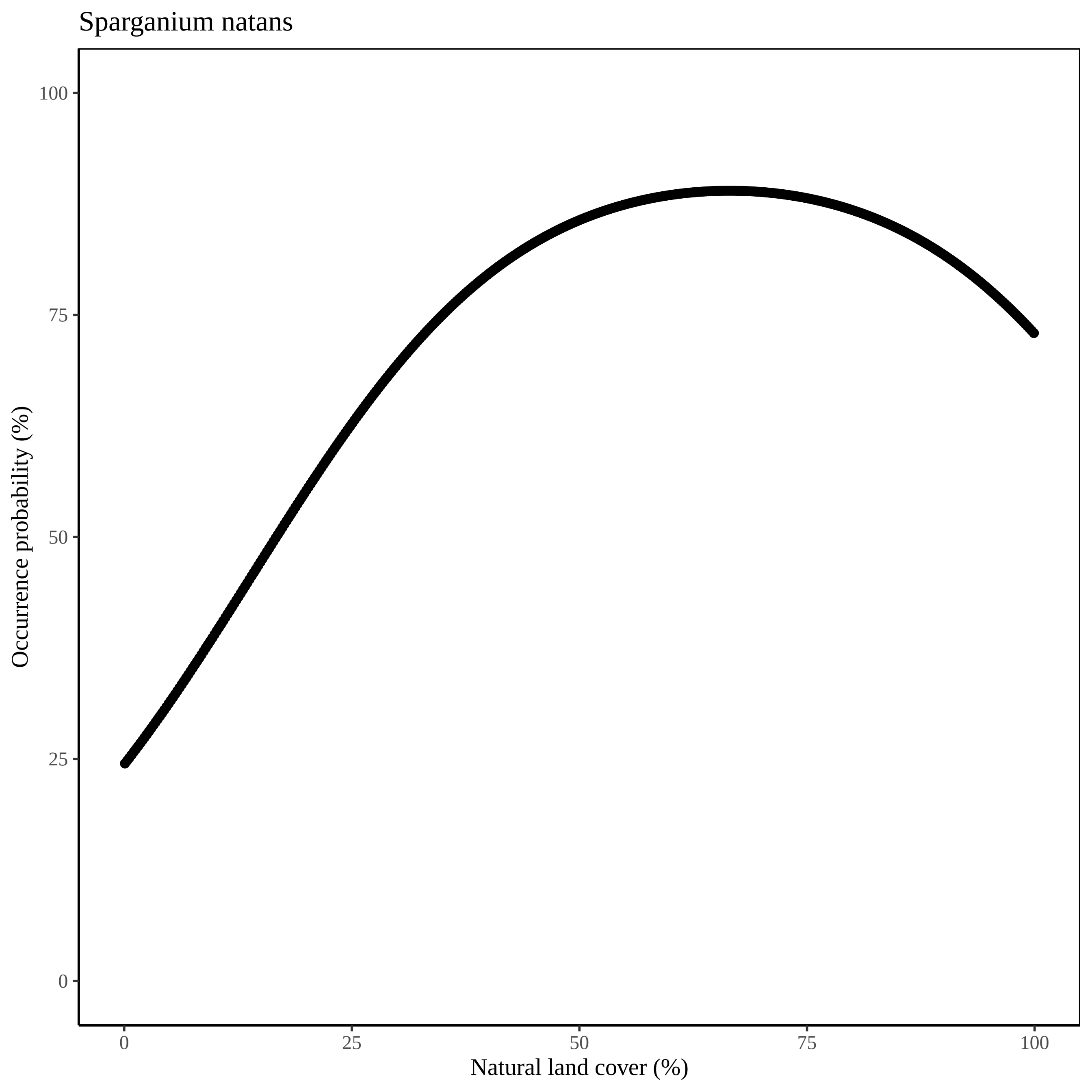

Sparganium natans
Occurrence probability (%)
100
75
50
25
0
0
25
50
75
100
Natural land cover (%)

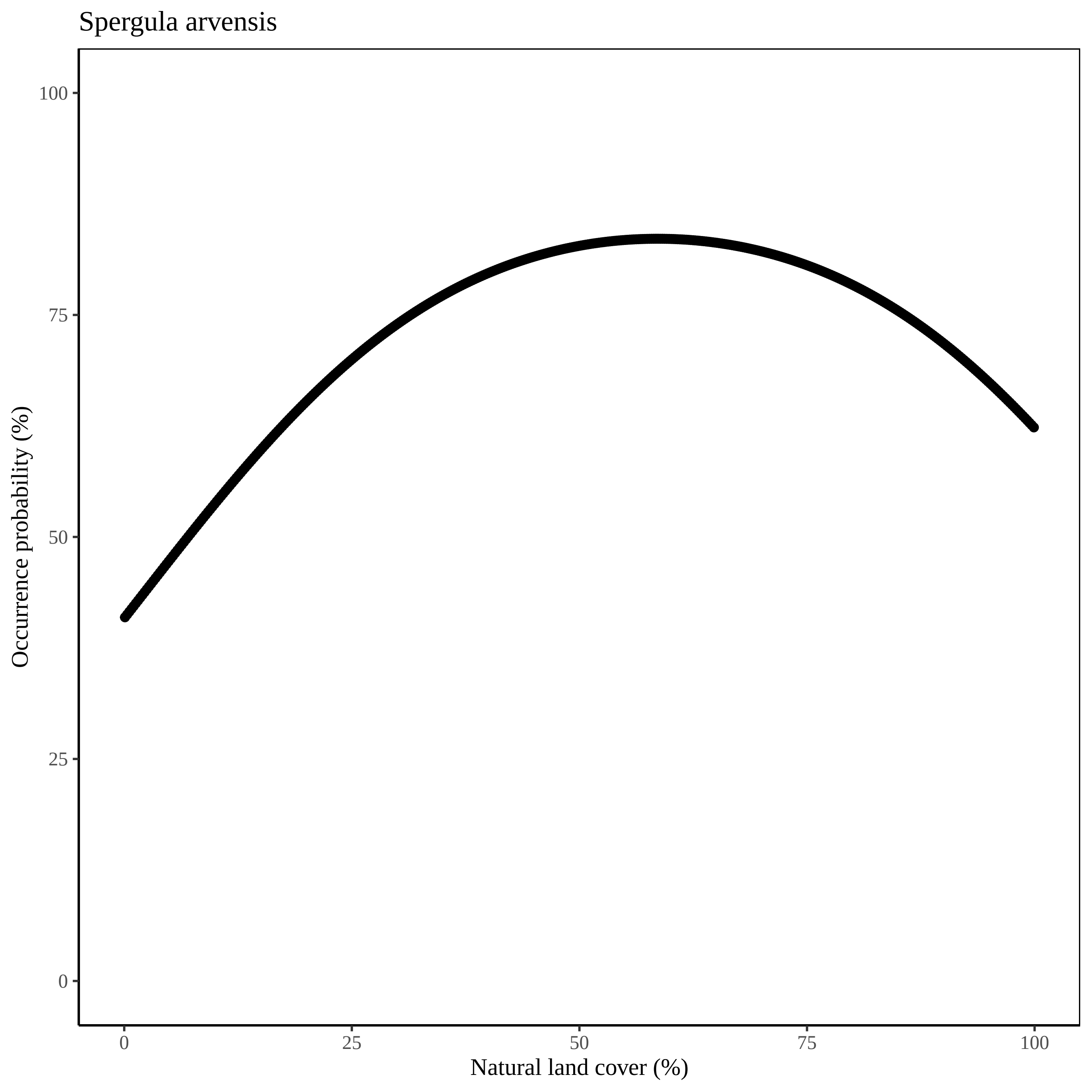
Spergula arvensis
100
75
50
25
0
Occurrence probability (%)
0
25
50
75
100
Natural land cover (%)

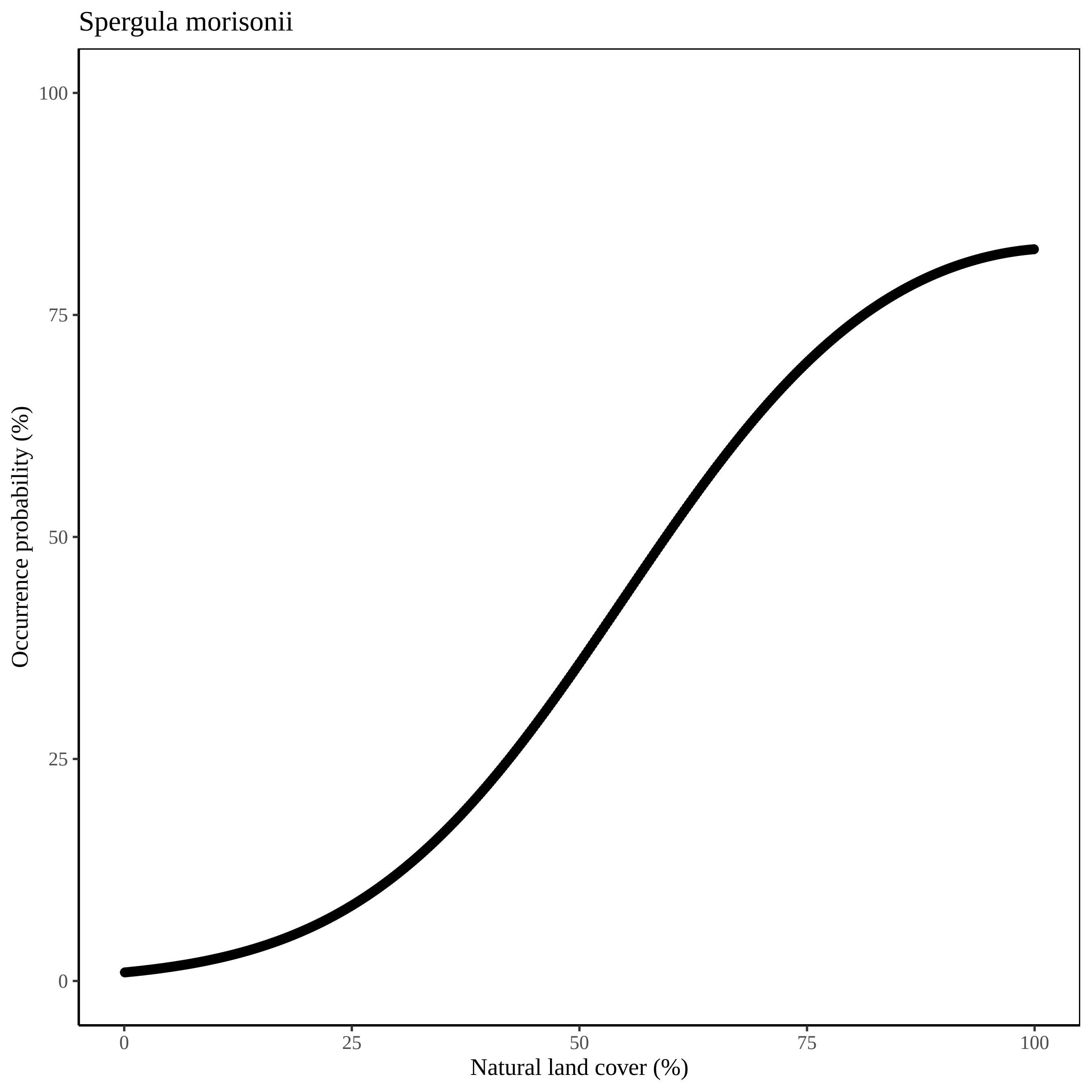
Spergula morisonii
Occurrence probability (%)
100
75
50
25
0
0
25
50
75
100
Natural land cover (%)

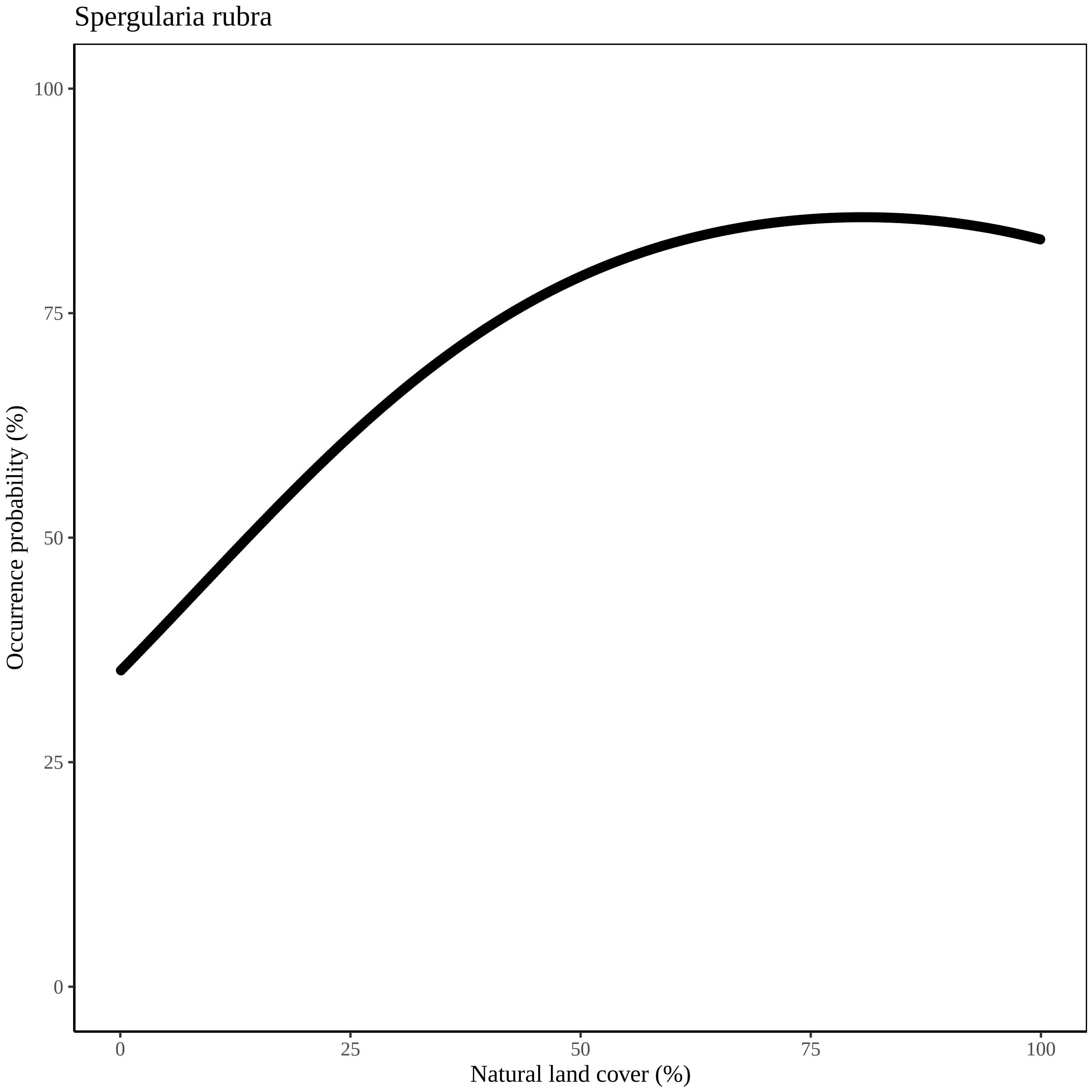
Spergularia rubra
Occurrence probability (%)
100
75
50
25
0
0
25
50
75
100
Natural land cover (%)

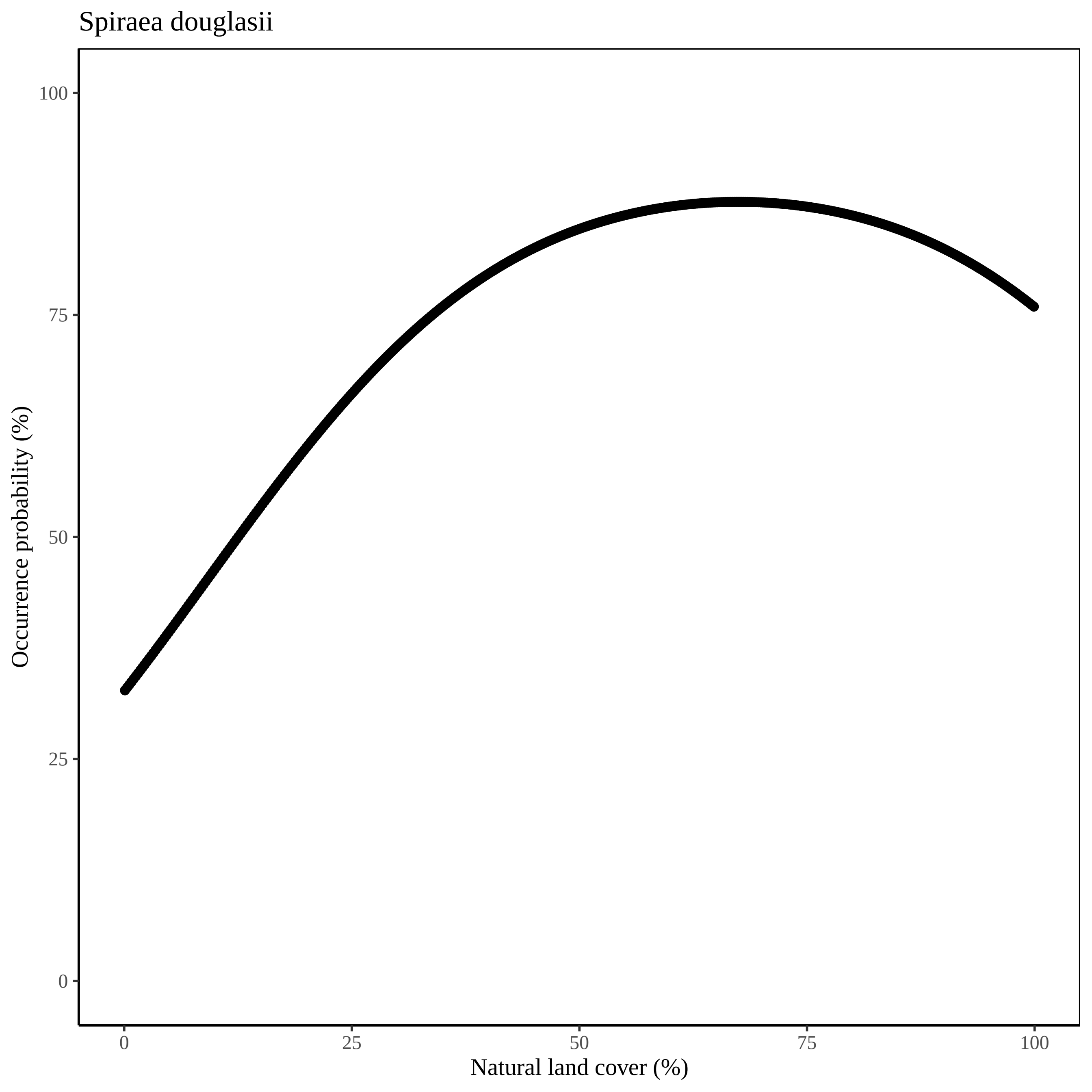
Spiraea douglasii
Occurrence probability (%)
100
75
50
25
0
0
25
50
75
100
Natural land cover (%)

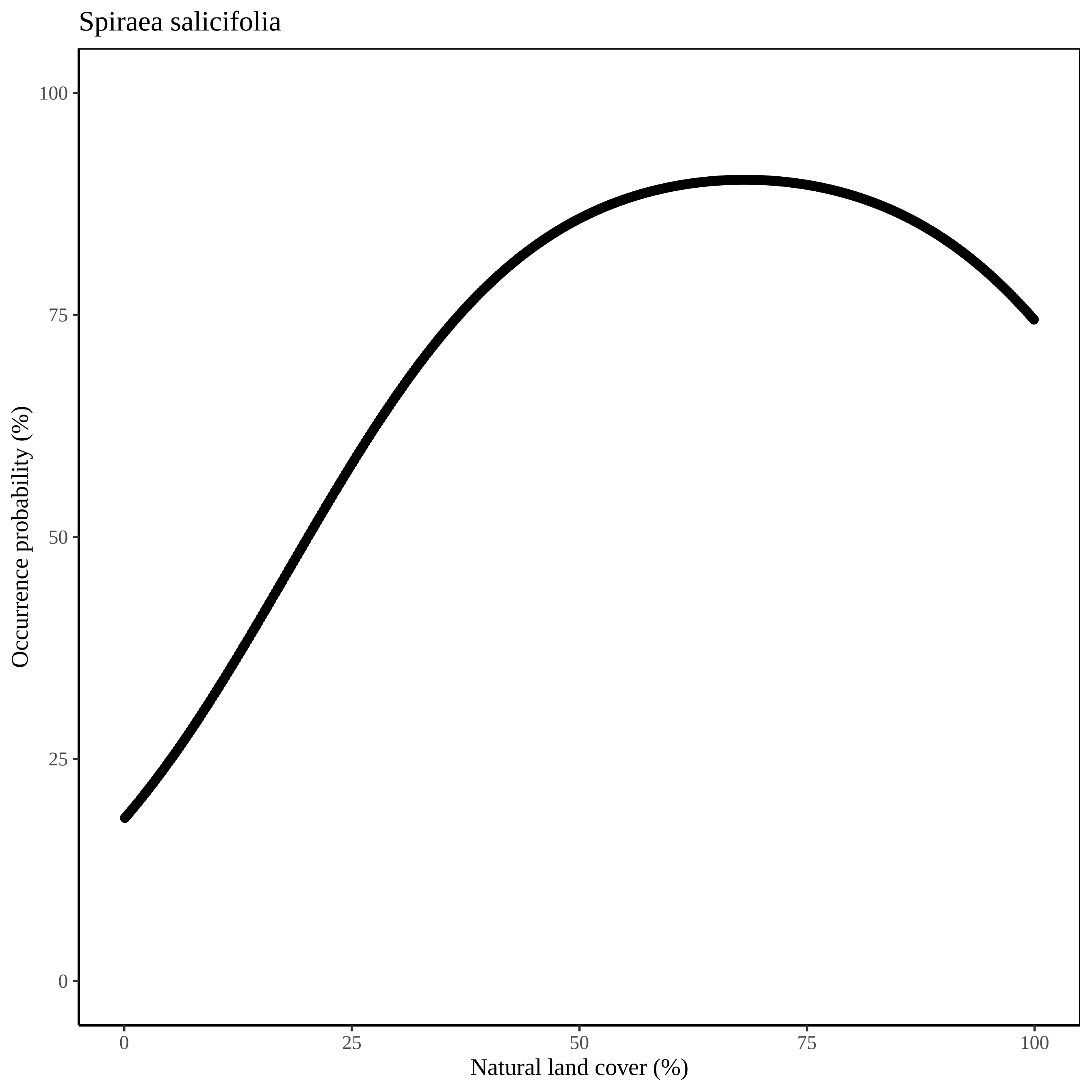
Spiraea salicifolia
Occurrence probability (%)
100
75
50
25
0
0
25
50
75
100
Natural land cover (%)

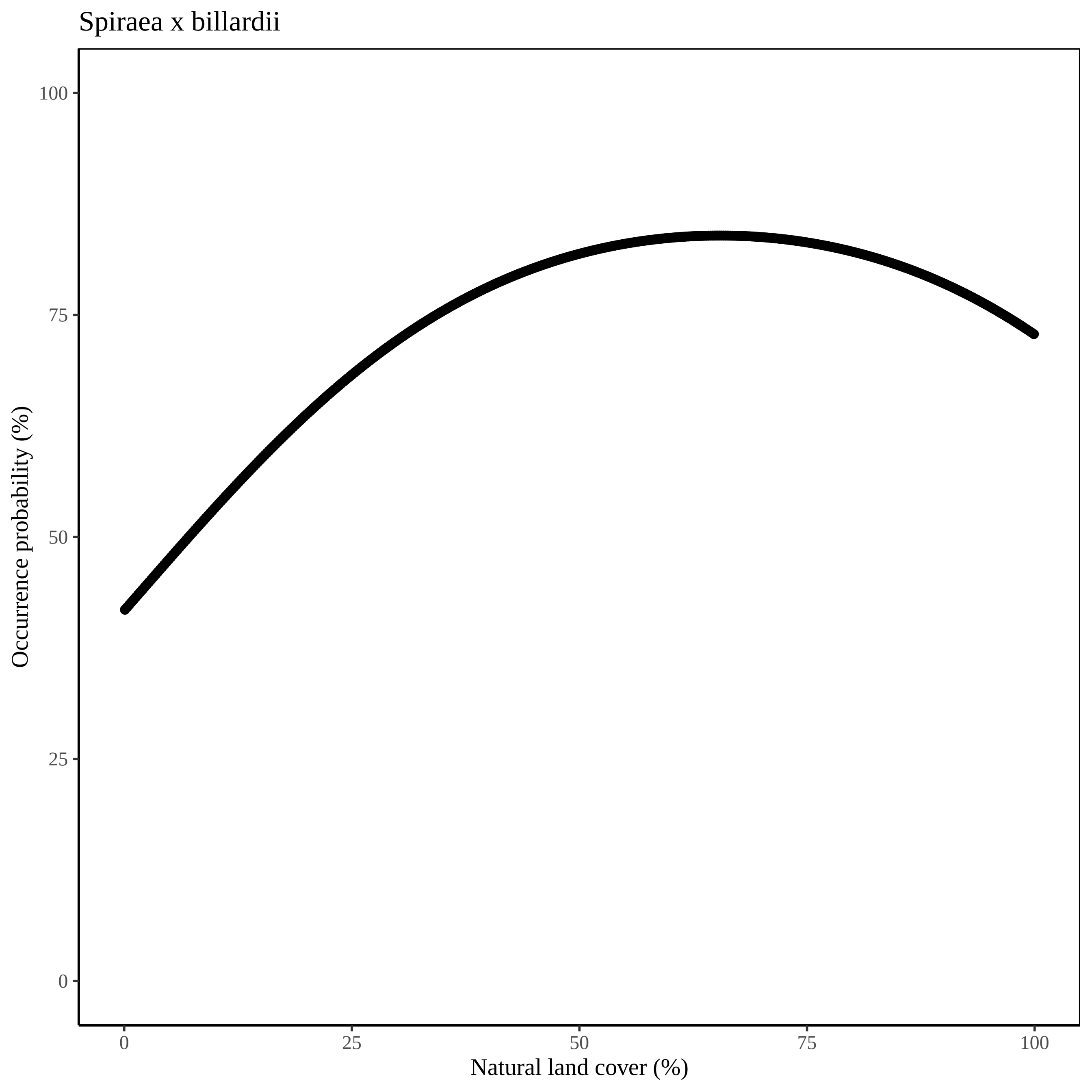
Spiraea x billardii
100
75
50
25
0
Occurrence probability (%)
0
25
50
75
100
Natural land cover (%)

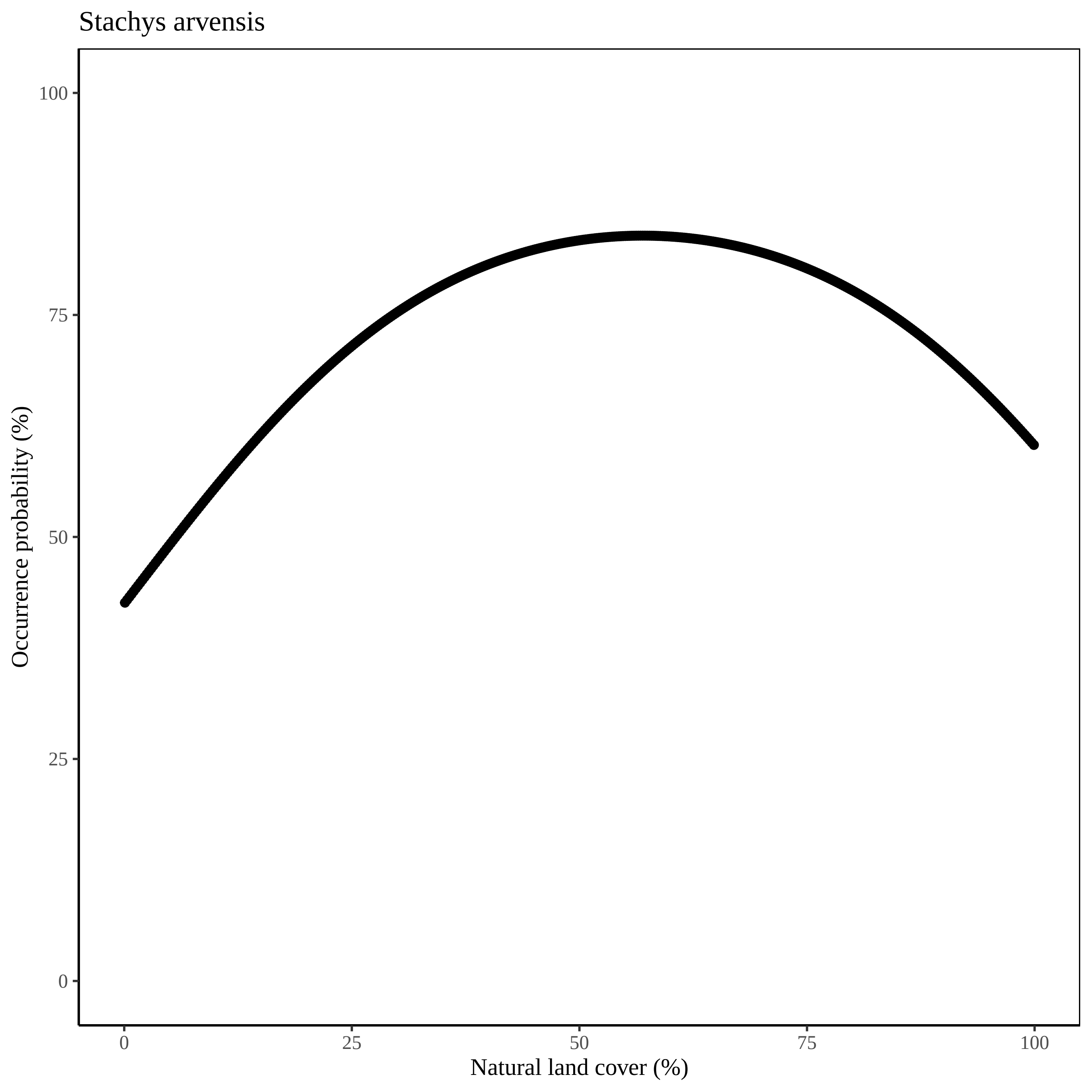
Stachys arvensis
Occurrence probability (%)
100
75
50
25
0
0
25
50
75
100
Natural land cover (%)

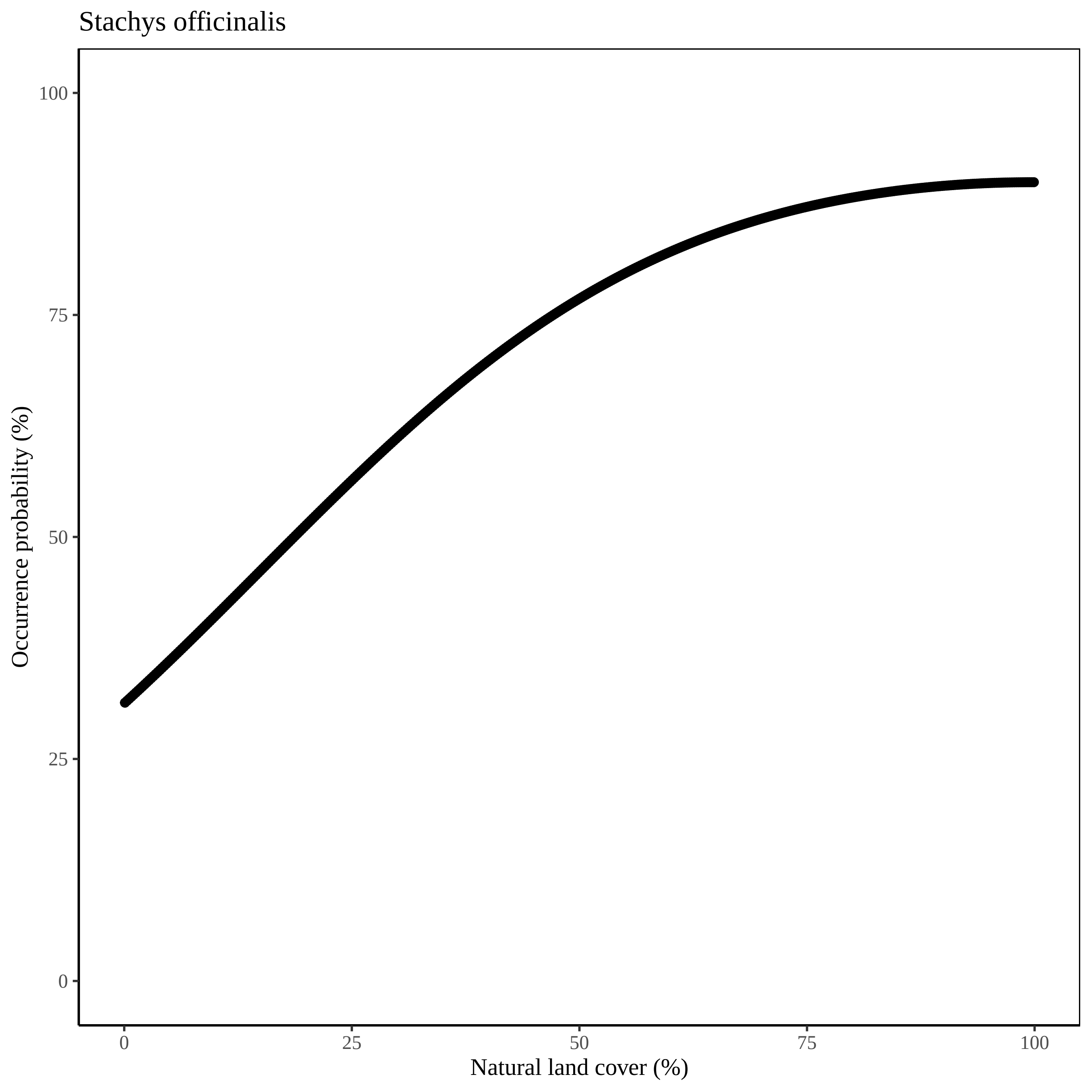
Stachys officinalis
100
75
50
25
0
Occurrence probability (%)
0
25
50
75
100
Natural land cover (%)

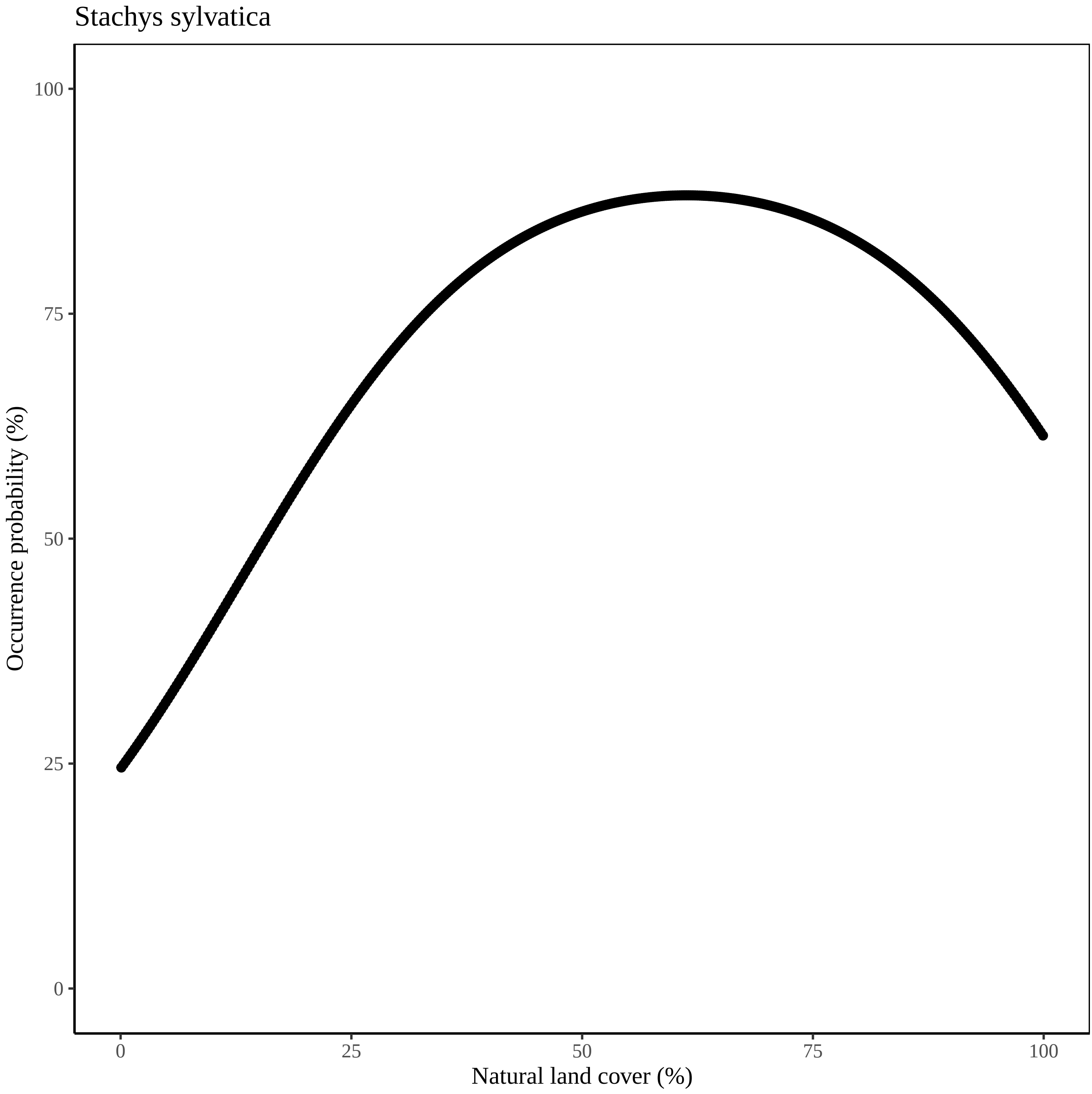
Stachys sylvatica
Occurrence probability (%)
100
75
50
25
0
0
25
50
75
100
Natural land cover (%)

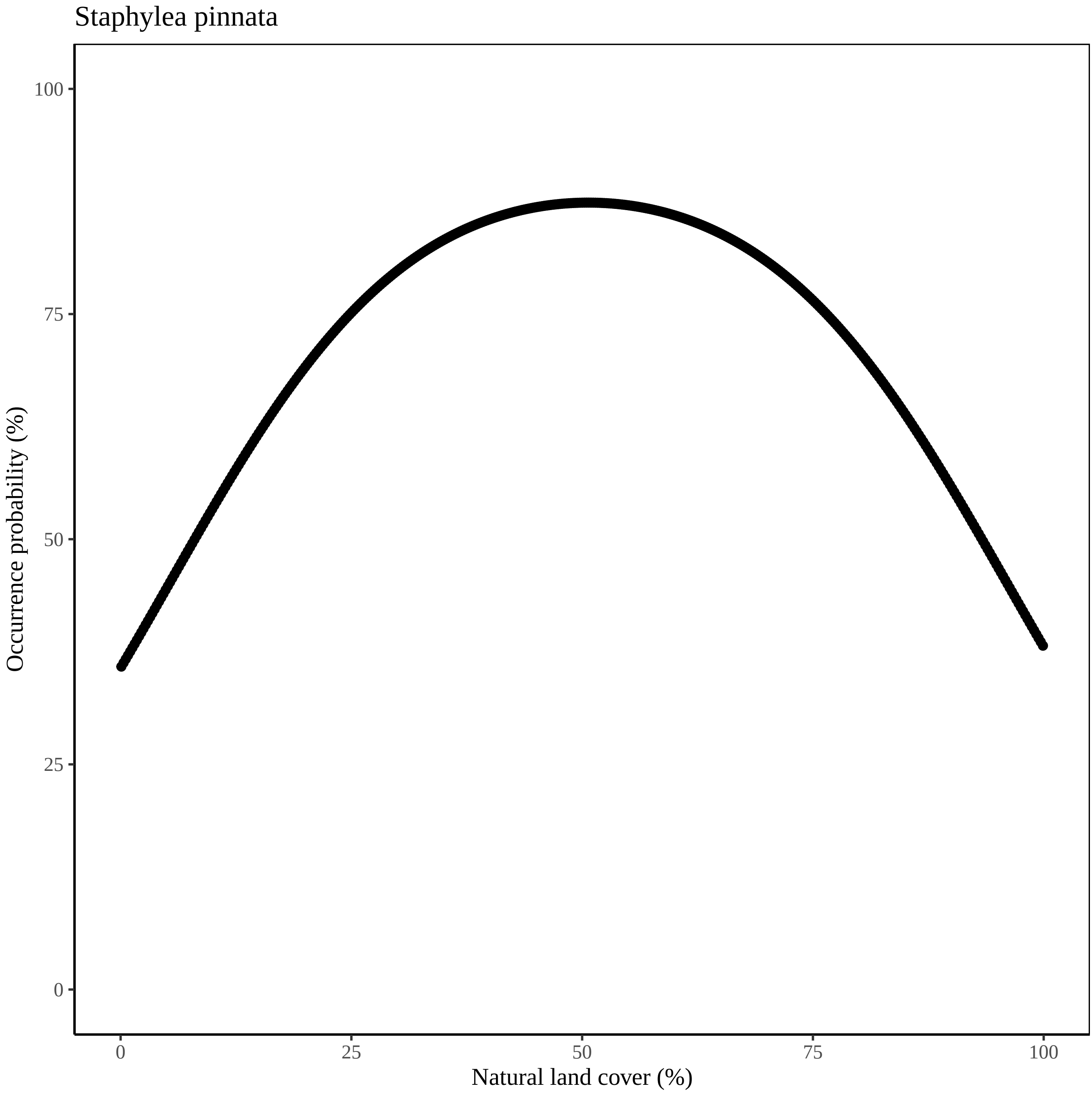

Staphylea pinnata
Occurrence probability (%)
100
75
50
25
0
0
25
50
75
100
Natural land cover (%)

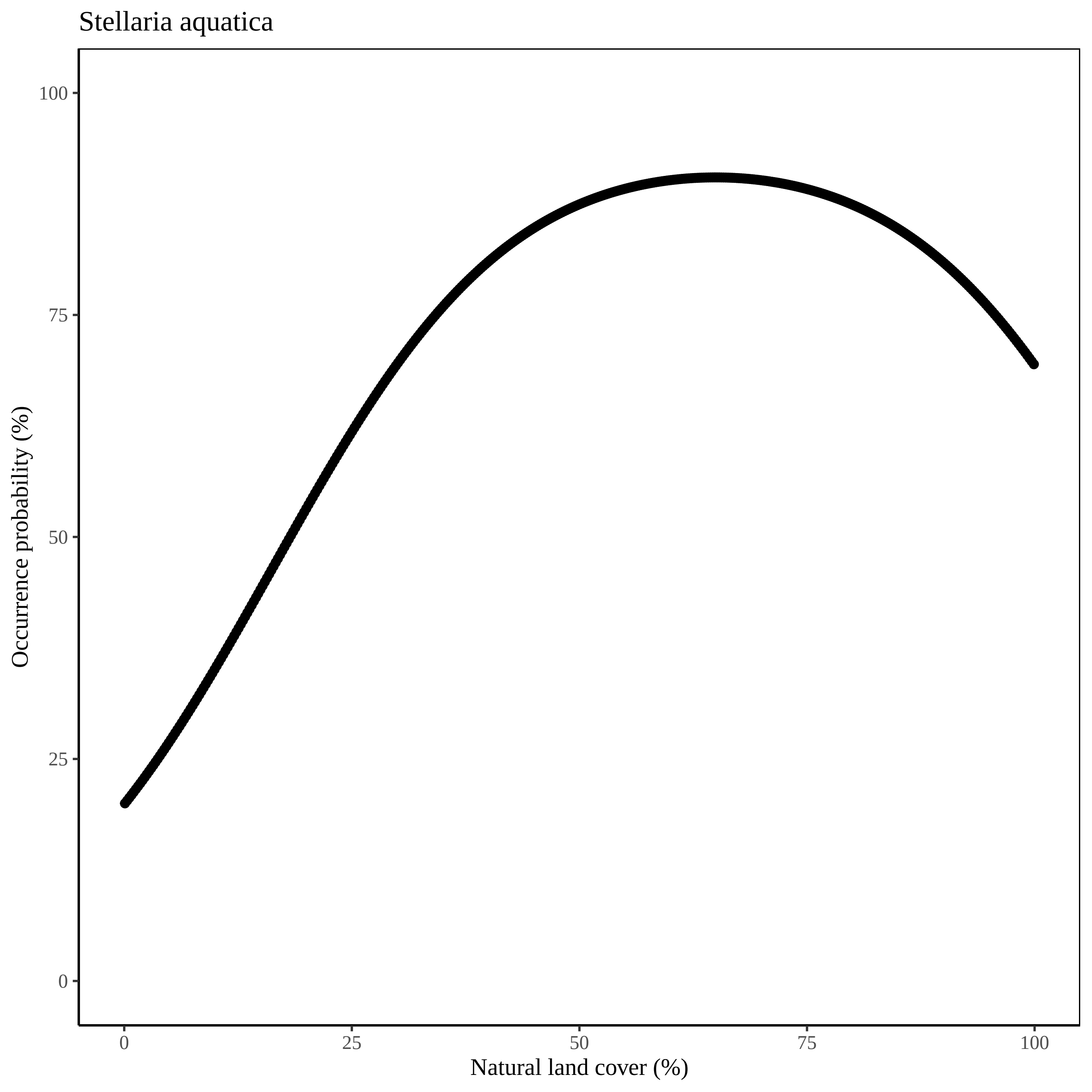
Stellaria aquatica
Occurrence probability (%)
100
75
50
25
0
0
25
50
75
100
Natural land cover (%)

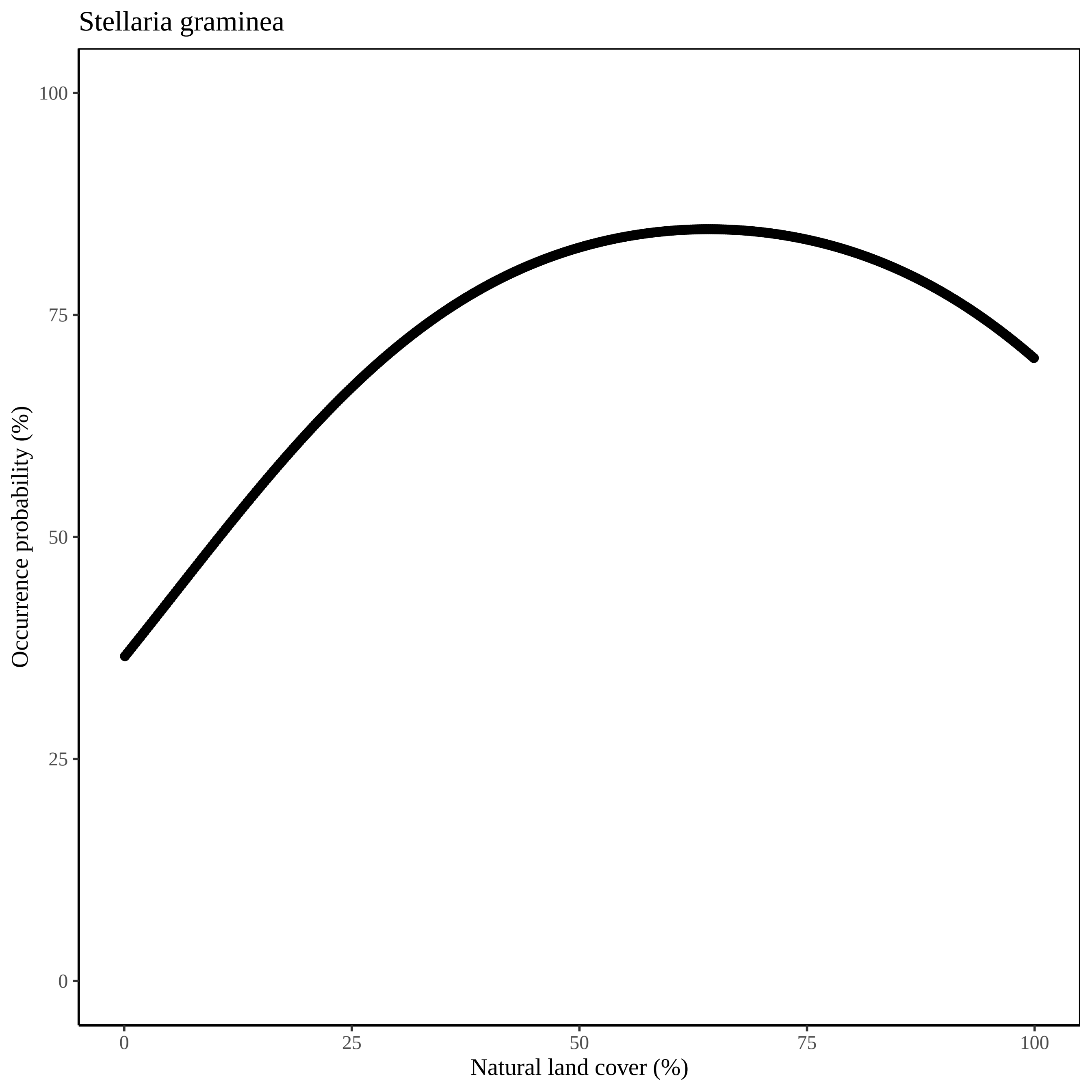
Stellaria graminea
Occurrence probability (%)
100
75
50
25
0
0
25
50
75
100
Natural land cover (%)

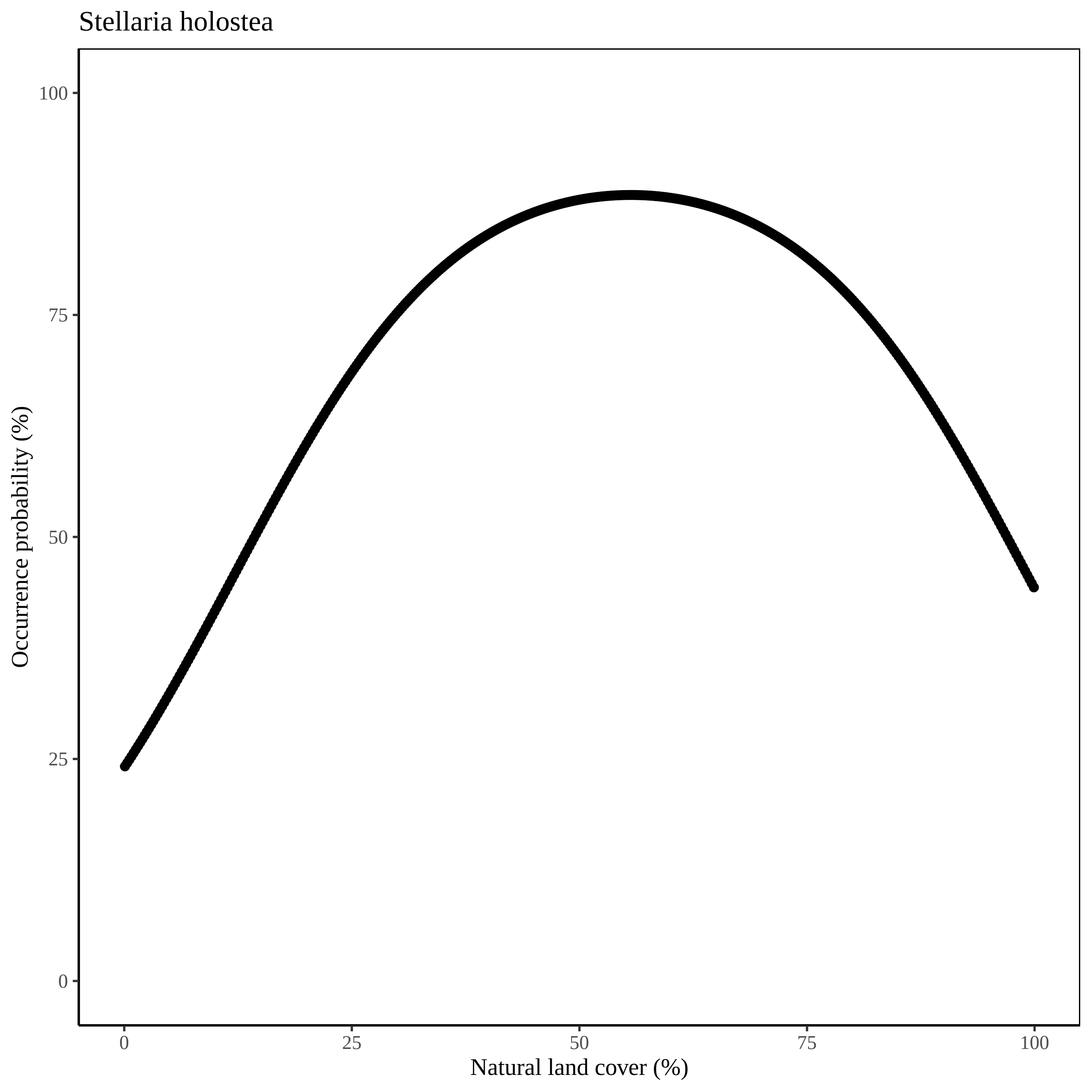
Stellaria holostea
Occurrence probability (%)
100
75
50
25
0
0
25
50
75
100
Natural land cover (%)

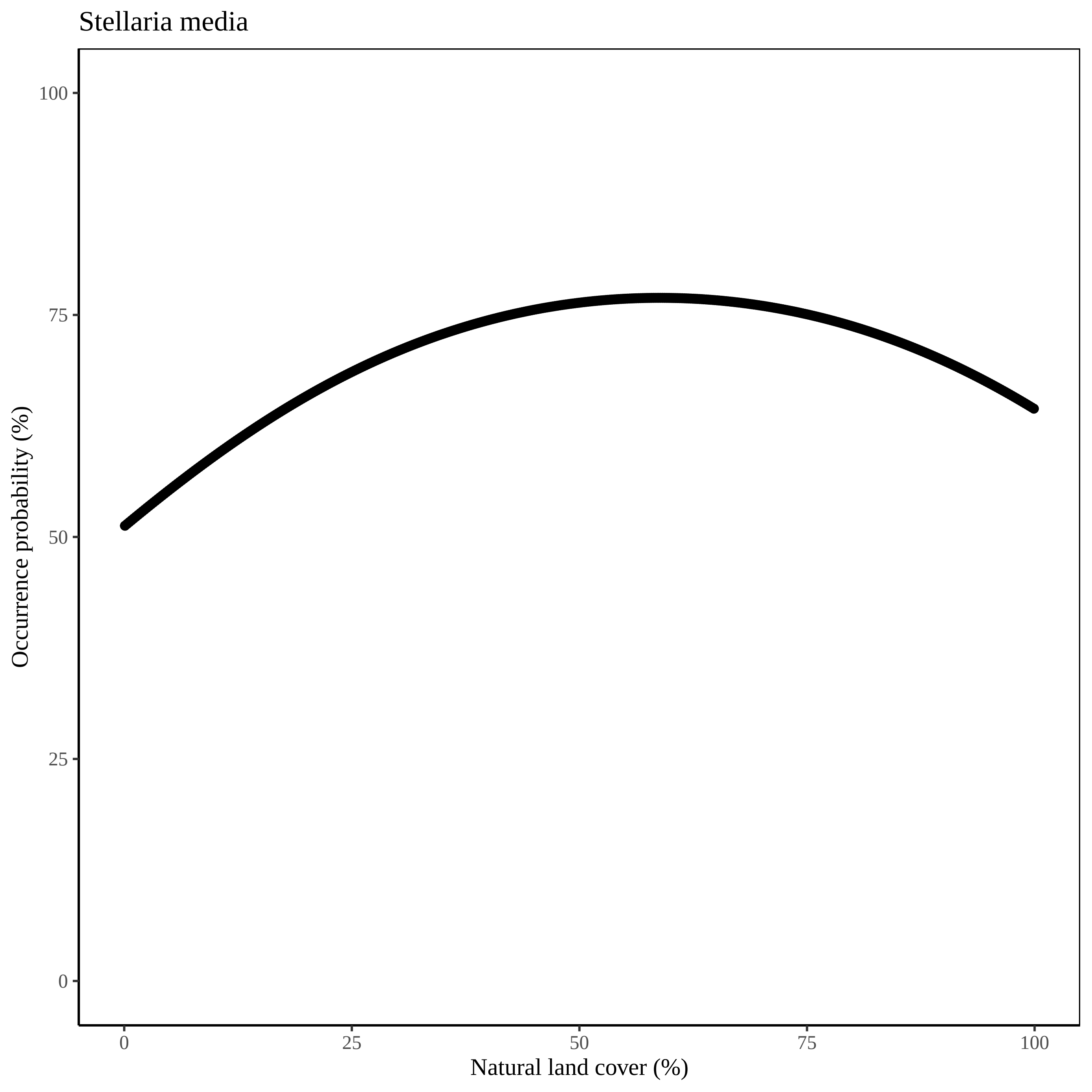
Stellaria media
Occurrence probability (%)
100
75
50
25
0
0
25
50
75
100
Natural land cover (%)

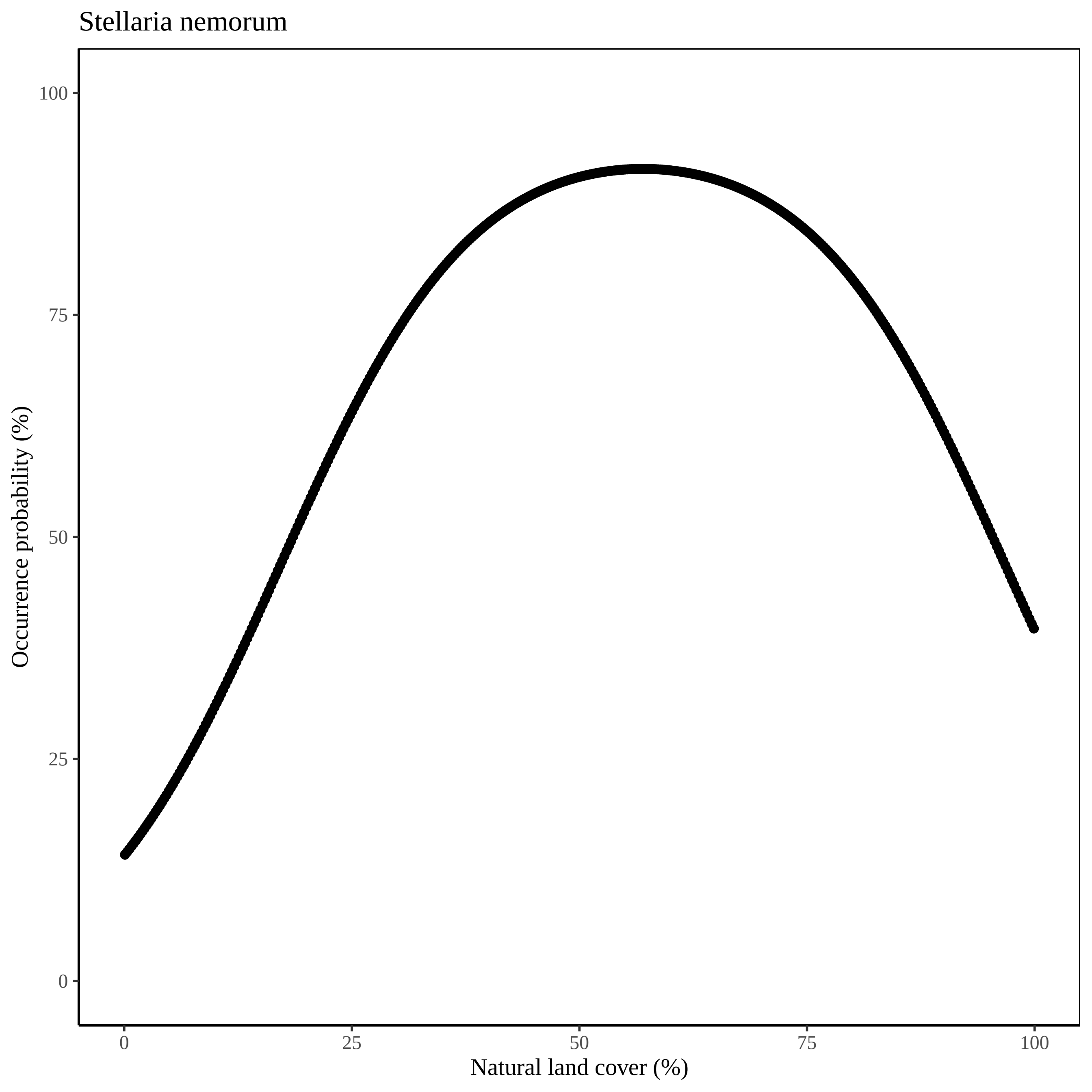

Stellaria nemorum
Occurrence probability (%)
100
75
50
25
0
0
25
50
75
100
Natural land cover (%)

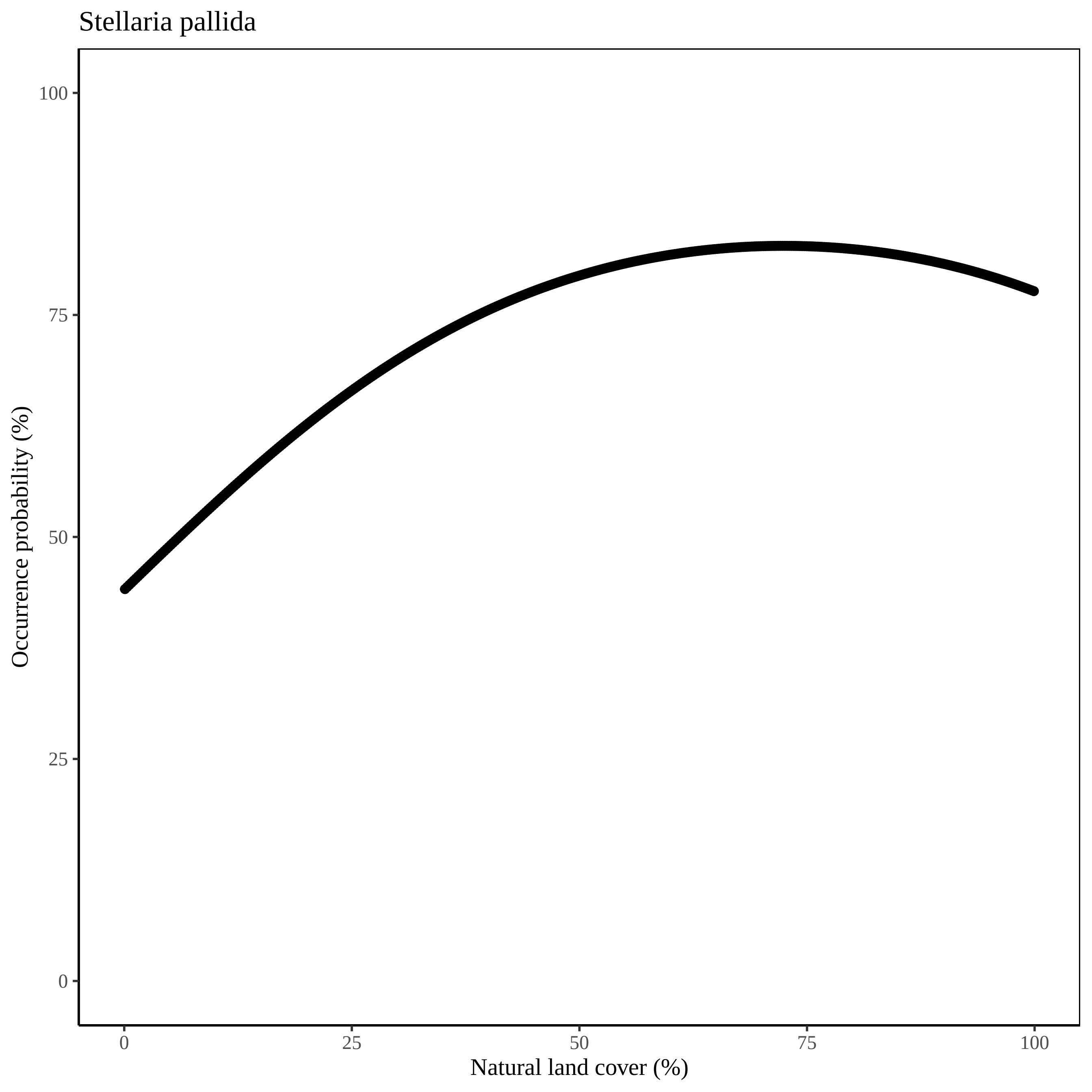
Stellaria pallida
Occurrence probability (%)
100
75
50
25
0
0
25
50
75
100
Natural land cover (%)

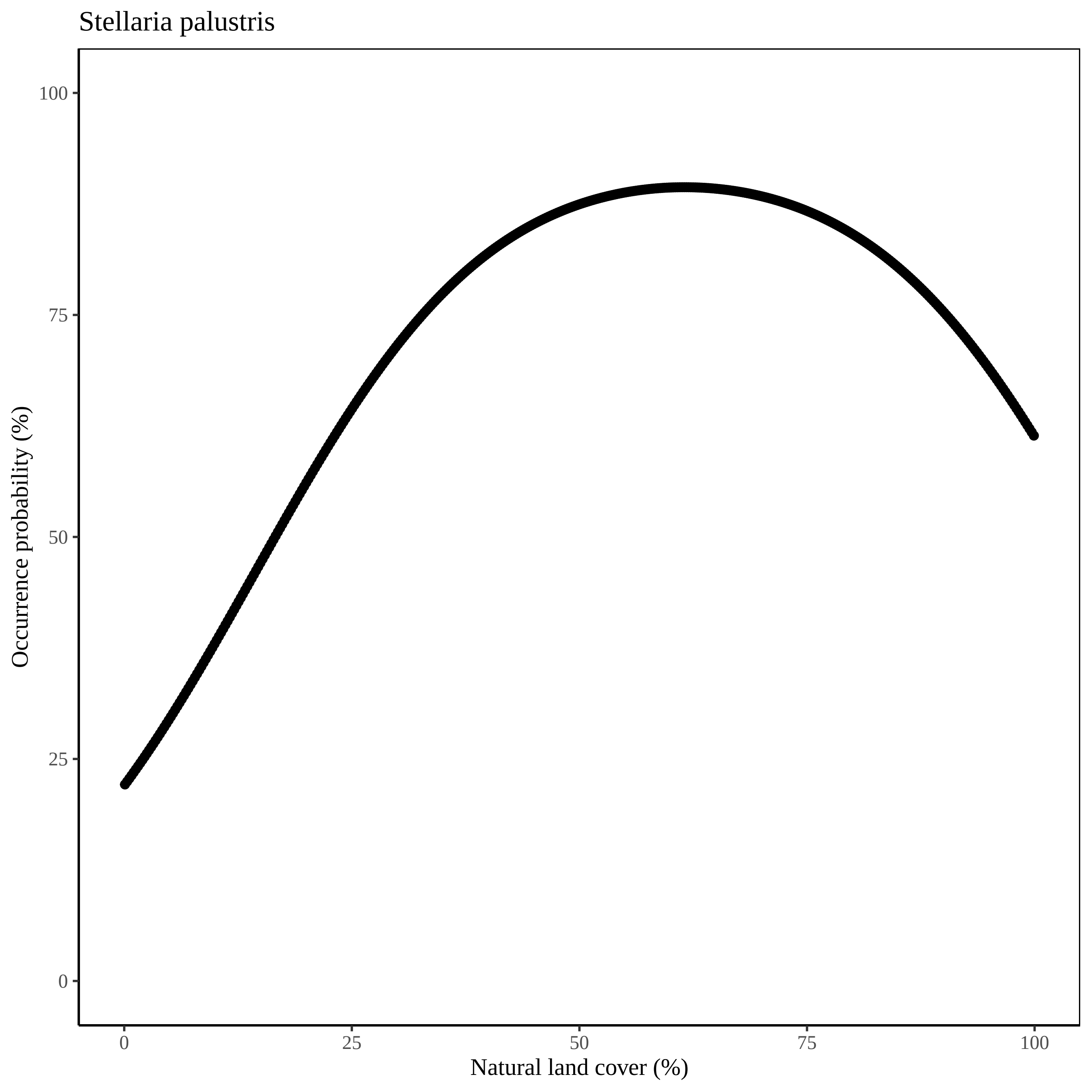
Stellaria palustris
100
75
50
25
0
Occurrence probability (%)
0
25
50
75
100
Natural land cover (%)

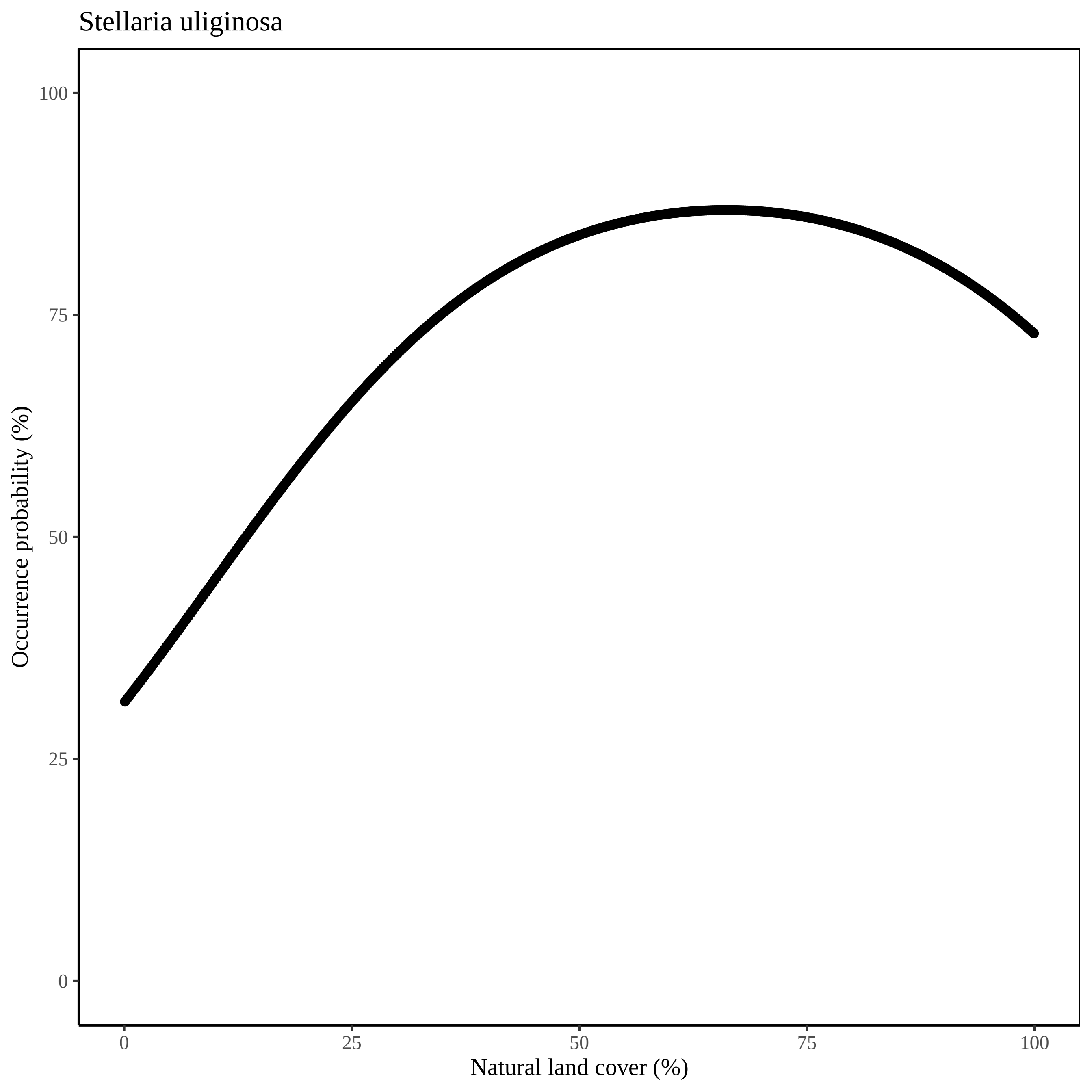
Stellaria uliginosa
Occurrence probability (%)
100
75
50
25
0
0
25
50
75
100
Natural land cover (%)

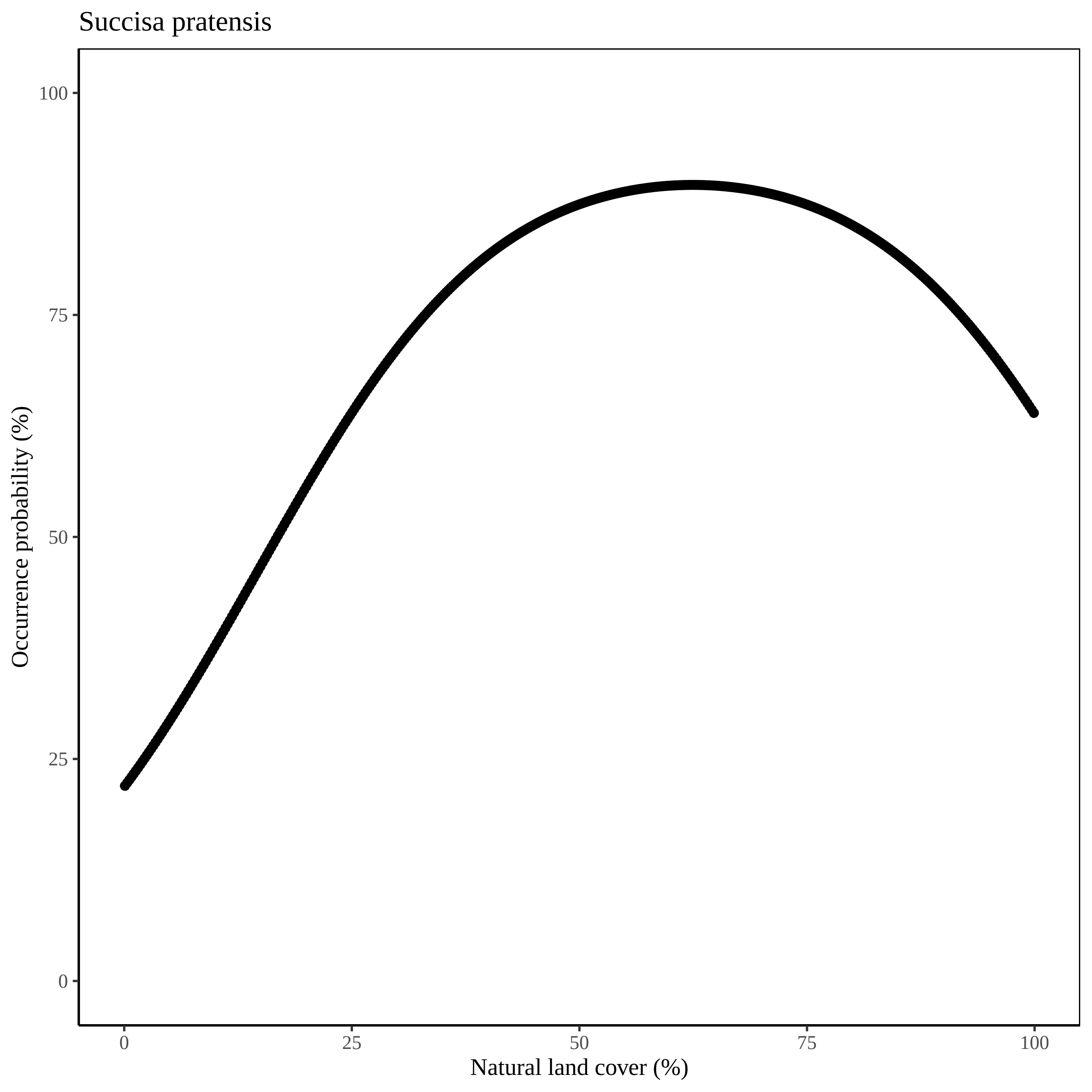

Succisa pratensis
100
75
50
25
0
Occurrence probability (%)
0
25
50
75
100
Natural land cover (%)

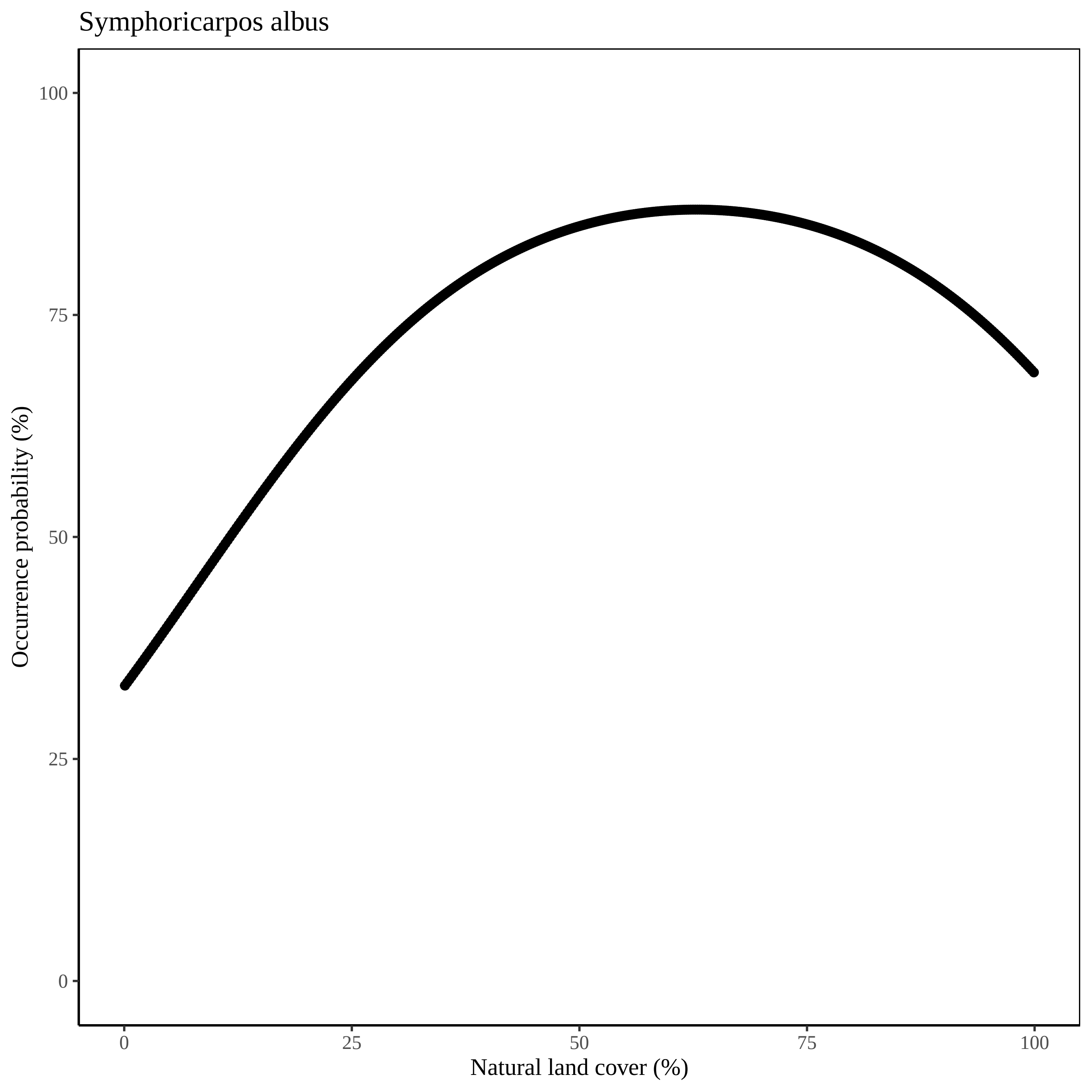
Symphoricarpos albus
Occurrence probability (%)
100
75
50
25
0
0
25
50
75
100
Natural land cover (%)

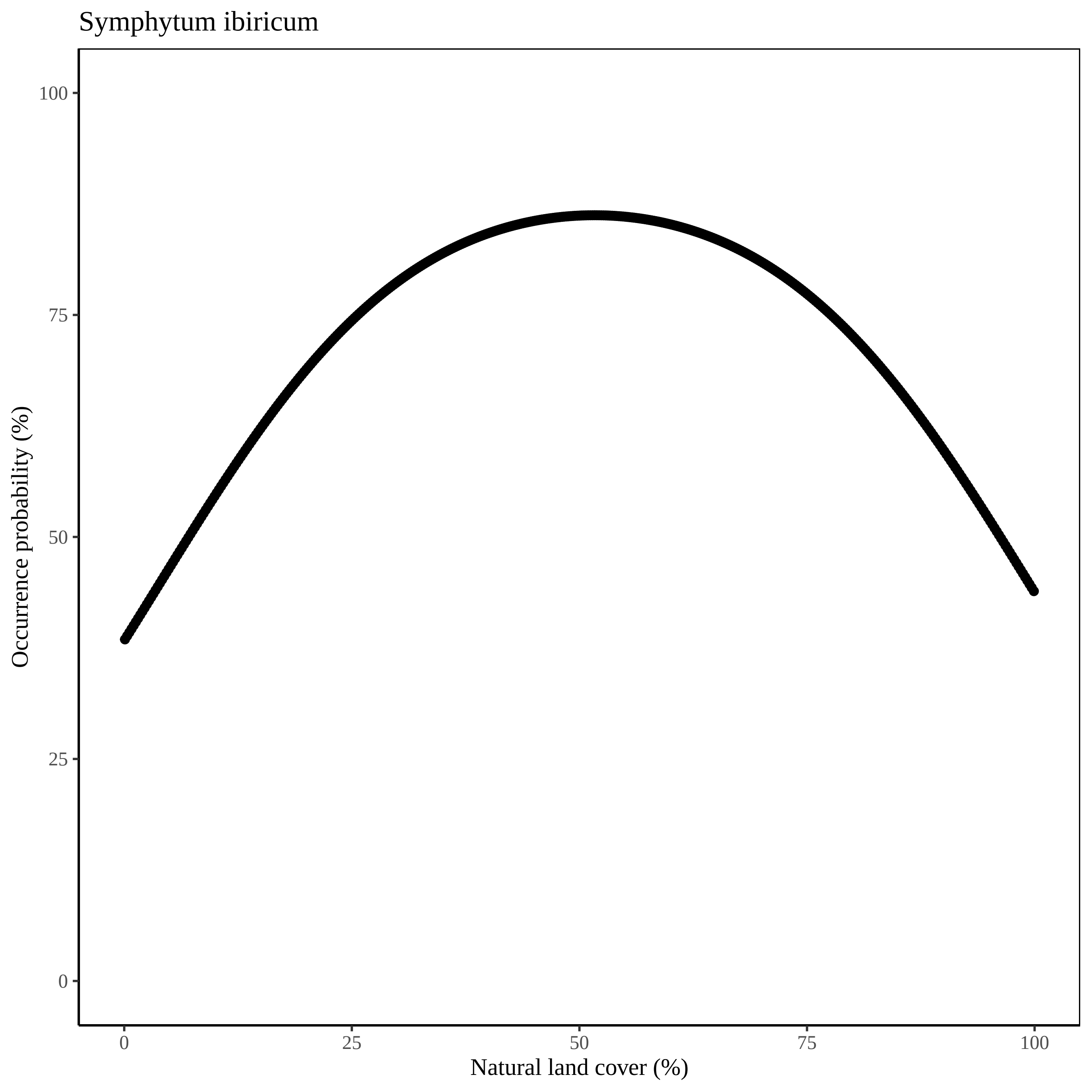
Symphytum ibiricum
Occurrence probability (%)
100
75
50
25
0
0
25
50
75
100
Natural land cover (%)

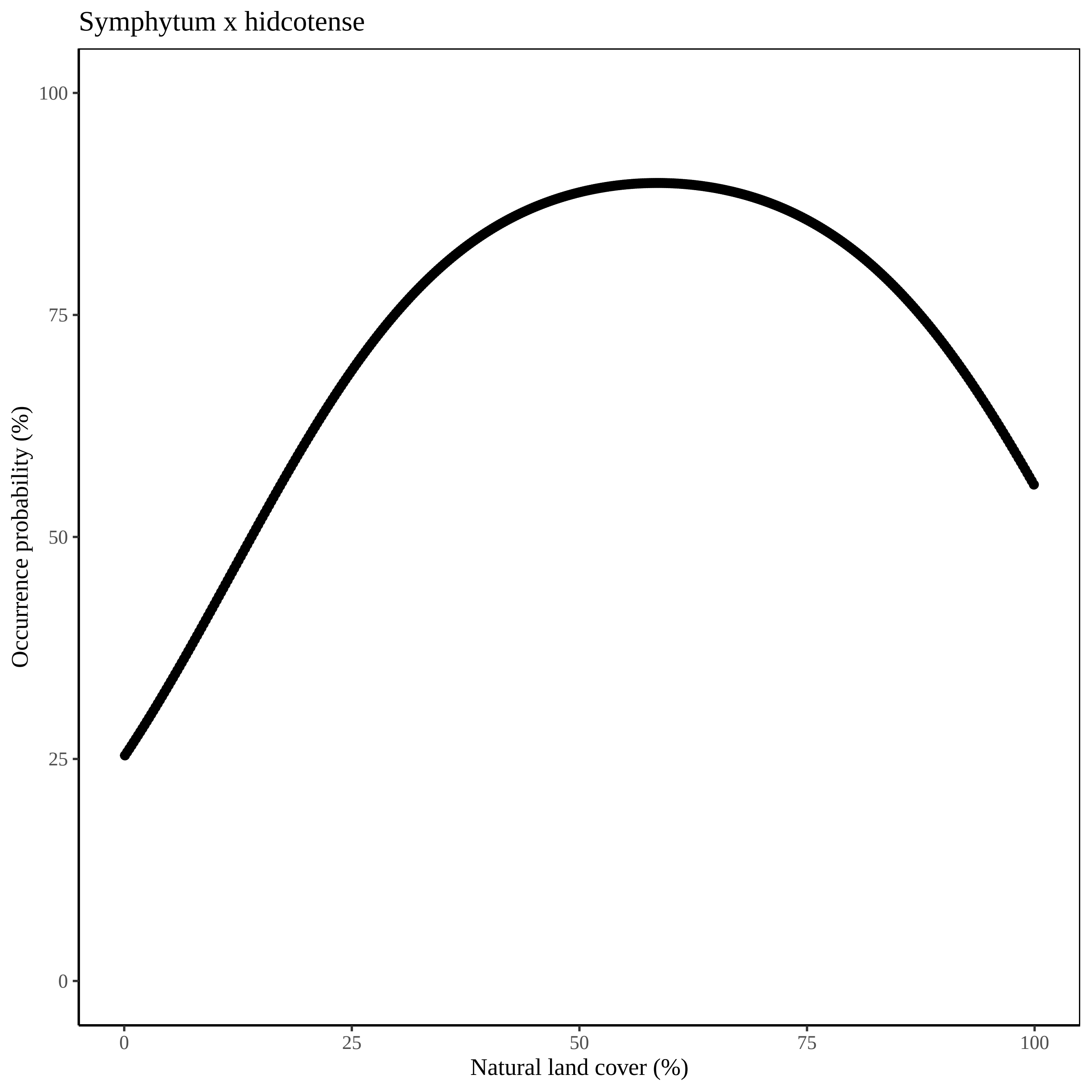
Symphytum x hidcotense
Occurrence probability (%)
100
75
50
25
0
0
25
50
75
100
Natural land cover (%)

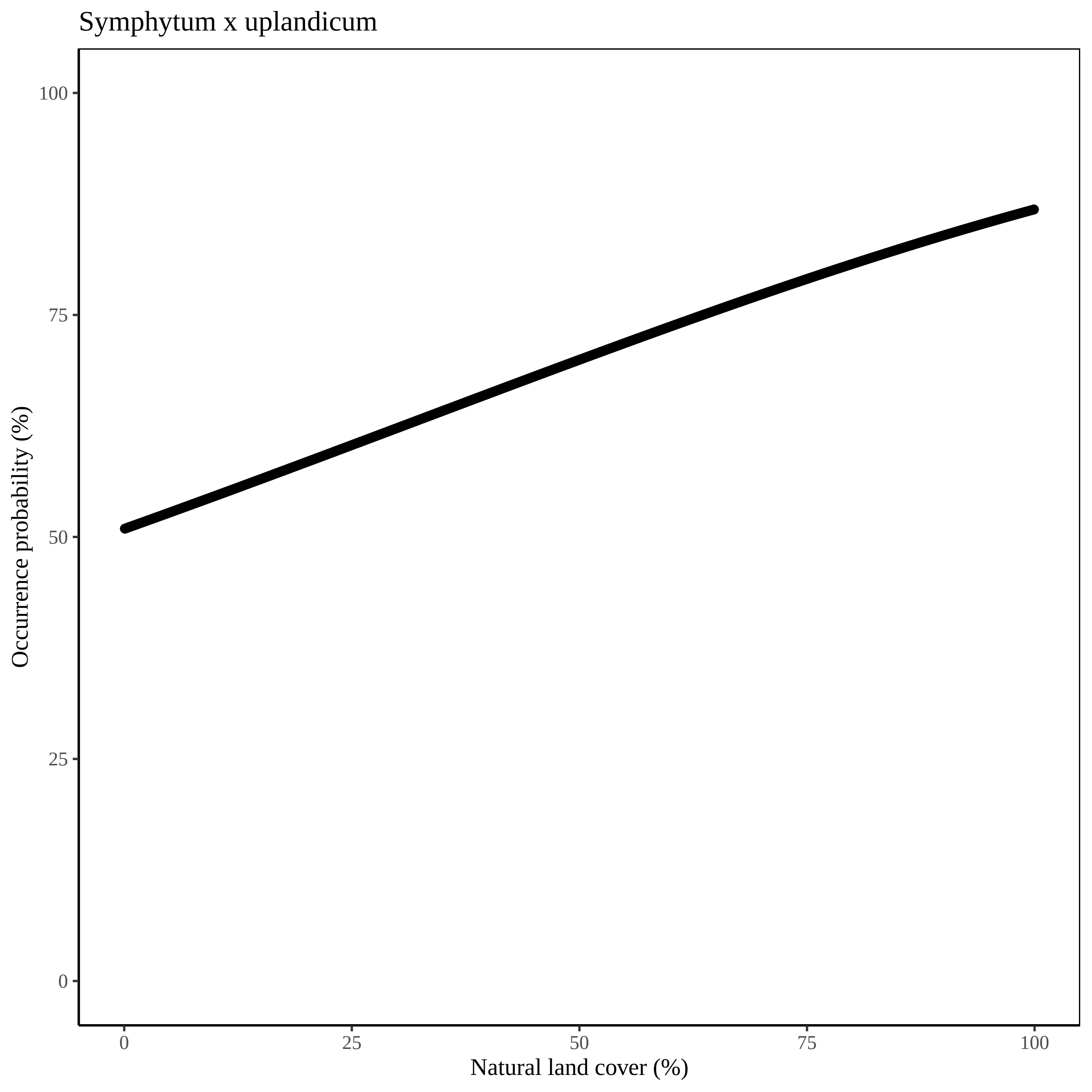
Symphytum x uplandicum
Occurrence probability (%)
100
75
50
25
0
0
25
50
75
100
Natural land cover (%)

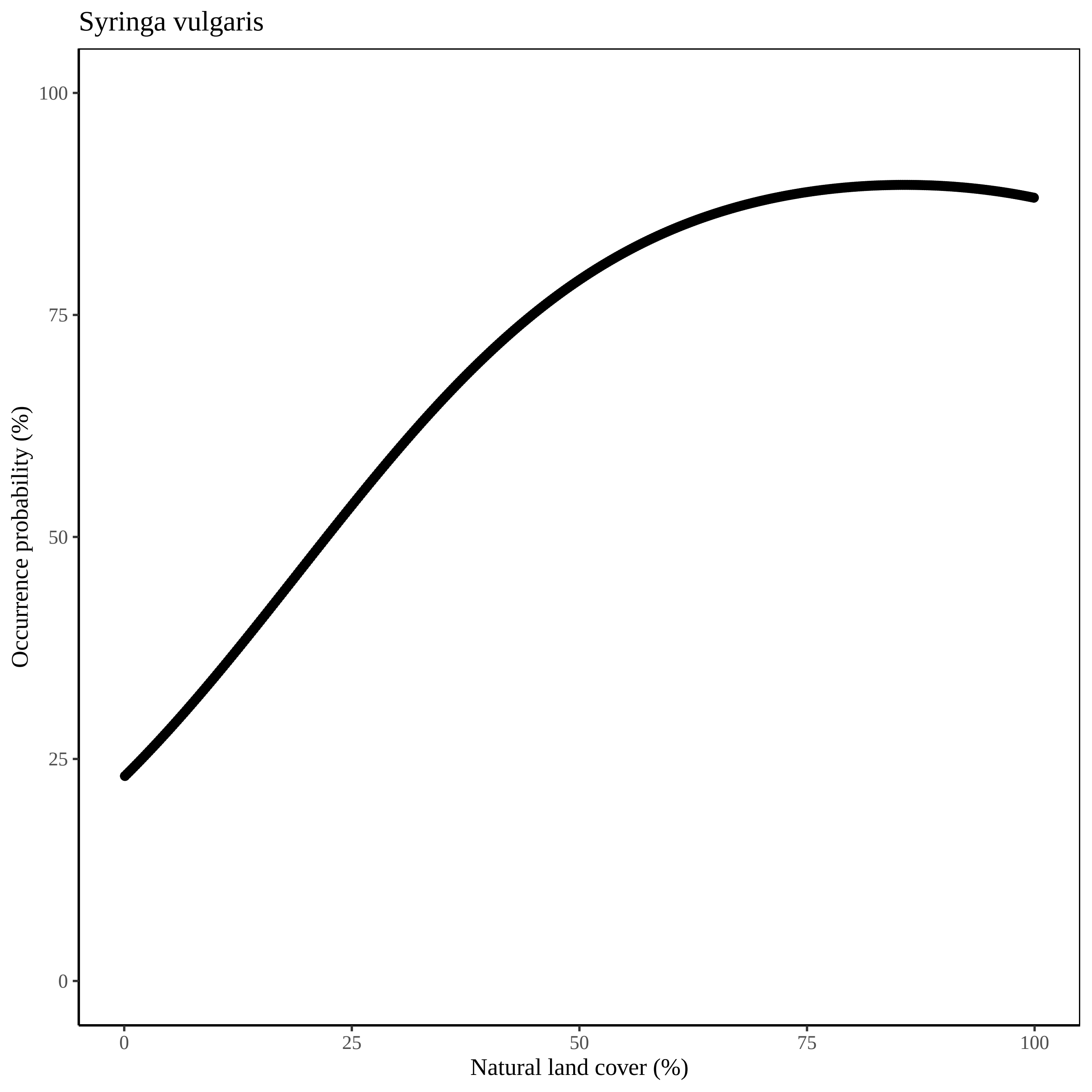
Syringa vulgaris
Occurrence probability (%)
100
75
50
25
0
0
25
50
75
100
Natural land cover (%)

Tamarix gallica

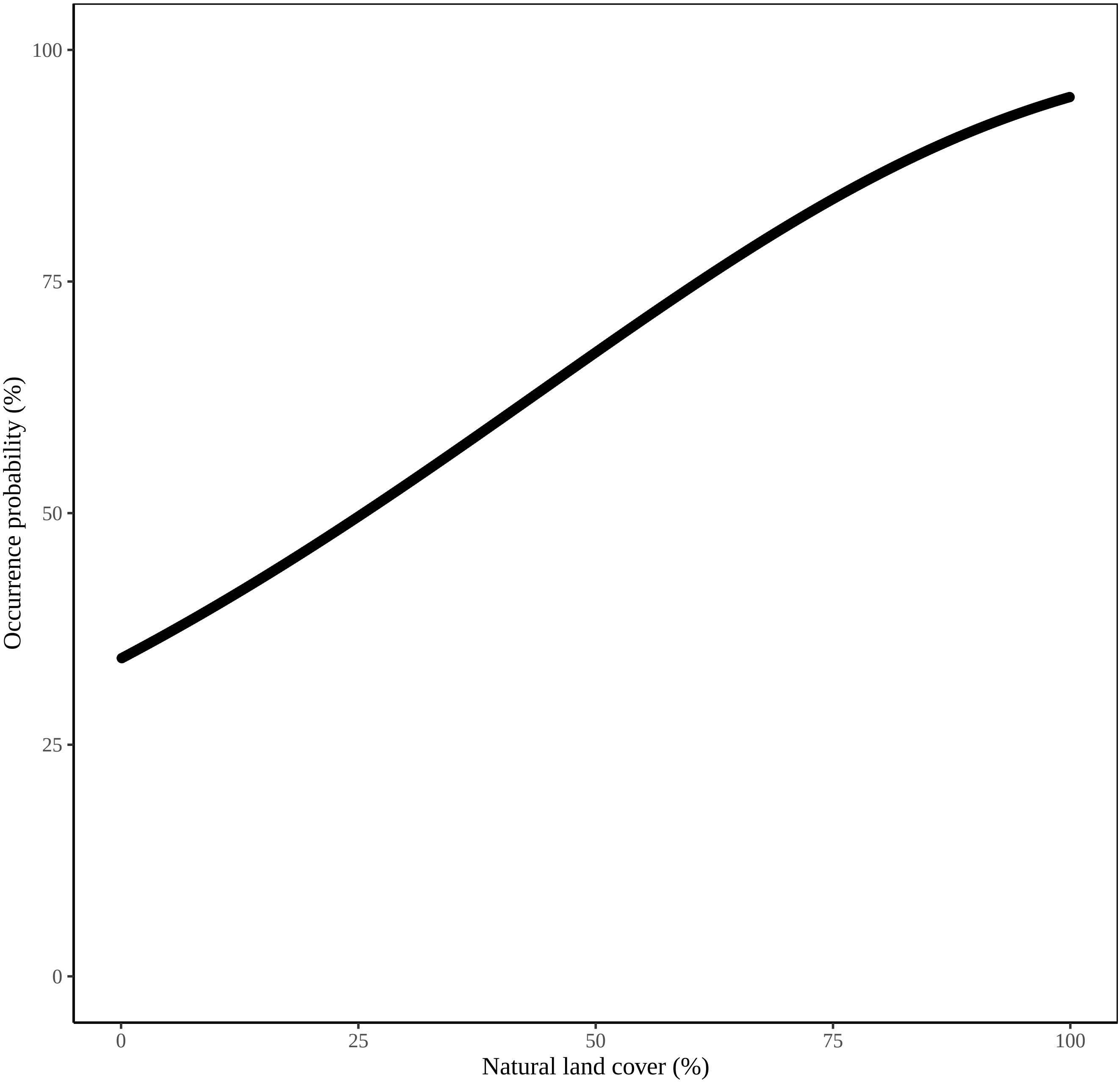

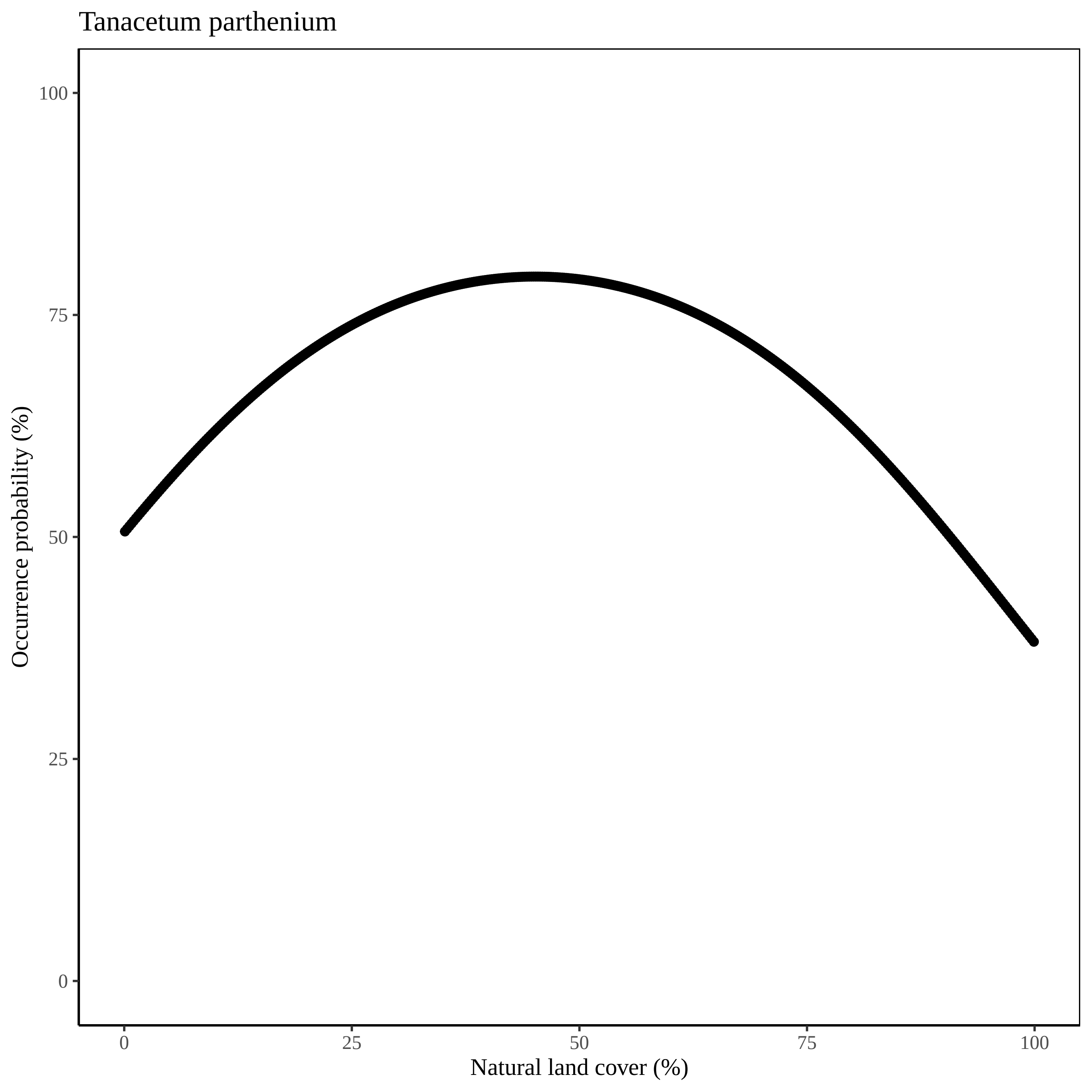
Tanacetum parthenium
Occurrence probability (%)
100
75
50
25
0
0
25
50
75
100
Natural land cover (%)

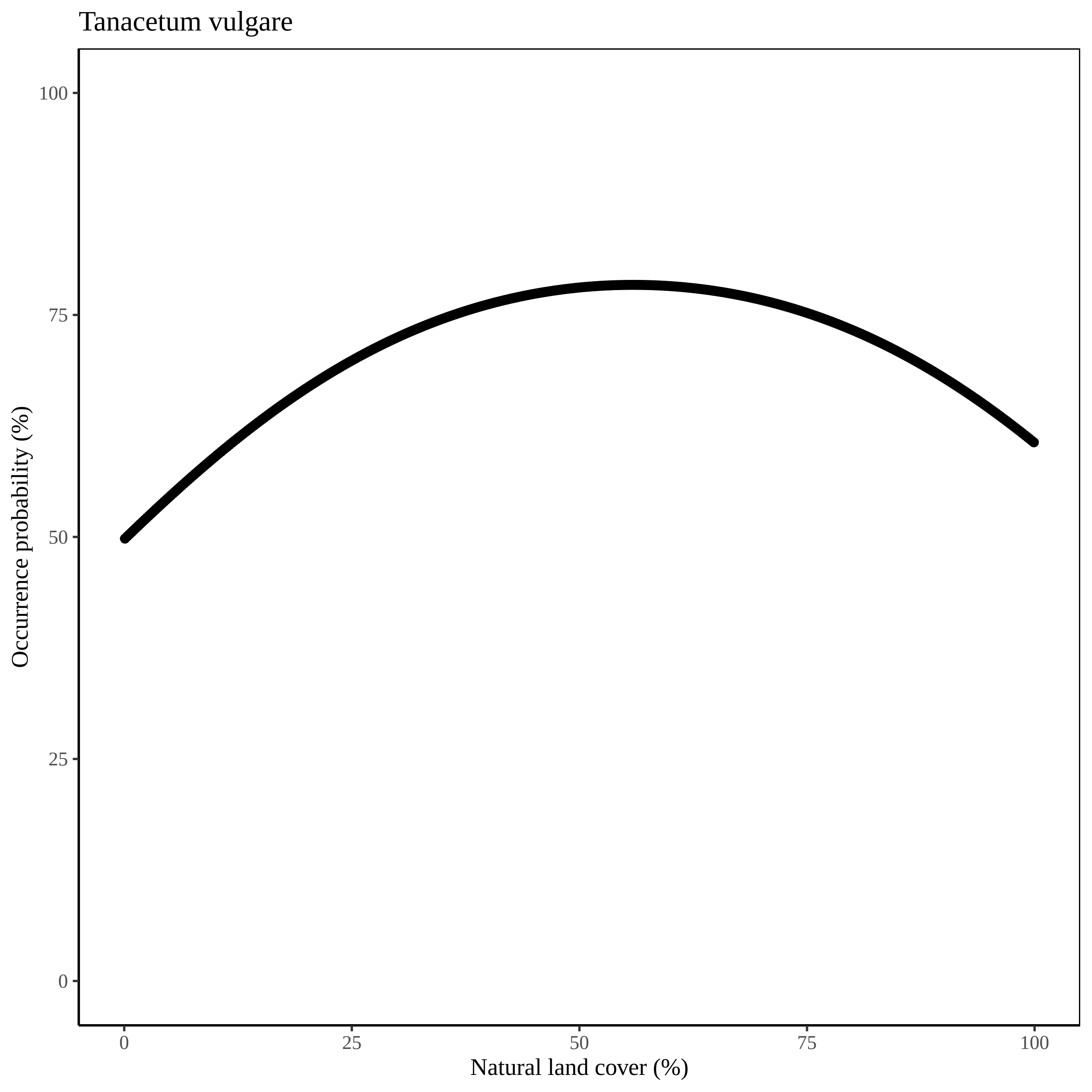
Tanacetum vulgare
100
75
50
25
0
Occurrence probability (%)
0
25
50
75
100
Natural land cover (%)

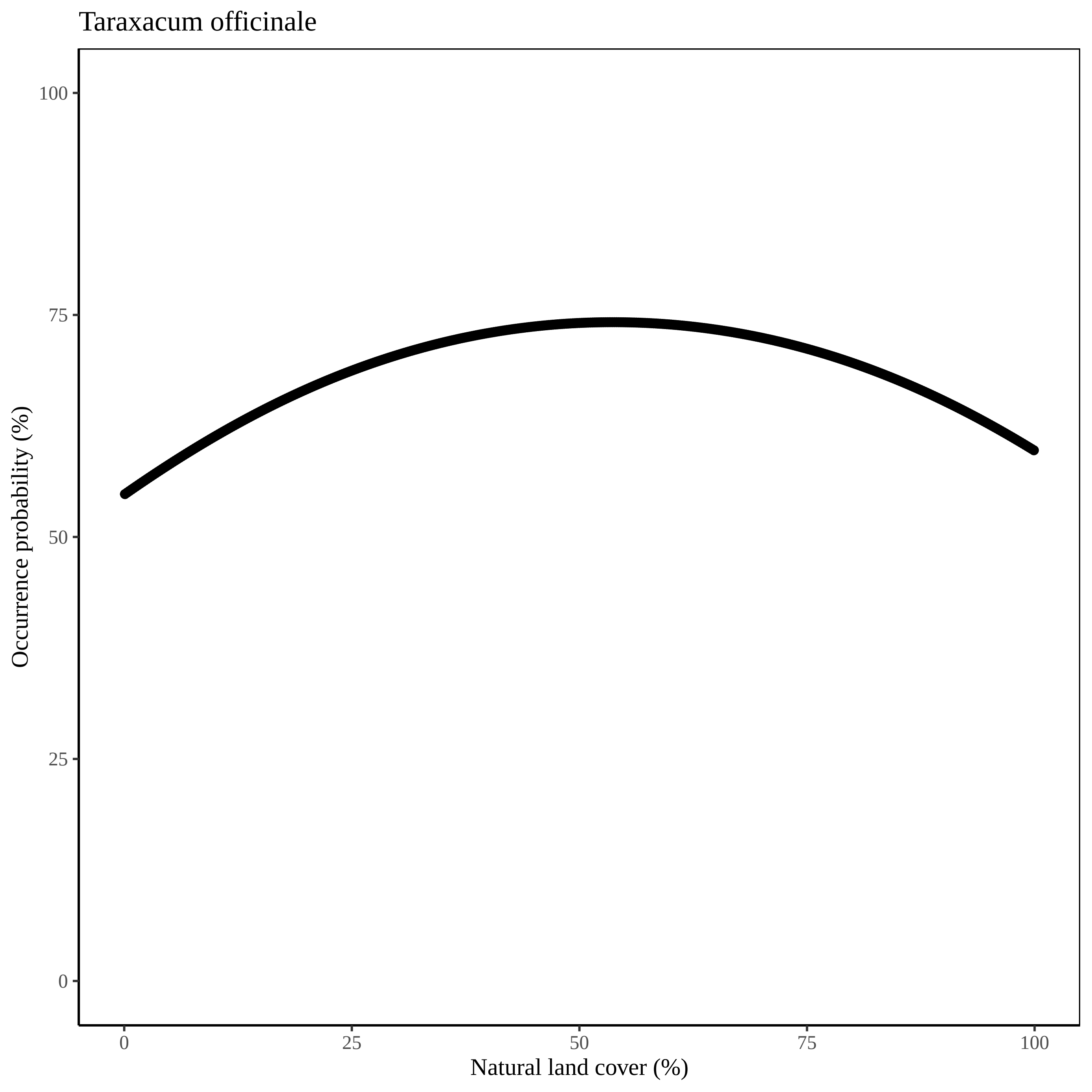
Taraxacum officinale
Occurrence probability (%)
100
75
50
25
0
0
25
50
75
100
Natural land cover (%)

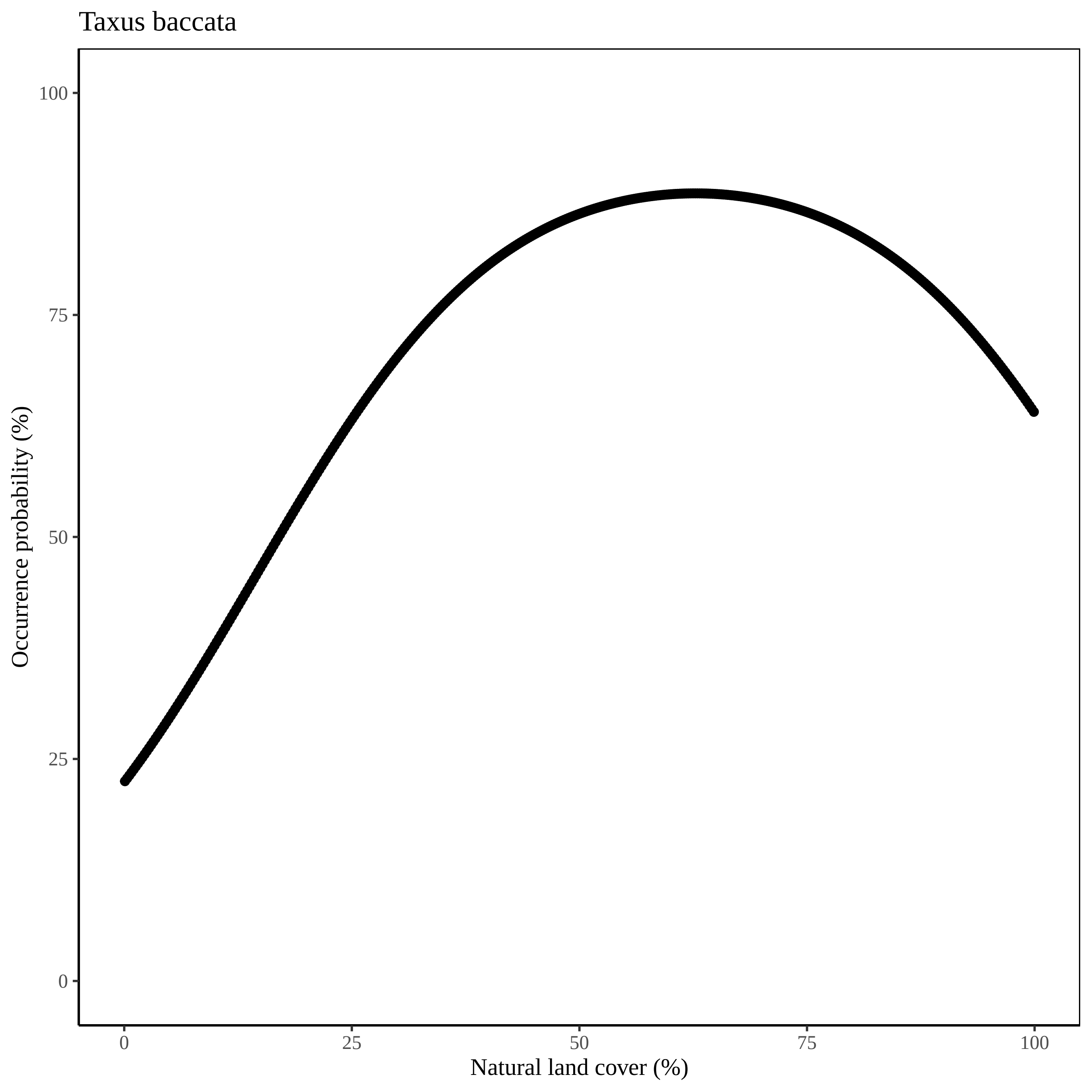
Taxus baccata
Occurrence probability (%)
100
75
50
25
0
0
25
50
75
100
Natural land cover (%)

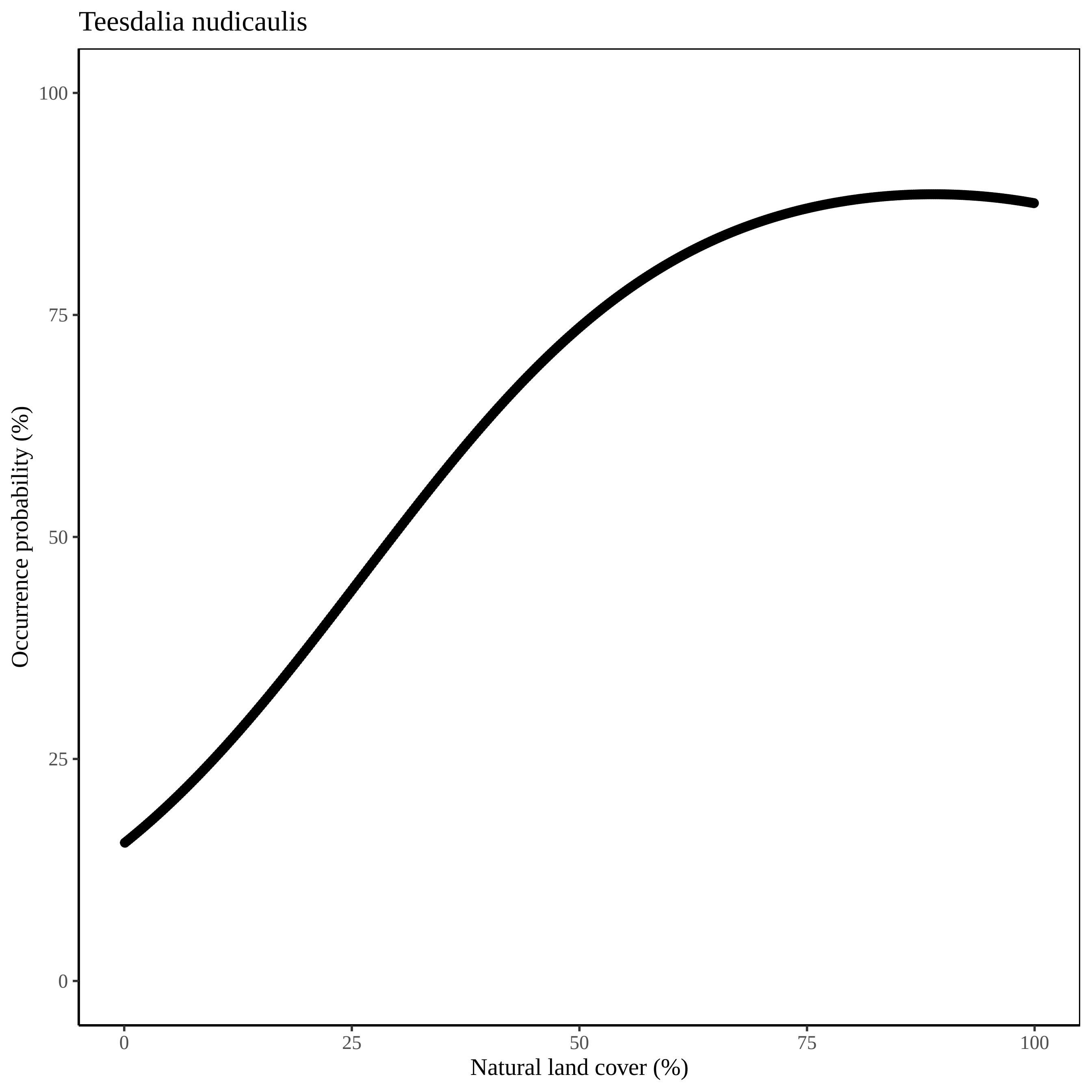
Teesdalia nudicaulis
Occurrence probability (%)
100
75
50
25
0
0
25
50
75
100
Natural land cover (%)

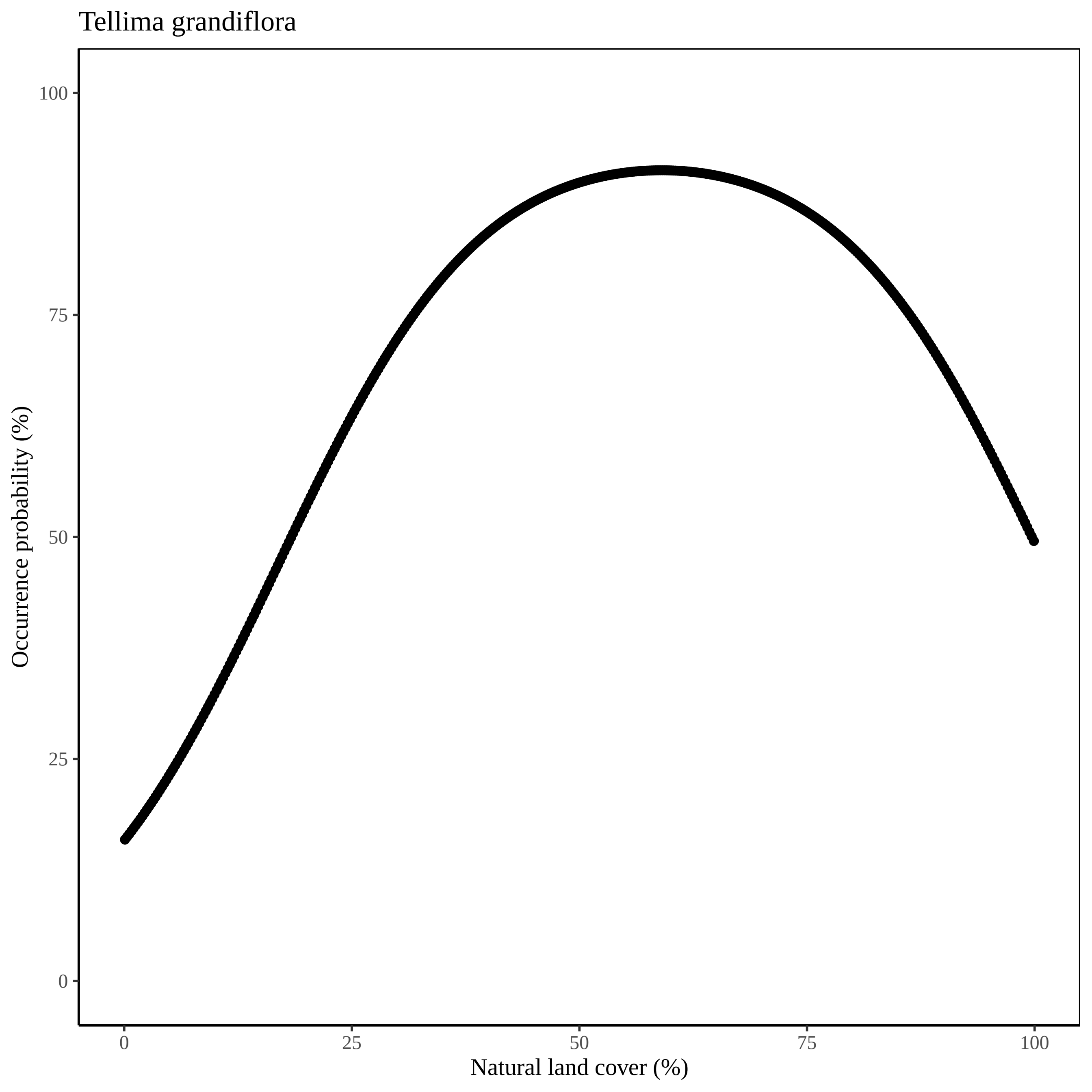
Tellima grandiflora
Occurrence probability (%)
100
75
50
25
0
0
25
50
75
100
Natural land cover (%)

Teucrium scorodonia

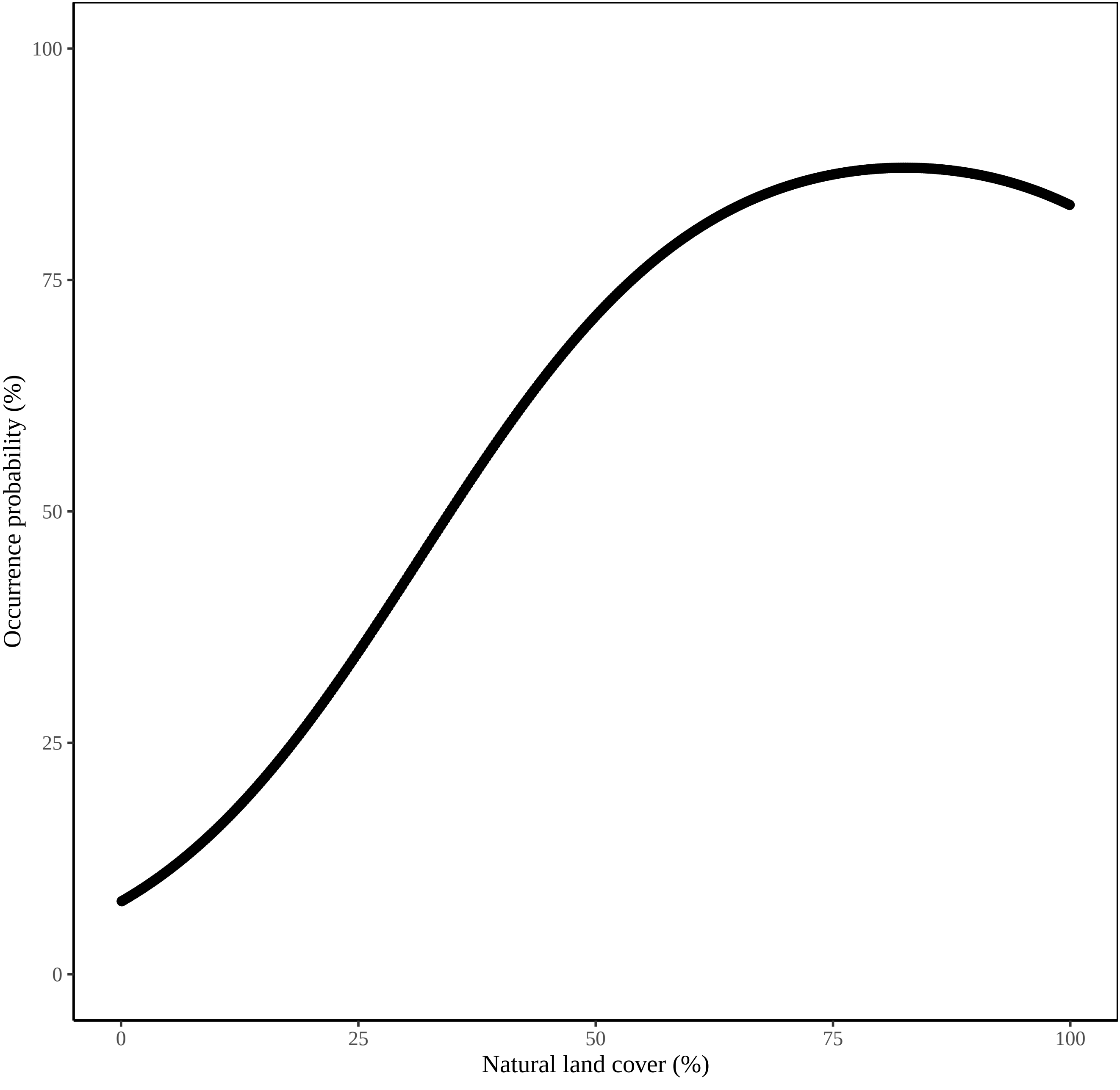

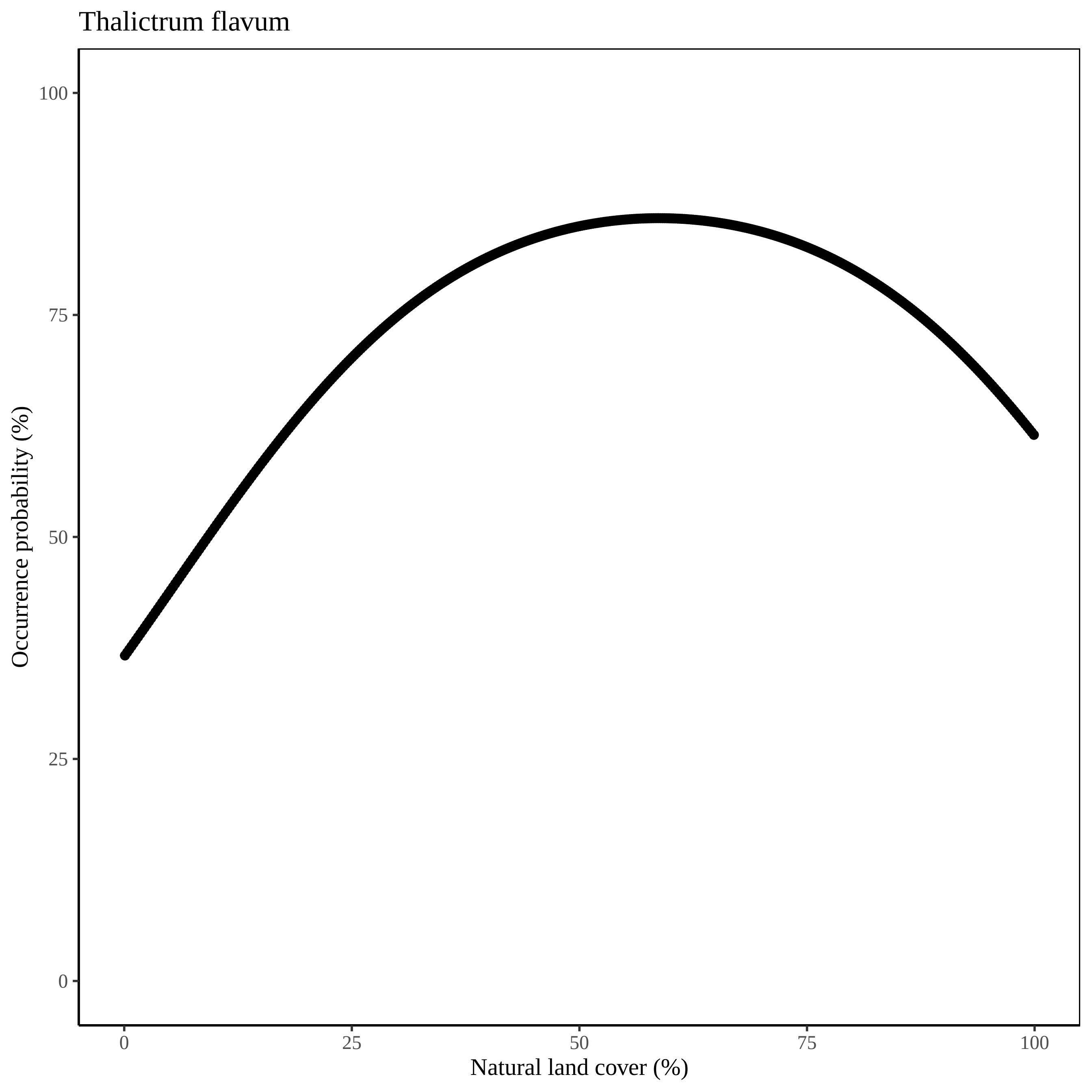
Thalictrum flavum
Occurrence probability (%)
100
75
50
25
0
0
25
50
75
100
Natural land cover (%)

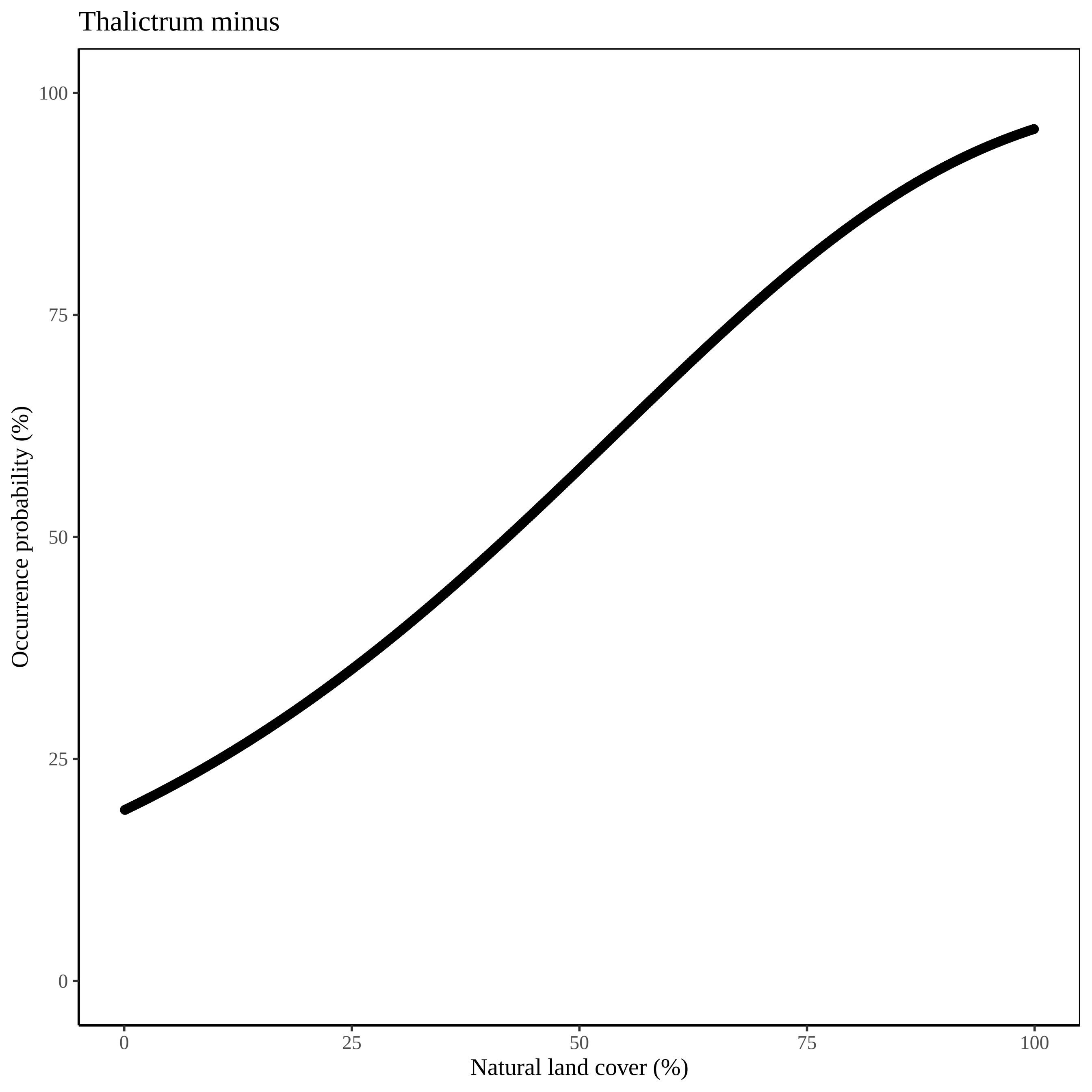
Thalictrum minus
Occurrence probability (%)
100
75
50
25
0
0
25
50
75
100
Natural land cover (%)

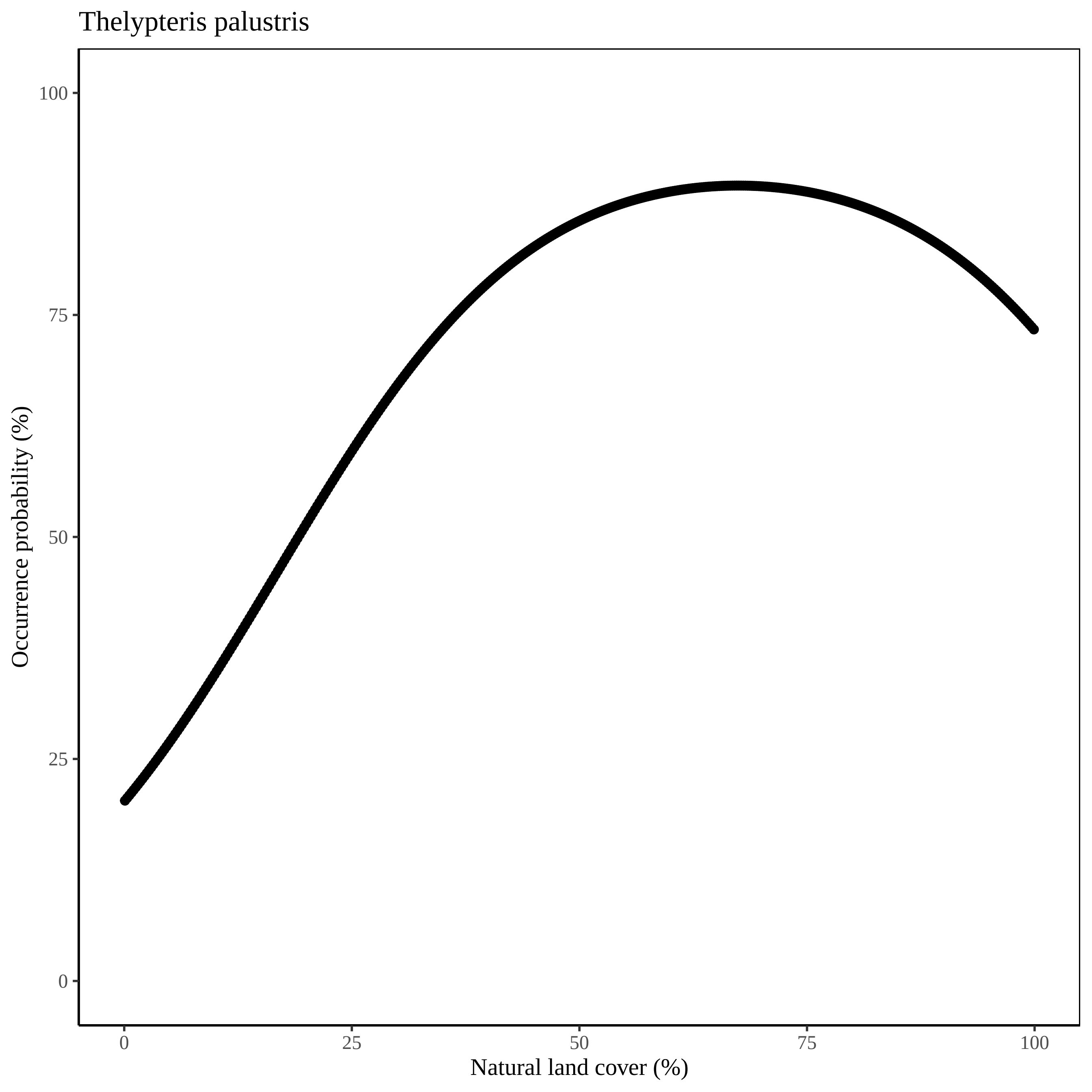
Thelypteris palustris
100
75
50
25
0
Occurrence probability (%)
0
25
50
75
100
Natural land cover (%)

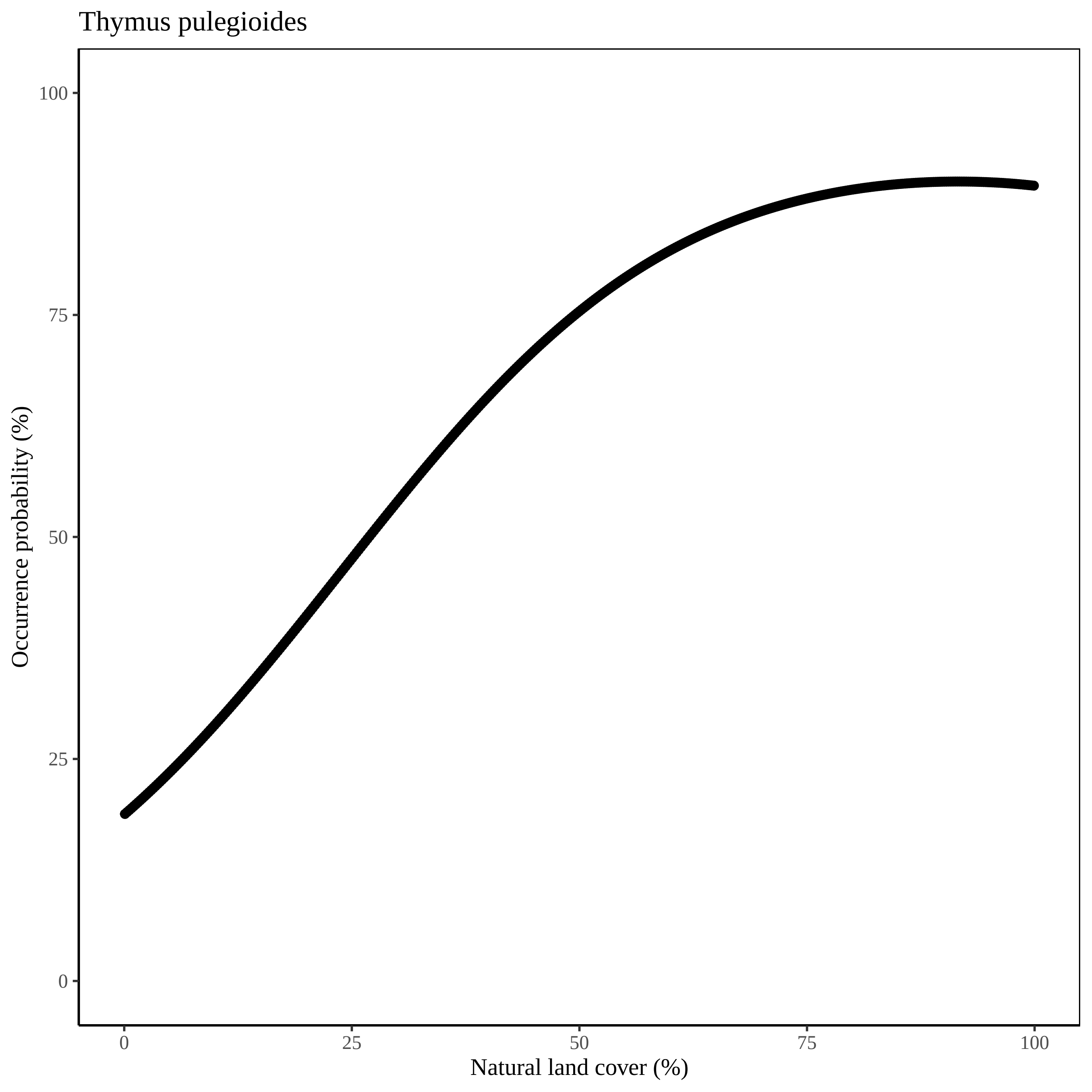
Thymus pulegioides
Occurrence probability (%)
100
75
50
25
0
0
25
50
75
100
Natural land cover (%)

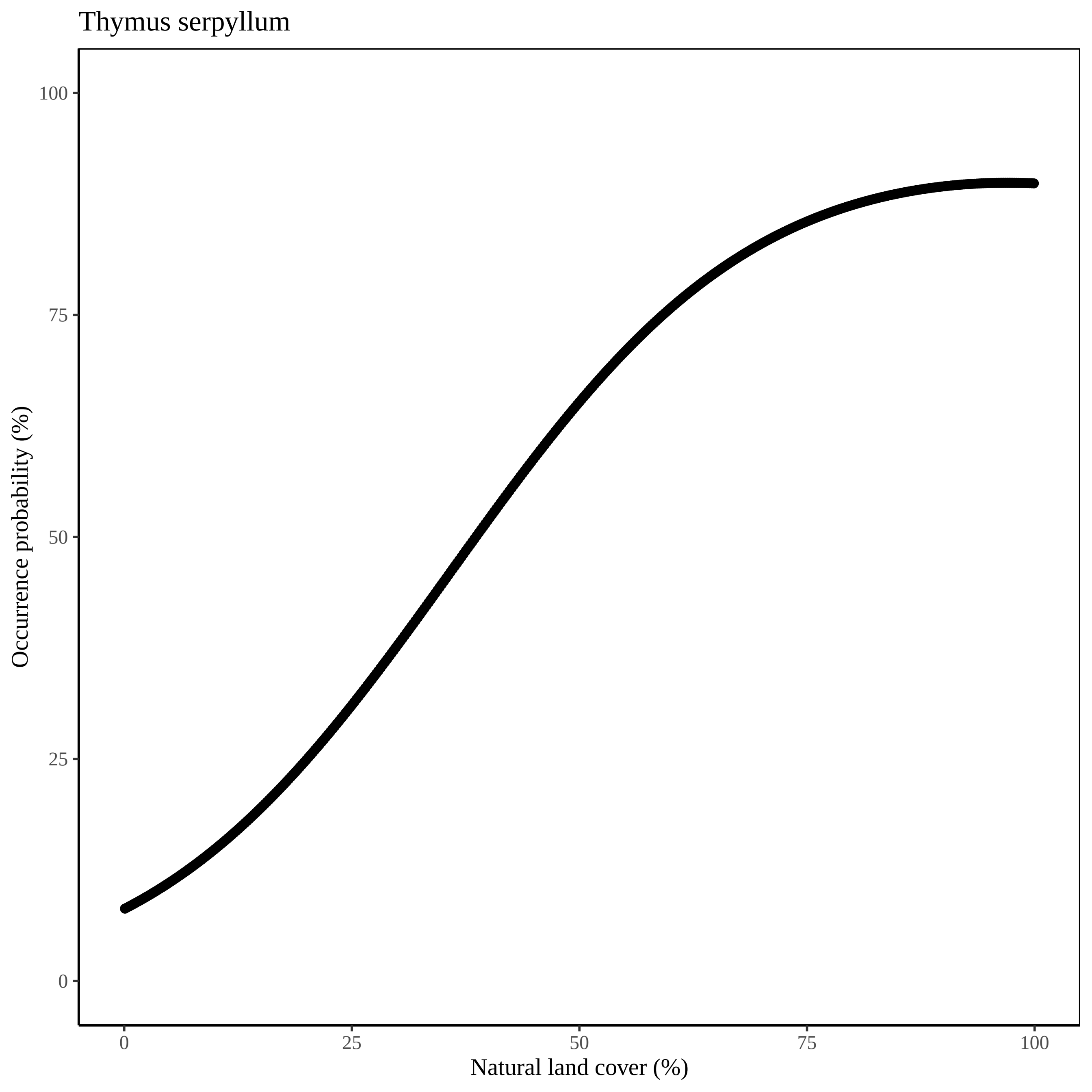
Thymus serpyllum
Occurrence probability (%)
100
75
50
25
0
0
25
50
75
100
Natural land cover (%)

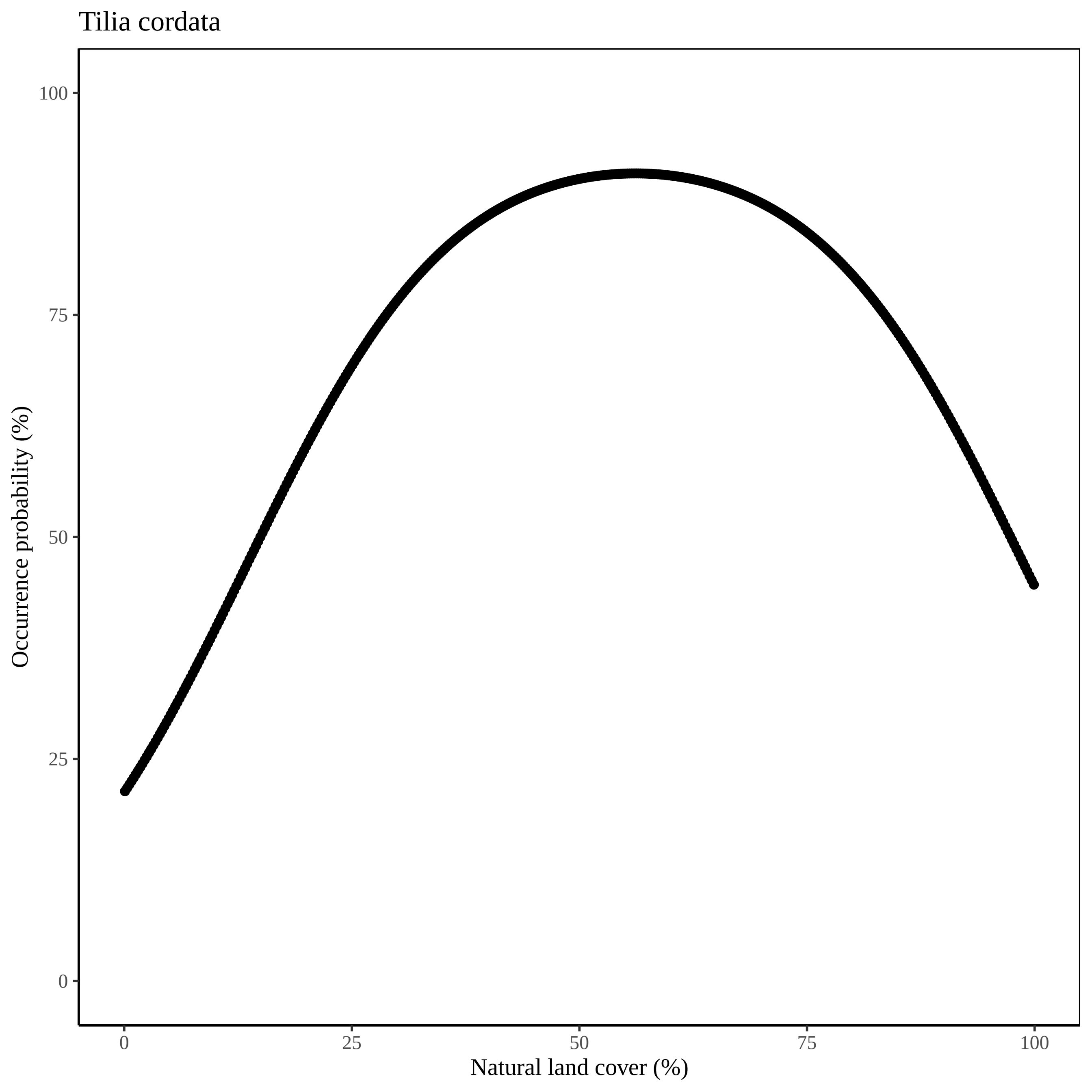
Tilia cordata
Occurrence probability (%)
100
75
50
25
0
0
25
50
75
100
Natural land cover (%)

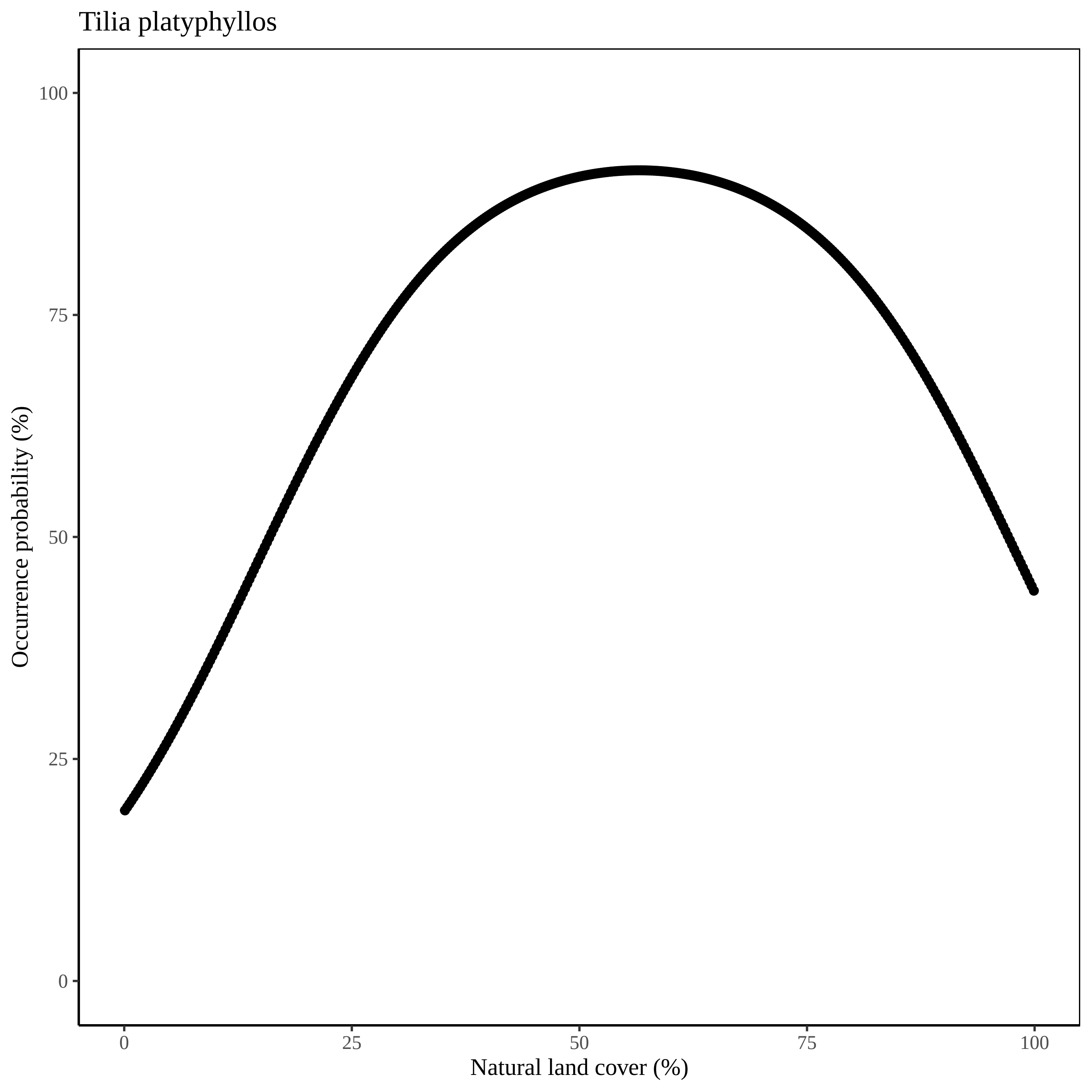
Tilia platyphyllos
Occurrence probability (%)
Natural land cover (%)
0
25
50
75
100

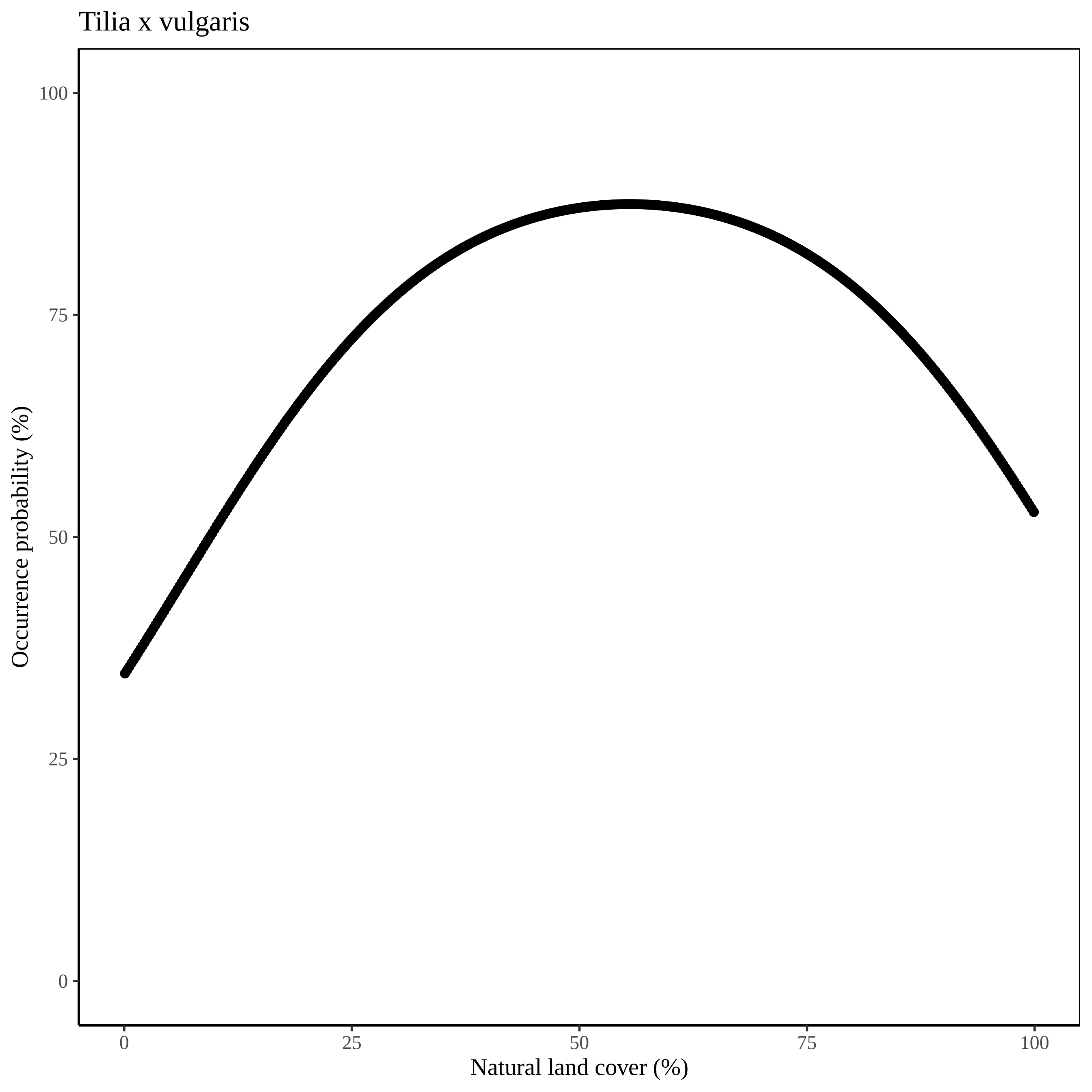
Tilia x vulgaris
100
75
50
25
0
0
25
50
75
100
Occurrence probability (%)
Natural land cover (%)

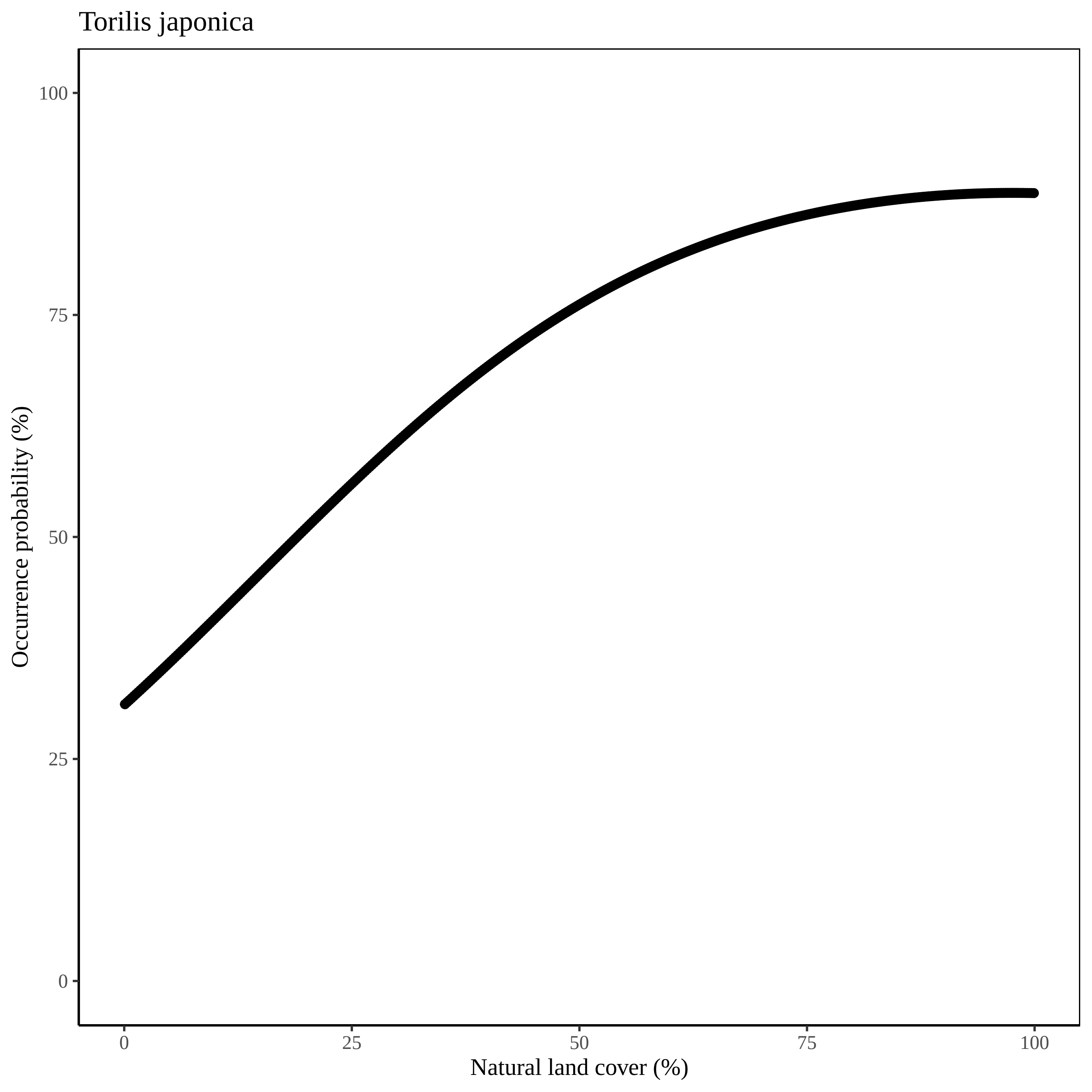
Torilis japonica
Occurrence probability (%)
100
75
50
25
0
0
25
50
75
100
Natural land cover (%)

Trichophorum cespitosum

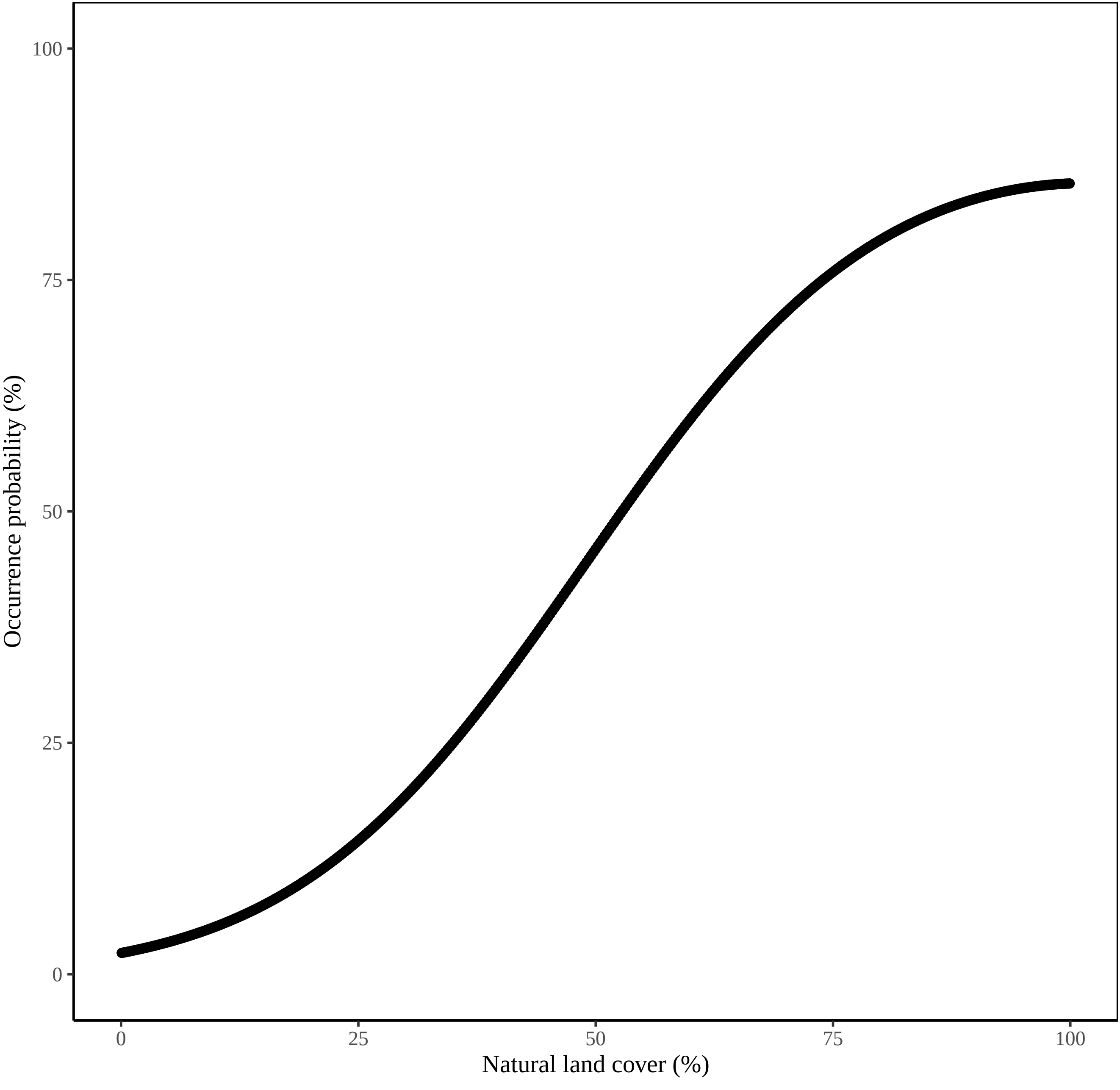

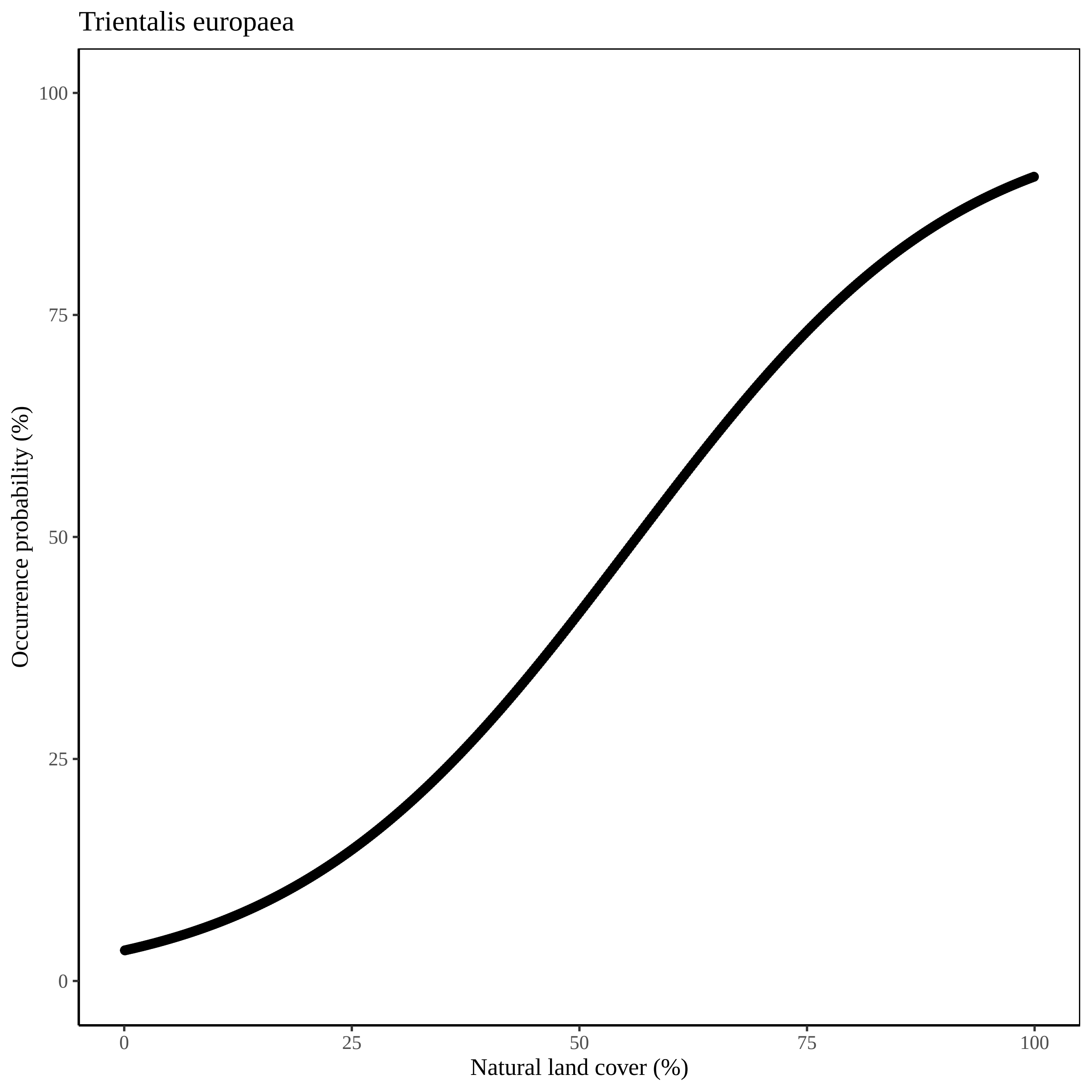

Trientalis europaea
Occurrence probability (%)
100
75
50
25
0
0
25
50
75
100
Natural land cover (%)

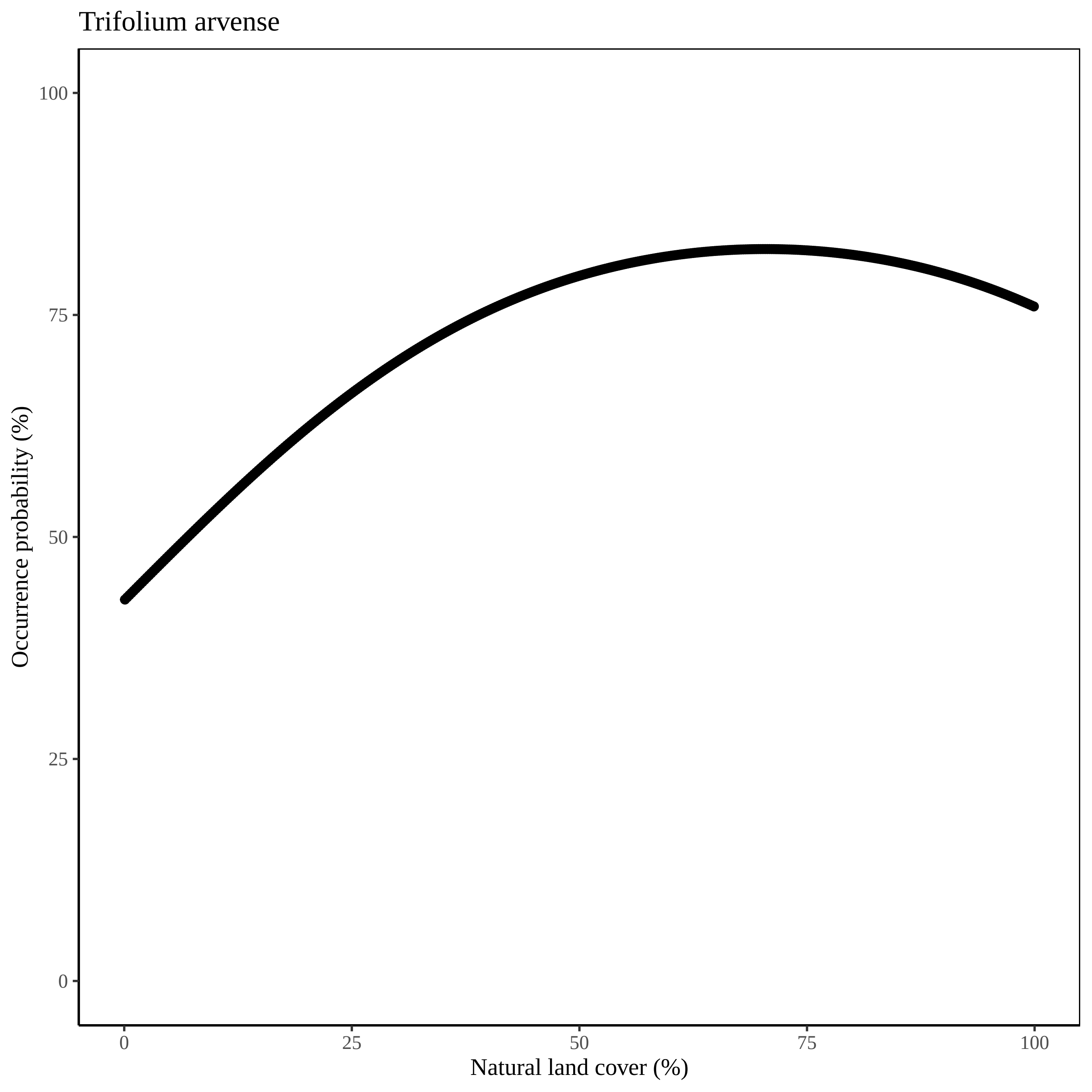
Trifolium arvense
Occurrence probability (%)
100
75
50
25
0
0
25
50
75
100
Natural land cover (%)

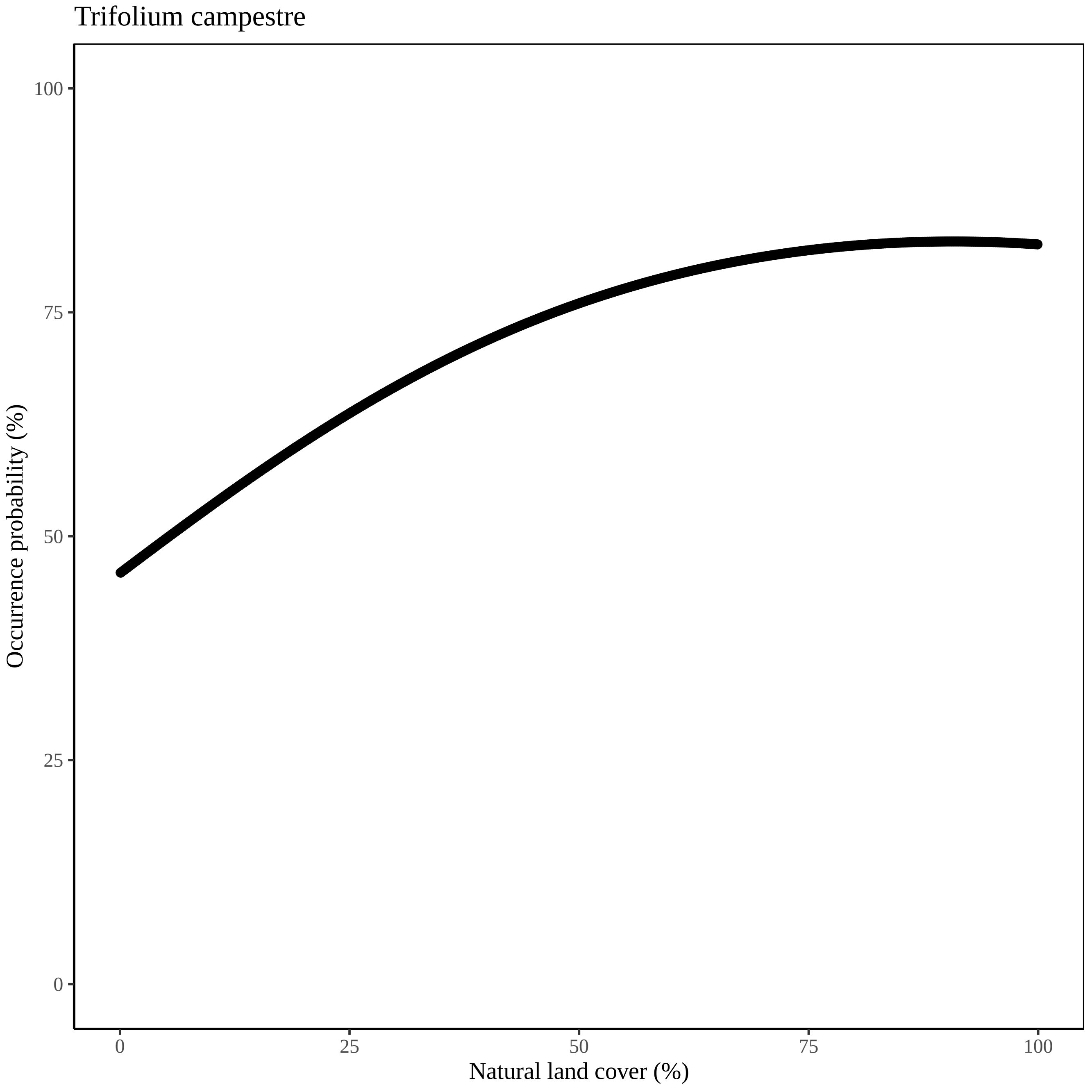
Trifolium campestre
Occurrence probability (%)
100
75
50
25
0
0
25
50
75
100
Natural land cover (%)

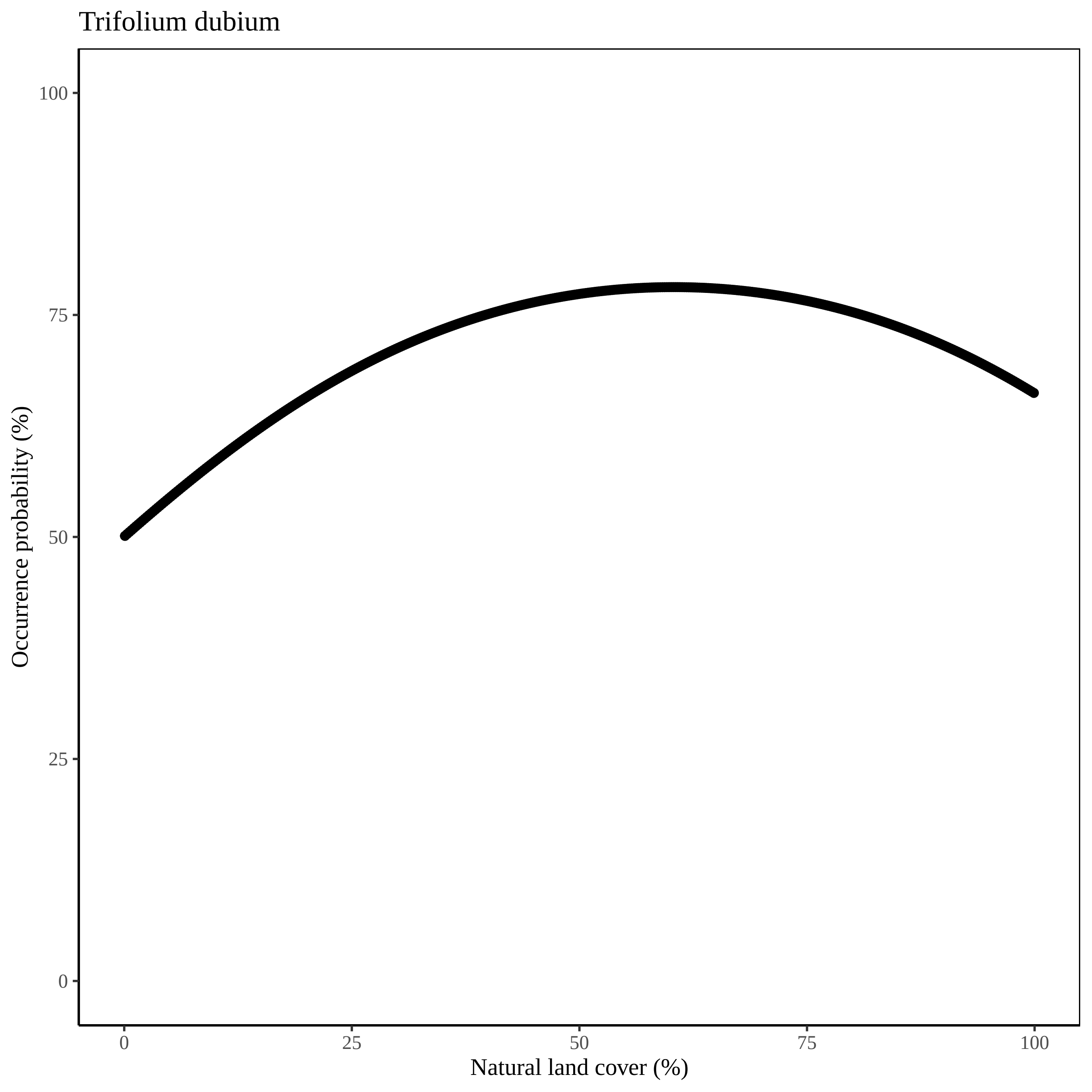
Trifolium dubium
Occurrence probability (%)
100
75
50
25
0
0
25
50
75
100
Natural land cover (%)

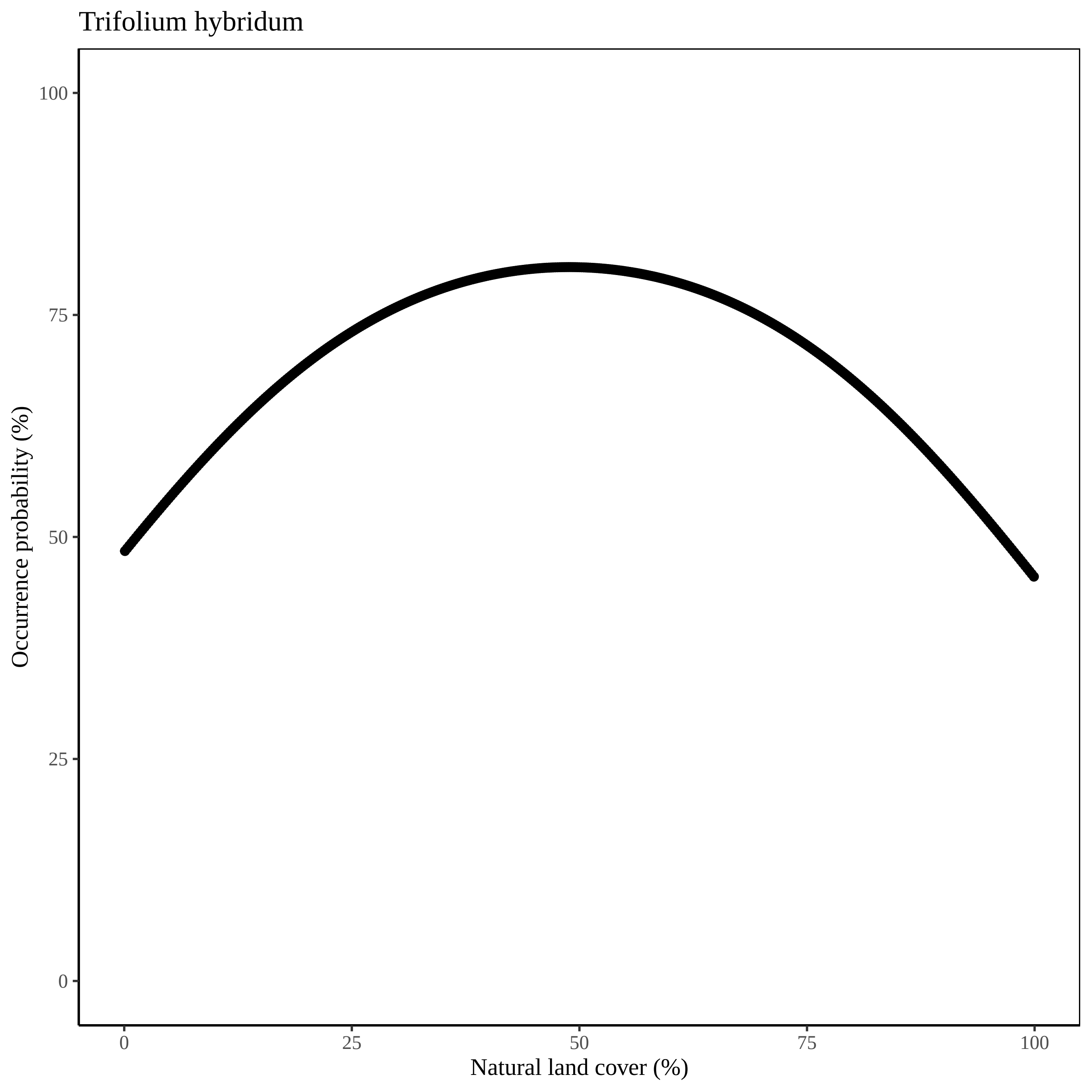
Trifolium hybridum
Occurrence probability (%)
100
75
50
25
0
0
25
50
75
100
Natural land cover (%)

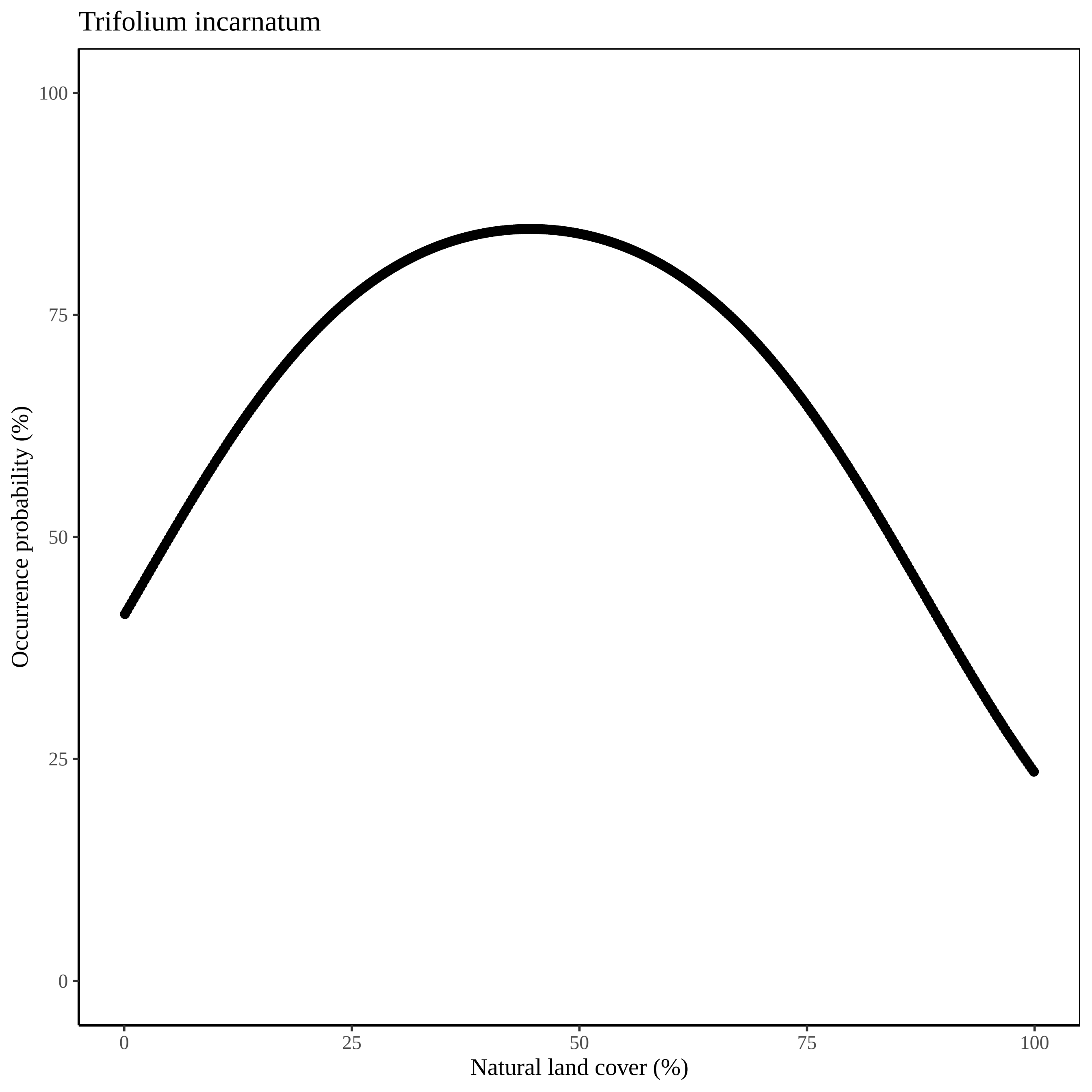
Trifolium incarnatum
Occurrence probability (%)
100
75
50
25
0
0
25
50
75
100
Natural land cover (%)

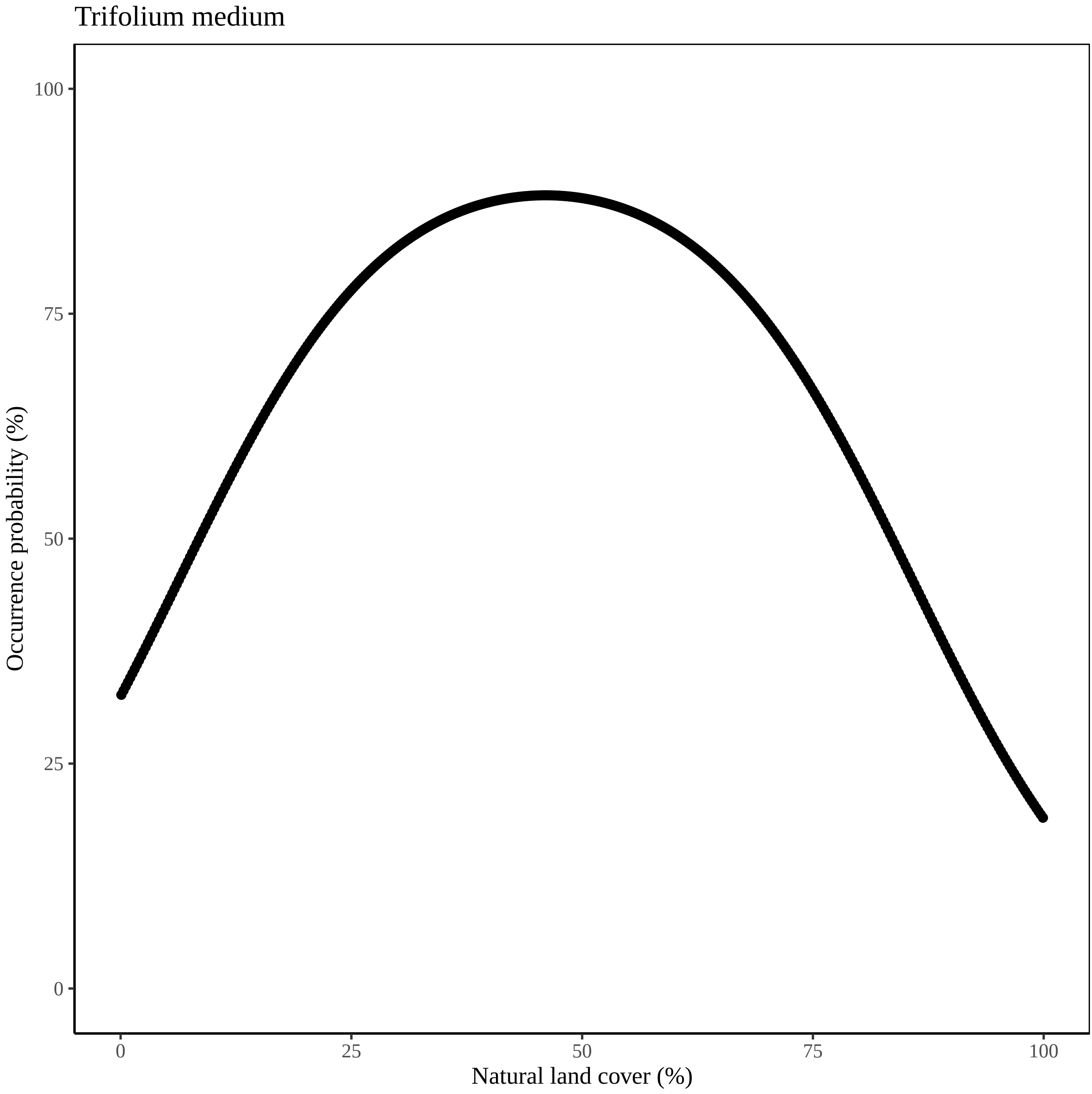
Trifolium medium
Occurrence probability (%)
100
75
50
25
0
0
25
50
75
100
Natural land cover (%)

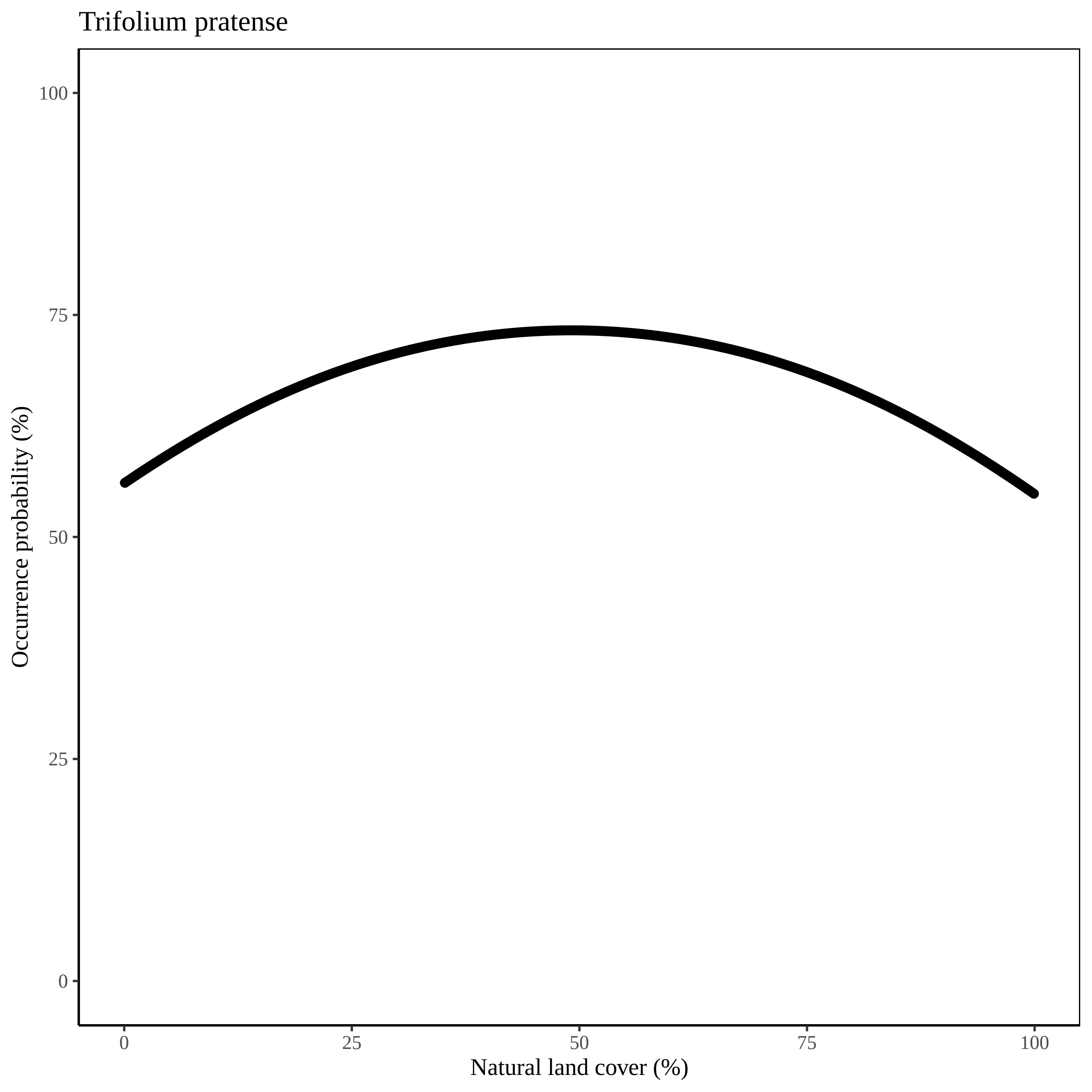

Trifolium pratense
Occurrence probability (%)
100
75
50
25
0
0
25
50
75
100
Natural land cover (%)

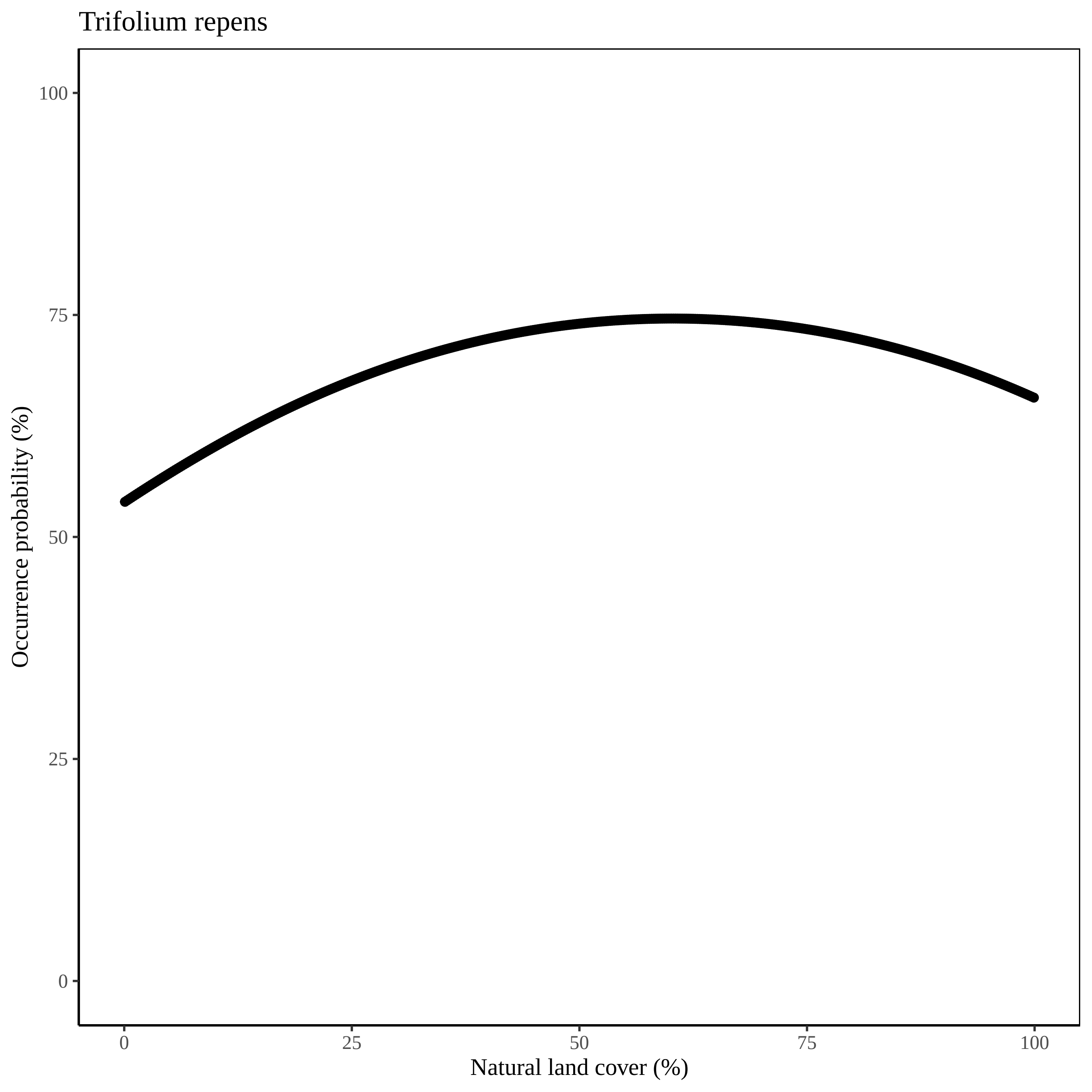
Trifolium repens
Occurrence probability (%)
100
75
50
25
0
0
25
50
75
100
Natural land cover (%)

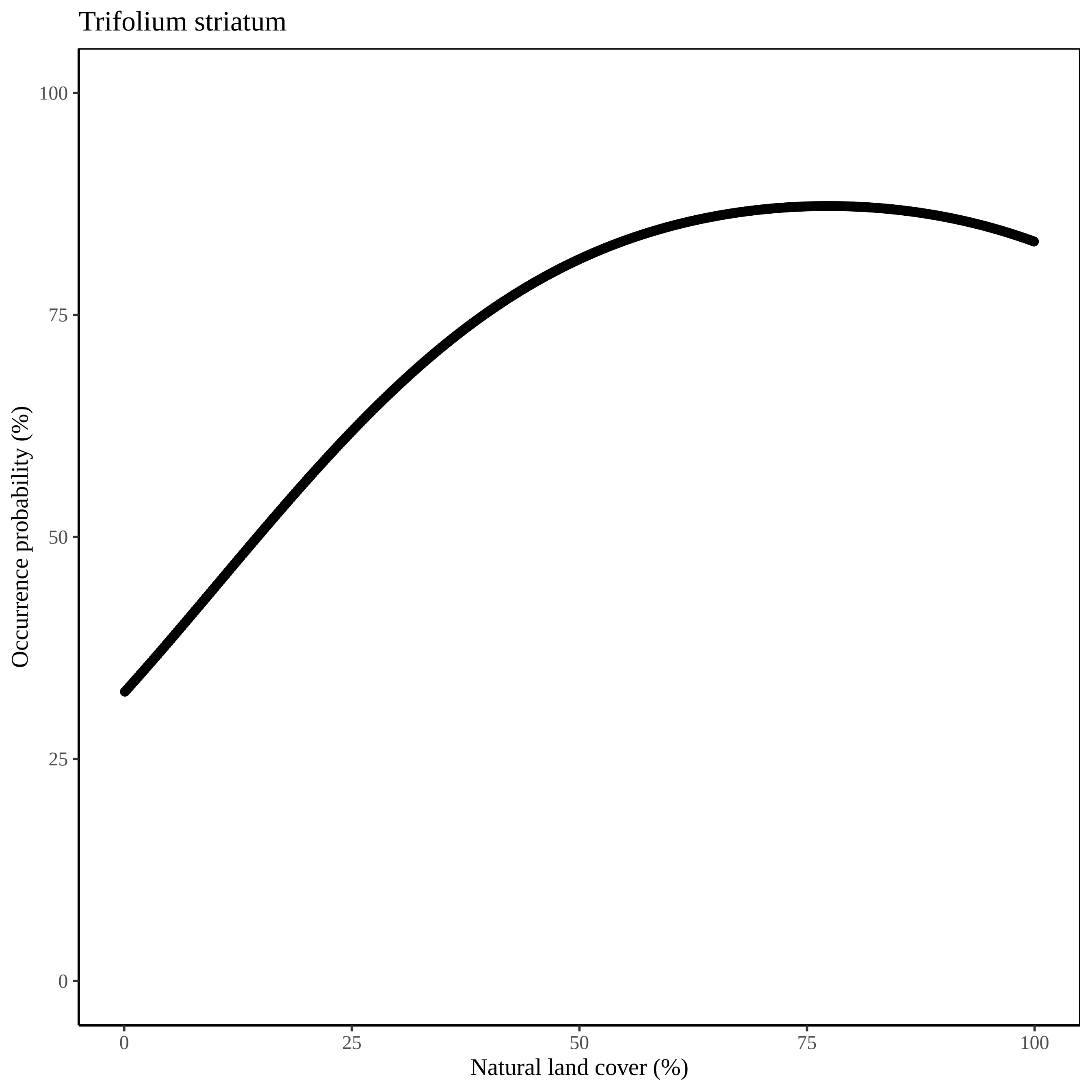
Trifolium striatum
Occurrence probability (%)
100
75
50
25
0
0
25
50
75
100
Natural land cover (%)

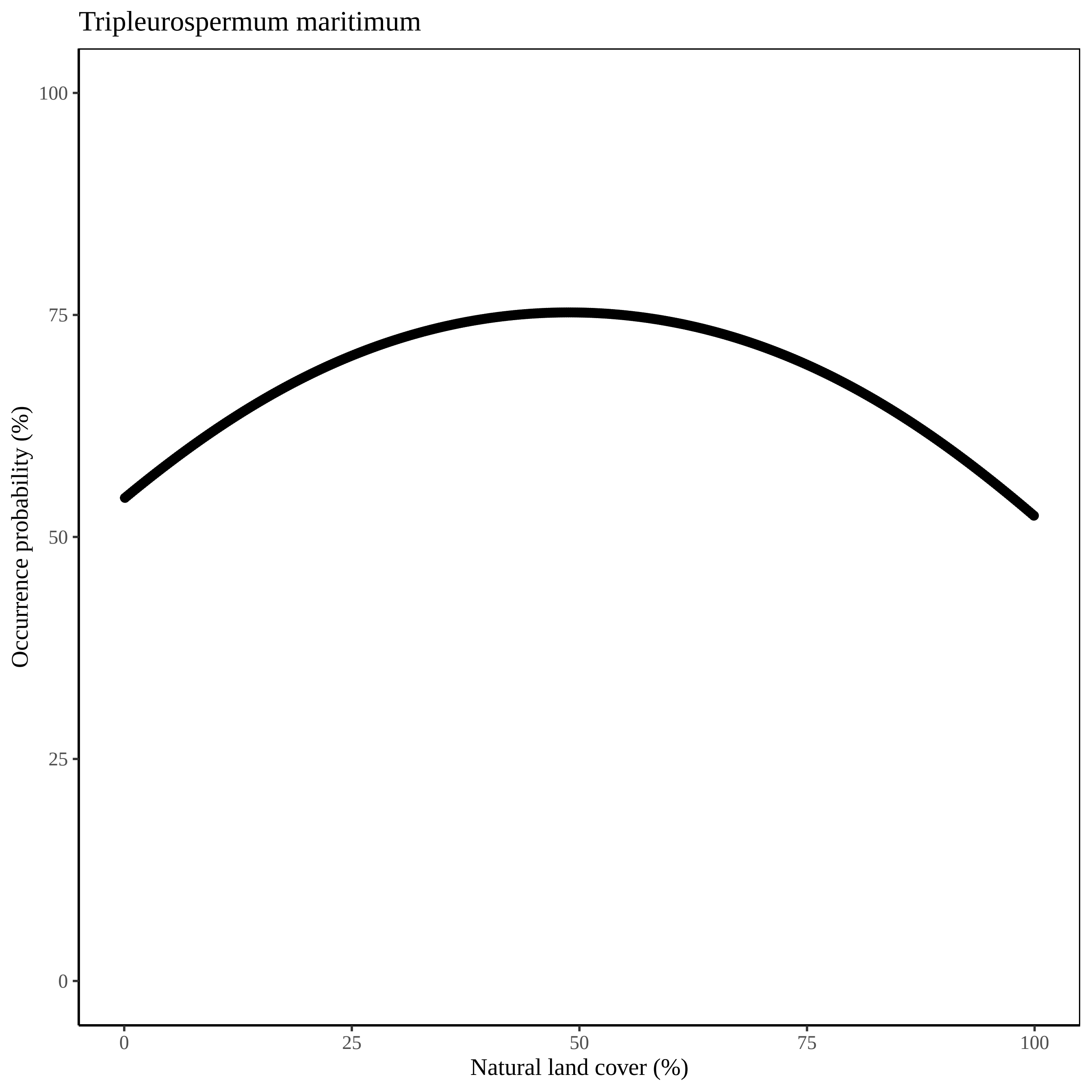
Tripleurospermum maritimum
100
75
50
25
0
Occurrence probability (%)
0
25
50
75
100
Natural land cover (%)

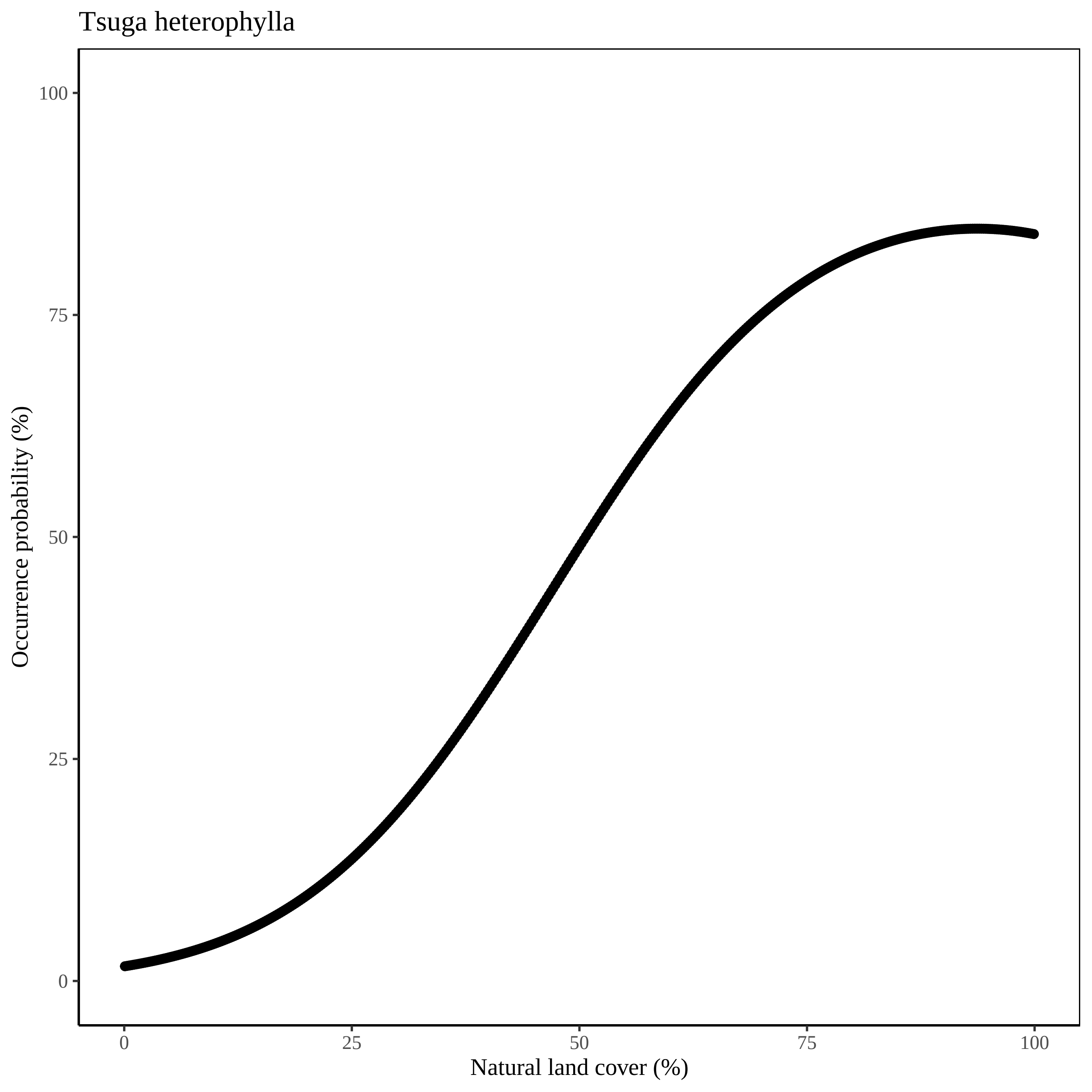
Tsuga heterophylla
Occurrence probability (%)
100
75
50
25
0
0
25
50
75
100
Natural land cover (%)

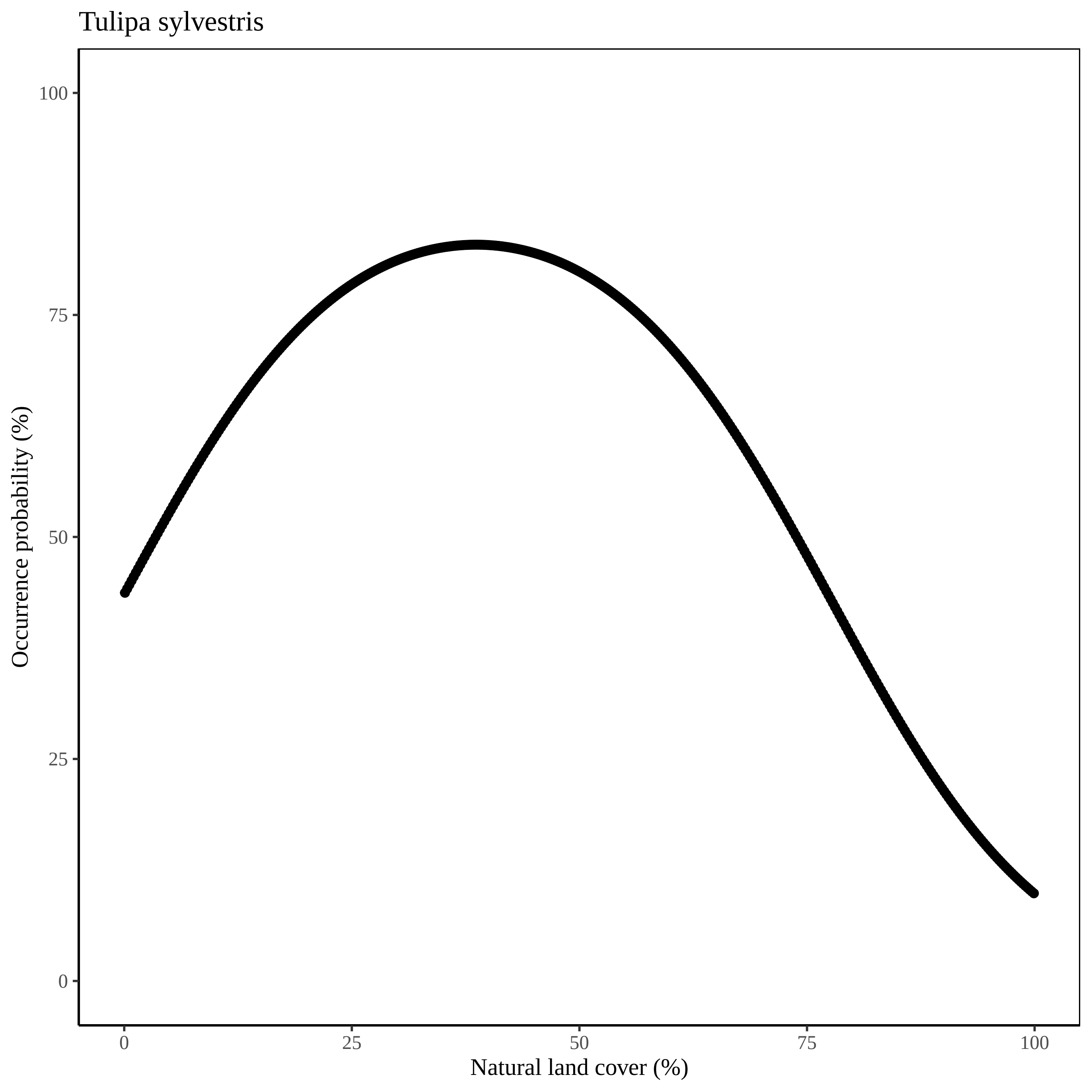
Tulipa sylvestris
Occurrence probability (%)
100
75
50
25
0
0
25
50
75
100
Natural land cover (%)

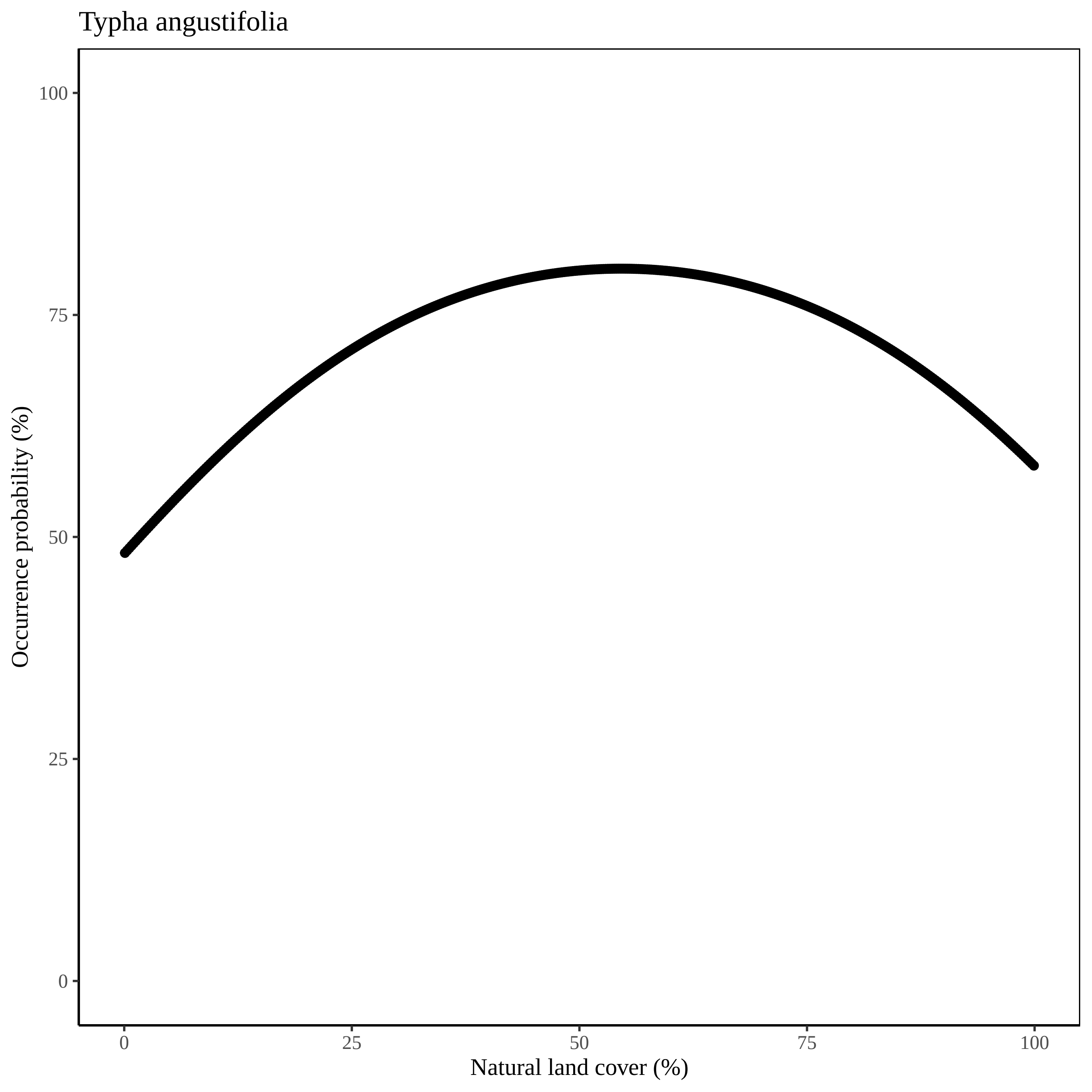
Typha angustifolia
100
75
50
25
0
Occurrence probability (%)
0
25
50
75
100
Natural land cover (%)

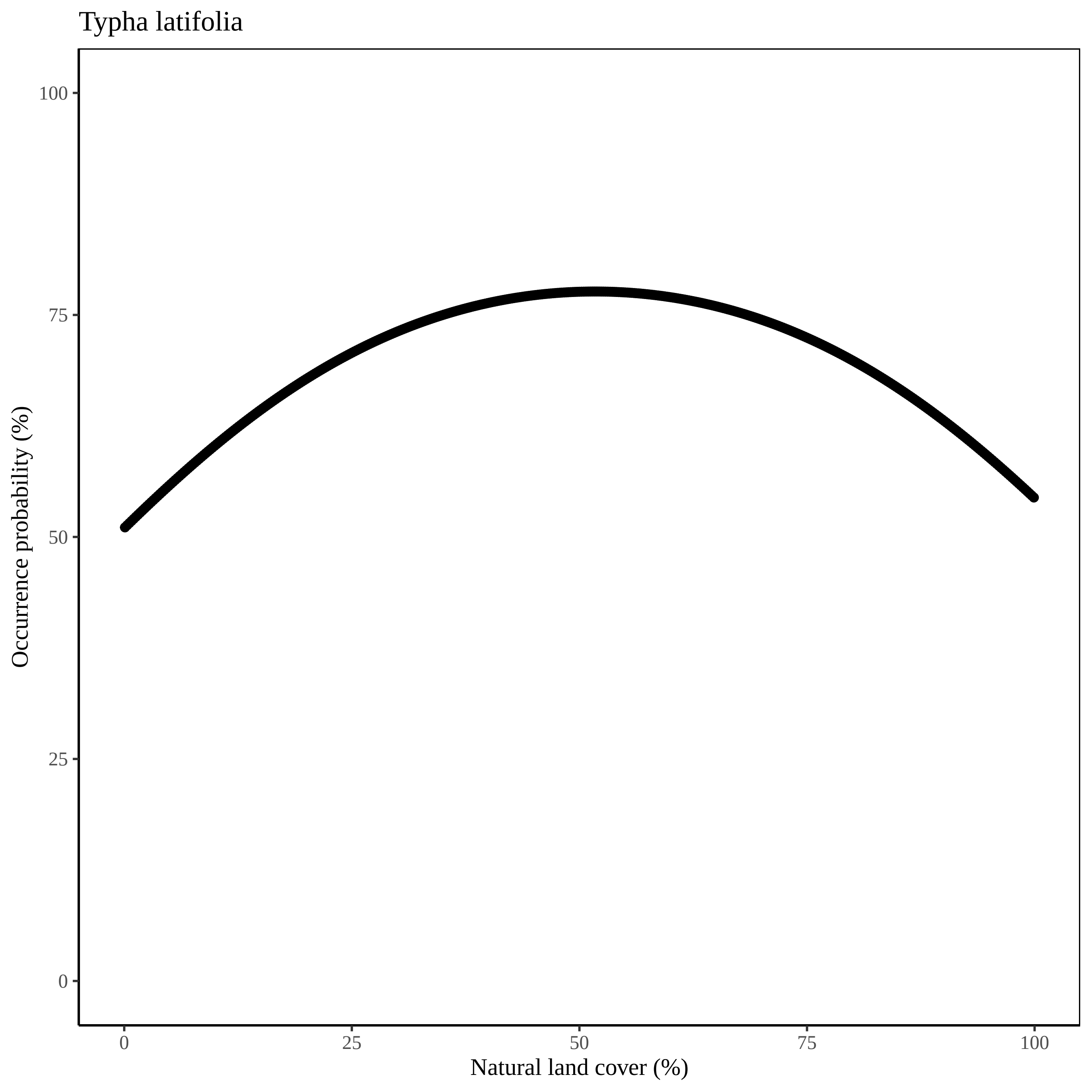
Typha latifolia
Occurrence probability (%)
100
75
50
25
0
0
25
50
75
100
Natural land cover (%)

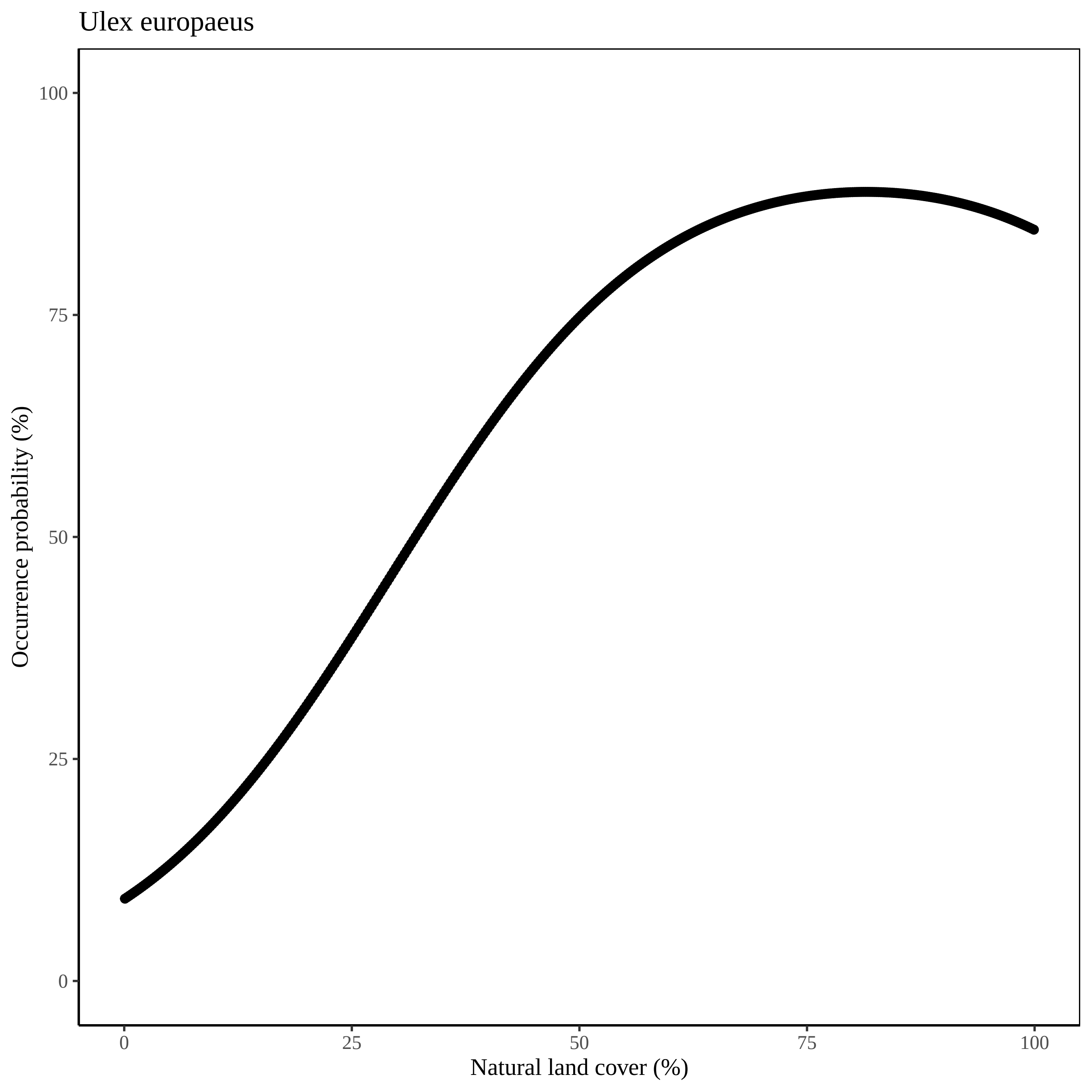

Ulex europaeus
Occurrence probability (%)
100
75
50
25
0
0
25
50
75
100
Natural land cover (%)

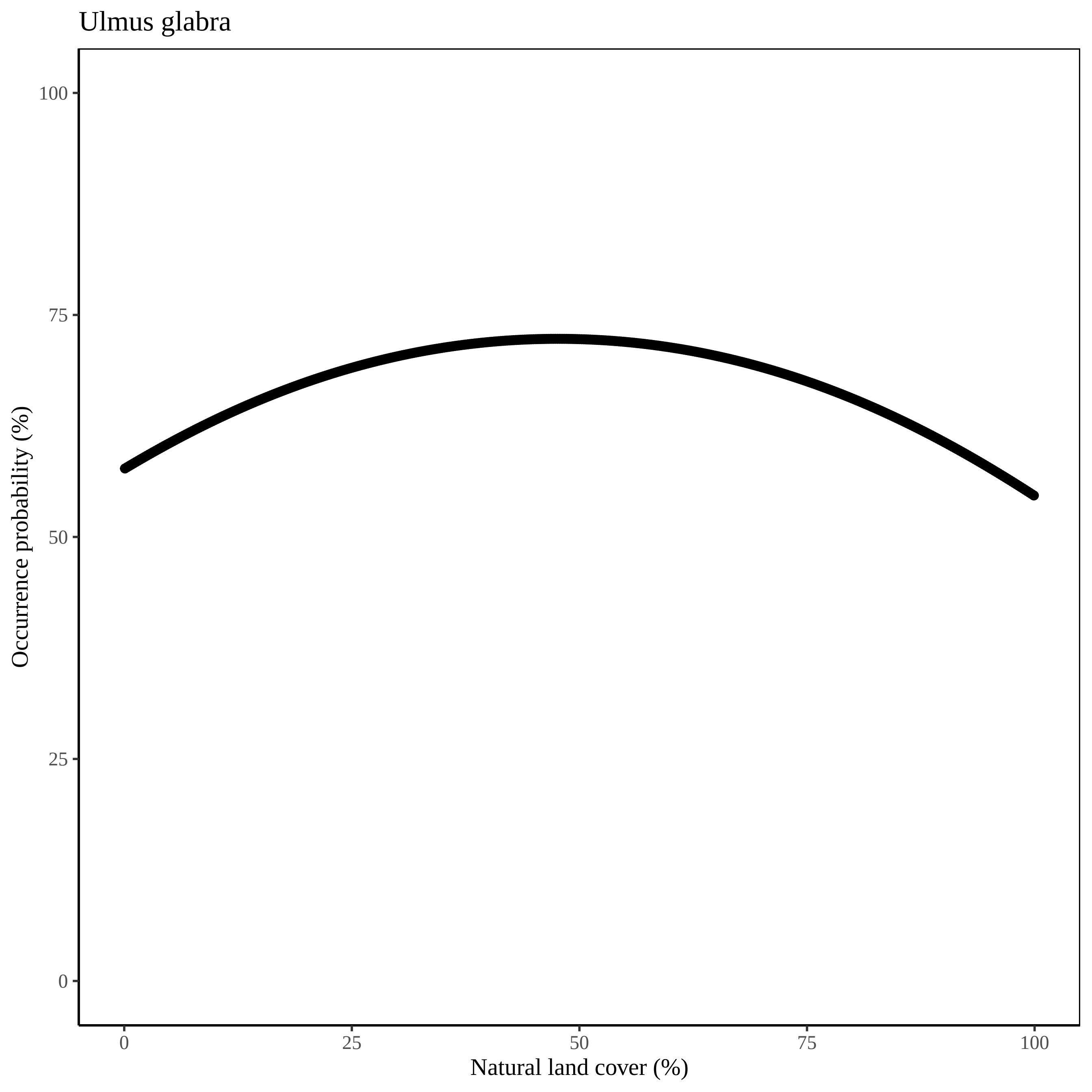
Ulmus glabra
100
75
50
25
0
Occurrence probability (%)
0
25
50
75
100
Natural land cover (%)

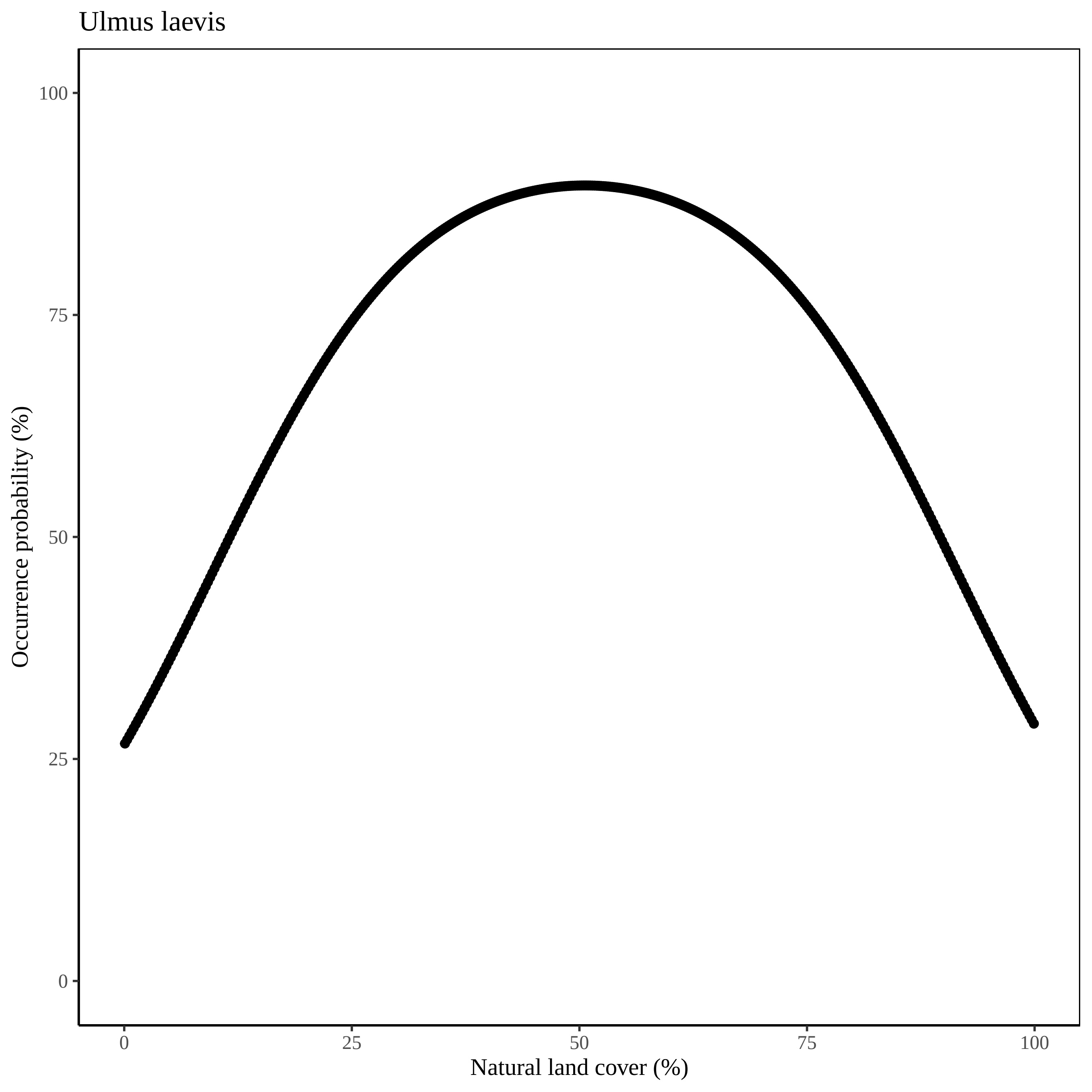
Ulmus laevis
Occurrence probability (%)
100
75
50
25
0
0
25
50
75
100
Natural land cover (%)

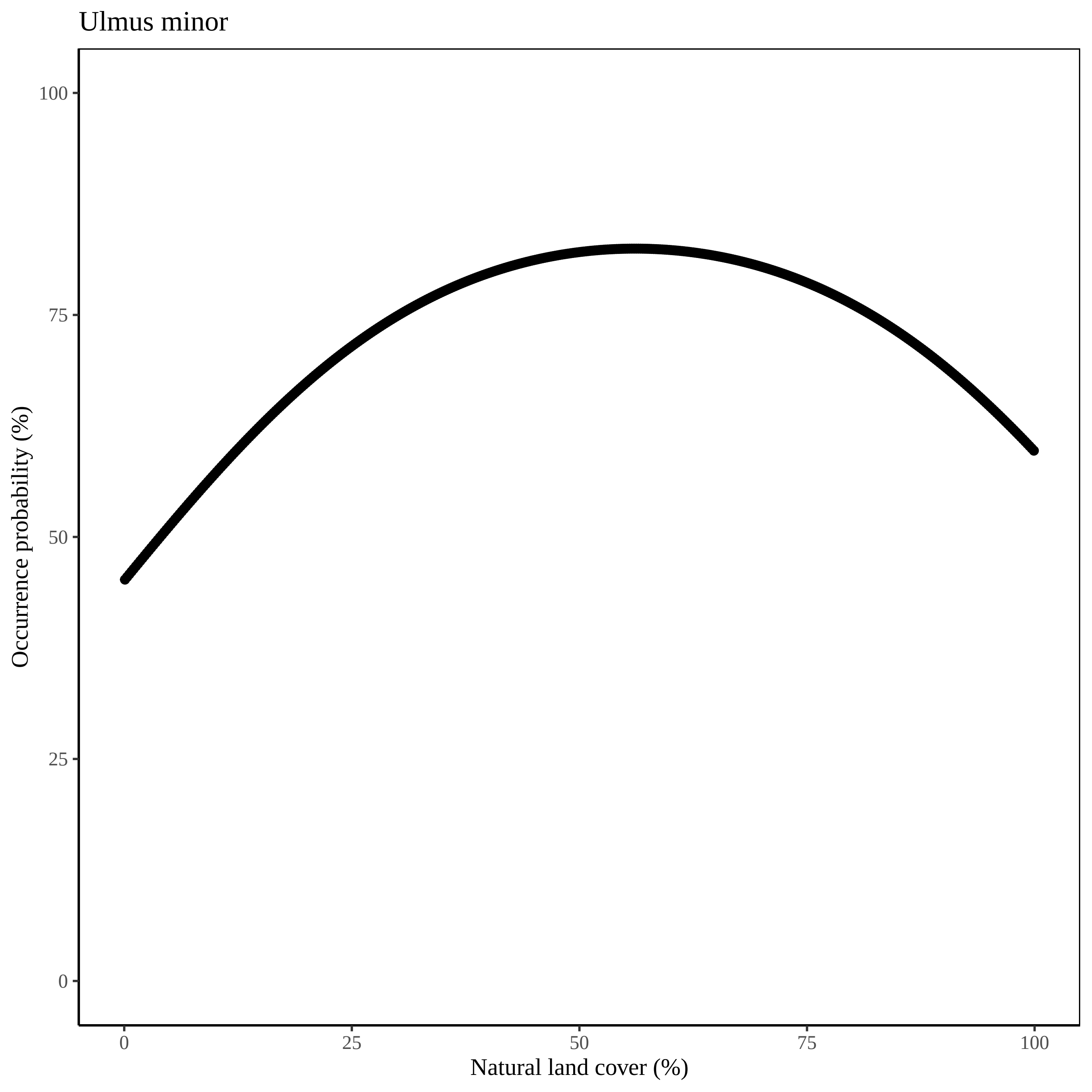
Ulmus minor
Occurrence probability (%)
100
75
50
25
0
0
25
50
75
100
Natural land cover (%)

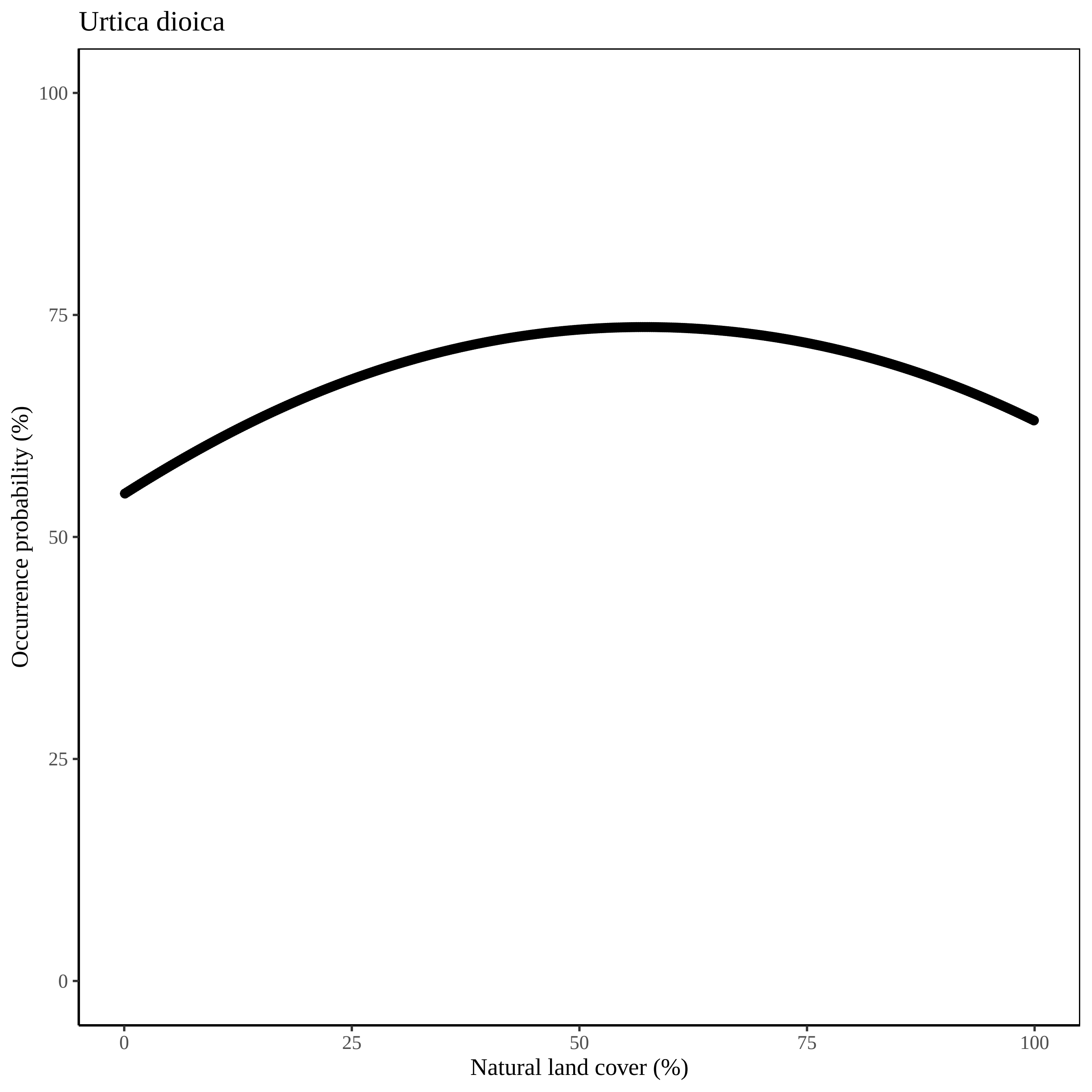
Urtica dioica
Occurrence probability (%)
100
75
50
25
0
0
25
50
75
100
Natural land cover (%)

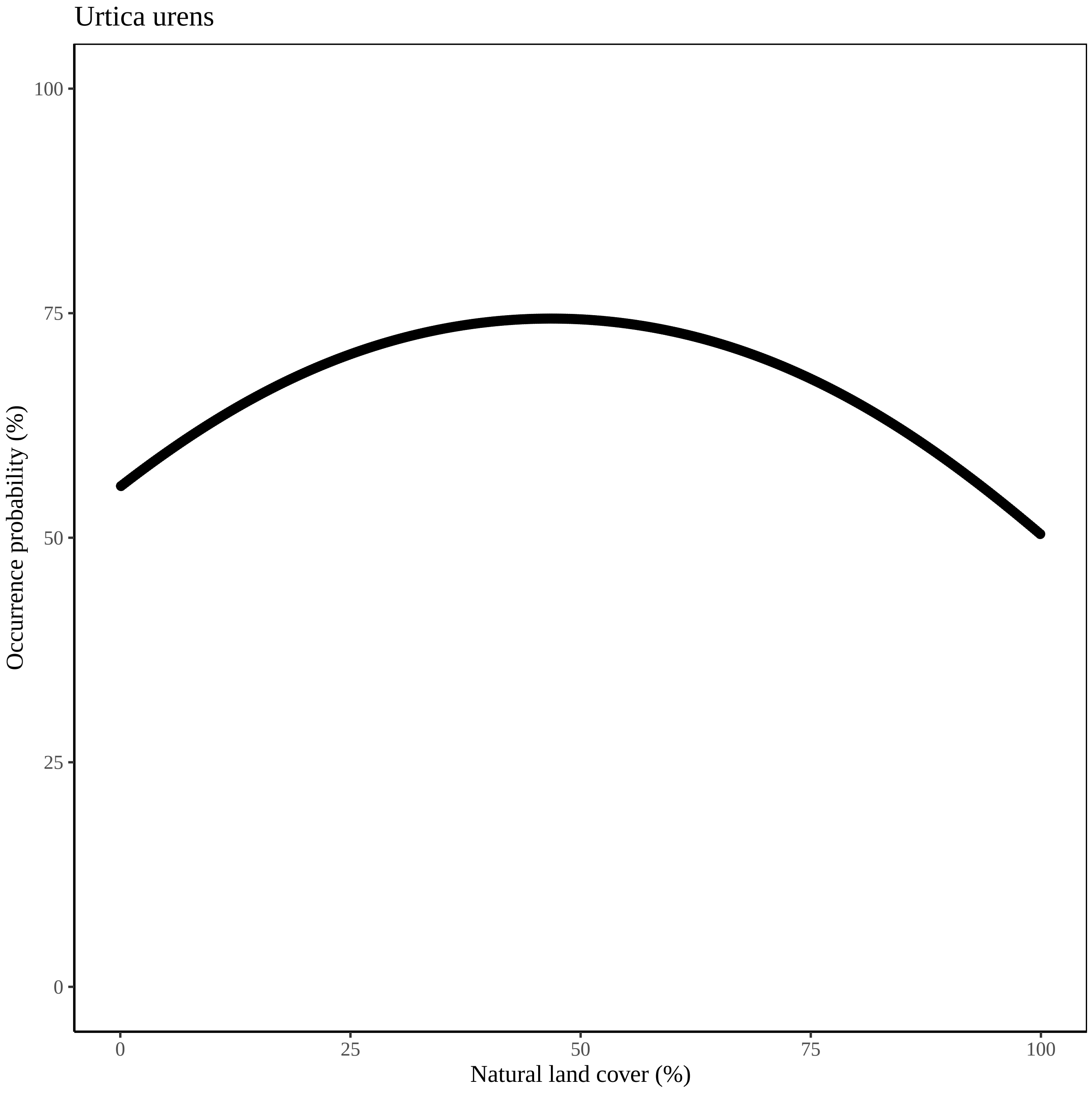

Urtica urens
Occurrence probability (%)
Natural land cover (%)
0
25
50
75
100
0
25
50
75
100

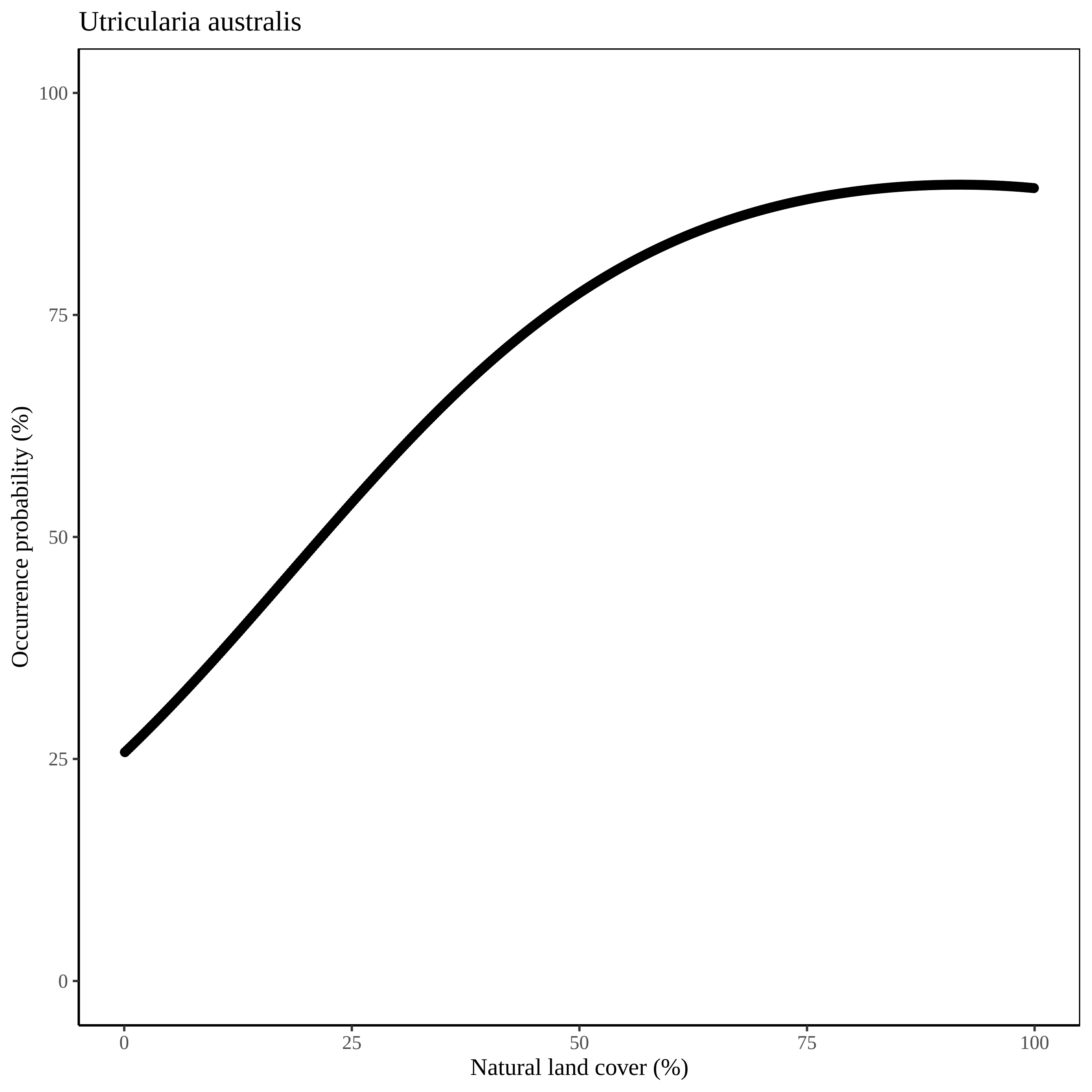

Utricularia australis
Occurrence probability (%)
100
75
50
25
0
0
25
50
75
100
Natural land cover (%)

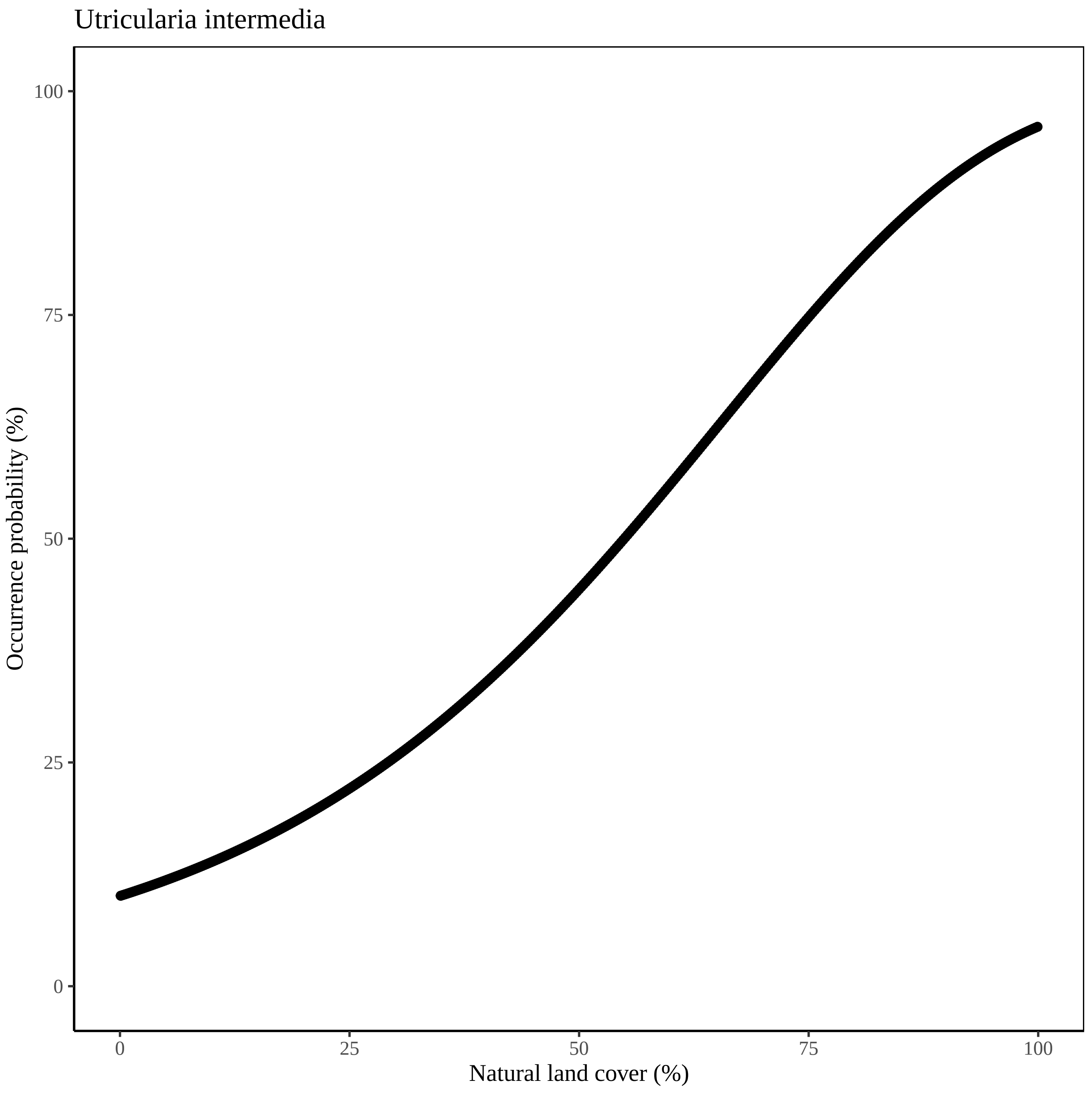
Utricularia intermedia
100
75
50
25
0
Occurrence probability (%)
0
25
50
75
100
Natural land cover (%)

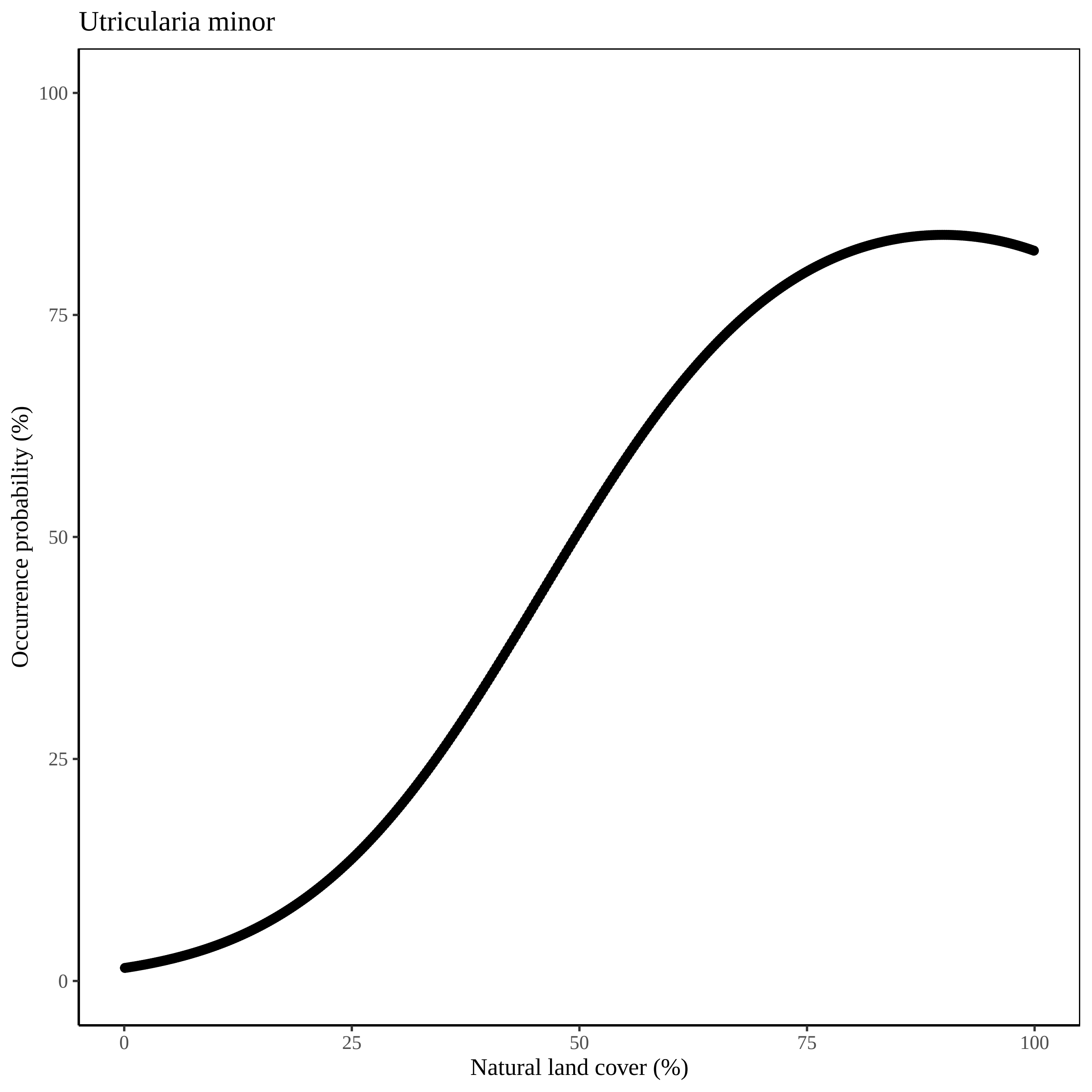
Utricularia minor
Occurrence probability (%)
100
75
50
25
0
0
25
50
75
100
Natural land cover (%)

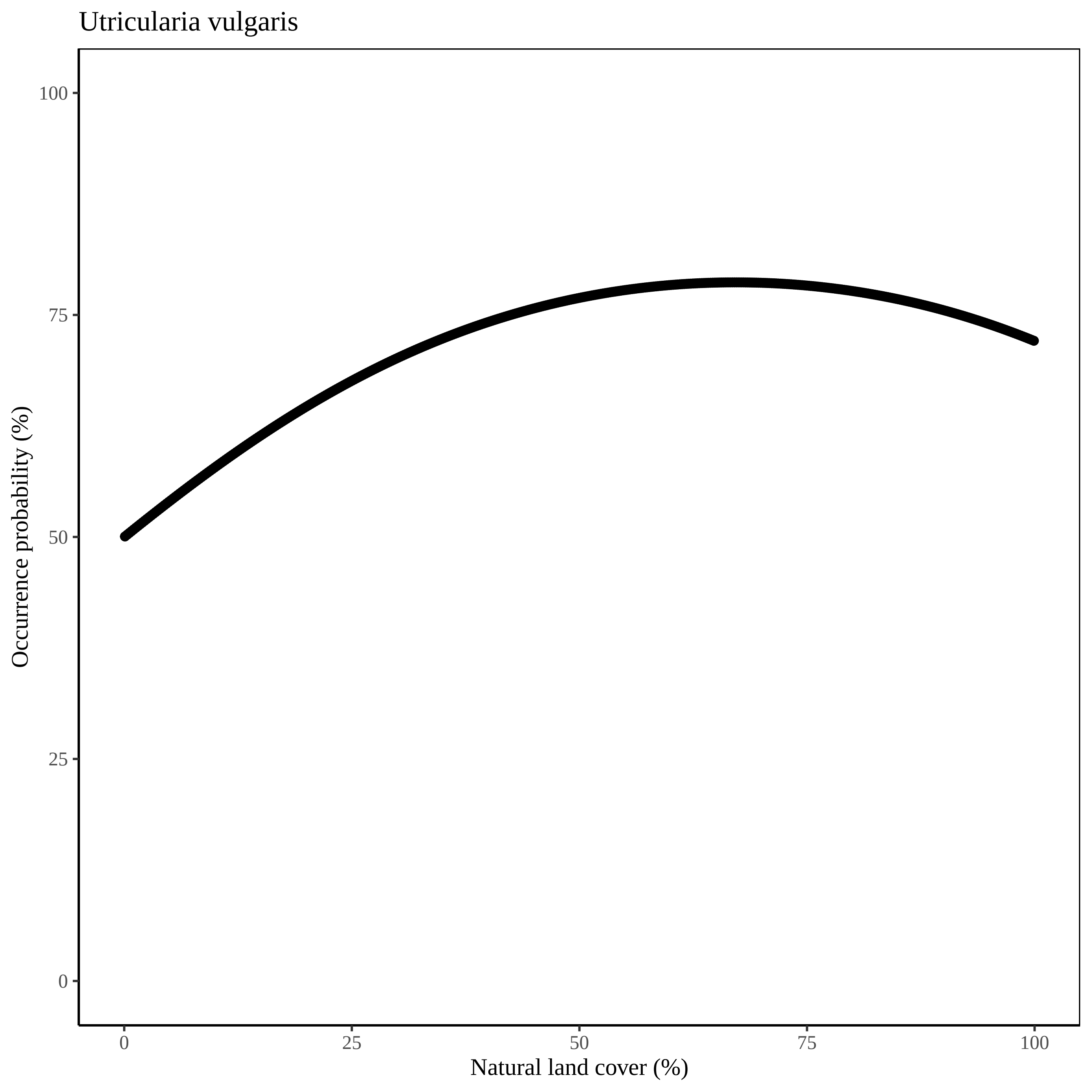
Utricularia vulgaris
Occurrence probability (%)
100
75
50
25
0
0
25
50
75
100
Natural land cover (%)

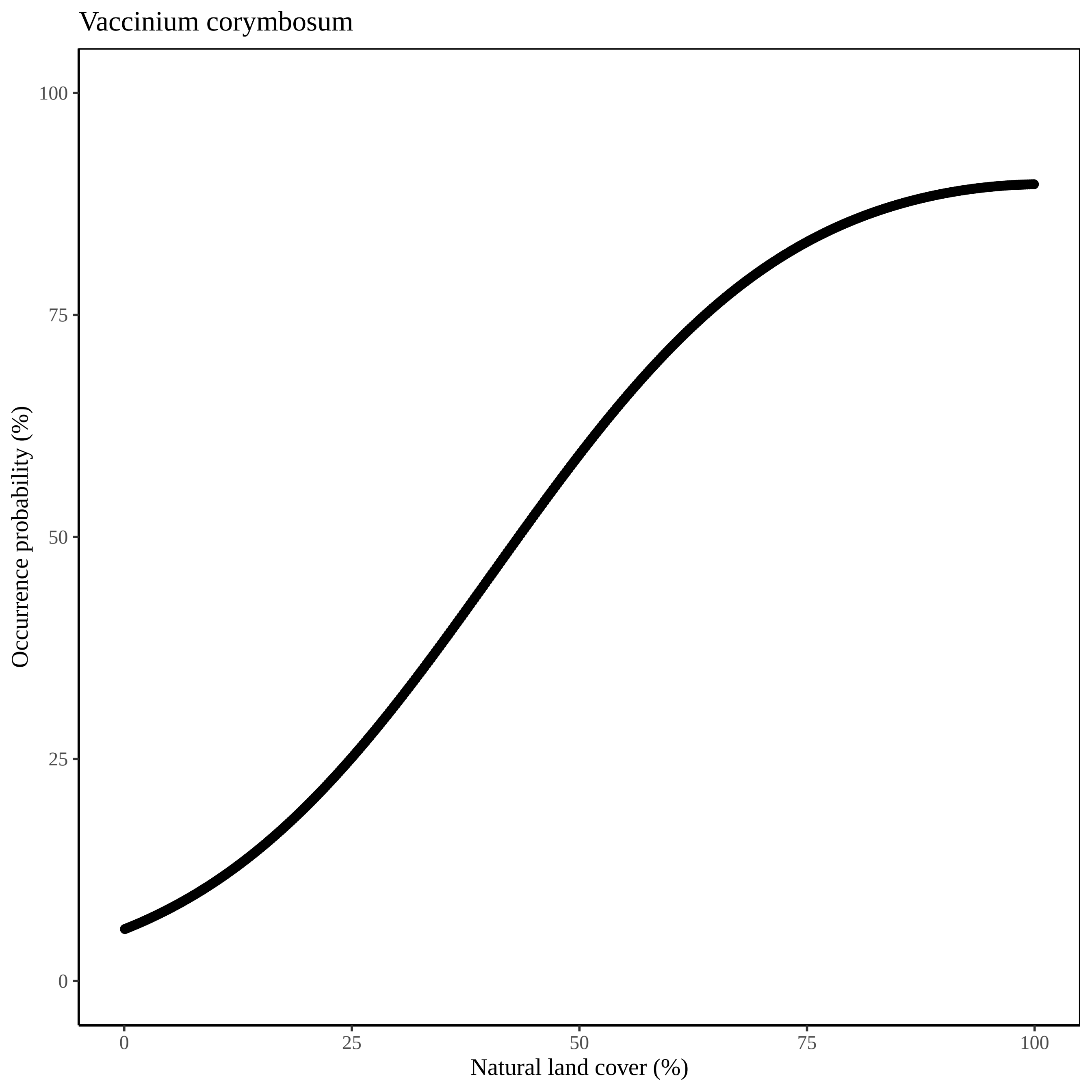
Vaccinium corymbosum
Occurrence probability (%)
100
75
50
25
0
0
25
50
75
100
Natural land cover (%)

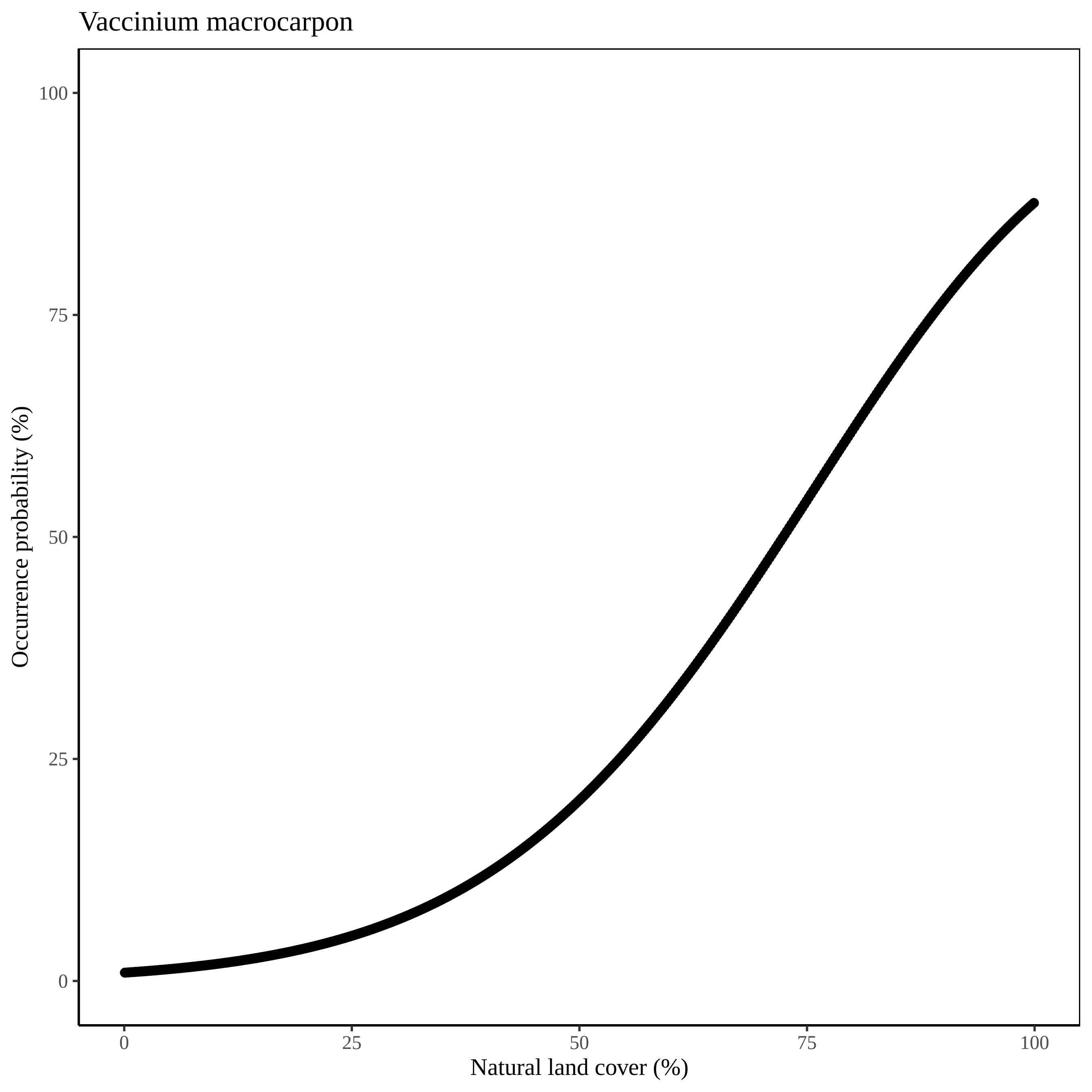
Vaccinium macrocarpon
Occurrence probability (%)
100
75
50
25
0
0
25
50
75
100
Natural land cover (%)

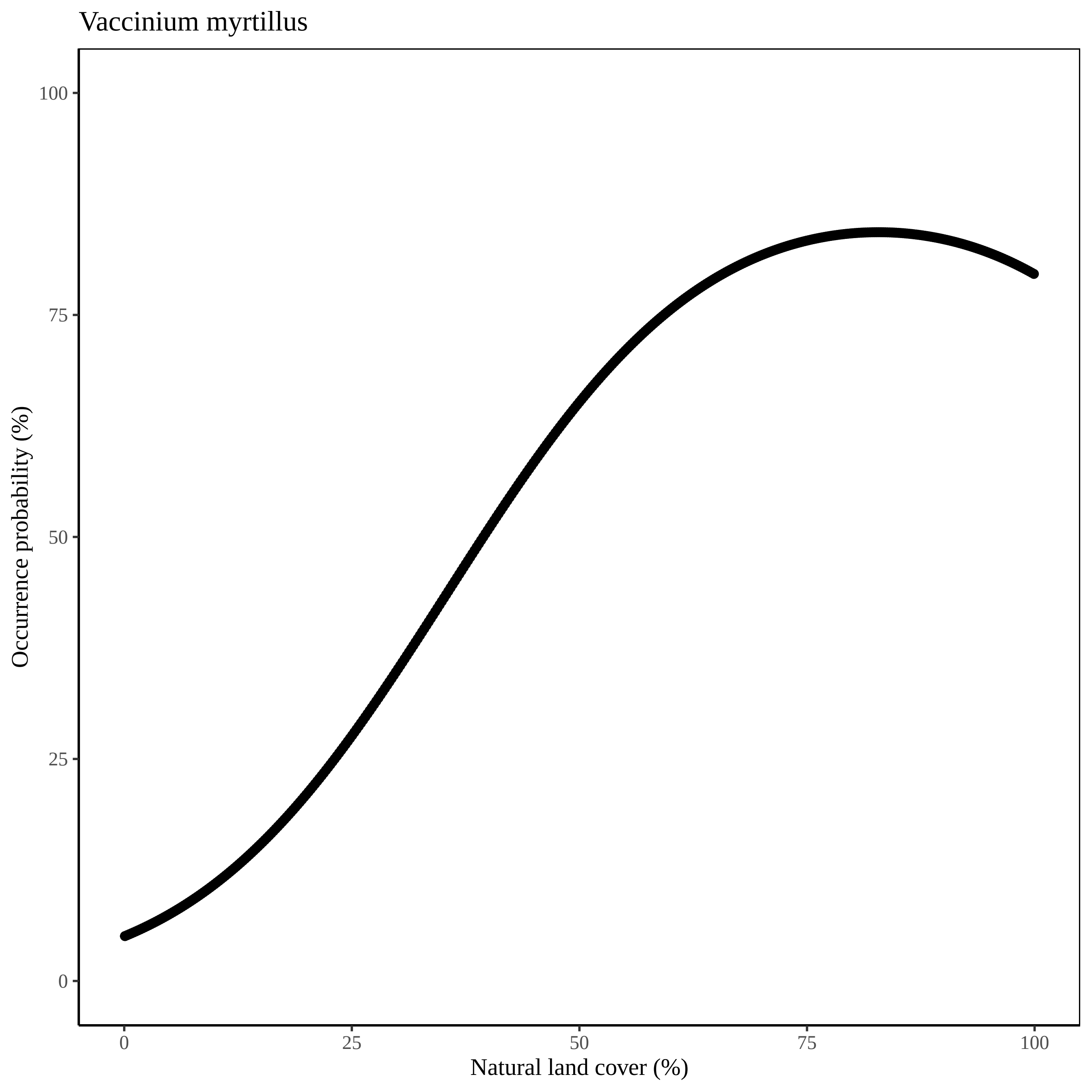
Vaccinium myrtillus
Occurrence probability (%)
100
75
50
25
0
0
25
50
75
100
Natural land cover (%)

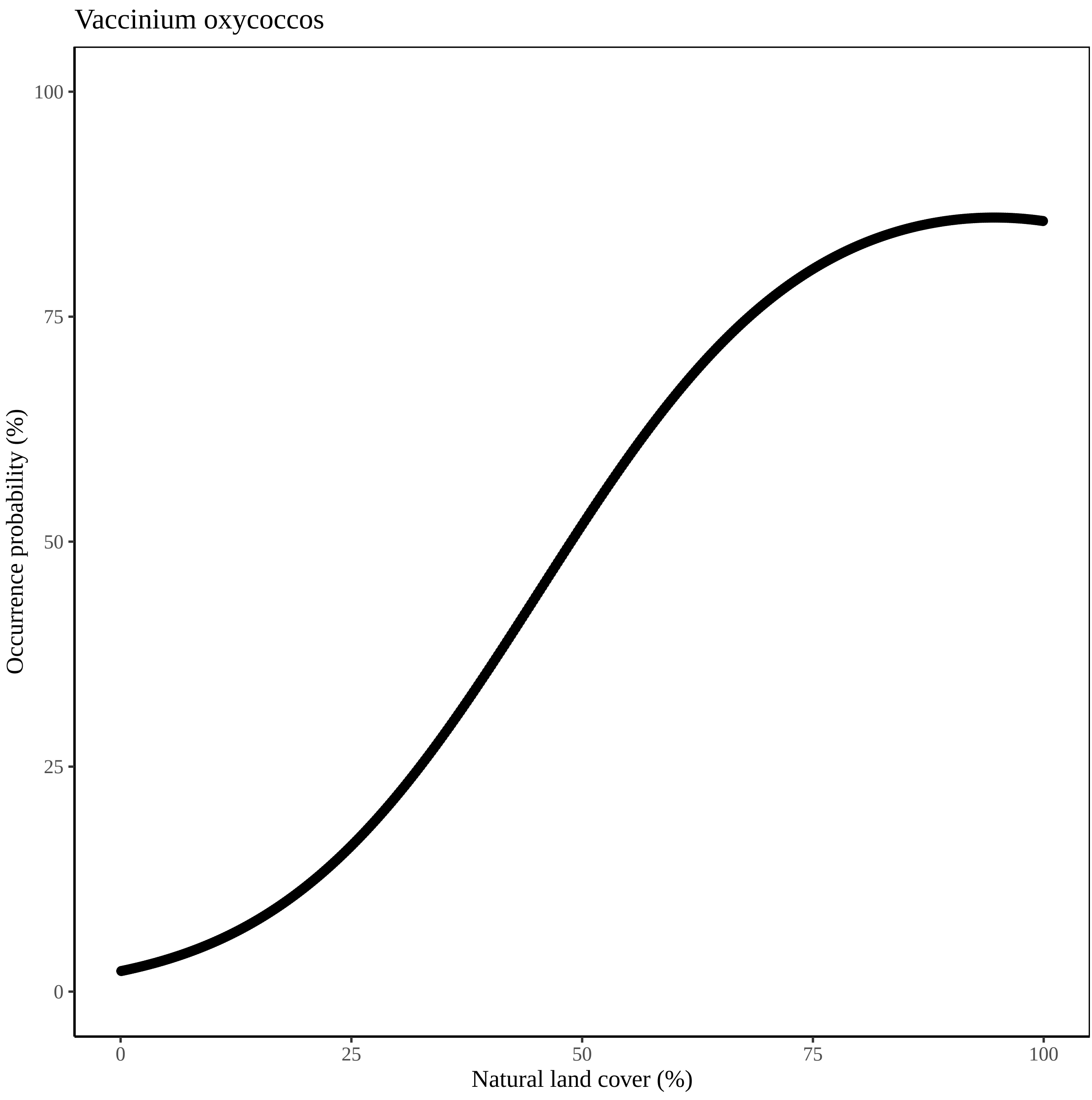
Vaccinium oxycoccos
Occurrence probability (%)
100
75
50
25
0
0
25
50
75
100
Natural land cover (%)

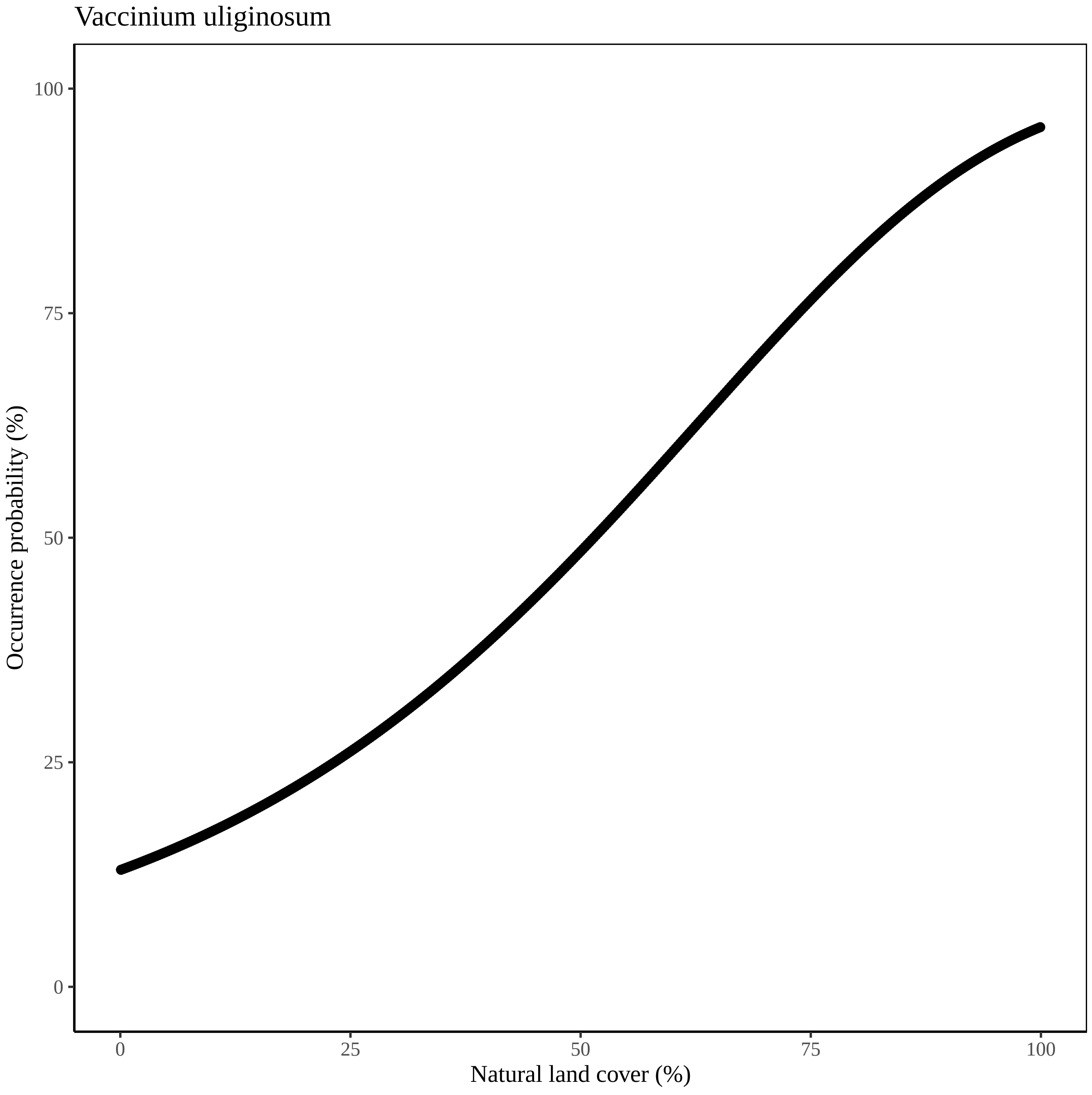
Vaccinium uliginosum
Occurrence probability (%)
100
75
50
25
0
0
25
50
75
100
Natural land cover (%)

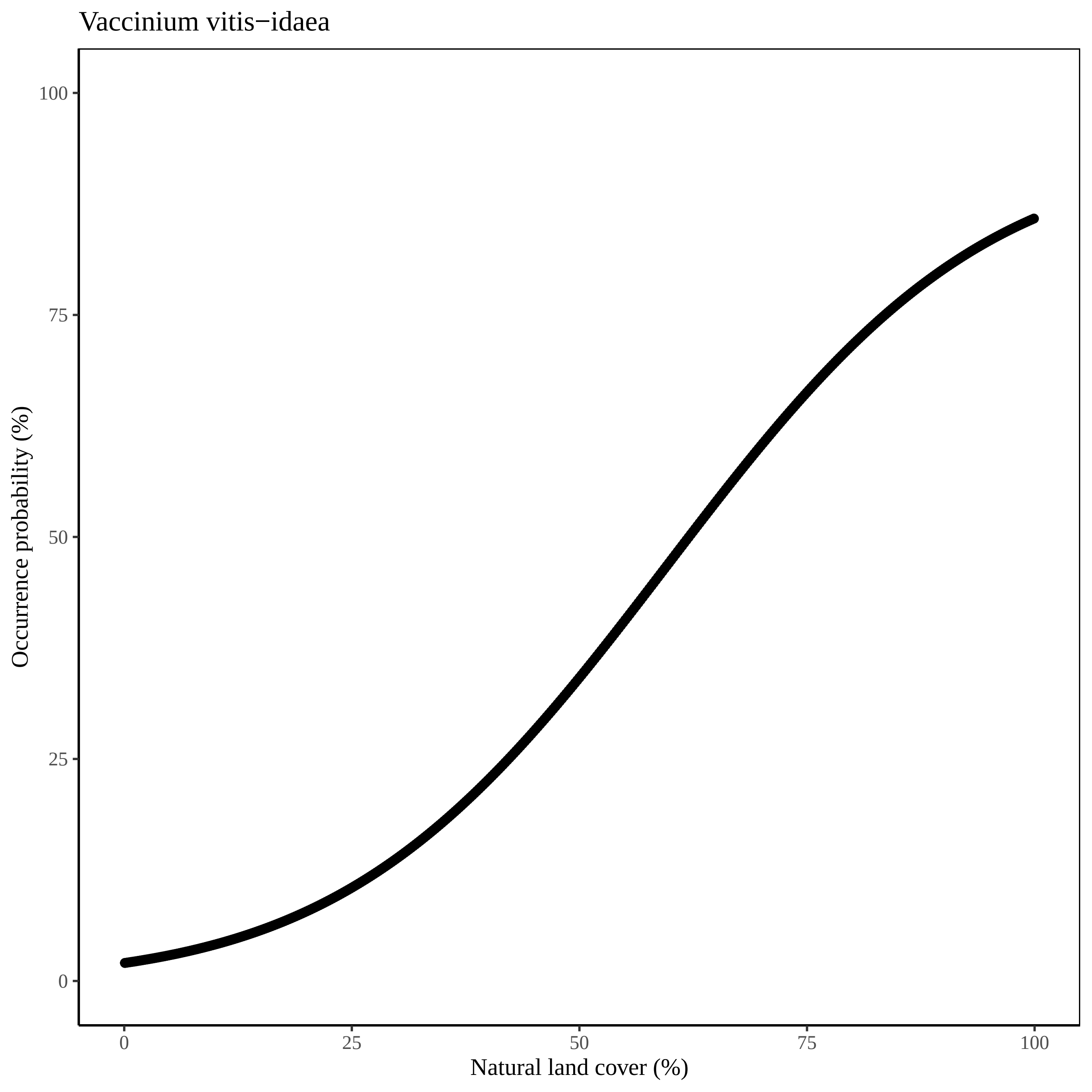
Vaccinium vitis−idaea
100
75
50
25
0
Occurrence probability (%)
0
25
50
75
100
Natural land cover (%)

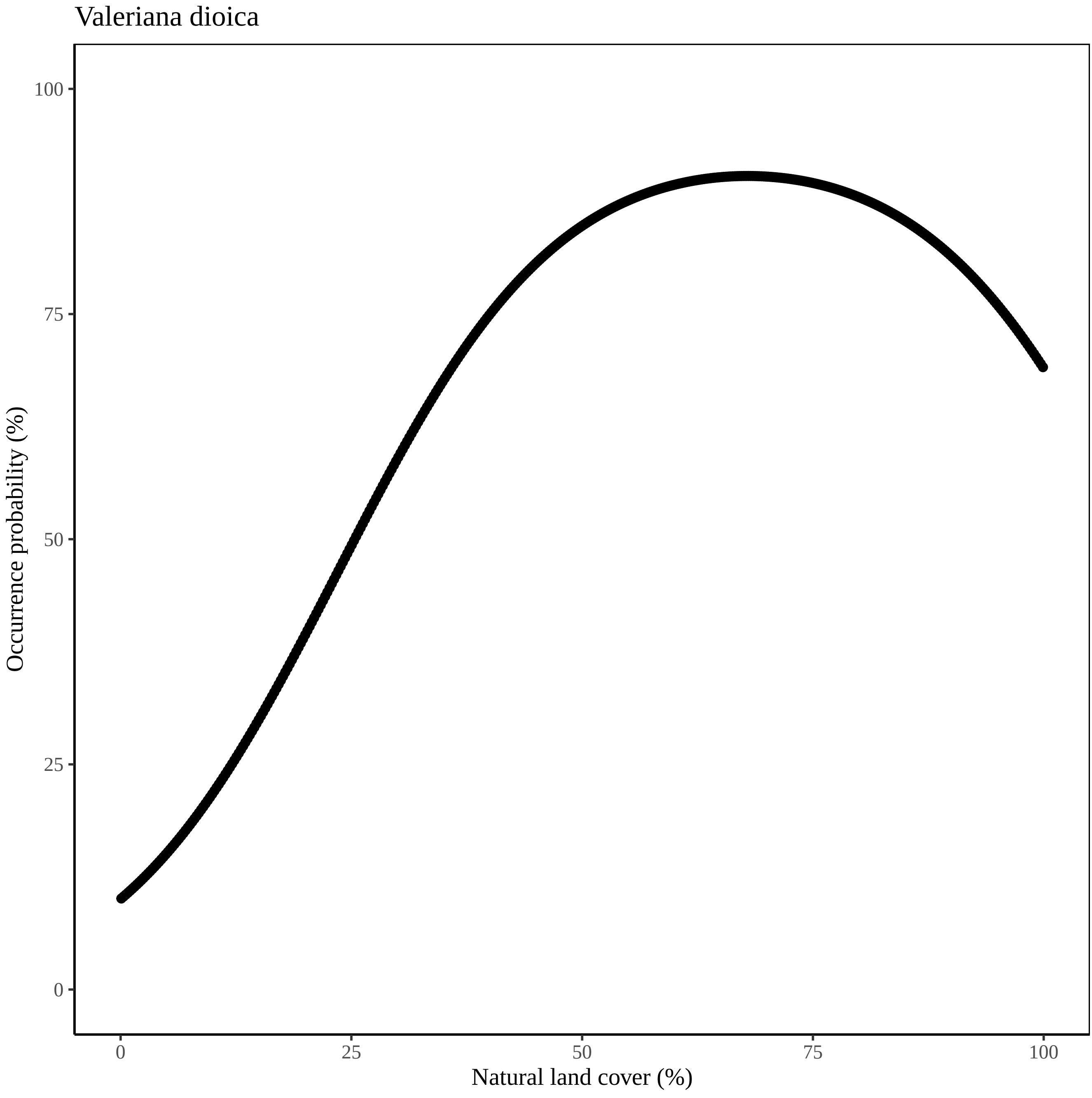

Valeriana dioica
Occurrence probability (%)
100
75
50
25
0
0
25
50
75
100
Natural land cover (%)

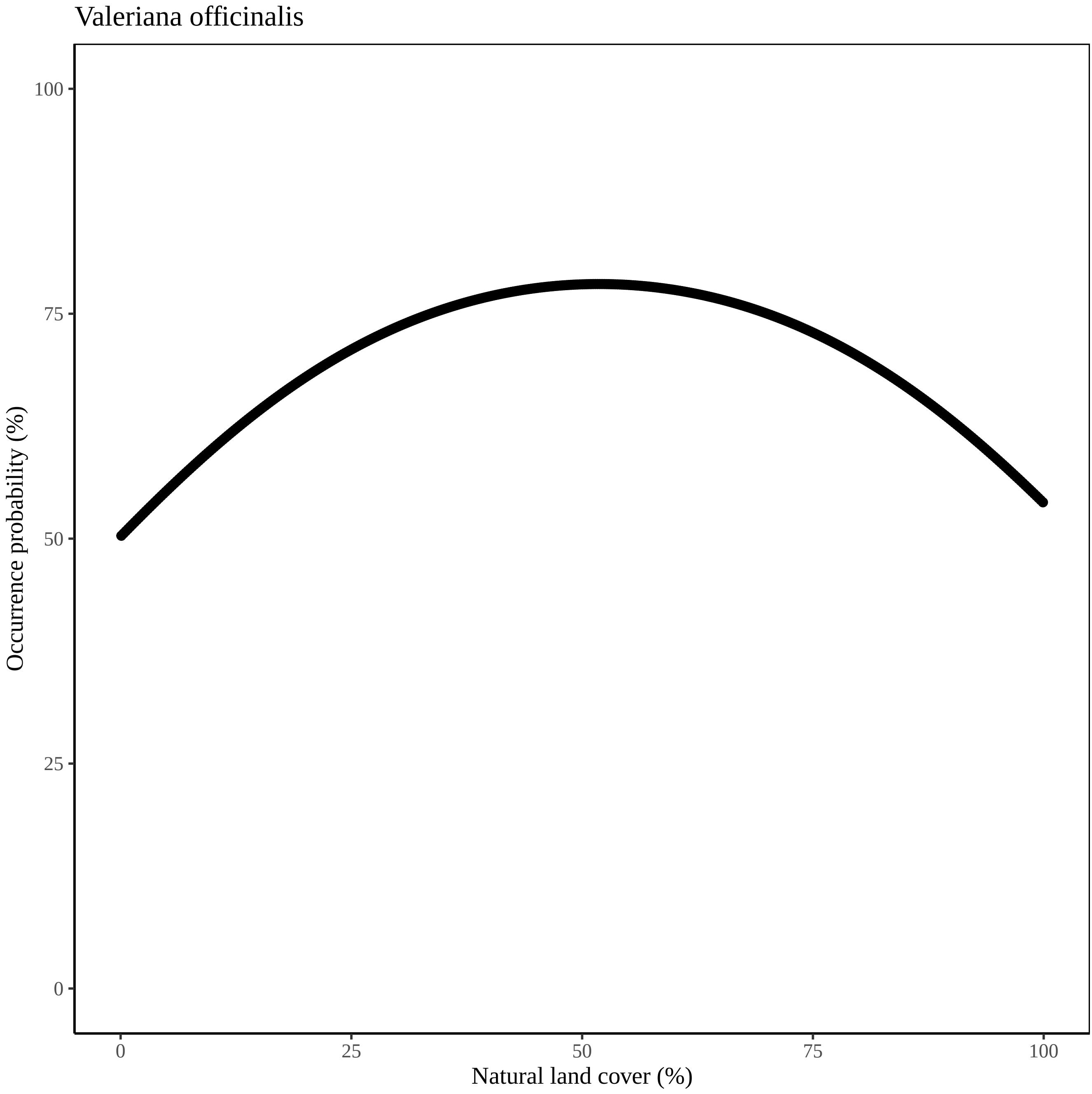

Valeriana officinalis
100
75
50
25
0
Occurrence probability (%)
0
25
50
75
100
Natural land cover (%)

Valerianella carinata

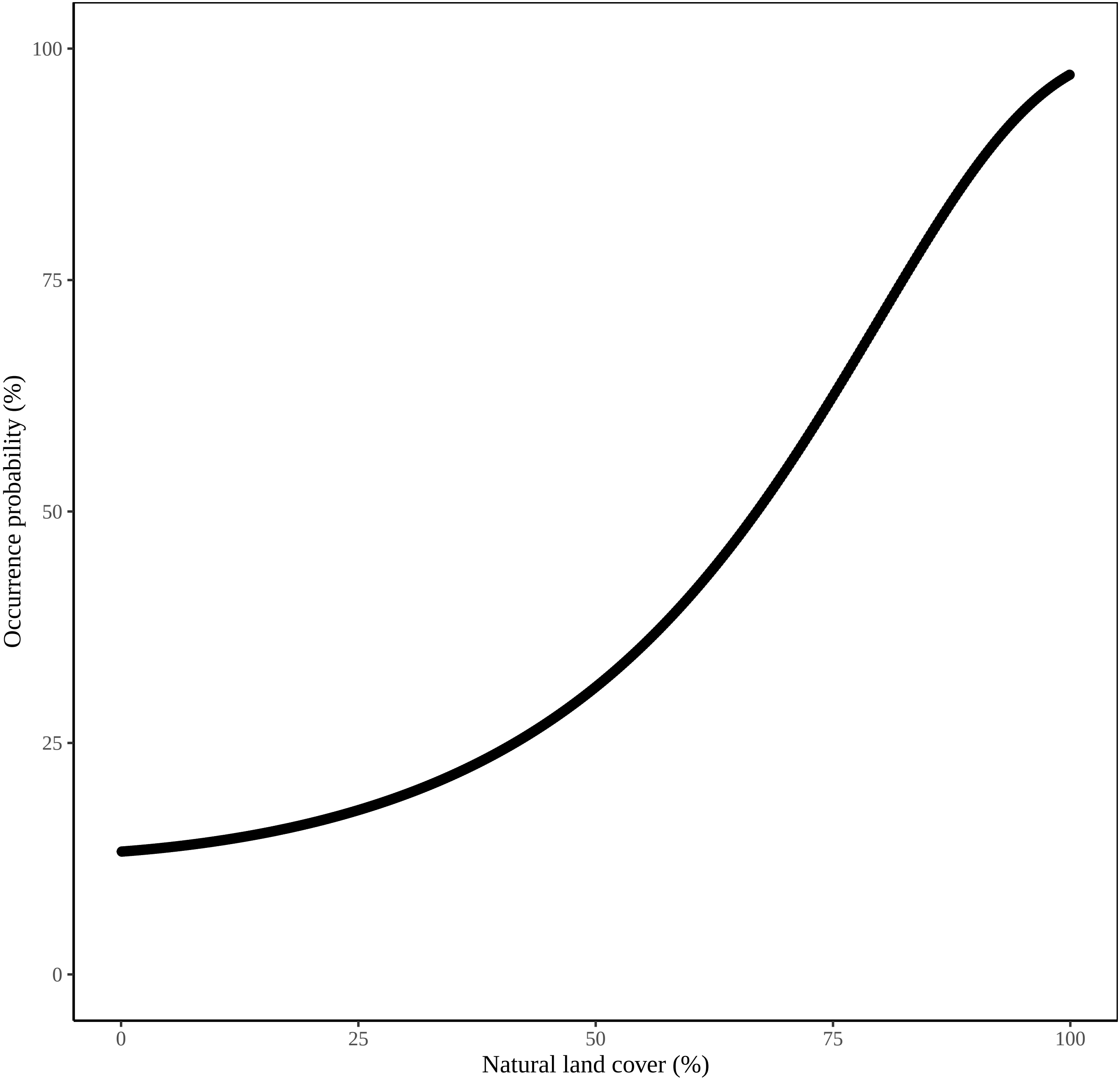

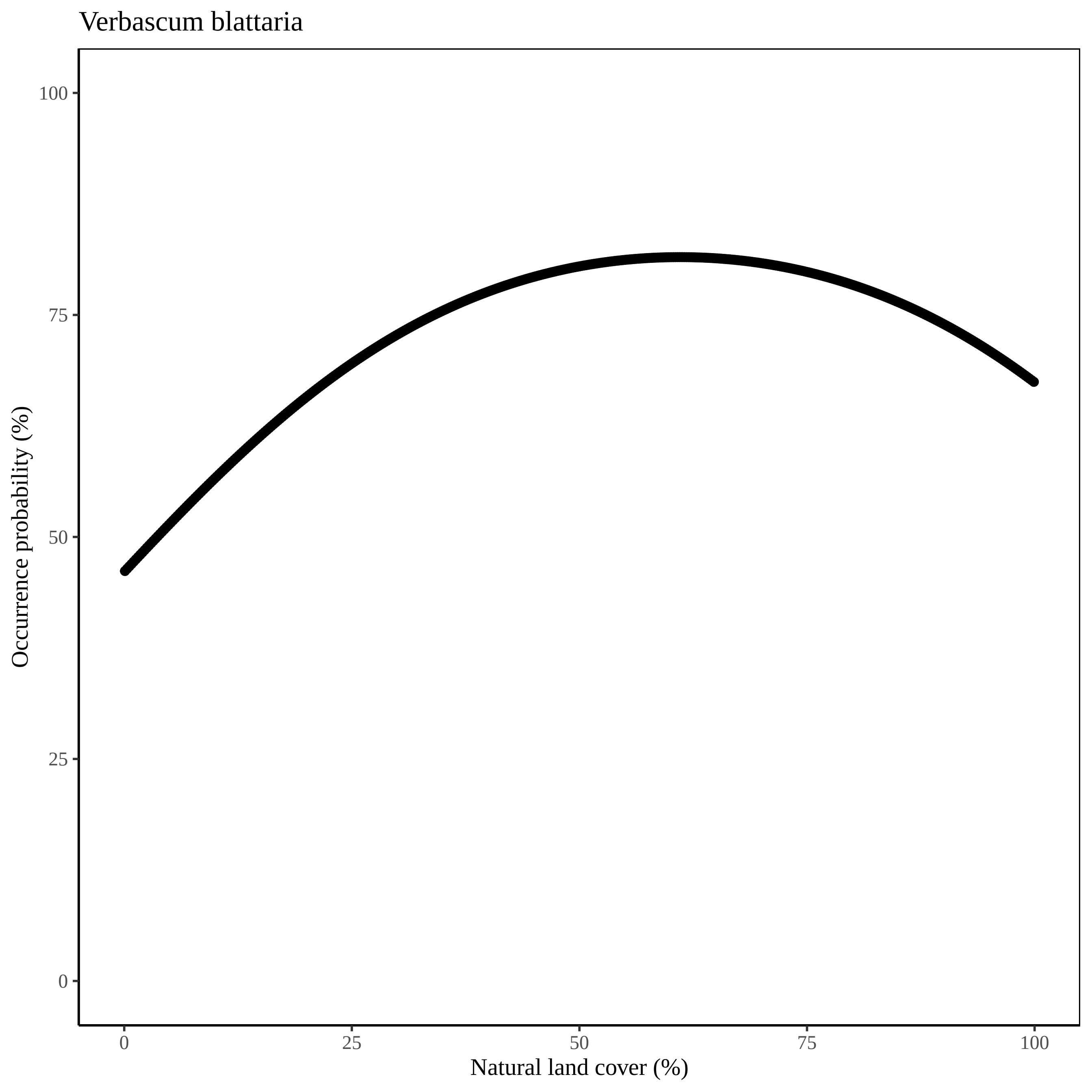
Verbascum blattaria
Occurrence probability (%)
100
75
50
25
0
0
25
50
75
100
Natural land cover (%)

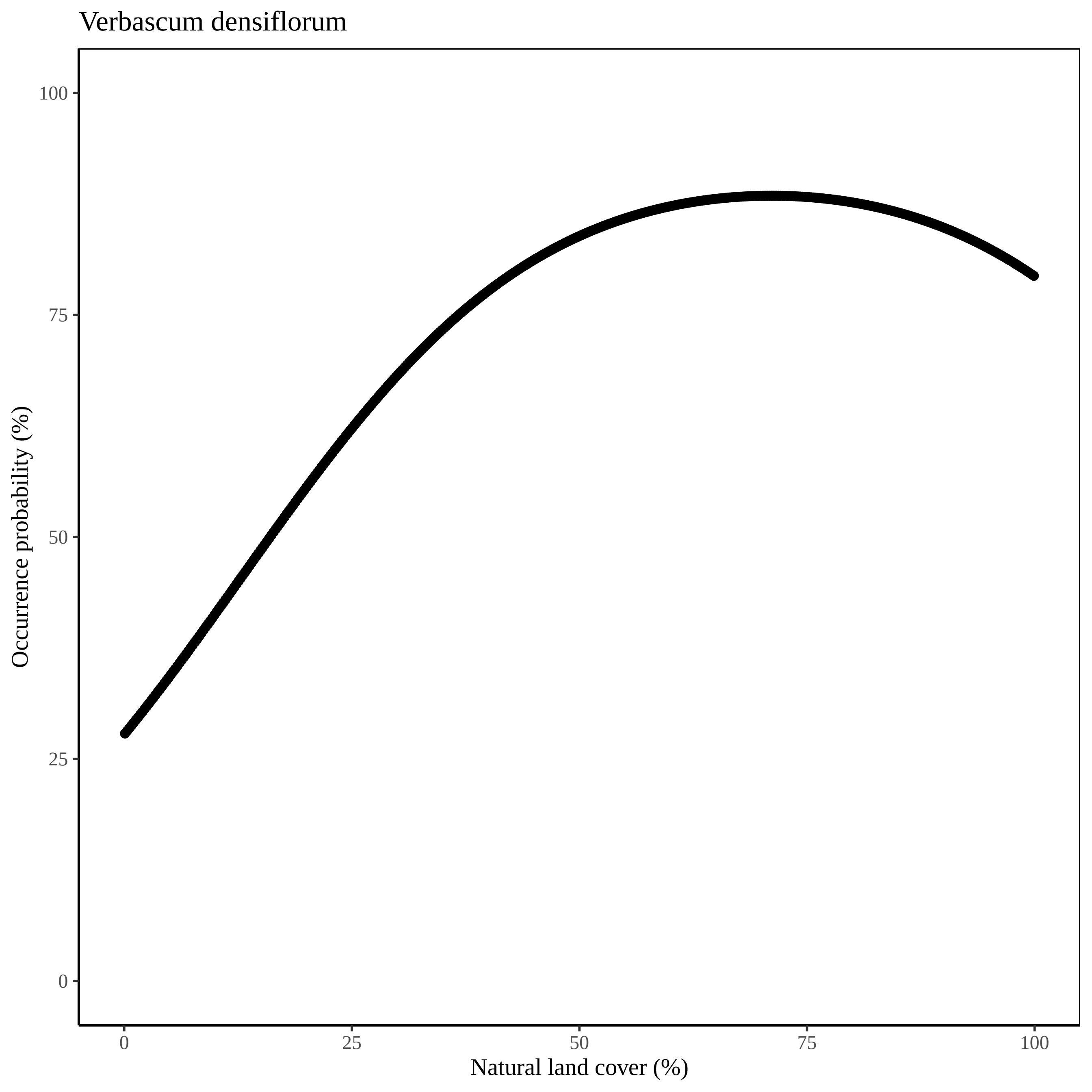
Verbascum densiflorum
Occurrence probability (%)
100
75
50
25
0
0
25
50
75
100
Natural land cover (%)

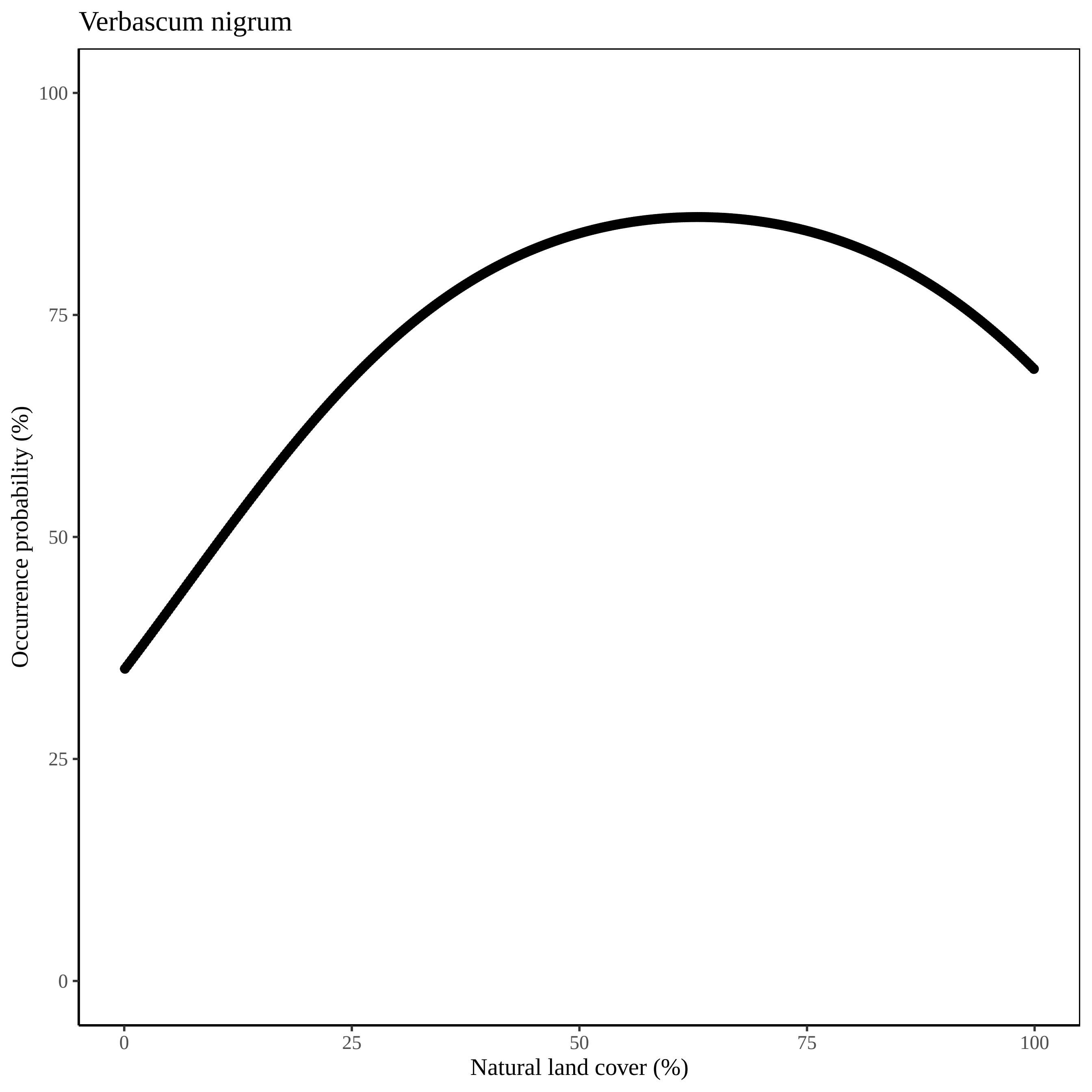
Verbascum nigrum
Occurrence probability (%)
100
75
50
25
0
0
25
50
75
100
Natural land cover (%)

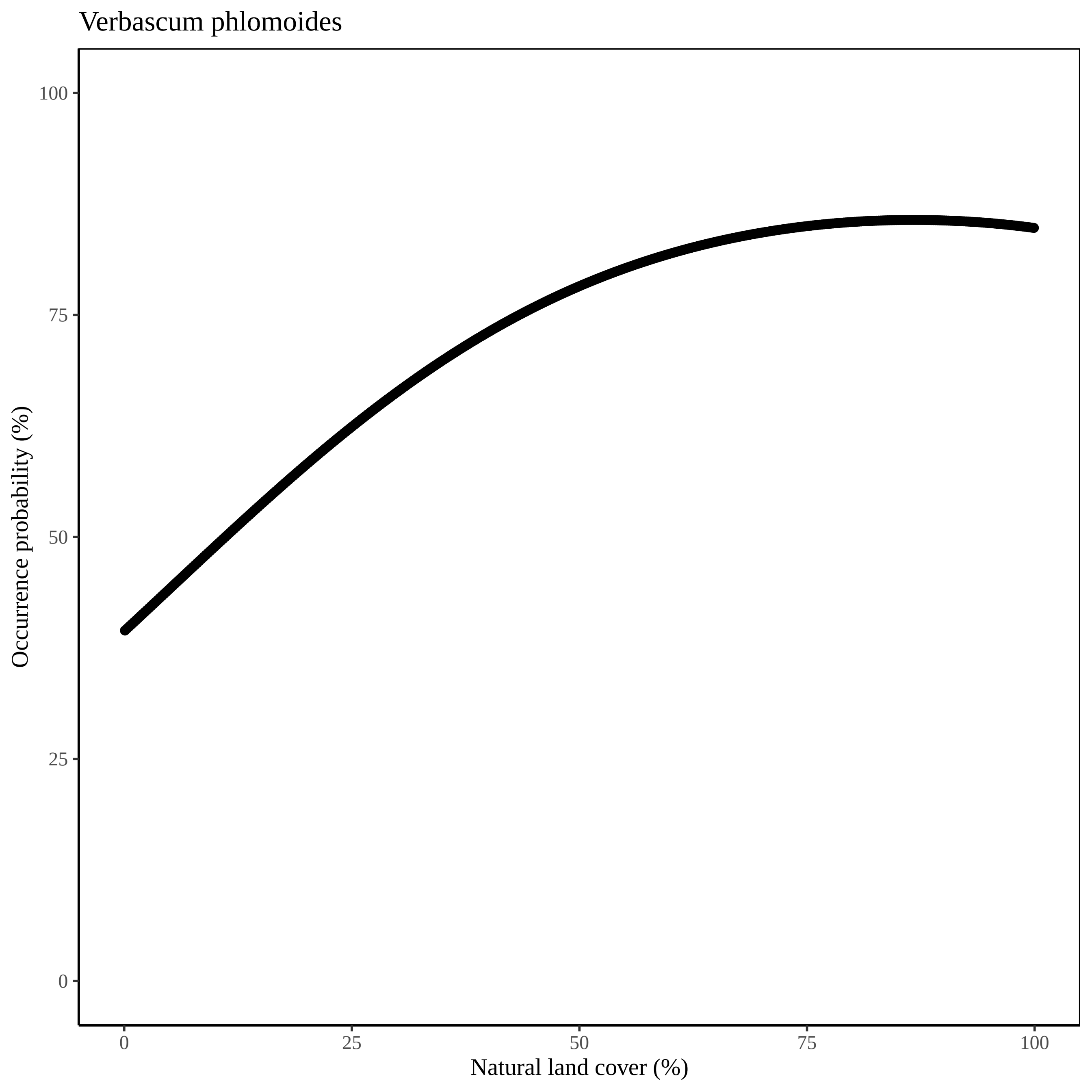

Verbascum phlomoides
Occurrence probability (%)
Natural land cover (%)
0
25
50
75
100

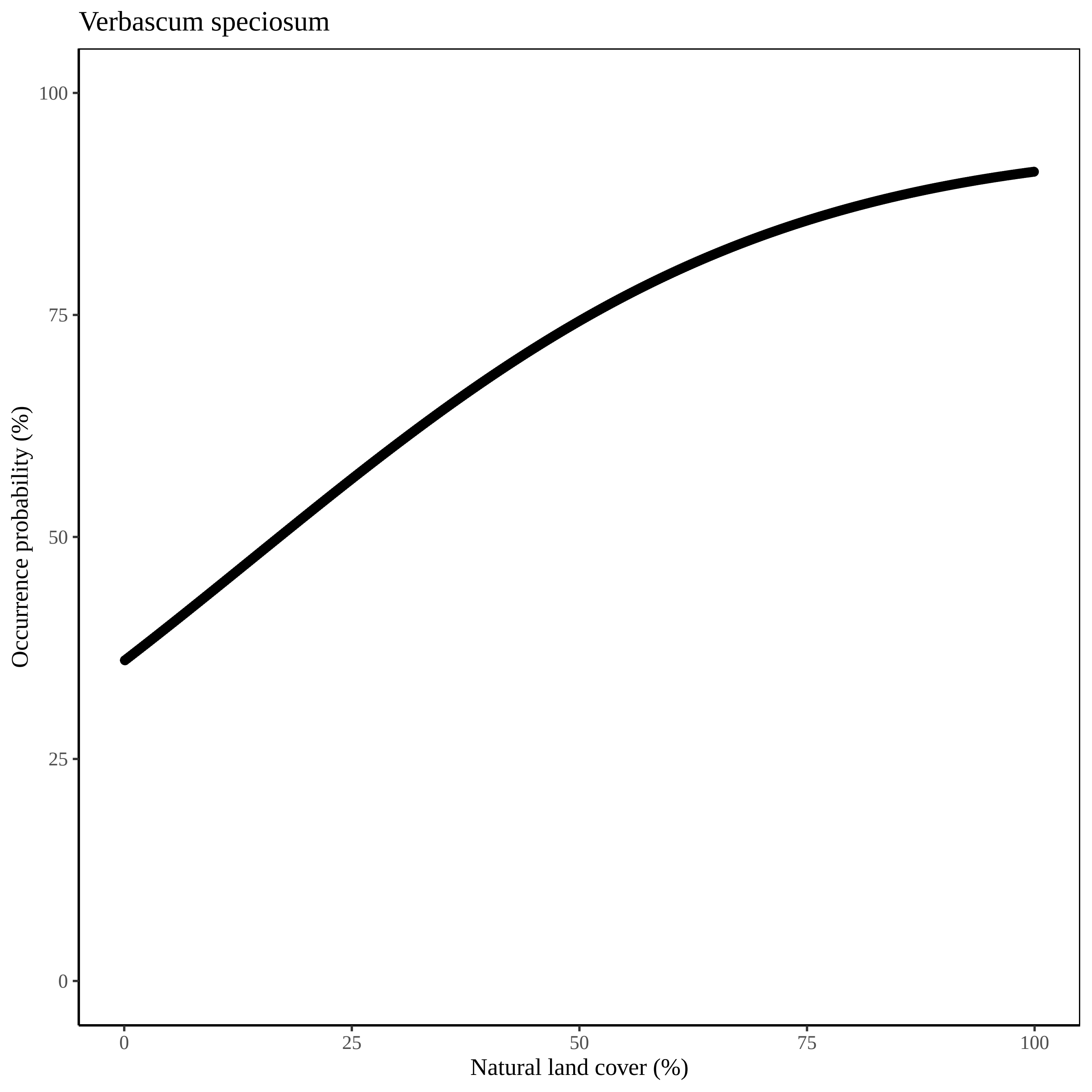
Verbascum speciosum
Occurrence probability (%)
100
75
50
25
0
0
25
50
75
100
Natural land cover (%)

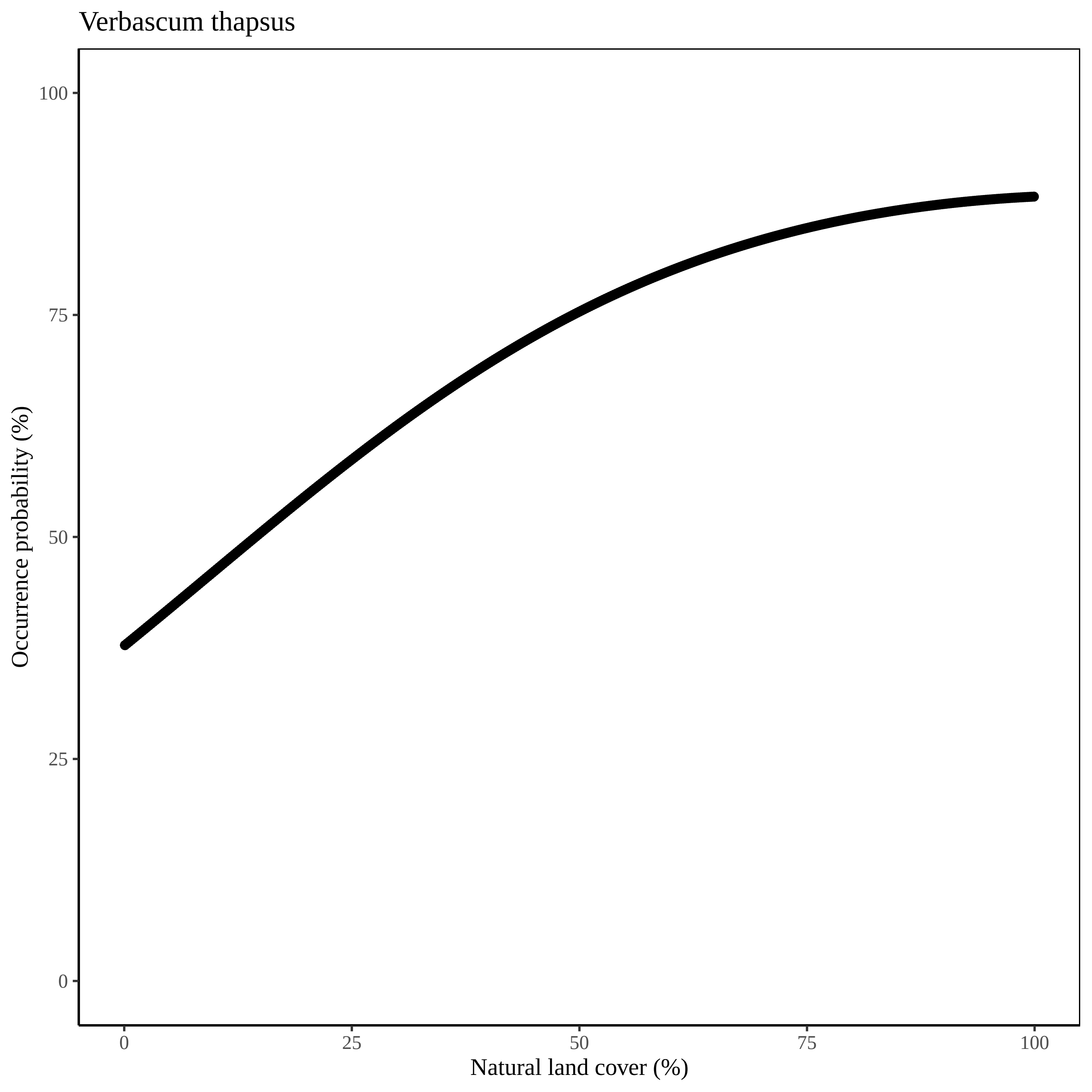
Verbascum thapsus
Occurrence probability (%)
100
75
50
25
0
0
25
50
75
100
Natural land cover (%)

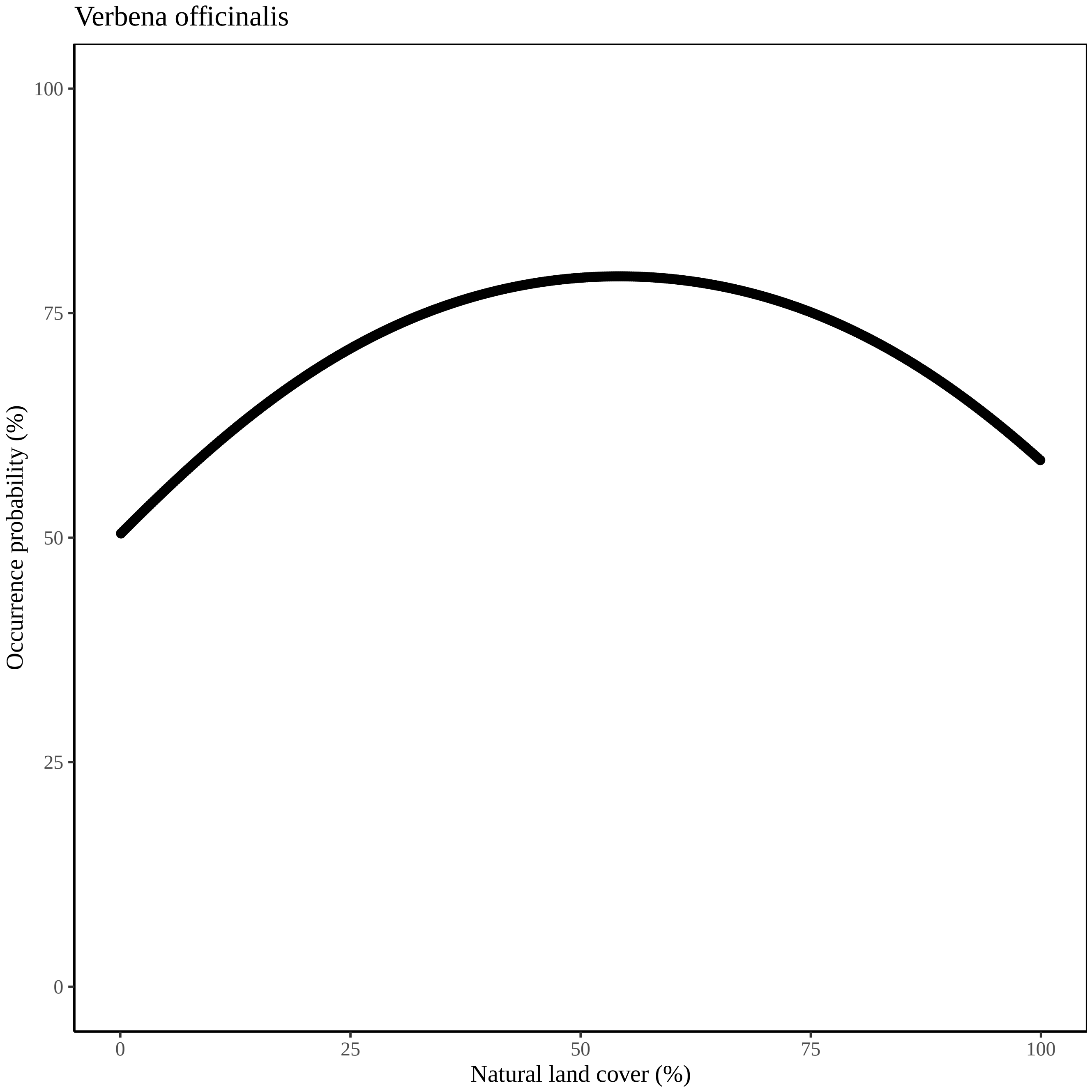
Verbena officinalis
Occurrence probability (%)
100
75
50
25
0
0
25
50
75
100
Natural land cover (%)

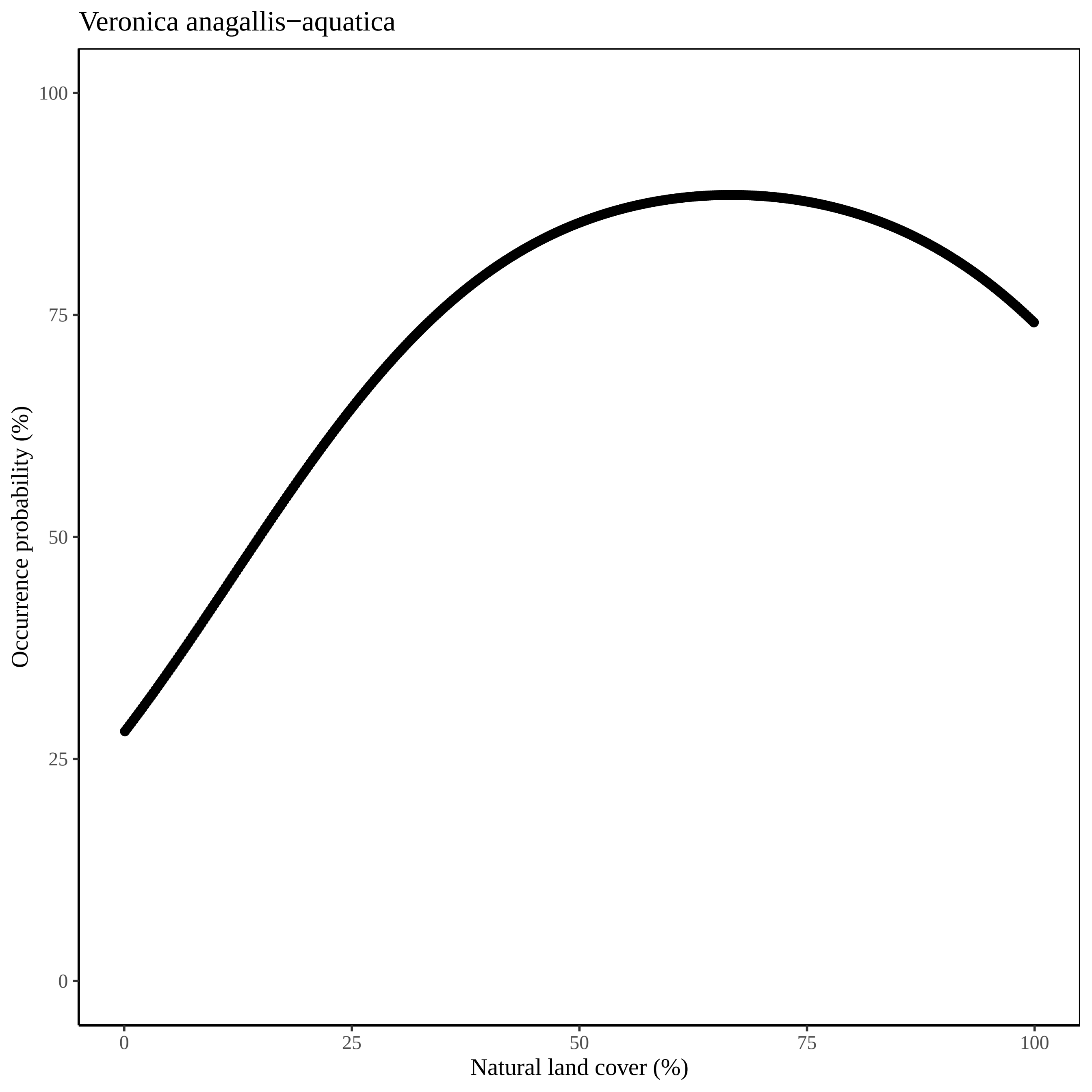
Veronica anagallis−aquatica
Occurrence probability (%)
100
75
50
25
0
0
25
50
75
100
Natural land cover (%)

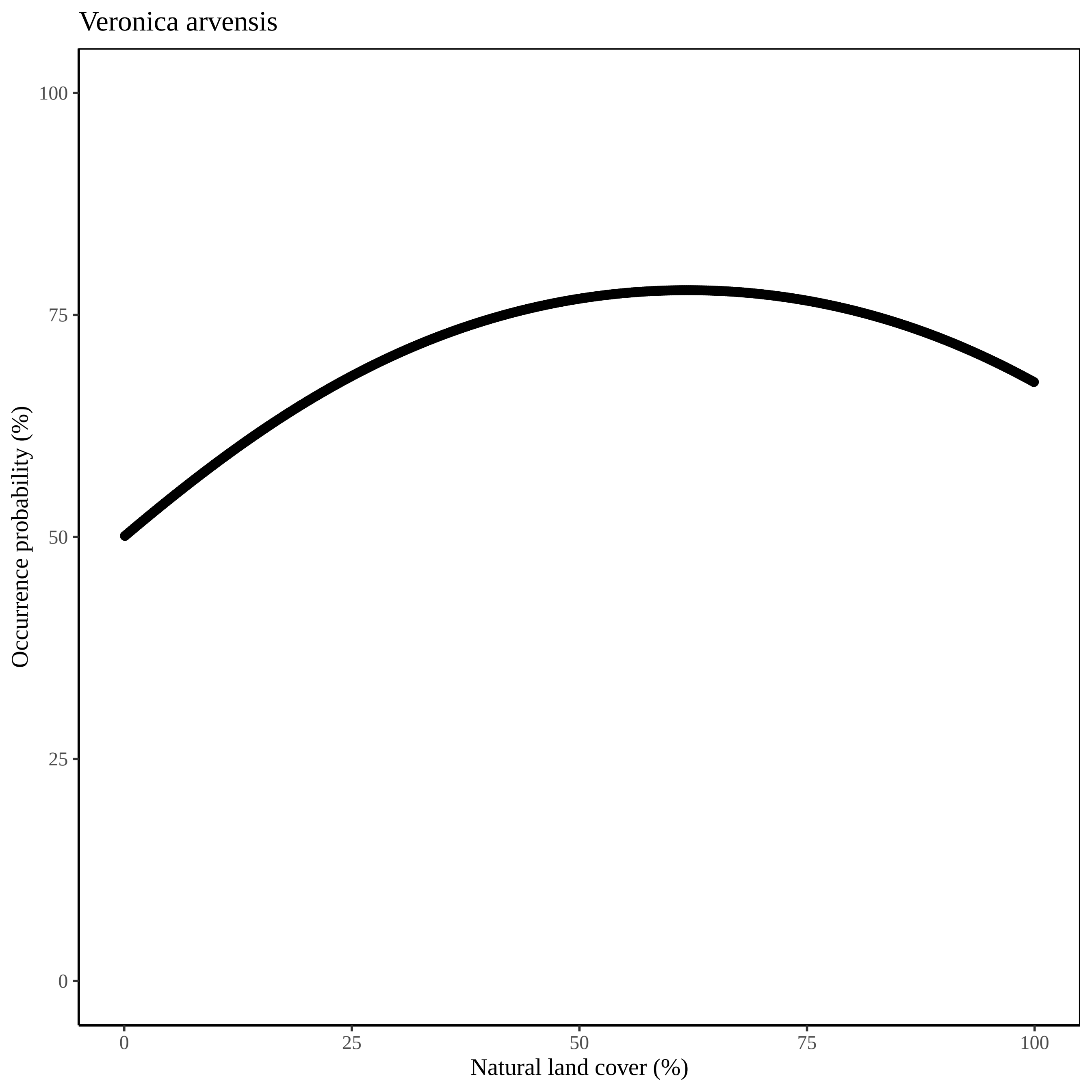
Veronica arvensis
Occurrence probability (%)
100
75
50
25
0
0
25
50
75
100
Natural land cover (%)

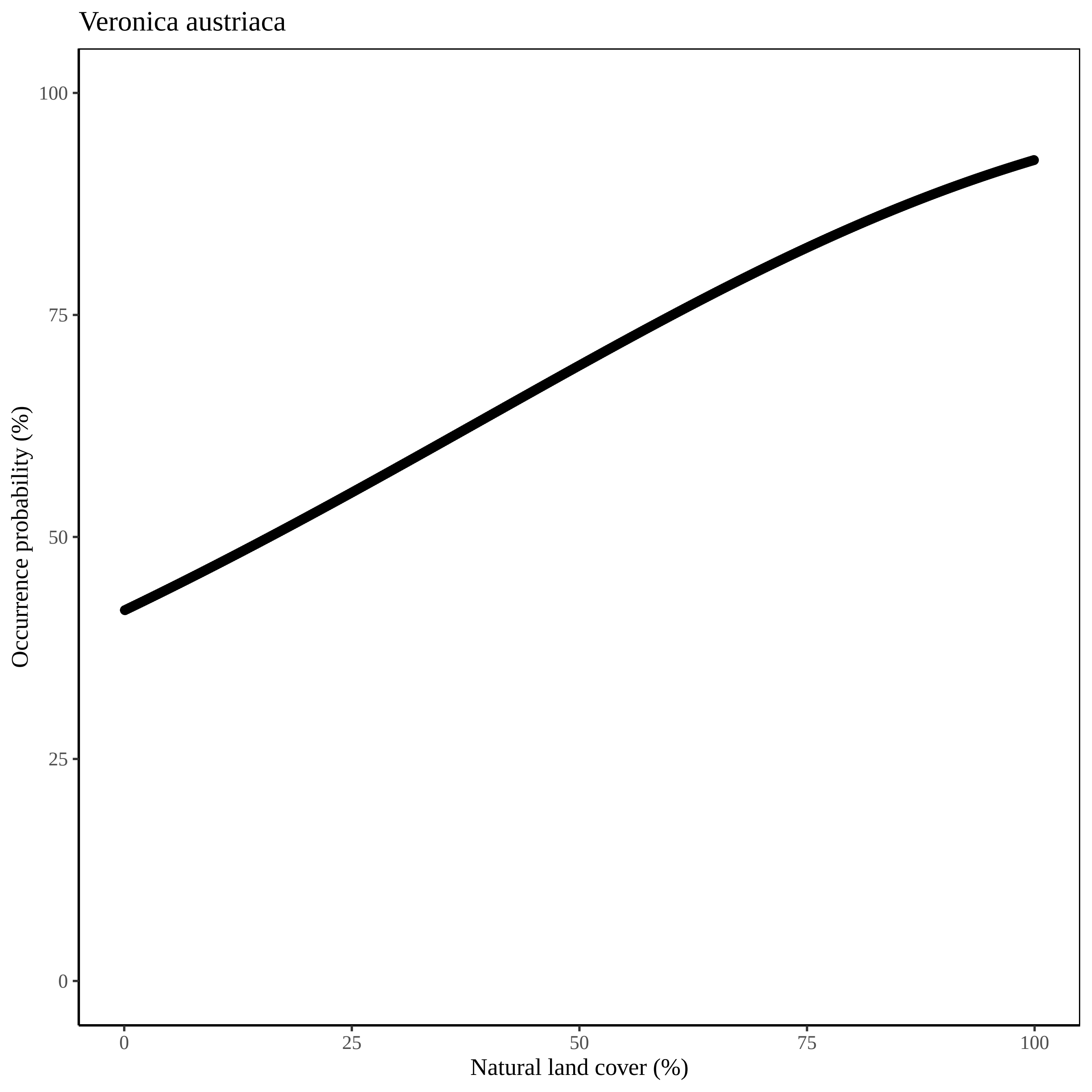
Veronica austriaca
100
75
50
25
0
Occurrence probability (%)
0
25
50
75
100
Natural land cover (%)

Veronica beccabunga

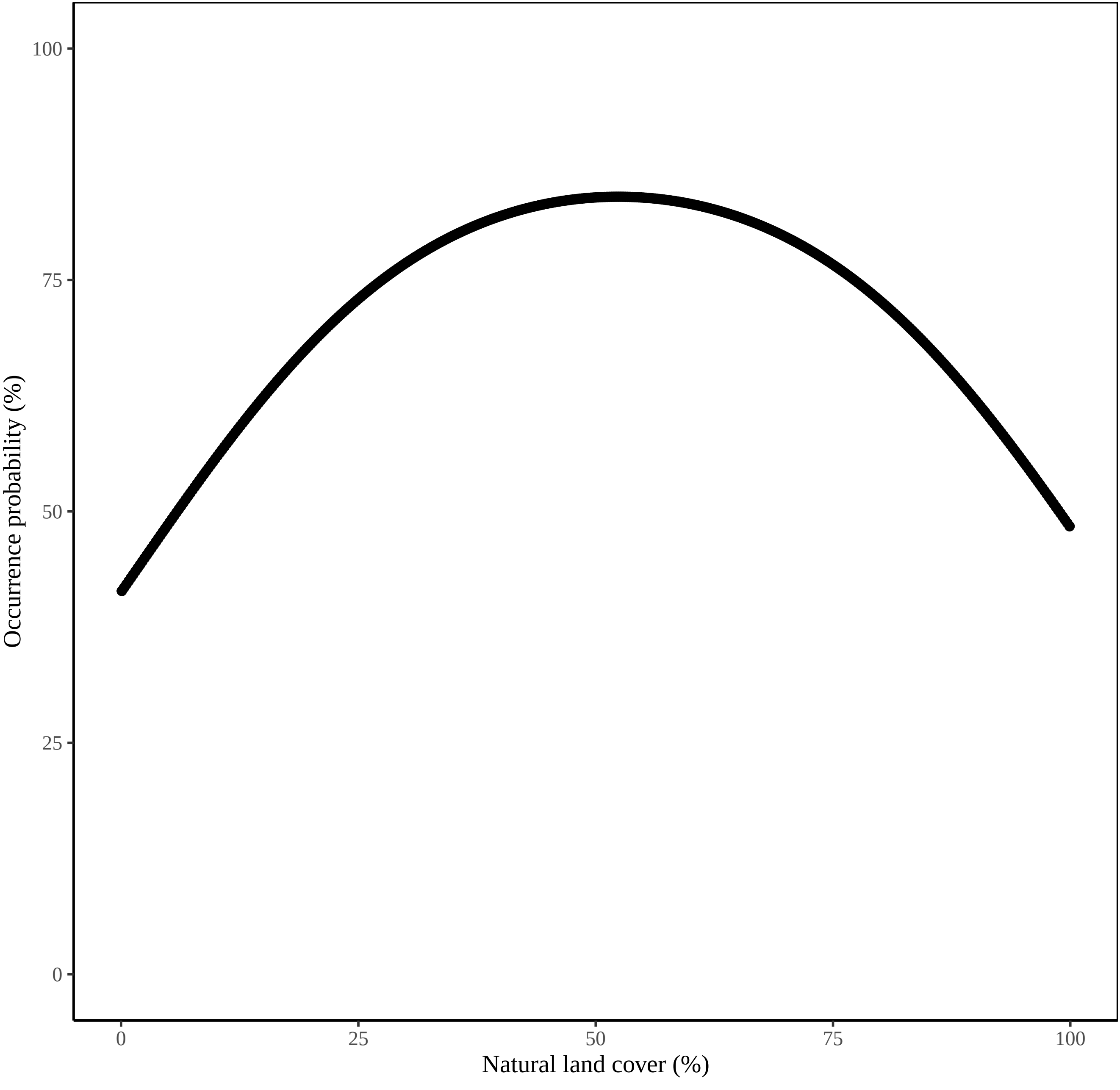

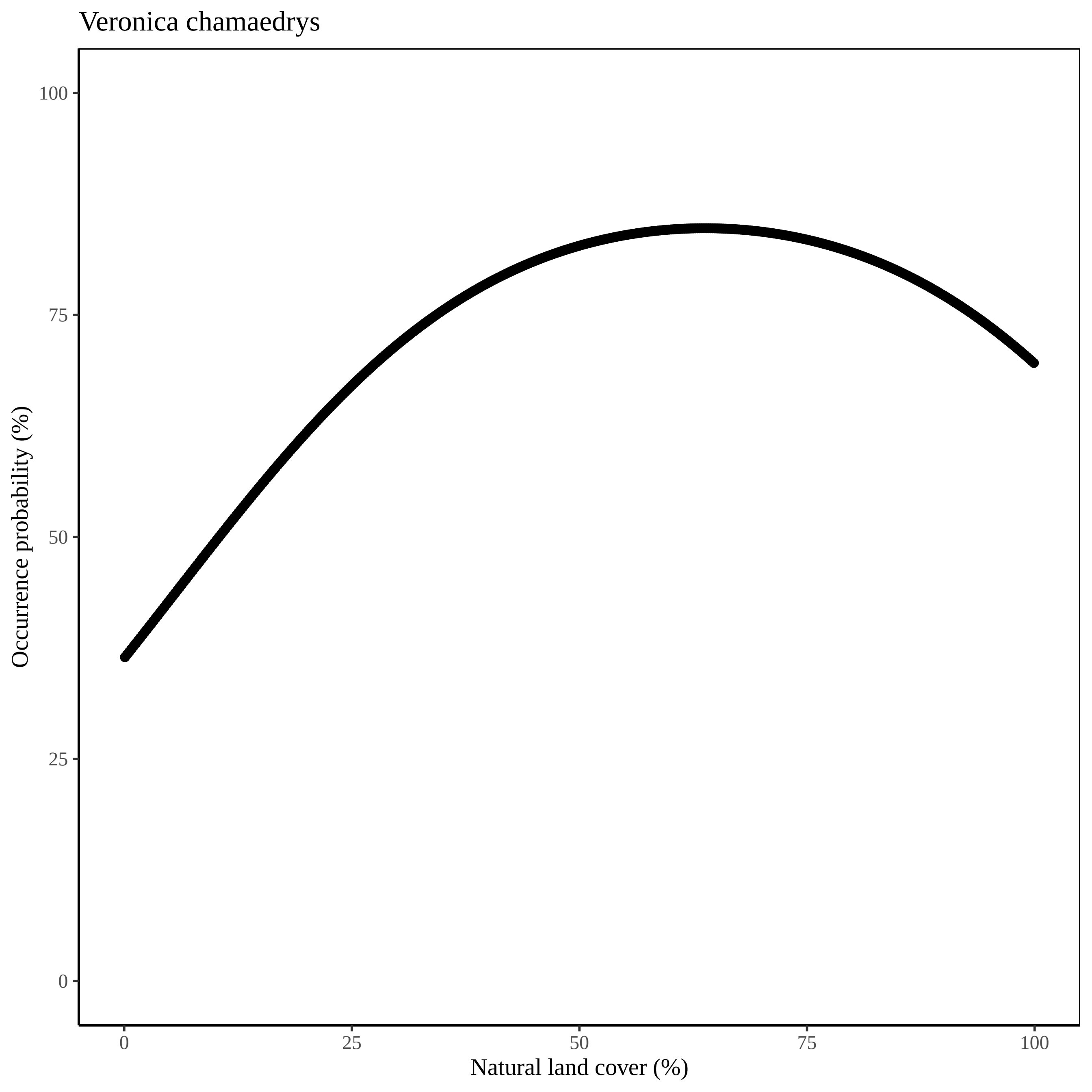
Veronica chamaedrys
Occurrence probability (%)
100
75
50
25
0
0
25
50
75
100
Natural land cover (%)

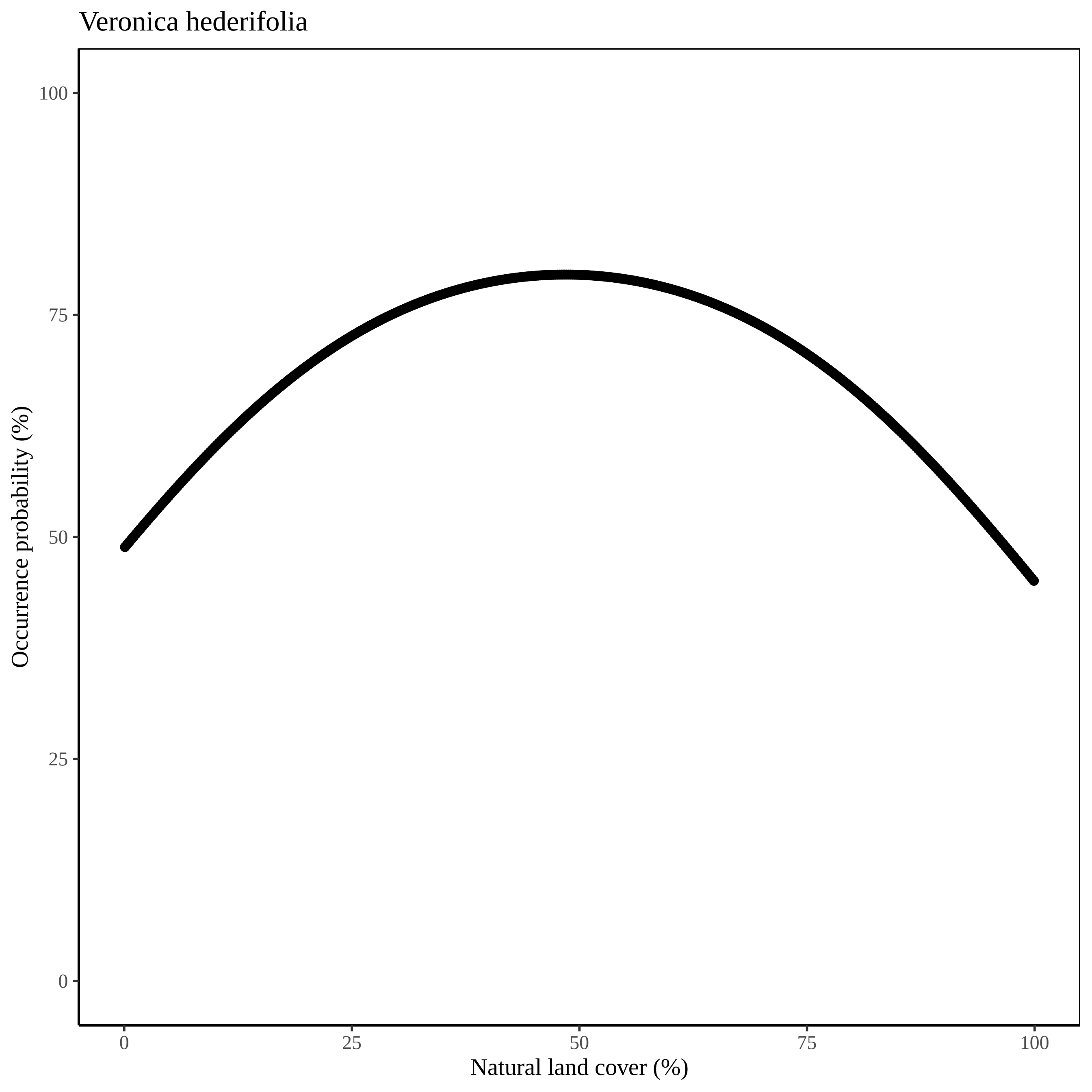

Veronica hederifolia
Occurrence probability (%)
100
75
50
25
0
0
25
50
75
100
Natural land cover (%)

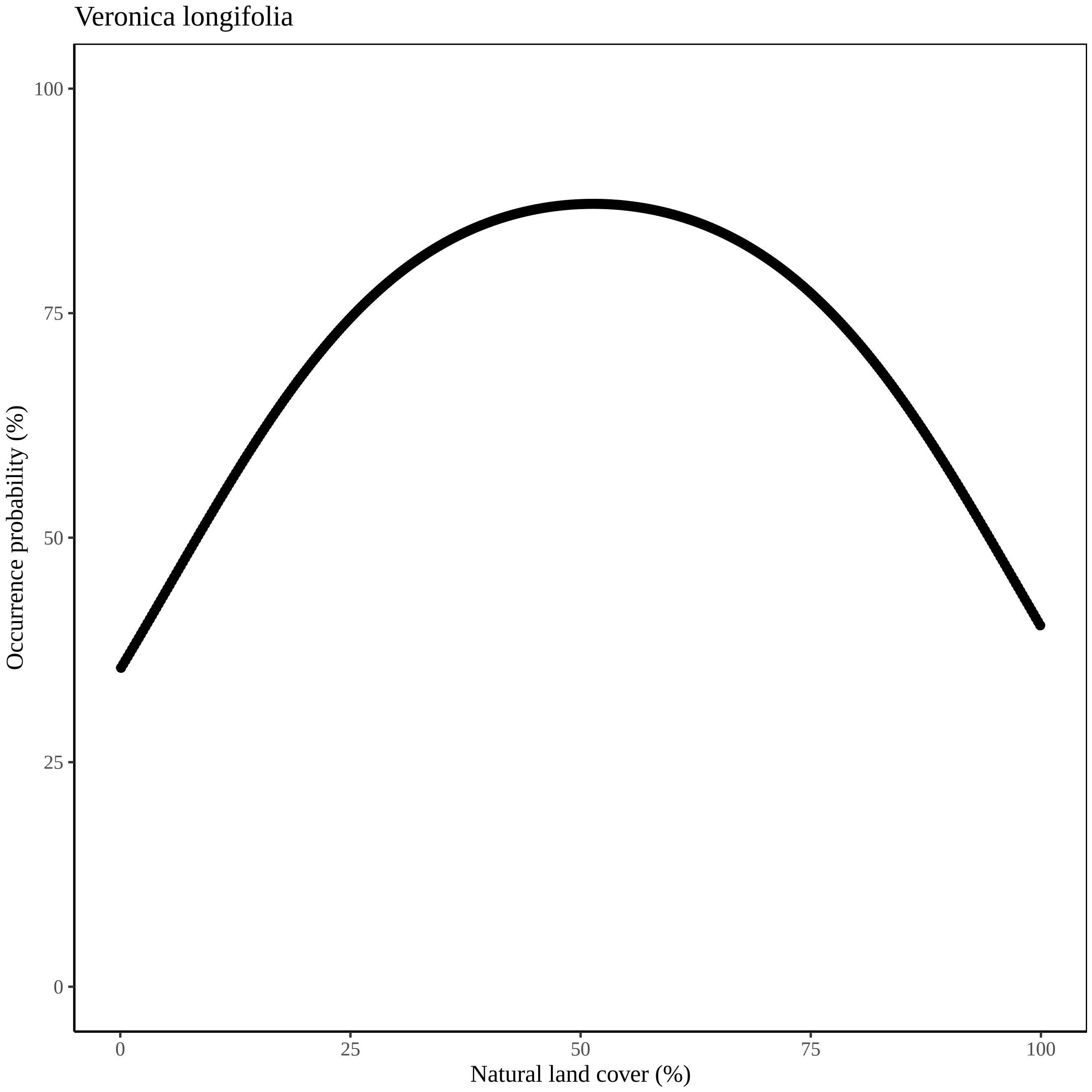
Veronica longifolia
100
75
50
25
0
Occurrence probability (%)
0
25
50
75
100
Natural land cover (%)

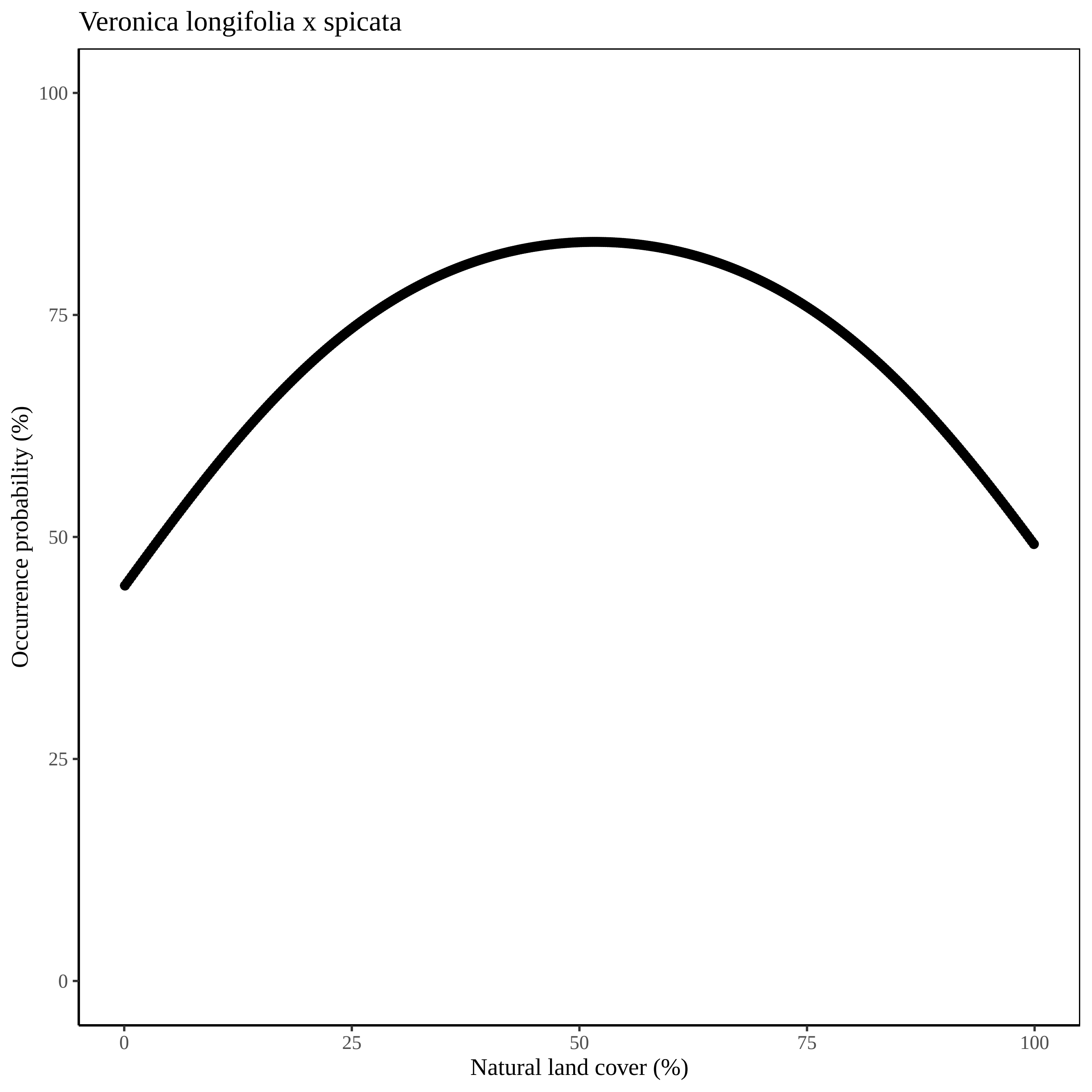
Veronica longifolia x spicata
Occurrence probability (%)
100
75
50
25
0
0
25
50
75
100
Natural land cover (%)

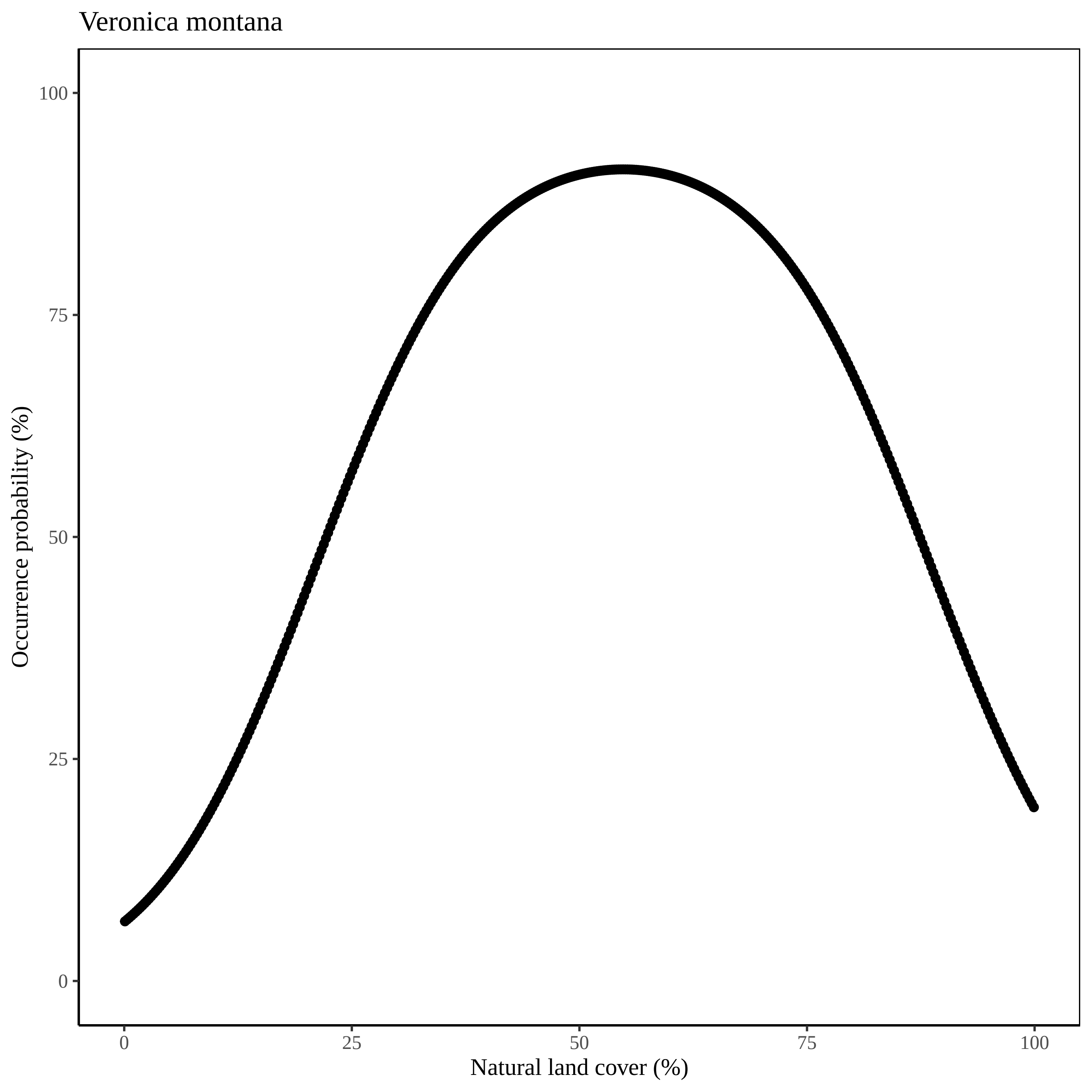
Veronica montana
Occurrence probability (%)
100
75
50
25
0
0
25
50
75
100
Natural land cover (%)

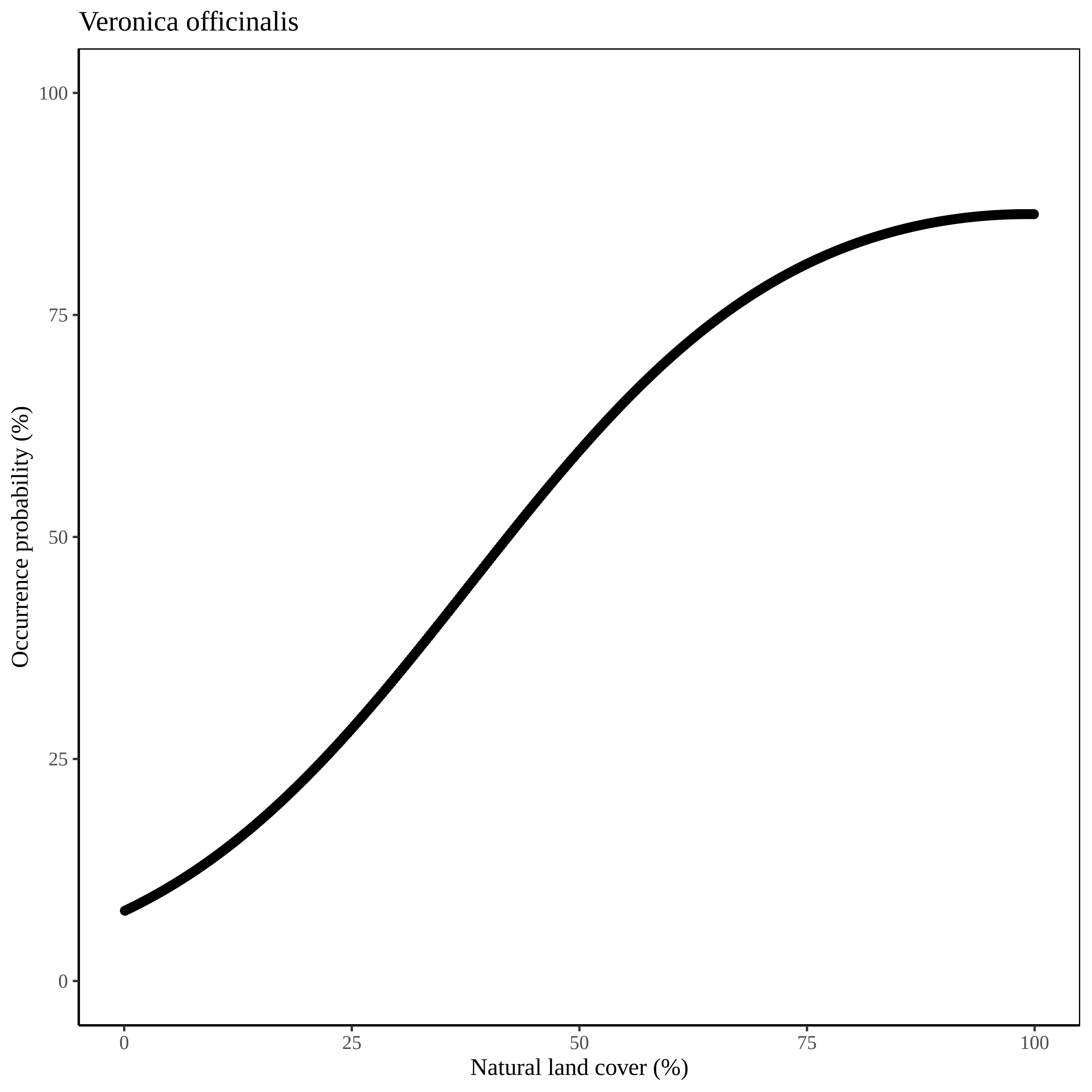
Veronica officinalis
Occurrence probability (%)
0
25
50
75
100
Natural land cover (%)

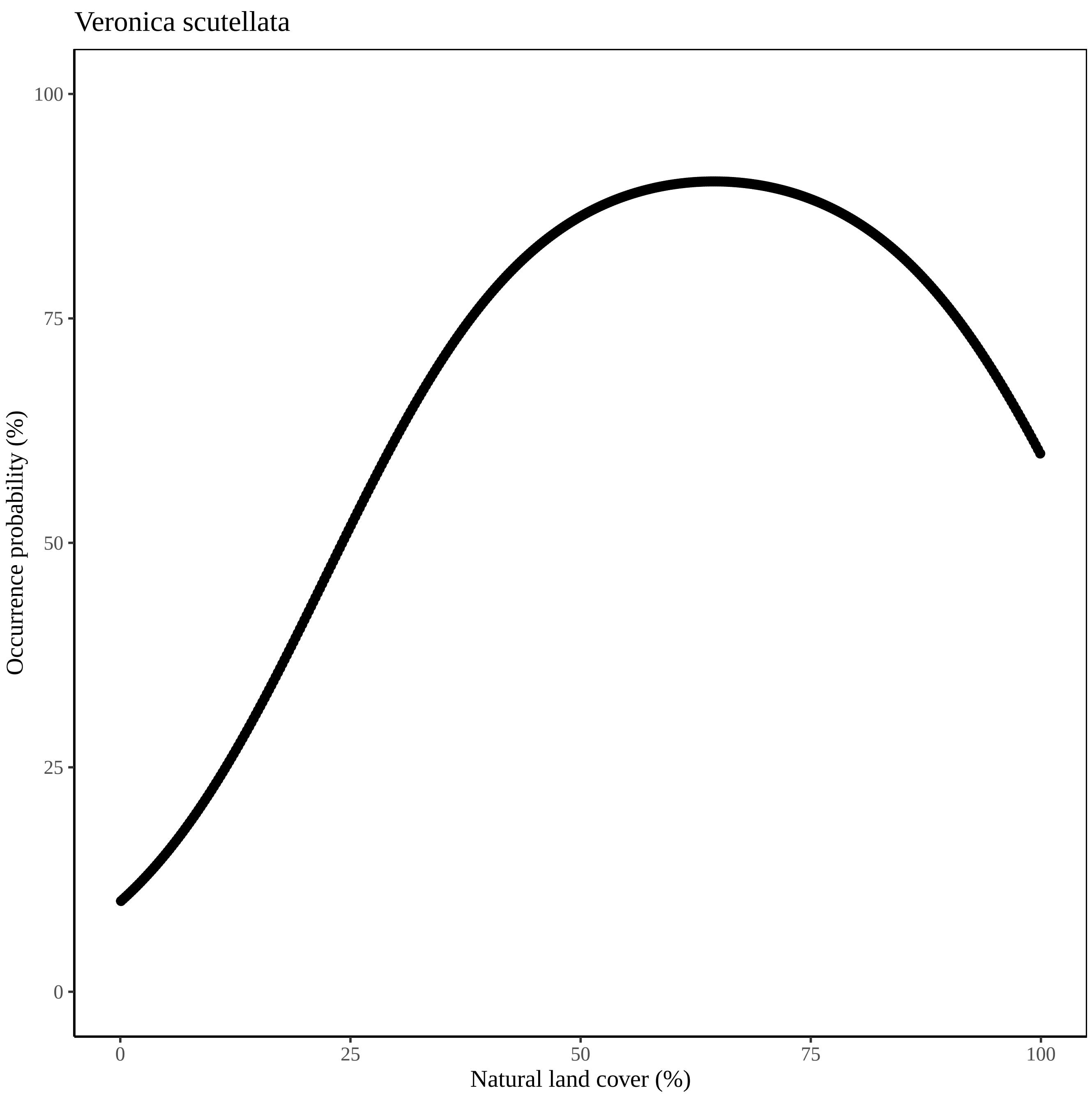

Veronica scutellata
100
75
50
25
0
Occurrence probability (%)
0
25
50
75
100
Natural land cover (%)

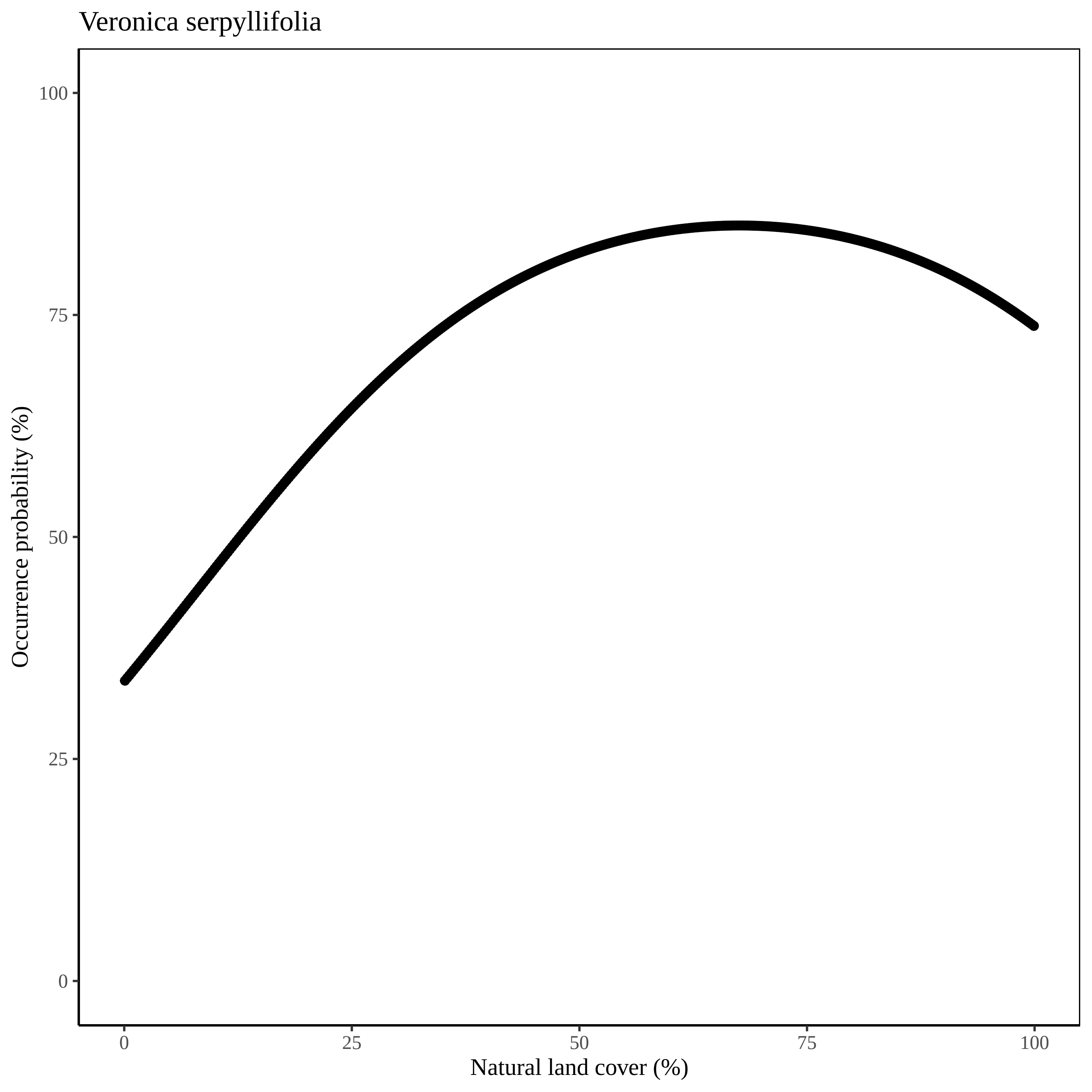
Veronica serpyllifolia
Occurrence probability (%)
100
75
50
25
0
0
25
50
75
100
Natural land cover (%)

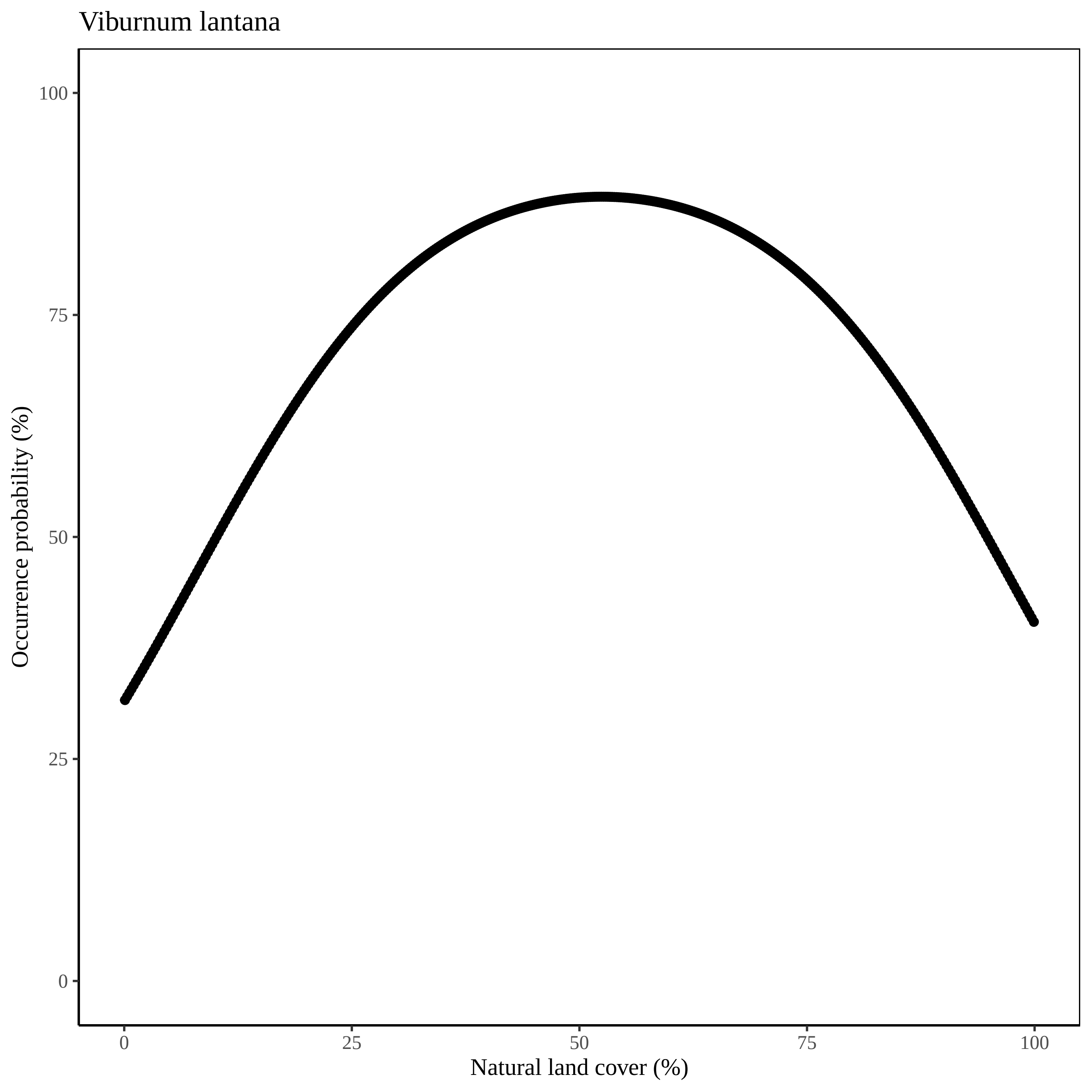
Viburnum lantana
Occurrence probability (%)
100
75
50
25
0
0
25
50
75
100
Natural land cover (%)

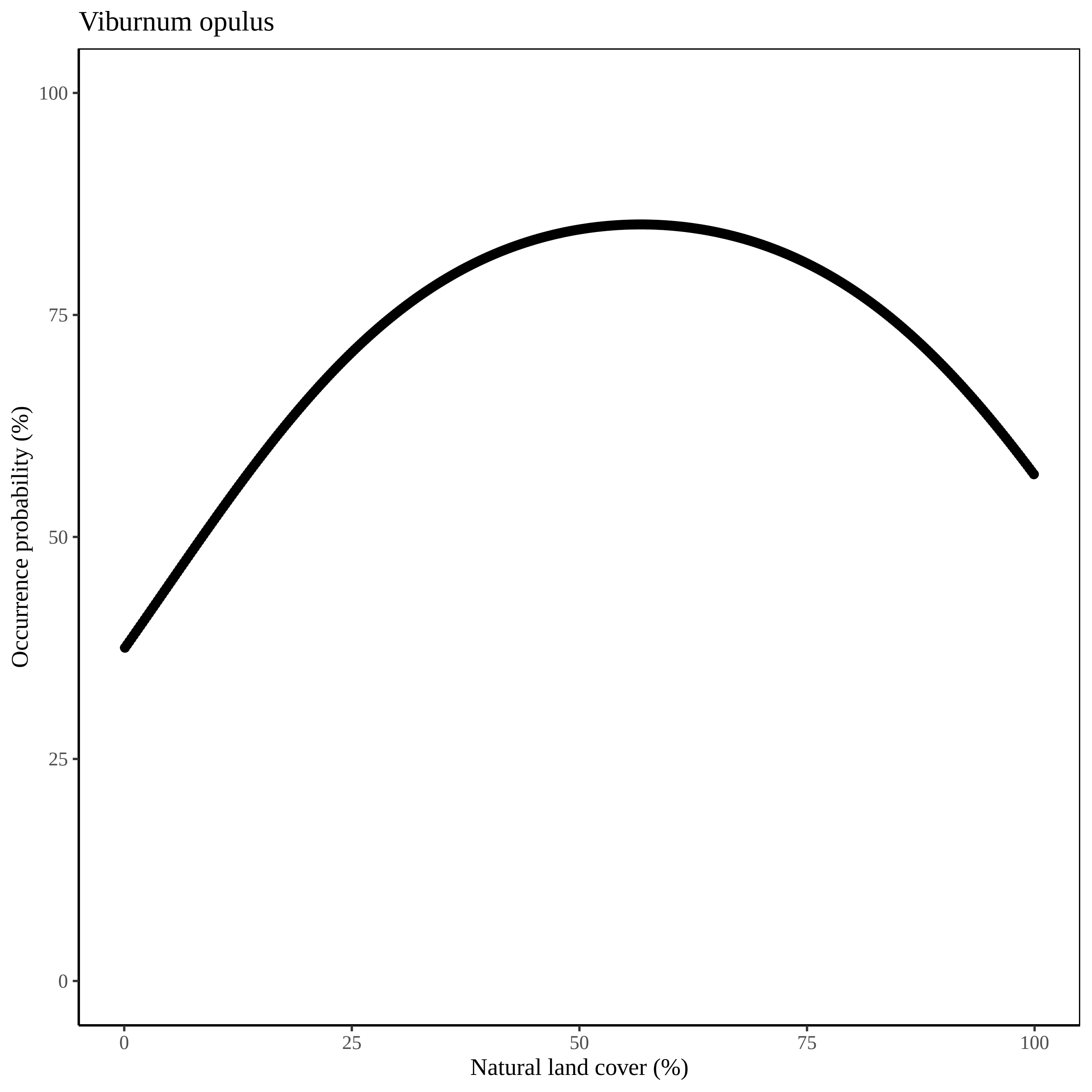
Viburnum opulus
Occurrence probability (%)
100
75
50
25
0
0
25
50
75
100
Natural land cover (%)

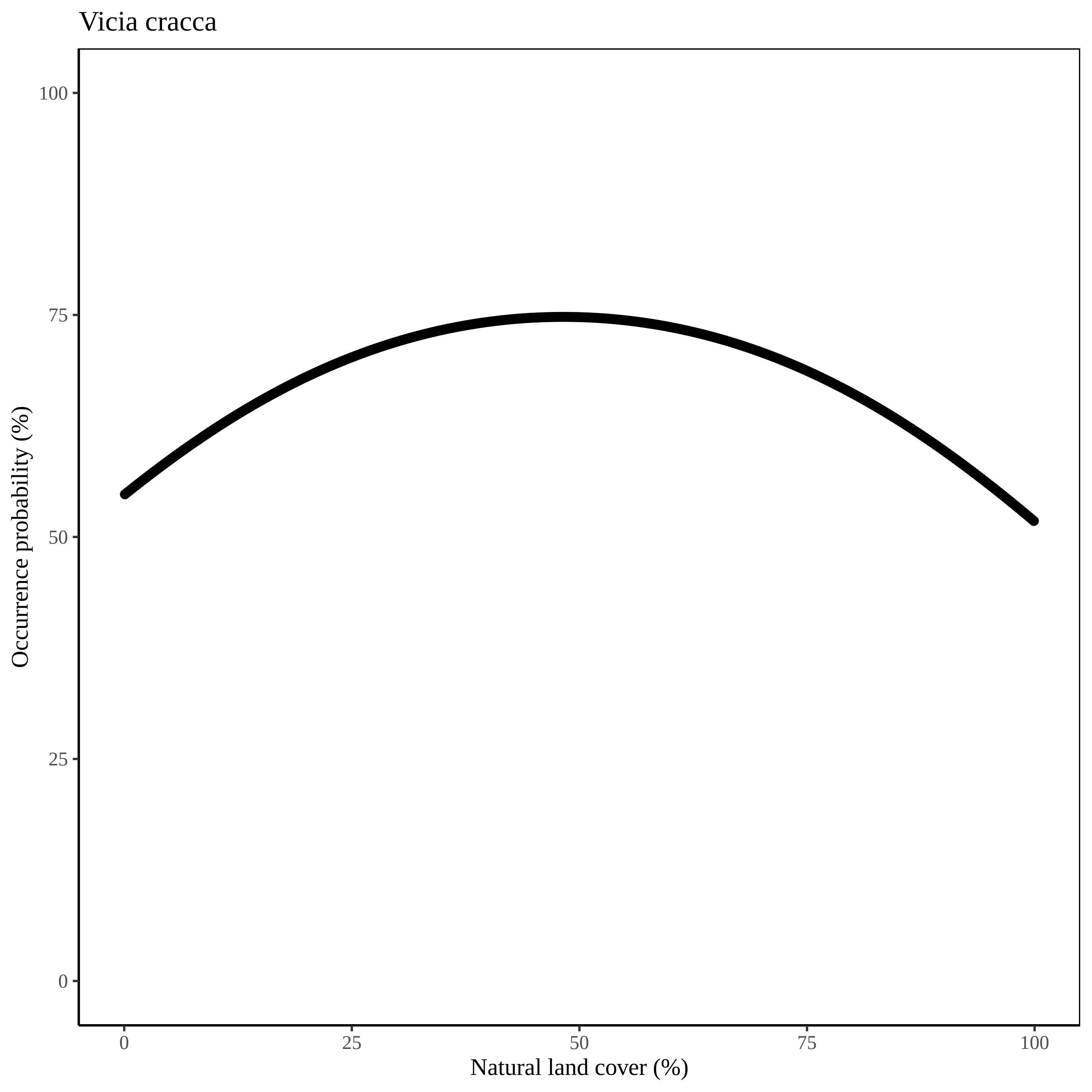
Vicia cracca
100
75
50
25
0
Occurrence probability (%)
0
25
50
75
100
Natural land cover (%)

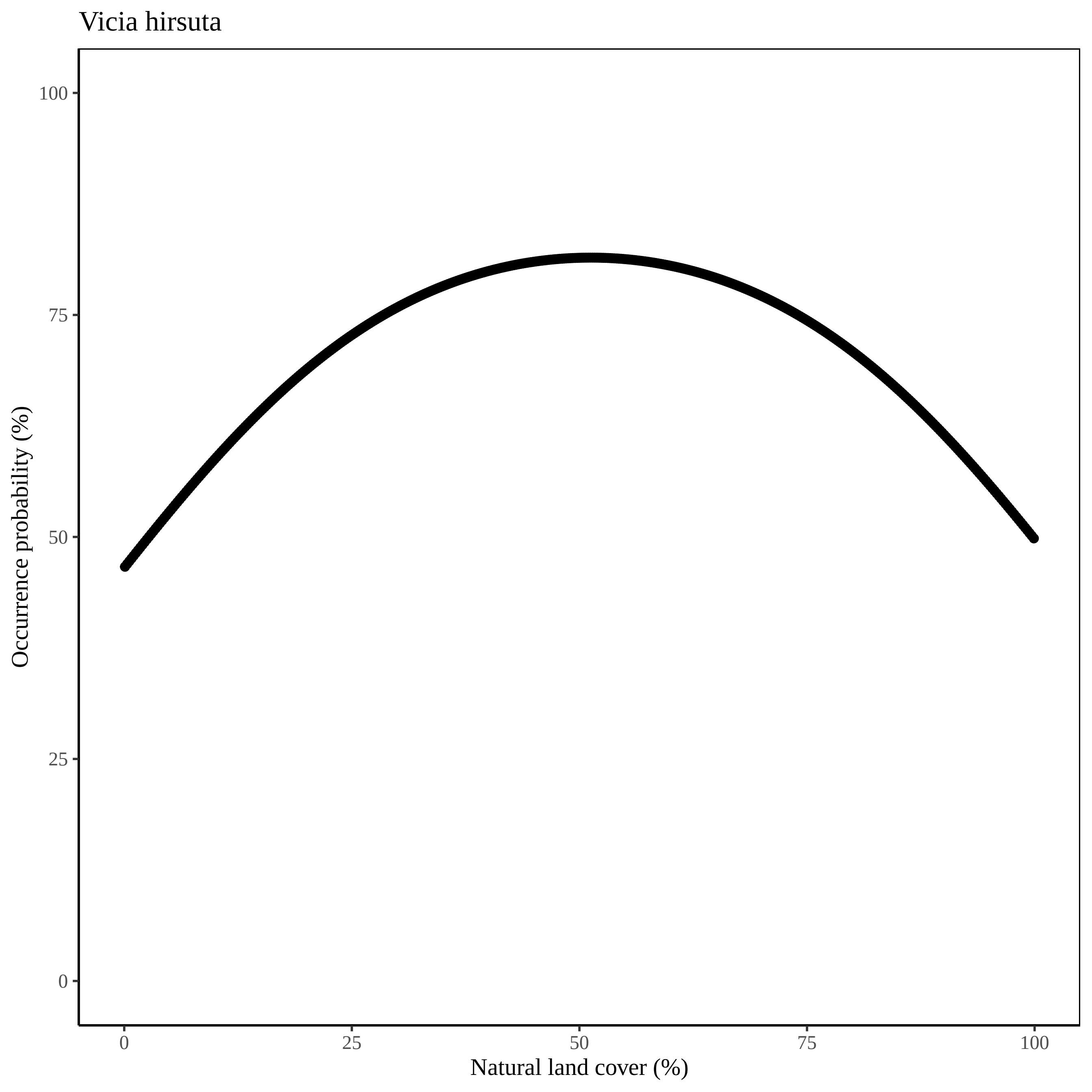
Vicia hirsuta
Occurrence probability (%)
100
75
50
25
0
0
25
50
75
100
Natural land cover (%)

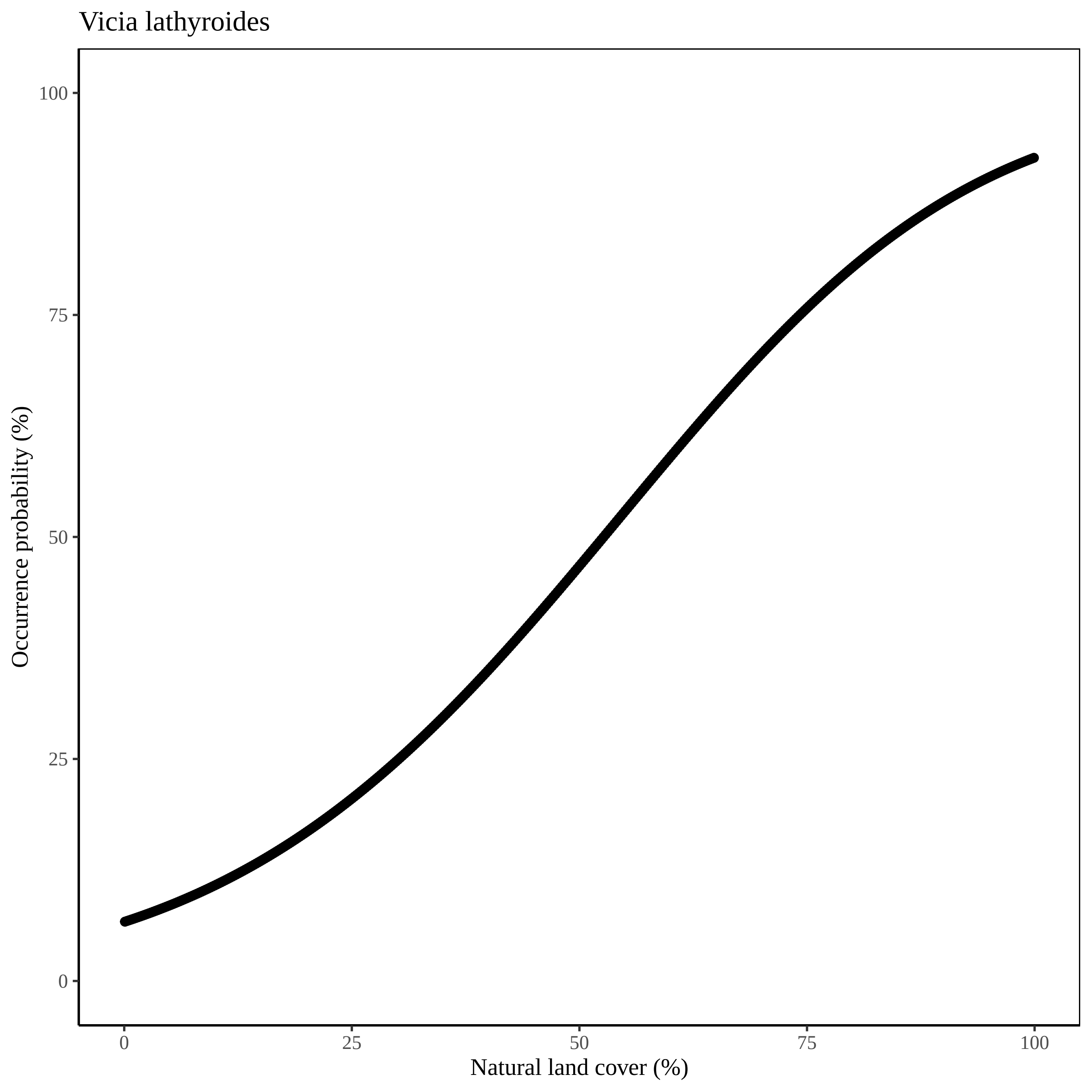

Vicia lathyroides
Occurrence probability (%)
100
75
50
25
0
0
25
50
75
100
Natural land cover (%)

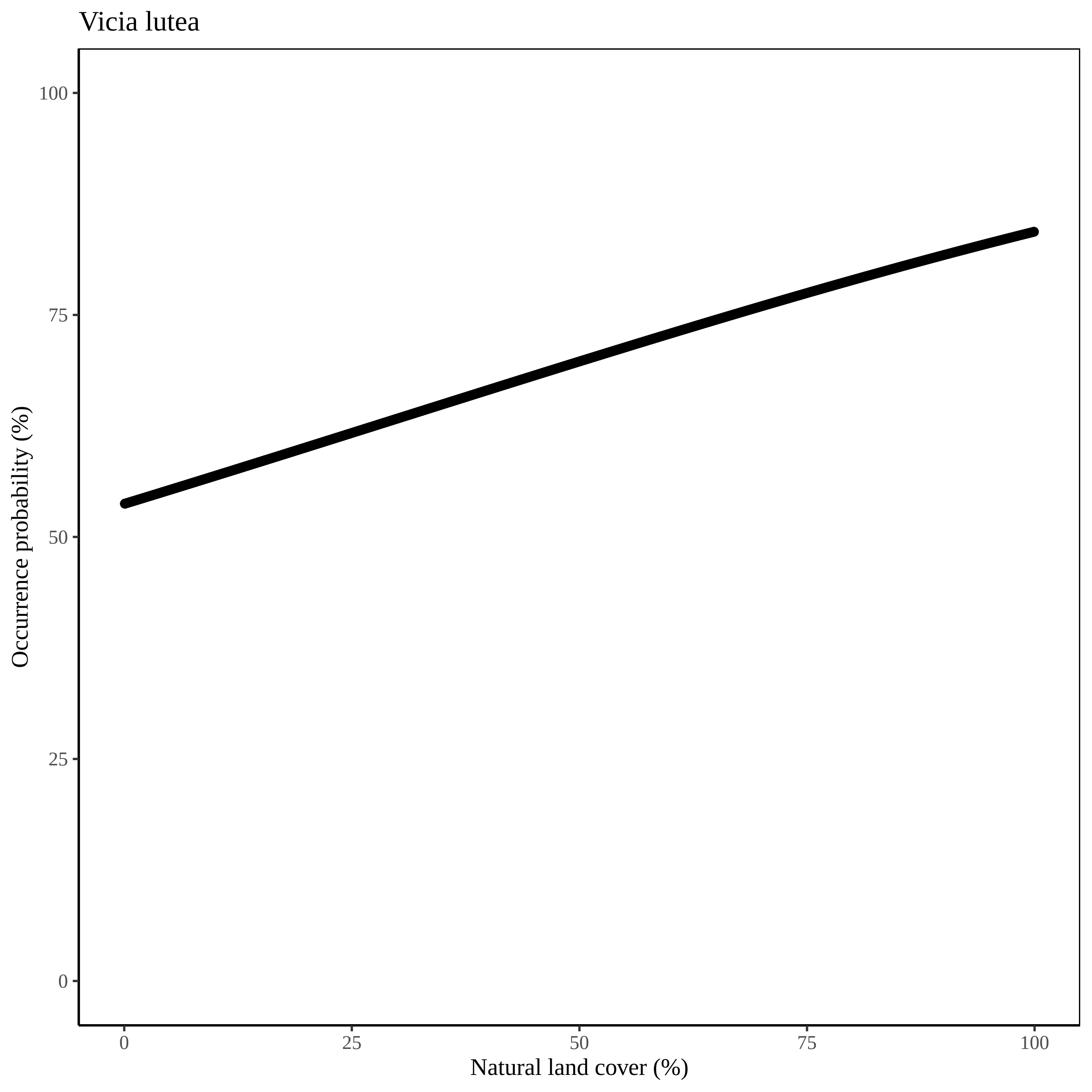
Vicia lutea
Occurrence probability (%)
100
75
50
25
0
0
25
50
75
100
Natural land cover (%)

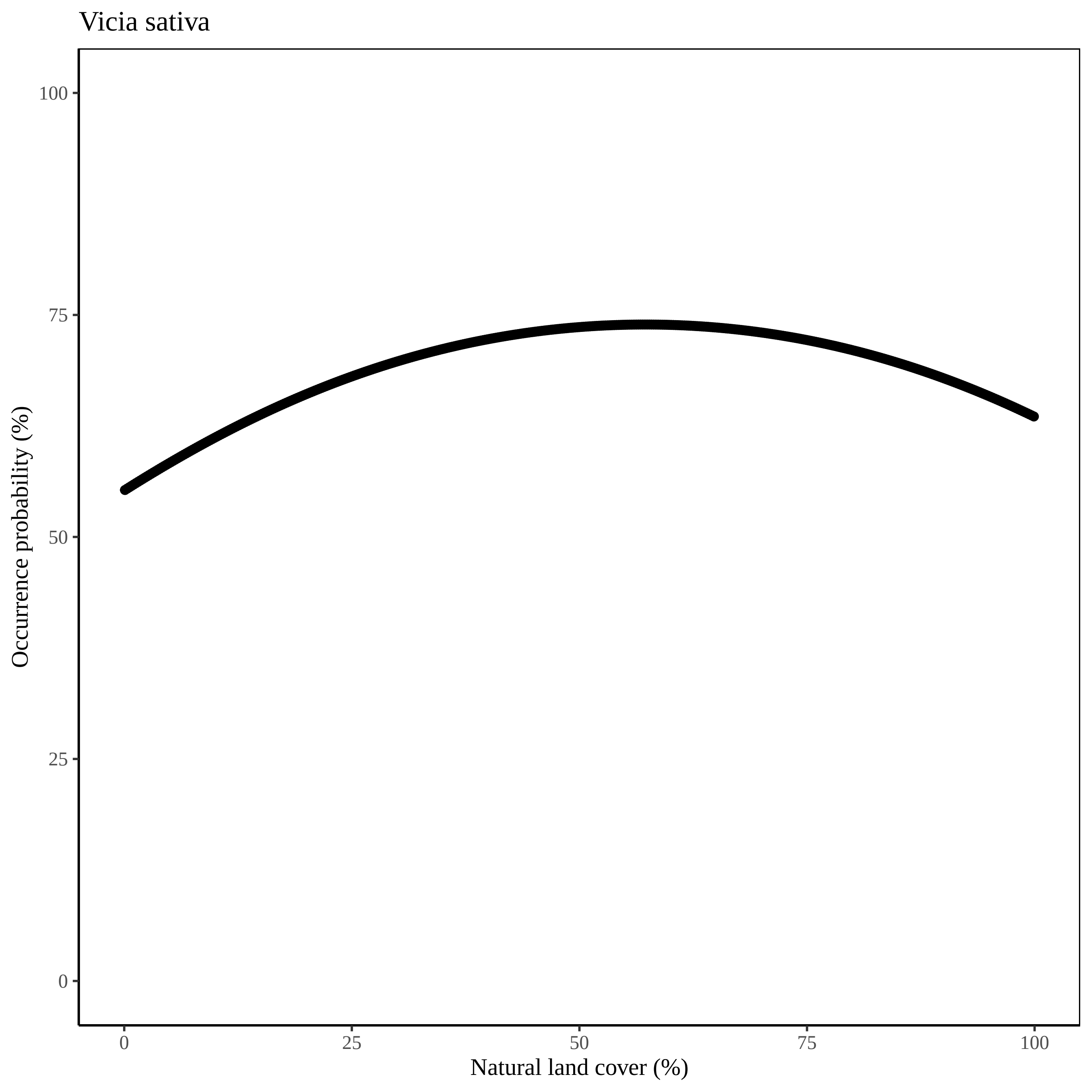
Vicia sativa
Occurrence probability (%)
100
75
50
25
0
0
25
50
75
100
Natural land cover (%)

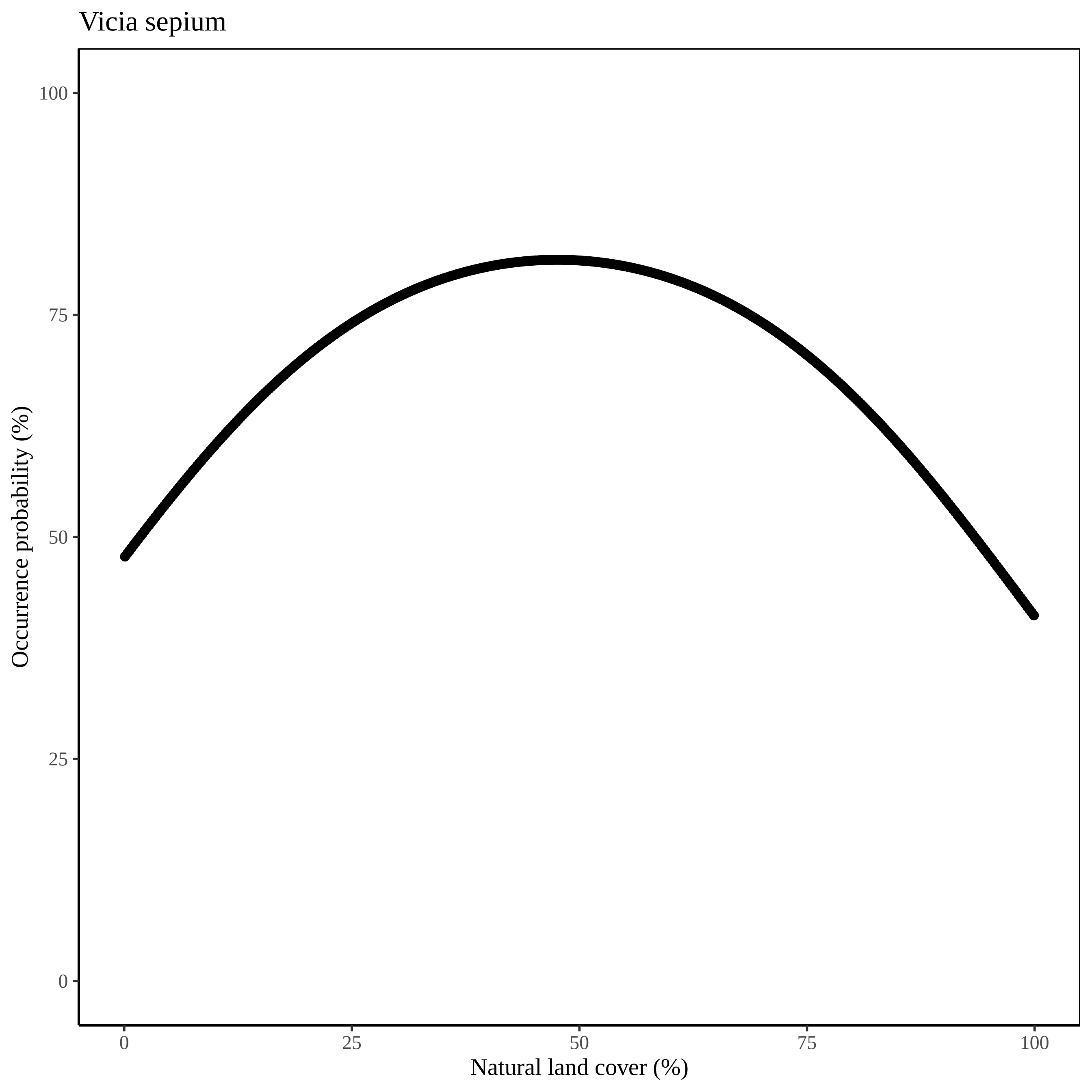
Vicia sepium
Occurrence probability (%)
100
75
50
25
0
0
25
50
75
100
Natural land cover (%)

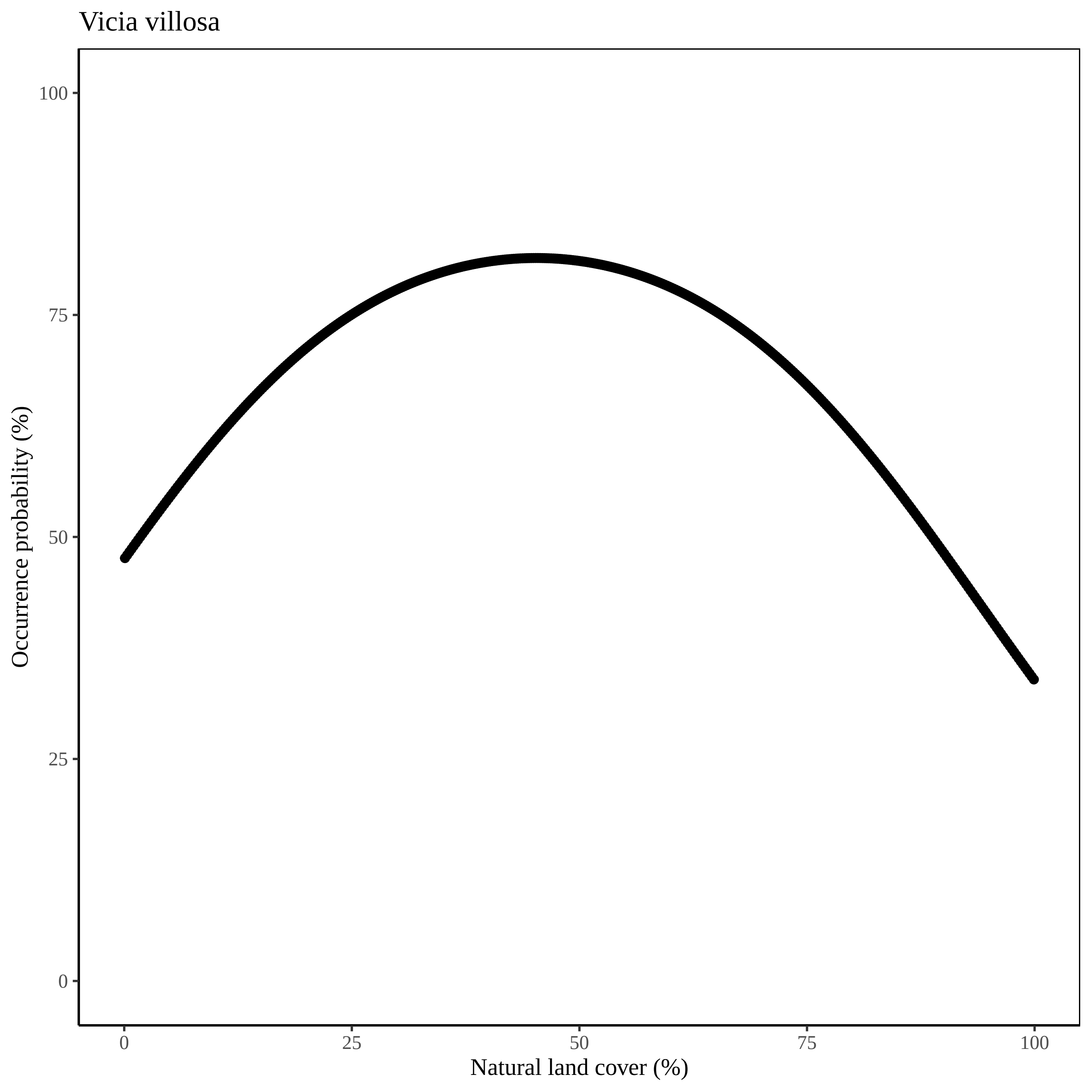
Vicia villosa
Occurrence probability (%)
100
75
50
25
0
0
25
50
75
100
Natural land cover (%)

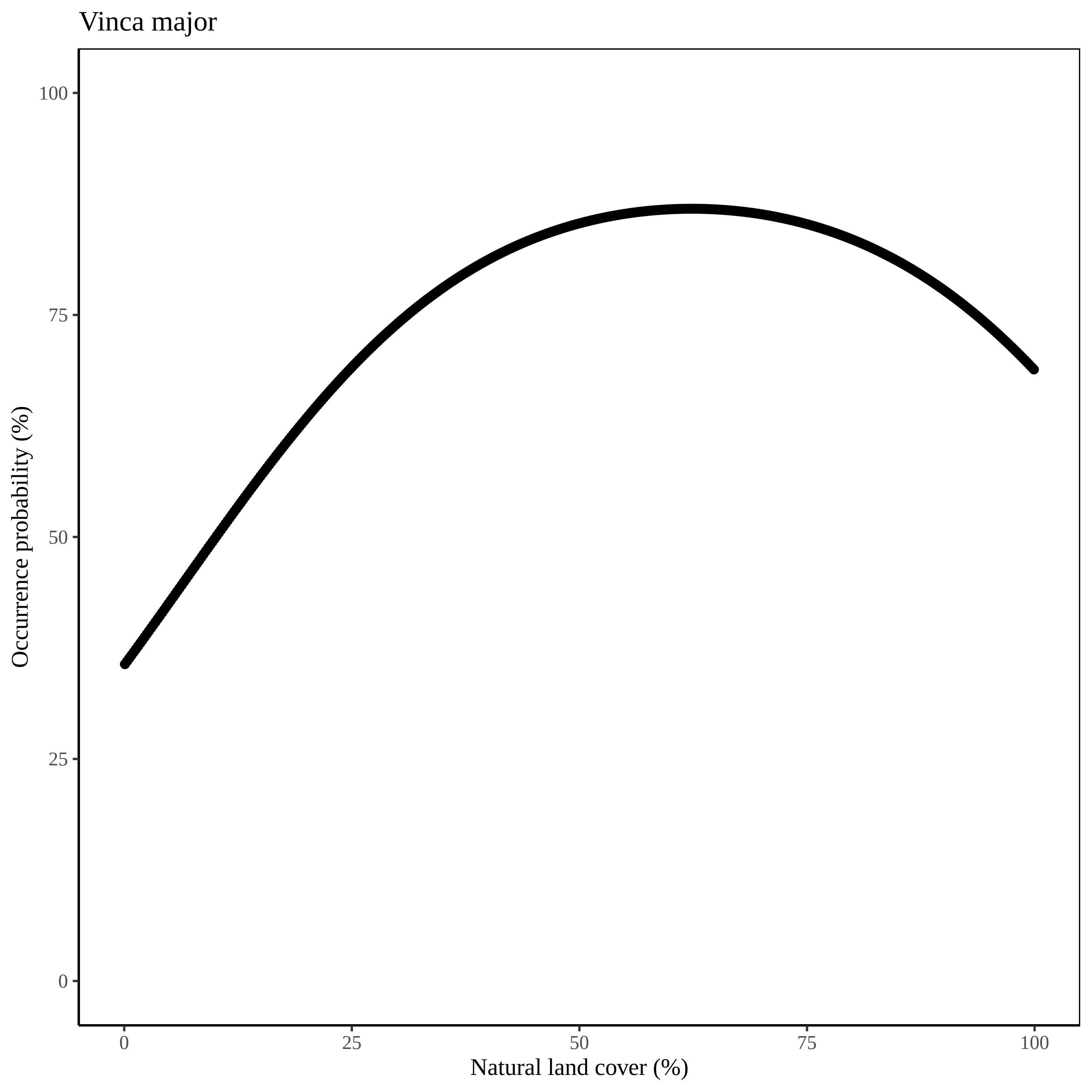

Vinca major
100
75
50
25
0
0
25
50
75
100
Natural land cover (%)
Occurrence probability (%)

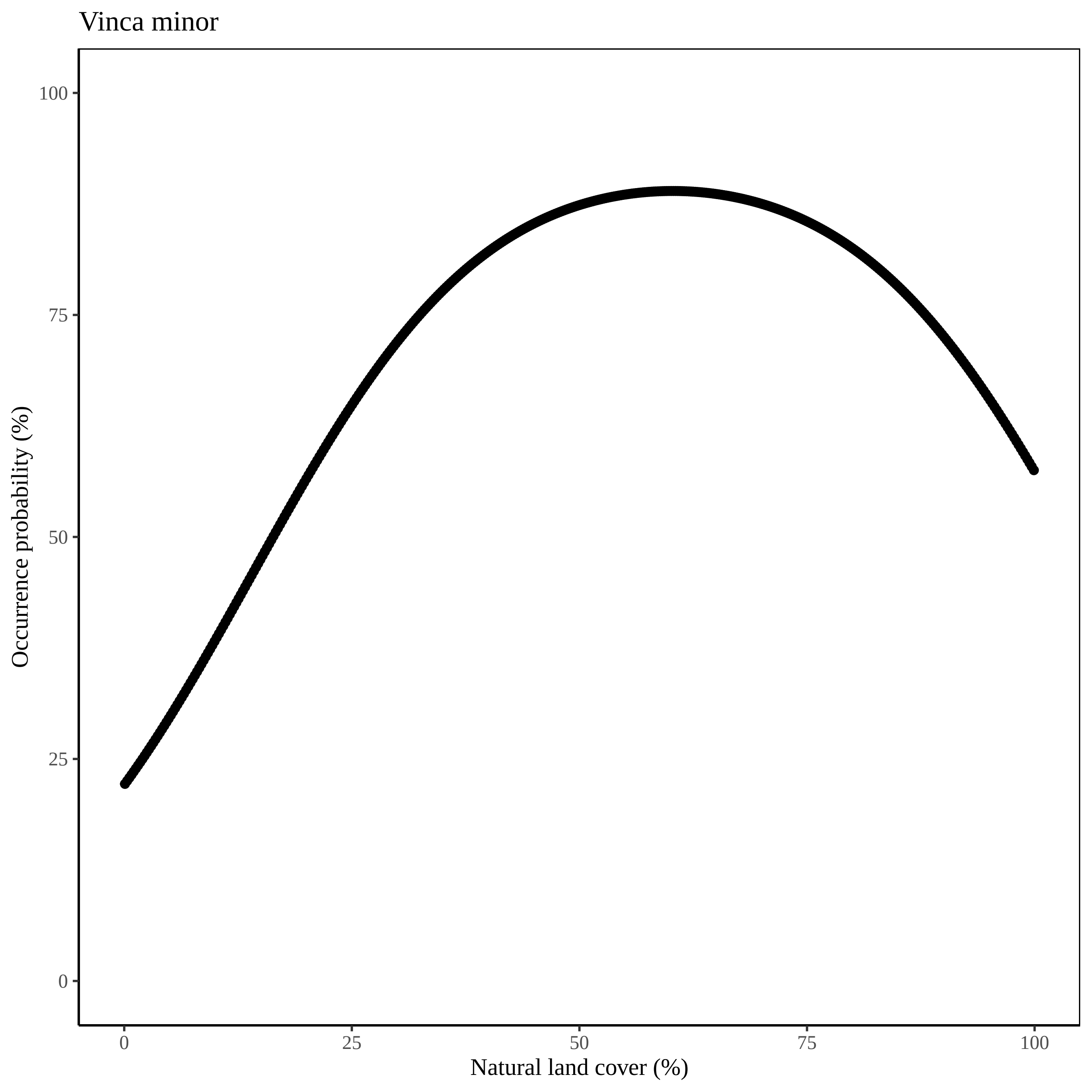
Vinca minor
100
75
50
25
0
Occurrence probability (%)
0
25
50
75
100
Natural land cover (%)

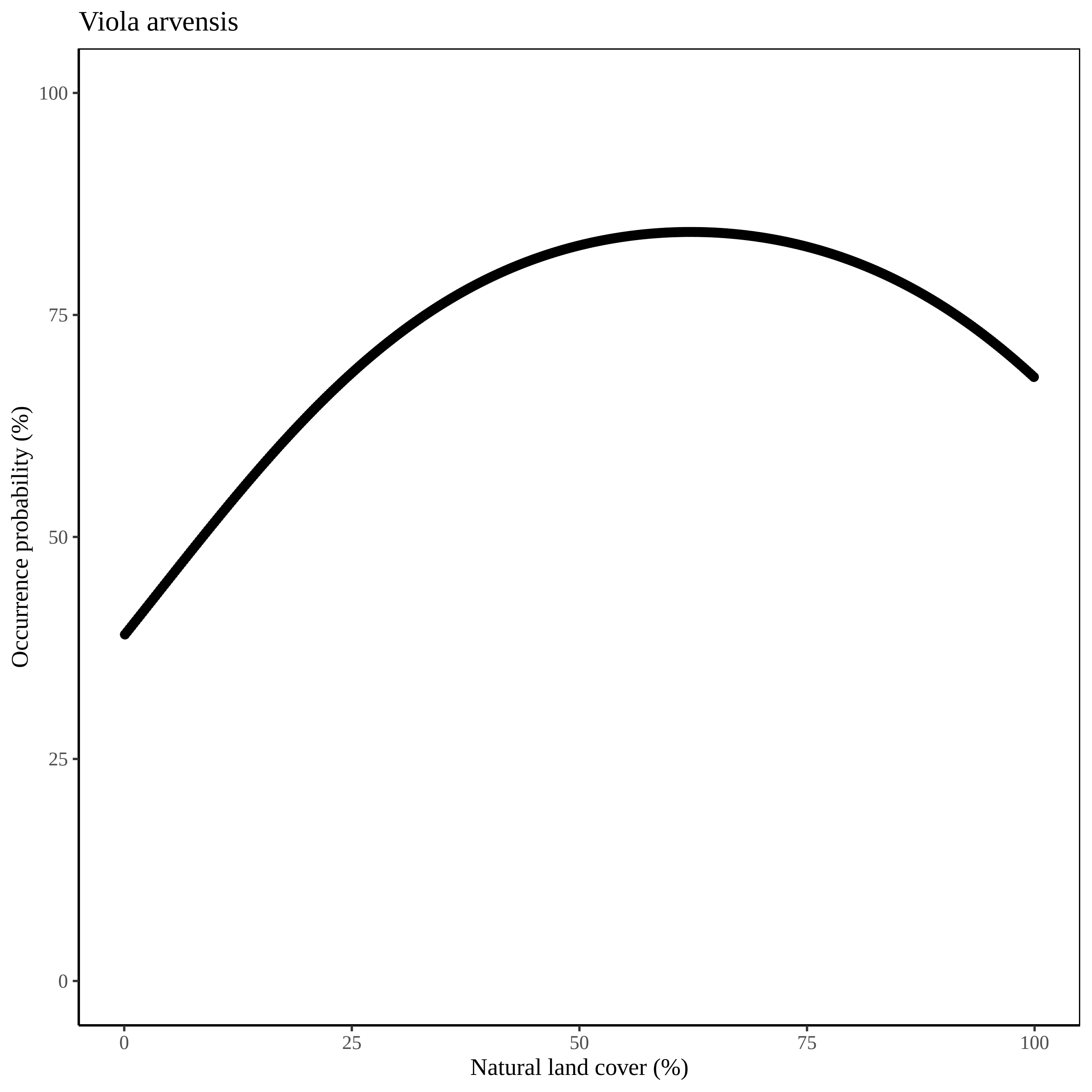
Viola arvensis
Occurrence probability (%)
100
75
50
25
0
0
25
50
75
100
Natural land cover (%)

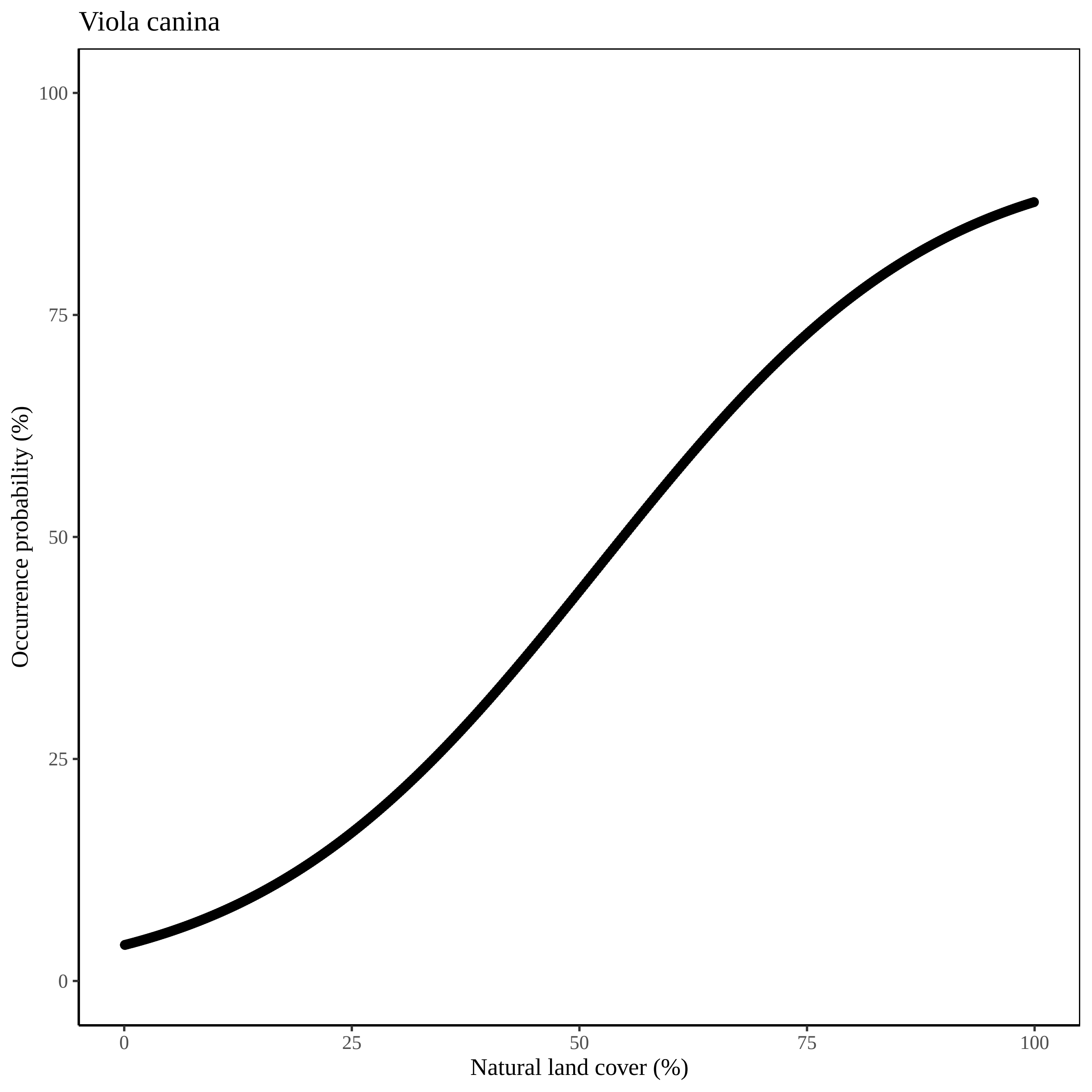
Viola canina
Occurrence probability (%)
100
75
50
25
0
0
25
50
75
100
Natural land cover (%)

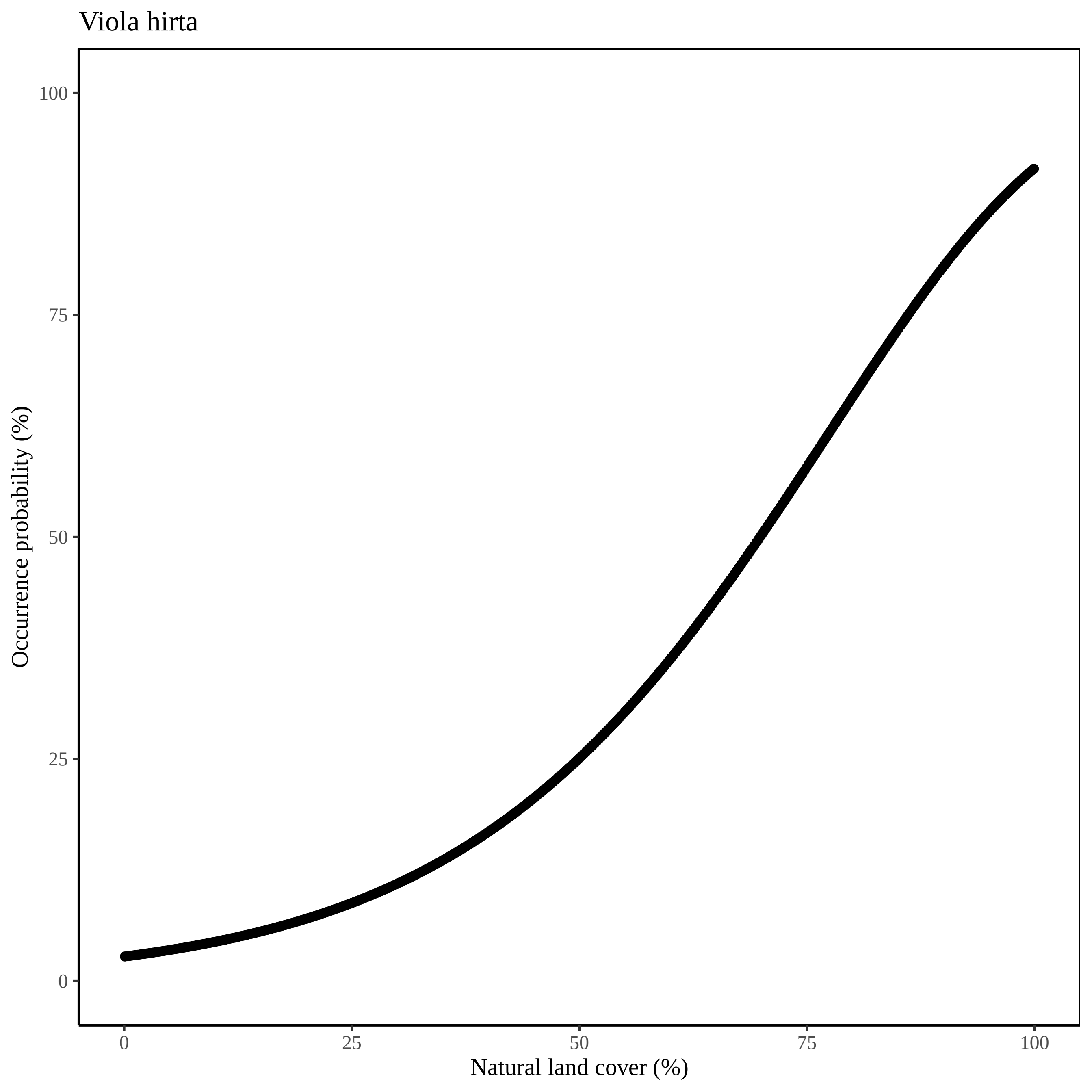
Viola hirta
Occurrence probability (%)
100
75
50
25
0
0
25
50
75
100
Natural land cover (%)

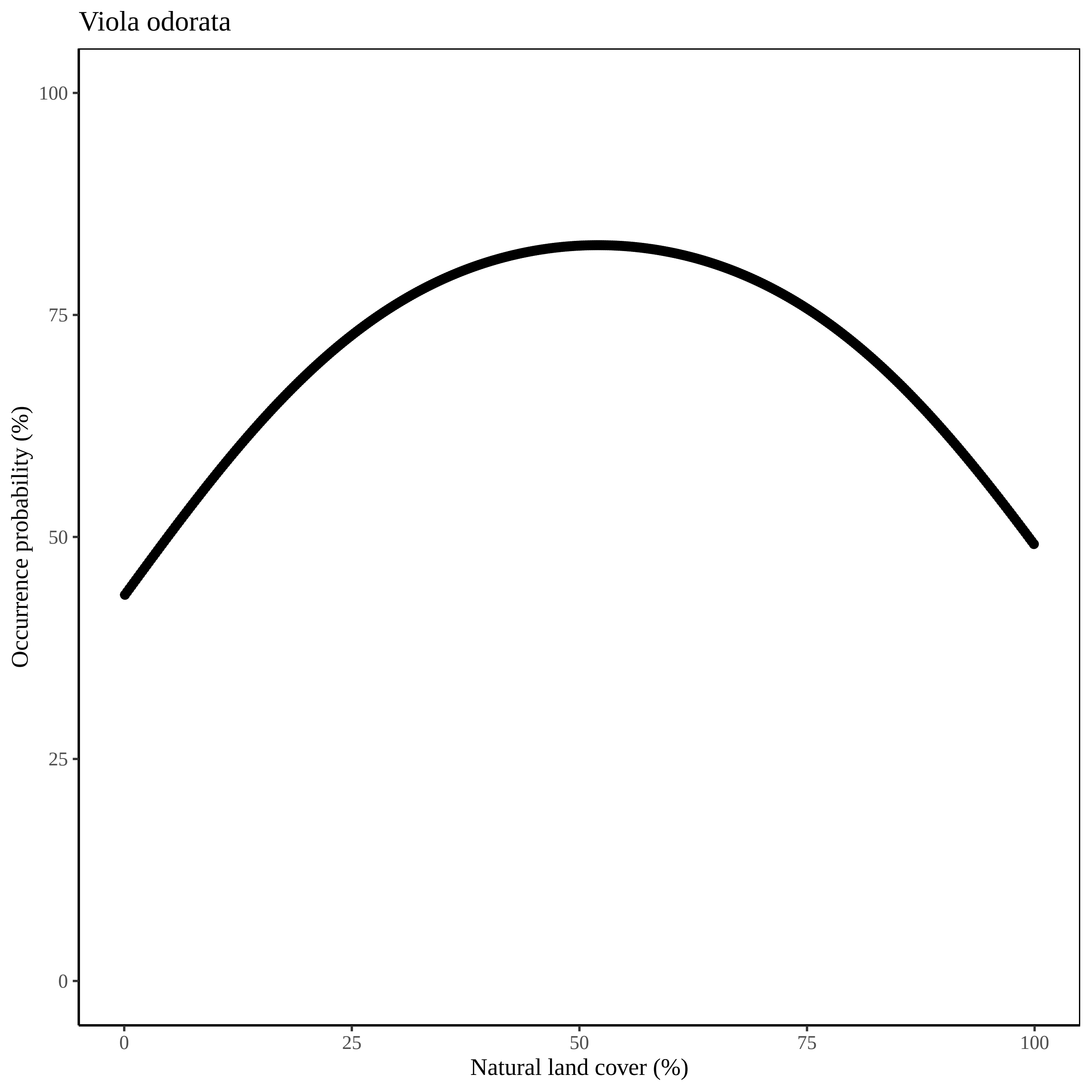
Viola odorata
Occurrence probability (%)
100
75
50
25
0
0
25
50
75
100
Natural land cover (%)

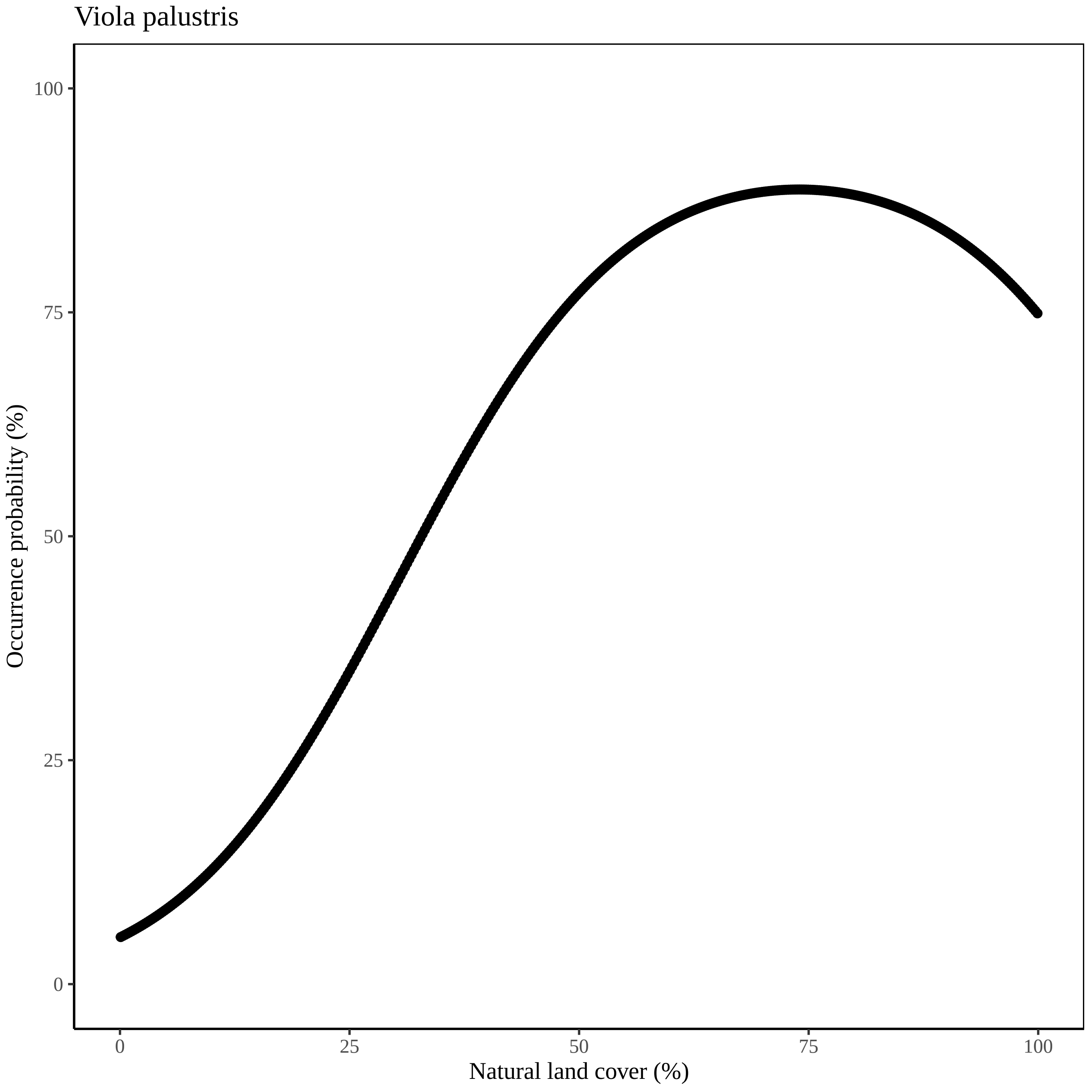
Viola palustris
Occurrence probability (%)
100
75
50
25
0
0
25
50
75
100
Natural land cover (%)

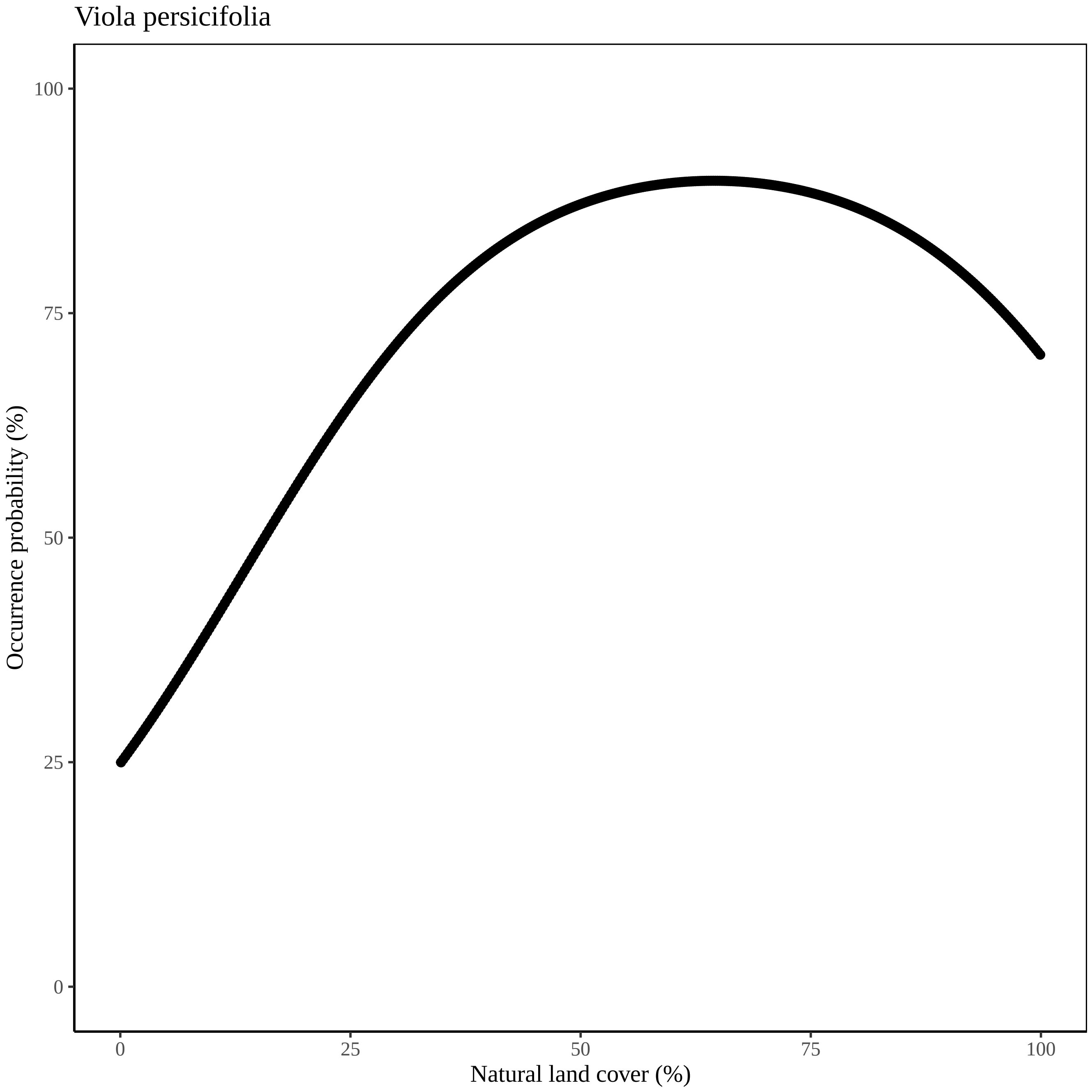
Viola persicifolia
Occurrence probability (%)
100
75
50
25
0
0
25
50
75
100
Natural land cover (%)

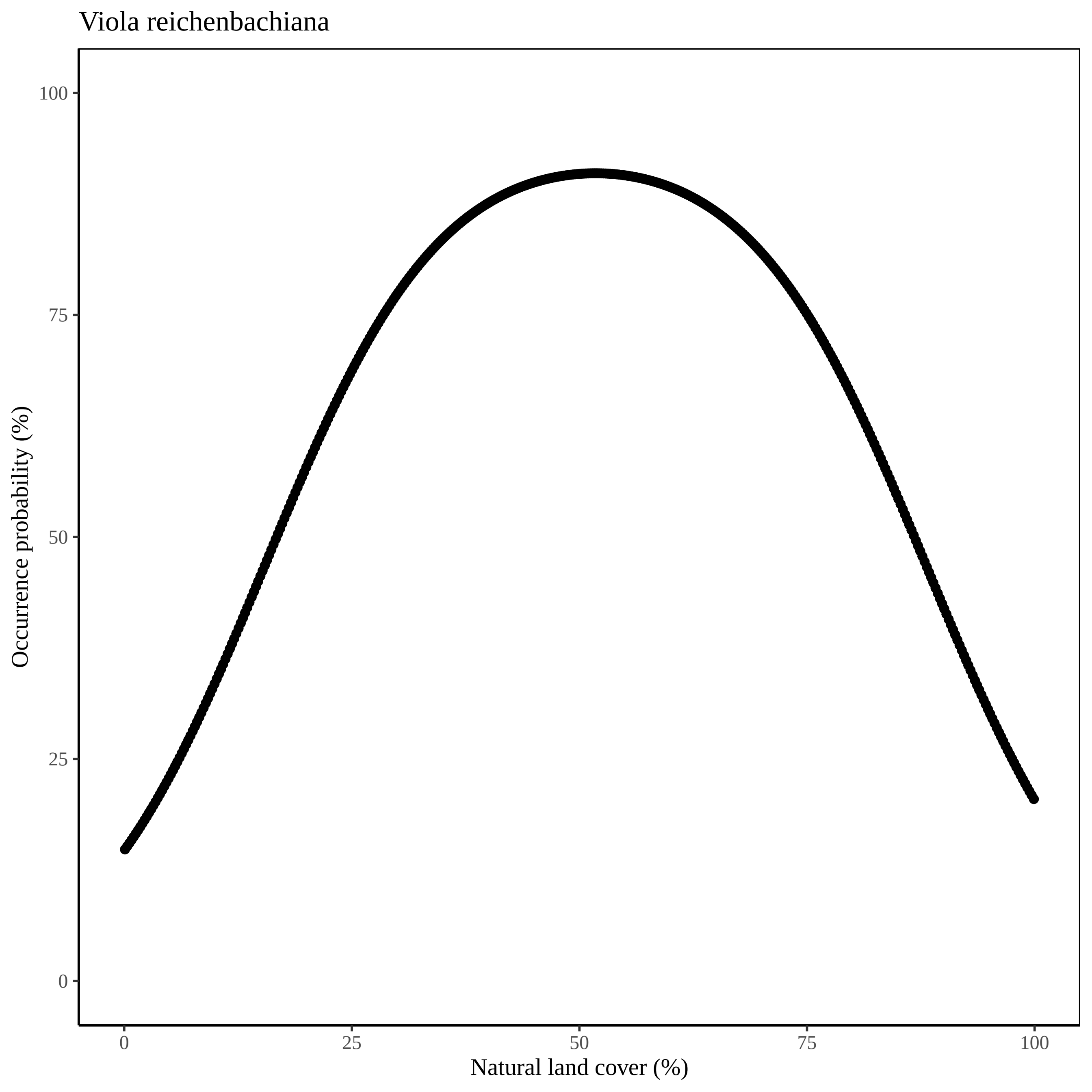
Viola reichenbachiana
Occurrence probability (%)
100
75
50
25
0
0
25
50
75
100
Natural land cover (%)

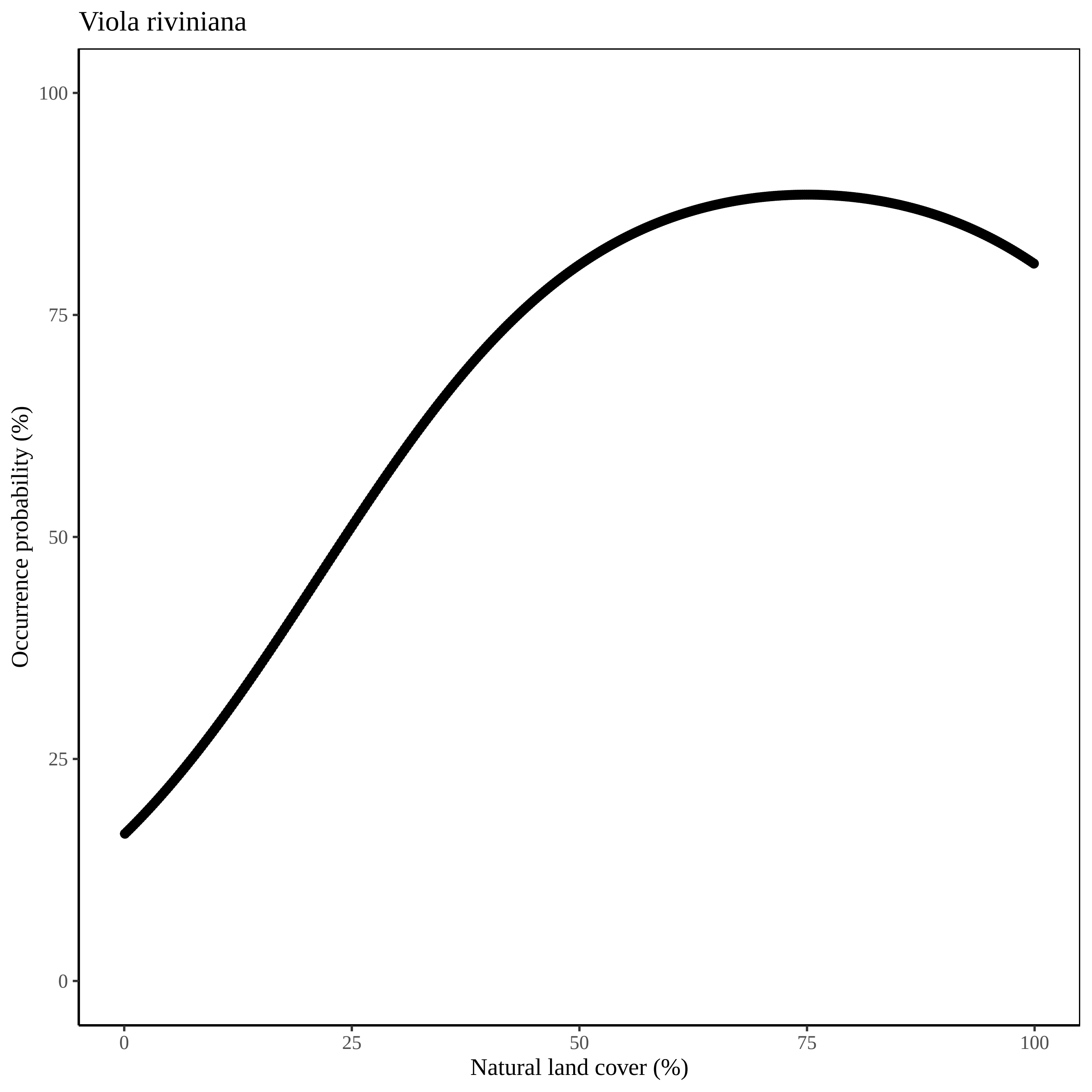
Viola riviniana
Occurrence probability (%)
100
75
50
25
0
0
25
50
75
100
Natural land cover (%)

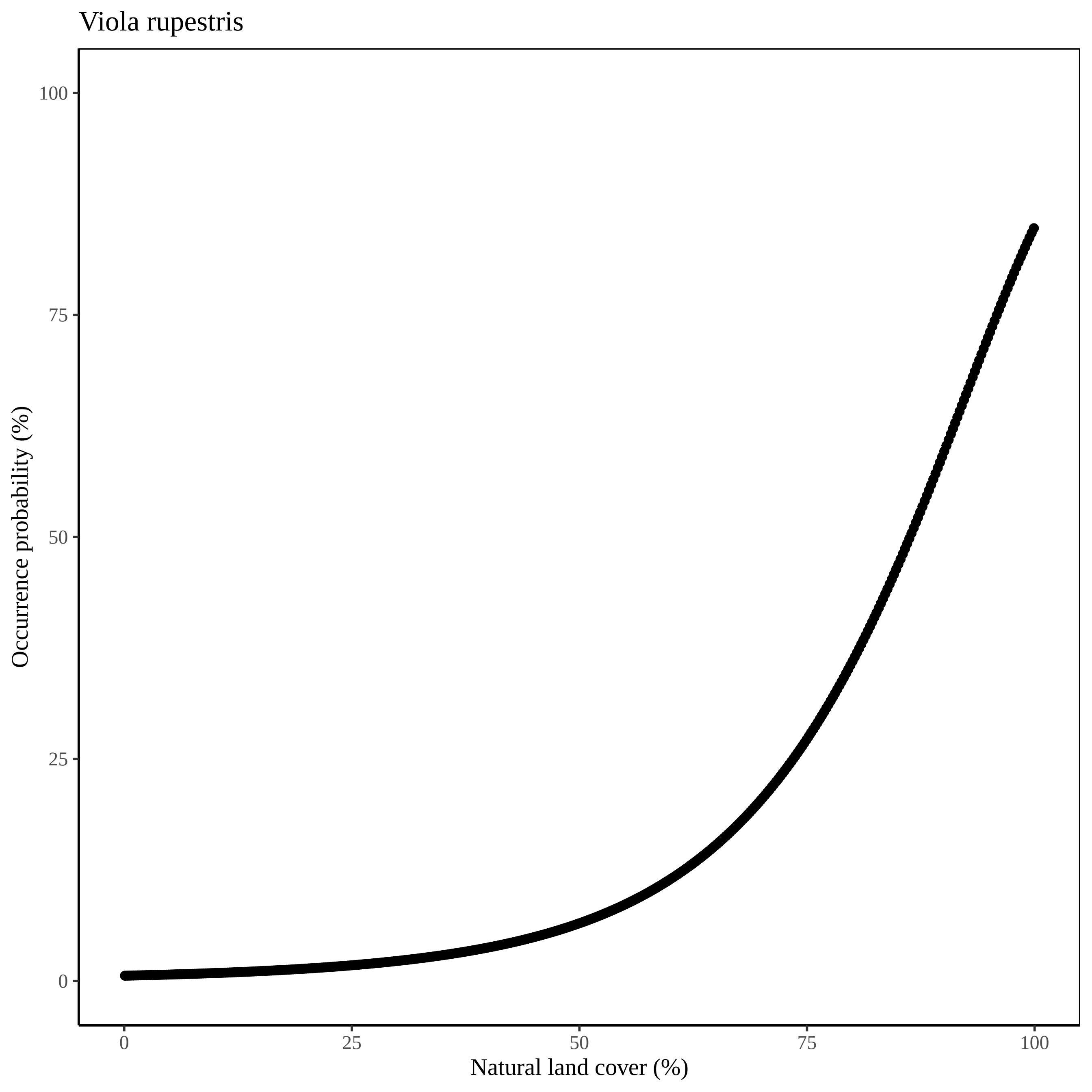
Viola rupestris
Occurrence probability (%)
100
75
50
25
0
0
25
50
75
100
Natural land cover (%)

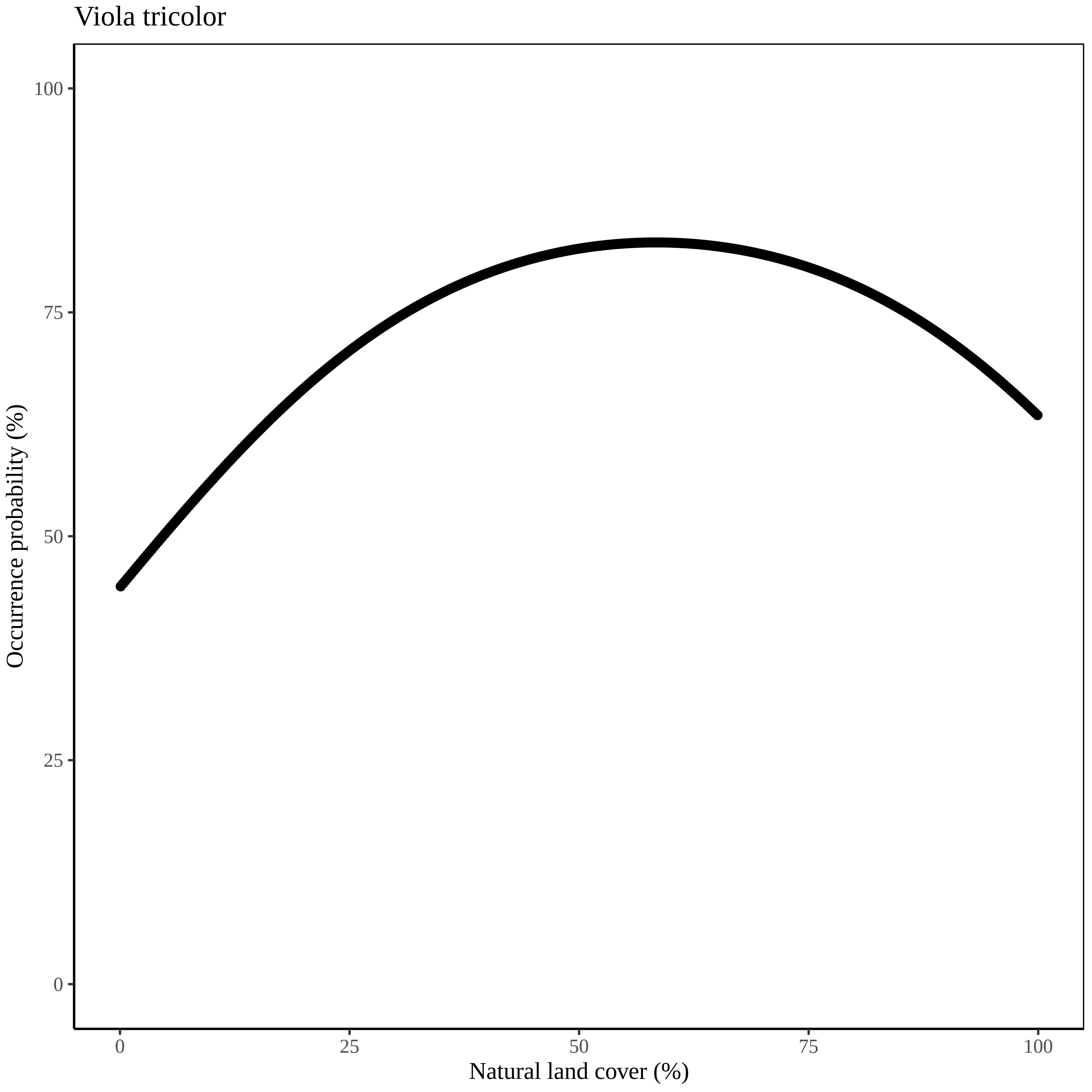
Viola tricolor
Occurrence probability (%)
100
75
50
25
0
0
25
50
75
100
Natural land cover (%)

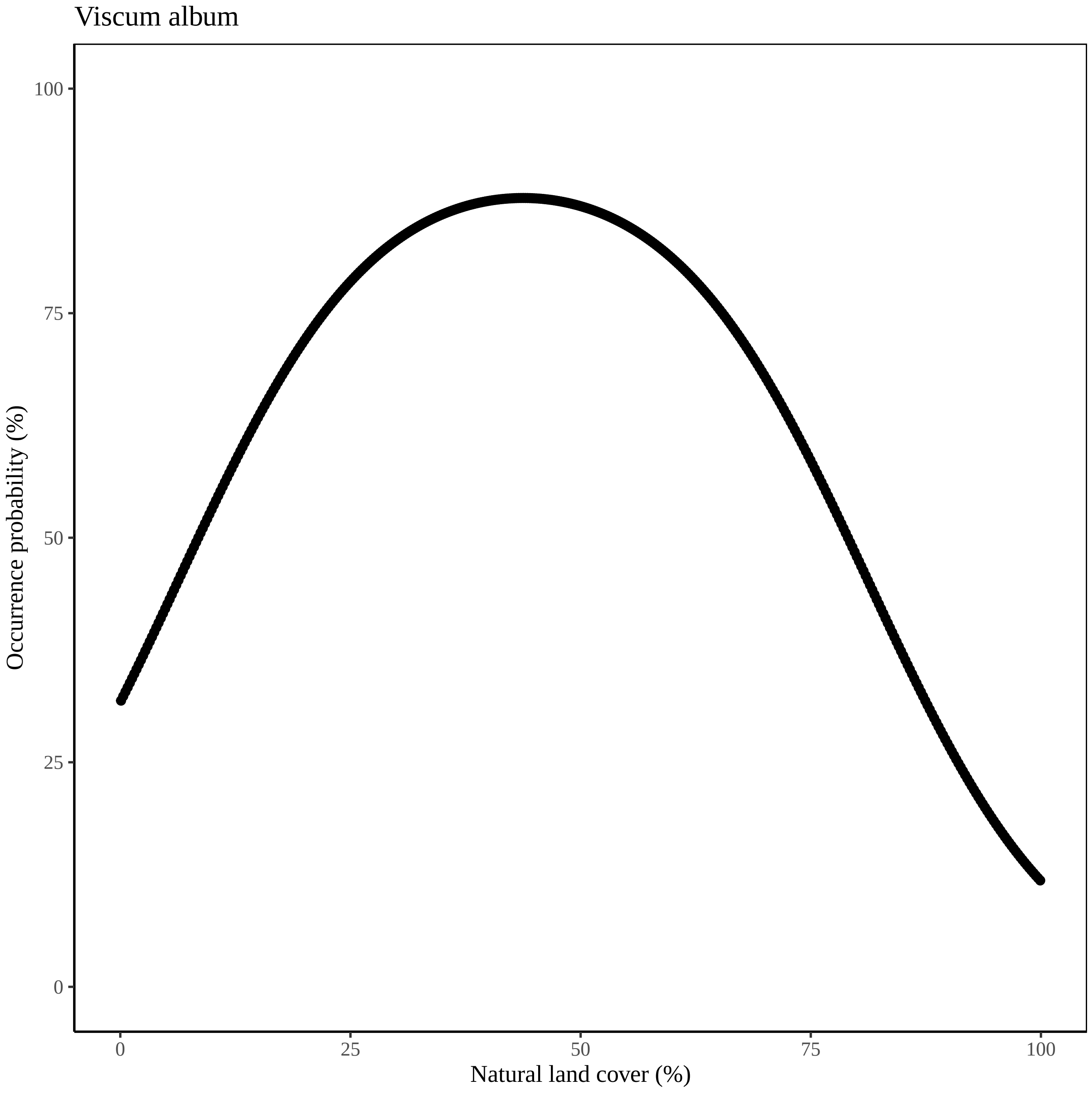
Viscum album
Occurrence probability (%)
100
75
50
25
0
0
25
50
75
100
Natural land cover (%)

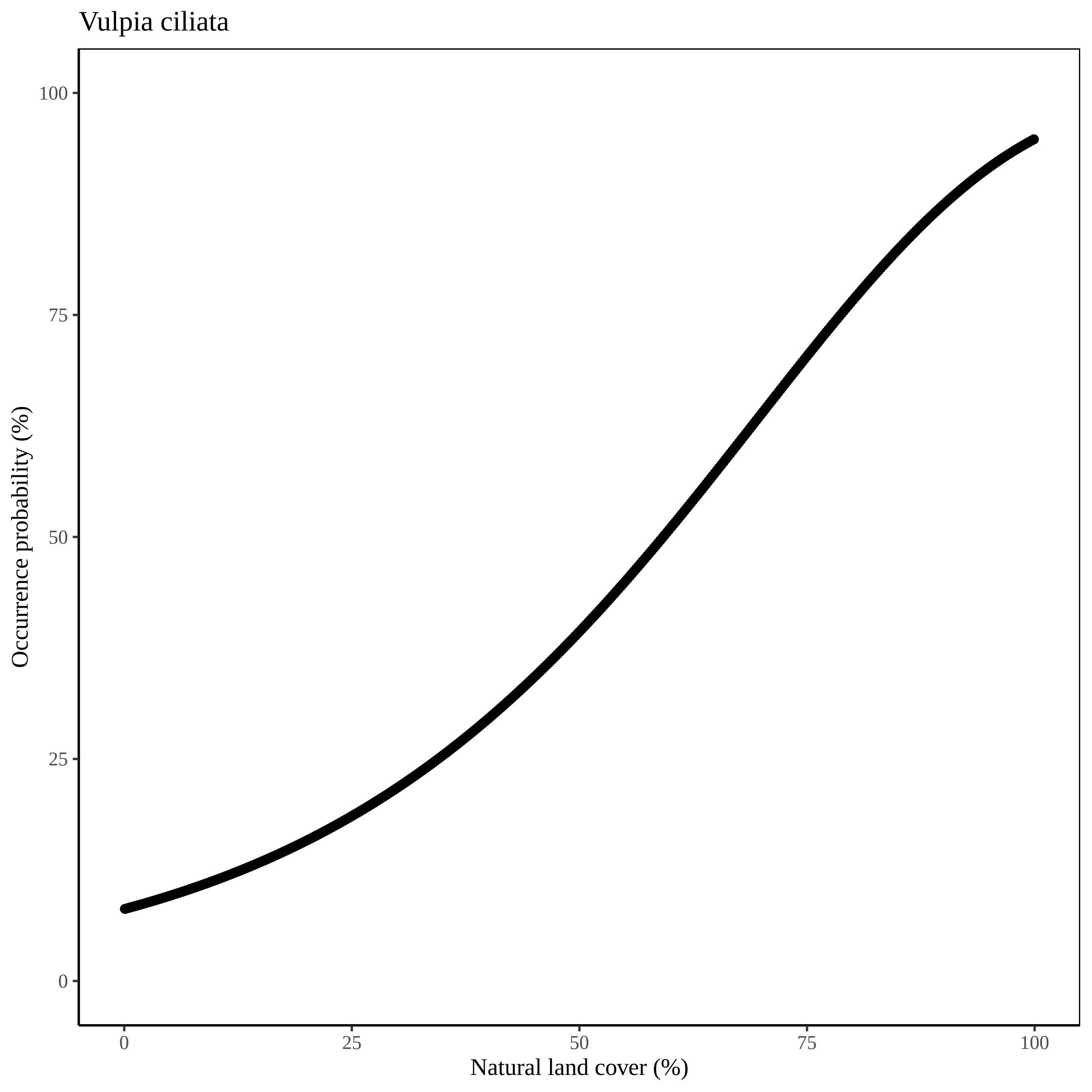
Vulpia ciliata
Occurrence probability (%)
100
75
50
25
0
0
25
50
75
100
Natural land cover (%)

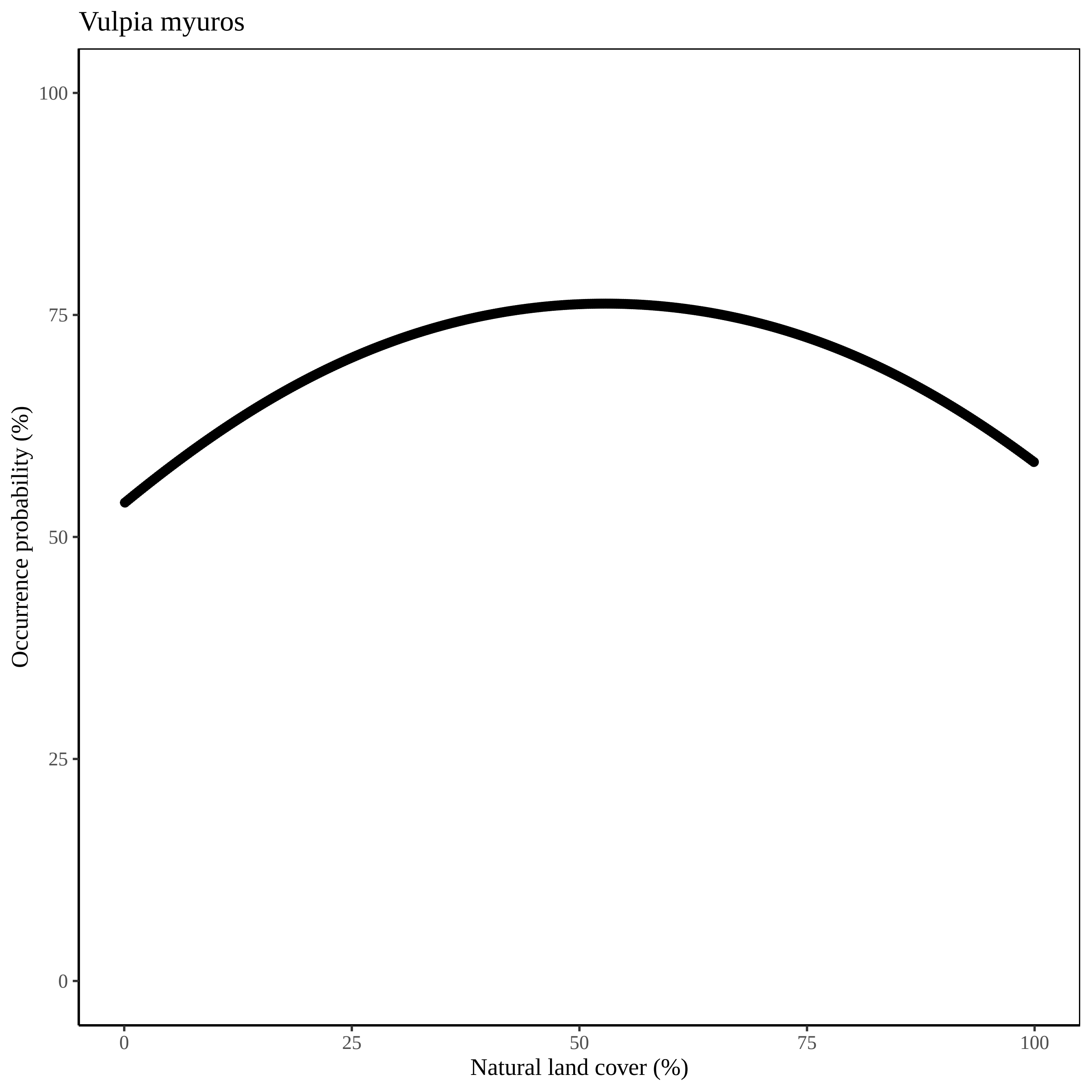

Vulpia myuros
Occurrence probability (%)
100
75
50
25
0
0
25
50
75
100
Natural land cover (%)

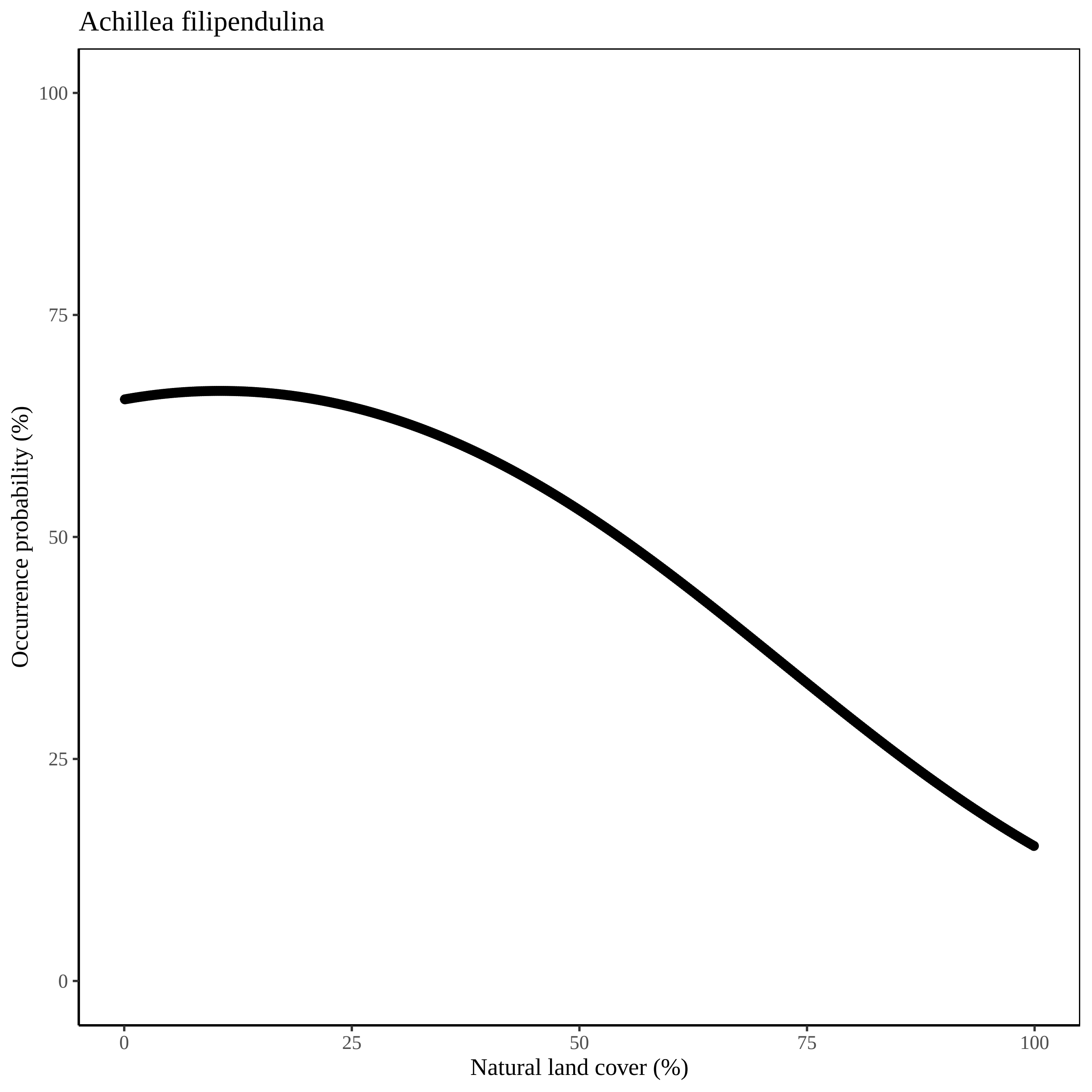
Achillea filipendulina
Occurrence probability (%)
100
75
50
25
0
0
25
50
75
100
Natural land cover (%)

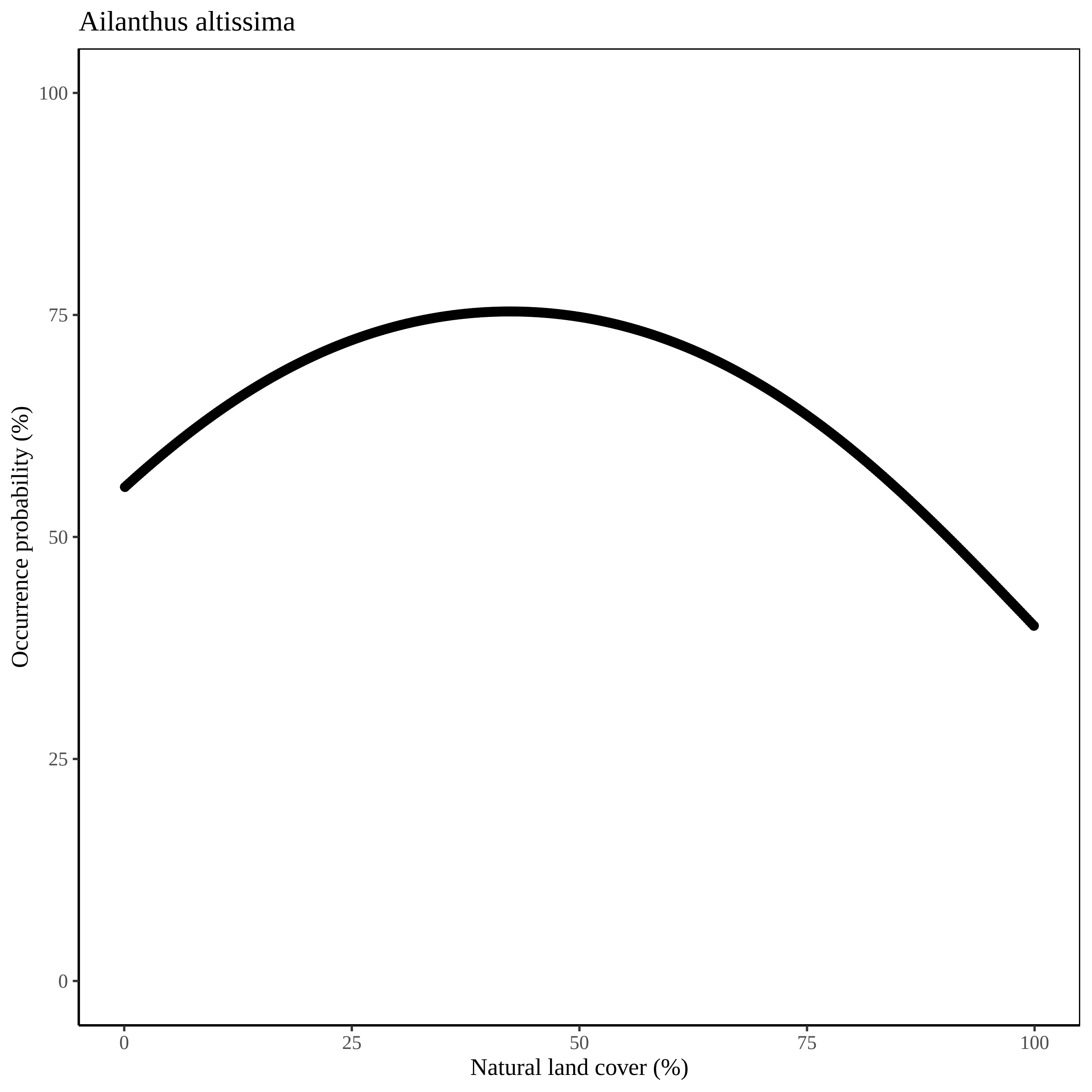
Ailanthus altissima
Occurrence probability (%)
100
75
50
25
0
0
25
50
75
100
Natural land cover (%)

Alcea rosea

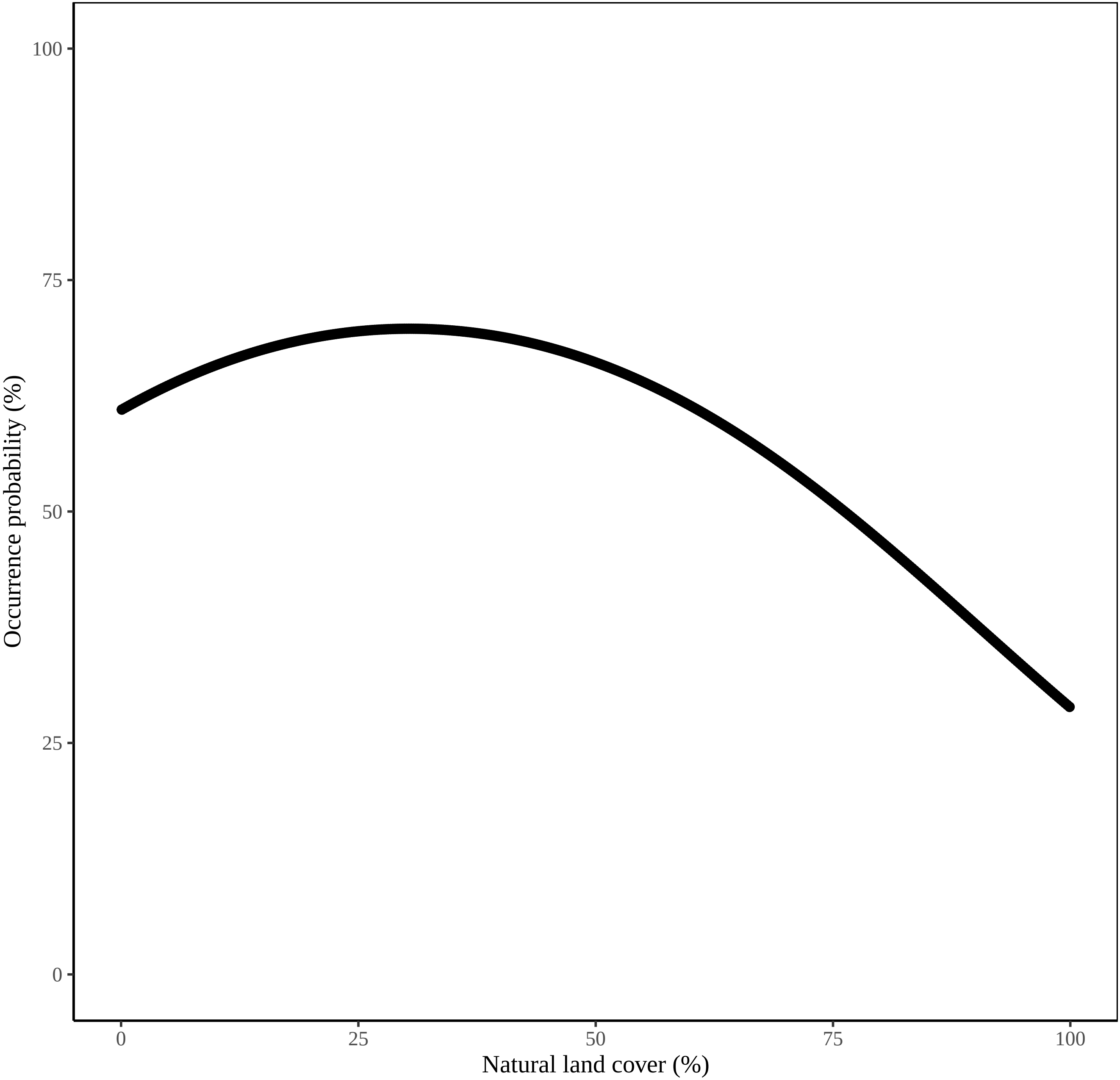

100
75
50
25
0
Occurrence probability (%)
0
25
50
75
100
Natural land cover (%)

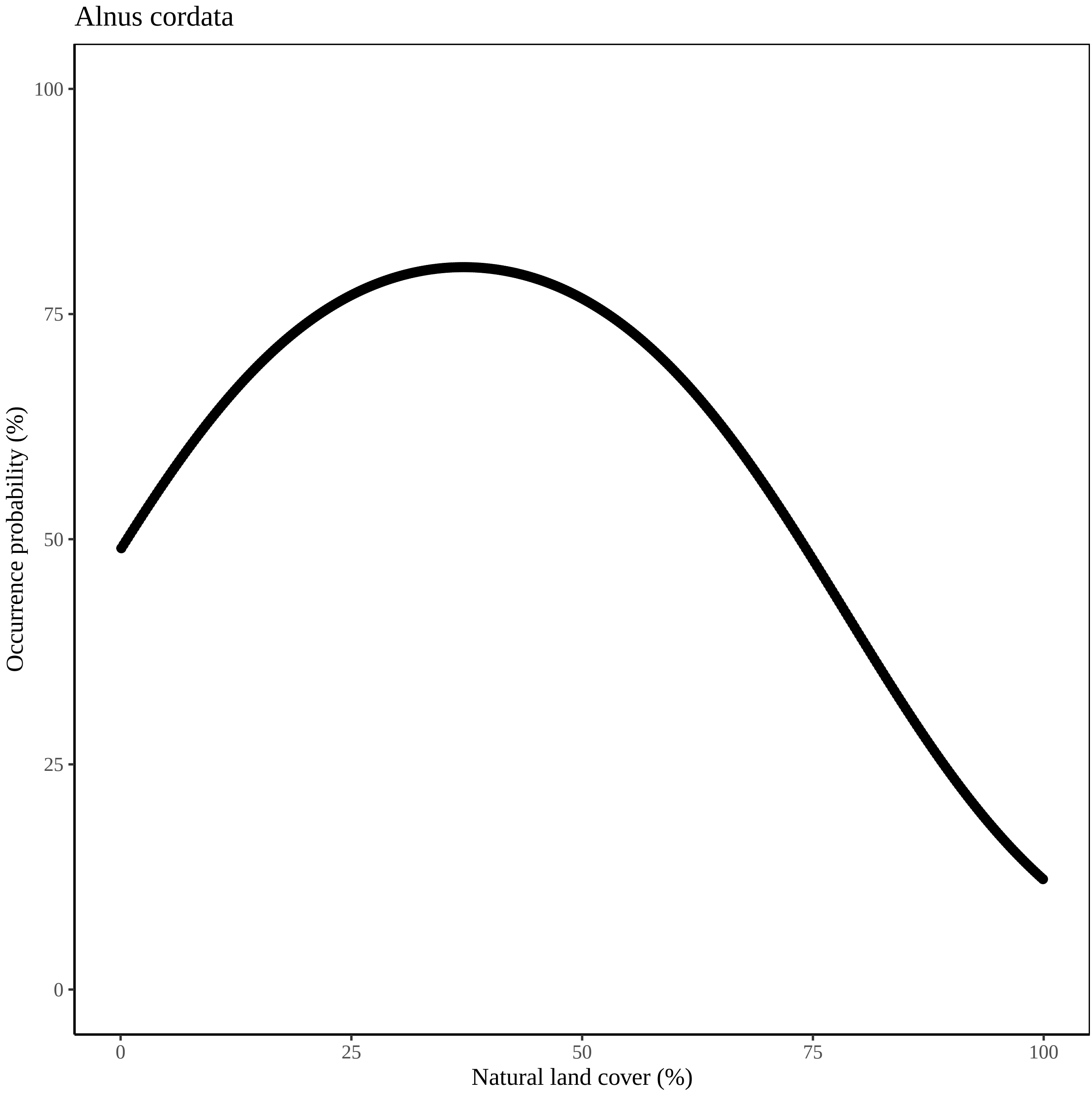

Alnus cordata
Occurrence probability (%)
100
75
50
25
0
0
25
50
75
100
Natural land cover (%)

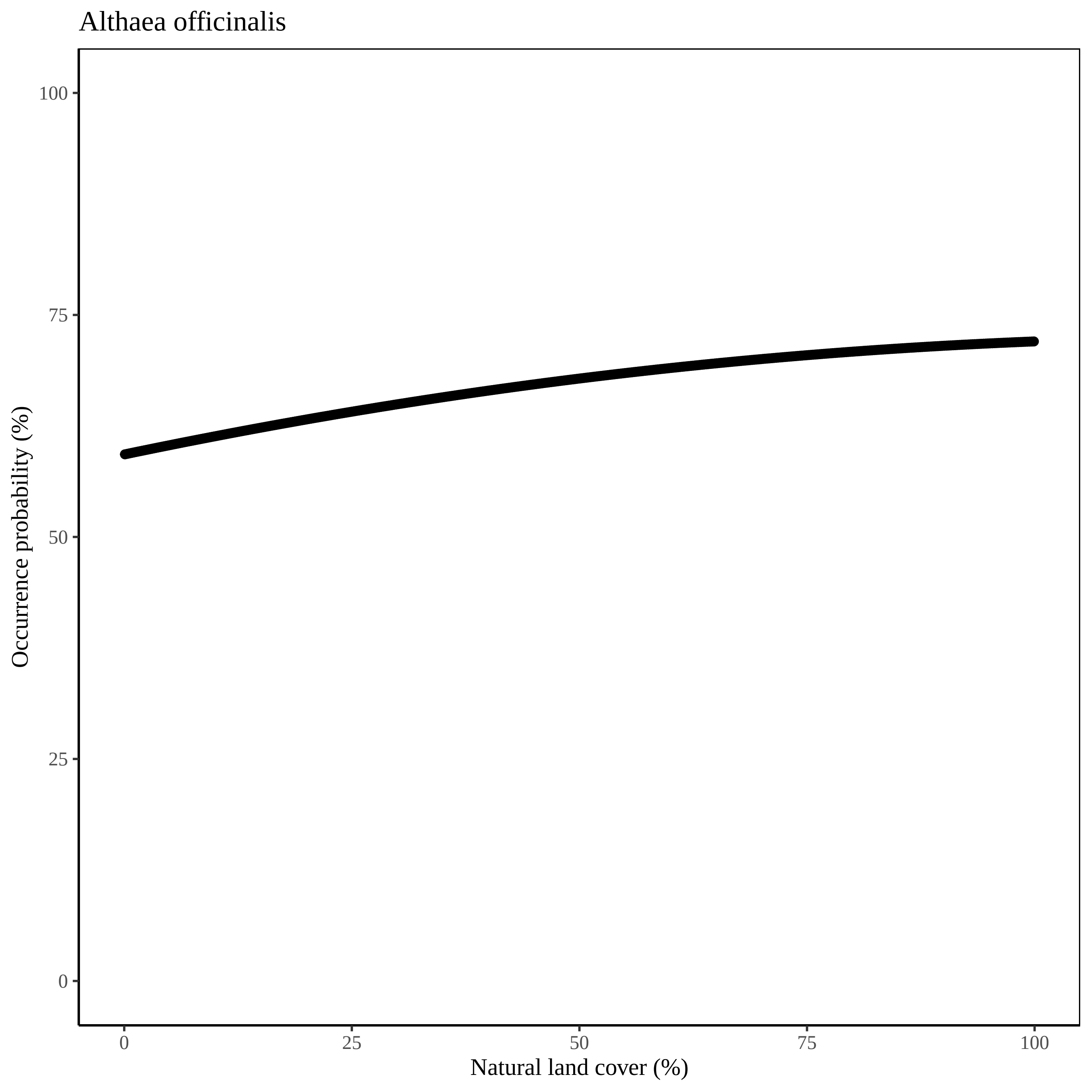

Althaea officinalis
Occurrence probability (%)
Natural land cover (%)
0
25
50
75
100

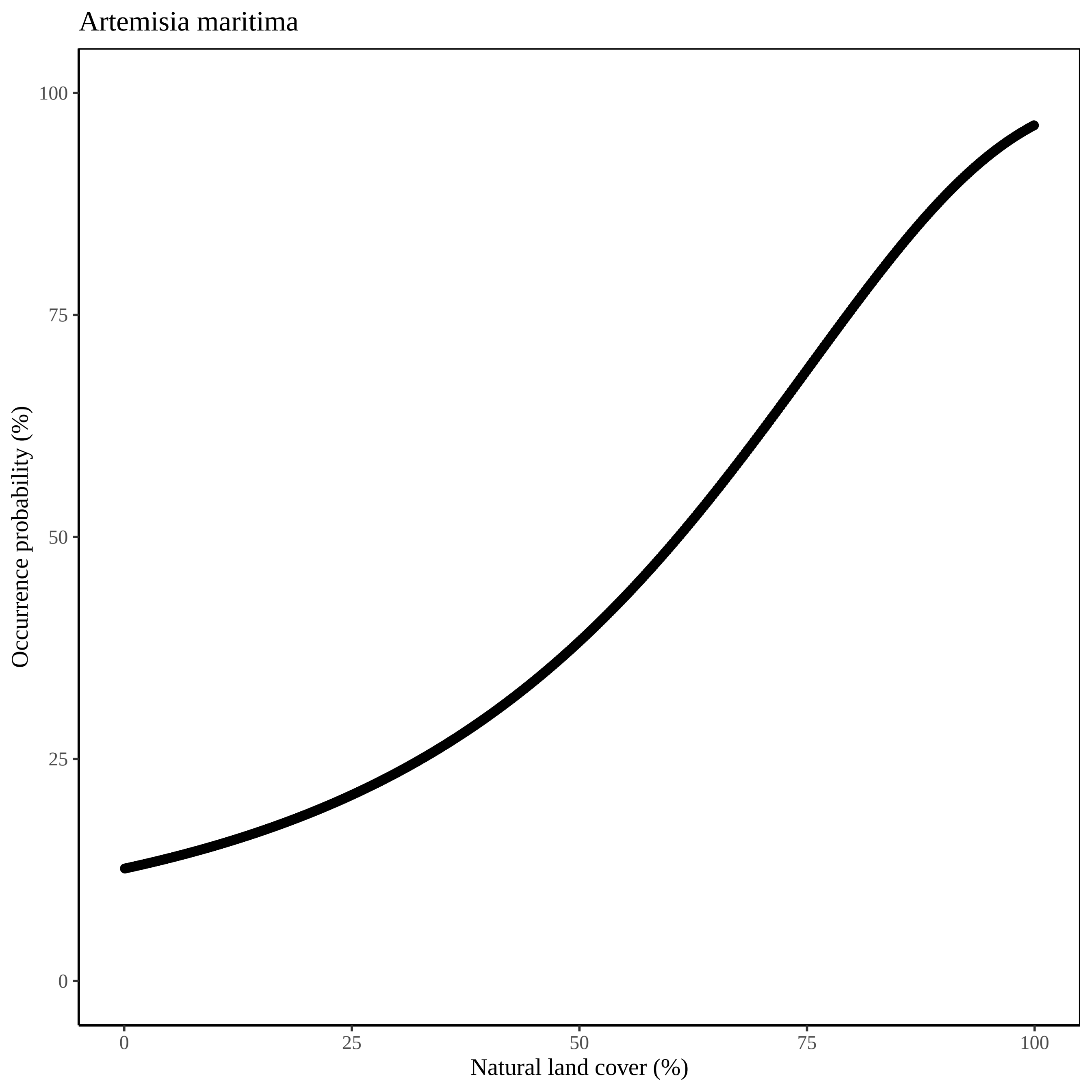

Artemisia maritima
Occurrence probability (%)
100
75
50
25
0
0
25
50
75
100
Natural land cover (%)

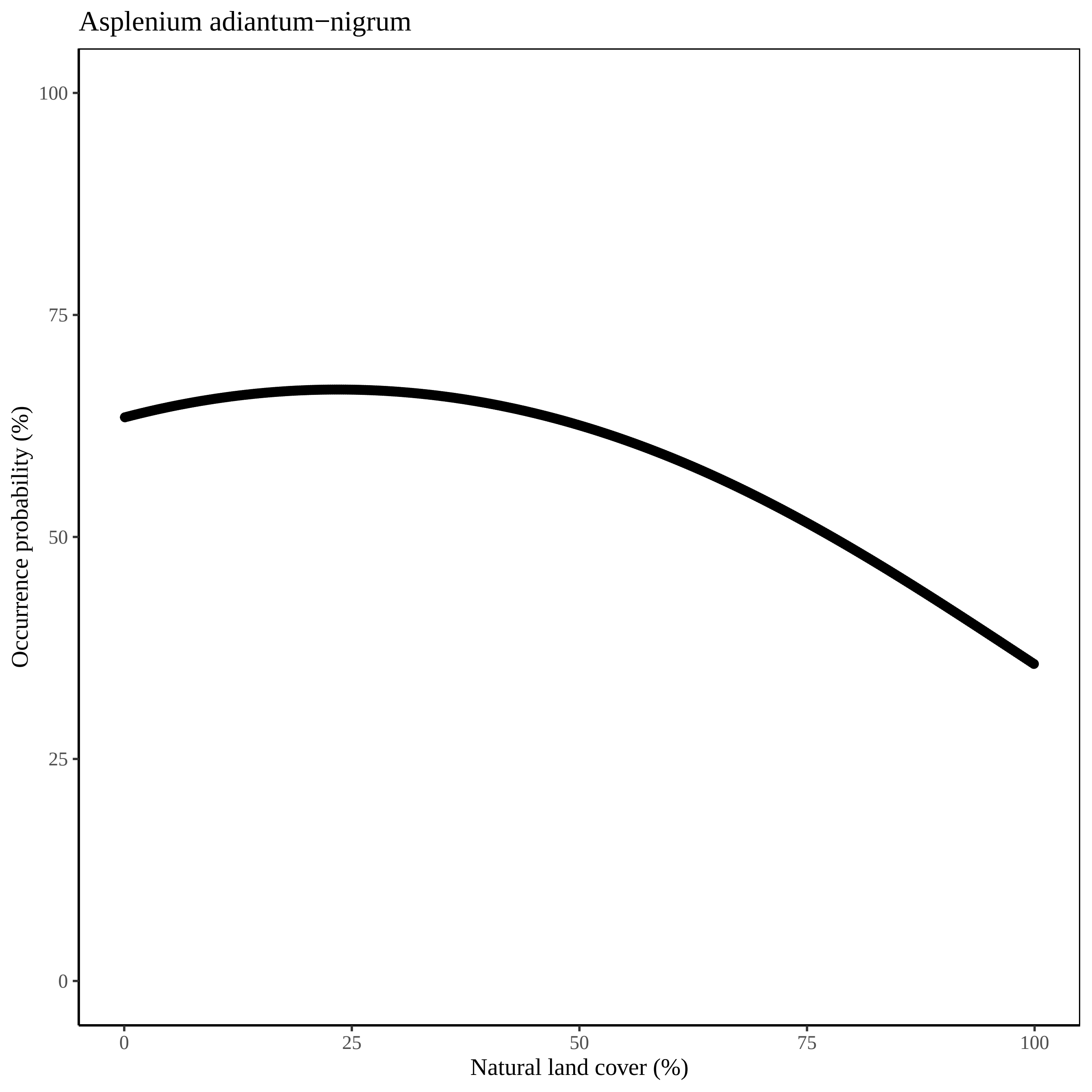
Asplenium adiantum−nigrum
100
75
50
25
0
Occurrence probability (%)
0
25
50
75
100
Natural land cover (%)

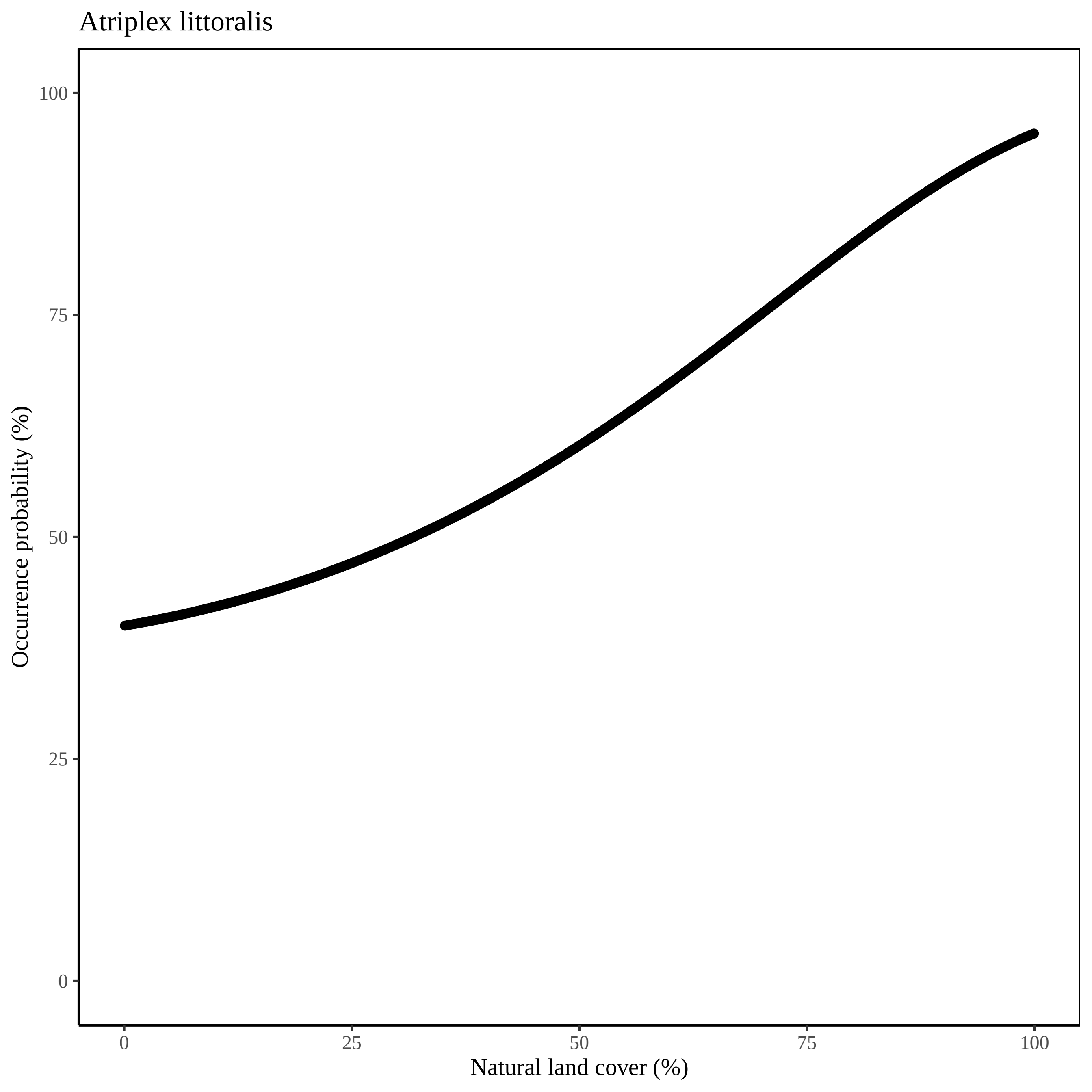
Atriplex littoralis
Occurrence probability (%)
100
75
50
25
0
0
25
50
75
100
Natural land cover (%)

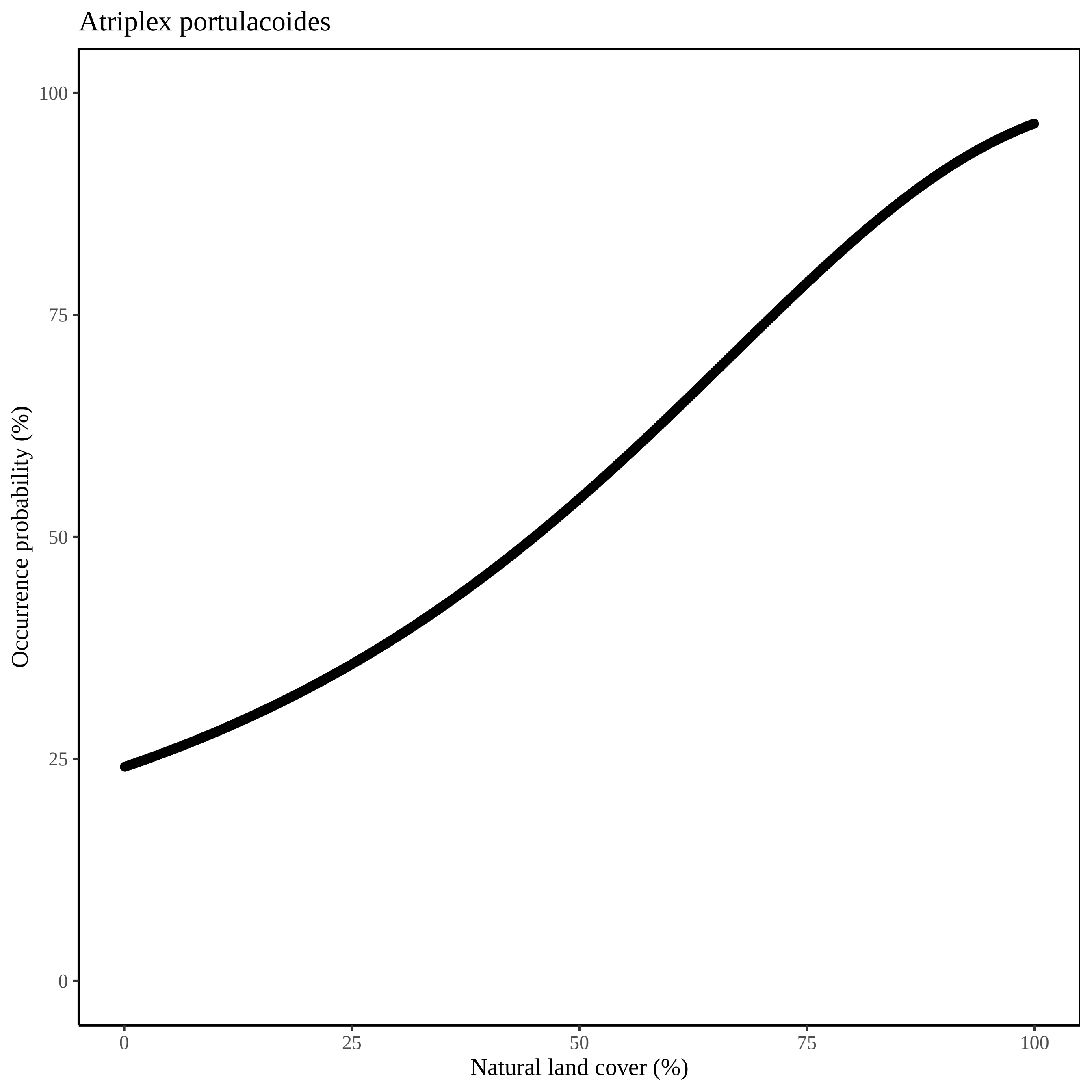
Atriplex portulacoides
Occurrence probability (%)
100
75
50
25
0
Natural land cover (%)
0
25
50
75
100

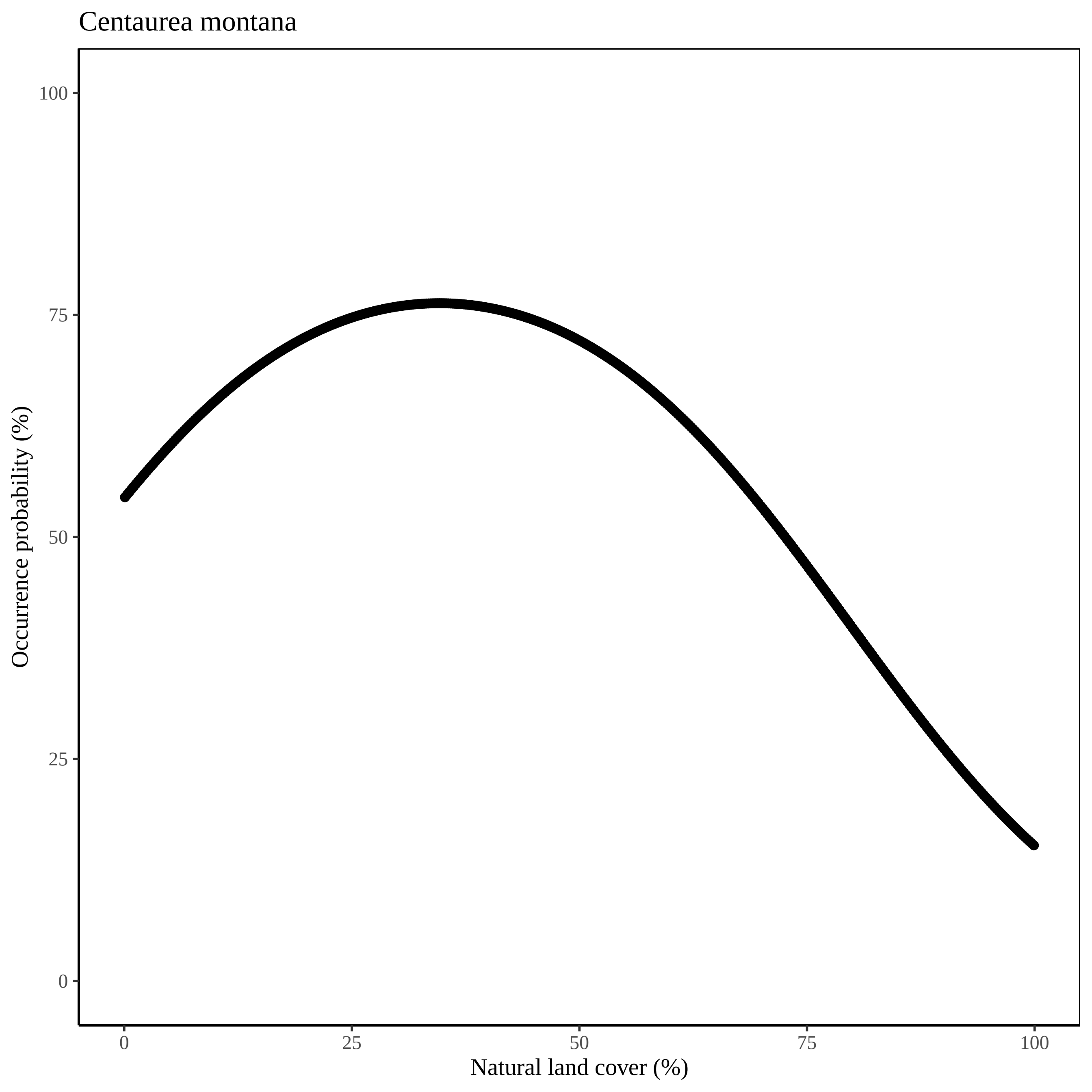
Centaurea montana
Occurrence probability (%)
100
75
50
25
0
0
25
50
75
100
Natural land cover (%)

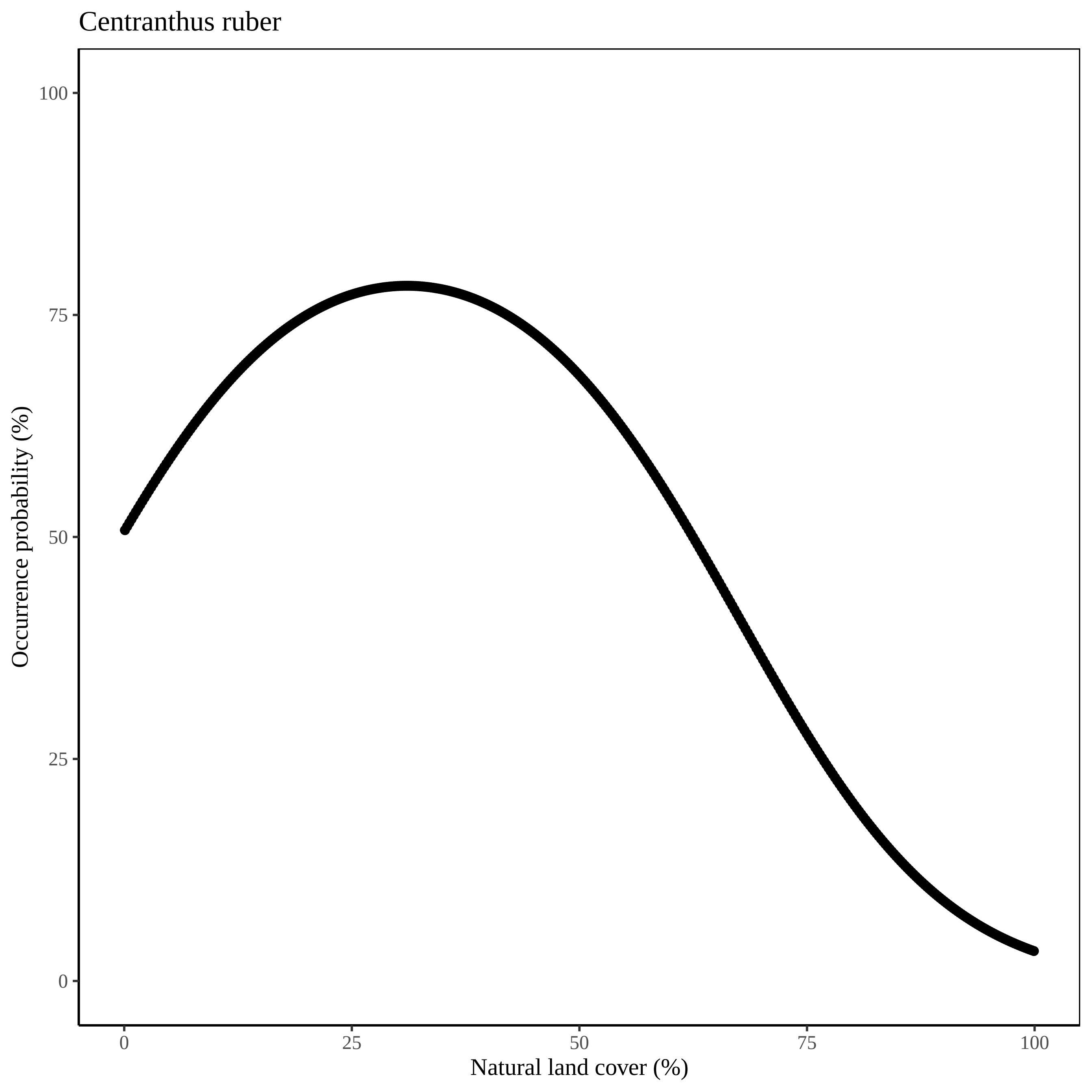
Centranthus ruber
Occurrence probability (%)
100
75
50
25
0
0
25
50
75
100
Natural land cover (%)

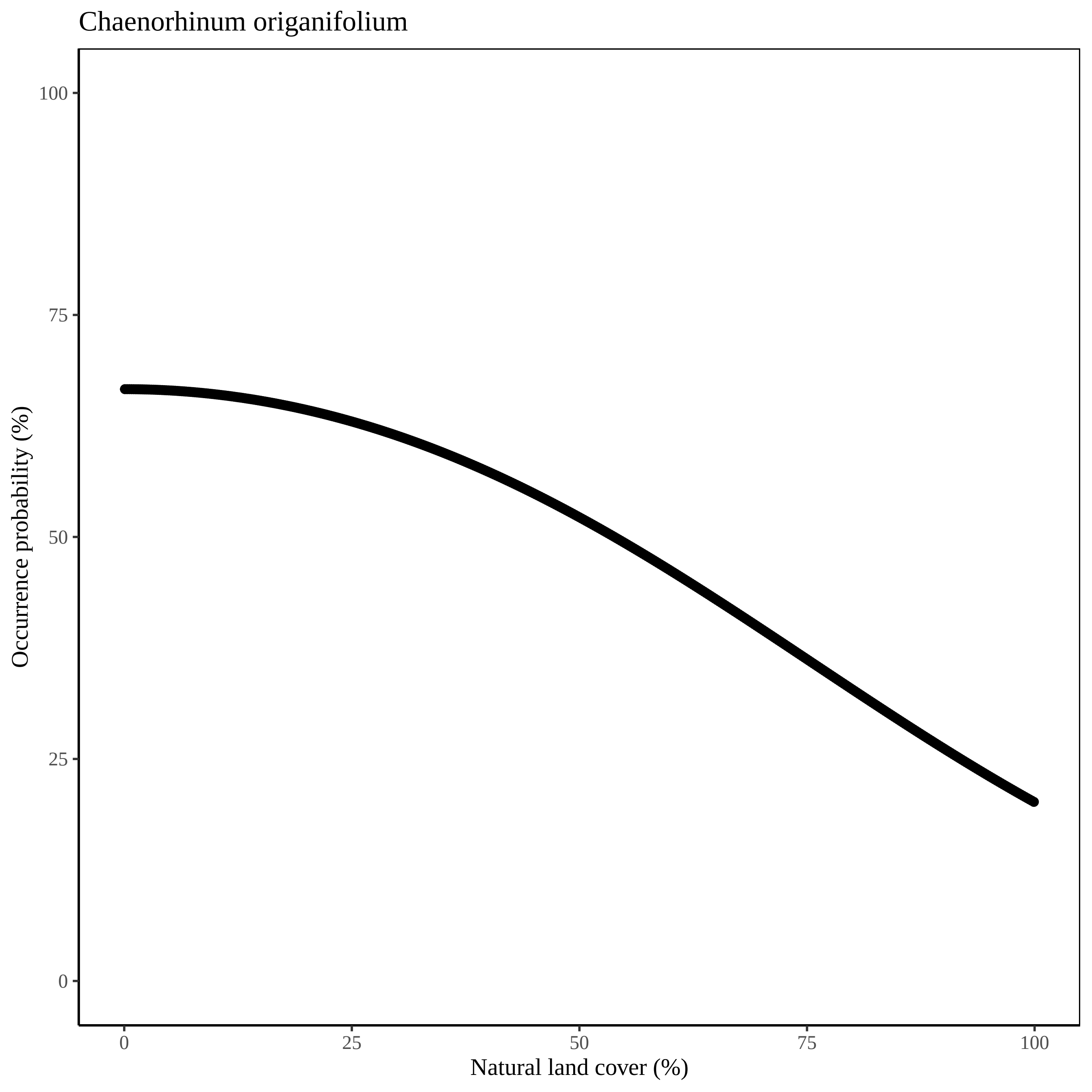

Chaenorhinum origanifolium
Occurrence probability (%)
100
75
50
25
0
0
25
50
75
100
Natural land cover (%)

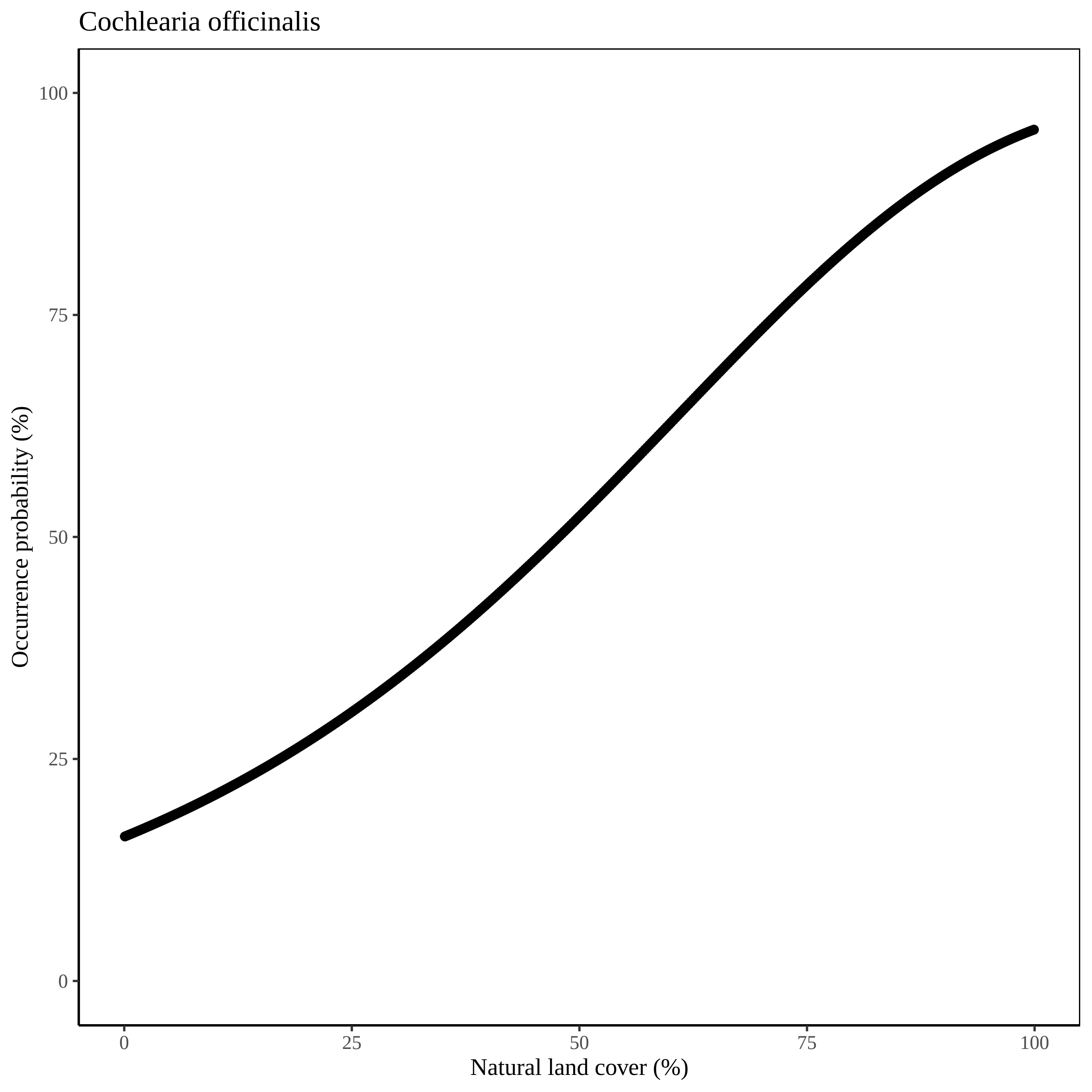
Cochlearia officinalis
Occurrence probability (%)
100
75
50
25
0
0
25
50
75
100
Natural land cover (%)

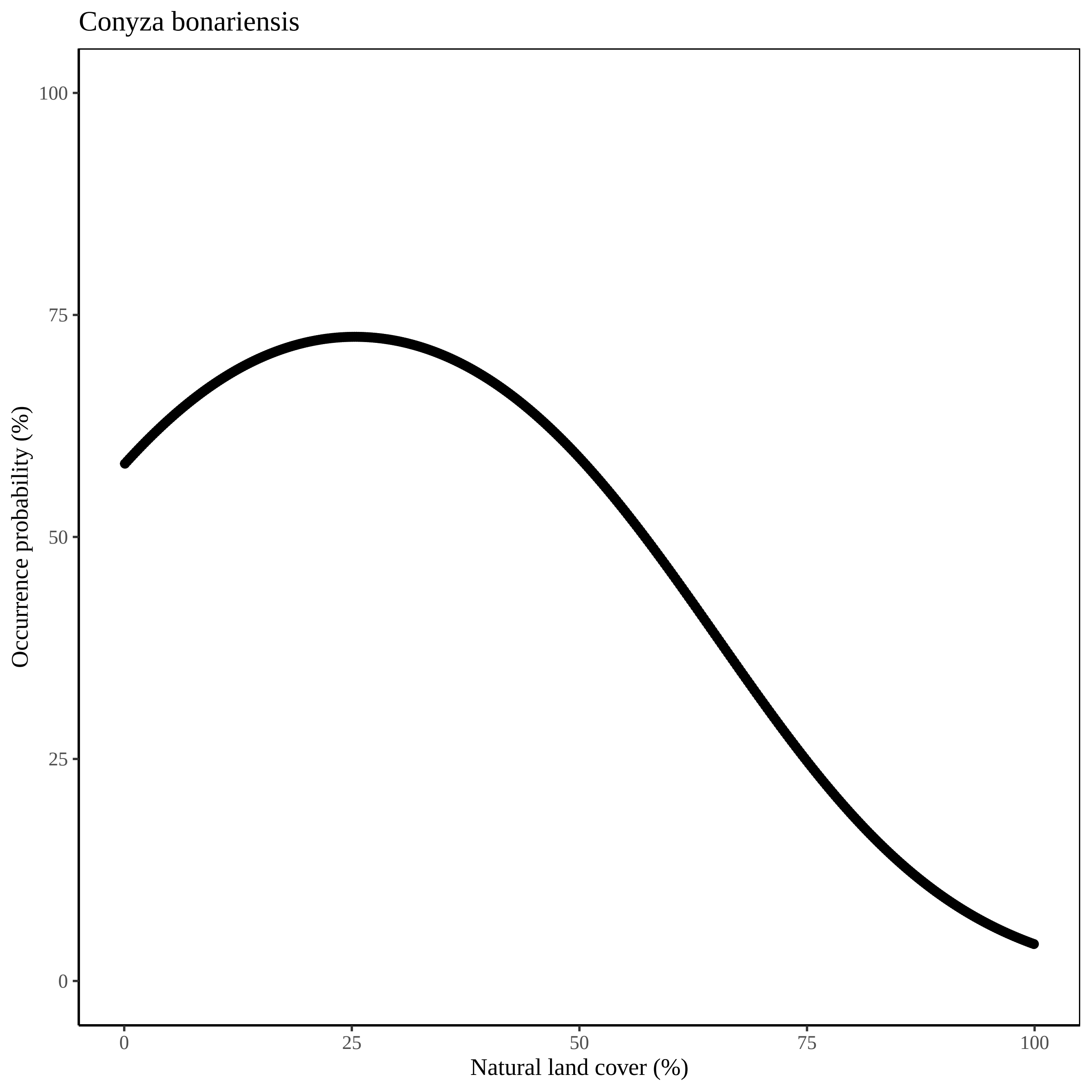
Conyza bonariensis
Occurrence probability (%)
100
75
50
25
0
0
25
50
75
100
Natural land cover (%)

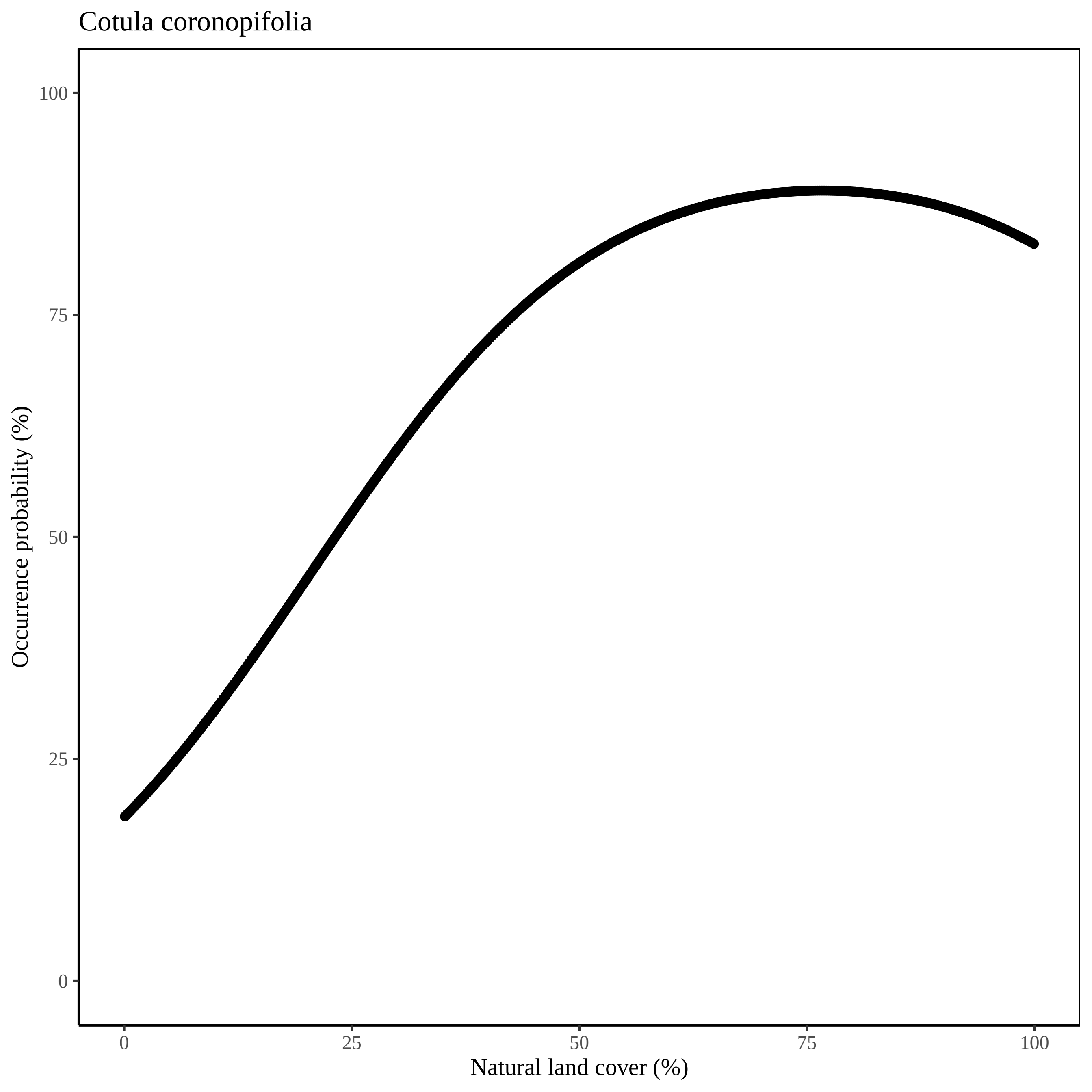

Cotula coronopifolia
Occurrence probability (%)
Natural land cover (%)
0
25
50
75
100
0
25
50
75
100

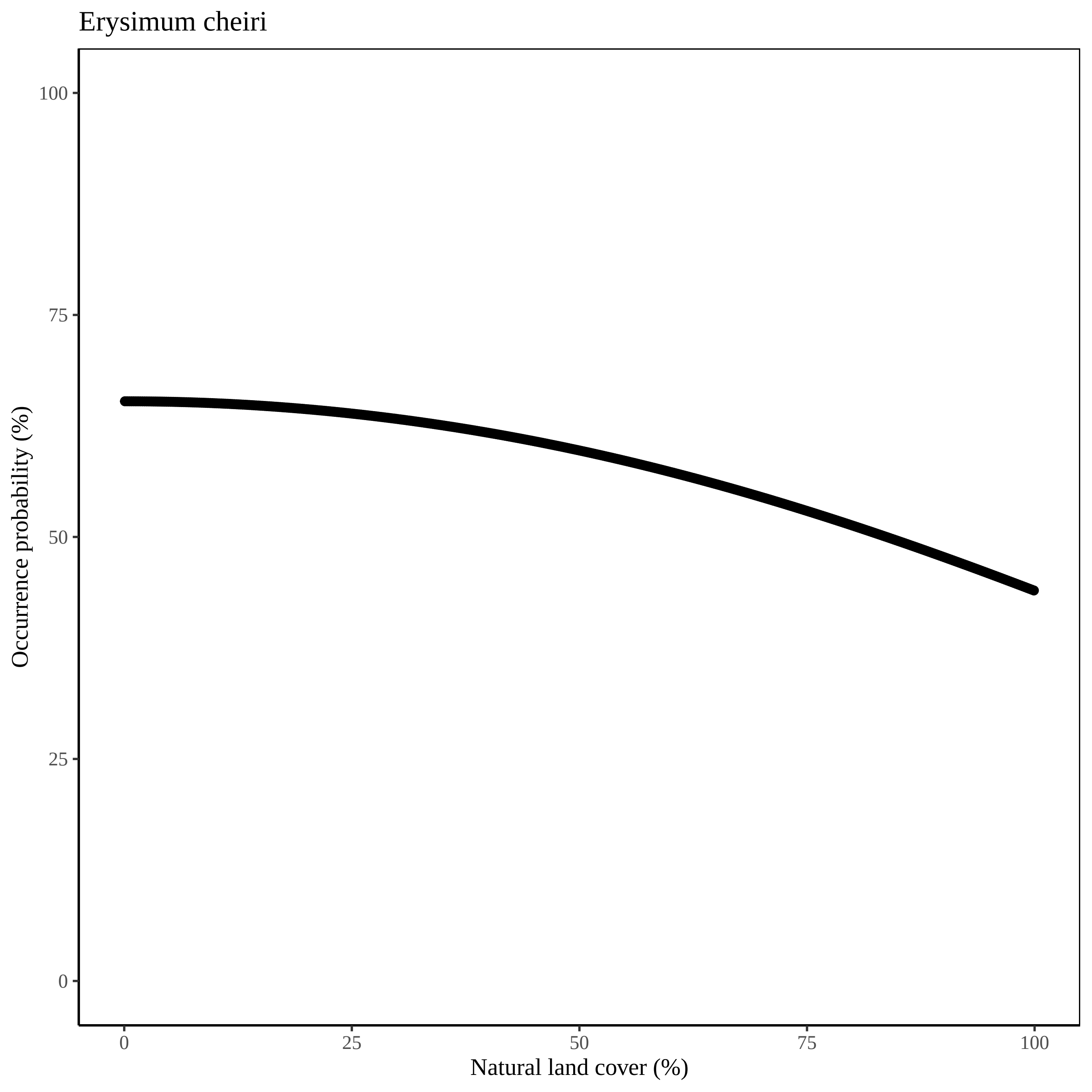
Erysimum cheiri
Occurrence probability (%)
100
75
50
25
0
0
25
50
75
100
Natural land cover (%)

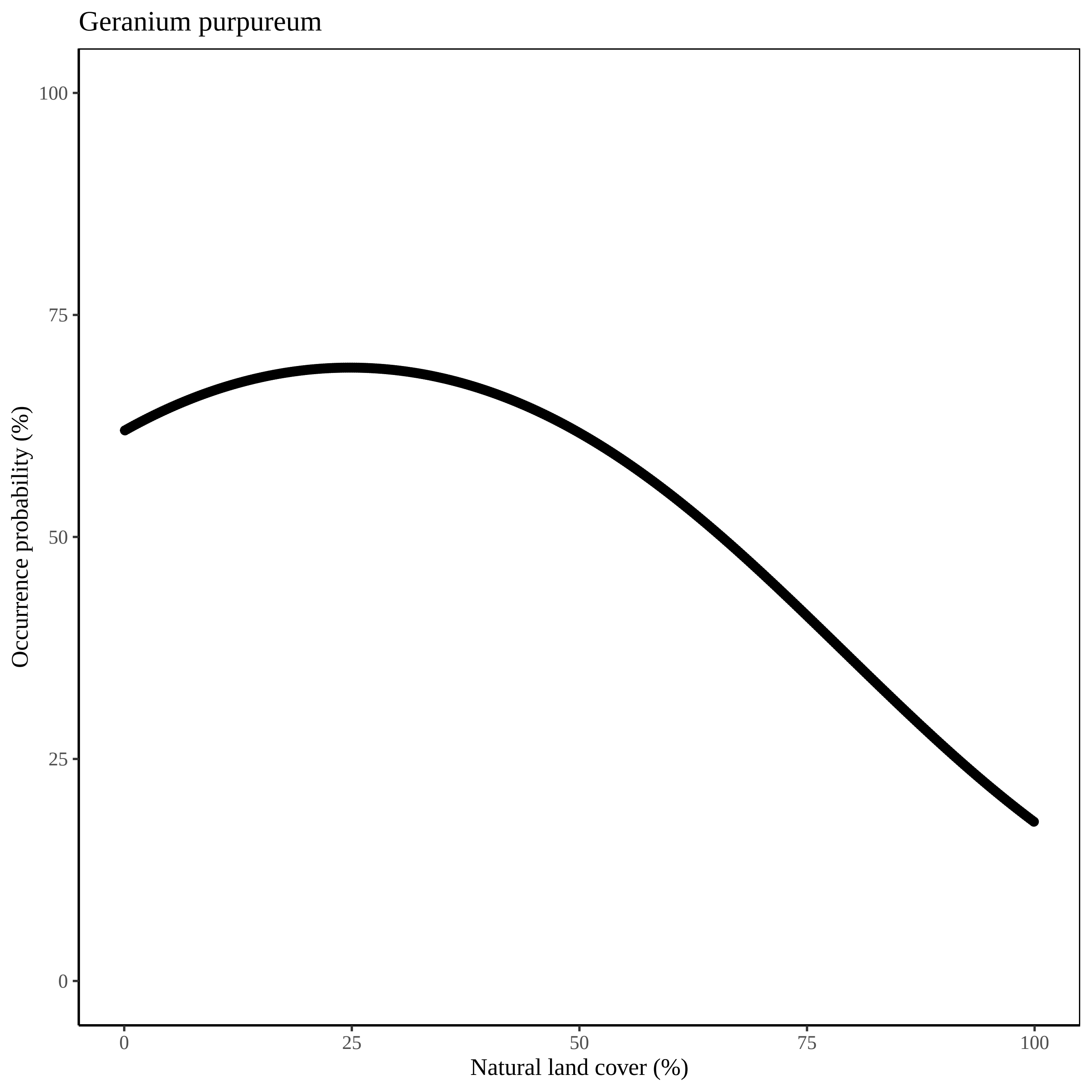
Geranium purpureum
Occurrence probability (%)
100
75
50
25
0
0
25
50
75
100
Natural land cover (%)

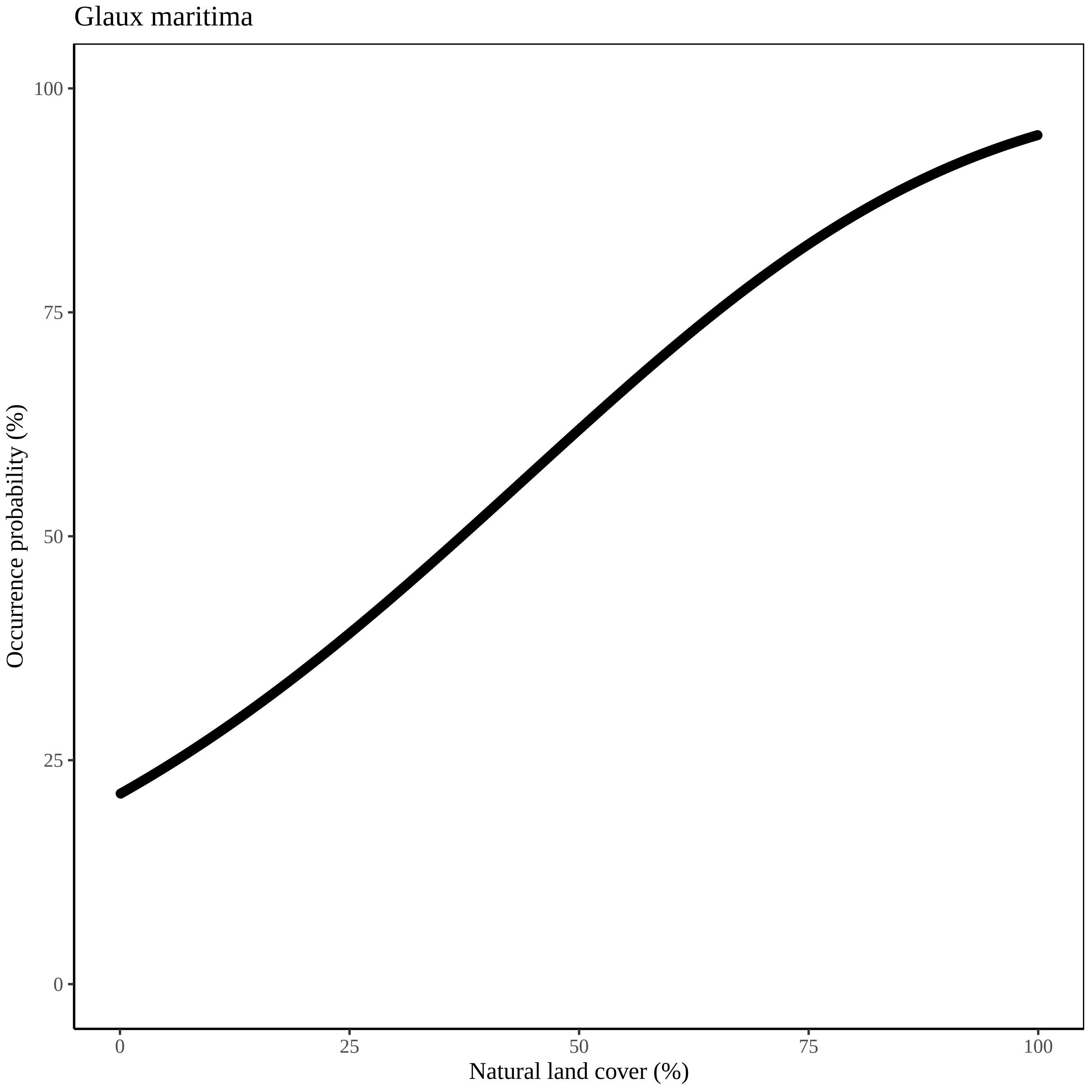

Glaux maritima
100
75
50
25
0
Occurrence probability (%)
0
25
50
75
100
Natural land cover (%)

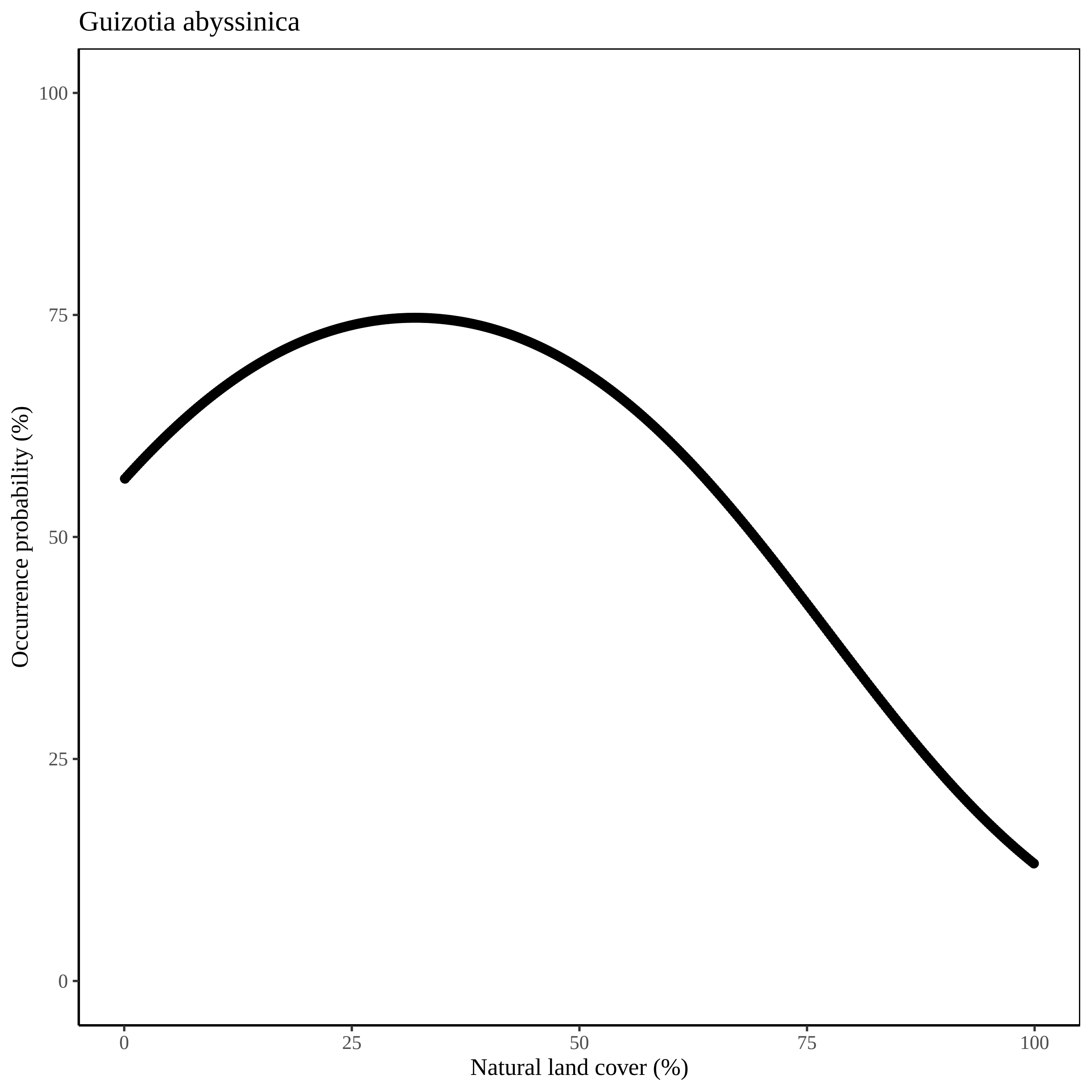
Guizotia abyssinica
Occurrence probability (%)
100
75
50
25
0
0
25
50
75
100
Natural land cover (%)

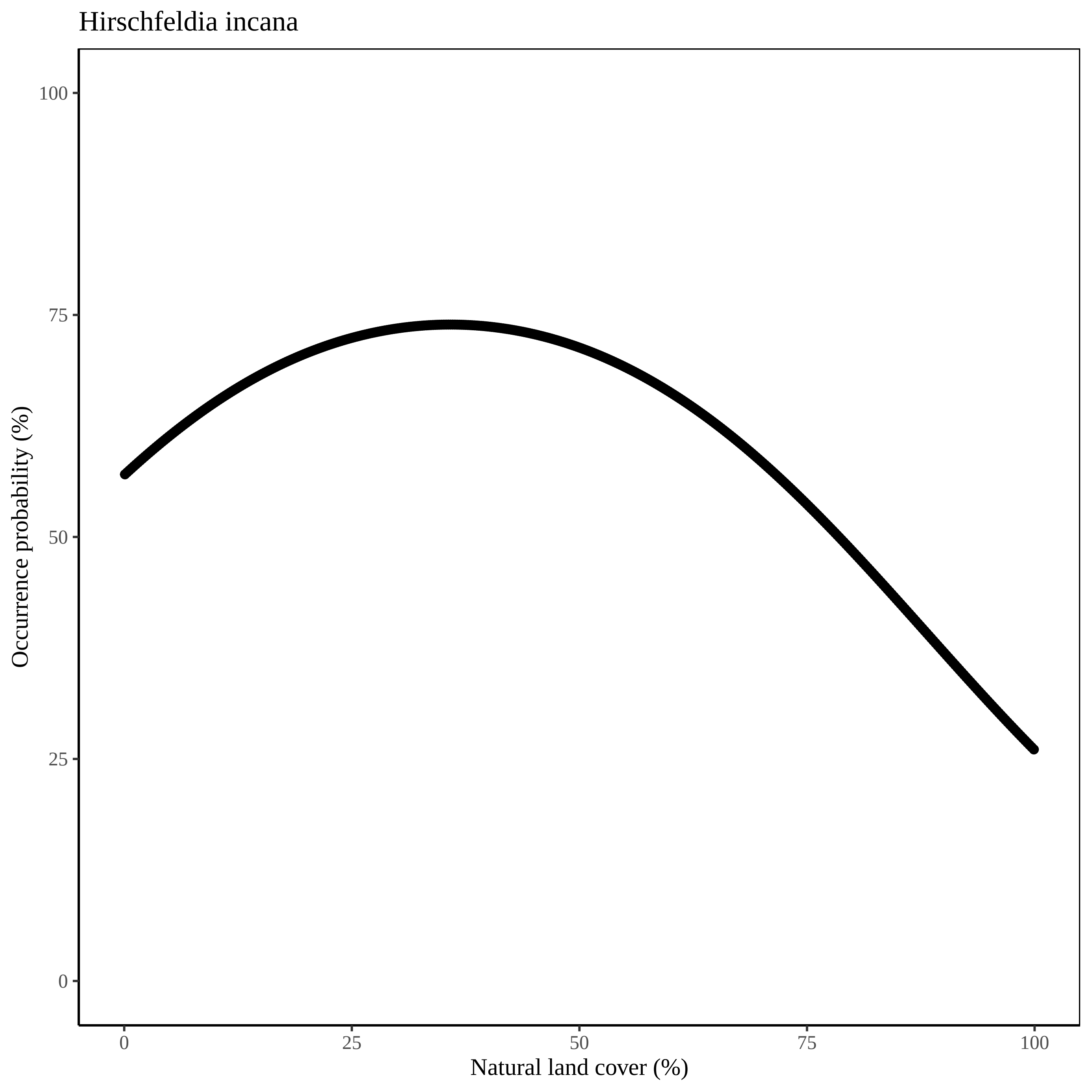
Hirschfeldia incana
Occurrence probability (%)
100
75
50
25
0
0
25
50
75
100
Natural land cover (%)

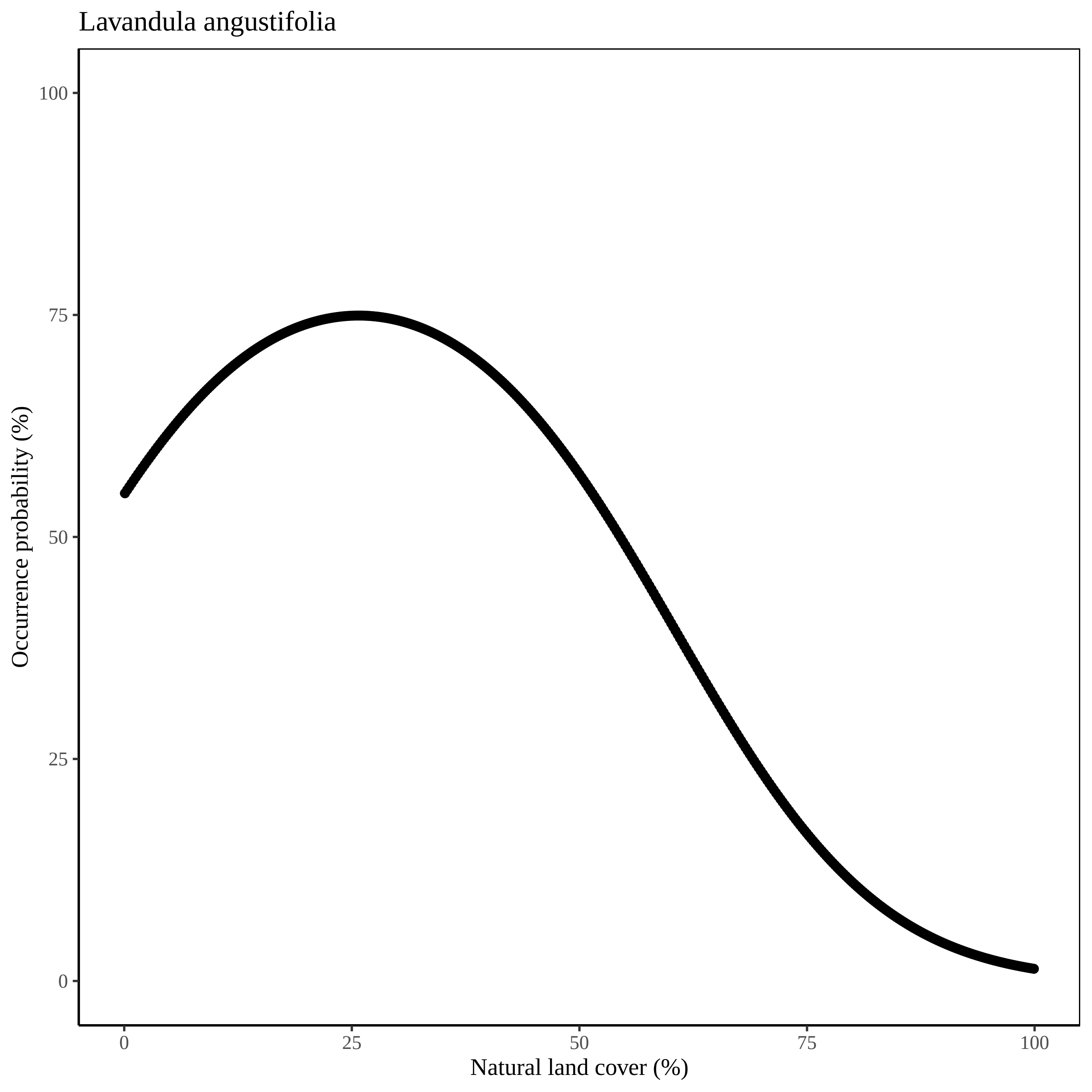
Lavandula angustifolia
Occurrence probability (%)
100
75
50
25
0
0
25
50
75
100
Natural land cover (%)

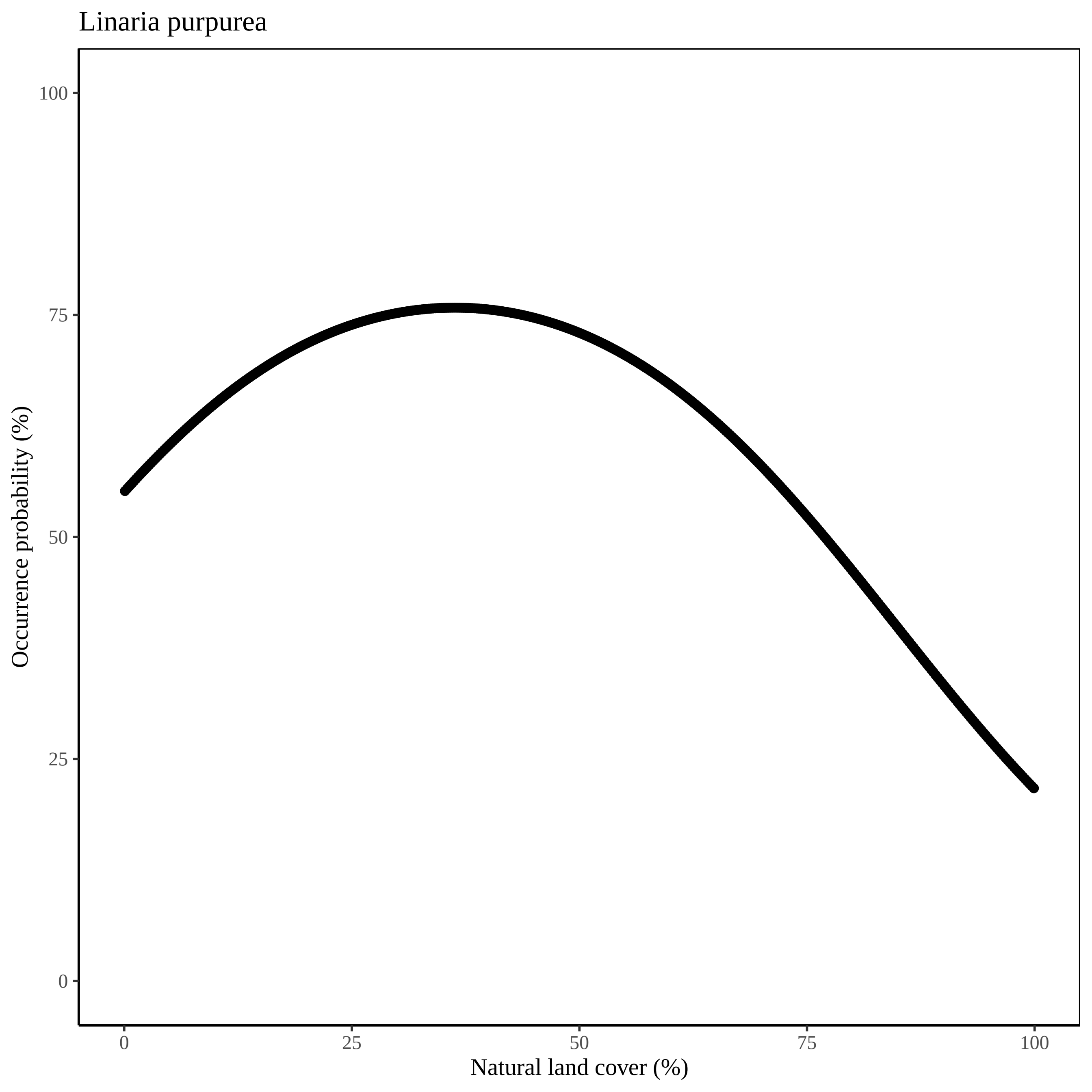
Linaria purpurea
Occurrence probability (%)
100
75
50
25
0
0
25
50
75
100
Natural land cover (%)

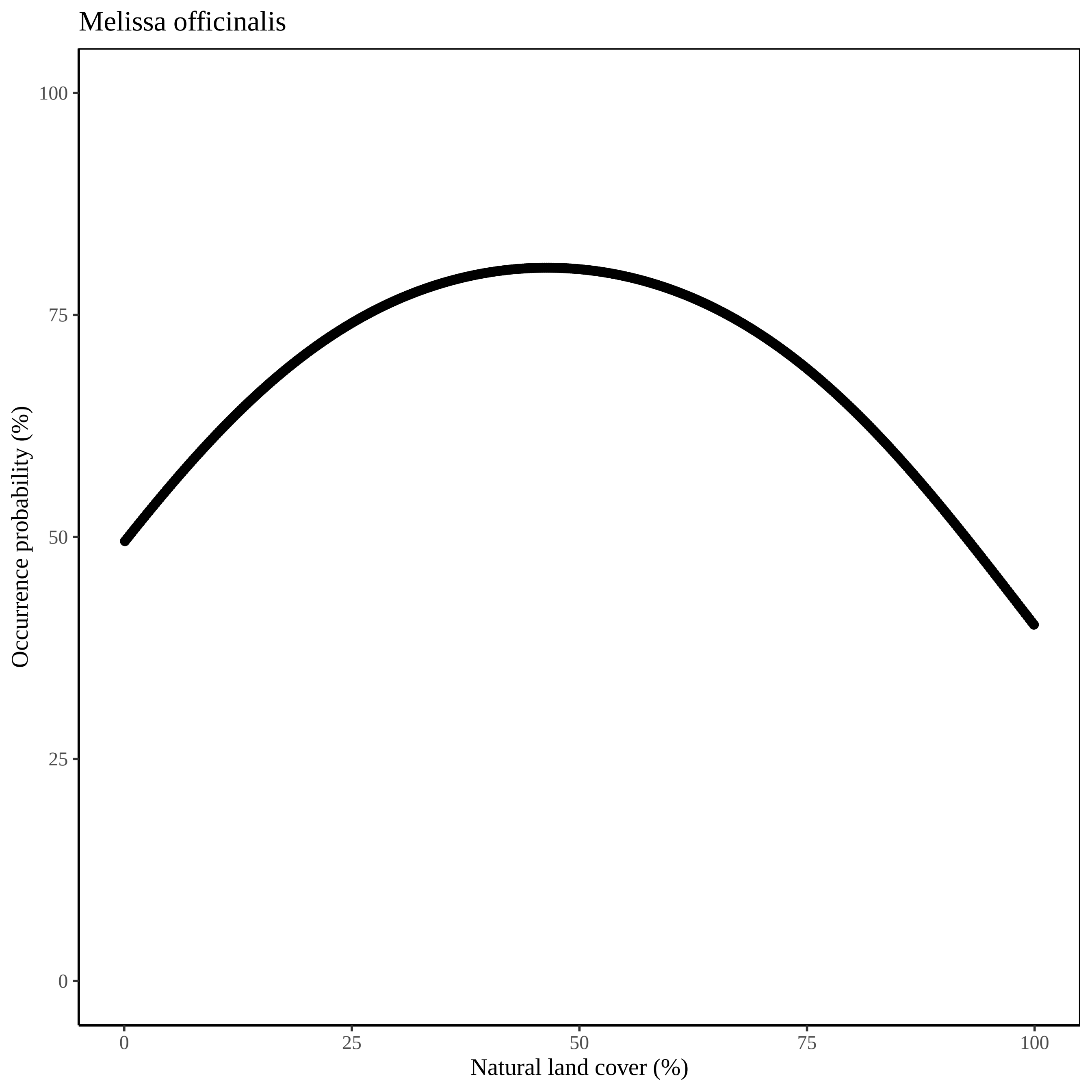
Melissa officinalis
Occurrence probability (%)
100
75
50
25
0
0
25
50
75
100
Natural land cover (%)

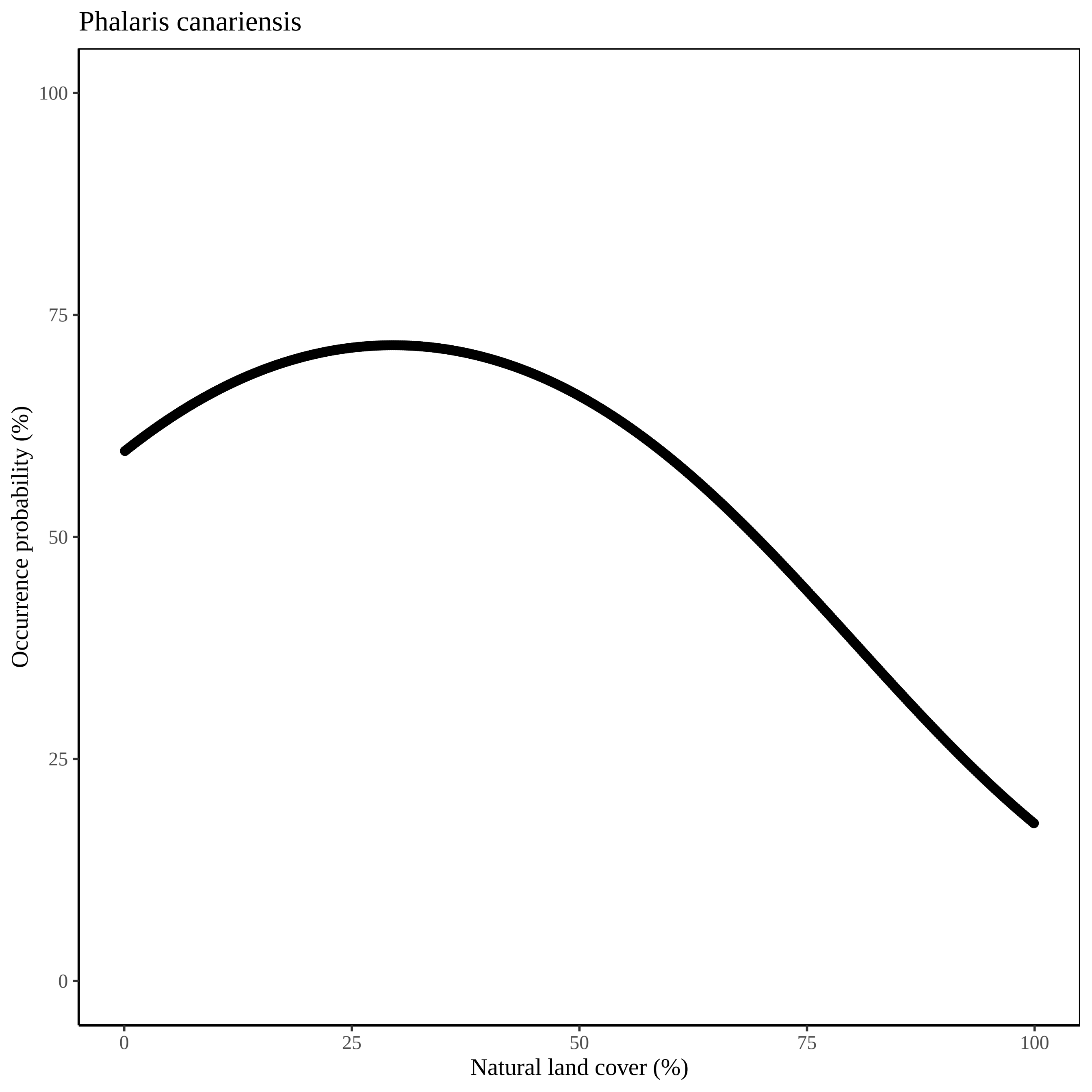
Phalaris canariensis
Occurrence probability (%)
0
25
50
75
100
0
25
50
75
100
Natural land cover (%)

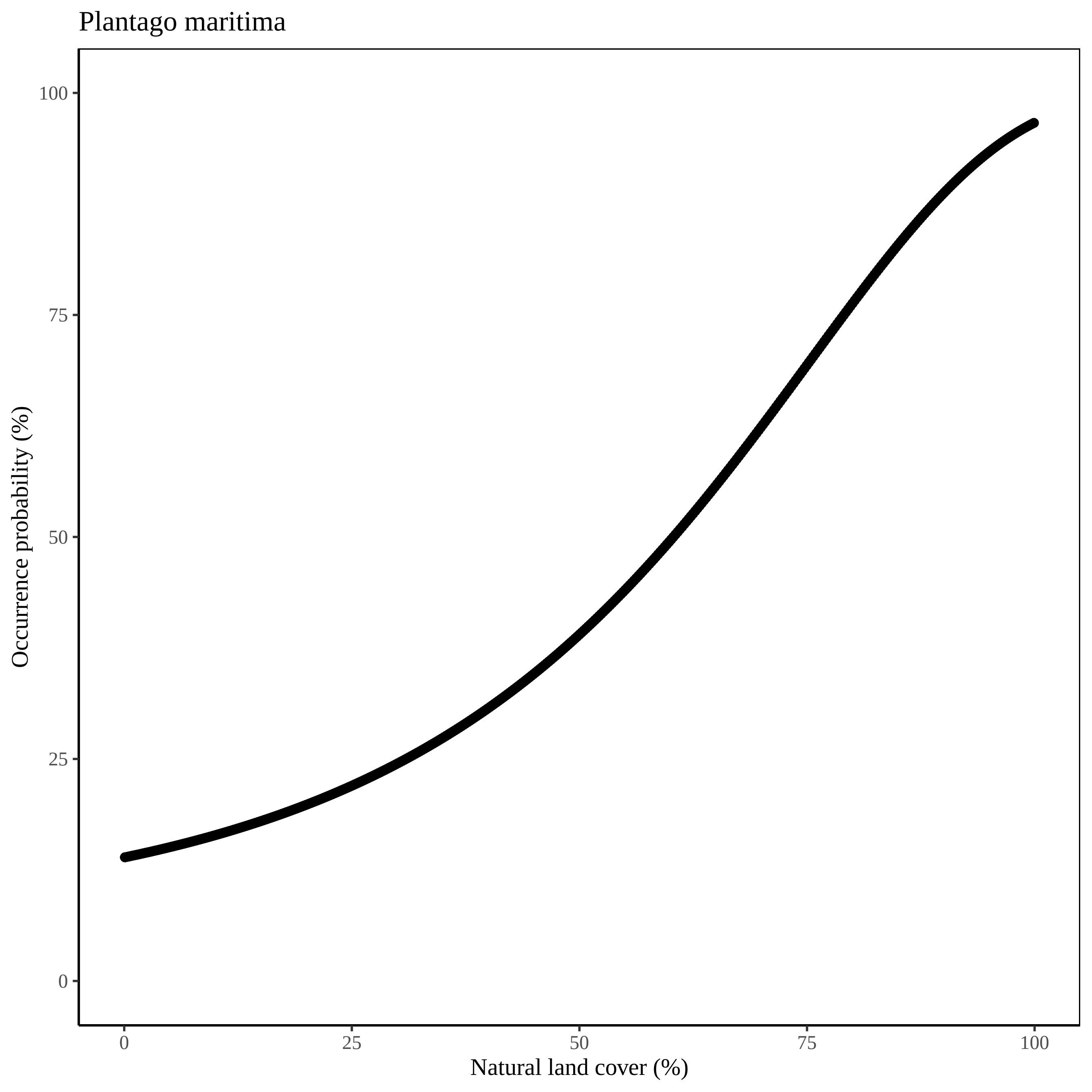

Plantago maritima
Occurrence probability (%)
100
75
50
25
0
0
25
50
75
100
Natural land cover (%)

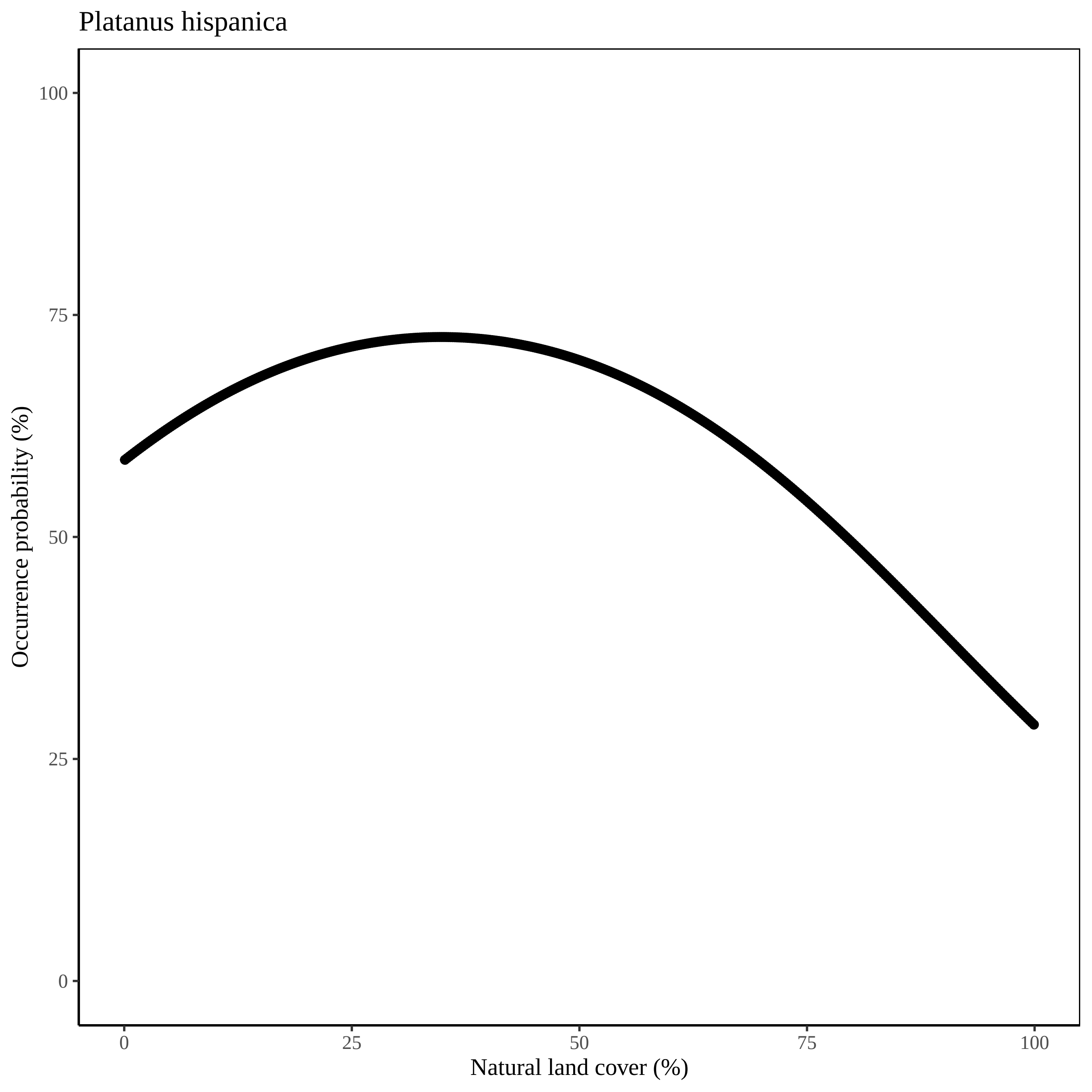

Platanus hispanica
Occurrence probability (%)
100
75
50
25
0
0
25
50
75
100
Natural land cover (%)

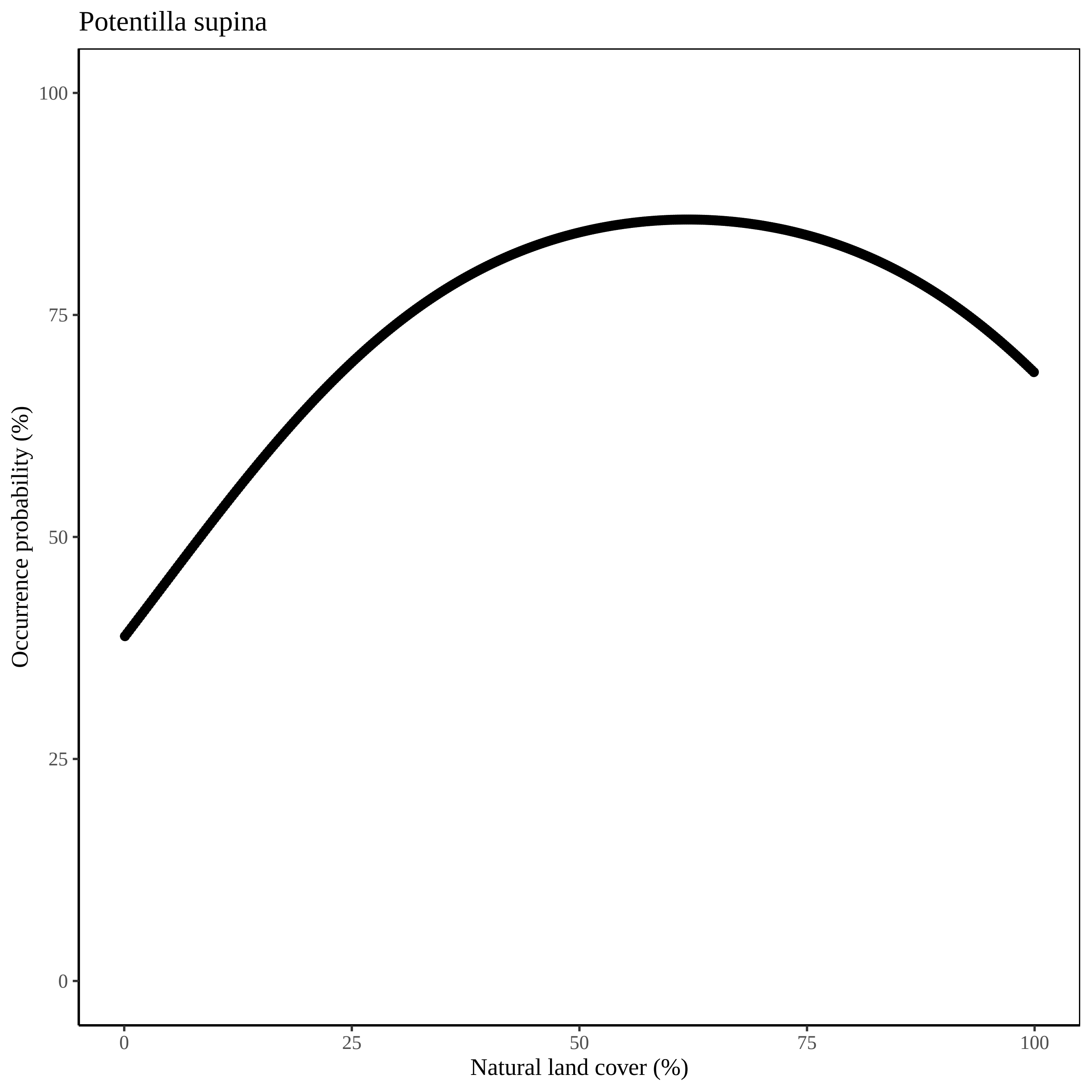
Potentilla supina
Occurrence probability (%)
100
75
50
25
0
0
25
50
75
100
Natural land cover (%)

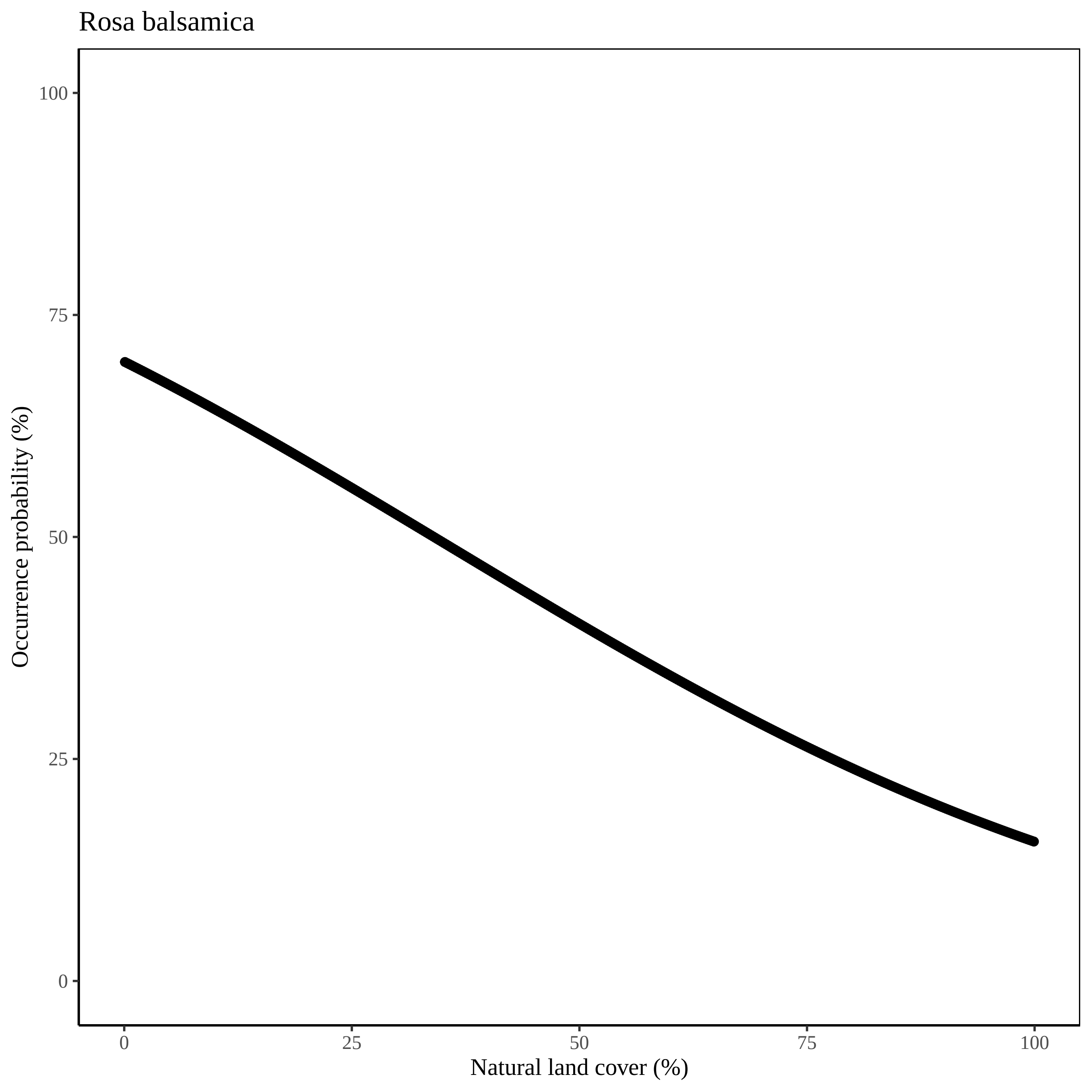

Rosa balsamica
100
75
50
25
0
Occurrence probability (%)
0
25
50
75
100
Natural land cover (%)

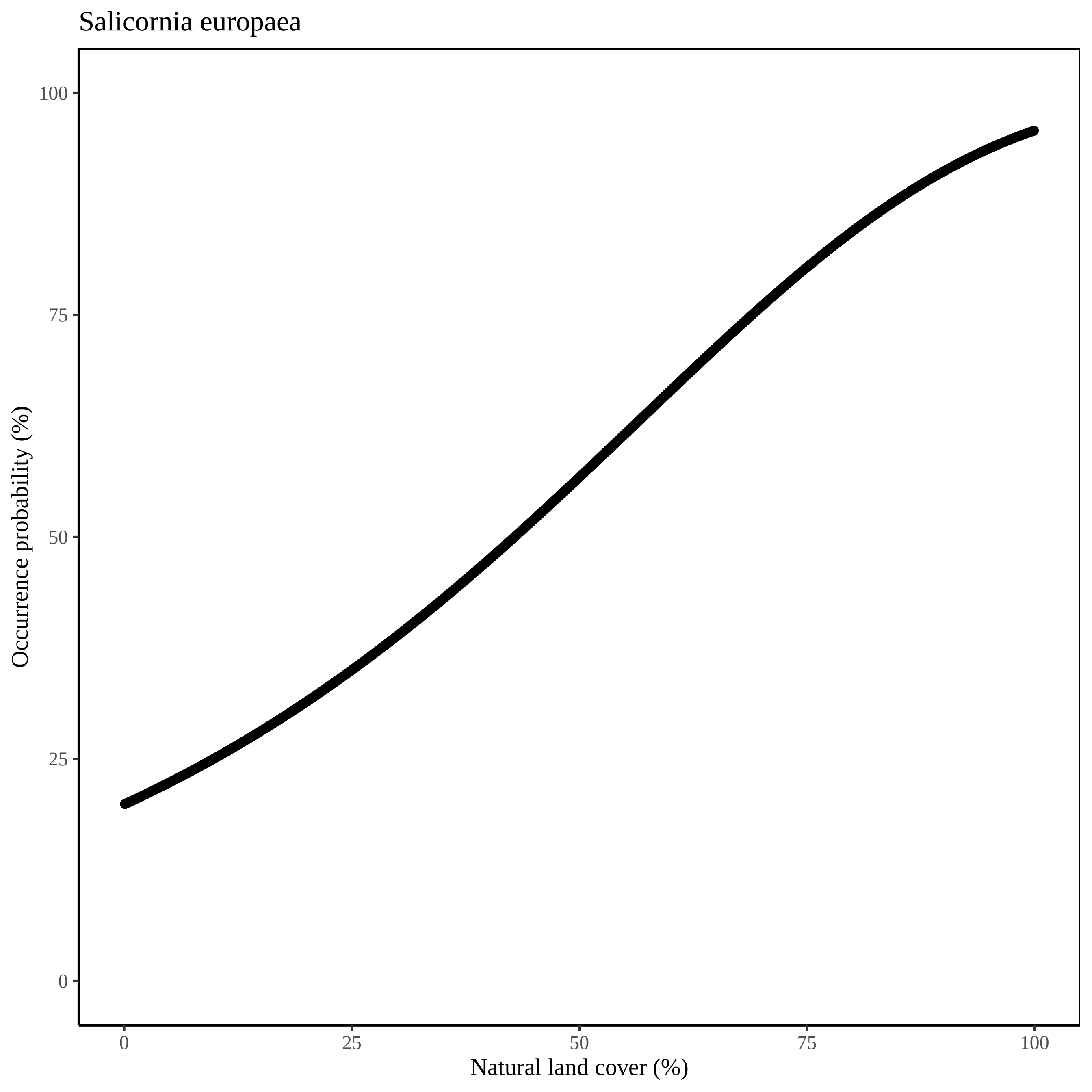
Salicornia europaea
Occurrence probability (%)
100
75
50
25
0
0
25
50
75
100
Natural land cover (%)

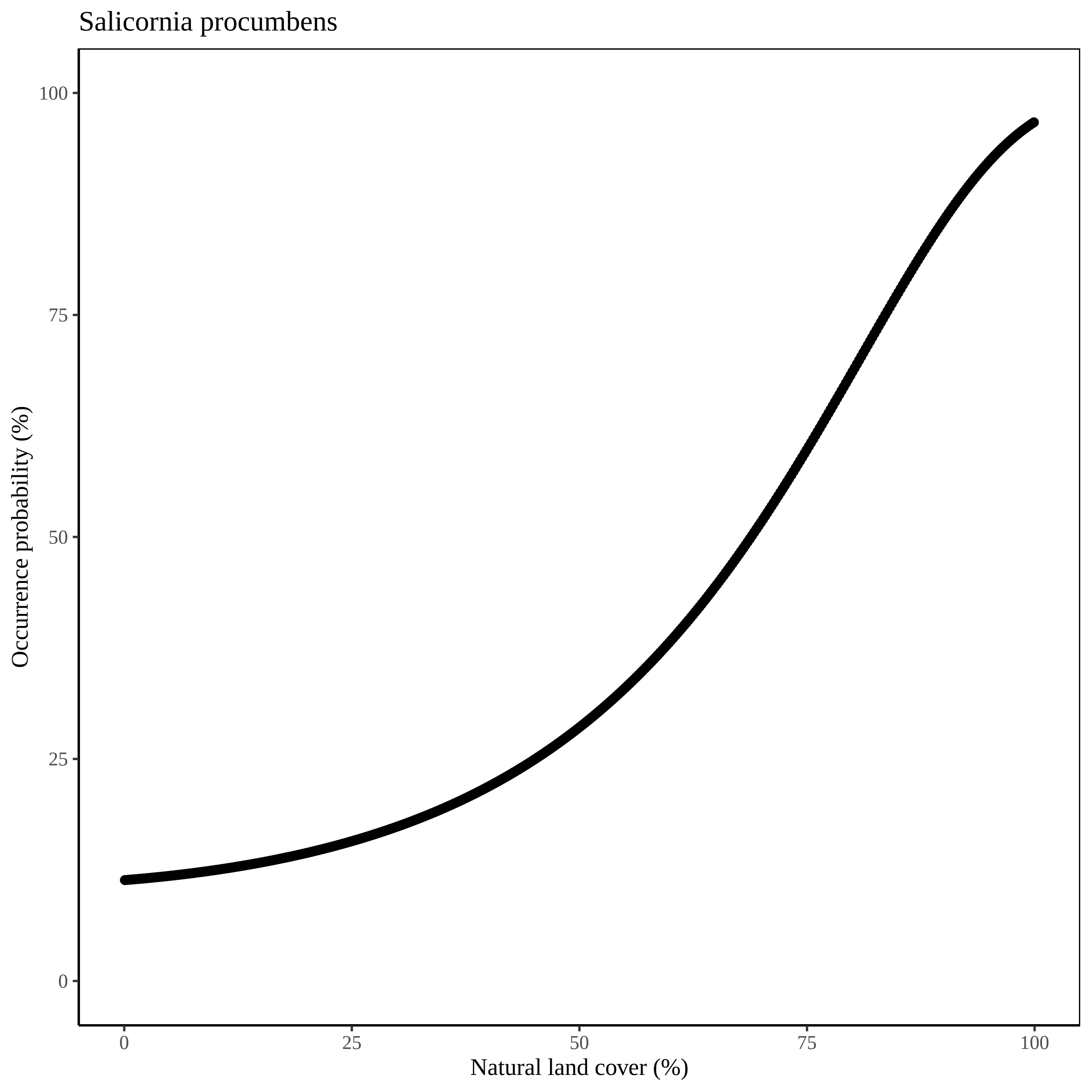
Salicornia procumbens
Occurrence probability (%)
100
75
50
25
0
0
25
50
75
100
Natural land cover (%)

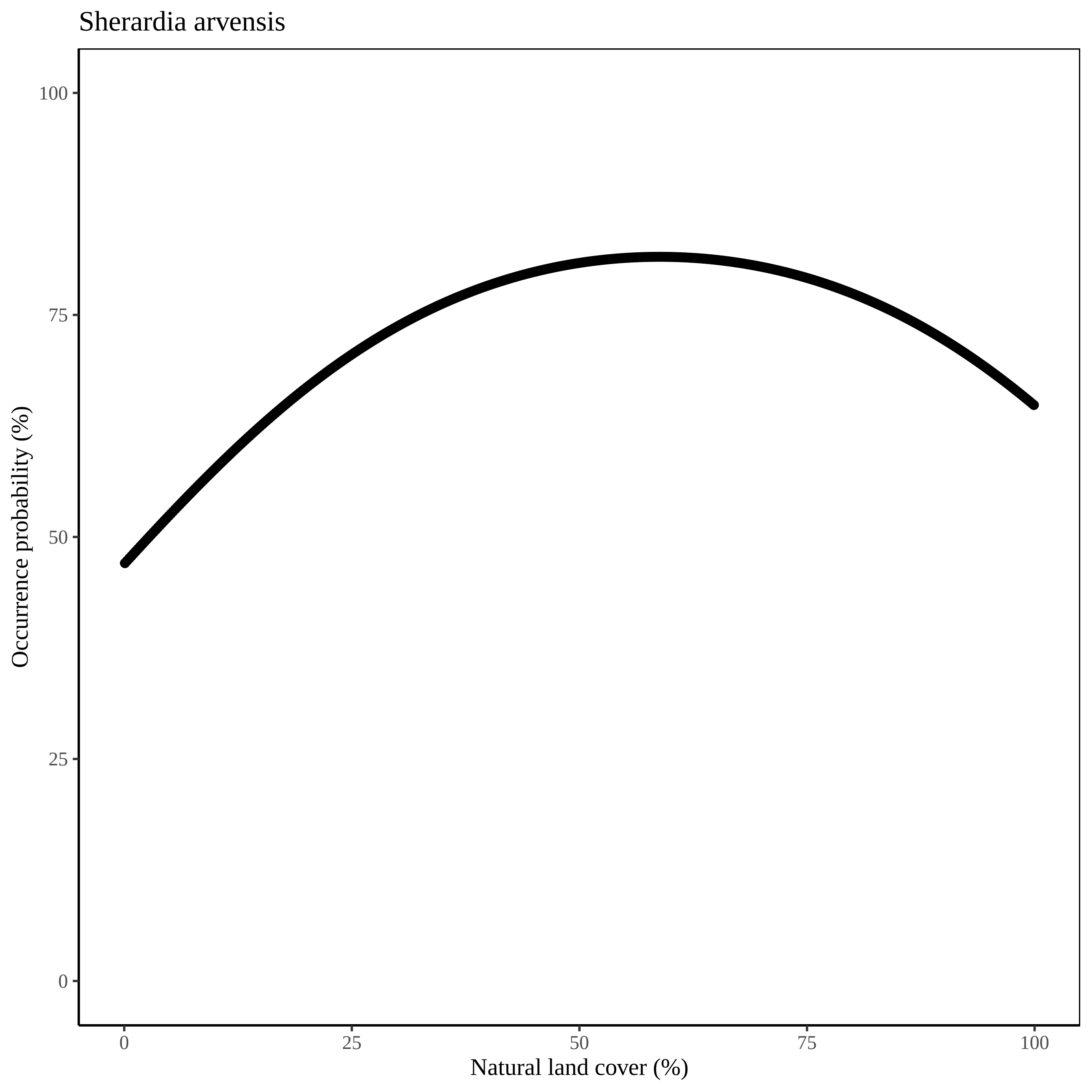

Sherardia arvensis
Occurrence probability (%)
100
75
50
25
0
0
25
50
75
100
Natural land cover (%)

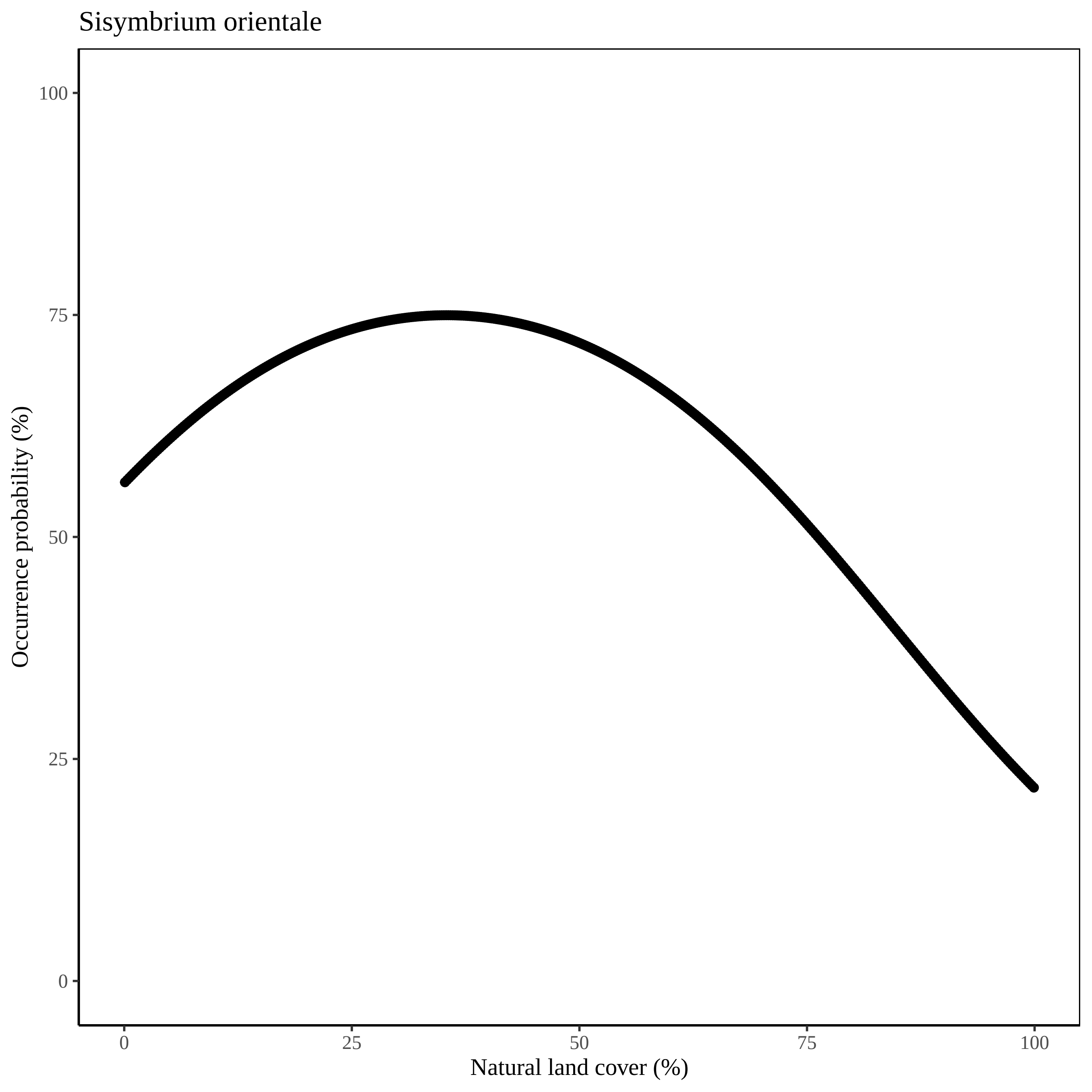
Sisymbrium orientale
100
75
50
25
0
Occurrence probability (%)
0
25
50
75
100
Natural land cover (%)

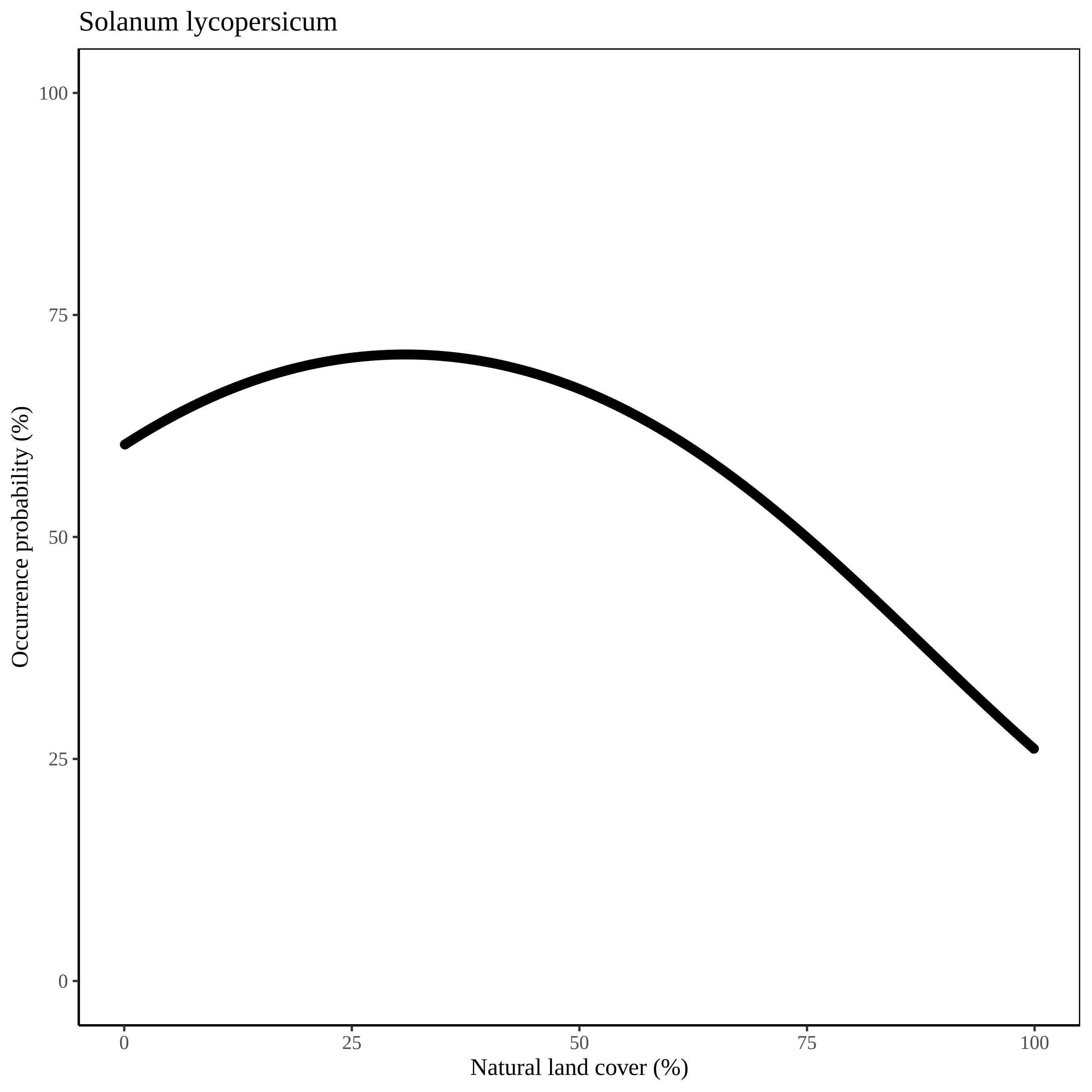
Solanum lycopersicum
100
75
50
25
0
Occurrence probability (%)
0
25
50
75
100
Natural land cover (%)

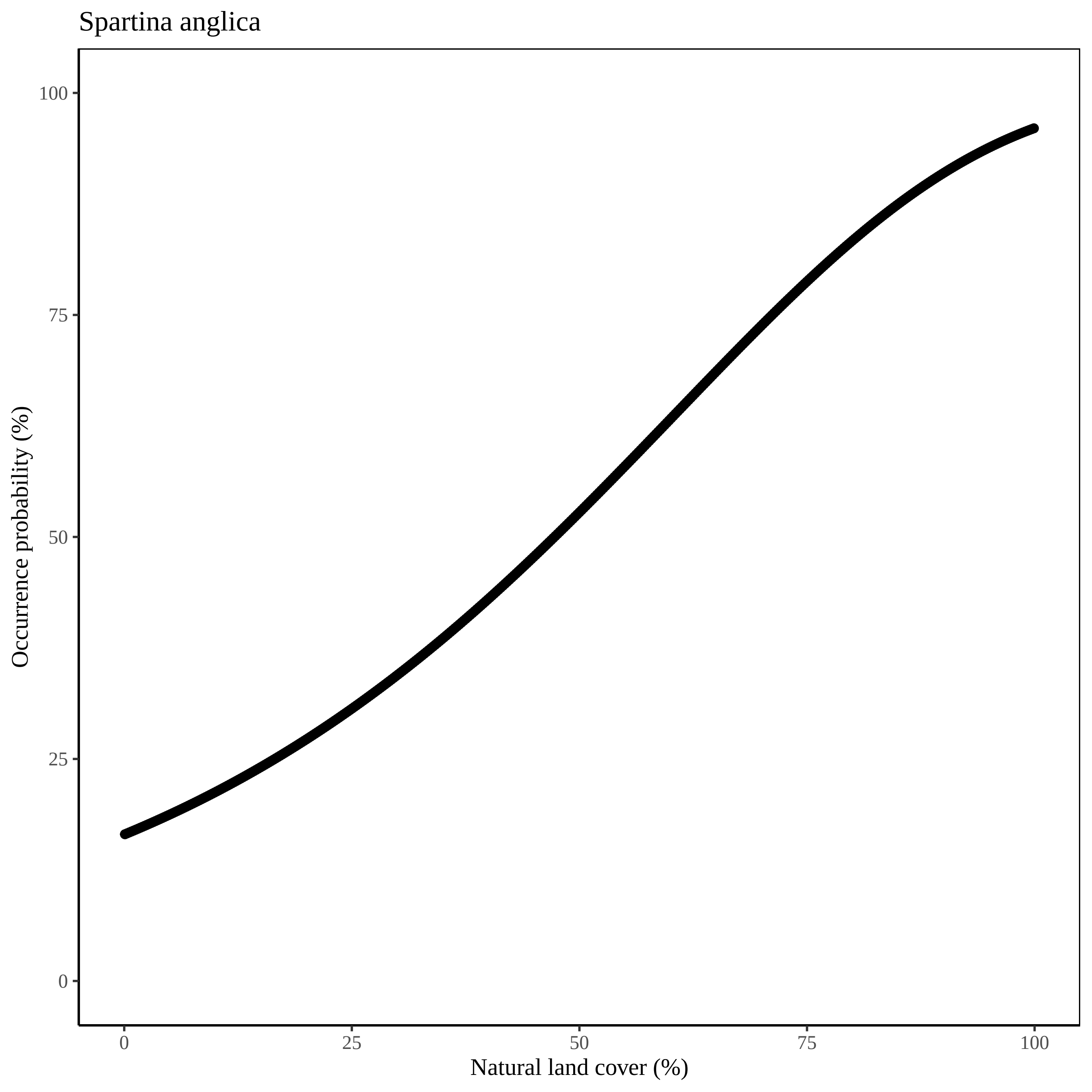
Spartina anglica
Occurrence probability (%)
100
75
50
25
0
0
25
50
75
100
Natural land cover (%)

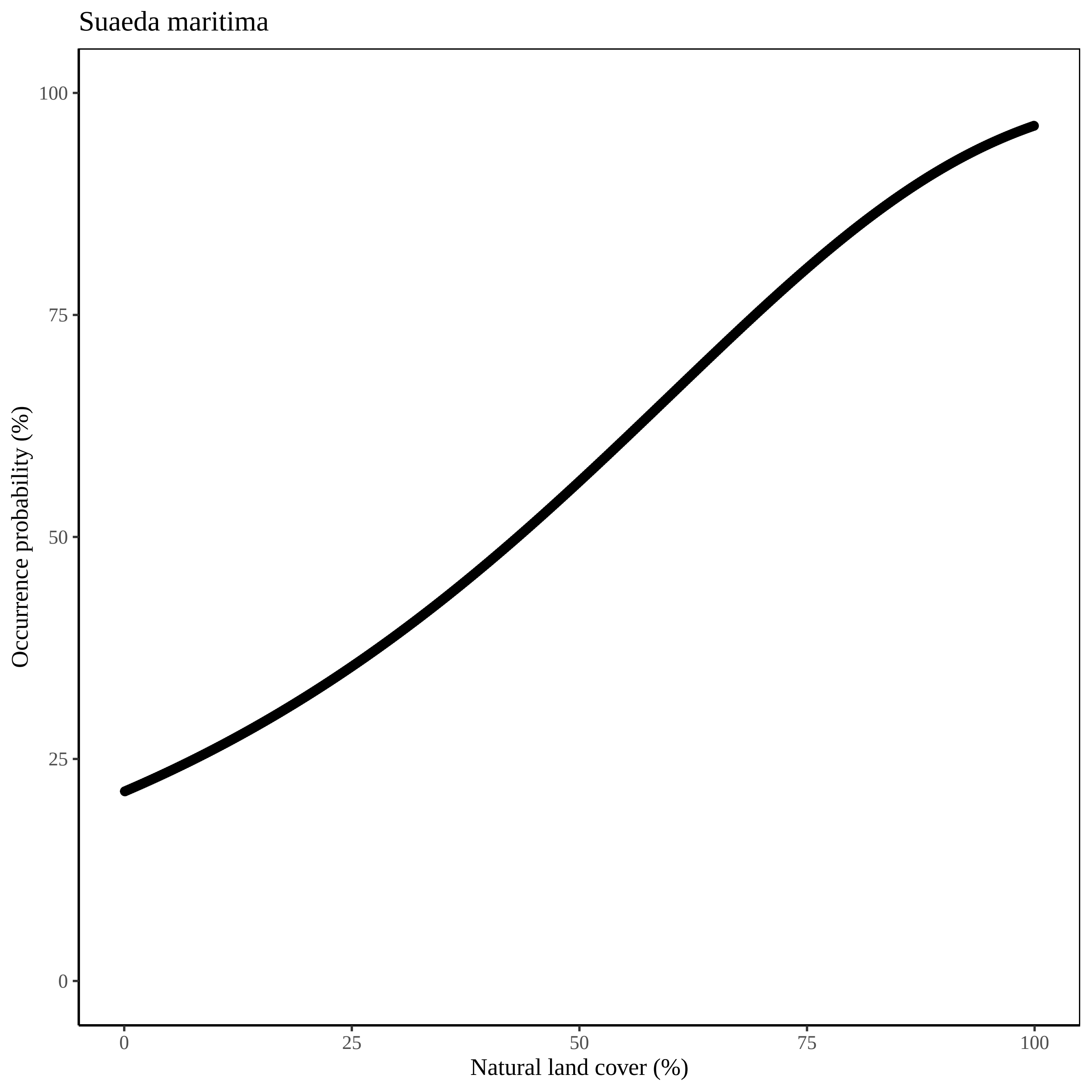
Suaeda maritima
100
75
50
25
0
0
25
50
75
100
Occurrence probability (%)
Natural land cover (%)

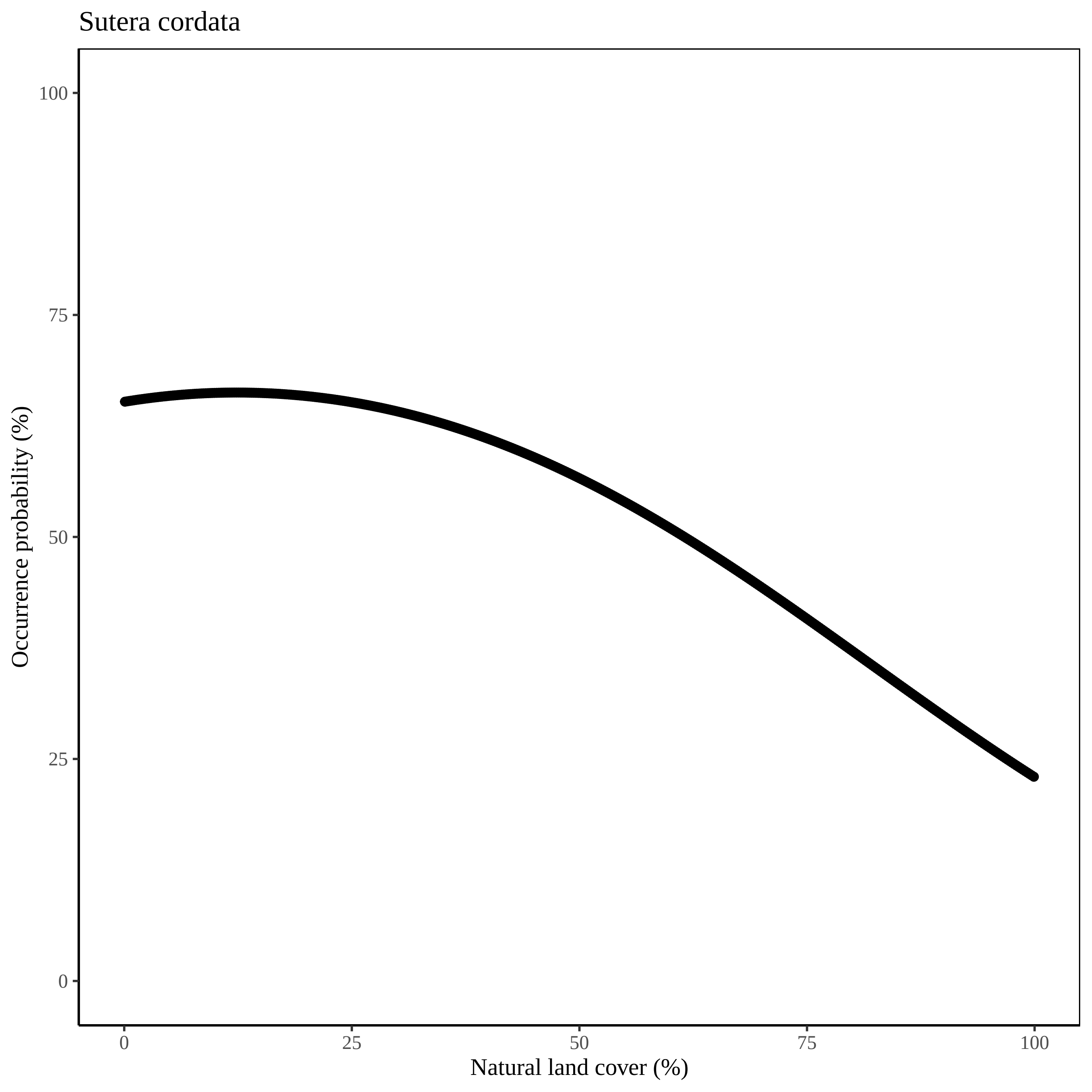
Sutera cordata
Occurrence probability (%)
100
75
50
25
0
0
25
50
75
100
Natural land cover (%)

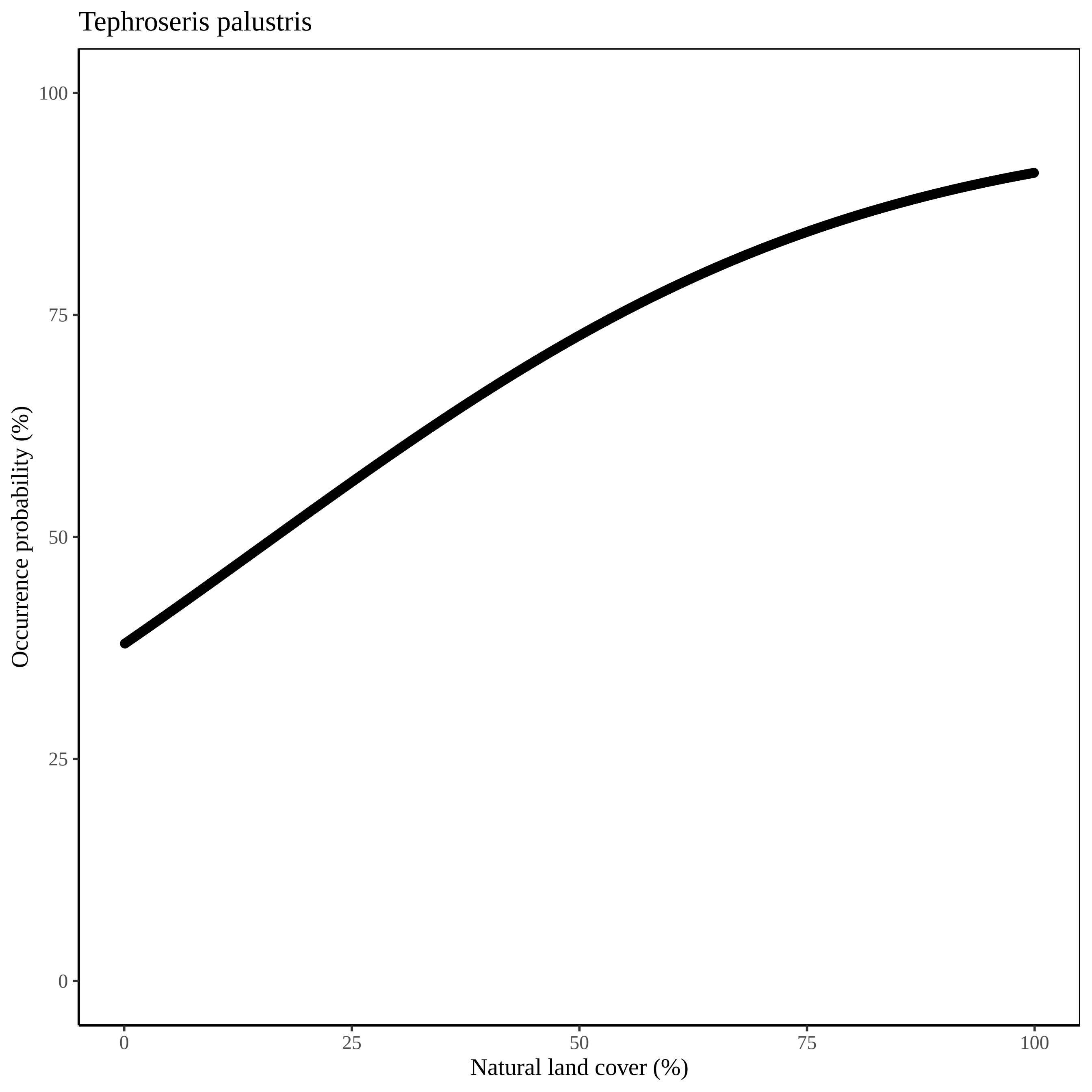

Tephroseris palustris
Occurrence probability (%)
100
75
50
25
0
0
25
50
75
100
Natural land cover (%)

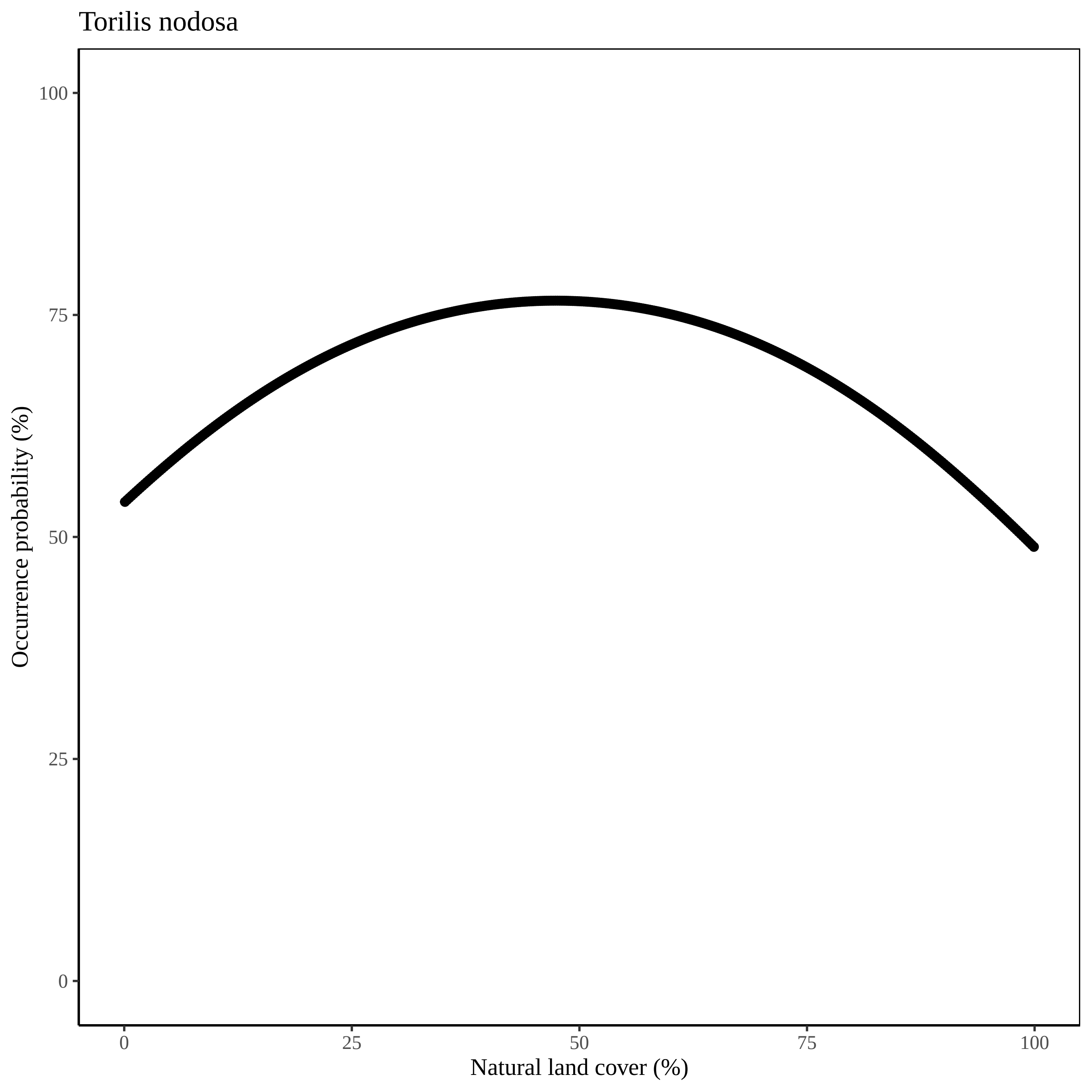
Torilis nodosa
Occurrence probability (%)
100
75
50
25
0
0
25
50
75
100
Natural land cover (%)

Tragopogon dubius

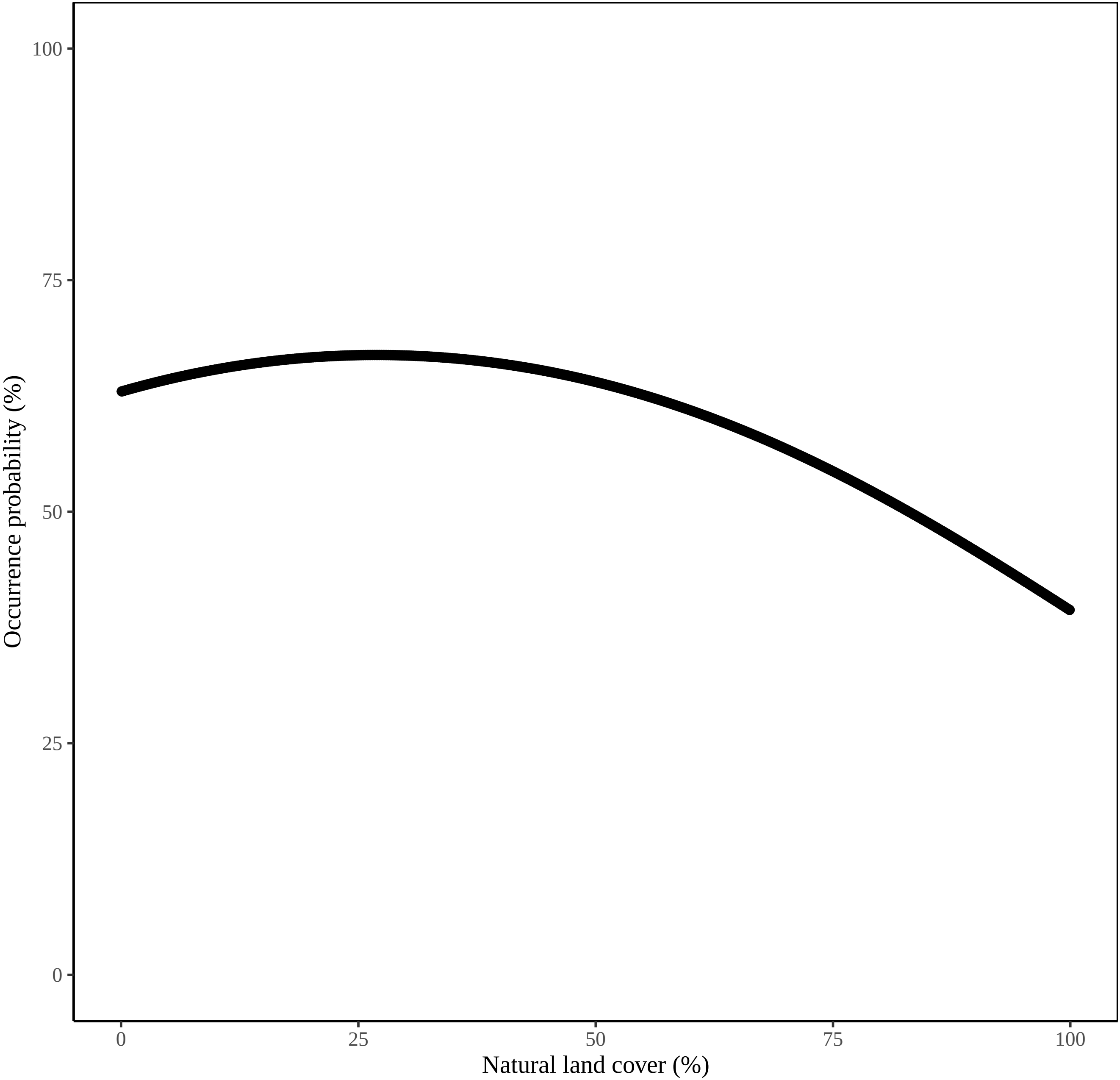

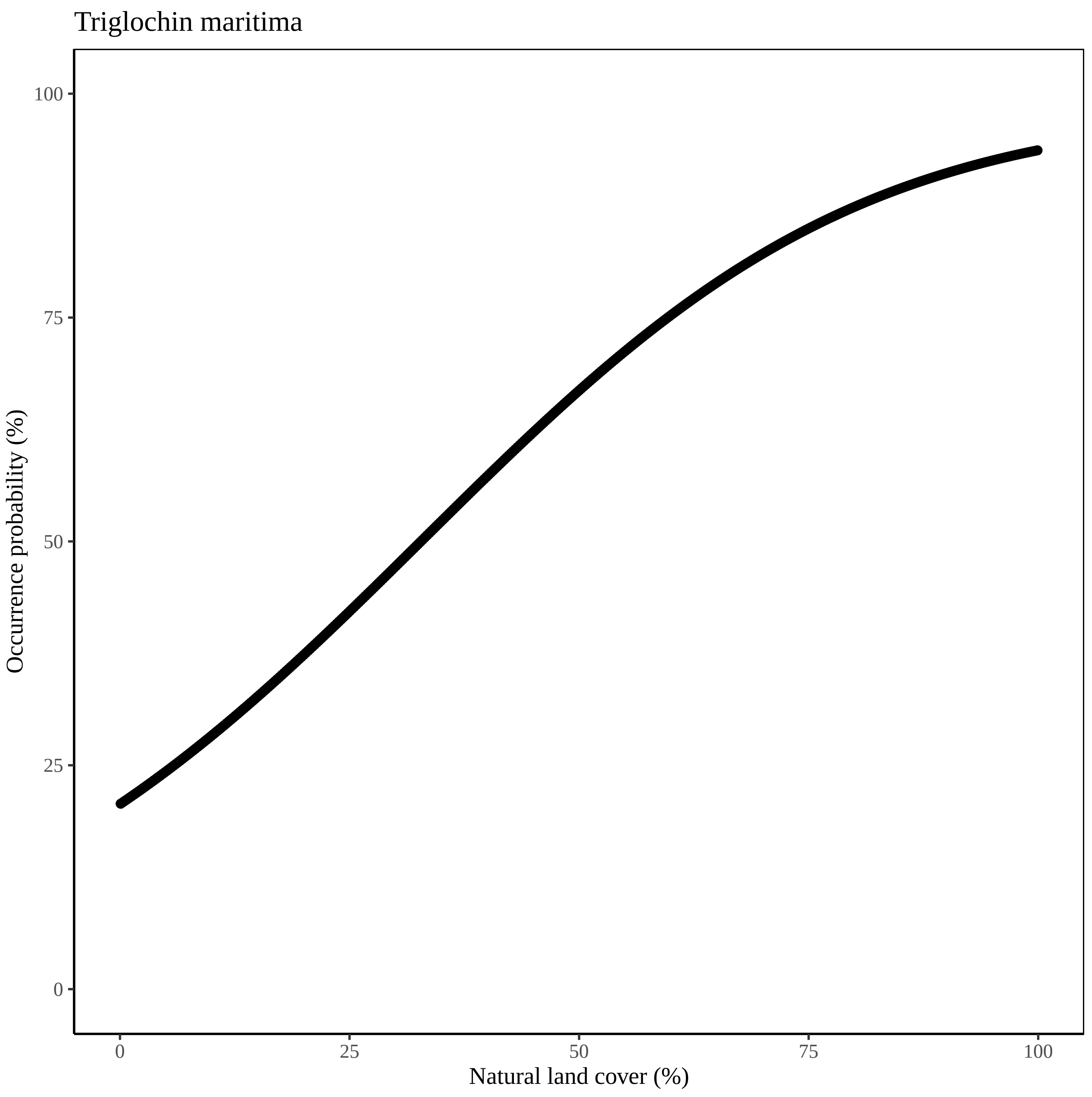
Triglochin maritima
Occurrence probability (%)
100
75
50
25
0
0
25
50
75
100
Natural land cover (%)

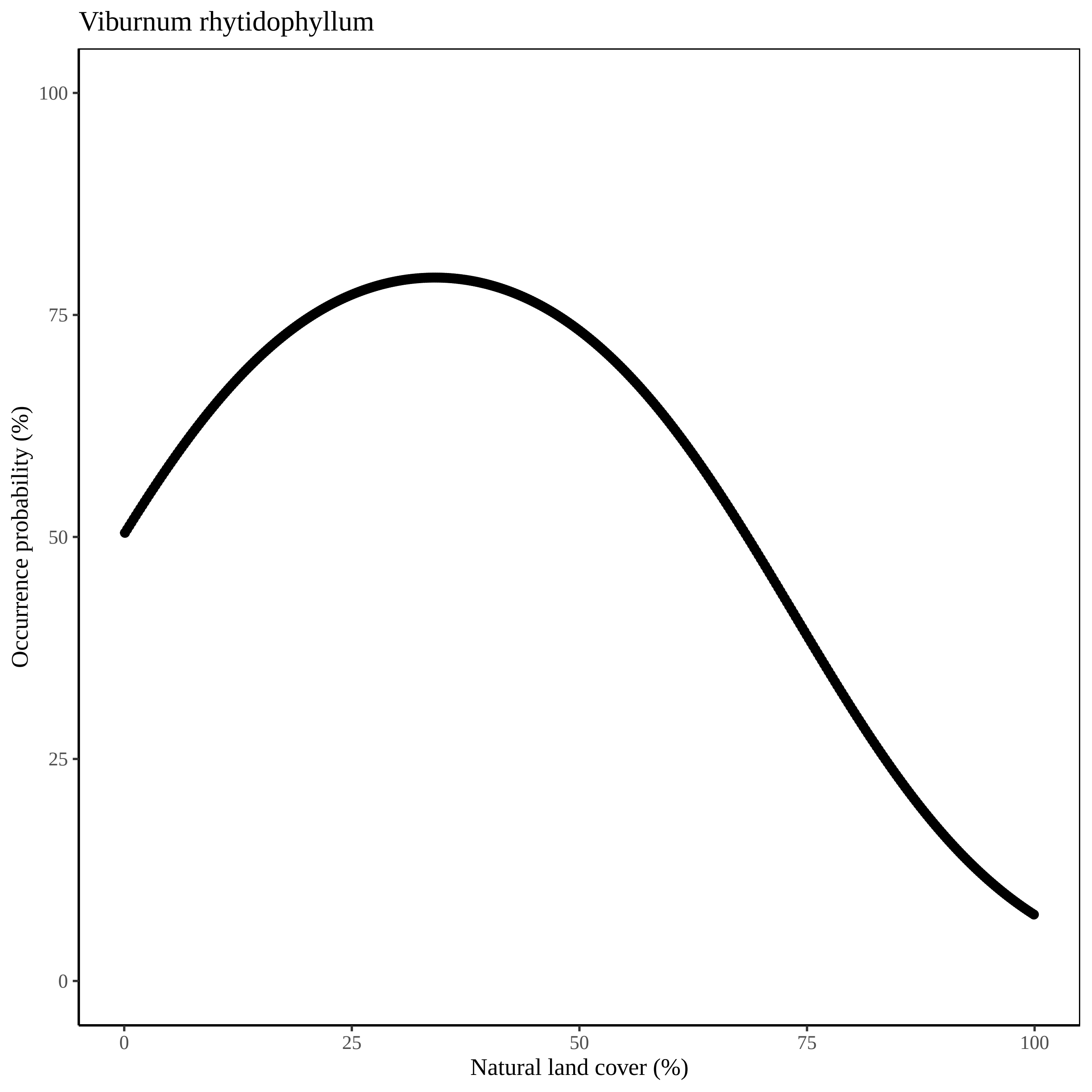

Viburnum rhytidophyllum
Occurrence probability (%)
100
75
50
25
0
0
25
50
75
100
Natural land cover (%)
