# Supplementary material for: Importance of natural land cover for plant species’ conservation: A nationwide study in The Netherlands
Source: PLoS One. 2021 Nov 16;16(11):e0259255. doi: 10.1371/journal.pone.0259255 (PMC8594855; doi:10.1371/journal.pone.0259255)
Supplement: Attachment — Submitted filename: Response to Reviewers_new.docx [file pone.0259255.s020.docx]

**Responses to reviewers**

**Journal Requirements:**

Please review your reference list to ensure that it is complete and correct. If you have cited papers that have been retracted, please include the rationale for doing so in the manuscript text, or remove these references and replace them with relevant current references. Any changes to the reference list should be mentioned in the rebuttal letter that accompanies your revised manuscript. If you need to cite a retracted article, indicate the article’s retracted status in the References list and also include a citation and full reference for the retraction notice.

***RE****: Thank you. We have checked it and all references have been cited correctly.*

-----------------------------------------------

**Reviewer #1:**

**Comment 3.** I infer that PLOS ONE is an international journal aiming to adhere to global standards, for a global audience. I don’t perceive that informal names like “ Salix ‘Sekka’ “ are good enough by international standards regardless of whether they are accepted by the Dutch government or not. Nonetheless, now that the authors have matched the Dutch names to World Flora Online names, authorities, and species concepts – I think this does partially resolve the issue for me. Although it does open a new can of worms… So let’s look at the changes…

b) “ Arenaria leptoclados “ is revealed as “ Arenaria serpyllifolia subsp. leptoclados (Rchb.) Nyman “

It is problematic to include this taxon in this analysis as World Flora Online recognises it only as a mere subspecies of Arenaria serpyllifolia L. which itself is included in the analysis already. I would recommend that the authors remove all taxa which World Flora Online only recognises as a subspecies, to ensure methodological ‘purity’ in only performing analyses on robust species-level taxa and not ‘species-level + subspecies-level taxa’. Needlessly including taxa that are considered to be only subspecies-level by some authorities (even if not necessarily Dutch authorities), weakens the robustness of the overall analysis. If there are only a few such taxa that are questionably subspecies-only, why not simply remove them, or perform additional/complementary analyses with them removed, to demonstrate the robustness of the results?

The data that the authors themselves present in column B (“Species name World Flora Online”) of Table S2, reveals that there are **eleven taxa in their dataset**, including Arenaria serpyllifolia subsp. leptoclados (Rchb.) Nyman that World Flora Online recognises **as subspecies only**. I understand the desire to perform analyses relative to Dutch accepted species-level taxa given the Dutch geographical context, but please also (or only) perform analyses in the global/international context – that is using full, robust species-level taxa only.

Those 11 subspecies taxa are:

Arenaria serpyllifolia subsp. leptoclados (Rchb.) Nyman
Clinopodium nepeta subsp. glandulosum (Req.) Govaerts
Ranunculus peltatus subsp. baudotii (Godr.) Meikle ex C.D.K.Cook
Raphanus raphanistrum subsp. sativus (L.) Domin
Salsola kali subsp. tragus (L.) Celak.
Montia fontana subsp. chondrosperma (Fenzl) Walters
Ononis spinosa subsp. procurrens (Wallr.) Briq.
Pilosella piloselloides subsp. praealta (Gochnat) S.Bräut. & Greuter
Tripolium pannonicum subsp. tripolium (L.) Greuter
Viola tricolor subsp. curtisii (E.Forst.) Syme
Amaranthus powellii subsp. bouchonii (Thell.) Costea & Carretero

With the addition of mappings to World Flora Online names and authorities, the data now becomes less cryptic, and more reproducible. Thank you.

***RE****: Thank you. We have removed these 11 species and reconducted analysis in the international context. Results have been updated in the main text.*

**Comment 4.**

**4a):** I apologise to authors for mistakenly alleging they did not include fern & clubmoss species in this analysis in my first-round review, I can see now they indeed do have some fern and clubmoss species. It would be nice to include a Class-level indication for each taxon in Table S2 e.g. “Lycopodiopsida” , “Polypodiopsida” , “Pinopsida” , “Magnoliopsida” to better illustrate the diversity of the data. If not Class-level then relative to the Order-level of the APG III system?

***RE****: Thank you for your suggestion! We have given the group, order and family according to the latest APG IV in the updated S2 Table.*

**4b):** with respect to _land cover_ and entirely aquatic plant species such as the duckweed species included in this work

I think I may have also made a mistake here in assuming that water could not be a type of ‘land cover’. I now see that by normal parlance water is an accepted type of land cover.

(1) Nevertheless, I can’t help but feel that strictly aquatic-only species might have considerably different properties to strictly terrestrial-only species. Does aquatic non-natural land cover even exist? Isn’t all aquatic land cover ‘natural’ or ‘semi-natural’, and thus aren’t aquatic-only species a possible source of bias in this analysis (assuming non-natural aquatic land cover does not exist).

***RE:*** *In the Netherlands water is ubiquitous and much of the land is neither natural nor semi-natural. Small parcels of aquatic non-natural land cover occur as part of urban green and grey (small ditches, ponds, etc), crop and pasture (small ditches, etc), in fact in all land uses. Thus explaining the results.*

(2) Relatedly how do aquatic-only species fit into testing the sub-hypothesis of “forests” versus “open habitats”. “Forests” and “open habitats” seem to me to be defined on the basis of terrestriality – I think the aquatic-only species should be excluded from this sub-hypothesis analysis, as I wouldn’t expect to find duckweed in areas of predominantly “forest” land-cover.

***RE****: Indeed as you can see from table S2 the preference of duckweed spp (e.g. Lemna and Wolffia, which was mentioned by the reviewer in the first round of review) for forest is 0 or very low. By the way, the reviewer may not realize that categorizing a location as forested versus open is not based on terrestriality, it is based on the major vegetation structure (=land use type). This does not mean that 100% of a location is dense forest or completely open, some locations have both, but one as the dominant type. Also many forests in the Netherlands include small streams, ponds or ditches which are habitats for all kinds of aquatic spp (incl. for example the duckweed spp that tend to occur in nutrient rich standing water). There are very few locations in the Netherlands without water.*

(3) I also note (~line 108) that cells containing >10% of open water were excluded from the analysis – isn’t that excluding the cells where duckweed and other aquatic-only species are quite likely to be found? Does this really not bias this sub-hypothesis analysis to be including aquatic-only vascular plant species in it?

***RE:*** *10% water is 10 ha, which means a decent amount of open water. Normally, no (or few) duckweeds and other aquatic-only species live in those open waters as we explained in the last response letter. It means those removed grid cells are not highly relevant to most species. In addition, as we explained, the Netherlands consists mostly of water. Most habitats where aquatic plants grow have small streams, ditches, ponds, marsh or wetland areas rather than larger areas of open water. In this way, removing open water (>10%) grid cells will not bias this hypothesis.*

**Comment 5.** I am satisfied with the explanation given in the reply to reviewers, but please explain this in the manuscript too.

***RE****: We have indicated it in the main text (Line 179-181). Thank you!*

**Comment** 6. I am pleased that the authors have matched Dutch names to World Flora Online names. As I mention in 3b) this reveals some further issues.

***RE****: We have addressed the problem in comment 3b).*

**Comment 8.** Hmmm… I guess it’s not strictly necessary to add this information but it would certainly be helpful

***RE****: A higher-level taxonomy for each species has been added to the S2 Table.*

**Comment** 9. Why not just upload the code to Zenodo? Zenodo just not charge, it is also a not for profit organisation. Whilst I understand the data has some proprietary protections on it imposed by NDFF, your code has no such encumberment.

***RE****: Thank you for your suggestion. We feel our response, making code available upon request is sufficient and fulfills the journal’s policy.*
